# A Systematic Review on the Development of Asthma and Allergic Diseases in Relation to International Immigration: The Leading Role of the Environment Confirmed

**DOI:** 10.1371/journal.pone.0105347

**Published:** 2014-08-20

**Authors:** Báltica Cabieses, Eleonora Uphoff, Mariona Pinart, Josep Maria Antó, John Wright

**Affiliations:** 1 Universidad del Desarrollo- Clínica Alemana, CAS-UDD, Lo Barnechea Santiago, Chile; 2 Bradford Institute for Health Research, BIHR, Bradford Royal Infirmary, Bradford, United Kingdom; 3 Department of Health Sciences University of York, Heslington, York, United Kingdom; 4 Centre for Research in Environmental Epidemiology (CREAL), Barcelona, Spain; 5 IMIM (Hospital del Mar Research Institute), Barcelona, Spain; 6 CIBER Epidemiología y Salud Pública (CIBERESP), Barcelona, Spain; 7 Universitat Pompeu Fabra, Departament de Ciències Experimentals i de la Salut, Barcelona, Spain; University of Vienna, Austria

## Abstract

**Background:**

The prevalence of asthma and allergic diseases is rising worldwide. Evidence on potential causal pathways of asthma and allergies is growing, but findings have been contradictory, particularly on the interplay between allergic diseases and understudied social determinants of health like migration status. This review aimed at providing evidence for the association between migration status and asthma and allergies, and to explore the mechanisms between migration status and the development of asthma and allergies.

**Methods and Findings:**

Systematic review on asthma and allergies and immigration status in accordance with the guidelines set by the Preferred Reporting Items for Systematic Reviews and Meta-Analyses (PRISMA) statement. The pooled odds ratio (OR) of the prevalence of asthma in immigrants compared to the host population was 0.60 (95% CI 0.45–0.84), and the pooled OR for allergies was 1.01 (95% CI 0.62–1.69). The pooled OR for the prevalence of asthma in first generation versus second generation immigrants was 0.37 (95% CI 0.25–0.58). Comparisons between populations in their countries of origin and those that emigrated vary depending on their level of development; more developed countries show higher rates of asthma and allergies.

**Conclusions:**

Our findings suggest a strong influence of the environment on the development of asthma and allergic diseases throughout the life course. The prevalence of asthma is generally higher in second generation than first generation immigrants. With length of residence in the host country the prevalence of asthma and allergic diseases increases steadily. These findings are consistent across study populations, host countries, and children as well as adults. Differences have been found to be significant when tested in a linear model, as well as when comparing between early and later age of migration, and between shorter and longer time of residence.

## Introduction

There is a global public health concern regarding a rising prevalence of asthma and allergies worldwide. [1] In some European countries, up to 50% of children are reported to have an IgE sensitisation to inhalant or food allergens. [2] Evidence on potential causal pathways of asthma and allergies is growing, but findings about the gene-environment influences have been contradictory. [3]–[7] Biomedical studies continue to identify genes associated with an increased susceptibility for atopy; however, environmental factors also appear to play a key role in the exposure to allergens and the risk of developing asthma and allergies. [8] The well-known hygiene hypothesis for example suggests that early exposure of children to infectious agents and parasites protects against development of allergic diseases. [9], [10] Although most of the variation in asthma and allergies remains unexplained, the multifactorial nature of these diseases is now widely acknowledged, [6] with a complex interplay between allergic diseases and multiple social determinants of health [11], [12].

One understudied social determinant of health that might be relevant to the development of asthma and allergies is migration status. [13], [14] The United Nations have defined an international immigrant a person that moves to a foreign country and stays in the new country for a year or longer [15]. Movement of people within and between countries is an essential part of contemporary society, [16] and migration has been recognised as an important determinant of social development and global health. [17], [18] Migrant populations are useful for studies linking genetic and environmental factors, as they can be followed as natural experiments for both individual and population-based epidemiological studies. It has been accepted that a comprehensive understanding of socio-demographics and health conditions of international immigrants before and after migration can make a valuable contribute to the study of causes of communicable and non-communicable diseases in general, and the study of asthma and allergic diseases in particular [18].

According to the international evidence, migrant populations show unique health features after arrival at the new country that might shed some light on the development of asthma and allergic diseases globally. The *healthy migrant effect* for example, resulting in immigrants being healthier than the host population upon arrival, may explain initial differences in prevalences of asthma and allergies. [19] Differences generally disappear over time as immigrants are influenced by the new environment, known as the *assimilation effect*. [20] A few years after arrival, for some immigrant groups health deteriorates further and particular health impairments are found to be more prevalent in these groups than in the host population. [21], [22] Which of these effects are relevant to the development of asthma and allergies in immigrants is unclear, and exploring the social pathways involved deserves more attention to help understand potential causal mechanisms.

The aim of this systematic review is to provide evidence for the association between migration status and asthma and allergic diseases, and to explore the mechanisms between migration status and these health conditions. Thus we aimed at providing a definitive summary of the published evidence and so inform current knowledge on social pathways involved in the development of asthma and allergic diseases related to international migration. We are not aware of any other previous systematic review on this topic. A non-systematic review on asthma and allergies in migrants was conducted by Rottem and colleagues in 2005, which included data on fourteen studies published before 2003. [14] The researchers concluded that immigrants from low-income countries migrating to Western countries have an increased risk of developing asthma and allergies, and they attributed this to lifestyle and environmental factors.

In this review we focused on addressing the following research objectives:

1. To examine whether there is any difference in the prevalence rate of asthma and allergic diseases between immigrants and the host populations when living in the foreign country
2. To examine whether there is any difference in the prevalence rate of asthma and allergic diseases between people in their country of origin and those that migrated to a new one
3. To examine the effect of length of stay in a foreign country in the rate of asthma and allergic diseases among immigrants living in a foreign country
4. To examine the effect of country of origin in the rate of asthma and allergic diseases among immigrants living in a foreign country

## Materials and Methods

### Search strategy

**Figure 1:**
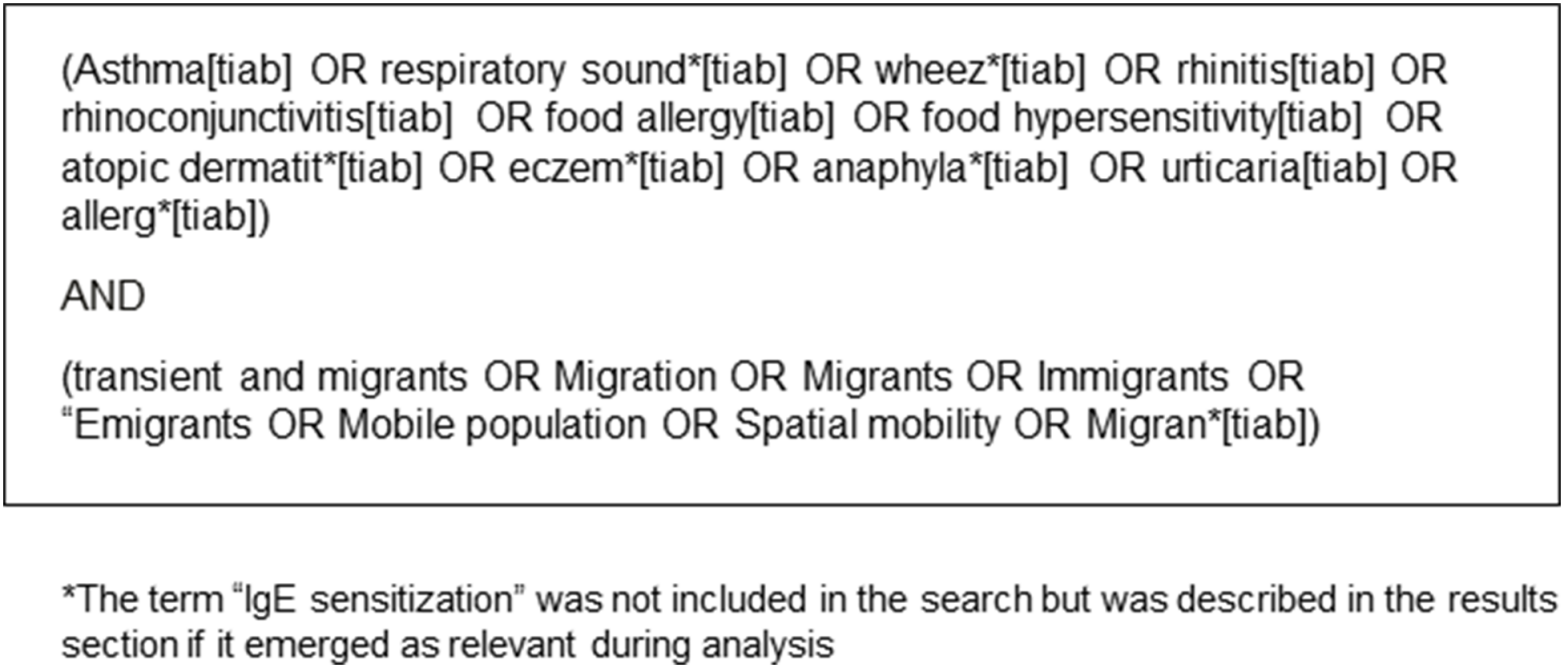
Search terms and equations used for this review in the PubMed database in March 2013.

We conducted a systematic review on asthma and allergic diseases and immigration status in accordance with the guidelines set by the Preferred Reporting Items for Systematic Reviews and Meta-Analyses (PRISMA) statement. [23] A protocol was designed before the review started and can be accessed at PROSPERO (http://www.crd.york.ac.uk/PROSPERO/, registration No. CRD42014008883) and BiB (http://www.borninbradford.nhs.uk/) web pages. This protocol has two main aims, one to explore the relationship between migration status and asthma and allergies, and a second one exploring the effect of socioeconomic status on the prevalence of these conditions. This manuscript refers to the first aim only, as the second one was being developed at the time this article was submitted for publication. In **step 1**, the search was conducted in the PubMed database in November 2012 and we chose broad string and MeSH terms in our search to include any definition of asthma and allergies, and migration status as follows in Figure 1. In order to include all relevant evidence available on this topic, we applied no timeframe or language filter. The titles and abstracts from all hits found during step 1 can be found in Figure S1.

### Papers selection and retrieval process

In **step 2** titles and abstracts of all hits were screened by two members of the research team (BC and EU) for key words related to asthma and/or allergies and international migration. A random sample of 20% of the studies was selected for double-checking. In **step 3** full-text papers were retrieved, and for those manuscripts not accessible via institutional licenses the authors were contacted via email. In **step 4**, all full-text papers were reviewed by two independent reviewers (BC and EU) and a final decision upon their inclusion or exclusion in this review was made based on the following inclusion and exclusion criteria:

- Inclusion criteria: Studies using any type of epidemiological design addressing the association between international immigration and the prevalence of asthma and/or allergies.
- Exclusion criteria: (a) Population: mixed sample of children and adults if results are not presented separately, (b) Objectives: (i) assessed biological aetiology of asthma and allergies (genomics, proteomics, transcriptomics and metabolomics), or (ii) severity, treatment and access to services on asthma and allergies, (c) Type of articles: experimental studies, genetic analysis.

Discussion papers and reviews were included to provide background to our systematic review, but were not included in the data extraction as they did not provide primary data. Studies which, upon extraction of the data in **step 5**, were found not to fit the inclusion criteria were not included in the results of this review.

### Data extraction

The data extraction form was designed in Microsoft Access, assessed by an independent reviewer (MP) and piloted with 10 articles, which lead to minor modifications (Figure S2). Assessment of the study quality was based on the guideline ‘Strengthening the Reporting of Observational Studies in Epidemiology’ (STROBE) Version 4 and the Mixed Methods Appraisal Tool (MMAT) Version 2011, and incorporated into the data extraction form. [24], [25]. Following these guidelines, quality assessment considered seven dimensions: data sources, representativeness of the sample, sample size, measures, method of analysis, adjustment for confounders, and any other comment (open-ended question). The six first dimensions were responded as binary variables (yes: it appears in the article; no: it does not appear in it) and the last one would provide additional input of the paper. We then a created an overall quality measure integrating the first six binary measures as follows: “+” would represent 1–3 dimensions were reported as yes; “++”would represent 4–5 dimensions were reported as yes; and “+++”would represent all 6 dimensions were properly reported in the paper. Good quality papers were then conceived as manuscripts with all 6 dimensions clearly available during data extraction process.

### Data analysis

We presented studies’ findings using Odds Ratios (OR) in forest plots as a descriptive approach to show the associations between being an immigrant and the prevalence of asthma and allergies, compared to the host population and the population in their countries of origin, respectively. A single descriptive pooled OR was estimated from the papers that had reported the prevalence of asthma and allergies as a synthesis of available data. Given the large heterogeneity between study settings, immigrant populations and comparison groups, and health measures, we did not perform a meta-analysis of selected studies [26]–[28]. Based on our four research questions we conducted narrative analysis and an extensive review of the quality of each study instead. Narrative analysis during data extraction was based on the unique dimensions of the PRISMA. It included study design, level of analysis, country in which the study was conducted, age category of the study sample (children, adults, both), recruitment period, any additional information about the sample (e.g. proportion of non-responders, random or convenience sample, etc.), health measure of the study and way of measurement (clinical diagnosis, lab test, etc.), migration status measure and its data source, reported significant and non-significant results and conclusions of the study.

## Results

**Figure 2:**
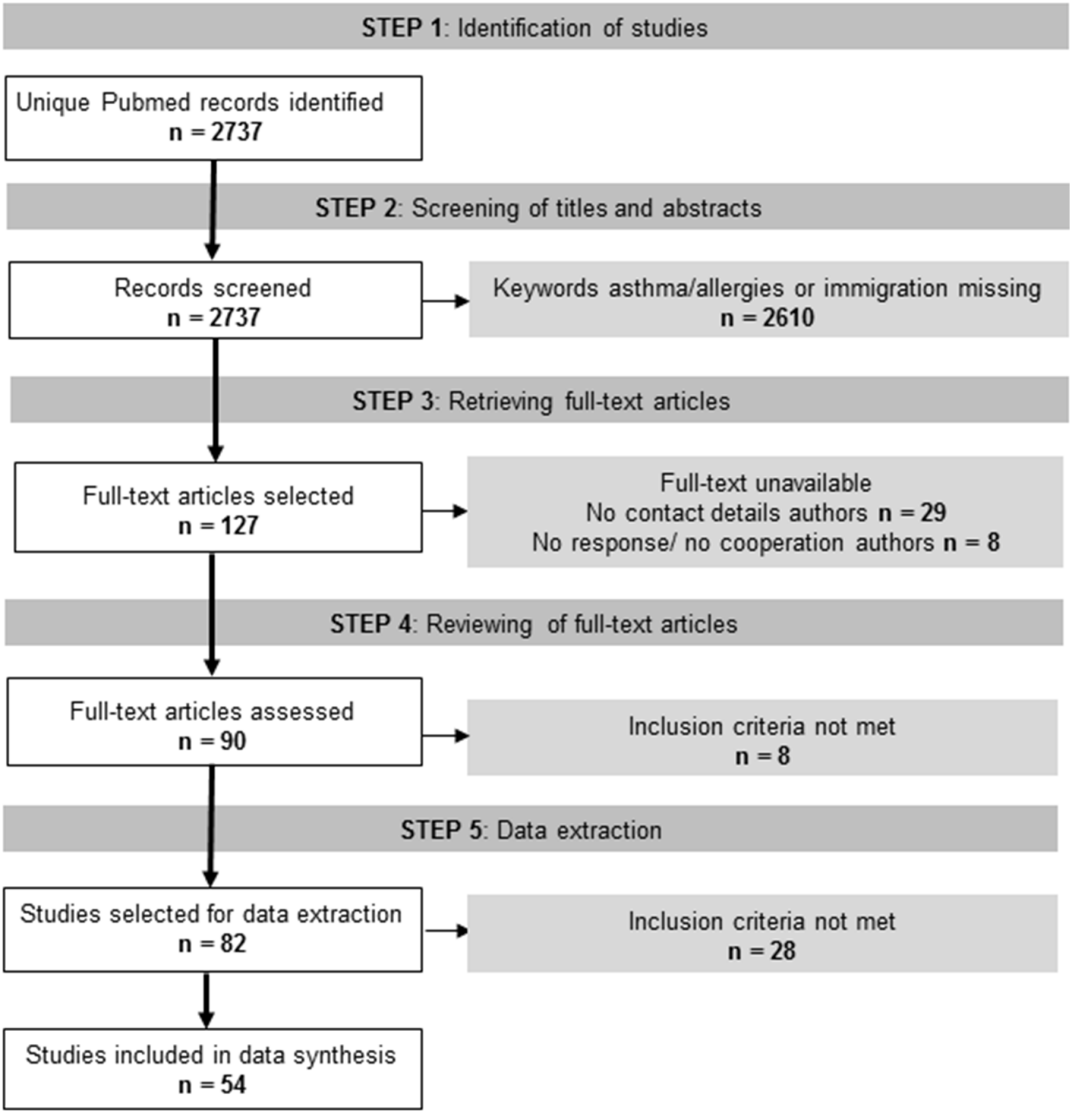
The PRISMA Flowchart.

Figure 2 shows the PRISMA flowchart of the process followed for the identification and selection of studies in detail. From the total of 2737 unique hits identified through the search, 127 studies were selected for a full-text review. There was agreement between researchers after double-checking for all but two abstracts. Initially 87 papers could be retrieved in full-text, and after contacting authors 90 papers were obtained in total. This excluded 37 papers, mainly representing older studies published before 1975 and back to 1954, for which no electronic version was available and authors’ contact details could not be traced. After reviewing full-text papers, 54 studies were selected for data extraction and synthesis. Table S1 displays a summary table of the 54 papers included in this review.

### Overview of studies

Of the included studies 76% (41/54) were published in the last decade. Together these 54 studies cover 11 483 094 observations, plus a large sample from the Israeli military registry for which a sample size was not reported. [29] Ten studies counted less than 200 observations, with the smallest one relying on only 25 observations. The majority based findings on more than 2000 observations, which includes six large cohort studies with sample sizes over 400 000. Studies were predominantly cross-sectional designs or longitudinal cohort studies analysed in a cross-sectional manner (n = 48), and six studies analysed longitudinal data retrospectively. Medical diagnoses extracted from medical records or results of a skin prick test (n = 27) were the commonest method for diagnosis followed by self-reported or parent-reported (n = 19) asthma. Other measures used were asthma severity questionnaires and use of medication.

**Figure 3:**
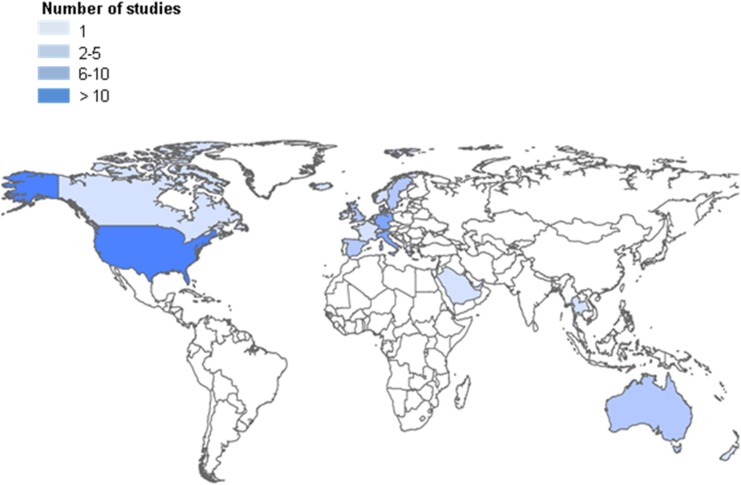
Map of countries in which included studies on asthma or allergies and migration were conducted.

**Figure 4:**
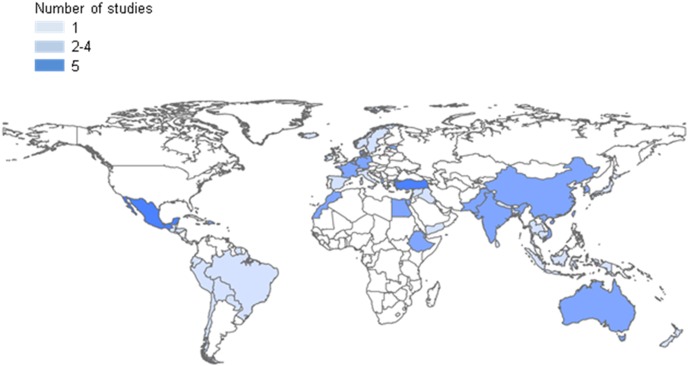
Map of countries of origin of immigrants, based on available data from included studies on the relationship between asthma or allergies and migration.

Figures 3 and 4 depict the countries immigrants moved to and countries of origin in a graphical representation. Most studies were set in Western countries, such as the United States (US) (n = 18), and European countries (n = 26). Germany was well represented with six studies; however, the United Kingdom, a country with a history of recent migration due to colonial relationships, is represented by only three of the studies included. The other 10 studies come from unique different countries. Immigrants from all regions of the world are included, representing the countries of Ethiopia, Albania, Korea, Japan, Vietnam, Turkey, Morocco, China, Mexico, and Egypt, and more globally studies focussed on the regions of South Asia, Northern Africa, Eastern Europe and Latin America.

### Quality of studies

Detailed information about the quality of each study can be found in Table S2. Six separate columns in the data extraction form assessed quality of data. In general, the quality of the studies was limited. Fair to poor quality of study design, analysis and reporting was found in older studies as well as more recent ones, in smaller and larger studies, and it was not associated with studies from any particular region or country.

Various papers missed what we consider basic elements of a study report. Some authors failed to discuss the generalizability of study findings (n = 8), and in older papers sources of funding were often not reported (n = 22). In addition, we argue that for some of the studies, the sample was not representative of the study population due to selection bias, a low response rate or drop-outs. One study title for example purported to describe allergic diseases in the general immigrant population, but only data from patients admitted to allergy clinics was used [30].

When looking at the risk of bias across studies, the measurement or identification of immigrant status was problematic in a number of cases. One study based in the United States used ‘Asian surname’ as an indication of immigration status, [31] while other studies did not differentiate between first- and second-generation immigrants or did not specify their ‘immigrant’ variable. [32]–[36] Regarding the measurement of asthma and allergic diseases, these were largely self-reported diagnosis or symptoms, with the risk of recall bias. Papers reporting skin prick test also face the risk of bias across studies due to differences in laboratory procedures between cities and countries.

### Differences in prevalence between immigrants and the host population

**Figure 5:**
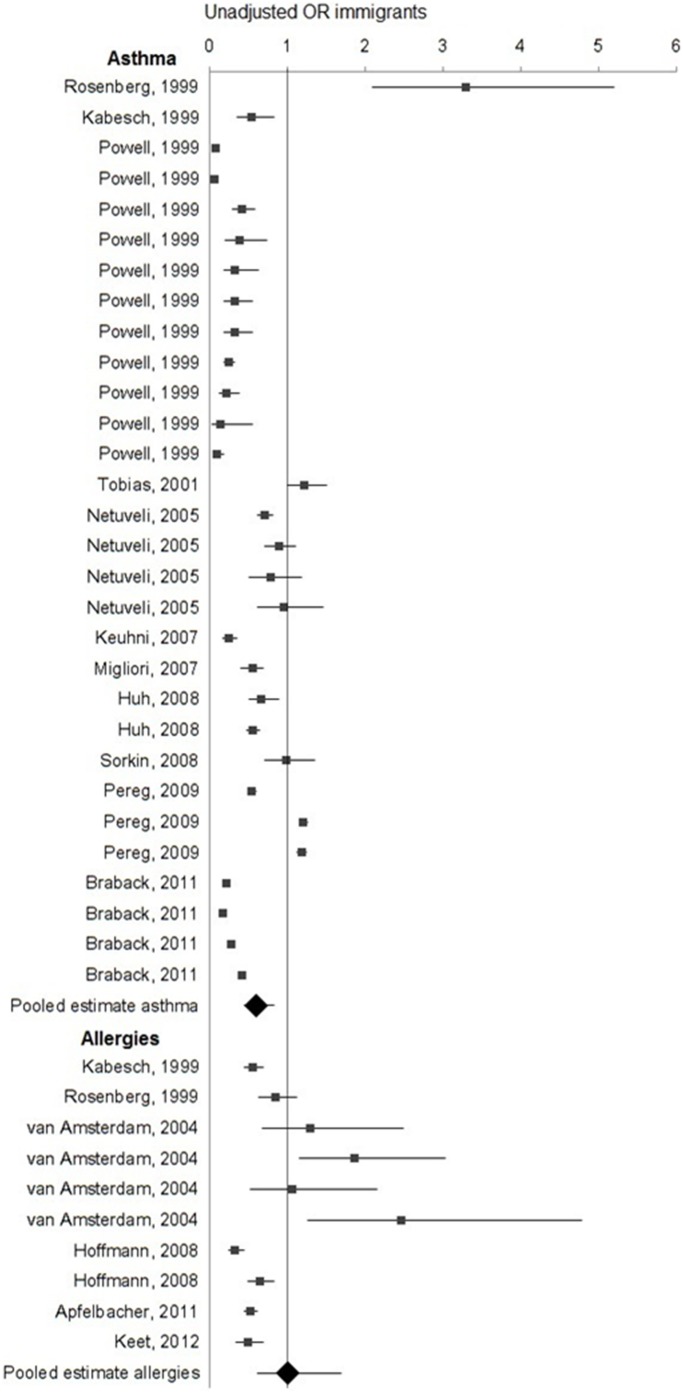
Forest plot estimating the difference in prevalence of asthma and allergic diseases between immigrants and the host population.

Most studies analysed the prevalence of asthma and allergies for immigrants from low to middle income countries to high income countries. Taking into consideration this setting of the research, results generally seem to indicate a lower rate for immigrants than for non-immigrants in the host population. Just as a descriptive summary of findings, Figure 5 illustrates the forest plot for all studies comparing the prevalence of asthma and/or allergies between immigrants and non-immigrants in the host population, for which Odds Ratios (ORs) could be identified in the paper or calculated from reported results (n = 15, Table 1). Regarding the prevalence of asthma, the summary pooled OR for immigrants showed this group is less likely to report asthma compared to the host population (pooled OR 0.60, 95% CI 0.45; 0.84), but there was no significant difference between the groups when looking at the prevalence of allergies (pooled OR 1.01, 95% CI 0.62; 1.69).

**Table 1:** Differences in prevalence of asthma and allergies between migrants and the host population.

Firstauthor	Year ofpublication	Studydesign	Sample	Healthmeasure	Crudeprevalence	Risk estimateimmigrants*
Rosenberg	1999	Cross-sectional case-	302 JewishEthiopianadults whomigratedto Israel as achild and 304	Doctor-diagnosedasthma andallergies;	*Asthma*Immigrants: 17.0%;Non-immigrants:5.8%	Asthma unadjusted:OR 3.30 (2.10–5.21);Allergies
		control	matched non-immigrantJewish Israeli.	medical records	*Allergies*Immigrants: 33.0%;Non-immigrants:37.0%	unadjusted: OR 0.85(0.63–1.13)
Kabesch	1999	Cross-sectional	5481 childrenliving inGermany; 451of Turkishorigin (first orsecond	Doctor-diagnosedasthma andatopic	*Asthma*Immigrants: 5.3%;Non-immigrants:9.4%	*Asthma* Unadjusted: OR0.54 (0.36–0.83); Adjusted:OR 0.53
		survey	generation)	sensitization;reported byparents	*Atopic sensitization* Immigrants:24.7%; Non-immigrants: 36.7%	(0.30–0.94). *Atopic* *sensitization* Unadjusted:OR 0.56 (0.45–0.70);Adjusted: OR 0.73(0.55–0.96)
Powell	1999	Cross-sectionalsurvey	Adolescents andyoungadults: 6682Australian,8496 immigrantsfromvarious regions.	Parent-reportedwheezeand asthmaattacksover 12 months	*Immigrants*West/NorthEurope/US/NewZealand:8.9%; Africa:6.8%;South East Asia:8.9%;Southern Europe:8.4%;South/CentralAmerica: 7.4%;Middle East/NorthAfrica:7.2%; South Asia:7.2%;Indochina: 5.5%;NorthEast Asia: 5.0%;Oceania:3.1%; EasternEurope: 2.1%	*Unadjusted:* West/NorthEurope/US/New Zealand:OR 0.08 (0.06–0.11);Africa: OR 0.07 (0.05–0.11);South East Asia: OR 0.41(0.29–0.58); Southern Europe:OR 0.39 (0.20–0.74);South/Central America:OR 0.33 (0.18–0.63);Middle East/North Africa:OR
					*Non-immigrants*19.2%	0.33 (0.19–0.56); South Asia:OR 0.33 (0.19–0.55); Indochina:OR 0.25 (0.19–0.33); North EastAsia: OR 0.22 (0.13–0.38);Oceania: OR 0.14 (0.03–0.56);Eastern Europe: OR 0.09 (0.04–0.19)
Tobias	2001	Cross-sectionalsurvey	Adults living inAustralia,Europe, USAand NewZealand. 17838non-immigrantsand1678 immigrants.	Self-reportedasthmasymptoms	*Immigrants* *** Australia16.8%; France 12.7%;Netherlands 5.8%;New Zealand 12.8%;Norway 9.8%; Sweden8.7%; Switzerland 12.0%;UK 8.1%; USA 8.6%;	*Unadjusted*Australia: OR 2.18(1.25–3.80); France:OR 1.33(0.90–2.00); Netherlands: OR1.16(0.35–3.87); NewZealand: OR 1.03(0.63–1.69); Norway: OR 1.71(0.64–4.58);Sweden:
					*Non-immigrants* Australia10.2%; France 11.0%;Netherlands 5.9%;New Zealand 14.1%;Norway 6.1%; Sweden8.2%; Switzerland 8.5%;UK 12.5%; USA 11.5%	OR 1.09 (0.65–1.83); Switzerland:OR 1.56 (0.93–2.63); UK: OR0.63 (0.28–1.38);USA: OR 0.67(0.20–2.28)
						Total immigrants: OR 1.21(1.00–1.51)
van Amsterdam	2004	Cross-sectional	School children livingin the Netherlands:241Dutch parents,271	Sensitization;positiveskin prick test	*Immigrants* *(2nd generation)*Turkish 23.6%;Moroccan30.6%; Surinam 60.0%;Other 37.0%	*Unadjusted* Turkish OR 1.30(0.68–2.49); Moroccan OR 1.87(1.15–3.04); Surinam OR 1.06
			2nd generationimmigrants: 68Turkish, 134Moroccan,20 Surinam, 49 other.		*Non-immigrants*19.1%	(0.52–2.15); Other OR 2.46(1.27–4.78)
Netuveli	2005	Cross-sectionalsurvey	405547 Whites, 5688South Asians,2508Afro-Caribbean and1785 ‘others’livingin the UK	Asthmaconsultation;medical records.	*Immigrants* **White: 19.8;South Asian: 24.6;Afro-Caribbean:22.0;Other: 27.8	*Unadjusted* White non-UK born:OR 0.71 (0.62–0.81); SouthAsian: OR 0.89 (0.71–1.11);Afro-Caribbean: OR 0.78
					*Non-immigrants*:27.1	(0.51–1.19); Other: OR 0.95(0.61–1.46)
						*Adjusted* White non-UK born:OR 0.82 (0.69–0.97);South Asian: OR 1.33(1.04–1.70); Afro-Caribbean:OR 0.96 (0.57–1.60); Other:OR 0.80 (0.40–1.61)
Kuehni	2007	Cross-sectional	4848 Whitenon-immigrantyoung women, 477South Asianwomen	Self-reportedasthmaor wheezing	Immigrants: 6.5%	Unadjusted: OR 0.25(0.17–0.36)
			immigratedaged 5 years orolder		Non-immigrants:21.8%	Adjusted: OR 0.24(0.16–0.35)
Migliore	2007	Cross-	Children andadolescentsliving in	Parent-reportedasthma	Immigrants: 5.4%	Unadjusted: OR 0.55(0.40-
		sectional	Italy: 26245non-immigrants,2058 childrenwith migrantparent (s),1012 1stgenerationimmigrants.		Non-immigrants:9.8%	0.70)
Huh	2008	Cross-sectional	46318 adultsliving in theUS: 35370US-bornnon-HispanicWhites, 1290	Self-reportedasthma	Asian immigrants:4.2%;Hispanicimmigrants: 3.5%	Unadjusted Asians:OR 0.67 (0.51–0.89)
		survey	Asianimmigrants, 250US-bornAsians, 5566Hispanicimmigrants,		Non-immigrants:6.1%	Unadjusted Hispanics: OR0.56 (0.48–0.65)
			3842 US-bornHispanics.			Adjusted Asians: RRR 0.58(0.44–0.77)
						Adjusted Hispanics: RRR0.51 (0.44–0.61)
Sorkin	2008	Cross-	Older adultsliving inthe US: 359	Doctor-diagnosed	Immigrants:20.9%	Unadjusted: OR 0.98(0.71-
		sectionalsurvey	Vietnamese and25177non-HispanicWhites.	asthma; self-reported	Non-immigrants:11.9%	1.36)
Pereg	2009	Cross-sectional	17 year oldsliving inIsrael: 1317556native-bornIsraeli, 16007Ethiopian,39109 fromWestern countries,	Doctor-diagnosedasthma andsymptoms;medical records	*Immigrants*Ethiopia: 2.6%;Western countries:5.6%;Former SovietUnion: 4.8%	*Unadjusted* Ethiopia OR 0.54(0.49–0.60); Western countries:OR 1.20 (1.15–1.26); Former
			93982 FormerSoviet Union.		Non-immigrants: 4.7%	Soviet Union:OR 1.18 (1.12–1.24)
Braback	2011	Cross-sectional	Adolescents/youngadults:1770092non-migrant,24252 internationaladoptees,40971 2^nd^generation	Purchase of‘inhaledcortisone’	*Immigrants* Eastern Europe:1.8%; East Asia: 1.4%;South Asia: 2.2%; LatinAmerica: 3.3%	*Unadjusted* Eastern Europe:OR 0.22 (0.20–0.25); EastAsia: OR 0.17 (0.15–0.20);South
			immigrants and479861st generationimmigrants		*Non-immigrants:*7.5%	Asia:OR 0.28 (0.24–0.32);Latin America: OR 0.42(0.38–0.47); *Age and* *sex-adjusted* Eastern Europe:OR 0.34 (0.25–0.57); EastAsia: OR 0.19 (0.16–0.22);South Asia: OR 0.31(0.27–0.36); Latin America:OR 0.48 (0.43–0.54)
Hoffmann	2008	Cross-sectional	965 childrenliving inGermany; 424childrenwith immigrantbackground	Sensitisation testsand doctor-diagnosed	*Allergic diseases* Immigrants:17.2%; Non-immigrants:38.5%	*Allergic diseases* Unadjusted:OR 0.33 (0.24–0.45)
			(1st or 2ndgeneration).	parent-reportedallergic diseases	*Sensitization*Immigrants:52.2%; Non-immigrants:63.0%	*Sensitisation* Unadjusted:OR 0.64 (0.49–0.83)
Apfelbacher	2011	Cross-	Children livingin Germany:14640	Eczema	Immigrants:8.0%	Unadjusted OR 0.52(0.45–0.61)
		sectionalsurvey	non-immigrants,2550immigrants.		Non-immigrants:14.3%	Adjusted OR 0.63(0.49–0.80)
Keet	2012	Cross-	Children andadolescentsliving in the	Foodsensitization;IgE	Immigrants:11%	Unadjusted
		sectionalsurvey	US: 2495 non-immigrants,714 2ndgenerationimmigrants,341 1stgenerationimmigrants.	tests	Non-immigrants:20%	OR 0.49 (0.34–0.69)

* Odds Ratios for first-generation immigrants compared to the host population unless stated otherwise.

** Mean number of new asthma consultations/1000 patient years.

*** Prevalence and risk estimates only reported for groups with ≥50 immigrants.

Studies that found a higher rate of asthma and allergies for immigrants than for the host population generally represented smaller samples or atypical settings. We identified great differences in the literature regarding types of immigrant communities across the world. Some high-skilled immigrants might migrate voluntarily in the search of a better life, whilst other might be involuntarily running away from civil war and corruption. The immigrants in the study by Rosenberg and colleagues for example were Jewish Ethiopians with a traumatic immigration experience, which involved living in primitive refugee camps prior to arrival. [37] Upon arrival, many immigrants suffered from anaemia and malnutrition. Another study compared immigrants to non-immigrants without specifying country of origin. [38] This is a complex challenge for data synthesis, since immigrants are well known for being a very heterogeneous group. Country of origin is a key variable to include in migration studies and the lack of information about this variable affects the external validity and limits the usefulness of its results. The third outlier is the study by van Amsterdam and colleagues, which reports on allergies in school children living in the Netherlands, with an immigrant group consisting mostly of Turkish and Moroccan children. [39] These children however were born in the Netherlands, and given that the Dutch prevalence of asthma and allergies seems low across studies compared to other Western countries, these findings may simply reflect the general prevalence in the Netherlands. Further results of variations between first and second generation immigrants are reported in the next section.

Six studies comparing asthma or allergies between immigrants and the host population are not shown in Figure 5 because crude prevalence figures were not reported, or sample sizes were missing, so no OR could be derived from the paper. For this reason, these papers were excluded from pooled estimates. Farfel and colleagues reported a lower prevalence of asthma in all immigrant groups compared to the Israeli population, but a higher prevalence of allergic rhinitis for immigrants from the Soviet Union. [29] A German study reported that there was no difference in rates of asthma, but did not specify whether the host population was compared to immigrants born in the country of origin or in Germany. [33] The remaining four studies found lower prevalences of asthma and allergies for immigrants than for the host populations [34], [40]–[42].

### Changes over time in prevalence of asthma and allergies

**Figure 6:**
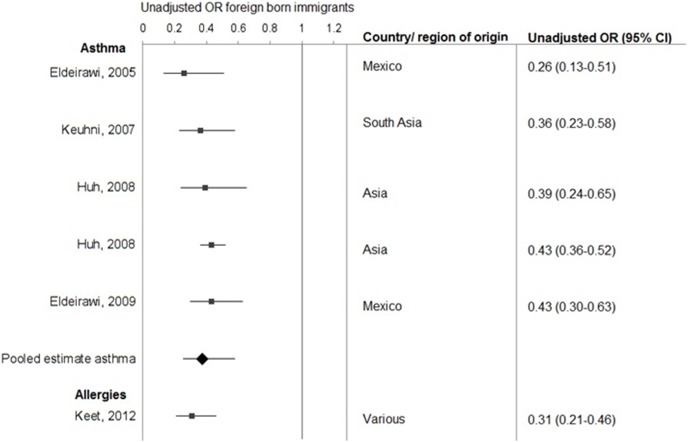
Forest plot estimating the difference in prevalence of asthma and allergic diseases between first generation immigrants and those born to foreign parents (second generation immigrants).

Studies on differences over time in the prevalence of asthma and allergies for immigrants typically rely on one of two methods or a combination of both: some compare prevalences between foreign born immigrants and people born to immigrant parents (Figure 6), and others study the difference in prevalence between immigrants in relation to time of residence in the host population (Table 2). Figure 6 shows that the studies comparing between first- and second-generation immigrants consistently find lower rates for first generation immigrants (pooled OR asthma 0.37; 95% CI 0.25–0.58). These studies seem to support an *assimilation effect* of second generation immigrants compared to their foreign-born parents, but this evidence is limited, particularly for allergic diseases. Three studies reporting on asthma in US Hispanic samples report very similar prevalence rates for US born and foreign-born Hispanics. [43]–[45] The prevalence of asthma and allergies in second generation immigrants is similar to the prevalence found in the host population.

**Table 2:** Studies comparing prevalences of asthma and allergies between immigrants with different duration of residence in host country.

Firstauthor	Year ofpublication	Studydesign	Sample*	Healthmeasure	Findings**	Conclusion:Higherlength of residenceassociated withhigherprevalence
Hjern	1999	Cross-sectional	1901 adoptedyoung men livingin Sweden.	Doctor diagnosedasthma, hayfever,eczema; medicalrecords.	Those adopted beforetwo years of age had ahigher risk of asthmathan those adopted laterin life (OR 2.04, 95% CI1.41–2.95), a higher riskof hayfever (OR 1.65, 95%CI 1.32–1.84) and eczema(OR 1.88, 95% CI 1.17–3.02).	Yes
Ventura	2004	Cross-sectional	152 Albanian immigrantsliving inSouthern Italy.	Self-reported asthma.Allergies; skin pricktest.	Duration of residence inItaly was positivelyassociated with an increasedrisk of hay fever (p<0.01),pollen sensitivity (p<0.05) andrhinitis-nasal allergy (p<0.05).	Yes
Pereg	2009	Cross-sectional	17 year oldsliving in Israel:16007 Ethiopian,39109 fromWesterncountries,93982 FormerSoviet Union.	Doctor-diagnosedasthma andsymptoms;medical records	Age of migration wasnegatively associated with theprevalence of asthma forimmigrants from Ethiopia(p<0.01) and the Former SovietUnion (p<0.0001), but not forimmigrants from Westerncountries.	Yes; somegroups
Johnson	2005	Cross-sectionalsurvey	618 Arab-AmericanUS residents.	Self-reportedasthma	Prevalence of asthma waslower for people who hadlived in the US for 1–10 yearscompared to those who hadlived there for over 10 years(OR 0.51, 95% CI 0.32–0.82).	Yes
Keet	2012	Cross-sectional survey	Children and adolescentsliving in the US: 341 1stgeneration immigrants.	Food sensitization;IgE tests	Children who had arrived inthe US before 2 years of agehad a higher odds of foodsensitization than those whoarrived later (OR 2.68, 95% CI1.19–6.08).	Yes
Keuhni	2007	Cross-sectional	89 South Asian womenimmigrated between age0–4, 135 between age 5–14,and 342≥15 years of age.	Self-reportedasthma or wheezing	Asthma prevalence seemed toincrease with duration ofresidence in the UK, althoughthe difference betweencategories was not significant.	Notsignificant
Migliore	2007	Cross-sectional	Children and adolescentsliving in Italy: 1012 1stgeneration immigrants.	Parent-reportedasthma	For each additional year ofresidence in Italy, there was a12% increase in the odds ofasthma among immigrantchildren (OR 1.12, 95% CI1.02–1.25).	Yes
Burastero	2011	Retro-spective	395 adult immigrants livingin Italy who attended thehospital.	Doctor-diagnosedasthma and allergy;medical diagnosis,skin prick test	A positive correlation wasfound between number ofsensitizations and time ofresidence in Milan (p<0.01).	Yes
Powell	1999	Cross-sectionalsurvey	8496 adolescent and youngadult immigrants fromvariousregions living in Australia.	Parent-reportedwheeze and asthmaattacks over 12months	Longer time of residence inAustralia was associated witha higher prevalence of wheeze(p<0.001).	Yes
Eldeirawi	2009	Cross-sectional	919 Mexican born childrenliving in the US.	Parent-reporteddoctor diagnosedasthma.	Children who moved to the USbefore the age of 2 were morelikely to report asthma thanthose who moved at an olderage (OR 2.08, 95% CI1.00–4.35).	Yes
Braback	2011	Cross-sectional	24252 international adoptees and 47986 other immigrants living in Sweden.	Purchase of‘inhaled cortisone’	Compared to those adopted atage 0, those adopted laterwere less likely to usecortisone. Age at adoption1–2 OR 0.72 (0.61–0.83),age 3–4 OR 0.52 (0.43–0.64)and age ≥5 OR 0.32(0.26–0.39). A similarcorrelation was found forage at migration. Comparedto age 0–4 years, the oddswere lower for 5–9 years OR0.77 (0.70–0.85), 10–14 yearsOR 0.49 (0.41–0.59) and ≥15years OR 0.33 (0.26–0.42).	Yes
Wang	2008	Cross-sectional	475 Chinese immigrantchildren living in Canada.	Doctor diagnosedasthma; medicalrecords.	Children who had lived inCanada less than 7 years wereless likely to be diagnosedwith asthma than those wholived in Canada for ≥7 years:OR 0.54 (0.28–1.05).	Yes

* Only subsamples of first-generation immigrants reported in this table.

** Significance levels reported for linear trends unless stated otherwise.

Table 2 shows that the overwhelming majority of studies assessing time trends in asthma prevalence after immigration find that a longer duration of residence in the host country is associated with a higher prevalence of asthma among immigrants when compared to similar age groups. This association has been demonstrated for various immigrant groups in Europe, the US, Australia and Israel, and none of the studies have showed a trend in the opposite direction. These findings could be explained by labelling bias in the foreign country (i.e. different ways or definitions to categorize people with asthma or allergy groups over time), changes in the availability or affordability of healthcare services in the foreign country over time, and increased accuracy of diagnosis over time, but they could also suggest that adaption to a new environment in the host country increases the rates of asthma and allergies, although it should be noted that all of these represent industrialized Western countries. Some but not all studies adjusted for potential confounders. One of the studies for example included maternal exposure to animals, parental asthma and allergies, and a history of infections, which did not eliminate the difference between immigrants and non-immigrants [45].

Several studies on time trends in the prevalence of asthma and allergies are not reported in the tables, because prevalence figures or rate estimates were missing. The study by Ormerod and colleagues showed a lack of association between asthma prevalence and duration of residence in the UK. [46] Johnson and colleagues did not report crude prevalences or unadjusted ORs, but their regression analysis showed that asthma prevalence was lower in Arab immigrants who arrived in the past 1–10 years than in American Arabs who had lived in the US for more than 10 years or where born in the US (OR 0.51, 95% CI 0.32–0.82). [47] Dumanovsky and Matte did not report details on the sample sizes for each subgroup and unadjusted ORs. [48] The prevalence of asthma attacks reported by foreign born Hispanics was lower than the prevalence reported by US born Hispanics living in New York City (4.8% versus 10.1%). Another study that did not specify country of birth for immigrants reported a higher rate of allergies for immigrants arriving after first grade compared to those who arrived earlier (OR 1.31, 95% CI 1.08–1.59) [49].

### Differences in prevalence between immigrants and non-immigrants living in the country of origin

Based on currently available data, as most of the international immigration analysed involves moving from low-income countries to Western countries, immigrants are found to have a lower prevalence of asthma and allergies - a healthy migrant effect. Also, a longer stay in the host country or being a child of immigrant parents is associated with increasing adaption to the new environment, and therefore the prevalence of asthma and allergies could over time converge with the prevalence in the non-immigrant host population. This idea of an assimilation effect of migrant populations in the host country assumes that other factors such as the availability of healthcare services and relevant socio-demographic factors remain constant.

A third aspect in the prevalence of asthma and allergies in international immigrants is to understand how and why the process of migration affects illness. The studies identified in this review are unable to answer this question. No longitudinal studies assessing the health of immigrants before, upon and after arrival were identified. The next best study design to address this question is a comparison between immigrants and non-immigrants living in the country of origin. The study by Sakai and colleagues is one of the few to be conducted in this way. [50] However, results of this study are difficult to interpret due to a very specific subpopulation of patients identified through hospitals and medical clinics. The prevalence of doctor-diagnosed asthma was very low for Japanese children in Thailand (1.5%) and very high for Japanese children living in Japan (22.6%), resulting in an OR of 0.07 (p<0.0001). The findings are contrary to those of the worldwide ISAAC survey Phase 3 outcomes, in which the prevalence of asthma symptoms among adolescents was estimated at 13.0% in Japan and 11.6% in Thailand. [51] Wang and colleagues compared the prevalence of asthma and wheezing in Chinese adolescents in China, Chinese adolescents born in Canada, and Chinese adolescents who immigrated to Canada. The prevalence of current wheeze and asthma was higher for those living in Canada than for those living in China, and differences persisted after adjusting for environmental variables [52].

## Discussion

### Summary of key findings

The main finding emerging from this systematic review of the literature is that the prevalence of asthma and allergic diseases in immigrants is lower than in the host country, and over time converges with the general prevalence in the local population. Our findings suggest a strong influence of the environment on the development of asthma and allergies throughout the life course, but findings could be, to some extent, limited by the quality of measurement of migration status and diagnosis of these conditions between populations and over time. The prevalence of asthma is generally higher in second generation than first generation immigrants, and with length of residence in the host country the prevalence of asthma and allergies increases steadily. These findings are consistent across study populations, host countries, and children as well as adults. Differences have been found to be statistically significant when tested in a linear model, as well as when comparing between early and later age of migration, and between shorter and longer time of residence.

### Comparison with international literature

Several different hypotheses of the development of asthma and allergies are currently available in the international scientific literature, from environmental hypotheses to genetic hypotheses. None of those include the effect of migration status as a risk factor or a moderator for asthma and allergies. This systematic review adds novel knowledge to the field as it explores how migration status and related measures like country of origin and length of stay in the country might determine the chance of developing these chronic and high-burden conditions globally. This study also identifies evidence gaps and a lack of high-quality studies in the field of migration and asthma and allergies research, particularly the lack of research describing the longitudinal trajectory of illness.

Findings from this study suggest there is a pervasive effect of the environment in the development of complex health conditions like asthma and allergies. As host populations in migration studies are in many cases found in an urban, populated Western setting, it is in this environment that the most important risk factors are likely to be found. Research has previously identified some of these factors, such as air pollution, heavy traffic, urban areas, and damp and mould at home, are associated with asthma and allergies. [53]–[55] These environmental influences seem to act across the life course, from the critical period of early life in which childhood asthma develops (i.e. the hygiene hypothesis) continuing into adulthood.

Whether the process of immigration itself increases the risk of asthma and allergic diseases remains unclear, as current evidence is scarce to fully support this hypothesis. One study suggests that traumatic experiences and distress during immigration may heighten the vulnerability of immigrants in a new environment leading to increased odds of developing asthma and allergies. [37] Evidence from longitudinal data included in this review generally indicates that the prevalence of asthma and allergic diseases in migrants rises to a level very similar to that of the host population, and does not exceed it. Only in the study by Tobias and colleagues were various immigrant groups found to have a higher prevalence of self-reported asthma, [38] but the prevalence reported by both immigrants and non-immigrants in this European survey was low compared to data from other studies, such as the worldwide ISAAC survey. [51] The authors point out the limitations of the study caused by low participation rates, and small sample sizes of immigrants below fifty observations for half of the immigrant groups [38].

Additionally, differences in availability, accessibility and affordability of healthcare services in home and host countries should be considered [56]. If immigrants are more likely to see a healthcare professional in a host country than in their home country due to available infrastructure and low cost services at accessible locations, then this is crucial for the likelihood of being diagnosed with either asthma or allergies preceding migration (particularly so for migrants from developing home and developed host countries), and this could somewhat affect the prevalences observed in this review.

### Strengths and limitations of this review

The wide search strategy and conservative selection process of studies reflect our aim to include all relevant papers published on the associations between international immigration and asthma and allergies in peer-reviewed journals to this date. We have deliberately chosen to include studies with any epidemiological design, and to distinguish between studies of higher and lower quality the data extraction was accompanied by a thorough examination of the quality of the studies.

Although PubMed was the only online database used for the identification of studies, a scoping search in Scopus and through cross-referencing has identified no additional papers. Findings of this study may have been biased by the unavailability of 37 potentially relevant papers. However, abstracts suggest that these mainly represent older studies with small sample sizes, and some of these seem to focus on access to health care and use of medication rather than the prevalence of asthma and allergies. The lack of access combines with difficulties in having proper medication control, which may lead to increase in prevalence of poorly controlled asthma that may in turn lead to other allergic comorbidities. In addition, many studies relied upon data from the same international survey (ISAAC), which means results are not independent between studies.

We carefully assessed quality of evidence in every paper included in this review. However, it is relevant to consider risk of bias across studies, too. In this sense, the choice of the measure for asthma or allergy is not always justified (i.e. self-reported status versus medical diagnosis or lab test) and migration status is too often poorly defined in many of reviewed studies, which complicates careful distinctions between first- and second-generation immigrants, or immigrants from different countries of origin.

Due to great heterogeneity between studies selected in this review, we did not conduct a meta-analysis. Forest plots were only presented to describe the prevalences reported by the selected articles. Papers differed in many ways, for example in their sample characteristics, countries, types of immigrants included for analysis, health outcome measures, and levels of analysis. Despite the existence of specific statistical techniques dealing with heterogeneity (e.g. moderator, sensitivity or subgroup analysis in meta-analysis), we intended to avoid the risk of residual confounding that exists when comparing diverse and mixed quality studies. Hence, we took the most pragmatic and conservative approach, presenting all data in a descriptive fashion and allowing readers to engage in the broad yet consistent patterns found in this unique review.

### Recommendations for future research

This systematic review identifies evidence gaps and a lack of high-quality studies in the field of migration and asthma and allergies research, particularly the lack of research describing the longitudinal trajectory of illness. This should be advanced in future research on this topic, as unique findings could emerge from following exposures and health status of immigrants before, during and after migration took place. Future studies should be clear about reporting valid measures of asthma and allergies and clear definitions of migration status. In reporting migration studies should also consider country of origin, length of stay, living in rural versus urban areas, reasons for migrating to a different country, and generation as minimum variables for adjustment [57].

Most of the studies identified made use of descriptive analysis, and only rarely were prevalence rates standardised for age- and sex, or were rate estimates adjusted for factors such as socioeconomic status, second-hand smoke, residential proximity to traffic or urban versus rural residence. Although some studies provide background information on the setting of the research and the socioeconomic situation of immigrants included in the study, more careful assessment of the environmental impacts on asthma and allergies is needed using multifactorial models to help understand mechanisms involved in the development of asthma and allergies. Future studies using a more comprehensive selection of high-quality and homogeneous papers could advance this study by conducting a meta-analysis of reported prevalence estimates.

Apart from providing an overview of the current state of the evidence on international immigration in relation to asthma and allergic diseases, we hope this systematic review highlights the potential of studying migration to improve our understanding of the aetiology of asthma and allergies. Migration status is an important social determinant of health that adds nuance to research in addition to measures such as ethnicity or country of birth, and it offers a natural experiment for understanding how chronic conditions develop in the context of the environment. Findings from this review suggests that the *healthy migrant effect* and the *assimilation effect* might be relevant to the development of asthma and allergies; and exploring the social pathways involved deserves more attention to help understand potential causal mechanisms. Studying immigration status and the process of international immigration as a dynamic variable affecting health throughout the life course improves our understanding of the gene-environment interaction on the aetiology of asthma and allergies.

## Supporting Information

Figure S1 **Hits retrieved from systematic search in step 1.**
(PDF)

Figure S2 **Data extraction form.**
(DOC)

Table S1 **Summary of 54 papers included for analysis in this review.**
(DOC)

Table S2 **Summary of quality of papers included in this review.**
(DOC)

Checklist S1 **PRISMA Checklist.**
(DOC)

Protocol S1 **Protocol.**
(PDF)
