# Supplementary material for: A Systematic Review on the Development of Asthma and Allergic Diseases in Relation to International Immigration: The Leading Role of the Environment Confirmed
Source: PLoS One. 2014 Aug 20;9(8):e105347. doi: 10.1371/journal.pone.0105347 (PMC4139367; doi:10.1371/journal.pone.0105347)
Supplement: Figure S2 — Data extraction form. (DOC) [file pone.0105347.s002.doc]

**Supplementary File 2** Data extraction form, dimensions included

***General description***

ID

PMID

First autor

Year of publication

Asthma keyword

Allergic diseases keyword

Migration keyword

***Criteria for exclusion***

Exclusion (yes/no)

Reason for exclusion

***Study description***

Study design

Other design

Level of analysis

Country

Sample Size

Age and age category (children, adults)

Recruitment period

Sample info other

Health measure

Way of measurement

Migration measure

Migration_data source

Significant results

Nonsignificant results

Conclusion (high risk, low risk, no difference in risk)

***Presence of relevant information in the article*** *(yes/no)*

Rationale

Objectives

Aim/hypothesis

Hypothesis

Design

Setting

Participants

Variables

Data source

Bias reduction

Statistics

Descriptives

Outcomes

Limitations

Interpretation

Generalisability

Funding

***Quality assessment*** *(good/poor)*

Data sources

Representativeness of the sample

Sample size

Measures

Method of analysis

Adjustment for confounders

Other comments (open question)
