# Supplementary material for: A Systematic Review on the Development of Asthma and Allergic Diseases in Relation to International Immigration: The Leading Role of the Environment Confirmed
Source: PLoS One. 2014 Aug 20;9(8):e105347. doi: 10.1371/journal.pone.0105347 (PMC4139367; doi:10.1371/journal.pone.0105347)
Supplement: Table S1 — Summary of 54 papers included for analysis in this review. (DOC) [file pone.0105347.s003.doc]

**Supplementary File 3** Summary of 54 papers included for analysis in this review

| **PMID*** | **First author** | **Year of publication** | **Aim/hypothesis** | **Study design** | **Level of analysis** | **Country where study was conducted** | | | **Country of origin** | **Sample size** | **Age** | **Health measure** | **Way of measurement** | **Migration measure** | **Significant results** | **Non significant results** |
| --- | --- | --- | --- | --- | --- | --- | --- | --- | --- | --- | --- | --- | --- | --- | --- | --- |
|  |  |  |  |  |  | **US (yes 1)** | **UK (yes 1)** | **Other country** |  |  |  |  |  |  |  |  |
| 10212770 | Rosenberg | 1999 | To evaluate the prevalence and characteristics of asthma in a population of Jews of Ethiopian origin who had been in Israel for 8–17 years. | Cross-sectional | Individual | 0 | 0 | Israel | Ethiopia | 906 | 20 and older | Asthma | Clinical criteria for asthma assessed from patient files. | Country of origin | The prevalence of asthma in the patients of Ethiopian origin was 17%, compared with 5.8% in the control group. | Eosinophilia was not correlated with asthma in the immigrant group. |
| 10232429 | Kabesch | 1999 | To evaluate the prevalence of asthma and atopy in Turkish children living in Germany and to investigate the role of ethnic origin on the development of asthma and atopy in this population. | Cross-sectional | Individual | 0 | 0 | Germany | Turkey | 5481 | 9-11 | asthma, atopy | physician diagnosed asthma, skin-prick test and bronchial hyperresponsiveness test | Child's nationality | The Turkish children showed a significantly lower prevalence of asthma, atopic sensitization and hyperresponsiveness in multivariate regression models controlling for potential explanatory factors. |  |
| 10464843 | Ormerod | 1999 | To study the prevalence of asthma and ‘probable’ asthma in the Asian population in Blackburn, U.K. | Cross-sectional | Individual | 0 | 1 |  | Asia (various countries) | 1783 | All ages | Asthma and probable asthma | Patient-reported symptoms and diagnosis of asthma | Country of birth, UK born, ethnicity | Those born in the U.K. were more likely to describe regular symptoms and to be on regular treatment, but for those born abroad there was an increasing rate of symptoms and medication use with increasing duration in the country. These observations are explained by the age/sex distribution of those born in the U.K. compared to immigrants. | Asthma in the Asian population was not related to social factors. |
| 10478611 | Hjern | 1999 | To study the influence of the early childhood environment on the risk of suffering from atopic disorders as young adults. | Cross-sectional | Individual | 0 | 0 | Sweden | Various countries | 1901 | 17 | asthma, hayfever, atopy | Medical diagnosis | Internationally adopted before age of 7 | The adopted young men who came to Sweden before 2 years of age suffered from asthma, hayfever and eczema significantly more often than those who came to Sweden between 2 and 6 years of age; the risk ratios (RR) were 1.6, 2.5 and 2.1, respectively. The young men who were born in the Far East were identified as being particularly susceptible to the development of hayfever and eczema, with RRs of 1.3 and 1.7. |  |
| 10846511 | Ledogar | 1999 | Measure asthma prevalence among Latinos in the US | Cross-sectional | Individual | 1 | 0 |  | Various Latin American countries | 3015 | all ages | asthma prevalence | Self-reported (household survey) | Ethnicity | Puerto Rican individuals had a higher chance of asthma than other Latino ethnic groups (OR=0.37). This difference is most pronounced for children up to 12 years of age and for Puerto Rican females aged 25 years and older the chance of asthma is exceptionally high compared to males and other ethnic groups. | The difference in the higher odds of asthma for Puerto Ricans was not explained by age, sex, insurance status, living environment, education or household size. For the age group 13-24 year old there was no significant difference between ethnic groups. |
| 15147448 | Ventura | 2004 | Determine the relative relevance of environmental vs genetic factors in causing the reported rapid increase of the prevalence of sensitization and allergic diseases in immigrants. | Cross-sectional | Individual | 0 | 0 | Italy | Albania | 152 | 20-44 | Respiratory allergy and skin sensitization | Skin prick test and self-reported asthma | Registration as immigrant | The incidence of reported asthma was very low (1.3%). The risk of developing hay fever increased with time spent in Italy. | There was a trend towards an increased risk of allergies with more time spent in Italy, although these findings did not reach significance. |
| 15900417 | Johnson | 2005 | To identify the demographic and environmental risk modifiers for asthma in Arab Americans living in the Detroit Metropolitan area. | Cross-sectional | Individual | 1 | 0 |  | Various Arabic countries | 618 | all ages | Asthma prevalence, severity, triggers | Household Survey | Self-reported country of origin | Asthma severity was worse for those born in the US, and for those that spoke Arabic. Asthma prevalence was highest among moderately acculturated immigrants compared to new immigrants and well-acculturated immigrants. The prevalence was also higher for those who had been in the US for 1-10 years. | There does not seem to be a linear relationship between time lived abroad and the risk of asthma. |
| 16242029 | Netuveli | 2005 | We investigated possible ethnic variations in incidence of asthma episodes and in addition explored the impact of migration on risk of developing asthma. | Longitudinal (prospective) data analyzed cross-sectionally | Individual | 0 | 0 | England and Wales | Various countries | 415528 | ? | Asthma | Consultation appointments | Born outside UK | After adjusting for confounding, White non-UK born were at lower risk of a first asthma consultation than UK born and South-Asian non-UK born were at higher risk. | There was no difference in OR for UK born and non-UK born for Afro-Caribbean or groups other than White and South-Asian. |
| 16598994 | Kim | 2006 | The objective of this study is to establish important contributing environmental factors and time until onset of development of allergic rhinitis in Korean immigrants to the United States. | Longitudinal (retrospective) | Individual | 1 | 0 |  | Korea | 178 | 10-83 | Allergic rhinitis | Diagnosis obtained from medical record + skin prick test | Birth and former residents in Korea | The most commonly identified allergens in the immigrants were ragweed pollen (59%), cat pelt (44%), cocklebur pollen (41%), house-dust mites (35%), and Penicillium. These findings are similar to previous findings among allergies in non-immigrant US citizens. |  |
| 16846455 | Cataldo | 2006 | To evaluate rates, distribution, clinical features and environmental risk factors for food intolerances and allergies in immigrant children. | Longitudinal (retrospective) | Individual | 0 | 0 | Italy | Various countries | 78 | 0.5-16 years | Food intolerances and allergies | Diagnosis in hospital medical record | Born outside Italy or parents born outside Italy | About 2% of the immigrant children had one or more allergies or intolerances. | Despite differences in their origin, the clinical features of immigrant children were similar to the ones of Italian patients and among each ethnic group. |
| 17402323 | Farfel | 2007 | To examine the current prevalence of common diseases, compare the results with those of previous cohorts, and assess the influence of the massive immigration during the 1990s. | Longitudinal (retrospective) | Individual | 0 | 0 | Israel | Various |  | 17 | Asthma, allergic rhinitis | Military medical examination | Country of birth | The prevalence of asthma is significantly higher among Israeli born men and lowest for immigrants from Africa and Ethiopia. Allergic rhinitis was most common among immigrants from the Soviet Union and rare among immigrants from Africa and Ethiopia. | There was no difference in rates of severe asthma due to very small prevalence numbers. |
| 17514453 | Kamtsiuris | 2007 | To present the descriptive data of a spectrum of acute and chronic childhood illnesses. | Cross-sectional | Individual | 0 | 0 | Germany | Not specified | 17641 | 0-17 | Asthma | Self-reported | ? |  | The incidence of asthma in immigrants is 4.4% [3.7:5.2], which is not significantly different from that in non-immigrants: 4.8% [4.4:5.2]. The incidence of hay fever in immigrants seemed higher but this difference was also non-significant. |
| 21745811 | Koinis-Mitchell | 2011 | to examine associations between immigration, acculturation, family cohesion and social support networks, and asthma morbidity in a sample of Dominican and Puerto Rican caregivers residing in the mainland U.S. | cross sectional | individual | 1 | 0 |  | Puerto Rico and Dominican Republic | 232 | Jul-16 | Asthma severity; Two indexes of child asthma morbidity were used in this study, asthma-related functional limitation and number of ED visits due to asthma in the previous year. | self reported; diagnosis | caregiver nativity and the caregiver’s country of upbringing, defined as the country in which the caregiver lived most of the time until age 16 | The frequency of Emergency Department use due to asthma may be higher for children of caregivers born in Puerto Rico. Acculturative stress levels were higher for Puerto Rican born caregivers residing in the mainland U.S. |  |
| 18594153 | Lombardi | 2007 | To assess the clinical characteristics of respiratory allergy in immigrants in Brescia, Italy. | cross sectional | Individual | 0 | 0 | Italy |  | 237 |  | diagnosis of asthma or allergy in a allergy unit in Italy | clinical or test-related diagnosis | Non Italian | None, descriptive study only | All patients were referred less than 1 month after the onset of symptoms. Family history for atopy was positive in 9% and clinical history of respiratory allergy was positive in 2%. The mean time of onset of symptoms after immigration was 5.21 years, and |
| 17546500 | Huh | 2008 | To examine the physical health status of immigrants with specific considerations of Asian and Hispanic populations and explores possible mechanisms through which health outcomes of interest can be explained. | cross sectional | household | 1 | 0 |  | Non US | 46.318 |  | self reported asthma | self reported | US versus Non US | foreign born individuals reported fewer chronic diseases (hypertension, heart disease, asthma, cancer and diabetes) and had lower prevalences of various chronic diseases compared with U.S.-born whites, controlling for possible confounders and mediators |  |
| 19038014 | Sakai | 2008 | To clarify the health-related conditions of Japanese expatriate children in Thailand. | cross sectional | Individual | 0 | 0 | Thailand | Japan | 2141 Japanese in Thailand versus 545000 Japanese in Japan | 0-14 | diagnosis of asthma | clinical medical records | Japanese in Thailand versus Japanese in Japan | In the subcategories of ‘diseases of the respiratory system’, ‘acute upper respiratory infections’ was frequent and asthma was infrequent in Thailand. Conversely, ‘acute upper respiratory infections’ showed a low percentage and asthma was the most frequently observed disease in Japan. |  |
| 18637981 | Sorkin | 2008 | Chronic medical conditions and self-rated health of older Vietnamese Americans were compared with those of non-Hispanic white adults living in California using the 2001 and 2003 California Health Interview Surveys (CHISs). | cross sectional | Individual | 1 | 0 |  | Vietnam | 359 Vietnamese and 25,177 non-Hispanic white adults | 55 and older | Chronic medical conditions and self-rated health | self reported | US white born versus Vietnamese americans | None, descriptive study only | No significant difference in the prevalence of asthma between these 2 groups (rate around 11%) |
| 18502174 | Hoffman | 2008 | We investigated the distribution of environmental exposures and health outcomes in preschool children and examined the role of social position on their associations | cross sectional | Individual | 0 | 0 | Germany | Turkish | CROSS sectional survey on 968 preschool children | 0-6 | asthma and allergies | face-to-face interviews for the assessment of the medical history, blood and urine sampling for bio-monitoring, and clinical examinations. | Immigrant background measured as both parents non-German versus one or both parents German nationality, or child or mother born outside Germany. | The odds ratio for the association between suspended particulate matter TSP and history of allergic diseases was 1.17 (95%CI 0.95–1.45) in children without immigration background and 0.71 (95%CI 0.53–0.95) with immigration background. |  |
| 21532645 | Domínguez-Ortega | 2011 | To present the clinical characteristics of respiratory allergy in immigrants in the central region of Spain | cross sectional | Individual | 0 | 0 | Spain | North of Africa, Latin America, and Eastern Europe | 62 immigrants and 32 Spanish |  | rhinitis, conjuntivitis y asthma | diagnosis | Immigrant or Spanish | More than 96% presented rhinitis, although persistent rhinitis was more prevalent amongst Latin Americans (76.9%) than in the Spanish population (48%). Sensitization to polcalcin was rare. Otherwise, 44.1% of the Spanish population was sensitized to profilin (only 4.88% among immigrants) although this sensitization did not associate allergy to fruits or other vegetables. | No differences were observed in asthma prevalence, although immigrants had higher rates of non-controlled and partially controlled asthma. The mean time of onset of symptoms after immigration was 43 months. |
| 21481023 | Braback | 2011 | we investigated the importance of exposure to a western lifestyle in different phases of development in Swedish residents with an origin in regions in the world where asthma usually is less prevalent. | cross sectional | Individual | 0 | 0 | Sweden | Unspecified | 24 252 international adoptees, 47 986 foreign-born and 40 971 Swedish-born with foreign-born parents and 1 770 092 Swedish-born residents with Swedish-born parents | Jun-25 | Asthma | Purchased prescribed inhaled corticosteroids (ICS) during 2006 were used as an indicator of asthma. | international adoptees, foreign-born, Swedish-born with foreign-born parents, Swedish-born residents with Swedish-born parents | International adoptees and children born in Sweden by foreign-born parents had three- to fourfold higher rates of asthma medication compared with foreign-born children. The odds ratios (ORs) of asthma medication declined persistently with age at immigration. For adoptees the ORs compared with infant adoptees were 0.78 [95% confidence interval (CI) 0.71–0.85] for those adopted at 1–2 years, 0.51 (0.42–0.61) at 3–4 years and 0.35 (0.27–0.44) after 5 or more years of age. Corresponding ORs for foreign-born children with foreign-born parents immigrating at 0–4 years, at 5–9 years, at 10–14 years and at 15 years or more were 0.73 (0.63–0.86), 0.56 (CI 0.46–0.68) and 0.35 (CI 0.28–0.43), respectively. | The ORs were only marginally affected by adjustment for region of birth and socio-economic indicators. |
| 10490527 | Powell | 1999 | Examination of the relation between respiratory symptoms and time since arrival in Australia in immigrant teenagers living in Melbourne. | Cross-sectional | Individual | 0 | 0 | Australia | Various countries | 9778 | 13-19 | Prevalence of wheeze during a 12 month period | Parent-reported wheeze and asthma attacks | Region of birth | The prevalence of asthma symptoms for Australian born subjects (19.2%; 95% CI, 18.2 to 20.2) was more than twice that among those born outside Australia (8.5%; 95% CI, 7.4 to 9.7). There was a strong trend to a higher 12 month prevalence of wheeze with longer time spent living in Australia (p < 0.001). |  |
| 11589342 | Tobias | 2001 | To compare prevalence rates of asthma symptoms, bronchial responsiveness, atopy and use of health services by those with asthma in first-generation immigrants and emigrants, and nonmigrants. | Cross-sectional | Individual | 0 | 0 | Various | Various | 19516 | 20-45 | asthma | Self-reported asthma attack, symptoms, medication | Country of birth + country of residence | Significantly higher prevalence rates of asthma symptoms were reported by immigrants (11.2%) than nonmigrants (8.6%). The corresponding OR adjusted by sex, age, and smoking was 1.21 (95% CI: 1.00–1.51) in immigrants compared to nonmigrants. For the US and Irish data only, immigrants had a significantly higher risk of atopy. | The prevalence of asthma in the host country was not a significant factor contributing to the risk of asthma in immigrants. Also, the prevalence of atopy did not differ amongst immigrants and non-immigrants for the total sample. |
| 12837867 | Klinnert | 2003 | To describe morbidity attributable to wheezing illness in a multi-ethnic sample of low-income infants younger than age 2, and examines biological, environmental, and psychosocial correlates of morbidity indexes. | Cross-sectional | Individual | 1 | 0 |  | Various Hispanic countries | 177 | 9-24 months | determinants of utilization for wheezing illness | hospitalization, ED visits, caregiver morbidity report, medications | Ethnicity (foreign born) | In the unadjusted analysis (only age-adjusted), foreign-born Hispanic families had significantly more ED visits for their children’s wheezing illness than US-born Hispanic families, whites, or blacks, although they used fewer controller medications and they reported less illness severity. |  |
| 14976393 | van Amsterdam | 2004 | To investigate the prevalence of allergic sensitisation in school children of Dutch, Turkish and Moroccan origin. | Cross-sectional | Individual | 0 | 0 | Netherlands | Turkey and Morocco | 512 | 7-14 | Allergic sensitisation | Skin prick test | Country of origin parents | Children from Moroccan parents had significantly higher rates of both indoor and outdoor sensitization than children from Dutch parents. | Rates of allergic sensitization seemed to be higher for Turkish children, although this difference was not significant. |
| 17027246 | van Dellen | 2007 | To find the predictors of asthma control among children from different ethnic origins as measured with the ACQ. | Cross-sectional | Individual | 0 | 0 | the Netherlands | Morocco, Turkey, Suriname | 278 | 7-17 | Asthma control | Asthma control questionnaire | Ethnic origin; child or parents born abroad. | In logistic regression analysis, Surinamese children showed a more than two-fold greater odds of having uncontrolled asthma. (OR 2.2; 95% CI 1.06–4.83). Children whose parents had an insufficient comprehension of the Dutch language also had a more then two fold greater odds of having uncontrolled asthma (OR 2.3; 95% CI 1.08–4.78). | A greater odds of having uncontrolled asthma was found for Moroccan children, although this was not statistically significant (OR 1.5; 95% CI 0.66–3.44). |
| 20472216 | Magzamen | 2010 | to determine the prevalence and predictors of possible undiagnosed asthma in a population of urban adolescents | cross sectional | Individual | 1 | 0 |  |  | 4017 |  | under- diagnosis of asthma | clinical diagnosis via structured questionnaire of signs and symptoms | US versus non US | 4.8% (95% confidence interval [CI]: 4.1, 5.5) were classified as possible undiagnosed asthma. Female students (odds ratio: 1.53, 95% CI: 1.07, 2.19) and students who resided in an urban residential area (odds ratio: 2.05, 95% CI: 1.05, 4.05) had significantly increased odds of classification as ‘‘possible undiagnosed asthma’’ compared to current asthma. Percentage of noncitizen recent immigrants in a census tract was related to increased odds of possible undiagnosed asthma. |  |
| 18384451 | Pereg | 2008 | to evaluate the importance and effect of immigration (country of birth and age at immigration to Israel) on the prevalence of asthma in a large group of Israeli adolescents. | cross sectional | Individual | 0 | 0 | Israel |  | 1 466 654 adolescents, including 1 317 556 (89.8%) NBI and 149 098 (10.2%) immigrants. | 17 years old | asthma | self reported sympstoms of asthma | Israeli versus non Israeli | The prevalence of asthma at age 17 was higher in native-born Israelis compared with Ethiopian immigrants [4.7% (61 921) vs 2.6% (418), respectively, P < 0.0005], lower compared with immigrants from Western countries [5.6% (2177), P < 0.0005], and similar to immigrants from the Former Soviet Union |  |
| 10565458 | Hjern | 1999 | To identify social and ethnic characteristics of children 2–18-y-old in need of improvement in disease management. | Cross-sectional | Individual | 0 | 0 | Sweden | Various countries | 780041 | 2-18 | Hospital admissions for asthma | Swedish Patient Discharge Register | Born outside Europe, born outside Sweden | Children born outside Western Europe, the USA and Australia were less commonly admitted to hospital because of asthma than other children in the population (adjusted OR: 0.1–0.5). | Children born in Sweden of mothers born in Western Europe had rates similar to those of Swedish-born children with Swedish-born mothers. |
| 10565562 | Hjern | 1999 | To describe and analyze the importance of ethnicity and migration for the development of asthma and allergic rhinitis among Swedish military conscripts. | Cross-sectional | Individual | 0 | 0 | Sweden | Various countries | 14630 | 17 | Asthma, allergic rhinitis | Clinical diagnosis | Born outside Sweden | Immigrants from Africa, Asia, Latin America and the Mediterranean had a significantly lower risk for asthma and allergic rhinitis than Swedish-born. The risk of atopic disorder among the foreign-born conscripts increased with time of residency in Sweden. | Results when taking into account country of birth of the mother and SES were not consistent. |
| 10950897 | Hijazi | 2000 | An investigation was undertaken of dietary and other risk factors for asthma in Saudi Arabia where major lifestyle differences and prevalences of allergic disease are found in different communities. | Cross-sectional | Individual | 0 | 0 | Saudi Arabia | Unknown | 316 | 12 | asthma | Self-reported wheezing/ asthma | Non-Saudi | Saudi children seemed to have a higher risk of asthma than non-Saudi children (p=0.07). |  |
| 12766216 | Lee | 2003 | To study the prevalence of asthma in inner-city Asian American immigrant children in the US. | Cross-sectional | Individual | 1 | 0 |  | Various Asian countries | 606 | ? | Asthma | Parent-reported asthma, wheezing | Asian vs. non-Asian | In an unadjusted analysis, those with Asian surnames were less likely than those with non-Asian surnames to have been previously diagnosed with asthma (p=0.036). |  |
| 15904513 | Greenfield | 2005 | To improve the methodology for asthma screening in Chinese-American immigrant children. | Cross-sectional | Individual | 1 | 0 |  | China | 152 | < 18 | Asthma | Brief Pediatric Asthma Screen + video aid | Birthplace | Foreign born children were less likely to be diagnosed with asthma. | Preferred language was not related to asthma diagnosis. |
| 15990771 | Eldeirawi | 2005 | To examine the associations of place of birth with doctor-diagnosed asthma, wheezing in the past 12 months, and other allergic conditions in Mexican American children. | Longitudinal (retrospective) | Individual | 1 | 0 |  | Mexico | 4121 | 2 months-16 years old | Asthma and allergies | Doctor-diagnosed asthma reported by parents, skin prick test | Country of birth | After correction for counfounding, asthma, wheezing and symptoms of allergies were higher in US-born than in Mexican-born children. The prevalence of cat, housemite and various pollen allergies was higher in US-born children, cockroaches sensitivity was more prevalent in Mexican-born children. | For children with a history of ear infection, there was no difference in asthma prevalence between US-born and Mexican-born children. There was also no significant difference in peanut, white oak, Russian tistle and perrenyial rye allergies. |
| 17210041 | Kuehni | 2007 | To cmpare the reported prevalence of asthma between young white and south Asian women in the UK, and to investigate associations with country of birth and age at immigration. | Cross-sectional | Individual | 0 | 1 |  | South-Asia | 6560 | ? | Asthma | Self-reported through survey | country of birth and ethnicity | The reported crude prevalence of asthma was 10.9% (95% CI 9.4–12.4) in south Asian women and 21.8% (20.6–22.9) in White Women (p<0.001). This difference remained significant after adjustment. Factors associated with an increased prevalence of asthma among south Asian women included being born in the United Kingdom or having migrated before age five, speaking English, eating mostly an English instead of an Asian diet and active smoking. Several indicators of low socio-economic background were associated with a lower prevalence. After adjustment, those who had migrated to the United Kingdom after the age of 5 years were less than half as likely to report asthma than those who had been born in the United Kingdom or had migrated o5 years old (OR 0.38; 95% CI 0.23–0.64, P<0.001. | The association of asthma with the Townsend deprivation score was marginal. Age, religion, exposure to pets and environmental tobacco smoke were not related to asthma. |
| 17298347 | Migliore | 2007 | To to compare the prevalence of respiratory symptoms in migrant and nonmigrant children resident in Italy, and to examine the effect of length of time living in Italy. | Cross-sectional | Individual | 0 | 0 | Italy | Various | 29305 | 6-7, 13-14 | Lifetime asthma, wheeze, persistent cough, bronchitis | Self-reported in survey | Country of birth, nationality | The prevalence of bronchitis (29.4% vs 21.1%, P < 0.001) and asthmatic bronchitis (7.1% vs 3.7%, P < 0.001) in the first 2 years of life, and the prevalence of lifetime asthma (9.7% vs 5.4%, P < 0.001) and current wheeze (6.9% vs 5.2%, p= 0.044), was significantly higher in children born in Italy than in migrants. Lower risks for lifetime asthma (POR2 = 0.39; 95% CI: 0.23–0.66) and current wheeze (POR2 = 0.72; 95% CI: 0.47–1.10) were found for children who had lived in Italy <5 years, while migrant children who had lived in Italy for 5 years or more had very similar risks to children born in Italy. For each additional year of residence in Italy, there was a 12% increase in the prevalence odds of lifetime asthma (POR = 1.12; 95% CI: 1.02–1.25, P = 0.023). | No effect modification by child’s age was found when the association between asthma or wheeze and child’s and parental nationality was evaluated separately in children and adolescents. No difference was found in asthma or wheeze prevalence between children born in Italy with, respectively, both parents Italian; only one parent Italian; both parents foreigners. |
| 17474985 | Brugge | 2007 | To assess the relationship between asthma and native or foreign place of birth. | Cross-sectional | Individual | 1 | 0 |  | Asia | 204 | 4-18 | Asthma | Brief Pediatric Asthma Screen questionnaire | Place of birth | Asthma prevalence was substantially higher in the US born group (33.1% vs. 9.2%; p < 0.001). | Possible undiagnosed asthma was found in a higher percentage of the foreign born children, but the difference was not statistically significant and was based on very small numbers of cases. Allergies were more common in the US born children, but the difference was modest and not statistically significant (29.2% vs. 20.3%; p = 0.18). |
| 17514454 | Schlaud | 2007 | To describe atopic illnesses and allergies in German children. | Cross-sectional | Individual | 0 | 0 | Germany | Not specified | 17641 | 0-17 | Asthma, hay fever, atopic eczema, allergies | Self-reported | ? | Atopic eczema, allergies and atopic diseases in general were significantly higher in non-immigrant than immigrant children. The incidence of atopic eczema was 8.0% [7.0:9.1] for immigrants compared to 14.3% [13.5:15.1] for non-immigrants. The incidence of allergies was 7.3% [6.2:8.7] in immigrants compared to 10.4 [9.8:11.1] in non-immigrants. Of the immigrants, 17.7% [16.3:19.2] had at least one atopic illness compared to 23.9% [23.0:24.9] of non-immigrants. | The incidence of asthma and hay fever seemed to be lower in immigrants compared to non-immigrants, but these differences were non-significant. |
| 17530529 | Dumanovsky | 2007 | To examine variations in asthma prevalence among Hispanic subpopulations by ancestry and place of birth. | Cross-sectional | Individual | 1 | 0 |  | South and middle America | 4800 | 18 and over | Asthma attack | Self-reported | Country of birth | Controlling only for sex and age, all Hispanic groups born in the US were significantly more likely to report an asthma attack in the past 12 months than groups born outside the US. After adjusting for confounders these differences remained significant. |  |
| 22905591 | Asero | 2012 | To investigate the pattern of airborne sensitization among allergic extra-European immigrants living in two areas of northern Italy. | Longitudinal (retrospective) | Individual | 0 | 0 | Italy | Non European countries | 95 | 6-95 | allergies (sensitization to airborne allergens) | skin prick test | Non european immigrants versus Italian local individuals | In Milan grass pollen allergy was more frequent among immigrants than in controls (75% vs 49%; p < 0.01), whereas ragweed allergy prevailed among Italians (56% vs 20%; p < 0.001). In Verona, immigrants were rarely multi-sensitized (21% vs 43%; p < 0.01), |  |
| 22250626 | Katsarou | 2012 | to characterize the spectrum of skin diseases affecting children in Greece. | Longitudinal (retrospective) | Individual | 0 | 0 | Greece | Albania, Egypt and others | 4071 | 0-12 | skin allergies | medical examination/diagnosis, lab tests | nationality | The most frequent disease was dermatitis/eczema (34.7%), with atopic dermatitis found in 20.7% of children, contact dermatitis in 6.9%, pityriasis alba in 2.1%, and seborrheic dermatitis in 1.8%. Infections (19.3%), nevi (5.6%), scabies (4.8%), and insect | Migration status was not a risk factor for skin allergies; bacterial infections and scabies were more prevalent on immigrants than eczema and skin allergies. |
| 22075329 | Keet | 2012 | to evaluate the relationship between personal and parental nativity and the risk of food sensitization. | cross sectional | household survey | 1 | 0 | Non US born |  | 3550 | 0-21 | food sensitization, which was defined by a specific IgE level of at least 0.35 kU/L to milk, egg, or peanut. | lab tests | US versus non US born | Compared with those born outside the United States (US), US-born children and adolescents had higher odds of ny food (OR, 2.05; 95% CI, 1.49-2.83; P <.001). sensitization to a Among the foreign-born group, those who arrived before 2 years of age had higher odds of food sensitization than those who arrived later (OR, 2.68; 95% CI, 1.19-6.08; P 5 .02). Within the US-born group, in contrast, children of immigrants were at the highest risk (OR, 1.53; 95% CI, 1.05-2.24; P 5.02). |  |
| 21920489 | Alvarez | 2012 | To determine the frequency of visits by immigrants to our dermatology clinic, to describe their skin complaints, and to compare them to those of the autochthonous Spanish population. | cross sectional | Individual | 0 | 0 | Spain |  | 86 |  | skin diseases; categories: infectious diseases, inflammatory diseases, tumors (benign and malignant), toxic skin reactions, mucosal disorders, nail disorders, pigmentation disorders, vascular disorders, hair disorders, and other. | diagnosis | country of origin, subsequently classified into the following geographic regions: Latin America, North Africa, the Middle East, sub-Saharan Africa, Eastern Europe, and Asia. | Visits by immigrants to the dermatology clinic accounted for 4.1% of the caseload. Their most frequent complaints were eczematous dermatitis (18.4%), viral warts (6.4%), and acne (6.3%). Comparison between the immigrant and autochthonous patient populations showed that eczematous dermatitis, alopecia, melasma, ringworm, scabies, Herpes simplex infection, keratosis pilaris, and xerosis were significantly more frequent among immigrant patients, whereas viral warts, actinic keratosis, hidradenitis suppurativa, lupus, melanoma, and squamous cell carcinoma were significantly less frequent (P < .05). |  |
| 21165526 | Mahmoud | 2010 | to assess how exposure to the wartime and postwar environment may have altered the fundamental patterns of immune reactivity among Kuwaitis in ways that affect pathogenesis of disease. | cross sectional | Individual | 0 | 0 | Kuwait | Unspecified | 25 | unclear | T Lymphocyte Activation Profile | Peripheral Blood | Long- Versus Short-Term Residents of Kuwait | Relative to healthy, long-term residents, signifi cantly elevated frequencies of all activated cell phenotypes were observed in the blood of the asthmatic group. The asthmatic group was also observed to have larger populations of CD3+ (pan-T cells), CD4+ (T helper cells) and CD8+ (cytotoxic T cells), CD3+CD56 (NKT-like cells) and CD56+CD16+ (NK cells) compared to healthy long-term residents. Compared to healthy recent immigrants, the blood of long-term residents contained elevated levels of CD3+CD56+ (NK-like), CD4+CD45RA+/CD45RO+ (Naive-to-Memory Transitional), but lower CD4+CD25+high. |  |
| 20934316 | Marcon | 2011 | To investigate whether the incidence of allergic and respiratory symptoms differed for Italian and immigrant children living in one area of Northern Italy. | cross sectional | Individual | 0 | 0 | Italy | Non Italian | 3854 | Mar-14 | Diagnosis of ashma ot allergy | clinical diagnosis by physician | Italian versus abroad | Parental asthma, allergic rhinitis and eczema were less frequent in immigrant children than in Italian children. Wheezing and eczema incidences were lower in children born to foreign parents (especially if born abroad, incidence rate ratio (IRR) Z 0.47, 95% CI: 0.26e0.82 and IRRZ0.43, 95% CI: 0.23e0.83, respectively),with respect to Italian children,while the occurrence of nasal allergieswas similar among the ethnic groups. The greatest incidence of persistent cough/ phlegmwas observed in children born in Italy to foreign parents (IRRZ1.98, 95%CI: 1.06e3.71) and in children whose parents had chronic bronchitis (IRRZ2.57, 95% CI: 1.52e4.33). |  |
| 20804468 | Apfelbache | 2011 | To investigate determinants of eczema in German children and adolescents. | cross sectional | Individual | 0 | 0 | Germany | non German | 17641 | 0-17 | broad set of environmental and lifestyle exposures with ever physician-diagnosed eczema | diagnosis of eczema | German versus non German | Being a migrant (OR 0.63, 95% CI 0.49–0.80) and keeping a dog (OR 0.78, 95% CI 0.64–0.96) showed significant inverse associations with eczema. | Other lifestyle (alcohol consumption during pregnancy) and environmental factors (mould on the walls, pets, origin from East/West Germany) were not significantly related to eczema. |
| 20561236 | Ru¨ hl | 2011 | Authors hypothesized that the previously reported lower prevalence of atopy among Turkish immigrant children in Germany might be related to a different pattern of serum carotenoids (diet related) | cross sectional | Individual | 0 | 0 | Germany | Turkish | 120 | 0-5 | atopy | blood tests | German versus Turkish | Median levels of pro-vitamin A carotenoids were lower in Turkish children if compared to German children: D 135lg/L, TR-D 100lg/L (p = 0.025), TR-TR 82lg/L | By contrast, median levels of non-pro-vitamin A carotenoids were not higher in German children. |
| 20484926 | Alsowaidi | 2009 | In a randomly selected, age-stratified cohort of adolescent school children and their caretakers in the United Arab Emirates (UAE), comorbidity of AR and asthma was calculated using multinomial regression to determine independent risk factors | cross sectional | Individual | 0 | 0 | United Arab Emirates | Any | 6543 |  | asthma and allergic rhinitis | clinical diagnosis | Arabic versus other | The standardized prevalence of concomitant asthma and AR was 7.3%. AR subjects had a 3-fold increased risk of asthma compared to subjects without AR (23.8 and 7.5%, respectively). Immigrants had a significantly lower prevalence of comorbidity of AR and asthma [adjusted odds ratio (OR) 0.53, 95% confidence interval (CI) 0.33–0.85] compared to UAE nationals |  |
| 19995440 | Svendsen | 2009 | To evaluate the impact of migration to the USA-Mexico border city of El Paso, Texas (USA), parental language preference, and Hispanic ethnicity on childhood asthma to differentiate between its social and environmental determinants | cross sectional | Indivdual | 1 | 0 |  | Mexico | 9797 | 9-10 years old | asthma or allergy | questionnaire and spirometry | Mexican | Asthma and allergy prevalence increased with longer duration of El Paso residency independent of ethnicity and preferred language. Compared with immigrants who arrived in El Paso after entering first grade (18%), lifelong El Paso residents (68%) had more prevalent allergy (OR, 1.72; 95% CI, 1.32 - 2.24), prevalent asthma (OR, 1.75; 95% CI, 1.24 - 2.46), and current asthma (OR, 2.01; 95% CI, 1.37 - 2.95). Spirometric measurements (FEV1/FVC and FEF25-75) also declined with increasing duration of El Paso residency (0.16% and 0.35% annual reduction, respectively). |  |
| 19863283 | Eldeirawi | 2009 | to examine the associations of doctor-diagnosed asthma with immigration-related variables and to investigate whether these associations could be explained by factors that may change with migration. | cross sectional | Indivdual | 1 | 0 |  | Mexico | 2023 | Jun-14 | doctor diagnosed asthma | clinical or tests | US born versus Mexica born | US-born children had a 2.42-fold (95% confidence interval [CI]: 1.52–3.83) increased odds of asthma compared with their Mexico-born peers. Mexico-born participants who moved to the US before 2 years of age were almost twice as likely to experience asthma compared with Mexico-born children who moved to the US ≥2 years of age. In addition, Mexico-born participants who lived in the US for 10 years or more were 2.37 times more likely to have asthma than Mexico-born students who lived in the US for less than 10 years. |  |
| 18972296 | Brugge | 2008 | To look at the effect of nativity on asthma prevalence among Black immigrants versus Black non immigrants in the US | cross sectional | Individual | 1 | 0 |  | Non US black immigrants | 350 |  | diagnosis of asthma or prevalence of signs and symptoms | self reported | Black non born in the US | For adult respondents (n = 290) there was a strong negative association between being born outside the United States and reporting asthma (OR = 0.39; p = 0.033) that was retained in our multivariate model. For children (n = 157, reported by their parents) there was also a strong association with being born outside the United States (p < 0.05 using χ2 tabular analysis); |  |
| 19449207 | Litt | 2009 | We conducted a population-based study of home environmental conditions among recently immigrated Mexican families (weighted n = 473), generally of low socioeconomic status, and the health conditions of their children, in an urban industrial area north of | cross sectional | household | 1 | 0 |  | Mexico | 250 households |  | diagnosis of atshma and allergies | Self reported via survey | Mexican families included in this study only | The majority of recent immigrants had low socioeconomic status; virtually all had household incomes below the Colorado median ($50,841). Approximately one quarter of homes were overcrowded. Adverse environmental conditions were present across recent immigrant homes. These conditions include dampness or mold (44%), pests (28%), and minimal to no ventilation potential (26%), all of which are associated with asthma and atopic diseases. | At least one of these three environmental hazards was found in 67% of homes; multiple hazards were present in 27% of homes. Children of recent immigrant families had active symptoms within the past 12 months suggestive of asthma (4%) and atopic disorders |
| 19015564 | Wang | 2008 | To compare the prevalence of asthma and wheezing among Chinese adolescents born in Canada, Chinese adolescents who had immigrated to Canada and Chinese adolescents living in China. | cross sectional | Individual | 0 | 0 | Canada | Chinese | Of 7794 Chinese adolescents who met the inclusion criteria, 3058 were from Guangzhou, 2824 were from Beijing, and 1912 were from Hong Kong. Of 2235 adolescents in Vancouver, Canada, 475 were Chinese immigrants, 617 were Canadian-born Chinese, and 1143 wer | 6-7 and 13-14 | diagnosis of asthma | clinical medical records and blood tests | Chinese in China vesus Chinese in Canada versus Canadian in Canada | The prevalence of current wheezing among boys ranged from 5.9% in Guangzhou to 11.2% in Canadian-born Chinese adolescents. For girls, the range was 4.3% in Guangzhou to 9.8% in Canadian-born Chinese adolescents. The prevalence of ever having had asthma ranged from 6.6% to 16.6% for boys and from 2.9% to 15.0% for girls. Prevalence gradients persisted after adjustment for other environmental variables (odds ratios for ever having had asthma among Canadian-born Chinese compared with native Chinese in Guangzhou: 2.72 [95% confidence interval 1.75–4.23] for boys and 5.50 [95% confidence interval 3.21–9.44] for girls; p < 0.001 for both). Among Chinese adolescents living in Vancouver, the prevalence of ever wheezing increased with duration of residence, from 14.5% among those living in Canada for less than 7 years to 20.9% among those living their entire life in Canada. The same pattern was observed for the prevalence of ever having had asthma, from 7.7% to 15.9%. |  |
| 21146915 | Burastero | 2011 | To perform the allergological assessment of 32,555 recent immigrants from different areas of the world to a polluted metropolitan area of Northern Italy. | cross sectional | Individual | 0 | 0 | Italy | extra european | 395 recent immigrants and 32160 non recent immigrants | 15-75 | allergic rhinitis and/or asthma | clinical diagnosis of allergic rhinitis and/or asthma | Recent extra-European immigrants to Milan versus long term immigrants and local populaiton | Immigrants with allergic rhinitis and/or asthma days since arrival in Italy correlated with number of sensitisations (p=0.0030). Moreover, personal (2.02%) or familial (2.78%) history of allergic diseases was lower in allergic immigrants as compared to allergic residents (37.77 and 29.39%, respectively; p<0.0001 for both comparisons). Finally, the frequency of allergic immigrants from South America (63.3%) was higher than expected from the overall proportion of individuals from this macro-area who sought medical help at the same facility (40.4%; p<0.0001, OR 2.289, CI 2.1670—3.255). |  |
| 19647902 | Esteban-Vasallo | 2009 | To estimate the prevalence rates of chronic disorders in immigrants and to compare them with those in the native population, based on electronic clinical records in primary care(ECRPC). | cross sectional | Individual | 0 | 0 | Spain | Non Spanish | 6 081 689 residents | 16 or over | diagnosis of atshma and allergies | diagnosis via medical records | Spanish versus abroad | After age-adjustment, a total of 36.8% immigrants had some chronic health problem (vs.55.3%natives). These disorders were more frequent among women and among the population from Africa and Latin America. The highest overall prevalence rates in the foreign population were allergy (10.2% crude rate). |  |

*PMIDs can be used for reference search
