# Supplementary material for: A Systematic Review on the Development of Asthma and Allergic Diseases in Relation to International Immigration: The Leading Role of the Environment Confirmed
Source: PLoS One. 2014 Aug 20;9(8):e105347. doi: 10.1371/journal.pone.0105347 (PMC4139367; doi:10.1371/journal.pone.0105347)
Supplement: Table S2 — Summary of quality of papers included in this review. (DOC) [file pone.0105347.s004.doc]

**Supplementary File 4** Summary of quality of the 54 papers included in this review. Poor quality has a value of 0 and good quality has a value of 1.

| **PMID*** | **First author** | **Year of publication** | **Aim/hypothesis** | **Study design** | **Quality_ data sources** | **Quality_ representiveness of the sample** | **Quality_ sample size** | **Quality_ measures** | **Quality_ method of analysis** | **Quality_ adjustment of counfounders** | **OVERALL QUALITY ASSESSMENT****  **(+ ++ +++)** |
| --- | --- | --- | --- | --- | --- | --- | --- | --- | --- | --- | --- |
| 10212770 | Rosenberg | 1999 | To evaluate the prevalence and characteristics of asthma in a population of Jews of Ethiopian origin who had been in Israel for 8–17 years. | Cross-sectional | 1 | 1 | 1 | 1 | 1 | 0 | **++** |
| 10232429 | Kabesch | 1999 | To evaluate the prevalence of asthma and atopy in Turkish children living in Germany and to investigate the role of ethnic origin on the development of asthma and atopy in this population. | Cross-sectional | 1 | 1 | 1 | 1 | 1 | 1 | **+++** |
| 10464843 | Ormerod | 1999 | To study the prevalence of asthma and ‘probable’ asthma in the Asian population in Blackburn, U.K. | Cross-sectional | 1 | 1 | 1 | 1 | 1 | 1 | **+++** |
| 10478611 | Hjern | 1999 | To study the influence of the early childhood environment on the risk of suffering from atopic disorders as young adults. | Cross-sectional | 1 | 1 | 1 | 1 | 1 | 1 | **+++** |
| 10846511 | Ledogar | 1999 | Measure asthma prevalence among Latinos in the US | Cross-sectional | 1 | 1 | 1 | 1 | 1 | 1 | **+++** |
| 15147448 | Ventura | 2004 | Determine the relative relevance of environmental vs genetic factors in causing the reported rapid increase of the prevalence of sensitization and allergic diseases in immigrants. | Cross-sectional | 1 | 1 | 1 | 1 | 1 | 1 | **+++** |
| 15900417 | Johnson | 2005 | To identify the demographic and environmental risk modifiers for asthma in Arab Americans living in the Detroit Metropolitan area. | Cross-sectional | 1 | 1 | 1 | 1 | 1 | 1 | **+++** |
| 16242029 | Netuveli | 2005 | We investigated possible ethnic variations in incidence of asthma episodes and in addition explored the impact of migration on risk of developing asthma. | Longitudinal (prospective) data analyzed cross-sectionally | 1 | 1 | 1 | 1 | 1 | 1 | **+++** |
| 16598994 | Kim | 2006 | The objective of this study is to establish important contributing environmental factors and time until onset of development of allergic rhinitis in Korean immigrants to the United States. | Longitudinal (retrospective) | 1 | 1 | 1 | 0 | 1 | 0 | **++** |
| 16846455 | Cataldo | 2006 | To evaluate rates, distribution, clinical features and environmental risk factors for food intolerances and allergies in immigrant children. | Longitudinal (retrospective) | 1 | 1 | 1 | 1 | 1 | 1 | **+++** |
| 17402323 | Farfel | 2007 | To examine the current prevalence of common diseases, compare the results with those of previous cohorts, and assess the influence of the massive immigration during the 1990s. | Longitudinal (retrospective) | 1 | 1 | 1 | 1 | 0 | 0 | **++** |
| 17514453 | Kamtsiuris | 2007 | To present the descriptive data of a spectrum of acute and chronic childhood illnesses. | Cross-sectional | 1 | 1 | 1 | 0 | 1 | 0 | **++** |
| 21745811 | Koinis-Mitchell | 2011 | To examine associations between immigration, acculturation, family cohesion and social support networks, and asthma morbidity in a sample of Dominican and Puerto Rican caregivers residing in the mainland U.S. | cross sectional | 1 | 1 | 1 | 1 | 0 | 1 | **++** |
| 18594153 | Lombardi | 2007 | To assess the clinical characteristics of respiratory allergy in immigrants in Brescia, Italy. | cross sectional | 1 | 0 | 0 | 1 | 1 | 0 | **++** |
| 17546500 | Huh | 2008 | To examine the physical health status of immigrants with specific considerations of Asian and Hispanic populations and explores possible mechanisms through which health outcomes of interest can be explained. | cross sectional | 1 | 0 | 1 | 1 | 1 | 1 | **++** |
| 19038014 | Sakai | 2008 | To clarify the health-related conditions of Japanese expatriate children in Thailand. | cross sectional | 1 | 0 | 1 | 1 | 1 | 0 | **++** |
| 18637981 | Sorkin | 2008 | Chronic medical conditions and self-rated health of older Vietnamese Americans were compared with those of non-Hispanic white adults living in California using the 2001 and 2003 California Health Interview Surveys (CHISs). | cross sectional | 1 | 0 | 1 | 1 | 1 | 0 | **++** |
| 18502174 | Hoffman | 2008 | We investigated the distribution of environmental exposures and health outcomes in preschool children and examined the role of social position on their associations | cross sectional | 1 | 0 | 1 | 1 | 1 | 1 | **++** |
| 21532645 | Domínguez-Ortega | 2011 | To present the clinical characteristics of respiratory allergy in immigrants in the central region of Spain | cross sectional | 0 | 1 | 1 | 0 | 1 | 1 | **++** |
| 21481023 | Braback | 2011 | We investigated the importance of exposure to a western lifestyle in different phases of development in Swedish residents with an origin in regions in the world where asthma usually is less prevalent. | cross sectional | 1 | 1 | 1 | 1 | 1 | 1 | **+++** |
| 10490527 | Powell | 1999 | Examination of the relation between respiratory symptoms and time since arrival in Australia in immigrant teenagers living in Melbourne. | Cross-sectional | 1 | 1 | 1 | 1 | 1 | 1 | **+++** |
| 11589342 | Tobias | 2001 | To compare prevalence rates of asthma symptoms, bronchial responsiveness, atopy and use of health services by those with asthma in first-generation immigrants and emigrants, and nonmigrants. | Cross-sectional | 1 | 1 | 1 | 1 | 1 | 1 | **+++** |
| 12837867 | Klinnert | 2003 | To describe morbidity attributable to wheezing illness in a multi-ethnic sample of low-income infants younger than age 2, and examines biological, environmental, and psychosocial correlates of morbidity indexes. | Cross-sectional | 1 | 1 | 1 | 1 | 0 | 0 | **++** |
| 14976393 | van Amsterdam | 2004 | To investigate the prevalence of allergic sensitisation in school children of Dutch, Turkish and Moroccan origin. | Cross-sectional | 1 | 1 | 1 | 1 | 0 | 0 | **++** |
| 17027246 | van Dellen | 2007 | To find the predictors of asthma control among children from different ethnic origins as measured with the ACQ. | Cross-sectional | 1 | 1 | 1 | 1 | 1 | 1 | **+++** |
| 20472216 | Magzamen | 2010 | To determine the prevalence and predictors of possible undiagnosed asthma in a population of urban adolescents | cross sectional | 1 | 1 | 1 | 1 | 1 | 1 | **+++** |
| 18384451 | Pereg | 2008 | To evaluate the importance and effect of immigration (country of birth and age at immigration to Israel) on the prevalence of asthma in a large group of Israeli adolescents. | cross sectional | 1 | 1 | 1 | 1 | 1 | 1 | **+++** |
| 10565458 | Hjern | 1999 | To identify social and ethnic characteristics of children 2–18-y-old in need of improvement in disease management. | Cross-sectional | 1 | 1 | 1 | 1 | 1 | 1 | **+++** |
| 10565562 | Hjern | 1999 | To describe and analyze the importance of ethnicity and migration for the development of asthma and allergic rhinitis among Swedish military conscripts. | Cross-sectional | 1 | 1 | 1 | 1 | 1 | 1 | **+++** |
| 10950897 | Hijazi | 2000 | An investigation was undertaken of dietary and other risk factors for asthma in Saudi Arabia where major lifestyle differences and prevalences of allergic disease are found in different communities. | Cross-sectional | 1 | 1 | 0 | 1 | 1 | 1 | **++** |
| 12766216 | Lee | 2003 | To study the prevalence of asthma in inner-city Asian American immigrant children in the US. | Cross-sectional | 0 | 1 | 1 | 0 | 0 | 0 | **+** |
| 15904513 | Greenfield | 2005 | To improve the methodology for asthma screening in Chinese-American immigrant children. | Cross-sectional | 1 | 1 | 1 | 1 | 1 | 1 | **+++** |
| 15990771 | Eldeirawi | 2005 | To examine the associations of place of birth with doctor-diagnosed asthma, wheezing in the past 12 months, and other allergic conditions in Mexican American children. | Longitudinal (retrospective) | 1 | 1 | 1 | 1 | 1 | 1 | **+++** |
| 17210041 | Kuehni | 2007 | To compare the reported prevalence of asthma between young white and south Asian women in the UK, and to investigate associations with country of birth and age at immigration. | Cross-sectional | 1 | 1 | 1 | 1 | 1 | 1 | **+++** |
| 17298347 | Migliore | 2007 | To compare the prevalence of respiratory symptoms in migrant and nonmigrant children resident in Italy, and to examine the effect of length of time living in Italy. | Cross-sectional | 1 | 1 | 1 | 1 | 1 | 1 | **+++** |
| 17474985 | Brugge | 2007 | To assess the relationship between asthma and native or foreign place of birth. | Cross-sectional | 1 | 1 | 1 | 1 | 1 | 0 | **++** |
| 17514454 | Schlaud | 2007 | To describe atopic illnesses and allergies in German children. | Cross-sectional | 1 | 1 | 1 | 0 | 1 | 0 | **++** |
| 17530529 | Dumanovsky | 2007 | To examine variations in asthma prevalence among Hispanic subpopulations by ancestry and place of birth. | Cross-sectional | 1 | 1 | 1 | 1 | 1 | 1 | **++** |
| 22905591 | Asero | 2012 | To investigate the pattern of airborne sensitization among allergic extra-European immigrants living in two areas of northern Italy. | Longitudinal (retrospective) | 1 | 0 | 0 | 1 | 1 | 1 | **++** |
| 22250626 | Katsarou | 2012 | To characterize the spectrum of skin diseases affecting children in Greece. | Longitudinal (retrospective) | 1 | 0 | 1 | 1 | 1 | 0 | **++** |
| 22075329 | Keet | 2012 | To evaluate the relationship between personal and parental nativity and the risk of food sensitization. | cross sectional | 1 | 1 | 1 | 1 | 1 | 1 | **+++** |
| 21920489 | Alvarez | 2012 | To determine the frequency of visits by immigrants to our dermatology clinic, to describe their skin complaints, and to compare them to those of the autochthonous Spanish population. | cross sectional | 0 | 1 | 1 | 0 | 1 | 1 | **++** |
| 21165526 | Mahmoud | 2010 | To assess how exposure to the wartime and postwar environment may have altered the fundamental patterns of immune reactivity among Kuwaitis in ways that affect pathogenesis of disease. | cross sectional | 0 | 0 | 0 | 1 | 1 | 1 | **++** |
| 20934316 | Marcon | 2011 | To investigate whether the incidence of allergic and respiratory symptoms differed for Italian and immigrant children living in one area of Northern Italy. | cross sectional | 1 | 1 | 1 | 1 | 1 | 1 | **+++** |
| 20804468 | Apfelbache | 2011 | To investigate determinants of eczema in German children and adolescents. | cross sectional | 1 | 1 | 1 | 1 | 1 | 1 | **+++** |
| 20561236 | Ru¨ hl | 2011 | Authors hypothesized that the previously reported lower prevalence of atopy among Turkish immigrant children in Germany might be related to a different pattern of serum carotenoids (diet related) | cross sectional | 0 | 0 | 0 | 1 | 1 | 0 | **++** |
| 20484926 | Alsowaidi | 2009 | In a randomly selected, age-stratified cohort of adolescent school children and their caretakers in the United Arab Emirates (UAE), comorbidity of AR and asthma was calculated using multinomial regression to determine independent risk factors | cross sectional | 1 | 1 | 1 | 1 | 1 | 1 | **+++** |
| 19995440 | Svendsen | 2009 | To evaluate the impact of migration to the USA-Mexico border city of El Paso, Texas (USA), parental language preference, and Hispanic ethnicity on childhood asthma to differentiate between its social and environmental determinants | cross sectional | 1 | 1 | 1 | 1 | 1 | 1 | **+++** |
| 19863283 | Eldeirawi | 2009 | To examine the associations of doctor-diagnosed asthma with immigration-related variables and to investigate whether these associations could be explained by factors that may change with migration. | cross sectional | 1 | 1 | 1 | 1 | 1 | 1 | **+++** |
| 18972296 | Brugge | 2008 | To look at the effect of nativity on asthma prevalence among Black immigrants versus Black non immigrants in the US | cross sectional | 1 | 0 | 0 | 1 | 1 | 0 | **++** |
| 19449207 | Litt | 2009 | We conducted a population-based study of home environmental conditions among recently immigrated Mexican families (weighted n = 473), generally of low socioeconomic status, and the health conditions of their children, in an urban industrial area | cross sectional | 1 | 1 | 0 | 1 | 1 | 0 | **++** |
| 19015564 | Wang | 2008 | To compare the prevalence of asthma and wheezing among Chinese adolescents born in Canada, Chinese adolescents who had immigrated to Canada and Chinese adolescents living in China. | cross sectional | 1 | 1 | 1 | 1 | 1 | 1 | **+++** |
| 21146915 | Burastero | 2011 | To perform the allergological assessment of 32,555 recent immigrants from different areas of the world to a polluted metropolitan area of Northern Italy. | cross sectional | 1 | 1 | 1 | 1 | 1 | 1 | **+++** |
| 19647902 | Esteba n-Vasallo | 2009 | To estimate the prevalence rates of chronic disorders in immigrants and to compare them with those in the native population, based on electronic clinical records in primary care(ECRPC). | cross sectional | 1 | 1 | 1 | 1 | 1 | 1 | **+++** |

*PMIDs can be used for reference search

**Overall quality assessment as follows: + 1-3 dimensions with good report, ++ 4-5 dimensions with good report, +++ all 6 dimensions with good report
