# Supplementary material for: A Systematic Review on the Development of Asthma and Allergic Diseases in Relation to International Immigration: The Leading Role of the Environment Confirmed
Source: PLoS One. 2014 Aug 20;9(8):e105347. doi: 10.1371/journal.pone.0105347 (PMC4139367; doi:10.1371/journal.pone.0105347)
Supplement: Protocol S1 — Protocol. (PDF) [file pone.0105347.s006.pdf]

BiB - MeDALL literature review: On the development of asthma and allergies: What are the proposed links to socioeconomic status and migration status?

Authors of this review work and their affiliations:

- Baltica Cabieses, Bradford Institute for Health Research, England
- Noortje Uphoff, University of York, England; Bradford Institute for Health Research, England
- Mariona Pinart, Fundació Centre de Recerca en Epidemiologia Ambiental, Barcelona, Spain
- John Wright, Bradford Institute for Health Research, England
- Josep Maria Antó, Fundació Centre de Recerca en Epidemiologia Ambiental, Barcelona, Spain

1. Background

There is a growing global public health concern on the increasing and dynamic rates of asthma and allergies all over the world. Causes explaining the epidemic of IgE-associated (allergic) diseases are unclear. The prediction of allergy and preventive strategies are currently insufficient to abate the epidemic. A wide variety of mechanisms are associated with allergic diseases. The most common diseases are linked with an IgE immune response. IgE-associated allergic diseases are complex multifactorial disorders, with both genetic and environmental interactions determining disease expression and leading to different (frequently co-existing) phenotypes. These interactions start *in utero* and during perinatal development, and develop in infancy and childhood. Adult allergic phenotypes are largely influenced by these earlier developments.

The different manifestations of allergic diseases involve the respiratory system (like **asthma, rhino-conjunctivitis**)**,** the skin (like **atopic eczema**), and the gastrointestinal tract, and may have a generalised expression involving different organs (**food allergy and anaphylaxis**). These are not separate diseases but are linked by complex and currently insufficiently defined inter-relationships which occur during childhood and persist throughout life. IgE-associated allergic diseases represent a global health problem which is increasing in frequency and severity. In some European countries, up to 50% of children have an IgE sensitisation to inhalant or food allergens. An epidemic of IgE-associated allergic diseases has occurred over the past decades in all parts of Europe and other continents in the world.

There is a large amount of evidence on potential causal pathways for the development of asthma and allergies worldwide. Two particularly challenging ones are the links between socioeconomic status (SES) and migration status. Knowledge from the social determinants of health informs us that "the social conditions in which people live powerfully influence their chances to be healthy. Indeed factors such as poverty, social exclusion and discrimination, poor housing, unhealthy early childhood conditions and low occupational status are important determinants of most diseases, deaths and health inequalities between and within countries" (WHO, 2004). The European Union Commission's plans to address health inequalities are set out in the Commission Communication - Solidarity in Health: Reducing Health Inequalities in the EU, published 20 October 2009 (http://ec.europa.eu/health).

Regarding SES, evidence indicates that even in the high- and middle-income countries of Europe, the possibilities for surviving and living a healthy life are still closely related to the socioeconomic background of individuals and families. These possibilities are reflected in substantial and even increasing social inequities in health within countries across Europe. These inequities in health are both unfair and avoidable, as they are caused by unhealthy public policies and lifestyles. Chronic diseases including allergic diseases hinder economic growth and reduce the development potential of countries, the poorest ones in particular. Chronic diseases and poverty are interconnected in a vicious circle. For example, in children with asthma, poverty aggravates asthma which, in turn, aggravates poverty (137). Thus, allergic diseases have a profound effect in the poorest countries, increasing inequities across Europe.

One understudied factor that might be relevant to the development of asthma and allergies is migration status. It is well-known that movement of people within and between countries has become a central and necessary part of contemporary society, and migration has been recognised as an important determinant of social development and global health. A comprehensive understanding of demographic and socioeconomic characteristics of international immigrants is essential to promote fair living and working conditions among them. The process of migration is inevitably selective and depends on both local and broader dynamics within a country and also at an international level.

The importance of economic migration to the search for a better life, especially in the context of globalisation and international industrialisation, may suggest that healthy, economically active young people are more likely to migrate. However, more complex and possibly hidden features, like migration of vulnerable women and children, ethnic groups and people in relative socioeconomic deprivation, might also result from specific national policies and market opportunities, which in addition might selectively discriminate against certain immigrant groups in the host countries. These dimensions of SES in the migration process requires further understanding worldwide, as the development and implementation of key social policies depends on accurate and robust indicators of migrant populations living in socioeconomic deprivation.

This protocol displays a detailed explanation of the sequential steps to be followed in the execution of a systematic review of international scientific evidence on the causal links between SES, migration status and the development of asthma and allergies. Findings from this review will directly inform researchers from the MeDALL study on the key evidence on such relationships and also the hypothesized underlying mechanisms. In order to understand the significance of this literature review to the mentioned studies, we explain their aims and context in the next few paragraphs.

1.a) The BiB study

Born in Bradford (BiB) was created in 2007 in response to rising concerns about the high rates of childhood morbidity and mortality in the city of Bradford, the sixth largest city in the UK with a population of about half a million and urban areas that are among the most deprived in the UK. BiB examines how genetic, nutritional, environmental and social factors impact on health and development during childhood and, with ongoing funding, will also examine long-term effects into adult life. The ultimate goal is to develop hypotheses, which can then be evaluated and tested for health and social interventions to improve childhood and adult health.

Around 20% of the population of Bradford is of South Asian origin (90% of whom are from Pakistan), and this constitutes a three-generation community that maintains close links with Pakistan. The relatively young age of the population of Pakistani origin and their higher fertility rates, compared with the White British majority population, explain why almost half of babies born in the city have parents of Pakistani origin. Sixty percent of the babies born in the city are born into the poorest 20% of the population of England and Wales based on the British government’s residential area Index of Multiple Deprivation. Infant mortality in Bradford has been

consistently above the national average, peaking at 9.4 deaths/1000 live births in 2003, when the national average was 5.5 deaths/1000 live births, and levels of congenital anomalies and childhood disability are among the highest in the UK.

The cohort was established in 2007 with the broad aims to:
1. describe health and ill health within a multi-ethnic (largely bi-ethnic) economically deprived population;
2. identify modifiable causal pathways that promote well-being or contribute to ill health;
3. develop, design and evaluate interventions to promote health;
4. provide a model for integrating operational, epidemiological and evaluative research into practice within the National Health Service and other health-related systems; and
5. build and strengthen local research capacity in Bradford.

BiB is a largely bi-ethnic cohort of families of White British and Pakistani origin, which has the advantage of relative homogeneity in the composition of each of these ethnic groups. This will allow a detailed assessment of the associations and causal analyses for differences between these ethnic groups in regard to key health outcomes such as obesity, diabetes and congenital anomalies. However, the cohort also includes large samples from other ethnic groups (e.g. Indian and Bangladeshi), and we hope our results will help to inform interventions aimed at reducing health inequalities and improving health in South Asian populations locally, nationally and internationally, as well as demonstrating that improving our understanding of disease processes benefits everyone irrespective of ethnic group. Recruitment for the study has been successful and the cohort population is representative of the population of Bradford, indicating minimal selection bias.

Bradford is unusual in the high levels of poverty and diversity; hence, it is not representative of the rest of the country, although there are similarities with other UK cities and with cities with high levels of ethnic minority and immigrant communities across the world. About half of all mothers of Pakistani origin in the cohort were born in Pakistan; therefore, the cohort provides the opportunity to compare populations by country of birth (Pakistan and UK) and by ethnicity. The cohort contains populations of particular interest and importance for health research, for example, in relation to infant growth and later health and in relation to community customs and practices and genetic illness.

1.b) The MeDALL study

The causes explaining the epidemic of IgE-associated (allergic) diseases are unclear. The prediction of allergy and preventive strategies are currently insufficient to abate the epidemic. One of the key substudies of BiB is the MeDALL study on early causes of asthma and allergies.

The MeDALL study (Mechanisms of the Development of ALLergy) aims to generate novel knowledge on the mechanisms of initiation of allergy from early childhood to young adulthoood, in order to propose early diagnosis, prevention and targets for therapy. A novel definition of phenotypes of allergic diseases and an integrative

translational approach are needed to understand how a network of molecular and environmental factors can lead to complex allergic phenotypes.

This study proposes a novel, stepwise, large-scale and integrative approach leaded by experts in allergy, epidemiology, allergen biochemistry, immunology, molecular biology, epigenetics and genomics, functional genomics, bioinformatics, computational and systems biology, combining the strengths of previous and ongoing EU projects. A feasible and achievable project links epidemiological and clinical research with experimental and animal models.

- Classical phenotypes (expert-based) and novel phenotypes of allergy (hypothesis-free statistical modelling) will be compared. Asthma and atopic dermatitis will be considered.
- Population-based data will include a large network of existing European birth-cohorts which will be followed using a common protocol and a “unique” cross-sectional study in Karelia, Finland-Russia.
- IgE to food and inhalant allergens will be tested using component-resolved diagnosis in populations across Europe.
- The discovery of biomarker profiles (“fingerprints”) of the classical and novel allergic phenotypes will be carried out in a multistep process using epigenetics, targeted proteomics and unbiased transcriptomics. Fingerprints will be extensively assessed in a subsample of the study populations and those associated with the allergic phenotypes validated in the study populations at large. Relevant fingerprints will be combined into network biomarker phenotype “handprints” using a systems biology approach and validated in a sufficiently powered sample. Animal studies and in vitro human immunology will reinforce the validation and confirmation processes.
- The role of risk and protective factors will be extensively assess both for the classical and novel phenotypes by means of modelling their interactions with epigenetic changes and biomarkers. Population groups at high risk of developing allergic diseases will be characterized.
- Results will be fitted into new integrative complex mathematical models to establish suitable biomarkers for the early diagnosis and prevention of allergy-associated diseases such as asthma and atopic dermatitis.

It is expected that MeDALL will contribute to a substantial improvement of the understanding of the allergic phenotypes and will expand the current knowledge of the genomic and environmental determinants of allergic diseases during childhood and adolescence, in an integrative way. It will lead also to novel diagnostic tools for the early diagnosis of allergy, molecular targets potentially useful for the development of novel treatment modalities, development of methods for the promotion of health and prevention of allergic diseases, particularly in children. It will reduce fragmentation of allergy research in Europe. This project ultimately aims at improving the health of European citizens and increasing the competitiveness and boosting the innovative capacity of Europe while addressing global health issues.

MeDALL outcomes will help to develop specific strategies for integrating equity-oriented health policies into economic and social policies. The inclusion of large birth cohorts in MeDALL offers also the opportunity to address socioeconomic differences in allergic diseases.

2. Rationale for the review of the literature on migration, socioeconomic status (SES) and asthma and allergies

An earlier synthesis of existing evidence on the causal pathways for asthma and allergies (unpublished) developed at BiB informed us that there is some interesting evidence on the relationship between socioeconomic status and development of asthma and allergies in childhood and adulthood (Table 1). However, little evidence seemed to be available on the effect of migration in the relationship between SES and asthma/allergies and this need to be further explored. As the previous literature review was not focused on this particular pathway for asthma and allergies, and given the huge and complex amount of evidence on the causes of asthma and allergies, we decided to develop a specific systematic review to answer the research question: what are the causal links between migration status, socioeconomic status (SES) and the development of asthma and allergies according to current available scientific evidence?

This question can be divided into the following four specific questions:

1. According to current evidence, what are the causal links between socioeconomic status and the development of asthma and allergies at the individual level?
2. According to current evidence, what are the causal links between socioeconomic (particularly income) inequality and the development of asthma and allergies at the population level?
3. According to current evidence, what are the causal links between migration, socioeconomic status and the development of asthma and allergies at the individual and the population level?

Table 1 Summary of evidence on the relationship between SES and asthma and allergies, results from a scoping literature search conducted in July 2012

Hypothesis	Sub-hypothesis	Authors	Related papers from further hand search	General overview of evidence	Research gaps?
Poor SES and high income inequality increases the chance of asthma and allergies and its severity	Positive correlation between nation´s GDP and rates of asthma and allergy	Arnedo-Pena 2011<br>Chen 2011<br>Asher 2007<br>Strachan 1997<br>ISAAC project 1998<br>Williams 1999	Stewart 2001<br>Lai 2009<br>Cope 2007<br>Cope 2008<br>Ungar 2011<br>Ungar 2010<br>Chen 2010<br>Valdivia 2009<br>Laurent 2008<br>Sole 2008<br>Shankardass 2007<br>Kim 2006<br>Lee 2006<br>Duran 1999<br>Suarez 1999<br>Bergmann 1999<br>Persky 1998<br>Ciccone 1997<br>Ernst 1995<br>Williesjacobo 1993	▪ Low socioeconomic status (SES) is a strong predictor of many health problems, including asthma impairment<br>▪ Low-SES children with asthma are significantly more likely to be hospitalized or visit an emergency department for asthma.<br>▪ Compared with higher-SES children with asthma, they also have more symptoms and more severe exacerbations<br>▪ Possible explanations: numerous environmental and behavioural factors, including heightened exposure to neighbourhood pollutants, a greater likelihood of engaging in risky behaviours (eg, smoking), and the experience of negative psychological states (eg, depression)<br>▪ Stress is known to be one of the primary psychological pathways linking low SES to poor health<br>▪ Regarding income inequality and asthma, there is a trend of increased rates worldwide:<br>o In countries with high GDP & GNI & GNP (positive correlation)<br>o And particularly among more unequal countries, irrespective of level of development or GDP, which needs further assessment as the amount of studies looking at this globally are fewer than those looking at SES and asthma within countries<br>▪ This findings suggest that environmental factors might matter more than genetic predisposition	▪ There is a relevant amount of evidence on the relative importance of SES in the appearance and severity of asthma and allergy, but this was weak in many ecological studies<br>▪ Further studies could explore how SES interacts with other individual, household and environmental factors<br>▪ Variations over the life span from antenatal SES and intergenerational effects could be explored<br>▪ No study on South Asian SES and asthma/ allergy in particular was found in this review

3. Methods

3.a) Overview

This systematic review of the international scientific literature is developed through a collaboration between BiB (England) and CREAL (Spain). Figure 1 shows the steps we will follow in the review process. We will conduct a systematic review in accordance with the guidelines set forth by the Preferred Reporting Items for Systematic Reviews and Meta-Analyses (PRISMA) statement from Cochrane collaboration guidelines for reporting systematic reviews.

Unlike most systematic reviews currently available in the medical literature, the present one will address a complex ill-defined question applied to a wide range of very common and often overlapping health conditions (asthma and allergies). The immediate consequence of the latter is that tens of thousands of documents may be considered eligible. However, papers on asthma and allergies will be limited to the measurement of both SES and migration status only. Consequently, the search strategy should be conducted in a way that specificity with regard to the general research question should be prioritized.

Thus, among other assumptions, we expect that publication bias (e.g. due to restriction to English, Spanish, French, Portuguese and German documents and limiting the search to few wide repositories like Pubmed, EMBASE, Cinahl, and Cochrane Library) is unlikely to result in a relevant bias in the identification of the relationship between migration, SES and asthma and allergies.

Figure 2 displays a conceptual model on how migration status, SES and socioeconomic inequality, and asthma and allergies might be related to each other over time. We developed this model based on expert knowledge on the topic. This conceptual model will be tested against current international evidence on this matter. This review exercise will inevitably lead to clear and transparent research gaps and hypotheses to test through the ongoing research collaboration between MeDALL and BiB.

Figure 1 Flowchart of the systematic literature search and the process of inclusion and exclusion criteria

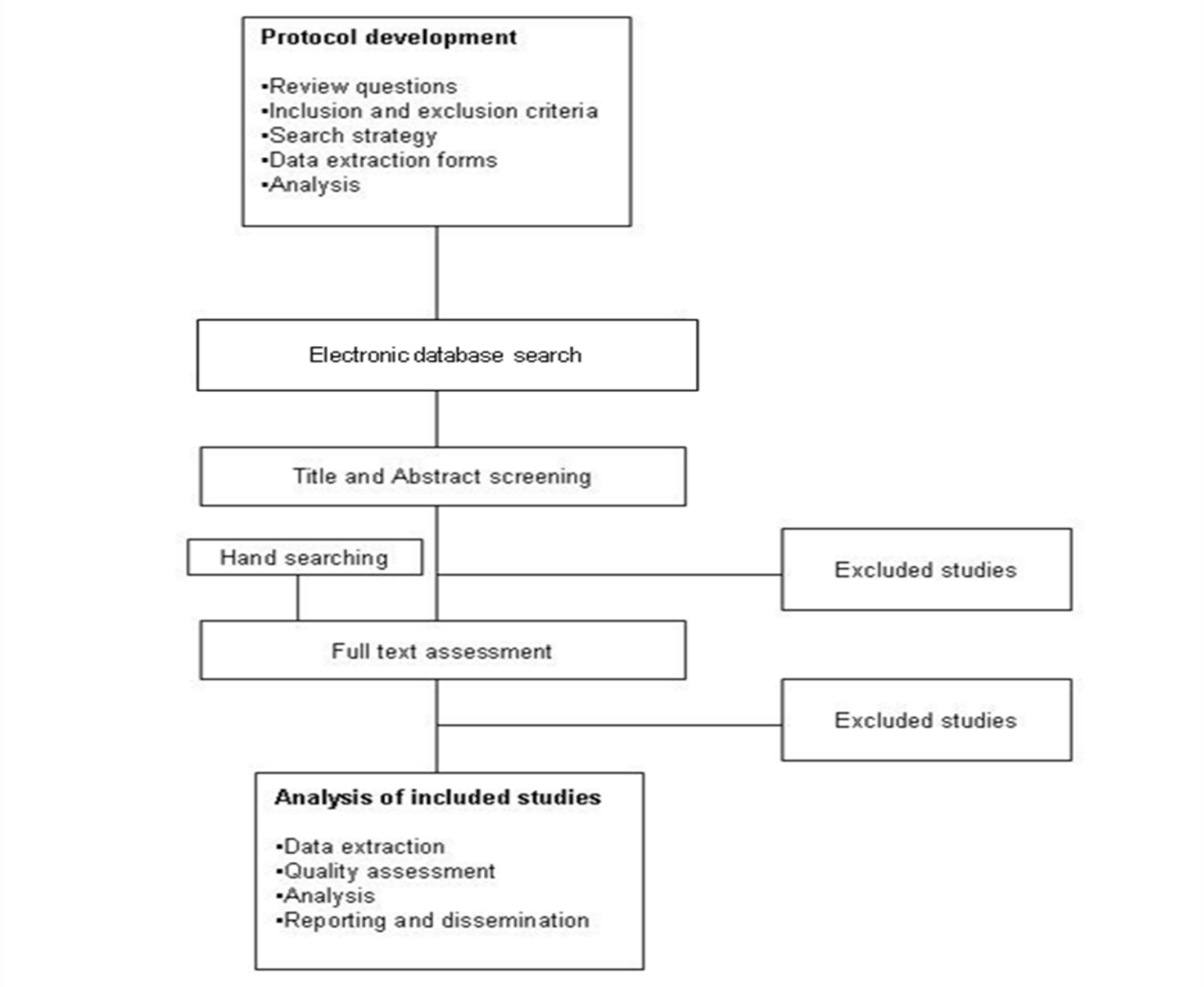

Figure 2 Proposed conceptual model used in this review on the relationship between migration status, SES and socioeconomic inequality, and asthma and allergies

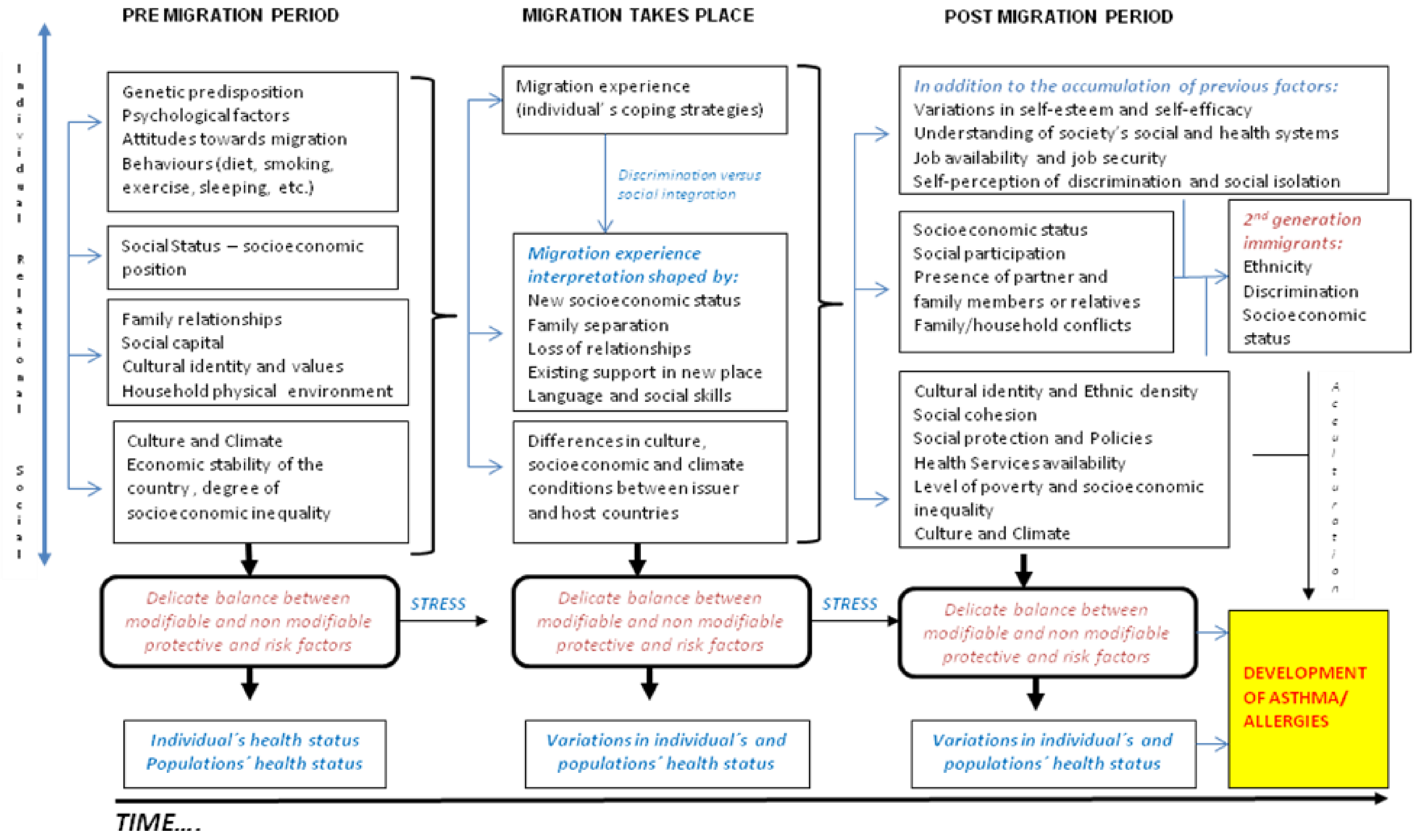

3.b) Objectives

i) Primary objective

To identify current international evidence on the causal links between migration, socioeconomic status (i.e. differences within societies) and income inequality (i.e. differences between societies) and the development of asthma and allergies worldwide.

This review will follow a preconceived order for articles search and data synthesis, starting with the relationship between asthma/allergies and SES and followed by a narrower search on migration, SES and these health outcomes. This will allow as capture all possible variations in SES and migration status between groups.

ii) Secondary objectives

1. To explore the causal links between the development of asthma and allergies, socioeconomic status and migration at the individual level within populations
    a. Variations in crude prevalence rates of asthma/allergies between different SES groups in a single population
    b. Variations in adjusted prevalence rates of asthma/allergies between different migrant groups in a population by SES and other potential confounders
    c. Variations in crude severity of asthma/allergies between different SES groups in a population
    d. Variations in adjusted severity of asthma/allergies between different migrant groups in a population by socioeconomic status and other potential confounders
    e. Variations in crude and adjusted rates of asthma/allergies between first and second generation immigrants, years living in the host country, country of origin, legal status of immigrants and other potentially relevant migration-related individual factors

2. To explore the causal links between the development of asthma and allergies, socioeconomic inequality (particularly income inequality) and migration through the comparison of different populations
    a. Variations in crude prevalence rates of asthma/ allergies between different populations depending on the degree of socioeconomic inequality of the country
    b. Variations in adjusted prevalence rates of asthma and allergies between different migrant populations depending on the degree of socioeconomic inequality of the country and adjusted by other potential confounders
    c. Variations in crude severity of asthma/ allergies between different populations depending on the degree of socioeconomic inequality of the country

   d. Variations in adjusted severity of asthma/allergies between different migrant populations depending on the degree of socioeconomic inequality of the country and adjusted by other potential confounders
3. To synthesize the causal links between socioeconomic status and the development of asthma and allergies at the individual level within populations
4. To synthesize the causal links between migration, country level socioeconomic inequality and the development of asthma and allergies through the comparison of different populations

3.c) Intervention(s), exposure(s)

This review will encompass any definition provided for each classical allergic disease studied (asthma/wheeze, rhinitis or rhinoconjunctivitis, eczema, urticaria, anaphylaxis and food allergy); information about any definition of migration status (migrants, immigrants, mobile populations, etc); and any measure of SES or socioeconomic inequality, at both individual and any aggregated level.

3.d) Comparator(s)/ control

As above

3.e) Key definitions

1. **Asthma:** The WHO has defined it as a disease characterized by recurrent attacks of breathlessness and wheezing, which vary in severity and frequency from person to person. In an individual, they may occur from hour to hour and day to day.Asthma attack symptoms occur particularly late at night or early in the morning. Asthma attacks all age groups but often starts in childhood. This condition is due to chronic inflammation of the airways in the lungs and is associated with airway hyperreactivity which lead in turn to recurrent episodes of wheezing, shortness of breath, often coughing and chest tightness. These recurrent episodes are associated to a widespread but variable airflow obstruction in the lungs which is often reversible either spontaneously or with treatment. In an attack, the lining of the bronchial tubes swell causing the airways to narrow and reducing the flow of air in and out of the lungs.

   Figure 3 displays one of the most frequently used criteria to diagnose asthma. As it can be observed in this figure, allergies and asthma are closely linked. Causes of asthma and allergies are to some extent overlapped, and at the same time one can be the precursor of the other. For the purpose of this review, we will allow all definitions considered in published papers in selected highest yield databases. Assessment of its nature, characteristics and usefulness will be reviewed afterwards in order to allow for a broad search and an exhaustive review of the international evidence.

   Figure 3 Diagnosis of Asthma, 3 components

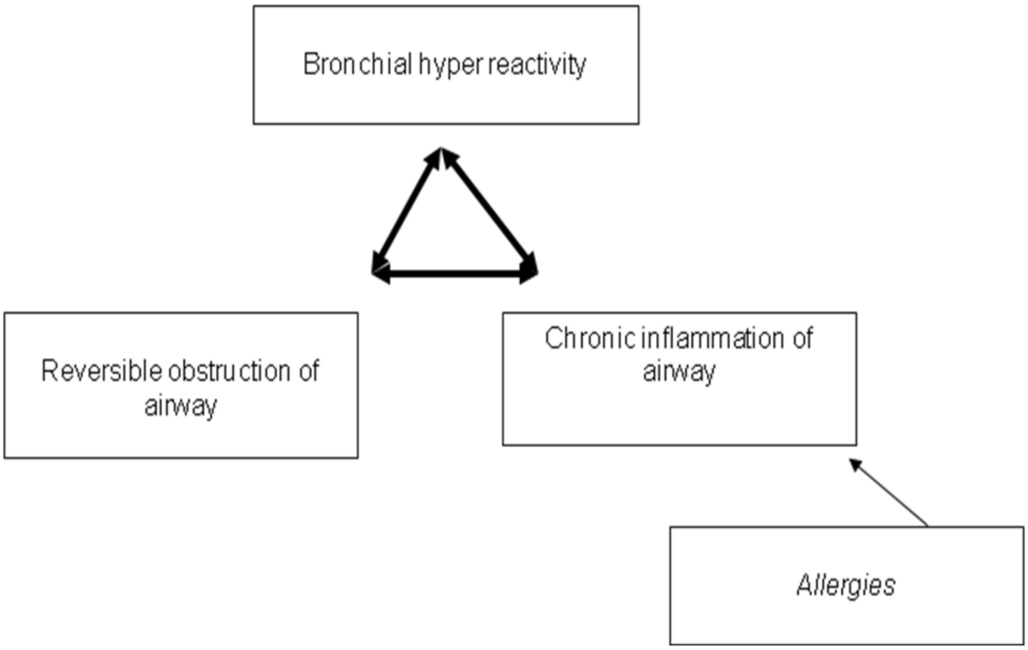

2. **Allergy:** It has been defined as a hypersensitivity disorder of the immune system. Allergic reactions usually occur when a person's immune system reacts to normally harmless substances in the environment. A substance that causes a reaction is called an allergen. These reactions are acquired, predictable, and

rapid. Allergy is one of four forms of hypersensitivity and is formally called *type I* (or *immediate*) hypersensitivity. Allergic reactions are distinctive because of excessive activation of certain white blood cells called mast cells and basophils by a type of antibody called Immunoglobulin E (IgE). This reaction results in an inflammatory response which can range from uncomfortable to dangerous.

Risk factors for allergy can be placed in two general categories, namely host and environmental factors. Host factors include heredity, gender, race, and age, with heredity being by far the most significant. However, there have been recent increases in the incidence of allergic disorders that cannot be explained by genetic factors alone. Four major environmental candidates are alterations in exposure to infectious diseases during early childhood, environmental pollution, allergen levels, and dietary changes. A scoping review of hypothesis for the development of asthma and allergies was conducted by the BiB team and can be found elsewhere. The CREAL team working on the MeDALL study has a long term research experience on causal factors for these diseases, particularly environmental factors. Similar to asthma, we will allow all definitions of allergies considered in published papers in selected highest yield databases. Assessment of its nature, characteristics and usefulness will be reviewed afterwards in order to allow for a broad search and an exhaustive review of the international evidence.

3. **Migration status:** Migration is the process of moving from one place to another. It has a rather obvious beginning, but the ending is not necessarily clear. There are variations in the definitions, theories, and evidence on migration and its association with health, but the topic remains controversial and challenging worldwide. The United Nations (UN) defines an international migrant as a person who stays outside their usual country of residence for at least one year. Attempts to define migration have been complex and diverse, involving a wide range of contexts, reasons and types of immigrant, from healthy migrants in search of better opportunities, to political refugees escaping civil wars in their countries of origin. More recently analysts have also highlighted the gendered dimensions of the migratory process which also have implications for health.

   Besides, the migration experience does not only imply the experience of *crossing frontiers*. Geographic boundaries are one part of the migration process. There are also symbolic frontiers between one community and another, which are separated by cultural differences. Experiences of crossing each frontier –the geographical and the cultural- may occur at different times and may involve different meanings that are difficult to measure and interpret. Usually, migration status is not defined in published papers but considered a relevant self-reported attribute to study. Nevertheless this limitation, we will allow all implicit and explicit definitions considered in published papers in selected highest yield databases. Assessment of its nature, characteristics and usefulness will be reviewed afterwards in order to allow for a broad search and an exhaustive review of the international evidence.

4. **Socioeconomic status (SES):** Socioeconomic status (SES) or socioeconomic position (SEP) is a commonly used concept in health research. Although researchers have an intuitive sense of what SES means, the numerous ways of measurement indicate the complexity of the construct. A variety of other terms, such as social class, social stratification, social or socioeconomic status, are often

used interchangeably despite their different theoretical bases and, therefore, interpretations.

As stated by Galobardes et al. in 2006, there is no single best indicator of SES suitable for all study aims and applicable at all time points in all settings. Each indicator measures different, often related aspects of socioeconomic stratification and may be more or less relevant to different health outcomes and at different stages in the life course. The choice of SES measure(s) should ideally be informed by consideration of the specific research question and the proposed mechanisms linking SES to the outcome.

For the purpose of this review, we will allow all definitions of SES considered in published papers in selected highest yield databases. Assessment of its nature, characteristics and usefulness will be reviewed afterwards in order to allow for a broad search and an exhaustive review of the international evidence. The key aspect of this concept will be its intra-population relevance, at the individual, the household or a small geographical area, but always within a single population. That is, different socioeconomic groups measured though various ways, are compared in their rates of immigrant people, diagnose of asthma and allergies, and related risk factors.

5. **Socioeconomic (particularly income) inequality:** There is strong scientific evidence that since the mid-1970s socioeconomic inequalities have increased significantly in the world including in Europe. Socioeconomic inequalities are nothing new and many EU citizens and policymakers at times feel that inequalities are an inevitable consequence of our modernity. However such a stance takes no account of the fact that research shows that between the 1930s and the 1970s socioeconomic inequalities were indeed reduced, and sometimes very significantly, in many parts of the World including Europe, mainly through the strong influence of “the Welfare State”. Thus socio-economic inequalities are not an automatic consequence of modernity they can be reduced and kept at bay.

   There is also ever more evidence that countries and regions with higher socio-economic inequalities experience the most acute socio-economic problems – whether we speak about lower economic growth, increases in violence, poorer educational achievement, declining civic or electoral participation or higher mortality rates. On the contrary, countries with a lower level of socio-economic inequalities fare better in all these domains. Socioeconomic inequality can be measured in many ways; the most frequent one on income inequality is the Gini coefficient and, in its relation to health, the concentration index.

   For the purpose of this review, we will allow all definitions of socioeconomic inequality considered in published papers in selected highest yield databases. Assessment of its nature, characteristics and usefulness will be reviewed afterwards in order to allow for a broad search and an exhaustive review of the international evidence. The key aspect of this concept will be its inter-population relevance, in opposition to measures of socioeconomic status. This will necessary require the inclusion of ecological studies that will compare rates of asthma and allergies between societies crude and adjusted by their degree of socioeconomic inequality.

3.f) Criteria for considering studies in this review

i) Inclusion criteria

Population

Subjects of all ages

Type of articles

- Original research
- Systematic reviews

Chapter of books, position articles, editorials, and guidelines, will be included in a separate folder as key background papers or documents

Language

We will consider only publications written in English or any other language

Source of publications

Publications will be obtained from:

- electronic databases: Pubmed/Medline, Scopus and Cochrane Library
- hand search of the first 50 articles listed in PubMed as “related citations” plus all references of selected papers
- expert criteria. In addition, evidence from the following guidelines will be included if not identified in the electronic search
    - Allergic Rhinitis and its Impact on Asthma (ARIA) www.whiar.org,
    - National Asthma Education Program (NAEPP) http://www.nhlbi.nih.gov/guidelines,
    - Global Initiative for Asthma (GINA) http://www.ginasthma.com/GuidelinesResources.asp,
    - Canadian Pediatric Asthma Consensus guidelines www.cmaj.ca ,
    - British Society for allergy and Clinical Immunology (BSACI) guidelines for the management of allergic and non-allergic rhinitis http://www.bsaci.org
    - Australasian Society of Clinical Immunology and Allergy (ASCIA), guidelines for prevention of food anaphylactic reactions in schools, preschools and childcare http://www.allergy.org.au/content/view/31/258/,

- National Institute of Allergy and Infectious Diseases (NIAID): food allergy guidelines http://www.niaid.nih.gov/topics/foodallergy/clinical/Pages/default.aspx,
- National Heart and Blood Institute (NHBLI), guidelines for asthma http://www.nhlbi.nih.gov/guidelines
- American College of Allergy, Asthma, and Immunology (ACAAI) , Guidelines for the Diagnosis and Management of Food Allergy in the United States www.acaai.org
- Guidelines for the Diagnosis and Management of Food Allergy in the United States: Report of the NIAID-Sponsored Expert Panel, http://www.jacionline.org/article/S0091-6749(10)01566-6/fulltext
- Guidelines for the management of atopic eczema, Primary Care Dermatology Society & British Association of Dermatologists http://www.eguidelines.co.uk/user/login_newsys.php

Study design

- Case-control studies
- Case-series
- Birth cohort studies
- Other prospective and retrospective longitudinal observational studies
- Cross-sectional studies
- Ecological studies
- Clinical trials, controlled trials, randomised controlled trials, randomised clinical trials and intervention studies comparing different socioeconomic groups AND immigrant versus native populations (if any)
- Systematic reviews of primary data on migration status, socioeconomic status and asthma and allergies

Non systematic reviews will be included in a separate folder as key background papers or documents

ii) Exclusion criteria

Population

- We will only exclude studies including mixed subjects (children and adults) if results are not presented separately for children and adults

Objective of articles

We will exclude:

- Articles whose primary aim is to assess mechanisms or aetiology of asthma and allergies i.e. those articles conducting omic studies (genomics, proteomics, transcriptomics and metabolomics) and that do not provide both of the following direct and relevant information about:
    - Socioeconomic status or socioeconomic inequality, and later on
    - Migration status

Type of article

- Experimental studies involving either animals or cell culture or both
- Studies whose primary aim is to analyse genes, non IgE biomarkers or performing epigenetic analysis in subjects without any input in SES or migration status relevant to this study
- Studies that identify, define and/or describe the relationship of interest in this review using unsupervised statistical methods

Study design

- Experimental studies involving either animals or cell culture or both
- Non systematic reviews, chapter books or reports (will be considered in a separate background folder)

3.g) Electronic search

Databases

We will conduct a systematic search in the following databases Pubmed/Medline, Scopus and Cochrane Library

We will choose MeSH terms according to the database (minimal variations) and a selection of string terms to increase the capture of relevant papers (see Figure 4)

We will follow a sequential search of the literature and clearly separated for individual and country level data (see Figure 5)

Figure 4 Search strategy for Medline (PubMed) *

[ON ASTHMA AND ALLERGIES….]

(Asthma[tiab] OR respiratory sound*[tiab] OR wheez*[tiab] OR rhinitis[tiab] OR rhinoconjunctivitis[tiab] OR food allergy[tiab] OR food hypersensitivity[tiab] OR atopic dermatit*[tiab] OR eczem*[tiab] OR anaphyla*[tiab] OR urticaria[tiab] OR allerg*[tiab])

{
AND

[ON SOCIOECONOMIC STATUS OR SOCIOECONOMIC INEQUALITY]

(health status disparities OR health inequality OR health inequity OR health inequalities OR health disparity OR health disparities OR Health Status Disparities[Mesh])

OR

(socioeconomic status OR social class OR poverty OR poor OR income OR disadvantaged OR deprivation OR deprived OR socioeconomic factors OR socioeconomic position OR deprivation OR deprived OR Socioeconomic Factors[Mesh])

}
AND

[ON MIGRATION STATUS….]

(transient and migrants OR Migration OR Migrants OR Immigrants OR “Emigrants OR Mobile population OR Spatial mobility OR Migran*[tiab])

* For EMBASE and CINAHL, appropriate Emtree and CINAHL subject headings were applied

Figure 5 Description of stages to follow during the articles search

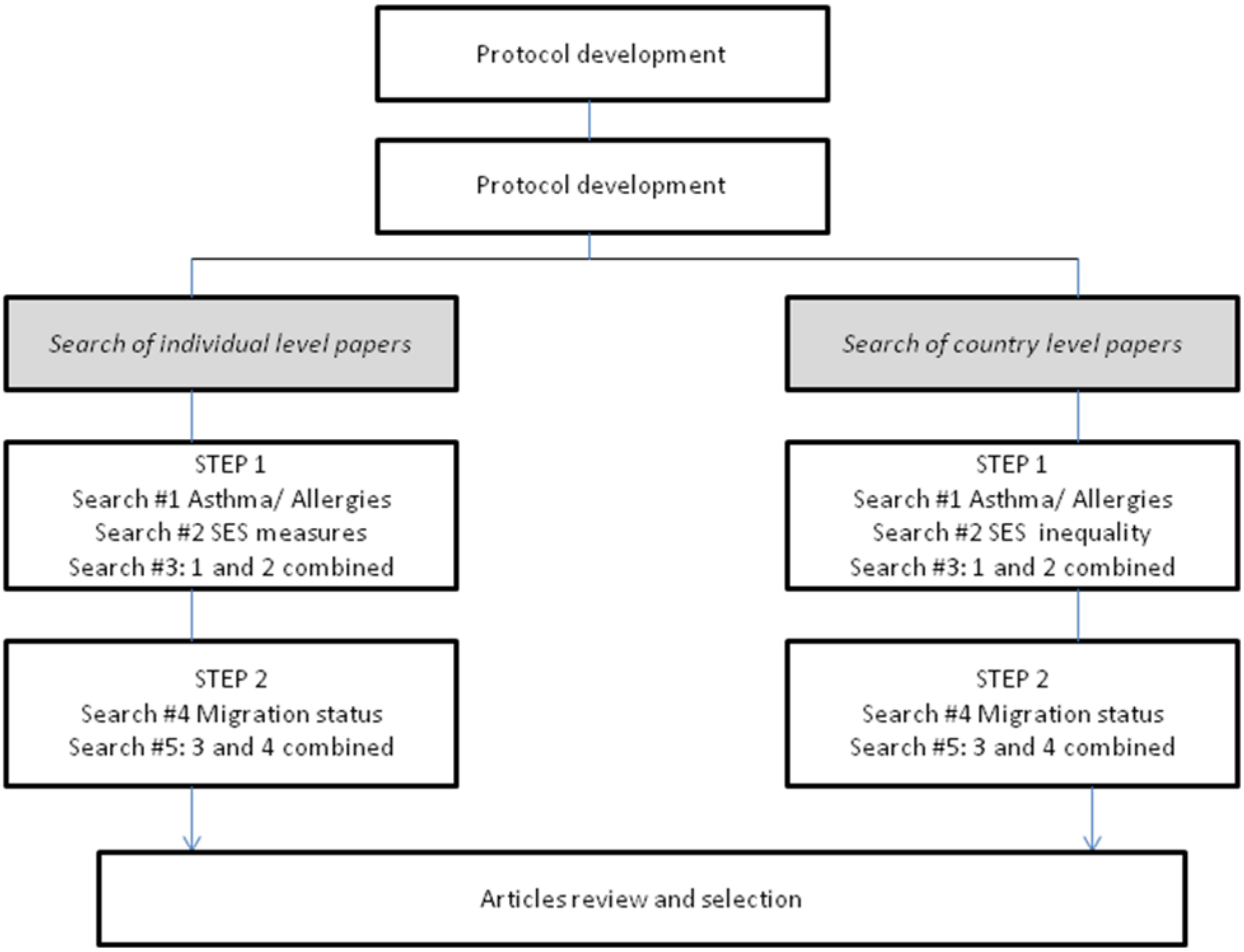

Management of references

For knowledge management (KM) (to gather, manage, store and share all kind of information, as well as generate citations and bibliographies), writing and collaboration between researchers, we will use Endnote reference manager. The formal structure and process of KM considered in this review will enable us the storage of all bibliographic details of retrieved articles and avoid out-of-date versions of the files. KM will thus provide a common up-to-date workspace where reviewers will be able to share and update references relevant to this review.

3.h) Study selection

The search strategy and selection of studies will allow for a wide variety of study designs and interpretations of social capital to be included. We reviewed studies published before March 2013 that could be located through online databases MEDLINE, Scopus and Cochrane Library.

In step one, two members of the research team will independently evaluate the retrieved full-text reports and make a decision on inclusion or exclusion according to the predefined selection criteria. They will record their decision about inclusion or exclusion in a KM file and will record the reason if they decide that the article should be excluded. Decisions of each reviewer will be recorded as follows:
0 = exclude;
1 = clearly eligible: order for full text assessment;
2 = unclear: reviewers should be conservative and order for full text assessment;
3 = related study, do not order but may be a useful reference

If it is clear that if the study is not eligible it will be labelled as “0” and excluded. If it is eligible it will be labelled as “1” and included. We will label as “2”, and thus request the full text for assessment, those studies where there are still doubts on its assignment of inclusion or exclusion.

Duplicates were removed from the initial results and in step 2, titles and abstracts will be independently screened by the first and second author. Studies will be considered eligible if the title or abstract contain terms related to asthma/allergies, SES or socioeconomic inequality, and migration status.

After step 2, studies agreed to include and studies on which no first agreement was reached will be reviewed in step 3. Abstracts of studies not agreed upon after this step will be discussed until complete agreement between the two researchers and will consider the inclusion of a third member of the team to reach agreement.

In step 3, a full-text review and data extraction of all selected papers will be performed by the first two authors by making a summary table of results. Given the great heterogeneity on migrant populations worldwide and hence the incomparability of dependent and independent variables used in the studies, no summary measures will be produced.

Dealing with lack of information

If after examining the full-text report the study does not provide enough information to ascertain suitability for inclusion and is classified as “unclear”, we will contact the authors of the studies by email. In case of failure to communicate with the primary investigators, or if there is no response within two weeks, we will list the particular study as “potentially relevant study”.

Dealing with duplication

Duplication can occur because there are identical manuscripts, translated reports or reports describing different numbers of participants and/or outcomes. Duplicates are likely to appear as the same paper might be available in more than one dataset included in the search. After excluding obvious duplicates for this reason, we will extract data from all other potential duplicated reports directly into a single data collection treating them as a single study but we will list all references from the same study.

Reporting study selection

We will use a flow chart to describe the study selection process according to the PRISMA STATEMENT (http://www.prisma-statement.org/) diagram.

Pilot study selection process

Two members of the team will conduct a pilot test to define the final search strategy by applying the inclusion and exclusion criteria to a sample of 10-20 randomly selected papers. Any disagreement or uncertainties over inclusion and exclusion will be discussed by the reviewers until they reach consensus. On the basis of this assessment, the search terms may then be modified. The abstract-title screening form will be also tested in a sample of 10-20 randomly selected abstracts. Then, the two reviewers will check consistency and change the form if necessary. The same process will be followed for the full text screening form.

3.i) Data extraction strategy

The data extraction form will include the following key characteristics: First author, year of publication, data source, aim of study, measure of asthma or allergy, SES or socioeconomic inequality measure, type of migration status measure, level of measurement, sample size and population, confounders or other factors included in analysis, results, and reported effect of SES and socioeconomic inequality on the rate and severity of asthma/allergies among immigrant and non immigrant populations.

Prior to data extraction of the study reports, two reviewers will test the extraction form to identify if the instructions are confusing or incomplete, so that the form can be further modified after consensus. These two reviewers will independently carry out the data extraction by using a pre-designed electronic data extraction form.

The reviewers will extract the information that must be explicit and detailed (using the words 'not reported' or 'unclear' if necessary) and the original information must be written in quotes. Missing information will be obtained from the original authors whenever possible.

Only after completion of the data extraction process, reviewers will compare their information. If disagreements occur, they will be resolved by consensus or referral to a third member of the review team when necessary.

3.j) Data extraction (selection and coding)

Two independent reviewers will conduct title scans in a parallel fashion. All abstracts will be reviewed by two reviewers independently. When two reviewers consider the abstract eligible for inclusion or one reviewer score the abstract as "included" and the other as "unclear", the study will be retrieved for full text assessment. Conversely, when the two reviewers consider an abstract to be "excluded", the full text will not be retrieved. Disagreements will be resolved through referral to a third reviewer. A pre-designed data extraction form will be developed and data from the texts in full will be abstracted by two reviewers independently with disagreements resolved by discussion among investigators.

4. Methods of analysis and synthesis

4.a) Risk of bias (quality) assessment

All studies included in the review will be grouped according to different focus topics and within each topic by study design. Two reviewers will assess independently the quality of the studies following recommended guidelines already available (i.e. the Strengthening the Reporting of Observational Studies in Epidemiology (STROBE) initiative and Mixed Methods Appraisal Tool (MMAT)-Version 2011 (MMAT-Version 2011)). Because we will group studies according to outcomes and diseases, we will assess specific methodological issues that may introduce potential bias following quality criteria previously established and agreed by the study group.

4.b) Strategy for data synthesis

The planned data synthesis will be narrative due to the heterogeneity of populations, methodologies, and measures of the studies included.

4.c) Analysis of subgroups or subsets

Given that this review encompasses several IgE-associated diseases, several levels of analysis, several measures of immigrants and SES, we will also explore the evidence of each of them in separate groups.

4.d) Dissemination plans

The results will be published in peer-reviewed literature.

5. Organization of review

1. **Lead**: Baltica Cabieses
2. **Protocol development**: Baltica Cabieses, Noortje Uphoff, Mariona Pinart, John Wright, Josep M Anto
3. **Screening of abstracts and full-text articles for possible inclusion and data extraction**: Baltica Cabieses, Noortje Uphoff
4. **Data analysis, interpretation and writing of the review**: Baltica Cabieses, Noortje Uphoff, Mariona Pinart

6. Relevant References

Wright J, Small N, Raynor P, Tuffnell D, Bhopal R, Cameron N, Fairley L, Lawlor DA, Parslow R, Petherick ES, Pickett KE, Waiblinger D, West J; on behalf of the Born in Bradford Scientific Collaborators Group. Cohort profile: The Born in Bradford multi-ethnic family cohort study. Int J Epidemiol. 2012 Oct 12. [Epub ahead of print]

Arnedo-Pena, A., et al., *Sunny hours and variations in the prevalence of asthma in schoolchildren according to the International Study of Asthma and Allergies (ISAAC) Phase III in Spain.* International Journal of Biometeorology, 2011. 55(3): p. 423-434.

Chen, E., et al., *Resilience in low-socioeconomic-status children with asthma: Adaptations to stress.* Journal of Allergy and Clinical Immunology, 2011. 128(5): p. 970-976.

Asher, M.I., et al., *Worldwide time trends in the prevalence of symptoms of asthma, allergic rhinoconjunctivitis, and eczema in childhood: ISAAC Phases One and Three repeat multicountry cross-sectional surveys (vol 368, pg 733, 2006).* Lancet, 2007. 370(9593): p. 1128-1128.

Worldwide variations in the prevalence of asthma symptoms: the International Study of Asthma and Allergies in Childhood (ISAAC). Eur Respir J, 1998. 12(2): p. 315-35.

Williams, H., et al., *Worldwide variations in the prevalence of symptoms of atopic eczema in the International Study of Asthma and Allergies in Childhood.* J Allergy Clin Immunol, 1999. 103(1 Pt 1): p. 125-38.

Stewart, A.W., et al., *The relationship of per capita gross national product to the prevalence of symptoms of asthma and other atopic diseases in children (ISAAC).* International Journal of Epidemiology, 2001. 30(1): p. 173-179.

Lai, C.K.W., et al., *Global variation in the prevalence and severity of asthma symptoms: Phase Three of the International Study of Asthma and Allergies in Childhood (ISAAC).* Thorax, 2009. 64(6): p. 476-483.

Cope, S.F., et al., *Socioeconomic factors related to asthma control in children.* Value in Health, 2007. 10(3): p. A116-A117.

Cope, S.F., W.J. Ungar, and R.H. Glazier, *Socioeconomic factors and asthma control in children.* Pediatric Pulmonology, 2008. 43(8): p. 745-752.

Ungar, W.J., et al., *Relationship of asthma management, socioeconomic status, and medication insurance characteristics to exacerbation frequency in children with asthma.* Annals of Allergy Asthma & Immunology, 2011. 106(1): p. 17-23.

Ungar, W.J., et al., *Socioeconomic Factors and Home Allergen Exposure in Children With Asthma.* Journal of Pediatric Health Care, 2010. 24(2): p. 108-115.

Ungar, W., et al., *Effects of Asthma Management, Socioeconomic Status and Medication Insurance Characteristics on Exacerbation Frequency in Children with Asthma.* Value in Health, 2010. 13(7): p. A326-A326.

Chen, E., et al., *Socioeconomic status associated with exhaled nitric oxide responses to acute stress in children with asthma.* Brain Behavior and Immunity, 2010. 24(3): p. 444-450.

Valdivia, G., et al., *Changes in asthma prevalence among school children during a 6-year period. Influence of socioeconomic status.* Revista Medica De Chile, 2009. 137(2): p. 215-225.

Laurent, O., et al., *Sales of Short-Acting Beta Agonist for Asthma, Air Pollution and Socioeconomic Deprivation: A Small-Area Case Crossover Study in Children and Adolescents.* Epidemiology, 2008. 19(6): p. S96-S97.

Sole, D., et al., *Is the prevalence of asthma and related symptoms among Brazilian children related to socioeconomic status?* Journal of Asthma, 2008. 45(1): p. 19-25.

Shankardass, K., et al., *The association between contextual socioeconomic factors and prevalent asthma in a cohort of Southern California school children.* Social Science & Medicine, 2007. 65(8): p. 1792-1806.

Kim, S.Y., H. Kim, and M.S. O'Neill, *Does socioeconomic position modify the effect of air pollution on asthma in children?* Epidemiology, 2006. 17(6): p. S258-S258.

Lee, J.T., et al., *Effect of air pollution on asthma-related hospital admissions for children by socioeconomic status associated with area of residence.* Archives of Environmental & Occupational Health, 2006. 61(3): p. 123-130.

Duran-Tauleria, E. and R.J. Rona, *Geographical and socioeconomic variation in the prevalence of asthma symptoms in English and Scottish children.* Thorax, 1999. 54(6): p. 476-481.

Suarez-Varela, M.M.M., A.L.G. Gonzalez, and M.I.M. Selva, *Socioeconomic risk factors in the prevalence of asthma and other atopic diseases in children 6 to 7 years old in Valencia, Spain.* European Journal of Epidemiology, 1999. 15(1): p. 35-40.

Bergmann, R.L., et al., *Socioeconomic status (SES) is a risk factor for asthma and hay fever in parents and for atopic dermatitis (AD) in their children.* Journal of Allergy and Clinical Immunology, 1999. 103(1): p. S17-S17.

Persky, V.W., et al., *Relationships of race and socioeconomic status with prevalence, severity, and symptoms of asthma in Chicago school children.* Annals of Allergy Asthma & Immunology, 1998. 81(3): p. 266-271.

Ernst, P., et al., *Socioeconomic-Status and Indicators of Asthma in Children.* American Journal of Respiratory and Critical Care Medicine, 1995. 152(2): p. 570-575.

Williesjacobo, L.J., et al., *Socioeconomic-Status and Allergy in Children with Asthma.* Journal of Allergy and Clinical Immunology, 1993. 92(4): p. 630-632.

Martinez FD. Genes, environments, development and asthma: a reappraisal. Eur Respir J. 2007 Jan;29(1):179-84.

Martinez FD. Heterogeneity of the association between lower respiratory illness in infancy and subsequent asthma. Proc Am Thorac Soc. 2005;2(2):157-61.

Spergel JM, Paller AS. Atopic dermatitis and the atopic march. J Allergy Clin Immunol. 2003 Dec;112(6 Suppl):S118-27.

Prevention and control of noncommunicable diseases: implementation of the global strategy (Report submitted to the 61st World Health Assembly). Geneva, World Health Organization. 2008.

Martinez FD. Gene-environment interaction in complex diseases: asthma as an illustrative case. Novartis Found Symp. 2008;293:184-92; discussion 92-7.

von Mutius E. Gene-environment interactions in asthma. J Allergy Clin Immunol. 2009 Jan;123(1):3-11; quiz 2-3.

Bousquet J, Jacot W, Yssel H, Vignola AM, Humbert M. Epigenetic inheritance of fetal genes in allergic asthma. Allergy. 2004Feb;59(2):138-47.
