# Supplementary material for: A Systematic Review on the Development of Asthma and Allergic Diseases in Relation to International Immigration: The Leading Role of the Environment Confirmed
Source: PLoS One. 2014 Aug 20;9(8):e105347. doi: 10.1371/journal.pone.0105347 (PMC4139367; doi:10.1371/journal.pone.0105347)
Supplement: Figure S1 — Hits retrieved from systematic search in step 1. (PDF) [file pone.0105347.s001.pdf]

1. Nat Rev Drug Discov. 2013 Jan 21. doi: 10.1038/nrd3838. [Epub ahead of print]

Targeting eosinophils in allergy, inflammation and beyond.

Fulkerson PC, Rothenberg ME.

Division of Allergy and Immunology, Department of Pediatrics, Cincinnati Children's Hospital Medical Center, 3333 Burnet Avenue, Cincinnati, Ohio 45229, USA.

Eosinophils can regulate local immune and inflammatory responses, and their accumulation in the blood and tissue is associated with several inflammatory and infectious diseases. Thus, therapies that target eosinophils may help control diverse diseases, including atopic disorders such as asthma and allergy, as well as diseases that are not primarily associated with eosinophils, such as autoimmunity and malignancy. Eosinophil-targeted therapeutic agents that are aimed at blocking specific steps involved in eosinophil development, migration and activation have recently entered clinical testing and have produced encouraging results and insights into the role of eosinophils. In this Review, we describe recent advances in the development of first-generation eosinophil-targeted therapies and highlight strategies for using personalized medicine to treat eosinophilic disorders.

PMID: 23334207 [PubMed - as supplied by publisher]

2. Vet Dermatol. 2013 Feb;24(1):32-e9. doi: 10.1111/j.1365-3164.2012.01082.x.

Innate immune defense system of the skin.

Afshar M, Gallo RL.

Division of Dermatology, Department of Medicine, University of California, San Diego, 8899 University Center Lane, San Diego, CA 92122, USA.

Background - Antimicrobial peptides (AMPs) have a pivotal role in cutaneous innate immunity. They are present in the skin of many animals, including mammals, and are both constitutively present and inducible by infection and injury. Functions - Antimicrobial peptides exhibit antimicrobial activity against bacteria, viruses, fungi and parasites, with different potencies depending on their peptide structure. They also act as multifunctional effector molecules that influence diverse cellular processes, including cell migration, proliferation and differentiation, cytokine production, angiogenesis and wound healing. Suppressed AMP production has been associated with increased susceptibility to microbial insults and the pathogenesis of atopic dermatitis. This review highlights recent observations on the expression and role of AMPs, particularly the AMPs cathelicidin and β-defensin, in healthy and diseased skin.

© 2013 The Authors. Veterinary Dermatology © 2013 ESVD and ACVD.

PMID: 23331677 [PubMed - in process]

3. Int J Dermatol. 2013 Jan 20. doi: 10.1111/j.1365-4632.2012.05777.x. [Epub ahead of print]

Clinical-epidemiological features of contact dermatitis in rural and urban communities in northern Ethiopia: correlation with environmental or occupational exposure.

Morrone A, Bordignon V, Barnabas GA, Dassoni F, Latini O, Padovese V, Ensoli F, Cristaudo A.

National Institute for Health, Migration and Poverty (NIHMP), Rome, Italy.

BACKGROUND: The widespread diffusion of low-quality products as well as the local cultural habits could be a relevant cause of allergic diseases in developing countries. In the present observational study, we explored the prevalence of allergic contact dermatitis in both rural and urban settings in northern Ethiopia, where skin diseases represent a frequent cause of morbidity. Clinical features and specific reactivities in association with environmental or occupational exposure were investigated. PATIENTS AND METHODS: We patch tested 480 consecutive patients, visited at the Mekele IDC, exhibiting symptoms of contact dermatitis. A detailed medical history of each patient was collected. RESULTS: A positive patch-test response was observed in 50% of subjects; nickel was the most frequent sensitizer (26.2%), followed by p-tert-butylphenol formaldehyde resin (10%), fragrance mix (7.1%), potassium dichromate (5.4%), cobalt chloride (4.6%), disperse blue (2.3%), and p-phenylenediamine (1.7%). Gender-related differences were analyzed for single allergen. Eczema represented the most common manifestation, affecting the head and neck as primary skin areas. While reactivity to nickel interested almost all the occupational categories, sensitization to other allergens could be ascribed to working habits or environmental exposure. CONCLUSIONS: The results gathered from this study, the first one conducted within the Tigray region in Ethiopia, confirm the need to take appropriate measures to limit the nickel rate in metal objects and may be useful to design allergenic series suitable for patch testing in those geographical settings.

© 2013 The International Society of Dermatology.

PMID: 23330703 [PubMed - as supplied by publisher]

4. J Fr Ophtalmol. 2013 Jan 11. pii: S0181-5512(12)00374-9. doi: 10.1016/j.jfo.2012.11.003. [Epub ahead of print]

[Anterior chamber migration of intravitreal dexamethasone implant (Ozurdex(®)) in pseudophakic eyes: Report of three cases.]

[Article in French]

Malclès A, Janin-Manificat H, Yhuel Y, Russo A, Agard E, El Chehab H, Ract Madoux G, Masse H, Burillon C, Dot C.

Service d'ophtalmologie, hôpital d'instruction des armées Desgenettes, 108, boulevard Pinel, 69003 Lyon, France. Electronic address: amalcles@yahoo.fr.

INTRODUCTION: Intravitreal implantation of Ozurdex(®) (Allergan Inc., Irvine, CA, USA) is being used widely for the treatment of macular edema secondary to retinal vein occlusion and in the setting of non-infectious posterior uveitis. We describe a complication little reported in the literature until now: migration of the dexamethasone implant into the anterior chamber. PATIENTS AND METHODS: We report three cases of migration in two pseudophakic patients with iris claw lenses (on the anterior and posterior aspects of the iris) and in one pseudophakic patient with a posterior chamber IOL and zonular rupture. DISCUSSION: The risk of anterior chamber migration of the Ozurdex(®) implant is increased in cases of prior vitrectomy (three cases), prone positioning and dilation of the pupil (mydriasis). Clinical tolerability of the implant in the anterior chamber is poor in all cases, with diffuse corneal edema. Endothelial

cell loss occurs, as demonstrated by specular microscopy performed in two of our patients. Removal or repositioning of the Ozurdex(®) implant into the posterior segment must be performed without delay because of the risk of endothelial toxicity. CONCLUSION: Patients without perfect zonular/posterior capsular integrity present a high risk of anterior chamber migration of the Ozurdex(®) implant. In such cases, anti-VEGF therapies should be discussed as an alternative.

Copyright © 2012 Elsevier Masson SAS. All rights reserved.

PMID: 23318000 [PubMed - as supplied by publisher]

5. J Allergy (Cairo). 2012;2012:245909. doi: 10.1155/2012/245909. Epub 2012 Dec 4.

Neutrophil Inhibitory Factor Selectively Inhibits the Endothelium-Driven Transmigration of Eosinophils In Vitro and Airway Eosinophilia in OVA-Induced Allergic Lung Inflammation.

Schnyder-Candrian S, Maillet I, Le Bert M, Brault L, Jacobs M, Ryffel B, Schnyder B, Moser R.

UMR7355, CNRS, Orleans, France ; Experimental and Molecular Immunology and Neurogenetics, University of Orleans, Orleans, France ; Institute for Clinical and Biomedical Research Thurgau, 9548 Matzingen, Switzerland.

Leukocyte adhesion molecules are involved in cell recruitment in an allergic airway response and therefore provide a target for pharmaceutical intervention. Neutrophil inhibitory factor (NIF), derived from canine hookworm (Ancylostoma caninum), binds selectively and competes with the A-domain of CD11b for binding to ICAM-1. The effect of recombinant NIF was investigated. Intranasal administration of rNIF reduced pulmonary eosinophilic infiltration, goblet cell hyperplasia, and Th(2) cytokine production in OVA-sensitized mice. In vitro, transendothelial migration of human blood eosinophils across IL-4-activated umbilical vein endothelial cell (HUVEC) monolayers was inhibited by rNIF (IC(50): 4.6 ± 2.6 nM; mean ± SEM), but not across TNF or IL-1-activated HUVEC monolayers. Treatment of eosinophils with rNIF together with mAb 60.1 directed against CD11b or mAb 107 directed against the metal ion-dependent adhesion site (MIDAS) of the CD11b A-domain resulted in no further inhibition of transendothelial migration suggesting shared functional epitopes. In contrast, rNIF increased the inhibitory effect of blocking mAbs against CD18, CD11a, and VLA-4. Together, we show that rNIF, a selective antagonist of the A-domain of CD11b, has a prominent inhibitory effect on eosinophil transendothelial migration in vitro, which is congruent to the in vivo inhibition of OVA-induced allergic lung inflammation.

PMCID: PMC3523160
PMID: 23304174 [PubMed]

6. BMC Genomics. 2012;13 Suppl 7:S10. doi: 10.1186/1471-2164-13-S7-S10. Epub 2012 Dec 13.

An analysis of the transcriptome of Teladorsagia circumcincta: its biological and biotechnological implications.

Menon R, Gasser RB, Mitreva M, Ranganathan S.

Department of Chemistry and Biomolecular Sciences, Macquarie University, Sydney, New South Wales 2109, Australia. shoba.ranganathan@mq.edu.au.

BACKGROUND: Teladorsagia circumcincta (order Strongylida) is an economically important parasitic nematode of small ruminants (including sheep and goats) in temperate climatic regions of the world. Improved insights into the molecular biology of this parasite could underpin alternative methods required to control this and related parasites, in order to circumvent major problems associated with anthelmintic resistance. The aims of the present study were to define the transcriptome of the adult stage of T. circumcincta and to infer the main pathways linked to molecules known to be expressed in this nematode. Since sheep develop acquired immunity against T. circumcincta, there is some potential for the development of a vaccine against this parasite. Hence, we infer excretory/secretory molecules for T. circumcincta as possible immunogens and vaccine candidates.
RESULTS: A total of 407,357 ESTs were assembled yielding 39,852 putative gene sequences. Conceptual translation predicted 24,013 proteins, which were then subjected to detailed annotation which included pathway mapping of predicted proteins (including 112 excreted/secreted [ES] and 226 transmembrane peptides), domain analysis and GO annotation was carried out using InterProScan along with BLAST2GO. Further analysis was carried out for secretory signal peptides using SignalP and non-classical sec pathway using SecretomeP tools.For ES proteins, key pathways, including Fc epsilon RI, T cell receptor, and chemokine signalling as well as leukocyte transendothelial migration were inferred to be linked to immune responses, along with other pathways related to neurodegenerative diseases and infectious diseases, which warrant detailed future studies. KAAS could identify new and updated pathways like phagosome and protein processing in endoplasmic reticulum. Domain analysis for the assembled dataset revealed families of serine, cysteine and proteinase inhibitors which might represent targets for parasite intervention. InterProScan could identify GO terms pertaining to the extracellular region. Some of the important domain families identified included the SCP-like extracellular proteins which belong to the pathogenesis-related proteins (PRPs) superfamily along with C-type lectin, saposin-like proteins. The 'extracellular region' that corresponds to allergen V5/Tpx-1 related, considered important in parasite-host interactions, was also identified.Six cysteine motif (SXC1) proteins, transthyretin proteins, C-type lectins, activation-associated secreted proteins (ASPs), which could represent potential candidates for developing novel anthelmintics or vaccines were few other important findings. Of these, SXC1, protein kinase domain-containing protein, trypsin family protein, trypsin-like protease family member (TRY-1), putative major allergen and putative lipid binding protein were identified which have not been reported in the published T. circumcincta proteomics analysis.Detailed analysis of 6,058 raw EST sequences from dbEST revealed 315 putatively secreted proteins. Amongst them, C-type single domain activation associated secreted protein ASP3 precursor, activation-associated secreted proteins (ASP-like protein), cathepsin B-like cysteine protease, cathepsin L cysteine protease, cysteine protease, TransThyretin-Related and Venom-Allergen-like proteins were the key findings.
CONCLUSIONS: We have annotated a large dataset ESTs of T. circumcincta and undertaken detailed comparative bioinformatics analyses. The results provide a comprehensive insight into the molecular biology of this parasite and disease manifestation which provides potential focal point for future research. We identified a number of pathways responsible for immune response. This type of large-scale computational scanning could be coupled with proteomic and metabolomic studies of this parasite leading to novel therapeutic intervention and disease control strategies. We have also successfully affirmed the use of bioinformatics tools, for the study of ESTs, which could now serve as a benchmark for the development of new computational EST analysis pipelines.

PMCID: PMC3521389
PMID: 23282110 [PubMed - in process]

7. J Leukoc Biol. 2013 Jan 4. [Epub ahead of print]

Respiratory syncytial virus infection modifies and accelerates pulmonary disease via DC activation and migration.

Jang S, Smit J, Kallal LE, Lukacs NW.

University of Michigan Medical School, Department of Pathology, Ann Arbor, Michigan, USA.

In the present studies, we have established that RSV can elicit a more pathogenic environment dependent on improper DC-associated sensitization. Our initial studies demonstrated that RSV, but not influenza, infection during an allergen exposure into the airway induced a more severe allergen response. The RSV-induced exacerbation included an increased Th2 cytokine response and pathophysiology as monitored by AHR and mucus overproduction. DCs played a central role in the allergen-induced responses, as instilling RSV-infected BMDC into the airway could recapitulate a live virus challenge. With the use of CCR6-/- mice that have a primary defect in the recruitment of mDC subsets, reduced exacerbation of disease was observed when RSV was administered along with allergen. Furthermore, sensitization of mice with RSV-infected BMDC into the airway produced a more severe immune response to a live virus challenge. Subsequently, using RSV-infected BMDC from CCR7-/- mice (that do not migrate efficiently to LNs) to sensitize the exacerbated response demonstrated that the response was dependent on DC migration to the LN. Finally, the ability of RSV-infected DCs to elicit an exacerbated, allergen-induced pathogenic response could be maintained for as long as 3 weeks, suggesting that RSV-infected DCs themselves created an altered immune environment that impacts off-target mucosal responses that could have prolonged effects.

PMID: 23293372 [PubMed - as supplied by publisher]

8. Clin Cancer Res. 2013 Jan 3. [Epub ahead of print]

Enhancement of human cancer cell motility and invasiveness by anaphylatoxin C5a via aberrantly expressed C5a-receptor (CD88).

Nitta H, Wada Y, Kawano Y, Murakami Y, Irie A, Taniguchi K, Kikuchi K, Yamada G, Suzuki K, Honda J, Wilson-Morifuji M, Araki N, Eto M, Baba H, Imamura T.

Gastroenterological Surgery, Kumamoto University School of Medicine.

PURPOSE: The anaphylatoxin C5a is a chemoattractant that induces leukocyte migration via C5a receptor (C5aR). There is emerging evidence that C5a is generated in the cancer microenvironment. We therefore sought C5aR expression and a direct influence of the C5a-C5aR axis on cancer cells. EXPERIMENTAL DESIGN: C5aR expression was investigated in human cancer tissues and cell lines. Effects of C5a stimulation on cancer cells were studied by cytoskeletal rearrangement, time-lapse analysis, Matrigel chamber assay and invasion in nude mouse in a comparison of C5aR-expressing cancer cells with control cells. RESULTS: C5aR was aberrantly expressed in various human cancers. Several cancer cell lines also expressed C5aR. C5a triggered cytoskeletal rearrangement and enhanced cell motility 3-fold and invasiveness 13-fold of C5aR-expressing cancer cells. Such enhancement by C5a was not observed in control cells. Cancer cell invasion was still enhanced in the absence of C5a concentration gradient and even after the removal of C5a stimulation, suggesting that random cell locomotion plays an important role in C5a-triggered cancer cell invasion. C5a increased the release of matrix metalloproteinases (MMPs) from cancer cells by 2 to 11-fold, and inhibition of MMP activity abolished the C5a enhancing effect on cancer cell invasion. Compared with control cells, C5aR-expressing cells spread 1.8-fold more

broadly at implanted nude mouse skin sites only when stimulated with C5a.
CONCLUSIONS: These results illustrate a novel activity of the C5a-C5aR axis that promotes cancer cell invasion through motility activation and MMP release. Targeting this signaling pathway may provide a useful therapeutic option for cancer treatment.

PMID: 23287562 [PubMed - as supplied by publisher]

9. Toxicol In Vitro. 2012 Dec 31. pii: S0887-2333(12)00363-3. doi: 10.1016/j.tiv.2012.12.025. [Epub ahead of print]

Development of an in vitro skin sensitization test based on ROS production in THP-1 cells.

Saito K, Miyazawa M, Nukada Y, Sakaguchi H, Nishiyama N.

Safety Science Research Laboratories, Kao Corporation, 2606 Akabane, Ichikai-Machi, Haga-Gun, Tochigi 321-3497, Japan. Electronic address: saitou.kazutoshi@kao.co.jp.

Recently, it has been reported that reactive oxygen species (ROS) produced by contact allergens can affect dendritic cell migration and contact hypersensitivity. The aim of the present study was to develop a new in vitro assay that could predict the skin sensitizing potential of chemicals by measuring ROS production in THP-1 (human monocytic leukemia cell line) cells. THP-1 cells were pre-loaded with a ROS sensitive fluorescent dye, 5-(and 6-)-chloromethyl-2', 7'-dichlorodihydrofluorescein diacetate, acetyl ester (CM-H(2)DCFDA), for 15min, then incubated with test chemicals for 30min. The fluorescence intensity was measured by flow cytometry. For the skin sensitizers, 25 out of 30 induced over a 2-fold ROS production at more than 90% of cell viability. In contrast, increases were only seen in 4 out of 20 non-sensitizers. The overall accuracy for the local lymph node assay (LLNA) was 82% for 50 chemicals tested. A correlation was found between the estimated concentration showing 2-fold ROS production in the ROS assay and the EC3 values (estimated concentration required to induce positive response) of the LLNA. These results indicated that the THP-1 cell-based ROS assay was a rapid and highly sensitive detection system able to predict skin sensitizing potentials and potency of chemicals.

Copyright © 2013. Published by Elsevier Ltd.

PMID: 23287046 [PubMed - as supplied by publisher]

10. J Biomed Sci. 2013 Jan 3;20(1):1. [Epub ahead of print]

Concurrent exposure to a dectin-1 agonist suppresses the Th2 response to epicutaneously introduced antigen in mice.

Lin JY, Chen JS, Chen PC, Chung MH, Liu CY, Miaw SC, Wang LF.

ABSTRACT: BACKGROUND: Epicutaneous sensitization with protein allergen that induces predominant Th2 responses is an important sensitization route in atopic dermatitis. Fungal components have been shown to modulate Th cell differentiation. However, the effects of fungal components on epicutaneous sensitization are unclear. RESULTS: In this study, we showed that co-administration of curdlan, a dectin-1 agonist, during epicutaneous ovalbumin sensitization of BALB/c mice decreased the IL-5 and IL-13 levels in supernatants of lymph node cell ovalbumin reactivation cultures. Mechanistically, curdlan co-administration decreased IL-4 and IL-1beta expressions in draining lymph

nodes. Curdlan co-administration also lower the migration of langerin+ CD103- epidermal Langerhans cells into draining lymph nodes at 96 hours post-sensitization which might be attributed to decreased expressions of IL-18 and IL-1beta in patched skin. Moreover, adoptive transfer of CFSE-labeled transgenic CD4 T cells confirmed that curdlan co-administration decreased the proliferation and IL-4-production of ovalbumin -specific T cells primed by epidermal Langerhans cells. CONCLUSIONS: These results indicated that concurrent exposure to a dectin-1 agonist suppresses the epicutaneously induced Th2 response by modulating the cytokine expression profiles in draining LNs and the migration of epidermal Langerhans cells. These results highlight the effects of fungal components on epicutaneous allergen sensitization in atopic diseases.

PMID: 23286586 [PubMed - as supplied by publisher]

11. Open Ophthalmol J. 2012;6:129-36. doi: 10.2174/1874364101206010129. Epub 2012 Dec 14.

Levocetirizine inhibits migration of immune cells to lymph nodes and induces treg cells in a murine type I allergic conjunctivitis model.

García-Zepeda S, Estrada-Muñiz E, Elizondo G, Terrazas LI, Rodríguez-Sosa M, Quintana-Hau JD, Tornero-Montaño R, Baiza-Durán L, Vega L.

Departamento de Toxicología, Centro de Investigación y de Estudios Avanzados-IPN. Av. IPN 2508, San Pedro Zacatenco, México D.F. 07360, México;

BACKGROUND #ENTITYSTARTX00026;PURPOSE: Levocetirizine is a histamine H(1) receptor antagonist. Here, we utilised DO11.10TCR transgenic mice to establish an antigen-specific T cell-dependent allergic conjunctivitis (AC) model to determine the effect of the topical application of an ophthalmic formulation of Levoceritizine as a treatment for AC.
EXPERIMENTAL APPROACH: DO11.10 mice (n=6/each) were exposed to ovalbumin (OVA, 50 μg) and treated with a Levocetirizine ophthalmic formulation (0.001-0.02% v/w) or placebo (vehicle) for 24-72 h. Serum, aqueous/vitreous humour and conjunctiva were obtained. Immunoglobulin (Ig)-E, interleukin (IL)-10 and lipoxin (LX)A(4) were determined by ELISA. Levels of tumour necrosis factor (TNF)-α, transforming growth factor (TGF)-β, interferon (IFN)-γ and 18rS expression were measured by RT-PCR. Proportions of total and activated antigen-presenting cells (APC), recruited T lymphocytes (CD4+), activated T lymphocytes (CD25+) and T regulatory cells (Treg) were measured by flow cytometry.
KEY RESULTS: OVA exposure induced AC in the animal model indicated by increased expression of LXA(4), TNF-α and TGF-β. Levocetirizine treatment (0.01-0.02% v/w) reduced LXA(4) in the eye humours. This treatment approach increased systemic IL-10 secretion and reduced TNF-α and TGF-β expression in conjunctiva without changing IFN-γ expression. Levocetirizine reduced APC levels in draining lymph nodes but increased the proportion of total lymphocytes recruited and their differentiation to Treg cells. CONCLUSIONS #ENTITYSTARTX00026;
IMPLICATIONS: Levocetirizine effectively reduces the activation and migration of APC to local draining lymph nodes and induces differentiation of Treg cells as one possible mechanism of its anti-inflammatory action.

PMCID: PMC3529389
PMID: 23284599 [PubMed]

12. Am J Physiol Lung Cell Mol Physiol. 2012 Dec 28. [Epub ahead of print]

Endothelial Cell PTP1B Regulates Leukocyte Recruitment During Allergic Inflammation.

Berdnikovs S, Abdala-Valencia H, Cook-Mills JM.

1Northwestern University Feinberg School of Medicine.

Pulmonary eosinophilia is a consistent hallmark of allergic lung inflammation. Infiltration of eosinophils into OVA-challenged lungs is dependent on the adhesion molecule vascular cell adhesion molecule-1 (VCAM-1) on endothelial cells. Ligation of VCAM-1 activates endothelial cell PTP1B, which is required for VCAM-1-dependent leukocyte migration in vitro. To examine whether nonhematopoietic PTP1B modulates eosinophil recruitment in vivo, mice deficient in PTP1B were irradiated and received wild-type hematopoietic cells to generate chimeric PTP1B-/- mice. In response to ovalbumin (OVA) challenge, the chimeric PTP1B-/- mice had reduced eosinophilia in the lung tissue and bronchoalveolar lavage, indicating a role for PTP1B in non-hematopoietic cells during leukocyte recruitment. To determine whether endothelial cell PTP1B modulates eosinophil recruitment, mice with an inducible endothelial cell-specific PTP1B deletion (iePTP1B mice) were generated and the PTP1B deletion was induced after antigen-sensitization before antigen-challenge. In response to OVA challenge, the iePTP1B mice with the endothelial cell PTP1B deletion had an increased accumulation of eosinophils bound to the luminal surface of the endothelium in the lung vasculature and had a decrease in leukocyte recruitment into the lung tissue. In the iePTP1B mice, expression of adhesion molecules, cytokines or chemokines that regulate leukocyte recruitment during inflammation was not altered, consistent with other studies that deletion of endothelial adhesion molecule signals does not alter lung cytokines and chemokines. In summary, these data suggest that VCAM-1 activation of PTP1B in the endothelium is necessary for eosinophil recruitment during allergic inflammation. Moreover, these studies provide a basis for targeting VCAM-1-dependent signaling pathways in allergy therapies.

PMID: 23275627 [PubMed - as supplied by publisher]

13. Mediators Inflamm. 2012;2012:475253. doi: 10.1155/2012/475253. Epub 2012 Oct 31.

CC-Chemokine CCL15 Expression and Possible Implications for the Pathogenesis of IgE-Related Severe Asthma.

Shimizu Y, Dobashi K.

Department of Pulmonary Medicine, Maebashi Red Cross Hospital, 3-21-36 Asahi-Cho, Gunma, Maebashi 371-0014, Japan ; Department of Medicine and Molecular Science, Gunma University Graduate School of Medicine, 3-39-15 Showa-Machi, Gunma, Maebashi 371-8511, Japan.

Airway inflammation is accompanied by infiltration of inflammatory cells and an abnormal response of airway smooth muscle. These cells secrete chemokines and express the cell surface chemokine receptors that play an important role in the migration and degranulation of inflammatory cells. Omalizumab is a monoclonal antibody directed against immunoglobulin E, and its blocking of IgE signaling not only reduces inflammatory cell infiltration mediated by the Th2 immune response but also inhibits other immune responses. The chemokine CCL15 is influenced by omalizumab, and the source of CCL15 has been reported to be airway smooth muscle cells and basophils. CCL15 binds to its receptor CCR1, which has been reported to be expressed by various inflammatory cells and also by airway smooth muscle cells. Therefore, CCL15/CCR1 signaling could be a target for the treatment of asthma. We review the role of CCL15 in the pathogenesis of asthma and also discuss the influence of IgE-mediated immunomodulation via CCL15 and its receptor CCR1.

PMCID: PMC3508751
PMID: 23258953 [PubMed - in process]

14. Circ Res. 2012 Dec 18. [Epub ahead of print]

Myeloid Cell 5-Lipoxygenase Activating Protein Modulates the Response to Vascular Injury.

Yu Z, Ricciotti E, Miwa T, Liu S, Ihida-Stansbury K, Landersberg G, Jones PL, Scalia R, Song W, Assoian RK, Fitzgerald GA.

1University of Pennsylvania, Philadelphia, Pennsylvania, UNITED STATES.

Rationale: Human genetics have implicated the 5- lipoxygenase (5-LO) enzyme in the pathogenesis of cardiovascular disease and an inhibitor of the 5-LO activating protein (FLAP) is in clinical development for asthma. Objective: Here we determined whether FLAP deletion modifies the response to vascular injury. Methods and Results: Vascular remodeling was characterized 4 weeks after femoral arterial injury in FLAP knockout (FLAP KO) mice and wild type (WT) controls. Both neointimal hyperplasia and the intima / media ratio of the injured artery were significantly reduced in the FLAP KOs while endothelial integrity was preserved. Lesional myeloid cells were depleted and vascular smooth muscle cell (VSMC) proliferation, as reflected by bromodeoxyuridine (BrdU) incorporation, was markedly attenuated by FLAP deletion. Inflammatory cytokine release from FLAP KO macrophages was depressed and their restricted ability to induce VSMC migration ex vivo was rescued with leukotriene B4 (LTB4). FLAP deletion restrained injury and attenuated upregulation of the extracellular matrix protein, tenascin C (TNC), which affords a scaffold for VSMC migration. Correspondingly, the phenotypic modulation of VSMC to a more synthetic phenotype, reflected by morphological change, loss of α-smooth muscle cell actin and upregulation of vascular cell adhesion molecule (VCAM) -1 was also suppressed in FLAP KO mice. Transplantation of FLAP replete myeloid cells rescued the proliferative response to vascular injury. Conclusions: Expression of lesional FLAP in myeloid cells promotes LTB4 dependent VSMC phenotypic modulation, intimal migration and proliferation.

PMID: 23250985 [PubMed - as supplied by publisher]

15. Inflammation. 2012 Dec 19. [Epub ahead of print]

Is the CCR5 Δ 32 Mutation Associated with Immune System-Related Diseases?

Ghorban K, Dadmanesh M, Hassanshahi G, Momeni M, Zare-Bidaki M, Arababadi MK, Kennedy D.

Department of Immunology, Faculty of Medicine, AJA University of Medical Sciences, Tehran, Iran.

Hypersensitivity and autoimmunity are the main features of immune system-related diseases such as type 2 diabetes (T2D), multiple sclerosis (MS), and asthma. It has been established that chemokines play key roles in the activation and regulation of immune cell migration which is important in the pathogenesis of the diseases mentioned. CC chemokines receptor 5 or CCR5 is a receptor for RANTES, MIP-1α, and MIP-1β and is expressed by several immune cells including NK cells, T lymphocytes, and macrophages. It plays key roles in the regulation of migration and activation of the immune cells during immune responses against microbe and self-antigens during autoimmunity and hypersensitivity disorders. Therefore, any

alteration in the sequence of CCR5 gene or in its expression could be associated with immune system-related diseases. Previous studies revealed that a 32-base pair deletion (Δ 32) in exon 1 of the CCR5 gene led to downregulation of the gene. Previous studies demonstrated that not only CCR5 expression was altered in autoimmune and hypersensitivity disorders, but also that the mutation is associated with the diseases. This review addresses the recent information regarding the association of the CCR5 Δ 32 mutation in immune-related diseases including T2D with and without nephropathy, MS, and asthma. Based on the collected data, it seems that the CCR5 Δ 32 mutation can be considered as a risk factor for MS, but not asthma and T2D with and without nephropathy.

PMID: 23250822 [PubMed - as supplied by publisher]

16. J Mol Med (Berl). 2012 Dec 19. [Epub ahead of print]

Myeloid cell HIF-1α regulates asthma airway resistance and eosinophil function.

Crotty Alexander LE, Akong-Moore K, Feldstein S, Johansson P, Nguyen A, McEachern EK, Nicatia S, Cowburn AS, Olson J, Cho JY, Isaacs H Jr, Johnson RS, Broide DH, Nizet V.

Pulmonary Critical Care Section, Veterans Affairs San Diego Healthcare System, La Jolla, CA, USA, lcrotty@ucsd.edu.

Hypoxia-inducible factor (HIF)-1α is a master regulator of inflammatory activities of myeloid cells, including neutrophils and macrophages. These studies examine the role of myeloid cell HIF-1α in regulating asthma induction and pathogenesis, and for the first time, evaluate the roles of HIF-1α and HIF-2α in the chemotactic properties of eosinophils, the myeloid cells most associated with asthma. Wild-type (WT) and myeloid cell-specific HIF-1α knockout (KO) C57BL/6 mice were studied in an ovalbumin (OVA) model of asthma. Administration of the pharmacological HIF-1α antagonist YC-1 was used to corroborate findings from the genetic model. WT, HIF-1α, and HIF-2α KO eosinophils underwent in vitro chemotaxis assays. We found that deletion of HIF-1α in myeloid cells and systemic treatment with YC-1 during asthma induction decreased airway hyperresponsiveness (AHR). Deletion of HIF-1α in myeloid cells in OVA-induced asthma also reduced eosinophil infiltration, goblet cell hyperplasia, and levels of cytokines IL-4, IL-5, and IL-13 in the lung. HIF-1α inhibition with YC-1 during asthma induction decreased eosinophilia in bronchoalveolar lavage, lung parenchyma, and blood, as well as decreased total lung inflammation, IL-5, and serum OVA-specific IgE levels. Deletion of HIF-1α in eosinophils decreased their chemotaxis, while deletion of the isoform HIF-2α led to increased chemotaxis. This work demonstrates that HIF-1α in myeloid cells plays a role in asthma pathogenesis, particularly in AHR development. Additionally, treatment with HIF-1α inhibitors during asthma induction decreases AHR and eosinophilia. Finally, we show that HIF-1α and HIF-2α regulate eosinophil migration in opposing ways.

PMID: 23250618 [PubMed - as supplied by publisher]

17. Evid Based Complement Alternat Med. 2012;2012:462383. doi: 10.1155/2012/462383. Epub 2012 Nov 27.

A Novel Herbal Medicine KIOM-MA Exerts an Anti-Inflammatory Effect in LPS-Stimulated RAW 264.7 Macrophage Cells.

Oh YC, Cho WK, Jeong YH, Im GY, Kim A, Hwang YH, Kim T, Song KH, Ma JY.

Korean Medicine (KM)-Based Herbal Drug Research Group, Korea Institute of

Oriental Medicine, 461-24 Jeonmin-Dong, Yuseong, Daejeon 305-811, Republic of Korea.

KIOM-MA was recently reported as a novel herbal medicine effective for atopic dermatitis and asthma. In this study, we have demonstrated the inhibitory effect of KIOM-MA on proinflammatory mediator produced in lipopolysaccharide (LPS)-stimulated RAW 264.7 cells. KIOM-MA significantly inhibited the expression of inducible nitric oxide synthase (iNOS) and cyclooxygenase-2 (COX-2) as well as nitric oxide (NO) and prostaglandin E(2) (PGE(2)). Consistent with the inhibitory effect on PGE(2), KIOM-MA suppresses the LPS-induced migration of macrophages and gelatinase activity and the expression of matrix metalloprotease-9 (MMP-9) in a dose-dependent manner. Additionally, KIOM-MA showed a strong suppressive effect on the inflammatory cytokines production such as tumor necrosis factor-α (TNF-α) and interleukin-6 (IL-6). We also found that KIOM-MA inhibits the activation of nuclear factor-κB (NF-κB) and represses the activity of extracellular signal-regulated kinase (ERK), p38, and c-Jun NH(2)-terminal kinase (JNK) mitogen-activated protein kinases (MAPKs). Taken together, we elucidated the mechanism of anti-inflammatory effect of KIOM-MA using RAW 264.7 cells stimulated by LPS.

PMCID: PMC3518860
PMID: 23243447 [PubMed]

18. J Immunol. 2013 Jan 1;190(1):324-33. doi: 10.4049/jimmunol.1201398. Epub 2012 Dec 5.

Gαi2 is the essential gαi protein in immune complex-induced lung disease.

Wiege K, Ali SR, Gewecke B, Novakovic A, Konrad FM, Pexa K, Beer-Hammer S, Reutershan J, Piekorz RP, Schmidt RE, Nürnberg B, Gessner JE.

Molecular Immunology Research Unit, Clinical Department of Immunology and Rheumatology, Hannover Medical School, 30625 Hannover, Germany;

Heterotrimeric G proteins of the Gα(i) family have been implicated in signaling pathways regulating cell migration in immune diseases. The Gα(i)-protein-coupled C5a receptor is a critical regulator of IgG FcR function in experimental models of immune complex (IC)-induced inflammation. By using mice deficient for Gα(i2) or Gα(i3), we show that Gα(i2) is necessary for neutrophil influx in skin and lung Arthus reactions and agonist-induced neutrophilia in the peritoneum, whereas Gα(i3) plays a less critical but variable role. Detailed analyses of the pulmonary IC-induced inflammatory response revealed several shared functions of Gα(i2) and Gα(i3), including mediating C5a anaphylatoxin receptor-induced activation of macrophages, involvement in alveolar production of chemokines, transition of neutrophils from bone marrow into blood, and modulation of CD11b and CD62L expression that account for neutrophil adhesion to endothelial cells. Interestingly, C5a-stimulated endothelial polymorphonuclear neutrophil transmigration, but not chemotaxis, is enhanced versus reduced in the absence of neutrophil Gα(i3) or Gα(i2), respectively, and knockdown of endothelial Gα(i2) caused decreased transmigration of wild-type neutrophils. These data demonstrate that Gα(i2) and Gα(i3) contribute to inflammation by redundant, overlapping, and Gα(i)-isoform-specific mechanisms, with Gα(i2) exhibiting unique functions in both neutrophils and endothelial cells that appear essential for polymorphonuclear neutrophil recruitment in IC disease.

PMID: 23225882 [PubMed - in process]

19. Am J Respir Cell Mol Biol. 2012 Dec 6. [Epub ahead of print]

Differential Regulation of Airway Smooth Muscle Cell Migration by Prostanoid EP Receptor Subtypes.

Aso H, Ito S, Mori A, Suganuma N, Morioka M, Takahara N, Kondo M, Hasegawa Y.

Department of Respiratory Medicine, Nagoya University Graduate School of Medicine, Nagoya, Japan.

Migration of airway smooth muscle (ASM) cells plays an important role in the pathophysiology of airway hyper-responsiveness and remodeling in asthma. It has been reported that prostaglandin E2 (PGE2) inhibits migration of ASM cells. Although PGE2 regulates cellular functions via binding to distinct prostanoid EP receptors, the role of EP receptor subtypes in mechanisms underlying cell migration has not been fully elucidated. We investigated the role of EP receptors in the inhibitory effects of PGE2 on the migration of human ASM cells. Migration induced by platelet-derived growth factor (PDGF)-BB (10 ng/mL, 6 h) was assessed by a chemotaxis chamber assay. PDGF-BB-induced cell migration was inhibited by PGE2, the specific EP2 agonist ONO-AE1-259-01, the specific EP4 agonist ONO-AE1-329, and cAMP mobilizing agents. The inhibition of cell migration by PGE2 was significantly reversed by a blockade of EP2 and EP4 receptors using antagonists or transfection with siRNAs. Moreover, PGE2, the EP2 agonist, and the EP4 agonist significantly increased phosphorylation of small heat shock protein 20 (HSP20), one of the protein substrates for protein kinase A (PKA), with depolymerization of actin. In contrast, the EP3 agonist ONO-AE-248 significantly promoted baseline cell migration without affecting PDGF-BB-induced cell migration. In summary, activation of EP2 and EP4 receptors and subsequent activation of the cAMP/PKA pathway are the main mechanisms of inhibition of ASM cell migration by PGE2. HSP20 phosphorylation by PKA is possibly involved in this mechanism. Conversely, EP3 is potent in promoting cell migration. EP receptor subtypes may be novel therapeutic target molecules in airway remodeling and asthma.

PMID: 23221043 [PubMed - as supplied by publisher]

20. Allergy. 2013 Feb;68(2):171-9. doi: 10.1111/all.12059. Epub 2012 Dec 4.

Interacting mast cells and eosinophils acquire an enhanced activation state in vitro.

Elishmereni M, Bachelet I, Ben-Efraim AH, Mankuta D, Levi-Schaffer F.

Institute for Drug Research, Faculty of Medicine, The Hebrew University of Jerusalem, Israel.

BACKGROUND: Mast cells (MCs) and eosinophils (Eos), the key effector cells in allergy, are abundantly co-localized particularly in the late and chronic stages of allergic inflammation. Recent evidence has outlined a specialized 'allergic effector unit' in which MCs and Eos communicate via both soluble mediators and physical contact. However, the functional impact of this bi-directional crosstalk on the cells' effector activities has not yet been revealed. We aimed to investigate whether MC/eosinophil interactions can influence the immediate and late activation phenotypes of these cells.
METHODS: Human and murine MCs and Eos were co-cultured under various conditions for 1-2 h or 1-3 days, and in selected experiments cell-cell contact was blocked. Cell migration and mediator release were examined, and flow cytometry was applied to stain intracellular signaling molecules and surface receptors.
RESULTS: Eosinophils enhanced basal MCs mediator release and co-stimulated IgE-activated MCs through physical contact involving CD48-2B4 interactions.

Reciprocally, resting and IgE-stimulated MCs led to eosinophil migration and activation through a paracrine-dependent mechanism. Increased phosphorylation of activation-associated signaling molecules, and enhanced release of tumor necrosis factor α, was observed in long-term co-cultures. Eosinophils also showed enhanced expression of intercellular adhesion molecule 1, which depended on direct contact with MCs.
CONCLUSIONS: Our findings reveal a new role for MC/eosinophil interplay in augmenting short- and long-term activation in both cells, in a combined physical/paracrine manner. This enhanced functional activity may thus critically contribute to the perpetuation of the inflammatory response in allergic conditions.

© 2012 John Wiley & Sons A/S.

PMID: 23205534 [PubMed - in process]

21. J Immunol. 2013 Jan 1;190(1):438-46. doi: 10.4049/jimmunol.1201827. Epub 2012 Nov 28.

YKL-40 Induces IL-8 Expression from Bronchial Epithelium via MAPK (JNK and ERK) and NF-κB Pathways, Causing Bronchial Smooth Muscle Proliferation and Migration.

Tang H, Sun Y, Shi Z, Huang H, Fang Z, Chen J, Xiu Q, Li B.

Department of Respiratory Medicine, Changzheng Hospital, Second Military Medical University, Shanghai 200003, People's Republic of China.

Recently, the serum levels of YKL-40, a chitinase-like glycoprotein, have been shown to be significantly elevated in asthmatics and are associated with asthma severity. Although these studies raise the possibility that YKL-40 may influence asthma, the mechanisms remain unknown. This study firstly investigated the mechanisms involved in YKL-40-mediated inflammation in human bronchial epithelial cells (HBECs) and analyzed the soluble factors secreted by bronchial epithelial cells exposed to YKL-40 that were responsible for increasing proliferation and migration of primary normal human bronchial smooth muscle cells (BSMCs). YKL-40-induced inflammation was assayed in two HBECs (BEAS-2B cell line and primary HBECs). In addition, we treated BEAS-2B cells and HBECs with YKL-40 and added the conditioned culture media to BSMCs. The proliferation and migration of BSMCs were determined by premixed WST-1 cell proliferation reagent (Clontech Laboratories) and QCM chemotaxis migration assay (Millipore), respectively. Bronchial epithelial cells treated with YKL-40 resulted in a significant increase of IL-8 production, which was dependent on MAPK (JNK and ERK) and NF-κB pathways activation. YKL-40-induced IL-8 was found to further stimulate proliferation and migration of BSMCs, and the effects were inhibited after neutralizing IL-8. Through investigating the interaction of airway epithelium and smooth muscle, our findings implicate that YKL-40 may be involved in the inflammation of asthma by induction of IL-8 from epithelium, subsequently contributing to BSMC proliferation and migration. Moreover, inhibition of IL-8 signaling is a potential therapeutic target for YKL-40-induced inflammation and remodeling of asthma.

PMID: 23197259 [PubMed - in process]

22. Eur J Pharmacol. 2013 Jan 5;698(1-3):463-9. doi: 10.1016/j.ejphar.2012.11.025. Epub 2012 Nov 23.

Hydrogen sulfide inhibits oxidative stress in lungs from allergic mice in vivo.

Benetti LR, Campos D, Gurgueira SA, Vercesi AE, Guedes CE, Santos KL, Wallace JL, Teixeira SA, Florenzano J, Costa SK, Muscará MN, Ferreira HH.

Laboratory of Inflammation Research, São Francisco University, Bragança Paulista, São Paulo 12 916 900, Brazil.

Recent studies show that endogenous hydrogen sulfide (H(2)S) plays an anti-inflammatory role in the pathogenesis of airway inflammation. This study investigated whether exogenous H(2)S may counteract oxidative stress-mediated lung damage in allergic mice. Female BALB/c mice previously sensitized with ovalbumin (OVA) were treated with sodium hydrosulfide (NaHS) 30min before OVA challenge. Forty eight hours after antigen-challenge, the mice were killed and leukocyte counting as well as nitrite plus nitrate concentrations were determined in the bronchoalveolar lavage fluid, and lung tissue was analysed for nitric oxide synthase (NOS) activity, iNOS expression, superoxide dismutase (SOD), catalase, glutathione reductase (GR) and glutathione peroxidase (GPx) activities, thiobarbituric acid reactive species and 3-nitrotyrosine containing proteins (3-NT). Pre-treatment of OVA-sensitized mice with NaHS resulted in significant reduction of both eosinophil and neutrophil migration to the lungs, and prevented the elevation of iNOS expression and activity observed in the lungs from the untreated allergic mice, although it did not affect 3-NT. NaHS treatment also abolished the increased lipid peroxidation present in the allergic mouse lungs and increased SOD, GPx and GR enzyme activities. These results show, for the first time, that the beneficial in vivo effects of the H(2)S-donor NaHS on allergic airway inflammation involve its inhibitory action on leukocyte recruitment and the prevention of lung damage by increasing endogenous antioxidant defenses. Thus, exogenous administration of H(2)S donors may be beneficial in reducing the deleterius impact of allergic pulmonary disease, and might represent an additional class of pharmacological agents for treatment of chronic pulmonary diseases.

Copyright © 2012 Elsevier B.V. All rights reserved.

PMID: 23183109 [PubMed - in process]

23. J Allergy Clin Immunol. 2012 Nov 19. pii: S0091-6749(12)01617-X. doi: 10.1016/j.jaci.2012.09.036. [Epub ahead of print]

IL-22 suppresses IFN-γ-mediated lung inflammation in asthmatic patients.

Pennino D, Bhavsar PK, Effner R, Avitabile S, Venn P, Quaranta M, Marzaioli V, Cifuentes L, Durham SR, Cavani A, Eyerich K, Chung KF, Schmidt-Weber CB, Eyerich S.

ZAUM-Center of Allergy and Environment, Technische Universität and Helmholtz Center Munich, Munich, Germany; Molecular Immunology, Department of Allergy and Clinical Immunology, National Heart and Lung Institute, Imperial College, London, United Kingdom. Electronic address: Davide.Pennino@lrz.tu-muenchen.de.

BACKGROUND: IL-22 controls tissue homeostasis by both proinflammatory and anti-inflammatory effects. However, the anti-inflammatory mechanisms of IL-22 remain poorly investigated. OBJECTIVE: We sought to investigate the anti-inflammatory role for IL-22 in human asthma. METHODS: T-cell lines derived from lung biopsy specimens of asthmatic patients were characterized by means of flow cytometry. Human bronchial epithelial cells from healthy and asthmatic subjects were stimulated with IL-22, IFN-γ, or the combination of both cytokines. Effects of cytokine stimulation were investigated by using whole-genome analysis, ELISA, and flow cytometry. The functional consequence of cytokine stimulation was evaluated in an in vitro wound repair model and T cell-mediated cytotoxicity

experiments. In vivo cytokine expression was measured by using immunohistochemistry and Luminex assays in bronchoalveolar lavage fluid of healthy and asthmatic patients. RESULTS: The current study identifies a tissue-restricted antagonistic interplay of IL-22 and the proinflammatory cytokine IFN-γ. On the one hand, IFN-γ antagonized IL-22-mediated induction of the antimicrobial peptide S100A7 and epithelial cell migration in bronchial epithelial cells. On the other hand, IL-22 decreased epithelial susceptibility to T cell-mediated cytotoxicity by inhibiting the IFN-γ-induced expression of MHC-I, MHC-II, and CD54/intercellular adhesion molecule 1 molecules. Likewise, IL-22 inhibited IFN-γ-induced secretion of the proinflammatory chemokines CCL5/RANTES and CXCL10/interferon-inducible protein 10 in vitro. Consistently, the IL-22 expression in bronchoalveolar lavage fluid of asthmatic patients inversely correlated with the expression of CCL5/RANTES and CXCL10/interferon-inducible protein 10 in vivo. CONCLUSIONS: IL-22 might control the extent of IFN-γ-mediated lung inflammation and therefore play a tissue-restricted regulatory role.

Copyright © 2012 American Academy of Allergy, Asthma & Immunology. Published by Mosby, Inc. All rights reserved.

PMID: 23174657 [PubMed - as supplied by publisher]

24. J Biol Chem. 2013 Jan 11;288(2):1374-84. doi: 10.1074/jbc.M112.422675. Epub 2012 Nov 20.

Novel Association between Vasoactive Intestinal Peptide and CRTH2 Receptor in Recruiting Eosinophils: A POSSIBLE BIOCHEMICAL MECHANISM FOR ALLERGIC EOSINOPHILIC INFLAMMATION OF THE AIRWAYS.

El-Shazly AE, Begon DY, Kustermans G, Arafa M, Dortu E, Henket M, Lefebvre PP, Louis R, Delvenne P.

From the Department of Oto-Rhino-Laryngology and Head and Neck Surgery and.

We explored the relation between vasoactive intestinal peptide (VIP), CRTH2, and eosinophil recruitment. It is shown that CRTH2 expression by eosinophils from allergic rhinitis (AR) patients and eosinophil cell line (Eol-1 cells) was up-regulated by VIP treatment. This was functional and resulted in exaggerated migratory response of cells against PGD2. Nasal challenge of AR patients resulted in a significant increase of VIP contents in nasal secretion (ELISA), and the immunohistochemical studies of allergic nasal tissues showed significant expression of VIP in association with intense eosinophil recruitment. Biochemical assays showed that VIP-induced eosinophil chemotaxis from AR patients and Eol-1 cells was mediated through the CRTH2 receptor. Cell migration against VIP was sensitive to protein kinase C (PKC) and protein kinase A (PKA) inhibition but not to tyrosine kinase or p38 MAPK inhibition or calcium chelation. Western blot demonstrated a novel CRTH2-mediated cytosol-to-membrane translocation of PKC-ε, PKC-δ, and PKA-α, -γ, and -IIαreg in Eol-1 cells upon stimulation with VIP. Confocal images and FACS demonstrated a strong association and co-localization between VIP peptide and CRTH2 molecules. Further, VIP induced PGD2 secretion from eosinophils. Our results demonstrate the first evidence of association between VIP and CRTH2 in recruiting eosinophils.

PMCID: PMC3543020 [Available on 2014/1/11]
PMID: 23168411 [PubMed - in process]

25. Mucosal Immunol. 2012 Nov 14. doi: 10.1038/mi.2012.109. [Epub ahead of print]

The C-C motif chemokine ligands CCL5, CCL11, and CCL24 induce the migration of

circulating fibrocytes from patients with severe asthma.

Isgrò M, Bianchetti L, Marini MA, Bellini A, Schmidt M, Mattoli S.

1] Avail Biomedical Research Institute, Basel, Switzerland [2] Laboratory of Cytopathology and Cytogenetics, ABR Operative Unit, Milan, Italy.

The C-C motif chemokine ligand 5 (CCL5), CCL11, and CCL24 are involved in the pathogenesis of asthma, and their function is mainly associated with the airway recruitment of eosinophils. This study tested their ability to induce the migration of circulating fibrocytes, which may contribute to the development of irreversible airflow obstruction in severe asthma. The sputum fluid phase (SFP) from patients with severe/treatment-refractory asthma (PwSA) contained elevated concentrations of CCL5, CCL11, and CCL24 in comparison with the SFP from patients with non-severe/treatment-responsive asthma (PwNSA). The circulating fibrocytes from PwSA expressed the receptors for these chemokines at increased levels and migrated in response to recombinant CCL5, CCL11, and CCL24. The SFP from PwSA induced the migration of autologous fibrocytes, and its activity was significantly attenuated by neutralization of endogenous CCL5, CCL11, and CCL24. These findings suggest that CCL5, CCL11, and CCL24 may contribute to the airway recruitment of fibrocytes in severe asthma.Mucosal Immunology advance online publication 14 November 2012; doi:10.1038/mi.2012.109.

PMID: 23149666 [PubMed - as supplied by publisher]

26. Recent Pat Inflamm Allergy Drug Discov. 2012 Nov 7. [Epub ahead of print]

Inflammation, Allergy and Asthma, Complex Immune Origin Diseases: Mechanisms and Therapeutic Agents.

Naik SR, Wala SM.

Department of Pharmacology, Sinhgad Institute of Pharmaceutical Sciences, Gat No. 309, off Mumbai-Pune Expressway, Kusgaon (Bk.), Lonavala-410401, Dist: Pune, Maharashtra, India. srnaik5@rediffmail.com.

Inflammation, Allergy and Asthma are the manifestation of multitude reactions of biological, cellular and immunological events. The various associated biological, cellular, and molecular events with inflammation, allergy and asthma participate to induce increased vascular permeability, vasodilatation, cellular migration, increased mucus secretion, broncho-constriction, structural changes of airway architecture, decline in pulmonary functions, release of intracellular mediators, increased formation of reactive oxygen species, cartilage degradation and loss of function. The participation of variety of effectors cells viz. leukocytes, neutrophils, eosinophils, basophils, monocytes, macrophages, mast cells, dendritic cells, T-cells, B-cells, NK-cells, lead to cascade of events trigger of intracellular mediators (cytokines, chemokines etc.) activating diverse biological effects and immune responses. Eicosanoids are major precursors in cyclooxygenase and lipooxygenase pathways and play an important role in inflammation, allergy and asthma. Such biological and cellular events are further enhanced by stress related effects. The wide varieties of synthetic and natural compounds have been showed to act on different molecular targets (receptor, enzymes, mediators, and cells) involved in inflammation, allergy and asthma and to alter produce specific/definite therapeutic activity. The present review describes pathogenesis and etiological aspects of inflammation, allergy and asthma with few relevant patents which would be immensely useful for researchers in the field of immunology and molecular pharmacology to explore new avenues/strategies for development of new generation of therapeutically active agents for treatment of inflammation and allergic disorders.

PMID: 23140569 [PubMed - as supplied by publisher]

27. Asia Pac Allergy. 2012 Oct;2(4):256-63. doi: 10.5415/apallergy.2012.2.4.256. Epub 2012 Oct 31.

Interferon-alpha inhibits airway eosinophila and hyperresponsiveness in an animal asthma model.

Kikkawa Y, Sugiyama K, Obara K, Hirata H, Fukushima Y, Toda M, Fukuda T.

Department of Pulmonary Medicine and Clinical Immunology, Dokkyo Medical University, 880 Kita-kobayashi, Mibu, Tochigi 321-0293, Japan.

BACKGROUND: Asthma is characterized by a chronic inflammatory process involving high numbers of inflammatory cells and mediators which have multiple inflammatory effects on the airway. Interferon (IFN)-alpha, which is used widely for treating chronic hepatitis C, is reported to have an effect on patients with Churg-Strauss syndrome. Therefore, it may also be suitable for patients with severe asthma.
OBJECTIVE: We studied the effect of IFN-alpha on airway eosinophilia in a guinea pig model of asthma and the expression of adhesion molecules on human eosinophils and vascular endothelial cells.
METHODS: After antigen challenge, airway hyperresponsiveness and airway eosinophilia were measured in a guinea pig asthma model with or without airway IFN-alpha administration. Expression of adhesion molecules on eosinophils and cultured human umbilical vein endothelial cells (HUVECs) was also evaluated with or without IFN-alpha.
RESULTS: IFN-alpha inhibited eosinophil recruitment into the tracheal wall and improved airway hyperresponsiveness in sensitized guinea pigs. IFN-alpha also significantly suppressed IL-1 beta-induced intercellular adhesion molecule-1 (ICAM-1) expression on HUVECs. However, IFN-alpha did not suppress platelet-activating factor-induced macrophage antigen-1 expression on human eosinophils. IFN-alpha significantly inhibited eosinophil adhesion to IL-1 beta-induced HUVECs and migration through IL-1 beta induced HUVECs.
CONCLUSION: The findings suggest that the modulation of ICAM-1 in lung with pre-existing inflammation following treatment with IFN-alpha may be a novel and selective treatment for control of chronic airway inflammation and hyperresponsiveness associated with asthma.

PMCID: PMC3486970
PMID: 23130331 [PubMed]

28. J Immunol. 2012 Dec 1;189(11):5194-205. doi: 10.4049/jimmunol.1101331. Epub 2012 Nov 2.

Mechanism of action of inhibition of allergic immune responses by a novel antedrug TLR7 agonist.

Matsui H, Tomizawa H, Eiho K, Kashiwazaki Y, Edwards S, Biffen M, Bell JP, Bahl A, Leishman AJ, Murray CM, Takaku H, Ueda Y.

Pharmacology Research Laboratory, Dainippon Sumitomo Pharma Co., Ltd., Osaka 541-0045, Japan. hiroyuki-matsui@ds-pharma.co.jp

Triggering innate immune responses through TLRs is expected to be a novel therapeutic strategy for the treatment of allergic diseases. TLR agonists are able to modulate Th2 immune responses through undefined mechanisms. We investigated the mechanism of action of the suppression of Th2 immune responses

with a novel antedrug TLR7 agonist. The antedrug is rapidly metabolized by plasma esterases to an acid with reduced activity to limit systemic responses. Topical administration of this compound inhibited features of the allergic airway inflammatory response in rat and murine allergic airways model. Type I IFN played a role in the suppression of Th2 cytokines produced from murine splenocytes. Inhibition of Th2 immune responses with the antedrug TLR7 agonist was shown to be via a type I IFN-dependent mechanism following short-term exposure to the compound, although there might be type I IFN-independent mechanisms following long-term exposure. We have demonstrated that local type I IFN signaling and plasmacytoid dendritic cells, but not Th1 immune responses, are required for in vivo efficacy against murine airway Th2-driven eosinophilia. Furthermore, migration of dendritic cell subsets into the lung was related to efficacy and is dependent on type I IFN signaling. Thus, the mechanism of action at the cytokine and cellular level involved in the suppression of Th2 allergic responses has been characterized, providing a potential new approach to the treatment of allergic disease.

PMID: 23125414 [PubMed - indexed for MEDLINE]

29. Allergy Asthma Immunol Res. 2012 Nov;4(6):332-40. doi: 10.4168/aair.2012.4.6.332. Epub 2012 Jun 25.

Most Highly Cytokinergic IgEs Have Polyreactivity to Autoantigens.

Kashiwakura J, Okayama Y, Furue M, Kabashima K, Shimada S, Ra C, Siraganian RP, Kawakami Y, Kawakami T.

Division of Cell Biology, La Jolla Institute for Allergy and Immunology, La Jolla, CA, USA. ; Division of Molecular Cell Immunology and Allergology, Advanced Medical Research Center, Nihon University Graduate School of Medical Science, Itabashi-ku, Tokyo, Japan.

PURPOSE: Monomeric IgE molecules, when bound to the high-affinity receptor, exhibit a vast heterogeneity in their ability to induce survival promotion and cytokine production in mast cells. At one end of this spectrum, highly cytokinergic (HC) IgEs can induce potent survival promotion, degranulation, cytokine production, migration, etc., whereas at the other end, poorly cytokinergic (PC) IgEs can do so inefficiently. In this study, we investigated whether IgEs recognize autoantigens and whether IgEs' binding of autoantigens correlates with difference s in HC versus PC properties.
METHODS: Enzyme-linked immunosorbent assays were performed to test whether IgEs bind antigens. Histamine-releasing factor in human sera was quantified by western blotting. Cultured mast cells derived from human cord blood were used to test the effects of human sera on cytokine production.
RESULTS: Most (7/8) of mouse monoclonal HC IgEs exhibited polyreactivity to double-stranded DNA (dsDNA), single-stranded DNA (ssDNA), β-galactosidase, thyroglobulin and/or histamine-releasing factor. By contrast, mouse PC IgEs failed to react with these antigens. A human monoclonal HC IgE also showed polyreactivity to histamine-releasing factor, dsDNA and ssDNA. Interestingly, sera from atopic dermatitis patients showed increased reactivity to ssDNA and β-galactosidase and increased levels of histamine-releasing factor. Some atopic dermatitis patients, but not healthy individuals, had substantial serum levels of HRF-reactive IgE. Sera from atopic dermatitis patients with high titers of DNA-reactive IgE could induce several fold more IL-8 secretion in human mast cells than sera from healthy individuals.
CONCLUSIONS: The results show that most HC, but not PC, IgEs exhibit polyreactivity to autoantigens, supporting the autoimmune mechanism in the pathogenesis of atopic dermatitis.

PMCID: PMC3479226
PMID: 23115729 [PubMed]

30. J Cardiovasc Pharmacol. 2012 Oct 26. [Epub ahead of print]

Endothelial Small- and Intermediate-Conductance KCa Channels: An Update on Their Pharmacology and Usefulness as Cardiovascular Targets.

Wulff H, Köhler R.

*Department of Pharmacology, University of California, Davis, USA †Cardiovascular and Renal Research, Institute of Molecular Medicine, University of Southern Denmark, Odense, Denmark §Aragon Institute of Health Sciences I+CS and ARAID, Zaragoza, Spain.

ABSTRACT:: Most cardiovascular researchers are familiar with intermediate-conductance KCa3.1 and small-conductance KCa2.3 channels because of their contribution to endothelium-derived hyperpolarization (EDH). However, to immunologists and neuroscientists these channels are primarily known for their role in lymphocyte activation and neuronal excitability. KCa3.1 is involved in the proliferation and migration of T cells, B cell, mast cells, macrophages, fibroblasts and dedifferentiated vascular smooth muscle cells and is, therefore, being pursued as a potential target for use in asthma, immunosuppression, and fibroproliferative disorders. In contrast, the three KCa2 channels (KCa2.1, KCa2.2 and KCa2.3) contribute to the neuronal medium afterhyperpolarization and, depending on the type of neuron, are involved in determining firing rates and frequencies or in regulating bursting. KCa2 activators are accordingly being studied as potential therapeutics for ataxia and epilepsy while KCa2 channel inhibitors like apamin have long been known to improve learning and memory in rodents. Given this background, we review the recent discoveries of novel KCa3.1 and KCa2.3 modulators and critically assess the potential of KCa activators for the treatment of diabetes and cardiovascular diseases by improving endothelium-derived hyperpolarizations.

PMID: 23107876 [PubMed - as supplied by publisher]

31. Physiol Rev. 2012 Oct;92(4):1619-49. doi: 10.1152/physrev.00046.2011.

Stem cell factor receptor/c-Kit: from basic science to clinical implications.

Lennartsson J, Rönnstrand L.

Ludwig Institute for Cancer Research, Uppsala University, Uppsala, Sweden.

Stem cell factor (SCF) is a dimeric molecule that exerts its biological functions by binding to and activating the receptor tyrosine kinase c-Kit. Activation of c-Kit leads to its autophosphorylation and initiation of signal transduction. Signaling proteins are recruited to activated c-Kit by certain interaction domains (e.g., SH2 and PTB) that specifically bind to phosphorylated tyrosine residues in the intracellular region of c-Kit. Activation of c-Kit signaling has been found to mediate cell survival, migration, and proliferation depending on the cell type. Signaling from c-Kit is crucial for normal hematopoiesis, pigmentation, fertility, gut movement, and some aspects of the nervous system. Deregulated c-Kit kinase activity has been found in a number of pathological conditions, including cancer and allergy. The observation that gain-of-function mutations in c-Kit can promote tumor formation and progression has stimulated the development of therapeutics agents targeting this receptor, e.g., the clinically used inhibitor imatinib mesylate. Also other clinically used multiselective

kinase inhibitors, for instance, sorafenib and sunitinib, have c-Kit included in their range of targets. Furthermore, loss-of-function mutations in c-Kit have been observed and shown to give rise to a condition called piebaldism. This review provides a summary of our current knowledge regarding structural and functional aspects of c-Kit signaling both under normal and pathological conditions, as well as advances in the development of low-molecular-weight molecules inhibiting c-Kit function.

PMID: 23073628 [PubMed - indexed for MEDLINE]

32. J Physiol Pharmacol. 2012 Aug;63(4):373-9.

Collagen receptors α(1)β(1) and α(2)β(1) integrins are involved in transmigration of peripheral blood eosinophils, but not mononuclear cells through human microvascular endothelial cells monolayer.

Bazan-Socha S, Zuk J, Plutecka H, Marcinkiewicz C, Zareba L, Musial J.

Second Department of Internal Medicine, Unit of Allergy and Clinical Immunology, Jagiellonian University Medical College, Cracow Poland. mmsocha@cyf-kr.edu.pl

Asthma development may be driven by T helper lymphocytes with eosinophils playing the role of major effector cells. Recruitment of the inflammatory cells from blood to the airways is mediated by adhesive molecules, e.g. selectins and integrins. The most important in cell trafficking are integrins containing α(4) and β(2) subunits. We hypothesized that also collagen receptors: α(1)β(1) and α(2)β(1), may be involved in cell migration to the inflammatory site in asthma. The aim of the study was to determine whether the inhibition of α(1)β(1) or α(2)β(1) integrins, affects transmigration of eosinophils and peripheral blood mononuclear cells (PBMC) through human microvascular endothelial cells monolayer (HMVEC) seeded on collagen IV coated wells in moderate persistent atopic asthmatics. Methods: PBMC from 9 asthmatics were separated by gradient centrifugation followed by negative magnetic separation of eosinophils. Snake venom derived anti-adhesive proteins: viperistatin and VP12 (potent and selective inhibitors of α(1)β(1) and α(2)β(1) integrins, respectively) as well as VLO4 (a non-selective inhibitor of α(4)β(1), α(5)β(1) and α(v)β(3) - used as a positive control), were used for inhibition studies. All anti-adhesive proteins studied, inhibited eosinophils, but only VLO4 affected PBMC transmigration through HMVEC. In bronchial asthma both collagen receptors α(1)β(1) and α(2)β(1) are likely to be involved in eosinophil transmigration to the inflammatory site. The role of α(2)β(1) on lymphocytes is probably different. As the α(2)β(1) integrin has been described as a stimulator of collagen accumulation, it might be, at least in part, responsible for asthma airway remodelling.

PMID: 23070086 [PubMed - in process]

33. Travel Med Infect Dis. 2012 Sep;10(5-6):247-56. doi: 10.1016/j.tmaid.2012.09.004. Epub 2012 Oct 11.

Demographics, health and travel characteristics of international travellers at a pre-travel clinic in Marseille, France.

Aubry C, Gaudart J, Gaillard C, Delmont J, Parola P, Brouqui P, Gautret P.

University Hospital Institute for Infectious and Tropical Diseases, Hôpital Nord, AP-HM, 13015 Marseille, France.

With the aim to identify at-risk individuals among a cohort of international

travellers, 3442 individuals who sought advice at Marseille travel health centre in 2009 were prospectively included. Demographics, travel characteristics, chronic medical conditions, vaccinations and antimalarial chemoprophylaxis were documented. Chronic medical conditions were reported by 11% of individuals, including hypertension (39%), asthma (20%), thyroid disease (15%) and depression (13%). 4% reported taking a daily medication, and psychotropic and cardiovascular medications were the most commonly used. Older travellers (≥60 years) accounted for 10% of the travellers and the prevalence of chronic medical conditions was 27% in this group. Individuals aged 15 years or less accounted for 13% of the travellers. Age, last minute travel (17%) and neurological and psychiatric diseases were the most frequent factors that influenced Yellow fever vaccination and malaria chemoprophylaxis, with more than one tenth of the travellers reporting at least one risk factor for which adjusted advice may be necessary. Migrants visiting their relatives in their origin country accounted for 14% of travellers and 73% of this group travelled with their family including young children. We demonstrate that a significant proportion of travellers are at-risk (43%) because of their travel conditions (VFR), their age, or their health status, and should be targeted for risk reduction strategies.

Copyright © 2012 Elsevier Ltd. All rights reserved.

PMID: 23062668 [PubMed - in process]

34. Int Arch Allergy Immunol. 2013;160(2):165-72. doi: 10.1159/000339743. Epub 2012 Sep 25.

Differences in systemic and skin migrating-specific CD4 T cells in papular urticaria by flea bite.

Dominguez-Amorocho O, Duarte S, González JM, Halpert E, Ortega MC, Rodríguez A, García E, Cuellar A.

Departamento de Microbiología, Facultad de Ciencias, Pontificia Universidad Javeriana, Bogotá, Colombia.

Background: Papular urticaria by flea bite is a chronic allergic condition in which clinical improvement may occur at the age of 7 years, thus representing a natural model of acquired immunologic tolerance in humans. The aim of this study was to characterize regulatory cells and specific responses to flea antigens of CD4(+) T lymphocytes expressing cutaneous migration markers in patients with papular urticaria caused by flea bite and with different disease evolution times. Methods: Cell populations were characterized by flow cytometry in samples from patients and healthy controls. Specific cell stimulation was performed with a complete flea body extract. The Mann-Whitney U test was used for comparisons. Results: Total dendritic cells were lower in patients than in healthy controls. No quantitative differences were found in CD4 regulatory T cells. CD4(+) T cells from patients produced more IL-4, lL-10, IL-17, and IFN-γ. Patients who experienced the onset of symptoms within the first 5 years of age showed a greater percentage of local (cutaneous lymphocyte antigen +) IL-4- and IL-17-producing cells, while patients who experienced the onset of symptoms after the age of 5 years had a higher percentage of systemic (cutaneous lymphocyte antigen -) IL-10-producing cells. Conclusion: Analysis of the cellular immune response against whole flea antigen in patients with papular urticaria by flea bites suggests a possible participation of inflammatory cytokines in the skin reaction (Th17) and a systemic control mechanism (IL-10). This pattern of cytokine production in patients could be a consequence of an impaired dendritic cell population.

Copyright © 2012 S. Karger AG, Basel.

PMID: 23018588 [PubMed - in process]

35. Thorax. 2012 Dec;67(12):1061-6. doi: 10.1136/thoraxjnl-2012-201634. Epub 2012 Sep 26.

Epithelial eotaxin-2 and eotaxin-3 expression: relation to asthma severity, luminal eosinophilia and age at onset.

Coleman JM, Naik C, Holguin F, Ray A, Ray P, Trudeau JB, Wenzel SE.

Division of Pulmonary, Allergy and Critical Care Medicine, Department of Medicine, University of Pittsburgh Asthma Institute at UPMC/UPSOM, Pittsburgh, PA 15213, USA.

BACKGROUND: Eosinophilic inflammation is implicated in asthma. Eotaxin 1-3 regulate eosinophil trafficking into the airways along with other chemotactic factors. However, the epithelial and bronchoalveolar lavage (BAL) cell expression of these chemokines in relation to asthma severity and eosinophilic phenotypes has not been addressed.
OBJECTIVE: To measure the expression of the three eotaxin isoforms in bronchoscopically obtained samples and compare them with clinically relevant parameters between normal subjects and patients with asthma.
METHODS: Normal subjects and patients with asthma of varying severity recruited through the Severe Asthma Research Program underwent clinical assessment and bronchoscopy with airway brushing and BAL. Eotaxin 1-3 mRNA/protein were measured in epithelial and BAL cells and compared with asthma severity, control and eosinophilic inflammation.
RESULTS: Eotaxin-2 and eotaxin-3 mRNA and eotaxin-2 protein were increased in airway epithelial brushings from patients with asthma and were highest in cases of severe asthma (p values 0.0155, 0.0033 and 0.0006, respectively), with eotaxin-2 protein increased with age at onset. BAL cells normally expressed high levels of eotaxin-2 mRNA/protein but BAL fluid levels of eotaxin-2 were lowest in severe asthma. Epithelial eotaxin-2 and eotaxin-3 mRNA/protein was associated with sputum eosinophilia, lower forced expiratory volume in 1 s and more asthma exacerbations. Airway epithelial cell eotaxin-2 protein differed by asthma severity only in those with late onset disease, and tended to be highest in those with late onset eosinophilic asthma.
CONCLUSIONS: Epithelial eotaxin-2 and 3 are increased in asthma and severe asthma. Their expression may contribute to luminal migration of eosinophils, especially in later onset disease, asthma control and severity.

PMID: 23015684 [PubMed - in process]

36. Pflugers Arch. 2012 Nov;464(5):481-92. doi: 10.1007/s00424-012-1160-5. Epub 2012 Sep 27.

Airway smooth muscle STIM1 and Orai1 are upregulated in asthmatic mice and mediate PDGF-activated SOCE, CRAC currents, proliferation, and migration.

Spinelli AM, González-Cobos JC, Zhang X, Motiani RK, Rowan S, Zhang W, Garrett J, Vincent PA, Matrougui K, Singer HA, Trebak M.

Center for Cardiovascular Sciences, Albany Medical College, Mail Code 8, 47 New Scotland Ave, Albany, NY 12208, USA.

Airway smooth muscle cell (ASMC) remodeling contributes to the structural changes in the airways that are central to the clinical manifestations of asthma. Ca(2+)

signals play an important role in ASMC remodeling through control of ASMC migration and hypertrophy/proliferation. Upregulation of STIM1 and Orai1 proteins, the molecular components of the store-operated Ca(2+) entry (SOCE) pathway, has recently emerged as an important mediator of vascular remodeling. However, the potential upregulation of STIM1 and Orai1 in asthmatic airways remains unknown. An important smooth muscle migratory agonist with major contributions to ASMC remodeling is the platelet-derived growth factor (PDGF). Nevertheless, the Ca(2+) entry route activated by PDGF in ASMC remains elusive. Here, we show that STIM1 and Orai1 protein levels are greatly upregulated in ASMC isolated from ovalbumin-challenged asthmatic mice, compared to control mice. Furthermore, we show that PDGF activates a Ca(2+) entry pathway in rat primary ASMC that is pharmacologically reminiscent of SOCE. Molecular knockdown of STIM1 and Orai1 proteins inhibited PDGF-activated Ca(2+) entry in these cells. Whole-cell patch clamp recordings revealed the activation of Ca(2+) release-activated Ca(2+) (CRAC) current by PDGF in ASMC. These CRAC currents were abrogated upon either STIM1 or Orai1 knockdown. We show that either STIM1 or Orai1 knockdown significantly inhibited ASMC proliferation and chemotactic migration in response to PDGF. These results implicate STIM1 and Orai1 in PDGF-induced ASMC proliferation and migration and suggest the potential use of STIM1 and Orai1 as targets for ASMC remodeling during asthma.

PMCID: PMC3489062 [Available on 2013/11/1]
PMID: 23014880 [PubMed - in process]

37. Proc Natl Acad Sci U S A. 2012 Oct 9;109(41):16660-5. doi: 10.1073/pnas.1208881109. Epub 2012 Sep 25.

β-Arrestin-2 mediates the proinflammatory effects of proteinase-activated receptor-2 in the airway.

Nichols HL, Saffeddine M, Theriot BS, Hegde A, Polley D, El-Mays T, Vliagoftis H, Hollenberg MD, Wilson EH, Walker JK, DeFea KA.

Division of Biomedical Sciences, University of California, Riverside, CA 92521, USA.

Proteinase-Activated receptor-2 (PAR(2)), a G-protein-coupled Receptor, activated by serine proteinases, is reported to have both protective and proinflammatory effects in the airway. Given these opposing actions, both inhibitors and activators of PAR(2) have been proposed for treating asthma. PAR(2) can signal through two independent pathways: a β-arrestin-dependent one that promotes leukocyte migration, and a G-protein/Ca(2+) one that is required for prostaglandin E(2) (PGE(2)) production and bronchiolar smooth muscle relaxation. We hypothesized that the proinflammatory responses to PAR(2) activation are mediated by β-arrestins, whereas the protective effects are not. Using a mouse ovalbumin model for PAR(2)-modulated airway inflammation, we observed decreased leukocyte recruitment, cytokine production, and mucin production in β-arrestin-2(-/-) mice. In contrast, PAR(2)-mediated PGE(2) production, smooth muscle relaxation, and decreased baseline airway resistance (measures of putative PAR(2) "protective" effects) were independent of β-arrestin-2. Flow cytometry and cytospins reveal that lung eosinophil and CD4 T-cell infiltration, and production of IL-4, IL-6, IL-13, and TNFα, were enhanced in wild-type but not β-arrestin-2(-/-) mice. Using the forced oscillation technique to measure airway resistance reveals that PAR(2) activation protects against airway hyperresponsiveness by an unknown mechanism, possibly involving smooth muscle relaxation. Our data suggest that the PAR(2)-enhanced inflammatory process is β-arrestin-2 dependent, whereas the protective anticonstrictor effect of bronchial epithelial PAR(2) may be β-arrestin independent.

PMCID: PMC3478622 [Available on 2013/4/9]
PMID: 23012429 [PubMed - indexed for MEDLINE]

38. Allergy. 2012 Dec;67(12):1501-10. doi: 10.1111/all.12019. Epub 2012 Sep 25.

Ursodeoxycholic acid suppresses eosinophilic airway inflammation by inhibiting the function of dendritic cells through the nuclear farnesoid X receptor.

Willart MA, van Nimwegen M, Grefhorst A, Hammad H, Moons L, Hoogsteden HC, Lambrecht BN, Kleinjan A.

Laboratory of Immunoregulation and Mucosal Immunology, Department of Molecular Biomedical Research, Flemish Interuniversity Institute of Biotechnology, Ghent, Belgium.

BACKGROUND: Ursodeoxycholic acid (UDCA) is the only known beneficial bile acid with immunomodulatory properties. Ursodeoxycholic acid prevents eosinophilic degranulation and reduces eosinophil counts in primary biliary cirrhosis. It is unknown whether UDCA would also modulate eosinophilic inflammation outside the gastrointestinal (GI) tract, such as eosinophilic airway inflammation seen in asthma. The working mechanism for its immunomodulatory effect is unknown.
METHODS: The immunosuppressive features of UDCA were studied in vivo, in mice, in an ovalbumin (OVA)-driven eosinophilic airway inflammation model. To study the mechanism of action of UDCA, we analyzed the effect of UDCA on eosinophils, T cells, and dendritic cell (DCs). DC function was studied in greater detail, focussing on migration and T-cell stimulatory strength in vivo and interaction with T cells in vitro as measured by time-lapse image analysis. Finally, we studied the capacity of UDCA to influence DC/T cell interaction.
RESULTS: Ursodeoxycholic acid treatment of OVA-sensitized mice prior to OVA aerosol challenge significantly reduced eosinophilic airway inflammation compared with control animals. DCs expressed the farnesoid X receptor for UDCA. Ursodeoxycholic acid strongly promoted interleukin (IL)-12 production and enhanced the migration in DCs. The time of interaction between DCs and T cells was sharply reduced in vitro by UDCA treatment of the DCs resulting in a remarkable T-cell cytokine production. Ursodeoxycholic acid-treated DCs have less capacity than saline-treated DCs to induce eosinophilic inflammation in vivo in Balb/c mice.
CONCLUSION: Ursodeoxycholic acid has the potency to suppress eosinophilic inflammation outside the GI tract. This potential comprises to alter critical function of DCs, in essence, the effect of UDCA on DCs through the modulation of the DC/T cell interaction.

© 2012 John Wiley & Sons A/S.

PMID: 23004356 [PubMed - in process]

39. PLoS One. 2012;7(9):e45056. doi: 10.1371/journal.pone.0045056. Epub 2012 Sep 11.

STIM1 regulates platelet-derived growth factor-induced migration and Ca2+ influx in human airway smooth muscle cells.

Suganuma N, Ito S, Aso H, Kondo M, Sato M, Sokabe M, Hasegawa Y.

Department of Respiratory Medicine, Nagoya University Graduate School of Medicine, Nagoya, Japan.

It is suggested that migration of airway smooth muscle (ASM) cells plays an important role in the pathogenesis of airway remodeling in asthma. Increases in

intracellular Ca(2+) concentrations ([Ca(2+)](i)) regulate most ASM cell functions related to asthma, such as contraction and proliferation. Recently, STIM1 was identified as a sarcoplasmic reticulum (SR) Ca(2+) sensor that activates Orai1, the Ca(2+) channel responsible for store-operated Ca(2+) entry (SOCE). We investigated the role of STIM1 in [Ca(2+)](i) and cell migration induced by platelet-derived growth factor (PDGF)-BB in human ASM cells. Cell migration was assessed by a chemotaxis chamber assay. Human ASM cells express STIM1, STIM2, and Orai1 mRNAs. SOCE activated by thapsigargin, an inhibitor of SR Ca(2+)-ATPase, was significantly blocked by STIM1 siRNA and Orai1 siRNA but not by STIM2 siRNA. PDGF-BB induced a transient increase in [Ca(2+)](i) followed by sustained [Ca(2+)](i) elevation. Sustained increases in [Ca(2+)](i) due to PDGF-BB were significantly inhibited by a Ca(2+) chelating agent EGTA or by siRNA for STIM1 or Orai1. The numbers of migrating cells were significantly increased by PDGF-BB treatment for 6 h. Knockdown of STIM1 and Orai1 by siRNA transfection inhibited PDGF-induced cell migration. Similarly, EGTA significantly inhibited PDGF-induced cell migration. In contrast, transfection with siRNA for STIM2 did not inhibit the sustained elevation of [Ca(2+)](i) or cell migration induced by PDGF-BB. These results demonstrate that STIM1 and Orai1 are essential for PDGF-induced cell migration and Ca(2+) influx in human ASM cells. STIM1 could be an important molecule responsible for airway remodeling.

PMCID: PMC3439366
PMID: 22984609 [PubMed - in process]

40. Nat Med. 2012 Oct;18(10):1560-9. doi: 10.1038/nm.2928. Epub 2012 Sep 16.

Endothelial PI3K-C2α, a class II PI3K, has an essential role in angiogenesis and vascular barrier function.

Yoshioka K, Yoshida K, Cui H, Wakayama T, Takuwa N, Okamoto Y, Du W, Qi X, Asanuma K, Sugihara K, Aki S, Miyazawa H, Biswas K, Nagakura C, Ueno M, Iseki S, Schwartz RJ, Okamoto H, Sasaki T, Matsui O, Asano M, Adams RH, Takakura N, Takuwa Y.

Department of Physiology, Kanazawa University School of Medicine, Kanazawa, Japan.

The class II α-isoform of phosphatidylinositol 3-kinase (PI3K-C2α) is localized in endosomes, the trans-Golgi network and clathrin-coated vesicles; however, its functional role is not well understood. Global or endothelial-cell-specific deficiency of PI3K-C2α resulted in embryonic lethality caused by defects in sprouting angiogenesis and vascular maturation. PI3K-C2α knockdown in endothelial cells resulted in a decrease in the number of PI3-phosphate-enriched endosomes, impaired endosomal trafficking, defective delivery of VE-cadherin to endothelial cell junctions and defective junction assembly. PI3K-C2α knockdown also impaired endothelial cell signaling, including vascular endothelial growth factor receptor internalization and endosomal RhoA activation. Together, the effects of PI3K-C2α knockdown led to defective endothelial cell migration, proliferation, tube formation and barrier integrity. Endothelial PI3K-C2α deficiency in vivo suppressed postischemic and tumor angiogenesis and diminished vascular barrier function with a greatly augmented susceptibility to anaphylaxis and a higher incidence of dissecting aortic aneurysm formation in response to angiotensin II infusion. Thus, PI3K-C2α has a crucial role in vascular formation and barrier integrity and represents a new therapeutic target for vascular disease.

PMID: 22983395 [PubMed - indexed for MEDLINE]

41. Exp Ther Med. 2012 Aug;4(2):205-210. Epub 2012 May 23.

Phosphodiesterase 4 regulates the migration of B16-F10 melanoma cells.

Watanabe Y, Murata T, Shimizu K, Morita H, Inui M, Tagawa T.

Department of Oral and Maxillofacial Surgery, Division of Reparative and Regenerative Medicine, Institute of Medical Science, Mie University Graduate School of Medicine, Tsu, Mie 514-8507, Japan.

Phosphodiesterases (PDEs) are important regulators of signal transduction processes. Eleven PDE gene families (PDE1-11) have been identified and several PDE isoforms are selectively expressed in various cell types. PDE4 family members specifically hydrolyze cyclic AMP (cAMP). Four genes (PDE4A-D) are known to encode PDE4 enzymes, with additional diversity generated by the use of alternative mRNA splicing and the use of different promoters. While PDE4 selective inhibitors show therapeutic potential for treating major diseases such as asthma and chronic obstructive pulmonary disease, little is known concerning the role of PDE4 in malignant melanoma. In this study, we examined the role of PDE4 in mouse B16-F10 melanoma cells. In these cells, PDE4 activity was found to be ~60% of total PDE activity. RT-PCR detected only PDE4B and PDE4D mRNA. Cell growth was inhibited by the cAMP analog, 8-bromo-cAMP, but not by the specific PDE4 inhibitors, rolipram and denbufylline, which increased intracellular cAMP concentrations. Finally, migration of the B16-F10 cells was inhibited by the PDE4 inhibitors and 8-bromo-cAMP, while migration was increased by a protein kinase A (PKA) inhibitor, PKI(14-22), and was not affected by 8-pCPT-2'-O-Me-cAMP, which is an analog of exchange protein activated by cAMP (Epac). The inhibitory effect of rolipram on migration was reversed by PKI(14-22). Based on these results, PDE4 appears to play an important role in the migration of B16-F10 cells, and therefore may be a novel target for the treatment of malignant melanoma.

PMID: 22970026 [PubMed]

42. Indian J Dermatol Venereol Leprol. 2012 Sep;78(5):664. doi: 10.4103/0378-6323.100519.

Oxidative stress and leukocyte migration inhibition response in cutaneous adverse drug reactions.

Verma P, Bhattacharya SN, Banerjee BD, Khanna N.

Department of Dermatology and STD, University College of Medical Sciences, University of Delhi, Delhi, India.

BACKGROUND: Cutaneous adverse drug reactions (CADRs) may either be immunological or non-immunological. The precise mechanisms, however, are largely obscure. Other concomitant mechanisms may amplify and/or contribute to the severity and duration of a reaction. One such mechanism could be oxidative stress, a state of imbalance between reactive oxygen species, and their subsequent detoxification by antioxidants.
AIMS: (a) to assess the oxidative stress status in the blood of cutaneous drug reaction patients by assaying for reduced glutathione (GSH) and malondialdehyde (MDA) levels, (b) to determine the leukocyte migration inhibition (LMI) response in these patients in response to the suspected drug (s), and (c) to look for the association between oxidative stress parameters and LMI.
METHODS: Ethical committee approval was obtained for this study. Fresh venous blood samples were obtained from the patients of CADRs (group A) during the acute phase of reaction and healthy control subjects (group B). MDA levels, a measure of oxidative lipid damage, and reduced GSH levels, a measure of anti-oxidant capacity, were assayed in the blood samples of both groups using

spectrophotometry. LMI response was measured by challenging the patients' peripheral blood mononuclear cells with the suspected drug to confirm immunological perturbation.
RESULTS: Totally 66 participants, 33 cases in group A and equal number of controls in group B, were studied. The mean MDA levels were found to be raised ($P < 0.001$), but GSH levels were significantly reduced in group A when compared with group B ($P = <0.001$). LMI response against drug(s) was performed in 33 cases (group A), out of which 25 cases showed a positive LMI response as follows: fixed drug eruption (10/25), SJS (5/25), urticaria (3/25), exfoliative dermatitis (2/25), morbilliform rash (2/25), erythroderma (1/25), vasculitis (1/25), and dapsone syndrome (1/25). The mean MDA levels were found to be significantly higher in the LMI positive CADRs ($P < 0.001$) when compared with LMI-negative ones, while no significant difference was seen for GSH ($P = 0.100$). Furthermore, there was a significant positive correlation between MDA levels and LMI response ($r = 0.831$, $P < 0.001$). On the other hand, a negative but statistically insignificant correlation was found between GSH and LMI response ($r = -0.248$, $P = 0.271$).
CONCLUSION: CADR patients were found to be under oxidative stress based on MDA and GSH levels in the peripheral blood. There is a significant positive correlation of LMI response (against the causative drug) with MDA levels, which strongly associates oxidative stress with the immunopathogenesis in CADRs.

PMID: 22960836 [PubMed - in process]

43. J Leukoc Biol. 2013 Jan;93(1):51-61. doi: 10.1189/jlb.0612293. Epub 2012 Sep 4.

The dual roles of inflammatory cytokines and chemokines in the regulation of autoimmune diseases and their clinical implications.

Shachar I, Karin N.

1.The Weizmann Institute of Science, Rehovot, Israel 76100. idit.shachar@weizmann.ac.il.

Cytokines and chemokines are secreted, small cell-signaling protein molecules, whose receptors are expressed on immune cells. These factors play a critical role in immune cell differentiation, migration, and polarization into functional subtypes and in directing their biological functions. Much attention has been devoted to exploring the role of key inflammatory cytokines and promigratory chemokines in autoimmune, autoinflammatory, and allergic diseases, leading to development of therapeutic strategies that are based on their targeted neutralization. Recent studies, including those coming from our groups, show that several major proinflammatory cytokines and chemokines, including IFN-γ, IL-2, CCL2, and CXCL12, may also function as anti-inflammatory mediators and therefore, may have potential as anti-inflammatory drugs. Likewise, major anti-inflammatory mediators, such as TGF-β, may under certain conditions, in combination with other cytokines, exhibit proinflammatory function and direct the polarization of the highly inflammatory CD4(+) Th17 cells. We show here that the biological function of pro- and anti-inflammatory cytokines is dependent on three key parameters: the local concentration of a given cytokine, the stage of disease in which it is administered, and its combination with other cytokines. The therapeutic implications of these findings are discussed, including two very recent studies summarizing clinical trials, in which low-dose administration of IL-2 was used to successfully suppress HCV and GVHD.

PMID: 22949334 [PubMed - in process]

44. J Intern Med. 2012 Nov;272(5):411-29. doi: 10.1111/j.1365-2796.2012.02588.x. Epub

2012 Oct 15.

Targeting T-cell migration in inflammatory bowel disease.

Marsal J, Agace WW.

Department of Gastroenterology, Skane University Hospital, Lund, Sweden.
jan.marsal@med.lu.se

Crohn's disease and ulcerative colitis are chronic inflammatory disorders of the gastrointestinal tract and are collectively referred to as inflammatory bowel disease (IBD). IBD is a major cause of lifetime morbidity, has a severe impact on quality of life of patients (equivalent to that of rheumatoid arthritis, asthma, migraine or diabetes) and constitutes a substantial economic burden to the healthcare system. The introduction of anti-tumour necrosis factor (TNF) antibodies has dramatically improved the treatment of IBD, but approximately one-third of patients are nonresponders and another 30-50% will eventually lose the therapeutic effect or become intolerant to these antibodies. Thus, there is an urgent and unmet need for new therapies. The aetiologies of the different forms of IBD have not been fully elucidated but there is strong evidence implicating T cells and T-cell migration to the gut in initiating and perpetuating the intestinal inflammatory process and tissue destruction. In recent years, progress in basic science has shed light on the mechanisms regulating T-cell migration to the gut and new therapeutics targeting these pathways have been developed. It is interesting that some of the factors directing the localization of T cells to the gut have been shown to be relatively organ specific, potentially enabling new T-cell-targeted treatments to demonstrate improved safety whilst preserving therapeutic efficacy. Here, fundamental aspects of the gut immune system, the generation of tissue-tropic effector T cells and the mechanisms of T-cell trafficking to the gut mucosa will be reviewed. In addition, the role of these processes in IBD and how they have been exploited for the development of novel therapies for IBD will be discussed.

© 2012 The Association for the Publication of the Journal of Internal Medicine.

PMID: 22946654 [PubMed - indexed for MEDLINE]

45. J Immunol. 2012 Oct 1;189(7):3641-52. Epub 2012 Aug 31.

Epithelial cell-derived IL-25, but not Th17 cell-derived IL-17 or IL-17F, is crucial for murine asthma.

Suzukawa M, Morita H, Nambu A, Arae K, Shimura E, Shibui A, Yamaguchi S, Suzukawa K, Nakanishi W, Oboki K, Kajiwara N, Ohno T, Ishii A, Körner H, Cua DJ, Suto H, Yoshimoto T, Iwakura Y, Yamasoba T, Ohta K, Sudo K, Saito H, Okumura K, Broide DH, Matsumoto K, Nakae S.

Department of Internal Medicine, Teikyo University School of Medicine, Tokyo 173-8605, Japan.

IL-17A, IL-17F, and IL-25 are ligands for IL-17RA. In the current study, we demonstrated that IL-25-deficient mice-but not IL-17A-, IL-17F-, IL-17A/F-, IL-23p19-, or retinoic acid-related orphan receptor (ROR)-γt-deficient mice-showed significant suppression of 1) the number of eosinophils and the levels of proinflammatory mediators in bronchoalveolar lavage fluids, 2) airway hyperresponsiveness to methacholine, and 3) OVA-specific IgG1 and IgE levels in the serum during OVA-induced Th2-type/eosinophilic airway inflammation. The IL-25 deficiency did not affect lung dendritic cell migration or Ag-specific memory-Th2 cell expansion during Ag sensitization. Adoptive transfer of T cells, mast cells,

or bone marrow cells from IL-25-deficient mice revealed that induction of Th2-type/eosinophilic airway inflammation was dependent on activation of lung epithelial cells and eosinophils by IL-25 produced by airway structural cells such as epithelial cells but not by such hematopoietic stem-cell-origin immune cells as T cells and mast cells. Therefore, airway structural cell-derived IL-25-rather than Th17 cell-derived IL-17A and IL-17F-is responsible for induction of local inflammation by promoting activation of lung epithelial cells and eosinophils in the elicitation phase of Th2-type/eosinophilic airway inflammation. It is not required for Ag-specific Th2 cell differentiation in the sensitization phase.

PMID: 22942422 [PubMed - indexed for MEDLINE]

46. Cell Mol Immunol. 2013 Jan;10(1):78-83. doi: 10.1038/cmi.2012.32. Epub 2012 Sep 3.

Staphylococcal enterotoxin B-derived haptens promote sensitization.

Yang SB, Li TL, Chen X, An YF, Zhao CQ, Wen JB, Tian DF, Wen Z, Xie MQ, Yang PC.

Department of Gastroenterology, Nan Lou Division, the PLA General Hospital, Beijing, China.

T helper 2 (Th2) polarization is a major pathological feature in allergic diseases; its etiology is not fully understood. This study aims to elucidate the adjuvant effect of the microbial product-derived small peptides in the initiation of antigen-specific Th2 polarization. In this study, a clinical survey of patients with chronic rhinosinusitis (CRS) and food allergy (FA) was carried out. The Staphylococcal enterotoxin B (SEB)-derived small peptides (Ssps) were examined in the human stool extracts. The formation of Ssp/antigen adducts was tested in a protein-protein combination assay. The bone marrow-derived dendritic cells (BMDCs) were employed to test the role of Ssp/ovalbumin (OVA) adducts in the dendritic cell (DC) maturation. A mouse model was developed to test the role of Ssp/OVA adducts in the initiation of Th2 polarization in the intestine. The results showed that 54 (18.2%) patients with FA were diagnosed among 296 patients with SEB(+) CRS; only eight (2.9%) FA patients were identified among 272 patients with SEB(-) CRS. Ssps were detected in the stool protein extracts from FA patients with SEB(+) CRS, but not in those with SEB(-) CRS. Ssp/OVA adducts induced DC maturation, speeded up DC migration, activated CD4(+) T cells in the regional lymph nodes and induced skewed Th2 polarization in the local tissue. We conclude that patients with SEB(+) CRS are prone to suffering from FA. SEB can be degraded to Ssps in the gastrointestinal tract. The Ssps can bind macromolecular antigens to form adducts to promote the antigenicity of the antigens and induction of the antigen-specific Th2 polarization and inflammation in the local tissue.

PMID: 22941409 [PubMed - in process]

47. Pulm Pharmacol Ther. 2012 Aug 23. pii: S1094-5539(12)00122-8. doi: 10.1016/j.pupt.2012.08.005. [Epub ahead of print]

Phenotype modulation of airway smooth muscle in asthma.

Wright DB, Trian T, Siddiqui S, Pascoe CD, Johnson JR, Dekkers BG, Dakshinamurti S, Bagchi R, Burgess JK, Kanabar V, Ojo OO.

Medical Research Council and Asthma UK Centre in Allergic Mechanisms of Asthma, King's College London, London, United Kingdom.

The biological responses of airway smooth muscle (ASM) are diverse, in part due to ASM phenotype plasticity. ASM phenotype plasticity refers to the ability of ASM cells to change the degree of a variety of functions, including contractility, proliferation, migration and secretion of inflammatory mediators. This plasticity occurs due to intrinsic or acquired abnormalities in ASM cells, and these abnormalities or predisposition of the ASM cell may alter the ASM response and in some cases recapitulate disease hallmarks of asthma. These phenotypic changes are ultimately determined by multiple stimuli and occur due to alterations in the intricate balance or reversible state that maintains ASM cells in either a contractile or synthetic state, through processes termed maturation or modulation, respectively. To elucidate the role of ASM phenotype in disease states, numerous in vitro studies have suggested a phenotypic switch in ASM primary cell cultures as an explanation for the plethora of responses mediated by ASM cells. Moreover, there is overwhelming evidence suggesting that the immunomodulatory response of ASM is due to the acquisition of a synthetic phenotype; however, whether this degree of plasticity is present in vivo as opposed to cell culture-based models remains speculative. Nonetheless, this review will give an overall scope of ASM phenotypic markers, triggers of ASM phenotype modulation and novel therapeutic approaches to control ASM phenotype plasticity.

Copyright © 2012 Elsevier Ltd. All rights reserved.

PMID: 22939888 [PubMed - as supplied by publisher]

48. Allergy Asthma Clin Immunol. 2012 Aug 31;8(1):15. doi: 10.1186/1710-1492-8-15.

IL-7Rα and L-selectin, but not CD103 or CD34, are required for murine peanut-induced anaphylaxis.

Maltby S, Debruin EJ, Bennett J, Gold MJ, Tunis MC, Jian Z, Marshall JS, McNagny KM.

The Biomedical Research Centre, University of British Columbia, Vancouver, BC, Canada. kelly@brc.ubc.ca.

BACKGROUND: Allergy to peanuts results in severe anaphylactic responses in affected individuals, and has dramatic effects on society and public policy. Despite the health impacts of peanut-induced anaphylaxis (PIA), relatively little is known about immune mechanisms underlying the disease. Using a mouse model of PIA, we evaluated mice with deletions in four distinct immune molecules (IL7Rα, L-selectin, CD34, CD103), for perturbed responses.
METHODS: PIA was induced by intragastric sensitization with peanut antigen and cholera toxin adjuvant, followed by intraperitoneal challenge with crude peanut extract (CPE). Disease outcome was assessed by monitoring body temperature, clinical symptoms, and serum histamine levels. Resistant mice were evaluated for total and antigen specific serum IgE, as well as susceptibility to passive systemic anaphylaxis.
RESULTS: PIA responses were dramatically reduced in IL7Rα-/- and L-selectin-/- mice, despite normal peanut-specific IgE production and susceptibility to passive systemic anaphylaxis. In contrast, CD34-/- and CD103-/- mice exhibited robust PIA responses, indistinguishable from wild type controls.
CONCLUSIONS: Loss of L-selectin or IL7Rα function is sufficient to impair PIA, while CD34 or CD103 ablation has no effect on disease severity. More broadly, our findings suggest that future food allergy interventions should focus on disrupting sensitization to food allergens and limiting antigen-specific late-phase responses. Conversely, therapies targeting immune cell migration following antigen challenge are unlikely to have significant benefits,

particularly considering the rapid kinetics of PIA.

PMCID: PMC3490721
PMID: 22935073 [PubMed]

49. Clin Exp Allergy. 2012 Sep;42(9):1302-12. doi: 10.1111/j.1365-2222.2012.03979.x.

Long-term macrolide treatment of chronic inflammatory airway diseases: risks, benefits and future developments.

Cameron EJ, McSharry C, Chaudhuri R, Farrow S, Thomson NC.

Respiratory Medicine, Institute of Infection, Immunity and Inflammation, University of Glasgow, Glasgow, UK. euancameron@nhs.net

Macrolide antibiotics were discovered over 50 years ago and following their use as antimicrobials it became apparent that this group of antibiotics also possessed anti-inflammatory properties. Subsequent clinical trials showed benefits of macrolides as long-term adjuncts in the treatment of a spectrum of chronic inflammatory respiratory diseases, particularly diffuse panbronchiolitis, cystic fibrosis, post-transplant bronchiolitis obliterans and more recently chronic obstructive pulmonary disease (COPD). The evidence for efficacy of macrolides in the long-term treatment of chronic asthma and bronchiectasis is less well established. The mechanism(s) of action of macrolides in the treatment of these diseases remains unexplained, but may be due to their antibacterial and/or anti-inflammatory actions, which include reductions in interleukin-8 production, neutrophil migration and/or function. Macrolides have additional potentially beneficial properties including anti-viral actions and an ability to restore corticosteroid sensitivity. The increased prescribing of macrolides for long-term treatment could result in the development of microbial resistance and adverse drug effects. New macrolides have been developed which do not possess any antimicrobial activity and hence lack the ability to produce microbial resistance, but which still retain immunomodulatory effects. Potentially novel macrolides may overcome a significant barrier to the use of this type of drug for the long-term treatment of chronic inflammatory airway diseases.

© 2012 Blackwell Publishing Ltd.

PMID: 22925316 [PubMed - indexed for MEDLINE]

50. Biochem Biophys Res Commun. 2012 Sep 14;426(1):116-21. doi: 10.1016/j.bbrc.2012.08.047. Epub 2012 Aug 17.

IL-33 promotes the migration and proliferation of circulating fibrocytes from patients with allergen-exacerbated asthma.

Bianchetti L, Marini MA, Isgrò M, Bellini A, Schmidt M, Mattoli S.

Avail Biomedical Research Institute, Basel, Switzerland. lbianchetti@avail-research.com

The release of IL-33 increases in the bronchial mucosa of asthmatic patients in relation to disease severity and several studies have demonstrated that IL-33 may enhance airway inflammation in asthma. This study tested the hypothesis that IL-33 may also contribute to the development of irreversible structural changes in asthma by favoring the airway recruitment and profibrotic function of circulating fibrocytes during episodes of allergen-induced asthma exacerbation. The circulating fibrocytes from patients with allergen-exacerbated asthma (PwAA)

showed increased expression of the specific IL-33 receptor component ST2L in comparison with the cells from non-asthmatic individuals (NAI). Recombinant IL-33 induced the migration of circulating fibrocytes from PwAA at clinically relevant concentrations and stimulated their proliferation in a concentration-dependent manner between 0.1 and 10 ng/ml, without affecting the constitutive release of type I collagen. The recombinant protein did not induce similar responses in circulating fibrocytes from NAI. This study uncovers an important mechanism through which fibrocytes may accumulate in the airways of allergic asthmatics when their disease is not adequately controlled by current treatment and provides novel information on the function of IL-33 in asthma.

Copyright © 2012 Elsevier Inc. All rights reserved.

PMID: 22921786 [PubMed - in process]

51. Mol Vis. 2012;18:2195-204. Epub 2012 Aug 8.

Ocular safety of cationic emulsion of cyclosporine in an in vitro corneal wound-healing model and an acute in vivo rabbit model.

Liang H, Baudouin C, Daull P, Garrigue JS, Brignole-Baudouin F.

INSERM, Paris, France.

PURPOSE: Topical preparations of cyclosporine (CsA) are common therapeutics for the treatment of dry eye. However, they are not devoid of side effects, such as allergy and irritation. The present study aimed at evaluating the safety profile of a new CsA formulation in cationic emulsion (CEm) in vitro with a dynamic corneal wound healing assay using human corneal epithelial (HCE) cells, and in vivo in a rabbit acute toxicity model.
METHODS: Three different csa formulations were tested: 1) 0.05%CsA-CEm, 2) commercial 0.05%CsA-Anionic emulsion (CsA-AEm, Restasis®), and 3) 0.05%CsA-Oil solution. Phosphate buffered saline (PBS) was used as negative control and 0.02% benzalkonium chloride (BAK) as the toxic control. In vitro, a wound was created by scratching through a confluent HCE cell layer and exposed 30 min to 1/10 dilutions of the different formulations. Cytotoxicity, cell migration, and proliferation were performed to analyze the recovery at days 1, 2, and 3. In vivo, the eye drops were applied to rabbit eyes 15 times at 5-min intervals. The ocular surface structures were examined with a slit-lamp and by corneal in vivo confocal microscopy (IVCM) for detailed examination of corneal epithelium, stroma, limbus, and conjunctiva-associated lymphoid tissue (CALT) structures.
RESULTS: The in vitro study confirmed that a 0.02% BAK solution delayed the corneal healing process (-57%) by severely damaging the remaining HCE cells. The other formulations maintained a normal healing rate with a similar behavior for CsA-CEm, CsA-AEm, and PBS with no significant differences (at D3, 66%-74% closure). In the rabbit, 0.02%BAK showed the highest toxicity, inducing redness, chemosis with damaged corneal epithelium, and inflammatory cell infiltrations. CsA-AEm and CsA-Oil induced moderate infiltrations of inflammatory cells around the CALT. CsA-CEm presented the lowest toxicity with patterns similar to PBS.
CONCLUSIONS: The combination of these in vitro and in vivo models evaluated the tolerance/cytotoxicity and the dynamic wound healing potential of CsA in different formulations. While CsA-AEm, CsA-CEm, and CsA-Oil are generally well tolerated, only CsA-CEm appeared to maintain the HCE cells' normal healing rate in vitro and low levels of inflammation in vivo.

PMCID: PMC3425577
PMID: 22919267 [PubMed - indexed for MEDLINE]

52. BMC Syst Biol. 2012 Aug 20;6:105. doi: 10.1186/1752-0509-6-105.

Chemoattraction of macrophages by secretory molecules derived from cells expressing the signal peptide of eosinophil cationic protein.

Liu YS, Tsai PW, Wang Y, Fan TC, Hsieh CH, Chang MD, Pai TW, Huang CF, Lan CY, Chang HT.

College of Medicine, China Medical University, Taichung, Taiwan.

BACKGROUND: Eosinophil cationic protein is a clinical asthma biomarker that would be released into blood, especially gathered in bronchia. The signal peptide of eosinophil cationic protein (ECPsp) plays an important role in translocating ECP to the extracellular space. We previously reported that ECPsp inhibits microbial growth and regulates the expression of mammalian genes encoding tumor growth factor-α (TGF-α) and epidermal growth factor receptor (EGFR).
RESULTS: In the present study, we first generated a DNA microarray dataset, which showed that ECPsp upregulated proinflammatory molecules, including chemokines, interferon-induced molecules, and Toll-like receptors. The levels of mRNAs encoding CCL5, CXCL10, CXCL11, CXCL16, STAT1, and STAT2 were increased in the presence of ECPsp by 2.07-, 4.21-, 7.52-, 2.6-, 3.58-, and 1.67-fold, respectively. We then constructed a functional linkage network by integrating the microarray dataset with the pathway database of Kyoto Encyclopedia of Genes and Genomes (KEGG). Follow-up analysis revealed that STAT1 and STAT2, important transcriptional factors that regulate cytokine expression and release, served as hubs to connect the pathways of cytokine stimulation (TGF-α and EGFR pathways) and inflammatory responses. Furthermore, integrating TGF-α and EGFR with the functional linkage network indicated that STAT1 and STAT2 served as hubs that connect two functional clusters, including (1) cell proliferation and survival, and (2) inflammation. Finally, we found that conditioned medium in which cells that express ECPsp had been cultured could chemoattract macrophages. Experimentally, we also demonstrated that the migration of macrophage could be inhibited by the individual treatment of siRNAs of STAT1 or STAT2. Therefore, we hypothesize that ECPsp may function as a regulator for enhancing the migration of macrophages through the upregulation of the transcriptional factors STAT1 and STAT2.
CONCLUSION: The increased expression and release of various cytokines triggered by ECPsp may attract macrophages to bronchia to purge damaged cells. Our approach, involving experimental and computational systems biology, predicts pathways and potential biological functions for further characterization of this novel function of ECPsp under inflammatory conditions.

PMCID: PMC3478170
PMID: 22906315 [PubMed - in process]

53. Eur Ann Allergy Clin Immunol. 2012 Jun;44(3):107-12.

Features of sensitization to airborne allergens among extra-European immigrants living in 2 distinct areas of Northern Italy.

Asero R, Conte M, Senna GE.

Ambulatorio di Allergologia, Clinica San Carlo, Paderno Dugnano (MI), Italy. r.asero@libero.it

BACKGROUND: Extra-European immigrants are increasingly seen in allergy clinics. In view of their different genetic background they represent an opportunity to investigate the dynamics of sensitization to airborne allergens.
OBJECTIVE: We investigated the pattern of airborne sensitization among allergic

extra-European immigrants living in two areas of northern Italy.
METHODS: Extra-European immigrants living in Milan and Verona were compared with age- and sex-matched or allergen-matched allergic Italians. Based on number of sensitizations to airborne allergens, patients and controls were divided into mono-/oligo-sensitized or multi-sensitized (1-3 or > 3, respectively).
RESULTS: In Milan grass pollen allergy was more frequent among immigrants than in controls (75% vs 49%; p < 0.01), whereas ragweed allergy prevailed among Italians (56% vs 20%; p < 0.001). In Verona, immigrants were rarely multi-sensitized (21% vs 43%; p < 0.01), more frequently sensitized to grass and birch. In both areas immigrants became allergic at a significantly older age than Italian controls (p < 0.0001), particularly to grass and mites. Allergy to HDM and ragweed was rare in Central & South Americans, whereas Africans showed the lowest proportion of multi-sensitizations. On average, immigrants became allergic after 2-4 years of stay in Italy.
CONCLUSION: Subjects genetically prone to become allergic to a particular allergen get eventually sensitized irrespective of their age when they are exposed to the "right" allergen for a sufficiently long time. The higher proportion of mono-/oligo-sensitized immigrants might reflect a shorter exposure to airborne allergen load in this group.

PMID: 22905591 [PubMed - indexed for MEDLINE]

54. Acta Clin Belg. 2012 May-Jun;67(3):198-200.

Sudden cardiac arrest during pregnancy: a rare complication of acquired maternal diaphragmatic hernia.

Jacobs R, Honore PM, Hosseinpour N, Nieboer K, Spapen HD.

ICU dept, Universitair Ziekenhuis Brussel, Belgium.

Acute cardiac arrest during pregnancy is a rare but devastating event. Major causes are haemorrhagic, septic or anaphylactic shock, trauma, pulmonary or amniotic fluid embolism, and congenital or acquired cardiac disease. We present a case of massive intrathoracic migration of viscera through a left diaphragmatic hernia in a pregnant multipara, causing acute obstructive shock and cardiac arrest. Complications of intrathoracic herniation occur when the intruding viscera cause left lung and cardiac compression or mediastinal "tamponade" with decreased venous return. Intrathoracic strangulation of viscera is also common and may cause ischaemia, gangrene and eventual perforation. Sudden cardiac arrest as first sign of left diaphragmatic rupture during pregnancy, however, has rarely been described. In contrast with our patient, this catastrophic event is mostly seen in nulli- and primipara with a known congenital left diaphragmatic defect. Management of a diaphragmatic hernia depends on the clinical presentation and the period of gestation during which it is detected. Despite prolonged resuscitation with more than 1 hour of chest compressions, our patient recovered completely.

PMID: 22897068 [PubMed - indexed for MEDLINE]

55. Pediatr Allergy Immunol. 2012 Dec;23(8):716-23. doi:
10.1111/j.1399-3038.2012.01342.x. Epub 2012 Aug 13.

Prevalence and risk factors of atopic diseases in German children and adolescents.

Schmitz R, Atzpodien K, Schlaud M.

Department of Epidemiology and Health Reporting, Robert Koch Institute, Berlin,

Germany. schmitzr@rki.de

BACKGROUND: Atopic diseases became an important health problem in affluent Western societies.
METHODS: To study the prevalence and factors associated with the risk of atopic diseases in Germany, data from the German Health Interview and Examination Survey for Children and Adolescents (KiGGS) were analysed (n = 17,450). Standardized, computer-assisted personal interviews with parents and parent-administered questionnaires provided physician diagnoses of allergic rhinoconjunctivitis, atopic dermatitis and asthma as well as data on demographic characteristics, migration background, birth order, age at the beginning of nursery, atopic diseases of parents, parents' smoking status, parents' occupation, breastfeeding and living environment.
RESULTS: The life-time prevalence of atopic dermatitis was 13.2% (95% confidence limit: 12.5-13.9%), 10.7% (10.1-11.3%) for allergic rhinoconjunctivitis and 4.7% (4.3-5.1%) for asthma. At least one atopic disease in parents was the strongest factor associated with atopic diseases in the offspring, with a prevalence ratio of up to 2.6. High and middle socio-economic status (prevalence ratio, 95% confidence limit: 1.28, 1.12-1.46; 1.15, 1.01-1.32) were associated with the risk of atopic dermatitis, whereas a two-sided background of migration reduced the risk (0.76, 0.65-0.88). Factors that reduced the risk of allergic rhinoconjunctivitis were parents working as self-employed farmers (0.48, 0.30-0.76) and older siblings (0.80, 0.71-0.89), whereas the beginning of nursery school at older age was associated with an increased risk in children who were cared for outside the family before school age (1.05, 1.00-1.10). Living in mould-infested rooms (1.64, 1.23-2.19), an urban living environment (1.20, 1.02-1.42) and a smoking mother and/or father (1.20, 1.02-1.40) were associated with the risk of asthma.
CONCLUSIONS: Our results are in line with the so-called 'hygiene hypothesis', which emphasizes the role of environmental factors in addition to a genetic predisposition in the development of atopic diseases. Research on factors associated with atopic diseases can facilitate decisions on preventive strategies. Further studies are needed to explore trends in prevalence and risk factors for atopic diseases.

© 2012 John Wiley & Sons A/S.

PMID: 22882467 [PubMed - in process]

56. J Neurochem. 2012 Oct;123(2):203-16. doi: 10.1111/j.1471-4159.2012.07900.x. Epub 2012 Sep 3.

Matrix metalloproteinase-3 in the central nervous system: a look on the bright side.

Van Hove I, Lemmens K, Van de Velde S, Verslegers M, Moons L.

Laboratory of Neural Circuit Development and Regeneration, Animal Physiology and Neurobiology Section, Department of Biology, KU Leuven, Leuven, Belgium.

Matrix metalloproteinases (MMPs) are a large family of proteases involved in many cell-matrix and cell-cell signalling processes through activation, inactivation or release of extracellular matrix (ECM) and non-ECM molecules, such as growth factors and receptors. Uncontrolled MMP activities underlie the pathophysiology of many disorders. Also matrix metalloproteinase-3 (MMP-3) or stromelysin-1 contributes to several pathologies, such as cancer, asthma and rheumatoid arthritis, and has also been associated with neurodegenerative diseases like Alzheimer's disease, Parkinson's disease and multiple sclerosis. However, based on defined MMP spatiotemporal expression patterns, the identification of novel

candidate molecular targets and in vitro and in vivo studies, a beneficial role for MMPs in CNS physiology and recovery is emerging. The main purpose of this review is to shed light on the recently identified roles of MMP-3 in normal brain development and in plasticity and regeneration after CNS injury and disease. As such, MMP-3 is correlated with neuronal migration and neurite outgrowth and guidance in the developing CNS and contributes to synaptic plasticity and learning in the adult CNS. Moreover, a strict spatiotemporal MMP-3 up-regulation in the injured or diseased CNS might support remyelination and neuroprotection, as well as genesis and migration of stem cells in the damaged brain.

© 2012 The Authors Journal of Neurochemistry © 2012 International Society for Neurochemistry.

PMID: 22862420 [PubMed - indexed for MEDLINE]

57. J Infect Dis. 2012 Oct 1;206(7):1059-68. Epub 2012 Jul 30.

Effectiveness of 1 dose of influenza A (H1N1) 2009 monovalent vaccines in preventing reverse-transcription polymerase chain reaction-confirmed H1N1 infection among school-aged children in maine.

Uzicanin A, Thompson M, Smith P, Chaves SS, Foster L, Dube N, Graitcer S, Jackson R, Ferdinands J, Gargiullo P, Mills D, Monto AS, Shay DK; Maine 2009 Influenza A (H1N1) Vaccine Effectiveness Evaluation Group.

Collaborators: Dube N, Mills D, Perkins K, Robinson S, Sears S, Smith P, Tipton M, Basurto-Davila R, Chen J, Cheng PY, Chaves SS, Jackson R, Ferdinands J, Gargiullo P, Graitcer S, King J, Shay D, Sokolow L, Uzicanin A, Zhou H, Monto AS.

Division of Global Migration and Quarantine, Centers for Disease Control and Prevention, Atlanta, Georgia 30333, USA. aau5@cdc.gov

BACKGROUND: In late October 2009, school-located pandemic vaccination was initiated in Maine before or concurrent with 2009 pandemic influenza A (H1N1) virus (pH1N1) peak activity.
METHODS: A case-control evaluation of 2009 H1N1 vaccine effectiveness was conducted in schools in Cumberland County, Maine. A case was a child who had an acute respiratory illness during 2 November-18 December 2009, and who tested positive for pH1N1 by real-time reverse-transcription polymerase chain reaction (rRT-PCR). For each case, ≥ 4 event time-matched controls were sampled among classmates present in school during the study period who did not have an influenza-like illness. Vaccine effectiveness was calculated as (1 - adjusted odds ratio [aOR])100%; aOR was estimated by using weighted logistic regression.
RESULTS: After adjusting for a diagnosis of asthma, 1 dose of 2009 H1N1 vaccine provided 69% protection (95% confidence interval (CI), 13-89) against rRT-PCR-confirmed H1N1 infection. Vaccine effectiveness estimates for live attenuated and inactivated vaccine were 81% (95% CI, -37 to 97), and 58% (95% CI: -39 to 87), respectively. Conclusions: One dose of monovalent pandemic vaccine provided substantial protection against pH1N1 infection among school-aged children.

PMID: 22850120 [PubMed - indexed for MEDLINE]

58. J Vis Exp. 2012 Jul 23;(65):e4014. doi: 10.3791/4014.

Analysis of pulmonary dendritic cell maturation and migration during allergic airway inflammation.

Kushwah R, Hu J.

Stem Cell and Cancer Research Institute, McMaster University, Hamilton.

Dendritic cells (DCs) are the key players involved in initiation of adaptive immune response by activating antigen-specific T cells. DCs are present in peripheral tissues in steady state; however in response to antigen stimulation, DCs take up the antigen and rapidly migrate to the draining lymph nodes where they initiate T cell response against the antigen. Additionally, DCs also play a key role in initiating autoimmune as well as allergic immune response. DCs play an essential role in both initiation of immune response and induction of tolerance in the setting of lung environment. Lung environment is largely tolerogenic, owing to the exposure to vast array of environmental antigens. However, in some individuals there is a break in tolerance, which leads to induction of allergy and asthma. In this study, we describe a strategy, which can be used to monitor airway DC maturation and migration in response to the antigen used for sensitization. The measurement of airway DC maturation and migration allows for assessment of the kinetics of immune response during airway allergic inflammation and also assists in understanding the magnitude of the subsequent immune response along with the underlying mechanisms. Our strategy is based on the use of ovalbumin as a sensitizing agent. Ovalbumin-induced allergic asthma is a widely used model to reproduce the airway eosinophilia, pulmonary inflammation and elevated IgE levels found during asthma. After sensitization, mice are challenged by intranasal delivery of FITC labeled ovalbumin, which allows for specific labeling of airway DCs which uptake ovalbumin. Next, using several DC specific markers, we can assess the maturation of these DCs and can also assess their migration to the draining lymph nodes by employing flow cytometry.

PMID: 22847488 [PubMed - indexed for MEDLINE]

59. Am J Physiol Lung Cell Mol Physiol. 2012 Oct 15;303(8):L642-60. doi: 10.1152/ajplung.00406.2011. Epub 2012 Jul 27.

Inhibition of allergic inflammation by supplementation with 5-hydroxytryptophan.

Abdala-Valencia H, Berdnikovs S, McCary CA, Urick D, Mahadevia R, Marchese ME, Swartz K, Wright L, Mutlu GM, Cook-Mills JM.

Division of Allergy-Immunology, Northwestern Univeristy Feinberg School of Medicine, Chicago, IL 60611, USA.

Clinical reports indicate that patients with allergy/asthma commonly have associated symptoms of anxiety/depression. Anxiety/depression can be reduced by 5-hydroxytryptophan (5-HTP) supplementation. However, it is not known whether 5-HTP reduces allergic inflammation. Therefore, we determined whether 5-HTP supplementation reduces allergic inflammation. We also determined whether 5-HTP decreases passage of leukocytes through the endothelial barrier by regulating endothelial cell function. For these studies, C57BL/6 mice were supplemented with 5-HTP, treated with ovalbumin fraction V (OVA), house dust mite (HDM) extract, or IL-4, and examined for allergic lung inflammation and OVA-induced airway responsiveness. To determine whether 5-HTP reduces leukocyte or eosinophil transendothelial migration, endothelial cells were pretreated with 5-HTP, washed and then used in an in vitro transendothelial migration assay under laminar flow. Interestingly, 5-HTP reduced allergic lung inflammation by 70-90% and reduced antigen-induced airway responsiveness without affecting body weight, blood eosinophils, cytokines, or chemokines. 5-HTP reduced allergen-induced transglutaminase 2 (TG2) expression and serotonylation (serotonin conjugation to proteins) in lung endothelial cells. Consistent with the regulation of endothelial serotonylation in vivo, in vitro pretreatment of endothelial cells

with 5-HTP reduced TNF-α-induced endothelial cell serotonylation and reduced leukocyte transendothelial migration. Furthermore, eosinophil and leukocyte transendothelial migration was reduced by inhibitors of transglutaminase and by inhibition of endothelial cell serotonin synthesis, suggesting that endothelial cell serotonylation is key for leukocyte transendothelial migration. In summary, 5-HTP supplementation inhibits endothelial serotonylation, leukocyte recruitment, and allergic inflammation. These data identify novel potential targets for intervention in allergy/asthma.

PMCID: PMC3469632 [Available on 2013/10/15]
PMID: 22842218 [PubMed - indexed for MEDLINE]

60. Adv Exp Med Biol. 2013;756:73-80. doi: 10.1007/978-94-007-4549-0_10.

Influence of sublingual immunotherapy on the expression of Mac-1 integrin in neutrophils from asthmatic children.

Ciepiela O, Zawadzka-Krajewska A, Kotula I, Pyrzak B, Demkow U.

Department of Laboratory Diagnostics and Clinical Immunology of Developmental Age, Warsaw Medical University, Warsaw, Poland. olga.ciepiela@wum.edu.pl

Asthma can be effectively treated with sublingual immunotherapy. The influence of -sublingual immunotherapy on the function of granulocytes in asthmatic patients is largely unknown. Mac-1 integrin is a transmembrane protein containing α (CD11b) and β (CD18) chains. High expression of the complex is found on the surface of neutrophils, NK cells, and macrophages. CD11b/CD18 may bind to CD23, ICAM-1, ICAM-2, and ICAM-4. It plays a crucial role in diapedesis of neutrophils. The aim of the present study was to assess Mac-1 expression on neutrophils from asthmatic children before and after sublingual immunotherapy. Twenty five children aged of 8.1 ± 3.1 suffering from atopic asthma and allergic rhinitis, shortlisted for specific immunotherapy, served as the study group. Fifteen healthy individuals, aged 9.8 ± 3.4, served as a control group. The assessment of CD11b and CD18 expression on cells from peripheral blood was performed with a flow cytometer. The tests were performed before and after 12 months of sublingual immunotherapy. In the asthmatic children, 98.08 (90.79-99.12)% of Mac-1 positive neutrophils were detected. The group was divided into two subgroups: of more than 98% and less than 95% of neutrophils with CD11b/CD18 expression in the sample. After immunotherapy, the percentage of Mac-1 positive granulocytes increased to 99.60 (99.29-99.68)%, $p = 0.01$. In the control group, 90.56 (87.08-88.86)% granulocytes were Mac-1 positive, $p = 0.002$. We conclude that sublingual immunotherapy strongly influences the function of the immunological system, including Mac-1 expression on neutrophils.

PMID: 22836621 [PubMed - indexed for MEDLINE]

61. J Cutan Pathol. 2012 Oct;39(10):901-10. doi: 10.1111/j.1600-0560.2012.01960.x. Epub 2012 Jul 26.

Melanocytic lesions with eczematous reaction (Meyerson's phenomenon) - a histopathologic analysis of 64 cases.

Pižem J, Stojanovič L, Luzar B.

Institute of Pathology, University of Ljubljana, Ljubljana, Slovenia. joze.pizem@mf.uni-lj.si

BACKGROUND: Eczematous (spongiotic) reaction in melanocytic lesions (Meyerson's

phenomenon) has not been systematically analyzed and has not been convincingly documented in melanoma.
METHODS: We analyzed 64 consecutive melanocytic lesions with spongiotic reaction, occurring in 57 patients (age range 14-81 years; mean, 39 years; 30 females, 27 males) including 16 common acquired nevi, 3 nevi with congenital features, 2 Spitz nevi, 29 dysplastic nevi, 6 in situ and 8 invasive melanomas.
RESULTS: The intensity of the spongiotic reaction was graded as mild in 24 (38%), moderate in 22 (34%) and marked in 18 (28%) lesions. It was moderate/marked in 6 of 14 (43%) in situ or invasive melanomas. Upward migration of melanocytes in the epidermis was noted in 7 (33%) non-dysplastic and 10 (34%) dysplastic nevi but was generally limited to the lower half of the epidermis. Moderate/severe cytologic atypia was found in 14 (48%) dysplastic nevi and all melanomas but not in non-dysplastic nevi.
CONCLUSIONS: Prominent spongiotic reaction with eosinophils in the inflammatory infiltrate can affect all types of melanocytic lesions. Upward migration involving the uppermost layers of the epidermis, especially when extensive and present in areas with a less pronounced spongiotic reaction, and moderate/severe cytologic atypia indicate a melanoma.

Copyright © 2012 John Wiley & Sons A/S.

PMID: 22830623 [PubMed - in process]

62. Recent Pat Inflamm Allergy Drug Discov. 2012 Sep;6(3):210-21.

Therapeutic strategies in allergic contact dermatitis.

Alase A, Wittmann M.

Institute for Molecular and Cellular Biology, Faculty of Biological Sciences, University of Leeds, UK.

Due to its high prevalence, allergic contact dermatitis (ACD) has an important economic and occupational health impact on society. ACD presents as an inflammatory response to small molecules and involves both skin resident cells and activated skin infiltrating T cells. Activation of skin resident cells plays an essential role in the initial sensitization phase. A number of different pathways are crucially involved in this phase including the activation of pattern recognition receptors such as TLR, inflammasome activation and production of reactive oxygen species all of which contribute to release of cellular mediators such as IL-1 family members. Chemokines regulate steps in elicitation of adaptive T cell responses including the migration to and presentation of the contact allergen by skin derived antigen presenting cells in the draining lymph node as well as the recruitment of these activated, allergen reactive CD4+ and CD8+ cells back into the skin. The current therapeutic regimens are largely restricted to the avoidance of the contact allergen and the topical use of anti-inflammatory drugs such as glucocorticosteroids. Recent research, as highlighted by current patents, focus on the use of anti-oxidants, the induction of immunological tolerance, interference with cell signaling molecules and blocking of cytokines actively involved in ACD.

PMID: 22827838 [PubMed - in process]

63. J Biol Regul Homeost Agents. 2012 Apr-Jun;26(2):193-201.

Role of mast cells in innate and adaptive immunity.

Tete S, Tripodi D, Rosati M, Conti F, Maccauro G, Saggini A, Salini V, Cianchetti

E, Caraffa A, Antinolfi P, Toniato E, Castellani ML, Pandolfi F, Frydas S, Conti
P, Theoharides TC.

Mast cells play a central role in inflammatory and immediate allergic reactions and are necessary for allergic reactions. Mast cells play a role in the pathophysiology of autoimmune diseases and appear to be especially important in inflamed tissues, because they infiltrate tissues and produce a variety of cytokines. Mast cells are important for both innate and adaptive immunity in tissues that are in close contact with the environment, i.e. the skin, the airways and the lung, and the lining of the intestine. However, there are still many unsolved issues of mast cell functions, including their regulatory mechanism on cell differentiation in bone marrow; for example, the cytokines and transcription factors necessary for their differentiation and expansion, as well as the molecular mechanism underlying basophil migration from the bloodstream to peripheral tissues such as lymph nodes still need to be clarified.

PMID: 22824746 [PubMed - indexed for MEDLINE]

64. Zhongguo Dang Dai Er Ke Za Zhi. 2012 Jul;14(7):521-3.

[Allergens and their relationships to childhood bronchial asthma in the Jiading District of Shanghai].

[Article in Chinese]

Dong W, Sheng J, Gu XM, Yan HJ, Zeng CY, Xu HT, Qian D, Huang QL, Shao J.

Department of Pediatrics, Nanxiang Hospital, Jiading District, Shanghai 201802, China.

OBJECTIVE: To study allergens and their relationship to the occurrence of childhood bronchial asthma in the Jiading District of Shanghai.
METHODS: Three hundred and eighty-two 4 to 12-year-old children with asthma in the remission stage from Nanxiang Hospital in the Jiading District of Shanghai were used as a case group (asthma group), and 402 children from two primary schools and two kindergartens in Jiading were enrolled by cluster sampling and served as control group. Parents of the children completed a questionnaire on living conditions and allergy-related disease history. Skin prick test (SPT) for 18 common allergens was carried out in both groups. In order to examine the effect of environment and living conditions on SPT results, children in the control group were further divided into two sub-groups according to birth place: migrant (219 cases) and resident (183 cases).
RESULTS: SPT results revealed that the main allergens identified in the Jiading region were dermatophagoides farinae, house dust mites, shrimps, cockroaches, and dog hair. The SPT positive rate was 67.9% in the asthma group, and this was significantly higher than in the control group (31.8%) ($P<0.01$). The environment and living conditions in the migrant group were significantly different from the resident group ($P<0.01$), whereas the SPT positive rate for this group was significantly lower than in the resident group ($P<0.01$).
CONCLUSIONS: Allergens in the Jiading region mainly originate from dermatophagoides farinae, household dust mites, shrimps, cockroaches and dog hair. Children with asthma are more susceptible to allergens. Environment and living conditions may be relevant, to a certain extent, to an SPT positive rate.

PMID: 22809606 [PubMed - indexed for MEDLINE]

65. Parasitol Int. 2012 Sep;61(3):425-30.

Dexamethasone reduces bronchial wall remodeling during pulmonary migration of Strongyloides venezuelensis larvae in rats.

Tefé-Silva C, Beneli CT, Celes MR, Machado ER, Ueta MT, Sorgi CA, Floriano EM, Faccioli LH, Ramos SG.

Department of Pathology, School of Medicine of Ribeirão Preto, University of São Paulo, Ribeirão Preto, Brazil. cristefe@usp.br

Strongyloidiasis is an intestinal parasitosis with an obligatory pulmonary cycle. A Th2-type immune response is induced and amplifies the cellular response through the secretion of inflammatory mediators. Although this response has been described as being similar to asthma, airway remodeling during pulmonary migration of larvae has not yet been established. The aim of this study was to identify the occurrence of airway remodeling during Strongyloides venezuelensis (S. v.) infection and to determine the ability of dexamethasone treatment to interfere with the mechanisms involved in this process. Rats were inoculated with 9,000 S. v. larvae, treated with dexamethasone (2 mg/kg) and killed at 1, 3, 5, 7, 14 and 21 days. Morphological and morphometric analyzes with routine stains and immunohistochemistry were conducted, and some inflammatory mediators were evaluated using ELISA. Goblet cell hyperplasia and increased bronchiolar thickness, characterized by edema, neovascularization, inflammatory infiltrate, collagen deposition and enlargement of the smooth muscle cell layer were observed. VEGF, IL1-β and IL-4 levels were elevated throughout the course of the infection. The morphological findings and the immunomodulatory response to the infection were drastically reduced in dexamethasone-treated rats. The pulmonary migration of S. venezuelensis larvae produced a transitory, but significant amount of airway remodeling with a slight residual bronchiolar fibrosis. The exact mechanisms involved in this process require further study.

PMID: 22808527 [PubMed - indexed for MEDLINE]

66. Cell Commun Signal. 2012 Jul 17;10(1):21. doi: 10.1186/1478-811X-10-21.

Functions of the Lyn tyrosine kinase in health and disease.

Ingley E.

Cell Signalling Group, Laboratory for Cancer Medicine, Western Australian Institute for Medical Research, Centre for Medical Research, The University of Western Australia, Rear 50 Murray Street, Perth, WA, 6000, Australia. evan.ingley@uwa.edu.au.

Src family kinases such as Lyn are important signaling intermediaries, relaying and modulating different inputs to regulate various outputs, such as proliferation, differentiation, apoptosis, migration and metabolism. Intriguingly, Lyn can mediate both positive and negative signaling processes within the same or different cellular contexts. This duality is exemplified by the B-cell defect in Lyn-/- mice in which Lyn is essential for negative regulation of the B-cell receptor; conversely, B-cells expressing a dominant active mutant of Lyn (Lynup/up) have elevated activities of positive regulators of the B-cell receptor due to this hyperactive kinase. Lyn has well-established functions in most haematopoietic cells, viz. progenitors via influencing c-kit signaling, through to mature cell receptor/integrin signaling, e.g. erythrocytes, platelets, mast cells and macrophages. Consequently, there is an important role for this kinase in regulating hematopoietic abnormalities. Lyn is an important regulator of autoimmune diseases such as asthma and psoriasis, due to its profound ability to influence immune cell signaling. Lyn has also been found to be important for maintaining the leukemic phenotype of many different liquid

cancers including acute myeloid leukaemia (AML), chronic myeloid leukaemia (CML) and B-cell lymphocytic leukaemia (BCLL). Lyn is also expressed in some solid tumors and here too it is establishing itself as a potential therapeutic target for prostate, glioblastoma, colon and more aggressive subtypes of breast cancer. LAY To relay information, a cell uses enzymes that put molecular markers on specific proteins so they interact with other proteins or move to specific parts of the cell to have particular functions. A protein called Lyn is one of these enzymes that regulate information transfer within cells to modulate cell growth, survival and movement. Depending on which type of cell and the source of the information input, Lyn can positively or negatively regulate the information output. This ability of Lyn to be able to both turn on and turn off the relay of information inside cells makes it difficult to fully understand its precise function in each specific circumstance. Lyn has important functions for cells involved in blood development, including different while blood cells as well as red blood cells, and in particular for the immune cells that produce antibodies (B-cells), as exemplified by the major B-cell abnormalities that mice with mutations in the Lyn gene display. Certain types of leukaemia and lymphoma appear to have too much Lyn activity that in part causes the characteristics of these diseases, suggesting it may be a good target to develop new anti-leukaemia drugs. Furthermore, some specific types, and even specific subtypes, of solid cancers, e.g. prostate, brain and breast cancer can also have abnormal regulation of Lyn. Consequently, targeting this protein in these cancers could also prove to be beneficial.

PMCID: PMC3464935
PMID: 22805580 [PubMed]

67. J Immunol. 2012 Aug 15;189(4):1699-707. doi: 10.4049/jimmunol.1200729. Epub 2012 Jul 16.

The HGF receptor/Met tyrosine kinase is a key regulator of dendritic cell migration in skin immunity.

Baek JH, Birchmeier C, Zenke M, Hieronymus T.

Department of Cell Biology, Institute for Biomedical Engineering, Medical Faculty, Rheinisch-Westfälische Technische Hochschule, 52074 Aachen, Germany.

The Met tyrosine kinase has a pivotal role in embryonic development and tissue regeneration, and deregulated Met signaling contributes to tumorigenesis. After binding of its cognate ligand hepatocyte growth factor, Met signaling confers mitogenic, morphogenic, and motogenic activity to various cells. Met expression in the hematopoietic compartment is limited to progenitor cells and their Ag-presenting progeny, including dendritic cells (DCs). In this study, we demonstrate that Met signaling in skin-resident DCs is essential for their emigration toward draining lymph nodes upon inflammation-induced activation. By using a conditional Met-deficient mouse model (Met(flox/flox)), we show that Met acts on the initial step of DC release from skin tissue. Met-deficient DCs fail to reach skin-draining lymph nodes upon activation while exhibiting an activated phenotype. Contact hypersensitivity reactions in response to various contact allergens is strongly impaired in Met-deficient mice. Inhibition of Met signaling by single-dose epicutaneous administration of the Met kinase-specific inhibitor SU11274 also suppressed contact hypersensitivity in wild-type mice. Additionally, we found that Met signaling regulates matrix metalloproteinase MMP2 and MMP9 activity, which is important for DC migration through extracellular matrix. These data unveil Met signaling in DCs as a critical determinant for the maintenance of normal immune function and suggest Met as a potential target for treatment of autoimmune skin diseases.

PMID: 22802413 [PubMed - indexed for MEDLINE]

68. OMICS. 2012 Oct;16(10):513-26. doi: 10.1089/omi.2011.0160. Epub 2012 Jul 9.

Identification of SRC as a potent drug target for asthma, using an integrative approach of protein interactome analysis and in silico drug discovery.

Randhawa V, Bagler G.

Biotechnology Division, Institute of Himalayan Bioresource Technology, Council of Scientific and Industrial Research (CSIR-IHBT), Palampur, India.

Network-biology inspired modeling of interactome data and computational chemistry have the potential to revolutionize drug discovery by complementing conventional methods. We consider asthma, a complex disease characterized by intricate molecular mechanisms, for our study. We aim to integrate prediction of potent drug targets using graph-theoretical methods and subsequent identification of small molecules capable of modulating activity of the best target. In this work, we construct the protein interactome underlying this disease: Asthma Protein Interactome (API). Using a strategy based on network analysis of the interactome, we identify a set of potential drug targets for asthma. Topologically and dynamically, v-src sarcoma (Schmidt-Ruppin A-2) viral oncogene homolog (SRC) emerges as the most central target in API. SRC is known to play an important role in promoting airway smooth muscle cell growth and facilitating migration in airway remodeling. From interactome analysis, and with the reported role in respiratory mechanisms, SRC emerges as a promising drug target for asthma. Further, we proceed to identify leads for SRC from a public database of small molecules. We predict two potential leads for SRC using ligand-based virtual screening methodology.

PMID: 22775150 [PubMed - in process]

69. Biochim Biophys Acta. 2012 Oct;1822(10):1638-42. doi: 10.1016/j.bbadis.2012.06.012. Epub 2012 Jul 3.

DHEA-S inhibits human neutrophil and human airway smooth muscle migration.

Koziol-White CJ, Goncharova EA, Cao G, Johnson M, Krymskaya VP, Panettieri RA Jr.

Department of Medicine, University of Pennsylvania, Philadelphia, PA 19104, USA. kcynthia@mail.med.upenn.edu

Airway diseases such as asthma, emphysema, and chronic bronchitis are, in part, characterized by reversible airflow obstruction and inflammation. In severe disease, marked decreases in lung function are associated with airway smooth muscle proliferation and airway neutrophilia. Inhaled glucocorticoids attenuate increased airflow obstruction and airway inflammation that occur, in part, due to increased smooth muscle migration and proliferation, as well as the airway neutrophilia. Glucocorticoids, however, have adverse side effects and, in some patients, are ineffective despite high doses. Recent research has explored the effects of non-traditional steroids on attenuation of inflammation associated with airway diseases. These non-traditional steroids have improved side effect profiles in comparison to glucocorticoid therapy. Our studies assessed effects of dehydroepiandrosterone-3-sulfate (DHEA-S) on migration of both human peripheral blood neutrophils (PMN) and human airway smooth muscle cells (HASM). DHEA-S dose-dependently inhibited chemotaxis of PMN and HASM while having no effect on the phosphorylation levels of Akt, ERK1/2, p38 MAPK or PKC, canonical positive regulators of cell migration. These studies demonstrate direct effects of DHEA-S

on cell migration, thereby suggesting that DHEA-S may attenuate airway inflammation and cell migration.

Copyright © 2012 Elsevier B.V. All rights reserved.

PMCID: PMC3418395 [Available on 2013/10/1]
PMID: 22771498 [PubMed - indexed for MEDLINE]

70. Eur Ann Allergy Clin Immunol. 2012 Apr;44(2):61-72.

T cell receptor excision circles (TREC) and recent thymic migrant cells in specific immunotherapy and respiratory allergy to Dermatophagoides pteronyssinus.

Pereira C, Loureiro G, Martinho A, Paiva A, Tavares B, Machado D, Nunes R, Pedreira S, Henriques A, Pais ML, Segorbe-Luís A.

Immunoallergy Department, Coimbra University Hospital, Coimbra Portugal. celsopereira.pt@gmail.com

INTRODUCTION: T cell receptor excision circles (TREC) on CD31+ T cells are related to recent thymic emigrant cells (RTEs). The involvement of the functional thymic tissue occurs early in the IgE-mediated allergic reaction, and in response to specific immunotherapy (SIT).
AIM: Evaluation of specific immunotherapy effects on TREC number in peripheral T cells in patients allergic to Dermatophagoides pteronyssinus (Dpt).
METHOD: 85 respiratory allergic patients (both genders), 41 of them (Group II) under maintenance treatment to Dpt SIT (21 sublingual-SLIT, and 20 subcutaneous-SCIT), were selected. The allergic patients (Group I) without specific treatment were submitted to an allergen challenge test (22 nasal and 22 conjunctival). Peripheral cell analysis was performed immediately before treatment and 60 or 240 minutes after allergenic extract administration. TREC quantification was performed in CD4+CD31+ and CD8+CD31+. The results were expressed per 100.000 cells related to RTEs. Samples from 10 healthy individuals (Control - Group III) were obtained with the same method.
RESULTS: The value of TRECs on RTEs was constant in control groups. For Group I patients (nasal or conjunctival test), TREC quantification in CD31+ T cells showed relevant individual changes, even in the patients tested earlier (60 minutes), and statistical significant at 240 minutes. Both SCIT and SLIT had also demonstrated enormous individual changes, particularly on TRECs/CD4+CD31+ cells assay. Basal values in Group III were significantly higher than those observed in active patients groups.
CONCLUSION: Thymic functional activity is earlier involved in the allergic reaction and SIT IgE-mediated allergy is able to induce RTEs in the periphery, particularly TRECs/CD4+CD31+ cells. Both SLIT and SCIT showed reduced RETs in the periphery, probably due to maturation of regulatory T cells. Our results suggest a crucial role of the functional thymic tissue on the central mechanism of this therapy.

PMID: 22768725 [PubMed - indexed for MEDLINE]

71. Allergy. 2012 Sep;67(9):1106-10. doi: 10.1111/j.1398-9995.2012.02870.x. Epub 2012 Jul 5.

CX3CL1 in allergic diseases: not just a chemotactic molecule.

Julia V.

Centre National de la Recherche Scientifique, Institut National de la Santé et de

la Recherche Médicale, Université de Nice-Sophia Antipolis, Valbonne, France.
julia@ipmc.cnrs.fr

Allergic asthma and atopic dermatitis (AD) are two allergic diseases that are primarily driven by the activation of T helper (Th)2 cells. Th2 cells produce cytokines that directly contribute to the symptoms of these diseases. The recruitment and maintenance of Th2 cells into the target tissues are two key events in the pathogenesis of allergic asthma and AD. While migration is mediated by both chemokines and lipid mediators such as leukotrienes and prostaglandins, very little is known about the molecules involved in lymphocyte survival and maintenance in inflamed tissues. However, chemokines could also play a role in this phenomenon. An example of this could be illustrated by CX3CL1, also known as fractalkine. CX3CL1 is a chemokine that is upregulated in some inflammatory diseases including allergic pathologies and that was recently demonstrated to provide a survival signal upon binding to its unique receptor CX3CR1.

© 2012 John Wiley & Sons A/S.

PMID: 22765026 [PubMed - indexed for MEDLINE]

72. Int Ophthalmol. 2012 Dec;32(6):583-4. doi: 10.1007/s10792-012-9604-7. Epub 2012 Jul 5.

Repositioning of dexamethasone intravitreal implant (Ozurdex) migrated into the anterior chamber.

Vela JI, Crespí J, Andreu D.

Department of Ophthalmology, Institut Condal d'Oftalmologia, C/Aragó 469 4° 5°, 08013 Barcelona, Spain. 34128jvs@gmail.com

Ozurdex (Allergan Inc., Irvine, CA, USA) is a biodegradable implant of dexamethasone (0.7 mg) injected into the vitreous cavity to treat macular oedema. We report a case of migration of Ozurdex into the anterior chamber in a patient who had a previous iris-claw lens implantation. Surgical removal of the implant was avoided due to the absence of anterior complications and the loss of effectiveness of the drug delivery system. Repositioning of the implant to the posterior segment is described. This case illustrates the efficacy of this technique in cases of migration into the anterior chamber in eyes without corneal decompensation.

PMID: 22763812 [PubMed - in process]

73. PLoS One. 2012;7(6):e39673. doi: 10.1371/journal.pone.0039673. Epub 2012 Jun 28.

IL-4 engagement of the type I IL-4 receptor complex enhances mouse eosinophil migration to eotaxin-1 in vitro.

Heller NM, Gwinn WM, Donnelly RP, Constant SL, Keegan AD.

Department of Microbiology and Immunology and the Center for Vascular and Inflammatory Diseases, The University of Maryland School of Medicine, Baltimore, Maryland, United States of America.

BACKGROUND: Previous work from our laboratory demonstrated that IL-4Rα expression on a myeloid cell type was responsible for enhancement of Th2-driven eosinophilic inflammation in a mouse model of allergic lung inflammation. Subsequently, we have shown that IL-4 signaling through type I IL-4 receptors on

monocytes/macrophages strongly induced activation of the IRS-2 pathway and a subset of genes characteristic of alternatively activated macrophages. The direct effect(s) of IL-4 and IL-13 on mouse eosinophils are not clear. The goal of this study was determine the effect of IL-4 and IL-13 on mouse eosinophil function.
METHODS: Standard Transwell chemotaxis assay was used to assay migration of mouse eosinophils and signal transduction was assessed by Western blotting.
RESULTS: Here we determined that (i) mouse eosinophils express both type I and type II IL-4 receptors, (ii) in contrast to human eosinophils, mouse eosinophils do not chemotax to IL-4 or IL-13 although (iii) pre-treatment with IL-4 but not IL-13 enhanced migration to eotaxin-1. This IL-4-mediated enhancement was dependent on type I IL-4 receptor expression: γC-deficient eosinophils did not show enhancement of migratory capacity when pre-treated with IL-4. In addition, mouse eosinophils responded to IL-4 with the robust tyrosine phosphorylation of STAT6 and IRS-2, while IL-13-induced responses were considerably weaker.
CONCLUSIONS: The presence of IL-4 in combination with eotaxin-1 in the allergic inflammatory milieu could potentiate infiltration of eosinophils into the lungs. Therapies that block IL-4 and chemokine receptors on eosinophils might be more effective clinically in reducing eosinophilic lung inflammation.

PMCID: PMC3386270
PMID: 22761864 [PubMed - indexed for MEDLINE]

74. Allergy Asthma Immunol Res. 2012 Jul;4(4):231-9. doi: 10.4168/aair.2012.4.4.231. Epub 2012 Jun 12.

Expression and Roles of MMP-2, MMP-9, MMP-13, TIMP-1, and TIMP-2 in Allergic Nasal Mucosa.

Mori S, Pawankar R, Ozu C, Nonaka M, Yagi T, Okubo K.

Department of Otorhinolaryngology, Nippon Medical School, Tokyo, Japan.

PURPOSE: Allergic rhinitis (AR) and asthma share many characteristics, but structural changes are observed far less often in AR. Matrix metalloproteinases (MMPs) constitute a family of Zn-dependent endopeptidases that can decompose the extracellular matrix and basement membrane, and regulate cell infiltration. We analyzed the expression of MMPs and their inhibitors, tissue inhibitors of metalloproteinases (TIMPs), in allergic nasal mucosa after nasal allergen challenge (NAC) and determined their relationship to inflammatory cells.
METHODS: Nasal mucosa specimens were obtained at surgery performed for hypertrophied turbinates. We performed NAC with house dust mite (HDM) allergen disks and control disks, and took biopsies at 30 minutes, 6 hours, and 12 hours after NAC. Cells expressing MMP-2, MMP-9, MMP-13, TIMP-1, and TIMP-2, as well as eosinophils and mast cells, were analyzed immunohistochemically. The MMPs and TIMPs in allergic nasal mucosa were quantified using enzyme-linked immunosorbent assays.
RESULTS: At 30 minutes post-NAC, HDM-exposed nasal mucosa exhibited significantly more MMP-2+, MMP-9+, MMP-13+, TIMP-1+, and TIMP-2+ cells compared with control mucosa, and the numbers of MMP-9+ and TIMP-1+ cells correlated strongly with the number of mast cells. At 6 hours post-NAC, the numbers of MMP+ and TIMP+ cells did not differ significantly between HDM-exposed mucosa and control mucosa, but the ratios of MMP+ cells to TIMP+ cells were higher in HDM-exposed mucosa. At 12 hours post-NAC, the number of MMP-13+ cells tended to be higher in HDM-exposed mucosa and was strongly correlated with the number of eosinophils.
Quantitatively, the levels of MMP-2 and MMP-13 were significantly higher than the MMP-9 level, and the TIMP-2 level was significantly higher than the TIMP-1 level in allergic nasal mucosa.
CONCLUSIONS: We demonstrated increased expression of MMP-2, MMP-9, and MMP-13 in allergic nasal mucosa, high MMPs-to-TIMP-1 ratios, and a strong correlation

between MMP-9 and mast cells and between MMP-13 and eosinophils. The imbalance between MMPs and TIMPs may contribute to the migration of inflammatory cells such as eosinophils and mast cells to the nasal mucosa of AR patients, suggesting a possible active role of MMPs in AR.

PMCID: PMC3378930
PMID: 22754717 [PubMed]

75. Int J Mol Sci. 2012;13(5):5952-71. doi: 10.3390/ijms13055952. Epub 2012 May 16.

Biological properties of acidic cosmetic water from seawater.

Liao WT, Huang TS, Chiu CC, Pan JL, Liang SS, Chen BH, Chen SH, Liu PL, Wang HC, Wen ZH, Wang HM, Hsiao SW.

Department of Biotechnology, Kaohsiung Medical University, 100, Shih-Chuan 1st Road, San-Ming District, Kaohsiung 807, Taiwan; E-Mails: wtliao@kmu.edu.tw (W.-T.L.); cchiu@kmu.edu.tw (C.-C.C.); l3893117@gmail.com (S.-S.L.); bhchen@kmu.edu.tw (B.-H.C.).

This current work was to investigate the biological effects of acidic cosmetic water (ACW) on various biological assays. ACW was isolated from seawater and demonstrated several bio-functions at various concentration ranges. ACW showed a satisfactory effect against Staphylococcus aureus, which reduced 90% of bacterial growth after a 5-second exposure. We used cultured human peripheral blood mononuclear cells (PBMCs) to test the properties of ACW in inflammatory cytokine release, and it did not induce inflammatory cytokine release from un-stimulated, normal PBMCs. However, ACW was able to inhibit bacterial lipopolysaccharide (LPS)-induced inflammatory cytokine TNF-α released from PBMCs, showing an anti-inflammation potential. Furthermore, ACW did not stimulate the rat basophilic leukemia cell (RBL-2H3) related allergy response on de-granulation. Our data presented ACW with a strong anti-oxidative ability in a superoxide anion radical scavenging assay. In mass spectrometry information, magnesium and zinc ions demonstrated bio-functional detections for anti-inflammation as well as other metal ions such as potassium and calcium were observed. ACW also had minor tyrosinase and melanin decreasing activities in human epidermal melanocytes (HEMn-MP) without apparent cytotoxicity. In addition, the cell proliferation assay illustrated anti-growth and anti-migration effects of ACW on human skin melanoma cells (A375.S2) indicating that it exerted the anti-cancer potential against skin cancer. The results obtained from biological assays showed that ACW possessed multiple bioactivities, including anti-microorganism, anti-inflammation, allergy-free, antioxidant, anti-melanin and anticancer properties. To our knowledge, this was the first report presenting these bioactivities on ACW.

PMCID: PMC3382787
PMID: 22754342 [PubMed]

76. Psychosom Med. 2012 Jul-Aug;74(6):635-41. doi: 10.1097/PSY.0b013e31825ac5e7. Epub 2012 Jun 28.

Prenatal stress and risk of asthma hospitalization in the offspring: a Swedish population-based study.

Khashan AS, Wicks S, Dalman C, Henriksen TB, Li J, Mortensen PB, Kenny LC.

Anu Research Center, Department of Obstetrics and Gynaecology, University College Cork, Cork University Maternity Hospital, Cork, Ireland. a.khashan@ucc.ie

OBJECTIVE: Recent research suggested that maternal stress and anxiety increase the risk of asthma and eczema in the offspring. In this study, we aimed to study whether maternal exposure to death of a spouse or a child is associated with risk of asthma hospitalization in the offspring using a very large population-based cohort.
METHODS: In a cohort of 3.2 million births in Sweden between January 1, 1973, and December 31, 2004, mothers were considered exposed if their spouse or child died up to 6 months before or during pregnancy. Offspring were followed up from birth to their death, migration, first hospitalization with asthma, or December 31, 2006, whichever came first; hospital admissions were identified by linkage of several national Swedish registers. Log-linear Poisson regression was used for data analysis.
RESULTS: Overall, the risk of offspring asthma was increased with any prenatal exposure to bereavement in any exposure period (adjusted relative risk [RR] = 1.20 [95% confidence interval {CI} = 1.03-1.39]). The risk was higher when the exposure period was restricted to pregnancy only (adjusted RR = 1.43 [95% CI = 1.06-1.92]). Furthermore, the risk of asthma was increased in relation to death of a spouse during pregnancy (adjusted RR = 1.59 [95% CI = 1.10-2.30]).
CONCLUSIONS: These findings suggest that prenatal exposure to severe life events increases the risk of hospitalization for asthma in the offspring. Fetal programming may be a plausible explanation for the association.

PMID: 22753636 [PubMed - indexed for MEDLINE]

77. J Cell Biochem. 2012 Nov;113(11):3567-75. doi: 10.1002/jcb.24235.

IL-6 augmented motility of airway epithelial cell BEAS-2B via Akt/GSK-3β signaling pathway.

Wang WC, Kuo CY, Tzang BS, Chen HM, Kao SH.

Institute of Biochemistry and Biotechnology, College of Medicine, Chung Shan Medical University, Taichung City, Taiwan.

Cell migration plays a pivotal role in airway repair and remodeling involved in respiratory diseases such as asthma. Interleukin-6 (IL-6) and fascin-1 are involved in cell migration upon stimulation; however, the roles of IL-6 and fascin-1 in migration of airway epithelial cell remain sketchy. The present study was aimed to investigate influence of IL-6 on cell motility with emphasis on the association with fascin-1. Wound healing assay and transmigration assay were performed to examine effect of IL-6 on migration and invasiveness of human bronchial epithelial cell BEAS-2B. Level of mRNA expression was determined by RT-PCR and quantitative real-time RT-PCR (Q-PCR). Involvement of kinase and transcription factor signaling in IL-6-induced cell migration was investigated using immunoblot and specific inhibitors. IL-6 significantly augmented cell migration and invasiveness in parallel with elevated fascin-1 expression. Further investigation showed that IL-6 dose-dependently upregulated fascin-1 expression in both mRNA and protein levels. We showed that IL-6 activated Akt and inhibited glycogen synthase kinase-3β (GSK-3β), highly associating with fascin-1 mRNA expression. Additionally, IL-6-induced migration was significantly diminished by phosphatidyl inositol 3-phosphate kinase (PI3K) inhibitor (wortamannin) and β-catenin inhibitor FH535. Moreover, LiCl and SB216763, inhibitors of GSK-3β augmented cell migration as well as fascin-1 mRNA expression. Conclusively, these findings reveal that IL-6-induced migration of BEAS-2B cell may be attributed to activation of Akt, inhibition of GSK-3β, and the associated increase of β-catenin and fascin-1 expression, indicating an important role of Akt/GSK-3β signaling and β-catenin/fascin-1 in IL-6 associated airway remodeling.

Copyright © 2012 Wiley Periodicals, Inc.

PMID: 22740511 [PubMed - in process]

78. Zhongguo Ying Yong Sheng Li Xue Za Zhi. 2012 Mar;28(2):103-6.

[Effect of TLR4 on the migration of asthmatic airway smooth muscle cells induced by airway epithelial cells].

[Article in Chinese]

Su HY, Mo BW, Wei JH, Huang JW, Wang CM, Zeng JR, Xu Q, Lin Y.

Department of Respiratory Medicine, Hospital Affiliated Guilin Medical College, Guilin 541001, China.

OBJECTIVE: To explore the effect of Toll-like receptor 4 (TLR4) activation on the migration of asthmatic airway smooth muscle cell (ASMCs) induced by airway epithelial cells.
METHODS: Primary ASMCs were cultured by the method of cell digestion. Cell culture supernatant of RTE cells were collected by TNF-alpha stimulation of epithelial cells. Detected the IL-8 and RANTES levels in the supernatant. The transmembrane migration of asthmatic ASMCs were detected by Modified Boyden chemotaxis chamber. The effect of TLR4 on the migration of asthmatic ASMCs induced by epithelial cells with TLR4 antibody drugs as a tool.
RESULTS: The levels of IL-8 and RANTES in the supernatant of TNF-alpha groups were significantly increased, and that in the 20 ng/ml group was significantly higher than other groups ($P < 0.01$). The transmembrane migration of asthmatic ASMCs groups was increased than that of control group. The transmembrane migration of asthmatic ASMCs from asthma group and TNF-alpha + TLR4 antibody group was significantly decreased compared with that in TNF-alpha group ($P < 0.01$). The migration of asthma ASMCs from TNF-alpha + TLR4 antibody group was increased than that of asthma group ($P < 0.05$).
CONCLUSION: TLR4 in the surface of asthmatic ASMCs may be activated by cytokines secreted by the airway epithelial cells and enhance the transmembrane migration of asthmatic ASMCs induced by airway epithelial cells so that it plays a role in airway remodeling of asthma.

PMID: 22737905 [PubMed - in process]

79. J Interferon Cytokine Res. 2012 Oct;32(10):450-61. doi: 10.1089/jir.2011.0111. Epub 2012 Jun 25.

Lactoferrin regulates an axis involving CD11b and CD49d integrins and the chemokines MIP-1α and MCP-1 in GM-CSF-treated human primary eosinophils.

Curran CS, Bertics PJ.

Department of Molecular Biology, University of Wisconsin, Madison, Wisconsin 53706, USA. colleen_curran@hotmail.com

Eosinophils are multifunctional immune cells that contribute to innate and adaptive immune/repair responses. Lactoferrin (LF) is an iron-binding protein indicated to alter cell adhesion and immune function by receptor-mediated interactions or by participating in redox mechanisms. The eosinophil adhesion molecules, αMβ2 and α4β1, are differentially expressed following exposure to the cytokine granulocyte-macrophage colony-stimulating factor (GM-CSF) and various redox agents. We hypothesized that LF can alter the function and production of

proteins involved in adhesion/migration. Utilizing eosinophil peroxidase activity or fluorescent labeling adhesion assays, LF reduced GM-CSF-induced eosinophil adhesion in the presence of fibronectin or vascular adhesion molecule-1 compared with GM-CSF treatment alone. Flow cytometric analysis of eosinophil αM (CD11b) and α4 (CD49d) integrins revealed that cotreatments (24 h) with LF plus GM-CSF induced a significant increase in CD11b compared with control and GM-CSF treatments but a significant decrease in CD49d compared with control and GM-CSF treatments. These changes in CD11b and CD49d levels were significantly correlated with the increased production of chemokines (macrophage inflammatory Protein-1α, monocyte chemotactic protein-1) and an identified increase in S100A9 production. Thus, LF release at sites of inflammation may alter eosinophil recruitment/activation and possibly the progression of diseases such as cancer and asthma where significant eosinophil influx has been described.

PMCID: PMC3464068 [Available on 2013/10/1]
PMID: 22731992 [PubMed - in process]

80. J Asthma. 2012 Jun;49(5):542-51. doi: 10.3109/02770903.2012.682125.

Effectiveness of educational interventions on asthma self-management in Punjabi and Chinese asthma patients: a randomized controlled trial.

Poureslami I, Nimmon L, Doyle-Waters M, Rootman I, Schulzer M, Kuramoto L, FitzGerald JM.

Division of Respiratory Medicine, Institute of Heart and Lung Health, The University of British Columbia, Vancouver, BC, Canada.

BACKGROUND: Asthma tends to be less well controlled among ethnic minority groups, and its prevalence in new immigrants increases significantly the longer they are in Canada; mainly due to their lack of familiarity with English and difficulty understanding information regarding the disease, health literacy, cultural issues, housing conditions, and lack of access to appropriate care services.
OBJECTIVE: To explore the effectiveness of different formats of culturally relevant information and its impact on asthma patients' self-management within the Punjabi, Mandarin, and Cantonese communities.
METHODS: Using a participatory approach, we developed and tested knowledge and community educational videos (with similar information, but used a different approach, i.e., scientific vs. colloquial) and a pictorial pamphlet. A total of 92 physician-diagnosed adult asthma patients (47 Chinese and 45 Punjabi) were assigned at random to three experimental groups (watched one or both videos) and one comparison group (read pictorial pamphlet) and participated in three in-person interviews and one telephone interview within a 9-month period. Patients received education on asthma self-management via videos and pamphlet and outcomes, including their knowledge of asthma triggers (environmental-related and behavioral-related triggers) and symptoms; inhaler use skills and patient-reported medication adherence were measured.
RESULTS: Knowledge of asthma symptoms, inhaler use, and understanding of physician's instructions improved significantly from pretest to 3 months post-intervention follow-up among all participants.
CONCLUSIONS: Participants performed significantly better at follow-up than they did at baseline assessment, with the most notable improvements observed in the group that watched both community and knowledge videos. The results suggest that short, simple, culturally, and linguistically appropriate interventions can promote knowledge gain about asthma and improve inhaler use that can be sustained over the short term. Such interventions that provide authentic learning materials that draw on patients' life experiences and sociocultural context can overcome certain limitations of conventional patient education approaches.

PMID: 22715910 [PubMed - indexed for MEDLINE]

81. Int Immunopharmacol. 2012 Sep;14(1):82-93. doi: 10.1016/j.intimp.2012.06.002. Epub 2012 Jun 15.

Gedunin, a natural tetranortriterpenoid, modulates T lymphocyte responses and ameliorates allergic inflammation.

Ferraris FK, Moret KH, Figueiredo AB, Penido C, Henriques Md.

Laboratório de Farmacologia Aplicada, Farmanguinhos, Fundação Oswaldo Cruz, Rio de Janeiro, Brazil.

T lymphocytes are critical cells involved in allergy. Here, we report that the natural tetranortriterpenoid gedunin impaired allergic responses primarily by modulating T lymphocyte functions. The intraperitoneal (i.p.) administration of gedunin inhibited pleural leukocyte accumulation triggered by intra-pleural (i.pl.) challenge with ovalbumin (OVA) in previously sensitized C57BL/6 mice; this inhibition was primarily due to the impairment of eosinophil and T lymphocyte influx. Likewise, i.pl. pre-treatment with gedunin inhibited eosinophil and T lymphocyte migration into mouse lungs 24 h after OVA intra-nasal (i.n.) instillation. Pre-treatment with gedunin diminished the levels of CCL2, CCL3, CCL5, CCL11, Interleukin-5 and leukotriene B(4) at the allergic site. In vitro pre-treatment with gedunin failed to inhibit T lymphocyte adhesion and chemotaxis towards pleural washes recovered from OVA-challenged mice, suggesting that gedunin inhibits T lymphocyte migration in vivo via the inhibition of chemotactic mediators in situ. In vivo pre-treatment with gedunin reduced the numbers of CD69(+) and CD25(+) T lymphocytes in the pleura and CD25(+) cells in the thoracic lymph nodes 24 h after OVA i.pl. challenge. In accordance, in vitro treatment of T lymphocytes with gedunin inhibited α-CD3 mAb-induced expression of CD69 and CD25, proliferation, Interleukin-2 production and nuclear translocation of NFκB and NFAT. Notably, post-treatment of mice with gedunin reverted OVA-induced lung allergic inflammation by decreasing the T lymphocyte and eosinophil counts and the levels of eosinophilotactic mediators in bronchoalveolar lavage fluid. Our results demonstrate a remarkable anti-allergic effect of gedunin due to its capability to modulate T cell activation and trafficking into the airways.

Copyright © 2012 Elsevier B.V. All rights reserved.

PMID: 22709475 [PubMed - indexed for MEDLINE]

82. J Immunol. 2012 Jul 15;189(2):980-7. doi: 10.4049/jimmunol.1200891. Epub 2012 Jun 15.

Defective macrophage migration in Gαi2- but not Gαi3-deficient mice.

Wiege K, Le DD, Syed SN, Ali SR, Novakovic A, Beer-Hammer S, Piekorz RP, Schmidt RE, Nürnberg B, Gessner JE.

Clinical Department of Immunology and Rheumatology, Hannover Medical School, Hannover, Germany.

Various heterotrimeric G(i) proteins are considered to be involved in cell migration and effector function of immune cells. The underlying mechanisms, how they control the activation of myeloid effector cells, are not well understood. To elucidate isoform-redundant and -specific roles for Gα(i) proteins in these processes, we analyzed mice genetically deficient in Gα(i2) or Gα(i3). First, we

show an altered distribution of tissue macrophages and blood monocytes in the absence of Gα(i2) but not Gα(i3). Gα(i2)-deficient but not wild-type or Gα(i3)-deficient mice exhibited reduced recruitment of macrophages in experimental models of thioglycollate-induced peritonitis and LPS-triggered lung injury. In contrast, genetic ablation of Gα(i2) had no effect on Gα(i)-dependent peritoneal cytokine production in vitro and the phagocytosis-promoting function of the Gα(i)-coupled C5a anaphylatoxin receptor by liver macrophages in vivo. Interestingly, actin rearrangement and CCL2- and C5a anaphylatoxin receptor-induced chemotaxis but not macrophage CCR2 and C5a anaphylatoxin receptor expression were reduced in the specific absence of Gα(i2). Furthermore, knockdown of Gα(i2) caused decreased cell migration and motility of RAW 264.7 cells, which was rescued by transfection of Gα(i2) but not Gα(i3). These results indicate that Gα(i2), albeit redundant to Gα(i3) in some macrophage activation processes, clearly exhibits a Gα(i) isoform-specific role in the regulation of macrophage migration.

PMID: 22706085 [PubMed - indexed for MEDLINE]

83. Clin Exp Allergy. 2012 Jul;42(7):1040-50. doi: 10.1111/j.1365-2222.2012.04019.x.

CCR3 induced-p42/44 MAPK activation protects against staurosporine induced-DNA fragmentation but not apoptosis in airway smooth muscle cells.

Markwick LJ, Clements D, Roberts ME, Ceresa CC, Knox AJ, Johnson SR.

Division of Therapeutics and Molecular Medicine and Nottingham NIHR Respiratory Biomedical Research Unit, University Hospital Queens Medical Centre, Nottingham, UK.

BACKGROUND: Chemokine receptors (CCRs) are expressed on airway smooth muscle (ASM) cells. As their ligands are present in the airways in asthma, we hypothesized that ASM CCR activation could promote the increase in ASM mass seen in patients with chronic asthma.
OBJECTIVE: To determine which CCRs are expressed by ASM cells and their potential functional relevance to the chronic airway changes seen in asthma.
METHODS: CCR expression in primary ASM cell cultures and airway biopsies from patients with and without asthma was examined by RT-PCR, fluorescence-activated cell sorting and immunohistochemistry. ASM p42/44 MAPK activity, proliferation, migration and apoptosis were examined by western blotting, thymidine incorporation, transwell assay and TUNEL assay respectively.
RESULTS: CCR3 was the most frequently expressed CCR protein and was present on 79 ± 14% of cells. CX3CR1 and CXCR6 were present on 6% and 11% of cells respectively. CCR3 ligands CCL11 and CCL24 caused rapid activation of p42/44 MAPK but not Akt. CCR3 activation did not affect ASM proliferation, migration or VEGF secretion. DNA fragmentation detected by TUNEL staining could be induced by staurosporine and Fas activation although only Fas activation resulted in caspase 3 cleavage. CCL11 and CCL24 protected ASM cells against DNA fragmentation dependent upon p42/44 MAPK activity only via caspase 3 independent pathways. CCR3 was expressed in the smooth muscle and epithelium in the airways of patients with and without asthma. Smooth muscle cell DNA fragmentation in the airways of patients with stable asthma and controls was very uncommon.
CONCLUSIONS AND CLINICAL RELEVANCE: CCR3 is strongly expressed by ASM cells in vitro and in vivo. Protection against cell death by CCR3 activation is dependent on p42/44 MAPK but does not affect caspase 3 mediated apoptosis.

© 2012 Blackwell Publishing Ltd.

PMID: 22702503 [PubMed - indexed for MEDLINE]

84. J Allergy Clin Immunol. 2012 Oct;130(4):977-85.e6. doi:
10.1016/j.jaci.2012.04.042. Epub 2012 Jun 13.

Autocrine-regulated airway smooth muscle cell migration is dependent on
IL-17-induced growth-related oncogenes.

Al-Alwan LA, Chang Y, Baglole CJ, Risse PA, Halayko AJ, Martin JG, Eidelman DH,
Hamid Q.

Meakins-Christie Laboratories and the Respiratory Division, Department of
Medicine, McGill University, Montreal, Quebec, Canada.

BACKGROUND: Airway smooth muscle cell (ASMC) migration is one of the proposed
mechanisms underlying the increased airway smooth muscle mass seen in airway
remodeling of patients with severe asthma. IL-17-related cytokines are a new
subgroup of inflammatory mediators that have been suggested to play a role in
regulating smooth muscle function. We hypothesized that IL-17-induced chemokine
production from smooth muscle cells can contribute to migration of additional
smooth muscle cells in the airways of asthmatic patients.
OBJECTIVE: We sought to investigate the effect of IL-17 on smooth muscle-derived
chemokines and to examine the mechanisms involved in their production and
contribution to the increase in airway smooth muscle migration.
METHODS: The effect of IL-17-induced supernatants on human ASMC migration was
investigated. IL-17-induced growth-related oncogene (GRO) production and mRNA
expression was assessed by using ELISA and RT-PCR, respectively. The direct
effect of GROs on ASMC migration and the involvement of the CXCR2 receptor were
also examined.
RESULTS: IL-17-induced supernatants promoted ASMC migration. After IL-17
stimulation, GROs were the most abundant chemokines produced from ASMCs, and
blocking their effect by using neutralizing antibodies significantly inhibited
ASMC migration. In addition, a combination of recombinant human GRO-α, GRO-β, and
GRO-γ was able to promote significant migration of ASMCs that was mediated
through the CXCR2 receptor.
CONCLUSION: These findings suggest that IL-17-induced GROs can be an important
mediator of ASMC migration and therefore might contribute to the pathogenesis of
airway remodeling in asthmatic patients.

Copyright © 2012 American Academy of Allergy, Asthma & Immunology. Published by
Mosby, Inc. All rights reserved.

PMID: 22698519 [PubMed - indexed for MEDLINE]

85. Allergy. 2012 Aug;67(8):998-1006. doi: 10.1111/j.1398-9995.2012.02851.x. Epub
2012 Jun 12.

Suppression of adrenomedullin contributes to vascular leakage and altered
epithelial repair during asthma.

Hagner S, Welz H, Kicic A, Alrifai M, Marsh LM, Sutanto EN, Ling KM, Stick SM,
Müller B, Weissmann N, Renz H.

Institute of Laboratory Medicine, Medical Faculty - Philipps University of
Marburg, Biomedical Research Center (BMFZ), Marburg, Germany.
hagnerbe@staff.uni-marburg.de

BACKGROUND: The anti-inflammatory peptide, adrenomedullin (AM), and its cognate
receptor are expressed in lung tissue, but its pathophysiological significance in
airway inflammation is unknown.

OBJECTIVES: This study investigated whether allergen-induced airway inflammation involves an impaired local AM response.
METHODS: Airway AM expression was measured in acute and chronically sensitized mice following allergen inhalation and in airway epithelial cells of asthmatic and nonasthmatic patients. The effects of AM on experimental allergen-induced airway inflammation and of AM on lung epithelial repair in vitro were investigated.
RESULTS: Adrenomedullin mRNA levels were significantly ($P < 0.05$) reduced in acute ovalbumin (OVA)-sensitized mice after OVA challenge, by over 60% at 24 h and for up to 6 days. Similarly, reduced AM expression was observed in two models of chronic allergen-induced inflammation, OVA- and house dust mite-sensitized mice. The reduced AM expression was restricted to airway epithelial and endothelial cells, while AM expression in alveolar macrophages was unaltered. Intranasal AM completely attenuated the OVA-induced airway hyperresponsiveness and mucosal plasma leakage but had no effect on inflammatory cells or cytokines. The effects of inhaled AM were reversed by pre-inhalation of the putative AM receptor antagonist, AM ((22-52)) . AM mRNA levels were significantly ($P < 0.05$) lower in human asthmatic airway epithelial samples than in nonasthmatic controls. In vitro, AM dose-dependently (10(-11) -10(-7) M) accelerated experimental wound healing in human and mouse lung epithelial cell monolayers and stimulated epithelial cell migration.
CONCLUSION: Adrenomedullin suppression in T(H) 2-related inflammation is of pathophysiological significance and represents loss of a factor that maintains tissue integrity during inflammation.

© 2012 John Wiley & Sons A/S.

PMID: 22686590 [PubMed - indexed for MEDLINE]

86. Toxicol In Vitro. 2012 Jun 7. [Epub ahead of print]

Dendritic cell migration assay: A potential prediction model for identification of contact allergens.

Gibbs S, Spiekstra S, Corsini E, McLeod J, Reinders J.

Department of Dermatology, VU University Medical Centre, Amsterdam, The Netherlands.

This manuscript describes methodology and a prediction model for the MUTZ-LC migration assay. The assay represents the physiological change in Langerhans cell (LC) behavior after exposure to a sensitizing chemical, resulting in LC migration from the epidermis to the dermis. MUTZ-LC are derived from the commercially available MUTZ-3 cell line. Upon exposure to a sensitizer MUTZ-LC migrate preferentially towards CXCL12 whereas upon exposure to a non-sensitizer MUTZ-LC migrate towards CCL5. A CXCL12/CCL5 ratio >1.10 in 2/3 independent experiments is indicative of a sensitizer, whereas a CXCL12/CCL5 ratio ≤1.10 is indicative of a non-sensitizer. At non cytotoxic chemical concentrations 9 sensitizers (2,4-dinitrochlorobenzene, paraphenylendiamine, cinnamaldehyde, isoeugenol, nickel-sulfate, tetramethylthiuram disulfide, eugenol, cinnamic-alcohol, ammonium-hexachloroplatinate) were distinguished from 4 non sensitizers (sodium lauryl sulfate, salicylic acid, phenol, octanoic acid). Critical points in assay performance are (i) MUTZ-3 passage number after thawing (p6-p40); (ii) cell viability (>80%); (iii) standard curve to optimize correlation of fluorescence with cell number; and (iv) optimization of the concentration of rhCXCL12 and rhCCL5 in transwell. The protocol has been tested in three European laboratories and results suggest that it may provide working conditions for performing the DC migration assay which is aimed at distinguishing sensitizers from non sensitizers.

Copyright © 2012. Published by Elsevier Ltd.

PMID: 22683935 [PubMed - as supplied by publisher]

87. Am J Physiol Lung Cell Mol Physiol. 2012 Aug 1;303(3):L189-98. doi: 10.1152/ajplung.00271.2011. Epub 2012 Jun 1.

Pulmonary intravascular macrophages as proinflammatory cells in heaves, an asthma-like equine disease.

Aharonson-Raz K, Lohmann KL, Townsend HG, Marques F, Singh B.

Department of Veterinary Biomedical Sciences, University of Saskatchewan, Saskatoon, Canada.

Heaves, an obstructive neutrophilic airway inflammation of horses, is triggered by dust components such as endotoxin and has similarities to human asthma. Pulmonary intravascular macrophages (PIMs) increase horses' sensitivity to endotoxin-induced lung inflammation; however, their role in an airborne pathology remains unknown. Therefore, we investigated the role of PIMs in the development of heaves in horses. Clinical and inflammatory responses were evaluated following induction of heaves by moldy hay exposure and PIM depletion with gadolinium chloride (GC). Mares (N = 9) were exposed to four treatments: alfalfa cubes (Cb), alfalfa cubes + GC (Cb-GC), moldy hay (MH), and moldy hay + GC (MH-GC). Clinical scores and neutrophil concentrations in bronchoalveolar lavage (BAL) fluid were higher when mares received MH compared with MH-GC. BAL cells from MH-GC-treated mares had significantly lower IL-8 and TLR4 mRNA expression compared with MH-treated mares. In vitro LPS challenge significantly increased IL-8 but not TLR4 mRNA expression in BAL cells recovered from horses fed with MH, but not from the MH-GC treatment. In summary, PIM depletion attenuated clinical scores, reduced the alveolar migration of neutrophils, and decreased the expression of proinflammatory molecules in BAL cells of heaves horses, suggesting a proinflammatory role of PIMs in the development of airborne pathology.

PMID: 22659880 [PubMed - indexed for MEDLINE]

88. Front Immunol. 2012;3:130. doi: 10.3389/fimmu.2012.00130. Epub 2012 May 25.

Cytoskeleton in mast cell signaling.

Dráber P, Sulimenko V, Dráberová E.

Department of Biology of Cytoskeleton, Institute of Molecular Genetics, Academy of Sciences of the Czech Republic Prague, Czech Republic.

Mast cell activation mediated by the high affinity receptor for IgE (FcεRI) is a key event in allergic response and inflammation. Other receptors on mast cells, as c-Kit for stem cell factor and G protein-coupled receptors (GPCRs) synergistically enhance the FcεRI-mediated release of inflammatory mediators. Activation of various signaling pathways in mast cells results in changes in cell morphology, adhesion to substrate, exocytosis, and migration. Reorganization of cytoskeleton is pivotal in all these processes. Cytoskeletal proteins also play an important role in initial stages of FcεRI and other surface receptors induced triggering. Highly dynamic microtubules formed by αβ-tubulin dimers as well as microfilaments build up from polymerized actin are affected in activated cells by kinases/phosphatases, Rho GTPases and changes in concentration of cytosolic Ca^{2+}. Also important are nucleation proteins; the γ-tubulin complexes in case

of microtubules or Arp 2/3 complex with its nucleation promoting factors and formins in case of microfilaments. The dynamic nature of microtubules and microfilaments in activated cells depends on many associated/regulatory proteins. Changes in rigidity of activated mast cells reflect changes in intermediate filaments build up from vimentin. This review offers a critical appraisal of current knowledge on the role of cytoskeleton in mast cells signaling.

PMCID: PMC3360219
PMID: 22654883 [PubMed]

89. Int Arch Allergy Immunol. 2012;159(2):171-8. doi: 10.1159/000336169. Epub 2012 May 31.

Mediators and cytokines in persistent allergic rhinitis and nonallergic rhinitis with eosinophilia syndrome.

Gröger M, Klemens C, Wendt S, Becker S, Canis M, Havel M, Pfrogner E, Rasp G, Kramer MF.

Department of Oto-Rhino-Laryngology, Head and Neck Surgery, Munich, Germany. moritz.groeger@med.uni-muenchen.de

BACKGROUND: Patients with nonallergic rhinitis with eosinophilia syndrome (NARES) show typical symptoms of persistent allergic rhinitis (PAR). The aim of the present study was to compare nasal cytokine patterns between NARES and PAR.
METHODS: Nasal secretions of 31 patients suffering from NARES, 20 patients with PAR to house dust mite and 21 healthy controls were collected using the cotton wool method and analyzed for interleukin (IL)-1β, IL-2, IL-4, IL-5, IL-6, IL-7, IL-8, IL-10, IL-12, IL-13, IL-17, granulocyte-macrophage colony-stimulating factor (GM-CSF), granulocyte colony-stimulating factor (G-CSF), interferon-γ (IFN-γ), tumor necrosis factor-α (TNF-α), monocyte chemoattractant protein-1 (MCP-1) and macrophage inflammatory protein-1β (MIP-1β) by Bio-Plex Cytokine Assay as well as eosinophil cationic protein (ECP) and tryptase by UniCAP-FEIA.
RESULTS: NARES and PAR presented elevated levels of tryptase, while ECP was markedly increased solely in NARES compared to both the controls and PAR. Elevated levels of IL-1β, IL-17, IFN-γ, TNF-α and MCP-1 were found in NARES compared to the controls as well as PAR. MIP-1β was elevated in NARES and PAR, while IL-4, IL-6 and G-CSF showed increased levels in NARES, and IL- 5 was elevated in PAR only.
CONCLUSIONS: In patients with NARES and PAR, eosinophils and mast cells appear to be the pivotal cells of inflammation, reflected by high levels of tryptase and ECP as well as IL-5 and GM-CSF as factors for eosinophil migration and survival. The elevated levels of proinflammatory cytokines in NARES may indicate the chronic, self-perpetuating process of inflammation in NARES which seems to be more pronounced than in PAR. IL-17 might be a factor for neutrophilic infiltration or be responsible for remodeling processes in NARES.

Copyright © 2012 S. Karger AG, Basel.

PMID: 22652688 [PubMed - indexed for MEDLINE]

90. Respir Res. 2012 May 31;13:39. doi: 10.1186/1465-9921-13-39.

Drug-induced interstitial lung disease: mechanisms and best diagnostic approaches.

Matsuno O.

Division of Medicine for Allergic Disease, Osaka Prefectural Medical Center for Respiratory and Allergic Diseases, Habikino City, Japan. matsunoo@ra.opho.jp

Drug-induced interstitial lung disease (DILD) is not uncommon and has many clinical patterns, ranging from benign infiltrates to life-threatening acute respiratory distress syndrome. There are two mechanisms involved in DILD, which are probably interdependent: one is direct, dose-dependent toxicity and the other is immune-mediated. Cytotoxic lung injury may result from direct injury to pneumocytes or the alveolar capillary endothelium. Drugs can induce all types of immunological reactions described by Gell and Coombs; however, most reactions in immune-mediated DILD may be T cell-mediated. DILD can be difficult to diagnose; diagnosis is often possible by exclusion alone. Identifying the causative drug that induces an allergy or cytotoxicity is essential for preventing secondary reactions. One method to confirm the diagnosis of a drug-induced disease is re-exposure or re-test of the drug. However, clinicians are reluctant to place patients at further risk of illness, particularly in cases with severe drug-induced diseases. Assessment of cell-mediated immunity has recently increased, because verifying the presence or absence of drug-sensitized lymphocytes can aid in confirmation of drug-induced disease. Using peripheral blood samples from drug-allergic patients, the drug-induced lymphocyte stimulation test (DLST) and the leukocyte migration test (LMT) can detect the presence of drug-sensitized T cells. However, these tests do not have a definite role in the diagnosis of DILD. This study explores the potential of these new tests and other similar tests in the diagnosis of DILD and provides a review of the relevant literature on this topic.

PMCID: PMC3426467
PMID: 22651223 [PubMed - in process]

91. Int J Food Microbiol. 2012 Jul 2;157(2):224-9. doi: 10.1016/j.ijfoodmicro.2012.05.005. Epub 2012 May 14.

Molecular identification and population dynamic of Anisakis pegreffii (Nematoda: Anisakidae Dujardin, 1845) isolated from the European anchovy (Engraulis encrasicolus L.) in the Adriatic Sea.

Mladineo I, Simat V, Miletić J, Beck R, Poljak V.

Institute of Oceanography and Fisheries, Split, Croatia. mladineo@izor.hr

Anchovy Engraulis encrasicolus (L.) is a coastal pelagic and euryhaline species that represents the only European species of the family Engraulidae, with a widespread distribution. In Croatia, it is marketed fresh, frozen, salted or marinated and mainly exported to Italy and Spain, however Anisakis sp. larval infection is frequently the reason for border rejection. Since it is known that the prevalence and intensity of Anisakis infection varies with fish species, fishing area and season, the aim of our study was to identify Anisakis sp. parasitizing European anchovy and infer its population dynamic through a 2.5-year period. Larvae were found coiled and encysted on the external wall of intestine (94%) and reproductive organs (6%), rarely in fillets. Prevalence was 76.1% (95% confidence limits 74.51-77.56%), mean abundance 6.59 (bootstrap 95% confidence limits 5.81-7.26) and mean intensity 8.67 (bootstrap 95% confidence limits 7.82-9.35). The partial CO2 mitochondrial DNA sequence of the isolated anisakids confirmed clustering of the anchovy parasite within A. pegreffii sister group. Parasite population structure showed plasticity inferred by fishing ground, sampling year and fish gender and size. Compared to anisakid prevalence/abundance in other fish, the European anchovy in the Adriatic Sea represents a moderately high-infected paratenic host, although in the Mediterranean and Atlantic waters, anchovies have shown strikingly lesser values of prevalence. Since this host

represents one of the most attractive Mediterranean fisheries products traditionally consumed without thermal preparation that in any case would not disrupt larval antigenicity and prevent human allergies, and given the high prevalence of the anisakid within the host, it is necessary to include anchovy into more firm risk assessment frames in order to develop measures that will support the safe alimentary production and consumption of seafood.

Copyright © 2012 Elsevier B.V. All rights reserved.

PMID: 22647674 [PubMed - indexed for MEDLINE]

92. Int Arch Allergy Immunol. 2012;158 Suppl 1:96-102. doi: 10.1159/000337801. Epub 2012 May 15.

Neutrophilic inflammation in severe asthma.

Nakagome K, Matsushita S, Nagata M.

Department of Respiratory Medicine, Saitama Medical University, Iruma-gun, Saitama, Japan. nakagomek-tky@umin.ac.jp

Neutrophils may play an important role in the pathogenesis of severe asthma. Their infiltration into the airway is increased. Interleukin (IL)-8 is involved in this process, and is actually upregulated in the airways of patients. We have observed that in the absence of eosinophil chemoattractants, neutrophils stimulated by IL-8 augment eosinophil trans-basement membrane migration by releasing superoxide anion, matrix metalloproteinase, leukotriene B(4) and platelet-activating factor. These findings suggest that IL-8-stimulated neutrophils could lead eosinophils to accumulate in the airways of asthmatic patients, which might be a mechanism for corticosteroid resistance in severe asthma. However, the mechanisms of IL-8 upregulation in the airway are not completely understood. Several studies suggest that IL-17 (or T helper 17 cells; Th17) is involved in the IL-8 upregulation observed in severe asthma. We clarified that dopamine induces Th17 differentiation through dopamine D1-like receptor (D1-like-R), and that the D1-like-R antagonist attenuates Th17-mediated diseases like experimental autoimmune encephalomyelitis. Furthermore, we demonstrated that a D1-like-R antagonist significantly suppressed ovalbumin (OVA)-induced neutrophilic airway inflammation in OVA T cell receptor-transgenic DO11.10 mice through inhibiting Th17-mediated immune responses. Therefore, dopamine D1-like-R antagonists could become useful for treating Th17-mediated neutrophil-dominant severe asthma. As inhaled corticosteroids are known to be less effective for controlling neutrophilic inflammation, a more effective therapeutic strategy for neutrophil-dominant asthma should still be elucidated.

Copyright © 2012 S. Karger AG, Basel.

PMID: 22627375 [PubMed - indexed for MEDLINE]

93. Int Arch Allergy Immunol. 2012;158 Suppl 1:87-91. doi: 10.1159/000337799. Epub 2012 May 15.

Obesity and eosinophilic inflammation: does leptin play a role.

Takeda M, Ueki S, Kato H, Konno Y, Chihara M, Itoga M, Kobayashi Y, Moritoki Y, Ito W, Kayaba H, Chihara J.

Department of Infection, Allergy, Clinical Immunology and Laboratory Medicine, Akita University Graduate School of Medicine, Akita, Japan.

It has been pointed out that obesity is a risk factor for, and is involved in the exacerbation of asthma. Mounting evidence about adipose tissue-derived proteins (adipokines) gave rise to the current understanding of obesity as a systemic inflammatory disorder. In this review, we summarized the involvement of leptin, focusing on eosinophil functions. Several studies have indicated that leptin can restrain eosinophil apoptosis, enhance migration, increase adhesion molecules and induce cytokine production. Since leptin also acts on a variety of immune cells related to allergic response, increased leptin in obese individuals potentially explains the mechanism by which obesity leads to an exacerbation of asthma. Further studies targeting adipokines will delineate the association between obesity and eosinophil-associated diseases.

Copyright © 2012 S. Karger AG, Basel.

PMID: 22627373 [PubMed - indexed for MEDLINE]

94. Int Arch Allergy Immunol. 2012;158 Suppl 1:51-7. doi: 10.1159/000337767. Epub 2012 May 15.

1,25-Dihydroxyvitamin D3 upregulates functional C-x-C chemokine receptor type 4 expression in human eosinophils.

Hiraguchi Y, Tanida H, Sugimoto M, Hosoki K, Nagao M, Tokuda R, Fujisawa T.

Institute for Clinical Research, Mie National Hospital, Tsu, Japan.

BACKGROUND: Epidemiological studies suggest that vitamin D may be protective against the inception and exacerbation of allergic diseases. However, the direct effect of vitamin D on eosinophils, the major effector cells in allergic inflammation, is not known. It has been reported that C-X-C chemokine receptor type 4 (CXCR4) in eosinophils is induced in non-Th2 cytokine milieu or in response to glucocorticoids, recruiting the cell to noninflammatory sites.
Objectives: To test whether 1,25-dihydroxyvitamin D(3) [1,25-(OH)(2)D(3) or calcitriol], the active metabolite of vitamin D, acts directly on eosinophils to induce upregulation of CXCR4.
METHODS: Peripheral blood eosinophils from normal volunteers were isolated by CD16 immunomagnetic beads. Vitamin D receptor (VDR) expression was detected by RT-PCR. Eosinophils were cultured with 1,25-(OH)(2)D(3) and the survival and expression of CXCR4 on eosinophils were measured by flowcytometry. Eosinophil migration by CXCL-12/SDF-1 in the presence of 1,25-(OH)(2)D(3) was also analyzed.
RESULTS: Eosinophils expressed VDR. 1,25-(OH)(2)D(3) prolonged eosinophil survival and upregulated eosinophil surface expression of CXCR4 in a concentration-dependent manner. Interleukin (IL)-5 significantly reduced CXCR4 expression and migration induced by the ligand CXCL-12/SDF-1. 1,25-(OH)(2)D(3) reversed the negative effects of IL-5 on the CXCR4-CXCL12 pathway.
CONCLUSION: 1,25-(OH)(2)D(3) regulates CXCR4 expression in eosinophils. The mechanism may be involved in eosinophil recruitment to noninflammatory sites where the ligand of CXCR4 is constitutively expressed.

Copyright © 2012 S. Karger AG, Basel.

PMID: 22627367 [PubMed - indexed for MEDLINE]

95. Sci Total Environ. 2012 Jul 15;430:101-8. doi: 10.1016/j.scitotenv.2012.04.067. Epub 2012 May 25.

Ragweed pollen observed in Turkey: detection of sources using back trajectory

models.

Zemmer F, Karaca F, Ozkaragoz F.

Fatih University, Department of Biology, 34500 Buyukcekmece, Istanbul, Turkey.
fzemmer@fatih.edu.tr

This paper discusses the pollen season and the source apportionment of ragweed (Ambrosia) grains detected in the atmosphere of Istanbul, Turkey. The dynamic migration of this invasive taxon is a serious environmental issue. Ragweed pollen is highly allergenic and causes sensitization in patients at low concentrations. At present, there is no floristic evidence of this taxon in the region. Aerobiological records presented here, though, indicate a local source. Moreover, we argue that ragweed pollen comes from distant sources through air mass movements. The analysis concerns the ragweed season 2007. Pollens were sampled with a Burkard trap and identified at a magnification of 400 ×. Grains were counted on 12 transverse traverses to estimate bi-hourly changes in concentrations. The peak day was on August 28 with 20 grainsm(-3). Ragweed was observed on 22 days during August and September 2007. On all days, except one, the daily average concentration was below 10 grainsm(-3). Diurnal bi-hourly ragweed concentrations reached a maximum at 11:00 EET. Relatively high concentrations were observed between 21:00 and 01:00 EET. This allowed for the assumption of a local and a remote ragweed pollen source. We used HYSPLIT backward trajectory ensembles to identify possible sources on peak day. A frequency analysis of back trajectories covering the entire ragweed season followed. Firstly, possible local sources were the Istanbul Province and Turkish Thrace; secondly, a likely over-regional source was Bulgaria; and lastly, remote sources of ragweed pollen were the Ukraine, the Russian coastal region of the Black Sea and Moldova. This study provides evidence that pollens detected on our receptor site stem from combined local and remote origins.

Copyright © 2012 Elsevier B.V. All rights reserved.

PMID: 22634556 [PubMed - indexed for MEDLINE]

96. Pulm Pharmacol Ther. 2012 May 24. [Epub ahead of print]

cAMP regulation of airway smooth muscle function.

Billington CK, Ojo OO, Penn RB, Ito S.

Division of Therapeutics and Molecular Medicine, The University of Nottingham, Nottingham NG7 2UH, UK.

Agonists activating β(2)-adrenoceptors (β(2)ARs) on airway smooth muscle (ASM) are the drug of choice for rescue from acute bronchoconstriction in patients with both asthma and chronic obstructive pulmonary disease (COPD). Moreover, the use of long-acting β-agonists combined with inhaled corticosteroids constitutes an important maintenance therapy for these diseases. β-Agonists are effective bronchodilators due primarily to their ability to antagonize ASM contraction. The presumed cellular mechanism of action involves the generation of intracellular cAMP, which in turn can activate the effector molecules cAMP-dependent protein kinase (PKA) and Epac. Other agents such as prostaglandin E(2) and phosphodiesterase inhibitors that also increase intracellular cAMP levels in ASM, can also antagonize ASM contraction, and inhibit other ASM functions including proliferation and migration. Therefore, β(2)ARs and cAMP are key players in combating the pathophysiology of airway narrowing and remodeling. However, limitations of β-agonist therapy due to drug tachyphylaxis related to β(2)AR desensitization, and recent findings regarding the manner in which β(2)ARs and

cAMP signal, have raised new and interesting questions about these well-studied molecules. In this review we discuss current concepts regarding β(2)ARs and cAMP in the regulation of ASM cell functions and their therapeutic roles in asthma and COPD.

Copyright © 2012. Published by Elsevier Ltd.

PMID: 22634112 [PubMed - as supplied by publisher]

97. J Int Med Res. 2012;40(2):740-7.

Climatic factors correlate with innate immune response in children with Dermatophagoides farinae-induced allergic asthma.

Tan YQ, Cao LF, Shen J, Yu Y.

Department of Paediatrics, Chongming Branching Hospital, Xinhua Hospital Affiliated to Shanghai Jiaotong University School of Medicine, 25 Nan men gang Road, Chongming County, Shanghai 202150, China. tanyq66@yahoo.cn

OBJECTIVE: To determine the effect of climatic factors on immune markers in children with Dermatophagoides farinae induced asthma.
METHODS: Serum concentrations of macrophage migration inhibitory factor (MIF), eosinophil cationic protein (ECP) and D. farinae-specific immunoglobulin E (DF-sIgE), together with peripheral blood eosinophil counts, were measured in children with D. farinae induced (n = 75) or non-D. farinae-induced asthma (n = 17), and in healthy controls (n = 30). Mean temperature and relative humidity in the month before enrolment were calculated from meteorological data.
RESULTS: MIF, ECP and eosinophil counts were significantly higher in children with D. farinae-induced asthma than in controls, but comparable with non-D. farinae-induced asthma. Children with D. farinae-induced asthma in a low temperature (< 16 °C) or low relative humidity (< 70%) climate had significantly lower DF-sIgE, MIF, ECP and eosinophil counts than those in a high temperature or high humidity climate. DF-sIgE correlated positively with MIF, ECP and eosinophil count in D. farinae-induced asthma.
CONCLUSIONS: Temperature and humidity influenced MIF, ECP, eosinophil count and DF-sIgE in D. farinae-induced asthma. Understanding this relationship may provide new strategies for asthma prevention and treatment.

PMID: 22613438 [PubMed - indexed for MEDLINE]

98. J Ethnopharmacol. 2012 Jun 26;142(1):253-8. doi: 10.1016/j.jep.2012.04.053. Epub 2012 May 9.

Effect of Sophora flavescens Aiton extract on degranulation of mast cells and contact dermatitis induced by dinitrofluorobenzene in mice.

Kim H, Lee MR, Lee GS, An WG, Cho SI.

School of Korean Medicine, Pusan National University, Pusan 626-870, South Korea. kronos7@pusan.ac.kr

ETHNOPHARMACOLOGICAL RELEVANCE: The dried root of Sophora flavescens Aiton (Sophorae radix, SR) has long been used in traditional medicine for the treatment of fever and swelling in eastern countries.
MATERIALS AND METHODS: The present study investigated the anti-allergic and anti-inflammatory effects of SR using 1-fluoro-2,4-dinitrofluorobenzene (DNFB)-induced contact dermatitis mouse model and in vitro using RBL-2H3 cells.

RESULTS: In mice, the topical application of 10 mg/mL of SR effectively inhibited enlargement of ear thickness and weight induced by repeated painting with DNFB. Topical application of SR also inhibited hyperplasia, edema, spongiosis and infiltration of mononuclear cells in ear tissue. In addition, production levels of interferon-gamma and tumor necrosis factor-alpha were decreased by SR in vivo. Finally, the release of histamine and β-hexosaminidase, and migration were inhibited by treatment with SR.
CONCLUSIONS: These data indicate the potential of SR in treating patients with allergic skin diseases and also suggest that related mechanisms are involved in anti-inflammatory action on the Th 1 skewing reaction and inhibition against recruitment and degranulation of mast cells.

Copyright © 2012 Elsevier Ireland Ltd. All rights reserved.

PMID: 22580027 [PubMed - indexed for MEDLINE]

99. Contemp Clin Dent. 2012 Jan;3(1):2-8. doi: 10.4103/0976-237X.94538.

Childhood caries as influenced by maternal and child characteristics in pre-school children of Kerala-an epidemiological study.

Retnakumari N, Cyriac G.

Department of Pedodontics, Government Dental College, Trivandrum, Kerala, India.

PURPOSE: The most common chronic disease of childhood is early childhood caries which is five times more prevalent than asthma and seven times higher than that of allergic rhinitis. Most children do not receive dental care until they are three years old, yet by the time more than thirty percent of children from lower socioeconomic groups already have caries. To determine the prevalence and severity of early childhood caries among pre-school children, to describe the child characteristics associated with the development of early childhood caries and to find the association of early childhood caries and maternal risk factors.
MATERIALS AND METHODS: This descriptive study was carried out among children attending the immunization clinic of Sree Avittam Thirunal Hospital, Medical College, Trivandrum and children attending the randomly selected Anganwadies and Day care centres in Trivandrum, the capital of Kerala, wherein there are migrants from all over the State. A total of 350 children aged 12-36 months and their mothers were studied. The mother was first interviewed by a structured questionnaire; then the child's and mothers clinical examination was carried out covering caries experience and oral hygiene status.
RESULTS: Among 350 children studied the prevalence of dental caries in this study population was found to be 50.6 %(177). Statistically significant associations were found between the severity of decay and the child's age(P<0.001), female gender(P<0.05),low socioeconomic status (P<0.05), feeding frequency (P<0.05), type of feeding(P<0.01), fell asleep with nipple in mouth (P<0.05), duration of breast feeding(P<0.001), consumption of cariogenic type of snacks(P<0.01), age of commencement of tooth brushing(P<0.05), brushing frequency(P<0.05), oral hygiene status of child(P<0.001), DMFS scores of mothers (P<0.001), and oral hygiene status of mother (P<0.001).

PMCID: PMC3341753
PMID: 22557889 [PubMed]

100. Nan Fang Yi Ke Da Xue Xue Bao. 2012 Apr;32(4):569-72.

[Effects of mustard seed on 2, 4-dinitrofluorobenzene-induced allergic contact dermatitis in BALB/c mice].

[Article in Chinese]

Wen C, Yang R, Hu J, Jiao Z, Yang Y, Yang J, Li H, Zhao H.

Department of Dermatology, General Hospital of PLA, Beijing 100853, China.
wenchunmiao8@163.com

OBJECTIVE: To investigate the therapeutic effect of mustard seed on allergic contact dermatitis (ACD) in mice and explore the mechanism.
METHODS: Eighteen BALB/c mice were randomly divided into normal control group, model group and mustard seed group. The mice in the normal control group and model group were fed with normal chow, and those in mustard seed group were given 5% mustard seed mixed in the chow. Three weeks later, ACD was induced on the ear using 2, 4-dinitrofluorobenzene. After 24 h, the swelling of the ear was examined, and the rats were sacrificed to collect the ear tissue ears and blood for histopathological and immunohistochemical examinations, RT-PCR and enzyme-linked immunosorbent assay.
RESULTS: In mice with ACD, feeding with mustard seeds significantly lessened the ear swelling, improved the tissue histopathology, lowered the number of infiltrating Langerhans cells, and reduced the expressions of IL-1β, TNF-α and IL-6 mRNA in the ear, but did not cause significant changes in serum levels of IL-4, IFN-γ and IL-17.
CONCLUSION: Mustard seed inhibits ACD in mice possibly by suppressing the expressions of IL-1β, TNF-α and IL-6 mRNA and inhibiting Langerhans cell migration in the epidermis.

PMID: 22543145 [PubMed - in process]

101. Eur J Immunol. 2012 May;42(5):1250-60. doi: 10.1002/eji.201142021.

CCL25 induces $\alpha_4\beta_7$ integrin-dependent migration of IL-17^+ γδ T lymphocytes during an allergic reaction.

Costa MF, Bornstein VU, Candéa AL, Henriques-Pons A, Henriques MG, Penido C.

Laboratório de Farmacologia Aplicada, Farmanguinhos, Fundação Oswaldo Cruz, Rio de Janeiro, Brazil.

Comment in
    Eur J Immunol. 2012 May;42(5):1097-101.

Herein, we provide evidence that during allergic inflammation, CCL25 induces the selective migration of IL-17(+) γδ T cells mediated by α(4) β(7) integrin. Intrapleural injection of CCL25 into ovalbumin (OVA)-immunized C57BL/6 mice triggered the accumulation of γδ T lymphocytes expressing CCR9 (CCL25 receptor) and α(4) β(7) integrin in the pleura, but failed to attract αβ T lymphocytes. CCL25 attracted CCR6(+) γδ T cells producing IL-17 (but not IFN-γ or IL-4). OVA challenge triggered increased production of CCL25 followed by the accumulation of CCR9(+) , α(4) β(7) (+) , and CCR6(+) /IL-17(+) γδ T cells into the pleural cavities of OVA-immunized mice, which was inhibited by the in vivo neutralization of CCL25. The in vivo blockade of α(4) β(7) integrin also inhibited the migration of IL-17(+) γδ T lymphocytes (but not of αβ T lymphocytes) into mouse pleura after OVA challenge, suggesting that the CCL25/α(4) β(7) integrin pathway is selective for γδ T cells. In addition, α(4) β(7) integrin blockade impaired the in vitro transmigration of γδ T cells across endothelium (which expresses α(4) β(7) ligands VCAM-1 and MadCAM-1), which was induced by CCL25 and by cell-free pleural washes recovered from OVA-challenged mice. Our results reveal that during an allergic reaction, CCL25 drives IL-17(+) γδ T-cell mobilization to inflamed

tissue via α(4) β(7) integrin and modulates IL-17 levels.

© 2012 WILEY-VCH Verlag GmbH & Co. KGaA, Weinheim.

PMID: 22539297 [PubMed - indexed for MEDLINE]

102. Eur J Immunol. 2012 May;42(5):1097-101. doi: 10.1002/eji.201242545.

Driving IL-17⁺ γδ T-cell migration in allergic reactions: a new "inflammatory" role for the "homeostatic" chemokine CCL25.

Silva-Santos B.

Instituto de Medicina Molecular, Faculdade de Medicina da Universidade de Lisboa, Lisboa, Portugal. bssantos@fm.ul.pt

Comment on
    Eur J Immunol. 2012 May;42(5):1250-60.

Chemokines are traditionally classified as homeostatic or inflammatory depending on whether they direct leukocyte migration in the absence or presence of inflammatory stimuli. CC chemokine ligand (CCL)25, a ligand for CC chemokine receptor (CCR)9, has mostly been characterized as a homeostatic chemokine that determines the migration pathway of T-cell progenitors within the thymus, and the recruitment of various lymphocyte subsets to the intestinal mucosa. In this issue of the European Journal of Immunology, Costa et al. [Eur. J. Immunol. 2012. 42: 1250-1260] describe a new inflammatory role for CCL25/CCR9 in controlling the migration of a subset of γδ T cells committed to IL-17 production (γδ17 cells) in a model of allergic pleurisy. Interestingly, the effect of CCL25 was selective for γδ17 cells, as it did not extend to other γδ or αβ T-cell subsets, and resulted in a specific increase of IL-17 (but not IL-4 or IFN-γ) levels in the allergic pleura. In this commentary, I discuss these results in the context of chemokine-mediated recruitment of γδ T cells to inflammatory sites, and the as yet unclear and controversial role of IL-17 in allergic reactions.

© 2012 WILEY-VCH Verlag GmbH & Co. KGaA, Weinheim.

PMID: 22539284 [PubMed - indexed for MEDLINE]

103. PLoS One. 2012;7(4):e34965. doi: 10.1371/journal.pone.0034965. Epub 2012 Apr 18.

Pentraxin 3 (PTX3) expression in allergic asthmatic airways: role in airway smooth muscle migration and chemokine production.

Zhang J, Shan L, Koussih L, Redhu NS, Halayko AJ, Chakir J, Gounni AS.

Department of Immunology, Faculty of Medicine, University of Manitoba, Winnipeg, Manitoba, Canada.

BACKGROUND: Pentraxin 3 (PTX3) is a soluble pattern recognition receptor with non-redundant functions in inflammation and innate immunity. PTX3 is produced by immune and structural cells. However, very little is known about the expression of PTX3 and its role in allergic asthma.
OBJECTIVES AND METHODS: We sought to determine the PTX3 expression in asthmatic airways and its function in human airway smooth muscle cells (HASMC). In vivo PTX3 expression in bronchial biopsies of mild, moderate and severe asthmatics was analyzed by immunohistochemistry. PTX3 mRNA and protein were measured by real-time RT-PCR and ELISA, respectively. Proliferation and migration were

examined using (3)H-thymidine incorporation, cell count and Boyden chamber assays.
RESULTS: PTX3 immunoreactivity was increased in bronchial tissues of allergic asthmatics compared to healthy controls, and mainly localized in the smooth muscle bundle. PTX3 protein was expressed constitutively by HASMC and was significantly up-regulated by TNF, and IL-1β but not by Th2 (IL-4, IL-9, IL-13), Th1 (IFN-γ), or Th-17 (IL-17) cytokines. In vitro, HASMC released significantly higher levels of PTX3 at the baseline and upon TNF stimulation compared to airway epithelial cells (EC). Moreover, PTX3 induced CCL11/eotaxin-1 release whilst inhibited the fibroblast growth factor-2 (FGF-2)-driven HASMC chemotactic activity.
CONCLUSIONS: Our data provide the first evidence that PTX3 expression is increased in asthmatic airways. HASMC can both produce and respond to PTX3. PTX3 is a potent inhibitor of HASMC migration induced by FGF-2 and can upregulate CCL11/eotaxin-1 release. These results raise the possibility that PTX3 may play a dual role in allergic asthma.

PMCID: PMC3329534
PMID: 22529962 [PubMed - indexed for MEDLINE]

104. Protein Sci. 2012 Jun;21(6):850-64. doi: 10.1002/pro.2072. Epub 2012 Apr 23.

Structural basis of interleukin-5 dimer recognition by its α receptor.

Kusano S, Kukimoto-Niino M, Hino N, Ohsawa N, Ikutani M, Takaki S, Sakamoto K, Hara-Yokoyama M, Shirouzu M, Takatsu K, Yokoyama S.

RIKEN Systems and Structural Biology Center, Tsurumi-ku, Yokohama, Japan.

Interleukin-5 (IL-5), a major hematopoietin, stimulates eosinophil proliferation, migration, and activation, which have been implicated in the pathogenesis of allergic inflammatory diseases, such as asthma. The specific IL-5 receptor (IL-5R) consists of the IL-5 receptor α subunit (IL-5RA) and the common receptor β subunit (βc). IL-5 binding to IL-5R on target cells induces rapid tyrosine phosphorylation and activation of various cellular proteins, including JAK1/JAK2 and STAT1/STAT5. Here, we report the crystal structure of dimeric IL-5 in complex with the IL-5RA extracellular domains. The structure revealed that IL-5RA sandwiches the IL-5 homodimer by three tandem domains, arranged in a "wrench-like" architecture. This association mode was confirmed for human cells expressing IL-5 and the full-length IL-5RA by applying expanded genetic code technology: protein photo-cross-linking experiments revealed that the two proteins interact with each other in vivo in the same manner as that in the crystal structure. Furthermore, a comparison with the previously reported, partial GM-CSF•GM-CSFRA•βc structure enabled us to propose complete structural models for the IL-5 and GM-CSF receptor complexes, and to identify the residues conferring the cytokine-specificities of IL-5RA and GM-CSFRA.

Copyright © 2012 The Protein Society.

PMCID: PMC3403420 [Available on 2013/6/1]
PMID: 22528658 [PubMed - indexed for MEDLINE]

105. Eur Ann Allergy Clin Immunol. 2012 Feb;44(1):5-11.

Prevalence of allergic disorders in Italy: the Cotignola population study.

Quercia O, Incorvaia C, Puccinelli P, Scurati S, Emiliani F, Frati F, Stefanini GF.

Allergology High Speciality Unit, General Medicine, Faenza Hospital, AUSL
Ravenna, Italy.

Erratum in
Eur Ann Allergy Clin Immunol. 2012 Jun;44(3):112.

BACKGROUND: The worldwide prevalence of allergic diseases such as rhinitis, asthma, and atopic dermatitis is continuously increasing, while other allergic disorders such as urticaria and angioedema are less investigated. We performed a population study evaluating the prevalence of any kind of allergic disorders.
METHODS: The entire population of 7,201 inhabitants of Cotignola (Ravenna, Italy) was surveyed by a questionnaire assessing symptoms related to rhinitis, asthma, anaphylaxis, skin symptoms and insect sting allergy as well as the features of clinical presentations, diagnosis, and treatment received.
RESULTS: Valid questionnaires were obtained by 6,676 inhabitants (92.7%). The sample was formed by 3,266 males and 3,495 females, the mean age was 45.6 years; 1,035 subjects (15.5%) were aged less than 18 years; 404 subjects (6%) had at least one episode of wheezing/breathlessness in their lifetime, and 243 of them (60.1%) had a diagnosis of asthma; 1,002 subjects (14.8%) had nose symptoms in their lifetime, and 375 of them (37.4%) had a diagnosis of allergic rhinitis or rhinoconjunctivitis. For other allergic manifestations, data were obtained from 5,730 subjects; of them, 178 (3.1%) had skin symptoms, 59 (1.1%) had oral symptoms, and 37 (0.6%) had anaphylaxis; 207 (3.6%) had reactions to insect stings. There were no significant differences in prevalence between Italians and immigrants. Only 51.7% of subjects with asthma, 46.5% of those with rhinitis, 22.7% of those with other allergies, but 97.1% of those with insect allergy, received treatment.
CONCLUSIONS: These findings confirm recent data on epidemiology of allergic diseases in Europe, particularly in Italy, and add some details on how such diseases are managed.

PMID: 22519126 [PubMed - indexed for MEDLINE]

106. Int J Sports Med. 2012 May;33(5):402-9. doi: 10.1055/s-0032-1301888. Epub 2012 Apr 17.

Exercise inhibits allergic lung inflammation.

Silva AC, Vieira RP, Nisiyama M, Santos AB, Perini A, Mauad T, Dolhnikoff M, Martins MA, Carvalho CR.

Physical Therapy, University of São Paulo, Sao Paulo, Brazil.

Aerobic conditioning (AC) performed either during or after sensitization reduces allergic inflammation in mice; however, the effects of AC performed before and during allergic sensitization on airway inflammation are unknown. Mice were divided into Control, AC, OVA, and AC + OVA groups. Mice were trained in a treadmill followed by either ovalbumin (OVA) sensitization or saline administration. Peribronchial inflammation, OVA-specific IgE and IgG1 titers, the expression of Th1 and Th2 cytokines, and airway remodeling were evaluated, as well as the expression of Eotaxin, RANTES, ICAM-1, VCAM-1, TGF-β and VEGF. Aerobic conditioning performed before and during allergic sensitization displayed an inhibitory effect on the OVA-induced migration of eosinophils and lymphocytes to the airways, a reduction of IgE and IgG1 titers and an inhibition of the expression of Th2 cytokines. The AC + OVA group also demonstrated reduced expression of ICAM-1, VCAM-1, RANTES, TGF-β and VEGF, as well as decreased airway remodeling ($p<0.05$). The effects of AC before and during the sensitization process inhibit allergic airway inflammation and reduce the production of Th2

cytokines and allergen-specific IgE and IgG1.

© Georg Thieme Verlag KG Stuttgart · New York.

PMID: 22510803 [PubMed - indexed for MEDLINE]

107. Mol Med Report. 2012 Jul;6(1):115-20. doi: 10.3892/mmr.2012.869. Epub 2012 Apr 12.

Effects of astragaloside IV on eosinophil activation induced by house dust mite allergen.

Gu X, Jiang D, Wang Y, Du Q, Cai J.

Department of Respiratory Medicine, The 454th Hospital of Chinese PLA, Nanjing, Jiangsu 210002, PR China.

Astragaloside IV (AS-IV) has been noted for its reduction of eosinophilic airway inflammation in a murine model of chronic asthma. To gain a better understanding of the mechanisms involved in this anti-inflammatory phenomenon, the effect of AS-IV on human blood eosinophils was studied in vitro. Eosinophils were isolated from the blood of patients with mild atopic asthma, preincubated with AS-IV for 1 h and stimulated in the presence or absence of the house dust mite allergen Dermatophagoides pteronyssinus (Der p) 1 for 4 h. The survival of the eosinophils at 48 h was investigated using trypan blue and the surface expression of CC chemokine receptor 3 (CCR3) and intercellular adhesion molecule-1 (ICAM-1) by the eosinophils was analyzed using flow cytometry. The secretion of cytokines in the supernatants and the chemotaxis of the eosinophils were measured by ELISA and the transwell system, respectively. Der p 1 was found to prolong the survival of the eosinophils. Similarly, the expression of CCR3 and ICAM-1, secretion of interleukin (IL)-1β, IL-5, tumor necrosis factor (TNF)-α and the granulocyte macrophage colony stimulating factor (GM-CSF) and transmigration of the eosinophils were increased in the presence of Der p 1. However, these inductive effects on the eosinophils were significantly inhibited by AS-IV (50 μg/ml). These findings suggest that AS-IV modulates eosinophil activation and trafficking in response to Der p 1 and may therefore be a useful therapeutic option in eosinophilic asthma.

PMID: 22505212 [PubMed - indexed for MEDLINE]

108. J Immunol. 2012 May 15;188(10):5123-31. doi: 10.4049/jimmunol.1101618. Epub 2012 Apr 9.

TLR3 but not TLR7/8 ligand induces allergic sensitization to inhaled allergen.

Reuter S, Dehzad N, Martin H, Böhm L, Becker M, Buhl R, Stassen M, Taube C.

III Department of Medicine, University Hospital Mainz, Mainz, Germany.

Epidemiological studies suggest that viral infections during childhood are a risk factor for the development of asthma. However, the role of virus-specific pattern recognition receptors in this process is not well defined. In the current study, we compare the effects of the inhaled viral TLR ligands polyinosinic-polycytidylic acid (TLR3) and resiquimod (TLR7/8) on sensitization to a model allergen (OVA) in a murine model. Both compounds enhance the migration, activation, and Ag-processing of myeloid dendritic cells from the lung to the draining lymph nodes comparable to the effects of LPS. Application of polyinosinic-polycytidylic acid [poly(I:C)] or LPS induces production of

allergen-specific IgE and IgG1, whereas resiquimod (R848) had no effect. In addition, rechallenge of mice with OVA resulted in airway inflammation and mucus production in animals that received either poly(I:C) or LPS but not after application of R848. In summary, these results show that activation of TLR3 in combination with inhaled allergen results in induction of dendritic cell activation and migration similar to the effects of LPS. This leads to the development of allergic airway disease after allergen rechallenge, whereas mice treated with R848 did not develop allergic airway disease. These findings give further insight into the effects of stimulation of different TLRs on the development of asthma.

PMID: 22491246 [PubMed - indexed for MEDLINE]

109. Environ Manage. 2012 Jun;49(6):1238-46. doi: 10.1007/s00267-012-9852-3. Epub 2012 Apr 7.

Responses of two invasive plants under various microclimate conditions in the Seoul metropolitan region.

Song U, Mun S, Ho CH, Lee EJ.

School of Biological Sciences, Seoul National University, Korea.

The possible consequences of global warming on plant communities and ecosystems have wide-ranging ramifications. We examined how environmental change affects plant growth as a function of the variations in the microclimate along an urban-suburban climate gradient for two allergy-inducing, invasive plants, Humulus japonicus and Ambrosia artemisiifolia var. elatior. The environmental factors and plant growth responses were measured at two urban sites (Gangbuk and Seongbuk) and two suburban sites (Goyang and Incheon) around Seoul, South Korea. The mean temperatures and CO(2) concentrations differed significantly between the urban (14.8 °C and 439 ppm CO(2)) and suburban (13.0 °C and 427 ppm CO(2)) sites. The soil moisture and nitrogen contents of the suburban sites were higher than those at the urban sites, especially for the Goyang site. The two invasive plants showed significantly higher biomasses and nitrogen contents at the two urban sites. We conducted experiments in a greenhouse to confirm the responses of the plants to increased temperatures, and we found consistently higher growth rates under conditions of higher temperatures. Because we controlled the other factors, the better performance of the two invasive plants appears to be primarily attributable to their responses to temperature. Our study demonstrates that even small temperature changes in the environment can confer significant competitive advantages to invasive species. As habitats become urbanized and warmer, these invasive plants should be able to displace native species, which will adversely affect people living in these areas.

PMID: 22484518 [PubMed - indexed for MEDLINE]

110. Clin Nurs Res. 2012 May;21(2):224-42. doi: 10.1177/1054773812438915. Epub 2012 Apr 3.

"I have to turn myself inside out": caring for immigrant families of children with asthma.

Dalheim Englund AC, Rydström I.

School of Health Sciences, University of Borås, Borås, Sweden.
lotta.englund@hb.se

In multicultural societies, health care professionals encounter immigrant families of children with asthma. They play an important role in supporting these families, but few studies have focused on this phenomenon. The aim of the present study is to gain a broader understanding of the challenges health care professionals face in their encounters with non-Western immigrant parents of children with asthma. Seventeen professional caregivers were interviewed, and their narratives were analyzed using qualitative content analysis. The results show that health care professionals' main challenges when encountering immigrant parents can be described by the theme, "Turning oneself inside out." This theme is characterized by five categories: gender and professional issues, impact on professional relationships, communication challenges, unfamiliar disease and treatment perceptions, and time issues. The results highlight the importance of providing health care professionals with support and organizational conditions that increase opportunities to understand the unique situation of these families.

PMID: 22473272 [PubMed - indexed for MEDLINE]

111. J Allergy Clin Immunol. 2012 May;129(5):1377-1386.e13. doi: 10.1016/j.jaci.2012.02.035. Epub 2012 Mar 27.

Naive T cells sense the cysteine protease allergen papain through protease-activated receptor 2 and propel TH2 immunity.

Liang G, Barker T, Xie Z, Charles N, Rivera J, Druey KM.

Molecular Signal Transduction Section, Laboratory of Allergic Diseases, National Institute of Allergy and Infectious Diseases, National Institutes of Health, Bethesda, MD, USA.

BACKGROUND: Sensitization to protease allergens, such as papain, or helminth infection is associated with basophil recruitment to draining lymph nodes (LNs). Basophils have the capacity to present antigen to naive T cells and promote T(H)2 differentiation directly or indirectly through IL-4 production.
OBJECTIVE: We studied how papain induces basophil migration to LNs and the contribution of various leukocytes to papain-induced immune responses.
METHODS: We immunized mice in the footpad with papain and studied leukocyte recruitment and inflammatory cytokine and chemokine production in the draining popliteal LNs.
RESULTS: Papain directly activated naive T cells through protease-activated receptor (PAR) 2 to initiate a chemokine/cytokine program that includes CCL17, CCL22, and IL-4. Papain-triggered innate immune responses were dependent on both CD4 T cells and PAR2 and were strongly reduced in the absence of CCR4, the primary receptor for CCL17/CCL22.
CONCLUSION: These results elucidate a novel innate allergen-recognition pathway mediated by naive T cells through PAR2, which provide an immediate source of chemokines and IL-4 upstream of basophils and antigen-restricted T(H)2 differentiation. PAR2 antagonism might thus hold promise for the treatment of allergic disease.

Published by Mosby, Inc.

PMCID: PMC3340436 [Available on 2013/5/1]
PMID: 22460072 [PubMed - indexed for MEDLINE]

112. Am J Respir Cell Mol Biol. 2012 Aug;47(2):245-52. doi: 10.1165/rcmb.2011-0412OC. Epub 2012 Mar 23.

Mycobacterium tuberculosis chaperonin 60.1 inhibits leukocyte diapedesis in a

murine model of allergic lung inflammation.

Riffo-Vasquez Y, Coates AR, Page CP, Spina D.

Sackler Institute of Pulmonary Pharmacology, Institute of Pharmaceutical Science, King's College London, UK. yanira.vasquez@kcl.ac.uk

Chaperonin 60.1 from Mycobacterium tuberculosis suppressed allergic lung inflammation and bronchial hyperresponsiveness in mice by a mechanism that is yet to be clarified. To investigate the possible antiinflammatory mechanism(s) of action of Cpn60.1 in a model of allergic lung inflammation, ovalbumin (OVA)-allergic mice were pretreated with Cpn60.1 intranasally 20 minutes before each OVA aerosol challenge in a total of three treatments. Readouts were performed 24 hours after last challenge. Pretreatment with Cpn60.1 (1.0-0.001 μg) significantly inhibited the number of eosinophils in bronchoalveolar lavage fluid (OVA, 49.2 ± 12.3 versus Cpn60.1 [1 μg dose], 90.4 ± 2.3 × 10(4) cells/ml) and IL-5 release (OVA, 43 ± 8.5 versus Cpn60.1 [1 μg dose], 3 ± 11 pg/ml) but increased IL-12 levels (OVA, 50 ± 24 versus Cpn60.1 [1 μg dose], 103 ± 13 pg/ml). The effect of Cpn60.1 on cell recruitment and IL-5, but not IL-12, release was abolished in TLR-4 knockout mice. Intravital microscopy demonstrated that Cpn60.1 reduced chemokine-mediated leukocyte rolling and transmigration across the vessel wall (rolling cells: eotaxin, 11.7 ± 1.1 versus Cpn60.1 [1 μg dose], 2.8 ± 1 cells in 30 s). Similarly, Cpn60.1 reduced eotaxin-induced leukocyte migration in vitro (eotaxin, 17.3 ± 3.3 versus Cpn60.1 [0.1 μg dose], 3.3 ± 0.4 cells × 10(4)/ml). Immunostaining demonstrated that Cpn60.1 inhibits VCAM-1 and increases vascular endothelial-cadherin expression in lung vascular tissue, suggesting that the antiinflammatory effect of Cpn60.1 is partly mediated by altering the expression of adhesion molecules. This study shows that Cpn60.1 inhibits leukocyte diapedesis by a TLR-4 and an adhesion molecule-dependent mechanism in allergic inflammation in mice.

PMID: 22447969 [PubMed - indexed for MEDLINE]

113. Am J Physiol Heart Circ Physiol. 2012 Jun 1;302(11):H2190-210. doi: 10.1152/ajpheart.01146.2011. Epub 2012 Mar 23.

Inositol trisphosphate receptors in smooth muscle cells.

Narayanan D, Adebiyi A, Jaggar JH.

Department of Physiology, University of Tennessee Health Science Center, Memphis, 38163, USA.

Inositol 1,4,5-trisphosphate receptors (IP(3)Rs) are a family of tetrameric intracellular calcium (Ca(2+)) release channels that are located on the sarcoplasmic reticulum (SR) membrane of virtually all mammalian cell types, including smooth muscle cells (SMC). Here, we have reviewed literature investigating IP(3)R expression, cellular localization, tissue distribution, activity regulation, communication with ion channels and organelles, generation of Ca(2+) signals, modulation of physiological functions, and alterations in pathologies in SMCs. Three IP(3)R isoforms have been identified, with relative expression and cellular localization of each contributing to signaling differences in diverse SMC types. Several endogenous ligands, kinases, proteins, and other modulators control SMC IP(3)R channel activity. SMC IP(3)Rs communicate with nearby ryanodine-sensitive Ca(2+) channels and mitochondria to influence SR Ca(2+) release and reactive oxygen species generation. IP(3)R-mediated Ca(2+) release can stimulate plasma membrane-localized channels, including transient receptor potential (TRP) channels and store-operated Ca(2+) channels. SMC IP(3)Rs also signal to other proteins via SR Ca(2+) release-independent mechanisms

through physical coupling to TRP channels and local communication with large-conductance Ca(2+)-activated potassium channels. IP(3)R-mediated Ca(2+) release generates a wide variety of intracellular Ca(2+) signals, which vary with respect to frequency, amplitude, spatial, and temporal properties. IP(3)R signaling controls multiple SMC functions, including contraction, gene expression, migration, and proliferation. IP(3)R expression and cellular signaling are altered in several SMC diseases, notably asthma, atherosclerosis, diabetes, and hypertension. In summary, IP(3)R-mediated pathways control diverse SMC physiological functions, with pathological alterations in IP(3)R signaling contributing to disease.

PMID: 22447942 [PubMed - indexed for MEDLINE]

114. PLoS One. 2012;7(3):e33329. doi: 10.1371/journal.pone.0033329. Epub 2012 Mar 19.

PGH1, the precursor for the anti-inflammatory prostaglandins of the 1-series, is a potent activator of the pro-inflammatory receptor CRTH2/DP2.

Schröder R, Xue L, Konya V, Martini L, Kampitsch N, Whistler JL, Ulven T, Heinemann A, Pettipher R, Kostenis E.

Molecular-, Cellular- and Pharmacobiology Section, Institute for Pharmaceutical Biology, University of Bonn, Bonn, Germany.

Prostaglandin H(1) (PGH(1)) is the cyclo-oxygenase metabolite of dihomo-γ-linolenic acid (DGLA) and the precursor for the 1-series of prostaglandins which are often viewed as "anti-inflammatory". Herein we present evidence that PGH(1) is a potent activator of the pro-inflammatory PGD(2) receptor CRTH2, an attractive therapeutic target to treat allergic diseases such as asthma and atopic dermatitis. Non-invasive, real time dynamic mass redistribution analysis of living human CRTH2 transfectants and Ca(2+) flux studies reveal that PGH(1) activates CRTH2 as PGH(2), PGD(2) or PGD(1) do. The PGH(1) precursor DGLA and the other PGH(1) metabolites did not display such effect. PGH(1) specifically internalizes CRTH2 in stable CRTH2 transfectants as assessed by antibody feeding assays. Physiological relevance of CRTH2 ligation by PGH(1) is demonstrated in several primary human hematopoietic lineages, which endogenously express CRTH2: PGH(1) mediates migration of and Ca(2+) flux in Th2 lymphocytes, shape change of eosinophils, and their adhesion to human pulmonary microvascular endothelial cells under physiological flow conditions. All these effects are abrogated in the presence of the CRTH2 specific antagonist TM30089. Together, our results identify PGH(1) as an important lipid intermediate and novel CRTH2 agonist which may trigger CRTH2 activation in vivo in the absence of functional prostaglandin D synthase.

PMCID: PMC3307725
PMID: 22442685 [PubMed - indexed for MEDLINE]

115. Neuroimmunomodulation. 2012;19(4):209-19. doi: 10.1159/000334098. Epub 2012 Mar 21.

3,4-methylenedioxymethamphetamine (ecstasy) decreases inflammation and airway reactivity in a murine model of asthma.

Stankevicius D, Ferraz-de-Paula V, Ribeiro A, Pinheiro ML, Ligeiro de Oliveira AP, Damazo AS, Lapachinske SF, Moreau RL, Tavares de Lima W, Palermo-Neto J.

Department of Pharmacology, Institute of Biomedical Sciences, University of São Paulo, São Paulo, Brazil.

OBJECTIVE: 3,4-Methylenedioxymethamphetamine (MDMA), or ecstasy, is a synthetic drug used recreationally, mainly by young people. It has been suggested that MDMA has a Th cell skewing effect, in which Th1 cell activity is suppressed and Th2 cell activity is increased. Experimental allergic airway inflammation in ovalbumin (OVA)-sensitized rodents is a useful model to study Th2 response; therefore, based on the Th2 skewing effect of MDMA, we studied MDMA in a model of allergic lung inflammation in OVA-sensitized mice.
METHODS: We evaluated cell trafficking in the bronchoalveolar lavage fluid, blood and bone marrow; cytokine production; L-selectin expression and lung histology. We also investigated the effects of MDMA on tracheal reactivity in vitro and mast cell degranulation.
RESULTS: We found that MDMA given prior to OVA challenge in OVA-sensitized mice decreased leukocyte migration into the lung, as revealed by a lower cell count in the bronchoalveolar lavage fluid and lung histologic analysis. We also showed that MDMA decreased expression of both Th2-like cytokines (IL-4, IL-5 and IL-10) and adhesion molecules (L-selectin). Moreover, we showed that the hypothalamus-pituitary-adrenal axis is partially involved in the MDMA-induced reduction in leukocyte migration into the lung. Finally, we showed that MDMA decreased tracheal reactivity to methacholine as well as mast cell degranulation in situ.
CONCLUSIONS: Thus, we report here that MDMA given prior to OVA challenge in OVA-sensitized allergic mice is able to decrease lung inflammation and airway reactivity and that hypothalamus-pituitary-adrenal axis activation is partially involved. Together, the data strongly suggest an involvement of a neuroimmune mechanism in the effects of MDMA on lung inflammatory response and cell recruitment to the lungs of allergic animals.

Copyright © 2012 S. Karger AG, Basel.

PMID: 22441537 [PubMed - indexed for MEDLINE]

116. ORL J Otorhinolaryngol Relat Spec. 2012;74(3):117-23. doi: 10.1159/000328587. Epub 2012 Mar 17.

Transforming growth factor-β1 promotes nasal mucosal mast cell chemotaxis in murine experimental allergic rhinitis.

Ouyang Y, Nakao A, Han D, Zhang L.

Key Laboratory of Otolaryngology, Head and Neck Surgery, Ministry of Education, Beijing Institute of Otolaryngology, Beijing, China.

BACKGROUND: Recent studies have shown that transforming growth factor-β(1) (TGF-β(1)) plays an important role in the progression of allergic diseases.
METHODS: Mouse models of allergic rhinitis were established by ovalbumin sensitization and challenge. Immunostaining was used to analyze the expression of TGF-β(1) in the mouse nasal mucosa. A chemotaxis assay was conducted to analyze the impact of TGF-β(1) stimulation on migration of mast cells differentiated from mouse bone marrow cells. Chemotaxis and Western blot analysis were further applied to investigate the pathways involved in mast cell migration induced by TGF-β(1) stimulation.
RESULTS: TGF-β(1) expression was induced in allergic rhinitis and phosphorylated Smad2 was expressed in mast cells present in the nasal mucosa. TGF-β(1) could induce migration of mast cells, but HTS466284, a TGF-β receptor 1 kinase inhibitor, inhibited this chemotactic activity. After TGF-β(1) stimulation, mast cell RhoA expression was significantly increased. TGF-β(1)-induced mast cell chemotaxis could be inhibited by the RhoA inhibitor Tat-C3 and myosin light chain kinase inhibitor ML-7.

CONCLUSION: TGF-β(1) plays a major role in inducing the accumulation of mast cells in allergic rhinitis.

Copyright © 2012 S. Karger AG, Basel.

PMID: 22433901 [PubMed - indexed for MEDLINE]

117. Chem Immunol Allergy. 2012;96:39-44. doi: 10.1159/000331870. Epub 2012 Mar 13.

Th17 and Th22 in skin allergy.

Cavani A, Pennino D, Eyerich K.

Laboratory of Experimental Immunology, IDI-IRCCS, Rome, Italy. Cavini@idi.it

Development of eczematous skin reactions depends on disease-specific and time-dependent recruitment of a variety of leukocytes affecting resident skin cells through cytotoxic mechanisms and release of cytokines. Th17 and Th22, defined as RORC+IL-17+ and IL-17-IFN-γ-IL-22+ cells, respectively, belong to a newly identified class of lymphocytes specifically involved in dialogue with non-immune cells. In line with this function, both Th17 and Th22 cells are enriched in many immune-mediated skin diseases, such as a topic dermatitis, allergic contact dermatitis and psoriasis. Both IL-17 and IL-22 activate keratinocyte innate immune defenses, thus protecting the skin from pathogen invasion. However, Th17 and Th22 differ in their proinflammatory functions, being prominent in the first T cell subset and occasional/opportunistic in the second T cell subset. Most of the proinflammatory functions of Th17 depend on the synergic activity of IFN-γ and IL-17 on target cells. Together with IFN-γ, IL-17 strongly enhances adhesion molecules on keratinocytes, thus promoting T cell-keratinocyte adhesion and T cell-mediated cytotoxicity, resulting in keratinocyte apoptosis. In contrast, Th22 cells guarantee skin integrity by inducing keratinocyte proliferation and migration. However, in inflamed skin, Th22 could contribute to the amplification of immune responses by enhancing the TNF-α-induced cytokines and chemokines released by keratinocytes.

Copyright © 2012 S. Karger AG, Basel.

PMID: 22433369 [PubMed - indexed for MEDLINE]

118. Chem Immunol Allergy. 2012;96:30-8. doi: 10.1159/000331868. Epub 2012 Mar 13.

Mechanisms of immune tolerance to allergens.

Fujita H, Meyer N, Akdis M, Akdis CA.

Swiss Institute of Allergy and Asthma Research, University of Zurich, Davos, Switzerland.

In allergic diseases, immune responses are induced by normally well-tolerated allergens, which result in chronic inflammation characterized by antibody secretion and T cell activation. For almost 100 years, allergen-specific immunotherapy (allergen-SIT) has been the potentially curative and antigen-specific method for the treatment of allergic diseases. Allergen-SIT alters the course of allergic diseases and can reduce allergic symptoms and medication use. The key mechanism behind allergen-SIT is the induction of peripheral T cell tolerance by altering the balance between Th cells and regulatory T cells. Both naturally occurring thymus-derived FOXP3(+)CD4(+)CD25(+) regulatory T cells and inducible type 1 regulatory T cells suppress the

development of allergic diseases via several mechanisms including suppression of dendritic cells, Th cells, mast cells, eosinophils and basophils; suppression of inflammatory cell migration to tissues; and decrease of the ratio between allergen-specific IgE and IgG4 antibodies. These effects are mainly mediated by the suppressive cytokines IL-10 and TGF-β. Knowledge of this molecular basis is crucial to understanding the regulation of the immune response and their possible therapeutic applications for allergic diseases.

Copyright © 2012 S. Karger AG, Basel.

PMID: 22433368 [PubMed - indexed for MEDLINE]

119. J Immunol. 2012 Apr 15;188(8):3784-90. doi: 10.4049/jimmunol.1102956. Epub 2012 Mar 16.

Lpa2 is a negative regulator of both dendritic cell activation and murine models of allergic lung inflammation.

Emo J, Meednu N, Chapman TJ, Rezaee F, Balys M, Randall T, Rangasamy T, Georas SN.

Division of Pulmonary and Critical Care Medicine, Department of Medicine, University of Rochester Medical Center, Rochester, NY 14610, USA.

Negative regulation of innate immune responses is essential to prevent excess inflammation and tissue injury and promote homeostasis. Lysophosphatidic acid (LPA) is a pleiotropic lipid that regulates cell growth, migration, and activation and is constitutively produced at low levels in tissues and in serum. Extracellular LPA binds to specific G protein-coupled receptors, whose function in regulating innate or adaptive immune responses remains poorly understood. Of the classical LPA receptors belonging to the Edg family, lpa2 (edg4) is expressed by dendritic cells (DC) and other innate immune cells. In this article, we show that DC from lpa2(-/-) mice are hyperactive compared with their wild-type counterparts and are less susceptible to inhibition by different LPA species. In transient-transfection assays, we found that lpa2 overexpression inhibits NF-κB-driven gene transcription. Using an adoptive-transfer approach, we found that allergen-pulsed lpa2(-/-) DC induced substantially more lung inflammation than did wild-type DC after inhaled allergen challenge. Finally, lpa2(-/-) mice develop greater allergen-driven lung inflammation than do their wild-type counterparts in models of allergic asthma involving both systemic and mucosal sensitization. Taken together, these findings identify LPA acting via lpa2 as a novel negative regulatory pathway that inhibits DC activation and allergic airway inflammation.

PMCID: PMC3324594 [Available on 2013/4/15]
PMID: 22427635 [PubMed - indexed for MEDLINE]

120. Am J Physiol Lung Cell Mol Physiol. 2012 Jun 1;302(11):L1179-91. doi: 10.1152/ajplung.00005.2012. Epub 2012 Mar 16.

The p110δ subunit of PI3K regulates bone marrow-derived eosinophil trafficking and airway eosinophilia in allergen-challenged mice.

Kang BN, Ha SG, Ge XN, Reza Hosseinkhani M, Bahaie NS, Greenberg Y, Blumenthal MN, Puri KD, Rao SP, Sriramarao P.

Laboratory of Allergic Diseases and Inflammation, Department of Veterinary and Biomedical Sciences, University of Minnesota, St. Paul, 55108, USA.

Trafficking and recruitment of eosinophils during allergic airway inflammation is mediated by the phosphatidylinositol 3-kinase (PI3K) family of signaling molecules. The role played by the p110δ subunit of PI3K (PI3K p110δ) in regulating eosinophil trafficking and recruitment was investigated using a selective pharmacological inhibitor (IC87114). Treatment with the PI3K p110δ inhibitor significantly reduced murine bone marrow-derived eosinophil (BM-Eos) adhesion to VCAM-1 as well as ICAM-1 and inhibited activation-induced changes in cell morphology associated with reduced Mac-1 expression and aberrant cell surface localization/distribution of Mac-1 and α4. Infused BM-Eos demonstrated significantly decreased rolling and adhesion in inflamed cremaster muscle microvessels of mice treated with IC87114 compared with vehicle-treated mice. Furthermore, inhibition of PI3K p110δ significantly attenuated eotaxin-1-induced BM-Eos migration and prevented eotaxin-1-induced changes in the cytoskeleton and cell morphology. Knockdown of PI3K p110δ with siRNA in BM-Eos resulted in reduced rolling, adhesion, and migration, as well as inhibition of activation-induced changes in cell morphology, validating its role in regulating trafficking and migration. Finally, in a mouse model of cockroach antigen-induced allergic airway inflammation, oral administration of the PI3K p110δ inhibitor significantly inhibited airway eosinophil recruitment, resulting in attenuation of airway hyperresponsiveness in response to methacholine, reduced mucus secretion, and expression of proinflammatory molecules (found in inflammatory zone-1 and intelectin-1). Overall, these findings indicate the important role played by PI3K p110δ in mediating BM-Eos trafficking and migration by regulating adhesion molecule expression and localization/distribution as well as promoting changes in cell morphology that favor recruitment during inflammation.

PMCID: PMC3379039 [Available on 2013/6/1]
PMID: 22427531 [PubMed - indexed for MEDLINE]

121. Curr Drug Targets. 2012 Jul;13(8):1107-18.

Leukotriene c4 synthase: upcoming drug target for inflammation.

Devi NS, Doble M.

Bioengineering and Drug Design Lab, Department of Biotechnology, Indian Institute of Technology Madras, Chennai, India.

Leukotrienes are important mediators of pain and inflammation and they are produced in the arachidonic acid pathway via 5-lipoxygenase. They have been shown to have important roles in pyresis following antigen attack and in aspirin-intolerant asthma. They promote inflammation processes including eosinophil migration, increase in vascular permeability and bronchoconstriction. Hence, targeting the enzymes involved in the synthesis of these mediators can lead to the development of novel anti-inflammatory drugs. However, no drugs have yet been developed targeting leukotriene C4 synthase, a key enzyme leading to the synthesis of cysteinyl leukotrienes. The recent elucidation of its crystal structure now opens up the possibility of drugs against it. The inhibitors developed for this enzyme until now and the structural features responsible for their activity are discussed in this review. This understanding could lead to the design of new chemical entities.

PMID: 22420309 [PubMed - indexed for MEDLINE]

122. Proc Natl Acad Sci U S A. 2012 Apr 10;109(15):E889-97. doi: 10.1073/pnas.1117674109. Epub 2012 Mar 12.

Cancer-associated epithelial cell adhesion molecule (EpCAM; CD326) enables epidermal Langerhans cell motility and migration in vivo.

Gaiser MR, Lämmermann T, Feng X, Igyarto BZ, Kaplan DH, Tessarollo L, Germain RN, Udey MC.

Dermatology Branch, Center for Cancer Research, National Cancer Institute, National Institutes of Health, Bethesda, MD 20892, USA.

After activation, Langerhans cells (LC), a distinct subpopulation of epidermis-resident dendritic cells, migrate from skin to lymph nodes where they regulate the magnitude and quality of immune responses initiated by epicutaneously applied antigens. Modulation of LC-keratinocyte adhesion is likely to be central to regulation of LC migration. LC express high levels of epithelial cell adhesion molecule (EpCAM; CD326), a cell-surface protein that is characteristic of some epithelia and many carcinomas and that has been implicated in intercellular adhesion and metastasis. To gain insight into EpCAM function in a physiologic context in vivo, we generated conditional knockout mice with EpCAM-deficient LC and characterized them. Epidermis from these mice contained increased numbers of LC with normal levels of MHC and costimulatory molecules and T-cell-stimulatory activity in vitro. Migration of EpCAM-deficient LC from skin explants was inhibited, but chemotaxis of dissociated LC was not. Correspondingly, the ability of contact allergen-stimulated, EpCAM-deficient LC to exit epidermis in vivo was delayed, and strikingly fewer hapten-bearing LC subsequently accumulated in lymph nodes. Attenuated migration of EpCAM-deficient LC resulted in enhanced contact hypersensitivity responses as previously described in LC-deficient mice. Intravital microscopy revealed reduced translocation and dendrite motility in EpCAM-deficient LC in vivo in contact allergen-treated mice. These results conclusively link EpCAM expression to LC motility/migration and LC migration to immune regulation. EpCAM appears to promote LC migration from epidermis by decreasing LC-keratinocyte adhesion and may modulate intercellular adhesion and cell movement within in epithelia during development and carcinogenesis in an analogous fashion.

PMCID: PMC3326512
PMID: 22411813 [PubMed - indexed for MEDLINE]

123. Subcell Biochem. 2012;58:111-81. doi: 10.1007/978-94-007-3012-0_5.

PI3Ks-Drug Targets in Inflammation and Cancer.

Wymann M.

Institute Biochemistry & Genetics, Department Biomedicine, University of Basel, Mattenstrasse 28, 4058, Basel, Switzerland, Matthias.Wymann@UniBas.CH.

Phosphoinositide 3-kinases (PI3Ks) control cell growth, proliferation, cell survival, metabolic activity, vesicular trafficking, degranulation, and migration. Through these processes, PI3Ks modulate vital physiology. When over-activated in disease, PI3K promotes tumor growth, angiogenesis, metastasis or excessive immune cell activation in inflammation, allergy and autoimmunity. This chapter will introduce molecular activation and signaling of PI3Ks, and connections to target of rapamycin (TOR) and PI3K-related protein kinases (PIKKs). The focus will be on class I PI3Ks, and extend into current developments to exploit mechanistic knowledge for therapy.

PMID: 22403076 [PubMed - in process]

124. J Chromatogr A. 2012 Apr 27;1235:141-8. doi: 10.1016/j.chroma.2012.02.039. Epub 2012 Feb 24.

Atmospheric pressure gas chromatography coupled to quadrupole-time of flight mass spectrometry as a powerful tool for identification of non intentionally added substances in acrylic adhesives used in food packaging materials.

Canellas E, Vera P, Domeño C, Alfaro P, Nerín C.

GUIA Group, Department of Analytical Chemistry, University of Zaragoza, I3A, María de Luna, 3, 50018 Zaragoza, Spain.

Acrylic adhesives are used to manufacture multilayer laminates that are used in food packaging to form the geometric shape of the package as well as to stick labels on the packages. Once applied on the packaging adhesives can supply potential migrants that could endanger the packaged food. Adhesives are complex matrices where intentionally and non intentionally added substances are present, but the identification of the migrants is required by law. In this study atmospheric pressure gas chromatography coupled to a quadrupole hyphenated to a time of flight mass spectrometer (APGC-MS/Q-TOF) has been explored for identification of unknowns coming from three different acrylic adhesives. The results are compared to those obtained by conventional GC-MS-Q (quadrupole). Sixteen compounds were identified by GC-MS/Q and five of them were confirmed by APGC-MS/Q-TOF as their molecular ions were found. Moreover, additional three new compounds were identified and their structure was elucidated working with the spectra obtained by APGC-MS/Q-TOF. This finding was very relevant as these compounds were biocides suspected to be allergenic and cytotoxic in humans. Migration studies were carried out using Tenax as solid food simulant and the results showed that the three acrylic adhesives tested in this work were safe for being used in food packaging materials since the migration of compounds previously identified was below the limit established in the current legislation.

Copyright © 2012 Elsevier B.V. All rights reserved.

PMID: 22402127 [PubMed - indexed for MEDLINE]

125. Am J Physiol Lung Cell Mol Physiol. 2012 May 15;302(10):L1118-27. doi: 10.1152/ajplung.00232.2011. Epub 2012 Mar 2.

Asthmatic airway smooth muscle CXCL10 production: mitogen-activated protein kinase JNK involvement.

Alrashdan YA, Alkhouri H, Chen E, Lalor DJ, Poniris M, Henness S, Brightling CE, Burgess JK, Armour CL, Ammit AJ, Hughes JM.

Respiratory Research Group, Faculty of Pharmacy, University of Sydney, Sydney, NSW 2006, Australia.

CXCL10 (IP10) is involved in mast cell migration to airway smooth muscle (ASM) bundles in asthma. We aimed to investigate the role of cytokine-induced MAPK activation in CXCL10 production by ASM cells from people with and without asthma. Confluent growth-arrested ASM cells were treated with inhibitors of the MAPKs ERK, p38, and JNK and transcription factor NF-κB, or vehicle, and stimulated with IL-1β, TNF-α, or IFN-γ, alone or combined (cytomix). CXCL10 mRNA and protein, JNK, NF-κB p65 phosphorylation, and Iκ-Bα protein degradation were assessed using real-time PCR, ELISA, and immunoblotting, respectively. Cytomix, IL-1β, and TNF-α induced CXCL10 mRNA expression more rapidly in asthmatic than nonasthmatic ASM cells. IL-1β and/or TNF-α combined with IFN-γ synergistically increased asthmatic ASM cell CXCL10 release. Inhibitor effects were similar in asthmatic and

nonasthmatic cells, but cytomix-induced release was least affected, with only JNK and NF-κB inhibitors halving it. Notably, JNK phosphorylation was markedly less in asthmatic compared with nonasthmatic cells. However, in both, the JNK inhibitor SP600125 reduced JNK phosphorylation and CXCL10 mRNA levels but did not affect CXCL10 mRNA stability or Iκ-Bα degradation. Together, the JNK and NF-κB inhibitors completely inhibited their CXCL10 release. We concluded that, in asthmatic compared with nonasthmatic ASM cells, JNK activation was reduced and CXCL10 gene expression was more rapid following cytomix stimulation. However, in both, JNK activation did not regulate early events leading to NF-κB activation. Thus JNK and NF-κB provide independent therapeutic targets for limiting CXCL10 production and mast cell migration to the ASM in asthma.

PMCID: PMC3362261 [Available on 2013/5/15]
PMID: 22387292 [PubMed - indexed for MEDLINE]

126. Vaccine. 2012 Apr 16;30(18):2848-58. doi: 10.1016/j.vaccine.2012.02.052. Epub 2012 Mar 2.

Combination of a TLR4 ligand and anaphylatoxin C5a for the induction of antigen-specific cytotoxic T cell responses.

Rudilla F, Fayolle C, Casares N, Durantez M, Arribillaga L, Lozano T, Villanueva L, Pio R, Sarobe P, Leclerc C, Prieto J, Lasarte JJ.

Gene Therapy and Hepatology Area and Oncology Area, Center for Applied Medical Research, University of Navarra, 31008 Pamplona, Spain.

The complement system and Toll-like receptors (TLR) are key innate defense systems which might interact synergistically on dendritic cells (DC) to reinforce adaptive immunity. In a previous work, we found that the extra domain A from fibronectin EDA (an endogenous ligand for TLR4) can favour antigen delivery to DC and induce their maturation. Given the potential of anaphylatoxins to cause inflammation and activation of myeloid cells, we hypothesized that a fusion protein between EDA, and anaphylatoxins C3a, C4a or C5a together with an antigen might improve the immunogenicity of the antigen. Naked DNA immunization with a construct expressing the fusion protein between C5a, EDA and the cytotoxic T cell epitope SIINFEKL from ovalbumin, induced strong antigen specific T cell responses. The purified recombinant fusion protein EDA-SIINFEKL-C5a induced activation of dendritic cells, the production of proinflammatory cytokines/chemokines and stimulated antigen presenting cell migration and NK cell activation. As compared to EDA-SIINFEKL, the fusion protein EDA-SIINFEKL-C5a did not induce the production of the immunosuppressive molecules IL-10, CCL17, CCL1, CXCL12 or XCL1 by DC. Moreover, EDA-SIINFEKL-C5a induced strong specific T cell responses in vivo and protected mice against E.G7-OVA tumor growth more efficiently than EDA-SIINFEKL or SIINFEKL-C5a recombinant proteins. Our results suggest that fusion proteins containing EDA, the anaphylatoxin C5a and the antigen may serve as a suitable strategy for the development of anti-tumor or anti-viral vaccines.

Copyright © 2012 Elsevier Ltd. All rights reserved.

PMID: 22387222 [PubMed - indexed for MEDLINE]

127. Clin Immunol. 2012 May;143(2):170-9. doi: 10.1016/j.clim.2012.01.008. Epub 2012 Jan 27.

Sublingual administration of Lactobacillus paracasei KW3110 inhibits Th2-dependent allergic responses via upregulation of PD-L2 on dendritic cells.

Inamine A, Sakurai D, Horiguchi S, Yonekura S, Hanazawa T, Hosokawa H, Matuura-Suzuki A, Nakayama T, Okamoto Y.

Department of Otolaryngology, Head and Neck Surgery (J2), Graduate School of Medicine, Chiba University, Chiba, Japan. ainamine@faculty.chiba-u.jp

Lactic acid bacteria have potential in immunomodulation therapy, but their clinical efficacy and underlying mechanisms are unclear. We aimed to clarify the anti-allergic immune responses induced by intragastric and sublingual administration of heat-killed Lactobacillus paracasei KW3110 and Lactobacillus acidophilus L-92. The KW3110 strain (but not the L-92 strain) enhanced ovalbumin (OVA)-induced expression of CCR-7 and PD-L2 in murine dendritic cells (DCs), and strongly inhibited IL-5 and IL-13 production in vitro in co-cultures with Th2-skewed CD4(+) T cells from DO11.10 transgenic mice. Sublingual administration of low-dose KW3110 (but not L-92) to OVA-sensitized mice selectively suppressed serum IgE production and Th2 cytokine expression in cervical lymph nodes, and significantly improved symptoms after OVA provocation in vivo. KW3110 probably accelerates DC migration into the regional lymph nodes and inhibits Th2 cytokine production through enhanced CCR-7 and PD-L2 expression. Thus, sublingual KW3110 administration may be effective in reducing allergic inflammation.

Copyright © 2012 Elsevier Inc. All rights reserved.

PMID: 22386865 [PubMed - indexed for MEDLINE]

128. Gut Liver. 2012 Jan;6(1):21-8. doi: 10.5009/gnl.2012.6.1.21. Epub 2012 Jan 12.

Evolutionary History of the Helicobacter pylori Genome: Implications for Gastric Carcinogenesis.

Correa P, Piazuelo MB.

Division of Gastroenterology, Department of Medicine, Vanderbilt University School of Medicine, Nashville, TN, USA.

The genome of the bacterium Helicobacter pylori has evolved over the millennia since its migration out of Africa along with its human host approximately 60,000 years ago. Human migrations, after thousands of years of permanent settlement in those lands, resulted in seven prototypes of genetic populations of H. pylori with distinct geographical distributions. In all continents, present day isolates of H. pylori have molecular markers that reflect population migrations. The colonization of the Americas as well as the slave trade introduced European and African strains to the New World. The relationship between H. pylori genome and gastric cancer rates is linked to the presence of the cagA gene, but the knowledge on this subject is incomplete because other genes may be involved in certain populations. A new situation for Homo sapiens is the absence of H. pylori colonization in certain, mostly affluent, populations, apparently brought about by improved home sanitation and widespread use of antibiotics during the last decades. The disappearance of H. pylori from the human microbiota may be linked to emerging epidemics of esophageal adenocarcinoma, some allergic diseases such as asthma and some autoimmune disorders.

PMCID: PMC3286735
PMID: 22375167 [PubMed]

129. Bundesgesundheitsblatt Gesundheitsforschung Gesundheitsschutz. 2012 Mar;55(3):318-28. doi: 10.1007/s00103-011-1440-9.

[Classification of specific IgE antibodies in children with hay fever and other atopic diseases in Germany. Results of the German Health Interview and Examination Survey for Children and Adolescents (KiGGS)].

[Article in German]

Langen U.

Epidemiologie nicht übertragbarer Krankheiten, Umweltmedizin, Robert Koch-Institut, General Pape Str. 62, 12101, Berlin, Deutschland. LangenU@rki.de

The dependencies between sensitization to common allergens (mono- and polysensitization, IgE level and patterns) and symptomatic hay fever and other atopic diseases, respectively, in children and adolescents are shown in this analysis. The evaluation was based on the KiGGS ("Kinder- und Jugendgesundheitssurvey") study. Our analysis was performed using complex samples methods with SPSS. Participants were interviewed by a physician using a validated questionnaire asking for atopic diseases and symptoms. Specific IgE levels were measured from the age of 3 years on by using the ImmunoCap® test system. The prevalences of hay fever and polysensitizations both significantly increase with increasing age of the participants, while boys are more often affected than girls and migrants less often regarding sensitizations. Prevalence of hay fever decreases with increasing number of older siblings and increases with atopy of one or both parents. Different positive correlations between increasing IgE levels and hay fever were identified, the greatest association was observed with herbal inhalative allergens and cross-reacting food allergens. Lowest IgE levels to nearly all of the tested allergens show a positive correlation with hay fever prevalence. In conclusion, the study indicates that the clinical definition of the lowest positive IgE levels as "marginal" should be discussed as well as indications for specific immunotherapy.

PMID: 22373844 [PubMed - indexed for MEDLINE]

130. Allergol Int. 2012 Jun;61(2):283-93. doi: 10.2332/allergolint.10-OA-0299. Epub 2012 Feb 25.

Possible Involvement of Sphingosine-1-Phosphate/G(i)/RhoA pathways in adherence of eosinophils to pulmonary endothelium.

Sashio T, Kume H, Takeda N, Asano T, Tsuji S, Kondo M, Hasegawa Y, Shimokata K.

Department of Respiratory Medicine, Nagoya University Graduate School of Medicine, Aichi, Japan.

BACKGROUND: Sphingosine-1-phosphate (S1P), a lysophospholipid released from inflammatory cells, causes cell migration by increasing cytokines and chemokines. This study was designed to determine whether S1P causes adherence of eosinophils to pulmonary endothelial cells via enhancement of adhesion molecule expression.
METHODS: Expression of VCAM-1 and ICAM-1 was assessed by RT-PCR and Western blot analysis in human pulmonary microvasucular endothelial cells (HPMVECs). The number of adherent eosinophils to HPMVECs was calculated according to adhesion assay.
RESULTS: Pre-treatment of HPMVECs with S1P increased mRNA and protein expression of VCAM-1, in contrast, did not dramatically increase those expression of ICAM-1. The maximal expression of these adhesion molecules in mRNA and protein was observed 4 and 8h after exposure to S1P, respectively. Pre-treatment with S1P also activated RhoA, a monomeric G protein; the ability of S1P to enhance the expression of VCAM-1 was attenuated by RhoA related inhibitors such as Y-27632,

C3 exoenzyme, and GGTI-286. The effects of S1P on VCAM-1 were attenuated by pre-incubation with pertussis toxin, which catalyzes the ADP-ribosylation of G(i), a heterotrimeric G protein. After HPMVECs were treated with S1P, adhesion of human eosinophilic leukemic cell line (EoL-1) cells to HPMVECs was enhanced in a concentration-dependent manner. Augmented adherence of EoL-1 cells by S1P was also attenuated by Y-27632 and pertussis toxin. S1P causes adherence of eosinophils to pulmonary endothelium via RhoA activation.
CONCLUSIONS: S1P may act as a lipid mediator in asthma. The RhoA/Rho-kinase pathway may be a therapeutic target for preventing eosinophil infiltration to the airway.

PMID: 22361510 [PubMed - indexed for MEDLINE]

131. Korean J Physiol Pharmacol. 2011 Dec;15(6):397-403. doi: 10.4196/kjpp.2011.15.6.397. Epub 2011 Dec 27.

Cigarette Smoke Extract-induced Reduction in Migration and Contraction in Normal Human Bronchial Smooth Muscle Cells.

Yoon CH, Park HJ, Cho YW, Kim EJ, Lee JD, Kang KR, Han J, Kang D.

Department of Rehabilitation Medicine, Gyeongsang National University School of Medicine, Jinju 660-751, Korea.

The proliferation, migration, cytokine release, and contraction of airway smooth muscle cells are key events in the airway remodeling process that occur in lung disease such as asthma, chronic obstruction pulmonary disease, and cancer. These events can be modulated by a number of factors, including cigarette smoke extract (CSE). CSE-induced alterations in the viability, migration, and contractile abilities of normal human airway cells remain unclear. This study investigated the effect of CSE on cell viability, migration, tumor necrosis factor (TNF)-α secretion, and contraction in normal human bronchial smooth muscle cells (HBSMCs). Treatment of HBSMCs with 10% CSE induced cell death, and the death was accompanied by the generation of reactive oxygen species (ROS). CSE-induced cell death was reduced by N-acetyl-l-cysteine (NAC), an ROS scavenger. In addition, CSE reduced the migration ability of HBSMCs by 75%. The combination of NAC with CSE blocked the CSE-induced reduction of cell migration. However, CSE had no effect on TNF-α secretion and NF-κB activation. CSE induced an increase in intracellular Ca(2+) concentration in 64% of HBSMCs. CSE reduced the contractile ability of HBSMCs, and the ability was enhanced by NAC treatment. These results demonstrate that CSE treatment induces cell death and reduces migration and contraction by increasing ROS generation in normal HBSMCs. These results suggest that CSE may induce airway change through cell death and reduction in migration and contraction of normal HBSMCs.

PMCID: PMC3282228
PMID: 22359478 [PubMed]

132. PLoS One. 2012;7(2):e30742. doi: 10.1371/journal.pone.0030742. Epub 2012 Feb 15.

Sphingosine kinase 1 mediation of expression of the anaphylatoxin receptor C5L2 dampens the inflammatory response to endotoxin.

Bachmaier K, Guzman E, Kawamura T, Gao X, Malik AB.

Department of Pharmacology, College of Medicine, Center of Lung and Vascular Biology, University of Illinois, Chicago, Illinois, United States of America.
kbachmai@uic.edu

The complement anaphylatoxin C5a has a pathogenetic role in endotoxin-induced lung inflammatory injury by regulating phagocytic cell migration and activation. Endotoxin and C5a activate the enzyme sphingosine kinase (Sphk) 1 to generate the signaling lipid sphingosine-1-phosphate (S1P), a critical regulator of phagocyte function. We assessed the function of Sphk1 and S1P in experimental lung inflammatory injury and determined their roles in anaphylatoxin receptor signaling and on the expression of the two C5a receptors, C5aR (CD88) and C5L2, on phagocytes. We report that Sphk1 gene deficient (Sphk1(-/-)) mice had augmented lung inflammatory response to endotoxin compared to wild type mice. Sphk1 was required for C5a-mediated reduction in cytokine and chemokine production by macrophages. Moreover, neutrophils from Sphk1(-/-) mice failed to upregulate the anaphylatoxin receptor C5L2 in response to LPS. Exogenous S1P restored C5L2 cell surface expression of Sphk1(-/-) mouse neutrophils to wild type levels but had no effect on cell surface expression of the other anaphylatoxin receptor, CD88. These results provide the first genetic evidence of the crucial role of Sphk1 in regulating the balance between expression of CD88 and C5L2 in phagocytes. S1P-mediated up-regulation of C5L2 is a novel therapeutic target for mitigating endotoxin-induced lung inflammatory injury.

PMCID: PMC3280265
PMID: 22355325 [PubMed - indexed for MEDLINE]

133. Praxis (Bern 1994). 2012 Feb 15;101(4):233-7. doi: 10.1024/1661-8157/a000835.

[COPD and Asthma: same same but different].

[Article in German]

Rothe T.

Abteilung für Innere Medizin und Pneumologie, Zürcher Höhenklinik Davos.
thomas.rothe@zhd.ch

In clinical practice, differentiation of COPD and asthma is difficult. A case report of an asthma patient with a drifter type of asthma imitating COPD is presented. In this context differences and similarities of both diseases are high-lighted. The definitions of asthma and COPD in international guidelines leave some space to misdiagnosing.

PMID: 22337513 [PubMed - indexed for MEDLINE]

134. Ther Umsch. 2012 Feb;69(2):107-9. doi: 10.1024/0040-5930/a000260.

[Advance directives in general practice].

[Article in German]

Harringer W.

Facharzt Allgemeine Medizin FMH, Psychosomatische Medizin SAPPM, Goldach.
harringer@hin.ch

View the fact of a doubling percentage of people aged 80 and over in the coming 20 years the topic of advance directives (AD) will play a more important role in general practice. It is the task of the GP as the integrating professional in any palliative care network to provide discussion and advice and to collaborate with the patient for a reliable version. Formulating AD's in principal means a life

long process respectfully accompanied by the GP. By this approach AD's facilitate the discussion about taboos and serve as a valuable instrument for decision making, both for the patient and the GP. There are many reasons to address AD's in our daily clinical practice and to document it routinely in our medical charts as systematic as "allergies" or "vaccinations". From a recent study we learn that the most simple version of AD's is preferred by patients with 77 % of pre-existing and 81 % of newly written AD's consisting of one page only. Longer and more complex versions may produce fears.

PMID: 22334201 [PubMed - indexed for MEDLINE]

135. Int Arch Allergy Immunol. 2012;158(2):168-74. doi: 10.1159/000330820. Epub 2012 Jan 25.

Prevalence of atopy and allergic diseases in Korean children: associations with a farming environment and rural lifestyle.

Lee SY, Kwon JW, Seo JH, Song YH, Kim BJ, Yu J, Park KS, Kim H, Kim EJ, Lee JS, Hong SJ.

Department of Pediatrics, Childhood Asthma Atopy Center, Asan Medical Center, University of Ulsan College of Medicine, School of Public Health, Seoul, Korea.

BACKGROUND: The results of recent studies suggest that factors in rural environments may protect against the development of allergic diseases, but the underlying mechanisms are not well understood. The aim of this study was to investigate the prevalence of allergic diseases, to establish if this prevalence is influenced by migration from rural to urban areas and to identify environmental risk factors associated with these diseases.
METHODS: A cross-sectional study of children aged 9-12 years from a rural village, a rural town and an urban city in Korea was conducted. Demographic and disease-related information was obtained via a detailed questionnaire, and skin prick tests were performed.
RESULTS: There were significant differences in lifestyle and environmental factors between children from the rural village, the rural town and the urban children. The prevalence of allergic diseases and atopy was higher in urban children. A lower prevalence of allergic diseases and atopy was associated with farming parents, contact with farm animals during pregnancy, owning pets or a stable, breast-feeding and having older siblings. A comparison of rural village and rural town children revealed no evidence of an association of allergic diseases and atopy with farming parents, contact with farm animals during pregnancy or owning a stable. On the other hand, having older siblings and antibiotic use during infancy were significantly associated with allergic diseases and atopy in these children.
CONCLUSIONS: Protective factors associated with a farming environment and/or rural lifestyle may influence the prevalence of allergic diseases and atopy in Korean children.

Copyright © 2012 S. Karger AG, Basel.

PMID: 22286539 [PubMed - indexed for MEDLINE]

136. Am J Respir Crit Care Med. 2012 Apr 1;185(7):715-22. doi: 10.1164/rccm.201105-0915OC. Epub 2012 Jan 26.

Role of YKL-40 in bronchial smooth muscle remodeling in asthma.

Bara I, Ozier A, Girodet PO, Carvalho G, Cattiaux J, Begueret H, Thumerel M,

Ousova O, Kolbeck R, Coyle AJ, Woods J, Tunon de Lara JM, Marthan R, Berger P.

Université Bordeaux, Centre de Recherche Cardio-Thoracique de Bordeaux, Bordeaux, France.

Comment in
Am J Respir Crit Care Med. 2012 Apr 1;185(7):692-4.
Am J Respir Crit Care Med. 2012 Aug 15;186(4):386; author reply 386-7.

RATIONALE: Bronchial remodeling, including increased bronchial smooth muscle (BSM) mass, contributes to bronchial obstruction in asthma. However, its mechanisms are complex and remain controversial. Recently, a role of the chitinase 3-like 1 protein (YKL-40) has been evoked in asthma. Indeed, YKL-40 concentration was increased in asthmatic serum, and correlated with asthma severity and subepithelial membrane thickness. Nevertheless, the role of YKL-40 on BSM cells remains to be investigated.
OBJECTIVES: To evaluate whether YKL-40 altered the physiologic properties of BSM cells in asthma in vitro and ex vivo.
METHODS: We enrolled 40 subjects with asthma, 13 nonsmokers, and 16 smokers. BSM cells were derived from bronchial specimens obtained by either fiberoptic bronchoscopy or lobectomy. We assessed cell proliferation using BrdU, flow cytometry, and cell count; signaling intermediates using Western blot; cell migration using inserts, wound healing, and phalloidin staining; and cell synthesis using ELISA and Western blot. The involvement of protease activated receptor (PAR)-2 was evaluated using blocking antibody and dedicated lentiviral small hairpin RNA. We also determined the BSM area and the YKL-40 staining ex vivo using immunohistochemistry on biopsies from subjects with asthma and control subjects.
MEASUREMENTS AND MAIN RESULTS: We demonstrated that YKL-40 increased BSM cell proliferation and migration through PAR-2-, AKT-, ERK-, and p38-dependent mechanisms. The increased cell migration was higher in BSM cells of subjects with asthma than that of control subjects. Furthermore, YKL-40 epithelial expression was positively correlated with BSM mass in asthma.
CONCLUSIONS: This study indicates that YKL-40 promotes BSM cell proliferation and migration through a PAR-2-dependent mechanism.

PMID: 22281830 [PubMed - indexed for MEDLINE]

137. Am J Respir Cell Mol Biol. 2012 Jun;46(6):781-9. doi: 10.1165/rcmb.2011-0152OC. Epub 2012 Jan 20.

Placental growth factor contributes to bronchial neutrophilic inflammation and edema in allergic asthma.

Bobic S, Seys S, De Vooght V, Callebaut I, Hox V, Dooms C, Vinckier S, Jonckx B, Saint-Remy JM, Stassen JM, Bullens DM, Ceuppens JL, Carmeliet P, Hellings PW.

Department of Otorhinolaryngology and Head and Neck Surgery, Catholic University Hospitals, Kapucijnevoer 33, Leuven, Belgium.

Placental growth factor (PlGF) and its receptor vascular endothelial growth factor receptor 1 (VEGFR1) play an important role in pathological conditions related to angiogenesis, vascular leakage, and inflammation. This study investigated their contributions to inflammation and the formation of edema in allergic asthma. The expression of PlGF and VEGFR1 was measured in induced sputum of patients with asthma (n = 11) and healthy subjects (n = 11), and in bronchial biopsies of house dust mite (HDM)-allergic patients stimulated with HDM allergens. The effects of the endonasal administration of human PlGF-2 and PlGF deficiency on inflammation and edema were evaluated in a murine model of allergic

asthma. The migration of human neutrophils in response to hPlGF-2 was tested in vitro. The expression of PlGF and VEGFR1 was significantly higher in the sputum of patients with asthma, and in Der p 1-induced PlGF in biopsies from HDM-allergic patients. PlGF was increased in the bronchi of ovalbumin (OVA)-challenged mice compared with control mice (65 ± 17 pg/mg versus 18 ± 1 pg/mg, respectively; $P < 0.01$), and VEGFR1 was expressed in bronchial epithelium, endothelium (control mice), and inflammatory cells (OVA-challenged mice). The endonasal instillation of hPlGF-2 in wild-type, OVA-challenged mice led to an increase in bronchial neutrophils, lung tissue wet/dry ratio, and IL-17. PlGF-deficient mice showed lower numbers of BAL-infiltrating neutrophils, a reduced lung wet/dry ratio, and lower production of IL-17, macrophage inflammatory protein-2, and granulocyte chemotactic protein-2/LPS-induced chemokine compared with wild-type, OVA-challenged mice. hPlGF-2 induced the migration of human neutrophils in vitro in a VEGFR1-dependent way. PlGF and its receptor VEGFR1 are up-regulated in allergic asthma and play a proinflammatory role by inducing tissue edema, and increasing tissue neutrophilia and the production of IL-17.

PMID: 22268141 [PubMed - indexed for MEDLINE]

138. Braz J Med Biol Res. 2012 Mar;45(3):264-72. Epub 2012 Jan 26.

Increased immunohistochemical expression of YKL-40 in the spleen of patients with portal hypertension.

Wang D, Lu JG, Wang Q, Du XL, Dong R, Wang P, Zhao L, Jiang X, Yuan LJ.

Department of General Surgery, The Second Affiliated Hospital, Fourth Military Medical University, 1 Xin Xi Road, Xi'an, China.

YKL-40 has been identified as a growth factor in connective tissue cells and also a migration factor in vascular smooth muscle cells. To a large extent, the increase of serum YKL-40 is attributed to liver fibrosis and asthma. However, the relationship of the expression and clinical/prognostic significance of YKL-40 to the splenomegaly of patients with portal hypertension is unclear. In the present study, the expression of YKL-40 was studied by immunohistochemistry in 48 splenomegaly tissue samples from patients with portal hypertension and in 14 normal spleen specimens. All specimens were quickly stored at -80°C after resection. Primary antibodies YKL-40 (1:150 dilution, rabbit polyclonal IgG) and MMP-9 (1:200 dilution, rabbit monoclonal IgG) and antirabbit immunoglobulins (HRP K4010) were used in this study. The relationship of clinicopathologic features with YKL-40 is presented. The expression of YKL-40 indicated by increased immunochemical reactivity was significantly up-regulated in splenomegaly tissues compared to normal spleen tissues. Overexpression of YKL-40 was found in 68.8% of splenomegaly tissues and was significantly associated with Child-Pugh classification ($P = 0.000$), free portal pressure (correlation coefficient = 0.499, $P < 0.01$) and spleen fibrosis (correlation coefficient = 0.857, $P < 0.01$). Further study showed a significant correlation between YKL-40 and MMP-9 (correlation coefficient = -0.839, $P < 0.01$), indicating that YKL-40 might be an accelerator of spleen tissue remodeling by inhibiting the expression of MMP-9. In conclusion, YKL-40 is an important factor involved in the remodeling of spleen tissue of portal hypertension patients and can be used as a therapeutic target for splenomegaly.

PMID: 22267006 [PubMed - in process]

139. J Immunol. 2012 Feb 15;188(4):1799-808. doi: 10.4049/jimmunol.1102481. Epub 2012 Jan 18.

Airway activation of formyl peptide receptors inhibits Th1 and Th17 cell responses via inhibition of mediator release from immune and inflammatory cells and maturation of dendritic cells.

Tae YM, Park HT, Moon HG, Kim YS, Jeon SG, Roh TY, Bae YS, Gho YS, Ryu SH, Kwon HS, Kim YK.

Department of Life Science, Pohang University of Science and Technology Biotech Center, Pohang 790-784, Republic of Korea.

Formyl peptide receptors (FPRs) are chemoattractant receptors that mediate inflammatory cell responses to infection. Recent evidence indicates that noneosinophilic asthma phenotypes can be developed by both Th1 and Th17 cell responses when exposed to LPS-containing allergens. In this study, we evaluated the effects of airway activation of FPRs by their synthetic agonist, Trp-Lys-Tyr-Met-Val-D-Met (W-peptide), on the development of Th1 and Th17 cell responses in a noneosinophilic asthma mouse model. A noneosinophilic asthma mouse model was generated by intranasal sensitization with 10 μg of LPS plus 75 μg of OVA on days 0, 1, 2, and 7. Mice were then challenged with 50 μg of OVA alone on days 14, 15, 21, and 22. W-peptide was administered during the sensitization period, and immune and inflammatory responses were evaluated after OVA challenge. Lung inflammation after OVA challenge was partly abolished by airway activation of FPRs during sensitization. Maturation of dendritic cells (DCs) and migration of DCs from the lung to lung-draining lymph nodes were inhibited by FPR activation. In addition, airway activation of FPRs inhibited allergen-specific T cell proliferation in the lymph nodes. Production of IL-12 and IL-6 (Th1- and Th17-polarizing cytokines) from lung DCs was decreased by airway activation of FPRs. This effect resulted in the inhibition of allergen-specific Th1 and Th17 cell responses. Airway activation of FPRs during sensitization effectively prevents the development of Th1 and Th17 cell responses induced by LPS-containing allergens via multiple mechanisms, such as inhibition of DC maturation and migration and the production of Th1- and Th7-polarizing cytokines.

PMID: 22262660 [PubMed - indexed for MEDLINE]

140. Int J Dermatol. 2012 Feb;51(2):173-7. doi: 10.1111/j.1365-4632.2011.04948.x.

Skin diseases in Greek and immigrant children in Athens.

Katsarou A, Armenaka M, Kosmadaki M, Lagogianni E, Vosynioti V, Tagka A, Stefanaki C, Katsambas A.

Department of Dermatology, University of Athens Medical School, Andreas Sygros Hospital, Athens, Greece. alkats.duoa@yahoo.gr

OBJECTIVES: This study aimed to characterize the spectrum of skin diseases affecting children in Greece.
METHODS: We retrospectively studied data for 4071 children, aged 0-12 years, who were examined and diagnosed with dermatoses at the outpatient clinic of a university dermatological hospital between December 2005 and August 2007. To evaluate changes in disease patterns, these data were compared with data for a cohort of 12,700 children diagnosed with skin diseases at the same clinic two to three decades earlier (in 1977, 1980, and 1983).
RESULTS: The most frequent disease was dermatitis/eczema (34.7%), with atopic dermatitis found in 20.7% of children, contact dermatitis in 6.9%, pityriasis alba in 2.1%, and seborrheic dermatitis in 1.8%. Infections (19.3%), nevi (5.6%), scabies (4.8%), and insect bites (4.3%) followed. More viral (12%) than bacterial (3.7%) and fungal (3.6%) infections were noted. Warts constituted 53.2% of viral

infections. Immigrants had an increased risk for bacterial infections and scabies.
CONCLUSIONS: Children diagnosed with skin diseases 24-30 years earlier were younger; exhibited lower prevalences of dermatitis/eczema ($P = 0.01$), viral infections ($P < 0.001$) and nevi ($P < 0.001$); higher prevalences of bacterial and fungal infections ($P < 0.001$) and insect bites ($P < 0.01$); and similar rates of scabies ($P = 0.17$). This study documents the high prevalence of atopic dermatitis in the region, the increasing incidence of viral infections and nevi, and the continuing problem of scabies, especially in immigrants.

© 2012 The International Society of Dermatology.

PMID: 22250626 [PubMed - indexed for MEDLINE]

141. FASEB J. 2012 Apr;26(4):1704-16. doi: 10.1096/fj.11-197061. Epub 2012 Jan 12.

Histamine may induce airway remodeling through release of epidermal growth factor receptor ligands from bronchial epithelial cells.

Hirota N, Risse PA, Novali M, McGovern T, Al-Alwan L, McCuaig S, Proud D, Hayden P, Hamid Q, Martin JG.

Meakins-Christie Laboratories, Department of Medicine, McGill University, 3626 St-Urbain, Montréal, QC, H2X 2P2 Canada.

Asthma is a chronic inflammatory disease that is associated with airway remodeling, including hyperplasia of airway epithelial cells and airway smooth muscle cells, and goblet cell differentiation. We wished to address the potential role of histamine, a key biogenic amine involved in allergic reactions, in airway remodeling through the epidermal growth factor receptor (EGFR) pathway. Here, we demonstrate that histamine releases 2 EGFR ligands, amphiregulin and heparin-binding epidermal growth factor-like growth factor (HB-EGF), from airway epithelial cells. Amphiregulin and HB-EGF were expressed in airway epithelium of patients with asthma. Histamine up-regulated their mRNA expression (amphiregulin 3.2-fold, $P<0.001$; HB-EGF 2.3-fold, $P<0.05$) and triggered their release (amphiregulin EC(50) 0.50 μM, 31.2 ± 2.7 pg/ml with 10 μM histamine, $P<0.01$; HB-EGF EC(50) 0.54 μM, 78.5 ± 1.8 pg/ml with 10 μM histamine, $P<0.001$) compared to vehicle control (amphiregulin 19.3 ± 0.9 pg/ml; HB-EGF 60.2 ± 1.0 pg/ml), in airway epithelial cells. Histamine increased EGFR phosphorylation (2.1-fold by Western blot analysis) and induced goblet cell differentiation (CLCA1 up-regulation by real-time qPCR) in normal human bronchial epithelial (NHBE) cells. Moreover, amphiregulin and HB-EGF caused proliferation and migration of both NHBE cells and human airway smooth muscle cells. These results suggest that histamine may induce airway remodeling via the epithelial-derived EGFR ligands amphiregulin and HB-EGF.

PMID: 22247333 [PubMed - indexed for MEDLINE]

142. Int J Immunopathol Pharmacol. 2011 Oct-Dec;24(4):949-60.

2B4 (CD244) is involved in eosinophil adhesion and chemotaxis, and its surface expression is increased in allergic rhinitis after challenge.

El-Shazly AE, Henket M, Lefebvre PP, Louis R.

Department of Oto-Rhino-Laryngology and Head and Neck Surgery, Liege University Hospitals, Liege, Belgium. amrel_shazly@hotmail.com

A role for the subtypes of CD2 Ig superfamily receptors has been recently demonstrated in eosinophilic inflammation in experimental asthma and atopic asthmatics. We investigated the functions of 2B4 (CD244) molecules in eosinophil adhesion and chemotaxis, and correlated the results to the pathophysiology of allergic rhinitis (AR). Herein, we show that agonistic stimulation of 2B4 by C1.7, the anti-human 2B4 functional grade purified antibody, resulted in significant increase of eosinophils and eosinophil cell line (Eol-1 cells) adhesion to collagen type IV, and random migration. These functions were associated with tyrosine kinase phosphorylation of several protein residues of low molecular weight. Flow cytometry (FACS) experiments demonstrated that Eol-1 cells, normal peripheral blood eosinophils and eosinophils from AR patients, express surface 2B4 molecules. In vitro AR model demonstrated that the CC-chemokine receptor CCR3 stimulation by eotaxin induced significant increase in the expression of surface 2B4 in eosinophils and Eol-1 cells. Immunofluorescence confocal microscopy images showed that eotaxin induces also redistribution of 2B4 molecules towards the pseudopods in eosinophils and Eol-1 cells, changing their shape. Blocking of 2B4 molecules by the corresponding neutralizing antibody inhibited eotaxin induced Eol-1-adhesion, chemotaxis and the cytoskeleton changes. Pretreatment of Eol-1 cells with 1 microM genistein blocked eotaxin-induced Eol-1 adhesion, chemotaxis and 2B4 up-regulated expression. In vivo correlation demonstrated the expression of 2B4 molecules in eosinophils from AR patients to be significantly increased, after nasal provocation challenge. These results identify a novel role for 2B4 molecules in eosinophil functional migratory response and may point to a novel tyrosine kinase-mediated ligation between CCR3 receptor and 2B4 co-receptor in eosinophil chemotaxis. If so, then 2B4 molecules would be a novel target for therapeutic modalities in diseases characterized by eosinophilia such as AR.

PMID: 22230401 [PubMed - indexed for MEDLINE]

143. Mol Ther. 2012 Jun;20(6):1270-9. doi: 10.1038/mt.2011.282. Epub 2012 Jan 3.

Foamy virus vector-mediated gene correction of a mouse model of Wiskott-Aldrich syndrome.

Uchiyama T, Adriani M, Jagadeesh GJ, Paine A, Candotti F.

Disorders of Immunity Section, Genetics and Molecular Biology Branch, National Human Genome Research Institute, National Institutes of Health, Bethesda, Maryland 20892-4442, USA.

Comment in
Mol Ther. 2012 Jun;20(6):1105-7.

The Wiskott-Aldrich syndrome (WAS) is an X-linked disorder characterized by eczema, thrombocytopenia and immunodeficiency. Hematopoietic cell transplantation can cure the disease and gene therapy is being tested as an alternative treatment option. In this study, we assessed the use of foamy virus (FV) vectors as a gene transfer system for WAS, using a Was knockout (KO) mouse model. Preliminary experiments using FV vectors expressing the green fluorescent protein under the transcriptional control of the endogenous WAS promoter or a ubiquitously acting chromatin opening element allowed us to define transduction conditions resulting in high (>40%) and long-term in-vivo marking of blood cells after transplantation. In following experiments, Was KO mice were treated with FV vectors containing the human WAS complementary DNA (cDNA). Transplanted animals expressed the WAS protein (WASp) in T and B lymphocytes, as well as platelets and showed restoration of both T-cell receptor-mediated responses and B-cell migration. We also observed recovery of platelet adhesion and podosome formation in dendritic cells (DCs) of treated mice. These data demonstrate that FV vectors

can be effective for hematopoietic stem cell (HSC)-directed gene correction of WAS.

PMCID: PMC3369282 [Available on 2013/6/1]
PMID: 22215016 [PubMed - indexed for MEDLINE]

144. Endocr Metab Immune Disord Drug Targets. 2012 Mar;12(1):33-44.

Toxocara infection and its association with allergic manifestations.

Pinelli E, Aranzamendi C.

Diagnostic Laboratory for Infectious Diseases and Perinatal Screening, Centre for Infectious Disease Control Netherlands, National Institute for Public Health and the Environment, Bilthoven, The Netherlands. elena.pinelli.ortiz@rivm.nl

Toxocara canis and Toxocara cati are roundworms of dogs and cats that can also infect humans worldwide. Although these parasites do not reach the adult stage in the human host the larvae migrate to different organs and can persist for many years. Migration of larvae through the lungs may result in respiratory distress such as wheezing, coughs, mucous production and hyper-reactivity of the airways. Epidemiological and experimental studies suggest that infection with this helminth contributes to the development of allergic manifestations, including asthma. These findings are however conflicting since in others studies no association between these two immunopathologies has been found. This article reviews information on Toxocara spp. and findings from epidemiological and experimental studies on the association between Toxocara infection and allergic manifestations. In addition, the immunological mechanisms and the factors involved in the helminth allergy-association are discussed.

PMID: 22214330 [PubMed - indexed for MEDLINE]

145. J Immunol. 2012 Feb 1;188(3):1479-90. doi: 10.4049/jimmunol.1102253. Epub 2011 Dec 30.

Regulation of eosinophil trafficking by SWAP-70 and its role in allergic airway inflammation.

Bahaie NS, Hosseinkhani MR, Ge XN, Kang BN, Ha SG, Blumenthal MS, Jessberger R, Rao SP, Sriramarao P.

Laboratory of Allergic Diseases and Inflammation, Department of Veterinary and Biomedical Sciences, University of Minnesota, St. Paul, MN 55108, USA.

Eosinophils are the predominant inflammatory cells recruited to allergic airways. In this article, we show that human and murine eosinophils express SWAP-70, an intracellular RAC-binding signaling protein, and examine its role in mediating eosinophil trafficking and pulmonary recruitment in a murine model of allergic airway inflammation. Compared with wild-type eosinophils, SWAP-70-deficient (Swap-70(-/-)) eosinophils revealed altered adhesive interactions within inflamed postcapillary venules under conditions of blood flow by intravital microscopy, exhibiting enhanced slow rolling but decreased firm adhesion. In static adhesion assays, Swap-70(-/-) eosinophils adhered poorly to VCAM-1 and ICAM-1 and exhibited inefficient leading edge and uropod formation. Adherent Swap-70(-/-) eosinophils failed to translocate RAC1 to leading edges and displayed aberrant cell surface localization/distribution of α4 and Mac-1. Chemokine-induced migration of Swap-70(-/-) eosinophils was significantly decreased, correlating with reduced intracellular calcium levels, defective actin

polymerization/depolymerization, and altered cytoskeletal rearrangement. In vivo, recruitment of eosinophils to the lungs of allergen-challenged Swap-70(-/-) mice, compared with wild-type mice, was significantly reduced, along with considerable attenuation of airway inflammation, indicated by diminished IL-5, IL-13, and TNF-α levels; reduced mucus secretion; and improved airway function. These findings suggest that regulation of eosinophil trafficking and migration by SWAP-70 is important for the development of eosinophilic inflammation after allergen exposure.

PMCID: PMC3267576 [Available on 2013/2/1]
PMID: 22210919 [PubMed - indexed for MEDLINE]

146. Am J Respir Cell Mol Biol. 2012 Jan;46(1):48-54. doi: 10.1165/rcmb.2011-0217OC.

β2-adrenergic receptor agonists modulate human airway smooth muscle cell migration via vasodilator-stimulated phosphoprotein.

Goncharova EA, Goncharov DA, Zhao H, Penn RB, Krymskaya VP, Panettieri RA Jr.

Airways Biology Initiative, Division of Pulmonary, Allergy, and Critical Care, Department of Medicine, University of Pennsylvania, Philadelphia, 19104, USA.

Severe asthma manifests as airway remodeling and irreversible airway obstruction, in part because of the proliferation and migration of human airway smooth muscle (HASM) cells. We previously reported that cyclic adenosine monophosphate-mobilizing agents, including β(2)-adrenergic receptor (β(2)AR) agonists, which are mainstay of asthma therapy, and prostaglandin E2 (PGE2), inhibit the migration of HASM cells, although the mechanism for this migration remains unknown. Vasodilator-stimulated phosphoprotein (VASP), an anticapping protein, modulates the formation of actin stress fibers during cell motility, and is negatively regulated by protein kinase A (PKA)-specific inhibitory phosphorylation at serine 157 (Ser157). Here, we show that treatment with β(2)AR agonists and PGE2 induces the PKA-dependent phosphorylation of VASP and inhibits the migration of HASM cells. The stable expression of PKA inhibitory peptide and the small interfering (si) RNA-induced depletion of VASP abolish the inhibitory effects of albuterol and PGE2 on the migration of HASM cells. Importantly, prolonged treatment with albuterol prevents the agonist-induced phosphorylation of VASP at Ser157, and reverses the inhibitory effects of albuterol and formoterol, but not PGE2, on the basal and PDGF-induced migration of HASM cells. Collectively, our data demonstrate that β(2)AR agonists selectively inhibit the migration of HASM cells via a β(2)AR/PKA/VASP signaling pathway, and that prolonged treatment with albuterol abolishes the inhibitory effect of β-agonists on the phosphorylation of VASP and migration of HASM cells because of β(2)AR desensitization.

PMCID: PMC3262659
PMID: 22210825 [PubMed - indexed for MEDLINE]

147. Biochem Pharmacol. 2012 Mar 15;83(6):778-87. doi: 10.1016/j.bcp.2011.12.021. Epub 2011 Dec 24.

Orally bioavailable allosteric CCR8 antagonists inhibit dendritic cell, T cell and eosinophil migration.

Connolly S, Skrinjar M, Rosendahl A.

AstraZeneca R&D Charnwood, Loughborough LE11 5RH, UK.

The chemokine receptor CCR8 is associated with asthma. Herein, we describe that both mature and immature dendritic cells (DC) express CCR8, whereas only mature DC migrate towards CCL1. Moreover, transient LPS challenge significantly down-regulates CCR8 expression hence attenuating CCL1 chemotaxis. To inhibit CCR8 pathophysiology, we recently developed a novel series of small molecule CCR8 antagonists containing a diazaspiroundecane scaffold, which had micromolar potency. However, these first generation antagonists had high lipophilicity that endowed the compounds with poor physicochemical properties, and were thus not suitable for further development. By introducing polar bicyclic groups on the N-benzyl substituent and building in further polar interactions on the amide group we now show second generation diazospiroundecane antagonists with significantly improved overall properties. Potency is substantially improved from micromolar to nanomolar potency in CCR8 binding and inhibition of chemotaxis in human primary T cells, DC and in an eosinophil cell line. In addition to high potency, the most attractive antagonist, AZ084 showed excellent selectivity, high metabolic stability in vitro and an attractive in vivo PK profile with a long half-life in rat. Interestingly, in ligand saturation experiments and in wash-off experiments, CCL1 was shown to have two binding sites to CCR8 with K(d) at 1.2/68pM respectively, and on-off rates of 0.004 and 0.0035/0.02pMmin, respectively. The lead antagonist, AZ084, appears to act as an allosteric inhibitor with a K(i) at 0.9nM. Taken together, we herein report a novel oral allosteric CCR8 antagonist with predicted low once-daily dosing capable of potent inhibition of both human T cell and DC functions.

Copyright © 2012 Elsevier Inc. All rights reserved.

PMID: 22209712 [PubMed - indexed for MEDLINE]

148. Mol Vis. 2011;17:3208-23. Epub 2011 Dec 14.

Eosinophil as a cellular target of the ocular anti-allergic action of mapracorat, a novel selective glucocorticoid receptor agonist.

Baiula M, Spartà A, Bedini A, Carbonari G, Bucolo C, Ward KW, Zhang JZ, Govoni P, Spampinato S.

Department of Pharmacology, University of Bologna, Bologna, Italy.

PURPOSE: Glucocorticoids can either suppress gene transcription (transrepression) or activate it (transactivation). This latter process may contribute to certain side effects caused by these agents. Mapracorat (also known as BOL-303242-X or ZK 245186) is a novel selective glucocorticoid receptor agonist that maintains a beneficial anti-inflammatory activity but seems to be less effective in transactivation, resulting in a lower potential for side effects; it has been proposed for the topical treatment of inflammatory skin disorders. This study assessed the anti-allergic activity of mapracorat at the ocular level and whether eosinophils and mast cells are targets of its action.
METHODS: With in vitro studies apoptosis was evaluated in human eosinophils by flow cytometry and western blot of caspase-3 fragments. Eosinophil migration toward platelet-activating factor was evaluated by transwell assays. Interleukin (IL)-6, IL-8, tumor necrosis factor-α (TNF-α), and the chemokine (C-C motif) ligand 5 (CCL5)/regulated upon activation normal T cell expressed, and presumably secreted (RANTES) were measured using a high-throughput multiplex luminex technology. Annexin I and the chemochine receptor C-X-C chemokine receptor 4 (CXCR4) were detected by flow cytometry. With in vivo studies, allergic conjunctivitis was induced in guinea pigs sensitized to ovalbumin by an ocular allergen challenge and evaluated by a clinical score. Conjunctival eosinophils were determined by microscopy or eosinophil peroxidase assay.
RESULTS: In cultured human eosinophils, mapracorat showed the same potency as

dexamethasone but displayed higher efficacy in increasing spontaneous apoptosis and in counteracting cytokine-sustained eosinophil survival. These effects were prevented by the glucocorticoid receptor antagonist mifepristone. Mapracorat inhibited eosinophil migration and IL-8 release from eosinophils or the release of IL-6, IL-8, CCL5/RANTES, and TNF-α from a human mast cell line with equal potency as dexamethasone, whereas it was clearly less potent than this glucocorticoid in inducing annexin I and CXCR4 expression on the human eosinophil surface; this was taken as a possible sign of glucocorticoid-dependent transactivation. In the guinea pig, mapracorat or dexamethasone eye drops induced an analogous reduction in clinical symptoms of allergic conjunctivitis and conjunctival eosinophil accumulation.
CONCLUSIONS: Mapracorat appears to be a promising candidate for the topical treatment of allergic eye disorders. It maintains an anti-allergic profile similar to that of dexamethasone but seems to have fewer transactivation effects in comparison to this classical glucocorticoid. Some of its cellular targets may contribute to eosinophil apoptosis and/or to preventing their recruitment and activation and to inhibiting the release of cytokines and chemokines.

PMCID: PMC3244483
PMID: 22194647 [PubMed - indexed for MEDLINE]

149. Cell Immunol. 2012;273(1):23-9. doi: 10.1016/j.cellimm.2011.11.010. Epub 2011 Dec 8.

PPAR-γ agonists, mainly 15d-PGJ(2), reduce eosinophil recruitment following allergen challenge.

Farnesi-de-Assunção TS, Alves CF, Carregaro V, de Oliveira JR, da Silva CA, Cheraim AB, Cunha FQ, Napimoga MH.

Laboratory of Immunology, Federal University of Triangulo Mineiro, Uberaba, MG, Brazil.

We evaluate the immunomodulation of Peroxisome proliferator-activated receptor-γ (PPAR-γ) agonists 15d-PGJ(2) and rosiglitazone (RGZ) in a model of chronic eosinophilia. 15d-PGJ(2) and RGZ significantly reduce eosinophil migration into the peritoneal cavity and down-regulate the eosinopoiesis. The synthesis of IL-5 was decreased after the treatment with 15d-PGJ(2) and RGZ corroborating with the eosinophil migration inhibition. However, IgE was decreased only after the administration of 15d-PGJ(2) in part due to B-cell inhibition. We also observed a decrease in the synthesis of IL-33, IL-17 and IL-23, suggesting that besides the modulation of Th2 pattern, there is a modulation via IL-23 and IL-17 suggesting a role of these cytokines in the eosinophil recruitment. In fact IL-17(-/-) mice failed to develop an eosinophilic response. Altogether, the results showed that PPAR-γ agonists mainly 15d-PGJ(2), have therapeutic efficacy in eosinophil-induced diseases with an alternative mechanism of control, via IL-23/IL-17 and IL-33.

Copyright © 2011 Elsevier Inc. All rights reserved.

PMID: 22192475 [PubMed - indexed for MEDLINE]

150. J Allergy (Cairo). 2012;2012:858306. doi: 10.1155/2012/858306. Epub 2011 Dec 1.

Demographic predictors of peanut, tree nut, fish, shellfish, and sesame allergy in Canada.

Ben-Shoshan M, Harrington DW, Soller L, Fragapane J, Joseph L, Pierre YS,

Godefroy SB, Elliott SJ, Clarke AE.

Division of Pediatric Allergy and Clinical Immunology, Department of Pediatrics, Montreal Children's Hospital, 2300 Tupper Street, Montreal, QC, Canada H3H 1P3.

Background. Studies suggest that the rising prevalence of food allergy during recent decades may have stabilized. Although genetics undoubtedly contribute to the emergence of food allergy, it is likely that other factors play a crucial role in mediating such short-term changes. Objective. To identify potential demographic predictors of food allergies. Methods. We performed a cross-Canada, random telephone survey. Criteria for food allergy were self-report of convincing symptoms and/or physician diagnosis of allergy. Multivariate logistic regressions were used to assess potential determinants. Results. Of 10,596 households surveyed in 2008/2009, 3666 responded, representing 9667 individuals. Peanut, tree nut, and sesame allergy were more common in children (odds ratio (OR) 2.24 (95% CI, 1.40, 3.59), 1.73 (95% CI, 1.11, 2.68), and 5.63 (95% CI, 1.39, 22.87), resp.) while fish and shellfish allergy were less common in children (OR 0.17 (95% CI, 0.04, 0.72) and 0.29 (95% CI, 0.14, 0.61)). Tree nut and shellfish allergy were less common in males (OR 0.55 (95% CI, 0.36, 0.83) and 0.63 (95% CI, 0.43, 0.91)). Shellfish allergy was more common in urban settings (OR 1.55 (95% CI, 1.04, 2.31)). There was a trend for most food allergies to be more prevalent in the more educated (tree nut OR 1.90 (95% CI, 1.18, 3.04)) and less prevalent in immigrants (shellfish OR 0.49 (95% CI, 0.26, 0.95)), but wide CIs preclude definitive conclusions for most foods. Conclusions. Our results reveal that in addition to age and sex, place of residence, socioeconomic status, and birth place may influence the development of food allergy.

PMCID: PMC3236463
PMID: 22187574 [PubMed]

151. Clin Dev Immunol. 2012;2012:721817. doi: 10.1155/2012/721817. Epub 2011 Nov 15.

Regulatory T cells accumulate in the lung allergic inflammation and efficiently suppress T-cell proliferation but not Th2 cytokine production.

Faustino L, Mucida D, Keller AC, Demengeot J, Bortoluci K, Sardinha LR, Carla Takenaka M, Basso AS, Faria AM, Russo M.

Departamento de Imunologia, Instituto de Ciências Biomédicas, Universidade de São Paulo, Brazil.

Foxp3(+)CD25(+)CD4(+) regulatory T cells are vital for peripheral tolerance and control of tissue inflammation. In this study, we characterized the phenotype and monitored the migration and activity of regulatory T cells present in the airways of allergic or tolerant mice after allergen challenge. To induce lung allergic inflammation, mice were sensitized twice with ovalbumin/aluminum hydroxide gel and challenged twice with intranasal ovalbumin. Tolerance was induced by oral administration of ovalbumin for 5 consecutive days prior to OVA sensitization and challenge. We detected regulatory T cells (Foxp3(+)CD25(+)CD4(+) T cells) in the airways of allergic and tolerant mice; however, the number of regulatory T cells was more than 40-fold higher in allergic mice than in tolerant mice. Lung regulatory T cells expressed an effector/memory phenotype (CCR4(high)CD62L(low)CD44(high)CD54(high)CD69(+)) that distinguished them from naive regulatory T cells (CCR4(int)CD62L(high)CD44(int)CD54(int)CD69(-)). These regulatory T cells efficiently suppressed pulmonary T-cell proliferation but not Th2 cytokine production.

PMCID: PMC3227414
PMID: 22162718 [PubMed - indexed for MEDLINE]

152. Ann Neurol. 2011 Nov;70(5):751-63. doi: 10.1002/ana.22519.

Role of Ninjurin-1 in the migration of myeloid cells to central nervous system inflammatory lesions.

Ifergan I, Kebir H, Terouz S, Alvarez JI, Lécuyer MA, Gendron S, Bourbonnière L, Dunay IR, Bouthillier A, Moumdjian R, Fontana A, Haqqani A, Klopstein A, Prinz M, López-Vales R, Birchler T, Prat A.

Neuroimmunology Research Unit, Centre de Recherche du Centre Hospitalier de l'Université de Montréal (CRCHUM), Faculté de Médecine, Université de Montréal, Montreal, Quebec, Canada.

OBJECTIVE: Blood-derived myeloid antigen-presenting cells (APCs) account for a significant proportion of the leukocytes found within lesions of multiple sclerosis (MS) and experimental allergic encephalomyelitis (EAE). These APCs along with activated microglia are thought to be pivotal in the initiation of the central nervous system (CNS)-targeted immune response in MS and EAE. However, the exact molecules that direct the migration of myeloid cells from the periphery across the blood-brain barrier (BBB) remain largely unknown.
METHODS: We identified Ninjurin-1 in a proteomic screen of human BBB endothelial cells (ECs). We assessed the expression of Ninjurin-1 by BBB-ECs and immune cells, and we determined the role of Ninjurin-1 in immune cell migration to the CNS in vivo in EAE mice.
RESULTS: Ninjurin-1 was found to be weakly expressed in the healthy human and mouse CNS but upregulated on BBB-ECs and on infiltrating APCs during the course of EAE and in active MS lesions. In human peripheral blood, Ninjurin-1 was predominantly expressed by monocytes, whereas it was barely detectable on T and B lymphocytes. Moreover, Ninjurin-1 neutralization specifically abrogated the adhesion and migration of human monocytes across BBB-ECs, without affecting lymphocyte recruitment. Finally, Ninjurin-1 blockade reduced clinical disease activity and histopathological indices of EAE and decreased infiltration of macrophages, dendritic cells, and APCs into the CNS.
INTERPRETATION: Our study uncovers an important cell-specific role for Ninjurin-1 in the transmigration of inflammatory APCs across the BBB and further emphasizes the importance of myeloid cell recruitment during the development of neuroinflammatory lesions.

Copyright © 2011 American Neurological Association.

PMID: 22162058 [PubMed - indexed for MEDLINE]

153. J Immunol. 2012 Jan 15;188(2):874-84. doi: 10.4049/jimmunol.1004108. Epub 2011 Dec 9.

PTP1B deficiency exacerbates inflammation and accelerates leukocyte trafficking in vivo.

Berdnikovs S, Pavlov VI, Abdala-Valencia H, McCary CA, Klumpp DJ, Tremblay ML, Cook-Mills JM.

Allergy-Immunology Division, Feinberg School of Medicine, Northwestern University, Chicago, IL 60611, USA.

It is reported that PTP1B limits cytokine signaling in vitro. However, PTP1B's function during inflammation in vivo is not known. In this report, we determined whether PTP1B deficiency affects allergic inflammation in vivo. Briefly, lungs of

OVA-challenged PTP1B(-/-) mice had elevated numbers of eosinophils and eosinophil progenitors at 6 h after one OVA challenge and at 24 h after a third OVA challenge as compared with OVA-challenged wild-type mice. There was also an increase in numbers of CD11b(+)SiglecF(+)CD34(+)IL-5Rα(+) eosinophil progenitors in the bone marrow, peripheral blood, and spleens of OVA-challenged PTP1B(-/-) mice. Intravital microscopy revealed that, in OVA-challenged PTP1B(-/-) mice, blood leukocytes rapidly bound to endothelium (5-30 min), whereas, in wild-type mice, blood leukocytes bound to endothelium at the expected 6-18 h. Consistent with early recruitment of leukocytes, lung eotaxin and Th2 cytokine levels were elevated early in the PTP1B(-/-) mice. Interestingly, spleen leukocytes from PTP1B(-/-) mice exhibited an increased chemotaxis, chemokinesis, and transendothelial migration in vitro. In summary, PTP1B functions as a critical negative regulator to limit allergic responses.

PMCID: PMC3253258
PMID: 22156494 [PubMed - indexed for MEDLINE]

154. J Asthma. 2012 Feb;49(1):10-5. doi: 10.3109/02770903.2011.637593. Epub 2011 Dec 7.

Macrophage migration inhibitory factor isolated from a parasite inhibited Th2 cytokine production in PBMCs of atopic asthma patients.

Park HK, Cho MK, Park HY, Kim KU, Kim YS, Lee MK, Park SK, Kim DH, Yu HS.

Department of Internal Medicine, School of Medicine, Pusan National University, Busan, Republic of Korea. hsyu@pusan.ac.kr

BACKGROUND: In a previous study, we demonstrated that the human macrophage migration inhibitory factor (MIF)-like protein (As-MIF) isolated from helminths could inhibit allergic airway inflammation via the recruitment of CD4(+)CD25(+)Foxp3(+) T cells.
OBJECTIVE: To evaluate the clinical importance of As-MIF as an antiasthma drug, we evaluated immune responses after recombinant As-MIF (rAs-MIF) treatment in peripheral blood mononuclear cell (PBMC) cultures.
METHODS: PBMC was isolated from 10 patients with atopic asthma, 8 patients with nonatopic asthma, and 12 nonatopic healthy subjects, and various concentrations of rAs-MIF were transferred into the PBMC culture medium. After 3 days, we measured the levels of T helper 2 and T helper 1 cytokines via ELISA.
RESULTS: In atopic asthma, IL-4 and IL-5 production was significantly reduced in the PBMC cultures after rAs-MIF treatment. These inhibitory effects were not observed in the nonatopic asthma group. By way of contrast, IL-10 production in the PMBC cultures was significantly increased after rAs-MIF treatment in all experimental groups.
CONCLUSION: The results of this study are similar to those previously reported in a mouse study, suggesting that As-MIF might be a candidate for the specific treatment of asthma.

PMID: 22149098 [PubMed - indexed for MEDLINE]

155. Int J Parasitol. 2012 Jan;42(1):63-70. doi: 10.1016/j.ijpara.2011.10.006. Epub 2011 Nov 22.

Molecular properties of a venom allergen-like protein suggest a parasitic function in the pinewood nematode Bursaphelenchus xylophilus.

Kang JS, Koh YH, Moon YS, Lee SH.

Research Institute for Agriculture and Life Science, Seoul National University, 151-921 Seoul, Republic of Korea.

The pinewood nematode, Bursaphelenchus xylophilus, is a destructive pest in several countries including Japan, China and Korea. Of three genes encoding the venom allergen-like protein in B. xylophilus, Bxvap-1 showed the highest transcript levels at the pine-grown propagative stage. In addition, western blot and immunohistochemical analyses using anti-BxVap-1 polyclonal antibody verified a specific increase in BxVap-1 expression levels at the pine-grown propagative stage. Using immunohistochemistry, BxVap-1 was detected around the putative oesophageal glands and metacarpus, suggesting that BxVap-1 is secreted into the host pine tree and is involved in the parasitic mechanism. To explain the parasitic role of BxVap-1, we measured the migration rate inside pine seedlings of B. xylophilus either with or without Bxvap-1 knockdown by RNA interference. Bxvap-1 knockdown resulted in a significantly lower migration rate in the >6cm region compared with the control B. xylophilus. These results suggest that BxVap-1 is involved in B. xylophilus migration, perhaps by suppressing the pine tree defence mechanism.

Copyright © 2011 Australian Society for Parasitology Inc. Published by Elsevier Ltd. All rights reserved.

PMID: 22142561 [PubMed - indexed for MEDLINE]

156. Curr Opin Gastroenterol. 2012 Mar;28(2):99-103. doi: 10.1097/MOG.0b013e32834e7b60.

Food allergy: a glimpse into the inner workings of gut immunology.

Kim JS, Sampson HA.

Department of Pediatrics, Mount Sinai School of Medicine, New York, New York, USA.

PURPOSE OF REVIEW: How food protein becomes recognized as an allergen remains a fundamental question. Previous studies indicated that the pathophysiology of food allergy is because of a skewed Th2 response to specific food glycoproteins. The focus has now shifted to understanding how a failure of regulatory mechanisms results in food allergy. This review summarizes the recent findings elucidating the small intestine's role in the pathophysiology of food allergy and the immune mechanisms of oral tolerance.
RECENT FINDINGS: Gut homeostasis and immunity occur via a complex interplay of innate and adaptive immune responses. Immune exclusion is performed mainly by secretory IgA, although there are back-up mechanisms in place to induce oral tolerance when secretory IgA is lacking. Oral tolerance cannot occur in murine models lacking T regulatory cells, for which Foxp3+ is a key marker. Migration of Foxp3+ T cells from the mesenteric lymph nodes (MLNs) to the lamina propria occurs via gut-homing signals. Also in the MLNs are CD103+ dendritic cells, which drive the differentiation of Foxp3+ T cells in the presence of TGF-β and retinoic acid produced from dietary vitamin A. Lastly, microenvironmental signals from the microbiome can serve to enhance these interactions.
SUMMARY: We have focused primarily on local immunologic variables that may affect the induction of oral tolerance in the gut and the mechanisms elucidated in animal models. However, many other variables such as genetics, commensal microbiota, and diet are likely to be important factors.

PMID: 22134223 [PubMed - indexed for MEDLINE]

157. Allergy Asthma Clin Immunol. 2011 Nov 29;7:19. doi: 10.1186/1710-1492-7-19.

VIP Regulates the Development & Proliferation of Treg in vivo in spleen.

Szema AM, Hamidi SA, Golightly MG, Rueb TP, Chen JJ.

Stony Brook University School of Medicine, Department of Medicine, 100 Nicolls Road, Stony Brook, NY 11794, USA. anthony.szema@sbumed.org.

BACKGROUND: Mounting evidence supports a key role for VIP as an anti-inflammatory agent and promoter of immune tolerance. It suppresses TNF-α and other inflammatory cytokines and chemokines, upregulates anti-inflammatory IL-10, and promotes immune tolerant cells called T regulatory (Treg) cells. VIP KO mice have recently been demonstrated to have spontaneous airway and pulmonary perivascular inflammatory responses, as part of asthma-like and pulmonary hypertension phenotypes, respectively. Both inflammatory responses are correctable with VIP. Focusing on this model, we have now investigated the influence of VIP not only on inflammatory cells but also on Treg cells.
METHODS: Using flow cytometric analysis, we examined the relative preponderance of CD25+CD4+ cells and anti-inflammatory Treg cells, in extracts of thymus and spleen from VIP KO mice (5 VIP KO; 5 VIP KO+ VIP; 10 wild-type). This method allowed antibody-based flow cytometric identification of Treg cells using surface markers CD25 and CD4, along with the: 1) intracellular activation marker FoxP3; and 2) Helios, which distinguishes cells of thymic versus splenic derivation.
CONCLUSIONS: Deletion of the VIP gene results in: 1) CD25+CD4- cell accumulation in the thymus, which is corrected by VIP treatment; 2) more Treg in thymus lacking Foxp3 expression, suggesting VIP is necessary for immune tolerance; and, 3) a tendency towards deficiency of Treg cells in the spleen, which is normalized by VIP treatment. Treg lacking Helios are induced by VIP intrasplenically rather than by migration from the thymus. These results confirm the dual role of VIP as an anti-inflammatory and immune tolerance-promoting agent.

PMCID: PMC3286388
PMID: 22126441 [PubMed]

158. Exp Biol Med (Maywood). 2011 Dec 1;236(12):1461-7. doi: 10.1258/ebm.2011.011221. Epub 2011 Nov 23.

Suppression of allergic airway inflammation in a mouse model of asthma by exogenous mesenchymal stem cells.

Ou-Yang HF, Huang Y, Hu XB, Wu CG.

Department of Respiratory Medicine, Xijing Hospital, Fourth Military Medical University, Xi’an 710032, China.

Mesenchymal stem cells (MSCs) have significant immunomodulatory effects in the development of acute lung inflammation and fibrosis. However, it is still unclear as to whether MSCs could attenuate allergic airway inflammation in a mouse model of asthma. We firstly investigated whether exogenous MSCs can relocate to lung tissues in asthmatic mice and analyzed the chemotactic mechanism. Then, we evaluated the in vivo immunomodulatory effect of exogenous MSCs in asthma. MSCs (2 × 10(6)) were administered through the tail vein to mice one day before the first airway challenge. Migration of MSCs was evaluated by flow cytometry. The immunomodulatory effect of MSCs was evaluated by cell counting in bronchoalveolar lavage fluid (BALF), histology, mast cell degranulation, airway hyperreactivity and cytokine profile in BALF. Exogenous MSCs can migrate to sites of inflammation in asthmatic mice through a stromal cell-derived factor-1α/CXCR4-dependent mechanism. MSCs can protect mice against a range of allergic airway inflammatory

pathologies, including the infiltration of inflammatory cells, mast cell degranulation and airway hyperreactivity partly via shifting to a T-helper 1 (Th1) from a Th2 immune response to allergens. So, immunotherapy based on MSCs may be a feasible, efficient therapy for asthma.

PMID: 22114062 [PubMed - indexed for MEDLINE]

159. J Invest Dermatol. 2012 Mar;132(3 Pt 1):626-34. doi: 10.1038/jid.2011.371. Epub 2011 Nov 24.

CXCL16 and CXCR6 are upregulated in psoriasis and mediate cutaneous recruitment of human CD8+ T cells.

Günther C, Carballido-Perrig N, Kaesler S, Carballido JM, Biedermann T.

Department of Dermatology, University Hospital, Technical University Dresden, Dresden, Germany. claudia.guenther@uniklinikum-dresden.de

Psoriatic skin lesions are characterized by an inflammatory infiltrate, consisting of dendritic cells, monocytes, and both CD4(+) and CD8(+) T lymphocytes. Although the chemokines involved in the migration of CD4(+) T cells into psoriatic skin are well characterized, those regulating CD8(+) T-cell recruitment are less understood. We found that the percentages of peripheral blood CD8(+) T cells expressing CXCR6 were higher in psoriatic patients than in healthy or atopic individuals. In addition, CXCR6 expression in psoriatic patients was more abundant in the CD8(+) than in the CD4(+) T-cell compartment. CXCR6 mRNA expression was also stronger in skin CD8(+) T cells than in the corresponding blood-derived counterparts. Immunofluorescence analysis revealed profound upregulation of the CXCR6 ligand CXCL16 by monocytes, keratinocytes, and dendritic cells in psoriatic skin compared with healthy or atopic dermatitis skin. In line with this, CXCR6(+) CD8(+) T cells also were most prevalent in psoriatic skin. Furthermore, CXCL16 induced Ca(2+) influx and chemotactic migration of psoriatic skin-derived CD8(+) T cells in vitro. Most importantly, CXCL16 potently recruited human CD8(+) T cells to human skin grafts previously transplanted onto SCID mice in vivo. These investigations indicate that CXCL16-CXCR6 interactions mediate homing of CD8(+) T cells into human skin, and thereby contribute to psoriasis pathogenesis.

PMID: 22113484 [PubMed - indexed for MEDLINE]

160. Inflamm Res. 2012 Mar;61(3):217-24. doi: 10.1007/s00011-011-0403-9. Epub 2011 Nov 24.

Anti-inflammatory effects of kaempferol-3-O-sophoroside in human endothelial cells.

Kim TH, Ku SK, Lee IC, Bae JS.

Department of Herbal Medicinal Pharmacology, College of Herbal Bio-Industry, Daegu Haany University, Gyeongsan 712-715, Republic of Korea.

BACKGROUND: Kaempferol-3-O-sophoroside (KPOS) was isolated from the leaves of cultivated mountain ginseng. Kaempferol (KP) has antitumor, anti-oxidative, anti-allergic and antidiabetic activities but the barrier protective effects and underlying mechanism are not fully identified. In this study, we attempted to determine whether pretreatment with KPOS induced significant barrier protective activities in lipopolysaccharide (LPS)-stimulated human umbilical vein endothelial cells (HUVECs).

METHODS: The anti-inflammatory activities of KPOS were determined by measuring solute flux, neutrophil adhesion and migration and activation of pro-inflammatory proteins in LPS-activated HUVECs.
RESULTS: We found that KPOS inhibited LPS-induced barrier disruption, expression of cell adhesion molecules, neutrophil adhesion and transendothelial migration of neutrophils to HUVECs. Further studies revealed that KPOS suppressed the production of tumor necrosis factor-α (TNF-α) and activation of nuclear factor-κB (NF-κB) by LPS, and that anti-inflammatory activities of KPOS were better than those of KP.
CONCLUSION: Collectively, these results suggest that KPOS possesses barrier integrity activity, inhibitory activity on cell adhesion and migration to endothelial cells by blocking the activation of NF-κB expression and production of TNF-α, thereby endorsing its usefulness as therapy for vascular inflammatory diseases.

PMID: 22113342 [PubMed - indexed for MEDLINE]

161. Clin Exp Allergy. 2012 Jan;42(1):131-43. doi: 10.1111/j.1365-2222.2011.03845.x. Epub 2011 Sep 1.

Recombinant DNA immunotherapy ameliorate established airway allergy in a IL-10 dependent pathway.

Fonseca DM, Wowk PF, Paula MO, Campos LW, Gembre AF, Turato WM, Ramos SG, Dias-Baruffi M, Barboza R, Gomes E, Silva CL, Russo M, Bonato VL.

Department of Biochemistry and Immunology, School of Medicine of Ribeirão Preto, University of São Paulo, Riberão Preto, Brazil.

Comment in
    Clin Exp Allergy. 2012 Jan;42(1):3-4.

BACKGROUND: Previous studies have established that mycobacterial infections ameliorate allergic inflammation. However, a non-infectious approach that controls allergic responses might represent a safer and more promising strategy. The 60-65 kDa heat shock protein (Hsp) family is endowed with anti-inflammatory properties, but it is still unclear whether and how single mycobacterial Hsp control allergic disorders.
OBJECTIVE: Therefore, in this study we determined whether the administration of Mycobacterial leprae Hsp65 expressed by recombinant a DNA plasmid could attenuate a previously established allergic response.
METHODS: We used an experimental model of airway allergic inflammation to test the effects of immunotherapy with DNA encoding Hsp65. Allergic mice, previously sensitized and challenged with ovalbumin, were treated with tree intramuscular doses of recombinant DNA encoding Hsp65. After treatment, mice received a second allergen challenge and the allergic response was measured.
RESULTS: We found that immunotherapy attenuated eosinophilia, pulmonary inflammation, Th2 cytokine and mucus production. Moreover, we showed that the inhibition of allergic response is dependent on IL-10 production. Both Hsp65 and allergen-specific IL-10-producing cells contributed to this effect. Cells transferred from DNA-immunized mice to allergic mice migrated to allergic sites and down-modulated the Th2 response.
CONCLUSIONS AND CLINICAL RELEVANCE: Our findings clearly show that immunotherapy with DNA encoding Hsp65 can attenuate an established Th2 allergic inflammation through an IL-10-dependent mechanism; moreover, the migration of allergen- and Hsp65-specific cells to the allergic sites exerts a fundamental role. This work represents a novel contribution to the understanding of immune regulation by Hsp65 in allergic diseases.

© 2011 Blackwell Publishing Ltd.

PMID: 22093133 [PubMed - indexed for MEDLINE]

162. Clin Exp Allergy. 2012 Feb;42(2):255-64. doi: 10.1111/j.1365-2222.2011.03889.x. Epub 2011 Oct 13.

Interleukin-4 and interleukin-13 prime migrational responses of haemopoietic progenitor cells to stromal cell-derived factor-1α.

Punia N, Smith S, Thomson JV, Irshad A, Nair P, Sehmi R.

Firestone Institute for Respiratory Health, Asthma Research Group, St Joseph's Healthcare, Hamilton, ON, Canada.

BACKGROUND: Lung-homing of progenitor cells is associated with inflammatory and remodelling changes in asthma. Factors that modulate the increased traffic of progenitor cells to the site of inflammation in asthma remain to be defined. Interleukin (IL)-4 and IL-13 are Th2 cytokines that are key regulators of asthma pathology.
OBJECTIVE: We investigated the role of IL-4 and IL-13 in modulating the trans-migrational responses of haemopoietic progenitor cells (HPC).
METHODS: HPC were enriched from cord blood (CB) and peripheral blood (PB) samples. Migration of HPC was assessed using transwell migration assays, and responding cells were enumerated by flow cytometry.
RESULTS: IL-4 and IL-13 primed migration of CB- and PB-derived HPC (CD34(+) 45(+) cells) to stromal cell-derived factor-1α (SDF-1α), in vitro. However, these cytokines had no effect on migrational responses of eosinophil-lineage committed progenitors (CD34(+) 45(+) IL-5Rα(+) cells) or mature eosinophils to SDF-1α. For HPC, priming effects of IL-4 (0.1 ng/mL) and IL-13 (0.1 ng/mL) were detectable within 1 h and optimal at 18-h post-incubation, and IL-4 was the more effective priming agent. Pre-incubation with IL-4 or IL-13 had no effect on the intensity of cell surface expression of SDF-1α receptor, CXCR4. Disruption of cell membrane cholesterol content by pre-incubation with polyene antibiotics inhibited IL-4 priming of SDF-1α stimulated migration of HPC indicating that increased incorporation of CXCR4 into membrane lipid rafts mediated the cytokine primed migrational response of HPC. This was confirmed by confocal fluorescent microscopy.
CONCLUSIONS AND CLINICAL RELEVANCE: IL-4 and IL-13 prime the migrational response of HPC to SDF-1α by enhancing the incorporation of CXCR4 into lipid rafts. The priming effect of these cytokines is specific to primitive HPC. These data suggest that increased local production of IL-4 and IL-13 within the lungs may promote increased SDF-1α mediated homing of HPC to the airways in asthma.

© 2011 Blackwell Publishing Ltd.

PMID: 22092872 [PubMed - indexed for MEDLINE]

163. Clin Exp Allergy. 2012 May;42(5):712-37. doi: 10.1111/j.1365-2222.2011.03854.x. Epub 2011 Sep 23.

Molecular and clinical rationale for therapeutic targeting of interleukin-5 and its receptor.

Molfino NA, Gossage D, Kolbeck R, Parker JM, Geba GP.

MedImmune, LLC, Gaithersburg, MD 20878, USA. molfinon@medimmune.com

Interleukin-5 is a Th2 homodimeric cytokine involved in the differentiation, maturation, migration, development, survival, trafficking and effector function of blood and local tissue eosinophils, in addition to basophils and mast cells. The IL-5 receptor (IL-5R) consists of an IL-5-specific α subunit that interacts in conformationally dynamic ways with the receptor's βc subunit, an aggregate of domains it shares with binding sites of IL-3 and granulocyte-macrophage colony-stimulating factor. IL-5 and IL-5R drive allergic and inflammatory immune responses characterizing numerous diseases, such as asthma, atopic dermatitis, chronic obstructive pulmonary disease, eosinophilic gastrointestinal diseases, hyper-eosinophilic syndrome, Churg-Strauss syndrome and eosinophilic nasal polyposis. Although corticosteroid therapy is the primary treatment for these diseases, a substantial number of patients exhibit incomplete responses and suffer side-effects. Two monoclonal antibodies have been designed to neutralize IL-5 (mepolizumab and reslizumab). Both antibodies have demonstrated the ability to reduce blood and tissue eosinophil counts. One additional monoclonal antibody, benralizumab (MEDI-563), has been developed to target IL-5R and attenuate eosinophilia through antibody-dependent cellular cytotoxicity. All three monoclonal antibodies are being clinically evaluated. Antisense oligonucleotide technology targeting the common βc IL-5R subunit is also being used therapeutically to inhibit IL-5-mediated effects (TPI ASM8). Small interfering RNA technology has also been used therapeutically to inhibit the expression of IL-5 in animal models. This review summarizes the structural interactions between IL-5 and IL-5R and the functional consequences of such interactions, and describes the pre-clinical and clinical evidence supporting IL-5R as a therapeutic target.

© 2011 MedImmune, LLC.

PMID: 22092535 [PubMed - indexed for MEDLINE]

164. J Allergy Clin Immunol. 2012 Jan;129(1):169-75.e1-5. doi:
10.1016/j.jaci.2011.10.002. Epub 2011 Nov 9.

Personal and parental nativity as risk factors for food sensitization.

Keet CA, Wood RA, Matsui EC.

Division of Allergy and Immunology, Department of Pediatrics, Johns Hopkins University School of Medicine, Baltimore, Md 21202, USA.

BACKGROUND: Immigrants to developed countries have low rates of aeroallergen sensitization and asthma, but less is known about both food allergy and the role of parental immigration status.
OBJECTIVE: We sought to evaluate the relationship between personal and parental nativity and the risk of food sensitization.
METHODS: Three thousand five hundred fifty subjects less than 21 years old from the Nation Health and Examination Survey 2005-2006 were included. Odds ratios (ORs) were generated by using logistic regression, which adjusted for race/ethnicity, sex, age, and household income and accounted for the complex survey design. Nativity was classified as US-born or foreign-born, and the age of immigration was estimated. Head-of-household nativity was used as a proxy for parental nativity. Food sensitization was defined as at least 1 specific IgE level of 0.35 kU/L or greater to milk, egg, or peanut. Aeroallergen-specific sensitizations and the presence of asthma, allergic rhinitis, or eczema were also assessed.
RESULTS: Compared with those born outside the United States (US), US-born children and adolescents had higher odds of sensitization to any food (OR, 2.05; 95% CI, 1.49-2.83; P < .001). Among the foreign-born group, those who arrived before 2 years of age had higher odds of food sensitization than those who

arrived later (OR, 2.68; 95% CI, 1.19-6.08; P = .02). Within the US-born group, in contrast, children of immigrants were at the highest risk (OR, 1.53; 95% CI, 1.05-2.24; P = .02).
CONCLUSION: Although foreign-born children and adolescents are at lower risk of food sensitization compared with those born in the US, among those born in the US, the children of immigrants are at the highest risk.

Copyright © 2011 American Academy of Allergy, Asthma & Immunology. Published by Mosby, Inc. All rights reserved.

PMCID: PMC3382052
PMID: 22075329 [PubMed - indexed for MEDLINE]

165. Biochem Biophys Res Commun. 2011 Dec 2;415(4):591-6. doi: 10.1016/j.bbrc.2011.10.111. Epub 2011 Nov 2.

Effects of micropatterned curvature on the motility and mechanical properties of airway smooth muscle cells.

Xu J, Chen C, Jiang X, Xu R, Tambe D, Zhang X, Liu L, Lan B, Cai K, Deng L.

Key Laboratory of Biorheological Science and Technology, Ministry of Education, and Bioengineering College, Chongqing University, 174 Shapingzhengjie Street, Chongqing 400044, China.

Geometric features such as size and shape of the microenvironment are known to alter cell behaviors such as growth, differentiation, apoptosis, and migration. Little is known, however, about the effect of curvature on cell behaviors despite that many cells reside in curved space of tubular organs such as the bronchial airways. To address this question, we fabricated micropatterned strips that mimic airway walls with varying curvature. Then, we cultured airway smooth muscle cells (ASMCs) on these strips and investigated the cells' motility and mechanical properties using time-lapse imaging microscopy and optical magnetic twisting cytometry (OMTC). We found that both motility and mechanical properties of the ASMCs were influenced by the curvature. In particular, when the curvature increased from 0 to 1/150 μm(-1), the velocity of cell migration first decreased (0-1/750 μm(-1)), and then increased (1/750-1/150 μm(-1)). In contrast, the cell stiffness increased and then decreased. Thus, at the intermediate curvature (1/750 μm(-1)) the ASMCs were the least motile, but most stiff. The contractility instead decreased consistently as the curvature increased. The level of F-actin, and vinculin expression within the ASMCs appeared to correlate with the contractility and motility, respectively, in relation to the curvature. These results may provide valuable insights to understanding the heterogeneity of airway constrictions in asthma as well as the developing and functioning of other tubular organs and tissue engineering.

Copyright © 2011 Elsevier Inc. All rights reserved.

PMID: 22074822 [PubMed - indexed for MEDLINE]

166. J Invest Dermatol. 2012 Mar;132(3 Pt 1):667-76. doi: 10.1038/jid.2011.349. Epub 2011 Nov 10.

Lymphatic dysfunction impairs antigen-specific immunization, but augments tissue swelling following contact with allergens.

Sugaya M, Kuwano Y, Suga H, Miyagaki T, Ohmatsu H, Kadono T, Okochi H, Blauvelt A, Tamaki K, Sato S.

Department of Dermatology, Faculty of Medicine, University of Tokyo, Tokyo, Japan. sugayam-der@h.u-tokyo.ac.jp

The lymph transports tissue-resident dendritic cells (DCs) to regional lymph nodes (LNs), having important roles in immune function. The biological effects on tissue inflammation following lymphatic flow obstruction in vivo, however, are not fully known. In this study, we investigated the role of the lymphatic system in contact hypersensitivity (CHS) responses using k-cyclin transgenic (kCYC(+/-)) mice, which demonstrate severe lymphatic dysfunction. kCYC(+/-) mice showed enhanced ear swelling to both DNFB and FITC, as well as stronger irritant responses to croton oil compared with wild-type littermates. Consistently, challenged ears of kCYC(+/-) mice exhibited massive infiltrates of inflammatory cells. In contrast, DC migration to regional LNs, drainage of cell-free antigen to LNs, antigen-specific IFN-γ production, and lymphocyte proliferation were impaired during the sensitization phase of CHS in kCYC(+/-) mice. Transfer experiments using lymphocytes from sensitized mice and real-time PCR analysis of cytokine expression using challenged ear revealed that ear swelling was enhanced because of impaired lymphatic flow. Collectively, we conclude that insufficient lymphatic drainage augments apparent inflammation to topically applied allergens and irritants. The findings add insight into the clinical problem of allergic and irritant contact dermatitis that commonly occurs in humans with peripheral edema of the lower legs.

PMID: 22071476 [PubMed - indexed for MEDLINE]

167. Heart Lung. 2012 May;41(3):226-37. doi: 10.1016/j.hrtlng.2011.09.014. Epub 2011 Nov 3.

Symptoms and functional performance in Korean immigrants with asthma or chronic obstructive pulmonary disease.

Park SK, Stotts NA, Douglas MK, Donesky-Cuenco D, Carrieri-Kohlman V.

School of Nursing, University of Michigan at Ann Arbor, 400 North Ingalls Street, Ann Arbor, MI 48109, USA. sookyung.park7@gmail.com

OBJECTIVES: People with chronic obstructive lung diseases often experience a variety of symptoms. Few studies, however, have described these symptoms in detail. This study sought to examine concurrent symptoms, symptom clusters, and the effects of symptoms on functioning in Korean immigrants with asthma or chronic obstructive pulmonary disease (COPD).
METHODS: Outpatients with asthma or COPD participated in this cross-sectional, correlational study. Symptoms, dyspnea, mood, and functional performance were assessed with questionnaires. Descriptive and inferential statistics were used to analyze the data.
RESULTS: The most frequently reported symptom was shortness of breath. Three factors emerged from 16 symptoms. Age, mean severity score of 7 symptoms, working status, level of acculturation, and level of education explained significant variance in functional performance.
CONCLUSION: The symptom cluster, consisting of 7 symptoms, showed the greatest effect on levels of functioning, which emphasizes the importance of assessment for coexisting symptoms in populations with these diseases.

Published by Mosby, Inc.

PMID: 22054719 [PubMed - indexed for MEDLINE]

168. Clin Cancer Res. 2011 Dec 15;17(24):7529-38. doi: 10.1158/1078-0432.CCR-11-1192. Epub 2011 Nov 2.

IL-22, but not IL-17, dominant environment in cutaneous T-cell lymphoma.

Miyagaki T, Sugaya M, Suga H, Kamata M, Ohmatsu H, Fujita H, Asano Y, Tada Y, Kadono T, Sato S.

Department of Dermatology, Faculty of Medicine, University of Tokyo, Tokyo, Japan.

PURPOSE: Both patients with cutaneous T-cell lymphoma (CTCL) and those with atopic dermatitis (AD) have pruritus, T(H)2-biased T cells, and a tendency to have bacterial infections, suggesting a common pathologic basis for these two diseases. Recently, interleukin (IL)-22-producing T cells were reported in skin of patients with AD. In this study, we investigated expression levels of T(H)22- and T(H)17-related molecules in lesional skin and sera isolated from patients with CTCL.
EXPERIMENTAL DESIGN: Skin biopsies and sera were collected from patients with CTCL or psoriasis and from healthy volunteers. Protein and mRNA expression levels of IL-22, IL-17A, IL-17F, IL-23p19, IL-10, IL-4, CCL20, CCR6, IL-8, and IL-20 were examined in lesional tissue and a subset of these molecules in sera. Phosphorylation of STAT3 was also assessed in lesional skin of CTCL and psoriasis by immunohistochemistry.
RESULTS: IL-22, IL-10, IL-4, CCL20, and CCR6 mRNA and protein levels, but not IL-17A, IL-17F, IL-23p19, IL-8, or IL-20, were significantly elevated in lesional skin of CTCL. Phosphorylation of STAT3 was detected in epidermis of CTCL skin. Moreover, serum IL-22, IL-10, and CCL20 levels were increased in CTCL and correlated with disease severity.
CONCLUSIONS: Our results suggest that IL-22 is important in establishing the tumor microenvironment for CTCL. Enhanced expression of CCL20 may explain epidermal hyperplasia and migration of CCR6(+) cells, such as Langerhans cells, into lesional skin. Relatively low expression of IL-17 may explain the lack of neutrophils in lesions of CTCL, which correlates with bacterial infections that commonly occur in skin affected by CTCL.

©2011 AACR.

PMID: 22048239 [PubMed - indexed for MEDLINE]

169. J Clin Med Res. 2010 Dec 11;2(6):247-55. doi: 10.4021/jocmr456w.

Parasites induced skin allergy: a strategic manipulation of the host immunity.

Bakiri AH, Mingomataj EC.

University of Tirana, Nursing Faculty, Dept. of Preclinical Disciplines, Albania.

The absence of a consistent link between parasitoses and skin allergic symptoms in the clinical investigations contrasts to the fact that some parasites are the most potent inducers of immunoglobulin E that exist in nature. To shed some light into this question, this review is focused on the actual knowledge regarding parasites life cycle, interactions with host immunity, the influence on host behavior, and finally the role of all these factors on the skin allergy. The collected data demonstrate that parasites could manipulate the host behavior for its own benefit in different ways, altering its (epi)genetic, biochemical, immunologic or physiologic functions as well as altering its behavior and activity. In this context, skin allergy may be associated with certain stages of the parasites' life cycle and migration into biological barriers, but not

necessarily with presence of the parasitosis in the host organism. As compared to T helper (Th) 1 response, the Th2 one, the eosinophilic infiltration and the complement inhibition could assure better conditions for the development of some parasites. Taken together, the suggested hypotheses could be a plausible explanation for the epidemiological puzzle regarding urticaria occurrence, Th2 response and parasitoses, but further studies are necessary to provide better-based conclusions. KEYWORDS: Eosinophilic Infiltration; Host behavior; Parasites life cycle; Skin allergy; Th1/Th2 response.

PMCID: PMC3194028
PMID: 22043257 [PubMed]

170. PLoS Negl Trop Dis. 2011 Oct;5(10):e1370. doi: 10.1371/journal.pntd.0001370. Epub 2011 Oct 25.

Proteomic analysis of excretory-secretory products of Heligmosomoides polygyrus assessed with next-generation sequencing transcriptomic information.

Moreno Y, Gros PP, Tam M, Segura M, Valanparambil R, Geary TG, Stevenson MM.

Institute of Parasitology and Centre for Host Parasite Interactions, McGill University, Ste-Anne de Bellevue, Quebec, Canada.

The murine parasite Heligmosomoides polygyrus is a convenient experimental model to study immune responses and pathology associated with gastrointestinal nematode infections. The excretory-secretory products (ESP) produced by this parasite have potent immunomodulatory activity, but the protein(s) responsible has not been defined. Identification of the protein composition of ESP derived from H. polygyrus and other relevant nematode species has been hampered by the lack of genomic sequence information required for proteomic analysis based on database searches. To overcome this, a transcriptome next generation sequencing (RNA-seq) de novo assembly containing 33,641 transcripts was generated, annotated, and used to interrogate mass spectrometry (MS) data derived from 1D-SDS PAGE and LC-MS/MS analysis of ESP. Using the database generated from the 6 open reading frames deduced from the RNA-seq assembly and conventional identification programs, 209 proteins were identified in ESP including homologues of vitellogenins, retinol- and fatty acid-binding proteins, globins, and the allergen V5/Tpx-1-related family of proteins. Several potential immunomodulators, such as macrophage migration inhibitory factor, cysteine protease inhibitors, galectins, C-type lectins, peroxiredoxin, and glutathione S-transferase, were also identified. Comparative analysis of protein annotations based on the RNA-seq assembly and proteomics revealed processes and proteins that may contribute to the functional specialization of ESP, including proteins involved in signalling pathways and in nutrient transport and/or uptake. Together, these findings provide important information that will help to illuminate molecular, biochemical, and in particular immunomodulatory aspects of host-H. polygyrus biology. In addition, the methods and analyses presented here are applicable to study biochemical and molecular aspects of the host-parasite relationship in species for which sequence information is not available.

PMCID: PMC3201918
PMID: 22039562 [PubMed - indexed for MEDLINE]

171. Br J Ophthalmol. 2012 Apr;96(4):485-9. doi: 10.1136/bjophthalmol-2011-300732. Epub 2011 Oct 27.

Indications and outcomes for revision of gold weight implants in upper eyelid loading.

Bladen JC, Norris JH, Malhotra R.

Corneo Plastic Unit, Queen Victoria Hospital NHS Trust, East Grinstead, UK.

Comment in
Br J Ophthalmol. 2012 Apr;96(4):471-2.

BACKGROUND: Gold weights are effective for upper eyelid loading in patients with lagophthalmos. Complications include poor cosmesis, migration, extrusion, allergy and astigmatism. The authors looked at indications for revision of primary gold weights inserted using a high pretarsal placement and outcomes following correction.
METHODS: A retrospective review of 107 consecutive primary gold weight implants in 95 patients with lagophthalmos in a single centre over a 5-year period. Implant placement utilised a combined high pretarsal placement, levator recession and fixation. Revision surgery included repositioning, removal or exchange. Blinded assessment of eyelid parameters, including cosmesis, was performed by an independent reviewer using photographs from each revision case taken preoperatively and 6 months postoperatively.
RESULTS: Mean follow-up 2.5 years (range 1-5) with 15/107 (14%) eyelids revised, the majority within 12 months of the primary procedure. Five eyelids required up to 3 further revisions, giving 21 revisions in total. Indications included prominent implants in 15/21 (71%) revisions; poor eyelid contour in 14/21 (67%, 9 drooped and 5 flattened eyelids); extrusion in 2/21 (10%); persistent erythema in 8/21 (29%, 5 gold allergies and 1 extrusion). Revisions consisted of platinum chain exchange (6), replacement (3), repositioning (8) and removal (4). Following final revision, eyelid contour returned to normal and five eyelids demonstrated mild prominence.
CONCLUSION: High pretarsal placement was successful in treating lagophthalmos, with a complication rate of 1 in 6 requiring a revision procedure, the majority within 12 months. Main indications were unsatisfactory cosmesis from prominence of implant and poor eyelid contour.

PMID: 22034542 [PubMed - indexed for MEDLINE]

172. PLoS One. 2011;6(10):e26133. doi: 10.1371/journal.pone.0026133. Epub 2011 Oct 12.

MiR-221 influences effector functions and actin cytoskeleton in mast cells.

Mayoral RJ, Deho L, Rusca N, Bartonicek N, Saini HK, Enright AJ, Monticelli S.

Institute for Research in Biomedicine, Bellinzona, Switzerland.

Mast cells have essential effector and immunoregulatory functions in IgE-associated allergic disorders and certain innate and adaptive immune responses, but the role of miRNAs in regulating mast cell functions is almost completely unexplored. To examine the role of the activation-induced miRNA miR-221 in mouse mast cells, we developed robust lentiviral systems for miRNA overexpression and depletion. While miR-221 favored mast cell adhesion and migration towards SCF or antigen in trans-well migration assays, as well as cytokine production and degranulation in response to IgE-antigen complexes, neither miR-221 overexpression, nor its ablation, interfered with mast cell differentiation. Transcriptional profiling of miR-221-overexpressing mast cells revealed modulation of many transcripts, including several associated with the cytoskeleton; indeed, miR-221 overexpression was associated with reproducible increases in cortical actin in mast cells, and with altered cellular shape and cell cycle in murine fibroblasts. Our bioinformatics analysis showed that this effect was likely mediated by the composite effect of miR-221 on many primary and

secondary targets in resting cells. Indeed, miR-221-induced cellular alterations could not be recapitulated by knockdown of one of the major targets of miR-221. We propose a model in which miR-221 has two different roles in mast cells: in resting cells, basal levels of miR-221 contribute to the regulation of the cell cycle and cytoskeleton, a general mechanism probably common to other miR-221-expressing cell types, such as fibroblasts. Vice versa, upon induction in response to mast cell stimulation, miR-221 effects are mast cell-specific and activation-dependent, contributing to the regulation of degranulation, cytokine production and cell adherence. Our studies provide new insights into the roles of miR-221 in mast cell biology, and identify novel mechanisms that may contribute to mast cell-related pathological conditions, such as asthma, allergy and mastocytosis.

PMCID: PMC3192147
PMID: 22022537 [PubMed - indexed for MEDLINE]

173. Immunol Lett. 2012 Jan 30;141(2):210-9. doi: 10.1016/j.imlet.2011.10.006. Epub 2011 Oct 14.

Sphingosine-kinase 1 and 2 contribute to oral sensitization and effector phase in a mouse model of food allergy.

Diesner SC, Olivera A, Dillahunt S, Schultz C, Watzlawek T, Förster-Waldl E, Pollak A, Jensen-Jarolim E, Untersmayr E, Rivera J.

Department of Pathophysiology and Allergy Research, Center of Pathophysiology, Infectiology and Immunology, Medical University of Vienna, Waehringer Guertel 18-20, 1090 Vienna, Austria.

Erratum in
    Immunol Lett. 2012 Aug 30;146(1-2):79.

BACKGROUND: Sphingosine-1-phosphate (S1P) influences activation, migration and death of immune cells. Further, S1P was proposed to play a major role in the induction and promotion of allergic diseases. However, to date only limited information is available on the role of S1P in food allergy.
OBJECTIVE: We aimed to investigate the role of sphingosine-kinase (SphK) 1 and 2, the enzymes responsible for endogenous S1P production, on the induction of food allergy.
METHODS AND RESULTS: Human epithelial colorectal CaCo2 cells stimulated in vitro with S1P revealed a decrease of transepithelial resistance and enhanced transport of FITC labeled OVA. We studied the effect of genetic deletion of the enzymes involved in S1P production on food allergy induction using a mouse model of food allergy based on intragastrically (i.g.) administered ovalbumin (OVA) with concomitant acid-suppression. Wild-type (WT), SphK1(-/-) and SphK2(-/-) mice immunized with OVA alone i.g. or intraperitoneally (i.p.) were used as negative or positive controls, respectively. SphK1- and SphK2-deficient mice fed with OVA under acid-suppression showed reduced induction of OVA specific IgE and IgG compared to WT mice, but had normal responses when immunized by the intraperitoneal route. Flow cytometric analysis of spleen cells revealed a significantly reduced proportion of CD4(+) effector T-cells in both SphK deficient animals after oral sensitization. This was accompanied by a reduced accumulation of mast cells in the gastric mucosa in SphK-deficient animals compared to WT mice. Furthermore, mouse mast cell protease-1 (mMCP-1) levels, an IgE-mediated anaphylaxis marker, were reliably elevated in allergic WT animals.
CONCLUSION: Modulation of the S1P homeostasis by deletion of either SphK1 or SphK2 alters the sensitization and effector phase of food allergy.

Copyright © 2011 Elsevier B.V. All rights reserved.

PMCID: PMC3243786 [Available on 2013/1/30]
PMID: 22020265 [PubMed - indexed for MEDLINE]

174. J Transl Med. 2011 Oct 21;9:181. doi: 10.1186/1479-5876-9-181.

Stem cell treatment for patients with autoimmune disease by systemic infusion of culture-expanded autologous adipose tissue derived mesenchymal stem cells.

Ra JC, Kang SK, Shin IS, Park HG, Joo SA, Kim JG, Kang BC, Lee YS, Nakama K, Piao M, Sohl B, Kurtz A.

Stem Cell Research Center, RNL BIO, Seoul, 153-768, Republic of Korea. jcra@rnl.co.kr

Prolonged life expectancy, life style and environmental changes have caused a changing disease pattern in developed countries towards an increase of degenerative and autoimmune diseases. Stem cells have become a promising tool for their treatment by promoting tissue repair and protection from immune-attack associated damage. Patient-derived autologous stem cells present a safe option for this treatment since these will not induce immune rejection and thus multiple treatments are possible without any risk for allogenic sensitization, which may arise from allogenic stem cell transplantations. Here we report the outcome of treatments with culture expanded human adipose-derived mesenchymal stem cells (hAdMSCs) of 10 patients with autoimmune associated tissue damage and exhausted therapeutic options, including autoimmune hearing loss, multiple sclerosis, polymyotitis, atopic dermatitis and rheumatoid arthritis. For treatment, we developed a standardized culture-expansion protocol for hAdMSCs from minimal amounts of fat tissue, providing sufficient number of cells for repetitive injections. High expansion efficiencies were routinely achieved from autoimmune patients and from elderly donors without measurable loss in safety profile, genetic stability, vitality and differentiation potency, migration and homing characteristics. Although the conclusions that can be drawn from the compassionate use treatments in terms of therapeutic efficacy are only preliminary, the data provide convincing evidence for safety and therapeutic properties of systemically administered AdMSC in human patients with no other treatment options. The authors believe that ex-vivo-expanded autologous AdMSCs provide a promising alternative for treating autoimmune diseases. Further clinical studies are needed that take into account the results obtained from case studies as those presented here.

PMCID: PMC3222617
PMID: 22017805 [PubMed - indexed for MEDLINE]

175. Z Orthop Unfall. 2012 Feb;150(1):40-7. doi: 10.1055/s-0031-1280249. Epub 2011 Oct 18.

[PVD-silicoating before cementation of zirconia-based knee prostheses effects better cement adhesion and lower aseptic loosening rates].

[Article in German]

Marx R, Faramarzi R, Oberbach T, Begand S, Grätz N, Wirtz DC.

CC&A Ltd., ZBMT Aachen, Pauwelsstraße 17, Aachen. marx@rwth-aachen.de

AIM: CoCrMo alloys are contraindicated for allergy patients. For these patients, cemented or uncemented prostheses made of titanium alloy are indicated.

Uncemented prostheses, however, have low primary retention, particularly the tibial components of knee joint prostheses because of the lack of a positive locking. Therefore, for knee replacement cemented CoCrMo prostheses may be suitable also for allergy sufferers if these are masked by ZrN or TiNbN layers. Alternatively the CoCrMo alloy may be replaced by high-strength oxide ceramics. For adhesion of bone cement to the ceramic surface, however, only inefficient mechanical retention spots are exposed as compared with a metal surface. Undercuts generated by corundum blasting, although highly efficient on a CoCrMo surface, are not such efficient centres on a ceramic surface due to its brittleness. Therefore, the mechanical component of retention is significantly reduced. When specific adhesion between bone cement and surface does not exist due to physical and chemical forces, the hydrolytic stability will be insufficient. Micromotions are promoted and early aseptic loosening is predictable. Silicoating of the ceramic surface will allow specific adhesion and can result in better hydrolytic stability of bonding.
METHODS: In order to evaluate the effectiveness of silicoating the bond strengths of blasted (mean size of corundum grains 50 µm) and silicate layered alumina-toughened zirconia (ATZ) surfaces were compared with "as fired" surfaces by utilising TiAlV probes (diameter 6 mm) for traction-adhesive strength testing. Samples machined out of CoCrMo alloy were utilised for reference. After preparing the samples for traction-adhesive strength testing (sequence: substrate, silicate and silane, protective lacquer [PolyMA], bone cement, TiAlV probe) they were aged up to 360 days at 37 °C in Ringer's solution.
RESULTS: The bond strengths observed for all ageing intervals were well above 20 MPa and much higher and more hydrolytically stable for blasted and silicate-layered compared with "as fired" ATZ samples.
CONCLUSION: Silicoating may be effective for achieving a high initial bond strength of bone cement on surfaces of oxide ceramics and also suitable to stabilise bond strength under hydrolytic conditions as present in the human body. Activation by low grain size corundum (mean grain size 50 µm) seems to be effective for activation without deteriorating the bending strength of the ceramics investigated. Due to the proposed layer system migration, micromotions and debonding should be widely reduced or even eliminated.

© Georg Thieme Verlag KG Stuttgart · New York.

PMID: 22009583 [PubMed - indexed for MEDLINE]

176. Toxicol In Vitro. 2011 Dec;25(8):2124-34. doi: 10.1016/j.tiv.2011.09.021. Epub 2011 Oct 4.

Inter-laboratory study of the in vitro dendritic cell migration assay for identification of contact allergens.

Rees B, Spiekstra SW, Carfi M, Ouwehand K, Williams CA, Corsini E, McLeod JD, Gibbs S.

Centre for Research in Biomedicine, Faulty of Health and Life Sciences, University of the West of England, Bristol, UK.

The aim of this study was to investigate the transferability of technology and reproducibility of MUTZ-3 derived Langerhans Cell (MUTZ-LC) migration assay. The protocol was transferred from the NL-lab to two Sens-it-iv project partners (UK-lab, Italy-lab). Intra- and inter-laboratory variation with regards to MUTZ-3 progenitor culture, differentiation to MUTZ-LC, maturation and migration assay were investigated. In the transwell-migration-assay, preferential migration of sensitizer-exposed MUTZ-LC towards CXCL12 was observed (three sensitizers), whereas non-sensitizer-exposed MUTZ-LC only migrated towards CCL5 (two non-sensitizers). Four pre-pro-haptens were also identified by UK-lab. When

taking the arbitrary criteria of at least two of three independent repetitions per laboratory having to have a CXCL12/CCL5 ratio>1.1 for classification as a sensitizer, all sensitizers tested in all labs were easily distinguished from all non-sensitizers. The number of repetitions giving false negative or false positive was very low (only 7 out of a total of 54 repetitions), indicating that both intra- and inter-laboratory variation was extremely low. Even though only a few chemicals were tested in this study, we show clearly that the in vitro DC migration assay is transferable between laboratories. The results were consistent between the laboratories, and the dose response data were reproduced in the three laboratories.

Copyright © 2011 Elsevier Ltd. All rights reserved.

PMID: 22001958 [PubMed - indexed for MEDLINE]

177. Cytokine. 2011 Dec;56(3):726-31. doi: 10.1016/j.cyto.2011.09.024. Epub 2011 Oct 12.

Study of gastric fluid induced cytokine and chemokine expression in airway smooth muscle cells and airway remodeling.

Chiu HY, Chen CW, Lin HT, Hsieh CC, Lin SS, Cheng CM.

Department of Pediatrics, Changhua Christian Hospital, Taiwan.

Asthma is a chronic airway inflammatory disease. Chronic aspiration by gastric fluid in gastroesophageal reflux disease (GERD) is considered a primary inflammatory factor exacerbating or predisposing patients to asthma. Airway smooth muscle cells (SMCs) are considered an important component in airway remodeling. To investigate the role of gastric fluid in airway SMC inflammation and airway remodeling, we examined gastric fluid-induced cytokine and chemokine profiles, airway SMC migration and matrix metalloproteinase expression in rat primary rat airway SMCs. The T helper cell type 2 (Th2) cytokines interleukin 4, interleukin 6 and tumor necrosis factor 2 (TNF-α) and the chemokines, lipopolysaccharide-induced CXC chemokine (LIX/CXCL5), cytokine-induced neutrophil chemoattractant 2 (CINC-2), CINC-3, fractalkine, ciliary neurotrophic factor (CNTF), and vascular endothelial growth factor were induced by gastric fluid in primary cultured rat airway SMCs. Migration of rat airway SMCs was enhanced by gastric fluid and conditioned medium. The migration of rat airway SMCs enhanced by gastric fluid was associated with actin polymerization and activation of focal adhesion kinase. Matrix metalloproteinase 2 expressions in airway SMCs was enhanced by gastric fluid and conditioned medium. The results suggest potential mechanisms by which gastric fluid aspiration might influence SMC-mediated airway remodeling.

Copyright © 2011 Elsevier Ltd. All rights reserved.

PMID: 21996013 [PubMed - indexed for MEDLINE]

178. Mol Cell Biochem. 2012 Feb;361(1-2):111-21. doi: 10.1007/s11010-011-1095-8. Epub 2011 Oct 11.

shRNA targeting β1-integrin suppressed proliferative aspects and migratory properties of airway smooth muscle cells.

Shi F, Qiu C, Qi H, Peng W.

Department of Respiratory, The Second Clinical College, Jinan University,

Shenzhen, China.

Dysfunction of airway smooth muscle (ASM) is an essential feature of airway remodeling in chronic asthma. However, the precise mechanisms of this pathological process have not been well studied. In previous study, we found that β1-integrin, which was dramatically upregulated in ASM cells in an asthmatic mouse model, was associated with the cell proliferation. In this study, we employed short hairpin RNA (shRNA) targeting β1-integrin to assess the effect of down-regulation of this receptor on the proliferative aspects and migratory properties of ASM cells in vitro. The cells were treated with shRNA expression vectors directed against β1-integrin, control vectors that included the blank control, empty vector without shRNA, and mismatched shRNA, respectively. The mRNA and protein expressions of β1-integrin were determined by real-time PCR and Western blotting. Cell proliferation was measured by BrdU ELISA and cell cycle by fluorescence-activated cell sorter. Cell apoptosis was detected by Annexin V-PE/7-AAD staining. Cell migration assays were evaluated by transwell assay and expression of IL-6 and IL-8 by ELISA. The results revealed that shRNA targeting β1-integrin significantly decreased the mRNA and protein expressions of β1-integrin, enhanced the proportion of cells in G0/G1 phase, decreased the proportion in S phase, promoted cell apoptosis, inhibited cell proliferation, migration, IL-6 and IL-8 secretion in vitro. In conclusion, the overexpression of β1-integrin in ASM cells is essential for airway dysfunction development because it promotes proliferative aspects and migratory properties of ASM cells. Importantly, shRNA targeting β1-integrin may provide a new approach to preventing airway remodeling in chronic asthma.

PMID: 21987234 [PubMed - indexed for MEDLINE]

179. Clin Exp Immunol. 2011 Nov;166(2):145-53. doi: 10.1111/j.1365-2249.2011.04464.x.

Up-regulation of CCL11 and CCL26 is associated with activated eosinophils in bullous pemphigoid.

Günther C, Wozel G, Meurer M, Pfeiffer C.

University Hospital for Dermatology, Technical University Dresden, Dresden, Germany. claudia.guenther@uniklinikum-dresden.de

Eosinophils contribute to the pathogenesis of bullous pemphigoid (BP) by secretion of proinflammatory cytokines and proteases. Trafficking of eosinophils into tissue in animal models and asthma depends on interleukin-5 and a family of chemokines named eotaxins, comprising CCL11, CCL24 and CCL26. Up-regulation of CCL11 has been described in BP, but the expression of the other two members of the eotaxin-family, CCL24 and CCL26, has not been investigated. In addition to these chemokines, expression of adhesion molecules associated with eosinophil migration to the skin should be analysed. We demonstrate that similar to CCL11, the concentration of CCL26 was up-regulated in serum and blister fluid of BP patients. In contrast, the concentration of CCL24 was not elevated in sera and blister fluid of the same BP patients. In lesional skin, CCL11 and CCL26 were detected in epidermis and dermis by immunohistochemistry. In contrast to CCL11, CCL26 was expressed strongly by endothelial cells. In line with these findings, eosinophils represented the dominating cell population in BP lesional skin outnumbering other leucocytes. The percentage of eosinophils expressing very late antigen (VLA): VLA-4 (CD49d) and CD11c correlated with their quantity in tissue. Macrophage antigen (MAC)-1 (CD11b/CD18) was expressed constitutively by tissue eosinophils. In conclusion, these data link the up-regulation of the eosinophil chemotactic factor CCL26 in BP to the lesional accumulation of activated eosinophils in the skin. Thereby they broaden the understanding of BP pathogenesis and might indicate new options for therapeutic intervention.

© 2011 The Authors. Clinical and Experimental Immunology © 2011 British Society for Immunology.

PMCID: PMC3219889
PMID: 21985360 [PubMed - indexed for MEDLINE]

180. Immunology. 2011 Nov;134(3):314-25. doi: 10.1111/j.1365-2567.2011.03492.x.

NOD-like receptors and RIG-I-like receptors in human eosinophils: activation by NOD1 and NOD2 agonists.

Kvarnhammar AM, Petterson T, Cardell LO.

Division of ENT Diseases, CLINTEC, Karolinska Institutet, Karolinska University Hospital, Stockholm, Sweden. anne.mansson@ki.se

NOD-like receptors (NLRs) and RIG-I-like receptors (RLRs) are newly discovered pattern-recognition receptors. They detect substructures of bacterial peptidoglycan and viral RNA, respectively, thereby initiating an immune response. However, their role in eosinophil activation remains to be explored. The aim of this study was to characterize the expression of a range of NLRs and RLRs in purified human eosinophils and assess their functional importance. Expression of NOD1, NOD2, NLRP3, RIG-I and MDA-5 was investigated using real-time reverse transcription PCR, flow cytometry and immunohistochemistry. The effects of the corresponding agonists iE-DAP (NOD1), MDP (NOD2), alum (NLRP3) and poly(I:C)/LyoVec (RIG-I/MDA-5) were studied in terms of cytokine secretion, degranulation, survival, expression of adhesion molecules and activation markers, and chemotactic migration. Eosinophils expressed NOD1 and NOD2 mRNA and protein. Low levels of RIG-I and MDA-5 were found, whereas expression of NLRP3 was completely absent. In accordance, stimulation with iE-DAP and MDP was found to induce secretion of interleukin-8, up-regulate expression of CD11b, conversely down-regulate CD62 ligand, increase expression of CD69 and induce migration. The MDP also promoted release of eosinophil-derived neurotoxin, whereas iE-DAP failed to do so. No effects were seen upon stimulation with alum or poly(I:C)/LyoVec. Moreover, the NOD1-induced and NOD2-induced activation was mediated via the nuclear factor-κB signalling pathway and augmented by interleukin-5 and granulocyte-macrophage colony-stimulating factor, but not interferon-γ. Taken together, the NLR system represents a novel pathway for eosinophil activation. The responses are enhanced in the presence of cytokines that regulate T helper type 2 immunity, suggesting that the NLRs constitute a link between respiratory infections and exacerbations of allergic disease.

© 2011 The Authors. Immunology © 2011 Blackwell Publishing Ltd.

PMCID: PMC3209571
PMID: 21978001 [PubMed - indexed for MEDLINE]

181. Health Aff (Millwood). 2011 Oct;30(10):1955-64. doi: 10.1377/hlthaff.2011.0635.

A regional health collaborative formed By NewYork-Presbyterian aims to improve the health of a largely Hispanic community.

Carrillo JE, Shekhani NS, Deland EL, Fleck EM, Mucaria J, Guimento R, Kaplan S, Polf WA, Carrillo VA, Pardes H, Corwin SJ.

NewYork-Presbyterian Hospital, New York City, NY, USA. ecarrill@nyp.org

Communities of poor, low-income immigrants with limited English proficiency and disproportionate health burdens pose unique challenges to health providers and policy makers. NewYork-Presbyterian Hospital developed the Regional Health Collaborative, a population-based health care model to improve the health of the residents of Washington Heights-Inwood. This area is a predominantly Hispanic community in New York City with high rates of asthma, diabetes, heart disease, and depression. NewYork-Presbyterian created an integrated network of patient-centered medical homes to form a "medical village" linked to other providers and community-based resources. The initiative set out to document the priority health needs of the community, target high-prevalence conditions, improve cultural competence among providers, and introduce integrated information systems across care sites. The first six months of the program demonstrated a significant 9.2 percent decline in emergency department visits for ambulatory care-sensitive conditions and a 5.8 percent decrease in hospitalizations that was not statistically significant. This initiative offers a model for other urban academic medical centers to better serve populations facing social and cultural barriers to care.

PMID: 21976340 [PubMed - indexed for MEDLINE]

182. Rehabilitation (Stuttg). 2011 Oct;50(5):284-91. doi: 10.1055/s-0031-1285889. Epub 2011 Oct 5.

[DGRW-update: patient education].

[Article in German]

Faller H, Reusch A, Meng K.

Institut für Psychotherapie und Medizinische Psychologie, Universität Würzburg, Klinikstraße 3, 97070 Würzburg.h.faller@uni-wuerzburg.de

Patient education programmes, i. e. standardized, manualized, interactive group programmes aiming to increase self-management and empowerment, are a core element of medical rehabilitation for chronic conditions. In an update of the evidence of the effectiveness of patient education, its effectiveness was proven for a broad spectrum of chronic disorders, such as diabetes mellitus, chronic low back pain, rheumatoid arthritis, coronary heart disease, chronic heart failure, bronchial asthma, COPD, and cancer, as well as for the modification of health behaviours, such as diet and exercise. To sustain effects, aftercare interventions, such as support provided by phone, were found to be successful. Interventions targeted to particular patient groups according to gender, age, or migration background are also being developed more frequently. When evaluating educational interventions not only distal outcomes, such as quality of life and participation, should be used but also proximal outcomes such as self-management skills. A recent survey of patient education practice in medical rehabilitation revealed a continuing potential for optimization relative to manualization, evaluation and didactics. However, the dissemination of innovative programmes into rehabilitation routine presents a major challenge.

© George Thieme Verlag KG Stuttgart · New York.

PMID: 21976261 [PubMed - indexed for MEDLINE]

183. J Ethnopharmacol. 2011 Nov 18;138(2):479-86. doi: 10.1016/j.jep.2011.09.037. Epub 2011 Sep 29.

Chemical composition, antinociceptive and anti-inflammatory effects in rodents of

the essential oil of Peperomia serpens (Sw.) Loud.

Pinheiro BG, Silva AS, Souza GE, Figueiredo JG, Cunha FQ, Lahlou S, da Silva JK, Maia JG, Sousa PJ.

Programa de Pós-Graduação em Ciências Farmacêuticas, Universidade Federal do Pará, 66075-900 Belém, Pará, Brazil.

ETHNOPHARMACOLOGICAL RELEVANCE: Peperomia serpens (Piperaceae), popularly known as "carrapatinho", is an epiphyte herbaceous liana grown wild on different host trees in the Amazon rainforest. Its leaves are largely used in Brazilian folk medicine to treat inflammation, pain and asthma.
AIM OF THE STUDY: This study investigated the effects of essential oil of Peperomia serpens (EOPs) in standard rodent models of pain and inflammation.
MATERIALS AND METHODS: The antinociceptive activity was evaluated using chemical (acetic acid and formalin) and thermal (hot plate) models of nociception in mice whereas the anti-inflammatory activity was evaluated by carrageenan- and dextran-induced paw edema tests in rats croton oil-induced ear edema, as well as cell migration, rolling and adhesion induced by carrageenan in mice. Additionally, phytochemical analysis of the EOPs has been also performed.
RESULTS: Chemical composition of the EOPs was analyzed by gas chromatography and mass spectrometry (GC/MS). Twenty-four compounds, representing 89.6% of total oil, were identified. (E)-Nerolidol (38.0%), ledol (27.1%), α-humulene (11.5%), (E)-caryophyllene (4.0%) and α-eudesmol (2.7%) were found to be the major constituents of the oil. Oral pretreatment with EOPs (62.5-500 mg/kg) significantly reduced the writhing number evoked by acetic acid injection, with an ED(50) value of 188.8 mg/kg that was used thereafter in all tests. EOPs had no significant effect on hot plate test but reduced the licking time in both phases of the formalin test, an effect that was not significantly altered by naloxone (0.4 mg/kg, s.c.). EOPs inhibited the edema formation induced by carrageenan and dextran in rats. In mice, EOPs inhibited the edema formation by croton oil as well as the leukocyte and neutrophil migration, the rolling and the adhesion of leukocytes.
CONCLUSIONS: These data show for the first time that EOPs has a significant and peripheral antinociceptive effect that seems unrelated to interaction with the opioid system. EOPs also displays a significant anti-inflammatory effect in acute inflammation models. This effect seems to be related to components which inhibit the production of several inflammatory mediators. These results support the widespread use of Peperomia serpens in popular medicine to treat inflammation and pain.

Copyright © 2011 Elsevier Ireland Ltd. All rights reserved.

PMID: 21971207 [PubMed - in process]

184. Thorax. 2012 Mar;67(3):229-37. doi: 10.1136/thoraxjnl-2011-200376. Epub 2011 Sep 28.

Aclidinium inhibits human lung fibroblast to myofibroblast transition.

Milara J, Serrano A, Peiró T, Gavaldà A, Miralpeix M, Morcillo EJ, Cortijo J.

Unidad de Investigación, Consorcio Hospital General, Universitario, Avenida Tres Vruces s/n, Valencia E-46014, Spain. xmilara@hotmail.com

BACKGROUND: Fibroblast to myofibroblast transition is believed to contribute to airway remodelling in lung diseases such as asthma and chronic obstructive pulmonary disease. This study examines the role of aclidinium, a new long-acting muscarinic antagonist, on human fibroblast to myofibroblast transition.

METHODS: Human bronchial fibroblasts were stimulated with carbachol (10(-8) to 10(-5) M) or transforming growth factor-β1 (TGF-β1; 2 ng/ml) in the presence or absence of aclidinium (10(-9) to 10(-7) M) or different drug modulators for 48 h. Characterisation of myofibroblasts was performed by analysis of collagen type I and α-smooth muscle actin (α-SMA) mRNA and protein expression as well as α-SMA microfilament immunofluorescence. ERK1/2 phosphorylation, RhoA-GTP and muscarinic receptors (M) 1, 2 and 3 protein expression were determined by western blot analysis and adenosine 3'-5' cyclic monophosphate levels were determined by ELISA. Proliferation and migration of fibroblasts were also assessed.
RESULTS: Collagen type I and α-SMA mRNA and protein expression, as well as percentage α-SMA microfilament-positive cells, were upregulated in a similar way by carbachol and TGF-β1, and aclidinium reversed these effects. Carbachol-induced myofibroblast transition was mediated by an increase in ERK1/2 phosphorylation, RhoA-GTP activation and cyclic monophosphate downregulation as well as by the autocrine TGF-β1 release, which were effectively reduced by aclidinium. TGF-β1 activated the non-neuronal cholinergic system. Suppression of M1, M2 or M3 partially prevented carbachol- and TGF-β1-induced myofibroblast transition. Aclidinium dose-dependently reduced fibroblast proliferation and migration.
CONCLUSION: Aclidinium inhibits human lung fibroblast to myofibrobast transition.

PMCID: PMC3282044
PMID: 21957094 [PubMed - indexed for MEDLINE]

185. Toxicol Appl Pharmacol. 2011 Dec 15;257(3):309-18. doi: 10.1016/j.taap.2011.09.001. Epub 2011 Sep 12.

Increased CCL24/eotaxin-2 with postnatal ozone exposure in allergen-sensitized infant monkeys is not associated with recruitment of eosinophils to airway mucosa.

Chou DL, Gerriets JE, Schelegle ES, Hyde DM, Miller LA.

California National Primate Research Center, UC Davis, Davis, CA 95616, USA.

Epidemiology supports a causal link between air pollutant exposure and childhood asthma, but the mechanisms are unknown. We have previously reported that ozone exposure can alter the anatomic distribution of CD25+ lymphocytes in airways of allergen-sensitized infant rhesus monkeys. Here, we hypothesized that ozone may also affect eosinophil trafficking to allergen-sensitized infant airways. To test this hypothesis, we measured blood, lavage, and airway mucosa eosinophils in 3-month old monkeys following cyclical ozone and house dust mite (HDM) aerosol exposures. We also determined if eotaxin family members (CCL11, CCL24, CCL26) are associated with eosinophil location in response to exposures. In lavage, eosinophil numbers increased in animals exposed to ozone and/or HDM. Ozone+HDM animals showed significantly increased CCL24 and CCL26 protein in lavage, but the concentration of CCL11, CCL24, and CCL26 was independent of eosinophil number for all exposure groups. In airway mucosa, eosinophils increased with exposure to HDM alone; comparatively, ozone and ozone+HDM resulted in reduced eosinophils. CCL26 mRNA and immunofluorescence staining increased in airway mucosa of HDM alone animals and correlated with eosinophil volume. In ozone+HDM animal groups, CCL24 mRNA and immunofluorescence increased along with CCR3 mRNA, but did not correlate with airway mucosa eosinophils. Cumulatively, our data indicate that ozone exposure results in a profile of airway eosinophil migration that is distinct from HDM mediated pathways. CCL24 was found to be induced only by combined ozone and HDM exposure, however expression was not associated with the presence of eosinophils within the airway mucosa.

Copyright © 2011 Elsevier Inc. All rights reserved.

PMCID: PMC3226879
PMID: 21945493 [PubMed - indexed for MEDLINE]

186. Actas Dermosifiliogr. 2012 Apr;103(3):214-22. doi: 10.1016/j.ad.2011.07.008. Epub 2011 Sep 13.

[Epidemiologic study of skin diseases among immigrants in Alicante, Spain].

[Article in Spanish]

Albares MP, Belinchón I, Ramos JM, Sánchez-Payá J, Betlloch I.

Servicio de Dermatología, Hospital General Universitario de Alicante, España. pityalte@hotmail.com

BACKGROUND: The influx of a large number of immigrants has altered the sociodemographic profile in Spain. To date, few studies of the skin diseases of immigrants to Spain have been done.
OBJECTIVE: To determine the frequency of visits by immigrants to our dermatology clinic, to describe their skin complaints, and to compare them to those of the autochthonous Spanish population.
PATIENTS AND METHODS: Prospective, descriptive, analytic study, with an observational substudy of cases and controls from a cross-section of the population. We included all immigrant patients seen at the dermatology clinic between February 2005 and February 2006.
RESULTS: Visits by immigrants to the dermatology clinic accounted for 4.1% of the caseload. Their most frequent complaints were eczematous dermatitis (18.4%), viral warts (6.4%), and acne (6.3%). Comparison between the immigrant and autochthonous patient populations showed that eczematous dermatitis, alopecia, melasma, ringworm, scabies, Herpes simplex infection, keratosis pilaris, and xerosis were significantly more frequent among immigrant patients, whereas viral warts, actinic keratosis, hidradenitis suppurativa, lupus, melanoma, and squamous cell carcinoma were significantly less frequent ($P < .05$).
CONCLUSIONS: The immigrant population consults the dermatologist about skin conditions that are already well represented in our routine practice. As the infectious skin diseases of immigrants are also common in our environment, these patients are unlikely to transmit serious tropical skin diseases to the local population.

Copyright Â© 2011 Elsevier España, S.L. and AEDV. All rights reserved.

PMID: 21920489 [PubMed - indexed for MEDLINE]

187. Trends Immunol. 2011 Oct;32(10):478-85. doi: 10.1016/j.it.2011.08.002. Epub 2011 Sep 13.

Mechanisms underlying the localisation of mast cells in tissues.

Collington SJ, Williams TJ, Weller CL.

Department of Pathology, School of Dental Medicine, University of Pennsylvania, Philadelphia, PA 19104, USA.

Mast cells are tissue-resident cells best known for their role in allergy and host defence against helminth parasites. They are involved in responses against other pathogenic infections, wound healing and inflammatory disease. Committed mast cell progenitors are released from the bone marrow into the circulation, from where they are recruited into tissues to complete their maturation under the

control of locally produced cytokines and growth factors. Directed migration occurs at distinct stages of the mast cell life-cycle and is associated with successive up- and downregulation of cell surface adhesion molecules and chemoattractant receptors as the cells mature. This article discusses some of the recent advances in our understanding of the mechanisms underlying mast cell recruitment.

Copyright © 2011. Published by Elsevier Ltd.

PMID: 21917522 [PubMed - indexed for MEDLINE]

188. Basic Clin Pharmacol Toxicol. 2011 Sep 14. doi: 10.1111/j.1742-7843.2011.00797.x. [Epub ahead of print]

Nonylphenol Induces Bronchial Epithelial Apoptosis via Fas-mediated Pathway and Stimulates Bronchial Epithelium to Secrete IL-6 and IL-8, causing Bronchial Smooth Muscle Proliferation and Migration.

Kuo PL, Hsu YL, Tsai MJ, Lien CT, Huang MS, Ko YC.

Institute of Clinical Medicine, College of Medicine, Kaohsiung Medical University, Kaohsiung, Taiwan Center of Excellence for Environmental Medicine, Kaohsiung Medical University, Kaohsiung, Taiwan Graduate Institute of Medicine, College of Medicine, Kaohsiung Medical University, Kaohsiung, Taiwan Division of Pulmonary and Critical Care Medicine, Kaohsiung Medical University Hospital, Kaohsiung, Taiwan.

Features of airway remodelling have been described using tissue obtained from fatal cases of asthma and bronchial biopsies from mildly, moderately and severely asthmatic patients. Epithelial detachment and smooth muscle mass enhancement are common features of asthmatic bronchial tissue. This study is the first to investigate the inhibitory effect of nonylphenol (NP) on human bronchial epithelial cell lines BEAS-2B and HBE135-E6E7 (HBE). The results show that NP inhibits bronchial epithelial proliferation via the Fas/Fas ligand apoptotic system. We also treated BEAS-2B and HBE with NP and harvested the condition medium (CM), which was then added to bronchial smooth muscle cells (BSMC). Cultures of BSMC with NP-BEAS-2B-CM and NP-HBE-CM increased BSMC proliferation and migration. Exposure of BEAS-2B and HBE to NP caused epithelial cells to produce inflammatory cytokines IL-6 and IL-8, which subsequently induced BSMC proliferation and migration. Depleting both IL-6 and IL-8 completely reversed the effect of NP-BEAS-2B-CM- and NP-HBE-CM-mediated BSMC proliferation and migration, suggesting that this effect is a synergistic influence of IL-6 and IL-8. This study is the first to demonstrate that NP not only induces bronchial epithelial apoptosis via the Fas-mediated pathway but also stimulates the bronchial epithelium to secrete IL-6 and IL-8, which cause bronchial smooth muscle proliferation and migration - major features in asthma remodelling.

© 2011 The Authors. Basic & Clinical Pharmacology & Toxicology © 2011 Nordic Pharmacological Society.

PMID: 21917119 [PubMed - as supplied by publisher]

189. J Immunol. 2011 Oct 15;187(8):3979-86. doi: 10.4049/jimmunol.1100531. Epub 2011 Sep 12.

T cell dynamics during induction of tolerance and suppression of experimental allergic encephalomyelitis.

Divekar RD, Haymaker CL, Cascio JA, Guloglu BF, Ellis JS, Tartar DM, Hoeman CM, Franklin CL, Zinselmeyer BH, Lynch JN, Miller MJ, Zaghouani H.

Department of Molecular Microbiology and Immunology, University of Missouri, Columbia, MO 65212, USA.

The cell dynamics associated with induction of peripheral T cell tolerance remain largely undefined. In this study, an in vivo model was adapted to two-photon microscopy imaging, and T cell behavior was analyzed on tolerogen-induced modulation. FcγR-deficient (FcγR(-/-)) mice were unable to resist or alleviate experimental allergic encephalomyelitis when treated with Ig-myelin oligodendrocyte glycoprotein (MOG) tolerogen, an Ig carrying the MOG35-55 peptide. However, when FcγR(+/+) dendritic cells (DCs) are adoptively transferred into FcγR(-/-) mice, uptake and presentation of Ig-MOG occurs and the animals were able to overcome experimental allergic encephalomyelitis. We then fluorescently labeled FcγR(+/+) DCs and 2D2 MOG-specific TCR-transgenic T cells, transferred them into FcγR(-/-) mice, administered Ig-MOG, and analyzed both T cell-DC contact events and T cell motility. The results indicate that tolerance takes place in lymphoid organs, and surprisingly, the T cells do not become anergic but instead have a Th2 phenotype. The tolerant Th2 cells displayed reduced motility after tolerogen exposure similar to Th1 cells after immunization. However, the Th2 cells had higher migration speeds and took longer to exhibit changes in motility. Therefore, both Th1 immunity and Th2 tolerance alter T cell migration on Ag recognition, but the kinetics of this effect differ among the subsets.

PMCID: PMC3186833
PMID: 21911603 [PubMed - indexed for MEDLINE]

190. J Biol Chem. 2011 Nov 4;286(44):38231-41. doi: 10.1074/jbc.M111.279554. Epub 2011 Sep 12.

N-Glycans differentially regulate eosinophil and neutrophil recruitment during allergic airway inflammation.

Bahaie NS, Kang BN, Frenzel EM, Hosseinkhani MR, Ge XN, Greenberg Y, Ha SG, Demetriou M, Rao SP, Sriramarao P.

Laboratory of Allergic Diseases and Inflammation, Department of Veterinary and Biomedical Sciences, University of Minnesota, St. Paul, Minnesota 55108, USA.

Allergic airway inflammation, including asthma, is usually characterized by the predominant recruitment of eosinophils. However, neutrophilia is also prominent during severe exacerbations. Cell surface-expressed glycans play a role in leukocyte trafficking and recruitment during inflammation. Here, the involvement of UDP-N-acetylglucosamine:α-6-D-mannoside β1,6-N-acetylglucosaminyltransferase V (MGAT5)-modified N-glycans in eosinophil and neutrophil recruitment during allergic airway inflammation was investigated. Allergen-challenged Mgat5-deficient (Mgat5(-/-)) mice exhibited significantly attenuated airway eosinophilia and inflammation (decreased Th2 cytokines, mucus production) compared with WT counterparts, attributable to decreased rolling, adhesion, and survival of Mgat5(-/-) eosinophils. Interestingly, allergen-challenged Mgat5(-/-) mice developed airway neutrophilia and increased airway reactivity with persistent elevated levels of proinflammatory cytokines (IL-17A, TNFα, IFNγ)). This increased neutrophil recruitment was also observed in LPS- and thioglycollate (TG)-induced inflammation in Mgat5(-/-) mice. Furthermore, there was significantly increased recruitment of infused Mgat5(-/-) neutrophils compared with WT neutrophils in the peritoneal cavity of TG-exposed WT mice. Mgat5(-/-) neutrophils demonstrated enhanced adhesion to P-selectin as well as

increased migration toward keratinocyte-derived chemokine compared with WT neutrophils in vitro along with increased calcium mobilization upon activation and expression of elevated levels of CXCR2, which may contribute to the increased neutrophil recruitment. These data indicate an important role for MGAT5-modified N-glycans in differential regulation of eosinophil and neutrophil recruitment during allergic airway inflammation.

PMCID: PMC3207438
PMID: 21911487 [PubMed - indexed for MEDLINE]

191. Allergy. 2012 Jan;67(1):41-9. doi: 10.1111/j.1398-9995.2011.02710.x. Epub 2011 Sep 12.

Macrophages from patients with atopic dermatitis show a reduced CXCL10 expression in response to staphylococcal α-toxin.

Kasraie S, Niebuhr M, Kopfnagel V, Dittrich-Breiholz O, Kracht M, Werfel T.

Division of Immunodermatology and Allergy Research, Department of Dermatology and Allergy, Hannover Medical School, Carl-Neuburg-Strasse 1, Hannover,Germany. kasraie.sadaf@mh-hannover.de

BACKGROUND: Patients with atopic dermatitis (AD) are frequently colonized with Staphylococcus aureus (S. aureus), one-third of them producing α-toxin, which is correlated with the severity of eczema in AD. Staphylococcus aureus colonizes in patients with psoriasis as well. Distinct expression of chemokine (C-C motif) ligand (CCL) and chemokine (C-X-C motif) ligand (CXCL) chemokines has been documented in both diseases. In this study, we investigated the effects of sublytic α-toxin concentrations on human macrophages that accumulate in the skin of patients with AD and psoriasis.
METHODS: IFN-γ-induced protein of 10-kDa (IP-10)/CXCL10 and macrophage-derived chemokine (MDC)/CCL22 production were evaluated at the mRNA or at the protein level using qRT-PCR or ELISA, respectively. Cell surface markers' expression and chemotaxis were determined by flow cytometry and Boyden chamber technique, respectively.
RESULTS: Sublytic concentrations of α-toxin strongly induced CXCL10 in macrophages at both the mRNA and the protein levels and significantly up-regulated MHC class II expression. Supernatants of α-toxin-stimulated macrophages induced the migration of human CD4+ lymphocytes via the CXCL10 receptor (CXCR3). Macrophages from patients with AD produced lower levels of CXCL10 compared to cells from patients with psoriasis as well as healthy controls in response to α-toxin. α-Toxin did not lead to a large variation in CCL22 production in macrophages from all three groups.
CONCLUSIONS: Staphylococcal α-toxin contributes to Th1 polarization by induction of CXCL10 in macrophages. Macrophages from patients with AD and psoriasis responded to α-toxin in the induction of Th1-related chemokine CXCL10 diversely, which could favour the recruitment of distinct leucocyte subsets into the skin.

© 2011 John Wiley & Sons A/S.

PMID: 21906079 [PubMed - indexed for MEDLINE]

192. J Sep Sci. 2011 Oct;34(19):2686-96. doi: 10.1002/jssc.201100360. Epub 2011 Aug 24.

Estimation of dermal and oral exposure of children to scented toys: analysis of the migration of fragrance allergens by dynamic headspace GC-MS.

Masuck I, Hutzler C, Luch A.

Department of Product Safety, German Federal Institute for Risk Assessment, Berlin, Germany. ines.masuck@bfr.bund.de

Fragrances capable of inducing contact allergy in skin potentially can migrate from the toy to the child via oral or dermal contacts. The goal of this work was the developing of an analytical method based on dynamic headspace GC-MS to determine the concentration of 24 fragrances in saliva or sweat simulant. Under optimized conditions, 5 mL of the migration simulant with 2 g sodium chloride were incubated for 10 min at 30°C. The headspace was purged at a flow rate of 50 mL/min. The compounds were quantified by internal calibration resulting in good linearity (>0.991). The recovery was greater than 66.3% for most of the compounds. The limits of detection ranged between 0.5 ng/mL for hydrophobic and 196.0 ng/mL for hydrophilic fragrances. The method was subsequently applied to seven real toys purchased from the market. The highest migration rate could be observed for benzyl benzoate with 268.0 ng/cm(2)/min. Based on the migration data measured, the ranges of dermal and oral exposure of children to fragrances in scented toys were calculated. The maximum oral and dermal exposure levels were estimated at 22.2 µg per kg body weight (BW) and day (d) for benzyl benzoate and 605.0 µg/kg BW/d for benzyl alcohol, respectively.

Copyright © 2011 WILEY-VCH Verlag GmbH & Co. KGaA, Weinheim.

PMID: 21898805 [PubMed - indexed for MEDLINE]

193. J Cell Physiol. 2012 Jun;227(6):2567-77. doi: 10.1002/jcp.22995.

Differential effect of CCL2 on constitutive neutrophil apoptosis between normal and asthmatic subjects.

Yang EJ, Choi E, Ko J, Kim DH, Lee JS, Kim IS.

Department of Biomedical Laboratory Science, School of Medicine, Eulji University, Daejeon, Repulbic of Korea.

In this study, we investigated the effects of CCL2 on constitutive apoptosis of normal and asthmatic neutrophils. CCL2 blocked the constitutive apoptosis of normal neutrophils through CCR2. CCL2 also induced elevation of the cytosolic Ca(2+) concentration but had no effect on normal neutrophil chemotaxis. Constitutive apoptosis, calcium influx, and cell migration of asthmatic neutrophils were not affected by CCL2 stimulation. Supernatant collected from CCL2-treated normal neutrophils inhibited the constitutive apoptosis of normal neutrophils. Anti-apoptotic signaling mediated by CCL2 was found to be associated with the PI3K/Akt/ERK/NF-κB cascade in normal neutrophils. Both the cleavage of procaspase 3 and procaspase 9 and the decrease of in Mcl-1 expression were delayed by CCL2 stimulation. Inhibition of NF-κB blocked constitutive apoptosis of neutrophils from asthmatic patients via inhibition of the cleavage of procaspase 3 and procaspase 9, in contrast to normal neutrophils. NF-κB was involved in CCL2-induced anti-apoptotic signaling in normal neutrophils, whereas NF-κB functioned as a basal pro-apoptotic factor in asthmatic neutrophils. A better understanding of the difference in the regulation of neutrophil apoptosis due to CCL2 between normal individuals and asthmatics will enable elucidation of the role of CC chemokine in neutrophils and a framework for understanding the pathogenesis of asthma.

Copyright © 2011 Wiley Periodicals, Inc.

PMID: 21898402 [PubMed - indexed for MEDLINE]

194. Leukemia. 2012 Jan;26(1):63-72. doi: 10.1038/leu.2011.242. Epub 2011 Sep 2.

A novel perspective on stem cell homing and mobilization: review on bioactive lipids as potent chemoattractants and cationic peptides as underappreciated modulators of responsiveness to SDF-1 gradients.

Ratajczak MZ, Kim CH, Abdel-Latif A, Schneider G, Kucia M, Morris AJ, Laughlin MJ, Ratajczak J.

Stem Cell Institute, James Graham Brown Cancer Center, University of Louisville, Louisville, KY 40202, USA. mzrata01@louisville.edu

Hematopoietic stem progenitor cells (HSPCs) respond robustly to α-chemokine stromal-derived factor-1 (SDF-1) gradients, and blockage of CXCR4, a seven-transmembrane-spanning G(αI)-protein-coupled SDF-1 receptor, mobilizes HSPCs into peripheral blood. Although the SDF-1-CXCR4 axis has an unquestionably important role in the retention of HSPCs in bone marrow (BM), new evidence shows that, in addition to SDF-1, the migration of HSPCs is directed by gradients of the bioactive lipids sphingosine-1 phosphate and ceramide-1 phosphate. Furthermore, the SDF-1 gradient may be positively primed/modulated by cationic peptides (C3a anaphylatoxin and cathelicidin) and, as previously demonstrated, HSPCs respond robustly even to very low SDF-1 gradients in the presence of priming factors. In this review, we discuss the role of bioactive lipids in stem cell trafficking and the consequences of HSPC priming by cationic peptides. Together, these phenomena support a picture in which the SDF-1-CXCR4 axis modulates homing, BM retention and mobilization of HSPCs in a more complex way than previously envisioned.

PMID: 21886175 [PubMed - indexed for MEDLINE]

195. J Allergy Clin Immunol. 2011 Dec;128(6):1303-1309.e2. doi: 10.1016/j.jaci.2011.07.047. Epub 2011 Aug 27.

Functional KCa3.1 K+ channels are required for human fibrocyte migration.

Cruse G, Singh SR, Duffy SM, Doe C, Saunders R, Brightling CE, Bradding P.

Department of Infection, Immunity and Inflammation, Institute for Lung Health, University of Leicester, Leicester, United Kingdom.

BACKGROUND: Fibrocytes are bone marrow-derived CD34(+) collagen I-positive cells present in peripheral blood that develop α-smooth muscle actin expression and contractile activity in tissue culture. They are implicated in the pathogenesis of tissue remodeling and fibrosis in both patients with asthma and those with idiopathic pulmonary fibrosis. Targeting fibrocyte migration might therefore offer a new approach for the treatment of these diseases. Ion channels play key roles in cell function, but the ion-channel repertoire of human fibrocytes is unknown.
OBJECTIVE: We sought to examine whether human fibrocytes express the K(Ca)3.1 K(+) channel and to determine its role in cell differentiation, survival, and migration.
METHODS: Fibrocytes were cultured from the peripheral blood of healthy subjects and patients with asthma. Whole-cell patch-clamp electrophysiology was used for the measurement of ion currents, whereas mRNA and protein were examined to confirm channel expression. Fibrocyte migration and proliferation assays were performed in the presence of K(Ca)3.1 ion-channel blockers.

RESULTS: Human fibrocytes cultured from the peripheral blood of both healthy control subjects and asthmatic patients expressed robust K(Ca)3.1 ion currents together with K(Ca)3.1 mRNA and protein. Two specific and distinct K(Ca)3.1 blockers (TRAM-34 and ICA-17043) markedly inhibited fibrocyte migration in transwell migration assays. Channel blockers had no effect on fibrocyte growth, apoptosis, or differentiation in cell culture.
CONCLUSIONS: The K(+) channel K(Ca)3.1 plays a key role in human fibrocyte migration. Currently available K(Ca)3.1-channel blockers might therefore attenuate tissue fibrosis and remodeling in patients with diseases such as idiopathic pulmonary fibrosis and asthma through the inhibition of fibrocyte recruitment.

Copyright © 2011 American Academy of Allergy, Asthma & Immunology. Published by Mosby, Inc. All rights reserved.

PMCID: PMC3526791
PMID: 21872912 [PubMed - indexed for MEDLINE]

196. Indian J Exp Biol. 2011 Aug;49(8):585-93.

Effects of beta-arrestin 2 on cytokine production of CD4+ T lymphocytes of mice with allergic asthma.

Wang G, Liu Y, Yang M, Liu S, Ma L, Gong S, Li K, Zhang L, Xiang X.

Department of Respiratory Medicine, the Second Xiangya Hospital of Central South University, Changsha, Hunan 410011, China.

Beta-arrestin 2 has been shown to participate in the pathogenesis of asthma by inducing Th2 cell migration to the lungs. Whether beta-arrestin 2 regulates cytokine production of CD4+ T cells is still unknown. The aim of the present study was to investigate the effect of beta-arrestin 2 on the cytokine production of CD4+ T lymphocytes and the mechanism involved in a mouse model for asthma. After silencing beta-arrestin 2 expression in CD4+ T lymphocytes from asthmatic mice by RNA interference (RNAi), the interleukin-4 (IL-4) and interferon-gamma (IFN-gamma) levels in CD4+ T lymphocyte culture supernatants with or without terbutaline stimulation were determined. Cell-surface beta2 adrenergic receptor (beta2AR) as well as GATA3 expression of CD4+ T lymphocytes were also measured. CD4+ T lymphocytes of mice with allergic asthma expressed higher levels of beta-arrestin 2 on both mRNA and protein levels. beta-arrestin 2 RNAi decreased IL-4 (43.16%) and GATA3 (protein 77.21%, mRNA 62.98%) expression after terbutaline stimulation. Cell-surface beta2AR of CD4+ T lymphocytes decreased (15.27%) after terbutaline treatment, but recovered after beta-arrestin 2 RNAi down-modulation. These findings demonstrate that beta-arrestin 2 regulates IL-4 production and GATA3 expression of CD4+ T lymphocytes partly through the beta2AR signaling pathway in an allergic asthma model.

PMID: 21870426 [PubMed - indexed for MEDLINE]

197. Int J Environ Res Public Health. 2011 Jul;8(7):2828-53. doi: 10.3390/ijerph8072828. Epub 2011 Jul 13.

Assessing environmental risks for established invasive weeds: Dalmatian (Linaria dalmatica) and yellow (L. vulgaris) toadflax in North America.

Sing SE, Peterson RK.

USDA Forest Service, Rocky Mountain Research Station, 1648 South 7th Avenue, MSU

Campus, Bozeman, MT 59717-2780, USA. ssing@fs.fed.us

Environmental risk assessments characterizing potential environmental impacts of exotic weeds are more abundant and comprehensive for potential or new invaders than for widespread and well-established species such as Dalmatian (Linaria dalmatica [L.] Mill.) and yellow (L. vulgaris Mill.) toadflax. Specific effects evaluated in our assessment of environmental risks posed by yellow and Dalmatian toadflax included competitive displacement of other plant species, reservoirs of plant disease, animal and insect use, animal toxicity, human toxicity and allergenicity, erosion, and wildfire. Effect and exposure uncertainties for potential impacts of toadflax on human and ecological receptors were rated. Using publicly available information we were able to characterize ecological and human health impacts associated with toadflax, and to identify specific data gaps contributing to a high uncertainty of risk. Evidence supporting perceived negative environmental impacts of invasive toadflax was scarce.

PMCID: PMC3155332
PMID: 21845161 [PubMed - indexed for MEDLINE]

198. J Inflamm (Lond). 2011 Aug 11;8:19. doi: 10.1186/1476-9255-8-19.

Triple selectin knockout (ELP-/-) mice fail to develop OVA-induced acute asthma phenotype.

Banerjee ER.

Division of Hematology, Department of Medicine, University of Washington, 1959 NE Pacific Street, Seattle, WA 98195, USA. enarb1@gmail.com.

OBJECTIVE: The recruitment of leukocytes from circulation to sites of inflammation requires several families of adhesion molecules among which are selectins expressed on a variety of cells. In addition, they have also been shown to play key roles in the activation of cells in inflammation.
METHODS: To explore the collective role of E-, L-, and P- selectins in OVA-induced Th2 mediated response in acute asthma pathophysiology, ELP-/- mice were used and compared with age-matched wildtype (WT).
RESULTS: Asthma phenotype was assessed by measuring pulmonary function, inflammation and OVA-specific serum IgE, which were completely abrogated in ELP-/- mice. Adoptive transfer of sensitized L selectin+CD4+ T cells into naïve ELP-/- mice which post-OVA challenge, developed asthma, suggesting that L-selectin may be critically involved in the onset of Th2 response in asthma. Tissue resident ELP-deficient cells were otherwise functionally competent as proved by normal proliferative response. Conclusions: Comparative studies between ELP-/- and WT mice uncovered functional roles of these three integrins in inflammatory response in allergic asthma. All three selectins seem to impede inflammatory migration while only L-selectin also possibly regulates activation of specific T cell subsets in lung and airways.

PMCID: PMC3170177
PMID: 21835035 [PubMed]

199. Eur J Immunol. 2011 Nov;41(11):3208-18. doi: 10.1002/eji.201141502. Epub 2011 Sep 26.

Induced Syk deletion leads to suppressed allergic responses but has no effect on neutrophil or monocyte migration in vivo.

Wex E, Bouyssou T, Duechs MJ, Erb KJ, Gantner F, Sanderson MP, Schnapp A,

Stierstorfer BE, Wollin L.

Department of Respiratory Diseases Research, Boehringer Ingelheim Pharma GmbH & Co. KG, Biberach, Germany.

The spleen tyrosine kinase (Syk) is a key mediator of immunoreceptor signaling in immune cells. Thus, interfering with the function of Syk by genetic deletion or pharmacological inhibition might influence a variety of allergic and autoimmune processes. Since conventional Syk knockout mice are not viable, studies addressing the effect of Syk deletion in adult animals have been limited. To further explore functions of Syk in animal models of allergy and to shed light on the role of Syk in the in vivo migration of neutrophils and monocytes, we generated inducible Syk knockout mice. These mice harbor a floxed Syk gene and a tamoxifen-inducible Cre recombinase under the control of the ubiquitously active Rosa26-promoter. Thus, treatment of mice with tamoxifen leads to the deletion of Syk in all organs. Syk-deleted mice were analyzed in mast cell-dependent models and in models focusing on neutrophil and monocyte migration. We show that Syk deletion in adult mice reduces inflammatory responses in mast cell-driven animal models of allergy and asthma but has no effect on the migration of neutrophils and monocytes. Therefore, the inducible Syk knockout mice presented here provide a valuable tool to further explore the role of Syk in disease-related animal models.

Copyright © 2011 WILEY-VCH Verlag GmbH & Co. KGaA, Weinheim.

PMID: 21830208 [PubMed - indexed for MEDLINE]

200. Zoonoses Public Health. 2012 Feb;59(1):8-15. doi: 10.1111/j.1863-2378.2011.01409.x. Epub 2011 May 17.

Seasonal dynamics of house dust mites in dust samples collected from sleeping places in north-western Poland.

Kosik-Bogacka DI, Kalisinska E, Henszel L, Kuzna-Grygiel W.

Department of Biology and Medical Parasitology, Pomeranian Medical University, Szczecin, Poland. kodan@sci.pam.szczecin.pl

The most common families of mites found in house dust are Pyroglyphidae, Glycyphagidae and Acaridae; all are a source of many antigens responsible for allergic diseases. The aim of this study was to examine the seasonal dynamics of allergenic mite populations in dust samples collected from sleeping places in apartments in north-western Poland. The mites were isolated from the dust using a saturated saline floating method. In 132 dust samples we determined: Dermatophagoides farinae, Dermatophagoides pteronyssinus, Euroglyphus maynei, Hirstia sp., Chortoglyphus arcuatus, Lepidoglyphus destructor, Gohieria fusca and Cheyletus sp. The greatest frequency was observed for D. farinae, D. pteronyssinus, Ch. arcuatus and Cheyletus sp., in the fourth quarter and D. farinae in the third quarter. Smaller coefficients of dominance were found for D. pteronyssinus, Ch. arcuatus and Cheyletus sp., and their greatest mean concentrations were found in the first and fourth quarters. Given the division of the year into heating and non-heating seasons, mites D. farinae and D. pteronyssinus achieved the highest mean concentration in the first season, and Cheyletus sp. in the second season. The analysis of the participation of developmental stages showed that the adults of D. farinae were more prevalent than juveniles in the first, second and third quarters, and imago stages of D. pteronyssinus were more numerous in relation to juveniles in the first, third and fourth quarters. The results confirm the high incidence of house dust mites in sleeping places in north-western Poland dwellings; the best conditions for the

development of these mites, mainly D. farinae and D. pteronyssinus, occur in the fourth quarter and are the least favourable in the second quarter. In many cases, these results are consistent with data from other parts of Poland collected by various authors.

© 2011 Blackwell Verlag GmbH.

PMID: 21824362 [PubMed - indexed for MEDLINE]

201. J Radiol. 2011 Jul-Aug;92(7-8):735-8. doi: 10.1016/j.jradio.2011.03.021. Epub 2011 Jul 7.

[Spontaneous rupture of a hydatid cyst and anaphylactic shock].

[Article in French]

Tonnelet R, Jausset F, Tissier S, Laurent V.

Service de radiologie adulte, CHU Nancy-Brabois, rue du Morvan, 54511 Vandœuvre, France. romain.tonnelet@gmail.com

PMID: 21819918 [PubMed - indexed for MEDLINE]

202. Aesthet Surg J. 2011 Aug;31(6):674-81. doi: 10.1177/1090820X11415393.

A comparative, long-term assessment of four soft tissue substitutes.

Cole PD, Stal D, Sharabi SE, Hicks J, Hollier LH Jr.

Division of Plastic Surgery, Baylor College of Medicine, Houston, Texas, USA.

Comment in
    Aesthet Surg J. 2012 Mar;32(3):355-8.

BACKGROUND: The ideal product for soft tissue replacement is durable, nonimmunogenic, and noninfectious. AlloDerm (LifeCell Corp., Branchburg, New Jersey), Enduragen (Stryker Corp., Kalamazoo, Michigan), and DermaMatrix (Synthes, Inc., West Chester, Pennsylvania) are frequently used for soft tissue replacement, but comparative analysis of these materials over an extended time period has not been reported. DuraMatrix (bovine tendon matrix; Stryker Corp.) is also promising, demonstrating desirable properties not only as a dural substitute but also for soft tissue replacement.
OBJECTIVES: The authors analyze in vivo gross and microscopic changes over time with four commercially available dermal matrices, utilizing the murine model for a controlled environment.
METHODS: AlloDerm, Enduragen, DermaMatrix, and DuraMatrix implants measuring 1 × 1 cm were each implanted in 40 adult mice, in individual dorsal submuscular pockets. The mice were then sacrificed in groups of 10 at three, six, nine, and 12 months. The implants and surrounding tissues were excised and evaluated for gross and microscopic appearance.
RESULTS: Histological analysis of the specimens demonstrated similar encapsulation, implant infiltration, and surrounding inflammation over time. Enduragen implants demonstrated the least amount of host cell infiltration, whereas AlloDerm demonstrated the most. Grossly, Enduragen maintained its original shape and became firmer over time, whereas AlloDerm became spherical and softer. DermaMatrix and DuraMatrix both maintained their original shape and consistency. Implant migration, explantation, infection, or allergic reactions were not noted.

CONCLUSIONS: All of the materials studied demonstrated high levels of host tolerance and tissue integration. AlloDerm demonstrated signs of resorption, whereas Enduragen maintained its size and became firmer in consistency. Together with the histological results, this suggests a proportional relationship between the amount of host cell integration and implant resorption.

PMID: 21813881 [PubMed - indexed for MEDLINE]

203. Ear Nose Throat J. 2011 Jul;90(7):306-12.

Cochlear implant fixation using resorbable mesh.

Lundy L, Karatayli-Ozgursoy S.

Department of Otolaryngology, Mayo Clinic Florida, Jacksonville, FL 32224, USA.
lundy.larry@mayo.edu

In this article we describe a new method of cochlear implant receiver-stimulator fixation using a resorbable poly (D,L) lactic acid mesh. We conducted a retrospective case review at a tertiary referral center; 10 pediatric and 4 adult patients had undergone cochlear implantation during the period from February to October 2008. Resorbable poly (D,L) lactic acid mesh and pins were used for fixation of the cochlear implant receiver stimulator. The receiver stimulator was assessed for stability/migration, and the scalp flap/incision were evaluated for allergic reactions, infections, and healing problems. With an average follow-up of 17.2 months, no patients had migration of the receiver stimulator, and there was no evidence of infection, wound dehiscence, or allergic reaction. Early results indicate that fixation of a cochlear implant receiver stimulator using resorbable mesh is well tolerated and provides good stability without device migration. Resorbable mesh fixation of the receiver stimulator is a reasonable alternative technique for cochlear implantation.

PMID: 21792798 [PubMed - indexed for MEDLINE]

204. Anestezjol Intens Ter. 2011 Jan-Mar;43(1):40-4.

[Pneumocephalus and pneumorrhachis after chest wall injury].

[Article in Polish]

Wośko J, Dabrowski W, Zadora P, Fijalkowska A, Grzycka-Kowalczyk L.

Klinika Anestezjologii i Intensywnej Terapii, Uniwersytet Medyczny w Lublinie.
anest@umlub.pl

Pneumocephalus and pneumorrhachis are rare findings, and may result from a variety of causes, including severe asthma or trauma. We describe a case, where intracranial and intraspinal air was found after trauma to the chest wall. CASE REPORT: A 24-yr-old patient suffered multiple trauma in a traffic accident, including a closed head injury and bursting fractures of theTh 7, 8 and 9 vertebral bodies with laceration of the spinal cord. Reposition of the spinal column was complicated by wound infection and septic shock. Intraoperatively, accidental extubation led to migration of gastric contents and was complicated by possible rupture of the oesophagus. Postoperative CT scan revealed the presence of air within the mediastinum, cranium and the entire spinal canal. The osteosynthetic material was removed, and the air quickly reabsorbed. The paraplegic patient was discharged from ITU in a satisfactory condition. DISCUSSION:The most probable cause of the complication was traumatic rupture of

the oesophagus and penetration of air via lacerated dura mater, to the spinal canal and the cranium. Conservative treatment was successful and led to complete (beside paraplegia) recovery.

PMID: 21786530 [PubMed - indexed for MEDLINE]

205. Allergol Int. 2011 Dec;60(4):497-504. doi: 10.2332/allergolint.10-OA-0288. Epub 2011 Jul 25.

A study of elevated interleukin-8 (CXCL8) and detection of leukocyte migration inhibitory activity in patients allergic to beta-lactam antibiotics.

Abe M, Yagi M, Wakasugi Y, Hattori H, Uno K.

Niigata University of Pharmacy and Applied Life Sciences, Niigata, Japan. abe@nupals.ac.jp

BACKGROUND: The leukocyte migration test (LMT) is effective in identifying the causative drug in drug allergies. Both leukocyte migration activating activity (LMAA) and leukocyte migration inhibitory activity (LMIA) are involved in the development of drug allergies. However, no cytokines associated with LMIA have been identified to date. Because CXCL8 played an important role in neutrophil infiltration and activation, we performed the LMT and measured CXCL8 levels in patients with hypersensitivity to beta-lactam antibiotics (beta-lactams) and antipyretic analgesics (APAs) and investigated the pathogenic mechanism of hypersensitivity to these drugs.
METHODS: The LMT was performed according to an improved version of the agarose plate method and CXCL8 levels in the reacted solution that had been stored as described were measured using a solid-phase sandwich enzyme-linked immunosorbent assay.
RESULTS: Migration index (MI) values for the LMT were 77.7 ± 11.7 for patients with hypersensitivity to beta-lactams and 83.6 ± 1.9 for those with hypersensitivity to APAs. The CXCL8 concentrations were significantly higher in patients after beta-lactams administration (175.9 ± 71.2 ng/mL) than those without beta-lactams administration (48.3 ± 34.9 ng/mL). The CXCL8 concentrations were significantly lower in patients after APAs administration (41.7 ± 24.3 ng/mL) than those without APAs administration (63.1 ± 30.2 ng/mL).
CONCLUSIONS: Increased CXCL8 levels produced by beta-lactams administration were accompanied by LMIA. CXCL8 may be involved in LMIA and play a role in beta-lactam allergies. In contrast, the LMIA detected in patients with allergies to APAs may be a cytokine or chemokine other than CXCL8.

PMID: 21778813 [PubMed - indexed for MEDLINE]

206. J Inflamm (Lond). 2011 Jul 21;8(1):18. doi: 10.1186/1476-9255-8-18.

The effect of substance P on asthmatic rat airway smooth muscle cell proliferation, migration, and cytoplasmic calcium concentration in vitro.

Li M, Shang YX, Wei B, Yang YG.

Department of Pediatrics, No. 2, Hospital of China Medical University, Shenyang 110004, China.

Airway remodeling and airway hyper-responsiveness are prominent features of asthma. Neurogenic inflammation participates in the development of asthma. Neurokinin substance P acts by binding to neurokinin-1 receptor (NK-1R). Airway smooth muscle cells (ASMC) are important effector cells in asthma. Increases in

ASMC proliferation, migration, and cytoplasmic Ca2+ concentration are critical to airway remodeling and hyper-responsiveness. The effects of substance P on ASMC were investigated in Wistar rats challenged with a previously described asthmatic rat model. To exclude possible influences from other factors, the role of substance P was also investigated in primary cultured rat ASMC. Substance P and WIN62577-induced changes in cytoplasmic Ca2+ concentration were observed by fluorescence microscopy, and expression of Ca2+ homeostasis-regulating genes was assessed with real-time PCR. We found that cytoplasmic Ca2+ concentration increased in normal rat ASMC treated with substance P, but decreased in asthmatic rat ASMC treated with WIN62577, an antagonist of NK-1R. Real-time PCR analysis revealed increased Serca2 mRNA expression but decreased Ip3r mRNA expression after WIN62577 treatment in asthmatic rat ASMC. Flow cytometric analysis (FCM) revealed that most asthmatic rat ASMC stayed at G1 phase after combined treatment with WIN62577 and IL-13 in vitro. Transwell analysis suggested that ASMC migration was reduced after WIN62577 treatment. Therefore, we conclude that NK-1R is related to asthma mechanisms and a NK-1R antagonist downregulates calcium concentration in asthmatic ASMC by increasing Serca2 mRNA and decreasing Ip3r mRNA expression. The NK-1R antagonist WIN62577 inhibited ASMC IL-13-induced proliferation and ASMC migration in vitro and therefore may be a new therapeutic option in asthma.

PMCID: PMC3148551
PMID: 21777465 [PubMed]

207. Anal Bioanal Chem. 2012 Jan;402(1):139-62. doi: 10.1007/s00216-011-5237-3. Epub 2011 Jul 20.

Challenges and trends in the determination of selected chemical contaminants and allergens in food.

Krska R, Becalski A, Braekevelt E, Koerner T, Cao XL, Dabeka R, Godefroy S, Lau B, Moisey J, Rawn DF, Scott PM, Wang Z, Forsyth D.

Food Research Division, Bureau of Chemical Safety, Food Directorate, Health Canada, Ottawa, ON, Canada.

This article covers challenges and trends in the determination of some major food chemical contaminants and allergens, which-among others-are being monitored by Health Canada's Food Directorate and for which background levels in food and human exposure are being analyzed and calculated. Eleven different contaminants/contaminant groups and allergens have been selected for detailed discussion in this paper. They occur in foods as a result of: use as a food additive or ingredient; processing-induced reactions; food packaging migration; deliberate adulteration; and/or presence as a chemical contaminant or natural toxin in the environment. Examples include acrylamide as a food-processing-induced contaminant, bisphenol A as a food packaging-derived chemical, melamine and related compounds as food adulterants and persistent organic pollutants, and perchlorate as an environmental contaminant. Ochratoxin A, fumonisins, and paralytic shellfish poisoning toxins are examples of naturally occurring toxins whereas sulfites, peanuts, and milk exemplify common allergenic food additives/ingredients. To deal with the increasing number of sample matrices and analytes of interest, two analytical approaches have become increasingly prevalent. The first has been the development of rapid screening methods for a variety of analytes based on immunochemical techniques, utilizing ELISA or surface plasmon resonance technology. The second is the development of highly sophisticated multi-analyte methods based on liquid chromatography coupled with multiple-stage mass spectrometry for identification and simultaneous quantification of a wide range of contaminants, often with much less requirement for tedious cleanup procedures. Whereas rapid screening methods enable testing of

large numbers of samples, the multi analyte mass spectrometric methods enable full quantification with confirmation of the analytes of interest. Both approaches are useful when gathering surveillance data to determine occurrence and background levels of both recognized and newly identified contaminants in foods in order to estimate human daily intake for health risk assessment.

PMID: 21773735 [PubMed - indexed for MEDLINE]

208. Trop Med Int Health. 2011 Nov;16(11):1457-64. doi: 10.1111/j.1365-3156.2011.02840.x. Epub 2011 Jul 18.

Skin disorders among travellers returning from tropical and non-tropical countries consulting a travel medicine clinic.

Herbinger KH, Siess C, Nothdurft HD, von Sonnenburg F, Löscher T.

Department of Infectious Diseases and Tropical Medicine, University Hospital, Ludwig-Maximilians University of Munich, Germany. herbinger@lrz.uni-muenchen.de

OBJECTIVE: To evaluate the causes and risks for imported skin disorders among travellers.
METHODS: Data of 34,162 travellers returning from tropical and non-tropical countries and presenting at the outpatient travel medicine clinic of the University of Munich, Germany, between 1999 and 2009 were analyzed for this study. Of these, 12.2% were diagnosed with skin disorders.
RESULTS: Main destinations visited were Asia (40%), Africa (27%) and Latin America (21%). Tourism in the form of adventure travel/backpacking (47%) and package holidays (23%) was the most common purpose of travel. The leading causes of skin disorders were arthropodal (23%), bacterial (22%), helminthic (11%), protozoan (6%), viral (6%), allergic (5%) and fungal (4%). The 10 most frequently diagnosed specific skin diseases associated with specific destinations were insect bites (17%, Southern Europe), cutaneous larva migrans (8%, Asia and Latin America), cutaneous leishmaniasis (2.4%, Mediterranean Region/Middle East), dengue fever (1.5%, Asia), rickettsioses (1.3%, Southern Africa), myiasis (0.8%, Central America), filarioses (0.7%, Africa), tick bites (0.6%, Central/Eastern Europe), schistosomiasis (0.6%, Africa) and tungiasis (0.6%, Africa). Travellers in sub-Saharan Africa had the highest relative risk of acquiring skin disorders.
CONCLUSION: As more than 20% of all skin disorders among returned travellers were caused by arthropods and about 50% by infectious pathogens, pre-travel consultations should include specific prophylaxis and consider the most important risk factor for the travel destination.

© 2011 Blackwell Publishing Ltd.

PMID: 21767336 [PubMed - indexed for MEDLINE]

209. J Allergy Clin Immunol. 2011 Oct;128(4):826-33. doi: 10.1016/j.jaci.2011.06.005. Epub 2011 Jul 16.

In vivo reprogramming of UV radiation-induced regulatory T-cell migration to inhibit the elicitation of contact hypersensitivity.

Schwarz A, Navid F, Sparwasser T, Clausen BE, Schwarz T.

Department of Dermatology and Allergology, University Kiel, Kiel, Germany.

BACKGROUND: Regulatory T (Treg) cells induced by UV radiation (UVR) inhibit only the induction and not the elicitation of contact hypersensitivity (CHS) because

they migrate into the lymph nodes but not the skin. The tissue-homing receptor expression and migratory behavior of Treg cells can be altered by means of in vitro coincubation with skin-derived antigen-presenting cells. On this in vitro treatment, Treg cells migrate into the skin and thus inhibit the elicitation of CHS.
OBJECTIVE: We attempted to determine whether Treg cells can be induced by UVR in sensitized mice and manipulated entirely in vivo in such a way that they suppress the elicitation of immune responses.
METHODS: Treg cells were induced by applying contact allergens onto UV-exposed skin in wild-type, langerin diphtheria toxin receptor knock-in, or depletion of Treg cell transgenic mice.
RESULTS: UVR-induced Treg cells inhibit the elicitation of CHS in sensitized mice when stimulated by means of an antigen-specific boost through the skin. This requires cutaneous antigen-presenting cells that alter the migratory behavior of Treg cells and drive them out of the lymph nodes into the skin.
CONCLUSIONS: The indication is that antigen-specific Treg cells can be induced in sensitized hosts and manipulated in such a way that they suppress the elicitation of specific immune reactions. Because this is achieved entirely in vivo without invasive interventions, our findings might have important implications for strategies aiming to induce and use Treg cells in a therapeutic setting.

Copyright © 2011 American Academy of Allergy, Asthma & Immunology. Published by Mosby, Inc. All rights reserved.

PMID: 21762977 [PubMed - indexed for MEDLINE]

210. J Allergy Clin Immunol. 2011 Dec;128(6):1251-1258.e2. doi: 10.1016/j.jaci.2011.06.007. Epub 2011 Jul 18.

Allergic sensitization can be induced via multiple physiologic routes in an adjuvant-dependent manner.

Dunkin D, Berin MC, Mayer L.

Division of Pediatric Gastroenterology, Mount Sinai School of Medicine, New York, NY 10029, USA. David.dunkin@mssm.edu

BACKGROUND: Oral exposure to food allergens may be limited in infancy, and the initial site of antigen exposure likely plays an important role in food allergy induction.
OBJECTIVE: To examine the impact of different routes of exposure by using milk allergens, with and without adjuvant, on sensitization.
METHODS: C3H/HeJ mice were repeatedly exposed to the milk allergen α-lactalbumin (ALA), with or without cholera toxin (CT). Sensitization routes used were intragastric, cutaneous, intranasal, and sublingual. Anaphylaxis severity was assessed by symptoms and body temperature in response to oral challenge. Antigen-specific serum antibodies were measured by ELISA. The mechanism of adjuvant activity of cutaneous CT was also determined.
RESULTS: Sensitization to ALA as measured by allergen-specific IgE occurred by all routes of sensitization and was maximal in response to cutaneous exposure. Sensitization was dependent on CT and did not occur to antigen alone by any route. Mucosal, but not cutaneous, exposure resulted in a robust allergen-specific IgA response. Anaphylaxis occurred in all sensitized groups when orally challenged with ALA. Topical CT induced migration of langerin(neg) dermal dendritic cells to the lymph node, resulting in enhanced proliferation and T(H)2 cytokine production from responder T cells.
CONCLUSIONS: Sensitization can occur via all physiologic routes when adjuvant is present. The skin is a potent and likely important physiologic route of sensitization whereby adjuvant induces an efflux of antigen-bearing dermal

dendritic cells to the lymph node that generate a proallergic T(H)2 response.

Copyright © 2011 American Academy of Allergy, Asthma & Immunology. Published by Mosby, Inc. All rights reserved.

PMCID: PMC3197891 [Available on 2012/12/1]
PMID: 21762973 [PubMed - indexed for MEDLINE]

211. Clin Exp Immunol. 2011 Oct;166(1):64-71. doi: 10.1111/j.1365-2249.2011.04442.x. Epub 2011 Jul 15.

Tumour necrosis factor-like weak inducer of apoptosis (TWEAK), an important mediator of endothelial inflammation, is associated with the pathogenesis of Henoch-Schonlein purpura.

Chen T, Guo ZP, Li MM, Li JY, Jiao XY, Zhang YH, Liu HJ.

Department of Dermatovenereology, West China Hospital of Sichuan University, Chengdu, Sichuan, China.

Tumour necrosis factor-like weak inducer of apoptosis (TWEAK), a member of the tumour necrosis factor (TNF) family, has been implicated as a proinflammatory cytokine in many types of autoimmune and infectious diseases. However, information about TWEAK in dermatological diseases is limited. Herein, we investigated the role of TWEAK in patients with Henoch-Schonlein purpura (HSP) and the ability of TWEAK on chemokine production in the human dermal microvascular endothelial cell line (HMEC-1). Serum TWEAK levels in patients with HSP, together with patients with psoriasis vulgaris (PV) and atopic dermatitis (AD), were detected by enzyme-linked immunosorbent assay (ELISA). HMEC-1 cells were treated with TWEAK at concentrations ranging from 1 ng/ml to 100 ng/ml. Serum levels of TWEAK were elevated in patients with HSP in the acute stage but not in patients with PV or AD. Moreover, TWEAK levels were correlated with the severity of HSP. TWEAK markedly induced CCL5 and CXCL8 production at both mRNA and protein levels in HMEC-1 cells. In addition, TWEAK-stimulated HMEC-1 supernatant enhanced HL-60 or human acute monocytic leukaemia cell line (THP-1) cell migration. Finally, Western blot data revealed that TWEAK can induce rapid phosphorylation of inhibitor of κB-α (IκBα) in HMEC-1 cells. In conclusion, we show that serum levels of TWEAK were elevated in patients with acute stage HSP. TWEAK may act as a regulator of nuclear factor-κB (NF-κB) activation and chemokine production in human dermal microvascular endothelial cells, thus promoting leucocyte migration in cutaneous vasculitis.

© 2011 The Authors. Clinical and Experimental Immunology © 2011 British Society for Immunology.

PMCID: PMC3193920
PMID: 21762126 [PubMed - indexed for MEDLINE]

212. Clin Exp Allergy. 2011 Aug;41(8):1054-8. doi: 10.1111/j.1365-2222.2011.03815.x.

Do migrant studies help to identify causes of asthma?

Kuehni CE.

Comment on
    Clin Exp Allergy. 2011 Aug;41(8):1108-15.

PMID: 21752114 [PubMed - indexed for MEDLINE]

213. J Pediatr Psychol. 2011 Nov-Dec;36(10):1130-43. doi: 10.1093/jpepsy/jsr041. Epub 2011 Jul 10.

Immigration and acculturation-related factors and asthma morbidity in Latino children.

Koinis-Mitchell D, Sato AF, Kopel SJ, McQuaid EL, Seifer R, Klein R, Esteban C, Lobato D, Ortega AN, Canino G, Fritz GK.

Child and Family Psychiatry, Bradley/Hasbro Research Center, Brown Medical School, 1 Hoppin Street, Coro West, 2nd Floor, Providence, RI, 02903, USA. dkoinismitchell@lifespan.org

OBJECTIVE: This article presents a summary of findings from asthma studies focusing on immigration and acculturation-related factors. A study examining associations between these processes, family cohesion and social support networks, and asthma morbidity in a sample of Dominican and Puerto Rican caregivers residing in the mainland U.S., is also described.
METHODS: Latino children with asthma (n = 232), ages 7-16 (49% female) and their caregivers completed interview-based questionnaires on immigration and acculturation-related processes, family characteristics, and asthma morbidity.
RESULTS: The frequency of ED use due to asthma may be higher for children of caregivers born in Puerto Rico. Acculturative stress levels were higher for Puerto Rican born caregivers residing in the mainland U.S.
CONCLUSION: Asthma-related educational and intervention programs for Latino children and families should be tailored to consider the effects that the immigration and acculturation experience can have on asthma management. Specific family-based supports focused on decreasing stress related to the acculturation process, and increasing social and family support around the asthma treatment process may help to reduce asthma morbidity in Latino children.

PMCID: PMC3247793
PMID: 21745811 [PubMed - indexed for MEDLINE]

214. Allergy Asthma Immunol Res. 2011 Jul;3(3):199-204. doi: 10.4168/aair.2011.3.3.199. Epub 2011 May 23.

The Role of Plasmacytoid and Myeloid Dendritic Cells in Induction of Asthma in a Mouse Model and the Effect of a TLR9 Agonist on Dendritic Cells.

Mo JH, Chung YJ, Hayashi T, Lee J, Raz E.

Department of Otorhinolaryngology-Head and Neck Surgery, Dankook University College of Medicine, Cheonan, Korea.

PURPOSE: To determine the role of plasmacytoid dendritic cells (pDC) and myeloid dendritic cells (mDC) in priming effector T cells to induce allergy, and to evaluate the effect of immunostimulatory sequences (ISS, TLR9 agonist) on dendritic cells.
METHODS: Cultured mDC and pDC with/without ISS were injected intratracheally into sensitized Balb/C mice. Mice were sacrificed, and then pulmonary function tests, bronchoalveolar lavage (BAL), cell counts, and cytokine levels were evaluated. Migration of dendritic cells was also evaluated after ISS administration.
RESULTS: In mice injected with mDC, airway hyperresponsiveness, eosinophil counts, and Th2 cytokine levels in BAL increased with increasing numbers of mDC injected. However, in mice injected with pDC, none of these changed, suggesting poor priming of T cells by pDC. In addition, mDC pulsed with ISS inhibited

asthmatic reactions, and ISS administration inhibited migration of DC to the lung.
CONCLUSIONS: We suggest that pDC played a limited role in priming T cells in this asthma model and that mDC played a major role in inducing asthma. In addition, ISS inhibited migration of DC to the lung.

PMCID: PMC3121062
PMID: 21738886 [PubMed]

215. Nutr Clin Pract. 2011 Aug;26(4):445-50. doi: 10.1177/0884533611406133. Epub 2011 Jul 1.

Consumption of DHA + EPA by low-income women during pregnancy and lactation.

Nochera CL, Goossen LH, Brutus AR, Cristales M, Eastman B.

Department of Biomedical Sciences, College of Liberal Arts and Sciences, Grand Valley State University, 1 Campus Drive, Allendale, MI 49401, USA.
nocherac@gvsu.edu

BACKGROUND: The ω-3-fatty acid docosahexaenoic acid (DHA) is important in infant brain development and maturation. The advisable intake of the ω-3 fatty acids DHA and eicosapentaenoic acid (EPA) for pregnant and lactating women is 300 mg/d or 9 g/month. The objective of this cross-sectional study was to test the hypothesis that low-income pregnant/or lactating women do not consume advisable amounts of DHA+EPA and to determine whether any of the measured demographic factors were related to DHA and EPA consumption.
METHODS: This study was conducted September 2007 to March 2008 and used the N-3 Fatty Acid Food Frequency Questionnaire for dietary assessment in a convenience sample of women (N = 68) enrolled in a local maternal infant health program. Women who reported fish or seafood allergies were excluded. The monthly consumption of DHA+EPA from food sources was measured, and participant race, ethnicity, country of origin, primary language, level of education, marital status, intake of prenatal vitamins containing DHA+EPA, and warnings of fish toxicity were assessed. The data were analyzed using 1-way analysis of variance and t tests.
RESULTS: The average reported DHA+EPA intake was 1.18 g/month across all race/ethnicities. African Americans consumed significantly more DHA+EPA, 2.79 g/month, compared with Hispanics (1.64 g) and Caucasians (0.93 g). United States natives consumed significantly more DHA+EPA than immigrants (2.45 g vs 1.55 g).
CONCLUSIONS: Low-income pregnant/and lactating women in the study consumed less than the advisable amounts of DHA+EPA. Both ethnicity and country of origin are related to DHA+EPA intake.

PMID: 21724916 [PubMed - indexed for MEDLINE]

216. World Allergy Organ J. 2011 Jul;4(7):120-5. doi: 10.1097/WOX.0b013e3182260a57. Epub 2011 Jul 14.

Climate change, migration, and allergic respiratory diseases: an update for the allergist.

D'Amato G, Rottem M, Dahl R, Blaiss M, Ridolo E, Cecchi L, Rosario N, Motala C, Ansotegui I, Annesi-Maesano I; WAO Special Committee on Climate Change and Allergy.

Division of Respiratory and Allergic Diseases, High Speciality Hospital A. Cardarelli, School of Specialization, Department of Respiratory Diseases,

University of Napoli, Napoli, Italy.

Local climate changes can impact on a number of factors, including air pollution, that have been shown to influence both the development and attacks of allergic respiratory diseases, and thus, they represent an important consideration for the allergist. Migration involves exposure to a new set of pollutants and allergens as well as changes in housing conditions, diet, and accessibility to medical services, all of which are likely to affect migrants' health. This review provides an update on climate change, migration, and allergy and discusses factors for consideration when making recommendations for local allergy service provision and for assessing an individual patient's environmental exposures.

PMCID: PMC3488916
PMID: 23268459 [PubMed]

217. Adv Exp Med Biol. 2011;716:123-42. doi: 10.1007/978-1-4419-9533-9_8.

An emerging role for the lipid mediator sphingosine-1-phosphate in mast cell effector function and allergic disease.

Olivera A, Rivera J.

Laboratory of Molecular Immunogenetics, National Institute of Arthritis and Musculoskeletal and Skin Diseases, National Institutes of Health, Bethesda, Maryland, USA. oliveraa@mail.nih.gov

Sphingosine-1-phosphate (S1P) plays important roles regulating functions of diverse biological systems, including the immune system. S1P affects immune cell function mostly by acting through its receptors at the cell membrane but it can also induce S1P receptor-independent responses in the cells where it is generated. S1P produced in allergically-stimulated mast cells mediates degranulation, cytokine and lipid mediator production and migration of mast cells towards antigen by mechanisms that are both S1P receptor-dependent and independent. Even in the absence of an antigen challenge, the differentiation and responsiveness of mast cells can be affected by chronic exposure to elevated S1P from a nonmast cell source, whichmay occur under pathophysiological conditions, potentially leading to the hyper-responsiveness of mast cells. The role of S1P extends beyond the regulation of the function of mast cells to the regulation of the surrounding or distal environment. S1P is exported out of antigen-stimulated mast cells and into the extracellular space and the resulting S1P gradient within the tissue may influence diverse surrounding tissue cells and several aspects of the allergic disease, such as inflammation or tissue remodeling. Furthermore, recent findings indicate that vasoactive mediators released systemically by mast cells induce the production of S1P in nonhematopoietic compartments, where it plays a role in regulating the vascular tone and reducing the hypotension characteristic of the anaphy lactic shock and thus helping the recovery. The dual actions of S1P, promoting the immediate response of mast cells, while controlling the systemic consequences of mast cell activity will be discussed in detail.

PMCID: PMC3214605
PMID: 21713655 [PubMed - indexed for MEDLINE]

218. Adv Exp Med Biol. 2011;716:14-28. doi: 10.1007/978-1-4419-9533-9_2.

Mast cell progenitor trafficking and maturation.

Hallgren J, Gurish MF.

Department of Medical Biochemistry and Microbiology, Uppsala University, Uppsala, Sweden.

Mast cells are derived from the hematopoietic progenitors found in bone marrow and spleen. Committed mast cell progenitors are rare in bone marrow suggesting they are rapidly released into the blood where they circulate and move out into the peripheral tissues. This migration is controlled in a tissue specific manner. Basal trafficking to the intestine requires expression of α4β7 integrin and the chemokine receptor CXCR2 by the mast cell progenitors and expression of MAdCAM-1 and VCAM-1 in the intestinal endothelium; and is also controlled by dendritic cells expressing the transcriptional regulatory protein T-bet. None of these play a role in basal trafficking to the lung. With the induction of allergic inflammation in the lung, there is marked recruitment of committed mast cell progenitors to lung and these cells must express α4β7 and α4β1 integrins. Within the lung there is a requirement for expression of VCAM-1 on the endothelium that is regulated by CXCR2, also expressed on the endothelium. There is a further requirement for expression of the CCR2/CCL2 pathways for full recruitment of the mast cell progenitors to the antigen-inflamed lung.

PMID: 21713649 [PubMed - indexed for MEDLINE]

219. BMC Public Health. 2011 Jun 28;11:509. doi: 10.1186/1471-2458-11-509.

Drug utilization patterns and reported health status in ethnic German migrants (Aussiedler) in Germany: a cross-sectional study.

Volodina A, Bertsche T, Kostev K, Winkler V, Haefeli WE, Becher H.

Institute of Public Health, University of Heidelberg, Germany.
anna.volodina@yahoo.com

BACKGROUND: Inadequate utilization of healthcare services by migrant populations is an important public health concern. Inadequate drug consumption and poor compliance to the therapeutic regimen are common manifestations of low health-care seeking behavior present in migrants even in the countries with well-established healthcare systems. There are few studies on the use of medicines among the different groups of migrants in Germany. The objective of this study is to investigate drug consumption patterns of ethnic German migrants (Aussiedler) and their current health status.
METHODS: A cross-sectional study nested into a cohort of 18,621 individuals aged 20-70 years who migrated to Germany from the former Soviet Union between 1990 and 2005 was conducted. Data on consumption of drugs, drug handling, major health risk factors, and one-year disease prevalence were obtained for 114 individuals through a self-administered questionnaire and phone interviews. Results were compared to the data on the German population derived from the Disease Analyzer database and Robert Koch Institute (RKI) annual reports. Direct age standardization, test of differences, Chi-square test, and descriptive statistics were applied as appropriate. For drug classification the Anatomical Therapeutic Chemical (ATC) system was used.
RESULTS: Of the respondents, 97% reported to have at least one disease within a 12-month period. The one-year prevalence of asthma (6.9%), hypertension (26.7%), chronic bronchitis (8.6%), and diabetes (4.9%) in migrants was similar to the general German population. 51% regularly took either over-the-counter (OTC) medication or prescription medicines. Six ATC groups were analyzed. The highest drug consumption was reported for the ATC cardiovascular (22%), nervous (9%), and muskulo-skeletal system (8%). 30% used OTC medicines obtained in the country of origin. Difficulties with drug handling were rare. Alcohol consumption did not differ from the German population ($p = 0.19$ males and 0.27 females), however smoking prevalence was lower ($p < 0.01$) in both sexes.

CONCLUSION: Ethnic German migrants seem to differ only slightly from Germans in health status, drug utilization, and disease risk factors, and if so, not in an extreme way. Country of origin remains a source of medicines for a substantial part of migrants. The study is limited by a small sample size and low response rate.

PMCID: PMC3141468
PMID: 21711531 [PubMed - indexed for MEDLINE]

220. Environ Manage. 2011 Sep;48(3):514-22. doi: 10.1007/s00267-011-9711-7. Epub 2011 Jun 28.

Paving the way for invasive species: road type and the spread of common ragweed (Ambrosia artemisiifolia).

Joly M, Bertrand P, Gbangou RY, White MC, Dubé J, Lavoie C.

École supérieure d'aménagement du territoire et de développement régional, Université Laval, Quebec City, QC, G1A 0V6, Canada.

Roads function as prime habitats and corridors for invasive plant species. Yet despite the diversity of road types, there is little research on the influence of these types on the spread of invaders. Common ragweed (Ambrosia artemisiifolia), a plant producing large amounts of allergenic pollen, was selected as a species model for examining the impact of road type on the spread of invasive plants. We examined this relationship in an agricultural region of Quebec, Canada. We mapped plant distribution along different road types, and constructed a model of species presence. Common ragweed was found in almost all sampling sites located along regional (97%) and local paved (81%) roads. However, verges of unpaved local roads were rarely (13%) colonized by the plant. A model (53% of variance explained), constructed with only four variables (paved regional roads, paved local roads, recently mown road verges, forest cover), correctly predicted (success rate: 89%) the spatial distribution of common ragweed. Results support the hypothesis that attributes associated with paved roads strongly favour the spread of an opportunistic invasive plant species. Specifically, larger verges and greater disturbance associated with higher traffic volume create propitious conditions for common ragweed. To date, emphasis has been placed on controlling the plant in agricultural fields, even though roadsides are probably a much larger seed source. Strategies for controlling the weed along roads have only focused on major highways, even though the considerable populations along local roads also contribute to the production of pollen. Management prioritizations developed to control common ragweed are thus questionable.

PMID: 21710219 [PubMed - indexed for MEDLINE]

221. Am J Pathol. 2011 Jul;179(1):302-14. doi: 10.1016/j.ajpath.2011.03.047. Epub 2011 May 10.

Dual functions of prostaglandin D2 in murine contact hypersensitivity via DP and CRTH2.

Yamamoto Y, Otani S, Hirai H, Nagata K, Aritake K, Urade Y, Narumiya S, Yokozeki H, Nakamura M, Satoh T.

Department of Dermatology, Graduate School, Tokyo Medical and Dental University, Tokyo, Japan.

Erratum in

Am J Pathol. 2012 Jan;180(1):429-30.

Prostaglandin D2 (PGD2) exerts its effects through two distinct receptors: the chemoattractant receptor-homologous molecule expressed on Th2 cells (CRTH2) and the D prostanoid (DP) receptor. Our previous study demonstrated that CRTH2 mediates contact hypersensitivity (CHS) in mice. However, the function of DP receptor remains to be fully established. In this study, we examine the pathophysiological roles of PGD2 using DP-deficient (DP(-/-)) and CRTH2/DP-deficient (CRTH2(-/-)/DP(-/-)) mice to elucidate receptor-mediated PGD2 action in CHS. We observed profound exacerbation of CHS in DP(-/-) mice. CRTH2(-/-)/DP(-/-) mice showed similar exacerbation, but to a lesser extent. These symptoms were accompanied by increased production of interferon-γ and IL-17. The increase in IL-17 producing γδ T cells was marked and presumably contributed to the enhanced CHS. DP deficiency promoted the in vivo migration of dendritic cells to regional lymph nodes. A DP agonist added to DCs in vitro was able to inhibit production of IL-12 and IL-1β. Interestingly, production of IL-10 in dendritic cells was elevated via the DP pathway, but it was lowered by the CRTH2 pathway. Collectively, PGD2 signals through CRTH2 to mediate CHS inflammation, and conversely, DP signals to exert inhibitory effects on CHS. Thus, we report opposing functions for PGD2 that depend on receptor usage in allergic reactions.

Copyright © 2011 American Society for Investigative Pathology. Published by Elsevier Inc. All rights reserved.

PMCID: PMC3123801
PMID: 21703412 [PubMed - indexed for MEDLINE]

222. Arterioscler Thromb Vasc Biol. 2011 Sep;31(9):1991-9. doi: 10.1161/ATVBAHA.111.230847. Epub 2011 Jun 23.

The oral spleen tyrosine kinase inhibitor fostamatinib attenuates inflammation and atherogenesis in low-density lipoprotein receptor-deficient mice.

Hilgendorf I, Eisele S, Remer I, Schmitz J, Zeschky K, Colberg C, Stachon P, Wolf D, Willecke F, Buchner M, Zirlik K, Ortiz-Rodriguez A, Lozhkin A, Hoppe N, von zur Muhlen C, zur Hausen A, Bode C, Zirlik A.

Department of Cardiology, University Hospital Freiburg, Freiburg, Germany.

OBJECTIVE: Spleen tyrosine kinase (SYK) has come into focus as a potential therapeutic target in chronic inflammatory diseases, such as rheumatoid arthritis and asthma, as well as in B-cell lymphomas. SYK has also been involved in the signaling of immunoreceptors, cytokine receptors, and integrins. We therefore hypothesized that inhibition of SYK attenuates the inflammatory process underlying atherosclerosis and reduces plaque development.
METHODS AND RESULTS: Low-density lipoprotein receptor-deficient mice consuming a high-cholesterol diet supplemented with 2 doses of the orally available SYK inhibitor fostamatinib for 16 weeks showed a dose-dependent reduction in atherosclerotic lesion size by up to 59±6% compared with the respective controls. Lesions of fostamatinib-treated animals contained fewer macrophages but more smooth muscle cells and collagen-characteristics associated with more stable plaques in humans. Mechanistically, fostamatinib attenuated adhesion and migration of inflammatory cells and limited macrophage survival. Furthermore, fostamatinib normalized high-cholesterol diet -induced monocytosis and inflammatory gene expression.
CONCLUSIONS: We present the novel finding that the SYK inhibitor fostamatinib attenuates atherogenesis in mice. Our data identify SYK inhibition as a potentially fruitful antiinflammatory therapeutic strategy in atherosclerosis.

PMID: 21700926 [PubMed - indexed for MEDLINE]

223. J Leukoc Biol. 2011 Nov;90(5):1027-33. doi: 10.1189/jlb.0610374. Epub 2011 Jun 22.

Technical advance: Langerhans cells derived from a human cell line in a full-thickness skin equivalent undergo allergen-induced maturation and migration.

Ouwehand K, Spiekstra SW, Waaijman T, Scheper RJ, de Gruijl TD, Gibbs S.

Department of Dermatology, VU University Medical Centre, Amsterdam, The Netherlands.

In this report, the construction of a functional, immunocompetent, full-thickness skin equivalent (SE) is described, consisting of an epidermal compartment containing keratinocytes, melanocytes, and human LCs derived from the MUTZ-3 cell line (MUTZ-LC) and a fibroblast-populated dermal compartment. The CD1a(+)Langerin(+)HLA-DR(+) MUTZ-LCs populate the entire epidermis at a similar density to that found in native skin. Exposure of the SE to subtoxic concentrations of the allergens NiSO(4) and resorcinol resulted in LC migration out of the epidermis toward the fibroblast-populated dermal compartment. A significant dose-dependent up-regulation of the DC maturation-related CCR7 and IL-1β transcripts and of CD83 at the protein level upon epidermal exposure to both allergens was observed, indicative of maturation and migration of the epidermally incorporated LC. We have thus successfully developed a reproducible and functional full-thickness SE model containing epidermal MUTZ-LC. This model offers an alternative to animal testing for identifying potential chemical sensitizers and for skin-based vaccination strategies and provides a unique research tool to study human LC biology in situ under controlled in vitro conditions.

PMID: 21697260 [PubMed - indexed for MEDLINE]

224. Br J Dermatol. 2011 Nov;165(5):976-84. doi: 10.1111/j.1365-2133.2011.10467.x. Epub 2011 Sep 15.

A functional promoter polymorphism of the human IL18 gene is associated with aspirin-induced urticaria.

Kim SH, Son JK, Yang EM, Kim JE, Park HS.

Department of Allergy and Clinical Immunology, Ajou University School of Medicine, Youngtonggu, Suwon 442-721, Korea.

BACKGROUND: Urticaria is the commonest cutaneous reaction caused by aspirin or other nonsteroidal anti-inflammatory drugs. The pathogenesis of aspirin-induced urticaria (AIU) is not fully understood, but appears to involve mast cell activation and neutrophil infiltration.
OBJECTIVES: To investigate the genetic contribution of interleukin (IL)-18, which can amplify acute inflammation by promoting mast cell activation, neutrophil migration and cytokine production, to the pathogenesis of AIU.
METHODS: A case-control association study was performed using 275 patients with AIU and 196 normal healthy controls in a Korean population. Two promoter polymorphisms of the IL18 gene (-607A/C and -137G/C) were genotyped using the primer extension method. The functional effect of the IL18 gene promoter polymorphism was investigated through in vitro studies including a luciferase reporter assay and electrophoretic mobility shift assays (EMSAs) and ex vivo

studies involving neutrophil chemotaxis assays.
RESULTS: A significant association was detected between both AIU in general and the aspirin-intolerant acute urticaria (AIAU) phenotype and the IL18 promoter polymorphism -607A/C. Patients with AIAU showed higher frequencies of the C(-607) G(-137) haplotype, ht1 [CG], compared with controls (P=0·02). Moreover, ht1 [CG] showed a high transcript haplotype by the luciferase activity assay, and EMSAs identified a -607C allele-specific DNA-binding protein as CREB2. Neutrophil chemotactic activity was highest in subjects with AIU exhibiting the high transcript haplotype, ht1 [CG] (P=0·019).
CONCLUSIONS: The high transcript haplotype ht1 [CG] of the IL18 gene may contribute to the development of acute cutaneous inflammation sensitive to aspirin, leading to the clinical presentation of AIAU.

© 2011 The Authors. BJD © 2011 British Association of Dermatologists.

PMID: 21692767 [PubMed - indexed for MEDLINE]

225. Nan Fang Yi Ke Da Xue Xue Bao. 2011 Jun;31(6):928-32.

[Localization and expression of Slingshot-1L in peripheral eosinophils from patients with acute asthma exacerbation].

[Article in Chinese]

Zhang WZ, Zhao HJ, Zhou F, Li WJ, Cai SX.

Department of Respiratory Medicine, Nanfang Hospital, Southern Medical University, Guangzhou 510515, China. zhangweizhen157@126.com

OBJECTIVE: Eosinophils play a pivotal role in asthmatic airway inflammation. We previously found a significantly high expression of Slingshot-1L (SSH-1L) in peripheral eosinophils in acute exacerbations of asthma. Objective To investigate the expression and localization patterns of SSH-1L in peripheral blood eosinophils of asthmatic patients and their changes after treatment with inhaled corticosteroids.
METHODS: We recruited 4 outpatients with acute exacerbations of asthma who received no previous corticosteroid treatment and 1 healthy volunteer. From all the subjects 30 ml peripheral venous blood samples were collected before and after a 3-month treatment with inhaled fluticasone. The eosinophils were isolated, purified and counted, and the expressions of SSH-1L in the eosinophils were examined by RT-PCR and Western blotting. The localization of SSH-1L phosphatases in the peripheral eosinophils was detected by immunofluorescence assay in one patient.
RESULTS: SSH-1L phosphatases distributed diffusely in the cytoplasm, especially dense near the membrane of the peripheral eosinophils. Glucocorticoids treatment resulted in a significant reduction in both the SSH-1L mRNA expression (0.7403∓0.1124 vs 0.4101∓0.0363, P=0.001) and SSH-1L protein expression (0.3410∓0.1337 vs 0.1543∓0.0551, P=0.039).
CONCLUSION: A high expression of SSH-1L in peripheral eosinophils in acute exacerbations of asthma may play a role in the activation and migration of eosinophils. The efficacy of inhaled corticosteroids in asthma control might be partly attributed to a down-regulated expression of SSH-1L.

PMID: 21690039 [PubMed - in process]

226. J Agric Food Chem. 2011 Jul 27;59(14):8028-35. doi: 10.1021/jf201747g. Epub 2011 Jun 21.

Pterostilbene suppresses benzo[a]pyrene-induced airway remodeling.

Kuo PL, Hsu YL, Tsai MJ, Huang MS.

Institute of Clinical Medicine, Kaohsiung Medical University, Kaohsiung 807, Taiwan.

This study has two novel findings: it is not only the first to demonstrate inflammatory cytokines, which are produced by the bronchial epithelium after exposure to benzo[a]pyrene (BaP) and contribute to airway remodeling by increasing human bronchial smooth muscle cells (BSMC) proliferation and migration, but also the first to reveal that pterostilbene, a constituent of grapes and berries, reverses BaP-mediated airway remodeling. Human bronchial epithelial cell lines BEAS-2B and HBE135-E6E7 (HBE) were treated with BaP, and then the condition medium (CM) was harvested, which was then added to BSMC. Cultures of BSMC with BaP-BEAS-2B-CM and -HBE-CM increased BSMC proliferation and migration, which are major features in asthma remodeling. Exposure of BEAS-2B and HBE to BaP caused epithelial cells to produce inflammatory cytokines IL-8, which subsequently induced BSMC proliferation and migration. Moreover, pterostilbene is more potent than resveratrol in suppressing BaP-mediated airway remodeling. This study suggests that pterostilbene is capable of preventing BaP-associated asthma.

PMID: 21675704 [PubMed - indexed for MEDLINE]

227. Soc Sci Med. 2011 Jul;73(1):13-21. doi: 10.1016/j.socscimed.2011.04.012. Epub 2011 May 18.

Hispanic immigrant women's perspective on healthy foods and the New York City retail food environment: A mixed-method study.

Park Y, Quinn J, Florez K, Jacobson J, Neckerman K, Rundle A.

Smith College School for Social Work, Northampton, MA, United States.

Much has been written about the role of dietary acculturation in the epidemic of obesity among Hispanic immigrants in the United States. Yet little is known about the role of beliefs and preferences in immigrants' dietary practices and their relationship to the retail food environment in which the practices occur. We conducted a mixed-methods convergence study of these issues. Twenty-eight foreign-born Hispanic adult women, recruited from families enrolled in a childhood asthma study and mainly living in New York City took part in 60-90 min, semi-structured interviews regarding their dietary beliefs, preferences, and practices. The findings were then used to formulate hypotheses for analyses of food frequency questionnaire (FFQ) data collected from the 345 New York Hispanic women enrolled in the asthma study. Generalized estimating equations were used to determine whether characteristics of the retail food environment within 0.5 km of the home predicted diet, adjusting for individual and neighborhood socio-demographic characteristics. In the interviews, healthy food was rarely discussed in terms of nutritional content. Instead, considerations of freshness, as indicated by time since harvest or slaughter and thus local sourcing; purity, as indicated by the absence of preservatives and processing; and naturalness, as indicated by chemical free farming practices, were the primary axes around which healthy food was defined. Quantitative results were consistent with the qualitative findings: 1) the presence of a farmers' market within the home neighborhood was associated with consumption of more total servings per day of fruit, vegetables, and juice, and 2) the presence of a farmers' market and/or a livestock market was associated with consumption of more servings per day of meat. Proximity to supermarkets or medium-sized grocery stores was not associated with consumption. The results suggest that the availability of fresh produce and

meat from local farms may influence diet among Hispanic women in urban neighborhoods.

Copyright © 2011 Elsevier Ltd. All rights reserved.

PMCID: PMC3126915
PMID: 21658831 [PubMed - indexed for MEDLINE]

228. Chest. 2011 Jun;139(6):1470-9. doi: 10.1378/chest.10-1914.

p38 mitogen-activated protein kinase pathways in asthma and COPD.

Chung KF.

National Heart and Lung Institute, Imperial College, and Biomedical Research Unit, Royal Brompton Hospital, London, England. f.chung@imperial.ac.uk

The mitogen-activated protein kinase (MAPK) family includes the p38 kinases, which consist of highly conserved proline-directed serine-threonine protein kinases that are activated in response to inflammatory signals. Of the four isoforms, p38α is the most abundant in inflammatory cells and has been the most studied through mainly the availability of small molecule inhibitors. The p38 substrates include transcription factors; other protein kinases, which in turn phosphorylate transcription factors; cytoskeletal proteins and translational components; and other enzymes. Both asthma and COPD are characterized by chronic airflow obstruction, airway and lung remodeling, and chronic inflammation. p38 is involved in the inflammatory responses induced by cigarette smoke exposure, endotoxin, and oxidative stress through activation and release of proinflammatory cytokines/chemokines, posttranslational regulation of these genes, and activation of inflammatory cell migration. Inhibition of p38 MAPK prevented allergen-induced pulmonary eosinophilia, mucus hypersecretion, and airway hyperresponsiveness, effects that may partly result from p38 activation on eosinophil apoptosis and on airway smooth muscle cell production of cytokines/chemokines. In addition, p38 regulates the augmented contractile response induced by oxidative stress. The activation of p38 observed in epithelial cells and macrophages also may underlie corticosteroid insensitivity of severe asthma and COPD. Therefore, p38 inhibitors present a potential attractive treatment of these conditions. Second-generation p38 inhibitors have been disappointing in the treatment of rheumatoid arthritis. In two 6-week studies in patients with COPD, the results were encouraging. Side effects such as liver toxicity remain a possibility, and whether the beneficial effects of p38 inhibitors are clinically significant and sustained need to be determined.

PMID: 21652557 [PubMed - indexed for MEDLINE]

229. Int Arch Allergy Immunol. 2011;155 Suppl 1:34-9. doi: 10.1159/000327263. Epub 2011 Jun 1.

Human eosinophils produce and release a novel chemokine, CCL23, in vitro.

Matsumoto K, Fukuda S, Hashimoto N, Saito H.

Department of Allergy and Immunology, National Research Institute for Child Health and Development, Tokyo, Japan.

BACKGROUND: CCL23 (MPIF1/CK-BETA-8) is a novel CC chemokine that plays important roles in the inhibition of myeloid progenitor cell development, the selective recruitment of resting T lymphocytes and monocytes, and the potentiation of

VEGF-induced proliferation and migration of human endothelial cells. Since eosinophils participate in the pathogenesis of airway remodeling, we examined CCL23 production and release by human eosinophils in vitro.
METHODS: Using Ficoll and antibody-coated immunomagnetic beads, eosinophils and other blood cells were purified from peripheral blood samples obtained from normal subjects and mildly allergic patients. Eosinophils were cultured in the presence of 10 ng/ml granulocyte-macrophage colony-stimulating factor (GM-CSF), 10 ng/ml IL-5, 100 ng/ml IFN-γ, 100 ng/ml IFN-α, or immobilized secretory IgA (sIgA). Total mRNA was extracted after 6 h of culture, and mRNA expression was measured using a microarray and RT-PCR. The CCL23 concentrations in the supernatants and cell lysates after 24 and 48 h of culture were measured by ELISA.
RESULTS: CCL23 mRNAs (both CK-β8-1 and CK-β8) were constitutively expressed in fresh eosinophils, and their expression levels were higher than in other types of blood cells. CCL23 mRNAs were significantly increased by stimulation with GM-CSF and IL-5 and slightly by IFN-α and immobilized sIgA. Fresh eosinophils contained trace amounts of CCL23 protein. CCL23 was significantly released into the supernatant when the eosinophils were stimulated with GM-CSF or IL-5 but not with IFN-γ or immobilized sIgA.
CONCLUSION: Our data suggest that eosinophils produce and release CCL23 and may be involved in some in vivo physiological and pathological conditions.

Copyright © 2011 S. Karger AG, Basel.

PMID: 21646793 [PubMed - indexed for MEDLINE]

230. Mol Immunol. 2011 Sep;48(15-16):1784-92. doi: 10.1016/j.molimm.2011.05.003. Epub 2011 Jun 6.

Interleukin(IL)-4 promotion of CXCL-8 gene transcription is mediated by ERK1/2 pathway in human pulmonary artery endothelial cells.

Yang DF, Huang H, Guan S, Zhu HF, Zhang CC, Zhao JP, Fang HJ, Lavoie JP.

Department of Respiratory Diseases, Tongji Hospital Affiliated to Tongji Medical College, Huazhong University of Science and Technology, 1095# Jiefang Ave., Wuhan 430030, China.

Interleukin-4 is central to allergic pulmonary inflammatory responses, but its contribution to airway neutrophilia remains controversial. The endothelium plays a critical role in regulating leukocyte recruitment and migration during inflammation. However, its response to IL-4 is reported to either increase or decrease the production of neutrophil chemotactic factors. We hypothesized that these conflicting findings may be due to the tissue and the size of the vessels from which endothelial cells have been derived. The expression of CXCL-8 by human primary culture umbilical veins endothelial cells (HUVECs), human pulmonary artery endothelial cells (HPAECs), and human pulmonary microvascular endothelial cells (HPMECs) when stimulated with recombinant human IL-4 (rhIL-4) was studied. The chemoattractant property of the cells' supernatants for neutrophils was evaluated using Boyden chambers. The role of the nuclear factor-κB (NF-κB), and mitogen-activated protein kinases (MAPK) in IL-4-induced HPAECs was studied using Western blotting and electrophoretic mobility shift assay (EMSA). We demonstrated that IL-4 increased the mRNA expression and the protein production of CXCL-8 in HPAECs, but not in HUVECs and HPMECs. The supernatants of HAPECs stimulated by IL-4 significantly promoted neutrophils migration in a dose-dependent manner, and was significantly attenuated by an inhibitor of CXCL-8. We also found that extracellular-regulated protein kinase1/2 (ERK1/2) is activated by IL-4 in HPAECs, but not JUN-N-terminal protein kinase (JNK) or p38 MAPK pathway. Furthermore, NF-κB-DNA binding activity, phosphorylation of IκBα and p65 levels

were not affected by rhIL-4 in HAPECs. These findings indicate marked functional differences in the response of micro and macro-ECs to IL-4. ERK1/2, rather than NF-κB, JNK and p38 MAPK signaling, plays a role in IL-4 induced chemokine activation. Our results suggest that inhibition of ERK1/2 may be a possible target for airway neutrophilia in allergic lung diseases.

Copyright © 2011 Elsevier Ltd. All rights reserved.

PMID: 21645924 [PubMed - indexed for MEDLINE]

231. J Clin Immunol. 2011 Aug;31(4):656-65. doi: 10.1007/s10875-011-9529-3. Epub 2011 Jun 4.

Dehydroepiandrosterone suppresses eosinophil infiltration and airway hyperresponsiveness via modulation of chemokines and Th2 cytokines in ovalbumin-sensitized mice.

Liou CJ, Huang WC.

Department of Nursing, Chang Gung Institute of Technology, 261 Wen-Hwa 1st Road, Kwei-Shan, Tao-Yuan, Taiwan, 333, Republic of China. ccliu@gw.cgit.edu.tw

In this study, we evaluated the anti-inflammatory response and the mechanism by which dehydroepiandrosterone modulates immunity in ovalbumin-sensitized asthmatic mice. Female BALB/c mice were sensitized and challenged with ovalbumin and then treated with oral administration of dehydroepiandrosterone on days 21 to 27. The results showed dehydroepiandrosterone could suppress airway hyperresponsiveness and decrease eosinophil infiltration of the lungs in ovalbumin-sensitized mice. Moreover, dehydroepiandrosterone inhibited chemokines, including CCL11/eotaxin-1 and CCL24/eotaxin-2, and Th2-associated cytokine levels in bronchoalveolar lavage fluid. After the inflammatory human bronchial epithelial cell line BEAS-2B was treated with dehydroepiandrosterone, levels of proinflammatory cytokines and chemokines were inhibited, including IL-6, IL-8, CCL11, and CCL24. We suggested that dehydroepiandrosterone inhibited inflammation in bronchial epithelial cells as indicated by the suppression of Th2-associated cytokines and chemokines. Dehydroepiandrosterone also suppressed eosinophil migration and infiltration into the lung to improve the symptoms of asthma in ovalbumin-sensitized mice.

PMID: 21643893 [PubMed - indexed for MEDLINE]

232. Arch Dermatol Res. 2011 Dec;303(10):715-25. doi: 10.1007/s00403-011-1150-z. Epub 2011 Jun 3.

CCL5, CXCL16, and CX3CL1 are associated with Henoch-Schonlein purpura.

Chen T, Guo ZP, Jiao XY, Jia RZ, Zhang YH, Li JY, Huang XL, Liu HJ, Lai T.

Department of Dermatovenereology, West China Hospital of Sichuan University, Chengdu, China.

Chemokines are involved in the pathogenesis of various vascular inflammations. However, information about chemokines in Henoch-Schonlein purpura (HSP) is limited. Herein, we investigated the serum CCL5, CXCL16, and CX3CL1 levels in HSP patients with controls and the ability of sera from HSP patients on chemokine production in human dermal microvascular endothelial cells. Enzyme-linked immunosorbent assay (ELISA) detected serum CCL5, CXCL16, and CX3CL1 levels in patients with HSP. Human dermal microvascular endothelial cell line (HMEC-1) was treated with sera from patients with HSP at different stages, patients with acute

spontaneous urticaria, or controls. Serum levels of CCL5, CXCL16, and CX3CL1 were elevated in HSP patients at acute stage, which correlated with the severity of this disease. Sera from patients with active HSP markedly induced CCL5, CXCL16, and CX3CL1 production at both mRNA and protein levels. In addition, patients' sera-stimulated HMEC-1 supernatants enhanced HL-60 or THP-1 cells migration. Furthermore, patients' sera increased the phosphorylation of inhibitor of κB-α (IκBα) and phosphorylation of extracellular signal-regulated kinase (ERK)1/2 protein levels, upregulated the translocation of nuclear factor-κB (NF-κB) p65 to the nucleus. Taken together, we show firstly that CCL5, CXCL16, and CX3CL1 may be involved in the pathogenesis of HSP. Factors present in sera from patients with active HSP may act as an inducer of inflammatory response in HMEC-1 cells and contribute to chemokine production through NF-κB and ERK 1/2 pathways.

PMID: 21638128 [PubMed - indexed for MEDLINE]

233. PLoS One. 2011;6(5):e20261. doi: 10.1371/journal.pone.0020261. Epub 2011 May 19.

IgE immune complexes stimulate an increase in lung mast cell progenitors in a mouse model of allergic airway inflammation.

Dahlin JS, Ivarsson MA, Heyman B, Hallgren J.

Department of Medical Biochemistry and Microbiology, Uppsala University, Uppsala, Sweden.

Mast cell numbers and allergen specific IgE are increased in the lungs of patients with allergic asthma and this can be reproduced in mouse models. The increased number of mast cells is likely due to recruitment of mast cell progenitors that mature in situ. We hypothesized that formation of IgE immune complexes in the lungs of sensitized mice increase the migration of mast cell progenitors to this organ. To study this, a model of allergic airway inflammation where mice were immunized with ovalbumin (OVA) in alum twice followed by three daily intranasal challenges of either OVA coupled to trinitrophenyl (TNP) alone or as immune complexes with IgE-anti-TNP, was used. Mast cell progenitors were quantified by a limiting dilution assay. IgE immune complex challenge of sensitized mice elicited three times more mast cell progenitors per lung than challenge with the same dose of antigen alone. This dose of antigen challenge alone did not increase the levels of mast cell progenitors compared to unchallenged mice. IgE immune complex challenge of sensitized mice also enhanced the frequency of mast cell progenitors per 10(6) mononuclear cells by 2.1-fold. The enhancement of lung mast cell progenitors by IgE immune complex challenge was lost in FcRγ deficient mice but not in CD23 deficient mice. Our data show that IgE immune complex challenge enhances the number of mast cell progenitors in the lung through activation of an Fc receptor associated with the FcRγ chain. This most likely takes place via activation of FcεRI, although activation via FcγRIV or a combination of the two receptors cannot be excluded. IgE immune complex-mediated enhancement of lung MCp numbers is a new reason to target IgE in therapies against allergic asthma.

PMCID: PMC3098291
PMID: 21625525 [PubMed - indexed for MEDLINE]

234. Clin Exp Allergy. 2011 Oct;41(10):1360-6. doi: 10.1111/j.1365-2222.2011.03775.x. Epub 2011 May 30.

Role of osteopontin, a multifunctional protein, in allergy and asthma.

Konno S, Kurokawa M, Uede T, Nishimura M, Huang SK.

First Department of Medicine, Hokkaido University School of Medicine, Sapporo, Japan. satkonno@med.hokudai.ac.jp

Osteopontin (OPN) is an extracellular matrix protein and immune modulator with a wide range of functions. OPN is recognized as a key cytokine in Th1 immune responses, yet its potential involvement in allergic/asthmatic responses has been investigated only recently. Current data from molecular and cellular studies and studies of OPN-deficient mice provide evidence that OPN plays multiple roles in the regulation of allergic responses, including regulation of IgE response, inflammatory cell migration, and the development of airway fibrosis and angiogenesis. These results suggest that OPN is a pleiotropic cytokine that functions both systemically and locally in tissue mucosa. Notably, OPN is able to exert its effects through different functional domains, and the secreted and intracellular forms of OPN may have distinct functions. Future research to elucidate all aspects of OPN function is needed to ultimately establish its role in the regulation of immune responses and various disease processes, including those critically involved in the development of allergies and asthma.

© 2011 Blackwell Publishing Ltd.

PMID: 21623969 [PubMed - indexed for MEDLINE]

235. Life Sci. 2011 Jul 4;89(1-2):15-9. doi: 10.1016/j.lfs.2011.04.023. Epub 2011 May 19.

Single early prenatal lipopolysaccharide exposure prevents subsequent airway inflammation response in an experimental model of asthma.

Kirsten TB, de Oliveira BP, de Oliveira AP, Kieling K, de Lima WT, Palermo-Neto J, Bernardi MM.

Neuroimmunomodulation Research Group, Department of Pathology, School of Veterinary Medicine, University of São Paulo, São Paulo, SP, Brazil. thik@hotmail.com

AIMS: There has been emerging interest in the prenatal determinants of respiratory disease. In utero factors have been reported to play a role in airway development, inflammation, and remodeling. Specifically, prenatal exposure to endotoxins might regulate tolerance to allergens later in life. The present study investigated whether prenatal lipopolysaccharide (LPS) administration alters subsequent offspring allergen-induced inflammatory response in adult rats.
MAIN METHODS: Pregnant Wistar rats were treated with LPS (100 µg/kg, i.p.) on gestation day 9.5 and their ovariectomized female offspring were sensitized and challenged with OVA later in adulthood. The bronchoalveolar lavage (BAL) fluid, peripheral blood, bone marrow leukocytes and passive cutaneous anaphylaxis were evaluated in these 75-day-old pups.
KEY FINDINGS: OVA sensitized pups of NaCl treated rats showed an increase of leucocytes in BAL after OVA challenge. This increase was attenuated, when mothers were exposed to a single LPS injection early in pregnancy. Thus, LPS prenatal treatment resulted in (1) lower increased total and differential (macrophages, neutrophils, eosinophils and lymphocytes) BAL cellularity count; (2) increased number of total, mononuclear and polymorphonuclear cells in the peripheral blood; and (3) no differences in bone marrow cellularity or passive cutaneous anaphylaxis.
SIGNIFICANCE: In conclusion, female pups treated prenatally with LPS presented an attenuated response to experimentally-induced asthma. We observed reduced immune cell migration from peripheral blood to the lungs, with no effect on the production of bone marrow cells or antibodies. It was suggested that inflammatory

events such as exposure to LPS in early fetal life can attenuate allergic inflammation in the lung, which is a common symptom in asthma.

Copyright © 2011 Elsevier Inc. All rights reserved.

PMID: 21620873 [PubMed - indexed for MEDLINE]

236. J Invest Dermatol. 2011 Aug;131(8):1668-76. doi: 10.1038/jid.2011.72. Epub 2011 May 26.

The histamine H4 receptor is highly expressed on plasmacytoid dendritic cells in psoriasis and histamine regulates their cytokine production and migration.

Gschwandtner M, Mommert S, Köther B, Werfel T, Gutzmer R.

Division of Immunodermatology and Allergy Research, Department of Dermatology and Allergy, Hannover Medical School, Hannover, Germany. maria_gschwandtner@gmx.at

Plasmacytoid dendritic cells (pDC) are present in inflammatory skin lesions, in particular, in psoriasis. In such lesions, the inflammatory mediator histamine is also detected in high amounts. We therefore investigated a possible interaction of pDC with histamine, especially via the most recently described histamine H(4) receptor (H(4)R). We detected the expression of the H(4)R on pDC in the blood and in lesional psoriasis skin. Interestingly, compared with healthy controls and patients with atopic dermatitis, pDC from the blood of psoriasis patients expressed the highest levels of the H(4)R, which was even more upregulated on stimulation with IFN-γ and CpG. After activation of the H(2)R and H(4)R on pDC, we observed downregulation of CpG-induced production of tumor necrosis factor α, IFN-α, and CXCL8, but not of the chemokine CXCL10. Histamine-induced downregulation of cytokine production was more pronounced in pDC derived from psoriasis patients. Furthermore, we observed F-actin polymerization and active migration of pDC in response to H(4)R agonist stimulation. Taken together, our results indicate that the H(4)R is highly expressed on pDC in psoriasis and influences cytokine production and migration of pDC. Therefore, the H(4)R alone or in combination with the H(2)R might be a promising therapeutic target in psoriasis.

PMID: 21614010 [PubMed - indexed for MEDLINE]

237. J Pediatr Gastroenterol Nutr. 2011 Jun;52(6):650-61. doi: 10.1097/MPG.0b013e3182128512.

Role of eosinophils in inflammatory bowel and gastrointestinal diseases.

Woodruff SA, Masterson JC, Fillon S, Robinson ZD, Furuta GT.

Digestive Health Institute, The Children's Hospital, Denver, CO, USA.

Inflammatory bowel diseases (IBD) are characterized by the invasion of leukocytes into the intestinal mucosa. However, a mixed inflammatory picture is observed that includes neutrophils, lymphocytes, monocytes, and eosinophils. To this day, the role of eosinophils in health and in disease remains unclear. Investigations into their function stem primarily from allergic diseases, asthma, and parasitic infections. This makes it even more difficult to discern a role for the fascinating eosinophil in IBDs because, unlike the lung or the skin, eosinophils reside in normal intestinal mucosa and increase in disease states; consequently, an intricate system must regulate their migration and numbers. These granulocytes are equipped with the machinery to participate in gastrointestinal (GI)

inflammation and in the susceptible microenvironment, they may initiate or perpetuate an inflammatory response. A significant body of literature characterizes eosinophils present in the GI microenvironment where they have the potential to interact with other resident cells, thus promoting intestinal remodeling, mucus production, epithelial barrier, cytokine production, angiogenesis, and neuropeptide release. A number of lines of evidence support both potential beneficial and deleterious roles of eosinophils in the gut. Although studies from the gut and other mucosal organs suggest eosinophils affect mucosal GI inflammation, definitive roles for eosinophils in IBDs await discovery.

PMID: 21593640 [PubMed - indexed for MEDLINE]

238. J Leukoc Biol. 2011 Aug;90(2):285-91. doi: 10.1189/jlb.0211087. Epub 2011 May 17.

Inhibitory effect of prostaglandin I2 on bone marrow kinetics of eosinophils in the guinea pig.

Sturm EM, Schuligoi R, Konya V, Sturm GJ, Heinemann A.

Institute for Experimental and Clinical Pharmacology, Medical University of Graz, Graz, Austria. eva.sturm@medunigraz.at

Enhanced eosinophil trafficking from bone marrow to the tissue is a hallmark of allergic diseases. We have shown previously that PGI(2) markedly attenuates the locomotion of human eosinophils in vitro. Here, we set out to determine the effect of PGI(2) on the trafficking of bone marrow eosinophils in the guinea pig. Shape change of bone marrow eosinophils was determined by flow cytometry, and chemotaxis assays were performed using a transwell migration system. Eosinophil release from bone marrow of guinea pigs was investigated in the isolated, perfused hind-limb preparation. We found that PGI(2) prevented the mobilization of eosinophils from bone marrow and attenuated the shape change and chemotactic responses of bone marrow eosinophils. These effects were mimicked by iloprost and were prevented by the IP antagonist CAY10441 and the adenylyl cyclase inhibitor SQ22536. Immunohistochemical staining of bone marrow confirmed the expression of IPs by bone marrow eosinophils. The rate-limiting enzyme of PGI(2) formation, PGIS, was found in large mononuclear cells. These data show that IP activation negatively modulates the mobilization and locomotion of bone marrow eosinophils and might therefore also protect against exaggerated recruitment of eosinophils to inflammatory sites.

PMID: 21586677 [PubMed - indexed for MEDLINE]

239. Subcell Biochem. 2011;55:139-57. doi: 10.1007/978-94-007-1217-1_6.

Purinergic signaling in wound healing and airway remodeling.

van der Vliet A, Bove PF.

Department of Pathology, University of Vermont, Burlington, VT, USA, albert.van-der-vliet@uvm.edu.

Airway epithelia are continuously damaged by airborne pollutants, pathogens and allergens, and they rely on intrinsic mechanisms to restore barrier integrity. Epithelial repair is a multi-step process including cell migration into the wounded area, proliferation, differentiation and matrix deposition. Each step requires the secretion of various molecules, including growth factors, integrins and matrix metalloproteinases. Evidence is emerging that purinergic signaling

promotes repair in human airway epithelia. An injury induces ATP release, which binds P2Y(2) receptors (P2Y(2)Rs) to initiate protein kinase C (PKC)-dependent oxidative activation of TNFα-converting enzyme (TACE), which then releases the membrane-bound ligands of the epidermal growth factor receptor (EGFR). The P2Y(2)R- and EGFR-dependent signaling cascades converge to induce mediator release, whereas the latter also induces cytoskeletal rearrangement for cell migration and proliferation. Similar roles for purinergic signaling are reported in pulmonary endothelial cells, smooth muscle cells and fibroblasts. In chronic airway diseases, the aberrant regulation of extracellular purines is implicated in the development of airway remodeling by mucus cell metaplasia and hypersecretion, excess collagen deposition, fibrosis and neovascularization. This chapter describes the crosstalk between these signaling cascades and discusses the impact of deregulated purinergic signaling in chronic lung diseases.

PMID: 21560047 [PubMed - in process]

240. J Clin Immunol. 2011 Aug;31(4):666-80. doi: 10.1007/s10875-011-9541-7. Epub 2011 May 11.

Role of macrophage migration inhibitory factor in the Th2 immune response to epicutaneous sensitization.

Das R, Moss JE, Robinson E, Roberts S, Levy R, Mizue Y, Leng L, McDonald C, Tigelaar RE, Herrick CA, Bucala R.

Department of Medicine, Yale University School of Medicine, The Anlyan Center Room S525, New Haven, CT 06520-8031, USA.

We examined the role of macrophage migration inhibitory factor (MIF) in the generation of the Th2 response using MIF-deficient mice in a model of epicutaneous sensitization to ovalbumin. Lymph node cells from sensitized MIF-deficient mice produce lower levels of Th2 cytokines after antigen challenge when compared to their wild-type counterparts. Sensitized mice lacking MIF show less pulmonary inflammation after intranasal antigen exposure. Mice deficient in CD74, the MIF receptor, also are unable to generate an inflammatory response to epicutaneous sensitization. Examination of the elicitation phase of the atopic response using DO11.10 OVA TCR transgenic animals shows that T cell proliferation and IL-2 production are strongly impaired in MIF-deficient T cells. This defect is most profound when both T cells and antigen-presenting cells are lacking MIF. These data suggest that MIF is crucial both for the sensitization and the elicitation phases of a Th2-type immune response in allergic disease.

PMID: 21559932 [PubMed - indexed for MEDLINE]

241. PLoS One. 2011 Apr 29;6(4):e19528. doi: 10.1371/journal.pone.0019528.

C-kit binding properties of hesperidin (a major component of KMP6) as a potential anti-allergic agent.

Jeong HJ, Choi Y, Kim KY, Kim MH, Kim HM.

Biochip Research Center, Hoseo University, Asan, Republic of Korea.

Accumulation of mast cells can be causally related to several allergic inflammations. Stem cell factor (SCF) as a mast cell chemotaxin induces mast cell migration. To clarify a new effect of Pyeongwee-San extract (KMP6, a drug for indigestion) for the treatment of allergy, we investigated the effects of KMP6 on SCF-induced migration of rat peritoneal mast cells (RPMCs). A molecular docking

simulation showed that hesperidin, a major component of KMP6, controls the SCF and c-kit binding by interaction with the active site of the c-kit. KMP6 and hesperidin significantly inhibited SCF-induced migration of RPMCs ($P<0.05$). The ability of the SCF to enhance morphological alteration and F-actin formation was also abolished by treatment with KMP6 or hesperidin. KMP6 and hesperidin inhibited SCF-induced p38 MAPK activation. In addition, SCF-induced inflammatory cytokine production was significantly inhibited by treatment with KMP6 or hesperidin ($P<0.05$). Our results show for the first time that KMP6 potently regulates SCF-induced migration, p38 MAPK activation and inflammatory cytokines production through hindrance of SCF and c-kit binding in RPMCs. Such modulation may have functional consequences during KMP6 treatment, especially mast cell-mediated allergic inflammation disorders.

PMCID: PMC3085475
PMID: 21559359 [PubMed - indexed for MEDLINE]

242. Aesthetic Plast Surg. 2011 Dec;35(6):1165-71. doi: 10.1007/s00266-011-9740-x. Epub 2011 May 10.

Lid loading for treatment of paralytic lagophthalmos.

Yu Y, Sun J, Chen L, Liu L.

Department of Oral & Maxillofacial Surgery, West China Hospital of Stomatology, Sichuan University, Chengdu, 610041, People's Republic of China.

Lagophthalmos secondary to facial palsy is a most clinically important condition that requires effective and early treatment because prolonged corneal exposure may cause corneal lesions, ranging from corneal spots to corneal ulceration and finally blindness. Lid loading is the therapy used most commonly to treat the condition. This method was first described in 1950, modified in 1966, and popularized in 1974. Since its inception, only several reviews have referred to the technology, but they talked about only parts of this technology and did not provide information on the technology overall. This review discusses lid loading in detail. This method now often uses gold and platinum as the material for the implant and should be done as early as possible in those patients whose paralytic lagophthalmos has little chance of being reversed. This method has shown good clinical results and given patients a better perspective. Of course, this method has its intrinsic complications such as allergic reactions, extrusion, and migration. However, with modification of the implant and the surgical procedure, the complication rate has decreased. In conclusion, although lid loading cannot solve all the problems associated with the paralyzed eyelid, it is a simple, reversible, and effective way to treat paralytic lagophthalmos.

PMID: 21556983 [PubMed - indexed for MEDLINE]

243. Arch Pharm Res. 2011 Mar;34(3):425-36. doi: 10.1007/s12272-011-0310-1. Epub 2011 May 6.

Anti-metastatic properties of the leaves of Eriobotrya japonica.

Cha DS, Shin TY, Eun JS, Kim DK, Jeon H.

College of Pharmacy, Woosuk University, Chonbuk, Korea.

The leaves of Eriobotrya japonica Lindl. have been widely used as a traditional medicine for the treatment of many diseases including gastroenteric disorders, diabetes mellitus, chronic bronchitis and asthma. In the present study, the

anti-metastatic action of the EtOAc fraction of the leaves of E. japonica (LEJ) was investigated. LEJ showed potent inhibitory effects on MMP-2 and MMP-9 activities and expressions via down-regulation of NF-κB translocation to the nucleus in B16F10 cells. In addition, the cell migration and invasion were down-regulated by LEJ. LEJ also significantly suppressed lung metastasis in vivo. Moreover, we isolated the compounds ursolic acid and 2α-hydroxyursolic acid from LEJ and both compounds also significantly suppressed MMP-2 and MMP-9 activities, indicating that they are the active components of LEJ. The present results demonstrate that LEJ may be used as valuable antimetastatic agent for the treatment of cancer metastasis.

PMID: 21547674 [PubMed - indexed for MEDLINE]

244. An Sist Sanit Navar. 2011 Jan-Apr;34(1):43-50.

[Allergic sensitization profile in the immigrant population living in the central region of Spain].

[Article in Spanish]

Dominguez-Ortega J, González de Olano D, Trujillo MJ, Henríquez A, Losada A, Rodríguez-Domínguez B; Grupo ESPLORA.

Collaborators: Trujillo MJ, Feliu A, Mancebo EG, Gandolfo M, González de Olano D, Ruiz-Hornillos J, Henríquez A, González E, Losada A, Bermejo S, Vidal C, Moreno A, Cervera JG, Domínguez BR, de la Torre F, Jimeno L, Huescar A, Jiménez BR, Ortega JD.

Servicio de Alergología, Hospital Universitario de Getafe, Madrid, Spain. alergologia.hugf@salud.madrid.org

INTRODUCTION: The prevalence of allergic diseases has increased worldwide in the last two decades, particularly in developed countries. Respiratory allergy is determined by genetic heredity, influenced by environmental factors. Migration is a good epidemiological model for assessing the influence of the environment. We present the clinical characteristics of respiratory allergy in immigrants in the central region of Spain.
METHODS: We prospectively collected data on all immigrants referred to the allergy units of 7 different hospitals in Madrid, Cuenca and Ciudad Real in March 2010. Respiratory Allergy was diagnosed using a standard study for allergic diseases. Results in immigrants were compared with data from a similar Spanish population.
RESULTS: Sixty-two immigrants and 32 Spanish patients were evaluated (63% female, mean age 28.4 years). Their countries of origin were uniformly distributed among 3 macroareas (North of Africa, Latin America, and Eastern Europe). More than 96% presented rhinitis, although persistent rhinitis was more prevalent amongst Latin Americans (76.9%) than in the Spanish population (48%). No differences were observed in asthma prevalence, although immigrants had higher rates of non-controlled and partially controlled asthma. The mean time of onset of symptoms after immigration was 43 months. Grass pollen was the most relevant allergen with the exception of Arab patients. Sensitization to polcalcin was rare. Otherwise, 44.1% of the Spanish population was sensitized to profilin (only 4.88% among immigrants) although this sensitization did not associate allergy to fruits or other vegetables.
CONCLUSIONS: In summary, a multicentric study is presented where significant differences have been observed in the sensitization pattern and clinical evolution of respiratory allergy in immigrants and the Spanish population.

PMID: 21532645 [PubMed - indexed for MEDLINE]

245. Ann Allergy Asthma Immunol. 2011 May;106(5):407-11. doi: 10.1016/j.anai.2011.01.023.

The possible influence of the environment on respiratory allergy: a survey on immigrants to Italy.

Lombardi C, Canonica GW, Passalacqua G; IGRAM, Italian Group on Respiratory Allergy in Migrants.

Collaborators: Antonicelli L, Ariano R, Asero R, Berra D, Bignardi D, Corsico A, Costantino MT, Crivellaro M, Gani F, Folletti I, Incorvaia C, Liccardi G, Marcer G, Marogna M, Milanese M, Minale P, Musarra A, Nebiolo F, Ridolo E, Rolla G, Senna G, Schiappoli M, Siracusa A, Voltolini S, Yacoub MR, Zanon P.

Allergy Unit, Department of Internal Medicine, Sant'Orsola Hospital, Brescia, Italy.

BACKGROUND: Respiratory allergy is influenced and determined by genetic and environmental factors. Migration is a good model to indirectly evaluate the possible influence of environment.
OBJECTIVE: To assess the clinical characteristics of respiratory allergy in immigrants to Italy, in comparison with the Italian population.
METHODS: The clinical/demographic data of those immigrants stably living in Italy and referred for the first time to allergy services for respiratory allergy were collected in a multicenter survey. All the patients underwent a standard diagnostic workup. A matched Italian population was also examined.
RESULTS: Six hundred ninety-eight immigrants and 859 Italians had at least one positive skin test and were analyzed. Most of the patients were referred to the allergy units by their general practitioners. In those patients, the demographic characteristics were not different, except for family size. Immigrants had less family history of atopy. Only 16% had a clinical history of allergy before migration. The time elapsed between migration and onset of symptoms was 5.3 ± 3.1 years, with a minimum of 0.5 and a maximum of 7 years. A higher rate of monosensitization was seen among immigrants, and the severity of their asthma/rhinitis was greater than in Italians. No difference was seen in the pattern of sensitizations.
CONCLUSION: In this population of immigrants, environmental factors play a relevant role in the onset of respiratory allergies.

Copyright © 2011 American College of Allergy, Asthma & Immunology. Published by Elsevier Inc. All rights reserved.

PMID: 21530873 [PubMed - indexed for MEDLINE]

246. Bioorg Med Chem Lett. 2011 Jun 1;21(11):3307-12. doi: 10.1016/j.bmcl.2011.04.021. Epub 2011 Apr 13.

Phosphodiesterase inhibitors. Part 1: Synthesis and structure-activity relationships of pyrazolopyridine-pyridazinone PDE inhibitors developed from ibudilast.

Allcock RW, Blakli H, Jiang Z, Johnston KA, Morgan KM, Rosair GM, Iwase K, Kohno Y, Adams DR.

Chemistry Department, School of Engineering and Physical Sciences, Heriot-Watt University, Riccarton, Edinburgh EH14 4AS, United Kingdom.

Ibudilast [1-(2-isopropylpyrazolo[1,5-a]pyridin-3-yl)-2-methylpropan-1-one] is a nonselective phosphodiesterase inhibitor used clinically to treat asthma. Efforts to selectively develop the PDE3- and PDE4-inhibitory activity of ibudilast led to replacement of the isopropyl ketone by a pyridazinone heterocycle. Structure-activity relationship exploration in the resulting 6-(pyrazolo[1,5-a]pyridin-3-yl)pyridazin-3(2H)-ones revealed that the pyridazinone lactam functionality is a critical determinant for PDE3-inhibitory activity, with the nitrogen preferably unsubstituted. PDE4 inhibition is strongly promoted by introduction of a hydrophobic substituent at the pyridazinone N(2) centre and a methoxy group at C-7' in the pyrazolopyridine. Migration of the pyridazinone ring connection from the pyrazolopyridine 3'-centre to C-4' strongly enhances PDE4 inhibition. These studies establish a basis for development of potent PDE4-selective and dual PDE3/4-selective inhibitors derived from ibudilast.

Copyright © 2011 Elsevier Ltd. All rights reserved.

PMID: 21530250 [PubMed - indexed for MEDLINE]

247. J Immunol. 2011 Jun 1;186(11):6485-96. doi: 10.4049/jimmunol.1001868. Epub 2011 Apr 27.

Priming of eosinophils by GM-CSF is mediated by protein kinase CbetaII-phosphorylated L-plastin.

Pazdrak K, Young TW, Straub C, Stafford S, Kurosky A.

Department of Biochemistry and Molecular Biology, University of Texas Medical Branch, Galveston, TX 77555, USA.

The priming of eosinophils by cytokines leading to augmented response to chemoattractants and degranulating stimuli is a characteristic feature of eosinophils in the course of allergic inflammation and asthma. Actin reorganization and integrin activation are implicated in eosinophil priming by GM-CSF, but their molecular mechanism of action is unknown. In this regard, we investigated the role of L-plastin, an eosinophil phosphoprotein that we identified from eosinophil proteome analysis. Phosphoproteomic analysis demonstrated the upregulation of phosphorylated L-plastin after eosinophil stimulation with GM-CSF. Additionally, coimmunoprecipitation studies demonstrated a complex formation of phosphorylated L-plastin with protein kinase CβII (PKCβII), GM-CSF receptor α-chain, and two actin-associated proteins, paxilin and cofilin. Inhibition of PKCβII with 4,5-bis(4-fluoroanilino)phtalimide or PKCβII-specific small interfering RNA blocked GM-CSF-induced phosphorylation of L-plastin. Furthermore, flow cytometric analysis also showed an upregulation of α(M)β(2) integrin, which was sensitive to PKCβII inhibition. In chemotaxis assay, GM-CSF treatment allowed eosinophils to respond to lower concentrations of eotaxin, which was abrogated by the above-mentioned PKCβII inhibitors. Similarly, inhibition of PKCβII blocked GM-CSF induced priming for degranulation as assessed by release of eosinophil cationic protein and eosinophil peroxidase in response to eotaxin. Importantly, eosinophil stimulation with a synthetic L-plastin peptide (residues 2-19) phosphorylated on Ser(5) upregulated α(M)β(2) integrin expression and increased eosinophil migration in response to eotaxin independent of GM-CSF stimulation. Our results establish a causative role for PKCβII and L-plastin in linking GM-CSF-induced eosinophil priming for chemotaxis and degranulation to signaling events associated with integrin activation via induction of PKCβII-mediated L-plastin phosphorylation.

PMCID: PMC3100773
PMID: 21525390 [PubMed - indexed for MEDLINE]

248. J Immunol. 2011 Jun 1;186(11):6129-35. doi: 10.4049/jimmunol.1004007. Epub 2011 Apr 22.

NK cells are effectors for resolvin E1 in the timely resolution of allergic airway inflammation.

Haworth O, Cernadas M, Levy BD.

Division of Pulmonary and Critical Care Medicine, Department of Internal Medicine, Brigham and Women's Hospital and Harvard Medical School, Harvard Institutes of Medicine, Boston, MA 02115, USA.

Immune responses are pathologically sustained in several common diseases, including asthma. To determine endogenous proresolving mechanisms for adaptive immune responses, we used a murine model of self-limited allergic airway inflammation. After cessation of allergen exposure, eosinophils and T cells were cleared concomitant with the appearance of increased numbers of NK cells in the lung and mediastinal lymph nodes. The mediastinal lymph node NK cells were activated, expressing CD27, CD11b, CD69, CD107a, and IFN-γ. NK cell depletion disrupted the endogenous resolution program, leading to delayed clearance of airway eosinophils and Ag-specific CD4(+) T cells. NK cell trafficking to inflamed tissues for resolution was dependent upon CXCR3 and CD62L. During resolution, eosinophils and Ag-specific CD4(+) T cells expressed NKG2D ligands, and a blocking Ab for the NKG2D receptor delayed clearance of these leukocytes. Of interest, NK cells expressed CMKLR1, a receptor for the proresolving mediator resolvin E1, and depletion of NK cells decreased resolvin E1-mediated resolution of allergic inflammation. Resolvin E1 regulated NK cell migration in vivo and NK cell cytotoxicity in vitro. Together, these findings indicate new functions in catabasis for NK cells that can also serve as targets for proresolving mediators in the resolution of adaptive immunity.

PMID: 21515793 [PubMed - indexed for MEDLINE]

249. Bull Acad Natl Med. 2010 Jun;194(6):1009-19.

[Severe asthma: new therapeutic targets].

[Article in French]

Aubier M.

Pneumologie A, Inserm U700, Hôpital Bichat, Faculté de médecine Denis Diderot, Université Paris 7. michel.aubier@bch.aphp.fr

Severe asthma is a highly incapacitating disease with no effective preventive or curative treatment. The 10% of patients with "refractory" or "difficult" asthma have chronic symptoms, episodic exacerbations and persistent airway obstruction, despite chronic? 2-agonist and steroid therapy. A major objective of current asthma research is to identify the underlying cellular and molecular mechanisms and thus to develop new treatments. Persistent airway eosinophilia is a hallmark of severe asthma. IL-5 is essential for terminal differentiation of committed eosinophil precursors and is also involved in eosinophily degranulation and priming. By releasing cytokines and cationic proteins, eosinophils contribute to airway inflammation and damage the bronchial mucosa. A monoclonal antibody against IL-5 has been shown to reduce exacerbations of refractory asthma. Interventions targeting eosinophil cationic proteins might have therapeutic potential. Structural changes in the bronchial wall, collectively known as airway

remodeling, are believed to play a prominent role in the persistent airflow obstruction associated with severe asthma. In this setting the airway epithelium shows major abnormalities, including loss of barrier function, phenotypic changes, and functional disorders. The abnormal respiratory epithelium is believed to orchestrate airway remodeling through aberrant production of extracellular matrix components, fibrogenic cytokines and chemokines, and growth factors responsible for the proliferation, migration and activation of smooth muscle cells and fibroblasts. Recently, increased ET-1 synthesis by the bronchial epithelium was observed in severe refractory asthma, and was found to correlate with airway remodeling and airway obstruction. ET-1 might represent a novel therapeutic target in severe steroid-refractory asthma.

PMID: 21513135 [PubMed - indexed for MEDLINE]

250. Klin Padiatr. 2011 Nov;223(6):352-5. doi: 10.1055/s-0031-1273752. Epub 2011 Apr 21.

Longitudinal study on specific IgE against natural rubber latex, banana and kiwi in patients with spina bifida.

Cremer R, Mennicken O.

Kliniken der Stadt Köln gGmbH, Kinderkrankenhaus, Klinik für Kinder- und Jugendmedizin, Köln, Germany. CremerR@kliniken-koeln.de

OBJECTIVE: Up to 2 out of 3 spina bifida (sb) patients with natural rubber latex (NRL) antibodies (ab) have crossreacting IgE-ab against tropical fruit, due to structural homologies between several NRL antigens and allergenic fruit proteins. It is essential to investigate whether the patients were first sensitized against NRL or fruit, to give recommendations for an evidence-based prophylaxis.
PATIENTS AND METHODS: We investigated sera of 96 sb patients for specific IgE ab against NRL, banana and kiwi as examples for crossreacting fruit by FEIA (ImmunoCAP System, Phadia). These tests were repeated up to 3 times (mean after 2 years, maximum after 7 years).
RESULTS: In the first testing only 2 of 50 NRL-IgE negative patients (4%) had ab against banana or kiwi. 4 of the 46 NRL-IgE positive patients (8%) showed ab against banana (2) or kiwi (2), 3 (7%) against both fruit. Symptoms of fruit allergy were presented by 3 patients, all symptomatic patients had high levels of specific fruit-ab. In the follow-up study 2 patients with low sensitization against NRL lost their NRL ab and their fruit ab, another 2 only the fruit ab, whereas 4 NRL-sensitized patients newly developed ab against banana and 1 against kiwi. Only 2 patients developed ab against fruit without being sensitized against NRL. 7 out of 10 patients with banana and kiwi ab were atopics.
CONCLUSIONS: In most cases the sensitization against fruit follows the NRL sensitization. There is no need to recommend sb patients without NRL sensitization to primarily avoid tropical fruit.

© Georg Thieme Verlag KG Stuttgart · New York.

PMID: 21512968 [PubMed - indexed for MEDLINE]

251. Acta Dermatovenerol Croat. 2011;19(1):51-68.

Immunology of allergic contact dermatitis.

Tončić RJ, Lipozenčić J, Martinac I, Gregurić S.

University Department of Dermatology and Venereology, University Hospital Center

Zagreb, School of Medicine University of Zagreb, Croatia.
jasna.lipozencic@zg.htnet.hr

Allergic contact dermatitis (ACD) is a T-cell mediated skin inflammation caused by repeated skin exposure to contact allergens. This review summarizes current knowledge on the immunology of ACD. Different phases in ACD are distinguished, i.e. sensitization, elicitation and resolution phases. We discuss contact allergen presentation and the central role of antigen presenting cells during sensitization phase. There is an extremely complex interaction of different kinds of immune cells, such as antigen presenting cells, T, B, NK lymphocytes, keratinocytes (KCs), endothelium, mast cells (MCs) and platelets, and this complex interaction is guided through orchestration of numerous cytokines and chemokines. The role of adaptive immunity has been recognized in contact hypersensitivity but we also discuss the important role of some parts of innate immunity such as natural killer T lymphocytes (NKT) and complement system. Cooperation of innate and adaptive immunity, in this case NK cells and B cells, initiates elicitation phase by complement cascade activation, vasoactive substance release and endothelial activation. KCs are not only innocent bystanders, on the contrary, they are involved in all phases of ACD, from the early phase of initiation through sending "danger" signals and activation of innate immunity, through their role in Langerhans cells (LCs) migration, T-cell trafficking, through the height of the inflammatory phase with direct interactions with epidermotropic T-cells, and finally through the resolution phase with the production of anti-inflammatory cytokines and tolerogenic presentation to effector T-cells. Th-1 and Th-17 cells are the main effector cells responsible for tissue damage. At the end, we point out several subsets of T regulatory cells, which exert down-regulatory function and regulate the magnitude and duration of inflammatory reaction.

PMID: 21489368 [PubMed - indexed for MEDLINE]

252. PLoS One. 2011 Mar 31;6(3):e18220. doi: 10.1371/journal.pone.0018220.

Decreased Th1-type inflammatory cytokine expression in the skin is associated with persisting symptoms after treatment of erythema migrans.

Sjöwall J, Fryland L, Nordberg M, Sjögren F, Garpmo U, Jansson C, Carlsson SA, Bergström S, Ernerudh J, Nyman D, Forsberg P, Ekerfelt C.

Division of Infectious Diseases, Department of Clinical and Experimental Medicine, Faculty of Health Sciences, University of Linköping, Linköping, Sweden.

BACKGROUND: Despite the good prognosis of erythema migrans (EM), some patients have persisting symptoms of various character and duration post-treatment. Several factors may affect the clinical outcome of EM, e.g. the early interaction between Borrelia (B.) burgdorferi and the host immune response, the B. burgdorferi genotype, antibiotic treatment as well as other clinical circumstances. Our study was designed to determine whether early cytokine expression in the skin and in peripheral blood in patients with EM is associated with the clinical outcome.
METHODS: A prospective follow-up study of 109 patients with EM was conducted at the Åland Islands, Finland. Symptoms were evaluated at 3, 6, 12 and 24 months post-treatment. Skin biopsies from the EM and healthy skin were immunohistochemically analysed for expression of interleukin (IL)-4, IL-10, IL-12p70 and interferon (IFN)-γ, as well as for B. burgdorferi DNA. Blood samples were analysed for B. burgdorferi antibodies, allergic predisposition and levels of systemic cytokines.
FINDINGS: None of the patients developed late manifestations of Lyme borreliosis. However, at the 6-month follow-up, 7 of 88 patients reported persisting symptoms

of diverse character. Compared to asymptomatic patients, these 7 patients showed decreased expression of the Th1-associated cytokine IFN-γ in the EM biopsies (p=0.003). B. afzelii DNA was found in 48%, B. garinii in 15% and B. burgdorferi sensu stricto in 1% of the EM biopsies, and species distribution was the same in patients with and without post-treatment symptoms. The two groups did not differ regarding baseline patient characteristics, B. burgdorferi antibodies, allergic predisposition or systemic cytokine levels.
CONCLUSION: Patients with persisting symptoms following an EM show a decreased Th1-type inflammatory response in infected skin early during the infection, which might reflect a dysregulation of the early immune response. This finding supports the importance of an early, local Th1-type response for optimal resolution of LB.

PMCID: PMC3069060
PMID: 21483819 [PubMed - indexed for MEDLINE]

253. Clin Exp Allergy. 2011 Aug;41(8):1108-15. doi: 10.1111/j.1365-2222.2011.03744.x. Epub 2011 Apr 11.

Migration and asthma medication in international adoptees and immigrant families in Sweden.

Bråbäck L, Vogt H, Hjern A.

Occupational & Environmental Medicine, Department of Public Health and Clinical Medicine, Umeå University, Umeå, Sweden. lennart.braback@lvn.se

Comment in
    Clin Exp Allergy. 2011 Aug;41(8):1054-8.

BACKGROUND: Studies of asthma in migrant populations illustrate the effects of environmental changes.
OBJECTIVE: In this register study we investigated the importance of exposure to a western lifestyle in different phases of development in Swedish residents with an origin in regions in the world where asthma usually is less prevalent.
METHODS: The study population comprised 24,252 international adoptees, 47,986 foreign-born and 40,971 Swedish-born with foreign-born parents and 1,770,092 Swedish-born residents with Swedish-born parents (age 6-25 years). Purchased prescribed inhaled corticosteroids (ICS) during 2006 were used as an indicator of asthma.
RESULTS: International adoptees and children born in Sweden by foreign-born parents had three- to fourfold higher rates of asthma medication compared with foreign-born children. The odds ratios (ORs) of asthma medication declined persistently with age at immigration. For adoptees the ORs compared with infant adoptees were 0.78 [95% confidence interval (CI) 0.71-0.85] for those adopted at 1-2 years, 0.51 (0.42-0.61) at 3-4 years and 0.35 (0.27-0.44) after 5 or more years of age. Corresponding ORs for foreign-born children with foreign-born parents immigrating at 0-4 years, at 5-9 years, at 10-14 years and at 15 years or more were 0.73 (0.63-0.86), 0.56 (CI 0.46-0.68) and 0.35 (CI 0.28-0.43), respectively. The ORs were only marginally affected by adjustment for region of birth and socio-economic indicators.
CONCLUSIONS AND CLINICAL RELEVANCE: Age at immigration is a more important determinant of purchased ICS than population of origin. This indicates the importance of environmental factors for asthma in schoolchildren and young adults.

© 2011 Blackwell Publishing Ltd.

PMID: 21481023 [PubMed - indexed for MEDLINE]

254. Eur J Immunol. 2011 Jun;41(6):1675-86. doi: 10.1002/eji.201041033. Epub 2011 May 25.

IL-33-activated dendritic cells are critical for allergic airway inflammation.

Besnard AG, Togbe D, Guillou N, Erard F, Quesniaux V, Ryffel B.

University of Orleans, Molecular Immunology and Embryology, Orleans, France.

IL-33, a new member of the IL-1 family cytokine, is involved in Th2-type responses in a wide range of diseases and signals through the ST2 receptor expressed on many immune cells. Since the effects of IL-33 on DCs remain controversial, we investigated the ability of IL-33 to modulate DC functions in vitro and in vivo. Here, we report that IL-33 activates myeloid DCs to produce IL-6, IL-1b, TNF, CCL17 and to express high levels of CD40, CD80 OX40L and CCR7. Importantly, IL-33-activated DCs prime naive lymphocytes to produce the Th2 cytokines IL-5 and IL-13, but not IL-4. In vivo, IL-33 exposure induces DC recruitment and activation in the lung. Using an OVA-induced allergic lung inflammation model, we demonstrate that the reduced airway inflammation in ST2-deficient mice correlates with the failure in DC activation and migration to the draining LN. Finally, we show that adoptive transfer of IL-33-activated DCs exacerbates lung inflammation in a DC-driven model of allergic airway inflammation. These data demonstrate for the first time that IL-33 activates DCs during antigen presentation and thereby drives a Th2-type response in allergic lung inflammation.

Copyright © 2011 WILEY-VCH Verlag GmbH & Co. KGaA, Weinheim.

PMID: 21469105 [PubMed - indexed for MEDLINE]

255. J Exp Med. 2011 Apr 11;208(4):853-67. doi: 10.1084/jem.20100023. Epub 2011 Apr 4.

Selective stimulation of IL-4 receptor on smooth muscle induces airway hyperresponsiveness in mice.

Perkins C, Yanase N, Smulian G, Gildea L, Orekov T, Potter C, Brombacher F, Aronow B, Wills-Karp M, Finkelman FD.

Department of Medicine, Cincinnati Veterans Affairs Medical Center, Cincinnati, OH 45220, USA.

Production of the cytokines IL-4 and IL-13 is increased in both human asthma and mouse asthma models, and Stat6 activation by the common IL-4/IL-13R drives most mouse model pathophysiology, including airway hyperresponsiveness (AHR). However, the precise cellular mechanisms through which IL-4Rα induces AHR remain unclear. Overzealous bronchial smooth muscle constriction is thought to underlie AHR in human asthma, but the smooth muscle contribution to AHR has never been directly assessed. Furthermore, differences in mouse versus human airway anatomy and observations that selective IL-13 stimulation of Stat6 in airway epithelium induces murine AHR raise questions about the importance of direct IL-4R effects on smooth muscle in murine asthma models and the relevance of these models to human asthma. Using transgenic mice in which smooth muscle is the only cell type that expresses or fails to express IL-4Rα, we demonstrate that direct smooth muscle activation by IL-4, IL-13, or allergen is sufficient but not necessary to induce AHR. Five genes known to promote smooth muscle migration, proliferation, and contractility are activated by IL-13 in smooth muscle in vivo. These observations demonstrate that IL-4Rα promotes AHR through multiple mechanisms and provide a model for testing smooth muscle-directed asthma therapeutics.

PMCID: PMC3135339
PMID: 21464224 [PubMed - indexed for MEDLINE]

256. Philos Transact A Math Phys Eng Sci. 2011 May 13;369(1942):1866-82. doi: 10.1098/rsta.2011.0007.

Global health and climate change: moving from denial and catastrophic fatalism to positive action.

Costello A, Maslin M, Montgomery H, Johnson AM, Ekins P.

Institute for Global Health, University College London, Gower Street, London WC1E 6BT, UK. a.costello@ich.ucl.ac.uk

The health effects of climate change have had relatively little attention from climate scientists and governments. Climate change will be a major threat to population health in the current century through its potential effects on communicable disease, heat stress, food and water security, extreme weather events, vulnerable shelter and population migration. This paper addresses three health-sector strategies to manage the health effects of climate change-promotion of mitigation, tackling the pathways that lead to ill-health and strengthening health systems. Mitigation of greenhouse gas (GHG) emissions is affordable, and low-carbon technologies are available now or will be in the near future. Pathways to ill-health can be managed through better information, poverty reduction, technological innovation, social and cultural change and greater coordination of national and international institutions. Strengthening health systems requires increased investment in order to provide effective public health responses to climate-induced threats to health, equitable treatment of illness, promotion of low-carbon lifestyles and renewable energy solutions within health facilities. Mitigation and adaptation strategies will produce substantial benefits for health, such as reductions in obesity and heart disease, diabetes, stress and depression, pneumonia and asthma, as well as potential cost savings within the health sector. The case for mitigating climate change by reducing GHGs is overwhelming. The need to build population resilience to the global health threat from already unavoidable climate change is real and urgent. Action must not be delayed by contrarians, nor by catastrophic fatalists who say it is all too late.

© 2011 Royal Society

PMID: 21464077 [PubMed - indexed for MEDLINE]

257. J Trauma. 2011 Oct;71(4):952-60. doi: 10.1097/TA.0b013e3181f8aa2d.

The anaphylatoxin receptor C5aR is present during fracture healing in rats and mediates osteoblast migration in vitro.

Ignatius A, Ehrnthaller C, Brenner RE, Kreja L, Schoengraf P, Lisson P, Blakytny R, Recknagel S, Claes L, Gebhard F, Lambris JD, Huber-Lang M.

Institute of Orthopaedic Research and Biomechanics, Department of Traumatology, Hand-, Plastic-, and Reconstructive Surgery, Center of Surgery, University of Ulm, Ulm, Germany. anita.ignatius@uni-ulm.de

BACKGROUND: There is evidence that complement components regulate cytokine production in osteoblastic cells, induce cell migration in mesenchymal stem cells, and play a regulatory role in normal enchondral bone formation. We proved the hypothesis that complement might be involved in bone healing after fracture.

METHODS: We investigated the expression of the key anaphylatoxin receptor C5aR during fracture healing in rats by immunostaining after 1, 3, 7, 14, and 28 days. C5aR expression was additionally analyzed in human mesenchymal stem cells (hMSC) during osteogenic differentiation, in human primary osteoblasts, and osteoclasts by reverse transcriptase polymerase chain reaction and immunostaining. Receptor functionality was proven by the migratory response of cells to C5a in a Boyden chamber.
RESULTS: C5aR was expressed in a distinct spatial and temporal pattern in the fracture callus by differentiated osteoblast, chondroblast-like cells in cartilaginous regions, and osteoclasts. In vitro C5aR was expressed by osteoblasts, osteoclasts, and hMSC undergoing osteogenic differentiation. C5aR was barely expressed by undifferentiated hMSC but was significantly induced after osteogenic differentiation. C5aR activation by C5a induced strong chemotactic activity in osteoblasts, and in hMSC, which had undergone osteogenic differentiation, being abolished by a specific C5aR antagonist. In hMSC, C5a induced less migration reflecting their low level of C5aR expression.
CONCLUSIONS: Our in vitro and in vivo results demonstrated the presence of C5aR in bone forming osteoblasts and bone resorbing osteoclasts. It is suggested that C5aR might play a regulatory role in fracture healing in intramembranous and in enchondral ossification, one possible function being the regulation of cell recruitment.

PMCID: PMC3186845
PMID: 21460748 [PubMed - indexed for MEDLINE]

258. Am J Physiol Lung Cell Mol Physiol. 2011 Jun;300(6):L951-7. doi: 10.1152/ajplung.00411.2010. Epub 2011 Apr 1.

Rhinovirus infection induces extracellular matrix protein deposition in asthmatic and nonasthmatic airway smooth muscle cells.

Kuo C, Lim S, King NJ, Johnston SL, Burgess JK, Black JL, Oliver BG.

Discipline of Pharmacology, University of Sydney, Camperdown, Australia.

Airway remodeling, which includes increases in the extracellular matrix (ECM), is a characteristic feature of asthma and is correlated to disease severity. Rhinovirus (RV) infections are associated with increased risk of asthma development in young children and are the most common cause of asthma exacerbations. We examined whether viral infections can increase ECM deposition and whether this increased ECM modulates cell proliferation and migration. RV infection of nonasthmatic airway smooth muscle (ASM) cells significantly increased the deposition of fibronectin (40% increase, n = 12) and perlecan (80% increase, n = 14), while infection of asthmatic ASM cells significantly increased fibronectin (75% increase, n = 9) and collagen IV (15% increase, n = 9). We then treated the ASM cells with the Toll-like receptor (TLR) agonists polyinosinic:polycytidylic acid, imiquimod, and pure RV RNA and were able to show that the mechanism through which RV induced ECM deposition was via the activation of TLR3 and TLR7/8. Finally, we assessed whether the virus-induced ECM was bioactive by measuring the amount of migration and proliferation of virus-naive cells that seeded onto the ECM. Basically, ECM from asthmatic ASM cells induced twofold greater migration of virus-naive ASM cells than ECM from nonasthmatic ASM cells, and these rates of migration were further increased on RV-modulated ECM. Increased migration on the RV-modulated ECM was not due to increased cell proliferation, as RV-modulated ECM decreased the proliferation of virus-naive cells. Our results suggest that viruses may contribute to airway remodeling through increased ECM deposition, which in turn may contribute to increased ASM mass via increased cell migration.

PMID: 21460120 [PubMed - indexed for MEDLINE]

259. Int Arch Allergy Immunol. 2011;156(1):51-61. doi: 10.1159/000322178. Epub 2011 Mar 29.

IL-17A induces CCL28, supporting the chemotaxis of IgE-secreting B cells.

Scanlon KM, Hawksworth RJ, Lane SJ, Mahon BP.

Cellular Immunology Laboratory, Institute of Immunology, National University of Ireland Maynooth, Maynooth, Ireland.

BACKGROUND: Atopic asthma is an allergic disease typically associated with T(H)2 cytokines. IL-17A is also associated with asthma, through the induction of chemokines. Mucosal CCL28 concentrations correlate with cellular recruitment to inflamed airways and support migration of IgA(+) B cells. Here, a link between IL-17A, CCL28 and IgE-secreting B cell chemotaxis is examined.
METHODS: Primary human airway cells and the airway epithelial line A549 were used to characterize IL-17A receptor expression and the effect of IL-17A on CCL28 transcription and translation. B cells, differentiated to IgE+ cells ex vivo, were assessed for CCR10 surface expression and chemotaxis to CCL28 by flow cytometry, transwell migration and ELISpot.
RESULTS: Human airway epithelium expressed both IL-17RA and IL-17RC, and was responsive to IL-17A stimulation. Cultured human IgE+ B cells expressed surface CCR10 and displayed CCR10-dependent chemotaxis towards recombinant CCL28. Enhanced levels of CCL28 were observed upon A549 cell incubation with IL-17A, and this up-regulation significantly increased the migration of IgE+ antibody-secreting B cells. The specificity of chemotaxis was confirmed by migration blockade in the presence of anti-CCL28 or anti-CCR10.
CONCLUSIONS: This work identifies a novel chemokine for the migration of IgE+ B cells, in addition to characterizing induction of CCL28 by IL-17A. Taken together the results presented here propose a new role for IL-17A in the allergic airways, linking this cytokine with the recruitment of IgE+ antibody-secreting B cells, via the induction of CCL28. These observations justify further in vivo studies of larger cohorts.

Copyright © 2011 S. Karger AG, Basel.

PMID: 21447959 [PubMed - indexed for MEDLINE]

260. J Leukoc Biol. 2011 Jul;90(1):167-76. doi: 10.1189/jlb.1110624. Epub 2011 Mar 29.

Chitin particles induce size-dependent but carbohydrate-independent innate eosinophilia.

Kogiso M, Nishiyama A, Shinohara T, Nakamura M, Mizoguchi E, Misawa Y, Guinet E, Nouri-Shirazi M, Dorey CK, Henriksen RA, Shibata Y.

Florida Atlantic University, 777 Glades Rd., Boca Raton, FL 33431-0991, USA.

Murine Mϕ that phagocytose CMP develop into M1; this response depends on the size and the chemical composition of the particles. In contrast, recent studies concluded that chitin particles induce M2 and eosinophil migration, promoting acquired Th2 immune responses against chitin-containing microbes or allergens. This study examined whether these apparently inconsistent responses to chitin could be induced by variation in the size and chemical composition of the chitin particles. We compared the responses of Mϕ with CMP, LCB, and Sephadex G-100 beads (>40 μm). Beads were given i.p. to WT mice and to mice deficient in a

CRTH2, a receptor for the eosinophil chemoattractant PGD(2). In contrast to the M1 activation induced by CMP, i.p. administration of LCB or Sephadex beads induced within 24 h a CRTH2-dependent peritoneal eosinophilia, as well as CRTH2-independent activation of peritoneal Mϕ that expressed Arg I, an M2 phenotype. LCB-induced Mϕ exhibited elevated Arg I and a surface MR, reduced surface TLR2 levels, and no change in the levels of CHI3L1 or IL-10 production. Our results indicate that the effects of chitin in vivo are highly dependent on particle size and that large, nonphagocytosable beads, independent of their chemical composition, induce innate eosinophilia and activate Mϕ expressing several M2, but not M1, phenotypes.

PMCID: PMC3114598
PMID: 21447645 [PubMed - indexed for MEDLINE]

261. Prog Community Health Partnersh. 2011 Spring;5(1):95-103. doi: 10.1353/cpr.2011.0004.

Evaluation of an asthma medication training program for immigrant Mexican community health workers.

Martin MA, Mosnaim GS, Rojas D, Hernandez O, Sadowski LS.

Rush University Medical Center, IL, USA.

BACKGROUND: Community health workers (CHWs) are frontline public health workers who connect immigrant communities with health care services. Although CHW asthma interventions have been shown to improve some outcomes, their ability to change medication adherence remains unclear.
OBJECTIVE: Our goal was to determine if intensive asthma medication training resulted in objective improvements in asthma medication instruction abilities for immigrant Mexican CHWs.
METHODS: Eleven CHWs participated in a 15-hour training course conducted in only Spanish. The course covered asthma pathophysiology, reliever and controller medications, medication technique, and self-management skills. Before and after the training, CHWs completed a written asthma knowledge test and were tested on medication delivery technique using a demonstrator metered dose inhaler (MDI), spacer, and dry powder inhaler (DPI). After the training, CHWs performed a standardized role play to assess their ability to deliver medication instruction. At follow-up evaluations, the CHWs described benefits and weaknesses of the training.
RESULTS: Before the training, the median correct medication technique scores were: MDI = 25%, spacer = 0%, and DPI = 0%. After the training, the median scores were: MDI = 69%, spacer = 64%, and DPI = 67% ($p < .01$). On the role plays, all CHWs were scored as "Demonstrates adequate understanding of a complicated skill" and four were "Ready for the field on a clinical trial." The CHWs described specific application of training skills during the subsequent delivery of an asthma intervention.
CONCLUSION: This training and follow-up evaluation provide objective evidence of improved asthma medication knowledge, delivery technique, and instruction abilities in immigrant Mexican CHWs. With proper training, CHWs can assist families to understand and correctly use complicated asthma medications.

PMID: 21441673 [PubMed - indexed for MEDLINE]

262. Int Immunopharmacol. 2011 Aug;11(8):1120-6. doi: 10.1016/j.intimp.2011.03.009. Epub 2011 Mar 23.

Mesenchymal stem cells ameliorate the histopathological changes in a murine model

of chronic asthma.

Firinci F, Karaman M, Baran Y, Bagriyanik A, Ayyildiz ZA, Kiray M, Kozanoglu I, Yilmaz O, Uzuner N, Karaman O.

Pediatric Allergy and Immunology, Faculty of Medicine, Dokuz Eylul University, Izmir, Turkey. fatih.firinci@deu.edu.tr

Asthma therapies are effective in reducing inflammation but airway remodeling is poorly responsive to these agents. New therapeutic options that have fewer side effects and reverse chronic changes in the lungs are essential. Mesenchymal stem cells (MSCs) are promising for the development of novel therapies in regenerative medicine. This study aimed to examine the efficacy of MSCs on lung histopathology in a murine model of chronic asthma. BALB/c mice were divided into four groups: Group 1 (control group, n=6), Group 2 (ovalbumin induced asthma only, n=10), Group 3 (ovalbumin induced asthma + MSCs, n=10), and Group 4 (MSCs only, n=10). Histological findings (basement membrane, epithelium, subepithelial smooth muscle thickness, numbers of goblet and mast cells) of the airways and MSC migration were evaluated by light, electron, and confocal microscopes. In Group 3, all early histopathological changes except epithelial thickness and all of the chronic changes were significantly ameliorated when compared with Group 2. Evaluation with confocal microscopy showed that no noteworthy amount of MSCs were present in the lung tissues of Group 4 while significant amount of MSCs was detected in Group 3. Serum NO levels in Group 3, were significantly lower than Group 2. The results of this study revealed that MSCs migrated to lung tissue and ameliorated bronchial asthma in murine model. Further studies are needed to evaluate the efficacy of MSCs for the treatment of asthma.

Copyright © 2011 Elsevier B.V. All rights reserved.

PMID: 21439399 [PubMed - indexed for MEDLINE]

263. Immunopharmacol Immunotoxicol. 2011 Dec;33(4):586-93. doi: 10.3109/08923973.2011.561861. Epub 2011 Mar 22.

Immunomodulation of experimental allergic encephalomyelitis by cinnamon metabolite sodium benzoate.

Pahan K.

Department of Neurological Sciences, Rush University Medical Center, Chicago, IL 60612, USA. Kalipada_Pahan@rush.edu

Experimental allergic encephalomyelitis (EAE) is an animal model of multiple sclerosis (MS), the most common human demyelinating disease of the central nervous system. Sodium benzoate (NaB), a metabolite of cinnamon and a FDA-approved drug against urea cycle disorders in children, is a widely used food additive, which is long known for its microbicidal effect. However, recent studies reveal that apart from its microbicidal effects, NaB can also regulate many immune signaling pathways responsible for inflammation, glial cell activation, switching of T-helper cells, modulation of regulatory T cells, cell-to-cell contact, and migration. As a result, NaB alters the neuroimmunology of EAE and ameliorates the disease process of EAE. In this review, we have made an honest attempt to analyze these newly-discovered immunomodulatory activities of NaB and associated mechanisms that may help in considering this drug for various inflammatory human disorders including MS as primary or adjunct therapy.

PMCID: PMC3206174
PMID: 21425926 [PubMed - indexed for MEDLINE]

264. PLoS One. 2011 Mar 15;6(3):e17766. doi: 10.1371/journal.pone.0017766.

An alternate STAT6-independent pathway promotes eosinophil influx into blood during allergic airway inflammation.

Wang W, Hansbro PM, Foster PS, Yang M.

Centre for Asthma and Respiratory Disease, School of Biomedical Sciences and Pharmacy, Faculty of Health, University of Newcastle and Hunter Medical Research Institute, Callaghan, New South Wales, Australia.

BACKGROUND: Enhanced eosinophil responses have critical roles in the development of allergic diseases. IL-5 regulates the maturation, migration and survival of eosinophils, and IL-5 and eotaxins mediate the trafficking and activation of eosinophils in inflamed tissues. CD4(+) Th2 cells are the main producers of IL-5 and other cells such as NK also release this cytokine. Although multiple signalling pathways may be involved, STAT6 critically regulates the differentiation and cytokine production of Th2 cells and the expression of eotaxins. Nevertheless, the mechanisms that mediate different parts of the eosinophilic inflammatory process in different tissues in allergic airway diseases remain unclear. Furthermore, the mechanisms at play may vary depending on the context of inflammation and microenvironment of the involved tissues.
METHODOLOGY/PRINCIPAL FINDINGS: We employed a model of allergic airway disease in wild type and STAT6-deficient mice to explore the roles of STAT6 and IL-5 in the development of eosinophilic inflammation in this context. Quantitative PCR and ELISA were used to examine IL-5, eotaxins levels in serum and lungs. Eosinophils in lung, peripheral blood and bone marrow were characterized by morphological properties. CD4(+) T cell and NK cells were identified by flow cytometry. Antibodies were used to deplete CD4(+) and NK cells. We showed that STAT6 is indispensible for eosinophilic lung inflammation and the induction of eotaxin-1 and -2 during allergic airway inflammation. In the absence of these chemokines eosinophils are not attracted into lung and accumulate in peripheral blood. We also demonstrate the existence of an alternate STAT6-independent pathway of IL-5 production by CD4(+) and NK cells that mediates the development of eosinophils in bone marrow and their subsequent movement into the circulation.
CONCLUSIONS: These results suggest that different points of eosinophilic inflammatory processes in allergic airway disease may be differentially regulated by the activation of STAT6-dependent and -independent pathways.

PMCID: PMC3057993
PMID: 21423619 [PubMed - indexed for MEDLINE]

265. Thorax. 2011 Sep;66(9):755-63. doi: 10.1136/thx.2010.152512. Epub 2011 Mar 21.

Bacterial-induced protection against allergic inflammation through a multicomponent immunoregulatory mechanism.

Nembrini C, Sichelstiel A, Kisielow J, Kurrer M, Kopf M, Marsland BJ.

Institute of Integrative Biology, Molecular Biomedicine, ETH Zürich, Switzerland.

Comment in
    Thorax. 2011 Sep;66(9):744-5.

BACKGROUND: Airborne microbial products have been reported to promote immune responses that suppress asthma, yet how these beneficial effects take place remains controversial and poorly understood.

METHODS: We exposed mice to the bacterium Escherichia coli and subsequently induced allergic airway inflammation through sensitization and intranasal challenge with ovalbumin.
RESULTS: Pulmonary exposure to the bacterium Escherichia coli leads to a suppression of allergic airway inflammation. This immune modulation was neither mediated by the induction of a T helper 1 (Th1) response nor regulatory T cells; however, it was dependent on Toll-like receptor 4 (TLR4) but did not involve TLR desensitisation. Dendritic cell migration to the draining lymph nodes and activation of T cells was unaffected by prior exposure to E. coli, while dendritic cells in the lung displayed a less activated phenotype and had impaired antigen presentation capacity. Consequently, in situ Th2 cytokine production was abrogated. The suppression of airway hyper-responsiveness was mediated through the recruitment of gd T cells; however, the suppression of dendritic cells and T cells was mediated through a distinct mechanism that could not be overcome by the local administration of activated dendritic cells, or by the in vivo administration of tumour necrosis factor a.
CONCLUSION: Our data reveal a localized immunoregulatory pathway that acts to protect the airways from allergic inflammation.

PMID: 21422039 [PubMed - indexed for MEDLINE]

266. J Immunol. 2011 May 1;186(9):5254-60. doi: 10.4049/jimmunol.1004054. Epub 2011 Mar 18.

Leptin enhances survival and induces migration, degranulation, and cytokine synthesis of human basophils.

Suzukawa M, Nagase H, Ogahara I, Han K, Tashimo H, Shibui A, Koketsu R, Nakae S, Yamaguchi M, Ohta K.

Division of Respiratory Medicine and Allergology, Department of Medicine, Teikyo University School of Medicine, Itabashi-ku, Tokyo 173-8605, Japan. fueta-tky@umin.ac.jp

Basophils are the rarest leukocytes in human blood, but they are now recognized as one of the most important immunomodulatory as well as effector cells in allergic inflammation. Leptin, a member of the IL-6 cytokine family, has metabolic effects as an adipokine, and it is also known to participate in the pathogenesis of inflammatory reactions. Because there is an epidemiologic relationship between obesity and allergy, we examined whether basophil functions are modified by leptin. We found that human basophils express leptin receptor (LepR) at both the mRNA and surface protein levels, which were upregulated by IL-33. Leptin exerted strong effects on multiple basophil functions. It induced a strong migratory response in human basophils, similar in potency to that of basophil-active chemokines. Also, leptin enhanced survival of human basophils, although its potency was less than that of IL-3. Additionally, CD63, a basophil activation marker expressed on the cell surface, was upregulated by leptin, an effect that was neutralized by blocking of LepR. Assessments of basophil degranulation and cytokine synthesis found that leptin showed a strong priming effect on human basophil degranulation in response to FcεRI aggregation and induced Th2, but not Th1, cytokine production by the cells. In summary, the present findings indicate that leptin may be a key molecule mediating the effects of adipocytes on inflammatory cells such as basophils by binding to LepR and activating the cellular functions, presumably exacerbating allergic inflammation.

PMID: 21421855 [PubMed - indexed for MEDLINE]

267. Basic Biology of Fungi.

Cole GT.
In: Baron S, editor. Medical Microbiology. 4th edition. Galveston (TX): University of Texas Medical Branch at Galveston; 1996. Chapter 73.

Macroscopic fungi such as morels, mushrooms, puffballs, and the cultivated agarics available in grocery stores represent only a small fraction of the diversity in the kingdom Fungi. The molds, for example, are a large group of microscopic fungi that include many of the economically important plant parasites, allergenic species, and opportunistic pathogens of humans and other animals. They are characterized by filamentous, vegetative cells called hyphae. A mass of hyphae forms the thallus (vegetative body) of the fungus, composed of mycelium. The more phylogenetically primitive molds (e.g., water molds, bread molds, and other sporangial—saclike—forms) produce cenocytic filaments (multinucleate cells without cross-walls), while the more advanced forms produce hyphae with cross-walls (septa) that subdivide the filament into uninucleate and multinucleate compartments. The septum, however, still provides for cytoplasmic communication, including intercellular migration of nuclei. Many fungi occur not as hyphae but as unicellular forms called yeasts, which reproduce vegetatively by budding. Some of the opportunistic fungal pathogens of humans are dimorphic, growing as a mycelium in nature and as a vegetatively reproducing yeast in the body. Candida is an example of such a dimorphic fungus (Fig. 73-1). It can undergo rapid transformation from the yeast to the hyphal phase in vivo, which partly contributes to its success in invading host tissue.
The true fungi obtain their carbon compounds from nonliving organic substrates (saprophytes) or living organic material (parasites) by absorption of nutrients through their cell wall. Small molecules (e.g., simple sugars and amino acids) accumulate in a watery film surrounding the hyphae or yeast and simply diffuse through the cell wall. Macromolecules and insoluble polymers (e.g., proteins, glycogen, starch, and cellulose), on the other hand, must undergo preliminary digestion before they can be absorbed by the fungal cell. This process involves release of specific proteolytic, glycolytic, or lipolytic enzymes from the hypha or yeast, extracellular breakdown of the substrate(s), and diffusion of the products of digestion through the fungal cell envelope (Fig. 73-2). Fungal pathogens rely on these digestive enzymes to penetrate natural host barriers.

PMID: 21413296 [PubMed]

268. Life Sci. 2011 May 9;88(19-20):830-8. doi: 10.1016/j.lfs.2011.03.001. Epub 2011 Mar 21.

G-CSF suppresses allergic pulmonary inflammation, downmodulating cytokine, chemokine and eosinophil production.

Queto T, Vasconcelos ZF, Luz RA, Anselmo C, Guiné AA, e Silva PM, Farache J, Cunha JM, Bonomo AC, Gaspar-Elsas MI, Xavier-Elsas P.

Dept. of Pediatrics, Instituto Fernandes Figueira, FIOCRUZ, Rio de Janeiro, Brazil.

AIMS: Granulocyte Colony-Stimulating Factor (G-CSF), which mobilizes hemopoietic stem cells (HSC), is believed to protect HSC graft recipients from graft-versus-host disease by enhancing Th2 cytokine secretion. Accordingly, G-CSF should aggravate Th2-dependent allergic pulmonary inflammation and the associated eosinophilia. We evaluated the effects of G-CSF in a model of allergic pulmonary inflammation.
MAIN METHODS: Allergic pulmonary inflammation was induced by repeated aerosol allergen challenge in ovalbumin-sensitized C57BL/6J mice. The effects of allergen challenge and of G-CSF pretreatment were evaluated by monitoring: a) eosinophilia

and cytokine/chemokine content of bronchoalveolar lavage fluid, pulmonary interstitium, and blood; b) changes in airway resistance; and c) changes in bone-marrow eosinophil production.
KEY FINDINGS: Contrary to expectations, G-CSF pretreatment neither induced nor enhanced allergic pulmonary inflammation. Instead, G-CSF: a) suppressed accumulation of infiltrating eosinophils in bronchoalveolar, peribronchial and perivascular spaces of challenged lungs; and b) prevented ovalbumin challenge-induced rises in airway resistance. G-CSF had multiple regulatory effects on cytokine and chemokine production: in bronchoalveolar lavage fluid, levels of IL-1 and IL-12 (p40), eotaxin and MIP-1a were decreased; in plasma, KC, a neutrophil chemoattractant, was increased, while IL-5 was decreased and eotaxin was unaffected. In bone-marrow, G-CSF: a) prevented the increase in bone-marrow eosinophil production induced by ovalbumin challenge of sensitized mice; and b) selectively stimulated neutrophil colony formation.
SIGNIFICANCE: These observations challenge the view that G-CSF deviates cytokine production towards a Th2 profile in vivo, and suggest that this neutrophil-selective hemopoietin affects eosinophilic inflammation by a combination of effects on lung cytokine production and bone-marrow hemopoiesis.

Copyright © 2011 Elsevier Inc. All rights reserved.

PMID: 21396376 [PubMed - indexed for MEDLINE]

269. Ther Adv Respir Dis. 2011 Aug;5(4):255-73. doi: 10.1177/1753465810396539. Epub 2011 Mar 3.

The airway epithelium: more than just a structural barrier.

Tam A, Wadsworth S, Dorscheid D, Man SF, Sin DD.

The UBC James Hogg Research Centre, Providence Heart and Lung Centre and Department of Medicine, University of British Columbia, UBC, Vancouver, BC, Canada.

The mammalian airway is lined by a variety of specialized epithelial cells that not only serve as a physical barrier but also respond to environment-induced damage through the release of biologically active factors and constant cellular renewal. The lung epithelium responds to environmental insults such as pathogens, cigarette smoke and pollution by secreting inflammatory mediators and antimicrobial peptides, and by recruiting immune cells to the site of infection or damage. When the epithelium is severely damaged, basal cells and Clara cells that have stem-cell-like properties are capable of self-renewal and proliferation in the affected area, to repair the damage. In order to effectively fight off infections, the epithelium requires the assistance of neutrophils recruited from the peripheral circulation through transendothelial followed by transepithelial migration events. Activated neutrophils migrate across the epithelium through a series of ligand-receptor interactions to the site of injury, where they secrete proteolytic enzymes and oxidative radicals for pathogen destruction. However, chronic activation and recruitment of neutrophils in airway diseases such as chronic obstructive pulmonary disease and asthma has been associated with tissue damage and disease severity. In this paper, we review the current understanding of the airway epithelial response to injury and its interaction with inflammatory cells, in particular the neutrophil.

PMID: 21372121 [PubMed - indexed for MEDLINE]

270. J Agric Food Chem. 2011 Apr 13;59(7):3429-38. doi: 10.1021/jf1049485. Epub 2011 Mar 14.

Ginger suppresses phthalate ester-induced airway remodeling.

Kuo PL, Hsu YL, Huang MS, Tsai MJ, Ko YC.

Institute of Clinical Medicine, College of Medicine, Kaohsiung Medical University, No. 100 Shih-Chuan first Road, Kaohsiung 807, Taiwan.

This study has two novel findings: it is not only the first to demonstrate inflammatory cytokines, which are produced by the bronchial epithelium after exposure to phthalate esters and contribute to airway remodeling by increasing human bronchial smooth muscle cells (BSMC) migration and proliferation, but it is also the first to reveal that ginger reverses phthalate ester-mediated airway remodeling. Human bronchial epithelial cell lines BEAS-2B and HBE135-E6E7 (HBE) were treated with butylbenzyl phthalate (BBP), bis(2-ethylhexyl) phthalate (BEHP), dibutyl phthalate (DBP), and diethyl phthalate (DEP), and the conditioned medium (CM) was harvested and then added to BSMC. Cultures of BSMC with BBP-, BEHP-, DBP-, and DEP-BEAS-2B-CM and DEP-HBE-CM increased BSMC proliferation and migration, which are major features in asthma remodeling. Exposure of BEAS-2B and HBE to DBP caused epithelial cells to produce inflammatory cytokines IL-8 and RANTES, which subsequently induced BSMC proliferation and migration. Depleting both IL-8 and RANTES completely reversed the effect of DBP-BEAS-2B-CM and DBP-HBE-CM-mediated BSMC proliferation and migration, suggesting this effect is a synergistic influence of IL-8 and RANTES. Moreover, [6]-shogaol, [6]-gingerol, [8]-gingerol, and [10]-gingerol, which are major bioactive compounds present in Zingiber officinale , suppress phthalate ester-mediated airway remodeling. This study suggests that ginger is capable of preventing phthalate ester-associated asthma.

PMID: 21370925 [PubMed - indexed for MEDLINE]

271. J Immunol. 2011 Apr 1;186(7):4156-63. doi: 10.4049/jimmunol.1001210. Epub 2011 Mar 2.

CC and CXC chemokines induce airway smooth muscle proliferation and survival.

Halwani R, Al-Abri J, Beland M, Al-Jahdali H, Halayko AJ, Lee TH, Al-Muhsen S, Hamid Q.

Prince Naif Center for Immunology Research, College of Medicine, King Saud University, Riyadh 11461, Saudi Arabia.

The increase in airway smooth muscle (ASM) mass is a major structural change in asthma. This increase has been attributed to ASM cell (ASMC) hyperplasia and hypertrophy. The distance between ASMC and the epithelium is reduced, suggesting migration of smooth muscle cells toward the epithelium. Recent studies have suggested a role of chemokines in ASMC migration toward the epithelium; however, chemokines have other biological effects. The objective of the current study is to test the hypothesis that chemokines (eotaxin, RANTES, IL-8, and MIP-1α) can directly influence ASMC mass by increasing the rate of proliferation or enhancing the survival of these cells. Human ASMCs were exposed to different concentrations of eotaxin, RANTES, IL-8, or MIP-1α. To test for proliferation, matched control and stimulated ASMC were pulsed with [(3)H]thymidine, or ASMCs were stained with BrdU and then analyzed with flow cytometry. Apoptosis was measured using Annexin V staining and flow cytometry. Expression of phosphorylated p42/p44 and MAPKs was assessed by Western blot. In a concentration-dependent manner, chemokines including eotaxin, RANTES, IL-8, and MIP-1α increased ASMC's [(3)H]thymidine incorporation and DNA synthesis. IL-8, eotaxin, and MIP-1α decreased the rate of apoptosis of ASMCs compared with the matched controls. A significant increase in

phosphorylated p42/p44 MAPKs was seen after treating ASMCs with RANTES and eotaxin. Moreover, inhibition of p42/p44 MAPK phosphorylation reduced the level of chemokine-induced ASM proliferation. We conclude that chemokines might contribute to airway remodeling seen in asthma by enhancing the number and survival of ASMCs.

PMID: 21368236 [PubMed - indexed for MEDLINE]

272. Cell Mol Life Sci. 2011 Nov;68(21):3573-87. doi: 10.1007/s00018-011-0642-5. Epub 2011 Mar 2.

EP4 receptor stimulation down-regulates human eosinophil function.

Luschnig-Schratl P, Sturm EM, Konya V, Philipose S, Marsche G, Fröhlich E, Samberger C, Lang-Loidolt D, Gattenlöhner S, Lippe IT, Peskar BA, Schuligoi R, Heinemann A.

Institute of Experimental and Clinical Pharmacology, Medical University of Graz, Universitaetsplatz 4, 8010 Graz, Austria.

Accumulation of eosinophils in tissue is a hallmark of allergic inflammation. Here we observed that a selective agonist of the PGE(2) receptor EP4, ONO AE1-329, potently attenuated the chemotaxis of human peripheral blood eosinophils, upregulation of the adhesion molecule CD11b and the production of reactive oxygen species. These effects were accompanied by the inhibition of cytoskeletal rearrangement and Ca(2+) mobilization. The involvement of the EP4 receptor was substantiated by a selective EP4 antagonist, which reversed the inhibitory effects of PGE(2) and the EP4 agonist. Selective kinase inhibitors revealed that the inhibitory effect of EP4 stimulation on eosinophil migration depended upon activation of PI 3-kinase and PKC, but not cAMP. Finally, we found that EP4 receptors are expressed by human eosinophils, and are also present on infiltrating leukocytes in inflamed human nasal mucosa. These data indicate that EP4 agonists might be a novel therapeutic option in eosinophilic diseases.

PMCID: PMC3192285
PMID: 21365278 [PubMed - indexed for MEDLINE]

273. Int Arch Allergy Immunol. 2011;155(4):335-44. doi: 10.1159/000321195. Epub 2011 Feb 22.

Leptin has a priming effect on eotaxin-induced human eosinophil chemotaxis.

Kato H, Ueki S, Kamada R, Kihara J, Yamauchi Y, Suzuki T, Takeda M, Itoga M, Chihara M, Ito W, Kayaba H, Chihara J.

Department of Infection, Allergy, Clinical Immunology and Laboratory Medicine, Akita University Graduate School of Medicine, Akita, Japan.

BACKGROUND: Tissue eosinophilia is one of the hallmarks of allergic diseases and Th2-type immune responses including asthma. Systemic inflammation caused by adipose tissue in obesity via production of adipokines such as leptin has been attracting attention recently as a contributor to exacerbation of allergic immune reactions. In this study, we examined whether leptin might affect eosinophil chemotactic responses.
METHODS: Peripheral blood eosinophils were purified, and the effect of leptin on eosinophil migration was investigated using in vitro systems.
RESULTS: High concentrations of leptin induced eosinophil chemotaxis and rapid phosphorylation of ERK1/2 and p38 mitogen-activated protein kinase but not

calcium mobilization. We also found that pretreatment of eosinophils with physiological concentrations of leptin amplified the chemotactic responses to eotaxin. This leptin-primed chemotaxis appears to be associated with increased calcium mobilization but not with ERK1/2 and p38 pathways.
CONCLUSIONS: These results indicate that leptin has both direct and indirect effects on eosinophil chemotaxis and intracellular signaling. In physiological settings, leptin may maintain eosinophil accumulation at allergic inflammatory foci.

Copyright © 2011 S. Karger AG, Basel.

PMID: 21346363 [PubMed - indexed for MEDLINE]

274. J Allergy Clin Immunol. 2011 Apr;127(4):1046-53.e1-2. doi: 10.1016/j.jaci.2010.12.1117. Epub 2011 Feb 22.

TH17 cytokines induce human airway smooth muscle cell migration.

Chang Y, Al-Alwan L, Risse PA, Roussel L, Rousseau S, Halayko AJ, Martin JG, Hamid Q, Eidelman DH.

Meakins-Christie Laboratories and Respiratory Division, Department of Medicine McGill University, Montreal, Quebec, Canada.

BACKGROUND: Migration of airway smooth muscle cells (ASMCs) might contribute to increased airway smooth muscle mass in asthma. T(H)17 cells and T(H)17-associated cytokines are involved in the pathogenesis of asthma and might also contribute to airway remodeling.
OBJECTIVE: We sought to explore the possibility that migration of ASMCs might contribute to airway remodeling through the action of T(H)17-related cytokines.
METHODS: The effect of exogenous T(H)17 cytokines on ex vivo human ASMC migration was investigated by using a chemotaxis assay. The involvement of signaling pathways, including p38 mitogen-activated protein kinase (MAPK), extracellular signal-regulated kinase 1/2 MAPK, nuclear factor κB, and phosphoinositide 3-kinase, was also examined.
RESULTS: We demonstrated that IL-17A, IL-17F, and IL-22 promote migration in a dose-dependent manner. We further demonstrated that ASMCs express receptors for IL-17RA, IL-17RC, and IL-22R1. Using mAbs directed against these receptors, we confirmed that T(H)17-associated cytokine-induced migration was dependent on selective receptor activation. Moreover, IL-17A and IL-17F exert their effects through signaling pathways that are distinct from those used by IL-22. The p38 MAPK inhibitor BIRB0796 inhibited the migration induced by IL-17A and IL-17F. PS1145, an inhibitor of nuclear factor κB, abolished the IL-22-induced migration.
CONCLUSION: These data raise the possibility that T(H)17-associated cytokines promote human ASMC migration in vivo and suggest an important new mechanism for the promotion of airway remodeling in asthma.

Copyright © 2011 American Academy of Allergy, Asthma & Immunology. Published by Mosby, Inc. All rights reserved.

PMID: 21345484 [PubMed - indexed for MEDLINE]

275. Auris Nasus Larynx. 2011 Oct;38(5):555-63. doi: 10.1016/j.anl.2011.01.011. Epub 2011 Feb 19.

Pathogenesis of airway inflammation in bronchial asthma.

Nakagome K, Nagata M.

Department of Respiratory Medicine, Saitama Medical University, 38 Morohongo,
Moroyama-cho, Iruma-gun, Saitama, Japan.

Bronchial asthma is a chronic disorder characterized by airway inflammation, reversible airway obstruction, and airway hyperresponsiveness. Eosinophils are believed to play important roles in the pathogenesis of asthma through the release of inflammatory mediators. In refractory eosinophilic asthma, anti-IL-5 mAb reduces exacerbations and steroid dose, indicating roles of eosinophils and IL-5 in the development of severe eosinophilic asthma. Even in the absence of IL-5, it is likely that the "Th2 network", including a cascade of vascular cell adhesion molecule-1/CC chemokines/GM-CSF, can sufficiently maintain eosinophilic infiltration and degranulation. Cysteinyl leukotrienes can also directly provoke eosinophilic infiltration and activation in the airways of asthma. Therefore, various mechanisms would be involved in the eosinophilic airway inflammation of asthma. In the pathogenesis of severe asthma, not only eosinophils but also mast cells or neutrophils play important roles. Mast cells are much infiltrated to smooth muscle in severe asthma and induce airway remodeling by release of inflammatory mediators such as amphiregulin. Treatment with anti-IgE Ab, which neutralizes circulating IgE and suppresses mast cell functions, reduces asthma exacerbations in severe asthmatic patients. Furthermore, infiltration of neutrophils in the airway is also increased in severe asthma. IL-8 plays an important role in the accumulation of neutrophils and is indeed upregulated in severe asthma. In the absence of chemoattractant for eosinophils, neutrophils stimulated by IL-8 augment the trans-basement membrane migration of eosinophils, suggesting that IL-8-stimulated neutrophils could lead eosinophils to accumulate in the airways of asthma. In view of these mechanisms, an effective strategy for controlling asthma, especially severe asthma, should be considered.

Copyright © 2011 Elsevier Ireland Ltd. All rights reserved.

PMID: 21334836 [PubMed - indexed for MEDLINE]

276. Colorectal Dis. 2011 Mar;13(3):e42-5. doi: 10.1111/j.1463-1318.2010.02476.x.

Novel use of an air-filled breast prosthesis to allow radiotherapy to recurrent colonic cancer.

O'Duffy F, Toomey DP, Fleming F, McNamara DA.

Department of Colorectal Surgery, Beaumont Hospital, Dublin, Ireland.
fergaloduffy@hotmail.com

AiM: The authors present the novel and successful use of an air-filled breast prosthesis for extra pelvic exclusion of small bowel to facilitate adjuvant radiotherapy following resection of recurrent adenocarcinoma of the ascending bowel. The therapeutic use of radiotherapy in colon cancer can cause acute or chronic radiation enteropathy. Mobile small bowel can be sequestered in 'dead space' or by adhesions exposing it to adjuvant radiotherapy. A variety of pelvic partitioning methods have been described to exclude bowel from radiation fields using both native and prosthetic materials.METHOD: In this case a 68 year old presented with ascending colon adenocarcinoma invading the peritoneum and underwent en bloc peritoneal resection. Thirty-seven months later surveillance CT identified a local recurrence. Subsequent resection resulted in a large iliacus muscle defect which would sequester small bowel loops thus exposing the patient to radiation enteropathy. The lateral position of the defect precluded the use of traditional pelvic partitioning methods which would be unlikely to remain in place long enough to allow radiotherapy. A lightweight air-filled breast prosthesis (Allergan 133 FV 750 cms) secured in place with an omentoplasty was

used to fill the defect.
RESULTS: Following well tolerated radiotherapy the prosthesis was deflated under ultrasound guidance and removed via a 7-cm transverse incision above the right iliac crest. The patient is disease free 18 months later with no evidence of treatment related morbidity.
CONCLUSION: The use of a malleable air-filled prosthesis for pelvic partitioning allows specific tailoring of the prosthesis size and shape for individual patient defects. It is also lightweight enough to be secured in place using an omentoplasty to prevent movement related prosthesis migration. In the absence of adequate omentum a mesh sling may be considered to allow fixation. In this case the anatomy of the prosthesis position allowed for its removal without the need for repeat laparotomy. Pre-operative deflation of the air-filled prosthesis under ultrasound guidance also reduces the size of the incision required for removal. This technique may be valuable to prevent collateral small bowel irradiation following resection of renal or retroperitoneal malignancy.

© 2011 The Authors. Colorectal Disease © 2011 The Association of Coloproctology of Great Britain and Ireland.

PMID: 21320268 [PubMed - indexed for MEDLINE]

277. Am J Respir Cell Mol Biol. 2011 Oct;45(4):753-60. doi: 10.1165/rcmb.2010-0404OC. Epub 2011 Feb 11.

Cigarette smoke differentially affects eosinophilia and remodeling in a model of house dust mite asthma.

Botelho FM, Llop-Guevara A, Trimble NJ, Nikota JK, Bauer CM, Lambert KN, Kianpour S, Jordana M, Stämpfli MR.

Department of Pathology and Molecular Medicine, Centre for Gene Therapeutics, McMaster University, 1200 Main Street West, Hamilton, ON, L8N 3Z5 Canada.

Although a similar prevalence of smoking is evident among patients with asthma and the general population, little is known about the impact of cigarette smoke on the immune inflammatory processes elicited by common environmental allergens. We investigated the impact of exposure to cigarette smoke on house dust mite (HDM)-induced allergic airway inflammation and its consequences for tissue remodeling and lung physiology in mice. BALB/c mice received intranasal HDMs daily, 5 days per week, for 3 weeks to establish chronic airway inflammation. Subsequently, mice were concurrently exposed to HDMs plus cigarette smoke, 5 days per week, for 2 weeks (HDMs + smoke). We observed significantly attenuated eosinophilia in the bronchoalveolar lavage of mice exposed to HDMs + smoke, compared with animals exposed only to HDMs. A similar activation of CD4 T cells and expression of IL-5, IL-13, and transforming growth factor-β was observed between HDM-treated and HDM + smoke-treated animals. Consistent with an effect on eosinophil trafficking, HDMs + smoke exposure attenuated the HDM-induced expression of eotaxin-1 and vascular cell adhesion molecule-1, whereas the survival of eosinophils and the numbers of blood eosinophils were not affected. Exposure to cigarette smoke also reduced the activation of B cells and the concentrations of serum IgE. Although the production of mucus decreased, collagen deposition significantly increased in animals exposed to HDMs + smoke, compared with animals exposed only to HDMs. Although airway resistance was unaffected, tissue resistance was significantly decreased in mice exposed to HDMs + smoke. Our findings demonstrate that cigarette smoke affects eosinophil migration without affecting airway resistance or modifying Th2 cell adaptive immunity in a murine model of HDM-induced asthma.

PMID: 21317378 [PubMed - indexed for MEDLINE]

278. Toxicology. 2011 Apr 28;283(1):32-40. doi: 10.1016/j.tox.2011.01.026. Epub 2011 Feb 17.

Slit2-N inhibits PDGF-induced migration in rat airway smooth muscle cells: WASP and Arp2/3 involved.

Ning Y, Sun Q, Dong Y, Xu W, Zhang W, Huang H, Li Q.

Department of Respiratory Medicine, Changhai Hospital, Second Military Medical University, 168 Changhai Road, Yangpu District, Shanghai 200433, PR China.

BACKGROUND: Slit2 has been reported to be implicated in many kinds of cell migration. However little is known about the effect of Slit2 on airway smooth muscle cell migration. This study was to detect the expression of Slit2 in rat airway smooth muscle (RASM) cells stimulated by platelet-derived growth factor (PDGF) and characterized the effect of Slit2-N on PDGF-induced migration of RASM cells in vitro.
METHODS: mRNAs of Slit-Robo in RASM cells were examined by RT-PCR, and the effect of exogenous Slit2-N at different doses on PDGF-induced migration of RASM cells were examined by transwell and scrape-wound assays. Actin filaments (F-actin) were stained with rhodamine-conjugated phalloidin and the levels of protein expression were detected by western blot.
RESULTS: RASM cells were identified to express Slit2, Slit3, Robo1, Robo2 and Robo4 in vitro. Slit2-N caused a time- and dose-dependent inhibition of cell proliferation, while had no significantly effect on cell apoptosis. Slit2-N pretreatment attenuated the elongated morphologic characteristics, reduced lamellipodia formation, inhibited actin rearrangement and cell migration induced by PDGF. PDGF-induced increase of WASP and Arp2/3 proteins were dramatically inhibited by 100 ng/ml Slit2-N.
CONCLUSION: Slit2-N inhibits RASM cells migration at least partly through attenuating the expressions of WASP and Arp2/3, inhibiting actin rearrangement in vitro. The results contribute to provide new insights into the pathogenesis of airway remodeling in asthma and may be helpful for development of effective treatments.

Copyright © 2011 Elsevier Ireland Ltd. All rights reserved.

PMID: 21315131 [PubMed - indexed for MEDLINE]

279. Exp Lung Res. 2011 Jun;37(5):279-90. doi: 10.3109/01902148.2010.540769. Epub 2011 Feb 11.

Chronic allergen challenge induces pulmonary extramedullary hematopoiesis.

Pandit TS, Hosseinkhani MR, Kang BN, Bahaie NS, Ge XN, Rao SP, Sriramarao P.

Laboratory of Allergic Diseases and Inflammation, Department of Veterinary and Biomedical Sciences, University of Minnesota, St. Paul, Minnesota 55108, USA.

Allergic inflammation is associated with increased generation and trafficking of inflammatory cells, especially eosinophils, to sites of inflammation. The effect of acute versus chronic airway allergen challenge on hematopoietic activity in the bone marrow (BM) and lungs was investigated using murine models of allergic airway inflammation. Acute allergen challenge induced proliferation of BM cells and significantly increased generation of eosinophil, but not multipotent, granulocyte-macrophage (GM), or B-lymphocyte progenitor cells. However, no hematopoietic activity was observed in the lungs. With chronic challenge, BM

cells failed to proliferate, but exhibited increased capacity to generate multipotent as well as eosinophil, GM, and B-lymphocyte progenitors. In addition, increased generation of eosinophil- and GM-specific progenitors was observed in the lungs. Although no differences were observed in their ability to roll on BM endothelium in vitro or in vivo, CD34-enriched hematopoietic/stem progenitor cells (HSPCs) from chronic-, but not acute-, challenged mice demonstrated reduced migration across BM endothelial cells associated with decreased CXCR4 expression. Overall, these studies demonstrate that chronic allergen exposure can alter BM homing due to decreased transendothelial migration enabling noninteracting HSPCs to egress out of the BM and recruit to sites of inflammation such as the airways, resulting in extramedullary hematopoiesis.

PMID: 21309736 [PubMed - indexed for MEDLINE]

280. Magn Reson Med. 2011 Jun;65(6):1738-49. doi: 10.1002/mrm.22757. Epub 2011 Feb 8.

Neural precursors exhibit distinctly different patterns of cell migration upon transplantation during either the acute or chronic phase of EAE: a serial MR imaging study.

Muja N, Cohen ME, Zhang J, Kim H, Gilad AA, Walczak P, Ben-Hur T, Bulte JW.

Russell H. Morgan Department of Radiology and Radiological Science, Division of MR Research, The Johns Hopkins University School of Medicine, Baltimore, Maryland 21205-2195, USA.

As the complex pathogenesis of multiple sclerosis contributes to spatiotemporal variations in the trophic micromilieu of the central nervous system, the optimal intervention period for cell-replacement therapy must be systematically defined. We applied serial, 3D high-resolution magnetic resonance imaging to transplanted neural precursor cells (NPCs) labeled with superparamagnetic iron oxide nanoparticles and 5-bromo-2-deoxyuridine, and compared the migration pattern of NPCs in acute inflamed (n = 10) versus chronic demyelinated (n = 9) brains of mice induced with experimental allergic encephalomyelitis (EAE). Serial in vivo and ex-vivo 3D magnetic resonance imaging revealed that NPCs migrated 2.5 ± 1.3 mm along the corpus callosum in acute EAE. In chronic EAE, cell migration was slightly reduced (2.3 ± 1.3 mm) and only occurred in the lateral side of transplantation. Surprisingly, in 6/10 acute EAE brains, NPCs were found to migrate in a radial pattern along RECA-1(+) cortical blood vessels, in a pattern hitherto only reported for migrating glioblastoma cells. This striking radial biodistribution pattern was not detected in either chronic EAE or disease-free control brains. In both acute and chronic EAE brain, Iba1(+) microglia/macrophage number was significantly higher in central nervous system regions containing migrating NPCs. The existence of differential NPC migration patterns is an important consideration for implementing future translational studies in multiple sclerosis patients with variable disease.

Copyright © 2011 Wiley-Liss, Inc.

PMCID: PMC3190231
PMID: 21305597 [PubMed - indexed for MEDLINE]

281. Clin Chem Lab Med. 2011 Apr;49(4):587-99. doi: 10.1515/CCLM.2011.094. Epub 2011 Feb 9.

Inflammatory markers in childhood asthma.

Dodig S, Richter D, Zrinski-Topić R.

Department of Clinical Laboratory Diagnosis, Srebrnjak Children's Hospital, Zagreb, Croatia. slavica.dodig@zg.t-com.hr

The major characteristic of asthma is persistent airway inflammation that fails to resolve spontaneously. Dysregulation of pro- and anti-inflammatory mechanisms is responsible for the development of chronic inflammation. The inflammatory reaction is mediated by numerous cells and their mediators. Detection and quantification of airway inflammation in children are subject to many requirements, e.g., use of biologic samples obtained in a non-invasive way; use of standardized analytical methods to determine biomarkers that can identify inflammation processes (inflammation itself, oxidative stress, apoptosis and remodelling); determining the role of systemic inflammation; assessment of correlation of various biomarkers of inflammation with clinical parameters and their diagnostic efficacy; providing a tool(s) to monitor diseases, and to evaluate adequacy of therapy; and predicting the clinical course of inflammation and prognosis of asthma. Using standardized analyses, it is now possible to determine direct markers of local inflammation, i.e., fractional nitric oxide (marker of oxidative stress) in exhaled breath, pH (marker of acid stress) in breath condensate, and indirect markers in blood/serum, i.e., eosinophil granulocytes (indicating migration), eosinophil cationic protein (marker of activated eosinophil granulocytes) and C-reactive protein (marker of systemic inflammation). However, none of these biomarkers are specific for asthma. Further standardization of the known pulmonary biomarkers of local inflammation and identification of new ones will allow for longitudinal follow-up of inflammation in children with asthma.

PMID: 21303302 [PubMed - indexed for MEDLINE]

282. B-ENT. 2010;6(4):299-301.

Maxillary sinusitis caused by the migration of a silastic implant used for an orbital floor reconstruction: a case report.

Saussez S, Filleul O, Mahillon V, Kamatchian M, Chantrain G.

Department of Otorhinolaryngology, Head and Neck Surgery, CHU Saint-Pierre, 322 Rue Haute, 1000 Brussels, Belgium. sven.saussez@umons.ac.be

OBJECTIVE: The authors report on a patient whose unilateral chronic maxillary sinusitis was caused by the migration of a silastic implant used for orbital floor repair.
CASE REPORT: A 32-year-old woman presented with a three-year history of right-sided maxillary discomfort that was associated with a purulent discharge. Her medical history included chronic allergic rhinitis and the placement of a silastic implant after a right orbital floor fracture at the age of 14. The silastic implant was then removed endoscopically in a right maxillary sinus meatotomy. The symptoms improved within three weeks after surgery.
CONCLUSION: Orbital implant migration is a rare cause of chronic unilateral sinusitis, and it must be suspected on the basis of a careful anamnesis, appropriate clinical examination, and sinus computed tomography. Misdiagnosing such a condition may increase patient morbidity by leading to inappropriate treatment.

PMID: 21302695 [PubMed - indexed for MEDLINE]

283. Toxicol Sci. 2011 May;121(1):177-90. doi: 10.1093/toxsci/kfr030. Epub 2011 Feb 4.

Bronchial epithelium-derived IL-8 and RANTES increased bronchial smooth muscle cell migration and proliferation by Krüppel-like factor 5 in areca nut-mediated airway remodeling.

Kuo PL, Hsu YL, Huang MS, Chiang SL, Ko YC.

Institute of Clinical Medicine, Kaohsiung Medical University Hospital, Kaohsiung 807, Taiwan.

This study is first to analyze the inflammatory cytokines, produced by the bronchial epithelium after exposure to areca nut extract (ANE), which contribute to airway remodeling by increasing human bronchial smooth muscle cells (BSMC) migration and proliferation. We treated human bronchial epithelial cell lines BEAS-2B and HBE135-E6E7 (HBE) with ANE, saliva-reacted ANE (sANE), and the areca alkaloids arecoline and then harvested the conditioned medium (CM) that was added to BSMC. Exposure of BEAS-2B and HBE to ANE, sANE, and arecoline increased interleukin 8 (IL-8) and Regulated upon Activation, Normal T-cell Expressed, and Secreted (RANTES) production. Cultures of BSMC with ANE-, sANE-, and arecoline-BEAS-2B-CM and -HBE-CM increased BSMC proliferation and migration. Induction of BSMC proliferation and migration by sANE-BEAS-2B-CM and -HBE-CM was associated with increased phosphorylation of Raf, MEK1/2, and extracellular signal regulated kinase (ERK)1/2 and the upregulation of krüppel-like factor 5 (KLF5), cyclin D, and integrin-linked kinase. Blocking ERK1/2 by a specific inhibitor significantly decreased BSMC proliferation and migration by inhibiting KLF5 enhancement. KLF5 knockdown also decreased sANE-BEAS-2B-CM, sANE-HBE-CM, and recombinant human interleukin 8/recombinant human RANTES-mediated BSMC proliferation and migration, suggesting that KLF5 was involved in the regulation of BSMC proliferation and migration. Our study suggests that inhibition of IL-8 and RANTES or IL-8/RANTES-mediated mitogen-activated protein kinase/KLF5 signaling is an attractive therapeutic target for areca nut-induced asthma.

PMID: 21297082 [PubMed - indexed for MEDLINE]

284. Curr Drug Targets. 2011 Jun;12(7):1056-81.

Phosphatidylinositol 3-kinase isoforms as novel drug targets.

Błajecka K, Borgström A, Arcaro A.

University of Bern, Department of Clinical Research, Bern, Switzerland.

Phosphatidylinositol 3-kinases (PI3Ks) are key molecules in the signal transduction pathways initiated by the binding of extracellular signals to their cell surface receptors. The PI3K family of enzymes comprises eight catalytic isoforms subdivided into three classes and control a variety of cellular processes including proliferation, growth, apoptosis, migration and metabolism. Deregulation of the PI3K pathway has been extensively investigated in connection to cancer, but is also involved in other commonly occurring diseases such as chronic inflammation, autoimmunity, allergy, atherosclerosis, cardiovascular and metabolic diseases. The fact that the PI3K pathway is deregulated in a large number of human diseases, and its importance for different cellular responses, makes it an attractive drug target. Pharmacological PI3K inhibitors have played a very important role in studying cellular responses involving these enzymes. Currently, a wide range of selective PI3K inhibitors have been tested in preclinical studies and some have entered clinical trials in oncology. However, due to the complexity of PI3K signaling pathways, developing an effective anti-cancer therapy may be difficult. The biggest challenge in curing cancer patients with various signaling pathway abnormalities is to target multiple components of different signal transduction pathways with mechanism-based

combinatorial treatments. In this article we will give an overview of the complex role of PI3K isoforms in human diseases and discuss their potential as drug targets. In addition, we will describe the drugs currently used in clinical trials, as well as promising emerging candidates.

PMID: 21291386 [PubMed - indexed for MEDLINE]

285. PLoS One. 2011 Jan 20;6(1):e16175. doi: 10.1371/journal.pone.0016175.

Chronic respiratory aeroallergen exposure in mice induces epithelial-mesenchymal transition in the large airways.

Johnson JR, Roos A, Berg T, Nord M, Fuxe J.

Division of Vascular Biology, Department of Medical Biochemistry and Biophysics, Karolinska Institutet, Stockholm, Sweden.

Chronic allergic asthma is characterized by Th2-polarized inflammation and leads to airway remodeling and fibrosis but the mechanisms involved are not clear. To determine whether epithelial-mesenchymal transition contributes to airway remodeling in asthma, we induced allergic airway inflammation in mice by intranasal administration of house dust mite (HDM) extract for up to 15 consecutive weeks. We report that respiratory exposure to HDM led to significant airway inflammation and thickening of the smooth muscle layer in the wall of the large airways. Transforming growth factor beta-1 (TGF-β1) levels increased in mouse airways while epithelial cells lost expression of E-cadherin and occludin and gained expression of the mesenchymal proteins vimentin, alpha-smooth muscle actin (α-SMA) and pro-collagen I. We also observed increased expression and nuclear translocation of Snail1, a transcriptional repressor of E-cadherin and a potent inducer of EMT, in the airway epithelial cells of HDM-exposed mice. Furthermore, fate-mapping studies revealed migration of airway epithelial cells into the sub-epithelial regions of the airway wall. These results show the contribution of EMT to airway remodeling in chronic asthma-like inflammation and suggest that Th2-polarized airway inflammation can trigger invasion of epithelial cells into the subepithelial regions of the airway wall where they contribute to fibrosis, demonstrating a previously unknown plasticity of the airway epithelium in allergic airway disease.

PMCID: PMC3024415
PMID: 21283768 [PubMed - indexed for MEDLINE]

286. Contact Dermatitis. 2011 Mar;64(3):142-50. doi: 10.1111/j.1600-0536.2010.01841.x.

Nickel allergy is still frequent in young German females - probably because of insufficient protection from nickel-releasing objects.

Schnuch A, Wolter J, Geier J, Uter W.

Information Network of Departments of Dermatology (IVDK), University of Göttingen, 37075 Göttingen, Germany. aschnuch@med.uni-goettingen.de

BACKGROUND: Nickel contact allergy is still frequent both in patch-tested patients and in the general population. Objectives. To explain this observation by relating clinical epidemiological data with recent chemical analyses of nickel release from costume jewellery.
METHODS: (i) The trend of nickel allergy was analysed using data registered between January 1994 and December 2009 in the Information Network of Departments of Dermatology. (ii) In 2008, different parts of items of costume jewellery

purchased at random on the German market (n = 609) were analysed for nickel release according to EN 1811:1998 + A1:2008 in five official German laboratories of food and non-food INVESTIGATION.
RESULTS: (i) Between 1994 and 2009, nickel allergy decreased in men (18-30 years) and in women (1-17 and 18-30 years); however, after 2000, there was no significant decrease in nickel allergy in the women aged 1-17 years. (ii) Of the post-assemblies, 28.0% exceeded the migration limit of ≥0.2 µg/cm(2) per week, and 5% released ≥26.8 µg/cm(2) per week. In articles with direct and prolonged contact with the skin, 12.8% of decorative parts and 17.1% of clasps exceeded the migration limit. If an adjustment factor was applied, according to the above norm, about half of the items otherwise rejected became acceptable.
CONCLUSION: Exposure to nickel-containing products exceeding the (unnecessarily relaxed) permitted limit may explain why nickel contact allergy remains a problem.

© 2011 John Wiley & Sons A/S.

PMID: 21272019 [PubMed - indexed for MEDLINE]

287. J Immunol. 2011 Mar 1;186(5):2719-28. doi: 10.4049/jimmunol.1000940. Epub 2011 Jan 26.

Basophils are the major producers of IL-4 during primary helminth infection.

van Panhuys N, Prout M, Forbes E, Min B, Paul WE, Le Gros G.

Malaghan Institute of Medical Research, Wellington 6012, New Zealand.
vanpanhuysn@niaid.nih.gov

IL-4 production by leukocytes is a key regulatory event that occurs early in the type 2 immune response, which induces allergic reactions and mediates expulsion of parasites. CD4(+) T cells and basophils are thought to be the key cell types that produce IL-4 during a type 2 response. In this study, we assessed the relative contribution of both CD4(+) T cell- and basophil-IL-4 production during primary and secondary responses to Nippostrongylus brasiliensis using a murine IL-4-enhanced GFP reporter system. During infection, IL-4-producing basophils were detected systemically, and tissue recruitment occurred independent of IL-4/STAT6 signaling. We observed that basophil recruitment to a tissue environment was required for their full activation. Basophil induction in response to secondary infection exhibited accelerated kinetics in comparison with primary infection. However, total basophil numbers were not enhanced, as predicted by previous models of protective immunity. Overall, the induction and migration of IL-4-producing basophils into peripheral tissues was found to be a prominent characteristic of the primary but not memory responses to N. brasiliensis infection, in which CD4(+) T cells were identified as the major source of IL-4. Whereas basophils were the major initial producers of IL-4, we determined that normal Th2 differentiation occurs independently of basophils, and depletion of basophils led to an enhancement of inflammatory cell recruitment to the site of infection.

PMCID: PMC3488853
PMID: 21270410 [PubMed - indexed for MEDLINE]

288. Biochem J. 2011 Feb 15;434(1):11-24. doi: 10.1042/BJ20101132.

Targeting chemokine receptors in allergic disease.

Pease JE.

Leukocyte Biology, and MRC and Asthma UK Centre in Allergic Mechanisms of Asthma, NHLI Division, Faculty of Medicine, Imperial College London, London, UK.
j.pease@imperial.ac.uk

The directed migration of cells in response to chemical cues is known as chemoattraction, and plays a key role in the temporal and spatial positioning of cells in lower- and higher-order life forms. Key molecules in this process are the chemotactic cytokines, or chemokines, which, in humans, constitute a family of approx. 40 molecules. Chemokines exert their effects by binding to specific GPCRs (G-protein-coupled receptors) which are present on a wide variety of mature cells and their progenitors, notably leucocytes. The inappropriate or excessive generation of chemokines is a key component of the inflammatory response observed in several clinically important diseases, notably allergic diseases such as asthma. Consequently, much time and effort has been directed towards understanding which chemokine receptors and ligands are important in the allergic response with a view to therapeutic intervention. Such strategies can take several forms, although, as the superfamily of GPCRs has historically proved amenable to blockade by small molecules, the development of specific antagonists has been has been a major focus of several groups. In the present review, I detail the roles of chemokines and their receptors in allergic disease and also highlight current progress in the development of relevant chemokine receptor antagonists.

PMID: 21269275 [PubMed - indexed for MEDLINE]

289. Respiration. 2011;82(2):169-76. doi: 10.1159/000323180. Epub 2011 Jan 21.

Eotaxin in exhaled breath condensate of allergic asthma patients with exercise-induced bronchoconstriction.

Zietkowski Z, Skiepko R, Tomasiak-Lozowska MM, Zietkowska E, Bodzenta-Lukaszyk A.

Department of Allergology and Internal Medicine, Medical University of Bialystok, Bialystok, Poland. z.zietkowski@wp.pl

BACKGROUND: Eosinophils are the key inflammatory cells in asthma, and more and more evidence suggests their crucial role in exercise-induced bronchoconstriction (EIB). Eotaxin, as the most important chemotactic factor for eosinophils, plays an important role in the pathogenesis of asthma.
OBJECTIVES: The aim of the study was to evaluate the changes in eotaxin levels in exhaled breath condensate (EBC) following intensive exercise in allergic asthmatics.
METHODS: The study was performed in a group of 27 asthmatics (17 with EIB, 13 without EIB) and 9 healthy volunteers. Changes induced by intensive exercise in the concentrations of eotaxin in EBC during the 24 h after an exercise test were assessed. The possible correlations of these measurements with the results of other tests commonly associated with eosinophilic airway inflammation were also determined.
RESULTS: In asthmatic patients with EIB, a statistically significant increase in eotaxin concentrations in EBC collected during the first 24 h after an exercise test - with maximal increase after 6 h - was revealed. A statistically significant correlation between the maximum increase in eotaxin concentrations in EBC after exercise, and an increase in either serum eosinophil cationic protein or F(ENO) 24 h after exercise in the group of asthmatics with EIB, was observed.
CONCLUSIONS: Our results confirm connections between EIB and airway eosinophilic inflammation. The increase of eotaxin in asthmatic airways, by promoting the migration and activation of eosinophils, may play an important role in upregulation and sustaining of the airway inflammation observed in EIB in

asthmatic patients.

Copyright © 2011 S. Karger AG, Basel.

PMID: 21252484 [PubMed - indexed for MEDLINE]

290. J Ethnopharmacol. 2011 Jul 14;136(3):399-405. doi: 10.1016/j.jep.2011.01.014. Epub 2011 Jan 18.

Inhibitory effects of casticin on migration of eosinophil and expression of chemokines and adhesion molecules in A549 lung epithelial cells via NF-κB inactivation.

Koh DJ, Ahn HS, Chung HS, Lee H, Kim Y, Lee JY, Kim DG, Hong M, Shin M, Bae H.

Department of Physiology, College of Oriental Medicine, Kyung Hee University, Seoul, Republic of Korea.

ETHNOPHARMACOLOGICAL RELEVANCE: The fruits of Vitex rotundifolia L. have long been used for the treatment of inflammation of the respiratory tract in East Asia.
AIM: To determine if casticin, one of the constituents of Vitex rotundifolia L., has anti-allergic and anti-inflammatory effects in asthma.
MATERIALS AND METHODS: The in vitro anti-inflammatory activity of casticin was studied in A549 human type II-like epithelial lung cells using an eotaxin inhibition assay. Additionally, its effects on eotaxin, regulated on activation normal T cell expressed and secreted (RANTES), vascular cell adhesion molecule (VCAM)-1, and inter-cellular adhesion molecule (ICAM)-1 expression were investigated by real time-polymerase chain reaction (real time-PCR). The inhibition of nuclear factor κB (NF-κB) activity in the presence of casticin was determined by analyzing confocal microscopy images of fluorescence immunocytochemical analysis while the suppression of inhibitory κB (IκB)-α phosphorylation was studied using Western blot analysis. Finally, the inhibitory effect of casticin on eosinophil migration toward prestimulated A549 cell media was measured using the human eosinophilic leukemia cell line.
RESULTS AND DISCUSSION: Casticin significantly suppressed eotaxin production in cytokine activated A549 lung epithelial cells. Casticin also suppressed the mRNA expression levels of eotaxin, RANTES, VCAM-1, and ICAM-1, which subsequently contributed to the inhibition of eosinophil migration. Furthermore, casticin inhibited IκB-α phosphorylation and nuclear translocation of p65 in A549 cells.
CONCLUSION: Casiticin inhibited the eosinophil migration and activity of chemokines and adhesion molecules involved in the inflammatory process of asthma by suppressing the NF-κB pathway. These results suggest that casticin has the potential for use in the treatment of allergic asthma.

Copyright © 2011 Elsevier Ireland Ltd. All rights reserved.

PMID: 21251967 [PubMed - indexed for MEDLINE]

291. Respir Res. 2011 Jan 21;12:14. doi: 10.1186/1465-9921-12-14.

Nogo-B regulates migration and contraction of airway smooth muscle cells by decreasing ARPC 2/3 and increasing MYL-9 expression.

Xu W, Hong W, Shao Y, Ning Y, Cai Z, Li Q.

Department of Respiratory Diseases, ChangHai Hospital, Second Military Medical University, Shanghai 200433, China.

BACKGROUND: Abnormal proliferation, apoptosis, migration and contraction of airway smooth muscle (ASM) cells in airway remodeling in asthma are basically excessive repair responses to a network of inflammatory mediators such as PDGF, but the mechanisms of such responses remain unclear. Nogo-B, a member of the reticulum family 4(RTN4), is known to play a key role in arteriogenesis and tissue repair. Further studies are needed to elucidate the role of Nogo-B in airway smooth muscle abnormalities.
METHODS: A mouse model of chronic asthma was established by repeated OVA inhalation and subjected to Nogo-B expression analysis using immunohistochemistry and Western Blotting. Then, primary human bronchial smooth muscle cells (HBSMCs) were cultured in vitro and a siRNA interference was performed to knockdown the expression of Nogo-B in the cells. The effects of Nogo-B inhibition on PDGF-induced HBSMCs proliferation, migration and contraction were evaluated. Finally, a proteomic analysis was conducted to unveil the underlying mechanisms responsible for the function of Nogo-B.
RESULTS: Total Nogo-B expression was approximately 3.08-fold lower in chronic asthmatic mice compared to naïve mice, which was obvious in the smooth muscle layer of the airways. Interference of Nogo-B expression by siRNA resulted nearly 96% reduction in mRNA in cultured HBSMCs. In addition, knockdown of Nogo-B using specific siRNA significantly decreased PDGF-induced migration of HBSMCs by 2.3-fold, and increased the cellular contraction by 16% compared to negative controls, but had limited effects on PDGF-induced proliferation. Furthermore, using proteomic analysis, we demonstrate that the expression of actin related protein 2/3 complex subunit 5 (ARPC 2/3) decreased and, myosin regulatory light chain 9 isoform a (MYL-9) increased after Nogo-B knockdown.
CONCLUSIONS: These data define a novel role for Nogo-B in airway remodeling in chronic asthma. Endogenous Nogo-B, which may exert its effects through ARPC 2/3 and MYL-9, is necessary for the migration and contraction of airway smooth muscle cells.

PMCID: PMC3037873
PMID: 21251247 [PubMed - indexed for MEDLINE]

292. Adv Exp Med Biol. 2010;681:107-16. doi: 10.1007/978-1-4419-6354-3_8.

Terminal signal: anti-inflammatory effects of α-melanocyte-stimulating hormone related peptides beyond the pharmacophore.

Brzoska T, Böhm M, Lügering A, Loser K, Luger TA.

Department of Dermatology, University of Münster, Von Esmarch-Str. 58, D-48149 Münster, Germany. brzoska@uni-muenster.de

During the last two decades a significant number of investigations has established the fact that α-Melanocyte-stimulating hormone (α-MSH) is a potent anti-inflammatory mediator. The anti-inflammatory effects of α-MSH can be elicited via melanocortin receptors (MC-Rs) broadly expressed in a number of tissues ranging from the central nervous system to cells of the immune system and on resident somatic cells of peripheral tissues. α-MSH affects various pathways regulating inflammatory responses such as NF-κB activation, expression of adhesion molecules, inflammatory cytokines, chemokine receptors, T-cell proliferation and activity and inflammatory cell migration. In vivo α-MSH has been shown to be anti-inflammatory as well in animal models of fever, irritant and allergic contact dermatitis, cutaneous vasculitis, fibrosis, in ocular, gastrointestinal, brain and allergic airway inflammation and arthritis. A broad range of effects of α-MSH exerted beyond the field of inflammation, its pigmentory capacity being only the most visible aspect, has been one of the major impediments limiting the use of α-MSH in human inflammatory disorders.

Interestingly KPV, C-terminal tripeptide of α-MSH, which lacks the entire sequence motif required for binding to any of the known MC-Rs, retains almost all of the anti-inflammatory capacity of the full hormone, but in its activities display a lack of any pigmentory action. While the exact signaling mechanism utilized by KPV and related peptides currently is unknown it has been demonstrated already that significant similarities between anti-inflammatory signaling of α-MSH and those short peptides exist. These α-MSH related tripeptides thus may be useful alternatives for anti-inflammatory peptide therapy. KdPT, a derivative of KPV corresponding to IL-1β(193-195), currently is emerging as another tripeptide with potent anti-inflammatory effects. A more limited spectrum of biologic activities, potentially advantageous physicochemical, pharmacokinetic and pharmacodynamic properties as well as the expectation of low costs for pharmaceutical production make these agents interesting candidates for the treatment of immune-mediated inflammatory skin and bowel diseases, allergic asthma and arthritis.

PMID: 21222263 [PubMed - indexed for MEDLINE]

293. Nat Immunol. 2011 Feb;12(2):178-85. doi: 10.1038/ni.1983. Epub 2011 Jan 9.

ECM1 controls T(H)2 cell egress from lymph nodes through re-expression of S1P(1).

Li Z, Zhang Y, Liu Z, Wu X, Zheng Y, Tao Z, Mao K, Wang J, Lin G, Tian L, Ji Y, Qin M, Sun S, Zhu X, Sun B.

Laboratory of Molecular Cell Biology, Institute of Biochemistry and Cell Biology, Shanghai Institutes for Biological Sciences, Chinese Academy of Sciences, Shanghai, China.

Comment in
    Nat Rev Immunol. 2011 Feb;11(2):77.

Type 2 helper T cells (T(H)2) are critically involved in allergies and asthma. Here we demonstrate that extracellular matrix protein-1 (ECM1) is highly and selectively expressed in T(H)2 cells. ECM1 deficiency caused impaired T(H)2 responses and reduced allergic airway inflammation in vivo. Functional analysis demonstrated that although the T(H)2 polarization of ECM1-deficient cells was unimpaired, these cells had a defect in migration and were retained in peripheral lymphoid organs. This was associated with reduced expression of KLF2 and S1P(1). We also found that ECM1 could directly bind the interleukin-2 (IL-2) receptor to inhibit IL-2 signaling and activate S1P(1) expression. Our data identify a previously unknown function of ECM1 in regulating T(H)2 cell migration through control of KLF2 and S1P(1) expression.

PMID: 21217760 [PubMed - indexed for MEDLINE]

294. J Allergy Clin Immunol. 2011 Jan;127(1):200-7, 207.e1-10. doi: 10.1016/j.jaci.2010.11.005.

TNF-like weak inducer of apoptosis (TWEAK) and TNF-α cooperate in the induction of keratinocyte apoptosis.

Zimmermann M, Koreck A, Meyer N, Basinski T, Meiler F, Simone B, Woehrl S, Moritz K, Eiwegger T, Schmid-Grendelmeier P, Kemeny L, Akdis CA.

Swiss Institute of Allergy and Asthma Research (SIAF), University of Zurich, Davos, Switzerland.

BACKGROUND: Activation of skin keratinocytes followed by their apoptotic death leads to eczema and spongiosis formations in patients with atopic dermatitis (AD). TNF-like weak inducer of apoptosis (TWEAK) binds to its receptor, fibroblast growth factor-inducible 14 (Fn14), and controls many cellular activities, including proliferation, migration, differentiation, apoptosis, angiogenesis, and inflammation.
OBJECTIVE: The aim of the study was to investigate the role of TWEAK and Fn14 in the formation of eczema in patients with AD.
METHODS: Primary keratinocytes were isolated from nonlesional skin from patients with AD and psoriasis and from normal skin of healthy donors. Apoptosis analysis was performed by using annexin V/7-aminoactinomycin D and terminal deoxynucleotidyl transferase-mediated dUTP nick end-labeling staining. The expression and regulation of TWEAK, TNF-α, Fn14, TNF receptor (TNFR) 1, and TNFR2 were measured by means of RT-PCR, flow cytometric analysis, and ELISA. TWEAK and Fn14 expression of lesional AD and psoriatic skin and normal control skin was analyzed by using immunohistochemistry and immunofluorescence.
RESULTS: TWEAK and TNF-α cooperate in the induction of apoptosis in primary keratinocytes obtained from patients with AD, patients with psoriasis, and healthy subjects and in artificial skin equivalents. TNFR1 and Fn14 were the main receptors involved. TWEAK upregulates TNF-α expression in primary keratinocytes, whereas TNF-α did not affect the expression of TWEAK and its receptors. High TWEAK expression was observed in AD lesions but not in psoriatic lesions or normal skin. Fn14 was highly expressed in the lesional skin of patients with AD and patients with psoriasis and in healthy control skin.
CONCLUSION: The high expression of TWEAK in lesional AD skin contributes to the difference in keratinocyte apoptosis and lesional formation between AD and psoriasis.

Copyright Â© 2010 American Academy of Allergy, Asthma & Immunology. Published by Mosby, Inc. All rights reserved.

PMID: 21211655 [PubMed - indexed for MEDLINE]

295. J Allergy Clin Immunol. 2011 Jan;127(1):194-9. doi: 10.1016/j.jaci.2010.11.002.

Eosinophil extracellular DNA traps in skin diseases.

Simon D, Hoesli S, Roth N, Staedler S, Yousefi S, Simon HU.

Department of Dermatology, Inselspital, Bern University Hospital, Bern, Switzerland. dagmar.simon@insel.ch

BACKGROUND: In the skin, eosinophils are found in a broad spectrum of diseases, including infectious diseases.
OBJECTIVE: In this study, we investigated whether eosinophil extracellular traps, structures containing DNA in association with eosinophil granule proteins able to bind and kill bacteria, are present in the skin under various pathologic conditions.
METHODS: Immunofluorescence staining was performed on sections of paraformaldehyde-fixed and paraffin-embedded skin biopsy tissues of 25 different eosinophilic skin diseases by using propidium iodide and an antibody to eosinophil cationic protein. Slides were evaluated by laser scanning microscopy.
RESULTS: Eosinophils releasing DNA together with eosinophil cationic protein were detected in infectious skin diseases such as ectoparasitosis and larva migrans. Further, we observed the extracellular DNA structures in allergic/reactive diseases (Wells syndrome, hypereosinophilic syndrome, positive reaction of atopy patch test, allergic contact dermatitis, drug hypersensitivity) and in autoimmune diseases (bullous pemphigoid, pemphigus foliaceus, dermatitis herpetiformis). The average number of eosinophils releasing DNA in the skin was usually below 10%,

although in Wells syndrome the proportion was up to 30%. In areas with clusters of eosinophils, up to 50% of the eosinophils were seen to generate eosinophil extracellular traps.
CONCLUSION: Eosinophil extracellular traps are seen in both infectious and noninfectious inflammatory skin diseases and are particularly common in Wells syndrome.

Copyright Â© 2010 American Academy of Allergy, Asthma & Immunology. Published by Mosby, Inc. All rights reserved.

PMID: 21211654 [PubMed - indexed for MEDLINE]

296. J Allergy Clin Immunol. 2011 Jan;127(1):18-27; quiz 28-9. doi: 10.1016/j.jaci.2010.11.030.

Mechanisms of allergen-specific immunotherapy.

Akdis CA, Akdis M.

Swiss Institute of Allergy and Asthma Research (SIAF), University of Zurich, Davos, Switzerland. akdisac@siaf.unizh.ch

Allergen-specific immunotherapy has been used for 100 years as a desensitizing therapy for allergic diseases and represents the potentially curative and specific method of treatment. The mechanisms of action of allergen-specific immunotherapy include the very early desensitization effects, modulation of T-and B-cell responses and related antibody isotypes, and migration of eosinophils, basophils, and mast cells to tissues, as well as release of their mediators. Regulatory T (Treg) cells have been identified as key regulators of immunologic processes in peripheral tolerance to allergens. Skewing of allergen-specific effector T cells to a regulatory phenotype appears as a key event in the development of healthy immune response to allergens and successful outcome in patients undergoing allergen-specific immunotherapy. Naturally occurring forkhead box protein 3-positive CD4(+)CD25(+) Treg cells and inducible T(R)1 cells contribute to the control of allergen-specific immune responses in several major ways, which can be summarized as suppression of dendritic cells that support the generation of effector T cells; suppression of effector T(H)1, T(H)2, and T(H)17 cells; suppression of allergen-specific IgE and induction of IgG4; suppression of mast cells, basophils, and eosinophils; and suppression of effector T-cell migration to tissues. New strategies for immune intervention will likely include targeting of the molecular mechanisms of allergen tolerance and reciprocal regulation of effector and Treg cell subsets.

Copyright Â© 2010 American Academy of Allergy, Asthma & Immunology. Published by Mosby, Inc. All rights reserved.

PMID: 21211639 [PubMed - indexed for MEDLINE]

297. J Allergy Clin Immunol. 2011 Feb;127(2):420-429.e1-6. doi: 10.1016/j.jaci.2010.10.043. Epub 2011 Jan 5.

Peroxisome proliferator-activated receptor γ-mediated suppression of dendritic cell function prevents the onset of atopic dermatitis in NC/Tnd mice.

Jung K, Tanaka A, Fujita H, Matsuda A, Oida K, Karasawa K, Okamoto N, Ohmori K, Jee Y, Shin T, Matsuda H.

Laboratory of Veterinary Molecular Pathology and Therapeutics, Division of Animal

Life Science, Graduate School, Institute of Agriculture, Tokyo University of Agriculture and Technology, Tokyo, Japan.

BACKGROUND: Dendritic cells (DCs) are one of the key regulators for the initiation of allergic responses in patients with atopic dermatitis (AD), being strongly triggered by epithelial cell-derived thymic stromal lymphopoietin (TSLP). Because peroxisome proliferator-activated receptor (PPAR) γ acts as a negative regulator in immune cells, suppressive properties of PPARγ in allergic responses have been proposed.
OBJECTIVE: Because pieces of evidence must be organized to identify the exact role of PPARγ in immune regulation, we explored the suppressive effects of a PPARγ agonist on various functions of DCs and the onset of AD in a murine model.
METHODS: Effects of rosiglitazone (RSG) on DCs that were derived from NC/Tnd mice, a model for human AD, were analyzed. RSG was administered to NC/Tnd mice to evaluate its preventive and therapeutic effects on the development of AD.
RESULTS: RSG inhibited TSLP-induced DC maturation through downregulation of costimulatory molecules. TSLP-promoted expressions of chemokines in DCs were also suppressed by RSG treatment. Moreover, we showed the necessity of matrix metalloproteinase 9 in TSLP-promoted DC migration by using DCs derived from matrix metalloproteinase 9-deficient NC/Tnd mice, as well as the suppressive effect of PPARγ in the process. Daily oral administration of RSG to NC/Tnd mice before the onset of AD revealed a significant reduction in severity of skin lesions and scratching behavior. In mice treated with RSG, both expression of TSLP in the skin and maturation and migration of DCs were markedly suppressed.
CONCLUSION: PPARγ can be provided as an inhibitory regulator of TSLP-stimulated DCs in the onset of allergic reactions.

Copyright © 2011 American Academy of Allergy, Asthma & Immunology. Published by Mosby, Inc. All rights reserved.

PMID: 21208653 [PubMed - indexed for MEDLINE]

298. Allergy. 2011 Feb;66(2):287-97. doi: 10.1111/j.1398-9995.2010.02478.x. Epub 2010 Sep 7.

The CKLF1-C19 peptide attenuates allergic lung inflammation by inhibiting CCR3- and CCR4-mediated chemotaxis in a mouse model of asthma.

Tian L, Li W, Wang J, Zhang Y, Zheng Y, Qi H, Guo X, Zhang Y, Ma D, Shen H, Wang Y.

Department of Medical Immunology, School of Basic Medical Science, Peking University Health Science Center, #38 Xueyuan Road, Beijing, China.

BACKGROUND: Human chemokine-like factor 1 (CKLF1) is a functional ligand for human CCR4, which is highly expressed on Th2 lymphocytes and plays an important role in the pathogenesis of asthma. The expression and function of CKLF1 are associated with asthma. The CKLF1 C-terminal peptides C19 and C27 also interact with human CCR4. Albeit with weaker chemotactic activity, C19 can inhibit chemotaxis induced by both CKLF1 and CCL17. Here, we explore whether C19 can act as an antagonist in the development of asthma.
METHODS: A mouse model of asthma and in vitro and in vivo chemotaxis assays were used.
RESULTS: Using a mouse model of asthma, we demonstrate here that C19 reduces airway eosinophilia, lung inflammation and airway hyperresponsiveness; in contrast, C27 has little effect on these parameters. The inhibitory effects of C19 on CCR4-mediated chemotaxis could be observed in human Th2 lymphocytes and in the splenocytes from ovalbumin-sensitized mice. Furthermore, we show that C19 can inhibit CCL11-induced chemotaxis of mouse eosinophils and human CCR3-transfected

or mouse Ccr3-transfected HEK293 cells. In vivo chemotaxis assays revealed that C19 and C27 can reduce CCL11-mediated recruitment of eosinophils into the peritoneal cavity and that this inhibitory effect is stronger for C19 than for C27.
CONCLUSIONS: Thus, C19 can attenuate airway eosinophilia and lung inflammation by inhibiting CCR3- and CCR4-mediated chemotaxis in a mouse model of asthma. Given its ability to inhibit human CCR3- and CCR4-meditated chemotaxis, C19 has great therapeutic potential for use in the treatment and control of allergic asthma.

© 2010 John Wiley & Sons A/S.

PMID: 21208220 [PubMed - indexed for MEDLINE]

299. J Vis Exp. 2010 Dec 23;(46). pii: 2315. doi: 10.3791/2315.

Real-time imaging of leukotriene B_4 mediated cell migration and BLT1 interactions with β-arrestin.

Jala VR, Haribabu B.

Microbiology and Immunology, James Graham Brown Cancer Center, University of Louisville, USA.

G-protein coupled receptors (GPCRs) belong to the seven transmembrane protein family and mediate the transduction of extracellular signals to intracellular responses. GPCRs control diverse biological functions such as chemotaxis, intracellular calcium release, gene regulation in a ligand dependent manner via heterotrimeric G-proteins(1-2). Ligand binding induces a series of conformational changes leading to activation of heterotrimeric G-proteins that modulate levels of second messengers such as cyclic adenosine monophosphate (cAMP), inositol triphosphate (IP3) and diacyl glycerol (DG). Concomitant with activation of the receptor ligand binding also initiates a series of events to attenuate the receptor signaling via desensitization, sequestration and/or internalization. The desensitization process of GPCRs occurs via receptor phosphorylation by G-protein receptor kinases (GRKs) and subsequent binding of β-arrestins(3). β-arrestins are cytosolic proteins and translocate to membrane upon GPCR activation, binding to phosphorylated receptors (most cases) there by facilitating receptor internalization (4-6). Leukotriene B(4;) (LTB(4;)) is a pro-inflammatory lipid molecule derived from arachidonic acid pathway and mediates its actions via GPCRs, LTB(4;) receptor 1 (BLT1; a high affinity receptor) and LTB(4;) receptor 2 (BLT2; a low affinity receptor)(7-9). The LTB(4;)-BLT1 pathway has been shown to be critical in several inflammatory diseases including, asthma, arthritis and atherosclerosis(10-17). The current paper describes the methodologies developed to monitor LTB(4;)-induced leukocyte migration and the interactions of BLT1 with β-arrestin and , receptor translocation in live cells using microscopy imaging techniques(18-19). Bone marrow derived dendritic cells from C57BL/6 mice were isolated and cultured as previously described (20-21). These cells were tested in live cell imaging methods to demonstrate LTB(4;) induced cell migration. The human BLT1 was tagged with red fluorescent protein (BLT1-RFP) at C-terminus and β-arrestin1 tagged with green fluorescent protein (β-arr-GFP) and transfected the both plasmids into Rat Basophilic Leukomia (RBL-2H3) cell lines(18-19). The kinetics of interaction between these proteins and localization were monitored using live cell video microscopy. The methodologies in the current paper describe the use of microscopic techniques to investigate the functional responses of G-protein coupled receptors in live cells. The current paper also describes the use of Metamorph software to quantify the fluorescence intensities to determine the kinetics of receptor and cytosolic protein interactions.

PMCID: PMC3159663
PMID: 21206474 [PubMed - indexed for MEDLINE]

300. Parasite Immunol. 2011 May;33(5):265-75. doi: 10.1111/j.1365-3024.2010.01276.x.

Amelioration of intestinal colitis by macrophage migration inhibitory factor isolated from intestinal parasites through toll-like receptor 2.

Cho MK, Lee CH, Yu HS.

Department of Parasitology, School of Medicine, Pusan National University, Yangsan, Korea.

In a previous study, we cloned type II MIFs (As-MIF) from Anisakis simplex 3rd stage larva and expressed a recombinant protein that suppressed allergic airway inflammation via regulatory T (CD4(+) CD25(+) Foxp3(+) T; T(reg))-cell recruitment. In this study, in an effort to evaluate the function of rAs-MIF on another immune disease, we induced intestinal inflammation in mice using dextran sodium sulphate (DSS) with or without the application of rAs-MIF treatment to the mice. As a consequence, weight losses were recovered, and the value of disease activity index (DAI) was reduced by rAs-MIF treatment during the experimental period. The levels of TGF-β and IL-10 in the spleens and mesenteric lymph nodes (MLN) from the rAs-MIF-treated mice were higher, but the levels of IFN-γ, IL-6 and IL-13 were lower than those of the mice treated with DSS but not with rAs-MIF. Additionally, the T(reg) cells observed were greatly increased in the MLNs of the rAs-MIF-treated mice than those of mice not treated with rAs-MIF. The results of our in vitro experiments showed that the elevated IL-10 production induced by rAs-MIF was generated via toll-like receptor 2. In conclusion, rAs-MIF appears to ameliorate DSS-induced colitis and may prove useful as a therapeutic agent for the treatment of intestinal inflammatory disease.

PMID: 21204854 [PubMed - indexed for MEDLINE]

301. BMJ Case Rep. 2011 Dec 6;2011. pii: bcr0920114812. doi: 10.1136/bcr.09.2011.4812.

A report on a rare case of Klebsiella ozaenae causing atrophic rhinitis in the UK.

Lee YJ, Moore LS, Almeyda J.

ENT Department, West Middlesex University Hospital, Middlesex, UK.
yjlee@doctors.org.uk

Ozena is a chronic disease of the nasal cavity characterised by atrophy of the mucosa and bone caused by Klebsiella ozaenae. It is endemic to subtropical and temperate regions affecting the lower socio-economic group, usually the poor who live in unhygienic conditions. It is a rare disease in the UK. There is usually a delay in diagnosis due to unfamiliarity of the disease. A 25-year-old Nigerian migrant presented with nasal obstruction with purulent nasal discharge. Isolation of the bacterium was found from cultures of nasal discharge, crusting and tissue biopsies. She was treated successfully with ciprofloxacin. It is important to consider this rare condition in cases of nasal obstruction even in non-endemic areas especially with the advances of modern travel.

PMID: 22669526 [PubMed - in process]

302. J Invest Dermatol. 2011 Apr;131(4):865-73. doi: 10.1038/jid.2010.395. Epub 2010

Dec 30.

Upregulation of aquaporin-3 is involved in keratinocyte proliferation and epidermal hyperplasia.

Nakahigashi K, Kabashima K, Ikoma A, Verkman AS, Miyachi Y, Hara-Chikuma M.

Department of Dermatology, Graduate School of Medicine, Kyoto University, Kyoto, Japan.

Aquaporin-3 (AQP3) is a water/glycerol-transporting protein expressed in keratinocytes of the epidermis. We previously showed that AQP3-mediated transport of water and glycerol is involved in keratinocyte migration and proliferation, respectively. However, the involvement of AQP3 in epidermal hyperplasia in skin diseases, such as atopic dermatitis (AD), is unknown. In this study, we found significantly increased AQP3 transcript and protein expression in the epidermis of human AD lesions. The upregulation of AQP3 expression in human keratinocytes by transfection with human AQP3 DNA plasmid was associated with increased cellular glycerol and ATP, as well as increased cell proliferation. Among several cytokines and chemokines produced in the skin, CCL17, which is highly expressed in AD, was found to be a strong inducer of AQP3 expression and enhanced keratinocyte proliferation. In mouse AD models, AQP3 was strongly overexpressed in the epidermis in wild-type mice. Epidermal hyperplasia was reduced in AQP3-deficient mice, with a decreased number of proliferating keratinocytes. These results suggest the involvement of AQP3 in epidermal hyperplasia by a mechanism involving upregulated AQP3 expression and consequent enhancement of keratinocyte proliferation.

PMID: 21191421 [PubMed - indexed for MEDLINE]

303. J Invest Dermatol. 2011 Apr;131(4):925-31. doi: 10.1038/jid.2010.418. Epub 2010 Dec 30.

Macrophage migration inhibitory factor is essential for eosinophil recruitment in allergen-induced skin inflammation.

Yoshihisa Y, Makino T, Matsunaga K, Honda A, Norisugi O, Abe R, Shimizu H, Shimizu T.

Department of Dermatology, Graduate School of Medicine and Pharmaceutical Sciences, University of Toyama, Sugitani, Toyama, Japan.

Macrophage migration inhibitory factor (MIF) is a pluripotent cytokine that has an essential role in the pathophysiology of experimental allergic inflammation. Recent findings suggest that MIF is involved in several allergic disorders, including atopic dermatitis (AD). In this study, the role of MIF in allergic skin inflammation was examined using a murine model of AD elicited by epicutaneous sensitization with ovalbumin (OVA). We observed the number of skin-infiltrating eosinophils to significantly increase in OVA-sensitized MIF transgenic (Tg) mice compared with their wild-type (WT) littermates. On the other hand, eosinophils were virtually absent from the skin of MIF knockout (KO) mice and failed to infiltrate their skin after repeated epicutaneous sensitization with OVA. The mRNA expression levels of eotaxin and IL-5 were significantly increased in OVA-sensitized skin sites of MIF Tg mice, but were significantly decreased in MIF KO mice in comparison with the levels in WT littermates. Eotaxin expression was induced by IL-4 stimulation in fibroblasts in MIF Tg mice, but not in MIF KO mice. These findings indicate that MIF can induce eosinophil accumulation in the skin. Therefore, the targeted inhibition of MIF might be a promising new therapeutic strategy for allergic skin diseases.

PMID: 21191413 [PubMed - indexed for MEDLINE]

304. J Immunol. 2011 Feb 1;186(3):1861-9. doi: 10.4049/jimmunol.1002568. Epub 2010 Dec 27.

Gene expression patterns of Th2 inflammation and intercellular communication in asthmatic airways.

Choy DF, Modrek B, Abbas AR, Kummerfeld S, Clark HF, Wu LC, Fedorowicz G, Modrusan Z, Fahy JV, Woodruff PG, Arron JR.

Immunology, Tissue Growth, and Repair Biomarker Discovery, Genentech, South San Francisco, CA 94080, USA.

Asthma is canonically thought of as a disorder of excessive Th2-driven inflammation in the airway, although recent studies have described heterogeneity with respect to asthma pathophysiology. We have previously described distinct phenotypes of asthma based on the presence or absence of a three-gene "Th2 signature" in bronchial epithelium, which differ in terms of eosinophilic inflammation, mucin composition, subepithelial fibrosis, and corticosteroid responsiveness. In the present analysis, we sought to describe Th2 inflammation in human asthmatic airways quantitatively with respect to known mediators of inflammation and intercellular communication. Using whole-genome microarray and quantitative real-time PCR analysis of endobronchial biopsies from 27 mild-to-moderate asthmatics and 13 healthy controls with associated clinical and demographic data, we found that asthmatic Th2 inflammation is expressed over a variable continuum, correlating significantly with local and systemic measures of allergy and eosinophilia. We evaluated a composite metric describing 79 coexpressed genes associated with Th2 inflammation against the biological space comprising cytokines, chemokines, and growth factors, identifying distinctive patterns of inflammatory mediators as well as Wnt, TGF-β, and platelet-derived growth factor family members. This integrated description of the factors regulating inflammation, cell migration, and tissue remodeling in asthmatic airways has important consequences for the pathophysiological and clinical impacts of emerging asthma therapeutics targeting Th2 inflammation.

PMID: 21187436 [PubMed - indexed for MEDLINE]

305. Dis Markers. 2010;29(3-4):157-75. doi: 10.3233/DMA-2010-0735.

The Wiskott-Aldrich syndrome: The actin cytoskeleton and immune cell function.

Blundell MP, Worth A, Bouma G, Thrasher AJ.

Molecular Immunology Unit, UCL Institute of Child Health, London, UK.

Wiskott-Aldrich syndrome (WAS) is a rare X-linked recessive primary immunodeficiency characterised by immune dysregulation, microthrombocytopaenia, eczema and lymphoid malignancies. Mutations in the WAS gene can lead to distinct syndrome variations which largely, although not exclusively, depend upon the mutation. Premature termination and deletions abrogate Wiskott-Aldrich syndrome protein (WASp) expression and lead to severe disease (WAS). Missense mutations usually result in reduced protein expression and the phenotypically milder X-linked thrombocytopenia (XLT) or attenuated WAS [1-3]. More recently however novel activating mutations have been described that give rise to X-linked neutropenia (XLN), a third syndrome defined by neutropenia with variable myelodysplasia [4-6]. WASP is key in transducing signals from the cell surface to

the actin cytoskeleton, and a lack of WASp results in cytoskeletal defects that compromise multiple aspects of normal cellular activity including proliferation, phagocytosis, immune synapse formation, adhesion and directed migration.

PMID: 21178275 [PubMed - indexed for MEDLINE]

306. Am J Respir Cell Mol Biol. 2011 Sep;45(3):542-9. doi: 10.1165/rcmb.2010-0238OC. Epub 2010 Dec 22.

Lys-des[Arg9]-bradykinin alters migration and production of interleukin-12 in monocyte-derived dendritic cells.

Gulliver R, Baltic S, Misso NL, Bertram CM, Thompson PJ, Fogel-Petrovic M.

Centre for Asthma, Allergy and Respiratory Research, The University of Western Australia, and Lung Institute of Western Australia, Perth, Western Australia, Australia.

This study tested the hypothesis that proinflammatory kinin peptides are involved in modulating human dendritic cell (DC) function. Inflammation is accompanied by an increased maturation of DCs and the generation of kinins, particularly Lys-des[Arg(9)]-bradykinin (Lda-BK). We assessed the role of Lda-BK in the activation and migration of human monocyte-derived DCs (hMo-DCs) matured through the use of LPS, TNF-α + IL-1β, or CD40 ligand. Kinin B(1) and B(2) receptor mRNA and protein expression were assessed by confocal microscopy, flow cytometry, and RT-PCR. The effects of Lda-BK on the migration of mature hMo-DCs were assessed in Transwell chambers, whereas the expression of costimulatory molecules and the secretion of IL-12 were assessed by flow cytometry and ELISA, respectively. The expression of the kinin B(1) receptor (B(1)R) was down-regulated during the maturation of hMo-DCs, whereas the expression of B(2)R was unchanged. The B(1)R agonist Lda-BK was not chemotactic for hMo-DCs matured using LPS, TNF-α + IL-1β, or CD40 ligand, but Lda-BK enhanced the secretion of IL-12p70 and inhibited the secretion of IL-12p40 by mature hMo-DCs. However, the exposure of hMo-DCs matured with TNF-α + IL-1β to Lda-BK for 6 hours decreased subsequent migration in response to Lda-BK, the chemokine CCL19, or Lda-BK combined with CCL19. The expression of B(1)R was increased in hMo-DCs from subjects with asthma compared with subjects without asthma, in keeping with a tendency toward increased in vitro migration of asthmatic hMo-DCs in response to Lda-BK. The increased formation of Lda-BK and the enhanced expression of B(1)R as a consequence of inflammation may alter the migration of mature, antigen-laden DCs to regional lymph nodes in response to CCL19, may modulate the secretion of cytokines by these DCs, and may contribute to the accumulation of mature DCs in the lungs of patients with asthma.

PMID: 21177981 [PubMed - indexed for MEDLINE]

307. J Immunol. 2011 Jan 15;186(2):848-55. doi: 10.4049/jimmunol.0903878. Epub 2010 Dec 20.

Substance P is a key mediator of stress-induced protection from allergic sensitization via modified antigen presentation.

Pavlovic S, Liezmann C, Blois SM, Joachim R, Kruse J, Romani N, Klapp BF, Peters EM.

University-Medicine Charité, Charité Center 12 for Internal Medicine and Dermatology, D-10117 Berlin, Germany.

Interaction between the nervous and immune systems greatly contributes to inflammatory disease. In organs at the interface between our body and the environment, the sensory neuropeptide substance P (SP) is one key mediator of an acute local stress response through neurogenic inflammation but may also alter cytokine balance and dendritic cell (DC) function. Using a combined murine allergic inflammation/noise stress model with C57BL/6 mice, we show in this paper that SP--released during repeated stress exposure--has the capacity to markedly attenuate inflammation. In particular, repeated stress exposure prior to allergen sensitization increases DC-nerve fiber contacts, enhances DC migration and maturation, alters cytokine balance, and increases levels of IL-2 and T regulatory cell numbers in local lymph nodes and inflamed tissue in a neurokinin 1-SP-receptor (neurokinin-1 receptor)-dependent manner. Concordantly, allergic inflammation is significantly reduced after repeated stress exposure. We conclude that SP/repeated stress prior to immune activation acts protolerogenically and thereby beneficially in inflammation.

PMID: 21172866 [PubMed - indexed for MEDLINE]

308. J Allergy Clin Immunol. 2011 Feb;127(2):439-446.e1-5. doi: 10.1016/j.jaci.2010.10.021. Epub 2010 Dec 17.

The extra domain A of fibronectin is essential for allergen-induced airway fibrosis and hyperresponsiveness in mice.

Kohan M, Muro AF, Bader R, Berkman N.

Institute of Pulmonology, Hadassah-Hebrew University Medical Center, Jerusalem, Israel.

BACKGROUND: Asthma is characterized by airway inflammation, airway remodeling, and airway hyperresponsiveness (AHR). Myofibroblast differentiation and subepithelial fibrosis are key features of airway remodeling. Extra domain A (EDA)-containing fibronectin (EDA-FN), an alternatively spliced form of the extracellular matrix protein fibronectin, has been implicated in fibroblast differentiation during wound healing and tissue fibrosis.
OBJECTIVES: We sought to investigate the role of EDA-FN in airway remodeling using a murine model of chronic allergen-induced experimental asthma.
METHODS: EDA(-/-) and wild-type (WT) mice were sensitized and exposed to inhaled ovalbumin (OVA) or saline for 5 weeks. EDA-FN expression was evaluated by means of PCR and immunostaining. Peribronchial fibrosis, smooth muscle area, mucus-producing cell numbers, bronchoalveolar cell counts, and lung function were assessed in WT and EDA(-/-) mice. Fibroblast activation and differentiation were evaluated ex vivo by using OVA-treated WT and EDA(-/-) lung fibroblasts.
RESULTS: Exposure to OVA increased EDA-FN expression in lung tissue and primary lung fibroblasts. OVA-treated EDA(-/-) mice showed reduced airway fibrosis and AHR and impaired expression of TGF-β1 and IL-13 without changes in airway inflammation or other aspects of remodeling. Lung fibroblasts from OVA-treated EDA(-/-) mice exhibited reduced proliferation, migration, α-smooth muscle actin expression, and collagen deposition and impaired TGF-β1 and IL-13 release compared with that seen in WT mice.
CONCLUSIONS: EDA-FN is essential for the development of OVA-induced airway fibrosis and AHR. The effect of the EDA domain on airway fibrosis after OVA challenge is through activation and differentiation of fibroblasts. Fibroblast activation and airway fibrosis are necessary for the development of AHR.

Copyright © 2010 American Academy of Allergy, Asthma & Immunology. Published by Mosby, Inc. All rights reserved.

PMID: 21167578 [PubMed - indexed for MEDLINE]

309. Ann Acad Med Singapore. 2010 Nov;39(11):854-60.

T lymphocyte activation profiles in peripheral blood of long- versus short-term residents of Kuwait: comparison with asthmatics.

Mahmoud F, Habeeb F, Arifhodzic N, Haines D, Novotny L.

Department of Medical Laboratory Sciences, Kuwait University, Kuwait.
fadia@hsc.edu.kw

INTRODUCTION: During the Arabian Gulf Wars of 1991 and 2003, the resident population of Kuwait sustained heavy exposure to environmental toxicants introduced by military activities. No comprehensive studies have been conducted to assess how exposure to the wartime and postwar environment may have altered the fundamental patterns of immune reactivity among Kuwaitis in ways that affect pathogenesis of disease. This present study addresses this issue by characterising immunological features of asthma and allergies in a Kuwaiti population that is unique and possibly correlates with toxicant exposures.
MATERIALS AND METHODS: Twenty-fi ve long-term residents of Kuwait afflicted with bronchial asthma concurrent with rhinitis; and 2 healthy control groups: 18 long-term residents and 10 newcomers to Kuwait were evaluated by 2- and 3-colour fl ow cytometry for peripheral blood T cell subpopulation frequencies.
RESULTS: Relative to healthy, long-term residents, significantly elevated frequencies of all activated cell phenotypes were observed in the blood of the asthmatic group (P <0.05 to P <0.001), except for CD8+HLA-DR+ cells and a presumed T-regulatory (Treg) subpopulation: CD4+CD25(high). The asthmatic group was also observed to have larger populations of CD3+ (pan-T cells), CD4+ (T helper cells) and CD8+ (cytotoxic T cells), CD3+CD56 (NKT-like cells) and CD56+CD16+ (NK cells) compared to healthy long-term residents. Compared to healthy recent immigrants, the blood of long-term residents contained elevated levels of CD3+CD56+ (NK-like), CD4+CD45RA+/ CD45RO+ (Naive-to-Memory Transitional), but lower CD4+CD25+(high) (Treg) (P <0.05).
CONCLUSIONS: Elevated representation of natural killer (NKT)-like and memory phenotypes may predispose long-term residents towards enhanced susceptibility for airway disease; while at the same time, reducing representation of Treg cells which are protective against airway disease, and this may increase vulnerability to these syndromes among the residents of Kuwait. These results may provide insight into the features of immunopathogenesis of asthma and allergies in Kuwait that arise as a result of the special environment of the country.

PMID: 21165526 [PubMed - indexed for MEDLINE]

310. Expert Rev Clin Immunol. 2011 Jan;7(1):93-109. doi: 10.1586/eci.10.82.

Osteopontin and allergic disease: pathophysiology and implications for diagnostics and therapy.

Frenzel DF, Weiss JM.

University of Ulm, Department of Dermatology and Allergy, Maienweg 12, D-89081 Ulm, Germany.

Osteopontin (OPN) is a phosphoglycoprotein that is expressed by various immune cells in a secreted and intracellular form. It has cytokine, chemotactic and cell signaling functions enhancing Th1 and Th17 immunity and protects against apoptosis. Recent studies found OPN to be modulatory in cell-mediated and immediate-type allergic diseases. In allergic asthma, OPN enhances sensitization

but downmodulates Th2-driven IL-4-dominated inflammation. The finding that OPN expression is augmented during specific immunotherapy supports a Th2 suppressive effect of OPN. In Th1-driven delayed-type allergy, such as allergic contact dermatitis, OPN supports dendritic cell migration and IL-12 expression and is secreted by T effector cells and keratinocytes, augmenting Th1-mediated allergy and supporting disease chronification. There are numerous missing links as to how OPN variants modulate allergic inflammation through different OPN receptors. OPN research in allergy is an interesting, rapidly expanding field that has high potential for translational research.

PMID: 21162653 [PubMed - indexed for MEDLINE]

311. Mult Scler. 2011 May;17(5):521-31. doi: 10.1177/1352458510392619. Epub 2010 Dec 15.

Chitinase effects on immune cell response in neuromyelitis optica and multiple sclerosis.

Correale J, Fiol M.

Department of Neurology, Raúl Carrea Institute for Neurological Research, FLENI, Buenos Aires, Argentina. jcorreale@fleni.org.ar

BACKGROUND: Recent studies conducted in arthritis, asthma, and inflammatory bowel disease suggest that chitinases are important in inflammatory processes and tissue remodeling.
OBJECTIVE: To investigate the role of chitinases in multiple sclerosis (MS) and neuromyelitis optica (NMO).
METHODS: Levels of chitotriosidase, acid mammalian chitinase (AMCase), and chitinase 3-like-1 (CHI3L1) were measured using ELISA, in cerebrospinal fluid (CSF) and in serum from 24 patients with relapsing remitting (RR) MS, 24 patients with secondary progressive (SP) MS, 12 patients with NMO, 24 patients with other inflammatory neurological diseases (OIND), and 24 healthy controls (HCs). The number of anti-MOG cytokine-secreting cells was studied using ELISPOT. Eotaxins, MCP-1, RANTES, and IL-8 were assessed using ELISA. Cell transmigration was determined using an in vitro blood-brain barrier (BBB) model, in the presence and absence of chitinases.
RESULTS: CSF chitinase levels were significantly increased in patients with RRMS and NMO compared with HCs and patients with SPMS and OIND. In contrast, no significant differences were detected in serum chitinase levels between groups. Chitinase CSF levels showed correlation with anti-MOG IL-13-producing cells, and eotaxin levels. In vitro experiments showed macrophage chitinase secretion was significantly increased by IL-13, but not by IL-5, IL-6, IL-12, or IFN-γ. Moreover, chitinases enhanced IL-8, RANTES, MCP-1, and eotaxin production, increasing migratory capacity in eosinophils, T cells, and macrophages across an in vitro BBB model.
CONCLUSIONS: Chitinases increased in the CSF from patients with NMO in response to IL-13. These enhanced levels could contribute to central nervous system inflammation by increasing immune cell migration across the BBB.

PMID: 21159721 [PubMed - indexed for MEDLINE]

312. Actas Dermosifiliogr. 2010 Dec;101(10):866-77.

[Comparative analysis of the expression of cell adhesion molecules in cutaneous T-cell lymphomas (mycosis fungoides/Sézary syndrome) and inflammatory skin diseases].

[Article in Spanish]

López-Lerma I, Estrach MT.

Hospital Clinic de Barcelona, Servicio de Dermatología, Barcelona, Spain.
ilopez@aedv.es

INTRODUCTION: Cell adhesion molecules play a pivotal role in the establishment of T-cell populations in the skin. In this study, we quantify the expression of cell adhesion molecules in patients with cutaneous T-cell lymphoma (CTCL) and compare it with the expression found in other skin diseases.
MATERIAL AND METHODS: Frozen material was obtained from 42 patients in 5 different groups: early CTCL, comprising patients with patch- and plaque-stage of mycosis fungoides (n=11); advanced CTCL (n=7), comprising patients with mycosis fungoides (n=3) and Sézary syndrome (n=4); inflammatory skin disease (n=12), comprising patients with psoriasis (n=9) and atopic dermatitis (n=3); chronic skin diseases with persistent plaques that do not fulfil the histological criteria for mycosis fungoides (pre-CTCL) (n=8); and healthy volunteers (n=4). Expression of the following cell adhesion molecules was analyzed: lymphocyte function-associated antigen 1, intercellular adhesion molecule 1 (ICAM-1), ICAM-3, cutaneous lymphocyte-associated antigen, E-selectin, very late antigen 4, vascular cell adhesion molecule 1, alphaEbeta7 integrin, and E-cadherin.
RESULTS: The immunohistochemical analyses used here revealed statistically significant differences between CTCL and other skin diseases but not between early and advanced CTCL. The expression of alphaEbeta7 integrin and ICAM-3 in the epidermis per high-power field (400× magnification) allowed the different groups to be distinguished from each other, except for advanced CTCL and pre-CTCL. There were statistically significant differences between advanced CTCL and pre-CTCL in terms of the expression of E-selectin at 400× magnification and the expression of ICAM-1 in a honeycomb pattern in epidermal keratinocytes.
CONCLUSIONS: The expression of cell adhesion molecules involved in the adhesion and migration of lymphocytes in the skin does not differ significantly between initial and advanced stages of CTCL.

PMID: 21159263 [PubMed - indexed for MEDLINE]

313. Exp Dermatol. 2011 Jan;20(1):1-6. doi: 10.1111/j.1600-0625.2010.01194.x.

MIF: a key player in cutaneous biology and wound healing.

Gilliver SC, Emmerson E, Bernhagen J, Hardman MJ.

Faculty of Life Sciences, University of Manchester, Manchester, UK.

Owing to its implication in a range of pathological conditions, including asthma, rheumatoid arthritis, atherosclerosis, inflammatory bowel disease and cancer, the pleiotropic cytokine macrophage migration inhibitory factor (MIF) has been the subject of intensive recent investigation. In the field of dermatology, MIF is believed to be a detrimental factor in diseases such as systemic sclerosis, atopic dermatitis, psoriasis, eczema and UV radiation damage. However, its contribution to other aspects of cutaneous biology is currently unclear. Although its expression in intact skin is well characterized, little is known about MIF's role in cutaneous homoeostasis. However, recent data do identify MIF as a key player in the immune privilege of hair follicles. Similarly, although MIF is rapidly released and its local expression significantly induced upon wounding, its primary role in the ensuing repair process remains a source of contention. MIF has been identified as being a key effector of the beneficial effects of estrogen on wound repair, yet studies employing Mif null mice, recombinant MIF, and neutralizing anti-MIF antibodies have failed to provide a consensus as to

whether it benefits or inhibits healing. In fact MIF appears to be able to exert both positive and negative effects, with the cell-specific relevancy of MIF in wound healing still unclear. Thus, if MIF and/or its downstream targets are to be therapeutically useful in the context of cutaneous repair, more needs to be done to establish the nature and mechanism of action of MIF and its receptors in healing wounds.

© 2010 John Wiley & Sons A/S.

PMID: 21158933 [PubMed - indexed for MEDLINE]

314. Allergol Immunopathol (Madr). 2011 Jul-Aug;39(4):232-9. doi: 10.1016/j.aller.2010.07.004. Epub 2010 Dec 13.

Early onset of allergic rhinitis and asthma in recent extra-European immigrants to Milan, Italy: the perspective of a non-governmental organisation.

Burastero SE, Masciulli A, Villa AM.

San Raffaele Scientific Institute, Milan, Italy. s.burastero@hsr.it

Erratum in
    Allergol Immunopathol (Madr). 2012 Jul-Aug;40(4):264.

BACKGROUND: Allergy is determined by genetic and environmental factors. People immigrating from under-developed to industrialised countries are at higher risk of developing allergic diseases and immigration is as a good epidemiological model to quantify the influence of the environment. We performed the allergological assessment of 32,555 recent immigrants from different areas of the world to a polluted metropolitan area of Northern Italy.
METHODS: We evaluated time of onset of allergic rhinitis and/or asthma, sensitisations and clinical characteristics of 395 subjects (3.74 ± 2.94 yrs, mean ± SD) from four macro-areas (Asia, Africa, East-Europe, South America) arriving to Milan, Italy from June 2005 to June 2009. Data were compared with immigrants having access to the same medical facility for any medical problem and with resident Italians living in the same area.
RESULTS: Immigrants with allergic rhinitis and/or asthma days since arrival in Italy correlated with number of sensitisations ($p=0.0030$). Moreover, personal (2.02%) or familial (2.78%) history of allergic diseases was lower in allergic immigrants as compared to allergic residents (37.77 and 29.39%, respectively; $p<0.0001$ for both comparisons). Finally, the frequency of allergic immigrants from South America (63.3%) was higher than expected from the overall proportion of individuals from this macro-area who sought medical help at the same facility (40.4%; $p<0.0001$, OR 2.289, CI 2.1670-3.255).
CONCLUSIONS: Environmental factors play a relevant role in the induction of allergies in immigrants to Northern Italy. Genetics appears as a further promoting factor in the case of immigrants from South America.

Copyright © 2010 SEICAP. Published by Elsevier Espana. All rights reserved.

PMID: 21146915 [PubMed - indexed for MEDLINE]

315. Antioxid Redox Signal. 2011 May 1;14(9):1589-600. doi: 10.1089/ars.2010.3583. Epub 2011 Feb 28.

S100A8 modulates mast cell function and suppresses eosinophil migration in acute asthma.

Zhao J, Endoh I, Hsu K, Tedla N, Endoh Y, Geczy CL.

Inflammation and Infection Research Centre, School of Medical Sciences, University of New South Wales, Sydney, Australia.

S100A8 is implicated in the pathogenesis of inflammatory diseases. S100A8 is upregulated in macrophages by Toll-like receptors (TLR)-3, 4, and 9 agonists in an IL-10-dependent manner, and by corticosteroids in vitro and in vivo, and scavenges oxidants generated by activated phagocytes. Because if its elevated expression in various lung disorders, we asked whether S100A8 was protective in allergic inflammation. S100A8, but not Cys(41)-Ala S100A8, in which the single reactive Cys residue was replaced by Ala, reduced mast cell (MC) degranulation and production of particular cytokines (IL-6, IL-4, and granulocyte macrophage colony-stimulating factor) in response to IgE-crosslinking in vitro, likely by inhibiting intracellular reactive oxygen species production, thereby reducing downstream linker for activation of T cells and extracellular signal regulated kinase/mitogen-activated protein kinase phosphorylation. In lungs of mice with acute asthma, S100A8, but not Cys(41)-Ala S100A8, reduced MC degranulation, production of eosinophil chemoattractants (IL-5, eotaxin, and monocyte chemoattractant protein-1), and eosinophil infiltration. Suppression of IL-6 and IL-13 could have contributed to reduced mucus production seen in lungs of S100A8-treated mice. IgE production was unaffected. In asthma, there is an imbalance of anti-oxidant systems that are generally protective. Our results strongly support a protective role for S100A8 in allergic inflammation by modulating MC activation and eosinophil recruitment, and by scavenging oxidants generated by activated leukocytes, in processes reliant on its thiol-scavenging capacity.

PMID: 21142608 [PubMed - indexed for MEDLINE]

316. G Ital Dermatol Venereol. 2010 Dec;145(6):747-62.

Langerhans cells in allergic contact dermatitis.

Tuchinda P, Gaspari AA.

Department of Dermatology, Faculty of Medicine Siriraj Hospital, Mahidol University, Bangkok Thailand.

Allergic contact dermatitis (ACD) is a common skin disease that has significant socio-economic impact. ACD is mediated by a T-cell mediated inflammatory reaction. Langerhans cells (LCs) are an epidermal DCs subset specialized in antigen presentation. After hapten exposure, LCs play a major role as in induction adaptive immune response against allergens. LCs recognize, take up and process haptens and migrate to the local draining lymph nodes. However, LCs specific functions and the LCs migration to local draining lymph nodes are not yet clearly defined. Recent advance in the knowledge of LCs function has increased in the past decades including the evidence for a tolerogenic function of LCs. The present review will focus on the role for LCs response to contact allergens.

PMID: 21139551 [PubMed - indexed for MEDLINE]

317. Mol Cell Proteomics. 2011 Mar;10(3):M110.000901. doi: 10.1074/mcp.M110.000901. Epub 2010 Dec 7.

S100A11 mediates hypoxia-induced mitogenic factor (HIMF)-induced smooth muscle cell migration, vesicular exocytosis, and nuclear activation.

Fan C, Fu Z, Su Q, Angelini DJ, Van Eyk J, Johns RA.

Department of Anesthesiology and Critical Care Medicine, School of Medicine, The Johns Hopkins University, Baltimore, MD 21205, USA.

Hypoxia-induced mitogenic factor (HIMF) is a newly discovered protein that is up-regulated in murine models of pulmonary arterial hypertension and asthma. Our previous study shows that HIMF is a potent mitogenic, angiogenic, and vasoconstrictive chemokine associated with pulmonary arterial hypertension. Two-dimensional gel electrophoresis was used to investigate downstream molecules in HIMF-induced cell signaling, demonstrating that S100A11, an EF-hand calcium-binding protein, was exclusively altered and was decreased (2.7±0.2-fold, $p<0.05$) in pulmonary artery smooth muscle cells (SMCs) treated with HIMF for 5 min compared with untreated cells (n=4). Immunofluorescence showed that in control cells S100A11 is a cytosolic protein, which then aggregates and translocates both to the plasma membrane with subsequent exocytosis and to the nucleus upon HIMF stimulation. Annexin A2, a known S100A11 binding partner, also colocalized with S100A11 during HIMF-induced membrane trafficking. To investigate the intracellular function of S100A11, siRNA was used to knock down S100A11 expression in SMCs. The S100A11 knockdown significantly reduced HIMF-induced SMC migration but did not affect the SMC mitogenic action of HIMF. Our data show that S100A11 mediates HIMF-induced smooth muscle cell migration, vesicular exocytosis, and nuclear activation.

PMCID: PMC3047144
PMID: 21139050 [PubMed - indexed for MEDLINE]

318. Clin Exp Allergy. 2011 Feb;41(2):149-59. doi: 10.1111/j.1365-2222.2010.03658.x. Epub 2010 Dec 1.

Mitogen-activated protein kinase signalling and ERK1/2 bistability in asthma.

Alam R, Gorska MM.

Department of Medicine, Division of Allergy & Immunology, National Jewish Health, Denver, CO 80206, USA. alamr@njhealth.org

Mitogen-activated protein kinases (MAPKs) integrate signals from numerous receptors and translate these signals into cell functions. MAPKs are critical for immune cell metabolism, migration, production of pro-inflammatory mediators, survival and differentiation. We provide a concise review of the involvement of MAPK in important cells of the immune system. Certain cell functions, e.g. production of pro-inflammatory mediators resolve quickly and may require a transient MAPK activation, other processes such as cell differentiation and long-term survival may require persistent MAPK signal. The persistent MAPK signal is frequently a consequence of positive feedback loops or double negative feedback loops which perpetuate the signal after removal of an external cell stimulus. This self-perpetuated activation of a signalling circuit is a manifestation of its bistability. Bistable systems can exist in 'on' and 'off' states and both states are stable. We have demonstrated the existence of self-perpetuated activation mechanism for ERK1/2 in bronchial epithelial cells. This sustained activation of ERK1/2 supports long-term survival of these cells and primes them for cytokine transcription. ERK1/2 bistability arises from repetitive stimulation of the cell. The repeated stimulation (e.g. repeated viral infection or repeated allergen exposure) seems to be a common theme in asthma and other chronic illnesses. We thus hypothesize that the self-perpetuated ERK1/2 signal plays an important role in the pathogenesis of asthma.

© 2010 Blackwell Publishing Ltd.

PMCID: PMC3115726
PMID: 21121982 [PubMed - indexed for MEDLINE]

319. Int Immunopharmacol. 2011 Feb;11(2):204-11. doi: 10.1016/j.intimp.2010.11.020. Epub 2010 Nov 24.

Role of the Mac-1 and VLA-4 integrins, and concomitant Th2-cytokine production, in nitric oxide modulated eosinophil migration from bone marrow to lungs in allergic mice.

Pelaquini EH, Guimarães Lde A, Benetti LR, Fernandes LG, Tamashiro WM, Conran N, Ferreira HH.

Laboratory of Inflammation Research, São Francisco University, Bragança Paulista, SP, Brazil.

Although numerous studies demonstrate the participation of nitric oxide (NO) in various inflammatory diseases, the precise function of NO in allergic asthma remains unclear. We investigated whether iNOS inhibition could interfere with the kinetics of VLA-4 and Mac-1 expression and adhesion properties of bone marrow and peripheral blood eosinophils of sensitized mice after antigen exposure. Treatment of allergic mice with 1400 W (iNOS inhibitor) increased the adhesion of bone marrow eosinophils to ICAM-1, but not blood eosinophils, at 24h and 48 h after OVA-challenge. Conversely, adhesion of blood eosinophils from 1400 W-treated mice to VCAM-1 diminished at 24h and was almost completely blocked at 48 h. 1400 W did not induce any change in the adhesion of bone marrow eosinophils to VCAM-1, at 24h, but cells collected 48 h after challenge showed significantly lower adherence. Flow cytometry demonstrated that 1400 W resulted in a significantly increased Mac-1 expression on bone marrow eosinophils at 24h, as compared to control mice. However, at 24h, 1400 W significantly decreased Mac-1 and VLA-4 expressions on blood eosinophils. At 48 h, the expressions of both Mac-1 and VLA-4 returned to previous levels. Results show a temporal effect of iNOS upon Mac-1 expression and function, the chief adhesion molecule involved in the eosinophil efflux from the bone marrow at 24h. In contrast, Mac-1 and VLA-4 were involved in eosinophil mobilization from blood to lungs at 48 h after antigen challenge. Data suggest an important role of the Mac-1 and VLA-4 in the iNOS-modulated migration of eosinophils to the lungs of allergic mice.

Copyright Â© 2010 Elsevier B.V. All rights reserved.

PMID: 21111080 [PubMed - indexed for MEDLINE]

320. Toxicol Lett. 2011 Jan 15;200(1-2):117-23. doi: 10.1016/j.toxlet.2010.11.004. Epub 2010 Nov 18.

Amphetamine modulates cellular recruitment and airway reactivity in a rat model of allergic lung inflammation.

de Oliveira AP, Lino-Dos-Santos-Franco A, Hamasato EK, Quinteiro-Filho W, Hebeda CB, Damazo AS, Farsky SH, Tavares-de-Lima W, Palermo-Neto J.

Department of Pharmacology, Institute of Biomedical Sciences, University of São Paulo, São Paulo, Brazil.

Asthma is characterized by pulmonary cellular infiltration, vascular exudation and airway hyperresponsiveness. Several drugs that modify central nervous system

(CNS) activity can modulate the course of asthma. Amphetamine (AMPH) is a highly abused drug that presents potent stimulating effects on the CNS and has been shown to induce behavioral, biochemical and immunological effects. The purpose of this study was to investigate the effects of AMPH on pulmonary cellular influx, vascular permeability and airway reactivity. AMPH effects on adhesion molecule expression, IL-10 and IL-4 release and mast cell degranulation were also studied. Male Wistar rats were sensitized with ovalbumin (OVA) plus alum via subcutaneous injection. One week later, the rats received another injection of OVA-alum (booster). Two weeks after this booster, the rats were subjected to AMPH treatment 12 h prior to the OVA airway challenge. In rats treated with AMPH, the OVA challenge reduced cell recruitment into the lung, the vascular permeability and the cellular expression of ICAM-1 and Mac-1. Additionally, elevated levels of IL-10 and IL-4 were found in samples of lung explants from allergic rats. AMPH treatment, in comparison, increased IL-10 levels but reduced those of IL-4 in the lung explants. Moreover, the tracheal responsiveness to methacholine (MCh), as well as to an in vitro OVA challenge, was reduced by AMPH treatment, and levels of PCA titers were not modified by the drug. Our findings suggest that single AMPH treatment down-regulates several parameters of lung inflammation, such as cellular migration, vascular permeability and tracheal responsiveness. These results also indicate that AMPH actions on allergic lung inflammation include endothelium-leukocyte interaction mechanisms, cytokine release and mast cell degranulation.

Copyright © 2010 Elsevier Ireland Ltd. All rights reserved.

PMID: 21093552 [PubMed - indexed for MEDLINE]

321. Biosci Rep. 2011 Apr;31(2):145-50. doi: 10.1042/BSR20100087.

Sphingosine kinase and sphingosine 1-phosphate in asthma.

Lai WQ, Wong WS, Leung BP.

Department of Physiology, Yong Loo Lin School of Medicine, National University of Singapore, 2 Medical Drive, Singapore 117597.

Sphingolipids are amphiphatic molecules ubiquitously expressed in all eukaryotic cell membranes. Initially characterized as structural components of cell membranes, sphingolipids have emerged as sources of important signalling molecules over the past decade. Sphingolipid metabolites, such as ceramide and S1P (sphingosine 1-phosphate), have been demonstrated to have roles as potent bioactive messengers involved in cell differentiation, proliferation, apoptosis, migration and angiogenesis. The importance of SphK (sphingosine kinase) and S1P in inflammation has been demonstrated extensively. The prevalence of asthma is increasing in many developed nations. Consequently, there is an urgent need for the development of new agents for the treatment of asthma, especially for patients who respond poorly to conventional therapy. Recent studies have demonstrated the important role of SphK and S1P in the development of asthma by regulating pro-inflammatory responses. These novel pathways represent exciting potential therapeutic targets in the treatment of asthma and are described in the present review.

PMID: 21091442 [PubMed - indexed for MEDLINE]

322. Cell Mol Life Sci. 2011 Feb;68(3):433-51. doi: 10.1007/s00018-010-0584-3. Epub 2010 Nov 16.

Emerging topics and new perspectives on HLA-G.

Fainardi E, Castellazzi M, Stignani M, Morandi F, Sana G, Gonzalez R, Pistoia V, Baricordi OR, Sokal E, Peña J.

Neuroradiology Unit, Department of Neurosciences and Rehabilitation, Azienda Ospedaliera-Universitaria, Arcispedale S. Anna, Corso della Giovecca 203, 44100 Ferrara, Italy. henryfai@tin.it

Following the Fifth International Conference on non-classical HLA-G antigens (HLA-G), held in Paris in July 2009, we selected some topics which focus on emerging aspects in the setting of HLA-G functions. In particular, HLA-G molecules could play a role in: (1) various inflammatory disorders, such as multiple sclerosis, intracerebral hemorrhage, gastrointestinal, skin and rheumatic diseases, and asthma, where they may act as immunoregulatory factors; (2) the mechanisms to escape immune surveillance utilized by several viruses, such as human cytomegalovirus, herpes simplex virus type 1, rabies virus, hepatitis C virus, influenza virus type A and human immunodeficiency virus 1 (HIV-1); and (3) cytokine/chemokine network and stem cell transplantation, since they seem to modulate cell migration by the downregulation of chemokine receptor expression and mesenchymal stem cell activity blocking of effector cell functions and the generation of regulatory T cells. However, the immunomodulatory circuits mediated by HLA-G proteins still remain to be clarified.

PMID: 21080027 [PubMed - indexed for MEDLINE]

323. J Invest Dermatol. 2011 Feb;131(2):418-25. doi: 10.1038/jid.2010.336. Epub 2010 Nov 11.

Irritant-induced migration of Langerhans cells coincides with an IL-10-dependent switch to a macrophage-like phenotype.

Ouwehand K, Oosterhoff D, Breetveld M, Scheper RJ, de Gruijl TD, Gibbs S.

Department of Dermatology, VU University Medical Centre, Amsterdam, The Netherlands.

Langerhans cells (LCs) migrate after topical exposure of the skin to irritants, despite the supposed independence of irritant contact dermatitis from adaptive immunity. Whereas allergen-activated LCs are known to migrate to the draining lymph nodes (LNs), the fate of migrated LCs upon topical irritant exposure is unknown. Here, we identified a phenotypic switch of LCs after their migration into the dermis upon irritant exposure. With the aid of ex vivo intact human skin and epidermal sheets, we show that dermal fibroblasts are necessary for an IL-10-dependent postmigrational phenotypic switch of LCs into macrophage-like cells. Exposure of ex vivo skin to a panel of seven irritants resulted in a decrease in the number of CD1a(+) cells and an increase in CD14(+)/CD68(+) cells in the dermis. Neutralizing antibodies against IL-10 totally inhibited the phenotypic LC-to-macrophage transition, but did not influence the migration of CD1a(+) cells. Exposure of epidermal sheets to irritants resulted in a fibroblast-dependent LC-to-CD14(+)/CD68(+) switch coinciding with migration, which could be totally inhibited by neutralizing antibodies against either IL-10 or CCL2/CCL5 (two chemokines responsible for epidermal-to-dermal migration). We have thus identified an IL-10-dependent phenotypic switch of LCs into macrophage-like cells upon irritant exposure and emigration from the epidermis.

PMID: 21068755 [PubMed - indexed for MEDLINE]

324. Peptides. 2011 Feb;32(2):368-73. doi: 10.1016/j.peptides.2010.10.028. Epub 2010

Nov 2.

Adrenomedullin level in the nasal discharge from allergic rhinitis cohort.

Fujikura T, Okubo K.

Department of Otorhinolaryngology, Nippon Medical School, Sendagi Bunkyo-ku, Tokyo, Japan. teru-fujik@nms.ac.jp

Adrenomedullin (AM) is a potent hypotensive and vasodilatory peptide. AM may exert protective actions against the development of many diseases by modulating the blood circulation and body fluid balance. In addition to these functions, it has recently been reported to play important roles in the development of allergy and infections. The purpose of the present study was to demonstrate the existence of AM in the human nasal mucosa and to discuss whether AM might contribute to the pathogenesis of nasal congestion. We measured the total AM concentrations in the nasal discharge. The total AM concentration in the nasal discharge was significantly higher in the non-allergy group (72.1 ± 55.5 fmol/ml) than in the allergy group (37.1 ± 44.2 fmol/ml). By immunohistochemical examination, we identified AM-containing cells in the nasal mucosa from both subjects with and without nasal allergy, and also in nasal polyps. Moreover, those cells were positive for anti-tryptase antibody which recognizes mast cells. In nasal allergy, vasodilatation and increase in vascular permeability are characteristic features of the immediate phase response. Reduced AM levels in the nasal discharge may be associated with attenuation of both of these factors. On the other hand, immunohistochemical analysis demonstrated AM-immunoreactive cells in the chronic phase of rhinosinusitis. In the late and inflammatory phase, mast cells produce AM, which possibly acts as an inhibitor of inflammatory cell migration. In conclusion, AM may be actively secreted into the nasal discharge. AM in the nasal discharge may have protective and anti-inflammatory effects in the nasal mucosa.

Copyright Â© 2010 Elsevier Inc. All rights reserved.

PMID: 21050876 [PubMed - indexed for MEDLINE]

325. Mucosal Immunol. 2011 May;4(3):343-53. doi: 10.1038/mi.2010.73. Epub 2010 Nov 3.

Langerin+ dendritic cells are responsible for LPS-induced reactivation of allergen-specific Th2 responses in postasthmatic mice.

Ortiz-Stern A, Kanda A, Mionnet C, Cazareth J, Lazzari A, Fleury S, Dombrowicz D, Glaichenhaus N, Julia V.

Université de Nice-Sophia Antipolis, Valbonne, France.

Allergic asthma is a T cell-dependent inflammatory lung disease that results from complex interactions between genetic predisposition and environmental factors, including exposure to lipopolysaccharide (LPS). In this study, we have shown that airway LPS exposure was sufficient to induce airway hyperreactivity (AHR) and eosinophil recruitment in mice that had previously experienced an acute episode of allergic asthma. LPS-induced disease reactivation depended on the activation of allergen-specific CD4(+) T cells by a subset of lung langerin(+) dendritic cells (DCs) that retained the allergen. Upon LPS exposure, migration of langerin(+) DCs from lungs to draining lymph nodes increased and LPS-exposed langerin(+) DCs instructed CD4(+) T cells toward a T helper (Th) 2 response. Selective depletion of langerin(+) DCs prevented LPS-induced eosinophil recruitment and T-cell activation, further demonstrating a critical role for langerin(+) DCs in disease reactivation. This finding provides a possible

explanation for the subclinical worsening of asthmatics following exposure to low-dose LPS.

PMID: 21048704 [PubMed - indexed for MEDLINE]

326. J Allergy Clin Immunol. 2010 Dec;126(6):1252-9.e12. doi: 10.1016/j.jaci.2010.08.014. Epub 2010 Nov 3.

Phosphodiesterase 4B is essential for T(H)2-cell function and development of airway hyperresponsiveness in allergic asthma.

Jin SL, Goya S, Nakae S, Wang D, Bruss M, Hou C, Umetsu D, Conti M.

Department of Life Sciences, National Central University, Jhongli.

BACKGROUND: Cyclic AMP (cAMP) signaling modulates functions of inflammatory cells involved in the pathogenesis of asthma, and type 4 cAMP-specific phosphodiesterases (PDE4s) are essential components of this pathway. Induction of the PDE4 isoform PDE4B is necessary for Toll-like receptor signaling in monocytes and macrophages and is associated with T cell receptor/CD3 in T cells; however, its exact physiological function in the development of allergic asthma remains undefined.
OBJECTIVES: We investigated the role of PDE4B in the development of allergen-induced airway hyperresponsiveness (AHR) and T(H)2-driven inflammatory responses.
METHODS: Wild-type and PDE4B(-/-) mice were sensitized and challenged with ovalbumin and AHR measured in response to inhaled methacholine. Airway inflammation was characterized by analyzing leukocyte infiltration and cytokine accumulation in the airways. Ovalbumin-stimulated cell proliferation and T(H)2 cytokine production were determined in cultured bronchial lymph node cells.
RESULTS: Mice deficient in PDE4B do not develop AHR. This protective effect was associated with a significant decrease in eosinophils recruitment to the lungs and decreased T(H)2 cytokine levels in the bronchoalveolar lavage fluid. Defects in T-cell replication, T(H)2 cytokine production, and dendritic cell migration were evident in cells from the airway-draining lymph nodes. Conversely, accumulation of the T(H)1 cytokine IFN-γ was not affected in PDE4B(-/-) mice. Ablation of the orthologous PDE4 gene PDE4A has no impact on airway inflammation.
CONCLUSION: By relieving a cAMP-negative constraint, PDE4B plays an essential role in T(H)2-cell activation and dendritic cell recruitment during airway inflammation. These findings provide proof of concept that PDE4 inhibitors with PDE4B selectivity may have efficacy in asthma treatment.

Copyright © 2010 American Academy of Allergy, Asthma & Immunology. Published by Mosby, Inc. All rights reserved.

PMCID: PMC3002752
PMID: 21047676 [PubMed - indexed for MEDLINE]

327. Scand J Immunol. 2010 Dec;72(6):491-503. doi: 10.1111/j.1365-3083.2010.02465.x.

PAS-1, an Ascaris suum protein, modulates allergic airway inflammation via CD8+γδTCR+ and CD4+CD25+FoxP3+ T cells.

de Araújo CA, Perini A, Martins MA, Macedo MS, Macedo-Soares MF.

Laboratory of Immunopathology, Butantan Institute, São Paulo, Brazil.

We have previously demonstrated that PAS-1, a 200 kDa protein from Ascaris suum,

has a potent immunomodulatory effect on humoral and cell-mediated responses induced by APAS-3 (an allergenic protein from A. suum) or unrelated antigens. In this study, we investigated the mechanisms by which PAS-1 is able to induce this effect on an allergic airway inflammation induced by OVA in mice. C57BL/6 mice were adoptively transferred on day 0 with seven different PAS-1-primed cell populations: PAS-1-primed CD19(+) or B220(+) or CD3(+) or CD4(+) or CD8(+) or CD4(+) CD25^{-} or CD4(+) CD25(+) lymphocytes. These mice were immunized twice with OVA and alum by intraperitoneal route (days 0 and 7) and challenged twice by intranasal route (days 14 and 21). Two days after the last challenge, the airway inflammation was evaluated by antibody levels, cellular migration, eosinophil peroxidase levels, cytokine and eotaxin production, and pulmonary mechanical parameters. Among the adoptively transferred primed lymphocytes, only CD4(+) CD25(+) , CD8(+) or the combination of both T cells impaired the production of total IgE and OVA-specific IgE and IgG1 antibodies, eosinophilic airway inflammation, Th2-type cytokines (IL-4, IL-5 and IL-13), eotaxin release and airway hyperreactivity. Moreover, airway recruited cells from CD4(+) CD25(+) and CD8(+) T-cell recipient secreted more IL-10/TGF-β and IFN-γ, respectively. Moreover, we found that PAS-1 expands significantly the number of CD4(+) CD25(+) FoxP3(+) and CD8(+) γδTCR(+) cells. In conclusion, these findings demonstrate that the immunomodulatory effect of PAS-1 is mediated by these T-cell subsets.

© 2010 The Authors. Scandinavian Journal of Immunology © 2010 Blackwell Publishing Ltd.

PMID: 21044123 [PubMed - indexed for MEDLINE]

328. J Asthma. 2011 Mar;48(2):139-46. doi: 10.3109/02770903.2010.529221. Epub 2010 Nov 3.

The feasibility and acceptability of a home-visitation, asthma education program in a rural, Latino/a population.

Postma JM, Smalley K, Ybarra V, Kieckhefer G.

Washington State University College of Nursing, Spokane, WA, USA. jpostma@wsu.edu

Comment in
J Asthma. 2011 Mar;48(2):210.

OBJECTIVES: The effectiveness of community health worker-delivered interventions to decrease environmental triggers for asthma in the home has been well documented in urban populations, but has had little evaluation in rural, Latino/a families. The purpose of this study was to evaluate the feasibility and acceptability of a home-visitation intervention designed to decrease environmental triggers for pediatric asthma in rural, Latino/a families.
METHODS: Data from a large community health clinic's pediatric asthma program (2002, 2003, 2004, 2005, 2006) were used to retrospectively explore associations between program participation and asthma-related health outcomes. Demographic data were collected on 866 patients. Behavioral outcomes were evaluated in 374 participants. A medical record abstraction was conducted in a subsample of 400 patients to evaluate asthma-related urgent care use. Nonparametric tests were used to compare outcomes before and after the intervention. Demographic attributes associated with program participation were examined using logistic regression.
RESULTS: Most (91%) participants were Hispanic, and 61% of participants' caregivers were either seasonal or migrant farmworkers. Over half (61%) of the participants did not complete the full intervention. A statistically significant improvement was found in caregivers' abilities to manage asthma medications and adopt behaviors to decrease triggers inside the home. Behaviors related to

decreasing outside triggers did not significantly change. Asthma-related urgent care use significantly decreased; however, there was no association between intervention dose and a decrease in urgent care use. Demographic attributes were generally not associated with program completion, having baseline and exit data on intermediate outcomes, and/or inclusion in the chart review.
CONCLUSIONS: Results suggest that the asthma intervention helped caregivers improve the air quality in their homes and reduce urgent care admissions among pediatric participants. The intervention dose may be less important than taking part in an intervention to the extent feasible or desired by the family. Findings suggest that policy-level interventions need to address reimbursement for home visitation and environmental exposures that are beyond caregiver control, such as support for healthy and affordable housing in farmworker communities.

PMID: 21043988 [PubMed - indexed for MEDLINE]

329. Int J Mol Med. 2010 Dec;26(6):893-9.

The PTEN tumor suppressor inhibits human airway smooth muscle cell migration.

Lan H, Zhong H, Gao Y, Ren D, Chen L, Zhang D, Lai W, Xu J, Luo Y.

Department of Respiratory Diseases, Nanfang Hospital, Southern Medical University, Guangzhou, Guangdong, P.R. China.

Airway remodeling in asthma is characterized by increased airway smooth muscle (ASM) mass, accompanied by cell migration. It is well known that the proliferation and migration of ASM cells (ASMCs) play a key role in airway remodeling, but the precise mechanism modulating these cellular events remains unclear. One of the genes most likely to be involved in this process is the phosphatase and tensin homolog (PTEN) gene, whose deletion from chromosome 10 can inhibit the proliferation and migration of many cell types. In this study, we investigated the effects of PTEN on human ASMCs. The cells were infected with recombinant adenovirus containing wild-type PTEN cDNA (Ad-PTEN), and the results were compared with those from the uninfected cells and those infected with the GFP-labeled adenovirus vector. Cell proliferation was measured using the MTT method. Cell migration was determined by wound-healing and transwell assays. The expressions of PTEN, phospho-Akt, Akt, phospho-ERK1/2, ERK1/2, phospho-focal adhesion kinase (FAK) and FAK, were examined by Western blot analysis. The results show that PTEN is expressed endogenously in ASMCs, and that Ad-PTEN inhibits the proliferation and migration of these cells. In addition, the Ad-PTEN treatment decreased the phosphorylation of Akt and FAK but not that of ERK1/2. In conclusion, this study demonstrates that PTEN overexpression inhibits the proliferation and migration of human ASMCs by down-regulating the activity of the Akt and FAK signaling pathways.

PMID: 21042784 [PubMed - indexed for MEDLINE]

330. Exp Mol Pathol. 2011 Feb;90(1):116-22. doi: 10.1016/j.yexmp.2010.10.012. Epub 2010 Oct 30.

Tranilast treatment decreases cell growth, migration and inhibits colony formation of human breast cancer cells.

Subramaniam V, Ace O, Prud'homme GJ, Jothy S.

Department of Laboratory Medicine and Keenan Research Centre of the Li Ka Shing Knowledge Institute, St Michael's Hospital, Toronto, Ontario, Canada.

In the treatment of breast cancer, although a wide of choice of drugs and treatment modalities are available, drug resistance or drug toxicity poses a considerable challenge. Tranilast is a well tolerated drug used in the treatment of allergic disorders. Previous works in various models have shown that tranilast has the potential to be used as an anti-cancer drug. Hence, in this study using human breast cancer cell lines BT-474 and MDA-MB-231, we studied the effect of tranilast on cell growth, migration and ability to prevent colony formation in vitro, properties that are relevant to a possible therapeutic effect in breast cancer. We found that tranilast inhibits the growth of both breast cancer cell lines. In the cell migration experiments, the tumor cells exhibit significantly slower wound closure after tranilast treatment, as well as reduced migration using an insert system. Downregulation of MRTF-A, a global cytoskeleton regulator was observed after tranilast treatment. Additionally, tranilast treatment increased levels of cleaved PARP in both cell lines tested indicating a stimulation of apoptosis. A significant reduction in colony size and number was observed in soft agar clonogenic assays in both cell lines after tranilast treatment. BT-474 cells were more responsive to tranilast treatment compared to MDA-MB-231 cells, suggesting a difference in modes of action, or sensitivity, possibly related to their different receptor status. Based on these changes in cancer cell lines, we conclude that tranilast exerts effects that set a rationale for future preclinical studies in animal models of breast cancer.

Copyright Â© 2010 Elsevier Inc. All rights reserved.

PMID: 21040720 [PubMed - indexed for MEDLINE]

331. Asian Pac J Allergy Immunol. 2010 Jun-Sep;28(2-3):95-106.

A revisit to cockroach allergens.

Sookrung N, Chaicumpa W.

Office for Research and Development, Faculty of Medicine Siriraj Hospital, Mahidol University, Bangkok 10700, Thailand.

Among cockroaches (CR) that live in people's homes, two species, i.e., German CR (Blattella germanica) and American CR (Periplaneta americana) predominate in temperate and tropical areas, respectively. CR is an important source of inhalant indoor allergens that sensitize atopic subjects to (localized) type I hypersensitivity or atopy including allergic rhinitis and atopic asthma. In Thailand the predominant CR species is P. americana. CR allergens are found throughout CR infested houses; the number found in kitchens correlates with the degree of CR infestation while sensitization and reactivation of the allergic morbidity are likely to occur in the living room and bedroom. Levels of the CR allergens in homes of CR allergic Thais, measured by using locally made quantification test kits, revealed that the highest levels occur in dust samples collected from the wooden houses of urban slums and in the cool and dry season. CR allergens are proteins that may be derived from any anatomical part of the insect at any developmental stage. The allergens may be also from CR secretions, excretions, body washes or frass. The proteins may be the insect structural proteins, enzymes or hormones. They may exist as dimers/multimers and/or in different isoforms. Exposure to CR allergens in infancy leads to allergic morbidity later in life. Clinical symptoms of CR allergy are usually more severe and prolonged than those caused by other indoor allergens. The mechanisms of acute and chronic airway inflammation and airway hyper-responsiveness (AHR) have been addressed including specific IgE- and non-IgE-mediated mechanisms, i.e., role of protease-activated receptor-2 (PAR2). Participation of various allergen activated-CD4+ T cells of different sublineages, i.e., Th2, Th17, Th22, Th9, Th25, Tregs/Th3 as well as invariant NKT cells, in asthma pathogenesis have been

mentioned. The diagnosis of CR allergy and the allergy intervention by CR population control are also discussed.

PMID: 21038777 [PubMed - indexed for MEDLINE]

332. PLoS One. 2010 Oct 13;5(10):e13360. doi: 10.1371/journal.pone.0013360.

Fibulin-1 is increased in asthma--a novel mediator of airway remodeling?

Lau JY, Oliver BG, Baraket M, Beckett EL, Hansbro NG, Moir LM, Wilton SD, Williams C, Foster PS, Hansbro PM, Black JL, Burgess JK.

Cooperative Research Centre for Asthma and Airways, Sydney, New South Wales, Australia.

BACKGROUND: The extracellular matrix is a dynamic and complex network of macromolecules responsible for maintaining and influencing cellular functions of the airway. The role of fibronectin, an extracellular matrix protein, is well documented in asthma. However, the expression and function of fibulin-1, a secreted glycoprotein which interacts with fibronectin, has not been reported. Fibulin-1 is widely expressed in basement membranes in many organs including the lung. There are four isoforms in humans (A-D) of which fibulin-1C and 1D predominate. The objective of this study was to study the expression of fibulin-1 in volunteers with and without asthma, and to examine its function in vitro.
METHODOLOGY/PRINCIPAL FINDINGS: We used immunohistochemistry and dot-blots to examine fibulin-1 levels in bronchial biopsies, bronchoalveolar lavage fluid and serum. Real-time PCR for fibulin-1C and 1D, and ELISA and western blotting for fibulin-1 were used to study the levels in airway smooth muscle cells. The function of fibulin-1C was determined by assessing its role, using an antisense oligonucleotide, in cell proliferation, migration and wound healing. A murine model of airway hyperresponsiveness (AHR) was used to explore the biological significance of fibulin-1. Levels of fibulin-1 were significantly increased in the serum and bronchoalveolar lavage fluid of 21 asthmatics compared with 11 healthy volunteers. In addition fibulin-1 was increased in asthma derived airway smooth muscle cells and fibulin-1C contributed to the enhanced proliferation and wound repair in these cells. These features were reversed when fibulin-1C was suppressed using an antisense oligomer. In a mouse model of AHR, treatment with an AO inhibited the development of AHR to methacholine.
CONCLUSIONS: Our data collectively suggest fibulin-1C may be worthy of further investigation as a target for airway remodeling in asthma.

PMCID: PMC2954173
PMID: 20967215 [PubMed - indexed for MEDLINE]

333. Int Immunopharmacol. 2011 Jan;11(1):1-11. doi: 10.1016/j.intimp.2010.09.010. Epub 2010 Oct 15.

Modulation of T lymphocyte and eosinophil functions in vitro by natural tetranortriterpenoids isolated from Carapa guianensis Aublet.

Ferraris FK, Rodrigues R, da Silva VP, Figueiredo R, Penido C, Henriques Md.

Laboratório de Farmacologia Aplicada, Farmanguinhos, Fundação Oswaldo Cruz, Rua Sizenando Nabuco 100, 21041-250, Rio de Janeiro, RJ, Brazil.

We have previously described the anti-allergic activities of a pooled fraction of tetranortriterpenoids (TNTPs) containing 6α-acetoxygedunin, 7-deacetoxy-7-oxogedunin, andirobin and methyl angolensate isolated from the

seeds of Carapa guianensis. In the present study, we performed in vitro studies in order to elucidate the mechanisms by which TNTPs present their anti-allergic effects and to identify the bioactive compound(s) present in such fraction. Here, we show that in vitro incubation of eosinophils with the pooled TNTP fraction, as well as with each one of the five isolated tetranortriterpenoids, impaired the adhesion of eosinophils to tumor necrosis factor-α (TNF-α)-primed tEND.1 endothelial cells. Furthermore, the individual or pooled TNTPs impaired CCL11/eotaxin-mediated chemotaxis. By contrast, pooled TNTPs failed to inhibit adhesion and chemotaxis of T lymphocytes. However, TNTPs were able to impair anti-CD3 monoclonal antibody-induced T cell proliferation and the expression of CD25 and CD69. These data suggest that TNTPs prevent T cell activation. Pretreatment of splenocytes with the pooled TNTP fraction, as well as with each one of the five isolated TNTPs, inhibited ovalbumin (OVA)-induced in vitro production of interleukin-2, chemokine (C-C motif) ligand 11 (CCL11) and regulated on activation normal T cell expressed and secreted (RANTES, also known as CCL5). TNTPs (except 6α-acetoxygedunin) also impaired nuclear factor-κB (NFκB) nuclear translocation in OVA-challenged splenocytes. Taken together, these results demonstrate that the anti-allergic effects of TNTPs isolated from C. guianensis might rely on their ability to inhibit eosinophil migration, as well as the activation of T lymphocytes, which is shared by the five isolated TNTPs.

Copyright Â© 2010 Elsevier B.V. All rights reserved.

PMID: 20951667 [PubMed - indexed for MEDLINE]

334. J Immunol. 2010 Nov 15;185(10):6294-305. doi: 10.4049/jimmunol.1001648. Epub 2010 Oct 13.

Slit2 regulates attractive eosinophil and repulsive neutrophil chemotaxis through differential srGAP1 expression during lung inflammation.

Ye BQ, Geng ZH, Ma L, Geng JG.

Laboratory of Molecular Cell Biology, Institute of Biochemistry and Cell Biology, Shanghai Institutes for Biological Sciences, Chinese Academy of Sciences, Shanghai, China.

Directional migration of leukocytes is an essential step in leukocyte trafficking during inflammatory responses. However, the molecular mechanisms governing directional chemotaxis of leukocytes remain poorly understood. The Slit family of guidance cues has been implicated for inhibition of leuocyte migration. We report that Clara cells in the bronchial epithelium secreted Slit2, whereas eosinophils and neutrophils expressed its cell-surface receptor, Robo1. Compared to neutrophils, eosinophils exhibited a significantly lower level of Slit-Robo GTPase-activating protein 1 (srGAP1), leading to activation of Cdc42, recruitment of PI3K to Robo1, enhancment of eotaxin-induced eosinophil chemotaxis, and exaggeration of allergic airway inflammation. Notably, OVA sensitization elicited a Slit2 gradient at so-called bronchus-alveoli axis, with a higher level of Slit2 in the bronchial epithelium and a lower level in the alveolar tissue. Aerosol administration of rSlit2 accelerated eosinophil infiltration, whereas i.v. administered Slit2 reduced eosinophil deposition. In contrast, Slit2 inactivated Cdc42 and suppressed stromal cell-derived factor-1α-induced chemotaxis of neutrophils for inhibiting endotoxin-induced lung inflammation, which were reversed by blockade of srGAP1 binding to Robo1. These results indicate that the newly identified Slit2 gradient at the bronchus-alveoli axis induces attractive PI3K signaling in eosinophils and repulsive srGAP1 signaling in neutrophils through differential srGAP1 expression during lung inflammation.

PMID: 20944010 [PubMed - indexed for MEDLINE]

335. Cell Signal. 2011 Apr;23(4):603-8. doi: 10.1016/j.cellsig.2010.10.002. Epub 2010 Oct 16.

Phosphoinositide 3-kinase delta (PI3Kδ) in leukocyte signaling and function.

Fung-Leung WP.

Department of Immunology, Johnson & Johnson Pharmaceutical Research & Development LLC, San Diego, CA 92121, USA. wleung@its.jnj.com

PI3Kδ is a lipid kinase of the PI3K class IA family involved in early signaling events of leukocytes responding to a wide variety of stimuli. The leukocyte specificity of PI3Kδ is defined by its expression, whereas its signaling function is via the production of phosphoinositide 3,4,5-triphosphates at the proximity of activated receptors for recruiting other signaling molecules. The importance of PI3Kδ in B cell development and function is most apparent, and its role in other leukocyte cell types can be easily demonstrated as well. PI3Kδ participates in the development, activation and migration of T cells and NK cells. The role of PI3Kδ in myeloid cell activities, such as inflammation driven cell infiltration, neutrophil oxidative burst, immune complex mediated macrophage activation, as well as mast cell maturation and degranulation, has been well illustrated in various studies. As a result of the broad effects of PI3Kδ in leukocyte functions, the disruption of PI3Kδ expression or activity leads to decreased inflammatory and immune responses in vivo. The protective role of PI3Kδ inactivation in animal models of arthritis, asthma or obstructive respiratory diseases has been demonstrated. These findings suggest the potential efficacy achievable with PI3Kδ inhibitors in the treatment of autoimmune and respiratory diseases.

Copyright © 2010 Elsevier Inc. All rights reserved.

PMID: 20940048 [PubMed - indexed for MEDLINE]

336. J Immigr Minor Health. 2011 Apr;13(2):315-22. doi: 10.1007/s10903-010-9405-x.

Health literacy, language, and ethnicity-related factors in newcomer asthma patients to Canada: a qualitative study.

Poureslami I, Rootman I, Doyle-Waters MM, Nimmon L, Fitzgerald JM.

UBC and VGH Divisions of Respiratory Medicine, Centre for Lung Health The Lung Centre, University of British Columbia, Gordon and Leslie Diamond Health Care Centre, Vancouver, BC, V5Z 1M9, Canada.

The objectives of this study were to investigate how asthma patients from new immigrant groups are being informed and educated about asthma and its management, and to identify barriers to knowledge transfer. Four focus groups (n = 29) from Latino, Chinese, Iranian and Punjabi cultural communities were conducted with asthmatic patients in the Greater Vancouver Area. Our results from the focus group discussions can be summarized in four broad areas (a) perceptions of and ways of coping with asthma, (b) perceptions of whether the healthcare system is culturally competent, (c) perceptions of language barriers in regards to accessing the healthcare system, and (d) perceptions of how to access reliable asthma information. The results of this study highlight the importance of diverse cultural beliefs and practices as factors that should be taken into consideration when tailoring interventions to improve asthma outcomes in vulnerable populations, including patients from ethno-cultural communities.

PMID: 20938742 [PubMed - indexed for MEDLINE]

337. Immunopharmacol Immunotoxicol. 2011 Jun;33(2):391-7. doi:
10.3109/08923973.2010.522195. Epub 2010 Oct 11.

Catechin inhibits adhesion and migration of peripheral blood B cells by blocking CD11b.

Kawai K, Tsuno NH, Kitayama J, Sunami E, Takahashi K, Nagawa H.

Department of Surgical Oncology, Faculty of Medicine, University of Tokyo, Tokyo, Japan. kz-kawai@mvd.biglobe.ne.jp

Context: Previously, we demonstrated that CD11b is expressed on peripheral blood memory B cells, and it plays an important role in the migration of B cells. And epigallocatechin gallate (EGCG), a bioactive component of green tea, by binding to CD11b, expressed on CD8(+) cytotoxic T cells, inhibited their migratory ability, one possible mechanism of the antiallergic activity of EGCG. Objective: Here, we investigated whether EGCG also affected CD11b expressed on B cells, similar to cytotoxic T cells. Materials and methods: Isolated peripheral blood CD19(+) B cells were treated with EGCG and the change in the expression of CD11b was analyzed using flow cytometry. The effects of EGCG on the ability of B cells to adhere to and to transmigrate through the endothelial cell layer were evaluated using the transwell assay. Results: EGCG significantly suppressed the apparent expression of CD11b on B cells, in the flow-cytometric analysis, and this apparent suppression was speculated to be dependent on the competitive binding of EGCG to CD11b. EGCG also significantly suppressed CD11b-mediated migration and adhesion of B cells to endothelial cells. Discussion and conclusion: EGCG has a strong suppressive activity on the adhesive and migratory abilities of peripheral blood B cells. This suppressive activity was mediated by the binding of EGCG to CD11b on B cells, and the consequent suppression of B-cell extravasation to the extravascular space. Because B cell plays an important role in the humoral immunity, EGCG could be a promising drug for the prevention and/or treatment of allergic and/or autoimmune diseases.

PMID: 20936888 [PubMed - indexed for MEDLINE]

338. Ann Allergy Asthma Immunol. 2010 Oct;105(4):323-4. doi:
10.1016/j.anai.2010.08.004. Epub 2010 Sep 1.

Challenges faced by expatriate children with food allergy in an Asian country.

Sivaraj H, Rajakulendran M, Lee BW, Shek L.

PMID: 20934635 [PubMed - indexed for MEDLINE]

339. Ann Allergy Asthma Immunol. 2010 Oct;105(4):249-55; quiz 256-8. doi:
10.1016/j.anai.2010.02.001. Epub 2010 Apr 14.

Local production of IgE in the respiratory mucosa and the concept of entopy: does allergy exist in nonallergic rhinitis?

Forester JP, Calabria CW.

Department of Allergy and Immunology, Wilford Hall Medical Center, Lackland AFB, Texas 78236, USA. joseph.forester@lackland.af.mil

OBJECTIVE: To review research regarding locally produced IgE and its impact on patients with chronic rhinitis.
DATA SOURCES: PubMed search with the following keywords: entopy, local IgE, nonallergic rhinitis, idiopathic rhinitis, vasomotor rhinitis, and allergic rhinitis.
STUDY SELECTION: Articles were selected based on their relevance to entopy and locally produced IgE and its clinical effect and relationship to idiopathic rhinitis (IR).
RESULTS: Local IgE has been found in a variety of tissues, including nasal and bronchial mucosa. IgE is produced in these local tissues and not simply the product of migration to the tissue from regional lymphoid tissue or blood. Local IgE has been identified in most of both atopic and nonatopic asthmatic patients and allergic rhinitis patients. Up to 40% of patients with IR and a positive nasal provocation test result have evidence of locally produced IgE, which has been coined entopy. Although patients with allergic rhinitis and IR show similar inflammatory patterns with increased activated mast cells, eosinophils, and T-cell subsets in some studies, other studies on IR patients are conflicting with regard to both inflammation and allergen-specific nasal provocation test results.
CONCLUSION: The concept of local allergy in IR patients is both intriguing and controversial. Studies have reported conflicting results, and currently there is no single best test to evaluate for entopy. It is known that there are a large number of IR patients for whom current treatment regimens are suboptimal. Therefore, further research elucidating the mechanisms of IR and the concept of localized IgE are needed to optimally diagnose this condition and treat this group of patients.

Copyright © 2010 American College of Allergy, Asthma & Immunology. Published by Elsevier Inc. All rights reserved.

PMID: 20934624 [PubMed - indexed for MEDLINE]

340. FEBS Lett. 2010 Nov 5;584(21):4469-74. doi: 10.1016/j.febslet.2010.10.007. Epub 2010 Oct 8.

Suppressor of cytokine signalling (SOCS) 1 and 3 enhance cell adhesion and inhibit migration towards the chemokine eotaxin/CCL11.

Stevenson NJ, McFarlane C, Ong ST, Nahlik K, Kelvin A, Addley MR, Long A, Greaves DR, O'Farrelly C, Johnston JA.

Centre for Infection & Immunity, School of Medicine, Dentistry & Biomedical Science, Queen's University of Belfast, United Kingdom. n.stevenson@tcd.ie

Suppressors of cytokine signalling (SOCS) proteins regulate signal transduction, but their role in responses to chemokines remains poorly understood. We report that cells expressing SOCS1 and 3 exhibit enhanced adhesion and reduced migration towards the chemokine CCL11. Focal adhesion kinase (FAK) and the GTPase RhoA, control cell adhesion and migration and we show the presence of SOCS1 or 3 regulates expression and tyrosine phosphorylation of FAK, while also enhancing activation of RhoA. Our novel findings suggest that SOCS1 and 3 may control chemotaxis and adhesion by significantly enhancing both FAK and RhoA activity, thus localizing immune cells to the site of allergic inflammation.

Copyright © 2010 Federation of European Biochemical Societies. Published by Elsevier B.V. All rights reserved.

PMID: 20934424 [PubMed - indexed for MEDLINE]

341. Respir Med. 2011 Feb;105(2):204-10. doi: 10.1016/j.rmed.2010.09.009. Epub 2010 Oct 8.

Incidence of respiratory and allergic symptoms in Italian and immigrant children.

Marcon A, Cazzoletti L, Rava M, Gisondi P, Pironi V, Ricci P, de Marco R.

Unit of Epidemiology and Medical Statistics, Department of Public Health and Community Medicine, University of Verona, Italy. alessandro.marcon@univr.it

BACKGROUND: Immigration usually implies a complete change of the environment where one lives. Hence, studies on immigrants may help to disentangle genetic and environmental determinants of disease. We investigated whether the incidence of allergic and respiratory symptoms differed for Italian and immigrant children living in one area of Northern Italy.
METHODS: In December 2006, all the children (3-14 years) living in the Viadana district were surveyed through a parental questionnaire (response rate = 99%, n = 3854). Retrospective incidences of several symptoms were compared across different ethnic groups.
RESULTS: Parental asthma, allergic rhinitis and eczema were less frequent in immigrant children than in Italian children. Wheezing and eczema incidences were lower in children born to foreign parents (especially if born abroad, incidence rate ratio (IRR) = 0.47, 95% CI: 0.26-0.82 and IRR = 0.43, 95% CI: 0.23-0.83, respectively), with respect to Italian children, while the occurrence of nasal allergies was similar among the ethnic groups. The greatest incidence of persistent cough/phlegm was observed in children born in Italy to foreign parents (IRR = 1.98, 95% CI: 1.06-3.71) and in children whose parents had chronic bronchitis (IRR = 2.57, 95% CI: 1.52-4.33).
CONCLUSIONS: Considering the distribution of parental atopic diseases and the low disease prevalence in the immigrants' countries of origin, we suggest that nasal allergies may be more sensitive than wheezing or eczema to the change in the environment related to migration. Genetic or environmental factors clustered into families seem to have a role on chronic bronchitis.

Copyright Â© 2010 Elsevier Ltd. All rights reserved.

PMID: 20934316 [PubMed - indexed for MEDLINE]

342. J Investig Med. 2010 Dec;58(8):987-90. doi: 10.231/JIM.0b013e3181fa02f7.

Airway platelet activation is associated with airway eosinophilic inflammation in asthma.

Benton AS, Kumar N, Lerner J, Wiles AA, Foerster M, Teach SJ, Freishtat RJ.

Center for Genetic Medicine Research, Children's National Medical Center, 111 Michigan Ave. NW, Washington, DC 20010, USA.

BACKGROUND: Allergic asthma is characterized by airway inflammation associated with recruitment and activation of eosinophils. In mice, allergen exposure induces platelet migration to the airways that is necessary for eosinophil recruitment and activation. We therefore hypothesized that in the airways of human subjects with asthma, platelet activation would be positively associated with eosinophil activation and platelet and eosinophil activation would both be associated with clinical asthma characteristics.
METHODS: Nasal wash levels of P-selectin (a measure of platelet activation) and eosinophil cationic protein (ECP) (a measure of eosinophil activation) were compared with each other and with clinical asthma characteristics in a

cross-sectional study of urban children and adolescents (age range, 6-20 years) with asthma.
RESULTS: Regression analysis revealed a significantly positive association between log10 P-selectin levels and log10 ECP levels (β = 0.50 ng/mL [95% confidence interval, 0.05-0.94 ng/mL]; P = 0.029). Additionally, ECP was significantly and negatively associated with 2 asthma-related quality of life measurements, and P-selectin was associated with one of these.
CONCLUSIONS: Our study shows the first significant association between platelet and eosinophil activation in airways of human subjects with asthma. These data provide a first step toward delineating what seems to be an important role for platelets in airway eosinophilia.

PMCID: PMC3324858
PMID: 20930644 [PubMed - indexed for MEDLINE]

343. J Immunol. 2010 Nov 1;185(9):5648-55. doi: 10.4049/jimmunol.1001713. Epub 2010 Oct 4.

Distinct roles of vascular endothelial growth factor receptor-1- and receptor-2-mediated signaling in T cell priming and Th17 polarization to lipopolysaccharide-containing allergens in the lung.

Kim YS, Choi SJ, Tae YM, Lee BJ, Jeon SG, Oh SY, Gho YS, Zhu Z, Kim YK.

Department of Life Science, Pohang University of Science and Technology, Pohang, Republic of Korea.

Vascular endothelial growth factor (VEGF) is a key mediator in the development of airway immune dysfunction to inhaled allergens. However, the exact role of its receptors-mediated signaling is controversial. In this study, we evaluated the role of VEGF receptor (VEGFR)-1- and VEGFR-2-mediated signaling in T cell priming and polarization in the context of inhalation of LPS-containing allergens. A murine asthma model of mixed Th1 and Th17 cell responses was generated using intranasal sensitization with LPS-containing allergens. Pharmacologic intervention was performed during sensitization. In vivo production of VEGF and Th1- and Th17-polarizing cytokines (IL-12p70 and IL-6, respectively) were upregulated by airway exposure to LPS. Pharmacological intervention with a VEGFR-2-neutralizing Ab (anti-Flk1 mAb) abolished the production of IL-6 (but not IL-12p70) and the subsequent development of allergen-specific Th17 cell response. On the other hand, blocking VEGFR-1 signaling with a VEGFR-1 antagonist (anti-Flt1 hexapeptide) did not affect the production of IL-12p70 and IL-6. However, blocking VEGFR-1 signaling resulted in T cell tolerance rather than priming, mainly by inhibiting the maturation of lung dendritic cells, and their migration into lung-draining lymph nodes. These results suggest that T cell priming to LPS-containing allergens depends on VEGFR-1-mediated signaling, and the subsequent Th17 polarization depends on VEGFR-2 signaling.

PMID: 20921519 [PubMed - indexed for MEDLINE]

344. J Inflamm (Lond). 2010 Sep 28;7:49. doi: 10.1186/1476-9255-7-49.

Salivary gland derived peptides as a new class of anti-inflammatory agents: review of preclinical pharmacology of C-terminal peptides of SMR1 protein.

Mathison RD, Davison JS, Befus AD, Gingerich DA.

Faculty of Medicine, University of Calgary, 3330 Hospital Drive NW, Calgary, Alberta, T2N 4N1, Canada. rmathiso@ucalgary.ca.

The limitations of steroidal and non steroidal anti-inflammatory drugs have prompted investigation into other biologically based therapeutics, and identification of immune selective anti-inflammatory agents of salivary origin. The traditional view of salivary glands as accessory digestive structures is changing as their importance as sources of systemically active immunoregulatory and anti-inflammatory factors is recognized. Salivary gland involvement in maintenance of whole body homeostasis is regulated by the nervous system and thus constitutes a "neuroendocrine axis". The potent anti-inflammatory activities, both in vivo and in vitro, of the tripeptide Phe-Glu-Gly (FEG) are reviewed. FEG is a carboxyl terminal peptide of the prohormone SMR1 identified in the rat submandibular salivary gland, The D-isomeric form (feG) mimics the activity of its L-isomer FEG. Macropharmacologically, feG attenuates the cardiovascular and inflammatory effects of endotoxemia and anaphylaxis, by inhibition of hypotension, leukocyte migration, vascular leak, and disruption of pulmonary function and intestinal motility. Mechanistically, feG affects activated inflammatory cells, especially neutrophils, by regulating integrins and inhibiting intracellular production of reactive oxygen species. Pharmacodynamically, feG is active at low doses (100 µg/kg) and has a long (9-12 hour) biological half life. As a therapeutic agent, feG shows promise in diseases characterized by over exuberant inflammatory responses such as systemic inflammatory response syndrome and other acute inflammatory diseases. Arthritis, sepsis, acute pancreatitis, asthma, acute respiratory inflammation, inflammatory bowel disease, and equine laminitis are potential targets for this promising therapeutic peptide. The term "Immune Selective Anti-Inflammatory Derivatives" (ImSAIDs) is proposed for salivary-derived peptides to distinguish this class of agents from corticosteroids and nonsteroidal anti-inflammatory drugs.

PMCID: PMC2955637
PMID: 20920210 [PubMed]

345. Allergy. 2010 Dec;65(12):1545-53. doi: 10.1111/j.1398-9995.2010.02426.x. Epub 2010 Sep 29.

The purinergic receptor P2Y2 receptor mediates chemotaxis of dendritic cells and eosinophils in allergic lung inflammation.

Müller T, Robaye B, Vieira RP, Ferrari D, Grimm M, Jakob T, Martin SF, Di Virgilio F, Boeynaems JM, Virchow JC, Idzko M.

Department of Pneumology, University Medical Centre Freiburg, Freiburg, Germany.

BACKGROUND: Extracellular ATP contributes to the pathogenesis of asthma via signalling at purinergic receptors. However, the precise purinergic receptors subtypes mediating the pro-asthmatic effects of ATP have not been identified, yet.
METHODS: In vivo studies were performed using the OVA-alum model. Functional expression of the P2Y(2) purinergic receptor subtype on human monocyte-derived dendritic cells and eosinophils was investigated using real-time PCR, migration assays, and production of reactive oxygen species.
RESULTS: Compared to wild-type animals P2Y(2) -/- mice showed reduced allergic airway inflammation which can be explained by defective migration of blood myeloid DCs towards ATP in vitro and in vivo, whereas the influence of ATP on maturation and cytokine production was not changed. Additionally, ATP failed to induce migration of bone marrow-derived eosinophils from P2Y(2) R-deficient animals. The relevance of our findings for humans was confirmed in functional studies with human monocyte-derived DCs and eosinophils. Interestingly, stimulation of human DCs derived from allergic individuals with house dust mite allergen induced functional up-regulation of the P2Y(2) R subtype. Furthermore,

eosinophils isolated from asthmatic individuals expressed higher levels of P2Y(2) R compared to healthy controls. This was of functional relevance as these eosinophils were more sensitive to ATP-induced migration and production of reactive oxygen metabolites.
CONCLUSIONS: In summary, P2Y(2) R appears to be involved in asthmatic airway inflammation by mediating ATP-triggered migration of mDCs and eosinophils, as well as reactive oxygen species production. Together our data suggest that targeting P2Y(2) R might be a therapeutic option for the treatment of asthma.

© 2010 John Wiley & Sons A/S.

PMID: 20880147 [PubMed - indexed for MEDLINE]

346. Immunology. 2011 Jan;132(1):144-54. doi: 10.1111/j.1365-2567.2010.03349.x. Epub 2010 Sep 28.

Local proliferation and mobilization of CCR3(+) CD34(+) eosinophil-lineage-committed cells in the lung.

Rådinger M, Bossios A, Sjöstrand M, Lu Y, Malmhäll C, Dahlborn AK, Lee JJ, Lötvall J.

Krefting Research Centre, Sahlgrenska Academy, University of Gothenburg, Gothenburg, Sweden. madeleine.radinger@gu.se

Emerging evidence suggests that haematopoietic CD34(+) progenitor cells migrate from bone marrow (BM) to sites of allergen exposure where they can undergo further proliferation and final maturation, potentially augmenting the degree of tissue inflammation. In the current study we used a well-characterized mouse model of allergen-induced airway inflammation to determine the role of CCR3 receptor-ligand interactions in the migration and function of CD34(+) cells. Allergen exposure significantly increased BM, blood and airway CD34(+) CCR3(+) cells as well as airway CD34(+) CCR3(+) stem cell antigen-1-positive (Sca-1(+)) and CD34(+) CD45(+) interleukin-5 receptor-α-positive (IL-5Rα(+)) cells. A portion of the newly produced CD34(+) CCR3(+), Sca-1(+) CCR3(+) and IL-5Ralpha(+) lung cells showed a significant proliferative capacity in response to allergen when compared with saline-treated animals. In addition, in vitro colony formation of lung CD34(+) cells was increased by IL-5 or eotaxin-2 whereas eotaxin-2 had no effect on BM CD34(+) cells. Furthermore, both eotaxin-1 and eotaxin-2 induced migration of BM and blood CD34(+) CCR3(+) cells in vitro. These data suggest that the CCR3/eotaxin pathway is involved in the regulation of allergen-driven in situ haematopoiesis and the accumulation/mobilization of eosinophil-lineage-committed progenitor cells in the lung. Hence, targeting both IL-5 and CCR3-mediated signalling pathways may be required to control the inflammation associated with allergen-induced asthma.

PMCID: PMC3015084
PMID: 20875077 [PubMed - indexed for MEDLINE]

347. J Immunol. 2010 Oct 15;185(8):4515-9. doi: 10.4049/jimmunol.1002227. Epub 2010 Sep 22.

Cutting edge: NLRP12 controls dendritic and myeloid cell migration to affect contact hypersensitivity.

Arthur JC, Lich JD, Ye Z, Allen IC, Gris D, Wilson JE, Schneider M, Roney KE, O'Connor BP, Moore CB, Morrison A, Sutterwala FS, Bertin J, Koller BH, Liu Z, Ting JP.

Department of Microbiology and Immunology, University of North Carolina at Chapel Hill, Chapel Hill, NC 27599, USA.

Nucleotide-binding domain leucine-rich repeat (NLR) proteins are regulators of inflammation and immunity. Although first described 8 y ago, a physiologic role for NLRP12 has remained elusive until now. We find that murine Nlrp12, an NLR linked to atopic dermatitis and hereditary periodic fever in humans, is prominently expressed in dendritic cells (DCs) and neutrophils. Nlrp12-deficient mice exhibit attenuated inflammatory responses in two models of contact hypersensitivity that exhibit features of allergic dermatitis. This cannot be attributed to defective Ag processing/presentation, inflammasome activation, or measurable changes in other inflammatory cytokines. Rather, Nlrp12(-/-) DCs display a significantly reduced capacity to migrate to draining lymph nodes. Both DCs and neutrophils fail to respond to chemokines in vitro. These findings indicate that NLRP12 is important in maintaining neutrophils and peripheral DCs in a migration-competent state.

PMID: 20861349 [PubMed - indexed for MEDLINE]

348. Epidemiol Prev. 2010 May-Jun;34(3):120-4.

[Health of immigrants in Italy: increasing evidences and forgotten issues in the epidemiological research].

[Article in Italian]

Fedeli U, Baussano I.

SER (Sistema epidemiologico regionale), via dei Carpani 16/Z, 31033 Castelfranco Veneto (TV). ugo.fedeli@ulssasolo.ven.it

To obtain a broad picture of epidemiological studies on health of immigrants in Italy, we analyzed abstracts presented at the last five annual meetings of the Italian Epidemiological Association (AIE), and abstracts on PubMed published in 2000-2009 (including also clinical studies). Studies at AIE meetings mainly used electronic archives of health records to investigate the overall health profile of immigrants, or specifically women and perinatal health; these surveys are then rarely published on scientific journals. By contrast, several areas addressed in the literature (infectious diseases, accidents, lifestyles, mental health, pediatric diseases, allergic diseases) are almost absent in recent AIE meetings. If Italian epidemiologists claim a role as technical support to policy makers, they should probably invest more in what has recently become the most important and debated issue in the Italian society.

PMID: 20852349 [PubMed - indexed for MEDLINE]

349. Exp Appl Acarol. 2011 Feb;53(2):103-19. doi: 10.1007/s10493-010-9394-4. Epub 2010 Sep 14.

Population growth and allergen accumulation of Dermatophagoides pteronyssinus cultured at 20 and 25 °C.

Yella L, Morgan MS, Arlian LG.

Department of Biological Sciences, Wright State University, 3640 Colonel Glenn Highway, Dayton, OH 45435, USA.

The house dust mites, Dermatophagoides pteronyssinus and D. farinae are cultured commercially and in research laboratories and material is harvested from these cultures to make extracts that are used for diagnosis, immunotherapy and research. Temperature and other climatic conditions can influence population growth rates, dynamics of allergen production, and the associated endotoxin, enzyme and protein levels of the mite material harvested from these cultures. Here we determined how temperature affected these parameters. Dermatophagoides pteronyssinus was cultured at 20 and 25 °C at 75% relative humidity, and at 2-week intervals the concentrations of mites, Der p 1 and Der p 2 allergens, endotoxin, and selected enzymes were determined. Mite density increased exponentially but growth rate and final population density were greater at 25 °C compared to 20 °C. The combined allergen (Der p 1 + Der p 2) concentrations accumulated in the cultures at about the same rate at both temperatures. However, individual Der p 1 and Der p 2 accumulation rates varied independently at the two temperatures. Der p 1 accumulated faster at 20 °C whereas Der p 2 accumulated faster at 25 °C. The amount of Der p 1 in whole cultures was greater than the amount of Der p 2. The concentration of allergen for washed mites harvested from the cultures was much less than for the whole cultures. Our study demonstrated that temperature is an important factor in population growth and the dynamics of allergen production in cultured mites.

PMID: 20838884 [PubMed - indexed for MEDLINE]

350. J Chromatogr B Analyt Technol Biomed Life Sci. 2010 Oct 15;878(28):2933-6. doi: 10.1016/j.jchromb.2010.08.027. Epub 2010 Aug 26.

Capillary electrophoresis with noncovalently bilayer-coated capillaries for stability study of allergenic proteins in simulated gastrointestinal fluids.

Zheng C, Liu Y, Zhou Q, Di X.

School of Pharmacy, Shenyang Pharmaceutical University, 103 Wenhua Road, Shenyang 110016, PR China.

A novel noncovalently bilayer-coated capillary using cationic polymer polybrene (PB) and anionic polymer (sodium 4-styrenesulfonate) (PSS) as coatings was prepared. This PB-PSS coating showed good migration-time reproducibility for proteins and high stability in the range of pH 2-10 and in the presence of 1M NaOH, acetonitrile and methanol. Capillary electrophoresis with PB-PSS coated capillaries was successfully applied to quantitatively investigate the stability of bovine serum albumin, ovomucoid, β-lactoglobulin and lysozyme in simulated gastrointestinal fluids. β-lactoglobulin A and β-lactoglobulin B were both stable in simulated gastric fluid with degradation percentages of 34.3% and 17.2% after 60min of incubation, respectively. Bovine serum albumin, ovomucoid and lysozyme were stable in simulated intestinal fluid with degradation percentages of 17.7%, 23.4% and 22.8% after 60min of incubation, respectively. The superiority of the proposed method over sodium dodecyl sulfate-polyacrylamide gel electrophoresis (SDS-PAGE) and capillary electrophoresis with untreated fused silica capillaries was demonstrated and emphasized.

Copyright © 2010 Elsevier B.V. All rights reserved.

PMID: 20837405 [PubMed - indexed for MEDLINE]

351. J Biol Chem. 2010 Sep 17;285(38):29101-10. doi: 10.1074/jbc.M109.0999952.

TNFα and IFNγ synergistically enhance transcriptional activation of CXCL10 in human airway smooth muscle cells via STAT-1, NF-κB, and the transcriptional

coactivator CREB-binding protein.

Clarke DL, Clifford RL, Jindarat S, Proud D, Pang L, Belvisi M, Knox AJ.

Nottingham Respiratory Biomedical Research Unit, City Hospital, University of Nottingham, Nottingham NG5 1PB, United Kingdom. ClarkeD@MedImmune.com

Asthmatic airway smooth muscle (ASM) expresses interferon-γ-inducible protein-10 (CXCL10), a chemokine known to mediate mast cell migration into ASM bundles that has been reported in the airways of asthmatic patients. CXCL10 is elevated in patients suffering from viral exacerbations of asthma and in patients with chronic obstructive pulmonary disease (COPD), diseases in which corticosteroids are largely ineffective. IFNγ and TNFα synergistically induce CXCL10 release from human ASM cells in a steroid-insensitive manner, via an as yet undefined mechanism. We report that TNFα activates the classical NF-κB (nuclear factor κB) pathway, whereas IFNγ activates JAK2/STAT-1α and that inhibition of the JAK/STAT pathway is more effective in abrogating CXCL10 release than the steroid fluticasone. The synergy observed with TNFα and IFNγ together, however, did not lie at the level of NF-κB activation, STAT-1α phosphorylation, or in vivo binding of these transcription factors to the CXCL10 promoter. Stimulation of human ASM cells with TNFα and IFNγ induced histone H4 but not histone H3 acetylation at the CXCL10 promoter, although no synergism was observed when both cytokines were combined. We show, however, that TNFα and IFNγ exert a synergistic effect on the recruitment of CREB-binding protein (CBP) to the CXCL10, which is accompanied by increased RNA polymerase II. Our results provide evidence that synergism between TNFα and IFNγ lies at the level of coactivator recruitment in human ASM and suggest that inhibition of JAK/STAT signaling may be of therapeutic benefit in steroid-resistant airway disease.

PMCID: PMC2937941
PMID: 20833730 [PubMed - indexed for MEDLINE]

352. Am J Rhinol Allergy. 2010 Jul-Aug;24(4):249-54. doi: 10.2500/ajra.2010.24.3499.

Allergic rhinitis and inflammatory airway disease: interactions within the unified airspace.

Marple BF.

Department of Otolaryngology-Head and Neck Surgery, UT Southwestern Medical Center at Dallas, Dallas, Texas 75390-9035, USA. Bradley.Marple@UTSoutwestern.edu

BACKGROUND: Allergic rhinitis (AR), the most common chronic allergic condition in outpatient medicine, is associated with immense health care costs and socioeconomic consequences. AR's impact may be partly from interacting of respiratory conditions via allergic inflammation. This study was designed to review potential interactive mechanisms of AR and associated conditions and consider the relevance of a bidirectional "unified airway" respiratory inflammation model on diagnosis and treatment of inflammatory airway disease.
METHODS: MEDLINE was searched for pathophysiology and pathophysiological and epidemiologic links between AR and diseases of the sinuses, lungs, middle ear, and nasopharynx.
RESULTS: Allergic-related inflammatory responses or neural and systemic processes fostering inflammatory changes distant from initial allergen provocation may link AR and comorbidities. Treating AR may benefit associated respiratory tract comorbidities. Besides improving AR outcomes, treatment inhibiting eosinophil recruitment and migration, normalizing cytokine profiles, and reducing asthma-associated health care use in atopic subjects would likely ameliorate other upper airway diseases such as acute rhinosinusitis, chronic rhinosinusitis

(CRS) with nasal polyposis (NP), adenoidal hypertrophy, and otitis media with effusion.
CONCLUSION: Epidemiological concordance of AR with several airway diseases conforms to a bidirectional "unified airway" respiratory inflammation model based on anatomic and histological upper and lower airway connections. Epidemiology and current understanding of inflammatory, humoral, and neural processes make links between AR and disorders including asthma, otitis media, NP, and CRS plausible. Combining AR with associated conditions increases disease burden; worsened associated illness may accompany worsened AR. AR pharmacotherapies include antihistamines, leukotriene antagonists, intranasal corticosteroids, and immunotherapy; treatments attenuating proinflammatory responses may also benefit associated conditions.

PMID: 20819460 [PubMed - indexed for MEDLINE]

353. J Immunol. 2010 Oct 1;185(7):4446-56. doi: 10.4049/jimmunol.1001254. Epub 2010 Sep 3.

Gene expression profiles in a rabbit model of systemic lupus erythematosus autoantibody production.

Rai G, Ray S, Milton J, Yang J, Ren P, Lempicki R, Mage RG.

Laboratory of Immunology, National Institute of Allergy and Infectious Diseases, National Institutes of Health, Bethesda, MD 20892, USA.

We previously reported the establishment of a rabbit (Oryctolagus cuniculus) model in which peptide immunization led to production of lupus-like autoantibodies including anti-Sm, -RNP, -SS-A, -SS-B, and -dsDNA characteristic of those produced in systemic lupus erythematosus (SLE) patients. Some neurologic symptoms in the form of seizures and nystagmus were observed. The animals used in the previous and in the current study were from a National Institute of Allergy and Infectious Diseases colony of rabbits that were pedigreed, Ig-allotype defined, but not inbred. Their genetic heterogeneity may correspond to that found among patients of a given ethnicity. We extended the information about this rabbit model by microarray-based expression profiling. We first demonstrated that human expression arrays could be used with rabbit RNA to yield information on molecular pathways. We then designed a study evaluating gene expression profiles in eight groups of control and treated rabbits (47 rabbits in total). Genes significantly upregulated in treated rabbits were associated with NK cytotoxicity, Ag presentation, leukocyte migration, cytokine activity, protein kinases, RNA spliceosomal ribonucleoproteins, intracellular signaling cascades, and glutamate receptor activity. These results link increased immune activation with upregulation of components associated with neurologic and anti-RNP responses, demonstrating the utility of the rabbit model to uncover biological pathways related to SLE-induced clinical symptoms, including neuropsychiatric lupus. Our finding of distinct gene expression patterns in rabbits that made anti-dsDNA compared with those that only made other anti-nuclear Abs should be further investigated in subsets of SLE patients with different autoantibody profiles.

PMCID: PMC2949067
PMID: 20817871 [PubMed - indexed for MEDLINE]

354. Eur Respir J. 2011 May;37(5):1226-36. doi: 10.1183/09031936.00192109. Epub 2010 Sep 3.

Signalling pathway of isophorone diisocyanate-responsive interleukin-8 in airway

smooth muscle cells.

Kuo PL, Huang MS, Huang SK, Ni WC, Hung JY, Ko YC, Hung CH, Tsai YM, Duh TH, Hsu YL.

Institute of Clinical Medicine, Kaohsiung Medical University, Taiwan.

This study is the first to analyse the soluble factors secreted by the bronchial epithelium after exposure to isophorone diisocyanate (IPDI) that are responsible for increasing migration and proliferation of primary normal human bronchial smooth muscle cells (BSMCs). We treated immortalised, nontumorigenic human bronchial epithelial cells (cell line BEAS-2B) and primary normal human bronchial epithelial cells (HBEC) with IPDI, and then collected the conditioned culture media (IPDI-BEAS-2B-CM and IPDI-HBEC-CM, respectively), which was added to BSMCs. Exposure of BEAS-2B cells and HBECs to IPDI increased interleukin (IL)-8 production. Culture of BSMCs with IPDI-BEAS-2B-CM and IPDI-HBEC-CM increased BSMC proliferation and migration, which are major features in asthma-related airway remodelling. Induction of BSMC proliferation and migration by IPDI-BEAS-2B-CM and IPDI-HBEC-CM was associated with increased focal adhesion kinase (FAK), Src, extracellular signal-regulated kinase (ERK)1/2 and AKT activation. Blocking FAK with a specific inhibitor significantly decreased BSMC migration and proliferation by inhibiting ERK1/2 activation. FAK and ERK1/2 inhibitor also decreased IPDI-BEAS-2B-CM-, IPDI-HBEC-CM- and recombinant human IL-8-mediated BSMC proliferation and migration, whereas blocking Rnd3 using small interfering RNA failed to affect BSMC proliferation, suggesting that Rnd3 was only involved in the regulation of BSMC migration. Our study suggests that inhibition of IL-8 or IL-8-mediated FAK/ERK/Rnd3 signalling is an attractive therapeutic target for IPDI-mediated asthma.

PMID: 20817708 [PubMed - indexed for MEDLINE]

355. Allergy. 2011 Feb;66(2):206-13. doi: 10.1111/j.1398-9995.2010.02464.x. Epub 2010 Aug 30.

Determinants of eczema: population-based cross-sectional study in Germany.

Apfelbacher CJ, Diepgen TL, Schmitt J.

Department of Clinical Social Medicine, University Hospital Heidelberg, Thibautstrasse 3, Heidelberg, Germany.
christian.apfelbacher@med.uni-heidelberg.de

BACKGROUND: Eczema is a chronic inflammatory skin disease and is among the most frequent chronic conditions in childhood and adolescence. It is the aim of this study to investigate determinants of eczema in German children and adolescents.
METHODS: Data were drawn from the public use files of the German Interview and Examination Survey for Children and Adolescents (KIGGS), a nationwide cross-sectional representative survey conducted between 2003 and 2006, including 17,641 children aged 0-17 (response rate: 66.6%). We investigated the association of a broad set of environmental and lifestyle exposures with ever physician-diagnosed eczema by means of univariable analyses and multivariable logistic regression modelling.
RESULTS: The weighted prevalence of ever physician-diagnosed eczema was 13.2% [95% confidence interval (CI) 12.5-13.9%]. In multivariable analysis, significant positive associations of parental allergies (OR 1.94, 95% CI 1.72-2.19), parent-reported infection after birth (OR 1.45, 95% CI 1.05-2.00) and parent-reported jaundice after birth (OR 1.27, 95% CI 1.04-1.54) were revealed. Being a migrant (OR 0.63, 95% CI 0.49-0.80) and keeping a dog (OR 0.78, 95% CI 0.64-0.96) showed significant inverse associations with eczema. Other lifestyle

(alcohol consumption during pregnancy) and environmental factors (mould on the walls, pets, origin from East/West Germany) were not significantly related to eczema.
CONCLUSIONS: This study suggests that a family history of allergies is the strongest determinant of eczema. Perinatal health problems were associated with eczema, pointing to the importance of early life factors in the manifestation of eczema.

© 2010 John Wiley & Sons A/S.

PMID: 20804468 [PubMed - indexed for MEDLINE]

356. Recent Pat Inflamm Allergy Drug Discov. 2010 Nov;4(3):210-3.

Antagonism of eosinophil accumulation in asthma.

Walsh GM.

School of Medicine & Dentistry, Institute of Medical Sciences, University of Aberdeen, Scotland, UK. g.m.walsh@abdn.ac.uk

There is considerable evidence that implicates eosinophils as important effector cells and immunomodulators in the inflammation characteristic of asthma. Numerous in vitro and animal studies have demonstrated essential roles for cell adhesion molecules in eosinophil adhesion and transendothelial migration including the selectins, ICAM-1, VCAM-1 together with many of the 1 and β2 integrins. A large body of evidence has also implicated several cytokines and chemokines in the selective recruitment of eosinophils to sites of asthmatic inflammation. Biopharmaceutical approaches have been used to identify inhibitory molecules that target key elements in the processes controlling eosinophil accumulation in asthma. This review will summarise, the problems and successes regarding recent patents and developments in adhesion-based therapeutic strategies aimed at reducing eosinophil-mediated inflammation in the asthmatic lung.

PMID: 20804449 [PubMed - indexed for MEDLINE]

357. Iran J Parasitol. 2010 Sep;5(3):40-7.

Strongyloides stercoralis: The Most Prevalent Parasitic Cause of Eosinophilia in Gilan Province, Northern Iran.

Ashrafi K, Tahbaz A, Rahmati B.

BACKGROUND: Eosinophilia occurs in a wide variety of situations such as parasitic infections, allergic disorders, and malignancies. Most cases of eosinophilia of parasitic origin, especially those with a tissue migration life cycles consists of human infections by helminth parasites. The aim of present study was to determine the parasitic causes of eosinophilia in patients in a major endemic area of human fascioliasis in Gilan Province, northern part of Iran.
METHODS: One hundred and fifty patients presenting with an elevated eosinophilia attending infectious disease clinics with or without clinical symptoms, were examined. After clinical history evaluation and physical examination, coprological examinations were performed using the formalin-ether and the Kato-Katz techniques for detection of Fasciola sp. and intestinal parasites.
RESULTS: Forty two percent of patients were infected with S. stercoralis, nine (6%) were found to be infected with Fasciola sp. while only a single patient (0.7%) were infected by Ttrichostrongylus sp.
CONCLUSION: Local clinicians in Gilan may consider eosinophilia as a suggestive

indication for diagnosis of human fascioliasis, especially when microscopic stool and/or serological tests are negative. Based on the results, local physicians should consider S. stercoralis as the potential causes of eosinophilia in patients with elevated eosinophilia.

PMCID: PMC3279844
PMID: 22347254 [PubMed]

358. J Allergy Clin Immunol. 2010 Nov;126(5):1032-40, 1040.e1-4. doi: 10.1016/j.jaci.2010.06.039. Epub 2010 Aug 21.

Exosomes from human macrophages and dendritic cells contain enzymes for leukotriene biosynthesis and promote granulocyte migration.

Esser J, Gehrmann U, D'Alexandri FL, Hidalgo-Estévez AM, Wheelock CE, Scheynius A, Gabrielsson S, Rådmark O.

Division of Physiological Chemistry II, Department of Medical Biochemistry and Biophysics, Karolinska Institutet, Stockholm, Sweden.

BACKGROUND: Leukotrienes (LTs) are potent proinflammatory lipid mediators with key roles in the pathogenesis of asthma and inflammation. Recently, nanovesicles (exosomes), released from macrophages and dendritic cells (DCs), have become increasingly appreciated as messengers in immunity.
OBJECTIVE: We investigated whether exosomes from human macrophages, DCs, and plasma contain enzymes for LT biosynthesis and studied potential roles for exosomes in transcellular LT metabolism and granulocyte chemotaxis.
METHODS: The presence of LT pathway enzymes and LT biosynthesis in exosomes and cells was analyzed by Western blot, immunoelectron microscopy, and enzyme activity assays. Surface marker expression was evaluated by flow cytometry, and granulocyte migration was assessed in a multiwell chemotaxis system.
RESULTS: Exosomes from macrophages and DCs contain functional enzymes for LT biosynthesis. After incubation of intact cells with the LT biosynthesis intermediate LTA(4), LTB(4) was the major product of macrophages, whereas DCs primarily formed LTC(4). However, in exosomes from both cell types, LTC(4) was the predominant LTA(4) metabolite. Exosomal LTC(4) formation (per milligram protein) exceeded that of cells. In macrophages and DCs, TGF-β1 upregulated LTA(4) hydrolase along with increased LTB(4) formation also in the exosomes. Moreover, TGF-β1 modified the expression of surface marker proteins on cells and exosomes and reduced the exosome yield from macrophages. On Ca(2+)-ionophore and arachidonic acid stimulation, exosomes produced chemotactic eicosanoids and induced granulocyte migration. Interestingly, active LTA(4) hydrolase and LTC(4) synthase were present also in exosomes from human plasma.
CONCLUSION: Our findings indicate that exosomes can contribute to inflammation by participation in LT biosynthesis and granulocyte recruitment.

Copyright © 2010 American Academy of Allergy, Asthma & Immunology. Published by Mosby, Inc. All rights reserved.

PMID: 20728205 [PubMed - indexed for MEDLINE]

359. Rheumatology (Oxford). 2010 Dec;49(12):2264-72. doi: 10.1093/rheumatology/keq224. Epub 2010 Aug 19.

Increased IL-17 production by peripheral T helper cells after tumour necrosis factor blockade in rheumatoid arthritis is accompanied by inhibition of migration-associated chemokine receptor expression.

Aerts NE, De Knop KJ, Leysen J, Ebo DG, Bridts CH, Weyler JJ, Stevens WJ, De Clerck LS.

Faculty of Medicine, Department of Immunology - Allergology - Rheumatology, University of Antwerp, University of Antwerp Hospital, Campus Drie Eiken, Universiteitsplein 1, B-2610 Antwerp, Belgium.

OBJECTIVES: The contribution of IL-17-producing Th17 cells to the pathogenesis of T-cell-mediated inflammatory disorders such as RA and atopic dermatitis (AD) has to be viewed in relation to the role of Th1/Th2 cells and long-recognized key cytokines like TNF. We aimed to study the frequency and migration-associated phenotype of peripheral Th17, Th1 and Th2 cells in healthy individuals, RA and AD patients, and to study the influence of anti-TNF therapy in RA.
METHODS: Intracellular IL-17, IFN-γ and IL-4 production and CC-chemokine receptor CCR4 and CCR6 expression were analysed flow cytometrically in peripheral memory Th cells from healthy individuals, AD and RA patients. The latter were grouped by disease activity and presence or absence of adalimumab therapy. In RA patients initiating anti-TNF therapy, cytokine production by in vitro-stimulated peripheral mononuclear cells was measured by cytometric bead array.
RESULTS: The peripheral Th17 cell frequency is elevated in AD but not in RA. In RA, Th17 cells and IL-17 production increase after anti-TNF therapy, irrespective of disease activity. Th1 cells and IFN-γ production are elevated in remission and under anti-TNF therapy. CCR6 expression is up-regulated in Th17 cells, but RA patients in remission under anti-TNF therapy have significantly lower expression than those with active disease.
CONCLUSIONS: The increase in peripheral Th17 cells in RA patients after anti-TNF therapy is accompanied by a decrease in Th17-specific CCR6 expression, which might prevent homing of these potentially pro-inflammatory cells to the synovium.

PMID: 20724433 [PubMed - indexed for MEDLINE]

360. Infect Immun. 2010 Nov;78(11):4936-43. doi: 10.1128/IAI.01118-09. Epub 2010 Aug 16.

Gelatinase contributes to the pathogenesis of endocarditis caused by Enterococcus faecalis.

Thurlow LR, Thomas VC, Narayanan S, Olson S, Fleming SD, Hancock LE.

Division of Biology, Kansas State University, Manhattan, KS 66506, USA.

The Gram-positive pathogen Enterococcus faecalis is a leading agent of nosocomial infections, including urinary tract infections, surgical site infections, and bacteremia. Among the infections caused by E. faecalis, endocarditis remains a serious clinical manifestation and unique in that it is commonly acquired in a community setting. Infective endocarditis is a complex disease, with many host and microbial components contributing to the formation of bacterial biofilm-like vegetations on the aortic valve and adjacent areas within the heart. In the current study, we compared the pathogenic potential of the vancomycin-resistant E. faecalis V583 and three isogenic protease mutants (ΔgelE, ΔsprE, and ΔgelE ΔsprE mutants) in a rabbit model of enterococcal endocarditis. The bacterial burdens displayed by GelE(-) mutants (ΔgelE and ΔgelE ΔsprE mutants) in the heart were significantly lower than those of V583 or the SprE(-) mutant. Vegetations on the aortic valve infected with GelE(-) mutants (ΔgelE and ΔgelE ΔsprE mutants) also showed a significant increase in deposition of fibrinous matrix layer and increased chemotaxis of inflammatory cells. In support of a role for proteolytic modulation of the immune response to E. faecalis, we also demonstrate that GelE can cleave the anaphylatoxin complement C5a and that this proteolysis leads to decreased neutrophil migration in vitro. In vivo, a decreased heterophil

(neutrophil-like cell) migration was observed at tissue sites infected with GelE-producing strains but not at those infected with SprE-producing strains. Taken together, these observations suggest that of the two enterococcal proteases, gelatinase is the principal mediator of pathogenesis in endocarditis.

PMCID: PMC2976315
PMID: 20713628 [PubMed - indexed for MEDLINE]

361. J Med Syst. 2011 Dec;35(6):1605-15. doi: 10.1007/s10916-010-9436-9. Epub 2010 Feb 2.

Experiences sharing of implementing Template-based Electronic Medical Record System (TEMRS) in a Hong Kong medical organization.

Ting SL, Kwok SK, Tsang AH, Lee WB, Yee KF.

Department of Industrial and Systems Engineering, The Hong Kong Polytechnic University, Kowloon, Hong Kong, China. jacky.ting@polyu.edu.hk.

This paper aims to investigate the efficacy and feasibility of Template-based Electronic Medical Record System (TEMRS) and factors for its successful implementation. A TEMRS was designed and implemented in one core clinic of a Hong Kong professional multi-disciplinary medical services provider with four core clinics located in different parts of Hong Kong. Eight doctors participated in the study. Surveys and interviews were conducted to acquire the users' feedback and satisfaction level. The design, development, and the factors related to the success of the implementation of TEMRS were analyzed. In the study period, 3,032 cases were collected. The most encountered diagnosis were upper respiratory tract infection (50.59%), gastroenteritis (10.19%), dermatitis (5.87%), dyspepsia (5.28%) and rhinitis (4.82%). The system gained an overall satisfaction by the users and the most satisfied areas were rapid retrieving the necessary information of patient (75%) and fasten the diagnostic selection (75%). TEMRS is an enabling system which can reduce the user resistance in new technology with its flexibility. The consideration of cost, security, human, technical, data migration and standardization issues are essential in the implementation of the TEMRS and further research should be conducted to expand the TEMRS's implementation in health care system.

PMID: 20703758 [PubMed - indexed for MEDLINE]

362. Am J Pathol. 2010 Sep;177(3):1244-54. doi: 10.2353/ajpath.2010.100191. Epub 2010 Aug 9.

CD34 is required for infiltration of eosinophils into the colon and pathology associated with DSS-induced ulcerative colitis.

Maltby S, Wohlfarth C, Gold M, Zbytnuik L, Hughes MR, McNagny KM.

The Biomedical Research Centre, University of British Columbia, Vancouver, British Columbia, Canada.

Eosinophil migration into the gut and the release of granular mediators plays a critical role in the pathogenesis of inflammatory bowel diseases, including ulcerative colitis. We recently demonstrated that eosinophil migration into the lung requires cell surface expression of the sialomucin CD34 on mast cells and eosinophils in an asthma model. Based on these findings, we investigated a similar role for CD34 in the migration of eosinophils and other inflammatory cells into the colon as well as explored the effects of CD34 ablation on disease

development in a dextran sulfate sodium-induced model of ulcerative colitis. Our findings demonstrate decreased disease severity in dextran sulfate sodium-treated Cd34(-/-) mice, as assessed by weight loss, diarrhea, bleeding, colon shortening and tissue pathology, compared with wild-type controls. CD34 was predominantly expressed on eosinophils within inflamed colon tissues, and Cd34(-/-) animals exhibited drastically reduced colon eosinophil infiltration. Using chimeric animals, we demonstrated that decreased disease pathology resulted from loss of CD34 from bone marrow-derived cells and that eosinophilia in Cd34(-/-)IL5(Tg) animals was sufficient to overcome protection from disease. In addition, we demonstrated a decrease in peripheral blood eosinophil numbers following dextran sulfate sodium treatment. These findings demonstrate that CD34 was expressed on colon-infiltrating eosinophils and played a role in eosinophil migration. Further, our findings suggest CD34 is required for efficient eosinophil migration, but not proliferation or expansion, in the development of ulcerative colitis.

PMCID: PMC2928958
PMID: 20696776 [PubMed - indexed for MEDLINE]

363. Curr Opin Allergy Clin Immunol. 2010 Oct;10(5):493-7. doi: 10.1097/ACI.0b013e32833dfa11.

High-risk corneal allografts and why they lose their immune privilege.

Niederkorn JY.

Department of Ophthalmology, University of Texas Southwestern Medical Center, Dallas, Texas 75390, USA. jerry.niederkorn@utsouthwestern.edu

PURPOSE OF REVIEW: Corneal allografts are routinely performed without HLA typing or systemic immunosuppressive drugs. However, certain conditions create high risks for immune rejection. This review discusses recent insights into the mechanisms that rob the corneal allograft of its immune privilege.
RECENT FINDINGS: Studies in mice have revealed that stimuli that induce new blood vessel growth in the cornea also elicit proliferation of lymph vessels. Lymph vessels facilitate migration of antigen-presenting cells to regional lymph nodes in which they induce alloimmune responses. The presence of blood vessels in the corneal graft bed creates a unique chemokine milieu that stimulates recruitment of sensitized lymphocytes into the corneal allograft. Other data indicate that although corneal allograft survival is closely associated with Foxp3 expression in CD4+CD25+Foxp3+ T regulatory cells (Tregs), reduced expression of Foxp3 in Tregs creates a high risk for graft rejection. Recent evidence indicates that allergic diseases have a profound impact on the immune response and produce a dramatic increase in corneal allograft rejection.
SUMMARY: Understanding the underlying mechanisms that create 'high-risk' hosts may provide important therapeutic targets for restoring immune privilege of corneal allografts and enhancing their survival.

PMCID: PMC3116237
PMID: 20689406 [PubMed - indexed for MEDLINE]

364. Int Immunopharmacol. 2010 Sep;10(9):1017-21. doi: 10.1016/j.intimp.2010.05.014. Epub 2010 Jun 4.

Phosphoinositide 3-kinase gamma mediates chemotactic responses of human eosinophils to platelet-activating factor.

Hasan AM, Mourtada-Maarabouni M, Hameed MS, Williams GT, Dent G.

Infection, Immunity and Inflammation Research Group, Institute of Science and Technology in Medicine, Keele University, Keele, Staffordshire, United Kingdom.

BACKGROUND: Eosinophils are characteristic participants in allergic inflammation. The intracellular signalling mechanisms involved in the migration of eosinophils to sites of allergic inflammation are poorly understood. Chemotactic responses of eosinophils to platelet-activating factor (PAF), but not eotaxin, have been demonstrated to be dependent upon the activation of phosphoinositide 3-kinase (PI3K) but the specific isoform of PI3K involved has not been identified.
OBJECTIVE: To determine the roles of the leukocyte-specific PI3K gamma and PI3K delta isoforms of PI3K in PAF-induced chemotaxis of human eosinophils.
METHODS: Chemotactic responses of the EoL-1 eosinophilic cell line and human peripheral blood eosinophils were measured. The effects of a PI3K gamma-selective inhibitor (5-[2,2-difluorobenzo(1,3)dioxol-5-ylmethylene]-thiazolidine-2,4-dione; AS604850) and gene knock-down of PI3K gamma and PI3K delta on chemotactic responses were determined.
RESULTS: AS604850 caused a concentration-dependent suppression of chemotactic responses of EoL-1 cells and blood eosinophils to PAF but not eotaxin. Specific siRNAs reduced the expression of PI3K gamma and PI3K delta in EoL-1 cells. Knock-down of PI3K gamma by siRNA resulted in a 75% inhibition of the chemotactic response to PAF but had no effect on the response to eotaxin. Knock-down of endogenous PI3K delta by siRNA resulted in a 38% inhibition of the chemotactic response to PAF but had no effect on the response to eotaxin.
CONCLUSION: PI3K gamma plays a major role in the induction of chemotaxis in PAF-stimulated eosinophils, while PI3K delta plays a lesser role. Interventions which reduce the activity of PI3K gamma may have therapeutic potential in allergic diseases.

(c) 2010 Elsevier B.V. All rights reserved.

PMID: 20685403 [PubMed - indexed for MEDLINE]

365. Toxicol Lett. 2010 May 31. [Epub ahead of print]

Differential effects of formaldehyde exposure on the cell influx and vascular permeability in a rat model of allergic lung inflammation.

Lino-Dos-Santos-Franco A, Domingos HV, Oliveira AP, Breithaupt-Faloppa AC, Peron JP, Bolonheis S, Muscará MN, Oliveira-Filho RM, Vargaftig BB, Tavares-de-Lima W.

Departament of Pharmacology, Institute of Biomedical Sciences, University of São Paulo, São Paulo, Brazil.

Exposure to air pollutants such as formaldehyde (FA) leads to inflammation, oxidative stress and immune-modulation in the airways and is associated with airway inflammatory disorders such as asthma. The purpose of our study was to investigate the effects of exposure to FA on the allergic lung inflammation. The hypothesized link between reactive oxygen species and the effects of FA was also studied. To do so, male Wistar rats were exposed to FA inhalation (1%, 90min daily) for 3 days, and subsequently sensitized with ovalbumin (OVA)-alum by subcutaneous route. One week later the rats received another OVA-alum injection by the same route (booster). Two weeks later the rats were challenged with aerosolized OVA. The OVA challenge of rats upon FA exposure induced an elevated release of LTB(4), TXB(2), IL-1beta, IL-6 and VEGF in lung cells, increased phagocytosis and lung vascular permeability, whereas the cell recruitment into lung was reduced. FA inhalation induced the oxidative burst and the nitration of proteins in the lung. Vitamins C, E and apocynin reduced the levels of LTB(4) in BAL-cultured cells of the FA and FA/OVA groups, but increased the cell influx

into the lung of the FA/OVA rats. In OVA-challenged rats, the exposure to FA was associated to a reduced lung endothelial cells expression of ICAM-1 (intercellular cell adhesion molecule 1). In conclusion, our findings suggest that FA down regulate the cellular migration into the lungs after an allergic challenge and increase the ability of resident lung cells likely macrophages to generate inflammatory mediators, explaining the increased lung vascular permeability. Our data are indicative that the actions of FA involve mechanisms related to endothelium-leukocyte interactions and oxidative stress, as far as the deleterious effects of this air pollutant on airways are concerned.

Copyright © 2010. Published by Elsevier Ireland Ltd.

PMID: 20685386 [PubMed - as supplied by publisher]

366. J Drugs Dermatol. 2010 Aug;9(8):921-7.

Statins in skin: research and rediscovery, from psoriasis to sclerosis.

Egesi A, Sun G, Khachemoune A, Rashid RM.

Baylor College of Medicine, Houston, TX, USA.

Statins, initially developed as antimicrobials, are primarily considered cholesterol-lowering agents. Recently, researchers discovered anti-inflammatory properties of statins. Studies on the effects of statins and the alterations noted include: bench work that supported a Th1/Th2 skew to Th1, altered lymphocyte migration, inhibition of MHC-II induction and cytokine release on antigen-presenting cells, inhibition of mast cell degranulation and inhibition of Th17 cells and IL-17 production. In addition to the anti-inflammatory properties, statins have been found to induce apoptosis in melanoma models. The potential therapeutic value of statins is illustrated in the management of alopecia areata, atopic dermatitis, psoriasis, systemic lupus erythematosus, cutaneous T-cell lymphomas, cutaneous melanoma, mastocytosis and more. This manuscript presents a comprehensive review of statins and their potential dermatologic application.

PMID: 20684142 [PubMed - indexed for MEDLINE]

367. Sleep Breath. 2011 Sep;15(3):543-8. doi: 10.1007/s11325-010-0398-4. Epub 2010 Jul 31.

Sleep apnea syndrome in a young cosmopolite urban adult population: risk factors for disease severity.

Bruyneel M, Ameye L, Ninane V.

Chest Service, Saint-Pierre University Hospital, Brussels, Belgium.
Marie_Bruyneel@stpierre-bru.be

PURPOSE: The purpose of this study is to investigate the risk factors for obstructive sleep apnea (OSA) in young patients from an urban European city with many migrants.
METHODS: The medical data of 2,343 patients referred for polysomnography from January 2007 till September 2009 were retrospectively reviewed in order to identify patients younger than 40 years with OSA and assess their characteristics.
RESULTS: One hundred twenty-one of the 2,093 patients (6%) referred for diagnostic polysomnography were younger than 40 years and had OSA. There were 17 women and 104 men. The race was Caucasian in 55% (67/121) and African in 42%

(51/121). The median apnea-hypopnea index (AHI) was 39 in men and 23 in women ($p < 0.01$), 30 in Caucasians and 39 in Africans ($p = 0.03$). BMI was positively correlated to the AHI (correlation of 0.19, $p = 0.04$). Multiple regression modeling showed that African origin ($p = 0.01$), current smoking ($p = 0.05$), and neck circumference ($p < 0.01$) were predictors of AHI, independently of BMI, but not the presence of upper airway abnormalities ($p = 0.75$). Co-morbidities were frequent (hypertension, 20%; diabetes, 13%; hypercholesterolemia, 27%; depression, 13%; reflux and gastric ulcer, 13%; hypothyroidism, 5%; asthma, 9%), and were related to BMI ($p = 0.02$), nocturnal desaturation time ($p = 0.02$), and African origin ($p = 0.024$).
CONCLUSIONS: In patients aged <40 years and suffering from OSA, disease severity was associated with high BMI, large neck circumference, male sex, and African origin. After adjustment for BMI, African origin, tobacco use, and neck circumference remained predictors of high AHI. Neither upper airway abnormalities nor co-morbidities were found to be a risk factor for higher AHI in this group of young patients from a European city.

PMID: 20680482 [PubMed - indexed for MEDLINE]

368. J Allergy Clin Immunol. 2010 Oct;126(4):836-844.e13. doi: 10.1016/j.jaci.2010.06.009. Epub 2010 Jul 31.

Interferon response factor 3 is essential for house dust mite-induced airway allergy.

Marichal T, Bedoret D, Mesnil C, Pichavant M, Goriely S, Trottein F, Cataldo D, Goldman M, Lekeux P, Bureau F, Desmet CJ.

Laboratory of Cellular and Molecular Physiology, GIGA-Research and Faculty of Veterinary Medicine, University of Liege, Liege, Belgium.

BACKGROUND: Pattern-recognition receptors (PRRs) are critically involved in the pathophysiology of airway allergy, yet most of the signaling pathways downstream of PRRs implicated in allergic airway sensitization remain unknown.
OBJECTIVE: We sought to study the effects of genetic depletion of interferon response factor (IRF) 3 and IRF7, important transcription factors downstream of various PRRs, in a murine model of house dust mite (HDM)-induced allergic asthma.
METHODS: We compared HDM-induced allergic immune responses in IRF3-deficient (IRF3(-/-)), IRF7(-/-), and wild-type mice.
RESULTS: Parameters of airway allergy caused by HDM exposure were strongly attenuated in IRF3(-/-), but not IRF7(-/-), mice compared with those in wild-type mice. Indeed, in HDM-exposed IRF3(-/-) mice HDM-specific T(H)2 cell responses did not develop. This correlated with impaired maturation and migration of IRF3(-/-) lung dendritic cells (DCs) on HDM treatment. Furthermore, adoptive transfer of HDM-loaded DCs indicated that IRF3(-/-) DCs had an intrinsic defect rendering them unable to migrate and to prime HDM-specific T(H)2 responses. Intriguingly, we also show that DC function and allergic airway sensitization in response to HDM were independent of signaling by type I interferons, the main target genes of IRF3.
CONCLUSION: Through its role in DC function, IRF3, mainly known as a central activator of antiviral immunity, is essential for the development of T(H)2-type responses to airway allergens.

Copyright © 2010 American Academy of Allergy, Asthma & Immunology. Published by Mosby, Inc. All rights reserved.

PMID: 20673978 [PubMed - indexed for MEDLINE]

369. BMC Pulm Med. 2010 Jul 28;10:39. doi: 10.1186/1471-2466-10-39.

Insulin modulates cytokine release and selectin expression in the early phase of allergic airway inflammation in diabetic rats.

Martins JO, Campos CA, Cruz JW, Manzolli S, Alves VA, Vianna EO, Jancar S, Sannomiya P.

Institute of Heart InCor, LIM-11, University of São Paulo Medical School, Av Dr Arnaldo, São Paulo 01246903, Brazil. martinsj@usp.br

BACKGROUND: Clinical and experimental data suggest that the inflammatory response is impaired in diabetics and can be modulated by insulin. The present study was undertaken to investigate the role of insulin on the early phase of allergic airway inflammation.
METHODS: Diabetic male Wistar rats (alloxan, 42 mg/Kg, i.v., 10 days) and controls were sensitized by s.c. injection of ovalbumin (OA) in aluminium hydroxide 14 days before OA (1 mg/0.4 mL) or saline intratracheal challenge. The following analyses were performed 6 hours thereafter: a) quantification of interleukin (IL)-1beta, tumor necrosis factor (TNF)-alpha and cytokine-induced neutrophil chemoattractant (CINC)-1 in the bronchoalveolar lavage fluid (BALF) by Enzyme-Linked Immunosorbent Assay, b) expression of E- and P- selectins on lung vessels by immunohistochemistry, and c) inflammatory cell infiltration into the airways and lung parenchyma. NPH insulin (4 IU, s.c.) was given i.v. 2 hours before antigen challenge.
RESULTS: Diabetic rats exhibited significant reduction in the BALF concentrations of IL-1beta (30%) and TNF-alpha (45%), and in the lung expression of P-selectin (30%) compared to non-diabetic animals. This was accompanied by reduced number of neutrophils into the airways and around bronchi and blood vessels. There were no differences in the CINC-1 levels in BALF, and E-selectin expression. Treatment of diabetic rats with NPH insulin, 2 hours before antigen challenge, restored the reduced levels of IL-1beta, TNF-alpha and P-selectin, and neutrophil migration.
CONCLUSION: Data presented suggest that insulin modulates the production/release of TNF-alpha and IL-1beta, the expression of P- and E-selectin, and the associated neutrophil migration into the lungs during the early phase of the allergic inflammatory reaction.

PMCID: PMC2916891
PMID: 20667094 [PubMed - indexed for MEDLINE]

370. Respir Res. 2010 Jul 26;11:102. doi: 10.1186/1465-9921-11-102.

NO2 inhalation induces maturation of pulmonary CD11c+ cells that promote antigenspecific CD4+ T cell polarization.

Hodgkins SR, Ather JL, Paveglio SA, Allard JL, LeClair LA, Suratt BT, Boyson JE, Poynter ME.

The Vermont Lung Center and Department of Medicine, University of Vermont, Burlington, VT 05405, USA.

BACKGROUND: Nitrogen dioxide (NO2) is an air pollutant associated with poor respiratory health, asthma exacerbation, and an increased likelihood of inhalational allergies. NO2 is also produced endogenously in the lung during acute inflammatory responses. NO2 can function as an adjuvant, allowing for allergic sensitization to an innocuous inhaled antigen and the generation of an antigen-specific Th2 immune response manifesting in an allergic asthma phenotype. As CD11c+ antigen presenting cells are considered critical for naïve T cell activation, we investigated the role of CD11c+ cells in NO2-promoted allergic

sensitization.
METHODS: We systemically depleted CD11c+ cells from transgenic mice expressing a simian diphtheria toxin (DT) receptor under of control of the CD11c promoter by administration of DT. Mice were then exposed to 15 ppm NO2 followed by aerosolized ovalbumin to promote allergic sensitization to ovalbumin and were studied after subsequent inhaled ovalbumin challenges for manifestation of allergic airway disease. In addition, pulmonary CD11c+ cells from wildtype mice were studied after exposure to NO2 and ovalbumin for cellular phenotype by flow cytometry and in vitro cytokine production.
RESULTS: Transient depletion of CD11c+ cells during sensitization attenuated airway eosinophilia during allergen challenge and reduced Th2 and Th17 cytokine production. Lung CD11c+ cells from wildtype mice exhibited a significant increase in MHCII, CD40, and OX40L expression 2 hours following NO2 exposure. By 48 hours, CD11c+MHCII+ DCs within the mediastinal lymph node (MLN) expressed maturation markers, including CD80, CD86, and OX40L. CD11c+CD11b- and CD11c+CD11b+ pulmonary cells exposed to NO2 in vivo increased uptake of antigen 2 hours post exposure, with increased ova-Alexa 647+ CD11c+MHCII+ DCs present in MLN from NO2-exposed mice by 48 hours. Co-cultures of ova-specific CD4+ T cells from naïve mice and CD11c+ pulmonary cells from NO2-exposed mice produced IL-1, IL-12p70, and IL-6 in vitro and augmented antigen-induced IL-5 production.
CONCLUSIONS: CD11c+ cells are critical for NO2-promoted allergic sensitization. NO2 exposure causes pulmonary CD11c+ cells to acquire a phenotype capable of increased antigen uptake, migration to the draining lymph node, expression of MHCII and co-stimulatory molecules required to activate naïve T cells, and secretion of polarizing cytokines to shape a Th2/Th17 response.

PMCID: PMC2918560
PMID: 20659336 [PubMed - indexed for MEDLINE]

371. Toxicol Lett. 2010 Sep 1;197(3):211-8.

Differential effects of formaldehyde exposure on the cell influx and vascular permeability in a rat model of allergic lung inflammation.

Lino-dos-Santos-Franco A, Domingos HV, de Oliveira AP, Breithaupt-Faloppa AC, Peron JP, Bolonheis S, Muscará MN, Oliveira-Filho RM, Vargaftig BB, Tavares-de-Lima W.

Department of Pharmacology, Institute of Biomedical Sciences, University of São Paulo, São Paulo, Brazil.

Exposure to air pollutants such as formaldehyde (FA) leads to inflammation, oxidative stress and immune-modulation in the airways and is associated with airway inflammatory disorders such as asthma. The purpose of our study was to investigate the effects of exposure to FA on the allergic lung inflammation. The hypothesized link between reactive oxygen species and the effects of FA was also studied. To do so, male Wistar rats were exposed to FA inhalation (1%, 90 min daily) for 3 days, and subsequently sensitized with ovalbumin (OVA)-alum by subcutaneous route. One week later the rats received another OVA-alum injection by the same route (booster). Two weeks later the rats were challenged with aerosolized OVA. The OVA challenge of rats upon FA exposure induced an elevated release of LTB 4, TXB 2, IL-1 beta, IL-6 and VEGF in lung cells, increased phagocytosis and lung vascular permeability, whereas the cell recruitment into lung was reduced. FA inhalation induced the oxidative burst and the nitration of proteins in the lung. Vitamins C, E and apocynin reduced the levels of LTB 4 in BAL-cultured cells of the FA and FA/OVA groups, but increased the cell influx into the lung of the FA/OVA rats. In OVA-challenged rats, the exposure to FA was associated to a reduced lung endothelial cells expression of intercellular cell adhesion molecule 1 (ICAM-1). In conclusion, our findings suggest that FA down

regulate the cellular migration into the lungs after an allergic challenge and increase the ability of resident lung cells likely macrophages to generate inflammatory mediators, explaining the increased lung vascular permeability. Our data are indicative that the actions of FA involve mechanisms related to endothelium-leukocyte interactions and oxidative stress, as far as the deleterious effects of this air pollutant on airways are concerned.

PMID: 20658762 [PubMed - indexed for MEDLINE]

372. Clin Exp Allergy. 2010 Aug;40(8):1214-21. doi: 10.1111/j.1365-2222.2010.03558.x.

Iloprost has potent anti-inflammatory properties on human monocyte-derived dendritic cells.

Müller T, Dürk T, Blumenthal B, Herouy Y, Sorichter S, Grimm M, Panther E, Cicko S, Norgauer J, Idzko M.

Department of Pneumology, University of Freiburg, Freiburg, Germany.

BACKGROUND: The stable prostaglandin I2 analogue (iloprost) iloprost has been shown to inhibit allergic airway inflammation in mice by modulating the function of myeloid dendritic cells (DCs).
OBJECTIVE: The aim of the current study was to investigate the biological activity of iloprost on human monocyte-derived DCs.
METHODS: I prostanoid (IP) receptor expression was analysed by RT-PCR. Cytokine secretion by DCs and CD4+ T cells was measured by ELISA. The expression of the transcription factor FoxP3 after co-culture of DCs with CD4+ CD45RA+ T cells was analysed by flow cytometry.
RESULTS: Human monocyte-derived DCs were found to express mRNA specific for the PGI2 receptor IP, and stimulation with iloprost resulted in increased cyclic AMP levels in both immature DCs (iDCs) and mature DCs (mDCs). Moreover, iloprost dose dependently inhibited the secretion of TNF-alpha, IL-6, IL-8 and IL-12p70 in mDCs, while it enhanced IL-10 production. Changes in cytokine secretion were paralleled by an altered T-cell priming capacity of DCs: in co-culture experiments of iloprost-treated mDC and naïve CD45RA+ T cells, an induction of regulatory T cells could be observed, as demonstrated by increased intracellular FoxP3 expression and IL-10 production. Additionally, iloprost inhibited the MIP-3beta-induced migration of mDCs.
CONCLUSION: In summary, our results provide evidence that iloprost profoundly affects the function of human myeloid DCs. Therefore, iloprost might also be a new therapeutical option for the treatment of asthma in humans.

PMID: 20649610 [PubMed - indexed for MEDLINE]

373. Acta Med Croatica. 2010 May;64(2):159-61.

[Salt and cancer].

[Article in Croatian]

Strnad M.

Akademija medicinskih znanosti Hrvatske, Zagreb, Hrvatska.
prof.marija.strnad@gmail.com

Besides cardiovascular disease, a high salt intake causes other adverse health effects, i.e., gastric and some other cancers, obesity (risk factor for many cancer sites), Meniere's disease, worsening of renal disease, triggering an

asthma attack, osteoporosis, exacerbation of fluid retention, renal calculi, etc. Diets containing high amounts of food preserved by salting and pickling are associated with an increased risk of cancers of the stomach, nose and throat. Because gastric cancer is still the most common cancer in some countries (especially in Japan), its prevention is one of the most important aspects of cancer control strategy. Observations among Japanese immigrants in the U.S.A. and Brazil based on the geographic differences, the trend in cancer incidence with time, and change in the incidence patterns indicate that gastric cancer is closely associated with dietary factors such as the intake of salt and salted food. The findings of many epidemiological studies suggest that high dietary salt intake is a significant risk factor for gastric cancer and this association was found to be strong in the presence of Helicobacter (H.) pylori infection with atrophic gastritis. A high-salt intake strips the lining of the stomach and may make infection with H. pylori more likely or may exacerbate the infection. Salting, pickling and smoking are traditionally popular ways of preparing food in Japan and some parts of Asia. In addition to salt intake, cigarette smoking and low consumption of fruit and vegetables increase the risk of stomach cancer. However, it is not known whether it is specifically the salt in these foods or a combination of salt and other chemicals that can cause cancer. One study identified a mutagen in nitrite-treated Japanese salted fish, and chemical structure of this mutagen suggests that it is derived from methionine and that salt and nitrite are precursors for its formation. Working under conditions of heat stress greatly increased the workers' salt excretion through perspiration. Workers exposed to heat stress consumed as much as 13-38 g salt daily. As salt strongly enhances and promotes chemical gastric carcinogenesis and H. pylori infection in both humans and animals, there is an association between work, salt intake, and development of stomach cancer. Reducing salt intake, especially during pregnancy, also reduces the risk of developing breast cancer and many other diseases, as well as obesity. The risk of most cancers is reduced by losing weight. The geographical data and analyses currently available suggest that road salt (road salting in winter) may be associated with elevated mortality from cancer of the breast, lung, esophagus, throat, larynx, large intestine, rectum and bladder. There is no available literature on the health impacts of road salt. The cause and effect relationships cannot be established without further studies.

PMID: 20649083 [PubMed - indexed for MEDLINE]

374. J Pharmacol Toxicol Methods. 2011 Jan-Feb;63(1):69-78. doi: 10.1016/j.vascn.2010.05.001. Epub 2010 May 26.

Validating 123I-metaiodobenzylguanidine as a platelet marker for non-invasive imaging in rabbits.

Saihkay HN, Rickards KJ, Page CP, Ballinger JR.

The Sackler Institute of Pulmonary Pharmacology, Waterloo Campus, King's College London, London SE1 9NH, UK. hom.saihkay@kcl.ac.uk

INTRODUCTION: Recent in vitro studies in our laboratory have demonstrated that platelets are necessary for leukocyte recruitment and airway remodelling in models of allergic airway inflammation, and also migrate to lung tissues in response to anti-IgE or relevant allergens in allergic asthma. Non-invasive imaging of platelet migration in vivo would provide a further insight into the roles of platelets in inflammatory diseases such as asthma, and metaiodobenzylguanidine (MIBG) was considered as a suitable platelet marker. METHODS: The kinetics of MIBG uptake into rabbit platelets, the effect of MIBG on platelet function and the effect of platelet activation on MIBG uptake and retention were investigated. MIBG-labelled platelets were administered intravenously into rabbits and the time course of radioactivity in the lung and

blood was monitored as a function of stimulation.
RESULTS: Following a 4h incubation of MIBG in rabbit PRP, a near maximal MIBG uptake (52.4 ± 20.2%) in platelets occurred. This time point was chosen for subsequent in vitro studies. In vitro platelet function studies showed that MIBG has no effect on ADP or PAF-induced platelet aggregation, PAF-induced thromboxane production or fMLP-induced platelet chemotaxis. However, serotonin showed a significant effect on MIBG uptake and retention, but only at high concentrations. Stimulation of rabbit platelets with ADP and PAF caused a significant release of stored MIBG in vitro. Following i.v. administration of MIBG labelled platelets, the response to i.v. ADP and PAF stimulation was small but significant.
DISCUSSION: The release of MIBG from platelets in vivo, particularly following stimulation, leads to high background levels. Therefore, MIBG may have limited utility as a label for imaging platelets in vivo using PET. However, it may be a useful marker in detecting pathological conditions where platelet migration is involved.

Copyright © 2010 Elsevier Inc. All rights reserved.

PMID: 20646985 [PubMed - indexed for MEDLINE]

375. Int J Immunopathol Pharmacol. 2010 Apr-Jun;23(2):437-47.

Role of effector cells (CCR7(-)CD27(-)) and effector-memory cells (CCR7(-)CD27(+)) in drug-induced maculopapular exanthema.

Fernandez TD, Torres MJ, Lopez S, Antunez C, Gomez E, Del Prado MF, Canto G, Blanca M, Mayorga C.

Research Laboratory for Allergic Diseases, Carlos Haya Hospital-Fundacion IMABIS, Malaga, Spain.

Maculopapular exanthema (MPE) induced by drugs is a T-cell mediated reaction and effector cells may play an important role in its development. We assessed the effector and cutaneous homing phenotype in peripheral blood cells from allergic patients after drug stimulation. This study included 10 patients and 10 controls. The effector phenotype (CCR7(-)CD27(+/-)), chemokine receptors (CCR4 and CCR10), and activation (CD25(low)) and regulatory markers (CD25(high)) were measured by flow cytometry in both peripheral blood mononuclear cells (PBMCs) and CD4-T-lymphocytes. Proliferation was determined by 5-(-6)-carboxyfluorescein diacetate succinimidyl ester (CFSE) assay and the migratory capacity by a chemotaxis assay using CCL17 and CCL27. Compared to controls, CCR7(-)CD27(-) cells were increased in patients without (p=0.003) and with drug stimulation (p less than 0.001) and had significantly higher proliferation (p=0.010). CCR10 expression was increased in patients after drug stimulation in total and memory CD27(+) T-cells. Lymphocyte migration with CCL27 was higher in patients with drug stimulation (p=0.048), with a decrease in CCR7(-)CD27(-) (p less than 0.0001) and an increase in CCR7(-)CD27(+) (p=0.017). In patients, CD4-T-lymphocytes were significantly activated after drug stimulation (p less than 0.001). In conclusion, we show that effector memory CD4(+) T-cells (CCR7(-)CD27(+)) respond specifically to the drug responsible for MPE and confirm previous data about the involvement of CCR10 in cell trafficking to the skin.

PMID: 20646339 [PubMed - indexed for MEDLINE]

376. Anti-ICAM-1 antibody-conjugated paramagnetic liposomes.

Zhang H.
In: Molecular Imaging and Contrast Agent Database (MICAD) [Internet]. Bethesda

(MD): National Center for Biotechnology Information (US); 2004-2013.
2008 Jan 03.

ICAM-1 (CD54) is a cell-surface glycoprotein belonging to the immunoglobulin superfamily of cell adhesion molecules (CAMs) (1). Upregulated by inflammatory mediators, ICAM-1 is expressed on various cells including leukocytes, epithelial cells, endothelial cells, and fibroblasts (2). The predominant function of ICAM-1 is the recruitment and trafficking of leukocytes through interactions with leukocyte-expressed integrins (2). Therefore, ICAM-1 plays a critical role in the development of the central nervous system (CNS), in immune and inflammatory responses, and in embryonic development (1). Many cerebrovascular and neurological diseases such as multiple sclerosis and its animal model (i.e., experimental allergic encephalomyelitis (EAE)), Alzheimer’s disease, and Parkinson’s disease have neuroinflammatory responses involving the migration of leukocytes into damaged or infected tissues (3). EAE, which is an ascending encephalomyelitis, is characterized by an intense perivascular inflammatory process in the white matter of the CNS, primarily on the spinal cord, brainstem, and cerebellum, which facilitates the attachment of leukocytes to inflamed endothelium (4). For example, a 10-fold increase in ICAM-1 expression has been found on the endothelium of capillaries and venules throughout the CNS at an early stage of EAE (4). Thus, ICAM-1 has been an attractive target for imaging the early and specific upregulation of vascular CAMs in diseases (4). Amphiphiles are made from molecules containing both a hydrophobic (non-polar tail) and a hydrophilic (polar head) section. Amphiphiles can self-associate into multiple concentric lipid bilayers (called lamellae) in different sizes and geometries (5). Liposomes are generally formed by a variety of modified amphiphiles and can deliver a substantial amount of encapsulated therapeutic or diagnostic agents to targeted sites (6). Traditional liposomes suffer from the drawbacks of fast elimination from blood and easy capture by the reticulo-endothelial system (7). As an alternative to traditional liposomes, paramagnetic polymerized liposomes (PPLs) are composed of a mixture of lipids with different functional groups on the aqueous exposed surface and a polymerizable group (diacetylene) in the lipid tail (8). A typical formulation consists of gadolinium III–diethylenetriamine pentaacetic acid (Gd-DTPA)–based lipids, amphiphilic carrier lipids, and biotinylated amphiphiles (9). PPLs possess increased physical stability, unique spectroscopic properties, and chemical modification durability (9). The diacetylene triple bonds in the PPLs are located in the fatty acyl chains. The polymerization of the triple bonds between lipids can be carried out by simple ultraviolet (UV) radiation to form a polydiacetylene backbone of alternating eneyne groups (8). A short polyethylene glycol (PEG) segment is covalently linked to the polar head of PPLs (9). The hydrophilicity of PEG allows for sterical stabilization of PPLs and protects against interactions with other molecular and biological components in blood stream, such as undesired lipid exchange or lipid fusion (9). Both polymerization and PEGylization prolong the in vivo circulation time of PPLs in blood. For instance, the half-life time of the PPLs is found to be ~19 h in rats (4). Because easy access to bulky water, the attachment of Gd-DTPA chelates on the surface of PPLs allows for greater relaxivity enhancement compared to Gd chelates entrapped in the traditional liposomes. The multiple Gd chelates on the surface of the liposomes further increase the sensitivity to detection by magnetic resonance imaging (MRI). The biotin groups in the PPLs allow for conjugation of biotinylated antibodies via an avidin-biotin linkage procedure (10). Avidin, a tetrameric protein with a molecular mass of 68 KDa, is capable of strongly binding four biotins (Ka = 1.7 × 1015 M-1) (10). Thus, avidins serve as a bridge between a biotinylated antibody against neural CAM (NCAM-1) and a biotinylated group on PPLs to form a NCAM-1–specific MRI contrast agent, anti-NCAM antibody-conjugated PPLs (ACPLs) (4).

PMID: 20641750 [PubMed]

377. Cy5.5-CGRRRQRRKKRG-Labeled T lymphocytes.

Zhang H.
In: Molecular Imaging and Contrast Agent Database (MICAD) [Internet]. Bethesda (MD): National Center for Biotechnology Information (US); 2004-2013.
2008 Jul 10.

T cells are responsible for regulating immune responses and maintaining immune tolerance via recognition of peptide antigens that are bound to human leukocytes (1). Some T cells possess autoimmunity or self-tolerance through recognition of self-antigens. Loss of this required self-tolerance can result in an autoimmune disorder. For instance, experimental allergic encephalomyelitis (EAE) is one of immune-mediated diseases in which immune cells become reactive against myelins, which leads to the destruction of myelin sheets (2). EAE can be induced in rodents by adoptive transfer of CD4+ T cells specific to myelin basic protein (MBP), an autoantigen of myelin (3). As an animal model for the inflammatory disease, EAE can reproduce many clinical neuropathological and immunological aspects of multiple sclerosis (MS) and thus has been widely used in therapeutic development for MS (4). The evolution of inflammatory lesions in EAE involves several steps (1, 3). After intravenous administration, the injected MBP-specific T cells cross the blood–brain barrier to recognize the T cell antigen located on perivascular microglia. This antigen-specific interaction produces a plethora of inflammatory cytokines and mediators, leading to amplification of the inflammatory reaction. The blood–brain barrier then opens to allow antigen-independent recruitment of various mononuclear inflammatory cells into the central nervous system, including additional T cells, macrophages, and granulocytes. Consequently, the inflammatory lesions evolve into severe neurological dysfunctions such as ascending paraparesis and paralysis. Because the activated T cells are involved in the entire process, the trafficking of the activated MBP-specific T cells in EAE can reflect their immune activity in every evolutionary phase (2). Labeling T cells with imaging probes will allow non-invasive tracking of the migration of T cells in vivo. The imaging probes can be internalized into cells with the use of cell-penetrating peptides (CPP) as vector/nuclear delivery vehicles of conjugated cargo (5). In general, CPP comprise a protein-transduction domain formed by small peptides (<20 amino acids) for cell membrane translocation. A commonly used CPP is a peptide truncated from the 86-mer transactivating transcriptional activator (Tat) protein in human immunodeficiency virus type 1 (HIV) (6). In particular, Tat(47-57) is widely used in cellular delivery of peptides, proteins, genetic material, antibodies, nanoparticles, and liposomes (5). Tat(47-57) contains an α-helical structure with a charged face formed by six arginine and two lysine residues (6). Tat-mediated internalization consists of multiple steps: the binding of Tat to the cell surface, stimulation of macropinocytotic uptake of Tat and transfer into macropinosomes, and finally endosomal escape into the cytoplasm (7). A T lymphocyte is labeled with Cy5.5-Cys-Gly-Arg-Arg-Arg-Gln-Arg-Arg-Lys-Lys-Arg-Gly (CGRRRQRRKKRG) (Cy5.5-Tat-T cell) for optical imaging (2). This agent contains a near-infrared fluorescence (NIRF) dye shuttled across the cell membrane of T cells via Tat(48-57). The fluorescence probe Cy5.5 is a cyanine dye consisting of two quaternized heteroaromatic bases (A and A’) joined by a polymethine chain with five carbons (8), and it is bound to cysteine-terminated Tat(48-57) via a maleimide group as a spacer (2). Cy5.5 has a delocalized positive charge in its chromophore and possesses high quantum yield (0.22 at 678 nm), good chemical stability, easy conjugation, and high sensitivity (mole extinction coefficient ~250,000 mol/cm) (9, 10). The excitation/emission wavelength is 674/692 nm for Cy5.5, where hemoglobin and water have their lowest absorption coefficient. The labeled cargo Cy5.5-Tat is translocated through the plasma membrane of cells by an energy-dependent process involving endocytosis (2). The produced Cy5.5-Tat-T cells can be adoptively transferred to EAE rats for visualization and quantification of T cells activity in the early inflammation stages of EAE.

PMID: 20641549 [PubMed]

378. Perfluoro-15-crown-5 ether-labeled dendritic cells.

Zhang H.
In: Molecular Imaging and Contrast Agent Database (MICAD) [Internet]. Bethesda (MD): National Center for Biotechnology Information (US); 2004-2013.
2008 Aug 01.

Dendritic cells (DCs), known as antigen-presenting cells, are found in almost all peripheral tissues and in primary/secondary lymphoid organs (1). DCs are the initiator and modulator in the adaptive immune responses against bacteria, viruses, allergens, and tumor antigens (2). DCs in peripheral tissues are responsible for the capture of antigens (3). In the absence of inflammation, DCs remain in an immature state. The captured antigens are transported with DCs to the lymph node, but no co-stimulatory activation to T cells occurs. In the presence of inflammation, numerous mature DCs migrate to the draining lymph nodes. During migration, the antigens are processed into small peptides bound to the major histocompatibility complex (MHC) on the surface of the DCs. At the lymph nodes, mature DCs present the MHC-peptide complex to naïve T cells (CD4+ T helper cells and cytolytic CD8+ T cells) to activate them. After activation, a series of immune responses are completed through the interactions of T cells with other cells and molecules such as B cells for antibody formation, macrophages for cytokine releases, and targets for lysis (1). Behaving as mobile sentinels, DCs bring antigens to T cells and express co-stimulators to induce immunity. DCs have been used in many clinical trials to treat cancers and immunological disorders (4). Because the migratory properties of DCs are directly related to their function (5), non-invasive tracking of DCs becomes very important to clinic applications (2). Being the only stable isotope of fluorine, with a natural abundance of ~100%, 19F has a nuclear spin 1/2 with a large gyromagnetic ratio (γ ~40.05 MHz/T) (6). The small γ difference between the 19F and 1H (~6%) allows the use of existing proton nuclear magnetic resonance (NMR) instrumentation with minor adjustments to detect fluorinated species at high sensitivity (~83% relative to 1H). Endogenous fluorine in vivo is found primarily in bones and teeth as solid fluorides, which have very short T2 relaxation times and result in an undetectable signal with NMR imaging. Therefore, exogenously administered fluorinated tracers can be used to track various biological processes in vivo. For example, perfluorocarbons (PFCs) are used to measure oxygen tensions in tissues and tumors (7). The lack of background 19F signal is advantageous in in vivo applications, but additional 1H images are required to provide anatomic interpretations. PFCs are extremely hydrophobic and do not dissolve in blood directly; they normally are formulated as biocompatible emulsions for intravenous administration (6). Inside the body, the PFC particles are cleared from circulation by phagocytes/macrophages or by respiration within several hours or days, depending on the administered dose, particle size, and PFCs (8). Many commercial PFC emulsions have been found to be nontoxic or do not cause any health problems other than tissue swelling (6). Perfluoro-15-crown-5 ether (perfluoropolyether, PFPE), a commonly used PFC, contains 20 equivalent 19F spins that generate a single resonance (-92.5 ppm) in NMR imaging (9). This singlet simplifies its images such that no chemical shift–induced artifact is expected. PFPE can be emulsified to form particles of ~100–200 nm in diameter, allowing for cellular uptake via endocytosis (4). DCs are labeled with PFPE (PFPE-DCs) for 19F magnetic resonance imaging (MRI).

PMID: 20641395 [PubMed]

379. Ann Allergy Asthma Immunol. 2010 Jul;105(1):96-7. doi: 10.1016/j.anai.2010.05.003.

The genetic association of the FPRL1 promoter polymorphism with chronic urticaria in a Korean population.

Yang EM, Kim SH, Kim NH, Park HS.

PMID: 20642210 [PubMed - indexed for MEDLINE]

380. Phytomedicine. 2011 Jan 15;18(2-3):230-3. doi: 10.1016/j.phymed.2010.05.012. Epub 2010 Jul 16.

A comparative chemical and pharmacological study of standardized extracts and vanillic acid from wild and cultivated Amburana cearensis A.C. Smith.

Leal LK, Pierdoná TM, Góes JG, Fonsêca KS, Canuto KM, Silveira ER, Bezerra AM, Viana GS.

Department of Pharmacy, Federal University of Ceará, Rua Capitão Francisco Pedro, 1210, 60430-370 Fortaleza, Brazil. kalyne@ufc.br

The objectives of this work were to carry out a comparative chemical study and to evaluate the antinociceptive and anti-inflammatory activities of ethanol extracts (EtOHE) and vanilic acid (VA) from cultivated and wild Amburana cearensis A.C. Smith (Fabaceae), an endangered species used in Northeast Brazil for the treatment of asthma. The HPLC analysis of EtOHE, showed that coumarin (CM) and VA were the major constituents from the cultivated plant, while in the extract from the wild plant the major constituents were amburoside A (AMB) and CM. Pharmacological tests were performed with male Swiss mice or male Wistar rats acutely administered with 100-400mg/kg, p.o. of EtOHEs or 12.5-50mg/kg, p.o. of VA. EtOHEs from A. cearensis with 4, 7 or 9 months of cultivation significantly inhibited, from 32 to 64%, both phases of the formalin test in mice. Similar results were observed with the EtOHE from the wild species. VA significantly reduced both phases of the formalin test. This effect was partially reversed by naloxone. EtOHE from cultivated or wild A. cearensis inhibited the carrageenan (Cg)-induced mice paw edema. Furthermore, VA inhibited the paw edema and the leukocyte migration in rat peritoneal cavity induced by Cg. On the other hand, it did not inhibit the edema and the increase of vascular permeability induced by dextran in the rat paw. All together, these results indicate that the EtOHE from cultivated A. cearensis exhibit similar chemical and pharmacological profiles, as related to the wild plant. VA is, at least partially, responsible for these pharmacological effects. Its antinociceptive effect occurs by a mechanism partly dependent upon the opioid system, while the anti-inflammatory action was manifested in inflammatory processes dependent on polymorphonuclear cells and are probably related to the VA inhibition of cytokines as observed by others.

2010 Elsevier GmbH. All rights reserved.

PMID: 20638258 [PubMed - indexed for MEDLINE]

381. Clin Chim Acta. 2010 Nov 11;411(21-22):1570-9. doi: 10.1016/j.cca.2010.07.006. Epub 2010 Jul 13.

MCP-1: chemoattractant with a role beyond immunity: a review.

Yadav A, Saini V, Arora S.

Department of Biochemistry, Lady Hardinge Medical College, New Delhi-110001, India.

BACKGROUND: Monocyte Chemoattractant Protein (MCP)-1, a potent monocyte attractant, is a member of the CC chemokine subfamily. MCP-1 exerts its effects through binding to G-protein-coupled receptors on the surface of leukocytes targeted for activation and migration. Role of MCP-1 and its receptor CCR2 in monocyte recruitment during infection or under other inflammatory conditions is well known.
METHOD: A comprehensive literature search was conducted from the websites of the National Library of Medicine (http://www.ncbl.nlm.nih.gov) and Pubmed Central, the US National Library of Medicine's digital archive of life sciences literature (http://www.pubmedcentral.nih.gov/). The data was assessed from books and journals that published relevant articles in this field.
RESULT: Recent and ongoing research indicates the role of MCP-1 in various allergic conditions, immunodeficiency diseases, bone remodelling, and permeability of blood - brain barrier, atherosclerosis, nephropathies and tumors.
CONCLUSION: MCP-1 plays an important role in pathogenesis of various disease states and hence MCP-1 inhibition may have beneficial effects in such conditions.

2010 Elsevier B.V. All rights reserved.

PMID: 20633546 [PubMed - indexed for MEDLINE]

382. J Biol Chem. 2010 Jul 14. [Epub ahead of print]

TNF{alpha} and IFN{gamma} synergistically enhance transcriptional activation of CXCL10 in human airway smooth muscle cells via STAT-1, NF-{kappa}B and the transcriptional coactivator CREB-binding protein.

Clarke DL, Clifford RL, Jindarat S, Proud D, Pang L, Belvisi MG, Knox AJ.

University of Nottingham, United Kingdom;

Asthmatic airway smooth muscle (ASM) express interferon-gamma-inducible protein-10 (CXCL10), a chemokine known to mediate mast cell migration into ASM bundles that has been reported in the airways of asthmatic patients. CXCL10 is elevated in patients suffering from viral exacerbations of asthma and in patients with chronic obstructive pulmonary disease (COPD), diseases in which corticosteroids are largely ineffective. IFNgamma and TNFalpha synergistically induce CXCL10 release from HASM cells in a steroid-insensitive manner, via an as yet undefined mechanism. We report that TNFalpha activates the classical NF-kappaB pathway whereas IFNgamma activates JAK2/STAT-1alpha and that inhibition of the JAK/STAT pathway is more effective in abrogating CXCL10 release than the steroid fluticasone. The synergy observed with TNFalpha and IFNgamma together, however, did not lie at the level of NF-kappaB activation, STAT-1alpha phosphorylation or in vivo binding of these transcription factors to the CXCL10 promoter. Stimulation of HASM cells with TNFalpha and IFNgamma induced histone H4 but not histone H3 acetylation at the CXCL10 promoter, although no synergism was observed when both cytokines were combined. We show, however, that TNFalpha and IFNgamma exert a synergistic effect on the recruitment of CREB-binding Protein (CBP) to the CXCL10 which is accompanied by increased RNA polymerase II. Our results provide evidence that synergism between TNFalpha and IFNgamma lies at the level of coactivator recruitment in human ASM and suggest that inhibition of JAK/STAT signalling may be of therapeutic benefit in steroid-resistant airway disease.

PMID: 20630866 [PubMed - as supplied by publisher]

383. Phytother Res. 2011 Jan;25(1):59-66. doi: 10.1002/ptr.3220.

Suppression of ovalbumin-induced airway inflammatory responses in a mouse model of asthma by Mimosa pudica extract.

Yang EJ, Lee JS, Yun CY, Ryang YS, Kim JB, Kim IS.

Department of Biomedical Laboratory Science, School of Medicine, Eulji University, Jung-gu, Daejeon, Republic of Korea.

Asthma is an inflammatory airway disease. The pathogenic mechanisms of asthma include the infiltration of leukocytes and release of cytokines. Mimosa pudica (Mp) has been used traditionally for the treatment of insomnia, diarrhea and inflammatory diseases. Although Mp extract has various therapeutic properties, the effect of this extract on asthma has not yet been reported. This study investigated the suppressive effects of Mp extract on asthmatic responses both in vitro and in vivo. Mp extract was acquired from dried and powdered whole plants of M. pudica using 80% ethanol. BALB/c mice were used for the mouse model of asthma induced by ovalbumin. Mp extract significantly inhibited the HMC-1 cell migration induced by stem cell factor and blocked the release of monocyte chemotactic protein-1 (MCP-1) and interleukin-6 (IL-6) in EoL-1 cells. Leukocytosis, eosinophilia and mucus hypersecretion in asthmatic lung were significantly suppressed by Mp extract. The release of ovalbumin-specific IgE in bronchoalveolar lavage fluid and serum was also decreased. Mp extract treatment resulted in no liver cytotoxicity. The Mp extract has inhibitory properties on asthma and may be used as a potent therapeutic agent for allergic lung inflammation.

Copyright © 2010 John Wiley & Sons, Ltd.

PMID: 20623591 [PubMed - indexed for MEDLINE]

384. Scand J Immunol. 2010 Aug;72(2):118-27. doi: 10.1111/j.1365-3083.2010.02419.x.

Natural killer cells accumulate in lung-draining lymph nodes and regulate airway eosinophilia in a murine model of asthma.

Ple C, Barrier M, Amniai L, Marquillies P, Bertout J, Tsicopoulos A, Walzer T, Lassalle P, Duez C.

INSERM, Institut National de la Santé et de la Recherche Médicale U774, Lille, France.

Increasing evidence suggests a key role for the innate immune system in asthma development. Although the role of Natural Killer (NK) cells in allergic asthma is poorly known, modifications of the blood NK cell populations have been found in asthmatic and/or allergic patients. Their repartition and activation status in the inflammatory (lungs) and the regulatory (draining lymph nodes) sites of the allergic reaction is unknown. The aim of our study was to monitor NK cell migration pattern and activation status and to investigate the consequences of NK cell depletion during allergic airway reaction in a mouse model. Ovalbumin sensitization and challenges of BALB/cByJ mice had no effect on the total number of lung NK cells but significantly decreased the number of most mature NK cells and increased the level of the activation marker CD86. In the lung-draining mediastinal lymph nodes, ovalbumin sensitization and challenges led to increased number of NK cells, and more precisely, immature NK cells and increased expression of CD86. Ovalbumin-sensitized mice also exhibited increased percentage of proliferating NK cells in lung-draining mediastinal lymph nodes. Anti-ASGM1 antibody treatment depleted most NK cells and decreased bronchoalveolar lavage eosinophilia but did not modify airway responsiveness. Altogether, our study

shows that pulmonary allergic sensitization induces modification in the NK cell compartment at the inflammatory and regulatory sites and suggests that NK cells may participate in the regulation of the asthmatic response and, more particularly, to the allergic airway eosinophilia.

PMID: 20618770 [PubMed - indexed for MEDLINE]

385. Rev Esp Enferm Dig. 2010 Jul;102(7):421-5.

Montelukast treatment (cysteinyl leukotriene receptor antagonist) in a model of food allergy: modifications in lymphatic cell population from rectal mucosa.

Vinuesa M, Bassan N, Cases AI, Krumrik G.

Histology and Embriology Department, School of Medical Sciences, Universidad Nacional de Rosario, Rosario, Argentina. vinuesamiguel@gmail.com

OBJECTIVE: The aim is to determine immunopathological modifications in rectal mucosa from rabbits after local challenge in ovalbumin (OVA) sensitized animals previously treated with montelukast.
MATERIAL AND METHODS: Experimental design: thirty two rabbits divided into four groups: G1: normal; G2: subcutaneously OVA sensitized; G3: sensitized, locally OVA challenged and sampled 4 hours after challenge; and G4: sensitized, locally OVA challenged and treated 4 hours before challenge with montelukast (0.15 mg/kg). Specific anti-OVA IgE levels were evaluated by passive cutaneous anaphylaxis test (PCA). In each group 200 high microscopical power fields (HPF) were counted. Results were expressed as arithmetic mean and SE. Anti -CD4, CD5, micro chain monoclonal antibodies were used. Avidin biotin horseradish peroxidase system was used.
RESULTS: CD 4: G1: 8.3 +/- 0.06; G2: 13.4 +/- 0.08, G3: 8.25 +/- 0.06, G4: 11.8 +/- 0.02. CD 5: G1: 7.3 +/- 0.05; G2: 9.4 +/- 0.05, G3: 11.3 +/- 0.06, G4: 8.1 +/- 0.06. mu chain: G1: 10.4 +/- 0.06; G2: 3.8 +/- 0.02, G3: 6.0 +/- 0.10, G4: 2.2 +/- 0.10. In all cases, experimental groups (G3 vs. G4) presented statistical significant differences (p < 0.05). CD4+, CD5+ cells and mu chain+ decrease in experimental group (G4), probably due to lymphocyte migration inhibition to challenged mucosa. mu chain+ cell decrease could be based on B cell activation and expression of different surface immunoglobulins. Cells expressing mu chain decreased in G2 and G3 likely due to activation of B cells and subsequent expression of other immunoglobulin chains in cell surface.
CONCLUSIONS: We conclude that obtained data are important to elucidate immunopathology of local anaphylactic reaction in rectal mucosa from systemic sensitized animals after treatment with montelukast.

PMID: 20617862 [PubMed - indexed for MEDLINE]

386. Eur Respir J. 2011 Jan;37(1):173-82. doi: 10.1183/09031936.00008109. Epub 2010 Jul 1.

Collagen remodelling by airway smooth muscle is resistant to steroids and β_2-agonists.

Bourke JE, Li X, Foster SR, Wee E, Dagher H, Ziogas J, Harris T, Bonacci JV, Stewart AG.

Dept of Pharmacology, University of Melbourne, Victoria, Australia. janeew@unimelb.edu.au

Bi-directional interactions between airway smooth muscle (ASM) and the altered

extracellular matrix (ECM) may influence airway wall remodelling and ASM function in asthma. We have investigated the capacity of cultured human ASM to reorganise the structure of three-dimensional collagen gels and the effects of endothelin (ET)-1 and agents used to treat asthma. Human ASM cells were cast in type I collagen gels. Reductions in gel area over 72 h were determined in the absence and presence of ET-1 and potential inhibitors, steroids and β_2-adrenoceptor agonists. Changes in gel wet weights and hydroxyproline content were measured and ASM gel morphology was examined by scanning electron microscopy. Cell density-dependent reductions in gel area were augmented by ET-1, mediated via ET(A) receptors. This process was not associated with ASM contraction or proliferation, but was consistent with ASM tractional remodelling and migration leading to collagen condensation rather than collagen degradation within gels. The collagen remodelling by ASM was unaffected by salbutamol and/or budesonide. This study demonstrates an additional potential role for ASM in ECM regulation and dysregulation in airways disease that is resistant to steroids and β_2-adrenoceptor agonists. Therapy-resistant collagen condensation within ASM bundles may facilitate ECM-ASM interactions and contribute to increased internal airways resistance.

PMID: 20595143 [PubMed - indexed for MEDLINE]

387. J Allergy Clin Immunol. 2010 Sep;126(3):638-45.e1. doi: 10.1016/j.jaci.2010.04.039. Epub 2010 Jun 26.

Phl p 5 resorption in human oral mucosa leads to dose-dependent and time-dependent allergen binding by oral mucosal Langerhans cells, attenuates their maturation, and enhances their migratory and TGF-beta1 and IL-10-producing properties.

Allam JP, Würtzen PA, Reinartz M, Winter J, Vrtala S, Chen KW, Valenta R, Wenghoefer M, Appel T, Gros E, Niederhagen B, Bieber T, Lund K, Novak N.

Department of Dermatology and Allergy, University of Bonn, Bonn, Germany.

Comment in
    J Allergy Clin Immunol. 2010 Sep;126(3):646-7.

BACKGROUND: Sublingual immunotherapy (SLIT) is safe and effective as treatment of allergic rhinitis and mild asthma. Oral mucosal Langerhans cells (oLCs) play a central role. However, little is known about allergen binding by oLCs during mucosal allergen resorption and its impact on oLC functions.
OBJECTIVE: Binding of Phl p 5 to oLCs was studied in a standardized ex vivo model to investigate mechanisms important for SLIT.
METHODS: Human oral mucosal biopsies were incubated with the grass pollen allergen Phl p 5. Migration, binding of Phl p 5, phenotype and cytokine production, and T-cell priming of Phl p 5-binding oLCs were analyzed.
RESULTS: Significant uptake required more than 5 minutes, and dose-dependent binding of Phl p 5 to oLCs was saturated at 100 microg/mL Phl p 5. Furthermore, Phl p 5 significantly increased the migratory capacity of oLCs but attenuated their maturation and strongly promoted the release of TGF-beta1 and IL-10 by oLCs themselves as well as by cocultured T cells.
CONCLUSION: Oral mucosal Langerhans cells bind Phlp5 in a dose-dependent and time-dependent manner, leading to an increased production of tolerogenic cytokines and an enhanced migratory capacity but decelerated maturation of oLCs.

Copyright (c) 2010 American Academy of Allergy, Asthma & Immunology. Published by Mosby, Inc. All rights reserved.

PMID: 20584546 [PubMed - indexed for MEDLINE]

388. Acta Pharmacol Sin. 2010 Jul;31(7):849-54. doi: 10.1038/aps.2010.63. Epub 2010 Jun 28.

Mast cell degranulation induced by chlorogenic acid.

Huang FH, Zhang XY, Zhang LY, Li Q, Ni B, Zheng XL, Chen AJ.

National Drug Screening Center, China Pharmaceutical University, Nanjing, China.

AIM: To investigate the mechanism of chlorogenic acid (CA)-induced anaphylactoid reactions.
METHODS: Degranulation of peritoneal mast cells was assayed by using alcian blue staining in guinea pigs, and the degranulation index (DI) was calculated. CA-induced degranulation of RBL-2H3 cells was also observed and assayed using light microscopy, transmission electron microscopy, flow cytometry, and beta-hexosaminidase release.
RESULTS: CA 0.2, 1.0, and 5.0 mmol/L was able to promote degranulation of peritoneal mast cells in guinea pigs in vitro, but it did not increase the degranulation of peritoneal mast cells in CA-sensitized guinea pigs compared with control ($P>0.05$). Treatment with CA 0.2, 1.0, and 5.0 mmol/L for 30, 60, and 120 min induced degranulation in RBL-2H3 cells in a dose- and time-dependent manner ($P<0.01$). Under transmission electron microscope typical characteristics of degranulation, including migration of granular vesicles toward the plasma membrane and integration combined with exocytosis, were observed, after CA or C48/80 treatment. Fluorescent microscopy and flow cytometric analysis showed that CA induced concentration-dependent translocation of phosphatidylserine in RBL-2H3 cells. beta-hexosaminidase release in RBL-2H3 cells was significantly increased after incubation with 1 mmol/L CA for 60 min and 5 mmol/L CA for 30 min ($P<0.01$).
CONCLUSION: CA induces degranulation of peritoneal mast cells and RBL-2H3 cells in guinea pigs, which might be one of the mechanisms of the generation of anaphylactoid reactions induced by CA.

PMID: 20581858 [PubMed - indexed for MEDLINE]

389. Morfologiia. 2010;137(2):44-7.

[Changes in mast cell composition in the hamster duodenum in experimental diphyllobothriosis].

[Article in Russian]

Kutyrev IA, Pronina SV.

The morphometric investigation was performed to study the reaction of mast cells (MC) in mucosal lamina propria of hamster duodenum to experimental infection with tapeworm Diphyllobothrium dendriticum. The invasion was found to result in a significant increase of MC cells absolute content, changes in the proportions of their functional groups, increased anaphylactic MC degranulation, enhanced MC precursor migration and their accelerated differentiation.

PMID: 20572394 [PubMed - indexed for MEDLINE]

390. Pediatr Allergy Immunol. 2010 Nov;21(7):1072-5. doi: 10.1111/j.1399-3038.2010.01017.x.

Serum carotenoids and atopy among children of different ethnic origin living in

Germany.

Rühl R, Taner C, Schweigert FJ, Wahn U, Grüber C.

Department of Biochemistry and Molecular Biology, University of Debrecen, Debrecen, Hungary. rruehl@dote.hu

The manifestation of atopy in early life is thought to be influenced by the diet. We hypothesized that the previously reported lower prevalence of atopy among Turkish immigrant children in Germany might be related to a different pattern of serum carotenoids. Serum carotenoid concentrations were measured in pre-school children of different ethnic origin from Berlin, D. German children (D, N = 49) were compared to Turkish children with well (TR-D, N = 32) or weak cultural adaptation (TR-TR, N = 41). Serum levels of pro-vitamin A carotenoids (α- and β-carotene, β-cryptoxanthin) and non-pro-vitamin A carotenoids (lutein, zeaxanthin, lycopene) were measured by high performance liquid chromatography. Serum IgE to common inhalant allergens was measured by immunoassay. Median levels of pro-vitamin A carotenoids were lower in Turkish children if compared to German children: D 135 μg/L, TR-D 100 μg/L ($p = 0.025$), TR-TR 82 μg/L ($p = 0.001$). By contrast, median levels of non-pro-vitamin A carotenoids were not higher in German children. The ratio of pro-vitamin A to non-pro-vitamin A carotenoid median levels was highest among D (2.05), lower among TR-D (1.32; $p = 0.001$) and lowest among TR-TR (1.26; $p < 0.001$)). A higher ratio was not significantly associated with atopy (atopic 1.79, non-atopic 1.36; $p = 0.067$). Pro-vitamin A carotenoids are higher in children originating from a cultural population with a higher prevalence of atopy, but atopy seems not to be directly related to the current carotenoid serum levels in children at school age. The distinct pattern of carotenoid levels among Turkish migrant and German children indicates changed nutrition patterns with acculturation.

© 2010 John Wiley & Sons A/S.

PMID: 20561236 [PubMed - indexed for MEDLINE]

391. J Appl Physiol. 2010 Sep;109(3):758-67. doi: 10.1152/japplphysiol.01086.2009. Epub 2010 Jun 17.

Circulating platelet-neutrophil complexes are important for subsequent neutrophil activation and migration.

Kornerup KN, Salmon GP, Pitchford SC, Liu WL, Page CP.

Sackler Institute of Pulmonary Pharmacology and Therapeutics, Division of Pharmaceutical Sciences, School of Biomedical and Health Sciences, King's College, London, UK. k.n.kornerup@qmul.ac.uk

Previous studies in our laboratory have shown that platelets are essential for the migration of eosinophils into the lungs of allergic mice, and that this is dependent on the functional expression of platelet P-selectin. We sought to investigate whether the same is true for nonallergic, acute inflammatory stimuli administered to distinct anatomic compartments. Neutrophil trafficking was induced in two models, namely zymosan-induced peritonitis and LPS-induced lung inflammation, and the platelet dependence of these responses investigated utilizing mice rendered thrombocytopenic. The relative contribution of selectins was also investigated. The results presented herein clearly show that platelet depletion (>90%) significantly inhibits neutrophil recruitment in both models. In addition, we show that P-selectin glycoprotein ligand-1, but not P-selectin, is essential for neutrophil recruitment in mice in vivo, thus suggesting the existence of different regulatory mechanisms for the recruitment of leukocyte

subsets in response to allergic and nonallergic stimuli. Further studies in human blood demonstrate that low-dose prothrombotic and pro-inflammatory stimuli (CCL17 or CCL22) synergize to induce platelet and neutrophil activation, as well as the formation of platelet-neutrophil conjugates. We conclude that adhesion between platelets and neutrophils in vivo is an important event in acute inflammatory responses. Targeting this interaction may be a successful strategy for inflammatory conditions where current therapy fails to provide adequate treatment.

PMID: 20558756 [PubMed - indexed for MEDLINE]

392. J Allergy Clin Immunol. 2010 Jul;126(1):150-7.e2. doi: 10.1016/j.jaci.2010.04.015. Epub 2010 Jun 11.

A single nucleotide polymorphism in the CCR3 gene ablates receptor export to the plasma membrane.

Wise EL, Bonner KT, Williams TJ, Pease JE.

Leukocyte Biology Section, National Heart and Lung Institute, Imperial College London, London, United Kingdom.

Comment in
    J Allergy Clin Immunol. 2010 Jul;126(1):158-9.

BACKGROUND: The chemokine receptor CCR3 orchestrates the migration of eosinophils, basophils, T(H)2 lymphocytes, and mast cells during the allergic response, with CCR3 blockade a potential means of therapeutic intervention. Non-synonymous single nucleotide polymorphisms (SNPs) within the ccr3 gene have previously been described, with little information regarding their effects on CCR3 function.
OBJECTIVE: To characterize the effects of nonsynonymous SNPs within the ccr3 gene.
METHODS: Site-directed mutagenesis was used to generate N-terminally tagged mutant CCR3 constructs corresponding to reported SNPs. Cell transfectants expressing either wild-type or mutant CCR3 were studied by flow cytometry, Western blotting, and confocal microscopy and examined for their ability to migrate to the CC chemokine ligand CCL11/eotaxin.
RESULTS: An L324P mutant CCR3 protein corresponding to the previously identified T971C SNP was not expressed at the cell surface, and cells remained unresponsive to CCL11 in chemotaxis assays. Confocal microscopy confirmed that L324P-CCR3 had a predominantly intracellular distribution compared with wild-type CCR3. A L324A variant of CCR3 had an identical phenotype to the L324P mutant, suggesting that L324 per se is critical for successful trafficking of nascent CCR3 to the cell membrane. The processes involved appear to be specific for CCR3, because an identical mutation in the homologous receptor CCR1 had minor effects.
CONCLUSION: Trafficking to the cell surface of nascent CCR3 is critically dependent on a C-terminal leucine residue, suggestive of specific mechanisms for CCR3 export. Manipulation of these mechanisms may suggest novel means of antagonizing CCR3 function in the treatment of allergy.

Copyright 2010 American Academy of Allergy, Asthma & Immunology. Published by Mosby, Inc. All rights reserved.

PMID: 20541248 [PubMed - indexed for MEDLINE]

393. Respir Res. 2010 Jun 11;11:75. doi: 10.1186/1465-9921-11-75.

Resolution of cell-mediated airways diseases.

Persson CG, Uller L.

Department of Clinical Pharmacology, Lund University Hospital, S-22185 Lund, Sweden. carl.persson@med.lu.se

"Inflammation resolution" has of late become a topical research area. Activation of resolution phase mechanisms, involving select post-transcriptional regulons, transcription factors, 'autacoids', and cell phenotypes, is now considered to resolve inflammatory diseases. Critical to this discourse on resolution is the elimination of inflammatory cells through apoptosis and phagocytosis. For major inflammatory diseases such as asthma and COPD we propose an alternative path to apoptosis for cell elimination. We argue that transepithelial migration of airway wall leukocytes, followed by mucociliary clearance, efficiently and non-injuriously eliminates pro-inflammatory cells from diseased airway tissues. First, it seems clear that numerous infiltrated granulocytes and lymphocytes can be speedily transmitted into the airway lumen without harming the epithelial barrier. Then there are a wide range of 'unexpected' findings demonstrating that clinical improvement of asthma and COPD is not only associated with decreasing numbers of airway wall inflammatory cells but also with increasing numbers of these cells in the airway lumen. Finally, effects of inhibition of transepithelial migration support the present hypothesis. Airway inflammatory processes have thus been much aggravated when transepithelial exit of leukocytes has been inhibited. In conclusion, the present hypothesis highlights risks involved in drug-induced inhibition of transepithelial migration of airway wall leukocytes. It helps interpretation of common airway lumen data, and suggests approaches to treat cell-mediated airway inflammation.

PMCID: PMC2900258
PMID: 20540713 [PubMed - indexed for MEDLINE]

394. Am J Respir Cell Mol Biol. 2011 Apr;44(4):509-16. doi: 10.1165/rcmb.2010-0004OC. Epub 2010 Jun 10.

Cross-talk between macrophage migration inhibitory factor and eotaxin in allergic eosinophil activation forms leukotriene C_4-synthesizing lipid bodies.

Vieira-de-Abreu A, Calheiros AS, Mesquita-Santos FP, Magalhães ES, Mourão-Sá D, Castro-Faria-Neto HC, Bozza MT, Bandeira-Melo C, Bozza PT.

Laboratório de Imunofarmacologia, Instituto Oswaldo Cruz, Fundação Oswaldo Cruz, Manguinhos, Rio de Janeiro, RJ, 21045-900 Brazil.

Recent studies have demonstrated an essential and nonredundant role for macrophage migration inhibitory factor (MIF) in asthma pathogenesis. Here we investigate the mechanisms involved in MIF-induced eosinophil activation. By using a model of allergic pulmonary inflammation, we observed that allergen challenge-elicited eosinophil influx, lipid body (also known as lipid droplets) biogenesis, and leukotriene (LT) C_4 synthesis are markedly reduced in Mif(-/-) compared with wild-type mice. Likewise, in vivo administration of MIF induced formation of new lipid bodies within eosinophils recruited to the inflammatory reaction site that corresponded to the intracellular compartment of increased LTC_4 synthesis. MIF-mediated eosinophil activation was at least in part due to a direct effect on eosinophils, because MIF was able to elicit lipid body assembly within human eosinophils in vitro, a phenomenon that was blocked by neutralization of the MIF receptor, CD74. MIF-induced eosinophil lipid body biogenesis, both in vivo and in vitro, was dependent on the cooperation of MIF and eotaxin acting in a positive-feedback loop, because anti-eotaxin and

anti-CCR3 antibodies inhibit MIF-elicited lipid body formation, whereas eotaxin-induced lipid body formation is affected by anti-CD74 and MIF expression deficiency. Therefore, allergy-elicited inflammatory MIF acts in concert with eotaxin as a key activator of eosinophils to form LTC_4-synthesizing lipid bodies via cross-talk between CD74 and CCR3. Due to the effect of MIF on eosinophils, strategies that inhibit MIF activity might be of therapeutic value in controlling allergic inflammation.

PMID: 20539011 [PubMed - indexed for MEDLINE]

395. Proc Natl Acad Sci U S A. 2010 Jun 22;107(25):11313-8. doi: 10.1073/pnas.1002716107. Epub 2010 Jun 8.

Allosteric inhibition of macrophage migration inhibitory factor revealed by ibudilast.

Cho Y, Crichlow GV, Vermeire JJ, Leng L, Du X, Hodsdon ME, Bucala R, Cappello M, Gross M, Gaeta F, Johnson K, Lolis EJ.

Department of Pharmacology, Yale University School of Medicine, New Haven, CT 06510, USA.

AV411 (ibudilast; 3-isobutyryl-2-isopropylpyrazolo-[1,5-a]pyridine) is an antiinflammatory drug that was initially developed for the treatment of bronchial asthma but which also has been used for cerebrovascular and ocular indications. It is a nonselective inhibitor of various phosphodiesterases (PDEs) and has varied antiinflammatory activity. More recently, AV411 has been studied as a possible therapeutic for the treatment of neuropathic pain and opioid withdrawal through its actions on glial cells. As described herein, the PDE inhibitor AV411 and its PDE-inhibition-compromised analog AV1013 inhibit the catalytic and chemotactic functions of the proinflammatory protein, macrophage migration inhibitory factor (MIF). Enzymatic analysis indicates that these compounds are noncompetitive inhibitors of the p-hydroxyphenylpyruvate (HPP) tautomerase activity of MIF and an allosteric binding site of AV411 and AV1013 is detected by NMR. The allosteric inhibition mechanism is further elucidated by X-ray crystallography based on the MIF/AV1013 binary and MIF/AV1013/HPP ternary complexes. In addition, our antibody experiments directed against MIF receptors indicate that CXCR2 is the major receptor for MIF-mediated chemotaxis of peripheral blood mononuclear cells.

PMCID: PMC2895110
PMID: 20534506 [PubMed - indexed for MEDLINE]

396. Int Arch Allergy Immunol. 2010;152 Suppl 1:96-100. doi: 10.1159/000312133. Epub 2010 Jun 4.

Effect of the hepatocyte growth factor on allergic inflammatory cells.

Ito W, Takeda M, Ueki S, Tanigai T, Kayaba H, Chihara J.

Department of Infection, Allergy, Clinical Immunology and Laboratory Medicine, Akita University Graduate School of Medicine, Akita, Japan.

Hepatocyte growth factor (HGF) has multiple activities in a variety of tissues, and is known to prevent the onset and progression of various diseases, but the mechanisms by which HGF exert its beneficial effects remain to be elucidated, although many studies have shown that HGF exerts anti-inflammatory effects in multiple animal models of diseases of the liver, kidney, lung and other organs.

Recently, we have reported that HGF also reduces allergic airway inflammation in a murine model of asthma by ovalbumin. Furthermore, HGF directly modulates various functions of eosinophils, which have been shown to play a pivotal role in the development of allergic airway inflammation. HGF influences a number of cell types, and regulates various biological activities, including cytokine production, cell migration, proliferation and survival. This review focuses on the effect of HGF on various inflammatory cells, e.g. eosinophils and dendritic cells, in allergic reactions.

(c) 2010 S. Karger AG, Basel.

PMID: 20523071 [PubMed - indexed for MEDLINE]

397. Int Arch Allergy Immunol. 2010;152 Suppl 1:90-5. doi: 10.1159/000312132. Epub 2010 Jun 4.

The pathophysiological roles of PI3Ks and therapeutic potential of selective inhibitors in allergic inflammation.

Takeda M, Ito W, Tanabe M, Ueki S, Kihara J, Kato H, Tanigai T, Kayaba H, Sasaki T, Chihara J.

Department of Infection, Allergy, Clinical Immunology and Laboratory Medicine, Akita University Graduate School of Medicine, Akita, Japan.

Phosphoinositide 3-kinases (PI3Ks) are known to be involved in a variety of cellular responses such as cell survival, proliferation, differentiation and cell migration. Recently, PI3Ks have been associated with the pathogenesis of asthma because various immune cells regulate allergic responses. Among the three classes of PI3Ks, the roles of PI3K gamma and PI3K delta in allergic responses have attracted particular attention. In a previous report, allergic airway hyperresponsiveness (AHR), inflammation and airway remodeling in an ovalbumin-induced asthma model were decreased in PI3K gamma-deficient mice compared with wild-type mice. In addition, AHR and inflammation were attenuated by administration of a selective PI3K delta inhibitor in a murine model of asthma. These results indicate that PI3K gamma and PI3K delta may be new therapeutic targets for asthma. However, PI3K gamma and PI3K delta may differ in terms of the mechanism of regulation. In this review, we focus on the roles of PI3K gamma and PI3K delta in the pathogenesis of asthma and discuss the mechanistic differences between PI3K gamma and PI3K delta.

(c) 2010 S. Karger AG, Basel.

PMID: 20523070 [PubMed - indexed for MEDLINE]

398. Thorax. 2010 Jun;65(6):547-52. doi: 10.1136/thx.2009.129296.

Ca(2+) homeostasis and structural and functional remodelling of airway smooth muscle in asthma.

Mahn K, Ojo OO, Chadwick G, Aaronson PI, Ward JP, Lee TH.

King's College London, MRC and Asthma UK Centre in Allergic Mechanisms of Asthma, London, UK.

Asthma is characterised by airway hyper-responsiveness and remodelling, and there is mounting evidence that alterations in the phenotype of airway smooth muscle (ASM) play a central role in these processes. Although the concept that

dysregulation of ASM Ca(2+) homeostasis may underlie at least part of these alterations has been around for many years, it is only relatively recently that the availability of ASM biopsies from subjects with mild and moderate asthma has allowed it to be properly investigated. In this article, critical components of the pathobiology of asthmatic ASM, including contractile function, proliferation, cell migration and secretion of proinflammatory cytokines and chemokines, are reviewed and related to associated changes in ASM Ca(2+) homeostasis. Based on this evidence, it is proposed that a unifying mechanism for the abnormal asthmatic phenotype is dysregulation of Ca(2+) homeostasis caused at least in part by a downregulation in expression and function of sarcoendoplasmic Ca(2+) ATPases (SERCAs).

PMID: 20522856 [PubMed - indexed for MEDLINE]

399. Clin Invest Med. 2010 Jun 1;33(3):E146-54.

Expression patterns of ClC-3 mRNA and protein in aortic smooth muscle, kidney and brain in diabetic rats.

Fu J, Fu J, Gao B, Wang L, Zhang N, Li X, Ji Q.

Department of Endocrinology, Xijing Hospital, Fourth Military Medical University, Xi'an, 710032, China.

ClC-3, a member of the ClC family of voltage-gated chloride channels, regulates cell proliferation of cultured rat aortic vascular smooth muscle cells, pathogenesis of allergic rhinitis and tumor cell migration. However, its role in diabetic animals is still unknown. To address this issue, we investigated the expression patterns of ClC-3 in diabetic rats. Five-week-old Sprague-Dawley rats were divided into two groups, 50 non-diabetic control rats (non-DM) and 50 diabetic model rats (DM). ClC-3 mRNA and protein expression in aortic smooth muscle, kidney and brain tissues were examined by fluorimeter-based quantitive RT-PCR assay and Western blot analysis, respectively. ClC-3 mRNA and protein were endogenously expressed in aortic smooth muscle, kidney (cortex and medulla) and brain tissues of both control and streptozotocin-induced diabetic rats. ClC-3 mRNA and protein expression levels were significantly higher in aortic smooth muscle and brain tissues of diabetic rats, but significantly decreased in kidney medulla tissue, relative to non-DM controls. There were no significant differences in ClC-3 mRNA and protein expression in kidney cortex between non-diabetic control and diabetic rats. Furthermore, the altered ClC-3 expression patterns in diabetic rat aortic smooth muscle, brain, and kidney medulla tissues all correlated with the changes in blood glucose levels ($p < 0.05$). In conclusion, our data show for the first time that diabetes alters both the gene and protein expression of ClC-3 channels. These changes may contribute to the impaired vascular, brain and kidney functions observed in diabetes.

PMID: 20519092 [PubMed - indexed for MEDLINE]

400. Clin Respir J. 2010 May;4 Suppl 1:9-14. doi: 10.1111/j.1752-699X.2010.00191.x.

The airway epithelium as regulator of inflammation patterns in asthma.

Erjefält JS.

Unit of Airway Inflammation, Department of Experimental Medical Science, BMC D12, Lund University, Lund, Sweden. jonas.erjefalt@med.lu.se

INTRODUCTION: Asthma is a complex, heterogeneous and mutifactorial disease and

represents a major health problem in Westernized countries. The airway epithelium, with its direct physical contact with luminal triggers, has a major role in determining the nature of inflammation that develops in asthmatic airways.
OBJECTIVE: The present review aims to provide a brief overview of the numerous ways the airway epithelium can affect and influence the histopathological picture in asthma.
RESULTS AND CONCLUSION: The ways the epithelium aggravates inflammation range from acute responses to luminal triggers such as allergens and infections to the multipathogenic events occurring as a consequence of repeated epithelial damage-repair responses. The airway epithelium also facilitates the selective migration of leukocytes into the airway lumen, a process that is important in regulating inflammatory cell homeostasis. The fact that only some of the important leukocyte subtypes participate in this process cause translational problems and difficulties in the interpretation of luminal samples. To further reveal the nature of the multifaceted involvement of the airway epithelium in inflamed asthmatic airways emerges as a promising goal for identifying new therapeutic strategies.

PMID: 20500604 [PubMed - indexed for MEDLINE]

401. Catheter Cardiovasc Interv. 2010 Jun 1;75(7):1056-9. doi: 10.1002/ccd.22409.

Wandering coronary stenoses: adrenaline-induced coronary artery spasm in a patient resuscitated from cardiac arrest.

Battaglia S, Nammas W.

Department of Cardiology, Hôpital Européen Georges Pompidou, Paris, France.
salvatore.battaglia@egp.aphp.fr

Comment in
    Catheter Cardiovasc Interv. 2010 Jun 1;75(7):1060-1.

A 68-year-old ex-smoker man with history of allergy, presented to the emergency department with progressive dyspnea one hour following self-medication with aspirin for troublesome headache. Examination revealed diffuse sibilant rhonchi over both lungs. Electrocardiogram showed signs of ischemia. In the intensive care unit, he received bronchodilators, nitroglycerin, and aspirin. Bronchospasm increased, and then the patient was shocked, and developed cardiac arrest. After resuscitation, he was kept on mechanical ventilation and adrenaline infusion. He was scheduled for coronary angiography. The left system demonstrated stenosis of the mid-segment of the left anterior descending artery (LAD), which was totally occluded distally, stenosis of the left circumflex (LCx) with a mild plaque in its marginal branch. The right system demonstrated stenosis of the mid-segment of the right coronary artery (RCA), with diffusely diseased posterior descending artery (PDA) and posterolateral left ventricular branch (PLLV). Successful direct stenting was performed to the RCA. Angiography demonstrated worsening of the distal stenosis in the PLLV and complete occlusion of the PDA. Balloon dilatation of the PLLV was adequate, but dilatation of the PDA failed. Repeat angiography of the left system revealed an occluded LCx with critical stenosis of its marginal branch; nevertheless, the LAD was as before. Balloon dilatation of the distal LAD was attempted without improvement, yet, angiography therein, demonstrated "migration" of the stenoses in the LCx. The procedure was halted, adrenaline infusion discontinued, and an intra-aortic balloon pump inserted. The patient was discharged one day later. Follow-up angiography 6 months later demonstrated mild atherosclerotic coronary irregularities.

PMID: 20517968 [PubMed - indexed for MEDLINE]

402. Electrophoresis. 2010 Jun;31(11):1805-12. doi: 10.1002/elps.201000077.

Successive multiple ionic polymer layer coated capillaries in the separation of proteins - recombinant allergen variants as a case study.

Weinbauer M, Stutz H.

Division of Chemistry and Bioanalytics, Department of Molecular Biology, University of Salzburg, Salzburg, Austria.

A successive multiple ionic polymer layer (SMIL) coating consisting of two pairs of poly(diallyldimethylammonium chloride) and dextran sulfate (DS) layers was applied for the separation of recombinant products of the major birch pollen allergen Betula verrucosa (Bet v 1a). The combination with volatile ammonium bicarbonate buffer at pH 6.70 offers the possibility for future MS hyphenation. The negative net charge of allergens required DS as terminal SMIL layer. The EOF was accelerated from 3.17x10(-8) m(2) V(-1) s(-1) in uncoated to 4.52x10(-8) m(2) V(-1) s(-1) in SMIL capillaries. Fresh prepared SMIL capillaries showed slight EOF acceleration due to gradual re-organization of SMIL structure until stabilization was achieved. Dry storage of SMIL capillaries prevented fluctuations in EOF and migration times and improved coating durability. However, the gradual reconstitution of entangled SMIL layers affected efficiency, but was cured by a 10 mmol/L NaOH rinsing step. Durability of SMIL capillaries in MS-applicable dimension was confirmed for > 70 runs and in total 42 h of voltage application with average intra-day precision of 0.22 and 0.79% and inter-day-precision of 0.91 and 1.17% for migration times of EOF and Bet v 1a, respectively. Final SMIL coating allowed for the separation of Bet v 1a, a hypoallergenic isoform and carbamylated variants with 150,000-685,000 plates.

PMID: 20506417 [PubMed - indexed for MEDLINE]

403. J Egypt Soc Parasitol. 2010 Apr;40(1):57-70.

A study on the prevalence of house dust mites in Al-Arish city, North Sinai Governorate, Egypt.

El-Sherbiny GT, El-Sherbini ET, Saled NM, Haridy FM, Morsy AT.

Department of Parasitology, Faculty of Pharmacy, October 6 University, 6th October, Egypt.

Free living mites comprise a huge and various groups of tiny arthropods in the class Arachida, mainly of the Pyroglyphidae family. Exposure to allergens derived from house dust mite (HDM) feces is a postulated risk factor for allergic sensitization, asthma development and asthma morbidity. However, practical and effective method to mitigate these allergens in low-income, urban home environments remains elusive. It well known that (HDM) physiology is greatly affected by hydrothermal microclimatic condition. El Arish has subtropical climate and warm humid summer, such situation are favourable to proliferate house dust mites. As no valid data are available for house dust mites fauna of El Arish, this study was carried out to determine the prevalence and contamination rates of homes in El Arish city. Samples of house dust collected in 2008 from 50 houses in El Arish city were subjected to acarological examination. Acri were found in (34.6 %) of the samples collected from these homes. Results indicated that dust mites were present in all humid environments. Also, hypersensitivity to dust mites was common among patients with asthma.

PMID: 20503586 [PubMed - indexed for MEDLINE]

404. Dev Ophthalmol. 2010;45:40-8. doi: 10.1159/000315018. Epub 2010 May 18.

Intravital multidimensional real-time imaging of the conjunctival immune system.

Gehlsen U, Hüttmann G, Steven P.

Department of Ophthalmology, Campus Lübeck, University Medical Center Schleswig-Holstein, University of Lübeck, Lübeck, Germany.

The conjunctiva, as a peripheral mucosal surface, is dependent on the migration of immune cells to facilitate an orchestrated immune response. So far, only limited data to visualize these dynamics directly have been obtained, mainly due to technical and experimental restrictions. To investigate migration on a cellular level, the following conditions need to be met: (1) intravital investigations need to be facilitated by suitable microscopic techniques; (2) tissues need to be investigated in three spatial dimensions and over time; (3) data need to contain detailed information about the tissue character. Whereas the use of confocal laser scanning microscopy allows high-resolution imaging of the superficial conjunctival immune system and enables the recording of rapid cellular migration, intravital two-photon microscopy further enables tracking of individual cells and characterization of cells and structures with unique optical features using autofluorescence detection, fluorescence lifetime measurements and second harmonic generation in deep tissue. Based on current results and experimental studies, two-photon microscopy has the potential for general use in basic research and clinical practice, and would greatly enhance possibilities for diagnosing and analyzing inflammatory processes of the ocular surface. In particular, inflammation in common diseases, such as allergy and dry eye, and its progress under treatment could be investigated in detail.

Copyright 2010 S. Karger AG, Basel.

PMID: 20502025 [PubMed - indexed for MEDLINE]

405. Postepy Hig Med Dosw (Online). 2010 May 25;64:244-50.

[The role of Th17 cells in anti-cancer immunity].

[Article in Polish]

Hus I, Maciag E, Roliński J.

Klinika Hematoonkologii i Transplantacji Szpiku Uniwersytetu Medycznego w Lublinie. iwonach.hus@gmail.com

Th17 cells are a newly identified population of CD4+ Th cells characterized by interleukin 17 production and are therefore named "Th17". Interleukin 17 (IL-17; IL-17A) is one of the key cytokines for the activation, recruitment, and migration of neutrophils, so Th17 cells have been suggested to play an important role in responses against extracellular bacteria and fungi, in which granulocyte infiltration is highly protective. Among the other cytokines secreted by Th17 cells are IL-22, IL-26, IL-6, and TNF-a. Th17 cells are considered to be involved in the pathogenesis of various inflammatory, autoimmune, and allergic diseases. Th17 cells were found in both mouse and human tumors; however, their role in the tumor microenvironment is still poorly understood IL-17, as a pleiotropic cytokine, may suppress or promote tumor growth since it was shown that it stimulated tumor growth by inducing tumor vascularization or enhancing

inflammation, but some other studies revealed also opposite roles for Th17 cells in human tumors. It seems that Th17 cells may play distinct roles in cancer depending of tumor immunogenicity, the stage of development, and the impact of inflammation and angiogenesis on tumor pathogenesis.

PMID: 20498501 [PubMed - indexed for MEDLINE]

406. J Food Sci. 2010 Apr;75(3):H87-92. doi: 10.1111/j.1750-3841.2010.01541.x.

Anti-inflammatory effects of limonene from yuzu (Citrus junos Tanaka) essential oil on eosinophils.

Hirota R, Roger NN, Nakamura H, Song HS, Sawamura M, Suganuma N.

Dept. of Environmental Medicine, Kochi Medical School, Kohasu, Oko, Nankoku, Kochi 783-8505, Japan. hirotar@kochi-u.ac.jp

Yuzu (Citrus junos Tanaka) has been used as a traditional medicine in Japan. We investigated in vitro anti-inflammatory effects of limonene from yuzu peel on human eosinophilic leukemia HL-60 clone 15 cells. To examine anti-inflammatory effects of limonene on the cells, we measured the level of reactive oxygen species (ROS), monocyte chemoattractant protein-1 (MCP-1), nuclear factor (NF) kappa B, and p38 mitogen-activated protein kinase (MAPK). We found that low concentration of limonene (7.34 mmol/L) inhibited the production of ROS for eotaxin-stimulated HL-60 clone 15 cells. 14.68 mmol/L concentration of limonene diminished MCP-1 production via NF-kappa B activation comparable to the addition of the proteasomal inhibitor MG132. In addition, it inhibited cell chemotaxis in a p38 MAPK dependent manner similar to the adding of SB203580. These results suggest that limonene may have potential anti-inflammatory efficacy for the treatment of bronchial asthma by inhibiting cytokines, ROS production, and inactivating eosinophil migration.

PMID: 20492298 [PubMed - indexed for MEDLINE]

407. BMJ. 2010 May 20;340:c2369. doi: 10.1136/bmj.c2369.

The health and socioeconomic impacts of major multi-sport events: systematic review (1978-2008).

McCartney G, Thomas S, Thomson H, Scott J, Hamilton V, Hanlon P, Morrison DS, Bond L.

Medical Research Council Social and Public Health Sciences Unit, Glasgow. gmccartney@nhs.net

Comment in
    BMJ. 2010;340:c2202.

OBJECTIVE: To assess the effects of major multi-sport events on health and socioeconomic determinants of health in the population of the city hosting the event.
DESIGN: Systematic review.
DATA SOURCES: We searched the following sources without language restrictions for papers published between 1978 and 2008: Applied Social Science Index and Abstracts (ASSIA), British Humanities Index (BHI), Cochrane database of systematic reviews, Econlit database, Embase, Education Resources Information Center (ERIC) database, Health Management Information Consortium (HMIC) database, International Bibliography of the Social Sciences (IBSS), Medline, PreMedline,

PsycINFO, Sociological Abstracts, Sportdiscus, Web of Knowledge, Worldwide Political Science Abstracts, and the grey literature. Review methods Studies of any design that assessed the health and socioeconomic impacts of major multi-sport events on the host population were included. We excluded studies that used exclusively estimated data rather than actual data, that investigated host population support for an event or media portrayals of host cities, or that described new physical infrastructure. Studies were selected and critically appraised by two independent reviewers.
RESULTS: Fifty four studies were included. Study quality was poor, with 69% of studies using a repeat cross-sectional design and 85% of quantitative studies assessed as being below 2+ on the Health Development Agency appraisal scale, often because of a lack of comparison group. Five studies, each with a high risk of bias, reported health related outcomes, which were suicide, paediatric health service demand, presentations for asthma in children (two studies), and problems related to illicit drug use. Overall, the data did not indicate clear negative or positive health impacts of major multi-sport events on host populations. The most frequently reported outcomes were economic outcomes (18 studies). The outcomes used were similar enough to allow us to perform a narrative synthesis, but the overall impact of major multi-sport events on economic growth and employment was unclear. Two thirds of the economic studies reported increased economic growth or employment immediately after the event, but all these studies used some estimated data in their models, failed to account for opportunity costs, or examined only short term effects. Outcomes for transport were also similar enough to allow synthesis of six of the eight studies, which showed that event related interventions--including restricted car use and public transport promotion--were associated with significant short term reductions in traffic volume, congestion, or pollution in four out of five cities.
CONCLUSIONS: The available evidence is not sufficient to confirm or refute expectations about the health or socioeconomic benefits for the host population of previous major multi-sport events. Future events such as the 2012 Olympic Games and Paralympic Games, or the 2014 Commonwealth Games, cannot be expected to automatically provide benefits. Until decision makers include robust, long term evaluations as part of their design and implementation of events, it is unclear how the costs of major multi-sport events can be justified in terms of benefits to the host population.

PMCID: PMC2874130
PMID: 20488915 [PubMed - indexed for MEDLINE]

408. J Health Care Poor Underserved. 2010 May;21(2 Suppl):125-39. doi: 10.1353/hpu.0.0286.

Self-reported lifetime asthma and nativity status in U.S. children and adolescents: results from the National Health and Nutrition Examination Survey 1999-2004.

Joseph SP, Borrell LN, Shapiro A.

The New York Children's Health Project, Community Pediatrics Programs, The Children's Hospital at Montefiore, Bronx, NY 10459, USA. sjoseph@montefiore.org

Nativity status seems to have a protective effect on asthma. However, there is little information in the literature on the effect of nativity status on asthma for non-Hispanic Black and White children and adolescents. This study investigates the association between nativity status and self-reported asthma in U.S. children and adolescents under 21 years of age after controlling for selected characteristics including education and income. Logistic regression was conducted using SUDAAN to estimate odds ratios from data of the National Health and Nutrition Examination Survey 1999-2004. The prevalence of asthma for U.S.

children and adolescents is 14.5%, with U.S.-born exhibiting a higher prevalence (15.1%) than foreign-born (7.3%, p<.0001). In the fully-adjusted analysis, foreign-born children and adolescents were almost twice less likely than U.S.-born counterparts to report asthma. This association differs by race/ethnicity, with the lowest odds being observed in Whites (OR: 0.29, 95% CI: 0.10-0.86), followed by Mexican Americans (OR: 0.38; 95% CI: 0.25-0.56) and Blacks (OR: 0.52; 95% CI: 0.29-0.94). Moreover, this association varies by education: Foreign-born children of survey respondents who had at least a high school education were more than twice less likely than their U.S.-born counterparts to have asthma. The findings of this study underscore the importance of inquiring about nativity status when studying asthma among U.S. children and adolescents.

PMID: 20453381 [PubMed - indexed for MEDLINE]

409. Mol Med. 2010 Sep-Oct;16(9-10):400-8. doi: 10.2119/molmed.2009.00128. Epub 2010 May 14.

ISO-1, a macrophage migration inhibitory factor antagonist, inhibits airway remodeling in a murine model of chronic asthma.

Chen PF, Luo YL, Wang W, Wang JX, Lai WY, Hu SM, Cheng KF, Al-Abed Y.

Departments of Respiratory Diseases and ShenZhen Third People Hospital, Guangdong, China.

Airway remodeling is the process of airway structural change that occurs in patients with asthma in response to persistent inflammation and leads to increasing disease severity. Drugs that decrease this persistent inflammation play a crucial role in managing asthma episodes. Mice sensitized (by intraperitoneal administration) and then challenged (by inhalation) with ovalbumin (OVA) develop an extensive eosinophilic inflammatory response, goblet cell hyperplasia, collagen deposition, airway smooth muscle thickening, and airway wall area increase, similar to pathologies observed in human asthma. We used OVA-sensitized/challenged mice as a murine model of chronic allergic airway inflammation with subepithelial fibrosis (i.e., asthma). In this OVA mouse model, mRNA and protein of macrophage migration inhibitory factor (MIF) are upregulated, a response similar to what has been observed in the pathogenesis of acute inflammation in human asthma. We hypothesized that MIF induces transforming growth factor-β1 (TGF-β1) synthesis, which has been shown to play an important role in asthma and airway remodeling. To explore the role of MIF in the development of airway remodeling, we evaluated the effects of an MIF small-molecule antagonist, (S,R)3-(4-hy-droxyphenyl)-4,5-dihydro-5-isoxazole acetic acid methyl ester (ISO-1), on pathologies associated with the airway-remodeling process in the OVA mouse model. We found that administration of ISO-1 significantly mitigated all symptoms caused by OVA treatment. In addition, the treatment of OVA-sensitized mice with the MIF antagonist ISO-1 significantly reduced TGF-β1 mRNA levels in pulmonary tissue and its protein level in bronchial alveolar lavage fluid supernatants. We believe the repression of MIF in the ISO-1 treatment group led to the significant suppression observed in the inflammatory responses associated with the allergen-induced lung inflammation and fibrosis in our murine asthma (OVA) model. Our results implicate a possible function of MIF in the pathogenesis of chronic asthma and suggest that MIF might be an important therapeutic target for airway remodeling.

PMCID: PMC2935952
PMID: 20485865 [PubMed - indexed for MEDLINE]

410. Int Arch Allergy Immunol. 2010;153(3):274-9. doi: 10.1159/000314368. Epub 2010 May 19.

Allergic rhinitis and asthma: a large cross-sectional study in the United Arab Emirates.

Alsowaidi S, Abdulle A, Bernsen R, Zuberbier T.

Department of Internal Medicine, Faculty of Medicine, United Arab Emirates University, Al-Ain, United Arab Emirates. shirina.alsowaidi@uaeu.ac.ae

BACKGROUND: Studies on comorbidity of allergic rhinitis (AR) and asthma have been carried out in developed countries; however, data from countries in development transition are lacking.
METHODS: In a randomly selected, age-stratified cohort of adolescent school children and their caretakers in the United Arab Emirates (UAE), comorbidity of AR and asthma was calculated using multinomial regression to determine independent risk factors.
RESULTS: A total of 6,543 subjects were included in the study; the median age was 30 years (range 8-93), and 52% were males. The standardized prevalence of concomitant asthma and AR was 7.3%. AR subjects had a 3-fold increased risk of asthma compared to subjects without AR (23.8 and 7.5%, respectively). Immigrants had a significantly lower prevalence of comorbidity of AR and asthma [adjusted odds ratio (OR) 0.53, 95% confidence interval (CI) 0.33-0.85] compared to UAE nationals, while greater age carried a lower risk (adjusted OR 0.58, 95% CI 0.44-0.78), but a family history of both AR (adjusted OR 3.03, 95% CI 2.31-3.98) and asthma (adjusted OR 4.65, 95% CI 3.53-6.12) was strongly associated with the co-occurrence of these 2 conditions, while gender and education were not. Asthma patients with AR had more severe symptoms than those without, i.e. 'dry cough at night' in 65 versus 36%, β-mimeticum use in 42 versus 30%, and steroid use in 25 versus 13%, respectively.
CONCLUSION: Comorbidity of asthma and AR in the UAE is high, with a prevalence of 7.3%. Both the underlying reasons and possibilities for better prevention now need to be focused on in future research.

Copyright © 2010 S. Karger AG, Basel.

PMID: 20484926 [PubMed - indexed for MEDLINE]

411. J Leukoc Biol. 2010 Aug;88(2):383-92. doi: 10.1189/jlb.0509366. Epub 2010 May 18.

CCR1- and CCR5-mediated inactivation of leukocytes by a nonglycosaminoglycan (non-GAG)-binding variant of n-nonanoyl-CCL14 (NNY-CCL14).

Gupta S, Rieder S, Richter R, Schulz-Maronde S, Manns J, Escher SE, Heitland A, Mack M, Forssmann WG, Elsner J, Forssmann U.

Center of Pharmacology and Toxicology, Clinic of Immunology and Rheumatology, Hannover Medical School, Feodor-Lynen-Strasse 31, 30625 Hannover, Germany. sgupta3@bidmc.harvard.edu

Intervention on chemokine receptors to prevent directional leukocyte migration is a potential therapeutic strategy. NNY-CCL14 is a CD26-resistant lead molecule, which exerts its effects on multiple chemokine receptors (CCR1, CCR2, CCR3, and CCR5). The inhibitory effects of NNY-CCL14 in murine models of allergic airway inflammation have been assigned to its interaction with CCR1 and CCR5. In this study, a non-GAG-binding variant of NNY-CCL14 was generated by mutating basic amino acids within the identified GAG-binding 49BBXB52 motif. This CD26-resistant, non-GAG binding variant, NNY-CCL14(G,A), does not promote

CCR1-dependent cell arrest on modeled endothelium. Its biological activity tested on human and murine chemokine receptors revealed distinguishing properties to NNY-CCL14. As suggested by EC50 values for intracellular calcium mobilization, NNY-CCL14(G,A) demonstrated a reduced ability to activate hCCR1, but internalization and desensitization of hCCR1 were unperturbed. Surprisingly, its activity on hCCR3 was strongly reduced, and it did not internalize mCCR3. A significantly reduced chemotactic activity of eosinophils and monocytes was observed. All biological effects mediated by NNY-CCL14(G,A) via hCCR5 and mCCR5 showed no difference to NNY-CCL14. In mice treated i.v. with NNY-CCL14(G,A), a sustained in vivo down-modulation of CCR5 was achieved over 3 h. Therefore, NNY-CCL14(G,A) inactivates leukocytes by desensitizing and internalizing multiple chemokine receptors, thus rendering them unresponsive to further stimulation by natural ligands. When administered systemically, NNY-CCL14(G,A) may modulate leukocyte functions prior to their interaction with other endothelium-bound chemokines expressed under pathophysiological conditions, such as allergic inflammation.

PMID: 20483925 [PubMed - indexed for MEDLINE]

412. J Phys Condens Matter. 2010 May 19;22(19):194117. doi: 10.1088/0953-8984/22/19/194117.

Neutrophil adhesion and chemotaxis depend on substrate mechanics.

Jannat RA, Robbins GP, Ricart BG, Dembo M, Hammer DA.

Department of Bioengineering University of Pennsylvania, Philadelphia, PA 19104, USA.

Neutrophil adhesion to the vasculature and chemotaxis within tissues play critical roles in the inflammatory response to injury and pathogens. Unregulated neutrophil activity has been implicated in the progression of numerous chronic and acute diseases such as rheumatoid arthritis, asthma, and sepsis. Cell migration of anchorage-dependent cells is known to depend on both chemical and mechanical interactions. Although neutrophil responses to chemical cues have been well characterized, little is known about the effect of underlying tissue mechanics on neutrophil adhesion and migration. To address this question, we quantified neutrophil migration and traction stresses on compliant hydrogel substrates with varying elasticity in a micro-machined gradient chamber in which we could apply either a uniform concentration or a precise gradient of the bacterial chemoattractant fMLP. Neutrophils spread more extensively on substrates of greater stiffness. In addition, increasing the stiffness of the substrate leads to a significant increase in the chemotactic index for each fMLP gradient tested. As the substrate becomes stiffer, neutrophils generate higher traction forces without significant changes in cell speed. These forces are often displayed in pairs and focused in the uropod. Increases in the mean fMLP concentration beyond the K(D) of the receptor lead to a decrease in chemotactic index on all surfaces. Blocking with an antibody against beta(2)-integrins leads to a significant reduction but not an elimination of directed motility on stiff materials, but no change in motility on soft materials, suggesting neutrophils can display both integrin-dependent and integrin-independent motility. These findings are critical for understanding how neutrophil migration may change in different mechanical environments in vivo and can be used to guide the design of migration inhibitors that more efficiently target inflammation.

PMCID: PMC2867619
PMID: 20473350 [PubMed - indexed for MEDLINE]

413. J Adolesc Health. 2010 Jun;46(6):583-91. doi: 10.1016/j.jadohealth.2009.11.203. Epub 2010 Feb 4.

Factors related to undiagnosed asthma in urban adolescents: a multilevel approach.

Magzamen S, Tager IB.

Division of Epidemiology, School of Public Health, University of California, Berkeley, California, USA. slm1@uw.edu

PURPOSE: We report the results of a school-based asthma case-identification survey to determine the prevalence and predictors of possible undiagnosed asthma in a population of urban adolescents.
METHODS: During school years 2006-2008, middle school students in Oakland, California, completed a brief survey adapted from the International Study of Asthma and Allergy in Children. Students were classified into one of three categories: no asthma, current asthma, or possible undiagnosed asthma. Students reported demographic information and residential address, which was geocoded and matched tract-level data from the US Census 2000, Oakland land use designations, public and assisted housing locations, and distance from closed-access roadways. Logistic regression was used to examine factors associated with possible undiagnosed asthma.
RESULTS: Of the 4,017 students who completed the survey, 4.8% (95% confidence interval [CI]: 4.1, 5.5) were classified as possible undiagnosed asthma. Female students (odds ratio: 1.53, 95% CI: 1.07, 2.19) and students who resided in an urban residential area (odds ratio: 2.05, 95% CI: 1.05, 4.05) had significantly increased odds of classification as "possible undiagnosed asthma" compared to current asthma. Percentage of noncitizen recent immigrants in a census tract was related to increased odds of possible undiagnosed asthma. Residence in a census tract with older residential units was significantly associated with decreased odds of undiagnosed asthma.
CONCLUSIONS: In urban settings, school-based asthma surveillance can aid in the identification of children with possible undiagnosed asthma. Implementation of a geographic information systems framework can enhance the identification of demographic and physical environmental factors associated with possible undiagnosed asthma.

Copyright 2010 Society for Adolescent Health and Medicine. Published by Elsevier Inc. All rights reserved.

PMID: 20472216 [PubMed - indexed for MEDLINE]

414. BMC Genet. 2010 May 7;11:36. doi: 10.1186/1471-2156-11-36.

Evaluating the transferability of Hapmap SNPs to a Singapore Chinese population.

Andiappan AK, Anantharaman R, Nilkanth PP, Wang de Y, Chew FT.

Department of Biological Sciences, National University of Singapore, Science Drive 4, Singapore.

BACKGROUND: The International Hapmap project serves as a valuable resource for human genome variation data, however its applicability to other populations has yet to be exhaustively investigated. In this paper, we use high density genotyping chips and resequencing strategies to compare the Singapore Chinese population with the Hapmap populations. First we compared 1028 and 114 unrelated Singapore Chinese samples genotyped using the Illumina Human Hapmap 550 k chip and Affymetrix 500 k array respectively against the 270 samples from Hapmap.

Secondly, data from 20 candidate genes on 5q31-33 resequenced for an asthma candidate gene based study was also used for the analysis.
RESULTS: A total of 237 SNPs were identified through resequencing of which only 95 SNPs (40%) were in Hapmap; however an additional 56 SNPs (24%) were not genotyped directly but had a proxy SNP in the Hapmap. At the genome-wide level, Singapore Chinese were highly correlated with Hapmap Han Chinese with correlation of 0.954 and 0.947 for the Illumina and Affymetrix platforms respectively with deviant SNPs randomly distributed within and across all chromosomes.
CONCLUSIONS: The high correlation between our population and Hapmap Han Chinese reaffirms the applicability of Hapmap based genome-wide chips for GWA studies. There is a clear population signature for the Singapore Chinese samples and they predominantly resemble the southern Han Chinese population; however when new migrants particularly those with northern Han Chinese background were included, population stratification issues may arise. Future studies needs to address population stratification within the sample collection while designing and interpreting GWAS in the Chinese population.

PMCID: PMC2877651
PMID: 20459637 [PubMed - indexed for MEDLINE]

415. Asia Pac J Public Health. 2010 Apr;22(2):159-93. doi: 10.1177/1010539509358193.

The health status of migrants in Australia: a review.

Anikeeva O, Bi P, Hiller JE, Ryan P, Roder D, Han GS.

The University of Adelaide, Australia.

This review summarizes the findings of studies conducted in Australia between 1980 and 2008 that focused on the health status of migrants in one or more of Australia's National Health Priority Areas (NHPAs), identifies gaps in knowledge, and suggests further research directions. Systematic literature searches were performed on CINAHL, MediText, PsycINFO, and MEDLINE. It was found that the majority of migrants enjoy better health than the Australian-born population in the conditions that are part of the NHPAs, with the exception of diabetes. Mediterranean migrants have particularly favorable health outcomes. The migrant health advantage appears to deteriorate with increasing duration of residence. Many of the analyzed studies were conducted more than 10 years ago or had a narrow focus. Little is known about the health status of migrants with respect to a number of NHPAs, including musculoskeletal conditions and asthma.The health status of recently arrived migrant groups from the Middle East and Africa has not been explored in detail.

PMID: 20457648 [PubMed - indexed for MEDLINE]

416. Eur J Immunol. 2010 Jul;40(7):2026-34. doi: 10.1002/eji.200940150.

Epidermis-to-dermis migration of immature Langerhans cells upon topical irritant exposure is dependent on CCL2 and CCL5.

Ouwehand K, Scheper RJ, de Gruijl TD, Gibbs S.

Department of Dermatology, VU University Medical Centre, Amsterdam, The Netherlands.

Skin irritation is generally not considered to be an immunological event; however, alterations in the density of Langerhans cells (LC) in the epidermis do occur, which is indicative of LC migration. In this study, we investigated the

migration of LC out of the epidermis after skin exposure to contact irritants and identified the chemokines involved. With the aid of ex vivo-intact human skin and epidermal sheets we show that dermal fibroblasts play a role in mediating LC migration towards the dermis. Exposure of ex vivo-intact human skin to a panel of seven irritants (SDS, salicylic acid, phenol, isopropanol, DMSO, TritonX, or benzalkonium chloride) resulted in decreased numbers of CD1a(+) cells in the epidermis and the accumulation of CD1a(+) cells in the dermis. In contrast to allergen exposure, neutralizing antibodies to either CXCL12 or CCL19/CCL21 did not inhibit LC migration out of the epidermis. Exposure of epidermal sheets to the prototypical irritant SDS resulted in a TNF-alpha-dependent LC migration towards dermal fibroblasts. This was a result of CCL2/MCP-1 and CCL5/RANTES chemokine secretion by fibroblasts: injection of CCL2- and CCL5-neutralizing antibodies into intact human skin totally inhibited LC migration into the dermis. We have thus identified a novel role for TNF-alpha-inducible dermis-derived CCL2 and CCL5 in initiating migration of irritant-exposed human LC out of the epidermis.

PMID: 20432237 [PubMed - indexed for MEDLINE]

417. J Immunol. 2010 Jun 1;184(11):6114-23. doi: 10.4049/jimmunol.0904177. Epub 2010 Apr 28.

The role of the CCL2/CCR2 axis in mouse mast cell migration in vitro and in vivo.

Collington SJ, Hallgren J, Pease JE, Jones TG, Rollins BJ, Westwick J, Austen KF, Williams TJ, Gurish MF, Weller CL.

Leukocyte Biology Section, Medical Research Council and Asthma UK Centre in Allergic Mechanisms of Asthma, National Heart and Lung Institute, Faculty of Medicine, Imperial College London, South Kensington, London, United Kingdom.

Tissue-resident mast cells (MCs) are important in allergic diseases. In a mouse model of allergic airways inflammation, an increase in peribronchiolar MCs was associated with increased concentrations of the chemokine CCL2 in lung lavage. MC progenitors (MCps) arising in bone marrow (BM) are recruited to tissues by transendothelial migration, and we found that CCL2 is chemotactic for MCps in freshly isolated BM in vitro. Immature, but not mature, BM-derived MCs migrated in response to CCL2 when cultured in IL-3+stem cell factor (SCF) but not when cultured in IL-3 alone. However, the cells under both culture conditions expressed mRNA for CCR2, the receptor for CCL2, and bound the radiolabeled chemokine with similar affinities, highlighting SCF as a key mediator in coupling CCR2 to downstream events, culminating in chemotaxis. Immature BM-derived MCs from IL-3 +SCF cultures, when administered i.v., accumulated at skin sites injected with CCL2 in vivo. MCp recruitment to the allergen-sensitized/challenged lung was significantly reduced in CCR2(-/-) and CCL2(-/-) mouse strains. However, reconstitution studies of sublethally irradiated and BM-reconstituted mice indicated that BM cells and stromal elements could provide CCL2, whereas the CCR2 function resided with stromal elements rather than BM cells. These experiments revealed a new function of SCF in chemokine receptor coupling, but they suggest a complex role of the CCL2/CCR2 axis in recruiting MCps during pulmonary inflammation.

PMCID: PMC2956277
PMID: 20427772 [PubMed - indexed for MEDLINE]

418. Proc Natl Acad Sci U S A. 2010 May 11;107(19):8736-41. doi: 10.1073/pnas.0906126107. Epub 2010 Apr 26.

Requirement of CCL17 for CCR7- and CXCR4-dependent migration of cutaneous dendritic cells.

Stutte S, Quast T, Gerbitzki N, Savinko T, Novak N, Reifenberger J, Homey B, Kolanus W, Alenius H, Förster I.

Molecular Immunology, Leibniz Institut fuer Umweltmedizinische Forschung, Düsseldorf, Germany.

Chemokines are known to regulate the steady-state and inflammatory migration of cutaneous dendritic cells (DCs). The beta-chemokine CCL17, a ligand of CCR4, is inducibly expressed in a subset of DCs and is strongly up-regulated in atopic diseases. Using an atopic dermatitis model, we show that CCL17-deficient mice develop acanthosis as WT mice, whereas dermal inflammation, T helper 2-type cytokine production, and the allergen-specific humoral immune response are significantly decreased. Notably, CCL17-deficient mice retained Langerhans cells (LCs) in the lesional skin after chronic allergen exposure, whereas most LCs emigrated from the epidermis of allergen-treated WT controls into draining lymph nodes (LNs). Moreover, CCL17-deficient LCs showed impaired emigration from the skin after exposure to a contact sensitizer. In contrast, the absence of CCR4 had no effect on cutaneous DC migration and development of atopic dermatitis symptoms. As an explanation for the major migratory defect of CCL17-deficient DCs in vivo, we demonstrate impaired mobility of CCL17-deficient DCs to CCL19/21 in 3D in vitro migration assays and a blockade of intracellular calcium release in response to CCR7 ligands. In addition, responsiveness of CCL17-deficient DCs to CXCL12 was impaired as well. We demonstrate that the inducible chemokine CCL17 sensitizes DCs for CCR7- and CXCR4-dependent migration to LN-associated homeostatic chemokines under inflammatory conditions and thus plays an important role in cutaneous DC migration.

PMCID: PMC2889308
PMID: 20421491 [PubMed - indexed for MEDLINE]

419. Clin Exp Immunol. 2010 Apr;160(1):98-105. doi: 10.1111/j.1365-2249.2010.04130.x.

99th Dahlem conference on infection, inflammation and chronic inflammatory disorders: controversial aspects of the 'hygiene hypothesis'.

Matricardi PM.

Department of Paediatric Pneumology and Immunology, Charité Medical University, Berlin, Germany. paolo.matricardi@charite.de

The 'hygiene hypothesis' proposes that the epidemic of allergic and autoimmune diseases is due to changes in the interactions between humans and the microbes of their ecosystem. This theory apparently does not explain (i) why allergic asthma is rising in 'unhygienic' American inner cities; (ii) why allergic diseases are less prevalent among migrants' children living in European big cities; (iii) why infections with airborne viruses do not 'protect' from allergic sensitization; (iv) why the inverse association between some infections (e.g. hepatitis A virus) and allergic diseases has been reproduced in some populations, but not in others; and (v) why probiotics are not effective in the prevention and therapy of allergic diseases. These challenging questions are useful starting points to improve our understanding of the hypothesis, and to identify among the infectious agents those really responsible for a protective influence against atopic and autoimmune diseases.

PMCID: PMC2841842
PMID: 20415858 [PubMed - indexed for MEDLINE]

420. Clin Exp Immunol. 2010 Apr;160(1):1-9. doi: 10.1111/j.1365-2249.2010.04139.x.

The 'hygiene hypothesis' for autoimmune and allergic diseases: an update.

Okada H, Kuhn C, Feillet H, Bach JF.

INSERM U1013, Necker-Enfants Malades Hospital, Paris, France.

According to the 'hygiene hypothesis', the decreasing incidence of infections in western countries and more recently in developing countries is at the origin of the increasing incidence of both autoimmune and allergic diseases. The hygiene hypothesis is based upon epidemiological data, particularly migration studies, showing that subjects migrating from a low-incidence to a high-incidence country acquire the immune disorders with a high incidence at the first generation. However, these data and others showing a correlation between high disease incidence and high socio-economic level do not prove a causal link between infections and immune disorders. Proof of principle of the hygiene hypothesis is brought by animal models and to a lesser degree by intervention trials in humans. Underlying mechanisms are multiple and complex. They include decreased consumption of homeostatic factors and immunoregulation, involving various regulatory T cell subsets and Toll-like receptor stimulation. These mechanisms could originate, to some extent, from changes in microbiota caused by changes in lifestyle, particularly in inflammatory bowel diseases. Taken together, these data open new therapeutic perspectives in the prevention of autoimmune and allergic diseases.

PMCID: PMC2841828
PMID: 20415844 [PubMed - indexed for MEDLINE]

421. Exp Eye Res. 2010 Jul;91(1):85-91. doi: 10.1016/j.exer.2010.04.006. Epub 2010 Apr 20.

Bepotastine besilate, a highly selective histamine H(1) receptor antagonist, suppresses vascular hyperpermeability and eosinophil recruitment in in vitro and in vivo experimental allergic conjunctivitis models.

Kida T, Fujii A, Sakai O, Iemura M, Atsumi I, Wada T, Sakaki H.

Research Laboratories, Senju Pharmaceutical Co., Ltd., 1-5-4, Murotani, Nishi-ku, Kobe, Hyogo 651-2241, Japan. tkida@senju.co.jp

To elucidate the ocular pharmacological properties of bepotastine besilate, a selective histamine H(1) receptor antagonist, when compared with other histamine H(1) receptor antagonists, using guinea pig allergic conjunctivitis models and in vitro models of eosinophil recruitment and mast cell membrane stabilization. Conjunctival vascular hyperpermeability was studied in guinea pigs passively sensitized with anti-ovalbumin antiserum or following subconjunctival injection of histamine. Modulation of eosinophil recruitment was evaluated for both platelet-activating factor (PAF)-induced eosinophil infiltration in guinea pigs and leukotriene B(4)-induced in vitro chemotaxis of guinea pig peritoneal eosinophils. Membrane-stabilizing effects of bepotastine also were studied with rat peritoneal mast cells stimulated with the ionophore A23187. Histamine H(1) receptor antagonists including bepotastine besilate were topically administered before ovalbumin, histamine or PAF challenges for in vivo experiments or were added directly to mast cell and eosinophil medium in vitro. Bepotastine besilate significantly inhibited conjunctival vascular hyperpermeability in a dose-dependent manner with maximal effect for bepotastine besilate 1.5%. In

separate in vivo experiments, bepotastine besilate 1.0% was significantly more effective than levocabastine 0.025% in the passive sensitization model or olopatadine 0.1% in the histamine-induced hyperpermeability model. Bepotastine besilate 1.0% further suppressed PAF-induced eosinophil infiltration into conjunctival tissue more effectively than ketotifen 0.05%. Chemotaxis of guinea pig peritoneal eosinophils and histamine release from rat peritoneal mast cells in vitro were also inhibited by addition of bepotastine. Olopatadine had a weak effect as compared to that of bepotastine on eosinophil chemotaxis and no effect on mast cell histamine release in our study conditions. Bepotastine besilate was more potent than olopatadine, ketotifen, or levocabastine in reducing vascular hyperpermeability in various animal models of allergic conjunctivitis. Mast cell function and eosinophil chemotaxis were also inhibited in vitro with bepotastine, suggesting bepotastine acts as an inhibitor of allergic response through multiple mechanisms: histamine H(1) receptor antagonism, mast cell stabilization, and inhibition of eosinophil migration to ocular inflammatory sites.

Copyright 2010 Elsevier Ltd. All rights reserved.

PMID: 20412793 [PubMed - indexed for MEDLINE]

422. Curr Drug Targets. 2010 Aug;11(8):957-70.

Potential therapeutic targets for steroid-resistant asthma.

Wang W, Li JJ, Foster PS, Hansbro PM, Yang M.

Centre for Asthma and Respiratory Disease, School of Biomedical Sciences and Pharmacy, University of Newcastle and Hunter Medical Research Institute, Callaghan, NSW 2300, Australia.

Glucocorticoids are the mainstay of asthma management and effectively treat acute exacerbations of asthma. However, a small subset of asthmatics, usually with severe asthma, respond poorly even to systemic administration of high-dose glucocorticoids and this condition is termed "steroid-resistant asthma". This cohort, although small, accounts for approximately 50% of total health care cost for asthma. New investigations into the mechanisms of glucocorticoid action have broadened and deepened our understanding of glucocorticoid resistance. Here we review the importance and characteristics of steroid resistant asthma, the mechanisms that mediate the function of glucocorticoids and that lead to the development of this disease and potential therapies to reverse resistance to treatment. Cellular and molecular factors, receptors and complex signalling pathways have all been implicated. Indeed, based on molecular biological studies, excessive activation of intracellular transcription factors, impaired histone deacetylase, and epigenetic (such as miR-18 and miR-124a) as well as other factors (e.g. vitamin D, P-glycoprotein 170, and macrophage migration inhibitory factor and T helper 17 cells and factors related to innate immunity (such as IFN-gamma and LPS)) may result in glucocorticoid resistance. A thorough understanding of the pathogenesis of steroid resistant asthma will help to develop more efficacious agents for the treatment of the disease.

PMID: 20412045 [PubMed - indexed for MEDLINE]

423. Basic Clin Pharmacol Toxicol. 2010 Oct;107(4):793-7. doi: 10.1111/j.1742-7843.2010.00578.x.

A novel CC chemokine receptor 4 antagonist RS-1269 inhibits ovalbumin-induced ear swelling and lipopolysaccharide-induced endotoxic shock in mice.

Nakagami Y, Kawashima K, Etori M, Yonekubo K, Suzuki C, Jojima T, Kuribayashi T, Nara F, Yamashita M.

Biological Research Laboratories, Daiichi Sankyo Co., Ltd, Tokyo, Japan.
nakagami.yasuhiro.y4@daiichisankyo.co.jp

There is growing evidence that chemokines recruit leukocytes in allergic, inflammatory and immune responses. CC chemokine receptor 4 (CCR4) is implicated as a preferential marker for T helper 2 cells, and the cells selectively respond to CC chemokine ligand 17 (CCL17) and CCL22. We searched for compounds having a profile as a CCR4 antagonist from an in-house library and have previously reported that
3-{2-[(2R)-2-phenyl-4-(4-pyridin-4-ylbenzyl)morpholin-2-yl]ethyl}quinazoline-2,4(1H,3H)-dione (named RS-1154) was capable of significantly inhibiting the binding of [(125) I]CCL17 to human CCR4-expressing CHO cells. From further synthesis of its derivatives, we newly focused on
3-(isobutyrylamino)-N-{2-[(2R)-2-phenyl-4-(4-pyridin-4-ylbenzyl)morpholin-2-yl]ethyl}benzamide (RS-1269), which showed potency comparable to RS-1154 in inhibiting CCL17-induced migration of DO11.10 mice-derived T helper 2 cells with an IC(50) value of 5.5 nM in vitro. We then investigated the pharmacological effects of RS-1269 on ovalbumin-induced ear swelling and lipopolysaccharide-induced endotoxic shock in mice. The ear thickness was significantly decreased by oral administration of RS-1269 at the dose of 30 mg/kg. Treatment with lipopolysaccharide significantly increased the serum level of tumour necrosis factor-α. Compared with an anti-CCL17 antibody, RS-1269 significantly inhibited the production at the dose of 100 mg/kg. These results raise the possibility that RS-1269 or one of its derivatives has potential to serve as a prototype compound to develop therapeutic agents for atopic dermatitis and inflammatory diseases.

© 2010 The Authors. © 2010 Nordic Pharmacological Society.

PMID: 20406201 [PubMed - indexed for MEDLINE]

424. J Innate Immun. 2010;2(2):144-59. doi: 10.1159/000254948. Epub 2009 Oct 30.

Intranasal administration of CpG induces a rapid and transient cytokine response followed by dendritic and natural killer cell activation and recruitment in the mouse lung.

Pesce I, Monaci E, Muzzi A, Tritto E, Tavarini S, Nuti S, De Gregorio E, Wack A.

Department of Immunology, Novartis Vaccines Research Center, Siena, Italy.

CpG-containing oligodeoxynucleotides are potent mucosal adjuvants and effective as stand-alone treatment of respiratory infections in mice. Although CpG is also used as a type 1 helper immunomodulator in the treatment of asthma and allergic disease, immune modulation following intranasal application has not been fully characterized yet. Using a B-type CpG, we monitored RNA expression profiles, cytokine production and cellular activation in lung tissue and bronchoalveolar lavages ex vivo and cytokine production of purified cell populations in vitro. CpG triggered the upregulation of many transcripts, including interferon response genes and proinflammatory cytokine genes, between 3 h and 4 days. Overlapping subsets of these cytokine proteins were induced in vitro in purified CD11c+ cells, B cells and alveolar macrophages from the lung, thus identifying these cells as direct targets of CpG. While lung B cells strongly respond to CpG in vitro, less activation is found ex vivo, suggesting efficient CpG sequestering or rapid B cell migration after activation. In contrast, a type II alveolar epithelial cell line did not respond to CpG in vitro. We noted selective recruitment of plasmacytoid dendritic cells (DCs) into the lung tissue, and of

conventional DCs and natural killer (NK) cells into the lung tissue and bronchoalveolar space. Furthermore, CpG induced activation of intrapulmonary DCs, NK and T cells. We hypothesize that CpG-linked adjuvanticity and clearance of respiratory pathogens are mediated by two major mechanisms: transient induction of the interferon pathway limiting microbial survival and selective recruitment of DCs and NK cells, which allows for better adaptive responses.

PMID: 20375632 [PubMed - indexed for MEDLINE]

425. Eur J Immunol. 2010 Jun;40(6):1590-602. doi: 10.1002/eji.200939907.

EGF receptor activation during allergic sensitization affects IL-6-induced T-cell influx to airways in a rat model of asthma.

Tsuchiya K, Jo T, Takeda N, Al Heialy S, Siddiqui S, Shalaby KH, Risse PA, Maghni K, Martin JG.

Meakins-Christie Laboratories, McGill University, Montreal, QC, Canada.

EGF receptor (EGFR) is involved in cell differentiation and proliferation in airways and may trigger cytokine production by T cells. We hypothesized that EGFR inhibition at the time of allergic sensitization may affect subsequent immune reactions. Brown Norway rats were sensitized with OVA, received the EGFR tyrosine kinase inhibitor, AG1478 from days 0 to 7 and OVA challenge on day 14. OVA-specific IgE in serum and cytokines and chemokines in BAL were measured 24 h after challenge. To evaluate effects on airway hyperresponsiveness (AHR), rats were sensitized, treated with AG1478, intranasally challenged, and then AHR was assessed. Furthermore chemotactic activity of BALF for CD4(+) T cells was examined. The eosinophils, neutrophils and lymphocytes in BAL were increased by OVA and only the lymphocytes were reduced by AG1478. OVA significantly enhanced IL-6 concentration in BAL, which was inhibited by AG1478. However AHR, OVA-specific IgE and IL-4 mRNA expression in CD4(+) T cells were not affected by AG1478. BALF from OVA-sensitized/challenged rats induced CD4(+) T-cell migration, which was inhibited by both AG1478 treatment in vivo and neutralization of IL-6 in vitro. EGFR activation during sensitization may affect the subsequent influx of CD4(+) T cells to airways, mainly mediated through IL-6.

PMID: 20373517 [PubMed - indexed for MEDLINE]

426. Am J Physiol Lung Cell Mol Physiol. 2010 Jun;298(6):L715-31. doi: 10.1152/ajplung.00361.2009. Epub 2010 Apr 2.

Epithelial repair mechanisms in the lung.

Crosby LM, Waters CM.

Departments of 1Physiology, University of Tennessee Health Science Center, Memphis, TN 38163-0001, USA.

The recovery of an intact epithelium following lung injury is critical for restoration of lung homeostasis. The initial processes following injury include an acute inflammatory response, recruitment of immune cells, and epithelial cell spreading and migration upon an autologously secreted provisional matrix. Injury causes the release of factors that contribute to repair mechanisms including members of the epidermal growth factor and fibroblast growth factor families (TGF-alpha, KGF, HGF), chemokines (MCP-1), interleukins (IL-1beta, IL-2, IL-4, IL-13), and prostaglandins (PGE(2)), for example. These factors coordinate processes involving integrins, matrix materials (fibronectin, collagen, laminin),

matrix metalloproteinases (MMP-1, MMP-7, MMP-9), focal adhesions, and cytoskeletal structures to promote cell spreading and migration. Several key signaling pathways are important in regulating these processes, including sonic hedgehog, Rho GTPases, MAP kinase pathways, STAT3, and Wnt. Changes in mechanical forces may also affect these pathways. Both localized and distal progenitor stem cells are recruited into the injured area, and proliferation and phenotypic differentiation of these cells leads to recovery of epithelial function. Persistent injury may contribute to the pathology of diseases such as asthma, chronic obstructive pulmonary disease, and pulmonary fibrosis. For example, dysregulated repair processes involving TGF-beta and epithelial-mesenchymal transition may lead to fibrosis. This review focuses on the processes of epithelial restitution, the localization and role of epithelial progenitor stem cells, the initiating factors involved in repair, and the signaling pathways involved in these processes.

PMCID: PMC2886606
PMID: 20363851 [PubMed - indexed for MEDLINE]

427. N Z Med J. 2010 Mar 19;123(1311):77-8.

Medical image. A case of rhinodynia and asthma. Nose piercing.

Darwish T.

Department of Internal Medicine IC1-A07, University of Missouri-Kansas City (UMKC), Truman Medical Center, 2301 Holmes Street, Kansas City, MO 64108, USA. darwisht@umkc.edu

PMID: 20360801 [PubMed - indexed for MEDLINE]

428. Allergy Asthma Immunol Res. 2010 Apr;2(2):87-101. doi: 10.4168/aair.2010.2.2.87. Epub 2010 Mar 24.

Eosinophil survival and apoptosis in health and disease.

Park YM, Bochner BS.

Department of Medicine, Division of Allergy and Clinical Immunology, Johns Hopkins University School of Medicine, Baltimore, MD, USA.

Eosinophilia is common feature of many disorders, including allergic diseases. There are many factors that influence the production, migration, survival and death of the eosinophil. Apoptosis is the most common form of physiological cell death and a necessary process to maintain but limit cell numbers in humans and other species. It has been directly demonstrated that eosinophil apoptosis is delayed in allergic inflammatory sites, and that this mechanism contributes to the expansion of eosinophil numbers within tissues. Among the proteins known to influence hematopoiesis and survival, expression of the cytokine interleukin-5 appears to be uniquely important and specific for eosinophils. In contrast, eosinophil death can result from withdrawal of survival factors, but also by activation of pro-apoptotic pathways via death factors. Recent observations suggest a role for cell surface death receptors and mitochondria in facilitating eosinophil apoptosis, although the mechanisms that trigger each of these death pathways remain incompletely delineated. Ultimately, the control of eosinophil apoptosis may someday become another therapeutic strategy for treating allergic diseases and other eosinophil-associated disorders.

PMCID: PMC2846745

PMID: 20358022 [PubMed]

429. Eur Respir J. 2010 Nov;36(5):1016-26. doi: 10.1183/09031936.00125809. Epub 2010 Mar 29.

Epidermal growth factor receptor signalling contributes to house dust mite-induced epithelial barrier dysfunction.

Heijink IH, van Oosterhout A, Kapus A.

Laboratory of Allergology and Pulmonary Diseases, Dept of Pathology and Medical Biology,University Medical Center Groningen, University of Groningen, Hanzeplein 1, NL-9713 GZ Groningen, The Netherlands. h.i.heijink@int.umcg.nl

Impaired airway epithelial barrier function has emerged as a key factor in the pathogenesis of allergic asthma. We aimed to discern the involvement of the epidermal growth factor receptor (EGFR) in allergen-induced epithelial barrier impairment, as we previously observed that house dust mite (HDM) signals through EGFR. We investigated the junctional integrity of human bronchial epithelial cells using electric cell-substrate impedance sensing and immunofluorescent staining. HDM induced a rapid, transient fall in epithelial resistance, concomitant with delocalisation of E-cadherin and zona occludens (ZO)-1, and proteolytic cleavage of the latter. EGFR inhibition by AG1478 reduced the HDM-triggered decrease in epithelial resistance and improved restoration of epithelial junctions. Similarly, AG1478 increased epithelial barrier recovery upon electroporation-induced injury, although it delayed the migration phase of the wound healing response. HDM-promoted redistribution of E-cadherin was mediated via EGFR-dependent activation of protease-activated receptor (PAR)-2, while the concomitant ZO-1 degradation was PAR-2/EGFR-independent. Importantly, the fibrogenic cytokine transforming growth factor (TGF)-β prolonged HDM-induced EGFR phosphorylation and inhibited ligand-induced EGFR internalisation/degradation, which resulted in sustained E-cadherin and ZO-1 redistribution. Thus, allergen-induced, PAR-2/EGFR-mediated signalling decreases epithelial resistance and promotes junction disassembly. The TGF-β-enhanced EGFR signalling may be an important contributor to barrier dysfunction and increased epithelial vulnerability in response to HDM.

PMID: 20351035 [PubMed - indexed for MEDLINE]

430. Pulm Pharmacol Ther. 2010 Aug;23(4):316-23. doi: 10.1016/j.pupt.2010.03.004. Epub 2010 Mar 20.

Effect of formoterol and budesonide on chemokine release, chemokine receptor expression and chemotaxis in human neutrophils.

Strandberg K, Blidberg K, Sahlander K, Palmberg L, Larsson K.

Lung and Allergy Research, The National Institute of Environmental Medicine, Karolinska Institutet, Box 287, SE-171 77 Stockholm, Sweden. karin.strandberg@ki.se

Severe persistent asthma and chronic obstructive pulmonary disease (COPD) are associated with neutrophil influx into the airways. It is not clear whether neutrophil chemotaxis is influenced by beta(2)-agonists and glucocorticoids, drugs commonly used in treatment of asthma and COPD. The effect of a long-acting beta(2)-agonist (formoterol), and a glucocorticosteroid (budesonide) on chemokine/cytokine release (CXCL8, CXCL1, IL-6), regulation of chemokine receptors (CXCR1, CXCR2), and migration were assessed in neutrophils from 10

non-allergic, healthy donors. Formoterol enhanced and budesonide inhibited IL-6, CXCL8 and CXCL1 release from LPS-stimulated neutrophils. Formoterol up-regulated both CXCR1 and CXCR2 expression, whereas budesonide up-regulated the expression of CXCR2 only. Despite the effects on chemokine release and drug-induced up-regulation of CXCR1 and CXCR2, no influence on neutrophil chemotaxis could be demonstrated. We conclude that a beta(2)-agonist and a glucocorticoid, commonly used in the treatment of obstructive lung diseases, influence chemokine release and receptor sensitivity but the functional consequences of these findings remain unclear.

Copyright (c) 2010 Elsevier Ltd. All rights reserved.

PMID: 20307681 [PubMed - indexed for MEDLINE]

431. J Immunol. 2010 May 1;184(9):4688-95. doi: 10.4049/jimmunol.0903477. Epub 2010 Mar 19.

IL-4 and TGF-beta 1 counterbalance one another while regulating mast cell homeostasis.

Macey MR, Sturgill JL, Morales JK, Falanga YT, Morales J, Norton SK, Yerram N, Shim H, Fernando J, Gifillan AM, Gomez G, Schwartz L, Oskeritzian C, Spiegel S, Conrad D, Ryan JJ.

Virginia Commonwealth University, Biology Department, Box 842012, Richmond, VA 23284-2012, USA.

Mast cell responses can be altered by cytokines, including those secreted by Th2 and regulatory T cells (Treg). Given the important role of mast cells in Th2-mediated inflammation and recent demonstrations of Treg-mast cell interactions, we examined the ability of IL-4 and TGF-beta1 to regulate mast cell homeostasis. Using in vitro and in vivo studies of mouse and human mast cells, we demonstrate that IL-4 suppresses TGF-beta1 receptor expression and signaling, and vice versa. In vitro studies demonstrated that IL-4 and TGF-beta1 had balancing effects on mast cell survival, migration, and FcepsilonRI expression, with each cytokine cancelling the effects of the other. However, in vivo analysis of peritoneal inflammation during Nippostrongylus brasiliensis infection in mice revealed a dominant suppressive function for TGF-beta1. These data support the existence of a cytokine network involving the Th2 cytokine IL-4 and the Treg cytokine TGF-beta1 that can regulate mast cell homeostasis. Dysregulation of this balance may impact allergic disease and be amenable to targeted therapy.

PMCID: PMC3339193
PMID: 20304823 [PubMed - indexed for MEDLINE]

432. J Cell Physiol. 2010 Jul;224(1):127-34. doi: 10.1002/jcp.22106.

Group V secreted phospholipase A2 contributes to LPS-induced leukocyte recruitment.

Lapointe S, Brkovic A, Cloutier I, Tanguay JF, Arm JP, Sirois MG.

Research Centre, Montreal Heart Institute, Université de Montréal, Montreal, QC, Canada.

Secreted phospholipases A(2) (sPLA(2)s) are well known for their contribution in the biosynthesis of inflammatory eicosanoids. These enzymes also participate in the inflammatory process by regulating chemokine production and protein

expression of adhesion molecules. The majority of sPLA(2) isoforms are up-regulated by proinflammatory stimuli such as bacterial lipopolysaccharide (LPS), which predominantly increases the expression of group V sPLA(2) (sPLA(2)-V). Furthermore, it has recently been shown that sPLA(2)-V is a critical messenger in the regulation of cell migration during allergic airway responsiveness. Herein, we investigated the effect of sPLA(2)-V on LPS-mediated leukocyte recruitment and its capacity to modulate adhesion molecule expression. We conducted our study in the murine air pouch model, using sPLA(2)-V null mice (sPLA(2)-V(-/-)) and control wild-type (WT) littermates. We observed that LPS (1 microg/ml)-mediated leukocyte emigration in sPLA(2)-V(-/-) was attenuated by 52% and 86% upon 6 and 12 h of treatment respectively, as compared to WT mice. In WT mice, treatment with the cell-permeable sPLA(2) inhibitor (12-epi-scalaradial; SLD) reduced LPS-mediated leukocyte recruitment by 67%, but had no additional inhibitory effect in sPLA(2)-V(-/-) mice. Protein analyses from the air pouch skin were carried out upon LPS-challenge, and the expression of intercellular adhesion molecule (ICAM)-1 and vascular cell adhesion molecule (VCAM)-1 were both significantly reduced in sPLA(2)-V(-/-) mice as compared to control WT mice. Together, our data demonstrate the role of sPLA(2)-V in LPS-induced ICAM-1 and VCAM-1 protein overexpression and leukocyte recruitment, supporting the contribution of sPLA(2)-V in the development of inflammatory innate immune responses.

(c) 2010 Wiley-Liss, Inc.

PMID: 20232296 [PubMed - indexed for MEDLINE]

433. Cell Tissue Res. 2010 May;340(2):381-8. doi: 10.1007/s00441-010-0947-y. Epub 2010 Mar 16.

Ultrastructural immunogold localization of nitric oxide synthase isoforms in rat and human eosinophils.

Saluja R, Saini R, Mitra K, Bajpai VK, Dikshit M.

Cardiovascular Pharmacology Unit, Central Drug Research Institute (Council of Scientific and Industrial Research), Lucknow, 226001, India.

Erratum in
Cell Tissue Res. 2010 Jun;340(3):613.

The involvement of nitric oxide (NO) as both pro and anti-inflammatory agent in allergic, airway inflammatory, and asthmatic diseases and the active participation of eosinophils in such ailments have been previously suggested. NO modulates eosinophil number, migration and their survival. The microenvironment of NO synthase (NOS) in subcellular organelles determines its rate and efficiency of catalysis, which in turn influences NO generation at distinct intracellular locales. The present study was undertaken to assess the intracellular distribution of NOS isoforms by transmission electron microscopy followed by morphometric analysis in human and rat eosinophils. Rat eosinophils were explored in parallel, and since they are widely used as model systems to mimic human diseases, a comparative study on NOS localization patterns might provide useful information in deciphering NO role in diverse aspects of eosinophil-related inflammatory ailments. The results demonstrated predominance of neuronal NOS (nNOS) in the eosinophilic granules and even distribution of inducible NOS (iNOS) and nNOS in the cytoplasm and nucleus of human eosinophils. In rat eosinophils, however, iNOS was mainly localized in the eosinophilic granules and nucleus, while nNOS was distributed evenly in cytoplasm and nucleus. Distribution of endothelial NOS (eNOS) in eosinophils was scanty. Differences in NOS isoforms and their localization in human and rat cells might have implications in differential

mode of catalysis and functional contribution to eosinophil physiology and pathology, warranting detailed investigations. The present study highlights species-specific differences in the relative abundance and distribution pattern of NOS isoforms in rat and human eosinophils, which should be considered cautiously in interpreting the rat data to humans.

PMID: 20232214 [PubMed - indexed for MEDLINE]

434. Clin Microbiol Infect. 2010 Mar;16(3):225-31. doi: 10.1111/j.1469-0691.2009.03131.x.

Acute schistosomiasis, a diagnostic and therapeutic challenge.

Jauréguiberry S, Paris L, Caumes E.

Department of Infectious, Tropical Diseases and Parasitology-Mycology, Groupe Hospitalier Pitié Salpêtrière, Assistance Publique des Hôpitaux de Paris, Paris, France. stephane.jaureguiberry@psl.aphp.fr

In non-endemic countries, acute (invasive) schistosomiasis (AS) is typically seen in non-immune travellers, whereas chronic schistosomiasis is more frequently diagnosed in immigrants. Travellers with AS initially present with non-specific signs such as fever, cough, headache, and urticaria. Life-threatening cardiac and neurological complications may occur. The positive diagnosis of AS relies on seroconversion, which appears together with hypereosinophilia approximately 3 weeks after the onset of symptoms. When prescribed during AS, praziquantel usually does not prevent the chronic phase of the disease and is associated with exacerbation of signs and symptoms in approximately 50% of cases. According to the published literature, corticosteroids may be recommended alone or in association with praziquantel. When associated with corticosteroids, pharmacokinetic interactions may impair the efficacy of praziquantel. We suggest that corticosteroids should be restricted to use in patients with systemic complications of AS, whereas praziquantel should be initiated only when ova are detected in either stools or urine, depending on the culprit species.

PMID: 20222897 [PubMed - indexed for MEDLINE]

435. Clin Exp Allergy. 2010 Jul;40(7):1079-90. doi: 10.1111/j.1365-2222.2010.03464.x. Epub 2010 Mar 1.

Staphylococcus aureus enterotoxin B facilitates allergic sensitization in experimental asthma.

Huvenne W, Callebaut I, Plantinga M, Vanoirbeek JA, Krysko O, Bullens DM, Gevaert P, Van Cauwenberge P, Lambrecht BN, Ceuppens JL, Bachert C, Hellings PW.

Upper Airways Research Laboratory, Department of Otorhinolaryngology, Ghent University Hospital, Ghent, Belgium. Wouter.Huvenne@UGent.be

BACKGROUND: Staphylococcus aureus Enterotoxin B (SEB) has immunomodulatory effects in allergic airway disease. The potential contribution of SEB to the sensitization process to allergens remains obscure.
OBJECTIVE: In order to study the effects of staphylococcal-derived toxins on the sensitization to ovalbumin (OVA) and induction of allergic airway inflammation, we have combined the nasal application of OVA with different toxins.
METHODS: Nasal applications of OVA and saline, SEA, SEB, toxic shock syndrome toxin (TSST)-1, protein A or lipopolysaccharide (LPS) were performed on alternate days from day 0 till 12. On day 14, mice were killed for the evaluation of

OVA-specific IgE, cytokine production by mediastinal lymph node (MLN) cells and bronchial hyperreactivity (BHR) to inhaled metacholine. The effect of SEB on dendritic cell (DC) migration and maturation, and on T cell proliferation was evaluated.
RESULTS: Concomitant endonasal application of OVA and SEB resulted in OVA-specific IgE production, whereas this was not found with SEA, TSST-1, protein A, LPS or OVA alone. Increased DC maturation and migration to the draining lymph nodes were observed in OVA/SEB mice, as well as an increased T cell proliferation. Bronchial inflammation with an influx of eosinophils and lymphocytes was demonstrated in OVA/SEB mice, together with hyperresponsiveness and the production of IL-4, IL-5, IL-10 and IL-13 by MLN stimulated with OVA.
CONCLUSIONS: Our data demonstrate that SEB facilitates sensitization to OVA and consecutive bronchial inflammation with features of allergic asthma. This is likely due to augmentation of DC migration and maturation, as well as the allergen-specific T cell proliferation upon concomitant OVA and SEB application.

PMID: 20214664 [PubMed - indexed for MEDLINE]

436. J Immunol. 2010 Apr 1;184(7):3331-5. doi: 10.4049/jimmunol.0903022. Epub 2010 Mar 3.

A critical role for the g protein-coupled receptor mFPR2 in airway inflammation and immune responses.

Chen K, Le Y, Liu Y, Gong W, Ying G, Huang J, Yoshimura T, Tessarollo L, Wang JM.

Laboratory of Molecular Immunoregulation, Center for Cancer Research, National Cancer Institute at Frederick, Maryland, USA.

The formylpeptide receptor-like 1, now officially termed FPR2, in human and its mouse homolog mFPR2 mediate leukocyte migration in response to agonists associated with inflammation and immune responses. To clarify the in vivo role of the receptor, we generated mice deficient in mFPR2. mFPR2(-/-) mice showed markedly reduced severity in OVA/alum-induced allergic airway inflammation. This was associated with diminished recruitment of CD11c(+) dendritic cells into the airway mucosa and secondary lymphoid organs, as well as reduced production of Type 2 cytokines and Igs. We also found that the bronchoalveolar lavage fluid from wild type mice with airway inflammation contained mFPR2 agonist activity. This study reveals a critical role for mFPR2 in the progression of allergic airway inflammation and immune responses.

PMID: 20200280 [PubMed - indexed for MEDLINE]

437. Immunol Cell Biol. 2010 Aug;88(6):658-66. doi: 10.1038/icb.2010.18. Epub 2010 Mar 2.

Lung homing T-cell generation is dependent on strength and timing of antigen delivery to lymph nodes.

Wikstrom ME, Batanero E, Judd SR, Wiqvist K, Holt PG, Stumbles PA.

Telethon Institute for Child Health Research and Centre for Child Health Research, School of Paediatrics and Child Health, University of Western Australia, Perth, Western Australia, Australia.

Inhaled allergens are known for their immediate and ongoing effects in the respiratory tract (RT). In this report, we track inhaled antigen in normal mice for 7 days and find that while it is cleared from the airways, inhaled antigen

persists in peripheral lung tissue and the draining lymph nodes (DLNs). The persistence of antigen led to ongoing presentation in the lymph nodes, but not the lungs, that decreased with time in direct proportion with the frequency of antigen-bearing RT dendritic cells (DCs). There was evidence of functional changes among the antigen-bearing DCs in the lymph nodes, as the expression of CD40, CD80 and CD86 were modulated over the course of 7 days. At the same time, there was a decrease in both CD4(+) T-cell proliferation in lymph nodes and the generation of recirculating CD4(+) T cells. However, early presentation of lower doses of inhaled antigen also resulted in a decrease in CD4(+) T-cell proliferation and recirculation. Thus, T-cell recirculation depends on the strength of stimulus in the DLNs and is produced by a combination of the dose of antigen delivered to the RT, DC migration and co-stimulatory molecule expression. These results provide an important insight into the fate of inhaled antigen in vivo and the influence of persistent antigen presentation on T-cell activation in the lymph nodes.

PMID: 20195280 [PubMed - indexed for MEDLINE]

438. Am J Respir Crit Care Med. 2010 Jun 15;181(12):1318-28. doi: 10.1164/rccm.200909-1396OC. Epub 2010 Mar 1.

The metalloprotease-disintegrin ADAM8 is essential for the development of experimental asthma.

Naus S, Blanchet MR, Gossens K, Zaph C, Bartsch JW, McNagny KM, Ziltener HJ.

Biomedical Research Centre, University of British Columbia, Vancouver, BC, Canada.

RATIONALE: Expression of the metalloprotease ADAM8 is increased in patients with asthma, but the functional significance of elevated ADAM8 expression in the context of asthma pathogenesis remains elusive.
OBJECTIVES: To study development of asthma in ADAM8-deficient mice.
METHODS: Ovalbumin-induced asthma was studied in wild-type, ADAM8-deficient, and ADAM8-chimeric mice. Lung inflammation was assessed by histology, analysis of bronchoalveolar lavage, and airway hyperresponsiveness.
MEASUREMENTS AND MAIN RESULTS: ADAM8-deficient mice are highly resistant to the development of ovalbumin-induced airway inflammation and hyperresponsiveness. ADAM8 expression was induced in both hematopoietic cells and the nonhematopoietic microenvironment after induction of asthma, and ADAM8 expression in both cell populations was required for the full manifestation of asthma. Interestingly, loss of ADAM8 on T cells alone was sufficient to significantly decrease the asthma response. The attenuated response was not due to an intrinsic defect in antigen presentation or cytokine production but reflected an impaired migration of T cells, eosinophils, CD11b(+) CD11c(-), and CD11c(+) cells from blood vessels to the lung and alveolar space, suggesting a general hematopoietic cell deficiency in the absence of ADAM8.
CONCLUSIONS: The results show that ADAM8 plays a proinflammatory role in airway inflammation. The milder disease outcome in the absence of ADAM8 suggests that this protein might be an interesting new target in treatment of this, and potentially other, inflammatory diseases in which recruitment of inflammatory cells is an essential part of pathogenesis.

PMID: 20194813 [PubMed - indexed for MEDLINE]

439. Vaccine. 2010 Feb 23;28(8):1997-2004. doi: 10.1016/j.vaccine.2009.10.092.

An immunotherapeutic treatment against flea allergy dermatitis in cats by

co-immunization of DNA and protein vaccines.

Jin J, Ding Z, Meng F, Liu Q, Ng T, Hu Y, Zhao G, Zhai B, Chu HJ, Wang B.

State Key Laboratory for Agro-Biotechnology, China Agricultural University, Beijing 100094, China.

Flea allergy dermatitis (FAD) is considered a harmful and persistent allergic disease in cats, dogs and humans. Effective and safe antigen-specific treatments are lacking. Previously we reported that the simultaneous co-immunization with a DNA vaccine and its cognate coded protein antigen could induce antigen-specific iTreg cells (inducible Treg cells); demonstrating its potential to protect animals from FAD in a murine model. Its clinical efficacy however, remains to be demonstrated. In this report, we clinically tested this protocol to treat established FAD in cats following flea infestations. We present data showing a profound therapeutic improvement of dermatitis in these FAD cats following two co-immunizations, not only in relieving clinical symptoms, but also the amelioration of the allergic responses, including antigen-induced wheal formation, elevated T cell proliferation, infiltration of lymphocytes and migration of mast cells to the sites. This study demonstrates that a co-immunization approach as described can be used to treat flea-induced allergic disease in animals, thus implicating its potential for a practical clinical application.

Copyright 2009 Elsevier Ltd. All rights reserved.

PMID: 20188255 [PubMed - indexed for MEDLINE]

440. Med Anthropol. 2009 Oct;28(4):336-67. doi: 10.1080/01459740903303944.

Childhood asthma, air quality, and social suffering among Mexican Americans in California's San Joaquin Valley: "Nobody talks to us here".

Schwartz NA, Pepper D.

Department of Population Studies, Colegio de la Frontera, Tijuana, Mexico. norah.schwartz@gmail.com

Nearly one in five Mexican American children residing in California's San Joaquin Valley (the Valley) in 2007 had an asthma attack at some point in their life. Numerous epidemiological studies have suggested that compared with other ethnic groups and Latino subgroups residing in the United States, Mexican origin children have the lowest rates of pediatric asthma. Ethnographic research conducted in central California, however, suggests otherwise. Known for its agricultural produce, extreme poverty, and poor air quality, the Valley is a magnet for the Mexican immigrant farm worker population. We conducted an exploratory ethnographic study to examine health disparities, social suffering, and childhood asthma in the Valley. Many Valley residents believe that their children's health concerns are being ignored. Open-ended interviews uncovered a largely rural community suffering not only from the effects of childhood asthma but the inability to have their experiences taken seriously.

PMID: 20182969 [PubMed - indexed for MEDLINE]

441. J Immunol. 2010 Mar 15;184(6):2974-84. doi: 10.4049/jimmunol.0803478. Epub 2010 Feb 19.

Dibutyl phthalate-induced thymic stromal lymphopoietin is required for Th2

contact hypersensitivity responses.

Larson RP, Zimmerli SC, Comeau MR, Itano A, Omori M, Iseki M, Hauser C, Ziegler SF.

Department of Immunology, University of Washington, Seattle, WA 98195, USA.

Thymic stromal lymphopoietin (TSLP) is an IL-7-related cytokine, produced by epithelial cells, that has been linked to atopic dermatitis and asthma; however, it remains unclear how TSLP shapes the adaptive immune response that causes these allergic disorders. In this study, we demonstrate a role for TSLP in a Th2 model of contact hypersensitivity in mice. TSLP is required for the development of Th2-type contact hypersensitivity induced by the hapten FITC in combination with the sensitizing agent dibutyl phthalate. TSLPR-deficient mice exhibited a dramatically reduced response, including markedly reduced local infiltration by eosinophils, Th2 cytokine production, and serum IgE levels, following FITC sensitization and challenge. The reduced response by TSLPR-deficient mice is likely due to decreased frequency and reduced T cell stimulatory function of skin-derived Ag-bearing FITC(+)CD11c(+) dendritic cells in draining lymph nodes following FITC sensitization. These data suggest that skin-derived dendritic cells are direct or indirect targets of TSLP in the development of type 2 immune responses in the skin, where TSLP drives their maturation, accumulation in skin draining lymph nodes, and ability to induce proliferation of naive allergen-specific T cells.

PMCID: PMC2922850
PMID: 20173025 [PubMed - indexed for MEDLINE]

442. Indian J Dermatol. 2009 Jul;54(3):229-36. doi: 10.4103/0019-5154.55630.

Serum mucosa-associated epithelial chemokine in atopic dermatitis: a specific marker for severity.

Ezzat MH, Shaheen KY.

Department of Pediatrics, Faculty of Medicine, Ain Shams University, Cairo, Egypt.

BACKGROUND: Mucosa-associated epithelial chemokine (MEC; CCL28) is considered pivotal in mediating migration of CCR3 and CCR10-expressing skin-homing memory CLA(+) T cells. CCL28 is selectively and continuously expressed by epidermal keratinocytes, but highly upregulated in inflammatory skin diseases such as atopic dermatitis (AD).
AIMS: This controlled longitudinal study was designed to evaluate the expression of CCL28 serum levels in childhood AD and bronchial asthma (BA) and its possible relations to disease severity and activity.
METHODS: Serum CCL28 levels were measured in 36 children with AD, 23 children with BA, and 14 children who had both conditions as well as in 21 healthy age and gender-matched subjects serving as controls. Sixteen patients in the AD group were followed-up and re-sampled for serum CCL28 after clinical remission. Serum CCL28 levels were correlated with some AD disease activity and severity variables.
RESULTS: Serum CCL28 levels in patients with AD whether during flare (median = 1530; mean +/- SD = 1590.4 +/- 724.3 pg/ml) or quiescence (median = 1477; mean +/- SD = 1575.2 +/- 522.1 pg/ml) were significantly higher than the values in healthy children (median = 301; mean +/- SD = 189.6 +/- 92.8 pg/ml). However, the levels during flare and quiescence were statistically comparable. The serum levels in BA (median = 340; mean +/- SD = 201.6 +/- 109.5 pg/ml) were significantly lower than the AD group and comparable with the healthy control

values. Serum CCL28 levels in severe AD were significantly higher as compared with mild and moderate cases and correlated positively to the calculated severity scores (LSS and SCORAD). CCL28 levels during exacerbation of AD could be positively correlated to the corresponding values during remission, the peripheral absolute eosinophil counts, and the serum lactate dehydrogenase levels. Serum CCL28 did not vary with the serum total IgE values in AD.
CONCLUSION: Our data reinforce the concept that CCL28 might share in the pathogenesis of AD probably through selective migration and infiltration of effector/memory Th2 cells into the skin. It may also represent an objective prognostic marker for disease severity. Further studies may pave the way for CCL28 antagonism among the adjuvant therapeutic strategies.

PMCID: PMC2810687
PMID: 20161852 [PubMed]

443. J Allergy Clin Immunol. 2010 May;125(5):1105-13. doi: 10.1016/j.jaci.2009.12.002. Epub 2010 Feb 11.

Endothelium-derived prostaglandin I(2) controls the migration of eosinophils.

Konya V, Sturm EM, Schratl P, Beubler E, Marsche G, Schuligoi R, Lippe IT, Peskar BA, Heinemann A.

Institute of Experimental and Clinical Pharmacology, Medical University Graz, A-8010 Graz, Austria.

BACKGROUND: Enhanced eosinophil migration from the blood into the tissue is a hallmark of allergic diseases. Prostaglandin (PG) I(2) is the major prostanoid released by endothelial cells. Mice deficient in PGI(2) receptors (IPs) show exaggerated eosinophilic inflammation in response to allergen.
OBJECTIVE: We set out to determine the role of PGI(2) in eosinophil trafficking.
METHODS: Human lung microvascular endothelial cells and purified human eosinophils were used to study adhesion and transendothelial migration. Morphologic studies were performed with fluorescence microscopy.
RESULTS: PGI(2) markedly attenuated the migration of eosinophils through cell-free filters but had no effect on neutrophil migration. The inhibitory effect of PGI(2) on eosinophils was prevented by the IP antagonist Cay10441 and the adenylyl cyclase inhibitor SQ22536. Similarly, PGI(2) prevented the adhesion of eosinophils to fibronectin and the rapid upregulation and activation of the adhesion molecule CD11b. IP expression on eosinophils was confirmed by means of flow cytometry and Western blotting. Furthermore, when endothelial cells were treated with the COX inhibitor diclofenac to abolish PGI(2) production, adhesion of eosinophils to endothelial monolayers and subsequent transendothelial migration were markedly enhanced. Similarly, the IP antagonist enhanced eosinophil adhesion to endothelial cells. Inhibition of PGI(2) biosynthesis decreased the electrical resistance of endothelial monolayers and compromised the texture of adherent junctions, as visualized by means of VE-cadherin and F-actin staining.
CONCLUSION: We propose that endothelium-derived PGI(2) might be fundamental for the maintenance of the endothelial barrier function against infiltrating cells. These results suggest that selective IP agonists might have beneficial effects in allergic inflammation.

Copyright 2010 American Academy of Allergy, Asthma & Immunology. Published by Mosby, Inc. All rights reserved.

PMID: 20153037 [PubMed - indexed for MEDLINE]

444. Eur J Immunol. 2010 May;40(5):1232-40. doi: 10.1002/eji.200940045.

Role of Treg in immune regulation of allergic diseases.

Palomares O, Yaman G, Azkur AK, Akkoc T, Akdis M, Akdis CA.

Swiss Institute of Allergy and Asthma Research (SIAF), University of Zurich, Davos, Switzerland.

Allergy is a Th2-mediated disease that involves the formation of specific IgE antibodies against innocuous environmental substances. The prevalence of allergic diseases has dramatically increased over the past decades, affecting up to 30% of the population in industrialized countries. The understanding of mechanisms underlying allergic diseases as well as those operating in non-allergic healthy responses and allergen-specific immunotherapy has experienced exciting advances over the past 15 years. Studies in healthy non-atopic individuals and several clinical trials of allergen-specific immunotherapy have demonstrated that the induction of a tolerant state in peripheral T cells represent a key step in healthy immune responses to allergens. Both naturally occurring thymus-derived CD4+CD25+FOXP3+ Treg and inducible type 1 Treg inhibit the development of allergy via several mechanisms, including suppression of other effector Th1, Th2, Th17 cells; suppression of eosinophils, mast cells and basophils; Ab isotype change from IgE to IgG4; suppression of inflammatory DC; and suppression of inflammatory cell migration to tissues. The identification of the molecules involved in these processes will contribute to the development of more efficient and safer treatment modalities.

PMID: 20148422 [PubMed - indexed for MEDLINE]

445. Arch Environ Occup Health. 2010 Jan-Mar;65(1):38-44. doi: 10.1080/19338240903390214.

Community-based participatory research in Boston's neighborhoods: A review of asthma case examples.

Brugge D, Rivera-Carrasco E, Zotter J, Leung A.

Department of Public Health and Family Medicine, Tufts University School of Medicine, Boston, MA, USA. dbrugge@aol.com

Three case examples of asthma studies that differ in terms of community and involvement are considered. The Boston Chinatown studies faced limited funding that restricted the level of community involvement, but resulted in some of the first published evidence about asthma in Asian American children. These studies led to an asthma education program grant to a local Asian clinic and elementary school. The public housing study was a well-funded multi-year study of asthma and pest management with city, university and community partners. Residents were trained to collect data and participated throughout the study. Follow up pest management and pesticide buy-back programs headed by the city and community partners have been funded. The Dorchester case had more limited funding, but had the greatest level of involvement of parents of asthmatic children in all phases of the research. This survey led to an interesting novel finding of lower asthma prevalence in foreign born black residents.

PMID: 20147002 [PubMed - indexed for MEDLINE]

446. Ann N Y Acad Sci. 2010 Jan;1183:158-82. doi: 10.1111/j.1749-6632.2009.05131.x.

Galectins: regulators of acute and chronic inflammation.

Liu FT, Rabinovich GA.

Department of Dermatology, University of California Davis, School of Medicine, Sacramento, California, USA. fliu@ucdavis.edu

Galectins, beta-galactoside-binding animal lectins, are differentially expressed by various immune cells as well as a wide range of other cell types. Extracellularly, galectins are able to exhibit bivalent or multivalent interactions with cell-surface glycans on various immune cells and exert various effects. These include cytokine and mediator production, cell adhesion, apoptosis, and chemoattraction. In addition, they can form lattices with cell-surface glycoprotein receptors, resulting in modulation of receptor functions, including clustering and endocytosis. Intracellularly, galectins can participate in signaling pathways and modulate biologic responses. These include apoptosis, cell differentiation, and cell migration. Thus, a large body of literature indicates that galectins play important roles in the immune and inflammatory responses through regulating the homeostasis and functions of immune cells. The use of mice deficient in individual galectins has provided additional evidence for the contributions of these proteins to these responses. Current research indicates that galectins play important roles in the development of acute inflammation as well as chronic inflammation associated with allergies, autoimmune diseases, atherosclerosis, infectious processes, and cancer. Thus, recombinant proteins or specific galectin inhibitors may be used as therapeutic agents for inflammatory diseases.

PMID: 20146714 [PubMed - indexed for MEDLINE]

447. Anticancer Drugs. 2010 Apr;21(4):351-61. doi: 10.1097/CAD.0b013e328334992c.

Tranilast inhibits cell proliferation and migration and promotes apoptosis in murine breast cancer.

Subramaniam V, Chakrabarti R, Prud'homme GJ, Jothy S.

Department of Laboratory Medicine and Keenan Research Centre of the Li Ka Shing Knowledge Institute, St Michael's Hospital, 30 Bond Street, Toronto, Ontario, Canada.

The malignant transformation of breast epithelium involves a number of cellular pathways, including those dependent on signaling from TGF beta. Tranilast [N-(3, 4-dimethoxycinnamonyl)-anthranilic acid] is a drug that is used in Japan to control allergic disorders in patients, and its mechanism of action involves TGF beta. In view of the multiple roles of TGF beta in tumor progression, we hypothesized in this study that tranilast impacts cell proliferation, apoptosis, and migration. Using the mouse breast cancer cell line 4T1, our studies showed that tranilast increases AKT1 phosphorylation and decreases ERK1/2 phosphorylation. Alterations in the cell cycle mediators' cyclin D1, p27, cyclin A, pRB, cyclin B, and Cdc2 were observed after exposure to tranilast, favoring cell arrest beyond the G1/S phase. Tranilast reduced tumor cell proliferation even when it was amplified by exogenous TGF beta. TGF beta-neutralizing antibody did not cause a significant decrease in cell proliferation. Tranilast treatment upregulates p53, induces PARP cleavage in vitro, consistent with a promotion of tumor cell apoptosis. TGF beta-neutralizing antibody downregulates endoglin and matrix metalloproteinases (MMP)-9 levels in vitro indicating that the tranilast effect is mediated through TGF beta modulation. Tranilast treatment results in the inhibition of cell migration and invasion. Western blot analysis of tumor lysates from tranilast-treated mice shows decreased levels of TGF beta1,

endoglin, and significantly higher levels of p53 and cleaved PARP. Cleaved caspase 3 expression is significantly elevated in tranilast-treated mouse breast tumors. To conclude, tranilast induces cellular and molecular changes in murine breast cancer that can be exploited in preclinical therapeutic trials.

PMID: 20145538 [PubMed - indexed for MEDLINE]

448. Ann Allergy Asthma Immunol. 2010 Jan;104(1):66-72. doi:
10.1016/j.anai.2009.11.003.

Eotaxin-2/CCL24 and eotaxin-3/CCL26 exert differential profibrogenic effects on human lung fibroblasts.

Kohan M, Puxeddu I, Reich R, Levi-Schaffer F, Berkman N.

Lung Cellular and Molecular Biology Laboratory, Institute of Pulmonology, Hadassah-Hebrew University Medical Center, Jerusalem, Israel.

BACKGROUND: Eotaxin-2/CCL24 and eotaxin-3/CCL26 play an important role in eosinophil chemotaxis and activation in asthma. We previously demonstrated that eotaxin/CCL11 is profibrogenic for human lung fibroblasts. The effect of eotaxin-2/ CCL24 and eotaxin-3/CCL26 on lung fibroblasts has not yet been investigated.
OBJECTIVE: To evaluate whether eotaxin-2/CCL24 and eotaxin-3/CCL26 modulate fibrotic properties of lung fibroblasts.
METHODS: Fibroblast proliferation was evaluated by means of 3-hydroxythymidine incorporation. Collagen production was assessed by means of 3-hydroxyproline incorporation and biochemical staining. Chemotaxis was determined using Boyden chambers. Expression of alpha-smooth muscle actin was evaluated by means of immunostaining. Transforming growth factor beta1 release was assessed using enzyme-linked immunosorbent assay. Parametric analysis of variance, followed by the Tukey-Kramer multiple comparisons test, was used to calculate statistical significance.
RESULTS: Eotaxin-2/CCL24 but not eotaxin-3/CCL26 stimulated human lung fibroblast proliferation and collagen synthesis. In contrast, eotaxin-3/CCL26 but not eotaxin-2/CCL24 promoted fibroblast migration. Neither eotaxin-2/CCL24 nor eotaxin-3/ CCL26 induced the expression of alpha-smooth muscle actin or transforming growth factor beta1 from lung fibroblasts.
CONCLUSIONS: Eotaxin-2/CCL24 and eotaxin-3/CCL26 have differential profibrogenic effects on human lung fibroblasts. These CC chemokines may, therefore, contribute to airway remodeling in asthma.

PMID: 20143648 [PubMed - indexed for MEDLINE]

449. J Egypt Soc Parasitol. 2009 Dec;39(3):731-44.

Seroprevalence of human toxocariasis (visceral larva migrans).

El-Shazly AM, Abdel Baset SM, Kamal A, Mohammed KA, Sakrs TI, Hammad SM.

Department of Parasitology, Faculty of Medicine, Mansoura University, Egypt.

A total of 455 patients who fulfilled the inclusion criteria were included in the study. The enrolled patients were subjected to a questionnaire (including sociodemographic and other risk factors) and thorough clinical examination was done for the patients. Sera were collected from patients and tested for anti-Toxocara IgG antibodies using ELISA. The overall anti- Toxocara seropositive was (7.7%). It was significantly higher than among the randomly selected 30

healthy controls. There were no significant differences between the seropositive and seronegative patients regarding age, sex, educational level and monthly family income of the patient. However, rural residence, poor house, pet's ownership and frequent contact with soil were found to be significant. Patients who had confirmed bronchial asthma were more than 2 times at higher risk of developing toxocariasis (OR, 2.33; 95% CI, 1.09-4.98) than those with other clinical diagnosis (PUO, hepatomegaly or heptosplenomegaly, lymphadenopathy, neurological disorders, gastrointestinal troubles and dermatitis). Patients with eosinophilia were at 149 times greater risk of being Toxocara seropositive compared to those without eosinophilia (OR, 148.7; 95% CI: 53.5-413.3). Multivariate regression analysis showed eosinophilia and contact with soil were the most important predictors of toxocariasis. OD of anti-Toxocara antibodies (ELISA) was significantly positive with eosinophilia level.

PMID: 20120741 [PubMed - indexed for MEDLINE]

450. Am J Respir Crit Care Med. 2010 May 1;181(9):889-98. doi: 10.1164/rccm.200907-1071OC. Epub 2010 Jan 28.

Decreased fibronectin production significantly contributes to dysregulated repair of asthmatic epithelium.

Kicic A, Hallstrand TS, Sutanto EN, Stevens PT, Kobor MS, Taplin C, Paré PD, Beyer RP, Stick SM, Knight DA.

Department of Respiratory Medicine, Princess Margaret Hospital for Children, Perth, 6001, Western Australia, Australia. anthonyk@ichr.uwa.edu.au

RATIONALE: Damage to airway epithelium is followed by deposition of extracellular matrix (ECM) and migration of adjacent epithelial cells. We have shown that epithelial cells from children with asthma fail to heal a wound in vitro.
OBJECTIVES: To determine whether dysregulated ECM production by the epithelium plays a role in aberrant repair in asthma. Methods: Airway epithelial cells (AEC) from children with asthma (n = 36), healthy atopic control subjects (n = 23), and healthy nonatopic control subjects (n = 53) were investigated by microarray, gene expression and silencing, transcript regulation analysis, and ability to close mechanical wounds.
MEASUREMENTS AND MAIN RESULTS: Time to repair a mechanical wound in vitro by AEC from healthy and atopic children was not significantly different and both were faster than AEC from children with asthma. Microarray analysis revealed differential expression of multiple gene sets associated with repair and remodeling in asthmatic AEC. Fibronectin (FN) was the only ECM component whose expression was significantly lower in asthmatic AEC. Expression differences were verified by quantitative polymerase chain reaction and ELISA, and reduced FN expression persisted in asthmatic cells over passage. Silencing of FN expression in nonasthmatic AEC inhibited wound repair, whereas addition of FN to asthmatic AEC restored reparative capacity. Asthmatic AEC failed to synthesize FN in response to wounding or cytokine/growth factor stimulation. Exposure to 5', 2'deoxyazacytidine had no effect on FN expression and subsequent analysis of the FN promoter did not show evidence of DNA methylation.
CONCLUSIONS: These data show that the reduced capacity of asthmatic epithelial cells to secrete FN is an important contributor to the dysregulated AEC repair observed in these cells.

PMCID: PMC2862303
PMID: 20110557 [PubMed - indexed for MEDLINE]

451. PLoS One. 2010 Jan 15;5(1):e8725. doi: 10.1371/journal.pone.0008725.

Therapeutic efficacy and immunological response of CCL5 antagonists in models of contact skin reaction.

Canavese M, Altruda F, Silengo L.

Department of Genetics, Biology and Biochemistry, University of Torino, Torino, Italy. miriamcanavese@yahoo.com

Skin-infiltrating T-cells play a predominant role in allergic and inflammatory skin diseases such as atopic dermatitis, psoriasis and allergic contact dermatitis. These T-cells are attracted by several chemotactic factors including the chemokine CCL5/RANTES, a CC chemokine inducing both the migration and activation of specific leukocyte subsets. CCL5 has been found to be associated with various cell-mediated hypersensitive disorders such as psoriasis, atopic dermatitis and irritant contact dermatitis. We have used two antagonists, the first, Met-CCL5, a dual CCR1/CCR5 antagonist and the second, a variant in which GAG binding is abrogated, (44)AANA(47)-CCL5, which acts as a dominant negative inhibitor of CCL5. The antagonists were tested in two models of contact skin reaction. The first, irritant contact dermatitis (ICD) is a pathological non-specific inflammatory skin condition arising from the release of pro-inflammatory cytokines by keratinocytes in response to haptens, usually chemicals. The second, contact hypersensitivity (CHS) is a T-cell dependent model, mimicking in part the T-cell-mediated skin diseases such as psoriasis. In both models, the CCL5 antagonists showed therapeutic efficacy by reducing swelling by 50% as well as the reduction of soluble mediators in homogenates derived from challenged ears. These results demonstrate that blocking the receptor or the ligand are both effective strategies to inhibit skin inflammation.

PMCID: PMC2806914
PMID: 20090949 [PubMed - indexed for MEDLINE]

452. J Cereb Blood Flow Metab. 2010 Apr;30(4):689-702. doi: 10.1038/jcbfm.2009.282. Epub 2010 Jan 20.

Mast cells as early responders in the regulation of acute blood-brain barrier changes after cerebral ischemia and hemorrhage.

Lindsberg PJ, Strbian D, Karjalainen-Lindsberg ML.

Department of Neurology, Helsinki University Central Hospital, Haartmaninkatu 8, 00290 Helsinki, Finland. perttu.lindsberg@hus.fi

The inflammatory response triggered by stroke has been viewed as harmful, focusing on the influx and migration of blood-borne leukocytes, neutrophils, and macrophages. This review hypothesizes that the brain and meninges have their own resident cells that are capable of fast host response, which are well known to mediate immediate reactions such as anaphylaxis, known as mast cells (MCs). We discuss novel research suggesting that by acting rapidly on the cerebral vessels, this cell type has a potentially deleterious role in the very early phase of acute cerebral ischemia and hemorrhage. Mast cells should be recognized as a potent inflammatory cell that, already at the outset of ischemia, is resident within the cerebral microvasculature. By releasing their cytoplasmic granules, which contain a host of vasoactive mediators such as tumor necrosis factor-alpha, histamine, heparin, and proteases, MCs act on the basal membrane, thus promoting blood-brain barrier (BBB) damage, brain edema, prolonged extravasation, and hemorrhage. This makes them a candidate for a new pharmacological target in attempts to even out the inflammatory responses of the neurovascular unit, and to

stabilize the BBB after acute stroke.

incidence confirms the importance of environment in its etiology. We aimed to assess the role of childhood and other environmental risk factors in IBD.
METHODS: A population-based case-control study was carried out in Canterbury, New Zealand. Participants comprised 638 prevalent Crohn's disease (CD) cases, 653 prevalent ulcerative colitis (UC) cases and 600 randomly-selected sex and age matched controls. Exposure rates to environmental risk factors were compared. Unadjusted and adjusted odds ratios (OR) with 95% confidence intervals (CI) are presented.
RESULTS: A family history of IBD (CD OR 3.06 [2.18-4.30], UC OR 2.52 [1.90-3.54]), cigarette smoking at diagnosis (CD OR 1.99 [1.48-2.68], UC OR 0.67 [0.48-0.94]), high social class at birth (CD and UC trend, P < 0.001) and Caucasian ethnicity (CD OR 2.04 [1.05-4.38], UC OR 1.47 [1.01-2.14]) were significantly associated with IBD. City living was associated with CD (P < 0.01). Being a migrant was associated with UC (UC OR 1.40 [1.14-2.01]). Having a childhood vegetable garden was protective against IBD (CD OR 0.52 [0.36-0.76], UC OR 0.65 [0.45-0.94]) as was having been breast-fed (CD OR 0.55 [0.41-0.74], UC OR 0.71 [0.52-0.96]) with a duration-response effect. Appendicectomy, tonsillectomy, infectious mononucleosis and asthma were more common in CD patients than controls (P < 0.01).
CONCLUSIONS: The importance of childhood factors in the development of IBD is confirmed. The duration-response protective association between breast-feeding and subsequent development of IBD requires further evaluation, as does the protective effect associated with a childhood vegetable garden.

PMID: 20074146 [PubMed - indexed for MEDLINE]

455. Rev Clin Esp. 2009 Dec;209(11):527-35.

[Skin diseases and tropical medicine. Results from a prospective study (2004-2007)].

[Article in Spanish]

Ramírez-Olivencia G, Bru Gorraiz FJ, Rivas González P, Lago Núñez M, Herrero Mendoza MD, Puente Puente S.

Unidad de Medicina Tropical. Servicio de Enfermedades Infecciosas. Hospital Carlos III. Madrid. España. germanramirezolivencia@hotmail.com

INTRODUCTION: An increase of international trips has been taken place in recent years, being Spain one of the principal issuing countries of internationl tourism. Dermatological diseases returning from tropical areas are frequent causes of medical consultation. Etiology is varied. OBJECTIVE. The aims of the present study are: to evaluate the importance of dermatological pathology in patients who come to a consultation of Tropical Medicine; to analyze the influence of duration, motive and the destination of the trip; and to describe the most frequent entities.
MATERIALS AND METHODS: An observational prospective study was realized, including all Spanish people older than 18 years-old who came to a consultation of Tropical Medicine. The period of study was between January 1st, 2004 and December 31st, 2007. Epidemiological and clinical items were collected from the group of patients with dermatological pathology.
RESULTS: There were attended 3,351 new consultations, with 660 cases of skin diseases. The infectious pathology constituted an almost the half (48.5%) of the dermatological pathology (320 cases). The injuries more frequently described were associated with stings arthropods (113 cases) and cutaneous larva migrans (CLM) (84), mycoses (52) and urticaria (43).
CONCLUSIONS: The appearance of dermatosis in the travelers seems to be determined by the motive, the duration and the destination. Given the heterogeneity of the

pathology, the recognition of the injuries is fundamental to initiate the suitable treatment.

PMID: 20067731 [PubMed - indexed for MEDLINE]

456. Immunology. 2010 Jan;129(1):115-24.

The function of CCR3 on mouse bone marrow-derived mast cells in vitro.

Collington SJ, Westwick J, Williams TJ, Weller CL.

Leukocyte Biology Section, MRC & Asthma UK Centre in Allergic Mechanisms of Asthma, National Heart and Lung Institute, Faculty of Medicine, Imperial College London, South Kensington, London, UK. sarah.collington@imperial.ac.uk

The mechanisms governing the population of tissues by mast cells are not fully understood, but several studies using human mast cells have suggested that expression of the chemokine receptor CCR3 and migration to its ligands may be important. In CCR3-deficient mice, a change in mast cell tissue distribution in the airways following allergen challenge was reported compared with wild-type mice. In addition, there is evidence that CCR3 is important in mast cell maturation in mouse. In this study, bone marrow-derived mast cells (BMMCs) were cultured and CCR3 expression and the migratory response to CCR3 ligands were characterized. In addition, BMMCs were cultured from wild-type and CCR3-deficient mice and their phenotype and migratory responses were compared. CCR3 messenger RNA was detectable in BMMCs, but this was not significantly increased after activation by immunoglobulin E (IgE). CCR3 protein was not detected on BMMCs during maturation and expression could not be enhanced after IgE activation. Resting and IgE-activated immature and mature BMMCs did not migrate in response to the CCR3 ligands eotaxin- 1 and eotaxin-2. Comparing wild-type and CCR3-deficient BMMCs, there were no differences in mast cell phenotype or ability to migrate to the mast cell chemoattractants leukotriene B4 and stem cell factor. The results of this study show that CCR3 may not mediate mast cell migration in mouse BMMCs in vitro. These observations need to be considered in relation to the findings of CCR3 deficiency on mast cells in vivo.

PMCID: PMC2807492
PMID: 20050333 [PubMed - indexed for MEDLINE]

457. Int J Emerg Med. 2010 Aug 21;3(4):227-32. doi: 10.1007/s12245-010-0198-4.

Utilization of a mobile medical van for delivering pediatric care in the bateys of the Dominican Republic.

Crouse HL, Macias CG, Cruz AT, Wilson KA, Torrey SB.

BACKGROUND: Bateys are impoverished areas of housing for migrant Haitian sugar cane workers in the Dominican Republic (DR). In these regions, preventative health care is almost non-existent, public service accessibility is limited, and geographic isolation prevents utilization of care even by those families with resources. Consequently, the development of a viable mobile system is vital to the delivery of acute and preventative health care in this region.
AIMS: This study evaluated an existing mobile medical system. The primary goal was to describe the population served, diseases treated, and resources utilized. A secondary goal was to determine qualitatively an optimal infrastructure for sustainable health care delivery within the bateys.
METHODS: Information on basic demographic data, diagnosis, chronicity of disease, and medications dispensed was collected on all pediatric patients seen in

conjunction with an existing mobile medical system over a 3-month period in the DR. Health statistics for the region were collected and interviews were conducted with health care workers (HCWs) and community members on existing and optimal health care infrastructure.
RESULTS: Five hundred eighty-four pediatric patients were evaluated and treated. Median age was 5 years (range 2 weeks to 20 years), and 53.7% of patients seen were 5 years of age or younger. The mean number of complaints per patient was 2.8 (range 0 to 6). Thirty-six percent (373) of all diagnoses were for acute complaints, and 64% (657) were chronic medical problems. The most common pediatric illnesses diagnosed clinically were gastrointestinal parasitic infection (56.6%), skin/fungal infection (46.2%), upper respiratory tract infections (URIs) (22.8%), previously undiagnosed asthma and allergies (8.2%), and symptomatic anemia (7.2%). Thirty HCWs and community members were interviewed, and all cited the need for similar resources: a community clinic and hospital referral site, health promoters within each community, and the initiation of pediatric training for community HCWs.
CONCLUSION: A mobile medical system is a sustainable, efficient mechanism for delivering acute and preventive care in the Haitian bateys of the Dominican Republic. The majority of patients served were 8 years of age or younger with multiple presenting symptoms. A pediatric protocol for identifying the most appropriate drugs and supplies for mobile units in the DR can be created based upon diseases evaluated. Qualitative data from HCWs and community members identified the need for an integrative health care delivery infrastructure and community health promoters versed in pediatric care who can aid in education of batey members and monitor chronic and acute illnesses. We are planning follow-up visits to implement these programs.

PMCID: PMC3047868
PMID: 21373288 [PubMed]

458. J Allergy Clin Immunol. 2010 Feb;125(2 Suppl 2):S314-23. doi: 10.1016/j.jaci.2009.08.018. Epub 2009 Dec 29.

Immunomodulator therapy: monoclonal antibodies, fusion proteins, cytokines, and immunoglobulins.

Lee SJ, Chinen J, Kavanaugh A.

Division of Rheumatology, Allergy, and Immunology, University of California, San Diego, CA 92093-0943, USA.

The immune system consists of a diverse array of immunocompetent cells and inflammatory mediators that exist in complex networks. These components interact through cascades and feedback circuits, maintaining physiologic inflammation (eg, tissue repair) and immunosurveillance. In various autoimmune and allergic diseases, a foreign antigen or autoantigen might upset this fine balance, leading to dysregulated immunity, persistent inflammation, and ultimately pathologic sequelae. In recent years, there has been tremendous progress delineating the specific components of the immune system that contribute to various aspects of normal immunity and specific disease states. With this greater understanding of pathogenesis coupled with advances in biotechnology, many immunomodulatory agents commonly called "biologic agents" have been introduced into the clinic for the treatment of various conditions, including immune globulins and cytokines. The 2 most common classes of approved biologic agents are mAbs and fusion proteins with exquisite specificity. These agents have the potential both to optimize outcomes through more thorough modulation of specific parts of the dysregulated immune response and to minimize toxicity compared with less specific methods of immunosuppression.

Copyright 2010 American Academy of Allergy, Asthma & Immunology. Published by Mosby, Inc. All rights reserved.

PMID: 20036416 [PubMed - indexed for MEDLINE]

459. Regul Pept. 2010 Feb 25;160(1-3):42-8. doi: 10.1016/j.regpep.2009.12.013. Epub 2009 Dec 24.

Sensory neuropeptides are potent chemoattractants for human basophils in vitro.

Cima K, Vogelsinger H, Kähler CM.

Pneumology, Internal Medicine I, Department of Internal Medicine, Medical University of Innsbruck, Anichstrasse 35, 6020 Innsbruck, Austria.

The sensory neuropeptides secretoneurin (SN) and substance P (SP) are involved in "neurogenic" inflammatory processes as they occur in bronchial asthma or allergic rhinitis. A possible interaction with basophils has not been reported to date. Basophils were isolated from healthy donors by magnetic cell sorting technique and migration was explored using Boyden microchemotaxis chambers. SN [10(-8)M] and SP [10(-6) to 10(-8)M] proved to be chemoattractants equally potent to FMLP [10(-8)M] or LPS [10 pg/ml]. Specific anti-SN antibodies and a trypsinization preparation of SN were used to determine the specificity of the SN effect on basophils. The preincubation of basophils with neurokinin-1 (NK-1) or -2 (NK-2) receptor antagonists revealed the SP effect to act via NK-1 receptors in basophils. In addition, we were able to show phosphodiesterases and phosphoinositide-3 kinases to be engaged in the downstream signalling pathway. Our observations reveal for the first time a link between basophils, which are engaged in allergic processes, and the neuropeptides SN and SP. Furthermore, our data might suggest phosphodiesterases or phosphoinositide-3 kinases to be new therapeutic targets for the treatment of allergic diseases such as asthma or allergic rhinitis.

Copyright 2009 Elsevier B.V. All rights reserved.

PMID: 20035805 [PubMed - indexed for MEDLINE]

460. J Allergy Clin Immunol. 2010 Feb;125(2):461-468.e6. doi: 10.1016/j.jaci.2009.09.016. Epub 2010 Jan 19.

Color-coded real-time cellular imaging of lung T-lymphocyte accumulation and focus formation in a mouse asthma model.

Hasegawa A, Hayashi K, Kishimoto H, Yang M, Tofukuji S, Suzuki K, Nakajima H, Hoffman RM, Shirai M, Nakayama T.

Department of Immunology, Graduate School of Medicine, Chiba University, Chiba 260-8670, Japan.

BACKGROUND: A critical role for CD4(+)T(H)2 cells in the pathogenesis of acute asthma has been demonstrated in the studies of human asthma as well as of animal models of asthma. T(H)2-cell migration into the lung is crucial for the initiation of asthma phenotype, but the dynamics of this process are poorly understood because it has been difficult to visualize this process.
OBJECTIVE: Our aim was to image the cellular dynamics of the migration of T(H)2 cells into the lung of living animals in a mouse model of asthma and identify the cellular processes required for the initiation of the asthma phenotype.
METHODS: We developed a color-coded real-time imaging model of cell migration

into the lung using green fluorescent protein (GFP) and red fluorescent protein (RFP) transgenic CD4 T cells.
RESULTS: Selective accumulation of antigen-specific CD4 T cells in the lungs was quantitatively imaged in a mouse model of asthma. The inhibition of accumulation by dexamethasone was imaged. Accumulating GFP(+) T(H)2 cells formed foci in the lungs from 6 to 20 hours after antigen inhalation. This process was also inhibited by the administration of anti-intercellular adhesion molecule 1 or anti-vascular cell adhesion molecule 1 mAbs. Two days after inhalation of antigen, GFP(+) T(H)2 cells were detected in the area of eosinophil infiltration.
CONCLUSION: Focus formation generated by accumulating antigen-specific T(H)2 cells in the lung appeared to be a critical process in the initiation of the asthma phenotype. This new model enables the study of in vivo cell biology of airway inflammation and novel drug discovery for lung inflammatory diseases.

Copyright 2010 American Academy of Allergy, Asthma & Immunology. Published by Mosby, Inc. All rights reserved.

PMID: 20031194 [PubMed - indexed for MEDLINE]

461. Clin Exp Allergy. 2010 Mar;40(3):427-34. doi: 10.1111/j.1365-2222.2009.03419.x. Epub 2009 Dec 16.

Interleukin-13 directly promotes oesophagus production of CCL11 and CCL24 and the migration of eosinophils.

Neilsen CV, Bryce PJ.

Division of Allergy-Immunology, Feinberg School of Medicine, Northwestern University, Chicago, IL 60610, USA.

BACKGROUND: Eosinophilic oesophagitis (EE) is a clinico-pathologically defined oesophageal disorder that is characterized by eosinophil migration into oesophageal tissues. There is growing support for EE being an allergic disease and for a contribution of T-helper type 2 (Th2)-associated cytokines in disease pathogenesis. The respiratory system has been shown to be critical in driving the development of EE in animal models. However, the mechanisms underlying the recruitment of eosinophils into the oesophagus remain unclear.
OBJECTIVE: We sought to investigate the influence of Th2-associated cytokines on the production of eosinophil-specific chemokines from the oesophagus directly.
METHODS: In order to eliminate the potential involvement of the lung, we utilized isolated oesophageal rings. These were treated in vitro with IL-4 or IL-13 and the expression and production of CCL11 and CCL24 were determined.
RESULTS: Our data demonstrate that IL-13 is a potent and direct inducer of both CCL11 and CCL24 production from the oesophagus, as is IL-4 also. The expression of CCL11 precedes CCL24 by several hours but then diminishes over time, as well as at high concentrations of IL-13. We demonstrate that there is an up-regulation of the inhibitory IL-13 receptor, IL-13Ralpha2 but that IL-13Ralpha1 remains unaltered. Oesophagus rings isolated from STAT6(-/-) mice were unable to produce CCL11 or CCL24 upon IL-13 treatment. Lastly, we demonstrate that oesophageal production of CCL11 and CCL24 upon IL-13 stimulation is sufficient to promote eosinophil migration.
CONCLUSIONS: IL-13 is capable of directly stimulating oesophageal tissue to produce eosinophil-attracting chemokines and drive eosinophil migration.

PMCID: PMC2835818
PMID: 20030665 [PubMed - indexed for MEDLINE]

462. Proc Natl Acad Sci U S A. 2009 Dec 29;106(52):22381-6. doi:

10.1073/pnas.0906372106. Epub 2009 Dec 16.

Mast cells regulate homeostatic intestinal epithelial migration and barrier function by a chymase/Mcpt4-dependent mechanism.

Groschwitz KR, Ahrens R, Osterfeld H, Gurish MF, Han X, Abrink M, Finkelman FD, Pejler G, Hogan SP.

Division of Allergy and Immunology, Department of Pediatrics, Cincinnati Children's Hospital Medical Center, University of Cincinnati College of Medicine, Cincinnati, OH 45229, USA.

Altered intestinal barrier function is postulated to be a central predisposing factor to intestinal diseases, including inflammatory bowel diseases and food allergies. However, the mechanisms involved in maintaining homeostatic intestinal barrier integrity remain undefined. In this study, we demonstrate that mice deficient in mast cells (Kit(W-sh/W-sh) [Wsh]) or mast cell chymase (Mcpt4(-/-)) have significantly decreased basal small intestinal permeability compared with wild-type (WT) mice. Altered intestinal barrier function was linked to decreased intestinal epithelial cell migration along the villus/crypt axis, altered intestinal morphology, and dysregulated claudin-3 crypt expression. Remarkably, engraftment of Wsh mice with WT but not Mcpt4(-/-) mast cells restored intestinal epithelial cell migration, morphology, and intestinal epithelial barrier function. Collectively, these findings identify a mechanism by which mast cells regulate homeostatic intestinal epithelial migration and barrier function.

PMCID: PMC2799737
PMID: 20018751 [PubMed - indexed for MEDLINE]

463. Zhonghua Yi Xue Za Zhi. 2009 Aug 4;89(29):2063-7.

[Experimental study of implantation of neurotropin-3 modified olfactory ensheathing cells in experimental allergic encephalomyelitis].

[Article in Chinese]

Guo SG, Qu CQ, Du YF, Wang MZ, Tang ZP, Zhang SM.

Department of Neurology, Provincial Hospital Affiliated to Shandong University,Jinan 250021, China.

OBJECTIVE: To investigate the therapeutic effect of neurotrophin-3 (NT-3) modified olfactory ensheathing cell (OEC) upon experimental allergic encephalomyelitis (EAE).
METHODS: OEC-NT-3 gene engineering cell, constructed by neurotrophin-3 transinfecting GEC inducted by retrovirus, was transplanted into lateral ventricle. The migration and distribution were observed and compared with control group and OEC transplantation group. Then myelin repairing and axon regeneration were evaluated from conical somatosensory evoked potential (CSEP), function score and ultrastructural morphology.
RESULTS: (1) OEC-NT-3 could survive, migrate within axons and spread diffusely away from the focus at Day 28 post-transplantation; (2) as compared with other two groups, more nerve fibers, better myelin repair and more distinct myelin structure were observed in the transgene group; (3) as compared with other two groups, the latent time was obviously shortened and the amplitude higher in the transplantation group ($P < 0.05$); (4) the transcription level of NT-3mRNA in the transgene group was significantly higher than the GEC group and the contrast group (212.32 +/- 16.14) x 10(-2) vs. (1.98 +/- 0.19) x 10(-2), (1.23 +/- 0.13) x 10(-2) ($P < 0.01$).

CONCLUSION: OEC-NT-3 cell expresses NT-3 stably and effectively in EAE. It may contribute to the repairing of myelin and the regeneration of axon.

PMID: 20017332 [PubMed - indexed for MEDLINE]

464. Eur J Immunol. 2010 Mar;40(3):787-96. doi: 10.1002/eji.200939724.

Thioredoxin suppresses airway inflammation independently of systemic Th1/Th2 immune modulation.

Torii M, Wang L, Ma N, Saito K, Hori T, Sato-Ueshima M, Koyama Y, Nishikawa H, Katayama N, Mizoguchi A, Shiku H, Yodoi J, Kuribayashi K, Kato T.

Department of Cellular and Molecular Immunology, Mie University Graduate School of Medicine, Tsu, Japan.

Oxidative stress plays an important role in the pathogenesis of asthma via the upregulation of local inflammatory mediators and/or promoting Th2-skewing during Ag sensitization. Thioredoxin (TRX), a 12 kDa redox-active protein with antioxidative property, has been recently shown to play a protective role in various inflammatory diseases. Using a mouse model of asthma, we show here that IL-13 and eotaxin production are decreased in TRX-Tg mice leading to reduced eosinophils recruitment and mucus metaplasia. The reduction in airway inflammation occurs without the attenuation of systemic Th2 immunity in that comparable levels of Th2-type cytokines and Ig were detected in LN and serum, respectively, from TRX-Tg and WT mice. Likewise, CD4(+) T cells from both strains of mice developed similar Th1 and Th2 responses in vitro. Asthmatic lungs of TRX-Tg and WT mice contained similar amounts of GATA-3(+) and Foxp3(+) T cells. Finally, production of MIF, an upstream modulator of airway inflammation, was significantly reduced in the lungs of TRX-Tg mice. Our data suggest that TRX suppresses airway inflammation by inhibiting MIF production thereby limiting the downstream recruitment of eosinophils to the lung independently of modulating systemic Th1/Th2 immunity.

PMID: 20017193 [PubMed - indexed for MEDLINE]

465. Clin Exp Immunol. 2010 Mar;159(3):303-14. doi: 10.1111/j.1365-2249.2009.04070.x. Epub 2009 Dec 4.

Systemic and local anti-C5 therapy reduces the disease severity in experimental autoimmune uveoretinitis.

Copland DA, Hussain K, Baalasubramanian S, Hughes TR, Morgan BP, Xu H, Dick AD, Nicholson LB.

Academic Unit of Ophthalmology, Department of Clinical Sciences South Bristol, Bristol, UK.

Activation of complement occurs during autoimmune retinal and intraocular inflammatory disease as well as neuroretinal degenerative disorders. The cleavage of C5 into fragments C5a and C5b is a critical event during the complement cascade. C5a is a potent proinflammatory anaphylatoxin capable of inducing cell migration, adhesion and cytokine release, while membrane attack complex C5b-9 causes cell lysis. Therapeutic approaches to prevent complement-induced inflammation include the use of blocking monoclonal antibodies (mAb) to prevent C5 cleavage. In these current experiments, the rat anti-mouse C5 mAb (BB5.1) was utilized to investigate the effects of inhibition of C5 cleavage on disease progression and severity in experimental autoimmune uveoretinitis (EAU), a model

of organ-specific autoimmunity in the eye characterized by structural retinal damage mediated by infiltrating macrophages. Systemic treatment with BB5.1 results in significantly reduced disease scores compared with control groups, while local administration results in an earlier resolution of disease. In vitro, contemporaneous C5a and interferon-gamma signalling enhanced nitric oxide production, accompanied by down-regulation of the inhibitory myeloid CD200 receptor, contributing to cell activation. These experiments demonstrate that C5 cleavage contributes to the full expression of EAU, and that selective C5 blockade via systemic and local routes of administration can suppress disease. This presents great therapeutic potential to protect against tissue damage during autoimmune responses in the retina or inflammation-induced degenerative disease.

PMCID: PMC2819496
PMID: 20002447 [PubMed - indexed for MEDLINE]

466. Respiration. 2010;80(6):463-71. doi: 10.1159/000264923. Epub 2009 Dec 7.

RANTES in exhaled breath condensate of allergic asthma patients with exercise-induced bronchoconstriction.

Zietkowski Z, Skiepko R, Tomasiak-Lozowska MM, Mroczko B, Szmitkowski M, Bodzenta-Lukaszyk A.

Departments of Allergology and Internal Medicine, Medical University of Bialystok, Bialystok, Poland. z.zietkowski@wp.pl

BACKGROUND: The response of asthmatics to exercise differs from that of healthy subjects, and the mechanisms responsible for exercise-induced bronchoconstriction (EIB) remain to be elucidated.
OBJECTIVES: The aim of this study was to evaluate changes in RANTES levels in exhaled breath condensate (EBC) following intensive exercise in allergic asthmatics.
METHODS: The study was conducted in a group of 19 asthmatics (11 with EIB and 8 without EIB) and 7 healthy volunteers. Changes in the concentrations of RANTES in EBC induced during the 24 h after intensive exercise were determined. Moreover, these measurements were tested for possible correlations with the results of other tests commonly associated with asthma as well as with changes in airway inflammation after exercise.
RESULTS: In contrast to asthmatic patients without EIB and healthy controls, in asthmatics with EIB RANTES concentrations were statistically significantly increased in EBC collected during the first 24 h after an exercise test. There was a statistically significant correlation between the maximum increase in RANTES concentrations in EBC after exercise and either baseline exhaled nitric oxide (F(ENO)) or bronchial hyperreactivity to histamine and an increase in serum eosinophil cationic protein or F(ENO) 24 h after exercise in the EIB asthmatics.
CONCLUSIONS: The increase in RANTES in asthmatic airways, promoting the migration and activation of inflammatory cells including eosinophils, may play an important role in the upregulation of airway inflammation after EIB in asthmatic patients.

Copyright © 2009 S. Karger AG, Basel.

PMID: 19996575 [PubMed - indexed for MEDLINE]

467. Environ Health. 2009 Dec 8;8:55. doi: 10.1186/1476-069X-8-55.

Variability in childhood allergy and asthma across ethnicity, language, and residency duration in El Paso, Texas: a cross-sectional study.

Svendsen ER, Gonzales M, Ross M, Neas LM.

University of South Carolina, Arnold School of Public Health, Columbia, SC, USA.
svendsee@mailbox.sc.edu

BACKGROUND: We evaluated the impact of migration to the USA-Mexico border city of El Paso, Texas (USA), parental language preference, and Hispanic ethnicity on childhood asthma to differentiate between its social and environmental determinants.
METHODS: Allergy and asthma prevalence was surveyed among 9797 fourth and fifth grade children enrolled in the El Paso Independent School District. Parents completed a respiratory health questionnaire, in either English or Spanish, and a sub-sample of children received spirometry testing at their school. Here we report asthma and allergy outcomes across ethnicity and El Paso residency duration.
RESULTS: Asthma and allergy prevalence increased with longer duration of El Paso residency independent of ethnicity and preferred language. Compared with immigrants who arrived in El Paso after entering first grade (18%), lifelong El Paso residents (68%) had more prevalent allergy (OR, 1.72; 95% CI, 1.32 - 2.24), prevalent asthma (OR, 1.75; 95% CI, 1.24 - 2.46), and current asthma (OR, 2.01; 95% CI, 1.37 - 2.95). Spirometric measurements (FEV1/FVC and FEF25-75) also declined with increasing duration of El Paso residency (0.16% and 0.35% annual reduction, respectively).
CONCLUSION: These findings suggest that a community-wide environmental exposure in El Paso, delayed pulmonary development, or increased health of immigrants may be associated with allergy and asthma development in children raised there.

PMCID: PMC2797777
PMID: 19995440 [PubMed - indexed for MEDLINE]

468. J Immunol. 2010 Jan 15;184(2):1003-13. doi: 10.4049/jimmunol.0900877. Epub 2009 Dec 4.

Inhibition of Th2 adaptive immune responses and pulmonary inflammation by leukocyte Ig-like receptor B4 on dendritic cells.

Breslow RG, Rao JJ, Xing W, Hong DI, Barrett NA, Katz HR.

Department of Medicine, Harvard Medical School and Division of Rheumatology, Immunology and Allergy, Brigham and Women's Hospital, Boston, MA 02115, USA.

We previously established that the inhibitory receptor LILRB4 mitigates LPS-induced, neutrophil-dependent pathologic effector mechanisms in inflammation. We now report that LILRB4 on dendritic cells (DCs) counterregulates development of an adaptive Th2 immune response and ensuing inflammation in a model of allergic pulmonary inflammation, initiated by inhalation sensitization with OVA and LPS followed by airway challenge with OVA. We found that Lilrb4(-/-) mice had significantly exacerbated eosinophilic pulmonary inflammation, as assessed in bronchoalveolar lavage and lung tissue, as well as elevated levels of OVA-specific IgE and Th2 cytokines produced by OVA-restimulated lymph node cells. LILRB4 was preferentially expressed on MHC class II(high)CD86(high) OVA-bearing DCs in lung-draining lymph nodes after sensitization or challenge. Moreover, the lymph nodes of Lilrb4(-/-) mice had significantly more of these mature DCs after challenge with OVA, which was accompanied by significantly more IL-4-producing lymphocytes, compared with Lilrb4(+/+) mice. Sensitization of naive Lilrb4(+/+) mice by transfer of OVA-LPS-pulsed Lilrb4(-/-) bone marrow-derived DCs was sufficient to confer exacerbated allergic lung pathology upon challenge with OVA, compared with mice that received Lilrb4(+/+) bone marrow-derived DCs. Our findings establish that maturation and migration of pulmonary DCs to lymph nodes

in response to Ag and an innate immune stimulus is associated with upregulated expression of LILRB4. In addition, this receptor attenuates the number of these mature DCs and attendant IL-4-producing lymphocytes in the lymph nodes, and accordingly, the ability of DCs to elicit pathologic Th2 pulmonary inflammation.

PMCID: PMC2836496
PMID: 19966208 [PubMed - indexed for MEDLINE]

469. Neuropeptides. 2010 Apr;44(2):107-13. doi: 10.1016/j.npep.2009.11.001. Epub 2009 Dec 3.

Bradykinin B(1) receptor antagonist R954 inhibits eosinophil activation/proliferation/migration and increases TGF-beta and VEGF in a murine model of asthma.

Vasquez-Pinto LM, Nantel F, Sirois P, Jancar S.

Department of Immunology, Institute of Biomedical Sciences, University of São Paulo, Brazil.

In the present study the effects of bradykinin receptor antagonists were investigated in a murine model of asthma using BALB/c mice immunized with ovalbumin/alum and challenged twice with aerosolized ovalbumin. Twenty four hours later eosinophil proliferation in the bone marrow, activation (lipid bodies formation), migration to lung parenchyma and airways and the contents of the pro-angiogenic and pro-fibrotic cytokines TGF-beta and VEGF were determined. The antagonists of the constitutive B(2) (HOE 140) and inducible B(1) (R954) receptors were administered intraperitoneally 30min before each challenge. In sensitized mice, the antigen challenge induced eosinophil proliferation in the bone marrow, their migration into the lungs and increased the number of lipid bodies in these cells. These events were reduced by treatment of the mice with the B(1) receptor antagonist. The B(2) antagonist increased the number of eosinophils and lipid bodies in the airways without affecting eosinophil counts in the other compartments. After challenge the airway levels of VEGF and TGF-beta significantly increased and the B(1) receptor antagonist caused a further increase. By immunohistochemistry techniques TGF-beta was found to be expressed in the muscular layer of small blood vessels and VEGF in bronchial epithelial cells. The B(1) receptors were expressed in the endothelial cells. These results showed that in a murine model of asthma the B(1) receptor antagonist has an inhibitory effect on eosinophils in selected compartments and increases the production of cytokines involved in tissue repair. It remains to be determined whether this effects of the B(1) antagonist would modify the progression of the allergic inflammation towards resolution or rather towards fibrosis.

Copyright 2009 Elsevier Ltd. All rights reserved.

PMID: 19962193 [PubMed - indexed for MEDLINE]

470. Allergy. 2010 Jul;65(7):840-9. doi: 10.1111/j.1398-9995.2009.02279.x. Epub 2009 Nov 26.

Murine and human Langerhans cells express a functional histamine H4 receptor: modulation of cell migration and function.

Gschwandtner M, Rossbach K, Dijkstra D, Bäumer W, Kietzmann M, Stark H, Werfel T, Gutzmer R.

Division of Immunodermatology and Allergy Research, Department of Dermatology and

Allergy, Hannover Medical School, Hannover, Germany.
gschwandtner.maria@mhhannover.de

BACKGROUND: Histamine is an important mediator of allergic reactions, and recent studies indicated that the function of different types of antigen presenting cells (APC) can be modulated by histamine, in particular via the newly described histamine H(4) receptor (H(4)R). Therefore, we investigated possible interactions of histamine via the H(4)R on Langerhans cells (LC), which represent the professional APC in the skin and therefore have an important role in the initiation and maintenance of allergic skin diseases.
METHODS: The expression of the H(4)R was evaluated by real-time PCR, flow cytometry and immunofluorescence staining. The function of the H(4)R was determined by intracellular flow cytometric measurement of chemokine production and LC migration assays.
RESULTS: Here, we show H(4)R expression on in vitro generated monocyte-derived LC (mRNA and protein) and on primary LC from murine and human skin samples (protein). The immunofluorescence staining in murine and human skin samples clearly proved that LC express the H(4)R in situ. Stimulation with histamine or a H(4)R agonist downregulated the chemokine (C-C motif) ligand 2 (CCL2) in human monocyte-derived LC and primary LC. Prestimulation with a selective H(4)R antagonist abolished this effect. Moreover, migration of LC from the epidermis was increased after H(4)R agonist stimulation in ex vivo migration assays using human epidermis and murine in vivo assays.
CONCLUSION: Our findings show that LC express a functional H(4)R and point towards a possible pathogenic relevance of the H(4)R in inflammatory and allergic diseases.

PMID: 19958313 [PubMed - indexed for MEDLINE]

471. PLoS One. 2009 Nov 26;4(11):e8045. doi: 10.1371/journal.pone.0008045.

Direct activation of RhoA by reactive oxygen species requires a redox-sensitive motif.

Aghajanian A, Wittchen ES, Campbell SL, Burridge K.

Department of Cell and Developmental Biology, University of North Carolina at Chapel Hill, Lineberger Comprehensive Cancer Center, Chapel Hill, North Carolina, United States of America. amir@med.unc.edu

BACKGROUND: Rho family GTPases are critical regulators of the cytoskeleton and affect cell migration, cell-cell adhesion, and cell-matrix adhesion. As with all GTPases, their activity is determined by their guanine nucleotide-bound state. Understanding how Rho proteins are activated and inactivated has largely focused on regulatory proteins such as guanine nucleotide exchange factors (GEFs) and GTPase activating proteins (GAPs). However, recent in vitro studies have indicated that GTPases may also be directly regulated by redox agents. We hypothesized that this redox-based mechanism occurs in cells and affects cytoskeletal dynamics, and in this report we conclude this is indeed a novel mechanism of regulating the GTPase RhoA.
METHODOLOGY/PRINCIPAL FINDINGS: In this report, we show that RhoA can be directly activated by reactive oxygen species (ROS) in cells, and that this requires two critical cysteine residues located in a unique redox-sensitive motif within the phosphoryl binding loop. First, we show that ROS can reversibly activate RhoA and induce stress fiber formation, a well characterized readout of RhoA activity. To determine the role of cysteine residues in this mechanism of regulation, we generated cysteine to alanine RhoA mutants. Mutation of these cysteines abolishes ROS-mediated activation and stress fiber formation, indicating that these residues are critical for redox-regulation of RhoA. Importantly, these mutants

maintain the ability to be activated by GEFs.
CONCLUSIONS/SIGNIFICANCE: Our findings identify a novel mechanism for the regulation of RhoA in cells by ROS, which is independent of classical regulatory proteins. This mechanism of regulation may be particularly relevant in pathological conditions where ROS are generated and the cellular redox-balance altered, such as in asthma and ischemia-reperfusion injury.

PMCID: PMC2778012
PMID: 19956681 [PubMed - indexed for MEDLINE]

472. Hum Genomics. 2009 Oct;4(1):43-55.

Evolutionary divergence and functions of the ADAM and ADAMTS gene families.

Brocker CN, Vasiliou V, Nebert DW.

Molecular Toxicology and Environmental Health Sciences Program, Department of Pharmaceutical Sciences, University of Colorado Denver, Aurora, CO 80045, USA.

The 'A-disintegrin and metalloproteinase' (ADAM) and 'A-disintegrin and metalloproteinase with thrombospondin motifs' (ADAMTS) genes make up two similar, yet distinct, gene families. The human and mouse genomes contain 21 and 24 putatively functional protein-coding ADAM genes, respectively, and 24 versus 32 putatively functional protein-coding ADAMTS genes, respectively. Analysis of evolutionary divergence shows that both families are unique. Each of the two families can be separated, if need be, into groups of more closely related members: six subfamilies for ADAM , four subfamilies for ADAMTS. The presence of both disintegrin and peptidase domains within the ADAM and ADAMTS proteins implies multiple biological roles within the cell. Membrane-anchored ADAM proteins are best known for their role in activating zymogens--including tumour necrosis factor-alpha, epidermal growth factor (EGF) and amyloid precursor protein (APP). ADAM proteins can also participate in cell adhesion via their interaction with integrins in neighbouring cells. ADAMTS are secreted proteins that participate in extracellular matrix maintenance by way of their cleavage of procollagen and proteoglycans. ADAMTS proteins also are involved in coagulation by cleaving von Willibrand factor precursor protein. ADAM and ADAMTS proteins participate in a wide range of cellular processes, including cell adhesion and migration, ectodomain shedding, proteolysis, development, ovulation and angiogenesis. Because these enzymes are believed to play an important role in a number of pathologies, including Alzheimer's disease, rheumatoid arthritis, atherosclerosis, asthma and cancer progression, the products of the ADAM and ADAMTS genes represent promising drug targets for the prevention and management of a number of human diseases.

PMID: 19951893 [PubMed - indexed for MEDLINE]

473. Methods Mol Biol. 2010;595:371-82. doi: 10.1007/978-1-60761-421-0_24.

Methods to study pulmonary dendritic cell migration.

Jakubzick C, Randolph GJ.

Department of Gene and Cell Medicine, Icahn Research Institute, Mount Sinai School of Medicine, New York, NY, USA.

Dendritic cell migration from the airway to the lymph nodes is a key event in the development of airway immunity during infection, allergy, and vaccination. With judicial selection of materials, there are two approaches to study dendritic cell

migration to the mediastinal lymph nodes without the induction of inflammation: airway administration of fluorescent OVA and latex beads. Our protocol describes how to label and track pulmonary dendritic cells from the airway and lung to the mediastinal lymph nodes and reveals how to avoid pitfalls and suboptimal assays.

PMID: 19941125 [PubMed - indexed for MEDLINE]

474. Clin Orthop Relat Res. 2010 Aug;468(8):2284-7. doi: 10.1007/s11999-009-1170-6. Epub 2009 Nov 21.

Case report: Fibroxanthoma: a complication of a biodegradable screw.

Sadat-Ali M, Azzam Q, Bluwi M, Al-Umran AS.

The University Department of Orthopaedic Surgery, College of Medicine, King Faisal University, Dammam, Saudi Arabia. drsadat@hotmail.com

Comment in
Clin Orthop Relat Res. 2011 Jan;469(1):300-1; author reply 302.

BACKGROUND: Biodegradable interference screws in anterior cruciate ligament (ACL) reconstruction have gained popularity because of their similar or superior fixation strength in comparison to metallic interference screws and because they do not cause imaging artifacts and do not need to be removed.
CASE DESCRIPTION: We report the case of a 23-year-old man who presented with slowly progressive firm swelling of 2 months' duration at the site of the tibial tunnel 3 years after ACL reconstruction using a biodegradable interference screw. After curettage and débridement, the material was sent for histopathologic examination, which was reported as a fibroxanthoma.
LITERATURE REVIEW: Reported complications are osteolysis around the screw, allergic reaction, sterile abscess formation, ganglion cyst formation, and intraarticular migration. A fibroxanthoma consists of fibroblasts and mononuclear or multinucleated cells with large lipid-filled histiocytes (foam cells). The cells are negative for S-100 and keratin and positive for anti-human macrophage marker HAM-56.
PURPOSES AND CLINICAL RELEVANCE: Use of biodegradable screws is associated with high healing rates and low complication rates; however, awareness of their potential complications may help in early recognition and prevention of associated morbidity.

PMCID: PMC2895830
PMID: 19936858 [PubMed - indexed for MEDLINE]

475. Trends Immunol. 2010 Jan;31(1):39-44. doi: 10.1016/j.it.2009.10.001. Epub 2009 Nov 18.

Eosinophils and allergic airway disease: there is more to the story.

Walsh ER, August A.

Center for Molecular Immunology & Infectious Disease and Department of Veterinary & Biomedical Sciences, The Pennsylvania State University, University Park, PA 16802, USA.

The eosinophil has been perceived as a terminal effector cell in allergic airway diseases. However, recent work has shown that this multifunctional cell could be more involved in the initial stages of allergic disease development than was previously thought, particularly with regard to the ability of the eosinophil to

modulate T-cell responses. In this review, we discuss recent advances that suggest that eosinophils can present antigen to naïve as well as to antigen-experienced T cells, induce T helper 2 cell development, cytokine production or both, and affect T-cell migration to sites of inflammation. These findings are changing the way that eosinophil function in disease is perceived, and represent a shift in the dogma of allergic disease development.

PMCID: PMC2846296
PMID: 19926338 [PubMed - indexed for MEDLINE]

476. J Immunol. 2009 Dec 15;183(12):8203-15. doi: 10.4049/jimmunol.0900646.

CD69 controls the pathogenesis of allergic airway inflammation.

Miki-Hosokawa T, Hasegawa A, Iwamura C, Shinoda K, Tofukuji S, Watanabe Y, Hosokawa H, Motohashi S, Hashimoto K, Shirai M, Yamashita M, Nakayama T.

Department of Immunology, Graduate School of Medicine, Chiba University, Chiba, Japan.

Airway inflammation and airway hyperresponsiveness are central issues in the pathogenesis of asthma. CD69 is a membrane molecule transiently expressed on activated lymphocytes, and its selective expression in inflammatory infiltrates suggests that it plays a role in the pathogenesis of inflammatory diseases. In CD69-deficient mice, OVA-induced eosinophilic airway inflammation, mucus hyperproduction, and airway hyperresponsiveness were attenuated. Cell transfer of Ag-primed wild-type but not CD69-deficient CD4 T cells restored the induction of allergic inflammation in CD69-deficient mice, indicating a critical role of CD69 expressed on CD4 T cells. Th2 responses induced by CD69-deficient CD4 T cells in the lung were attenuated, and the migration of CD4 T cells into the asthmatic lung was severely compromised. The expression of VCAM-1 was also substantially altered, suggesting the involvement of VCAM-1 in the CD69-dependent migration of Th2 cells into the asthmatic lung. Interestingly, the administration of anti-CD69 Ab inhibited the induction of the OVA-induced airway inflammation and hyperresponsiveness. This inhibitory effect induced by the CD69 mAb was observed even after the airway challenge with OVA. These results indicate that CD69 plays a crucial role in the pathogenesis of allergen-induced eosinophilic airway inflammation and hyperresponsiveness and that CD69 could be a possible therapeutic target for asthmatic patients.

PMID: 19923457 [PubMed - indexed for MEDLINE]

477. Immunol Cell Biol. 2010 Feb;88(2):220-3. doi: 10.1038/icb.2009.86. Epub 2009 Nov 17.

Inhibition of eosinophil migration by lactoferrin.

Bournazou I, Mackenzie KJ, Duffin R, Rossi AG, Gregory CD.

Medical Research Council (MRC) Centre for Inflammation Research, Queen's Medical Research Institute, The University of Edinburgh, Edinburgh, UK.

Eosinophilic granulocytes are innate effector cells that are important in immune responses against helminth parasitic infections and contribute towards the pathology associated with allergic inflammatory conditions, including allergic rhinitis and asthma. Their recruitment to inflammatory sites occurs in response to chemotactic and activation signals, such as eotaxin and interleukin-5, and is a tightly controlled process. However, the mechanisms that counterbalance these

positive chemoattractive processes, thereby preventing excessive eosinophil infiltration, have received little attention. Here, we show that, lactoferrin (LTF), a pleiotropic 80-kDa glycoprotein with iron-binding properties, acts as a powerful inhibitor of eosinophil migration. Irrespective of its source (milk or neutrophil derived), LTF inhibits eotaxin-stimulated eosinophil migration with no effects on eosinophil viability. Transferrin, a closely related cationic glycoprotein, failed to produce an analogous effect. Furthermore, the iron-saturation status of LTF did not influence the observed inhibitory effect on migration, proving that LTF exerts its effect on eosinophil chemotaxis independent of its iron-chelating activity. These results highlight LTF as one of the few molecules reported to negatively regulate eosinophil migration. Thus, through its ability to inhibit eosinophil migration, LTF has potential as an effective therapeutic in the control of eosinophil infiltration in atopic inflammatory conditions.

PMID: 19918259 [PubMed - indexed for MEDLINE]

478. J Immunol. 2009 Dec 1;183(11):7531-8. doi: 10.4049/jimmunol.0901341. Epub 2009 Nov 16.

Fms-like tyrosine kinase 3 ligand regulates migratory pattern and antigen uptake of lung dendritic cell subsets in a murine model of allergic airway inflammation.

Shao Z, Makinde TO, McGee HS, Wang X, Agrawal DK.

Department of Biomedical Sciences, Creighton University of School of Medicine, Omaha, NE 68178, USA.

Fms-like tyrosine kinase 3 ligand (Flt3L) reverses the features of allergic airway inflammation and increases a Th2-suppressive regulatory lung CD11c(high)CD11b(low) dendritic cell (DC) subset in a mouse model. We examined the migratory pattern and Ag uptake efficiency of lung DC subsets in the therapeutic effect of Flt3L. Lung CD11c(high)CD11b(low) and CD11c(low)CD11b(high) DCs from PBS-treated, OVA-sensitized, and Flt3L-treated/OVA-sensitized BALB/c mice were sorted using MACS and FACS for phenotype analysis. Lymphatic chemokine expression in thoracic lymph nodes was determined by immunohistochemistry. Migration of two lung DC subsets to lymphatic chemokines was examined in vitro using a Transwell chemotaxis assay. Labeled Ag was intranasally delivered into mouse lung to track the migration and Ag uptake of lung DCs. The in vitro cytokine secretion of mediastinal lymph node cells was determined using ELISA. CD11c(low)CD11b(high) DCs have higher expression of CCR5, CCR6, and CCR7, but lower expression of CCR2 than CD11c(high)CD11b(low) DCs. CD11c(low)CD11b(high) DCs in Flt3L-treated/OVA-sensitized mice demonstrated a less mature phenotype, inefficiency in Ag uptake, and impaired migration in vitro to lymphatic chemokine than those in OVA-sensitized mice. Administration of Flt3L decreased the expression of CCR5 and CCR7 in CD11c(low)CD11b(high) DCs in OVA-sensitized mice. Fewer Ag-carrying cells were detected in the lungs and lymph nodes in Flt3L-treated/OVA-sensitized mice than OVA-sensitized mice with a greater decrease in CD11c(low)CD11b(high) DCs. Mediastinal lymph node cells from Flt3L-treated mice secreted higher levels of Th1 cytokines and IL-10 than OVA-sensitized mice in vitro. In conclusion, Flt3L-generated lung immunogenic CD11c(low)CD11b(high) DCs have a less mature phenotype, impaired Ag uptake, and impaired migration to draining lymph nodes.

PMID: 19917684 [PubMed - indexed for MEDLINE]

479. Br J Pharmacol. 2009 Dec;158(7):1705-12. doi: 10.1111/j.1476-5381.2009.00452.x.

Epigallocatechin gallate reduces human monocyte mobility and adhesion in vitro.

Melgarejo E, Medina MA, Sánchez-Jiménez F, Urdiales JL.

Departamento de Biología Molecular y Bioquímica, Facultad de Ciencias, Universidad de Málaga, and CIBER de Enfermedades Raras (CIBERER), Málaga, Spain.

BACKGROUND AND PURPOSE: Monocytes/macrophages are an important population of immune inflammatory cells that have diverse effector functions in which their mobility and adhesion play a very relevant role. Epigallocatechin gallate (EGCG), a major component of green tea, has been reported to have anti-allergic and anti-inflammatory activities, but its effects on monocytes remain to be determined. Here we investigated the effects of EGCG on the migration and adhesion of monocytes.
EXPERIMENTAL APPROACH: We used a human monocyte cell line (THP-1) to analyse the effects of treatment with EGCG under non-cytotoxic conditions on the expression levels of the monocyte chemotactic protein-1 (MCP-1) and of the MCP-1 receptor (CCR2) and on the activation of beta1 integrin. A functional validation was carried out by evaluating the inhibitory effect of EGCG on monocyte adhesiveness and migration in vitro.
KEY RESULTS: Treatment of THP-1 cells with EGCG decreased MCP-1 and CCR2 gene expression, together with MCP-1 secretion and CCR2 expression at the cell surface. EGCG also inhibited beta1 integrin activation. The effects on these molecular targets were in agreement with the EGCG-induced inhibition of THP-1 migration in response to MCP-1 and adhesion to fibronectin.
CONCLUSIONS AND IMPLICATIONS: Under our experimental conditions, EGCG treatment inhibited the migration and adhesion of monocytes. These inhibitory effects of EGCG on monocyte function should be considered as a promising new anti-inflammatory response with a potential therapeutic role in the treatment of inflammation-dependent diseases.

PMCID: PMC2801211
PMID: 19912233 [PubMed - indexed for MEDLINE]

480. J Invest Dermatol. 2010 Apr;130(4):1023-33. doi: 10.1038/jid.2009.358. Epub 2009 Nov 12.

The histamine H4 receptor mediates inflammation and pruritus in Th2-dependent dermal inflammation.

Cowden JM, Zhang M, Dunford PJ, Thurmond RL.

Department of Immunology, Johnson & Johnson Pharmaceutical Research & Development, LLC, San Diego, California 92121, USA.

The role of histamine H(4) receptor (H(4)R) was investigated in a T-helper type 2 (Th2)-cell-mediated mouse skin inflammation model that mimics several of the features of atopic dermatitis. Treatment with two specific H(4)R antagonists before challenge with FITC led to a significant reduction in ear edema, inflammation, mast cell, and eosinophil infiltration. This was accompanied by a reduction in the levels of several cytokines and chemokines in the ear tissue. Upon ex vivo antigen stimulation of lymph nodes, H(4)R antagonism reduced lymphocyte proliferation and IL-4, IL-5, and IL-17 levels. One explanation for this finding is that lymph nodes from animals dosed with the H(4)R antagonist, JNJ 7777120, contained a lower number of FITC-positive dendritic cells. The effect of H(4)R antagonism on dendritic cell migration in vivo may be an indirect result of the reduction in tissue cytokines and chemokines or a direct effect on chemotaxis. In addition to anti-inflammatory effects, JNJ 7777120 also significantly inhibited the pruritus shown in the model. Therefore, the dual

effects of H(4)R antagonists on pruritus and Th2-cell-mediated inflammation point to their therapeutic potential for the treatment of Th2-mediated skin disorders, including atopic dermatitis.

PMID: 19907432 [PubMed - indexed for MEDLINE]

481. J Clin Invest. 2009 Dec;119(12):3723-38. doi: 10.1172/JCI39717. Epub 2009 Nov 9.

Lung interstitial macrophages alter dendritic cell functions to prevent airway allergy in mice.

Bedoret D, Wallemacq H, Marichal T, Desmet C, Quesada Calvo F, Henry E, Closset R, Dewals B, Thielen C, Gustin P, de Leval L, Van Rooijen N, Le Moine A, Vanderplasschen A, Cataldo D, Drion PV, Moser M, Lekeux P, Bureau F.

Laboratory of Cellular and Molecular Physiology, GIGA-Research, University of Liège, Liège, Belgium.

The respiratory tract is continuously exposed to both innocuous airborne antigens and immunostimulatory molecules of microbial origin, such as LPS. At low concentrations, airborne LPS can induce a lung DC-driven Th2 cell response to harmless inhaled antigens, thereby promoting allergic asthma. However, only a small fraction of people exposed to environmental LPS develop allergic asthma. What prevents most people from mounting a lung DC-driven Th2 response upon exposure to LPS is not understood. Here we have shown that lung interstitial macrophages (IMs), a cell population with no previously described in vivo function, prevent induction of a Th2 response in mice challenged with LPS and an experimental harmless airborne antigen. IMs, but not alveolar macrophages, were found to produce high levels of IL-10 and to inhibit LPS-induced maturation and migration of DCs loaded with the experimental harmless airborne antigen in an IL-10-dependent manner. We further demonstrated that specific in vivo elimination of IMs led to overt asthmatic reactions to innocuous airborne antigens inhaled with low doses of LPS. This study has revealed a crucial role for IMs in maintaining immune homeostasis in the respiratory tract and provides an explanation for the paradox that although airborne LPS has the ability to promote the induction of Th2 responses by lung DCs, it does not provoke airway allergy under normal conditions.

PMCID: PMC2786798
PMID: 19907079 [PubMed - indexed for MEDLINE]

482. Curr Eye Res. 2009 Jun;34(6):426-37.

Cyclosporin A eye drops inhibit fibrosis and inflammatory cell infiltration in murine type I allergic conjunctivitis without affecting the early-phase reaction.

Shii D, Nakagawa S, Shinomiya K, Yoshimi M, Katsuta O, Oda T, Nakamura M.

Research and Development Center, Santen Pharmaceutical Co., Ltd., 8916-16 Takayama-cho, Ikoma-shi, Nara 630-0101, Japan. daisuke.shii@santen.co.jp

PURPOSE: To understand the mechanisms of action of cyclosporin A eye drops in severe allergic diseases such as vernal keratoconjunctivitis, the inhibitory effects of cyclosporin A eye drops on fibrosis and inflammatory cell infiltration in murine allergic conjunctivitis were evaluated.
METHODS: BALB/c mice that had been actively sensitized with ovalbumin were challenged with ovalbumin on days 10-14 after initial sensitization. Cyclosporin A (0.1%) or betamethasone (0.1%) eye drops were instilled 1, 4, and 7 hours after

each challenge. Ocular tissue was harvested for histological evaluation 24 hours after the last challenge, and conjunctival tissue was collected for the measurement of collagen content and quantitative PCR 8 hours after the last challenge.
RESULTS: Scores for fibrosis and inflammatory cell infiltration and collagen content in the conjunctiva were higher after 5 days of antigen challenge than in normal non-challenged conjunctiva. Instillation of cyclosporin A or betamethasone reduced the antigen-induced increases in scores for fibrosis and inflammatory cell infiltration in the conjunctiva, and cyclosporin A significantly reduced the antigen-induced increase in conjunctival collagen content. Betamethasone also showed a tendency to reduce the increase in collagen content. Cyclosporin A and betamethasone decreased the numbers of CD3(+) and CD4(+) T-cells and eosinophils in the conjunctiva, but did not affect the number of mast cells. Neither type of eye drop suppressed the increase in vascular permeability that occurred for 30 minutes after the last antigen challenge. In quantitative PCR, cyclosporin A suppressed the expression of IL-4 and IL-5 mRNA but did not suppress the expression of transforming growth factor (TGF)-beta 1, whereas betamethasone suppressed the expression of IL-4, IL-5, and TGF-beta 1.
CONCLUSION: The results suggest that cyclosporin A eye drops inhibited fibrosis and inflammatory cell infiltration by the suppression of Th2 cytokine production in repeatedly antigen-challenged conjunctiva without affecting the early-phase reaction.

PMID: 19899977 [PubMed - indexed for MEDLINE]

483. Can J Physiol Pharmacol. 2009 Oct;87(10):743-55. doi: 10.1139/Y09-054.

S100A8/A9: a mediator of severe asthma pathogenesis and morbidity?

Halayko AJ, Ghavami S.

Department of Physiology, University of Manitoba, Respiratory Hospital, Winnipeg, MB R3A 1R8, Canada. ahalayk@cc.umanitoba.ca

Nearly 12% of children and 6% of adults in Canada have been diagnosed with asthma. Although in most patients symptoms are controlled by inhaled steroids, a subpopulation (approximately 10%) characterized by excessive airway neutrophilia, is refractory to treatment; these patients exhibit severe disease, and account for more than 50% of asthma health care costs. These numbers underscore the need to better understand the biology of severe asthma and identify pro-asthma mediators released by cells, such as neutrophils, that are unresponsive to common steroid therapy. This review focuses on a unique protein complex consisting of S100A8 and S100A9. These subunits belong to the large Ca2+-binding S100 protein family and are some of the most abundant proteins in neutrophils and macrophages. S100A8/A9 is a damage-associated molecular pattern (DAMP) protein complex released in abundance in rheumatoid arthritis, inflammatory bowel disease, and cancer, but there are no definitive studies on its role in inflammation and obstructive airways disease. Two receptors for S100A8/A9, the multiligand receptor for advanced glycation end products (RAGE) and Toll-like receptor 4 (TLR4), are expressed in lung. TLR4 is linked with innate immunity that programs local airway inflammation, and RAGE participates in mediating fibroproliferative remodeling in idiopathic pulmonary fibrosis. S100A8/A9 can induce cell proliferation, or apoptosis, inflammation, collagen synthesis, and cell migration. We hypothesize that this capacity suggests S100A8/A9 could underpin chronic airway inflammation and airway remodeling in asthma by inducing effector responses of resident and infiltrating airway cells. This review highlights some key issues related to this hypothesis and provides a template for future research.

PMID: 19898558 [PubMed - indexed for MEDLINE]

484. Allergy. 2010 Mar;65(3):319-26. doi: 10.1111/j.1398-9995.2009.02240.x. Epub 2009 Nov 2.

Histamine H(4) receptor antagonist ameliorates chronic allergic contact dermatitis induced by repeated challenge.

Seike M, Furuya K, Omura M, Hamada-Watanabe K, Matsushita A, Ohtsu H.

Department of Food and Nutrition Science, Sagami Women's Junior College, Bunkyo, Sagamihara, Kanagawa, Japan.

BACKGROUND: The present study observed effects of the histamine H(4) receptor on chronic allergic contact dermatitis induced by repeated challenge in mice.
METHODS: Acute contact dermatitis was induced by single epicutaneous challenge of 2,4,6-trinitro-1-chlorobenzene (TNCB) to the ear. Chronic allergic contact dermatitis was developed by repeated epicutaneous challenge using TNCB on the dorsal back skin. H(4) receptor antagonist JNJ7777120 was administered to wild-type mice, while H(4) receptor agonist 4-methylhistamine was administered to histidine decarboxylase (HDC) (-/-) mice that synthesized no histamine.
RESULTS: HDC (-/-) mice did not differ phenotypically from HDC (+/+) mice, and H(4) receptor antagonist/agonist did not have clinical effects in terms of acute contact dermatitis reactions. H(4) receptor antagonist ameliorated skin eczematous lesions induced by repeated TNCB challenge in HDC (+/+) mice. On the contrary, H(4) receptor agonist exacerbated skin lesions exclusively in HDC (-/-) mice. Application of H(4) receptor agonist induced migration of mast cells and eosinophils in skin lesions, and H(4) receptor antagonist suppressed these changes. H(4) receptor was immunohistochemically detected on mast cells in eczematous lesions. Levels of interleukin (IL)-4, -5, and -6 in lesions were decreased, whereas levels of interferon-gamma and IL-12 were increased by H(4) receptor antagonistic activity. Serum Immunoglobulin E levels rapidly increased with repeated challenge, but decreased with H(4) receptor antagonist.
CONCLUSION: Because chronic allergic contact dermatitis is developed by H(4) receptor stimulation, H(4) receptor antagonists might represent new candidate drugs for treating chronic allergic contact dermatitis.

PMID: 19886918 [PubMed - indexed for MEDLINE]

485. Pediatrics. 2009 Nov;124 Suppl 3:S224-36. doi: 10.1542/peds.2009-1100K.

Health care quality-improvement approaches to reducing child health disparities.

Chin MH, Alexander-Young M, Burnet DL.

University of Chicago, Department of Medicine, Section of General Internal Medicine, 5841 S Maryland Ave, MC 2007, Chicago, IL 60637, USA. mchin@medicine.bsd.uchicago.edu

Comment in
Pediatrics. 2009 Nov;124 Suppl 3:S255-6.

Relatively few quality-improvement efforts have been aimed at reducing differences in children's care and outcomes across race and ethnicity, socioeconomic status, and insurance status. To inform quality-improvement efforts to reduce child health disparities, we summarize lessons learned from the adult disparities-intervention literature, identify interventions that have reduced disparities in pediatric asthma outcomes and immunization rates, and outline

special considerations for child disparity interventions. Key recommendations for providers, health care organizations, and researchers include: (1) examine your performance data stratified according to insurance status, race/ethnicity, language, and socioeconomic status; (2) measure and improve childhood health-related quality of life, development, and condition-specific targets (such as asthma and immunizations); (3) measure and improve anticipatory guidance for early prevention of conditions (such as injuries, violence, substance abuse, and sexually transmitted diseases) and efforts to promote positive growth (such as readership programs to improve low literacy); (4) measure and improve structural aspects of care that affect child health outcomes and can reduce disparities, such as patient-centered medical-home elements; (5) incorporate families into interventions; (6) use multidisciplinary teams with close tracking and follow-up of patients; (7) integrate non-health care partners into quality-improvement interventions; and (8) culturally tailor quality improvement. A key recommendation for payers is to align financial incentives to reduce disparities. The National Institutes of Health and other funders should support (1) disparity-intervention studies on these recommendations that analyze clinical outcomes, intervention-implementation processes, and costs, and (2) creation of new child health services researchers who can find effective quality-improvement approaches for reducing disparities.

PMCID: PMC3132074
PMID: 19861474 [PubMed - indexed for MEDLINE]

486. J Asthma. 2009 Oct;46(8):796-802.

Associations of doctor-diagnosed asthma with immigration status, age at immigration, and length of residence in the United States in a sample of Mexican American School Children in Chicago.

Eldeirawi K, McConnell R, Furner S, Freels S, Stayner L, Hernandez E, Amoruso L, Torres S, Persky VW.

Health Systems Science, College of Nursing, University of Illinois at Chicago, IL 60612, USA. Keldei1@uic.edu

OBJECTIVES: Among Mexican Americans in the United States, children who were born in the US had higher rates of asthma than their Mexico-born peers. The purpose of this study was to examine the associations of doctor-diagnosed asthma with immigration-related variables and to investigate whether these associations could be explained by factors that may change with migration.
METHODS: We surveyed parents of 2,023 school children of Mexican descent and examined the associations of asthma with nativity, age at immigration, and length of residence in the US after adjusting for potential confounding variables.
RESULTS: In multivariate analyses, US-born children had a 2.42-fold (95% confidence interval [CI]: 1.52-3.83) increased odds of asthma compared with their Mexico-born peers. Mexico-born participants who moved to the US before 2 years of age were almost twice as likely to experience asthma compared with Mexico-born children who moved to the US >or=2 years of age. In addition, Mexico-born participants who lived in the US for 10 years or more were 2.37 times more likely to have asthma than Mexico-born students who lived in the US for less than 10 years. These associations were not explained by a wide variety of factors such as place of residence in infancy; exposure to animals/pets; history of infections, Tylenol use, and antibiotic use in infancy; breastfeeding; exposure to environmental tobacco smoke; daycare attendance and number of siblings; and language use.
CONCLUSIONS: Our findings point to the effects of nativity, age at immigration, and duration of residence in the US on the risk of asthma in Mexican American children, suggesting that potentially modifiable factors that change with

migration may be linked with the disease. The findings of this study should stimulate further research to explain factors that may be responsible for the observed differentials in the risk of asthma among Mexican Americans.

PMID: 19863283 [PubMed - indexed for MEDLINE]

487. Curr Opin Allergy Clin Immunol. 2010 Feb;10(1):82-6. doi: 10.1097/ACI.0b013e32833363b2.

Evidence for airway remodeling in chronic asthma.

Bai TR.

UBC James Hogg Research Centre, Providence Heart and Lung Institute, St Paul's Hospital and Department of Medicine, University of British Columbia, Vancouver, Canada. tbai@mrl.ubc.ca

PURPOSE OF REVIEW: This review focuses on recent findings in relation to potential functional consequences of structural changes in the asthmatic airway.
RECENT FINDINGS: Increases in smooth muscle mass have been shown to be an early finding in childhood asthma, related to clinical severity and predictive of greater airflow obstruction. Both hyperplasia and hypertrophy contribute to the increase in smooth muscle mass. A phenotypic shift in the epithelium of asthmatic airways related to stress and injury is suggested by recent data, with likely direct transformation of epithelial cells into mesenchymal cells. Fibrocyte in-migration from the vasculature may be an additional source of increased smooth muscle mass. The increased smooth muscle may contribute to neovascularization via vascular endothelial growth factor. Computed tomography studies continue to show some correlations between wall thickness and airway physiology. Exacerbations are predictive of greater lung function decline and hence remodeling.
SUMMARY: On balance, recent evidence continues to show that structural changes contribute to asthma persistence, airflow obstruction, lung function decline, and clinical severity, though there is increased recognition of the heterogeneity of asthma and in some phenotypes inflammatory cell influx or vascular effects may be more important than structural effects.

PMID: 19858714 [PubMed - indexed for MEDLINE]

488. Allergol Immunopathol (Madr). 2009 Nov-Dec;37(6):302-8. doi: 10.1016/j.aller.2009.06.003.

Evaluation of phagocytes in atopic dermatitis.

Forte WC, Guardian VC, Mantovani PA, Dionigi PC, Menezes MC.

Santa Casa Medical School and Hospital, São Paulo, Brazil. wilmanevesforte@yahoo.com.br

BACKGROUND: Patients with atopic dermatitis frequently present recurrent infections by pyogenic bacteria or by intracellular microorganisms, suggesting an immune disorder.
OBJECTIVE: Laboratorial investigation of phagocyte activity and chemotactic response by neutrophilic polymorphonuclear and mononuclear phagocytes in the peripheral blood of patients with atopic dermatitis from moderate to severe.
METHODS: Through a transversal study, patients with atopic dermatitis from moderate to severe were selected. The neutrophilic and mononuclear phagocytes were separated and the phagocytic ingestion of zymosan particles was analysed, in addition to migration distance to the bacterial lipopolysaccharide chemotactic

factor, comparing the results to the values obtained from healthy individuals within the same age group.
RESULTS: Nineteen patients were selected, 11 female and 8 male. The mean age was 6.47 years (+/-4.65). Among the 19 patients studied, 14 (73.68%) presented a reduction in the neutrophilic and mononuclear phagocyte activity, with two (1.53%) patients presenting a reduction in the activity of both phagocytes.
CONCLUSION: Our results demonstrated a reduction in chemotactic response and phagocytic activity by neutrophilic and/or mononuclear phagocytes in the majority of patients with atopic dermatitis from moderate to severe. Our results were coherent with the clinical data concerning the higher incidence of infections by pyogenic bacteria and fungi in patients with atopic dermatitis, which are microorganisms that require defence by the phagocytes researched in the present study.

PMID: 19853354 [PubMed - indexed for MEDLINE]

489. Mol Cell Biochem. 2010 Apr;337(1-2):77-81. doi: 10.1007/s11010-009-0287-y. Epub 2009 Oct 23.

Histamine stimulates human lung fibroblast migration.

Kohyama T, Yamauchi Y, Takizawa H, Kamitani S, Kawasaki S, Nagase T.

Department of Respiratory Medicine, Graduate School of Medicine, The University of Tokyo, Tokyo, Japan. koyama-tky@umin.ac.jp

Histamine is a potent mediator in allergic inflammatory processes and is released by basophils and mast cells. The aim of this study was to investigate the effect of histamine on in vitro migration of human fetal lung fibroblasts (HFL-1) to human plasma fibronectin (HFn), a chemoattractant. Using the blindwell chamber technique, histamine alone had no chemotactic activity. However, histamine augmented HFn-induced HFL-1 migration at concentrations ranging between 0 and 10(-7) M (290.6 +/- 20.8%) (P < 0.05). The concentration-response was bell-shaped. The effect of histamine increased with time. The stimulatory effect of histamine on HFL-1 migration was inhibited by an H4 receptor antagonist, JNJ7777120 (10(-5) M). Histamine's effect was also inhibited by pertussis toxin (50 ng/ml), showing that the effect was mediated by the H4 receptor. This study demonstrated that histamine has the potential to stimulate human lung fibroblast migration, and thus may contribute to regulation of wound healing and the development of fibrotic disorders of the lung.

PMID: 19851834 [PubMed - indexed for MEDLINE]

490. Br J Dermatol. 2010 Mar;162(3):487-96. doi: 10.1111/j.1365-2133.2009.09552.x. Epub 2009 Oct 15.

Visualizing CD4 T-cell migration into inflamed skin and its inhibition by CCR4/CCR10 blockades using in vivo imaging model.

Wang X, Fujita M, Prado R, Tousson A, Hsu HC, Schottelius A, Kelly DR, Yang PA, Wu Q, Chen J, Xu H, Elmets CA, Mountz JD, Edwards CK 3rd.

Department of Medicine, Division of Clinical Immunology and Rheumatology, University of Alabama at Birmingham, Birmingham, AL 35294, USA.

BACKGROUND: Chemokines are critical mediators of T-cell homing into inflamed skin. The complex nature of this multicellular response makes it difficult to analyse mechanisms mediating the early responses in vivo.

OBJECTIVES: To visualize directly T-cell homing into inflamed skin and its inhibition by blockades using a unique noninvasive confocal microscopy.
MATERIALS AND METHODS: A mouse model of allergic contact dermatitis was used. T cells from oxazolone-sensitized and -challenged Balb/c mice were first analysed phenotypically in vitro. CD4 T cells were then labelled with a tracker dye and transferred into Balb/c-SCID mice. The recipient mice were challenged with oxazolone and CD4 T-cell homing into inflamed skin was visualized.
RESULTS: T cells with the skin homing receptors CCR4 and CCR10 were increased in the affected skin and draining lymph nodes, and effectively attracted by their specific chemokines CCL17, CCL22 and CCL27 in vitro. Using in vivo imaging, T-cell migration into the inflamed skin was observed at 2 h after application, peaking at 12 h and continuing for 48 h. Simultaneous systemic administration of neutralizing antibodies against CCR4 ligands (CCL17 and CCL22) and CCR10 ligand (CCL27) led to a significant suppression of T-cell migration and skin inflammation.
CONCLUSIONS: Our data indicate that these tissue-selective adhesion molecules and chemokine/receptor pathways act in concert to attract specialized T-cell populations to mediate cutaneous inflammation. The in vivo imaging technique can be applicable to other models of cutaneous diseases to help with better understanding of the pathogenesis and monitoring the therapeutic effects.

PMCID: PMC3037861
PMID: 19832835 [PubMed - indexed for MEDLINE]

491. Cases J. 2009 May 11;2:6881. doi: 10.1186/1757-1626-2-6881.

The co-occurrence of Toxocara ocular and visceral larva migrans syndrome: a case series.

Paul M, Stefaniak J, Twardosz-Pawlik H, Pecold K.

Department and Clinic of Tropical and Parasitic Diseases, University of Medical Sciences, Przybyszewskiego 49, 60-355 Poznań, Poland. mpaul@am.poznan.p

INTRODUCTION: Ocular toxocarosis associated with high peripheral eosinophilia and together with systemic signs of visceral damage has been reported sporadically. Eye infections caused by numerous migrating larvae of Toxocara parasites, probably due to re-invasion or delayed reactivation, and leading to a progressive loss of vision is relatively rare. We report three atypical cases of toxocarosis with the co-existence of ocular larva migrans syndrome and generalized signs of Toxocara infection in schoolboys.
CASE PRESENTATION: Two children aged 8 and 14 years respectively, with symptomatic ocular and visceral larva migrans syndromes, and one 16-year-old adolescent with chronic multifocal eye invasion, characterized by severe granulomatous retinochoroiditis with unilateral blindness, chronic abdominal pain and generalized synthesis of total immunoglobulin E antibody are described. The three patients, heavily infected with Toxocara species were boys of Polish origin. Ocular location of the parasite was confirmed by the detection of intraocular synthesis of specific anti-Toxocara immunoglobulin G antibody in aqueous humour samples from the affected eyes. Immunological parameters of tissue eosinophilia, allergy or hypersensitivity reactions to the presence of the migrating Toxocara parasites were analysed. Irreversible eye complications were observed in the patients with high level of exposure to Toxocara species in a contaminated environment, with a suggestion of possible re-activation or re-infection by different species or strains of the parasite.
CONCLUSIONS: Wide promotion of sanitary education is strongly justified in children and adolescents in Toxocara endemic areas in order to reduce the potential risk of primary invasion or re-infection with the parasites, which can lead to a severe course or progression of the disease. A long-term clinical

follow-up and more intensive anti-parasitic treatment is recommended in patients with subclinical and overt forms of toxocarosis to prevent later reactivation of the migrating larvae in tissues.

PMCID: PMC2740066
PMID: 19829876 [PubMed]

492. Int J Immunopathol Pharmacol. 2009 Jul-Sep;22(3):591-603.

A new compound, 1H,8H-pyrano[3,4-c]pyran-1,8-dione, suppresses airway epithelial cell inflammatory responses in a murine model of asthma.

Lee H, Han AR, Kim Y, Choi SH, Ko E, Lee NY, Jeong JH, Kim SH, Bae H.

Department of Physiology, College of Oriental Medicine, Kyung Hee University, Seoul, Republic of Korea.

Clinical and experimental studies have established eosinophilia as a sign of allergic disorders. Activation of eosinophils in the airways is believed to cause epithelial tissue injury, contraction of airway smooth muscle and increased bronchial responsiveness. As part of the search for new antiasthmatic agents produced by medicinal plants, the effects of 270 standardized medicinal plant extracts on cytokine-activated A549 human lung epithelial cells were evaluated. After several rounds of activity-guided screening, the new natural compound, 1H,8H-Pyrano[3,4-c]pyran-1,8-dione (PPY), was isolated from Vitex rotundifolia L. To elucidate the mechanism by which the anti-asthmatic responses of PPY occurred in vitro, lung epithelial cells (A549 cell) were stimulated with TNF-alpha, IL-4 and IL-1beta to induce the expression of chemokines and adhesion molecules involved in eosinophil chemotaxis. PPY treatments reduced the expression of eotaxin, IL-8, IL-16 and VCAM-1 mRNA significantly. Additionally, PPY reduced eotaxin secretion in a dose-dependent manner and significantly inhibited eosinophil migration toward A549 medium. In addition, PPY treatment suppressed the phosphorylation of p65 and ERK1/2, suggesting that it can inhibit the MAPK/NF-KB pathway. To clarify the anti-inflammatory and antiasthmatic effects of PPY in vivo, we examined the influence of PPY on the development of pulmonary eosinophilic inflammation in a murine model of asthma. To accomplish this, mice were sensitized and challenged with ovalbumin (OVA) and then examined for the following typical asthmatic reactions: an increase in the number of eosinophils in BALF; the presence of Th2 cytokines such as IL-4 and IL-5 in the BALF; the presence of allergen-specific IgE in the serum; and a marked influx of inflammatory cells into the lung. Taken together, our results revealed that PPY exerts profound inhibitory effects on the accumulation of eosinophils into the airways while reducing the levels of IL-4, IL-5, and IL-13 in the BALF. Therefore, these results suggest that PPY may be useful as a new therapeutic drug for the treatment of allergic asthma.

PMID: 19822076 [PubMed - indexed for MEDLINE]

493. Eur J Pharmacol. 2009 Dec 10;624(1-3):38-44. doi: 10.1016/j.ejphar.2009.09.058. Epub 2009 Oct 8.

Novel CC chemokine receptor 4 antagonist RS-1154 inhibits ovalbumin-induced ear swelling in mice.

Nakagami Y, Kawashima K, Yonekubo K, Etori M, Jojima T, Miyazaki S, Sawamura R, Hirahara K, Nara F, Yamashita M.

Biological Research Laboratories, Daiichi Sankyo Co., Ltd., 1-16-13, Kitakasai,

Edogawa-ku, Tokyo 134-8630, Japan. nakagami.yasuhiro.y4@daiichisankyo.co.jp

CC chemokine ligand 17 (CCL17/thymus and activation-regulated chemokine: TARC) and CCL22 (macrophage-derived chemokine: MDC) selectively bind to CC chemokine receptor 4 (CCR4). The CCR4 system is considered to be responsible for the pathology of allergic diseases such as atopic dermatitis. To find and develop potential medicines against allergic diseases, we screened an in-house library to search for compounds having a profile as a CCR4 antagonist. From among the screening hits, we focused on 3-{2-[(2R)-2-phenyl-4-(4-pyridin-4-ylbenzyl)morpholin-2-yl]ethyl}quinazoline-2,4(1H,3H)-dione (named RS-1154), which had been newly synthesized in our laboratory. This compound inhibited the binding of [(125)I]CCL17 to human CCR4-expressing CHO cells with an IC(50) value of 27.7 nM and moreover inhibited CCL17-induced migration of DO11.10 mice-derived T helper 2 cells with an IC(50) value of 1.5 nM in vitro. We then examined the effect of RS-1154 in an ovalbumin-induced ear swelling assay. The ear thickness was decreased by intravenous administration of anti-CCL17 or anti-CCL22 antibodies, suggesting that the CCR4 system is involved in the ear swelling. Though partially, the oral administration of RS-1154 also significantly ameliorated the ear swelling at the doses of 30 and 100 mg/kg. Furthermore, the serum level of interleukin-4 decreased after the administration of RS-1154. In this study, we succeeded in obtaining a newly-synthesized compound, RS-1154, which has a potential to inhibit the chemotaxis of T helper 2 cells in vitro and to ameliorate ovalbumin-induced ear swelling in vivo. These results raise the possibility that RS-1154 or one of derivatives might become a therapeutic agent for atopic dermatitis patients.

PMID: 19818758 [PubMed - indexed for MEDLINE]

494. Am J Respir Crit Care Med. 2010 Jan 1;181(1):7-16. doi: 10.1164/rccm.200902-0190OC. Epub 2009 Oct 8.

Specific CD8 T cells in IgE-mediated allergy correlate with allergen dose and allergic phenotype.

Aguilar-Pimentel JA, Alessandrini F, Huster KM, Jakob T, Schulz H, Behrendt H, Ring J, de Angelis MH, Busch DH, Mempel M, Ollert M.

Department of Dermatology and Allergy, Biederstein Technische Universität München, Munich, Germany.

RATIONALE: Studies in humans and rodents have indicated a causative role for CD8(+) T cells in IgE-mediated allergic inflammation, but their function is still controversial.
OBJECTIVES: To analyze the role of allergen-specific CD8(+) T cells during the development of allergic airway inflammation in two parallel but diverging outcome models.
METHODS: We used H2-Kb SIINFEKL (OVA(257-264)) multimers to analyze induction, natural distribution, and phenotype of allergen-specific CD8(+) T cells in a murine C57BL/6 model of ovalbumin (OVA)-induced allergic airway inflammation using low-dose or high-dose OVA sensitization.
MEASUREMENTS AND MAIN RESULTS: The low-dose protocol was characterized by a significant induction of total and OVA-specific IgE, eosinophilic airway inflammation, IL-4 levels in bronchoalveolar lavage fluid. And significant alterations in lung function. The high dose protocol was characterized by a significant reduction of the allergic phenotype. Using OVA(257-264) H2-Kb multimers, we observed lung and airway infiltrating OVA-specific CD8(+) T cells showing an effector/effector-memory phenotype. The high-dose protocol caused significantly higher infiltration of allergen-specific CD8(+) cells to the airways and enhanced their cytotoxicity. Adoptive transfer with CD8(+) T cells

from transgenic OT-I mice to TAP1(-/-) or wild-type mice showed their migration to the lungs and TAP1-dependent proliferation after OVA-aerosol exposure. TAP1(-/-) mice defective in CD8(+) T cells showed exacerbated symptoms in the low-dose sensitization model.
CONCLUSIONS: Allergen-specific CD8(+) T cells seem to protect from allergic inflammation in the lungs. Their number, which is dependent on the sensitization dose, appears to be a critical predictor for the severity of the allergic phenotype.

PMID: 19815810 [PubMed - indexed for MEDLINE]

495. Autoimmunity. 2009 May;42(4):376-9.

Pathogenesis of Churg-Strauss syndrome: recent insights.

Zwerina J, Axmann R, Jatzwauk M, Sahinbegovic E, Polzer K, Schett G.

Department of Internal Medicine 3, Institute for Clinical Immunology, University of Erlangen-Nuremberg, Erlangen, Germany. jochen.zwerina@uk-erlangen.de

Churg-Strauss syndrome (CSS) is a rare systemic necrotizing vasculitis associated with granuloma formation and severe blood and tissue eosinophilia. CSS occurs almost exclusively in patients with asthma. Its pathogenesis remains largely unknown, as triggering factors for CSS development have not been identified so far. AAb, such as anti-neutrophil cytoplasmic autoantibodies, are found in less than half of patients and possibly constitute a subtype of CSS with different clinical behaviour. On a cellular level, CSS is characterized by a strong Th2-type immune response. Th2-associated cytokines such as IL-4, IL-13 and IL-5 may precipitate the severe eosinophilia in CSS, while migration of Eos to inflammatory sites is possibly mediated by eotaxin-3. This review summarizes recent advances in the knowledge on epidemiology, clinical features, and pathogenesis of CSS.

PMID: 19811306 [PubMed - indexed for MEDLINE]

496. Ann Neurol. 2009 Sep;66(3):390-402. doi: 10.1002/ana.21748.

Preferential recruitment of interferon-gamma-expressing TH17 cells in multiple sclerosis.

Kebir H, Ifergan I, Alvarez JI, Bernard M, Poirier J, Arbour N, Duquette P, Prat A.

Neuroimmunology Research Unit, Center for Excellence in Neuromics, Montreal, Quebec, Canada.

OBJECTIVE: There is substantial evidence supporting the role of interferon (IFN)-gamma-producing T helper (T(H)) 1 and interleukin (IL)-17-expressing T(H)17 lymphocytes in multiple sclerosis (MS) and its animal model, experimental allergic encephalomyelitis (EAE). However, to date little is known about the potential cooperative interplay between these 2 cytokines. In the current study, we sought to evaluate the frequency of IFN-gamma-expressing T(H)17 lymphocytes in MS and EAE, and study their recruitment into the central nervous system (CNS).
METHODS: Human T(H)17 lymphocytes were expanded in vitro from the blood of healthy controls and relapsing MS patients using IL-23. Immune cell migration to the CNS was assessed in vitro with primary cultures of human blood-brain barrier (BBB)-derived endothelial cells, and in vivo in EAE mice.
RESULTS: We demonstrate that in response to IL-23, human memory lymphocytes

expand into a T(H)17 phenotype, with a subpopulation of cells simultaneously expressing IFN-gamma and IL-17. We note that lymphocytes obtained from the blood of relapsing MS patients have an increased propensity to expand into IFN-gamma-producing T(H)17 cells and identify numerous T lymphocytes coexpressing IL-17 and IFN-gamma in brain tissue of MS patients. We also find lymphocytes expressing both the T(H)1- and the T(H)17-associated transcription factors ROR gamma t and T-bet, in situ and in vitro. We further provide in vitro and in vivo evidence that IFN-gamma(+) T(H)17 lymphocytes preferentially cross the human BBB and accumulate in the CNS of mice during the effector phase of EAE.
INTERPRETATION: Our data underscore the involvement of IFN-gamma(+) T(H)17 lymphocytes in the pathology of MS and EAE and their preferential recruitment into the CNS during inflammatory events.

PMID: 19810097 [PubMed - indexed for MEDLINE]

497. Am J Respir Cell Mol Biol. 2010 Sep;43(3):269-75. doi: 10.1165/rcmb.2009-0198OC. Epub 2009 Oct 5.

Both hematopoietic-derived and non-hematopoietic-derived {beta}-arrestin-2 regulates murine allergic airway disease.

Hollingsworth JW, Theriot BS, Li Z, Lawson BL, Sunday M, Schwartz DA, Walker JK.

Duke University Medical Center, Durham, NC 27710, USA.

Allergic asthma, a major cause of morbidity and leading cause of hospitalizations, is an inflammatory disease orchestrated by T helper cells and characterized by the lung migration of eosinophils, which are important asthma effector cells. Lung migration of inflammatory cells requires, among other events, the chemokine receptor transduction of lung-produced inflammatory chemokines. Despite the widespread prevalence of this disease, the molecular mechanisms regulating chemokine production and receptor regulation in asthma are poorly understood. Previous work from our laboratory demonstrated that beta-arrestin-2 positively regulates the development of allergic airway disease in a mouse model, partly through positive regulation of T-lymphocyte chemotaxis to the lung. However, beta-arrestin-2 is expressed in many cell types, including other hematopoietic cells and lung structural cells, which are involved in the development and manifestation of allergic airway disease. To determine the cell types required for beta-arrestin-2-dependent allergic inflammation, we generated bone marrow chimera mice. Using the ovalbumin murine model of allergic airway disease, we show that eosinophilic and lymphocytic inflammation is restored in chimeric mice, with expression of beta-arrestin-2 exclusively on hematopoietic-derived cell types. In contrast, airway hyperresponsiveness is dependent on the expression of beta-arrestin-2 in structural cells. Our data demonstrate that the expression of beta-arrestin-2 in at least two divergent cell types contributes to the pathogenesis of allergic airway disease.

PMCID: PMC2933545
PMID: 19805483 [PubMed - indexed for MEDLINE]

498. Allergy. 2010 Mar;65(3):338-46. doi: 10.1111/j.1398-9995.2009.02158.x. Epub 2009 Oct 1.

Genetic variability in CRTH2 polymorphism increases eotaxin-2 levels in patients with aspirin exacerbated respiratory disease.

Palikhe NS, Kim SH, Cho BY, Ye YM, Choi GS, Park HS.

Department of Allergy & Rheumatology, Ajou University School of Medicine, Suwon, South Korea.

INTRODUCTION: CRTH2 is expressed on the surface of eosinophils and has been shown to mediate PGD2-induced eosinophil migration in vitro. Eosinophilic infiltration in the upper and lower airways is the key feature of asthma. Considering the fact that eosinophil infiltration is prominent in the upper and lower airways of aspirin exacerbated respiratory disease (AERD) compared to aspirin-tolerant asthma (ATA) patients, we hypothesized that activation of eosinophils via dysregulation of the CRTH2 gene may play an important role and be an important marker for AERD.
METHODS: The three study groups - 107 with AERD, 115 with ATA and 133 normal healthy controls (NC) - were recruited from Ajou University Hospital, South Korea. Two polymorphisms of the CRTH2 gene at -466T>C and -129C>A were genotyped using primer extension methods.
RESULTS: AERD patients had significantly higher serum eotaxin-2 levels than did those with ATA ($P = 0.034$). A significant difference in the genotype frequencies of CRTH2 -466T>C was detected between AERD and ATA patients ($P < 0.05$). The serum eotaxin-2 level was significantly higher in AERD patients carrying the TT genotype of CRTH2 -466T>C than those with the CT and CC ($P < 0.05$). In vitro functional study demonstrated that the -466T allele had lower luciferase activity ($P < 0.001$) and lower mRNA expression with higher production of eotaxin-2 ($P = 0.003$) in human lung epithelial cells. EMSA showed that CRTH2 -466T produced a specific band with a higher affinity than CRTH2 -466C had.
CONCLUSION: The CRTH2 -466T>C polymorphism increases serum and cellular eotaxin-2 production through lowered CRTH2 expression, leading to eosinophilic infiltration in AERD patients.

PMID: 19796209 [PubMed - indexed for MEDLINE]

499. World Allergy Organ J. 2009 Oct;2(10):224-32. doi: 10.1097/WOX.0b013e3181c2a95e.

Mast cell regulation of the immune response.

Ryan JJ, Morales JK, Falanga YT, Fernando JF, Macey MR.

From the Department of Biology, Virginia Commonwealth University, Richmond, VA.

Mast cells are well known as principle effector cells of type I hypersensitivity responses. Beyond this role in allergic disease, these cells are now appreciated as playing an important role in many inflammatory conditions. This review summarizes the support for mast cell involvement in resisting bacterial infection, exacerbating autoimmunity and atherosclerosis, and promoting cancer progression. A commonality in these conditions is the ability of mast cells to elicit migration of many cell types, often through the production of inflammatory cytokines such as tumor necrosis factor. However, recent data also demonstrates that mast cells can suppress the immune response through interleukin-10 production. The data encourage those working in this field to expand their view of how mast cells contribute to immune homeostasis.

PMID: 23283207 [PubMed]

500. Int Arch Allergy Immunol. 2010;151(3):214-22. doi: 10.1159/000242359. Epub 2009 Sep 29.

Mast cells induce migration of dendritic cells in a murine model of acute allergic airway disease.

Reuter S, Dehzad N, Martin H, Heinz A, Castor T, Sudowe S, Reske-Kunz AB, Stassen M, Buhl R, Taube C.

Department of Pulmonary Medicine, Third Medical Clinic, Johannes Gutenberg University Mainz, Mainz, Germany.

Background: The migration of dendritic cells (DCs) from the lungs to the regional lymph nodes is necessary for the development of allergic airway disease. Following activation, mast cells release a variety of stored or de novo-produced inflammatory mediators, several of them being capable of activating DCs. In this study, the role of mast cells on DC migration from the lungs to the thoracic lymph nodes was investigated in sensitized mice. Methods: Mast cell-deficient mice (Kit(W-sh/W-sh)) and their wild-type counterparts were sensitized intraperitoneally with ovalbumine (OVA) in saline and challenged by a single intranasal administration of OVA labeled with a fluorescent dye (OVA-Alexa). Results: Following challenge, the relative and absolute amount of OVA-Alexa-positive DCs was clearly increased in sensitized wild-type mice compared to nonsensitized mice. In contrast, sensitized Kit(W-sh/W-sh) showed no increase in OVA-Alexa-positive DCs compared to nonsensitized mast cell-deficient animals. In sensitized Kit(W-sh/W-sh) mice reconstituted with bone marrow-derived mast cells (BMMCs), the number of OVA- Alexa-positive DCs was comparable to that in sensitized wild-type animals. However, transfer of allergen-exposed BMMCs to sensitized mice prior to airway challenge augmented airway inflammation similarly in wild-type and mast cell-deficient mice. In line with this, sensitization with allergen-pulsed DCs induced allergic airway disease independently of mast cells. Conclusions: This study shows an interaction between mast cells and DCs following allergen challenge in sensitized hosts. However, the function of mast cells can be bypassed in models utilizing activated allergen-exposed DCs to initiate the development of allergic airway disease.

PMID: 19786802 [PubMed - indexed for MEDLINE]

501. Stem Cells. 2009 Nov;27(11):2824-32. doi: 10.1002/stem.225.

Complement-derived anaphylatoxin C3a regulates in vitro differentiation and migration of neural progenitor cells.

Shinjyo N, Ståhlberg A, Dragunow M, Pekny M, Pekna M.

Department of Medical Chemistry and Cell Biology, Institute of Biomedicine, Sahlgrenska Academy at University of Gothenburg, SE-405 30 Gothenburg, Sweden.

Anaphylatoxin C3a is a third complement component (C3)-derived peptide, the multiple functions of which range from stimulation of inflammation to neuroprotection. In a previous study, we have shown that signaling through C3a receptor positively regulates in vivo neurogenesis in adult mouse brain. Here, we studied the direct effects of C3a on adult mouse whole brain-derived neural progenitor cells (NPCs) in vitro. Our results demonstrate that NPCs bind C3a in a specific and reversible manner and that C3a stimulates neuronal differentiation of NPCs. Furthermore, C3a stimulated the migration of NPCs induced by low concentrations of stromal cell-derived factor (SDF)-1alpha, whereas it inhibited NPC migration at high concentration of SDF-1alpha. In the same manner, C3a modulated SDF-1alpha-induced extracellular-signal-regulated kinases 1 and 2 (ERK1/2) phosphorylation in these cells. In addition, C3a had inhibitory effect on SDF-1alpha-induced neuronal differentiation of NPCs. These data show that C3a modulates SDF-1alpha-induced differentiation and migration of these cells, conceivably through the regulation of ERK1/2 phosphorylation. Our results provide the first evidence that C3a regulates neurogenesis by directly affecting the fate and properties of NPCs.

PMID: 19785034 [PubMed - indexed for MEDLINE]

502. Transl Res. 2009 Oct;154(4):165-74. doi: 10.1016/j.trsl.2009.06.008. Epub 2009 Jul 14.

Targeting the airway smooth muscle for asthma treatment.

Camoretti-Mercado B.

Section of Pulmonary and Critical Care Medicine, Department of Medicine, University of Chicago, Chicago, IL, USA. bcamoret@medicine.bsd.uchicago.edu

Asthma is a complex respiratory disease whose incidence has increased worldwide in the last decade. Currently there is no cure for asthma. Although bronchodilator and anti-inflammatory medications are effective medicines in some asthmatic patients, it is clear that an unmet therapeutic need persists for a subpopulation of individuals with severe asthma. This chronic lung disease is characterized by airflow limitation, lung inflammation, and remodeling that includes increased airway smooth muscle (ASM) mass. In addition to its contractile properties, the ASM also contributes to the inflammatory process by producing active mediators, which modify the extracellular matrix composition and interact with inflammatory cells. These undesirable functions make interventions aimed at reducing ASM abundance an attractive strategy for novel asthma therapies. The following three mechanisms could limit the accumulation of smooth muscle: decreased cell proliferation, augmented cell apoptosis, and reduced cell migration into the smooth muscle layer. Inhibitors of the mevalonate pathway or statins hold promise for asthma treatment, because they exhibit anti-inflammatory, antimigratory, and antiproliferative effects in preclinical and clinical studies, and they can target the smooth muscle. This review will discuss current knowledge of ASM biology and identify gaps in the field to stimulate future investigations of the cellular mechanisms that control ASM overabundance in asthma. Targeting ASM has the potential to be an innovative venue of treatment for patients with asthma.

PMCID: PMC2764304
PMID: 19766960 [PubMed - indexed for MEDLINE]

503. Immunity. 2009 Sep 18;31(3):412-24. doi: 10.1016/j.immuni.2009.08.008.

Biology of lung dendritic cells at the origin of asthma.

Lambrecht BN, Hammad H.

Department of Respiratory Diseases, Laboratory of Immunoregulation and Mucosal Immunology, University Hospital Gent, Gent 9000, Belgium. bart.lambrecht@ugent.be

Dendritic cells (DCs) initiate and maintain adaptive T helper 2 (Th2) cell responses to inhaled allergens in asthma. Various functions like antigen uptake, migration to the draining LNs, and induction of tolerance and adaptive immunity are not equally shared by all subsets of DCs, adding considerable complexity to understanding the immunology of allergic sensitization. Whereas the epithelium was initially considered solely as a physical barrier, it is now seen as a central player in controlling the function of lung DCs through release of Th2 cell-promoting cytokines. Although DCs are sufficient and necessary for induction of Th2 cell responses to many antigens, some allergens might require antigen presentation by basophils. Clinically relevant allergens, as well as environmental and genetic risk factors for allergy and asthma, often interfere

directly or indirectly with the innate immune functions of airway epithelial cells, basophils, and DCs. This review summarizes the recent progress on our understanding how DCs control Th2 cell immunity in the lung.

PMID: 19766084 [PubMed - indexed for MEDLINE]

504. J Org Chem. 2009 Oct 16;74(20):7998-8000. doi: 10.1021/jo901707x.

[1,2]-Anionic rearrangement of 2-benzyloxypyridine and related pyridyl ethers.

Yang J, Dudley GB.

Department of Chemistry and Biochemistry, Florida State University, Tallahassee, Florida 32306-4390, USA.

An anionic rearrangement of 2-benzyloxypyridine is described. Pyridine-directed metalation of the benzylic carbon leads to 1,2-migration of pyridine via a postulated associative mechanism (addition/elimination). Several aryl pyridyl carbinols were obtained in high yields. A formal synthesis of carbinoxamine, an antihistamine drug used for the treatment of seasonal allergies and hay fever, emerges from this methodology.

PMID: 19761204 [PubMed]

505. Adv Exp Med Biol. 2009;647:186-94. doi: 10.1007/978-0-387-89520-8_13.

Tumor necrosis factor receptor superfamily member 21: TNFR-related death receptor-6, DR6.

Benschop R, Wei T, Na S.

Lilly Research Laboratories, Eli Lilly and Co, Indianapolis, IN, 46285, USA.

TNFRSF21 (death receptor-6, DR6) is an orphan TNF receptor superfamily member and belongs to a subgroup of receptors called death receptors. DR6 is expressed ubiquitously with high expression in lymphoid organs, heart, brain and pancreas. Ectopic expression of DR6 in some cell lines leads to apoptosis and activation of the JNK and NF-kappaB pathways. Some tumor cells overexpress DR6, typically in conjunction with elevated anti-apoptosis molecules. DR6 deficient mice (DR6(-/-)) show normal development with no gross pathology in any major organs. In the absence of DR6, ligation of the TCR results in enhanced T-cell proliferation, activation and skewed Th2 cytokine production. Similarly, B-cells lacking Dr6 show increased proliferation, cell division and cell survival upon mitogenic stimulation (anti-CD40 and LPS) or BCR ligation. As a result, DR6(-/-) mice show increased Th2 immune responses to both T-dependent and -independent antigens. All those data indicate that DR6 plays an important regulatory role for the generation of adaptive immunity. More importantly, DR6(-/-) mice are resistant to EAE and allergic airway hypersensitivity, possibly as a result of a deficiency in the migration of antigen specific T-cells. Therefore, DR6 is a potential therapeutic target for treating inflammatory and autoimmune disease by means of biological intervention. In addition, DR6 is highly expressed in many tumor cell lines and tumor samples. Interestingly, both of its transcriptional and cell surface expression are regulated by the NF-kappaB pathway and metalloproteinase in some tumor cell lines, respectively. The role of DR6 as an apoptosis-inducing receptor is less clear and perhaps cell type dependent. Therefore, in addition to its roles in regulating immune responses, DR6 may also be involved in tumor cell survival and immune evasion, which is subject to future investigations.

PMID: 19760075 [PubMed - indexed for MEDLINE]

506. J Biol Chem. 2009 Nov 6;284(45):31463-72. doi: 10.1074/jbc.M109.030213. Epub 2009 Sep 15.

Evidence that integrin alpha IIb beta 3-dependent interaction of mast cells with fibrinogen exacerbates chronic inflammation.

Oki T, Eto K, Izawa K, Yamanishi Y, Inagaki N, Frampton J, Kitamura T, Kitaura J.

Division of Cellular Therapy, Advanced Clinical Research Center, The Institute of Medical Science, The University of Tokyo, 4-6-1 Shirokanedai, Minato-ku, Tokyo 108-8639, Japan.

Integrin alpha IIb beta 3 is expressed in mast cells as well as in megakaryocytes/platelets. A recent study has shown that surface expression levels of integrin alpha V beta 3 are elevated in integrin alpha IIb-deficient bone marrow-derived mast cells (BMMCs) as compared with wild-type (WT) counterparts, but the underlying mechanism remains obscure. Here we demonstrate by transducing integrin alpha IIb into integrin alpha IIb-deficient BMMCs that surface expression levels of integrin alpha V beta 3 are inversely related to those of integrin alpha IIb beta 3. Thus, competitive association of integrin beta 3 with integrin alpha IIb or integrin alpha V determines surface expression levels of integrin alpha IIb beta 3 or alpha V beta 3 in mast cells. We compared WT and integrin alpha IIb-deficient BMMCs as well as integrin alpha IIb-deficient BMMCs transduced with integrin alpha IIb(WT) or non-functional alpha IIb(D163A) mutant and found that enhancement of proliferation, degranulation, cytokine production, and migration of BMMCs through interaction with fibrinogen (FB) depended on integrin alpha IIb beta 3. In addition, elevated surface expression of integrin alpha V beta 3 failed to compensate for loss of FB-associated functions in integrin alpha IIb-deficient BMMCs while enhancing adhesion to vitronectin or von Willebrand factor. Importantly, integrin alpha IIb deficiency strongly suppressed chronic inflammation with the remarkable increase of mast cells induced by continuous intraperitoneal administration of FB, although it did not affect acute allergic responses or mast cell numbers in tissues in steady states. Interestingly, soluble FB promoted cytokine production of BMMCs in response to Staphylococcus aureus with FB-binding capacity, through integrin alpha IIb beta 3-dependent recognition of this pathogen. Collectively, integrin alpha IIb beta 3 in mast cells plays an important part in FB-associated, chronic inflammation and innate immune responses.

PMCID: PMC2781542
PMID: 19755424 [PubMed - indexed for MEDLINE]

507. J Allergy Clin Immunol. 2009 Dec;124(6):1333-42.e1. doi: 10.1016/j.jaci.2009.07.021.

Selective control of SIRP-alpha-positive airway dendritic cell trafficking through CD47 is critical for the development of T(H)2-mediated allergic inflammation.

Raymond M, Rubio M, Fortin G, Shalaby KH, Hammad H, Lambrecht BN, Sarfati M.

Immunoregulation Laboratory, Centre Hospitalier de l'Université de Montréal Research Center, Hôpital Notre-Dame, Montreal, Quebec, Canada.

BACKGROUND: Dendritic cells (DCs) are essential for the initiation and maintenance of T(H)2 responses to inhaled antigen that lead to the establishment

of allergic diseases. Two subpopulations of nonplasmacytoid DCs (ie, CD11b(low)CD103+ and CD11b(high)CD103(-)) are found in lung/airway tissues. Yet the identification and migratory properties of the DC subset that contributes to T(H)2-mediated responses remain to be clarified. CD47, a signal regulatory protein (SIRP)-alpha partner, reportedly governed skin DC migration.
OBJECTIVE: We here thought to investigate the role of CD47/SIRP-alpha interactions in airway DC trafficking and the development of allergic airway inflammation.
METHODS: We characterized the DC influx into lungs and mediastinal lymph nodes in CD47(-/-) and CD47(+/+) BALB/c mice by using experimental models of allergic asthma. Mice were systemically (intraperitoneal ovalbumin/alum) or locally (intratracheal ovalbumin-loaded bone marrow-derived DCs) immunized and challenged by ovalbumin aerosol. We also evaluated the consequences of SIRP-alpha-Fc fusion molecule administration on the induction of airway disease in BALB/c mice.
RESULTS: SIRP-alpha selectively identified the CD11b(high)CD103(-) DC subset that predominantly accumulated in mediastinal lymph nodes during airway inflammation. However, CD103(-)SIRP-alpha+ DC trafficking, T(H)2 responses, and airway disease were impaired in CD47(-/-) mice. Importantly, the adoptive transfer of CD103(-) SIRP-alpha+CD47(+/+) but not CD47(-/-) DCs elicited a strong T(H)2 response in CD47(-/-) mice. Finally, the administration of SIRP-alpha-Fc molecule protected BALB/c mice from allergic airway inflammation.
CONCLUSION: Lung CD11b(high)CD103(-)SIRP-alpha+ DC migration is governed by self-CD47 expression, and manipulation of the CD47/SIRP-alpha pathway suppresses CD103(-)SIRP-alpha(+) DC-driven pathogenic T(H)2 responses and airway inflammation.

PMID: 19748659 [PubMed - indexed for MEDLINE]

508. Ann Allergy Asthma Immunol. 2009 Aug;103(2):152-9. doi: 10.1016/S1081-1206(10)60169-X.

Inhibition effects of Vitex rotundifolia on inflammatory gene expression in A549 human epithelial cells.

Sohn SH, Ko E, Oh BG, Kim SH, Kim Y, Shin M, Hong M, Bae H.

Department of Physiology, College of Oriental Medicine, Kyung Hee University, Seoul, Republic of Korea.

BACKGROUND: Vitex rotundifolia has long been used in traditional medicine to treat asthma and other allergic diseases.
OBJECTIVE: To evaluate the anti-inflammatory mechanisms of V rotundifolia in cultured A549 human alveolar epithelial cells.
METHODS: In the present study, A549 cells were stimulated with tumor necrosis factor alpha, interleukin 4, and interleukin 1beta to induce expression of chemokines and adhesion molecules involved in eosinophil chemotaxis. The anti-inflammatory effects of V rotundifolia on stimulated A549 cells were then evaluated by analyzing eotaxin secretion and eosinophil migration. In addition, the effects of V rotundifolia on gene expression profiles in stimulated A549 cells were evaluated by oligonucleotide microarray and real-time reverse transcription-polymerase chain reaction (RTRP).
RESULTS: The V rotundifolia-treated A549 cells had significantly suppressed eotaxin secretion and eosinophil migration in a dose-dependent manner. In addition, the results of the microarray analysis and RTRP revealed that inflammation-related genes and cell adhesion-related genes were down-regulated in V rotundifolia-treated A549 cells. Furthermore, several genes related to the mitogen-activated protein kinase pathway were down-regulated in V rotundifolia-treated A549 cells.
CONCLUSIONS: The mechanism responsible for the effects of V rotundifolia on A549

cells is closely associated with regulation of the mitogen-activated protein kinase pathway. Thus, V rotundifolia may be useful in the treatment of asthma and other allergic diseases.

PMID: 19739429 [PubMed - indexed for MEDLINE]

509. Clin Exp Allergy. 2009 Nov;39(11):1684-92. doi: 10.1111/j.1365-2222.2009.03310.x. Epub 2009 Sep 7.

Airway smooth muscle chemokine receptor expression and function in asthma.

Saunders R, Sutcliffe A, Kaur D, Siddiqui S, Hollins F, Wardlaw A, Bradding P, Brightling C.

Department of Infection, Immunity and Inflammation, Institute for Lung Health, University of Leicester, Leicester, UK.

BACKGROUND: Chemokine receptors play an important role in cell migration and wound repair. In asthma, CCR3 and 7 are expressed by airway smooth muscle (ASM) and CCR7 has been implicated in the development of ASM hyperplasia. The expression profile of other chemokine receptors by ASM and their function needs to be further explored.
OBJECTIVE: We sought to investigate ASM chemokine receptor expression and function in asthma.
METHODS: ASM cells were derived from 17 subjects with asthma and 36 non-asthmatic controls. ASM chemokine receptor expression was assessed by flow cytometry and immunofluorescence. The function of chemokine receptors expressed by more than 10% of ASM cells was investigated by intracellular calcium measurements, chemotaxis, wound healing, proliferation and survival assays.
RESULTS: In addition to CCR3 and 7, CXCR1, 3 and 4 were highly expressed by ASM. These CXC chemokine receptors were functional with an increase in intracellular calcium following ligand activation and promotion of wound healing [CXCL10 (100 ng/mL) 34 +/- 2 cells/high-powered field (hpf) vs. control 29 +/- 1; P=0.03; n=8]. Spontaneous wound healing was inhibited by CXCR3 neutralizing antibody (mean difference 7 +/- 3 cells/hpf; P=0.03; n=3). CXC chemokine receptor activation did not modulate ASM chemotaxis, proliferation or survival. No differences in chemokine receptor expression or function were observed between ASM cells derived from asthmatic or non-asthmatic donors.
CONCLUSIONS: Our findings suggest that the chemokine receptors CXCR1, 3 and 4 modulate some aspects of ASM function but their importance in asthma is uncertain.

PMCID: PMC2774481
PMID: 19735481 [PubMed - indexed for MEDLINE]

510. Ann Otolaryngol Chir Cervicofac. 2009 Nov-Dec;126(5-6):264-8. doi: 10.1016/j.aorl.2009.07.001. Epub 2009 Sep 2.

[Study of a case of cochlear implant with recurrent cutaneous extrusion].

[Article in French]

Poncet-Wallet C, Ormezzano Y, Ernst E, Toffin C, Dhote R, Harboun-Cohen E, Frachet B.

Service ORL, hôpital Avicenne, 125, route de Stalingrad, 93009 Bobigny, France. christine.poncet@avc.aphp.fr

OBJECTIVE: An observation of a difficult-to-diagnose complication of the cochlear implant: recurring cutaneous extrusion of a cochlear implant is reported.
PATIENTS AND METHODS: The history of an adult patient with total deafness is reported. She received an implant in her right ear in 1988, which was then explanted because of absence of hearing results. She received a second implant in her left ear in 2002, which was then explanted in 2007 because of cutaneous extrusion. In 2008, a second implant of a different brand was placed in her left ear, with the central part placed away from the first site, but extrusion recurred. A new attempt to encapsulate the central part with a hydroxyapatite box also ended in extrusion and was explanted in 2008.
RESULTS: Various diagnoses to explain these cutaneous problems were suggested during this clinical progression: infection, allergy, and a reaction to a foreign body. No hypothesis could be clearly ruled out.
CONCLUSION: Cutaneous complications after cochlear implant are exceptional. As soon as cutaneous disorders appear, a rigorous diagnostic process must be followed so that the patient can be recommended a long-lasting solution to restore quality hearing.

PMID: 19729148 [PubMed - indexed for MEDLINE]

511. Inflammation. 2009 Dec;32(6):387-92. doi: 10.1007/s10753-009-9147-x.

Procaterol inhibits lung fibroblast migration.

Kohyama T, Yamauchi Y, Takizawa H, Itakura S, Kamitani S, Desaki M, Kawasaki S, Nagase T.

Department of Respiratory Medicine, Graduate School of Medicine, The University of Tokyo, Tokyo, Japan. koyama-tky@umin.ac.jp

Fibroblasts are important cells that are involved in modulation of fibrosis after injuries. In some uncontrollable inflammatory processes, excess fibroblasts migrate around the small airway. The pathogenesis of chronic obstructive pulmonary disease is related to fibrosis around the small airways. The aim of the current study was to investigate the effect of procaterol, a second-generation beta (2)-agonist, on migration of human fetal lung fibroblasts (HFL-1) induced by human plasma fibronectin (HFn). Using the blindwell chamber technique, 10(-8) M procaterol inhibited migration of HFL-1 (control, 100%; 10(-8) M, 73.2 +/- 4.9%; n = 6, p < 0.05). The inhibitory effect of procaterol was concentration-dependent. Although a beta 2-receptor inhibitor, ICI 181551, blocked the inhibitory effect of procaterol, a beta 1-receptor inhibitor, atenolol, did not. Because a cyclic AMP-dependent protein kinase (PKA) inhibitor, KT5720, blocked the effect of procaterol, the cyclic AMP-PKA pathway may be involved in the migration inhibitory process. Procaterol, which is prescribed mainly for treatment of bronchial asthma, might be a useful drug for inhibiting lung fibrosis following injuries to the lung.

PMID: 19728063 [PubMed - indexed for MEDLINE]

512. Am J Otolaryngol. 2009 Sep-Oct;30(5):356-9. doi: 10.1016/j.amjoto.2008.07.001. Epub 2009 Mar 6.

Foreign body granulomas after injection of Bio-alcamid for lip augmentation.

Akrish S, Dayan D, Taicher S, Adam I, Nagler RM.

Department Oral Pathology and Medicine, School of Dental Medicine, Tel Aviv University, Tel Aviv, Israel.

Bio-alcamid is one of the newest agents on the market for soft tissue augmentation. Seven studies were documented in the medical literature that examined the safety of Bio-alcamid (Polymekon, Brindisy, Italy); all reported no cases of tissue migration, foreign body granulomas, allergenicity, or interference with the control of cell proliferation. On 2 separate occasions, a woman who had recently undergone lip augmentation presented at our hospital with submucosal nodules of the lip. Histologic examination revealed multiple foreign body-type granulomas composed of giant cells, epithelioid cells, and chronic inflammation of the lip. Efforts to produce a cosmetic material that fulfills all the criteria as an "ideal" agent has not yet been found because all injectable foreign agents have the potential to induce adverse reactions. Caution must be exercised in all cases and the risks explained to the patient before its use.

PMID: 19720259 [PubMed - indexed for MEDLINE]

513. Pharmacology. 2009;84(3):171-82. doi: 10.1159/000235748. Epub 2009 Aug 27.

Inhibitory effect of the new orally active CCR4 antagonist K327 on CCR4+CD4+ T cell migration into the lung of mice with ovalbumin-induced lung allergic inflammation.

Sato T, Komai M, Iwase M, Kobayashi K, Tahara H, Ohshima E, Arai H, Miki I.

Drug Discovery Research Laboratories, Kyowa Hakko Kirin Co., Ltd., Shizuoka 411-8731, Japan.

CC chemokine receptor 4 (CCR4) is expressed on Th2 cells, found in inflamed tissues of allergic diseases, and is therefore suspected to be involved in the pathogenesis of allergic diseases by controlling Th2 cell migration into inflamed tissues. The aim of the present study was to investigate the inhibitory effect of a selective CCR4 antagonist, K327 [6-cyclopropancarbonyl-4-(2,4-dichlorobenzylamino)-2-(4-[2-(piperidin-1-yl)ethyl] piperazin-1-yl)-7,8-dihydro-5H-pyrido (4,3-d)pyrimidine], on the recruitment of CCR4+CD4+ T cells to the airway of mice with ovalbumin-induced allergic airway inflammation. K327 was administered to mice in which CCR4+CD4+ T cell accumulation was elicited by multiple inhalations of aerosolized ovalbumin. K327 significantly and dose-dependently inhibited the recruitment of CCR4+CD4+ T cells with an ID(50)value of 44 mg/kg, p.o. twice daily. The antiasthmatic potential of K327 was also demonstrated by the fact that K327 suppressed the elevation of Th2 cytokines and airway eosinophilia. These results indicate that CCR4 antagonists can control in vivo migration of Th2 cells which express CCR4 and, presumably, serve as a new class of therapeutic agent for allergy.

Copyright 2009 S. Karger AG, Basel.

PMID: 19713720 [PubMed - indexed for MEDLINE]

514. J Biol Chem. 2009 Oct 16;284(42):28599-606. doi: 10.1074/jbc.M109.047282. Epub 2009 Aug 27.

Regulation of immature dendritic cell migration by RhoA guanine nucleotide exchange factor Arhgef5.

Wang Z, Kumamoto Y, Wang P, Gan X, Lehmann D, Smrcka AV, Cohn L, Iwasaki A, Li L, Wu D.

Program for Vascular Biology and Therapeutics and Department of Pharmacology,

Yale University School of Medicine, New Haven, Connecticut 06520,, USA.

There are a large number of Rho guanine nucleotide exchange factors, most of which have no known functions. Here, we carried out a short hairpin RNA-based functional screen of Rho-GEFs for their roles in leukocyte chemotaxis and identified Arhgef5 as an important factor in chemotaxis of a macrophage phage-like RAW264.7 cell line. Arhgef5 can strongly activate RhoA and RhoB and weakly RhoC and RhoG, but not Rac1, RhoQ, RhoD, or RhoV, in transfected human embryonic kidney 293 cells. In addition, Gbetagamma interacts with Arhgef5 and can stimulate Arhgef5-mediated activation of RhoA in an in vitro assay. In vivo roles of Arhgef5 were investigated using an Arhgef-5-null mouse line. Arhgef5 deficiency did not affect chemotaxis of mouse macrophages, T and B lymphocytes, and bone marrow-derived mature dendritic cells (DC), but it abrogated MIP1alpha-induced chemotaxis of immature DCs and impaired migration of DCs from the skin to lymph node. In addition, Arhgef5 deficiency attenuated allergic airway inflammation. Therefore, this study provides new insights into signaling mechanisms for DC migration regulation.

PMCID: PMC2781403
PMID: 19713215 [PubMed - indexed for MEDLINE]

515. Expert Rev Mol Med. 2009 Aug 20;11:e25. doi: 10.1017/S146239940900115X.

Molecular mechanisms of leukocyte trafficking in T-cell-mediated skin inflammation: insights from intravital imaging.

Deane JA, Hickey MJ.

Centre for Inflammatory Diseases, Monash University Department of Medicine, Monash Medical Centre, Clayton, Australia.

Infiltration of T cells is a key step in the pathogenesis of the inflammatory skin diseases atopic dermatitis, allergic contact dermatitis and psoriasis. Understanding the mechanisms of T cell recruitment to the skin is therefore of fundamental importance for the discovery and application of novel therapies for these conditions. Studies of both clinical samples and experimental models of skin inflammation have implicated specific adhesion molecules and chemokines in lymphocyte recruitment. In particular, recent studies using advanced in vivo imaging techniques have greatly increased our understanding of the kinetics and molecular basis of this process. In this review, we summarise the current understanding of the cellular immunology of antigen-driven dermal inflammation and the roles of adhesion molecules and chemokines. We focus on results obtained using intravital microscopy to examine the dermal microvasculature and interstitium to determine the mechanisms of T cell recruitment and migration in experimental models of T-cell-mediated skin inflammation.

PMID: 19691914 [PubMed - indexed for MEDLINE]

516. Curr Top Med Chem. 2009;9(8):738-53.

Small molecule inhibitors of phosphoinositide 3-kinase (PI3K) delta and gamma.

Ameriks MK, Venable JD.

Johnson & Johnson Pharmaceutical Research & Development LLC San Diego, CA 92121, USA. mameriks@its.jnj.com

In recent years, pharmaceutical companies have increasingly focused on

phosphoinositide 3-kinases delta (PI3Kdelta) and gamma (PI3Kgamma) as therapeutic targets for the treatment of inflammatory and autoimmune diseases. All class 1 PI3-kinases (alpha/beta/gamma/delta) generate phospholipid second messengers that help govern cellular processes such as migration, proliferation, and apoptosis. PI3K delta/ gamma lipid kinases are mainly restricted to the hematopoetic system whereas PI3K alpha/beta are ubiquitously expressed, thus raising potential toxicity concerns for chronic indications such as asthma and rheumatoid arthritis. Therefore, the challenge in developing a small molecule inhibitor of PI3K is to define and attain the appropriate isoform selectivity profile. Significant advances in the design of such compounds have been achieved by utilizing x-ray crystal structures of various inhibitors bound to PI3Kgamma in conjunction with pharmacophore modeling and high-throughput screening. Herein, we review the history and challenges involved with the discovery of small molecule isoform-specific PI3K inhibitors. Recent progress in the design of selective PI3Kdelta, PI3Kgamma, and PI3Kdelta/gamma dual inhibitors will be presented.

PMID: 19689378 [PubMed - indexed for MEDLINE]

517. Br J Pharmacol. 2009 Aug;157(8):1330-9. doi: 10.1111/j.1476-5381.2009.00362.x.

The K+ channels K(Ca)3.1 and K(v)1.3 as novel targets for asthma therapy.

Bradding P, Wulff H.

Department of Infection, Immunity and Inflammation, Institute for Lung Health, University of Leicester, UK. pbradding@hotmail.com

Asthma affects 10% of the UK population and is an important cause of morbidity and mortality at all ages. Current treatments are either ineffective or carry unacceptable side effects for a number of patients; in consequence, development of new approaches to therapy are important. Ion channels are emerging as attractive therapeutic targets in a variety of non-excitable cells. Ion channels conducting K(+) modulate the activity of several structural and inflammatory cells which play important roles in the pathophysiology of asthma. Two channels of particular interest are the voltage-gated K(+) channel K(v)1.3 and the intermediate conductance Ca(2+)-activated K(+) channel K(Ca)3.1 (also known as IK(Ca)1 or SK4). K(v)1.3 is expressed in IFNgamma-producing T cells while K(Ca)3.1 is expressed in T cells, mast cells, macrophages, airway smooth muscle cells, fibroblasts and epithelial cells. Both channels play important roles in cell activation, migration, and proliferation through the regulation of membrane potential and calcium signalling. We hypothesize that K(Ca)3.1- and/or K(v)1.3-dependent cell processes are one of the common denominators in asthma pathophysiology. If true, these channels might serve as novel targets for the treatment of asthma. Emerging evidence lends support to this hypothesis. Further validation through the study of the role that these channels play in normal and asthmatic airway cell (patho)physiology and in vivo models will provide further justification for the assessment of small molecule blockers of K(v)1.3 and K(Ca)3.1 in the treatment of asthma.

PMCID: PMC2765317
PMID: 19681865 [PubMed - indexed for MEDLINE]

518. Int Arch Allergy Immunol. 2010;151(1):46-55. doi: 10.1159/000232570. Epub 2009 Aug 6.

Impact of urbanization on the proteome of birch pollen and its chemotactic activity on human granulocytes.

Bryce M, Drews O, Schenk MF, Menzel A, Estrella N, Weichenmeier I, Smulders MJ, Buters J, Ring J, Görg A, Behrendt H, Traidl-Hoffmann C.

Division of Environmental Dermatology and Allergy, Helmholtz Center Munich/TUM, ZAUM - Center of Allergy and Environment, Technische Universität, Munich, Germany.

BACKGROUND: Epidemiologic studies reveal a dramatic increase in allergies in the last decades. Air pollution is considered to be one of the factors responsible for this augmentation. The aim of this study was to analyze the impact of urbanization on birch pollen. The birch pollen proteome was investigated in order to identify differences in protein abundance between pollen from rural and urban areas. The allergenicity of birch pollen from both areas was evaluated by assessing its chemotactic potency as well as its protein and allergen contents.
METHODS: Difference gel electrophoresis (DIGE) was used to analyze the pollen proteome. The chemotactic activity of aqueous pollen extracts was determined by migration assays of human neutrophils.
RESULTS: DIGE revealed 26 differences in protein spot intensity between pollen from urban and rural areas. One of these proteins was identified by de novo sequencing as the 14-3-3 protein, which resembles a stress-induced factor in other plant species. Furthermore, extracts from pollen collected in urban areas had higher chemotactic activity on human neutrophils compared to pollen from rural sites.
CONCLUSIONS: The present study points to an impact of air pollution on allergen carrier proteome and release of chemotactic substances. The increment in proinflammatory substances such as pollen-associated lipid mediators might contribute to the described urban-rural gradient of allergy prevalence. Furthermore, our study suggests that allergenicity is determined by more than the sole allergen content.

PMID: 19672096 [PubMed - indexed for MEDLINE]

519. Ulus Travma Acil Cerrahi Derg. 2009 Jul;15(4):311-6.

Volume replacement in trauma.

Ribeiro MA Jr, Epstein MG, Alves LD.

Department of Surgery, Universidade Cidade De Sao Paulo Medical School, Sao Paulo, Brazil. mribeiro@cwaynet.com.br

We aimed to compare different fluids indicated in volume replacement in multiple trauma patients, enlightening the indications, mechanisms of action and side effects. An extensive review of references (indexed journals) between 1997 and 2008 was performed. There is not yet a consensus about which fluids should be used in trauma patients. The systematic reviews available did not show a benefit of colloid solutions over crystalloid fluids. Crystalloids intensify physiological internal dilution, furthered by water migration from interstitial and intracellular spaces into intravascular space due to hypovolemia. The most recent hypertonic solutions used in resuscitation have a large role in expanding blood volume and making blood pressure rise. The hyperoncotic effect of dextran solution produces an initial expansion of intravascular volume that is bigger than the administered volume. Gelatins are no longer used in developed countries due to their insignificant ability regarding volume expansion when compared to crystalloids and the potential risks of anaphylactic reactions. The crystalloids are used more in trauma, even if some authors prefer the use of colloids, which can produce a quicker restoration of the intravascular volume. No convincing evidence shows a clear superiority of colloids over crystalloids for restoration of the volume depletion.

PMID: 19669957 [PubMed - indexed for MEDLINE]

520. J Immunol. 2009 Sep 1;183(5):3443-53. doi: 10.4049/jimmunol.0804223. Epub 2009 Aug 10.

Impaired lung dendritic cell migration and T cell stimulation induced by immunostimulatory oligonucleotides contribute to reduced allergic airway inflammation.

Constabel H, Stankov MV, Hartwig C, Tschernig T, Behrens GM.

Clinic for Immunology and Rheumatology, Hannover Medical School, Hannover, Germany.

CpG-containing oligonucleotides (CpG) have been shown to reduce key features of allergic airway inflammation in mouse models. Given the inhibitory effects of CpG treatment on Ag presentation of subsequently encountered Ags via MHC class I and II molecules by dendritic cells (DC), we hypothesized that intranasal CpG treatment would lead to reduced Ag-specific T cell stimulation in the lung-draining lymph nodes, thereby reducing the inflammatory response in sensitized mice. Intranasal CpG administration led to phenotypic maturation of lung and mediastinal lymph node DC as determined by expression of MHC class II, CD80, and CD86. This was accompanied by a significant reduction in the proliferation of adoptively transferred Ag-specific CD4(+) and CD8(+) T cells in mediastinal lymph nodes, when CpG was given before inhalative OVA challenges. DC obtained from mediastinal lymph nodes of CpG-treated mice before OVA inhalation led to reduced T cell stimulation via MHC class I and II molecules. In addition, CpG diminished airway eosinophilia and pulmonary infiltration after sensitization or following adoptive transfer of Ag-specific Th2 cells. These results were explained by reduced CCL21 expression and inhibition of lung DC migration following CpG administration, which could be restored by transfer of bone marrow-derived DC, because CpG had no major impact on the constitutive MHC class II Ag presentation of protein-derived Ag by lung tissue-derived DC. We conclude that CpG treatment can effectively impair the DC-mediated Ag transport from the lungs to the lymph nodes, resulting in reduced T cell activation and blunted airway inflammation.

PMID: 19667097 [PubMed - indexed for MEDLINE]

521. Int J Dermatol. 2009 Aug;48(8):822-9. doi: 10.1111/j.1365-4632.2009.04069.x.

Serum mucosa-associated epithelial chemokine (MEC/CCL28) in atopic dermatitis: a specific marker for severity.

Ezzat MH, Sallam MA, Shaheen KY.

Faculty of Medicine, Ain Shams University, Cairo, Egypt. ezzatmhm@hotmail.com

BACKGROUND: Mucosa-associated epithelial chemokine (MEC; CCL28) is considered to be pivotal in mediating the migration of CC chemokine receptor 3- and 10- (CCR3- and CCR10)-expressing, skin-homing, memory, cutaneous lymphocyte-associated antigen-positive (CLA(+)) T cells. CCL28 is selectively and continuously expressed by epidermal keratinocytes, but highly upregulated in inflammatory skin diseases, such as atopic dermatitis (AD).
AIM: This controlled longitudinal study was designed to evaluate the expression of CCL28 in serum in childhood AD and bronchial asthma (BA), and its possible relationship to disease severity and activity.

METHODS: Serum CCL28 levels were measured in 36 children with AD, 23 with BA, 14 with both conditions, and 21 healthy age- and sex-matched controls. Sixteen patients in the AD group were followed up and resampled for serum CCL28 after clinical remission. Serum CCL28 levels were correlated with some AD disease activity and severity variables.
RESULTS: Serum CCL28 levels in AD, whether during flare [median, 1530 pg/mL; mean +/- standard deviation (SD) = 1590.4 +/- 724.3 pg/mL] or quiescence (median, 1477 pg/mL; mean +/- SD = 1575.2 +/- 522.1 pg/mL), were significantly higher than those in healthy children (median, 301 pg/mL; mean +/- SD = 189.6 +/- 92.8 pg/mL); however, the levels during flare and quiescence were statistically comparable. The serum levels in BA (median, 340 pg/mL; mean +/- SD = 201.6 +/- 109.5 pg/mL) were significantly lower than those in the AD group, and comparable with those in healthy controls. Serum CCL28 levels in severe AD were significantly higher than those in mild and moderate cases, and correlated positively with the calculated severity scores [Leicester Sign Score (LSS) and Scoring Atopic Dermatitis (SCORAD)]. CCL28 levels during the exacerbation of AD were positively correlated with the corresponding values during remission, the peripheral absolute eosinophil counts, and serum lactate dehydrogenase levels. Serum CCL28 levels were not correlated with the serum total immunoglobulin E values in AD.
CONCLUSIONS: Our data reinforce the concept that CCL28 might contribute to the pathogenesis of AD, probably through the selective migration and infiltration of effector/memory T-helper-2 cells in the skin. CCL28 may also represent an objective prognostic marker for disease severity. Further studies may pave the way for CCL28 antagonism among adjuvant therapeutic strategies.

PMID: 19659860 [PubMed - indexed for MEDLINE]

522. Gac Sanit. 2009 Nov-Dec;23(6):548-52. doi: 10.1016/j.gaceta.2009.05.009. Epub 2009 Aug 3.

[Prevalence of diagnosed chronic disorders in the immigrant and native population].

[Article in Spanish]

Esteban-Vasallo MD, Domínguez-Berjón MF, Astray-Mochales J, Gènova-Maleras R, Pérez-Sania A, Sánchez-Perruca L, Aguilera-Guzmán M, González-Sanz FJ.

Dirección General de Atención Primaria, Consejería de Sanidad, Comunidad de Madrid, España. maria.estebanv@salud.madrid.org

OBJECTIVE: To estimate the prevalence rates of chronic disorders in immigrants and to compare them with those in the native population, based on electronic clinical records in primary care (ECRPC).
METHODS: We performed a descriptive cross-sectional study in patients aged 16 and over included in the Madrid Regional Public Health System. Age-adjusted prevalence rates for each sex and region were estimated on the basis of medically examined cases registered in the ECRPC with any new data entry made in 2005 or 2006.
RESULTS: After age-adjustment, a total of 36.8% immigrants had some chronic health problem (vs. 55.3% natives). These disorders were more frequent among women and among the population from Africa and Latin America. The highest overall prevalence rates in the foreign population were allergy (10.2% crude rate), low-back pain (9.1%), chronic skin problems (6.8%) and mental disorders (6.4%).
CONCLUSIONS: The prevalence rate of chronic disease is lower in the foreign population and differs according to sex and country of origin.

PMID: 19647902 [PubMed - indexed for MEDLINE]

523. Mol Biol Rep. 2010 Apr;37(4):1993-2001. doi: 10.1007/s11033-009-9650-z. Epub 2009 Jul 31.

Molecular regulation of mast cell development and maturation.

Liu C, Liu Z, Li Z, Wu Y.

Allergy and Immunology Institute, School of Medicine, Shenzhen University, Shenzhen, China.

Mast cells play a crucial role in the pathogenesis of allergic diseases. In recent years, tremendous progresses have been made in studies of mast cell origination, migration, proliferation, maturation and survival, and the cytokines regulating these activities. These advances have significantly improved our understandings to mast cell biology and to the molecular mechanisms of mast cells in the pathogenesis of allergic diseases.

PMID: 19644767 [PubMed - indexed for MEDLINE]

524. Eur J Clin Microbiol Infect Dis. 2009 Nov;28(11):1327-34. doi: 10.1007/s10096-009-0785-6. Epub 2009 Jul 31.

Prevalence of antibodies against Ascaris suum and its association with allergic manifestations in 4-year-old children in The Netherlands: the PIAMA birth cohort study.

Pinelli E, Willers SM, Hoek D, Smit HA, Kortbeek LM, Hoekstra M, de Jongste J, van Knapen F, Postma D, Kerkhof M, Aalberse R, van der Giessen JW, Brunekreef B.

Department of Parasitology and Mycology, Diagnostic Laboratory for Infectious Diseases and Perinatal Screening, Centre for Infectious Disease Control Netherlands, National Institute of Public Health and the Environment (RIVM), P.O. Box 1, 3720 BA, Bilthoven, The Netherlands. elena.pinelli.ortiz@rivm.nl

The association between helminth infections and childhood atopic diseases remains controversial. The majority of studies have been carried out in tropical areas, whereas less information is available from western countries with low intensity of helminth infections. In the Netherlands, the infection of pigs with Ascaris suum is very common, particularly on pig farms with outdoor facilities. This helminth can also infect humans, causing visceral larva migrans. This study aims at determining the prevalence of antibodies against A. suum and its association with allergic symptoms and sensitisation in a population of 4-year-old children living in The Netherlands. Blood samples from 629 children from the prospective birth cohort Prevention and Incidence of Asthma and Mite Allergy (PIAMA) study were examined for Ascaris antibodies. Data on allergic symptoms and sensitisation were collected using questionnaires and radioallergosorbent tests (RAST). A total of 45 out of 629 (7%) were found to be Ascaris-seropositive. In addition, a positive association between Ascaris seropositivity and wheeze in the last year, doctor-diagnosed asthma and food and aero-allergen sensitisation was found. These results support the hypothesis that low-level or transient infection with helminths enhances allergic reactivity.

PMID: 19644714 [PubMed - indexed for MEDLINE]

525. BMC Mol Biol. 2009 Jul 28;10:75. doi: 10.1186/1471-2199-10-75.

Characterisation of urokinase plasminogen activator receptor variants in human airway and peripheral cells.

Stewart CE, Sayers I.

Division of Therapeutics and Molecular Medicine, Nottingham Respiratory Biomedical Research Unit, University of Nottingham, Queen's Medical Centre, Nottingham, NG7 2UH, UK. ceri.stewart@nottingham.ac.uk

BACKGROUND: Expression of the urokinase plasminogen activator receptor (UPAR) has been shown to have clinical relevance in various cancers. We have recently identified UPAR as an asthma susceptibility gene and there is evidence to suggest that uPAR may be upregulated in lung diseases such as COPD and asthma. uPAR is a key receptor involved in the formation of the serine protease plasmin by interacting with uPA and has been implicated in many physiological processes including proliferation and migration. The current aim was to determine key regulatory regions and splice variants of UPAR and quantify its expression in primary human tissues and cells (including lung, bronchial epithelium (HBEC), airway smooth muscle (HASM) and peripheral cells).
RESULTS: Using Rapid Amplification of cDNA Ends (RACE) a conserved transcription start site (-42 to -77 relative to ATG) was identified and multiple transcription factor binding sites predicted. Seven major splice variants were identified (>5% total expression), including multiple exon deletions and an alternative exon 7b (encoding a truncated, soluble, 229aa protein). Variants were differentially expressed, with a high proportion of E7b usage in lung tissue and structural cells (55-87% of transcripts), whereas classical exon 7 (encoding the GPI-linked protein) was preferentially expressed in peripheral cells (approximately 80% of transcripts), often with exon 6 or 5+6 deletions. Real-time PCR confirmed expression of uPAR mRNA in lung, as well as airway and peripheral cell types with ~50-100 fold greater expression in peripheral cells versus airway cells and confirmed RACE data. Protein analysis confirmed expression of multiple different forms of uPAR in the same cells as well as expression of soluble uPAR in cell supernatants. The pattern of expression did not directly reflect that seen at the mRNA level, indicating that post-translational mechanisms of regulation may also play an important role.
CONCLUSION: We have identified multiple uPAR isoforms in the lung and immune cells and shown that expression is cell specific. These data provide a novel mechanism for uPAR regulation, as different exon splicing may determine uPAR function e.g. alternative E7b results in a soluble isoform due to the loss of the GPI anchor and exon deletions may affect uPA (ligand) and/or integrin binding and therefore influence downstream pathways. Expression of different isoforms within the lung should be taken into consideration in studies of uPAR in respiratory disease.

PMCID: PMC2724484
PMID: 19638192 [PubMed - indexed for MEDLINE]

526. Hautarzt. 2009 Aug;60(8):663-71; quiz 672-3. doi: 10.1007/s00105-008-1502-6.

[Ectoparasites. Part 1: lice and fleas].

[Article in German]

Nenoff P, Handrick W, Krüger C, Herrmann J, Schmoranzer B, Paasch U.

Haut- und Laborarzt/Allergologie, Andrologie, Laboratorium für medizinische Mikrobiologie, Str. des Friedens 8, 04579 Mölbis. nenoff@mykologie-experten.de

Ectoparasites and epidermal parasitic skin diseases are a heterogeneous group of

infections of the external layer of the skin. The most common forms world-wide are scabies, lice (Pediculosis capitis, corporis, vestimentorum and pubis), tungiasis and the hookworm-associated Larva migrans cutanea. The head louse is the most widespread parasite in children in Germany. The symptoms, apart from pruritus, eczematous skin eruptions and ictus reactions of the skin, are often unspecific and many differential diagnoses must be considered. Treatment of ectoparasites includes manual procedures, such as repeated cleansing and combing out of lice-infected hair and also local antiparasitic treatment with permethrin, pyrethrum extract, allethrin and dimeticon. Lindan which has been used for decades can no longer be used in medications after 2008 after a decision of the EU Commission. Failure of treatment of head lice can be a result of errors in the treatment which favor survival of the eggs, larvae or adults. This can be a result of too short reaction times and too economical use or unequal distribution of medications, excessive dilution due to wet hair or omitting repeated treatment stages. Additionally resistance of head lice to pyrethrum is a known phenomenon and has been reported in several countries.

PMID: 19633823 [PubMed - indexed for MEDLINE]

527. Immunobiology. 2009;214(9-10):748-60. doi: 10.1016/j.imbio.2009.06.003. Epub 2009 Jul 21.

Regulation of macrophage function by sphingosine-1-phosphate.

Weigert A, Weis N, Brüne B.

Institute of Biochemistry I/ZAFES, Goethe-University Frankfurt am Main, Germany.

The bioactive lipid sphingosine-1-phosphate (S1P) fulfils manifold tasks in the immune system acting in auto- and/or paracrine fashion. This includes regulation of apoptosis, migration and proliferation. Upon its generation by sphingosine kinases from plasma membrane sphingolipids, S1P can either act as a second messenger within cells or can be released from cells to occupy a family of specific G-protein-coupled receptors (S1P1-5). This diversity is reflected by the impact of S1P on macrophage biology and function. Over the last years it became apparent that the sphingosine kinase/S1P/S1P-receptor signalling axis in macrophages might play a central role in the pathogenesis of inflammatory diseases such as atherosclerosis, asthma, rheumatoid arthritis and cancer. Here, we summarize the current knowledge of the function of S1P in macrophage biology and discuss potential implications for pathology.

PMID: 19625101 [PubMed - indexed for MEDLINE]

528. Mycol Res. 2009 Oct;113(Pt 10):1127-36. doi: 10.1016/j.mycres.2009.07.010. Epub 2009 Jul 18.

Characterization of a Plasmopara species on Ambrosia artemisiifolia, and notes on P. halstedii, based on morphology and multiple gene phylogenies.

Choi YJ, Kiss L, Vajna L, Shin HD.

Division of Environmental Science and Ecological Engineering, College of Life Sciences and Biotechnology, Korea University, Seoul 136-701, Republic of Korea.

Common ragweed (Ambrosia artemisiifolia) is an invasive and highly allergenic plant species, on which two species, Plasmopara halstedii and Plasmopara angustiterminalis, have been recognized to cause downy mildew disease. In this study, morphological and molecular patterns of seven Plasmopara specimens

collected from A. artemisiifolia in Canada, Hungary, and USA were compared with those of P. halstedii and P. angustiterminalis from Helianthus and Xanthium, respectively. Analyses of partial sequences of three genes, namely those for the large subunit (28S) of rDNA, cytochrome c oxidase subunit II (COX2), and NADH dehydrogenase subunit I (ND1) of mtDNA, were carried out to examine the phylogenetic relationships among these specimens using both Bayesian and maximum parsimony methods. All the phylogenetic analyses revealed that the downy mildew pathogens infecting A. artemisiifolia in Hungary and North America clearly represent a lineage distinct from other Plasmopara taxa investigated. The shape of sporangia and the width of trunks and branches also allowed the separation of the specimens parasitic to A. artemisiifolia from P. halstedii on Helianthus annuus and P. angustiterminalis on Xanthium strumarium. Surprisingly, the Hungarian and the Canadian specimens were more closely related to each other than to those from the USA based on COX2 and ND1 mtDNA data, although the D1/D2/D3 sequences of 28S rDNA were identical in all these Plasmopara specimens. The regional distribution of the mtDNA haplotypes seen in this study suggests a transatlantic migration has occurred and would be interesting to follow up with a more detailed sampling. To investigate the diversity within P. halstedii sensu lato, infecting different host plant species, specimens from six asteraceous genera, Ambrosia, Flaveria, Helianthus, Siegesbeckia, Solidago, and Xanthium, were also included in molecular analyses. These represented six distinct lineages according to the host plant genera. These findings might serve as a basis for a taxonomical reassessment of the P. halstedii complex and also for the delimitation of several well-defined species within this complex.

PMID: 19619650 [PubMed - indexed for MEDLINE]

529. J Allergy Clin Immunol. 2009 Sep;124(3):397-403. doi: 10.1016/j.jaci.2009.05.029. Epub 2009 Jul 16.

Efficacy of leukotriene receptor antagonists and synthesis inhibitors in asthma.

O'Byrne PM, Gauvreau GM, Murphy DM.

Department of Medicine, McMaster University, Firestone Institute for Respiratory Health, St Joseph's Healthcare, Hamilton, Ontario, Canada. obyrnep@mcmaster.ca

Cysteinyl leukotrienes are important mediators of asthmatic responses. They are the most potent bronchoconstrictors known; their release is triggered by exposure to inhaled allergens after exercise and after aspirin ingestion by subjects with aspirin-sensitive asthma. The cysteinyl leukotrienes promote inflammatory cell migration into the airways, as well as bone marrow eosinophilopoiesis after allergen inhalation. Leukotriene inhibitors are effective at attenuating asthmatic responses to all of these stimuli and are also effective at treating persistent asthma. These drugs are a viable alternative to low-dose inhaled corticosteroid (ICS) treatment but should be reserved for patients who cannot or will not use ICSs, often because of concerns about potential side effects of ICS treatment, which limits their use, particularly in children. Leukotriene receptor antagonists are also alternatives to long-acting inhaled beta(2)-agonists as add-on therapy to ICSs, but their efficacy together with ICSs is less than that of ICS/long-acting inhaled beta(2)-agonist combinations. Leukotriene receptor antagonists have an excellent safety profile.

PMID: 19608262 [PubMed - indexed for MEDLINE]

530. Am J Respir Cell Mol Biol. 2010 May;42(5):595-603. doi: 10.1165/rcmb.2009-0016OC. Epub 2009 Jul 2.

Macrophages: regulators of sex differences in asthma?

Melgert BN, Oriss TB, Qi Z, Dixon-McCarthy B, Geerlings M, Hylkema MN, Ray A.

University of Pittsburgh Medical Center, Division of Pulmonary, Allergy, and Critical Care Medicine, Pittsburgh, Pennsylvania, USA. bnmelgert@mac.com

Females are more susceptible to development of asthma than are males. In a mouse model of ovalbumin-induced airway inflammation, with aggravated disease in females compared with males, we studied interactions between immune and resident lung cells during asthma development to elucidate which processes are affected by sex. We studied numbers of regulatory T cells (Tregs), effector T cells, myeloid dendritic cells (mDCs), and alternatively activated macrophages (AAMPhi), and their functional capabilities. Male and female mice had comparable Treg numbers in lung tissue and comparable Treg function, but effector T cells had expanded to a greater extent in lungs of females after ovalbumin exposure. This difference in T cell expansion was therefore not the result of lack of Treg control, but appeared to be driven by a greater number of inflammatory mDCs migrating from the lungs to lymph nodes in females. Resident lung cells can influence mDC migration, and AAMPhi in lung tissue were found to be involved. Artificially elevating the number of AAMPhi in lung tissue increased the migration of mDCs and airway inflammation. We found greater numbers of AAMPhi in female lungs than in males; we therefore postulate that AAMPhi are involved in increased airway inflammation found in female mice.

PMCID: PMC2874445
PMID: 19574533 [PubMed - indexed for MEDLINE]

531. J Toxicol Sci. 2009;34 Suppl 2:SP349-61.

Children's immunology, what can we learn from animal studies (3): Impaired mucosal immunity in the gut by 2,3,7,8-tetraclorodibenzo-p-dioxin (TCDD): a possible role for allergic sensitization.

Ishikawa S.

Dept. of Molecular Preventive Medicine, Graduate School of Medicine, The Univ. of Tokyo, Japan. yamasho@m.u-tokyo.ac.jp

We have recently reported breakdown of mucosal immunity in the gut by tetraclorodibenzo-p-dioxin (TCDD). That is, single oral administration of low dose 2,3,7,8-TCDD resulted in a marked decrease in IgA secretion in AhR-dependent manner and impaired oral tolerance in the gut. In the present study, we found TCDD exposure by breast feeding also resulted in decreased level of IgA in the gut. Ig production by B cells by LPS stimulation was not affected by TCDD administration. Instead, particular chemokine receptor expression on B1 cells, a major cell source for intestinal IgA antibody, was decreased in mice treated with TCDD. In consistence with this observation, B1, but not B2 cells from TCDD treated mice showed impaired chemotaxis towards B lymphocyte chemokine (BLC)/CXCL13. In contrast, chemotaxis of intestinal dendritic cells (DCs) towards secondary lymphoid-tissue chemokine (SLC)/CXCL19 was not impaired in mice treated with TCDD. Furthermore, there was no change in the number and profile of intestinal microflora in TCDD-treated mice. These results indicate that TCDD exposure by breast feeding results in breakdown of intestinal mucosal immunity of pups and that it may be attributed in part to impaired B1 cell migration from the peritoneal cavity to intestinal mucosa.

PMID: 19571490 [PubMed - indexed for MEDLINE]

532. Br J Pharmacol. 2009 Oct;158(4):982-93. doi: 10.1111/j.1476-5381.2009.00281.x. Epub 2009 Jun 25.

Sphingolipids in inflammation: pathological implications and potential therapeutic targets.

Nixon GF.

School of Medical Sciences, University of Aberdeen, Foresterhill, UK. g.f.nixon@abdn.ac.uk

Sphingolipids are formed via the metabolism of sphingomyelin, a constituent of the plasma membrane, or by de novo synthesis. Enzymatic pathways result in the formation of several different lipid mediators, which are known to have important roles in many cellular processes, including proliferation, apoptosis and migration. Several studies now suggest that these sphingolipid mediators, including ceramide, ceramide 1-phosphate and sphingosine 1-phosphate (S1P), are likely to have an integral role in inflammation. This can involve, for example, activation of pro-inflammatory transcription factors in different cell types and induction of cyclooxygenase-2, leading to production of pro-inflammatory prostaglandins. The mode of action of each sphingolipid is different. Increased ceramide production leads to the formation of ceramide-rich areas of the membrane, which may assemble signalling complexes, whereas S1P acts via high-affinity G-protein-coupled S1P receptors on the plasma membrane. Recent studies have demonstrated that in vitro effects of sphingolipids on inflammation can translate into in vivo models. This review will highlight the areas of research where sphingolipids are involved in inflammation and the mechanisms of action of each mediator. In addition, the therapeutic potential of drugs that alter sphingolipid actions will be examined with reference to disease states, such as asthma and inflammatory bowel disease, which involve important inflammatory components. A significant body of research now indicates that sphingolipids are intimately involved in the inflammatory process and recent studies have demonstrated that these lipids, together with associated enzymes and receptors, can provide effective drug targets for the treatment of pathological inflammation.

PMCID: PMC2785521
PMID: 19563535 [PubMed - indexed for MEDLINE]

533. Exp Dermatol. 2009 Sep;18(9):750-9. doi: 10.1111/j.1600-0625.2009.00926.x. Epub 2009 Jun 23.

Osteopontin and the skin: multiple emerging roles in cutaneous biology and pathology.

Buback F, Renkl AC, Schulz G, Weiss JM.

Department of Dermatology and Allergology, University of Ulm, Ulm, Germany.

Osteopontin (OPN) is a glycoprotein expressed by various tissues and cells. The existence of variant forms of OPN as a secreted (sOPN) and intracellular (iOPN) protein and its modification through post-translational modification and proteolytic cleavage explain its broad range of functions. There is increasing knowledge which receptors OPN isoforms can bind to and which signaling pathways are activated to mediate different OPN functions. sOPN interacts with integrins and CD44, mediates cell adhesion, migration and tumor invasion, and has T helper 1 (Th1) cytokine functions and anti-apoptotic effects. iOPN has been described to regulate macrophage migration and interferon-alpha secretion in plasmacytoid

dendritic cells. Both sOPN and iOPN, through complex functions for different dendritic cell subsets, participate in the regulation of Th cell lineages, among them Th17 cells. For skin disease, OPN from immune cells and tumor cells is of pathophysiological relevance. OPN is secreted in autoimmune diseases such as lupus erythematosus, and influences inflammation of immediate and delayed type allergies and granuloma formation. We describe that OPN is overexpressed in psoriasis and propose a model to study OPN function in psoriatic inflammation. Through cytokine functions, OPN supports immune responses against Mycobacteria and viruses such as herpes simplex virus. OPN is also implicated in skin tumor progression. Overexpression of OPN influences invasion and metastasis of melanoma and squamous cell carcinoma cells, and OPN expression in melanoma is a possible prognostic marker. As OPN protein preparations and anti-OPN antibodies may be available in the near future, in-depth knowledge of OPN functions may open new therapeutic approaches for skin diseases.

PMID: 19558497 [PubMed - indexed for MEDLINE]

534. Clin Immunol. 2009 Sep;132(3):371-84. doi: 10.1016/j.clim.2009.05.016. Epub 2009 Jun 24.

Lung vascular endothelial growth factor expression induces local myeloid dendritic cell activation.

Chapoval SP, Lee CG, Tang C, Keegan AD, Cohn L, Bottomly K, Elias JA.

Section of Pulmonary and Critical Care Medicine, Department of Internal Medicine, Yale University School of Medicine, 300 Cedar Street, 441-C TAC, New Haven, CT 06520-8057, USA. schapoval@som.umaryland.edu

We previously demonstrated that vascular endothelial growth factor (VEGF) expression in the murine lung increases local CD11c+MHCII+ DC number and activation. In this study, employing a multicolor flow cytometry, we report increases in both myeloid (mDC) and plasmacytoid (pDC) DC in the lungs of VEGF transgenic (tg) compared to WT mice. Lung pDC from VEGF tg mice exhibited higher levels of activation with increased expression of MHCII and costimulatory molecules. As VEGF tg mice display an asthma-like phenotype and lung mDC play a critical role in asthmatic setting, studies were undertaken to further characterize murine lung mDC. Evaluations of sorted mDC from VEGF tg lungs demonstrated a selective upregulation of cathepsin K, MMP-8, -9, -12, and -14, and chemokine receptors as compared to those obtained from WT control mice. They also had increased VEGFR2 but downregulated VEGFR1 expression. Analysis of chemokine and regulatory cytokine expression in these cells showed an upregulation of macrophage chemotactic protein-3 (MCP-3), thymus-expressed chemokine (TECK), secondary lymphoid organ chemokine (SLC), macrophage-derived chemokine (MDC), IL-1beta, IL-6, IL-12 and IL-13. The antigen (Ag) OVA-FITC uptake by lung DC and the migration of Ag-loaded DC to local lymph nodes were significantly increased in VEGF tg mice compared to WT mice. Thus, VEGF may predispose the lung to inflammation and/or repair by activating local DC. It regulates lung mDC expression of innate immunity effector molecules. The data presented here demonstrate how lung VEGF expression functionally affects local mDC for the transition from the innate response to a Th2-type inflammatory response.

PMCID: PMC2780370
PMID: 19553159 [PubMed - indexed for MEDLINE]

535. Acta Biochim Pol. 2009;56(2):355-60. Epub 2009 Jun 18.

Chemerin and the recruitment of NK cells to diseased skin.

Skrzeczyńska-Moncznik J, Stefańska A, Zabel BA, Kapińska-Mrowiecka M, Butcher EC, Cichy J.

Department of Immunology, Faculty of Biochemistry, Biophysics and Biotechnology, Jagiellonian University, Kraków, Poland.

Natural killer (NK) cells play a major role in the initial control of many viral pathogens and in the rejection of tumors. Consistent with their roles as immune sentinels, NK cells are found in inflamed skin, including lichen planus, psoriasis and atopic dermatitis (AD) lesions. In oral lichen planus lesions, the recruitment as well as intradermal colocalization of NK cells and pDC (plasmacytoid dendritic cells) appear to be mediated by chemerin, a recently identified protein ligand for chemokine-like receptor 1 (CMKLR1), a chemoattractant receptor expressed by both cell types. Dendritic cells can regulate NK cell activity, and NK cells can regulate DC-mediated responses. Since chemerin was recently implicated in recruitment of pDC to psoriatic skin, in this work we determined whether chemerin facilitates interactions between NK and pDC in psoriatic plaques through controlling influx of NK cells to diseased skin. We demonstrate that circulating NK cells from normal donors as well as psoriasis and AD patients respond similarly in functional migration assays to chemerin. However, differences in the distribution of NK cells and pDC in skin lesions suggest that recruitment of both NK cells and pDC is unlikely to be controlled solely by chemerin.

PMID: 19543554 [PubMed - indexed for MEDLINE]

536. J Immunol. 2009 Jul 1;183(1):209-20. doi: 10.4049/jimmunol.0802712.

IFN-gamma attenuates antigen-induced overall immune response in the airway as a Th1-type immune regulatory cytokine.

Nakagome K, Okunishi K, Imamura M, Harada H, Matsumoto T, Tanaka R, Miyazaki J, Yamamoto K, Dohi M.

Department of Allergy and Rheumatology, University of Tokyo, Japan.

Allergic inflammation in the airway is generally considered a Th2-type immune response. However, recent studies demonstrated that Th1- and Th17-type immune responses also play important roles in this process. IFN-gamma is a Th1-type cytokine that generally counteracts the Th2 response. Although previous studies suggest that exogenous IFN-gamma suppresses allergic airway inflammation, the mechanism of suppression has not been fully clarified. In this study, we elucidated whether IFN-gamma suppresses Ag-induced immune responses including the production of Th1- and Th17-type cytokines in the lung, and examined its mechanism of action. BALB/c mice were sensitized and challenged with OVA-Ag to induce airway inflammation. An IFN-gamma-producing plasmid vector was delivered before systemic Ag sensitization. IFN-gamma suppressed indicators of Th2-type immune responses such as airway eosinophilia, IL-5 and IL-13 production in the lung, and bronchial mucus production. Moreover, IFN-gamma also suppressed the production of IL-17 and IFN-gamma itself. The suppression was not mediated by inducing regulatory T cells or by inducing apoptosis in immunocytes. Instead, IFN-gamma suppressed the Ag-presenting capacity and cytokine production of splenic dendritic cells and thus subsequently suppressed OVA-induced activation of CD4(+) T cells. Furthermore, IFN-gamma also attenuated allergic airway inflammation when delivered during the OVA challenge. Various functions of lung CD11c(+) APCs and their migration to regional lymph nodes were also suppressed. These results suggest that the Th1 cytokine IFN-gamma has broad immune regulatory

potential through suppressing APC functions. They also suggest that delivery of IFN-gamma could be an effective strategy for regulating Ag-induced immune responses in the lung.

PMID: 19542432 [PubMed - indexed for MEDLINE]

537. FASEB J. 2009 Oct;23(10):3506-15. doi: 10.1096/fj.08-128900. Epub 2009 Jun 17.

Sphingosine-1-phosphate induces development of functionally mature chymase-expressing human mast cells from hematopoietic progenitors.

Price MM, Kapitonov D, Allegood J, Milstien S, Oskeritzian CA, Spiegel S.

Department of Biochemistry and Molecular Biology, Virginia Commonwealth University, School of Medicine, 1101 E. Marshall St., Richmond, VA 23298, USA.

Mast cells (MCs) play a critical role in both acute and chronic inflammation and mature in peripheral tissues from bone marrow-derived progenitors that circulate in the blood as immature precursors. MCs developed from cord blood-derived progenitors cultured with stem cell factor (SCF) alone express intragranular tryptase (MC(T)s), the phenotype predominant in the lung. MC progenitors are likely to encounter the serum-borne bioactive sphingolipid metabolite, sphingosine-1-phosphate (S1P), during migration to target tissues. S1P accelerated the development of cord blood-derived MCs (CB-MCs) and strikingly increased the numbers of MC-expressing chymase. These MCs have functional Fc epsilonRIs, and similar to skin MC(TC)s that express both tryptase and chymase, also express CD88 and are activated by anaphylatoxin C5a and the secretagogue compound 48/80. S1P induced release of IL-6, a cytokine known to promote development of functionally mature MC(TC)s, from cord blood cultures containing adherent macrophages, and from highly purified macrophages, but not from macrophage-depleted CB-MCs. In contrast, S1P stimulated secretion of the chemokine, monocyte chemoattractant protein 1 (MCP-1/CCL2), from these macrophage-depleted and purified CB-MCs. These results suggest crucial roles for S1P in regulating development of human MCs and their functions and reveal a complex interplay between macrophages and MC progenitors in the development of mature human MCs.

PMCID: PMC3236593
PMID: 19535686 [PubMed - indexed for MEDLINE]

538. Vet Res Commun. 2009 Dec;33(8):831-7. doi: 10.1007/s11259-009-9231-6.

Pharmacokinetics of masitinib in cats.

Bellamy F, Bader T, Moussy A, Hermine O.

AB Science, 3 Avenue George V, 75008 Paris, France.

Masitinib is the first veterinary drug recently approved in Europe to treat mast cell tumours in dogs (Hahn et al. JVIM, Masivet). This inhibitor is selective and highly efficient in blocking c-Kit, PDGFR, and Lyn tyrosine kinase activities. It showed good efficacy and acceptable toxicity in several animal studies such as mice, rats, rabbits and dogs (Dubreuil P, et al. submitted, and Hahn et al. (J Vet Intern Med 22(6):8, 2008)). C-kit is a tyrosine kinase receptor that plays a critical role in the biology of mast cells including differentiation, survival, migration and cytokine/mediator release. Mast cells are involved in a number of allergy-and immune-related diseases in cats such as asthma (Reinero Carol et al. Vet Immunol Immunopathol 121(3-4):9, 2008), inflammatory bowel disease, (Janeczko

et al. Vet Mic 128(1-2):15, 2008), and feline mast cell tumours (Rassnick et al. J Am Vet Med Assoc 232(8):1200-1205, 2008). Therefore, there might be a strong rationale to use masitinib in these indications. Here, we report the results of a preliminary pharmacokinetic study of masitinib in cats which showed a good bioavailability of ~60% in both sexes. We propose that an oral dose of 10-15 mg/kg masitinib is appropriate to achieve adequate plasma concentrations.

PMID: 19533403 [PubMed - indexed for MEDLINE]

539. Clin Exp Allergy. 2009 Sep;39(9):1370-80. doi: 10.1111/j.1365-2222.2009.03293.x. Epub 2009 Jun 11.

Platelet-derived growth factor and transforming growth factor-beta modulate the expression of matrix metalloproteinases and migratory function of human airway smooth muscle cells.

Ito I, Fixman ED, Asai K, Yoshida M, Gounni AS, Martin JG, Hamid Q.

Meakins-Christie Laboratories, Department of Medicine, McGill University, Montreal, QC, Canada.

BACKGROUND: Matrix metalloproteinases (MMPs) and tissue inhibitors of metalloproteinase (TIMPs) have been suggested to be involved in the pathogenesis of asthma. Their expression in airway smooth muscle (ASM) cells could be involved in collagen turnover and migration of these cells and thus may contribute to airway remodelling.
OBJECTIVE: To examine the effect of pro-fibrotic growth factors TGF-beta and platelet-derived growth factor (PDGF) on the expression of MMPs/TIMPs in cultured human ASM cells and to examine the role of MMP in the migration of ASM cells.
METHODS: ASM cells were stimulated with TGF-beta and/or PDGF. Expression and activity of MMP-1, MMP-2, MMP-3, TIMP-1 and TIMP-2 were evaluated by quantitative RT-PCR, Western blot and zymography. Modified Boyden-chamber migration assay was performed to investigate the effect of secreted MMP-3 and TIMP-1 on ASM-cell migration.
RESULTS: PDGF strongly up-regulated the expression of MMP-1 at mRNA and protein levels. PDGF, when combined with TGF-beta, caused synergistic up-regulation of MMP-3. TIMP-1 was additively up-regulated by TGF-beta and PDGF. These growth factors had no effect on the expression of MMP-2 and TIMP-2. U0126, an extracellular signal-regulated kinase (ERK) pathway inhibitor, inhibited the up-regulation of MMP-1 by PDGF. The synergistic/additive up-regulation of MMP-3 and TIMP-1 was inhibited by U0126 and SB431542, a Smad pathway inhibitor. Supernatant from ASM cells in which MMP-3 production was knocked down by RNA interference showed a decreased migratory effect on ASM cells, whereas supernatant from cells with suppressed TIMP-1 expression resulted in increased migration.
CONCLUSION: Our results suggest that PDGF with/without TGF-beta could facilitate migration of ASM cells by modification of MMP-TIMP balance through the ERK pathway.

PMID: 19522858 [PubMed - indexed for MEDLINE]

540. Am J Respir Cell Mol Biol. 2010 Apr;42(4):491-7. doi: 10.1165/rcmb.2008-0469OC. Epub 2009 Jun 11.

Inhibition of Pyk2 blocks airway inflammation and hyperresponsiveness in a mouse model of asthma.

Duan Y, Learoyd J, Meliton AY, Clay BS, Leff AR, Zhu X.

Section of Pulmonary and Critical Care Medicine, Department of Medicine, The University of Chicago, 5841 South Maryland Avenue, Chicago, IL 60637, USA.

The objective of this investigation was to determine the role of Pyk2, an intracellular nonreceptor protein tyrosine kinase for postadhesive inflammatory cell migration, on airway inflammation and hyperresponsiveness in immune-sensitized mice. Blockade of Pyk2 was effected by intraperitoneal administration of dominant-negative C-terminal Pyk2 fused to a TAT protein transduction domain (TAT-Pyk2-CT). Ovalbumin challenge elicited infiltration of both eosinophils and lymphocytes into airways, increased mucus-containing epithelial cells, and caused increased airway hyperresponsiveness to methacholine in immune-sensitized mice. Pretreatment with 10 mg/kg TAT-Pyk2-CT intraperitoneally blocked all of these effects and further decreased secretion of Th2 cytokine IL-4, IL-5, and IL-13 into the bronchoalveolar lavage fluid. Intranasal administration of IL-5 caused eosinophil migration into the airway lumen, which was attenuated by systemic pretreatment with TAT-Pyk2-CT. In each paradigm, treatment with control protein TAT-GFP had no blocking effect. We conclude that Pyk2, which is essential for inflammatory cell migration in vitro, regulates airway inflammation, Th2 cytokine secretion, and airway hyperresponsiveness in the ovalbumin-sensitized mice during antigen challenge in vivo.

PMCID: PMC2848740
PMID: 19520918 [PubMed - indexed for MEDLINE]

541. Curr Gene Ther. 2009 Jun;9(3):185-91.

Gene therapy for allergic diseases.

Chuang YH, Yang YH, Wu SJ, Chiang BL.

Department of Clinical Laboratory Sciences and Medical Biothechnology, National Taiwan University, Taipei, Taiwan.

Allergic diseases, such as allergic asthma, allergic rhinitis, atopic dermatitis, conjunctivitis, urticaria, food allergy, and/or anaphylaxis, are associated with the skewing of immune responses towards a T helper 2 (TH2) phenotype, resulting in eosinophilic inflammation. TH2 cytokines, such as interleukin (IL)-4, IL-5 and IL-13, promote IgE production, mast cell differentiation, and eosinophil growth, migration and activation which then lead to the pathologic abnormalities in allergic diseases. Moreover, the impaired function of regulatory T cells has been noted in allergic diseases. To date, treatments for allergic diseases, such as antihistamines, corticosteroids, bronchodilators and some allergen-specific immunotherapy, are effective but costly and require long-term and recurrent drug administration. Gene therapy has been shown to be an easy, effective, and convenient treatment by delivering the allergen or the therapeutic protein in the form of plasmid DNA in vivo to modulate allergic immune responses. We summarize here the recent advances of gene therapy in allergic diseases and discuss the challenges in clinical application.

PMID: 19519363 [PubMed - indexed for MEDLINE]

542. Int Arch Allergy Immunol. 2009;149 Suppl 1:31-8. doi: 10.1159/000210651. Epub 2009 Jun 3.

Eosinophil superoxide anion generation induced by adhesion molecules and leukotriene D4.

Mori M, Takaku Y, Kobayashi T, Hagiwara K, Kanazawa M, Nagata M.

Department of Respiratory Medicine, Saitama Medical University, Saitama 350-0495, Japan.

RATIONALE: Eosinophils preferentially accumulate at sites of inflammation in the asthmatic airway. Participation of circulating eosinophils in the airway inflammation in asthma involves their interaction with adhesion molecules expressed on the endothelial cell surface and exposure to inflammatory mediators, such as cysteinyl leukotrienes (cysLTs).
OBJECTIVE: To investigate whether interaction of eosinophils with adhesion molecules modifies the functions of these cells induced by cysLTs. Methods: Eosinophils were isolated from the blood of healthy donors, incubated in the EIA plates coated with adhesion proteins, and then exposed to LTD4. The generation of superoxide anion (O2-), adhesion to the plates, and release of eosinophil-derived neutrotoxin (EDN) were evaluated.
RESULTS: Neither VCAM-1 nor LTD4 (100 nM) independently induced eosinophil O2- generation, however, combined exposure to the two molecules synergistically induced eosinophil O2- generation. ICAM-1 by itself induced eosinophil O2- generation, which was enhanced by LTD4. On the contrary, P-selectin did not induce O2- generation, either in the presence or absence of LTD4. LTD4 significantly enhanced eosinophil adhesion to rh-VCAM-1 and rh-ICAM-1, but not to rh-P-selectin. Finally, we observed that combined exposure of eosinophils to LTD4 and VCAM-1 induced the release of EDN.
CONCLUSION: Combined exposure to VCAM-1 or ICAM-1 and cysLT effectively induces the effector functions of eosinophils. Eosinophil adhesion to and migration across endothelial cells via these specific adhesion proteins and subsequent exposure to cysLTs may be mechanisms underlying activation of the effector functions of eosinophils in the asthmatic airway.

Copyright 2009 S. Karger AG, Basel.

PMID: 19494502 [PubMed - indexed for MEDLINE]

543. Sci Signal. 2009 Jun 9;2(74):ra27. doi: 10.1126/scisignal.2000259.

PI3Kgamma adaptor subunits define coupling to degranulation and cell motility by distinct PtdIns(3,4,5)P3 pools in mast cells.

Bohnacker T, Marone R, Collmann E, Calvez R, Hirsch E, Wymann MP.

Institute of Biochemistry and Genetics, Department of Biomedicine, University of Basel, Mattenstrasse 28, Basel, Switzerland.

Comment in
    Sci Signal. 2009;2(74):pe35.

Phosphoinositide 3-kinase gamma (PI3Kgamma) plays a major role in chronic inflammation and allergy. It is a heterodimer of a catalytic p110gamma subunit and an adaptor protein, either p101 or the p101 homolog p84 (p87(PIKAP)). It is unclear whether both PI3Kgamma complexes specifically modulate responses such as chemotaxis and degranulation. In mast cells, the p84:p110gamma complex synergizes with immunoglobulin E (IgE)- and antigen-clustered FcepsilonRI receptor signaling and is required to achieve maximal degranulation. During this process, PI3Kgamma is activated by ligands of heterotrimeric guanine nucleotide-binding protein (G protein)-coupled receptors (GPCRs), in particular adenosine receptors, through autocrine and paracrine pathways. Here, we show that p110gamma needs p84 to relay signals from GPCRs to formation of phosphatidylinositol 3,4,5-trisphosphate

[PtdIns(3,4,5)P(3)], phosphorylation of Akt, migration of cells, and synergistic adenosine-enforced degranulation. Furthermore, the absence of adaptor subunits could not be compensated for by increased p110gamma abundance. Differentiated, p110gamma null cells also lost adaptor proteins. Complementation of p110gamma null mast cells with p101 and p110gamma restored the activation of Akt and cell migration, but failed to support degranulation. Lack of degranulation was attributed to a change in the spatiotemporal localization of PI3Kgamma-derived PtdIns(3,4,5)P(3); although both p84:p110gamma and p101:p110gamma complexes initially deposited PtdIns(3,4,5)P(3) at the plasma membrane, p101:p110gamma-derived PtdIns(3,4,5)P(3) was rapidly endocytosed to motile, microtubule-associated vesicles. In addition, p84:p110gamma, but not p101:p110gamma signaling was sensitive to disruption of lipid rafts. Our results demonstrate a nonredundant function for the p101 and p84 PI3Kgamma adaptor proteins and show that distinct pools of PtdIns(3,4,5)P(3) at the plasma membrane can elicit specific cell responses.

PMID: 19509406 [PubMed - indexed for MEDLINE]

544. Vet Immunol Immunopathol. 2009 Dec 15;132(2-4):116-21. doi: 10.1016/j.vetimm.2009.05.003. Epub 2009 May 18.

Cholinergic stimulation attenuates the IL-4 induced expression of E-selectin and vascular endothelial growth factor by equine pulmonary artery endothelial cells.

Huang H, Lavoie-Lamoureux A, Lavoie JP.

Department of Clinical Sciences, Faculty of Veterinary Medicine, Université de Montréal, C.P. 5000, 3200 Sicotte, Saint-Hyacinthe, Québec, Canada, J2S 7C6. huanghong@tjh.tjmu.edu.cn

The endothelium plays a critical role in regulating leukocyte recruitment and migration during inflammation. Recent studies provide evidence that acetylcholine (ACh) and other cholinergic mediators block endothelial cells activation and leukocyte recruitment during inflammation. We thus postulated that the non-neuronal cholinergic system might modulate the recruitment of neutrophils during allergic pulmonary inflammation. In the present study, we examined the effects of cholinergic stimulation on the expression of neutrophil chemokines and adhesion molecules by endothelial cells stimulated by recombinant equine (re) IL-4. Using primary equine pulmonary artery endothelial cells culture and real-time RT-PCR method, we observed that ACh, nicotine, and muscarine inhibit the expression of E-selectin and vascular endothelial growth factor by endothelial cells stimulated by reIL-4. The expression of CXCL-8, a potent neutrophil chemotactic cytokine, remained unaffected however. These findings suggest that the cholinergic anti-inflammatory pathway may modulate pulmonary allergic inflammation and remodeling by the inhibition of selected adhesion molecules and growth factors.

PMID: 19501920 [PubMed - indexed for MEDLINE]

545. J Allergy Clin Immunol. 2009 Jul;124(1):90-9. doi: 10.1016/j.jaci.2009.04.025. Epub 2009 May 30.

DNA vaccination against macrophage migration inhibitory factor improves atopic dermatitis in murine models.

Hamasaka A, Abe R, Koyama Y, Yoshioka N, Fujita Y, Hoshina D, Sasaki M, Hirasawa T, Onodera S, Ohshima S, Leng L, Bucala R, Nishihira J, Shimizu T, Shimizu H.

Department of Dermatology, Hokkaido University Graduate School of Medicine,
Sapporo 060-8638, Japan.

BACKGROUND: Atopic dermatitis (AD) is a common chronic inflammatory skin disease. Macrophage migration inhibitory factor (MIF) is a proinflammatory cytokine that has been implicated in the pathogenesis of AD. Recently, we developed a novel DNA vaccine that generates neutralizing endogenous anti-MIF antibodies.
OBJECTIVE: This study explores the preventive and therapeutic effects of this MIF-DNA vaccine in mouse models of AD.
METHODS: Two different AD model mice (DS-Nh and NC/Nga) received MIF-DNA vaccination to analyze preventive and therapeutic effects, as assessed by clinical skin scores, histologic findings, and serum IgE levels.
RESULTS: In murine models of AD, MIF-DNA vaccination prevented the occurrence of the AD skin phenotype. Furthermore, administration of MIF-DNA vaccine to mice that had already developed AD produced a rapid improvement in AD skin manifestation. There were reduced histologic signs of inflammation and lower serum IgE levels in treated mice compared with those seen in control animals. Finally, passive transfer of IgG from MIF-DNA vaccinated mice to AD mice also produced a significant therapeutic effect. These results demonstrate that MIF-DNA vaccination not only prevents the development of AD but also improves the symptoms of pre-existing AD.
CONCLUSION: Taken together, the induction of an anti-MIF autoantibody response using MIF-DNA vaccination appears to be a useful approach in the treatment of AD.

PMID: 19482347 [PubMed - indexed for MEDLINE]

546. Lancet. 2009 May 30;373(9678):1905-17. doi: 10.1016/S0140-6736(09)60326-3.

Glucocorticoid resistance in inflammatory diseases.

Barnes PJ, Adcock IM.

National Heart and Lung Institute, Imperial College, London, UK.
p.j.barnes@imperial.ac.uk

Glucocorticoid resistance or insensitivity is a major barrier to the treatment of several common inflammatory diseases-including chronic obstructive pulmonary disease and acute respiratory distress syndrome; it is also an issue for some patients with asthma, rheumatoid arthritis, and inflammatory bowel disease. Several molecular mechanisms of glucocorticoid resistance have now been identified, including activation of mitogen-activated protein (MAP) kinase pathways by certain cytokines, excessive activation of the transcription factor activator protein 1, reduced histone deacetylase-2 (HDAC2) expression, raised macrophage migration inhibitory factor, and increased P-glycoprotein-mediated drug efflux. Patients with glucocorticoid resistance can be treated with alternative broad-spectrum anti-inflammatory treatments, such as calcineurin inhibitors and other immunomodulators, or novel anti-inflammatory treatments, such as inhibitors of phosphodiesterase 4 or nuclear factor kappaB, although these drugs are all likely to have major side-effects. An alternative treatment strategy is to reverse glucocorticoid resistance by blocking its underlying mechanisms. Some examples of this approach are inhibition of p38 MAP kinase, use of vitamin D to restore interleukin-10 response, activation of HDAC2 expression by use of theophylline, antioxidants, or phosphoinositide-3-kinase-delta inhibitors, and inhibition of macrophage migration inhibitory factor and P-glycoprotein.

PMID: 19482216 [PubMed - indexed for MEDLINE]

547. J Allergy Clin Immunol. 2009 Jul;124(1):59-65.e1-3. doi:
10.1016/j.jaci.2009.03.024. Epub 2009 May 27.

Expression of functional leukotriene B4 receptors on human airway smooth muscle cells.

Watanabe S, Yamasaki A, Hashimoto K, Shigeoka Y, Chikumi H, Hasegawa Y, Sumikawa T, Takata M, Okazaki R, Watanabe M, Yokogawa T, Yamamura M, Hayabuchi T, Gerthoffer WT, Halayko AJ, Shimizu E.

Division of Medical Oncology and Molecular Respirology, Department of Multidisciplinary Internal Medicine, Faculty of Medicine, Tottori University, Tottori, Japan.

BACKGROUND: Leukotriene B4 (LTB4) increases in induced sputum and exhaled breath condensate in people with asthma. Furthermore, the T(H)2-type immune response and airway hyperresponsiveness induced by ovalbumin sensitization is markedly suppressed in LTB4 receptor (BLT) 1 null mice. These studies suggest that LTB4 may contribute to asthma pathophysiology. However, the direct effects of LTB4 on human airway smooth muscle (ASM) have not been studied.
OBJECTIVES: We sought to determine the expression of LTB4 receptors on human ASM and its functional role in mediating responses of human ASM cells, and the effect of LTB4 on these cells.
METHODS: Immunohistochemistry, RT-PCR, Western blotting, and flow cytometry were used to determine the expression of LTB4 receptors. To determine the effect of LTB4 on human ASM cells, cell proliferation was assessed by counting cells, and chemokinesis was assessed by gold particle phagokinesis assay.
RESULTS: We confirmed expression of both BLT1 and BLT2 in human ASM cells in bronchial tissue and in cell culture. LTB4 markedly induced cyclin D1 expression, proliferation, and chemokinesis of human ASM cells. LTB4 also induced phosphorylation of both p42/p44 mitogen-activated protein kinase (MAPK) and downstream PI3 kinase effector, Akt1. However, we observed no induction of c-Jun N-terminal kinase or p38 MAPK. Notably, LTB4-induced migration and proliferation of ASM cells were inhibited by the BLT1 specific antagonist, U75302, and by inhibitors of p42/p44 MAPK phosphorylation (U1026), and PI3 kinase (LY294002).
CONCLUSIONS: These observations are the first to suggest a role for a LTB4-BLT1 signaling axis in ASM responses that may contribute to the pathogenesis of airway remodeling in asthma.

PMID: 19477492 [PubMed - indexed for MEDLINE]

548. J Invest Dermatol. 2009 Nov;129(11):2552-66. doi: 10.1038/jid.2009.122. Epub 2009 May 28.

Chemokine receptors in T-cell-mediated diseases of the skin.

Lonsdorf AS, Hwang ST, Enk AH.

Department of Dermatology, University Hospital Heidelberg, Heidelberg, Germany.
anke.lonsdorf@med.uni-heidelberg.de

The chemokine/chemokine receptor network is an integral element of the complex system of homeostasis and immunosurveillance. Initially studied because of their role in coordinating tissue-specific migration and activation of leucocytes, chemokines have been implicated in the pathogenesis of various malignancies and diseases with strong inflammatory components. We discuss recent findings suggesting a critical involvement of chemokine receptor interactions in the immunopathogenesis of classical inflammatory skin disorders such as psoriasis and atopic dermatitis, as well as neoplastic diseases with a T-cell origin, such as

mycosis fungoides. A deeper understanding of the underlying contribution of the chemokine network in the disease processes is key for the development of selective targeted immunotherapeutics that may meet the delicate balance between efficacy and safety.

PMID: 19474804 [PubMed - indexed for MEDLINE]

549. J Biol Chem. 2009 Jul 17;284(29):19445-51. doi: 10.1074/jbc.M109.002550. Epub 2009 May 27.

Synaptotagmin-2 controls regulated exocytosis but not other secretory responses of mast cells.

Melicoff E, Sansores-Garcia L, Gomez A, Moreira DC, Datta P, Thakur P, Petrova Y, Siddiqi T, Murthy JN, Dickey BF, Heidelberger R, Adachi R.

Department of Pulmonary Medicine, The University of Texas M. D. Anderson Cancer Center, Houston, Texas 77030, USA.

Mast cell degranulation is a highly regulated, calcium-dependent process, which is important for the acute release of inflammatory mediators during the course of many pathological conditions. We previously found that Synaptotagmin-2, a calcium sensor in neuronal exocytosis, was expressed in a mast cell line. We postulated that this protein may be involved in the control of mast cell-regulated exocytosis, and we generated Synaptotagmin-2 knock-out mice to test our hypothesis. Mast cells from this mutant animal conferred an abnormally decreased passive cutaneous anaphylaxis reaction on mast cell-deficient mice that correlated with a specific defect in mast cell-regulated exocytosis, leaving constitutive exocytosis and nonexocytic mast cell effector responses intact. This defect was not secondary to abnormalities in the development, maturation, migration, morphology, synthesis, and storage of inflammatory mediators, or intracellular calcium transients of the mast cells. Unlike neurons, the lack of Synaptotagmin-2 in mast cells was not associated with increased spontaneous exocytosis.

PMCID: PMC2740570
PMID: 19473977 [PubMed - indexed for MEDLINE]

550. Exp Hematol. 2009 Jun;37(6):715-727.e3. doi: 10.1016/j.exphem.2009.03.010.

Absence of alpha 4 but not beta 2 integrins restrains development of chronic allergic asthma using mouse genetic models.

Banerjee ER, Jiang Y, Henderson WR Jr, Latchman Y, Papayannopoulou T.

Department of Medicine, Division of Allergy and Infectious Diseases, University of Washington, Seattle, WA 98195-7710, USA.

OBJECTIVE: Chronic asthma is characterized by ongoing recruitment of inflammatory cells and airway hyperresponsiveness leading to structural airway remodeling. Although alpha 4 beta 1 and beta2 integrins regulate leukocyte migration in inflammatory diseases and play decisive roles in acute asthma, their role has not been explored under the chronic asthma setting. To extend our earlier studies with alpha 4(Delta/Delta) and beta2(-/-) mice, which showed that both alpha 4 and beta2 integrins have nonredundant regulatory roles in acute ovalbumin (OVA)-induced asthma, we explored to what extent these molecular pathways control development of structural airway remodeling in chronic asthma.
MATERIALS AND METHODS: Control, alpha 4(Delta/Delta), and beta2(-/-) mouse

groups, sensitized by intraperitoneal OVA as allergen, received intratracheal OVA periodically over days 8 to 55 to induce a chronic asthma phenotype. Post-OVA assessment of inflammation and pulmonary function (airway hyperresponsiveness), together with airway modeling measured by goblet cell metaplasia, collagen content of lung, and transforming growth factor beta1 expression in lung homogenates, were evaluated.
RESULTS: In contrast to control and beta2(-/-) mice, alpha 4(Delta/Delta) mice failed to develop and maintain the composite chronic asthma phenotype evaluated as mentioned and subepithelial collagen content was comparable to baseline. These data indicate that beta2 integrins, although required for inflammatory migration in acute asthma, are dispensable for structural remodeling in chronic asthma.
CONCLUSION: alpha 4 integrins appear to have a regulatory role in directing transforming growth factor beta-induced collagen deposition and structural alterations in lung architecture likely through interactions of Th2 cells, eosinophils, or mast cells with endothelium, resident airway cells, and/or extracellular matrix.

PMID: 19463772 [PubMed - indexed for MEDLINE]

551. J Immunol. 2009 Jun 1;182(11):6907-14. doi: 10.4049/jimmunol.0803533.

Macrophage migration inhibitory factor homologs of anisakis simplex suppress Th2 response in allergic airway inflammation model via CD4+CD25+Foxp3+ T cell recruitment.

Park SK, Cho MK, Park HK, Lee KH, Lee SJ, Choi SH, Ock MS, Jeong HJ, Lee MH, Yu HS.

Department of Parasitology, School of Medicine, Pusan National University Busan, Busan, Korea.

We have cloned the macrophage migration inhibitory factor (MIF)-like protein (Anisakis simplex (As)-MIF) from larvae of the whale worm (Anisakis simplex third-stage larvae). Asthma was induced in the mice using OVA/alum, with or without various concentrations of rAs-MIF treatment before OVA/alum challenge. Treatment with rAs-MIF coupled with OVA/alum during the challenge period induced a complete inhibition of eosinophilia and goblet cell hyperplasia within the lung and profoundly ameliorated the development of lung hyperreactivity. Also, rAs-MIF was shown to reduce profoundly the quantity of Th2-related cytokines (IL-4, IL-5, and IL-13) in the bronchial alveolar lavage fluid and allergen-specific IgG2a in sera. IL-10 and TGF-beta levels in the bronchoalveolar lavage fluid of the rAs-MIF-treated group were significantly higher than in the other groups. Additionally, CD4(+)CD25(+)Foxp3(+) T cells (regulatory T) were recruited to the spleen and lungs of the rAs-MIF-treated mice, but this recruitment was inhibited by anti-rAs-MIF Ab.

PMID: 19454687 [PubMed - indexed for MEDLINE]

552. Aesthetic Plast Surg. 2010 Aug;34(4):413-22. doi: 10.1007/s00266-009-9359-3. Epub 2009 May 19.

Unacceptable results with an accepted soft tissue filler: polyacrylamide hydrogel.

Manafi A, Emami AH, Pooli AH, Habibi M, Saidian L.

Department of Plastic and Reconstructive Surgery, Fatemeh Zahra Hospital, Iran University of Medical Sciences, Street No. 21, Seyed Jamaladin Asad-Abadi Avenue,

Tehran, Iran. manafimd@gmail.com

Comment in
    Aesthetic Plast Surg. 2010 Aug;34(4):423.

BACKGROUND: Polyacrylamide hydrogel, considered a safe and biocompatible soft tissue filler, is widely used in cosmetic procedures. Its use for facial contouring and breast augmentation in Iran has increased dramatically in recent years. Most patients and many doctors are unaware of possible and reported adverse effects related to its administration.
METHODS: This study enrolled 98 patients experiencing unsatisfactory results and complications of polyacrylamide hydrogel. Adverse effects related to gel administration were documented for all the patients. Lab values were requested together with related medical care and surgical treatments, and gel was extracted by incision, milking, and irrigation.
RESULTS: The most common findings at the time of presentation were inflammation (n = 51), asymmetry (n = 31), irregularity (n = 18), infection and abscess formation (n = 11), and gel migration (n = 8). In one patient, severe anaphylactoid reaction was observed 1 week after gel injection, which led to significant complications for the patient. Histologic findings showed granuloma formation (n = 17), fat necrosis (n = 9), and fibrosis (n = 17). Macroscopic gel-related complications resolved after extraction of the injected material, except for skin necrosis and hyperpigmentation, which remained unchanged. For eight patients, the gel could not be extracted by squeezing and irrigation entirely. Three patients experienced gel reaccumulation after seemingly complete removal of the gel.
CONCLUSIONS: A wide range of complications seen among our patients showed that polyacrylamide hydrogel may not be as safe and biocompatible as it was thought previously. Both patients and physicians must be aware of the potential side effects of polyacrylamide hydrogel before gel administration.

PMID: 19452201 [PubMed - indexed for MEDLINE]

553. J Immigr Minor Health. 2010 Oct;12(5):617-25. doi: 10.1007/s10903-009-9261-8.

Housing environments and child health conditions among recent Mexican immigrant families: a population-based study.

Litt JS, Goss C, Diao L, Allshouse A, Diaz-Castillo S, Bardwell RA, Hendrikson E, Miller SL, DiGuiseppi C.

Department of Environmental & Occupational Health, Colorado School of Public Health, University of Colorado Denver, Denver, CO 80045, USA.
jill.litt@ucdenver.edu

The influx of immigrants to urban areas throughout the United States has raised concerns about accessibility of safe, affordable housing and the health consequences of poor-quality housing, particularly among immigrant children. We conducted a population-based study of home environmental conditions among recently immigrated Mexican families (weighted n = 473), generally of low socioeconomic status, and the health conditions of their children, in an urban industrial area north of Denver, Colorado. The majority of recent immigrants had low socioeconomic status; virtually all had household incomes below the Colorado median ($50,841). Approximately one quarter of homes were overcrowded. Adverse environmental conditions were present across recent immigrant homes. These conditions include dampness or mold (44%), pests (28%), and minimal to no ventilation potential (26%), all of which are associated with asthma and atopic diseases. At least one of these three environmental hazards was found in 67% of homes; multiple hazards were present in 27% of homes. Children of recent

immigrant families had active symptoms within the past 12 months suggestive of asthma (4%) and atopic disorders (10%); however, fewer than 2% had been diagnosed with these conditions. The prevalence of asthma and atopic symptoms among Mexican immigrant children, albeit lower than in other low income and minority communities, is partially explained by housing conditions. Many of the conditions identified (e.g., pest infestation, mold resulting from plumbing leaks, and lack of exhaust fans) are amenable to low cost interventions. Solutions to address unhealthy housing conditions among recent immigrants must be multi-faceted and include strategies that target household-level improvements and access to health care.

PMID: 19449207 [PubMed - indexed for MEDLINE]

554. Am J Physiol Lung Cell Mol Physiol. 2009 Jul;297(1):L164-73. doi: 10.1152/ajplung.90453.2008. Epub 2009 May 15.

Insulin receptor substrate-1/2 mediates IL-4-induced migration of human airway epithelial cells.

White SR, Martin LD, Abe MK, Marroquin BA, Stern R, Fu X.

Department of Medicine, University of Chicago, Chicago, Illinois 60637, USA. swhite@medicine.bsd.uchicago.edu

Migration of airway epithelial cells (AEC) is an integral component of airway mucosal repair after injury. The inflammatory cytokine IL-4, abundant in chronic inflammatory airways diseases such as asthma, stimulates overproduction of mucins and secretion of chemokines from AEC; these actions enhance persistent airway inflammation. The effect of IL-4 on AEC migration and repair after injury, however, is not known. We examined migration in primary human AEC differentiated in air-liquid interface culture for 3 wk. Wounds were created by mechanical abrasion and followed to closure using digital microscopy. Concurrent treatment with IL-4 up to 10 ng/ml accelerated migration significantly in fully differentiated AEC. As expected, IL-4 treatment induced phosphorylation of the IL-4 receptor-associated protein STAT (signal transducer and activator of transcription)6, a transcription factor known to mediate several IL-4-induced AEC responses. Expressing a dominant negative STAT6 cDNA delivered by lentivirus infection, however, failed to block IL-4-stimulated migration. In contrast, decreasing expression of either insulin receptor substrate (IRS)-1 or IRS-2 using a silencing hairpin RNA blocked IL-4-stimulated AEC migration completely. These data demonstrate that IL-4 can accelerate migration of differentiated AEC after injury. This reparative response does not require STAT6 activation, but rather requires IRS-1 and/or IRS-2.

PMCID: PMC2711809
PMID: 19447894 [PubMed - indexed for MEDLINE]

555. Clin Ther. 2009 Apr;31(4):728-40. doi: 10.1016/j.clinthera.2009.04.019.

Two first-in-human, open-label, phase I dose-escalation safety trials of MEDI-528, a monoclonal antibody against interleukin-9, in healthy adult volunteers.

White B, Leon F, White W, Robbie G.

MedImmune, LLC, Gaithersburg, Maryland 20878, USA. whiteb@medimmune.com

BACKGROUND: Interleukin-9 (IL-9) is involved in pathogenic aspects of the

asthmatic response, including induction of the proliferation of T-helper type 2 lymphocytes, mucus production, and mast-cell differentiation, proliferation, and recruitment to the lung. In preclinical studies in mice, inhibition of IL-9 through neutralizing monoclonal antibody (mAb) treatment partially reduced airway hyperresponsiveness and mast-cell progenitor migration to the lung.
OBJECTIVE: The goal of the present studies was to determine the safety and pharmacokinetic profiles and immunogenicity of MEDI-528, a humanized immunoglobulin G1k anti-IL-9 mAb, in healthy adult volunteers.
METHODS: In separate open-label, Phase I dose-escalation studies, single doses of MEDI-528 0.3, 1.0, 3.0, or 9.0 mg/kg were administered as an intravenous infusion (20 mg/min administered over 1-40 minutes, depending on dose) and by subcutaneous injection. All subjects were followed for 84 days. Any laboratory test value outside the normal reference range was considered an adverse event (AE).
RESULTS: Twenty-four subjects were enrolled in the intravenous study, and 29 subjects were enrolled in the subcutaneous study. No deaths or serious or severe AEs occurred in either study. The most frequently reported AEs in the intravenous study were laboratory test abnormalities; the most frequently reported AEs in the subcutaneous study were pharyngolaryngeal pain, palpable lymph nodes, and laboratory test abnormalities. The single-dose pharmacokinetics of MEDI-528 were linear and dose proportional over the dose range studied with both routes of administration. The mean t((1/2)) after intravenous administration was approximately 26 days (range, 25-28 days); the mean t((1/2)) after subcutaneous administration ranged from 33 to 87 days across doses. A low titer (1:80) of antibodies to MEDI-528 was detected on day 84 in a single volunteer receiving intravenous MEDI-528 3.0 mg/kg. No antibody titers were detected in any of the volunteers receiving subcutaneous MEDI-528.
CONCLUSIONS: Administered intravenously or subcutaneously, MEDI-528 had an acceptable safety profile and exhibited linear pharmacokinetics over the dose range studied in healthy adults in these Phase I studies. The findings support further investigation of MEDI-528 in multiple-dose trials in patients with asthma. ClinicalTrials.gov Identification numbers: NCT00192296 (intravenous study); NCT00116168 (subcutaneous study).

PMID: 19446146 [PubMed - indexed for MEDLINE]

556. Clin Exp Allergy. 2009 Jul;39(7):1009-17. doi: 10.1111/j.1365-2222.2009.03246.x. Epub 2009 May 3.

Human airway smooth muscle promotes eosinophil differentiation.

Fanat AI, Thomson JV, Radford K, Nair P, Sehmi R.

Asthma Research Group, Firestone Institute for Respiratory Health, St Joseph's Healthcare, Department of Medicine, McMaster University, Hamilton, ON, Canada.

Comment in
    Clin Exp Allergy. 2009 Jul;39(7):938-9.

INTRODUCTION: Human airway smooth muscle (HASM) cells in culture synthesize cytokines and chemokines that may orchestrate the tissue homing and in situ differentiation of haemopoietic progenitor cells from the peripheral circulation.
OBJECTIVE: To study the effect of a supernatant from cultured HASM cells on the differentiative and transmigrational responses of haemopoietic progenitor cells.
METHODS: HASM cells were grown to confluence and stimulated with a cytomix of TNF-alpha, IL-1beta and IFN-gamma. Peripheral blood-derived progenitors from atopic asthmatics (n=12) and non-atopic controls (n=11) were grown in a methylcellulose culture with a supernatant from stimulated HASM cells to assess clonogenic potential. The ability of HASM cells to stimulate directional migration and adhesion to fibronectin of blood progenitors was also investigated.

RESULTS: HASM cells stimulated significant growth of eosinophil/basophil colony forming units (Eo/B CFUs) from blood progenitor cells from both groups of subjects. This activity was significantly attenuated in the presence of anti-IL-5 and anti-granulocyte macrophage-colony forming factor blocking antibodies and by pre-treatment with SB202190 [p38 mitogen-activated protein kinase (MAPK) inhibitor]. An src kinase (srcK) inhibitor (Pyrazolopyrimidine 1) was less effective at attenuating IL-5- and HASM-stimulated Eo/B CFU growth from both groups of subjects. Examination of the phosphorylation of these kinases in CD34(+) cells following co-incubation with the major constituents of HASM showed activation of p38 MAPK but not that of the srcK pathway. The HASM supernatant had no significant effect on the migrational and adhesive responses of haemopoietic progenitor cells in vitro.
CONCLUSION: We have shown that HASM cell-derived cytokines promote eosinophil differentiation that is dependent on p38 MAPK but not on the srcK pathway. This study shows that a major structural cell of the lungs, airway smooth muscle, has the capability to direct eosinophil differentiation and maturation from progenitor cells, which in turn may perpetuate an eosinophilic inflammation and consequently tissue remodelling in patients with chronic asthma.

PMID: 19438586 [PubMed - indexed for MEDLINE]

557. Prostate. 2009 Aug 1;69(11):1222-34. doi: 10.1002/pros.20975.

Tranilast inhibits hormone refractory prostate cancer cell proliferation and suppresses transforming growth factor beta1-associated osteoblastic changes.

Izumi K, Mizokami A, Li YQ, Narimoto K, Sugimoto K, Kadono Y, Kitagawa Y, Konaka H, Koh E, Keller ET, Namiki M.

Department of Integrative Cancer Therapy and Urology, Kanazawa University Graduate School of Medical Science, Kanazawa, Ishikawa 920-8641, Japan.

BACKGROUND: Tranilast is a therapeutic agent used in treatment of allergic diseases, although it has been reported to show anti-tumor effects on some cancer cells. To elucidate the effects of tranilast on prostate cancer, we investigated the mechanisms of its anti-tumor effect on prostate cancer.
METHODS: The anti-tumor effects and related mechanisms of tranilast were investigated both in vitro on prostate cancer cell lines and bone-derived stromal cells, and in vivo on severe combined immunodeficient (SCID) mice. We verified its clinical effect in patients with advanced hormone refractory prostate cancer (HRPC).
RESULTS: Tranilast inhibited the proliferation of LNCaP, LNCaP-SF, and PC-3 cells in a dose-dependent manner and growth of the tumor formed by inoculation of LNCaP-SF in the dorsal subcutis and in the tibia of castrated SCID mice. Flow cytometry and TUNEL assay revealed induction of cell cycle arrest and apoptosis by tranilast. Tranilast increased expression of proteins involved in induction of cell cycle arrest and apoptosis. Coculture with bone-derived stromal cells induced proliferation of LNCaP-SF cells. Tranilast also suppressed secretion of transforming growth factor beta1 (TGF-beta1) from bone-derived stromal cells, which induced their differentiation. Moreover, tranilast inhibited TGF-beta1-mediated differentiation of bone-derived stromal cells and LNCaP-SF cell migration induced by osteopontin. In the clinical investigation, PSA progression was inhibited in 4 of 16 patients with advanced HRPC.
CONCLUSIONS: These observations suggest that tranilast may be a useful therapeutic agent for treatment of HRPC via the direct inhibitory effect on cancer cells and suppression of TGF-beta1-associated osteoblastic changes in bone metastasis.

PMID: 19434660 [PubMed - indexed for MEDLINE]

558. BMC Pediatr. 2009 May 8;9:31. doi: 10.1186/1471-2431-9-31.

Antibiotic use among 8-month-old children in Malmö, Sweden--in relation to child characteristics and parental sociodemographic, psychosocial and lifestyle factors.

Mangrio E, Wremp A, Moghaddassi M, Merlo J, Bramhagen AC, Rosvall M.

Department of Clinical Sciences, Social Epidemiology, Malmö University Hospital, Lund University, Malmö, Sweden. Elisabeth.mangrio@skane.se

BACKGROUND: In the county of Scania, Sweden, antibiotic use among small children is among the highest in the country. The aim of this study was to investigate the associations between antibiotic use among 8-month-old children in Malmö and characteristics of the child as well as parental sociodemographic characteristics, lifestyle factors, and psychosocial support.
METHODS: The study was a population-based cross-sectional survey. The study population consisted of children who visited the Child Health Care (CHC) centres in Malmö for their 8-month health checkup during 2003-2006 and whose parents answered a self-administered questionnaire (n = 7266 children). The questionnaire was distributed to parents of children registered with the CHC and invited for an 8-month checkup during the study period.
RESULTS: The odds of using antibiotics increased as parental educational level decreased. Using high educational level as a reference group, low maternal educational level was associated with an increased antibiotic use for the child, odds ratio (OR) = 1.61 (95% CI: 1.34-1.93). Furthermore, children whose parents were born outside Sweden showed higher antibiotic use, OR = 1.43 (95% CI: 1.24-1.65), in comparison with children whose parents were born in Sweden. Exposure to environmental smoking, parental experience of economic stress, and a low level of emotional support increased the odds for antibiotic use. Boys had higher odds of use of antibiotics than girls, OR = 1.40 (95% CI: 1.25-1.57). Having a low birth weight, having an allergy and having siblings also increased the odds for early antibiotic use, while breastfeeding seemed to have a protective role.
CONCLUSION: There were clear associations between parental factors such as sociodemographic, psychosocial and lifestyle factors and antibiotic use at this early stage of life. Several characteristics of the child also affected the use of antibiotics.

PMCID: PMC2685137
PMID: 19426489 [PubMed - indexed for MEDLINE]

559. J Immunol. 2009 May 15;182(10):6136-42. doi: 10.4049/jimmunol.0802878.

Impaired mast cell-driven immune responses in mice lacking the transcription factor NFATc2.

Becker M, Heib V, Klein M, Doener F, Bopp T, Taube C, Radsak M, Schild H, Schmitt E, Stassen M.

Institute for Immunology, University of Mainz, Mainz, Germany.

The three calcium-dependent factors NFATc1, c2, and c3 are expressed in cells of the immune system and play pivotal roles in modulating cellular activation. With regard to NFATc2, it was reported that NFATc2-deficient mice display increased immune responses in several models for infection and allergy in vivo. This led to the assumption that NFATc2 is involved in the maintenance of immune homeostasis.

Using the synthetic TLR7 agonist imiquimod as an adjuvant in epicutaneous peptide immunization, we observed that both the inflammatory reaction and the peptide-specific CTL response are severely impaired in NFATc2-deficient mice. Detailed analyses revealed that early production of proinflammatory cytokines, lymph node hypertrophy, and migration of Langerhans cells are strongly reduced in NFATc2-deficient animals. With the aid of mast cell-deficient mice and reconstitution experiments using mast cells derived from either NFATc2-deficient mice or wild-type controls, we were able to show that NFATc2 expressed in mast cells is critical for the initiation of inflammation, migration of Langerhans cells, and the development of full-blown CTL responses following epicutaneous immunization. Thus, NFATc2 is an important factor controlling mast cell accessory function at the interface of innate and adaptive immunity.

PMID: 19414766 [PubMed - indexed for MEDLINE]

560. Travel Med Infect Dis. 2009 May;7(3):125-46. doi: 10.1016/j.tmaid.2009.03.003. Epub 2009 Apr 11.

A practical approach to common skin problems in returning travellers.

O'Brien BM.

Worldwise Traveller's Health, Newmarket, Auckland, New Zealand.
brigido@adhb.govt.nz

Skin diseases are the third most common cause of morbidity in returning travellers and may affect 8% of travellers during travel. Classic tropical diseases account for one quarter and the remainder are cosmopolitan diseases. The majority are of infectious origin, and of these bacterial infections are the most common and lead to the most hospitalisations. The ten most frequently encountered diagnoses comprise four classical tropical infections (cutaneous larva migrans, myiasis, tungiasis and cutaneous leishmaniasis) and six nontropical diseases (bacterial skin infections, arthropod bites, allergic reactions, scabies, animal bites and superficial fungal infections). Other notable skin problems include swimmer's itch, dengue fever presenting with a rash and rickettsial infections presenting with a rash or eschar. Delayed diagnosis, especially of tropical diseases, is common and may be reduced by improved knowledge and a systematic approach to skin problems. This involves a thorough travel specific, traveller specific and skin problem based history, combined with targeted examination and investigations. A frequency weighted differential diagnosis of the most common skin lesions is presented. An increased emphasis on preventative advice in relation to skin disease is encouraged during pre-travel consultations.

PMID: 19411040 [PubMed - indexed for MEDLINE]

561. Immunol Lett. 2009 Jun 4;124(2):81-7. doi: 10.1016/j.imlet.2009.04.007. Epub 2009 May 3.

Unimpaired immune functions in the absence of Mrp4 (Abcc4).

van de Ven R, de Groot J, Reurs AW, Wijnands PG, van de Wetering K, Schuetz JD, de Gruijl TD, Scheper RJ, Scheffer GL.

Department of Pathology, VU University Medical Center/Cancer Center Amsterdam, de Boelelaan 1117, 1081 HV Amsterdam, The Netherlands.

Dendritic cell (DC) migration to draining lymph nodes is important for the initiation of an effective immune response. Recently we reported that the human

ATP-binding cassette (ABC) transporter multidrug resistance protein 4 (MRP4 and ABCC4) is required for the migration of human DC. Since the ABC transporter MRP1 (ABCC1) was previously shown to play a role in both human and mouse DC migration, we here studied whether Mrp4 is similarly required for DC migration in mice and whether the absence of Mrp4 interferes with the generation of an immune response. Immunological responses were compared in wild-type FVB (FVBwt), FVB Mrp4 knockout (KO) or FVB Mrp4/5 double knockout (dKO) mice. Skin, a preferred immunization site, was analyzed for DC markers, as well as for Mrp1 and Mrp4 expression. Whereas Mrp1 was abundantly present within FVBwt skin, only few Mrp4 expressing cells were detected. In addition, no Mrp4 protein expression was detected on in vitro cultured FVBwt bone marrow-derived DC (BM-DC). DC migration from murine ear skin was unaltered between FVBwt and MRP4/5 dKO animals. The absence of Mrp4 also had no effect on immune responses upon allergen sensitization, immunization or oral tolerance induction. We thus conclude that in contrast to its human counterpart, murine Mrp4 is not involved in DC migration, nor indeed, in the generation of an effective immune response. These data reveal disparities in the physiological role of ABC transporters between species, which may derive from differences in substrate specificity.

PMID: 19406153 [PubMed - indexed for MEDLINE]

562. Pediatr Int. 2009 Apr;51(2):241-5. doi: 10.1111/j.1442-200X.2008.02687.x.

Toxocara seropositivity in Sri Lankan children with asthma.

Fernando D, Wickramasinghe P, Kapilananda G, Dewasurendra RL, Amarasooriya M, Dayaratne A.

Department of Parasitology, Faculty of Medicine, University of Colombo, Colombo, Sri Lanka. deepfern@slt.lk

BACKGROUND: Toxocariasis occurs in humans due to infection with Toxocara canis or T. cati, the nematode parasites of dogs and cats, respectively. The relationship between toxocariasis and asthma is complex, with some studies demonstrating that children with asthma were more likely to be Toxocara seropositive as compared to non-asthmatic children, and other studies indicating no such significant relationship. The aim of the present study was to investigate Toxocara seropositivity and its association with asthma in a selected group of Sri Lankan children.
METHODS: Two groups of children were studied: group 1 included 100 children with confirmed bronchial asthma who were on regular inhaler steroid treatment for asthma; group 2 included 96 children who did not have physician-diagnosed asthma or upper respiratory tract infections, attending the same hospital. Diagnosis of Toxocara seropositivity was based on IgG Toxocara Microwell Serum Elisa Kits. Enzyme-linked immunosorbent assay was regarded as positive for a reading of 0.3 optical density units. Stool samples were examined for helminth ova.
RESULTS: Toxocara seropositivity in children with asthma was 29% and this was significantly more than Toxocara seropositivity among non-asthmatic children ($P < 0.001$). Toxocara seropositivity was identified as a significant risk factor of asthma in a univariate model. Eosinophilia was seen in a significantly higher proportion of non-asthmatic and asthmatic children who were Toxocara seropositive. Toxocara seropositivity, however, was not identified as a significant risk factor in a multivariate model.
CONCLUSIONS: The analysis confirmed previously identified risk factors for asthma but there was no association between the helminth parasitic infection, toxocariasis and bronchial asthma in children.

PMID: 19405924 [PubMed - indexed for MEDLINE]

563. Blood. 2009 Jul 2;114(1):64-73. doi: 10.1182/blood-2009-02-204297. Epub 2009 Apr 28.

Identification of crassin acetate as a new immunosuppressant triggering heme oxygenase-1 expression in dendritic cells.

Matsushima H, Tanaka H, Mizumoto N, Takashima A.

Department of Dermatology, University of Texas Southwestern Medical Center, Dallas, TX, USA.

By screening 720 natural compounds in a standard 2-way allogeneic mixed leukocyte reaction assay, we identified a potent immunosuppressive capacity of crassin acetate (CRA), a coral-derived cembrane diterpenoid. CRA efficiently inhibited allogeneic mixed leukocyte reaction as well as antigen-specific activation of CD4 T cells by bone marrow-derived dendritic cells (DCs). With regard to cellular targets, CRA suppressed not only mitogen-triggered T-cell activation, but also lipopolysaccharide-induced DC maturation, indicating dual functionality. Treatment with CRA at nontoxic doses induced heme oxygenase-1 (HO-1) mRNA/protein expression and HO-1 enzymatic activity in DCs, suggesting a unique mechanism of action. In fact, lipopolysaccharide-induced DC maturation was also inhibited by structurally unrelated compounds known to induce HO-1 expression or carbon monoxide (CO) release. Allergic contact hypersensitivity response to oxazolone and oxazolone-induced Langerhans cell migration from epidermis were both prevented almost completely by systemic administration of CRA. Not only do our results support the recent concept that HO-1/CO system negatively regulates immune responses, they also form both conceptual and technical frameworks for a more systematic, large-scale drug discovery effort to identify HO-1/CO-targeted immunosuppressants with dual target specificity.

PMCID: PMC2710955
PMID: 19401559 [PubMed - indexed for MEDLINE]

564. Clin Exp Allergy. 2009 Aug;39(8):1152-9. doi: 10.1111/j.1365-2222.2009.03249.x. Epub 2009 Apr 23.

Osteopontin is involved in migration of eosinophils in asthma.

Takahashi A, Kurokawa M, Konno S, Ito K, Kon S, Ashino S, Nishimura T, Uede T, Hizawa N, Huang SK, Nishimura M.

First Department of Medicine, Hokkaido University School of Medicine, Sapporo, Hokkaido, Japan.

BACKGROUND: Osteopontin (OPN) is an extracellular matrix protein with a wide range of functions, and is involved in various inflammatory diseases. However, the role of OPN in eosinophilic airway inflammation is unclear.
OBJECTIVE: To elucidate the role of OPN in eosinophilic airway inflammation.
METHODS: OPN protein levels in induced sputum from asthmatic patients and healthy controls were measured. Eosinophil migration assays were performed in the presence or absence of OPN, a blocking antibody (Ab) recognizing its integrin-binding domain (2K1) and an anti-integrin alpha 4 Ab (P1H4). In the mouse asthma model, the levels of eosinophilia were examined in bronchoalveolar lavage fluids (BALFs) from ovalbumin (OVA)-sensitized and -challenged mice with or without administration of an Ab (M5) corresponding to human 2K1.
RESULTS: Levels of OPN in induced sputum were significantly higher in asthmatic patients when compared with healthy controls. In addition, levels of OPN were correlated with the percentage of sputum eosinophils. OPN induced significant

migration of human eosinophils and this effect was inhibited by 2K1 and P1H4. M5 significantly attenuated OVA-induced eosinophilia in BALFs.
CONCLUSION: These results indicate that OPN plays a role in the migration of eosinophils into the airways and may be involved in the pathogenesis of asthma.

PMID: 19400906 [PubMed - indexed for MEDLINE]

565. Clin Exp Allergy. 2009 Jul;39(7):1060-8. doi: 10.1111/j.1365-2222.2009.03187.x. Epub 2009 Apr 20.

Safety of hookworm infection in individuals with measurable airway responsiveness: a randomized placebo-controlled feasibility study.

Feary J, Venn A, Brown A, Hooi D, Falcone FH, Mortimer K, Pritchard DI, Britton J.

Division of Epidemiology and Public Health, University of Nottingham, UK. johanna.feary@nottingham.ac.uk

BACKGROUND: Epidemiological evidence suggests that hookworm infection protects against asthma. However, for ethical and safety reasons, before testing this hypothesis in a clinical trial in asthma it is necessary to establish whether experimental hookworm infection might exacerbate airway responsiveness during larval lung migration.
OBJECTIVE: To determine whether hookworm larval migration through the lungs increases airway responsiveness in allergic individuals with measurable airway responsiveness but not clinical asthma, and investigate the general tolerability of infection and effect on allergic symptoms.
METHODS: Thirty individuals with allergic rhinoconjunctivitis and measurable airway responsiveness to adenosine monophosphate (AMP) but not clinically diagnosed asthma were randomized, double-blind to cutaneous administration of either 10 hookworm larvae or histamine placebo, and followed for 12 weeks. The primary outcome was the maximum fall from baseline in provocative dose of inhaled AMP required to reduce 1-s forced expiratory volume by 10% (PD(10)AMP) measured at any time over the 4 weeks after active or placebo infection. Secondary outcomes included peak flow variability in the 4 weeks after infection, rhinoconjunctivitis symptom severity and adverse effect diary scores over the 12-week study period, and change in allergen skin test responses between baseline and 12 weeks.
RESULTS: Mean maximum change in PD(10)AMP from baseline was slightly but not significantly greater in the hookworm than the placebo group (-1.67 and -1.16 doubling doses; mean difference -0.51, 95% confidence interval -1.80 to 0.78, P=0.42). Symptom scores of potential adverse effects were more commonly reported in the hookworm group, but infection was generally well tolerated. There were no significant differences in peak-flow variability, rhinoconjunctivitis symptoms or skin test responses between groups.
CONCLUSION: Hookworm infection did not cause clinically significant exacerbation of airway responsiveness and was well tolerated. Suitably powered trials are now indicated to determine the clinical effectiveness of hookworm infection in allergic rhinoconjunctivitis and asthma.

PMCID: PMC2728895
PMID: 19400893 [PubMed - indexed for MEDLINE]

566. J Biol Chem. 2009 Jun 19;284(25):16922-30. doi: 10.1074/jbc.M808560200. Epub 2009 Apr 24.

Inter-alpha-trypsin inhibitor promotes bronchial epithelial repair after injury

through vitronectin binding.

Adair JE, Stober V, Sobhany M, Zhuo L, Roberts JD, Negishi M, Kimata K, Garantziotis S.

National Institute of Environmental Health Sciences, Research Triangle Park, North Carolina 27709, USA.

Pulmonary epithelial injury is central to the pathogenesis of many lung diseases, such as asthma, pulmonary fibrosis, and the acute respiratory distress syndrome. Regulated epithelial repair is crucial for lung homeostasis and prevents scar formation and inflammation that accompany dysregulated healing. The extracellular matrix (ECM) plays an important role in epithelial repair after injury. Vitronectin is a major ECM component that promotes epithelial repair. However, the factors that modify cell-vitronectin interactions after injury and help promote epithelial repair are not well studied. Inter-alpha-trypsin inhibitor (IaI) is an abundant serum protein. IaI heavy chains contain von Willebrand A domains that can bind the arginine-glycine-aspartate domain of vitronectin. We therefore hypothesized that IaI can bind vitronectin and promote vitronectin-induced epithelial repair after injury. We show that IaI binds vitronectin at the arginine-glycine-aspartate site, thereby promoting epithelial adhesion and migration in vitro. Furthermore, we show that IaI-deficient mice have a dysregulated response to epithelial injury in vivo, consisting of decreased proliferation and epithelial metaplasia. We conclude that IaI interacts not only with hyaluronan, as previously reported, but also other ECM components like vitronectin and is an important regulator of cellular repair after injury.

PMCID: PMC2719329
PMID: 19395377 [PubMed - indexed for MEDLINE]

567. J AAPOS. 2009 Apr;13(2):161-5. doi: 10.1016/j.jaapos.2008.10.017.

Actinic conjunctivitis in children: Clinical features, relation to sun exposure, and proposed staging and treatment.

Engel JM, Molinari A, Ostfeld B, Deen M, Croxatto O.

Department of Ophthalmology, Robert Wood Johnson Medical School, New Brunswick, New Jersey, USA. engel@uceyecenter.com

BACKGROUND: Actinic conjunctivitis is an ocular photosensitivity reaction found mainly in children in certain populations in the Andean regions of South America, Mexico, and in the southwestern United States. Its clinical features, treatment, and possible relation to duration of sun exposure have not been fully described in the ophthalmologic literature.
METHODS: A 20-member ophthalmic team traveled to an Andean region of Ecuador to provide ophthalmic care to children. All children with conjunctivitis were examined. A novel 3-stage classification of actinic conjunctivitis, devised by one of the authors, was used to stage the disease. The parents of each child with actinic conjunctivitis were asked how much time the child spent outside. Histopathological evaluations were performed on children who underwent surgery.
RESULTS: A total of 206 children were examined, of whom 36 had changes consistent with actinic conjunctivitis. Stage 1 disease was diagnosed in 17 children, stage 2 in 9, and stage 3 in 10 in the most severely affected eye. The amount of time the child spent outside correlated with disease severity ($r = 0.77$, $p < 0.001$). Histopathologic samples showed an intense inflammatory response with hyperplasia of the vascular endothelium, pigmentary migration, and occasional eosinophilia.
CONCLUSIONS: Actinic conjunctivitis is prevalent among children of the highlands of Ecuador. Although it has an allergic component, our data suggest that the

severity of the disease is significantly associated with sun exposure. The finding that the lesions are found only in the exposed conjunctiva supports the hypothesis that UV exposure is the main cause of the disease.

PMID: 19393514 [PubMed - indexed for MEDLINE]

568. Pol Merkur Lekarski. 2009 Mar;26(153):184-7.

[Analysis of L, E, and P selectins concentration in infants and young children with spastic bronchitis].

[Article in Polish]

Gasiorowska J, Czerwionka-Szaflarska M, Swincow G, Stefańska A, Odrowaz-Sypniewska G.

Department of Pediatrics, Allergology and Gastroenterology, Nicolaus Copernicus University of Torun, Collegium Medicum in Bydgoszcz, Poland. klped@cm.umk.pl

Spastic bronchitis in infants and young children is one of the most frequent causes of hospitalization in this age group. Both infectious and allergic inflammations lead to inflammation cells activation and their flow to the place of inflammation by the activation of adhesive molecules. One of groups of adhesive molecules are L, E and P selectins responsible for leucocytes migration through the vessel wall. The aim of the study was to assess the concentration of solved fractions of L, P and E selectins in infants and young children in the course of spastic bronchitis.MATERIAL AND METHODS: Fifty four patients with spastic bronchitis (group I) including 32 with the first bronchitis (group IA) and 22 patients with recurrent bronchitis (at last the third one--group IB) were included into the study. Comparative group (group II) was consisted of 26 patients hospitalized due to other causes and with no bronchitis in the past. Patients were from 1 to 36 months of age. Among all patients solved fractions of selectins L, E and P were analyzed by ELISA tests.
RESULTS: Mean sL-selectin concentration in group I was 4126.3 ng/ml and in group II 4222.31 ng/ml and was not statistically significant. Concentrations of sL-selectin in the group of patients with the first episode of spastic bronchitis was 4099.37 ng/ml and in the group of patients with recurrent bronchitis was 4166 ng/ml and had no statistical difference. Mean sE-selectin concentration in group I was 205.49 ng/ml and in group II 214.50 ng/ml and was not statistically significant. Concentrations of sE-selectin in the group of patients with the first episode of spastic bronchitis was 195.22 ng/ml and in the group of patients with recurrent bronchitis was 220.43 ng/ml and had no statistical difference. Concentration of sP-selectin was assessed among 51 patients with bronchitis and among 26 patients from comparative group. Because of the lack of normal distribution values of sP-selectin concentrations were changed by decimal logarithm. Mean sP-selectin concentration in group I was 235.95 ng/ml and in group II 164.70 ng/ml. After logarithm change values of concentrations were: 2.249 and 2.005 and had statistical difference ($p = 0.0221$). Concentrations of sP-selectin in the group of patients with the first episode of spastic bronchitis was 234.0 ng/ml and in the group of patients with recurrent bronchitis was 238.20 ng/ml and after logarithm change concentrations were 2.26 in the group of patients with the first episode of spastic bronchitis and 2.24 in the group of patients with recurrent bronchitis and had no statistical difference.
CONCLUSION: On the ground of our study in infants and young children with spastic bronchitis increase of sP-selectin concentration was observed and sE-selectin and sL-selectin concentrations were the same.

PMID: 19388529 [PubMed - indexed for MEDLINE]

569. Med J Malaysia. 2008 Oct;63(4):331-2.

A case of cutaneous larva migrans acquired from soiled toilet floors in urban Kuala Lumpur.

Robson NZ, Othman S.

Department of Primary Care Medicine, Faculty of Medicine, University of Malaya, 50603 Kuala Lumpur, Malaysia. noorzurani@um.edu.my

Despite being a common skin dermatosis in the tropics, physicians in the tropics may miss the diagnosis of cutaneous larva migrans for other pruritic skin manifestation. This is especially in those who live in urban housing with no history of travel. Cutaneous larva migrans, an intensely pruritic skin pathology is mainly contracted by people with history of beach holiday or contact with moist soft sand which had been contaminated with dog or cat faeces. This article reports a patient who presented with intensely itchy papular spots over the dorsum of his foot after walking barefooted in an urban toilet soiled with cat faeces. The patient had initially seen an urban general practitioner who diagnosed the papular skin lesion as an allergic reaction, and prescribed antihistamines. The patient subsequently developed creeping skin lesions and was seen by the author who prescribed albendazole 400 mg twice daily for three days. The patient reported reduction in itching after two days of albendazole treatment and a follow up at ten days revealed a healed infection.

PMID: 19385496 [PubMed - indexed for MEDLINE]

570. J Exp Med. 2009 May 11;206(5):1037-46. doi: 10.1084/jem.20082179. Epub 2009 Apr 13.

Mast cells mediate neutrophil recruitment and vascular leakage through the NLRP3 inflammasome in histamine-independent urticaria.

Nakamura Y, Kambe N, Saito M, Nishikomori R, Kim YG, Murakami M, Núñez G, Matsue H.

Department of Dermatology, Chiba University Graduate School of Medicine, Chuo-ku, Chiba 260-8670, Japan.

Erratum in
    J Exp Med. 2009 May 11;206(5):1205. Nishikomiri, Ryuta [corrected to Nishikomori, Ryuta].

Urticarial rash observed in cryopyrin-associated periodic syndrome (CAPS) caused by nucleotide-binding oligomerization domain-leucine-rich repeats containing pyrin domain 3 (NLRP3) mutations is effectively suppressed by anti-interleukin (IL)-1 treatment, suggesting a pathophysiological role of IL-1beta in the skin. However, the cellular mechanisms regulating IL-1beta production in the skin of CAPS patients remain unclear. We identified mast cells (MCs) as the main cell population responsible for IL-1beta production in the skin of CAPS patients. Unlike normal MCs that required stimulation with proinflammatory stimuli for IL-1beta production, resident MCs from CAPS patients constitutively produced IL-1beta. Primary MCs expressed inflammasome components and secreted IL-1beta via NLRP3 and apoptosis-associated speck-like protein containing a caspase recruitment domain when stimulated with microbial stimuli known to activate caspase-1. Furthermore, MCs expressing disease-associated but not wild-type NLRP3 secreted IL-1beta and induced neutrophil migration and vascular leakage, the histological hallmarks of urticarial rash, when transplanted into mouse skin. Our

findings implicate MCs as IL-1beta producers in the skin and mediators of histamine-independent urticaria through the NLRP3 inflammasome.

PMCID: PMC2715029
PMID: 19364881 [PubMed - indexed for MEDLINE]

571. Clin Exp Allergy. 2009 Jul;39(7):1018-26. doi: 10.1111/j.1365-2222.2009.03238.x. Epub 2009 Apr 2.

Epithelium-derived chemokines induce airway smooth muscle cell migration.

Takeda N, Sumi Y, Préfontaine D, Al Abri J, Al Heialy N, Al-Ramli W, Michoud MC, Martin JG, Hamid Q.

Meakins-Christie Laboratories, McGill University, Montreal, Quebec, Canada.

BACKGROUND: The remodelling of airway smooth muscle (ASM) associated with asthma severity may involve the migration of ASM cells towards the epithelium. However, little is known about the mechanisms of cell migration and the effect of epithelial-derived mediators on this process.
OBJECTIVE: The main objective of the current study is to assess the effects of epithelial-derived chemokines on ASM cell migration.
METHODS: Normal human ASM cells were incubated with supernatants from cells of the bronchial epithelial cell line BEAS-2B and normal human bronchial epithelial (NHBE) cells. To induce chemokine production, epithelial cells were treated with TNF-alpha. Chemokine expression by epithelial cells was evaluated by quantitative real-time PCR, ELISA and membrane antibody array. To identify the role of individual chemokines in ASM cell migration, we performed migration assays with a modified Boyden chamber using specific neutralizing antibodies to block chemokine effects.
RESULTS: Supernatants from BEAS-2B cells treated with TNF-alpha increased ASM cell migration; migration was increased 1.6 and 2.5-fold by supernatant from BEAS-2B cells treated with 10 and 100 ng/mL TNF-alpha, respectively. Protein levels in supernatants and mRNA expression by BEAS-2B cells of regulated on activation, normal T cell expressed and secreted (RANTES) and IL-8 were significantly increased by 100 ng/mL TNF-alpha treatment. The incubation of supernatant with antibodies to RANTES or IL-8 significantly reduced ASM cell migration, and the combined antibodies further inhibited the cell migration. The migratory effects of supernatants and inhibiting effects of RANTES and/or IL-8 were confirmed also using NHBE cells.
CONCLUSION: The results show that chemokines from airway epithelial cells cause ASM cell migration and might potentially play a role in the process of airway remodelling in asthma.

PMID: 19364333 [PubMed - indexed for MEDLINE]

572. Blood. 2009 Jun 25;113(26):6638-47. doi: 10.1182/blood-2009-02-204321. Epub 2009 Apr 10.

Lymphoid-tissue-specific homing of bone-marrow-derived dendritic cells.

Creusot RJ, Yaghoubi SS, Chang P, Chia J, Contag CH, Gambhir SS, Fathman CG.

Department of Medicine, Division of Immunology and Rheumatology, Stanford University School of Medicine, 300 Pasteur Drive, Stanford, CA 94305, USA.

Because of their potent immunoregulatory capacity, dendritic cells (DCs) have been exploited as therapeutic tools to boost immune responses against tumors or

pathogens, or dampen autoimmune or allergic responses. Murine bone marrow-derived DCs (BM-DCs) are the closest known equivalent of the blood monocyte-derived DCs that have been used for human therapy. Current imaging methods have proven unable to properly address the migration of injected DCs to small and deep tissues in mice and humans. This study presents the first extensive analysis of BM-DC homing to lymph nodes (and other selected tissues) after intravenous and intraperitoneal inoculation. After intravenous delivery, DCs accumulated in the spleen, and preferentially in the pancreatic and lung-draining lymph nodes. In contrast, DCs injected intraperitoneally were found predominantly in peritoneal lymph nodes (pancreatic in particular), and in omentum-associated lymphoid tissue. This uneven distribution of BM-DCs, independent of the mouse strain and also observed within pancreatic lymph nodes, resulted in the uneven induction of immune response in different lymphoid tissues. These data have important implications for the design of systemic cellular therapy with DCs, and in particular underlie a previously unsuspected potential for specific treatment of diseases such as autoimmune diabetes and pancreatic cancer.

PMCID: PMC2710920
PMID: 19363220 [PubMed - indexed for MEDLINE]

573. Z Orthop Unfall. 2009 Mar-Apr;147(2):175-82. doi: 10.1055/s-0029-1185456. Epub 2009 Apr 8.

[Silicate coating of cemented titanium-based shafts in hip prosthetics reduces high aseptic loosening].

[Article in German]

Marx R, Faramarzi R, Jungwirth F, Kleffner BV, Mumme T, Weber M, Wirtz DC.

Klinik für Zahnärztliche Prothetik, Lehr- und Forschungsgebiet Werkstoffkunde, Universitätsklinikum Aachen. marx@rwth-aachen.de

AIM: For cemented hip prostheses, all requirements can be fulfilled by using forged Co/Cr/Mo stems. Co/Cr/Mo alloys, however, are contraindicated for allergy sufferers. For these patients, a cemented prosthesis made of titanium (alloy) would be indicated. Cemented stems from titanium (alloy), depending on the geometry of the prosthesis and its specific surface texture, however, may have loosening rates which are clinically not tolerable. In comparison to Co/Cr/Mo alloys, the greater roughness in conjunction with lesser abrasion resistance of titanium-based alloys leads to high loosening rates caused by abrasion. On the other hand, the greater surface roughness permits good mechanical retention of bone cement to the surface. Good mechanical retention enhances migration behaviour and reduces micromotions. However, there is no stable hydrolytic bond between bone cement and metallic surface; intermediate-term debonding between metal and bone cement is predictable. This debonding results in relative movements, consequently in wear particles which have their origin both from the rough metallic surface and from the PMMA cement. The roughness of the metallic surface operates as emery and with that, a rubbing wear from the PMMA.
METHOD: For the above reasons, a low or moderate roughness is essential for easily abradable implants such as shafts made of titanium (alloy) because low roughness provides a fail-safe running function in case of debonding. Thus, one must allow for inappropriate migration behaviour accompanied by greater micromotions due to insufficient mechanical retention in the case of low roughness. This can be accomplished by a silicate layer coating applied to the metal shaft surface via electrochemical "ECD" or physical vapour deposition "PVD". For analysis, specimens (screws for pull-out, cones for push-out tests) were sand-blasted, so that roughnesses between Ra = 0.8 microm (Rz = 4 microm) and Ra = 2.0 microm (Rz = 9 microm) were generated.

RESULTS: The bond strengths observed in tensile tests for roughnesses of Ra = 1.7 mm were always well above 25 MPa for all periods of hydrolytic load. Therefore, the investigation shows that surfaces of moderate roughness (Ra = 1.7 microm), however coated, provide a steady retention. Cave-in and micromotions should widely be prevented.
CONCLUSION: The abrasion, which is a consequence of and reason for debonding and loosening at the same time, should be avoidable if the bonding of cement on the metallic shaft is stabilised with the help of a suitable chemical bond system.

PMID: 19358071 [PubMed - indexed for MEDLINE]

574. G Ital Dermatol Venereol. 2009 Apr;144(2):173-85.

Innate and adaptive immune responses in contact dermatitis: analogy with infections.

Freudenberg MA, Esser PR, Jakob T, Galanos C, Martin SF.

Max-Planck-Institute for Immunobiology, Freiburg, Germany.
freudenberg@immunbio.mpg.de

Allergic contact dermatitis (ACD) is an inflammatory skin disease of great and steadily increasing importance as an occupational health problem. The disease is induced by chemicals and metal ions which penetrate the skin and form complexes with host proteins. This process is accompanied by a strong, allergen-induced inflammatory reaction and leads to the migration of allergen-carrying dendritic cells (DC) from the skin to regional lymph nodes, where they promote generation of allergen-specific T cells. The latter are the ultimate effector cells of the disease. Re-exposure to the causative agent leads to the recruitment of the T effector cells, which then elicit the typical skin inflammatory reaction at the site of contact. Although DC and effector T cells play a protagonistic role in the sensitization and elicitation phase of ACD, respectively, other cell types including keratinocytes, NK cells, mast cells and B cells contribute to the pathogenesis of the disease. In this review the authors summarize recent findings that identify stress responses and innate immune pathways triggered by contact allergens and review recent data regarding the adaptive T cell response. The new data were collected mainly from studies on contact hypersensitivity (CHS), the corresponding experimental mouse model of human ACD. The elucidation of the molecular events involved in contact allergen-induced innate responses will help to design new treatment strategies and may allow to develop predictive in vitro assays for the identification of contact allergens.

PMID: 19357624 [PubMed - indexed for MEDLINE]

575. Eur Respir J. 2009 Oct;34(4):958-66. doi: 10.1183/09031936.00165308. Epub 2009 Apr 8.

Anti-angiogenic activity of carebastine: a plausible mechanism affecting airway remodelling.

De Luisi A, Mangialardi G, Ria R, Acuto G, Ribatti D, Vacca A.

Dept of Internal Medicine and Clinical Oncology, Unit of Allergology and Clinical Immunology, Bari, Italy.

Ebastine is a well-known selective second-generation histamine H(1) receptor antagonist, which is used for seasonal and perennial allergic rhinitis and chronic urticaria. Angiogenesis plays a crucial role in the development of airway

inflammation and remodelling in allergic rhinitis and asthmatic patients, in whom, indeed, the mucosa displays increased vascularity and overexpression of vascular endothelial growth factor (VEGF). The aim of the present study was to evaluate the anti-angiogenic properties of carebastine, the active metabolite of ebastine. The effects of carebastine on human umbilical vein endothelial cell (EC) (HUVEC) and human pulmonary artery EC (HPAEC) proliferation, migration and capillary-like tube formation were investigated in vitro, and in the chick embryo chorioallantoic membrane (CAM) assay in vivo. Moreover, the effect of carebastine on phosphorylation of the cell VEGF receptor fetal liver kinase-1, or VEGF receptor 2 (VEGFR-2), and Akt kinase (Akt) was evaluated by Western blotting. Carebastine inhibited VEGF-induced HUVEC and HPAEC proliferation, migration and angiogenesis in a dose-dependent manner in vitro. Cell proliferation was inhibited by 42 and 64% in HUVECs and 62 and 75% in HPAECs upon exposure for 48 and 72 h, respectively, to 20 microM carebastine (p < or = 0.03), and even more with 30 microM carebastine. Cell migration was inhibited by 37 and 70% in HUVECs (p < or = 0.03) and 60 and 78% in HPAECs (p < or = 0.01) in the presence of 10 and 30 microM carebastine, respectively. Carebastine (20 microM) caused a significant reduction (70-86%; p<0.01) in topological parameters of the capillary network produced in vitro by both EC lines on a basement membrane extract. Carebastine (30 and 50 microM) inhibited the VEGF-induced angiogenesis in the CAM assay in vivo two- and three-fold, respectively (p<0.001). Finally, both EC lines, on exposure to 10 and 20 microM carebastine, showed a four- to six-fold reduction (p < or = 0.01) in both VEGF- and H1 receptor-induced VEGFR-2 and Akt phosphorylation. Overall, these data provide the first evidence regarding the anti-angiogenic activity of ebastine, and suggest its potential use as an anti-angiogenic molecule, besides its antihistaminic activity for the treatment of allergic diseases in which angiogenesis takes place.

PMID: 19357149 [PubMed - indexed for MEDLINE]

576. J Allergy Clin Immunol. 2009 Apr;123(4):735-46; quiz 747-8. doi: 10.1016/j.jaci.2009.02.030.

Mechanisms and treatment of allergic disease in the big picture of regulatory T cells.

Akdis CA, Akdis M.

Swiss Institute of Allergy and Asthma Research, University of Zurich, Davos, Switzerland. akdisac@siaf.unizh.ch

Various populations of regulatory T (Treg) cells have been shown to play a central role in the maintenance of peripheral homeostasis and the establishment of controlled immune responses. Their identification as key regulators of immunologic processes in peripheral tolerance to allergens has opened an important era in the prevention and treatment of allergic diseases. Both naturally occurring CD4+CD25+ Treg cells and inducible populations of allergen-specific, IL-10-secreting Treg type 1 (T(R)1) cells inhibit allergen-specific effector cells in experimental models. Skewing of allergen-specific effector T cells to a regulatory phenotype appears to be a key event in the development of healthy immune response to allergens and successful outcome in allergen-specific immunotherapy. Forkhead box protein 3-positive CD4+CD25+ Treg cells and T(R)1 cells contribute to the control of allergen-specific immune responses in several major ways, which can be summarized as suppression of dendritic cells that support the generation of effector T cells; suppression of effector T(H)1, T(H)2, and T(H)17 cells; suppression of allergen-specific IgE and induction of IgG4; suppression of mast cells, basophils, and eosinophils; interaction with resident tissue cells and remodeling; and suppression of effector T-cell migration to tissues. Current

strategies for drug development and allergen-specific immunotherapy exploit these observations, with the potential for preventive therapies and cure for allergic diseases.

PMID: 19348912 [PubMed - indexed for MEDLINE]

577. J Proteome Res. 2009 Jun;8(6):2720-32. doi: 10.1021/pr800984e.

The human eosinophil proteome. Changes induced by birch pollen allergy.

Woschnagg C, Forsberg J, Engström A, Odreman F, Venge P, Garcia RC.

Department of Medical Sciences, Clinical Chemistry, Uppsala University, 75185 Uppsala, Sweden.

Proteins from human eosinophils were separated bidimensionally and identified by mass spectrometry (336 spots/bands, 98 different proteins). Of these, 24.7% belonged to the cytoskeleton/migration group. Highly basic proteins (11.3%) were concentrated in the granule-containing cell fraction. We detected novel hyperacidic forms of cofilin-1, profilin-1 and adenylyl cyclase-associated protein, and hyperbasic forms of eosinophil-derived neurotoxin/eosinophil protein X and major basic protein homologue. We also found evidence of the triglycosylation of the heavy chain of eosinophil peroxidase. In addition, through comparative 2D image analysis, spot quantification and MS, it was found that hsc70, actin-capping protein and hyperacidic forms of eosinophil peroxidase heavy chain are overexpressed in cells from birch pollen allergic subjects, at the peak of a season. The link between these findings and an increased cellular antigen-presenting capacity and motility are discussed.

PMID: 19348509 [PubMed - indexed for MEDLINE]

578. FASEB J. 2009 Aug;23(8):2412-24. doi: 10.1096/fj.09-130542. Epub 2009 Apr 3.

Resveratrol attenuates C5a-induced inflammatory responses in vitro and in vivo by inhibiting phospholipase D and sphingosine kinase activities.

Issuree PD, Pushparaj PN, Pervaiz S, Melendez AJ.

S.P., Department of Physiology, Yong Loo Lin School of Medicine, National University of Singapore, Singapore 117597.

The anti-inflammatory activity of the phytoalexin resveratrol (RSV) was evaluated in C5 anaphylatoxin (C5a)-stimulated primary neutrophils and in a mouse model of acute peritonitis. Pretreatment of human and mouse neutrophils with RSV significantly blocked oxidative burst, leukocyte migration, degranulation, and inflammatory cytokine production. The anti-inflammatory activity of RSV was a function of inhibition of sphingosine kinase (SphK) activity (IC(50) approximately 20 microM) within 5 min of exposure, its membrane localization, and SphK1-mediated Ca(2+) release. As an experimental control, the SphK1 pharmacological inhibitor N,N-dimethyl sphingosine (DMS) was used to compare the inhibitory effect of RSV. We also provide evidence that the SphK inhibitory effect of RSV was mediated via its ability to block phospholipase D (PLD) activity and membrane recruitment. Furthermore, RSV blocked ERK1/2 phosphorylation, which functioned independently of SphK1 in this study. To provide in vivo relevance to these data, C5a-induced model of acute peritonitis was established, and the effects of prior injection of RSV were investigated. Indeed, prior injection of RSV virtually completely attenuated the effects of C5a on vascular permeability, neutrophil migration, release of interleukin 1beta,

tumor necrosis factor alpha, interleukin 6, and the chemokine MIP-1alpha. Taken together, these data demonstrate strong anti-inflammatory activity of RSV in vitro and in vivo and highlight SphK1 as a potential target of this remarkable phytoalexin. These data could have tremendous implications for the clinical use of RSV in inflammatory pathologies.

PMID: 19346296 [PubMed - indexed for MEDLINE]

579. Int Immunol. 2009 Jun;21(6):621-32. doi: 10.1093/intimm/dxp031. Epub 2009 Apr 3.

Murine bone marrow-derived mast cells express chemoattractant receptor-homologous molecule expressed on T-helper class 2 cells (CRTh2).

Boehme SA, Franz-Bacon K, Chen EP, Ly TW, Kawakami Y, Bacon KB.

Actimis Pharmaceuticals, Inc., 10835 Road to the Cure, San Diego, CA 92121, USA. sboehme@actimis.com

Mast cells are bone marrow-derived effector cells that can initiate inflammatory responses to infectious organisms or allergens by releasing a multitude of pro-inflammatory factors including prostaglandin (PG) D(2). We demonstrate that primary murine bone marrow-derived mast cells (BMMCs) express the PGD(2) receptor; chemoattractant receptor-homologous molecule expressed on T(h) class 2 cells (CRT(h)2). Activation of CRT(h)2 on BMMC by PGD(2) or the CRT(h)2-specific agonist, 13,14-dihydro-15-keto-prostaglandin D(2) (DK-PGD(2)), resulted in signaling response including Ca(2+) mobilization and phosphorylation of the p42/p44 extracellular signal-regulated kinases (ERKs) kinases. Phosphorylation of the ERKs could be blocked by pertussis toxin, as well as a small molecule antagonist of CRT(h)2, Compound A. Activation of CRT(h)2 on BMMC also resulted in the up-regulation of CD23 and CD30 on the cell surface, as well as CD62L shedding. Finally, PGD(2) and DK-PGD(2) induced the migration of BMMC in vitro and in vivo in response to an intra-dermal DK-PGD(2) injection. Both these processes were inhibited by the CRT(h)2 antagonist. These results raise the possibility that the functional consequences of the PGD(2)-CRT(h)2 interaction on mast cells may be relevant in allergic inflammation.

PMID: 19346259 [PubMed - indexed for MEDLINE]

580. Phytomedicine. 2009 Sep;16(9):814-22. doi: 10.1016/j.phymed.2009.01.009. Epub 2009 Mar 25.

Inhibitory effects of Schizandrae Fructus on eotaxin secretion in A549 human epithelial cells and eosinophil migration.

Oh BG, Lee H, Kim Y, Shin M, Hong M, Jung SK, Kim J, Bae H.

Department of Physiology, College of Oriental Medicine, Kyung Hee University, Seoul, Republic of Korea.

Eosinophilia have been implicated in a broad range of diseases, most notably allergic conditions (e.g. asthma, rhinitis and atopic dermatitis) and inflammatory diseases. These diseases are characterized by an accumulation of eosinophils in the affected tissue. Defining the mechanisms that control the recruitment of eosinophil is fundamental to understanding how these diseases progress and identifying a novel target for drug therapy. Accordingly, this study was conducted to evaluate the regulatory effect of Schizandrae Fructus (SF) on the expression of eotaxin, an eosinophil-specific chemokine released in respiratory epithelium following allergic stimulation, as well as its effects on

eosinophil migration. To accomplish this, human epithelial lung cells (A549 cell) were stimulated with a combination of TNF-alpha (100ng/ml) and IL-4 (100ng/ml) for 24h. The cells were then restimulated with TNF-alpha (100ng/ml) and IL-1beta (10ng/ml) to induce the expression of chemokines and adhesion molecules involved in eosinophil chemotaxis for another 24h. Next, the samples were treated with various concentrations of Schizandrae Fructus (SF) (1, 10, 100, 1000microg/ml) or one of the major constituents of SF, schizandrin (0.1, 1, 10, 100microg/ml), after which following inhibition effect assay was performed triplicates in three independence. The levels of eotaxin in secreted proteins were suppressed significantly by SF (100 and 1000microg/ml, p<0.01) and schizandrin (10 and 100microg/ml, p<0.01). In addition, SF (1, 10, 100 and 1000microg/ml) decreased mRNA expression levels in A549 cells significantly (p<0.01). Eosinophil recruitment to lung epithelial cells was also reduced by SF, which indicates that eotaxin plays a role in eosinophil recruitment. Furthermore, treatment with SF suppressed the expression of another chemokine, IL-8 (0.1 and 1microg/ml SF, p<0.01), as well as intercellular adhesion molecule-1 (10 and 100microg/ml SF, p<0.01) and vascular cell adhesion molecule-1 (0.1 and 1microg/ml SF, p<0.05), which are all related to eosinophil migration. Taken together, these findings indicate that SF may be a desirable medicinal plant for the treatment of allergic diseases.

PMID: 19324539 [PubMed - indexed for MEDLINE]

581. Tissue Antigens. 2009 Apr;73(4):302-6. doi: 10.1111/j.1399-0039.2008.01206.x.

Association of MIF promoter polymorphisms with childhood asthma in a northeastern Chinese population.

Wu J, Fu S, Ren X, Jin Y, Huang X, Zhang X, Bai J, Fu S.

Laboratory of Medical Genetics, Harbin Medical University, Harbin, China.

Macrophage migration inhibitory factor (MIF) is a proinflammatory cytokine that plays an important role in pathogenesis of asthma. A high level of MIF has been detected in bronchoalveolar lavage fluid, serum and sputum in asthma. Polymorphisms associated with inflammatory diseases exist in the promoter region of MIF, which alter its expression. The aim of this study was to evaluate the potential relationship between functional polymorphisms of MIF and childhood asthma in a northeastern Chinese population. The study consisted of a set of 41 trios and an independent sample set of 189 childhood asthma patients and 261 healthy controls. We genotyped MIF-173G/C using polymerase chain reaction-restriction fragment length polymorphism (PCR-RFLP). Additionally, MIF-794CATT(5-8) microsatellite polymorphism was genotyped by polyacrylamide gel electrophoresis (PAGE). A statistically significant difference in the distribution of the MIF-173C allele between patients and controls [P = 0.01, odds ratio (OR) = 1.55, 95% confidence interval (CI) = 1.13-2.18] was observed. In addition, the frequency of the MIF-173CC genotype was higher in asthmatic children (P < 0.01, OR = 3.37, 95% CI = 1.27-8.93). No difference in the distribution of CATT(5-8) was found between patients and healthy controls. Haplotype analysis showed that only the MIFCATT(7)-173C haplotype was associated with greater susceptibility to childhood asthma (P = 0.03, OR = 1.54, 95% CI 1.03-2.28). However, the transmission disequilibrium test confirmed a positive association between MIF-173G/C and childhood asthma (P = 0.005), and the absence of an association between the MIF-794CATT(5-8) and the disease. These preliminary results suggest an association between the MIF-173C allele and childhood asthma.

PMID: 19317738 [PubMed - indexed for MEDLINE]

582. Br J Pharmacol. 2009 May;157(1):24-33. doi: 10.1111/j.1476-5381.2009.00151.x. Epub 2009 Mar 20.

The role of histamine H4 receptor in immune and inflammatory disorders.

Zampeli E, Tiligada E.

Department of Pharmacology, Medical School, National and Kapodistrian University of Athens, Athens, Greece.

Since its discovery at the beginning of the 20th century, histamine has been established to play a pathophysiological regulatory role in cellular events through binding to four types of G-protein-coupled histamine receptors that are differentially expressed in various cell types. The discovery, at the turn of the millennium, that the histamine H4 receptor is largely expressed in haemopoietic cells as well as its chemotactic properties designate its regulatory role in the immune system. H4 receptors modulate eosinophil migration and selective recruitment of mast cells leading to amplification of histamine-mediated immune responses and eventually to chronic inflammation. H4 receptor involvement in dendritic cell activation and T cell differentiation documents its immunomodulatory function. The characterization of the H4 as the immune system histamine receptor directed growing attention towards its therapeutic exploitation in inflammatory disorders, such as allergy, asthma, chronic pruritus and autoimmune diseases. The efficacy of a number of H4 receptor ligands has been evaluated in in vivo and in vitro animal models of disease and in human biological samples. However, before reaching decisive conclusions on H4 receptor pathophysiological functions and therapeutic exploitation, identification of genetic polymorphisms and interspecies differences in its relative actions and pharmacological profile need to be addressed and taken into consideration. Despite certain variations in the reported findings, the available data strongly point to the H4 receptor as a novel target for the pharmacological modulation of histamine-transferred immune signals and offer an optimistic perspective for the therapeutic exploitation of this promising new drug target in inflammatory disorders.

PMCID: PMC2697784
PMID: 19309354 [PubMed - indexed for MEDLINE]

583. Int Arch Allergy Immunol. 2009;149(4):315-22. doi: 10.1159/000205577. Epub 2009 Mar 17.

IL-16 variability and modulation by antiallergic drugs in a murine experimental allergic rhinitis model.

Akiyama K, Karaki M, Kobayshi R, Dobashi H, Ishida T, Mori N.

Department of Otorhinolaryngology, Faculty of Medicine, Kagawa University, Kagawa 761-0793, Japan. kakaka@med.kagawa-u.ac.jp

BACKGROUND: Interleukin-16 (IL-16) is a cytokine that induces selective migration of CD4+ cells and participates in inflammatory diseases including allergic rhinitis. Histamine and prostaglandin D(2) are important chemical mediators of allergic inflammation, and antiallergic drugs are commonly used for the treatment of allergic rhinitis. It remains unknown whether treatment with the drugs affects IL-16.
OBJECTIVE: We evaluated the variability of IL-16 and the effects of the antiallergic drugs fexofenadine (40 mg/kg/day) and ramatroban (30 mg/kg/day) on IL-16 in an OVA-sensitized BALB/c murine experimental allergic rhinitis model.
METHODS: We measured the expression level of IL-16 protein in the mouse nasal

septal mucosa by immunohistochemistry, and the serum level of IL-16 by ELISA. Several other parameters associated with allergic rhinitis (nasal symptoms, OVA-specific IgE, eosinophil and T cell infiltration) were also measured.
RESULTS: Local and systemic expressions of IL-16 were significantly increased in OVA-sensitized mice when compared to the nonsensitized group. Fexofenadine and ramatroban significantly inhibited the following OVA-induced allergic features when compared to the nontreated sensitized group: sneezing, nasal rubbing, eosinophil infiltration, IL-16 expressions in nasal tissue, and serum IL-16 level. Serum OVA-specific IgE and local T cell infiltration were reduced, but they did not reach significant values.
CONCLUSIONS: These results suggest that IL-16 was both systemically and locally upregulated in the murine allergic rhinitis model and that IL-16 changed in parallel to allergic state by treatment with the drugs.

Copyright (C) 2009 S. Karger AG, Basel.

PMID: 19295235 [PubMed - indexed for MEDLINE]

584. Int J Oncol. 2009 Apr;34(4):995-1003.

Inhibition of calcineurin by cyclosporine A exerts multiple effects on human melanoma cell lines HT168 and WM35.

Juhász T, Matta C, Veress G, Nagy G, Szíjgyártó Z, Molnár Z, Fodor J, Zákány R, Gergely P.

Department of Anatomy, Histology and Embryology, Medical and Health Science Centre, University of Debrecen, Hungary.

The immunosuppressant cyclosporine A (CsA) is a specific pharmacological inhibitor of calcineurin, the Ca2+-calmodulin activated phospho-Ser/Thr-specific protein phosphatase. Although calcineurin-inhibiting compounds are applied for local treatment of psoriasis or atopic dermatitis in dermatological practice, little is known about the functions of calcineurin in epidermis-derived malignancies. We investigated the effects of CsA on two human melanoma cell lines, the metastasis forming HT168 and WM35 established from an RGP primary lesion. CsA of 2 microM lowered the enzyme activity by 50% and caused elevation in both mRNA and protein expression of calcineurin. Cell proliferation was diminished, as well as the cellular morphology and the actin organization were altered in both cell lines. CsA increased cell death moderately in both cell lines and reduced the metabolic activity of HT168 cells, but not that of WM35 cells. CsA also elevated the expressions of both Bcl-2 and ERK1/2. Fibronectin guided migration of HT168 cells was stimulated under the effect of CsA, while that of WM35 cells was reduced, moreover, HT168 cells switched from the expression of beta3 to beta1 integrin, but WM35 cells continued to express beta3. Based on our results we propose a multiple, partly malignancy-dependent role of calcineurin in these melanoma cell lines.

PMID: 19287956 [PubMed - indexed for MEDLINE]

585. Am J Physiol Lung Cell Mol Physiol. 2009 May;296(5):L763-70. doi: 10.1152/ajplung.90638.2008. Epub 2009 Mar 13.

A severe deficiency of coagulation factor VIIa results in attenuation of the asthmatic response in mice.

Shinagawa K, Ploplis VA, Castellino FJ.

University of Notre Dame, Notre Dame, IN 46556, USA.

Eosinophil counts in the bronchoalveolar lavage fluid of wild-type (WT) mice increased after ovalbumin (OVA) challenge, a response that was diminished in comparably challenged low-expressing coagulation factor VII (FVII(tTA/tTA)) mice. Levels of T helper type 2 (Th2) cytokines, IL-4, IL-5, and IL-13, and eosinophil-attracting chemokines, eotaxin and RANTES, were also lower in the OVA-challenged FVII(tTA/tTA) mice. Eosinophils purified from low-FVII mice underwent apoptosis at a faster rate compared with WT eosinophils, and eosinophil migration in response to eotaxin was reduced in eosinophils obtained from FVII(tTA/tTA) mice. Airway hyperresponsiveness and mucous layer thickness were reduced in OVA-treated FVII(tTA/tTA) mice, and addition of exogenous coagulation factor X (FX) enhanced mucin production in human epithelial NCI-H292 cells. Correspondingly, incubation of FX with NCI-H292 cells resulted in activated (a) FX production, suggesting that the components required for FX activation were present on NCI-H292 cells. These results demonstrate that FVIIa functions in the asthmatic response to an allergen by stimulating lung eosinophilia, airway hyperresponsiveness, and mucin production, this latter effect through its ability to activate FX in conjunction with tissue factor.

PMCID: PMC2681354
PMID: 19286924 [PubMed - indexed for MEDLINE]

586. Scand J Immunol. 2009 Apr;69(4):381-6. doi: 10.1111/j.1365-3083.2009.02233.x.

Eosinophil cationic protein stimulates migration of human lung fibroblasts in vitro.

Zagai U, Lundahl J, Klominek J, Venge P, Sköld CM.

Department of Medicine, Division of Respiratory Medicine, Karolinska Institutet, Stockholm, Sweden. ulrika.zagai@cardoz.se

Asthma is characterized by eosinophilic inflammation and remodelling of the airways. Eosinophil cationic protein (ECP) is a protein released by activated eosinophils and the hypothesis that ECP contributes to the development of structural changes in the airways of asthmatics has been posed. Fibroblast recruitment is an important step in the remodelling process, and we therefore put the question whether ECP stimulates migration of human lung fibroblasts. Human peripheral eosinophils isolated from buffycoats from healthy individuals were cultured and conditioned media (CM) were collected. Native ECP was extracted from human peripheral eosinophils by gel filtration, ion-exchange and chelating chromatography. The ability of eosinophil CM and ECP to stimulate fibroblast migration was determined using the 48-well Boyden chamber. ECP concentrations in CM were assayed by ECP-CAP-FEIA. Both CM and ECP significantly stimulated fibroblast migration (48.4+/-cells/field versus 33+/-2 and 36+/-6 versus 25+/-4; P<0.001 and 0.05 respectively) in a time- and concentration-dependent manner. Adding neutralizing ECP antibodies attenuated fibroblast migration induced by both ECP as well as CM. ECP stimulates migration of human lung fibroblasts, suggesting a potential mechanism for eosinophils in the fibrotic response. This may be an important mechanism by which ECP promotes remodelling of extracellular matrix leading to airway fibrosis in asthmatics.

PMID: 19284504 [PubMed - indexed for MEDLINE]

587. Am J Respir Crit Care Med. 2009 May 15;179(10):894-902. doi: 10.1164/rccm.200807-1081OC. Epub 2009 Feb 20.

Osteopontin deficiency protects against airway remodeling and hyperresponsiveness in chronic asthma.

Simoes DC, Xanthou G, Petrochilou K, Panoutsakopoulou V, Roussos C, Gratziou C.

G.P. Livanos and M. Simou Laboratories, Evangelismos Hospital, Department of Critical Care and Pulmonary Services, University of Athens, Medical School, Athens, Greece. davinasimoes@yahoo.co.uk

RATIONALE: Osteopontin (OPN) is a cytokine that is upregulated in epithelial cells and macrophages in the lungs of mice during chronic allergen challenge and airway remodeling and also in lungs of patients with asthma. However, it remains unclear whether OPN has an in vivo effect on lung remodeling in allergic asthma. Based on its ability to induce smooth muscle and fibroblast proliferation and migration we hypothesize that OPN regulates lung remodeling and also affects subsequent airway hyperresponsiveness (AHR).
OBJECTIVES: Study the role of OPN in airway remodeling using OPN-knockout (KO) mice and a reversal approach administering recombinant mouse OPN (rOPN) in KO mice before challenge.
METHODS: A chronic allergen-challenge model of airway remodeling with OPN KO mice, KO mice treated with rOPN, and human bronchial smooth muscle were used.
MEASUREMENTS AND MAIN RESULTS: OPN deficiency protected mice against ova-induced AHR, which was associated with lower collagen and mucus production, gob-5 mRNA expression, submucosal cell area infiltration, and proliferation. Administration of rOPN to KO mice, just at the final five allergen challenges, exacerbated AHR and all the remodeling characteristics measured. In addition, rOPN increased the expression of IL-13 and pro-matrix metalloproteinase-9 in the lungs. Moreover, we demonstrated that rOPN induces proliferation of human BSM through binding to its alpha(v)beta3 integrin receptor and activation of PI3K/Akt downstream signaling pathway.
CONCLUSIONS: We conclude that OPN deficiency protects against remodeling and AHR. Thus our data reveal OPN as a novel therapeutic target for airway remodeling and associated AHR in chronic asthma.

PMID: 19234104 [PubMed - indexed for MEDLINE]

588. Exp Cell Res. 2009 Jun 10;315(10):1745-58. doi: 10.1016/j.yexcr.2009.02.005. Epub 2009 Feb 20.

Opposite effects of PDGF-BB and prostaglandin E1 on cell-motility related processes are paralleled by modifications of distinct actin-binding proteins.

van Wieringen T, Kimani SG, Hultgård-Ekwall AK, Forsberg J, Reyhani V, Engström A, Rubin K.

Department of Medical Biochemistry and Microbiology, Uppsala University, BMC, Uppsala, Sweden.

Prostaglandin E(1) (PGE(1)) lowers dermal interstitial fluid pressure (IFP) in vivo and inhibits fibroblast-mediated collagen gel contraction in vitro. PDGF-BB, in contrast, stimulates contraction and normalizes IFP lowered as a result of anaphylaxis. Human diploid AG1518 fibroblasts expressed EP2, EP3 and IP prostaglandin receptors. The inhibitory effect of PGE(1) on contraction depended on cAMP. Short-term stimulation with PDGF-BB transiently induced formation of actin-containing membrane and circular ruffles and breakdown of stress fibers. PGE(1) had no effect on stress fibers nor did it modulate the effects of PDGF-BB. PGE(1) alone or in combination with PDGF-BB inhibited initial adhesion and spreading to collagen. PDGF-BB had no effect on adhesion but stimulated cell spreading. Two-dimensional gel electrophoresis and MALDI TOF analyses of

SDS/Triton X-100-soluble proteins revealed changes in migration pattern of actin-binding proteins. Interestingly, PDGF-BB and PGE(1) affected both similar and different sets of actin-binding proteins. PDGF-BB and PGE(1) did not trans-modulate their respective effects on actin-binding proteins, cytoskeletal organization or initial adhesion. Our data show that PDGF-BB stimulates actin cytoskeleton dynamics, whereas PGE(1) inhibits processes dependent on cytoskeletal motor functions. We suggest that these different activities may partly explain the contrasting effects of PGE(1) and PDGF-BB on contraction and IFP.

PMID: 19233168 [PubMed - indexed for MEDLINE]

589. J Allergy Clin Immunol. 2009 Apr;123(4):805-12. doi: 10.1016/j.jaci.2008.11.047. Epub 2009 Feb 20.

Allergic airway hyperresponsiveness, inflammation, and remodeling do not develop in phosphoinositide 3-kinase gamma-deficient mice.

Takeda M, Ito W, Tanabe M, Ueki S, Kato H, Kihara J, Tanigai T, Chiba T, Yamaguchi K, Kayaba H, Imai Y, Okuyama K, Ohno I, Sasaki T, Chihara J.

Department of Clinical and Laboratory Medicine, Akita University School of Medicine, Akita, Japan.

BACKGROUND: Bronchial asthma is characterized by chronic airway inflammation caused by inflammatory cells. Phosphoinositide 3-kinases (PI3Ks) are known to play a prominent role in fundamental cellular responses of various inflammatory cells, including proliferation, differentiation, and cell migration. PI3Ks therefore are expected to have therapeutic potential for asthma. Although some investigations of the involvement between the pathogenesis of asthma and PI3K have been performed, it is unknown whether PI3Kgamma, a PI3K isoform, is involved in the pathogenesis of asthma.
OBJECTIVE: We investigated the role of PI3Kgamma in allergen-induced allergic airway inflammation, airway hyperresponsiveness (AHR), and airway remodeling with PI3Kgamma-deficient mice.
METHODS: After ovalbumin (OVA) sensitization, wild-type (WT) and PI3Kgamma-deficient mice were exposed to aerosolized OVA 3 days per week for 5 weeks.
RESULTS: In OVA-sensitized and OVA-challenged (OVA/OVA) PI3Kgamma-deficient mice, levels of airway inflammation, AHR, and airway remodeling were significantly decreased compared with those in OVA/OVA WT mice. On the other hand, no significant differences were detected in serum OVA-specific IgE and IgG1 levels and CD4/CD8 balance in bronchoalveolar lavage fluid between OVA/OVA WT mice and OVA/OVA PI3Kgamma-deficient mice. To determine in which phase of allergic responses PI3Kgamma plays a role, we transferred splenocytes from OVA-sensitized WT or PI3Kgamma-deficient mice to naive mice of either genotype. Similar increased levels of eosinophils were induced in both WT recipient mice but not in both PI3Kgamma-deficient recipient mice.
CONCLUSION: PI3Kgamma might be involved in allergic airway inflammation, AHR, and airway remodeling by regulating the challenge/effector phase of allergic responses.

PMID: 19232703 [PubMed - indexed for MEDLINE]

590. Int J Infect Dis. 2009 Sep;13(5):e288-91. doi: 10.1016/j.ijid.2008.11.019. Epub 2009 Feb 23.

Strongyloides stercoralis hyperinfection presenting as acute respiratory failure

and Gram-negative sepsis in a patient with astrocytoma.

Shorman M, Al-Tawfiq JA.

Internal Medicine Department, King Fahad Specialist Hospital, Dammam, Saudi Arabia. mashorman@yahoo.com

In developing countries, Strongyloides stercoralis infection is a common cause of morbidity and mortality. Death from strongyloidosis can result from hyperinfection or disseminated disease. Infections due to S. stercoralis are unusual in Saudi Arabia and are usually diagnosed in immigrants from endemic areas. We report a case in which S. stercoralis was isolated from the sputum of a patient with Gram-negative sepsis and respiratory failure, and review the salient features of this disease. A high index of suspicion should be maintained by clinicians treating patients in endemic areas presenting with new-onset wheezing, acute respiratory distress and/or Gram-negative sepsis to prevent the serious complications of Strongyloides hyperinfection and dissemination.

PMID: 19231269 [PubMed - indexed for MEDLINE]

591. Am J Respir Cell Mol Biol. 2009 Oct;41(4):449-58. doi: 10.1165/rcmb.2008-0124OC. Epub 2009 Feb 12.

Role of A disintegrin and metalloprotease-12 in neutrophil recruitment induced by airway epithelium.

Estrella C, Rocks N, Paulissen G, Quesada-Calvo F, Noël A, Vilain E, Lassalle P, Tillie-Leblond I, Cataldo D, Gosset P.

INSERM U774, Biomolecules and Pulmonary Inflammation, Institut Pasteur de Lille, 1 rue du Pr Calmette, BP245, 59019 Lille Cedex, France.

Among proteases, metalloproteases are implicated in tissue remodeling, as shown in numerous diseases including allergy. ADAMs (A Disintegrin And Metalloprotease) metalloproteases are implicated in physiologic processes such as cytokine and growth factor shedding, cell migration, adhesion, or repulsion. Our aim was to measure ADAM-12 expression in airway epithelium and to define its role during the allergic response. To raise this question, we analyzed the ADAM-12 expression ex vivo after allergen exposure in patients with allergic rhinitis and in vitro in cultured primary human airway epithelial cells (AEC). Clones of BEAS-2B cells transfected with the full-length form of ADAM-12 were generated to study the consequences of ADAM-12 up-regulation on AEC function. After allergen challenge, a strong increase of ADAM-12 expression was observed in airway epithelium from patients with allergic rhinitis but not from control subjects. In contrast with the other HB-epidermal growth factor sheddases, ADAM-10 and -17, TNF-alpha in vitro increased the expression of ADAM-12 by AEC, an effect amplified by IL-4 and IL-13. Up-regulation of ADAM-12 in AEC increased the expression of alpha3 and alpha4 integrins and to the modulation of cell migration on fibronectin but not on collagen. Moreover, overexpression of ADAM-12 in BEAS-2B enhanced the secretion of CXCL1 and CXCL8 and their capacity to recruit neutrophils. CD47 was strongly decreased by ADAM-12 overexpression, a process associated with a reduced adhesion of neutrophils. These effects were mainly dependent on epidermal growth factor receptor activation. In summary, ADAM-12 is produced during allergic reaction by AEC and might increase neutrophil recruitment within airway mucosa.

PMID: 19213876 [PubMed - indexed for MEDLINE]

592. Allergy. 2009 May;64(5):718-24. doi: 10.1111/j.1398-9995.2008.01871.x. Epub 2009

Feb 11.

Soluble vascular cell adhesion molecule-1 induces human eosinophil migration.

Ueki S, Kihara J, Kato H, Ito W, Takeda M, Kobayashi Y, Kayaba H, Chihara J.

Department of Clinical and Laboratory Medicine, Akita University School of Medicine, Akita, Japan.

BACKGROUND: Tissue eosinophilia is one of the hallmarks of allergic diseases and Th2-type immune responses including asthma. Adhesion molecules are known to play an important role in the accumulation of eosinophils in allergic inflammatory foci, and they contribute to eosinophil activation. Elevated levels of the soluble forms of adhesion molecules in the body fluid of asthmatic patients have been observed, although their pathophysiological significance remains to be fully elucidated.
METHODS: Peripheral blood eosinophils were purified, and the effect of soluble vascular cell adhesion molecule-1 (sVCAM-1) on eosinophil migration was investigated using in vitro systems.
RESULTS: We found that sVCAM-1 (1 to 10 mug/ml) induced eosinophil chemotaxis, rather than chemokinesis, in a concentration-dependent fashion. In addition, sVCAM-1 induced cell shape change and actin polymerization, which are necessary for cell movement. Manipulations with very late antigen (VLA)-4-neutralizing antibody and signal inhibitors indicated that the sVCAM-1-induced chemotaxis was mediated through ligand-dependent activation of tyrosine kinase Src, p38 mitogen-activated protein kinase (MAPK), and extracellular signal-regulated kinase (ERK) MAPK. Rapid phosphorylation of these signaling molecules was observed using a bead-based multiplex assay.
CONCLUSION: Our results raise the possibility of sVCAM-1 in the fluid phase as a significant contributor to the heightened eosinophilic inflammatory response.

PMID: 19210349 [PubMed - indexed for MEDLINE]

593. Ther Clin Risk Manag. 2008 Oct;4(5):1009-22.

Effectiveness of twice daily azelastine nasal spray in patients with seasonal allergic rhinitis.

Horak F.

Medical University Vienna, ENT - Univ. Clinic, Vienna, Austria.

Azelastine nasal spray (Allergodil((R)), Lastin((R)), Afluon((R)); Meda AB, Stockholm, Sweden) is a fast-acting, efficacious and well-tolerated H1-receptor antagonist for the treatment of rhinitis. In addition it also has mast-cell stabilizing and anti-inflammatory properties, reducing the concentration of leukotrienes, kinins and platelet activating factor in vitro and in vivo, as well as inflammatory cell migration in rhinitis patients. Well-controlled studies in patients with seasonal allergic rhinitis (SAR), perennial rhinitis (PR) or vasomotor rhinitis (VMR) confirm that azelastine nasal spray has a rapid onset of action, and improves nasal symptoms associated with rhinitis such as nasal congestion and post-nasal drip. Azelastine nasal spray is effective at the lower dose of 1 spray as well at a dose of 2 sprays per nostril twice daily, but with an improved tolerability profile compared to the 2-spray per nostril twice daily regimen. Compared with intranasal corticosteroids, azelastine nasal spray has a faster onset of action and a better safety profile, showing at least comparable efficacy with fluticasone propionate (Flonase((R)); GSK, USA), and a superior efficacy to mometasone furoate (Nasonex((R)); Schering Plough, USA). In combination with fluticasone propionate, azelastine nasal spray exhibits greater

efficacy than either agent used alone, and this combination may provide benefit for patients with difficult to treat seasonal allergic rhinitis. In addition, azelastine nasal spray can be used on an as-needed basis without compromising clinical efficacy. Compared with oral antihistamines, azelastine nasal spray also demonstrates superior efficacy and a more rapid onset of action, and is effective even in patients who did not respond to previous oral antihistamine therapy. Unlike most oral antihistamines, azelastine nasal spray is effective in alleviating nasal congestion, a particularly bothersome symptom for rhinitis sufferers. Azelastine nasal spray is well tolerated in both adults and children with allergic rhinitis. Bitter taste which seems to be associated with incorrect dosing technique is the most common side effect reported by patients, but this problem can be minimized by correct dosing technique.

PMCID: PMC2621402
PMID: 19209282 [PubMed]

594. Allergy. 2009 Aug;64(8):1130-5. doi: 10.1111/j.1398-9995.2009.01969.x. Epub 2009 Feb 7.

Thioredoxin reduces C-C chemokine-induced chemotaxis of human eosinophils.

Kobayashi N, Yamada Y, Ito W, Ueki S, Kayaba H, Nakamura H, Yodoi J, Chihara J.

Department of Clinical and Laboratory Medicine, Akita University School of Medicine, Akita 010-8543, Japan.

BACKGROUND: Human thioredoxin (TRX) is one of redox-active proteins that regulate reactive oxidative metabolisms. In recent study, we found that serum levels of TRX were elevated in asthmatic patients with exacerbation; however, few details are known about the physiological role of TRX in allergic inflammation, involving eosinophil infiltration.
OBJECTIVE: In the present study, we examined whether TRX modulated C-C chemokine-induced chemotaxis of human eosinophils. Methods: Eosinophils were isolated from subjects with mild eosinophilia by modified CD16 negative selection. After incubation with or without recombinant TRX, chemotaxis of human eosinophils was measured using Boyden chamber.
RESULTS: Preincubation with TRX suppressed eotaxin- and regulated on activation, normal T-cell expressed and secreted (RANTES)-induced chemotaxis of eosinophils. Although, TRX had no effect on the expression of C-C chemokine receptor 3, which is a receptor of eotaxin and RANTES, we demonstrated that the activation of extracellular signal-regulated kinase 1/2 and p38 mitogen-activated protein kinases, which play an important role in eosinophil migration, was attenuated by the treatment with TRX.
CONCLUSION: Our results suggest that the elicited TRX is beneficial to reduce allergic inflammation through negative regulation of eosinophil functions and has potential in the treatment of allergic diseases, such as asthma.

PMID: 19208085 [PubMed - indexed for MEDLINE]

595. J Interferon Cytokine Res. 2009 Apr;29(4):227-39. doi: 10.1089/jir.2008.0051.

CCL26-targeted siRNA treatment of alveolar type II cells decreases expression of CCR3-binding chemokines and reduces eosinophil migration: implications in asthma therapy.

Errahali YJ, Taka E, Abonyo BO, Heiman AS.

College of Pharmacy and Pharmaceutical Sciences, Florida A&M University,

Tallahassee, Florida 32307, USA.

The underlying inflammation present in chronic airway diseases is orchestrated by increased expression of CC chemokines that selectively recruit leukocyte populations into the pulmonary system. Human CCL26 signals through CC chemokine receptor 3 (CCR3), is dramatically upregulated in challenged asthmatics, and stimulates recruitment of eosinophils (EOSs) and other leukocytes. CCL26 participates in regulation of its receptor CCR3 and modulates expression of a variety of chemokines in alveolar type II cells. Utilizing the A549 alveolar type II epithelial cell culture model, we carried out studies to test the hypothesis that CCL26-siRNA treatment of these cells would ameliorate Th2-driven release of the eotaxins and other CCR3 ligands that would, in turn, decrease recruitment and activation of EOSs. Results demonstrate that CCL26-siRNA treatments decreased interleukin-4-induced CCL26 and CCL24 expression by >70%. CCL26-directed small-interfering RNA (siRNA) treatments significantly decreased release of CCL5 (RANTES), CCL15 (MIP-1δ), CCL8 (MCP-2), and CCL13 (MCP-4). In bioactivity assays it was shown that EOS migration and activation were reduced up to 80% and 90%, respectively, when exposed to supernatants of CCL26-siRNA-treated cells. These results provide evidence that CCL26 may be an appropriate target for development of new therapeutic agents designed to alleviate the underlying inflammation associated with chronic diseases of the airways.

PMCID: PMC2941665
PMID: 19203252 [PubMed - indexed for MEDLINE]

596. J Invest Dermatol. 2009 Aug;129(8):1954-62. doi: 10.1038/jid.2008.454. Epub 2009 Feb 5.

Topical application of sphingosine-1-phosphate and FTY720 attenuate allergic contact dermatitis reaction through inhibition of dendritic cell migration.

Reines I, Kietzmann M, Mischke R, Tschernig T, Lüth A, Kleuser B, Bäumer W.

Department of Pharmacology, Toxicology, and Pharmacy, University of Veterinary Medicine Hannover, Hannover, Germany.

Comment in
    J Invest Dermatol. 2009 Aug;129(8):1852-3.

Migration of Langerhans cells (LCs) from the skin to the lymph node is an essential step in the pathogenesis of allergic contact dermatitis (ACD). Therefore, inhibition of LC-migration could be a promising strategy to improve this skin disease. Effects of sphingosine-1-phosphate (S1P) and the immunomodulator FTY720 on LC trafficking is not well defined, yet. Thus, we investigated the action of topically administered S1P and FTY720 in a murine model of ACD. Most interestingly, FTY720 as well as S1P inhibited the inflammatory reaction in the elicitation phase of ACD. In the sensitization phase, FTY720, and S1P reduced the weight and cell count of the draining auricular lymph node, as well as immigrated dendritic cells provoked by repetitive topical administration of the hapten. Correspondingly, the density of LCs in the epidermis was higher in FTY720- and S1P-treated mice compared to vehicle treatment. A skin dendritic cell migration assay confirmed the significant inhibition of dendritic cell migration by FTY720 and S1P. These data supply conclusive evidence that the strategy of targeting the migratory response of LCs with locally acting S1P or FTY720 represents an emerging option in the treatment of allergic skin diseases like contact hypersensitivity and atopic dermatitis.

PMID: 19194476 [PubMed - indexed for MEDLINE]

597. Indoor Air. 2009 Feb;19(1):45-57. doi: 10.1111/j.1600-0668.2008.00560.x.

Health evaluation of volatile organic compound (VOC) emission from exotic wood products.

Kirkeskov L, Witterseh T, Funch LW, Kristiansen E, Mølhave L, Hansen MK, Knudsen BB.

Department of Occupational and Environmental Medicine, Viborg Hospital, Resenvej 25, Skive, Denmark. lilli.kirkeskov.jensen@sygehusviborg.dk

The purpose of this study was to measure and evaluate the impact of the emissions of selected products of exotic wood on health. Ten products were screened for chemical compounds, and five of the most used products which emitted more than 800 microg/kg were selected for further quantitative analyses by climate chamber measurement (iroko, ramin, sheesham, merbau, and rubber tree). Samples of exotic wood (rubber tree and belalu) were further analyzed for emission of chemical compounds by migration into artificial saliva and for content of pesticides and allergenic natural rubber latex (NR latex) (rubber tree). The toxicological effects of all substances identified were evaluated and the lowest concentrations of interest (LCI) assessed. An R-value was calculated for each wood product (R-value below 1 is considered to be unproblematic as regards health). Emission from the evaluated exotic wood only took place to a very limited extent. None of the selected products, under the chosen rating system, is likely to cause adverse health effects. Products with surface treatment might pose a problem if used as kitchen utensils, as children's toys, or when they are in close contact with the skin for a long time. PRACTICAL IMPLICATIONS: The authors investigated the chemical emissions from selected products from exotic wood by climate chamber measurement. Quantitative chemical analyses of emissions from the five most used exotic products in Denmark were performed, and all chemical compounds found were evaluated toxicologically. Emission from the evaluated exotic wood was very limited. None of the products is likely, under our exposure conditions, to cause health problems in relation to indoor air.

PMID: 19191927 [PubMed - indexed for MEDLINE]

598. Glia. 2009 Aug 15;57(11):1192-203. doi: 10.1002/glia.20841.

Human bone marrow-derived mesenchymal stem cells induce Th2-polarized immune response and promote endogenous repair in animal models of multiple sclerosis.

Bai L, Lennon DP, Eaton V, Maier K, Caplan AI, Miller SD, Miller RH.

Case Western Reserve University, Centers for Stem Cells and Regenerative Medicine, Translational Neuroscience, Department of Neurosciences, Case School of Medicine, Cleveland, Ohio 44106, USA.

Cell-based therapies are attractive approaches to promote myelin repair. Recent studies demonstrated a reduction in disease burden in mice with experimental allergic encephalomyelitis (EAE) treated with mouse mesenchymal stem cells (MSCs). Here, we demonstrated human bone marrow-derived MSCs (BM-hMSCs) promote functional recovery in both chronic and relapsing-remitting models of mouse EAE, traced their migration into the injured CNS and assayed their ability to modulate disease progression and the host immune response. Injected BM-hMSCs accumulated in the CNS, reduced the extent of damage and increased oligodendrocyte lineage cells in lesion areas. The increase in oligodendrocytes in lesions may reflect BM-hMSC-induced changes in neural fate determination, since neurospheres from

treated animals gave rise to more oligodendrocytes and less astrocytes than nontreated neurospheres. Host immune responses were also influenced by BM-hMSCs. Inflammatory T-cells including interferon gamma producing Th1 cells and IL-17 producing Th17 inflammatory cells and their associated cytokines were reduced along with concomitant increases in IL-4 producing Th2 cells and anti-inflammatory cytokines. Together, these data suggest that the BM-hMSCs represent a viable option for therapeutic approaches.

PMCID: PMC2706928
PMID: 19191336 [PubMed - indexed for MEDLINE]

599. World Allergy Organ J. 2009 Feb;2(2):13-6. doi: 10.1097/WOX.0b013e3181971b89.

"United airways disease" and phenotypic peculiarities of respiratory allergy in immigrants.

Lombardi C, Passalaqua G, Canonica GW.

From the Allergy and Respiratory Diseases, Department of Internal Medicine, University of Genoa, Genoa, Italy.

ABSTRACT: Allergy is the result of a complex interaction between genetic background and environmental factors, including exposure to allergens and lifestyle. Migration is a process that involves many radical changes in the environment, including diet, pollutants, allergens, different housing conditions, and patterns of infections. Thus, studies in immigrants may provide important information about the role of environmental factors in the development of allergic respiratory diseases. Several studies addressed this aspect and consistently found that migrants develop allergies at different rates from the local population, and very often the symptoms appear with a delay of 3 to 5 years after migration. More recent data showed that the severity of allergic diseases is greater in migrants, and that usually the onset is with associated asthma and rhinitis. The immigration model strongly suggests that environmental factors overcome the genetic background, and that the clinical phenotype of respiratory allergy in migrants has some peculiarities.

PMID: 23282932 [PubMed]

600. Prim Care Respir J. 2009 Jun;18(2):69-75. doi: 10.3132/pcrj.2009.00009.

Systems for the management of respiratory disease in primary care--an international series: South Africa.

Bateman E, Feldman C, Mash R, Fairall L, English R, Jithoo A.

Division of Pulmonology, Department of Medicine, University of Cape Town, and University of Cape Town Lung Institute, Cape Town, South Africa.
eric.bateman@uct.ac.za

INTRODUCTION: Progress to democracy in South Africa in 1994 was followed by the adoption of a primary health care approach with free access for all. State health facilities serve 80% of the population, and a private sector comprising general practitioners, specialists and private hospitals, serves the remainder. NATIONAL POLICIES AND MODELS: There are national prescribing guidelines for common diseases, and these specify the medicines on the Essential Drugs List that are available at primary care facilities for respiratory diseases including asthma, COPD, pneumonia and tuberculosis.
EPIDEMIOLOGY: Asthma prevalence is average among children (13%) but morbidity is

high. COPD rates are high owing to concurrent risk factors of smoking (in both men and women), occupational exposures, biomass fuel use and previous lung infections including tuberculosis. Tuberculosis and HIV are rampant, and together with pneumococcal co-infection account for considerable mortality. ACCESS TO CARE: Primary care facilities are within reach of most communities, but major barriers to care include loss of income, waiting times in clinics, cost of transportation, and inconvenient hours. FACILITIES AVAILABLE: The country is divided into districts each served by a hospital, several community health centres and many fixed or mobile clinics. The latter provide predominantly nurse-led care by nurse practitioners with additional qualifications. Some clinics and most community health centres are served by doctors. Referrals are made to secondary and tertiary hospitals served by specialists. FUTURE: Innovations to address staff shortages include the creation of the specialty of family medicine for physicians and development of the clinical associate who is trained to perform a limited clinical role, as well as in-service on-site training of nurses through programmes of integrated care for infectious and chronic diseases. There is an urgent need to address low staff morale and medical migration resulting from a decade of poor leadership and AIDS denialism. CONCLUSIONS: The structures and policies for primary care in South Africa provide some grounds for optimism that services may begin to match the promise of quality care for all, but the burden of disease and resource constraints - particularly in terms of qualified personnel - mitigate against an early delivery of this promise.

PMID: 19173089 [PubMed - indexed for MEDLINE]

601. Arerugi. 2008 Dec;57(12):1317-24.

[Eosinophil chemotaxis assay using novel device EZ-TAXIScan].

[Article in Japanese]

Kato H, Ueki S, Ito W, Takeda M, Chiba T, Yamaguchi K, Kayaba H, Chihara J.

Department of Clinical and Laboratory Medicine, Akita University School of Medicine.

BACKGROUND: Eosinophils are major effector cells in the pathogenesis of allergic inflammation such as bronchial asthma, and eosinophil migration to sites of inflammation is an important step. To date, several approaches have been developed to study eosinophil chemotaxis. Among them, the Boyden chamber method has been widely used, although this system requires a relatively large number of cells, and it usually provides no longitudinal information. In this study, we investigated real-time eosinophil chemotaxis using EZ-TAXIScan, a novel horizontal microchannel device.
METHODS: Eosinophils were isolated from subjects with mild eosinophilia by modified CD16-negative selection. Eosinophil chemotaxis and migration speed induced by various chemoattractants including eotaxin, RANTES, PAF, and prostaglandin (PGD2) were measured by EZ-TAXIScan. We also determined the time course of chemotaxis using Boyden chambers.
RESULT: By using EZ-TAXIScan, rapid (a few minutes after stimulation) and fast (20-30 microm/min) eosinophil chemotactic responses were observed by stimulation with PAF or PGD2, although eosinophils stimulated with eotaxin or RANTES showed relatively late (60 minutes after stimulation) and slow (15 microm/min) responses. In contrast, using a Boyden Chamber, the chemotactic responses we tested showed a similar time course peaking at 20-60 min.
CONCLUSION: The availability of EZ-TAXIScan for investigation of eosinophil chemotaxis was confirmed. However, it should be noted that EZ-TAXIScan showed a different response to certain chemoattractants compared with the conventional

method.

PMID: 19169086 [PubMed - indexed for MEDLINE]

602. Biochem Biophys Res Commun. 2009 Mar 6;380(2):323-7. doi: 10.1016/j.bbrc.2009.01.071. Epub 2009 Jan 23.

Potential role of chemerin in recruitment of plasmacytoid dendritic cells to diseased skin.

Skrzeczyńska-Moncznik J, Wawro K, Stefańska A, Oleszycka E, Kulig P, Zabel BA, Sułkowski M, Kapińska-Mrowiecka M, Czubak-Macugowska M, Butcher EC, Cichy J.

Department of Immunology, Faculty of Biochemistry, Biophysics and Biotechnology, Jagiellonian University, Gronostajowa 7 St., 30-387 Krakow, Poland.

Interferon alpha-producing plasmacytoid dendritic cells (pDC) are crucial contributors to pro-inflammatory or tolerogenic immune responses and are important in autoimmune diseases such as psoriasis. pDC accumulate in the lesional skin of psoriasis patients, but are rarely found in the affected skin of patients with atopic dermatitis (AD). While homeostatic chemokine CXCL12 and inducible pro-inflammatory CXCR3 chemokine ligands may regulate pDC influx to psoriatic skin, the mechanism responsible for selective pDC recruitment in psoriasis vs. AD remains unknown. Circulating pDC from normal donors express a limited number of chemoattractant receptors, including CXCR3 and CMKLR1 (chemokine-like receptor 1). In this work, we demonstrate that circulating pDC from normal donors as well as psoriasis and AD patients express similar levels of CXCR3 and responded similarly in functional migration assays to CXCL10. We next found that blood pDC from normal, AD, and psoriasis patients express functional CMKLR1. In contrast to normal skin, however, lesional skin from psoriasis patients contains the active form of the CMKLR1 ligand chemerin. Furthermore, in affected skin from psoriatic patients the level of active chemerin was generally higher than in AD skin. Taken together, these results indicate that local generation of active chemerin may contribute to pDC recruitment to psoriatic skin.

PMID: 19168032 [PubMed - indexed for MEDLINE]

603. J Leukoc Biol. 2009 Apr;85(4):656-63. doi: 10.1189/jlb.0808492. Epub 2009 Jan 21.

Crucial implication of protein kinase C (PKC)-delta, PKC-zeta, ERK-1/2, and p38 MAPK in migration of human asthmatic eosinophils.

Langlois A, Chouinard F, Flamand N, Ferland C, Rola-Pleszczynski M, Laviolette M.

Unité de Recherche en Pneumologie, Centre de Recherche de l'Hôpital Laval, Institut Universitaire de Cardiologie et de Pneumologie de l'Université Laval, Québec City, Quebec, Canada.

Asthma is associated with an eosinophil infiltration into the bronchial mucosa. 5-Oxo-6,8,11,14(E,Z,Z,Z)-eicosatetraenoic acid (5-oxo-ETE), a potent eosinophil chemotactic factor, activates cell motility, adherence, and proteolysis, notably, by promoting CD11b expression, matrix metalloproteinase (MMP)-9 secretion, and plasmin generation. We investigated the intracellular signaling pathways implicated in these various steps by using different, selective inhibitors. Human eosinophil migration through a reconstituted basement membrane in response to 5-oxo-ETE was greatly inhibited (>or=72%) by the protein kinase C (PKC)-delta, PKC-zeta, ERK-1/2, and p38 inhibitors. Our findings indicate that PKC-delta

mediates cell motility, CD11b expression, and MMP-9 granule release. PKC-zeta is also largely involved in eosinophil migration, although its specific targets remain undefined. ERK-1/2 and p38 modulate CD11b expression; ERK-1/2 is also involved in long-term MMP-9 secretion and p38 in the plasmin activation system. We demonstrated the crucial implication of PKC-delta, PKC-zeta, ERK-1/2, and p38 in human blood eosinophil migration through extracellular matrix components. Targeting specific pathways may have therapeutic potential for the treatment of allergic airway inflammation.

PMID: 19164129 [PubMed - indexed for MEDLINE]

604. J Allergy Clin Immunol. 2009 Apr;123(4):933-9.e10. doi: 10.1016/j.jaci.2008.11.037. Epub 2009 Jan 18.

Selective deregulation in chemokine signaling pathways of CD4+CD25(hi)CD127(lo)/(-) regulatory T cells in human allergic asthma.

Nguyen KD, Vanichsarn C, Fohner A, Nadeau KC.

Department of Pediatrics, Stanford University, Stanford, CA 94305, USA.

BACKGROUND: CD4+CD25(hi)CD127(lo)/(-) regulatory T cells have been suggested to be critical regulators of inflammatory processes in allergic asthma. Recent studies reported a selective decrease in the frequency of regulatory T cells in the bronchoalveolar lavage fluid of allergic asthmatic (AA) subjects, prompting the possibility of defective recruitment of these cells to the airway in response to chemokines produced during asthmatic inflammation.
OBJECTIVES: This study aimed to characterize the chemotactic profile of circulating regulatory T cells in AA subjects in response to chemokines abundantly produced in airway inflammation, such as CCL1, CCL17, and CCL22.
METHODS: The study was performed in a cohort of 26 AA, 16 healthy control, and 16 non-AA subjects. We used chemotaxis assays to evaluate cell migration, flow cytometry to examine chemokine receptor expression, and phospho-ELISA to study consequent signaling pathways in regulatory T cells.
RESULTS: Regulatory T cells, but not CD4+CD25(-)T cells, from AA subjects showed decreased chemotactic responses, specifically to CCL1, in comparison with their healthy control and non-AA counterparts. Decreased CCL1-mediated chemotaxis in AA regulatory T cells was associated with decreased phosphorylation of protein kinase B (AKT), a protein involved in chemokine intracellular signaling. Furthermore, the decreased chemotactic response to CCL1 in AA regulatory T cells significantly correlated with asthma severity and decreased pulmonary function in AA subjects.
CONCLUSIONS: These results provide the first evidence of dysfunction in the chemokine signaling pathway in AA regulatory T cells.

PMID: 19152963 [PubMed - indexed for MEDLINE]

605. Am J Respir Cell Mol Biol. 2009 Sep;41(3):290-6. doi: 10.1165/rcmb.2008-0307OC. Epub 2009 Jan 16.

Osteopontin induces airway remodeling and lung fibroblast activation in a murine model of asthma.

Kohan M, Breuer R, Berkman N.

Lung Cellular and Molecular Biology Laboratory, Institute of Pulmonology, Hadassah-Hebrew University Medical Center, Jerusalem, Israel.

Airway remodeling is a central feature of asthma; however, the mechanisms underlying its development have not been fully elucidated. We have demonstrated that osteopontin, an inflammatory cytokine and an extracellular matrix glycoprotein with profibrotic properties, is up-regulated in a murine model of allergen-induced airway remodeling. In the present study, we determined whether osteopontin plays a functional role in airway remodeling. Osteopontin (OPN)-deficient (OPN(-/-)) and wild-type mice were sensitized and exposed to inhaled ovalbumin (OVA) or saline for 5 weeks. Collagen production, peribronchial smooth muscle area, mucus-producing cell number, and bronchoalveolar cell counts were assessed. The functional behavior and phenotype of lung fibroblasts from OVA-treated OPN(-/-) and from wild-type mice were studied using ex vivo cultures. OVA-treated OPN(-/-) mice exhibited reduced lung collagen content, smooth muscle area, mucus-producing cells, and inflammatory cell accumulation as compared with wild-type mice. Reduced matrix metalloproteinase-2 activity and expression of transforming growth factor-beta1 and vascular endothelial growth factor were observed in OVA-treated OPN(-/-) mice. Lung fibroblasts from OVA-treated OPN(-/-) mice showed reduced proliferation, migration, collagen deposition, and alpha-smooth muscle actin expression in comparison with OVA-treated wild-type lung fibroblasts. Thus, OPN is key for the development of allergen-induced airway remodeling in mice. In response to allergen, OPN induces the switching of lung fibroblasts to a pro-fibrogenic myofibroblast phenotype.

PMID: 19151319 [PubMed - indexed for MEDLINE]

606. Int Immunopharmacol. 2009 Mar;9(3):303-12. doi: 10.1016/j.intimp.2008.12.004. Epub 2009 Jan 7.

Requirement of L-selectin for gammadelta T lymphocyte activation and migration during allergic pleurisy: co-relation with eosinophil accumulation.

Costa MF, Nihei J, Mengel J, Henriques MG, Penido C.

Laboratório de Farmacologia Aplicada, Departamento de Farmacologia Aplicada, Farmanguinhos, Fundação Oswaldo Cruz, Rio de Janeiro, Brazil.

Intra-thoracic antigenic challenge (ovalbumin, 12.5 microg/cavity) led to increased numbers of gammadelta T lymphocytes in pleural cavities, blood and thoracic lymph nodes in sensitized mice within 48 h. Part of these cells expressed CD62L, which increased on gammadelta T cell surfaces obtained from lymph nodes after ovalbumin (OVA) challenge. Selectin blockade by fucoidan pre-treatment (10 mg/kg, i.v.) impaired in vivo increase in CD25(+) and c-fos(+) gammadelta T cell numbers in lymph nodes, indicating a role for selectins on gammadelta T lymphocyte activation and proliferation. In vivo selectin blockade by fucoidan or alpha-CD62L mAb (200 microg/mice, i.p.) also inhibited OVA-induced gammadelta T cell accumulation in pleural cavities. Confirming the direct effect of CD62L on gammadelta T cell transmigration, the migration of i.v. adoptively-transferred CFSE-labeled gammadelta T lymphocytes into pleural cavities of challenged recipient mice was impaired by fucoidan ex vivo treatment. It is noteworthy that eosinophil influx was also impaired in those mice, indicating that reduced eosinophil migration by CD62L in vivo blockade depended on gammadelta T cell migration via CD62L molecules. Accordingly, pleural gammadelta T lymphocytes from fucoidan-treated mice presented reduced OVA-induced IL-5 and CCL11 production. Supporting these data, the depletion of Vgamma4 T lymphocytes, which are pulmonary gammadelta T cells, decreased OVA-induced eosinophil influx into allergic site. Such results demonstrate that CD62L is crucial for the activation of gammadelta T cells in lymph nodes, for their migration into inflamed tissue and for the modulation of eosinophil influx during allergic response.

PMID: 19135179 [PubMed - indexed for MEDLINE]

607. J Leukoc Biol. 2009 Apr;85(4):719-27. doi: 10.1189/jlb.0808494. Epub 2009 Jan 7.

Role of atopic status in Toll-like receptor (TLR)7- and TLR9-mediated activation of human eosinophils.

Månsson A, Cardell LO.

Laboratory of Clinical and Experimental Allergy Research, Department of Otorhinolaryngology, Malmö University Hospital, Lund University, Malmö, Sweden.

Viral respiratory infections are increasingly implicated in allergic exacerbations. The mechanisms behind this are not known, but a virus-induced activation of eosinophils through TLRs could be involved. Herein, we investigated the expression and function of TLR7 and TLR9 in purified eosinophils from peripheral blood and assessed their role in allergic airway inflammation. Eosinophils expressed TLR7 and TLR9 proteins. Stimulation with the cognate ligands R-837 and CpG was found to prolong survival, up-regulate expression of CD11b and conversely down-regulate L-selectin expression, increase expression of the activation marker CD69, facilitate the chemotactic migration, and enhance IL-8 secretion by eosinophils. Also, CpG induced release of eosinophil-derived neurotoxin (EDN), and R-837 failed to do so. Analogously, eosinophils activated by CpG, but not R-837, promoted airway epithelial cell death and cytokine release. Priming with the allergic mediators histamine, IL-4, and most prominently IL-5, augmented the TLR-induced IL-8 and EDN secretion, revealing an ability to sensitize eosinophils for TLR7 and TLR9 activation. Moreover, the TLR responses of eosinophils were higher in allergic as compared with healthy subjects, manifested by an increase in IL-8 and EDN release. Correspondingly, allergic subjects displayed an elevated serum level of IL-5, suggesting increased IL-5-mediated priming. This study shows that activation via TLR7 and TLR9 affects several eosinophil functions and that the atopic status of the donor and the presence of a Th2-like cytokine milieu affect the outcome of the response. Thus, eosinophil activation via TLR7 and TLR9 might engender a link between viral infection and allergic exacerbations.

PMID: 19129482 [PubMed - indexed for MEDLINE]

608. Adv Immunol. 2009;104:51-113. doi: 10.1016/S0065-2776(08)04003-0. Epub 2009 Dec 1.

Pathogenic mechanisms of allergic inflammation: atopic asthma as a paradigm.

Holt PG, Strickland DH, Bosco A, Jahnsen FL.

Faculty of Medicine and Dentistry, Telethon Institute for Child Health Research and Centre for Child Health Research, The University of Western Australia, Perth, Western Australia, Australia.

Prospective studies tracking birth cohorts over periods of years indicate that the seeds for atopic asthma in adulthood are sewn during early life. The key events involve programming of functional phenotypes within the immune and respiratory systems which determine long-term responsiveness to ubiquitous environmental stimuli, particularly respiratory viruses and aeroallergens. A crucial component of asthma pathogenesis is early sensitization to aeroallergens stemming from a failure of mucosal tolerance mechanisms during the preschool years, which is associated with delayed postnatal maturation of a range of adaptive and innate immune functions. These maturational defects also increase

risk for severe respiratory infections, and the combination of sensitization and infections maximizes risk for early development of the persistent asthma phenotype. Interactions between immunoinflammatory pathways stimulated by these agents also sustain the disease in later life as major triggers of asthma exacerbations. Recent studies on the nature of these interactions suggest the operation of an infection-associated lung:bone marrow axis involving upregulation of FcERlalpha on myeloid precursor populations prior to their migration to the airways, thus amplifying local inflammation via IgE-mediated recruitment of bystander atopic effector mechanisms. The key participants in the disease process are airway mucosal dendritic cells and adjacent epithelial cells, and transiting CD4(+) effector and regulatory T-cell populations, and increasingly detailed characterization of their roles at different stages of pathogenesis is opening up novel possibilities for therapeutic control of asthma. Of particular interest is the application of genomics-based approaches to drug target identification in cell populations of interest, exemplified by recent findings discussed below relating to the gene network(s) triggered by activation of Th2-memory cells from atopics.

2009 Elsevier Inc. All rights reserved.

PMID: 20457116 [PubMed - indexed for MEDLINE]

609. Pulm Pharmacol Ther. 2009 Oct;22(5):426-35. doi: 10.1016/j.pupt.2008.12.003. Epub 2008 Dec 25.

Lipid metabolites as regulators of airway smooth muscle function.

Clarke DL, Dakshinamurti S, Larsson AK, Ward JE, Yamasaki A.

Respiratory Pharmacology, National Heart and Lung Institute, Imperial College, London, UK.

Compelling evidence identifies airway smooth muscle (ASM) not only as a target but also a cellular source for a diverse range of mediators underlying the processes of airway narrowing and airway hyperresponsiveness in diseases such as asthma. These include the growing family of plasma membrane phospholipid-derived polyunsaturated fatty acids broadly characterised by the prostaglandins, leukotrienes, lipoxins, isoprostanes and lysophospholipids. In this review, we describe the enzymatic and non-enzymatic biosynthetic pathways of these lipid mediators and how these are influenced by drug treatment, oxidative stress and airways disease. Additionally, we outline their cognate receptors, many of which are expressed by ASM. We describe potential deleterious and protective roles for these lipid mediators in airway inflammatory and remodelling processes by describing their effects on diverse functions of ASM in asthma that have the potential to contribute to asthma pathogenesis and symptoms. These functions include contractile tone development, cytokine and extracellular matrix production, and cellular proliferation and migration.

PMID: 19114116 [PubMed - indexed for MEDLINE]

610. Inflamm Res. 2008 Nov;57(11):535-41. doi: 10.1007/s00011-008-8047-0.

The allergic inflammatory reaction in neonatal streptozotocininduced diabetic rats: evidence of insulin resistance and microvascular dysfunction.

Cavalher-Machado SC, Cuman RK, Sartoretto JL, Martins JO, de Lima WT, Martins MA, Silva PM, Sannomiya P.

Research Division, Institute of Heart (InCor), LIM-11, University of São Paulo
Medical School, São Paulo, SP, Brazil. simachado@far.fiocruz.br

OBJECTIVE: To investigate the allergic reaction in neonatal streptozotocin (nSTZ)-induced diabetes mellitus.
MATERIAL: Male newborn Wistar rats were made diabetic by the injection of streptozotocin (160 mg/kg, i. p.) and used 8 weeks thereafter.
TREATMENT: Animals were sensitized against ovalbumin (OA, 50 microg and Al(OH)3, 5 mg, s. c.) and challenged 14 or 21 days thereafter.
METHODS: OA-induced airway inflammation and OA-induced pleurisy models were used to investigate leukocyte migration (total and differential leukocyte counts) and lung vascular permeability (Evans blue dye extravasation).
RESULTS: nSTZ-diabetic rats presented glucose intolerance and insulin resistance. Relative to controls, nSTZ rats exhibited a 30% to 50% reduction in lung vascular permeability. Leukocyte infiltration in both models of allergen-induced inflammation, and number of pleural mast cells did not differ between groups.
CONCLUSIONS: Data suggest that the reduction of allergic inflammatory reactions in nSTZ rats is restricted to microvascular dysfunctions and associated, probably, with insulin resistance in lung microvascular endothelium.

PMID: 19109747 [PubMed - indexed for MEDLINE]

611. Eur J Dermatol. 2009 Mar-Apr;19(2):157-62. doi: 10.1684/ejd.2008.0600. Epub 2008 Dec 23.

Dermatoses in Latin American immigrants seen in a tertiary hospital.

Albares Tendero MP, Belinchón Romero I, Ramos Rincón JM, Sánchez Payá J, Costa AL, Pérez Crespo M, Silvestre Salvador JF.

Servicio de Dermatología, Hospital General Universitario de Alicante, 03010 Alicante, Spain. pityalte@hotmail.com

Europe, and in particular Spain, has become the destination of a considerable number of immigrants, 50% come from Latin America. The purpose of this study was to describe the cases of dermatoses seen in the immigrant Latin American population and compare them with those found in the control Spanish population. Over a year all the visits of economic immigrants seen in the Dermatology Section of the Hospital General Universitario de Alicante were prospectively recorded. During the study period 706 Latin American patients were seen. The most frequent dermatoses were eczema (18.2%), acne (6.5%) and non-genital viral warts (6.3%). The comparative study of dermatoses adjusted for age and sex, found a greater frequency of eczema, alopecia, melasma, herpes simplex, pilar keratosis, xerosis, and scabies ($p < 0.01$) in the Latin American population. On the other hand, melanocytic nevi and melanoma were less frequent in these patients ($p < 0.05$). We may say that the skin type and socio-sanitary conditions of the Latin American immigrant population lead to a greater frequency of eczema, melasma and scabies. In addition, the skin type and younger age favour a lower frequency of skin tumours.

PMID: 19106052 [PubMed - indexed for MEDLINE]

612. Toxicon. 2009 Mar 1;53(3):323-9. doi: 10.1016/j.toxicon.2008.12.002. Epub 2008 Dec 10.

Mechanisms involved in the rat peritoneal leukocyte migration induced by a Kunitz-type inhibitor isolated from Dimorphandra mollis seeds.

Mello GC, Desouza IA, Mariano NS, Ferreira T, Macedo ML, Antunes E.

Department of Pharmacology of Faculty of Medical Sciences, State University of Campinas (UNICAMP), P.O. Box 6111, 13084-971 Campinas (SP), Brazil. glaumello@yahoo.com.br

DMTI-II is a Kunitz-type inhibitor isolated from Dimorphandra mollis seeds that causes rat inflammatory edema by mechanisms involving activation of mast cells and sensory C-fibers. The present study aimed to further explore the inflammatory mechanisms involved in DMTI-II-induced inflammation, focusing to the leukocyte migration in vivo. Male Wistar rats (250-280 g) were injected with DMTI-II (1-100microg/cavity), and at 4-24h thereafter the leukocyte counts in peritoneal lavage were evaluated. DMTI-II caused dose- and time-dependent accumulation of neutrophils and eosinophils. The peritoneal neutrophil influx initiated at 4h, achieving maximal responses at 16 h after DMTI-II injection (16- and 22-fold increase, respectively). The DMTI-II-induced eosinophil recruitment was observed as early as 4h achieving the maximal responses at 16 h (12- and 17-fold increase, respectively). The mononuclear cell number increased at 4h and 16 h (1.5-fold and 1.6-increase, respectively). Prior treatments with dexamethasone, the cyclooxygenase (COX) inhibitors indomethacin and celecoxib, as well as the PAF receptor antagonist PCA4248 largely reduced the neutrophil and eosinophil accumulation. The selective lypoxygenase inhibitor AA861, the tachykinin NK(1) antagonist SR-140333 and the nitric oxide inhibitor L-NAME reduced only the eosinophil number. The eotaxin levels were significantly higher in DMTI-II-injected rats compared with control animals. In conclusion, DMTI-II causes an early migration of eosinophils and neutrophils by mechanisms involving COX-2- and lipoxygenase-derived metabolites, PAF, substance P and NO. The capacity of DMTI-II to recruit eosinophils at early times is likely to reflect the allergen properties of proteinase inhibitors belonging to Kunitz family.

PMID: 19103216 [PubMed - indexed for MEDLINE]

613. Ann Fr Anesth Reanim. 2009 Jan;28(1):61-73. doi: 10.1016/j.annfar.2008.09.022. Epub 2008 Dec 18.

[Mastocytosis and anaesthesia].

[Article in French]

Dewachter P, Mouton-Faivre C, Cazalaà JB, Carli P, Lortholary O, Hermine O.

Service d'anesthésie-réanimation chirurgicale, Samu de Paris, hôpital Necker-Enfants-Malades, AP-HP, université Paris-Descartes, 149, rue de Sèvres, 75443 Paris, France. pascale.dewachter@nck.aphp.fr

Mastocytosis are characterized by an accumulation of abnormal mast cells in various tissues. Their incidence is estimated at 1/150,000 patients. Pure cutaneous mastocytosis which are mainly observed during childhood may resolve spontaneously during adolescence, whereas systemic mastocytosis involving one or more organs or tissues are more observed in adults. The initial event leading to mastocytosis is believed to be related to activating mutations in c-kit receptor, thus resulting in increased proliferation of mast cells precursors, migration in various tissues and degranulation leading to clinical signs. This nosologic entity does not belong to allergic diseases. Their perioperative management involves a multidisciplinary approach. The degranulation of mast cells with subsequent clinical symptoms can be triggered by psychological, chemical, traumatic, physical (rubbing, extreme temperatures...) agents. Avoiding these triggers should be realized whenever possible according to each patient. The premedication has not proven its efficiency. Tryptase measurement is part of the

preoperative biological assessment. The clinical signs severity is related to the cardiovascular homeostasis disturbances (arterial hypotension, cardiovascular collapse, cardiac arrest). The cardiovascular symptoms do not correlate to the cutaneous versus systemic description of the disease. The drug of choice for the treatment of the severe cardiovascular signs is epinephrine associated to vascular loading. The aim of this literature review is to suggest the different modalities of perioperative care of patients with mastocytosis.

PMID: 19097849 [PubMed - indexed for MEDLINE]

614. FASEB J. 2009 Apr;23(4):1262-71. doi: 10.1096/fj.08-124248. Epub 2008 Dec 16.

Macrophage migration inhibitory factor is critical to interleukin-5-driven eosinophilopoiesis and tissue eosinophilia triggered by Schistosoma mansoni infection.

Magalhães ES, Paiva CN, Souza HS, Pyrrho AS, Mourão-Sá D, Figueiredo RT, Vieira-de-Abreu A, Dutra HS, Silveira MS, Gaspar-Elsas MI, Xavier-Elsas P, Bozza PT, Bozza MT.

Departamento de Imunologia, Instituto de Microbiologia, Universidade Federal do Rio de Janeiro, Rio de Janeiro, RJ, Brazil.

Macrophage migration inhibitory factor (MIF) participates in the pathogenesis of inflammatory diseases, including asthma, in which it enhances airway hypersensitivity and tissue eosinophilia. Herein, we investigated the role of MIF in eosinophilopoiesis and tissue eosinophilia using Schistosoma mansoni infection. MIF-deficient (Mif(-/-)) mice had similar numbers of adult worms, eggs, and granulomas compared to wild-type mice, but the size of granulomas was strikingly reduced due to smaller numbers of eosinophils. MIF did not affect the acquired response to infection, as Mif(-/-) mice produced normal amounts of Th2 cytokines and IgE. Nevertheless, recombinant MIF (rMIF) behaved as a chemoattractant for eosinophils, what could partially explain the reduced eosinophilia in infected Mif(-/-) mice. Moreover, the percentage of eosinophils was reduced in bone marrows of Mif(-/-) mice chronically infected with S. mansoni compared to wild type. Mif(-/-) had impaired eosinophilopoiesis in response to interleukin (IL)-5 and addition of rMIF to bone marrow cultures from IL-5 transgenic mice enhanced the generation of eosinophils. In the absence of MIF, eosinophil precursors were unable to survive the IL-5-supplemented cell culture, and were ingested by macrophages. Treatment with pancaspase inhibitor z-VAD or rMIF promoted the survival of eosinophil progenitors. Together, these results indicate that MIF participates in IL-5-driven maturation of eosinophils and in tissue eosinophilia associated with S. mansoni infection.

PMID: 19088181 [PubMed - indexed for MEDLINE]

615. J Allergy Clin Immunol. 2009 Feb;123(2):376-84. doi: 10.1016/j.jaci.2008.10.048. Epub 2008 Dec 10.

Fibrocyte localization to the airway smooth muscle is a feature of asthma.

Saunders R, Siddiqui S, Kaur D, Doe C, Sutcliffe A, Hollins F, Bradding P, Wardlaw A, Brightling CE.

Institute for Lung Health, Department of Infection, Immunity and Inflammation, University of Leicester, Leicester, United Kingdom.

BACKGROUND: Airway smooth muscle (ASM) hyperplasia is a hallmark of asthma that

is associated with disease severity and persistent airflow obstruction.
OBJECTIVES: We sought to investigate whether fibrocytes, a population of peripheral blood mesenchymal progenitors, are recruited to the ASM compartment in asthma.
METHODS: We assessed the number of fibrocytes in bronchial biopsy specimens and peripheral blood from subjects with mild-to-severe refractory asthma versus healthy control subjects. In vitro we investigated potential mechanisms controlling fibrocyte migration toward the ASM bundle.
RESULTS: Fifty-one subjects with asthma and 33 control subjects were studied. In bronchial biopsy specimens, the number of fibrocytes was increased in the lamina propria of subjects with severe refractory asthma (median [interquartile range] number, 1.9/mm(2) [1.7/mm(2)]) versus healthy control subjects (median [interquartile range] number, 0/mm(2) [0.3/mm(2)], P < .0001) and in the ASM bundle of subjects with asthma of all severities (subjects with severe asthma, median [interquartile range] number, 3.8/mm(2) [9.4/mm(2)]; subjects with mild-to-moderate asthma, median [interquartile range] number, 1.1/mm(2) [2.4/mm(2)]); healthy control subjects, (median [interquartile range] number, 0/mm(2) [0/mm(2)]); P = .0004). In the peripheral blood the fibrocyte number was also increased in subjects with severe refractory asthma (median [interquartile range] number, 1.4 x 10(4)/mL [2.6 x 10(4)/mL]) versus healthy control subjects (median [interquartile range] number, 0.4 x 10(4)/mL [1.0 x 10(4)/mL], P = .002). We identified that in vitro ASM promotes fibrocyte chemotaxis and chemokinesis (distance of migration after 4.5 hours, 31 microm [2.9 microm] vs 17 microm [2.4 microm], P = .0001), which was in part mediated by platelet-derived growth factor (mean inhibition by neutralizing antibody, 16% [95% CI, 2% to 32%], P = .03) but not by activation of chemokine receptors.
CONCLUSION: This study provides the first evidence that fibrocytes are present in the ASM compartment in asthma and that ASM can augment fibrocyte migration. The importance of fibrocytes in the development of ASM hyperplasia and airway dysfunction in asthma remains to be determined.

PMID: 19081612 [PubMed - indexed for MEDLINE]

616. Clin Exp Immunol. 2009 Jan;155(1):107-16. doi: 10.1111/j.1365-2249.2008.03800.x.

An investigation of the impact of the location and timing of antigen-specific T cell division on airways inflammation.

Hutchison S, Choo-Kang BS, Gibson VB, Bundick RV, Leishman AJ, Brewer JM, McInnes IB, Garside P.

Centre for Biophotonics, Strathclyde Institute of Pharmacy and Biomedical Sciences, University of Strathclyde, Glasgow, UK. s.hutchison@strath.ac.uk

It is widely accepted that allergic asthma is orchestrated by T helper type 2 lymphocytes specific for inhaled allergen. However, it remains unclear where and when T cell activation and division occurs after allergen challenge, and whether these factors have a significant impact on airways inflammation. We therefore employed a CD4-T cell receptor transgenic adoptive transfer model in conjunction with laser scanning cytometry to characterize the location and timing of T cell division in asthma in vivo. Thus, for the first time we have directly assessed the division of antigen-specific T cells in situ. We found that accumulation of divided antigen-specific T cells in the lungs appeared to occur in two waves. The first very early wave was apparent before dividing T cells could be detected in the lymph node (LN) and coincided with neutrophil influx. The second wave of divided T cells accumulating in lung followed the appearance of these cells in LN and coincided with peak eosinophilia. Furthermore, accumulation of antigen-specific T cells in the draining LN and lung tissue, together with accompanying pathology, was reduced by intervention with the sphingosine

1-phosphate receptor agonist FTY720 2 days after challenge. These findings provide greater insight into the timing and location of antigen-specific T cell division in airways inflammation, indicate that distinct phases and locations of antigen presentation may be associated with different aspects of pathology and that therapeutics targeted against leukocyte migration may be useful in these conditions.

PMCID: PMC2665686
PMID: 19076834 [PubMed - indexed for MEDLINE]

617. Allergy. 2009 Jan;64(1):5-17. doi: 10.1111/j.1398-9995.2008.01902.x. Epub 2008 Nov 28.

Asthma in Latin America: a public heath challenge and research opportunity.

Cooper PJ, Rodrigues LC, Cruz AA, Barreto ML.

Centro de Investigaciones FEPIS, Quininde, Esmeraldas Province Ecuador.

Asthma has emerged as an important public health problem in many Latin American countries over the past decade. In Brazil and Costa Rica, the prevalence of asthma and associated morbidity is as great or greater as reported in traditional high prevalence countries such as the US, but remains neglected as a public health priority. Asthma in Latin America is associated particularly with underprivileged populations living in cities but remains relatively rare in many rural populations. The causes of asthma in Latin America are likely to be associated with urbanization, migration, and the adoption of a modern 'Westernized' lifestyle and environmental changes that follow these processes that include changes in diet, physical activity, hygiene, and exposures to allergens, irritants, and outdoor and indoor pollutants. Because of the enormous social, genetic, and environmental contrasts within and between Latin American countries, and the large differences in prevalence associated with these differences, the investigation of asthma in Latin America provides important research opportunities to identify the social and biological mechanisms that underlie asthma development. Asthma in Latin America poses enormous challenges for health policy makers, health services, and researchers to respond to and alleviate the growing burden of asthma disability, particularly among marginalized urban populations.

PMID: 19076533 [PubMed - indexed for MEDLINE]

618. Recent Pat Inflamm Allergy Drug Discov. 2008 Jan;2(1):1-10.

Isoform selective phosphoinositide 3-kinase gamma and delta inhibitors and their therapeutic potential.

Ghigo A, Hirsch E.

Molecular Biotechnology Center, University of Torino, Torino, Italy. galessandra@interfree.it

Phosphoinositide 3-kinases (PI3Ks) represent a family of dual specificity kinases that by acting as both lipid and protein kinases regulate numerous biological processes, including cell growth, differentiation, survival, proliferation, migration and metabolism. The availability of genetically modified mice has recently allowed the functional characterization of class I PI3Ks, which are the most well studied PI3Ks. Whereas PI3Kalpha and PI3Kbeta are ubiquitously expressed, PI3Kdelta and PI3Kgamma are mainly restricted to leukocytes and

represent key modulators of innate and adaptive immune responses. Therefore, PI3Kdelta and PI3Kgamma have become attractive drug targets for the treatment of disorders of both innate and adaptive immune system, causing inflammatory and allergic diseases. The lack of specificity, isoform selectivity and biopharmaceutical properties of the initially available pharmacological inhibitors have provided impetus to the development of novel compounds that, by exhibiting improved isoform selectivity, potency and pharmacokinetic profile, might be more safely employed. Here, we describe recently published patent specifications disclosing new PI3K inhibitors, with a main focus on compounds displaying some selectivity for PI3Kdelta and gamma isoforms and their potential therapeutic applications.

PMID: 19075988 [PubMed - indexed for MEDLINE]

619. Pediatr Allergy Immunol. 2009 Sep;20(6):571-7. doi: 10.1111/j.1399-3038.2008.00829.x. Epub 2008 Dec 5.

Allergic rhinitis in children: incidence and treatment in Dutch general practice in 1987 and 2001.

de Bot CM, Moed H, Schellevis FG, de Groot H, van Wijk RG, van der Wouden JC.

Department of General Practice, Erasmus MC - University Medical Center Rotterdam, 3000 CA Rotterdam, The Netherlands. c.debot@erasmusmc.nl

Allergic rhinitis is a common chronic disorder in children, mostly diagnosed in primary health care. This study investigated the national incidence and treatment of allergic rhinitis among children aged 0-17 yr in Dutch general practice in 1987 and 2001 to establish whether changes have occurred. A comparison was made with data from the first (1987) and second (2001) Dutch national surveys of general practice on children aged 0-17 yr. Incidence rates were compared by age, sex, level of urbanization and season. The management of the general practitioner was assessed regarding drug prescriptions and referrals to medical specialists, and compared with the clinical guideline issued in 1996. The incidence rate of allergic rhinitis increased from 6.6 (1987) to 9.2 (2001) per 1000 person-years. We found a male predominance with a switch in adolescence to a female predominance at both time points. The increase in incidence was the highest in rural (<30,000 inhabitants) and suburban areas (30,000-50,000 inhabitants). Compared to 1987, there was a significant increase in incidence in the central part of the Netherlands in 2001. In both years, the incidence was higher in spring compared with the other seasons. In 2001, children of natives and western immigrants visited the general practitioner more often with complaints of allergic rhinitis compared to 1987. In 1987, prescribed medication consisted mainly of nasal corticosteroids (36%) and in 2001 of oral antihistamines (45%). Although a clinical guideline was not issued until 1996, overall, the treatment of allergic rhinitis by general practitioners was in both years in accordance with the current clinical guideline, but with a stronger adherence in 2001. The results show an increased incidence in the past decades of allergic rhinitis in children in Dutch general practice. The shift to a smaller spectrum of prescriptions in 2001 may be a result of the 1996 clinical guideline.

PMID: 19067886 [PubMed - indexed for MEDLINE]

620. Ann Agric Environ Med. 2008 Dec;15(2):317-22.

Respiratory disorders in two workers of customs depositories occupationally exposed to mouldy tobacco.

Mackiewicz B, Skórska C, Krysińska-Traczyk E, Larsson L, Szponar B, Milanowski J, Czekajska-Chehab E, Sitkowska J, Cholewa G, Szymańska J, Dutkiewicz J.

Department of Pneumonology, Oncology and Allergology, Medical University of Lublin, Jaczewskiego 8, Lublin, Poland. b.mack@wp.pl

Work-related respiratory symptoms, including dyspnoea, cough, fever, tiredness and malaise, were recorded in two customs officers employed in 2 depositories of confiscated cigarettes, of which one showed signs of dampness. Microbiological sampling of the air and the cigarettes stored in a damp depository revealed the presence of potentially pathogenic fungi and bacteria and the biochemical markers of bacterial lipopolysaccharide and fungal biomass. The Penicillium species (P. simplicissimum, P. inflatum, P. commune) dominated in the damp depository, while in the other one Aspergillus fumigatus was prevalent. The patients under study did not show a specific sensitization to microbial allergens in the precipitin test, the test for inhibition of leukocyte migration and the bronchial provocation challenge, except for a weak reaction to fungal allergens in the test for inhibition of leukocyte migration. Moreover, one patient responded with subjective symptoms after exposure to inhalation of increased doses of Penicillium simplicissimum antigen. Both cases were diagnosed as a specific form of organic dust toxic syndrome (ODTS). It is hypothesized that the symptoms were evoked most probably by the non-specific action of low molecular fungal metabolites, such as mycotoxins or VOCs (volatile organic compounds), with the possible contribution of bacterial endotoxin. However, as there is no a direct proof to support this presumption, and the effects of nicotine and other tobacco constituents cannot be excluded, further studies are needed to elucidate etiopathogenesis of the disorders associated with the exposure to stored tobacco.

PMID: 19061269 [PubMed - indexed for MEDLINE]

621. Pharmacol Ther. 2009 Feb;121(2):174-84. doi: 10.1016/j.pharmthera.2008.10.008. Epub 2008 Nov 21.

Eosinophil progenitors in allergy and asthma - do they matter?

Rådinger M, Lötvall J.

Mast Cell Biology Section, Laboratory of Allergic Diseases, National Institute of Allergy and Infectious Diseases, National Institutes of Health, Bethesda, Maryland, USA. radingerm@niaid.nih.gov

Allergic inflammation is associated with marked infiltration of eosinophils in affected tissues. The eosinophil is believed to be a key effector cells in allergen induced asthma pathogenesis. However, the role of eosinophils in the clinical manifestation of asthma has recently been questioned, since therapies directed against eosinophil infiltration (i.e. anti-interleukin-5) failed to improve clinical symptoms such as airways hyper-responsiveness (AHR) in patients with asthma. Although eosinophils in peripheral blood and the airways were largely depleted after anti-IL-5 treatment, residual eosinophilia in lung tissue persisted, which permits speculation that the remaining eosinophils may be sufficient to drive the asthma symptomatology. Furthermore, recent findings suggest that primitive eosinophil progenitor cells traffic from the bone marrow to sites of inflammation in response to allergen exposure. These progenitors may then differentiate in situ and thus provide an ongoing supply of mature pro-inflammatory cells and secretory mediators that augment the inflammatory response. In the present article, we will review the evidence for these findings, and discuss the rationale for targeting hematopoiesis and their migration pathways in the treatment of allergic diseases. Furthermore, this review will highlight the hypothesis that both IL-5- and CCR3-mediated signaling pathways may

need to be targeted in order to control the inflammation and AHR associated with asthma.

PMID: 19059433 [PubMed - indexed for MEDLINE]

622. Exp Dermatol. 2009 Apr;18(4):409-13. doi: 10.1111/j.1600-0625.2008.00803.x. Epub 2009 Oct 23.

The human skin/chick chorioallantoic membrane model accurately predicts the potency of cosmetic allergens.

Slodownik D, Grinberg I, Spira RM, Skornik Y, Goldstein RS.

Department of Dermatology, Hadassah-Hebrew University Medical Center, Jerusalem, Israel. gbds@netvision.net.il

The current standard method for predicting contact allergenicity is the murine local lymph node assay (LLNA). Public objection to the use of animals in testing of cosmetics makes the development of a system that does not use sentient animals highly desirable. The chorioallantoic membrane (CAM) of the chick egg has been extensively used for the growth of normal and transformed mammalian tissues. The CAM is not innervated, and embryos are sacrificed before the development of pain perception. The aim of this study was to determine whether the sensitization phase of contact dermatitis to known cosmetic allergens can be quantified using CAM-engrafted human skin and how these results compare with published EC3 data obtained with the LLNA. We studied six common molecules used in allergen testing and quantified migration of epidermal Langerhans cells (LC) as a measure of their allergic potency. All agents with known allergic potential induced statistically significant migration of LC. The data obtained correlated well with published data for these allergens generated using the LLNA test. The human-skin CAM model therefore has great potential as an inexpensive, non-radioactive, in vivo alternative to the LLNA, which does not require the use of sentient animals. In addition, this system has the advantage of testing the allergic response of human, rather than animal skin.

PMID: 19054059 [PubMed - indexed for MEDLINE]

623. Respir Res. 2008 Dec 1;9:76. doi: 10.1186/1465-9921-9-76.

The effects of interleukin-8 on airway smooth muscle contraction in cystic fibrosis.

Govindaraju V, Michoud MC, Ferraro P, Arkinson J, Safka K, Valderrama-Carvajal H, Martin JG.

Seymour Heisler Laboratory of the Montreal Chest Institute Research Center and Meakins Christie Laboratories, McGill University, Montreal, Quebec, Canada. Vasanthi.govindaraju@mcGill.ca

BACKGROUND: Many cystic fibrosis (CF) patients display airway hyperresponsiveness and have symptoms of asthma such as cough, wheezing and reversible airway obstruction. Chronic airway bacterial colonization, associated with neutrophilic inflammation and high levels of interleukin-8 (IL-8) is also a common occurrence in these patients. The aim of this work was to determine the responsiveness of airway smooth muscle to IL-8 in CF patients compared to non-CF individuals.
METHODS: Experiments were conducted on cultured ASM cells harvested from subjects with and without CF (control subjects). Cells from the 2nd to 5th passage were studied. Expression of the IL-8 receptors CXCR1 and CXCR2 was assessed by flow

cytometry. The cell response to IL-8 was determined by measuring intracellular calcium concentration ([Ca2+](i)), cell contraction, migration and proliferation. RESULTS: The IL-8 receptors CXCR1 and CXCR2 were expressed in both non-CF and CF ASM cells to a comparable extent. IL-8 (100 nM) induced a peak Ca2+ release that was higher in control than in CF cells: 228 +/- 7 versus 198 +/- 10 nM (p < 0.05). IL-8 induced contraction was greater in CF cells compared to control. Furthermore, IL-8 exposure resulted in greater phosphorylation of myosin light chain (MLC20) in CF than in control cells. In addition, MLC20 expression was also increased in CF cells. Exposure to IL-8 induced migration and proliferation of both groups of ASM cells but was not different between CF and non-CF cells. CONCLUSION: ASM cells of CF patients are more contractile to IL-8 than non-CF ASM cells. This enhanced contractility may be due to an increase in the amount of contractile protein MLC20. Higher expression of MLC20 by CF cells could contribute to airway hyperresponsiveness to IL-8 in CF patients.

PMCID: PMC2633308
PMID: 19046427 [PubMed - indexed for MEDLINE]

624. Altern Ther Health Med. 2008 Nov-Dec;14(6):46-53.

A possible central mechanism in autism spectrum disorders, part 1.

Blaylock RL.

Belhaven College, Jackson, Mississippi, USA.

The autism spectrum disorders (ASD) are a group of related neurodevelopmental disorders that have been increasing in incidence since the 1980s. Despite a considerable amount of data being collected from cases, a central mechanism has not been offered. A careful review of ASD cases discloses a number of events that adhere to an immunoexcitotoxic mechanism. This mechanism explains the link between excessive vaccination, use of aluminum and ethylmercury as vaccine adjuvants, food allergies, gut dysbiosis, and abnormal formation of the developing brain. It has now been shown that chronic microglial activation is present in autistic brains from age 5 years to age 44 years. A considerable amount of evidence, both experimental and clinical, indicates that repeated microglial activation can initiate priming of the microglia and that subsequent stimulation can produce an exaggerated microglial response that can be prolonged. It is also known that one phenotypic form of microglia activation can result in an outpouring of neurotoxic levels of the excitotoxins, glutamate and quinolinic acid. Studies have shown that careful control of brain glutamate levels is essential to brain pathway development and that excesses can result in arrest of neural migration, as well as dendritic and synaptic loss. It has also been shown that certain cytokines, such as TNF-alpha, can, via its receptor, interact with glutamate receptors to enhance the neurotoxic reaction. To describe this interaction I have coined the term immunoexcitotoxicity, which is described in this article.

PMID: 19043938 [PubMed - indexed for MEDLINE]

625. Exp Dermatol. 2009 Jan;18(1):88-90. doi: 10.1111/j.1600-0625.2008.00796.x. Epub 2008 Nov 19.

Transcellular migration of neutrophils is a quantitatively significant pathway across dermal microvascular endothelial cells.

Marmon S, Cammer M, Raine CS, Lisanti MP.

Neutrophil extravasation is central to inflammatory skin diseases like psoriasis and atopic dermatitis. In vivo, neutrophils have been shown to migrate through cell-to-cell junctions (paracellular pathway) or directly through the body of the endothelial cell (transcellular pathway). In vitro, however, neutrophil migration is a largely paracellular process where cells preferentially cross at tricellular corners devoid of tight junctions. To approximate the type of cells encountered by extravasating neutrophils in vivo, we developed a neutrophil-migration assay using primary human dermal microvascular endothelial cells. We show here that a large proportion of migrating neutrophils traverse a monolayer of microvascular endothelium using a purely transcellular pathway. In addition, we demonstrate that F-actin is rearranged similarly in neutrophils undergoing diapedesis along either route. This in vitro model closely simulates the physiological process of neutrophil extravasation in vivo and can be further utilized to evaluate the relative contribution of distinct migratory pathways to the pathophysiology of inflammatory skin disease.

PMID: 19040450 [PubMed - indexed for MEDLINE]

626. Ann Dermatol Venereol. 2008 Dec;135 Suppl 7:S326-34. doi: 10.1016/S0151-9638(08)75485-1.

[What's new in dermatological research?].

[Article in French]

Humbert P.

Service de Dermatologie, CHU Saint-Jacques, Université de Franche-Comté, INSERM U645, Besançon, France. philippe.humbert@univ-fcomte.fr

Medical literature is rich with new and relevant information, resulting from basic or applied research. Some strong arguments are presented in this document. Firstly, the discovery and role of a virus, the polyomavirus, in the development of Merkel tumours. It is a small virus with double bit DNA strand, coding for a oncoprotein. If the polyomavirus plays a causal role in the tumorigenesis, it acts by various mechanisms. The micro-RNAs represent an abundant class of small RNA not coding for proteins, but which control the gene expression coding for proteins on a post-transcriptional level. The first obvious sign of the role of the micro-RNAs in the inflammatory dermatoses appeared recently, in particular when these micro-RNAs associated with psoriasis and atopic dermatitis were identified through a broad genomic analysis of the expression of these micro-RNAs. A new giant virus strain sheltering another unknown tiny virus to date has just been discovered. This virus infinitely small called Sputnik enables to deteriorate a much larger virus baptized Mama, at the point of preventing it to manufacture normal viral particles and also preventing it from reproducing. This discovery raises a crucial question: Is Sputnik a new system of transfer of genes of a species of one virus to another? A group of blood cells expressing E-cadherin, the dot cells, found in the fetal blood of the dermis, contributes to tissue repair through the mechanisms of cellular differentiation and their action allows healing without scar. CD4+ T helper lymphocytes producing interleukin 17 (IL17) play a pathogenic part in atopic dermatitis. The genes of the beta defensins could be involved in the genetic susceptibility of the psoriatic disease. The autoimmune origin of the alopecia areata is supported by a great number of observations, the role of neuropeptides in the initiation of the autoimmunity during alopecia areata has just been demonstrated. The dendritic cells are cells presenting antigens which play a crucial role in the adaptive immunological response. It was shown that activation of the proliferation of the lymphocytes T after the migration of dendritic cells on the level of the lymphatic ganglion depended not on Langerhans cell, but of the dendritic cell. A

new way appears to control the autoimmunity in the psoriasis and involves the plasmacytoid dendritic cells which are sensitized with the DNA itself when it is coupled with an antibacterial peptide. Mast cells express cathelicidin, which acts like an antibiotic with broad spectrum and influences the defence system of the epitheliums. We have perhaps found a new therapeutic target for rosacea by disclosing high rates of cathelicidin and a series of associated proteases in skin lesions. The sebocytes express antibacterial functional peptides deriving from cathelicidin which can have a bactericidal effect against P. Acnes. A vast genomic study in the androgenetic alopecia highlighted the existence of new loci localized on the 20p11 chromosome, associated with the risk of androgenetic alopecia. New alleles to determine the color of hair and the cutaneous pigmentation were identified. Two loci (IRF 4 and SLC24A4) are highly associated with the color of hair, like three other areas. The blue color of the eyes could be due to a change of an element located in gene HERC2 preventing of the expression of OCA2. Thus, many fields of dermatology were the object of research which opens new prospects for diagnosis and treatment.

PMID: 19264208 [PubMed - indexed for MEDLINE]

627. Acta Paediatr. 2009 Mar;98(3):573-8. doi: 10.1111/j.1651-2227.2008.01130.x. Epub 2008 Nov 22.

Disease patterns of outpatient visits by Japanese expatriate children in Thailand.

Sakai R, Wongkhomthong SA, Marui E.

Department of Public Health, Juntendo University School of Medicine, Tokyo, Japan. riesakai@juntendo.ac.jp

AIM: To clarify the health-related conditions of Japanese expatriate children in Thailand.
METHODS: Records of Japanese children who consulted outpatient clinics at Bangkok Hospital in 2005 and 2006 (n = 2141) were analysed, and then compared with data from the patient survey conducted by the Ministry of Health, Labour and Welfare of Japan in 2005 (n = 575 400).
RESULTS: 'diseases of the respiratory system', categorized as chapter X under ICD-10 was the most frequent category in both Thailand and Japan. Although 'certain infectious and parasitic diseases' (chapter I) was the second most frequent category in Thailand, it was infrequent in Japan. In the subcategories of 'diseases of the respiratory system', 'acute upper respiratory infections' was frequent and asthma was infrequent in Thailand. Conversely, 'acute upper respiratory infections' showed a low percentage and asthma was the most frequently observed disease in Japan.
CONCLUSION: This study examined Japanese children having the same genetic background but divided into two groups according to different living environments. Results demonstrate that children living in Japan contract asthma more frequently than infectious diseases, whereas those living in Thailand show the opposite trend, which supports the hygiene hypothesis that infections may protect against the development of allergic diseases, such as asthma.

PMID: 19038014 [PubMed - indexed for MEDLINE]

628. Proc Natl Acad Sci U S A. 2008 Dec 9;105(49):19402-7. doi: 10.1073/pnas.0810035105. Epub 2008 Nov 26.

CD4 cell-secreted, posttranslationally modified cytokine GIF suppresses Th2 responses by inhibiting the initiation of IL-4 production.

Kim-Saijo M, Janssen EM, Sugie K.

Division of Developmental Immunology, La Jolla Institute for Allergy and Immunology, La Jolla, CA 92037, USA.

T helper 2 (Th2) cells are critical to the induction of IgE antibody and allergic inflammation, but how the pathological pathways are controlled in nonallergic individuals remains unclear. Here we report that glycosylation-inhibiting factor (GIF) suppresses Th2 effector generation. GIF is a cytokine encoded by the same gene that codes for macrophage migration inhibitory factor (MIF). GIF-deficient mice demonstrated enhanced T-dependent antibody formation especially of IgE isotype and allergic airway inflammation with the generation of regulatory T cells unaffected. GIF-deficient macrophages and dendritic cells revealed normal responsiveness to toll-like receptor (TLR) ligands. GIF undergoes a unique posttranslational modification, cysteinylation. The modified GIF, mainly secreted by activated T cells derived from CD4(+)CD25(-) cells, inhibited IL-4 production by the same cells whereas the unmodified GIF showed no effect. Bone marrow chimera experiment demonstrated that T cell-derived GIF suppressed the generation of Th effectors that secrete IL-4. During the first 24 h of CD3/CD28 stimulation in vitro, GIF secreted from naïve CD4 cells acted on the same cells, maintained nuclear factor of activated T cells (NFAT)c2 in the nucleus, and repressed IL-4 mRNA levels. Thus, GIF represents a self-regulatory mechanism of Th2 cell generation from naïve CD4 cells, in which the posttranslational modification plays a crucial role.

PMCID: PMC2614773
PMID: 19036925 [PubMed - indexed for MEDLINE]

629. Pulm Pharmacol Ther. 2009 Oct;22(5):446-54. doi: 10.1016/j.pupt.2008.10.006. Epub 2008 Oct 31.

The effect of asthma therapeutics on signalling and transcriptional regulation of airway smooth muscle function.

Ammit AJ, Burgess JK, Hirst SJ, Hughes JM, Kaur M, Lau JY, Zuyderduyn S.

Respiratory Research Group, Faculty of Pharmacy, University of Sydney, NSW, Australia. ajammit@pharm.usyd.edu.au

SCOPE OF THE REVIEW: Our knowledge of the multifunctional nature of airway smooth muscle (ASM) has expanded rapidly in the last decade, but the underlying molecular mechanisms and how current therapies for obstructive airway diseases, such as asthma and chronic obstructive pulmonary disease (COPD), affect these are still being elucidated. Our current knowledge has built on the pharmacology of human ASM contraction and relaxation established prior to that and which is reviewed in detail elsewhere in this issue. The advent of methods to isolate and culture ASM cells, especially human ASM cells, has made it possible to study how they may contribute to airway remodelling through their synthetic, proliferative, and migratory capacities. Now the underlying molecular mechanisms of ASM growth factor secretion, extracellular matrix (ECM) production, proliferation and migration, as well as contraction and relaxation, are being determined. A complex network of signalling pathways leading to gene transcription in ASM cells permits this functional plasticity in healthy and diseased airways. This review is an overview of the effects of current therapies, and some of those in development, on key signalling pathways and transcription factors involved in these ASM functions.

PMID: 19022391 [PubMed - indexed for MEDLINE]

630. J Endocrinol. 2008 Dec;199(3):417-24. doi: 10.1677/JOE-08-0110.

Complement C5a receptors in the pituitary gland: expression and function.

Francis K, Lewis BM, Monk PN, Ham J.

Centre for Endocrine and Diabetes Sciences, Cardiff University, Cardiff, CF14 4XN, UK.

Communication between the immune and endocrine system is important for the control of inflammation that is primarily mediated through the hypothalamic-pituitary-adrenal axis. The innate immune system rapidly responds to pathogens by releasing complement proteins that include the anaphylatoxins C3a and C5a. We previously reported the existence of C3a receptors in the anterior pituitary gland and now describe the presence of C5a receptors in the gland. C5a and its less active derivative (C5adR) can bind to its own receptor and to another receptor called C5L2. Using RT-PCR and immunocytochemistry, C5a receptors and C5L2 were demonstrated in the rat anterior pituitary gland and in several rodent anterior pituitary cell lines. Western blotting analysis showed that C5a stimulated the phosphorylation of MAPK and AKT but not p38; C5adR on the other hand, had no effect on any of the signal molecules investigated. The effects of C5a and C5adR on the secretion of the inflammatory molecule, macrophage migration inhibitory factor (MIF) were investigated by ELISA. Both compounds showed a dose-dependent inhibition of MIF release, 30-40% inhibition at around 35-70 nM agonist with IC50 values of around 20 nM. C5a and C5adR also stimulated ACTH secretion (up to 25%) from AtT-20DV16 cells. These data show that functional C5a receptors (C5a and C5L2) are present in the anterior pituitary gland and they may play a role in dampening down inflammation by inhibiting the release of MIF and stimulating the release of ACTH.

PMID: 19020281 [PubMed - indexed for MEDLINE]

631. CMAJ. 2008 Nov 18;179(11):1133-42. doi: 10.1503/cmaj.071797.

Prevalence of asthma among Chinese adolescents living in Canada and in China.

Wang HY, Wong GW, Chen YZ, Ferguson AC, Greene JM, Ma Y, Zhong NS, Lai CK, Sears MR.

Guangzhou Institute of Respiratory Diseases, First Affiliated Hospital of Guangzhou Medical College, Guangzhou, China.

BACKGROUND: Studies of the prevalence of asthma among migrating populations may help in identifying environmental risk factors.
METHODS: We analyzed data from Vancouver, Canada, and from Guangzhou, Beijing and Hong Kong, China, collected during phase 3 of the International Study of Asthma and Allergies in Childhood. We subdivided the Vancouver adolescents according to whether they were Chinese immigrants to Canada, Canadian-born Chinese or Canadian-born non-Chinese. We compared the prevalence of asthma and wheezing among Chinese adolescents born in Canada, Chinese adolescents who had immigrated to Canada and Chinese adolescents living in China.
RESULTS: Of 7794 Chinese adolescents who met the inclusion criteria, 3058 were from Guangzhou, 2824 were from Beijing, and 1912 were from Hong Kong. Of 2235 adolescents in Vancouver, Canada, 475 were Chinese immigrants, 617 were Canadian-born Chinese, and 1143 were Canadian-born non-Chinese. The prevalence of current wheezing among boys ranged from 5.9% in Guangzhou to 11.2% in Canadian-born Chinese adolescents. For girls, the range was 4.3% in Guangzhou to

9.8% in Canadian-born Chinese adolescents. The prevalence of ever having had asthma ranged from 6.6% to 16.6% for boys and from 2.9% to 15.0% for girls. Prevalence gradients persisted after adjustment for other environmental variables (odds ratios for ever having had asthma among Canadian-born Chinese compared with native Chinese in Guangzhou: 2.72 [95% confidence interval 1.75-4.23] for boys and 5.50 [95% confidence interval 3.21-9.44] for girls; p < 0.001 for both). Among Chinese adolescents living in Vancouver, the prevalence of ever wheezing increased with duration of residence, from 14.5% among those living in Canada for less than 7 years to 20.9% among those living their entire life in Canada. The same pattern was observed for the prevalence of ever having had asthma, from 7.7% to 15.9%.
INTERPRETATION: Asthma symptoms in Chinese adolescents were lowest among residents of mainland China, were greater for those in Hong Kong and those who had immigrated to Canada, and were highest among those born in Canada. These findings suggest that environmental factors and duration of exposure influence asthma prevalence.

PMCID: PMC2582762
PMID: 19015564 [PubMed - indexed for MEDLINE]

632. J Allergy Clin Immunol. 2008 Oct;122(4):700-9. doi: 10.1016/j.jaci.2008.07.048.

New insights into mechanisms of immunoregulation in 2007.

Akdis CA.

Swiss Institute of Allergy and Asthma Research (SIAF), Davos, Switzerland.
akdisac@siaf.uzh.ch

Substantial progress in understanding the mechanisms of immune regulation in allergic diseases and asthma has been made during the last year. In asthma, rhinitis, and atopic dermatitis the immune system is activated by allergens, autoantigens, and components of superimposed infectious agents. Immune regulation in the lymphatic organs and in the tissue has an important role in the control and suppression of allergic disease in all stages of the inflammatory process, such as cell migration to tissues, cells gaining an inflammatory and tissue-destructive phenotype in the tissues, and their interaction with resident tissue cells to augment the inflammation. After the discovery of regulatory T cells, the importance of their unique suppressive capacity was strongly emphasized for the suppression of effector T-cell responses. However, it seems that all 3 subsets of effector T(H)1, T(H)2, and T(H)17 cells, as well as regulatory T cells, regulate each other at the level of transcription, major cytokines, and surface molecules. This review highlights key advances in immune regulation that were published in the Journal of Allergy and Clinical Immunology.

PMID: 19014761 [PubMed - indexed for MEDLINE]

633. Acta Derm Venereol. 2008;88(6):566-72. doi: 10.2340/00015555-0550.

T-cell receptor excision circles (TREC) in CD4+ and CD8+ T-cell subpopulations in atopic dermatitis and psoriasis show major differences in the emission of recent thymic emigrants.

Just HL, Deleuran M, Vestergaard C, Deleuran B, Thestrup-Pedersen K.

Department of Dermatology, Aarhus University Hospital, Aarhus, Denmark.

We used T-cell receptor excision circles (TREC) to evaluate thymic function in

adult patients with atopic dermatitis and psoriasis. We observed that men, but not women, with atopic dermatitis had a significantly faster decline in TREC content with increasing age compared with healthy men. In contrast, both men and women with psoriasis had significantly reduced TREC levels, which were, on average, only 30% of that of healthy persons. In atopic dermatitis the levels of TREC declined with increasing levels of IgE, disease intensity and extent of eczema. Furthermore, patients with atopic dermatitis showed signs of altered thymus function, as they had a significantly greater variation in TREC content measured over time than healthy controls, especially within the CD8+ T-cell subpopulation. Because both atopic dermatitis and psoriasis patients have an increased number of T-cells, this indicates that atopic dermatitis patients can have compensatory emissions of thymic emigrants, whereas psoriatic patients do not, thus supporting different thymic function in these two diseases.

PMID: 19002340 [PubMed - indexed for MEDLINE]

634. Curr Med Chem. 2008;15(27):2876-85.

RhoA/Rho-kinase as a therapeutic target in asthma.

Kume H.

Department of Respiratory Medicine, Nagoya University Graduate School of Medicine, 65 Tsurumai-cho, Showa-ku, Nagoya, Japan. hkume@med.nagoya-u.ac.jp

Rho-kinase is an effector molecule of RhoA, a monomeric GTP-binding protein, and causes Ca(2+) sensitization via inactivation of myosin phosphatase. The major physiological functions of Rho-kinase include contraction, migration, and proliferation in cells. These actions are thought to be related to the pathophysiological features of asthma, i.e., airflow limitation, airway hyperresponsiveness, beta-adrenergic desensitization, eosinophil recruitment and airway remodeling. Here, the roles of RhoA/Rho-kinase in the pathophysiology and treatment of asthma were investigated. In airway smooth muscle, pre-exposure to chemical mediators released from inflammatory cells markedly enhances methacholine-induced contraction without elevating intracellular concentrations of Ca(2+). This augmented responsiveness to methacholine involves the phosphorylation of myosin phosphatase targeting protein 1 (MYPT1) via Rho-kinase, however, it is attenuated by pre-treatment with Rho-kinase inhibitors such as Y-27632 and HA-1077. Airway smooth muscle contraction due to asthma-related substances such as contractile agonists and reactive oxygen species is suppressed by these Rho-kinase inhibitors. Reduced responsiveness to beta-adrenergic receptor agonists occurs via Ca(2+) sensitization, after exposure to lysophospholipids and proteases released from inflammatory cells. This beta-adrenergic desensitization is also attenuated in the presence of Y-27632. Furthermore, the proliferation of airway smooth muscle cells is elevated by Rho-kinase, however, it is markedly suppressed by Y-27632. Antigen challenges cause hyperresponsiveness and eosinophilia in the airways; however, these reactions are markedly suppressed by these Rho-kinase inhibitors. These findings indicate that RhoA/Rho-kinase is involved in the pathophysiology of asthma, and suggest that Rho-kinase inhibitors have therapeutic potential for prohibiting these features. In conclusion, RhoA/Rho-kinase is a novel target molecule for the treatment of asthma.

PMID: 18991642 [PubMed - indexed for MEDLINE]

635. J Immunol. 2008 Nov 15;181(10):7273-83.

Prostaglandin E2 inhibits eosinophil trafficking through E-prostanoid 2

receptors.

Sturm EM, Schratl P, Schuligoi R, Konya V, Sturm GJ, Lippe IT, Peskar BA, Heinemann A.

Institute of Experimental and Clinical Pharmacology, Medical University of Graz, Graz, Austria.

The accumulation of eosinophils in lung tissue is a hallmark of asthma, and it is believed that eosinophils play a crucial pathogenic role in allergic inflammation. Prostaglandin (PG) E(2) exerts anti-inflammatory and bronchoprotective mechanisms in asthma, but the underlying mechanisms have remained unclear. In this study we show that PGE(2) potently inhibits the chemotaxis of purified human eosinophils toward eotaxin, PGD(2), and C5a. Activated monocytes similarly attenuated eosinophil migration, and this was reversed after pretreatment of the monocytes with a cyclooxygenase inhibitor. The selective E-prostanoid (EP) 2 receptor agonist butaprost mimicked the inhibitory effect of PGE(2) on eosinophil migration, whereas an EP2 antagonist completely prevented this effect. Butaprost, and also PGE(2), inhibited the C5a-induced degranulation of eosinophils. Moreover, selective kinase inhibitors revealed that the inhibitory effect of PGE(2) on eosinophil migration depended upon activation of PI3K and protein kinase C, but not cAMP. In animal models, the EP2 agonist butaprost inhibited the rapid mobilization of eosinophils from bone marrow of the in situ perfused guinea pig hind limb and prevented the allergen-induced bronchial accumulation of eosinophils in OVA-sensitized mice. Immunostaining showed that human eosinophils express EP2 receptors and that EP2 receptor expression in the murine lungs is prominent in airway epithelium and, after allergen challenge, in peribronchial infiltrating leukocytes. In summary, these data show that EP2 receptor agonists potently inhibit eosinophil trafficking and activation and might hence be a useful therapeutic option in eosinophilic diseases.

PMID: 18981149 [PubMed - indexed for MEDLINE]

636. J Immunol. 2008 Nov 15;181(10):6889-97.

Differential regulatory function of resting and preactivated allergen-specific CD4+ CD25+ regulatory T cells in Th2-type airway inflammation.

Saito K, Torii M, Ma N, Tsuchiya T, Wang L, Hori T, Nagakubo D, Nitta N, Kanegasaki S, Hieshima K, Yoshie O, Gabazza EC, Katayama N, Shiku H, Kuribayashi K, Kato T.

Department of Cellular and Molecular Immunology, Mie University Graduate School of Medicine, Tsu, Japan.

Although CD4(+)CD25(+) regulatory T (Treg) cells are known to suppress Th1 cell-mediated immune responses, their effect on Th2-type immune responses remains unclear. In this study we examined the role of Treg cells in Th2-type airway inflammation in mice. Depletion and reconstitution experiments demonstrated that the Treg cells of naive mice effectively suppressed the initiation and development of Th2-driven airway inflammation. Despite effective suppression of Th2-type airway inflammation in naive mice, adoptively transferred, allergen-specific Treg cells were unable to suppress airway inflammation in allergen-presensitized mice. Preactivated allergen-specific Treg cells, however, could suppress airway inflammation even in allergen-presensitized mice by accumulating in the lung, where they reduced the accumulation and proliferation of Th2 cells. Upon activation, allergen-specific Treg cells up-regulated CCR4, exhibited enhanced chemotactic responses to CCR4 ligands, and suppressed the

proliferation of and cytokine production by polarized Th2 cells. Collectively, these results demonstrated that Treg cells are capable of suppressing Th2-driven airway inflammation even in allergen-presensitized mice in a manner dependent on their efficient migration into the inflammatory site and their regulation of Th2 cell activation and proliferation.

PMID: 18981108 [PubMed - indexed for MEDLINE]

637. J Cutan Pathol. 2008 Nov;35(11):979-88. doi: 10.1111/j.1600-0560.2008.01045.x.

Mucocutaneous Splendore-Hoeppli phenomenon.

Hussein MR.

Department of Pathology, Assir Central Hospital, King Khalid University, Abha, Kingdom of Saudi Arabia. mrcpath17@gmail.com

Splendore-Hoeppli phenomenon (asteroid bodies) is the in vivo formation of intensely eosinophilic material (radiate, star-like, asteroid or club-shaped configurations) around microorganisms (fungi, bacteria and parasites) or biologically inert substances. This study presents a literature review concerning Splendore-Hoeppli reaction in the mucocutaneous diseases. It examines the histopathological features, nature and differential diagnosis of this reaction. It also discusses the mucocutaneous infections and the non-infective diseases associated with it. Available studies indicate that several mucocutaneous infections can generate Splendore-Hoeppli reaction. The fungal infections include sporotrichosis, pityrosporum folliculitis, zygomycosis, candidiasis, aspergillosis and blastomycosis. The bacterial infections include botryomycosis, nocardiosis and actinomycosis. The parasitic conditions include orbital pythiosis, strongyloidiasis, schistosomiasis and cutaneous larva migrans. In addition, Splendore-Hoeppli reaction may be seen with non-infective pathology such as hypereosinophilic syndrome and allergic conjunctival granulomas. The Splendore-Hoeppli reaction material comprises antigen-antibody complex, tissue debris and fibrin. Although the exact nature of this reaction is unknown, it is thought to be a localized immunological response to an antigen-antibody precipitate related to fungi, parasites, bacteria or inert materials. The characteristic formation of the peribacterial or perifungal Splendore-Hoeppli reaction probably prevents phagocytosis and intracellular killing of the insulting agent leading to chronicity of infection. To conclude, Splendore-Hoeppli reaction is a tell tale of a spectrum of infections and reactive conditions. The molecular pathways involved in the development of this reaction are open for future investigations.

PMID: 18976399 [PubMed - indexed for MEDLINE]

638. J Asthma. 2008 Nov;45(9):785-9. doi: 10.1080/02770900802179957.

Community-level data suggest that asthma prevalence varies between U.S. and foreign-born black subpopulations.

Brugge D, Woodin M, Schuch TJ, Salas FL, Bennett A, Osgood ND.

School of Medicine, Public Health and Family Medicine, Tufts University, Boston, MA 02155, USA. dbrugge@aol.com

For Mexican and Chinese immigrants it has been reported that foreign born children have a lower prevalence of asthma than U.S.-born children. Inner-city black populations have a high prevalence of asthma. However, despite growing

populations of black immigrants, we are aware of no previous studies that have looked at the effect of nativity on their asthma prevalence. We report data collected from a convenience sample in the Dorchester neighborhood of Boston for black respondents. The survey was conducted by medical students and community residents using a community-based participatory research approach. For adult respondents (n = 290) there was a strong negative association between being born outside the United States and reporting asthma (OR = 0.39; p = 0.033) that was retained in our multivariate model. For children (n = 157, reported by their parents) there was also a strong association with being born outside the United States (p < 0.05 using chi(2) tabular analysis); however, there were no foreign-born children with asthma so an OR could not be calculated and this association could not be carried forward into multivariate analyses. For children, but not adults, there were also strong associations between asthma and environmental factors. These findings point to the need for further research into nativity and asthma in black U.S. populations. Future studies should seek to obtain a representative sample, gather more demographic data than we did and seek a larger sample of children. It makes sense to ask about nativity in asthma prevalence studies in order to distinguish these two apparently very different subpopulations.

PMID: 18972296 [PubMed - indexed for MEDLINE]

639. PLoS One. 2008;3(10):e3562. doi: 10.1371/journal.pone.0003562. Epub 2008 Oct 29.

Trans-epithelial immune cell transfer during suckling modulates delayed-type hypersensitivity in recipients as a function of gender.

Ma LJ, Walter B, Deguzman A, Muller HK, Walker AM.

Division of Biomedical Sciences, University of California Riverside, Riverside, California, USA.

INTRODUCTION: Breast feeding has long term effects on the developing immune system which outlive passive immunization of the neonate. We have investigated the transfer of milk immune cells and examined the result of transfer once the recipients were adult.
METHODS: Non-transgenic mouse pups were foster-nursed by green fluorescent protein (GFP) transgenic dams for 3 weeks and the fate of GFP+ cells was followed by FACS analysis, immunohistochemistry and RT-PCR for GFP and appropriate immune cell markers. Pups suckled by non-transgenic dams served as controls.
RESULTS: Despite a preponderance of B cells and macrophages in the stomach contents of the pups, most cells undergoing trans-epithelial migration derived from the 3-4% of milk cells positive for T lymphocyte markers. These cells homed to the spleen and thymus, with maximal accumulation at 3-4 weeks. By sensitizing dams with an antigen which elicits a T cell-mediated delayed-type-hypersensitivity (DTH) response, we determined that nursing by a sensitized dam (compared to a non-sensitized dam) amplified a subsequent DTH response in females and yet suppressed one in males.
DISCUSSION: These results suggest that clinical evaluation weighing the pros and cons of nursing male versus female children by mothers with genetically-linked hypersensitivity diseases, such as celiac disease and eczema, or those in regions of the world with endemic DTH-eliciting diseases, such as tuberculosis, may be warranted.

PMCID: PMC2569205
PMID: 18958163 [PubMed - indexed for MEDLINE]

640. J Immunol. 2008 Nov 1;181(9):5981-9.

An IL-1 cytokine member, IL-33, induces human basophil activation via its ST2 receptor.

Suzukawa M, Iikura M, Koketsu R, Nagase H, Tamura C, Komiya A, Nakae S, Matsushima K, Ohta K, Yamamoto K, Yamaguchi M.

Department of Allergy and Rheumatology, University of Tokyo Graduate School of Medicine, Japan.

Basophils are thought to play pivotal roles in allergic inflammation through rapid release of chemical mediators in addition to sustained production of Th2 cytokines, including IL-4. A newly identified cytokine, IL-33, has been recognized as one of the key cytokines enhancing Th2-balanced immune regulation through its receptor, ST2. The present study was conducted to elucidate whether IL-33 acts directly on, and affects the functions of, human basophils. Real-time PCR analysis showed that basophils express transcripts for ST2. The expression levels were significantly higher compared with eosinophils and neutrophils, and treatment with IL-33 significantly up-regulated basophil ST2 mRNA expression. Expressions of IL-4 and IL-13 mRNA were also up-regulated by IL-33, and there was also enhanced secretion of IL-4 protein. IL-33 increased the surface levels of basophil CD11b expression and enhanced basophil adhesiveness. Although IL-33 failed to directly induce degranulation or attract basophils, it exerted priming effects on basophils. It enhanced degranulation in response to IgE-crosslinking stimulus and also enhanced basophil migration toward eotaxin without changing surface CCR3. Also, IL-33 synergistically enhanced IL-4 production and CD11b expression by IL-3-stimulated basophils. Neutralization using Ab specific for ST2 significantly diminished the enhancing effects of IL-33 on both basophil CD11b expression and migration toward eotaxin, indicating that IL-33 signals via ST2 expressed on basophils. This study revealed that IL-33 potently regulates migration and activation of human basophils. IL-33 may be a key cytokine in the pathogenesis of Th2-dominant inflammation by acting not only on lymphocytes but also on effector cells such as basophils.

PMID: 18941187 [PubMed - indexed for MEDLINE]

641. Eur J Immunol. 2008 Nov;38(11):3050-9. doi: 10.1002/eji.200838384.

CXCL12 is essential for migration of activated Langerhans cells from epidermis to dermis.

Ouwehand K, Santegoets SJ, Bruynzeel DP, Scheper RJ, de Gruijl TD, Gibbs S.

Department of Dermatology, VU University Medical Centre, Amsterdam, The Netherlands.

The initial step in Langerhans cell (LC) migration from the epidermis to the lymph node involves migration of maturing LC into the dermis. Here, we investigated the migration of LC out of the epidermis after exposure of the skin to contact allergens. Ex vivo intact human skin, epidermal sheets, and LC derived from the MUTZ-3 cell line (MUTZ-LC) were used to determine whether dermal fibroblasts play a role in mediating LC migration towards the dermis. Exposure of epidermal sheets or MUTZ-LC to allergens (nickel sulphate, 2,4-dinitrochlorobenzene, and cinnamaldehyde) or a cytokine maturation cocktail resulted in LC migration towards dermal fibroblasts. This was due to upregulation of CXCR4 on maturing LC and secretion of CXCL12/stromal derived factor-1 chemokine by fibroblasts. Neutralizing antibodies to either CXCL12 or CXCR4 completely blocked migration. Injection of CXCL12 neutralizing antibodies into intact human skin totally inhibited LC migration into the dermis. In contrast,

neutralizing antibodies to CCL19/CCL21 did not inhibit migration into the dermis. We describe a novel and essential role of dermis-derived CXCL12 in initiating migration of maturing human LC to the dermis thus permitting their further journey to the draining lymph nodes.

PMID: 18924211 [PubMed - indexed for MEDLINE]

642. Mol Biol (Mosk). 2008 Jul-Aug;42(4):639-44.

[Cloning of rat TARC cDNA and analysis of tissue-specific mRNA expression].

[Article in Russian]

Chae JI, Ju SK, Lee MK, Park JH, Shim JH, Lee KK, Lee DS.

Thymus and activation-regulated chemokine (TARC) is one that selectively controls the migration of type 2-helper T lymphocytes into inflammatory lesions. TARC is a CC chemokine, and plays an essential role in recruiting CC chemokine receptor 4-positive Th2 cells to allergic lesions. We cloned TARC cDNA from rat thymus using RT-PCR. The rat TARC clone contained a full-length open reading frame encoding 93 amino acids that showed 83% and 66% homology with mouse and human homologs, respectively. The expression of TARC mRNA was mainly in the lymphoid organs, for example, the thymus, spleen, and lymph node. The recombinant TARC was expressed in Escherichia coli and purified in an active form. In addition, the purified rat TARC with S-tagged specifically binds to human CCR4 in CD4.CCR4-transfected HOS cells by Cell-binding assay using flow-cytometry. The TARC cDNA clones obtained in this study will be valuable for future studies on allergic diseases in rats.

PMID: 18856064 [PubMed - indexed for MEDLINE]

643. Int Arch Allergy Immunol. 2009;148(3):219-27. doi: 10.1159/000161582. Epub 2008 Oct 10.

Immunoglobulin E-dependent regulation of the CCR3 chemokine receptor by interferon-gamma in atopic asthmatics.

García-Vega Y, Rodríguez-Perez J, Bermúdez-Badell C, Blanco-Garcés E, Valenzuela-Silva C, López-Saura P, Bello-Rivero I.

Centro de Investigaciones Biológicas, Clinical Trials Division, La Habana, Cuba.

BACKGROUND: The chemokine receptor CCR3 mediates the migration of cells that play an important role in the pathogenesis of asthma to inflammatory foci. Interferon (IFN)-gamma is known to downregulate the expression of some chemokine receptors. Therefore, we decided to analyze the regulation of CCR3 by IFN-gamma in asthmatics and to characterize the dependence of this process on immunoglobulin E (IgE) levels.
METHODS: Atopic asthmatics were treated with IFN-gamma or placebo, and the IgE concentration in the blood was measured using an ultra-micro-ELISA for total IgE. Mononuclear cells from patients and controls were isolated by Ficoll-Hypaque gradient and incubated in the absence or presence of IFN-gamma for different periods of time. After incubation, the cells were washed and lysed for RT-PCR analysis, which was performed using a Perkin-Elmer kit.
RESULTS: IFN-gamma treatment apparently improved the evaluated clinical variables; however, the differences were not significant compared to the placebo group. We found that IFN-gamma downregulated CCR3 mRNA expression ex vivo and in vivo in those patients with IgE levels higher than 500 IU/ml, whereas IFN-gamma

upregulated CCR3 mRNA expression in patients with IgE levels lower than 500 IU/ml. Correspondence between ex vivo and in vivo results was observed using this approach. There was found to be a direct correlation between total serum IgE and CCR3 mRNA expression.
CONCLUSIONS: In those asthmatic patients with high levels of IgE, who are thus susceptible to downregulation of CCR3 by IFN-gamma, a significant therapeutic effect with systemic IFN-gamma might be expected.

(c) 2008 S. Karger AG, Basel.

PMID: 18849613 [PubMed - indexed for MEDLINE]

644. Blood. 2009 Jan 15;113(3):575-84. doi: 10.1182/blood-2008-04-151803. Epub 2008 Oct 7.

Dexamethasone augments CXCR4-mediated signaling in resting human T cells via the activation of the Src kinase Lck.

Ghosh MC, Baatar D, Collins G, Carter A, Indig F, Biragyn A, Taub DD.

Laboratory of Immunology, Intramural Research Program, National Institute of Aging, National Institutes of Health, Baltimore, MD 21224-6825, USA.

Dexamethasone (DM) is a synthetic member of the glucocorticoid (GC) class of hormones that possesses anti-inflammatory and immunosuppressant activity and is commonly used to treat chronic inflammatory disorders, severe allergies, and other disease states. Although GCs are known to mediate well-defined transcriptional effects via GC receptors (GCR), there is increasing evidence that GCs also initiate rapid nongenomic signaling events in a variety of cell types. Here, we report that DM induces the phosphorylation of Lck and the activation of other downstream mediators, including p59Fyn, Zap70, Rac1, and Vav in resting but not activated human T cells. DM treatment also augments CXCL12-mediated signaling in resting T cells through its cell surface receptor, CXCR4 resulting in the enhanced actin polymerization, Rac activation, and cell migration on ligand exposure. Lck was found to be a critical intermediate in these DM-induced signaling activities. Moreover, DM-mediated Lck phosphorylation in T cells was dependent on the presence of both the GCR and the CD45 molecule. Overall, these results elucidate additional nongenomic effects of DM and the GCR on resting human T cells, inducing Lck and downstream kinase activation and augmenting chemokine signaling and function.

PMCID: PMC2628365
PMID: 18840710 [PubMed - indexed for MEDLINE]

645. Eur J Clin Invest. 2008 Sep;38(9):663-71. doi: 10.1111/j.1365-2362.2008.01989.x.

A novel antagonist of prostaglandin D2 blocks the locomotion of eosinophils and basophils.

Royer JF, Schratl P, Carrillo JJ, Jupp R, Barker J, Weyman-Jones C, Beri R, Sargent C, Schmidt JA, Lang-Loidolt D, Heinemann A.

Institute of Experimental and Clinical Pharmacology, Medical University Graz, Graz, Austria.

BACKGROUND: Chemoattractant receptor homologous molecule of Th2 cells (CRTH2) has been shown to mediate the chemotaxis of eosinophils, basophils and Th2-type T lymphocytes. The major mast cell product prostaglandin (PG) D(2) is considered to

be the principal ligand of CRTH2.
MATERIALS AND METHODS: We developed a novel CRTH2 antagonist, AZ11665362 [2,5-dimethyl-3-(8-methylquinolin-4-yl)-1H-indole-1-yl]acetic acid, and characterized its efficacy in binding assay in HEK293 cells, eosinophil and basophil shape change assay and migration assay, platelet aggregation and eosinophil release from guinea pig bone marrow. The effects were compared with ramatroban, the sole CRTH2 antagonist clinically available to date.
RESULTS: AZ11665362 bound with high affinity to human and guinea pig CRTH2 expressed in HEK293 cells and antagonized eosinophil and basophil shape change responses to PGD(2). AZ11665362 was without effect on the PGD(2)-induced inhibition of platelet aggregation. In contrast, AZ11665362 effectively inhibited the in vitro migration of human eosinophils and basophils towards PGD(2). The release of eosinophils from the isolated perfused hind limb of the guinea pig was potently stimulated by PGD(2), and this effect was prevented by AZ11665362. In all assays tested, AZ11665362 was at least 10 times more potent than ramatroban.
CONCLUSIONS: AZ11665362 is a potent CRTH2 antagonist that is capable of blocking the migration of eosinophils and basophils, and the rapid mobilization of eosinophils from bone marrow. AZ11665362 might hence be useful for the treatment of allergic diseases.

PMID: 18837743 [PubMed - indexed for MEDLINE]

646. J Leukoc Biol. 2009 Jan;85(1):136-45. doi: 10.1189/jlb.0608387. Epub 2008 Oct 3.

Prostaglandin H2 induces the migration of human eosinophils through the chemoattractant receptor homologous molecule of Th2 cells, CRTH2.

Schuligoi R, Sedej M, Waldhoer M, Vukoja A, Sturm EM, Lippe IT, Peskar BA, Heinemann A.

Institute of Experimental and Clinical Pharmacology, Medical University of Graz, Universitaetsplatz 4. A-8010 Graz, Austria.

The major mast cell product PGD2 is released during the allergic response and stimulates the chemotaxis of eosinophils, basophils, and Th2-type T lymphocytes. The chemoattractant receptor homologous molecule of Th2 cells (CRTH2) has been shown to mediate the chemotactic effect of PGD2. PGH2 is the common precursor of all PGs and is produced by several cells that express cyclooxygenases. In this study, we show that PGH2 selectively stimulates human peripheral blood eosinophils and basophils but not neutrophils, and this effect is prevented by the CRTH2 receptor antagonist (+)-3-[[(4-fluorophenyl)sulfonyl] methyl amino]-1,2,3,4-tetrahydro-9H-carbazole-9-acetic acid (Cay10471) but not by the hematopoietic PGD synthase inhibitor 4-benzhydryloxy-1-[3-(1H-tetrazol-5-yl)-propyl]piperidine (HQL79). In chemotaxis assays, eosinophils showed a pronounced migratory response toward PGH2, but eosinophil degranulation was inhibited by PGH2. Moreover, collagen-induced platelet aggregation was inhibited by PGH2 in platelet-rich plasma, which was abrogated in the presence of the D-type prostanoid (DP) receptor antagonist 3-[(2-cyclohexyl-2-hydroxyethyl)amino]-2,5-dioxo-1-(phenylmethyl)-4-imidazolidine-heptanoic acid (BWA868c). Each of these effects of PGH2 was enhanced in the presence of plasma and/or albumin. In eosinophils, PGH2-induced calcium ion (Ca^{2+}) flux was subject to homologous desensitization with PGD2. Human embryo kidney (HEK)293 cells transfected with human CRTH2 or DP likewise responded with Ca^{2+} flux, and untransfected HEK293 cells showed no response. These data indicate that PGH2 causes activation of the PGD2 receptors CRTH2 and DP via a dual mechanism: by interacting directly with the receptors and/or by giving rise to PGD2 after catalytic conversion by plasma proteins.

PMID: 18835884 [PubMed - indexed for MEDLINE]

647. Stem Cells. 2009 Jan;27(1):259-65. doi: 10.1634/stemcells.2008-0283.

IFATS collection: Immunomodulatory effects of adipose tissue-derived stem cells in an allergic rhinitis mouse model.

Cho KS, Park HK, Park HY, Jung JS, Jeon SG, Kim YK, Roh HJ.

Department of Otorhinolaryngology, Medical Research Institute, Pusan National University School of Medicine, Busan, Korea.

Adipose tissue-derived stem cells (ASCs) exhibit immunosuppressive effects in allogeneic transplantation. However, there is no report that evaluates the in vivo immune-modulating effect of ASCs in an experimental allergic rhinitis (AR) model. We investigated whether ASCs migrate to the nasal mucosa in an AR mouse model and evaluated the immune-modulating effect of ASCs in the AR mouse model. Cultured ASCs (2 x 10(6)) were injected i.v. before the first allergen challenge in the AR mouse model. Migration of ASCs to the nasal mucosa was evaluated by immunofluorescence. The immunomodulatory effects of ASCs were evaluated by nasal symptoms, histology, serum ovalbumin (OVA)-specific antibody, and the cytokine profile of the spleen. ASCs migrated to the nasal mucosa in the AR mouse model. ASCs significantly reduced allergic symptoms and inhibited eosinophilic inflammation in the nasal mucosa. ASCs significantly decreased the serum allergen-specific IgE level and the IgG(1)/IgG(2a) ratio and significantly increased the IgG(2a) level in the AR mouse model. ASCs inhibited interleukin (IL)-4 and IL-5 production from OVA-incubated splenocytes, but enhanced interferon-gamma production. In conclusion, ASCs can migrate to the nasal mucosa in the AR mouse model and inhibit eosinophilic inflammation partly via shifting to a T-helper 1 (Th1) from a Th2 immune response to allergens.

PMID: 18832595 [PubMed - indexed for MEDLINE]

648. J Leukoc Biol. 2009 Jan;85(1):124-31. doi: 10.1189/jlb.0208135. Epub 2008 Sep 25.

The extracellular matrix protein mindin regulates trafficking of murine eosinophils into the airspace.

Li Z, Garantziotis S, Jia W, Potts EN, Lalani S, Liu Z, He YW, Foster WM, Hollingsworth JW.

Department of Immunology, Duke University Medical Center, Box 103004, Durham, NC 27710, USA.

Asthma remains a major cause of morbidity and hospitalizations in developed nations. Despite the widespread prevalence of this disease, the genetic and environmental factors that mediate development and progression of allergic airways disease remain poorly understood. Pulmonary recruitment of eosinophils is believed to contribute to many cardinal features of allergic airways disease. Therefore, it is paramount to understand host factors that contribute to pulmonary eosinophil recruitment into the lungs. Mindin is a component of pulmonary extracellular matrix, which can regulate inflammatory cell recruitment. We characterized the role of mindin in the severity of allergic airways disease using established murine models. There were no baseline differences in wild-type and mindin-deficient animals in cell counts or airway physiology. Using the OVA murine model of allergic airways disease, we observed that mindin-deficient animals have less-severe allergic airways disease with fewer airspace eosinophils and lower lung-lavage levels of inflammatory Th2 cytokines such as IL-13 and IL-4. Furthermore, mindin-deficient animals have reduced airway

hyper-responsiveness after methacholine challenge. To determine the role of mindin in eosinophil trafficking, independent of antigen immunization or T lymphocyte activation, we instilled IL-13 directly into the lungs of mice. In this model, mindin regulates eosinophil recruitment into the airspace. In vitro experiments demonstrate that mindin can enhance eotaxin-mediated eosinophil adhesion and migration, which are dependent on the expression of integrins alphaMbeta2 and alpha4beta1. In conclusion, these data suggest that mindin participates in integrin-dependent trafficking of eosinophils and can contribute to the severity of allergic airways disease.

PMCID: PMC2626769
PMID: 18818374 [PubMed - indexed for MEDLINE]

649. Respir Med. 2009 Jan;103(1):50-8. doi: 10.1016/j.rmed.2008.08.004. Epub 2008 Sep 24.

Influenza vaccination among Canadians with chronic respiratory disease.

Vozoris NT, Lougheed MD.

Division of Respirology, Department of Medicine, University of Toronto, Medical Sciences Building, 1 King's College Circle, Toronto, Ontario, Canada.
nick.vozoris@utoronto.ca

BACKGROUND: The purposes of this study were to estimate the prevalence of recent influenza vaccination, to identify sociodemographic risk factors and reasons for non-vaccination, and to examine the association between influenza vaccination and respiratory medication use, among individuals with asthma and COPD in Canada.
METHODS: Data from the 2003 Statistics Canada Canadian Community Health Survey (n=134,072, age 12-80+ years) were analyzed. All data were based on self-report.
RESULTS: An estimated 36.3% and 47.9% of individuals with asthma and COPD, respectively, were immunized for influenza within the last year in 2003. These vaccination rates were relatively lower than those seen with other non-respiratory health conditions. Respondents thinking that influenza vaccination was not necessary was the most frequent reason cited for non-vaccination among individuals with asthma and COPD. Men, non-Ontarians, younger age groups, current smokers, and those without a family doctor were less likely to be vaccinated among individuals with asthma and COPD. After controlling for potential sociodemographic confounders, among individuals with asthma, those vaccinated for influenza had significantly greater odds of using inhalers/nebulizers (OR=1.8, 95% CI=1.3-2.4). No other significant medication use associations were observed among individuals with asthma and COPD.
CONCLUSIONS: Recent self-reported influenza vaccination rates among individuals with asthma and COPD were relatively low. Influenza vaccination was not associated with decreased respiratory medication use among individuals with asthma and COPD, suggesting that vaccination may not help prevent exacerbations. More research is needed to fully clarify the role of influenza vaccination in chronic respiratory disease, particularly asthma, to justify policies of mass-immunization.

PMID: 18818066 [PubMed - indexed for MEDLINE]

650. J Laryngol Otol. 2008 Dec;122(12):1309-17. doi: 10.1017/S0022215108003241. Epub 2008 Sep 23.

Role of sphingosine 1-phosphate receptor expression in eosinophils of patients with allergic rhinitis, and effect of topical nasal steroid treatment on this receptor expression.

Mackle T, Gendy SS, Walsh M, McConn-Walsh R, Costello RW, Walsh MT.

Department of Otorhinolaryngology, Beaumont Hospital, Dublin, Ireland.
Mackle_tara@hotmail.com

OBJECTIVE: Recent research has indicated that sphingosine 1-phosphate plays a role in allergy. This study examined the effect of allergen challenge on the expression of sphingosine 1-phosphate receptors on the eosinophils of allergic rhinitis patients, and the effect of steroid treatment on this expression.
STUDY DESIGN: A prospective, non-randomised study.
METHODS: The study had three parts. Firstly, sphingosine 1-phosphate receptor expression on the eosinophils of allergic rhinitis patients and control patients was determined. Secondly, sphingosine 1-phosphate receptor expression was quantified pre- and post-allergen challenge, before and after a short course of fluticasone propionate; all patients underwent symptom scoring and peak nasal inspiratory flow measurement pre- and post-allergen challenge, both before and after steroid or saline treatment. Thirdly, the effect of sphingosine 1-phosphate on eosinophil migration was examined.
RESULTS: The eosinophils of both allergic rhinitis patients and controls expressed sphingosine 1-phosphate1, 3, 4, and 5. Eosinophils from all allergic rhinitis patients demonstrated up-regulation in sphingosine 1-phosphate expression after allergen challenge. These changes were statistically very significant for sphingosine 1-phosphate1, 4, and 5, and moderately significant for sphingosine 1-phosphate3. Sphingosine 1-phosphate receptor expression up-regulation was abolished in the steroid-treated group after allergen challenge; however, the saline-treated group showed no change in sphingosine 1-phosphate receptor expression after allergen challenge. Peak nasal inspiratory flow scores were significantly diminished after allergen challenge prior to treatment, but not after a course of topical nasal steroids. Sphingosine 1-phosphate induced eosinophil chemotaxis was increased following allergen challenge in allergic rhinitis subjects.
CONCLUSIONS: Local intranasal steroid therapy acts directly to block allergen-induced up-regulation of sphingosine 1-phosphate receptors on the peripheral eosinophils of allergic rhinitis patients, and this is coincident with post-challenge peak nasal inspiratory flow measurement improvements. These observations support the idea that such an increase in sphingosine 1-phosphate receptor expression is clinically relevant in allergic rhinitis, with potential consequences for eosinophil migration and survival.

PMID: 18808729 [PubMed - indexed for MEDLINE]

651. J Am Acad Dermatol. 2008 Dec;59(6):1031-42. doi: 10.1016/j.jaad.2008.06.031. Epub 2008 Sep 14.

Cutaneous manifestations of human toxocariasis.

Gavignet B, Piarroux R, Aubin F, Millon L, Humbert P.

Department of Parasitology and Mycology, Jean Minjoz University Hospital, Besançon, France.

Human toxocariasis is a parasitic disease characterized by the presence of larvae of the genus Toxocara in human tissues. T canis and T cati, the adult roundworms of which are found in dog and cat intestines, respectively, are the most common causative agents of the disease. Toxocaral larvae usually cause two severe syndromes: visceral larva migrans and ocular larva migrans, depending on the location of the larvae. Two other syndromes, covert toxocariasis and common toxocariasis, which are less typical and not as severe, have also been described.

During the last two decades, cutaneous manifestations such as chronic urticaria, chronic pruritus, and miscellaneous eczema, in patients with Toxocara antibodies, have been studied by different authors. In some cases, these cutaneous manifestations are the only signs indicating the presence of the disease, and they are cured after antihelmintic treatment when there is good patient compliance. In this review, we focus on these particular skin manifestations regarding their clinical description, diagnosis, and treatment.

PMID: 18793816 [PubMed - indexed for MEDLINE]

652. Allergy Asthma Clin Immunol. 2008 Sep 15;4(3):95-105. doi: 10.1186/1710-1492-4-3-95. Epub 2008 Sep 15.

Chitosan Interferon-gamma Nanogene Therapy for Lung Disease: Modulation of T-Cell and Dendritic Cell Immune Responses.

Kong X, Hellermann GR, Zhang W, Jena P, Kumar M, Behera A, Behera S, Lockey R, Mohapatra SS.

Division of Allergy and Immunology, Culverhouse Airway Disease Research and Nanomedicine Center, University of South Florida College of Medicine, Tampa, FL.

: The use of chitosan nanoparticles as carriers for expression plasmids represents a major improvement in gene expression technology. We demonstrated previously that treatment with chitosan interferon-gamma (IFN-gamma) plasmid deoxyribonucleic acid (DNA) nanoparticles (chitosan interferon-gamma nanogene [CIN]) led to in situ production of IFN-gamma and a reduction in inflammation and airway reactivity in mice, but the mechanism underlying the immunomodulatory effects of CIN remains unclear. In this report, the effect of CIN treatment on the immune responses of CD8+ T cells and dendritic cells was examined in a BALB/c mouse model of ovalbumin (OVA)-induced allergic asthma. OT1 mice (OVA-T cell receptor [TCR] transgenic) were also used to test the effects of CIN on OVA-specific CD8+ T cells. CIN treatment caused a reduction in IFN-gamma production in a subpopulation of OVA-specific CD8+ T cells cultured in vitro in the presence of OVA. CIN also reduced apoptosis of the CD8+ T cells. Examination of dendritic cells from lung and lymph nodes indicated that CIN treatment decreased their antigen-presenting activity, as evident from the reduction in CD80 and CD86 expression. Furthermore, CIN treatment significantly decreased the number of CD11c+b+ dendritic cells in lymph nodes, suggesting that endogenous IFN-gamma expression may immunomodulate dendritic cell migration and activation. CIN therapy results in a reduction in proinflammatory CD8+ T cells and decreases the number and antigen-presenting activity of dendritic cells.

PMCID: PMC2868869
PMID: 20525130 [PubMed]

653. Allergy. 2008 Oct;63(10):1317-23. doi: 10.1111/j.1398-9995.2008.01787.x.

n-Nonanoyl-CCL14 (NNY-CCL14), a novel inhibitor of allergic airway inflammation is a partial agonist of human CCR2.

Gupta S, Erdmann G, Schulz-Maronde S, Escher SE, Richter R, Forssmann WG, Elsner J, Forssmann U.

Clinic of Immunology and Rheumatology, Hannover Medical School, Germany.

BACKGROUND: Modulation of leukocyte recruitment through blocking of chemokine receptors has been proposed as an attractive therapeutic strategy. We have

previously demonstrated that n-Nonanoyl-CC chemokine ligand 14 (NNY-CCL14), a modified analog of the naturally occurring chemokine CCL14(9-74) internalizes and desensitizes human CCR3 resulting in the inactivation of eosinophils. However, inhibitory effects of NNY-CCL14 in murine models of allergic airway inflammation are assigned to its interaction with CCR1 and CCR5.
AIM OF THE STUDY: As CCL2 and its receptor CCR2 have been shown to play important roles in the development of Th2 inflammation, we further evaluated the effects of NNY-CCL14 treatment on CCL2-mediated activation of CCR2.
METHODS: Effects of NNY-CCL14 treatment were studied on cell lines transfected with human CCR2 and primary leukocytes. Functional effects were assessed by calcium efflux assays, flow cytometry and chemotaxis.
RESULTS: Prestimulation with NNY-CCL14 desensitized CCR2-mediated responses to further stimulation with its selective ligand CCL2. No significant internalization of CCR2 was observed when the cells were stimulated with NNY-CCL14, even at concentrations eliciting maximal [Ca(2+)]i mobilization. Above all, NNY-CCL14 pretreatment blocked CCL2-induced chemotaxis of monocytes.
CONCLUSIONS: This study demonstrates that NNY-CCL14 is a partial agonist of CCR2, inhibiting responses of monocytes to the CCR2-selective ligand CCL2. NNY-CCL14 attenuates CCR2-mediated responses by rapidly desensitizing the receptor and preventing chemotaxis, although it is able to induce calcium mobilization but does not lead to CCR2 internalization. Hence this study provides further insights into the possible mechanisms of action of NNY-CCL14, which interacts with multiple chemokine receptors inhibiting the migration and activation of different cell populations involved, thus acting as a potential therapeutic compound to alleviate allergic inflammation.

PMID: 18782110 [PubMed - indexed for MEDLINE]

654. Am J Epidemiol. 2008 Oct 15;168(8):906-14. doi: 10.1093/aje/kwn207. Epub 2008 Sep 8.

Methods and software for estimating health disparities: the case of children's oral health.

Cheng NF, Han PZ, Gansky SA.

Division of Oral Epidemiology and Dental Public Health, University of California, San Francisco, 3333 California Street, Suite 495, San Francisco, CA 94143-1361, USA.

The National Center for Health Statistics recently issued a monograph with 11 guidelines for reporting health disparities. However, guidelines on confidence intervals (CIs) cannot be readily implemented with the complex sample surveys often used for disease surveillance. In the United States, dental caries (decay) is the most common chronic childhood disease-5 times more common than asthma. Racial/ethnic minorities, immigrants, and persons of lower socioeconomic position (SEP) have a greater prevalence of caries. The authors provide methods for applying National Center for Health Statistics guidelines to complex sample surveys (health disparity indices and absolute and relative difference measures assessing associations of race/ethnicity and SEP to health outcomes with CIs); illustrate the application of those methods to children's untreated caries; provide relevant software; and report results from a simulation varying prevalence. They use data on untreated caries from the California Oral Health Needs Assessment of Children 2004-2005 and school percentage of participation in free/reduced-price lunch programs to illustrate the methods. Absolute and relative measures, the Slope Index of Inequality, the Relative Index of Inequality (mean and ratio), and the Health Concentration Index were estimated. Taylor series linearization and rescaling bootstrap methods were used to estimate CIs. Oral health differed significantly between White children and all non-White

children and was significantly related to SEP.

PMCID: PMC2597673
PMID: 18779387 [PubMed - indexed for MEDLINE]

655. Immunology. 2009 Apr;126(4):579-87. doi: 10.1111/j.1365-2567.2008.02916.x. Epub 2008 Sep 2.

Signalling mechanisms regulating the activation of human eosinophils by mast-cell-derived chymase: implications for mast cell-eosinophil interaction in allergic inflammation.

Wong CK, Ng SS, Lun SW, Cao J, Lam CW.

Department of Chemical Pathology, The Chinese University of Hong Kong, Prince of Wales Hospital, Shatin, Hong Kong. ck-wong@cuhk.edu.hk

Allergic diseases such as asthma and allergic dermatitis are associated with the degranulation of mast cells. Chymase, a mast-cell-specific protease, is the major component in mast cell granules that can induce eosinophil infiltration into inflammatory sites. We examined the immunopathological mechanisms for the activation of eosinophils by chymase in allergic inflammation. Cytokines were measured by cytometric bead array Flex Sets multiplex assay using flow cytometry and enzyme-linked immunosorbent assay. Adhesion molecules, migration and intracellular signalling pathways were assessed by flow cytometry, Boyden chamber assay and Western blot, respectively. Chymase suppressed the apoptosis of eosinophils and induce the release of the cytokine interleukin-6 (IL-6) and chemokines CXCL8, CCL2 and CXCL1 by eosinophils dose-dependently. It also up-regulated the surface expression of adhesion molecule CD18 and stimulated the chemokinetic migration of eosinophils. The expressions of adhesion molecules, cytokines and chemokines, and chemokinetic migration were differentially regulated by the activation of extracellular signal-regulated kinase, p38 mitogen-activated protein kinase, Akt, Janus-activated kinase and nuclear factor-kappaB pathways. Chymase therefore plays a pivotal immunological role in the interaction between mast cells and eosinophils in allergic diseases such as allergic dermatitis by inducing adhesion molecule-mediated chemokinetic migration and inflammatory cytokines and chemokines of eosinophils, through multiple intracellular signalling molecules and transcription factor. Our results therefore provide a further biochemical basis for the pathogenesis of allergic inflammation consequent on the interaction between mast cells and eosinophils, and give insight for the development of new therapies.

PMCID: PMC2673369
PMID: 18771439 [PubMed - indexed for MEDLINE]

656. Acta Otolaryngol. 2009 Jul;129(7):741-4. doi: 10.1080/00016480802398954.

Surgical and medical complications in different cochlear implant devices.

Migirov L, Dagan E, Kronenberg J.

Department of Otolaryngology Head and Neck Surgery, Sheba Medical Center, Tel Hashomer, 52621, Israel. sabim@bezeqint.net

CONCLUSION: There were no surgery-related complications among the patients in the current study. Positioner and removable magnets have been associated with postoperative problems, and the silicone devices were the only ones found by us to cause foreign body and allergic reactions.

OBJECTIVES: To evaluate the complication rate in patients who were implanted with cochlear devices manufactured by different companies.
PATIENTS AND METHODS: This retrospective study included all the patients who underwent cochlear implantation (138 Nucleus, 105 Med-El and 14 Clarion devices) via the suprameatal approach in our department during 2001-2007 and followed up for at least 18 months. Complications such as magnet displacement, foreign body reaction and protrusion of the positioner were considered as being implant-related. Allergy to implant, cholesteatoma, perforated tympanic membrane, intraoperative cerebrospinal fluid (CSF) leakage, wound breakdown, haematoma or seroma, and vestibular disturbances were considered to be patient-related.
RESULTS: Vestibular and wound problems emerged as the most common complications, but there were no significant differences in their rate of occurrence among the various devices. Explantation of the device was required in one case of foreign body reaction, one case of allergy to implant and one case of extrusion of the positioner followed by device failure.

PMID: 18763176 [PubMed - indexed for MEDLINE]

657. Mol Aspects Med. 2008 Oct;29(5):258-89. doi: 10.1016/j.mam.2008.08.001. Epub 2008 Aug 15.

The ADAM metalloproteinases.

Edwards DR, Handsley MM, Pennington CJ.

Biomedical Research Centre, School of Biological Sciences, University of East Anglia, Norwich NR4 7TJ, UK. dylan.edwards@uea.ac.uk

The ADAMs (a disintegrin and metalloproteinase) are a fascinating family of transmembrane and secreted proteins with important roles in regulating cell phenotype via their effects on cell adhesion, migration, proteolysis and signalling. Though all ADAMs contain metalloproteinase domains, in humans only 13 of the 21 genes in the family encode functional proteases, indicating that at least for the other eight members, protein-protein interactions are critical aspects of their biological functions. The functional ADAM metalloproteinases are involved in "ectodomain shedding" of diverse growth factors, cytokines, receptors and adhesion molecules. The archetypal activity is shown by ADAM-17 (tumour necrosis factor-alpha convertase, TACE), which is the principal protease involved in the activation of pro-TNF-alpha, but whose sheddase functions cover a broad range of cell surface molecules. In particular, ADAM-17 is required for generation of the active forms of Epidermal Growth Factor Receptor (EGFR) ligands, and its function is essential for the development of epithelial tissues. Several other ADAMs have important sheddase functions in particular tissue contexts. Another major family member, ADAM-10, is a principal player in signalling via the Notch and Eph/ephrin pathways. For a growing number of substrates, foremost among them being Notch, cleavage by ADAM sheddases is essential for their subsequent "regulated intramembrane proteolysis" (RIP), which generates cleaved intracellular domains that translocate to the nucleus and regulate gene transcription. Several ADAMs play roles in spermatogenesis and sperm function, potentially by effecting maturation of sperm and their adhesion and migration in the uterus. Other non-catalytic ADAMs function in the CNS via effects on guidance mechanisms. The ADAM family are thus fundamental to many control processes in development and homeostasis, and unsurprisingly they are also linked to pathological states when their functions are dysregulated, including cancer, cardiovascular disease, asthma, Alzheimer's disease. This review will provide an overview of current knowledge of the human ADAMs, discussing their structure, function, regulation and disease involvement.

PMID: 18762209 [PubMed - indexed for MEDLINE]

658. Lung. 2008 Nov-Dec;186(6):375-80. doi: 10.1007/s00408-008-9099-1. Epub 2008 Aug 29.

Found in inflammatory zone 1 induces angiogenesis in murine models of asthma.

Sun Y, Wang J, Li H, Han X.

Department of Pediatrics, Shandong Provincial Hospital, Shandong University, 44 Wenhua Xi Road, Jinan, 250012, People's Republic of China.

Angiogenesis plays an important role in airway remodeling that occurs in asthma, while the mechanisms underlying this process are not fully elucidated. Found in inflammatory zone 1 (FIZZ1), a novel cysteine-rich secreted protein, is able to promote proliferation and migration of pulmonary endothelial cells, and to upregulate the expression of vascular endothelial growth factor (VEGF). However, the role of FIZZ1 in angiogenesis of asthma has not been fully investigated. Murine models of asthma were sensitized on days 1 and 14 by ovalbumin (OVA) and challenged with 2% OVA beginning from day 21. Mice were divided into four groups: asthmatic mice challenged with OVA for 7 days, 14 days, and 28 days, respectively, and healthy control mice that were sensititized and challenged with PBS. The expressions of FIZZ1, VEGF, and von Willebrand factor (vWF)-stained vascular area were measured in asthmatic mice and healthy controls (n = 10). Histologic examination was also performed on airway inflammation. Levels of FIZZ1 were increased significantly after allergic challenge, reached the peak by 7 days, declined by 14 days, and were reduced further by 28 days after OVA challenge. Similarly, percentages of vWF-stained vascular area (percentage of vascularity) were largely increased within 7 days and then reduced from day 14 of challenge. The expression of FIZZ1 in asthma was positively correlated with vWF-stained vascular area and VEGF expression in a time-dependent manner. FIZZ1 expression was significantly correlated with the percentage of vascularity and VEGF expression, suggesting that FIZZ1 and VEGF have crucial roles in angiogenesis of asthma.

PMID: 18758859 [PubMed - indexed for MEDLINE]

659. Clin Immunol. 2008 Nov;129(2):202-10. doi: 10.1016/j.clim.2008.07.015. Epub 2008 Aug 30.

Local blockade of TSLP receptor alleviated allergic disease by regulating airway dendritic cells.

Shi L, Leu SW, Xu F, Zhou X, Yin H, Cai L, Zhang L.

Department of Immunology, Hangzhou Normal University, Hangzhou, PR China.

Thymic stromal lymphopoietin (TSLP) emerges as a central mediator of T helper cell (Th)2-dominant allergic diseases. However, the role of TSLP receptor (TSLPR) in allergen-induced Th2 priming, and the effects of TSLP signaling blocking on the development of asthma remain unclear. Here we showed that allergen challenge caused a rapid accumulation of TSLP in the airways of asthmatic mice, correlating well with eosinophils counts and interleukin (IL)-5 productions. When TSLP signaling was blocked by intratracheal administration of anti-TSLPR antibody before sensitization, eosinophilic airway inflammation, goblet cell hyperplasia and Th2 cytokines productions were significantly reduced. The alleviating effects of TSLPR blocking were achieved by inhibition of maturation and migration of airway dendritic cells (DCs), as well as their abilities of initiating CD4+T cell responses. Thus, local application of anti-TSLPR prevented Th2-mediated airway

inflammation, at least partly, by regulating DCs function, which might be exploited to develop novel treatments for asthma.

PMID: 18757241 [PubMed - indexed for MEDLINE]

660. Int Arch Allergy Immunol. 2009;148(1):31-40. doi: 10.1159/000151503. Epub 2008 Aug 21.

Changes in RANTES and beta-thromboglobulin after intensive exercise in patients with allergic asthma.

Zietkowski Z, Bodzenta-Lukaszyk A, Tomasiak MM, Skiepko R, Mroczko B, Szmitkowski M.

Department of Allergology and Internal Medicine, Medical University of Białystok, Białystok, Poland. z.zietkowski@wp.pl

BACKGROUND: There is increasing evidence that exercise-induced bronchoconstriction (EIB) is associated with eosinophilic airway inflammation. In the pathogenesis of EIB the role of chemokines - responsible for promoting the migration and activation of inflammatory cells - as well as blood platelets, a potential source of those chemokines, remains unclear.
METHODS: The study was conducted in a group of 19 asthmatics (11 with EIB, 8 without EIB) and 8 healthy volunteers. Changes in the plasma concentrations of RANTES and beta-thromboglobulin (beta-TG) induced by intensive exercise were determined. Moreover, the possible correlation of these measurements with the results of other tests used in the diagnosis of asthma as well as laboratory tests commonly associated with asthma were investigated.
RESULTS: A comparison of the concentrations of beta-TG in all groups studied at rest did not reveal any significant differences. In all groups studied, 30 min after exercise elevated beta-TG concentrations were observed; the most significant increase was revealed in asthmatics with EIB. The baseline concentrations of RANTES before exercise in both groups of asthmatics were significantly higher in comparison to the group of healthy volunteers. After exercise, in the group of patients with EIB, a significant increase in RANTES concentrations was observed. These changes correlated with an increase in other markers of airway inflammation 24 h after exercise.
CONCLUSIONS: We suggest that platelet activation, resulting in elevated RANTES release, could be one of the factors responsible for the increase of airway inflammation observed in consequence of EIB in asthmatics.

(c) 2008 S. Karger AG, Basel.

PMID: 18716401 [PubMed - indexed for MEDLINE]

661. Allergy. 2008 Sep;63(9):1164-76. doi: 10.1111/j.1398-9995.2008.01747.x.

Treatment with anti-CC chemokine receptor 3 monoclonal antibody or dexamethasone inhibits the migration and differentiation of bone marrow CD34 progenitor cells in an allergic mouse model.

Ben S, Li X, Xu F, Xu W, Li W, Wu Z, Huang H, Shi H, Shen H.

Department of Respiratory Disease, Second Affiliated Hospital, Zhejiang University College of Medicine, The Institute of Respiratory Diseases of Zhejiang University, Hangzhou, China.

BACKGROUND: The migration and in situ differentiation of CD34(+) progenitors

contribute to inflammatory eosinophilia in asthma and corticosteroids have been widely used in asthma. However, little is know about whether and how corticosteroids modulate the migration and differentiation of CD34(+) progenitors. This study was aimed to investigate the impact of anti-CC chemokine receptor 3 (CCR3) or dexamethasone on inflammatory eosinophilia in asthma and possible mechanism(s) underlying the action of dexamethasone or anti-CCR3 on migration and differentiation of CD34(+) progenitors in asthmatic context.
METHODS: Using an asthmatic mouse model, airway inflammation of anti-CCR3- or dexamethasone-treated mice and that of controls were characterized. And the migration and differentiation of CD34(+) progenitor cells were analyzed in vivo, ex vivo or in vitro.
RESULTS: Treatment with anti-CCR3 or dexamethasone significantly inhibited allergen-induced eosinophilia and CD34(+) progenitor cell infiltration in the lung, which was accompanied by lower levels of airway hyper-responsiveness and mucus production. Moreover, anti-CCR3 inhibited the eotaxin-mediated migration and IL-5/eotaxin-induced differentiation of CD34(+) progenitors in vitro. Dexamethasone was also shown to mitigate eotaxin-mediated migration and IL-5 or eotaxin-promoted differentiation of CD34(+) progenitor cells ex vivo, which were associated with the down-regulation of CCR3 expression on bone marrow progenitor cells.
CONCLUSIONS: Treatment with anti-CCR3 or dexamethasone can inhibit the migration and differentiation of CD34(+) progenitor cells by regulating the eotaxin/CCR3 axis in asthmatic mice. Our findings provide new insights into understanding the mechanism(s) underlying the action of dexamethasone and CCR3-mediated signaling in allergic inflammation and aid in the design of new immunotherapy for intervention of human asthma.

PMID: 18699933 [PubMed - indexed for MEDLINE]

662. Allergy. 2008 Sep;63(9):1148-55. doi: 10.1111/j.1398-9995.2008.01684.x.

The airway smooth muscle CCR3/CCL11 axis is inhibited by mast cells.

Saunders R, Sutcliffe A, Woodman L, Kaur D, Siddiqui S, Okayama Y, Wardlaw A, Bradding P, Brightling C.

Institute for Lung Health, Department of Infection, Immunity and Inflammation, University of Leicester, Leicester, UK.

BACKGROUND: Airway smooth muscle hyperplasia is a feature of asthma, and increases with disease severity. CCR3-mediated recruitment of airway smooth muscle progenitors towards the airway smooth muscle bundle has been proposed as one possible mechanism involved in airway smooth muscle hyperplasia. Mast cells are microlocalized to the airway smooth muscle bundle and whether mast cells influence CCR3-mediated migration is uncertain.
METHODS: We examined the expression of CCR3 by primary cultures of airway smooth muscle cells from asthmatics and nonasthmatics. CCR3 function was examined using intracellular calcium measurements, chemotaxis, wound healing, cell proliferation and survival assays. We investigated the recovery and function of both recombinant and airway smooth muscle-derived CCL11 (eotaxin) after co-culture with beta-tryptase and human lung mast cells.
RESULTS: Airway smooth muscle expressed CCR3. Airway smooth muscle CCR3 activation by CCL11 mediated intracellular calcium elevation, concentration-dependent migration and wound healing, but had no effect on proliferation or survival. Co-culture with beta-tryptase or mast cells degraded recombinant and airway smooth muscle-derived CCL11, and beta-tryptase inhibited CCL11-mediated airway smooth muscle migration.
CONCLUSIONS: CCL11 mediates airway smooth muscle migration. However co-culture with beta-tryptase or mast cells degraded recombinant and airway smooth

muscle-derived CCL11 and inhibited CCL11-mediated airway smooth muscle migration. Therefore these findings cast doubt on the importance of the CCL11/CCR3 axis in the development of airway smooth muscle hyperplasia in asthma.

PMID: 18699931 [PubMed - indexed for MEDLINE]

663. Inflamm Allergy Drug Targets. 2008 Jun;7(2):85-93.

Anti-selectin therapy for the treatment of inflammatory diseases.

Rossi B, Constantin G.

Department of Pathology, Section of General Pathology, University of Verona, Strada le Grazie 8, Verona, Italy.

Leukocyte migration into the tissues represents a key process in the pathogenesis of inflammatory diseases. Data obtained in clinical trials have convincingly shown that inhibition of leukocyte migration into the target organs represents an effective therapeutic approach for diseases in which inflammation has a noxious effect. Leukocyte tethering and rolling are the earliest steps of leukocyte adhesion cascade in inflamed vessels. Selectins are type I transmembrane glycoproteins that bind sialylated carbohydrate structures in a calcium-dependent manner and are involved in the tethering and rolling of leukocytes under physiological and pathological conditions. Three selectins have been identified: L-, P- and E-selectin. Current understanding of the glycosylation-dependent selectin function reveals a complex role for selectins and their ligands during inflammatory diseases. Among selectin ligands, mucin P-selectin glycoprotein ligand-1 (PSGL-1) binds all three selectins and has a well-documented role in organ targeting during inflammation in animal models. However, although inhibition of selectins and their ligands in animal models of inflammatory diseases has proven the validity of this approach in vivo, only a limited number of anti-selectin drugs have been tested in humans. Recent results obtained in clinical trials for asthma and psoriasis show that, although very challenging, the development of selectin antagonists holds concrete promise for the therapy of inflammatory diseases.

PMID: 18691137 [PubMed - indexed for MEDLINE]

664. Curr Mol Med. 2008 Aug;8(5):401-7.

Chloride channel expression and functional diversity in the immune cells of allergic diseases.

Cheng G, Ramanathan A, Shao Z, Agrawal DK.

Department of Biomedical Sciences, Creighton University School of Medicine, Omaha, NE 68178, USA.

Chloride channels are involved in many different physiological processes such as cell migration, proliferation and apoptosis. The importance of the CLC family of chloride channels in these cellular functions has been recognized only recently. Infiltration of inflammatory cells, such as eosinophils, T cells, mast cells and neutrophils, is a hallmark of allergy and asthma. Indeed, chronic asthma is associated with widespread damage to the bronchial epithelium, due to excessive apoptosis, and with defective epithelial repair. However, the relationship between the immune cells of allergic airway diseases and chloride channels has not been clearly elucidated. In this review, characteristics of CLC channels are mainly discussed based on their function and presence in different immune cells

in airway diseases. Not only are chloride channels involved in the recruitment of immune cells, they also play a role in the activation of these cells. Thus, understanding the role of CLC channels in the immune cells would provide unique insights to the pathophysiologic process of chronic asthma and the means to prevent or reverse the disease.

PMID: 18691067 [PubMed - indexed for MEDLINE]

665. Curr Mol Med. 2008 Aug;8(5):365-75.

Prostaglandin D2 receptors DP and CRTH2 in the pathogenesis of asthma.

Arima M, Fukuda T.

Department of Developmental Genetics (H2), Graduate School of Medicine, Chiba University, Japan.

Prostaglandin D2 (PGD2) is a major prostanoid produced mainly by mast cells in allergic diseases, including bronchial asthma. However, its role in the pathogenesis of asthma remains unclear. PGD2-induced vasodilatation and increased permeability are well-known classical effects that may facilitate transendothelial migration of inflammatory cells, such as eosinophils, mast cells, lymphocytes, and monocytes in allergic inflammation. These effects are initiated via a PGD2 receptor, D prostanoid receptor (DP), and are referred to as DP-mediated vasodilation-extravasation. Recently, novel functions of DP have been identified. Furthermore, a novel and different receptor of PGD2, CRTH2, has been discovered. To date, DP and CRTH2 have been shown to be major PGD(2)-related receptors that have pivotal roles in mediating allergic diseases by effects such as directly regulating the migration of inflammatory cells and controlling the production of cytokines and lipid mediators. Available evidence suggests that CRTH2 and DP may collaborate in allergic inflammation. This review focuses on the novel roles of DP and CRTH2 in the initiation and maintenance of allergy.

PMID: 18691063 [PubMed - indexed for MEDLINE]

666. Proc Am Thorac Soc. 2008 Aug 15;5(6):689-94. doi: 10.1513/pats.200801-010AW.

Epithelial cell-extracellular matrix interactions and stem cells in airway epithelial regeneration.

Coraux C, Roux J, Jolly T, Birembaut P.

INSERM UMRS 903, Centre Hospitalier Universitaire Maison Blanche, 45 rue Cognacq Jay, 51092 Reims Cedex, France. christelle.coraux@univ-reims.fr

In healthy subjects, the respiratory epithelium forms a continuous lining to the airways and to the environment, and plays a unique role as a barrier against external deleterious agents to protect the airways from the insults. In respiratory diseases such as cystic fibrosis (CF), chronic obstructive pulmonary disease (COPD), chronic bronchitis, or asthma, the airway epithelium is frequently remodeled and injured, leading to the impairment of its defense functions. The rapid restoration of the epithelial barrier is crucial for these patients. The complete regeneration of the airway epithelium is a complex phenomenon, including not only the epithelial wound repair but also the epithelial differentiation to reconstitute a fully well differentiated and functional epithelium. The regeneration implies two partners: the epithelial stem/progenitor cells and factors able to regulate this process. Among these factors, epithelial cells-extracellular matrix (ECM) interactions play a crucial

role. The secretion of a provisional ECM, the cell-ECM relationships through epithelial receptors, and the remodeling of the ECM by proteases (mainly matrix metalloproteinases) contribute not only to airway epithelial repair by modulating epithelial cell migration and proliferation, but also to the differentiation of repairing cells leading to the complete restoration of the wounded epithelium. A better characterization of resident stem cells and of effectors of the regeneration process is an essential prerequisite to propose new regenerative therapeutics to patients suffering from infectious/inflammatory respiratory diseases.

PMID: 18684718 [PubMed - indexed for MEDLINE]

667. J Immunol Methods. 2008 Sep 15;337(2):121-31. doi: 10.1016/j.jim.2008.07.005. Epub 2008 Jul 26.

Optimization of methods to study pulmonary dendritic cell migration reveals distinct capacities of DC subsets to acquire soluble versus particulate antigen.

Jakubzick C, Helft J, Kaplan TJ, Randolph GJ.

Department of Gene and Cell Medicine, Icahn Research Institute, Mount Sinai School of Medicine, NY, New York 10029, United States. claudia.jakubzick@mssm.edu

Dendritic cell migration from the airway to lymph nodes is a key event in the development of airway immunity during infection, allergy, and vaccination. To identify the best approaches to investigate DC migration to lung-draining lymph nodes, we directly compared three methods previously used to track DC migration: airway administration of fluorescent OVA, latex beads, or carboxyfluorescein succinimidyl ester (CFSE). We show that two of the methods employed in optimal conditions-administration of fluorescent OVA or latex particles-have broadly relevant utility in studies of pulmonary DC migration, both in the presence and absence of inflammatory mediators. However, CFSE was of limited value because it induced a robust airway inflammatory response upon instillation. Unexpectedly, antigen-loaded tracers with distinct physical properties differently affected the populations that acquired the tracers and the overall T cell response. Specifically, soluble OVA and OVA formulated as a particulate after conjugation to latex beads were acquired in different proportions in vivo by the two characterized subsets of pulmonary DCs: CD11b(hi)CD103(-) and CD11b(lo)CD103(+)langerin(+) DC populations. Consequently, and in line with recent studies that these two subsets of DCs respectively activate CD4(+) and CD8(+) lymphocyte populations, the physical nature of the antigen delivery vehicle strongly influenced the degree of CD4(+) versus CD8(+) OVA-specific T cell activation. This finding suggests that changes in the physical presentation of the same antigen delivered to the airway during natural immune responses or vaccinations may markedly affect the character of the T cell response that ensues.

PMCID: PMC3537501
PMID: 18662693 [PubMed - indexed for MEDLINE]

668. Parasitol Res. 2008 Oct;103(5):1191-6. doi: 10.1007/s00436-008-1115-0. Epub 2008 Jul 24.

Aelurostrongylus abstrusus in a feline colony from central Italy: clinical features, diagnostic procedures and molecular characterization.

Traversa D, Di Cesare A, Milillo P, Iorio R, Otranto D.

Department of Comparative Biomedical Sciences, Faculty of Veterinary Medicine, Piazza Aldo Moro 45, 64100, Teramo, Italy. dtraversa@unite.it

Clinical features and conventional and molecular diagnostic procedures have been investigated and evaluated for the infection caused by the lungworm Aelurostrongylus abstrusus (Nematoda, Strongylida). Individual fecal samples from all cats living in a colony with suspected lungworm infection underwent coprological flotation with sugar and zinc sulfate solution and the Baermann migration method. Also, pharyngeal swabs collected for each animal were subjected to a diagnostic nested PCR assay specific for a region internal to the ribosomal Internal Transcribed Spacer 2 of A. abstrusus. Eighteen animals were positive at the Baermann method, while 12 and ten out of them were negative when feces were subjected to the flotation with sugar and zinc sulfate solution, respectively. The nested PCR assay yielded positive results when using the pharyngeal swabs from the 18 coprologically positive cats and from six more cats which were coprologically negative, thus indicating an overall infection rate of 24.4%. Twenty-two out of 24 infected cats showed clinical respiratory symptoms and the most common were general respiratory distress, cough, wheezing, sneezing, and nasal discharge. These results indicate that cat aelurostrongylosis is of clinical importance and, thus, needs to be included in differential diagnosis of feline respiratory diseases. The importance of the disease is discussed together with pros and cons of different conventional and innovative diagnostic approaches.

PMID: 18651179 [PubMed - indexed for MEDLINE]

669. Dtsch Med Wochenschr. 2008 Jul;133 Suppl 3:S88-94. doi: 10.1055/s-2008-1067327.

[What are the changes in the nasal mucosa caused by allergic rhinitis?].

[Article in German]

Klimek L, Böttcher I.

Zentrum für Rhinologie und Allergologie, Wiesbaden.
Ludger.Klimek@HNO-Wiesbaden.de

Allergic rhinitis is one of the most common illnesses of mankind. It is important to be aware that it is an immunological disease associated with extensive changes in the mucosa of the respiratory tract. The cardinal symptoms are nasal obstruction and/or discharge, as well as itching and sneezing urge. The mechanisms involved are complex and include activation and migration of inflammatory cells, vascular dilatation, increase and changes of glandular activity, activation of nerve endings, onset of a neurogenic inflammation and morphologically demonstrable alterations in the mucosa. All this makes it clear that allergic rhinitis is a disease that has to be taken seriously and treated thoroughly, rather than be treated lightly, as has been the case--perhaps because it is so frequent.

PMID: 18642237 [PubMed - indexed for MEDLINE]

670. J Am Geriatr Soc. 2008 Aug;56(8):1543-8. doi: 10.1111/j.1532-5415.2008.01805.x. Epub 2008 Jul 15.

Self-reported health status of vietnamese and non-Hispanic white older adults in california.

Sorkin D, Tan AL, Hays RD, Mangione CM, Ngo-Metzger Q.

Division of General Internal Medicine and Primary Care, Center for Health Policy Research, University of California at Irvine, Irvine, California 92697, USA.

Vietnamese Americans are a rapidly growing minority group in the United States, yet little is known about their health status. Chronic medical conditions and self-rated health of older Vietnamese Americans were compared with those of non-Hispanic white adults living in California using the 2001 and 2003 California Health Interview Surveys (CHISs). The CHIS employed a random-digit-dial telephone survey, and its sample is representative of California's noninstitutionalized population. The sample included 359 Vietnamese and 25,177 non-Hispanic white adults aged 55 and older. Vietnamese and non-Hispanic white adults were compared in terms of limitations in activities of daily living, chronic medical conditions (diabetes mellitus, hypertension, heart disease, asthma), mental health care, and self-reported health, adjusting for age, sex, and education. Vietnamese were more likely than white participants to report needing help for mental health problems (adjusted odds ratio (aOR)=2.1, 95% confidence interval (CI)=1.4-3.1) but less likely to have had their medical providers discuss their mental health problems with them (aOR=0.3, 95% CI=0.1-0.5). In addition, Vietnamese participants reported significantly worse health than white adults on five of eight domains of the Medical Outcomes Survery 12-item Short Form survey (P<.006). Clinicians caring for older Vietnamese individuals should be aware of the high risk for mental health needs in this population and should initiate discussions about mental health with their patients. Further research is needed to better understand why older Vietnamese Americans are at higher risk for worse self-reported health than older white adults.

PMID: 18637981 [PubMed - indexed for MEDLINE]

671. Mol Immunol. 2008 Aug;45(14):3767-74. doi: 10.1016/j.molimm.2008.05.027. Epub 2008 Jul 16.

Role of complement anaphylatoxin receptors (C3aR, C5aR) in the development of the rat cerebellum.

Bénard M, Raoult E, Vaudry D, Leprince J, Falluel-Morel A, Gonzalez BJ, Galas L, Vaudry H, Fontaine M.

Inserm U413, Laboratory of Cellular and Molecular Neuroendocrinology, 76821 Mont-Saint-Aignan, France. magalie.benard@univ-rouen.fr

There is now strong evidence for non-immune or inflammatory functions of complement, notably in the central nervous system. In particular, it has been recently reported that the anaphylatoxin receptors C3aR and C5aR are transiently expressed in the cerebellar cortex of newborn rat, suggesting that anaphylatoxins are involved in the histogenesis of the cerebellum. In the present study, we have investigated the effects of C3aR and C5aR agonists and antagonists on the development of the cerebellum of 11-12-day-old rats in vivo and in vitro. Sub-dural injection of C3aR and C5aR agonists at the surface of the cerebellum transiently modified the thickness of the cortical layers. The C5aR agonist provoked an enlargement of the external granule cell layer (EGL) that was due to increased proliferation of immature granule neurons. Conversely, the C3aR agonist decreased the thickness of the EGL and increased the thickness of the internal granule cell layer (IGL), suggesting that C3a accelerates the migration process of granule cells from the EGL to the IGL. Video-microscopy examination of cultured granule neurons confirmed the role of C3aR in cell motility. These results provide clear evidence for the involvement of anaphylatoxin receptors in the histogenesis of the cerebellar cortex.

PMID: 18635264 [PubMed - indexed for MEDLINE]

672. Int J Biol Markers. 2008 Apr-Jun;23(2):115-20.

Protein biochip array of adhesion molecule expression in peripheral blood of patients with nasal polyposis.

Corsi MM, Pagani D, Dogliotti G, Perona F, Sambataro G, Pignataro L.

Institute of General Pathology, Medical Faculty, University of Milan - Italy. mmcorsi@unimi.it

Nasal polyposis is a chronic non-infectious inflammatory disease of the nasal and paranasal cavity mucosa of unknown multifactorial origin in which inflammatory cells, and in particular eosinophils, seem to play a pivotal role. Eosinophil migration from the bloodstream to nasal polyps is considered to be specific and is a complex process involving several different molecules such as ICAM-1, VCAM-1, and L-, P- and E-selectins. The aim of this study was to investigate, using a protein biochip array technology, the concentrations of these molecules in the peripheral blood of a group of patients affected by nasal polyposis. Patients exhibited a significantly higher expression of VCAM-1, E-selectin, and L-selectin compared to healthy controls, and Spearman's rank correlation test limited to the molecules with significant betweengroup differences demonstrated a significant correlation between VCAM-1 and E-selectin, VCAM-1 and L-selectin, and Eselectin and L-selectin. The results of this investigation are in line with those coming from various imunohistochemical analyses, and seem to confirm the role of inflammation in the pathogenesis of nasal polyposis. These molecules may also represent novel therapeutic targets in the treatment of nasal polyps, and may allow the selection of pharmacological prophylactics that would allow effective inhibition of the inflammation induced by a given allergen.

PMID: 18629784 [PubMed - indexed for MEDLINE]

673. Endocr Rev. 2008 Aug;29(5):581-602. doi: 10.1210/er.2007-0027. Epub 2008 Jul 8.

Alpha-melanocyte-stimulating hormone and related tripeptides: biochemistry, antiinflammatory and protective effects in vitro and in vivo, and future perspectives for the treatment of immune-mediated inflammatory diseases.

Brzoska T, Luger TA, Maaser C, Abels C, Böhm M.

Department of Dermatology, University of Münster, Von Esmarch-Strasse 58, D-48149 Münster, Germany.

Alpha-MSH is a tridecapeptide derived from proopiomelanocortin. Many studies over the last few years have provided evidence that alpha-MSH has potent protective and antiinflammatory effects. These effects can be elicited via centrally expressed melanocortin receptors that orchestrate descending neurogenic antiinflammatory pathways. alpha-MSH can also exert antiinflammatory and protective effects on cells of the immune system and on peripheral nonimmune cell types expressing melanocortin receptors. At the molecular level, alpha-MSH affects various pathways implicated in regulation of inflammation and protection, i.e., nuclear factor-kappaB activation, expression of adhesion molecules and chemokine receptors, production of proinflammatory cytokines and mediators, IL-10 synthesis, T cell proliferation and activity, inflammatory cell migration, expression of antioxidative enzymes, and apoptosis. The antiinflammatory effects of alpha-MSH have been validated in animal models of experimentally induced fever; irritant and allergic contact dermatitis, vasculitis, and fibrosis;

ocular, gastrointestinal, brain, and allergic airway inflammation; and arthritis, but also in models of organ injury. One obstacle limiting the use of alpha-MSH in inflammatory disorders is its pigmentary effect. Due to its preserved antiinflammatory effect but lack of pigmentary action, the C-terminal tripeptide of alpha-MSH, KPV, has been delineated as an alternative for antiinflammatory therapy. KdPT, a derivative of KPV corresponding to amino acids 193-195 of IL-1beta, is also emerging as a tripeptide with antiinflammatory effects. The physiochemical properties and expected low costs of production render both agents suitable for the future treatment of immune-mediated inflammatory skin and bowel disease, fibrosis, allergic and inflammatory lung disease, ocular inflammation, and arthritis.

PMID: 18612139 [PubMed - indexed for MEDLINE]

674. J Immunol. 2008 Jul 15;181(2):1170-8.

Leukotriene B4 receptor 1 expression on dendritic cells is required for the development of Th2 responses and allergen-induced airway hyperresponsiveness.

Miyahara N, Ohnishi H, Matsuda H, Miyahara S, Takeda K, Koya T, Matsubara S, Okamoto M, Dakhama A, Haribabu B, Gelfand EW.

Department of Pediatrics, Division of Cell Biology, National Jewish Medical and Research Center, Denver, CO 80206, USA.

Dendritic cells (DC) are important APCs that control allergen-induced airway responses by interacting directly with T cells. Leukotriene B(4) (LTB(4)), interacting with its high-affinity receptor, LTB(4) receptor 1 (BLT1), is known to attract and activate leukocytes during inflammation. We have previously shown that BLT1 expression on Ag-primed T cells is required for the development of airway hyperresponsiveness (AHR; Miyahara et al. 2005. Am. J. Respir. Crit. Care Med. 172: 161-167). However, the role for the LTB(4)-BLT1 pathway in DC function in allergen-induced airway responses has not been defined. Bone marrow-derived DCs (BMDC) were generated. Naive BALB/c mice received OVA-pulsed BLT1-deficient (BLT1(-/-)) BMDCs or wild-type BMDCs intratracheally and were then challenged with OVA for 3 days. Airway responses were monitored 48 h after the last allergen challenge. BLT1(-/-) BMDCs showed normal maturation judged from surface expression of CD markers. Compared with recipients of wild-type BMDCs, mice that received BLT1(-/-) BMDCs developed significantly lower AHR to inhaled methacholine, lower goblet cell metaplasia, and eosinophilic infiltration in the airways and decreased levels of Th2 type cytokines in the bronchoalveolar lavage fluid. Migration of BLT1(-/-) BMDCs into peribronchial lymph nodes was significantly impaired compared with BLT1(+/+) BMDCs after intratracheal instillation. These data suggest that BLT1 expression on DCs is required for migration of DCs to regional lymph nodes as well as in the development of AHR and airway inflammation.

PMID: 18606670 [PubMed - indexed for MEDLINE]

675. J Immunol. 2008 Jul 15;181(2):1019-24.

ICOS costimulation expands Th2 immunity by augmenting migration of lymphocytes to draining lymph nodes.

Tesciuba AG, Shilling RA, Agarwal MD, Bandukwala HS, Clay BS, Moore TV, Weinstock JV, Welcher AA, Sperling AI.

Department of Medicine, Committee on Immunology, University of Chicago, Chicago,

IL 60637, USA.

The T cell costimulatory molecule ICOS regulates Th2 effector function in allergic airway disease. Recently, several studies with ICOS(-/-) mice have also demonstrated a role for ICOS in Th2 differentiation. To determine the effects of ICOS on the early immune response, we investigated augmenting ICOS costimulation in a Th2-mediated immune response to Schistosoma mansoni Ags. We found that augmenting ICOS costimulation with B7RP-1-Fc increased the accumulation of T and B cells in the draining lymph nodes postimmunization. Interestingly, the increased numbers were due in part to increased migration of undivided Ag-specific TCR transgenic T cells and surprisingly B cells, as well as non-TCR transgenic T cells. B7RP-1-Fc also increased the levels of the chemokines CCL21 and CXCL13 in the draining lymph node, suggesting ICOS costimulation contributes to migration by direct or indirect effects on dendritic cells, stromal cells and high endothelial venules. Further, the effects of B7RP-1-Fc were not dependent on immunization. Our data support a model in which ICOS costimulation augments the pool of lymphocytes in the draining lymph nodes, leading to an increase in the frequency of potentially reactive T and B cells.

PMCID: PMC2560985
PMID: 18606653 [PubMed - indexed for MEDLINE]

676. Biosci Biotechnol Biochem. 2008 Jul;72(7):1901-7. Epub 2008 Jul 7.

Dietary alpha-linked galacto-oligosaccharide suppresses ovalbumin-induced allergic peritonitis in BALB/c mice.

Pirapatdit S, Kishino E, Fujita K, Hashimoto H, Mori S, Saito S, Sato T, Watanabe J, Sonoyama K.

Laboratory of Food Biochemistry, Division of Applied Bioscience, Graduate School of Agriculture, Hokkaido University, Sapporo, Japan.

To determine whether alpha-linked galacto-oligosaccharide (alpha-GOS) prevents allergic peritonitis, BALB/c mice were fed a synthetic diet with and without alpha-GOS supplementation for 7 d, and were then subcutaneously immunized with ovalbumin on days 0 and 7. The mice were challenged by intraperitoneal injection with ovalbumin on day 14, followed by peritoneal lavage on day 15. The total number of peritoneal exudate cells was significantly lower in the mice fed the alpha-GOS diet than in those fed the control diet. Peritoneal lavage fluid from mice fed the alpha-GOS diet not only had less potency to attract peripheral blood leukocytes and peritoneal exudate cells ex vivo, but also had lower concentrations of monocyte chemoattractant protein-1 (MCP-1) and eotaxin. Preincubation of the cells with alpha-GOS failed to affect the migration to peritoneal lavage fluid. We propose that dietary alpha-GOS reduces cell infiltration in allergic peritonitis by reducing antigen-induced elicitation of MCP-1 and eotaxin in mice.

PMID: 18603777 [PubMed - indexed for MEDLINE]

677. Respir Med. 2008 Aug;102(8):1198-202. doi: 10.1016/j.rmed.2008.03.010. Epub 2008 Jul 7.

RANTES in exhaled breath condensate of stable and unstable asthma patients.

Zietkowski Z, Tomasiak MM, Skiepko R, Bodzenta-Lukaszyk A.

Department of Allergology and Internal Medicine, Medical University of Bialystok,

Poland. z.zietkowski@wp.pl

RANTES has been implicated in the allergic inflammation of asthma by promoting the migration and activation of the inflammatory cells, including eosinophils. The study was undertaken to evaluate RANTES levels in the exhaled breath condensate (EBC) of asthmatics with different degrees of asthma severity. EBC was collected from 33 patients with allergic asthma (11 with steroid-naïve mild asthma, 10 with ICS-treated, stable mild-to-moderate asthma, 12 with ICS-treated unstable, severe asthma) and seven healthy volunteers. In the three groups of asthmatics, RANTES concentrations in EBC were significantly higher compared with healthy volunteers. RANTES levels were significantly higher in patients with unstable asthma than in the two groups with stable disease. We observed statistically significant correlations between the concentrations of RANTES in EBC and F(ENO) in the three studied groups of asthmatics; notably, the correlation between the parameters described above was strong positive in the group of unstable and steroid-naïve stable asthmatics. We also discovered a significantly positive correlation between RANTES in EBC and the serum ECP or blood eosinophil count in the groups of asthmatics with severe, unstable asthma and between RANTES and serum ECP in the group of steroid-naïve stable asthmatics. Measurements of RANTES in EBC may provide another useful diagnostic tool for detecting and monitoring inflammation in patients with asthma.

PMID: 18603420 [PubMed - indexed for MEDLINE]

678. Int Immunopharmacol. 2008 Sep;8(9):1172-82. doi: 10.1016/j.intimp.2008.04.002. Epub 2008 May 15.

Epigallocatechin-3-gallate improves Dermatophagoides pteronissinus extract-induced atopic dermatitis-like skin lesions in NC/Nga mice by suppressing macrophage migration inhibitory factor.

Noh SU, Cho EA, Kim HO, Park YM.

Department of Dermatology, Kangnam St. Mary's Hospital, College of Medicine, The Catholic University of Korea, Seoul, Republic of Korea.

Epigallocatechin-3-gallate (EGCG) has been shown to exert anti-inflammatory effects on the inflammatory skin conditions. However, little is known about its effect on atopic dermatitis (AD). We first attempted to assess the anti-inflammatory effect of topical application of EGCG in vivo AD model using NC/Nga mice and to determine whether EGCG exerts the anti-inflammatory effect by inhibiting macrophage migration inhibitory factor (MIF) and other cytokines that are related to immune dysregulation in the pathogenesis of AD. Murine AD-like skin lesions were made by painting Dermatophagoides pteronissinus extract (DPE). The effects of EGCG treatment were assessed by total clinical severity score and ear thickness, and by histological grading. In addition, the mRNA and protein expression of the cytokines including MIF were measured by real-time RT-PCR and immunohistochemistry. The serum levels of MIF and IgE were measured by ELISA. In the AD mouse model, EGCG significantly reduced the total clinical severity score and ear thickness ($p<0.05$). The histological grading was also markedly improved. The mRNA expression of MIF, TNF-alpha, IFN-gamma, IL-2 and IL-12 p40, but not of IL-4, IL-5 and IL-13 in the lesions was significantly reduced by EGCG ($p<0.05$). On the immunohistochemistry, EGCG also markedly diminished the expression of MIF, TNF-alpha and IFN-gamma. The serum MIF and IgE production was significantly reduced by EGCG ($p<0.05$). These results demonstrate that topical application of EGCG may improve the AD-like skin lesions by suppressing MIF and T helper 1 cytokines. Taken together, it is suggested that EGCG may be a potential therapeutic modality for AD.

PMID: 18602062 [PubMed - indexed for MEDLINE]

679. Int Immunopharmacol. 2008 Sep;8(9):1164-71. doi: 10.1016/j.intimp.2008.03.013. Epub 2008 Apr 11.

Effects of single or repeated amphetamine treatment and withdrawal on lung allergic inflammation in rats.

Ligeiro de Oliveira AP, Lazzarini R, Cavriani G, Quinteiro-Filho WM, Tavares de Lima W, Palermo-Neto J.

Department of Pharmacology, Biomedical Sciences Institute, University of São Paulo, São Paulo, Brazil.

The effects of single or repeated amphetamine (AMPH) treatment and those of AMPH withdrawals on immune-mediated lung inflammatory response were studied in rats. Two experiments were done. In the first, rats egg-albumin (OVA) sensitized were singularly or repeatedly (21 days, once daily) treated with AMPH (1.0 mg/kg) or with a similar number and volume of 0.9% NaCl. The OVA aerosol challenge was performed 12 h after the single or last repeated AMPH treatment and also 72 and 120 h after AMPH withdrawal. In the second experiment, the effects of reserpine (1.0 mg/kg/day for 5 consecutive days) on single AMPH actions on lung allergic response of rats were analyzed. Single and repeated AMPH treatment induced opposite actions on Bronchoalveolar lavage fluid (BAL) cellularity of allergic rats: single treatment decreased and repeated treatment increased the total number of cells as well as those of macrophages, neutrophils and eosinophils. Our data also showed that single but not repeated AMPH treatment decreased the number of neutrophils, monocytes and lymphocytes in the peripheral blood, and increased the total number of bone marrow cells in rats sensitized and challenged with OVA. Furthermore, it was shown that reserpine treatment precluded the effects of single AMPH treatment on cellular migration to the lung of OVA-sensitized and challenged rats. It was concluded that AMPH effects on lung inflammatory response and cell recruitment to the lung in allergic rats rely at least partially on corticosterone serum levels. The possible involvement of vesicular monoamine transporter type 2 (VMAT2) with these observed effects was discussed.

PMID: 18602061 [PubMed - indexed for MEDLINE]

680. Int Arch Allergy Immunol. 2008;147(3):231-4. doi: 10.1159/000142046. Epub 2008 Jul 2.

The clinical characteristics of respiratory allergy in immigrants in northern Italy.

Lombardi C, Penagos M, Senna G, Canonica GW, Passalacqua G.

Allergy Unit, Department of Internal Medicine, Sant'Orsola Hospital, Brescia, Italy.

BACKGROUND: Respiratory allergy is influenced and determined by genetic and environmental factors. Migration is a good epidemiological model to indirectly assess the influence of the environment. We assessed the clinical characteristics of respiratory allergy in immigrants in Brescia, Italy.
METHODS: We prospectively collected the data of all immigrants referred to our allergy service for respiratory complaints since 1992. All patients underwent a standard diagnostic workup. The records of a matched Italian population of 1,534 patients were examined for comparison.
RESULTS: Two hundred and thirty-seven patients were evaluated (108 male, mean age

36.3 years). Their countries of origin were uniformly distributed among 4 macroareas (Asia, Africa, South America, Eastern Europe). All patients were referred less than 1 month after the onset of symptoms. Family history for atopy was positive in 9% and clinical history of respiratory allergy was positive in 2%. The mean time of onset of symptoms after immigration was 5.21 years, and the onset symptoms were rhinitis and asthma in 68% patients. Twenty-five percent were monosensitized subjects and 20% of patients had cockroach positivity. Some characteristics (family history, previous clinical history and severity of rhinitis) were clearly different from those of the Italian control population. CONCLUSION: In this population of immigrants, it seems that environmental factors, more than genetic ones, play a role in the onset of respiratory allergy.

Copyright (c) 2008 S. Karger AG, Basel.

PMID: 18594153 [PubMed - indexed for MEDLINE]

681. J Food Prot. 2008 Jun;71(6):1273-6.

Anisakis antigens detected in fish muscle infested with Anisakis simplex L3.

Solas MT, García ML, Rodriguez-Mahillo AI, Gonzalez-Munoz M, de las Heras C, Tejada M.

Department of Cellular Biology, Faculty of Biology, Universidad Complutense de Madrid, Madrid, Spain.

Anisakis simplex is a fish parasite that is a public health risk to those consuming raw or poorly cooked marine fish and cephalopods because of the possibility of becoming infested with live larvae. In humans, penetration of the larvae into the gastrointestinal track can cause acute and chronic symptoms and allergic anisakiasis. Excretion and secretion products released by the larvae are thought to play a role in migration through the tissues and induce an immunoglobulin E-mediated immune response. The aim of this preliminary study was to detect parasite antigens and allergens in fish tissues surrounding the migrating larvae. Hake and anchovy fillets were artificially parasitized with Anisakis larvae and stored in chilled conditions for 5 days. Larvae were evaluated for fluorescence, fish muscle tissue was examined with transmission electron microscopy, and immunohistochemical reactions of two rabbit polyclonal antisera against a parasite crude extract and the allergen Ani s 4 were recorded. Larvae immediately migrated into the fish muscle, and no emission of bluish fluorescence was observed. Fish muscle areas in contact with the parasite showed disruptions in the structure and inclusion of granules within sarcomeres. Both parasite antigens and the Ani s 4 allergen were located in areas close to the larvae and where sarcomere structure was preserved. These findings indicate that parasite antigens and allergens are dispersed into the muscle and might cause allergic symptoms such as dyspnea, vomiting, diarrhea, urticaria, angioedema, or anaphylaxis in some individuals sensitive to A. simplex.

PMID: 18592760 [PubMed - indexed for MEDLINE]

682. J Ethnopharmacol. 2008 Sep 2;119(1):41-6. doi: 10.1016/j.jep.2008.05.036. Epub 2008 Jun 6.

Anti-allergic effect of bee pollen phenolic extract and myricetin in ovalbumin-sensitized mice.

Medeiros KC, Figueiredo CA, Figueredo TB, Freire KR, Santos FA, Alcantara-Neves NM, Silva TM, Piuvezam MR.

Laboratório de Tecnologia Farmacêutica, Centro de Ciências da Saúde, Universidade Federal da Paraíba, UFPB, Brazil.

ETHNOPHARMACOLOGICAL RELEVANCE: The bee pollen is used in folk medicine to alleviate allergic reactions. The bee pollen phenolic extract (BPPE) consists in phenolic compounds (flavonoids) from plants picked by Apis mellifera bee.
AIM OF THIS STUDY: Here we evaluated the anti-allergic property of the BPPE and the flavonoid myricetin (MYR) in murine model of ovalbumin (OVA)-induced allergy.
MATERIALS AND METHODS: The study focused on the BPPE or myricetin treatment of OVA-sensitized BALB/c mice and their effects on the IgE and IgG1 production, pulmonary cell migration, eosinophil peroxidase (EPO) activity and anaphylactic shock reaction.
RESULTS: The BPPE treatment (200mg/kg) showed inhibition of the paw edema, IgE and IgG(1) OVA-specific production, leukocyte migration to the bronchoalveolar lavage (BAL) and EPO activity in lungs. In addition, BPPE treatment showed partial protection on the anaphylactic shock reaction induced by OVA. Treatment with myricetin (5 mg/kg) also inhibited pulmonary cell migration and IgE and IgG(1) OVA-specific production.
CONCLUSIONS: These results support the hypothesis the myricetin is one of the flavonoids of BPPE responsible for the anti-allergic effect and a potential tool to treat allergies.

PMID: 18588965 [PubMed - indexed for MEDLINE]

683. Pharmacol Ther. 2008 Jul;119(1):44-54. doi: 10.1016/j.pharmthera.2008.04.005. Epub 2008 May 16.

Small heat shock proteins in smooth muscle.

Salinthone S, Tyagi M, Gerthoffer WT.

Department of Neurology, Oregon Health Sciences University, Portland, OR, USA.

The small heat shock proteins (HSPs) HSP20, HSP27 and alphaB-crystallin are chaperone proteins that are abundantly expressed in smooth muscles are important modulators of muscle contraction, cell migration and cell survival. This review focuses on factors regulating expression of small HSPs in smooth muscle, signaling pathways that regulate macromolecular structure and the biochemical and cellular functions of small HSPs. Cellular processes regulated by small HSPs include chaperoning denatured proteins, maintaining cellular redox state and modifying filamentous actin polymerization. These processes influence smooth muscle proliferation, cell migration, cell survival, muscle contraction and synthesis of signaling proteins. Understanding functions of small heat shock proteins is relevant to mechanisms of disease in which dysfunctional smooth muscle causes symptoms, or is a target of drug therapy. One example is that secreted HSP27 may be a useful marker of inflammation during atherogenesis. Another is that phosphorylated HSP20 which relaxes smooth muscle may prove to be highly relevant to treatment of hypertension, vasospasm, asthma, premature labor and overactive bladder. Because small HSPs also modulate smooth muscle proliferation and cell migration they may prove to be targets for developing effective, novel treatments of clinical problems arising from remodeling of smooth muscle in vascular, respiratory and urogenital systems.

PMCID: PMC2581864
PMID: 18579210 [PubMed - indexed for MEDLINE]

684. Int Arch Allergy Immunol. 2008;146 Suppl 1:82-7. doi: 10.1159/000126067. Epub

2008 May 27.

Anti-allergic inflammatory effects of hepatocyte growth factor.

Ito W, Takeda M, Tanabe M, Kihara J, Kato H, Chiba T, Yamaguchi K, Ueki S, Kanehiro A, Kayaba H, Chihara J.

Department of Clinical and Laboratory Medicine, Akita University School of Medicine, Akita, Japan.

Hepatocyte growth factor (HGF) is known to influence a number of cell types and regulate various biological activities including cytokine production, cell migration, proliferation and survival. Thus, HGF is now recognized to be a key factor in the prevention and attenuation of disease progression. We have reported that HGF reduces allergic airway inflammation, airway hyperresponsiveness, remodeling and development of Th2 cytokines as well as growth factors such as transforming growth factor-beta in vivo. In vitro, HGF directly attenuates chemotaxis of eosinophils in the absence of Th2 cytokines and modulates mitogen-activated protein kinases, which play an important role in eosinophil migration. In this review, we discuss the physiological role of HGF in allergic inflammation and its mechanism of anti-inflammatory effects, including the regulation of eosinophil functions.

Copyright 2008 S. Karger AG, Basel.

PMID: 18504413 [PubMed - indexed for MEDLINE]

685. Food Addit Contam Part A Chem Anal Control Expo Risk Assess. 2008 Jul;25(7):911-20. doi: 10.1080/02652030701837399.

Bisphenol A diglycidyl ether (BADGE) migrating from packaging material 'disappears' in food: reaction with food components.

Petersen H, Biereichel A, Burseg K, Simat TJ, Steinhart H.

Central Institute of Medical Service German Bundeswehr, Kiel, Germany. h_petersen@gmx.net

Bisphenol A diglycidyl ether (BADGE) is widely used as a monomer for coatings and adhesives for food-contact applications. Previous publications indicate that, after migration from packaging into foodstuffs, BADGE undergoes various reactions with unidentified food components. In order to elucidate the fate of BADGE, losses were determined after incubation with different foodstuffs and food components. Food proteins were identified as the main reaction partner with BADGE. Adduct formation was found with nucleophilic side-chains of amino acids. In vitro, cysteine exhibited significant activity. The previously reported occurrence of methylthio-derivatives of BADGE in foodstuffs was shown to originate from the reaction of BADGE with methionine. BADGE-methylthio derivatives can, therefore, be used as marker substances in foodstuffs for protein reactions with BADGE. The reported results offer a new viewpoint on the evaluation of BADGE migration. The hydrolysis and hydrochlorination derivatives subject to European legislation make up only a fraction of the totally migrated BADGE, and a further concern is that the toxic or allergenic potential of the protein adducts are unknown.

PMID: 18569010 [PubMed - indexed for MEDLINE]

686. Korean J Parasitol. 2008 Jun;46(2):95-9. doi: 10.3347/kjp.2008.46.2.95.

A 27 kDa cysteine protease secreted by newly excysted Paragonimus westermani metacercariae induces superoxide anion production and degranulation of human eosinophils.

Chung YB, Kita H, Shin MH.

Department of Parasitology, Cheju National University College of Medicine, Jeju 690-756, Korea.

Eosinophil degranulation plays a crucial role in tissue inflammatory reactions associated with helminth parasitic infections and allergic diseases. Paragonimus westermani, a lung fluke causing human paragonimiasis, secretes a large amount of cysteine proteases, which are involved in nutrient uptake, tissue invasion, and modulation of hos's immune responses. There is, however, limited information about the response of eosinophils to direct stimulation by cysteine proteases (CP) secreted by P. westermani. In the present study, we tested whether degranulation and superoxide production from human eosinophils can be induced by stimulation of the 2 CP (27 kDa and 28 kDa) purified from excretory-secretory products (ESP) of P. westermani newly excysted metacercariae (PwNEM). A large quantity of eosinophil-derived neurotoxin (EDN) was detected in the culture supernatant when human eosinophils isolated from the peripheral blood were incubated with the purified 27 kDa CP. Furthermore, the 27 kDa CP induced superoxide anion production by eosinophils in time- and dose-dependent manners. In contrast, the purified 28 kDa CP did not induce superoxide production and degranulation. These findings suggest that the 27 kDa CP secreted by PwNEM induces superoxide production and degranulation of human eosinophils, which may be involved in eosinophil-mediated tissue inflammatory responses during the larval migration in human paragonimiasis.

PMCID: PMC2532610
PMID: 18552546 [PubMed - indexed for MEDLINE]

687. Pharmacol Rev. 2008 Jun;60(2):181-95. doi: 10.1124/pr.107.07113. Epub 2008 Jun 13.

"Inside-out" signaling of sphingosine-1-phosphate: therapeutic targets.

Takabe K, Paugh SW, Milstien S, Spiegel S.

Department of Surgery, Division of Surgical Oncology, Virginia Commonwealth University School of Medicine, Richmond, VA 23298, USA.

Sphingosine 1-phosphate (S1P) is a bioactive sphingolipid metabolite involved in many critical cellular processes including proliferation, survival, and migration, as well as angiogenesis and allergic responses. S1P levels inside cells are tightly regulated by the balance between its synthesis by sphingosine kinases and degradation. S1P is interconvertible with ceramide, which is a critical mediator of apoptosis. It has been postulated that the ratio between S1P and ceramide determines cell fate. Activation of sphingosine kinase by a variety of agonists increases intracellular S1P, which in turn can function intracellularly as a second messenger or be secreted out of the cell and act extracellularly by binding to and signaling through S1P receptors in autocrine and/or paracrine manners. Recent studies suggest that this "inside-out" signaling by S1P may play a role in many human diseases, including cancer, atherosclerosis, inflammation, and autoimmune disorders such as multiple sclerosis. In this review we summarize metabolism of S1P, mechanisms of sphingosine kinase activation, and S1P receptors and their downstream signaling pathways and examine relationships to multiple disease processes. In particular, we describe recent preclinical and

clinical trials of therapies targeting S1P signaling, including
2-amino-2-propane-1,3-diol hydrochloride (FTY720, fingolimod), S1P receptor
agonists, sphingosine kinase inhibitors, and anti-S1P monoclonal antibody.

PMCID: PMC2695666
PMID: 18552276 [PubMed - indexed for MEDLINE]

688. Ned Tijdschr Geneeskd. 2008 May 17;152(20):1157-63.

[Prevalence of and health care consumption for asthma and COPD in relation to
ethnicity].

[Article in Dutch]

Uijen AA, Schermer TR, van den Hoogen HJ, Mulder J, Zantinge EM, Bottema BJ.

Universitair Medisch Centrum St Radboud, Afd. Huisartsgeneeskunde, Postbus 9101,
6500 HB Nijmegen. a.uijen@hag.umcn.nl

Comment in
    Ned Tijdschr Geneeskd. 2008 May 17;152(20):1136-7.

OBJECTIVE: To determine whether there are differences in prevalence of and health
care consumption for asthma and COPD between Dutch people of Turkish, Moroccan
and Surinamese origin and indigenous Dutch people.
DESIGN: Retrospective.
METHOD: Based on data from the 'Second Dutch national study into morbidity and
interventions in general practice', we compared the prevalence of asthma and COPD
in the different ethnic groups. In addition, we compared the use of various
airway medications and the number of general practice contacts between these
ethnic groups.
RESULTS: We analysed data of 240,067 indigenous Dutch, 2,942 Turkish, 2,416
Moroccan and 3,320 Surinamese subjects. Asthma is more prevalent among Surinamese
and seems less prevalent among Moroccans. COPD seems less prevalent among
immigrants than among the indigenous Dutch population. Immigrants tend to have
less prescriptions of prophylactic maintenance airway medication and they also
tend to have less airway-related general practice contacts than indigenous Dutch
patients.
CONCLUSION: Differences exist in the prevalence of and health care consumption
for asthma and COPD between the different ethnic groups in the Netherlands. There
seems to be underdiagnosis of COPD in immigrants. Moreover, immigrant asthma and
COPD patients are probably undertreated.

PMID: 18549142 [PubMed - indexed for MEDLINE]

689. J Invest Dermatol. 2008 Dec;128(12):2783-91. doi: 10.1038/jid.2008.153. Epub 2008
Jun 12.

Histamine upregulates keratinocyte MMP-9 production via the histamine H1
receptor.

Gschwandtner M, Purwar R, Wittmann M, Bäumer W, Kietzmann M, Werfel T, Gutzmer R.

Department of Dermatology, Hannover Medical School, Hannover, Germany.
gschwandtner.maria@mh-hannover.de

Comment in
    J Invest Dermatol. 2008 Dec;128(12):2748-50.

Skin inflammation and the migration of cells at the site of the immune response play an important role in allergic skin diseases. It has already been described that matrix metalloproteinase 9 (MMP-9) influences tissue remodeling and facilitates cell migration by proteolytic degradation of basal membrane components. The aim of this study was to investigate MMP-9 expression on human primary keratinocytes (KCs) upon stimulation with histamine, a potent mediator in allergic responses. With ELISA and zymography, we could show that histamine induced dose-dependent upregulation of MMP-9 in cultured KCs and in punch biopsies of human skin. The histamine H(1) receptor (H(1)R) agonist beta-histine-but not agonists for H(2)R, H(3)R, and H(4)R-induced MMP-9, whereas the H(1)R antagonist clemastine blocked the effect in a dose-dependent manner. Immunohistological staining showed that histamine-induced MMP-9 led to destruction of type IV collagen at the basement membrane in healthy skin. In a coculture system of KCs and T cells, migration of T cells through an artificial basement membrane was increased after histamine stimulation of KCs. Our findings demonstrate enhanced MMP-9 production and cell migration after histamine stimulation and may represent a new mechanism by which KCs contribute to the pathology of skin diseases.

PMID: 18548114 [PubMed - indexed for MEDLINE]

690. Scand J Public Health. 2008 Jun;36(4):353-60. doi: 10.1177/1403494808089062.

The association between mental health problems and inflammatory conditions across gender and immigrant status: a population-based cross-sectional study among 10th-grade students.

Lien L.

Institute of Psychiatry, University of Oslo, Oslo, Norway.
lars.lien@medisin.uio.no

AIM: The aim of this study was to describe the prevalence of and investigate the association between mental health problems, asthma, allergy and eczema in Norwegian and immigrant youths.
METHODS: A cross-sectional study was performed of all 10th-grade students in Oslo, Norway, in two school years; 1999-2000 and 2000-2001. Of the 8316 eligible students, 7345 (88.3%) participated. Internalized mental problems were measured using the Hopkins Symptom Check List 10-version, and two subscales of the Strength and Difficulties Questionnaire were used to study externalizing mental health problems. All questions are based on self-report, and 25% of the sample had an immigrant background.
RESULTS: Immigrant boys had higher scores on internalizing problems than Norwegian boys. First-generation immigrants reported less asthma and eczema than Norwegians. The strongest association between mental health problems and inflammatory conditions was for allergy and internalizing problems for boys (odds ratio (OR)=2.5 for immigrants and OR=1.8 for Norwegians). For girls, it was allergy in Norwegians (OR=1.6) and asthma for immigrants (OR=2.2). For externalizing problems, the association was strongest for asthma in boys and eczema in girls. Immigrant boys had stronger associations between number of inflammatory conditions and internalizing mental health problems than Norwegians (OR=3.2 vs. OR=2.4). Among girls, the figures were 1.7 for Norwegians and 1.8 for immigrants.
CONCLUSIONS: There is a strong association between number of inflammatory conditions and internalizing mental health problems, especially among boys with an immigrant background. The association with externalizing mental health problems was less prominent.

PMID: 18539689 [PubMed - indexed for MEDLINE]

691. J Immunol. 2008 Jun 15;180(12):8354-60.

CCL11 and GM-CSF differentially use the Rho GTPase pathway to regulate motility of human eosinophils in a three-dimensional microenvironment.

Muessel MJ, Scott KS, Friedl P, Bradding P, Wardlaw AJ.

Institute for Lung Health, Department of Infection, Immunity and Inflammation, University of Leicester, Leicester, UK.

Asthma is a common disease that causes considerable morbidity. Increased numbers of airway eosinophils are a hallmark of asthma. Mechanisms controlling the entry of eosinophils into asthmatic lung have been intensively investigated, but factors regulating migration within the tissue microenvironment are less well understood. We modeled this by studying chemoattractant and growth factor-mediated human eosinophil migration within a three-dimensional collagen matrix. Stimulation with GM-CSF induced dose-dependent, random migration with a maximum of 77 +/- 4.7% of cells migrating. In contrast, CCL11 and C5a caused a more modest although significant degree of migration (19 +/- 1.8% and 20 +/- 2.6%, respectively). Migration to GM-CSF was partially dependent on Ca(2+) and alpha(M)beta(2) integrins. The Rho family of small GTPases regulates intracellular signaling of cell migration. GM-CSF-induced migration was only partially dependent on Rho kinase/Rho-associated kinase (ROCK) and was independent of RhoA activation. In contrast, CCL11-induced migration was fully dependent on both RhoA and ROCK. Activation of RhoA was therefore neither necessary nor sufficient to cause eosinophil migration in a three-dimensional collagen environment. This study suggests that eosinophil growth factors are likely to be required for eosinophil migration within the bronchial mucosa, and this involves signal transduction pathways distinct from those used by G protein-associated chemoattractants.

PMID: 18523302 [PubMed - indexed for MEDLINE]

692. Psychiatr Serv. 2008 Jun;59(6):641-7. doi: 10.1176/appi.ps.59.6.641.

Prevalence, diagnosis, and treatment of depression and generalized anxiety disorder in a diverse urban community.

Gwynn RC, McQuistion HL, McVeigh KH, Garg RK, Frieden TR, Thorpe LE.

Mailman School of Public Health, Columbia University, New York, NY 10032, USA. crg2128@columbia.edu

OBJECTIVE: This study assessed the prevalence, diagnosis, and treatment of major depressive disorder and generalized anxiety disorder among New York City adults.
METHODS: As part of the first community-specific Health and Nutrition Examination Survey in the United States, depression and anxiety were assessed in a representative sample of 1,817 noninstitutionalized adults in 2004.
RESULTS: A total of 8% had major depressive disorder and 4% had generalized anxiety disorder. Respondents with depression were more likely to be formerly married, publicly insured, younger, and U.S. born. Only 55% of adults with depression were diagnosed, and 38% of those with depression or anxiety were in treatment; individuals with a diagnosis of depression were more likely to receive treatment than those without a diagnosis (61% versus 7%; p<.001). Immigrants with depression were 60% less likely to be diagnosed than their U.S.-born counterparts; immigrants arriving in this country ten or more years ago had

slightly more anxiety than immigrants arriving less than ten years ago (3% versus 2%, not significant). Among respondents with anxiety, 23% reported disability compared with 15% of those with depression. Compared with adults with neither diagnosis, adults with depression or anxiety were twice as likely to smoke tobacco (p<.05), adults with depression were twice as likely to have diabetes (p<.01), and those with anxiety were twice as likely to have asthma (p<.01).
CONCLUSIONS: Mental disorders are often disabling and inadequately diagnosed and treated. Foreign-born adults experience barriers to diagnosis and treatment despite having less depression; anxiety may increase with time since immigration. Increased awareness of and linkage to mental health services are needed, especially in larger, more diverse urban communities.

PMID: 18511584 [PubMed - indexed for MEDLINE]

693. Zhonghua Er Bi Yan Hou Tou Jing Wai Ke Za Zhi. 2008 Feb;43(2):130-3.

[Role of aquaporin 1 in the migration of eosinophils from asthmatic guinea pigs].

[Article in Chinese]

Lei F, Zhu DD, Wang XR, Guan GM, Jiang YP, Dong Z.

Department of Otorhinolaryngology Head and Neck Surgery, China-Japan Union Hospital, Jilin University, Changchun 130033, China.

OBJECTIVE: To investigate the role of aquaporin 1 (AQP1) in the migration of eosinophils (EOS) and to determine if AQP-1 can be viewed as the chemotactic activity marker of EOS.
METHODS: Asthma model of guinea pigs were developed and EOS were purified from both peripheral blood and bronchoalveolar lavage fluid (BALF). The smears of EOS were studied by in situ hybridization for determining AQP1 mRNA and immunofluorescence under laser scanning confocal microscope for determining AQP1 protein.
RESULTS: AQP1 was found expressed in EOS both from peripheral blood and from BALF. Compared with the expression of AQP1 mRNA (mean grey value 109.200 +/- 5.756, x +/- s) and protein (average fluorescence intensity 279.926 +/- 11.293) in EOS from BALF, there was stronger expression of AQP1 mRNA (92.904 +/- 3.290) and protein (425.081 +/- 17.474) in EOS from peripheral blood. The difference both of AQP1 mRNA (t = 9.519, P < 0.05) and protein(t = 27.020, P < 0.05) were considered statistically significant respectively.
CONCLUSIONS: AQP1 plays a crucial role in EOS movement. It is possible that EOS produce more AQP1 protein to accelerate its migration to inflammatory tissues under allergic disease and EOS with AQP1 highly expressed are activated. AQP1 can be viewed as the chemotactic activity marker of EOS.

PMID: 18510218 [PubMed - indexed for MEDLINE]

694. Int J Hyg Environ Health. 2009 Mar;212(2):146-56. doi: 10.1016/j.ijheh.2008.04.002. Epub 2008 May 23.

Childhood social position and associations between environmental exposures and health outcomes.

Hoffmann B, Kolahgar B, Rauchfuss K, Eberwein G, Franzen-Reuter I, Kraft M, Wilhelm M, Ranft U, Jöckel KH.

Institute for Medical Informatics, Biometry and Epidemiology, University Hospital Essen, University of Duisburg-Essen, Germany. barbara.hoffmann@uk-essen.de

BACKGROUND: Growing evidence indicates that environmental exposures are more prevalent among socially disadvantaged groups. We investigated the distribution of environmental exposures and health outcomes in preschool children, and examined the role of social position on their associations.
METHODS: We analysed data from a cross-sectional study on 968 preschool children from three districts in the Ruhr Area and one rural community in North Rhine-Westphalia in 2000. Parents filled in a questionnaire on socio-demographic characteristics, environmental exposures, respiratory infections and allergic diseases. Residential annual total suspended particulate matter (TSP) mass concentrations were derived from a small-scale interpolation model. Lung function, allergic sensitisation and immunologic function were assessed. We analysed the associations between environmental exposures and health outcomes in social subgroups with logistic regression.
RESULTS: High TSP concentrations at the home address and unfavourable living conditions were more prevalent in the socially disadvantaged groups, while allergic and respiratory infectious diseases were reported more frequently in the privileged groups. The odds ratio for the association between TSP and history of allergic diseases was 1.17 (95% CI 0.95-1.45) in children without immigration background and 0.71 (95% CI 0.53-0.95) with immigration background. Heterogeneity for exposure-outcome associations was also seen between TSP and lung function as well as unfavourable living conditions and allergic diseases.
CONCLUSIONS: We found evidence for an influence of social position on environment-health associations. Possible explanations for heterogeneity include social group-specific over- and underreporting and effect measure modification, which need to be taken into account when designing and analysing environmental health studies.

PMID: 18502174 [PubMed - indexed for MEDLINE]

695. Clin Exp Allergy. 2008 Jun;38(6):957-67. doi: 10.1111/j.1365-2222.2008.02982.x.

Human innate immune responses to hexamethylene diisocyanate (HDI) and HDI-albumin conjugates.

Wisnewski AV, Liu Q, Liu J, Redlich CA.

Department of Internal Medicine, Yale School of Medicine, New Haven, CT 06520, USA. adam.wisnewski@yale.edu

BACKGROUND: Isocyanates, a leading cause of occupational asthma, are known to induce adaptive immune responses; however, innate immune responses, which generally precede and regulate adaptive immunity, remain largely uncharacterized.
OBJECTIVE: The aim of the study was to identify and characterize the cellular, molecular and systemic innate immune responses induced by hexamethylene diisocyanate (HDI).
METHODS: Human peripheral blood mononuclear cells (PBMCs) were stimulated in vitro with HDI-albumin conjugates or control antigen, and changes in phenotype, gene and protein expression were characterized by flow cytometry, microarray, Western blot and ELISA. Cell uptake of isocyanate was visualized microscopically using HDI-albumin conjugates prepared with fluorescently labelled albumin. In vivo, human HDI exposure was performed via a specific inhalation challenge, and subsequent changes in PBMCs and serum proteins were measured by flow cytometry and ELISA. Genotypes were determined by PCR.
RESULTS: Human monocytes take up HDI-albumin conjugates and undergo marked changes in morphology and gene/protein expression in vitro. The most significant (P-values 0.007-0.05) changes in microarray gene expression were noted in lysosomal genes, especially peptidases and proton pumps involved in antigen processing. Chemokines that regulate monocyte/macrophage trafficking (MIF, MCP-1)

and pattern-recognition receptors that bind chitin (chitinases) and oxidized low-density lipoprotein (CD68) were also increased following isocyanate-albumin exposure. In vivo, HDI-exposed subjects exhibited a drastic increase in the percentage of PBMCs with the same HDI-albumin responsive phenotype characterized in vitro (HLA-DR(+)/CD11c(+) with altered light scatter properties). An exposure-dependent decrease (46+/-11%; P<0.015) in serum concentrations of chitinase 3-like-1 was also observed in individuals who lack the major (type 1) human chitinase (due to genetic polymorphism), but not in individuals possessing at least one functional chitinase-1 allele.
CONCLUSIONS: Previously unrecognized innate immune responses to HDI and HDI-albumin conjugates could influence the clinical spectrum of exposure reactions.

PMCID: PMC3131007
PMID: 18498542 [PubMed - indexed for MEDLINE]

696. Arch Esp Urol. 2008 Mar;61(2):263-8.

Vantris, a biocompatible, synthetic, non-biodegradable, easy-to-inject bulking substance. Evaluation of local tissular reaction, localized migration and long-distance migration.

Ormaechea M, Paladini M, Pisano R, Scagliotti M, Sambuelli R, Lopez S, Guidi A, Muñoz J, Rossetti V, Carnerero M, Beltramo D, Alasino R, Bianco I, Griguol O, Valladares D, De Badiola F.

Universidad Católica de Córdoba, School of Medicine, Córdoba, Argentina.
maria.ormaechea@hospitalitaliano.org.ar

Biodegradable injectable bulking agents of animal origin present a fast rate of bio-reabsorption and may cause an allergic reaction. Biodegradable elements of synthetic origin have a high rate of reabsorption after a year. Non-biodegradable agents of synthetic origin lead to the formation of a fibrotic capsule, giving stability and long-term permanence. VANTRIS is categorized into this last group; it belongs to the family of Acrylics, particles of polyacrylate polyalcohol copolymer immersed in a glycerol and physiological solution carrier. Molecular mass is very high. When injected in soft tissues, this material causes a bulkiness that remains stable through time. The carrier is a 40% glycerol solution with a pH of 6. Once injected, the carrier is eliminated by the reticular system through the kidneys, without metabolizing. Particles of this polyacrylate polyalcohol with glycerol are highly deformable by compression, and may be injected using a 23-gauge needle. The average of particles size is 320 mm. Once implanted, particles are covered by a fibrotic capsule of up to 70 microns. Particles of this new material are anionic with high superficial electronegativity, thus promoting a low cellular interaction and low fibrotic growth. The new polyacrylate polyalcohol copolymer with glycerol was tested for biocompatibility according to ISO 10993-1:2003 in vitro, showing that they are not mutagenic for the Salmonella T. strains analyzed. The extract turned out to be non-cytotoxic for cell lines in culture and non-genotoxic for mice. In in vivo studies, acrylate did not cause sensitization in mice. The macroscopic reaction of tissue irritation was not significant in subcutaneous implants and in urethras of rabbits. Seven female dogs were injected transurethrally with VANTRIS to evaluate short and long-term migration (13 weeks and 12 months respectively). No particles or signs of inflammation or necrosis are observed in any of the organs examined 13 weeks and 12 months after implantation. To conclude, this new material meets the conditions of ideal tissue bulking material.

PMID: 18491745 [PubMed - indexed for MEDLINE]

697. J Immunol. 2008 Jun 1;180(11):7597-603.

IL-10 inhibits cysteinyl leukotriene-induced activation of human monocytes and monocyte-derived dendritic cells.

Woszczek G, Chen LY, Nagineni S, Shelhamer JH.

Critical Care Medicine Department, Clinical Center, National Institutes of Health, Bethesda, MD 20892, USA.

The immunoregulatory cytokine IL-10 plays an essential role in down-modulating adaptive and innate immune responses leading to chronic inflammatory diseases. In contrast, cysteinyl leukotrienes (cysLTs), important proinflammatory mediators of cell trafficking and innate immune responses, are thought to enhance immune reactions in the pathogenesis of diseases, such as bronchial asthma, atherosclerosis, and pulmonary fibrosis. The aim of this study was to determine the IL-10 regulatory role in cysLT-induced activation of human monocytes and monocyte-derived dendritic cells. Herein we show that cysLT-induced activation and chemotaxis of human monocytes and monocyte-derived immature dendritic cells (iDC) are inhibited by IL-10 pretreatment. IL-10 down-regulated cysLT type 1 and 2 receptors' mRNA in a time- and concentration-dependent fashion. cysLT-induced activation of monocytes and iDCs measured by intracellular calcium flux and immediate-early gene expression (FBJ murine osteosarcoma viral oncogen homolog B and early growth response-2) was potently decreased by IL-10 and by the cysLT antagonist MK571. Chemotaxis of monocytes and iDCs to increasing concentrations of leukotriene D(4) (LTD(4)) was also inhibited by IL-10. LTD(4) enhanced iDC migration in response to CCL5. IL-10 selectively inhibited LTD(4)-induced chemotaxis without affecting migration to CCL5. These data indicate that cysLT-induced activation of human monocytes and dendritic cells may be specifically inhibited by IL-10, suggesting a direct link between the 5-lipoxygenase proinflammatory pathway and IL-10 regulatory mechanisms. Antileukotriene therapies may reproduce some regulatory mechanisms played by IL-10 in inflammatory processes.

PMCID: PMC2435075
PMID: 18490762 [PubMed - indexed for MEDLINE]

698. J Immunol. 2008 Jun 1;180(11):7423-30.

Chronically inflamed human tissues are infiltrated by highly differentiated Th17 lymphocytes.

Pène J, Chevalier S, Preisser L, Vénéreau E, Guilleux MH, Ghannam S, Molès JP, Danger Y, Ravon E, Lesaux S, Yssel H, Gascan H.

Institut National de la Santé et de la Recherche Médicale, Unité 844, Montpellier, France.

Chronic inflammatory diseases are characterized by local tissue injury caused by immunocompetent cells, in particular CD4(+) T lymphocytes, that are involved in the pathogenesis of these disorders via the production of distinctive sets of cytokines. Here, we have characterized single CD4(+) T cells that infiltrate inflamed tissue taken from patients with psoriasis, Crohn's disease, rheumatoid arthritis, or allergic asthma. Results from a cytokine production and gene profile analysis identified a population of in vivo differentiatedretinoid-related orphan receptor gamma-expressing T cells, producing high levels of IL-17, that can represent up to 30% of infiltrating T lymphocytes. Activated Th17 cells produced IL-26, TNF-alpha, lymphotoxin-beta,

and IL-22. IL-17 and IL-22 concentrations secreted by tissue infiltrating Th17 cells could reach up to 100 nM and were inversely correlated with the production of Th1- and Th2-associated cytokines. In addition, tissue-infiltrating Th17 cells are also characterized by high cell surface expression of CCR6, a chemokine receptor that was not expressed by Th1 and Th2 cells, isolated from the same lesions, and by the production of CCL20/MIP3alpha, a CCR6 ligand, associated with tissue infiltration. Culture supernatants of activated Th17 cells, isolated from psoriatic lesions, induced the expression of gene products associated with inflammation and abnormal keratinocyte differentiation in an IL-17 and IL-22-dependent manner. These results show that tissue-infiltrating Th17 cells contribute to human chronic inflammatory disease via the production of several inflammatory cytokines and the creation of an environment contributing to their migration and sequestration at sites of inflammation.

PMID: 18490742 [PubMed - indexed for MEDLINE]

699. Int J Paediatr Dent. 2008 Jul;18(4):243-50. doi: 10.1111/j.1365-263X.2008.00921.x. Epub 2008 May 16.

Oral health in preschool children with asthma.

Stensson M, Wendt LK, Koch G, Oldaeus G, Birkhed D.

Department of Paediatric Dentistry, Institute for Postgraduate Dental Education, Jönköping, Sweden. malin.stensson@lj.se

OBJECTIVE: The aim of this study was to investigate oral health and its determinants in 3-year-old and 6-year-old children with asthma.
METHODS AND SUBJECTS: Caries and gingivitis were examined in 127 asthmatic (all children with asthma in a selected area and born during a specific time period) and 117 matched, healthy control children. The parents were interviewed regarding various oral-health-related factors.
RESULTS: The mean dfs (+/- standard deviation) in the 3-year-old with asthma was 1.4 +/- 3.2 compared with 0.5 +/- 1.2 in the controls (P < 0.05). The corresponding figures for the 6-year-old were 2.5 +/- 3.9 and 1.8 +/- 2.8. The 3-year-old asthmatic children had more gingival bleeding than the healthy controls (P < 0.05). There were no significant differences in gingivitis in the 6-year-old children. Asthmatic children reported higher consumption of sugar-containing drinks and were more frequently mouthbreathers than healthy children (P < 0.05). In 3-year-old children with asthma and immigrant background, the mean dfs was higher compared with immigrant children in the control group (P < 0.01).
CONCLUSION: The results indicate that preschool children with asthma have higher caries prevalence than healthy children. The factors discriminating for caries in asthmatic children are higher intake of sugary drinks, mouth breathing, and immigrant background.

PMID: 18489575 [PubMed - indexed for MEDLINE]

700. Int Immunopharmacol. 2008 Jul;8(7):1039-48. doi: 10.1016/j.intimp.2008.03.009. Epub 2008 Apr 11.

Mycophenolate mofetil and triptolide alleviating airway inflammation in asthmatic model mice partly by inhibiting bone marrow eosinophilopoiesis.

Mao H, Chen XR, Yi Q, Li SY, Wang ZL, Li FY.

Department of Respiratory Medicine, West China Hospital of Sichuan University,

Chengdu, PR China.

The bone marrow eosinophilopoiesis makes a major contribution to the chronic airway inflammation in asthmatic animals and patients. Some anti-asthmatic medicines alleviated the asthmatic airway inflammation by inhibiting the bone marrow eosinophilopoiesis. Immunosuppressive agents have been commonly used in patients with glucocorticoid refractory asthma and have been proved to be effective. However, the research on the effect of the immunosuppressive agents on the bone marrow eosinophilopoiesis has seldom been reported. The purpose of the study was to explore the effect of mycophenolate mofetil (MMF) and triptolide (TP) on the bone marrow eosinophilopoiesis and to further investigate the mechanisms of the immunosuppressive agents involved in the anti-asthmatic effect. Balb/c mice were sensitized and challenged by OVA to establish the asthmatic model, and respectively administered orally with sterile saline, MMF, and TP once daily for 2 weeks. Airway inflammation, and inflammatory mediators IL-5 and eotaxin in the peripheral blood and bone marrow were measured by histology and ELISA. Immunocytochemistry combined with in situ hybridization technique and Western blot analysis was performed to estimate the amount of CD34+ IL-5R mRNA+ cells and IL-5R expression in the bone marrow. The count of new produced eosinophils in the bone marrow was detected by anti-BrdU immunocytochemistry. We found that MMF and TP attenuated OVA-induced eosinophil (EOS) recruitment in bronchoalveolar lavage fluid (BALF), inflammatory mediator expression of IL-5 and eotaxin in the peripheral blood, inflammatory cells expressing eotaxin in the lung tissues and the number of new produced EOS in the bone marrow. Also, MMF abated the migration of CD34+ cells from the bone marrow to the peripheral blood, which was associated with a decreased eotaxin expression in the bone marrow and a decreased CCR3 expression on bone marrow cells. While, MMF or TP failed to decrease the amount of CD34+ IL-5R mRNA+ cells (EOS progenitors), and IL-5R expression in the bone marrow of asthmatic model mice. These results demonstrated that MMF and TP reduce the eosinophilopoiesis of the bone marrow; this is associated with a decrease of IL-5 produced by T cells, which contribute to alleviate the allergic airway inflammation in asthma. In addition, MMF decreased the CD34+ cells migration from the bone marrow to the peripheral blood by the reduction of the level of eotaxin in the bone marrow and the expression of CCR3 on the bone marrow cells.

PMID: 18486916 [PubMed - indexed for MEDLINE]

701. Travel Med Infect Dis. 2008 May;6(3):130-6. doi: 10.1016/j.tmaid.2008.03.004.

Simultaneous vaccination of Chinese applicants for a United States immigrant visa.

Hua L, Hongtao H, Shunqin W, Jinping G, Jiandong C, Zhaoliang L, Xinwen F.

Guangdong International Travel Healthcare Center, Guangzhou 510130, China.

BACKGROUND: Simultaneous vaccination is still uncommon in China, and many Chinese people are quite concerned about the adverse reactions because few data regarding the adverse reactions of simultaneous vaccination in Chinese people have been reported. The objective of this study was to evaluate the safety of simultaneous vaccination and the frequency of adverse reactions following simultaneous vaccinations in Chinese applicants for a United States immigrant visa.
METHODS: We conducted a prospective observational study in 772 applicants receiving required vaccination in Guangdong International Travel Healthcare Center. The vaccines required for vaccination included diphtheria and tetanus toxoids and acellular pertussis vaccine (DTaP), adult formulation tetanus and diphtheria toxoids (Td), haemophilus influenzae type-b conjugate vaccine (Hib), oral polio vaccine (OPV), hepatitis B vaccine (HepB), combined measles mumps

rubella vaccine (MMR), varicella vaccine (Var), and influenza vaccine (Inf), pneumococcal polysaccharide vaccine (PPV). Data on adverse reactions were collected by questionnaires.
RESULTS: Seven hundred and seventy-two participants have received a total of 2533 doses of different vaccines, and 49.6% of the participants reported adverse reactions within 7 days following vaccination, with 39.8%(307/772) local reactions and 20.2%(156/772) systemic reactions. There were no allergic reactions. Only one vaccinee visited hospital seeking treatment due to fever, and recovered well. The most frequent local reaction was pain at the injection site (260/772, 33.7%), especially in the case of PPV vaccination, 61.2% (63/103) vaccinees who received PPV complained of pain at the site of injection, while the most frequent systemic reaction was fever (84/772, 10.9%). Pain and fever were all temporary reactions and resolved within 72h. Logistic regression analysis found that females experienced adverse reactions more frequently than males [(local reactions: female:male=41.7%(187/448):37%(120/324), p=0.04; systemic reactions: female:male=23%(103/448):16.4%(53/324), p=0.026)]; vaccinees given PPV developed local reactions more frequently than those receiving the other vaccines. The number of vaccines has no significant influence on adverse reactions.
CONCLUSIONS: Simultaneous vaccination is feasible for Chinese applicants for a United States immigrant visa because the adverse reactions are mostly mild and temporary. Our data suggest that more Chinese people should be encouraged to receive simultaneous vaccination if the time is limited so as to reduce the risk of vaccine-preventable diseases.

PMID: 18486068 [PubMed - indexed for MEDLINE]

702. Pediatr Allergy Immunol. 2009 Feb;20(1):19-29. doi: 10.1111/j.1399-3038.2008.00754.x. Epub 2008 May 15.

Maternal country of birth and previous pregnancies are associated with breast milk characteristics.

Amoudruz P, Holmlund U, Schollin J, Sverremark-Ekström E, Montgomery SM.

Department of Immunology, Wenner-Gren Institute, Stockholm University, Sweden. petra.amoudruz@imun.su.se

Populations in high infectious exposure countries are at low risk of some immune-mediated diseases such as Crohn's disease and allergy. This low risk is maintained upon immigration to an industrialized country, but the offspring of such immigrants have a higher immune-mediated disease risk than the indigenous population. We hypothesize that early life exposures in a developing country shape the maternal immune system, which could have implications for the offspring born in a developed country with a low infectious load. The aim of this study was to investigate if exposures in childhood (indicated by country of origin) and subsequent exposures influence immunologic characteristics relevant to stimulation of offspring. Breast milk components among 64 mothers resident in Sweden, 32 of whom immigrated from a developing country, were examined using the ELISA and Cytometric Bead Array methods. Immigrants from a developing country had statistically significantly higher levels of breast milk interleukin-6 (IL-6), IL-8 and transforming growth factor-beta1. A larger number of previous pregnancies were associated with down-regulation of several substances, statistically significant for soluble CD14 and IL-8. The results suggest that maternal country of birth may influence adult immune characteristics, potentially relevant to disease risk in offspring. Such a mechanism may explain the higher immune-mediated disease risk among children of migrants from a developing to developed country. Older siblings may influence disease risk through the action of previous pregnancies on maternal immune characteristics.

PMID: 18484963 [PubMed - indexed for MEDLINE]

703. J Leukoc Biol. 2008 Aug;84(2):357-67. doi: 10.1189/jlb.1207841. Epub 2008 May 13.

Pivotal advance: IgE accelerates in vitro development of mast cells and modifies their phenotype.

Kashiwakura J, Xiao W, Kitaura J, Kawakami Y, Maeda-Yamamoto M, Pfeiffer JR, Wilson BS, Blank U, Kawakami T.

Division of Cell Biology, La Jolla Institute for Allergy and Immunology, 9420 Athena Circle, La Jolla, CA 92037, USA.

Antigen-dependent activation of IgE-bound mast cells is critical for immediate hypersensitivity and other allergic disorders. Recent studies have revealed the effects of monomeric IgEs on mast cell survival and activation. Furthermore, IgE molecules exhibit a wide range of heterogeneity in the ability to induce mast cell activation in the absence of antigen. Highly cytokinergic (HC) IgEs can induce a variety of activation events including cell survival, degranulation, cytokine production, and migration, whereas poorly cytokinergic (PC) IgEs can do so inefficiently. Here, we show that culture of bone marrow cells in the presence of monomeric IgEs results in an increased number of mast cells compared with cultures grown without IgE. Furthermore, time in culture required to generate > or =80% pure mast cells is decreased. IgE molecules can directly influence mast cell progenitors to differentiate into mast cells. mRNA expression of several mast cell proteases and mast cell-related transcription factors is higher in mast cells cultured with an HC IgE than those cultured with a PC IgE or without IgE. Expression of early growth response factor-1, a transcription factor that is involved in the production of TNF-alpha in mast cells, is enhanced in cultures containing high and low concentrations of HC IgE and a high concentration of PC IgE. Consistent with this, expression of TNF-alpha is higher in mast cells cultured with HC IgE than PC IgE. Therefore, our results suggest that monomeric IgEs, especially HC IgEs, not only promote mast cell development but also modulate the mast cell phenotype.

PMCID: PMC2516357
PMID: 18477690 [PubMed - indexed for MEDLINE]

704. Curr Pharm Des. 2008;14(12):1231-9.

Structure-based rationale for interleukin 5 receptor antagonism.

Ishino T, Harrington AE, Gopi H, Chaiken I.

Drexel University College of Medicine, Department of Biochemistry and Molecular Biology, Philadelphia, PA 19102-1192, USA.

Human interleukin 5 (IL5) is the major hematopoietin that stimulates the proliferation, migration and activation of eosinophils and is implicated in the pathogenesis of inflammatory and other myeloproliferative diseases. IL5 functions through the signaling of a common receptor subunit beta (beta c), in a receptor activation process that requires initial recruitment of an IL5 specific receptor subunit alpha (IL5Ralpha), for cytokine presentation to beta c. Important advances have been made to understand molecular mechanisms of cytokine recognition and receptor antagonism. Mutational studies indicate that a pair of charge complementary regions play an essential role in specific interaction between IL5Ralpha and IL5. Moreover, peptide studies with the IL5 system have

identified a cyclic peptide inhibitor, AF17121, which binds specifically to IL5Ralpha by mimicking the cytokine. A key receptor-recognition pharmacophore has been identified in this peptide inhibitor, and sites of inhibitor recognition can be proposed in the homology-deduced structural model of IL5Ralpha. These results provide an experimental platform to derive enhanced-potency peptidomimetic inhibitors. Such inhibitors have potential use as tools to evaluate the role of eosinophilia in disease and as potential leads to antagonists to treat hyper-eosinophilic diseases such as eosinophilic esophagitis, asthma and chronic myeloproliferative leukemias.

PMID: 18473871 [PubMed - indexed for MEDLINE]

705. Ther Clin Risk Manag. 2007 Oct;3(5):885-92.

Montelukast: its role in the treatment of childhood asthma.

Harmanci K.

Department of Allergy, Ministry of Health, Ankara Diskapi Children's Diseases Training and Research Hospital Ankara, Turkey.

The cysteinyl leukotrienes, LTC(4), LTD(4), and LTE(4), play an integral role in the pathophysiology of asthma. Acting via the type 1 leukotriene (CysLT(1)) receptor, these proinflammatory mediators have numerous effects in the lungs, including decreased activity of respiratory cilia, increased mucus secretion, increased venopermeability, and promotion of eosinophil migration into airway mucosa. Blocking studies show that Cys-LTs are pivotal mediators in the pathophysiology of asthma. Cys-LTs are key components in the early and late allergic airway response and also contribute to bronchial obstruction after exercise and hyperventilation of cold, dry air in asthmatics. Effects of the cysteinyl leukotrienes are blocked by leukotriene receptor antagonists; these agents inhibit bronchoconstriction in normal subjects provoked with inhaled cysteinyl leukotrienes, as well as in patients with asthma undergoing allergen, exercise, cold air, or aspirin challenge. Montelukast is a potent and selective blocker of the CysLT(1) receptor. For treatment of chronic asthma, montelukast is administered once daily to adults as a 10-mg film-coated tablet, to children aged 6-14 years as a 5-mg chewable tablet, and to children aged 2-5 years as a 4-mg chewable tablet form. Given their efficacy, antiinflammatory activity, oral administration, and safety, leukotriene modifiers will play an important role in the treatment of asthmatic children.

PMCID: PMC2376066
PMID: 18473012 [PubMed]

706. Mol Ther. 2008 Jul;16(7):1323-30. doi: 10.1038/mt.2008.91. Epub 2008 May 6.

Topical application of cream-emulsified CD86 siRNA ameliorates allergic skin disease by targeting cutaneous dendritic cells.

Ritprajak P, Hashiguchi M, Azuma M.

Department of Molecular Immunology, Graduate School, Tokyo Medical and Dental University, Bunkyo-ku, Tokyo, Japan.

Induction of the co-stimulatory molecule CD86 on dendritic cells (DCs) in the peripheral tissues is a critical event in triggering antigen-specific immune responses. In this study, we propose a new small interfering RNA (siRNA)-based therapy using cream-emulsified CD86 siRNA, targeting DCs for murine contact

hypersensitivity (CH) and atopic dermatitis (AD)-like disease. Topical application of CD86 siRNA efficiently inhibited CH and markedly decreased the numbers of infiltrating CD86(+) or major histocompatibility complex (MHC) class II(+) cells in murine ear skin. The total number of cells, the percentage of hapten-carrying DCs, and their CD86 expression in the regional lymph nodes (RLNs) also significantly decreased. These results suggest that the silencing of CD86 in local DCs inhibits the recruitment and migration of DCs into the skin and RLNs, respectively, resulting in reduced antigen-specific local inflammation. The therapeutic efficacy of the CD86 siRNA was confirmed in AD-prone NC/Nga mice. Treatment produced marked amelioration in the clinical manifestations of AD and reduced the antigen-specific production of interleukin-4 (IL-4) and serum immunoglobulin E (IgE) and IgG1. Our results suggest that the targeting of cutaneous DCs by CD86 siRNA may be a promising strategy in the treatment of allergic skin disease.

PMID: 18461054 [PubMed - indexed for MEDLINE]

707. Curr Dir Autoimmun. 2008;10:1-26. doi: 10.1159/000131410.

Allergic contact dermatitis.

Gober MD, Gaspari AA.

University of Maryland School of Medicine, Baltimore, MD 21030, USA.

Allergic contact dermatitis is a classic example of a cell mediated hypersensitivity reaction in the skin. This occurs as a result of xenobiotic chemicals penetrating into the skin, chemically reacting with self proteins, eventually resulting in a hapten-specific immune response. It is precisely because of this localized immune response that allergic signs and symptoms occur (redness, edema, warmth and pruritus). It has been known for years that conventional T-cells (CD4+ or CD8+ T-cells that express a T-cell receptor alpha/Beta) are critical effectors for this reaction. There is emerging evidence that innate immune lymphocytes such as invariant Natural killer T-cells and even Natural killer cells may play important role. Other T-cell types such as Tregulatory cells and the IL-10 secreting Tregulatory cells type I are likely to be important in the control (resolution) of allergic contact dermatitis. Other cell types that may contribute include B-cells and hapten-specific IgM. Additionally, epidermal Langerhans cells have been ascribed an indispensable role as an antigen presenting cell to educate T-cells of the skin immune system. Studies of mice that lack this cell type suggest that Langerhans cells may be dispensible, and may even play a regulatory role in allergic contact dermatitis. The identity of the antigen presenting cells that complement Langerhans cells has yet to be identified. Lastly, Keratinocytes play a role in all phases of allergic contact dermatitis, from the early initiation phase with the elaboration of inflammatory cytokines, that plays a role in Langerhans cell migration, and T-cell trafficking, through the height of the inflammatory phase with direct interactions with epidermotrophic T-cells, through the resolution phase of allergic contact dermatitis with the production of anti-inflammatory cytokines and tolerogenic antigen presentation to effector T-cells. As the understanding of allergic contact dermatitis continues to improve, this will provide novel therapeutic targets for immune modulating therapy.

PMID: 18460878 [PubMed - indexed for MEDLINE]

708. J Immunol. 2008 May 1;180(9):6317-24.

TLR2-mediated activation of neutrophils in response to German cockroach frass.

Page K, Lierl KM, Hughes VS, Zhou P, Ledford JR, Wills-Karp M.

Division of Critical Care Medicine, Cincinnati Children's Hospital Medical Center and Research Foundation, 3333 Burnet Avenue, Cincinnati, OH 45229, USA. kristen.page@cchmc.org

It is becoming increasingly clear that innate immune mediators play a role in regulating adaptive immune responses in asthma pathogenesis. Cockroach exposure is a major risk factor for the development of asthma. In this study we asked whether German cockroach (GC) feces (frass) could initiate an innate immune response. Naive BALB/c mice were challenged with a single intratracheal inhalation of GC frass. Proinflammatory cytokines were significantly increased in the bronchoalveolar lavage fluid at 3 h and were maintained at higher than baseline levels for at least 24 h. Neutrophil migration into the airways was evident as early as 3 h but was maximal between 6 and 24 h postinhalation. The early increase in cytokine expression was independent of TLR2 or TLR4. Newly infiltrated airway neutrophils were responsible for maintaining high levels of cytokines in the airways. Using neutrophils as an early marker of the innate immune response, we show that show that neutrophils isolated from the airways following GC frass inhalation express TLR2 and release cytokines. GC frass directly affected neutrophil cytokine production via TLR2, but not TLR4, as evidenced by the use of TLR-neutralizing Abs and neutrophils from TLR-deficient mice. Activation of cytokine expression occurred via GC frass-induced NF-kappaB translocation and DNA binding. These data show that GC frass contains a TLR2 agonist and, to our knowledge, this is the first report of an allergen directly activating cells of the innate immune system via TLR2 and suggests an important link between innate and adaptive immunity.

PMID: 18424755 [PubMed - indexed for MEDLINE]

709. Am J Respir Cell Mol Biol. 2008 Oct;39(4):475-81. doi: 10.1165/rcmb.2007-0091OC. Epub 2008 Apr 17.

The effects of leptin on airway smooth muscle responses.

Nair P, Radford K, Fanat A, Janssen LJ, Peters-Golden M, Cox PG.

Firestone Institute for Respiratory Health, St. Joseph's Healthcare, Hamilton, Ontario, Canada. parames@mcmaster.ca

Obesity is associated with asthma and airway hyperresponsiveness. Leptin modulates some of the proinflammatory effects observed in obesity. The objective of this study was to determine the effects of leptin on airway smooth muscle responses. The effect of leptin (0.1-100 ng/ml) on migration (toward platelet-derived growth factor [PDGF], 10 ng/ml, across collagen-coated membrane in Transwell culture plates), proliferation (by BrDU incorporation), and cytokine production (by Bioplex bead assay) of cultured human airway smooth muscle cells from nine nonasthmatic donors was assessed. Effects of leptin on the contractile responses were studied in bovine tracheal smooth muscle rings. Leptin receptor expression and activation of STAT-3, Src kinase, Suppressor of Cytokine Signaling-3 (SOCS-3), and COX were evaluated by Western blotting and PCR. PGE(2) levels in supernatant were assessed by enzyme immunoassay. Human airway smooth muscle cells express leptin receptor, which, when engaged, phosphorylated STAT-3. Leptin inhibited PDGF-induced human airway smooth muscle migration and proliferation and IL-13-induced eotaxin production. Leptin did not stimulate cytokine synthesis and did not evoke contractile responses or inhibit isoproterenol-induced relaxation of carbachol-induced contraction of bovine tracheal rings. The inhibitory effects on migration and eotaxin production are

not due to activation of SOCS-3 but are partly due to increased production of PGE(2) because they were attenuated by indomethacin. In conclusion, leptin inhibited human airway smooth muscle proliferation, migration toward PDGF, and IL-13-induced eotaxin production. This is partly mediated by PGE(2) secretion from smooth muscle cells induced by leptin. The association between obesity and asthma is unlikely to be due to a direct effect of leptin on airway smooth muscle.

PMID: 18421018 [PubMed - indexed for MEDLINE]

710. Am J Respir Cell Mol Biol. 2008 Oct;39(4):390-9. doi: 10.1165/rcmb.2008-0044OC. Epub 2008 Apr 17.

Pulmonary mastocytosis and enhanced lung inflammation in mice heterozygous null for the Foxf1 gene.

Kalin TV, Meliton L, Meliton AY, Zhu X, Whitsett JA, Kalinichenko VV.

Division of Pulmonary Biology, Cincinnati Children's Hospital Research Foundation, Cincinnati, OH 45229, USA.

The Forkhead Box f1 (Foxf1) transcriptional factor (previously known as HFH-8 or Freac-1) is expressed in endothelial and smooth muscle cells in the embryonic and adult lung. To assess effects of Foxf1 during lung injury, we used CCl(4) and butylated hydroxytoluene (BHT) injury models. Foxf1(+/-) mice developed severe airway obstruction and bronchial edema, associated with increased numbers of pulmonary mast cells and increased mast cell degranulation after injury. Pulmonary inflammation in Foxf1(+/-) mice was associated with diminished expression of Foxf1, increased mast cell tryptase, and increased expression of CXCL12, the latter being essential for mast cell migration and chemotaxis. After ovalbumin (OVA) sensitization and OVA challenge, increased lung inflammation, airway hyperresponsiveness to methacholine, and elevated expression of CXCL12 were observed in Foxf1(+/-) mice. During lung development, Foxf1(+/-) embryos displayed a marked increase in pulmonary mast cells before birth, and this was associated with increased CXCL12 levels in the lung. Expression of a doxycycline-inducible Foxf1 dominant-negative transgene in primary cultures of lung endothelial cells increased CXCL12 expression in vitro. Foxf1 haploinsufficiency caused pulmonary mastocytosis and enhanced pulmonary inflammation after chemically induced or allergen-mediated lung injury, indicating an important role for Foxf1 in the pathogenesis of pulmonary inflammatory responses.

PMCID: PMC2551700
PMID: 18421012 [PubMed - indexed for MEDLINE]

711. Nihon Ganka Gakkai Zasshi. 2008 Mar;112(3):179-212; discussion 213.

[The cornea: stasis and dynamics].

[Article in Japanese]

Nishida T.

Department of Ophthalmology, Yamaguchi University Graduate School of Medicine, Ube-City, Japan.

The physiological roles of the cornea are to conduct external light into the eye, focus it, together with the lens, onto the retina, and to provide rigidity to the

entire eyeball. Good vision thus requires maintenance of the transparency and proper refractive shape of the cornea. Although the cornea appears to be a relatively static structure, dynamic processes operate within and around the cornea at the tissue, cell, and molecular level. In this article, I review the mechanisms responsible for maintenance of corneal homeostasis as well as the development of new modes of treatment for various corneal diseases. I. The static cornea: structure and physiological functions. The cornea is derived from ectoderm, so that it can be considered as transparent skin. It is devoid of blood vessels and manifests the highest sensitivity in the entire body. The surface of the cornea is covered by tear fluid, which serves both as a lubricant and as a conduit for regulatory molecules. The cornea is also supplied with oxygen and various nutrients by the aqueous humor and a loop vascular system in addition to tear fluid. The cornea interacts with its surrounding tissues directly as well as indirectly through tear fluid or aqueous humor, with such interactions playing an important role in the regulation of corneal structure and functions. The resident cells of the cornea-epithelial cells, fibroblasts (keratocytes), and endothelial cells--also engage in mutual interactions through network systems. These interactions as well as those with infiltrated cells and regulation by nerves contribute to the maintenance of the normal structure and functions of the cornea as well as to the repair of corneal injuries. II. The dynamic cornea: maintenance of structure and functions by network systems. Developments in laser and computer technology have allowed observation of the cells and collagen fibers within the cornea. Furthermore, progress in cell and molecular biology has allowed characterization of dynamic network systems-including cell-cell and cell-extracellular matrix interactions as well as cytokines and neural factors-that contribute to the maintenance of corneal transparency and shape. III. Disruption of network systems: persistent corneal epithelial defects and corneal ulcer. Selection of the appropriate treatment for pathologic lesions of the cornea and the accompanying decrease in visual acuity requires localization of the lesion with regard to the epithelium, stroma, or endothelium of the cornea. In certain instances, however, it is not possible to determine the cause of the problem within the cornea. In such cases, the cause of the pathologic lesion and the target for treatment may lie in the surrounding tissues or environment. For example, corneal epithelial wound healing may be delayed, leading to the development of persistent epithelial defects, as a result of disruption of intercellular junctions between epithelial cells, an abnormality of the corneal basement membrane, altered concentrations of various cytokines in tear fluid, a lowered corneal sensation, or allergic reactions in the lid conjunctiva. Loss of corneal epithelial barrier function can further allow inflammatory cytokines present in tear fluid, together with infiltrated cells, to activate keratocytes and elicit excessive degradation of collagen in the stroma, thereby giving rise to corneal ulcer. IV. Development of new drugs for corneal diseases. We have attempted to apply the results of basic scientific research to the development of new drugs for corneal diseases that remain difficult to treat. The process of authorization for new drugs from the Ministry of Health, Labor, and Welfare takes more than two decades, however. The path from the bench to clinical practice is thus a long one. 1. Development of eyedrops for treatment of persistent corneal epithelial defects. We demonstrated the clinical efficacy of fibronectin eyedrops for the treatment of persistent epithelial defects of the cornea. However, the possibility of blood-borne infections has interfered with the development of serum-derived fibronectin as a drug. An automated machine for the preparation of autologous fibronectin eyedrops has therefore recently been developed. Furthermore, in seeking an alternative to fibronectin eyedrops, we are investigating the effects of a peptide corresponding to the second cell-binding domain of fibronectin on corneal epithelial wound healing. Considering that urokinase-type plasminogen activator may be expressed at the site of corneal epithelial defects and facilitates epithelial migration, the potential clinical application of annexin V, which stimulates the secretion of urokinase-type plasminogen activator for the treatment of persistent corneal epithelial defects is also now under investigation in Japan. 2. Development of eyedrops for

treatment of neurotrophic keratopathy. Substance P, a neurotransmitter, stimulates corneal epithelial migration in a synergistic manner with insulin-like growth factor (IGF)--1. We have shown that eyedrops containing both the substance P-derived peptide FGLM-amide and the IGF-1--derived peptide SSSR are effective for the treatment of persistent corneal epithelial defects in individuals with diabetic keratopathy or neurotrophic keratopathy, both of which are associated with a reduction in corneal sensation. 3. Development of drugs for corneal ulcer. Treatment of corneal infection with antibiotics does not necessarily halt the process of corneal ulceration, which is characterized by excessive degradation of stromal collagen, or resolve persistent corneal epithelial defects. In addition to eyedrops for the treatment of persistent corneal epithelial defects, we have therefore also been working on the development of new drugs for the treatment of corneal ulcer. To this end, we have established an experimental system in which corneal fibroblasts are cultured in a three-dimensional collagen gel. With this system, we have shown that triptolide and steroids inhibit collagen degradation by corneal fibroblasts. Triptolide or its derivatives are thus potential drugs for the treatment of corneal ulcer and would work by acting directly on corneal fibroblasts rather than by inhibiting the secreted enzymes(matrix metalloproteinases) responsible for collagen degradation.

PMID: 18411711 [PubMed - indexed for MEDLINE]

712. Mol Ther. 2008 May;16(5):836-44. doi: 10.1038/mt.2008.43. Epub 2008 Mar 18.

Improvement of migratory defects in a murine model of Wiskott-Aldrich syndrome gene therapy.

Blundell MP, Bouma G, Calle Y, Jones GE, Kinnon C, Thrasher AJ.

Molecular Immunology Unit, Wolfson Centre for Gene Therapy of Childhood Disease, University College London Institute of Child Health, London, UK.

Wiskott-Aldrich syndrome (WAS) is an X-linked hematological disease characterized by immunodeficiency, eczema, and thrombocytopaenia, and shows promise for treatment with hematopoietic stem cell gene therapy. The immunopathology of WAS is attributable at least in part to defects of cell migration and localization as a result of chemotactic, adhesive, and chemokinetic defects. Whereas previous studies using either gammaretroviral or lentiviral vectors have demonstrated variable correction of T-cell proliferation and dendritic cell (DC) cytoarchitecture, we have used a lentiviral vector expressing an eGFP-WASp fusion protein to test the potential for restoration of cell migratory defects. Multilineage expression of the fusion transgene was present for up to 10 months after primary engraftment, and also in secondary recipients analyzed after a further 9 months. Transduced bone marrow-derived dendritic cells (BMDCs) demonstrated recovery of podosome numbers and turnover, while B cells, BMDCs, and Langerhans cells (LCs) exhibited enhanced chemotactic responses to specific stimuli. As an indication of functionality in vivo, splenic marginal zone B cells and a cutaneous contact hypersensitivity (CHS) response to dinitrofluorobenzene (DNFB) were both partially restored. These proof of principle experiments demonstrate that WAS protein (WASp) transgene expression can be successfully maintained long term in primary and secondary recipients, and that it is associated with a significant repair of migratory defects.

PMID: 18388921 [PubMed - indexed for MEDLINE]

713. Allergy. 2008 Aug;63(8):1040-5. doi: 10.1111/j.1398-9995.2008.01661.x. Epub 2008 Mar 31.

Prevalence of asthma in a large group of Israeli adolescents: influence of country of birth and age at migration.

Pereg D, Tirosh A, Lishner M, Goldberg A, Shochat T, Confino-Cohen R.

Department of Internal Medicine A, Meir Medical Center, Kfar Saba, and Sackler Faculty of Medicine, Tel Aviv University, Tel Aviv, Israel.

BACKGROUND: The occurrence of asthma has geographic variations and is lower in developing compared with industrialized countries. Both environmental and genetic factors may influence its prevalence. We aimed to evaluate the importance and effect of immigration (country of birth and age at immigration to Israel) on the prevalence of asthma in a large group of Israeli adolescents.
METHODS: Computerized medical records of 17-year-old adolescents, who underwent routine examination before military recruitment, were studied. The sample comprised both native-born Israelis (NBI) and immigrants from Ethiopia, the Former Soviet Union (FSU), and Western countries (WC). Asthma was defined as clinical symptoms and signs compatible with the disease accompanied by abnormal spirometry or documented chronic use of inhaled steroids.
RESULTS: Our cohort consisted of 1 466 654 adolescents, including 1 317 556 (89.8%) NBI and 149 098 (10.2%) immigrants. The prevalence of asthma at age 17 was higher in NBI compared with Ethiopian immigrants [4.7% (61 921) vs 2.6% (418), respectively, P < 0.0005], lower compared with immigrants from WC [5.6% (2177), P < 0.0005], and similar to immigrants from the FSU. Further analysis of the association between age at immigration and the risk for developing asthma showed that the younger immigrants from the FSU and Ethiopia arrived to Israel, the higher their prevalence of asthma at the age of 17 was.
CONCLUSIONS: Both environmental and genetic factors seem to influence the prevalence of asthma in 17-year-old adolescents. However, the higher risk for developing asthma associated with young age of immigration points toward an environmental predominance.

PMID: 18384451 [PubMed - indexed for MEDLINE]

714. Gene Expr Patterns. 2008 May;8(5):291-6. doi: 10.1016/j.gep.2008.02.002. Epub 2008 Feb 19.

Expression of trpC1 and trpC6 orthologs in zebrafish.

Möller CC, Mangos S, Drummond IA, Reiser J.

Nephrology Division and Program in Glomerular Disease, Massachusetts General Hospital and Harvard Medical School, Suite 8214, CNY 149 13th Street, Boston, MA 02129, USA.

Transient receptor potential (TRP) genes encode subunits that form cation-selective ion channels in a variety of organisms and cell types. TRP channels serve diverse functions ranging from thermal, tactile, taste, and osmolar sensing to fluid flow sensing. TRPC1 and TRPC6 belong to the TRPC subfamily, members of which are thought to contribute to several cellular events such as regulated migration of neuronal dendrites, contractile responses of smooth muscle cells and maintenance of the structural integrity of kidney podocytes. Pathogenic roles have been suggested for TRPC1 in asthma and chronic obstructive pulmonary disease, and TRPC6 dysfunction was recently linked to proteinuric kidney disease. To explore the potential roles for TRPC channels in zebrafish organ function, we cloned zebrafish trpC1 and trpC6 cDNAs, and investigated their expression during zebrafish development. We detected trpC1 expression in the head, in cells surrounding the outflow tract of the heart, and in the ganglion cells as well as the inner nuclear layer of the eye. trpC6

expression was detected in the head, pectoral fins, aortic endothelial cells, and gastrointestinal smooth muscle cells. Our results point to roles of TRPC channels in several tissues during zebrafish development, and suggest that the zebrafish may be a suitable model system to study the pathophysiology of TRPC1 and TRPC6 in specific cell types.

PMCID: PMC2431112
PMID: 18378501 [PubMed - indexed for MEDLINE]

715. Chin Med J (Engl). 2008 Mar 5;121(5):424-9.

Effect of basic fibroblast growth factor on the proliferation, migration and phenotypic modulation of airway smooth muscle cells.

Zou H, Nie XH, Zhang Y, Hu M, Zhang YA.

Cell Therapy Centre, Xuanwu Hospital, Capital Medical University, Beijing 100053, China.

BACKGROUND: Proliferation, cell migration and phenotypic modulation of airway smooth muscle cells (ASMCs) are important features of airway remodelling in asthma. The precise cellular and molecular mechanisms that regulate ASMCs proliferation, migration and phenotypic modulation in the lung remain unknown. Basic fibroblast growth factor (bFGF), a highly specific chemotactic and mitogenic factor for many cell types, appears to be involved in the development of airway remodelling. Our study assessed whether bFGF directly stimulates the proliferation, migration and phenotypic modulation of ASMCs.
METHODS: Confluent and growth arrested human ASMCs were treated with human recombinant FGF. Proliferation was measured by BrdU incorporation and cell counting. Migration was examined using Boyden chamber apparatus. Expressions of smooth muscle (sm)-alpha-actin and sm-myosin heavy chain (MHC) isoform 1 were determined by RT-PCR and Western blot analysis.
RESULTS: It was found that hrbFGF (10 ng/ml), when added to ASMCs, induced a significant increase in BrdU uptake and cell number by ASMCs as compared to controls and a significant increase in ASMCs migration with respect to controls. The mRNA and protein expressions of sm-alpha-actin and sm-MHC in ASMCs that were stimulated with hrbFGF decreased with respect to controls.
CONCLUSION: It appears that bFGF can directly stimulate proliferation and migration of ASMCs, however, the expressions of cells' contractive phenotype decreased.

PMID: 18364115 [PubMed - indexed for MEDLINE]

716. Dtsch Med Wochenschr. 2008 Apr;133(14):727-32. doi: 10.1055/s-2008-1067317.

[Immunomodulation therapy for allergic asthma. What is already possible, what is to come?].

[Article in German]

Taube C, Buhl R.

III. Medizinische Klinik, Schwerpunkt Pneumologie, Johannes Gutenberg Universität, Mainz. taube@3-med.klinik.uni-mainz.de

Allergic asthma is an immunological disease characterized by certain inflammatory changes in the airways and the lung. Different approaches for immune modulation have been developed to treat this disease. Clinical approved immune modulators

include the specific immune therapy (SIT) and treatment with monoclonal antibodies against IgE. Further approaches, like inhibition or modulation of T cell responses, inhibition of effector cytokines or inhibition of cell migration are very interesting but still in development and so far not established for treatment of patients.

PMID: 18363195 [PubMed - indexed for MEDLINE]

717. Int J Mol Med. 2008 Apr;21(4):447-52.

Influence of interleukin-13 on beta-catenin levels in eosinophilic chronic rhinosinusitis cell culture.

Sauter A, Stern-Straeter J, Chang RC, Hörmann K, Naim R.

Universitäts-HNO-Klinik, Theodor-Kutzer-Ufer, D-68135 Mannheim, Germany. alexander.sauter@googlemail.com

Chronic rhinosinusitis (CRS) is one of the most common chronic diseases. The etiology and classification of CRS, with and without nasal polyps, remain unclear. Eosinophils and their products are important in the pathophysiology of allergic diseases and in host immunity to certain organisms. Interleukin 13 (IL-13) plays a pivotal role in eosinophilic inflammation. The migration of epithelial cells requires permanent re-establishment of the intercellular connection. Intercellular connections are maintained by the modulation of adherens junctions consisting of an E-cadherin/beta-catenin complex. In our study we examined the eosinophilic and non-eosinophilic paranasal mucosa obtained from two patients undergoing functional endoscopic sinus surgery. Cell cultures were incubated with human recombinant IL-13 for up to 72 h and beta-catenin concentration was determined with ELISA techniques. Furthermore, immunostaining for beta-catenin was used for the semi-quantitative description of specimens. We were able to ascertain a significant increase in beta-catenin expression in the eosinophilic paranasal cell culture after IL-13 administration compared to the non-eosinophilic culture. Immunostaining for beta-catenin was restricted to the membrane of the cells. Concerning the increased mural expression of beta-catenin, we presume that a fibrotic reaction similar to asthma and chronic obstructive pulmonary disease occurs in patients suffering from CRS. Furthermore, beta-catenin overexpression might be responsible for mucosal thickening and IL-13 seems to be an important marker in eosinophilic CRS.

PMID: 18360690 [PubMed - indexed for MEDLINE]

718. Toxicol In Vitro. 2008 Jun;22(4):988-94. doi: 10.1016/j.tiv.2008.02.004. Epub 2008 Feb 15.

In vitro toxicity evaluation of diesel exhaust particles on human eosinophilic cell.

Hirota R, Akimaru K, Nakamura H.

Department of Environmental Medicine, Kochi Medical School, Kohasu, Oko, Nakoku, Kochi 783-8505, Japan. hirotar@kochi-u.ac.jp <hirotar@kochi-u.ac.jp>

Diesel exhaust particles (DEPs), comprised mainly of particles less than 2.5 microm (PM 2.5) in aerodynamic diameter, have been assumed to enhance the response of asthma to allergen inhalation. Although eosinophilic infiltration is remarkable in the event of bronchial asthma induced by DEPs, the precise mechanisms leading to eosinophilia are unknown. To examine the effect of DEPs on

eosinophils, we measured the cytokine products and activity of nuclear factor-kappa B (NF-kappa B) after addition of the proteasomal inhibitor MG132 in HL-60 clone 15 cells differentiated into eosinophils. We measured eotaxin-induced chemotaxis of cells and their activity of p38 mitogen-activated protein (MAP) kinase was analysed. Interleukin (IL)-8 and monocyte chemoattractant protein-1 (MCP-1) were increased markedly in DEPs-treated cells. The active form of NF-kappaB in cells treated with DEPs was increased, and this effect was significantly decreased by the administration of MG132. Cell migration in the presence of DEPs was significantly greater, and inhibited by adding N-acetyl l-cysteine. P38 MAP kinase activity was highly influenced by DEPs-treatment. DEPs induce MCP-1 and IL-8 production by up-regulating NF-kappa B activity, which is inhibited in the presence of an inhibitor of proteasomal degradation. DEP also promotes eotaxin-induced chemotaxis in a p38-dependent manner.

PMID: 18359185 [PubMed - indexed for MEDLINE]

719. J Immunol. 2008 Apr 1;180(7):4441-50.

Gastrointestinal dendritic cells promote Th2 skewing via OX40L.

Blázquez AB, Berin MC.

Division of Pediatric Allergy and Immunology, and the Immunology Institute, Mount Sinai School of Medicine, New York, NY 10029, USA.

Mice can be sensitized to food proteins by oral administration with the adjuvant cholera toxin (CT), such that they undergo anaphylaxis when rechallenged with the sensitizing allergen. In contrast, feeding of Ags alone leads to oral tolerance. Our aim was to define the mechanisms by which gastrointestinal dendritic cells (DCs) participate in the deviation of tolerance to allergic sensitization in the gut in response to CT. BALB/c mice were fed with CT or PBS. The impact of CT on DC subsets in the mesenteric lymph node (MLN) was assessed by flow cytometry. Ag presentation assays were performed with DCs isolated from the MLN of PBS- or CT-fed mice, using OVA-specific CD4(+) T cells as responder cells. Gene expression in MLN DCs was determined by real-time PCR, and neutralizing Abs were used to test the function of OX40 ligand (OX40L) in Th2 skewing. Oral administration of CT induced an increase in the total CD11c(+) population in the MLN. CT induced a selective increase in migration of the CD11c(+)CD11b(-)CD8alpha(-) DC subset and the maturation of all DC subsets. Maturation of DCs in vivo enhanced T cell proliferation and cytokine secretion. Oral CT induced up-regulation of Jagged-2 and OX40L by MLN DCs. Neutralizing anti-OX40L Abs completely abrogated the CT-induced Th2 cytokine response. We show that oral CT induces selective DC migration, maturation, and T cell priming activity in the MLN. Th2 skewing is mediated by OX40L, and we speculate that this molecule may be an important inducer of allergic sensitization to food allergens.

PMID: 18354165 [PubMed - indexed for MEDLINE]

720. BioDrugs. 2008;22(2):85-100.

Cell adhesion antagonists: therapeutic potential in asthma and chronic obstructive pulmonary disease.

Woodside DG, Vanderslice P.

Department of Drug Discovery, Biological Sciences, Encysive Pharmaceuticals Inc., Houston, Texas, USA. dwoodside@encysive.com

Chronic obstructive pulmonary disease (COPD) and asthma are inflammatory diseases of the lung where a hallmark feature is excessive leukocyte infiltration that leads to tissue injury. Cell adhesion molecules (e.g. selectins and integrins) play a key role in cell trafficking, and in the lung they regulate leukocyte extravasation, migration within the interstitium, cellular activation, and tissue retention. All selectin family members (including L-selectin, P-selectin, and E-selectin) and many of the beta1 and beta2 integrins appear to be important therapeutic targets, as numerous animal studies have demonstrated essential roles for these cell adhesion molecules in lung inflammation. Not surprisingly, these families of adhesion molecules have been under intense investigation by the pharmaceutical industry for the development of novel therapeutics. Integrins are validated drug targets, as drugs that antagonize integrin alphaIIbbeta3 (e.g. abciximab), integrin alphaLbeta2 (efalizumab), and integrin alpha4beta1 (natalizumab) are currently US FDA-approved for acute coronary syndromes, psoriasis, and multiple sclerosis, respectively. However, none has been approved for indications related to asthma or COPD. Here, we provide an overview of roles played by selectins and integrins in lung inflammation. We also describe recent clinical results (both failures and successes) in developing adhesion molecule antagonists, with specific emphasis on those targets that may have potential benefit in asthma and COPD. Early clinical trials using selectin and integrin antagonists have met with limited success. However, recent positive phase II clinical trials with a small-molecule selectin antagonist (bimosiamose) and a small-molecule integrin alpha4beta1 antagonist (valategrast [R411]), have generated enthusiastic anticipation that novel strategies to treat asthma and COPD may be forthcoming.

PMID: 18345706 [PubMed - indexed for MEDLINE]

721. Int J Infect Dis. 2008 Nov;12(6):593-602. doi: 10.1016/j.ijid.2007.12.008. Epub 2008 Mar 14.

Dermatologic conditions of the ill returned traveler: an analysis from the GeoSentinel Surveillance Network.

Lederman ER, Weld LH, Elyazar IR, von Sonnenburg F, Loutan L, Schwartz E, Keystone JS; GeoSentinel Surveillance Network.

Collaborators: Barnett E, Brown G, Torresi J, Carosi G, Castelli F, Chen L, Wilson M, Connor B, Delmont J, Parola P, Freedman D, Gurtman A, Hale D, Gelman S, Piper-Jenks N, Jong E, Haulman J, Keystone J, Kain K, Kozarsky P, Franco-Paredes C, Licitra C, Loutan L, Lynch M, MacDonald S, McLellan S, Müller R, Nutman T, Klion A, Pandey P, Perez CP, Sagara H, Sack B, McKenzie R, Schwartz E, Shaw M, Stauffer W, Walker P, Steffen R, Schlagenhauf P, von Sonnenburg F, Wilder-Smith A, Lim PL, Wittner M.

U.S. Naval Medical Research Unit No. 2, Jakarta, Indonesia. edith.lederman@med.navy.mil

BACKGROUND: Skin disorders are common in travelers. Knowledge of the relative frequency of post-travel-related skin disorders, including their geographic and demographic risk factors, will allow for effective pre-travel counseling, as well as improved post-travel diagnosis and therapeutic intervention.
METHODS: We performed a retrospective study using anonymous patient demographic, clinical, and travel-related data from the GeoSentinel Surveillance Network clinics from January 1997 through February 2006. The characteristics of these travelers and their itineraries were analyzed using SAS 9.0 statistical software.
RESULTS: A skin-related diagnosis was reported for 4594 patients (18% of all patients seen in a GeoSentinel clinic after travel). The most common skin-related diagnoses were cutaneous larva migrans (CLM), insect bites including

superinfected bites, skin abscess, and allergic reaction (38% of all diagnoses). Arthropod-related skin diseases accounted for 31% of all skin diagnoses. Ill travelers who visited countries in the Caribbean experienced the highest proportionate morbidity due to dermatologic conditions. Pediatric travelers had significantly more dog bites and CLM and fewer insect bites compared with their adult counterparts; geriatric travelers had proportionately more spotted fever and cellulitis.
CONCLUSIONS: Clinicians seeing patients post-travel should be alert to classic travel-related skin diseases such as CLM as well as more mundane entities such as pyodermas and allergic reactions. To prevent and manage skin-related morbidity during travel, international travelers should avoid direct contact with sand, soil, and animals and carry a travel kit including insect repellent, topical antifungals, and corticosteroids and, in the case of extended and/or remote travel, an oral antibiotic with ample coverage for pyogenic organisms.

PMID: 18343180 [PubMed - indexed for MEDLINE]

722. J Sep Sci. 2008 Apr;31(6-7):1137-43. doi: 10.1002/jssc.200700591.

Determination of montelukast sodium by capillary electrophoresis.

Shakalisava Y, Regan F.

School of Chemical Science, Dublin City University, Dublin, Ireland.

This work verifies the potential of CE in the analysis of significant impurities of montelukast sodium - an active ingredient for the treatment of bronchial asthma. Using 20 mM borate buffer pH 9.2 with 10 mM SDS and 10 mM (2-hydroxypropyl)-gamma-CD (2HP-gamma-CD) it was possible to separate montelukast and several impurities, including its cis-isomer, after exposure to light and oxygen. The obtained method surpasses a chromatographic method for montelukast sodium in terms of time of analysis (9 min of CE analysis vs. 35 min HPLC) and efficiency (CE offered over 900 000 theoretical plates for montelukast). Good repeatability of the method was supported by the low % RSD for the migration time of montelukast (0.53%). For the first time, the capillary electrophoretic method was employed for temporal study of the degradation of montelukast. The results showed that degradation of montelukast and the formation of the cis-isomer mainly occurred during the first 2 days of exposure, and occurred to a higher degree when there was no contact with the air (oxygen) in the exposed sample.

PMID: 18338403 [PubMed - indexed for MEDLINE]

723. J Ethnopharmacol. 2008 Apr 17;117(1):34-40. doi: 10.1016/j.jep.2008.01.013. Epub 2008 Jan 20.

Suppressive effects of Houttuynia cordata Thunb (Saururaceae) extract on Th2 immune response.

Lee JS, Kim IS, Kim JH, Kim JS, Kim DH, Yun CY.

Department of Biology, Daejeon University, Daejeon 300-716, South Korea.

ETHNOPHARMACOLOGICAL RELEVANCE: Houttuynia cordata Thunb (Saururaceae), known as 'E-Sung-Cho' in Korea, has been traditionally used for the treatment of herpes simplex, chronic sinusitis, and allergy.
AIM OF THE STUDY: To investigate the inhibitory activity of Houttuynia cordata Thunb fractions (HcFs) on the T helper 2 (Th2) immune response, we evaluated the alternation of the release of Th2-type cytokines and chemokines such as

interleukin (IL)-4 and IL-5, and thymus and activation-regulated chemokine (TARC/CCL17).
MATERIALS AND METHODS: Ethanol fraction was obtained from dried and powdered whole plants of Houttuynia cordata Thunb using ethanol. The residue was diluted with water and was then successively partitioned with n-hexane, EtOAc and BuOH. HcFs include ethanol, n-hexane, EtOAc, BuOH and water fractions. RT-PCR and ELISA were performed to measure mRNA and protein expression of cytokines.
RESULTS: HcFs inhibited the expression of IL-4 and IL-5 in response to phorbol 12-myristate 13-acetate (PMA) and calcium ionophore (CaI) in Jurkat T cells and the human mast cell line, HMC-1. IL-4- and tumor necrosis factor-alpha (TNF-alpha)-induced TARC production was blocked by HcFs in skin fibroblast CCD-986sk cells, particularly by the ethanol extract of Hc. Stimulants included in PMA, phytohemagglutinin (PHA) and CaI, increased the mRNA level of CC chemokine receptor 4 (CCR4), a receptor of TARC, in Jurkat T cells, and the ethanol extract of HcF weakly blocked the increased mRNA level. However, the stimulants and ethanol extract had no effect on the CCR4 protein level. The ethanol extract inhibited TARC-induced migration, as well as basal migration of Jurkat T cells.
CONCLUSIONS: This study may show the usefulness of HcFs in the ethnopharmacological treatment of Th2-mediated or allergic inflammation, through the down-regulation of the production of Th2 cytokines and TARC, as well as cell migration.

PMID: 18325701 [PubMed - indexed for MEDLINE]

724. Prim Care Respir J. 2008 Mar;17(1):19-25. doi: 10.3132/pcrj.2008.00015.

Systems for the management of respiratory disease in primary care - an international series: Australia.

Glasgow N.

The Australian National University - Australian National University Medical School, Australia. nicholas.glasgow@anu.edu.au

Comment in
    Prim Care Respir J. 2008 Mar;17(1):5-6.

INTRODUCTION: Australia has a complex health system with policy and funding responsibilities divided across federal and state/territory boundaries and service provision split between public and private providers. General practice is largely funded through the federal government. Other primary health care services are provided by state/territory public entities and private allied health practitioners. Indigenous health services are specifically funded by the federal government through a series of Aboriginal Community Controlled Organisations.
NATIONAL POLICY AND MODELS: The dominant primary health care model is federally-funded private "small business" general practices. Medicare reimbursement items have incrementally changed over the last decade to include increasing support for chronic disease care with both generic and disease specific items as incentives. Asthma has received a large amount of national policy attention. Other respiratory diseases have not had similar policy emphasis.
EPIDEMIOLOGY: Australia has a high prevalence of asthma. Respiratory-related encounters in general practice, including acute and chronic respiratory illness and influenza immunisations, account for 20.6% of general practice activity. Lung cancer is a rare disease in general practice. Tuberculosis is uncommon and most often found in people born outside of Australia. Aboriginal and Torres Strait Islanders have higher rates of asthma, smoking and tuberculosis. ACCESS TO CARE: Access to care is positively influenced by substantial public funding

underpinning both the private and public sectors through Medicare. Access to general practice care is negatively influenced by workforce shortages, the ongoing demands of acute care, and the incremental way in which system redesign is occurring in general practice. FACILITIES AVAILABLE: Most general practice operates from privately-owned rooms. The Australian Government requires general practice facilities to be accredited against certain standards in order for the practice to receive income from a number of government programs. These standards require GPs to have ready access to spirometry, but do not require every practice to have a spirometer. FUTURE: The initial assessment and management of acute respiratory illnesses currently seen in primary health care settings will continue, but for this to occur the sector may have to adapt traditional workforce roles because of workforce shortages. In the longer term, climate change and migration patterns may result in changes in the epidemiology of regions and populations. The health system will continue to reform incrementally in order to deliver improved chronic disease care, including care of people with asthma and COPD. The incoming Labor Government's National Primary Health Care Strategy provides the high level policy opportunity to drive reform. CONCLUSIONS: Australia's complex primary health care system is incrementally changing from one of exclusive acute- and episodic-care orientation in both the public and private sectors to a system that delivers effective anticipatory chronic disease care as well. From a national policy perspective, asthma has received most attention. COPD and possibly other respiratory diseases may now receive focus.

PMID: 18322632 [PubMed - indexed for MEDLINE]

725. J Immunol. 2008 Mar 15;180(6):4323-9.

The role of sphingosine kinase in a murine model of allergic asthma.

Lai WQ, Goh HH, Bao Z, Wong WS, Melendez AJ, Leung BP.

Department of Physiology, Yong Loo Lin School of Medicine, National University of Singapore, Singapore, Republic of Singapore.

Asthma is an allergic disease characterized by chronic airway eosinophilia and pulmonary infiltration of lymphocytes, particularly of the Th2 subtype, macrophages and mast cells. Previous studies have shown a pivotal role for sphingosine kinase (SphK) on various proinflammatory cells, such as lymphocyte and eosinophil migration and mast cell degranulation. We therefore examined the roles of SphK in a murine model of allergic asthma. In mice previously sensitized to OVA, i.p. administration of N,N-dimethylsphingosine (DMS), a potent SphK inhibitor, significantly reduced the total inflammatory cell infiltrate and eosinophilia and the IL-4, IL-5, and eotaxin levels in bronchoalveolar lavage fluid in response to inhaled OVA challenge. In addition, DMS significantly suppressed OVA-induced inflammatory infiltrates and mucus production in the lungs, and airway hyperresponsiveness to methacholine in a dose-dependent manner. OVA-induced lymphocyte proliferation and IL-4 and IL-5 secretion were reduced in thoracic lymph node cultures from DMS-treated mice. Moreover, similar reduction in inflammatory infiltrates, bronchoalveolar lavage, IL-4, IL-5, eotaxin, and serum OVA-specific IgE levels was observed in mice with SphK1 knock-down via small interfering RNA approach. Together, these data demonstrate the therapeutic potential of SphK modulation in allergic airways disease.

PMID: 18322246 [PubMed - indexed for MEDLINE]

726. J Immunol. 2008 Mar 15;180(6):3833-8.

Differential sensitivity to Itk kinase signals for T helper 2 cytokine production and chemokine-mediated migration.

Sahu N, Mueller C, Fischer A, August A.

Center for Molecular Immunology & Infectious Disease, The Pennsylvania State University, University Park, PA 16802, USA.

Erratum in
J Immunol. 2008 May 15;180(10):7047-8.

Allergic asthma is dependent on chemokine-mediated Th2 cell migration and Th2 cytokine secretion into the lungs. The inducible T cell tyrosine kinase Itk regulates the production of Th2 cytokines as well as migration in response to chemokine gradients. Mice lacking Itk are resistant to developing allergic asthma. However, the role of kinase activity of Itk in the development of this disease is unclear. In addition, whether distinct Itk-derived signals lead to T cell migration and secretion of Th2 cytokines is also unknown. Using transgenic mice specifically lacking Itk kinase activity, we show that active kinase signaling is required for control of Th2 responses and development of allergic asthma. Moreover, dominant suppression of kinase Itk activity led to normal Th2 responses, but significantly reduced chemokine-mediated migration, resulting in prevention of allergic asthma. These observations indicate that signals required for Th2 responses and migration are differentially sensitive to Itk activity. Manipulation of Itk's activity can thus provide a new strategy to treat allergic asthma by differentially affecting migration of T cells into the lungs, leaving Th2 responses intact.

PMCID: PMC2913463
PMID: 18322190 [PubMed - indexed for MEDLINE]

727. Cell Mol Immunol. 2008 Feb;5(1):9-21. doi: 10.1038/cmi.2008.2.

The classical and regulatory functions of C1q in immunity and autoimmunity.

Lu JH, Teh BK, Wang Ld, Wang YN, Tan YS, Lai MC, Reid KB.

Department of Microbiology, Yong Loo Lin School of Medicine and 2NUS Immunology Program, National University of Singapore, Blk MD4, 5 Science Drive 2, Singapore 117597. miclujh@nus.edu.sg

A classical function of C1q is to bind immune complexes and initiate complement activation producing membrane lytic complexes, opsonins and anaphylatoxins. This classical pathway of complement activation is also elicited when C1q binds some other ligands. Besides complement activation, C1q also regulates cell differentiation, adhesion, migration, activation and survival. C1q deficiency is associated with autoimmunity as well as increased susceptibility to infections. In this article, we discuss the basic properties of C1q, its expression, and classical and regulatory functions.

PMID: 18318990 [PubMed - indexed for MEDLINE]

728. Electrophoresis. 2008 Feb;29(4):777-82. doi: 10.1002/elps.200700495.

Cationic surfactants for micellar electrokinetic chromatography: 2. Representative applications to acidic, basic, and hydrophobic analytes.

Schnee VP, Palmer CP.

Department of Chemistry, University of Montana, Missoula, MT 59812, USA.

Changes in MEKC chemical selectivity that are induced by changes in the headgroup structure of cationic surfactants are examined. Separations of acidic, basic, and hydrophobic solutes are examined. The acidic analytes are comprised of methoxyphenols, which are of interest due to their prevalence in wood smoke. The basic solutes consist of compounds often found in forensic urine analysis, and represent typical basic pharmaceuticals. The hydrophobic solutes are six pharmaceutical corticosteroids used in replacement therapy of adrenocortical insufficiency and nonspecific treatment of inflammatory and allergic conditions. The role of the headgroup was found to be quite significant when analyzing acidic compounds with not all the surfactants being able to resolve all of the analytes. The headgroup also induced migration order switches among the acidic analytes. All of the surfactants examined here in were found to be suitable for the analysis of basic analytes with each surfactant providing unique selectivity. The hydrophobic solutes were separated best with the larger more hydrophobic surfactant headgroups. The steroid separation with these two surfactants was achieved without the use of organic modifiers or a mixed micellar phase.

PMID: 18297645 [PubMed - indexed for MEDLINE]

729. Circ J. 2008 Mar;72(3):469-75.

IL-13 attenuates vascular tube formation via JAK2-STAT6 pathway.

Nishimura Y, Nitto T, Inoue T, Node K.

Department of Cardiovascular and Renal Medicine, Faculty of Medicine, Saga University, Saga, Japan.

BACKGROUND: Interleukin (IL)-13, which is a cytokine produced by type 2 helper T cells, has pathophysiological roles in allergic inflammation and fibrosis formation. IL-13 shares many functional properties with IL-4, which is known to inhibit angiogenesis.
METHODS AND RESULTS: The effects of IL-13 on angiogenesis were examined using human coronary artery endothelial cells (HCAECs), in addition to investigating the mechanism(s) of this action. Using an in vitro assay of angiogenesis it was demonstrated that IL-13, as well as IL-4, significantly inhibited capillary-like tube formation. Migration of HCAECs, considered to be a process of new capillary tube formation, was also significantly inhibited by IL-13. IL-13 activated signal transduction and transcription 6 (STAT6) as a result of the activation of Janus kinase 2 (JAK2). The inhibitory effect of IL-13 on angiogenesis was abolished by depletion of JAK2 and STAT6 by RNA interference.
CONCLUSION: IL-13 has anti-angiogenic activity as a result of activation of JAK2 and subsequent activation of STAT6.

PMID: 18296848 [PubMed - indexed for MEDLINE]

730. Pharmacol Ther. 2008 Mar;117(3):393-405. doi: 10.1016/j.pharmthera.2007.11.001. Epub 2007 Dec 15.

Macrolide antibiotics as immunomodulatory medications: proposed mechanisms of action.

Shinkai M, Henke MO, Rubin BK.

Ministry of Defense and National Defense Medical College, Japan.

Macrolide antibiotics administered in sub-antimicrobial doses improve pulmonary function and decrease exacerbation frequency for persons with diffuse panbronchiolitis or cystic fibrosis. Data also suggest a beneficial effect of macrolide antibiotics in the treatment of steroid dependent asthma. Many potential immunomodulatory effects of macrolide antibiotics have been reported including the ability to down-regulate prolonged inflammation, decreasing airway mucus secretion, inhibiting bacterial biofilm, decreasing the production of reactive oxygen species, inhibiting neutrophil activation and mobilization, accelerating neutrophil apoptosis, and blocking the activation of nuclear transcription factors. Macrolides initially decrease, then increase, and have finally a sustained suppression of cytokine secretions from normal human bronchial epithelial cells through inhibition and activation of extracellular signal-regulated kinases (ERK) and then reversibly retard cell proliferation probably through ERK. Consistent with this, macrolide antibiotics possibly reduce mucin production as well as neutrophil migration by interfering with ERK signal transduction.

PMID: 18289694 [PubMed - indexed for MEDLINE]

731. Scand J Immunol. 2008 Apr;67(4):377-84. doi: 10.1111/j.1365-3083.2008.02081.x. Epub 2008 Feb 12.

Responsiveness of eosinophils to aeroallergens may be independent of atopic status.

Redvall E, Bengtsson U, Wennerås C.

Department of Clinical Bacteriology, Göteborg University, Göteborg, Sweden.

It has been reported that extracts from common aeroallergens directly activate eosinophils from non-allergic individuals, eliciting chemotaxis and degranulation. The aims of this study were to compare the reactivity of eosinophils from non-atopic and atopic individuals to airborne allergens, and to assess if this reactivity was modulated by natural exposure to birch pollen. Blood-derived eosinophils were stimulated with allergen extracts from birch pollen, cat dander, house dust mite and timothy grass, and their capacity to degranulate (eosinophil peroxidase, EPO; major basic protein, MBP) and produce T helper type 1 and 2 cytokines were evaluated as well as their capacity to migrate in vitro, in and out of the birch pollen season. Eosinophils from atopic and non-atopic individuals responded similarly to stimulation with allergen extracts with respect to directed migration, EPO and MBP release, which was independent of the season when the samples were collected. Interestingly, eosinophils from both study groups were incapable of producing tumour necrosis factor-alpha (TNF-alpha) during the birch pollen season, but could generate interleukin-4. Innate responsiveness of eosinophils to aeroallergens is independent of the atopic status of the individual. In vivo exposure to birch allergen as seen during the birch pollen season downregulates the capacity of eosinophils to produce the cytokine TNF-alpha.

PMID: 18282233 [PubMed - indexed for MEDLINE]

732. Klin Oczna. 2007;109(7-9):345-8.

[The role of chemokines in ocular diseases. Part II. Participation of chemokines in ocular diseases].

[Article in Polish]

Malesiński R, Mrugacz M, Bakunowicz-Łazarczyk A.

Oddziału Chorób Oczu Wojewódzkiego Szpitala Zespolonego w Białymstoku.

The chemokines are family of ca. 50 chemotactic cytokines produced by leucocytes and tissue cells, which regulate and stimulate leucocytes migration. Dysregulation of this process is apparent in chronic inflammation, allergic eye disease, corneal graft rejection, proliferative vitreoretinopathies and age-related macular degeneration. Antagonists of chemokine receptors have demonstrated anti-inflammatory and antiviral activity and may represent new generations of therapeutic agents.

PMID: 18260295 [PubMed - indexed for MEDLINE]

733. J Cell Sci. 2008 Feb 15;121(Pt 4):487-95. doi: 10.1242/jcs.016980.

A role for membrane-bound CD147 in NOD2-mediated recognition of bacterial cytoinvasion.

Till A, Rosenstiel P, Bräutigam K, Sina C, Jacobs G, Oberg HH, Seegert D, Chakraborty T, Schreiber S.

Institute for Clinical Molecular Biology, University Hospital Schleswig-Holstein, Campus Kiel, Schittenhelmstr 12, Kiel, Germany.

NOD2 is an intracellular receptor for the bacterial cell wall component muramyl dipeptide. Mutations in the leucine-rich repeat region of NOD2, which lead to an impaired recognition of muramyl dipeptide, have been associated with chronic inflammatory diseases of barrier organs such as Crohn disease, asthma and atopic eczema. In this study we identify CD147 (also known as BSG and EMMPRIN), a membrane-bound regulator of cellular migration, differentiation and inflammatory processes, as a protein interaction partner of NOD2. We demonstrate a complex influence of the CD147-NOD2 interaction on NOD2-dependent signaling responses. We show that CD147 itself acts as an enhancer of the invasion of Listeria monocytogenes, an intracellular bacterial pathogen. We propose that the CD147-NOD2 interaction serves as a molecular guide to regulate NOD2 function at sites of pathogen invasion.

PMID: 18256385 [PubMed - indexed for MEDLINE]

734. J Cosmet Dermatol. 2008 Mar;7(1):50-4. doi: 10.1111/j.1473-2165.2008.00361.x.

Different formulations of botulinum toxin type A have different migration characteristics: a double-blind, randomized study.

Cliff SH, Judodihardjo H, Eltringham E.

Epsom and St. Helier University NHS Trust, and Surrey and Sussex NHS Trust, UK. sandeep_cliff@hotmail.com

Comment in
    J Cosmet Dermatol. 2008 Sep;7(3):230.

OBJECTIVE: Different formulations of botulinum toxin type A (BoNTA) are not identical and may behave differently in clinical practice. The reportedly lower incidence of adverse effects with one formulation (from Allergan, Ltd.) relative to another (from Ipsen, Ltd.) may be due to differences in the degree of

migration of the neurotoxin-protein complex from its injection site. A double-blind, randomized, within-subject pilot study was performed to compare the migration characteristics of each formulation.
METHODS: Twelve healthy volunteers were randomly assigned to receive three 0.1 mL intradermal injections in their forehead: 4 U BoNTA (Allergan) on one side, 12 U BoNTA (Ipsen) on the contralateral side, and saline in the center. At day 14, Minor's iodine starch test was performed, and the subjects walked around a hot room to induce sweating. The appearance of each forehead was documented using Canfield photography and the area of each anhidrotic halo calculated using software.
RESULTS: Overall, the area of anhidrosis was significantly larger with BoNTA (Ipsen) than BoNTA (Allergan) - mean +/- SD of 2.7 +/- 0.78 cm(2) vs. 1.8 +/- 0.65 cm(2) (P = 0.005) - with the area of anhidrosis being greater with BoNTA (Ipsen) than BoNTA (Allergan) in 11 of the 12 subjects. Across all subjects, the area of anhidrosis was greater with BoNTA (Ipsen) than BoNTA (Allergan) by a mean of 77%.
CONCLUSIONS: BoNTA (Ipsen) migrates more than BoNTA (Allergan) under the conditions described. The lower potential of BoNTA (Allergan) to migrate promotes more precise localization of clinical effects, thereby helping to optimize the risk/benefit ratio.

PMID: 18254812 [PubMed - indexed for MEDLINE]

735. J Immunol. 2008 Feb 15;180(4):2669-78.

Inhibitory ITAM signaling by Fc alpha RI-FcR gamma chain controls multiple activating responses and prevents renal inflammation.

Kanamaru Y, Pfirsch S, Aloulou M, Vrtovsnik F, Essig M, Loirat C, Deschênes G, Guérin-Marchand C, Blank U, Monteiro RC.

INSERM Unité 699, Universite Paris 7-Denis Diderot, Faculte de Medecine, Site Xavier Bichat, 16 rue Henri Huchard, Paris, France.

Inhibitory signaling is an emerging function of ITAM-bearing immunoreceptors in the maintenance of homeostasis. Monovalent targeting of the IgA Fc receptor (FcalphaRI or CD89) by anti-FcalphaRI Fab triggers potent inhibitory ITAM (ITAM(i)) signaling through the associated FcRgamma chain (FcalphaRI-FcRgamma ITAM(i)) that prevents IgG phagocytosis and IgE-mediated asthma. It is not known whether FcalphaRI-FcRgamma ITAM(i) signaling controls receptors that do not function through an ITAM and whether this inhibition requires Src homology protein 1 phosphatase. We show in this study that FcalphaRI-Fcgamma ITAM(i) signals depend on Src homology protein 1 phosphatase to target multiple non-ITAM-bearing receptors such as chemotactic receptors, cytokine receptors, and TLRs. We found that anti-FcalphaRI Fab treatment in vivo reduced kidney inflammation in models of immune-mediated glomerulonephritis and nonimmune obstructive nephropathy by a mechanism that involved decreased inflammatory cell infiltration and fibrosis development. This treatment also prevented ex vivo LPS activation of monocytes from patients with lupus nephritis or vasculitis, as well as receptor activation through serum IgA complexes from IgA nephropathy patients. These findings point to a crucial role of FcalphaRI-FcRgamma ITAM(i) signaling in the control of multiple heterologous or autologous inflammatory responses. They also identify anti-FcalphaRI Fab as a new potential therapeutic tool for preventing progression of renal inflammatory diseases.

PMID: 18250479 [PubMed - indexed for MEDLINE]

736. J Immunol. 2008 Feb 15;180(4):2600-7.

Granulocyte-macrophage colony-stimulating factor is required for bronchial eosinophilia in a murine model of allergic airway inflammation.

Su YC, Rolph MS, Hansbro NG, Mackay CR, Sewell WA.

Immunology and Inflammation Research Program, Garvan Institute of Medical Research, 384 Victoria Street, Darlinghurst, New South Wales 2010, Australia.

GM-CSF plays an important role in inflammation by promoting the production, activation, and survival of granulocytes and macrophages. In this study, GM-CSF knockout (GM-CSF(-/-)) mice were used to investigate the role of GM-CSF in a model of allergic airway inflammation. In allergic GM-CSF(-/-) mice, eosinophil recruitment to the airways showed a striking pattern, with eosinophils present in perivascular areas, but almost completely absent in peribronchial areas, whereas in wild-type mice, eosinophil infiltration appeared in both areas. In the GM-CSF(-/-) mice, mucus production in the airways was also reduced, and eosinophil numbers were markedly reduced in the bronchoalveolar lavage (BAL)(3) fluid. IL-5 production was reduced in the lung tissue and BAL fluid of GM-CSF(-/-) mice, but IL-4 and IL-13 production, airway hyperresponsiveness, and serum IgE levels were not affected. The presence of eosinophils in perivascular but not peribronchial regions was suggestive of a cell migration defect in the airways of GM-CSF(-/-) mice. The CCR3 agonists CCL5 (RANTES) and CCL11 (eotaxin-1) were expressed at similar levels in GM-CSF(-/-) and wild-type mice. However, IFN-gamma mRNA and protein were increased in the lung tissue and BAL fluid in GM-CSF(-/-) mice, as were mRNA levels of the IFN-gamma-inducible chemokines CXCL9 (Mig), CXCL10 (IP-10), and CXCL11 (I-Tac). Interestingly, these IFN-gamma-inducible chemokines are natural antagonists of CCR3, suggesting that their overproduction in GM-CSF(-/-) mice contributes to the lack of airway eosinophils. These findings demonstrate distinctive abnormalities to a model of allergic asthma in the absence of GM-CSF.

PMID: 18250471 [PubMed - indexed for MEDLINE]

737. J Invest Dermatol. 2008 Jul;128(7):1696-703. doi: 10.1038/sj.jid.5701250. Epub 2008 Jan 31.

Human inflammatory dendritic epidermal cells express a functional histamine H4 receptor.

Dijkstra D, Stark H, Chazot PL, Shenton FC, Leurs R, Werfel T, Gutzmer R.

Department of Dermatology and Allergology, Hannover Medical School, Hannover, Germany. dijkstra.dorothea@mh-hannover.de

Comment in
J Invest Dermatol. 2008 Jul;128(7):1615-6.

Expression of histamine H(4) receptor (H(4)R) on leukocytes suggests an immunomodulatory role of this receptor. Here we investigated the expression and function of H(4)R on human inflammatory dendritic epidermal cells (IDECs). H(4)R is expressed by IDEC of the skin. On monocyte-derived IDECs (Mo-IDECs), H(4)R is also expressed and upregulated by IFN-gamma. Functionally, histamine and H(4)R agonists clobenpropit and 4-methylhistamine downregulated the production of the Th2-linked chemokine CCL2 and the Th1 cytokine IL-12 on Mo-IDEC, whereas agonists for the other histamine receptors did not. An H(4)R-selective antagonist (JNJ7777120) blocked the effect of H(4)R agonists. Downregulation of CCL2 also led to a decreased migration of monocytes. Thus, IDEC express a functionally active H(4)R, which upon stimulation leads to downregulation of CCL2 and IL-12.

This might have implications for the treatment of atopic dermatitis, since H(4)R agonists may have beneficial effects in downregulating inflammation.

PMID: 18239617 [PubMed - indexed for MEDLINE]

738. Biol Pharm Bull. 2008 Feb;31(2):299-304.

Comparative study of the usefulness of the drug-induced lymphocyte stimulation test and the leukocyte migration test in drug allergies.

Saito M, Yagi M, Uno K, Takanaka K.

The Shibata Center Pharmacy, Kyoeido Ltd, Shibata, Niigata, Japan.
mikiohs@ybb.ne.jp

In 133 patients suspected of hypersensitivity to drugs and 102 control patients without hypersensitivity to drugs, the identification of allergenic drugs was performed by the drug-induced lymphocyte stimulation test (DLST) and the leukocyte migration test (LMT) to compare their usefulness in identifying drug allergies. In the 133 subject patients, the positive rate was 24.8% on the DLST and 60.9% on the LMT (agreement rate; 77.4%); thus, the LMT showed a significantly higher positive rate than the DLST (p<0.000001, chi(2)-test). In the 102 control patients, the positive rates on the DLST and LMT were 6.9%. In addition, the LMT showed a higher positive rate than the DLST for many hypersensitivity symptoms such as skin eruptions and hepatic injury, and for many drug efficacy categories of the suspected drugs such as antibacterial drugs, etc. Furthermore, the positive rate of the DLST did not change when adjusted for the patients' serum and sex, while that of the LMT increased when adjusted for the patients' serum and was found to be higher in females than in males. Our findings indicate that the LMT may be more useful than the DLST in identifying the causative drug in drug allergies and that its interpretation is influenced by the patient's serum and sex.

PMID: 18239291 [PubMed - indexed for MEDLINE]

739. J Cosmet Laser Ther. 2007 Dec;9(4):249-52. doi: 10.1080/14764170701545657.

The 'Nefertiti lift': a new technique for specific re-contouring of the jawline.

Levy PM.

Faculté de Médecine et Pharmacologie de Besançon, Besançon, France.
philliplevy@bluewin.ch

Botulinum toxin type A (BoNTA) is now used extensively for rejuvenation of the forehead, glabellar and periocular regions and there is increasing focus on treatment of the lower face. Although there is well-documented evidence for the efficacy of botulinum toxin in the correction of platysmal bands, little work has been done to explore its potential role in rejuvenation of the jawline. To date, effects in this area have been reported as a consequence of platysmal banding treatment and are inconsistent. Hesitancy to explore treatment may be due to evidence of a greater, more durable response to the toxin in the lower facial muscles as well as reports of increased potential migration and subsequent side effects. This paper describes a new technique using BoNTA (Vistabel); Allergan, Irvine, CA, USA) to drape the skin of the jawline contour and provide the visual effect of a 'mini lift'. Experience with 130 patients with doses of BoNTA up to 20 U is described. Patient satisfaction is extremely high and the specificity of dosing and technique has led to a low incidence of adverse effects. The

'Nefertiti lift' is a minimally invasive, effective and acceptable alternative for those patients seeking an effective way to push back surgery.

PMID: 18236245 [PubMed - indexed for MEDLINE]

740. Pediatr Neurosurg. 2008;44(2):169-71. doi: 10.1159/000113123. Epub 2008 Jan 24.

Transoral protrusion of a peritoneal catheter: a rare complication of ventriculoperitoneal shunt.

Berhouma M, Messerer M, Houissa S, Khaldi M.

Department of Neurosurgery, National Institute of Neurology, Tunis, Tunisia. berhouma.moncef@laposte.net

Ventriculoperitoneal (VP) shunt surgery is the most used technique for the treatment of hydrocephalus. This procedure is associated with a large amount of complications. Bowel perforation caused by a peritoneal shunt catheter is one of these complications, sometimes fatal, and is usually difficult to recognize, except when protrusion of the peritoneal catheter through a natural orifice occurs. This report presents the case of a 2-year-old boy who had undergone a VP shunt and later presented with protrusion of the peritoneal catheter through his mouth. The shunt device was removed and an external shunt procedure was achieved, using the original ventricular catheter kept in place. The diagnosis of bacterial meningitis was retained and an antibiotic therapy was started. The evolution was fatal in 15 days secondary to a bacterial ventriculitis. Through the reported cases of bowel perforation, many risk factors were individualized, such as age, congenital etiology of the hydrocephalus, silicon allergy or the length of the peritoneal catheter. Bowel perforation is a serious complication of VP shunt surgery, leading sometimes to a fatal outcome.

PMID: 18230935 [PubMed - indexed for MEDLINE]

741. Eur J Immunol. 2008 Feb;38(2):338-41. doi: 10.1002/eji.200738131.

Osteopontin as a new player in mast cell biology.

Bulfone-Paus S, Paus R.

Department of Immunology and Cell Biology, Research Center Borstel, Borstel, Germany. sbulfone@fz-borstel.de

Comment on
    Eur J Immunol. 2008 Feb;38(2):489-99.

The secreted glycoprotein osteopontin (OPN) sets into motion an astounding variety of activities that range from bone remodeling via immunomodulation to the inhibition of apoptosis. In the current issue of the European Journal of Immunology, OPN now also enters mast cell biology and the regulation of IgE-dependent immune responses since it is reported that connective tissue-type mast cells from fetal murine skin constitutively secrete biologically active OPN. Moreover, it is shown that, in vitro, OPN augments IgE-mediated mast cell degranulation and migration via ligand binding to cognate OPN receptors on the mast cell surface (CD44, alpha v integrin) and that the magnitude of an IgE-mediated passive cutaneous anaphylaxis reaction is augmented by OPN in vivo. Here, we discuss why this newly discovered property of OPN fits well into the emerging concept that OPN may serve as a multi-purpose environmental damage-response protein.

PMID: 18228246 [PubMed - indexed for MEDLINE]

742. Inflamm Allergy Drug Targets. 2007 Dec;6(4):236-44.

Innate and adaptive immune responses in allergic contact dermatitis and autoimmune skin diseases.

Edele F, Esser PR, Lass C, Laszczyk MN, Oswald E, Strüh CM, Rensing-Ehl A, Martin SF.

Allergy Research Group, Department of Dermatology, University Medical Center Freiburg, D-79104 Freiburg, Germany.

Allergic contact dermatitis is induced by chemicals or metal ions. A hallmark of this T cell mediated skin disease is the activation of the innate immune system by contact allergens. This immune response results in inflammation and is a prerequisite for the activation of the adaptive immune system with tissue-specific migration of effector and regulatory T cells. Recent studies have begun to address in detail the innate immune cells as well as the innate receptors on these cells and the associated signaling pathways which lead to skin inflammation. We review here recent findings regarding innate and adaptive immune responses and immune regulation of contact dermatitis and other skin diseases as well as recent developments towards an in vitro assessment of the allergenic potential of chemicals. The elucidation of the innate inflammatory pathways, cellular components and mediators will help to identify new drug targets for more efficient treatment of allergic contact dermatitis and hopefully also for its prevention.

PMID: 18220958 [PubMed - indexed for MEDLINE]

743. BMC Health Serv Res. 2008 Jan 24;8:22. doi: 10.1186/1472-6963-8-22.

Suboptimal asthma care for immigrant children: results of an audit study.

Urbanus-van Laar JJ, de Koning JS, Klazinga NS, Stronks K.

Department of Social Medicine, Academic Medical Center-University of Amsterdam, The Netherlands. Nathalie.urbanus@xs4all.nl

BACKGROUND: Little is known on the scope and nature of ethnic inequalities in suboptimal asthma care for children. This study aimed to assess (1) ethnic differences in suboptimal asthma care for children with an asthma exacerbation who consulted a physician, and (2) ethnic differences in the nature of suboptimal care.
METHODS: All children aged 6-16 years who during a period of six months consulted the paediatric department of the Academic Medical Centre-University of Amsterdam or one of the six regional primary care centres with an asthma exacerbation were included. Clinical guidelines were systematically converted to review criteria following the strategy as proposed by the Agency for Health Care Policy and Research. Based upon these review criteria and their experience experts of two multidisciplinary panels retrospectively assessed the quality of care and its (possible) failure to prevent the occurrence of asthma exacerbation.
RESULTS: Only a small number of children (n = 35) were included in the analysis as a result of which the ethnic differences in suboptimal care were not significant. However, the results do indicate immigrant children, in particular 'other non-Western' children (n = 11), more frequently to receive suboptimal care related to the asthma exacerbation when compared to ethnic Dutch children.

Furthermore, we found the nature of suboptimal care to differ with under-prescribing in the 'other non-Western' group (n = 11), lack of information exchange between physicians in the Surinamese/Antillean group (n = 12) and lack of education, and counselling of patients and parents in the ethnic Dutch (n = 12) as the most relevant factor.
CONCLUSION: Ethnic inequalities in the scope and nature of suboptimal asthma care for children in the Netherlands seem to exist. For the non-western immigrant groups the results indicate the importance of the prescription behaviour of the medical doctor, as well as the supervision by one health care provider.

PMCID: PMC2254382
PMID: 18218104 [PubMed - indexed for MEDLINE]

744. Allergol Int. 2008 Mar;57(1):73-8. doi: 10.2332/allergolint.O-06-481. Epub 2008 Mar 1.

Contribution of lung fibroblast migration in the fibrotic process of airway remodeling in asthma.

Yamauchi E, Shoji S, Nishihara M, Shimoda T, Nishima S.

Fukuoka National Hospital, Fukuoka, Japan.

BACKGROUND: The fibrotic process in airway remodeling of asthma may be characterized by an exaggerated deposition of extracellular matrix (ECM) components such as fibronectin and type I, III and IV collagen. In the present study, we established airway remodeling model mice and examined the mechanism of fibrotic change by measuring chemotactic activity of lung fibroblasts and quantifying collagen content in lung tissues.
METHODS: Airway remodeling model mice were made by ovalbumin (OA) sensitization and inhalation. Bronchoalveolar lavage (BAL) and bronchial biopsy were performed. Cell migration was assessed by the Boyden's chamber technique. The collagen content of lung tissue was measured using ELISA.
RESULTS: The chemotactic activity in lung fibroblasts toward the mouse BAL fluid (BALF) was significantly increased in OA-inhaled mice. Total soluble collagen content was significantly increased in OA-inhaled mice. We observed markedly increased collagen deposition around the airway wall in OA-inhaled mice, which was not shown in saline-inhaled mice. Furthermore, fibronectin in the BALF of OA-inhaled mice was significantly higher than that in the control mice.
CONCLUSIONS: The total soluble collagen content increased during the fibrotic change of airway remodeling in asthma. Furthermore, migration of fibroblasts may play a key role in this remodeling process, and fibronectin and type I and IV collagen seem to be chemotactic factors for the fibroblasts.

PMID: 18209507 [PubMed - indexed for MEDLINE]

745. Eur J Immunol. 2008 Feb;38(2):489-99. doi: 10.1002/eji.200737057.

Osteopontin is produced by mast cells and affects IgE-mediated degranulation and migration of mast cells.

Nagasaka A, Matsue H, Matsushima H, Aoki R, Nakamura Y, Kambe N, Kon S, Uede T, Shimada S.

Department of Dermatology, University of Yamanashi, Interdisciplinary Graduate School of Medicine and Engineering, Yamanashi, Japan.

Comment in

Eur J Immunol. 2008 Feb;38(2):338-41.

Osteopontin (OPN), originally discovered in bone as an extracellular matrix protein, was identified in many cell types in the immune system, presumably being involved in many aspects of pathogenesis of inflammatory and immune diseases. Mast cells are also involved in such pathological aspects by secreting multiple mediators. However, it has not been determined whether mast cells produce OPN and whether it affects their function. To test this, we used murine fetal skin-derived cultured mast cells (FSMC) and bone marrow-derived cultured mast cells. We found that OPN was spontaneously produced by FSMC and inducible by ionomycin and FcepsilonRI aggregation in bone marrow-derived cultured mast cells. In the presence of mast cell growth factors, FSMC were similarly generated from both OPN-deficient (OPN(-/-)) and -sufficient (OPN(+/+)) mice without significant differences in yield, purity, granularity, and viability. Using OPN(-/-) FSMC, we found that recombinant OPN augmented IgE-mediated degranulation and induced FSMC chemotaxis. Both effects were mediated by OPN receptors (i.e. CD44 and integrin alphav). IgE-mediated passive cutaneous anaphylaxis was significantly reduced in OPN(-/-) mice compared with OPN(+/+) mice, indicating physiological relevance of OPN. These results indicate that OPN is a mast cell mediator, enhances mast cell responses to antigen, and thus may influence mast cell-related pathological conditions.

PMID: 18200503 [PubMed - indexed for MEDLINE]

746. Int Immunol. 2008 Mar;20(3):353-63. doi: 10.1093/intimm/dxm146. Epub 2008 Jan 8.

FAK-mediated activation of ERK for eosinophil migration: a novel mechanism for infection-induced allergic inflammation.

Cheung PF, Wong CK, Ip WK, Lam CW.

Department of Chemical Pathology, Chinese University of Hong Kong, Prince of Wales Hospital, Shatin, NT, Hong Kong.

Bacterial and viral infections often induce the exacerbation of allergic diseases. In this study, we investigated the activation of human eosinophils by different microbial products via Toll-like receptors (TLRs). The underlying intracellular mechanism involving activation of extracellular signal-regulated kinase (ERK) and focal adhesion kinase (FAK), an integrin-associated focal adhesion molecule, was also examined. Seven TLR ligands were studied for their abilities in promoting survival, modulating the expression of adhesion molecules and facilitating chemotactic migration of eosinophils. While peptidoglycan (PGN) (TLR2 ligand) showed the most prominent effects, flagellin (TLR5 ligand) and imiquimod R837 (TLR7 ligand) were also effective in activating eosinophils. However, little or no effect was observed for double-stranded polyinosinic-polycytidylic acid (TLR3 ligand), ultra-purified LPS (TLR4 ligand), single-stranded RNA (ssRNA) (TLR8 ligand) and CpG-DNA (TLR9 ligand). Further investigation confirmed that PGN, flagellin and R837 commonly transmitted signals through ERK activation that required prior phosphorylation of tyrosine 925, but not tyrosine 577, on FAK. Moreover, the inhibition of ERK activation by selective inhibitor PD98059 and FAK expression by FAK-specific RNA interference could significantly abolish the stimulatory effects induced by PGN, flagellin and R837. Taken together, our findings indicate the involvement of FAK-dependent activation of ERK1 in TLR-mediated eosinophil stimulation. A potential role of eosinophils was also suggested in exacerbating allergic inflammation in response to microbial infections.

PMID: 18182379 [PubMed - indexed for MEDLINE]

747. Blood. 2008 Apr 15;111(8):4193-200. doi: 10.1182/blood-2007-09-115451. Epub 2008 Jan 4.

Distinct roles of sphingosine kinases 1 and 2 in human mast-cell functions.

Oskeritzian CA, Alvarez SE, Hait NC, Price MM, Milstien S, Spiegel S.

Department of Biochemistry and Molecular Biology, Virginia Commonwealth University School of Medicine, 1101 E Marshall St, Richmond, VA 23298-0614, USA.

Sphingosine-1-phosphate (S1P) is now emerging as a potent lipid mediator produced by mast cells that contributes to inflammatory and allergic responses. In contrast to its weak effect on degranulation of murine mast cells, S1P potently induced degranulation of the human LAD2 mast-cell line and cord blood-derived human mast cells (hMCs). S1P also stimulated production and secretion of cytokines, TNF-alpha and IL-6, and markedly enhanced secretion of a chemokine, CCL2/MCP-1, important modulators of inflammation. S1P is produced in mast cells by the 2 sphingosine kinases, SphK1 and SphK2. SphK1 but not SphK2 plays a critical role in IgE/Ag-induced degranulation, migration toward antigen, and CCL2 secretion from hMCs, as determined by specifically down-regulating their expression. However, both isoenzymes were required for efficient TNF-alpha secretion. Taken together, our data suggest that differential formation of S1P by SphK1 and SphK2 has distinct and important actions in hMCs.

PMCID: PMC2971746
PMID: 18178871 [PubMed - indexed for MEDLINE]

748. Cytometry A. 2008 Apr;73(4):299-304. doi: 10.1002/cyto.a.20514.

Histamine-induced actin polymerization in human eosinophils: an imaging approach for histamine H4 receptor.

Barnard R, Barnard A, Salmon G, Liu W, Sreckovic S.

Biomarkers & Translational Biology, Pfizer, Sandwich, Kent, United Kingdom. ruth.barnard@pfizer.com

Image-based screening, a new and flexible tool in the drug discovery cascade, is amenable to many different targets. This article describes a particular use of the Cellomics ArrayScan in developing a functional screen for histamine H(4) receptor (H(4)R) antagonists that have potential utility in inflammatory diseases of the airways such as asthma, with H(4)R being expressed on a wide variety of immune cells including eosinophils. Exposure to histamine causes eosinophils to migrate from the bloodstream into the tissue where they contribute to inflammation. Migration is manifested through rearrangements of the actin cytoskeleton and phalloidin, a biological peptide, selectively binds F-actin over G-actin and can be used to detect these cytoskeletal changes mediating inflammatory function. A fluorescent conjugate of phalloidin was used to visualize histamine-induced actin polymerization in human eosinophils on the Cellomics ArrayScan. Inhibition of this phenomenon by commercially available histamine receptor antagonists was measured. The selective H(4)R antagonist JNJ7777120 inhibited histamine-induced actin polymerization in eosinophils most potently. This assay illustrates that this phenomenon is mediated through the H(4)R and that the image-based format has enhanced screening utility for identifying selective H(4)R antagonists over traditional flow cytometry methods.

(c) 2007 International Society for Analytical Cytology.

PMID: 18163465 [PubMed - indexed for MEDLINE]

749. Wien Klin Wochenschr. 2008;120(19-20 Suppl 4):101-3. doi: 10.1007/s00508-008-1039-9.

Primary cystic echinococcosis in the subcutaneous gluteal region - a case report.

Steurer S, Auer H.

Pathologisch-bakteriologisches Institut am Otto Wagner Spital, Vienna, Austria.

Cystic echinococcosis (hydatidosis, hydatid disease) belongs to the group of the most severe parasitic diseases of man and is caused by metacestodes (larvae) of Echinococcus granulosus. Dogs and other canid mammals are the definitive hosts harbouring the adult stages of the tape worm in their intestines. Proglottids containing eggs are passed in the faeces of the definitive host. The eggs are ingested by intermediate hosts (i.e. sheep, cattle, swine etc.), in which the metacestode stage develops. Humans are accidental hosts and are infected by ingestion of eggs upon contact with contaminated water, vegetables, fur (of the dogs) or contaminated hands, the subsequently developing metacestodes are usually localized in the liver, the lungs and many other organs. We report on a case of a 57 year old female patient of Turkish origin suffering from a multicystic gluteal lesion diagnosed by CT- and MRT scan. Surgical intervention revealed a subcutaneous hydatid cyst of 15 cm in diameter. Parasitological-serological examinations revealed a highly positive specific antibody level against E. granulosus arc 5 antigen. The surgically resected cysts could be identified as E. granulosus sheep strain (genotype G1). Additionally to the surgical treatment the patient received albendazole postoperatively. Primary subcutaneous cystic echinococcosis without involving thoracic or abdominal organs is extremely rare and it has not been reported so far. Since soft tissue tumors may resemble hydatid cysts, careful preoperative evaluation is critical for proper handling during surgery to avoid possible anaphylactic reactions or spillage of protoscoleces, particularly when patients are deriving from endemic areas.

PMID: 19066783 [PubMed - indexed for MEDLINE]

750. Int Immunol. 2008 Feb;20(2):259-66. Epub 2007 Dec 21.

CpG-ODN inhibits airway inflammation at effector phase through down-regulation of antigen-specific Th2-cell migration into lung.

Ashino S, Wakita D, Zhang Y, Chamoto K, Kitamura H, Nishimura T.

Division of Immunoregulation, Institute for Genetic Medicine, Hokkaido University, Sapporo 060-0815, Japan.

Allergic airway inflammation is one of the most typical characteristic features of bronchial asthma. T(h)2 cells, which produce IL-4, IL-5 and IL-13, are well known as major effector lymphocytes of the inflammation. In the present work, we found that subcutaneous injection of Toll-like receptor-9-ligand, CpG-oligodeoxynucleotides (CpG-ODN), remarkably suppressed eosinophilia and mucus hyper-production in T(h)2 cell-dependent airway inflammation model at the effector phase. The injection of CpG-ODN significantly blocked T(h)2 cell migration into lung. The inhibitory effects of CpG-ODN were observed even when IFN-gamma-deficient T(h)2 cells were transferred into IFN-gamma(-/-) mice. In contrast, the administration of neutralizing mAbs against type I cytokines such as IFN-alpha, IFN-beta and IL-12 significantly suppressed the inhibitory effect of CpG-ODN on airway inflammation and T(h)2 cell migration into the lung. We

further demonstrated that the production of T(h)2 chemokines, thymus and activation-regulated chemokine (TARC) and macrophage-derived chemokine (MDC), was significantly reduced by the CpG-ODN. The reduction of both TARC and MDC was also inhibited by the blockade of IFN-alpha, IFN-beta and IL-12 with mAbs. Thus, we revealed here that IFN-alpha, IFN-beta and IL-12, but not IFN-gamma, were required for the inhibitory effect of CpG-ODN in T(h)2 cell-mediated allergic airway inflammation. The present evidence strongly suggest that induction of type I cytokines would be promising therapeutic targets in T(h)2-dependent allergic diseases such as bronchial asthma.

PMID: 18156622 [PubMed - indexed for MEDLINE]

751. Am J Respir Crit Care Med. 2008 Mar 15;177(6):604-12. Epub 2007 Dec 20.

Allergen induces the migration of platelets to lung tissue in allergic asthma.

Pitchford SC, Momi S, Baglioni S, Casali L, Giannini S, Rossi R, Page CP, Gresele P.

Department of Internal Medicine, Section of Internal and Cardiovascular Medicine, University of Perugia, Via E. dal Pozzo, I-06126 Perugia, Italy.

RATIONALE: Platelets are essential for pulmonary leukocyte recruitment, airway hyperresponsiveness, and bronchial remodeling in animals with allergic inflammation and can be found in bronchoalveolar lavage of sensitized animals. No studies, however, have explored the direct migration of platelets to lungs.
OBJECTIVES: To assess whether platelets migrate into lung parenchyma in response to inhaled allergen in ovalbumin-sensitized mice; to assess the role of the FcepsilonRI receptor in this phenomenon; and to evaluate whether platelets from patients with asthma, or from sensitized mice, undergo chemotaxis in vitro in response to relevant antigens.
METHODS: Ovalbumin-sensitized wild-type (WT) mice, or FcRgamma(-/-) mice lacking the FcepsilonRIgamma, were challenged with aerosolized allergen and lungs analyzed by platelet-specific immunohistochemistry. In some experiments, mice were depleted of platelets and cross-transfused with either WT or FcRgamma(-/-) platelets to assess the role of platelet FcRgamma(-/-). Chemotaxis of platelets from patients with asthma or from sensitized mice was studied in vitro.
MEASUREMENTS AND MAIN RESULTS: Histology of lungs revealed isolated platelets, migrating out of vessels and localizing underneath the airways after allergen challenge in WT but not in FcRgamma(-/-) mice. Platelets from patients with asthma and from sensitized WT mice, but not from sensitized FcRgamma(-/-) mice, migrated in vitro toward the relevant allergen or an anti-IgE. Platelets from normal mice were found to express FcepsilonRIgamma and platelet-bound IgEs were increased in sensitized mice.
CONCLUSIONS: Platelets migrate extravascularly in response to a sensitizing allergen via a mechanism dependent on the interaction among allergen, allergen-specific IgE, and the FcepsilonRI, and this may allow them to participate directly in allergic tissue inflammation.

PMID: 18096710 [PubMed - indexed for MEDLINE]

752. Proc Am Thorac Soc. 2008 Jan 1;5(1):89-96.

Airway smooth muscle growth in asthma: proliferation, hypertrophy, and migration.

Bentley JK, Hershenson MB.

Department of Pediatrics and Communicable Diseases, University of Michigan,

Medical Science Research Building II, 1150 W. Medical Center Drive, Room 3570B, Ann Arbor, MI 48109-0688, USA.

Increased airway smooth muscle mass is present in fatal and non-fatal asthma. However, little information is available regarding the cellular mechanism (i.e., hyperplasia vs. hypertrophy). Even less information exists regarding the functional consequences of airway smooth muscle remodeling. It would appear that increased airway smooth muscle mass would tend to increase airway narrowing and airflow obstruction. However, the precise effects of increased airway smooth muscle mass on airway narrowing are not known. This review will consider the evidence for airway smooth muscle cell proliferation and hypertrophy in asthma, potential functional effects, and biochemical mechanisms.

PMCID: PMC2645305
PMID: 18094090 [PubMed - indexed for MEDLINE]

753. Am J Clin Dermatol. 2008;9(1):15-31.

Peroxisome proliferator-activated receptors (PPARs) and the human skin: importance of PPARs in skin physiology and dermatologic diseases.

Sertznig P, Seifert M, Tilgen W, Reichrath J.

Department of Dermatology, The Saarland University Hospital, Homburg/Saar, Germany.

Peroxisome proliferator-activated receptors (PPARs) are members of the nuclear receptor superfamily that regulate lipid, glucose, and amino acid metabolism. More recently, PPARs and corresponding ligands have been shown in skin and other organs to regulate important cellular functions, including cell proliferation and differentiation, as well as inflammatory responses. These new functions identify PPARs and corresponding ligands as potential targets for the treatment of various skin diseases and other disorders. It has been shown that in inflammatory skin disorders, including hyperproliferative psoriatic epidermis and the skin of patients with atopic dermatitis, the expression of both PPARalpha and PPARgamma is decreased. This observation suggests the possibility that PPARalpha and PPARgamma activators, or compounds that positively regulate PPAR gene expression, may represent novel NSAIDs for the topical or systemic treatment of common inflammatory skin diseases such as atopic dermatitis, psoriasis, and allergic contact dermatitis. Moreover, recent findings indicate that PPAR-signaling pathways may act as a promising therapeutic target for the treatment of hyperproliferative skin diseases including skin malignancies. Studies in non-diabetic patients suggest that oral thiazolidinediones, which are synthetic ligands of PPARgamma, not only exert an antidiabetic effect but also may be beneficial for moderate chronic plaque psoriasis by suppressing proliferation and inducing differentiation of keratinocytes; furthermore, they may even induce cell growth arrest, apoptosis, and terminal differentiation in various human malignant tumors. It has been reported that PPARalpha immunoreactivity is reduced in human keratinocytes of squamous cell carcinoma (SCC) and actinic keratosis (AK), while PPARdelta appears to be upregulated. Additionally, the microvessel density is significantly higher in AK and SCC that express high levels of PPARdelta. PPARdelta has been demonstrated to have an anti-apoptotic role and to maintain survival and differentiation of epithelial cells, whereas PPARalpha and PPARgamma activators induce differentiation and inhibit proliferation and regulate apoptosis. In melanoma, the growth inhibitory effect of PPARgamma activation is independent of apoptosis and seems to occur primarily through induction of cell cycle arrest in the G1 phase of the cell cycle or induction of re-differentiation. PPARalpha activation causes inhibition of migration of melanoma cells and anchorage-independent growth, whereas primary tumor growth

remains unaltered. In clinical trials of gemfibrozil, a PPARalpha ligand, significantly fewer patients treated with this lipid-lowering drug were diagnosed with melanoma as compared to those in the control group. In conclusion, an increasing body of evidence indicates that PPAR signaling pathways may represent interesting therapeutic targets for a broad variety of skin disorders, including inflammatory skin diseases such as psoriasis and atopic dermatitis, and skin malignancies.

PMID: 18092840 [PubMed - indexed for MEDLINE]

754. MedGenMed. 2007 Aug 21;9(3):40.

A systematic review of asthma and health literacy: a cultural-ethnic perspective in Canada.

Poureslami IM, Rootman I, Balka E, Devarakonda R, Hatch J, Fitzgerald JM.

Institute for Health Promotion, University of British Columbia, Vancouver.

BACKGROUND: Asthma is one of the most common inflammatory lung diseases and its prevalence and incidence have increased in many developed and developing countries. Asthma places a heavy burden on healthcare expenditures and productivity, which in turn diminishes the quality of life of the individuals involved as well as their families. The goal of improving a patient's knowledge about asthma management should include the enhancement of the individual's skills with the hopeful outcome of improving how the individual manages the condition. However, when health professionals prepare a training program, they are faced with the challenging cosmopolitan reality of individuals with different ethnic backgrounds.
METHODS: In order to find links between asthma and health literacy in a cultural/ethnicity perspective, we performed a systematic review of all publications on the topic of asthma, health, and literacy among cultural groups from 1980 to 2006 using the Internet and journals: Medline (Ovid), ERIC, EMBASE, PsycINFO, Google, Google Scholar, Sociological Abstracts, and Anthropology Plus. Key words included the following: "asthma," "culture," "ethnicity," "literacy," "health," "health literacy," "health beliefs," "adults," "disease management," "chronic condition," "ethnocultural groups," "minority groups," and "newcomers/immigrants."
RESULTS: More than 650 articles were initially identified in our review; 65 met our inclusion criteria. From these, we examined the factors related to asthma and literacy/health literacy with a cultural lens. All of these are categorized and summarized below. We chose what we considered to be the most relevant and important articles/documents in the research literature to date. Because many of the studies were qualitative, a formal meta-analytic review was not undertaken. We found that current asthma management techniques - including patient education - are not culturally sensitive, linguistically sensitive, or relevant, which creates further difficulties for ethnocultural communities and minority groups in many Western countries. In this systematic review, several themes were identified, including: approaches to language limitation and cultural barriers; the recognition of healthcare system bias (in terms of culturally competent care); and relationship-building to facilitate participatory decision-making by both provider and patient. This review provides further understanding and considerations regarding the beliefs and perspectives of care providers and populations in relation to health and illness, literacy and health literacy, and their association with asthma among ethnocultural communities.
CONCLUSIONS: There is an urgent need to better define the impact of cultural and ethnic issues in the management of asthma in Canada. Appropriately designed studies should better define the barriers in the optimal delivery of asthma care influenced by these parameters.

PMCID: PMC2100106
PMID: 18092046 [PubMed - indexed for MEDLINE]

755. Gastroenterology. 2007 Dec;133(6):1905-15. Epub 2007 Sep 25.

Allergen-IgE complexes trigger CD23-dependent CCL20 release from human intestinal epithelial cells.

Li H, Chehade M, Liu W, Xiong H, Mayer L, Berin MC.

Immunology Institute, Mount Sinai School of Medicine, New York, New York, USA.

BACKGROUND & AIMS: In food allergic individuals, exposure to food allergens by the oral route can trigger immediate (within minutes) local hypersensitivity reactions in the intestine followed by a late-phase inflammatory response. Previous work has shown that CD23 is constitutively expressed by human intestinal epithelial cells and mediates the uptake of allergen-IgE complexes. We hypothesized that allergen-IgE complexes could also signal via CD23 to trigger an inflammatory cascade in the local environment.
METHODS: Caco-2 monolayers were stimulated with human IgE-antigen (Ag) complexes. IL-8 and CCL20 mRNA and protein were determined by RT-PCR and ELISA, respectively. Signaling pathways were assessed by immunoblotting. Endogenous CD23 expression was knocked down by stable transfection with CD23 shRNA retroviral plasmid. Migration assays were performed using human monocyte-derived dendritic cells.
RESULTS: Stimulation of Caco-2 cells with IgE-Ag complexes triggered upregulation of IL-8 and CCL20 at the mRNA and protein level. Allergen complexes induced phosphorylation of ERK and JNK, but not p38 MAP kinase or NK-kappaB, and resulted in AP-1 activation. Cross-linking of CD23 replicated the findings with IgE-Ag complexes, and silencing of CD23 expression abrogated the response to allergen-IgE complexes. Supernatant from IgE-Ag-stimulated epithelial cells induced migration of dendritic cells in a CCL20-dependent manner. Finally, immunostaining of duodenal biopsies demonstrated that CCL20 was constitutively expressed by epithelial cells in vivo.
CONCLUSIONS: Signaling via epithelial CD23 may participate in the late-phase inflammatory response by the release of chemokines capable of recruiting antigen presenting cells and effector cells of allergic inflammation.

PMCID: PMC2174915
PMID: 18054562 [PubMed - indexed for MEDLINE]

756. Zhonghua Er Bi Yan Hou Tou Jing Wai Ke Za Zhi. 2007 Sep;42(9):654-9.

[Differential gene expression profile in seasonal allergic rhinitis with and without asthma].

[Article in Chinese]

Xue JM, Zhao CQ, Zhao HL, Liang AH, Xie J.

Department of Otorhinolaryngology, Second Hospital of Shanxi Medical University, Taiyuan 030001, China.

OBJECTIVE: To screen the differential expression gene profile in nasal mucosa of seasonal allergic rhinitis (SAR) and SAR with asthma, oligonucleotide microarray (Affymetrix HG-U133-plus2) was employed to analyze the changes of gene expressions with GeneSpring software.

METHODS: Inferior turbinate mucosa was obtained from five SAR patients and four SAR with asthma patients. Total RNA was extracted from the nasal mucosal biopsies and pooled into one SAR control pool and one SAR with asthma patient pool, and biotin-labeled cRNA probes were hybridized with Affymetrix HG-U133-plus2 array. The hybridization results were confirmed by RT-PCR analysis. The analysis of differential expression profiles were performed by GeneSpring software 7.3.
RESULTS: Out of 47,000 analysed transcripts, 1,900 genes were differentially expressed at least 2-fold in which 849 genes were up-regulated and 1,051 genes were down-regulated in nasal mucosa of SAR with asthma patients compared with that in SAR patients. These genes were involved in cell metabolism, gene transcription, cell proliferation, signal transduction, immune response, enzyme activity, transmembrane receptor activity, cytoskeletal protein binding, and many other aspects. Pathway analysis displayed 161 groups, of which including more than 20 genes were as follow: cytokine-cytokine receptor interaction, focal adhesion, cell adhesion molecules (CAMs), regulation of actin cytoskeleton, cell communication, gap junction, MAPK signaling pathway, calcium signaling pathway, leukocyte transendothelial migration, and purine metabolism.
CONCLUSIONS: The data suggested that multigentic expression and regulation changes were involved in the development of SAR and SAR complicated with asthma, whose molecular mechanisms might be elucidated by identification of these differential genes.

PMID: 18051562 [PubMed - indexed for MEDLINE]

757. Int Immunopharmacol. 2007 Dec 20;7(14):1862-8. Epub 2007 Aug 6.

Comparison of kinin B(1) and B(2) receptor expression in neutrophils of asthmatic and non-asthmatic subjects.

Bertram C, Misso NL, Fogel-Petrovic M, Figueroa C, Thompson PJ, Bhoola KD.

Lung Institute of Western Australia and Centre for Asthma, Allergy & Respiratory Research, The University of Western Australia, Perth, Australia.

Kinins have been implicated in the pathophysiology of asthma and activation of kinin receptors stimulates neutrophil chemotaxis. However, the expression of kinin receptors on neutrophils of asthmatic subjects has not been assessed. The aim of this study was to compare the expression of kinin B(1) and B(2) receptor mRNA and proteins in neutrophils of asthmatic and non-asthmatic subjects, and to assess whether inhaled corticosteroid treatment may influence expression of the kinin receptors. Neutrophils were isolated from peripheral blood of asthmatic (n=27) and non-asthmatic subjects (n=14). The presence of kinin B(1) and B(2) receptor protein on neutrophils was confirmed by immunolabeling with specific antibodies followed by immunoperoxidase, immunofluorescence and FACS detection. Kinin B(1) and B(2) receptor mRNA expression was assessed by RT-PCR. Quantitative image analysis of fluorescence immunolabeled neutrophils showed no differences in kinin B(1) or B(2) receptor protein expression between asthmatic and non-asthmatic subjects. Similarly, quantitative real time RT-PCR analysis demonstrated no differences in expression of mRNA for the kinin B(1) or B(2) receptors between asthmatic and non-asthmatic subjects. However, B(1) receptor mRNA expression was significantly lower in asthmatic subjects using > or =2000 microg of inhaled corticosteroid per day ($p<0.05$) and B(1) receptor protein levels also tended to be lower in these subjects. Corticosteroids may have a beneficial anti-inflammatory effect in asthma by down-regulating B(1) receptor expression on neutrophils, thereby decreasing the migration of these inflammatory cells into the airways.

PMID: 18039523 [PubMed - indexed for MEDLINE]

758. Am J Clin Dermatol. 2007;8(6):329-36.

Sphingosine-1-phosphate signaling and the skin.

Herzinger T, Kleuser B, Schäfer-Korting M, Korting HC.

Klinik und Poliklinik für Dermatologie und Allergologie,
Ludwig-Maximilian-University, Munich, Germany.
Thomas.Herzinger@lrz.uni-muenchen.de

Erratum in
Am J Clin Dermatol.2008;9(4):277.

Sphingolipids have long been viewed as rather passive structural components of cellular membranes. More recently, it has become evident that metabolism of sphingomyelin yields several lipid mediators that evoke diverse and specific responses in different cell types. One sphingomyelin derivate, sphingosine-1-phosphate (S1P), has attracted particular attention for its effect on epidermal cells, which differs from those on most other cell types. S1P inhibits keratinocyte proliferation and induces keratinocyte differentiation and migration, suggesting a role for S1P in the re-epithelialization of wounds. The migratory response involves the phosphorylation and activation of Smad3. In epithelial tumors, S1P signaling has been linked with potential oncogenic effects, but has also been found to inhibit metastasis in a mouse melanoma model. S1P promotes endothelial cell survival, acts as a chemoattractant for vascular cells, and exerts a protective effect on the endothelial barrier. Conversely, S1P receptor knockout leads to embryonic lethality mainly due to impaired vascular maturation. S1P presumably modulates peripheral T-lymphocyte levels by stimulating their egress from lymphoid organs rather than by promoting T-cell proliferation. The S1P analog FTY720 (fingolimod) acts as a functional antagonist by inhibiting lymphocyte egress, and thus holds great promise as an immunosuppressant drug for the prevention of allograft rejection and treatment of T-lymphocyte-driven inflammatory skin diseases, such as lupus erythematosus, psoriasis, and atopic dermatitis. Topical use of S1P and other sphingosine compounds is also under investigation, particularly for the treatment of acne vulgaris.

PMID: 18039015 [PubMed - indexed for MEDLINE]

759. Med Sci (Paris). 2007 Nov;23(11):980-4.

[Multiple talents of the chemokine receptor-CXCR4].

[Article in French]

Desjardins SF, Berchiche YA, Haddad E, Heveker N.

Département de Biochimie, Université de Montréal, Montréal (Québec), H3T 1J4 Canada.

CXCR4 is a clinically relevant chemokine receptor that has first gained attention as one of the cofactors for HIV entry into target cells. Moreover, the receptor is involved in cancer cell migration to distant metastatic sites and immune effector recruitment in inflammatory diseases such as asthma and rheumatoid arthritis. Unfortunately, pharmacologic intervention is complicated by the vital function of CXCR4 in the organism. The most prominent of these functions is its role in stem cell homing. The CXCR4 chemokine ligand, produced by bone marrow stromal cells, leads both to migration of hematopoietic stem cells towards this

niche and their retention in this compartment. As models of G-protein coupled receptor (GPCR) activation evolve, it becomes clear that multiple factors modulate the functional outcome of ligand binding to a receptor. Modulation of GPCR activity, for example by allosteric ligands, may permit more subtle therapeutic approaches adapted to long term treatment. In addition, GPCR signalling can be altered by hetero-oligomerization of GPCRs. In this perspective, it might be possible to achieve modulation of GPCR signalling by also targeting the oligomerization partner of a given receptor. This approach is described using the example of strategies that aim at the optimization of stem cell homing in the context of cord blood-derived hematopoietic stem cell transplantation.

PMID: 18021711 [PubMed - indexed for MEDLINE]

760. Paediatr Perinat Epidemiol. 2007 Nov;21 Suppl 3:15-22.

Latino populations: a unique opportunity for epidemiological research of asthma.

Salari K, Burchard EG.

Department of Genetics, Stanford University, Stanford, CA, USA.

Asthma is a significant health problem among Latinos, the largest minority population in the US. Asthma prevalence, morbidity and mortality are highest in Puerto Ricans, intermediate in Dominicans and Cubans, and lowest in Mexicans and Central Americans. From a cultural and social perspective, Latinos represent a wide variety of national origins and ethnic and cultural groups, with a full spectrum of social class. From a genetic perspective, Latinos have descended from Native American, European and African populations. Here, we review results from recent genetic and clinical studies to illustrate the unique opportunity Latino groups offer for studying the interaction between racial, genetic and environmental contributions to asthma and drug responsiveness.

PMID: 17935571 [PubMed - indexed for MEDLINE]

761. Ann Rheum Dis. 2007 Nov;66 Suppl 3:iii52-5.

alpha-MSH related peptides: a new class of anti-inflammatory and immunomodulating drugs.

Luger TA, Brzoska T.

Department of Dermatology, University Clinics Münster, Von-Esmarch-Str. 58, D-48149 Münster, Germany. luger@uni-muenster.de

alpha-Melanocyte-stimulating hormone (alpha-MSH) is a tridecapeptide derived from the proopiomelanocortin by post-translational processing. In addition to its effects on melanocytes, alpha-MSH has potent anti-inflammatory effects when administered systemically or locally. The anti-inflammatory effects of alpha-MSH are mediated by direct effects on cells of the immune system as well as indirectly by affecting the function of resident non-immune cells. alpha-MSH affects several pathways implicated in regulation of inflammatory responses such as NF-kappaB activation, expression of adhesion molecules and chemokine receptors, production of pro-inflammatory cytokines and other mediators. Thus alpha-MSH may modulate inflammatory cell proliferation, activity and migration. The anti-inflammatory effects of alpha-MSH have been confirmed by means of animal models of inflammation such as irritant and allergic contact dermatitis, cutaneous vasculitis, asthma, inflammatory bowel disease, rheumatoid arthritis,

ocular and brain inflammation. Most of the anti-inflammatory activities of alpha-MSH can be attributed to its C-terminal tripeptide KPV. K(D)PT, a derivative of KPV corresponding to the amino acid 193-195 of IL-1beta, is currently emerging as another tripeptide with potent anti-inflammatory effects. The anti-inflammatory potential together with the favourable physiochemical properties most likely will allow these agents to be developed for the treatment of inflammatory skin, eye and bowel diseases, allergic asthma and arthritis.

PMCID: PMC2095288
PMID: 17934097 [PubMed - indexed for MEDLINE]

762. Biochim Biophys Acta. 2008 Jan;1784(1):159-85. Epub 2007 Oct 12.

Targeting phosphoinositide 3-kinase: moving towards therapy.

Marone R, Cmiljanovic V, Giese B, Wymann MP.

Institute of Biochemistry and Genetics, Department of Biomedicine, University of Basel, Mattenstrasse 28, CH-4058, Basel, Switzerland.

Phosphoinositide 3-kinases (PI3K) orchestrate cell responses including mitogenic signaling, cell survival and growth, metabolic control, vesicular trafficking, degranulation, cytoskeletal rearrangement and migration. Deregulation of the PI3K pathway occurs by activating mutations in growth factor receptors or the PIK3CA locus coding for PI3Kalpha, by loss of function of the lipid phosphatase and tensin homolog deleted in chromosome ten (PTEN/MMAC/TEP1), by the up-regulation of protein kinase B (PKB/Akt), or the impairment of the tuberous sclerosis complex (TSC1/2). All these events are linked to growth and proliferation, and have thus prompted a significant interest in the pharmaceutical targeting of the PI3K pathway in cancer. Genetic targeting of PI3Kgamma (p110gamma) and PI3Kdelta (p110delta) in mice has underlined a central role of these PI3K isoforms in inflammation and allergy, as they modulate chemotaxis of leukocytes and degranulation in mast cells. Proof-of-concept molecules selective for PI3Kgamma have already successfully alleviated disease progress in murine models of rheumatoid arthritis and lupus erythematosus. As targeting PI3K moves forward to therapy of chronic, non-fatal disease, safety concerns for PI3K inhibitors increase. Many of the present inhibitor series interfere with target of rapamycin (TOR), DNA-dependent protein kinase (DNA-PK(cs)) and activity of the ataxia telangiectasia mutated gene product (ATM). Here we review the current disease-relevant knowledge for isoform-specific PI3K function in the above mentioned diseases, and review the progress of >400 recent patents covering pharmaceutical targeting of PI3K. Currently, several drugs targeting the PI3K pathway have entered clinical trials (phase I) for solid tumors and suppression of tissue damage after myocardial infarction (phases I,II).

PMID: 17997386 [PubMed - indexed for MEDLINE]

763. Ambul Pediatr. 2007 Nov-Dec;7(6):421-30.

The relationship of immigrant status with access, utilization, and health status for children with asthma.

Javier JR, Wise PH, Mendoza FS.

Department of Pediatrics, Division of General Pediatrics, Stanford University School of Medicine, Palo Alto, CA, USA. jo.javier@chla.usc.edu

OBJECTIVE: Despite their high levels of poverty and less access to health care,

children in immigrant families have better than expected health outcomes compared with children in nonimmigrant families. However, this observation has not been confirmed in children with chronic illness. The objective of this study was to determine whether children with asthma in immigrant families have better than expected health status than children with asthma in nonimmigrant families.
METHODS: Data from the 2001 and 2003 California Health Interview Survey (CHIS) were used to identify 2600 children, aged 1 to 11, with physician-diagnosed asthma. Bivariate analyses and logistic regression were performed to examine health care access, utilization, and health status measures by our primary independent variable, immigrant family status.
RESULTS: Compared with children with asthma in nonimmigrant families, children with asthma in immigrant families are more likely to lack a usual source of care (2.6% vs 1.0%; P < .05), report a delay in medical care (8.9% vs 5.2%; P < .01), and report no visit to the doctor in the past year (7.0% vs 3.8%; P < .05). They are less likely to report asthma symptoms (60.8% vs 74.4%; P < .01) and an emergency room visit in the past year (14.1% vs 21.1%; P < .01), yet more likely to report fair or poor perceived health status (25.0% vs 10.5%; P < .01). Multivariate models revealed that the relationship of immigrant status with health measures was complex. These models suggested that lack of insurance and poverty was associated with reduced access and utilization. Children in immigrant families were less likely to visit the emergency room for asthma in the past year (odds ratio 0.58, confidence interval, 0.36-0.93). Poverty was associated with having a limitation in function and fair or poor perceived health, whereas non-English interview language was associated with less limitation in function but greater levels of fair or poor perceived health.
CONCLUSIONS: Clinicians should be aware of important barriers to care that may exist for immigrant families who are poor, uninsured, and non-English speakers. Reduced health care access and utilization by children with asthma in immigrant families requires policy attention. Further research should examine barriers to care as well as parental perceptions of health for children with asthma in immigrant families.

PMID: 17996835 [PubMed - indexed for MEDLINE]

764. J Leukoc Biol. 2008 Feb;83(2):361-7. Epub 2007 Nov 5.

IL-18 is a key proximal mediator of contact hypersensitivity and allergen-induced Langerhans cell migration in murine epidermis.

Antonopoulos C, Cumberbatch M, Mee JB, Dearman RJ, Wei XQ, Liew FY, Kimber I, Groves RW.

St. John's Institute of Dermatology, King's College London, London, United Kingdom.

Langerhans cells (LC) migrate rapidly from epidermis to lymph node following epicutaneous application of antigen. In this study, we have explored the role of IL-18, a cytokine with structural similarities to IL-1 beta, in murine LC migration and contact hypersensitivity (CHS), which to oxazolone (OX) and 2-4,dinitrofluorobenzene (DNFB) was suppressed significantly in IL-18 knockout (IL-18-/-) mice and could be rescued by local intradermal administration of IL-18 prior to sensitization, suggesting that the defect in these mice was in the afferent phase of CHS. To determine the effect of IL-18 on LC migration, mice were treated topically with OX or DNFB, and remaining LC numbers were assessed. A significant decline in remaining epidermal LC occurred in wild-type (WT) mice but did not occur in IL-18-/- mice. Sodium lauryl sulfate, a nonantigenic LC migratory stimulus, induced equivalent LC migration in IL-18-/- and WT mice. In IL-18-/- mice, IL-1 beta and TNF-alpha were equally able to mobilize LC from epidermis, indicating that migration in response to these cytokines is not

dependent on IL-18 and suggesting that IL-18 acts upstream of these cytokines in the initiation of antigen-induced LC migration. Moreover, IL-1 beta but not IL-18 was able to rescue the defective CHS response observed in caspase-1-/- mice, which have no functional IL-1 beta or IL-18. These data indicate that IL-18 is a key proximal mediator of LC migration and CHS, acting upstream of IL-1 beta and TNF-alpha, and may play a central role in regulation of cutaneous immune responses.

PMID: 17984289 [PubMed - indexed for MEDLINE]

765. Curr Top Med Chem. 2007;7(17):1743-52.

NPY and receptors in immune and inflammatory diseases.

Wheway J, Herzog H, Mackay F.

Autoimmune Research Unit, Neuroscience Research Program, Garvan Institute of Medical Research, 384 Victoria St, Darlinghurst, NSW, 2010, Australia. j.wheway@garvan.org.au

Growing evidence suggests that neuropeptide Y (NPY) plays an important role in the immune system. NPY is produced by the central and peripheral nervous system but also by immune cells in response to activation. NPY has pleiotropic effects on both the innate and adaptive arms of the immune system, with effects ranging from the modulation of cell migration to macrophage, T helper (Th) cell cytokine release, and antibody production. Subsequent studies have confirmed the importance of this system in immunity in particular via the demonstration that Y1, a receptor for NPY, plays a fundamental role in autoimmunity and inflammation using Y1-deficient animals. Furthermore, clinical studies have suggested a role for NPY in other immune disorders such as asthma and arthritis. This review provides the latest information on the role of NPY and Y1 in the immune system, and discusses the potential new opportunities of this work for the development of a new generation of immuno-modulatory treatments of autoimmune and inflammatory diseases.

PMID: 17979783 [PubMed - indexed for MEDLINE]

766. Curr Med Chem. 2007;14(25):2716-9.

TFF (trefoil factor family) peptides and their potential roles for differentiation processes during airway remodeling.

Hoffmann W.

Institute of Molecular Biology and Medical Chemistry, Otto-von-Guericke-University Magdeburg, Leipziger Strasse 44, D-39120 Magdeburg, Germany. Werner.Hoffmann@med.ovgu.de

Several lines of defense maintain the surface integrity of the delicate airway epithelium which is regularly subjected to severe trauma. These defense mechanisms include protection by the mucus layer, rapid repair by restitution (cell migration) and regeneration via proliferation and differentiation. Luminal surveillance peptides such as epidermal growth factor (EGF) and trefoil factor family (TFF) peptides support synergistically these processes. TFFs are well known particularly for their key role in mucosal restitution and there is an increasing body of evidence that TFFs also support mucosal differentiation processes. Mucus overproduction during inflammatory and obstructive airway diseases is a partial consequence of an increase in the number of goblet cells

due to cell division (goblet cell hyperplasia) or differentiation (goblet cell metaplasia). Particularly the latter process reflects the plasticity of the airway epithelium and causes intense airway remodeling. Goblet cells are derived, at least in part, from Clara cells, which trans-differentiate from a serous into a mucous phenotype. This process is critically dependent upon IL-13. In a recent report (Kouznetsova et al. AJRCMB 36:286-297, 2007) using a murine asthma model it was shown that trans-differentiating Clara cells specifically express Tff1 which is stored in a specific subset of secretory granules. This points to a role for Tff1 as an autocrine factor for the trans-differentiation of Clara cells toward goblet cells. Such a role of TFFs for differentiation processes of the airways is supported by another recent study (LeSimple et al. AJRCMB 36:296-303, 2007) where induction of TFF3 synthesis was shown with differentiation in in vivo humanized tracheal xenograft and in vitro air-liquid interface culture models. Furthermore, exogenous TFF3 promoted differentiation of ciliated cells in an EGF receptor-dependent manner. Taken together, both studies imply that TFFs may play key roles for various differentiation processes of the airways and they could be promising novel targets in order to treat severe chronic and acute airway diseases.

PMID: 17979720 [PubMed - indexed for MEDLINE]

767. Transl Res. 2007 Nov;150(5):303-10. Epub 2007 May 29.

Effects of neurotrophins on human bronchial smooth muscle cell migration and matrix metalloproteinase-9 secretion.

Dagnell C, Kemi C, Klominek J, Eriksson P, Sköld CM, Eklund A, Grunewald J, Olgart Höglund C.

Department of Medicine Solna, Division of Respiratory Medicine, Karolinska Institutet and Karolinska University Hospital, Stockholm, Sweden.

The neurotrophins nerve growth factor (NGF), brain-derived neurotrophic factor (BDNF), and neurotrophin-3 (NT-3) have been found to be upregulated in inflammatory pulmonary diseases, including asthma. The functional role for the neurotrophins in the airways is still not known, but it has been proposed that neurotrophins induce airway hyperreactivity and tissue remodeling. Bronchial smooth muscle cells have been suggested to be involved in the remodeling process through their capacity to proliferate, migrate, and secrete inflammatory mediators and matrix metalloproteinases (MMPs). Therefore, we studied the effect of NGF, BDNF, and NT-3 on human bronchial smooth muscle cell (HBSMC) migration and MMP-2 and MMP-9 secretion. Immunocytochemistry studies showed that HBSMCs expressed the neurotrophin receptors TrkA, TrkB, and TrkC. BDNF, NT-3, and NGF increased MMP-9, but not MMP-2, secretion as shown by zymography. BDNF and NT-3, but not NGF, stimulated HBSMC migration as evaluated by Boyden chamber. Taken together, our data indicate that the neurotrophins may stimulate events important for airway remodeling.

PMID: 17964519 [PubMed - indexed for MEDLINE]

768. Drugs R D. 2007;8(6):400-2.

MN 001: KCA 757, KCA-757, MN-001.

[No authors listed]

MN 001 is an orally bioavailable anti-inflammatory agent, originated by Kyorin Pharmaceutical, which is being developed in clinical trials by the US company

MediciNova for the treatment of interstitial cystitis and asthma. Kyorin is developing the drug as KCA 757 for these indications. The actions of the drug are described by MediciNova as consisting of eosinophil migration inhibition, leukotriene antagonism, and phosphodiesterase IV inhibition. Other mechanisms described for MN 001 include the inhibition of phosphodiesterases III, 5-lipoxy-genase, phospholipase C as well as thromboxane A2. Development of an immediate-release formulation of MN 001 has been discontinued. An extended-release formulation remains in development. MediciNova is looking for partnering/outlicensing opportunities for MN 001 in North America and Europe. MediciNova licensed MN 001 from Kyorin Pharmaceutical in March 2002, and now holds exclusive worldwide rights, excluding Japan, China, Taiwan and South Korea, to develop and commercialise the drug. The phase III clinical programme of immediate-release MN 001 was initiated by Medicinova in the US in November 2006. In the first phase III trial, 705 patients with mild to moderate asthma were to be randomised to receive MN 001 (750 mg twice daily, 500 mg three times daily) or placebo for 12 weeks. The change from baseline in mean forced expiratory volume in 1 second (FEV(1)) will be the primary endpoint. The primary endpoint was met in a phase II study of MN 001 in patients with mild to moderate asthma. The trial evaluated the efficacy of three different doses of MN 001 for the treatment of asthma. Results have been reported. MediciNova has received Notices of Allowance from the US Patent and Trademark Office for three patent applications covering certain compositions, uses and manufacturing processes associated with MN 001. MN 001 has received patent protection through at least 2023.

PMID: 17963431 [PubMed - indexed for MEDLINE]

769. J Immunol. 2007 Nov 1;179(9):6193-203.

Prostaglandin I2-IP signaling blocks allergic pulmonary inflammation by preventing recruitment of CD4+ Th2 cells into the airways in a mouse model of asthma.

Jaffar Z, Ferrini ME, Buford MC, Fitzgerald GA, Roberts K.

Center for Environmental Health Sciences, Biomedical and Pharmaceutical Sciences, University of Montana, Missoula, MT 59812, USA.

PGI(2) plays a key role in limiting Th2-mediated airway inflammation. In studies to investigate the mechanism underlying such regulation, we found that the PGI(2) receptor, IP, is preferentially expressed by effector CD4(+) Th2 cells, when compared with Th1 cells. Adoptive transfer of DO11.10 Th2 cells pretreated with PGI(2) resulted in considerably attenuated pulmonary inflammation and airway hyperreactivity in BALB/c recipient mice in response to OVA inhalation. This suppression was independent of increased cAMP levels, because pretreatment of Th2 cells with dibutyryl cAMP before transfer had no effect on airway inflammation. Moreover, PGI(2) pretreatment of Th2 cells suppressed the ability of the cells to infiltrate the lungs but not the spleen. In vitro studies showed that PGI(2) did not affect IL-4 and IL-5 production or the level of IFN-gamma by the T cells. However, the prostanoid strongly inhibited CCL17-induced chemotaxis of CD4(+) Th2 but not Th1 cells. The IP was implicated in this process since migration of wild-type Th2 cells in response to CCL17 was markedly reduced following treatment with PGI(2), whereas IP-deficient Th2 cells were unaffected and migrated effectively. Collectively, these experiments suggest that PGI(2), which is generated by endothelial cells during lung inflammatory response, serves to limit the influx of Th2 cells to the airways. Our results identify PGI(2)-IP as an important pathway for inhibiting allergic pulmonary inflammation by controlling recruitment of CD4(+) Th2 cells into the inflammatory site.

PMID: 17947695 [PubMed - indexed for MEDLINE]

770. Curr Opin Pulm Med. 2007 Sep;13(5):428-33.

Tropical pulmonary eosinophilia: pathogenesis, diagnosis and management.

Vijayan VK.

Vallabhbhai Patel Chest Institute, University of Delhi, India.
vijayanvk@hotmail.com

PURPOSE OF REVIEW: Tropical pulmonary eosinophilia is predominantly seen in the tropical and subtropical regions of the world. It is being increasingly reported from other parts of world, however, due to increases in global travel and migration. This review focuses attention on recent developments.
RECENT FINDINGS: Tropical pulmonary eosinophilia is an occult form of human filariasis. The gamma-glutaryl transpeptidase found in the infective L3 stage larvae of Brugia malayi has been found to have similarities with the gamma-glutaryl transpeptidase present on the surface of human pulmonary epithelium. It has, therefore, been proposed that filarial gamma-glutaryl transpeptidase may play an important role in the pathogenesis of tropical eosinophilia. Airway hyperresponsiveness, manifesting as asthma-like syndrome, has been reported in tropical pulmonary eosinophilia and it has been suggested that interleukin-4 induces and interferon-gamma suppresses filarial-induced airway hyperresponsiveness. The intense eosinophilic alveolitis seen in acute tropical pulmonary eosinophilia is suppressed by 3 weeks of treatment with diethylcarbamazine citrate. A mild eosinophilic alveolitis along with radiological, physiological and hematological abnormalities, though with reduced intensity, persists in some patients however.
SUMMARY: A chronic mild interstitial lung disease has been found to persist in tropical pulmonary eosinophilia despite treatment.

PMID: 17940489 [PubMed - indexed for MEDLINE]

771. J Allergy Clin Immunol. 2007 Nov;120(5):1031-5. Epub 2007 Oct 24.

Is vitamin D deficiency to blame for the asthma epidemic?

Litonjua AA, Weiss ST.

Channing Laboratory, Department of Medicine, Brigham and Women's Hospital, Boston, MA, USA. augusto.litonjua@channing.harvard.edu

Comment in
    J Allergy Clin Immunol. 2008 Mar;121(3):782-3; author reply 783-4.
    J Allergy Clin Immunol. 2008 Apr;121(4):1065-6; author reply 1066.

In the 1960s, the prevalence of asthma and allergic diseases began to increase worldwide. Currently, the burden of the disease is more than 300 million people affected. We hypothesize that as populations grow more prosperous, more time is spent indoors, and there is less exposure to sunlight, leading to decreased cutaneous vitamin D production. Coupled with inadequate intake from foods and supplements, this then leads to vitamin D deficiency, particularly in pregnant women, resulting in more asthma and allergy in their offspring. Vitamin D has been linked to immune system and lung development in utero, and our epidemiologic studies show that higher vitamin D intake by pregnant mothers reduces asthma risk by as much as 40% in children 3 to 5 years old. Vitamin D deficiency has been associated with obesity, African American race (particularly in urban, inner-city settings), and recent immigrants to westernized countries, thus reflecting the

epidemiologic patterns observed in the asthma epidemic. Providing adequate vitamin D supplementation in pregnancy may lead to significant decreases in asthma incidence in young children.

PMID: 17919705 [PubMed - indexed for MEDLINE]

772. Clin Immunol. 2007 Dec;125(3):291-8. Epub 2007 Oct 29.

The role of CCL22/macrophage-derived chemokine in allergic rhinitis.

Yanai M, Sato K, Aoki N, Takiyama Y, Oikawa K, Kobayashi H, Kimura S, Harabuchi Y, Tateno M.

Department of Pathology, Asahikawa Medical College, Hokkaido, Japan.
mitsuru@asahikawa-med.ac.jp

Dendritic cells (DCs) are considered to be the most powerful antigen-presenting cells (APCs). DCs are thought to be associated with Th1 or Th2 polarization and with polarization-induced disease such as atopic dermatitis, asthma and allergic rhinitis, but its mechanism is not well known. In this study, we analyzed the mRNA expression of DCs between birch pollen allergic rhinitis and healthy controls by using cDNA array. We found that the expressions of CCL22/macrophage-derived chemokine (MDC) differed significantly. We also revealed that CCL22/MDC production was higher in patients than in healthy donors. By chemotaxis assay, CCL22/MDC can enhance the migration of patient's T cells rather than those of healthy controls. Surface marker analysis of migrated cells revealed that the most of migrated cells expressed CCR4, which were considered to be Th2 cells. Furthermore, CD1a(+) CD83(+) cells located in the nasal mucosa expressed CCL22/MDC in vivo. To the best of our knowledge, this is the first report clearly indicating the role of CCL22/MDC in allergic rhinitis.

PMID: 17911044 [PubMed - indexed for MEDLINE]

773. Biochem Biophys Res Commun. 2007 Nov 30;363(4):948-53. Epub 2007 Sep 29.

Characterization of the promoter of human CRTh2, a prostaglandin D2 receptor.

Quapp R, Madsen N, Cameron L.

Department of Medicine, Division of Pulmonary Medicine, Pulmonary Research Group, 574B Heritage Medical Research Centre, University of Alberta, Edmonton, AB, Canada T6G 2S2.

Chemoattractant-receptor homologous molecule expressed on Th2 cells (CRTh2) is a receptor for prostaglandin (PG)D2, a lipid mediator involved in allergic inflammation. CRTh2 is expressed by Th2 cells, eosinophils and basophils and PDG(2)-CRTh2 signaling induces calcium mobilization, cell migration and expression of the Th2 cytokines IL-4, IL-5, and IL-13. Despite the role of CRTh2 in allergic inflammation, transcriptional regulation of this gene has not been studied. Here, we demonstrated that a reporter construct of the CRTh2 promoter was induced following T cell stimulation. This activity could be further enhanced by over-expression of GATA-3, but not NFAT2 or STAT6. Electromobility shift assay demonstrated GATA-3 binding to a probe from the CRTh2 promoter. This study provides the first detailed analysis of transcriptional regulation of the human CRTh2 promoter. These findings may help identify strategies to attenuate expression of this gene and influence the maintenance and proliferation of Th2 cells in allergic inflammation.

PMID: 17910949 [PubMed - indexed for MEDLINE]

774. Thyroid. 2007 Oct;17(10):1005-11.

Chemokine orchestration of autoimmune thyroiditis.

Kimura H, Caturegli P.

Department of Pathology, the Johns Hopkins School of Medicine, Baltimore, Maryland, USA.

Chemokines are low-molecular-weight proteins that attract leukocytes and other cell types, via interaction with G protein-coupled receptors. Chemokines control leukocyte migration not only during inflammatory processes, but also throughout ontogeny and differentiation of lymphoid tissues. They have been involved in the pathogenesis of numerous diseases, such as human immunodeficiency virus infection, allergy, atherosclerosis, cancer, and autoimmunity. The number of studies focusing on chemokine biology is expanding exponentially. For example, searching PubMed for the terms "thyroid" and "chemokine" retrieved 1 article in 1980s, 18 articles in 1990s, and 81 articles from 2000 to July 2007. This review will focus on studies analyzing the role of chemokine in autoimmune thyroiditis (Graves' disease and Hashimoto's thyroiditis), performed in both patients and experimental animals. The goal is to emphasize how a better understanding of chemokine biology has advanced our knowledge of the pathogenesis of autoimmune thyroiditis.

PMID: 17910527 [PubMed - indexed for MEDLINE]

775. Ann Allergy Asthma Immunol. 2007 Sep;99(3):236-43.

Associations of physician-diagnosed asthma with country of residence in the first year of life and other immigration-related factors: Chicago asthma school study.

Eldeirawi KM, Persky VW.

Division of Epidemiology and Biostatistics, School of Public Health, University of Illinois at Chicago, Chicago, Illinois 60612, USA. keldei1@uic.edu

BACKGROUND: Among Mexican Americans in the United States, US-born children have higher rates of asthma than their Mexico-born peers. Objective: To evaluate the associations of immigration-related variables with physician-diagnosed asthma in a sample of Mexican American children.
METHODS: We analyzed data from the ongoing Chicago Asthma School Study, a population-based cross-sectional study, for 10,106 Mexican American schoolchildren in Chicago, Illinois.
RESULTS: Mexican American children who lived in the United States in the first year of life were more likely to have physician-diagnosed asthma than their peers who lived in Mexico in the first year of life, independent of age, sex, income, language, and country of birth (odds ratio [OR], 1.79; 95% confidence interval [CI], 1.09-2.94). The risk of asthma in US-born children was higher (but not significantly) than that observed in Mexico-born children after accounting for covariates, including country of residence in the first year of life (OR, 1.37; 95% CI, 0.86-2.18). Long-term immigrants (lived in the United States for 10 years) had an increased risk of asthma compared with short-term immigrants (lived in the United States for <10 years), independent of country of residence in the first year of life (OR, 1.93; 95% CI, 1.00-3.73).
CONCLUSION: These findings confirm the importance of early childhood exposures and environmental factors that are modified with migration and acculturation in

asthma development.

PMID: 17910327 [PubMed - indexed for MEDLINE]

776. Trends Endocrinol Metab. 2007 Oct;18(8):300-7. Epub 2007 Sep 29.

Autocrine and paracrine roles of sphingosine-1-phosphate.

Alvarez SE, Milstien S, Spiegel S.

Department of Biochemistry and Molecular Biology, Virginia Commonwealth
University School of Medicine, Richmond, VA 23298, USA.

Sphingosine-1-phosphate (S1P) is a bioactive sphingolipid metabolite that has been implicated in many biological processes, including cell migration, survival, proliferation, angiogenesis and immune and allergic responses. S1P levels inside cells are regulated tightly by the balance between its synthesis by sphingosine kinases and degradation by S1P lyases and S1P phosphatases. Activation of sphingosine kinase by any of a variety of agonists increases S1P levels, which in turn can function intracellularly as a second messenger or in an autocrine and/or paracrine fashion to activate and signal through S1P receptors present on the surface of the cell. This review summarizes recent findings on the roles of S1P as a mediator of the actions of cytokines, growth factors and hormones.

PMID: 17904858 [PubMed - indexed for MEDLINE]

777. Dermatol Clin. 2007 Oct;25(4):643-53, x.

Beta adrenergic receptors in keratinocytes.

Sivamani RK, Lam ST, Isseroff RR.

Department of Dermatology, University of California, Davis, School of Medicine,
Dermatology Research, TB 192, One Shields Avenue, Davis, CA 95616, USA.

Beta2 adrenergic receptors were identified in keratinocytes more than 30 years ago, but their function in the epidermis continues to be elucidated. Abnormalities in their expression, signaling pathway, or in the generation of endogenous catecholamine agonists by keratinocytes have been implicated in the pathogenesis of cutaneous diseases such as atopic dermatitis, vitiligo, and psoriasis. New studies also indicate that the beta2AR also modulates keratinocyte migration, and thus can function to regulate wound reepithelialization. This review focuses on the function of these receptors in keratinocytes and their contribution to cutaneous physiology and disease.

PMCID: PMC2169297
PMID: 17903623 [PubMed - indexed for MEDLINE]

778. Inflamm Allergy Drug Targets. 2007 Sep;6(3):183-90.

Macrophage migration inhibitory factor: a therapeutic target across inflammatory
diseases.

Hoi AY, Iskander MN, Morand EF.

Centre for Inflammatory Diseases, Monash Institute of Medical Research, Monash
University, Melbourne, Australia.

Macrophage migration inhibitory factor (MIF), a cytokine originally reported in the 1960s as the prototypic T lymphokine, has emerged in recent years as a key factor regulating inflammatory responses. Both by directly activating immune cells, and by participating in activation entrained by other stimuli, MIF is important in innate and adaptive immune responses as well as tissue-specific mechanisms of damage. As a consequence of its involvement in multiple stages of the immune-inflammatory response, MIF has the potential to be involved in the pathogenesis of a range of immune-mediated inflammatory diseases affecting multiple organ systems. Diseases in which a role for MIF has been strongly validated include rheumatoid arthritis, inflammatory bowel disease, multiple sclerosis, atherosclerosis, asthma, inflammatory liver disease, and most recently systemic lupus erythematosus. Recent data have provided mechanisms of action for MIF which further support its suitability as a therapeutic target. Finally, MIF has a unique relationship with glucocorticoids, acting to counter-regulate their anti-inflammatory effects, such that MIF antagonist therapy may be a direct route to 'steroid-sparing'. Methods of targeting MIF therapeutically have also emerged in recent years, based on the unique protein structure of MIF which affords opportunities for direct antagonism by small molecules, as well as by protein therapeutics such as monoclonal antibodies. Clinical trials of MIF antagonist therapies are likely before the end of the current decade.

PMID: 17897055 [PubMed - indexed for MEDLINE]

779. Anticancer Agents Med Chem. 2007 Sep;7(5):504-14.

Targeting aberrant TGF-beta signaling in pre-clinical models of cancer.

Mourskaia AA, Northey JJ, Siegel PM.

Department of Medicine, McGill University, Montréal, Québec, Canada.

The TGF-beta signaling pathway is central to the control of diverse biological processes including cellular proliferation, cell survival, apoptosis, extracellular matrix deposition/remodeling, migration, invasion and immune regulation/inflammation. Given the pleiotropic effects of this cytokine, it comes as no surprise that numerous pathological conditions are associated with alterations in the TGF-beta pathway, including chronic fibrosis, airway remodeling (asthma), cardiovascular disease and cancer. Thus, there are increasing efforts to develop reagents and therapeutic strategies to impair TGF-beta signaling. Here we review several classes of inhibitors, including knock-down strategies aimed at signaling components of the TGF-beta pathway, TGF-beta neutralizing antibodies, TGF-beta receptor extracellular domains that function as ligand traps and small molecule kinase inhibitors. Strategies with potential for application as anti-cancer therapeutics that have been evaluated in pre-clinical animal models will be discussed. TGF-beta action is complex, shifting from a tumor suppressor to a promoter of tumor cell invasion and metastasis in several types of cancer. This raises important issues regarding not only the status of the TGF-beta pathway in the individual patient but also the precise stage during disease progression that such inhibitors should be employed. Potential consequences of targeting the TGF-beta pathway will also be considered.

PMID: 17896911 [PubMed - indexed for MEDLINE]

780. Am J Respir Cell Mol Biol. 2008 Mar;38(3):276-82. Epub 2007 Sep 20.

Epithelium expression and function of retinoid receptors in asthma.

Druilhe A, Zahm JM, Benayoun L, El Mehdi D, Grandsaigne M, Dombret MC, Mosnier I, Feger B, Depondt J, Aubier M, Pretolani M.

Inserm Unité 700, Université Paris 7, Faculté de Médecine Denis Diderot, site Xavier Bichat, 16, rue Henri Huchard, 75018 Paris, France.

Erratum in
Am J Respir Cell Mol Biol.2008 Nov;39(5):628-9.

Abnormal epithelial repair to damage participates in airway remodeling in asthma by the paracrine regulation of mesenchymal cell functions. Retinoids control epithelial functions through nuclear retinoic acid receptor (RAR) and retinoid X receptor (RXR) activation, yet their expression and contribution to epithelial repair and to airway remodeling in asthma are unknown. We determined the plasma levels of retinol and the immunohistochemical expression of retinoid receptors in damaged and repaired bronchial epithelium from 9 control subjects, 10 subjects with intermittent asthma, 8 subjects with mild-to-moderate asthma, and 8 subjects with severe asthma. In addition, the effect of the retinoid receptor ligands, all-trans-retinoic acid, and 9-cis retinoic acid, on the synthesis of 38 factors potentially involved in epithelial repair and in airway remodeling was determined in human cultured airway epithelial cells and correlated with cell migration and proliferation. Circulating retinol was similar in the three patient groups. In contrast, the epithelial expression of RARgamma, RXRalpha, and RXRgamma was greater in subjects with severe asthma, as compared with patients with milder disease and to control subjects. Retinoid receptor expression correlated positively with the proportion of morphologically intact epithelium. In vitro, retinoids up-regulated the expression of the transcripts encoding transforming growth factor (TGF)-beta1, metalloproteinase-9, beta1-integrin, and hepatocyte growth factor receptor, and promoted wound repair and chemokinesis of human airway epithelial cells without altering proliferation. Cell treatment with an anti-TGF-beta1 monoclonal antibody partially reduced retinoid-induced effects. Persistent interaction between retinoids and some of their receptors, which are overexpressed by the bronchial epithelium of individuals with severe asthma, may contribute to an abnormal repair and to airway remodeling, partly through TGF-beta1 production.

PMID: 17884991 [PubMed - indexed for MEDLINE]

781. J Ethnopharmacol. 2007 Nov 1;114(2):186-93. Epub 2007 Aug 15.

Inhibition effects of Moutan Cortex Radicis on secretion of eotaxin in A549 human epithelial cells and eosinophil migration.

Kim J, Lee H, Lee Y, Oh BG, Cho C, Kim Y, Shin M, Hong M, Jung SK, Bae H.

Department of Oriental Physiology, College of Pharmacy, Kyung Hee University, #1 Hoeki-dong Dongdaemoon-gu, Seoul 130-701, Republic of Korea.

Eosinophils have been implicated in a broad range of diseases, most notably allergic conditions (e.g. asthma, rhinitis and atopic dermatitis) and inflammatory diseases. These diseases are characterized by an accumulation of eosinophils in the tissue. Defining the mechanisms that control eosinophil recruitment is fundamental to understanding how these diseases progress and may identify a novel target for drug therapy. Eotaxin is a potent eosinophil-specific chemokine that is released in the respiratory epithelium after allergic stimulation.AIM OF THE STUDY: In this study, we determined whether Moutan Cortex Radicis (MCR), a plant extract, effects eotaxin secretion from A549 epithelial cells and eosinophil chemotaxis, and then examined the mechanism involved. MATERIALS AND METHODS: Prior to assaying MCR's effects, A549 cells were

stimulated with tumor necrosis factor-alpha (TNF-alpha), interleukin-4 (IL-4) and IL-1beta to induce expression of chemokines and adhesion molecules involved in eosinophil chemotaxis. In the presence of MCR, eotaxin, regulated on activation in normal T cells expressed and secreted (RANTES), IL-8, IL-16, intercellular adhesion molecule-1 (ICAM-1) and vascular cell adhesion molecule-1 (VCAM-1) transcripts were quantitated by real-time RT-PCR.
RESULTS: As a result, 0.01, 1, and 100 microg/ml of MCR treatments reduced eotaxin expression significantly and 0.01, 0.1, 1, 10, and 100 microg/ml of MCR reduced significantly eotaxin secretion. In addition, MCR treatment significantly inhibited eosinophil migration toward A549 medium. And 100 microg/ml of MCR suppressed the activated of nuclear factor (NF)-kappaB.
CONCLUSIONS: These findings indicate that suppressed eotaxin secretion by MCR treatment is due to the inhibition of NF-kappaB activation. Therefore, MCR might be of therapeutic value in treating asthma.

PMID: 17881168 [PubMed - indexed for MEDLINE]

782. J Immunol. 2007 Oct 1;179(7):4800-7.

Deletion of secretory group V phospholipase A2 attenuates cell migration and airway hyperresponsiveness in immunosensitized mice.

Muñoz NM, Meliton AY, Arm JP, Bonventre JV, Cho W, Leff AR.

Section of Pulmonary and Critical Care Medicine, Department of Medicine, University of Chicago, Chicago, IL 60637, USA.

We investigated the role of group V phospholipase A2 (gVPLA2) in OVA-induced inflammatory cell migration and airway hyperresponsiveness (AHR) in C57BL/6 mice. Repeated allergen challenge induced biosynthesis of gVPLA2 in airways. By aerosol, gVPLA2 caused dose-related increase in airway resistance in saline-treated mice; in allergic mice, gVPLA2 caused persistent airway narrowing. Neither group IIa phospholipase A2, a close homolog of gVPLA2, nor W31A, an inactive gVPLA2 mutant with reduced activity, caused airway narrowing in immune-sensitized mice. Pretreatment with MCL-3G1, a blocking Ab against gVPLA2, before OVA challenge blocked fully gVPLA2-induced cell migration and airway narrowing as marked by reduction of migrating leukocytes in bronchoalveolar lavage fluid and decreased airway resistance. We also assessed whether nonspecific AHR caused by methacholine challenge was elicited by gVPLA2 secreted from resident airway cells of immune-sensitized mice. MCL-3G1 also blocked methacholine-induced airway bronchoconstriction in allergic mice. Blockade of bronchoconstriction by MCL-3G1 was replicated in allergic pla2g5-/- mice, which lack the gene encoding gVPLA2. Bronchoconstriction caused by gVPLA2 in pla2g4-/- mice was comparable to that in pla2g4+/+ mice. Our data demonstrate that gVPLA2 is a critical messenger enzyme in the development of AHR and regulation of cell migration during immunosensitization by a pathway that is independent of group IVa phospholipase A2.

PMID: 17878379 [PubMed - indexed for MEDLINE]

783. J Immunol. 2007 Oct 1;179(7):4766-74.

Analysis of eosinophil turnover in vivo reveals their active recruitment to and prolonged survival in the peritoneal cavity.

Ohnmacht C, Pullner A, van Rooijen N, Voehringer D.

Institute for Immunology, University of Munich, Munich, Germany.

Eosinophils are potent effector cells associated with allergic inflammation and parasite infections. However, limited information exists about their turnover, migration, and survival in vivo. To address these important questions, we determined murine eosinophil turnover under steady state and inflammatory conditions by flow cytometric analysis of BrdU incorporation and analyzed their migration pattern and survival in different tissues after adoptive transfer into recipient mice. In naive mice approximately 50% of bone marrow eosinophils were labeled with BrdU during a 15-h pulse, whereas only 10% of splenic eosinophils were labeled within this time frame. Unexpectedly, the rate of eosinophil production did not change during acute infection with the helminth parasite Nippostrongylus brasiliensis despite massive eosinophilia in several tissues. Eosinophils present in lung and peritoneum remained largely BrdU negative, indicating that eosinophilia in end organs was mainly caused by increased survival of already existing eosinophils rather than increased production of new eosinophils in the bone marrow. Adoptive transfer experiments revealed that eosinophils preferentially migrated to the peritoneum in a macrophage-independent and pertussis toxin-sensitive manner, where they survived for several days. Peritoneal eosinophils expressed high levels of the inhibitory receptor Siglec-F, released less eosinophil peroxidase compared with eosinophils from the spleen, and could recirculate to other organs. These results demonstrate that the peritoneum serves as reservoir for eosinophils.

PMID: 17878375 [PubMed - indexed for MEDLINE]

784. J Ayub Med Coll Abbottabad. 2007 Jan-Mar;19(1):13-5.

Analysis of tracheobronchial foreign bodies with respect to sex, age, type and presentation.

Asif M, Shah SA, Khan F, Ghani R.

Department of ENT, Gomal Medical College, DI Khan.

BACKGROUND: Foreign body inhalation is one of the life threatening emergencies. It may happen at any age, however, most of these accidents occur in children especially below the age of five.
METHODS: This prospective study was done at Department of Otolaryngology, Head and Neck Surgery, Ayub Teaching Hospital, Abbottabad, from 1 January 2003 to 30 June 2005. A total of Eighty one patients, referred from the casualty and Paediatric unit with suspicion of tracheobronchial foreign body were included in the study.
RESULTS: Eighty one were studied. Fifty (61.7%) were male and thirty one (38.3%) were female. Sixty three (77.8%) were below five years, thirteen (16%) were between five and fifteen years and five (6.2%) were above fifteen years. Sixty seven patients (82.7%), presented mainly with chocking, while fifty nine patients (72.8%) had stridor and forty five patients (55.6%) had cough at initial presentation. Seventy two (88.9%) patients had decreased air entry and forty two (51.9%) had wheeze on auscultation, whereas cyanosis was noticed in five (6.2%) patients. Peanut was the commonest foreign body, retrieved in forty five patients (55.6%). Other foreign bodies were whistle (18.5%), maize seed (13.6%), bean seed (6.2 %), nuts (2.5%), sewing needle with thread, dice and denture (1.2%) each.
CONCLUSIONS: Foreign body inhalation is more common in male patients, mostly below five years of age. Chocking is the commonest symptom and decreased air entry on auscultation is the typical examination finding. Peanut has been found to be the commonest type of foreign body.

PMID: 17867472 [PubMed - indexed for MEDLINE]

785. Int Arch Allergy Immunol. 2008;145(2):111-21. Epub 2007 Sep 10.

Effect of ciclesonide treatment on allergen-induced changes in T cell regulation in asthma.

Heijink IH, Kauffman HF, Vellenga E, Veltman-Starkenburg CA, Postma DS, de Monchy JG.

Department of Allergology, University Medical Centre Groningen, University of Groningen, Groningen, The Netherlands. h.i.heijink@int.umcg.nl

BACKGROUND: The allergen-induced release of CCL17/thymus and activation-regulated chemokine (TARC) may be crucial in asthmatic airway inflammation by recruitment of Th2 cells. In addition, it might lead to aberrant Th2 cell activity through impairment of beta2-adrenergic receptor (beta2-AR) control. We questioned how chemokine patterns change upon allergen challenge and whether treatment with the inhaled steroid ciclesonide can reduce chemokine release and subsequently prevent allergen-induced changes in Th2 cell regulation and migration.
METHODS: Asthma patients were double-blindly treated with placebo or 80 microg ciclesonide for 7 days. We studied allergen-induced changes in sputum chemokines, migration of peripheral blood T cells and control of beta2-agonist fenoterol over T cell migration and alpha-CD3/alpha-CD28-induced cytokine production.
RESULTS: Treatment with 80 microg ciclesonide significantly diminished the late asthmatic response. The late asthmatic response was associated with increased sputum levels of CCL17 and CCL4 (but none of the other chemokines measured) and loss of beta2-AR control over T cell migration and Th2-type cytokine production. Although ciclesonide treatment did not prevent chemokine release nor altered beta2-AR function in circulating T cells, it exerted an inhibitory effect on TARC-induced T cell migration and alpha-CD3/alpha-CD28-induced cytokine production.
CONCLUSION: Our data support the hypothesis that CCL17 is involved in allergen-induced dysregulation of Th2 cell migration and cytokine production. Ciclesonide treatment inhibits T cell migration and cytokine production upon allergen inhalation, which is regulated independently from reducing CCL17 release, but may contribute to beneficial effects of ciclesonide on Th2-mediated airway inflammation.

2007 S. Karger AG, Basel

PMID: 17848804 [PubMed - indexed for MEDLINE]

786. J Pediatr Health Care. 2007 Sep-Oct;21(5):307-14.

The environmental health of Latino children.

Carter-Pokras O, Zambrana RE, Poppell CF, Logie LA, Guerrero-Preston R.

Department of Epidemiology and Biostatistics, University of Maryland, College Park, MD, USA.

Representing 1 in 6 children in the United States, Latino children incur disproportionate exposures to air pollutants, pesticides, and toxic industrial chemicals, as well as lead and mercury from candy, traditional folk remedies, religious practices, and other sources. Latino children also have higher rates of asthma, lead and mercury poisoning, behavioral and developmental disorders, and certain cancers. Concurrent exposure to multiple pollutants, pre-existing disease, poor nutrition, substandard housing, limited access to health care, and other factors related to their lower socioeconomic status increase Latino

children's susceptibility to environmental contaminants. Targeted research, education, prevention and intervention efforts, and economic development initiatives are needed.

PMCID: PMC2967224
PMID: 17825728 [PubMed - indexed for MEDLINE]

787. Harefuah. 2007 Jun;146(6):420-4, 503.

[Adolescent health status: new immigrants from the former Soviet Union and Israeli-born].

[Article in Hebrew]

Kaplan G, Fraimovich M, Sikron F.

The Gertner Institute for Epidemiology and Health Policy Research, Haifa University. giorak@gertner.health.gov.il

BACKGROUND: The relationship between immigration and health has unique aspects during adolescence because of the psychosocial aspects of health status at this age.
AIMS: To provide data on the physical and emotional health, risk behavior and services utilization of the new immigrant youth from the Former Soviet Union, compared with long-term Israeli residents and Israeli-born youth.
METHODS: A survey was performed among Israeli-born and former Soviet Union-born pupils from middle and high schools living in Rishon-le-Zion. A self-reported anonymous questionnaire was completed by 861 adolescents, 29% of them new immigrants.
RESULTS: No relationship was found between the immigration status or the number of years in Israel and physical or emotional health. Overall, 82% of the entire sample reported at least one health symptom such as pain or fatigue; 5% reported a chronic disease (mostly asthma) and 9% were overweight; 80% reported to have at least one emotional problem; however the immigration status had no influence on this situation. A total of 11% of the new immigrants reported smoking (5% of the Israeli-born), but in the multivariable analysis the immigration status was not significant. Furthermore, 40% of the new immigrants reported consuming alcoholic beverages compared to 25% of the Israeli-born. One quarter of the sample stated that they know someone who uses narcotic drugs. The percent of girls reporting they were involved in fights was higher among the new immigrants (21% compared to 12%). New immigrants report less visits to dentists even when controlling for other variables.
CONCLUSIONS: In general, the authors found many health problems among the youth but no significant differences between the two groups were found. New immigrants reported higher rates of risk behavior.

PMID: 17760393 [PubMed - indexed for MEDLINE]

788. Curr Med Res Opin. 2007 Oct;23(10):2441-52.

Azelastine hydrochloride: a review of pharmacology, pharmacokinetics, clinical efficacy and tolerability.

Bernstein JA.

Bernstein Clinical Research Center, Cincinnati, OH, USA.
jonathan.bernstein@uc.edu

INTRODUCTION: Azelastine hydrochloride (Astelin) nasal spray 0.1% solution is a second-generation intranasal antihistamine available in the US for treatment of both seasonal allergic rhinitis (SAR) and nonallergic vasomotor rhinitis (VMR).
SCOPE: Searches of journal articles including the title word 'azelastine' from 1979 through the present were conducted by the product manufacturer primarily through Medline and EMBASE but also included, at various times, Dialog, Biosis, Toxline, and Diogenes (an adverse-event database). One limitation of the present review is that it could not exclude the possibility of publication bias, whereby findings from smaller studies and/or trials with negative findings may not have been published.
FINDINGS: Azelastine is a phthalazinone derivative with H(1)-receptor binding approximately tenfold greater than chlorpheniramine on a milligram-per-milligram basis. Azelastine has demonstrated a wide range of pharmacologic effects on chemical mediators of inflammation including leukotrienes, kinins, and platelet activating factor in vitro and in vivo. The molecule also has been shown to downregulate intercellular adhesion molecule-1 expression and to reduce inflammatory cell migration in patients with rhinitis. Well-controlled studies in SAR and VMR demonstrated that azelastine nasal spray improves nasal symptoms of rhinitis, including congestion and postnasal drip, and has a rapid onset of action that appears likely due to topical activity. Azelastine nasal spray has demonstrated greater efficacy when used in combination with fluticasone propionate nasal spray when compared to either agent alone, and this combination may provide benefit for patients with moderate-to-severe rhinitis. Bitter taste is the most common side effect associated with azelastine nasal spray and this problem can be mitigated by the dosing technique recommended by the manufacturer in the product labeling. The incidence of somnolence also may be reduced with the recommended administration technique.
CONCLUSIONS: Azelastine is an effective, rapid-acting, and well-tolerated second-generation antihistamine that improves nasal symptoms associated with SAR and VMR. Clinical studies demonstrated that azelastine nasal spray can improve symptoms of SAR in patients who remained symptomatic after treatment with oral antihistamines and that azelastine nasal spray in combination with fluticasone nasal spray provided significantly ($p < 0.05$) greater relief than either agent alone in patients with SAR.

PMID: 17723160 [PubMed - indexed for MEDLINE]

789. Am J Physiol Lung Cell Mol Physiol. 2007 Oct;293(4):L1059-68. Epub 2007 Aug 10.

Angiogenesis is induced by airway smooth muscle strain.

Hasaneen NA, Zucker S, Lin RZ, Vaday GG, Panettieri RA, Foda HD.

The Department of Medicine and Research, Veterans Affairs Medical Center, Northport, New York, USA. Hussein.Foda@med.va.gov

Angiogenesis is an important feature of airway remodeling in both chronic asthma and chronic obstructive pulmonary disease (COPD). Airways in those conditions are exposed to excessive mechanical strain during periods of acute exacerbations. We recently reported that mechanical strain of human airway smooth muscle (HASM) led to an increase in their proliferation and migration. Sustained growth in airway smooth muscle in vivo requires an increase in the nutritional supply to these muscles, hence angiogenesis. In this study, we examined the hypothesis that cyclic mechanical strain of HASM produces factors promoting angiogenic events in the surrounding vascular endothelial cells. Our results show: 1) a significant increase in human lung microvascular endothelial cell (HMVEC-L) proliferation, migration, and tube formation following incubation in conditioned media (CM) from HASM cells exposed to mechanical strain; 2) mechanical strain of HASM cells induced VEGF expression and release; 3) VEGF neutralizing antibodies inhibited

the proliferation, migration, and tube formations of HMVEC-L induced by the strained airway smooth muscle CM; 4) mechanical strain of HASM induced a significant increase in hypoxia-inducible factor-1alpha (HIF-1alpha) mRNA and protein, a transcription factor required for VEGF gene transcription; and 5) mechanical strain of HASM induced HIF-1alpha/VEGF through dual phosphatidylinositol 3-kinase (PI3K)/Akt/mammalian target of rapamycin (mTOR) and ERK pathways. In conclusion, exposing HASM cells to mechanical strain induces signal transduction pathway through PI3K/Akt/mTOR and ERK pathways that lead to an increase in HIF-1alpha, a transcription factor required for VEGF expression. VEGF release by mechanical strain of HASM may contribute to the angiogenesis seen with repeated exacerbation of asthma and COPD.

PMID: 17693481 [PubMed - indexed for MEDLINE]

790. Int Arch Allergy Immunol. 2007;143 Suppl 1:44-9. Epub 2007 May 1.

Theophylline attenuates the neutrophil-dependent augmentation of eosinophil trans-basement membrane migration.

Kikuchi I, Kikuchi S, Kobayashi T, Takaku Y, Hagiwara K, Kanazawa M, Nagata M.

Department of Respiratory Medicine, Saitama Medical University, Saitama, Japan.

BACKGROUND: Recent evidence suggests that both neutrophilic and eosinophilic inflammation persist in the airways of patients with severe asthma. Neutrophils can secrete a variety of mediators which may augment the migration of eosinophils. We have reported that activated neutrophils augment the trans-basement membrane migration (TBM) of eosinophils in vitro. Theophylline has been shown to modulate some functions of both neutrophils and eosinophils. The objective of this study was to evaluate whether theophylline modulates the neutrophil-dependent augmentation of eosinophil TBM.
METHODS: Eosinophils and neutrophils were isolated from peripheral blood collected from healthy donors and were then preincubated with either 0.1 mM theophylline or the medium control. The TBM of eosinophils in response to IL-8 was evaluated in the presence or absence of neutrophils by using the chambers with a Matrigel-coated Transwell insert. The generation of O(2)(-) was evaluated by the cytochrome c reduction assay.
RESULTS: As previously reported, IL-8-stimulated neutrophils significantly augmented the TBM of eosinophils. Theophylline significantly attenuated the neutrophil-dependent augmentation of eosinophil TBM ($p < 0.001$) and did not directly modify the TBM of neutrophils in response to IL-8 or LTB4. Similarly, the LTB4-induced TBM of eosinophils was not modified by theophylline. Finally, theophylline attenuated the superoxide anion generation from IL-8-stimulated neutrophils on the Matrigel-coated plates.
CONCLUSIONS: Our results show that theophylline can attenuate the neutrophil-dependent augmentation of eosinophil TBM. This effect is possibly attributable to the suppression of neutrophil activation provoked by the combination of basement membrane and IL-8.

PMID: 17541276 [PubMed - indexed for MEDLINE]

791. Int Arch Allergy Immunol. 2007;143 Suppl 1:38-43. Epub 2007 May 1.

Eosinophils do not enhance the trans-basement-membrane migration of neutrophils.

Kobayashi T, Takaku Y, Kikuchi I, Soma T, Hagiwara K, Kanazawa M, Nagata M.

Department of Respiratory Medicine, Saitama Medical University, Iruma-gun,

Saitama, Japan.

BACKGROUND: There is increasing evidence that both neutrophilic and eosinophilic inflammation persist in the airways of patients with severe asthma. We have reported a positive relationship between the concentrations of eosinophils and neutrophils in sputum from severe asthmatics, suggesting a possible role of eosinophils in regulating neutrophilic inflammation. The aim of this study was to investigate whether activated eosinophils modify the trans-basement membrane migration (TBM) of neutrophils.
METHODS: Eosinophils and neutrophils were isolated from peripheral blood drawn from healthy donors. The TBM of neutrophils in response to a variety of chemoattractants was evaluated in the presence or absence of eosinophils by using the chambers with a Matrigel-coated Transwell insert.
RESULTS: As expected, eotaxin (10 nM) and RANTES (10 nM), but not IL-8 (10 nM), induced the TBM of eosinophils. On the contrary, only IL-8 induced the TBM of neutrophils. When eosinophils were coincubated with neutrophils and stimulated with IL-8, the TBM of eosinophils was significantly augmented. On the other hand, when neutrophils were coincubated with eosinophils and stimulated with eotaxin or RANTES, the TBM of neutrophils was not modified.
CONCLUSIONS: Neutrophils migrated by IL-8 may lead eosinophils to accumulate in the airways of patients with severe asthma. On the other hand, it is unlikely that eosinophils migrated by chemoattractants such as CC chemokines regulate neutrophilic inflammation.

PMID: 17541275 [PubMed - indexed for MEDLINE]

792. J Allergy Clin Immunol. 2007 Oct;120(4):827-34. Epub 2007 Aug 6.

H4 histamine receptor mediates optimal migration of mast cell precursors to CXCL12.

Godot V, Arock M, Garcia G, Capel F, Flys C, Dy M, Emilie D, Humbert M.

Institut National de la Santé et de la Recherche Médicale, Université Paris-Sud 11, Faculté de Médecine Paris Sud, Institut Fédératif de Recherche 13, Clamart, France. veronique.godot@gmail.com

BACKGROUND: CXCL12, a constitutive chemokine (ligand of CXCR4 and CXCR7), is expressed in the skin and airway epithelium and plays a significant role in allergic airway diseases. The pleiotropic effects of CXCL12 are enhanced by cofactors specific to the target cell.
OBJECTIVE: We hypothesized that histamine, a major mediator of allergic reactions, could interact with CXCL12 to promote human mast cell (MC) migration.
METHODS: The chemotactic effects of CXCL12 alone or in combination with histamine were evaluated on human precursor and mature MCs by using in vitro migration assays.
RESULTS: CXCL12 exerts a chemotactic activity on both precursor and fully mature MCs. Histamine and supernatants from IgE-activated MCs enhanced CXCL12 chemotactic activity on the precursor MC population. The synergy between histamine and CXCL12 was not observed with mature MCs, CD4(+) T cells, and monocytes. Inhibition of histamine receptors pharmacologically or with specific small interfering RNA (siRNA) indicated that synergy between histamine and CXCL12 required the H(4) receptor.
CONCLUSION: Histamine released by allergen-activated mature MCs might promote MC-rich allergic inflammation by enhancing recruitment of their precursors in tissues constitutively expressing CXCL12, including skin and airways.
CLINICAL IMPLICATIONS: This work highlights a novel role of the H(4) receptor in the perpetuation of allergic responses and provides evidence for the utility of H(4) receptor antagonists in the treatment of allergic diseases.

PMID: 17681365 [PubMed - indexed for MEDLINE]

793. Inflammation. 2007 Dec;30(6):198-204. Epub 2007 Jul 31.

Acute immune and non-immune inflammatory response in spontaneously hypertensive rats and normotensive rats. Role of endogenous nitric oxide.

Ferreira AA, Kwasniewski FH, Delani TC, Torres MG, Silva MA, Caparroz-Assef SM, Cuman RK, Bersani-Amado CA.

Laboratory of Inflammation, Department of Pharmacy and Pharmacology, University of Maringá, Maringá, Paraná, Brazil.

The present study investigated the acute inflammatory response (increase in vascular permeability and leukocytes migration) in the pleura of spontaneously hypertensive rats (SHR) and normotensive rats (NTR), using two different stimulus: carrageenan and active anaphylaxis. In addition, the role of endogenous nitric oxide in these responses was investigated. RESULTS: The inflammatory response induced by intrapleural carrageenan injection in SHR developed similarly to that in NTR. Treatment with L-NAME, reduced the intensity of this response in both groups of rats. The inflammatory response induced by active anaphylaxis in SHR and NTR was different. The increase in vascular permeability occurred later in the SHR compared to NTR. The number of leukocyte present in inflammatory exudates was increased at 4 h in both groups of rats. L-NAME treatment did not inhibit exudation at the intervals under analysis, however, reduced the number of mononuclear cells in the inflammatory exudate of SHR. CONCLUSION: The development of the inflammatory response in SHR differs from that in NTR, depending on the nature of the inflammatory stimulus. Endogenous NO plays a clear role in carrageenan-induced inflamma-tion, but not in immunologically mediated inflammation in the analyzed period.

PMID: 17665293 [PubMed - indexed for MEDLINE]

794. Ann Agric Environ Med. 2007;14(1):1-4.

Cathelicidin LL-37: LPS-neutralizing, pleiotropic peptide.

Golec M.

Department of Occupational Biohazards, Institute of Agricultural Medicine, Jaczewskiego 2, 20-090 Lublin, Poland. msgolec@yahoo.com

Human organism, constantly exposed to a large variety of pathogenic microorganisms and their products, such as lipopolysaccharide (LPS), developed innate immunity as a first line of defence. One of the compartments of our organism well equipped with these defence mechanisms is the respiratory system. The cells lining the airways respond to the presence of virulent microorganisms by producing natural antimicrobial peptides, including the only member of the cathelicidins family found to date in humans, peptide LL-37. LL-37 is a small peptide of 37 amino acid residues. The peptide, in addition to its bactericidal effect, plays numerous roles in inflammatory and tissue remodelling processes. It stimulates angiogenesis, induces proliferation of lung epithelial cells, accelerates wound closure of the airway epithelium, and provokes cytokine release (e.g. IL-8) and cell migration. LL-37 is also able to neutralize LPS, a heteropolymer associated with organic dust, produced by Gram-negative bacteria. LPS (commonly referred to as endotoxin) plays an important role in pathogenesis of many respiratory diseases caused by organic dust, including organic dust toxic

syndrome and chronic illnesses such as chronic obstructive pulmonary disease (COPD), asthma or allergic alveolitis (hypersensitivity pneumonitis). LPS is a strong pro-inflammatory stimulus, inducing in respiratory airways expression of antimicrobial peptides, including LL-37, which is in turn a potent LPS-neutralizing factor. The article discusses the complex interplay between endotoxin and the LPS-neutralizing, pleiotropic peptide LL-37 in pathogenic mechanisms of lung diseases, with regard to closer perspectives of using LL-37 and its derivatives as therapeutic agents.

PMID: 17655171 [PubMed - indexed for MEDLINE]

795. Allergol Int. 2007 Sep;56(3):201-8. Epub 2007 Aug 1.

Dendritic cells: importance in allergy.

Aiba S.

Department of Dermatology, Tohoku University Graduate School of Medicine, Miyagi, Japan. aiba@mail.tains.tohoku.ac.jp

In this review we discuss the role of dendritic cells (DC) in the pathogenesis of allergic contact hypersensitivity (ACH) and atopic disorders, such as asthma and atopic eczema. In ACH patients, DC recognize the invasion of simple chemicals such as haptens, and trigger antigen-specific T cell responses leading to the characteristic histological and clinical changes such as spongiosis and papulovesicular eruptions. During atopic disorders, it is well known that the Th2-deviated immune response plays a crucial role in their pathogenesis. DC provide T cells with antigen and costimulatory signals (signals 1 and 2, respectively), as well as with a polarizing signal (signal 3). When studying ACH, it is important to understand how simple chemicals induce the activation of DC and their migration to the draining lymph nodes where they supply signals 1 and 2 to naive T cells. The mechanisms by which DC induce the Th2-deviated immune response, namely via the Th2-deviated signal 3, are central topics in the pathogenesis of atopic disorders.

PMID: 17646737 [PubMed - indexed for MEDLINE]

796. J Immunol. 2007 Aug 1;179(3):1577-85.

Sphingosine 1-phosphate-mediated trafficking of pathogenic Th2 and mast cells for the control of food allergy.

Kurashima Y, Kunisawa J, Higuchi M, Gohda M, Ishikawa I, Takayama N, Shimizu M, Kiyono H.

Division of Mucosal Immunology, Department of Microbiology and Immunology, The Institute of Medical Science, The University of Tokyo, Tokyo, Japan.

Sphingosine 1-phosphate (S1P) has been proposed as a regulator of lymphocyte trafficking, but its role in mucosa-associated diseases, such as in food allergies, remains to be elucidated. To examine the role of S1P in allergic diseases in the intestine, we used a Th2 cell-mediated Ag-specific allergic diarrhea model and demonstrated that type 1 S1P receptor (S1P(1)) expression was preferentially associated with pathogenic CD4(+) T cells for the development of allergic reactions. Consistent with this demonstration, treatment with FTY720, a modulator of the S1P(1), prevented allergic diarrhea by inhibiting the migration of systemically primed pathogenic CD4(+) T cells induced by oral challenge with allergen into the large intestine. In addition, FTY720 hampered mast cell

infiltration into the large intestine, whereas eosinophil infiltration into the large intestine and total and allergen-specific serum IgE production were comparable between mock- and FTY720-treated groups. These results suggest that modulation of the S1P-mediated pathway to inhibit the migration of pathogenic CD4(+) T cells and mast cells into the large intestine could be a novel strategy for preventing allergic diarrhea.

PMID: 17641024 [PubMed - indexed for MEDLINE]

797. J Immunol. 2007 Aug 1;179(3):1438-48.

Controls for lung dendritic cell maturation and migration during respiratory viral infection.

Grayson MH, Ramos MS, Rohlfing MM, Kitchens R, Wang HD, Gould A, Agapov E, Holtzman MJ.

Division of Allergy and Immunology, Washington University School of Medicine, St. Louis, MO 63110, USA. wheeze@allergist.com

Dendritic cells are ideally suited to orchestrate the innate and adaptive immune responses to infection, but we know little about how these cells respond to infection with common respiratory viruses. Paramyxoviral infections are the most frequent cause of serious respiratory illness in childhood and are associated with an increased risk of asthma. We therefore used a high-fidelity mouse model of paramyxoviral respiratory infection triggered by Sendai virus to examine the response of conventional and plasmacytoid dendritic cells (cDCs and pDCs, respectively) in the lung. We found that pDCs are scarce at baseline but become the predominant population of lung dendritic cells during infection. This recruitment allows for a source of IFN-alpha locally at the site of infection. In contrast, cDCs rapidly differentiate into myeloid cDCs and begin to migrate from the lung to draining lymph nodes within 2 h after viral inoculation. These events cause the number of lung cDCs to decrease rapidly and remain decreased at the site of viral infection. Maturation and migration of lung cDCs depends on Ccl5 and Ccr5 signals because these events are significantly impaired in Ccl5(-/-) and Ccr5(-/-) mice. cDCs failure to migrate to draining lymph nodes in Ccl5(-/-) or Ccr5(-/-) mice is associated with impaired up-regulation of CCR7 that would normally direct this process. Our results indicate that pDCs and cDCs respond distinctly to respiratory paramyxoviral infection with patterns of movement that should serve to coordinate the innate and adaptive immune responses, respectively.

PMID: 17641009 [PubMed - indexed for MEDLINE]

798. Eur J Immunol. 2007 Aug;37(8):2337-48.

Leptin-mediated cytokine release and migration of eosinophils: implications for immunopathophysiology of allergic inflammation.

Wong CK, Cheung PF, Lam CW.

Department of Chemical Pathology, The Chinese University of Hong Kong, Prince of Wales Hospital, Shatin, Hong Kong.

Leptin is a pleiotropic adipocyte-derived cytokine used in hypothalamic regulation of body weight and modulation of immune response by stimulating T cells, macrophages and neutrophils. Leptin has been shown to be an eosinophil survival factor. We examined the immunopathological mechanisms for the activation

of human eosinophils from healthy volunteers by leptin in allergic inflammation. Adhesion molecules, cytokines and cell migration were assessed by flow cytometry, ELISA and Boyden chamber assay, respectively. Intracellular signaling molecules were investigated by membrane array and Western blot. Leptin could up-regulate cell surface expression of adhesion molecule ICAM-1 and CD18 but suppress ICAM-3 and L-selectin on eosinophils. Leptin could also stimulate the chemokinesis of eosinophils, and induce the release of inflammatory cytokines IL-1beta and IL-6, and chemokines IL-8, growth-related oncogene-alpha and MCP-1. We found that leptin-mediated induction of adhesion molecules, release of cytokines and chemokines, and chemokinesis were differentially regulated by the activation of ERK, p38 MAPK and NF-kappaB. In view of the above results and elevated production of leptin in patients with allergic diseases such as atopic asthma and atopic dermatitis, leptin could play crucial immunopathophysiological roles in allergic inflammation by activation of eosinophils via differential intracellular signaling cascades.

PMID: 17634954 [PubMed - indexed for MEDLINE]

799. Eur J Immunol. 2007 Aug;37(8):2257-67.

Quinacrine inhibits the epidermal dendritic cell migration initiating T cell-mediated skin inflammation.

Gorbachev AV, Gasparian AV, Gurova KV, Gudkov AV, Fairchild RL.

Department of Immunology, Cleveland Clinic, Cleveland, OH 44195-0001, USA. gorbaca@ccf.org

Quinacrine (QC) is an anti-inflammatory drug that has been used for the treatment of malaria and rheumatoid diseases. The mechanism(s) underlying the anti-inflammatory activity of QC remains poorly understood. We recently reported the QC-mediated inhibition of the NF-kappaB pathway using an in vitro model. To test this potential mechanism in vivo, we used the contact hypersensitivity response (CHS) to chemical allergen sensitization and challenge in mice as a model of skin inflammation. The results indicated that QC treatment inhibited NF-kappaB activation in the skin during allergen sensitization. This inhibition was reflected by decreased mRNA expression and protein production of the NF-kappaB-dependent cytokines TNF-alpha and IL-1beta and the chemokine CCL21 in the skin. The decreases in these cytokines resulted in reduced migration of allergen-presenting dendritic cells from the skin into skin-draining lymph nodes and markedly decreased activation of effector CD8+ T cells for the CHS response to allergen challenge (inhibitory concentration 50% or IC50 was 55 mg/kg). These findings reveal a previously unrecognized mechanism of QC-mediated inhibition of inflammation.

PMID: 17634953 [PubMed - indexed for MEDLINE]

800. Int Immunopharmacol. 2007 Sep;7(9):1160-9. Epub 2007 May 29.

Antiinflammatory, antioxidant and cytotoxic actions of beta-glucan-rich extract from Geastrum saccatum mushroom.

Guerra Dore CM, Azevedo TC, de Souza MC, Rego LA, de Dantas JC, Silva FR, Rocha HA, Baseia IG, Leite EL.

Laboratório de Glicobiologia, Departamento de Bioquímica, Universidade Federal do Rio Grande do Norte-UFRN, Natal-RN, Brazil.

The Geastrum saccatum a mushroom, native to Brazil, is produced under natural conditions in the unexplored reserve of Mata da Estrela-RN. This species has curative properties for eye infections and diseases such as asthma. The tissues of this mushroom contain carbohydrates, proteins, lipids, moisture and ashes in amounts of 42.3%, 37.05%, 9.01, 1.4% and 10.2%, respectively. An extract from this mushroom was characterized by chemical analyses and (13)C and (1)H NMR spectroscopy. It contains high amount of glucose and traces of galactose. The signal appearing at 103.5 ppm was assigned to C1 of beta-glucose. The signals observed between 20 and 40 ppm suggest the presence of a glucan-protein compound. This glucan inhibited the lipid peroxidation at the dose of 0.27 mg/mL (59.1%) and it can protect cells against oxidative stress by scavenging of the hydroxyl (77%) and superoxide (88.4%) radicals at 0.27 mg/mL. The glucan (30 mg/kg) reduces the polymorphonuclear cell migration (57.6%). The ear edema induced by croton oil was inhibited by glucan (60.4% at 10 mg/kg) and by its association with diclofenac (5 mg/kg) (89.2%) or L-NAME (60 mg/kg) (86.23%). Histological analyses of the ear edema induced by croton oil in the presence of glucan (10, 30 or 50 mg/kg) showed a reduced degree of the polymorphonuclear cell migration. We concluded that the glucan has antioxidant, and antiinflammatory properties as well as its antiinflammatory effect are mediated by inhibition of both nitric oxide synthase (NOS) and cyclooxygenase (COX).

PMID: 17630194 [PubMed - indexed for MEDLINE]

801. Vitam Horm. 2007;76:393-418.

Vitamin E and mast cells.

Zingg JM.

Institute of Biochemistry and Molecular Medicine, University of Bern, 3012 Bern, Switzerland.

Mast cells play an important role in the immune system by interacting with B and T cells and by releasing several mediators involved in activating other cells. Hyperreactivity of mast cells and their uncontrolled accumulation in tissues lead to increased release of inflammatory mediators contributing to the pathogenesis of several diseases such as rheumatoid arthritis, atherosclerosis, multiple sclerosis, and allergic disorders such as asthma and allergic rhinitis. Interference with mast cell proliferation, survival, degranulation, and migration by synthetic or natural compounds may represent a preventive strategy for the management of these diseases. Natural vitamin E covers a group of eight analogues-the alpha-, beta-, gamma-, and delta-tocopherols and the alpha-, beta-, gamma-, and delta-tocotrienols, but only alpha-tocopherol is efficiently retained by the liver and distributed to peripheral tissues. Mast cells preferentially locate in the proximity of tissues that interface with the external environment (the epithelial surface of the skin, the gastrointestinal mucosa, and the respiratory system), what may render them accessible to treatments with inefficiently retained natural vitamin E analogues and synthetic derivatives. In addition to scavenging free radicals, the natural vitamin E analogues differently modulate signal transduction and gene expression in several cell lines; in mast cells, protein kinase C, protein phosphatase 2A, and protein kinase B are affected by vitamin E, leading to the modulation of proliferation, apoptosis, secretion, and migration. In this chapter, the possibility that vitamin E can prevent diseases with mast cells involvement by modulating signal transduction and gene expression is evaluated.

PMID: 17628183 [PubMed - indexed for MEDLINE]

802. J Immunol. 2007 Jul 15;179(2):1264-73.

Eotaxin-1/CC chemokine ligand 11: a novel eosinophil survival factor secreted by human pulmonary artery endothelial cells.

Farahi N, Cowburn AS, Upton PD, Deighton J, Sobolewski A, Gherardi E, Morrell NW, Chilvers ER.

Division of Respiratory Medicine, Department of Medicine, University of Cambridge School of Clinical Medicine, Addenbrooke's and Papworth Hospitals, Cambridge, UK.

Airway eosinophilia plays a major role in the pathogenesis of asthma with the inhibition of apoptosis by GM-CSF and IL-5 proposed as a mechanism underlying prolonged eosinophil survival. In vivo and ex vivo studies have indicated the capacity of interventions that drive human eosinophil apoptosis to promote the resolution of inflammation. Far less is known about the impact of transendothelial migration on eosinophil survival, in particular, the capacity of endothelial cell-derived factors to contribute toward the apoptosis-resistant phenotype characteristic of airway-resident eosinophils. We examined the effects of conditioned medium from human pulmonary artery endothelial cells (HPAEC-CM) on eosinophil apoptosis in vitro. HPAEC-CM inhibited eosinophil, but not neutrophil apoptosis. This effect was specific to HPAECs and comparable in efficacy to the survival effects of GM-CSF and IL-5. The HPAEC survival factor was shown, on the basis of GM-CSF, IL-5, and IL-3 detection assays, Ab neutralization, and sensitivity to PI3K inhibition, to be clearly discrete from these factors. Gel filtration of HPAEC-CM revealed a peak of eosinophil survival activity at 8-12 kDa, and PCR confirmed the presence of mRNA for CCL5, CCL11, CCL24, CCL26, and CCL27 in the HPAECs. The CCR3 antagonist GW782415 caused a major inhibition of the HPAEC-CM-induced survival effect, and Ab neutralization of individual CCR3 chemokines revealed CCL11 as the major survival factor present in the HPAEC-CM. Furthermore, chemokine Ab arrays demonstrated up-regulation of CCL11 in HPAEC-CM. These data demonstrate the capacity of HPAECs to generate CCR3 agonists and the ability of CCL11 to inhibit human eosinophil apoptosis.

PMID: 17617619 [PubMed - indexed for MEDLINE]

803. Proc Am Thorac Soc. 2007 Jul;4(3):226-33.

Dissecting complex diseases in complex populations: asthma in latino americans.

Choudhry S, Seibold MA, Borrell LN, Tang H, Serebrisky D, Chapela R, Rodriguez-Santana JR, Avila PC, Ziv E, Rodriguez-Cintron W, Risch NJ, Burchard EG.

Department of Medicine, University of California, San Francisco, San Francisco, CA 94143-2911, USA.

Asthma is a common but complex respiratory ailment; current data indicate that interaction of genetic and environmental factors lead to its clinical expression. In the United States, asthma prevalence, morbidity, and mortality vary widely among different Latino ethnic groups. The prevalence of asthma is highest in Puerto Ricans, intermediate in Dominicans and Cubans, and lowest in Mexicans and Central Americans. Independently, known socioeconomic, environmental, and genetic differences do not fully account for this observation. One potential explanation is that there may be unique and ethnic-specific gene-environment interactions that can differentially modify risk for asthma in Latino ethnic groups. These gene-environment interactions can be tested using genetic ancestry as a surrogate for genetic risk factors. Latinos are admixed and share varying proportions of African, Native American, and European ancestry. Most Latinos are unaware of

their precise ancestry and report their ancestry based on the national origin of their family and their physical appearance. The unavailability of precise ancestry and the genetic complexity among Latinos may complicate asthma research studies in this population. On the other hand, precisely because of this rich mixture of ancestry, Latinos present a unique opportunity to disentangle the clinical, social, environmental, and genetic underpinnings of population differences in asthma prevalence, severity, and bronchodilator drug responsiveness.

PMCID: PMC2647623
PMID: 17607004 [PubMed - indexed for MEDLINE]

804. J Invest Dermatol. 2008 Jan;128(1):59-66. Epub 2007 Jun 28.

Modulation of keratinocyte-derived MMP-9 by IL-13: a possible role for the pathogenesis of epidermal inflammation.

Purwar R, Kraus M, Werfel T, Wittmann M.

Department of Dermatology and Allergology, Hannover Medical School, Hannover, Germany.

Skin inflammation and remodeling are important pathophysiological features of chronic eczematous skin diseases. Matrix metalloproteinases (MMP) have been described to influence tissue remodeling and to facilitate cell migration through their ability to proteolyse the extracellular matrix. The aim of this study was to investigate the influence of IL-13 on the modulation of MMPs in human primary keratinocytes (KCs). IL-13 stimulation of KCs induced the expression of MMP-9 but not of MMP-3 or MMP-2 at mRNA level. A major substrate of MMP-9 is the type IV collagen of the basement membrane. IL-13 induced the release of active MMP-9 in KCs as detected by an ELISA-based assay. Moreover, migration of lymphocytes cultured with IL-13-activated KC showed increased migration through a basement membrane equivalent. The MMP-9 expression was prominent near the basement membrane of IL-13-treated skin biopsies. Collagen type IV staining pointed to a loss of this major basement membrane constituent in IL-13-treated skin. Finally, we demonstrated the concomitant mRNA expression of MMP-9 and IL-13 in biopsies from lesional, acutely inflamed eczematous skin. Our results suggest that release of active MMP-9 by IL-13-stimulated KCs may play a crucial role in skin inflammation by facilitating migration of leukocytes into the epidermis.

PMID: 17597813 [PubMed - indexed for MEDLINE]

805. Eur J Immunol. 2007 Aug;37(8):2156-63.

HIV-1 induced generation of C5a attracts immature dendriticcells and promotes infection of autologous T cells.

Soederholm A, Bánki Z, Wilflingseder D, Gassner C, Zwirner J, López-Trascasa M, Falkensammer B, Dierich MP, Stoiber H.

Department of Hygiene, Microbiology and Social Medicine, Innsbruck Medical University, and Central Institute for Blood Transfusion and Division for Immunology, University Hospital, Austria.

For the recruitment of dendritic cells (DC) to the site of infection, DC express several sensors for danger signals, such as receptors for C5a. This anaphylatoxin is generated upon complement activation. As HIV-1 triggers the complement cascade, we determined whether C5a is generated by the virus and tested the

functional activity of C5a in migration and infection assays. The immature (i)DC responded in migration assays to recombinant C5a and native C5a, which was generated in situ upon activation of the complement system by HIV-1. In combined migration and infection assays, a C5a-dependent enhancement of HIV-1 infection in DC-T cell cocultures was observed. These results indicate that HIV induces generation of C5a and thereby attracts iDC, which in turn promote the productive infection of autologous primary T cells.

PMID: 17595678 [PubMed - indexed for MEDLINE]

806. Philos Trans R Soc Lond B Biol Sci. 2007 Aug 29;362(1484):1281-91.

Designed triple-helical peptides as tools for collagen biochemistry and matrix engineering.

Koide T.

Faculty of Pharmaceutical Sciences, Niigata University of Pharmacy and Applied Life Sciences, Niigata 956-8603, Japan. koi@waseda.jp

Collagens, characterized by a unique triple-helical structure, are the predominant component of extracellular matrices (ECMs) existing in all multicellular animals. Collagens not only maintain structural integrity of tissues and organs, but also regulate a number of biological events, including cell attachment, migration and differentiation, tissue regeneration and animal development. The specific functions of collagens are generally triggered by specific interactions of collagen-binding molecules (membrane receptors, soluble factors and other ECM components) with certain structures displayed on the collagen triple helices. Thus, synthetic triple-helical peptides that mimic the structure of native collagens have been used to investigate the individual collagen-protein interactions, as well as collagen structure and stability. The first part of this article illustrates the design of various collagen-mimetic peptides and their recent applications in matrix biology. Collagen is also acknowledged as one of the most promising biomaterials in regenerative medicine and tissue engineering. However, the use of animal-derived collagens in human could put the recipients at risks of pathogen transmission or allergic reactions. Hence, the production of safe artificial collagen surrogates is currently of considerable interest. The latter part of this article reviews recent attempts to develop artificial collagens as novel biomaterials.

PMCID: PMC2440396
PMID: 17581806 [PubMed - indexed for MEDLINE]

807. Respir Med. 2007 Sep;101(9):1947-53. Epub 2007 Jun 15.

Serum and bronchial lavage fluid concentrations of IL-8, SLPI, sCD14 and sICAM-1 in patients with COPD and asthma.

Hollander C, Sitkauskiene B, Sakalauskas R, Westin U, Janciauskiene SM.

Department of Otolaryngology and Head and Neck Surgery, University Hospital, 20502 Malmö, Sweden.

BACKGROUND: Airway inflammation is associated with an increased expression and release of inflammatory reactants that regulate processes of cell migration, activation and degranulation. The purpose of this study was to quantify bronchial lavage (BAL) fluid and serum levels of chemokine (IL-8), secretory leukocyte protease inhibitor (SLPI), soluble intracellular adhesion molecules-1 (sICAM-1)

and sCD14, as surrogate markers of inflammatory and immune response in asthma and chronic obstructive pulmonary disease (COPD) patients with similar disease duration time.
METHODS: Biomarkers in serum and BAL fluid from asthma (n=13) and COPD (n=25) patients were measured using commercially available ELISA kits.
RESULTS: We found that in asthma and COPD groups the concentrations of IL-8 and SLPI are significantly higher in BAL fluid than in serum, while levels of sICAM-1 and sCD14 in BAL fluid are significantly lower than in serum. Of these 4 measured biomarkers, only the BAL IL-8 was higher in COPD patients when compared to asthma (P<0.05). In both groups, BAL IL-8 correlated with SLPI (r=0.577, P<0.01 and r=0.589, P<0.05, respectively). In patients with COPD the BAL sICAM-1 correlated with sCD14 (r=0.576, P<0.01), while in asthma patients BAL sICAM-1 correlated with FEV(1)/FVC (r=0.418, P<0.01). Moreover, in asthma patients the serum SLPI correlated with sCD14 (r=0.688, P<0.01) and serum sICAM-1 negatively correlated with FEV(1)/FVC (r=-0.582, P<0.05).
CONCLUSION: Our findings point to the importance of selecting a correct biological fluid when analyzing specific biomarkers, and also show that of 4 measured biomarkers, only the BAL IL-8 was higher in COPD patients when compared to asthma.

PMID: 17574828 [PubMed - indexed for MEDLINE]

808. J Allergy Clin Immunol. 2007 Jun;119(6):1423-9.

Integrin beta 3 genotype influences asthma and allergy phenotypes in the first 6 years of life.

Thompson EE, Pan L, Ostrovnaya I, Weiss LA, Gern JE, Lemanske RF Jr, Nicolae DL, Ober C.

Department of Human Genetics, University of Chicago, Chicago, Ill., USA. eethomps@uchicago.edu

BACKGROUND: The integrin beta3 gene (ITGB3) encodes a subunit of the platelet and monocyte-specific fibrinogen receptor and the widely expressed vitronectin receptor, which have diverse roles in cell migration, adhesion, and signaling. Previous work from our laboratory reported associations between single nucleotide polymorphisms (SNPs) in ITGB3 and asthma and allergic sensitization in 4 populations.
OBJECTIVE: To examine whether SNPs in ITGB3 are associated with the development of asthma and allergic phenotypes in early life.
METHODS: We typed 13 SNPs in 206 children participating in a birth cohort study and tested for associations with asthma and allergy phenotypes in the first 6 years of life.
RESULTS: Our study revealed significant associations between SNPs in ITGB3 and asthma, wheezing, and IgE levels, suggesting an early role for this gene in the development of asthma and allergy. In particular, SNPs at the 3' end of the gene were significantly associated with IgE levels beginning at 1 year of age, whereas a SNP in intron 1 showed significant interaction effects with viral respiratory illness in infancy on asthma susceptibility.
CONCLUSION: Our results suggest that genetic variation in ITGB3 contributes to asthma susceptibility and allergic sensitization, and that the effects of this gene begin early in life. Similar to our earlier study, different SNPs in the gene are associated with asthma and IgE.
CLINICAL IMPLICATIONS: ITGB3 may play an important role in the development of asthma and allergy and may represent a potential therapeutic target for the treatment of these disorders.

PMID: 17556058 [PubMed - indexed for MEDLINE]

809. Exp Cell Res. 2007 Jul 15;313(12):2695-702. Epub 2007 Mar 21.

Interleukin-13 interferes with CFTR and AQP5 expression and localization during human airway epithelial cell differentiation.

Skowron-zwarg M, Boland S, Caruso N, Coraux C, Marano F, Tournier F.

Laboratoire de Cytophysiologie et Toxicologie Cellulaire, Université Paris 7, Tour 53-54, Paris cedex 05, France.

Interleukin-13 (IL-13) is a central regulator of Th2-dominated respiratory disorders such as asthma. Lesions of the airway epithelial barrier frequently observed in chronic respiratory inflammatory diseases are repaired through proliferation, migration and differentiation of epithelial cells. Our work is focused on the effects of IL-13 in human cellular models of airway epithelial cell regeneration. We have previously shown that IL-13 altered epithelial cell polarity during mucociliary differentiation of human nasal epithelial cells. In particular, the cytokine inhibited ezrin expression and interfered with its apical localization during epithelial cell differentiation in vitro. Here we show that CFTR expression is enhanced in the presence of the cytokine, that two additional CFTR protein isoforms are expressed in IL-13-treated cells and that part of the protein is retained within the endoplasmic reticulum. We further show that aquaporin 5 expression, a water channel localized within the apical membrane of epithelial cells, is completely abolished in the presence of the cytokine. These results show that IL-13 interferes with ion and water channel expression and localization during epithelial regeneration and may thereby influence mucus composition and hydration.

PMID: 17553491 [PubMed - indexed for MEDLINE]

810. J Immunol. 2007 Jun 15;178(12):8081-9.

Histamine 4 receptor activation induces recruitment of FoxP3+ T cells and inhibits allergic asthma in a murine model.

Morgan RK, McAllister B, Cross L, Green DS, Kornfeld H, Center DM, Cruikshank WW.

Pulmonary Center, Boston University School of Medicine, 715 Albany Street, Boston, MA 02118, USA.

Histamine has an important role in regulation of immune response which is mediated by differential expression of four distinct receptors, H1R-H4R. H1R and HR2 have previously been shown to be involved with modulation of lung inflammation. H4R is also expressed on inflammatory cells; therefore, we investigated the potential role of H4R in development of allergic asthma in a murine model. We determined that the H4R agonist 4-methylhistamine when delivered intratracheally before Ag challenge mitigated airway hyperreactivity and inflammation. This was associated with an increase in IL-10 and IFN-gamma, but not TGF-beta or IL-16, as well as a decrease in IL-13 in the bronchoalveolar lavage fluid. We also observed that H4R agonist instillation resulted in accumulation of FoxP3(+) T cells suggesting a direct effect on T regulatory cell recruitment. To investigate this further, we determined the in vitro effect of H4R stimulation on human T cell migration. The H4R agonist induced a 2- to 3-fold increase in T cell migration, similar to that seen for H1R agonists. Cells transmigrating to the H4R agonist, but not H1R, were skewed toward a CD4 cell expressing CD25 and intracellular FoxP3. H4R-responsive cells suppressed proliferation of autologous T cells, an effect that was dependent on IL-10

production. We conclude that H4R stimulation enriches for a regulatory T cell with potent suppressive activity for proliferation. These findings identify a novel function for H4R and suggest a potential therapeutic approach to attenuation of asthmatic inflammation.

PMID: 17548646 [PubMed - indexed for MEDLINE]

811. J Immigr Minor Health. 2008 Apr;10(2):103-18.

The impact of nativity on chronic diseases, self-rated health and comorbidity status of Asian and Hispanic immigrants.

Huh J, Prause JA, Dooley CD.

Department of Psychology and Social Behavior, University of California at Irvine, 3340 Social Ecology Building II, Irvine, CA 92697-7085, USA. huhj@uci.edu

This study examines the physical health status of immigrants with specific considerations of Asian and Hispanic populations and explores possible mechanisms through which health outcomes of interest can be explained. Analyses of the National Health Interview Surveys (NHIS) of 2000 and 2001 revealed that foreign-born individuals reported fewer chronic diseases (hypertension, heart disease, asthma, cancer and diabetes) and had lower prevalences of various chronic diseases compared with U.S.-born whites, controlling for possible confounders and mediators. However, U.S-born minority groups did not show the health advantage seen in foreign-born immigrants, reflecting the importance of nativity distinctions in studying immigrant health. Despite having fewer chronic diseases, foreign-born Asians were more likely to rate their health negatively relative to their U.S.-born counterparts and to U.S.-born whites. In addition, our findings provide evidence that failure to consider comorbid status may attenuate the nativity effect on certain chronic diseases.

PMID: 17546500 [PubMed - indexed for MEDLINE]

812. Cell Mol Biol (Noisy-le-grand). 2006 Dec 31;52(4):8-16.

Hydrogen peroxide activation of endothelial cell-associated MMPs during VCAM-1-dependent leukocyte migration.

Cook-Mills JM.

Allergy-Immunology Division, Northwestern University Feinberg School of Medicine, Chicago, IL 60611, USA. j-cook-mills@northwestern.edu

Leukocyte migration from the blood into tissues is vital for immune surveillance and inflammation. Specificity for the site of leukocyte migration is determined by the combination and concentration of adhesion molecules, cytokines and chemokines in the microenvironment. Leukocytes bound at sites of extravasation migrate within minutes. We have focused on the function of the adhesion molecule VCAM-1 and have reported an active function for the endothelium during VCAM-1-dependent leukocyte migration. VCAM-1 activates endothelial cell NADPH oxidase followed by the generation of 1 microM H2O2. This stimulates endothelial cell-associated matrix metalloproteinase (MMP) activity in minutes, consistent with the time for lymphocyte migration. The endothelial cell NADPH oxidase and endothelial cell MMP activities are required for VCAM-1-dependent lymphocyte migration as determined by scavenging of ROS, by pharmacologic or antisense inhibition of NADPH oxidase and by pharmacologic inhibition of endothelial cell MMPs. Furthermore, antioxidants block VCAM-1 activation of MMPs. In vivo,

administration of the antioxidant bilirubin blocks VCAM-1-dependent leukocyte migration into the lung in experimental asthma. In summary, endothelial cells are not simply a scaffold for leukocyte adhesion. Instead, endothelial cells have an active function during VCAM-1-dependent leukocyte transendothelial migration.

PMCID: PMC2749564
PMID: 17543193 [PubMed - indexed for MEDLINE]

813. Am J Respir Crit Care Med. 2007 Aug 15;176(4):333-42. Epub 2007 May 31.

Plasminogen is an important regulator in the pathogenesis of a murine model of asthma.

Swaisgood CM, Aronica MA, Swaidani S, Plow EF.

Cleveland Clinic, Department of Molecular Cardiology, NB50, 9500 Euclid Avenue, Cleveland, OH 44195, USA.

RATIONALE: Asthma is a syndrome whose common pathogenic expression is inflammation of the airways. Plasminogen plays an important role in cell migration and is also implicated in tissue remodeling, but its role in asthma has not been defined.
OBJECTIVES: To test whether plasminogen is a critical component in the development of asthma.
METHODS: We used a mouse model of ovalbumin-induced pulmonary inflammation in Plg(+/+), Plg(+/-), and Plg(-/-) mice.
MEASUREMENTS AND MAIN RESULTS: The host responses measured included lung morphometry, and inflammatory mediators and cell counts were assessed in bronchoalveolar lavage fluid. Bronchoalveolar lavage demonstrated a marked increase in eosinophils and lymphocytes in ovalbumin-treated Plg(+/+) mice, which were reduced to phosphate-buffered saline-treated control levels in Plg(+/-) or Plg(-/-) mice. Lung histology revealed peribronchial and perivascular leukocytosis, mucus production, and increased collagen deposition in ovalbumin-treated Plg(+/+) but not in Plg(+/-) or Plg(-/-) mice. IL-5, tumor necrosis factor-alpha, and gelatinases, known mediators of asthma, were detected in bronchoalveolar lavage fluid of ovalbumin-treated Plg(+/+) mice, yet were reduced in Plg(-/-) mice. Administration of the plasminogen inhibitor, tranexamic acid, reduced eosinophil and lymphocyte numbers, mucus production, and collagen deposition in the lungs of ovalbumin-treated Plg(+/+) mice.
CONCLUSIONS: The decreased inflammation in the lungs of Plg(-/-) mice and its blockade with a plasminogen inhibitor indicate that plasminogen plays an important role in orchestrating the asthmatic response and suggests that plasminogen may be a therapeutic target for the treatment of asthma.

PMCID: PMC1994216
PMID: 17541016 [PubMed - indexed for MEDLINE]

814. Malays J Pathol. 2007 Jun;29(1):1-18.

Dendritic cell immunobiology and potential roles in immunotherapy.

Fadilah SA, Cheong SK.

Clinical Haematology & Stem Cell Transplantation Services, Department of Medicine, Faculty of Medicine, National University of Malaysia.

Owing to the importance of dendritic cells (DC) in the induction and control of immunity, an understanding of their biology is central to the development of

potent immunotherapies for cancer, chronic infections, autoimmune disease, and induction of transplantation tolerance. This review surveys the heterogeneity of DC with regards to their phenotype and developmental origin, and how they initiate, modify and regulate the immune response, with emphasis on their maturation, migration, antigen-presentation and interaction with T cells and other immune cells. Much of this knowledge is obtained through research on murine DC. Research on human DC has been hampered by limitations associated with in vitro assays and limited access to human tissues. New approaches on human DC research are required in order to develop novel strategies for the treatment of microbial infections, the control of graft rejection, and the improvement of DC-based immunotherapeutic protocols for autoimmunity, allergy, and cancer.

PMID: 19108040 [PubMed - indexed for MEDLINE]

815. J Asthma. 2007 May;44(4):297-303.

Variation in adult asthma prevalence in Hispanic subpopulations in New York City.

Dumanovsky T, Matte TD.

Division of Health Promotion and Disease Prevention, New York City Department of Health and Mental Hygiene, New York, New York, USA. tdumanov@health.nyc.gov

BACKGROUND: We compared asthma prevalence among New York City Hispanics-Puerto Rican, Dominican, and other Hispanics-in relation to nativity, socioeconomic status, and asthma risk factors.
METHODS: Weighted logistic regression analyses on telephone survey data for New York City (NYC) adults in 2003/2004.
RESULTS: Asthma prevalence was highest among Puerto Ricans (11.8%) compared with Dominicans and other Hispanics. Non-US-born Dominicans and other Hispanics were significantly less likely to report current asthma than were Puerto Ricans (OR = 0.27, 95% CI 0.18-0.41 and OR = 0.17, 95% CI 0.11-0.26, respectively). In multivariate analyses, US-born Dominicans and other Hispanics had rates comparable to Puerto Ricans.
CONCLUSIONS: Puerto Ricans, both mainland- and native-born, report the highest rates of adult asthma. Non-US-born Hispanics report lower rates. Acculturation and patterns of residential settlement may account for this variation.

PMID: 17530529 [PubMed - indexed for MEDLINE]

816. Am J Med. 2007 Jun;120(6):545.e1-8.

Maltreatment of Strongyloides infection: case series and worldwide physicians-in-training survey.

Boulware DR, Stauffer WM, Hendel-Paterson BR, Rocha JL, Seet RC, Summer AP, Nield LS, Supparatpinyo K, Chaiwarith R, Walker PF.

Division of Infectious Disease and International Medicine, Department of Medicine, University of Minnesota, Minneapolis 55455, USA. boulw001@umn.edu

BACKGROUND: Strongyloidiasis infects hundreds of millions of people worldwide and is an important cause of mortality from intestinal helminth infection in developed countries. The persistence of infection, increasing international travel, lack of familiarity by health care providers, and potential for iatrogenic hyperinfection all make strongyloidiasis an important emerging infection.
METHODS: Two studies were performed. A retrospective chart review of

Strongyloides stercoralis cases identified through microbiology laboratory records from 1993-2002 was conducted. Subsequently, 363 resident physicians in 15 training programs worldwide were queried with a case scenario of strongyloidiasis, presenting an immigrant with wheezing and eosinophilia. The evaluation focused on resident recognition and diagnostic recommendations.
RESULTS: In 151 strongyloidiasis cases, stool ova and parasite sensitivity is poor (51%), and eosinophilia (>5% or >400 cells/microL) commonly present (84%). Diagnosis averaged 56 months (intra-quartile range: 4-72 months) after immigration. Presenting complaints were nonspecific, although 10% presented with wheezing. Hyperinfection occurred in 5 patients prescribed corticosteroids, with 2 deaths. Treatment errors occurred more often among providers unfamiliar with immigrant health (relative risk of error: 8.4; 95% confidence interval, 3.4-21.0; P <.001). When presented with a hypothetical case scenario, US physicians-in-training had poor recognition (9%) of the need for parasite screening and frequently advocated empiric corticosteroids (23%). International trainees had superior recognition at 56% (P <.001). Among US trainees, 41% were unable to choose any parasite causing pulmonary symptoms.
CONCLUSIONS: Strongyloidiasis is present in US patients. Diagnostic consideration should occur with appropriate exposure, nonspecific symptoms including wheezing, or eosinophilia (>5% relative or >400 eosinophils/microL). US residents' helminth knowledge is limited and places immigrants in iatrogenic danger. Information about Strongyloides should be included in US training and continuing medical education programs.

PMCID: PMC1950578
PMID: 17524758 [PubMed - indexed for MEDLINE]

817. Zh Mikrobiol Epidemiol Immunobiol. 2007 Jan-Feb;(1):83-9.

[Methods of assessment and control of house dust mites population and mite allergen exposure].

[Article in Russian]

Zheltikova TM, Petrova-Nikitina AD, Mitereva DE, Antropova AB, Arkhipova NV.

The work is devoted to analysis of various methods of assessment and control of house dust mites population and mite allergens in city dwellings using own results and the literature data. Data about in-house dust mites biology and ecology, mechanisms of their migration and circulation in modern city conditions are presented. The comparison of several methods of mites number assessment and mite allergens exposure (classical acarological analysis, colorimetric method of guanine detection in house dust, immunochemical methods) has been performed and their advantages and disadvantages analyzed. As choice of adequate avoidance measures is one of the key question, various such measures (mechanical, physical and chemical) have been compared.

PMID: 17523439 [PubMed - indexed for MEDLINE]

818. Clin Exp Allergy. 2007 Jun;37(6):856-64.

Maternal allergy influences the proliferation of neonatal T cells expressing CCR4, CXCR5 or CD103.

Haddeland U, Brandtzaeg P, Nakstad B.

Laboratory for Immunohistochemistry and Immunopathology (LIIPAT), Department and Institute of Pathology, University of Oslo, Rikshospitalet-Radiumhospitalet

Medical Centre, Oslo, Norway.

BACKGROUND: Elevated proliferative response to allergen in cord blood mononuclear cells (CBMCs) is related to subsequent allergy development of the neonate and has been suggested as a screening marker for high allergy risk.
OBJECTIVE: To characterize the proliferating cells in CBMCs from a neonatal group influenced by maternal allergy compared with a control group without known allergic heredity.
METHODS: CBMCs were stimulated with bovine beta-lactoglobulin (beta-LG) and proliferation was analysed by radioactive thymidine incorporation and expressed both as the traditional stimulation index (SI) and SI corrected by eliminating non-specific proliferation. After beta-LG combined with endotoxin stimulation, cellular expression of IL-4 and IFN-gamma mRNA was determined by quantitative RT-PCR and adhesion as well as chemokine receptors were analysed by three-colour flow cytometry in proliferating T cells (CD3+ Ki-67+).
RESULTS: The percentage of CCR4+ cells correlated weakly with concurrent IL-4 expression (r(S)=0.5, P<0.05), while CXCR3 correlated strongly with IFN-gamma expression (r(S)=0.83, P<0.001). In the allergy risk group, the percentage of proliferating T cells expressing CCR4 or integrin alphaE (CD103) was significantly reduced compared with the control group, while CXCR5 and the corrected SI were relatively increased (CCR4: P=0.01; integrin alphaE: P=0.03; CXCR5: P=0.04; SI: P=0.04).
CONCLUSION: Our results implied delayed maturation of immune functions involved in cellular migration, cell-cell interaction and immunoregulatory functions in neonates with hereditary allergy risk. The alterations observed in this subject group suggested that the corrected SI as well as proliferation of CCR4+, CXCR5+ or CD103+ T cells in allergen-stimulated CBMCs might serve as early screening markers for allergy risk.

PMID: 17517099 [PubMed - indexed for MEDLINE]

819. Bundesgesundheitsblatt Gesundheitsforschung Gesundheitsschutz. 2007 May-Jun;50(5-6):701-10.

[Allergic diseases. Results from the German Health Interview and Examination Survey for Children and Adolescents (KiGGS)].

[Article in German]

Schlaud M, Atzpodien K, Thierfelder W.

Robert Koch-Institut, Berlin, BRD. SchlaudM@rki.de

In the German Health Interview and Examination Survey for Children and Adolescents (KiGGS) from 2003-2006 information was collected on allergic diseases (asthma, atopic dermatitis, hay fever, allergic contact eczema) from a population-based sample of 17,641 0-to 17-year-olds, and blood samples were studied for specific IgE antibodies to 20 common allergens. The lifetime prevalence (LTP) of at least one atopic disease was 22.9 % (95 % CI: 22.0-23.7 %), the 12-month prevalence (12MP) was 16.1 % (15.4-16.8 %); boys (17.3; 16.3-18.2 %) were more frequently affected than girls (14.9; 14.0-15.8 %). Children with a background of migration were less often currently affected by an atopic disease, as were children from families of low social status. According to parents' accounts, the LTP of allergic contact eczema was 9.9 % (9.4-10.5 %); girls (13.8;12.9-14.8) were more frequently affected than boys (6.2; 5.6-6.9 %). Of the 3- to 17-year-olds, 40.8 % (39.6-42.0 %) were sensitised to at least one of the allergens tested; boys (45.0; 43.5-46.5 %) more frequently so than girls (36.4; 35.0-37.9 %). In the states of the former FRG, the LTP of allergic contact eczema was higher (10.2; 9.6-10.9 %) than those in the former GDR (8.4 % 7.4-9.6

%); otherwise there were no East-West differences. The KiGGS data provide the first nationally representative data on allergic diseases and sensitisation. The differences in prevalence observed correspond to a great extent with previous studies and may support the hygiene hypothesis. The prevalences in East and West Germany now seem to have equalised.

PMID: 17514454 [PubMed - indexed for MEDLINE]

820. Bundesgesundheitsblatt Gesundheitsforschung Gesundheitsschutz. 2007 May-Jun;50(5-6):686-700.

[Prevalence of somatic diseases in German children and adolescents. Results of the German Health Interview and Examination Survey for Children and Adolescents (KiGGS)].

[Article in German]

Kamtsiuris P, Atzpodien K, Ellert U, Schlack R, Schlaud M.

Robert Koch-Institut, Berlin, BRD. KamtsiurisP@rki.de

In the German Health Interview and Examination Survey for Children and Adolescents (KiGGS), which was conducted from 2003 to 2006, data on acute/infectious and chronic diseases were collected from a population-based sample of 17,641 subjects aged 0 to 17 years. The annual prevalence rates among acute diseases vary widely. Children and adolescents are most frequently affected by acute (infectious) respiratory conditions. 88.5 % of the surveyed children and adolescents experienced at least one episode of common cold within the last 12 months. Among the other acute respiratory infections, bronchitis and tonsillitis were the most frequently encountered conditions with 19.9 % and 18.5 %, respectively. The 12-month prevalence of otitis media and pseudocroup was 11 % and 6.6 %, respectively. 1.5 % of the children and adolescents experienced an episode of pneumonia. Apart from respiratory infections, gastrointestinal infections were very frequently stated as reasons for acute illness. Furthermore, 12.8 % of the children and adolescents experienced a herpetic infection, 7.8 % a conjunctivitis and 4.8 % a urinary tract infection. Lifetime prevalence rates of infectious diseases were as follows: pertussis 8.7 %, measles 7.4 %, mumps 4.0 %, rubella 8.5 %, varicella 70.6 %, scarlet fever 23.5 %. The various chronic somatic diseases in children and adolescents had different lifetime prevalence rates. Most frequently, children and adolescents were affected by obstructive bronchitis (13.3 %), neurodermatitis/atopic eczema (13.2 %) and hay fever (10.7 %). Scoliosis and asthma had been diagnosed by a doctor in 5.2 % and 4.7 % of subjects aged 0-17 years, respectively. The lifetime prevalence rates of the remaining diseases varied between 0.14 % for diabetes mellitus and 3.6 % for convulsions/epileptic fits. For the first time ever, these survey results provide nationwide representative information on the prevalence rates of acute/infectious and chronic diseases in children and adolescents which is based on a population-representative sample.

PMID: 17514453 [PubMed - indexed for MEDLINE]

821. Life Sci. 2007 May 30;80(24-25):2352-60. Epub 2007 Apr 29.

The cholinergic system is involved in regulation of the development of the hematopoietic system.

Serobyan N, Jagannathan S, Orlovskaya I, Schraufstatter I, Skok M, Loring J, Khaldoyanidi S.

La Jolla Institute for Molecular Medicine, 4570 Executive Drive, San Diego, CA 92121, USA.

Gene expression profiling demonstrated that components of the cholinergic system, including choline acetyltransferase, acetylcholinesterase and nicotinic acetylcholine receptors (nAChRs), are expressed in embryonic stem cells and differentiating embryoid bodies (EBs). Triggering of nAChRs expressed in EBs by nicotine resulted in activation of MAPK and shifts of spontaneous differentiation toward hemangioblast. In vivo, non-neural nAChRs are detected early during development in fetal sites of hematopoiesis. Similarly, in vivo exposure of the developing embryo to nicotine resulted in higher numbers of hematopoietic progenitors in fetal liver. However postpartum, the number of hematopoietic stem/progenitor cells (HSPC) was decreased, suggesting an impaired colonization of the fetal bone marrow with HSPCs. This correlated with increased number of circulating HSPC and decreased expression of CXCR4 that mediates migration of circulating cells into the bone marrow regulatory niche. In addition, protein microarrays demonstrated that nicotine changed the profile of cytokines produced in the niche. While the levels of IL1alpha, IL1beta, IL2, IL9 and IL10 were not changed, the production of hematopoiesis-supportive cytokines including G-CSF, GM-CSF, IL3, IL6 and IGFBP-3 was decreased. This correlated with the decreased repopulating ability of HSPC in vivo and diminished hematopoietic activity in bone marrow cultures treated with nicotine. Interestingly, nicotine stimulated the production of IL4 and IL5, implying a possible role of the cholinergic system in pathogenesis of allergic diseases. Our data provide evidence that the nicotine-induced imbalance of the cholinergic system during gestation interferes with normal development and provides the basis for negative health outcomes postpartum in active and passive smokers.

PMCID: PMC2873871
PMID: 17512954 [PubMed - indexed for MEDLINE]

822. Am J Respir Cell Mol Biol. 2007 Sep;37(3):322-9. Epub 2007 May 16.

Glutathione redox regulates airway hyperresponsiveness and airway inflammation in mice.

Koike Y, Hisada T, Utsugi M, Ishizuka T, Shimizu Y, Ono A, Murata Y, Hamuro J, Mori M, Dobashi K.

Department of Medicine and Molecular Science, Gunma University Graduate School of Medicine, Maebashi, Gunma, Japan.

Glutathione is the major intracellular redox buffer. We have shown that glutathione redox status, which is the balance between intracellular reduced (GSH) and oxidized (GSSG) glutathione, in antigen-presenting cells (APC) regulates the helper T cell type 1 (Th1)/Th2 balance due to the production of IL-12. Bronchial asthma is a typical Th2 disease. Th2 cells and Th2 cytokines are characteristic of asthma and trigger off an inflammation. Accordingly, we studied the effects of the intracellular glutathione redox status on airway hyperresponsiveness (AHR) and allergen-induced airway inflammation in a mouse model of asthma. We used gamma-Glutamylcysteinylethyl ester (gamma-GCE), which is a membrane-permeating GSH precursor, to elevate the intracellular GSH level and GSH/GSSG ratio of mice. In vitro, gamma-GCE pretreatment of human monocytic THP-1 cells elevated the GSH/GSSG ratio and enhanced IL-12(p70) production induced by LPS. In the mouse asthma model, intraperitoneal injection of gamma-GCE elevated the GSH/GSSG ratio of lung tissue and reduced AHR. gamma-GCE reduced levels of IL-4, IL-5, IL-10, and the chemokines eotaxin and RANTES (regulated on activation, normal T cell expressed and secreted) in bronchoalveolar lavage

fluid, whereas it enhanced the production of IL-12 and IFN-gamma. Histologically, gamma-GCE suppressed eosinophils infiltration. Interestingly, we also found that gamma-GCE directly inhibited chemokine-induced eosinophil chemotaxis without affecting eotaxin receptor chemokine receptor 3 (CCR3) expressions. Taken together, these findings suggest that changing glutathione redox balance, increase in GSH level, and the GSH/GSSG ratio by gamma-GCE, ameliorate bronchial asthma by altering the Th1/Th2 imbalance through IL-12 production from APC and suppressing chemokine production and eosinophil migration itself.

PMID: 17507665 [PubMed - indexed for MEDLINE]

823. Int Arch Allergy Immunol. 2007;144(1):91-4. Epub 2007 May 15.

CX3CR1 polymorphisms are associated with atopy but not asthma in German children.

Depner M, Kormann MS, Klopp N, Illig T, Vogelberg C, Weiland SK, von Mutius E, Combadière C, Kabesch M.

University Children's Hospital, Ludwig Maximilians University Munich, Munich, Germany.

Chemokines and their receptors are involved in many aspects of immunity. Chemokine CX3CL1, acting via its receptor CX3CR1, regulates monocyte migration and macrophage differentiation as well as T cell-dependent inflammation. Two common, nonsynonymous polymorphisms in CX3CR1 have previously been shown to alter the function of the CX3CL1/CX3CR1 pathway and were suggested to modify the risk for asthma. Using matrix-assisted laser desorption/ionization time-of-flight technology, we genotyped polymorphisms Val249Ile and Thr280Met in a cross-sectional population of German children from Munich (n = 1,159) and Dresden (n = 1,940). For 249Ile an odds ratio of 0.77 (95% confidence interval 0.63-0.96; p = 0.017) and for 280Met an odds ratio of 0.71 (95% confidence interval 0.56-0.89; p = 0.004) were found with atopy in Dresden but not in Munich. Neither polymorphism was associated with asthma. Thus, amino acid changes in CX3CR1 may influence the development of atopy but not asthma in German children. Potentially, other factors such as environmental effects may modify the role of CX3CR1 polymorphisms.

PMID: 17505143 [PubMed - indexed for MEDLINE]

824. Int Immunopharmacol. 2007 Jul;7(7):937-44. Epub 2007 Apr 2.

Gab2 antisense oligonucleotide blocks rat basophilic leukemic cell functions.

Chan JH, Liao W, Lau HY, Wong WS.

Department of Pharmacology, Yong Loo Lin School of Medicine, National University of Singapore, Singapore.

Adapter molecule Grb2-associated binder-like protein 2 (Gab2) plays a critical role in FcepsilonRI-induced mast cell degranulation and activation. The present study aimed to investigate the pharmacological effects of an antisense oligonucleotide (ASO) targeted at Gab2 on the immune responses of rat basophilic leukemic (RBL)-2H3 cells. Gab2 ASOs were rationally designed and transfected into RBL-2H3 cells. Gab2 mRNA and protein knockdown was confirmed by real-time RT-PCR and immunoblotting, respectively. Effects of Gab2 ASO on FcepsilonRI-induced release of histamine and beta-hexosaminidase was measured by EIA and an enzymatic assay, respectively; signaling events by immunoblotting; and cytokine mRNA expression by RT-PCR. Effects of Gab2 ASO on cell adhesion and migration were

performed on fibronectin-coated 96-well plate and transwells cell culture
chambers, respectively. We have characterized a phosphorothioate-modified ASO
targeted at Gab2 mRNA that was able to knockdown Gab2 mRNA and protein in RBL-2H3
cells. Gab2 ASO significantly blocked IgE-mediated mast cell release of
beta-hexosaminidase and histamine; phosphorylation of Akt, p38 mitogen-activated
protein kinase and PKCdelta; and up-regulation of cytokine mRNA levels (e.g.
IL-4, -6, -9 and -13, and TNF-alpha). In addition, Gab2 ASO markedly prevented
mast cell adhesion to fibronectin-coated plates and restrained random migration
of RBL-2H3 cells in cell culture chambers. Our findings show that Gab2 knockdown
in RBL-2H3 cells by ASO strategy can suppress many aspects of the mast cell
functions and, therefore, a selective Gab2 ASO may have therapeutic potential for
mast cell-dependent allergic disorders.

PMID: 17499196 [PubMed - indexed for MEDLINE]

825. Am J Physiol Lung Cell Mol Physiol. 2007 May;292(5):L1147-54.

IL-4 stimulates the expression of CXCL-8, E-selectin, VEGF, and inducible nitric
oxide synthase mRNA by equine pulmonary artery endothelial cells.

Huang H, Lavoie-Lamoureux A, Moran K, Lavoie JP.

Department of Clinical Sciences, Faculty of Veterinary Medicine, Université de
Montréal, Saint-Hyacinthe, Québec, Canada.

Little is known concerning the possible contribution of T helper 2 (Th2)-type
cytokines to the recruitment of neutrophils into the lung tissue. In the present
study, endothelial cells from equine pulmonary arteries were cultured in the
presence of recombinant equine (re) IL-4 and reIL-5, and the cytokine mRNA
expression of molecules implicated in the chemotaxis and migration of neutrophils
was studied using real-time RT-PCR. The functional response of reIL-4-induced
endothelial cell stimulation on neutrophil migration was also studied using a
chemotaxis chamber. ReIL-4 either increased the expression of CXCL-8, E-selectin,
vascular endothelial growth factor (VEGF), and inducible nitric oxide synthase
(iNOS), or potentiated the coeffects of lipopolysaccharide (LPS) and tumor
necrosis factor-alpha (TNF-alpha) on CXCL-8. Supernatants collected from cultured
endothelial cells stimulated with reIL-4 significantly promoted neutrophil
migration in a dose-dependent manner. Dexamethasone (DXM) decreased the
expression of CXCL-8, VEGF, and iNOS induced by reIL-4, while 1400W
dihydrochloride (1400W), a selective inhibitor of iNOS, decreased the expression
of E-selectin, VEGF, and iNOS. DXM and 1400W attenuated the mRNA expression of
E-selectin and iNOS induced by the costimulation of reIL-4, reTNF-alpha, and LPS.
Neither equine nor human recombinant IL-5 influenced the mRNA expression of
CXCL-8, E-selectin, or VEGF. These findings suggest that Th2-type cytokines may
contribute to pulmonary neutrophilia during allergic inflammation by the
increased expression of neutrophil chemokines and adhesion molecules by
endothelial cells. DXM and the iNOS inhibitors may decrease pulmonary
neutrophilia due, in part, to a direct inhibition of some of these factors.

PMID: 17494951 [PubMed - indexed for MEDLINE]

826. Chest. 2007 May;131(5):1331-8.

Area of residence, birthplace, and asthma in Puerto Rican children.

Cohen RT, Canino GJ, Bird HR, Shen S, Rosner BA, Celedón JC.

Channing Laboratory, Department of Medicine, Brigham and Women's Hospital, 181

Longwood Avenue, Boston, MA 02115, USA. robyn.cohen@childrens.harvard.edu

RATIONALE: Puerto Ricans have the highest prevalence of asthma among all ethnic groups in the United States. There have been no studies that directly compare the burden of asthma between Puerto Ricans living in Puerto Rico and those living in the mainland United States.
OBJECTIVE: To examine the relation between birthplace, area of residence, and asthma in Puerto Rican children.
METHODS: Multistage population-based probability sample of children in the San Juan and Caguas metropolitan areas in Puerto Rico and in the Bronx, NY. Information was collected in a household survey of 2,491 children and their primary caretakers.
RESULTS: The overall prevalence of asthma among Puerto Rican children in this study was very high (38.6%). Although children from Puerto Rico had higher socioeconomic status and lower rates of premature birth and prenatal smoke exposure, the prevalence of lifetime asthma was higher in Puerto Rican children living in Puerto Rico than in Puerto Rican children living in the South Bronx (41.3% vs 35.3%, p = 0.01). In multivariable analysis, residence in Puerto Rico was associated with increased odds of lifetime asthma (odds ratio [OR], 1.27; 95% confidence interval [CI], 1.03 to 1.57) and lifetime hospitalization for asthma (OR, 1.47; 95% CI, 1.04-2.07).
CONCLUSIONS: Puerto Rican children in Puerto Rico had a higher risk of asthma than Puerto Rican children in the South Bronx, highlighting the need for further examination of the roles of migration, acculturation, and environmental and psychosocial factors on the development of asthma in this high-risk population.

PMID: 17494783 [PubMed - indexed for MEDLINE]

827. Toxicol Appl Pharmacol. 2007 Jun 15;221(3):268-77. Epub 2007 Mar 31.

Immunotoxicity assessment for the novel Spleen tyrosine kinase inhibitor R406.

Zhu Y, Herlaar E, Masuda ES, Burleson GR, Nelson AJ, Grossbard EB, Clemens GR.

Rigel Pharmaceuticals, Inc., 1180 Veterans Blvd., South San Francisco, CA 94080, USA.

Spleen tyrosine kinase (Syk) is a novel pharmaceutical target for treatment of allergic, autoimmune, and neoplastic disorders. Previous studies have indicated that Syk signaling plays critical roles in regulating the lymphohematopoietic system. These observations prompted us to investigate whether inhibition of Syk would promote immunotoxicity. In a series of studies, rats were treated orally with R406, at dose levels up to and including 100 mg/kg/day (or its prodrug R788 at dose levels up to and including 100 mg/kg/day, reduced to 50 mg/kg/day for females as MTD was exceeded), a potent Syk inhibitor, twice daily for 28 days. In addition to standard toxicological assessments, immunophenotyping by flow cytometric analysis, and a study of humoral immune response measuring anti-KLH IgM and IgG levels, were undertaken. Other immunotoxicity studies included three host resistance models in female Balb/c mice to further ascertain effects of R406 on innate and acquired immunity. Following R406 treatment, expected immunomodulating effects (e.g., decreased thymic and spleen weight, hypocellularity of bone marrow, and reduced lymphocyte counts, including T and B cells) were observed in the rat studies. These changes essentially resolved during a 14-day treatment-free recovery period. A KLH challenge in rats demonstrated no adverse effects on IgG or IgM response. R788/406, administered orally at dose levels up to and including 80 mg/kg/day for 28 days, did not affect bacterial or viral clearance in the Listeria, Streptococcal, or Influenza host resistance mouse models, respectively. This correlated with previous in vitro macrophage and neutrophil function assays (assessing migration,

phagocytosis, oxidative burst and microbicidal activity), which revealed that R406 did not adversely affect macrophage or neutrophil function in innate immune responses. Collectively, these results demonstrate that R406 has minimal functional immunotoxicity notwithstanding its lymphocytopenic effect, suggesting that inhibition of Syk might not lead to unacceptable mechanism-based adverse effects.

PMID: 17490694 [PubMed - indexed for MEDLINE]

828. Exp Appl Acarol. 2007;42(1):37-46. Epub 2007 May 4.

Temperature-dependent population growth of three species of stored product mites (Acari: Acaridida).

Aspaly G, Stejskal V, Pekár S, Hubert J.

College of Science Elmarje, Gariunes University, Bengasi, Libya. galasbaly@yahoo.com

The pest potential of stored product mites depends on the reproduction rate that is affected by the environmental conditions. In this study we investigated the effect of temperature, ranging from 5 to 35 degrees C, on the population growth of three important mite species, Acarus siro, Tyrophagus putrescentiae and Auleroglyphus ovatus at 85% r.h. Starting with 10 individuals the population increase of mites was observed after 3 weeks of cultivation, or after 6 weeks for those kept at low temperatures (5, 10, 12.5, and 15 degrees C). The rate of increase was calculated for each temperature and species. The obtained data were fitted with polynomial models. The mite population growth rates increased with increasing moderate temperatures until 25 degrees C, when r (m)-values were 0.179, 0.177 and 0.190 for A. siro, A. ovatus and T. putrescentiae, respectively. The lower development threshold was 10.2 degrees C in all three species. Estimated upper temperature threshold was higher in T. putrescentiae (49 degrees C) than in A. siro and A. ovatus (38 degrees C). Simulation of the rate of population increase under ideal conditions, using real temperature records obtained from Czech grain stores, showed that the pest mite populations increase only during 3.5 months within a typical 9-month storage season in Central Europe. These results indicate that control of mites, be it chemical, physical or biological, is recommended during the months when allergens and pests are produced, i.e. from September to mid November and in May.

PMID: 17479350 [PubMed - indexed for MEDLINE]

829. Environ Health. 2007 May 2;6:13.

Native and foreign born as predictors of pediatric asthma in an Asian immigrant population: a cross sectional survey.

Brugge D, Lee AC, Woodin M, Rioux C.

Department of Public Health and Family Medicine, Tufts University School of Medicine, Boston, MA, USA. dbrugge@aol.com

BACKGROUND: Asthma prevalence is lower in less developed countries and among some recent immigrant populations in the US, but the reasons for this are not clear. One possibility is that early childhood infections are protective against asthma. METHODS: We surveyed Asian immigrant children (n = 204; age 4-18) to assess the relationship between asthma and native or foreign place of birth. We included questions about environmental exposures, demographic variables and family history

of asthma to test whether they might explain effects of place of birth on asthma. RESULTS: The native and foreign born groups were similar in most respects. Analysis of association with diagnosed asthma for all ages together resulted in two logistic regression models. Both retained born in the US (ORs were 3.2 and 4.3; $p < 0.01$) and family history of asthma (ORs were 6.4 and 7.2; $p < 0.001$). One model retained living near heavy motor traffic (OR = 2.6; $p = 0.012$). The other retained language (OR = 3.2; $p = 0.003$). However, for older children (11-18 years of age) being born in the US lost some of its predictive power. CONCLUSION: Our findings are consistent with early childhood infections that are prevalent outside the US protecting against asthma.

PMCID: PMC1876214
PMID: 17474985 [PubMed - indexed for MEDLINE]

830. Br J Pharmacol. 2007 Jul;151(6):725-36. Epub 2007 Apr 30.

Chemokines and their receptors as potential targets for the treatment of asthma.

Palmqvist C, Wardlaw AJ, Bradding P.

Department of Respiratory Medicine, Glenfield Hospital, Leicester, UK.

Asthma is a chronic and sometimes fatal disease, which affects people of all ages throughout the world. Important hallmarks of asthma are airway inflammation and remodelling, with associated bronchial hyperresponsiveness and variable airflow obstruction. These features are orchestrated by cells of both the innate (eosinophils, neutrophils and mast cells) and the adaptive (T(H)2 T cells) immune system, in concert with structural airway cells. Chemokines are important for the recruitment of both immune and structural cells to the lung, and also for their microlocalisation within the lung tissue. Specific blockade of the responses elicited by chemokines and chemokine receptors responsible for the pathological migration of airway cells could therefore be of great therapeutic interest for the treatment of asthma.

PMCID: PMC2014125
PMID: 17471178 [PubMed - indexed for MEDLINE]

831. J Econ Entomol. 2007 Apr;100(2):586-90.

Suppressive potential of bean (Phaseolus vulgaris) flour against five species of stored-product mites (Acari: Acarididae).

Hubert J, Stejskal V, Aspaly G, Münzbergová Z.

Research Institute of Crop Production, Drnovská 507, Praha 6, Ruzyne, CZ-16106, Czech Republic. hubert@vurv.cz

Previous research has demonstrated that legume proteins have insecticidal activity against stored-product pests, but activity against stored-product mites has not been tested. A study was therefore conducted to explore the potential of bean, Phaseolus vulgaris L., flour as novel botanical acaricide against five species of storage and dust mites: Acarus siro L., Aleuroglyphus ovatus (Troupeau), Caloglyphus redickorzevi (Zachvatkin), Lepidoglyphus destructor (Schrank), and Tyrophagus putrescentiae (Schrank). The effect of wheat, Triticum aestivum L., grain enriched with bean flour to eight concentrations (0, 0.01, 0.1, 0.5, 1, 2.5, 5, and 10%) on population growth initiating from the density of 50 mites per 100 g of wheat was recorded for 21 d under laboratory conditions (grain moisture 14.6% moisture content and 25 degree C in darkness). The

enrichment of grain with bean flour suppressed the population growth of all tested species: 0.01% concentration reduced population growth of all tested species to >50% in comparison with the control population. The most sensitive species were A. siro and L. destructor, followed by T. putrescentiae and C. redickorzevi. The least sensitive species was A. ovatus. The terminal (i.e., after 21 d) density of mites positively correlated with bean flour concentration. The suppressive effect of bean flour was not linear but rather asymptotic. The results of this study are discussed in the context of the application of bean flour in integrated control of stored-product mites and the elimination of stored-product mite allergens.

PMID: 17461087 [PubMed - indexed for MEDLINE]

832. Allergol Int. 2007 Jun;56(2):165-9. Epub 2007 May 1.

Effects of respiratory syncytial virus infection on dendritic cells and cysteinyl leukotrienes in lung tissues of a murine model of asthma.

Matsuse H, Hirose H, Tsuchida T, Fukahori S, Fukushima C, Mizuta Y, Kohno S.

Second Department of Internal Medicine, Nagasaki University School of Medicine, Nagasaki, Japan. hmatsuse@nagasaki-u.ac.jp

BACKGROUND: Pulmonary dendritic cells (DCs) play critical roles in both allergy and in viral infection. Levels of cysteinyl leukotrienes (cysLTs) increase after allergen sensitization and viral infection and can modulate the migration and functions of DCs. The present study examines the effects of respiratory syncytial virus (RSV) infection on numbers of DCs and cysLT concentrations in lung tissues of mice sensitized with mite allergen.
METHODS: We examined Control, Dermatophagoides farinae allergen sensitized (Df), RSV infected (RSV) and Df allergen sensitized and RSV infected (Df-RSV) Balb/c mice. We then determined the number of CD11c-positive DCs and the LT concentration in lung tissues of the mice and examined lung pathology and cytokine profiles in thoracic lymph nodes.
RESULTS: Infection with RSV significantly enhanced allergic airway inflammation in Df mice with concomitant increases in Th1 and Th2 immunity. The number of DCs and the cysLT concentrations were significantly increased in the lungs of Df and RSV mice and more so in Df-RSV, than in Df mice.
CONCLUSIONS: The present findings suggest that RSV infection increases the number of DCs and the cysLT concentrations in lung tissues of asthma patients, both of which could result in enhanced allergic airway inflammation.

PMID: 17460444 [PubMed - indexed for MEDLINE]

833. Am J Respir Cell Mol Biol. 2007 Aug;37(2):248-53. Epub 2007 Apr 19.

CD44 regulates macrophage recruitment to the lung in lipopolysaccharide-induced airway disease.

Hollingsworth JW, Li Z, Brass DM, Garantziotis S, Timberlake SH, Kim A, Hossain I, Savani RC, Schwartz DA.

Division of Pulmonary, Allergy, and Critical Care Medicine, DUMC 3136, Duke University Medical Center, Durham, NC 27710, USA. holli017@mc.duke.edu

LPS from bacteria is ubiquitous in the environment and can cause airway disease and modify allergic asthma. Identification of gene products that modulate the biologic response to inhaled LPS will improve our understanding of inflammatory

airways disease. Previous work has identified quantitative trait loci for the response to inhaled LPS on chromosomes 2 and 11. In these regions, 28 genes had altered RNA expression after inhalation of LPS, including CD44, which was associated with differences in both TNF-alpha levels and neutrophil recruitment into the lung. It has previously been shown that CD44 can modulate macrophage recruitment in response to Mycobacterium tuberculosis, as well as clearance of neutrophils after lung injury with both bleomycin and live Escherichia coli bacteria. In this study, we demonstrate that the biologic response to inhaled LPS is modified by CD44. Macrophages failed to be recruited to the lungs of CD44-deficient animals at all time points after LPS exposure. CD44-deficient macrophages showed reduced motility in a Transwell migration assay, reduced ability to secrete the proinflammatory cytokine TNF-alpha, reduced in vivo migration in response to monocyte chemotactic protein-1, and diminished adhesion to vascular endothelia in the presence of TNF-alpha. In addition, CD44-deficient animals had 150% fewer neutrophils at 24 h and 50% greater neutrophils 48 h after LPS exposure. These results support the role of CD44 in modulating the biologic response to inhaled LPS.

PMCID: PMC1976546
PMID: 17446529 [PubMed - indexed for MEDLINE]

834. Blood. 2007 Aug 1;110(3):946-53. Epub 2007 Apr 19.

Mast cells are crucial for early inflammation, migration of Langerhans cells, and CTL responses following topical application of TLR7 ligand in mice.

Heib V, Becker M, Warger T, Rechtsteiner G, Tertilt C, Klein M, Bopp T, Taube C, Schild H, Schmitt E, Stassen M.

Institute for Immunology, University of Mainz, Mainz, Germany.

Until recently, IgE-activated mast cells have been regarded merely as effector cells of adaptive immune responses, involved in allergic reactions and mucosal immunity to parasites. Herein, we report that murine dermal mast cells, activated by local administration of a cream containing the synthetic TLR7 ligand imiquimod, are essential to initiate an early inflammatory reaction. The mast-cell-derived cytokines TNF-alpha and IL-1beta play an important role in this process. Furthermore, TLR7-activated mast cells are also able to promote the emigration of Langerhans cells, which partly depends on the expression of mast-cell-derived IL-1beta. We have previously shown that TLR7 ligation enhances transcutaneous immunization evoked by topical application of vaccine antigens to the skin, a procedure that directly targets skin-resident antigen-presenting cells. Consequently, we now demonstrate here that the capacity to mount a peptide-specific cytotoxic T-lymphocyte response following transcutaneous immunization using imiquimod as adjuvant is severely impaired in mast-cell-deficient mice. Thus, these findings demonstrate the potent versatility of alternatively activated mast cells at the interface of innate and adaptive immunity.

PMID: 17446350 [PubMed - indexed for MEDLINE]

835. Am J Respir Crit Care Med. 2007 Jul 1;176(1):27-35. Epub 2007 Apr 19.

Galectin-9 inhibits CD44-hyaluronan interaction and suppresses a murine model of allergic asthma.

Katoh S, Ishii N, Nobumoto A, Takeshita K, Dai SY, Shinonaga R, Niki T, Nishi N, Tominaga A, Yamauchi A, Hirashima M.

Department of Cell Regulation, Faculty of Medicine, Kagawa University, Kagawa, Japan. kshigeki@med.kagawa-u.ac.jp

RATIONALE: Galectin-9 (Gal-9) belongs to the galectin family, which exhibits affinity for beta-galactosides. Gal-9 has a variety of biological activities; however, its role in allergic inflammation is unknown.
OBJECTIVES: We evaluated the effect of a stable form of the human protein on allergic airway inflammation in a mite allergen-induced asthma model.
METHODS: Human stable Gal-9 was given by intravenous injection to mice during antigen challenge. The effect of Gal-9 on airway inflammation and airway hyperresponsiveness (AHR) was then evaluated.
MEASUREMENTS AND MAIN RESULTS: Gal-9 reduced AHR as well as Th2-associated airway inflammation. Furthermore, administration of Gal-9 as well as anti-CD44 monoclonal antibody inhibited the infiltration of peripheral blood Th2 cells into the airway. Interestingly, Gal-9 directly bound the CD44 adhesion molecule and inhibited interactions with hyaluronan (HA). Consistent with the concept that CD44-HA interactions mediate the migration of T cells into the lung, Gal-9 blocked CD44-dependent adhesion of BW5147 mouse T cells to HA.
CONCLUSIONS: We conclude that Gal-9 inhibits allergic inflammation of the airway and AHR by modulating CD44-dependent leukocyte recognition of the extracellular matrix.

PMID: 17446336 [PubMed - indexed for MEDLINE]

836. Dis Manag. 2007 Apr;10(2):51-60.

The boomers are coming: a total cost of care model of the impact of population aging on the cost of chronic conditions in the United States.

Garrett N, Martini EM.

Health Care Informatics, Blue Cross and Blue Shield of Minnesota, Eagan, Minnesota, USA.

The purpose of this study is to estimate the impact of population aging on medical costs over the next five decades in the United States. Specifically, we focus on the impact of aging on the chronic and/or expensive conditions most often included in disease management programs: coronary artery disease (CAD), congestive heart failure (CHF), diabetes, asthma, pregnancy, psychiatry, and chemical dependency. We apply estimated age-, gender-, and condition-specific annualized costs to the projected US population in each age and gender group for future years, through 2050, to provide an estimate of future healthcare costs. The primary data sources are pooled claims and membership for 2002 and 2003 for HealthPartners, a large midwestern health plan. Secondary sources are US annualized medical costs for 2003 and US Census Bureau demographic projections for the next five decades. Using the Episodes Treatment Group (ETG) grouper from Symmetry, we grouped HealthPartners data into 574 clinically meaningful episodes of care units. We then aggregate selected ETGs into the conditions reported in this study. Using data for all types of health services, we find that aging will have a greater impact on per capita costs for diseases where the ratio of costs for older versus younger ages is greater, such as CHF, CAD, and diabetes. In addition, we project that aging of the US population will actually reduce per capita costs for pregnancy and infertility, chemical dependency, and psychiatric conditions. Aging will have more of an impact on care for specific chronic diseases. These projections can inform health policy and planning as providers of health care, health plans, disease management vendors, and the government anticipate meeting future US healthcare needs.

PMID: 17444790 [PubMed - indexed for MEDLINE]

837. J Immunol. 2007 May 1;178(9):5912-20.

Scavenger Receptors SR-AI/II and MARCO limit pulmonary dendritic cell migration and allergic airway inflammation.

Arredouani MS, Franco F, Imrich A, Fedulov A, Lu X, Perkins D, Soininen R, Tryggvason K, Shapiro SD, Kobzik L.

Department of Environmental Health, Harvard School of Public Health, 665 Huntington Avenue, Boston, MA 02115, USA.

The class A scavenger receptors (SR-A) MARCO and SR-AI/II are expressed on lung macrophages (MPhis) and dendritic cells (DCs) and function in innate defenses against inhaled pathogens and particles. Increased expression of SR-As in the lungs of mice in an OVA-asthma model suggested an additional role in modulating responses to an inhaled allergen. After OVA sensitization and aerosol challenge, SR-AI/II and MARCO-deficient mice exhibited greater eosinophilic airway inflammation and airway hyperresponsiveness compared with wild-type mice. A role for simple SR-A-mediated Ag clearance ("scavenging") by lung MPhis was excluded by the observation of a comparable uptake of fluorescent OVA by wild-type and SR-A-deficient lung MPhis and DCs. In contrast, airway instillation of fluorescent Ag revealed a significantly higher traffic of labeled DCs to thoracic lymph nodes in SR-A-deficient mice than in controls. The increased migration of SR-A-deficient DCs was accompanied by the enhanced proliferation in thoracic lymph nodes of adoptively transferred OVA-specific T cells after airway OVA challenge. The data identify a novel role for SR-As expressed on lung DCs in the down-regulation of specific immune responses to aeroallergens by the reduction of DC migration from the site of Ag uptake to the draining lymph nodes.

PMID: 17442975 [PubMed - indexed for MEDLINE]

838. J Leukoc Biol. 2007 Jul;82(1):57-71. Epub 2007 Apr 18.

Anti-CD44-mediated blockade of leukocyte migration in skin-associated immune diseases.

Zöller M, Gupta P, Marhaba R, Vitacolonna M, Freyschmidt-Paul P.

Department of Tumor Progression and Tumor Defense, German Cancer Research Center, Heidelberg, Germany. m.zoeller@dkfz.de

CD44 plays an important role in leukocyte extravasation, which is fortified in autoimmune diseases and delayed-type hypersensitivity (DTH) reactions. There is additional evidence that distinct CD44 isoforms interfere with the extravasation of selective leukocyte subsets. We wanted to explore this question in alopecia areata (AA), a hair-follicle centric autoimmune disease, and in a chronic eczema. The question became of interest because AA is treated efficiently by topical application of a contact sensitizer, such that a mild DTH reaction is maintained persistently. Aiming to support the therapeutic efficacy of a chronic eczema in AA by anti-CD44 treatment, it became essential to control whether a blockade of migration, preferentially of AA effector cells, could be achieved by CD44 isoform-specific antibodies. Anti-panCD44 and anti-CD44 variant 10 isoform (CD44v10) inhibited in vitro migration of leukocytes from untreated and allergen-treated, control and AA mice. In vivo, both antibodies interfered with T cell and monocyte extravasation into the skin; only anti-panCD44 prevented T cell homing into lymph nodes. Contributing factors are disease-dependent alterations

in chemokine/chemokine receptor expression and a blockade of CD44 on endothelial cells and leukocytes. It is important that CD44 can associate with several integrins and ICAM-1. Associations depend on CD44 activation and vary with CD44 isoforms and leukocyte subpopulations. CD44 standard isoform preferentially associates with CD49d in T cells and CD44v10 with CD11b in monocytes. Accordingly, anti-panCD44 and anti-CD49d inhibit T cell, anti-CD11b, and anti-CD44v10 macrophage migration most efficiently. Thus, allergen treatment of AA likely can be supported by targeting AA T cells selectively via a panCD44-CD49d-bispecific antibody.

PMID: 17442857 [PubMed - indexed for MEDLINE]

839. Eur Arch Otorhinolaryngol. 2007 Aug;264(8):887-93. Epub 2007 Apr 13.

The role of interleukin-16 in eosinophilic chronic rhinosinusitis.

Lackner A, Raggam RB, Stammberger H, Beham A, Braun H, Kleinhappl B, Buzina W, Kittinger C, Reinisch S, Berghold A, Freudenschuss K, Barth S, Marth E.

Department of Otorhinolaryngology, Head and Neck Surgery, Medical University Graz, Auenbruggerplatz 26-28, 8036, Graz, Austria. andreas.lackner@meduni-graz.at

Eosinophilic granulocytes (Eos) are found in great numbers both in the tissue and in the mucus of patients suffering from chronic rhinosinusitis with polyposis (ECRS). Interleukin-16 (IL-16) is known as a highly potent chemotactic and chemoattractant molecule (ED 10-11) for Eos. In an open, explorative, controlled study we examined the presence of IL-16 in mucosa tissue, mucus and serum in patients suffering from ECRS and its association to Eos activation. Tissue and nasal mucus specimen from 10 previously untreated, non allergic ECRS-patients undergoing paranasal sinus surgery and from 10 healthy non sinusitis subjects, undergoing nasal surgery because of anatomic nasal obstruction were investigated by real-time (RT-) PCR targeting human IL-16 mRNA. Haematoxylin-eosin (HE) staining and immunohistochemistry of formalin embedded tissue and mucus were applied for detection and determination of the proportion of activated Eos (aEos) and IL-16. Serum IL-16 was analyzed by enzyme-linked-immunosorbent assay (ELISA). IL-16 mRNA and IL-16 protein levels were elevated in nasal mucus, polyp tissue and in the serum of ECRS patients compared to healthy controls. There was a high proportion of aEos in ECRS patients compared to healthy subjects. Serum IL-16, IL-16 mRNA expression and IL-16 protein in mucus and tissue specimens were significantly associated with the presence of aEos in polyps of ECRS patients. Immunohistochemically IL-16 protein was mainly expressed in aEos, mast cells, lymphocytes and epithelial cells. In conclusion our data indicate that IL-16 may stimulate the migration and persistence of activated Eos in ECRS. IL-16 production in ECRS patients is not mediated by Immunglobuline-E (IgE).

PMID: 17431659 [PubMed - indexed for MEDLINE]

840. Am J Respir Cell Mol Biol. 2007 Aug;37(2):240-7. Epub 2007 Apr 12.

Modulation of human airway smooth muscle migration by lipid mediators and Th-2 cytokines.

Parameswaran K, Radford K, Fanat A, Stephen J, Bonnans C, Levy BD, Janssen LJ, Cox PG.

Firestone Institute for Respiratory Health, St. Joseph's Healthcare, 50 Charlton Avenue East, Hamilton, ON, L8N 4A6 Canada. parames@mcmaster.ca

Cysteinyl leukotrienes and the T helper (Th)-2 cytokines IL-5 and IL-13 directly modulate human airway smooth muscle functions such as contraction and proliferation. We studied the effects of other lipid mediators involved in asthma pathophysiology such as prostaglandin D(2) (PGD(2)), lipoxin, and isoprostanes, and the cytokines, IL-5, IL-4, and IL-13 on human airway smooth muscle cell migration. Chemotaxis and chemokinesis of cultured airway smooth muscle cells from humans without asthma (second to fifth passages, n = 6) were studied using collagen-I-coated polycarbonate membranes in Transwell culture plates. Receptor expression and kinase activation were studied by flow cytometry, polymerase chain reaction, and Western blotting techniques. In contrast to LTE(4)- stimulated (10(-6) M) chemokinesis and LTE(4)-primed migration toward platelet-derived growth factor (PDGF), isoprostane 15-F(2t)-IsoP, and IL-5 were neither chemotactic nor chemokinetic. PGD(2) (10(-10)-10(-6) M) was a chemoattractant and primed migration toward PDGF through the DP(2)/CRTh(2) receptor. Although airway smooth muscle cells did not express the lipoxin A(4) cognate receptor, LTE(4)-primed migration toward PDGF was blocked by lipoxin A(4) (10(-6) M), suggesting that this is mediated through CysLT(1)R antagonism. IL-13 (10 ng/ml), but not IL-4 (0.1-100 ng/ml), augmented migration toward PDGF. This was associated with increased Src-kinase phosphorylation and up-regulation of PDGF-alpha and -beta receptors, and was attenuated by IL-13Ralpha- and IL-4Ralpha-neutralizing antibodies, an Src-kinase antagonist (PP1, 3 muM), a CysLT(1)R antagonist, montelukast (10(-6) M), and by lipoxin A(4) (10(-6) M). PGD(2) and IL-13 promote human airway smooth muscle migration. IL-13 can promote airway smooth muscle migration through Src-kinase and leukotriene-dependent pathways. This may contribute to the accumulation of smooth muscle cells in remodeled airway submucosa.

PMID: 17431098 [PubMed - indexed for MEDLINE]

841. Clin Exp Allergy. 2007 Apr;37(4):599-607.

Effects of Rho-kinase inactivation on eosinophilia and hyper-reactivity in murine airways by allergen challenges.

Taki F, Kume H, Kobayashi T, Ohta H, Aratake H, Shimokata K.

Department of Respiratory Medicine, Nagoya University Graduate School of Medicine, Nagoya, Japan.

BACKGROUND: A small GTPase, Rho, and its target molecule, Rho-kinase, play an important role in the cell functions, including contractility, chemotaxis, adhesion, and migration. It is generally considered that eosinophilic inflammation and hyper-reactivity to methacholine in airways are fundamental to the pathophysiology of bronchial asthma.
OBJECTIVE: This study was designed to determine whether the Rho/Rho-kinase pathways are involved in the eosinophil recruitment and airway hyper-reactivity. We investigated inhibitory effects of fasudil, a specific inhibitor of Rho-kinase, on acute allergic inflammation in mice.
METHODS: BALB/c mice were sensitized and challenged with ovalbumin (OVA). OVA-challenged mice were treated orally with fasudil (3, 10, 30 mg/kg) or saline before each OVA challenge. Total cell counts, differential cell counts, cytokines, and chemokines levels were measured in bronchoalveolar lavage (BAL), and lungs were examined histologically. Moreover, respiratory resistance in response to methacholine was measured.
RESULTS: When fasudil was administrated to OVA-challenged mice, increased cell numbers of total cells and eosinophils were significantly attenuated in a dose-dependent manner. However, inflammatory cells other than eosinophils were not affected by fasudil. Fasudil caused a dose-dependent inhibition in increased levels of IL-5, IL-13, and eotaxin in BAL fluid by OVA challenges. Histological

analysis of the airways revealed that both infiltration of inflammatory cells and goblet cell hyperplasia were significantly suppressed in fasudil treatment. Furthermore, fasudil significantly suppressed the augmented responsiveness to methacholine induced by OVA challenges.
CONCLUSION: Oral administration of fasudil inhibits eosinophil recruitment, goblet cell hyperplasia and airway hyper-reactivity by allergen challenges. These effects of this agent may be mediated by suppressing a chemokine and cytokines related to the pathophysiology of bronchial asthma such as eotaxin, IL-5, and IL-13. Our findings provide evidence that inhibition of the Rho/Rho-kinase pathway may be beneficial for bronchial asthma.

PMID: 17430358 [PubMed - indexed for MEDLINE]

842. Inflamm Res. 2007 Mar;56(3):112-7.

A simple preparation method for mouse eosinophils and their responses to anti-allergic drugs.

Ebihara S, Kurachi H, Watanabe Y.

Japan Tobacco Inc., Central Pharmaceutical Research Institute,1-1 Murasaki-cho, Takatsuki, Osaka 569-1125, Japan. shin.ebihara@ims.jti.co.jp

OBJECTIVE AND DESIGN: A simple method for preparing mouse eosinophils was established, and the characteristics of the eosinophils were assessed including their responses to anti-allergic drugs.
MATERIALS OR SUBJECTS: Mouse eosinophils were prepared from peritoneal exudate cells of BALB/c mice primed and boosted with antigen ovalbumin (OVA).
METHODS: Surface phenotypes, migration activities and leukotriene C(4) (LTC(4)) production abilities of these eosinophils were examined. In addition, the effects of anti-allergic drugs, oxatomide and tranilast, on generation of LTC(4) from mouse eosinophils were examined.
RESULTS: Eosinophils of mice boosted with OVA were phenotypically and functionally identical with human eosinophils. Around 1 x 10(7) eosinophils were obtained from mouse peritoneal exudate. It was found that these mouse eosinophils enabled to migrate in response to eotaxin as well as platelet-activating factor (PAF), and generated LTC(4) by IL-5 stimulation. Moreover, it was revealed that clinically used anti-allergic drugs inhibited LTC(4)-production dose-dependently.
CONCLUSIONS: The present study provides a convenient method to obtain fully functional mouse eosinophils that are useful for drug screening and for evaluating implications of eosinophils in allergic responses.

PMID: 17406808 [PubMed - indexed for MEDLINE]

843. Nat Protoc. 2006;1(6):2933-9.

Assays for in vitro monitoring of human airway smooth muscle (ASM) and human pulmonary arterial vascular smooth muscle (VSM) cell migration.

Goncharova EA, Goncharov DA, Krymskaya VP.

Department of Medicine, Pulmonary, Allergy & Critical Care Division, University of Pennsylvania, Philadelphia, Pennsylvania 19104, USA.

Migration of human pulmonary vascular smooth muscle (VSM) cells contributes to vascular remodeling in pulmonary arterial hypertension and atherosclerosis. Evidence also indicates that, in part, migration of airway smooth muscle (ASM) cells may contribute to airway remodeling associated with asthma. Here we

describe migration of VSM and ASM cells in vitro using Transwell or Boyden chamber assays. Because dissecting signaling mechanisms regulating cell migration requires molecular approaches, our protocol also describes how to assess migration of transfected VSM and ASM cells. Transwell or Boyden chamber assays can be completed in approximately 8 h and include plating of serum-deprived VSM or ASM cell suspension on membrane precoated with collagen, migration of cells toward chemotactic gradient and visual (Transwell) or digital (Boyden chamber) analysis of membrane. Although the Transwell assay is easy, the Boyden chamber assay requires hands-on experience; however, both assays are reliable cell-based approaches providing valuable information on how chemotactic and inflammatory factors modulate VSM and ASM migration.

PMID: 17406553 [PubMed - indexed for MEDLINE]

844. Mol Endocrinol. 2007 Jun;21(6):1324-34. Epub 2007 Apr 3.

Liver X receptor stimulates cholesterol efflux and inhibits expression of proinflammatory mediators in human airway smooth muscle cells.

Delvecchio CJ, Bilan P, Radford K, Stephen J, Trigatti BL, Cox G, Parameswaran K, Capone JP.

Department of Biochemistry and Biomedical Sciences, McMaster University, Hamilton, Ontario, Canada.

Human (h) airway smooth muscle (ASM) cells are important mediators of the inflammatory process observed in asthma and other respiratory diseases. We show here that primary hASM cells express liver X receptor (LXR; alpha and beta subtypes), an oxysterol-activated nuclear receptor that controls expression of genes involved in lipid and cholesterol homeostasis, and inflammation. LXR was functional as determined by transient assays using LXR-responsive reporter genes and by analysis of mRNA and protein expression of endogenous LXR target genes in cells exposed to LXR agonists. LXR activation induced expression of the ATP-binding cassette transporters ABCA1 and ABCG1 and increased efflux of cholesterol to apolipoprotein AI and high-density lipoprotein acceptors, pointing to a role for hASM cells in modulating cholesterol homeostasis in the airway. Under inflammatory conditions, hASM cells release a variety of chemokines and cytokines that contribute to inflammatory airway diseases. Activation of LXR inhibited the expression of multiple cytokines in response to proinflammatory mediators and blocked the release of both granulocyte macrophage colony-stimulating factor and granulocyte colony stimulating factor. LXR activation also inhibited proliferation of hASM cells and migration toward platelet-derived growth factor chemoattractant, two important processes that contribute to airway remodeling. Our findings reveal biological roles for LXR in ASM cells and suggest that modulation of LXR activity offers prospects for new therapeutic approaches in the treatment of asthma and other inflammatory respiratory diseases.

PMID: 17405904 [PubMed - indexed for MEDLINE]

845. Isr Med Assoc J. 2007 Mar;9(3):149-52.

Trends in specific morbidity prevalence in male adolescents in Israel over a 50 year period and the impact of recent immigration.

Farfel A, Green MS, Shochat T, Noyman I, Levy Y, Afek A.

Medical Corps, Israel Defense Forces, Israel.

BACKGROUND: Most Israeli males aged 16-17 undergo a thorough medical examination prior to recruitment into the army. During the last 50 years, extensive data have been gathered enabling a study of time trends in the prevalence of common diseases in this age group.
OBJECTIVES: To examine the current prevalence of common diseases, compare the results with those of previous cohorts, and assess the influence of the massive immigration during the 1990s.
METHODS: The health examination at the recruitment centers includes a medical history, complete physical examination, and review of medical documentation provided by the family physician. If needed, additional tests and referral to specialists are ordered. The prevalence of selected diseases and severity was drawn from the computerized database of the classification board. Two cohorts, 1992-94 and 2003-04, were examined and compared with three previous cohort studies in 1957-61, 1977-78 and 1982-84. Data were stratified according to origin and country of birth.
RESULTS: The prevalence of asthma increased dramatically during the years from 10.2 per 1000 examinees in 1957-61 to 111.6 per 1000 examinees in 2003-04. The prevalence of tuberculosis declined and then increased from 0.6 per 1000 adolescents in 1982-84 to 2.4 per 1000 adolescents in 2003-04. The prevalence of type 1 diabetes mellitus increased from 0.2 cases per 1000 examinees in 1957-61 to 0.8 cases in 1977-78 and 1982-84 and 0.9 cases per 1000 examinees in 2003-04. The prevalence of severe heart defects and severe epilepsy declined in the last 20 years (1.4 and 1.7 cases per 1000 examinees in the 1982-84 cohort to 0.4 and 0.3 cases per 1000 examinees in the 2003-4 cohort respectively). The patterns of disease prevalence were different for immigrants: tuberculosis was more common while asthma and allergic rhinitis were less prevalent.
CONCLUSIONS: The prevalence of common diseases among adolescents in Israel has changed over the last 50 years. There is a different pattern for immigrants and for those born in Israel.

PMID: 17402323 [PubMed - indexed for MEDLINE]

846. FASEB J. 2007 Aug;21(10):2335-42. Epub 2007 Mar 28.

PKC-dependent regulation of the receptor locus dominates functional consequences of cysteinyl leukotriene type 1 receptor activation.

Deshpande DA, Pascual RM, Wang SW, Eckman DM, Riemer EC, Funk CD, Penn RB.

Department of Internal Medicine and Center for Human Genomics, Wake Forest University Health Science, Winston-Salem, NC 27157, USA.

Leukotrienes are important lipid mediators of asthma that contribute to airway inflammation and bronchoconstriction. Critical mechanisms for physiological regulation of the main G protein-coupled receptor (GPCR) mediating the leukotriene responses in asthma, cysteinyl leukotriene type 1 receptor (CysLT1R), have not been delineated. Although desensitization of GPCRs is a well-established phenomenon, studies demonstrating its physiological relevance are lacking. Here, we demonstrate that relief of PKC-mediated desensitization of endogenous CysLT1Rs augments multiple LTD4-stimulated cellular functions, with associated increases in intracellular signaling events. In analyses of airway smooth muscle contraction ex vivo, PKC inhibition augmented LTD4-stimulated contraction, and increased phosphoinositide hydrolysis and calcium flux in both murine and human airway smooth muscle cells. Similarly, for human monocytes, PKC inhibition augmented LTD4-stimulated calcium flux and cell migration assessed in transwell chamber experiments and also augmented LTD4-induced production of monocyte chemotactic protein assessed by ELISA. In contrast, PKC inhibition had no effect or slightly attenuated these cell functions and signaling events promoted by

other receptor agonists, suggesting that despite antithetical effects on downstream events, desensitization of the CysLT1R is the principal mechanism by which PKC regulates the functional consequences of CysLT1R activation.

PMID: 17392478 [PubMed - indexed for MEDLINE]

847. Respir Res. 2007 Mar 29;8:28.

Nitric oxide induces MUC5AC mucin in respiratory epithelial cells through PKC and ERK dependent pathways.

Song JS, Kang CM, Yoo MB, Kim SJ, Yoon HK, Kim YK, Kim KH, Moon HS, Park SH.

Department of Internal Medicine, St Mary's hospital, Catholic University Medical College, Yeoi-Do Dong, Young Dung Po Gu, Seoul, Korea. jssong@catholic.ac.kr

BACKGROUND: Nitric oxide (NO) is generally increased during inflammatory airway diseases. This increased NO stimulates the secretion of mucin from the goblet cell and submucosal glands but the mechanism is still unknown precisely. In this study, we investigated potential signaling pathways involving protein kinase C (PKC) and mitogen-activated protein kinase (MAPK) in the NO-induced MUC5AC mucin gene and protein expression in A549 cells.
METHODS: Nitric oxide was donated to the A549 cells by NOR-1. MUC5AC mucin levels were assayed by enzyme-linked immunosorbent assay (ELISA). MUC5AC promoter activity was determined by measuring luciferase activity after the lysing the transfected cells. Activation of PKC isoforms were measured by assessing the distribution of the enzyme between cytosolic and membrane fractions using immunoblotting. Immunoblotting experiments using a monoclonal antibody specific to PKC isoforms were performed in the cytosol and membrane fractions from A549 cells. Western blot analysis for pERK and p38 were performed using the corresponding antibodies from the cell lysates after donating NO to the A549 cells by NOR-1.
RESULTS: The transcriptional activity of MUC5AC promoter was maximal at the concentration of 0.1 mM NOR-1 for 1 hour incubation in transfected A549 cells. (+/-)-(E)-methyl-2-((E)-hydroxyimino)-5-nitro-6-methoxy-3-hexenamide (NOR-1) markedly displaced the protein kinase C (PKC)alpha and PKCdelta from the cytosol to the membrane. Furthermore, the PKC-alpha,betainhibitors, GO6976 (10 nM) and PKCdelta inhibitors, rottlerin (4 muM) inhibited the NOR-1 induced migration of PKCalpha and PKCdelta respectively. NOR-1 also markedly increased the MUC5AC promoter activity and mRNA expression, mucin synthesis and ERK1/2 phosphorylation. The PKC inhibitors also inhibited the NOR-1 induced MUC5AC mRNA and MUC5AC protein synthesis by inhibiting the activation of PKCalpha and PKCdelta with ERK1/2 pathways.
CONCLUSION: Exogenous NO induced the MUC5AC mucin gene and protein through the PKCalpha and PKCdelta-ERK pathways in A549 cells. Inhibition of PKC attenuated NO-mediated MUC5AC mucin synthesis. In view of this findings, PKC inhibitors might be useful in the treatment of bronchial asthma and chronic bronchitis patients where NO and mucus are increased in the bronchial airways.

PMCID: PMC1847818
PMID: 17391532 [PubMed - indexed for MEDLINE]

848. Allergy Asthma Proc. 2007 Jan-Feb;28(1):35-9.

Mucosal immunization application to allergic disease: sublingual immunotherapy.

Frati F, Moingeon P, Marcucci F, Puccinelli P, Sensi L, Di Cara G, Incorvaia C.

Department of Obstetric, Gynecologic, and Pediatric Sciences, University of Perugia, Italy. frati.f@stallergenes.it

Sublingual immunotherapy (SLIT) is an effective and safe treatment for respiratory allergy, and its mechanism of action currently is investigated with increasing attention. Studies of pharmacokinetics showed that allergen extracts administered via the sublingual route are not directly absorbed by the oral mucosa but are long retained at mucosal level, where the allergen molecules are captured by dendritic cells and, following their migration in the draining lymph nodes, presented to T cells. This seems to be the pivotal factor underlying the mechanisms of action of SLIT, at least for the long-term effects, and for the short-term efficacy, observed with ultrarush or coseasonal treatment, a down-regulation of mast cells resulting in hyporeactivity at the peak of the pollen season may be suggested. Regarding the clinically established long-lasting effects, the core mechanism is likely to consist of T regulatory (Treg) cell activation. In particular, Treg cells differentiate from naive T cells after application of soluble antigens to the mucosae, a crucial factor being the tolerogenic function of dendritic cells, and exert a suppressive effect on both Th1 and Th2 responses. Moreover, at least for the type 1 cells (Treg1), a production of IL-10 with consequent down-modulation of the immune response has been reported. Another characteristic of sublingual immunization is the absence of effectors cells, viz., mast cells, basophils, and eosinophils, in the oral mucosa of allergic subjects, which account for the excellent tolerability of SLIT.

PMID: 17390755 [PubMed - indexed for MEDLINE]

849. J Immigr Minor Health. 2007 Oct;9(4):245-54.

A Comparison of knowledge about asthma between Asians and non-Asians at two pediatric clinics.

Lee AC, Brugge D, Phan L, Woodin M.

School of Arts and Sciences, Tufts University, Medford, MA 02111, USA.

Little is known about the relative knowledge of asthma in recent immigrant Asian populations in the United States (US). To comparatively assess asthma knowledge for Asian and non-Asian populations, 333 parents and children were surveyed at two geographically close urban clinics that had a large percentage of Asian patients, most of whom were Chinese. The Asian respondents scored lower compared to the non-Asian respondents on 4 of the 6 knowledge questions ($p < 0.001$). Subcategories of non-Asians (white, African-American, Hispanic) were more similar to each other than they were to Asians. In multivariate analysis we found that SES (measured as parental occupation) and being Asian were independent predictors of less asthma knowledge. Having family members with asthma did not improve knowledge scores. A single focus group of Cantonese-speaking parents of asthmatic children suggested that a combination of cultural factors and lack of knowledge contribute to lower knowledge scores in this Asian population. Asthma education programs need to be developed, tailored to recent Asian immigrants and tested for efficacy.

PMID: 17387614 [PubMed - indexed for MEDLINE]

850. Biochem Biophys Res Commun. 2007 May 11;356(3):745-9. Epub 2007 Mar 16.

Intrinsic pro-angiogenic status of cystic fibrosis airway epithelial cells.

Verhaeghe C, Tabruyn SP, Oury C, Bours V, Griffioen AW.

Unit of Human Genetics, GIGA-Research, University of Liège, Belgium.

Cystic fibrosis is a common genetic disorder characterized by a severe lung inflammation and fibrosis leading to the patient's death. Enhanced angiogenesis in cystic fibrosis (CF) tissue has been suggested, probably caused by the process of inflammation, as similarly described in asthma and chronic bronchitis. The present study demonstrates an intrinsic pro-angiogenic status of cystic fibrosis airway epithelial cells. Microarray experiments showed that CF airway epithelial cells expressed several angiogenic factors such as VEGF-A, VEGF-C, bFGF, and PLGF at higher levels than control cells. These data were confirmed by real-time quantitative PCR and, at the protein level, by ELISA. Conditioned media of these cystic fibrosis cells were able to induce proliferation, migration and sprouting of cultured primary endothelial cells. This report describes for the first time that cystic fibrosis epithelial cells have an intrinsic angiogenic activity. Since excess of angiogenesis is correlated with more severe pulmonary disease, our results could lead to the development of new therapeutic applications.

PMID: 17382901 [PubMed - indexed for MEDLINE]

851. Exp Hematol. 2007 Apr;35(4):605-17.

Alpha4 and beta2 integrins have nonredundant roles for asthma development, but for optimal allergen sensitization only alpha4 is critical.

Banerjee ER, Jiang Y, Henderson WR Jr, Scott LM, Papayannopoulou T.

Divisions of Allergy and Infectious Diseases, University of Washington, Seattle, WA, USA.

Erratum in
Exp Hematol. 2007 Jul;35(7):1153-61.

OBJECTIVE: Recruitment of effector cell subsets to inflammatory lung, together with airway resident cells responsive to secreted products, play pivotal roles in developing and maintaining asthma. Differential use of adhesion molecules dictates the recruitment patterns of specific cell subsets, yet a clear understanding of the distinctive adhesive molecular pathways guiding them to lung is lacking. To provide further insight into the role of alpha4beta1/VCAM-1 pathway and to compare this to the role of beta2 integrin in the development of acute asthma phenotype, we used genetically deficient mice, in contrast to previous studies with anti-functional antibodies yielding ambiguous results.
METHODS: Allergen-dependent airway inflammation and hyperresponsiveness was induced in conditional alpha4(Delta/Delta), VCAM-1(-/-), and beta2(-/-) mice. Cytology, immunocytochemistry, cytokine and immunoglobulin measurements, and cell type accumulation in lung, BAL fluid, plasma, and hemopoietic tissues were carried out.
RESULTS: Asthma phenotype was totally abrogated in alpha4- or beta2-deficient mice. Adoptive transfer of sensitized alpha4(Delta/Delta) CD4(+) cells into challenged normal mice failed to induce asthma, whereas alpha4(+/+) CD4(+) cells were able to induce asthma in challenged alpha4(Delta/Delta) mice. Parallel studies with beta2(-/-) or VCAM-1(-/-) mice uncovered novel mechanistic insights in primary sensitization and into redundant or unique functional roles of these adhesion pathways in allergic asthma.
CONCLUSIONS: The lack of alpha4 integrin not only impedes the migration of all white cell subsets to lung and airways, but also prevents upregulation of vascular cell adhesion molecule-1 (VCAM-1) in inflamed lung vasculature and, unlike beta2, attenuates optimal sensitization and ovalbumin-specific IgE

production in vivo. As VCAM-1 deficiency did not protect mice from asthma, interactions of alpha4beta1(+) or alpha4beta7(+) cells with other ligands are suggested.

PMID: 17379071 [PubMed - indexed for MEDLINE]

852. Eur J Immunol. 2007 Apr;37(4):1097-106.

Macrophage migration inhibitory factor is essential for allergic asthma but not for Th2 differentiation.

Magalhães ES, Mourao-Sa DS, Vieira-de-Abreu A, Figueiredo RT, Pires AL, Farias-Filho FA, Fonseca BP, Viola JP, Metz C, Martins MA, Castro-Faria-Neto HC, Bozza PT, Bozza MT.

Departamento de Imunologia, Instituto de Microbiologia, UFRJ, Rio de Janeiro, Brazil.

Macrophage migration inhibitory factor (MIF) is increased in asthmatic patients and plays a critical role in the pathogenesis of asthma. We show here that mice lacking MIF failed to develop airway hyper-responsiveness (AHR), tissue eosinophilia, and mucus metaplasia. Analysis of the bronchoalveolar fluids revealed a substantial reduction of IL-13, eotaxin and cysteinyl-leukotrienes. The lack of these cardinal features of asthma in MIF(-/-) mice occurs regardless of high concentrations of IL-4 in the lung and OVA-specific IgE in the serum. Antigen-specific lymphocyte proliferation and IL-13 production were similarly increased in the draining lymph nodes of OVA-immunized and challenged MIF(-/-) mice compared to WT, but were reduced in the spleen of MIF(-/-), thus indicating differential roles of MIF in these compartments. Stimulation of naive CD4(+) cells with anti-CD3 antibody demonstrated that MIF(-/-) cells produced increased amounts of IFN-gamma and IL-4 compared to WT CD4(+) cells. Finally, treatment of sensitized BALB/c mice with neutralizing anti-MIF antibody abrogated the development of ARH and airway inflammation without affecting the production of Th2 cytokines or IgE. The present study demonstrates that MIF is required for allergic inflammation, adding important elements to our knowledge of asthma pathogenesis and suggesting that neutralization of MIF might be of therapeutic value in asthma.

PMID: 17373669 [PubMed - indexed for MEDLINE]

853. J Immunol. 2007 Apr 1;178(7):4615-22.

Oncostatin M secreted by skin infiltrating T lymphocytes is a potent keratinocyte activator involved in skin inflammation.

Boniface K, Diveu C, Morel F, Pedretti N, Froger J, Ravon E, Garcia M, Venereau E, Preisser L, Guignouard E, Guillet G, Dagregorio G, Pène J, Moles JP, Yssel H, Chevalier S, Bernard FX, Gascan H, Lecron JC.

UPRES-EA 3806, Centre Hospitalier de l'Université de Poitiers, Poitiers, France.

Cutaneous inflammatory diseases such as psoriasis vulgaris and atopic dermatitis are associated with altered keratinocyte function, as well as with a particular cytokine production profile of skin-infiltrating T lymphocytes. In this study we show that normal human epidermal keratinocytes express a functional type II oncostatin-M (OSM) receptor (OSMR) consisting of the gp130 and OSMRbeta components, but not the type I OSMR. The type II OSMR is expressed in skin lesions from both psoriatic patients and those with atopic dermatitis. Its

ligand, OSM, induces via the recruitment of the STAT3 and MAP kinase pathways a gene expression profile in primary keratinocytes and in a reconstituted epidermis that is characteristic of proinflammatory and innate immune responses. Moreover, OSM is a potent stimulator of keratinocyte migration in vitro and increases the thickness of a reconstituted epidermis. OSM transcripts are enhanced in both psoriatic and atopic dermatitic skin as compared with healthy skin and mirror the enhanced production of OSM by T cells isolated from diseased lesions. Results from a microarray analysis comparing the gene-modulating effects of OSM with those of 33 different cytokines indicate that OSM is a potent keratinocyte activator similar to TNF-alpha, IL-1, IL-17, and IL-22 and that it acts in synergy with the latter cytokines in the induction of S100A7 and beta-defensin 2 expression, characteristic of psoriatic skin. Taken together, these results demonstrate that OSM and its receptor play an important role in cutaneous inflammatory responses in general and that the specific effects of OSM are associated with distinct inflammatory diseases depending on the cytokine environment.

PMID: 17372020 [PubMed - indexed for MEDLINE]

854. J Ethnopharmacol. 2007 May 30;112(1):90-5. Epub 2007 Feb 13.

The inhibitory effect of Houttuynia cordata extract on stem cell factor-induced HMC-1 cell migration.

Kim IS, Kim JH, Kim JS, Yun CY, Kim DH, Lee JS.

Department of Biomedical Laboratory Science, School of Medicine, Eulji University, 143-5 Yeuongdu-dong, Jung-gu, Daejeon 301-832, Republic of Korea. orientree@eulji.ac.kr

Hottuynia cordata Thunb (Saururaceae; HC) is known as a therapeutic drug that has been used in traditional oriental medicine for the treatment of allergy. Mast cells play an important role in a variety of inflammatory diseases, and specifically asthma and atopy. In the present study, we investigated the effect of HC extracts on the migration of the human mast cell line, HMC-1, in response to stem cell factor (SCF). Treatment with HC extracts at a concentration of 10mug/ml for 24h showed no significant decrease in the survival rate of the HMC-1 cells. SCF showed the typical bell-shape curve for the HMC-1 cell chemoattraction with the peak of the curve at the SCF concentration of 100ng/ml. HC-1, which was the whole plant (Houttuynia cordata) extracted with 80% EtOH, and HC-3, which was the residue successively partitioned with EtOAc, both had inhibitory effects on HMC-1 cell movement. After the treatment with 10mug/ml HC-1 extract for 6 and 24h, the chemotactic index (CI) of HMC-1 cells decreased up to 74 and 63%, respectively. HC-3 extract treatment for 6 and 24h lowered the CI to 72 and 44%, respectively. The HC-1 and HC-3 extracts had no inhibitory effect on the mRNA and surface protein expressions of c-kit, SCF receptor. SCF mediated the chemotaxis signaling via NF-kappaB activation, and both extracts inhibited the activation. Therefore, our results indicate that HC-1 and HC-3 extracts decrease the chemotactic ability of HMC-1 cells in response to SCF by inhibiting the NF-kappaB activation, and these substances may be useful for treating mast cell-induced inflammatory diseases.

PMID: 17368775 [PubMed - indexed for MEDLINE]

855. Allergy. 2007 Apr;62(4):415-22.

Hepatocyte growth factor attenuates eotaxin and PGD2-induced chemotaxis of human eosinophils.

Ito W, Chiba T, Kanehiro A, Kato H, Yamaguchi K, Ueki S, Kayaba H, Chihara J.

Department of Clinical and Laboratory Medicine, Akita University School of Medicine, Akita, Japan.

BACKGROUND: Hepatocyte growth factor (HGF) is known to influence a number of cell types, and regulate various biologic activities including cell migration, proliferation, and survival. In a recent study, we found that, in vivo, HGF suppresses allergic airway inflammation, i.e. the infiltration of inflammatory cells including eosinophils into the airway, and further, that HGF reduces Th2 cytokine levels; however, the directly physiologic role of HGF with eosinophils remains unclear. In this study, we investigate the potential of recombinant HGF to regulate the factor-induced chemotaxis of human eosinophils.
METHODS: Eosinophils were isolated from subjects with mild eosinophilia by modified CD16-negative selection. After culture with or without recombinant HGF, esoinophil chemotaxis was measured by Boyden chamber and KK chamber.
RESULTS: Treatment with HGF prevented eotaxin or prostaglandin D(2) (PGD(2))-induced chemotaxis of eosinophils. Moreover, we demonstrated that extracellular signal-regulated kinase (ERK) 1/2 and p38 mitogen-activated protein kinases as well as the enhancement of Ca(2+) influx, which are indispensable for eosinophil chemotaxis, were attenuated by HGF treatment.
CONCLUSION: Taken together, these data suggest that in allergic diseases, HGF not only mediates eosinophils through the inhibition of Th2 cytokines, but also regulates the function of eosinophils directly, provides further insight into the cellular and molecular pathogenesis of allergic reactions.

PMID: 17362253 [PubMed - indexed for MEDLINE]

856. Arterioscler Thromb Vasc Biol. 2007 May;27(5):1014-22. Epub 2007 Mar 8.

cAMP signaling in leukocyte transendothelial migration.

Lorenowicz MJ, Fernandez-Borja M, Hordijk PL.

Sanquin Research and Landsteiner Laboratory, Academic Medical Centre, University of Amsterdam, Plesmanlaan 125, 1066 CX Amsterdam, The Netherlands.

The migration of leukocytes across the vascular endothelium is crucial for immunosurveillance as well as for inflammatory responses. Uncontrolled leukocyte transendothelial migration results in pathologies such as asthma, rheumatoid arthritis, and atherosclerosis. The molecular mechanisms that regulate leukocyte transendothelial migration involve signaling downstream of intracellular messengers such as cAMP, calcium, phosphoinositol lipids, or reactive oxygen species. Among these, cAMP is particularly intriguing because it is generated in both leukocytes and endothelial cells and regulates leukocyte chemotaxis as well as endothelial barrier function. In addition, physiological stimuli that induce cAMP production generate both pro- and antiinflammatory signals, underscoring the complexity of cAMP-driven signaling. This review discusses our current knowledge of the control of leukocyte transendothelial migration by two main cAMP effectors: protein kinase A and the Rap exchange factor Epac (Exchange protein directly activated by cAMP).

PMID: 17347487 [PubMed - indexed for MEDLINE]

857. Orv Hetil. 2007 Jan 7;148(1):21-6.

[Adulthood atopic dermatitis: epidemiology, clinical symptoms, provoking and

prognostic factors].

[Article in Hungarian]

Pónyai G, Temesvári E, Kárpáti S.

Semmelweis Egyetem, Altalános Orvostudományi Kar Bor- és Nemikórtani és Boronkológiai Klinika Budapest Mária u. 41. 1085. gyorgyi.ponyai@freemail.hu

The prevalence of atopic diseases, including allergic rhinitis, asthma bronchiale and atopic dermatitis is increasing both in children and adults at different parts of the world. Atopic dermatitis is a chronic inflammatory skin disease affecting mostly children, but the atopic trait continues, not only for later respiratory allergies, but also for skin symptoms in adulthood. In this form dry skin, flexural lichenification, head and neck dermatitis, hand dermatitis are typical. The exact etiology of atopic dermatitis is unknown, in the background interactions of genetical predisposition, skin barrier defects and immunological and environmental factors can be verified. In the complex approach of atopic dermatitis, a pivotal role is ascribed to the evaluation and possibly the elimination of provoking factors, like gender, family structure, clothing, aero-, alimentary and contact allergens, psychosocial stress, migration, infections, and personal home environment. Authors review clinical manifestations, triggering and prognostic factors of the adulthood atopic dermatitis.

PMID: 17344114 [PubMed - indexed for MEDLINE]

858. Inflamm Res. 2007 Jan;56(1):24-31.

Blockade of macrophage migration inhibitory factor (MIF) prevents the antigen-induced response in a murine model of allergic airway inflammation.

Amano T, Nishihira J, Miki I.

Pharmaceutical Research Center, Kyowa Hakko Kogyo Co., Ltd., Shizuoka, 411-8731, Japan.

OBJECTIVE AND DESIGN: The role of macrophage migration inhibitory factor (MIF), a proinflammatory cytokine, was tested using a mouse asthma model.
MATERIALS: One hundred and four male BALB/c mice were used in this study.
TREATMENT: Mice were actively sensitized with an intraperitoneal injection of ovalbumin (OVA) and challenged with repeated nebulization of 1 w/v% OVA. Polyclonal anti-MIF antibody was intraperitoneally injected at 10 mg/kg during the antigen challenge period.
METHODS: Bronchoalveolar lavage (BAL) was performed 8 h after the last challenge. Airway hyperresponsiveness to inhaled methacholine was measured 24 h after the last challenge.
RESULTS: Antigen challenge to immunized mice induced increase in inflammatory cells and concentration of Th2 cytokines in BAL fluid (BALF), and caused the development of airway hyperresponsiveness. Anti-MIF antibody significantly decreased the numbers of inflammatory cells including macrophages, eosinophils, lymphocytes and neutrophils in BALF from OVA-challenged mice. Prednisolone decreased the numbers of eosinophils, lymphocytes and neutrophils but not macrophages. Anti-MIF antibody reduced airway hyperresponsiveness. Anti-MIF antibody affected neither the cytokine levels in BALF nor the IgE levels in serum.
CONCLUSION: MIF was involved in the antigen-induced inflammatory cell accumulation in the lung and airway hyperresponsiveness without affecting immune responses.

PMID: 17334667 [PubMed - indexed for MEDLINE]

859. Toxicol Appl Pharmacol. 2007 Apr 15;220(2):138-45. Epub 2007 Jan 12.

The regulatory effect of SC-236
(4-[5-(4-chlorophenyl)-3-(trifluoromethyl)-1-pyrazol-1-l]benzenesulfonamide) on stem cell factor induced migration of mast cells.

Kim SJ, Jeong HJ, Park RK, Lee KM, Kim HM, Um JY, Hong SH.

VestibuloCochlear Research Center of Wonkwang University, Iksan, Jeonbuk 570-749, Republic of Korea.

SC-236,
(4-[5-(4-chlorophenyl)-3-(trifluoromethyl)-1-pyrazol-1-]benzenesulfonamide; C(16)H(11)ClF(3)N(3)O(2)S), is a highly selective cyclooxygenase (COX)-2 inhibitor. Recently, there have been reports that SC-236 protects against cartilage damage in addition to reducing inflammation and pain in osteoarthritis. However, the mechanism involved in the inflammatory allergic reaction has not been examined. Mast cells accumulation can be related to inflammatory conditions, including allergic rhinitis, asthma, and rheumatoid arthritis. The aim of the present study is to investigate the effects of SC-236 on stem cell factor (SCF)-induced migration, morphological alteration, and cytokine production of rat peritoneal mast cells (RPMCs). We observed that SCF significantly induced the migration and morphological alteration. The ability of SCF to enhance migration and morphological alteration was abolished by treatment with SC-236. In addition, production of tumor necrosis factor (TNF)-alpha, interleukin (IL)-1beta, and vascular endothelial growth factor (VEGF) production induced by SCF was significantly inhibited by treatment with SC-236. Previous work has demonstrated that SCF-induced migration and cytokine production of mast cells require p38 MAPK activation. We also showed that SC-236 suppresses the SCF-induced p38 MAPK activation in RPMCs. These data suggest that SC-236 inhibits migration and cytokine production through suppression of p38 MAPK activation. These results provided new insight into the pharmacological actions of SC-236 and its potential therapeutic role in the treatment of inflammatory allergic diseases.

PMID: 17320132 [PubMed - indexed for MEDLINE]

860. Allergy. 2007 Mar;62(3):293-300.

Prevalence of respiratory symptoms in migrant children to Italy: the results of SIDRIA-2 study.

Migliore E, Pearce N, Bugiani M, Galletti G, Biggeri A, Bisanti L, Caranci N, Dell'Orco V, De Sario M, Sestini P, Piffer S, Viegi G, Forastiere F, Galassi C, Ciccone G; SIDRIA-2 Collaborative Group.

Cancer Epidemiology Unit, San Giovanni Battista Hospital - Center for Cancer Prevention Piemonte (CPO), Turin, Italy.

Comment in
    Allergy. 2007 Mar;62(3):213-5.

BACKGROUND: Epidemiological studies have documented large international variations in the prevalence of asthma, and 'westernization' seems to play an important role in the development of the disease. The aims of this study were to compare the prevalence of respiratory symptoms in migrant and nonmigrant children resident in Italy, and to examine the effect of length of time living in Italy.

METHODS: Data were collected in a large cross-sectional study (SIDRIA-2) performed in 12 Italian centres, using standardized parental questionnaires. For the 29 305 subjects included in the analysis (6-7 and 13-14 years old), information about place of birth and parental nationality was available.
RESULTS: There were 1012 children (3%) born outside of Italy, mainly in East Europe. Lifetime asthma and current wheeze were generally significantly less common among children born abroad than among children born in Italy (lifetime asthma: 5.4% and 9.7% respectively, P < 0.001; current wheeze: 5.2% and 6.9%, respectively, P = 0.04). Lower risks for lifetime asthma (prevalence odds ratio, POR = 0.39; 95% CI: 0.23-0.66) and current wheeze (POR = 0.72; 95% CI: 0.47-1.10) were found for children who had lived in Italy <5 years, while migrant children who had lived in Italy for 5 years or more had risks very similar to Italian children.
CONCLUSIONS: Migrant children have a lower prevalence of asthma symptoms than children born in Italy. Prevalence increased with the number of years of living in Italy, suggesting that exposure to environmental factors may play an important role in the development of asthma in childhood.

PMID: 17298347 [PubMed - indexed for MEDLINE]

861. Allergy. 2007 Mar;62(3):272-80.

The protective effect of rural living against atopy in Mongolia.

Viinanen A, Munhbayarlah S, Zevgee T, Narantsetseg L, Naidansuren Ts, Koskenvuo M, Helenius H, Terho EO.

Department of Pulmonary Diseases and Clinical Allergology, University of Turku, Turku, Finland.

BACKGROUND: Farm environment in childhood protects against atopy. We investigated in a population-based study in Mongolia the effects of rural living and migration from rural to urban areas on the risk of atopy.
METHODS: The screening study data of 9453 subjects, aged 10-60 years, were used for taking the sample for the clinical study in which 869 subjects were examined. Asthma, allergic rhinoconjunctivitis and sensitization were clinically defined and their risk factors analysed by logistic regression.
RESULTS: The risks of allergic rhinoconjunctivitis [adjusted odds ratio (OR) 0.43, 95% confidence interval (CI) 0.19-0.98] and allergic sensitization (OR 0.26, 95% CI 0.13-0.55) were the lowest in subjects living in a village from birth and intermediate in subjects who had relocated from a village to a town (OR for rhinoconjunctivitis 0.68, 95% CI 0.36-1.27, OR for sensitization 0.62, 95% CI 0.35-1.12) compared with subjects living in a town from birth. Simultaneous exposure to herd animals and dung heating decreased the risk of atopy. Keeping animals was a risk-factor for asthma only in Ulaanbaatar city.
CONCLUSIONS: Continuing farm exposure after childhood may be important in reducing the risk of atopy.

PMID: 17298344 [PubMed - indexed for MEDLINE]

862. Allergy. 2007 Mar;62(3):237-46.

Allergic disorders in African countries: linking immunology to accurate phenotype.

van Ree R, Yazdanbakhsh M.

Department of Experimental Immunology, Academic Medical Center, Amsterdam, the

Netherlands.

Identification and characterization of risk and protective factors for allergy is important for developing strategies for prevention or treatment. The prevalence of allergy is clearly higher in affluent countries than in developing countries like, e.g. Africa. Especially in urban areas of developing countries, allergy is however on the increase. In Africa, we have the unique opportunity to investigate risk and protective factors and the influence of urbanization and westernization, i.e. almost to take a look at Europe, Australia or the USA as they were before their allergy epidemics. Moreover, migrants from developing to affluent countries experiencing an increased burden of allergy provide new insights into risk and protective factors. Allergen exposure, diet and infections are the major exogenous influences playing a role as risk and protective factors. Depending on the nature, timing, chronicity and level of exposure, each of them can promote or inhibit allergy. Perhaps with the exception of infections, availability of data from Africa on their role in the development of allergy is limited. Detailed epidemiological studies in rural and urban Africa combined with basic immunological research are needed to unravel mechanisms of increase in allergy and of protection. The maturation of the immune system at young age under influence of exogenous factors results in differences in T-cell-skewing (Th1/Th2/Treg) and humoral responses. It is essential to perform studies from a 'non-Eurocentric' angle (e.g. local allergens, locally validated questionnaires and diagnostic procedures). Such studies will provide the affluent countries with new leads to combat the allergy epidemic and more importantly help prevent it in Africa.

PMID: 17298340 [PubMed - indexed for MEDLINE]

863. Sheng Li Xue Bao. 2007 Feb 25;59(1):94-102.

[Role of extracellular signal-regulated kinase 1/2 signaling pathway in migration of bronchial smooth muscle cells of chronic asthmatic rats].

[Article in Chinese]

Xie M, Liu XS, Xu YJ, Zhang ZX, Bai J, Ni W, Chen SX.

Department of Respiratory Medicine, Tongji Medical College, Huazhong University of Science and Technology, Wuhan 430030, China.

This work was designed to explore the role of extracellular signal-regulated kinase 1/2 (ERK1/2) signaling pathway in migration of bronchial smooth muscle cells (BSMCs) of chronic asthmatic rats. To make chronic asthma model, Wistar rats underwent ovabumin (OVA) injection and eight-week inhalation. BSMCs were cultured in vitro. The expression of ERK1/2 in BSMCs was analyzed by immunocytochemistry, Western blot and RT-PCR. Migration of BSMCs was detected by both plate test and Boyden cell test. Results showed: (1) With Western blot technique, the ratio of p-ERK1/2 to total ERK1/2 in chronic asthmatic group was obviously higher than that in the control group (0.55 +/- 0.05 vs 0.48 +/- 0.04, n=10, P<0.01). (2) With RT-PCR, the relative A values of ERK1 and ERK2 mRNA in airways of chronic asthmatic rats were 1.83 +/- 0.24 and 1.07 +/- 0.11, respectively, which were significantly increased compared with that in the control group (0.58 +/- 0.14 and 0.51 +/- 0.12, n=10, P<0.01). (3) In plate test, the migration of BSMCs of chronic asthmatic rats was 2.9 times of that in the control group and reached 5.0 times by epidermal growth factor (EGF) stimulation, but decreased to 1.7 times by 30 mumol/L PD98059. (4) In Boyden cell test, the migration of BSMCs of chronic asthmatic rats was 1.9 times of that in the control group, and reached 3.1 times by EGF stimulation, but decreased to 1.45 times by 30 mumol/L PD98059. Our results indicate that the migration ability of BSMCs of

chronic asthmatic rats increases, and ERK1/2 signaling pathway may play an important role in this process.

PMID: 17294048 [PubMed - indexed for MEDLINE]

864. Am J Physiol Lung Cell Mol Physiol. 2007 May;292(5):L1111-25. Epub 2007 Feb 9.

Nonhematopoietic NADPH oxidase regulation of lung eosinophilia and airway hyperresponsiveness in experimentally induced asthma.

Abdala-Valencia H, Earwood J, Bansal S, Jansen M, Babcock G, Garvy B, Wills-Karp M, Cook-Mills JM.

Allergy-Immunology Division, Northwestern University Feinberg School of Medicine, Chicago, Illinois 60611, USA.

Pulmonary eosinophilia is one of the most consistent hallmarks of asthma. Infiltration of eosinophils into the lung in experimental asthma is dependent on the adhesion molecule vascular cell adhesion molecule-1 (VCAM-1) on endothelial cells. Ligation of VCAM-1 activates endothelial cell NADPH oxidase, which is required for VCAM-1-dependent leukocyte migration in vitro. To examine whether endothelial-derived NADPH oxidase modulates eosinophil recruitment in vivo, mice deficient in NADPH oxidase (CYBB mice) were irradiated and received wild-type hematopoietic cells to generate chimeric CYBB mice. In response to ovalbumin (OVA) challenge, the chimeric CYBB mice had increased numbers of eosinophils bound to the endothelium as well as reduced eosinophilia in the lung tissue and bronchoalveolar lavage. This occurred independent of changes in VCAM-1 expression, cytokine/chemokine levels (IL-5, IL-10, IL-13, IFNgamma, or eotaxin), or numbers of T cells, neutrophils, or mononuclear cells in the lavage fluids or lung tissue of OVA-challenged mice. Importantly, the OVA-challenged chimeric CYBB mice had reduced airway hyperresponsiveness (AHR). The AHR in OVA-challenged chimeric CYBB mice was restored by bypassing the endothelium with intratracheal administration of eosinophils. These data suggest that VCAM-1 induction of NADPH oxidase in the endothelium is necessary for the eosinophil recruitment during allergic inflammation. Moreover, these studies provide a basis for targeting VCAM-1-dependent signaling pathways in asthma therapies.

PMCID: PMC2710034
PMID: 17293377 [PubMed - indexed for MEDLINE]

865. Immunol Lett. 2007 Mar 15;109(1):56-63. Epub 2007 Jan 30.

Normal neutrophil functions in sphingosine kinase type 1 and 2 knockout mice.

Zemann B, Urtz N, Reuschel R, Mechtcheriakova D, Bornancin F, Badegruber R, Baumruker T, Billich A.

Novartis Institutes for BioMedical Research, Vienna, Brunnerstrasse 59, A-1235 Wien, Austria.

Sphingosine kinase (SPHK) has been implicated as an important element in neutrophil responses to diverse stimulatory agents. To get more insight into the role of the type 1 and 2 isoforms of SPHK in neutrophil functions, we made use of the respective SPHK knockout mice. Neutrophils isolated from the bone marrow of these mice showed normal increase of intracellular Ca(2+) when stimulated in vitro by fMLP, platelet-activating factor, the anaphylatoxin C5a, or ATP, and normal migration towards fMLP and C5a. Also, recruitment of neutrophils into the peritoneum towards the chemokines KC and MIP-2 or to LPS, and into the peripheral

blood after fMLP injection was similar in SPHK knockout strains and wild-type animals. An in vivo model of bacterial lung infection revealed an accelerated progression of disease in SPHK2 (but not SPHK1) knockout mice as compared to wild-type controls. However, effector functions of SPHK-deficient neutrophils, such as superoxide production, beta-glucuronidase release and their capacity to kill bacteria were unchanged as compared to wild-type cells. To conclude, the data derived from SPHK knockout mice do not support the hypothesis that any of the two lipid kinases plays a crucial role in signalling downstream of various neutrophil stimuli; SPHKs appear not to be essential for neutrophil recruitment and effector functions.

PMID: 17292973 [PubMed - indexed for MEDLINE]

866. J Leukoc Biol. 2007 May;81(5):1245-51. Epub 2007 Feb 8.

Modulation of eosinophil activation in vitro by a nicotinic receptor agonist.

Blanchet MR, Langlois A, Israël-Assayag E, Beaulieu MJ, Ferland C, Laviolette M, Cormier Y.

Centre de Recherche, Hôpital Laval, Institut Universitaire de Cardiologie et de Pneumologie de l'Université Laval, 2725 Chemin Ste-Foy, Ste-Foy, Québec, Canada, G1V 4G5.

Nicotinic receptor agonists decreased the infiltration of eosinophils into the lung and airways in a mouse model of asthma. To better understand the mechanisms implicated in this anti-inflammatory phenomenon, the expression of nicotinic acetylcholine receptors (nAChRs) and the effect of dimethylphenylpiperazinium (DMPP), a nonselective nAChR agonist, on human blood eosinophils were studied. The expression of alpha-3, -4, and -7 nAChR subunits on human blood eosinophils was measured by cell ELISA and immunocytochemistry. mRNA expression for all three subunits was evaluated by quantitative RT-PCR. The effect of DMPP on leukotriene C4 (LTC4) and matrix metalloproteinase-9 (MMP-9) production, eosinophil migration, and intracellular calcium mobilization was measured. The results show that the alpha-3, -4, and -7 nAChR subunits and mRNAs are expressed by blood eosinophils. In vitro treatment of these cells with various concentrations of DMPP reduced platelet-activating factor (PAF)-induced LTC4 production significantly. DMPP (160 microM) decreased eotaxin, and 5-oxo-6,8,11,14-eicosatetranoic acid induced eosinophil migration through Matrigel by 40.9% and 55.5%, respectively. This effect was reversed by the nAChR antagonist mecamylamine. In addition, DMPP reduced MMP-9 release and the inositol 1,4,5-triphosphate-dependent intracellular calcium increase provoked by PAF. Taken together, these results indicate that functional nAChRs are expressed on eosinophils and that nAChR agonists down-regulate eosinophil function in vitro. These anti-inflammatory effects could be of interest in the treatment of allergic asthma.

PMID: 17289799 [PubMed - indexed for MEDLINE]

867. J Immunol Methods. 2007 Mar 30;320(1-2):155-63. Epub 2007 Jan 22.

Quantitative analysis of eosinophil chemotaxis tracked using a novel optical device -- TAXIScan.

Nitta N, Tsuchiya T, Yamauchi A, Tamatani T, Kanegasaki S.

Central Lab, Effector Cell Institute, Inc., 4-7-7, Aobadai, Meguro-ku, Tokyo 153-0042, Japan. nitta@effectorcell.co.jp

We have reported previously the development of an optically accessible, horizontal chemotaxis apparatus, in which migration of cells in the channel from a start line can be traced with time-lapse intervals using a CCD camera (JIM 282, 1-11, 2003). To obtain statistical data of migrating cells, we have developed quantitative methods to calculate various parameters in the process of chemotaxis, employing human eosinophil and CXCL12 as a model cell and a model chemoattractant, respectively. Median values of velocity and directionality of each cell within an experimental period could be calculated from the migratory pathway data obtained from time-lapse images and the data were expressed as Velocity-Directionality (VD) plot. This plot is useful for quantitatively analyzing multiple migrating cells exposed to a certain chemoattractant, and can distinguish chemotaxis from random migration. Moreover precise observation of cell migration revealed that each cell had a different lag period before starting chemotaxis, indicating variation in cell sensitivity to the chemoattractant. Thus lag time of each cell before migration, and time course of increment of the migrating cell ratio at the early stages could be calculated. We also graphed decrement of still moving cell ratio at the later stages by calculating the duration time of cell migration of each cell. These graphs could distinguish different motion patterns of chemotaxis of eosinophils, in response to a range of chemoattractants; PGD(2), fMLP, CCL3, CCL5 and CXCL12. Finally, we compared parameters of eosinophils from normal volunteers, allergy patients and asthma patients and found significant difference in response to PGD(2). The quantitative methods described here could be applicable to image data obtained with any combination of cells and chemoattractants and useful not only for basic studies of chemotaxis but also for diagnosis and for drug screening.

PMID: 17289072 [PubMed - indexed for MEDLINE]

868. Am J Physiol Lung Cell Mol Physiol. 2007 Jun;292(6):L1414-21. Epub 2007 Feb 2.

Raf-1 kinase mediates adenylyl cyclase sensitization by TNF-alpha in human airway smooth muscle cells.

Osawa Y, Yim PD, Xu D, Panettieri RA, Emala CW.

Department of Anesthesiology, College of Physicians and Surgeons of Columbia University, New York, New York 10032, USA.

Tumor necrosis factor (TNF)-alpha is a potent inflammatory cytokine implicated in the exacerbation of asthma. Chronic exposure to TNF-alpha has been reported to induce G protein-coupled receptor desensitization, but adenylyl cyclase sensitization, in airway smooth muscle cells by an unknown mechanism. Cyclic AMP, which is synthesized by adenylyl cyclases in response to G protein-coupled receptor signals, is an important second messenger involved in the regulation of the airway muscle proliferation, migration, and tone. In other cell types, TNF-alpha receptors transactivate the EGF receptor, which activates raf-1 kinase. Further studies in transfected cells show that raf-1 kinase can phosphorylate and activate some isoforms of adenylyl cyclase. Cultured human airway smooth muscle cells were treated with TNF-alpha in the presence or absence of inhibitors of prostaglandin signaling, protein kinases, or G(i) proteins. TNF-alpha caused a significant dose- (1-10 ng/ml) and time-dependent (24 and 48 h) increase in forskolin-stimulated adenylyl cyclase activity, which was abrogated by pretreatment with GW5074 (a raf-1 kinase inhibitor), was partially inhibited by an EGF receptor inhibitor, but was unaffected by pertussis toxin. TNF-alpha also increased phosphorylation of Ser(338) on raf-1 kinase, indicative of activation. IL-1beta and EGF sensitization of adenylyl cyclase activity was also sensitive to raf-1 kinase inhibition by GW5074. Taken together, these studies link two signaling pathways not previously characterized in human airway smooth muscle

cells: TNF-alpha transactivation of the EGF receptor, with subsequent raf-1 kinase-mediated activation of adenylyl cyclase.

PMID: 17277048 [PubMed - indexed for MEDLINE]

869. J Clin Invest. 2007 Feb;117(2):464-72.

Inhaled iloprost suppresses the cardinal features of asthma via inhibition of airway dendritic cell function.

Idzko M, Hammad H, van Nimwegen M, Kool M, Vos N, Hoogsteden HC, Lambrecht BN.

Department of Pulmonary Medicine, Erasmus University Medical Center, Rotterdam, The Netherlands.

Inhalation of iloprost, a stable prostacyclin (PGI(2)) analog, is a well-accepted and safe treatment for pulmonary arterial hypertension. Although iloprost mainly acts as a vasodilator by binding to the I prostanoid (IP) receptor, recent evidence suggests that signaling via this receptor also has antiinflammatory effects through unclear mechanisms. Here we show in a murine model of asthma that iloprost inhalation suppressed the cardinal features of asthma when given during the priming or challenge phase. As a mechanism of action, iloprost interfered with the function of lung myeloid DCs, critical antigen-presenting cells of the airways. Iloprost treatment inhibited the maturation and migration of lung DCs to the mediastinal LNs, thereby abolishing the induction of an allergen-specific Th2 response in these nodes. The effect of iloprost was DC autonomous, as iloprost-treated DCs no longer induced Th2 differentiation from naive T cells or boosted effector cytokine production in primed Th2 cells. These data should pave the way for a clinical effectiveness study using inhaled iloprost for the treatment of asthma.

PMCID: PMC1783814
PMID: 17273558 [PubMed - indexed for MEDLINE]

870. Nan Fang Yi Ke Da Xue Xue Bao. 2006 Dec;26(12):1698-700.

[Role of Slingshot-1L in peripheral eosinophils of asthmatic patients].

[Article in Chinese]

Zhang WZ, Cai SX, Zou F, Zhao HJ, Li WJ.

Department of Respiratory Diseases, Nanfang Hospital, Southern Medical University, Guangzhou 510515, China. zhangweizhen157@126.com

OBJECTIVE: To investigate the expression of Slingshot-1L (SSH-1L) in peripheral blood eosinophils of asthmatic patients.
METHODS: Peripheral vein blood sample of 30 ml was collected from 15 healthy volunteers, 15 asthmatic patients with glucocorticoid treatment and 15 asthmatic patients without the treatment. The eosinophils were isolated, purified and counted for each sample, and SSH-1L/beta-actin gene fragments were amplified simultaneously by RT-PCR for the total RNA. SSH-1L protein was detected by Western blotting from the total protein of the peripheral eosinophils. The expressions of SSH-1L at both mRNA and protein levels are compared between different groups.
RESULTS: SSH-1L/beta-actin ratio significantly increased in untreated patients with asthma attacks in comparison with healthy volunteers ($P<0.05$), which did not occur in patients treated with glucocorticoids ($P>0.05$). The optical density of

SSH-1L protein significantly increased in untreated asthmatic patients ($P<0.05$), but not in patients treated with glucocorticoids ($P>0.05$), as compared with the healthy volunteers.
CONCLUSION: Significantly increased SSH-1L expression in peripheral eosinophils may play an important role in the activation and migration of eosinophils.

PMID: 17259100 [PubMed - indexed for MEDLINE]

871. Eur J Pharmacol. 2007 Mar 22;559(2-3):202-9. Epub 2006 Dec 12.

Inhibition of eosinophilia in vivo by a small molecule inhibitor of very late antigen (VLA)-4.

Okigami H, Takeshita K, Tajimi M, Komura H, Albers M, Lehmann TE, Rölle T, Bacon KB.

Bayer Yakuhin, Ltd., Research Center Kyoto, Respiratory Disease Research, Japan. dah.okigami@cc.nara-wu.ac.jp

The alpha4beta1 integrin (very late antigen-4, VLA-4) plays an important role in the migration of lymphocytes, monocytes, and eosinophils, but not neutrophils, to sites of inflammation. Pharmacological antagonism of VLA-4 is an attractive prospect for the treatment of predominantly eosinophil mediated diseases such as asthma and allergic rhinitis. We report here on a potent and selective, small molecule VLA-4 inhibitor, (2S)-3-(2',
5'-dichlorobiphenyl-4-yl)-2-({[1-(2-methoxybenzoyl)piperidin-3-yl]carbonyl}amino)propanoic acid, compound 1, and characterize the antagonist activities of this molecule in various cell-based assays and in an animal model of eosinophil migration. Compound 1 inhibited VLA-4/ vascular cell adhesion molecule-1(VCAM-1) interactions with in vitro potencies (IC50 value of 210 nM) in VLA-4-expressing Ramos cells, although the compound did not inhibit cell adhesion to fibronectin via alpha5beta1 integrin (very late antigen-5, VLA-5). Blockade of phorbol-12-myristate-13-acetate (PMA)- or Mn2+-stimulated VLA-4 interactions with compound 1 was observed in human T lymphocytes (IC50 value of 230 nM), human eosinophils (IC50 value of 4.0 microM) and mouse eosinophils (IC50 value of 1.6 microM). Furthermore, compound 1 administered by intraperitoneal injection inhibited eosinophil infiltration in a dose-dependent manner by up to 80% in an air pouch model. These data support the use of small molecule VLA-4 antagonists in the treatment of relevant diseases, such as asthma, atopic dermatitis, or allergic rhinitis.

PMID: 17234179 [PubMed - indexed for MEDLINE]

872. Ann Allergy Asthma Immunol. 2007 Jan;98(1):83-8.

A role for endothelial selectins in allergic and nonallergic inflammatory disease.

Jiao A, Fish SC, Mason LE, Schelling SH, Goldman SJ, Williams CM.

Department of Respiratory Diseases, Wyeth Research, Cambridge, Massachusetts 02140, USA.

BACKGROUND: Several studies indicate that selectin-mediated leukocyte migration may depend on the types of initiating inflammatory stimuli or on the vascular beds involved in the inflammatory response. Thus, targeting selectin interactions to treat inflammation may have variable effects depending on the site and origin of the inflammatory response.

OBJECTIVE: To address whether selectin-mediated leukocyte recruitment is stimulus or tissue dependent.
METHODS: We examined pulmonary and cutaneous allergic inflammatory responses and silica-induced nonallergic lung inflammation and fibrosis in wild-type and P- and E-selectin-deficient (P/E-/-) double knockout mice. Allergen-sensitized wild-type and P/E-/- double knockout mice were challenged either intradermally or via the airways to induce allergic responses in the skin or lung, respectively. Other animals were subjected to intranasal silica administration to induce a nonallergic lung inflammatory/fibrotic response.
RESULTS: The P/E-/- mice exhibited significantly reduced allergic inflammation in the skin and lung. Allergic late-phase ear swelling and allergic lung airway hyperresponsiveness were also significantly attenuated in the P/E-/- mice compared with identically treated wild-type animals. In contrast, pulmonary inflammation and fibrosis induced by intranasal administration of silica particles resulted in a more severe phenotype in the P/E-/- mice.
CONCLUSIONS: Selectin interactions drive allergic inflammation in the lung and skin. Silica-induced pulmonary inflammation and fibrosis, however, was more pronounced in the absence of selectin interactions, suggesting that selectin-mediated leukocyte migration may depend on the types of initiating inflammatory stimuli.

PMID: 17225725 [PubMed - indexed for MEDLINE]

873. Handchir Mikrochir Plast Chir. 2006 Dec;38(6):354-69.

[Complications after dermal fillers and their treatment].

[Article in German]

Lemperle G, Gauthier-Hazan N, Wolters M.

Division of Plastic Surgery, University of California, San Diego, CA 92103-8220, USA. glemperle@aol.com

All dermal fillers are associated with the risk of both early and late complications. Early side effects such as swelling, redness, and bruising occur after intradermal or subdermal injections. The patient has to be aware of these risks and be prepared to accept them. Adverse events that last longer than 2 weeks can be attributable to technical shortcomings (e.g., the implantation of a long-lasting filler substance was too superficial). Such adverse events can be treated with intradermal 5-fluorouracil and steroid injections, vascular lasers, or intense pulsed light, and later with dermabrasion or shaving. Late adverse events also include immunological phenomena such as late-onset allergy and non-allergic foreign body granulomas. Both react well to intralesional steroid injections, which often have to be repeated to establish the right dose. Surgical excisions should remain the last option and are indicated for hard lumps in the lips and visible hard nodules or hard granulomas in the subcutaneous fat.

PMID: 17219319 [PubMed - indexed for MEDLINE]

874. Obes Surg. 2006 Dec;16(12):1685-8.

Long-term pulmonary complications after laparoscopic adjustable gastric banding.

Alamoudi OS.

Department of Medicine, King Abdulaziz University Hospital, Jeddah, Saudi Arabia. dramoudi@yahoo.com

Early pulmonary complications following laparoscopic adjustable gastric banding (LAGB) have been rare, while long-term pulmonary complications have not been reported. Herein, we report two patients who presented 2 and 3 years after LAGB with unexpected pulmonary complications. The first patient had aspiration pneumonia secondary to stomal obstruction and esophageal reflux. The second patient had left lobar pneumonia, in which the connecting catheter appeared as a linear structure within the consolidation. This may be due to migration of the connecting catheter through the diaphragm, piercing lung parenchyma. Both complications presented as asthma-like symptoms. Diagnosis could have been missed if not evaluated properly. A high index of suspicion and long-term follow-up are important for diagnosing such complications after LAGB.

PMID: 17217649 [PubMed - indexed for MEDLINE]

875. Vet Res Commun. 2007 Feb;31(2):197-206. Epub 2006 Dec 23.

Stimulation of the in vitro migration of ovine eosinophils by factors derived from the sheep scab mite, Psoroptes ovis.

Wildblood LA, Jones DG.

Moredun Research Institute, Pentlands Science Park, Penicuik, Scotland, UK.

The ectoparasitic astigmatid mite Psoroptes ovis causes sheep scab, a highly contagious, severe allergic dermatitis associated with damage to the fleece and hide, loss of condition and occasional mortality. The scab lesion is characterized by a massive infiltration of eosinophils that begins very rapidly after infection. This paper reports the finding that mite-derived factors directly enhance the migration of ovine eosinophils in vitro. Significant (p < 0.01) and dose-dependent (r = 0.972 +/- 0.018 (SD)) activity was initially identified in whole mite extracts, by comparison with medium controls in an assay based on modified Boyden chambers and ovine bone marrow target cells. Similar pro-migratory activity (p < 0.005; r = 0.928 +/- 0.069 (SD)) was detected in washes containing mite excretory/secretory material. By direct comparison with migration ratios (n = 3) for defined chemotactic (rmeotaxin = 3.430 +/- 0.360 (SD)) and chemokinetic (rminterleukin-5 = 0.982 +/- 0.112 (SD)) stimuli it was determined that the activity in both mite extracts (0.992 +/- 0.038 (SD)) and mite washes (0.969 +/- 0.071 (SD)) was chemokinetic. Subsequent experiments (n = 3) in which live mites were incorporated directly into the in vitro assay system indicated that they produced factors that significantly (p < 0.001) enhanced eosinophil migration to a degree directly related to mite numbers (r = 0.993 +/- 0.005 (SD)). The identity of the factor(s) responsible is uncertain, but their presence suggests that mites may be capable of directly activating eosinophils in vivo, and raises the possibility that mites could directly influence, perhaps even initiate, the rapid early tissue eosinophilic response observed in experimental sheep scab infections.

PMID: 17216315 [PubMed - indexed for MEDLINE]

876. Clin Exp Allergy. 2007 Jan;37(1):47-53.

Asthma in young south Asian women living in the United Kingdom: the importance of early life.

Kuehni CE, Strippoli MP, Low N, Silverman M.

Swiss Paediatric Respiratory Research Group, Department of Social and Preventive

Medicine, University of Berne, Berne, Switzerland. kuehni@ispm.unibe.ch

BACKGROUND: Studies of immigrants suggest that the environment during fetal life and duration of residence in the host country might influence the development of asthma. Little is known about the importance of the timing of the exposure in the host country and whether migrants might be especially vulnerable in certain age windows.
OBJECTIVE: We compared the reported prevalence of asthma between young white and south Asian women in the United Kingdom, and investigated associations with country of birth and age at immigration.
METHODS: A questionnaire on atopic disorders was posted to 2380 south Asian and 5796 white young mothers randomly sampled in Leicestershire. Data on ethnicity were also available from maternity records. Data were analysed using multivariable logistic regression and a propensity score approach. Results The reported prevalence of asthma was 10.9% in south Asian and 21.8% in white women. South Asian women who migrated to the United Kingdom aged 5 years or older reported less asthma (6.5%) than those born in the United Kingdom or who migrated before age 5 (16.0%), with an adjusted odds ratio of 0.38 [95% Confidence Interval 0.23-0.64, P<0.001]. For those who migrated aged over 5 years, the prevalence did not alter with the duration of residence in the United Kingdom. Current exposure to common environmental risk factors had relatively little effect on prevalence estimates.
CONCLUSION: These data from a large population-based study support the hypothesis that early life environmental factors influence the risk of adult asthma.

PMID: 17210041 [PubMed - indexed for MEDLINE]

877. Przegl Lek. 2006;63(7):554-6.

[Mast cells, their adenosine receptors and reactive oxygen species in chronic inflammatory pathologies of childhood].

[Article in Polish]

Renke J, Popadiuk S, Wozniak M, Szlagatys-Sidorkiewicz A, Hansdorfer-Korzon R.

Klinika Pediatrii, Gastroenterologii i Onkologii, Dzieciecej Akademii Medycznej w Gdańsku. jrenke@amg.gda.pl

Mast cells were described by Erhlich at the end of XIX-th century. Their role was deeply investigated in asthma and allergy. The massive degranulation of mast cells in allergy can lead to anaphylactic shock. Recently, mast cells have been recognized again as a very interesting topic for investigation, due to their possible role in chronic inflammation. Moreover, through adenosine receptors, mast cells can be activated or inactivated. That is why these cells are regarded as a potential target of new drugs. It has been reported, that mast cells generate intracellular reactive oxygen species (ROS) in response to stimulation with divergent physiologically relevant stimulants. The intensification of ROS production may be measured by the level of carbonyl groups, as a marker of protein peroxidation. However, the role of mast cells in other than asthma diseases with chronic inflammation needs further investigation. It was found out that the information about mast cell distribution in colonic mucosa may serve as help in differentiation between inflammatory bowel disease and collagenous colitis. Moreover, its accumulation in focal active gastritis was confirmed in patients with Crohn's disease. An important role in regulation of inflammatory process seems to be reserved for adenosine receptors present on mastocytes. The activation of mast cells through the adenosine receptor is connected with 11-8 release, which stimulate the migration of leukocytes and oxidation reactions. The detection of mast cells in tissues should not be limited only to the simple

histologic examination. It should be completed by the detection of products of degranulation, e.g. tryptase. This is the way to find out their actual function and state of activation.

PMID: 17203808 [PubMed - indexed for MEDLINE]

878. Pathol Int. 2007 Jan;57(1):1-11.

Roles of the ribosomal protein S19 dimer and the C5a receptor in pathophysiological functions of phagocytic leukocytes.

Yamamoto T.

Department of Molecular Pathology, Faculty of Medical and Pharmaceutical Sciences, Kumamoto University Graduate School, Honjo, Kumamoto, Japan.
tetsu@gpo.kumamoto-u.ac.jp

Monocytes and neutrophils, the major phagocytic leukocytes, migrate to inflammatory sites by sensing chemoattractants such as anaphylatoxin C5a with membrane receptors such as C5a receptor. Upon stimulation, the leukocytes increase cytoplasmic Ca(2+) concentration and generate radical oxygen species. These leukocytes have different functions in inflammation. Neutrophils migrate more rapidly and induce vascular plasma leakage upon infiltration. Monocytes infiltrate tissue more slowly but have superior capacities of phagocytosis and antigen presentation. There must be mechanisms to separately recruit the leukocyte species at an inflammatory site. Ribosomal protein S19 (RP S19) is a component of ribosome. During apoptosis, RP S19 is dimerized and obtains a ligand capacity to C5a receptor. The RP S19 dimer attracts monocytes to phagocytically clear the apoptotic cells that released the dimer molecules. The phagocytic monocytes/macrophages then translocate to regional lymph nodes and present apoptotic cell-derived antigens. Oppositely, the RP S19 dimer inhibits C5a-induced neutrophil migration and promotes apoptosis of neutrophils via the C5a receptor. The RP S19 dimer seems to prevent excessive tissue destruction induced by neutrophils. Skp is a molecular chaperon of Gram-negative bacteria. Skp also attracts monocytes and neutrophils as a ligand of C5a receptor. However, it promotes neither cytoplasmic Ca(2+) enhancement nor radical oxygen generation.

PMID: 17199736 [PubMed - indexed for MEDLINE]

879. Biomark Insights. 2007 May 3;2:128-46.

Human chitinases and chitinase-like proteins as indicators for inflammation and cancer.

Kzhyshkowska J, Gratchev A, Goerdt S.

Department of Dermatology and Allergology, University Medical Centre Mannheim, Ruprecht-Karls, University of Heidelberg, Mannheim D-68167, Germany.

Human Glyco_18 domain-containing proteins constitute a family of chitinases and chitinase-like proteins. Chitotriosidase and AMCase are true enzymes which hydrolyse chitin and have a C-terminal chitin-binding domain. YKL-40, YKL-39, SI-CLP and murine YM1/2 proteins possess solely Glyco_18 domain and do not have the hydrolytic activity. The major sources of Glyco_18 containing proteins are macrophages, neutrophils, epithelial cells, chondrocytes, synovial cells, and cancer cells. Both macrophages and neutrophils use the regulated secretory mechanism for the release of Glyco_18 containing proteins. Glyco_18 containing proteins are established biomarkers for human diseases. Chitotriosidase is

overproduced by lipid-laden macrophages and is a major marker for the inherited lysosomal storage Gaucher disease. AMCase and murine lectin YM1 are upregulated in Th2-environment, and enzymatic activity of AMCase contributes to asthma pathogenesis. YKL proteins act as soluble mediators for the cell proliferation and migration, and are also involved in rheumatoid arthritis, inflammatory bowel disease, hepatic fibrosis and cirrhosis. Chitotriosidase and YKL-40 reflect the macrophage activation in atherosclerotic plaques. Serum level of YKL-40 is a diagnostic and prognostic marker for numerous types of solid tumors. YKL-39 is a marker for the activation of chondrocytes and the progression of the osteoarthritis in human. Recently identified SI-CLP is upregulated by Th2 cytokine IL-4 as well as by glucocorticoids. This unique feature of SI-CLP makes it an attractive candidate for the examination of individual sensitivity of patients to glucocorticoid treatment and prediction of side effects of glucocorticoid therapy. Human chitinases and chitinase-like proteins are found in tissues and circulation, and can be detected by non-invasive technologies.

PMCID: PMC2717817
PMID: 19662198 [PubMed]

880. Recent Pat Inflamm Allergy Drug Discov. 2007 Feb;1(1):1-8.

VEGF, angiopoietin-1 and -2 in bronchial asthma: new molecular targets in airway angiogenesis and microvascular remodeling.

Kanazawa H.

Department of Respiratory Medicine, Graduate School of Medicine, Osaka City University, 1-4-3, Asahi-machi, Abenoku, Osaka, 545-8585, Japan.
kanazawa-h@med.osaka-cu.ac.jp

Airway angiogenesis and microvascular remodeling are known features of bronchial asthma, but the mechanisms of these structural alterations are just beginning to be elucidated. Vascular endothelial growth factor (VEGF), one of the most potent angiogenic factors, stimulates endothelial cell proliferation and induces the angiogenesis. Recently, considerable attentions have been devoted to the physiological roles of angiopoietin (Ang)-1 and -2 as regulatory factors of VEGF. Ang-1 has been shown to induce the migration and sprouting of endothelial cells, and coexpression of Ang-1 and VEGF enhanced angiogenesis. In the presence of high levels of VEGF, Ang-2 also promotes rapid increase in capillary diameter, remodeling of the basal lamina, proliferation and migration of endothelial cells, and stimulates sprouting of new blood vessels. Thus, VEGF, Ang-1 and -2 may play complementary and coordinated roles in airway angiogenesis and microvascular remodeling, and these structural changes are potentially reversible by therapeutic intervention. The scope of the present review is to discuss from a clinical point of view the potential interactions between VEGF and angiopoietins in the asthmatic airways, and focus on the therapeutic implications targeting for these angiogenic factors. Recently, there is an increasing number of patents which have been focused on the inhibitors of VEGF action. These inhibitors are directed towards the receptors of VEGF or intracellular substrates for the receptors. We will also discuss several patents regarding inhibitors of VEGF action in the present review.

PMID: 19075960 [PubMed - indexed for MEDLINE]

881. Biomed Pharmacother. 2007 Jan;61(1):29-33. Epub 2006 Dec 5.

Mechanisms of immunotherapy in allergic rhinitis.

Jayasekera NP, Toma TP, Williams A, Rajakulasingam K.

Department of Respiratory Medicine/Allergy, Homerton University Hospital NHS Foundation Trust, Homerton Row, London E9 6SR, UK.

Allergic rhinitis is a common condition, but many people still experience suboptimal control of symptoms despite measures such as allergen avoidance, intra-nasal steroids and antihistamines. Specific immunotherapy (SIT) has been used for many years, but though many studies show clinical efficacy, its mechanism of action is still not clearly understood. Earlier studies showed changes in antibodies and it may be that SIT works through mechanisms that alter the ratio of 'protective' IgG4 to 'pro-allergenic' IgE. Other studies have shown a reduction in eosinophil migration to nasal mucosa as well as a reduction in inflammatory mediator release including basophil histamine release. More recent studies have proposed that SIT works through inhibition of T-helper 2 lymphocytes (Th2) which preferentially produce cytokines that promote allergic responses. SIT may cause a deviation from Th2 to Th1 (T-helper 1 lymphocytes) or may induce T-regulatory cells (T-regs) which inhibit Th2 responses directly or through inhibitory cytokines.

PMID: 17189678 [PubMed - indexed for MEDLINE]

882. Am J Physiol Lung Cell Mol Physiol. 2007 Apr;292(4):L1030-8. Epub 2006 Dec 22.

Collagen I and thrombin activate MMP-2 by MMP-14-dependent and -independent pathways: implications for airway smooth muscle migration.

Henderson N, Markwick LJ, Elshaw SR, Freyer AM, Knox AJ, Johnson SR.

Division of Therapeutics and Molecular Medicine, University Hospital, Queens Medical Centre, Nottingham, United Kingdom.

Increased proinflammatory mediators and ECM deposition are key features of the airways in asthma. Matrix metalloproteinases (MMPs) are produced by airway smooth muscle (ASM) cells and have multiple roles in inflammation and tissue remodeling. We hypothesized that components of the asthmatic airway would stimulate MMP production and activation by ASM and contribute to airway remodeling. We measured human ASM-derived MMP mRNA, protein, and activity by real-time RT-PCR, zymography, Western blotting, and MMP activity assay. Collagen I and thrombin caused a synergistic increase in MMP-2 protein and total MMP activity but paradoxically decreased MMP-2 mRNA. Additionally, collagen I activated MMP-2 in culture supernatants independent of the cell surface. Together, collagen I and thrombin strongly enhanced MMP-14 mRNA and protein but had no effect individually, suggesting increased MMP-14, the activating protease for MMP-2, may be partially responsible for MMP-2 activation. Furthermore, collagen I reduced tissue inhibitor of metalloproteinase-2 protein (TIMP-2). We examined the role of MMPs in functions of ASM related to airway remodeling and found migration and proliferation were MMP dependent, whereas adhesion and apoptosis were not. Ilomastat inhibited migration by 25%, which was also inhibited by TIMPs 1-4 and increased by the MMP-2 activator thrombin. These in vitro findings suggest that the environment within the airways of patients with asthma enhances MMP-2 and -14 protein and activity by a complex interaction of transcriptional and posttranscriptional mechanisms, which may contribute to ASM migration.

PMID: 17189319 [PubMed - indexed for MEDLINE]

883. Treat Respir Med. 2006;5(6):419-28.

Pulmonary manifestations of malaria : recognition and management.

Taylor WR, Cañon V, White NJ.

Travel and Migration Medicine Unit, Department of Community Medicine, Geneva University Hospital, Geneva, Switzerland.

Lung involvement in malaria has been recognized for more than 200 hundred years, yet our knowledge of its pathogenesis and management is limited. Pulmonary edema is the most severe form of lung involvement. Increased alveolar capillary permeability leading to intravascular fluid loss into the lungs is the main pathophysiologic mechanism. This defines malaria as another cause of acute lung injury (ALI) and acute respiratory distress syndrome (ARDS).Pulmonary edema has been described most often in non-immune individuals with Plasmodium falciparum infections as part of a severe systemic illness or as the main feature of acute malaria. P.vivax and P.ovale have also rarely caused pulmonary edema.Clinically, patients usually present with acute breathlessness that can rapidly progress to respiratory failure either at disease presentation or, interestingly, after treatment when clinical improvement is taking place and the parasitemia is falling. Pregnant women are particularly prone to developing pulmonary edema. Optimal management of malaria-induced ALI/ARDS includes early recognition and diagnosis. Malaria must always be suspected in a returning traveler or a visitor from a malaria-endemic country with an acute febrile illness. Slide microscopy and/or the use of rapid antigen tests are standard diagnostic tools. Malaria must be treated with effective drugs, but current choices are few: e.g. parenteral artemisinins, intravenous quinine or quinidine (in the US only). A recent trial in adults has shown that intravenous artesunate reduces severe malaria mortality by a third compared with adults treated with intravenous quinine. Respiratory compromise should be managed on its merits and may require mechanical ventilation.Patients should be managed in an intensive care unit and particular attention should be paid to the energetic management of other severe malaria complications, notably coma and acute renal failure. ALI/ARDS may also be related to a coincidental bacterial sepsis that may not be clinically obvious. Clinicians should employ a low threshold for starting broad spectrum antibacterials in such patients, after taking pertinent microbiologic specimens. Despite optimal management, the prognosis of severe malaria with ARDS is poor.ALI/ARDS in pediatric malaria appears to be rare. However, falciparum malaria with severe metabolic acidosis or acute pulmonary edema may present with a clinical picture of pneumonia, i.e. with tachypnea, intercostal recession, wheeze or inspiratory crepitations. This results in diagnostic confusion and suboptimal treatment. Whilst this is increasingly being recognized in malaria-endemic countries, clinicians in temperate zones should be aware that malaria may be a possible cause of 'pneumonia' in a visiting or returning child.

PMID: 17154671 [PubMed - in process]

884. Eur Respir J. 2006 Dec;28(6):1258-63.

Evolution and respiratory genetics.

Le Souëf PN, Candelaria P, Goldblatt J.

School of Paediatrics and Child Health, Princess Margaret Hospital for Children, GPO Box D184, Perth WA, Australia 6001. plesouef@cyllene.uwa.edu.au

Comment in
    Eur Respir J. 2006 Dec;28(6):1079-80.

Evolution is a plausible explanation for between-population differences in

particular allele frequencies if: the genes involved have related functions; the heterogeneous alleles involved have similar functional consequences; the involved genes are not linked chromosomally; and the patterns observed would result in a biologically plausible, survival-enhancing gene-environment interaction. However, possible evolutionary effects have to be differentiated from founder effects and random genetic drift. The current authors have noted the existence of a consistent pattern of allelic frequencies in genes related to T-helper 2 (Th2) immune responses in humans of different ancestral backgrounds, residing in climatically similar regions. Th2 responses are thought to have evolved in mammals to resist infection by parasites, particularly helminths. Modern man arose in tropical Africa where helminths thrived. Relatively recently, humans migrated to cooler or drier climates where most helminths struggled to reproduce. The genetic tendency to strong Th2 responses may have become a health liability, the reduction in risk from parasites being counterbalanced by an increased inherited propensity to atopic or allergic diseases. The pattern noted by the present authors includes specific alleles of interleukin-4 and its receptor, interleukin-13, interleukin-10, the beta chain of the high-affinity receptor for immunoglobulin E, the beta(1)-adrenergic receptor, and the alpha chain of tumour necrosis factor. These population-specific polymorphism profiles are likely to be relevant in current disease patterns. The high incidence of asthma in migrants from tropical locations to affluent temperate countries is likely to be related to these patterns. Of even more concern is the possibility that increasing westernisation among the approximately 2 billion people living in the tropics will produce rapidly increasing levels of asthma, as these populations have a high genetic predisposition to allergic disease.

PMID: 17138680 [PubMed - indexed for MEDLINE]

885. J Biol Chem. 2007 Feb 2;282(5):3213-20. Epub 2006 Nov 29.

Airway epithelial cell migration and wound repair by ATP-mediated activation of dual oxidase 1.

Wesley UV, Bove PF, Hristova M, McCarthy S, van der Vliet A.

Department of Microbiology and Molecular Genetics, University of Vermont, Burlington, Vermont 05405, USA.

The airway epithelium is continuously subjected to environmental pollutants, airborne pathogens, and allergens and relies on several intrinsic mechanisms to maintain barrier integrity and to promote epithelial repair processes following injury. Here, we report a critical role for dual oxidase 1 (Duox1), a newly identified NADPH oxidase homolog within the tracheobronchial epithelium, in airway epithelial cell migration and repair following injury. Activation of Duox1 during epithelial injury is mediated by cellular release of ATP, which signals through purinergic receptors expressed on the epithelial cell surface. Purinergic receptor stimulation by extracellular ATP is a critical determinant of epithelial cell migration and repair following injury and is associated with activation of extracellular signal-regulated kinases (ERK1/2) and matrix metalloproteinase-9 (MMP-9). Stimulation of these integral features of epithelial cell migration and repair processes was found to require the activation of Duox1. Our findings demonstrate a novel role for Duox1 in the tracheobronchial epithelium, in addition to its proposed role in antimicrobial host defense, by participating in epithelial repair processes to maintain epithelial integrity and barrier function in the face of environmental stress.

PMID: 17135261 [PubMed - indexed for MEDLINE]

886. Rev Med Chil. 2006 Jun;134(6):703-12. Epub 2006 Aug 14.

[Changes in the patterns of disease after the epidemiological transition in health in Chile, 1950-2003].

[Article in Spanish]

Luque C, Cisternas FA, Araya M.

Programa de especialización en Gastroenterología Pediátrica, Universidad de Chile, Santiago, Chile.

BACKGROUND: During the twentieth century there was a change in the pattern of diseases in Europe, with an increase in the incidence of allergies and autoimmune disorders, that paralleled a decrease of infectious conditions. The Hygiene hypothesis proposes that these phenomena are causally related. Aim: To evaluate the epidemiological changes of allergic, autoimmune, and infectious diseases in Chile between 1950 and 2003.
MATERIAL AND METHODS: Search for the incidence and prevalence of these diseases in the national records published by the Ministry of Health, as well as through a systematic search of national literature using PubMed and Scielo as search engines.
RESULTS: The annual incidence of tuberculosis, rheumatic fever, measles, and typhoid fever has progressively diminished in Chile since 1970. Figures for the national prevalence for asthma, rheumatoid arthritis, and type I diabetes are scarce and difficult to compare, but clearly show an increasing epidemiological trend in the last 20 years.
CONCLUSIONS: The national figures suggest that, although the country has only recently gone through an epidemiological transition in health problems, there are detectable changes that show the same trends described in Europe.

PMID: 17130944 [PubMed - indexed for MEDLINE]

887. Rev Med Chil. 2006 Jun;134(6):675-8. Epub 2006 Aug 14.

[Epidemiological transition: the other side of the coin].

[Article in Spanish]

Valdivia G.

The model of epidemiological transition proposed by Omram explains the changes in disease patterns in communities. In societies with a high level of development this model has been complemented with the study of the post transition process. In this context, the emergence of allergic diseases, asthma and subsequently, of autoimmune diseases, has reached worrisome proportions in some countries, and no model can explain these changes. The hygiene theory supported by Strachan gives a reasonable explanation to this phenomenon. It postulates that the reduction of early exposure to biological agents, along with an improvement of sanitation conditions, immunizations and medical therapies, causes an asymmetrical immunological response, favoring the expression of Th2 response. The hygiene theory does not fully explain by itself what is happening in developed countries and it is not universally accepted. Chile is experiencing an epidemiological transition from a high burden of infectious diseases to a growing prevalence of non communicable diseases. In a previous similar setting in developed countries, there is some evidence to suspect that asthma, allergic and autoimmune diseases are becoming part of the epidemiological situation of Chile.

PMID: 17130940 [PubMed - indexed for MEDLINE]

888. Trends Immunol. 2007 Jan;28(1):33-8. Epub 2006 Nov 28.

IL-12p40: an inherently agonistic cytokine.

Cooper AM, Khader SA.

Trudeau Institute Inc., 154 Algonquin Ave., Saranac Lake, NY 12983, USA.
acooper@trudeauinstitute.org

IL-12p40 is known as a component of the bioactive cytokines interleukin (IL)-12 and IL-23 but it is not widely recognized as having intrinsic functional activity. Recent publications have altered this perception and support an independent role for IL-12p40. IL-12p40 is induced in excess over the other subunits of IL-12 and IL-23 and can exist in a monomeric or homodimeric form. Its most widely appreciated function is to provide a negative feedback loop by competitively binding to the IL-12 receptor. However, IL-12p40 acts as a chemoattractant for macrophages and promotes the migration of bacterially stimulated dendritic cells. It is associated with several pathogenic inflammatory responses such as silicosis, graft rejection and asthma but it is also protective in a mycobacterial model. An appreciation of the independent function of IL-12p40 is important for improving our understanding of both protective and pathogenic immune responses.

PMID: 17126601 [PubMed - indexed for MEDLINE]

889. Sao Paulo Med J. 2006 May 4;124(3):135-40.

Antipyretic efficacy and tolerability of oral ibuprofen, oral dipyrone and intramuscular dipyrone in children: a randomized controlled trial.

Prado J, Daza R, Chumbes O, Loayza I, Huicho L.

San Bartolomé Mother-Child National Teaching Hospital, Lima, Peru.

CONTEXT AND OBJECTIVE: Dipyrone is a widely used over-the-counter antipyretic in Latin America, and elsewhere among Latin immigrants. Despite limited evidence, physicians often prescribe oral ibuprofen or intramuscular dipyrone as the most effective antipyretics. Our aim was to compare the antipyretic efficacy and tolerability of a single dose of oral ibuprofen, oral dipyrone or intramuscular dipyrone in febrile children.
DESIGN AND SETTING: Randomized, single-blind clinical trial, at San Bartolomé Mother-Child National Teaching Hospital, Lima, Peru.
METHODS: Children from six months to six years old with fever (rectal temperature: 38.3 to 39.8 degrees C) in the emergency ward between February and June 2003 were eligible. Seventy-five children were randomly assigned to receive a single dose of oral ibuprofen (10 mg/kg), oral dipyrone (15 mg/kg) or intramuscular dipyrone (15 mg/kg). The primary outcome was mean temperature reduction after 30, 45, 60, 90 and 120 minutes. Secondary outcomes were fever-associated symptoms and clinical adverse events.
RESULTS: Fever decreased by about 0.5 degrees C after 45 minutes and by about 1.0 degrees C after 120 minutes in all three groups. Mean temperatures were similar for the three groups at all times. There was a significant decrease in fever-associated symptoms for all groups. Six patients (four receiving oral dipyrone and two receiving ibuprofen) were withdrawn because of vomiting within 20 minutes after first dose of study medication. One patient assigned to oral ibuprofen presented transient urticaria.
CONCLUSIONS: Antipyretic efficacy and tolerability were similar for oral

ibuprofen, oral dipyrone and intramuscular dipyrone. Oral antipyretics seem more appropriate for feverish children.

PMID: 17119689 [PubMed - indexed for MEDLINE]

890. Acta Otolaryngol. 2006 Dec;126(12):1286-94.

Protease-dependent activation of nasal polyp epithelial cells by airborne fungi leads to migration of eosinophils and neutrophils.

Shin SH, Lee YH, Jeon CH.

Department of Otolaryngology, School of Medicine, Catholic University of Daegu, Daegu, Korea. hsseung@cu.ac.kr

CONCLUSIONS: Proteases in fungi interact with nasal epithelial cells and enhance the production of inflammatory cytokines in vitro. These cytokines induced the migration of eosinophils and neutrophils. Protease-activated receptors (PARs) might also play a role in the process of epithelial cell activation.
OBJECTIVE: The nasal epithelium is the first barrier encountered by airborne allergens and an active participant in airway inflammation. Fungi have been increasingly recognized as important pathogens in sinusitis and consist of several allergenic proteins.
MATERIALS AND METHODS: Nasal polyp epithelial cells were obtained from patients and stimulated with Alternaria, Aspergillus, and Cladosporium. Interleukin-8 (IL-8), granulocyte-macrophage colony stimulating factor (GM-CSF), and regulated on activation normal T expressed and secreted (RANTES) were measured to determine the activation of epithelial cells. Reverse transcriptase-polymerase chain reaction test (RT-PCR) for PAR mRNA expression in nasal epithelial cells was performed. Eosinophil and neutrophil migration was induced with nasal polyp epithelial cells conditioned media (HPECM).
RESULTS: Fungi enhanced the production of chemical mediators from nasal epithelial cells. When nasal epithelial cells were activated with fungi, PAR2 and PAR3 mRNAs were more strongly expressed than in nonactivated cells. Eosinophil migration was induced by RANTES and eotaxin, and neutrophil migration was induced by IL-8 in HPECM.

PMID: 17101590 [PubMed - indexed for MEDLINE]

891. J Am Acad Dermatol. 2007 Feb;56(2):222-7. Epub 2006 Oct 13.

Efalizumab for severe atopic dermatitis: a pilot study in adults.

Takiguchi R, Tofte S, Simpson B, Harper E, Blauvelt A, Hanifin J, Simpson E.

Department of Dermatology at Oregon Health & Science University, Portland, OR 97239-3098, USA.

BACKGROUND: Severe atopic dermatitis (AD) often cannot be adequately controlled with topical agents. The continuous use of current systemic therapies for AD is limited by end-organ toxicities. A safe and effective systemic therapy for patients with recalcitrant AD is greatly needed.
OBJECTIVE: To evaluate the potential safety and efficacy of efalizumab, an inhibitor of T cell activation and migration, in adults with severe AD.
METHODS: An investigator-initiated, prospective, open-label, pilot study was conducted involving ten subjects with severe AD. Subjects received an initial conditioning subcutaneous dose of efalizumab of 0.7 mg/kg followed by 1.0 mg/kg weekly for another 11 weeks for a total of 12 doses. The primary efficacy outcome

was the change in the mean Eczema Area and Severity Index (EASI) score from baseline as measured at week 12. Monitoring of adverse events continued for 8 weeks after discontinuation of therapy.
RESULTS: EASI scores improved from a mean baseline score of 37.1 +/- 13.5 to 17.6 +/- 14.5 at week 12 (52.3% improvement; P < .0001). Six out of ten subjects reached at least a 50% improvement in EASI score by week 12. Pruritus levels decreased from 6.9 cm +/- 1.8 cm to 4.9 cm +/- 2.5 cm utilizing a visual analogue score (P < .015). Overall, efalizumab was well tolerated. There were three significant adverse events during the course of this study, including thrombocytopenia, viral gastroenteritis, and a subject with worsening of disease beyond baseline levels after drug discontinuation.
LIMITATIONS: It is difficult to apply these findings to larger populations of patients with AD because this study lacked a control group and involved a small number of subjects with very severe disease. Long-term efficacy and safety of efalizumab in this population is not known.
CONCLUSIONS: Efalizumab therapy resulted in significant clinical improvements in six of ten subjects with severe AD. Efalizumab may serve as a good alternative to current systemic immunosuppressants used for AD; however, double-blind placebo-controlled studies are needed to test its efficacy and safety.

PMID: 17097386 [PubMed - indexed for MEDLINE]

892. Protein Sci. 2006 Dec;15(12):2805-15. Epub 2006 Nov 6.

Enzymatic processing of collagen IV by MMP-2 (gelatinase A) affects neutrophil migration and it is modulated by extracatalytic domains.

Monaco S, Sparano V, Gioia M, Sbardella D, Di Pierro D, Marini S, Coletta M.

Department of Experimental Medicine and Biochemical Sciences, University of Roma Tor Vergata, I-00133 Roma, Italy.

Proteolytic degradation of basement membrane influences the cell behavior during important processes, such as inflammations, tumorigenesis, angiogenesis, and allergic diseases. In this study, we have investigated the action of gelatinase A (MMP-2) on collagen IV, the major constituent of the basement membrane. We have compared quantitatively its action on the soluble forms of collagen IV extracted with or without pepsin (from human placenta and from Engelbreth-Holm-Swarm [EHS] murine sarcoma, respectively). The catalytic efficiency of MMP-2 is dramatically reduced in the case of the EHS murine sarcoma with respect to the human placenta, probably due to the much tighter packing of the network which renders very slow the speed of the rate-limiting step. We have also enquired on the role of MMP-2 domains in processing collagen IV. Addition of the isolated collagen binding domain, corresponding to the fibronectin-like domain of whole MMP-2, greatly in hibits the cleavage process, demonstrating that MMP-2 interacts with collagen type IV preferentially through its fibronectin-like domain. Conversely, the removal of the hemopexin-like domain, using only the catalytic domain of MMP-2, has only a limited effect on the catalytic efficiency toward collagen IV, indicating that the missing domain does not have great relevance for the overall mechanism. Finally, we have investigated the effect of MMP-2 proteolytic activity ex vivo. MMP-2 action negatively affects the neutrophils' migration across type IV coated membranes and this is likely related to the production of lower molecular weight fragments that impair the cellular migration.

PMCID: PMC2242443
PMID: 17088321 [PubMed - indexed for MEDLINE]

893. J Ethnopharmacol. 2007 Mar 21;110(2):323-33. Epub 2006 Nov 3.

Anti-inflammatory and anti-allergic properties of the essential oil and active compounds from Cordia verbenacea.

Passos GF, Fernandes ES, da Cunha FM, Ferreira J, Pianowski LF, Campos MM, Calixto JB.

Department of Pharmacology, Centre of Biological Sciences, Universidade Federal de Santa Catarina, 88049-900 Florianópolis, SC, Brazil.

The anti-inflammatory and anti-allergic effects of the essential oil of Cordia verbenacea (Boraginaceae) and some of its active compounds were evaluated. Systemic treatment with the essential oil of Cordia verbenacea (300-600mg/kg, p.o.) reduced carrageenan-induced rat paw oedema, myeloperoxidase activity and the mouse oedema elicited by carrageenan, bradykinin, substance P, histamine and platelet-activating factor. It also prevented carrageenan-evoked exudation and the neutrophil influx to the rat pleura and the neutrophil migration into carrageenan-stimulated mouse air pouches. Moreover, Cordia verbenacea oil inhibited the oedema caused by Apis mellifera venom or ovalbumin in sensitized rats and ovalbumin-evoked allergic pleurisy. The essential oil significantly decreased TNFalpha, without affecting IL-1beta production, in carrageenan-injected rat paws. Neither the PGE(2) formation after intrapleural injection of carrageenan nor the COX-1 or COX-2 activities in vitro were affected by the essential oil. Of high interest, the paw edema induced by carrageenan in mice was markedly inhibited by both sesquiterpenic compounds obtained from the essential oil: alpha-humulene and trans-caryophyllene (50mg/kg, p.o.). Collectively, the present results showed marked anti-inflammatory effects for the essential oil of Cordia verbenacea and some active compounds, probably by interfering with TNFalpha production. Cordia verbenacea essential oil or its constituents might represent new therapeutic options for the treatment of inflammatory diseases.

PMID: 17084568 [PubMed - indexed for MEDLINE]

894. J Immunol. 2006 Nov 15;177(10):7346-54.

Induction of tolerance to innocuous inhaled antigen relies on a CCR7-dependent dendritic cell-mediated antigen transport to the bronchial lymph node.

Hintzen G, Ohl L, del Rio ML, Rodriguez-Barbosa JI, Pabst O, Kocks JR, Krege J, Hardtke S, Förster R.

Institute of Immunology, Hannover Medical School, Hannover, Germany.

Allergic airway diseases such as asthma are caused by a failure of the immune system to induce tolerance against environmental Ags. The underlying molecular and cellular mechanisms that initiate tolerance are only partly understood. In this study, we demonstrated that a CCR7-dependent migration of both CD103+ and CD103- lung dendritic cells (DC) to the bronchial lymph node (brLN) is indispensable for this process. Although inhaled Ag is amply present in the brLN of CCR7-deficient mice, T cells cannot be tolerized because of the impaired migration of Ag-carrying DC and subsequent transport of Ag from the lung to the draining lymph node. Consequently, the repeated inhalation of Ag protects wild-type but not CCR7-deficient mice from developing allergic airway diseases. Thus, the continuous DC-mediated transport of inhaled Ag to the brLN is critical for the induction of tolerance to innocuous Ags.

PMID: 17082654 [PubMed - indexed for MEDLINE]

895. J Immunol. 2006 Nov 15;177(10):7312-21.

Proteomic identification of in vivo substrates for matrix metalloproteinases 2 and 9 reveals a mechanism for resolution of inflammation.

Greenlee KJ, Corry DB, Engler DA, Matsunami RK, Tessier P, Cook RG, Werb Z, Kheradmand F.

Department of Medicine, Baylor College of Medicine, Houston, TX 77030, USA.

Clearance of allergic inflammatory cells from the lung through matrix metalloproteinases (MMPs) is necessary to prevent lethal asphyxiation, but mechanistic insight into this essential homeostatic process is lacking. In this study, we have used a proteomics approach to determine how MMPs promote egression of lung inflammatory cells through the airway. MMP2- and MMP9-dependent cleavage of individual Th2 chemokines modulated their chemotactic activity; however, the net effect of complementing bronchoalveolar lavage fluid of allergen-challenged MMP2(-/-)/MMP9(-/-) mice with active MMP2 and MMP9 was to markedly enhance its overall chemotactic activity. In the bronchoalveolar fluid of MMP2(-/-)/MMP9(-/-) allergic mice, we identified several chemotactic molecules that possessed putative MMP2 and MMP9 cleavage sites and were present as higher molecular mass species. In vitro cleavage assays and mass spectroscopy confirmed that three of the identified proteins, Ym1, S100A8, and S100A9, were substrates of MMP2, MMP9, or both. Function-blocking Abs to S100 proteins significantly altered allergic inflammatory cell migration into the alveolar space. Thus, an important effect of MMPs is to differentially modify chemotactic bioactivity through proteolytic processing of proteins present in the airway. These findings provide a molecular mechanism to explain the enhanced clearance of lung inflammatory cells through the airway and reveal a novel approach to target new therapies for asthma.

PMCID: PMC2580826
PMID: 17082650 [PubMed - indexed for MEDLINE]

896. J Immunol. 2006 Nov 15;177(10):6852-8.

CD8+ IL-17-producing T cells are important in effector functions for the elicitation of contact hypersensitivity responses.

He D, Wu L, Kim HK, Li H, Elmets CA, Xu H.

Department of Dermatology, University of Alabama at Birmingham, Birmingham, AL 35294, USA.

Allergen-induced contact hypersensitivity (CHS) is a T cell-mediated delayed-type immune response which has been considered to be primarily mediated by CD8+ T cytotoxic type I (Tc1) cells. IFN-gamma, the prototype Tc1 (Th1) cytokine, has been implicated as the primary inflammatory cytokine for CHS. In this study, we demonstrate that neutralization of IL-17 rather than IFN-gamma suppresses the elicitation of CHS. The suppression does not result from inhibition of the proliferation of allergen-activated T cells. Allergen sensitization induces the development of distinct CD8+ T cell subpopulations that produce IFN-gamma or IL-17. Although CD8+ IL-17-producing cells are stimulated by IL-23, they are inhibited by IL-12, a prototypical stimulator of IFN-gamma-producing Tc1 cells. This indicates that CD8+ IL-17-producing cells are distinct from Tc1 cells and are important in effector functions at the elicitation of CHS. These studies provide insights into a novel mechanism for CHS.

PMCID: PMC3179908

PMID: 17082599 [PubMed - indexed for MEDLINE]

897. J Clin Invest. 2006 Nov;116(11):2935-44.

Local application of FTY720 to the lung abrogates experimental asthma by altering dendritic cell function.

Idzko M, Hammad H, van Nimwegen M, Kool M, Müller T, Soullié T, Willart MA, Hijdra D, Hoogsteden HC, Lambrecht BN.

Department of Pulmonary Medicine, Erasmus University Medical Center, Rotterdam, The Netherlands.

Airway DCs play a crucial role in the pathogenesis of allergic asthma, and interfering with their function could constitute a novel form of therapy. The sphingosine 1-phosphate receptor agonist FTY720 is an oral immunosuppressant that retains lymphocytes in lymph nodes and spleen, thus preventing lymphocyte migration to inflammatory sites. The accompanying lymphopenia could be a serious side effect that would preclude the use of FTY720 as an antiasthmatic drug. Here we show in a murine asthma model that local application of FTY720 via inhalation prior to or during ongoing allergen challenge suppresses Th2-dependent eosinophilic airway inflammation and bronchial hyperresponsiveness without causing lymphopenia and T cell retention in the lymph nodes. Effectiveness of local treatment was achieved by inhibition of the migration of lung DCs to the mediastinal lymph nodes, which in turn inhibited the formation of allergen-specific Th2 cells in lymph nodes. Also, FTY720-treated DCs were intrinsically less potent in activating naive and effector Th2 cells due to a reduced capacity to form stable interactions with T cells and thus to form an immunological synapse. These data support the concept that targeting the function of airway DCs with locally acting drugs is a powerful new strategy in the treatment of asthma.

PMCID: PMC1626118
PMID: 17080194 [PubMed - indexed for MEDLINE]

898. Blood. 2007 Mar 1;109(5):2023-31. Epub 2006 Oct 31.

IL-21 receptor signaling is integral to the development of Th2 effector responses in vivo.

Fröhlich A, Marsland BJ, Sonderegger I, Kurrer M, Hodge MR, Harris NL, Kopf M.

Institute of Integrative Biology, Molecular Biomedicine, Swiss Federal Institute of Technology (ETH) Zürich, Switzerland.

Interleukin 21 (IL-21) is a member of the common gamma-chain family of cytokines, which influence a broad spectrum of immunologic responses. A number of studies have examined the function of IL-21, but its specific role in Th1/Th2-cell differentiation and related effector responses remains to be clarified. Thus, we generated IL-21R-deficient mice and have investigated the role of IL-21R signaling using a series of in vivo experimentally induced disease models. We first addressed the role of IL-21R signaling in Th2 immune responses by examining allergic airway inflammation, and Nippostrongylus brasiliensis and Heligmosomoides polygyrus antihelminth responses. In each of these systems, IL-21R signaling played a clear role in the development of Th2 responses. Comparatively, IL-21R signaling was not required for the containment of Leishmania major infection or the development of experimental autoimmune myocarditis, indicative of competent Th1 and Th17 responses, respectively.

Adoptive transfer of T cells and analysis of IL-21R+/+/IL-21R-/- chimera mice revealed that IL-21R-signaling was central to Th2-cell survival or migration to peripheral tissues. Overall, our data show IL-21 plays a crucial role in supporting polarized Th2 responses in vivo, while appearing superfluous for Th1 and Th17 responses.

PMID: 17077330 [PubMed - indexed for MEDLINE]

899. Allergol Int. 2006 Jun;55(2):121-9.

Fibroblasts as local immune modulators in ocular allergic disease.

Fukuda K, Kumagai N, Fujitsu Y, Nishida T.

Department of Ocular Pathophysiology, Yamaguchi University School of Medicine, Ube, Yamaguchi, Japan. k.fukuda@yamaguchi-u.ac.jp

Vernal keratoconjunctivitis (VKC), a severe form of ocular allergic disease, is characterized by the formation of giant papillae at the upper tarsal conjunctiva and corneal lesions that threaten vision. Recent evidence indicates that resident fibroblasts function as immune modulators in the pathogenesis of the chronic allergic inflammation associated with VKC. The T helper 2 (Th2) cell-derived cytokines interleukin (IL)-4 and IL-13 stimulate the migration and proliferation of conjunctival fibroblasts as well as protecting these cells from apoptotic cell death, effects that likely underlie the hyperplasia of fibroblasts that contributes to the formation of giant papillae. Conjunctival fibroblasts also synthesize extracellular matrix proteins and tissue inhibitors of metalloproteinases as well as down-regulate the expression of matrix metalloproteinases in response to these cytokines, effects that likely contribute to the excessive deposition of extracellular matrix that is characteristic of giant papillae. Stimulation of fibroblasts in the corneal stroma with the combination of a proinflammatory cytokine and either IL-4 or IL-13 results in up-regulation of the expression of the chemokine eotaxin and thymus- and activation-regulated chemokine as well as of vascular cell adhesion molecule-1, which together mediate the infiltration and activation of eosinophils and Th2 cells. Fibroblasts therefore appear to play a central role in the induction and amplification of ocular allergic inflammation and the consequent development of giant papillae and corneal disorders in individuals with VKC. Fibroblasts and fibroblast-derived factors thus represent new and potentially important therapeutic targets for treatment of the giant papillae and corneal disorders associated with VKC.

PMID: 17075248 [PubMed - indexed for MEDLINE]

900. Cytokine. 2006 Sep;35(5-6):270-4. Epub 2006 Oct 27.

Macrophage migration inhibitory factor in zinc-allergic systemic contact dermatitis.

Yanagi T, Kodama K, Yoshihisa Y, Shimizu H, Shimizu T.

Department of Dermatology, Hokkaido University Graduate School of Medicine, Kita-ku, Sapporo 060-8638, Japan. yanagi@med.hokudai.ac.jp

Macrophage migration inhibitory factor (MIF) is a proinflammatory cytokine whose expression has been found to be critical to the generation of the antigen-specific immune response. Recent studies suggested that MIF plays a role in the initiation and maintenance of allergic disease. The aim of this study was

to investigate whether MIF is involved in the pathogenesis of zinc-allergic systemic contact dermatitis. A 49-year-old Japanese woman developed facial edema, blepharedema and pruritic edematous erythema with papules over the entire body. Based of the results of a metal patch test, drug lymphocyte stimulating test and drug challenge test, diagnosis of zinc-allergic systemic contact dermatitis was made. Serum MIF and TNF-alpha levels of the patient, 20 healthy controls and other 6 patients who showed positive reaction to metal patch test were measured by an ELISA. Moreover we examined MIF production of peripheral blood mononuclear cells (PBMCs) from our patient, 3 healthy controls and other 2 patients who showed positive reaction to metal patch test at various metal concentrations. The patient's serum showed high MIF and TNF-alpha levels compared to healthy controls and other metal allergy patients. Furthermore, zinc stimulation of patient's PBMC showed higher MIF and TNF-alpha secretion compared with healthy subjects. The MIF content of 2 patients with other metal allergy was not significantly increased after metal stimulation. Our data suggest that zinc in the peripheral blood of zinc-allergic patients induce PBMCs to produce increased MIF levels, which could lead to systemic contact dermatitis.

PMID: 17070066 [PubMed - indexed for MEDLINE]

901. Ann Allergy Asthma Immunol. 2006 Oct;97(4):477-83.

Coping with asthma in immigrant Hispanic families: a focus group study.

Mosnaim G, Kohrman C, Sharp LK, Wolf ME, Sadowski LS, Ramos L, Grammer LC.

Department of Immunology and Microbiology, Rush Medical College, Chicago, Illinois 60612, USA. gmosnaim@rush.edu

BACKGROUND: Little is known about how childhood asthma affects immigrant Hispanic families in the United States. Qualitative research is effective for understanding the social, cultural, functional, and structural aspects of asthma in the family context. Furthermore, such knowledge is necessary to develop culturally appropriate interventions for these families.
OBJECTIVES: To describe participants' perceptions of their roles in caring for an asthmatic child, to compare family patterns of caring for an asthmatic child by parents' country of origin, to identify barriers to caring for an asthmatic child, and to evaluate specific coping needs of low-income immigrant Hispanic families caring for an asthmatic child.
METHODS: Five focus groups were conducted with low-income, immigrant, Spanish-speaking Hispanic adults caring for an asthmatic child, including community health workers, mothers, fathers, and grandparents, along with women with asthma. Audiotaped focus groups were transcribed verbatim in Spanish, forward translated into English, and back translated into Spanish. Data analysis was performed using qualitative analytic methods.
RESULTS: Forty-one participants represented a range of countries of origin. Different themes emerged for community health workers vs parents and grandparents and for women vs men caring for a child with asthma. All the participants reported strong beliefs in using folk medicines. Barriers identified included language, culture, poverty, lack of health insurance, and poor living conditions.
CONCLUSIONS: Results highlight the lack of asthma self-management skills, diagnostic uncertainty, and the use of folk medicine as factors that should be taken into consideration when tailoring interventions to improve asthma outcomes in this vulnerable population.

PMID: 17069102 [PubMed - indexed for MEDLINE]

902. J Immunol. 2006 Nov 1;177(9):6108-14.

Rapid selective priming of FcalphaR on eosinophils by corticosteroids.

ten Hove W, Houben LA, Raaijmakers JA, Koenderman L, Bracke M.

Department of Pulmonary Diseases, University Medical Center Utrecht, Heidelberglaan 100, 3584 CX Utrecht, The Netherlands.

Preactivation or priming of eosinophils by (proinflammatory) cytokines is important in the pathogenesis of allergic diseases. Several priming-dependent eosinophil responses, such as migration and adhesion, are reduced by treatment with corticosteroids. Many inhibitory effects of corticosteroids are mediated by the glucocorticoid receptor via genomic mechanisms, which are evident only after prolonged interaction (>30 min). However, also faster actions of corticosteroids have been identified, which occur in a rapid, nongenomic manner. In this study, fast effects of corticosteroids were investigated on the function of eosinophil opsonin receptors. Short term corticosteroid treatment of eosinophils for maximal 30 min with dexamethasone (Dex) did not influence eosinophil cell surface CD11b/CD18 expression, adhesion, and/or chemokinesis. In marked contrast, incubation with Dex resulted in a rapid increase in binding of IgA-coated beads to human eosinophils, showing that Dex can up-regulate the activation of FcalphaR (CD89). This priming response by Dex was dose dependent and optimal between 10(-8) and 10(-6) M and was mediated via the glucocorticoid receptor as its selective antagonist RU38486 (10(-6) M) blocked the priming effect. In contrast to FcalphaR, eosinophil FcgammaRII (CD32) was not affected by Dex. Further characterization of the Dex-induced inside-out regulation of FcalphaR revealed p38 MAPK as the central mediator. Dex dose dependently enhanced p38 MAPK phosphorylation and activation in situ as measured by phosphorylation of its downstream target mitogen-activated protein kinase-activated protein kinase 2. The dose responses of the Dex-induced activation of these kinases were similar as seen for the priming of FcalphaR. This work demonstrates that corticosteroids selectively activate the FcalphaR on eosinophils by activation of p38 MAPK.

PMID: 17056537 [PubMed - indexed for MEDLINE]

903. J Immunol. 2006 Nov 1;177(9):5779-84.

Cutting edge: Deficiency of macrophage migration inhibitory factor impairs murine airway allergic responses.

Wang B, Huang X, Wolters PJ, Sun J, Kitamoto S, Yang M, Riese R, Leng L, Chapman HA, Finn PW, David JR, Bucala R, Shi GP.

Department of Medicine, Brigham and Women's Hospital and Harvard Medical School, 77th Avenue Louis Pasteur, Boston, MA 02115, USA.

Increased levels of macrophage migration inhibitory factor (MIF) in serum, sputum, and bronchioalveolar lavage fluid (BALF) from asthmatic patients and time/dose-dependent expression of MIF in eosinophils in response to phorbol myristate acetate suggest the participation of MIF in airway inflammation. In this study, we examined inflammation in OVA-sensitized mouse lungs in wild-type and MIF-deficient mice (MIF(-/-)). We report increased MIF in the lung and BALF of sensitized wild-type mice. MIF(-/-) mice demonstrated significant reductions in serum IgE and alveolar inflammatory cell recruitment. Reduced Th1/Th2 cytokines and chemokines also were detected in serum or BALF from MIF(-/-) mice. Importantly, alveolar macrophages and mast cells, but not dendritic cells or splenocytes, from MIF(-/-) mice demonstrated impaired CD4+ T cell activation, and the reconstitution of wild-type mast cells in MIF(-/-) mice restored the phenotype of OVA-induced airway inflammation, revealing a novel and essential

role of mast cell-derived MIF in experimentally induced airway allergic diseases.

PMID: 17056501 [PubMed - indexed for MEDLINE]

904. Proc Natl Acad Sci U S A. 2006 Oct 31;103(44):16394-9. Epub 2006 Oct 18.

Role of ABCC1 in export of sphingosine-1-phosphate from mast cells.

Mitra P, Oskeritzian CA, Payne SG, Beaven MA, Milstien S, Spiegel S.

Department of Biochemistry, Virginia Commonwealth University School of Medicine, Richmond, VA 23298, USA.

Mast cells play a pivotal role in inflammatory and immediate-type allergic reactions by secreting a variety of potent inflammatory mediators, including sphingosine-1-phosphate (S1P). However, it is not known how S1P is released from cells. Here, we report that S1P is exported from mast cells independently of their degranulation and demonstrate that it is mediated by ATP binding cassette (ABC) transporters. Constitutive and antigen-stimulated S1P release was inhibited by MK571, an inhibitor of ABCC1 (MRP1), but not by inhibitors of ABCB1 (MDR-1, P-glycoprotein). Moreover, down-regulation of ABCC1 with small interfering RNA, which decreased its cell surface expression, markedly reduced S1P export from both rat RBL-2H3 and human LAD2 mast cells. Transport of S1P by ABCC1 influenced migration of mast cells toward antigen but not degranulation. These findings have important implications for S1P functions in mast cell-mediated immune responses.

PMCID: PMC1637593
PMID: 17050692 [PubMed - indexed for MEDLINE]

905. Acta Med Croatica. 2006 Sep;60(4):379-84.

[The impact of some bad habits and environmental factors on the somatic status of male adolescents].

[Article in Croatian]

Lalić H, Kalebota N, Kabalin M.

Dom zdravlja Primorsko-goranske zupanije, ispostava Rijeka, Medicina rada, Medicinski fakultet Sveucilista u Rijeci, Rijeka, Hrvatska. hlalic@inet.hr

AIM: The aim of the study was to investigate whether some bad habits of sedentary lifestyle influence the occurrence of diagnoses in male adolescents, divided according to their environment into urban, rural and and island groups.
PATIENTS AND METHODS: A total of 437 male adolescents underwent physical examination and functional diagnostic tests at Occupational Medicine Clinic in Rijeka, in order to evaluate their fitness for military service. The method of physical examination and anthropologic measurements was used. The results were analyzed by the Stat Soft, Statistics 6.0 software. Pearson chi-squared-test test and correlation matrices were used.
RESULTS: The results showed obesity to be present in a relatively high percentage of subjects from urban area (10.94%) and rural area (13.19%), whereas asthenia was more pronounced in islanders (8.69%), yielding a statistically significant between-group difference ($p<0.05$). In contrast to asthenia, the islanders had the lowest percentage of flatfoot, with a significant difference from the two other groups ($p<0.05$). Refraction errors, primarily myopia, were not influenced by the place of residence. The incidence of myopia in all three groups was slightly over 20%. Contrary to our expectation, bronchial asthma was most common in the

islanders (5.43%), however, there data could not be considered representative because of the rather big rate of migration from the inland to prevent relapses of respiratory diseases. The incidence of mild kyphoscoliosis ranged from 5.55% in the subjectss from rural settings up to 11.95% in the islanders, without a statistically significant between-group difference.
DISCUSSION: It is difficult to identify the causes of differences in body weight among adolescents from urban, rural and island settings. It is not so easy to criticize the former for predominantly sedentary life, watching TV, video or Internet. Physical activity cannot be readily performed in towns because of the increasing presence of pollutants in the atmosphere. The prevalence of bronchial asthma was slightly higher than expected, which was explained by the permanent residence of atopics on the islands in order to prevent disease relapses. The lowest incidence of flatfoot among islanders was explained by their free lifestyle, barefoot walking along the rocky seaside, and high level of physical activity. Refraction errors including myopia as the leading diagnosis were equally present in the three groups, exceeding 20%. Besides heredity, the sight is influenced by intensified effects of ultraviolet radiation that causes changes of the eye structure. Also, neon signs and lights as well as too strong night streetlights lead to phototoxic vision damage in adolescents. Study results showed that male adolescents who lead physically inactive life should not to be blamed for the occurrence of the mentioned diagnoses. Like all of us they are daily affected by harmful pollutants that cause damage to the eyes, cardiovascular, respiratory and other organ systems. This study has helped identify the causes of the mentioned diseases in the group of male adolescents, emphasizing the role of occupational medicine. Specialists in occupational medicine should be involved in the monitoring of somatic and other parameters in adolescents from the early school age. In this period, it is still possible to reduce or and even prevent the occurrence of the mentioned diagnoses by examinations, education, exercises and diets. In this way, the candidates for military service would be healthier and fit.

PMID: 17048794 [PubMed - indexed for MEDLINE]

906. J Allergy Clin Immunol. 2006 Oct;118(4):885-91. Epub 2006 Aug 28.

Biophysical determinants of toluene diisocyanate antigenicity associated with exposure and asthma.

Ye YM, Kim CW, Kim HR, Kim HM, Suh CH, Nahm DH, Park HS, Redlich CA, Wisnewski AV.

Department of Allergy and Rheumatology, Ajou University School of Medicine, Suwon, Korea.

BACKGROUND: Toluene diisocyanate (TDI), a widely used aromatic diisocyanate with the potential to cause asthma, reacts with albumin in the airway fluid, which acts as a carrier protein for chemical presentation to the immune system. Structural elucidation of TDI-albumin conjugates is crucial to understanding the human immune response to TDI exposure.
OBJECTIVE: Investigate the dependence of TDI's antigenicity on the biophysics of exposure and its association with TDI asthma.
METHODS: Toluene diisocyanate-albumin conjugates were generated by exposing albumin to TDI in liquid or vapor phase (liquid or vapor TDI-albumin, respectively). Conjugates were characterized by native gel electrophoresis and matrix-assisted laser desorption/ionization-mass spectrometry, and used as antigens in ELISA assays for serum specific-IgE and IgG.
RESULTS: The physical phase of TDI (vapor vs liquid) affects the formation of TDI-albumin conjugates, with measurable differences in the amount of TDI per albumin molecule, migration in native gels, matrix-assisted laser

desorption/ionization-mass spectrometry mass/charge spectra, and antigenicity. Vapor TDI-albumin conjugates were recognized by IgE from 44% of subjects with TDI asthma, whereas liquid TDI-albumin conjugates are recognized by IgE from only 17% of these patients. A significant (P < .05) association between TDI exposure and vapor TDI-albumin specific serum IgG was also observed.
CONCLUSION: Biophysics of TDI exposure substantially affects formation of TDI-albumin conjugates recognized by the immune system in association with exposure and asthma.
CLINICAL IMPLICATIONS: The data suggest that serology may help identify TDI asthmatics and exposed workers if the appropriate form of TDI is used as the antigenic basis for analysis.

PMID: 17030242 [PubMed - indexed for MEDLINE]

907. Respir Med. 2007 Apr;101(4):779-85. Epub 2006 Oct 4.

Predictors of asthma control in children from different ethnic origins living in Amsterdam.

van Dellen QM, Stronks K, Bindels PJ, Ory FG, Bruil J, van Aalderen WM; PEACE Study Group.

Department of Paediatric Pulmonology, Emma Children's Hospital, Academic Medical Center, University of Amsterdam, The Netherlands. q.vandellen@amc.uva.nl

To identify factors associated with asthma control in a multi-ethnic paediatric population. We interviewed 278 children with paediatrician diagnosed asthma (aged 7-17 years) and one of their parents. Asthma control was assessed with the Asthma Control Questionnaire (ACQ). Detailed information about sociodemographic variables, asthma medication, knowledge of asthma, inhalation technique and environmental factors were collected. Turkish and Moroccan parents were interviewed in their language of choice. Logistic regression analyses were used to identify correlates of asthma control. Of the 278 children, 85 (30.6%) were Dutch, 84 (30.2%) were Moroccan, 58 (20.9%) were Turkish and 51 (18.3%) were Surinamese. Overall, almost 60% had a status of well-controlled asthma, as indicated by the ACQ. Only 51 of the 142 (35.9%) Moroccan and Turkish parents had a good comprehension of the Dutch language. In logistic regression analyses the risk of having uncontrolled asthma was significantly higher among Surinamese children (OR 2.3; 95% CI 1.06-4.83), respondents with insufficient comprehension of the Dutch language (OR 2.3; 95% CI 1.08-4.78), children using woollen blankets (OR 9.8; 95% CI 1.52-63.42), and significantly lower among male (OR 0.5; 95% CI 0.31-0.91) and non-daily users of inhaled corticosteroids (OR 0.6; 95% CI 0.38-1.07). In conclusion, ethnicity as well as insufficient comprehension of the Dutch language appeared to be independent risk factors for uncontrolled asthma. Special attention should be given to children from immigrants groups for example by calling in an interpreter by physicians when comprehension is insufficient.

PMID: 17027246 [PubMed - indexed for MEDLINE]

908. Int J Immunopathol Pharmacol. 2006 Jul-Sep;19(3):629-38.

Relationship between specific serum IgE to Ascaris lumbricoides and onset of respiratory symptoms in Bangladesh immigrants.

Di Lorenzo G, Pacor ML, Mansueto P, Esposito-Pellitteri M, Scichilone N, Ditta V, Lo Bianco C, Leto-Barone MS, Di Fede G, Corrocher R, Mansueto S, Rini GB.

Dipartimento di Medicina Clinica e delle Patologie Emergenti, Università degli

Studi di Palermo, Italy. dilo601@unipa.it

The role of helminths in asthma and/or rhinitis and in allergic sensitization is still unclear. We assessed the relationship between Ascaris-specific IgE, respiratory symptoms and allergic sensitization in Bangladesh immigrants. 246 individuals were examined from 1996 to 2001. Serum total IgE, Ascaris IgE, specific IgE to inhalant allergens, skin prick tests (SPT) and parasitological evaluation of the stool were performed. Total serum IgE were significantly higher in Ascaris-IgE positive (> 0.35 kU/L) individuals (806.5 [409.0-1436.0] kU/L vs. 207.0 [127.0-332.5] kU/L; P < 0.0001) and in subjects with respiratory symptoms (413.0 [239.0-1096.0] kU/L vs. 259.5 [147.0-387.0] kU/L), (P < 0.0001), but not in SPT positive subjects (413.0 [179.0-894.0] kU/L vs. 404.6 [305.0-1201.0] kU/L (P = 0.5). Ascaris-specific IgE were detected in 48 subjects with respiratory symptoms (40.0%) and in 46 subjects without respiratory symptoms (36.5%) (P = 0.5). The SPT positivity was similar between Ascaris-IgE seropositive (38.2%) and Ascaris-IgE seronegative (38.1%) subjects (P = 0.9). Total IgE and length of stay in Italy correlated with SPT positivity (OR 5.6 [CI 95% 1.5-19.8], P = 0.007, and OR 1.5 [CI 95% 1.3-1.7], P< 0.0001), and with respiratory symptoms (OR 13.7 [CI 95% 3.0-62.4];, P = 0.0007, and OR 2.4 [CI 95% 1.9-3.0], P < 0.0001). Ascaris-IgE were negatively associated with SPT positivity (OR 0.3 [CI 95% 0.1-0.8], P = 0.02) and with respiratory symptoms (OR 0.1 [CI 95% 0.04-0.7], P = 0.01). Our findings favour the role of environmental factors in the development of respiratory symptoms in immigrants, irrespective of Ascaris-IgE.

PMID: 17026848 [PubMed - indexed for MEDLINE]

909. FASEB J. 2006 Nov;20(13):2408-10. Epub 2006 Oct 3.

Induction of a gene expression program in dendritic cells with a cross-linking IgM antibody to the co-stimulatory molecule B7-DC.

Blocki FA, Radhakrishnan S, Van Keulen VP, Heckman KL, Ciric B, Howe CL, Rodriguez M, Kwon E, Pease LR.

Department of Immunology, Mayo Clinic, College of Medicine, 200 First St. S.W., Rochester, MN 55905, USA.

Retraction in
    Pease LR. FASEB J. 2010 Jun;24(6):2135-6.

While bivalent antibodies can block ligand-receptor interactions, IgM pentamers efficiently cross-link cell surface targets and evoke physiological responses. We have described one such interaction between an IgM antibody (Ab) and the B7-DC costimulatory molecule expressed by dendritic cells that induces strong antitumor immunity and modulates pathogenic responses associated with allergic asthma. Progressive changes in gene expression in dendritic cells activated by an IgM B7-DC cross-linking Ab resulted in the increased expression in 350 genes and decreased expression of more than 200 genes over the course of 24 h following Ab treatment. In particular, up-regulation of the caspase inhibitor FLIP and the chemokine receptor CCR7, and the down-regulation of the CXCR4 receptor provide a mechanistic basis of Ab-induced survival and enhanced migration into draining lymph nodes. Increased expression of both cell surface and secreted molecules known to be mediators of the immunomodulatory properties of dendritic cells was detected at both the levels of RNA and protein expression. This analysis documents the ability of IgM Ab to activate a gene expression cascade leading to important biological changes in cellular function and provides mechanistic insight into the potent immunomodulatory properties attributed to this Ab.

PMID: 17023390 [PubMed - indexed for MEDLINE]

910. Med Lav. 2006 Jul-Aug;97(4):586-91.

[A particular case of hard metal disease].

[Article in Italian]

Todaro A, Vitelli N, Riboldi L, Maggi L, Belotti L, Mosconi G.

Fondazione IRCCS, Ospedale Maggiore Policlinico, Mangiagalli e Regina Elena, Dipartimento di Medicina del Lavoro, Clinica del Lavoro L. Devoto, Milano. aldo.todro@unimi.it

BACKGROUND: The production and the use of hard metal tools have becoming increasingly widespread since the second half of the last century also thanks to the great variety of applications that extends from DIY to the aeronautical industry. It has already been known for many years that occupational exposure to hard metals dusts (which occurs especially in people employed in production of the metals or in the sharpening of tools that contain them) can determine the onset of pulmonary fibrosis, bronchial asthma and contact dermatitis.
OBJECTIVES: clinical evaluation of a peculiar case of hard metal disease. Descriptions of cases with single pathological pictures due to hard metals, are, in fact, common in the literature, neither are cases with two different clinical pictures (more frequently asthma and pulmonary fibrosis) rare. However, cases in which all the signs and symptoms appeared simultaneously have never been reported.
METHODS: a male worker aged 41 years, employed i a hard metal factory for seven years in sintering, and then in grinding. A year later he developed dry cough, wheeze, and eczematous patches. The diagnosis of hard metal disease was based on the work-related symptoms, clinical evaluation, spirometry, chest x-ray, HRCT and patch tests.
RESULTS: during the working period, ventilatory function decreased substantially, and then normalized one month after the patient stopped working. Patch tests confirmed sensitivity to cobalt, and skin lesions improved, as did ventilatory function. Chest x-ray and HRCT showed a pulmonary fibrosis that, at the last radiological examination, was still unchanged.
CONCLUSIONS: The particular susceptibility to the development of the diseases, could, in our opinion, be in relationship with the race of the subject, many studies (particularly American) have shown that allergic diseases are more frequent, and often more serious, in African subjects, particularly in immigrants from Africa. In the case of occupational diseases attention also needs to be given to the fact that immigrants are often employed in duties that involve a greater exposure to harmful or sensitizing agents. The occurrence of a whole series of occupational allergic diseases among these workers is therefore to be expected.

PMID: 17017385 [PubMed - indexed for MEDLINE]

911. Int Arch Allergy Immunol. 2007;142(1):51-8. Epub 2006 Oct 2.

Genetic impact of functional single nucleotide polymorphisms in the 3'-UTR region of the chemoattractant receptor expressed on Th2 cells (CRTH2) gene on asthma and atopy in a Japanese population.

Maeda Y, Hizawa N, Takahashi D, Fukui Y, Konno S, Nishimura M.

First Department of Medicine, Hokkaido University School of Medicine, Sapporo, Japan.

BACKGROUND: The human chemoattractant receptor expressed on Th2 cells (CRTH2), the receptor for prostaglandin D2, induces cell migration in eosinophils, basophils, and Th2 cells. The gene encoding CRTH2 is located on chromosome 11q13. Several groups, including ours, have reported significant associations between this region and various traits associated with allergic diseases such as asthma and atopy. Two single nucleotide polymorphisms in the 3'-UTR of the CRTH2 gene (1544G-->C and 1651G-->A) are associated with the mRNA stability of the gene; they have also been associated with asthma in both African American and Chinese populations.
METHODS: Because CRTH2 is a biologically important candidate gene on chromosome 11q13, we conducted a case-control analysis using 787 Japanese subjects (384 asthmatics and 403 controls) to evaluate the genetic impact of the CRTH2 gene on asthma and asthma-related traits. Four polymorphisms [1544G-->C (rs11571288), 1651G-->A (rs545659), 11336T-->C (rs2074422), and 12375G-->T (rs561285)] were studied.
RESULTS: The allele, genotype, or haplotype frequencies for 2 functional polymorphisms in our Japanese population were significantly different from those in the Chinese or African American populations. No association was found between any polymorphisms or haplotypes in the CRTH2 gene and asthma, atopy, or total serum IgE levels in a Japanese population.
CONCLUSIONS: Our data failed to support previous associations of functional polymorphisms at the 3'-UTR of the CRTH2 gene implicated in asthma. We did show a significant difference in the allele and genotype frequencies as well as different haplotype frequencies among African American, Chinese, and Japanese populations, suggesting that the genetic impacts of these functional polymorphisms on asthma and asthma-related phenotypes may vary in different populations.

Copyright 2007 S. Karger AG, Basel.

PMID: 17016057 [PubMed - indexed for MEDLINE]

912. Am J Clin Dermatol. 2006;7(5):273-9.

Role of bacterial superantigens in atopic dermatitis : implications for future therapeutic strategies.

Cardona ID, Cho SH, Leung DY.

Department of Pediatrics, National Jewish Medical and Research Center, Denver, CO 80206, USA.

The role of staphylococcal superantigens in the pathophysiology of atopic dermatitis (AD) has been the focus of intense interest during the past decade. Although the increased prevalence of Staphylococcus aureus and its bacterial toxins in AD skin is well established, exploitation of the known mechanisms of superantigens in this disease for the development of novel therapies remains an active area of research. With the emergence of multi-drug resistant S. aureus, the need for a better understanding of the pathophysiology of bacterial superantigens in AD has become increasingly important. This review examines the mechanisms of S. aureus colonization and infection, of which the most important are defective skin barrier function, increased S. aureus adherence, and the decreased innate immune responses found in AD skin. The contribution of superantigens to the pathophysiology of AD is then discussed. Important immunologic mechanisms in this context include the role of superantigens in promoting T helper-2 skin inflammation, IgE production, T-regulatory cell subversion, expansion and migration of skin-homing T cells, and IgE anti-superantigen production. Lastly, these findings are discussed with reference

to current therapeutic approaches, of which the most important include anti-inflammatory and antimicrobial medications, and future strategies, which are expected to consist of immune-modulators and synthetic antibacterials.

PMID: 17007538 [PubMed - indexed for MEDLINE]

913. Int Immunol. 2006 Nov;18(11):1575-83. Epub 2006 Sep 19.

Trans-basement membrane migration of human basophils: role of matrix metalloproteinase-9.

Suzukawa M, Komiya A, Iikura M, Nagase H, Yoshimura-Uchiyama C, Yamada H, Kawasaki H, Ohta K, Matsushima K, Hirai K, Yamamoto K, Yamaguchi M.

Department of Allergy and Rheumatology, University of Tokyo Graduate School of Medicine, 7-3-1 Hongo, Tokyo 113-8655, Japan.

In allergic disorders, basophils migrate from the blood stream to inflamed tissue sites. Since trans-basement membrane migration is an important step for local basophil accumulation, we performed a human basophil transmigration assay using a model basement membrane, Matrigel. IL-3 in the upper chamber was critical for basophil trans-basement membrane migration over baseline levels, since none of the chemoattractants placed in the lower chambers induced migration. RANTES, IL-8, 5-oxo-6E,8Z,11Z,14Z-eicosatetraenoic acid (5-oxo-ETE) and platelet-activating factor (PAF) significantly up-regulated the transmigration of IL-3-treated basophils. Neutralizing experiments indicated the involvement of beta2 integrin and matrix metalloproteinase (MMP)-2/9 in basophil transmigration. Real-time quantitative PCR revealed that basophils constitutively expressed transcripts for MMP-9, and at lower levels, MMP-2, but cell-surface expression was only detected for MMP-9. MMP-9 was also detected in the cytoplasm and culture supernatant of the basophils. Treatment with IL-3 up-regulated the surface level of MMP-9 on the basophils. Our results suggest that basophils possess a unique regulatory mechanism for trans-basement membrane migration which is affected by cytokines, chemoattractants, beta2 integrin and MMPs, especially MMP-9. MMP-9 may be critically involved in the pathogenesis of local basophil influx in allergic diseases.

PMID: 16985079 [PubMed - indexed for MEDLINE]

914. Eur J Immunol. 2006 Oct;36(10):2700-14.

Membrane-bound eotaxin-3 mediates eosinophil transepithelial migration in IL-4-stimulated epithelial cells.

Yuan Q, Campanella GS, Colvin RA, Hamilos DL, Jones KJ, Mathew A, Means TK, Luster AD.

Center for Immunology and Inflammatory Diseases, Division of Rheumatology, Allergy and Immunology, Massachusetts General Hospital and Harvard Medical School, Boston, USA.

Epithelial cells play an important role in orchestrating mucosal immune responses. In allergic-type inflammation, epithelial cells control the recruitment of eosinophils into the mucosa. Th2-type cytokine-driven release of eosinophil-active chemokines from epithelial cells directs eosinophil migration into the mucosal epithelium. CCR3, the main eosinophil chemokine receptor, regulates this process; however, the respective contribution of individual CCR3 ligands in eosinophil transepithelial migration is less well understood. Using an

in vitro transepithelial chemotaxis system, we found that eotaxin-3 produced by IL-4-stimulated airway epithelial cells and CCR3 on eosinophils exclusively mediate eosinophil transepithelial migration. Eotaxin-3 protein levels were also increased in the nasal mucosal epithelium recovered from allergic patients as compared to non-allergic patients. Surprisingly, eotaxin-3 in IL-4-stimulated airway epithelial cells was predominantly cell surface bound, and the cell surface form was critical for eosinophil transepithelial migration. Eotaxin-3 cell surface association was partially glycosaminoglycan (GAG) dependent, but was completely protein dependent, suggesting that eotaxin-3 associates with both GAG and cell surface proteins. We thus provide evidence that cell surface-associated eotaxin-3 is the critical IL-4-dependent chemotactic signal mediating eosinophil transepithelial migration in the setting of allergic inflammation.

PMID: 16983721 [PubMed - indexed for MEDLINE]

915. Vet Immunol Immunopathol. 2006 Dec 15;114(3-4):238-46. Epub 2006 Sep 18.

Distribution of CCR3 mRNA expression in horse tissues.

Weston MC, Cunningham FM, Collins ME.

Department of Veterinary Basic Sciences, The Royal Veterinary College, Hawkshead Lane, North Mymms, Hertfordshire AL9 7TA, UK. marie.weston@piramed.com

CCL11 (also known as eotaxin) is a very potent and selective mediator of eosinophil migration which exerts its effects through its receptor, CCR3. In this study we report the cloning of an equine CCR3 cDNA sequence and investigation of the localization of CCR3 mRNA expression in horse tissues. Equine CCR3 displayed high levels of sequence identity with CCR3 sequences in other species. RT-PCR analysis revealed the expression of CCR3 in colon, lung and spleen of normal horses. In situ hybridisation experiments indicated that expression of CCR3 mRNA in colon was predominantly in eosinophils and to a lesser extent in mast cells, whereas CCR3 was seen mainly in lymphocytes of the lung and spleen. In view of the role of CCR3 in the recruitment of cells into sites of allergic inflammation, equine-specific CCR3 sequence data and information on tissue localization will be of potential benefit in the development of CCR3-targeted anti-inflammatory therapies in the horse.

PMID: 16979246 [PubMed - indexed for MEDLINE]

916. J Pharmacol Exp Ther. 2006 Dec;319(3):1395-404. Epub 2006 Sep 14.

A potent human anti-eotaxin1 antibody, CAT-213: isolation by phage display and in vitro and in vivo efficacy.

Main S, Handy R, Wilton J, Smith S, Williams L, Fou LD, Andrews J, Conroy LA, May R, Anderson I, Vaughan TJ.

Cambridge Antibody Technology, Granta Park CB1 6GH, UK.

The CC chemokine, eotaxin1 (CCL11) is an important regulator of eosinophil function. A marked accumulation of eosinophils in tissues has been correlated with the up-regulation of eotaxin1 expression in several diseases. The potential therapeutic value of neutralizing the effects of eotaxin1 in inflammatory conditions (including asthma) is under investigation. A human single-chain fragment variable antibody that neutralizes human eotaxin1 (CAT-212) was produced using antibody phage display and converted to whole antibody IgG4 format (CAT-213). A novel approach to lead optimization in which the length of the

variable heavy chain complementarity-determining region 3 was reduced by one amino acid resulted in an increase in potency of >1000-fold compared with the parent anti-eotaxin1 antibody. The optimized antibody binds eotaxin1 with high affinity (80.4 pM) and specificity. CAT-213 and CAT-212 do not bind or neutralize a range of other human proteins including human monocyte chemoattractant protein-1, a structurally similar chemokine. CAT-213 neutralizes the ability of eotaxin1 to cause an increase in intracellular calcium signaling (with an IC(50) value of 2.86 nM), migration of CCR3-expressing L1.2 cells (with an IC(50) value of 0.48 nM), and inhibition of the eotaxin1-evoked shape change of human eosinophils in vitro (with an IC(50) of 0.71 nM). Local administration of CAT-213 to mice (1-100 microg kg(-1)) attenuates dermal eosinophilia induced by human eotaxin1, achieving >90% inhibition of eosinophil influx. CAT-213 may therefore be of therapeutic value in inhibiting diseases in which eotaxin1 and eosinophils play a major role, for example, severe asthma.

PMID: 16973884 [PubMed - indexed for MEDLINE]

917. BMC Pulm Med. 2006 Sep 13;6:24.

Risk factors for asthma and allergy associated with urban migration: background and methodology of a cross-sectional study in Afro-Ecuadorian school children in Northeastern Ecuador (Esmeraldas-SCAALA Study).

Cooper PJ, Chico ME, Vaca MG, Rodriguez A, Alcântara-Neves NM, Genser B, de Carvalho LP, Stein RT, Cruz AA, Rodrigues LC, Barreto ML.

Instituto de Microbiologia, Universidad San Francisco de Quito, Quito, Ecuador. pcooper@ecnet.ec

BACKGROUND: Asthma and allergic diseases are becoming increasingly frequent in children in urban centres of Latin America although the prevalence of allergic disease is still low in rural areas. Understanding better why the prevalence of asthma is greater in urban migrant populations and the role of risk factors such as life style and environmental exposures, may be key to understand what is behind this trend.
METHODS/DESIGN: The Esmeraldas-SCAALA (Social Changes, Asthma and Allergy in Latin America) study consists of cross-sectional and nested case-control studies of school children in rural and urban areas of Esmeraldas Province in Ecuador. The cross-sectional study will investigate risk factors for atopy and allergic disease in rural and migrant urban Afro-Ecuadorian school children and the nested case-control study will examine environmental, biologic and social risk factors for asthma among asthma cases and non-asthmatic controls from the cross-sectional study. Data will be collected through standardised questionnaires, skin prick testing to relevant aeroallergen extracts, stool examinations for parasites, blood sampling (for measurement of IgE, interleukins and other immunological parameters), anthropometric measurements for assessment of nutritional status, exercise testing for assessment of exercise-induced bronchospasm and dust sampling for measurement of household endotoxin and allergen levels.
DISCUSSION: The information will be used to identify the factors associated with an increased risk of asthma and allergies in migrant and urbanizing populations, to improve the understanding of the causes of the increase in asthma prevalence and to identify potentially modifiable factors to inform the design of prevention programmes to reduce the risk of allergy in urban populations in Latin America.

PMCID: PMC1578586
PMID: 16970809 [PubMed - indexed for MEDLINE]

918. Br J Pharmacol. 2006 Oct;149(4):365-73. Epub 2006 Sep 11.

Collagen impairs glucocorticoid actions in airway smooth muscle through integrin signalling.

Bonacci JV, Schuliga M, Harris T, Stewart AG.

Department of Pharmacology, The University of Melbourne, Victoria, Australia.

BACKGROUND AND PURPOSE: Airway wall remodelling in asthma is characterised by a number of structural changes, including an increase in the volume of airway smooth muscle (ASM), and the abundance of the extracellular matrix (ECM) protein, collagen, is increased. We have investigated the mechanism of collagen-induced glucocorticoid resistance of proliferation, and migration of ASM.
EXPERIMENTAL APPROACH: ASM cultured from human airways has been seeded on to either type I monomeric collagen or a laminin pentapeptide, YIGSR. The role of alpha2beta1 integrin in the collagen-induced glucocorticoid resistance was investigated using a function blocking monoclonal antibody.
KEY RESULTS: Culture of ASM on collagen I, but not laminin, led to a greater proliferative response that was insensitive to regulation by dexamethasone (100 nM). The anti-migratory effects of the glucocorticoid, fluticasone propionate (1 nM) were also impaired by contact of ASM with collagen. The impaired anti-mitogenic action of dexamethasone was associated with a failure to reduce the levels of the rate-limiting cell cycle regulatory protein, cyclin D1. When signalling through the alpha2beta1 integrin was reduced, dexamethasone-mediated reductions in proliferation and cyclin D1 levels were restored.
CONCLUSIONS AND IMPLICATIONS: In the collagen-rich microenvironment of the inflamed and fibrotic asthmatic airway, integrin/ECM interactions may contribute to glucocorticoid resistance.

PMCID: PMC1978431
PMID: 16967051 [PubMed - indexed for MEDLINE]

919. Am J Respir Crit Care Med. 2006 Dec 1;174(11):1179-88. Epub 2006 Sep 7.

Airway smooth muscle and mast cell-derived CC chemokine ligand 19 mediate airway smooth muscle migration in asthma.

Kaur D, Saunders R, Berger P, Siddiqui S, Woodman L, Wardlaw A, Bradding P, Brightling CE.

Institute for Lung Health, and Department of Infection, Inflammation and Immunity, University of Leicester, Leicester, United Kingdom.

RATIONALE: Airway smooth muscle (ASM) hyperplasia is a feature of asthma, and increases with disease severity. We hypothesized that this results from migration of ASM or progenitors in response to chemokines derived from ASM or mast cells within the ASM bundle.
OBJECTIVES: To examine expression of the chemokine receptor, CC chemokine receptor (CCR) 7, in vivo by ASM in patients with asthma and healthy control subjects, and by primary cultures of ASM and fibroblasts; to define expression of its ligands, CC chemokine ligand (CCL) 19 and CCL21, in bronchial biopsies, and primary cultures of ASM and mast cells; and to investigate CCR7's role in ASM migration and repair.
METHODS: ASM was isolated from bronchoscopy and resection tissue. Receptor and chemokine expression was examined by immunohistochemistry, immunofluorescence, flow cytometry, ELISA, and reverse transcriptase-polymerase chain reaction. CCR7 function was examined by intracellular calcium measurements, chemotaxis, wound healing assays, and measurement of cell proliferation.
MEASUREMENTS AND MAIN RESULTS: ASM, myofibroblasts, and fibroblasts expressed

CCR7. CCL19, but not CCL21, was highly expressed in bronchial biopsies by mast cells and vessels in asthma of all severities, ASM in severe disease, and ex vivo ASM and mast cells. ASM CCR7 activation by CCL19-mediated intracellular calcium elevation and concentration-dependent migration, but not proliferation. Importantly, mast cell and ASM-derived CCL19 mediated ASM migration and repair. CONCLUSIONS: The CCL19/CCR7 axis may play an important role in the development of ASM hyperplasia in asthma.

PMID: 16959919 [PubMed - indexed for MEDLINE]

920. Nihon Naika Gakkai Zasshi. 2006 Aug 10;95(8):1501-6.

[Analysis of asthma-related genes].

[Article in Japanese]

Hizawa N, Kawaguchi M.

PMID: 16955937 [PubMed - indexed for MEDLINE]

921. Acta Derm Venereol. 2006;86(5):393-8.

Glucocorticoid-induced tumour necrosis factor receptor (GITR) and its ligand (GITRL) in atopic dermatitis.

Baumgartner-Nielsen J, Vestergaard C, Thestrup-Pedersen K, Deleuran M, Deleuran B.

Department of Dermatology, Aarhus University Hospital, P.P Oerumsgade 11, DK-8000 Aarhus C, Denmark. jaban@as.aaa.dk

The glucocorticoid-induced tumour necrosis factor receptor-related gene (GITR) is expressed on regulatory T-cells (Treg), which are CD4+CD25+ lymphocytes. Binding of the GITR-ligand (GITRL) leads to downregulation of the regulatory function of Tregs. Patients suffering from a defect in their Tregs exhibit a condition in their skin resembling atopic dermatitis. GITR also exists in a soluble form, and increased levels of this lead to decreased levels of GITRL and thereby increased Treg activity. We have measured the levels of GITR and GITRL in plasma from atopic dermatitis patients and found it not to be increased. Furthermore, plasma levels of GITR and GITRL did not correlate with SCORAD. Both GITR and GITRL correlated with the levels of thymus- and activation-regulated chemokine/CCL17 and cutaneous T-cell-attracting chemokine/CCL27, two chemokines believed to play a major role in the pathogenesis of atopic dermatitis and the migration of Tregs and skin-homing T-cells. Immunohistochemistry showed GITR and GITRL were present in few dermal cells of both patients with atopic dermatitis, and normal healthy volunteers, and often localized in close proximity to each other. Since regulatory T-cells are localized in the vicinity of GITRL-expressing cells in atopic dermatitis skin, the GITR/GITRL interaction may serve to perpetuate the inflammation locally.

PMID: 16955181 [PubMed - indexed for MEDLINE]

922. J Immunol. 2006 Sep 15;177(6):3763-70.

IL-12 instructs skin homing of human Th2 cells.

Biedermann T, Lametschwandtner G, Tangemann K, Kund J, Hinteregger S,

Carballido-Perrig N, Rot A, Schwärzler C, Carballido JM.

Novartis Institutes for Biomedical Research, Vienna, Austria.

Distinct pattern of homing receptors determines the tissue preference for T cells to exert their effector functions. This homing competence is mostly determined early during T cell activation of naive T cells. In contrast, mechanisms governing the acquisition of particular homing receptors by T cells of the memory phenotype remain enigmatic. Th2 cell-mediated allergic diseases tend to flare during infections despite that these infections prime APCs to produce the prototypic Th1 cell-differentiating cytokine IL-12. In this study, we investigate the effect of IL-12 on the regulation of cutaneous lymphocyte Ag (CLA) on differentiated Th2 cells and consequences of this expression for allergic inflammation. Upon activation with IL-12, CLA- Th2 cells rapidly up-regulated IL-12Rbeta2 chain, alpha(1-3)-fucosyltransferase VII, and CLA molecules. IL-12-mediated CLA expression on Th2 cells was functional because it mediated rolling of these Th2 cells on E-selectin in vitro and migration into human skin grafts in SCID mice. CLA induction occurred immediately after exposure to IL-12 and was independent of IFN-gamma expression. In accordance, the transcription factor mediating IFN-gamma expression, T-bet, does not directly affect CLA expression. However, CLA expression was further enhanced after IL-12 treatment of T-bet+ -transfected Th2 cells in agreement with an increased IL-12 responsiveness of these cells caused by T-bet. The finding that IL-12 conferred skin-homing potential to already differentiated Th2 cells before inducing a switch in their cytokine production profile may explain the observed exacerbation of allergic skin diseases following bacterial infections.

PMID: 16951337 [PubMed - indexed for MEDLINE]

923. J Leukoc Biol. 2006 Dec;80(6):1214-21. Epub 2006 Aug 30.

GRKs and arrestins: regulators of migration and inflammation.

Vroon A, Heijnen CJ, Kavelaars A.

Department of Psychoneuroimmunology, Room KC03.063.0, Lundlaan 6, 3584 EA, Utrecht, The Netherlands.

In the immune system, signaling by G protein-coupled receptors (GPCRs) is crucial for the activity of multiple mediators, including chemokines, leukotrienes, and neurotransmitters. GPCR kinases (GRKs) and arrestins control GPCR signaling by mediating desensitization and thus, regulating further signal propagation through G proteins. Recent evidence suggests that the GRK-arrestin desensitization machinery fulfills a vital role in regulating inflammatory processes. First, GRK/arrestin levels in immune cells are dynamically regulated in response to inflammation. Second, in animals with targeted deletion of GRKs or arrestins, the progression of various acute and chronic inflammatory disorders, including autoimmunity and allergy, is profoundly affected. Third, chemokine receptor signaling in vitro is known to be tightly regulated by the GRK/arrestin machinery, and even small changes in GRK/arrestin expression can have a marked effect on cellular responses to chemokines. This review integrates data about the role of GRKs and arrestins in inflammation, with results on the molecular mechanism of action of GRKs/arrestins, and describes the pivotal role of GRKs/arrestins in inflammatory processes, with a special emphasis on regulation of chemokine responsiveness.

PMID: 16943386 [PubMed - indexed for MEDLINE]

924. Curr Top Med Chem. 2006;6(13):1335-44.

Antagonists of CCR4 as immunomodulatory agents.

Purandare AV, Somerville JE.

Discovery Chemistry, Pharmaceutical Research Institute, Bristol-Myers Squibb, P.O. Box 4000, Princeton, NJ 08543-4000, USA. ashok.purandare@bms.com

The chemokine receptor CCR4 is broadly expressed on cells of the immune system. It is known to play a central role in T cell migration to the thymus, and T cell maturation and education. In addition, CCR4 is known to modulate T cell migration to several sites of inflammation in the body, including the skin, and lungs. It is best known as a drug target for airway inflammation and atopic dermatitis, but cells expressing CCR4 are found in many inflammatory diseases. CCR4 small molecule antagonists have not yet reached the clinic, but at least one has been validated in an in vivo model. Here we review the current status of structurally novel CCR4 receptor antagonists.

PMID: 16918452 [PubMed - indexed for MEDLINE]

925. Parasite Immunol. 2006 Sep;28(9):453-61.

Early stages of Ascaris suum induce airway inflammation and hyperreactivity in a mouse model.

Enobe CS, Araújo CA, Perini A, Martins MA, Macedo MS, Macedo-Soares MF.

Laboratory of Immunopathology, Butantan Institute, São Paulo, Brazil.

The inflammatory and functional changes that occur in murine lung after infection with 2500 infective Ascaris suum eggs were studied in this work. A sequential influx of neutrophils, mononuclear cells and eosinophils occurred into airways concomitantly with migration of larvae from liver to the lungs. Histological analysis of the lung showed a severe intra-alveolar haemorrhage at the peak of larval migration (day 8) and the most intense inflammatory cell infiltrate on day 14. Ascaris L3 were found in alveolar spaces and inside bronchioles on day 8. The number of eosinophils was elevated in the blood on days 8 and 14. The peak of eosinophil influx into the lung was at day 14, as indicated by the high levels of eosinophil peroxidase activity, followed by their migration into the airways. The antibody response against egg and larval antigens consisted mainly of IgG1 and IgM, and also of IgE and anaphylactic IgG1, that cross-reacted with adult worm antigens. Total IgE levels were substantially elevated during the infection. Measurement of lung mechanical parameters showed airway hyperreactivity in infected mice. In conclusion, the murine model of A. suum infection mimics the Th2-induced parameters observed in pigs and humans and can be used to analyse the immunoregulatory properties of this helminth.

PMID: 16916369 [PubMed - indexed for MEDLINE]

926. Hosp Health Netw. 2006 Jul;80(7):59-60, 62, 64 passim.

Nova Awards 2006. The American Hospital Association 2006 Award winners.

American Hospital Association.

Whether their projects target children with asthma; those whose working parents cannot afford health insurance, but do not quality for federal aid; or immigrants

or poor elderly, the hospitals and health systems that won the 2006 NOVA Awards understand that collaboration with other organizations is the most effective way to achieve a community's health care goals.

PMID: 16915973 [PubMed - indexed for MEDLINE]

927. J Invest Dermatol. 2006 Sep;126(9):1948-65.

Adrenergic and cholinergic control in the biology of epidermis: physiological and clinical significance.

Grando SA, Pittelkow MR, Schallreuter KU.

Department of Dermatology, University of California, Davis, 95817, USA.
sagrando@ucdavis.edu

The presence of an autocrine adrenergic and cholinergic intra/intercellular signal transduction network in the human epidermis contributes significantly to homeostatic and compensatory responses regulating vital functions in keratinocytes and melanocytes. The ligands produced control autocrine and paracrine loops to initiate responses through cognate receptors expressed within the same or adjacent cells. The epidermal adrenergic signal controls calcium homeostasis, cell growth, differentiation, motility, and pigmentation via the beta2 and alpha1 adrenoceptors. The cholinergic system is highly complex comprising both nicotinic and muscarinic receptors with multiple subtypes and this system plays an important role in keratinocyte cell cycle progression, differentiation, directional migration, adhesion, and apoptotic secretion. Moreover, lymphocytes also express adrenergic and cholinergic receptors. Both types of signal transduction receptors are coupled to classical intracellular second messenger pathways, including cAMP-, cGMP-, and calcium-mediated downstream responses. To date, it has been recognized that several dermatoses such as psoriasis, atopic dermatitis, Mal de Meleda, vitiligo, palmoplantar pustulosis, and pemphigus may be mediated, in part, by the non-neuronal adrenergic/cholinergic systems. A detailed understanding of the physiology and pathophysiology of the adrenergic/cholinergic network in the skin could offer the development of specific drugs for novel treatment modalities.

PMID: 16912692 [PubMed - indexed for MEDLINE]

928. Croat Med J. 2006 Aug;47(4):627-34.

Chronic respiratory symptoms in Croatian Adriatic island metapopulations.

Zuskin E, Smolej Narancić N, Skarić-Jurić T, Barbalić M, Rudan P, Kujundzić-Tiljak M, Pucarin-Cvetković J, Schachter EN.

Department of Environmental and Occupational Health, Andrija Stampar School of Public Health, Zagreb University School of Medicine, Zagreb, Croatia.
ezuskin@snz.hr

AIM: To investigate the prevalence of chronic respiratory symptoms in 9 metapopulations on Adriatic islands in Croatia, and the relationship between respiratory symptoms and individual genetic background.
METHODS: We obtained random sample of 1001 adult inhabitants of 9 Adriatic island villages in Croatia, that also included immigrants to these villages. European Union respiratory health questionnaire and World Health Organization non-communicable diseases questionnaire were used. Personal genetic histories were reconstructed, based on the two-generation ancestral pedigrees. Bivariate

and multivariate methods were used in the analysis.
RESULTS: Women reported the occurrence of acute dyspnea (P=0.017), cough (P=0.002), and asthma (P=0.002) more often than men. Gender was the strongest predictor for acute and/or chronic cough (odds ratio [OR], 1.69; 95% confidence interval [CI], 1.23-2.33) and asthma (OR, 2.00; 95% CI, 1.00-4.01), whereas smoking was the strongest risk factor for acute and chronic dyspnea (OR, 1.90; 95% CI, 1.21-2.99) and airway narrowing (OR, 1.84; 95% CI, 1.18-2.87). Residence on the northern islands increased the odds of allergy, whereas the highest odds ratio of 3.20 was associated with the interaction of northern residence and immigrant background. Genetic background was a significant predictor only for the occurrence of allergy symptoms.
CONCLUSION: Differences in respiratory findings among the island inhabitants were often associated with smoking prevalence. Interaction of residence on northern Adriatic islands and immigrant background proved to be the strongest predictor for the occurrence of allergy symptoms. This study indicated that environmental factors played a very important role in the occurrence of respiratory symptoms.

PMCID: PMC2080458
PMID: 16909461 [PubMed - indexed for MEDLINE]

929. Ann Otol Rhinol Laryngol. 2006 Jul;115(7):507-17.

Airway manifestations of pediatric eosinophilic esophagitis: a clinical and histopathologic report of an emerging association.

Dauer EH, Ponikau JU, Smyrk TC, Murray JA, Thompson DM.

Department of Otorhinolaryngology-Head and Neck Surgery, Mayo Eugenio Litta Children's Hospital Rochester, Minnesota, USA.

OBJECTIVES: Pediatric eosinophilic esophagitis (EE) typically presents with dysphagia, vomiting, dyspepsia, or food impaction. The purpose of this study was to highlight the emerging association of pediatric EE and airway disease. An additional goal of this study was to describe the unique histopathologic findings found in EE and specifically explore the potential role of the cytotoxic protein called eosinophil major basic protein (MBP) in the pathophysiology of the disease.
METHODS: A retrospective review of 3 children with EE and airway symptoms included symptom presentation, aerodigestive tract endoscopic findings, ambulatory 24-hour dual pH-metry, allergy tests, treatment modalities, and treatment response. Esophageal tissue obtained from biopsies of each patient was evaluated by hematoxylin and eosin to determine the number of eosinophils per high-power field, by immunofluorescent anti-MBP staining to determine the presence of MBP, and by standard light and transmission electron microscopy to evaluate eosinophil migration patterns.
RESULTS: All patients had airway inflammation that included nonspecific laryngeal edema and grade I or II subglottic stenosis. Allergy testing was positive in the 2 patients who were tested. All patients had symptoms refractory to standard reflux therapy. Ambulatory pH-metry findings were normal in 2 patients and abnormal in 1 patient despite maximum treatment. Two patients had visual abnormalities seen during esophageal examination. The number of eosinophils ranged from 20 to 45 per high-power field. Intracellular and extracellular MBP deposition was found in all esophageal biopsy specimens. All patients were treated with swallowed fluticasone, and 2 had symptom relapses that required repeat treatment.
CONCLUSIONS: The spectrum of pediatric EE can include upper airway disease. Intracellular and extracellular MBP deposition is present in EE, which potentially releases cytotoxic mediators that explain the esophageal and airway clinical symptoms seen in those with the disease. Eosinophilic esophagitis should

be considered in patients with a history of atopic diseases and unexplained upper airway findings refractory to reflux treatment. Treatment with swallowed fluticasone is successful; however, relapses are common and require repeat treatment and close follow-up.

PMID: 16900805 [PubMed - indexed for MEDLINE]

930. Chest. 2006 Aug;130(2):371-8.

Chemokine concentrations and mast cell chemotactic activity in BAL fluid in patients with eosinophilic bronchitis and asthma, and in normal control subjects.

Woodman L, Sutcliffe A, Kaur D, Berry M, Bradding P, Pavord ID, Brightling CE.

Insitute for Lung Health, University of Leicester, Glenfield Hospital, UK.

BACKGROUND: Asthma and eosinophilic bronchitis share many immunopathologic features including increased numbers of eosinophils and mast cells in the superficial airway. The mast cell chemotactic activity of airway secretions has not been assessed in patients with eosinophilic bronchitis.
OBJECTIVES: To investigate the concentration of chemokines in bronchial wash samples and BAL fluid, and the mast cell chemotactic activity in BAL fluid from subjects with asthma and eosinophilic bronchitis, and from healthy control subjects.
METHODS: We measured the concentrations of CCL11, CXCL8, and CXCL10 in bronchial wash samples and BAL fluid from 14 subjects with eosinophilic bronchitis, 14 subjects with asthma, and 15 healthy control subjects. Mast cell chemotaxis to BAL fluid from these subjects was examined using the human mast cell line HMC-1.
RESULTS: The bronchial wash sample and BAL fluid concentrations of CXCL10 and CXCL8 was increased in subjects with eosinophilic bronchitis compared to those in subjects with asthma and healthy control subjects ($p < 0.05$). The CCL11 concentration was below the limit of detection in most subjects. BAL fluid from subjects with eosinophilic bronchitis was chemotactic for mast cells (1.4-fold migration compared to a control, 95% confidence interval, 1.1 to 1.9; $p = 0.04$) and was inhibited by blocking CXCR1 (45% inhibition; $p = 0.002$), CXCR3 (38% inhibition; $p = 0.034$), or both (65% inhibition; $p = 0.01$). BAL fluid from the subjects with asthma and healthy control subjects was not chemotactic for mast cells. Mast cell migration to BAL fluid was correlated with the concentration of CXCL8 ($r = 0.42$; $p = 0.031$) and CXCL10 ($r = 0.52$; $p = 0.007$).
CONCLUSION: In subjects with eosinophilic bronchitis, CXCL8 and CXCL10 concentrations were elevated in airway secretions. These chemokines may play a key role in mast cell recruitment to the superficial airway in this condition.

PMID: 16899834 [PubMed - indexed for MEDLINE]

931. Gastroenterology. 2006 Aug;131(2):402-9.

Allergy controls the population density of Necator americanus in the small intestine.

Croese J, Wood MJ, Melrose W, Speare R.

The Department of Gastroenterology, The Townsville Hospital, Townsville, Australia. jcroese@bigpond.com

BACKGROUND & AIMS: Nearly 700 million people remain infected with hookworms. Although allergy is intuitively linked to immunity against helminths, few positive examples have been characterized. Larval migration through the lungs has

been considered the likely interface at which hookworm attrition occurs. As part of a study evaluating a potential role for hookworms in the modulation of human autoimmunity, we examined parasite migration and intestinal colonization.
METHODS: Capsule and conventional endoscopies supplemented the evaluation of healthy volunteers and Crohn's disease patients recently inoculated with larvae of the human hookworm Necator americanus. Two healthy volunteers with a previously established and stable hookworm infection were inoculated with 50 larvae and had serial capsule endoscopies performed.
RESULTS: Eosinophilic enteritis developed in all subjects after the initial inoculation. Newly inoculated larvae in the 2 subjects with an established infection reliably reached the intestine within 4 weeks. Thereafter, the colony diminished to the host's constitutive status quo because mostly immature worms failed to attach. The intensity of the eosinophilic response correlated negatively with the time available for hookworms to feed and positively with hookworm attrition.
CONCLUSIONS: Necator larval migration to the intestine is uncontested. We propose that allergic inflammation purposefully degrades the hookworm's bite, causing premature detachment, restricted feeding, and expulsion. This novel biological dynamic suggests a new paradigm of hookworm resistance.

PMID: 16890593 [PubMed - indexed for MEDLINE]

932. J Affect Disord. 2007 Feb;98(1-2):99-108. Epub 2006 Aug 4.

Psychological distress in migrants in Australia over 50 years old: a longitudinal investigation.

Chou KL.

Department of Politics and Sociology, Lingnan University, Tuen Mun, Hong Kong, China. klchou@hku.hk

BACKGROUND: Although it is a well-known fact that migration is a risk factor contributing to psychopathology, little is known in migrants who migrated in their old age. The present study examined whether origin of countries and visa types predicted psychological distress over a period of 1 year and whether their association changed after factors in health, social roles, cohort effect and social support were adjusted.
METHODS: A nationwide representative sample of 431 migrants who aged 50 and above were interviewed in 2000-2001 and 359 of them were re-interviewed 1 year after the baseline assessment. 12-item General Health Questionnaire (GHQ-12) was used measure psychological distress and a series of questions regarding socio-demographic characteristics (age, gender, living alone), days in Australia, origin of countries, visa types, health, social role, cohort effect, and social support were also included.
RESULTS: GHQ-12 scores did deteriorate over a period of 1 year among older migrants to Australia. In multiple regression analyses, origin of countries and visa types were significant predictors of future GHQ-12 scores. Baseline GHQ-12 scores, age, gender, living alone, days in Australia, poor self-rated health, the presence of heart disease, diabetes, and asthma, being a student or economically inactive, widowhood or divorce, as well as education were also significant predictors of GHQ-12 scores at 1-year follow-up.
CONCLUSIONS: The status of refugees predicts future psychological distress in older migrants even when other known correlates of psychological distress are controlled.

PMID: 16890296 [PubMed - indexed for MEDLINE]

933. J Immunol. 2006 Aug 15;177(4):2630-7.

Lipoic acid affects cellular migration into the central nervous system and stabilizes blood-brain barrier integrity.

Schreibelt G, Musters RJ, Reijerkerk A, de Groot LR, van der Pol SM, Hendrikx EM, Döpp ED, Dijkstra CD, Drukarch B, de Vries HE.

Department of Molecular Cell Biology and Immunology, VU University Medical Center, 1007 MB Amsterdam, The Netherlands.

Reactive oxygen species (ROS) play an important role in various events underlying multiple sclerosis (MS) pathology. In the initial phase of lesion formation, ROS are known to mediate the transendothelial migration of monocytes and induce a dysfunction of the blood-brain barrier (BBB). In this study, we describe the beneficial effect of the antioxidant alpha-lipoic acid (LA) on these phenomena. In vivo, LA dose-dependently prevented the development of clinical signs in a rat model for MS, acute experimental allergic encephalomyelitis (EAE). Clinical improvement was coupled to a decrease in leukocyte infiltration into the CNS, in particular monocytes. Monocytes isolated from the circulation of LA-treated rats revealed a reduced migratory capacity to cross a monolayer of rat brain endothelial cells in vitro compared with monocytes isolated from untreated EAE controls. Using live cell imaging techniques, we visualized and quantitatively assessed that ROS are produced within minutes upon the interaction of monocytes with brain endothelium. Monocyte adhesion to an in vitro model of the BBB subsequently induced enhanced permeability, which could be inhibited by LA. Moreover, administration of exogenous ROS to brain endothelial cells induced cytoskeletal rearrangements, which was inhibited by LA. In conclusion, we show that LA has a protective effect on EAE development not only by affecting the migratory capacity of monocytes, but also by stabilization of the BBB, making LA an attractive therapeutic agent for the treatment of MS.

PMID: 16888025 [PubMed - indexed for MEDLINE]

934. J Immunol. 2006 Aug 15;177(4):2621-9.

Prostaglandin D2 plays an essential role in chronic allergic inflammation of the skin via CRTH2 receptor.

Satoh T, Moroi R, Aritake K, Urade Y, Kanai Y, Sumi K, Yokozeki H, Hirai H, Nagata K, Hara T, Utsuyama M, Hirokawa K, Sugamura K, Nishioka K, Nakamura M.

Department of Dermatology, Graduate School, Tokyo Medical and Dental University, 1-5-45 Yushima, Bunkyo-ku, Tokyo 113-8519, Japan. tasa1688.derm@tmd.ac.jp

PGD(2) plays roles in allergic inflammation via specific receptors, the PGD receptor designated DP and CRTH2 (chemoattractant receptor homologous molecule expressed on Th2 cells). We generated mutant mice carrying a targeted disruption of the CRTH2 gene to investigate the functional roles of CRTH2 in cutaneous inflammatory responses. CRTH2-deficent mice were fertile and grew normally. Ear-swelling responses induced by hapten-specific IgE were less pronounced in mutant mice, giving 35-55% of the responses of normal mice. Similar results were seen in mice treated with a hemopoietic PGD synthase inhibitor, HQL-79, or a CRTH2 antagonist, ramatroban. The reduction in cutaneous responses was associated with decreased infiltration of lymphocytes, eosinophils, and basophils and decreased production of macrophage-derived chemokine and RANTES at inflammatory sites. In models of chronic contact hypersensitivity induced by repeated hapten application, CRTH2 deficiency resulted in a reduction by approximately half of skin responses and low levels (63% of control) of serum IgE production, although

in vivo migration of Langerhans cells and dendritic cells to regional lymph nodes was not impaired in CRTH2-deficient mice. In contrast, delayed-type hypersensitivity to SRBC and irritation dermatitis in mutant mice were the same as in wild-type mice. These findings indicate that the PGD(2)-CRTH2 system plays a significant role in chronic allergic skin inflammation. CRTH2 may represent a novel therapeutic target for treatment of human allergic disorders, including atopic dermatitis.

PMID: 16888024 [PubMed - indexed for MEDLINE]

935. Wiad Parazytol. 1999;45(3):381-6.

[Own clinical observations of toxocara infections].

[Article in Polish]

Jeske J, Kamerys J, Malinowska B, Lupa S, Seneczko F, Kotłowski A.

Wojewódzki Szpital Specjalistyczny im. dr. WI. Biegańskiego 91-347 Lódź.

Toxocarosis clinical forms wers diagnosed according to Kraus et. al. (1995). There were three forms: 1) asymptomatic - marked by hypereosynophilia and ELISA positive serologic reaction, 2) minor - with cutaneous, pulmomary and pseudorheumatic lesions, 3) major - with hepatosplenomegaly and multifocal inflammatory lesions of organ of sight. Over the years of 1994-1997 in Infectious, Parasitic and Tropical Diseases clinic of the Voivodship Specialist Hospital of Lódź we found and treated 137 Toxocara canis cases. Among them 63 (46:0%) asymptomatic, 57 (42.6%) minor and 17 (12.4%) major forms were diagnosed. The disease was detected in 80 (58.4%) adults and 57 (41.6%) children. Seventy six patients lived in towns and 61 in country. Minor form patients had the symptoms as follows: skin allergy, large joints lesions, augmentation in lymph nodes. Radiology examinations revealed pulmonary lesions. Using ultrasonography there was found hepatosplenomegaly and changes echogenity in patients with major form. Biochemical tests showed elevation in hepatic enzymes activity. Lesions of organ of sight were multifocal and usually included uveitis, retinitis, inflammation of anterior chamber and inflammation of vitreous body.

PMID: 16886380 [PubMed - indexed for MEDLINE]

936. Wiad Parazytol. 2000;46(1):3-20.

[Regulation of defence and allergic reaction in infections with parasites].

[Article in Polish]

Doligalska M.

Zakład Parazytologii Instytutu Zoologii, Uniwersytet Warszawski.
mdolig@eco1.biol.uw.edu.pl

Cytokines regulate development, differentiation and expression of effector function of the immune system. The profile of immune reactions depends on cytokine contents at the recognition of parasite. However, parasites themselves can modify immunological events and favourite these, which allow them to survive. The host immune defence can be transformed into the chronic reaction. Inflammatory reactions result from the recruitment of different cells, to the site where worms are localised. The eosinophil and mast cell migration preferentially is induced. These cells play also a major role in immediate

allergic responses. Mast cells can bind allergen-specific IgE to their FcepsilonRI, the high affinity receptor for IgE. Eosinophils, through their receptors for IgG1, IgG2, IgA and IgE are mainly involved in the cytotoxic reaction directed against parasites. Crosslinking of these receptors by antigen binding will lead to subsequent release of stored mediators and cytokines. Granular materials released from mast cell accelerate inflammatory reaction and in the case of intestinal worm parasites may be involved in the expulsion phenomenon. However these cells may also induce Th2 related immunological response because they produce and release of IL-4. Eosinophils are required into the tissue and release cytotoxic and stress proteins including reactive oxygen species. Parasites are destroyed but accelerated reaction results in the destruction of host proteins and cells. Antibodies, cytokines, chemokines and adhesion molecules are essential for elevation of defence against parasites. The role of cytokines, emphasizing IL-5, and function of eosinophils, mast cells and IgE are discussed in terms of induction and effector mechanisms during parasite infections.

PMID: 16886349 [PubMed - indexed for MEDLINE]

937. Parassitologia. 2006 Jun;48(1-2):121-4.

Mycetes and urban areas.

Tampieri MP.

Department of Veterinary Public Health and Animal Pathology, Alma Mater Studiorum, Bologna University, Bologna, Italy.

Mycetes are ubiquitous organisms that can cause mycoses in human and animals. The role of animals in the epidemiology of human mycoses in urban areas is multiform, but here will be discuss only two features: A) animals as vectors of mycoses and B) animal substrates as growth factor of pathogenic fungi. A) Animals as vectors of mycoses: this role is important as zoofilic dermatophytes are very important agents of zoonosis; the urban dermatophytozoonoses are prevalent caused by Microsporum canis which is prevalent in cats and dogs. Cats are often asymptomatic carriers. The pattern of human dermatomycoses has changed in Italy during the past century: at the beginning of the century anthropophilic fungi were prevalent while at present the zoophilic fungi are the most important causes. B) Animal substrata as growth factor of pathogenic fungi: soil "animalization" (i.e., the addition of such debris as hair, skin scales, dropping and other organic matters) creates an optimal substratum for the growth and the multiplication of geophilic or saprophyitic fungi, such as Microsporum gypseum and Cryptococcus neoformans. The present human lifestyle, which favours a an overpopulation of birds, wild animals, domestic mammals and sinanthropic together with man in crowded areas seems to favour the formation of environments adapted to the abundant growth of some pathogenic fungi with consequent infection for man and animals. Finally, an environment heavily populated by fungi can cause allergic pulmonary reactions as well as reactions in other organs and tissues. The control of human and animal fungi, and the efficient use of a monitoring system require ample knowledge of mycological problems both in human and veterinary medicine and of efficient laboratories capable of resolving the needs of both disciplines. Close collaboration between veterinarians, doctors and mycologists is necessary in order to resolve health problems linked to mycosis.

PMID: 16881412 [PubMed - indexed for MEDLINE]

938. Mol Immunol. 2007 Jan;44(1-3):95-102. Epub 2006 Jul 27.

Complement-related molecular events in sepsis leading to heart failure.

Hoesel LM, Niederbichler AD, Ward PA.

Department of Pathology, University of Michigan Medical School, Ann Arbor, MI 48109, USA.

Despite intensive ongoing research efforts, the mortality of patients with sepsis remains unacceptably high. A significant number of clinical trials have failed to produce sufficient therapeutic strategies despite showing promising results in animal models. So far, many studies have focused on deterioration of the humoral and cellular components of the immune system, the main cause of death in septic patients being multi-organ failure. However, not much is known about the effects of the complement system on parenchymal cells of organs such as the heart. Recently, septic cardiomyopathy has been recognized as one of the major complications during sepsis, often determining the clinical outcome. In this review, we describe molecular events which are thought to be related to cardiac dysfunction during sepsis. A special emphasis will be placed on the complement system, which generates powerful anaphylatoxins (such as C5a) and which has recently been associated with septic cardiomyopathy. Together with the impact on cardiac function of various cytokines we will provide a synopsis of the current knowledge regarding the pathophysiology underlying cardiac failure during sepsis with a special emphasis on C5a and C5aR.

PMID: 16875736 [PubMed - indexed for MEDLINE]

939. Int Immunopharmacol. 2006 Sep;6(9):1515-23. Epub 2006 May 17.

Modulation of eosinophil generation and migration by Mangifera indica L. extract (Vimang).

Sá-Nunes A, Rogerio AP, Medeiros AI, Fabris VE, Andreu GP, Rivera DG, Delgado R, Faccioli LH.

Departamento de Análises Clínicas, Toxicológicas e Bromatológicas, Faculdade de Ciências Farmacêuticas de Ribeirão Preto, Universidade de São Paulo, Ribeirão Preto, 14040-903 SP, Brazil. anunes@mail.nih.gov

The effects of Vimang, an aqueous extract of the stem bark of Mangifera indica L. (Anacardiaceae), on cell migration in an experimental model of asthma was investigated. In vivo treatment of Toxocara canis-infected BALB/c mice for 18 days with 50 mg/kg Vimang reduced eosinophil migration into the bronchoalveolar space and peritoneal cavity. Also, eosinophil generation in bone marrow and blood eosinophilia were inhibited in infected mice treated with Vimang. This reduction was associated with inhibition of IL-5 production in serum and eotaxin in lung homogenates. In all these cases the effects of Vimang were more selective than those observed with dexamethasone. Moreover, Vimang treatment is not toxic for the animals, as demonstrated by the normal body weight increase during infection. These data confirm the potent anti-inflammatory effect of Vimang and support its potential use as an alternative therapeutic drug to the treatment of eosinophilic disorders including those caused by nematodes and allergic diseases.

PMID: 16846846 [PubMed - indexed for MEDLINE]

940. Pediatr Allergy Immunol. 2006 Aug;17(5):364-9.

Are food intolerances and allergies increasing in immigrant children coming from developing countries ?

Cataldo F, Accomando S, Fragapane ML, Montaperto D; SIGENP and GLNBI Working Groups on Food Intolerances.

Department of Paediatrics, University of Palermo, Palermo, Italy.

There are not available data concerning the occurrence, the clinical features and the environmental risk factors for food intolerances and allergies in immigrant children. The aim of the study was to evaluate rates, distribution, clinical features and environmental risk factors for food intolerances and allergies in immigrant children. Hospital records of 4,130 patients with celiac disease (CD), cow milk protein intolerance (CMPI) and food allergies (FA) diagnosed in 24 Italian Centres from 1999 to 2001 were retrospectively reviewed, comparing immigrant patients with Italian ones. 78/4,130 (1.9%) patients were immigrant: 36/1,917 (1.9%) had CD, 24/1,370 (1.75%) CMPI and 18/843 (2.1%) FA. They were evenly distributed across Italy and their native areas were: East Europe (23/78), Northern Africa (23/78), Southern Asia (14/78), Saharan and Sub-Saharan Africa (9/78), Southern America (4/78), Far East (3/7), Middle East (2/78). Despite differences in their origin, the clinical features of immigrant children were similar to the ones of Italian patients and among each ethnic group. The majority of them were born in Italy (57/78) or have been residing in Italy since several years (19/78). All of them had lost dietary habits of the native countries and had acquired those of the Italian childhood population. Food intolerances and allergies are present also in children coming from developing countries, and paediatricians will need to have a full awareness of them because the number of immigrant children in Italy is quickly increasing. The clinical features of food intolerances and allergies appear the same in each ethnic group, despite differences in races. Sharing of dietary habits with the Italian childhood population seems to be an important environmental risk factor.

PMID: 16846455 [PubMed - indexed for MEDLINE]

941. Hautarzt. 2007 Feb;58(2):133-4, 136-41.

[Parasites as a cause of urticaria. Helminths and protozoa as triggers of hives?].

[Article in German]

Ronellenfitsch U, Bircher A, Hatz C, Blum J.

Abteilung für Medizin und Diagnostik, Schweizerisches Tropeninstitut, Socinstrasse 57, 4002 Basel.

Urticaria is one of the most prevalent disorders in dermatological practice. The disease can be incapacitating. There are clear pathophysiological and epidemiological hints that helminths and protozoa are rare but treatable causes of acute and chronic urticaria. Doctors and patients are often not aware that parasitic diseases are increasingly common even in industrialized countries due to a steep rise in migration and international travel. This review presents the most important parasitic causes of urticaria and provides relevant details regarding personal history, clinical presentation, diagnosis and therapy.

PMID: 16832670 [PubMed - indexed for MEDLINE]

942. Am J Physiol Cell Physiol. 2006 Nov;291(5):C957-65. Epub 2006 Jul 5.

Interleukin-8: novel roles in human airway smooth muscle cell contraction and

migration.

Govindaraju V, Michoud MC, Al-Chalabi M, Ferraro P, Powell WS, Martin JG.

Seymoure Heisler Laboratory of the Montreal Chest Institute Research Center, McGill University, Montreal, Quebec, Canada H2X 2P2.

In patients with cystic fibrosis (CF) and asthma, elevated levels of interleukin-8 (IL-8) are found in the airways. IL-8 is a CXC chemokine that is a chemoattractant for neutrophils through CXCR1 and CXCR2 G protein-coupled receptors. We hypothesized that IL-8 acts directly on airway smooth muscle cells (ASMC) in a way that may contribute to the enhanced airway responsiveness and airway remodeling observed in CF and asthma. The aim of this study was to determine whether human ASMC (HASMC) express functional IL-8 receptors (CXCR1 and CXCR2) linked to cell contraction and migration. Experiments were conducted on cells harvested from human lung specimens. Real-time PCR and fluorescence-activated cell sorting analysis showed that HASMC expressed mRNA and protein for both CXCR1 and CXCR2. Intracellular Ca(2+) concentration ([Ca(2+)](i)) increased from 115 to 170 nM in response to IL-8 (100 nM) and decreased after inhibition of phospholipase C (PLC) with U-73122. On blocking the receptors with specific neutralizing antibodies, changes in [Ca(2+)](i) were abrogated. IL-8 also contracted the HASMC, decreasing the length of cells by 15%, and induced a 2.5-fold increase in migration. These results indicate that HASMC constitutively express functional CXCR1 and CXCR2 that mediate IL-8-triggered Ca(2+) release, contraction, and migration. These data suggest a potential role for IL-8 in causing abnormal airway structure and function in asthma and CF.

PMID: 16822944 [PubMed - indexed for MEDLINE]

943. Mol Cancer Ther. 2006 Jun;5(6):1434-45.

Withanolides potentiate apoptosis, inhibit invasion, and abolish osteoclastogenesis through suppression of nuclear factor-kappaB (NF-kappaB) activation and NF-kappaB-regulated gene expression.

Ichikawa H, Takada Y, Shishodia S, Jayaprakasam B, Nair MG, Aggarwal BB.

Cytokine Research Laboratory, Department of Experimental Therapeutics, The University of Texas M.D. Anderson Cancer Center, 1515 Holcombe Boulevard, Houston, TX 77030, USA.

The plant Withania somnifera Dunal (Ashwagandha), also known as Indian ginseng, is widely used in the Ayurvedic system of medicine to treat tumors, inflammation, arthritis, asthma, and hypertension. Chemical investigation of the roots and leaves of this plant has yielded bioactive withanolides. Earlier studies showed that withanolides inhibit cyclooxygenase enzymes, lipid peroxidation, and proliferation of tumor cells. Because several genes that regulate cellular proliferation, carcinogenesis, metastasis, and inflammation are regulated by activation of nuclear factor-kappaB (NF-kappaB), we hypothesized that the activity of withanolides is mediated through modulation of NF-kappaB activation. For this report, we investigated the effect of the withanolide on NF-kappaB and NF-kappaB-regulated gene expression activated by various carcinogens. We found that withanolides suppressed NF-kappaB activation induced by a variety of inflammatory and carcinogenic agents, including tumor necrosis factor (TNF), interleukin-1beta, doxorubicin, and cigarette smoke condensate. Suppression was not cell type specific, as both inducible and constitutive NF-kappaB activation was blocked by withanolides. The suppression occurred through the inhibition of inhibitory subunit of IkappaB alpha kinase activation, IkappaB alpha phosphorylation, IkappaB alpha degradation, p65 phosphorylation, and subsequent

p65 nuclear translocation. NF-kappaB-dependent reporter gene expression activated by TNF, TNF receptor (TNFR) 1, TNFR-associated death domain, TNFR-associated factor 2, and IkappaB alpha kinase was also suppressed. Consequently, withanolide suppressed the expression of TNF-induced NF-kappaB-regulated antiapoptotic (inhibitor of apoptosis protein 1, Bfl-1/A1, and FADD-like interleukin-1beta-converting enzyme-inhibitory protein) and metastatic (cyclooxygenase-2 and intercellular adhesion molecule-1) gene products, enhanced the apoptosis induced by TNF and chemotherapeutic agents, and suppressed cellular TNF-induced invasion and receptor activator of NF-kappaB ligand-induced osteoclastogenesis. Overall, our results indicate that withanolides inhibit activation of NF-kappaB and NF-kappaB-regulated gene expression, which may explain the ability of withanolides to enhance apoptosis and inhibit invasion and osteoclastogenesis.

PMID: 16818501 [PubMed - indexed for MEDLINE]

944. J Allergy Clin Immunol. 2006 Jul;118(1):113-9. Epub 2006 May 19.

Montelukast regulates eosinophil protease activity through a leukotriene-independent mechanism.

Langlois A, Ferland C, Tremblay GM, Laviolette M.

Unité de Recherche en Pneumologie, Centre de Recherche de l'Hôpital Laval, Institut Universitaire de Cardiologie et de Pneumologie de l'Université Laval, 2725 Chemin Sainte-Foy, Quebec G1V 4G5, Canada.

BACKGROUND: Migration of eosinophils into bronchial mucosa requires proteolysis. Montelukast, a cysteinyl leukotriene (CysLT) 1 receptor antagonist used in asthma treatment, decreases eosinophil infiltration into the asthmatic airways, suggesting that CysLTs modulate eosinophil protease activity.
OBJECTIVE: We sought to determine whether CysLTs and montelukast regulate eosinophil protease activity.
METHODS: Purified blood eosinophils were treated with or without montelukast; MK-0591, a 5-lipoxygenase-activating protein inhibitor; or leukotriene (LT) D(4). Migration assays through Matrigel were performed in the presence of 5-oxo-6,8,11,14-eicosatetraenoic acid (5-oxo-ETE), a potent eosinophil chemotactic factor, or LTD(4). Expression of molecules implicated in plasmin generation and matrix metalloproteinase (MMP) 9 release were also evaluated.
RESULTS: Montelukast and MK-0591 decreased eosinophil migration promoted by 5-oxo-ETE, whereas LTD(4) failed to induce eosinophil migration. However, LTD(4) significantly boosted the migration rate obtained with a suboptimal concentration of 5-oxo-ETE and partially reversed the inhibition obtained with MK-0591. Montelukast significantly reduced the maximal rate of activation of plasminogen into plasmin by eosinophils obtained with 5-oxo-ETE. 5-Oxo-ETE increased the number of eosinophils expressing urokinase plasminogen activator receptor and stimulated secretion of MMP-9. Montelukast, but neither MK-0591 nor LTD(4), reduced the expression of urokinase plasminogen activator receptor and the secretion of MMP-9 and increased total cellular activity of urokinase plasminogen activator and the expression of plasminogen activator inhibitor 2 mRNA.
CONCLUSION: Montelukast inhibits eosinophil protease activity in vitro through a mechanism that might be independent of its antagonist effect on CysLT 1 receptor.
CLINICAL IMPLICATIONS: This could partially explain montelukast's anti-inflammatory effect in asthma and eventually amplify to improve its therapeutic efficacy.

PMID: 16815146 [PubMed - indexed for MEDLINE]

945. Pharmacol Ther. 2006 Nov;112(2):489-500. Epub 2006 Jul 11.

Strategies for targeting T-cells in allergic diseases and asthma.

Heijink IH, Van Oosterhout AJ.

Laboratory of Allergology and Pulmonary Diseases, Groningen University Medical Center, Groningen University, 9700 RB Groningen, The Netherlands.

T helper (Th) 2 lymphocytes play a crucial role in the initiation, progression and persistence of allergic diseases, including asthma. Drugs that interfere with the activation of T-cells or more selectively Th2-specific signaling molecules and drugs that prevent the selective migration into lung tissue are promising novel strategies for the treatment of allergic asthma. Although the mainstay asthma therapy of inhaled glucocorticoids is rather effective, targeting Th2 cells may be an important alternative in childhood. Regulatory T-cells (Treg cells) have a physiological role in protection of unwanted immune responses to auto-antigens and allergens. Literature data indicate that an imbalance between Th2 and Treg cells may underlie development and disease expression of allergic asthma. Drugs or immunotherapies that stimulate these counter-Treg cells may limit aberrant Th2 responses leading to suppression of symptoms. Furthermore, these types of treatments may offer the perspective of disease modification and long-term relief of symptoms.

PMID: 16814862 [PubMed - indexed for MEDLINE]

946. Thorax. 2006 Oct;61(10):880-5. Epub 2006 Jun 29.

Functional KCa3.1 K+ channels are required for human lung mast cell migration.

Cruse G, Duffy SM, Brightling CE, Bradding P.

Institute for Lung Health, Department of Infection, Immunity and Inflammation, University of Leicester, Glenfield Hospital, Groby Road, Leicester LE3 9QP, UK. glenncruse@hotmail.com

BACKGROUND: Mast cell recruitment and activation are critical for the initiation and progression of inflammation and fibrosis. Mast cells infiltrate specific structures in many diseased tissues such as the airway smooth muscle (ASM) in asthma. This microlocalisation of mast cells is likely to be key to disease pathogenesis. Human lung mast cells (HLMC) express the Ca2+ activated K+ channel K(Ca)3.1 which modulates mediator release, and is proposed to facilitate the retraction of the cell body during migration of several cell types. A study was undertaken to test the hypothesis that blockade of K(Ca)3.1 would attenuate HLMC proliferation and migration.
METHODS: HLMC were isolated and purified from lung material resected for bronchial carcinoma. HLMC proliferation was assessed by cell counts at various time points following drug exposure. HLMC chemotaxis was assayed using standard Transwell chambers (8 microm pore size). Ion currents were measured using the single cell patch clamp technique.
RESULTS: K(Ca)3.1 blockade with triarylmethane-34 (TRAM-34) did not inhibit HLMC proliferation and clotrimazole had cytotoxic effects. In contrast, HLMC migration towards the chemokine CXCL10, the chemoattractant stem cell factor, and the supernatants from tumour necrosis factor alpha stimulated asthmatic ASM was markedly inhibited with both the non-selective K(Ca)3.1 blocker charybdotoxin and the highly specific K(Ca)3.1 blocker TRAM-34 in a dose dependent manner. Although K(Ca)3.1 blockade inhibits HLMC migration, K(Ca)3.1 is not opened by the chemotactic stimulus, suggesting that it must be involved downstream of the initial receptor-ligand interactions.

CONCLUSIONS: Since modulation of K(Ca)3.1 can inhibit HLMC chemotaxis to diverse chemoattractants, the use of K(Ca)3.1 blockers such as TRAM-34 could provide new therapeutic strategies for mast cell mediated diseases such as asthma.

PMCID: PMC2104766
PMID: 16809411 [PubMed - indexed for MEDLINE]

947. Exp Lung Res. 2006 Jan-Feb;32(1-2):15-26.

Doxycycline decreases monocyte chemoattractant protein-1 in human lung epithelial cells.

Raza M, Ballering JG, Hayden JM, Robbins RA, Hoyt JC.

Research Service, Carl T. Hayden VA Medical Center, Phoenix, Arizona 85012, USA.

Certain antibiotics possess anti-inflammatory properties and could potentially be used to treat inflammatory lung diseases associated with an influx of monocytes such as panbronchiolitis, asthma, cystic fibrosis, and bronchitis. Doxycycline is reported to possess anti-inflammatory effects. Monocyte chemoattractant protein-1 (MCP-1) is a major inflammatory cytokine and a powerful chemoattractant for monocytes. The authors hypothesized that doxycycline exerts its anti-inflammatory effects, in part, by reducing MCP-1 production. To test this hypothesis, A549 human lung epithelial cells were stimulated with cytomix in the presence or absence of doxycycline. In stimulated cells doxycycline decreased MCP-1 production by 95% and in monocyte chemotaxis assays migration decreased by 55%. However, doxycycline did decrease expression of MCP-1 mRNA and did not effect its stability. These data demonstrate that doxycycline modulates MCP-1 production and suggest that doxycycline may provide a new anti-inflammatory therapy for chronic lung diseases.

PMID: 16809218 [PubMed - indexed for MEDLINE]

948. Curr Drug Targets. 2006 Jun;7(6):691-8.

Targeting mitogen-activated protein kinases for asthma.

Duan W, Wong WS.

Molecular Immunology Division, La Jolla Institute of Allergy and Immunology, San Diego, CA 92121, USA.

Allergic asthma is a chronic airway inflammatory disorder attributable to T-helper 2 cell responses together with other inflammatory cells such as mast cells, B cells and eosinophils, and pro-inflammatory cytokines and chemokines. Mitogen-activated protein kinase (MAPK) signaling cascades have been shown to be important in the differentiation, activation, proliferation, degranulation and migration of various immune cells, and airway smooth muscle and epithelial cells. In mammal, MAPK signaling modules are divided into at least 3 groups: extracellular signal-regulated kinase (ERK), p38 MAPK, and c-Jun NH2-terminal kinase (JNK). Each MAPK module plays a discrete yet complementary role in accentuating allergic airway inflammation. Cumulative evidence reveals potential anti-inflammatory activities of MAPK inhibitors in a variety of in vitro models of inflammation. Recently, the anti-inflammatory effects of MAPK kinase inhibitor (U0126), p38 MAPK inhibitors (SB239063 and respirable p38alpha MAPK antisense oligonucleotide) and JNK inhibitor (SP600125) have been demonstrated in in vivo animal models of asthma. Development of inhibitors targeting at MAPK could be an attractive strategy for the treatment of asthma.

PMID: 16787171 [PubMed - indexed for MEDLINE]

949. J Clin Immunol. 2006 Jul;26(4):376-87. Epub 2006 Jun 20.

Glucocorticoids increase repair potential in a novel in vitro human airway epithelial wounding model.

Wadsworth SJ, Nijmeh HS, Hall IP.

Division of Therapeutics and Molecular Medicine, D floor, South Block, Queens Medical Centre, University Hospital, Nottingham, NG7 2UH, United Kingdom. Sam.Wadsworth@nottingham.ac.uk

Airway epithelial damage is a cardinal feature of chronic asthma. Agents which enhance epithelial repair without triggering uncontrolled fibrosis of the mesenchyme would be predicted to be useful in the management of asthma. We have developed a repeat wound model using mucociliated human bronchial epithelial cell (HBEC) cultures to define the key pathways involved in airway epithelial repair, and to study the effects of potential therapeutic agents on epithelial repair in a chronic setting. We show that repair occurs primarily by cell migration to close a defect; this process requires activation of the EGF receptor (EGFR) and subsequent tyrosine kinase signalling. Migration is accompanied by up-regulation of CD44 in motile cells at the wound margins with proliferation of non-migrating cells adjacent to the wound area. In long-term studies beta2 adrenoceptor agonists and phosphodiesterase (PDE) inhibitors have no effect on repair potential, in contrast chronic treatment with the glucocorticoid dexamethasone extends the lifespan of repeatedly wounded differentiated cultures. We suggest part of the beneficial effects of glucocorticoids in asthma is related to this ability to prolong repair potential following repeated episodes of epithelial injury.

PMID: 16786432 [PubMed - indexed for MEDLINE]

950. J Invest Dermatol. 2006 Nov;126(11):2422-9. Epub 2006 Jun 15.

Human plasmacytoid dendritic cells express receptors for anaphylatoxins C3a and C5a and are chemoattracted to C3a and C5a.

Gutzmer R, Köther B, Zwirner J, Dijkstra D, Purwar R, Wittmann M, Werfel T.

Department of Dermatology and Allergology, Hannover Medical School, Hannover, Germany. gutzmer.ralf@mh-hannover.de

The presence of plasmacytoid dendritic cells (pDC) was recently demonstrated in lesions of inflammatory skin diseases. Since anaphylatoxins or their precursors were also found in such lesions, we investigated a possible interaction between pDC and anaphylatoxins C3a and C5a. pDC precursors isolated from peripheral blood did not express the receptors for C3a and C5a, complement C3a receptor (C3aR) and complement C3a receptor (C5aR). If these pDC precursors were cultured with IL-3, the resultant immature pDC expressed both receptors. Expression of C3aR and C5aR could also be demonstrated on pDC in lesions of cutaneous lupus erythematosus and allergic contact dermatitis. Such pDC were immature since they lacked the expression of the maturation marker CD83. Blood-derived pDC matured with CpG oligonucleotides downregulated the receptors. Immature pDC responded to C3a and C5a (but not C3adesArg) stimulation with increased F-actin polymerization and chemotactic migration. In contrast, interferon alpha production, surface molecule expression, and T-cell stimulatory capacity were not significantly modulated by

C3a or C5a. Thus, immature pDC represent another type of antigen-presenting cell that express C3aR and C5aR, and respond to anaphylatoxins with chemotaxis. This might be relevant in the direction of pDC to cutaneous lesions of inflammation, for example, in lupus erythematosus or contact dermatitis.

PMID: 16778800 [PubMed - indexed for MEDLINE]

951. Int Arch Allergy Immunol. 2006;140 Suppl 1:55-8. Epub 2006 Jun 9.

Th2-cell-mediated chemokine synthesis is involved in allergic airway inflammation in mice.

Mori A, Ogawa K, Kajiyama Y, Suko M, Kaminuma O.

Clinical Research Center for Allergy and Rheumatology, National Hospital Organization, Sagamihara National Hospital, Kanagawa, Japan. mori-kkr@umin.ac.jp

Eosinophilic inflammation in the bronchial mucosa has been recognized as a prominent pathological feature of bronchial asthma. Th2 cells have been implicated in the local infiltration and activation of eosinophils. The migration of eosinophils as well as Th2 cells is controlled by chemokines, suggesting a crucial role of chemokines in the pathogenesis of bronchial asthma. To elucidate the mechanism by which Th2 cells induce eosinophilic inflammation, a Th2-cell-dependent murine model of asthma was employed in this study. Along with the infiltration of eosinophils and antigen-specific Th2 cells, CC chemokine receptor-3-active eotaxin, monocyte chemoattractant protein (MCP)-3 and RANTES, as well as CC chemokine receptor-3-inactive MCP-1 were produced in the lungs of Th2-cell-transferred mice after antigen provocation in vivo. On the other hand, differentiated antigen-specific Th2 cells produced MCP-3 and RANTES but not eotaxin or MCP-1 upon stimulation in vitro. Chemokines synthesized by Th2 cells and other cell types are involved in the development of eosinophilic inflammation in bronchial asthma.

Copyright 2006 S. Karger AG, Basel.

PMID: 16772728 [PubMed - indexed for MEDLINE]

952. Int Arch Allergy Immunol. 2006;140 Suppl 1:3-7.

Chemotaxis of human peripheral blood eosinophils to 2-arachidonoylglycerol: comparison with other eosinophil chemoattractants.

Kishimoto S, Oka S, Gokoh M, Sugiura T.

Faculty of Pharmaceutical Sciences, Teikyo University, Kanagawa, Japan.

BACKGROUND: 2-Arachidonoylglycerol (2-AG), an endogenous ligand for the cannabinoid receptors (CB1 and CB2), has been shown to exhibit a variety of cannabimimetic activities in vitro and in vivo. Recently, we found that human eosinophilic leukemia EoL-1 cells and human peripheral blood eosinophils express the CB2 receptor. We also found that 2-AG induces the migration of these cells in a CB2 receptor-dependent manner. In this study, we investigated whether the 2-AG-induced migration of human eosinophils is due to chemotaxis or chemokinesis. We also compared the ability of 2-AG to induce the migration of eosinophils with those of other eosinophil chemoattractants.
METHODS: Eosinophils were separated from the peripheral blood of healthy donors. The migration of eosinophils to various stimulants was examined using Transwell inserts. In view of the fact that 2-AG is rapidly metabolized by cells, we

employed 2-AG ether, an ether-linked nonhydrolyzable analog of 2-AG, instead of 2-AG to determine whether the 2-AG-induced migration is due to chemotaxis or chemokinesis.
RESULTS: 2-AG ether induced the migration of human eosinophils, like 2-AG. The 2-AG ether-induced migration was reduced by the coincubation of eosinophils with 2-AG ether in the upper compartment of the Transwell inserts, indicating that the migration is attributable to chemotaxis. The concentration of 2-AG required to induce the eosinophil migration appears to be pathophysiologically relevant, although the order of the pharmacologically effective concentration of 2-AG was approximately ten times lower than those of platelet-activating factor, RANTES and eotaxin.
CONCLUSION: These results strongly suggest that 2-AG is involved in the infiltration of eosinophils during allergic inflammation.

Copyright 2006 S. Karger AG, Basel.

PMID: 16772720 [PubMed - indexed for MEDLINE]

953. Pediatr Allergy Immunol. 2006 Jun;17(4):250-8.

Effects of leukotriene receptor antagonists on monocyte chemotaxis, p38 and cytoplasmic calcium.

Hung CH, Li CY, Hua YM, Chen CJ, Yang KD, Jong YJ.

Department of Pediatrics, Tri-service General Hospital, National Defense Medical Center, Taipei, and Department of Pediatrics, Faculty of Pediatrics, College of Medicine, Kaohsiung Medical University, Taiwan, Republic of China.
pedhung@kmu.edu.fw

Montelukast and zafirlukast, two cysteinyl leukotriene receptor antagonists (LTRAs), have been shown to have a beneficial effect on the clinical symptoms of asthma. LTRAs can inhibit eosinophil recruitment; however, little is known about their role in monocyte migration. We investigated whether montelukast and zafirlukast could suppress chemokine-induced chemotaxis of monocytes and signaling. Chemotaxis of monocytes from peripheral blood mononuclear cells (PBMCs), cord blood mononuclear cells (CBMCs), and THP-1 cells was evaluated using a 24-well transwell microchamber. [Ca2+]i was measured with the fluorescence calcium indicator fura-2/AM photometry system. p38 MAPK expression was measured by Western blotting. Results showed that montelukast (1-100 microm) and zafirlukast (100 microm) significantly down-regulated monocyte chemoattractant protein-1(MCP-1)-induced chemotaxis of THP-1 cells and human primary monocytes from PBMCs and CBMCs (p<0.05, each comparison). Montelukast also abolished MCP-1-induced [Ca2+]i and pp38 MAPK expression in THP-1 cells in a dose-dependent manner. These data demonstrate that montelukast is effective in down-regulating human monocyte chemotaxis induced by MCP-1. This effect may involve the down-regulation of MCP-1-induced [Ca2+]i and p38 MAPK expression.

PMID: 16771778 [PubMed - indexed for MEDLINE]

954. Catheter Cardiovasc Interv. 2006 Jul;68(1):145-52.

Transcatheter closure of perimembranous ventricular septal defects using amplatzer asymmetric ventricular septal defect occluder: preliminary experience with 18-month follow up.

Pinto RJ, Dalvi BV, Sharma S.

Glenmark Cardiac Centre, Matunga (E), Mumbai, India.

BACKGROUND: This study reports our experience in the nonsurgical closure of perimembranous ventricular septal defects in children and adolescents with the Amplatzer asymmetric ventricular septal defect occluder and the outcome of an 18-month follow up.
METHODS AND RESULTS: Twenty patients (median age:10 years; median weight:32 kg) with perimembranous ventricular septal defect were selected for transcatheter closure with the Amplatzer device. The prosthesis diameter chosen was 1-2 mm larger than the largest measured diameter of the defect on transesophageal echo (TEE). All patients were put on oral aspirin (5 mg/kg/day in children and 150 mg/day in adults) five days prior to and for six months after closure. Follow-up evaluation at 48 hr and 1, 6, 12 and 18 months included clinical examination, electrocardiogram, and a transthoracic echocardiogram. The mean defect diameter on color flow mapping on TEE was 7.1 +/- 2.3 mm. The device diameter ranged from 6-14 mm (median = 8 mm). One patient developed an anaphylactic reaction to contrast. The procedure was successful in 17 out of 19 patients where it was attempted (89.4%). In two patients with associated significant aortic valve prolapse and mild aortic regurgitation the device could not be successfully deployed. A trivial residual shunt observed during postdeployment left ventricular angiogram in 7 of 17 patients (41.2%) completely disappeared at one month follow-up. Three patients had right bundle branch block (2 complete and 1 incomplete) whereas one developed junctional escape rhythm with a right bundle branch block morphology. One patient had clinically silent thromboembolism to the left vertebral artery and another patient had hemolysis which resolved spontaneously within 48 hr. Follow-up at 13.5 +/- 5.3 months (range 1-18 months) revealed no residual shunt. The left ventricular internal dimension in diastole decreased significantly from 45 +/- 6 mm to 40 +/- 6 mm (P < 0.01) at the time of the last follow up. The baseline tricuspid regurgitation (n = 4) and aortic regurgitation (n = 3) remained unchanged during the follow up period. None of the patients developed left ventricular outflow tract obstruction or new aortic or tricuspid regurgitation. There were no other device related complications such as device migration, systemic thromboembolism, infective endocarditis, pericardial effusion or delayed conduction disturbances.
CONCLUSIONS: In carefully selected children and young adults, the Amplatzer asymmetric ventricular septal defect occluder is a promising device for transcatheter closure of perimembranous ventricular septal defect with encouraging results on short term follow up.

Copyright 2006 Wiley-Liss, Inc.

PMID: 16763998 [PubMed - indexed for MEDLINE]

955. Immunol Res. 2006;34(2):97-115.

Development, migration, and survival of mast cells.

Okayama Y, Kawakami T.

Research Unit for Allergy Transcriptome, Research Center for Allergy and Immunology, RIKEN Yokohama Institute, Yokohama, Japan.

Mast cells play a pivotal role in immediate hypersensitivity and chronic allergic reactions that can contribute to asthma, atopic dermatitis, and other allergic diseases. Because mast cell numbers are increased at sites of inflammation in allergic diseases, pharmacologic intervention into the proliferation, migration, and survival (or apoptosis) of mast cells could be a promising strategy for the management of allergic diseases. Mast cells differentiate from multipotent hematopoietic progenitors in the bone marrow. Stem cell factor (SCF) is a major

chemotactic factor for mast cells and their progenitors. SCF also elicits cell-cell and cell-substratum adhesion, facilitates the proliferation, and sustains the survival, differentiation, and maturation, of mast cells. Therefore, many aspects of mast cell biology can be understood as interactions of mast cells and their precursors with SCF and factors that modulate their responses to SCF and its signaling pathways. Numerous factors known to have such a capacity include cytokines that are secreted from activated T cells and other immune cells including mast cells themselves. Recent studies also demonstrated that monomeric IgE binding to FcepsilonRI can enhance mast-cell survival. In this review we discuss the factors that regulate mast cell development, migration, and survival.

PMCID: PMC1490026
PMID: 16760571 [PubMed - indexed for MEDLINE]

956. Am J Physiol Lung Cell Mol Physiol. 2006 Oct;291(4):L794-801. Epub 2006 Jun 2.

Effects of corticosteroid-induced apoptosis on airway epithelial wound closure in vitro.

Dorscheid DR, Patchell BJ, Estrada O, Marroquin B, Tse R, White SR.

James Hogg iCAPTURE Centre for Cardiovascular and Pulmonary Research, University of British Columbia, Vancouver, Canada.

Damage to the airway epithelium is common in asthma. Corticosteroids induce apoptosis in and suppress proliferation of airway epithelial cells in culture. Whether apoptosis contributes to impaired epithelial cell repair after injury is not known. We examined whether corticosteroids would impair epithelial cell migration in an in vitro model of wound closure. Wounds (approximately 0.5-1.3 mm2) were created in cultured 1HAEo- human airway epithelial cell monolayers, after which cells were treated with up to 10 microM dexamethasone or budesonide for 24 h. Cultured cells were pretreated for 24 or 48 h with dexamethasone to observe the effect of long-term exposure on wound closure. After 12 h, the remaining wound area in monolayers pretreated for 48 h with 10 microM dexamethasone was 43+/-18% vs. 10+/-8% for untreated control monolayers. The addition of either corticosteroid immediately after injury did not slow closure significantly. After 12 h the remaining wound area in monolayers treated with 10 microM budesonide was 39+/-4% vs. 43+/-3% for untreated control monolayers. The proportion of apoptotic epithelial cells as measured by terminal deoxynucleotidyltransferase-mediated dUTP biotin nick end labeling both at and away from the wound edge was higher in monolayers treated with budesonide compared with controls. However, wound closure in the apoptosis-resistant 1HAEo-.Bcl-2+ cell line was not different after dexamethasone treatment. We demonstrate that corticosteroid treatment before mechanical wounding impairs airway epithelial cell migration. The addition of corticosteroids after injury does not slow migration, despite their ability to induce apoptosis in these cells.

PMID: 16751221 [PubMed - indexed for MEDLINE]

957. J Allergy Clin Immunol. 2006 Jun;117(6):1285-91.

Mast cells: ontogeny, homing, and recruitment of a unique innate effector cell.

Gurish MF, Boyce JA.

Division of Rheumatology, Immunology and Allergy, Department of Medicine, Brigham and Women's Hospital, MA 02115-6007, USA. mgurish@rics.bwh.harvard.edu

Mast cells (MCs) are found principally in peripheral tissues yet are of bone marrow origin. Recent studies in mice trace the MC lineage from the common myeloid progenitor through the granulocyte-macrophage progenitor in the bone marrow to a committed MC progenitor (MCP). Additionally, at least in the mouse, a bipotent basophil-MC progenitor has been identified in the spleen, suggesting a physiologic role for this organ in MC development. MCPs are especially abundant in the mouse intestine, likely ensuring the capacity for a rapid expansion of MCs in the intestinal epithelium during the effector response to helminth infection and perhaps providing a pool of committed cells capable of redistribution to other tissues. Migration of MCPs to the intestine is constitutive and controlled by alpha chemokine receptor 2 and alpha4beta7 integrins expressed on the MCPs, with the latter integrin interacting with endothelial vascular cell adhesion molecule 1 and mucosal addressin cell adhesion molecule 1. In contrast, normal mouse lung tissue contains few MCPs and MCs, and these cellular reservoirs are not affected by the lack of alpha chemokine receptor 2 or alpha4beta7 integrin. Nonetheless, robust recruitment of MCPs to the lung occurs during experimentally induced allergic pulmonary inflammation and requires alpha4beta7 and alpha4beta1 integrins interacting with vascular cell adhesion molecule 1 but not with mucosal addressin cell adhesion molecule 1. Thus although MCs are present in all organs, the pathways responsible for the trafficking of MCPs from the circulation are organ specific and include both constitutive and inducible systems, ensuring both resident MCs and the potential for incremental recruitment in accord with the requirements of the immune response. These findings in mice await confirmation in human subjects.

PMID: 16750988 [PubMed - indexed for MEDLINE]

958. Int Immunol. 2006 Aug;18(8):1233-42. Epub 2006 May 30.

CCL27 is a critical factor for the development of atopic dermatitis in the keratin-14 IL-4 transgenic mouse model.

Chen L, Lin SX, Agha-Majzoub R, Overbergh L, Mathieu C, Chan LS.

Department of Dermatology, University of Illinois at Chicago, College of Medicine, MC624, 808 South Wood Street, Room 376, 60612, USA.

The keratin-14 IL-4 transgenic (Tg) mouse model of atopic dermatitis (AD) is characterized by skin infiltration of T cells, early up-regulation of T(h)2 cytokines and late surge of T(h)1 cytokines. In the present study, we investigated the role of CCL27, a T cell skin-homing chemokine known to be elevated in sera of human AD patients, in disease development in our animal model of AD. The results showed that the mRNA and protein levels of CCL27 in the skin and serum were significantly increased in IL-4 Tg mice. The percentage of T cells expressing CCR10 in skin draining lymph nodes of IL-4 Tg mice was increased, consistent with the findings of >80% of skin-infiltrating T cells in Tg mice expressing CCR10. Chemotaxis transmigration assay demonstrated that CCL27 promotes a greater degree of migration of T cells in diseased Tg mice. Subcutaneous injection of neutralizing anti-CCL27 to IL-4 Tg mice with early skin lesions resulted in reduced clinical progression of inflammation, accompanied with decreased T cell and mast cell infiltration in the skin, and down-regulation of inflammatory cytokines. In conclusion, CCL27 and CCR10 interaction is important for the development of skin inflammation in our AD model.

PMID: 16735375 [PubMed - indexed for MEDLINE]

959. Am J Respir Cell Mol Biol. 2006 Oct;35(4):503-11. Epub 2006 May 25.

Urokinase-type plasminogen activator modulates airway eosinophil adhesion in asthma.

Brooks AM, Bates ME, Vrtis RF, Jarjour NN, Bertics PJ, Sedgwick JB.

Department of Medicine, Allergy, Immunology and Pulmonary Unit, University of Wisconsin, Madison, USA.

Eosinophils migrate from the vascular circulation to the inflamed airways during asthma exacerbations. While the mechanism(s) of this process is not known, the expression of urokinase-type plasminogen activator receptor (uPAR) has been found to modulate neutrophil adhesion and migration to inflammatory sites. We hypothesized that increased expression of uPAR and its ligand, uPA, enhance eosinophil adhesion in patients with asthma. Patients with allergic asthma underwent segmental bronchoprovocation with allergen; 48 h later, peripheral blood and airway (from bronchoalveolar lavage fluid) eosinophils were isolated. uPA and uPAR protein expression were measured by flow cytometry and Western blot; mRNA was quantified by real-time PCR. Eosinophil adhesion to intercellular adhesion molecule (ICAM)-1 and vascular cell adhesion molecule (VCAM)-1 was assessed by eosinophil peroxidase activity. Airway eosinophils expressed significantly more uPA and uPAR protein and uPAR mRNA than peripheral blood eosinophils. Removal of cell-bound uPA and/or addition of exogenous uPA had no effect on blood eosinophil adhesion to ICAM-1 or VCAM-1. In contrast, exogenous uPA stimulated ICAM and VCAM adhesion of airway eosinophils. N-formyl-methionyl-leucyl-phenylalanine-activated airway eosinophil adherence to VCAM-1 and ICAM-1 (VCAM-1, 52.8 +/- 4.7%; ICAM-1, 49.2 +/- 5.3%) was increased over blood eosinophil adhesion (VCAM-1, 38.4 +/- 3.6%; ICAM-1, 27.7 +/- 4.9%; $P < 0.05$). Removal of cell-bound uPA from airway eosinophils decreased adhesion to blood cell levels; reintroduction of exogenous uPA completely restored adhesion levels. These data suggest that constitutive uPA primes, and exogenous uPA can activate, airway eosinophil adhesion following segmental allergen challenge and that increased uPA expression may be a mechanism of increased eosinophil infiltration and function in asthma.

PMCID: PMC2643268
PMID: 16728704 [PubMed - indexed for MEDLINE]

960. J Agric Saf Health. 2006 May;12(2):155-63.

Skin disease among Latino farmworkers in North Carolina.

Krejci-Manwaring J, Schulz MR, Feldman SR, Vallejos QM, Quandt SA, Rapp SR, Arcury TA.

Department of Dermatology, Wake Forest University School of Medicine, Winston-Salem, North Carolina 27157-1084, USA.

An estimated 4.2 million seasonal and migrant farmworkers and their dependents live in the U.S. Most of these farmworkers are Latino. These workers are exposed to numerous occupational and environmental risk factors that can result in skin disease. Few data exist on the prevalence of skin disease in this population. The purpose of this study was to estimate the prevalence and predictors of skin disease in a sample of Latino farmworkers in North Carolina. A sample of 59 farmworkers was recruited and interviewed at two camps during the 2004 agricultural season. A dermatologist completed a skin exam of each worker and recorded any skin disease present. Forty-two (77.7%) of the 54 men, and all five of the women examined had a diagnosed skin disease. For the men, onychomycosis (nail fungus, 31.5%), tinea pedis (foot fungus, 27.8%), and acne (24.1 %) were

the most commonly diagnosed skin diseases, with contact dermatitis diagnosed in 5.6% of the sample. Other diagnoses included scars, sunburn, and atopic dermatitis. Among the women, diagnoses included melasma (dark patches on the face, 2 cases), xerosis (excessively dry skin, 1 case), tinea pedis (2 cases), onychomycosis (1 case), acne (1 case), and insect bites (1 case). There were no statistically significant differences between workers in the two camps despite different growing seasons and different crops harvested. Skin disease is prevalent among the North Carolina Latino farmworkers who participated in this study, with fungal disease being the most prevalent.

PMID: 16724791 [PubMed - indexed for MEDLINE]

961. Curr Drug Targets. 2006 May;7(5):567-77.

Extracellular matrix, integrins, and mesenchymal cell function in the airways.

Fernandes DJ, Bonacci JV, Stewart AG.

Department of Pharmacology, University of Melbourne, Parkville 3010, Australia. fernandesdj@yahoo.com.au

Subepithelial fibrosis is one of the characteristic features of asthmatic airways. The fibrotic response includes an increase in volume occupied by extracellular matrix (ECM) tissue, and a change in the ECM composition favouring wound type collagens, fibronectin and a number of glycoproteins and proteoglycans normally associated with development. The altered ECM is likely to be deposited by the mesenchymal cells (including (myo) fibroblasts and smooth muscle) that are increased in number in asthmatic airways. In turn, the altered asthmatic ECM is likely to influence the function of the resident airway cells, and may be directly responsible for increasing proliferation, migration, ECM synthesis, inflammatory mediator release, and survival of resident mesenchymal cells. Therefore, the deposited ECM may perpetuate the disease phenotype. The different components of the ECM bi-directionally communicate with cells through a family of transmembrane receptors called integrins. Current research has begun to characterize: 1) the particular ECM components altered in airways disease; 2) the breadth of activity of different ECM components on airway cell function; and 3) the particular integrins responsible for mediating these effects. Further understanding of the role of integrins in transmitting responses of ECM in healthy or diseased airways may lead to novel targets for anti-asthma therapy.

PMID: 16719767 [PubMed - indexed for MEDLINE]

962. Gac Med Mex. 2006 Mar-Apr;142(2):139-44.

[The role of eotaxins in bronchial asthma and nasal polyposis].

[Article in Spanish]

Terán LM, Ledesma-Soto Y, Krengel S, Lezcano-Meza D.

Instituto Nacional de Enfermedades Respiratorias, SSA, Mexico DF, México.

Over the last few years, three specific eosinophil activating peptides, eotaxin-1, -2 and -3, members of the chemokine family have been identified. These cytokines exert a number of functions on eosinophils including chemotaxis, transendothelial migration and induction of the release of reactive oxygen species. Eosinophils are considered to play an important role in allergic disease by causing tissue damage through the release of toxic proteases, lipid mediators,

cytokines and oxygen free radicals. This article reviews the role of eotaxins in asthma and nasal polyps. Discussion focuses on therapeutic guidelines, particularly as it has been shown that CCR3, the major chemokine receptor in eosinophils, serves as a eotaxin receptor.

PMID: 16711548 [PubMed - indexed for MEDLINE]

963. Exp Dermatol. 2006 Jun;15(6):432-40.

Skin irritants and contact sensitizers induce Langerhans cell migration and maturation at irritant concentration.

Jacobs JJ, Lehé CL, Hasegawa H, Elliott GR, Das PK.

Department of Pathobiology, Utrecht University, Utrecht, The Netherlands.

Skin irritants and contact allergens reduce the number of Langerhans cells (LCs). It has been assumed that this reduction is due their migration to the draining lymph node (LN) for initiating immune sensitization in a host. Skin irritation, however, as opposed to contact allergy is not considered to be an immunological disease. Nevertheless, skin irritants are also known for their adjuvant-like effects on contact allergy, resulting in skin hypersensitivity reactions like toxic dermatitis. The human organotypic skin explant culture (hOSEC) model is used to study the characteristics of chemical-induced migration of CD1a(+) LCs out of the epidermis in relation to irritancy or toxicity. We analysed cells emigrating out of hOSEC for CD1a(+) LCs, CD83(+) mature dendritic cells (DCs) and CCR7(+) LN homing cells. After exposure to a toxic concentration of a non-immunogenic irritant, an increase of CD1a(+)CD83(+) LCs was found in the culture medium. A non-toxic concentration of an sensitizer induced an increase of CD1a(+) cells. About 50% of skin emigrating CD1a(+) LCs were CD83(-) (immature) but all were CCR7(+). Skin irritation by both non-allergenic and allergenic compounds induces LC migration and maturation. In contrast, only allergenic compounds induced LC migration with partial maturation at subtoxic concentration. This effectively demonstrates that irritation is physiologically needed stimuli for inducing LC maturation.

PMID: 16689859 [PubMed - indexed for MEDLINE]

964. Exp Dermatol. 2006 Jun;15(6):421-31.

Subtoxic concentrations of allergenic haptens induce LC migration and maturation in a human organotypic skin explant culture model: a novel method for identifying potential contact allergens.

Lehé CL, Jacobs JJ, Hua CM, Courtellemont P, Elliott GR, Das PK.

Department of Pathology, UvA-AMC, Amsterdam, The Netherlands.

The accelerated migration of Langerhans cells (LCs) out of the epidermis and up-regulation of maturation markers, upon treatment with subtoxic concentrations of chemicals, were used as the criteria to determine the potential of allergenic chemicals capable of inducing a hapten-specific delayed-type hypersensitivity reaction. Here we report the findings of a study in which seven chemicals, coded and tested in a blind fashion, were classified as contact allergens or non-allergens using the human organotypic skin explant culture (hOSEC) model. All chemicals that were identified as a contact sensitizer on decoding induced a definite decrease in the number of CD1a and HLA-DR-positive epidermal LCs in the epidermis of the skin explants, as determined by both semiquantitative

immunohistochemistry and quantitative flow cytometric analysis. A significant increase in the number of CD83(+) cells was accompanied by up-regulation of activation molecules in the epidermis of hOSEC exposed specifically to contact allergens. In contrast, there were only minor alterations in epidermal LC numbers, expression of CD83 and other activation markers by LCs when the biopsies were treated with non-toxic concentrations of non-allergenic irritants and vehicles. The data suggest that an increased epidermal LC migration and maturation accompanied by increased expression of activation markers could be used as end-point determinants to screen allergens in a non-animal alternative hOSEC model.

PMID: 16689858 [PubMed - indexed for MEDLINE]

965. J Clin Invest. 2006 Jun;116(6):1624-32. Epub 2006 May 4.

The H1 histamine receptor regulates allergic lung responses.

Bryce PJ, Mathias CB, Harrison KL, Watanabe T, Geha RS, Oettgen HC.

Division of Immunology, Children's Hospital, Harvard Medical School, Boston, Massachusetts, USA. p-bryce@northwestern.edu

Histamine, signaling via the type 1 receptor (H1R), has been shown to suppress Th2 cytokine production by in vitro cultured T cells. We examined the role of H1R in allergic inflammation in vivo using a murine asthma model. Allergen-stimulated splenic T cells from sensitized H1R-/- mice exhibited enhanced Th2 cytokine production. Despite this Th2 bias, allergen-challenged H1R-/- mice exhibited diminished lung Th2 cytokine mRNA levels, airway inflammation, goblet cell metaplasia, and airway hyperresponsiveness (AHR). Restoration of pulmonary Th2 cytokines in H1R-/- mice by intranasal IL-4 or IL-13 restored inflammatory lung responses and AHR. Further investigation revealed that histamine acts as a T cell chemotactic factor and defective T cell trafficking was responsible for the absence of lung inflammation. Cultured T cells migrated in response to histamine in vitro, but this was ablated by blockade of H1R but not H2R. In vivo, allergen-specific WT but not H1R-/- CD4+ T cells were recruited to the lungs of naive recipients following inhaled allergen challenge. H1R-/- T cells failed to confer airway inflammation or AHR observed after transfer of WT T cells. Our data establish a role for histamine and H1R in promoting the migration of Th2 cells into sites of allergen exposure.

PMCID: PMC1448167
PMID: 16680192 [PubMed - indexed for MEDLINE]

966. J Invest Dermatol. 2006 Jul;126(7):1559-73. Epub 2006 May 4.

A chronic contact eczema impedes migration of antigen-presenting cells in alopecia areata.

Gupta P, Freyschmidt-Paul P, Vitacolonna M, Kiessling S, Hummel S, Hildebrand D, Marhaba R, Zöller M.

Department of Tumor Progression and Tumor Defense, German Cancer Research Center, Heidelberg, Germany.

Long-lasting allergen treatment is the most efficient therapy in alopecia areata (AA). The underlying mechanism is unknown. We here asked whether treatment with a contact sensitizer influences leukocyte migration such that dendritic cell (DC) migration or the recruitment of activated T-cells towards the skin become

hampered. Allergen treatment of AA mice was not accompanied by a decrease in skin-infiltrating leukocytes or draining lymph node cells (LNC). However, the distribution of leukocyte subsets was changed with a dominance of monocytes in the skin and a reduced percentage of DCs in draining nodes. Chemokine and chemokine receptor expression in skin and draining nodes was strikingly increased and LNC from untreated and allergen-treated AA mice showed high migratory activity in vitro and readily homed in draining nodes and skin after intravenous injection. However, FITC labelling of the skin and subcutaneous transfer of dye-labelled DC revealed that allergen treatment created a chemokine milieu severely hampering DC migration from the skin towards the draining node. An allergic eczema-induced reduction in DC migration and antigen transfer could well contribute to insufficient T-cell activation and the recovery of hair follicle in AA and possibly be of relevance for other skin-related autoimmune diseases.

PMID: 16675965 [PubMed - indexed for MEDLINE]

967. J Allergy Clin Immunol. 2006 May;117(5):1155-62. Epub 2006 Feb 21.

Toll-like receptor agonists differentially regulate cysteinyl-leukotriene receptor 1 expression and function in human dendritic cells.

Thivierge M, Stankova J, Rola-Pleszczynski M.

Immunology Division, Department of Pediatrics, Faculty of Medicine, Université de Sherbrooke, Quebec, Canada.

BACKGROUND: Dendritic cells (DCs) acquire, during their maturation, the expression of the chemokine receptor CCR7 and the ability to migrate to lymph nodes in response to CC chemokine ligand 19 (CCL19). This migration is impaired in mice lacking the leukotriene (LT) C4 transporter and restored by addition of exogenous LTC4.
OBJECTIVE: To define the role of LT in human DC function, we studied the expression and function of the cysteinyl-leukotriene (CysLT) receptors during DC differentiation from monocytes and subsequent maturation.
METHODS: Receptor expression was measured by flow cytometry and real-time PCR. Responsiveness to LTD4 stimulation was assessed by calcium flux and chemotaxis.
RESULTS: Maturation of DC with LPS, a classic Toll-like receptor 4 agonist, reduced CysLT receptor 1 (CysLT1) expression by 50%, whereas CysLT receptor 2 expression was increased. In contrast, the Toll-like receptor 3 agonist poly inosinic and cytidylic acid (polyI:C) had no effect on receptor expression. Downregulation of CysLT1 expression by LPS could not be mimicked by TNF-alpha alone or in combination with IL-1beta or IL-6. It was, however, prevented by inhibitors of COX and could be reproduced by a combination of TNF-alpha and prostaglandin E2. Immature DCs and DCs matured with polyI:C, but not with LPS, responded to LTD4 with a robust cytosolic calcium flux, which was prevented by the CysLT1 antagonist montelukast. LTD4 induced DC chemotaxis and enhanced DC migration in response to CCL19 in DCs matured with polyI:C, but only weakly in DCs matured with LPS.
CONCLUSION: Our data suggest that human DCs may differentially respond to leukotriene, depending on their maturational stimuli.
CLINICAL IMPLICATIONS: Our study demonstrates that some microbial agents can reduce the migration of dendritic cells in response to leukotrienes, with potential for differential involvement of these cells in allergic inflammation.

PMID: 16675346 [PubMed - indexed for MEDLINE]

968. Clin Exp Allergy. 2006 Apr;36(4):475-82.

Inhibitory effects of fluvastatin on cytokine and chemokine production by peripheral blood mononuclear cells in patients with allergic asthma.

Samson KT, Minoguchi K, Tanaka A, Oda N, Yokoe T, Yamamoto Y, Yamamoto M, Ohta S, Adachi M.

First Department of Internal Medicine, Showa University School of Medicine, Tokyo, Japan.

BACKGROUND: Statins have anti-inflammatory effects on immune cells.
OBJECTIVE: To investigate the immunomodulatory effects of fluvastatin on peripheral blood mononuclear cells (PBMCs) after allergen-specific and non-allergen-specific stimulation in patients with asthma and in healthy subjects.
METHODS: PBMCs from seven patients with asthma who showed elevated immunoglobulin (Ig)E to house dust mite were isolated and stimulated with Dermatofagoides farinae, purified protein derivative, and phytohaemagglutinin (PHA) in the presence or absence of fluvastatin. PBMCs from seven healthy subjects were stimulated with PHA. The effects of fluvastatin on cell proliferation and production of cytokines (interferon [IFN]-gamma and interleukin [IL]-5) and chemokines (chemokine CXC motif, ligand [CXCL10], and CC chemokine ligand [CCL17]) were measured. Migration of T helper (Th)1 and Th2 cell lines was also investigated. The expression of CXCR3 and CCR4 was analysed with flow cytometry. Steroid-insensitive PBMCs induced by preculture with IL-2 and IL-4 were also evaluated. Some experiments were performed in the presence of mevalonic acid.
RESULTS: Fluvastatin inhibited the proliferation of PBMCs and decreased the production of IL-5, IFN-gamma, CCL17, and CXCL10 after allergen-specific and non-allergen-specific stimulation; all these effects, except for decreased CXCL10 production, were partially reversed by mevalonic acid. Culture supernatants obtained in the presence of fluvastatin prevented the migration of Th1 and Th2 cell lines in a dose-dependent manner. In addition, CCR4 and CXCR3 expression on CD4(+) T cells was not affected by the presence of fluvastatin. Fluvastatin inhibited the proliferative response of steroid-insensitive PBMCs to phytohaemagglutinin.
CONCLUSION: Fluvastatin has inhibitory effects on cytokine and chemokine production, and thus might be used as a potential therapeutic agent in severe asthma.

PMID: 16630152 [PubMed - indexed for MEDLINE]

969. J Pharmacol Exp Ther. 2006 Jul;318(1):411-7. Epub 2006 Apr 13.

Selective inhibition of eosinophil influx into the lung by small molecule CC chemokine receptor 3 antagonists in mouse models of allergic inflammation.

Das AM, Vaddi KG, Solomon KA, Krauthauser C, Jiang X, McIntyre KW, Yang XX, Wadman E, Welch P, Covington M, Graden D, Yeleswaram K, Trzaskos JM, Newton RC, Mandlekar S, Ko SS, Carter PH, Davies P.

Department of Immunology, Bristol Myers Squibb Co., P.O. Box 4000, Mail code K24-09, Princeton, NJ 08543-4000, USA.

CC chemokine receptor (CCR) 3 is a chemokine receptor implicated in recruiting cells, particularly eosinophils (EPhi), to the lung in episodes of allergic asthma. To investigate the efficacy of selective, small molecule antagonists of CCR3, we developed a murine model of EPhi recruitment to the lung. Murine eotaxin was delivered intranasally to mice that had previously received i.p. injections of ovalbumin (OVA), and the effects were monitored by bronchoalveolar lavage. A selective eosinophilic influx was produced in animals receiving eotaxin but not

saline. Furthermore, the number of EPhi was concentration- and time-dependent. Although anti-CCR3 antibody reduced the number of EPhi, the effect of eotaxin in OVA-sensitized mice was not a direct chemotactic stimulus because mast cell deficiency (in WBB6F1-Kitw/Kitw-v mice) significantly reduced the response. Two representative small molecule CCR3 antagonists from our program were characterized as being active at mouse CCR3. They were administered p.o. to wild-type mice and found to reduce eotaxin-elicited EPhi selectively in a dose-dependent manner. Pump infusion of one of the inhibitors to achieve steady-state levels showed that efficacy was not achieved at plasma concentrations equivalent to the in vitro chemotaxis IC90 but only at much higher concentrations. To extend the results from our recruitment model, we tested one of the inhibitors in an allergenic model of airway inflammation, generated by adoptive transfer of OVA-sensitive murine T helper 2 cells and aerosolized OVA challenge of recipient mice, and found that it inhibited EPhi recruitment. We conclude that small molecule CCR3 antagonists reduce pulmonary eosinophilic inflammation elicited by chemokine or allergenic challenge.

PMID: 16614169 [PubMed - indexed for MEDLINE]

970. Semin Respir Crit Care Med. 2006 Apr;27(2):171-84.

Parasitic pulmonary eosinophilia.

Chitkara RK, Krishna G.

Division of Pulmonary and Critical Care Medicine, Veterans Affairs Palo Alto Healthcare System, Stanford University School of Medicine, Palo Alto, California 94304, USA. rkc@stanford.edu

Parasitic infections, although common in tropical and subtropical regions, are prevalent worldwide because of changing immigration patterns and in international travel. The burden of worm infection is enormous and the intensity of infection is usually high among the poor and in immunocompromised individuals. Pulmonary eosinophilia occurs in almost all metazoan infections. In the Western world, the most common infections are caused by Strongyloides, Ascaris, Toxocara, and Ancylostoma species. Most of the nematodes multiply within the human host and cause pulmonary eosinophilia during larval migration through the lungs. Despite larval migration through the lungs, there is usually no permanent lung damage. The result is an increased number of eosinophils in the airways or lung parenchyma with or without peripheral eosinophilia. Löffler's syndrome, visceral larva migrans, and tropical pulmonary eosinophilia are the most common infections that cause pulmonary eosinophilia. The most serious parasitic eosinophilic lung disease is tropical pulmonary eosinophilia, a disorder caused by the filarial worms Wuchereria bancrofti and Brugia malayi, in which cases have typically been reported to masquerade acute or refractory bronchial asthma. Increasing awareness, newer diagnostic techniques, preventative measures, and antiparasitic drugs are important in reducing the worldwide morbidity and mortality from parasitic helminths and protozoa. This review focuses on common and some uncommon causes of pulmonary parasitic eosinophilia and their manifestations, diagnosis, and management.

PMID: 16612768 [PubMed - indexed for MEDLINE]

971. Thorax. 2006 Aug;61(8):657-62. Epub 2006 Apr 6.

Mast cell migration to Th2 stimulated airway smooth muscle from asthmatics.

Sutcliffe A, Kaur D, Page S, Woodman L, Armour CL, Baraket M, Bradding P, Hughes

JM, Brightling CE.

Institute for Lung Health, Department of Infection, University of Leicester, Leicester, UK.

BACKGROUND: Mast cell microlocalisation within the airway smooth muscle (ASM) bundle is an important determinant of the asthmatic phenotype. We hypothesised that mast cells migrate towards ASM in response to ASM derived chemokines.
METHODS: Primary ASM cultures from subjects with and without asthma were stimulated with interleukin (IL)-1beta, IL-4, and IL-13 alone and in combination. Mast cell chemotaxis towards these ASM supernatants was investigated, and the chemotaxins mediating migration by using specific blocking antibodies for stem cell factor (SCF) and the chemokine receptors CCR3, CXCR1, 3 and 4 as well as the Gi inhibitor pertussis toxin and the tyrosine kinase inhibitor genistein were defined. The concentrations of CCL11, CXCL8, CXCL10, TGF-beta, and SCF in the supernatants were measured and the effect of non-asthmatic ASM supernatants on the mast cell chemotactic activity of asthmatic ASM was examined.
RESULTS: Human lung mast cells and HMC-1 cells migrated towards Th2 stimulated ASM from asthmatics but not non-asthmatics. Mast cell migration was mediated through the combined activation of CCR3 and CXCR1. CCL11 and CXCL8 expression by ASM increased markedly after stimulation, but was similar in those with and without asthma. ASM supernatants from non-asthmatics inhibited mast cell migration towards the asthmatic ASM supernatant.
CONCLUSION: Th2 stimulated ASM from asthmatics is chemotactic for mast cells. Non-asthmatic ASM releases a mediator or mediators that inhibit mast cell migration towards stimulated asthmatic ASM. Specifically targeting mast cell migration into the ASM bundle may provide a novel treatment for asthma.

PMCID: PMC2104682
PMID: 16601090 [PubMed - indexed for MEDLINE]

972. Allergy Asthma Proc. 2006 Jan-Feb;27(1):59-62.

Allergic rhinitis in Korean immigrants to the United States.

Kim AS, Majmudar SP, Yu BH.

Rush University Medical Center, Chicago, Illinois 60612, USA.

The prevalence of allergic rhinitis among Korean immigrants to the United States is unknown. However, after arrival in the United States, many develop allergic rhinitis for the first time. This study is undertaken to investigate and establish some contributing environmental factors and the time until onset of allergic rhinitis in Korean immigrants to the United States living in Chicago. Information regarding 246 patients of Korean origin who presented to a Chicago allergy/immunology clinic from 1993 to 1998 were analyzed by retrospective chart review. The diagnosis of allergic rhinitis was established by history, physical examination, and skin testing for immediate hypersensitivity to airborne allergens. The mean residential time in the United States of our patients was 13.5 years (range, 2-38 years). The mean interval from arrival in the United States to onset of symptoms was 8 years (range, 0-24 years) The most commonly identified allergens were ragweed pollen (59%), cat pelt (44%), cocklebur pollen (41%), house-dust mite (35%), and Penicillium (29%). We conclude that the spectrum of responsible allergens in Korean immigrants closely resembles that seen in native citizens of the United States and that environmental factors play an important role in the pathogenesis of allergic rhinitis in this population.

PMID: 16598994 [PubMed - indexed for MEDLINE]

973. Neuroscience. 2006 Jul 7;140(3):1011-22. Epub 2006 Apr 3.

The tripeptide phenylalanine-(D) glutamate-(D) glycine modulates leukocyte infiltration and oxidative damage in rat injured spinal cord.

Bao F, John SM, Chen Y, Mathison RD, Weaver LC.

Spinal Cord Injury Team, Laboratory of Spinal Cord Injury, BioTherapeutics Research Group, Robarts Research Institute, 100 Perth Drive, London, Ontario, Canada N6A 5K8.

The tripeptide, phenylalanine-glutamate-glycine (FEG) and its d-isomeric form phenylalanine-(D) glutamate-(D) glycine (feG), derived from submandibular gland peptide-T, significantly reduce the allergic inflammatory response and leukocyte trafficking and neutrophil migration into intestine, heart and lungs. Due to these actions, we hypothesized that feG would attenuate the early inflammatory response to spinal cord injury, reduce free radical production and improve neurological outcomes, like other leukocyte-limiting strategies we have used previously. We tested this using a clip compression model of spinal cord injury in rats. Following spinal cord injury at the 4th thoracic cord segment, we quantified leukocyte infiltration, free radical formation and oxidative damage at the lesion site after feG or control peptide phenylalanine-(D) aspartate-(D) glycine treatment. In rats treated with feG at 2 and 12 h, or 6 and 12 h after spinal cord injury, mean myeloperoxidase activity and ED-1 expression were significantly lower (approximately 40%) than in controls at 24 h. Free radical formation generated in injured spinal cord was detected using 2',7'-dichlorofluorescin-diacetate as a fluorescent probe. Free radical production in the injured cord increased significantly after spinal cord injury and feG treatment significantly reduced this free radical production. Oxidative enzymes, lipid peroxidation and cell death were also significantly (approximately 40%), gp91 (approximately 30%), thiobarbituric acid reactive substance levels (approximately 35%), 4-hydroxynonenal-bound protein (approximately 35%) and caspase-3 (approximately 32%). Early administration of feG decreases infiltration of inflammatory cells into the injured spinal cord and intraspinal free radical formation, thereby reducing oxidative damage and secondary cell death after spinal cord injury.

PMID: 16581192 [PubMed - indexed for MEDLINE]

974. J Neuroimmunol. 2006 Jun;175(1-2):12-8. Epub 2006 Mar 31.

Cytokine-mediated modulation of MMPs and TIMPs in multipotential neural precursor cells.

Ben-Hur T, Ben-Yosef Y, Mizrachi-Kol R, Ben-Menachem O, Miller A.

Department of Neurology, The Agnes Ginges Center for Human Neurogenetics, Hadassah-Hebrew University Medical Center, Jerusalem, Israel.
tamir@hadassah.org.il

Recent studies have implicated the inflammatory process during experimental allergic encephalomyelitis (EAE) in triggering migration and differentiation of transplanted neural precursors cells (NPCs) into the inflamed white matter. The pro-inflammatory cytokines tumor necrosis factor (TNF)-alpha and interferon (IFN)-gamma are key factors in the pathogenesis of brain inflammation in EAE and were shown to enhance NPCs migration in vitro. As cell migration is dependent on extracellular matrix remodeling, involving proteolytic enzyme members of the matrix metalloproteinase (MMPs) family, we characterized the profile of

expression of MMPs and their endogenous inhibitors (TIMPs) in rat NPCs, and evaluated the effects of TNF-alpha, IFN-gamma and IFN-beta, a clinically proven modulator of brain inflammation, on the expression of these molecules. Newborn rat striatal NPCs were expanded in spheres as nestin+, PSA-NCAM+ and NG2(-) cells, which can differentiate into astrocytes, oligodendrocytes and neurons. NPCs' gelatinase activities of MMP-2 and MMP-9, as determined by zymography, were increased by TNF-alpha, and to a lesser extent by IFN-gamma. Semi-quantitative RT-PCR indicated that TNF-alpha also upregulated MMP-9 mRNA levels. IFN-beta suppressed the TNF-alpha-induced levels of secreted MMP-9 and MMP-2, while enhancing the expression of TIMP-1 and TIMP-2 mRNA. These results suggest that MMPs activity is induced in NPCs by pro-inflammatory cytokines to mobilize them for promoting reparative processes. IFN-beta, on the other hand, appears to have an anti-proteolytic influence that may attenuate such NPC-mediated repair processes.

PMID: 16580738 [PubMed - indexed for MEDLINE]

975. Lin Chuang Er Bi Yan Hou Ke Za Zhi. 2006 Jan;20(2):73-7.

[The significance and effect of substance P on the expression of RANTES mRNA in allergic rhinitis].

[Article in Chinese]

Zhang R, Liu G, Wen W, Yan Z, Wu G.

Department of Otolaryngology, Changhai Hospital, Second Military Medical University, Shanghai 200433, China. rxzhang@x263.net

OBJECTIVE: To study the significance and effect of SP on the expression of RANTES mRNA in allergic rhinitis (AR), and deeply understand the pathogenesis of AR.
METHOD: The model of AR was established in randomly selected healthy guinea pigs by using ovalbumin. Then the nasal dripping of SP was used to challenge the AR groups, as compared with the normal controls, in observation of the symptoms and histopathologic changes in the nasal mucosa. The number of eosinophil in the nasal lavage fluid was also counted. By means of RT-PCR, the expressions of RANTES mRNA in the nasal mucosa of the different groups were quantitatively compared.
RESULT: SP challenge induced similar AR symptoms in the normal group and aggravated AR symptoms and inflammation in the nasal mucosa in the model of AR groups. The expression level of RANTES mRNA ($P < 0.05$) and the number of eosinophil ($P < 0.01$) in AR groups increased significantly after challenged with SP.
CONCLUSION: In the pathogenesis of AR, SP induces the expression of chemokine RANTES in nasal mucosa, promotes eosinophil chemotaxis and migration, induces and aggravates the allergic inflammatory reaction in AR.

PMID: 16570818 [PubMed - indexed for MEDLINE]

976. J Exp Med. 2006 Apr 17;203(4):953-60. Epub 2006 Mar 27.

Impaired Langerhans cell migration in psoriasis.

Cumberbatch M, Singh M, Dearman RJ, Young HS, Kimber I, Griffiths CE.

Syngenta Central Toxicology Laboratory, Macclesfield, Cheshire, SK10 4TJ, England, UK.

We have examined whether psoriasis is associated with systemic effects on epidermal Langerhans cell (LC) function and, specifically, the migration of LCs from the skin. Compared with normal skin, the frequency and morphology of epidermal LCs in uninvolved skin from patients with psoriasis was normal. However, mobilization of these cells in response to stimuli that normally induce migration (chemical allergen, tumor necrosis factor alpha [TNF-alpha], and interleukin-1beta [IL-1beta]) was largely absent, despite the fact that treatment with TNF-alpha and IL-1beta was associated with comparable inflammatory reactions in patients and controls. The failure of LC migration from uninvolved skin was not attributable to altered expression of receptors for IL-1beta or TNF-alpha that are required for mobilization, nor was there an association with induced cutaneous cytokine expression. Although a role for altered dynamics of LC migration/turnover has not been formally excluded, these data reveal a very consistent decrement of LC function in psoriasis that may play a decisive role in disease pathogenesis.

PMCID: PMC2118293
PMID: 16567387 [PubMed - indexed for MEDLINE]

977. Curr Allergy Asthma Rep. 2006 Mar;6(2):91-6.

T-cell subsets in the pathogenesis of human asthma.

Meiler F, Zimmermann M, Blaser K, Akdis CA, Akdis M.

Genetic predisposition and environmental instructions tune thresholds for activation of T cells, other inflammatory cells, and resident tissue cells in asthmatic inflammation. Selective migration of peripheral-blood T cells to the lungs, their survival and reactivation in the submucosa, and their effector functions represent sequential immunologic events. Activation-induced T-cell death and peripheral T-cell tolerance are critical events in disease pathogenesis. As a mechanism for peripheral Th2 response in atopic diseases, particularly, the high interferon (IFN)-gamma-producing Th1 compartment of activated effector T cells shows increased activation-induced cell death, skewing the immune response toward surviving Th2 cells in allergic asthma. After migration to asthmatic lung, these cells switch on effector cytokines and induce bronchial epithelial apoptosis with mainly IFN-gamma, tumor necrosis factor (TNF)-alpha, and Fas-ligand. In addition, skewing of allergen-specific effector T cells to T-regulatory cells appears to be an essential event in the control of harmful immune response induced by allergens as a possible means for remedy.

PMID: 16566857 [PubMed - indexed for MEDLINE]

978. BMC Public Health. 2006 Mar 27;6:78.

Differences in avoidable mortality between migrants and the native Dutch in The Netherlands.

Stirbu I, Kunst AE, Bos V, Mackenbach JP.

Department of Public Health, Erasmus MC, University Medical Center Rotterdam, PO Box 1738, 3000 DR Rotterdam, The Netherlands. i.stirbu@erasmusmc.nl

BACKGROUND: The quality of the healthcare system and its role in influencing mortality of migrant groups can be explored by examining ethnic variations in 'avoidable' mortality. This study investigates the association between the level of mortality from 'avoidable' causes and ethnic origin in the Netherlands and identifies social factors that contribute to this association.

METHODS: Data were obtained from cause of death and population registries in the period 1995-2000. We compared mortality rates for selected 'avoidable' conditions for Turkish, Moroccan, Surinamese and Antillean/Aruban groups to native Dutch.
RESULTS: We found slightly elevated risk in total 'avoidable' mortality for migrant populations (RR = 1.13). Higher risks of death among migrants were observed from almost all infectious diseases (most RR > 3.00) and several chronic conditions including asthma, diabetes and cerebro-vascular disorders (most RR > 1.70). Migrant women experienced a higher risk of death from maternity-related conditions (RR = 3.37). Surinamese and Antillean/Aruban population had a higher mortality risk (RR = 1.65 and 1.31 respectively), while Turkish and Moroccans experienced a lower risk of death (RR = 0.93 and 0.77 respectively) from all 'avoidable' conditions compared to native Dutch. Control for demographic and socioeconomic factors explained a substantial part of ethnic differences in 'avoidable' mortality.
CONCLUSION: Compared to the native Dutch population, total 'avoidable' mortality was slightly elevated for all migrants combined. Mortality risks varied greatly by cause of death and ethnic origin. The substantial differences in mortality for a few 'avoidable' conditions suggest opportunities for quality improvement within specific areas of the healthcare system targeted to disadvantaged groups.

PMCID: PMC1435889
PMID: 16566833 [PubMed - indexed for MEDLINE]

979. Curr Opin Pharmacol. 2006 Jun;6(3):244-50. Epub 2006 Mar 23.

Pulmonary and vascular pharmacology of sphingosine 1-phosphate.

Brinkmann V, Baumruker T.

Autoimmunity & Transplantation, Novartis Institutes for BioMedical Research, WSJ-386.101, Lichtstrasse 35, CH-4002 Basel, Switzerland.
volker.brinkmann@novartis.com

Dysregulation of vasomotor tone, endothelial barrier function and immune cell trafficking are central to the pathology of many lung diseases, including acute lung injury, adult respiratory distress syndrome, chronic obstructive pulmonary disease and asthma. There is increasing evidence that the serum sphingolipid sphingosine 1-phosphate and its G-protein-coupled receptors are pivotal not only in the regulation of lymphocyte migration, but also in the maintenance of vascular homeostasis and the preservation of permeability barriers that separate discrete compartments in the lung.

PMID: 16563863 [PubMed - indexed for MEDLINE]

980. BMC Dermatol. 2006 Mar 21;6:4.

Increasing incidence of skin disorders in children? A comparison between 1987 and 2001.

Mohammedamin RS, van der Wouden JC, Koning S, van der Linden MW, Schellevis FG, van Suijlekom-Smit LW, Koes BW.

Department of General Practice, Room FF 304, Erasmus MC-University Medical Center Rotterdam, PO Box 1738, 3000 DR Rotterdam, The Netherlands.
s.mohammedamin@erasmusmc.nl

BACKGROUND: The increasing proportion of skin diseases encountered in general practice represents a substantial part of morbidity in children. Only limited

information is available about the frequency of specific skin diseases. We aimed to compare incidence rates of skin diseases in children in general practice between 1987 and 2001.
METHODS: We used data on all children aged 0-17 years derived from two consecutive surveys performed in Dutch general practice in 1987 and 2001. Both surveys concerned a longitudinal registration of GP consultations over 12 months. Each disease episode was coded according to the International Classification of Primary Care. Incidence rates of separate skin diseases were calculated by dividing all new episodes for each distinct ICPC code by the average study population at risk. Data were stratified for socio-demographic characteristics.
RESULTS: The incidence rate of all skin diseases combined in general practice decreased between 1987 and 2001. Among infants the incidence rate increased. Girls presented more skin diseases to the GP. In the southern part of the Netherlands children consulted their GP more often for skin diseases compared to the northern part. Children of non-Western immigrants presented relatively more skin diseases to the GP. In general practice incidence rates of specific skin diseases such as impetigo, dermatophytosis and atopic dermatitis increased in 2001, whereas warts, contact dermatitis and skin injuries decreased.
CONCLUSION: The overall incidence rate of all skin diseases combined in general practice decreased whereas the incidence rates of bacterial, mycotic and atopic skin diseases increased.

PMCID: PMC1435925
PMID: 16551358 [PubMed - indexed for MEDLINE]

981. Clin Chest Med. 2006 Mar;27(1):53-69, vi.

Airway smooth muscle as a regulator of immune responses and bronchomotor tone.

Lazaar AL, Panettieri RA Jr.

Pulmonary, Allergy, and Critical Care Division, University of Pennsylvania Medical Center, BRB II/III, 421 Curie Boulevard, Philadelphia, PA 19104-6160, USA. Aili.L.Lazaar@gsk.com

The traditional view of airway smooth muscle (ASM) in asthma, as a purely contractile tissue, seems to be inadequate. Compelling evidence now suggests that ASM plays an important role in regulating bronchomotor tone, in perpetuating airway inflammation, and in remodeling of the airways. This article reviews three distinct functions of ASM cells: the process of excitation-contraction coupling, with a particular focus on the role of cytokines in modulating calcium responses; the processes of smooth muscle cell proliferation and migration; and the synthetic and immunomodulatory function of ASM cells. This article also discusses how altered synthetic function contributes to airway remodeling.

PMID: 16543052 [PubMed - indexed for MEDLINE]

982. J Health Care Poor Underserved. 2006 Feb;17(1 Suppl):26-43.

The impact of community health worker training and programs in NYC.

Perez M, Findley SE, Mejia M, Martinez J.

Alianza Dominicana, Inc., USA.

The Northern Manhattan Community Voices Collaborative is committed to improving health care in Harlem, Washington Heights, Inwood, and low-income communities in New York City, large parts of which are home to many immigrants to the U.S. The

collaborative developed a program to train and integrate community health workers (CHWs) into ongoing programs at partner community organizations. We report on our 2000-2005 experiences with CHWs for health insurance, child immunizations, and asthma management. A total of 1,504 CHWs were trained, with 16%-200% increase in CHW competency for selected skills. The CHWs facilitated health insurance enrollment for about 30,000 individuals, assisted 8,000 children to become completely immunized, and supported 4,000 families improving asthma management. Integration of CHW training into community programs is effective for empowering health promotion in underserved communities.

PMID: 16520505 [PubMed - indexed for MEDLINE]

983. J Med Assoc Thai. 2005 Dec;88(12):1966-72.

Update on nasal polyps: etiopathogenesis.

Kirtsreesakul V.

Division of Allergy and Rhinology, Department of Otolaryngology, Faculty of Medicine, Prince of Songkla University, Hat Yai, Songkhla, 90110, Thailand. kvirat2002@yahoo.com

PURPOSE OF REVIEW: Nasal polyps is a common ENT disease with high medical failure and recurrence rate, reflecting unknown pathogenesis. The present review is an update on the etiopathogenesis of nasal polyps.
RECENT FINDING: Several mechanisms have been proposed for the formation of nasal polyps, including allergy, mucosal allergy, autonomic imbalance, nitric oxide, superantigens, infection, abnormal transepithelial ion transport, mucopolysaccharide abnormality, mechanical obstruction and epithelial rupture. Eosinophils comprises more than 60% of the cell population. Activated T cells, mast cells and plasma cells are also increased compared with the normal nasal mucosa. The stroma has numerous mediators, including cytokines, growth factors, adhesion molecules, and immunoglobulins. Both Th1 and Th2 types of cytokines are upregulated independent of the atopic status. An increased production of GM-CSF, IL5, RANTES and eotaxin can contribute to chronic eosinophilic inflammation by regulating the migration, survival and activation of eosinophils.
CONCLUSION: Nasal polyps is a multifactorial disease, with infectious, noninfectious, inflammation, anatomic and genetic abnormalities. Chronic inflammation remains the central major factor in nasal polyps.

PMID: 16519003 [PubMed - indexed for MEDLINE]

984. J Immunol. 2006 Mar 15;176(6):3788-95.

Mast cell beta-tryptase selectively cleaves eotaxin and RANTES and abrogates their eosinophil chemotactic activities.

Pang L, Nie M, Corbett L, Sutcliffe A, Knox AJ.

Division of Respiratory Medicine, City Hospital, University of Nottingham, Hucknall Road, Nottingham NG5 1PB, United Kingdom. linhua.pang@nottingham.ac.uk

Recent studies have shown that a lack of eosinophils in asthmatic airway smooth muscle (ASM) bundles in contrast to the large number of mast cells is a key feature of asthma. We hypothesized that this is caused by beta-tryptase, the predominant mast cell-specific protease, abrogating the eosinophil chemotactic activities of ASM cell-derived eosinophil chemoattractants such as eotaxin and RANTES. We studied the effect of beta-tryptase on the immunoreactivities of human

ASM cell-derived and recombinant eotaxin and other recombinant chemokines that are known to be produced by human ASM cells. We report in this study that purified beta-tryptase markedly reduced the immunoreactivity of human ASM cell-derived and recombinant eotaxin, but had no effect on eotaxin mRNA expression. The effect was mimicked by recombinant human beta-tryptase in the presence of heparin and was reversed by heat inactivation and the protease inhibitor leupeptin, suggesting that the proteolytic activity of tryptase is required. beta-Tryptase also exerted similar effects on recombinant RANTES, but not on the other chemokines and cytokines that were screened. Furthermore, a chemotaxis assay revealed that recombinant eotaxin and RANTES induced eosinophil migration concentration-dependently, which was abrogated by pretreatment of these chemokines with beta-tryptase. Another mast cell protease chymase also markedly reduced the immunoreactivity of eotaxin, but had no effect on RANTES and other chemokines and did not affect the influence of beta-tryptase on RANTES. These findings suggest that mast cell beta-tryptase selectively cleaves ASM-derived eotaxin and RANTES and abrogates their chemotactic activities, thus providing an explanation for the eosinophil paucity in asthmatic ASM bundles.

PMID: 16517749 [PubMed - indexed for MEDLINE]

985. FEBS Lett. 2006 Mar 20;580(7):1883-90. Epub 2006 Feb 28.

Epigallocatechin-3-gallate protects toluene diisocyanate-induced airway inflammation in a murine model of asthma.

Kim SH, Park HJ, Lee CM, Choi IW, Moon DO, Roh HJ, Lee HK, Park YM.

Department of Pediatrics, Pusan National University College of Medicine, Pusan 602-739, Republic of Korea.

Epigallocatechin-3-gallate (EGCG), a major form of tea catechin, has anti-allergic properties. To elucidate the anti-allergic mechanisms of EGCG, we investigated its regulation of matrix metalloproteinase (MMP-9) expression in toluene diisocyanate (TDI)-inhalation lung tissues as well as TNF-alpha and Th2 cytokine (IL-5) production in BAL fluid. Compared with untreated asthmatic mice those administrated with EGCG had significantly reduced asthmatic reaction. Also, increased reactive oxygen species (ROS) generation by TDI inhalation was diminished by administration of EGCG in BAL fluid. These results suggest that EGCG regulates inflammatory cell migration possibly by suppressing MMP-9 production and ROS generation, and indicate that EGCG may be useful as an adjuvant therapy for bronchial asthma.

PMID: 16516891 [PubMed - indexed for MEDLINE]

986. Exp Dermatol. 2006 Apr;15(4):322-9.

Non-steroidal and steroidal anti-inflammatory drugs vary in their modulation of dendritic cell function in the elicitation phase of allergic contact dermatitis.

Bäumer W, Krekeler S, DeVries VC, Niedorf F, Tschernig T, Kietzmann M.

Department of Pharmacology, Toxicology, and Pharmacy, University of Veterinary Medicine Hannover, Germany. wolfgang.baeumer@tiho-hannover.de

The role of dendritic cells (DCs) in allergic contact dermatitis has been clearly demonstrated for the induction phase. However, the situation during the elicitation phase is very complex within a distinct inflammatory response. This study was performed to exploit DC migration in the elicitation phase in a mouse

model of allergic contact dermatitis and to evaluate the effects of steroidal and non-steroidal anti-inflammatory drugs (NSAIDs) on DC migration through skin in the elicitation phase of allergic contact dermatitis. Topically and systemically administered acetylsalicylic acid (ASA) did not reduce the inflammatory response. However, systemically administered ASA significantly reduced the DC migration to the draining lymph node. In contrast, topically administered indomethacin reduced the inflammatory response, but had only minor effects on DC migration, whereas diflorasone diacetate reduced both inflammatory reaction and DC migration. Thus, NSAIDs may differ in their inhibitory action in immunological inflammation.

PMID: 16512880 [PubMed - indexed for MEDLINE]

987. J Chromatogr B Analyt Technol Biomed Life Sci. 2006 Jul 24;839(1-2):19-29. Epub 2006 Feb 28.

Profiling preparations of recombinant birch pollen allergen Bet v 1a with capillary zone electrophoresis in pentamine modified fused-silica capillaries.

Punzet M, Ferreira F, Briza P, van Ree R, Malissa H Jr, Stutz H.

Department of Molecular Biology, Division of Chemistry, University of Salzburg, Salzburg, Austria.

Three preparation batches of the recombinant birch pollen allergen Bet v 1a have been analyzed by capillary zone electrophoresis (CZE) using a separation electrolyte consisting of 100 mmol L(-1) phosphate at pH 6.50 with 2.0 mmol L(-1) tetraethylenepentamine (TEPA) added. TEPA improved the resolution by wall shielding and selective attachment to allergens, but reduced migration repeatability at concentrations >2.0 mmol L(-1). Heterogeneity of preparations determined by CZE and electrospray ionization-quadrupole-time-of flight-MS were in accordance and revealed chemically modified (carbamylated) allergens in one of the preparations. The method was validated according to the ICH-guidelines. Repeatability of effective electrophoretic mobility (mu(eff)) was <0.55% R.S.D. (n = 5). Migration time corrected peak areas were used for quantification. Limit of quantification (LOQ) was 25 microg mL(-1) for the major isoform Bet v 1a, based on a signal-to-noise ratio of 10, and detector response was linear between LOQ and 0.90 mg mL(-1). Purity of the different rBet v 1a preparations was determined to be between 40 and 92% depending on the manufacturing protocol.

PMID: 16504605 [PubMed - indexed for MEDLINE]

988. Clin Mol Allergy. 2006 Feb 17;4:2.

Impact of IL8 and IL8-receptor alpha polymorphisms on the genetics of bronchial asthma and severe RSV infections.

Puthothu B, Krueger M, Heinze J, Forster J, Heinzmann A.

University Children's Hospital, University of Freiburg, Mathildenstrasse 1, D-79106 Freiburg, Germany. beenamary@hotmail.com

BACKGROUND: Interleukin 8 (IL8) belongs to the family of chemokines. It mediates the activation and migration of neutrophils from peripheral blood into tissue and hereby plays a pivotal role in the initiation of inflammation. Thus it is important in inflammatory lung diseases like bronchial asthma or severe infections by Respiratory Syncytial Virus (RSV). IL8 acts through binding to the IL8-Receptor alpha (IL8RA). For both genes association with asthma has been described. In addition, IL8 has been found in association with RSV bronchiolitis.

The aim of our study was to test both genes for association with asthma and severe RSV infections. In addition we were interested in whether a common genetic background of both diseases exists in regards to these genes.
METHODS: We genotyped the two IL8 promotor polymorphisms -251A/T and -781C/T and the three amino acid variants M31R, S276T and R335C in IL8RA on 322 children with asthma, 131 infants with severe RSV associated diseases and 270 controls. Statistical analyses made use of the Armitage's trend test for single polymorphisms and FAMHAP for calculations of haplotypes.
RESULTS: We found association of the IL8 polymorphism -781C/T as well as IL8 haplotypes with asthma (p = 0.011 and p = 0.036, respectively). In addition, direct comparison of the asthmatic population with the RSV population revealed significant differences, both for -781C/T alone (p = 0.034) and IL8 haplotypes (p = 0.005). The amino acid variants in IL8RA were evenly distributed in between all three populations.
CONCLUSION: We conclude from our data that IL8 might play a role in the genetic predisposition to asthma and that these effects are different or even opposite to the effects on severe RSV diseases. Furthermore, IL8RA is unlikely to play a major role in the genetics of either disease.

PMCID: PMC1388233
PMID: 16503988 [PubMed]

989. J Immunol. 2006 Mar 1;176(5):3127-40.

Acetyl-11-keto-beta-boswellic acid potentiates apoptosis, inhibits invasion, and abolishes osteoclastogenesis by suppressing NF-kappa B and NF-kappa B-regulated gene expression.

Takada Y, Ichikawa H, Badmaev V, Aggarwal BB.

Cytokine Research Section, Department of Experimental Therapeutics, University of Texas M. D. Anderson Cancer Center, Houston, TX 77030, USA.

Acetyl-11-keto-beta-boswellic acid (AKBA), a component of an Ayurvedic therapeutic plant Boswellia serrata, is a pentacyclic terpenoid active against a large number of inflammatory diseases, including cancer, arthritis, chronic colitis, ulcerative colitis, Crohn's disease, and bronchial asthma, but the mechanism is poorly understood. We found that AKBA potentiated the apoptosis induced by TNF and chemotherapeutic agents, suppressed TNF-induced invasion, and inhibited receptor activator of NF-kappaB ligand-induced osteoclastogenesis, all of which are known to require NF-kappaB activation. These observations corresponded with the down-regulation of the expression of NF-kappaB-regulated antiapoptotic, proliferative, and angiogenic gene products. As examined by DNA binding, AKBA suppressed both inducible and constitutive NF-kappaB activation in tumor cells. It also abrogated NF-kappaB activation induced by TNF, IL-1beta, okadaic acid, doxorubicin, LPS, H2O2, PMA, and cigarette smoke. AKBA did not directly affect the binding of NF-kappaB to the DNA but inhibited sequentially the TNF-induced activation of IkappaBalpha kinase (IKK), IkappaBalpha phosphorylation, IkappaBalpha ubiquitination, IkappaBalpha degradation, p65 phosphorylation, and p65 nuclear translocation. AKBA also did not directly modulate IKK activity but suppressed the activation of IKK through inhibition of Akt. Furthermore, AKBA inhibited the NF-kappaB-dependent reporter gene expression activated by TNFR type 1, TNFR-associated death domain protein, TNFR-associated factor 2, NF-kappaB-inducing kinase, and IKK, but not that activated by the p65 subunit of NF-kappaB. Overall, our results indicated that AKBA enhances apoptosis induced by cytokines and chemotherapeutic agents, inhibits invasion, and suppresses osteoclastogenesis through inhibition of NF-kappaB-regulated gene expression.

PMID: 16493072 [PubMed - indexed for MEDLINE]

990. J Invest Dermatol. 2006 May;126(5):1043-51.

IL-13-stimulated human keratinocytes preferentially attract CD4+CCR4+ T cells: possible role in atopic dermatitis.

Purwar R, Werfel T, Wittmann M.

Department of Dermatology and Allergology, Hannover Medical School, Hannover, Germany. purwarrahul@yahoo.com

Skin inflammation in atopic dermatitis (AD) is characterized by the predominant infiltration of T-helper (Th)2-cells in lesional skin. However, the mechanism of recruitment of these cells in lesional skin of AD is not yet fully elucidated. In this study, we investigated the role of IL-13-stimulated human primary keratinocytes (HPKs) in the recruitment of lymphocytes and further delineated the mechanism of enrichment of these cells. In the migration assays, we observed preferential enrichment of CD4(+)CCR4(+) T cells towards IL-13-stimulated HPKs. Interestingly, CD4(+)CCR4(+) T cells from AD showed a higher chemotactic response than those from healthy individuals. We observed a significant increase in the expression of CCL22 in IL-13-stimulated HPKs as compared to unstimulated cells. Blocking of CCL22 in IL-13-stimulated HPKs by a neutralizing antibody resulted in 70-90% inhibition in migration of CD4(+)CCR4(+) T cells. Moreover, IL-13 upregulated IFN-gamma-induced chemokines, CCL2 and CCL5, in HPKs. Taken together, our data suggest that IL-13-stimulated HPKs participate in a positive feedback loop by preferentially enriching Th2-cells in lesional skin of acute AD patients. However, in chronic phase, IL-13 may act in synergy with IFN-gamma resulting in lymphocytes recruitment of a mixed phenotype at the site of inflammation, thus contributing to the chronification of eczema.

PMID: 16484990 [PubMed - indexed for MEDLINE]

991. Respir Res. 2006 Feb 15;7:25.

Alterations in vasodilator-stimulated phosphoprotein (VASP) phosphorylation: associations with asthmatic phenotype, airway inflammation and beta2-agonist use.

Hastie AT, Wu M, Foster GC, Hawkins GA, Batra V, Rybinski KA, Cirelli R, Zangrilli JG, Peters SP.

Department of Internal Medicine, Center for Human Genomics, Wake Forest University School of Medicine, Winston-Salem, NC, USA. ahastie@wfubmc.edu

BACKGROUND: Vasodilator-stimulated phosphoprotein (VASP) mediates focal adhesion, actin filament binding and polymerization in a variety of cells, thereby inhibiting cell movement. Phosphorylation of VASP via cAMP and cGMP dependent protein kinases releases this "brake" on cell motility. Thus, phosphorylation of VASP may be necessary for epithelial cell repair of damage from allergen-induced inflammation. Two hypotheses were examined: (1) injury from segmental allergen challenge increases VASP phosphorylation in airway epithelium in asthmatic but not nonasthmatic normal subjects, (2) regular in vivo beta2-agonist use increases VASP phosphorylation in asthmatic epithelium, altering cell adhesion.
METHODS: Bronchial epithelium was obtained from asthmatic and non-asthmatic normal subjects before and after segmental allergen challenge, and after regularly inhaled albuterol, in three separate protocols. VASP phosphorylation was examined in Western blots of epithelial samples. DNA was obtained for beta2-adrenergic receptor haplotype determination.

RESULTS: Although VASP phosphorylation increased, it was not significantly greater after allergen challenge in asthmatics or normals. However, VASP phosphorylation in epithelium of nonasthmatic normal subjects was double that observed in asthmatic subjects, both at baseline and after challenge. Regularly inhaled albuterol significantly increased VASP phosphorylation in asthmatic subjects in both unchallenged and antigen challenged lung segment epithelium. There was also a significant increase in epithelial cells in the bronchoalveolar lavage of the unchallenged lung segment after regular inhalation of albuterol but not of placebo. The haplotypes of the beta2-adrenergic receptor did not appear to associate with increased or decreased phosphorylation of VASP.
CONCLUSION: Decreased VASP phosphorylation was observed in epithelial cells of asthmatics compared to nonasthmatic normals, despite response to beta-agonist. The decreased phosphorylation does not appear to be associated with a particular beta2-adrenergic receptor haplotype. The observed decrease in VASP phosphorylation suggests greater inhibition of actin reorganization which is necessary for altering attachment and migration required during epithelial repair.

PMCID: PMC1388207
PMID: 16480498 [PubMed - indexed for MEDLINE]

992. Curr Allergy Asthma Rep. 2006 Feb;6(1):1-3.

Changing paradigms in the immunologic science of allergy.

Goetzl EJ.

Department of Medicine, University of California, San Francisco 94143-0711, USA. Edward.Goetzl@ucsf.edu

PMID: 16476187 [PubMed - indexed for MEDLINE]

993. Am J Physiol Cell Physiol. 2006 Jul;291(1):C34-9. Epub 2006 Feb 8.

CXCR3 chemokine receptor-induced chemotaxis in human airway epithelial cells: role of p38 MAPK and PI3K signaling pathways.

Shahabuddin S, Ji R, Wang P, Brailoiu E, Dun N, Yang Y, Aksoy MO, Kelsen SG.

Department of Medicine, Temple University School of Medicine, Philadelphia, Pennsylvania, USA.

Human airway epithelial cells (HAEC) constitutively express the CXC chemokine receptor CXCR3, which regulates epithelial cell movement. In diseases such as chronic obstructive pulmonary disease and asthma, characterized by denudation of the epithelial lining, epithelial cell migration may contribute to airway repair and reconstitution. This study compared the potency and efficacy of three CXCR3 ligands, I-TAC/CXCL11, IP-10/CXCL10, and Mig/CXCL9, as inducers of chemotaxis in HAEC and examined the underlying signaling pathways involved. Studies were performed in cultured HAEC from normal subjects and the 16-HBE cell line. In normal HAEC, the efficacy of I-TAC-induced chemotaxis was 349 +/- 88% (mean +/- SE) of the medium control and approximately one-half the response to epidermal growth factor, a highly potent chemoattractant. In normal HAEC, Mig, IP-10, and I-TAC induced chemotaxis with similar potency and a rank order of efficacy of I-TAC = IP-10 > Mig. Preincubation with pertussis toxin completely blocked CXCR3-induced migration. Of interest, intracellular [Ca(2+)] did not rise in response to I-TAC, IP-10, or Mig. I-TAC induced a rapid phosphorylation (5-10 min) of two of the three MAPKs, i.e., p38 and ERK1/2. Pretreatment of HAEC with

the p38 inhibitor SB 20358 or the PI3K inhibitor wortmannin dose-dependently inhibited the chemotactic response to I-TAC. In contrast, the ERK1/2 inhibitor U0126 had no effect on chemotaxis. These data indicate that in HAEC, CXCR3-mediated chemotaxis involves a G protein, which activates both the p38 MAPK and PI3K pathways in a calcium-independent fashion.

PMID: 16467404 [PubMed - indexed for MEDLINE]

994. Ann Agric Environ Med. 2005;12(2):247-52.

Health effects of exposure to herb dust in valerian growing farmers.

Skórska C, Golec M, Mackiewicz B, Góra A, Dutkiewicz J.

Department of Occupational Biohazards, Institute of Agricultural Medicine, Jaczewskiego 2, 20-090 Lublin, Poland.

The aim of the present study was to determine the health status of farmers cultivating valerian (Valeriana officinalis L.) and occupationally exposed to dust from this plant. A group of 75 valerian growing farmers were examined. As a reference group, 50 urban dwellers, not exposed to any kind of organic dust were examined. All people were interviewed for the presence of work-related symptoms and subjected to physical and spirometric examinations. Skin prick tests were conducted with 4 microbial antigens associated with organic dust and 3 herbal extracts, precipitin tests with 12 microbial antigens and 4 herbal extracts and tests for specific inhibition of leukocyte migration with 4 microbial antigens. 30.7 % of the valerian farmers reported occurrence of work-related symptoms. No significant differences were found between the spirometric values in the group of valerian farmers and the reference group. Valerian farmers showed a low frequency of positive skin reactions to all tested antigens (0-4.0 %), not significantly greater compared to reference group. The frequency of positive precipitin reactions to the antigen of Gram-negative bacterium Pantoea agglomerans was very high in valerian farmers (45.5 %) with 3-fold concentrated sera and significantly greater compared to the reference group ($p < 0.001$). The positive precipitin response of valerian farmers to other microbial and herbal antigens was much lower or absent and did not show any difference compared to reference group. In the test for specific inhibition of leukocyte migration, the highest frequencies of positive reactions in valerian farmers were noted with Pantoea agglomerans and Saccharopolyspora rectivirgula (15.0 % each), in both cases significantly greater compared to reference group ($p < 0.05$). In conclusion, the farmers growing valerian showed a moderate frequency of work-related symptoms and low reactivity to most microbial and herbal allergens. They exhibited an increased immunologic response to Gram-negative bacterium Pantoea agglomerans which appears to be the most important risk factor associated with valerian dust.

PMID: 16457481 [PubMed - indexed for MEDLINE]

995. Cell Biochem Biophys. 2006;44(1):139-46.

Role of extracellular matrix and its regulators in human airway smooth muscle biology.

Parameswaran K, Willems-Widyastuti A, Alagappan VK, Radford K, Kranenburg AR, Sharma HS.

Firestone Institute for Respiratory Health, McMaster University and St. Joseph's Healthcare, Hamilton, Ontario, Canada.

Altered extracellular matrix (ECM) deposition contributing to airway wall remodeling is an important feature of asthma and chronic obstructive pulmonary disease (COPD). The molecular mechanisms of this process are poorly understood. One of the key pathological features of these diseases is thickening of airway walls. This thickening is largely to the result of airway smooth muscle (ASM) cell hyperplasia and hypertrophy as well as increased deposition of ECM proteins such as collagens, elastin, laminin, and proteoglycans around the smooth muscle. Many growth factors and cytokines, including fibroblast growth factor (FGF)-1, FGF-2, and transforming growth factor (TGF)-beta1, that are released from the airway wall have the potential to contribute to airway remodeling, revealed by enhanced ASM proliferation and increased ECM protein deposition. TGF-beta1 and FGF-1 stimulate mRNA expression of collagen I and III in ASM cells, suggesting their role in the deposition of extracellular matrix proteins by ASM cells in the airways of patients with chronic lung diseases. Focus is now on the bidirectional relationship between ASM cells and the ECM. In addition to increased synthesis of ECM proteins, ASM cells can be involved in downregulation of matrix metalloproteinases (MMPs) and upregulation of tissue inhibitors of metalloproteinases (TIMPs), thus eventually contributing to the alteration in ECM. In turn, ECM proteins promote the survival, proliferation, cytokine synthesis, migration, and contraction of human airway smooth muscle cells. Thus, the intertwined relationship of ASM and ECM and their response to stimuli such as chronic inflammation in diseases such as asthma and COPD contribute to the remodeling seen in airways of patients with these diseases.

PMID: 16456242 [PubMed - indexed for MEDLINE]

996. Am J Respir Cell Mol Biol. 2006 Jun;34(6):760-5. Epub 2006 Feb 2.

Eosinophil trans-basement membrane migration induced by interleukin-8 and neutrophils.

Kikuchi I, Kikuchi S, Kobayashi T, Hagiwara K, Sakamoto Y, Kanazawa M, Nagata M.

Department of Respiratory Medicine, Saitama Medical School, Saitama, Japan.

Comment in
    Am J Respir Cell Mol Biol. 2006 Jun;34(6):645-6.

Neutrophilic inflammation observed with severe asthma is often associated with interleukin-8 (IL-8). Neutrophils can secrete a variety of mediators that may augment the migration of eosinophils. We have reported a positive correlation between the concentrations of neutrophils and eosinophils in sputum from subjects with severe asthma, suggesting a possible role of neutrophils in regulating eosinophilic inflammation. The aim of this study was to investigate whether neutrophils stimulated with IL-8 modify the trans-basement membrane migration (TBM) of eosinophils. Eosinophils and neutrophils were isolated from peripheral blood drawn from healthy donors or subjects with mild asthma. The TBM of eosinophils in response to IL-8 was evaluated in the presence or absence of neutrophils using the chambers with a Matrigel-coated transwell insert. Neither IL-8 alone nor the presence of neutrophils alone induced the TBM of eosinophils. However, when eosinophils were coincubated with neutrophils and stimulated with IL-8, the TBM of eosinophils was significantly augmented. This augmented TBM of eosinophils was inhibited by a matrix metalloproteinase-9 inhibitor, a leukotriene B4 receptor antagonist, platelet-activating factor antagonists, or an anti-TNF-alpha monoclonal antibodies. These results suggest that neutrophils migrated in response to IL-8 may lead eosinophils to accumulate in the airways of asthma and possibly aggravate this disease.

PMID: 16456187 [PubMed - indexed for MEDLINE]

997. Am J Respir Cell Mol Biol. 2006 Jun;34(6):695-703. Epub 2006 Feb 2.

Retinoic acid inhibits airway smooth muscle cell migration.

Day RM, Lee YH, Park AM, Suzuki YJ.

Department of Pharmacology, Georgetown University Medical Center, NW403 Medical-Dental Building, Washington, DC 20057, USA.

Airway remodeling in chronic asthma is characterized by increased smooth muscle mass that is associated with the reduction of the bronchial lumen as well as airway hyperresponsiveness. The development of agents that inhibit smooth muscle growth is therefore of interest for therapy to prevent asthma-associated airway remodeling. All-trans retinoic acid (ATRA) suppresses growth of vascular smooth muscle cells (SMCs) from the systemic and pulmonary circulation. The present study investigated the effects of ATRA on human bronchial (airway) SMCs. Human bronchial SMCs were found to express mRNAs for retinoic acid receptor (RAR)-alpha, -beta, -gamma, and retinoid X receptor (RXR)-alpha, -beta, but not RXR-gamma. Although ATRA was not effective in inhibiting proliferation or in inducing apoptosis in airway SMCs, we found that ATRA (0.2-2 microM) inhibited the SMC migration in response to platelet-derived growth factor (PDGF), as determined in a modified Boyden chamber assay. Both RAR and RXR agonists also blocked PDGF-induced airway SMC migration. ATRA also inhibited PDGF-induced actin reorganization associated with migration. PDGF-induced actin reorganization and migration were blocked by inhibitors of phosphatidylinositol 3 kinase (PI3K) and Akt. However, migration was blocked by inhibitors of the MEK/ERK pathway, with no effect on cytoskeletal reorganization. ATRA suppressed PDGF-induced Akt activation without influencing ERK activation. RAR was found to form protein-protein interactions with the p85 PI3K subunit. These results suggest that retinoic acid inhibits airway SMC migration through the modulation of the PI3K/Akt pathway.

PMCID: PMC1831536
PMID: 16456186 [PubMed - indexed for MEDLINE]

998. Eur Respir J. 2006 Apr;27(4):726-34. Epub 2006 Feb 2.

Role of macrophage migration inhibitory factor in ovalbumin-induced airway inflammation in rats.

Kobayashi M, Nasuhara Y, Kamachi A, Tanino Y, Betsuyaku T, Yamaguchi E, Nishihira J, Nishimura M.

First Department of Medicine, Hokkaido University School of Medicine, Sapporo, and Department of Respiratory Medicine, Oji Municipal General Hospital, Tomakomai, Japan.

Macrophage migration inhibitory factor (MIF) is a pro-inflammatory cytokine that reportedly counteracts the anti-inflammatory effect of endogenous glucocorticoids. There have only been a few reports that demonstrate a potential link between MIF and bronchial asthma. In an attempt to further clarify the precise role of MIF in asthma, the present authors examined the effect of anti-MIF antibody (Ab) on airway inflammation and airway hyperresponsiveness in an ovalbumin-immunised rat asthma model. Actively immunised Brown Norway rats received ovalbumin inhalation with or without treatment of anti-MIF Ab. The levels of MIF in bronchoalveolar lavage fluid were significantly elevated after the ovalbumin challenge. An immunohistochemical study revealed positive

immunostaining for MIF in bronchial epithelium, even in nonsensitised rats, and the MIF staining in bronchial epithelium was enhanced after the ovalbumin challenge. Anti-MIF Ab significantly decreased the number of total cells, neutrophils and eosinophils in the bronchoalveolar lavage fluid of the ovalbumin-challenged rats, and also attenuated the ovalbumin-induced airway hyperresponsiveness to ovalbumin and methacholine. However, anti-MIF Ab did not affect the level of serum ovalbumin-specific IgE, suggesting that anti-MIF Ab did not suppress immunisation itself. The results indicate that macrophage migration inhibitory factor plays a crucial role in airway inflammation and airway hyperresponsiveness in asthma.

PMID: 16455830 [PubMed - indexed for MEDLINE]

999. Int Arch Allergy Immunol. 2006;139(3):245-57. Epub 2006 Jan 30.

Increased expression of RANTES, CCR3 and CCR5 in the lesional skin of patients with atopic eczema.

Kato Y, Pawankar R, Kimura Y, Kawana S.

Department of Dermatology, Nippon Medical School, Tokyo, Japan.

BACKGROUND: Atopic eczema (AE) is a relapsing inflammatory disease based on IgE sensitization and characterized by peripheral blood eosinophilia and eosinophil infiltration into the lesional skin. In the patch test reaction of AE by allergens, an increased infiltration of activated eosinophils has been demonstrated peaking at 24-48 h. Regulated on activation normal T cell expressed and secreted (RANTES/CCL5) is a chemokine that induces eosinophil migration, and CCR3 and CCR5 are the receptors of RANTES.
OBJECTIVE: In order to further clarify the pathomechanisms of eosinophil infiltration in ongoing chronic inflammation in the skin of patients with AE and its relation to disease severity, we examined the expression of RANTES and its receptors CCR3 and CCR5 in challenged and unchallenged lesional skin of AE.
METHODS: We examined the number of RANTES+ cells, CCR3+ cells, CCR5+cells, activated (EG2+) eosinophils and CD3+ T cells in normal skin of healthy volunteers, and in challenged lesional skin (24 h after mite patch test) as well as unchallenged lesional skin of AE patients by immunohistochemistry. The cellular source of RANTES, CCR3 and CCR5 was analyzed by double immunohistochemistry using specific antibodies to RANTES, CCR3 or CCR5, and antibodies to ECP (EG2) or CD3.
RESULTS: The numbers of RANTES+ cells, CCR3+ cells, CCR5+ cells, EG2+ cells and CD3+ cells were all significantly increased in challenged (mite patch-tested) lesional skin of AE patients as compared to those in unchallenged lesional skin and normal skin. The numbers of these cells in unchallenged lesional skin were greater than those in normal skin. The number of EG2+ cells in the unchallenged lesional skin correlated with both the peripheral blood eosinophil count and the SCORAD index. The number of EG2+ cells in challenged lesional skin correlated with the number of CCR5+ cells. Activated eosinophils and T cells expressed RANTES and various proportions of these cells were CCR3+ and CCR5+ in both challenged and unchallenged lesional skin.
CONCLUSION: Taken together, these results suggest that RANTES as well as its receptors CCR3 and CCR5 may play important roles in the orchestration of eosinophil infiltration in ongoing chronic inflammation in AE, and also reflect the severity of disease.

Copyright (c) 2006 S. Karger AG, Basel.

PMID: 16449815 [PubMed - indexed for MEDLINE]

1000. Respir Res. 2006 Jan 23;7:11.

Functional and phenotypical comparison of myofibroblasts derived from biopsies and bronchoalveolar lavage in mild asthma and scleroderma.

Larsen K, Malmström J, Wildt M, Dahlqvist C, Hansson L, Marko-Varga G, Bjermer L, Scheja A, Westergren-Thorsson G.

Experimental Medical Science, Division of Vascular and Airway Research, Lund University, S-221 84 Lund, Sweden. kristoffer.larsen@med.lu.se

BACKGROUND: Activated fibroblasts, which have previously been obtained from bronchoalveolar lavage fluid (BALF), are proposed to be important cells in the fibrotic processes of asthma and scleroderma (SSc). We have studied the motility for BALF derived fibroblasts in patients with SSc that may explain the presence of these cells in the airway lumen. Furthermore, we have compared phenotypic alterations in activated fibroblasts from BALF and bronchial biopsies from patients with mild asthma and SSc that may account for the distinct fibrotic responses.
METHODS: Fibroblasts were cultured from BALF and bronchial biopsies from patients with mild asthma and SSc. The motility was studied using a cell migration assay. Western Blotting was used to study the expression of alpha-smooth muscle actin (alpha-SMA), ED-A fibronectin, and serine arginine splicing factor 20 (SRp20). The protein expression pattern was analyzed to reveal potential biomarkers using two-dimensional electrophoresis (2-DE) and sequencing dual matrix-assisted laser desorption ionization time-of-flight mass spectrometry (MALDI-TOF-TOF). The Mann-Whitney method was used to calculate statistical significance.
RESULTS: Increased migration and levels of ED-A fibronectin were observed in BALF fibroblasts from both groups of patients, supported by increased expression of RhoA, Rac1, and the splicing factor SRp20. However, these observations were exclusively accompanied by increased expression of alpha-SMA in patients with mild asthma. Compared to BALF fibroblasts in mild asthma, fibroblasts in SSc displayed a differential protein expression pattern of cytoskeletal- and scavenger proteins. These identified proteins facilitate cell migration, oxidative stress, and the excessive deposition of extracellular matrix observed in patients with SSc.
CONCLUSION: This study demonstrates a possible origin for fibroblasts in the airway lumen in patients with SSc and important differences between fibroblast phenotypes in mild asthma and SSc. The findings may explain the distinct fibrotic processes and highlight the motile BALF fibroblast as a potential target cell in these disorders.

PMCID: PMC1386661
PMID: 16430780 [PubMed - indexed for MEDLINE]

1001. J Immunol. 2006 Feb 1;176(3):1860-8.

Fraktalkine produced by airway smooth muscle cells contributes to mast cell recruitment in asthma.

El-Shazly A, Berger P, Girodet PO, Ousova O, Fayon M, Vernejoux JM, Marthan R, Tunon-de-Lara JM.

Université Victor Segalen Bordeaux 2, Laboratoire de Physiologie Cellulaire Respiratoire, Institut National de la Santé et de la Recherche Médicale, E356, Bordeaux, France.

Human airway smooth muscle cells (HASMC) secrete fractalkine (FKN), a chemokine

the concentration of which is increased in asthmatic patients. HASMC also induce mast cell chemotaxis, as a component of asthma inflammation. We therefore evaluated the role of smooth muscle-derived FKN in mast cell migration. We assessed the capacity of recombinant FKN to induce human mast cell chemotaxis. This effect implicates a calcium-independent pathway involving actin reorganization and protein kinase C-delta. We found that HASMC constitutively produce FKN, the synthesis of which is reinforced upon proinflammatory stimulation. Under basal experimental conditions, FKN production by HASMC is not sufficient to induce mast cell chemotaxis. However, pretreatment of mast cells with the neuropeptide vasoactive intestinal peptide (VIP) increases FKN potency to attract mast cells. Since we observed, in asthmatic patients, an increase in both FKN and VIP expression by airway smooth muscle and a positive correlation between VIP staining and mast cell infiltration of the smooth muscle layer, we conclude that HASMC-derived FKN may contribute to mast cell recruitment in asthma.

PMID: 16424217 [PubMed - indexed for MEDLINE]

1002. J Invest Dermatol. 2006 Jan;126(1):91-6.

Bullous allergic hypersensitivity to bed bug bites mediated by IgE against salivary nitrophorin.

Leverkus M, Jochim RC, Schäd S, Bröcker EB, Andersen JF, Valenzuela JG, Trautmann A.

Department of Dermatology, University of Würzburg, Würzburg, Germany.
leverkus@medizin.uni-magdeburg.de

In Central Europe, bites from the common bed bug (Cimex lectularius) are nowadays rather uncommon. Nevertheless, infestations are sometimes observed in old framehouses and by immigration due to international travel and migration. The clinical picture of bug bites substantially varies between individuals, depending upon previous exposure and the degree of an immune response. The host immune response and potential protein antigens present in the saliva of C. lectularius or specific antibodies have not been characterized thus far. We describe a patient with bullous bite reactions after sequential contact with C. lectularius over a period of 1 year. In skin tests, we observed immediate reactions to the salivary gland solution of C. lectularius, which were followed by a pronounced partially blistering late-phase response. Immunoblot analysis of the patient's serum with salivary gland extracts and recombinant C. lectularius saliva proteins revealed specific IgE antibodies against the 32 kDa C. lectularius nitrophorin, but not to 37 kDa C. lectularius apyrase. Our data demonstrate that bullous cimicosis may be the late-phase response of an allergic IgE-mediated hypersensitivity to C. lectularius nitrophorin.

PMID: 16417223 [PubMed - indexed for MEDLINE]

1003. Clin Exp Pharmacol Physiol. 2005 Nov;32(11):988-94.

Connective tissue growth factor: a role in airway remodelling in asthma?

Burgess JK.

Department of Pharmacology, University of Sydney and Woolcock Institute of Medical Research, Sydney, New South Wales, Australia.
janette@pharmacol.usyd.edu.au

1. Severe persistent asthma is accompanied by structural changes in the airway, referred to as remodelling. The mechanisms driving airway remodelling are poorly understood. 2. Transforming growth factor (TGF)-beta is increased in the airways of patients with asthma. Many of the effects of TGF-beta are mediated by connective tissue growth factor (CTGF). 3. Overexpression of CTGF is linked to many fibrotic diseases, but its exact role in airway remodelling is unknown. 4. Connective tissue growth factor mediates cell adhesion, migration, proliferation, survival, extracellular matrix synthesis and has a role in angiogenesis. 5. Current asthma therapies do not inhibit CTGF induction. 6. Understanding the mechanisms underlying the role of CTGF in airway remodelling may lead to new therapeutic strategies for asthma.

PMID: 16405457 [PubMed - indexed for MEDLINE]

1004. Clin Exp Allergy. 2005 Dec;35(12):1572-80.

Expression of CXCR6 and its ligand CXCL16 in the lung in health and disease.

Morgan AJ, Guillen C, Symon FA, Huynh TT, Berry MA, Entwisle JJ, Briskin M, Pavord ID, Wardlaw AJ.

Institute for Lung Health, Department of Infection, Immunity and Inflammation, Leicester University, Glenfield Hospital, Leicester, UK.

BACKGROUND: Chemokine receptors (CR) play an important role in T cell migration, but their contribution to lung trafficking is unclear.
OBJECTIVE: We hypothesized that if a particular CR was involved in T cell homing its expression would be enriched on lung T cells compared with peripheral blood T cells (PBT).
METHODS: We have measured the CR expression on BAL T cells from patients with sarcoid, other interstitial lung diseases (ILD), asthma and healthy volunteers.
RESULTS: Of 14 CR studied in sarcoid, CXCR6 expression was the most markedly increased in the lung compared with the blood, a finding that was also seen in ILD patients. A striking although lesser increase was also seen in asthmatics and healthy controls. Analysis of expression of the CXCR6 ligand, CXCL16, by immunohistochemistry suggested that alveolar macrophages (AM) were the major source of CXCL16 in the lung. AM expressed mRNA for CXCL16 and released nanogram quantities after adhesion to plastic as shown by RT-PCR, Western blotting and ELISA. Bronchoalveolar lavage (BAL) fluid from all subjects contained large amounts of CXCL16. The full-length CXCL16 was the predominant isoform in AM lysates, supernatants and BAL.
CONCLUSION: This data suggests that CXCR6 and CXCL16 may play a role in T cell recruitment to the lung.

PMID: 16393323 [PubMed - indexed for MEDLINE]

1005. J Allergy Clin Immunol. 2006 Jan;117(1):103-10. Epub 2005 Dec 2.

The CC chemokine eotaxin/CCL11 has a selective profibrogenic effect on human lung fibroblasts.

Puxeddu I, Bader R, Piliponsky AM, Reich R, Levi-Schaffer F, Berkman N.

Department of Pharmacology, School of Pharmacy, Faculty of Medicine, The Hebrew University of Jerusalem, Jerusalem 91120, Israel.

BACKGROUND: Eotaxin/CCL11 plays an important role in asthma. It acts through the chemokine receptor CCR3 expressed on hematopoietic and nonhematopoietic cells in

the lung.
OBJECTIVE: To determine whether eotaxin/CCL11 modulates lung and bronchial fibroblast properties and thereby might contribute to airway remodeling.
METHODS: CCR3 expression was characterized on a lung fibroblast line (MRC-5; flow cytometry, fluorescent microscopy, RT-PCR, and Northern blotting), on primary bronchial fibroblasts (flow cytometry), and on fibroblasts in human lung tissue (confocal laser microscopy). The effects of eotaxin/CCL11 on lung fibroblast migration (Boyden chamber), proliferation (tritiated thymidine incorporation), alpha-smooth muscle actin expression (ELISA), 3-dimensional collagen gel contraction (floating gel), pro-alpha1(I) collagen mRNA (Northern blotting), total collagen synthesis (tritiated proline incorporation), matrix metalloproteinase activity (gelatin zymography), and TGF-beta(1) release (ELISA) were evaluated. The contribution of eotaxin/CCL11/CCR3 binding on lung fibroblasts was also investigated by neutralizing experiments.
RESULTS: CCR3 is constitutively expressed in cultured lung and primary bronchial fibroblasts and colocalizes with specific surface markers for human fibroblasts in lung tissue. Eotaxin/CCL11 selectively modulates fibroblast activities by increasing their proliferation, matrix metalloproteinase 2 activity, and collagen synthesis but not their differentiation into myofibroblasts, contractility in collagen gel, or TGF-beta(1) release. Eotaxin/CCL11 enhances migration of lung fibroblasts in response to nonspecific chemoattractants, and this effect is completely inhibited by anti-CCR3-neutralizing antibodies.
CONCLUSION: These data demonstrate that eotaxin/CCL11 has a direct and selective profibrogenic effect on lung and bronchial fibroblasts, providing a novel mechanism whereby eotaxin/CCL11 can participate in airway remodeling in asthma.

PMID: 16387592 [PubMed - indexed for MEDLINE]

1006. Chang Gung Med J. 2005 Oct;28(10):716-23.

Leprosy in the Department of Dermatology, Chang Gung Memorial Hospital at Kaohsiung from 1988 to 2004: a clinical and histopathologic study of 13 cases.

Wang CY, Huang PH, Cheng YW, Ho JC, Chen WC.

Department of Dermatology, Chang Gung Memorial Hospital, Kaohsiung, Taiwan.

BACKGROUND: Leprosy has long been in Taiwan, but it has never been eradicated. Incidental cases are easily overlooked nowadays because most younger dermatologists are unfamiliar with this disease.
METHODS: We review and analyze 13 cases diagnosed as leprosy at the Department of Dermatology, Chang Gung Memorial Hospital at Kaohsiung from 1988 to 2004, all of which were histopathologically proven.
RESULTS: The ages of the 13 recruited patients ranged from 31 to 73 (mean, 58.6) years, without a gender preference (male: female, 7:6). Two male patients were under 40 years old; one was a foreign worker from Thailand and the other was a local person in Penghu working as the chief officer on a fishing boat. The most-common clinical subtype was lepromatous leprosy (5/13), followed by borderline lepromatous leprosy, borderline tuberculoid leprosy, and tuberculoid leprosy (each 2/13), and then borderline leprosy and indeterminate leprosy (each 1/13). The initial clinical impression before the histopathological diagnosis included granuloma annulare, generalized eczema, lymphoma, syphilis, papular urticaria, cutaneous tuberculous infection, Sweet's syndrome, erythema annulare centrifugum, and hematoma. Most of these patients only received irregular treatment after the diagnosis was made and were soon lost to follow-up.
CONCLUSIONS: With increasing numbers of foreign workers and immigrants living in Taiwan in recent years, leprosy seems to have become a challenging diagnosis for younger dermatologists. Dermatologists should keep this ancient disease in mind and not overlook it. Because of the necessity of prolonged medication, complete

treatment and long-term follow-up of leprosy cases will remain a major problem in public health.

PMID: 16382756 [PubMed - indexed for MEDLINE]

1007. Isr Med Assoc J. 2005 Dec;7(12):799-802.

Serum immunoglobulin E levels in Israeli-Ethiopian children: environment and genetics.

Iancovici Kidon M, Stein M, Geller-Bernstein C, Weisman Z, Steinberg S, Greenberg Z, Handzel ZT, Bentwich Z.

Allergy and Clinical Immunology Unit, Kaplan Medical Center, Rehovot, Israel. drkidon@bezeqint.net

BACKGROUND: Since 1984, several waves of Ethiopian immigrants have settled in Israel. On arrival they were found to be highly infected with intestinal parasites and to have increased serum immunoglobulin E and eosinophilia.
OBJECTIVES: To study serum IgE levels in Ethiopian children growing up in the environment of Israel.
METHODS: We assessed four groups of children of Ethiopian origin: a) adolescents examined on their arrival to Israel (group 1, n=11); b) adolescents born in Ethiopia and living in Israel for more than 7 years (group 2, n=10); c) children of Ethiopian origin born in Israel, without a history of allergy or asthma (group 3, n=15); and d) asthmatic children of Ethiopian origin born in Israel (group 4, n=8). A thorough clinical interview and examination as well as laboratory work up (including serum IgE levels, stool parasites and absolute eosinophil count) were performed.
RESULTS: Group 1 (11 newly arrived Ethiopian adolescents) had a mean eosinophil count of 688 cells/ml (0-1739) and a mean serum IgE of 1043 IU/ml (253-2932), P< 0.0009 as compared to group 2. Helminthic parasites were observed in 8/11 individuals; after 1 year of follow-up and anti-parasitic treatment, serum IgE levels did not change significantly. Group 2 (10 Ethiopian born adolescents living in Israel for on average 10 years, 7-15 years) had a normal leukocyte count, MEC 192 cells/ml (range 54-289), serum IgE 142 IU/ml (range 14-399 IU/ml) and no parasites in stool. Group 3 (15 Ethiopian children born in Israel) had a normal leukocyte count, MEC 128 cells/ml (0-324), serum IgE 55 IU/ml (7-189 IU/ml), similar to age-matched Israeli controls. In group 4 (8 Israeli born children of Ethiopian descent diagnosed with asthma), serum IgE showed significant elevation compared to Israeli age-matched asthmatic children (P< 0.005).
CONCLUSIONS: High levels of IgE found in Ethiopian children on arrival to Israel declined to Israeli control levels after several years of living in the new environment. Ethiopian children born in Israel had normal levels of IgE, suggesting that environment is the main factor affecting IgE levels in this population. Israeli born Ethiopian children with asthma had significantly increased serum IgE levels compared to asthmatics of Israeli origin. These findings suggest that both environmental and genetic factors determine the level of serum IgE in these children.

PMID: 16382704 [PubMed - indexed for MEDLINE]

1008. FEBS Lett. 2006 Jan 23;580(2):373-9. Epub 2005 Dec 13.

9alpha,11beta-PGF2 and its stereoisomer PGF2alpha are novel agonists of the chemoattractant receptor, CRTH2.

Sandig H, Andrew D, Barnes AA, Sabroe I, Pease J.

Leukocyte Biology Section, NHLI Division, Imperial College Faculty of Medicine, South Kensington, London SW7 2AZ, UK.

CRTH2 is a recently described chemoattractant receptor for the prostaglandin, PGD(2), expressed by Th2 cells, eosinophils and basophils, and believed to play a role in allergic inflammation. Here we describe the potency of several PGD(2) metabolites at the receptor to induce cell migration and activation. We report for the first time that the PGD(2) metabolite, 9alpha,11beta-PGF(2), and its stereoisomer, PGF(2alpha), are CRTH2 agonists. 9alpha,11beta-PGF(2) is a major metabolite produced in vivo following allergen challenge, whilst PGF(2alpha) is generated independently of PGD synthetase, with implications for CRTH2 signalling in the presence or absence of PGD(2) production.

PMID: 16378605 [PubMed - indexed for MEDLINE]

1009. J Immunol. 2006 Jan 1;176(1):557-66.

Chemotactic responses of IL-4-, IL-10-, and IFN-gamma-producing CD4+ T cells depend on tissue origin and microbial stimulus.

Debes GF, Dahl ME, Mahiny AJ, Bonhagen K, Campbell DJ, Siegmund K, Erb KJ, Lewis DB, Kamradt T, Hamann A.

Experimental Rheumatology, Medical Clinic, Charité University Medicine Berlin, Berlin, Germany. debes@stanford.edu

Erratum in
J Immunol. 2006 Feb 15;176(4):2671.

Th1- and Th2-polarized immune responses are crucial in the defense against pathogens but can also promote autoimmunity and allergy. The chemokine receptors CXCR3 and CCR4 have been implicated in differential trafficking of IFN-gamma- and IL-4-producing T cells, respectively, but also in tissue and inflammation-specific homing independent of cytokine responses. Here, we tested whether CD4+ T cells isolated from murine tissues under homeostatic or inflammatory conditions exhibit restricted patterns of chemotactic responses that correlate with their production of IFN-gamma, IL-4, or IL-10. In uninfected mice, IL-4-producing T cells preferentially migrated to the CCR4 ligand, CCL17, whereas IFN-gamma-expressing T cells as well as populations of IL-4+ or IL-10+ T cells migrated to the CXCR3 ligand, CXCL9. All cytokine-producing T cell subsets strongly migrated to the CXCR4 ligand, CXCL12. We assessed chemotaxis of T cells isolated from mice infected with influenza A virus or the nematode Nippostrongylus brasiliensis, which induce a strong Th1 or Th2 response in the lung, respectively. Unexpectedly, the chemotactic responses of IL-4+ T cells and T cells expressing the immunosuppressive cytokine IL-10 were influenced not only by the strongly Th1- or Th2-polarized environments but also by their anatomical localization, i.e., lung or spleen. In contrast, IFN-gamma+ T cells exhibited robust chemotaxis toward CXCL9 and had the most consistent migration pattern in both infection models. The results support a model in which the trafficking responses of many effector and regulatory T cells are regulated as a function of the infectious and tissue environments.

PMID: 16365450 [PubMed - indexed for MEDLINE]

1010. Int Arch Allergy Immunol. 2006;139(2):114-21. Epub 2005 Dec 19.

Expression of vascular adhesion protein-1 in atopic eczema.

Madej A, Reich A, Orda A, Szepietowski JC.

Department of Dermatology, Venereology and Allergology, University of Medicine, Wroclaw, Poland.

BACKGROUND: Vascular adhesion protein-1 (VAP-1) is an adhesion molecule with an enzymatic activity which partakes in the migration process of lymphocytes. The aim of this study was to investigate VAP-1 expression in atopic eczema (AE) in comparison with healthy controls and psoriatic individuals.
MATERIAL AND METHODS: Forty adult patients suffering from AE aged between 18 and 56 years were included in the study. The control group consisted of 35 healthy volunteers aged between 19 and 49 years and of 71 psoriatic patients aged between 23 and 89 years. The serum concentration of soluble VAP-1 (sVAP-1) was evaluated by ELISA and VAP-1 expression in the skin by immunohistochemistry.
RESULTS: Serum level of sVAP-1 in AE patients before treatment was significantly higher compared with healthy volunteers. Similarly, a higher mean number of VAP-1-positive vessels was found in both lesional and nonlesional atopic skin compared with healthy skin. Treatment of AE resulted in a significant reduction in the serum level of sVAP-1. On the other hand, both the serum level of sVAP-1 and the number of dermal vessels with expression of VAP-1 were significantly lower in AE patients compared with psoriatic individuals.
CONCLUSION: This study indicates the important role of VAP-1 in the pathogenesis of chronic inflammatory cutaneous disorders, including AE.

Copyright (c) 2006 S. Karger AG, Basel.

PMID: 16361866 [PubMed - indexed for MEDLINE]

1011. Am J Physiol Lung Cell Mol Physiol. 2006 May;290(5):L909-18. Epub 2005 Dec 9.

CXCR3 surface expression in human airway epithelial cells: cell cycle dependence and effect on cell proliferation.

Aksoy MO, Yang Y, Ji R, Reddy PJ, Shahabuddin S, Litvin J, Rogers TJ, Kelsen SG.

Department of Medicine, Division of Pulmonary Disease and Critical Care Medicine, Temple University School of Medicine, Temple University Hospital, 762 Parkinson Pavilion, 3401 N. Broad St., Philadelphia, PA 19140, USA. mark.aksoy@temple.edu

We recently demonstrated that human bronchial epithelial cells (HBEC) constitutively express the CXC chemokine receptor CXCR3, which when activated, induces directed cell migration. The present study in HBEC examined the relative expression of the CXCR3 splice variants CXCR3-A and -B, cell cycle dependence of CXCR3 expression, and the effects of the CXCR3 ligand, the interferon-gamma-inducible CXC chemokine I-TAC/CXCL11, on DNA synthesis and cell proliferation. Both CXCR3-A and -B mRNA, assessed by real-time RT-PCR, were expressed in normal HBEC (NHBEC) and the HBEC line 16-HBE. However, CXCR3-B mRNA was 39- and 6-fold greater than CXCR3-A mRNA in NHBEC and 16-HBE, respectively. Although most HBEC (>80%) assessed by flow cytometry and immunofluorescence microscopy contained intracellular CXCR3, only a minority (<40%) expressed it on the cell surface. In this latter subset of cells, most (>75%) were in the S + G(2)/M phases of the cell cycle. Stimulation of CXCR3 with I-TAC enhanced thymidine incorporation and cell proliferation and increased p38 and ERK1/2 phosphorylation. These data indicate that 1) human airway epithelial cells primarily express CXCR3-B mRNA, 2) surface expression of CXCR3 is largely confined to the S + G(2)/M phases of the cell cycle, and 3) activation of CXCR3 induces DNA synthesis, cell proliferation, and activation of MAPK pathways. We

speculate that activation of CXCR3 exerts a mitogenic effect in HBEC, which may be important during airway mucosal injury in obstructive airway diseases such as asthma and chronic obstructive pulmonary disease.

PMID: 16339779 [PubMed - indexed for MEDLINE]

1012. Graefes Arch Clin Exp Ophthalmol. 2006 Jul;244(7):825-8. Epub 2005 Dec 6.

Increase in macrophage migration inhibitory factor levels in lacrimal fluid of patients with severe atopic dermatitis.

Kitaichi N, Shimizu T, Honda A, Abe R, Ohgami K, Shiratori K, Shimizu H, Ohno S.

Department of Ophthalmology and Visual Sciences, Hokkaido University Graduate School of Medicine, 060-8638, Sapporo, Japan. nobukita@med.hokudai.ac.jp

BACKGROUND AND AIMS OF THE STUDY: Atopic dermatitis is a chronic inflammatory skin disorder that often involves some ophthalmic features. Macrophage migration inhibitory factor (MIF) is a proinflammatory cytokine that is associated with the generation of cell-mediated immune responses. Although serum MIF levels may be elevated in severe atopic dermatitis, the quantity of MIF in regional ocular fluid remains unknown. We measured MIF levels in tears (lacrimal fluid) of patients with atopic dermatitis.
PATIENTS AND METHODS: Tear samples were collected from 16 patients with atopic dermatitis, 10 patients with allergic conjunctivitis, and 15 healthy control subjects. The clinical severity of atopic dermatitis was evaluated according to the Scoring Atopic Dermatitis (SCORAD) index. The index was calculated by summing the following scores: extent criteria, intensity criteria, and subjective symptoms. Macrophage migration inhibitory factor levels were determined by a human MIF enzyme-linked immunosorbent assay. All comparisons were two-tailed, and P values <0.01 were considered as statistically significant.
RESULTS: The mean MIF concentration in lacrimal fluid collected from healthy control subjects was 0.69+/-0.2 ng/ml. The mean tear MIF levels were 17.87+/-6.3 ng/ml in moderate-to-severe atopic dermatitis (SCORAD> or =15, P=0.002), 0.93+/-0.08 ng/ml in mild atopic dermatitis (SCORAD<15), and 2.76+/-0.86 ng/ml in allergic conjunctivitis (P=0.008).
CONCLUSIONS: A proinflammatory cytokine MIF level was elevated in tears as well as serum in cases of severe atopic dermatitis. These results suggest that MIF may play an important role in the induction or enhancement of ophthalmic features related to severe atopic dermatitis.

PMID: 16331484 [PubMed - indexed for MEDLINE]

1013. Med Sci (Paris). 2005 Dec;21(12):1063-9.

[Repair and regeneration of the airway epithelium].

[Article in French]

Coraux C, Hajj R, Lesimple P, Puchelle E.

Inserm UMRS 514, IFR 53, CHU Maison Blanche, 45, rue Cognacq- Jay, 51092 Reims Cedex, France.

Despite an efficient defence system, the airway surface epithelium, in permanent contact with the external milieu, is frequently injured by inhaled pollutants, microorganisms and viruses. The response of the airway surface epithelium to an acute injury includes a succession of cellular events varying from the loss of

the surface epithelium integrity to partial shedding of the epithelium or even to complete denudation of the basement membrane. The epithelium has then to repair and regenerate to restore its functions, through several mechanisms including basal cell spreading and migration, followed by proliferation and differentiation of epithelial cells. The cellular and molecular factors involved in wound repair and epithelial regeneration are closely interacting and imply extracellular matrix proteins, matrix metalloproteinases (MMPs) and their inhibitors as well as cytokines and growth factors secreted by airway epithelial and mesenchymal cells. The development of in vitro and in vivo models of airway epithelium wound repair allowed the study of the spatio-temporal modulation of these factors during the different steps of epithelial repair and regeneration. In this context, several studies have demonstrated that the matrix and secretory environment are markedly involved in these mechanisms and that their dysregulation may induce remodelling of the airway mucosa. A better knowledge of the mechanisms involved in airway epithelium regeneration may pave the way to regenerative therapeutics allowing the reconstitution of a functional airway epithelium in numerous respiratory diseases such as asthma, chronic obstructive pulmonary diseases, cystic fibrosis and bronchiolitis.

PMID: 16324647 [PubMed - indexed for MEDLINE]

1014. Eur Respir J. 2005 Dec;26(6):1040-6.

Macrophages induce an allergen-specific and long-term suppression in a mouse asthma model.

Vissers JL, van Esch BC, Hofman GA, van Oosterhout AJ.

Department of Pharmacology and Pathophysiology, Utrecht University, The Netherlands.

Increasing evidence suggests that macrophages (Mphi) play a crucial downregulatory role in the initiation and progression of allergic asthma. Recently, the current authors demonstrated that ovalbumin (OVA)-loaded Mphi (OVA-Mphi) suppress subsequent OVA-induced airway manifestations of asthma and that this effect could be potentiated upon selective activation. In the present study, the authors further delineated the underlying pathway by which Mphi exert this immunosuppressive effect. To examine the migration of OVA-Mphi, cells were labelled with 5'chloromethylfluorescein diacetate (CMFDA) and were administered (i.v.) into OVA-sensitised BALB/c mice. After 20 h, the relevant organs were dissected and analysed using fluorescent microscopy. Allergen-specificity was investigated by treating OVA-sensitised mice with keyhole limpet haemocyanin (KLH)-Mphi activated with immunostimulatory sequence oligodeoxynucleotide (ISS-ODN). By lengthening the period between treatment and challenge to 4 weeks it was examined whether OVA-Mphi exerted an immunosuppressive memory response. Strikingly, CMFDA-labelled Mphi were not trapped in the lungs, but migrated to the spleen. ISS-ODN-stimulated KLH-Mphi failed to suppress OVA-induced airway manifestations of asthma. Moreover, treatment with ISS-ODN-stimulated OVA-Mphi was still effective after lengthening the period between treatment and challenge. These data demonstrate that allergen-loaded macrophages can induce an indirect immunosuppressive response that is allergen-specific and long lasting, which are both hallmarks of a memory lymphocyte response.

PMID: 16319333 [PubMed - indexed for MEDLINE]

1015. Mol Ecol. 2005 Dec;14(14):4275-85.

High genetic diversity in French invasive populations of common ragweed, Ambrosia

artemisiifolia, as a result of multiple sources of introduction.

Genton BJ, Shykoff JA, Giraud T.

Laboratoire Ecologie, Systématique et Evolution, UMR CNRS-UPS-ENGREF 8079, Université Paris-Sud, Bâtiment 360, 91 405 Orsay cedex, France.

Ambrosia artemisiifolia is an aggressive North American annual weed, found particularly in sunflower and corn fields. Besides its economic impact on crop yield, it represents a major health problem because of its strongly allergenic pollen. Ragweed was imported inadvertently to Europe in the 18th century and has become invasive in several countries, notably in the Rhône Valley of France. It has recently expanded in both the Provence-Alpes-Côte-d'Azur and Bourgogne regions. As first steps towards understanding the causes and mechanisms of ragweed invasion, genetic variability of French and North American populations was analysed using microsatellites. Overall genetic variability was similar in North America and in the Rhône-Alpes region, but within-population levels of genetic variability were surprisingly lower in native than in invasive French populations. French populations also exhibited lower among-population differentiation. A significant pattern of isolation by distance was detected among North American populations but not among French populations. Assignment tests and distribution of rare alleles did not point to a single origin for all French populations, nor for all individuals within populations and private alleles from different North American populations were found in the same French populations. Indeed, within all French populations, individual plants were roughly equally assigned to the different North American populations. Altogether, these results suggest that the French invasive populations include plants from a mixture of sources. Reduced diversity in populations distant from the original area of introduction indicated that ragweed range expansion probably occurred through sequential bottlenecks from the original populations, and not from subsequent new introductions.

PMID: 16313592 [PubMed - indexed for MEDLINE]

1016. J Biol Chem. 2006 Jan 20;281(3):1489-94. Epub 2005 Nov 22.

Mast cell and neutrophil peptidases attack an inactivation segment in hepatocyte growth factor to generate NK4-like antagonists.

Raymond WW, Cruz AC, Caughey GH.

Department of Medicine and Cardiovascular Research Institute, The University of California at San Francisco, San Francisco, California 94143, USA.

Hepatocyte growth factor (HGF) is a plasminogen-like protein with an alpha chain linked to a trypsin-like beta chain without peptidase activity. The interaction of HGF with c-met, a receptor tyrosine kinase expressed by many cells, is important in cell growth, migration, and formation of endothelial and epithelial tubes. Stimulation of c-met requires two-chain, disulfide-linked HGF. Portions of an alpha chain containing an N-terminal segment and four kringle domains (NK4) antagonize HGF activity. Until now, no physiological pathway for generating NK4 was known. Here we show that chymases, which are chymotryptic peptidases secreted by mast cells, hydrolyze HGF, thereby abolishing scatter factor activity while generating an NK4-like antagonist of HGF scatter factor activity. Thus, chymase interferes with HGF directly by destroying active protein and indirectly by generating an antagonist. The site of hydrolysis, Leu480, lies in the alpha chain on the N-terminal side of the cysteine linking the alpha and beta chains. This site appears to be specific for HGF because chymase does not hydrolyze other plasminogen-like proteins, such as macrophage-stimulating protein and plasminogen

itself. Mast cell/neutrophil cathepsin G and neutrophil elastase generate similar fragments of HGF by cleaving near the chymase site. Mast cell and neutrophil peptidases are secreted during tissue injury, infection, ischemia, and allergic inflammation, where they may oppose HGF effects on epithelial repair. Thus, HGF possesses an "inactivation segment" that serves as an Achilles' heel attacked by inflammatory proteases. This work reveals a potential physiological pathway for inactivation of HGF and generation of NK4-like antagonists.

PMCID: PMC2271111
PMID: 16303761 [PubMed - indexed for MEDLINE]

1017. J Invest Dermatol. 2005 Nov;125(5):969-76.

Junctional adhesion molecules (JAM)-B and -C contribute to leukocyte extravasation to the skin and mediate cutaneous inflammation.

Ludwig RJ, Zollner TM, Santoso S, Hardt K, Gille J, Baatz H, Johann PS, Pfeffer J, Radeke HH, Schön MP, Kaufmann R, Boehncke WH, Podda M.

Department of Dermatology, Klinikum der J.W. Goethe Universität, Frankfurt am Main, Germany. r.ludwig@em.uni-frankfurt.de

Leukocyte extravasation is a finely tuned process, in which transmigration is the final step. Transmigration depends on molecules located at borders of endothelial cells; e.g., junctional adhesion molecules (JAM-A, -B and -C). In vivo blockade of JAM-A lead to decreased migration of monocytes into the skin. In contrast, the role of JAM-B and -C in development of cutaneous inflammation is unknown. We therefore elicited an allergic contact dermatitis in mice using 2,4-dinitro-1-fluorobenzene. RT-PCR and immunofluorescent staining of healthy skin revealed a constitutive JAM-B (66.4%+/-6.7% of all vessels) and -C expression (88.6+/-13.2%), which remained constant after induction of contact dermatitis. Functional studies, in which either JAM-B or -C neutralizing antibodies were injected into sensitized mice prior to allergen challenge showed a concentration-dependent reduction of the contact dermatitis. Decreased ear swelling was accompanied by reduction of leukocyte infiltration as analyzed by hematoxylin and eosin (H&E) histology and enzyme activity. Combined antibody treatment at doses of 1.25 mg per kg bodyweight lead to additive inhibition of allergic contact dermatitis, indicating that JAM-B and -C may have distinct functions. In conclusion, interactions with JAM-B and -C are essential for development of cutaneous inflammation.

PMID: 16297198 [PubMed - indexed for MEDLINE]

1018. Pulm Pharmacol Ther. 2006;19(1):32-8. Epub 2005 Apr 25.

Regulation of human airway mesenchymal cell proliferation by glucocorticoids and beta2-adrenoceptor agonists.

Bonacci JV, Stewart AG.

Department of Pharmacology, The University of Melbourne, Vic. 3010, Australia.

Altered rates of cell proliferation play important roles in the pathogenesis of a variety of conditions, including cancer, inflammation and several airway and cardiovascular diseases. One of the most consistently observed changes in asthmatic airways is an increased volume of airway smooth muscle (ASM), that has been explained by proliferation, hypertrophy, extracellular matrix deposition within the smooth muscle bundles, and more recently, the migration of mesenchymal

precursor cells to the airways. The best characterised of these is proliferation of ASM cells. In vitro studies suggest that the proliferation is driven by various mitogens, and ECM proteins found in asthma, such as collagen type I. Therefore, we compared the anti-mitogenic actions of two classes of anti-asthma agents, the glucocorticoids and the beta2-adrenoceptor agonists, in ASM cells grown on collagen type I. Culture on collagen type I prevented the anti-mitogenic actions of glucocorticoids, but not beta2-adrenoceptor agonists. In contrast, glucocorticoids are efficacious in regulating ASM production of GM-CSF, whereas beta2-adrenoceptor agonists are without effect. Therefore, combination therapy may have increased efficacy over glucocorticoids alone in controlling remodelling events due to complementary actions of the two classes of compounds.

PMID: 16286235 [PubMed - indexed for MEDLINE]

1019. J Dtsch Dermatol Ges. 2003 Jan;1(1):8-11.

[How does eczema arise?].

[Article in German]

Trautmann A, Disch R, Bröcker EB, Akdis CA, Gillitzer R.

Klinik und Poliklinik für Hautkrankheiten der Universität Würzburg.
trautmann_a@klinik.uni-wuerzburg.de

New experimental results on the role of T cells and keratinocytes have led to a better understanding of eczematous inflammation and can help explain both the clinical and histological pictures of eczema. Besides activated endothelial cells and adhesion molecules, a complex interaction of numerous chemokines controls the recruitment of T cells from the blood vessels and their migration into the dermis and epidermis. Activated T cells damage the epidermis by pro-inflammatory cytokines and can induce apoptosis of individual keratinocytes through "killer molecules". Cleavage of adhesion molecules on keratinocytes leads to spongiotic changes. Keratinocytes then activate repair mechanisms, which cause acanthosis and parakeratosis in chronic eczema.

PMID: 16285287 [PubMed - indexed for MEDLINE]

1020. Chron Respir Dis. 2004;1(3):153-60.

Crosscultural communication in those with airway diseases.

Car J, Partridge MR.

Department of Primary Care and Social Medicine, Imperial College, London, UK.

Transcultural consultations are becoming commonplace. Such consultations arise because patients from ethnic groups consult doctors, but also because patients consult doctors from other ethnic backgrounds. Such consultations require a cultural awareness and sensitivity which may be particularly necessary when concerning those with respiratory illnesses which are often long-term and about which there may be considerable stigma. The prevalence of respiratory disease can vary between ethnic groups, most noticeably in tuberculosis and smoking; and in diseases such as asthma, health service usage and treatment can vary significantly with ethnicity. Some of this may represent cultural, rather than disease specific differences. Good communication is essential throughout medical practice, but in transcultural consultations it is especially important that the doctor pays appropriate attention to likely patient beliefs and approaches to

shared decision making. Usual negotiation regarding goals and outcomes first requires the clinician to understand how a patient's understanding of illness may vary from a traditional western scientific approach. Special attention needs to be paid to the optimal way of using interpreters and more time is often needed for crosscultural consultations. Specific training is necessary for health practitioners to enable them to acquire the skills for crosscultural care and this involves learning about other cultures and an appreciation of how a change in attitude often needs to be incorporated into the clinical approach. Acquiring these skills and understandings to facilitate optimal transcultural consultation enables transfer of these skills to other similar clinical scenarios such as the approach to those with disability. The global burden of long-term respiratory disease, both infectious and noncommunicable, coupled with increased migration and geographical mobility means that a successful crosscultural approach is now a priority area for attention.

PMID: 16281657 [PubMed - indexed for MEDLINE]

1021. Int Immunopharmacol. 2005 Dec;5(13-14):1966-74. Epub 2005 Jun 20.

Anti-allergic properties of the bromeliaceae Nidularium procerum: inhibition of eosinophil activation and influx.

Vieira-de-Abreu A, Amendoeira FC, Gomes GS, Zanon C, Chedier LM, Figueiredo MR, Kaplan MA, Frutuoso VS, Castro-Faria-Neto HC, Weller PF, Bandeira-Melo C, Bozza PT.

Laboratório de Imunofarmacologia, Departamento de Fisiologia e Farmacodinâmica, Instituto Oswaldo Cruz, Fundação Oswaldo Cruz, Avenida Brasil 4365, Manguinhos, 21045-900 Rio de Janeiro, Brazil.

New therapeutic approaches for the treatment of allergic diseases can be aided by the development of agents capable of regulating eosinophilic leukocytes. Here, we evaluated the anti-allergic properties of a crude extract of the Brazilian bromeliaceae Nidularium procerum, focusing on its effects on allergic eosinophilia. By studying allergic pleurisy in actively sensitized C57Bl/6 mice, we observed that pretreatment with N. procerum (2 mg/kg; i.p.) reduced pleural eosinophil influx triggered by allergen challenge. N. procerum was also able to reduce lipid body numbers found within infiltrating eosinophils, indicating that N. procerum in vivo is able to affect both migration and activation of eosinophils. Consistently, pretreatment with N. procerum blocked pleural eosinophil influx triggered by PAF or eotaxin, key mediators of the development of allergic pleural eosinophilia. The effect of N. procerum was not restricted to eosinophils, since N. procerum also inhibited pleural neutrophil and mononuclear cell influx. Of note, N. procerum failed to alter the acute allergic reaction, characterized by mast cell degranulation, oedema, and cysteinyl leukotriene release. N. procerum also had direct effects on murine eosinophils, since it inhibited both PAF- and eotaxin-induced eosinophil chemotaxis on an in vitro chemotactic assay. Therefore, N. procerum may be a promising anti-allergic therapy, inasmuch as it presents potent anti-eosinophil activity.

PMID: 16275631 [PubMed - indexed for MEDLINE]

1022. Epidemiol Infect. 2005 Dec;133(6):979-84.

Past mortality from infectious diseases and current burden of allergic diseases in England and Wales.

Arkwright PD, David TJ.

Academic Unit of Child Health, University of Manchester, Manchester, UK.

This study documents the changes in mortality in England and Wales over the last 100 years as a possible explanation for our increasingly allergy-prone Western society. A total of 53 million computerized recorded deaths, which occurred from 1901 to 2000 were analysed retrospectively. Childhood mortality decreased by 98%, from 40.6% of total annual deaths in 1901 to 0.9% in 2000. In 1901, 36.2% of all deaths and 51.5% of childhood deaths were from infectious diseases. By contrast in 2000, 11.6% of all deaths and only 7.4% of childhood deaths were from infectious diseases. Infectious diseases were a significant cause of childhood mortality in British cities until about 40 years ago. Several factors, including vaccination, antibiotics and improved sanitation have contributed to this trend. Survival of individuals with heightened immunity to infections may have led to natural selection of allergy-prone individuals in England and Wales. However, the relationship between changes in rates of infection and allergy is complex and not fully understood.

PMCID: PMC2870331
PMID: 16274494 [PubMed - indexed for MEDLINE]

1023. Inflammopharmacology. 2005;13(1-3):91-101.

Polyamines regulate expression of E-cadherin and play an important role in control of intestinal epithelial barrier function.

Wang JY.

Surgical Service, Baltimore Veterans Affairs Medical Center, 10 North Greene Street, Baltimore, MD 21201, USA. jwang@smail.umaryland.edu

Epithelial cells line the gastrointestinal mucosa and form an important barrier that protects the subepithelial tissue against a wide array of noxious substances, allergens, viruses and luminal microbial pathogens. Restoration of mucosal integrity following injury and various environmental stresses requires epithelial cell decisions that regulate signaling networks controlling gene expression, survival, migration and proliferation. Recently, it has been shown that polyamines play an important role in the regulation of cell-cell interactions and are critical for maintenance of intestinal epithelial integrity. Both the function of polyamines in expression of adherens junction proteins and their possible mechanisms, especially in implication of intracellular Ca2+ and c-Myc transcription factor, are the subject of this review article.

PMID: 16259731 [PubMed - indexed for MEDLINE]

1024. J Pharmacol Exp Ther. 2006 Feb;316(2):780-8. Epub 2005 Oct 28.

A novel cannabinoid peripheral cannabinoid receptor-selective inverse agonist blocks leukocyte recruitment in vivo.

Lunn CA, Fine JS, Rojas-Triana A, Jackson JV, Fan X, Kung TT, Gonsiorek W, Schwarz MA, Lavey B, Kozlowski JA, Narula SK, Lundell DJ, Hipkin RW, Bober LA.

New Lead Discovery, Schering-Plough Research Institute, Kenilworth, New Jersey, USA. charles.lunn@spcorp.com.

The expression of the cannabinoid peripheral cannabinoid receptor (CB(2)) receptor on peripheral immune cells suggests that compounds specific for CB(2)

might be effective anti-inflammatory agents. In this report, we present the initial biological profiling of a novel triaryl bis-sulfone, Sch.336 (N-[1(S)-[4-[[4-methoxy-2-[(4-methoxyphenyl)sulfonyl]phenyl]-sulfonyl]phenyl]ethyl]methanesulfonamide), which is selective for the human cannabinoid CB(2) receptor (hCB(2)). Sch.336 is an inverse agonist at hCB(2), as shown by its ability to decrease guanosine 5'-3-O-(thio)triphosphate (GTPgammaS) binding to membranes containing hCB(2), by the ability of GTPgammaS to left-shift Sch.336 binding to hCB(2) in these membranes, and by the compound's ability to increase forskolin-stimulated cAMP levels in CHO cells expressing hCB(2). In these systems, Sch.336 displays a greater potency than that reported for the CB(2)-selective dihydropyrazole, SR144528 (N-[(1S)-endo-1,3,3-trimethylbicyclo [2.2.1]heptan2-yl]-5-(4-chloro-3-methylphenyl)-1-[(4-methylphenyl)methyl]-1H-pyrazole-3-carboxamide). In vitro, Sch.336 impairs the migration of CB(2)-expressing recombinant cell lines to the cannabinoid agonist 2-arachidonylglycerol. In vivo, the compound impairs migration of cells to cannabinoid agonist HU210 [(6aR)-trans-3-(1,1-dimethylheptyl)-6a,7,10,10a-tetrahydro-1-hydroxy-6,6-dimethyl-6H-dibenzo [b,d] pyran-9-methanol]. Oral administration of the Sch.336 significantly inhibited leukocyte trafficking in several rodent in vivo models, induced either by specific chemokines or by antigen challenge. Finally, oral administration of Sch.336 blocked ovalbumin-induced lung eosinophilia in mice, a disease model for allergic asthma. We conclude that selective cannabinoid CB(2) inverse agonists may serve as novel immunomodulatory agents in the treatment of a broad range of acute and chronic inflammatory disorders in which leukocyte recruitment is a hallmark of disease pathology.

PMID: 16258021 [PubMed - indexed for MEDLINE]

1025. IDrugs. 2005 Oct;8(10):783-6.

Inflammation 2005 - Seventh World Congress. Respiratory inflammation.

Parnham MJ.

PLIVA Research Institute Ltd, Prilaz baruna Filipovica 25, Zagreb, Croatia. michael.parnham@pliva.com

In this conference, unlike previous conferences in this series, relatively little time was given to new drug therapy approaches for the treatment of inflammation. Instead, the emphasis was on basic mechanisms with a strong bias toward Australian research. The etiology of chronic obstructive pulmonary disease (COPD) and asthma was discussed in detail with an intensive consideration of mechanisms involved in the development of corticosteroid resistance, particularly the role of histone deacetylase. Various rodent model systems for testing potential inhibitors of COPD and asthma were also presented.

PMID: 16254789 [PubMed - indexed for MEDLINE]

1026. Biochem Soc Trans. 2005 Dec;33(Pt 6):1327-9.

Microtubule-associated proteins (MAPs) regulate cAMP signalling through exchange protein directly activated by cAMP (EPAC).

Yarwood SJ.

Division of Biochemistry and Molecular Biology, IBLS (Institute of Biomedical and Life Sciences), Davidson Building, University of Glasgow, Glasgow G12 8QQ, UK. S.Yarwood@bio.gla.ac.uk

cAMP is an essential signalling molecule whose concentration in cells is regulated by a wide range of hormones. A large number of diseases, including cancer and asthma, are linked to improper regulation of the cAMP signalling system, and manipulation of cAMP levels by pharmaceutical agents has proven therapeutic benefit. The action of cAMP in cells is mediated through the signalling enzymes PKA (protein kinase A) and EPAC (exchange protein directly activated by cAMP). The study of the function of these proteins is essential to understand the role of cAMP in controlling disease. We have found that EPAC interacts with an ancillary protein, called LC2 (light chain 2), and this interaction enhances EPAC's ability to activate its substrate protein, Rap1 GTPase. This is an important finding because Rap1 is involved in the control of cell migration and cell shape, functions that are disrupted in diseases like cancer. LC2 appears to enhance EPAC activity towards Rap1 by increasing the ability of EPAC to interact with cAMP, so that EPAC activation occurs at lower concentrations of cAMP. The design of inhibitors that disrupt or enhance EPAC1-LC2 interaction may therefore form the basis of future therapeutics for diseases where cAMP signalling through Rap1 is improperly regulated.

PMID: 16246110 [PubMed - indexed for MEDLINE]

1027. Parasitol Int. 2006 Mar;55(1):31-2. Epub 2005 Oct 21.

Outward migration of Gnathostoma spinigerum in interferon alpha treated hepatitis C patient.

Sangchan A, Sawanyawisuth K, Intapan PM, Mahakkanukrauh A.

Department of Medicine, Faculty of Medicine, Khon Kaen University, Khon Kaen, 40002, Thailand.

After the first dose injection of pegylated interferon alpha-2b (Peg-IFN alpha-2b) to a HCV infected Thai woman, she developed cyclic painful swelling nodules on right upper quadrant of abdomen and right anterior lower chest wall. The nodules subsided spontaneously within 1-2 days but were recurrent after every Peg-IFN alpha-2b injection. She also experienced acute urticaria. After nine months of therapy, an immature male of G. spinigerum migrated out from the skin nodule shortly after a Peg-IFN alpha-2b injection as scheduled. The worm showed a head-bulb bearing 8 transverse rows of spines which indicated immature stage. It had well defined four pairs of caudal papillae on posterior body part which were used to identify male gender. Painful migratory swelling and urticaria disappeared after the parasite was removed. She was continually treated and had sustained both virological and biochemical responses to HCV treatment. This case demonstrates that the outward migration of G. spinigerum may be stimulated by the injection of Peg-IFN alpha-2b.

PMID: 16243581 [PubMed - indexed for MEDLINE]

1028. Respir Res. 2005 Oct 21;6:120.

Ethnic variations in incidence of asthma episodes in England & Wales: national study of 502,482 patients in primary care.

Netuveli G, Hurwitz B, Sheikh A.

Department of Primary Care & Social Medicine, Imperial College London, London W6 8RP, UK. g.netuveli@imperial.ac.uk

BACKGROUND: Recent studies have demonstrated marked international variations in

the prevalence of asthma, but less is known about ethnic variations in asthma epidemiology within individual countries and in particular the impact of migration on risk of developing asthma. Recent within country comparisons have however revealed that despite originating from areas of the world with a low risk for developing asthma, South Asian and Afro-Caribbean people in the UK are significantly (3x and 2x respectively) more likely to be admitted to hospital for asthma related problems than Whites.
METHODS: Using data from the Fourth National Study of Morbidity Statistics in General Practice, a one-percent broadly representative prospective cohort study of consultations in general practice, we investigated ethnic variations in incident asthma consultations (defined as new or first consultations), and compared consultation rates between those born inside and outside the UK (migrant status). Logistic regression models were used to examine the combined effects of ethnicity and migration on asthma incident consultations.
RESULTS: Results showed significantly lower new/first asthma consultation rates for Whites than for each of the ethnic minority groups studied (mean age-adjusted consultation rates per 1000 patient-years: Whites 26.4 (95%CI 26.4, 26.4); South Asians 30.4 (95%CI 30.3, 30.5); Afro-Caribbeans 35.1 (95%CI 34.9, 35.3); and Others 27.8 (27.7, 28.0). Within each of these ethnic groups, those born outside of the UK showed consistently lower rates of incident asthma consultations. Modelling the combined effects of ethnic and migrant status revealed that UK-born South Asians and Afro-Caribbeans experienced comparable risks for incident GP consultations for asthma to UK-born Whites. Non-UK born Whites however experienced reduced risks (adjusted OR 0.82, 95%CI 0.69, 0.97) whilst non-UK born South Asians experienced increased risks (adjusted OR 1.33, 95%CI 1.04, 1.70) compared to UK-born Whites.
CONCLUSION: These findings strongly suggest that ethnicity and migration have significant and independent effects on asthma incidence. The known poorer asthma outcomes in UK South Asians and Afro-Caribbeans may in part be explained by the offspring of migrants experiencing an increased risk of developing asthma when compared to UK-born Whites. This is the first study to find heterogeneity for incident asthma consultations in Whites by migrant status.

PMCID: PMC1276818
PMID: 16242029 [PubMed - indexed for MEDLINE]

1029. Clin Infect Dis. 2005 Nov 15;41 Suppl 7:S513-9.

Macrophage migration inhibitory factor: gene polymorphisms and susceptibility to inflammatory diseases.

Renner P, Roger T, Calandra T.

Department of Internal Medicine, Centre Hospitalier Universitaire Vaudois, Lausanne, Switzerland.

The cytokine macrophage migration inhibitory factor (MIF) is a constitutive element of the host antimicrobial defenses and stress response that promotes proinflammatory function of the innate and acquired immune systems. MIF plays an important role in the pathogenesis of acute and chronic inflammatory or autoimmune disorders, such as sepsis, acute respiratory distress syndrome, asthma, rheumatoid arthritis, and inflammatory bowel diseases. Polymorphisms of the human MIF gene (that is, guanine-to-cytosine transition at position -173 or CATT-tetranucleotide repeat at position -794) have been associated with increased susceptibility to or severity of juvenile idiopathic and adult rheumatoid arthritis, ulcerative colitis, atopy, or sarcoidosis. Whether these MIF polymorphisms affect the susceptibility to and outcome of sepsis has not yet been examined. Analyses of MIF genotypes in patients with sepsis may help to classify patients into risk categories and to identify those patients who may benefit from

anti-MIF therapeutic strategies.

PMID: 16237655 [PubMed - indexed for MEDLINE]

1030. Georgian Med News. 2005 Sep;(126):55-8.

[Radiological methods in the diagnostics of extrinsic allergic alveolitis].

[Article in Russian]

Makhmudova S.

Research Institute for Lung Diseases, Ministry of Health, Baku, Azerbaijan.

There were 77 patients with EAA under our observation with the following X-ray symptom groups: emphysematous-interstitial; parenchymatous-interstitial; pneumonic. The emphysematous-interstitial X-ray symptom group is the most non-specific for the X-ray diagnostics. The changes indicate to symptoms of impaired bronchial conductance of different expression and are relevant to clinical EAA options characteristic for the obstructive syndrome. The parenchymatous-interstitial X-ray symptom group is more characteristic for occupational EAA. In such cases the most important are changes in the lung parenchyma that have a diffusive character and are accompanied by general symptoms indicating to bronchial impairments of different severity. In pneumatic X-ray symptom group, the major X-ray symptom is presence of local sites of lung tissue consolidation of hypoventilation-infiltrative character that can be bilateral, multiple with a trend to migration.

PMID: 16234596 [PubMed - indexed for MEDLINE]

1031. Cornea. 2005 Nov;24(8 Suppl):S66-S70.

Prostaglandin D2 induces chemotaxis in eosinophils via its receptor CRTH2 and eosinophils may cause severe ocular inflammation in patients with allergic conjunctivitis.

Fujishima H, Fukagawa K, Okada N, Takano Y, Tsubota K, Hirai H, Nagata K, Matsumoto K, Saito H.

Department of Ophthalmology, Keio University School of Medicine, Tokyo, Japan. fhiroshi@sc.itc.keio.ac.jp

PURPOSE: Eosinophils are known to have important roles in the pathogenesis of allergic conjunctivitis. Prostaglandin (PG) D2, which has been implicated as a factor in allergic diseases, is known to have chemotactic activity for eosinophils. Its receptor, chemoattractant receptor homologous molecule expressed on TH2 (CRTH2), serves as a receptor for PGD2 and has been reported to mediate PGD2-dependent migration of eosinophils. In the present study, both eosinophil toxic activity for corneal epithelial cells and chemotaxis induced by PGD2 in normal volunteers were investigated. Expression of CRTH2 in normal subjects was also measured.
METHODS: Primary cultured corneal epithelial cells and eosinophils in serum from normal volunteers were used and a human corneal epithelial cell line was established. Studies were performed with/without amniotic membrane. CRTH2 expression on eosinophils was assessed by flow cytometry. Chemotaxis experiments were performed using a modified Boyden chamber technique.
RESULTS: Corneal epithelial cells cultured with eosinophils showed higher floating epithelial cells and epithelial defect than those cultured in the

absence of eosinophils. Flow cytometry analysis revealed that eosinophils expressed CRTH2. PGD2 induced chemotaxis of eosinophils.
CONCLUSIONS: Corneal epithelial damage might be caused by eosinophils, which are recruited by PGD2 secretion via CRTH2 expressed on eosinophils.

PMID: 16227827 [PubMed - indexed for MEDLINE]

1032. J Am Acad Dermatol. 2005 Nov;53(5 Suppl 1):S221-4.

Atopic dermatitis with increased severity along a line of Blaschko.

Hladik F, Jurecka W, Hayek B, Stingl G, Volc-Platzer B.

Division of Immunology, Department of Dermatology, Allergy and Infectious Diseases, University of Vienna Medical School, Vienna, Austria. fhladik@fhcrc.org

Several primarily symmetric skin diseases may, on rare occasions, manifest themselves more prominently along the embryonic migration pathways of cutaneous cell clones. Loss of heterozygosity along these lines of Blaschko resulting in hemizygosity or homozygosity of alleles predisposing for the disease is the most likely explanation for this phenomenon. Here, we report a case of severe Blaschko linear atopic dermatitis superimposed on a milder symmetric eruption of atopic eczema in a 36-year-old man with personal and familial history of allergy. Continuous transition of linear atopic eczema to linear vesicular (dyshidrotic) plantar eczema demonstrates the relationship between these two entities. Individuals such as our patient offer an opportunity to identify intraindividual genetic variations marking loci involved in the pathogenesis of atopy and atopic eczema.

PMID: 16227095 [PubMed - indexed for MEDLINE]

1033. Int Arch Occup Environ Health. 2006 Feb;79(2):169-75. Epub 2005 Oct 11.

The effects of long-term occupational exposure to dust from herbs.

Golec M.

Department of Occupational Biohazards, Institute of Agricultural Medicine, Jaczewskiego 2, 20-950, Lublin, Poland. msgolec@yahoo.com

INTRODUCTION: Herbs are a heterogeneous group of many species with several thousand plants, which are used in large quantities in the pharmaceutical and food industries. The aim of the study was to analyse the health effects of long-term occupational exposure to dust from herbs.
METHODS: A group of 150 people occupationally exposed to dust from herbs, consisting of farmers and workers from herbs processing industry, was examined. As a reference group, 50 urban dwellers not exposed to any kind of organic dust were examined. Examined people were interviewed with the help of the ATS questionnaire compiled by Ferris and by the questionnaire developed in the Institute of Agricultural Medicine in Lublin, Poland for examination of work-related symptoms caused by organic dust. The lung function examination (vital capacity (VC), forced expiratory volume in the first second (FEV(1)), and FEV(1)/VC (%) of normal ranges) and allergological tests (skin prick test, precipitin test and inhibition of leukocyte migration (MIF) test) with microbial antigens were conducted.
RESULTS: 71.3% (95% CI 64.1-78.6%) of the exposed people reported occurrence of work-related symptoms. A post-shift decrease of spirometric values (VC, VC%) was observed in the exposed group (mean decrease 2.6%, P<0.01). A significant

relationship was found between the number of work-related symptoms and decrease of FEV(1) values, both before (Spearman correlation coefficient r=-0.21, P<0.05) and after work (r=-0.31, P<0.01). In allergological tests, the frequencies of positive reactions in the exposed group were significantly higher than in the reference group. Precipitins specific to Pantoea agglomerans were found in 30.6% (95% CI 23.2-38.1%) of exposed, compared to 12.0% among unexposed (P=0.01). The frequency of positive results in the migration inhibition test was significantly higher among exposed workers for all antigens tested.
CONCLUSION: Long-term exposure to dust from herbs causes work-related symptoms and decrease of lung function parameters, which, finally, may lead to occupational disease.

PMID: 16217676 [PubMed - indexed for MEDLINE]

1034. Chem Immunol Allergy. 2006;90:14-28.

The mutual influence of nematode infection and allergy.

Negrao-Correa D, Teixeira MM.

Department of Parasitologia, Instituto de Ciencias Biológicas, Universidade Federal de Minas Gerais, Belo Horizonte, MG, Brazil.

Several studies have now shown that the prevalence of helminth infections is negatively correlated with the prevalence and/or severity of allergic diseases. Here, we describe studies in rodents infected with Strongyloides venezuelensis examining the mutual influence of nematode infection and allergy. S. venezuelensis has a lung cycle, much akin to the human hookworm and Strongyloidiasis, and induces airway eosinophilia, local IgE and mucus production, and airway hyperreactivity. Both the Th2 and functional responses are relevant for the ability of rodents to deal with S. venezuelensis infection. Nevertheless, the parasite elicits the release of cytokines, such as IL-10, which are capable of regulating immune and functional manifestations. In infected animals, allergic inflammation prevents parasite migration and establishment. Nevertheless, the parasite is capable of regulating the allergic response, preventing part of the tissue damage and functional changes induced by allergy. Understanding the mechanisms by which helminths regulate inflammation may potentially lead to the development of strategies aimed at controlling unwanted inflammation in allergic and autoimmune diseases.

PMID: 16210900 [PubMed - indexed for MEDLINE]

1035. J Allergy Clin Immunol. 2005 Oct;116(4):820-6. Epub 2005 Aug 2.

Stem cell factor stimulates the chemotaxis, integrin upregulation, and survival of human basophils.

Heinemann A, Sturm GJ, Ofner M, Sturm EM, Weller C, Peskar BA, Hartnell A.

Department of Experimental and Clinical Pharmacology, Medical University of Graz, Austria. akos.heinemann@meduni-graz.at

BACKGROUND: Little is known about the mechanisms that regulate the selective recruitment of basophils to sites of allergic inflammation.
OBJECTIVE: Here we examine the role of stem cell factor (SCF) in the regulation of basophil function.
METHODS: Human basophils were isolated from peripheral blood, and their migration was investigated in chemotaxis assays. Apoptosis was detected by means of annexin

V and propidium iodide staining. The expression of cell-surface molecules was measured by means of flow cytometry.
RESULTS: SCF amplified the chemotactic responsiveness of human peripheral blood basophils to the chemoattractants eotaxin, monocyte chemotactic protein 2 and macrophage inflammatory protein 1alpha, and C5a, without being chemotactic or chemokinetic by itself. SCF synergized with chemoattractants in causing basophil upregulation of the integrin CD11b, and this effect was inhibited by a c-kit antibody, the tyrosine kinase inhibitor imatinib mesylate (STI-571), and a phosphatidylinositol 3 kinase inhibitor but not by inhibitors of p38 mitogen-activated protein kinase or mitogen-activated protein kinase/extracellular signal-regulated kinase kinase. Basophils bound fluorescence-labeled SCF and expressed its receptor, c-kit, which was markedly upregulated in culture for 24 to 48 hours in the presence of IL-3. Moreover, SCF prolonged basophil survival in concert with IL-3 by delaying apoptosis. These effects of SCF were selective for basophils because chemotaxis and CD11b upregulation of eosinophils or neutrophils were unchanged.
CONCLUSION: SCF might be an important selective modulator of basophil function through a phosphatidylinositol 3 kinase-dependent pathway.

PMID: 16210056 [PubMed - indexed for MEDLINE]

1036. J Allergy Clin Immunol. 2005 Oct;116(4):812-9. Epub 2005 Aug 24.

Effects of the very late adhesion molecule 4 antagonist WAY103 on human peripheral blood eosinophil vascular cell adhesion molecule 1-dependent functions.

Sedgwick JB, Jansen KJ, Kennedy JD, Kita H, Busse WW.

Allergy and Immunology Unit, Department of Medicine, University of Wisconsin, Madison, WI, USA. jxs@medicine.wisc.edu

BACKGROUND: Eosinophil infiltration to the lung in allergic inflammation can be initiated by the tethering of circulating cells through very late adhesion molecule 4 (VLA-4; alpha4beta1, CD49d/CD29) to vascular cell adhesion molecule 1 (VCAM-1) expressed on pulmonary vascular endothelium. Small-molecule VLA-4 antagonists have been proposed as a therapeutic mechanism to prevent eosinophil infiltration in asthma; however, they might affect other eosinophil functions.
OBJECTIVE: The small-molecule VLA-4 antagonist (2S)-3-(4-Dimethylcarbamoyloxyphenyl)-2-{[(4R)-5,5-dimethyl-3-(1-methyl-1H-pyrazole-4 sulfonyl)thiazolidine-4-carbonyl]amino}propionic acid (WAY103) was assessed for its effects on eosinophil VLA-4-dependent functions, including adhesion, migration, respiratory burst, and degranulation.
METHODS: Human peripheral blood eosinophils were preincubated with WAY103, anti-alpha4, and/or anti-beta2 integrin mAbs and then assessed for adhesion to recombinant VCAM-1, intercellular adhesion molecule 1, and endothelial cell monolayers. Transmigration was measured by using human pulmonary microvascular endothelial cell monolayers and Transwell filters. Superoxide anion generation was determined by means of cytochrome C reduction and degranulation by means of eosinophil-derived neurotoxin release.
RESULTS: WAY103 inhibition of eosinophil adhesion to recombinant VCAM-1 was dose dependent (63% inhibition with 100 nM WAY103, $P < .04$) and comparable with inhibition caused by anti-alpha4 mAb (60.1% inhibition). Although pretreatment with WAY103 also decreased eosinophil adhesion to TNF-alpha plus IL-4-activated human pulmonary microvascular endothelial cell monolayers, it did not prevent eosinophil transendothelial migration in response to RANTES. Finally, WAY103 inhibited VCAM-1-stimulated superoxide generation but enhanced cytokine-activated eosinophil-derived neurotoxin degranulation.
CONCLUSION: Although small-molecule VLA-4 antagonists, such as WAY103, might

reduce eosinophil adhesion, this approach might not be sufficient to eliminate this cell from in vivo allergic airway inflammatory participation and could even promote specific cell activation.

PMID: 16210055 [PubMed - indexed for MEDLINE]

1037. Proc Natl Acad Sci U S A. 2005 Oct 4;102(40):14410-5. Epub 2005 Sep 26.

Role for macrophage migration inhibitory factor in asthma.

Mizue Y, Ghani S, Leng L, McDonald C, Kong P, Baugh J, Lane SJ, Craft J, Nishihira J, Donnelly SC, Zhu Z, Bucala R.

Yale University School of Medicine, New Haven, CT 06520-8031, USA.

Macrophage migration inhibitory factor (MIF) is an immunologic regulator that is expressed in inflammatory and autoimmune disorders. We investigated MIF's role in asthma using genetic approaches in a mouse model and in a cohort of asthma patients. Mice genetically deficient in MIF that were primed and aerosol-challenged with ovalbumin showed less pulmonary inflammation and lower airway hyperresponsiveness than genetically matched, wild-type controls. MIF deficiency also resulted in lower titers of specific IgE, IgG(1), and IgG(2a), and decreased pulmonary, T(H)2 cytokine levels. IL-5 concentrations were lower and corresponded to decreased eosinophil numbers in bronchoalveolar lavage fluid. T cell studies also showed a lower level of antigen-specific responses in MIF-KO versus wild-type mice. In an analysis of 151 white patients with mild, moderate, or severe asthma (Global Initiative for Asthma criteria), a significant association was found between mild asthma and the low-expression, 5-CATT MIF allele. Pharmacologic inhibition of MIF may be beneficial and could be guided by the MIF genotype of affected individuals.

PMCID: PMC1242335
PMID: 16186482 [PubMed - indexed for MEDLINE]

1038. Mini Rev Med Chem. 2005 Sep;5(9):805-24.

Potential clinical applications of the CXCR4 antagonist bicyclam AMD3100.

De Clercq E.

Rega Institute for Medical Research, K.U.Leuven, B-3000 Leuven, Belgium.
declercq@rega.kuleuven.ac.be

The bicyclam AMD3100 (originally called JM3100), in which the two cyclam rings are tethered by an aromatic bridge, emanated from JM2763, where the two cyclam moieties are tethered by an aliphatic linker - JM2763 in turn originated from JM1657, where the cyclam rings are directly linked to one another via a C-C bridge, and which was identified as an impurity, showing anti-HIV activity, in a commercial cyclam preparation. AMD3100 proved very effective against HIV-1 and HIV-2, inhibiting virus replication within the nM range, without toxicity for the host cells at concentrations that were > 100,000-fold higher than those required to inhibit HIV replication. The anti-HIV activity of AMD3100 appeared to be confined to the T-lymphotropic (X4) HIV strains, i.e. those strains that use the CXCR4 receptor to enter their target cells, and AMD3100 as of today still stands as one of the most potent and selective CXCR4 antagonists ever discovered. Hence, AMD3100 was found to interfere with a number of (patho)physiological processes which depend on the interaction of CXCR4 with its natural ligand, stromal derived factor (SDF-1) and which play an important role in rheumatoid, allergic and

malignant diseases. AMD3100 has been shown to mobilize CD34+ stem cells from the bone marrow into the bloodstream and has also been shown to augment migration of bone marrow-derived endothelial progenitor cells into sites of neovascularization after myocardial infarction. Currently, AMD3100 is actively pursued as a stem cell mobilizer for transplantation in patients with multiple myeloma and non-Hodgkin's lymphoma.

PMID: 16178723 [PubMed - indexed for MEDLINE]

1039. J Neuroimmunol. 2005 Nov;168(1-2):168-74.

Macrophage migration inhibitory factor (MIF) seems crucially involved in Guillain-Barré syndrome and experimental allergic neuritis.

Nicoletti F, Créange A, Orlikowski D, Bolgert F, Mangano K, Metz C, Di Marco R, Al Abed Y.

Department of Biomedical Sciences, School of Medicine, University of Catania, Italy.

Macrophage migration inhibitory factor (MIF) is a proinflammatory type 1 cytokine that plays a pathogenic role in several inflammatory and autoimmune diseases. The role of this cytokine in peripheral nerve inflammatory disease has not been evaluated. Therefore, to evaluate the role of macrophage migration inhibitory factor (MIF) in Guillain-Barré syndrome (GBS) and experimental allergic neuritis (EAN), we determined MIF circulating levels in a series of patients with GBS and healthy subjects with ELISA and evaluated the effect of two specific inhibitors of MIF, a neutralizing monoclonal antibody or a chemical inhibitor ISO1 on the course of murine EAN. The data show increased MIF plasma levels in GBS patients as compared to healthy controls ($p<0.0001$) and a progressive increase of MIF circulating concentration with patient's disability ($p<0.0001$). Both anti-MIF mAb and ISO1 favorably influenced the course of EAN. Treated mice had a lower cumulative severity score ($p=0.001$) and reduced disease duration than the control mice ($p<0.03$). MIF may promote immune reaction in GBS. Therapeutic effects of both anti-MIF mAb and ISO1 in EAN suggest that MIF could be a promising therapeutic target in inflammatory demyelinating peripheral nerve disorders.

PMID: 16171874 [PubMed - indexed for MEDLINE]

1040. J Allergy Clin Immunol. 2005 Sep;116(3):578-85.

5-Lipoxygenase products regulate basophil functions: 5-Oxo-ETE elicits migration, and leukotriene B(4) induces degranulation.

Iikura M, Suzukawa M, Yamaguchi M, Sekiya T, Komiya A, Yoshimura-Uchiyama C, Nagase H, Matsushima K, Yamamoto K, Hirai K.

Department of Allergy and Rheumatology, University of Tokyo School of Medicine, Tokyo, Japan.

BACKGROUND: 5-Lipoxygenase (5-LO) products have been strongly implicated in the pathogenesis of allergic diseases. In addition to their physiologic effects on residential cells, 5-LO products are capable of stimulating various eosinophil functions. However, little is known regarding the effects of 5-LO products on basophil functions.
OBJECTIVE: This study was designed to elucidate the effects of the main 5-LO products (ie, leukotriene [LT] B(4), LTD(4), and 5-oxo-6,8,11,14-eicosatetraenoic acid [5-oxo-ETE]), as well as their receptor expression on human basophils.

METHODS: We studied the effects of 5-LO products on Ca(2+) mobilization,
migration, CD 11b expression, and degranulation of human basophils. Expression of the receptors for LTC(4)/D(4)/E(4) (cysteinyl leukotriene 1 [CysLT(1)] and CysLT(2)), LTB4 (BLT(1) and BLT(2)), and 5-oxo-ETE (oxoeicosanoid [OXE]) was assessed by means of real-time PCR and flow cytometry.
RESULTS: At the mRNA level, basophils strongly expressed OXE and predominantly expressed CysLT(1) and BLT(2). The expression level of OXE mRNA in basophils was approximately 20-fold higher than in neutrophils and similar to that in eosinophils. At the protein level, basophils expressed CysLT(1), CysLT(2), BLT(1), and OXE, but not BLT(2). All products elicited a transient increase of cytosolic calcium, with the order of magnitude being LTB(4)>5-oxo-ETE>LTD(4). 5-Oxo-ETE induced a strong basophil migratory response that was almost equivalent to that of prostaglandin D(2). LTB(4) elicited significant degranulation of IL-3-primed basophils. In contrast, no functional significance was observed for LTD(4).
CONCLUSION: Among 5-LO products, 5-oxo-ETE induces a potent basophil migratory response, and LTB(4) elicits degranulation under certain conditions. Our results strongly suggest that 5-oxo-ETE might afford opportunities for therapeutic targeting in allergic inflammation.

PMID: 16159627 [PubMed - indexed for MEDLINE]

1041. Cytokine. 2005 Oct 21;32(2):67-70. Epub 2005 Sep 8.

Cytokines and Langerhans cell mobilisation in mouse and man.

Griffiths CE, Dearman RJ, Cumberbatch M, Kimber I.

Dermatopharmacology Unit, The Dermatology Centre, The University of Manchester, Hope Hospital, Salford M6 8HD, UK.

A critical event during the development of cutaneous immune responses, including those provoked by contact allergens, is the mobilisation of epidermal Langerhans cells (LC). These cells act as sentinels of the immune system in the skin, responding to a variety of local insults with migration and delivery of potentially foreign signals to draining lymph nodes. Experimental studies have revealed that the regulation of mobilisation and migration of LC display striking similarities in man and mouse. In both species it has been found that the successful induction of migration requires that LC receive (at least) 2 independent cytokine signals; provided by tumour necrosis factor-alpha (TNF-alpha) and interleukin 1beta. In addition, a similar heterogeneity in man and mouse is apparent with regard to the fraction of LC responding rapidly to mobilisation signals, with the same proportion of cells (20%-30%) being stimulated to migrate in each case. Other similarities exist between mice and humans with respect to LC function, including an age-related decrement in both LC frequency and responsiveness to TNF-alpha. Collectively these studies demonstrate that the mouse provides a valuable experimental surrogate for the human skin immune system, particularly with respect to LC biology, and suggest that it is possible to perform extrapolations between species with some confidence.

PMID: 16153855 [PubMed - indexed for MEDLINE]

1042. J Immunol. 2005 Sep 15;175(6):3698-706.

Complement C3a enhances CXCL12 (SDF-1)-mediated chemotaxis of bone marrow hematopoietic cells independently of C3a receptor.

Honczarenko M, Ratajczak MZ, Nicholson-Weller A, Silberstein LE.

Joint Program in Transfusion Medicine, Children's Hospital Boston, Harvard
Medical School, Boston, MA 02115, USA.

Complement C3a promotes CXCL12-induced migration and engraftment of human and murine hemopoietic progenitor cells, suggesting a cross-influence between anaphylatoxin and chemokine axes. Here we have explored the underlying mechanism(s) of complement anaphylatoxin and chemokine cooperation. In addition to C3a, C3a-desArg and C4a but not C5a, are potent enhancers of CXCL12-induced chemotaxis of human and murine bone marrow (BM) stem/progenitor cells and B lineage cells. C3a enhancement of chemotaxis is chemokine specific because it is also observed for chemotaxis to CCL19 but not to CXCL13. The potentiating effect of C3a on CXCL12 is independent of the classical C3a receptor (C3aR). First, human BM CD34(+) and B lineage cells do not express C3aR by flow cytometry. Second, the competitive C3aR inhibitor SB290157 does not affect C3a-mediated enhancement of CXCL12-induced chemotaxis. Third, enhancement of chemotaxis of hemopoietic cells is also mediated by C3a-desArg, which does not bind to C3aR. Finally, C3a enhances CXCL12-induced chemotaxis of BM cells from C3aR knockout mice similar to BM cells from wild-type mice. Subsequent studies revealed that C3a increased the binding affinity of CXCL12 to human CXCR4(+)/C3aR(-), REH pro-B cells, which is compatible with a direct interaction between C3a and CXCL12. BM stromal cells were able to generate C3a, C3a-desArg, C4a, as well as CXCL12, suggesting that this pathway could function in vivo. Taken together, we demonstrate a C3a-CXCL12 interaction independent of the C3aR, which may provide a mechanism to modulate the function of CXCL12 in the BM microenvironment.

PMID: 16148115 [PubMed - indexed for MEDLINE]

1043. J Rheumatol. 2005 Sep;32(9):1765-74.

Roxithromycin specifically inhibits development of collagen induced arthritis and production of proinflammatory cytokines by human T cells and macrophages.

Urasaki Y, Nori M, Iwata S, Sasaki T, Hosono O, Kawasaki H, Tanaka H, Dang NH, Ikeda E, Morimoto C.

Division of Clinical Immunology, Advanced Clinical Research Center, Institute of Medical Science, University of Tokyo, Japan.

OBJECTIVE: Roxithromycin (RXM) is a macrolide antibiotic that is effective in treatment of chronic lower respiratory tract diseases including diffuse panbronchiolitis and bronchial asthma. Its mechanism of action apart from its antibacterial action remains unclear. To determine the mechanism of action of RXM, we evaluated the effect of RXM on T cell functions and the inflammatory responses in mice with collagen induced arthritis (CIA).
METHODS: T cell proliferation, cytokine production by T cells stimulated through CD28, CD26, or PMA with or without anti-CD3 Mab, cytokine production by macrophages stimulated with lipopolysaccharide, and transendothelial migration of T cells were analyzed in the presence or absence of various concentrations of RXM. We evaluated the effect of RXM treatment in collagen induced arthritis in mice.
RESULTS: RXM did not affect the production of Th1-type and Th2-type cytokines, whereas it specifically inhibited production of proinflammatory cytokines such as tumor necrosis factor-a and interleukin 6 (IL-6) by T cells and macrophages. RXM inhibited T cell migration. We found that RXM treatment of mice with CIA reduced the severity of arthritis and serum level of IL-6, as well as leukocyte migration into the affected joints and destruction of bones and cartilage.
CONCLUSION: Our findings strongly suggest that RXM may be useful for the therapy of rheumatoid arthritis as well as other inflammatory diseases such as Crohn's

disease.

PMID: 16142877 [PubMed - indexed for MEDLINE]

1044. Epidemiol Prev. 2005 Mar-Apr;29(2 Suppl):36-41.

[Prevalence of asthma and allergies among migrant children and adolescents in Italy].

[Article in Italian]

Migliore E, Bugiani M, Berti G, Ciccone G, Russo A, Galassi C, Talassi F, Pistelli R; Gruppo Collaborativo SIDRIA-2.

CPO Piemonte, Torino.

Prevalence of asthma and allergies in children shows large variations among different populations, but there is limited evidence about these conditions in immigrants from developing countries. The aim of this study was to evaluate the prevalence of asthma symptoms and allergies in immigrant children and adolescents living in Italy and to investigate the possible role of genetic and environmental factors in the development of such diseases. There were 1340 (4.0%) immigrants in Italy, mainly from East Europe (31.7%); 532 were born in Italy to foreign parents and 808 were born abroad. Asthma and other atopic diseases were on the whole significantly less common among immigrant children than among Italians, while some infectious diseases in the first two years of life resulted more present in immigrant children than in Italians.

PMID: 16128552 [PubMed - indexed for MEDLINE]

1045. Epidemiol Prev. 2005 Mar-Apr;29(2 Suppl):14-23.

[Environmental, social and demographic characteristics of children and adolescents, resident in different Italian areas].

[Article in Italian]

Chellini E, Talassi F, Corbo G, Berti G, De Sario M, Rusconi F, Piffer S, Caranci N, Petronio MG, Sestini P, Dell'Orco V, Bonci E, Armenio L, La Grutta S; Gruppo Collaborativo SIDRIA-2.

CSPO, Firenze.

The second phase of the SIDRIA study provides important information regarding the family characteristics of Italian children (6-7 years old) and adolescents (13-14 years old), and the frequency of risk factors for asthma and allergies, allowing comparisons between study areas according to differences in latitude (North, Centre, South) and urbanization level (metropolitan areas, with at least 500,000 inhabitants, and other areas). Parental education level was higher in metropolitan and central areas. The frequency of children and adolescents born abroad, and the percentage of mothers and fathers employed were higher outside metropolitan areas and there was an increase from the South to the North of Italy. This trend was paralleled by an increase in maternal age at child's birth and in the frequency of low birth weight and day care attendance. The frequency of breastfeeding was greater in children than in adolescents; the opposite was registered for passive smoking, with a frequency of exposure higher in adolescents than in children, especially in the Northern and Central areas, even if the proportion of subjects having at least one parent who smokes was still

high in both age groups. The prevalence of overweight children was striking, especially in the South where physical activity was less frequent and children had the unhealthy habit of consuming a lot of snacks and carbonated beverages. Frequency of exposure to traffic in the area of residence was particularly high, especially in the metropolitan areas.

PMID: 16128549 [PubMed - indexed for MEDLINE]

1046. Chem Immunol Allergy. 2005;87:130-44.

Chemokine receptor expression by mast cells.

Juremalm M, Nilsson G.

Department of Genetics and Pathology, Uppsala University, Uppsala, Stockholm, Sweden. Gunnar.Nilsson@medks.ki.se

There is a growing interest in the role of chemokines and their receptors in the determination of mast cell tissue localization and how chemokines regulate mast cell function. At least nine chemokine receptors (CXCR1, CXCR2, CXCR3, CXCR4, CX3CR1, CCR1, CCR3, CCR4 and CCR5) have been described to be expressed by human mast cells of different origins. Seven chemokines (CXCL1, CXCL5, CXCL8, CXCL14, CX3CL1, CCL5 and CCL11) have been shown to act on some of these receptors and to induce mast cell migration. Mast cells have a unique expression pattern of CCR3, CXCR1 and CXCR2. These receptors are mainly expressed intracellularly on cytoplasmic membranes. Upon an allergic activation, CCR3 expression is increased on the cell surface and the cell becomes vulnerable for CCL11 treatment. Chemokines do not induce mast cell degranulation but CXCL14 causes secretion of de novo synthesized CXCL8. Because of the expression of CCR3, CCR5 and CXCR4 on mast cell progenitors, these cells are susceptible to HIV infection and mast cells might therefore be a persistent HIV reservoir in AIDS. In this review, we summarize the knowledge about chemokine receptor expression and function on mast cells.

PMID: 16107768 [PubMed - indexed for MEDLINE]

1047. Mediators Inflamm. 2005 Aug 14;2005(3):150-9.

Suppression of matrix metalloproteinase production in nasal fibroblasts by tranilast, an antiallergic agent, in vitro.

Shimizu T, Kanai K, Asano K, Hisamitsu T, Suzaki H.

Department of Otolaryngology, School of Medicine, Showa University, Tokyo, Japan.

Allergic rhinitis is an inflammatory disease characterized by nasal wall remodeling with intense infiltration of eosinophils and mast cells/basophils. Matrix metalloproteinases (MMPs), MMP-2 and MMP-9, are the major proteolytic enzymes that induce airway remodeling. These enzymes are also important in the migration of inflammatory cells through basement membrane components. We evaluated whether tranilast (TR) could inhibit MMP production from nasal fibroblasts in response to tumor necrosis factor-alpha (TNF-alpha) stimulation in vitro. Nasal fibroblasts (NF) were established from nasal polyp tissues taken from patients with allergic rhinitis. NF (2 x 10(5) cells/mL) were stimulated with TNF-alpha in the presence of various concentrations of TR. After 24 hours, the culture supernatants were obtained and assayed for MMP-2, MMP-9, TIMP-1, and TIMP-2 levels by ELISA. The influence of TR on mRNA expression of MMPs and TIMPs in cells cultured for 12 hours was also evaluated by RT-PCR. TR at more than 5 x

10(-5) M inhibited the production of MMP-2 and MMP-9 from NF in response to TNF-alpha stimulation, whereas TIMP-1 and TIMP-2 production was scarcely affected. TR also inhibited MMP mRNA expression in NF after TNF-alpha stimulation. The present data suggest that the attenuating effect of TR on MMP-2 and MMP-9 production from NF induced by inflammatory stimulation may underlie the therapeutic mode of action of the agent in patients with allergic diseases, including allergic rhinitis.

PMCID: PMC1526475
PMID: 16106101 [PubMed - indexed for MEDLINE]

1048. Int Immunol. 2005 Sep;17(9):1249-55. Epub 2005 Aug 15.

IgE- and FcepsilonRI-mediated migration of human basophils.

Suzukawa M, Hirai K, Iikura M, Nagase H, Komiya A, Yoshimura-Uchiyama C, Yamada H, Ra C, Ohta K, Yamamoto K, Yamaguchi M.

Department of Allergy and Rheumatology and.

Local accumulation of basophils at inflammatory sites is observed in experimental antigen challenge and in allergic diseases. It is not fully known what factor(s) regulates local basophil influx in tissues, and it has not been determined whether antigens belong in a panel of basophil chemoattractants. This study was designed to elucidate whether IgE- and high-affinity receptor for IgE (FcepsilonRI)-mediated stimulation can induce human basophil migration. The migration-inducing potency of an anti-FcepsilonRI alpha-chain mAb, CRA-1, was examined on human basophils. CRA-1 mAb elicited significant migration of basophils. The migration-inducing potency of this mAb was maximal at 100 ng ml-1, and CRA-1 mAb at 100 ng ml-1 attracted approximately 10% of total inoculated basophils above baseline levels after incubation for 2.5 h. Checkerboard analysis indicated that basophil migration induced by this mAb was mainly chemotactic and partially chemokinetic. An antigen, Der f 2, also induced migration of basophils from Der f-sensitive subjects. Basophils mixed with 1 ng ml-1 of CRA-1 mAb showed an exaggerated migration response to eotaxin, indicating that FcepsilonRI cross-linkage enhances basophil migration to other chemoattractants. Induction of basophil migration by IgE- and FcepsilonRI-cross-linking stimulation may, at least in part, explain the pathogenesis of local basophil accumulation clinically observed in allergic diseases such as asthma.

PMID: 16103029 [PubMed - indexed for MEDLINE]

1049. Tissue Antigens. 2005 Sep;66(3):163-72.

Mechanisms of lymphocyte migration in autoimmune disease.

Norman MU, Hickey MJ.

Immunology Research Group, University of Calgary, Calgary, AB, Canada.

The recruitment of leukocytes to inflamed tissues plays an essential role in combating infection and promoting wound healing. However, in autoimmune diseases such as multiple sclerosis and diabetes, leukocytes enter tissues and contribute to inappropriate inflammatory responses, which cause tissue injury and dysfunction. In diseases of this type, lymphocytes play critical roles in initiating and maintaining these aberrant inflammatory responses. The aim of this review is to examine the mechanisms whereby T-lymphocytes enter tissues in autoimmune diseases and to compare these mechanisms between various organs and

diseases. An overview of the mechanisms of leukocyte recruitment and the techniques used to study leukocyte trafficking is provided, focusing on the use of intravital microscopy as a tool to assess the functional microvasculature in vivo. We also discuss the series of tissue homing events which allow naïve lymphocytes to first enter lymph nodes and undergo activation, then subsequently to home to the peripheral organ where their cognate antigen is present. Finally, we examine mechanisms of leukocyte recruitment in diseases such as multiple sclerosis, autoimmune diabetes, systemic lupus erythematosus, rheumatoid arthritis, inflammatory bowel disease and asthma.

PMID: 16101827 [PubMed - indexed for MEDLINE]

1050. Curr Drug Targets Inflamm Allergy. 2005 Aug;4(4):481-6.

Control of eosinophil toxicity in the lung.

Walsh GM, Al-Rabia M, Blaylock MG, Sexton DW, Duncan CJ, Lawrie A.

Allergic and Asthmatic Inflammation Group, School of Medicine, University of Aberdeen, Scotland, UK. g.m.walsh@abdn.ac.uk

The inappropriate accumulation of eosinophils and the subsequent release of their potent pro-inflammatory mediator arsenal are thought to be important contributors to the pathogenesis of asthma and other allergic diseases. It is also becoming apparent that eosinophils may play a role in the orchestration of immune responses in the asthmatic lung. There is therefore much interest in the development of strategies to limit or prevent eosinophil-induced toxicity. The mechanisms by which eosinophils accumulate in the peribronchial tissues of the lung are complex and include enhanced differentiation and release from the bone marrow, selective adhesion and transendothelial migration, directed movement in response to specific chemotactic mediators and finally prolonged survival as a consequence of delayed apoptosis. Thus it can be appreciated that there are many points at which the toxicity of eosinophils can be limited or even prevented. Important areas for potential advances in glucocorticoid (GC) development include efforts to dissociate their anti-inflammatory effects from unwanted side effects. Other areas include the development of humanized monoclonal antibodies against IL-4, IL-13 and IL-5 together with the inhibition of adhesion pathways and/or chemokines responsible for eosinophil accumulation in the asthmatic lung. Several avenues of research are currently underway in an attempt to define mechanisms by which pro-inflammatory cells such as eosinophils can be safely removed from the asthmatic lung through apoptosis induction and their subsequent ingestion by phagocytes. This review will discuss both the potential and shortcomings of these diverse approaches to limit eosinophil toxicity in the asthmatic lung.

PMID: 16101525 [PubMed - indexed for MEDLINE]

1051. Exp Dermatol. 2005 Sep;14(9):700-8.

CD44, but not l-selectin, is critically involved in leucocyte migration into the skin in a murine model of allergic dermatitis.

Gonda A, Gál I, Szántó S, Sarraj B, Glant TT, Hunyadi J, Mikecz K.

Leukocyte Migration Research Group, Department of Orthopedic Surgery, Rush University Medical Center, Chicago, IL 60612, USA.

CD44 and l-selectin (CD62L) are major adhesion receptors that mediate leucocyte recruitment at inflammatory sites and lymph nodes, by supporting cell rolling

under blood flow. Both CD44 and CD62L have been implicated in inflammatory skin disorders, but their specific involvement in an immediate-type allergic reaction remains uncertain. We used mice deficient in CD44 or CED62L or both in order to determine whether one or both of these molecules were required for leucocyte extravasation in an atopic dermatitis-like allergic response. Wild-type (WT) mice and mice deficient in CD44, CD62L or both were immunized with ovalbumin (OVA). Inflammatory reaction in the ear was elicited once by means of intradermal injection of OVA. Effective sensitization of CD62L knockout (KO) mice required intraperitoneal antigen injection; however, OVA-specific T helper 2 (Th2)-type immune responses and IgE production in mice lacking CD44, CD62L or both were comparable to those in WT mice following intraperitoneal immunization. We employed intravital videomicroscopy to monitor the recruitment of fluorescence-labelled leucocytes to the ear tissue following challenge with OVA. The number of adherent leucocytes was significantly reduced in CD44 KO and CD44/CD62L double KO mice, indicating that CD44 was involved in firm adhesion, the committed step of leucocyte extravasation. Histology of the OVA-challenged ears showed a diminished leucocyte infiltration in the ears of CD44 KO and double KO mice. The results of our study demonstrate that CD44, but not CD62L, is required for leucocyte extravasation during a Th2-type inflammatory response.

PMID: 16098130 [PubMed - indexed for MEDLINE]

1052. Exp Dermatol. 2005 Sep;14(9):641-66.

Experimental approaches to lymphocyte migration in dermatology in vitro and in vivo.

Radeke HH, Ludwig RJ, Boehncke WH.

Pharmazentrum Frankfurt, Dr-Hans-Schleussner-Foundation Immune Pharmacology, Frankfurt, Germany. Radeke@em.uni-frankfurt.de

Lymphocyte trafficking through the dermal compartment is part of the physiological surveillance process of the adaptive immune system. On the other hand, persistent or recurrent lymphocyte infiltrates are hallmarks of both types of chronic inflammatory skin diseases, Th1-type such as psoriasis or Th2/allergic-type like atopic dermatitis. A better understanding of the mechanisms underlying lymphocyte movements is one of the key prerequisites for developing more effective therapies. In this review, we introduce a range of simple-to-sophisticated experimental in vitro and in vivo approaches to analyze lymphocyte migration. These methods start from static in vitro adhesion and chemotaxis assays, include dynamic endothelial flow chamber, intravital dual photon, and transcutaneous live-video microscopy, and finally encompass specific genetically deficient or engineered animal models. Discussing pros and cons of these assay systems hopefully generates both state-of-the-art knowledge about the factors involved in most common chronic skin diseases as well as an improved understanding of the limitations and chances of new biologic pharmaceuticals that are currently introduced into clinical practice.

PMID: 16098125 [PubMed - indexed for MEDLINE]

1053. J Immunol. 2005 Aug 15;175(4):2702-8.

CCR3 expression and function in asthmatic airway smooth muscle cells.

Joubert P, Lajoie-Kadoch S, Labonté I, Gounni AS, Maghni K, Wellemans V, Chakir J, Laviolette M, Hamid Q, Lamkhioued B.

Meakins-Christie Laboratories, McGill University, Montréal, Québec, Canada.

Asthma is characterized by an increase in airway smooth muscle mass and a decreased distance between the smooth muscle layer and the epithelium. Furthermore, there is evidence to indicate that airway smooth muscle cells (ASMC) express a wide variety of receptors involved in the immune response. The aims of this study were to examine the expression of CCR3 on ASMC, to compare this expression between asthmatic and nonasthmatic subjects, and to determine the implications of CCR3 expression in the migration of ASMC. We first demonstrated that ASMC constitutively express CCR3 at both mRNA and protein levels. Interestingly, TNF-alpha increases ASMC surface expression of CCR3 from 33 to 74%. Furthermore, using FACS analysis, we found that ASMC CCR3 is expressed to a greater degree in asthmatic vs control subjects (95 vs 75%). Functionality of the receptor was demonstrated by calcium assay; the addition of CCR3 ligand eotaxin to ASMC resulted in an increase in intracellular calcium production. Interestingly, ASMC was seen to demonstrate a positive chemotactic response to eotaxin. Indeed, ASMC significantly migrated toward 100 ng/ml eotaxin (2.2-fold increase, compared with control). In conclusion, the expression of CCR3 by ASMC is increased in asthmatics, and our data show that a CCR3 ligand such as eotaxin induces migration of ASMC in vitro. These results may suggest that eotaxin could be involved in the increased smooth muscle mass observed in asthmatics through the activation of CCR3.

PMID: 16081847 [PubMed - indexed for MEDLINE]

1054. Pharmacol Ther. 2006 Jan;109(1-2):107-36. Epub 2005 Aug 2.

Targeting memory Th2 cells for the treatment of allergic asthma.

Epstein MM.

Medical University of Vienna, Department of Dermatology, Lazarettgasse 19, Vienna A-1090, Austria. Michelle.Epstein@meduniwien.ac.at

Th2 memory cells play an important role in the pathogenesis of allergic asthma. Evidence from patients and experimental models indicates that memory Th2 cells reside in the lungs during disease remission and, upon allergen exposure, become activated effectors involved in disease exacerbation. The inhibition of memory Th2 cells or their effector functions in allergic asthma influence disease progression, suggesting their importance as therapeutic targets. They are allergen specific and can potentially be suppressed or eliminated using this specificity. They have distinct activation, differentiation, cell surface phenotype, migration capacity, and effector functions that can be targeted singularly or in combination. Furthermore, memory Th2 cells residing in the lungs can be treated locally. Capitalizing on these unique attributes is important for drug development for allergic asthma. The aim of this review is to present an overview of therapeutic strategies targeting Th2 memory cells in allergic asthma, emphasizing Th2 generation, differentiation, activation, migration, effector function, and survival.

PMID: 16081161 [PubMed - indexed for MEDLINE]

1055. Allergy. 2005 Sep;60(9):1204-7.

Differential dependence of eosinophil chemotactic responses on phosphoinositide 3-kinase (PI3K).

Mishra RK, Scaife JE, Harb Z, Gray BC, Djukanovic R, Dent G.

Division of Infection, Inflammation and Repair, University of Southampton School of Medicine, Southampton, UK.

BACKGROUND: Control of eosinophil migration to sites of inflammatory responses is a potentially therapeutic intervention in diseases such as bronchial asthma. Chemoattractants, their receptors and the associated signalling pathways may, therefore, be important targets for novel therapeutics. While several potentially important chemoattractants have been identified, the signalling pathways mediating their actions are incompletely understood.
AIMS OF THE STUDY: The role of phosphoinositide 3-kinase (PI3K) in responses of human eosinophils to two important eosinophil chemoattractants -- platelet-activating factor (PAF) and eotaxin (CCL11) -- was studied to determine whether this enzyme activity might be crucial for eosinophil migration.
METHODS: Eosinophils were isolated from atopic donor blood by immunomagnetic selection. Chemotaxis was assayed in a 96-well blind-chamber cell fluorescence assay. Respiratory burst and leukotriene C(4) secretion were also assayed.
RESULTS: Two PI3K inhibitors, wortmannin and LY294002, caused concentration-dependent inhibition of PAF-induced eosinophil chemotaxis (IC(50) = 0.54 nM and 0.15 microM, respectively) but exhibited at least 100-fold lower potency against eotaxin-induced responses (IC(50) = 48 nM and >100 microM, respectively), indicating that these responses were not dependent upon PI3K. Wortmannin and LY294002 also inhibited PAF induced respiratory burst but not PAF-induced LTC(4) secretion.
CONCLUSIONS: We conclude that PI3K-dependence varies with stimulus and response, and that eotaxin-induced eosinophil migration is not controlled by PI3K. This may indicate a limit to the potential of PI3K inhibitors to suppress tissue eosinophilia in diseases such as asthma.

PMID: 16076309 [PubMed - indexed for MEDLINE]

1056. Am J Pathol. 2005 Aug;167(2):545-54.

A role for the plasminogen activator system in inflammation and neurodegeneration in the central nervous system during experimental allergic encephalomyelitis.

East E, Baker D, Pryce G, Lijnen HR, Cuzner ML, Gverić D.

Department of Neuroinflammation, Institute of Neurology, University College London, 1 Wakefield St., London, WC1N 1PJ, UK. e.east@ion.ucl.ac.uk

Early signs of inflammatory demyelination include entry of fibrin(ogen) into the central nervous system (CNS), which is normally excluded by the blood-brain barrier, and up-regulation of components of the plasminogen activator system. Using mice deficient in tissue-type plasminogen activator (tPA-/-) and urokinase plasminogen activator receptor (uPAR-/-), we investigated the involvement of the PA system on the clinical and pathological features of experimental allergic encephalomyelitis, an animal model of multiple sclerosis. tPA-/- mice suffered an early and a more severe acute disease characterized by incomplete recovery when compared to wild-type controls, with significantly higher CNS levels of plasminogen activator inhibitor-1. This correlated with fibrin accumulation, which co-localized with nonphosphorylated neurofilament on thickened axons in experimental allergic encephalomyelitis tissue. In contrast, uPAR-/- mice had a delayed, less acute disease reflected in delayed infiltration of inflammatory cells. These animals developed chronic disease as a result of steadily increased inflammation, increased levels of urokinase-type plasminogen activator (uPA), and greater degree of demyelination. Thus, the plasminogen activator system can modulate both inflammatory and degenerative events in the CNS through the respective effects of tPA and uPAR on fibrinolysis and cell adhesion/migration,

manipulation of which may have therapeutic implications for multiple sclerosis.

PMCID: PMC1603566
PMID: 16049338 [PubMed - indexed for MEDLINE]

1057. Clin Exp Immunol. 2005 Sep;141(3):459-66.

Interleukin-4 and interleukin-13 enhance CCL26 production in a human keratinocyte cell line, HaCaT cells.

Kagami S, Saeki H, Komine M, Kakinuma T, Tsunemi Y, Nakamura K, Sasaki K, Asahina A, Tamaki K.

Department of Dermatology, Faculty of Medicine, University of Tokyo, Tokyo, Japan. kagamis-tky@umin.ac.jp

Eotaxin-2/CCL24 and eotaxin-3/CCL26 are CC chemokines and their receptor, CC chemokine receptor 3 is preferentially expressed on eosinophils. It was reported that vascular endothelial cells and dermal fibroblasts produced CCL26. However, the regulation of CCL24 and CCL26 production in keratinocytes has not been well documented. We investigated the expression and production of CCL24 and CCL26 in the human keratinocyte cell line, HaCaT cells. Reverse transcription and polymerase chain reaction was performed using these cells and Enzyme-linked immunosorbent assay was carried out using supernatant of these cells. The production of CCL24 in HaCaT cells was slightly enhanced by IL-4 and that of CCL26 was strongly enhanced by IL-4 and IL-13. Furthermore, TNF-alpha generated a synergistic effect on IL-4 enhanced CCL26 production. Dexamethasone, IFN-gamma and the p38 mitogen-activated protein kinase inhibitor SB202190 inhibited IL-4 enhanced CCL26 production. IL-4 enhanced production of CCL26 was inhibited by leflunomide and JAK inhibitor 1, but not by JAK3 inhibitor, which indicates that it is mediated by JAK1-STAT6-dependent pathway. This result also strongly suggests the involvement of the type 2 IL-4 receptor in IL-4 enhanced production of CCL26. These results suggest that keratinocytes are involved in the migration of CC chemokine receptor 3 positive cells such as eosinophils in a Th2-dominant situation like atopic dermatitis.

PMCID: PMC1809447
PMID: 16045735 [PubMed - indexed for MEDLINE]

1058. J Dermatol. 2005 May;32(5):346-53.

Beneficial effects of tissue inhibitor of metalloproteinases-2 (TIMP-2) on chronic dermatitis.

Miyoshi H, Kanekura T, Aoki T, Kanzaki T.

Department of Dermatology, Kagoshima University Graduate School of Medical and Dental Sciences, Kagoshima, Japan.

Chronic dermatitis, such as contact dermatitis (CD) or atopic dermatitis (AD), is a longstanding inflammatory skin disease with cutaneous damage such as erosion, ulceration, and lichenification due to itch-induced scratching. The resultant lesion can be considered to be a kind of wound. The tissue inhibitor metalloproteases-2 (TIMP-2) accelerates wound healing by enhancing the proliferation and migration of epidermal keratinocytes and dermal fibroblasts; it is also a physiologic inhibitor of matrix metalloproteinases. The aim of this study was to test the effect of TIMP-2 on chronic dermatitis. NC/Kuj mice were sensitized with Dermatophagoides farinae (Df) extract. Eczema was induced by

repeated applications of this mite allergen to the skin of 20 sensitized mice that were maintained under specific pathogen-free conditions. One group of 10 mice was then treated with topical TIMP-2 solution (0.1 ml, 0.5%) for 28 days, and the other with vehicle alone and the effects of TIMP-2 were evaluated macro- and microscopically. The effect on skin barrier function was estimated by measuring transepidermal water loss (TEWL). Scoring of gross skin findings showed that TIMP-2 significantly reduced the severity of eczema (P<0.05) on days 12-28. Histological examination revealed that TIMP-2 treated mice manifested lower degrees of hyperkeratosis, acanthosis, and spongiosis in the epidermis and fewer inflammatory cells in the dermis than vehicle-treated mice. There were significant reductions in the epidermal thickness and dermal inflammatory cells in the TIMP-2 treated animals (P<0.01); their TEWL was significantly decreased on day 28 (P<0.05). Our results suggest that NC/Kuj mice with Df extract-induced chronic eczema may be a useful model for investigating chronic dermatitis, and that TIMP-2 may be a good agent for treating chronic dermatitis as well as chronic ulcers.

PMID: 16043896 [PubMed - indexed for MEDLINE]

1059. Proteomics. 2005 Aug;5(12):3137-41.

Proteome profiling of interleukin-12 treated human T helper cells.

Rosengren AT, Nyman TA, Lahesmaa R.

Turku Centre for Biotechnology, University of Turku and Abo Akademi University, PO Box 123, FIN-20521 Turku, Finland. arsi.rosengren@btk.fi

Selective activation of T helper subsets 1 (Th1) and 2 (Th2) plays a crucial role in different pathological conditions. Th1 cell response is involved in pathogenesis of autoimmune diseases, such as type II diabetes and multiple sclerosis, and Th2 cell response in pathogenesis of allergy and asthma. Cytokine interleukin-12 (IL-12) is one of the key factors in the differentiation of naïve CD4(+) T cells into Th1 cells. In this study we used 2-DE and MS to find and identify IL-12 regulated proteins in human CD4(+) T cells. In total, 42 protein spots were found to be differentially expressed following IL-12 stimulation, of which 22 were up- and 20 down-regulated. Among the upregulated proteins there are a multifunctional cytokine macrophage migration inhibitory factor and a known IL-12 target gene Programmed cell death 4. Downregulated proteins include p21-activated kinase 2 and its upstream GTPase Cdc42. Compared to previous reports our analysis provides a new view on the IL-12 induced changes on CD4(+) T cells underscoring the importance of creating and combining the data generated at various levels to build a comprehensive view of a given biological process of the cell.

PMID: 16038020 [PubMed - indexed for MEDLINE]

1060. Br J Dermatol. 2005 Jul;153(1):136-44.

Cilomilast, tacrolimus and rapamycin modulate dendritic cell function in the elicitation phase of allergic contact dermatitis.

Bäumer W, Sülzle B, Weigt H, De Vries VC, Hecht M, Tschernig T, Kietzmann M.

Department of Pharmacology, Toxicology and Pharmacy, University of Veterinary Medicine Hannover, Hannover, Germany. wolfgang.baeumer@tiho-hannover.de

BACKGROUND: Cilomilast and tacrolimus as well as rapamycin are potential drugs

for the treatment of allergic skin diseases like atopic dermatitis and allergic contact dermatitis.
OBJECTIVES: To compare the in vitro and in vivo immunomodulatory effects of the phosphodiesterase 4 inhibitor cilomilast with those of tacrolimus and rapamycin.
METHODS: The in vitro action of cilomilast, tacrolimus and rapamycin were tested in a mixed leucocyte reaction (MLR). In vivo, the inhibitory action of the immunomodulatory drugs was compared in the toluene-2,4-diisocyanate (TDI)-induced allergic inflammatory response with particular focus on dendritic cell (DC) function.
RESULTS: Cilomilast, tacrolimus and rapamycin were all able to inhibit DC-mediated T-cell activation in a MLR. But it was demonstrated for cilomilast that the target cells are T cells rather than DC. In vivo, a combination of systemic and topical administration of each of these three substances significantly inhibited swelling in the murine ear 16 h after TDI challenge. There was also a reduction in the weight of the draining auricular lymph node, in lymphocyte cell count, and in the number of emigrated DC. The density of Langerhans cells in the epidermis was correspondingly higher in mice treated with cilomilast, tacrolimus and rapamycin than in those treated with vehicle. All three substances were found to inhibit DC migration ex vivo in a skin DC migration assay performed on ear tissue after TDI challenge.
CONCLUSIONS: DC migration into the draining lymph node also takes place in the elicitation phase of allergic contact dermatitis and this migration can be influenced by tacrolimus and rapamycin, and, to a lesser extent, by cilomilast.

PMID: 16029339 [PubMed - indexed for MEDLINE]

1061. Proc Natl Acad Sci U S A. 2005 Jul 26;102(30):10598-603. Epub 2005 Jul 18.

Accelerated chemokine receptor 7-mediated dendritic cell migration in Runx3 knockout mice and the spontaneous development of asthma-like disease.

Fainaru O, Shseyov D, Hantisteanu S, Groner Y.

Department of Molecular Genetics, The Weizmann Institute of Science, Rehovot 76100, Israel.

The Runx3 transcription factor is a key regulator of lineage-specific gene expression in several developmental pathways and could also be involved in autoimmunity. We report that, in dendritic cells (DC), Runx3 regulates TGFbeta-mediated transcriptional attenuation of the chemokine receptor CCR7. When Runx3 is lost, i.e., in Runx3 knockout mice, expression of CCR7 is enhanced, resulting in increased migration of alveolar DC to the lung-draining lymph nodes. This increased DC migration and the consequent accumulation of activated DC in draining lymph nodes is associated with the development of asthma-like features, including increased serum IgE, hypersensitivity to inhaled bacterial lipopolysaccharide, and methacholine-induced airway hyperresponsiveness. The enhanced migration of DC in the knockout mice could be blocked in vivo by anti-CCR7 antibodies and by the drug Ciglitazone, known to inhibit CCR7 expression. The data indicate that Runx3 transcriptionally regulates CCR7 and that, when absent, the dysregulated expression of CCR7 in DC plays a role in the etiology of asthmatic conditions that recapitulate clinical symptoms of the human disease. Interestingly, human RUNX3 resides in a region of chromosome 1p36 that contains susceptibility genes for asthma and hypersensitivity against environmental antigens. Thus, mutations in RUNX3 may be associated with increased sensitivity to asthma development.

PMCID: PMC1180803
PMID: 16027362 [PubMed - indexed for MEDLINE]

1062. Med Hypotheses. 2005;65(5):966-9.

Immunity to tetanus is protective against the development of multiple sclerosis.

Verstraeten T, Davis R, DeStefano F.

Epidemic Intelligence Service (EIS) Program, Epidemiology Program Office, Centers for Disease Control and Prevention, Altanta, GA, USA. tvbelgium@hotmail.com

Following allegations that Hepatitis B vaccination causes or triggers multiple sclerosis (MS), several epidemiological studies have been conducted to evaluate the association between MS and vaccination. In one study conducted in the US, a significant protective effect on the development of MS was observed for tetanus immunization. We reviewed the medical literature and found two additional recent studies, as well as several older studies, which also observed a significant protective effect of tetanus immunization on the development or progression of MS. Furthermore, decreased humoral and cellular immunity to tetanus toxoid has been observed among MS patients. We postulate that naturally acquired or vaccine-induced immunity to tetanus has a protective effect against the development and progression of MS. We also postulate that this link to tetanus is in part responsible for the gender, age, geographic and socio-economic distribution of MS, as well as its pattern among migrants. The biological basis for this protective effect could be an unspecific boost of bystander suppression of auto-immunity as shown for other infections. Our hypothesis can be tested in several ways. The simplest approach would be to compare tetanus exposure and MS occurrence on a population level. Stronger support would come from the re-analysis of previous studies that have information at the individual level on both tetanus exposure, whether induced or natural, and on the development of MS. Laboratory evidence could be sought by testing the effect of tetanus toxoid on experimental allergic encephalomyelitis, the experimental animal model of MS.

PMID: 16023300 [PubMed - indexed for MEDLINE]

1063. Pneumonol Alergol Pol. 2004;72(9-10):375-82.

[Use of selected parameters of nasal lavage fluid in asthma severity monitoring].

[Article in Polish]

Fal AM, Nowak AA, Nowak MT, Litwa M.

Katedra i Klinika Chorób Wewnetrznych i Alergologii AM we Wrocławiu. amfal@pro.onet

Allergic inflammation is a chronic disorder underlying asthma and allergic rhinitis. There is a theory "one system--one disease" connecting these two atopic diseases. Eosinophil is a major effector cell in both processes. The possible correlations between spirometric parameters and concentrations of selected mediators in peripheral blood and nasal lavage (NLF) fluid were estimated. 14 asthmatic patients entered the study, all of them symptoms free. In NLF supernatants and in blood ECP and sVCAM-1 concentrations were measured and cells in NLF were counted. RESULTS: Number of eosinophils in NLF correlated with sVCAM-1 concentration in NLF (R2 = 0.68; $p < 0.05$). In serum ECP and sVCAM-1 concentrations correlated with each other (R2 = 0.73; $p < 0.05$). Correlation between ECP concentration in serum or NLF and FEV1 was: R2ser = 0.49, R2NLF = 0.50 ($p < 0.05$), respectively. ECP concentration concentration correlated in serum and NLF correlated with MEF25: R2ser = 0.77, R2NLF = 0.45 ($p < 0.05$), and sVCAM-1 spirometric parameters: R2FEV1 = 0.26, R2MEF25 = 0.67, $p < 0.05$.

CONCLUSION: VCAM-1 is active in selective eosinophil migration to peripheral tissue. It can also activate these cells. Concentration of ECP and sVCAM-1 in blood serum and NLF correlate with the course of bronchial asthma described by spirometric parameters, and therefore can be used in disease treatment monitoring.

PMID: 16021990 [PubMed - indexed for MEDLINE]

1064. FASEB J. 2005 Sep;19(11):1507-9. Epub 2005 Jul 13.

Cyclic mechanical strain-induced proliferation and migration of human airway smooth muscle cells: role of EMMPRIN and MMPs.

Hasaneen NA, Zucker S, Cao J, Chiarelli C, Panettieri RA, Foda HD.

Department of Medicine and Research, VAMC Northport, Northport, New York, USA.

Airway smooth muscle (ASM) proliferation and migration are major components of airway remodeling in asthma. Asthmatic airways are exposed to mechanical strain, which contributes to their remodeling. Matrix metalloproteinase (MMP) plays an important role in remodeling. In the present study, we examined if the mechanical strain of human ASM (HASM) cells contributes to their proliferation and migration and the role of MMPs in this process. HASM were exposed to mechanical strain using the FlexCell system. HASM cell proliferation, migration and MMP release, activation, and expression were assessed. Our results show that cyclic strain increased the proliferation and migration of HASM; cyclic strain increased release and activation of MMP-1, -2, and -3 and membrane type 1-MMP; MMP release was preceded by an increase in extracellular MMP inducer; Prinomastat [a MMP inhibitor (MMPI)] significantly decreased cyclic strain-induced proliferation and migration of HASM; and the strain-induced increase in the release of MMPs was accompanied by an increase in tenascin-C release. In conclusion, cyclic mechanical strain plays an important role in HASM cell proliferation and migration. This increase in proliferation and migration is through an increase in MMP release and activation. Pharmacological MMPIs should be considered in the pursuit of therapeutic options for airway remodeling in asthma.

PMID: 16014803 [PubMed - indexed for MEDLINE]

1065. J Sep Sci. 2005 Jun;28(9-10):941-7.

Analysis of trace amounts of bovine beta-lactoglobulin in infant formulas by capillary electrophoresis with on-capillary derivatization and laser-induced fluorescence detection.

Veledo MT, de Frutos M, Diez-Masa JC.

Institute of Organic Chemistry, Juan de la Cierva 3, 28006 Madrid, Spain.

Bovine beta-lactoglobulin (betaLG) has been described by several authors as the main allergen present in cow's milk. It can induce allergic reactions even at the low concentration existing in hypoallergenic formulas based on hydrolyzed cow's milk proteins (generally lower than microM). In this paper, the usefulness of a capillary electrophoresis method with on-capillary derivatization and laser-induced fluorescence detection for the analysis of trace amounts of betaLG in a commercial hypoallergenic formula has been demonstrated. To confirm the identity of the peak of betaLG based on migration time, an immunorecognition step employing an anti-betaLG antibody was performed. BetaLG was quantitated in the whey and casein fractions of the hypoallergenic formula. The concentration of

betaLG in the whey fraction of the formula was about 3 orders of magnitude lower than the average value present in cow's milk. In the casein fraction of the formula, the concentration of betaLG was about 1 order of magnitude lower than in the whey fraction. The method developed was also used for the quality control of three cereal-based infant foods formulated without milk to test the presence or absence of betaLG as an indicator of milk contamination during the fabrication process. BetaLG in a concentration of 10(-7) M or higher was not observed in any of the cereal-based infant formulas analyzed.

PMID: 16013820 [PubMed - indexed for MEDLINE]

1066. Clin Exp Allergy. 2005 Jul;35(7):873-9.

Modulation of murine experimental asthma by Ascaris suum components.

Itami DM, Oshiro TM, Araujo CA, Perini A, Martins MA, Macedo MS, Macedo-Soares MF.

Laboratory of Immunopathology, Butantan Institute, São Paulo, Brazil.

BACKGROUND: We have recently isolated two distinct components from Ascaris suum adult worms with different effects on the immune system: the allergenic protein of A. suum (APAS-3), which induces IgE antibody production, and suppressive protein of A. suum (PAS-1), which inhibits humoral and cellular immune responses induced by unrelated antigens. In this study, we investigated the immunomodulatory effect of PAS-1 on a murine model of asthma induced by APAS-3.
METHODS: BALB/c mice were immunized twice with APAS-3 or APAS-3 plus PAS-1 by the intraperitoneal and subcutaneous route (on days 0 and 7) and challenged twice with the same antigens intranasally (days 14 and 21). Two days after the last challenge, the allergic airway inflammation was evaluated by cellular migration, eosinophil peroxidase (EPO) activity, cytokine and chemokine production and pulmonary mechanical parameters.
RESULTS: The allergenic properties of APAS-3 were confirmed by the stimulation of anaphylactic IgE and IgG1 antibody production and eosinophilic airway inflammation and hyper-responsiveness. On the other hand, PAS-1-treated mice showed a marked suppression of cellular migration and EPO activity that correlated well with a significant reduction in the levels of IL-4, IL-5, eotaxin and RANTES in the bronchoalveolar lavage (BAL) fluid. In contrast, considerable amounts of IL-10 were observed in the BAL fluid of PAS-1-treated mice. Airway hyper-responsiveness was obtained in APAS-3-immunized mice, but the conductance of the respiratory system was restored to normal values in the presence of PAS-1.
CONCLUSION: These results indicate that A. suum allergenic protein APAS-3 induces a T helper 2-type immune response and, consequently, eosinophilic airway inflammation and hyper-responsiveness. Moreover, the modulatory protein PAS-1 has a marked suppressive effect on this response, and the inhibition of cytokine (IL-4, IL-5) and chemokine (eotaxin and RANTES) release, probably because of the presence of IL-10, may contribute to this effect.

PMID: 16008672 [PubMed - indexed for MEDLINE]

1067. J Immunol. 2005 Jul 15;175(2):1276-85.

Mucosal B cell deficiency in IgA-/- mice abrogates the development of allergic lung inflammation.

Arnaboldi PM, Behr MJ, Metzger DW.

Center for Immunology and Microbial Disease, Albany Medical College, 47 New

Scotland Avenue, Albany, NY 12208, USA.

We have investigated the consequence of lack of IgA on host immunity using a murine model of allergic lung inflammation. Mice with a targeted disruption of the alpha-switch region and 5' H chain gene (IgA(-/-) mice), which lack total IgA, developed significantly reduced pulmonary inflammation with fewer inflammatory cells in lung tissue and bronchoalveolar lavage fluids, as well as reduced levels of total and IgG1 OVA-specific Abs and decreased IL-4 and IL-5 in bronchoalveolar lavage fluids compared with IgA(+/+) controls, following allergen sensitization and challenge. This defect was attributable to fewer B cells in the lungs of IgA(-/-) mice. Polymeric IgR-deficient (pIgR(-/-)) mice, which lack the receptor that transports polymeric IgA across the mucosal epithelium where it is cleaved to form secretory IgA, were used to assess the contribution of secretory IgA vs total IgA in the induction of allergic lung inflammation. pIgR(-/-) and pIgR(+/+) mice had comparable levels of inflammation, demonstrating that IgA bound to secretory component is not necessary for the development of allergic lung inflammation, although this does not necessarily rule out a role for transudated IgA in lung secretions because of "mucosal leakiness" in these mice. The results indicate that Ag-specific B cells are required at mucosal surfaces for induction of inflammation and likely function as major APCs in the lung for soluble protein Ags.

PMID: 16002732 [PubMed - indexed for MEDLINE]

1068. Toxicol Appl Pharmacol. 2005 Sep 1;207(2 Suppl):142-8.

In vitro assessment of sensitizing activity of low molecular weight compounds.

Vandebriel RJ, Van Och FM, van Loveren H.

Laboratory for Toxicology, Pathology and Genetics, National Institute of Public Health and the Environment, P.O. Box 1, 3720 BA Bilthoven, The Netherlands. r.vandebriel@rivm.nl

Predictive tests to identify the sensitizing properties of chemicals are carried out using animals. There is as yet, no accepted in vitro method for the identification of skin sensitizing chemicals. Such in vitro tests should encompass (parts of) the sensitization phase of contact hypersensitivity. Two cell types are predominantly involved in this process, keratinocytes (KC) and Langerhans cells, the latter being a specialized type of skin dendritic cells (DC). Low molecular weight chemicals act as haptens; KC respond to allergen contact by, among others, producing proinflammatory cytokines, while DC take up the haptenized protein, migrate, and present antigen to T-cells. During migration, DC mature, resulting in a loss of antigen uptake capacity, thereby increasing expression of certain surface molecules. Thus, both cytokine production by KC and surface marker expression by DC may be used as in vitro models for the identification of sensitizers. Several reports have shown that intracellular IL-1 alpha is a promising candidate to identify sensitizers using KC. We have recently shown that the potency of sensitizers may be determined by dose-response analysis of intracellular IL-1 alpha and IL-18 using a murine KC cell line. The ranking of potency using this in vitro method was similar to the ranking previously established using the local lymph node assay. Using DC, effects on the expression of various cell surface markers, cytokines, and molecules involved in antigen uptake have been shown to identify sensitizers. One study showed that also the potency of sensitizers may be determined using DC. Additional studies are required to establish whether KC or DC, or combinations thereof, are most suitable for in vitro identification and potency assessment of sensitizers.

PMID: 16000204 [PubMed - indexed for MEDLINE]

1069. Nat Rev Immunol. 2005 Jul;5(7):560-70.

Sphingosine 1-phosphate and its receptors: an autocrine and paracrine network.

Rosen H, Goetzl EJ.

Department of Immunology, The Scripps Research Institute, La Jolla, CA 92037, USA.

Sphingosine 1-phosphate (S1P) is a biologically active lysophospholipid that transmits signals through a family of G-protein-coupled receptors to control cellular differentiation and survival, as well as the vital functions of several types of immune cell. In this Review article, we discuss recent results that indicate that S1P and its receptors are required for the emigration of thymocytes from the thymus, the trafficking of lymphocytes in secondary lymphoid organs and the migration of B cells into splenic follicles. In an autocrine manner, through interactions with different G-protein-coupled receptors, S1P also enhances optimal mast-cell migration and release of pro-inflammatory mediators in allergic reactions. S1P-S1P-receptor regulatory systems might therefore be novel targets for the therapy of diverse immunological diseases.

PMID: 15999095 [PubMed - indexed for MEDLINE]

1070. Expert Opin Ther Targets. 2001 Feb;5(1):109-23.

Sphingosine-1-phosphate: an emerging therapeutic target.

Toman RE, Milstien S, Spiegel S.

Interdisciplinary Program in Neuroscience, Georgetown University Medical Center, Washington, DC 20007, USA.

Sphingosine-1-phosphate (SPP) is a polar sphingolipid metabolite that has received increasing attention as both an extracellular mediator and an intracellular second messenger. SPP is the ligand of a family of specific cell surface G-protein coupled receptors (GPCR), known as the endothelial differentiation gene-1 (EDG-1) family. These receptors, which include EDG-1, -3, -5, -6 and -8, regulate diverse processes including cell migration, angiogenesis, vascular maturation, heart development, neurite retraction and soma rounding. In addition, abundant evidence indicates that SPP also acts as an intracellular lipid messenger, regulating calcium mobilisation, cell growth and survival. The relative intracellular level of SPP and ceramide, another sphingolipid metabolite associated with cell death and cell growth arrest, is an important factor in determining cell fate. Changes in SPP and ceramide have been implicated in a number of pathological conditions in which apoptosis plays an important role, including cancer and neurodegenerative disorders, as well as in atherosclerosis and allergic responses. This review will examine the biosynthesis, metabolism and potential functions of SPP in diverse diseases in order to illuminate targets for the pharmaceutical and therapeutic manipulation of SPP levels.

PMID: 15992170 [PubMed]

1071. Expert Opin Investig Drugs. 1998 Jan;7(1):19-25.

The link between inflammatory disease and patterns of leukocyte recruitment.

Newman W, Mirabelli CK.

LeukoSite, Inc., 215 First St., Cambridge, MA 02142, USA.

Leukocytes participate in different ways in inflammatory diseases. The nature of the disease can be seen as a reflection, in part, of the migration patterns of different leukocyte types in asthma versus inflammatory bowel disease (IBD). The molecular basis that underlies the selective recruitment of distinct leukocytes to unique microenvironments is a result of the interplay of selectins, integrins and chemoattractants. The ability to understand and dissect these pathways in disease processes will be an important tool for the development of novel therapeutics with highly effective suppressive potential and greatly reduced side-effects.

PMID: 15991916 [PubMed]

1072. J Allergy Clin Immunol. 2005 Jul;116(1):42-8.

Associations of place of birth with asthma and wheezing in Mexican American children.

Eldeirawi K, McConnell R, Freels S, Persky VW.

Division of Epidemiology and Biostatistics, School of Public Health, University of Illinois at Chicago, IL 60612-7260, USA. Keldei1@uic.edu

BACKGROUND: There are wide global variations in the prevalence of asthma and wheezing.
OBJECTIVES: We examined the associations of place of birth with doctor-diagnosed asthma, wheezing in the past 12 months, and other allergic conditions in Mexican American children.
METHODS: The study used data on 4121 Mexican American children age 2 months to 16 years who participated in the Third National Health and Nutrition Examination Survey.
RESULTS: The risk of asthma was associated with being born in the United States after adjusting for sex, age, history of ear infection, and having a regular place for health care (odds ratio, 2.19; 95% CI, 1.09-4.40). Among children with no previous history of ear infection, US-born children were more likely to report wheezing in the past 12 months than their peers born in Mexico after controlling for confounding variables (odds ratio, 2.05; 95% CI, 1.09-3.87). Mexican American children born in the United States were more likely to have positive skin reaction to cat, house mite, Alternaria alternata , peanut, Bermuda grass, and short ragweed but were less likely to have a positive skin test to German cockroaches after adjusting for sex, age, ear infection, having a regular place for health care, and area of residence.
CONCLUSION: Our study indicated significant associations of place of birth with respiratory symptoms and allergic conditions in Mexican American children. These findings highlight the need for further studies to examine environmental factors that change by migration and explain the observed differential in the risk of asthma or wheezing.

PMID: 15990771 [PubMed - indexed for MEDLINE]

1073. J Allergy Clin Immunol. 2005 Jul;116(1):38-41.

Immigration to the United States and acculturation as risk factors for asthma and allergy.

Gold DR, Acevedo-Garcia D.

Channing Laboratory, Department of Medicine, Brigham and Women's Hospital and Harvard Medical School. diane.gold@channing.harvard.edu

PMID: 15990770 [PubMed - indexed for MEDLINE]

1074. Ann Allergy Asthma Immunol. 2005 Jun;94(6):609-18; quiz 618-20, 669.

The role of leukotrienes in allergic rhinitis.

Peters-Golden M, Henderson WR Jr.

Department of Internal Medicine, University of Michigan Health System, Ann Arbor, Michigan 48109-0642, USA. petersm@umich.edu

OBJECTIVE: To review the role of cysteinyl leukotrienes (cysLTs) in allergic rhinitis and the scientific rationale for therapy with leukotriene receptor antagonists (LTRAs).
DATA SOURCES: Relevant basic science and clinical articles were identified by a search of the PubMed database for articles published from 1984 to 2004 using the following keywords: allergic rhinitis; nose; immune response; allergen challenge; leukotrienes C, D, and E; cysteinyl leukotriene; cysteinyl leukotriene receptor; cytokine; leukocyte; montelukast; zafirlukast; and pranlukast.
STUDY SELECTION: The authors' expert opinion was used to select studies for inclusion in this review.
RESULTS: CysLTs are synthesized via 5-lipoxygenase metabolism of arachidonic acid by mast cells and basophils during the early-phase response to antigen and by eosinophils and macrophages during the late phase. The cysLT levels in nasal secretions are elevated after short-term allergen instillation and in allergy season in patients with allergic rhinitis. These lipid mediators act locally and systemically by interacting with receptors, particularly the cysLT1 receptor, on target cells. Evidence derived from topical application of cysLTs in the nose and from the effects of LTRAs indicates that cysLTs contribute to nasal mucous secretion, congestion, and inflammation. CysLTs promote allergic inflammation by enhancing immune responses and the production, adhesion, migration, and survival of inflammatory cells such as eosinophils. They also increase the generation of an array of other proinflammatory mediators, such as cytokines, which in turn increase the production of and receptors for cysLTs. Clinical trials have demonstrated that LTRAs have significant but modest efficacy as single agents but additive efficacy when used with other classes of agents.
CONCLUSIONS: CysLTs fulfill the criteria for relevant mediators of allergic rhinitis via their diverse effects on immune, inflammatory, and local structural components of disease. By blocking the cysLT1 receptor responsible for most of these effects, LTRAs represent a useful approach to treatment of this important and prevalent disorder.

PMID: 15984591 [PubMed - indexed for MEDLINE]

1075. Contact Dermatitis. 2005 Jul;53(1):1.

Lymphocyte transformation test in patients with allergic contact dermatitis.

Basketter D, Menné T.

PMID: 15982223 [PubMed - indexed for MEDLINE]

1076. Curr Top Microbiol Immunol. 2005;293:43-71.

Thymic commitment of regulatory T cells is a pathway of TCR-dependent selection that isolates repertoires undergoing positive or negative selection.

Coutinho A, Caramalho I, Seixas E, Demengeot J.

Laboratoire Européen Associé au CNRS, Instituto Gulbenkian de Ciência, Oeiras, Portugal.

The seminal work of Le Douarin and colleagues (Ohki et al. 1987; Ohki et al. 1988; Salaun et al. 1990; Coutinho et al. 1993) first demonstrated that peripheral tissue-specific tolerance is centrally established in the thymus, by epithelial stromal cells (TEC). Subsequent experiments have shown that TEC-tolerance is dominant and mediated by CD4 regulatory T cells (Treg) that are generated intrathymically by recognition of antigens expressed on TECs (Modigliani et al. 1995; Modigliani et al. 1996a). From these and other observations, in 1996 Modigliani and colleagues derived a general model for the establishment and maintenance of natural tolerance (MM96) (Modigliani et al. 1996b), with two central propositions: (1) T cell receptor (TCR)-dependent sorting of emergent repertoires generates TEC-specific Treg displaying the highest TCR self-affinities below deletion thresholds, thus isolating repertoires undergoing positive and negative selection; (2) Treg are intrathymically committed (and activated) for a unique differentiative pathway with regulatory effector functions. The model explained the embryonic/perinatal time window of natural tolerance acquisition, by developmental programs determining (1) TCR multireactivity, (2) the cellular composition in the thymic stroma (relative abundance of epithelial vs hemopoietic cells), and (3) the dynamics of peripheral lymphocyte pools, built by accumulation of recent thymic emigrants (RTE) that remain recruitable to regulatory functions. We discuss here the MM96 in the light of recent results demonstrating the promiscuous expression of tissue-specific antigens by medullary TECs (Derbinski et al. 2001; Anderson et al. 2002; Gotter et al. 2004) and indicating that Treg represent a unique differentiative pathway (Fontenot et al. 2003; Hori et al. 2003; Khattri et al. 2003), which is adopted by CD4 T cells with high avidity for TEC-antigens (Bensinger et al. 2001; Jordan et al. 2001; Apostolou et al. 2002). In the likelihood that autoimmune diseases (AID) result from Treg deficits, some of which might have a thymic origin, we also speculate on therapeutic strategies aiming at selectively stimulating their de novo production or peripheral function, within recent findings on Treg responses to inflammation (Caramalho et al. 2003; Lopes-Carvalho et al., submitted, Caramalho et al., submitted). In short, the MM96 argued that natural tolerance is dominant, established and maintained by the activity of Treg, which are selected upon high-affinity recognition of self-ligands on TECs, and committed intrathymically to a unique differentiative pathway geared to anti-inflammatory and antiproliferative effector functions. By postulating the intrathymic deletion of self-reactivities on hemopoietic stromal cells (THC), together with the inability of peripheral resident lymphocytes to engage in the regulatory pathway, the MM96 simultaneously explained the maintenance of responsiveness to non-self in a context of suppression mediating dominant self-tolerance. The major difficulty of the MM96 is related to the apparent tissue specificity of Treg repertoires generated intrathymically. This difficulty has now been principally solved by the work of Hanahan, Kyewski and others (Jolicoeur et al. 1994; Derbinski et al. 2001; Anderson et al. 2002; Gotter et al. 2004), demonstrating the selective expression of a variety of tissue-specific antigens by TECs, in topological patterns that are compatible with the MM96, but difficult to conciliate with recessive tolerance models (Kappler et al. 1987; Kisielow et al. 1988). While the developmentally regulated multireactivity of TCR repertoires (Gavin and Bevan 1995), as well as the peripheral recruitment of Treg among RTE (Modigliani et al. 1996a) might add to this process, it would seem that

the establishment of tissue-specific tolerance essentially stems from the "promiscuous expression of tissue antigens" by TEC. The findings of AID resulting from natural mutations (reviewed in Pitkanen and Peterson 2003) or the targeted inactivation (Anderson et al. 2002; Ramsey et al. 2002) of the AIRE transcription factor that regulates promiscuous gene expression on TECs support this conclusion. The observations on the correlation of natural or forced expression of the Foxp3 transcription factor in CD4 T cells with Treg phenotype and function (Fontenot et al. 2003; Hori et al. 2003; Khattri et al. 2003) provided support for the MM96 contention that Treg represent a unique differentiative pathway that is naturally established inside the thymus. Furthermore, Caton and colleagues (Jordan et al. 2001), as well as several other groups (Bensinger et al. 2001; Apostolou et al. 2002), have provided direct evidence for our postulate that Treg are selected among differentiating CD4 T cells with high affinity for ligands expressed on TECs (Modigliani et al. 1996b). Finally, the demonstration by Caramalho et al. that Treg express innate immunity receptors (Caramalho et al. 2003) and respond to pro-inflammatory signals and products of inflammation (Caramalho et al., submitted) brought about a new understanding on the peripheral regulation of Treg function. Together with the observation that Treg also respond to ongoing activities of "naïve/effector" T cells--possibly through the IL-2 produced in these conditions--these findings explain the participation of Treg in all immune responses (Onizuka et al. 1999; Shimizu et al. 1999; Annacker et al. 2001; Curotto de Lafaille et al. 2001; Almeida et al. 2002; Shevach 2002; Bach and Francois Bach 2003; Wood and Sakaguchi 2003; Mittrucker and Kaufmann 2004; Sakaguchi 2004), beyond their fundamental role in ensuring self-tolerance (e.g., Modigliani et al. 1996a; Shevach 2000; Hori et al. 2003; Sakaguchi 2004; Thompson and Powrie 2004). Thus, anti-inflammatory and anti-proliferative Treg are amplified by signals that promote or mediate inflammation and proliferation, accounting for the quality control of responses (Coutinho et al. 2001). In turn, such natural regulation of Treg by immune responses to non-self may well explain the alarming epidemiology of allergic and AID in wealthy societies (Wills-Karp et al. 2001; Bach 2002; Yazdanbakhsh et al. 2002), where a variety of childhood infections have become rare or absent. Thus, it is plausible that Treg were evolutionarily set by a given density of infectious agents in the environment. With hindsight, it is not too surprising that natural Treg performance falls once hygiene, vaccination, and antibiotics suddenly (i.e., 100 years) plunged infectious density to below some critical physiological threshold. As the immune system is not adapted to modern clean conditions of postnatal development, clinical immunologists must now deal with frequent Treg deficiencies (allergies and AID) for which they have no curative or rational treatments. It is essential, therefore, that basic immunologists concentrate on strategies to selectively stimulate the production, survival, and activity of this set of lymphocytes that is instrumental in preventing immune pathology. We have argued that the culprit of this inability of basic research to solve major clinical problems has been the self-righteousness of recessive tolerance champions, from Ehrlich to some of our contemporaries. It is ironical, however, that none of us--including the heretic opponents of horror autotoxicus--had understood that self-tolerance, or its robustness at least, is in part determined by the frequency and intensity of the responses to non-self. In the evolution of ideas on immunological tolerance, the time might be ripe for some kinds of synthesis. First, conventional theory reduced self-tolerance to negative selection and microbial defense to positive selection, while the MM96 solution was the precise opposite: positive selection of autoreactivities for self-tolerance (Treg) and negative selection (of Treg) for ridding responses. In contrast, it would now appear that positive and negative selection of autoreactive T cells are both necessary to establish either self-tolerance or competence to eliminate microbes, two processes that actually reinforce each other in the maintenance of self-integrity. Second, V-region recognition has generally been held responsible for specific discrimination between what should be either tolerated or eliminated from the organism. In contrast again, it would now seem that both processes of self-tolerance and microbial defense (self/non-self discrimination) also operate on the basis of

evolutionarily ancient, germ-line-encoded innate, nonspecific receptors (Medzhitov and Janeway 2000) capable of a coarse level of self/non-self discrimination (Coutinho 1975). It could thus be interesting to revisit notions of cooperativity between V-regions and such mitogen receptors, both in single cell functions (Coutinho et al. 1974) and in the system's evolution (Coutinho 1975, 1980) as well. After all, major transitions in evolution were cooperative (Maynard-Smith and Szathmary 1995).

PMID: 15981475 [PubMed - indexed for MEDLINE]

1077. J Immunol. 2005 Jul 1;175(1):43-50.

Impact of cutaneous IL-10 on resident epidermal Langerhans' cells and the development of polarized immune responses.

Cumberbatch M, Clelland K, Dearman RJ, Kimber I.

Syngenta Central Toxicology Laboratory, Alderley Park, Macclesfield, Cheshire, United Kingdom. marie.cumberbatch@syngenta.com

Prolonged topical exposure of BALB/c mice to chemical contact and respiratory allergens stimulates, respectively, preferential Th1- and Th2-type responses with respect to serum Ab isotype and cytokine secretion phenotypes displayed by draining lymph node cells. We now report that differential cytokine secretion patterns are induced rapidly in the skin following first exposure to the contact allergen 2,4-dinitrochlorobenzene (DNCB) and the respiratory sensitizer trimellitic anhydride (TMA). TMA induced early expression of IL-10, a cytokine implicated in the negative regulation of Langerhans cell (LC) migration, whereas exposure to DNCB resulted in production of the proinflammatory cytokine IL-1beta. Associated with this, TMA provoked LC migration with delayed kinetics compared with DNCB, and local neutralization of IL-10 caused enhanced LC mobilization in response to TMA with concomitant up-regulation of cutaneous IL-1beta. We hypothesize that these differential epidermal cytokine profiles contribute to the polarization of immune responses to chemical allergens via effects on the phenotype of activated dendritic cells arriving in the draining lymph node. Thus, TMA-exposed dendritic cells that have been conditioned in vivo with IL-10 (a potent inhibitor of the type 1-polarizing cytokine IL-12) are effective APCs for the development of a Th2-type response.

PMID: 15972630 [PubMed - indexed for MEDLINE]

1078. Clin Exp Allergy. 2005 Jun;35(6):708-12.

CCL17 and CCL22 attenuate CCL5-induced mast cell migration.

Juremalm M, Olsson N, Nilsson G.

Research Group on Mast Cell Biology, Department of Genetics and Pathology, The Rudbeck Laboratory, Uppsala University, Uppsala, Sweden.

BACKGROUND: Mast cells (MCs) accumulate at sites of allergic mucosal inflammation where they act as central effectors and regulatory cells. Chemokines are believed to be crucial for the recruitment of MCs to sites of inflammation. We recently reported that human umbilical cord blood MCs (CBMCs) expresses the CC chemokine receptors, CCR1 and CCR4. We found a unique response profile to ligands of the respective receptors in which, of all tested ligands, only CCL5/RANTES-induced migration.
OBJECTIVE: To further investigate the function of CCR4 in MCs.

METHODS: CBMCs were used for competition binding experiments, migration, and intracellular calcium mobilization and release response studies.
RESULTS: The natural ligands for CCR4, CCL17/TARC and CCL22/MDC could both compete for binding with radiolabelled CCL5. Further, both CCL17 and CCL22 act as CCR4 antagonists by inhibiting CCL5-induced migration. Although both CCL17 and CCL22 caused mobilization of intracellular calcium, none of them induced migration or histamine release.
CONCLUSIONS: These results suggest that CCL5-induced migration of MCs via CCR4 can be regulated by the natural agonists CCL17 and CCL22, which are up-regulated at sites of allergic inflammation.

PMID: 15969658 [PubMed - indexed for MEDLINE]

1079. Trends Mol Med. 2005 Jun;11(6):266-70.

The OXE receptor: a new therapeutic approach for asthma?

Jones CE.

Respiratory Diseases Therapeutic Area, Novartis Institutes for Biomedical Research, Horsham, RH12 5AB, UK. carol-e.jones@novartis.com

The eicosanoid 5-oxo-6E,8Z,11Z,14Z-eicosatetraenoic acid (5-oxo-ETE) has recently been identified as the ligand for the oxoeicosanoid (OXE) receptor. In vitro and in vivo studies have suggested that 5-oxo-ETE has a role in the asthmatic inflammatory response and it has been shown to stimulate eosinophil migration to the airways. New data suggest that eosinophils have an important role in the pathogenesis of asthma, being required for mucus accumulation, airway hyperresponsiveness and remodelling of the airways. However, there are several mediators that can stimulate the recruitment of eosinophils to the airways and the development of antagonists against the OXE receptor is required to evaluate the potential of the OXE receptor as a new therapeutic approach for asthma.

PMID: 15949767 [PubMed - indexed for MEDLINE]

1080. Int Arch Allergy Immunol. 2005;137 Suppl 1:2-6. Epub 2005 Jun 2.

Effect of the cysteinyl leukotriene antagonist pranlukast on transendothelial migration of eosinophils.

Nagata M, Saito K, Kikuchi I, Hagiwara K, Kanazawa M.

Department of Respiratory Medicine, Saitama Medical School, Japan. favre4mn@saitama-med.ac.jp

BACKGROUND: Evidence shows that leukotriene receptor antagonist (LTRA) can cause a partial reduction of eosinophils in the asthmatic airway. Although cysteinyl leukotrienes (CysLTs) can regulate the development of eosinophilic inflammation, LTRA might modulate the eosinophilic response to other inflammatory molecules involved in allergic inflammation. Montelukast is an LTRA that inhibits eosinophil transendothelial migration (TEM) in response to platelet-activating factor (PAF). The present study evaluates whether pranlukast (an LTRA) modifies eosinophil TEM in response to chemoattractants including PAF and C-C chemokines.
METHODS: Eosinophils isolated from the blood of healthy individuals were incubated with or without pranlukast. We then evaluated eosinophil transmigration across human umbilical vein endothelial cells in response to LTD(4), eotaxin, RANTES and PAF.
RESULTS: Pranlukast did not modify the spontaneous transmigration of eosinophils

(n = 5). As reported, eosinophil TEM was significantly augmented by 0.1 microM LTD(4) and this enhancement was blocked by 1 microM pranlukast (p < 0.001; n = 6). On the other hand, pranlukast did not modify eosinophil transmigration in response to eotaxin, RANTES, or PAF (p > 0.1; n = 5).
CONCLUSION: The inhibitory effect of pranlukast on eosinophil transmigration is highly specific for the CysLT1-dependent pathway.

Copyright 2005 S. Karger AG, Basel.

PMID: 15947477 [PubMed - indexed for MEDLINE]

1081. Expert Opin Ther Targets. 2005 Apr;9(2):225-43.

Lymphocyte trafficking to inflamed skin--molecular mechanisms and implications for therapeutic target molecules.

Schön MP, Ludwig RJ.

Department of Dermatology and Venereology, Rudolf Virchow Center, DFG Research Center for Experimental Biomedicine, Bayerische Julius-Maximilians University, Würzburg, Germany. michael.schoen@virchow.uni-wuerzburg.de

Tissue-selective recruitment of lymphocytes to peripheral organs, such as the skin, is crucial for spatial compartmentalisation within the immune system as well as immune surveillance under normal conditions. In addition, this process plays a key role for the pathogenesis of various diseases including common inflammatory disorders such as atopic dermatitis or psoriasis, but also malignancies such as cutaneous T cell lymphomas. Recruitment of lymphocytes to the skin is a highly complex process that involves adhesion to the endothelial lining, extravasation, migration through the connective tissue, and, finally, localisation of a subpopulation of lymphocytes to the epithelial compartment, the epidermis. An intertwined network of constitutively expressed and inducible cytokines, chemokines and other mediators provides guidance for lymphocyte migration, and a large number of adhesion receptors mediate sequential steps of cell-cell- and cell-substrate-interactions resulting in tissue-specific localisation of immune cells. Selectively targeting the functions of one or several key molecules involved in this complex cascade promises exciting new therapeutic options for treating inflammatory disorders, but at the same time, bears considerable imponderables which will be discussed in this article.

PMID: 15934912 [PubMed - indexed for MEDLINE]

1082. Cytokine. 2005 Jul 21;31(2):103-8.

Macrophage migration inhibitory factor (MIF) contributes to the development of allergic rhinitis.

Nakamaru Y, Oridate N, Nishihira J, Takagi D, Furuta Y, Fukuda S.

Department of Otolaryngology and Head and Neck Surgery, Hokkaido University Graduate School of Medicine, West 7 North 15, Sapporo 060-8638, Japan.
nmaru@med.hokudai.ac.jp

Macrophage migration inhibitory factor (MIF) is a proinflammatory cytokine whose expression has been found to be critical to the generation of antigen-specific immune responses. Recent studies suggested that MIF played a role in the initiation and maintenance of allergic diseases. To elucidate MIF's role in the pathogenesis of allergic rhinitis (AR), we sensitized MIF-deficient gene knockout

(KO) mice and wild-type (WT) mice intraperitoneally with ovalbumin (OVA) and compared their clinical symptoms and allergic responses after intranasal challenge. Antigen-induced nasal symptoms were significantly reduced in MIF KO mice compared to WT mice. Histological examination of nasal mucosa showed that the number of infiltrating eosinophils in MIF KO mice was significantly lower than that in WT mice ($P < 0.05$). The concentration of TNF-alpha in nasal mucosa was also significantly lower in MIF KO mice than in WT mice ($P < 0.05$). We have demonstrated that the absence of MIF affects several aspects of experimental AR. One mechanism by which these effects might be mediated is by down regulating TNF-alpha. The block of allergic inflammation in MIF KO mice suggests that MIF may play a role in the allergic response.

PMID: 15922619 [PubMed - indexed for MEDLINE]

1083. Pharmacol Res. 2005 Jul;52(1):83-92.

Poly(ADP-ribosyl)ation in asthma and other lung diseases.

Virág L.

Department of Medical Chemistry, Research Center for Molecular Medicine, Medical and Health Science Center, University of Debrecen, H-4026 Debrecen, Bem tér 18/B, Hungary. lvirag@dote.hu

Inhibition of poly(ADP-ribosyl)ation in oxidative stress-related pathologies has recently emerged as a very effective anti-inflammatory intervention in animal models of arthritis, colitis, diabetes and shock. Recent data from three laboratories also support the role of poly(ADP-ribose) polymerase-1 (PARP-1) activation in asthma. Similarly to other inflammatory conditions, the protective effects of PARP inhibition and the PARP-1 knock out phenotype in asthma models have been attributed to inhibition of inflammatory signal transduction (mainly via NF-kappaB) and of oxidative stress-induced cell dysfunction and tissue injury. Here I discuss the complex role of poly(ADP-ribosyl)ation in the regulation of inflammatory cell migration, chemokine and cytokine production and expression of other inflammatory mediators (inducible nitric oxide synthase, matrix metalloproteinases) in asthma. The role of PARP-1 in other oxidative stress-related lung diseases such as asbestosis, silicosis, acute respiratory distress syndrome and ischemia-reperfusion injury is also reviewed.

PMID: 15911336 [PubMed - indexed for MEDLINE]

1084. Basic Clin Pharmacol Toxicol. 2005 Jun;96(6):429-35.

Safety of blocking vascular adhesion protein-1 in patients with contact dermatitis.

Vainio PJ, Kortekangas-Savolainen O, Mikkola JH, Jaakkola K, Kalimo K, Jalkanen S, Veromaa T.

Biotie Therapies Corp., Turku FIN-20520, Finland. petri.vainio@biotie.com

Vascular adhesion protein-1 mediates leukocyte binding to vascular endothelia and migration to tissues. It is upregulated in inflammatory conditions. We studied the safety of vascular adhesion protein-1 blockade by a single dose of the mouse monoclonal antibody vepalimomab in patients with nickel-induced allergic contact dermatitis lesions. Vepalimomab, 0.05-0.50 mg kg(-1) was safe and well tolerated. Four of nine patients reported adverse events of mild to moderate intensity. Human antimouse antibodies were detected after infusion in all the patients and

they remained above the basal level for at least one month. Vepalimomab dose-dependently labelled vascular adhesion protein-1 in the inflamed skin. Luminal upregulation of vascular adhesion protein-1 on the endothelium upon inflammation was demonstrated for the first time in patients in vivo. Vepalimomab was found on the endothelium up to 24 hr after dosing whilst it was cleared from the circulation with an apparent half-life of 25-50 min. The results provide in vivo support for the concept of blocking vascular adhesion protein-1 in human disease states and support previous proposals that vascular adhesion protein-1 is a potential target molecule for inhibition of inflammatory reactions.

PMID: 15910406 [PubMed - indexed for MEDLINE]

1085. Curr Opin Otolaryngol Head Neck Surg. 2005 Jun;13(3):171-5.

The role of the eosinophil in nasal diseases.

Eliashar R, Levi-Schaffer F.

Department of Otolaryngology/Head and Neck Surgery, The Hebrew University School of Medicine, Hadassah Medical Center, Jerusalem, Israel. ron@eliashar.com

PURPOSE OF REVIEW: The eosinophil is involved in physiologic and pathologic processes, such as asthma, parasitic diseases, granulomatous disorders, fibrosis, malignant tumors and several sino-nasal diseases.
RECENT DEVELOPMENTS: Recent data on the structure and function of the eosinophil provides additional information regarding the pathophysiology and the treatment options of these diseases. In this paper the most recently acquired data on the role of the eosinophil in allergic rhinitis (with or without bronchial asthma), chronic sinusitis (with or without nasal polyposis) and allergic fungal sinusitis are reviewed.
SUMMARY: The data provides evidence regarding the pivotal role of the eosinophil in sino-nasal diseases. Possible ways to target the eosinophils are discussed.

PMID: 15908816 [PubMed - indexed for MEDLINE]

1086. Curr Opin Pharmacol. 2005 Jun;5(3):227-31.

Targeting T cells for asthma.

Heijink IH, Van Oosterhout AJ.

Laboratory of Allergology and Pulmonary Diseases and Department of Pathology, University Medical Center Groningen (UMCG), Groningen University, P.O. Box 30.001, 9700 RB, Groningen, The Netherlands.

The type 2 T-helper (Th2) lymphocyte can be regarded as an important target cell for the treatment of allergic asthma as it plays a crucial role in the initiation, progression and persistence of disease. Several strategies to target Th2 cells can be envisioned. Drugs that prevent Th2-cells from migrating into the lung tissue, such as antibodies to the chemokine receptor CCR4 and inhibitors of the adhesion molecule VLA-4, are promising for the treatment of asthma. To inhibit Th2-cell activation, novel asthma drugs that act on Th2-selective transciption factors such as GATA3 are being developed. Although initial strategies aimed to block the action of Th2-derived cytokines, the generation of counter-regulatory Th1 lymphocytes and regulatory T cells is currently being explored.

PMID: 15907907 [PubMed - indexed for MEDLINE]

1087. Clin Mol Allergy. 2005 May 20;3:6.

Research upregulation of CD23 (FcepsilonRII) expression in human airway smooth muscle cells (huASMC) in response to IL-4, GM-CSF, and IL-4/GM-CSF.

Belleau JT, Gandhi RK, McPherson HM, Lew DB.

Department of Pediatrics, Children's Foundation Research Center at the Le Bonheur Children's Medical Center, University of Tennessee Health Science Center, 50 North Dunlap Street, Rm401, WPT, Memphis, TN 38103, USA. jcbelleau@yahoo.com

BACKGROUND: Airway smooth muscle cells play a key role in remodeling that contributes to airway hyperreactivity. Airway smooth muscle remodeling includes hypertrophy and hyperplasia. It has been previously shown that the expression of CD23 on ASMC in rabbits can be induced by the IgE component of the atopic serum. We examined if other components of atopic serum are capable of inducing CD23 expression independent of IgE.
METHODS: Serum starved huASMC were stimulated with either IL-4, GM-CSF, IL-13, IL-5, PGD2, LTD4, tryptase or a combination of IL-4, IL-5, IL-13 each with GM-CSF for a period of 24 h. CD23 expression was analyzed by flow cytometry, western blot, and indirect immunofluorescence.
RESULTS: The CD23 protein expression was upregulated in huASMC in response to IL-4, GM-CSF, and IL-4/GM-CSF. The percentage of cells with increased fluorescence intensity above the control was 25.1 +/- 4.2% (IL-4), 15.6 +/- 2.7% (GM-CSF) and 32.9 +/- 13.9% (IL-4/GMCSF combination)(n = 3). The protein content of IL-4/GMCSF stimulated cells was significantly elevated. Expression of CD23 in response to IL-4, GM-CSF, IL-4/GM-CSF was accompanied by changes in cell morphology including depolymerization of isoactin fibers, cell spreading, and membrane ruffling. Western blot revealed abundant expression of the IL-4Ralpha and a low level expression of IL-2Rgammac in huASMC. Stimulation with IL-4 resulted in the phosphorylation of STAT-6 and an increase in the expression of the IL-2Rgammac.
CONCLUSION: CD23 on huASMC is upregulated by IL-4, GM-CSF, and IL-4/GM-CSF. The expression of CD23 is accompanied by an increase in cell volume and an increase in protein content per cell, suggesting hypertrophy. Upregulation of CD23 by IL-4/GM-CSF results in phenotypic changes in huASMC that could play a role in cell migration or a change in the synthetic function of the cells. Upregulation of CD23 in huASMC by IL-4 and GM-CSF can contribute to changes in huASMC and may provide an avenue for new therapeutic options in asthma targeting ASMC.

PMCID: PMC1173127
PMID: 15907205 [PubMed]

1088. J Immunol. 2005 Jun 1;174(11):6955-66.

In vivo IL-10 gene delivery suppresses airway eosinophilia and hyperreactivity by down-regulating APC functions and migration without impairing the antigen-specific systemic immune response in a mouse model of allergic airway inflammation.

Nakagome K, Dohi M, Okunishi K, Komagata Y, Nagatani K, Tanaka R, Miyazaki J, Yamamoto K.

Department of Allergy and Rheumatology, Graduate School of Medicine, University of Tokyo, Japan.

IL-10 is an immunosuppressive cytokine. Although previous studies have reported

that exogenous delivery of IL-10 reduced airway inflammation in experimental allergic airway inflammation, the mechanism of action has not been fully clarified. In this report, we elucidated a mechanism of action of IL-10 in vivo. BALB/c mice were immunized and aerosol challenged with OVA-Ag. We delivered the IL-10 gene to the mice before systemic sensitization or during aerosol Ag challenge by administering an IL-10-producing plasmid vector. Not only presensitization delivery of IL-10, as reported, but also delivery during inflammation strongly suppressed the development of airway eosinophilia and hyperreactivity. Presensitization delivery suppressed the Ag-specific Th2-type immune response in both the lung and spleen. In contrast, delivery in the effector phase suppressed the Th2 response only in the lung, whereas that in the spleen was not affected. IL-10 gene delivery did not induce the development of a regulatory phenotype of T cells or dendritic cells; rather, it suppressed the overall functions of CD11c(+) APCs of the lung such as Ag-presenting capacity, cytokine production, and transportation of OVA-Ag to lymph nodes, thus attenuating Th2-mediated allergic airway inflammation. Further, IL-10 revealed a distinct immunosuppressive effect in the presence of Ag and APCs. These results suggest that suppression of APC function in the lung, the site of immune response, played a critical role in the IL-10-mediated suppression of Ag-induced airway inflammation and hyperreactivity. Therefore, if delivered selectively, IL-10 could site specifically suppress the Ag-specific immune response without affecting systemic immune responses.

PMID: 15905538 [PubMed - indexed for MEDLINE]

1089. BMC Public Health. 2005 May 17;5:48.

Screening for asthma in Cantonese-speaking immigrant children.

Greenfield RO, Lee AC, Tang R, Brugge D.

Department of Public Health and Family Medicine, Tufts University School of Medicine, Boston, USA. robyn.greenfield@tufts.edu

BACKGROUND: Asthma prevalence among Chinese immigrant children is poorly understood and attempts to screen these children have produced varied outcomes. We sought to learn how to improve screening for asthma in Chinese immigrant children.
METHODS: Children (n = 152) were administered the Brief Pediatric Asthma Screen in either Cantonese or English, they then viewed and reacted to a video showing people wheezing and subsequently took a pulmonary function test.
RESULTS: The diagnosed asthma prevalence for our study population was 27.0%, with another 5.3% having possible undiagnosed asthma. Very few children had spirometry findings below normal. In multivariate analysis, being native born (p = 0.002) and having a family history of asthma (p = 0.003) were statistically associated with diagnosis of asthma. After viewing the video, 35.6% of respondents indicated that the images differed from their conception of wheezing. Of four translations of the word "wheeze" no single word was chosen by a majority.
CONCLUSION: Our findings suggest that asthma diagnoses are higher for Chinese children who were born in the US suggesting that desegregation of data might reveal at risk subpopulations. Care needs to be taken when diagnosing asthma for Cantonese speakers because of the centrality of the word wheeze and the challenges of translation.

PMCID: PMC1168903
PMID: 15904513 [PubMed - indexed for MEDLINE]

1090. J Clin Invest. 2005 Jun;115(6):1590-600. Epub 2005 May 12.

Role of C5 in the development of airway inflammation, airway hyperresponsiveness, and ongoing airway response.

Peng T, Hao L, Madri JA, Su X, Elias JA, Stahl GL, Squinto S, Wang Y.

Alexion Pharmaceuticals Inc., Cheshire, Connecticut 06410, USA.

The role of complement component C5 in asthma remains controversial. Here we examined the contribution of C5 at 3 critical checkpoints during the course of disease. Using an mAb specific for C5, we were able to evaluate the contribution of C5 during (a) the initiation of airway inflammation, (b) the maintenance of airway hyperresponsiveness (AHR), and (c) sustainment of an ongoing airway response to allergen provocation. Our results indicate that C5 is probably activated intrapulmonarily after infections or exposures to allergen and C5 inhibition has profound effects at all 3 critical checkpoints. In contrast to an earlier report, C5-deficient mice with established airway inflammation did not have elevated AHR to nonspecific stimuli. In the presence of airway inflammation, C5a serves as a direct link between the innate immune system and the development of AHR by engaging directly with its receptors expressed in airways. Through their powerful chemotactic and cell activation properties, both C5a and C5b-9 regulate the downstream inflammatory cascade, which results in a massive migration of inflammatory cells into the bronchial airway lumen and triggers the release of multiple harmful inflammatory mediators. This study suggests that targeting C5 is a potential clinical approach for treating patients with asthma.

PMCID: PMC1090470
PMID: 15902311 [PubMed - indexed for MEDLINE]

1091. Am J Respir Cell Mol Biol. 2005 Sep;33(3):248-53. Epub 2005 May 18.

Interleukin-17 as a recruitment and survival factor for airway macrophages in allergic airway inflammation.

Sergejeva S, Ivanov S, Lötvall J, Lindén A.

The Lung Pharmacology Group, Department of Respiratory Medicine and Allergology, Institute of Internal Medicine, Sahlgrenska Academy at Göteborg University, Gothenburg, Sweden. Svetlana.Sergejeva@lungall.gu.se

Recent data indicate that the proinflammatory cytokine, interleukin (IL)-17, stimulates certain effector functions of human macrophages. We evaluated whether IL-17 mediates allergen-induced accumulation of airway macrophages and, if so, whether such an effect relates to the control of macrophage recruitment and survival. BALB/c mice were sensitized and challenged with ovalbumin. Three hours before challenge an anti-mouse IL-17 mAb (a-IL-17) was administered. Sampling was conducted 24 h after the allergen challenge. In vitro chemotaxis assay for blood monocytes and culture of airway macrophages, immunocytochemistry for Fas-antigen, and matrix metalloproteinase-9 (MMP-9) were used to determine the effect of IL-17 on the recruitment, survival, and activity of airway macrophages. A-IL-17 reduced the number of airway neutrophils and macrophages after allergen challenge. In vitro, recombinant IL-17 induced migration of blood monocytes and prolonged survival of airway macrophages. A-IL-17 also increased the expression of Fas-antigen in airway macrophages in vivo. Finally, the expression of MMP-9 by airway neutrophils and macrophages in vivo was downregulated by a-IL-17. This study indicates that endogenous IL-17 mediates the accumulation of macrophages during allergen-induced airway inflammation. IL-17 exerts its effects by acting directly on airway macrophages by promoting their recruitment and survival. Furthermore, IL-17 is involved in controlling the proteolytic activity of

macrophages and neutrophils in allergen-induced airway inflammation.

PMID: 15901616 [PubMed - indexed for MEDLINE]

1092. J Immigr Health. 2005 Jul;7(3):165-78.

Asthma prevalence and severity in Arab American communities in the Detroit area, Michigan.

Johnson M, Nriagu J, Hammad A, Savoie K, Jamil H.

Department of Environmental Health Sciences, School of Public Health, University of Michigan, Ann Arbor, Michigan, USA.

Immigrant populations provide a unique intersection of cultural and environmental risk factors implicated in asthma etiology. This study focuses on asthma prevalence and severity in 600 Arab American households in metro Detroit, the largest immigrant reception zone for Arab Americans in North America. The survey method introduced a number of novel features: (a) a ranking scheme for the key environmental risk factors for asthma was used to derive an aggregated environmental risk index (ERI) for each household, and (b) an aggregate measure of asthma severity based on symptom frequency and intensity. Environmental risk factors and surrogates for socioeconomic status (SES) were found to be stronger predictors of asthma prevalence than asthma severity, while demographic variables such as English fluency and birth in the United States were better predictors of asthma severity than asthma prevalence. These results suggest that SES variables may be more reflective of environmental exposures in communities involved in this study, while English fluency and birth in the United States may be linked to health care access and utilization behavior that can influence the asthma management. We also found a significant relationship between asthma prevalence and degree of acculturation. Asthma prevalence was highest among moderately acculturated immigrants compared with new immigrants and those who were well acculturated, suggesting that among Arab Americans in the Detroit area, risk factors associated with new immigrant status are replaced by "western" risk factors as the population becomes more acculturated.

PMID: 15900417 [PubMed - indexed for MEDLINE]

1093. Contact Dermatitis. 2005 May;52(5):268-72.

Potency and risk assessment of a skin-sensitizing disperse dye using the local lymph node assay.

Betts CJ, Dearman RJ, Kimber I, Maibach HI.

Syngenta Central Toxicology Laboratory, Alderley Park, Macclesfield, Cheshire SK1 4TJ, UK. catherine.betts@syngenta.com

Disperse Blue 106 is an acknowledged skin-sensitization hazard. However, information about the relative sensitization potency of this chemical is lacking, and to provide this information was the purpose of the investigations described here. The approach taken was to measure dose-response relationships for C.I. Disperse Blue in the local lymph node assay, a method for the assessment of skin-sensitization potential in which activity is measured as a function of lymphocyte proliferative responses induced in draining lymph nodes. From these data, it was possible to derive EC3 values (such being the estimated concentration of chemical required to elicit a 3-fold increase in proliferation) that have been shown previously to reflect the relative sensitizing potency of

contact allergens. These analyses revealed that Disperse Blue 106 had a relatively low EC3 value (0.01%), comparable to that measured concurrently for 2,4-dinitrochlorobenzene, a potent contact allergen. Collectively, these data reveal that Disperse Blue 106 represents a significant skin-sensitization hazard, and, in combination with information on dye migration and percutaneous penetration from various types of fabric and use conditions provide a basis for the development of effective and accurate risk assessments.

PMID: 15899000 [PubMed - indexed for MEDLINE]

1094. Blood Cells Mol Dis. 2005 Jul-Aug;35(1):11-6.

Phenotypic and functional characterization of intestinal epithelial exosomes.

Mallegol J, van Niel G, Heyman M.

INSERM EMI 0212, Faculté Necker-Enfants Malades, 156 rue de Vaugirard, 75730 Paris, France.

Intestinal epithelial cells (IEC) are located at a strategic position between the external environment and the most extended lymphoid tissue in the body. Besides their central role in the absorption of nutrients, IEC also provide antigenic information to the immune system and are involved in the balance tolerance/allergy to food antigens. Like professional antigen presenting cells, IEC have been shown to secrete 30- to 90-nm diameter vesicles named exosomes, in a polarized way, either from their apical or basolateral side. These vesicles carry molecules involved in adhesion and antigen presentation, comprising major histocompatibility complex (MHC) class I and class II molecules, tetraspan proteins, CD26/dipeptidyl-peptidase IV, and A33 antigen, a molecule essentially restricted to the intestinal epithelium. Invariant chain, transferrin receptor, and Na-K-ATPase are not expressed on epithelial exosomes. In vivo, in mice, epithelial exosomes carrying MHC/ovalbumin peptide complexes induce specific immune responses when injected intraperitoneally. A33 antigen, an Ig-like molecule highly specific for intestinal epithelial cells and enriched in epithelial exosomes, is found at the surface of cells entering mesenteric lymph nodes suggesting exosome migration from the epithelial layer to the gut associated lymphoid system. Taken together, intestinal epithelial exosomes released at the basolateral surface of enterocytes could be antigen-carrying structures constituting a link between luminal antigens and the local immune system and acting as sensors of the antigenic information present in the intestinal lumen.

PMID: 15893486 [PubMed - indexed for MEDLINE]

1095. Am J Respir Crit Care Med. 2005 May 15;171(10):1103-8. Epub 2005 Feb 4.

The CXCL10/CXCR3 axis mediates human lung mast cell migration to asthmatic airway smooth muscle.

Brightling CE, Ammit AJ, Kaur D, Black JL, Wardlaw AJ, Hughes JM, Bradding P.

Institute for Lung Health, Department of Infection, Inflammation and Immunity, Leicester-Warwick Medical School and University Hospitals of Leicester, Leicester, United Kingdom. ceb17@le.ac.uk

Mast cell microlocalization within the airway smooth muscle bundle is an important determinant of the asthmatic phenotype. We hypothesized that mast cells migrate toward airway smooth muscle in response to smooth muscle-derived

chemokines. In this study, we investigated (1) chemokine receptor expression by mast cells in the airway smooth muscle bundle in bronchial biopsies from subjects with asthma using immunohistology, (2) the concentration of chemokines in supernatants from stimulated ex vivo airway smooth muscle cells from subjects with and without asthma measured by enzyme-linked immunosorbent assay, and (3) mast cell migration toward these supernatants using chemotaxis assays. We found that CXCR3 was the most abundantly expressed chemokine receptor on human lung mast cells in the airway smooth muscle in asthma and was expressed by 100% of these mast cells compared with 47% of mast cells in the submucosa. Human lung mast cell migration was induced by airway smooth muscle cultures predominantly through activation of CXCR3. Most importantly, CXCL10 was expressed preferentially by asthmatic airway smooth muscle in bronchial biopsies and ex vivo cells compared with those from healthy control subjects. These results suggest that inhibition of the CXCL10/CXCR3 axis offers a novel target for the treatment of asthma.

PMID: 15879427 [PubMed - indexed for MEDLINE]

1096. J Invasive Cardiol. 2005 Mar;17(3):142-8.

Use of a tacrolimus-eluting stent to inhibit neointimal hyperplasia in a porcine coronary model.

Huang Y, Salu K, Wang L, Liu X, Li S, Lorenz G, Wnendt S, Verbeken E, Bosmans J, Van de Werf F, De Scheerder I.

Department of Cardiology, University of Leuven, U.Z. Gasthuisberg O/N, Herestraat 49, Leuven 3000, Belgium. yanming.huang@med.kuleuven.ac.be

In-stent restenosis remains an unresolved problem which occurs in 5-20% of patients undergoing coronary stenting within the first 3-6 months. Neointimal formation is the main contributor to in-stent restenosis. Stent-induced arterial injury and peri-strut inflammation are involved in the process of neointimal formation by activating cytokines and growth factors which induce smooth muscle cell dedifferentiation, migration, and proliferation. Histopathological studies found that neointimal hyperplasia is principally composed of smooth muscle cells, inflammatory cells, and extracellular matrix. Stent-based delivery of anti-proliferative and/or anti-inflammatory agents have shown beneficial effects on neointimal hyperplasia in experimental studies and clinical trials. Tacrolimus (FK506) is a water-insoluble macrolide immunosuppressant discovered in 1984. It has been widely used in reducing the incidence and severity of allograft rejection after organ transplantation. It has also been used to treat other inflammatory conditions such as atopic dermatitis. In this study, we evaluated the efficacy of stent-based delivery of tacrolimus on inflammation and neointimal formation in an overstretched coronary stent model.

PMID: 15867441 [PubMed - indexed for MEDLINE]

1097. J Leukoc Biol. 2005 May;77(5):800-10.

Phosphoinositide-3 kinases critically regulate the recruitment and survival of eosinophils in vivo: importance for the resolution of allergic inflammation.

Pinho V, Souza DG, Barsante MM, Hamer FP, De Freitas MS, Rossi AG, Teixeira MM.

Immunopharmacology, Departamento de Bioquímica e Imunologia, Universidade Federal de Minas Gerais, Belo Horizonte, Brazil.

The phosphatidylinositol-3 kinase (PI3K) family of signaling enzymes plays a crucial role in leukocyte recruitment and activation and hence, likely regulates the induction and propagation phases of inflammation. However, little data have emerged showing a role for these processes in the resolution phase in models of in vivo inflammation. Here, we have evaluated the role of PI3K for the migration and survival of eosinophils in a model of allergic pleurisy in mice. Eosinophil accumulation in PI3Kgamma-deficient mice was inhibited at 48 h, as compared with wild-type mice but not at earlier time-points (6 and 24 h). Experiments with adoptive transfer of bone marrow showed that PI3Kgamma in eosinophils but not in non-bone marrow-derived cells was required for their accumulation. Systemic treatment with PI3K inhibitors before antigen challenge prevented the recruitment of eosinophils. This was associated with decreased Akt phosphorylation, interleukin-5 production, and eosinophil release from the bone marrow. Treatment with PI3K inhibitors 24 h after antigen challenge markedly cleared the accumulated eosinophils, an effect associated with inhibition of Akt phosphorylation and an increased number of apoptotic events. Altogether, our data demonstrate an important role of PI3Kgamma for the maintenance of eosinophilic inflammation in vivo, whereas other isoforms of PI3K may be relevant for the recruitment process.

PMID: 15860799 [PubMed - indexed for MEDLINE]

1098. Biochem Biophys Res Commun. 2005 Jun 3;331(2):640-7.

Sphingosine 1-phosphate inhibits migration and RANTES production in human bronchial smooth muscle cells.

Kawata T, Ishizuka T, Tomura H, Hisada T, Dobashi K, Tsukagoshi H, Ishiwara M, Kurose H, Mori M, Okajima F.

Department of Medicine and Molecular Science, Gunma University Graduate School of Medicine, 3-39-15, Showa-machi, Maebashi 371-8511, Japan.

Sphingosine 1-phosphate (S1P), a bioactive lipid mediator, has been shown to be increased in bronchoalveolar lavage fluid after allergen challenge in asthmatic patients. Here, we examined S1P actions and their intracellular signalings in cultured human bronchial smooth muscle cells (BSMCs). Expression of mRNAs of three subtypes of S1P receptors, including S1P(1), S1P(2), and S1P(3), was detected in BSMCs, and exposure of the cells to S1P inhibited platelet-derived growth factor (PDGF)-induced migration and tumor necrosis factor-alpha-induced RANTES production. S1P also inhibited PDGF-induced Rac1 activation, and dominant negative Rac1 inhibited PDGF-induced migration. On the other hand, dominant negative Galpha(q) attenuated the S1P-induced inhibition of RANTES production. Finally, an S1P(2)-selective antagonist, JTE-013, suppressed the S1P-induced inhibition of migration response and RANTES production. These results suggest that S1P attenuates cell migration by inhibiting a Rac1-dependent signaling pathway and decreases RANTES production by stimulating a Galpha(q)-dependent mechanism both possibly through the S1P(2) receptors.

PMID: 15850807 [PubMed - indexed for MEDLINE]

1099. J Leukoc Biol. 2005 Jul;78(1):210-9. Epub 2005 Apr 21.

TNF-alpha promotes a stop signal that inhibits neutrophil polarization and migration via a p38 MAPK pathway.

Lokuta MA, Huttenlocher A.

Department of Pediatrics, University of Wisconsin, Madison, 53706, USA.
malokuta@facstaff.wisc.edu

Neutrophils are a major component of the inflammatory response in patients with asthma and other inflammatory conditions. Proinflammatory cytokines, such as tumor necrosis factor alpha (TNF-alpha), are increased in the airway of patients with severe asthma and have been implicated in the recruitment of neutrophils into areas of inflammation. Here, we show that TNF-alpha induces a stop signal that promotes firm neutrophil adhesion and inhibits neutrophil polarization and chemotaxis to chemoattractants including interleukin-8 and C5a. TNF-alpha treatment of neutrophils plated on a fibrinogen-coated surface promotes firm neutrophil adhesion and the formation of vinculin-containing focal complexes. TNF-alpha induces a more than tenfold increase in p38 mitogen-activated protein kinase (MAPK) but not extracellular signal-regulated kinase phosphorylation. Inhibition of p38 MAPK in neutrophils treated with TNF-alpha causes neutrophil polarization and motility. These findings suggest that TNF-alpha initiates a stop signal through a p38 MAPK pathway, which may promote the retention of neutrophils in inflammatory sites. Together, our data suggest that inhibition of p38 MAPK may be an attractive target to limit inflammatory responses that are mediated by TNF-alpha.

PMID: 15845648 [PubMed - indexed for MEDLINE]

1100. J Immunol. 2005 May 1;174(9):5758-65.

Endogenous attenuation of allergic lung inflammation by syndecan-1.

Xu J, Park PW, Kheradmand F, Corry DB.

Department of Medicine, Baylor College of Medicine, Houston, TX 77030, USA.

The airway plays a vital role in allergic lung diseases by responding to inhaled allergens and initiating allergic inflammation. Various proinflammatory functions of the airway epithelium have been identified, but, equally important, anti-inflammatory mechanisms must also exist. We show in this study that syndecan-1, the major heparan sulfate proteoglycan of epithelial cells, attenuates allergic lung inflammation. Our results show that syndecan-1-null mice instilled with allergens exhibit exaggerated airway hyperresponsiveness, glycoprotein hypersecretion, eosinophilia, and lung IL-4 responses. However, administration of purified syndecan-1 ectodomains, but not ectodomain core proteins devoid of heparan sulfate, significantly inhibits these inflammatory responses. Furthermore, syndecan-1 ectodomains are shed into the airway when wild-type mice are intranasally instilled with several biochemically distinct inducers of allergic lung inflammation. Our results also show that syndecan-1 ectodomains bind to the CC chemokines (CCL7, CCL11, and CCL17) implicated in allergic diseases, inhibit CC chemokine-mediated T cell migration, and suppress allergen-induced accumulation of Th2 cells in the lung through their heparan sulfate chains. Together, these findings uncover an endogenous anti-inflammatory mechanism of the airway epithelium where syndecan-1 ectodomains attenuate allergic lung inflammation via suppression of CC chemokine-mediated Th2 cell recruitment to the lung.

PMID: 15843578 [PubMed - indexed for MEDLINE]

1101. J Immunol. 2005 May 1;174(9):5224-32.

Histamine H4 receptor stimulation suppresses IL-12p70 production and mediates chemotaxis in human monocyte-derived dendritic cells.

Gutzmer R, Diestel C, Mommert S, Köther B, Stark H, Wittmann M, Werfel T.

Department of Dermatology and Allergology, Hannover Medical University, Hannover, Germany. rgutzmer@gmx.de

There is increasing evidence that histamine as an important mediator of immediate type allergic reactions also effects professional APCs. Recent reports showed effects of histamine on human monocyte-derived dendritic cells (MoDC) mediated primarily via histamine H1 receptors (H1R) and H2R. We show here that MoDC also express H3R and H4R at the mRNA and protein level. mRNA of the H3R is down-regulated and mRNA of the H4R is up-regulated during the differentiation from monocytes to MoDC. H4R or H2R stimulation suppressed IL-12p70 production in MoDC. Induction of cAMP was necessary for IL-12p70 inhibition mediated via the H2R. In contrast, H4R stimulation did not affect cAMP production but induced the transcription factor AP-1, and U0126, an inhibitor of AP-1 transactivation and MEK, rescued H4R mediated IL-12p70 suppression. Moreover, MoDC responded to a H4R agonist (and also to a H2R agonist) with increased F-actin polymerization and migration in modified Boyden chamber assays, suggesting a chemotactic effect of histamine via the H2R and the H4R. Thus, H4R stimulation on MoDC results in immunomodulatory and chemotactic effects. Histamine induces chemotaxis and IL-12p70 suppression via different receptors using different signaling pathways, which might be important for the pathogenesis of and therapeutic interventions in allergic diseases.

PMID: 15843518 [PubMed - indexed for MEDLINE]

1102. Med Hypotheses. 2005;64(6):1097-9.

Site specific therapy: an integrative approach to treating melanoma.

Kavekos M.

Department of Immunology, Center for Molecular Cell Biology, Florida Atlantic University, 777 Glades Road, P.O. Box 3091 Boca Raton, FL 33431-0991, USA. mannykav@netzero.com

There have been many proposed theories for effectively treating melanoma, especially through the regulation of histamine. Histamine has been proven to be a major regulator of the immune system's T-helper cell subset balance and major shifts in this balance towards TH2 cytokines have contributed to diseases such as asthma, lupus and cancer. Histamine also causes suppression of interferon-induced proteins needed for anti-tumor response and activates T-suppressor cell function in cancers such as squamous cell carcinoma and melanoma. Scientific evidence has suggested the possibility of an anthistamine approach as treatment to these diseases and for melanoma, there has been great promise. This is due to the fact that melanotic cells have been elucidated to express histamine receptors and as a result, regulation of histamine could occur specifically at the site of these epidermal growths. Another factor to consider is how effective an inflammatory response can be when combined with regulation of histamine. Inflammation is a very powerful tool against pathogenic environments by causing cytokine recruitment and migration of dendritic cells to infected sites. Adequate stimulation of an inflammatory response at the specific site of any cancerous region would greatly weaken its evasive mechanisms. However, there are no reports showing high efficacy utilizing the benefits of regulating inflammation and histamine that could cause TH1 subset levels to predominate, down-regulate T-suppressor cells, up-regulate interferon-induced proteins and properly sustain migration of dendritic cells concurrently. These benefits have been proven in separate instances for a range of diseases but have not been assessed as a

combined modality for melanoma therapy. Therefore successful melanoma treatment should integrate these principles involving: the use of H2 antagonists for preventing the negative effects of histamine, monoclonal antibodies to ensure an effective dendritic cell response, and routine pro-inflammatory induction at the specific site of the melanotic tissue to ensure recognition of the cancer that has evaded immunity.

PMID: 15823692 [PubMed - indexed for MEDLINE]

1103. Allergy. 2005 May;60(5):693-6.

Anti-IL-5 recombinant humanized monoclonal antibody (mepolizumab) for the treatment of atopic dermatitis.

Oldhoff JM, Darsow U, Werfel T, Katzer K, Wulf A, Laifaoui J, Hijnen DJ, Plötz S, Knol EF, Kapp A, Bruijnzeel-Koomen CA, Ring J, de Bruin-Weller MS.

Department of Dermatology and Allergology, University Medical Centre Utrecht, Utrecht, The Netherlands.

BACKGROUND: Eosinophils may play an important role in the pathogenesis of atopic dermatitis (AD). Interleukin-5 is essential for eosinophil growth, differentiation and migration. A monoclonal antibody to human interleukin-5 (mepolizumab) was developed for atopic diseases. This study was designed to study the effect of mepolizumab in AD.
METHODS: Two single doses of 750 mg mepolizumab, given 1 week apart, were studied in patients with moderate to severe AD using a randomized, placebo-controlled parallel group design. The primary endpoint of 'success' to treatment was defined as the percentage of patients with at least 'marked improvement' after 2 weeks as assessed by the Physician's Global Assessment of Improvement (PGA). Furthermore, SCORing AD (SCORAD), pruritus scoring, number of blood eosinophils and serum thymus and activation-regulated chemokine (TARC) values served as secondary endpoints. Fluticasone propionate cream 0.05%, once daily could be used as rescue medication from day 16 if no improvement was recorded.
RESULTS: Eighteen patients received mepolizumab and 22 placebo treatment. Peripheral blood eosinophil numbers were significantly reduced in the treatment group compared with placebo ($P < 0.05$). No clinical success was reached by PGA assessment ($P = 0.115$), SCORAD ($P = 0.293$), pruritus scoring and TARC values in the mepolizumab-treated group compared with placebo. However, modest improvement (<50% improvement) assessed by PGA was scored significantly more in the mepolizumab-treated group compared with placebo ($P < 0.05$).
CONCLUSION: Two single doses of 750 mg mepolizumab did not result in clinical success in patients with AD, despite a significant decrease in peripheral blood eosinophils.

PMID: 15813818 [PubMed - indexed for MEDLINE]

1104. Int J Dermatol. 2005 Mar;44(3):203-5.

Pathergy in atypical eosinophilic pustular folliculitis.

Hsu PJ, Huang CJ, Wu MT.

Department of Dermatology, China Medical College Hospita, Taichung, Taiwan. rickspri@ms62.hinet.net

A 15-year-old, unmarried female presented to our dermatology department for an intensely pruritic skin rash that had appeared abruptly 3 days earlier. She had a

remarkable medical history for a case of allergic rhinitis and several attacks of asthma in her early childhood. The condition waxed and waned initially but had improved in recent years. Physical examination revealed several erythematous plaques, papules studded with scattered pustules having diameters less than 0.3 mm. Conspicuous scratch marks had caused erythematous wheal-like indurations also studded with pustules in a linear distribution across the waist, forearms (Fig. 1), and back (Fig. 2). Discrete papulopustules were present on the face, nape and neck. The patient was otherwise healthy. There were no other symptoms such as fever, malaise, weakness, or lymphadenopathy Laboratory results were normal for hepatic and renal functions, serum electrolytes, glucose, protein, erythrocyte sedimentation rate (8 mm/h), and C-reactive protein (0.355 mg/l). A human immunodeficiency virus (HIV) antibody screen test was negative. Serum was positive for herpes simplex virus (HSV)-1 and HSV-2 IgG (in low titers), but negative for HSV-1 and HSV-2 IgM. White blood cell count revealed leukocytosis (11.2 x 10(3)/l), with a differential count of 68% neutrophils, 27% lymphocytes, and 8% eosinophils. Serum IgA, IgG, and IgM were within normal limits, but the IgE level was elevated (677 mg/dl). Cultures from peripheral blood and pustules were negative. A Tzank smear performed on the pustules showed no multinucleated giant cells. Fungal testing of skin scrapings from the initial lesion site gave negative results. Routine stool tests, including common pathogen and parasite screens, were negative, and urinalysis results were unremarkable. A biopsy specimen obtained from a skin pustule showed subcorneal eosinophilic and neutrophilic pustules in the follicular infundibulum with marked spongiosis of the follicular epithelium. (Fig. 3). There was a moderately dense superficial and deep perivascular mixed inflammatory cell infiltrate comprising eosinophils, neutrophils and lymphocytes. Migration of eosinophils and neutrophils through the vessel wall with variable luminal intramural fibrin deposition, histologically indicative of vasculopathy, was seen. There was concomitant slight perivascular dermal necrosis. (Fig. 4). Based on the clinical presentation and light microscopic findings on biopsy, a diagnosis of eosinophilic pustular folliculitis with pathergy was made. Systemic prednisolone 30 mg in divided doses was given. After 1 week of systemic corticosteroid therapy, the patient's condition was significantly improved and the patient was subsequently discharged. Two months later she had a relapse, upon which corticosteroid therapy was commenced leading to lesional resolution. The foci of eosinophilic folliculitis healed with areas of hyperpigmentation with variable scarring.

PMID: 15807726 [PubMed - indexed for MEDLINE]

1105. J Allergy Clin Immunol. 2005 Apr;115(4):771-8.

Asthmatic bronchial epithelium activated by the proteolytic allergen Der p 1 increases selective dendritic cell recruitment.

Pichavant M, Charbonnier AS, Taront S, Brichet A, Wallaert B, Pestel J, Tonnel AB, Gosset P.

INSERM U416, IFR 17, Institut Pasteur, 1 rue du Professeur Calmette, BP 245, 59019 Lille Cedex, France.

BACKGROUND: Airway dendritic cells (DCs) are crucial for allergen-induced sensitization and inflammation in allergic asthma. After allergen challenge, an increased number of DCs is observed in airway epithelium from patients with allergy.
OBJECTIVE: Because Der p 1, a cysteine protease allergen from Dermatophagoides pteronyssinus , induces chemokine production by bronchial epithelial cells (BECs), the purpose of this investigation was to evaluate the capacity of BEC exposed to Der p 1 to recruit DCs.
METHODS: Chemotactic activity of BEAS-2B, a bronchial epithelial cell line, and

BECs from nonatopic controls and patients with allergic asthma was evaluated on the migration of precursors, immature and mature monocyte-derived DCs (MDDCs), and CD34 + -derived Langerhans cells (LCs).
RESULTS: C-C chemokine ligand (CCL)-2, CCL5, and C-X-C chemokine ligand 10 production by BEAS-2B and BEC was increased after Der p 1 exposure, whereas the proenzyme proDer p 1 devoid of enzymatic activity had no effect. Der p 1 stimulation of BEAS-2B and BEC from both groups increased significantly the recruitment of MDDC precursors, depending on CCL2, CCL5, and C-X-C chemokine ligand 10 production. In a reconstituted polarized epithelium, apical application of Der p 1 enhanced MDDC precursor migration into the epithelial layer. Moreover, Der p 1 stimulation of BEC from patients with asthma but not from controls increased the migration of LC precursors, mainly dependent on CCL20 secretion. No migration of immature and mature DCs was observed.
CONCLUSION: These data confirmed that BECs participate in the homeostasis of the DC network present within the bronchial epithelium through the secretion of chemokines. In allergic asthma, upregulation of CCL20 production induced LC recruitment, the role of which remains to be determined.

PMID: 15805997 [PubMed - indexed for MEDLINE]

1106. J Pharmacol Exp Ther. 2005 Jul;314(1):244-51. Epub 2005 Mar 29.

Inhibitory effect of the 4-aminotetrahydroquinoline derivatives, selective chemoattractant receptor-homologous molecule expressed on T helper 2 cell antagonists, on eosinophil migration induced by prostaglandin D2.

Mimura H, Ikemura T, Kotera O, Sawada M, Tashiro S, Fuse E, Ueno K, Manabe H, Ohshima E, Karasawa A, Miyaji H.

Pharmaceutical Research Center, Kyowa Hakko Kogyo Co., Ltd., 1188 Shimotogari, Nagaizumi-cho, Sunto-gun, Shizuoka 411-8731, Japan. hideki.mimura@kyowa.co.jp

Prostaglandin (PG) D2, a major cyclooxygenase metabolite generated from immunologically stimulated mast cells, is known to induce activation and chemotaxis in eosinophils, basophils, and T helper 2 (Th2) lymphocytes via a newly identified PGD2 receptor, chemoattractant receptor-homologous molecule expressed on Th2 cells (CRTH2). CRTH2 is hypothesized to play an important role in the outcome of allergic responses. However, the absence of selective CRTH2 antagonists has prevented the elucidation of the role of CRTH2 in pathogenesis of allergic diseases. We now report compounds discovered as selective CRTH2 antagonists,
(2R*,4S*)-N-(1-benzoyl-2-methyl-1,2,3,4-tetrahydroquinolin-4-yl)-N-phenylisobutyramide (K117) and
(2R*,4S*)-N-(1-benzoyl-2-methyl-1,2,3,4-tetrahydroquinolin-4-yl)-N-phenylcyclopropanecarboxamide (K604). K117 and K604 have inhibitory effects on human CRTH2 with Ki values of 5.5 and 11 nM, respectively. The effect of these compounds is CRTH2-specific with no cross-reactivity against 15 other receptors and four arachidonic acid-metabolizing enzymes. K117 and K604 has no effect on the basal Ca2+ level and inhibited the Ca2+ response induced by PGD2 in 293EBNA cells expressing human CRTH2. Also, K117 and K604 inhibit PGD2-induced human eosinophil chemotaxis with IC50 values of 7.8 and 42.2 nM, respectively, but they do not inhibit the CC-chemokine receptor 3 agonist eotaxin-induced chemotaxis. These results indicate that K117 and K604 are highly potent and selective antagonists for human CRTH2. These compounds have possibilities to become useful tools to explore CRTH2 functions in allergic diseases.

PMID: 15798001 [PubMed - indexed for MEDLINE]

1107. Invest Ophthalmol Vis Sci. 2005 Apr;46(4):1364-70.

Urokinase plasminogen activator, uPa receptor, and its inhibitor in vernal keratoconjunctivitis.

Leonardi A, Brun P, Sartori MT, Cortivo R, Dedominicis C, Saggiorato G, Abatangelo G, Secchi AG.

Department of Neuroscience, Ophthalmology Unit, University of Padua, Padua, Italy. andrea.leonardi@unipd.it

PURPOSE: Plasminogen activators play a role, not only in fibrinolysis but also in events such as chemotaxis, collagen degradation, and cell spreading. The serine protease urokinase (uPA) is a potent chemoattractant for leukocytes that may be involved in the pathogenesis of severe forms of allergic conjunctivitis such as vernal keratoconjunctivitis (VKC).
METHODS: Tear and peripheral blood samples were obtained from 20 patients with active VKC and from 19 normal subjects who formed the control group. Levels of plasminogen activity, uPA, tissue plasminogen activator (tPA), and their inhibitor, plasminogen activator inhibitor type-1 (PAI-1) were measured in tears and plasma of patients with VKC. The presence of tPA, uPA, and urokinase receptor (uPAR) in conjunctival tissues were evaluated by immunohistochemistry. uPA, uPAR, and PAI-1 expression and production were measured in conjunctival epithelial cell and fibroblast cultures treated with cytokines.
RESULTS: Tear levels of uPA and tPA and tear plasminogen activity levels were significantly greater in patients with VKC than in control subjects. Increased staining for uPA and uPAR was found in VKC tissues compared with normal conjunctiva. Both conjunctival epithelial cells and fibroblasts demonstrated an increased expression of uPAR after exposure to IL-4 or -13, whereas uPA was highly expressed by epithelial cells exposed to IL-4. PAI-1 levels in culture medium were increased in IL-4-exposed epithelial cells compared to nonstimulated cells and were decreased in fibroblast culture.
CONCLUSIONS: Increased expression of fibrinolytic system components and imbalance between plasminogen activators and PAI may be involved in the pathogenesis of severe allergic conjunctivitis, thus contributing to inflammatory cell migration and tissue remodeling.

PMID: 15790903 [PubMed - indexed for MEDLINE]

1108. Br J Dermatol. 2005 Mar;152(3):409-17.

The immunomodulatory effects of regulatory T cells: implications for immune regulation in the skin.

Birch KE, Vukmanovic-Stejic M, Reed JR, Akbar AN, Rustin MH.

Department of Dermatology, Royal Free Hospital, Pond Street, London NW3 2QG, UK. Kebirch@aol.com

Regulatory T cells are thought to have a critical role in the suppression of immune responses. In addition to the prevention of the development of autoimmunity, they are also thought to have a role in the prevention of allergic responses to environmental allergens, immune responses to tumours and the development of memory responses to chronic infections. They have been isolated within the skin and have been shown to express surface markers that enable skin-specific migration, suggesting that regulatory T cells have a functional role in the skin. There is accumulating evidence to suggest that regulatory T cells may be involved in numerous skin disorders and may also be modified by various therapeutic agents used to treat these disorders. We review the evidence

for the presence of this T-cell subset in humans, the suppressive effects of regulatory T cells, and their role in the skin.

PMID: 15787808 [PubMed - indexed for MEDLINE]

1109. Clin Exp Allergy. 2005 Mar;35(3):382-7.

Percutaneous application of peptidoglycan from Staphylococcus aureus induces an increase in mast cell numbers in the dermis of mice.

Matsui K, Nishikawa A.

Department of Immunobiology, Meiji Pharmaceutical University, Tokyo 204-8588, Japan. kmatsui@my-pharm.ac.jp

BACKGROUND: Atopic dermatitis (AD) is a chronic inflammatory skin disease with immunopathologic features that vary depending on the duration of the lesion. The dermis of lesional skin of AD patients shows an increased number of inflammatory cells such as mast cells, eosinophils and mononuclear cells and superficial Staphylococcus aureus colonization.
OBJECTIVE: The purpose of this study was to determine the effects of peptidoglycan (PEG) from S. aureus on mast cell induction in murine skin.
METHODS: PEG was applied to barrier-disrupted abdominal skin of mice every 5 days and the number of mast cells in the abdominal skin was counted 20 days after the first application. The cytokine response was investigated by RT-PCR and immunohistologic analysis.
RESULTS: The number of mast cells in the skin of mice treated with PEG was increased significantly compared with that of mice given phosphate-buffered saline. In addition, application of PEG to the abdominal skin increased the expression of mRNA for transforming growth factor-beta(1) (TGF-beta(1)), which supports mast cell migration, but not that for IL-3 or stem cell factor, which support both mast cell proliferation and mast cell migration. Immunohistologic analysis demonstrated that levels of TGF-beta(1) transcripts corresponded with those of protein synthesis in the epidermis. TGF-beta(1) was found to be highly expressed in keratinocytes of the basal epidermis of PEG-treated skin. Furthermore, intraperitoneal injection of anti-TGF-beta(1) antibodies neutralized the induction of mast cells into the skin.
CONCLUSION: These results suggest that PEG may have the ability to induce an increase in mast cell numbers in the skin through TGF-beta(1) production by epidermal keratinocytes. Skin inflammation might therefore be linked to colonization with S. aureus in AD patients.

PMID: 15784119 [PubMed - indexed for MEDLINE]

1110. J Leukoc Biol. 2005 Jun;77(6):993-8. Epub 2005 Mar 17.

WASP deficiency leads to global defects of directed leukocyte migration in vitro and in vivo.

Snapper SB, Meelu P, Nguyen D, Stockton BM, Bozza P, Alt FW, Rosen FS, von Andrian UH, Klein C.

Gastrointestinal Unit and the Center for Inflammatory Bowel Diseases, Massachusetts General Hospital, Boston, 02114, USA. ssnapper@partners.org

Intact cellular migration is critically important for the induction and regulation of the immune response. The Wiskott-Aldrich syndrome protein (WASP) regulates surface receptor signaling to the actin cytoskeleton in hematopoietic

cells and thus plays a pivotal role in cellular locomotion. WASP deficiency causes the Wiskott-Aldrich syndrome (WAS), characterized by immunodeficiency, thrombocytopenia, and eczema. Cell migration defects may contribute to the pathophysiology of WAS. In this study, we used a variety of in vitro and in vivo assays to comprehensively analyze migration properties of lymphocytes, dendritic cells (DC), and neutrophils from WASP-deficient mice. We provide evidence that WASP-deficient lymphocytes show a marked reduction in tethering in an in vitro flow chamber assay as well as decreased migration of T cells in response to the CC chemokine ligand 19 (CCL19). In vivo, compared with wild-type lymphocytes, WASP-deficient lymphocytes showed significantly impaired homing to Peyer's patches upon adoptive transfer into recipient mice. In addition, bone marrow-derived DC migrated less efficiently in response to CCL19. In vivo studies showed decreased migration of DC from skin to draining lymph nodes in WASP-deficient animals. Finally, we also document decreased neutrophil migration in vitro and in vivo. In summary, our studies suggest that WASP plays an important role in the locomotion of lymphocytes, DC, and granulocytes in vitro and in vivo and thus, reveal a crucial role of WASP in physiological trafficking of various hematopoietic cell lineages. These results further delineate immunological abnormalities in WASP-deficient mice, which will be useful to assess preclinical gene therapy studies.

PMID: 15774550 [PubMed - indexed for MEDLINE]

1111. Genes Immun. 2005 May;6(3):225-30.

Association of interleukin-8 receptor alpha polymorphisms with chronic obstructive pulmonary disease and asthma.

Stemmler S, Arinir U, Klein W, Rohde G, Hoffjan S, Wirkus N, Reinitz-Rademacher K, Bufe A, Schultze-Werninghaus G, Epplen JT.

Department of Human Genetics, Ruhr-University, Bochum, Germany. susanne.stemmler@rub.de

Chronic obstructive pulmonary disease (COPD) and asthma are common complex diseases characterized by airflow obstruction and inflammatory processes in the small airways. lnterleukin 8 (IL-8) is a potent proinflammatory cytokine which interacts with the IL-8 receptor alpha (IL8RA, CXCR1) and beta (IL8RB, CXCR2), leading to activation and migration of leukocytes. In order to evaluate the role of the IL8RA gene in the pathogenesis of COPD and asthma, we screened the coding region of IL8RA for mutations by means of single-strand conformation polymorphism analysis in 50 COPD patients and identified three exchanges (M31R, S276T and R335C). These three polymorphisms were subsequently genotyped in 182 adult patients with COPD, 68 adult patients and 130 children with asthma as well as 454 healthy controls. The frequencies of the IL8RA 31R and 335C alleles were significantly increased in patients with COPD and in children with asthma compared to healthy controls (P=0.0073 and 0.023, respectively). Thus, these polymorphisms may play a role in the pathogenesis of COPD and asthma.

PMID: 15772681 [PubMed - indexed for MEDLINE]

1112. Inj Control Saf Promot. 2004 Sep;11(3):153-7.

The magnitude of child injuries in Bangladesh: a major child health problem.

Rahman F, Rahman A, Linnan M, Giersing M, Shafinaz S.

Institute of Child and Mother Health, Matuail, Dhaka-1362, Bangladesh.

fazlur@citechco.net

In recent times, many developing countries including Bangladesh not only have to cope with infectious diseases and malnutrition but also with new health problems, such as asthma, cancer and accidents. The emergence of chronic diseases and injuries has not been seen as an important health issue to date. The work presented here has the objectives of conceptualizing the dynamic changes in child mortality within the framework of the health transition, to provide a basis for projection of future mortality and disability in children in Bangladesh. This paper reviews a number of reports and published articles related to the causes of child deaths in Bangladesh. These include: 1) Year books of Bangladesh Bureau of Statistics; 2) UNICEF reports; 3) Reports of International Centre for Diarrhoeal Disease and Research, Bangladesh; and 4) Reports of Institute of Child and Mother Health. Bangladesh clearly has been progressing along its epidemiological transition. At the current stage, chronic diseases and injuries have overtaken infectious diseases as leading causes of child death. Injury has been identified as a major cause of child death in Bangladesh, and is emerging as the leading cause of child mortality, similar to what is occurring in other developing countries. For these countries, in the advancing stages of their health transition, more research aimed at understanding the dynamic change of child health priorities is urgently needed for appropriate policy and planning.

PMID: 15764101 [PubMed - indexed for MEDLINE]

1113. Pneumonol Alergol Pol. 2004;72(1-2):14-8.

[Antieosinophil action of IL-12 in human polyp culture].

[Article in Polish]

Fal AM, Rabczyński J, Gerber A, Mikulewicz W, Barg W, Obojski A.

Kliniki Chorób Wewnetrznych i Alergologii, AM we Wrocławiu.

RATIONALE: Tissue eosinophilia is an important pathophysiological issue in bronchial asthma and nasal polyps. Its magnitude is regulated by several mechanisms, including selective migration of eosinophils to peripheral tissues and prolongation of survival. A key role in this processes is assigned to Th2 originating cytokines: IL-3, IL-5, GM-CSF. It has been previously demonstrated that IL-12 diminishes tissue eosinophilia in an animal model, and Hofstra showed that IL-12 together with IL-18 prevents allergen-induced increase bronchial hyperresponsiveness, BAL eosinophilia and the development of allergen-specific Th2 cells.
METHODS: Nasal polyps were obtained during routine surgery and were cultured in fragments of approximately 30 mg for 2, 6, and 15 days in RPMI 1640 in the absence or presence of IL-12. Afterwards a dose-dependency was tested at day 2 of culture. Polyp tissue from cultures was than processed to slides, stained with Giemsa and cells were counted in light microscopy (400x).
RESULTS: Eosinophils represented 62.8+/-21.3% of residing cells in nasal polyps at the day 0. IL-12 (1 microg/ml) caused a significant time-dependent decrease in the percentage of Eos after 2 and 6 days. The effect of IL-12 at day 2 was concentration-dependent: control, 28.2+/-2.9; at 10 ng/ml, 13.9+/-6.4 (n=4, p<0.05); at 100 ng/ml, 11.6+/-2.1 (p<0.01); at 1 microg/ml, 7.5+/-1.5 (p<0.005).
CONCLUSION: IL-12 acts as potent topical antieosinophilic agent. Its action can be seen in a cultured polyp environment. It is visible already after two days and is concentration-dependent. Further study is needed to elucidate tissue mechanisms of this action.

PMID: 15757285 [PubMed - indexed for MEDLINE]

1114. J Allergy Clin Immunol. 2005 Mar;115(3):501-7.

Progenitor egress from the bone marrow after allergen challenge: role of stromal cell-derived factor 1alpha and eotaxin.

Dorman SC, Babirad I, Post J, Watson RM, Foley R, Jones GL, O'Byrne PM, Sehmi R.

Asthma Research Group, Firestone Institute for Respiratory Health, St Joseph's Healthcare, and Department of Medicine, McMaster University, Hamilton, Ontario, Canada.

BACKGROUND: CCR3 expression on CD34+ cells mediates migration to eotaxin in vitro. CXCR4 and stromal cell-derived factor (SDF)-1alpha are important for stem cell homing to hemopoietic compartments.
OBJECTIVE: To study chemokine-mediated progenitor cell traffic in allergic inflammation.
METHODS: Bone marrow (BM) aspirates were obtained at baseline from normal subjects; atopic subjects without asthma; and subjects with asthma before, 5 hours after, and 24 hours after allergen inhalation (dual and early responders). Changes in chemokine receptor expression and migration were assessed.
RESULTS: Expression of CXCR4, but not CCR3, on BM CD34+ cells was greater in normal subjects compared with atopic subjects with asthma. Likewise, SDF-1alpha, but not eotaxin, stimulated a greater migrational response by BM CD34+ cells from normal subjects compared with subjects with asthma. For all subjects, a positive correlation was found between intensity of CXCR4 expression and magnitude of CD34+ cell response to SDF-1alpha. Allergen inhalation attenuated both intensity of CXCR4 expression and SDF-1alpha levels in marrow from dual compared with early responders 24 hours postallergen. In contrast, the intensity of CCR3 expression on BM CD34+ cells increased in dual compared with early responders at 24 hours postallergen. In addition, an increase in migrational responsiveness of BM CD34+ cells to eotaxin and a decrease to SDF-1alpha 24 hours postallergen was found in dual responder subjects with asthma.
CONCLUSION: After allergen inhalation in subjects with asthma, a downregulation in CXCR4 intensity on BM CD34+ cells and a reduction in BM SDF-1alpha levels may reduce progenitor retention to marrow stroma promoting peripheral egress, possibly mediated by the CCR3/eotaxin axis.

PMID: 15753896 [PubMed - indexed for MEDLINE]

1115. J Immunol. 2005 Mar 15;174(6):3709-18.

Unconjugated bilirubin inhibits VCAM-1-mediated transendothelial leukocyte migration.

Keshavan P, Deem TL, Schwemberger SJ, Babcock GF, Cook-Mills JM, Zucker SD.

Division of Digestive Diseases, University of Cincinnati, Cincinnati, OH 45267, USA. keshavpa@ucmail.uc.edu

During lymphocyte migration, engagement of VCAM-1 stimulates the generation of endothelial cell-derived reactive oxygen species (ROS) and activation of matrix metalloproteinases, facilitating endothelial retraction. Because bilirubin is a potent antioxidant, we examined the hypothesis that this bile pigment inhibits VCAM-1-dependent cellular events. The migration of isolated murine splenic lymphocytes across monolayers of murine endothelial cell lines (which constitutively express VCAM-1) is significantly inhibited by physiological concentrations of bilirubin, in the absence of an effect on lymphocyte adhesion.

Bilirubin administration also suppresses VCAM-1-stimulated ROS generation and reduces endothelial cell matrix metalloproteinase activity. In a murine asthma model characterized by VCAM-1-dependent airway inflammation, treatment of C57BL6/J mice with i.p. bilirubin decreases the total leukocyte count in the lung parenchyma and lavage fluid, through specific inhibition of eosinophil and lymphocyte infiltration. Blood eosinophil counts were increased in bilirubin-treated animals, while VCAM-1 expression in the capillary endothelium and cytokine levels in both lung lavage and supernatants from cultured lymph node lymphocytes were unchanged, suggesting that bilirubin inhibits leukocyte migration. Conclusion: bilirubin blocks VCAM-1-dependent lymphocyte migration in vitro and ameliorates VCAM-1-mediated airway inflammation in vivo, apparently through the suppression of cellular ROS production. These findings support a potential role for bilirubin as an endogenous immunomodulatory agent.

PMID: 15749910 [PubMed - indexed for MEDLINE]

1116. J Immunol. 2005 Mar 15;174(6):3626-32.

Stem cell factor has a suppressive activity to IgE-mediated chemotaxis of mast cells.

Sawada J, Shimizu S, Tamatani T, Kanegasaki S, Saito H, Tanaka A, Kambe N, Nakahata T, Matsuda H.

Laboratory of Molecular Pathology and Therapeutics, Division of Animal Life Science, Graduate School, Institute of Symbiotic Science and Technology, Tokyo University of Agriculture and Technology, Tokyo, Japan.

Stem cell factor (SCF), which is well known as a cytokine capable of amplifying development and functions of mast cells, is mainly released from fibroblasts in the peripheral tissue. To investigate whether SCF controlled chemotactic migration of mast cells induced by IgE-specific Ag, murine bone marrow-derived cultured mast cells (BMCMC) and human cord blood-derived cultured mast cells (HuCMC) were preincubated with SCF. Although BMCMC and HuCMC sensitized with IgE directly moved toward specific Ag, preincubation for even 1 h with an optimal dose of SCF suppressed the IgE-mediated chemotactic movement. No or little inhibitory effect of SCF was detected in BMCMC derived from c-kit receptor-defect WBB6F1-W/Wv mice. In contrast, preincubation of BMCMC and HuCMC with SCF enhanced beta-hexosaminidase release and Ca2+ mobilization in response to Ag after sensitization with IgE. Using the real-time record of chemotactic migration, BMCMC preincubated with SCF manifested motionless without degranulation. These results suggest that locally produced SCF may have an inhibitory effect on chemotaxis of mast cells, contributing to their accumulation and enhancement of functions at the peripheral site in allergic and nonallergic conditions.

PMID: 15749900 [PubMed - indexed for MEDLINE]

1117. FASEB J. 2005 Mar;19(3):428-30. Epub 2004 Dec 20.

Src is necessary and sufficient for human airway smooth muscle cell proliferation and migration.

Krymskaya VP, Goncharova EA, Ammit AJ, Lim PN, Goncharov DA, Eszterhas A, Panettieri RA Jr.

Pulmonary, Allergy and Critical Care Division, Department of Medicine, University of Pennsylvania, 421 Curie Blvd., BRB II/III, Philadelphia, PA 19104-6160, USA. krymskay@mail.med.upenn.edu

Airway smooth muscle (ASM) hypertrophy and hyperplasia, important pathological features in chronic severe asthma, likely contribute to irreversible airflow obstruction. Despite considerable research effort, the precise cellular mechanisms that modulate ASM growth remain unknown. Src, a nonreceptor tyrosine kinase proto-oncogene, reportedly modulates cell proliferative responses to growth factors, contractile agonists, and inflammatory mediators. Here, we show that Src activation is required for human ASM mitogenesis and motility. Platelet-derived growth factor (PDGF), epidermal growth factor (EGF), and thrombin induce rapid activation of Src, and inhibition of Src induces a concentration-dependent abrogation of PDGF-, EGF-, and thrombin-induced ASM cell proliferation. Src immunoprecipitates had associated phosphatidylinositol 3-kinase, or PI3K, activation in response to PDGF and thrombin but not EGF. Further, Src activation is both necessary and sufficient for the stimulation of DNA synthesis as demonstrated by dominant negative Src inhibition of PDGF-, EGF-, and thrombin-induced DNA synthesis. Human ASM cell migration was also attenuated by transfection of cells with dominant negative Src. Further, expression of constitutively active Src promoted cell migration. Collectively, these data demonstrate that Src modulates human ASM cell proliferation and migration, suggesting that Src may play an important role in promoting ASM cell growth and migration that occur in airway remodeling found in asthma and chronic obstructive pulmonary disease, or COPD.

PMID: 15746183 [PubMed - indexed for MEDLINE]

1118. Blood. 2005 Jun 15;105(12):4736-42. Epub 2005 Mar 1.

Expression of SphK1 impairs degranulation and motility of RBL-2H3 mast cells by desensitizing S1P receptors.

Jolly PS, Bektas M, Watterson KR, Sankala H, Payne SG, Milstien S, Spiegel S.

Department of Biochemistry, Virginia Commonwealth University Medical Center, Richmond, VA 23298-0614, USA.

Mast cells play a central role in inflammatory and immediate-type allergic reactions by secreting a variety of biologically active substances, including sphingosine-1 phosphate (S1P). Sphingosine kinase 1 (SphK1) and formation of S1P, which leads to transactivation of S1P receptors and their downstream signaling pathways, regulates mast-cell functions initiated by cross-linking of the high-affinity immunoglobulin E (IgE) receptor FcepsilonRI. Surprisingly, overexpression of SphK1 in rat basophilic leukemia (RBL)-2H3 mast cells impaired degranulation as well as migration toward antigen. These effects were reversed by serum withdrawal, yet the increased formation and secretion of S1P were the same as in the presence of serum. Nonetheless, serum increased localization of SphK1 at the plasma membrane. This restricted formation of S1P induced internalization and desensitization of S1P receptors on the surface of mast cells as determined by confocal immunofluorescence microscopy, aberrant S1P receptor signaling, and lack of S1P receptor coupling to G proteins. Serum starvation, which significantly reduced membrane-associated SphK1 activity, restored S1P receptor functions. Our results have important implications for mast-cell migration and degranulation as well as for the biologic functions of the S1P receptors on cells that are circulating in the bloodstream.

PMCID: PMC1894993
PMID: 15741218 [PubMed - indexed for MEDLINE]

1119. Eur J Immunol. 2005 Apr;35(4):1301-10.

The carboxyl terminus of the chemokine receptor CCR3 contains distinct domains which regulate chemotactic signaling and receptor down-regulation in a ligand-dependent manner.

Sabroe I, Jorritsma A, Stubbs VE, Xanthou G, Jopling LA, Ponath PD, Williams TJ, Murphy PM, Pease JE.

Leukocyte Biology Section, Biomedical Sciences Division, Imperial College London, UK.

The chemokine receptor CCR3 regulates the chemotaxis of leukocytes implicated in allergic disease, such as eosinophils. Incubation of eosinophils with CCL11, CCL13 or CCL5 resulted in a rapid decrease of cell-surface CCR3 which was replicated using CCR3 transfectants. Progressive truncation of the CCR3 C terminus by 15 amino acids produced three constructs, Delta340, Delta325 and Delta310. Delta340 and Delta325 were able to bind CCL11 with affinities similar to wild-type CCR3. Delta340 transfectants exhibited enhanced migration and reduced receptor down-regulation in response to CCL11 and CCL13. Delta325 transfectants displayed chemotactic responses to CCL11 and CCL13 similar to wild-type CCR3, and had impaired down-regulation when stimulated with CCL13 but not CCL11. In contrast, neither the Delta325 nor Delta340 truncation affected chemotaxis or receptor down-regulation induced by CCL5. Delta310 transfectants bound CCL11 poorly and were biologically inactive. Inhibitors of p38 mitogen-activated protein kinase and PI3-kinase antagonized eosinophil shape change responses and chemotaxis of transfectants to CCL11 and CCL13. In contrast, shape change but not chemotaxis was sensitive to inhibition of the extracellular signal-regulated kinase kinase pathway suggesting differential regulation of the two responses. Thus, the CCR3 C terminus contains distinct domains responsible for the regulation of receptor desensitization and for coupling to chemotactic responses.

PMID: 15739168 [PubMed - indexed for MEDLINE]

1120. Epidemiol Prev. 2004 Jul-Oct;28(4-5):258-64.

[Breathing difficulties and asthma prevalence in children from zero to five years of age in five Rom settlements].

[Article in Italian]

Monasta L.

Centro de Investigación de enfermedades tropicales (CIET), Universidad autónoma del Guerrero, Acapulco, México. lmonasta@ciet.org

OBJECTIVE: To study the relation between living conditions in temporary Rom settlements and asthma prevalence in children.
DESIGN: Purposive cluster (Rom settlements) sampling.
SETTING: Five settlements of Rom from Macedonia and Kosovo located in five cities in northern and central Italy. These settlements were chosen to represent the different types of existing living arrangements.
PARTICIPANTS: 137 Rom families residing in the selected settlements. In total 737 people were covered, of which 167 were children from zero to five years of age.
MAIN OUTCOME MEASURES: Respiratory wheezing, breathing difficulties during lifetime and number of episodes in the last 12 months. Asthma diagnosis and period prevalence.
RESULTS: The comparison with the data of the SIDRIA study for children aged 6-7 confirms the reliability of the results we are presenting. However, the study

reveals a higher incidence of asthma symptoms in Rom children which could lead to a worse asthma prognosis in later years. Moreover, the prevalence of asthma and the incidence of asthma symptoms in the last year vary significantly according to the settlement of origin and the state of the housing in which the child lives.
CONCLUSION: The health of Rom children who live in settlements is put under great strain by the poor state of repair of the majority of these structures. It is therefore necessary to overcome the "emergency" approach and the "temporary" nature of the measures taken so far to deal with the issue of Rom from Eastern Europe, and plan serious reception and integration policies.

PMID: 15732680 [PubMed - indexed for MEDLINE]

1121. Vet Clin Pathol. 2005;34(1):17-22.

Frequency and severity of tracheal wash hemosiderosis and association with underlying disease in 96 cats: 2002-2003.

DeHeer HL, McManus P.

Department of Pathobiology, University of Pennsylvania, School of Veterinary Medicine, Philadelphia, PA, USA. deheer@vet.upenn.edu

BACKGROUND: Hemosiderophages may be found in feline tracheal wash specimens in association with various disease conditions, including heart failure, trauma, infection, foreign body migration, lung lobe torsion, pulmonary embolism or infarction, neoplasia, and bleeding diathesis. Based on observations in our laboratory, we hypothesized that tracheal wash specimens from cats with asthma also frequently contain hemosiderophages, even in the absence of other known causes of pulmonary hemorrhage.
OBJECTIVES: The purpose of this study was to determine the frequency and severity of hemosiderosis in tracheal wash fluid from cats with various diseases, including asthma.
METHODS: Feline tracheal wash fluid specimens submitted for cytologic evaluation between March 2002 and August 2003 were included in the study. One hundred and one specimens from 96 cats were examined with both Wright's-Giemsa and Prussian blue stains. Cats were assigned to 6 disease categories: feline asthma, pneumonia, pulmonary neoplasia, rhinitis, heart disease, and other disorders. Based on the percentage of Prussian blue-positive macrophages, hemosiderosis was categorized as negative (0%), mild (<20%), moderate (21-50%), or marked (>50%).
RESULTS: The frequency of tracheal wash hemosiderosis in the study population was 63.5% (61/96); hemosiderosis was mild (29/96, 30.2%), moderate (22/96, 22.9%), or marked (10/96, 10.4%). Hemosiderosis was found in 85.7% (6/7) of cats with rhinitis, 78.6% (11/14) of cats with pulmonary neoplasia, 75.0% (27/36) of cats with asthma, 71.4% (5/7) of cats with primary or concurrent heart disease, 25.0% (5/20) of cats with pneumonia, and 66.7% (12/18) of cats with other disorders. In cats with asthma, hemosiderosis was usually mild to moderate and frequently was accompanied by increased eosinophils.
CONCLUSIONS: The results of this study confirm that hemosiderosis is a common finding in tracheal wash specimens collected from cats with diverse disease conditions, including feline asthma syndrome.

PMID: 15732012 [PubMed - indexed for MEDLINE]

1122. Allergy. 2005 Apr;60(4):538-9.

Hypothesis: urbanization and the allergy epidemic--a reverse case of immunotherapy?

Linneberg A.

Research Centre for Prevention and Health, Glostrup, Denmark.
linneberg@dadlnet.dk

PMID: 15727592 [PubMed - indexed for MEDLINE]

1123. Int Arch Allergy Immunol. 2005 Mar;136(3):295-302. Epub 2005 Feb 16.

Interferon-gamma inhibits in vitro mobilization of eosinophils by interleukin-5.

Park CS, Choi EN, Kim JS, Choi YS, Rhim TY, Chang HS, Chung IY.

Genome Research Center for Asthma and Allergic Diseases, Division of Allergy and Respiratory Medicine, Soonchunhyang University Hospital, Seoul and Bucheon, Korea.

BACKGROUND: Th2 cytokines play pivotal roles in allergic inflammation, including eosinophilia, and their actions are antagonized by Th1 cytokines, conferring them therapeutic potential.
METHODS: In this study, we examined the ability of a number of cytokines to suppress the activation of eosinophils that function as effector cells for allergic airway diseases.
RESULTS: Interleukin (IL)-5, IL-6, and tumor necrosis factor (TNF) induced an eosinophil shape change, whereas interferon (IFN)-gamma significantly inhibited the shape change. Other cytokines, including IL-1beta, IL-4, IL-10 and IL-13, had little or only slightly enhancing or reducing effects on the shape change. We further analyzed the IFN-gamma effect, showing that pretreatment with IFN-gamma strongly suppressed IL-5-induced eosinophil shape change, and cycloheximide (CHX) abrogated the suppression by IFN-gamma, suggesting that new protein synthesis is required for the inhibitory effect by this cytokine. In agreement with these results, IFN-gamma blocked the eosinophil migration and ERK phophorylation induced by IL-5, and the addition of CHX restored eosinophil chemotaxis.
CONCLUSIONS: Collectively, IFN-gamma may attenuate eosinophilic inflammation by directly negating eosinophil mobilization.

Copyright (c) 2005 S. Karger AG, Basel.

PMID: 15722640 [PubMed - indexed for MEDLINE]

1124. Int Arch Allergy Immunol. 2005 Feb;136(2):198-204.

Atopy and asthma in migrants.

Rottem M, Szyper-Kravitz M, Shoenfeld Y.

Division of Allergy, Asthma and Immunology, Ha'Emek Medical Center, Afula, Israel. menachem@rottem.net

Atopy and asthma result from the effects of environmental factors on genetically susceptible persons, and different prevalence rates have been documented worldwide. In developed and industrialized countries a higher prevalence of atopy and asthma is observed as compared with undeveloped and less affluent countries. Migration involves exposure to a new set of pollutants and allergens. In addition, it involves several socioeconomic and cultural issues such as housing conditions, diet and accessibility to medical services, all of which are likely to affect migrants' health. Migration studies provide information on the role of environmental factors in the development of atopy and asthma. Immigration to

allergy-prevalent countries causes more allergies and asthma in immigrants as compared to the prevalence of atopy in their countries of origin. The increase in allergy and asthma is usually not related to ethnicity, but in certain populations may play an important role. Studies on migrants support the notion that lifestyle and environmental factors in western industrialized countries facilitate atopy and asthma. The effect is time-dependent. Acquiring allergy is influenced by the age at the time of immigration. Migrants, in general, are more prone to the development of allergies than the local population. Low hygiene prior to immigration does not seem to protect against the development of atopy or asthma. Vaccinations do not affect the development of atopy or asthma in the general population and in migrants. Migrants should be aware of the potential of developing allergies and/or asthma. Strategies for primary prevention in high-risk atopic individuals and secondary prevention guidelines should be developed both for populations in developing countries as well as for immigrants from such countries to atopy-prevalent developed countries.

Copyright (c) 2005 S. Karger AG, Basel.

PMID: 15711097 [PubMed - indexed for MEDLINE]

1125. Rev Pneumol Clin. 2004 Nov;60(5 Pt 1):255-64.

[Etiological work-up for bronchectasia in adults].

[Article in French]

Lioté H.

Service de Pneumologie, Hôpital Tenon, 4 rue de la Chine, 75020 Paris.
huguette.liote@tnn.ap-hop-paris.fr

Search for an etiology of bronchiectasis consists in identifying constitutional or acquired defense mechanisms of the respiratory mucosa. The question is timely because causes change. In developing countries, presumed sequelae of infection account for about 30% of the cases despite vaccination campaigns, control of endemic tuberculosis, and widespread use of antibiotics. Genetic diseases account for 20% of the causes when identified by high-performance prospective diagnostic tests (CFTR mutation). Computed tomography enables the identification of frequent associations between bronchiectasis and rheumatoid disease or ulcerative colitis. Recent diseases such as HIV infection or GVHD can also lead to bronchiectasis. Nevertheless, the cause remains unknown in 30-50% of patients. After a detailed analysis of the clinical presentation and diagnostic criteria specific for each etiology, we propose a two-phase diagnostic procedure. The first step, used for all patients (careful history taking, physical examination, imaging, bronchofibroscopy, limited blood tests) enables detecting localized bronchial obstacles and obvious etiologies (situs inversus of primary ciliary dyskinesia, known systemic disease, HIV...). If the first step is negative, the second phase is oriented by the clinical context. Sequelae of infection (tuberculosis...) in older subjects or migrants, a genetic cause in younger subjects, particularly if there is a familial history and/or infertility, a systemic disease or allergic bronchopulmonary aspergillosis if there is an extra-respiratory context. This etiological search should help improve patient management and provide a better prognosis and prevention of bronchiectasis.

PMID: 15687908 [PubMed - indexed for MEDLINE]

1126. Curr Allergy Asthma Rep. 2005 Mar;5(2):155-60.

New chemokine targets for asthma therapy.

Garcia G, Godot V, Humbert M.

Service de Pneumologie et Réanimation respiratoire, Hôpital Antoine Béclère, 157 rue de la Porte de Trivaux, 92140 Clamart, France.

Chemokines and chemokine receptors are part of a complex network of molecules that play a key role in leukocyte migration and activation. The chemokine family role is crucial in the immune system, orchestrating innate and acquired immune responses, but also in allergic inflammation. A subset of chemokines, including CCL11, CCL24, CCL26, CCL7, CCL13, CCL17, and CCL22 is highly expressed by the three main cell types involved in allergic inflammation: eosinophils, basophils, and Th2 lymphocytes. In vitro and in vivo experimental studies in murine models of asthma as well as evidence from patients with asthma confirm the role of these chemokines and their receptors, including CCR3, CCR4, and CCR8, establishing a subset of chemokine/chemokine receptor that is potentially important in allergic inflammation. Recent data support the concept that interfering with chemokines or chemokine receptors represents a new approach in allergy therapy. However, even if some of them have been shown to be effective in animal models, none is as yet used in human patients.

PMID: 15683617 [PubMed - indexed for MEDLINE]

1127. Wien Klin Wochenschr. 2004;116 Suppl 4:7-18.

[Nosology and epidemiology of human toxocarosis--the recent situation in Austria].

[Article in German]

Auer H, Aspöck H.

Abteilung für Medizinische Parasitologie, Klinisches Institut für Hygiene und Medizinische Mikrobiologie, Medizinische Universität Wien, Wien, Osterreich. herbert.auer@meduniwien.ac.at

Toxocara canis and T. cati are not only ubiquitously distributed parasites of dogs, foxes and cats, but may also infest humans, causing a great variety of symptoms and sometimes also severe diseases: the visceral larva migrans syndrome, the ocular larva migrans syndrome, covert toxocarosis, common toxocarosis, neurological toxocarosis, and some other clinical pictures (asthma bronchiale, epilepsy, rheumatoid arthritis) are considered to be induced by Toxocara species. Both Toxocara species are also widely distributed in Austrian dog, fox and cat populations; seroepidemiological studies carried out in Austria revealed seroprevalence rates of 3.7% among the normal human population and up to 44% among persons particularly exposed to those parasites (i.e. veterinarians, farmers). Although many Toxocara infestations do not cause severe clinical manifestations, a few dozens of toxocarosis patients have been registered every year during the last years; in reality, however, we have to assume that several hundreds of patients suffer from toxocarosis. This paper tries to give a synoptic overview of the nosology of this (still) largely almost unknown helminthozoonosis, moreover it summarizes the most important epidemiologic parameters, and presents the diagnostic and therapeutic possibilities available today.

PMID: 15683037 [PubMed - indexed for MEDLINE]

1128. J Leukoc Biol. 2005 May;77(5):759-66. Epub 2005 Jan 26.

Differential expression of CCR3 and CXCR3 by human lung and bone marrow-derived mast cells: implications for tissue mast cell migration.

Brightling CE, Kaur D, Berger P, Morgan AJ, Wardlaw AJ, Bradding P.

Institute for Lung Health, Department of Infection, Immunity and Inflammation, Leicester-Warwick Medical School and University Hospitals of Leicester, Groby Road, Leicester, LE3 9QP, UK. ceb17@le.ac.uk

The selective microlocalization of mast cells within specific airway structures, such as the airway smooth muscle and submucosal glands, in asthma is important in the pathophysiology of inflammatory lung disease. Chemokines are likely candidates mediating mast cell migration into these tissue compartments. In this study, we have defined the chemokine receptor profile of human lung mast cells (HLMC) compared with mast cells derived from human bone marrow (BM) and the human mast cell line HMC-1. CXC chemokine receptor 3 (CXCR3) was the most highly expressed chemokine receptor on ex vivo HLMC analyzed by flow cytometry, and CXCR3 expression by mast cells in the bronchial mucosa was confirmed by immuno-histochemistry. CXCR3 was functional, inducing a rise in cytosolic-free Ca^{2+}, actin reorganization, and chemotaxis in response to the CXC ligands CXCL9, -10, and -11. CXCR3 activation did not induce degranulation or cytokine synthesis. In addition, more than 10% of ex vivo HLMC expressed CC chemokine receptor 3, CXCR1, and CXCR4. It is interesting that CXCR3 was not expressed by human BM-derived mast cells, suggesting its expression is induced during tissue maturation. As CXCR3 ligands are elevated in many pulmonary diseases, CXCR3 may be important for determining the anatomical microlocalization of mast cells within the human lung.

PMID: 15673545 [PubMed - indexed for MEDLINE]

1129. Regul Pept. 2005 Mar 30;126(3):195-201.

Effects of the neuropeptide secretoneurin on natural killer cell migration and cytokine release.

Feistritzer C, Mosheimer BA, Colleselli D, Wiedermann CJ, Kähler CM.

Intensive Care Unit, Clinical Division of General Internal Medicine, Department of Internal Medicine, Innsbruck Medical University, Anichstrasse 35, 6020 Innsbruck, Austria.

Secretoneurin has a widespread occurrence in airway mucosal innervation of patients with allergic diseases and may play an important role in the local traffic of immune cells in human airway mucosa. Whether secretoneurin affects natural killer cell migration and cytokine release in vitro was tested. Natural killer cells were obtained from venous blood of healthy donors. Cell migration was studied by micropore filter assays. Signalling mechanisms required for secretoneurin-dependent migration were tested using signalling enzyme blockers. Cytokine release was measured in natural killer cell supernatants by ELISA. Secretoneurin significantly stimulated natural killer cell chemotaxis via activation of phosphatidylinositol 3'-kinase and protein kinase C. IL-2 stimulated natural killer cells showed a stronger response toward secretoneurin than unstimulated cells. Moreover, secretoneurin increased the release of interleukin-5 in a dose-dependent manner but did not affect Th1 cytokine release by natural killer cells. Data suggest that secretoneurin stimulates directed migration of natural killer cells and may modulate Th1/Th2-response via affecting chemokine release. Thus, secretoneurin may play an important role in the early

stages of allergic inflammation.

PMID: 15664667 [PubMed - indexed for MEDLINE]

1130. Clin Exp Allergy. 2004 Dec;34(12):1890-8.

Suppressive activity of fexofenadine hydrochloride on metalloproteinase production from nasal fibroblasts in vitro.

Asano K, Kanai KI, Suzaki H.

Department of Physiology, School of Medicine, Showa University, Tokyo 142-8555, Japan. asanok@med.showa-u.ac.jp

BACKGROUND: Allergic rhinitis (AR) is an inflammatory disease characterized by nasal wall remodelling with intense infiltration of eosinophils and mast cells/basophils. Matrix metalloproteinases (MMPs), MMP-2 and MMP-9, are the major proteolytic enzymes that induce airway remodelling. These enzymes are also important in the migration of inflammatory cells through basement membrane components.
OBJECTIVE: We evaluated whether fexofenadine hydrochloride (FEX), the carboxylic acid metabolite of terfenadine with selective H(1)-receptor antagonist activity, could inhibit MMP production from nasal fibroblasts (NFs) in response to TNF-alpha stimulation in vitro.
METHODS: NFs were established from nasal polyp-derived fibroblasts (PFs) taken from patients with AR. Nasal mucosal fibroblasts (MFs) were also induced from nasal mucosal tissues from septal deformity patients without allergy. PF and MF (2 x 10(5) cells/mL, each) were stimulated with TNF-alpha in the presence of various concentrations of FEX. After 24 h, culture supernatants were obtained and assayed for MMP-2, MMP-9, tissue inhibitor of metalloproteinase (TIMP)-1 and TIMP-2 levels by ELISA. The influence of FEX on mRNA expression of MMPs and TIMPs in 4 h-cultured cells was also evaluated by real-time RT-PCR. Furthermore, nuclear factor-kappa B (NF-kappa B) activation in fibroblasts treated with FEX for 4 h was examined by ELISA.
RESULTS: FEX at more than 350 ng/mL inhibited the production of MMP-2 and MMP-9 from both PF and MF in response to TNF-alpha stimulation, whereas TIMP-1 and TIMP-2 production was scarcely affected by FEX. FEX also inhibited MMP mRNA expression and NF-kappa B activation in PF and MF after TNF-alpha stimulation.
CONCLUSION: The present data suggest that the attenuating effect of FEX on MMP-2 and -9 production from NFs induced by inflammatory stimulation may underlie the therapeutic mode of action of the agent on allergic diseases, including AR.

PMID: 15663564 [PubMed - indexed for MEDLINE]

1131. Clin Exp Allergy. 2004 Dec;34(12):1868-75.

Identification of anti-inflammatory drugs according to their capacity to suppress type-1 and type-2 T cell profiles.

Moed H, Stoof TJ, Boorsma DM, von Blomberg BM, Gibbs S, Bruynzeel DP, Scheper RJ, Rustemeyer T.

Departments of Dermatology and Pathology, VU University Medical Center Amsterdam, 1007 MB Amsterdam, The Netherlands.

BACKGROUND: Down-regulation or modulation of T cell activity by immunosuppressive drugs is an effective treatment in diseases where exaggerated T cell responses play a role. A primary effect of the anti-inflammatory drugs (AIDs) is inhibition

of the synthesis of growth factors, such as IL-2, thereby down-regulating T cell proliferation. However, it is still largely unknown to what extent these AIDs are able to down-regulate specifically type-1 or type-2 T cell cytokine production, and whether they can down-modulate chemokine receptor expression, thereby preventing migration of T cells to the site of inflammation.
OBJECTIVE: We investigated the suppressive effect of dermatologically used AID (cyclosporin A (CsA), lactoferrin (LF), 1 alpha, 25-dihydroxyvitamin D(3) (VD(3)), hydrocortisone (HC), di-methyl-fumarate (DMF), diclofenac (DF)) on both type-1 and type-2 T cells. Since allergic contact dermatitis is a skin disorder in which an exaggerated T cell response of both types of T cell subsets can be observed, we used this disorder as a model to study the capacity of AID to suppress type-1 or type-2 T cell responses.
METHODS: Peripheral blood mononuclear cells of nickel allergic patients were cultured in the presence of allergen and increasing concentrations of AID. Proliferation was determined by measuring (3)H thymidine incorporation; chemokine receptor (CCR10, CCR4, CXCR3) expression was studied by flow cytometric analysis and IFN-gamma or IL-5 cytokine production was measured by ELISA.
RESULTS: Three major patterns can be distinguished regarding the effect of AID on T cell responses. The first group, including CsA and LF, inhibited non-selectively T cell proliferation, chemokine receptor expression and cytokine production, with CsA as the most potent drug tested. A second group of AID, which included VD(3), HC and DMF, suppressed mainly type-1 T cell responses, as revealed by strong interference with IFN-gamma production and CXCR3 expression, and limited effects on either or both IL-5 and CCR4 expression. The third pattern was displayed by DF, which down-regulated IL-5 production and CCR4 expression, whereas IFN-gamma and CXCR3 were unaltered.
CONCLUSIONS: Using a contact allergy model, we have demonstrated that various AIDs show distinct pharmacological profiles in that either type-1 or type-2 or both T cell responses are suppressed. These results should contribute to a more rational selection of AID in treating inflammatory skin diseases mediated by either or both of these T cell subsets.

PMID: 15663561 [PubMed - indexed for MEDLINE]

1132. Clin Exp Allergy. 2004 Dec;34(12):1834-40.

Differential capacity of CD8+ alpha or CD8- alpha dendritic cell subsets to prime for eosinophilic airway inflammation in the T-helper type 2-prone milieu of the lung.

Hammad H, de Vries VC, Maldonado-Lopez R, Moser M, Maliszewski C, Hoogsteden HC, Lambrecht BN.

Department of Pulmonary Medicine, Erasmus Medical Center, 3015 GE Rotterdam, The Netherlands.

BACKGROUND: Different subsets of dendritic cells (DCs), identified in mouse spleen by their differential expression of CD8 alpha, can induce different T-helper (Th) responses after systemic administration. CD8 alpha(-) DCs have been shown to preferentially induce Th type 2 (Th2) responses whereas CD8 alpha(+) DCs induce Th1 responses.
OBJECTIVE: To study if these DC subsets can still induce different Th responses in the Th2-prone milieu of the lung and differentially prime for eosinophilic airway inflammation, typical of asthma.
METHODS: Donor mice first received daily Flt3L injections to expand DC numbers. Purified CD8 alpha(+) or CD8 alpha(-) splenic DCs were pulsed with ovalbumin (OVA) or phosphate-buffered saline and injected intratracheally into recipient mice in which carboxyfluorescein diacetate succinimidyl ester-labelled OVA-specific T cell receptor transgenic T cells had been injected intravenously 2

days earlier. T cell proliferation and cytokine production of Ag-specific T cells were evaluated in the mediastinal lymph nodes (MLNs) 4 days later. The capacity of both subsets of DCs, to prime for eosinophilic airway inflammation was determined by challenging the mice with OVA aerosol 10 days later.
RESULTS: CD8 alpha(-) DCs migrated to the MLN and induced a vigorous proliferative T cell response accompanied by high-level production of IL-4, IL-5, IL-10 and also IFN-gamma during the primary response and during challenge with aerosol, leading to eosinophilic airway inflammation. In the absence of migration to the MLN, CD8 alpha(+) DCs still induced a proliferative response with identical levels of IFN-gamma but reduced Th2 cytokines compared with CD8 alpha(-) DCs, which led to weak eosinophilic airway inflammation upon OVA aerosol challenge. Unpulsed DCs did not induce proliferation or cytokine production in Ag-specific T cells.
CONCLUSION: CD8 alpha(-) DCs are superior compared with CD8 alpha(+) DCs in inducing Th2 responses and eosinophilic airway inflammation in the Th2-prone environment of the lung.

PMID: 15663556 [PubMed - indexed for MEDLINE]

1133. J Immunol. 2005 Feb 1;174(3):1723-8.

CCL18 is expressed in atopic dermatitis and mediates skin homing of human memory T cells.

Günther C, Bello-Fernandez C, Kopp T, Kund J, Carballido-Perrig N, Hinteregger S, Fassl S, Schwärzler C, Lametschwandtner G, Stingl G, Biedermann T, Carballido JM.

Novartis Institutes for Biomedical Research, Vienna, Austria.

CCL18 is a human chemokine secreted by monocytes and dendritic cells. The receptor for CCL18 is not yet known and the functions of this chemokine on immune cells are not fully elucidated. In this study, we describe that CCL18 is present in skin biopsies of atopic dermatitis (AD) patients but not in normal or psoriatic skin. CCL18 was specifically expressed by APCs in the dermis and by Langerhans and inflammatory dendritic epidermal cells in the epidermis. In addition, the serum levels of CCL18 and the percentages of CCL18-producing monocyte/macrophages and dendritic cells were significantly increased in AD patients compared with healthy controls. Furthermore, we demonstrate that CCL18 binds to CLA(+) T cells in peripheral blood of AD patients and healthy individuals and induces migration of AD-derived memory T cells in vitro and in human skin-transplanted SCID mice. These findings highlight a unique role of CCL18 in AD and reveal a novel function of this chemokine mediating skin homing of a subpopulation of human memory T cells.

PMID: 15661937 [PubMed - indexed for MEDLINE]

1134. Clin Exp Immunol. 2005 Feb;139(2):189-201.

Correlation of disease evolution with progressive inflammatory cell activation and migration in the IL-4 transgenic mouse model of atopic dermatitis.

Chen L, Martinez O, Venkataramani P, Lin SX, Prabhakar BS, Chan LS.

Department of Dermatology, University of Illinois at Chicago, College of Medicine, Chicago, IL 60612, USA.

Atopic dermatitis is a chronic inflammatory skin disease characterized by inflammatory cell infiltration in the skin. In order to assess the roles of

inflammatory cells in this disease, we analysed the activation status and surface markers of various leucocytes in the IL-4 transgenic mouse model of atopic dermatitis, by flow cytometry, immuofluorescence microscopy, and T cell proliferation assays. The studies were performed with a nontransgenic mouse control and transgenic mice at three disease stages: before disease onset, early skin disease, and late skin disease, so that we can delineate the immunological sequence of events. As the skin disease evolves, the skin draining lymph node cells from IL-4-Tg mice show a spontaneous proliferation and a progressively enhanced proliferative response to stimulants including anti-CD3, Con A, PHA, and Staphylococcus enterotoxins A and B. As the disease evolves, the percent of lymphoid organ T cells expressing activation molecules (CD44 and CD69) and costimulatory molecules (ICOS and PD-1) are progressively increased; the percent and total number of T cells are reduced in an incremental manner in the secondary lymphoid organs while the number of T cells infiltrating the skin increases in an incremental fashion; the total number of dendritic antigen presenting cells, macrophages, and NK cells gradually increases in the lymphoid organs. Collectively, our results suggest that there is a continued and progressive migration of activated inflammatory cells from the secondary lymphoid organs into the skin where they participate in immune responses resulting in the pathology associated with inflammation.

PMCID: PMC1809278
PMID: 15654817 [PubMed - indexed for MEDLINE]

1135. Clin Exp Allergy. 2005 Jan;35(1):26-33.

Chemokine-receptor expression on T cells in lung compartments of challenged asthmatic patients.

Kallinich T, Schmidt S, Hamelmann E, Fischer A, Qin S, Luttmann W, Virchow JC, Kroczek RA.

Department of Pediatric Pneumology and Immunology, Charité Campus Virchow Klinikum, Humboldt University, Berlin, Germany. tilmann.kallinich@charite.de

Comment in
    Clin Exp Allergy. 2005 Jan;35(1):4-7.

BACKGROUND: The interaction of chemokines with their receptors strongly influences the migration of leucocytes.
OBJECTIVE: In order to assess the contribution of these molecules to the local recruitment of T cells in bronchial asthma, we analysed the expression of 14 chemokine receptors on lung-derived T cells.
METHODS: Chemokine-receptor expression by T cells derived from the peripheral blood, the bronchoalveolar lavage fluid and the bronchial mucosa was analysed by flow cytometry and immunohistochemistry. Expression profiles in healthy and mildly asthmatic individuals were compared, the latter prior and after segmental allergen provocation.
RESULTS: Compared with peripheral blood, alveolar T cells expressed significantly more CCR2, CCR5, CCR6, CXCR3 and CCR4. However, no differences were observed between healthy controls and unchallenged asthmatics. In patients developing significant inflammatory responses following specific allergen challenge, a marked increase in the percentage of CCR4+ and CCR7+, and reduced numbers of CXCR3-bearing alveolar T cells were detected. Following specific allergen challenge, chemokine-receptor expression profiles of T cells from the alveolar space and the mucosa or the submucosa were similar, excluding a particular subcompartmentalization of the chemokine/chemokine-receptor system.
CONCLUSION: The expression of certain chemokine receptors by lung T cells suggests a contribution to the physiological recruitment of T cells to the lungs,

both in healthy controls and unchallenged mild asthmatics. However, strong allergen-induced airway responses were associated with a specific chemokine-receptor profile, suggesting the involvement of certain chemokine receptors in the pathogenesis of allergic bronchial inflammation.

PMID: 15649262 [PubMed - indexed for MEDLINE]

1136. Blood. 2005 Apr 15;105(8):3222-9. Epub 2005 Jan 6.

IgE- and IgE+Ag-mediated mast cell migration in an autocrine/paracrine fashion.

Kitaura J, Kinoshita T, Matsumoto M, Chung S, Kawakami Y, Leitges M, Wu D, Lowell CA, Kawakami T.

Division of Cell Biology, La Jolla Institute for Allergy and Immunology, 10 355 Science Center Dr, San Diego, CA 92121, USA.

Mast cells are the major effector cells for immediate hypersensitivity and chronic allergic reactions. These cells accumulate in mucosal tissues of allergic reactions, where immunoglobulin E (IgE) is produced locally. Here we provide evidence that, in addition to antigen that can attract IgE-bound mast cells, the type of IgE molecules that efficiently activate mast cells can promote the migration of mast cells in the absence of antigen. IgE- and IgE+Ag-mediated migration involves an autocrine/paracrine secretion of soluble factors including adenosine, leukotriene B4, and several chemokines. Their secretion depends on 2 tyrosine kinases, Lyn and Syk, and they are agonists of G-protein-coupled receptors and signal through phosphatidylinositol 3-kinase gamma, leading to mast cell migration. In mouse experiments, naive mast cells are attracted to IgE, and IgE-sensitized mast cells are attracted to antigen. Therefore, IgE and antigen are implicated in mast cell accumulation at allergic tissue sites with local high IgE levels.

PMCID: PMC1464406
PMID: 15637135 [PubMed - indexed for MEDLINE]

1137. J Thromb Haemost. 2005 Jan;3(1):35-45.

Pleiotropic functions of plasminogen activator inhibitor-1.

Lijnen HR.

Center for Molecular and Vascular Biology, KU, Leuven, Belgium.
roger.lijnen@med.kuleuven.ac.be

Plasminogen activator inhibitor-1 (PAI-1), a 45-kDa serine proteinase inhibitor with reactive site peptide bond Arg345-Met346, is the main physiological plasminogen activator inhibitor. It occurs in human plasma at an antigen concentration of about 20 ng mL(-1). Besides the active inhibitory form of PAI-1 that spontaneously converts to a latent form, also a substrate form exists that is cleaved at the P1-P1' site by its target enzymes, but does not form stable complexes. Besides its role in regulating hemostasis, PAI-1 plays a role in several biological processes dependent on plasminogen activator or plasmin activity. Studies with transgenic mice have revealed a functional role for PAI-1 in wound healing, atherosclerosis, metabolic disturbances such as obesity and insulin resistance, tumor angiogenesis, chronic stress, bone remodeling, asthma, rheumatoid arthritis, fibrosis, glomerulonephritis and sepsis. It is not always clear if these functions depend on the antiproteolytic activity of PAI-1, on its binding to vitronectin or on its intereference with cellular migration or matrix

binding.

PMID: 15634264 [PubMed - indexed for MEDLINE]

1138. Eur Ann Allergy Clin Immunol. 2004 Nov;36(9):337-41.

[Ambrosia pollen under high surveillance].

[Article in French]

Thibaudon M, Lachasse C.

RNSA, Chemin des Gardes, BP 8, 69610 Saint-Genis-l'Argentière.

The pollinic season of ambrosia in 2003 present strong disturbances due to the meteorology of the end of this summer. The areas with the allergenic risk are concentrated on the area of Rhône-Alpes (more than 40 days with a risk equal to or higher than 3) and on the peripheral areas: the area Centre Auvergne with Saint Etienne, Clermont-Ferrand and Montlucon with approximately 10 days with a the allergenic risk equal to or higher than 3; the Saône-Savoie area with the towns of Macon and Grenoble with about twenty days with a allergenic risk equal to or higher than 3 and Dijon, Châlon-sur-Saône with 6 days with a allergic risk equal to or higher than 3; the Mediterranean area with the towns of Avignon and Aix-in-Provence presenting ten days with allergenic risk equal to or higher than 3, Marseille and Toulon with 7 days with a allergenic risk equal to or higher than 3. The allergic risk related to the ambrosia in 2003 had peaks overall less high, but the season is spread out and had during until September 20. The daily peaks for the Rhône-Dauphiné-Drôme area were noted between 6 h and 8 h or 8 h and 10 h, for 2003. On the peripheral areas, we remark a diversity of the daily peaks (Châlon-sur-Saône between 18 h and 20 h). Are these pollens local grains or immigrants grains? On Lyon I (Gerland), the follow-up of the data of the ambrosia since 1987 permit to remark a stagnation of the number of days with an allergic risk, despite a reduction of the number of pollens.

PMID: 15633371 [PubMed - indexed for MEDLINE]

1139. Ont Health Technol Assess Ser. 2005;5(6):1-57. Epub 2005 Apr 1.

Osteogenic protein-1 for long bone nonunion: an evidence-based analysis.

Health Quality Ontario.

OBJECTIVE: To assess the efficacy of osteogenic protein-1 (OP-1) for long bone nonunion. CLINICAL NEED: Although most fractures heal within a normal period, about 5% to 10% do not heal and are classified as delayed or nonunion fractures. Nonunion and segmental bone loss after fracture, reconstructive surgery, or lesion excision can present complex orthopedic problems, and the multiple surgical procedures often needed are associated with patient morbidity and reduced quality of life. Many factors contribute to the pathogenesis of a delayed union or nonunion fractures, including deficiencies of calcium, vitamin D, or vitamin C, and side effects of medications such as anticoagulants, steroids, some anti-inflammatory drugs, and radiation. It has been shown that smoking interferes with bone repair in several ways. INCIDENCE OF NONUNION AND DELAYED UNION CASES: An estimated 5% to 10% of fractures do not heal properly and go on to delayed union or nonunion. If this overall estimate of incidence were applied to the Ontario population, the estimated number of delayed union or nonunion in the province would be between 3,863 and 7,725. TREATMENT OF NONUNION CASES: The treatment of nonunion cases is a challenge to orthopedic surgeons. However, the

basic principle behind treatment is to provide both mechanical and biological support to the nonunion site. Fracture stabilization and immobilization is frequently used with the other treatment modalities that provide biological support to the fractured bone. Biological support includes materials that could be served as a source of osteogenic cells (osteogenesis), a stimulator of mesenchymal cells (osteoinduction), or a scaffold-like structure (osteoconduction). The capacity to heal a fracture is a latent potential of the stromal stem cells, which synthesize new bone. This process has been defined as osteogenesis. Activation of the stem cells to initiate osteogenic response and to differentiate into bone-forming osteoblasts is called osteoinduction. These 2 properties accelerate the rate of fracture healing or reactivate the ineffective healing process. Osteoconduction occurs when passive structures facilitate the migration of osteoprogenitor cells, the perivascular tissue, and capillaries into these structures. BONE GRAFTS AND BONE GRAFT SUBSTITUTES: Bone graft and bone graft substitutes have one or more of the following components: Undifferentiated stem cellsGrowth factorsStructural latticeUndifferentiated stem cells are unspecialized, multipotential cells that can differentiate into a variety of specialized cells. They can also replicate themselves. The role of stem cells is to maintain and repair the tissue in which they are residing. A single stem cell can generate all cell types of that tissue. Bone marrow is a source of at least 2 kinds of stem cells. Hematopoietic stem cells that form all types of blood cells, and bone marrow stromal stem cells that have osteogenic properties and can generate bone, cartilage, and fibrous tissue. Bone marrow has been used to stimulate bone formation in bone defects and cases of nonunion fractures. Bone marrow can be aspirated from the iliac crest and injected percutaneously with fluoroscopic guidance into the site of the nonunion fracture. The effectiveness of this technique depends on the number and activity of stem cells in the aspirated bone marrow. It may be possible to increase the proliferation and speed differentiation of stem cells by exposing them to growth factor or by combining them with collagen. Many growth factors and cytokines induced in response to injury are believed to have a considerable role in the process of repair. Of the many bone growth factors studied, bone morphogenetics (BMPs) have generated the greatest attention because of their osteoinductive potential. The BMPs that have been most widely studied for their ability to induce bone regeneration in humans include BMP-2 and BMP-7 (osteogenic protein). Human osteogenic protein-1 (OP-1) has been cloned and produced with recombinant technology and is free from the risk of infection or allergic reaction. The structural lattice is osteoconductive; it supports the ingrowth of developing capillaries and perivascular tissues. Three distinct groups of structural lattice have been identified: collagen, calcium sulphate, and calcium phosphate. These materials can be used to replace a lost segment of bone. GRAFTS USED FOR NONUNION: Autologous bone graft is generally considered the gold standard and the best material for grafting because it contains several elements that are critical in promoting bone formation, including osteoprogenitor cells, the matrix, and bone morphogenetic proteins. The osteoconductive property of cancellous autograft is related to the porosity of bone. The highly porous, scaffold-like structure of the graft allows host osteoblasts and host osteoprogenitor cells to migrate easily into the area of the defect and to begin regeneration of bone. Sources of cancellous bone are the iliac crest, the distal femur, the greater trochanter, and the proximal tibia. However, harvesting the autologous bone graft is associated with postoperative pain at the donor site, potential injury to the surrounding arteries, nerves, and tissues, and the risk of infection. Thus the development of synthetic materials with osteoconductive and osteoinductive properties that can eliminate the need for harvesting has become a major goal of orthopedic research. Allograft is the graft of tissue between individuals who are of the same species but are of a disparate genotype. Allograft has osteoconductive and limited osteoinductive properties. Demineralized bone matrix (DBM) is human cortical and cancellous allograft. These products are prepared by acid extraction of allograft bone, resulting in the loss of most of the mineralized component while collagen and noncollagenous proteins, including

growth factors, are retained. Figures 1 to 5 demonstrate the osteogenic, osteoinduction, and osteoconduction properties of autologous bone graft, allograft, OP-1, bone graft substitutes, and bone marrow. Figure 1.Autologous Bone GraftFigure 2.Osteogenic Protein-1Figure 3.Allograft bone and Demineralized Bone MatrixFigure 4.Bone Graft SubstitutesFigure 5.Autologous Bone Marrow Graft NEW TECHNOLOGY BEING REVIEWED: OSTEOGENIC PROTEIN-1 Health Canada issued a Class IV licence for OP-1 in June 2004 (licence number 36320). The manufacturer of OP-1 is Stryker Biotech (Hapkinton, MA). The United States Food and Drug Administration (FDA) issued a humanitarian device exemption for the application of the OP-1 implant as an "alternative to autograft in recalcitrant long bone nonunions where use of autograft is unfeasible and alternative treatments have failed." Regulatory agencies in Europe, Australia, and New Zealand have permitted the use of this implant in specific cases, such as in tibial nonunions, or in more general cases, such as in long bone nonunions. According to the manufacturer, OP-1 is indicated for the treatment of long bone nonunions. It is contraindicated in the patient has a hypersensitivity to the active substance or collagen, and it should not be applied at the site of a resected tumour that is at or near the defect or fracture. Finally, it should not be used in patients who are skeletally immature (< 18 years of age), or if there is no radiological evidence of closure of epiphysis. REVIEW STRATEGY:
OBJECTIVE: To summarize the safety profile and effectiveness of OP-1 in the treatment of cases of long bone nonunion and bone defectsTo compare the effectiveness and cost effectiveness of OP-1 in the treatment of long bone nonunions and bone defects with the alternative technologies, particularly the gold standard autologous bone graft.
LITERATURE SEARCH: International Network of Agencies for Health Technology Assessments (INAHTA), the Cochrane Database of Systematic Reviews and the CCTR (formerly Cochrane Controlled Trials Register) were searched for health technology assessments. MEDLINE, EMBASE, Medline In Process and Other Non-Indexed Citations were searched from January 1, 1996 to January 27, 2004 for studies on OP-1. The search was limited to English-language articles and human studies. The search yielded 47 citations. Three studies met inclusion criteria (2 RCTs and 1 Ontario-based study presented at an international conference.
SUMMARY OF FINDINGS: Friedlaender et al. conducted a prospective, randomized, partially blinded clinical trial on the treatment tibial nonunions with OP-1. Tibial nonunions were chosen for this study because of their high frequency, challenging treatment requirements, and substantial morbidity. All of the nonunions were at least 9 months old and had shown no progress toward healing over the previous 3 months. The patients were randomized to receive either treatment with autologous bone grafting or treatment with OP-1 in a type-1 collagen carrier. Both groups received reduction and fixation with an intramedullary rod. Table 1 summarizes the clinical outcomes of this study. Table 1:Outcomes in a Randomized Clinical Trial on Tibial Nonunions: Osteogenic Protein-1 versus Autologous Bone GraftingClinical Indicator at 9 monthsSuccess by ProcedureOP-1 % (range)Autograft % (range)PWeight-bearing*8685not significantPain on Weight-bearing*8990not significantBridging seen on radiograph (at least 1 view)7584not significantBridging seen on radiograph (at least 3 views)6274not significantRepeated surgery*510not significantPhysician satisfaction8690not significantMean operative time in minutes (range)169 (58 - 420)178 (58 - 420)not significantMean operative blood loss in ml (range)254 (10-1,150)345 (35 - 1,200).049Mean length of stay in days (range)3.7 (0 - 18)4.1 (1 - 24)not significantPain at the donor siteN/A80N/AAt 6 months postsurgery20At 12 months postsurgery13Osteomyelitis % (number)3 (2/61)21 (13/61). (ABSTRACT TRUNCATED)

PMCID: PMC3382627
PMID: 23074475 [PubMed]

1140. Eur Ann Allergy Clin Immunol. 2004 Oct;36(8):313-6.

Allergies in immigrants.

Geller-Bernstein C, Kenett R.

Kaplan medical Centre, Rehovot - Israel.

We studied the influence of environmental factors on allergy disease in immigrants that came to Israel during the last 20 years from A) Ethiopia and B) former Soviet Union. Immigrants who came from Ethiopia had no allergies upon arrival; they suffered from severe parasitic infections and had extremely elevated IgE levels. They got thorough anti parasitic treatment and were gradually integrated in the old timer Israeli population. After 5-10 years from arrival, follow up assessments showed a significant drop in IgE levels while respiratory allergies with positive skin tests Respiratory allergies with positive skin tests) to aero allergens appeared at a prevalence of 11%. Israeli born newborns and children from Ethiopian descent had no stool parasites and their total IgE levels were similar to those of the indigenous population. Immigrants from former Soviet Union who had respiratory allergies upon arrival, showed skin tested hypersensitivity to pollen common in their Russian, homelands while they were not sensitive to the Mediterranean pollen common in Israel. At yearly follow up testing over the first 10 years in Israel, odds for sensitization to Russian pollen decreased while odds for sensitization to Israeli pollen increased significantly. The results of our studies plead the case for the very important role played by the environment in the dynamics of allergy diseases.

PMID: 15623247 [PubMed - indexed for MEDLINE]

1141. J Exp Med. 2004 Dec 20;200(12):1667-72.

Flavonoids influence monocytic GTPase activity and are protective in experimental allergic encephalitis.

Hendriks JJ, Alblas J, van der Pol SM, van Tol EA, Dijkstra CD, de Vries HE.

Dept. of Molecular Cell Biology and Immunology, VUMC, FdG, PO Box 7057, 1007 MB Amsterdam, Netherlands.

In the chronic disabling disease multiple sclerosis (MS), migration of monocytes across the blood-brain barrier is a crucial step in the formation of new lesions in the central nervous system (CNS). Infiltrating monocyte-derived macrophages secrete inflammatory mediators such as oxygen radicals, which contribute to axonal demyelination and damage, resulting in neurological deficits. Flavonoids are compounds occurring naturally in food, which scavenge oxygen radicals and have antiinflammatory properties. To investigate whether they might suppress clinical symptoms in MS, we treated rats sensitized for acute and chronic experimental allergic encephalomyelitis, an experimental model of MS, with flavonoids. We demonstrated that the flavonoid luteolin substantially suppressed clinical symptoms and prevented relapse when administered either before or after disease onset. Luteolin treatment resulted in reduced inflammation and axonal damage in the CNS by preventing monocyte migration across the brain endothelium. Luteolin influenced migration by modulating the activity of Rho GTPases, signal transducers involved in transendothelial migration. Oral administration of luteolin also significantly reduced clinical symptoms.

PMCID: PMC2212002
PMID: 15611292 [PubMed - indexed for MEDLINE]

1142. J Pharmacol Sci. 2004 Dec;96(4):367-75. Epub 2004 Dec 15.

New perspectives in the studies on endocannabinoid and cannabis: 2-arachidonoylglycerol as a possible novel mediator of inflammation.

Sugiura T, Oka S, Gokoh M, Kishimoto S, Waku K.

Faculty of Pharmaceutical Sciences, Teikyo University, Sagamiko, Tsukui-gun, Kanagawa 199-0195, Japan. sugiurat@pharm.teikyo-u.ac.jp

2-Arachidonoylglycerol is an endogenous ligand for the cannabinoid receptors. To date, two types of cannabinoid receptors (CB1 and CB2) have been identified. The CB1 receptor is assumed to be involved in the attenuation of synaptic transmission. On the other hand, the physiological roles of the CB2 receptor, which is abundantly expressed in several types of inflammatory cells and immunocompetent cells, have not yet been fully elucidated. Recently, we investigated in detail possible physiological roles of the CB2 receptor and 2-arachidonoylglycerol in inflammation. We found that 2-arachidonoylglycerol induces the activation of p42/44 and p38 mitogen-activated protein kinases and c-Jun N-terminal kinase; actin rearrangement and morphological changes; augmented production of chemokines in HL-60 cells; and the migration of HL-60 cells differentiated into macrophage-like cells, human monocytes, natural killer cells, and eosinophils. We also found that the level of 2-arachidonoylglycerol in mouse ear is markedly elevated following treatment with 12-O-tetradecanoylphorbol 13-acetate, which induces acute inflammation. Notably, the inflammation induced by 12-O-tetradecanoylphorbol 13-acetate was blocked by treatment with SR144528, a CB2-receptor antagonist. Similar results were obtained with an allergic inflammation model in mice. These results strongly suggest that 2-arachidonoylglycerol plays essential roles in the stimulation of various inflammatory reactions in vivo.

PMID: 15599096 [PubMed - indexed for MEDLINE]

1143. Cytokine. 2005 Jan 21;29(2):49-55.

Tumor necrosis factor-alpha-induced CTACK/CCL27 (cutaneous T-cell-attracting chemokine) production in keratinocytes is controlled by nuclear factor kappaB.

Vestergaard C, Johansen C, Otkjaer K, Deleuran M, Iversen L.

Department of Dermatology, Research Laboratory D, Marselisborg Centret, Aarhus Amtssygehus, University of Aarhus, PP Ørumsgade 11, DK 8000 Aarhus C, Denmark. chr-vest@post9.tele.dk

CTACK/CCL27 is pivotal in mediating the migration of lymphocytes into the skin, through the binding to the chemokine receptor CCR10. CCL27 is continuously expressed by keratinocytes, but highly upregulated in inflammatory skin diseases such as atopic dermatitis and psoriasis. CCL27 can be induced in cultured keratinocytes by tumor necrosis factor (TNF)-alpha, which is also known to induce activity of the transcription factor NF-kappaB. NF-kappaB plays a vital role in controlling inflammation through its regulation of transcription of chemokines and pro-inflammatory cytokines. We show here that inhibition of NF-kappaB with the non-specific NF-kappaB inhibitors SSC (sodium salicylate), DCIC (3,4-dichloroisocoumarin) and PAO (phenylarsine oxide) results in a downregulation of TNF-alpha-induced CCL27 production. To substantiate the result and to investigate the role of NF-kappaB we investigated if specific antisense oligonucleotides against the p50 and p65 subunits of NF-kappaB had the same effect. Inhibition of either p50 or p65 production with antisense oligonucleotides resulted in a significant downregulation of TNF-alpha-induced

CCL27 production. These results indicate that CCL27 expression is under the control of NF-kappaB, and that NF-kappaB, as indicated by others, may be an attractive target for therapy in inflammatory skin diseases.

PMID: 15598438 [PubMed - indexed for MEDLINE]

1144. J Immunol. 2004 Dec 15;173(12):7531-8.

Nonredundant function of phosphodiesterases 4D and 4B in neutrophil recruitment to the site of inflammation.

Ariga M, Neitzert B, Nakae S, Mottin G, Bertrand C, Pruniaux MP, Jin SL, Conti M.

Division of Reproductive Biology, Department of Obstetrics and Gynecology, Stanford University School of Medicine, Stanford, CA 94305, USA.

Neutrophils have been implicated in the pathogenesis of many inflammatory lung diseases, including chronic obstructive pulmonary disease and asthma. With this study, we investigated how disruption of cAMP signaling impacts the function of neutrophil recruitment to the lung. Four genes code for type 4 phosphodiesterases (PDE4s), enzymes critical for regulation of cAMP levels and cell signaling. Ablation of two of these genes, PDE4B and PDE4D, but not PDE4A, has profound effects on neutrophil function. In a paradigm of mouse lung injury induced by endotoxin inhalation, the number of neutrophils recovered in the bronchoalveolar lavage was markedly decreased in PDE4D(-/-) and PDE4B(-/-) mice 4 and 24 h after exposure to LPS. Acute PDE4 inhibition with rolipram had additional inhibitory effects on neutrophil migration in PDE4B(-/-) and, to a lesser extent, PDE4D(-/-) mice. This decreased neutrophil recruitment occurred without major changes in chemokine accumulation in bronchoalveolar lavage, suggesting a dysfunction intrinsic to neutrophils. This hypothesis was confirmed by investigating the expression of adhesion molecules on the surface of neutrophils and chemotaxis in vitro. CD18 expression was decreased after ablation of both PDE4B and PDE4D, whereas CD11 expression was not significantly affected. Chemotaxis in response to KC and macrophage inflammatory protein-2 was markedly reduced in PDE4B(-/-) and PDE4D(-/-) neutrophils. The effect of PDE4 ablation on chemotaxis was comparable, but not additive, to the effects of acute PDE4 inhibition with rolipram. These data demonstrate that PDE4B and PDE4D play complementary, but not redundant, roles in the control of neutrophil function.

PMID: 15585880 [PubMed - indexed for MEDLINE]

1145. Cell Tissue Res. 2004 Dec;318(3):473-82. Epub 2004 Nov 3.

Vascular adhesion and transendothelial migration of eosinophil leukocytes.

Gonlugur U, Efeoglu T.

Department of Chest Diseases, Cumhuriyet University Medical School Gogus, Hastaliklari Klinigi, 58140, Sivas, Turkey.

Tissues respond to injury with inflammation in an effort to protect and repair the damaged site. During inflammation, leukocytes typically accumulate in response to certain chemicals produced within the tissue itself. The passage of leukocytes through the vascular lumen into tissues occurs in several phases, including rolling, activation, firm adhesion, transendothelial migration, and subendothelial migration. Although infiltration of eosinophil leukocytes is one of the most important aspects of allergic inflammatory reactions, eosinophils also participate in nonallergic inflammation. Eosinophil accumulation is

regulated not only by endothelial adhesion molecules, but also by interactions between eosinophil adhesion molecules and extracellular matrix elements. This review summarizes the regulation of eosinophil leukocyte adhesion and migration. A better understanding of eosinophil recruitment responses may lead to the development of novel therapeutics for chronic allergic diseases.

PMID: 15578268 [PubMed - indexed for MEDLINE]

1146. Mol Pharmacol. 2005 Mar;67(3):640-7. Epub 2004 Nov 24.

Structural determinants of arylacetic acid nonsteroidal anti-inflammatory drugs necessary for binding and activation of the prostaglandin D2 receptor CRTH2.

Hata AN, Lybrand TP, Marnett LJ, Breyer RM.

Department of Pharmacology, Vanderbilt University School of Medicine, Nashville, TN 37232-2372, USA.

The chemoattractant receptor-homologous molecule expressed on Th2 cells (CRTH2) receptor, a G protein-coupled receptor that mediates chemotaxis of inflammatory cells in response to prostaglandin D2 (PGD2), is hypothesized to play a role in Th2-mediated allergic disease. In addition to PGD2, CRTH2 can be activated by indomethacin, a nonselective cyclooxygenase inhibitor and widely used nonsteroidal anti-inflammatory drug (NSAID). To evaluate the structural features that confer CRTH2 binding selectivity, structure-activity relationship analysis of arylacetic acid class NSAIDs as CRTH2 receptor ligands was performed. Indomethacin, sulindac sulfide, and zomepirac displaced [3H]PGD2 binding at the mouse CRTH2 receptor (mCRTH2) with comparable affinity (Ki = 1.5 +/- 0.1, 2.5 +/- 0.4, and 3.3 +/- 0.3 microM, respectively). The indomethacin metabolite 5'-O-desmethyl indomethacin (5'-DMI) possessed binding affinity similar to indomethacin; however, elimination of the 2-methyl substituent on the indole ring resulted in a 10-fold decrease in binding affinity. No binding was detected for indole acetic acid and indole derivatives such as tryptophan, serotonin, and 5-hydroxy indole acetic acid, demonstrating the importance of the N-acyl moiety of indomethacin. Neutral derivatives of indomethacin also failed to bind to mCRTH2, suggesting that the negatively charged carboxylate moiety participates in a key ligand-receptor interaction. Despite similar binding affinities, NSAID-type mCRTH2 ligands exhibited variable potencies as mCRTH2 agonists. Sulindac sulfide and 5'-DMI inhibited intracellular cyclic AMP ([cAMP]i) generation and stimulated cell migration comparable with indomethacin. In contrast, zomepirac did not inhibit [cAMP]i generation or stimulate cell migration but weakly antagonized the effects of indomethacin on [cAMP]i. Together, these results reveal structural features of arylacetic acid NSAIDs that may be exploited for the development of selective CRTH2 ligands.

PMID: 15563582 [PubMed - indexed for MEDLINE]

1147. Altern Lab Anim. 2003 Dec;31(6):553-61.

A two-centre evaluation of the human organotypic skin explant culture model for screening contact allergens.

Lehé CL, Jacobs JJ, Elliott GR, Das PK.

Department of Pathology, UvA-AMC, Meibergdreef 9, 1105 AZ Amsterdam, The Netherlands.

Animal models are considered to be the "gold standard" for determining the

potential contact allergenicity of low molecular weight chemicals. However, governmental regulations and ethical considerations limit the use of animals for such purposes. There is therefore a need for in vitro alternative models. The human organotypic skin explant culture (HOSEC) model is reported to be a promising alternative method for the predictive testing of contact allergens. The accelerated migration of Langerhans cells from the epidermis upon exposure to contact allergens is used to identify chemicals that are potentially capable of inducing a delayed-type hypersensitivity. In the study described in this paper, the model was further refined, and used, in two independent laboratories, to screen 23 low molecular weight compounds of known classification for their allergenicity. Each laboratory was able to accurately detect the contact allergens, despite small variations in the protocols used. However, the classification of dermal irritants, which have often been falsely classified as allergens, varied between the two laboratories. Despite the current limitations of the HOSEC model, the accuracy of the predictions made (sensitiser or non-sensitiser) compare favourably with classifications obtained with commonly used animal models. The HOSEC model has the potential to be developed further as an in vitro alternative to animal models for screening for contact allergens.

PMID: 15560745 [PubMed - indexed for MEDLINE]

1148. J Allergy Clin Immunol. 2004 Nov;114(5):1077-84.

Expression and function of the angiopoietin receptor Tie-2 in human eosinophils.

Feistritzer C, Mosheimer BA, Sturn DH, Bijuklic K, Patsch JR, Wiedermann CJ.

Division of General Internal Medicine, Department of Internal Medicine, Medical University of Innsbruck, Anichstrasse 35, A-6020 Innsbruck, Austria.

BACKGROUND: Increased vascularity of bronchial mucosa is closely related to the expression of angiogenic factors, which contribute to the pathogenesis of diseases such as asthma bronchiale.
OBJECTIVE: Here we examine the effects of the angiogenic growth factors angiopoietin 1 and angiopoietin 2 on eosinophil function in vitro and possible involvement of the angiopoietin receptor Tie-2.
METHODS: Eosinophil migration was studied by micropore filter assays. Signaling mechanisms required for angiopoietin-dependent migration were tested by using signaling enzyme blockers. Tie-2 mRNA and receptor expression on the cell surface of eosinophils was demonstrated in RT-PCR and by fluorescence-activated cell sorting analysis.
RESULTS: Angiopoietin 1 significantly stimulated eosinophil chemotaxis via activation of phosphodiesterase, phosphatidylinositol 3'-kinase, and tyrosine kinases. The effect on eosinophil migration of angiopoietin 1 was reversed by an antibody against the Tie-2 receptor and by angiopoietin 2. Incubation of eosinophils with angiopoietin 1 abolished the chemotactic effects of vascular endothelial growth factor on human eosinophils via the Tie-2 receptor. Finally, Tie-2 expression by human eosinophils was demonstrated on the transcriptional and protein level.
CONCLUSIONS: Data suggest that angiopoietin 1 stimulates directed migration and possibly inhibits vascular endothelial growth factor-induced eosinophil chemotaxis via its Tie-2 receptor, which is expressed by eosinophils. Thus, angiopoietin 1 may play an important role in the modulation of eosinophilic inflammation.

PMID: 15536413 [PubMed - indexed for MEDLINE]

1149. Curr Opin Immunol. 2004 Dec;16(6):724-31.

Chemoattractants and their receptors in homeostasis and inflammation.

Sallusto F, Mackay CR.

Institute for Research in Biomedicine, Via Vincenzo Vela 6, CH-6500 Bellinzona, Switzerland. federica.sallusto@irb.unisi.ch

The study of leukocyte migration continues to provide new insights into the regulation of lymphocyte priming in secondary lymphoid organs and effector responses in inflamed tissues. Chemoattractant receptors have always been viewed as facilitators of cell movement into a tissue. This whole concept must now be revised with the discovery of sphingosine 1 phosphate receptors, which control cell exit from lymphoid tissues. The chemoattractants that regulate lymphoid tissue homing are usually different to those that regulate leukocyte recruitment to inflamed tissues. There is evidence, however, of inflammatory pathways of leukocyte recruitment in lymph nodes and, conversely of constitutive pathways in peripheral tissues. Finally, antagonists (or agonists) of chemoattractant receptors and their signalling pathways represent the most attractive strategy for the treatment of a wide range of inflammatory diseases, including allergy.

PMID: 15511664 [PubMed - indexed for MEDLINE]

1150. Asian Am Pac Isl J Health. 2003 Summer-Autumn;10(2):114-9.

The health status of Vietnamese immigrants in Hawaii from chart records.

Nguyen LT, Withy K.

Department of Surgery, John A. Burns School of Medicine, University of Hawaii, Manoa, HI 96813, USA. nguyenl2002@yahoo.com

OBJECTIVES: We determined the most common diseases among adult Vietnamese men and women in Hawaii.
METHODS: A chart review of 952 adult Vietnamese patients was conducted between January and February 2000. A data collection form with 39 categories of diagnosed chronic illnesses or problems was used. The study was conducted at a Vietnamese internist's private medical office in Honolulu, HI.
RESULTS: Chart review revealed that the five most prevalent diagnostic conditions seen in Vietnamese men, from the highest to lowest frequency, were gastrointestinal disorders (39%), cutaneous conditions (31%), lower back pain (23%), headache (18%), and allergies (18%). In women, gastrointestinal disorders (38%), cutaneous conditions (34%), headache (32%), gynecologic conditions (30%), and arthritic diseases (24%) were most common. Gender, years of U.S. arrival, and types of occupation were significantly correlated with certain diseases (p< or =0.05).
CONCLUSIONS: This is the first study to examine frequency of diseases diagnosed in an ambulatory care setting in a cohort of Vietnamese patients in Hawaii.

PMID: 15509152 [PubMed - indexed for MEDLINE]

1151. Nature. 2004 Oct 21;431(7011):1007-11.

Essential role for the p110delta phosphoinositide 3-kinase in the allergic response.

Ali K, Bilancio A, Thomas M, Pearce W, Gilfillan AM, Tkaczyk C, Kuehn N, Gray A, Giddings J, Peskett E, Fox R, Bruce I, Walker C, Sawyer C, Okkenhaug K, Finan P,

Vanhaesebroeck B.

Ludwig Institute for Cancer Research, 91 Riding House Street, London W1W 7BS, UK.

Inflammatory substances released by mast cells induce and maintain the allergic response. Mast cell differentiation and activation are regulated, respectively, by stem cell factor (SCF; also known as Kit ligand) and by allergen in complex with allergen-specific immunoglobulin E (IgE). Activated SCF receptors and high-affinity receptors for IgE (FcvarepsilonRI) engage phosphoinositide 3-kinases (PI(3)Ks) to generate intracellular lipid second messenger signals. Here, we report that genetic or pharmacological inactivation of the p110delta isoform of PI(3)K in mast cells leads to defective SCF-mediated in vitro proliferation, adhesion and migration, and to impaired allergen-IgE-induced degranulation and cytokine release. Inactivation of p110delta protects mice against anaphylactic allergic responses. These results identify p110delta as a new target for therapeutic intervention in allergy and mast-cell-related pathologies.

PMID: 15496927 [PubMed - indexed for MEDLINE]

1152. Br J Ophthalmol. 2004 Nov;88(11):1467-74.

Autologous serum eye drops for ocular surface disorders.

Geerling G, Maclennan S, Hartwig D.

Department of Ophthalmology, University of Lübeck, Ratzeburger Allee 160, D-23538, Germany. ggeerling@ophtha.mu-luebeck.de

Tears have antimicrobial, nourishing, mechanical, and optical properties. They contain components such as growth factors, fibronectin, and vitamins to support proliferation, migration, and differentiation of the corneal and conjunctival epithelium. A lack of these epitheliotrophic factors--for example, in dry eye, can result in severe ocular surface disorders such as persistent epithelial defects. Recently, the use of autologous serum in the form of eye drops has been reported as a new treatment for severe ocular surface disorders. Serum eye drops may be produced as an unpreserved blood preparation. They are by nature non-allergenic and their biomechanical and biochemical properties are similar to normal tears. In vitro cell culture experiments showed that corneal epithelial cell morphology and function are better maintained by serum than by pharmaceutical tear substitutes. Clinical cohort studies have reported its successful use for severe dry eyes and persistent epithelial defects. However, the protocols to prepare and use autologous serum eye drops varied considerably between the studies. As this can result in different biochemical properties protocol variations may also influence the epitheliotrophic effect of the product. Before the definitive role of serum eye drops in the management of severe ocular surface disease can be established in a large randomised controlled trial this has to be evaluated in more detail. In view of legislative restrictions and based upon the literature reviewed here a preliminary standard operating procedure for the manufacture of serum eye drops is proposed.

PMCID: PMC1772389
PMID: 15489495 [PubMed - indexed for MEDLINE]

1153. J Allergy Clin Immunol. 2004 Oct;114(4):820-5.

Migration and accumulation of eosinophils toward regional lymph nodes after airway allergen challenge.

Duez C, Dakhama A, Tomkinson A, Marquillies P, Balhorn A, Tonnel AB, Bratton DL, Gelfand EW.

Department of Pediatrics, Division of Basic Sciences, National Jewish Medical and Research Center, Denver, CO 80206, USA.

BACKGROUND: Eosinophils play a major role in allergic airway inflammation because of their ability to release toxic mediators. In addition, they are able to migrate toward draining thoracic lymph nodes (TLNs) after intratracheal administration, where they can function as antigen-presenting cells.
OBJECTIVE: In this study, we evaluated in vivo eosinophil migration toward the TLN after allergen sensitization and analyzed expression of molecules involved in antigen presentation.
METHODS: Mice were sensitized by intraperitoneal injection of ovalbumin on days 1 and 10 and challenged once intranasally with ovalbumin on day 20. The kinetics of eosinophilia was evaluated in blood, lung tissue homogenate, bronchoalveolar lavage fluid, and TLN. Cell surface staining was analyzed by flow cytometry.
RESULTS: The kinetics of eosinophil recruitment was similar in TLN, lung tissue, and blood, beginning at 12 hours and peaking at 48 hours after allergen challenge. Approximately 70% of TLN eosinophils expressed MHC class II molecules, compared with less than 25% in blood and lungs. Moreover, TLN eosinophils expressed higher levels of MHC class II and CD86 compared with blood and lung eosinophils. Most eosinophils expressed CD80 and CD54, whereas only a few eosinophils expressed CD40. Eosinophils in lungs and TLN appeared to be activated with lower CD62-ligand expression compared with blood eosinophils.
CONCLUSION: The presence of eosinophils with a different phenotype in the TLN at early time points after allergen challenge of sensitized mice supports their capacity to serve as antigen-presenting cells, sustaining allergic/inflammatory responses in the airways.

PMID: 15480321 [PubMed - indexed for MEDLINE]

1154. Clin Exp Allergy. 2004 Oct;34(10):1621-6.

Red blood cells regulate eosinophil chemotaxis by scavenging RANTES secreted from endothelial cells.

Kanda A, Adachi T, Kayaba H, Yamada Y, Ueki S, Yamaguchi K, Hamada K, Fujita M, Chihara J.

Department of Clinical and Laboratory Medicine, Akita University School of Medicine, Akita, Japan.

BACKGROUND: Eosinophils play a critical role in the pathogenesis of allergic diseases. CC chemokines, such as regulated on activation, normal, T cell expressed, and secreted (RANTES), are key regulators of eosinophil locomotion. Although eosinophils migrate from the bloodstream into tissues, mechanisms that generate a chemogradient across the endothelium remain to be fully elucidated.
OBJECTIVE: We first examined the polar secretion of RANTES by endothelial cells. We also studied the functional scavenging effect of red blood cells (RBCs) on RANTES secreted into the intravascular side. METHODS and
RESULTS: Endothelial cells were cultured in a transwell chamber with a membrane pore size of 0.45, 3.0, and 8.0 microm and stimulated with TNF-alpha, IL-1beta, or IFN-gamma from the apical or basolateral side for 16 h. The measurement of RANTES in the supernatant was performed by ELISA. We did not see any difference in the amount of RANTES secreted from the cytokine-stimulated endothelium between inner (intravascular side) and outer (extravascular side) wells separated by the 8.0-microm membrane, although apical polarization was observed with the

0.45-microm membrane. The addition of RBCs (hemoglobin (Hb): 0.5-15 g/dL) to the apical supernatant of TNF-alpha-stimulated endothelial cells reduced the RANTES level in a concentration-dependent manner. The treatment of supernatant on the intravascular side with RBCs significantly enhanced the migration of eosinophils. CONCLUSION: RBCs possess a scavenging effect on intravascular RANTES, and thereby regulate transendothelial migration of eosinophils. Our findings suggest a new role of RBCs in allergic inflammation.

PMID: 15479279 [PubMed - indexed for MEDLINE]

1155. FEMS Immunol Med Microbiol. 2004 Nov 1;42(3):321-31.

Migration and maturation of human dendritic cells infected with Toxoplasma gondii depend on parasite strain type.

Diana J, Persat F, Staquet MJ, Assossou O, Ferrandiz J, Gariazzo MJ, Peyron F, Picot S, Schmitt D, Vincent C.

EA3732, Université Claude Bernard LYON I, Pavillon R, Hôpital Edouard Herriot, 5 place d'Arsonval, 69437 Lyon 03, France.

Migration and maturation of human dendritic cells derived from CD34+ progenitor cells (DC) infected by Toxoplasma gondii were studied in an in vitro model. We demonstrated that infection with virulent type I strains RH and ENT or type II low virulent strains PRU and CAL induced DC migration towards MIP-3beta. However, type II strains induced a higher percentage of migrating cells than that induced by type I strains or positive controls (chemical allergen or lipopolysaccharides). Type II strains produced soluble factors responsible of the high migration whereas heat killed tachyzoites did not induced a migration higher than positive controls. We also demonstrated that infection by virulent strains and not by type II stains or heat killed tachyzoites triggers DC maturation. A soluble factor released by type II strains was responsible of the absence of DC maturation. Taken together, these results demonstrated that the interference of T. gondii in the behaviour of DC functions is related to the strain types and can be supported by secretion of soluble factors by the parasite.

PMID: 15477046 [PubMed - indexed for MEDLINE]

1156. J Immunol. 2004 Oct 15;173(8):5189-95.

Transendothelial migration of human basophils.

Iikura M, Ebisawa M, Yamaguchi M, Tachimoto H, Ohta K, Yamamoto K, Hirai K.

Department of Allergy and Rheumatology, University of Tokyo Graduate School of Medicine, Hongo, Bunkyo-ku, Japan. iikura-tky@umin.ac.jp

During allergic reactions, basophils migrate from the blood compartment to inflammatory sites, where they act as effector cells in concert with eosinophils. Because transendothelial migration (TEM) represents an essential step for extravasation of cells, for the first time we have studied basophil TEM using HUVEC. Treatment of HUVEC with IL-1beta significantly enhanced basophil TEM, which was further potentiated by the presence of a CCR3-specific ligand, eotaxin/CCL11. In addition to CCR3 ligands, MCP-1/CCL2 was also active on basophil TEM. Although stromal cell-derived factor-1/CXCL12, a CXCR4 ligand, failed to induce TEM in freshly isolated basophils, it caused strong TEM in 24-h cultured cells. IL-3 enhanced basophil TEM by increasing the chemokinetic response. Spontaneous TEM across activated HUVEC was inhibited by treatment of

cells with anti-CD18 mAb, but not with anti-CD29 mAb, and also by treatment of HUVEC with anti-ICAM-1 mAb. Anti-VCAM-1 mAb alone failed to inhibit TEM, but showed an additive inhibitory effect in combination with anti-ICAM-1 mAb. In contrast, eotaxin- and IL-3-mediated TEM was significantly inhibited by anti-CD29 mAb as well as anti-CD18 mAb. These results indicate that beta2 integrins play the primary role in basophil TEM, but beta1 integrins are also involved, especially in TEM of cytokine/chemokine-stimulated basophils. In conclusion, the regulatory profile of basophil TEM is very similar to that reported for eosinophils. Our results thus support the previous argument for a close relationship between basophils and eosinophils and suggest that the in vivo kinetics of these two cell types are similar.

PMID: 15470064 [PubMed - indexed for MEDLINE]

1157. Salud Publica Mex. 2004 Jul-Aug;46(4):286-93.

[Health differences between male and female migrant agricultural workers in Sinaloa, Mexico].

[Article in Spanish]

Palacios-Nava ME, Moreno-Tetlacuilo LM.

Departamento de Salud Pública, Facultad de Medicina, Universidad Nacional Autónoma de México, México, DF, México. pnme@servidor.unam.mx

OBJECTIVE: To assess the differences in the prevalence of muscarinic and nicotinic type symptoms and the level of erythrocytic cholinesterase, prior to pesticide exposure, in male and female migrant agricultural workers.
MATERIAL AND METHODS: A cross-sectional study was carried out in 2001 in Sinaloa State, Mexico, among 488 migrant workers. A questionnaire was applied and erythrocytic cholinesterase levels were measured before the beginning of the agricultural season. The differences by sex were compared using the "t" test for continuous variables and chi2 test for categorical variables. Prevalence odds ratios were also estimated. Statistical significance was assessed using p-values <0.05 and 95% confidence intervals.
RESULTS: Significant differences were found by age, migration type, place of origin, education, and migration time (p=0.000). Women were six times more likely to have anemia and asthma, twice more likely to have parasites and respiratory and gastrointestinal diseases and 38% more likely to suffer from heart disease. They were also at greater prevalence in thirteen of nineteen investigated symptoms. The average cholinesterase level was within normal limits (4.22 U/ml+/-0.77) and it was similar to the levels reported using the Magnotti method.
CONCLUSIONS: The prevalence of symptoms, illnesses and cholinesterase levels found in this study may serve as baseline values for future comparisons of the health effects of pesticide exposure. The English version of this paper is available at: http://www.insp.mx/salud/index.html.

PMID: 15468569 [PubMed - indexed for MEDLINE]

1158. J Leukoc Biol. 2005 Jan;77(1):59-66. Epub 2004 Oct 5.

The anti-inflammatory effects of a selectin ligand mimetic, TBC-1269, are not a result of competitive inhibition of leukocyte rolling in vivo.

Hicks AE, Abbitt KB, Dodd P, Ridger VC, Hellewell PG, Norman KE.

Cardiovascular Research Unit, University of Sheffield, United Kingdom.

Selectins and their ligands support leukocyte rolling, facilitating the subsequent firm adhesion and migration that occur during inflammation. TBC-1269 (Bimosiamose), a structural mimetic of natural selectin ligands, inhibits P-, E-, and L-selectin in vitro, has anti-inflammatory effects in vivo, and recently underwent phase II clinical trials for childhood asthma and psoriasis. We studied whether the anti-inflammatory effects of TBC-1269 could be related to leukocyte rolling in vivo. Although TBC-1269 inhibited rolling of a murine leukocyte cell line on murine P-selectin in vitro and thioglycollate-induced peritonitis in vivo, it did not alter leukocyte rolling in mouse cremaster venules. TBC-1269 reduced neutrophil recruitment in thioglycollate-induced peritonitis in wild-type and P-selectin-/- mice but not in E-selectin-/- mice. We suggest that the in vivo effects of TBC-1269 may be mediated through E-selectin but do not appear to involve leukocyte rolling.

PMID: 15466915 [PubMed - indexed for MEDLINE]

1159. Am J Pathol. 2004 Oct;165(4):1331-41.

Hyaluronan participates in the epidermal response to disruption of the permeability barrier in vivo.

Maytin EV, Chung HH, Seetharaman VM.

Biomedical Engineering, Cleveland Clinic Foundation, 9500 Euclid Avenue, Cleveland, OH 44195, USA. maytine@ccf.org.

Hyaluronan (hyaluronic acid, HA) is a glycosaminoglycan in the extracellular matrix of tissues that plays a role in cellular migration, proliferation and differentiation. Injury to the stratum corneum elicits an epidermal hyperproliferative response, a pathogenic feature in many cutaneous diseases including eczema and psoriasis. Because HA is abundant in the matrix between keratinocytes, we asked whether the presence of HA is required for epidermal hyperplasia to occur in response to barrier injury. Disruption of the stratum corneum, by acetone application on the skin of hairless mice, led to a marked accumulation of HA in the matrix between epidermal basal and spinous keratinocytes, and also within keratinocytes of the upper epidermis. To test whether HA may have a functional role in epidermal hyperplasia, we used Streptomyces hyaluronidase (StrepH), delivered topically, to degrade epidermal HA and blunt the accumulation of epidermal HA after acetone. StrepH signficantly reduced epidermal HA levels, and also significantly inhibited the development of epidermal hyperplasia. This reduction in epidermal thickness was not attributable to any decrease in keratinocyte proliferation, but rather to an apparent acceleration in terminal differentiation (ie, increased keratin 10 and filaggrin expression). Overall, the data show that HA is a significant participant in the epidermal response to barrier injury.

PMCID: PMC1618628
PMID: 15466397 [PubMed - indexed for MEDLINE]

1160. Eur J Pharmacol. 2004 Oct 1;500(1-3):453-65.

Nerve growth factor and its receptors in asthma and inflammation.

Frossard N, Freund V, Advenier C.

EA 3771 "Inflammation and Environment in Asthma" Université Louis Pasteur-Strasbourg-I, Faculté de Pharmacie, Illkirch, France.

frossard@pharma.u-strasbg.fr

Nerve growth factor (NGF) is a high molecular weight peptide that belongs to the neurotrophin family. It is synthesized by various structural and inflammatory cells and activates two types of receptors, the TrkA (tropomyosin-receptor kinase A) receptor and the p75(NTR) receptor, in the death receptor family. NGF was first studied for its essential role in neuronal growth and survival. Recent reports indicate that it may also help mediate inflammation, especially in the airways. Several studies in animals have reported that NGF may induce bronchial hyperresponsiveness, an important feature of asthma, by increasing sensory innervation. It may also induce migration and activation of inflammatory cells, which infiltrate the bronchial mucosa, and of structural cells, including epithelial, smooth muscle cells and pulmonary fibroblasts. Increased NGF expression and release is observed in asthma patients after bronchial provocation with allergen. Taken together, the data from the literature suggest that NGF may play a role in inflammation, bronchial hyperresponsiveness and airway remodelling in asthma and may help us to understand the neuro-immune cross-talk involved in chronic inflammatory airway diseases.

PMID: 15464052 [PubMed - indexed for MEDLINE]

1161. Eur Respir J. 2004 Oct;24(4):545-51.

Extracellular matrix regulates human airway smooth muscle cell migration.

Parameswaran K, Radford K, Zuo J, Janssen LJ, O'Byrne PM, Cox PG.

Asthma Research Group, Firestone Institute for Respiratory Health, St. Joseph's Healthcare, McMaster University, Hamilton, Canada.

Extracellular matrix proteins regulate the survival and proliferation of smooth muscle cells. Their effect on airway smooth muscle cell migration is not known. Their role in leukotriene-primed (0.1 microM leukotriene E4) chemotaxis of cultured human airway smooth muscle cells towards platelet-derived growth factor BB (1 ng.mL(-1)) was investigated. Migration of cells was greater on membranes coated with collagens III and V and fibronectin compared to collagen I, elastin and laminin (all 10 microg.mL(-1)). Concentration-dependent promotion of migration was observed on collagen I (1,000>10 microg.mL(-1)), which was associated with increased phosphorylation of Src kinase. This was not observed on laminin or elastin. The role of Src kinase was further confirmed by demonstrating that its inhibitor, PP1 analogue (1 microM), inhibited chemotaxis. Collagen I itself was not a chemoattractant; however, haptokinesis was observed when cells were primed with leukotriene E4, and haptotaxis when cells were primed with platelet-derived growth factor. The priming effect of leukotrienes on chemotaxis was not elicited by promoting adhesion, increasing surface expression of beta1, alphav and alpha5 integrin, or Src kinase phosphorylation. These experiments demonstrate that the extracellular matrix, along with growth factors and cysteinyl leukotrienes, can regulate human airway smooth muscle cell migration. This may be relevant in the remodelling process in chronic airway diseases, such as asthma.

PMID: 15459131 [PubMed - indexed for MEDLINE]

1162. Int Immunopharmacol. 2004 Dec 15;4(13):1687-96.

Concentration-dependent activity of mometasone furoate and dexamethasone on blood eosinophils isolated from atopic children: modulation of Mac-1 expression and chemotaxis.

Sale R, Sabatini F, Silvestri M, Serpero L, Petecchia L, Rossi GA.

Pulmonary Disease Unit, G. Gaslini Institute, Genoa, 16147 Genoa, Italy.

Treatment of asthma with corticosteroids results in downregulation of eosinophilic airway inflammation. We evaluated in vitro the activity of an "inhaled" corticosteroid, mometasone furoate (MF), and of a "systemic" corticosteroid, dexamethasone (DEX), on eosinophil functions, i.e. adhesion molecule expression and cell chemotaxis. Partially purified blood eosinophils were obtained from 18 asthmatic subjects sensitized to house dust mites. The expression of the macrophage antigen (Mac)-1 (CD11b/CD18) was measured by specific monoclonal antibody (mAb) staining and flow cytometry analysis at baseline or after stimulation with N-formyl-methionyl-leucyl-phenylalanine (fMLP) or with recombinant human (rh) granulocyte macrophage-colony stimulating factor (GM-CSF) plus a mAb anti-human (ah) IgE low affinity receptor [FcepsilonRII or CD23]. Cell chemotaxis toward the complement fragment 5a (C5a) or rh interleukin (IL)-5 was evaluated in Boyden microchambers by light microscopy. Eosinophils showed a significant increase in Mac-1 expression after activation with fMLP or with rh GM-CSF plus ah CD23 mAbs ($p<0.05$, each comparison) and a remarkable chemotactic response to both C5a or rh IL-5 ($p<0.001$, each comparison). To test the inhibitory activity of MF and DEX on eosinophil functions, the cells were preincubated for 3 h with four concentrations (0.1, 1, 10 and 100 nM) of each of the two drugs, before being activated by fMLP or by rh GM-CSF plus ah CD23 mAbs or tested with C5a or with rh IL-5. Independently of the stimulus used, both Mac-1 expression and eosinophil migration were effectively downregulated by preincubation with MF or DEX at 1, 10 and 100 nM ($p<0.05$). The inhibitory activity on cell chemotaxis in response to both C5a or with rh IL-5 was higher for MF than DEX, but only at the highest concentration tested ($p<0.05$, each comparison). These data demonstrate that concentrations of MF similar to those obtained in vivo are highly effective in inhibiting eosinophil functions involved in airway inflammation.

PMID: 15454120 [PubMed - indexed for MEDLINE]

1163. Curr Drug Targets Inflamm Allergy. 2004 Sep;3(3):257-61.

Chemokine receptor inhibitors as a novel option in treatment of asthma.

Adcock IM, Caramori G.

Department of Thoracic Medicine, National Heart and Lung Institute, Imperial College London, UK. ian.adcock@imperial.ac.uk

The migration of cells towards and into the site of an inflammatory insult is critical for maintenance of the inflammatory response and its resolution. This is particularly so in the case of asthma where recruitment of key effector cells may control disease severity, responsiveness to current therapies and the airway remodelling associated with the disease. Chemokine receptor antagonists have the hope of preventing inflammatory cell recruitment to the airway and perhaps as a consequence affect the resolution of airway remodelling. A number of selective antagonists directed at various CC and CXC receptors thought to be important in asthma are currently at various stages of clinical development. Results from these studies will determine whether chemokine receptor antagonists will prove beneficial in severe glucocorticoid-dependent and -resistant asthmatic subjects. Furthermore, it is possible that early treatment with these agents may prevent the disease from becoming established.

PMID: 15379593 [PubMed - indexed for MEDLINE]

1164. Am J Ind Med. 2004 Oct;46(4):371-4.

Exposure to bioaerosols: allergic reactions and respiratory function in Polish hop growers.

Góra A, Skórska C, Prazmo Z, Krysińska-Traczyk E, Sitkowska J, Dutkiewicz J.

Institute of Agricultural Medicine, Lublin, Poland. annagora@galen.imw.lublin.pl

BACKGROUND: The aim of the study was to assess the prevalence of work-related symptoms in hop growers and their relation to bioaerosols exposure. The study group comprised 69 hop growers and 58 office workers as controls. The examination included: physician-administrated questionnaire, PEF measurements, skin prick test, agar-gel precipitation test, and migration inhibition test. Microbiological air sampling was performed on all farms.
RESULTS: The concentrations of total airborne microflora ranged from 2.08 to 129.6 x 10(3) CFU/m3. Airborne endotoxin and dust concentrations ranged from 26 to 6250 ng/m3 and 0.2-31.7 mg/m3, respectively. Altogether 52.2% of farmers complained of work-related symptoms. Positive skin reactions to microbial allergens were significantly more frequent in a group of hop growers with work-related respiratory symptoms compared to the rest of the farmers (18% vs 2%, P <0.05). Positive reactions in agar-gel precipitation test and in the leukocyte migration inhibition test were not correlated with the occurrence of work-related symptoms. The mean daily PEF values in farmers were lower compared to controls (469.7 +/- 127.5 vs 562.9 +/- 123.8; P <0.001). PEF (amp%mean) was higher in farmers compared to controls (9.3% vs 8.1%; P <0.05).
CONCLUSION: Despite relatively lower exposure to bioaerosols, compared to farmers in other branches of agriculture, over 50% of hop growers complained of work-related symptoms. This may be partly due to the effects of microbial allergens and toxins and partly to the irritant or allergic properties of hop plant itself.

PMID: 15376215 [PubMed - indexed for MEDLINE]

1165. Am J Ther. 2004 Sep-Oct;11(5):344-53.

Urinary tract diseases revealed after DTP vaccination in infants and young children: cytokine irregularities and down-regulation of cytochrome P-450 enzymes induced by the vaccine may uncover latent diseases in genetically predisposed subjects.

Prandota J.

Faculty of Medicine and Dentistry, University Medical School, Wroclaw, Poland. Jzef.854735@pharmanet.com.pl

Prophylactic vaccinations may sometimes shorten the incubation period of some illnesses and/or convert a latent infection/inflammation into a clinically apparent disease. Cytokines play a major role in mediating the inflammatory process in various clinical entities and represent a potential source of tissue damage if their production is not sufficiently well controlled. It seems that irregularities in production of proinflammatory cytokines may be responsible for the abnormalities associated with full-blown clinical symptoms of various urinary tract diseases observed after DTP vaccination in 13 infants and young children hospitalized over the past 24 years. On admission, upper respiratory tract diseases, atopic dermatitis, and/or latent urinary tract infection/inflammation were found in these children. It is suggested that the whole-cell pertussis

present in DTP vaccine, acting as an excessive stimulus in these patients, produced symptoms reminiscent of biologic responses to circulating proinflammatory monokines such as IL-1beta, TNF-alpha, and IL-6 because earlier it was reported that in vitro the whole-cell vaccine induced significantly more such cytokine production than did the acellular pertussis or diphtheria-tetanus-only vaccine. Analysis of the cellular immune disturbances previously reported in urinary tract infection/inflammation (increased serum and/or urinary IL-1alpha, IL-1 receptor antagonist, IL-6 and IL-8), steroid-sensitive nephrotic syndrome (increased IL-2, IFN-gamma, TNF-alpha, and decreased or increased IL-4, depending on the cells studied), and atopic dermatitis (decreased IFN-gamma and increased IL-4 production), may suggest that similar subclinical chronic cytokine-mediated abnormalities produced in the course of latent diseases revealed in our patients, combined with those caused by DTP vaccination stimulus, were responsible for the pathomechanism of these clinical entities. This speculation is in agreement with the reports on the long-lasting induction of cytokine release and down-regulation of hepatic cytochrome P-450 isoenzyme activities after administration of DTP vaccine to mice and may be supported by the fact that TH1 phenotype is associated with the up-regulation of intercellular adhesion molecule-1 and RANTES, whereas TH2 phenotype is associated with the up-regulation of the vascular cell adhesion molecule and P-selectin, which are key players in the migration into inflamed tissues and localization of lymphocytes and other allergic effector and inflammatory cells. Because several inflammatory cytokines down-regulate gene expression of major cytochrome P-450 and/or other enzymes with the specific effects on mRNA levels, protein expression, and enzyme activity, thus affecting the metabolism of several endogenous lipophilic substances such as steroids, lipid-soluble vitamins, prostaglandins, leukotrienes, thromboxanes, and exogenous substances, their irregularities in the body may eventually lead to the flare of latent diseases in some predisposed subjects. Also, interleukin genetic polymorphisms, especially the constellation of TNF-alpha and IL-6 genetic variants, might predispose some infants with infection to a more than usually intense inflammatory response in the kidneys after vaccination. It seems that the aforementioned pathomechanism may also be responsible for some cases of sudden infant death syndrome, which is often preceded by infection/inflammation.

PMID: 15356430 [PubMed - indexed for MEDLINE]

1166. J Immunol. 2004 Sep 15;173(6):3599-603.

Cutting edge: serotonin is a chemotactic factor for eosinophils and functions additively with eotaxin.

Boehme SA, Lio FM, Sikora L, Pandit TS, Lavrador K, Rao SP, Sriramarao P.

La Jolla Institute for Molecular Medicine, San Diego, CA 92121, USA.

Elevated levels of serotonin (5-hydroxytryptamine, 5-HT) are observed in the serum of asthmatics. Herein, we demonstrate that 5-HT functions independently as an eosinophil chemoattractant that acts additively with eotaxin. 5-HT2A receptor antagonists (including MDL-100907 and cyproheptadine (CYP)) were found to inhibit 5-HT-induced, but not eotaxin-induced migration. Intravital microscopy studies revealed that eosinophils roll in response to 5-HT in venules under conditions of physiological shear stress, which could be blocked by pretreating eosinophils with CYP. OVA-induced pulmonary eosinophilia in wild-type mice was significantly inhibited using CYP alone and maximally in combination with a CCR3 receptor antagonist. Interestingly, OVA-induced pulmonary eosinophilia in eotaxin-knockout (Eot-/-) mice was inhibited by treatment with the 5-HT2A but not CCR3 receptor antagonist. These results suggest that 5-HT is a potent eosinophil-active chemoattractant that can function additively with eotaxin and a dual CCR3/5-HT2A

receptor antagonist may be more effective in blocking allergen-induced eosinophil recruitment.

Copyright 2004 The American Association of Immunologists, Inc.

PMID: 15356103 [PubMed - indexed for MEDLINE]

1167. Clin Exp Allergy. 2004 Sep;34(9):1356-63.

Altered beta2-adrenergic regulation of T cell activity after allergen challenge in asthma.

Heijink IH, van den Berge M, Vellenga E, de Monchy JG, Postma DS, Kauffman HF.

Department of Allergology, University Hospital Groningen, Groningen, The Netherlands.

BACKGROUND: Airway inflammation in asthma is orchestrated by recruitment of T helper (Th)2 lymphocytes to the lung and subsequent production of Th2-like cytokines upon allergen challenge.
OBJECTIVE: To examine whether allergen-induced dysfunction of the beta2-adrenergic receptor (beta2-AR) contributes to the enhanced T(h2) cell activity in asthma.
METHODS: Beta2-adrenergic regulation of cytokine mRNA expression was studied in alpha-CD3/alpha-CD28-activated peripheral blood lymphocytes from seven asthma patients before and 6 h after allergen challenge, in conjunction with the effects of beta2-agonist fenoterol on T cell chemotaxis and signalling pathways.
RESULTS: A complete loss of beta2-AR control over expression of the Th2 cytokines IL-4, IL-5 and IL-13, but not of the Th1 cytokine IFN-gamma, was observed after allergen challenge. Furthermore, we found impaired beta2-AR regulation of T cell migration as well as signal transduction pathways, i.e. the phosphorylation of cyclic adenosine monophosphate-responsive element binding protein and the inhibition of the mitogen-activated protein kinase pathway. The loss of beta2-AR control was associated with increased beta-adrenergic receptor kinase expression, which might be involved in beta2-AR desensitization. In addition, we demonstrate for the first time that T cells exposed to the chemokine thymus and activation-regulated chemokine show hyporesponsiveness to fenoterol.
CONCLUSION: Our results suggest that allergen-induced loss of beta2-AR control, possibly mediated by chemokine release, plays an important role in enhanced Th2-like activity in asthma.

PMID: 15347367 [PubMed - indexed for MEDLINE]

1168. Nat Rev Immunol. 2004 Sep;4(9):711-24.

T-cell trafficking in asthma: lipid mediators grease the way.

Luster AD, Tager AM.

Center for Immunology and Inflammatory Diseases, Division of Rheumatology, Allergy and Immunology, Massachusetts General Hospital, Building 149-8301, 13th Street, Charlestown, Massachusetts 02129, USA. aluster@partners.org

Recruitment of T cells to the airways is crucial in the pathogenesis of asthma, and it is thought to be mediated mainly by peptide chemokines. By contrast, lipid mediators such as leukotrienes and prostaglandins have classically been thought to contribute to asthma pathogenesis by other mechanisms. However, as we discuss here, the recent molecular identification of leukotriene and prostaglandin

receptors, as well as the generation of mice that are genetically deficient in them, has revealed that two of these lipids - leukotriene B(4) and prostaglandin D(2) - also direct T-cell migration and seem to cooperate with chemokines in a non-redundant, sequential manner to recruit T cells to the airways in asthma.

PMID: 15343370 [PubMed - indexed for MEDLINE]

1169. Immunol Lett. 2004 Aug 15;95(1):37-44.

Absence of interleukin-3 does not affect the severity of local and systemic anaphylaxis but does enhance eosinophil infiltration in a mouse model of allergic peritonitis.

Neel NF, Creasy BM, Rankin JN, Pierce EM, McCoy ME, Daner RH, Fowler JA, Daniel JC, Lantz CS.

Department of Biology, James Madison University, Harrisonburg, VA 22807, USA.

Interleukin-3 (IL-3), which is derived from T cells and other sources, can promote the differentiation, proliferation, and migration of mast cells, basophils, and eosinophils. However, little is known about the ability of IL-3 to regulate the function of these cells in IgE-dependent and -independent allergic responses in vivo. Therefore, we sought to investigate the extent to which endogenously produced IL-3 can influence mast cell secretory function, the expression of local and systemic anaphylactic responses, and ragweed-induced eosinophilic peritonitis. We found that peritoneal mast cells from IL-3 deficient (IL-3 -/-) mice released less serotonin following challenge with low doses of anti-IgE antibody or antigen ex vivo than do cells isolated from corresponding wild-type (IL-3 +/+) mice. Both IL-3 -/- and +/+ mice expressed equivalent IgE-dependent passive cutaneous anaphylaxis responses following challenge with specific antigen and exhibited equivalent active systemic anaphylaxis responses to ovalbumin as assessed by changes in body temperature, death rates, total IgE production, and histamine release. In contrast, ragweed allergen immunization and peritoneal allergen challenge resulted in eosinophil recruitment that was greater in IL-3 -/- mice than in IL-3 +/+ mice. Our data demonstrates that IL-3 does not appear to be essential for local or systemic anaphylaxis. However, IL-3 production in vivo was found to enhance the mediator release from freshly isolated peritoneal mast cells stimulated ex vivo, and, unexpectedly, to inhibit the accumulation of eosinophils associated with a ragweed-induced allergic peritonitis model.

PMID: 15325796 [PubMed - indexed for MEDLINE]

1170. J Immunol. 2004 Sep 1;173(5):3084-92.

Functional maturation of CD4+CD25+CTLA4+CD45RA+ T regulatory cells in human neonatal T cell responses to environmental antigens/allergens.

Thornton CA, Upham JW, Wikström ME, Holt BJ, White GP, Sharp MJ, Sly PD, Holt PG.

School of Medicine, University of Wales, Swansea, United Kingdom.

A number of laboratories have reported cord blood T cell responses to ubiquitous environmental Ags, including allergens, by proliferation and cytokine secretion. Moreover, the magnitude of these responses has been linked with risk for subsequent expression of allergy. These findings have been widely interpreted as evidence for transplacental priming and the development of fetal T memory cells against Ags present in the maternal environment. However, we present findings

below that suggest that neonatal T cell responses to allergens (and other Ags) differ markedly from those occurring in later life. Notably, in contrast to allergen-responsive adult CD4(+) T cell cultures, responding neonatal T cell cultures display high levels of apoptosis. Comparable responses were observed against a range of microbial Ags and against a parasite Ag absent from the local environment, but not against autoantigen. A notable finding was the appearance in these cultures of CD4(+)CD25(+)CTLA4(+) T cells that de novo develop MLR-suppressive activity. These cells moreover expressed CD45RA and CD38, hallmarks of recent thymic emigrants. CFSE-labeling studies indicate that the CD4(+)CD25(+) cells observed at the end of the culture period were present in the day 0 starting populations, but they were not suppressive in MLR responses. Collectively, these findings suggest that a significant component of the reactivity of human neonatal CD4(+) T cells toward nominal Ag (allergen) represents a default response by recent thymic emigrants, providing an initial burst of short-lived cellular immunity in the absence of conventional T cell memory, which is limited in intensity and duration via the parallel activation of regulatory T cells.

PMID: 15322168 [PubMed - indexed for MEDLINE]

1171. J Leukoc Biol. 2004 Nov;76(5):1002-9. Epub 2004 Aug 17.

2-arachidonoylglycerol, an endogenous cannabinoid receptor ligand, induces the migration of EoL-1 human eosinophilic leukemia cells and human peripheral blood eosinophils.

Oka S, Ikeda S, Kishimoto S, Gokoh M, Yanagimoto S, Waku K, Sugiura T.

Faculty of Pharmaceutical Sciences, Teikyo University, Sagamiko, Kanagawa, Japan.

2-arachidonoylglycerol (2-AG) is an endogenous cannabinoid receptor ligand. To date, two types of cannabinoid receptors have been identified: the CB1 receptor, abundantly expressed in the brain, and the CB2 receptor, expressed in various lymphoid tissues such as the spleen. The CB1 receptor has been assumed to play an important role in the regulation of synaptic transmission, whereas the physiological roles of the CB2 receptor remain obscure. In this study, we examined whether the CB2 receptor is present in human eosinophils and found that the CB2 receptor is expressed in human peripheral blood eosinophils. In contrast, human neutrophils do not contain a significant amount of the CB2 receptor. We then examined the effect of 2-AG on the motility of eosinophils. We found that 2-AG induces the migration of human eosinophilic leukemia EoL-1 cells. The migration evoked by 2-AG was abolished in the presence of SR144528, a CB2 receptor antagonist, or by pretreatment of the cells with pertussis toxin, suggesting that the CB2 receptor and Gi/o are involved in the 2-AG-induced migration. The migration of EoL-1 cells induced by 2-AG was suggested to be a result of chemotaxis. In contrast to 2-AG, neither anandamide nor free arachidonic acid elicited the migration. Finally, we examined the effect of 2-AG on human peripheral blood eosinophils and neutrophils and found that 2-AG induces migration of eosinophils but not neutrophils. These results suggest that the CB2 receptor and its endogenous ligand 2-AG may be closely involved in allergic inflammation accompanied by the infiltration of eosinophils.

PMID: 15316028 [PubMed - indexed for MEDLINE]

1172. Ann Univ Mariae Curie Sklodowska Med. 2003;58(1):459-65.

Health effects of inhalation exposure to organic dust in hops farmers.

Skórska C, Mackiewicz B, Góra A, Golec M, Dutkiewicz J.

Pulmonary Department, Medical University of Lublin.

Medical examinations were performed in a group of 23 hops farmers exposed to organic dust from hop (Humulus lupulus). The examinations took place in individual farms during harvesting, sorting and transporting of hop cones. As a reference group, 50 urban dwellers not exposed to organic dust were examined. There were conducted physical examinations, interviews concerning the occurrence of respiratory disorders and work-related symptoms, lung function tests, determination of cytokines concentrations, and allergological tests comprising skin prick test with 4 microbial antigens associated with organic dust, precipitin test with 12 microbial antigens, and a test for inhibition of leukocyte migration. Five farmers (21.7%) reported occurrence of work-related symptoms, including dry cough and dyspnoea. Eight farmers (34.8%) reported symptoms of chronic bronchitis. Mean spirometric values were within normal ranges. The farmers showed positive responses in precipitin test and test for inhibition of leukocyte migration to antigens of environmental microbes, mainly to the antigen of Gram-negative bacterium Pantoea agglomerans. The results showed a potential risk of occupational respiratory diseases in the population of hops farmers.

PMID: 15315032 [PubMed - indexed for MEDLINE]

1173. J Allergy Clin Immunol. 2004 Aug;114(2 Suppl):S32-50.

Synthetic responses in airway smooth muscle.

Howarth PH, Knox AJ, Amrani Y, Tliba O, Panettieri RA Jr, Johnson M.

Respiratory Cell and Molecular Biology, Southampton General Hospital, Southampton, United Kingdom. phh1@soton.ac.uk

Human airway smooth muscle (ASM) has several properties and functions that contribute to asthma pathogenesis, and increasing attention is being paid to its synthetic capabilities. ASM can promote the formation of the interstitial extracellular matrix, and in this respect, ASM from asthmatic subjects compared with normal subjects responds differently, both qualitatively and quantitatively. Thus, ASM cells are important regulating cells that potentially contribute to the known alterations within the extracellular matrix in asthma. In addition, through integrin-directed signaling, extracellular matrix components can alter the proliferative, survival, and cytoskeletal synthetic function of ASM cells. ASM also functions as a rich source of biologically active chemokines and cytokines that are capable of perpetuating airway inflammation in asthma and chronic obstructive pulmonary disease by promoting recruitment, activation, and trafficking of inflammatory cells in the airway milieu. Emerging evidence shows that airway remodeling may also be a result of the autocrine action of secreted inflammatory mediators, including T(H)2 cytokines, growth factors, and COX-2-dependent prostanoids. Finally, ASM cells contain both beta(2)-adrenergic receptors and glucocorticoid receptors and may represent a key target for beta(2)-adrenergic receptor agonist/corticosteroid interactions. Combinations of long-acting beta(2)-agonists and corticosteroids appear to have additive and/or synergistic effects in inhibiting inflammatory mediator release and the migration and proliferation of ASM cells.

PMID: 15309017 [PubMed - indexed for MEDLINE]

1174. J Allergy Clin Immunol. 2004 Aug;114(2 Suppl):S2-17.

Proliferative aspects of airway smooth muscle.

Hirst SJ, Martin JG, Bonacci JV, Chan V, Fixman ED, Hamid QA, Herszberg B, Lavoie JP, McVicker CG, Moir LM, Nguyen TT, Peng Q, Ramos-Barbón D, Stewart AG.

Department of Asthma, Allergy and Respiratory Science, Guy's, King's and St. Thomas' School of Medicine, Guy's Hospital Campus, King's College London, United Kingdom. stuart.hirst@kcl.ac.uk

Increased airway smooth muscle (ASM) mass is perhaps the most important component of the airway wall remodeling process in asthma. Known mediators of ASM proliferation in cell culture models fall into 2 categories: those that activate receptors with intrinsic receptor tyrosine kinase activity and those that have their effects through receptors linked to heterotrimeric guanosine triphosphate-binding proteins. The major candidate signaling pathways activated by ASM mitogens are those dependent on extracellular signal-regulated kinase and phosphoinositide 3'-kinase. Increases in ASM mass may also involve ASM migration, and in culture, the key signaling mechanisms have been identified as the p38 mitogen-activated protein kinase and the p21-activated kinase 1 pathways. New evidence from an in vivo rat model indicates that primed CD4(+) T cells are sufficient to trigger ASM and epithelial remodeling after allergen challenge. Hyperplasia has been observed in an equine model of asthma and may account for the increase in ASM mass. Reduction in the rate of apoptosis may also play a role. beta(2)-Adrenergic receptor agonists and glucocorticoids have antiproliferative activity against a broad spectrum of mitogens, although it has become apparent that mitogens are differentially sensitive. Culture of ASM on collagen type I has been shown to enhance proliferative activity and prevent the inhibitory effect of glucocorticoids, whereas beta(2)-agonists are minimally affected. There is no evidence that long-acting beta(2)-agonists are more effective than short-acting agonists, but persistent stimulation of the beta(2)-adrenergic receptor probably helps suppress growth responses. The maximum response of fluticasone propionate against thrombin-induced proliferation is increased when it is combined with salmeterol.

PMID: 15309015 [PubMed - indexed for MEDLINE]

1175. Clin Exp Allergy. 2004 Aug;34(8):1299-306.

Allergic airway inflammation is exacerbated during acute influenza infection and correlates with increased allergen presentation and recruitment of allergen-specific T-helper type 2 cells.

Marsland BJ, Scanga CB, Kopf M, Le Gros G.

Malaghan Institute of Medical Research, Wellington School of Medicine, Wellington, New Zealand. marsland@env.ethz.ch

Comment in
    Clin Exp Allergy. 2004 Aug;34(8):1153-5.

BACKGROUND: Respiratory viral infections are a leading cause of the hospitalization of asthmatics, however, the cellular immunological interactions which underlie these two diseases remain elusive.
OBJECTIVE: We sought to characterize the effect influenza viral infection has on allergic airway inflammation and to identify the cellular pathways involved.
METHODS: We have used an ovalbumin (OVA) model of allergic airway inflammation, which involves sensitization of animals with OVA adsorbed in alum adjuvant followed by an intranasal challenge with OVA in phosphate-buffered saline. To

study T cell recruitment into the lung, we adoptively transferred in vitro activated T cell receptor-transgenic T cells, which were subsequently identified by fluorescence-activated cell sorting (FACS) analysis. In addition, to study in vivo dendritic cell (DC) migration, we administered fluorescently labelled dextran and identified DCs that had phagocytosed it by FACS analysis.
RESULTS: We found that different stages of influenza infection had contrasting effects upon the outcome of OVA-induced allergic airway inflammation. The allergic response against OVA was exacerbated during the acute stage of influenza infection; however, mice were protected against the development of airway eosinophilia at late time-points following infection. We investigated the mechanisms responsible for the virus-induced exacerbation and found that the response was partially independent of IL-4 and that there was increased delivery of inhaled allergens to the draining lymph node during the acute stage of the infection. In addition, virus-induced inflammation in the lung and draining lymph node resulted in the non-specific recruitment of circulating allergen-specific effector/memory cells.
CONCLUSION: In addition to virus-mediated damage to the lung and airways, influenza viral infection can also enhance unrelated local allergic responses.

PMID: 15298573 [PubMed - indexed for MEDLINE]

1176. Clin Exp Allergy. 2004 Aug;34(8):1283-90.

Differential modulation of human basophil functions through prostaglandin D2 receptors DP and chemoattractant receptor-homologous molecule expressed on Th2 cells/DP2.

Yoshimura-Uchiyama C, Iikura M, Yamaguchi M, Nagase H, Ishii A, Matsushima K, Yamamoto K, Shichijo M, Bacon KB, Hirai K.

Department of Allergy and Rheumatology, University of Tokyo Graduate School of Medicine, Tokyo, Japan.

BACKGROUND: Both prostaglandin (PG) D receptor (DP) and CRTH2 (chemoattractant receptor-homologous molecule expressed on Th2 cells)/DP2 are high-affinity receptors for PGD2. Previous studies have demonstrated that PGD2 enhances releasability and induces CRTH2/DP2-mediated migration in human basophils, but the precise effects of PGD2 on basophils as well as receptor usage have not been fully clarified.
OBJECTIVE: We comprehensively explored the roles of DP and CRTH2/DP2 in basophil functions by using selective agonists and antagonists for each receptor.
METHODS: DP and CRTH2/DP2 transcripts were quantified by real-time PCR. We studied the effects of selective agonists (DP: BW245C; CRTH2/DP2: 13,14-dihydro-15-keto (DK)-PGD2) and/or antagonists (DP: BWA868C; CRTH2/DP2: ramatroban) on Ca2+ mobilization, migration, degranulation, CD11b expression and survival of human basophils.
RESULTS: Basophils expressed transcripts of both DP and CRTH2/DP2, but the levels of CRTH2/DP2 transcripts were ca. 100-fold higher compared with DP transcripts. Ca2+ influx was induced in basophils by either PGD2 or DK-PGD2/CRTH2 agonist but not by BW245C/DP agonist. Basophils treated with PGD2 were completely desensitized to subsequent stimulation with DK-PGD2, but not vice versa. DK-PGD2 as well as PGD2 up-regulated CD11b expression, induced migration and enhanced degranulation, and those effects were completely antagonized by ramatroban/CRTH2 antagonist. In contrast, BW245C/DP agonist exhibited an inhibitory effect on basophil migration and IgE-mediated degranulation, and the migration inhibitory effect was effectively antagonized by BWA868C/DP antagonist. On the other hand, while PGD2 significantly shortened the basophil life-span, neither DK-PGD2/CRTH2 agonist nor BW245C/DP agonist did.
CONCLUSION: CRTH2/DP2 is primarily responsible for the pro-inflammatory effects

of PGD2 on human basophils, while DP introduces negative signals capable of antagonizing the effects of CRTH2/DP2 in these cells. The effects of PGD2 on longevity imply a mechanism(s) other than via DP or CRTH2/DP2. CRTH2/DP2 on basophils may afford opportunities for therapeutic targeting in allergic inflammation.

PMID: 15298571 [PubMed - indexed for MEDLINE]

1177. Clin Exp Allergy. 2004 Aug;34(8):1276-82.

Interactions between eotaxin, histamine and mast cells in early microvascular events associated with eosinophil recruitment to the site of allergic skin reactions in humans.

Menzies-Gow A, Ying S, Phipps S, Kay AB.

Department of Allergy and Clinical Immunology, National Heart and Lung Institute, Imperial College, London, UK.

BACKGROUND: The mechanism whereby allergen induces eotaxin expression at the site of allergic inflammation is incompletely understood. Structural cells, including endothelial cells, are a major source of eotaxin.
OBJECTIVE: We have investigated, in vivo and in vitro, the relationship between mast cell activation and the expression of eotaxin (eotaxin 1) by endothelial cells.
METHODS: The effects of intradermal allergen challenge and histamine injection on eotaxin mRNA and protein generation were studied in atopic subjects using immunofluorescence, immunohistochemistry and in situ hybridization. Histamine-induced expression of eotaxin mRNA and protein by endothelial cells was also measured, as was histamine-induced eosinophil adhesion to cultured endothelial cells.
RESULTS: A rapid increase in degranulating cutaneous mast cells, together with a concomitant increase in eosinophils, was observed 60 min after allergen challenge. This was accompanied by the appearance of immunoreactive eotaxin that peaked at 1 h around blood vessels and at 3 h within the tissue. Intradermal histamine injection produced an increase in the number of eotaxin+ cells in the tissues, which was maximal at the 3-h time-point. In vitro, endothelial cells produced eotaxin mRNA and protein product in a dose- and time-dependent fashion following incubation with histamine, an effect that was blocked by levocetirizine. Pre-incubation of endothelial cells with histamine also induced a significant increase in eosinophil adherence, an effect that was inhibited with an anti-eotaxin blocking monoclonal antibody.
CONCLUSION: The antigen-induced expression of eotaxin by endothelial cells and the adherence and subsequent migration of eosinophils from the microvasculature to the tissues are rapid events partially under the control of histamine released from degranulating mast cells.

PMID: 15298570 [PubMed - indexed for MEDLINE]

1178. Int Arch Allergy Immunol. 2004 Sep;135(1):12-6. Epub 2004 Jul 29.

Thrombin affects eosinophil migration via protease-activated receptor-1.

Feistritzer C, Mosheimer BA, Kaneider NC, Riewald M, Patsch JR, Wiedermann CJ.

Division of General Internal Medicine, Department of Internal Medicine, Medical University of Innsbruck, Anichstrasse 35, AT-6020 Innsbruck, Austria.

BACKGROUND: Protease-activated receptors (PARs) are a unique class of G-protein-coupled receptors, which are activated by proteolytic cleavage of the amino terminus of the receptor itself. Although expression of the PAR1, which is typically activated by thrombin, on human eosinophils has been demonstrated, no effect of thrombin on eosinophil function has been shown yet. Thus we investigated whether thrombin affects eosinophil migration in vitro.
METHODS: Eosinophils were obtained from venous blood of healthy donors. Cell migration was studied by micropore filter assays. Involvement of PARs in thrombin-dependent migration was tested functionally using selective agonist peptides for PARs and a cleavage blocking PAR1 antibody.
RESULTS: Thrombin significantly stimulated eosinophil chemotaxis in a dose-dependent manner. This effect was mimicked by the PAR1 but not the PAR2 agonist and was reversed by the cleavage blocking PAR1 antibody. Checkerboard experiments indicated that eosinophil migration depends on the presence of thrombin in a concentration gradient.
CONCLUSIONS: Data suggest that activation of PAR1 by thrombin stimulates directed migration of human eosinophils and thereby may affect eosinophils in tissue and allergic inflammation.

Copyright 2004 S. Karger AG, Basel

PMID: 15286440 [PubMed - indexed for MEDLINE]

1179. Fish Shellfish Immunol. 2004 Sep;17(3):293-303.

Production of recombinant C5a from rainbow trout (Oncorhynchus mykiss): role in leucocyte chemotaxis and respiratory burst.

Boshra H, Peters R, Li J, Sunyer JO.

Department of Pathobiology, School of Veterinary Medicine, University of Pennsylvania, 413 Rosenthal, 3800 Spruce Street, Philadelphia, PA 19104, USA.

Activation of the complement system can lead to the formation of the membrane attack complex, in which the component C5 is cleaved into C5a and C5b fragments. The C5a anaphylatoxin is a very potent pro-inflammatory molecule that induces chemotaxis and respiratory burst processes in a variety of mammalian leucocytes. While C5a has been well studied in mammals, little is known about the structure and function of C5a in teleost fish or other non-mammalian species. In the present study, we have produced and purified recombinant rainbow trout C5a (rtC5a), and we have shown that it plays an important role in inducing leucocyte migration as well as in triggering the respiratory burst of peripheral blood (PBLs) and head kidney leucocytes (HKLs). When the carboxy-terminal Arg was removed from rtC5a, its ability to induce cell migration and superoxide production remained intact. Interestingly, we show that leucocytes migrating towards rtC5a attached to the plate with a well-spread circular morphology, whereas those migrating towards activated trout serum displayed more irregular and dendritic-like shapes. Our data suggest that the basic mechanisms of action of the C5a anaphylotoxin have remained conserved for more than 300 million years.

PMID: 15276608 [PubMed - indexed for MEDLINE]

1180. Toxicol Appl Pharmacol. 2004 Aug 1;198(3):434-43.

Arsenic-induced alterations in the contact hypersensitivity response in Balb/c mice.

Patterson R, Vega L, Trouba K, Bortner C, Germolec D.

Laboratory of Molecular Toxicology, National Institute of Environmental Health Sciences, Research Triangle Park, NC 27709, USA.

Previous studies in our laboratory indicate that arsenic alters secretion of growth promoting and inflammatory cytokines in the skin that can regulate the migration and maturation of Langerhans cells (LC) during allergic contact dermatitis. Therefore, we hypothesized that arsenic may modulate hypersensitivity responses to cutaneous sensitizing agents by altering cytokine production, LC migration, and T-cell proliferation. To investigate this hypothesis, we examined the induction and elicitation phases of dermal sensitization. Mice exposed to 50 mg/l arsenic in the drinking water for 4 weeks demonstrated a reduction in lymph node cell (LNC) proliferation and ear swelling following sensitization with 2,4-dinitrofluorobenzene (DNFB), compared to control mice. LC and T-cell populations in the draining lymph nodes of DNFB-sensitized mice were evaluated by fluorescence-activated cell sorting; activated LC were reduced in cervical lymph nodes, suggesting that LC migration may be altered following arsenic exposure. Lymphocytes from arsenic-treated animals sensitized with fluorescein isothiocyanate (FITC) exhibited reduced proliferative responses following T-cell mitogen stimulation in vitro; however, lymphocyte proliferation from nonsensitized, arsenic-treated mice was comparable to controls. Arsenic exposure also reduced the number of thioglycollate-induced peritoneal macrophages and circulating neutrophils. These studies demonstrate that repeated, prolonged exposure to nontoxic concentrations of sodium arsenite alters immune cell populations and results in functional changes in immune responses, specifically attenuation of contact hypersensitivity.

PMID: 15276424 [PubMed - indexed for MEDLINE]

1181. Biochem Pharmacol. 2004 Aug 15;68(4):631-9.

Modulation of eosinophil migration from bone marrow to lungs of allergic rats by nitric oxide.

Ferreira HH, Costa RA, Jacheta JM, Martins AR, Medeiros MV, Macedo-Soares MF, De Luca IM, Antunes E, De Nucci G.

Laboratory of Inflammation Research, São Francisco University, Bragança Paulista, São Paulo, Brazil. heloisaferreira@saofrancisco.edu.br

Chronic blockade of nitric oxide (NO) synthesis attenuates the eosinophil infiltration into airways of allergic rats. This study was designed to investigate whether the inhibition of eosinophil influx to the lung of allergic rats reflects modifications in the pattern of cell mobilization from the bone marrow to peripheral blood and/or to lung. Male Wistar rats were treated with N(omega)-nitro-l-arginine methyl ester (l-NAME; 20mg/rat per day) for 4 weeks and sensitized with ovalbumin (OVA). In control rats, the pulmonary OVA-challenge promoted an early (24h) increase in the bone marrow eosinophil population that normalized at 48 h after OVA-challenge, at which time the eosinophils disappeared from the blood and reached the lungs in mass. In l-NAME-treated rats, an accumulation of eosinophils in bone marrow was observed at 24 and 48 h post-OVA-challenge. No variation in this cell type number was observed in peripheral blood and bronchoalveolar lavage throughout the time-course studied. In control rats, the adhesion of bone marrow eosinophils to fibronectin-covered wells was significantly increased at 24h after OVA-challenge, whereas in l-NAME-treated rats the increased adhesion was detected at 48 h. A 32% decrease in the expression of inducible nitric oxide synthase (iNOS) (but not endothelial nitric oxide synthase; eNOS) in eosinophils from l-NAME-treated rats was observed. The levels of IgE, IgG(1) and IgG(2a) were not affected by the l-NAME

treatment. Our findings suggest that inhibition of NO synthesis upregulates the binding of eosinophils to extracellular matrix proteins such as fibronectin, producing a delayed efflux of eosinophils from bone marrow to peripheral blood and lungs.

PMID: 15276070 [PubMed - indexed for MEDLINE]

1182. J Immigr Health. 2004 Jul;6(3):137-44.

The health of the California region bordering Mexico.

Garza A, Rodriguez-Lainz A, Ornelas IJ.

Latino Center for Medical Education and Research, University of California, San Francisco-Fresno, 93710-7702, USA. agarza@ucsfresno.edu

Healthy Border (HB) 2010 is the health promotion and disease prevention agenda through the year 2010 of the United States-Mexico Border Health Commission (BHC). On the United States side, it draws from the Healthy People (HP) 2010 objectives, identifying those most important and relevant for the border. The BHC has harmonized the list of objectives from both countries into a set of 19 that will be monitored and addressed in a collaborative manner. HB provides a framework for describing the border region's health and comparing with others. For this report, available data were collected for the HB indicators for San Diego and Imperial counties, and for California. Data on Latino populations were considered a proxy for Mexican-Americans and people of Mexican origin in California, because more specific data are not available. Results are presented on the 14 indicators for which the data were most complete. Those of most concern include access to health care and tuberculosis in both counties, plus motor vehicle crash injury deaths and asthma hospitalizations in Imperial. These issues should be given priority attention. Conversely, the region's and Latinos' experience with breast cancer mortality and infant mortality is favorable. Recommendations include binational collaborations in assessing and improving the health of our border communities.

PMID: 15269517 [PubMed - indexed for MEDLINE]

1183. J Asthma. 2004;41(3):289-304.

Association between traffic volume and health care use for asthma among residents at a U.S.-Canadian border crossing point.

Lwebuga-Mukasa JS, Oyana T, Thenappan A, Ayirookuzhi SJ.

Department of Internal Medicine, University at Buffalo School of Medicine and Biomedical Sciences, Kaleida Health Buffalo General Division, Buffalo, New York 14203, USA. jlwebuga@acsu.buffalo.edu

Little information is available about health impacts of the North American Free Trade Agreement (NAFTA) traffic-related pollution on residents near the major traffic corridors along the U.S.-Canadian border. Here we report on a 10 year (1991-2000) retrospective study of commercial traffic volumes across the Peace Bridge and health care use for asthma in a residential community, which serves as a conduit for traffic crossing between Fort Erie, Ontario, Canada, and Buffalo, New York. We hypothesized that commercial traffic pollution was impacting on residents in close proximity to the trade corridor. Commercial traffic volumes, hospital discharges for asthma, and outpatient visits to area hospitals and clinics were analyzed before and after implementation of NAFTA. Results showed a positive association between increased commercial traffic volume and increased

health care use for asthma. Zip codes 14201 and 14213, which surround the Peace Bridge Plaza Complex (PBC), had the highest prevalence rates and health care use rates for asthma. Statistical analysis showed the findings to be significant ($p < 0.05$) in that residential proximity to the PBC was associated with greater hospital discharge rates for asthma. The findings were strongest ($p < 0.000$) in the zip codes where the PBC was located (14213) and the major highway I-190 passed through (14201). A yearly excess of 230.2 adult asthma hospital discharges was associated with an increase in traffic volume during the period from 1991 to 1996 in the study area. This is in contrast to an overall decrease in the national rate of hospitalizations for asthma by 7.5% in the same period. The results suggest that NAFTA-related commercial traffic has a negative health impact on asthmatics living in close proximity to the trade corridor. Health and social costs due to traffic pollution need to be included in cost estimates of transport decisions related to the NAFTA corridors. Similar health effects due to NAFTA traffic need to be studied at other U.S.-Canada border crossing points.

PMID: 15260462 [PubMed - indexed for MEDLINE]

1184. World J Gastroenterol. 2004 Aug 1;10(15):2254-8.

Pathophysiological significance of a reaction in mouse gastrointestinal tract associated with delayed-type hypersensitivity.

Yu WG, Lin P, Pan H, Xiao L, Gong EC, Mei L.

Department of Physiology, Medical College of Yangtze University, Jingzhou, Hubei Province, China.

AIM: To explore the pathophysiological significance of delayed type hypersensitivity (DTH) reaction in mouse gastrointestinal tract induced by an allergen 2,4-dinitrochlorobenzene (DNCB).
METHODS: BALB/c mice were randomly divided into control and DTH(1-6) groups. After sensitized by DNCB smeared on the abdominal skin, the mice were challenged with DNCB by gavage or enema. The weight, stool viscosity and hematochezia were observed and accumulated as disease active index (DAI) score; the gastrointestinal motility was represented by active charcoal propulsion rate; the colon pathological score was achieved by macropathology and HE staining of section prepared for microscopy; and the leukocyte migration inhibitory factor (LMIF) activity was determined by indirect capillary assay of the absorbance (A) of migrated leukocytes.
RESULTS: Active charcoal propulsion rates of small intestine in the DNCB gavages groups were significantly higher than that in the control group ($P<0.01$). The DAI scores and pathological score in DNCB enema groups were also higher than that in the control group ($P<0.05$), and there were significant rises in LMIF activity in DNCB enema groups as compared with control groups ($P<0.01$).
CONCLUSION: Mouse gastrointestinal DTH reaction could be induced by DNCB, which might facilitate the mechanism underlying the ulcerative colitis.

PMID: 15259076 [PubMed - indexed for MEDLINE]

1185. Eur J Immunol. 2004 Aug;34(8):2100-9.

Disruption of the stratum corneum allows potent epicutaneous immunization with protein antigens resulting in a dominant systemic Th2 response.

Strid J, Hourihane J, Kimber I, Callard R, Strobel S.

Immunobiology Unit, Institute of Child Health, University College London, 30

Guilford Street, London WC1N 1EH, GB. j.strid@ich.ucl.ac.uk

The skin is an important immunological organ with an outer protective layer, the stratum corneum forming a barrier between the skin-associated lymphoid tissue and the environment. We show that gently removing the stratum corneum with adhesive tape permits potent epicutaneous immunization to protein antigens. IL-4 secretion by T cells from draining lymph nodes and high levels of specific IgE and IgG1 with no IgG2a showed that the immune responses induced following epicutaneous antigen exposure are strongly Th2 biased. Similar responses were obtained with different antigens and mouse strains. In contrast, subcutaneous immunization with antigen delivery into the dermis was less potent and gave predominantly Th1 responses. Removal of the stratum corneum increased expression of MHC class II, CD86, CD40, CD54 and CD11c on Langerhans cells, but did not cause them to migrate. Rapid migration from epidermis to draining lymph node was obtained, however, by exposure to antigen after removal of the stratum corneum, suggesting that maturation and migration of Langerhans cells are independently regulated events. These results suggest that antigen presentation by Langerhans cells gives predominantly Th2 responses. This may provide an explanation for allergic sensitization to some antigens. It may also be a useful non-invasive, non-adjuvant-dependent method of vaccination.

PMID: 15259007 [PubMed - indexed for MEDLINE]

1186. Am J Respir Cell Mol Biol. 2004 Nov;31(5):528-37. Epub 2004 Jul 15.

Epidermal growth factor and trefoil factor family 2 synergistically trigger chemotaxis on BEAS-2B cells via different signaling cascades.

Chwieralski CE, Schnurra I, Thim L, Hoffmann W.

Institut für Molekularbiologie und Medizinische Chemie, Otto-von-Guericke-Universität, Magdeburg, Germany.

Injured areas of the respiratory epithelium are subject to rapid repair by the migration of adjacent epithelial cells, a process termed "restitution". Rapid re-epithelialization is promoted by interactions between migrating cells and the extracellular matrix proteins. Furthermore, epidermal growth factor (EGF) as well as trefoil factor family (TFF) peptides are well known regulators of epithelial restitution due to their motogenic effects. Migration of the human bronchial epithelial cell line BEAS-2B in modified Boyden chambers was used as a model system for airway restitution. EGF or recombinant human TFF2 or TFF3 showed mainly chemotactic activity. The motogenic response was strictly dependent upon a haptotactic substrate, but to different degrees. EGF induced phosphorylation of extracellular signal-regulated kinases (ERK) 1/2, c-Jun-N-terminal kinase, p38, Akt, and p70S6K in BEAS-2B cells. Using specific inhibitors, the signaling cascades responsible for the motogenic response were shown to differ drastically when EGF was compared with TFF2. The motogenic effect of TFF2 was previously demonstrated to depend on ERK1/2 and protein kinase C activation; whereas the EGF-triggered motogenic response was completely independent of ERK1/2 activation but sensitive to the inhibition of phosphoinositide 3-kinase, p38, protein kinase C, or nuclear factor kappaB. However, the motogenic effects of EGF and TFF2 are additive. These data suggest that luminal EGF and TFF peptides can act synergistically in the human respiratory epithelium to enhance rapid repair processes in the course of diseases such as asthma.

PMID: 15256384 [PubMed - indexed for MEDLINE]

1187. Proc Natl Acad Sci U S A. 2004 Jul 27;101(30):11170-5. Epub 2004 Jul 14.

Human eosinophil chemotaxis and selective in vivo recruitment by sphingosine 1-phosphate.

Roviezzo F, Del Galdo F, Abbate G, Bucci M, D'Agostino B, Antunes E, De Dominicis G, Parente L, Rossi F, Cirino G, De Palma R.

Dipartimento di Scienze Farmaceutiche, Università di Salerno, 84084 Salerno, Italy.

Sphingosine 1-phosphate (S1P) is a sphingolipid mediator that is involved in diverse biological functions. Local administration of S1P causes inflammation coupled to a large eosinophil (EO) recruitment in the rat-paw tissue. The inflammatory response is accompanied by an increase in S1P receptors, namely S1P(1), S1P(2), S1P(3), and by an enhanced expression of CCR3, which is the main chemokine receptor known to be involved in EO function. Human EOs constitutively express S1P(1) and, at a lower extent, S1P(2), S1P(3) receptors. S1P in vitro causes cultured human EO migration and an increase in S1P receptor mRNA copies and strongly up-regulates CCR3 and RANTES (regulated on activation, normal T cell-expressed and secreted) message levels; in particular CCR3 is up-regulated 18,000-fold by S1P. A blocking anti-CCR3 Ab inhibits S1P-induced chemotaxis, implying that S1P acts as specific recruiting signal for EOs not only through its own receptors but also through CCR3. These results show that S1P is involved in EO chemotaxis and contribute to shed light on the complex mechanisms underlying EO recruitment in several diseases such as asthma and some malignancies.

PMCID: PMC503758
PMID: 15254297 [PubMed - indexed for MEDLINE]

1188. Toxicol In Vitro. 2004 Oct;18(5):681-90.

Assessment of contact allergens by dissociation of irritant and sensitizing properties.

Jacobs JJ, Lehé CL, Cammans KD, Das PK, Elliott GR.

AmCOGG, AMC, Meibergdreef 9, Amsterdam 1105 AZ, The Netherlands. jjljacobs@yahoo.com

The human organotypic skin explant culture (hOSEC) model is a promising alternative in vitro model for screening contact allergens. In this model, the chemical-induced migration of Langerhans cells (LCs) out of the epidermis, evaluated after a 24-h exposure period, is used as a measure of sensitizer potential. As skin irritants can also induce LC migration it is essential that concentrations of test chemicals are used that are not even weakly irritant. Using the hOSEC irritation model chemicals are classified as weak irritants if they are toxic after a 48-h exposure period. Toxicity is determined by methyl green-pyronine (MGP) staining of hOSEC. We studied three frequently used non-sensitizing skin irritants and six potent or frequent human sensitizers in a dose-response. A complete discrimination between non-sensitizers and contact sensitizers was obtained for the chemicals tested when the concentrations used were lower than the weak irritant concentrations. Frequency of positive allergen reactions in patch test of human populations correlated with the difference between weak irritant concentrations and the lowest concentration inducing significant LC migration. Sensitizer potency correlated with chemical irritancy as determined by keratinocyte death. For the compounds tested, the hOSEC model predicted allergenicity in humans better than the guinea pig maximization test and the mouse local lymph node assay.

PMID: 15251187 [PubMed - indexed for MEDLINE]

1189. J Immunol. 2004 Jul 15;173(2):1380-9.

A3 adenosine receptor signaling contributes to airway inflammation and mucus production in adenosine deaminase-deficient mice.

Young HW, Molina JG, Dimina D, Zhong H, Jacobson M, Chan LN, Chan TS, Lee JJ, Blackburn MR.

Department of Biochemistry and Molecular Biology, University of Texas-Houston Medical School, Houston, TX 77030, USA.

Adenosine signaling has been implicated in chronic lung diseases such as asthma and chronic obstructive pulmonary disease; however, the specific roles of the various adenosine receptors in processes central to these disorders are not well understood. In this study, we have investigated the role(s) of the A(3) adenosine receptor in adenosine-dependent pulmonary inflammation observed in adenosine deaminase (ADA)-deficient mice. The A(3) receptor (A(3)R) was found to be expressed in eosinophils and mucus-producing cells in the airways of ADA-deficient mice. Treatment of ADA-deficient mice with MRS 1523, a selective A(3)R antagonist, prevented airway eosinophilia and mucus production. Similar findings were seen in the lungs of ADA/A(3) double knockout mice. Although eosinophils were decreased in the airways of ADA-deficient mice following antagonism or removal of the A(3)R, elevations in circulating and lung interstitial eosinophils persisted, suggesting signaling through the A(3)R is needed for the migration of eosinophils into the airways. These findings identify an important role for the A(3)R in regulating lung eosinophilia and mucus production in an environment of elevated adenosine.

PMID: 15240734 [PubMed - indexed for MEDLINE]

1190. J Agric Food Chem. 2004 Jul 14;52(14):4571-6.

Inhibitory effect of (-)-epigallocatechin 3-gallate, a polyphenol of green tea, on neutrophil chemotaxis in vitro and in vivo.

Takano K, Nakaima K, Nitta M, Shibata F, Nakagawa H.

Department of Physiological Chemistry, Faculty of Pharmaceutical Sciences, Toyama Medical and Pharmaceutical University, 2630 Sugitani, Toyama 930-0194, Japan. takanok@ms.toyama-mpu.ac.jp

Comment in
    J Agric Food Chem. 2005 Feb 23;53(4):1306; author reply 1307-8.

The effect of (-)-epigallocatechin 3-gallate (EGCG), a major polyphenol of green tea, on neutrophil migration has been studied using multiwell-type Boyden chambers in vitro and a fluorescein isothiocyanate-labeled ovalbumin (FITC-OVA)-induced rat allergic inflammation model in vivo. EGCG inhibited rat neutrophil chemotaxis toward cytokine-induced neutrophil chemoattractant-1 (CINC-1) in a concentration-dependent manner. In addition, CINC-1-induced neutrophil chemotaxis was suppressed by the pretreatment of rat neutrophils with EGCG at the concentration over 15 microg/mL. EGCG caused concentration-dependent suppression of the transient increase in CINC-1-induced intracellular free calcium level in both rat neutrophils and rat CXC chemokine receptor 2 (CXCR2)-transfected HEK 293 cells. EGCG inhibited CINC-1 production by IL-1beta-stimulated rat fibroblasts (NRK-49F cells) and

lipopolysaccharide-stimulated rat macrophages at the concentration over 50 microg/mL, a comparatively high concentration. Oral administration of EGCG (1.0 mg or 1.5 mg/rat) at 1 h before the challenge with FITC-OVA suppressed neutrophil infiltration into the air pouch (inflammatory site) in the air-pouch type FITC-OVA-induced allergic inflammation in rats. Chemokine levels in the pouch fluids, however, were not influenced by EGCG administration. The results suggest that EGCG suppressed neutrophil infiltration by a direct action on neutrophils, but not by indirect actions, including the suppression of chemokine production at the inflammatory site.

PMID: 15237969 [PubMed - indexed for MEDLINE]

1191. Ann Agric Environ Med. 2004;11(1):121-7.

Immunologic reactivity to work-related airborne allergens in people occupationally exposed to dust from herbs.

Golec M, Skórska C, Mackiewicz B, Dutkiewicz J.

Department of Occupational Biohazards, Institute of Agricultural Medicine, Jaczewskiego 2, 20-090 Lublin, Poland. msgolec@yahoo.com

A group of 150 people occupationally exposed to dust from herbs were examined. The examined group consisted of 47 thyme farmers, 32 chamomile farmers, 31 sage farmers and 40 workers of herbs processing industry. As a reference group, 50 urban dwellers, not exposed to any kind of organic dust, were examined. Skin prick tests and precipitin tests were conducted with, respectively, 4 and 11 microbial antigens associated with organic dust. Both skin and precipitin tests were also conducted with herbal extracts of chamomile and sage. Precipitin tests were carried out with sera not concentrated and sera 3-fold concentrated. Tests for inhibition of leukocyte migration (MIF) were also conducted with 4 microbial antigens. People occupationally exposed to dust from herbs showed a higher frequency of positive skin reactions to microbial antigens compared to the reference group. The results of precipitin test also revealed greater reactivity to the environmental microbial antigens in the examined group, compared to the reference group. The highest frequency of positive results was noted with the antigen of Pantoea agglomerans (30.6 % with sera not concentrated and 48.3 % with sera 3-fold concentrated) - the difference compared to the reference group (12.0 %) was highly significant ($p < 0.01$). The frequencies of positive results of MIF test in the examined group were high with all antigens tested: Arthrobacter globiformis (12.6 %), Pantoea agglomerans (11.1 %), Saccharopolyspora rectivirgula (17.0 %), Aspergillus fumigatus (13.3 %), and, compared to the reference group with no positive result for any antigen, all the differences were significant ($p < 0.05$). In conclusion, the frequency of positive allergological reactions to airborne microorganisms was high in people occupationally exposed to dust from herbs and suggests a potential role of microbial allergens in the pathogenesis of work-related health disorders among herb workers. The risk of sensitization seems to be greatest among thyme farmers, who showed the highest positive response. The results confirmed the particular allergenic importance of Gram-negative bacterium Pantoea agglomerans.

PMID: 15236509 [PubMed - indexed for MEDLINE]

1192. Aviakosm Ekolog Med. 2004;38(2):24-8.

[Outcome of the long-term isolation on the development of allergic reactions in humans].

[Article in Russian]

Rykova MP, Gertsik IuG, Antropova EN, Buravkova LB, Meshkov DO.

Studied were consequences of long-term isolation in airtight environment for development of the type-I (IgE-antibodies production) and type-IV (involvement of sensibilized T-lymphocytes) allergic reactions in humans. No significant changes in total IgE, specific IgE-antibodies for domestic, epidermal, fungal, grass pollen and food-borne allergens or serum IL-4 level were found in the period of the 240-d isolation with the microclimate and atmospheric parameters within their normal variations, and on completion of the experiment. Yet, after 3 mos. in isolation all subjects exhibited inhibited leukocyte migration in the presence of tuberculin. These findings bear witness to an activation of sensibilized lymphocytes known as effectors of delayed hypersensitivity.

PMID: 15233033 [PubMed - indexed for MEDLINE]

1193. Mediators Inflamm. 2004 Jun;13(3):195-9.

Effect of influenza vaccinations on immune response and serum eotaxin level in patients with allergic bronchial asthma.

Jahnz-Rózyk K, Brydak LB, Targowski T, Machała M, Plusa T.

Department of Internal Medicine, Pneumonology and Allergology, Military Institute of Medicine, 128 Szaserów Street 00-909 Warsaw, Poland. krozyk@poczta.onet.pl

BACKGROUND: One of the most promising markers of allergic inflammation is eotaxin, which has a selective influence on the migration of eosinophils. Its serum content significantly correlates with the intensity of allergic symptoms, so it might be interesting to know whether vaccination has any influence on serum expression of this chemokine.
AIMS: Comparison of the humoral response to influenza vaccine and post-vaccination changes in the serum eotaxin level in patients with allergic bronchial asthma and healthy controls.
METHODS: Forty-two asthmatics and 45 healthy individuals were vaccinated with a single dose of influenza subunit vaccine (Influvac). The serum eotaxin level and the antibody response to haemagglutinin (HI) and neuraminidase (NI) glycoproteins were measured before and after vaccination.
RESULTS: A significant increase of geometric mean titres of HI and NI was observed in both groups. There were no significant differences between the groups in meanfold increase of HI and NI titres, response rate and protective level of HI. After vaccination, a significant decrease of the mean serum eotaxin value was observed in patients with asthma (149.4 +/- 71.0 versus 125.1 +/- 67.0, p= 0.0017), while no similar effect was present in healthy individuals (153.4 +/- 56.9 versus 159.3 +/- 54.4, p= 0.5).
CONCLUSIONS: The results indicate that in patients with allergic bronchial asthma influenza vaccinations assure efficient protective antibody level and modulate the serum level of eotaxin.

PMCID: PMC1781554
PMID: 15223611 [PubMed - indexed for MEDLINE]

1194. Prog Retin Eye Res. 2004 Jul;23(4):435-48.

The role of chemokines and their receptors in ocular disease.

Wallace GR, John Curnow S, Wloka K, Salmon M, Murray PI.

The Birmingham and Midland Eye Centre, Sandwell and West Birmingham Hospitals NHS Trust, The University of Birmingham, City Hospital, Dudley Road, Birmingham B18 7QU, UK. g.r.wallace@bham.ac.uk

The migration and infiltration of cells into the eye whether blood-borne leucocytes, endothelial or epithelial cells occurs in many ocular diseases. Dysregulation of this process is apparent in chronic inflammation, corneal graft rejection, allergic eye disease and other sight-threatening conditions. Under normal and inflammatory conditions, chemokines and their receptors are important contributors to cell migration. To date, 47 chemokines and 19 chemokine receptors have been identified and characterised. In recent years, investigations into the role of chemokines and their receptors in ocular disease have generated an increasing number of publications. In the eye, the best understood action of these molecules has arisen from the study of their ability to control the infiltration of leucocytes in uveitis. However, the involvement of chemokines in angiogenesis in several ocular conditions and in the survival of corneal transplants demonstrates the multifaceted nature of their effects. Interestingly, the constitutive expression of chemokines and their receptors in ocular tissues suggests that certain chemokines have a homeostatic function. In this review, we discuss the nature and function of chemokines in health and disease, and describe the role of chemokines in the pathogenesis of different ocular conditions.

PMID: 15219876 [PubMed - indexed for MEDLINE]

1195. Exp Dermatol. 2004 Jul;13(7):452-60.

Measurement of cytokine expression and Langerhans cell migration in human skin following suction blister formation.

Dearman RJ, Bhushan M, Cumberbatch M, Kimber I, Griffiths CE.

Syngenta Central Toxicology Laboratory, Alderley Park, Macclesfield, Cheshire SK10 4TJ, UK. rebecca.dearman@syngenta.com

Contact allergen-induced migration of epidermal Langerhans cells (LCs) to draining lymph nodes is dependent upon receipt by LCs of at least two cytokine signals provided by tumor necrosis factor-alpha (TNF-alpha) and interleukin (IL)-1beta. It has been reported previously that intradermal injection of healthy human volunteers with homologous TNF-alpha or IL-1beta each induces a significant reduction in LC frequency, as measured in epidermal sheets prepared from 6-mm punch biopsies. In the current experiments, we have compared the frequency of LCs in punch biopsies with those obtained concurrently in epidermal sheets from the roofs of suction blisters isolated from the sun-protected buttock skin of healthy adult volunteers. There was a significant, approximately 30%, reduction in CD1a(+) LC numbers in suction blister roofs compared with punch biopsies. Injection of homologous recombinant IL-1beta, a stimulus that provokes measurable epidermal LC mobilization in punch biopsy sites, failed to provoke further LC migration in suction blister sites. These data suggest that the mechanical trauma to the skin caused by the creation of suction blisters provokes the degree of cutaneous inflammation necessary for LC mobilization. The responsive cells (only a proportion of resident LCs, approximately 30%) have already migrated, thus addition of an exogenous cytokine signal (IL-1beta) is without further effect. It is not possible therefore to measure the regulation of LC mobilization by exogenous cytokines in suction blister roofs. However, this technique provides an opportunity to profile induced changes in the cutaneous cytokine environment, with cytokine expression measured by a multiple cytokine array system. Using this technique, intradermal injection of IL-1beta was found to cause a marked upregulation of proinflammatory cytokines including TNF-alpha, IL-6, IL-8,

monocyte chemotactic protein-1 (MCP-1) and the anti-inflammatory cytokine IL-10 in fluid from suction blisters raised at the site of injection. In conclusion, the suction blister technique appears to be a powerful tool for measurement of induced changes in cutaneous cytokines.

PMID: 15217366 [PubMed - indexed for MEDLINE]

1196. Med Wieku Rozwoj. 2003 Oct-Dec;7(4 Pt 2):557-63.

[Detection of the T-lymphocyte-specific sensitivity in the diagnosis of food allergies].

[Article in Polish]

Gołebiowska-Wawrzyniak M, Markiewicz K, Kozar A, Siwińska-Gołebiowska H.

Zaklad Immunologii Klinicznej, Instytut Matki i Dziecka, Kasprzaka 17a, 01-211 Warszawa, Poland. immunolog@imid.med.pl

BACKGROUND: The possibility of detection of lymphocytes T specific sensitivity after contact with the allergen (mainly food allergen) even in early age of children has been reported in this paper. METHODS/DATA BASE: This is the literature review of basic applied methods such as: lymphocyte blastic transformation in allergen stimulated culture, macrophage migration inhibition test and flow cytometric analysis of in vivo and in vitro lymphocyte specific activation by using activation markers (CD69, PCNA - proliferating cell nuclear antigen and others). RESULTS/CONCLUSIONS: The significance of such investigation is underlined especially for young children during early diagnosis of food allergy and monitoring of the treatment.

PMID: 15213370 [PubMed - indexed for MEDLINE]

1197. J Biochem. 2004 Jun;135(6):673-81.

Sphingosine 1-phosphate inhibits migration of RBL-2H3 cells via S1P2: cross-talk between platelets and mast cells.

Yokoo E, Yatomi Y, Takafuta T, Osada M, Okamoto Y, Ozaki Y.

Departments of Otolaryngology and Laboratory Medicine, University of Yamanashi Faculty of Medicine, 1110 Tamaho, Nakakoma, Yamanashi 409-3898, Japan.

To analyze the involvement in allergic reactions of platelets and sphingosine 1-phosphate (Sph-1-P), a lysophospholipid mediator released from activated platelets, the effects of Sph-1-P and a supernatant prepared from activated platelets on mast cell line RBL-2H3 were examined. Sph-1-P strongly inhibited the migration of both non-stimulated and fibronectin-stimulated RBL-2H3 cells, which was reversed by JTE-013, a specific antagonist of G protein-coupled Sph-1-P receptor S1P(2); S1P(2) was confirmed to be expressed in these cells. A similar anti-motility effect of Sph-1-P was observed in a phagokinetic assay. Consistent with these results, treatment of RBL-2H3 cells with Sph-1-P resulted in a rounded cell morphology, which was blocked by JTE-013. Under the present conditions, Sph-1-P failed to induce intracellular Ca(2+) mobilization or histamine degranulation, responses postulated to be elicited by intracellular Sph-1-P. Importantly, the Sph-1-P effect, i.e., the regulation of RBL-2H3 cell motility, was mimicked by the supernatant (both with and without boiling) prepared from activated platelets, and this effect of the supernatant was also blocked by JTE-013. Our results suggest that the motility of mast cells can be regulated by

Sph-1-P and also platelets (which release Sph-1-P), via cell surface receptor S1P(2) (not through intracellular Sph-1-P actions, postulated previously in the same cells).

PMID: 15213242 [PubMed - indexed for MEDLINE]

1198. J Allergy Clin Immunol. 2004 Jun;113(6):1152-60.

Chemotaxis and activation of human peripheral blood eosinophils induced by pollen-associated lipid mediators.

Plötz SG, Traidl-Hoffmann C, Feussner I, Kasche A, Feser A, Ring J, Jakob T, Behrendt H.

Division of Environmental Dermatology and Allergy GSF/TUM, Center for Allergy and Environment, Technical University Munich, Biedersteiner Strasse 29, 80802 Munich, Germany.

BACKGROUND: Eosinophil accumulation at sites of allergic inflammation is largely regulated by chemokines and lipid mediators released by a variety of cells of the local microenvironment. Recent studies have shown that pollen grains, apart from their function as allergen carriers, are a rich exogenous source of eicosanoid-like lipid mediators that are rapidly released on contact with the aqueous phase and thus may contribute to the generation of local inflammatory responses.
OBJECTIVE: Here we analyze the biological activity of pollen-associated lipid mediators (PALMs) on peripheral human blood eosinophils.
METHODS: Human eosinophils were coincubated with pollen grains and analyzed by electron microscopy. The lipid mediator composition of aqueous pollen extracts (APEs) was analyzed by HPLC. Human eosinophils were exposed to APEs or lipid fractions from pollen. Effects on eosinophils were tested by transwell migration and surface expression of CD11b.
RESULTS: In vitro experiments showed adhesion of eosinophils to Phleum pratense pollen. In chemotaxis assays eosinophils displayed significant directed migration to APEs. HPLC analysis of APEs from Phleum pratense and Betula alba pollen demonstrated the occurrence of linoleic and alpha-linolenic acid as well as their monohydroxylated derivatives. Moreover, total lipid extracts from pollen and RP-HPLC fractions containing monohydroxylated derivatives of linoleic and alpha-linolenic acid induced similar migratory responses, although to a lesser degree than APEs. In addition, APEs and lipid extracts induced up-regulation of CD11b surface expression and secretion of eosinophil cationic protein. APE-induced chemotaxis was blocked by the leukotriene B(4) receptor antagonist LY293111, suggesting that PALMs may serve as ligands for LTB(4) receptors.
CONCLUSION: Pollen grains release lipid mediators that recruit and activate eosinophils in vitro. Similar mechanisms may be effective under natural exposure conditions, in which PALMs may play a role in the recruitment of eosinophils to the site of allergic inflammation.

PMID: 15208598 [PubMed - indexed for MEDLINE]

1199. Med J Malaysia. 2003 Oct;58(4):579-86.

Utilisation of ophthalmic services by foreign nationals in Johor: a review of 452 patients.

Hooi SH, Hooi ST.

Department of Ophthalmology, Hospital Sultanah Aminah, 80100 Johor Bahru.

Between 1st January 1999 and 31st December 2000, 452 foreign nationals were treated at the Department of Ophthalmology, Hospital Sultanah Aminah, Johor Bahru. Eighty-five percent were male. The peak age range was from 21 to 30 years old. The patients were predominantly Indonesians (61%). A history of trauma was present in 63% of patients. Eight percent of eyes had severe visual impairment. Six patients (1.3%) were blind by WHO standards. Traumatic eye conditions, inflammatory/allergic eye conditions and degenerative eye conditions comprised 66%, 13% and 10% respectively of ocular pathology seen. The commonest ocular findings were corneal foreign body, corneal abrasion and subconjunctival haemorrhage.

PMID: 15190634 [PubMed - indexed for MEDLINE]

1200. Cardiovasc Drug Rev. 2004 Summer;22(2):71-90.

Ramatroban (BAY u 3405): a novel dual antagonist of TXA2 receptor and CRTh2, a newly identified prostaglandin D2 receptor.

Ishizuka T, Matsui T, Okamoto Y, Ohta A, Shichijo M.

Department of Medical Engineering, National Defense Medical College, Namiki 3-2, Tokorozawa, Saitama 359, Japan. tir2@hotmail.com.

It is known that thromboxane A2 (TXA2) contributes to various diseases such as bronchial asthma, ischemic heart disease, cerebrovascular disorders and allergic rhinitis. A number of TXA2 synthase inhibitors and TXA2 receptor (TP receptor) antagonists have been developed to treat these diseases. Ramatroban (BAY u 3405) was developed as a potent TP receptor antagonist with excellent efficacy against allergic rhinitis in many animal models and patients. Recent studies also revealed that ramatroban can block the newly identified PGD2 receptor, chemoattractant receptor-homologous molecule expressed on Th2 cells (CRTh2). PGD2 induces migration and degranulation of eosinophils through CRTh2 and contributes to late-phase inflammation and cell damage. Accordingly, it was considered that ramatroban suppresses the late-phase inflammation via TP receptor and CRTh2 blockade. In terms of the efficacy on vascular systems, it was revealed that ramatroban can suppress the expression of monocyte chemoattractant protein-1 (MCP-1) and adhesion molecules in endothelial cells and prevent exacerbation of inflammation by blocking these responses. According to our recent studies in hypercholesterolemic rabbits ramatroban prevents macrophage infiltration through MCP-1 downregulation and neointimal formation after balloon injury and attenuates vascular response to acetylcholine. Therefore, ramatroban may be beneficial in the treatment of atherosclerosis.

PMID: 15179446 [PubMed - indexed for MEDLINE]

1201. Pol Merkur Lekarski. 2004 Feb;16(92):179-82.

[Role of intra cellular adhesion molecule-1 (ICAM-1) and its soluble form (sICAM) in chronic airway inflammation].

[Article in Polish]

Grzelewska-Rzymowska I, Pietrzkowicz M.

Klinika Gruźlicy i Chorób Płuc Instytutu Medycyny Wewnetrznej UM w Łodzi.

Mucosal inflammation is the feature of both bronchial asthma and allergic

rhinitis with evident of tissue eosinophilia, mast cells, eosinophils and T-lymphocytes activation. The initial phase of cell recruitment is the margination and adhesion of leucocytes to the endothelium, prior to their transendothelial migration under a directed chemotactic stimulus. This adhesion occurs through specific ligand-receptor couplets involving leucocyte-endothelial adhesion molecules. One of these cell adhesion molecules is ICAM-1, an important early marker of immune activation and response. Its ligand, leukocyte function-associated antigen one (LFA-1) is expressed on neutrophils, eosinophils and T-cells. ICAM-1 was found to be expressed on epithelial and endothelial cells in rhinitis patients and in bronchial biopsies obtained from asthmatics also after allergen challenge. Circulating forms of these adhesion molecules have been identified in the peripheral blood, bronchoalveolar lavage (BAL) fluid and nasal lavage in patients with asthma and rhinitis. Systemic and local up-regulation of sICAM-1 suggests a function role for this soluble form of ICAM in the allergic inflammation.

PMID: 15176306 [PubMed - indexed for MEDLINE]

1202. Pol Merkur Lekarski. 2004 Feb;16(92):173-8.

[Role of tumor necrosis factor-alpha in allergic inflammation and airway hyperresponsiveness].

[Article in Polish]

Grzelewska-Rzymowska I, Pietrzkowicz M.

Klinika Gruźlicy i Chorób Płuc Instytutu Medycyny Wewnetrznej UM w Łodzi.

TNF-alpha is a potent multifunctional cytokine that plays a central role in the pathogenesis of many inflammatory diseases such as asthma and allergic rhinitis. This proinflammatory cytokine is produced by a variety of cells in the airways and is released via the IgE-dependent activation of mast cells. The elevated levels of TNF-alpha can be detected in the airways, bronchoalveolar lavage (BAL) fluid and nasal lavage from asthmatic and rhinitic patients. Leucocytes and monocytes isolated from BAL fluid of asthmatics were shown to release more TNF-alpha than cells from control subjects. Recent research indicates that TNF-alpha maybe associated with acquired airway hyperresponsiveness a pathophysiological hallmark of asthma. It was suggested that TNF-alpha upregulates adhesion molecules and is directly responsible for transendothelial migration of inflammatory cells a central feature underlying the inflammatory response. A direct chemotactic effect has also been attributed to TNF-alpha. This cytokine is a chemoatractant for neutrophils and monocytes, can also induce transepithelial migration of neutrophils through production of IL-8.

PMID: 15176305 [PubMed - indexed for MEDLINE]

1203. Pol Merkur Lekarski. 2004 Feb;16(92):101-3.

[Role of neurotrophin and neuropeptides in bronchial asthma].

[Article in Polish]

Pałgan K, Dziedziczko A.

Asthma represents a chronic inflammatory process of the airways. The neurothrophin (NGF) and neuropeptides such as substance P (SP), neurokinin A (NKA), and calcitonin gene-related peptide (CGRP) play important role in

stimulation of airways inflammation in asthmatics. NGF stimulates the differentiation and the migration of mast cells to bronchi epithelium. Furthermore, NGF stimulates mast cell degranulation and mediator upregulation and release. It also influences activity of basophils, eosinophils, neurophils, macrophages and T-cells. In addition, its important role in releasing of hyperresponsiveness has been proved. Neuropeptides such as CGRP and SP stimulate migration and degranulation of eosinophils and influence on airway responsiveness in asthmatics. This review article discusses the neuropeptides and NGF actions and mechanisms in the pathogenesis of asthma.

PMID: 15176288 [PubMed - indexed for MEDLINE]

1204. Bioessays. 2004 Jun;26(6):646-55.

Synthesis of immune modulators by smooth muscles.

Singer CA, Salinthone S, Baker KJ, Gerthoffer WT.

Department of Pharmacology, University of Nevada School of Medicine, Reno, NV 89557-0270, USA.

The primary function of smooth muscle cells is to contract and alter the stiffness or diameter of hollow organs such as blood vessels, the airways and the gastrointestinal and urogenital tracts. In addition to purely structural functions, smooth muscle cells may play important metabolic roles, particularly in various inflammatory responses. In cell culture, these cells have been shown to be metabolically dynamic, synthesizing and secreting extracellular matrix proteins, glycosaminoglycans and a wide variety of cell-cell signaling proteins, such as interleukins, chemokines and peptide growth factors. Secreted cell signaling proteins participate in the inflammatory response of smooth muscle-containing organs, and some can also stimulate smooth muscle migration, proliferation and contraction. The cellular signaling pathways controlling synthesis of these signaling proteins are similar to those used by cells mediating innate immunity and may contribute to pathogenesis of diverse diseases including atherosclerosis, asthma, inflammatory bowel diseases and preterm labor. Appreciating the role of smooth muscle cells in these diseases may lead to better understanding of the beneficial effects of anti-inflammatory drugs as well as identification of new targets for anti-inflammatory therapy.

Copyright 2004 Wiley Periodicals, Inc.

PMID: 15170862 [PubMed - indexed for MEDLINE]

1205. Int Arch Allergy Immunol. 2004 Jun;134 Suppl 1:21-4.

Immunotherapy attenuates eosinophil transendothelial migration induced by the supernatants of antigen-stimulated mononuclear cells from atopic asthmatics.

Nagata M, Saito K, Kikuchi I, Tabe K, Hagiwara K, Kanazawa M, Sakamoto Y.

Department of Respiratory Medicine, Saitama Medical School, Iruma-gun, Japan. favre4mn@saitama-medd.ac.jp

BACKGROUND: Eosinophil transendothelilal migration across vascular endothelial cells is an initial step of eosinophil accumulation in allergic inflammation. There is increasing evidence that specific immunotherapy (SIT) modulates the production of inflammatory molecules from mononuclear cells.
OBJECTIVE: The present study was undertaken to examine whether SIT modifies

eosinophil transendothelial migration induced by the supernatants of
antigen-stimulated mononuclear cells from atopic asthmatics.
METHODS: Dermatophagoides farinae (Df)-sensitive mild persistent asthmatics were divided into a SIT-treated group and a control group. Peripheral blood mononuclear cells (PBMC) were isolated before and after SIT using the rush protocol, and cultured for 96 h at 37 degrees C in the presence or absence of Df antigen. Eosinophils were isolated from the blood of healthy subjects, and put on transwell filters coated with pulmonary microvascular endothelial cell monolayers stimulated with IL-4 plus TNF-alpha. The supernatants of PBMC were applied to the lower compartment and the transmigration of eosinophils was examined.
RESULTS: Df stimulation of PBMC resulted in an augmentation of eosinophil transendothelial migration. This enhancement was abrogated following SIT. In the control group, the antigen-induced effect on eosinophil transmigration did not show an interval change.
CONCLUSION: SIT attenuates eosinophil transendothelial migration induced by antigen-stimulated mononuclear cells.

Copyright 2004 S. Karger AG, Basel

PMID: 15166479 [PubMed - indexed for MEDLINE]

1206. Mt Sinai J Med. 2004 May;71(3):191-6.

Adolescent school-based health care: a description of two sites in their 20th year of service.

Pastore DR, Techow B.

Mount Sinai Adolescent Health Care Center, Division of Adolescent Medicine, New York, NY, USA.

PURPOSE: While there are currently nearly 1,400 school-based health centers (SBHC) nationwide, only 20% have been in operation for more than 10 years. The Mount Sinai Adolescent SBHC Program is now in its 20th year of service. The purpose of this study is to: (a) present the demographic data for 2003 high school SBHC medical visits, including age, sex and insurance status; (b) describe the current prevalence of medical and psychosocial risk factors of the students seen for examination; and (c) present general distributions for psychosocial risk factors found in 1988 archival information and note differences from current risk factors.
METHODS: A retrospective chart review was conducted in high school A, whose SBHC serves students mainly interested in going to college, and in high school B, whose SBHC has a heterogeneous population with a large proportion of recent immigrants. Data collected included demographic variables as well as reports of risk factors such as: considering oneself to be overweight, history of sexual activity, history of sexually transmitted diseases, same-sex attraction, use of alcohol, cigarette smoking, use of marijuana, suicidal ideation and exposure to violence.
RESULTS: For those participating from high school A (n=231): 78% female, mean age 15.75; asthma (17%); think oneself overweight (30%); family member with HIV (11%); sexually active (35%); same-sex attraction (3%); cigarette use (14%); marijuana use (13%); alcohol use (38%); suicide ideation (14%); witnessed violence (37%); and overweight and obese (33%). For those participating from high school B (n=241): 64% female; mean age 16; asthma (16%); think oneself overweight (32%); family member with HIV (9%); sexually active (43%); same-sex attraction (7%); cigarette use (38%); marijuana use (24%); alcohol use (53%); suicide ideation (23%); witnessed violence (33%); and overweight and obese (31%). In 1988, students at these schools reported: sexually active status (41%); marijuana use (13%); cocaine use (12%); alcohol use (20%); and sadness/depression (43%).

CONCLUSIONS: While a snapshot of the risk factors in 2003 might indicate that sexual activity has decreased somewhat, substance use, as well as eating-related and AIDS-related issues have come to the forefront. SBHCs continue to serve students with intense medical and psychological needs. It remains crucial that SBHCs provide comprehensive medical and mental health services.

PMID: 15164134 [PubMed - indexed for MEDLINE]

1207. Immunol Lett. 2004 May 15;93(2-3):97-108.

Commensal bacteria (normal microflora), mucosal immunity and chronic inflammatory and autoimmune diseases.

Tlaskalová-Hogenová H, Stepánková R, Hudcovic T, Tucková L, Cukrowska B, Lodinová-Zádníková R, Kozáková H, Rossmann P, Bártová J, Sokol D, Funda DP, Borovská D, Reháková Z, Sinkora J, Hofman J, Drastich P, Kokesová A.

Department of Immunology and Gnotobiology, Institute of Microbiology, Academy of Sciences of the Czech Republic, Vídenská 1083, 142 20 Prague 4, Czech Republic. tlaskalo@biomed.cas.cz

Commensal microflora (normal microflora, indigenous microbiota) consists of those micro-organisms, which are present on body surfaces covered by epithelial cells and are exposed to the external environment (gastrointestinal and respiratory tract, vagina, skin, etc.). The number of bacteria colonising mucosal and skin surfaces exceeds the number of cells forming human body. Commensal bacteria co-evolved with their hosts, however, under specific conditions they are able to overcome protective host responses and exert pathologic effects. Resident bacteria form complex ecosystems, whose diversity is enormous. The most abundant microflora is present in the distal parts of the gut; the majority of the intestinal bacteria are Gram-negative anaerobes. More than 50% of intestinal bacteria cannot be cultured by conventional microbiological techniques. Molecular biological methods help in analysing the structural and functional complexity of the microflora and in identifying its components. Resident microflora contains a number of components able to activate innate and adaptive immunity. Unlimited immune activation in response to signals from commensal bacteria could pose the risk of inflammation; immune responses to mucosal microbiota therefore require a precise regulatory control. The mucosal immune system has developed specialised regulatory, anti-inflammatory mechanisms for eliminating or tolerating non-dangerous, food and airborne antigens and commensal micro-organisms (oral, mucosal tolerance). However, at the same time the mucosal immune system must provide local defense mechanisms against environmental threats (e.g. invading pathogens). This important requirement is fulfilled by several mechanisms of mucosal immunity: strongly developed innate defense mechanisms ensuring appropriate function of the mucosal barrier, existence of unique types of lymphocytes and their products, transport of polymeric immunoglobulins through epithelial cells into secretions (sIgA) and migration and homing of cells originating from the mucosal organised tissues in mucosae and exocrine glands. The important role of commensal bacteria in development of optimally functioning mucosal immune system was demonstrated in germ-free animals (using gnotobiological techniques). Involvement of commensal microflora and its components with strong immunoactivating properties (e.g. LPS, peptidoglycans, superantigens, bacterial DNA, Hsp) in etiopathogenetic mechanism of various complex, multifactorial and multigenic diseases, including inflammatory bowel diseases, periodontal disease, rheumatoid arthritis, atherosclerosis, allergy, multiorgan failure, colon cancer has been recently suggested. Animal models of human diseases reared in defined gnotobiotic conditions are helping to elucidate the aetiology of these frequent disorders. An improved understanding of commensal bacteria-host interactions employing germ-free animal models with selective

colonisation strategies combined with modern molecular techniques could bring new insights into the mechanisms of mucosal immunity and also into pathogenetic mechanisms of several infectious, inflammatory, autoimmune and neoplastic diseases. Regulation of microflora composition (e.g. by probiotics and prebiotics) offers the possibility to influence the development of mucosal and systemic immunity but it can play a role also in prevention and treatment of some diseases.

PMID: 15158604 [PubMed - indexed for MEDLINE]

1208. J Leukoc Biol. 2004 Aug;76(2):388-98. Epub 2004 May 20.

Agonists of proteinase-activated receptor-2 modulate human neutrophil cytokine secretion, expression of cell adhesion molecules, and migration within 3-D collagen lattices.

Shpacovitch VM, Varga G, Strey A, Gunzer M, Mooren F, Buddenkotte J, Vergnolle N, Sommerhoff CP, Grabbe S, Gerke V, Homey B, Hollenberg M, Luger TA, Steinhoff M.

Department of Dermatology, University of Münster, von-Esmarch-Str. 58, 48149 Münster, Germany.

Proteinase-activated receptor-2 (PAR2) belongs to a novel subfamily of G-protein-coupled receptors with seven-transmembrane domains. PAR2 can be activated by serine proteases such as trypsin, mast cell tryptase, and allergic or bacterial proteases. This receptor is expressed by various cells and seems to be crucially involved during inflammation and the immune response. As previously reported, human neutrophils express functional PAR2. However, the precise physiological role of PAR2 on human neutrophils and its implication in human diseases remain unclear. We demonstrate that PAR2 agonist-stimulated human neutrophils show significantly enhanced migration in 3-D collagen lattices. PAR2 agonist stimulation also induced down-regulation of L-selectin display and up-regulation of membrane-activated complex-1 very late antigen-4 integrin expression on the neutrophil cell surface. Moreover, PAR2 stimulation results in an increased secretion of the cytokines interleukin (IL)-1beta, IL-8, and IL-6 by human neutrophils. These data indicate that PAR2 plays an important role in human neutrophil activation and may affect key neutrophil functions by regulating cell motility in the extracellular matrix, selectin shedding, and up-regulation of integrin expression and by stimulating the secretion of inflammatory mediators. Thus, PAR2 may represent a potential therapeutic target for the treatment of diseases involving activated neutrophils.

PMID: 15155775 [PubMed - indexed for MEDLINE]

1209. Allergy. 2004 Jun;59(6):632-6.

Allergy, asthma and markers of infections among Albanian migrants to Southern Italy.

Ventura MT, Munno G, Giannoccaro F, Accettura F, Chironna M, Lama R, Hoxha M, Panetta V, Ferrigno L, Rosmini F, Matricardi PM, Barbuti S, Priftanji A, Bonini S, Tursi A.

Department of Internal Medicine, Immunology and Infectious Diseases, University of Bari Medical School, Policlinico, Piazza Giulio Cesare no. 11, 70124 Bari, Italy.

BACKGROUND: Studies of immigrants represent an useful tool to determine the

relative relevance of environmental vs genetic factors in causing the reported rapid increase of the prevalence of sensitization and allergic diseases.
METHODS: A total of 152 Albanian migrants to Southern Italy responded to a questionnaire based on the European Community Respiratory Health Survey (ECRHS) and 139 of them underwent skin prick test, and 61 serological assays for total IgE and IgG antibodies against Toxoplasma gondii (TG), herpes simplex virus 1 (HSV-1), hepatitis A virus (HAV) and Helicobacter pylori (HP).
RESULTS: Reported asthma was rare (2/152; 1.3%) and reported nasal allergies rather frequent (24/152; 15.8%). Sensitization to common inhalant allergens occurred in 27/139 (19.4%) subjects. The frequency of skin sensitization to pollen (P = 0.003) and that of hay fever (P = 0.004) increased with the time spent in Apulia. All the 61 sera had antibodies against HAV, 59/61 (96.7%) against HSV-1, 48/61 (78.7%) against HP and 34/61 (55.7%) against TG. The prevalence of skin sensitization and hay fever symptoms were correlated to the duration of residence in Southern Italy.
CONCLUSIONS: Data presented indicate that Albanian migrants to Italy, in spite of the low prevalence of allergic diseases and sensitization in their country of origin, manifest with time an increasing prevalence of sensitization to local allergens and nasal symptoms after immigration to Italy. This would suggest a permanent role of allergen exposure and lifestyle factors in influencing the appearance of sensitization and symptoms of allergic diseases.

PMID: 15147448 [PubMed - indexed for MEDLINE]

1210. Sci Total Environ. 2004 Jun 29;326(1-3):123-41.

Airborne viable, non-viable, and allergenic fungi in a rural agricultural area of India: a 2-year study at five outdoor sampling stations.

Adhikari A, Sen MM, Gupta-Bhattacharya S, Chanda S.

Division of Palynology and Environmental Biology, Department of Botany, Bose Institute, 93/1 Acharya Prafulla Chandra Road, Kolkata (Calcutta) 700 009, India. adhikaa@email.uc.edu

The information on airborne allergenic fungal flora in rural agricultural areas is largely lacking. Adequate information is not available to the bioaerosol researchers regarding the choice of single versus multiple sampling stations for the monitoring of both viable and non-viable airborne fungi. There is no long-term study estimating the ratios of viable and non-viable fungi in the air and earlier studies did not focus on the fractions of airborne allergenic fungi with respect to the total airborne fungal load. To fill these knowledge gaps, volumetric paired assessments of airborne viable and non-viable fungi were performed in five outdoor sampling stations during two consecutive years in a rural agricultural area of India. Samples were collected at 10-day intervals by the Burkard Personal Slide Sampler and the Andersen Two-Stage Viable Sampler. The data on the concentrations of total and individual fungal types from five stations and 2 different years were analyzed and compared by statistical methods. The allergenicity of the prevalent airborne viable fungi was estimated by the skin-prick tests of >100 rural allergy patients using the antigenic fungal extracts from isolates collected with the Andersen sampler. The ranges of total fungal spore concentration were 82-2365 spores per cubic meter of air (spores/m3) in the first sampling year and 156-2022 spores/m3 in the second sampling year. The concentration ranges of viable fungi were 72-1796 colony-forming units per cubic meter of air (CFU/m3) in the first sampling year and 155-1256 CFU/m3 in the second sampling year. No statistically significant difference was observed between the total spore data of the 2 years, however, the data between five stations showed a significant difference (P<0.0001). No statistically significant difference existed between stations and years with respect to the concentration

of viable fungi. When the data of individual allergenic fungal concentrations were compared between stations and years, no statistically significant difference was observed in all cases except for Aspergillus japonicus and Rhizopus nigricans, which showed significant difference in case of stations and years, respectively. The ratios between the total fungal spores collected by the Burkard sampler and the viable fungi collected by the Andersen sampler from all sampling stations ranged between 0.29 and 7.61. The antigenic extracts of eight prevalent viable airborne fungi (A. flavus, A. japonicus, A. fumigatus, Alternaria alternata, Cladosporium cladosporioides, Curvularia pallescens, Fusarium roseum, and R. nigricans) demonstrated >60% positive reactions in the skin prick test. These selected allergenic fungi collectively represented 31.7-63.2% of the total airborne viable fungi in different stations. The study concluded that: (i) a rich fungal airspora existed in the rural study area, (ii) to achieve representative information on the total airborne fungal spores of an area, the monitoring in multiple sampling stations is preferable over a single sampling station; for viable fungi, however, one station can be considered, (iii) the percentage of airborne fungal viability is higher in rural agricultural areas, and (iv) approximately 52% of the viable airborne fungi in the rural study area were allergenic.

Copryright 2003 Elsevier B.V.

PMID: 15142771 [PubMed - indexed for MEDLINE]

1211. Am Fam Physician. 2004 Apr 15;69(8):1935-7.

Identifying the vector of Lyme disease.

Lo Re V 3rd, Occi JL, MacGregor RR.

Division of Infectious Diseases, University of Pennsylvania School of Medicine, Philadelphia 19104, USA. vincent.lore@uphs.upenn.edu

Lyme disease is the most common vector-borne illness in the United States. It is caused by the spirochete Borrelia burgdorferi, which is transmitted by the deer tick. Deer ticks have a four-stage life cycle (egg, larva, nymph, and adult), and nymphal ticks transmit B. burgdorferi to humans more frequently than adult ticks. Transmission of this spirochete typically requires a minimum of 24 to 48 hours of tick attachment. Early stages of Lyme disease are characterized by a hallmark rash, erythema migrans. The overall risk of acquiring Lyme disease is low in a person who has a deer tick bite. If erythema migrans develops at the site of the bite, treatment may include doxycycline in persons who are at least eight years of age. Administration of amoxicillin is appropriate for pregnant women or children younger than eight years. For those who are allergic to these medications, cefuroxime axetil may be used.

PMID: 15117014 [PubMed - indexed for MEDLINE]

1212. J Dermatol Sci. 2004 May;34(3):237-40.

Determination of thymus and activation-regulated chemokine (TARC)-contents in scales of atopic dermatitis.

Morita E, Hiragun T, Mihara S, Kaneko S, Matsuo H, Zhang Y, Dekio S.

BACKGROUND: Thymus and activation-regulated chemokine (TARC) is a cytokine which selectively controls the migration of type 2-helper T lymphocytes into inflammatory lesions, and the serum level is strongly associated with disease

severity of atopic dermatitis (AD).
OBJECTIVE: To examine the role of TARC in the pathogenesis of AD, we determined TARC-contents in the scales obtained from lesional skin of the patients with AD.
RESULTS: High amount of TARC was detected in the scales of lesional skin obtained from the patients with AD, and the amount was well correlated with the serum IgE levels but not with the blood eosinophil counts. The TARC-content in the lesional scales was not correlated with a-431C/T polymorphism of TARC promotor gene, suggesting other regulating mechanisms in TARC production in the lesion.
CONCLUSION: High amount of TARC is produced in the kesion of AD, and analysis of cytokine content in lesional scales may provide some tools to clarify the pathogenesis of AD.

Copyright 2004 Japanese Society for Investigative Dermatology

PMID: 15113595 [PubMed - indexed for MEDLINE]

1213. Toxicol Lett. 2004 May 2;150(3):301-16.

Gene expression changes in peripheral blood-derived dendritic cells following exposure to a contact allergen.

Ryan CA, Gildea LA, Hulette BC, Dearman RJ, Kimber I, Gerberick GF.

Miami Valley Laboratories, Central Product Safety Department, The Procter & Gamble Company, P.O. Box 538707, Cincinnati, OH 45253, USA. ryan.ca@pg.com

A critical step in the induction of allergic contact allergy is the activation and subsequent migration of Langerhans cells (LC), an important antigen presenting dendritic cell (DC) of the skin. As the Langerhans cells migrate, they undergo a maturation process. It has been proposed that contact allergen exposure can induce DC maturation. While changes in DC gene expression profiles induced by various maturation stimuli have been explored, there are no published reports describing genomic-scale analysis of the changes induced by chemical allergen exposure. Therefore, to explore the concept of chemical allergen-induced DC maturation and to identify genes that are regulated by exposure to allergens we examined, at the transcriptional level, the effects of exposure to a contact allergen on DC. Peripheral blood-derived DC were exposed for 24 h to either 1mM or 5 mM dinitrobenzenesulfonic acid (DNBS). Changes in gene expression were analyzed using Affymetrix U95Av2 GeneChip. Comparison of mean signal values from replicate cultures revealed 173 genes that were significantly different (P < or = 0.001) between 1 mM DNBS treated and untreated control DC and 1249 significant gene changes between 5 mM DNBS treated and control DC. Real-time reverse-transcriptase polymerase chain reaction (RT-PCR) was used to evaluate the observed transcript changes for selected genes in DC derived from a second donor. Comparison of the fold-changes in transcript levels between the two platforms and donors revealed a good correlation in both direction and magnitude. RT-PCR analysis was also used to assess the allergen specificity of a selected number of genes in DC derived from a third donor. Many of the gene expression changes were found to be induced only by exposure to the allergen, DNBS, and not by exposure to a structurally similar non-allergen, benzenesulfonic acid. A number of gene expression changes induced by allergen exposure were found to be consistent with what is known of the DC maturation process, and thus provide support for the theory of contact allergen-induced DC maturation. Additionally, it is hoped that some of the transcript changes identified through this approach will be shown to be suitable for use in the development of an in vitro predictive assay for contact sensitization.

PMID: 15110082 [PubMed - indexed for MEDLINE]

1214. Chin Med J (Engl). 2004 Apr;117(4):592-7.

Effects of glucocorticoid and cysteinyl leukotriene 1 receptor antagonist on CD(34+) hematopoietic cells in bone marrow of asthmatic mice.

Mao H, Yin KS, Wang ZL, Li FY, Zhang XL, Liu CT, Lei S.

Department of Respiratory Medicine, First Affiliated Hospital, Nanjing Medical University, Nanjing 210029, China. fuyulirose@hotmail.com

BACKGROUND: Corticosteroids remain the most effective therapy available for asthma. They have widespread effects on asthmatic airway inflammation. However, little is known about the effects of corticosteroids on the production of bone marrow inflammatory cells in asthma. This study observed the effects of glucocorticoid and cysteinyl leukotriene 1 receptor antagonist on CD34+ hematopoietic cells, so as to explore the possible effectiveness of a bone marrow-targeted anti-inflammatory strategy.
METHODS: Balb/c mice were sensitized and challenged with ovalbumin (OVA) to establish an asthmatic model. For two consecutive weeks, asthmatic mice were challenged with OVA while being given either prednisone, montelukast, prednisone plus montelukast, or sterile saline solution. The mice were killed 24 hours after the last challenge with OVA, and bronchoalveolar lavage fluid (BALF), peripheral blood, and bone marrow were collected. Eosinophils in peripheral blood and BALF, and nucleated cells in BALF, peripheral blood, and bone marrow were counted. The percentages of CD34+ cells, CD4+ T lymphocytes and CD8+ T lymphocytes among nucleated cells in peripheral blood and bone marrow were counted by flow cytometry. Immunocytochemistry and in situ hybridization were employed to detect expression of CD34 and interleukin (IL)-5Ralpha mRNA (CD34+ IL-5Ralpha mRNA+ cells) among bone marrow hematopoietic cells.
RESULTS: Compared with the sterile saline solution group, the number of eosinophils in BALF and peripheral blood, CD34+ cells in peripheral blood and bone marrow, and CD34+ IL-5Ralpha mRNA+ cells in bone marrow of mice from the prednisone and prednisone plus montelukast groups were significantly lower (P < 0.01). The number of eosinophils in BALF from the montelukast group was also significantly lower (P < 0.05).
CONCLUSIONS: The results suggest that, in this asthmatic mouse model, prednisone probably inhibits proliferation, differentiation, and migration of CD34+ cells in bone marrow, blocks eosinophilopoiesis in bone marrow, and interferes with eosinophil migration into peripheral blood and subsequent recruitment in the airway. In addition, montelukast may suppress eosinophil infiltration into the lungs of asthmatic mice. However, a significant inhibitory effect of montelukast on the proliferation and migration of CD34+ cells and a cooperating effect with prednisone on bone marrow of asthmatic mice were not observed.

PMID: 15109455 [PubMed - indexed for MEDLINE]

1215. Curr Opin Allergy Clin Immunol. 2004 Feb;4(1):5-10.

Prognostic value of asymptomatic skin sensitization to aeroallergens.

Bodtger U.

Allergy Clinic, National University Hospital, Copenhagen, Denmark. uffechristianheitmanrh07946boedger@rh.dk

PURPOSE OF REVIEW: Asymptomatic skin sensitization to aeroallergens is frequently encountered in epidemiological studies and in everyday clinical life. Correct management of the condition is essential to avoid both progression into allergic

disease and unnecessary intervention. Understanding immunological mechanisms in asymptomatic skin sensitization might provide new insights into the natural history and treatment of respiratory allergy.
RECENT FINDINGS: Research on asymptomatic skin sensitization is rare, and the present review unites previous studies with recent findings. It is a common condition affecting 8-30% of the population when using a local standard panel of aeroallergens. Clinically, immediate but not late-phase reactions are induced by allergen challenge. Absent eosinophil stimulation and migration and low IL-5 levels appear to be sentinel mechanisms. Prospective studies show that 30-60% become allergic, depending on allergens and follow-up period. No prospective intervention studies have been performed; however, allergen avoidance seems efficacious in reducing allergy development to occupational and domestic allergens. Asymptomatic skin sensitization due to an erroneously positive skin test must be ruled out before allergen avoidance measures are initiated.
SUMMARY: Surprisingly few papers exist on asymptomatic skin sensitization epidemiology and immunology, despite the intriguing question as to why symptoms do not develop in IgE-sensitized patients. It is a common condition and a risk factor for later development of respiratory allergic disease. Cross-sectional intervention studies suggest that allergy development is reduced by allergen avoidance. Immunologically, control of eosinophil stimulation and migration seems to be pivotal. How this control is maintained remains to be elucidated.

PMID: 15090912 [PubMed - indexed for MEDLINE]

1216. J Invest Dermatol. 2004 Mar;122(3):673-84.

Corticosteroids but not pimecrolimus affect viability, maturation and immune function of murine epidermal Langerhans cells.

Hoetzenecker W, Meingassner JG, Ecker R, Stingl G, Stuetz A, Elbe-Bürger A.

DIAID, Department of Dermatology, Medical University of Vienna, Austria.

Given the importance of dendritic cells in the immune response, we investigated the effect of corticosteroids (CS) on the integrity, survival, and function of murine Langerhans cells (LC) in comparison with pimecrolimus, a novel anti-inflammatory drug for the topical treatment of atopic dermatitis. BALB/c mice were treated twice on one day with ethanolic solutions of the compounds. At 24-72 h after the last application, we observed fragmented DNA, caspase-3 activity, and an upregulation of CD95 expression in LC from mice treated with CS but not in LC of pimecrolimus- or vehicle-treated animals. CS-epidermal cell (EC) supernatants but not pimecrolimus-EC supernatants contained significantly lower amounts of soluble factors (GM-CSF, TNF-alpha, IL-1alpha) required for LC survival and maturation than EC supernatants from vehicle-treated mice. With regard to LC maturation, CS but not pimecrolimus inhibited the expression of CD25, CD205, and costimulatory molecules. In line with this, LC from pimecrolimus-treated mice were similar to LC from vehicle-treated mice in their capacity to stimulate antigen-presenting function and migration, whereas LC from CS-treated mice were greatly impaired in these abilities. In summary, our data show for the first time that CS but not pimecrolimus induce apoptosis in LC in situ, implying that the prolonged use of CS could have adverse effects on the skin immune system.

PMID: 15086553 [PubMed - indexed for MEDLINE]

1217. Bioorg Med Chem Lett. 2004 May 3;14(9):2323-6.

Amidines as amide bond replacements in VLA-4 antagonists.

Kamenecka TM, Park YJ, Lin LS, de Laszlo S, McCauley ED, Van Riper G, Egger L, Kidambi U, Mumford RA, Tong S, Tang W, Colletti A, Teffera Y, Stearns R, MacCoss M, Schmidt JA, Hagmann WK.

Department of Medicinal Chemistry, Merck Research Laboratories, Rahway, NJ 07065, USA. theodore.kamenecka@merck.com

VLA-4 (alpha(4)beta(1), very late activating antigen-4), a key cell surface integrin plays an important role in inflammation by promoting leukocyte attachment and extravasation from the vasculature into the peripheral tissues. As such, VLA-4 antagonists may be useful in the treatment, prevention, and suppression of diseases where cell adhesion and migration are important such as asthma, rheumatoid arthritis, and multiple sclerosis. Herein, we report on the discovery, synthesis, and biological evaluation of amidines as small molecule antagonists of VLA-4.

PMID: 15081033 [PubMed - indexed for MEDLINE]

1218. Proc Natl Acad Sci U S A. 2004 Apr 20;101(16):6116-21. Epub 2004 Apr 12.

Bystander suppression of allergic airway inflammation by lung resident memory CD8+ T cells.

Marsland BJ, Harris NL, Camberis M, Kopf M, Hook SM, Le Gros G.

Malaghan Institute of Medical Research, Wellington School of Medicine, 6002 Wellington, New Zealand. marsland@env.ethz.ch

CD8+ memory T cells have recently been recognized as playing a key role in natural immunity against unrelated viral infections, a phenomenon referred to as "heterologous antiviral immunity." We now provide data that the cellular immunological interactions that underlie such heterologous immunity can play an equally important role in regulating T helper 2 immune responses and protecting mucosal surfaces from allergen-induced inflammation. Our data show that CD8+ T cells, either retained in the lung after infection with influenza virus, or adoptively transferred via the intranasal route can suppress allergic airway inflammation. The suppression is mediated by IFN-gamma, which acts to reduce the activation level, T helper 2 cytokine production, airways hyperresponsiveness, and migration of allergen-specific CD4+ T cells into the lung, whereas the systemic and draining lymph node responses remain unchanged. Of note, adoptive transfer of previously activated transgenic CD8+ T cells conferred protection against allergic airway inflammation, even in the absence of specific-antigen. Airway resident CD8+ T cells produced IFN-gamma when directly exposed to conditioned media from activated dendritic cells or the proinflammatory cytokines IL-12 and IL-18. Taken together these data indicate that effector/memory CD8+ T cells present in the airways produce IFN-gamma after inflammatory stimuli, independent of specific-antigen, and as a consequence play a key role in modifying the degree and frequency of allergic responses in the lung.

PMCID: PMC395932
PMID: 15079067 [PubMed - indexed for MEDLINE]

1219. J Leukoc Biol. 2004 Jul;76(1):185-94. Epub 2004 Apr 9.

Synergy between proinflammatory ligands of G protein-coupled receptors in neutrophil activation and migration.

Gouwy M, Struyf S, Catusse J, Proost P, Van Damme J.

Rega Institute for Medical Research, University of Leuven, Minderbroedersstraat 10, B-3000 Leuven, Belgium.

The chemokine dose and the time period during which the chemotactic gradient is established determine the number of leukocytes that infiltrate inflamed tissues. At suboptimal chemokine concentrations, neutrophils may require a priming agent or a second stimulus for full activation. An interesting mode of cooperative action to reach maximal migration is synergy between chemokines. This was first observed between the plasma CC chemokine regakine-1 and the tissue CXC chemokine ligand interleukin-8 (IL-8/CXCL8) in neutrophil chemotaxis. Addition of antibodies against IL-8 or regakine-1 in the Boyden microchamber assay abrogated this synergy. Other CC chemokines, such as CC chemokine ligand-2 monocyte chemotactic protein-1 (MCP-1/CCL2), MCP-2 (CCL8), and MCP-3 (CCL7) as well as the CXC chemokine receptor-4 (CXCR4) agonist stromal cell-derived factor-1alpha (SDF-1alpha/CXCL12), also dose-dependently enhanced neutrophil chemotaxis toward a suboptimal concentration of IL-8. These chemokines synergized equally well with the anaphylatoxin C5a in neutrophil chemotaxis. Alternatively, IL-8 and C5a did not synergize with an inactive precursor form of CXCL7, connective tissue-activating peptide-III/CXCL7, or the chemoattractant neutrophil-activating peptide-2/CXCL7. In the chemotaxis assay under agarose, MCP-3 dose-dependently increased the migration distance of neutrophils toward IL-8. In addition, the combination of IL-8 and MCP-3 resulted in enhanced neutrophil shape change. AMD3100, a specific CXCR4 inhibitor, reduced the synergistic effect between SDF-1alpha and IL-8 significantly. SDF-1alpha, but not MCP-1, synergized with IL-8 in chemotaxis with CXCR1-transfected, CXCR4-positive Jurkat cells. Thus, proinflammatory chemokines (IL-8, MCP-1), coinduced during infection in the tissue, synergize with each other or with constitutive chemokines (regakine-1, SDF-1alpha) to enhance the inflammatory response.

PMID: 15075362 [PubMed - indexed for MEDLINE]

1220. J Telemed Telecare. 2004;10(2):118-20.

Use of interactive videoconferencing to deliver asthma education to inner-city immigrants.

Reznik M, Sharif I, Ozuah PO.

Albert Einstein College of Medicine, Children's Hospital at Montefiore, Bronx, New York 10467, USA.

Videoconferencing to deliver asthma health education to patients or their parents has not been widely used in the USA. We used two 90 min interactive videoconferences to deliver an asthma education programme to a convenience sample of mainly Latino immigrant parents in the Bronx, New York. In these sessions short presentations were given on asthma triggers, medications and the prevention of asthma exacerbations. To test gains in knowledge, we administered a self-completion questionnaire consisting of true/false statements before and after the videoconferences. Sixty subjects participated in the first videoconference and 56 in the second. Asthma knowledge improved significantly after both. Three months later, knowledge retention from the first videoconference was good. The majority of subjects asked questions and commented on their personal experiences of asthma. Interactive videoconferencing allowed asthma education to be delivered to a large immigrant population, elicited concerns from the audience and was effective in improving knowledge.

PMID: 15068650 [PubMed - indexed for MEDLINE]

1221. J Exp Med. 2004 Apr 5;199(7):959-70.

Transactivation of sphingosine-1-phosphate receptors by FcepsilonRI triggering is required for normal mast cell degranulation and chemotaxis.

Jolly PS, Bektas M, Olivera A, Gonzalez-Espinosa C, Proia RL, Rivera J, Milstien S, Spiegel S.

Department of Biochemistry, Virginia Commonwealth University Medical Center, Richmond 23298, USA.

Mast cells secrete various substances that initiate and perpetuate allergic responses. Cross-linking of the high-affinity receptor for IgE (FcepsilonRI) in RBL-2H3 and bone marrow-derived mast cells activates sphingosine kinase (SphK), which leads to generation and secretion of the potent sphingolipid mediator, sphingosine-1-phosphate (S1P). In turn, S1P activates its receptors S1P1 and S1P2 that are present in mast cells. Moreover, inhibition of SphK blocks FcepsilonRI-mediated internalization of these receptors and markedly reduces degranulation and chemotaxis. Although transactivation of S1P1 and Gi signaling are important for cytoskeletal rearrangements and migration of mast cells toward antigen, they are dispensable for FcepsilonRI-triggered degranulation. However, S1P2, whose expression is up-regulated by FcepsilonRI cross-linking, was required for degranulation and inhibited migration toward antigen. Together, our results suggest that activation of SphKs and consequently S1PRs by FcepsilonRI triggering plays a crucial role in mast cell functions and might be involved in the movement of mast cells to sites of inflammation.

PMCID: PMC2211871
PMID: 15067032 [PubMed - indexed for MEDLINE]

1222. Acta Pharmacol Sin. 2004 Apr;25(4):503-7.

Inhibitory effects of cryptoporus polysaccharide on airway constriction, eosinophil release, and chemotaxis in guinea pigs.

Zhao XY, Xie QM, Chen JQ, Ke CK.

Zhejiang Respiratory Drugs Research Laboratory of State Drugs Administration of China, Medical School of Zhejiang University, Hangzhou 310031, China.

AIM: To study effects of cryptoporus polysaccharide (CP) on antigen-induced bronchoconstriction, eosinophil peroxidase (EPO) release in vivo, and on platelet activating factor (PAF)-induced eosinophil chemotaxis in vitro in guinea pig. METHODS: The asthma model of guinea pig was formed with ovalbumin (OVA). The changes of lung resistance (R(L)) and dynamic lung compliance (C(dyn)), EPO level in bronchoalveolar lavage fluids (BALF) and eosinophil migration were determined. RESULTS: Pretreatment of CP at doses of 3, 9, and 27 mg/kg by intragastric gavage (i.g.), qd for 10 d, inhibited early asthma response in a dose-dependent manner. Inhibitory rates of mean increase value from 1 to 30 min of R(L) were 34.8 %, 74.4 % ($P<0.05$), and 79.6 % ($P<0.05$), respectively. Inhibitory rate of mean reduction value of C(dyn) were 22.9 %, 40.5 % ($P<0.01$), and 66.5 % ($P<0.01$), respectively. Pretreatment of CP at doses of 3, 9, and 27 mg/kg also inhibited late asthma response, and the reduction of EPO level in BALF were 3.1 %, 16.9 % ($P<0.01$), and 20.1 % ($P<0.01$), respectively. The inhibitory rates of CP at concentrations of 0.13, 1.3, 13, 130 nmol/L to eosinophil migration induced by PAF were 6.8 %, 17.2 % ($P<0.05$), 29.6 % ($P<0.01$), and 35.9 % ($P<0.01$). CONCLUSION: CP protects lung against increase of R(L) and reduction of C(dyn),

decreases EPO level in the asthma model, and inhibits eosinophil chemotaxis induced by PAF. The results suggest that CP may be a novel antiinflammatory agent for the treatment of asthma and allergic diseases.

PMID: 15066221 [PubMed - indexed for MEDLINE]

1223. Contact Dermatitis. 2004 Jan;50(1):31-8.

Characterization of lymphocyte subpopulations and cytokine profiles in peripheral blood of nickel-sensitive individuals with systemic contact dermatitis after oral nickel exposure.

Jensen CS, Lisby S, Larsen JK, Veien NK, Menné T.

Department of Dermatology, Gentofte Hospital, Hellerup, Denmark.
chstje01@gentoftehosp.kbhamt.dk

Several studies have shown that oral nickel exposure can elicit systemic contact dermatitis (SCD) in nickel-sensitive individuals. The current study describes some of the immunological mechanisms underlying such nickel-allergic reactions elicited by oral exposure to nickel. Following oral exposure to graded concentrations of nickel or placebo, blood samples were taken from nickel-sensitive individuals and from non-nickel-sensitive controls. T-cell subtypes (CD3+, CD4+, CD8+ and CD45RO+), expression of skin-homing receptor, cutaneous lymphocyte-associated antigen (CLA) and cytokine profiles [interleukin (IL)-2, IL-4, IL-5, IL-6, IL-10, interferon-gamma and tumour necrosis factor-alpha] were investigated. A definite dose-response reaction pattern to oral nickel exposure was observed among nickel-sensitive individuals. Nickel-sensitive individuals whose dermatitis flared after oral challenge with nickel showed significant decreases in fractions of CD3+ CD45RO+ CLA+ and CD8+ CD45RO+ CLA+ blood lymphocytes, suggesting migration of CD8+ 'memory' CLA+ T lymphocytes from the blood to peripheral tissues. Only those nickel-sensitive individuals who clinically reacted to oral challenge with nickel (4 mg) had elevated levels of IL-5 in the serum, indicating an activation of type 2 T lymphocytes in the peripheral blood. In conclusion, the study indicates that CD8+ CD45RO+ CLA+ T lymphocytes and T lymphocytes with a type 2 cytokine profile are involved in SCD elicited by nickel.

PMID: 15059101 [PubMed - indexed for MEDLINE]

1224. Immunology. 2004 Apr;111(4):435-43.

Human monocyte-derived dendritic cells are chemoattracted to C3a after up-regulation of the C3a receptor with interferons.

Gutzmer R, Lisewski M, Zwirner J, Mommert S, Diesel C, Wittmann M, Kapp A, Werfel T.

Department of Dermatology and Allergology, Hannover Medical University, Hannover, Germany. rgutzmer@gmx.de

The anaphylatoxin C3a is an important inflammatory mediator in the innate and adaptive immune systems. Recent reports in various animal models have fostered the role of C3a in mediating allergic reactions such as pulmonary allergies. However, data in humans are limited and the cellular targets for C3a are not fully understood. We sought to explore human dendritic cells as a new target for C3a, because C3a receptor (C3aR) expression has been described on myeloid cells, and dendritic cells are likely make contact with C3a at sites of inflammatory

reactions. In this study, we demonstrated the expression of the C3aR on human monocyte-derived dendritic cells (MoDC) and its up-regulation by interferon (IFN)-alpha, IFN-gamma and prostaglandin E2 (PGE2). The strongest up-regulation was yielded by the combination of IFN-alpha+ IFN-gamma. Tumour necrosis factor-alpha (TNF-alpha) down-regulated the C3aR. After up-regulation of the C3aR by IFN-alpha+ IFN-gamma, C3a significantly up-regulated the surface expression of CD54, CD83 and CD86, but not of CD40, CD80 or human leucocyte antigen (HLA)-DR. C3a had no effect on the production of interleukin (IL)-10 or IL-12p70, or on the capacity of MoDC to stimulate autologous T-cell proliferation. However, C3a had a direct migratory effect on MoDC, as indicated by the induction of F-actin polymerization and migration in Boyden chamber experiments, which was pronounced after up-regulation of the C3aR with IFN-alpha+ IFN-gamma. Therefore, dendritic cells represent another group of target cells that might be recruited by C3a to areas of inflammation, in particular under conditions where IFNs are increased in the surrounding environment.

PMCID: PMC1782440
PMID: 15056381 [PubMed - indexed for MEDLINE]

1225. Ann Otol Rhinol Laryngol. 2004 Mar;113(3 Pt 1):205-9.

Macrophage migration inhibitory factor in allergic rhinitis: its identification in eosinophils at the site of inflammation.

Nakamaru Y, Oridate N, Nishihira J, Takagi D, Furuta Y, Fukuda S.

Department of Otolaryngology-Head and Neck Surgery, Hokkaido University Graduate School of Medicine, Sapporo, Japan.

The aim of this study was to assess the potential role of macrophage migration inhibitory factor (MIF) in the pathogenesis of allergic rhinitis (AR). Serum MIF concentrations were measured by a specific enzyme-linked immunosorbent assay. In order to elucidate the cellular source of MIF, we performed double immunostaining of biopsy specimens of the nasal mucous membrane with markers for MIF and for inflammatory cells. The mean MIF level in sera from patients with AR was significantly higher than that in sera from healthy controls. Moreover, the levels were significantly correlated with the severity of the clinical symptoms. The majority of the MIF-positive cells at the site of allergic inflammation were eosinophils. These data suggest that MIF plays a role in the initiation and maintenance of AR. Eosinophils formed the largest population of MIF-producing cells; this finding suggests that they may be a major source of MIF at inflammatory sites in atopic disease.

PMID: 15053202 [PubMed - indexed for MEDLINE]

1226. Eur J Immunol. 2004 Apr;34(4):1068-76.

The anti-inflammatory effects of 1,25-dihydroxyvitamin D3 on Th2 cells in vivo are due in part to the control of integrin-mediated T lymphocyte homing.

Topilski I, Flaishon L, Naveh Y, Harmelin A, Levo Y, Shachar I.

Department of Immunology, The Weizmann Institute of Science, Rehovot, Israel.

The fat soluble vitamin D3 metabolite 1,25-dihydroxyvitamin D3 [1,25(OH)(2)D(3)], and its nuclear receptor play an important role in regulating immune responses. While 1,25(OH)(2)D(3)is known to inhibit transcription of cytokine genes that are required for Th1 differentiation or are products of differentiated Th1 cells,

its role in regulating differentiation of Th2 cells is less clear. In this study, we show that 1,25(OH)(2)D(3) has anti-inflammatory effects in an in vivo Th2-dependent asthma model. In addition, we demonstrate that 1,25(OH)(2)D(3)down-regulates the cytoskeleton rearrangement required for promoting integrin-mediated adhesion of naive and effector CD4(+) T cells. Finally, 1,25(OH)(2)D(3)inhibits chemokine-induced migration of naive cells and their homing to the lymph nodes. Thus, in addition to its regulation of cytokine transcription, 1,25(OH)(2)D(3)regulates migration of cells and thus controls the skewing of various Th subsets in the secondary lymphoid organs and inhibits Th function at sites of inflammation.

PMID: 15048717 [PubMed - indexed for MEDLINE]

1227. Biochem Soc Trans. 2004 Apr;32(Pt 2):366-77.

Chemokine inhibition--why, when, where, which and how?

Johnson Z, Power CA, Weiss C, Rintelen F, Ji H, Ruckle T, Camps M, Wells TN, Schwarz MK, Proudfoot AE, Rommel C.

Serono Pharmaceutical Research Institute, Serono International, 14 Chemin des Aulx, CH 1228 Geneva, Switzerland.

Chemokines are small chemoattractant cytokines that control a wide variety of biological and pathological processes, ranging from immunosurveillance to inflammation, and from viral infection to cancer. Genetic and pharmacological studies have shown that chemokines are responsible for the excessive recruitment of leucocytes to inflammatory sites and damaged tissue. In the present paper, we discuss the rationale behind interfering with the chemokine system and introduce various points for therapeutic intervention using either protein-based or small-molecule inhibitors. Unlike other cytokines, chemokines signal via seven-transmembrane GPCRs (G-protein-coupled receptors), which are favoured targets by the pharmaceutical industry, and, as such, they are the first cytokines for which small-molecule-receptor antagonists have been developed. In addition to the high-affinity receptor interaction, chemokines have an in vivo requirement to bind to GAGs (glycosaminoglycans) in order to mediate directional cell migration. Prevention of the GAG interaction has been shown to be a viable therapeutic strategy. Targeting chemokine intracellular signalling pathways offers an alternative small-molecule approach. One of the key signalling targets downstream of a variety of chemokine receptors identified to date is PI3Kgamma (phosphoinositide 3-kinase gamma), a member of the class I PI3K family. Thus the chemokine system offers many potential entry points for innovative anti-inflammatory therapies for autoimmune diseases, such as multiple sclerosis, rheumatoid arthritis and allergic contact dermatitis.

PMID: 15046611 [PubMed - indexed for MEDLINE]

1228. J Immunol. 2004 Apr 1;172(7):4381-90.

Cloning, expression, cellular distribution, and role in chemotaxis of a C5a receptor in rainbow trout: the first identification of a C5a receptor in a nonmammalian species.

Boshra H, Li J, Peters R, Hansen J, Matlapudi A, Sunyer JO.

Department of Pathobiology, School of Veterinary Medicine, University of Pennsylvania, Philadelphia, PA 19104, USA.

C3a, C4a, and C5a anaphylatoxins generated during complement activation play a key role in inflammation. C5a is the most potent of the three anaphylatoxins in eliciting biological responses. The effects of C5a are mediated by its binding to C5a receptor (C5aR, CD88). To date, C5aR has only been identified and cloned in mammalian species, and its evolutionary history remains ill-defined. To gain insights into the evolution, conserved structural domains, and functions of C5aR, we have cloned and characterized a C5aR in rainbow trout, a teleost fish. The isolated cDNA encoded a 350-aa protein that showed the highest sequence similarity to C5aR from other species. Genomic analysis revealed the presence of one continuous exon encoding the entire open reading frame. Northern blot analysis showed significant expression of the trout C5a receptor (TC5aR) message in PBLs and kidney. Flow cytometric analysis showed that two Abs generated against two different areas of the extracellular N-terminal region of TC5aR positively stained the same leukocyte populations from PBLs. B lymphocytes and granulocytes comprised the majority of cells recognized by the anti-TC5aR. More importantly, these Abs inhibited chemotaxis of PBLs toward a chemoattractant fraction purified from complement-activated trout serum. Our data suggest that the split between C5aR and C3aR from a common ancestral molecule occurred before the emergence of teleost fish. Moreover, we demonstrate that the overall structure of C5aR as well as its role in chemotaxis have remained conserved for >300 million years.

PMID: 15034053 [PubMed - indexed for MEDLINE]

1229. Int Arch Allergy Immunol. 2004 Apr;133(4):348-56. Epub 2004 Mar 17.

Novel protein kinase C and matrix metalloproteinase inhibitors of vegetable origin as potential modulators of Langerhans cell migration following hapten-induced sensitization.

Staquet MJ, Piccardi N, Piccirilli A, Vincent C, Schmitt D, Msika P.

INSERM 346, Lyon, France.

BACKGROUND: Migration and maturation of epidermal dendritic cells, the Langerhans cells (LC), are central events in the initiation of the cutaneous immune response. LC migration from skin to draining lymph nodes is regarded as an indispensable step for the early phase of antigen-specific sensitization. Among the several agents which influence the ability of LC to migrate, previous studies have revealed that matrix metalloproteinases (MMPs) and protein kinase C (PKC) contribute to promoting LC migration. In this work, we studied the effect of two recently developed PKC and MMPs inhibitors of vegetable origin on the migration of in vitro activated LC.
METHODS: The migratory capacity of epidermal and in vitro generated LC was assessed using a reconstituted basement membrane assay (Matrigel), mimicking the prerequisite passage through the dermal-epidermal basement membrane on the way to the lymph nodes.
RESULTS: Contact with chemical allergens, Bandrowski's base or 2,4-dinitrobenzenesulfonic acid (DNBS), triggered migration. In the presence of PKC inhibitors, D-erythro-sphingosine and OX100, or an inhibitor of MMPs, LU105, allergen-induced migration of LC was strongly decreased. The association between OX100 and LU105 was more efficient in modulating the migration of activated LC compared to each molecule tested separately.
CONCLUSIONS: These results showed that PKC and MMPs inhibitors act in synergy to inhibit the migration of activated epidermal dendritic cells in vitro. They underscore the role of PKC and MMPs inhibitors and suggest they may be of relevance for therapeutically regulating epidermal dendritic cell migration in inflammatory dermatoses.

Copyright 2004 S. Karger AG, Basel

PMID: 15031608 [PubMed - indexed for MEDLINE]

1230. Scand J Immunol. 2004 Mar;59(3):267-72.

Regulation of mast cell migration by T and T cytokines: identification of tumour necrosis factor-alpha and interleukin-4 as mast cell chemotaxins.

Olsson N, Taub DD, Nilsson G.

Department of Genetics and Pathology, The Rudbeck Laboratory, Uppsala University, Uppsala, Sweden.

Mast cells act as central effector and regulatory cells in many inflammatory disorders, including T helper 1 (T(H1))-mediated inflammations such as autoimmunity and T(H2)-mediated inflammations such as allergy and parasite infections. One characteristic for mast cell-mediated inflammations is the accumulation of mast cells in the inflamed tissue. The factors regulating mast cell recruitment in these inflammations are still not fully characterized. We have investigated the potency of T(H1)- and T(H2)-secreted cytokines to mediate mast cell migration. Supernatants from six different T(H1) and T(H2) clones were tested for mast cell-chemotactic activity using the human mast cell line (HMC-1) as a responder cell. All six clones produced factors that induced mast cell migration. Using blocking antibodies to a broad range of cytokines, we found that anti-tumour necrosis factor-alpha (anti-TNF-alpha) reduced the migration of mast cells to supernatants from T(H1) clones. In contrast, the main mast cell chemoattractants secreted by T(H2) clones were found to be interleukin-4 (IL-4) and IL-8. The potency of these cytokines to act as mast cell chemoattractants was confirmed by using recombinant IL-4, IL-8 and TNF-alpha. Our results suggest that TNF-alpha can be involved in the recruitment of mast cells in T(H1)-mediated inflammations, whereas IL-4 and IL-8 might play a similar role in T(H2)-mediated inflammations.

PMID: 15030577 [PubMed - indexed for MEDLINE]

1231. J Immunol. 2004 Mar 15;172(6):3822-9.

Activation of the D prostanoid receptor 1 regulates immune and skin allergic responses.

Angeli V, Staumont D, Charbonnier AS, Hammad H, Gosset P, Pichavant M, Lambrecht BN, Capron M, Dombrowicz D, Trottein F.

Unité 547, Institut National de la Santé et de la Recherche Médicale, Institut Fédératif de Recherche 17, Institut Pasteur de Lille, Lille, France.

The mobilization of Langerhans cells (LCs) from epithelia to the draining lymph nodes is an essential process to initiate primary immune responses. We have recently shown that in mice, PGD2 is a potent inhibitor of epidermal LC emigration. In this study, we demonstrate that activation of the D prostanoid receptor 1 (DP1) impedes the TNF-alpha-induced migration of human LCs from skin explants and strongly inhibits the chemotactic responses of human LC precursors and of maturing LCs to CC chemokine ligands 20 and 19, respectively. Using a murine model of atopic dermatitis, a chronic Th2-type allergic inflammatory disease, we demonstrate that the potent DP1 agonist BW245C dramatically decreases the Ag-specific T cell activation in the skin draining lymph nodes and markedly prevents the skin lesions following repeated epicutaneous sensitization with OVA.

Interestingly, analysis of the local response indicates that BW245C treatment strongly reduces the recruitment of inflammatory cells into the dermis and disrupts the Th1/Th2 balance, probably through the increased production of the immunoregulatory cytokine IL-10, in the skin of sensitized mice. Taken together, our results suggest a new function for DP1 in the regulation of the immune and inflammatory responses. We propose that DP1 activation by specific agonists may represent a strategy to control cutaneous inflammatory Th2-associated diseases.

PMID: 15004188 [PubMed - indexed for MEDLINE]

1232. J Pharmacol Exp Ther. 2004 Jul;310(1):90-7. Epub 2004 Mar 2.

Investigation into the potential anti-inflammatory effects of endothelin antagonists in a murine model of experimental monosodium urate peritonitis.

Getting SJ, Di Filippo C, Lam CW, Rossi F, D'Amico M.

Department of Biochemical Pharmacology, The William Harvey Research Institute, Charterhouse Square, London, UK. s.j.getting@qmul.ac.uk

Endothelin (ET)-1 has been detected in many inflammatory pathologies, including rheumatoid arthritic patients, asthma, and ischemic-reperfusion injury. In this study, we have investigated the effect of a panel of different ET-1 antagonists displaying different selectivities for the receptors in a murine model of experimental inflammatory peritonitis. Systemic treatment of mice with the ETA antagonist C33H44N6O5,
N-[N-[-N(hexahydro-1H-azepin-1-yl)carbonyl]-L-leucyl]-1-methyl-D-tryptophyl]-3-(2-pyridinyl)-D-alanine (FR139317) inhibited neutrophil accumulation. However, a greater degree of inhibition was observed with the ETB antagonist C34H51N5O7,
N-cis-2,6-dimethylpiperidinocarbonyl-b-tBu-Ala-D-Trp(1-methoxycarbonyl)-D-Nle-OH (BQ-788) and the ET(A and B) antagonist C52H65N7O10,
N-acetyl-alpha-[10,11-dihydro-5H-dibenzo-[a,d]cycloheptadien-5-yl]-D-Gly-Leu-Asp-lle-lle-Trp (PD145065); all these effects occurred without altering peripheral blood cell counts. Release of the CXC chemokine KC was significantly reduced by the FR139317 and PD145065 but not by BQ-788. Evaluation of the therapeutic potential of these antagonists showed that PD145065 inhibited neutrophil migration and KC release, whereas the others caused a nonsignificant reduction in these parameters. Parameters of endothelial cell activation showed that urate-stimulated interleukin-1beta release was inhibited by BQ-788 and PD145065 but not by FR139317, whereas ET-1 was only inhibited by the mixed antagonist. A different scenario was observed with respect to release of the CXC chemokine KC with FR139317 and PD145065 being effective, whereas with a marker of polymorphonuclear activation the ETA and mixed antagonist inhibited adhesion molecule expression. These data show that ET-1 antagonists elicit different mechanisms of actions in the way they display their antimigratory effects in a murine model of monosodium urate crystal peritonitis.

PMID: 14996949 [PubMed - indexed for MEDLINE]

1233. Ann Thorac Surg. 2004 Mar;77(3):1033-7; discussion 1037-8.

Preoperative imaging of the lung sentinel lymphatic basin with computed tomographic lymphography: a preliminary study.

Ueda K, Suga K, Kaneda Y, Li TS, Ueda K, Hamano K.

First Department of Surgery, Yamaguchi University School of Medicine, Ube Yamaguchi, Japan. kaueda@yamaguchi-u.ac.jp

BACKGROUND: Preoperative localization of the sentinel node basin would guide selective lymph node dissection. We tried to identify these nodal stations with indirect computed tomographic lymphography using a conventional extracellular contrast agent, iopamidol.
METHODS: Eleven consecutive patients scheduled to undergo anatomic resection of suspected lung cancer, without lymphadenopathy, were given a peritumoral injection of undiluted iopamidol under computed tomography guidance, and lymphatic migration was assessed by multidetector-row helical computed tomography.
RESULTS: There were no complications such as bleeding, pneumothorax, or allergic reactions. Enhanced nodes were detected in all but 1 patient who had diffuse lymph nodal calcification. Enhanced nodes were identified at 32 ipsilateral intrathoracic nodal stations (20 hilar stations and 12 mediastinal stations). The average length of the longer axis of the enhanced nodes was 4.8 mm (range, 3 to 8 mm), and the average attenuation of the enhanced nodes was 132 (range, 46 to 261) Hounsfield units. In 9 patients with confirmed lung cancer, enhanced nodes appeared at 26 nodal stations, and all apparent enhanced nodes were identified as actual lymph nodes at appropriate position during lymphadenectomy. None of the resected lymph nodes had metastatic involvement.
CONCLUSIONS: Indirect computed tomographic lymphography with the peritumoral injection of iopamidol effectively depicts the drainage nodes unless they are diffusely calcified. Although further study is required, this method could guide selective lymph node dissection.

PMID: 14992921 [PubMed - indexed for MEDLINE]

1234. Biochem Biophys Res Commun. 2004 Mar 19;315(4):782-7.

Endothelin-1 peptides and IL-5 synergistically increase the expression of IL-13 in eosinophils.

Cui P, Sharmin S, Okumura Y, Yamada H, Yano M, Mizuno D, Kido H.

Division of Enzyme Chemistry, Institute for Enzyme Research, The University of Tokushima, Tokushima 770-8503, Japan.

Erratum in
    Biochem Biophys Res Commun. 2004 Jun 4;318(3):802.

The 21-amino acid-length endothelin-1 (ET-1)(1-21) and its novel derivative, 31 amino acid-length ET-1(1-31), have proinflammatory properties and induce significant eosinophil migration mediated by an increase in the local levels of eotaxin and IL-5. We have analyzed by reverse transcription polymerase chain reaction and enzyme immunoassay the effects of ETs on the expression of IL-13 mRNA and protein in eosinophils with or without cell priming with IL-5. The expression of the ETA receptor (ETAR) and its membrane localization were detected in the eosinophils, whereas the ETB receptor was undetectable. ET peptides synergistically increased the expression of IL-13 in eosinophils after priming with IL-5, and the increase was blocked by the ETAR antagonist BQ123, though these peptides did not directly influence the expression. These results may explain the presence of eosinophilia in the airways' epithelium of patients suffering from asthma, along with an increase in immunoreactive ETs.

PMID: 14985080 [PubMed - indexed for MEDLINE]

1235. Med Sci Monit. 2004 Mar;10(3):BR77-83. Epub 2004 Mar 1.

Effects of poly(ADP-ribose) polymerase inhibition on inflammatory cell migration in a murine model of asthma.

Virág L, Bai P, Bak I, Pacher P, Mabley JG, Liaudet L, Bakondi E, Gergely P, Kollai M, Szabó C.

Department of Medical Chemistry, Research Center for Molecular Medicine, Medical and Health Science Center, University of Debrecen, Debrecen, Hungary.

BACKGROUND: Poly(ADP-ribose) polymerase-1 (PARP-1), a monomeric nuclear enzyme present in eukaryotes, plays a role in cell death, inflammatory mediator expression, and mononuclear cell recruitment in various experimental models of inflammation and reperfusion injury. Part of the molecular mechanism of this function involves the regulation of cytokine and chemokine production. Since chemokines are principal regulators of mononuclear and polymorphonuclear cell trafficking in asthma, we investigated the possibility whether PARP modulates chemokine production and cell recruitment in a murine model of asthma.
MATERIAL/METHODS: We studied ovalbumin-sensitized mice challenged with a single dose of ovalbumin.
RESULTS: PARP inhibition with the phenanthridinone-based PARP inhibitor PJ34 suppressed inflammatory cell migration. These effects were associated with downregulation of the CC chemokine MIP-1alpha, but not the CXC chemokine MIP-2. The production of TNF- alpha and IL-12, but not IL-5 or IL-13, was also suppressed by PARP inhibition.
CONCLUSIONS: Our results demonstrate the pathogenetic role of PARP activation in a murine model of asthma. PARP selectively regulates the production of certain chemokines and cytokines in this experimental model, which may be responsible for some of the observed protective effects seen in the current murine asthma model.

PMID: 14976461 [PubMed - indexed for MEDLINE]

1236. Int Arch Allergy Immunol. 2004 Mar;133(3):248-54. Epub 2004 Feb 16.

The prevalence of allergic sensitisation in immigrant children in The Netherlands.

van Amsterdam JG, Bischoff EW, Hady M, Opperhuizen A, Steerenberg PA.

Laboratory for Toxicology, Pathology and Genetics, National Institute for Public Health and the Environment, Bilthoven, The Netherlands. van.Amsterdam@rivm.nl

BACKGROUND: Differences in the prevalence of allergic sensitisation have been reported in immigrant children living in the same urban environment. The purpose of this study is to investigate the prevalence of allergic sensitisation in school children of Dutch, Turkish and Moroccan origin.
METHODS: The prevalence of sensitisation to aero-allergens was assessed using the skin prick test in a non-selected sample of 512 children (response rate 54%) living in the same inner city district of Utrecht. In addition, exhaled nitric oxide (FeNO) was determined.
RESULTS: The prevalence of allergic sensitisation was dependent on the ethnic origin. As compared with Dutch children (19.1%), a higher prevalence of allergic sensitisation was observed in immigrant children for whom both parents were born in Turkey (23.6%, not significant) or Morocco (30.6%, $p<0.05$). The prevalence of allergic sensitisation in Dutch children was nearly 2 times lower than the reported prevalence in German children. In all sensitised children, the mean FeNO value was significantly ($p<0.05$) higher than in non-sensitised children, and the mean FeNO level was highest in Moroccan children sensitised to indoor allergens.
CONCLUSION: In The Netherlands, immigrant children show a higher prevalence of allergic sensitisation as compared to Dutch children.

Copyright 2004 S. Karger AG, Basel

PMID: 14976393 [PubMed - indexed for MEDLINE]

1237. J Histochem Cytochem. 2004 Mar;52(3):401-10.

Localization and enhanced mRNA expression of the orphan chemokine receptor L-CCR in the lung in a murine model of ovalbumin-induced airway inflammation.

Oostendorp J, Hylkema MN, Luinge M, Geerlings M, Meurs H, Timens W, Zaagsma J, Postma DS, Boddeke HW, Biber K.

Department of Molecular Pharmacology, University of Groningen, Groningen, The Netherlands. j.oostendorp@farm.rug.nl

Various CC chemokine receptors are expressed on effector cells in allergic inflammation and their distinct expression pattern may dictate, to a large extent, the migration of inflammatory cells to sites of airway inflammation. The lipopolysaccharide (LPS)-inducible CC chemokine receptor (L-CCR) is an orphan chemokine receptor that has previously been identified in the murine macrophage cell line RAW 264.7 and in murine brain glial cells. In this study we investigated the induction and localization of L-CCR mRNA expression in mouse lung after ovalbumin (OVA)-induced airway inflammation. Both RT-PCR experiments and in situ hybridization (ISH) experiments in whole lung sections revealed a rapid upregulation of L-CCR mRNA expression as early as 1 hr and 3 hr after OVA challenge. Expression was found predominantly in MAC3(+) macrophages and in bronchial epithelium, as shown by ISH and immunohistochemistry (IHC). We demonstrated that L-CCR mRNA expression is strongly upregulated in mouse lung after OVA challenge and is localized in macrophages and bronchial epithelium. Regarding the likely role of L-CCR as a chemokine receptor with the putative ligand monocyte chemotactic protein-1 (MCP-1, CCL2), this receptor may have an important function in the early phase of airway inflammation.

PMID: 14966207 [PubMed - indexed for MEDLINE]

1238. Curr Top Med Chem. 2004;4(5):539-52.

Molecules of parasites as immunomodulatory drugs.

Imai S, Fujita K.

Section of Environmental Parasitology, Department of International Health Development, Division of Public Health, Graduate School, Tokyo Medical and Dental University, 1-5-45 Yushima Bunkyo-ku, Tokyo 113-8519, Japan. sitimai@ac.mbn.or.jp

Parasite molecules offer unique advantages for the treatment of immunologicical disorders, and several candidate molecules have been shown to be effective. In our studies, it was shown that a factor inducing immunoglobulin E from filarial nematode parasites was suppressive in animal models of immunological disorders such as allergy and insulin dependent diabetes mellitus (IDDM). The Th1/Th2 paradigm of CD4+ T helper cell subsets can provide the basis for the development of new types of drugs and of novel strategies for the treatment of allergic and autoimmune disorders by parasite molecules. In our experimental system, parasite molecules from a filarial nematode parasite led to the down-regulation of the allergic reaction in animal models. In the majority of hosts, infection with helminths is associated with markedly reduced cellular immune reactions and polarization of T cell responses to Th2 and Th3 types. Some studies have

suggested that the stimulation of host immunoregulatory networks with parasite molecules leading to the synthesis of anti-inflammatory cytokines (interleukin10, transforming growth factor-beta (TGF-beta and others) can provide new therapy for immunological disorders. It is known that parasites produce some types of molecule that mimic host molecules such as CD40 ligand, TGF-beta and macrophage migration inhibitory factor. These molecules are also candidates for medicinal agents. This review describes many of the latest possibilities in this field and shows how they can be best put to use for the development of medicinal agents, molecular target identification, and for prioritization.

PMID: 14965305 [PubMed - indexed for MEDLINE]

1239. Am J Respir Crit Care Med. 2004 May 1;169(9):1014-8. Epub 2004 Feb 12.

Functional polymorphisms in the promoter region of macrophage migration inhibitory factor and atopy.

Hizawa N, Yamaguchi E, Takahashi D, Nishihira J, Nishimura M.

First Department of Medicine, Hokkaido University School of Medicine, Kita-Ku N-15 W-7, Sapporo, Japan 060-8638. nhizawa@med.hokudai.ac.jp

Macrophage migration inhibitory factor (MIF) is a pleiotrophic lymphocyte and macrophage cytokine; it is likely to play an important role in innate immunity. Genome-wide search for atopy susceptibility genes recently identified human chromosome 22q11, where the gene encoding MIF resides, as a region of interest for atopic traits. Both the -173G/C and -794 [CATT]5-8 repeat polymorphisms in the MIF promoter region are associated with altered levels of MIF gene transcription in vitro. We, therefore, hypothesized that these potentially functional polymorphisms may influence susceptibility to atopy and asthma. A case-control analysis examined the genetic influence of these promoter polymorphisms on the development of atopy and asthma in a Japanese population (n = 584). Evidence for significant association between the -173G/C and -794 [CATT]5-8 repeat polymorphisms and atopy was found; odds ratio for homozygotes of -173C allele was 3.67 (compared with homozygotes of -173G allele, 95% confidence interval = 1.43-9.46, $p < 0.01$), and odds ratio for noncarriers of the -794 [5-CATT] allele was 3.51 (compared with 5-CATT repeat homozygotes, 95% confidence interval = 1.82-6.78, $p < 0.0005$). No associations with asthma were detected. These results indicate that promoter polymorphisms in the MIF promoter region are risk factors for atopy and implicate MIF in the pathogenesis of atopy in a Japanese population.

PMID: 14962818 [PubMed - indexed for MEDLINE]

1240. J Invest Dermatol. 2004 Jan;122(1):119-24.

Modulation of skin norepinephrine turnover by allergen sensitization: impact on contact hypersensitivity and T helper priming.

Maestroni GJ.

Center for Experimental Pathology, Istituto Cantonale di Patologia, Locarno, Switzerland. georges.maestroni@ti.ch

The information gathered by dendritic cells during the innate immune response is determinant for the type and strength of the adaptive response. We showed that the sympathetic neurotransmitter norepinephrine influences dendritic cell migration and T helper priming via alpha- and beta-adrenoceptors. Others have

shown that Langerhans cells also express mRNA for beta 1-, beta 2-, and alpha 1A-adrenoceptors and that catecholamines may inhibit the antigen-presenting capability via beta 2-adrenoceptors. Here we report that oxazolone, which induces a predominant T-helper-1-type contact hypersensitivity response, but not fluorescein isothiocyanate, which induces a prevailing T-helper-2-type response, inhibits the local norepinephrine turnover in the skin of mice during the first 8 h of sensitization. Oxazolone also induced higher expression of the inflammatory cytokines interleukin-1 and interleukin-6 mRNA in the skin. Lack or blockade of these cytokines as well as inhibition of prostaglandin synthesis, however, did not influence the oxazolone effect. Only the nonspecific anti-inflammatory steroid dexamethasone could neutralize the effect of oxazolone. Furthermore, fluorescein isothiocyanate but not oxazolone sensitization in the presence of the specific beta 2-adrenoceptor antagonist ICI 118,551 enhanced the consequent contact hypersensitivity response as well as the production of T helper 1 cytokines in draining lymph nodes; conversely T helper 2 cytokines were not affected. Thus, the extent of T helper 1 priming in the adaptive response to a sensitizing agent seems to depend also on its ability to modulate the local sympathetic nervous activity during the innate immune response.

PMID: 14962099 [PubMed - indexed for MEDLINE]

1241. Curr Allergy Asthma Rep. 2004 Mar;4(2):109-15.

Factors controlling airway smooth muscle proliferation in asthma.

Stewart AG, Bonacci JV, Quan L.

Department of Pharmacology, University of Melbourne, Grattan Street, Victoria, Australia 3010. astew@clyde.its.unimelb.edu.au

Airway smooth muscle proliferation has been the focus of considerable attention, as it is a quantitatively important component of the airway wall remodeling response in asthma and has been suggested as a suitable target for the development of novel anti-asthma agents. Such agents are considered likely to reduce airway hyperresponsiveness and, consequently, airway obstruction, resulting in fewer symptoms and exacerbations. Identifying suitable drug targets has proved an elusive goal, as no dominant molecular mechanism for remodeling has emerged. Moreover, recent findings raise some doubt as to whether smooth muscle proliferation per se is the explanation of the increase in smooth muscle cell number in asthma, with alternative explanations including the proposal that cells migrate either from the interstitial compartment or from a circulating precursor stem cell population. Therefore, drug targeting of migration responses should be considered as an alternative approach to regulating the smooth muscle component of airway wall remodeling.

PMID: 14769259 [PubMed - indexed for MEDLINE]

1242. Curr Allergy Asthma Rep. 2004 Mar;4(2):102-8.

Airway smooth muscle and fibroblasts in the pathogenesis of asthma.

Johnson PR, Burgess JK.

Department of Pharmacology, The University of Sydney, NSW Australia 2006. pjohno@pharmacol.usyd.edu.au

Asthma is a disease characterized by marked structural changes within the airway wall. These changes include deposition of extracellular matrix proteins and an

increase in the numbers of airway smooth muscle cells and subepithelial fibroblasts. Both these cell types possess properties that would enable them to be involved in remodeling and inflammation. These properties include the production of a variety of cytokines; growth factors and fibrogenic mediators; proliferation, migration and release of extracellular matrix proteins; matrix metalloproteinases; and their tissue inhibitors. Airway smooth muscle and subepithelial fibroblasts are likely to be key players in the asthmatic airway pathophysiology through their interaction with each other, inflammatory cells, and other mesenchymal cells, such as the epithelium. Current asthma therapies lack the ability to completely prevent or reverse the remodeling of the airways, therefore indicating the need for new therapeutic strategies to counter this important aspect of asthma.

PMID: 14769258 [PubMed - indexed for MEDLINE]

1243. Front Biosci. 2004 Jan 1;9:457-63.

Role of chemokines and their receptors in the pathogenesis of multiple sclerosis.

Sindern E.

Department of Neurology, BG-Kliniken Bergmannsheil, Ruhr-University, Burkle-de-la-Camp-Platz 1, 44789 Bochum, Germany.
eckhart.sindern@ruhr-uni-bochum.de

Multiple sclerosis (MS) is an autoimmune disease of the human central nervous system (CNS) of unknown etiology that causes demyelination and associated tissue injury. Trafficking of inflammatory T cells into the CNS is a crucial event in the pathogenesis of MS, a process in which chemokines and their receptors have been demonstrated to play an important role. Chemokines are key mediators of inflammation and have major effects on migration of cells to the sites of inflammation as well as activation of recruited and resident CNS cells. This paper summarizes recent and new information about the expression and function of elements of the chemokine system in MS and its animal model experimental allergic encephalomyelitis. Analysis of the chemokine system provides insights into mechanisms of CNS inflammatory reactions and may lead to new targets of therapeutic intervention in MS.

PMID: 14766382 [PubMed - indexed for MEDLINE]

1244. J Biomed Mater Res A. 2004 Mar 1;68(3):411-22.

Evaluation of biocompatibility of polypyrrole in vitro and in vivo.

Wang X, Gu X, Yuan C, Chen S, Zhang P, Zhang T, Yao J, Chen F, Chen G.

Jiangsu Key Laboratory of Neuron Regeneration, Nantong Medical College, Nantong 226001, People's Republic of China. wxdzw@hotmail.com

In this study, the biocompatibility of the electrically conductive polymer polypyrrole (PPy) with nerve tissue was evaluated in vitro and in vivo. The extraction solution of PPy powder, which was synthesized chemically, was tested for acute toxicity, subacute toxicity, pyretogen, quantitative measure of cell viability, hemolysis, allergen, and micronuclei. The PPy membrane was synthesized electrochemically on the indium tin oxide conductive borosilicate glass. The dorsal root ganglia from 1-3-day-old Sprague-Dawley rats were cultured above PPy membrane and observed by light or scanning electron microscopy. The PPy-silicone tube (PPy membrane on the inner surface of the silicone tube) also synthesized

electrochemically was used to bridge across 10-mm sciatic nerve gap in rats. Twenty-four weeks after the operation to rats, the regenerated tissues were observed by electrophysiological and histological techniques. PPy extraction solution showed no evidence of acute and subacute toxicity, pyretogen, hemolysis, allergen, and mutagenesis, and the Schwann cells from the PPy extraction solution group showed better survival rate and proliferation rate as compared with the saline solution control group. The migration of the Schwann cells and the neurite extension from dorsal root ganglia on the surface of PPy membrane-coated glass was better than those of bare glass. There was only lightly inflammation during 6 months of the postoperation, when the PPy-silicone tube bridged across the gap of the transected sciatic nerve. The regeneration of nerve tissue in the PPy-silicone tube was slightly better than that in the plain silicone tube by means of electrophysiological and histological examination. The results of this study indicate that PPy has a good biocompatibility with rat peripheral nerve tissue and that PPy might be a candidate material for bridging the peripheral nerve gap.

Copyright 2003 Wiley Periodicals, Inc.

PMID: 14762920 [PubMed - indexed for MEDLINE]

1245. Thorax. 2004 Feb;59(2):136-43.

Rapid and efficient clearance of airway tissue granulocytes through transepithelial migration.

Erjefält JS, Uller L, Malm-Erjefält M, Persson CG.

Department of Physiological Sciences, Lund University, Lund, Sweden.
jonas.erjefalt@mphy.lu.se

BACKGROUND: Clearance of tissue granulocytes is central to the resolution of airway inflammation. To date the focus has been on apoptotic mechanisms of cell removal and little attention has been given to alternative processes. The present study explores transepithelial migration as a mechanism of cell clearance.
METHOD: Guinea pig tracheobronchial airways where eosinophils are constitutively present in the mucosal tissue were studied. A complex topical stimulus (allergen challenge) was applied and the fate of the eosinophils was determined by selective tracheobronchial lavage and histological examination of the tissue.
RESULTS: Within 10 minutes of the allergen challenge, massive migration of eosinophils into the airway lumen occurred together with a reduction in tissue eosinophil numbers. Cell clearance into the lumen continued at high speed and by 30 and 60 minutes the tissue eosinophilia had been reduced by 63% and 73%, respectively. The marked transepithelial migration (estimated maximal speed 35,000 cells/min x cm2 mucosal surface) took place ubiquitously between epithelial cells without affecting epithelial integrity as assessed by transmission and scanning electron microscopy. Eosinophil apoptosis was not detected but occasional cytolytic eosinophils occurred.
CONCLUSION: This study shows that luminal entry has a remarkably high capacity as a granulocyte elimination process. The data also suggest that an appropriate stimulus of transepithelial migration may be used therapeutically to increase the resolution of inflammatory conditions of airway tissues.

PMCID: PMC1746951
PMID: 14760154 [PubMed - indexed for MEDLINE]

1246. Curr Pharm Des. 2004;10(2):163-70.

Modulation of PMN-endothelial cells interactions by cyclic nucleotides.

Sato Y.

Division of Thoracic Surgery, Department of Surgery, Jichi Medical School, Japan. tcvysato@jichi.ac.jp

Polymorphonuclear leukocytes (PMN) interact with endothelial cells (EC) under normal and diseased conditions. Common Mechanisms exist regulating PMN-EC interactions in the systemic and pulmonary circulations and adhesion molecules play significant roles in both circulations, however there are important differences. Alterations in PMN deformability appear to be important in the pulmonary circulation because of the unique geometric and hydrodynamic conditions that exist in the pulmonary microvasculature. PMN work as the host's first line of defence against invading pathogens. Under certain circumstances, however, dysregulation of PMN-EC interactions may contribute to local or global tissue injury in diseases such as acute respiratory syndrome and multiple organ failure syndrome. Therefore, a thorough understanding of the regulation of PMN-EC interactions is important to understand the pathogenesis of this type of tissue injury, and modulation of PMN-EC interactions could be applicable to prevent or treat injury. cGMP and cAMP are cyclic nucleotides that work as second messengers and control numerous functions in PMN. This review covers the modulation of PMN-EC interactions with cGMP and cAMP. Recent studies have shown that both cGMP and cAMP have inhibitory effects on events such as rolling, adhesion, migration and deformability change of PMN that are essential to PMN-EC interactions. Therefore, it is expected that the modulation of cyclic nucleotides is applicable for the treatment not only of local inflammatory diseases such as asthma but also of global tissue injury such as acute respiratory distress syndrome.

PMID: 14758814 [PubMed - indexed for MEDLINE]

1247. Clin Rev Allergy Immunol. 2004 Feb;26(1):61-72.

The innate allergenicity of helminth parasites.

Falcone FH, Loukas A, Quinnell RJ, Pritchard DI.

Immune Modulation Research Group, Boots Science Building, School of Pharmacy, University of Nottingham, Nottingham, UK. Franco.Falcone@nottingham.ac.uk

Helminth parasites are well known to induce an immune response in their hosts characterised by elevated IgE, peripheral blood or local tissue eosinophilia, and in some cases, intestinal mastocytosis. This immunological response has a strong T-helper 2 (Th2) cytokine bias and is reminiscent of the immunological constellation found in allergic diseases. However, the molecular forces driving the Th2 response to helminth parasites are still not understood. By using the human hookworm parasite Necator americanus as an example, the authors of the current article propose that in the course of its life cycle, this parasite becomes innately allergenic through the secretion of a molecular array designed to promote tissue migration and homing, feeding and survival against immunological attack. This complex array comprises proteases, lectins and other classes of molecules. Subsequent immunological and physiological events seemingly protect the host from both the allergic sequelae of exposure to environmental allergens and, moreover, from the parasite itself.

PMID: 14755076 [PubMed - indexed for MEDLINE]

1248. J Cosmet Laser Ther. 2003 Dec;5(3-4):226-30.

Bio-Alcamid in drug-induced lipodystrophy.

Protopapa C, Sito G, Caporale D, Cammarota N.

Department of Plastic Surgery, University of Parma, Parma, Italy.

The development of new drugs to counter human immunodeficiency virus (HIV) infection has led to an increase in lipodystrophic syndrome among HIV-infected individuals receiving combination therapy. Bio-Alcamid(TM) is a recently developed polymeric substance that can be implanted to compensate for adipose effects. We have implanted this substance in 73 patients with up to three years' follow-up. The aesthetic results were deemed excellent by both physicians and patients. No implant dislocation, implant migration, granuloma, allergic reaction or intolerance were recorded.

PMID: 14741840 [PubMed - indexed for MEDLINE]

1249. J Asthma. 2003 Dec;40(8):883-6.

Endothelin-1 (ET-1) decreases human bronchial epithelial cell migration and proliferation: implications for airway remodeling in asthma.

Dosanjh A, Zuraw B.

Molecular and Experimental Medicine, The Scripps Research Institute, La Jolla, California 92037, USA. dosamr@scripps.edu

The respiratory epithelium is a protective barrier that also functions as an interactive metabolically active component of the lung. The healing and repair of the epithelium involves initial migration of epithelial cells, and subsequent proliferation. The purpose of our study was to assess the effect of inflammatory mediators, in particular endothelin-1 (ET-1), on bronchial epithelial cell proliferation and migration. Under the conditions studied, ET-1 slows proliferation of human bronchial epithelial cells, compared to control ($p < 0.01$). The presence of ET-1 results in slower migration of epithelial cells compared to control ($p < 0.04$). Based on these in vitro findings, ET-1 could potentially lead to inhibition of repair of the lung epithelium and enhanced remodeling.

PMID: 14736087 [PubMed - indexed for MEDLINE]

1250. Clin Exp Allergy. 2004 Jan;34(1):103-9.

Cetirizine, an H1-receptor antagonist, suppresses the expression of macrophage migration inhibitory factor: its potential anti-inflammatory action.

Shimizu T, Nishihira J, Watanabe H, Abe R, Ishibashi T, Shimizu H.

Departments of Dermatology Molecular Biochemistry, Hokkaido University Graduate School of Medicine, Sapporo, Japan.

BACKGROUND: H1-receptor antagonists are often effective in the treatment of allergic disorders such as atopic dermatitis. Cetirizine, a putative H1-receptor antagonist, has recently been shown to have anti-inflammatory properties through the inhibition of leucocyte recruitment and activation, and by the reduction of ICAM-1 expression on keratinocytes.
OBJECTIVE: To further elucidate the anti-inflammatory properties of cetirizine,

we first examined its effects on antigen-induced eosinophilia and neutrophila in vivo. We then examined the anti-inflammatory effects of cetirizine on a human keratinocyte A431cell line.
METHODS: Mice were sensitized subcutaneously with ragweed pollen and were challenged intraperitoneally with the allergen. Cetirizine diluted in sterile water (0-20 mg/kg) or only sterile water was administered orally. Peritoneal cells were obtained at 8 and 24 h after challenge. The eosinophilia and neutrophilia induced by ragweed pollen extract were quantitated. Macrophage migration inhibitory factor (MIF), macrophage inflammatory protein 2 (MIP-2) and eotaxin contents of peritoneal fluid were also measured by mouse ELISA. The effects of cetirizine on MIF-induced IL-8 production in A431 cells were examined by ELISA. The effects of cetirizine on MIF expression and production in A431 cells were examined by human MIF ELISA and Northern blot analysis.
RESULTS: Eosinophilia and neutrophilia induced by ragweed pollen extract were found to be significantly reduced in cetirizine-treated mice (20 mg/kg). MIF, a pleuripotent cytokine, was significantly decreased at 8 and 24 h in the peritoneal fluid by cetirizine treatment. MIP-2 and eotaxin were also decreased 8 and 24 h, respectively, after challenge in the peritoneal fluid with cetirizine treatment. MIF stimulates IL-8 production in A431 cells. We found that MIF production in A431 cells was inhibited by 10 microm cetirizine. Consistent with this, cetirizine significantly inhibited MIF-induced IL-8 production.
CONCLUSION: These results suggest that cetirizine exerts its anti-inflammatory effects by inhibiting MIF as well as IL-8 production, such as those involved in inflammatory allergic skin disease, suggesting a broad spectrum of action beyond its mere H1-receptor-antagonistic function.

PMID: 14720269 [PubMed - indexed for MEDLINE]

1251. Clin Exp Allergy. 2004 Jan;34(1):77-84.

Effect of a single dose of the selectin inhibitor TBC1269 on early and late asthmatic responses.

Avila PC, Boushey HA, Wong H, Grundland H, Liu J, Fahy JV.

Department of Medicine and Cardiovascular Research Institute, University of California at San Francisco, San Francisco, CA 94143-0130, USA.
avila@itsa.ucsf.edu

BACKGROUND: Selectins participate in the initial phase of leucocyte migration from circulation to inflamed tissues and may play a role in inflammatory cellular influx into airways in asthma. In the sheep asthma model, TBC1269, a pan-selectin antagonist, reduced late allergen response by 74%.
OBJECTIVE: To determine whether a single dose of TBC1269 inhibits early (EAR) and late (LAR) asthmatic responses, and whether it inhibits sputum leucocyte influx after inhalation allergen challenge in atopic asthmatic subjects treated with bronchodilators only.
METHODS: Twenty-one asthmatic subjects (mean+/-SD, age=32.5+/-6.7 years, 8 males, FEV1 percent predicted=84+/-15%) with known late asthmatic response based on a screening inhalation allergen challenge were randomly assigned to receive intravenous treatment with either placebo (n=11) or TBC1269 (n=10, 30 mg/kg) infused over 15 min immediately prior to a second (post-treatment) allergen challenge at least 4 weeks after the screening challenge. After each challenge, EAR and LAR were monitored for 7 h. In addition, sputum was induced 1 day before and 1 day after each allergen challenge.
RESULTS: TBC1269 did not attenuate the EAR compared with placebo (largest fall in FEV1 within 1 h of 34.1+/-13.9% vs. 31.8+/-12.2% for TBC1269 and placebo groups respectively, P=0.61) or the LAR (largest fall in FEV1 between 3 and 7 h of 39.3+/-15.3% vs. 32.6+/-13.8%, P=0.24). TBC1269 had only minor effects on

allergen-induced sputum eosinophilia.
CONCLUSION: We conclude that TBC1269 administered before allergen challenge as a single intravenous dose does not attenuate early or late asthmatic responses to allergen in asthmatic subjects.

PMID: 14720266 [PubMed - indexed for MEDLINE]

1252. J Invest Dermatol. 2003 Nov;121(5):951-62.

The molecular basis of lymphocyte recruitment to the skin: clues for pathogenesis and selective therapies of inflammatory disorders.

Schön MP, Zollner TM, Boehncke WH.

Department of Dermatology, Otto-von-Guericke-University, Magdeburg, Germany. Michael.Schoen@virchow.uni-wuerzburg.de

Spatial compartmentalization and tissue-selective localization of T lymphocytes to the skin are crucial for immune surveillance and the pathogenesis of various disorders including common inflammatory diseases such as atopic dermatitis or psoriasis, but also malignancies such as cutaneous T cell lymphomas. Cutaneous recruitment of lymphocytes is a highly complex process that involves extravasation, migration through the dermal connective tissue, and eventually, localization to the epidermis. An intertwined network of cytokines and chemokines provides the road signs for leukocyte migration, while various adhesion receptors orchestrate the dynamic events of cell-cell and cell-substrate interactions resulting in cutaneous localization of T cells. Selectively targeting the functions of molecules involved in this interplay promises exciting new therapeutic options for treating inflammatory skin disorders.

PMID: 14708592 [PubMed - indexed for MEDLINE]

1253. Exp Dermatol. 2003 Oct;12(5):682-91.

Comparison of two in vitro dendritic cell maturation models for screening contact sensitizers using a panel of methacrylates.

Rustemeyer T, Preuss M, von Blomberg BM, Das PK, Scheper RJ.

Department of Pathology, Free University Medical Centre, Amsterdam, The Netherlands. t.rustemeyer@vumc.nl

Allergen-induced emigration and maturation of dendritic cells (DC) are pivotal steps in sparking off allergic contact dermatitis. In vitro models, reflecting these steps, may provide tools for assessment of sensitizing capacities of putative contact allergens. Here, we evaluated the applicability of such models for a panel of methacrylate congeners, the sensitizing properties of which were established previously in clinical and experimental animal studies. First, using interleukin-4 (IL-4)/granulocyte-macrophage colony-stimulating factor (GM-CSF)-induced, blood monocyte-derived DC, hapten-induced up-regulation of maturation/ activation markers, including CD80, CD83, CD86, chemokine receptors CXCR4 and CCR5, as well as the drug resistance related molecules P-glycoprotein (Pgp) and lung resistance protein (LRP), were monitored by flow cytometry. Of note, whereas CD86 and CXCR4 were most sensitive in discriminating between the contact sensitizers and irritants included in the panel, i.e. sodium dodecyl sulphate (SDS) and croton oil (CO), assessment of CD83 and LRP expression reflected the relatively lower sensitizing capacity of methyl methacrylate. Second, using ex vivo skin explant cultures, allergen-induced LC migration from

epidermal to basal membranous and dermal skin structures was most reliably monitored by CD1a, as compared with Pgp, LRP, HLA-DR or CD54 staining. The extent of CD1a+ LC migration was found to closely correlate with the sensitizing capacities of the panel of test compounds. These results support the view that both in vitro models can provide valuable data on contact sensitizing properties, and add chemokine receptors and drug resistance related molecules to the list of DC membrane markers revealing allergenic signaling.

PMID: 14705810 [PubMed - indexed for MEDLINE]

1254. Nihon Yakurigaku Zasshi. 2004 Jan;123(1):15-22.

[Prostaglandin D2 in allergy: PGD2 has dual receptor systems].

[Article in Japanese]

Hirai H.

Department of Advanced Medicine and Development, BML, Inc., Kawagoe, Saitama, Japan.

Allergic inflammations feature an accumulation of T helper 2 (Th2) cells, eosinophils, and basophils into the inflamed sites and are often triggered by antigen-IgE mediated activation of mast cells that secret a variety of mediators. Therefore, the mast cell is known as a conductor cell in allergic inflammations. Prostaglandin (PG) D(2) is the major prostanoid secreted from the activated mast cell and has long been implicated in allergic diseases. The involvement of PGD(2) in allergic inflammation has been corroborated by several studies. Two PGD(2) receptors are known as the DP receptor and CRTH2. CRTH2 differs from DP in its signal pathways: CRTH2 is coupled with Gi-type G protein and DP is coupled with Gs-type G protein. It was reported that DP-deficient mice subjected to ovalbumin-induced asthma model systems showed suppressed allergic reactions. Functions of CRTH2 in vivo have not been clear, but CRTH2 mediates PGD(2)-dependent cell migration and the activation of Th2 cells, eosinophils, and basophils. Therefore, the CRTH2 signal seems to promote allergic disease. The findings from these in vivo and vitro studies suggest that PGD(2) secreted from activated mast cells may be involved in the formation and/or maintenance of allergic inflammations through its dual receptor systems.

PMID: 14695454 [PubMed - indexed for MEDLINE]

1255. J Immunol. 2004 Jan 1;172(1):349-55.

A functional C5a anaphylatoxin receptor in a teleost species.

Holland MC, Lambris JD.

Department of Pathology and Laboratory Medicine, University of Pennsylvania, 401 Stellar Chance, Philadelphia, PA 19104, USA.

The anaphylatoxins are potent, complement-derived low m.w. proteins that bind to specific seven-transmembrane receptors to elicit and amplify a variety of inflammatory reactions. C5a is the most potent of these phlogistic peptides and is a strong chemoattractant for neutrophils and macrophages/monocytes. Although lower vertebrates possess complement systems that are believed to function similarly to those of mammals, anaphylatoxin receptors have not previously been characterized in any nonmammalian vertebrate. To study the functions of C5a in teleost fish, we generated recombinant C5a of the rainbow trout, Oncorhynchus

mykiss (tC5a), and used fluoresceinated tC5a (tC5aF) and flow cytometry to identify the C5a receptor (C5aR) on trout leukocytes. Granulocytes/Macrophages present in cell suspensions of the head kidney (HKL), the main hemopoietic organ in teleosts, showed a univariate type of receptor expression, whereas those from the peripheral blood demonstrated either a low or high level of expression. The binding of tC5aF was inhibited by excess amounts of unlabeled tC5a or tC5a(desArg), demonstrating that sites other than the C-terminal of tC5a interact with the C5aR. Both tC5a and tC5a(desArg) were able to induce chemotactic responses in granulocytes in a concentration-dependent manner, but the desArg derivative was at least 10-fold less active. Homologous desensitization occurred after HKL were exposed to continuous or high concentrations of tC5a, with a loss of tC5aF binding and an 80% reduction in chemotactic responses toward tC5a. Pertussis toxin reduced the migration of HKL toward tC5a by 40%, suggesting only a partial involvement of pertussis toxin-sensitive G(i) proteins in tC5a-mediated chemotaxis.

PMID: 14688343 [PubMed - indexed for MEDLINE]

1256. J Immunol. 2004 Jan 1;172(1):268-73.

CXCR1+CD4+ T cells in human allergic disease.

Francis JN, Jacobson MR, Lloyd CM, Sabroe I, Durham SR, Till SJ.

Upper Respiratory Medicine, National Heart and Lung Institute, Imperial College, Dovehouse Street, London SW3 6LY, United Kingdom.

Chemokine receptors play an important role in the migration of leukocytes to sites of allergic inflammation in humans. In this study, we have identified increased expression of the chemokine receptor CXCR1 on CD4+ T lymphocytes derived from patients with atopic disease compared with normal donors. Enhanced expression of CXCR1 by atopic donors was identified on freshly isolated peripheral blood cells and on expanded cell populations derived from nasal mucosal biopsies and from the periphery. Identification of CXCR1 expression on CD4 cells in the nasal mucosa was confirmed by double immunofluorescence. In addition, expression of CXCR1 was dramatically decreased in patients undergoing successful treatment of allergic rhinitis by specific immunotherapy. CXCR1 provided a functional receptor capable of regulating T cells in the context of allergic disease, since expression of CXC chemokine ligand 8 was up-regulated at the site of allergic inflammation and freshly isolated CXCR1+CD4+ cells from atopic donors showed an enhanced functional response to this ligand. CXCR1 expression on CD4+ T cells was increased in vitro in response to the pro-Th2 cytokine IL-4. Phenotypic analysis reveals that IFN-gamma expression was lower in the CXCR1+CD4+ cells. The identification of CXCR1 as a marker of allergic rhinitis reveals a possible target for therapeutic intervention in atopic disease.

PMID: 14688334 [PubMed - indexed for MEDLINE]

1257. Am J Physiol Gastrointest Liver Physiol. 2004 May;286(5):G702-10. Epub 2003 Dec 11.

Chronic allergy to dietary ovalbumin induces lymphocyte migration to rat small intestinal mucosa that is inhibited by MAdCAM-1.

Ogawa T, Miura S, Tsuzuki Y, Ogino T, Teramoto K, Inamura T, Watanabe C, Hokari R, Nagata H, Ishii H.

School of Medicine, Keio Univ., 35 Shinanomichi, Shijuku-Ku, Tokyo 160-8582, Japan.

Few models have described a chronic food allergy with morphological changes in the intestinal mucosa. Here we established an ovalbumin (OVA)-induced, cell-mediated, allergic rat model and examined lymphocyte migration in the gut. Brown Norway rats were intraperitoneally sensitized to OVA and then given 10 mg OVA/day by gastric intubation for 6 wk. Lymphocyte subsets and adhesion molecules were examined immunohistochemically, and the migration of T lymphocytes to microvessels of Peyer's patches and villus mucosa was observed by using an intravital microscope. Serum OVA-specific IgG and IgE levels were increased in animals repeatedly exposed to OVA. Significant villus atrophy and increased crypt depth was accompanied by increased infiltration of T lymphocytes in the small intestinal mucosa of the group given OVA. Expression of rat mast cell protease II and of mucosal addressin cell adhesion molecule-1 (MAdCAM-1) was also increased in these groups. The administration of anti-MAdCAM-1 antibody significantly attenuated the OVA-induced changes in the mucosal architecture and in CD4 T lymphocyte infiltration. Intravital observation demonstrated that in rats with a chronic allergy, T lymphocytes significantly accumulated in villus microvessels as well as in Peyer's patches via a MAdCAM-1-dependent process. Our model of chronic food allergy revealed that lymphocyte migration was increased with MAdCAM-1 upregulation.

PMID: 14670821 [PubMed - indexed for MEDLINE]

1258. Mol Cell Neurosci. 2003 Nov;24(3):623-31.

Effects of proinflammatory cytokines on the growth, fate, and motility of multipotential neural precursor cells.

Ben-Hur T, Ben-Menachem O, Furer V, Einstein O, Mizrachi-Kol R, Grigoriadis N.

Department of Neurology, The Agnes Ginges Center for Human Neurogenetics, Hadassah-Hebrew University Hospital, Jerusalem, Israel. tamir@hadassah.org.il

We have recently shown that the inflammatory process during experimental allergic encephalomyelitis (EAE), the animal model of MS, attracts transplanted NPC migration into the inflamed white matter. Here we studied how the proinflammatory cytokines tumor necrosis factor-alpha (TNFalpha) and interferon-gamma (IFNgamma) affect NPC growth, survival, differentiation, and migration. Newborn rat striatal NPCs were expanded in spheres as nestin+, PSA-NCAM+, NG2(-) cells, which differentiated into astrocytes, oligodendrocytes, and neurons. NPCs expressed receptors of TNFalpha and IFNgamma but not interleukin-1. TNFalpha and IFNgamma inhibited sphere cell proliferation, determined by [(3)H]thymidine and BrdU incorporation. IFNgamma increased apoptotic cell death (determined by TUNEL stains); this effect partially blocked by TNFalpha. Neither cytokine affected NPC lineage fate, determined by percentage of GFAP+, neurofilament+, and GalC+ cells after differentiation. TNFalpha and IFNgamma increased outward migration of cells from spheres in vitro. Thus, TNFalpha and IFNgamma, key players in MS and EAE, inhibit NPC proliferation and induce their migration.

PMID: 14664813 [PubMed - indexed for MEDLINE]

1259. J Immunol. 2003 Dec 15;171(12):6519-26.

Neuronal repellent Slit2 inhibits dendritic cell migration and the development of immune responses.

Guan H, Zu G, Xie Y, Tang H, Johnson M, Xu X, Kevil C, Xiong WC, Elmets C, Rao Y, Wu JY, Xu H.

Department of Dermatology, University of Alabama, Birmingham, AL 35294, USA.

One of the essential functions of dendritic cells is to take up Ags in peripheral tissues and migrate into secondary lymphoid organs to present Ags to lymphocytes for the induction of immune responses. Although many studies have demonstrated that the migration of dendritic cells is closely associated with the development of immune responses, little is known about factors that inhibit dendritic cell migration and control the extent of immune responses to Ag stimulation. We show that Slit2, a neuronal repellent factor, is up-regulated in the skin by allergen sensitization and down-regulates the migration of Langerhans cells. The effect is mediated by direct interaction of Slit2 with cells that express a Slit-specific receptor, Robo1. Slit2-mediated inhibition of Langerhans cell migration results in suppression of contact hypersensitivity responses. These findings provide insights into a novel mechanism by which Slit2 functions as an anti-inflammatory factor for the initiation of immune responses.

PMID: 14662852 [PubMed - indexed for MEDLINE]

1260. J Biol Chem. 2004 Feb 20;279(8):7098-104. Epub 2003 Dec 2.

Structural insights into the interaction of ROCKI with the switch regions of RhoA.

Dvorsky R, Blumenstein L, Vetter IR, Ahmadian MR.

Max-Planck-Institute fuer Molekulare Physiologie, Abteilung Strukturelle Biologie, Otto-Hahn-Strasse 11, 44227 Dortmund, Germany.

The Rho-ROCK pathway modulates the phosphorylation level of a variety of important signaling proteins and is thereby involved in miscellaneous cellular processes including cell migration, neurite outgrowth, and smooth muscle contraction. The observation of the involvement of the Rho-ROCK pathway in tumor invasion and in diseases such as hypertension and bronchial asthma makes it an interesting target for drug development. We herein present the crystal structure of the complex between active RhoA and the Rho-binding domain of ROCKI. The Rho-binding domain structure forms a parallel alpha-helical coiled-coil dimer and, in contrast to the published Rho-protein kinase N structure, binds exclusively to the switch I and II regions of the guanosine 5'-(beta,gamma-imido)triphosphate-bound RhoA. The switch regions of two different RhoA molecules form a predominantly hydrophobic patch, which is complementarily bound by two identical short helices of 13 residues (amino acids 998-1010). The identified ROCK-binding site of RhoA strikingly supports the assumption of a common consensus-binding site for effector recognition.

PMID: 14660612 [PubMed - indexed for MEDLINE]

1261. J Allergy Clin Immunol. 2003 Dec;112(6):1139-46.

The CX3C chemokine fractalkine in allergic asthma and rhinitis.

Rimaniol AC, Till SJ, Garcia G, Capel F, Godot V, Balabanian K, Durand-Gasselin I, Varga EM, Simonneau G, Emilie D, Durham SR, Humbert M.

Service de Pneumologie et Réanimation Respiratoire, Hôpital Antoine Béclére, Université Paris-Sud, Clamart, France.

BACKGROUND: Unlike other chemokines, fractalkine is expressed as a membrane-bound form, mainly on endothelial and epithelial cells, and can be shed as a soluble chemotactic form. Fractalkine can capture leukocytes expressing its receptor (CX(3)CR(1)), including T lymphocytes, rapidly and firmly in an integrin-independent manner. Because of its dual activity, fractalkine plays a major role in the transendothelial and transepithelial migration of leukocytes during inflammation.
OBJECTIVE: We sought to study the fractalkine-CX(3)CR(1) axis in patients with allergic airways diseases.
METHODS: Plasma fractalkine levels were measured by means of ELISA in 19 control subjects and 55 patients with symptomatic allergic rhinitis, asthma, or both, and CX(3)CR(1) function was studied by using triple-color flow cytometry in circulating T-lymphocyte subpopulations. Segmental allergen challenge was performed in 16 allergic asthmatic patients to analyze fractalkine expression and inflammatory cell recruitment in bronchoalveolar lavage fluid and bronchial biopsy specimens.
RESULTS: Compared with control subjects, patients with symptomatic allergic rhinitis and asthmatic patients had increased circulating fractalkine levels, and CX(3)CR(1) function was upregulated in circulating CD4(+) T lymphocytes. Twenty-four hours after segmental allergen challenge, bronchoalveolar lavage fluid soluble fractalkine concentrations increased and correlated with the total number of recruited cells. Bronchial epithelial and endothelial cells expressed high levels of the membrane-bound form of fractalkine before and after challenge.
CONCLUSION: Allergic asthma and rhinitis are associated with systemic and bronchial upregulation of the chemotactic axis fractalkine-CX(3)CR(1). This might contribute to the rapid recruitment of circulating CD4(+) T lymphocytes in the airways after allergen stimulation.

PMID: 14657873 [PubMed - indexed for MEDLINE]

1262. J Allergy Clin Immunol. 2003 Dec;112(6 Suppl):S118-27.

Atopic dermatitis and the atopic march.

Spergel JM, Paller AS.

Division of Allergy and Immunology, The Children's Hospital of Philadelphia, University of Pennsylvania School of Medicine, Philadelphia, PA, USA.

Atopic dermatitis (AD), one of the most common skin disorders seen in infants and children, usually has its onset during the first 6 months of life. The prevalence of AD is similar in the United States, Europe, and Japan and is increasing, similar to that of other atopic disorders, particularly asthma. AD has been classified into 3 sequential phases: infantile, childhood, and adult, each with characteristic physical findings. AD has a tremendously negative effect on the quality of life of patients as well as family, most commonly disturbing sleep. The condition also creates a great financial burden for both the family and society. The cutaneous manifestations of atopy often represent the beginning of the atopic march. On the basis of several longitudinal studies, approximately half of AD patients will develop asthma, particularly with severe AD, and two thirds will develop allergic rhinitis. Epicutaneous sensitization has been thought to be responsible, with subsequent migration of sensitized T cells into the nose and airways, causing upper and lower airway disease. Animal models and human observation concur with this theory. Preliminary prevention studies with oral antihistamines provide evidence that early intervention might slow the atopic march.

PMID: 14657842 [PubMed - indexed for MEDLINE]

1263. Pulm Pharmacol Ther. 2004;17(1):1-10.

Development of cell adhesion molecule antagonists as therapeutics for asthma and COPD.

Vanderslice P, Biediger RJ, Woodside DG, Berens KL, Holland GW, Dixon RA.

Department of Biology, Encysive Pharmaceuticals, 7000 Fannin, 19th Floor, Houston, TX 77030, USA. pvanderslice@encysive.com

Airway inflammation is a hallmark of respiratory diseases such as asthma and chronic obstructive pulmonary disease. Cell adhesion molecules play critical roles in the recruitment and migration of cells to sites of inflammation. Not surprisingly, these receptors have garnered the attention of the pharmaceutical industry as targets for the development of drugs to treat inflammatory and autoimmune diseases. Although several potential cell adhesion targets exist, development of compounds for pulmonary indications has centered around the selectins and the integrin VLA-4. In vitro and in vivo studies have implicated these receptors in the recruitment of inflammatory cells to the lung as well as to key cellular activation pathways. Several first generation compounds are currently in clinical development for asthma. Positive data from a phase II clinical trial using an inhaled formulation of a selectin antagonist has recently been reported. Initial results from clinical trials using first generation VLA-4 antagonists have been less promising but additional trials with more fully optimized compounds are underway. Results from these trials will provide insight into what the future holds for this exciting new class of drugs to treat pulmonary diseases.

PMID: 14643165 [PubMed - indexed for MEDLINE]

1264. Eur J Pharmacol. 2003 Nov 28;481(2-3):271-9.

Effects of cilomilast on dendritic cell function in contact sensitivity and dendritic cell migration through skin.

Bäumer W, Tschernig T, Sülzle B, Seegers U, Lührmann A, Kietzmann M.

Department of Pharmacology, Toxicology and Pharmacy, School of Veterinary Medicine, Buenteweg 17, D-30559 Hannover, Germany.
wolfgang.baeumer@tiho-hannover.de

The phosphodiesterase 4 inhibitor cilomilast demonstrated strong inhibitory effects in a model of allergic contact dermatitis. In this study, we examined whether this inhibitory effect is at least partly due to modulation of dendritic cell function. Bone marrow-derived dendritic cells were pulsed with the sensitizer toluene-2,4-diisocyanate and administered subcutaneously to nonsensitized mice. Five days later, the mice were challenged with a low dose of toluene-2,4-diisocyanate onto the ears. In contrast to sham-treated mice, mice obtaining toluene-2,4-diisocyanate pulsed dendritic cells showed a significant increase in ear swelling. This swelling was not influenced when the dendritic cells were pre-incubated with cilomilast. When cilomilast was administered systemically simultaneously to the application of toluene-2,4-diisocyanate pulsed cells, there was an impaired allergic reaction provoked 5 days later. Additionally, a topical treatment with cilomilast resulted in a significant inhibition of skin dendritic cell migration. These results indicate that the antigen-presenting function of dendritic cells is not influenced by cilomilast but the dendritic cell T cell interaction and dendritic cell migration is

modulated.

PMID: 14642795 [PubMed - indexed for MEDLINE]

1265. Acta Trop. 2003 Dec;89(1):85-9.

Cross-reactivity between Anisakis simplex sensitization and visceral larva migrans by Toxocara canis.

Perteguer MJ, Cuéllar C, Guillén JL, Aguila C, Fenoy S, Chivato T, Laguna R.

Departamento de Parasitología, Facultad de Farmacia, Universidad Complutense, 28040 Madrid, Spain.

The aim of this work was to study cross-reactivity in the diagnosis of two related ascaridosis. Nineteen patients diagnosed with recidivous acute urticaria (RAU) caused by Anisakis simplex and 26 patients diagnosed with visceral larva migrans (VLM) caused by Toxocara canis were studied employing commercial diagnostic kits and "in house" assay kits. Cross-reactivity observed was greater when using "in house" assay kits, suggesting that T. canis excretory-secretory antigens were not only recognized by antibodies from patients with RAU but with greater intensity compared to the A. simplex excretory-secretory antigens.

PMID: 14636986 [PubMed - indexed for MEDLINE]

1266. J Immunol. 2003 Dec 1;171(11):6206-14.

Inhibition of Th1- and Th2-mediated airway inflammation by the sphingosine 1-phosphate receptor agonist FTY720.

Sawicka E, Zuany-Amorim C, Manlius C, Trifilieff A, Brinkmann V, Kemeny DM, Walker C.

Novartis Horsham Research Centre, Horsham, U.K.

The sphingosine 1-phosphate receptor agonist FTY720 is a novel immunomodulator that sequesters lymphocytes in secondary lymphoid organs and thereby prevents their migration to sites of inflammation. However, there is currently no information available on whether this drug affects Th1 or Th2 cell-mediated lung-inflammatory responses. The effect of FTY720 was therefore investigated in a murine airway inflammation model using OVA-specific, in vitro differentiated, and adoptively transferred Th1 and Th2 cells. Both Th1 and Th2 cells express a similar pattern of FTY720-targeted sphingosine 1-phosphate receptors. The OVA-induced Th1-mediated airway inflammation characterized by increased numbers of lymphocytes and neutrophils in bronchoalveolar lavage fluid was significantly inhibited by oral FTY720 treatment. Similarly, FTY720 suppressed the Th2 cell-induced bronchoalveolar lavage fluid eosinophilia and the infiltration of T lymphocytes and eosinophils into the bronchial tissue. Moreover, the Ag-induced bronchial hyperresponsiveness to inhaled metacholine was almost completely blocked. The inhibitory effect of FTY720 on airway inflammation, induction of bronchial hyperresponsiveness, and goblet cell hyperplasia could be confirmed in an actively Ag-sensitized murine asthma model, clearly indicating that Th2 cell-driven allergic diseases such as asthma could benefit from such treatment.

PMID: 14634137 [PubMed - indexed for MEDLINE]

1267. Water Res. 2004 Jan;38(1):225-35.

Distribution of bacteria in a domestic hot water system in a Danish apartment building.

Bagh LK, Albrechtsen HJ, Arvin E, Ovesen K.

Environmental & Resources DTU, Technical University of Denmark, Building 115, DK-2800, Lyngby, Denmark. lkb@rovesta.dk

Bacterial growth in hot water systems seems to cause problems such as bad odor of the water, skin allergies and increased heat transfer resistance in heating coils. In order to establish a basis for long-term suppression of bacterial growth, we studied the distribution of bacteria in a Danish domestic hot water system. Heterotrophic plate counts (HPC) were measured in both water and biofilm samples from various sampling sites in the system. In hot water samples, where the temperature was 55-60 degrees C, the HPC were 10(3)-10(4)CFU/mL at incubation temperatures of 25 degrees C or 37 degrees C and 10(5)CFU/mL at 55 degrees C or 65 degrees C. In the cold water (10 degrees C) supplying the hot water system, the HPC at 25 degrees C or 37 degrees C was lower than in the hot water, and no bacteria were found after incubation at 55 degrees C or 65 degrees C. HPC constituted from 38% to 84% of the AODC results in hot water but only 2% in cold water, which showed a high ratio of culturable bacteria in hot water. Biofilm samples from the hot water tank and the inner surface of the pipes in the cold and hot water distribution system were collected by specially designed sampling devices, which were exposed in the system for 42 days. The quasi-steady-state number of bacteria in the biofilm, measured as the geometric mean of the HPC obtained between 21 and 42 days, was five-fold higher in the hot water pipe (13x10(5)CFU/cm(2) at 55 degrees C) than in the cold water pipe (2.8x10(5)CFU/cm(2) at 25 degrees C). There was no significant difference between the number of bacteria in the biofilm samples from the top, middle and bottom of the hot water tank, and the number of bacteria in the biofilm counted at 55 degrees C ranged from 0.6x10(4) to 1.7x10(4)CFU/cm(2). The surfaces of the sacrificial aluminum anodes and the heating coils in the hot water tank also contained high bacterial numbers. The measured number of bacteria in water and biofilm samples was related to the dimensions of the hot water system, and calculations showed that the majority of bacteria (72%) were located in the biofilm especially in the distribution system, which accounts for the greatest surface area. Free-living bacteria accounted for 26% and only a minor part of the bacteria were in the sludge in the hot water tank (2%).

PMID: 14630121 [PubMed - indexed for MEDLINE]

1268. J Exp Med. 2003 Nov 17;198(10):1573-82.

Blockade of inflammation and airway hyperresponsiveness in immune-sensitized mice by dominant-negative phosphoinositide 3-kinase-TAT.

Myou S, Leff AR, Myo S, Boetticher E, Tong J, Meliton AY, Liu J, Munoz NM, Zhu X.

Section of Pulmonary and Critical Care Medicine, Department of Medicine, The University of Chicago, 5841 South Maryland Avenue, Chicago, IL 60637, USA.

Phosphoinositide 3-kinase (PI3K) is thought to contribute to the pathogenesis of asthma by effecting the recruitment, activation, and apoptosis of inflammatory cells. We examined the role of class IA PI3K in antigen-induced airway inflammation and hyperresponsiveness by i.p. administration into mice of Deltap85 protein, a dominant negative form of the class IA PI3K regulatory subunit, p85alpha, which was fused to HIV-TAT (TAT-Deltap85). Intraperitoneal administration of TAT-Deltap85 caused time-dependent transduction into blood

leukocytes, and inhibited activated phosphorylation of protein kinase B (PKB), a downstream target of PI3K, in lung tissues in mice receiving intranasal FMLP. Antigen challenge elicited pulmonary infiltration of lymphocytes, eosinophils and neutrophils, increase in mucus-containing epithelial cells, and airway hyperresponsiveness to methacholine. Except for modest airway neutrophilia, these effects all were blocked by treatment with 3-10 mg/kg of TAT-Deltap85. There was also significant reduction in IL-5 and IL-4 secretion into the BAL. Intranasal administration of IL-5 caused eosinophil migration into the airway lumen, which was attenuated by systemic pretreatment with TAT-Deltap85. We conclude that PI3K has a regulatory role in Th2-cell cytokine secretion, airway inflammation, and airway hyperresponsiveness in mice.

PMCID: PMC2194122
PMID: 14623911 [PubMed - indexed for MEDLINE]

1269. Clin Exp Allergy. 2003 Nov;33(11):1544-54.

Ebastine inhibits T cell migration, production of Th2-type cytokines and proinflammatory cytokines.

Nori M, Iwata S, Munakata Y, Kobayashi H, Kobayashi S, Umezawa Y, Hosono O, Kawasaki H, Dang NH, Tanaka H, Shiohara T, Morimoto C.

Division of Clinical Immunology, Advanced Clinical Research Center, Institute of Medical Science, University of Tokyo, 4-6-1 Shirokanedai, Minato-ku, Tokyo, Japan.

Comment in
    Clin Exp Allergy. 2003 Nov;33(11):1476-8.

BACKGROUND: Cytokine imbalance and cellular migration to inflammatory sites are critical components of allergic diseases. Redirecting cytokine imbalance and inhibiting cell migration therefore represent important therapeutic strategies for the treatment of these disorders.
OBJECTIVES: To study the in vitro effect of ebastine, a novel non-sedating H1 receptor antagonist, on cytokine secretion and migration of activated T cells, as well as production of pro-inflammatory cytokines by macrophages.
METHODS: Peripheral T cells obtained from healthy volunteers were cultured in wells coated with the combination of anti-CD3 monoclonal antibody (mAb) and anti-CD26 mAb, anti-CD3 mAb and anti-CD28 mAb, or anti-CD3 mAb with PMA, in the presence or absence of ebastine. T cell proliferation and the production of cytokines were measured by [3H]thymidine incorporation assay and ELISA, respectively. In addition, transendothelial migration of T cells and production of pro-inflammatory cytokines by macrophages were examined.
RESULTS: Ebastine inhibited T cell proliferation and the production of IL-4, IL-5, IL-6, and TNF-alpha by T cells under each co-stimulatory condition tested, whereas it exhibited no effect on the production of IL-2 or IFN-gamma. In addition, T cell migration and the production of such pro-inflammatory cytokines as TNF-alpha and IL-6 by macrophages were inhibited by ebastine.
CONCLUSIONS: These results indicate that ebastine has a specific inhibitory effect on Th2-type cytokine production. Moreover, ebastine inhibited T cell migration and pro-inflammatory cytokine production by T cells and macrophages, suggesting that ebastine might be useful for the treatment of T cell-mediated allergic inflammatory disorders, including asthma, atopic dermatitis, and Th2-type autoimmune diseases.

PMID: 14616867 [PubMed - indexed for MEDLINE]

1270. J Ethnopharmacol. 2003 Dec;89(2-3):181-4.

Immunostimulant principles from Curculigo orchioides.

Lakshmi V, Pandey K, Puri A, Saxena RP, Saxena KC.

Medicinal Chemistry Division, Central Drug Research Institute, Lucknow-226001, India.

Curculigo orchioides Gaerten belongs to the family Amaryllidaceae. The rhizomes of the plants are used for the treatment of decline in strength, jaundice and asthma. Its methanolic extract has been shown to enhance phagocytic activity of macrophages. Present studies have led to the isolation of two phenolic glycosides and a purified glycoside fraction. These were studied for their effect on macrophage migration index (MMI), haemagglutination (HA) titre, plaque forming cell (PFC), PHA-induced blast transformation of lymphocytes (BTL) and delayed type hypersensitivity (DTH). Significant immunostimulant activity was found in purified glycoside-rich fraction isolated from the ethyl acetate extract. The exact structure of the active glycoside is yet to be determined. The enhancement of HA titre and PFC count on one hand and that of DTH response on the other indicates that glycoside fraction stimulates both humoral and Cell-mediated immune responses. Glycoside fraction stimulates immune response by acting both on macrophages and the lymphocytes.

PMID: 14611880 [PubMed - indexed for MEDLINE]

1271. J Immunol. 2003 Nov 15;171(10):5602-10.

Local somatic hypermutation and class switch recombination in the nasal mucosa of allergic rhinitis patients.

Coker HA, Durham SR, Gould HJ.

The Randall Centre, King's College London, London, United Kingdom.

Immunoglobulin E is produced by nasal B cells in response to allergen. We have analyzed IgE V(H) region sequences expressed in the nasal mucosa of patients suffering from allergic rhinitis. V(H) region sequences were amplified by RT-PCR from IgE(+) B cells from nasal biopsies. In two of six patients, sequence analysis clearly demonstrated the presence of closely related IgE(+) B cell clones: cells displaying identical signature regions across CDR3/FWR4, indicating a common clonal ancestry, but a mixture of shared and diverse somatic mutations across the V(H) region. Furthermore, in one of the two patients exhibiting related IgE(+) B cell clones, five IgA(+) B cell clones, related to the IgE(+) B cell family, were also isolated from the patient's nasal mucosa. This evidence, combined with the local expression of mRNA transcripts encoding activation-induced cytidine deaminase, suggests that local somatic hypermutation, clonal expansion, and class switch recombination occur within the nasal mucosa of allergic rhinitics. The presence of related B cells in the nasal mucosa does not appear to result from the random migration of IgE(+) cells from the systemic pool, as analysis of a nonatopic subject with highly elevated serum IgE did not exhibit any detectable V(H)-Cepsilon transcripts in the nasal mucosa. We have provided evidence that suggests for the first time that the nasal mucosa of allergic rhinitics is an active site for local somatic hypermutation, clonal expansion, and class switch recombination, making it of major significance for the targeting of future therapies.

PMID: 14607969 [PubMed - indexed for MEDLINE]

1272. J Immunol. 2003 Nov 15;171(10):5498-506.

Inhibition of airway inflammation by amino-terminally modified RANTES/CC chemokine ligand 5 analogues is not mediated through CCR3.

Chvatchko Y, Proudfoot AE, Buser R, Juillard P, Alouani S, Kosco-Vilbois M, Coyle AJ, Nibbs RJ, Graham G, Offord RE, Wells TN.

Serono Pharmaceutical Research Institute, Plan les Ouates, Geneva, Switzerland.

Chemokines play a key role in the recruitment of activated CD4(+) T cells and eosinophils into the lungs in animal models of airway inflammation. Inhibition of inflammation by N-terminally modified chemokines is well-documented in several models but is often reported with limited dose regimens. We have evaluated the effects of doses ranging from 10 ng to 100 micro g of two CC chemokine receptor antagonists, Met-RANTES/CC chemokine ligand 5 (CCL5) and aminooxypentane-RANTES/CCL5, in preventing inflammation in the OVA-sensitized murine model of human asthma. In the human system, aminooxypentane-RANTES/CCL5 is a full agonist of CCR5, but in the murine system neither variant is able to induce cellular recruitment. Both antagonists showed an inverse bell-shaped inhibition of cellular infiltration into the airways and mucus production in the lungs following allergen provocation. The loss of inhibition at higher doses did not appear to be due to partial agonist activity because neither variant showed activity in recruiting cells into the peritoneal cavity at these doses. Surprisingly, neither was able to bind to the major CCR expressed on eosinophils, CCR3. However, significant inhibition of eosinophil recruitment was observed. Both analogues retained high affinity binding for murine CCR1 and murine CCR5. Their ability to antagonize CCR1 and CCR5 but not CCR3 was confirmed by their ability to prevent RANTES/CCL5 and macrophage inflammatory protein-1beta/CCL4 recruitment in vitro and in vivo, while they had no effect on that induced by eotaxin/CCL11. These results suggest that CCR1 and/or CCR5 may be potential targets for asthma therapy.

PMID: 14607956 [PubMed - indexed for MEDLINE]

1273. Am J Respir Cell Mol Biol. 2004 May;30(5):729-35. Epub 2003 Nov 7.

Expression and function of the vascular endothelial growth factor receptor FLT-1 in human eosinophils.

Feistritzer C, Kaneider NC, Sturn DH, Mosheimer BA, Kähler CM, Wiedermann CJ.

Department of Internal Medicine, University of Innsbruck, Anichstrasse 35, A-6020 Innsbruck, Austria.

Vascular endothelial growth factor (VEGF) is highly expressed in the airway of patients with asthma. Whether VEGF affects eosinophil function in vitro and if VEGF receptors are involved was tested. Eosinophils were from venous blood of healthy donors. Cell migration was studied by micropore filter assays. Signaling mechanisms required for VEGF-dependent migration were tested using signaling enzyme blockers. Expression of flt-1 and KDR/flk-1 mRNA in eosinophils was demonstrated in reverse transcriptase-polymerase chain reaction, and receptor expression was investigated by fluorescence-activated cell sorting analysis. Eosinophil cationic protein release was measured in eosinophil supernatants by enzyme-linked immunosorbent assay. VEGF significantly stimulated eosinophil chemotaxis via activation of protein kinase C and phosphatidylinositol 3'-kinase. The effect on migration was reversed by an antibody against VEGF receptor flt-1, but not by an antibody against KDR/flk-1. Expression of VEGF receptor flt-1 mRNA

was shown and synthesis of VEGF receptor in eosinophils is suggested by detection of VEGF receptor immunoreactivity on the cell surface. Data suggest that VEGF receptor flt-1 is expressed by eosinophils whose activation with VEGF stimulates directed migration and release of eosinophil cationic protein. Thus, VEGF may play an important role in the modulation of eosinophilic inflammation.

PMID: 14607815 [PubMed - indexed for MEDLINE]

1274. J Cataract Refract Surg. 2003 Oct;29(10):1974-9.

Postoperative change in effective lens position of a 3-piece acrylic intraocular lens.

Koeppl C, Findl O, Kriechbaum K, Buehl W, Wirtitsch M, Menapace R, Drexler W.

Department of Ophthalmology, University of Vienna, Vienna, Austria.

PURPOSE: To study the change in postoperative position of an angulated, 3-piece, foldable acrylic intraocular lenses (IOLs) and evaluate the effect of a sharp posterior optic edge compared with that of a round optic edge on the change in postoperative anterior chamber depth (ACD).
SETTING: Department of Ophthalmology, University of Vienna, Vienna, Austria.
METHODS: This randomized intraindividual-comparison study comprised 104 eyes of 52 patients with age-related cataract. Patient received a sharp-edged Sensar OptiEdge AR40e IOL (Allergan Surgical) in 1 eye and a round-edged Sensar AR40 IOL in the other eye. Postoperative follow-up included ACD measurement by partial coherence interferometry and evaluation of the capsulorhexis area by standardized retroillumination photography at 1 day, 1 week, and 1 and 6 months.
RESULTS: A decrease in ACD during the first postoperative week was followed by a small increase in ACD during the first 6 months. There was no significant difference between the sharp-edged group and the round-edged group.
CONCLUSIONS: The angulated 3-piece acrylic IOLs showed significant forward movement over the first postoperative 6 months. Although the change in refraction was small, there was variability among patients. The sharp posterior optic edge design did not affect the IOL's movement in the capsular bag.

PMID: 14604720 [PubMed - indexed for MEDLINE]

1275. Regul Pept. 2003 Nov 15;116(1-3):119-26.

Natural killer cell functions mediated by the neuropeptide substance P.

Feistritzer C, Clausen J, Sturn DH, Djanani A, Gunsilius E, Wiedermann CJ, Kähler CM.

Intensive Care Unit, Division of General Internal Medicine, Department of Internal Medicine, Faculty of Medicine, University of Innsbruck, Anichstrasse 35, 6020, Innsbruck, Austria.

The neuropeptide substance P (SP) can modulate a number of immunological functions in vitro and in vivo. Here, we investigated if SP boosts migration and cytotoxicity of natural killer cells, thus providing a further link between "innate immunity" and neurogenic inflammatory processes like asthma bronchiale. We demonstrate a dose-dependent effect of SP on natural killer cell migration with a maximal response at 10(-8) M SP. SP was shown to stimulate unstimulated as well as interleukin-2 (IL-2)-activated natural killer cells. Stimulation of natural killer cell migration was neurokinin-1 receptor dependent. Furthermore, mRNA encoding the neurokinin-1 receptor was demonstrated as being present in

natural killer cells using RT-PCR while mRNA of the neurokinin-2 receptor was not detectable. Additionally, SP seems to influence specific cytotoxicity against Raji and K567 effector cells by a receptor-independent mechanism. In conclusion, our data indicate that functionally active neurokinin-1 receptors can be expressed by human natural killer cells. Substance P might therefore be a novel link between neural structures and innate immunity.

PMID: 14599723 [PubMed - indexed for MEDLINE]

1276. J Pediatr Adolesc Gynecol. 2003 Oct;16(5):269-83.

Common pediatric and adolescent skin conditions.

Sanfilippo AM, Barrio V, Kulp-Shorten C, Callen JP.

University of Louisville School of Medicine, Louisville, Kentucky 40202, USA.

Skin lesions are encountered in all areas of medicine, and it is therefore important for physicians to understand the fundamentals of explaining and diagnosing common skin conditions. This article begins with a discussion of description and documentation of skin lesions based on color, size, morphology, and distribution. Pigmentation disorders such as vitiligo are depicted. Cutaneous growths that are found in the pediatric and adolescent population include acrochordons, dermatofibromas, keloids, milia, neurofibromas, and pyogenic granulomas. Treatment of these growths usually involves observation or curettage with electrodessication.Psoriasis, atopic dermatitis, poison ivy, and eczema are comprised of scaling patches and plaques; poison ivy and atopic dermatitis may also present with bullous and vesicular changes. Therapy typically consists of topical emollients and corticosteroids; phototherapy is reserved for refractory cases. Acne vulgaris is the most common skin disease of the pediatric and adolescent population. This condition can be psychologically debilitating and, therefore, proper treatment is of paramount importance. Therapeutic options include topical as well as oral antibiotics and retinoids. Extreme caution must be used when prescribing retinoids to post-pubescent females, as these agents are teratogenic. Vascular anomalies are most commonly exemplified as port wine stains and hemangiomas. Port wine stains may be treated with pulsed dye laser or may be observed if they are not of concern to the patient or physician. Hemangiomas typically spontaneously regress by age ten; however, there has been recent concern that certain cases may need to be treated. Dermal rashes may be localized or generalized. Treatment of generalized drug eruptions involves elimination of the inciting agent, topical antipruritics, and systemic corticosteroids for severe reactions. Infectious etiologic agents of skin disease include bacteria, fungi, and viruses. Many sexually transmitted diseases are bacterial or viral in origin and present as a rash or ulcer. Impetigo is a bacterial infection which may present as a bullous eruption or as an erosion with a honey colored crust. Other bacterial infections include erythema chronicum migrans, folliculitis, and cellulitis. Fungal infections include the various forms of tinea and are usually treated with topical antifungals; if the infection is located in a hair-bearing area, systemic antifungals are necessary. Viral infections include warts, varicella, molluscum contagiosum, and herpes. Treatment varies from observation or antivirals for varicella to cryosurgery and topical imiquimod for warts. Finally, scabies and lice are infectious agents that can be treated with permethrin and pyrethrin solutions.

PMID: 14597015 [PubMed - indexed for MEDLINE]

1277. Biochem J. 2004 Mar 1;378(Pt 2):539-47.

Calcium mobilization and Rac1 activation are required for VCAM-1 (vascular cell adhesion molecule-1) stimulation of NADPH oxidase activity.

Cook-Mills JM, Johnson JD, Deem TL, Ochi A, Wang L, Zheng Y.

Department of Pathology, University of Cincinnati, Cincinnati, OH 45267-0529, USA. joan.cook@uc.edu

VCAM-1 (vascular cell adhesion molecule-1) plays an important role in the regulation of inflammation in atherosclerosis, asthma, inflammatory bowel disease and transplantation. VCAM-1 activates endothelial cell NADPH oxidase, and this oxidase activity is required for VCAM-1-dependent lymphocyte migration. We reported previously that a mouse microvascular endothelial cell line promotes lymphocyte migration that is dependent on VCAM-1, but not on other known adhesion molecules. Here we have investigated the signalling mechanisms underlying VCAM-1 function. Lymphocyte binding to VCAM-1 on the endothelial cell surface activated an endothelial cell calcium flux that could be inhibited with anti-alpha4-integrin and mimicked by anti-VCAM-1-coated beads. VCAM-1 stimulation of calcium responses could be blocked by an inhibitor of intracellular calcium mobilization, a calcium channel inhibitor or a calcium chelator, resulting in the inhibition of NADPH oxidase activity. Addition of ionomycin overcame the calcium channel blocker suppression of VCAM-1-stimulated NADPH oxidase activity, but could not reverse the inhibitory effect imposed by intracellular calcium blockage, indicating that both intracellular and extracellular calcium mobilization are required for VCAM-1-mediated activation of NADPH oxidase. Furthermore, VCAM-1 specifically activated the Rho-family GTPase Rac1, and VCAM-1 activation of NADPH oxidase was blocked by a dominant negative Rac1. Thus VCAM-1 stimulates the mobilization of intracellular and extracellular calcium and Rac1 activity that are required for the activation of NADPH oxidase.

PMCID: PMC1223954
PMID: 14594451 [PubMed - indexed for MEDLINE]

1278. Braz J Med Biol Res. 2003 Nov;36(11):1455-63. Epub 2003 Oct 22.

The role of chemokines and chemokine receptors in eosinophil activation during inflammatory allergic reactions.

Oliveira SH, Lukacs NW.

Departamento de Ciências Básicas, Faculdade de Odontologia de Araçatuba, Universidade Estadual Paulista, Araçatuba, SP, Brasil. shpoliv@foa.unesp.br

Chemokines are important chemotactic cytokines that play a fundamental role in the trafficking of leukocytes to sites of inflammation. They are also potent cell-activating factors, inducing cytokine and histamine release and free radical production, a fact that makes them particularly important in the pathogenesis of allergic inflammation. The action of chemokines is regulated at the level of agonist production and processing as well as at the level of receptor expression and coupling. Therefore, an analysis of the ligands must necessarily consider receptors. Eosinophils are target cells involved in the allergic inflammatory response since they are able to release a wide variety of mediators including CC and CXC chemokines and express their receptors. These mediators could damage the airway epithelial cells and might be important to stimulate other cells inducing an amplification of the allergic response. This review focuses on recently emerging data pertaining to the importance of chemokines and chemokine receptors in promoting eosinophil activation and migration during the allergic inflammatory process. The analysis of the function of eosinophils and their chemokine receptors during allergic inflammation might be a good approach to understanding

the determinants of asthma severity and to developing novel therapies.

PMID: 14576899 [PubMed - indexed for MEDLINE]

1279. Curr Drug Targets Inflamm Allergy. 2002 Mar;1(1):109-16.

Chemokines in allergy.

Chantry D, Burgess LE.

Icos Corporation, 22021 20th Ave., Bothell, WA 98021, USA.
lburgess@arraybiopharma.com

Allergic diseases such as atopic dermatitis, asthma, and allergic rhinitis represent a significant healthcare problem. Understanding these diseases as dysregulated inflammatory responses has led to many new targets for therapeutic intervention. Recent data concerning soluble IL-4 receptor, monoclonal antibodies against IL-5 and an antibody toward IgE have lead to an appreciation of the crucial role played by Th2 subset of CD4(+) T cells and their corresponding cytokines. While these potential drugs are presently in clinical trials and may be valuable therapeutics, orally bioavailable small molecule inhibitors of Th2 cell responses would be desirable for treatment of these chronic diseases. One strategy is to prevent effector cell migration (Th2 cells, mast cells, and eosinophils) via chemokine receptor antagonism with a suitable small molecule. Chemokine receptors are a subset of the seven transmembrane-spanning family, which mediate their effects through interaction with heterotrimeric G-proteins. The ligands are a structurally related set of proteins that are selectively expressed in certain disease settings. Three chemokine receptors CCR3, CCR4, and CCR8 are preferentially expressed by Th2 cells, mast cells and eosinophils and therefore represent therapeutic targets for allergy. This mini-review will focus on new research involving CCR3, CCR4 and CCR8. The cellular distribution of each receptor, the corresponding chemokine ligands, and various validation studies are discussed. Recent drug discovery advances concerning pharmacological tools and small molecule receptor antagonists will also be presented.

PMID: 14561210 [PubMed - indexed for MEDLINE]

1280. Curr Drug Targets Inflamm Allergy. 2003 Mar;2(1):81-94.

Eotaxins and CCR3 receptor in inflammatory and allergic skin diseases: therapeutical implications.

Amerio P, Frezzolini A, Feliciani C, Verdolini R, Teofoli P, De Pità O, Puddu P.

Department of Immunodermatology and Allergology, Istituto Dermopatico dell' Immacolata-IRCCS, Rome, Italy. p.amerio@idi.it

Cell migration is mediated by a group of chemotactic cytokines called chemokines: low molecular weight molecules that have been shown as important leukocyte chemical attractants to sites of inflammation and infection. Eotaxin-1, also called CCL11, was first described in 1994, as a highly specific eosinophils chemokine. Many cell types including lymphocytes, macrophages, bronchial smooth muscle cells, endothelial cells and eosinophils, are able to produce this chemokine, predominantly after cytokine stimulation, however little is known about its expression in human skin in vivo. Eotaxin-1 also regulates the chemiotaxis and, in some conditions, activation of basophils, mast cells and T lymphocytes. Chemokine receptors are named from their ligand families, thus the CC chemokine eotaxin-1 binds to the CCR3 receptor which is expressed on

eosinophis, mast cells, Th2 type lymphocytes and even on keratinocytes. It seems that eotaxin-1 is one of the most important cytokines involved in tissue inflammation playing a central role in the pathogenesis of allergic airway diseases (asthma and rhinitis), in inflammatory bowel disease and gastrointestinal allergic hypersensitivity and recently it has been proposed as a therapeutical target for these conditions. Our group has studied the role of eotaxin-1 in the pathogenesis of two skin conditions: dermatitis herpetiformis and AIDS-associated eosinophilic folliculitis, demonstrating that this chemokine, together with Th2 type cytokines (IL-13 and IL-4) is important in cell recruitment, inflammation and tissue damage; moreover eotaxin has proven to paly an important role in other skin conditions such as, bullous pemphigoid, pemphigoid gestationis, atopic dermatitis and allergic drug reactions Recent advances in the understanding of eotaxin-1-mediated mechanisms of chemotaxis in allergic and inflammatory conditions may predict that therapeutic antagonism is achievable. This paper will focus on the role that eotaxin and its receptor play in the pathogenetical mechanism in a number of dermatologic diseases, some of which, like atopic dermatitis, may benefit from the introduction of novel and more selective therapeutic options.

PMID: 14561178 [PubMed - indexed for MEDLINE]

1281. Curr Drug Targets Inflamm Allergy. 2003 Jun;2(2):169-74.

Regulation of eosinophil migration and Th2 cell function by IL-5 and eotaxin.

Mattes J, Foster PS.

Department of Pediatrics and Adolescent Medicine, University of Freiburg, Mathildenstr. 1, 79106 Freiburg, Germany. mattes@kkl200.ukl.uni-freiburg.de

Asthma is characterized by elevated production of IgE, Th2 cytokines, chemokines, mucus hypersecretion, globlet cell metaplasia/hyperplasia, airway obstruction, eosinophilia and enhanced bronchial hyperresponsiveness. These hallmark features of asthma have all been linked to the effector functions of Th2 cytokines (e.g., interleukin-(IL)-4,5,9,10, and 13) in clinical and experimental investigations. This article will detail some of the pathogenic effects regulated by IL-13, IL-5 and the eotaxin subfamily of chemokines to regulate certain aspects of allergic disease. In particular, the potency of IL-13 in inducing enhanced bronchial responsiveness to spasmogenic stimuli and mucus hypersecretion suggests a key role of this molecule in the induction of airways obstruction. Recent studies also indicate that IL-5 and eotaxin, through eosinophils, may regulate Th2 cell function and IL-13 production from this lymphocyte. Therefore, IL-5 and IL-13 signaling systems are not necessarily mutually exclusive effector mechanisms, but may also be integrated through eosinophils to regulate certain aspects of allergic diseases. Blocking IL-13, or pathways that may promote IL-13-associated allergic lung responses (IL-5 and eotaxin) could provide an important strategy to improve the specificity of asthma therapy.

PMID: 14561170 [PubMed - indexed for MEDLINE]

1282. Curr Drug Targets Inflamm Allergy. 2003 Sep;2(3):199-205.

CpG oligodeoxynucleotides: a novel therapeutic approach for atopic disorders.

Hussain I, Kline JN.

Roy J. and Lucille A. Carver College of Medicine, University of Iowa, C33GH UIHC, 200 Hawkins Drive, Iowa City, IA 52242, USA.

Atopic disorders such as allergic rhinitis, asthma and atopic dermatitis are associated with skewing of immune responses towards a TH2 phenotype, resulting in eosinophilic inflammation. TH2 cytokines promote eosinophil growth, migration and activation, mast cell differentiation, and IgE production, and are candidate mediators of pathologic abnormalities in asthma and other atopic diseases. There has been a significant increase in the prevalence of allergic disorders over the past several decades. Recent epidemiological studies suggest that reduced early-life exposure to strong TH1 stimuli in industrialized counties has skewed the TH1/TH2 balance towards TH2 responses. Improved hygiene, vaccination, and use of antibiotics may contribute to this imbalance. In the last half of the twentieth century we have seen the use of multiple agents to treat atopic disorders, ranging from antihistamines, steroids and leukotriene modifiers to anti-IgE antibodies. All these agents can block symptoms but do not significantly modify the course of the disease. Recent attempts to restore TH1/TH2 balance by blocking TH2 cytokines or inducing TH1 cytokines, have not only failed to alter the outcome of atopic diseases but, in some cases, have caused significant adverse effects. An alternate method of suppressing TH2 responses takes advantage of the innate immune response to bacterial DNA. Oligodeoxynucleotides (ODN) containing sequence motifs centered on unmethylated CG dinucleotides (CpG ODN) resemble bacterial DNA, and like bacterial DNA are immunostimulatory; we and others have shown that CpG ODN can suppress TH2-mediated atopic inflammation without requiring the induction of TH1-type cytokines. These agents may represent a novel therapeutic approach toward restoring immune tolerance in atopic individuals.

PMID: 14561154 [PubMed - indexed for MEDLINE]

1283. Neuroimmunomodulation. 2004;11(1):20-7.

Regulation of allergic lung inflammation in rats: interaction between estradiol and corticosterone.

Ligeiro de Oliveira AP, Oliveira-Filho RM, da Silva ZL, Borelli P, Tavares de Lima W.

Department of Pharmacology, Institute of Biomedical Sciences, University of São Paulo, São Paulo, Brazil.

OBJECTIVE: One third of asthmatic women report a decreased expiratory peak flow during menses. Since asthma is characterized by lung inflammation and bronchopulmonary hyperresponsiveness, we investigated the role played by estradiol in allergic lung inflammation.
METHODS: Cell migration to the lungs of allergic female rats subjected to oophorectomy (OVx) was compared to that in their sham-operated (sham) control counterparts. Seven days after OVx or sham operation, the rats were sensitized intraperitoneally with ovalbumin (OA, 1 mg/kg) suspended in aluminum hydroxide (day 0). At day 7, a subcutaneous booster of OA was performed and an aerosolized OA challenge was carried out at day 14. One day later (day 15), the rats were killed and cell counts were performed in bronchoalveolar lavages (BAL), in peripheral blood and in bone marrow lavages.
RESULTS: After the antigen challenge, OVx rats showed a significant decrease in cell migration to the lung as compared to sham-operated rats. Differential analyses of BAL revealed a reduced number of eosinophils, mononuclear cells and neutrophils. In contrast, in bone marrow as well as in the peripheral blood the numbers of eosinophils, mononuclear cells and neutrophils were increased relative to sham controls. Mast cell numbers were similar in both groups. The estradiol receptor antagonist tamoxifen decreased the allergic lung inflammation in intact rats down to levels similar to those found in untreated OVx rats. In contrast,

17beta-estradiol replacement in OVx rats reestablished the allergic lung inflammation, as observed by an elevated number of eosinophils, mononuclear cells and neutrophils recovered in BAL. Similarly, an elevated number of inflammatory cells were quantified in BAL from allergic OVx rats when corticosterone effects were blocked with metyrapone or RU-486.
CONCLUSION: Our results suggest that estradiol has proinflammatory actions on the allergic lung response, and these actions seem to be mediated, at least in part, by endogenous glucocorticoids.

Copyright 2004 S. Karger AG, Basel

PMID: 14557675 [PubMed - indexed for MEDLINE]

1284. FEBS Lett. 2003 Oct 9;553(1-2):11-7.

The nuclear protein HMGB1, a new kind of chemokine?

Degryse B, de Virgilio M.

Department of Cell Biology, Division of Vascular Biology/VB5, The Scripps Research Institute, La Jolla, CA 92130, USA.

The chromosomal protein HMGB1 is now regarded as a proinflammatory cytokine. Importantly, HMGB1 has chemotactic activity suggesting its involvement in the early and late events of the inflammatory reaction. Therefore, HMGB1 has all the hallmarks of a chemokine (chemotactic cytokine). We propose to classify HMGB1 into a new group of proteins unrelated structurally to chemokines but having chemokine-like functions, and to name this class CLF (chemokine-like functions). The CLF class should include other unrelated molecules such as urokinase and its receptor, cytokines macrophage migration inhibitory factor (MIF) and interleukin (IL)-6, anaphylatoxin C5a, ribosomal protein S19, and thioredoxin that have similar chemokine-like activities. This innovative concept may lead to the identification of new therapeutic targets.

PMID: 14550538 [PubMed - indexed for MEDLINE]

1285. J Allergy Clin Immunol. 2003 Oct;112(4 Suppl):S78-82.

Effects of fexofenadine and other antihistamines on components of the allergic response: adhesion molecules.

Ciprandi G, Tosca MA, Cosentino C, Riccio AM, Passalacqua G, Canonica GW.

Allergy and Respiratory Diseases, Department of Internal Medicine, University of Genoa, Genoa, Italy.

Intercellular adhesion molecules (ICAMs), in particular ICAM-1, appear to play a crucial role in the recruitment and migration of inflammatory cells to the site of an allergic reaction. Glucocorticoids and allergen-specific immunotherapy have been shown to exert effects on selected components of this system, both in vitro and in vivo, but further research is required to better understand the effects of these therapies. Nasal and conjunctival challenge models (including natural and experimental allergen exposure) represent useful and safe tools for studying the activity of antiallergy drugs in vivo. These tests allow the investigation of a wide variety of parameters including inflammatory infiltrate, ICAM-1 expression, and changes in the concentration of soluble inflammatory mediators. With these tools, anti-inflammatory activity related to the modulation of epithelial cell adhesion molecules has been demonstrated in vivo for several H(1)-receptor

antagonists (azelastine, cetirizine, loratadine, levocabastine, oxatomide, and terfenadine). Fexofenadine is a nonsedating, long-acting antihistamine with highly selective H(1)-receptor antagonist activity and a particularly favorable safety profile. In addition, fexofenadine has proven anti-inflammatory activity and has been shown to inhibit a number of mediators at clinically relevant concentrations, including in vitro inhibition of ICAM-1 expression on conjunctival and nasal epithelial cells.

PMID: 14530792 [PubMed - indexed for MEDLINE]

1286. J Immunol. 2003 Oct 15;171(8):4073-80.

Psychological stress exerts an adjuvant effect on skin dendritic cell functions in vivo.

Saint-Mezard P, Chavagnac C, Bosset S, Ionescu M, Peyron E, Kaiserlian D, Nicolas JF, Bérard F.

Institut National de la Santé et de la Recherche Médicale Unité 503, IFR 128, and Department of Clinical Immunology and Allergy, CHU Lyon-Sud, Lyon, France.

Psychological stress affects the pathophysiology of infectious, inflammatory, and autoimmune diseases. However, the mechanisms by which stress could modulate immune responses in vivo are poorly understood. In this study, we report that application of a psychological stress before immunization exerts an adjuvant effect on dendritic cell (DC), resulting in increased primary and memory Ag-specific T cell immune responses. Acute stress dramatically enhanced the skin delayed-type hypersensitivity reaction to haptens, which is mediated by CD8(+) CTLs. This effect was due to increased migration of skin DCs, resulting in augmented CD8(+) T cell priming in draining lymph nodes and enhanced recruitment of CD8(+) T cell effectors in the skin upon challenge. This adjuvant effect of stress was mediated by norepinephrine (NE), but not corticosteroids, as demonstrated by normalization of the skin delayed-type hypersensitivity reaction and DC migratory properties following selective depletion of NE. These results suggest that release of NE by sympathetic nerve termini during a psychological stress exerts an adjuvant effect on DC by promoting enhanced migration to lymph nodes, resulting in increased Ag-specific T cell responses. Our findings may open new ways in the treatment of inflammatory diseases, e.g., psoriasis, allergic contact dermatitis, and atopic dermatitis.

PMID: 14530328 [PubMed - indexed for MEDLINE]

1287. J Immunol. 2003 Oct 15;171(8):3936-40.

Prostaglandin D2 inhibits airway dendritic cell migration and function in steady state conditions by selective activation of the D prostanoid receptor 1.

Hammad H, de Heer HJ, Soullie T, Hoogsteden HC, Trottein F, Lambrecht BN.

Department of Pulmonary Medicine, Erasmus Medical Center, Rotterdam, The Netherlands. h.hammad@erasmusmc.nl

PGD(2) is the major mediator released by mast cells during allergic responses, and it acts through two different receptors, the D prostanoid receptor 1 (DP1) and DP2, also known as CRTH2. Recently, it has been shown that PGD(2) inhibits the migration of epidermal Langerhans cells to the skin draining lymph nodes (LNs) and affects the subsequent cutaneous inflammatory reaction. However, the role of PGD(2) in the pulmonary immune response remains unclear. Here, we show

that the intratracheal instillation of FITC-OVA together with PGD(2) inhibits the migration of FITC(+) lung DC to draining LNs. This process is mimicked by the DP1 agonist BW245C, but not by the DP2 agonist DK-PGD(2). The ligation of DP1 inhibits the migration of FITC-OVA(+) DCs only temporarily, but still inhibits the proliferation of adoptively transferred, OVA-specific, CFSE-labeled, naive T cells in draining LNs. These T cells produced lower amounts of the T cell cytokines IL-4, IL-10, and IFN-gamma compared with T cells from mice that received FITC-OVA alone. Taken together, our data suggest that the activation of DP receptor by PGD(2) may represent a pathway to control airway DC migration and to limit the activation of T cells in the LNs under steady state conditions, possibly contributing to homeostasis in the lung.

PMID: 14530310 [PubMed - indexed for MEDLINE]

1288. Immunol Allergy Clin North Am. 2003 Aug;23(3):519-31.

Assessment of the indoor environment: evaluation of mold growth indoors.

Horner WE.

Air Quality Sciences, 1337 Capital Circle, Atlanta, GA 30067, USA.
ehorner@aqs.com

Much attention has been focused on indoor molds; resulting in modest amounts of new research. There is strong evidence of respiratory effects. Although mechanisms are disputed, some of the effect (but not all) is likely to be allergy related. There is some evidence that atopic individuals may be more affected, but many nonatopic individuals also are affected. This area needs more general research and specific research on exposure measures (such as what fungal components should be measured) and on health-effect mechanisms. It is worthwhile to emphasize the practical knowledge that is readily available. Buildings should be designed, built, operated, and occupied so that the buildings stay dry. When this situation does not occur, the environmental and clinical aspects that are observed by competent professionals should both be considered when determining causal relationships.

PMID: 14524389 [PubMed - indexed for MEDLINE]

1289. Immunol Allergy Clin North Am. 2003 Aug;23(3):501-18.

Indoor fungal exposure.

Rogers CA.

Environmental Science and Engineering Program, Department of Environmental Health, Harvard School of Public Health, Landmark Center, PO Box 15677, 401 Park Drive, Boston, MA 02215, USA. crogers@hsph.harvard.edu

Fungi affect humans in complex ways and are capable of eliciting a number of disease responses, such as infectious, allergic, and irritant and toxic effects. Fungal exposure is unequivocally associated with exacerbations of asthma, although the role of fungi in causing the disease is yet to be determined. The association between home dampness and respiratory health effects is strong, and fungal exposure is suspected to be associated with this linkage. Fear of toxin exposures has generated debate over the possible toxic health effects of airborne fungi; however, several recent reviews discount the health impacts of mycotoxin through indoor exposures. Nevertheless, fungal contamination of indoor environments is undesirable. Knowledge of sources and characteristics of fungal

spore release and dispersal are important for understanding the processes of exposure. Environmental monitoring for fungi and their disease agents are important aspects of exposure assessment, but few guidelines exist for interpreting their health impacts. Much work is needed in isolating, characterizing and standardizing fungal disease agents to properly assess the prevalence of fungal health effects.

PMID: 14524388 [PubMed - indexed for MEDLINE]

1290. Immunol Allergy Clin North Am. 2003 Aug;23(3):443-68.

Biology, ecology, and prevalence of dust mites.

Arlian LG, Morgan MS.

Department of Biological Sciences, Wright State University, 3640 Colonel Glenn Highway, Dayton, OH 45435-0001, USA. larry.arlian@wright.edu

The house dust mites D. farinae, D. pteronyssinus, and E. maynei are sources of multiple potent allergens in the indoor environment. They are common inhabitants in homes worldwide. Many biologically significant studies have revealed how well adapted these mites are to the microhabitats in homes. Ambient RH is a key factor in determining where these mites are found. Many aspects of the biology of house dust mites are not understood. A greater understanding of the biology of dust mites may reveal new strategies for controlling dust mites and their allergens in homes.

PMID: 14524385 [PubMed - indexed for MEDLINE]

1291. Pol Merkur Lekarski. 2003 Jun;14(84):605-8.

[Allergic contact dermatitis].

[Article in Polish]

Gliński W.

Klinika Dermatologiczna Akademii Medycznej w Warszawie.

Clinical definition of eczema is based on the presence of multiform erythematous, papular and vesicular lesions which are followed by marked desquamation. When the underlying mechanism is allergic, skin lesions are mediated by inflammatory Th1 lymphocytes recognizing hapten determinants, i.e. IVth allergic mechanism in allergic contact dermatitis. This mechanism is responsible also for allergy to bacterial proteins in nummular eczema, or to fungal and bacterial proteins, atopens and nickel in dyshidrotic eczema. Eczematous lesions can develop after damage to the skin barrier by toxic chemical or irritating substances, as non-allergic irritation eczema. These substances non-specifically stimulate the Langerhans cells and keratinocytes to produce numerous cytokines inducing expression of adhesion molecules on the endothelial cells. This is responsible for non-specific mobilization of Th1 cells to the skin. A model example is dermatitis induced by mercury salts and/or sodium lauryl sulfate. Intermediate position between allergic and non-allergic eczema is occupied by atopic dermatitis/eczema. In addition to classical IgE-dependent mechanism leading to degranulation of mast cells, there is generation of specific population of Th2 lymphocytes recognizing food and air-borne atopens. This reaction is responsible for skin inflammation in the late-phase response, in which allergic process started by Th2 cells switches to non-specific migration of Th1 cells attracted by

cytokines released from eosinophils, keratinocytes and Th2 lymphocytes.

PMID: 14524282 [PubMed - indexed for MEDLINE]

1292. Pol Merkur Lekarski. 2003 Jun;14(84):519-21.

[Contemporary views on the pathological mechanism of asthma].

[Article in Polish]

Kuna P.

Klinika Pneumonologii i Alergologii Instytutu Medycyny Wewnetrznej Uniwersytetu Medycznego w Łodzi.

Asthma is a chronic inflammatory disease of the airways. Genetic and environmental factors contribute to the development of asthma. Both inflammation and remodelling are essential mechanisms in the development of subepithelial fibrosis and smooth muscle hypertrophy leading to progressing decrease of FEV1. Inflammatory changes in asthma are characterised by cellular infiltration of airway epithelium, bronchial wall and smooth muscle layer. The most important cells participating in asthma include T lymphocytes, mast cells, airway epithelial cells, eosinophils, antigen-presenting cells, neutrophils, fibroblasts, myofibroblasts and macrophages. They produce many cytokines responsible for the development of allergen-specific clones of Th2 lymphocytes, for production of IgE by B-cells, for increased expression of adhesion molecules, for migration of cells, their activation and release of inflammatory mediators. The mediators coming mainly from mast cells and eosinophils that include leukotrienes, prostaglandins, histamine, alkaline proteins and enzymes are responsible for airway obturation that results from bronchospasm, bronchial mucosal oedema and excessive amount of abnormal mucous secretion. An important role in the obturation is played by smooth muscle hypertrophy and deposition of proteins under the basal membranes of respiratory epithelium.

PMID: 14524260 [PubMed - indexed for MEDLINE]

1293. Pol Merkur Lekarski. 2003 Jun;14(84):497-9.

[Cytokines in allergic inflammation].

[Article in Polish]

Kuna P.

Klinika Pneumonologii i Alergologii Instytutu Medycyny Wewnetrznej Uniwersytetu Medycznego w Łodzi.

Cytokines play a central role in the pathogenesis of allergic diseases and allergic inflammation. Therefore, an understanding of mechanisms which regulate production and function of cytokines is very important and may result in the development of more effective methods of treatment of allergic diseases. Recent studies have demonstrated that the induction of allergic inflammation requires genetic background and environmentak factors. The most important cytokines and chemokines are IL-4, IL-5, IL-3, IL-13, GM-CSF and TNF-alpha. Many other cytokines are responsible for the growth, maturity, migration activation and apoptosis of all cells involved in allergic inflammation, among them are IL-2, IL-5, IL-6, IL-9, MIP-1 alpha, RANTES, IL-8, IL-12, IL-18, IFN-alpha i gamma, TGF-beta, sIL-4, IL-1Ra. Recently it has been proven that IL-10 and other

cytokines from the IL-10 family, and TGF-beta have anti-inflammatory properties in allergy.

PMID: 14524254 [PubMed - indexed for MEDLINE]

1294. J Clin Invest. 2003 Oct;112(7):1008-18.

Prevention of leukocyte migration to inflamed skin with a novel fluorosugar modifier of cutaneous lymphocyte-associated antigen.

Dimitroff CJ, Kupper TS, Sackstein R.

Harvard Skin Disease Research Center, Department of Dermatology, Brigham and Women's Hospital, Harvard Medical School, Boston, Massachusetts, USA. cdimitroff@rics.bwh.harvard.edu

Comment in
J Clin Invest. 2003 Oct;112(7):980-3.

E-selectin and P-selectin on dermal postcapillary venules play critical roles in the migration of effector T cells into inflamed skin. P-selectin glycoprotein ligand-1 (PSGL-1) modified by alpha1,3-fucosyltransferase is the principal selectin ligand on skin-homing T cells and is required for effector T cell entry into inflamed skin. We have previously shown that a fluorinated analog of N-acetylglucosamine peracetylated-4-fluorinated-d-glucosamine (4-F-GlcNAc), inhibits selectin ligand expression on human T cell PSGL-1. To analyze 4-F-GlcNAc efficacy in dampening effector T cell migration to inflamed skin, we elicited allergic contact hypersensitivity (CHS) reactions in mice treated with 4-F-GlcNAc. We also investigated 4-F-GlcNAc efficacy on lymphocyte E-selectin ligand expression in LNs draining antigen-sensitized skin and on other immunological processes requisite for CHS responses. Our results showed that 4-F-GlcNAc treatment attenuated lymphocyte E-selectin ligand expression in skin-draining LNs and prevented CHS reactions. Significant reductions in inflammatory lymphocytic infiltrate were observed, while pathways related to antigenic processing and presentation and naive T cell recognition within skin-draining LNs were unaffected. These data indicate that 4-F-GlcNAc prevents CHS by inhibiting selectin ligand activity and the capacity of effector T cells to enter antigen-challenged skin without affecting the afferent phase of CHS.

PMCID: PMC198531
PMID: 14523038 [PubMed - indexed for MEDLINE]

1295. Clin Exp Allergy. 2003 Oct;33(10):1368-73.

Expression of intercellular adhesion molecules on circulating lymphocytes in relation to different manifestations of cow's milk allergy.

Osterlund P, Smedberg T, Schröder J, Järvinen KM.

Skin and Allergy Hospital, Department of Dermatology, Helsinki University Central Hospital, Helsinki, Finland. pamela.osterlund@helsinki.fi

BACKGROUND: The complex interactions between immune cells are partly mediated by different adhesion molecules, but little is known about their role in the systemic immunoinflammatory process following sensitization to food antigens in early infancy.
OBJECTIVE: The aim of this study was to investigate the expression of intercellular adhesion molecule-1 (ICAM-1or CD54) and the alpha subunits of its

ligands' lymphocyte function-associated antigen-1 (LFA-1) (alphaL subunit or CD11a) and Mac-1 (alphaM subunit or CD11b) on peripheral blood leucocytes in infants with cow's milk allergy (CMA) and in healthy controls.
METHODS: Thirty-nine breastfed infants, aged from 0.6 to 8.3 months, and their lactating mothers were included in the study from delivery onwards. During follow-up, 25 infants developed CMA and 14 remained healthy. Expressions of CD54 and CD11b on peripheral blood leucocytes were evaluated by flow cytometry. In addition, the expression of CD11a on peripheral blood leucocytes was analysed by immunocytochemistry. Mothers' milk samples were collected and their leucocyte content was evaluated using a light microscope.
RESULTS: The frequency of ICAM-1 expressing peripheral blood lymphocytes was significantly higher in patients with CMA than in healthy infants (P=0.03, Mann-Whitney U-test). Furthermore, the high proportion of ICAM-1-expressing cells was associated with gastrointestinal and multiorgan symptoms in the CMA infants. There was no significant difference in the expression of Mac-1 alphaM on lymphocytes in our study groups, but the LFA-1 alphaL expression seemed to be higher in the IgE-mediated CMA.
CONCLUSION: We suggest that the high expression of ICAM-1 on peripheral blood lymphocytes may reflect enhanced stimulation of T cells in vivo and their migration to the effector tissues in an early-phase of developing CMA. Furthermore, high ICAM-1 expression may be associated with the presence of multiorgan manifestations of CMA, whereas high LFA-1 expression may reflect the IgE-mediated disease.

PMID: 14519142 [PubMed - indexed for MEDLINE]

1296. Respir Physiol Neurobiol. 2003 Sep 16;137(2-3):237-50.

MAPK regulation of gene expression in airway smooth muscle.

Gerthoffer WT, Singer CA.

Department of Pharmacology, University of Nevada School of Medicine, Reno, NV 89557-0270, USA. wtg@med.unr.edu

Mitogen-activated protein kinases (MAPK) are important components of signaling modules activated by neurotransmitters, cytokines, and growth factors, as well as chemical and mechanical stressors. In the airway, these external signals produce acute responses that modify smooth muscle contraction and may also induce chronic responses that modify airway structure. Both acute and chronic events in airway remodeling result from altered expression of multiple genes encoding protein mediators of cell-cell signaling, extracellular matrix remodeling, cell cycle control and intracellular signaling pathways. This review will focus on inflammatory and growth factor mediators of cell-cell signaling regulated by the ERK and p38 MAPK pathways in airway smooth muscle (ASM). These signaling mediators affect ASM tissue mechanics, cell migration, and gene expression patterns in a paracrine and autocrine fashion, although the relative importance of each MAPK pathway varies with the stimulus. These events thereby contribute to normal airway function and participate in pathological changes in ASM that accompany symptoms of asthma.

PMID: 14516729 [PubMed - indexed for MEDLINE]

1297. Br J Dermatol. 2003 Sep;149(3):475-83.

Induction of cutaneous delayed hypersensitivity reactions in mice sensitized with intragastrically administered hapten: activation of Langerhans cells in the sensitization and elicitation phases.

Fujita N, Kobayashi H, Tsuruta D, Sakaguchi I, Yamanaka K, Kutsuna H, Teramae H, Mizuno N, Ikeda N, Kato Y, Minamino M, Kaneda K, Ishii M.

Departments of Dermatology and Anatomy, Graduate School of Medicine, Osaka City University, 1-4-3 Asahimachi, Abeno-ku, Osaka 545-8585, Japan.
nao@clubcosmetics.co.jp

BACKGROUND: As seen in atopic dermatitis, allergic diseases often produce lesions both in the gastrointestinal tract and the skin, suggesting the involvement of an immunological relationship between the two organs in the pathogenesis.
OBJECTIVES: To study the role of gastric and epidermal Langerhans cells (LCs) in the sensitization and elicitation phases, respectively, of cutaneous delayed-type hypersensitivity (DTH) reactions to intragastrically administered hapten.
METHODS: BALB/c mice, which were subjected to intragastric administration of trinitrochlorobenzene 5 days previously, received an elicitative challenge of the same hapten to the ear skin. Sections of the ear were immunostained for CD4 and CD8. Epidermal sheets of the ear and epithelial sheets of the forestomach were immunostained for I-A and observed under a confocal laser scanning microscope.
RESULTS: Cutaneous DTH reactions were induced in mice, as demonstrated by an increase in ear thickness and a prominent infiltration of CD4+ and CD8+ lymphocytes at 24-36 h after the elicitative challenge. In the elicitation phase, epidermal LCs showed a significant increase in size, indicating in vivo activation, at 24 h. In the sensitization phase, gastric LCs increased in size at 2 h, became round at 6 h, and decreased in number at 24 h, possibly representing the sequential events of LC activation and migration from the epithelium.
CONCLUSIONS: The present study demonstrated that gastric LCs and epidermal LCs were activated in vivo in the sensitization and elicitation phases, respectively, of cutaneous DTH reactions in orally sensitized mice.

PMID: 14510978 [PubMed - indexed for MEDLINE]

1298. J Immunol. 2003 Oct 1;171(7):3372-8.

Airway eosinophils accumulate in the mediastinal lymph nodes but lack antigen-presenting potential for naive T cells.

van Rijt LS, Vos N, Hijdra D, de Vries VC, Hoogsteden HC, Lambrecht BN.

Department of Pulmonary and Critical Care Medicine, Erasmus University Medical Center, Rotterdam, The Netherlands. L.vanrijit@erasmusmc.nl

Asthma is characterized by infiltration of the airway wall with eosinophils. Although eosinophils are considered to be effector cells, recent studies have reported their ability to activate primed Th2 cells. In this study, we investigated whether eosinophils are capable of presenting Ag to unprimed T cells in draining lymph nodes (DLN) of the lung and compared this capacity with professional dendritic cells (DC). During development of eosinophilic airway inflammation in OVA-sensitized and challenged mice, CCR3(+) eosinophils accumulated in the DLN. To study their function, eosinophils were isolated from the bronchoalveolar lavage fluid of mice by sorting on CCR3(+)B220(-)CD3(-)CD11c(dim) low autofluorescent cells, avoiding contamination with other APCs, and were intratracheally injected into mice that previously received CFSE-labeled OVA TCR-transgenic T cells. Eosinophils did not induce divisions of T cells in the DLN, whereas DC induced on average 3.7 divisions in 45.7% of T cells. To circumvent the need for Ag processing or migration in vivo, eosinophils were pulsed with OVA peptide and were still not able to induce T cell priming in vitro, whereas DC induced vigorous proliferation. This lack of Ag-presenting ability was explained by the very weak expression of MHC class II

on fresh eosinophils, despite expression of the costimulatory molecules CD80 and ICAM-1. This investigation does not support any role for airway eosinophils as APCs to naive T cells, despite their migration to the DLN at times of allergen exposure. DC are clearly superior in activating T cells in the DLN of the lung.

PMID: 14500630 [PubMed - indexed for MEDLINE]

1299. APMIS. 2003 Jul-Aug;111(7-8):797-804.

Epidermal Langerhans cell migration and sensitisation to chemical allergens.

Cumberbatch M, Dearman RJ, Griffiths CE, Kimber I.

Syngenta Central Toxicology Laboratory, Alderley Park, Macclesfield, Cheshire and The Dermatology Centre, University of Manchester, Hope Hospital, Salford, Manchester, UK. marie.cumberbatch@syngenta.com

Epidermal Langerhans cells (LC) form part of the wider family of dendritic cells (DC; professional antigen-processing and antigen-presenting cells). LC are considered to serve in the skin as sentinels of the adaptive immune system, surveying the local environment and transporting foreign antigen for presentation to responsive T lymphocytes in regional lymph nodes. As such, LC play pivotal roles in the initiation of cutaneous immune responses, including immune responses to chemical allergens encountered at skin surfaces. Here we explore two aspects of LC function in the context of sensitisation to chemical allergens. The first is consideration of the cytokine and chemokine signals that regulate and counter-regulate the mobilisation and migration of LC from the epidermis to skin-draining lymph nodes following topical sensitisation. The second is examination of the ways in which LC may influence the polarity of induced T lymphocytes, and thereby the quality of immune responses.

PMID: 12974781 [PubMed - indexed for MEDLINE]

1300. Mem Inst Oswaldo Cruz. 2003 Jul;98(5):593-7. Epub 2003 Sep 8.

Prevalence of Toxocara infection in schoolchildren from the Butantã region, São Paulo, Brazil.

Alderete JM, Jacob CM, Pastorino AC, Elefant GR, Castro AP, Fomin AB, Chieffi PP.

Unidade de Alergia e Imunologia, Departamento de Pediatria, Faculdade de Medicina, Universidade de São Paulo, São Paulo, SP, 05403-900, Brasil.

Visceral larva migrans syndrome by Toxocara affects mainly children between 2 and 5 years of age, it is generally asymptomatic, and the seroprevalence varies from 3 to 86% in different countries. A total of 399 schoolchildren from 14 public schools of the Butantã region, São Paulo city, Brazil, were evaluated by Toxocara serology (enzyme-linked immunosorbent assay). Epidemiological data to the Toxocara infection obtained from a protocol were submitted to multiple logistic regression analysis for a risk profile definition. Blood was collected on filter paper by finger puncture, with all samples tested in duplicate. Considering titers > or = 1/160 as positive, the seroprevalence obtained was 38.8%. Among infected children, the mean age was 9.4 years, with a similar distribution between genders. A significant association was observed with the presence of onychophagia, residence with a dirty backyard, living in a slum, previous wheezing episodes, school attended, and family income ($p < 0.05$). All data, except "living in a slum", were considered to be determinant of a risk profile for the acquisition of Toxocara infection. A monthly income > or = 5 minimum

salaries represented a protective factor, although of low relevance. Toxocara eggs were found in at least one of the soil samples obtained from five schools, with high prevalence of Toxocara infections, indicating the frequent soil contamination by this agent.

PMID: 12973524 [PubMed - indexed for MEDLINE]

1301. Chem Res Toxicol. 2003 Sep;16(9):1145-54.

Beryllium chemical speciation in elemental human biological fluids.

Sutton M, Burastero SR.

Analytical and Nuclear Chemistry Division, Lawrence Livermore National Laboratory, P.O. Box 808, Livermore, California 94551, USA. Sutton18@llnl.gov

The understanding of beryllium chemistry in human body fluids is important for understanding the prevention and treatment of chronic beryllium disease. Thermodynamic modeling has traditionally been used to study environmental contaminant migration and rarely in the examination of metal (particularly beryllium) toxicology. In this work, a chemical thermodynamic speciation code (MINTEQA2) has been used to model and understand the chemistry of beryllium in simulated human biological fluids such as intracellular, interstitial, and plasma fluids, a number of airway surface fluids for patients with lung conditions, saliva, sweat, urine, bile, gastric juice, and pancreatic fluid. The results show that predicted beryllium solubility and speciation vary markedly between each simulated biological fluid. Formation of beryllium hydroxide and/or phosphate was observed in most of the modeled fluids, and results support the postulation that beryllium absorption in the gastrointestinal tract may be limited by the formation of beryllium phosphate solids. It is also postulated that beryllium is potentially 13% less soluble in the airway surface fluid of a patient with asthma when compared to a "normal" case. The results of this work, supported by experimental validation, can aid in the understanding of beryllium toxicology. Our results can potentially be applied to assessing the feasibility of biological monitoring or chelation treatment of beryllium body burden.

PMID: 12971803 [PubMed - indexed for MEDLINE]

1302. Int J Biochem Cell Biol. 2003 Dec;35(12):1667-73.

Expression of membrane type-4 matrix metalloproteinase (metalloproteinase-17) by human eosinophils.

Gauthier MC, Racine C, Ferland C, Flamand N, Chakir J, Tremblay GM, Laviolette M.

Institut Universitaire de Cardiologie et de Pneumologie de l'Université Laval, Que., G1V 4G5, Sainte-Foy, Canada.

Circulating eosinophils need proteinases to mediate a spatially limited and orientated digestion of the extracellular matrix and to migrate into tissue. Moreover, proteinases are likely involved in tissue remodeling, a crucial feature of chronic diseases including asthma. Eosinophils express matrix metalloproteinase (MMP)-9, which is increased upon stimulation with TNF-alpha. Other MMPs, the membrane type (MT)-MMPs, likely play a major role in cell invasion and tissue remodeling. MT4-MMP was identified in peripheral blood leukocyte preparations, but it is not known whether eosinophils express MT4-MMP. We investigated the expression of MT4-MMP and its modulation by TNF-alpha in purified human blood eosinophils. The constitutive expression of MT4-MMP mRNA was

detected by RT-PCR in unstimulated eosinophils, lymphocytes, and monocytes, but not neutrophils. Stimulation of eosinophils with TNF-alpha increased MT4-MMP mRNA expression. This effect appeared at 4h and reached a maximum at 8h of incubation. MT4-MMP protein was detected in freshly isolated blood eosinophils by Western blotting and immunocytochemistry. TNF-alpha increased expression of the MT4-MMP protein. MT4-MMP protein was also detected in nasal polyp eosinophils by immunohistochemistry. In conclusion, eosinophils constitutively express MT4-MMP, which is increased upon stimulation with TNF-alpha. Consequently, MT4-MMP may be directly involved in the degradation of extracellular matrix components and/or modulate the activity of other proteins implicated in eosinophil migration and tissue remodeling.

PMID: 12962706 [PubMed - indexed for MEDLINE]

1303. Nat Immunol. 2003 Oct;4(10):974-81. Epub 2003 Aug 31.

Mast cell-dependent migration of effector CD8+ T cells through production of leukotriene B4.

Ott VL, Cambier JC, Kappler J, Marrack P, Swanson BJ.

Integrated Department of Immunology, National Jewish Medical and Research Center, 1400 Jackson Street, and University of Colorado Health Sciences Center, Denver, Colorado 80206, USA. ottv@njc.org

Comment in
    Nat Immunol. 2003 Oct;4(10):937-9.

Studies in both humans and rodents indicate that CD8+ T cells may be important in allergic inflammation. However, neither the mechanisms that mediate CD8+ T cell recruitment to inflamed tissues nor the relative participation of effector and central memory CD8+ T cells is known. Here we report that activated mast cells induced chemotaxis of effector, but not central memory, CD8+ T cells through production of leukotriene B4 (LTB4). These studies indicate that LTB4 production by activated peripheral leukocytes could be important for the recruitment of effector CD8+ T cells to sites of inflammation.

PMID: 12949532 [PubMed - indexed for MEDLINE]

1304. Sheng Li Xue Bao. 2003 Aug 25;55(4):365-72.

Cellular signaling in rapid intestinal epithelial restitution: implication of polyamines and K+ channels.

Wang JY.

Department of Surgery, University of Maryland School of Medicine and Baltimore Veterans Affairs Medical Center, Baltimore, Maryland 21201, USA. jwang@smail.umaryland.edu

Epithelial cells line the gastrointestinal (GI) mucosa and form an important barrier that protects the subepithelial tissue against a wide array of noxious substances, allergens, viruses, and luminal microbial pathogens. Restoration of mucosal integrity following injury requires epithelial cell decisions that regulate signaling networks controlling gene expression, survival, migration, and proliferation. Over the past few years, polyamines have been shown to play a critical role in GI mucosal repair, and the control of cellular polyamines is a central convergence point for the multiple signaling pathways. Both the function

of polyamines in rapid intestinal mucosal epithelial restitution and the underlying mechanism, especially the implication of K(+) channel activity, are the subject of this mini-review article.

PMID: 12937813 [PubMed - indexed for MEDLINE]

1305. Endocrinology. 2003 Sep;144(9):4080-6.

Inhibition of the stem cell factor-induced migration of mast cells by dexamethasone.

Jeong HJ, Na HJ, Hong SH, Kim HM.

Department of Pharmacology, Kyung Hee University College of Oriental Medicine, 130-701 Seoul, South Korea.

Mast cell accumulation can be causally related to several allergic inflammations. Previous work has demonstrated that glucocorticoids decreased tissue mast cell number, and stem cell factor (SCF)-induced migration of mast cells required p38 MAPK activation. In the present study we investigated the effects of dexamethasone on SCF-induced migration of rat peritoneal mast cells (RPMCs). SCF significantly induced the migration of RPMCs at 4 h. Dexamethasone dose-dependently inhibited SCF-induced migration of RPMCs (approximately 90.1% at 100 nM; $P < 0.05$). The MAPK p38 inhibitor SB203580 (20 microM) also inhibited the SCF-induced migration. The ability of SCF to enhance morphological alteration and filamentous actin formation was also abolished by treatment with dexamethasone. Dexamethasone inhibited SCF-induced p38 MAPK activation to near-basal levels and induced MAPK phosphatase-1 expression. In addition, SCF-induced inflammatory cytokine production was significantly inhibited by treatment with dexamethasone or SB203580 ($P < 0.01$). Our results show that dexamethasone potently regulates SCF-induced migration, p38 MAPK activation, and inflammatory cytokine production through the expression of MKP-1 protein in RPMCs. Such modulation may have functional consequences during dexamethasone treatment, especially mast cell-mediated allergic inflammation disorders.

PMID: 12933682 [PubMed - indexed for MEDLINE]

1306. Am J Trop Med Hyg. 2003 Jul;69(1):67-73.

An outbreak of gnathostomiasis among Korean emigrants in Myanmar.

Chai JY, Han ET, Shin EH, Park JH, Chu JP, Hirota M, Nakamura-Uchiyama F, Nawa Y.

Department of Parasitology and Tropical Medicine, Seoul National University College of Medicine, and Institute of Endemic Diseases, Seoul National University Medical Research Center, Seoul, Republic of Korea. cjy@plaza.snu.ac.kr

Thirty-eight (designated as cases) of 60 Korean emigrants who consumed raw fresh water fish in Yangon, Myanmar developed migratory swellings and creeping eruptions on the back, abdomen, flank, and other cutaneous areas 1-10 weeks later. The symptoms included itching, nodule formation, fatigue, urticaria, fever, pain on the skin, and erythematous plaques. Skin biopsies of two cases revealed no parasites. However, the mean +/- SD peripheral blood eosinophilia among the cases was 6.3 +/- 6.5% (n = 29) and 9.0 +/- 9.8% (n = 26) in two examinations. An enzyme-linked immunosorbent assay of their serum samples, using Gnathostoma doloresi adult worms as the antigen, showed mean +/- SD optical densities of 0.47 +/- 0.29 (n = 28) and 0.32 +/- 0.20 (n = 30) in two examinations and 0.12 +/- 0.09 (n = 50) in healthy controls. Two advanced

third-stage larvae of G. spinigerum were found in two of six catfish purchased at a local market in Yangon. The outbreak of the human infection is suggested to have been due to G. spinigerum, which is known to live out its life cycle in the Yangon area of Myanmar.

PMID: 12932100 [PubMed - indexed for MEDLINE]

1307. Arerugi. 2003 Jun;52(6):522-9.

[In vitro evaluation of allergenicity of dried food powders manufactured for food provocation test].

[Article in Japanese]

Shimakura K, Nagashima Y, Shiomi K, Kunou M, Ebisawa M, Akasawa A, Iikura Y.

Department of Food Science and Technology, Tokyo University of Fisheries.

Food provocation test (FPT) is one of important diagnostic methods for food allergy, but no standard antigens for FPT have yet been developed. In this study, dried powders were manufactured from five kinds of foods (cow's milk, hen egg, chicken, soybean and wheat) by spray-drying or freeze-drying and examined in vitro for their usefulness as antigens for FPT. In SDS-PAGE, the migration pattern of the extract from each powder was the same as or closely similar to that of the extract from its material. When analyzed by ELISA, a good correlation (r=0.853-0.978) in the reactivity with sera from food-allergic patients was observed between the extracts from each powder and its material. Moreover, in cow's milk, hen egg and soybean, almost the same ELISA inhibition curves were drawn, regardless of whether the extracts from each powder and its material were used as immobilized antigens or inhibitors. These results demonstrated that each powder contains the same allergens as its material at almost the same levels, being useful as an antigen for FPT. Favorably, the powders were found to be stored without significant changes in IgE reactivity at -20 degrees C or 5 degrees C for more than 18 months, although their storage at room temperature was suggested to be avoided.

PMID: 12928611 [PubMed - indexed for MEDLINE]

1308. J Clin Invest. 2003 Aug;112(4):566-74.

Beta-arrestin-2 regulates the development of allergic asthma.

Walker JK, Fong AM, Lawson BL, Savov JD, Patel DD, Schwartz DA, Lefkowitz RJ.

Department of Medicine, Duke University Medical Center, Box 3821, Durham, North Carolina 27710, USA.

Asthma is a chronic inflammatory disorder of the airways that is coordinated by Th2 cells in both human asthmatics and animal models of allergic asthma. Migration of Th2 cells to the lung is key to their inflammatory function and is regulated in large part by chemokine receptors, members of the seven-membrane-spanning receptor family. It has been reported recently that T cells lacking beta-arrestin-2, a G protein-coupled receptor regulatory protein, demonstrate impaired migration in vitro. Here we show that allergen-sensitized mice having a targeted deletion of the beta-arrestin-2 gene do not accumulate T lymphocytes in their airways, nor do they demonstrate other physiological and inflammatory features characteristic of asthma. In contrast, the airway inflammatory response to LPS, an event not coordinated by Th2 cells, is fully

functional in mice lacking beta-arrestin-2. beta-arrestin-2-deficient mice demonstrate OVA-specific IgE responses, but have defective macrophage-derived chemokine-mediated CD4+ T cell migration to the lung. This report provides the first evidence that beta-arrestin-2 is required for the manifestation of allergic asthma. Because beta-arrestin-2 regulates the development of allergic inflammation at a proximal step in the inflammatory cascade, novel therapies focused on this protein may prove useful in the treatment of asthma.

PMCID: PMC171386
PMID: 12925697 [PubMed - indexed for MEDLINE]

1309. Clin Exp Allergy. 2003 Aug;33(8):1141-6.

Increased expression of surface activation markers on neutrophils following migration into the nasal lumen.

Kinhult J, Egesten A, Benson M, Uddman R, Cardell LO.

Laboratory for Clinical and Experimental Allergy Research, Department of Otorhinolaryngology Department of Medical Microbiology, Malmö University Hospital, Malmö, Sweden.

BACKGROUND: The sequence of events following the recruitment of a free-flowing neutrophil in the peripheral circulation, via adhesion, migration and release of mediators, to a neutrophil on the surface of the nasal epithelium is a co-ordinated process. Little is known about the state of neutrophil activation following this course of events.
OBJECTIVES: To investigate the expression of surface activation markers on neutrophils, reflecting activation during their recruitment to the nose, and to see whether the inflammatory process during allergic rhinitis influences this process.
METHOD: Nine healthy controls and 12 patients with grass pollen-induced intermittent allergic rhinitis were investigated during the peak of the pollen season. The expression of CD11b, CD66b and CD63 on the neutrophil cell surface, as a reflection of activation, was analysed using flow cytometry. Neutrophils were derived from peripheral blood and nasal lavage fluid. In addition, eosinophil cationic protein (ECP) and myeloperoxidase (MPO) as well as L-, P- and E-selectins in the nasal lavage fluid were analysed using RIA and ELISA, respectively.
RESULTS: A marked increase in the expression of all three CD markers on the neutrophil cell surface was noticed following migration from the bloodstream to the surface of the nasal mucosa. At the peak of the grass pollen season, the MPO levels increased, reflecting an increase in the total number of nasal fluid neutrophils. In parallel, the expression of CD11b was further augmented. The expression of the CDb11b was reduced on neutrophils remaining in the circulation. In addition, the level of L-selectin was reduced on neutrophils derived from the blood during allergic inflammation.
CONCLUSION: Neutrophils might become activated during their transfer from the blood to the surface of the nasal mucosa, but these changes may also be due to depletion of activated neutrophils in the blood via activated endothelial/epithelial adhesion and chemoattractant measures. The increased expression of surface activation markers during allergic rhinitis suggests roles for neutrophils in the inflammatory process.

PMID: 12911790 [PubMed - indexed for MEDLINE]

1310. Clin Exp Allergy. 2003 Aug;33(8):1118-24.

Expression of CS-1 fibronectin precedes monocyte chemoattractant protein-1 production during elicitation of allergic contact dermatitis.

Martín AP, Gagliardi J, Baena-Cagnani CE, Eberhard Y, Uguccioni M, Gallino N, Mariani AL, Serra HM.

Inmunología, Departamento de Bioquímica Clínica, Facultad de Ciencias Químicas, Universidad Nacional de Córdoba, Córdoba, Argentina.

BACKGROUND: Leucocyte migration within inflammatory skin compartments in allergic contact dermatitis (ACD) is the result of a sophisticated multi-step event where multiple molecules are involved.
OBJECTIVE: Since non-antigen-specific mechanisms have been described as an early participant in elicitation of ACD, we investigated the kinetics of the expression of monocyte chemoattractant protein-1 (MCP-1/CCL2) and the type of infiltrating cells. We compared the time course production of MCP-1/CCL2 with connecting segment-1 (CS-1) fibronectin and thymus and activation-regulated chemokine (TARC/CCL17) expression.
METHODS: Biopsies from 10 individuals challenged in their back with the antigen responsible for their contact dermatitis and an irrelevant antigen were taken at different times and histology, immunohistochemistry for CS-1 fibronectin, TARC/CCL17, CD3, CD68, CXCR3, CCR4 and in situ hybridization for MCP-1/CCL2 were performed.
RESULTS: At positive antigen stimulated sites expression of MCP-1/CCL2 by basal keratinocytes and isolated cells in dermis started at 10 h. CS-1 fibronectin and TARC/CCL17 expression by blood endothelial cells was found at 2 and 10 h, respectively. This was followed by dermal accumulation of mononuclear cells with a significant increase of CD3+ and CD68+cells. At 48 h, approximately 58% of infiltrating cells were CXCR3+, and 35% CCR4+.
CONCLUSIONS: We showed evidence of the fact that CS-1 fibronectin expression precedes the production of MCP-1/CCL2 and TARC/CCL17 in the skin of patients with ACD, suggesting that these molecules participate in the early complex process of migrating mononuclear cells during elicitation of ACD.

PMID: 12911787 [PubMed - indexed for MEDLINE]

1311. Ann Ig. 2003 May-Jun;15(3):261-70.

[Immigration and health: observational study concerning the foreign children attending the Bologna community pediatric service].

[Article in Italian]

Stampi S, Matteini P, Zanetti F, Baldi E.

Dip. di Medicina e Sanità Pubblica, Sezione di Igiene, Università degli Studi di Bologna. serena.stampi@unibo.it

An analysis of the health situation of 2583 immigrant children examined by the Community Paediatric Service in the city of Bologna in 1999 and 2000 was made. The data were obtained from health records, from medical notes made in the districts or during school readmissions. Our investigation showed that: 68.8% of the children were of immigrant couples, 21.6% had at least one Italian parent, 6.0% had lost one or both parents and 3.6%, had been adopted by Italian couples; 68.8% were born in EU countries (1620 in Bologna and 133 in other regions). 99.6% of the children had received all compulsory vaccinations; 1853 subjects had also had one or more optional vaccinations: against whooping-cough 45.2%, measles 46.9% and mumps 33.8%. Only 38.5% of children had pathologies, and those most frequently encountered involved the respiratory and digestive systems. Diseases

are more often infectious and allergic as in Italian population. Parasitic infections occurred more frequently than in Italian children; amoebiasis and cutaneous diseases were seen in 1.9% of the children (especially Indians). Cases of tuberculosis were limited. The children more often undergoing diagnostic investigations involving hospitalisation were from Asia and Africa. Only 10.1 of mothers and 7.6% of fathers reported medical problems; allergic pathologies were prevalent.

PMID: 12910879 [PubMed - indexed for MEDLINE]

1312. Pediatr Pulmonol. 2003 Sep;36(3):209-15.

Migration to a western country increases asthma symptoms but not eosinophilic airway inflammation.

Gibson PG, Henry RL, Shah S, Powell H, Wang H.

Hunter Medical Research Institute, University of Newcastle, John Hunter Hospital, Newcastle, New South Wales, Australia. mdpgg@mail.newcastle.edu.au

The prevalence of asthma symptoms varies markedly throughout the world. However, the asthma mechanisms involved are not defined. Studying the effects of migration can help identify the reasons for this geographic variation. The aims of this study were to examine the prevalence of asthma symptoms, airway hyperresponsiveness (AHR), and induced sputum eosinophils in adolescents who migrate to Australia. The study was conducted in Sydney, Australia, where adolescent students completed a video symptom questionnaire, hypertonic saline challenge, sputum induction, and allergy skin testing. The 211 students had widely different cultural backgrounds, including Asian, South Pacific, Middle Eastern, European, and African countries. Among adolescents who were migrants to Australia, the prevalence of asthma symptoms was higher than that reported using a similar methodology in their country of origin. Asthma symptom prevalence was related to residence time in Australia. The prevalence of wheeze was 17.2% in recent arrivals, 20.5% in adolescents living in Australia for >2 years, and 36.3% in those living all their lifetime in Australia (P = 0.013). For every year of residence in Australia, there was an 11% increase in prevalence of current wheeze (odds ratio, 1.11; P = 0.02). This effect was not related to atopy, AHR, or eosinophilic airway inflammation. Sputum neutrophils were elevated in recent arrivals. In conclusion, adolescents who migrate to Australia report increased asthma symptoms, compared to their country of origin, and asthma symptoms are further increased for every additional year of residence in Australia. The development of wheeze after migration to Australia was independent of eosinophilic inflammation and consistent with noneosinophilic asthma mechanisms.

Copyright 2003 Wiley-Liss, Inc.

PMID: 12910582 [PubMed - indexed for MEDLINE]

1313. J Allergy Clin Immunol. 2003 Aug;112(2):375-81.

Endothelial protein C receptor-dependent inhibition of human eosinophil chemotaxis by protein C.

Feistritzer C, Sturn DH, Kaneider NC, Djanani A, Wiedermann CJ.

Division of General Internal Medicine, Department of Internal Medicine, University of Innsbruck, Innsbruck, Austria.

BACKGROUND: Eosinophil infiltration is a characteristic feature of allergic inflammation. Allergic responses are associated with local activation of the coagulation pathway and accumulation of fibrin.
OBJECTIVE: We tested whether protein C and activated protein C (APC), which are endogenous anti-inflammatory coagulation inhibitors, affect eosinophil function.
METHODS: Eosinophils were from venous blood of healthy donors. Cell migration and apoptosis were studied by using micropore filter assays and fluorometry, respectively. Receptor expression was investigated by means of RT-PCR and SDS-PAGE of immunoprecipitated protein.
RESULTS: Protein C and APC had no significant chemotactic effects on eosinophils. Eosinophils pretreated with protein C or APC showed significantly reduced migration toward chemoattractants. No effect of either protein C preparation was seen in eosinophil apoptosis assays. The inhibiting effect on migration was reversed by an antibody against the endothelial protein C receptor (EPCR). Synthesis of EPCR by eosinophils is suggested by demonstration of receptor mRNA expression and detection of metabolically labeled receptor protein.
CONCLUSIONS: Data suggest that an EPCR is expressed by eosinophils whose activation with protein C or APC arrests directed migration. Protein C-affected eosinophil chemotaxis is a novel thrombin-independent component of the protein C pathway.

PMID: 12897745 [PubMed - indexed for MEDLINE]

1314. J Allergy Clin Immunol. 2003 Aug;112(2):369-74.

Cysteinyl leukotrienes induce nuclear factor kappa b activation and RANTES production in a murine model of asthma.

Kawano T, Matsuse H, Kondo Y, Machida I, Saeki S, Tomari S, Mitsuta K, Obase Y, Fukushima C, Shimoda T, Kohno S.

Second Department of Internal Medicine, Nagasaki University School of Medicine, 1-7-1 Sakamoto, Nagasaki 852-8501, Japan.

BACKGROUND: It has been demonstrated that both cysteinyl leukotrienes (cysLTs) and cytokines are involved in the pathophysiology of bronchial asthma. Nonetheless, the exact mechanism involved in the interaction between these 2 molecules has yet to be determined.
OBJECTIVE: The aim of the present study was to determine the effects of cysLTs on allergic airway inflammation and allergen-specific cytokine production in a murine model of asthma.
METHODS: Four groups of BALB/c mice (control mice, Dermatophagoides farinae allergen-sensitized mice, pranlukast cysLT receptor antagonist-treated allergen-sensitized mice, and dexamethasone-treated allergen-sensitized mice) were examined.
RESULTS: Allergen-sensitized mice exhibited increased airway responsiveness and inflammation. Pranlukast-treated mice showed significant attenuation of these changes concomitant with reduction of T(H)2 cytokine and IFN-gamma production by isolated lung mononuclear cells (MNCs). A much stronger inhibition of all cytokines was noted in dexamethasone-treated mice. Pranlukast also significantly inhibited production of RANTES and activation of nuclear factor kappa B (NF-kappa B) in the isolated lung MNCs. Leukotriene D(4) stimulated isolated lung MNCs to produce RANTES but not any other cytokines and also activated NF-kappa B in these cells.
CONCLUSIONS: Our results suggest that cysLTs activate NF-kappa B and induce RANTES production from isolated lung MNCs, which in turn might cause migration of eosinophils and activated T lymphocytes into the airway.

PMID: 12897744 [PubMed - indexed for MEDLINE]

1315. Biochem Biophys Res Commun. 2003 Aug 8;307(4):797-802.

Gene structure and functional properties of mouse CRTH2, a prostaglandin D2 receptor.

Hirai H, Abe H, Tanaka K, Takatsu K, Sugamura K, Nakamura M, Nagata K.

Department of Advanced Medicine and Development, R&D Center, BML, Inc., Saitama 350-1101, Japan. hirai@alk.co.jp

CRTH2, the second receptor for prostaglandin D(2) (PGD(2)), is thought to play a role in allergic inflammations through the induction of chemotactic migration and/or the activation of Th2, eosinophils, and basophils, in humans. We previously identified the mouse CRTH2 homolog of human CRTH2 and suggest that animal models would provide a clear understanding on the precise function of CRTH2 in allergic disorders. To this end we have confirmed that mouse CRTH2 is similar in gene structure to human CRTH2 and revealed that mouse CRTH2 is predominantly expressed in the eosinophils derived from IL-5-transgenic mice. Moreover, mouse CRTH2 harbors the ability to bind PGD(2) with high affinity and intracellular Ca(2+) mobilization in a Gi-dependent manner and chemotactic responses in several transfected cell lines. The results demonstrated here indicate that mouse CRTH2 is the functional ortholog of human CRTH2 and paves the way for future analysis of the in vivo functions of CRTH2.

PMID: 12878180 [PubMed - indexed for MEDLINE]

1316. J Immunol. 2003 Aug 1;171(3):1542-55.

Role of regulator of G protein signaling 16 in inflammation-induced T lymphocyte migration and activation.

Lippert E, Yowe DL, Gonzalo JA, Justice JP, Webster JM, Fedyk ER, Hodge M, Miller C, Gutierrez-Ramos JC, Borrego F, Keane-Myers A, Druey KM.

Molecular Signal Transduction Section, National Institute of Allergy and Infectious Diseases, National Institutes of Health, Rockville, MD 20852, USA.

Chemokine-induced T lymphocyte recruitment to the lung is critical for allergic inflammation, but chemokine signaling pathways are incompletely understood. Regulator of G protein signaling (RGS)16, a GTPase accelerator (GTPase-activating protein) for Galpha subunits, attenuates signaling by chemokine receptors in T lymphocytes, suggesting a role in the regulation of lymphocyte trafficking. To explore the role of RGS16 in T lymphocyte-dependent immune responses in a whole-organism model, we generated transgenic (Tg) mice expressing RGS16 in CD4(+) and CD8(+) cells. rgs16 Tg T lymphocytes migrated to CC chemokine ligand 21 or CC chemokine ligand 12 injection sites in the peritoneum, but not to CXC chemokine ligand 12. In a Th2-dependent model of allergic pulmonary inflammation, CD4(+) lymphocytes bearing CCR3, CCR5, and CXCR4 trafficked in reduced numbers to the lung after acute inhalation challenge with allergen (OVA). In contrast, spleens of sensitized and challenged Tg mice contained increased numbers of CD4(+)CCR3(+) cells producing more Th2-type cytokines (IL-4, IL-5, and IL-13), which were associated with increased airway hyperreactivity. Migration of Tg lymphocytes to the lung parenchyma after adoptive transfer was significantly reduced compared with wild-type lymphocytes. Naive lymphocytes displayed normal CCR3 and CXCR4 expression and cytokine responses, and compartmentation in secondary lymphoid organs was normal without allergen challenge. These results suggest that RGS16 may regulate T lymphocyte activation in response to

inflammatory stimuli and migration induced by CXCR4, CCR3, and CCR5, but not CCR2 or CCR7.

PMID: 12874248 [PubMed - indexed for MEDLINE]

1317. Immunology. 2003 Aug;109(4):536-46.

Allergen-induced fluctuation in CC chemokine receptor 3 expression on bone marrow CD34+ cells from asthmatic subjects: significance for mobilization of haemopoietic progenitor cells in allergic inflammation.

Sehmi R, Dorman S, Baatjes A, Watson R, Foley R, Ying S, Robinson DS, Kay AB, O'Byrne PM, Denburg JA.

Firestone Institute of Respiratory Health, Asthma Research Group, St. Joseph's Healthcare, Divisions of Clinical Immunology, McMaster University, Hamilton, Ontario, Canada. sehmir@mcmaster.ca

There is increasing evidence that primitive progenitors migrate from the bone marrow (BM) via the peripheral circulation to tissue sites where they undergo in situ differentiation to provide a continued source of effector cells, such as eosinophils, during an allergic inflammatory response. To study mechanisms of progenitor cell mobilization in allergic reactions, we investigated fluctuations in the expression of the eotaxin receptor, CC chemokine receptor 3 (CCR3), on CD34+ cells from stable asthmatics following allergen (i.e. antigen) challenge. BM aspirates were taken from seven early responder (ER) and 10 dual responder (DR) asthmatics who, following antigen challenge developed only an early bronchoconstrictor response and an early and late- bronchoconstrictor response, respectively. Expression of CCR3 was detected on primitive (CD34+ cells) and eosinophil-lineage committed progenitors (CD34+ interleukin-5 receptor alpha-subunit+ cells) by flow cytometry and confirmed by co-localization of CCR3 messenger RNA to CD34 immunopositive cells using in situ hybridization. When preantigen levels were compared to 24-hr postantigen levels, significant increases in BM CD34+ CCR3+ cells were detected in DR, who also developed a significant sputum and blood eosinophilia and increased methacholine airway responsiveness. In contrast, a significant attenuation of BM CD34+ CCR3+ cells was observed in ER. In a dose-dependent manner eotaxin, but not interleukin (IL)-5, stimulated CD34+ progenitor cell migration in vitro. This migrational response to eotaxin was abrogated by anti-CCR3 monoclonal antibody and primed by preincubation with IL-5. We propose that fluctuations in CCR3 expression on human BM CD34+ cells may facilitate chemokine-mediated progenitor cell mobilization to the peripheral circulation and the resultant development of pulmonary eosinophilia, a cardinal feature of asthma.

PMCID: PMC1782995
PMID: 12871220 [PubMed - indexed for MEDLINE]

1318. Pharmacol Res. 2003 Sep;48(3):267-71.

Effect of Poncirus fructus on stem cell factor-induced mast cell migration.

Na HJ, Jeong HJ, Hwang WJ, Cho KH, Kim HM.

Department of Pharmacology, College of Oriental Medicine, Kyung Hee University, 1 Hoegi-dong, Dongdaemun-gu, Seoul 130-701, South Korea.

Mast cell hyperplasia can be causally related with chronic inflammation. Stem cell factor (SCF), the ligand of the c-kit protooncogene product, is a major

regulator and chemoattractant of mast cells. Poncirus fructus (PF) has been used against allergic diseases for generations in South Korea. PF (1 mg ml(-1)) significantly inhibited the SCF-induced migration of rat peritoneal mast cells (RPMCs). RPMCs exposed to SCF (50 ng ml(-1)) resulted in a drastic shape change with a polarized morphology while the cells exposed to PF (1 mg ml(-1)) remained resting, with little or no shape alteration. The drastic morphological alteration and distribution of polymerized actin were blocked by pretreatment with PF. In addition, PF inhibited both TNF-alpha and IL-6 secretion from RPMCs stimulated with SCF. Our findings provide evidence that PF inhibits chemotactic response and inflammatory cytokines secretion to SCF in mast cells.

PMID: 12860445 [PubMed - indexed for MEDLINE]

1319. Allergy. 2003 Aug;58(8):795-801.

The effect of recombinant interleukin-8 on eosinophils' and neutrophils' migration in vivo and in vitro.

Bocheńska-Marciniak M, Kupczyk M, Górski P, Kuna P.

Department of Pneumonology and Allergology, Medical University of Lodz, Lodz, Poland.

BACKGROUND: Interleukin-8 (IL-8) is a chemokine that causes chemotaxis of neutrophils, eosinophils and lymphocytes in vitro; however, its role as a chemoattractant in allergic inflammation is unclear. The objective of this study was to investigate the effect of nasal instillation of IL-8 on the influx of inflammatory cells.
METHODS: Twelve patients suffering from seasonal allergic rhinitis hypersensitive to grass pollens, with average age 30.1 +/- 2.67 years were challenged both with diluent for IL-8 and IL-8 on a subsequent day, in two phases: before the pollen season (unprimed mucosa) and during the season (primed mucosa). The number of neutrophils, eosinophils and myeloperoxydase (MPO) levels in the nasal fluid collected after IL-8 or placebo challenge were determined.
RESULTS: Challenge with IL-8 of primed nasal mucosa induced a significant influx of neutrophils (29 x 10(4) cells/ml at 0.5 h, 251 x 10(4) at 2 h and 334 x 10(4) at 3 h). Number of eosinophils in comparison with diluent challenge was not significant. There was no difference in MPO levels in the nasal lavage between IL-8 and diluent challenge of unprimed and primed mucosa. We did not find the relationship between MPO levels and the neutrophils number in the lavage (rank Spearman factor, RS = 0.258, P = 0.42).
CONCLUSION: We have demonstrated that IL-8 causes influx of neutrophils but not eosinophils into nasal mucosa in vivo. MPO level seems to be of little value as a marker of neutrophil influx into nasal mucosa.

PMID: 12859561 [PubMed - indexed for MEDLINE]

1320. J Biomol Screen. 2003 Jun;8(3):324-31.

Identification and characterization of novel antagonists of the CCR3 receptor.

Warrior U, McKeegan EM, Rottinghaus SM, Garcia L, Traphagen L, Grayson G, Komater V, McNally T, Helfrich R, Harris RR, Bell RL, Burns DJ.

Abbott Laboratories, Global Pharmaceutical Products Division, Abbott Park, IL, USA. Usha.Warrior@abbott.com

Eotaxin, an inducer of eosinophil migration and activation, exerts its activity

by binding to CCR3, the C-C chemokine receptor 3. An inhibitor of the eotaxin-CCR3 binding interaction may have potential as an anti-inflammatory drug for treatment of asthma, parasitic infections, and allergic disorders. A radioligand binding assay was developed using HEK cells transfected with CCR3, with (125)I eotaxin as the ligand. Whole cells grown on polylysine-coated plates were used as the receptor source for the screen. Screening of more than 200,000 compounds with this assay yielded a number of screening hits, and of these, 2 active novel antagonists were identified. These compounds showed inhibitory effects on eosinophil chemotaxis in both in vitro and in vivo assays.

PMID: 12857386 [PubMed - indexed for MEDLINE]

1321. Int J Tissue React. 2003;25(1):1-8.

Cetirizine modulates adhesion molecule expression in a double-blind controlled study conducted in psoriatic patients.

Pestelli E, Floriani I, Fabbri P, Caproni M.

Department of Dermatologic Science, University of Florence, Florence, Italy.

Erratum in
    Int J Tissue React. 2003;25(2):79.

Psoriasis is a chronic inflammatory T-cell-mediated immune dermatosis, characterized by the cutaneous expression of adhesion molecules belonging to the beta1 and beta2 integrin subfamilies, such as intracellular adhesion molecule (ICAM)-1, ICAM-3, lymphocyte function associated antigen (LFA)-1, vascular cell adhesion molecule (VCAM)-1 and endothelial adhesion molecule (ELAM)-1. Cetirizine is a nonsedating, selective H1-receptor antagonist, whose therapeutic efficacy is probably the result of its effect on both the immediate allergic reaction and the late-phase allergic response. The aim of this study was to investigate adhesion molecule expression (ICAM-1, ICAM-3, VCAM-1, LFA-1 and ELAM-1) by using an immunophosphatase alkaline (APAAP) technique in a double-blind controlled study. Nineteen patients with active psoriasis vulgaris minima were randomized into two groups: group A (two men and six women, aged 22-59 years) was treated with cetirizine (30 mg a day, 3 times a day for 15 days) and group B (three men and eight women, aged 24-72 years) were administered placebo. Positive cells were counted by two independent and blinded observers and at least three adjacent high-power fields (250 X) were analyzed. In group A, ICAM-1-positive cells decreased from 75.8 (SE +/- 15.12) to 38.8 (SE +/- 7.57) ICAM-3-positive cells decreased from 61.7 (SE +/- 12.72) to 45.2 (SE +/- 9.44) and LFA-1 decreased from 103.9 (SE +/- 17.34) to 66.5 (SE +/- 8.63) after cetirizine treatment (p = 0.02). In group B, a nonsignificant reduction was found after placebo administration in the expression of adhesion molecules except for ELAM-1, which showed a slight variation, from 23.4 (SE +/- 3.56) to 21.5 (SE +/- 3.26). The reduction in the expression of adhesion molecules in psoriasis after cetirizine treatment suggests a possible inhibitory effect of this drug on some cell surface proteins and subsequently on the migration of inflammatory cells in psoriatic skin lesions. Our findings support its antiinflammatory effect in addition to its H1-blocking activity.

PMID: 12854881 [PubMed - indexed for MEDLINE]

1322. Curr Eye Res. 2003 May;26(5):271-6.

The effect of topically applied secretory leukocyte protease inhibitor on the eosinophil response in the late phase of allergic conjunctivitis.

Murata E, Sharmin S, Shiota H, Shiota M, Yano M, Kido H.

Department of Ophthalmology and Visual Neuroscience, School of Medicine, The University of Tokushima, Tokushima 770-8503, Japan.

PURPOSE: This study examined the effects of secretory leukocyte protease inhibitor (SLPI), a protease inhibitor in tears, in allergic conjunctivitis.
METHODS: Conjunctiva of male Hartley guinea pigs sensitized with ovalbumin were treated with SLPI or the vehicle 10 min before antigen challenge or simultaneously. The animals were sacrificed after antigen challenges of 0-24 h duration, and the inhibition of eosinophil conjunctival migration and degranulation by SLPI was analyzed histochemically. The effects of SLPI on mast cell chymase and tryptase were also examined.
RESULTS: Treatment of sensitized guinea pigs with SLPI suppressed the conjunctival recruitment and degranulation of eosinophils after antigen challenge for 6 h, inhibiting the development of allergic conjunctivitis. The effects of SLPI were observed at concentrations > or =0.1 microM, with a peak at 5 microM. SLPI inhibited chymase in a dose-dependent manner, but had no effect on tryptase.
CONCLUSION: The topical SLPI application may be therapeutic in allergic conjunctivitis.

PMID: 12854054 [PubMed - indexed for MEDLINE]

1323. J Immunol. 2003 Jul 15;171(2):1078-84.

A second step of chemotaxis after transendothelial migration: keratinocytes undergoing apoptosis release IFN-gamma-inducible protein 10, monokine induced by IFN-gamma, and IFN-gamma-inducible alpha-chemoattractant for T cell chemotaxis toward epidermis in atopic dermatitis.

Klunker S, Trautmann A, Akdis M, Verhagen J, Schmid-Grendelmeier P, Blaser K, Akdis CA.

Swiss Institute of Allergy and Asthma Research, Davos, Switzerland.

Activation and skin-selective homing of T cells and their effector functions in the skin represent sequential immunological events in the pathogenesis of atopic dermatitis (AD). Apoptosis of keratinocytes, induced mainly by T cells and mediated by IFN-gamma and Fas, is the essential pathogenetic event in eczema formation. Keratinocyte apoptosis appears as activation-induced cell death in AD. By IFN-gamma stimulation, chemokines such as IFN-gamma-inducible protein 10, monokine induced by IFN-gamma, and IFN-gamma-inducible alpha-chemoattractant are strongly up-regulated in keratinocytes. These chemokines attract T cells bearing the specific receptor CXCR3, which is highly expressed on T cells isolated from skin biopsies of AD patients. Accordingly, an increased T cell chemotaxis was observed toward IFN-gamma-treated keratinocytes. Supporting these findings, enhanced IFN-gamma-inducible protein 10, monokine induced by IFN-gamma, and IFN-gamma-inducible alpha-chemoattractant expression was observed in lesional AD skin by immunohistochemical staining. These results indicate a second step of chemotaxis inside the skin after transendothelial migration of the inflammatory cells. Keratinocytes undergoing apoptosis in acute eczematous lesions release chemokines that attract more T cells toward the epidermis, which may further augment the inflammation and keratinocyte apoptosis.

PMID: 12847282 [PubMed - indexed for MEDLINE]

1324. Laryngoscope. 2003 Jul;113(7):1199-205.

The epidemiology of chronic rhinosinusitis in Canadians.

Chen Y, Dales R, Lin M.

Department of Epidemiology and Community Medicine, Faculty of Medicine, University of Ottawa, 451 Smyth Road, Ottawa, Ontario, Canada K1H 8M5. ychen@uottawa.ca

OBJECTIVE: To study the prevalence of chronic rhinosinusitis and its risk factors among Canadians.
STUDY DESIGN: Complex survey design incorporating stratification, multiple stages of selection, and unequal probabilities of selection of respondents.
METHODS: We used the cross-sectional data from 73,364 subjects (34,241 male and 39,123 female subjects) 12 years of age or older who participated in the second cycle of the National Population Health Survey, which was conducted from 1996 to 1997. All these individuals were asked whether they had certain chronic health conditions that had lasted or were expected to last 6 months or longer, including rhinosinusitis.
RESULTS: The prevalence of rhinosinusitis was higher in female (5.7%) than in male (3.4%) subjects. The sex difference was consistent across age groups. The prevalence increased with age and leveled off after the age of 60 years. In female but not in male subjects, the prevalence was slightly higher among those living the eastern region or among native Canadians as compared with those living in the central or western regions or immigrants. Cigarette smoking and low income were associated with a higher prevalence of rhinosinusitis in both sexes. The smoking effect was modified by allergy history in male subjects. Rhinosinusitis was more common among subjects with allergy history, asthma, or chronic obstructive pulmonary disease. The prevalence of rhinosinusitis was similar in subjects with or without reporting regular alcohol drinking and exercise.
CONCLUSION: Previous data indicating an increased susceptibility of women to asthma and chronic obstructive pulmonary disease, together with the similar finding for rhinosinusitis, suggest that women have a general increase in susceptibility to respiratory tract disease.

PMID: 12838019 [PubMed - indexed for MEDLINE]

1325. Pediatrics. 2003 Jul;112(1 Pt 1):49-57.

Morbidity patterns among low-income wheezing infants.

Klinnert MD, Price MR, Liu AH, Robinson JL.

Department of Pediatrics, National Jewish Medical and Research Center, Denver, Colorado 80206, USA. klinnertm@njc.org

OBJECTIVE: This study describes morbidity attributable to wheezing illness in a multi-ethnic sample of low-income infants younger than age 2, and examines biological, environmental, and psychosocial correlates of morbidity indexes.
METHOD: Infants 9 to 24 months old, considered at risk for developing asthma on the basis of having had 3 or more health care contacts with documented wheezing, received comprehensive evaluations as part of an environmental intervention study. Baseline evaluations with the infants, their families, and their home environments focused on biological, environmental, and psychosocial factors that would potentially increase asthma risk for the children. At study entry, prior morbidity attributable to wheezing illness was assessed with caregiver reports of symptom frequency and severity and medication use, caregiver quality of life, and medical record documentation of hospitalizations and emergency department (ED) visits.

RESULTS: Forty-six percent of the infants had 1 or more hospitalizations and 59% had 2 or more ED visits since birth for wheezing illness. Foreign-born Hispanic families had significantly more ED visits for their children's wheezing illness than US-born Hispanic families, whites, or blacks, although they used fewer controller medications and they reported less illness severity. Multivariate analyses showed 3 biological factors, respiratory syncytial virus, elevated child IgE, and cockroach allergen in the home, were independently associated with hospitalizations within this sample. Similar analyses showed that ED visits were not associated with biological variables, but rather with caregivers with single parent status and smokers. Caregiver reports of wheezing illness severity were correlated with ED visits, but not with hospitalizations. Severity ratings were higher for children of mothers with asthma and for those whose caregivers had higher anxiety and stress. The only correlate of caregiver ratings of poor quality of life was high caregiver anxiety.
CONCLUSIONS: Ethnic and immigrant status was an important factor in morbidity attributable to infant wheezing illness. In addition to respiratory infection, both allergic processes and social variables were associated with morbidity as measured by health care utilization. Caregiver reports of illness severity were significantly correlated with psychosocial factors.

PMID: 12837867 [PubMed - indexed for MEDLINE]

1326. J Exp Med. 2003 Jul 7;198(1):19-30. Epub 2003 Jun 30.

Bidirectional interactions between antigen-bearing respiratory tract dendritic cells (DCs) and T cells precede the late phase reaction in experimental asthma: DC activation occurs in the airway mucosa but not in the lung parenchyma.

Huh JC, Strickland DH, Jahnsen FL, Turner DJ, Thomas JA, Napoli S, Tobagus I, Stumbles PA, Sly PD, Holt PG.

Telethon Institute for Child Health Research and Centre for Child Health Research, Faculty of Medicine and Dentistry, The University of Western Australia, Perth, Western, Australia 6008.

The airway mucosal response to allergen in asthma involves influx of activated T helper type 2 cells and eosinophils, transient airflow obstruction, and airways hyperresponsiveness (AHR). The mechanism(s) underlying transient T cell activation during this inflammatory response is unclear. We present evidence that this response is regulated via bidirectional interactions between airway mucosal dendritic cells (AMDC) and T memory cells. After aerosol challenge, resident AMDC acquire antigen and rapidly mature into potent antigen-presenting cells (APCs) after cognate interactions with T memory cells. This process is restricted to dendritic cells (DCs) in the mucosae of the conducting airways, and is not seen in peripheral lung. Within 24 h, antigen-bearing mature DCs disappear from the airway wall, leaving in their wake activated interleukin 2R+ T cells and AHR. Antigen-bearing activated DCs appear in regional lymph nodes at 24 h, suggesting onward migration from the airway. Transient up-regulation of CD86 on AMDC accompanies this process, which can be reproduced by coculture of resting AMDC with T memory cells plus antigen. The APC activity of AMDC can be partially inhibited by anti-CD86, suggesting that CD86 may play an active role in this process and/or is a surrogate for other relevant costimulators. These findings provide a plausible model for local T cell activation at the lesional site in asthma, and for the transient nature of this inflammatory response.

PMCID: PMC2196086
PMID: 12835476 [PubMed - indexed for MEDLINE]

1327. Environ Health Perspect. 2003 Jun;111(8):1114-21.

Clinical and laboratory investigation of allergy to genetically modified foods.

Bernstein JA, Bernstein IL, Bucchini L, Goldman LR, Hamilton RG, Lehrer S, Rubin C, Sampson HA.

Department of Medicine, University of Cincinnati, Cincinnati, Ohio, USA.

Technology has improved the food supply since the first cultivation of crops. Genetic engineering facilitates the transfer of genes among organisms. Generally, only minute amounts of a specific protein need to be expressed to obtain the desired trait. Food allergy affects only individuals with an abnormal immunologic response to food--6% of children and 1.5-2% of adults in the United States. Not all diseases caused by food allergy are mediated by IgE. A number of expert committees have advised the U.S. government and international organizations on risk assessment for allergenicity of food proteins. These committees have created decision trees largely based on assessment of IgE-mediated food allergenicity. Difficulties include the limited availability of allergen-specific IgE antisera from allergic persons as validated source material, the utility of specific IgE assays, limited characterization of food proteins, cross-reactivity between food and other allergens, and modifications of food proteins by processing. StarLink was a corn variety modified to produce a (Italic)Bacillus thuringiensis(/Italic) (Bt) endotoxin, Cry9C. The Centers for Disease Control and Prevention investigated 51 reports of possible adverse reactions to corn that occurred after the announcement that StarLink, allowed for animal feed, was found in the human food supply. Allergic reactions were not confirmed, but tools for postmarket assessment were limited. Workers in agricultural and food preparation facilities have potential inhalation exposure to plant dusts and flours. In 1999, researchers found that migrant health workers can become sensitized to certain Bt spore extracts after exposure to Bt spraying.

PMCID: PMC1241560
PMID: 12826483 [PubMed - indexed for MEDLINE]

1328. Magn Reson Med. 2003 Jul;50(1):201-5.

MR microscopy of magnetically labeled neurospheres transplanted into the Lewis EAE rat brain.

Bulte JW, Ben-Hur T, Miller BR, Mizrachi-Kol R, Einstein O, Reinhartz E, Zywicke HA, Douglas T, Frank JA.

Department of Radiology and Radiological Sciences, Johns Hopkins University School of Medicine, Baltimore, Maryland 21205-2195, USA. jwmbulte@mri.jhu.edu

Stem cell transplantation is being explored as a new paradigm for the treatment of demyelinating diseases. Magnetically labeled multipotential neural precursor cells were transplanted into the ventricles of rats with acute experimental allergic encephalomyelitis (EAE) and high-resolution (microscopic) MR images were obtained ex vivo. Migration patterns of live cells into periventricular white matter structures could be easily visualized, with a good correlation of the corresponding histopathology. The present results confirm that MR cell tracking can be used to guide the development of successful transplantation protocols.

Copyright 2003 Wiley-Liss, Inc.

PMID: 12815696 [PubMed - indexed for MEDLINE]

1329. Otol Neurotol. 2003 May;24(3):353-7.

Detection of Chlamydia pneumoniae in cholesteatoma tissue: any pathogenetic role?

Ronchetti F, Ronchetti R, Guglielmi F, Chiappini I, Contini C, Filipo R, Santino I, Cerruto R, Bernardeschi D, Barbara M.

Departments of Neurology and Otorhinolaryngology, University "La Sapienza" I, Rome, Italy.

BACKGROUND: Acquired cholesteatoma is a complication of chronic otitis media that is usually associated with an intense local inflammatory reaction. Cholesteatoma probably arises from epithelial migration close to an ongoing host inflammatory response attributable to a chronic bacterial infection. Chlamydia pneumoniae is an intracellular microorganism associated with several pathologic conditions originally considered noninflammatory, including asthma, atherosclerosis, and Alzheimer disease. To investigate a possible relationship between C. pneumoniae and the development of cholesteatoma, tissue was studied in three different layers by polymerase chain reaction analysis. The results were compared with those relative to other two common middle-ear pathogens, Mycoplasma pneumoniae and Haemophilus influenzae.
METHODS: Cholesteatoma specimens were collected from 32 patients undergoing middle ear surgery. A series of 5 microm-thick specimens were obtained at three different tissue levels, internal (matrix), intermediate (perimatrix), and external (granulation tissue), and processed by polymerase chain reaction for detection of C. pneumoniae, H. influenzae, and M. pneumoniae. Fragmentation and polymerase chain reaction amplification were carried out using two substantially different techniques.
RESULTS: C. pneumoniae was detected with either polymerase chain reaction techniques in the internal layers in 16 of the 32 cholesteatomas (50%), associated with a positive finding in the intermediate layer in two cases and in the external layer in one case. Four specimens contained H. influenzae, always in the external layer, whereas none contained M. pneumoniae.
CONCLUSIONS: The close relationship between cholesteatoma and C. pneumoniae demonstrated by the findings of this study could suggest a direct cause and effect link between the pathogen action and the clinical manifestations. Otherwise, a facilitated colonization by C. pneumoniae and chronic pathology of the ear could both take origin from a peculiar immunologic background of the host.

PMID: 12806283 [PubMed - indexed for MEDLINE]

1330. Arerugi. 2003 May;52(5):477-80.

[Migration of cells in allergic inflammation].

[Article in Japanese]

Tanaka Y.

The First Department of Internal Medicine, University of Occupational & Environmental Health, School of Medicine. tanaka@med.uoeh-u.ac.jp

PMID: 12799499 [PubMed - indexed for MEDLINE]

1331. Eur Respir J. 2003 Jun;21(6):918-24.

Endothelial cells modulate eosinophil surface markers and mediator release.

Dallaire MJ, Ferland C, Pagé N, Lavigne S, Davoine F, Laviolette M.

Unité de Recherche en Pneumologie, Centre de Recherche de l'Hôpital Laval, Institut Universitaire de Cardiologie et de Pneumologie de l'Université Laval, Quebec, Canada.

Migration from blood to tissue modulates eosinophil function, possibly through interactions with endothelial cells. The effects of contact with and migration through endothelial cells on eosinophil expression of surface markers and release of leukotriene C4 were evaluated. A small proportion (2.6%) of eosinophils spontaneously migrated through endothelial cell monolayers. Activation of endothelial cells by interleukin (IL)-4 or IL-1beta slightly increased this migration (to 12.4%), which became much greater when a chemoattractant was placed in the lower chamber (84.3%). However, the chemotactic effect was downregulated by pretreating endothelial cells with interferon gamma (IFN-gamma; 63.1%). At baseline, 5% of eosinophils expressed CD69; this increased to 30.7% in culture on untreated endothelial cells and to 50.9% on IL-1beta-pretreated endothelial cells. This effect was mediated through intercellular adhesion molecule-1/CD11b interaction. Eosinophil migration through endothelial cells further increased CD69 expression to 63.9% and also increased CD35 expression from 83.3 to 91.3%. Upon stimulation, eosinophils that had migrated through endothelial cells produced more leukotriene C4 than control cells (872.4 and 103.9 pg x mL(-1), respectively). Endothelial cell pretreatment with IL-4 or IL-1beta further increased leukotriene C4 release (1,789.1 and 2,895.1 pg x mL(-1), respectively), whereas pretreatment with IFN-gamma decreased it (293.7 pg x mL(-1)). These data show that in vitro interactions with endothelial cells upregulate eosinophil membrane receptor expression and mediator release and that these effects are differently modulated by T-helper cell type 1 and 2 cytokines. These eosinophil modulations may play an important role in asthma pathogenesis.

PMID: 12797482 [PubMed - indexed for MEDLINE]

1332. Am J Respir Crit Care Med. 2003 Aug 1;168(3):342-7. Epub 2003 Jun 5.

Prenatal cigarette smoke decreases lung cAMP and increases airway hyperresponsiveness.

Singh SP, Barrett EG, Kalra R, Razani-Boroujerdi S, Langley RJ, Kurup V, Tesfaigzi Y, Sopori ML.

Respiratory Immunology and Asthma Program, Lovelace Respiratory Research Institute, Albuquerque, New Mexico, 87108, USA.

Epidemiologic studies suggest that in utero exposure to tobacco smoke, primarily through maternal smoking, increases the risk for asthma in children; however, the mechanism of this phenomenon is not clear. Cyclic adenosine monophosphate relaxes airway smooth muscles in the lung and acts as an antiasthmatic. In this study, we examined the effects of in utero cigarette smoke exposure of Balb/c mice on airway responsiveness, as determined by Penh measurements. Animals exposed prenatally but not postnatally to cigarette smoke exhibited increased airway hyperresponsiveness after a single intratracheal injection of Aspergillus fumigatus extract. The increased airway hyperresponsiveness was not associated with increased leukocyte migration or mucous production in the lung but was causally related to decreased lung cyclic adenosine monophosphate levels, increased phosphodiesterase-4 enzymatic activity, and phosphodiesterase-4D (PDE4D) isoform-specific messenger ribonucleic acid expression in the lung. Exposure of adult mice to cigarette smoke did not significantly alter airway

responsiveness, cyclic adenosine monophosphate levels, or the phosphodiesterase activity. These results suggest that prenatal exposure to cigarette smoke affects lung airway reactivity by modulating the lung cyclic adenosine monophosphate levels through changes in phosphodiesterase-4D activity, and these effects are independent of significant mucous production or leukocyte recruitment into the lung.

PMID: 12791581 [PubMed - indexed for MEDLINE]

1333. J Allergy Clin Immunol. 2003 Jun;111(6):1278-84.

Matrix metalloproteinase inhibitor regulates inflammatory cell migration by reducing ICAM-1 and VCAM-1 expression in a murine model of toluene diisocyanate-induced asthma.

Lee KS, Jin SM, Kim HJ, Lee YC.

Department of Internal Medicine, Research Center for Allergic Immune Diseases, Chonbuk National University Medical School, 634-18 Keumamdong, Chonju 561-712, South Korea.

BACKGROUND: Matrix metalloproteinase-9 (MMP-9) has been reported to play a crucial role in the transmigration of neutrophils, lymphocytes, and eosinophils. Neutrophils, eosinophils, and lymphocytes migrate from the blood to the lungs in response to inflammatory mediators produced in the airways and are subsequently released into the circulation. This traffic is mediated by adhesion molecules. However, little is known about the migration of inflammatory cells through the endothelial and epithelial basement membranes in toluene diisocyanate (TDI)-induced asthma.
OBJECTIVES: An aim of this study was to evaluate the effect of MMP inhibitors on the expression of ICAM-1 and VCAM-1 in the migration of inflammatory cells in a murine model of TDI-induced asthma.
METHODS: We used a murine model to investigate TDI-induced asthma to examine the possible involvement of ICAM-1 and VCAM-1 in the pathogenesis of that disease and the effect of MMP inhibitors on the expression of ICAM-1 and VCAM-1.
RESULTS: In mice, the following typical pathophysiologic features develop in the lungs: increased numbers of inflammatory cells and increased expression of MMP-9, ICAM-1, and VCAM-1 mRNA and protein. Administration of MMP inhibitors reduced the increased numbers of inflammatory cells and the increased expression of ICAM-1 and VCAM-1 mRNA expression and protein. In addition, MMP inhibitors significantly abrogated the increased expression of IL-1beta, IL-4, and TNF-alpha mRNA in lung tissues and levels of IL-1beta, IL-4, and TNF-alpha in bronchoalveolar lavage fluids after TDI inhalation.
CONCLUSIONS: These results suggest that MMP inhibitors regulate inflammatory cell migration by reducing ICAM-1 and VCAM-1 expression and possibly also by suppressing IL-1beta, IL-4, and TNF-alpha expression.

PMID: 12789230 [PubMed - indexed for MEDLINE]

1334. Int Immunopharmacol. 2003 Jun;3(6):845-52.

Inhibitory effects of glucocorticoids on rat eosinophil superoxide generation and chemotaxis.

Sugimoto Y, Ogawa M, Tai N, Kamei C.

Department of Pharmacology, Faculty of Pharmaceutical Sciences, Okayama University, 1-1-1 Tsushima-naka, 700-8530, Okayama, Japan.

Eosinophil infiltration into inflammatory tissues and the subsequent release of inflammatory mediators are the hallmarks of several inflammatory allergic diseases. Although there have been a considerable number of publications on anti-inflammatory effects of glucocorticoids, little is known about whether glucocorticoids affect the activation of eosinophils directly. We studied the effects of three glucocorticoids, mometasone furoate, dexamethasone and beclomethasone dipropionate, on superoxide generation and the chemotaxis of rat eosinophils. Highly purified rat eosinophils were treated for 6 h with mometasone furoate, dexamethasone or beclomethasone dipropionate. Eosinophils were stimulated with phorbol myristate acetate (PMA) for superoxide generation, while for induction of chemotaxis, platelet-activating factor (PAF) or leukotriene B(4) (LTB(4)) was used. None of the glucocorticoids used in the present study caused significant suppressive effects on superoxide generation induced by PMA. On the other hand, both PAF- and LTB(4)-induced migration of rat eosinophils were inhibited in a concentration-dependent manner by glucocorticoids. Mometasone furoate showed a significant effect at concentrations higher than 10(-11) M. Dexamethasone and beclomethasone dipropionate also caused a significant inhibition at concentrations higher than 10(-8) and 10(-7) M, respectively. These results indicated that the anti-inflammatory effects of glucocorticoids were mediated by direct inhibition of eosinophil migration. Furthermore, mometasone furoate was suggested to be more useful than the other drugs in the treatment of allergic diseases responsible for eosinophil chemotaxis.

PMID: 12781701 [PubMed - indexed for MEDLINE]

1335. Am J Respir Cell Mol Biol. 2003 Dec;29(6):702-9. Epub 2003 May 30.

Oxidized low-density lipoprotein activates migration and degranulation of human granulocytes.

Sedgwick JB, Hwang YS, Gerbyshak HA, Kita H, Busse WW.

University of Wisconsin, H6/355 CSC-3244 600 Highland Ave., Madison, WI 53792, USA. jxs@medicine.wisc.edu

Oxidized low-density lipoprotein (oxLDL) has been reported as a major participant in the pathogenesis of atherosclerosis. We hypothesized that oxLDL can also interact with granulocytes during inflammatory airway diseases, such as asthma. To test the chemotactic effect of oxLDL, isolated human peripheral granulocytes were added to the upper chambers of Transwell filters and migration in response to oxLDL was determined. Cu+2-oxidized LDL stimulated neutrophil (23.4 +/- 3.2% for 100 microg/ml oxLDL versus 2.9 +/- 1.1% for buffer, P < 0.05) and eosinophil (19.3 +/- 3.5% versus 0.6 +/- 0.02% for buffer, P < 0.05) chemotaxis in a concentration-dependent manner. The magnitude of chemotaxis was dependent on the degree of LDL oxidation. Granulocyte transmigration across IL-1beta-activated human pulmonary microvascular endothelial cell monolayers was similarly stimulated by oxLDL. OxLDL activated significant degranulation of both neutrophils (100.9 +/- 9.8 versus 49.6 +/- 8.4 ng lactoferrin released/5 x 105 neutrophils for buffer, P < 0.05) and eosinophils (342 +/- 115.4 versus 85.8 +/- 30.4 ng eosinophil-derived neurotoxin/1 x 106 eosinophils for buffer, P < 0.05). Therefore, in vivo influx and oxidation of LDL may be an important mediator for the initiation of bronchial inflammation where granulocytes are recruited to the lung.

PMID: 12777245 [PubMed - indexed for MEDLINE]

1336. Immunobiology. 2003;207(3):187-96.

Adenoviral-mediated delivery of a viral chemokine binding protein blocks CC-chemokine activity in vitro and in vivo.

Bursill CA, Cai S, Channon KM, Greaves DR.

Department of Cardiovascular Medicine, University of Oxford, John Radcliffe Hospital, Oxford OX3 9DU, UK.

Chemokines are important mediators of leukocyte recruitment and activation that play critical roles in the pathology of inflammatory diseases such as atherosclerosis, rheumatoid arthritis and asthma. The vaccinia virus (strain Lister) expresses a 35 kDa soluble protein ('35K') that binds and inactivates a wide range of CC chemokines. We generated a recombinant adenovirus encoding soluble 35K (Ad35K). Ad35K-infected cell culture medium, containing recombinant 35K, potently reduced migration of CCR5-transfected 293 cells by 95% in response to the CC-chemokine RANTES, but had no effect on cells transfected with the CX3CR1 fractalkine receptor. Delivery of Ad35K to mice in vivo via tail vein injection resulted in expression of recombinant 35K in plasma and increased serum RANTES and MIP-1alpha levels when quantified by ELISA. However, chemotaxis of both CCR5-transfected cells and primary macrophages was inhibited by more than 90% by plasma from Ad35K-infected animals compared with control plasma from animals injected with AdGFP. Furthermore, 35K delivered by intra-peritoneal injection more than halved biogel-induced inflammatory cell recruitment in peritoneal exudates compared to AdGFP medium. These studies identify broad-spectrum CC-chemokine blockade using in vivo adenoviral-mediated recombinant 35K expression as a promising strategy to reduce local and systemic inflammation.

PMID: 12777060 [PubMed - indexed for MEDLINE]

1337. Rev Bras Anestesiol. 2003 Jun;53(3):401-18.

[Anesthesia in the afro-american population].

[Article in Portuguese]

Vale NB, Delfino J.

Universidade Federal do Rio Grande do Norte, Brazil.

BACKGROUND AND OBJECTIVES: A significant percentage of the 12 million Afro-Americans may present physiological, pathophysiological and pharmacological changes able to impact the success of anesthesia; Brazilian Afro-American population (40%) is subject to those changes for having the same ethnic and geographic origin. This review aimed at re-evaluating racial differences bias on potential anesthetic drug and adjuvant effect changes during anesthesia.
CONTENTS: The analysis of pathophysiological studies inherent to the historical migration of the African gene as compared to Caucasians shows significant racial differences between Afro-American and African populations, suggesting a close interface between genetics and environment able to affect anesthesia. Unfavorable Afro-American socio-economic conditions, as a result of 400 years of slavery, are still influencing the preservation of cultural and physiological differences beyond the color of the skin: organic system dysfunctions are related to CNS, CVS, respiratory and renal systems. However, different effects of anesthetic drugs and adjuvants, such as decreased local analgesic effect of the anesthetic ointment EMLA, increased propofol hypnotic effect and paracetamol toxicity, less anti-hypertensive effects of renin-decreasing drugs (ACEI, beta2 blockers and AT1), decreased beta2-vasodilator effects and less t-PA fibrinolysis, may affect

pre and postanesthetic approaches, especially in hypertensive, renal, asthma or stroke Afro-American patients.
CONCLUSIONS: Drug response may vary among different populations due to biological (age, gender, disease), genetic, cultural and environmental factors. Race should be taken into account during preanesthetic evaluation to prevent perioperative idiosyncratic reactions and assure the anesthetic-surgical success.

PMID: 19475293 [PubMed]

1338. Mediators Inflamm. 2003 Apr;12(2):71-8.

Dexamethasone prevents granulocyte-macrophage colony-stimulating factor-induced nuclear factor-kappaB activation, inducible nitric oxide synthase expression and nitric oxide production in a skin dendritic cell line.

Vital AL, Gonçalo M, Cruz MT, Figueiredo A, Duarte CB, Lopes MC.

Faculdade de Farmácia, Universidad de Coimbra, Coimbra, Portugal.

AIMS: Nitric oxide (NO) has been increasingly implicated in inflammatory skin diseases, namely in allergic contact dermatitis. In this work, we investigated the effect of dexamethasone on NO production induced by the epidermal cytokine granulocyte-macrophage colony-stimulating factor (GM-CSF) in a mouse fetal skin dendritic cell line.
METHODS: NO production was assessed by the method of Griess. Expression of the inducible isoform of nitric oxide synthase (iNOS) protein was evaluated by western blot analysis and immunofluorescence microscopy. Western blot analysis was also performed to evaluate cytosolic IkappaB-alpha (IkappaB-alpha) protein levels. The electrophoretic mobility shift assay was used to evaluate the activation or inhibition of nuclear factor kappa B (NF-kappaB).
RESULTS: GM-CSF induced iNOS expression and NO production, and activated the transcription factor NF-kappaB. Dexamethasone inhibited, in a dose-dependent manner, NO production induced by GM-CSF. Addition of dexamethasone to the culture, 30 min before GM-CSF stimulation, significantly inhibited the cellular expression of iNOS. Dexamethasone also inhibited GM-CSF-induced NF-kappaB activation by preventing a significant decrease on the IkappaB-alpha protein levels, thus blocking NF-kappaB migration to the nucleus.
CONCLUSIONS: The corticosteroid dexamethasone inhibits GM-CSF-induced NF-kappaB activation, iNOS protein expression and NO production. These results suggest that dexamethasone is a potent inhibitor of intracellular events that are involved on NO synthesis, in skin dendritic cells.

PMCID: PMC1781603
PMID: 12775356 [PubMed - indexed for MEDLINE]

1339. Public Health Rep. 2003 May-Jun;118(3):215-20.

Asthma prevalence among inner-city Asian American schoolchildren.

Lee T, Brugge D, Francis C, Fisher O.

West Suburban Family Practice Residency Program, Oak Park, IL, USA.

OBJECTIVE: Though asthma has been studied in many inner-city populations in the United States, there have been no studies specifically on Asian American immigrants. The authors conducted a cross-sectional survey of the prevalence of asthma among schoolchildren at the Josiah Quincy Elementary School, located in Boston Chinatown. Roughly 62% of the students in the school are Asian American.

METHODS: The authors utilized the Brief Asthma Pediatric Screen (BAPS), a five-question instrument that was validated through the Chicago public schools. The survey was administered to kindergarten through fifth grade students.
RESULTS: Of the 606 respondents (69.9% of the students), 16% had previously diagnosed asthma and 3% had possible undiagnosed asthma. Asthma was more prevalent in boys than in girls (relative risk [RR] 1.75; 95% confidence interval [CI] 1.20, 2.56). In addition, the respondents who lived in Chinatown were less likely to have been diagnosed with asthma (RR 0.59; 95% CI 0.39, 0.90), as were those with Asian surnames (RR 0.65; 95% CI 0.44, 0.97).
CONCLUSION: Although this study was preliminary, our results suggest that asthma rates are substantial among inner-city Asian immigrant children, but possibly lower than for other inner-city children.

PMCID: PMC1497544
PMID: 12766216 [PubMed - indexed for MEDLINE]

1340. Am J Respir Cell Mol Biol. 2003 Jun;28(6):713-21.

Priming of eosinophil migration across lung epithelial cell monolayers and upregulation of CD11b/CD18 are elicited by extracellular Ca2+.

Liu L, Hakansson L, Ridefelt P, Garcia RC, Venge P.

Department of Medical Sciences, Clinical Chemistry, Uppsala University, Uppsala, Sweden. lixliu@ucalgary.ca

In patients with asthma, eosinophils are primed and massively infiltrate lung tissues and migrate across epithelia into airways. Using blocking monoclonal antibodies, we found that eosinophil transmigration across a lung epithelial cell monolayer depended on the functions of alphaMbeta2 integrin CD11b/CD18. To study the role of Ca2+ in eosinophil priming and transepithelial migration, we treated eosinophils with eotaxin or thapsigargin (TG), reagents that increase cytoplasmic free Ca2+ concentrations by receptor- or nonreceptor-mediated mechanisms, respectively. Pretreatment of eosinophils with TG enhanced CD11b/CD18-dependent transmigration across lung epithelium. Within minutes, TG time- and dose-dependently upregulated the expression of CD11b/CD18 but did not upregulate the expression of alphaL (CD11a) or beta1 (CD29) integrin. The upregulation of CD11b/CD18 expression by eotaxin or TG was prevented when Ca2+ entry was blocked. The priming of eosinophil transmigration by TG was also abrogated by the blockade of Ca2+ entry. Our results indicate that induction of Ca2+ entry by the depletion of Ca2+ from intracellular stores upregulates CD11b/CD18 expression on eosinophils and primes eosinophil transmigration across lung epithelium. Both responses are therefore elicited by extracellular Ca2+. We suggest that, as an important priming signal for human eosinophil functional responses, store-operated Ca2+ entry may be one of the underlying mechanisms of eosinophilic inflammation in asthma.

PMID: 12760968 [PubMed - indexed for MEDLINE]

1341. Am J Respir Cell Mol Biol. 2003 Jun;28(6):648-54.

Airway epithelial cells release MIP-3alpha/CCL20 in response to cytokines and ambient particulate matter.

Reibman J, Hsu Y, Chen LC, Bleck B, Gordon T.

Division of Pulmonary and Critical Care Medicine, Department of Medicine, New York University School of Medicine, New York, NY 10016, USA.

reibmj01@gcrc.med.nyu.edu

The initiation and maintenance of airway immune responses in Th2 type allergic diseases such as asthma are dependent on the specific activation of local airway dendritic cells (DCs). The cytokine microenvironment, produced by local cells, influences the recruitment of specific subsets of immature DCs and their subsequent maturation. In the airway, DCs reside in close proximity to airway epithelial cells (AECs). We examined the ability of primary culture human bronchial epithelial cells (HBECs) to synthesize and secrete the recently described CC-chemokine, MIP-3alpha/CCL20. MIP-3alpha/CCL20 is the unique chemokine ligand for CCR6, a receptor with a restricted distribution. MIP-3alpha/CCL20 induces selective migration of DCs because CCR6 is expressed on some immature DCs but not on CD14+ DC precursors or mature DCs. HBECs were stimulated with pro-inflammatory cytokines tumor necrosis factor-alpha and interleukin (IL)-1beta or, because of their critical role in allergic diseases, IL-4 and IL-13. Cells were also exposed to small size-fractions of ambient particulate matter. Each of these stimuli induced MIP-3alpha/CCL20 gene and protein expression. Moreover, these agents upregulated mitogen-activated protein kinase pathways in HBECs. Inhibition of the ERK1/2 pathway or p38 reduced cytokine-induced MIP-3alpha/CCL20 expression. These data suggest a mechanism by which AEC may facilitate recruitment of DC subsets to the airway.

PMID: 12760962 [PubMed - indexed for MEDLINE]

1342. J Infect Dis. 2003 Jun 1;187(11):1809-17.

Rhinovirus induction of the CXC chemokine epithelial-neutrophil activating peptide-78 in bronchial epithelium.

Donninger H, Glashoff R, Haitchi HM, Syce JA, Ghildyal R, van Rensburg E, Bardin PG.

Lung and Allergy Unit and Medical Virology, University of Stellenbosch, Cape Town, South Africa.

Epithelial-neutrophil activating peptide-78 (ENA-78) induces neutrophil migration, an early response to viral infection. Rhinovirus serotype 16 (RV16) was used to infect primary bronchial epithelial cells and a cell line (BEAS-2B). Release of ENA-78 protein was measured by enzyme-linked immunosorbent assay, ENA-78 mRNA production was quantified by reverse-transcription polymerase chain reaction, and ENA-78 promoter activity was assessed by use of a promoter construct. After infection with RV16, ENA-78 protein and mRNA increased significantly, and RV16 induced 3-fold increases in ENA-78 gene transcription. Nasal ENA-78 measured in patients with asthma with and without RV infection was more elevated in patients with RV infection present. Our study demonstrates that ENA-78 is produced in bronchial epithelial cells in response to RV16 infection. With other chemokines, it may be an important initiator of neutrophil airway inflammation during RV common colds and thus may play a role in the development of virus-associated airway pathologies.

PMID: 12751040 [PubMed - indexed for MEDLINE]

1343. Pulm Pharmacol Ther. 2003;16(3):121-30.

Calcitonin gene-related peptide as inflammatory mediator.

Springer J, Geppetti P, Fischer A, Groneberg DA.

Division of Allergy Research, Department of Pediatric Pneumology and Immunology, Charité Campus-Virchow, Humboldt-University Berlin, 13353 Berlin, Germany.

Sensory neuropeptides have been proposed to play a key role in the pathogenesis of a number of respiratory diseases such as asthma, chronic obstructive pulmonary disease or chronic cough. Next to prominent neuropeptides such as tachykinins or vasoactive intestinal polypeptide (VIP), calcitonin gene-related peptide (CGRP) has long been suggested to participate in airway physiology and pathophysiology. CGRP is a 37 amino-acid peptide which is expressed by nerve fibers projecting to the airways and by pulmonary neuroendocrine cells. The most prominent effects of CGRP in the airways are vasodilatation and in a few instances bronchoconstriction. A further pulmonary effect of CGRP is the induction of eosinophil migration and the stimulation of beta-integrin-mediated T cell adhesion to fibronectin at the site of inflammation. By contrast, CGRP inhibits macrophage secretion and the capacity of macrophages to activate T-cells, indicating a potential anti-inflammatory effect. Due to the complex pulmonary effects of CGRP with bronchoconstriction and vasodilatation and diverse immunomodulatory actions, potential anti-asthma drugs based on this peptide have not been established so far. However, targeting the effects of CGRP may be of value for future strategies in nerve modulation.

PMID: 12749828 [PubMed - indexed for MEDLINE]

1344. J Immunol. 2003 May 15;170(10):5295-301.

Peroxisome proliferator-activated receptor gamma inhibits the migration of dendritic cells: consequences for the immune response.

Angeli V, Hammad H, Staels B, Capron M, Lambrecht BN, Trottein F.

Unité 547, Institut National de la Santé et de la Recherche Médicale, Institut Pasteur de Lille, Lille, France.

The migration of dendritic cells (DCs) from the epithelia to the lymphoid organs represents a tightly regulated multistep event involved in the induction of the immune response. In this process fatty acid derivatives positively and negatively regulate DC emigration. In the present study we investigated whether activation of peroxisome proliferator-activated receptors (PPARs), a family of nuclear receptors activated by naturally occurring derivatives of arachidonic acid, could control DC migration from the peripheral sites of Ag capture to the draining lymph nodes (DLNs). First, we show that murine epidermal Langerhans cells (LCs) express PPAR gamma, but not PPAR alpha, mRNA, and protein. Using an experimental murine model of LC migration induced by TNF-alpha, we show that the highly potent PPAR gamma agonist rosiglitazone specifically impairs the departure of LCs from the epidermis. In a model of contact allergen-induced LC migration, PPAR gamma activation not only impedes LC emigration, and their subsequent accumulation as DCs in the DLNs, but also dramatically prevents the contact hypersensitivity responses after challenge. Finally, after intratracheal sensitization with an FITC-conjugated Ag, PPAR gamma activation inhibits the migration of DCs from the airway mucosa to the thoracic LNs and also profoundly reduces the priming of Ag-specific T lymphocytes in the DLNs. Our results suggest a novel regulatory pathway via PPAR gamma for DC migration from epithelia that could contribute to the initiation of immune responses.

PMID: 12734379 [PubMed - indexed for MEDLINE]

1345. J Immunol. 2003 May 15;170(10):4943-52.

Prostaglandin D2 affects the maturation of human monocyte-derived dendritic cells: consequence on the polarization of naive Th cells.

Gosset P, Bureau F, Angeli V, Pichavant M, Faveeuw C, Tonnel AB, Trottein F.

Institut National de la Santé et de la Recherche Médicale, Unité 416 and Institut Fédératif de Recherche 17, Institut Pasteur de Lille, Lille, France.
Philippe.Gosset@pasteur-lille.fr

Among the factors produced at inflammatory sites and those capable of modulating dendritic cell (DC) functions, PGD(2) may be important in the outcome of immune responses. The biological roles for PGD(2) are in part effected through two plasma membrane G protein-coupled receptors: the D prostanoid (DP) receptor and the chemoattractant receptor-homologous molecule expressed on Th2 lymphocytes (CRTH2). In this report, we studied the effects of PGD(2) and of its major physiological metabolite, 15-deoxy-Delta(12,14)-PGJ(2) (15d-PGJ(2)), on the functions of human monocyte-derived DC. First, we show that PGD(2) exerts in vitro chemotactic effects on monocytes via CRTH2 activation while it inhibits the chemokine-driven migration of monocyte-derived DC through DP. We also report that PGD(2) and 15d-PGJ(2) alter the LPS- and allergen-induced DC maturation and enhance the CD80/CD86 ratio on mature DC in a DP- and CRTH2-independent manner. Moreover, PGD(2) and 15d-PGJ(2) strongly reduce the secretion of the Th1 promoting cytokine IL-12 and affect the synthesis of chemokines involved in Th1 cell chemotaxis, particularly CXCL10. Inhibition of cytokine/chemokine secretion implicates at least in part DP, but not CRTH2. The effects exerted by PGD(2) are associated with the phosphorylation of CREB, but do not parallel with the deactivation of the NF-kappa B and mitogen-activated protein kinase pathways. In contrast, 15d-PGJ(2) seems to target other cellular proteins. Finally, in a model of Th CD45RA(+) differentiation induced by allergen- and superantigen-pulsed DC, PGD(2) impacts on the orientation of the immune response by favoring a Th2 response.

PMID: 12734337 [PubMed - indexed for MEDLINE]

1346. Am J Respir Crit Care Med. 2003 Jul 15;168(2):215-21. Epub 2003 Apr 30.

Effect of diesel on chemokines and chemokine receptors involved in helper T cell type 1/type 2 recruitment in patients with asthma.

Sénéchal S, de Nadai P, Ralainirina N, Scherpereel A, Vorng H, Lassalle P, Tonnel AB, Tsicopoulos A, Wallaert B.

U-416, Institut Pasteur de Lille, BP 245, 59 019 Lille Cedex, France.

The objective of this study was to evaluate if diesel exhausts could favor helper T cell type (Th) 2-associated allergic reactions either through an increased production of Th2-associated chemokines and of their associated receptors or through a decrease of Th1-attracting chemokines and chemokine receptors. Diesel but not allergen exposure of peripheral blood mononuclear cells from subjects with allergy induced a release of I-309, whereas both diesel and Der p 1 induced an early but transient release of monokine induced by IFN-gamma and a late release of pulmonary and activation-regulated chemokine. Although both Th1- and Th2-attracting chemokines were induced, the resulting effect was an increased chemotactic activity on Th2 but not Th1 cells. Surprisingly, diesel induced a late increase in the expression of the Th1-associated CXC receptor 3 and CC receptor 5. T cell CXC receptor 3 upregulation was not associated with an increased migration to its ligands. These two antagonistic effects have been previously reported as a scavenger mechanism to clear chemokines. Altogether, these results suggest that diesel, even without allergen, may amplify a type 2

immune response but that it can also increase late Th1-associated chemokine receptor expression, perhaps as a scavenger mechanism to clear pro-Th1 chemokines and promote the Th2 pathway.

PMID: 12724126 [PubMed - indexed for MEDLINE]

1347. Br J Pharmacol. 2003 Apr;138(8):1589-97.

Implication of the bradykinin receptors in antigen-induced pulmonary inflammation in mice.

Eric J, Gabra BH, Sirois P.

Institute of Pharmacology of Sherbrooke, School of Medicine, University of Sherbrooke, Sherbrooke, PQ, Canada J1H 5N4.

1. The involvement of bradykinin (BK) receptors in the allergic inflammation associated with airway hyper-reactivity (AHR) was evaluated by means of the selective bradykinin B(1) receptor (BKB(1)-R) antagonists R-715 (Ac-Lys-[D-betaNal(7), Ile(8)]desArg(9)-BK) and R-954 (Ac-Orn[Oic(2), alpha-MePhe(5), D-betaNal(7), Ile(8)]desArg(9)-BK) or the selective bradykinin B(2) receptor (BKB(2)-R) antagonist HOE-140 (D-Arg(0)-Hyp(3)-Thi(5)-D-Tic(7)-Oic(8)-BK). Cellular migration and AHR were examined 24 h after the second ovalbumin (OA) challenge. 2. R-715 (10-500 microg kg(-1)) and R-954 (1-100 microg kg(-1)) injected intravenously (i.v.), 5 min prior to aerosol OA challenges, decreased by approximately 50% the induced lung eosinophilia in OA-sensitized mice but did not reduce AHR. 3. HOE-140 (1 microg kg(-1)) administered in the same manner, decreased mononuclear cell and eosinophil infiltration in the bronchoalveolar lavage fluid (BALF) of OA-sensitized mice. Moreover, treatment of OA-sensitized mice with HOE-140 (100 microg kg(-1)) completely abolished the AHR to carbachol. 4. The BKB(1)-R agonist desArg(9)-BK (DBK; 10-1000 microg kg(-1)) administered intratrachealy to normal mice had no effect on the basal cell counts recovered in BALF nor on the plasma extravasation, while the BKB(2)-R selective agonist BK (20 microg kg(-1)) stimulated mononuclear cell migration, neutrophilia and plasma extravasation in normal mouse lungs. Such effects were inhibited by HOE-140 (10 microg kg(-1)). 5. Our results suggest that the airway inflammatory response induced by antigen challenge in mice is mediated by stimulation of both BKB(1)-R and BKB(2)-R.

PMCID: PMC1573809
PMID: 12721115 [PubMed - indexed for MEDLINE]

1348. J Leukoc Biol. 2003 May;73(5):657-64.

Ligand density modulates eosinophil signaling and migration.

Holub A, Byrnes J, Anderson S, Dzaidzio L, Hogg N, Huttenlocher A.

Department of Pediatrics, University of Wisconsin, Madison 53706, USA.

Eosinophils are a major component of the inflammatory response in persistent airway inflammation in asthma. The factors that determine the retention of eosinophils in the airway remain poorly understood. Elevated levels of fibronectin have been observed in the airway of patients with asthma, and the levels correlate with eosinophil numbers. To determine if fibronectin density modulates eosinophil function, we investigated the effect of fibronectin and vascular cell adhesion molecule 1 (VCAM-1) density on eosinophil migration and signaling via the p38 and extracellular regulated kinase (ERK)-mitogen-activated

protein kinase (MAPK) signaling pathways. There was a dose-dependent inhibition of eosinophil spreading and migration on increasing concentrations of fibronectin but not VCAM-1. In addition, activation of p38 MAPK was inhibited at high fibronectin but not high VCAM-1 concentrations, and ERK activity was slightly reduced at high VCAM-1 and fibronectin concentrations. Together, the results demonstrate that fibronectin but not VCAM-1 inhibits eosinophil migration and signaling.

PMID: 12714581 [PubMed - indexed for MEDLINE]

1349. J Immunol. 2003 May 1;170(9):4810-7.

CC chemokine ligand 1 promotes recruitment of eosinophils but not Th2 cells during the development of allergic airways disease.

Bishop B, Lloyd CM.

Leukocyte Biology Section, Division of Biomedical Sciences, Faculty of Medicine, Imperial College, London, United Kingdom.

One of the characteristic features of allergic asthma is recruitment of large numbers of inflammatory cells including eosinophils and Th2 lymphocytes to the lung. This influx of inflammatory cells is thought to be a controlled and coordinated process mediated by chemokines and their receptors. It is thought that distinct, differential expression of chemokine receptors allows selective migration of T cell subtypes in response to the chemokines that bind these receptors. Th2 cells preferentially express CCR8 and migrate selectively to its ligand, CC chemokine ligand (CCL)1. We studied the role of the CCR8 ligand, CCL1, in the specific recruitment of Th2 cells and eosinophils to the lung in a murine model of allergic airway disease. We have demonstrated for the first time that CCL1 is up-regulated in the lung following allergen challenge. Moreover, a neutralizing Ab to CCL1 reduced eosinophil migration to the lung, but had no effect on recruitment of Th2 cells following allergen challenge. In addition, there was no change in airway hyperresponsiveness or levels of Th2 cytokines. In a Th2 cell transfer system of pulmonary inflammation, anti-CCL1 also failed to affect recruitment of Th2 cells to the lung following allergen challenge. Significantly, intratracheal instillation of rCCL1 increased recruitment of eosinophils but not Th2 cells to the lung in allergen-sensitized and -challenged mice. In summary, our results indicate that CCL1 is important for the pulmonary recruitment of eosinophils, rather than allergen-specific Th2 cells, following allergen challenge.

PMID: 12707363 [PubMed - indexed for MEDLINE]

1350. J Immunol. 2003 May 1;170(9):4752-8.

Delta 12-prostaglandin J2, a plasma metabolite of prostaglandin D2, causes eosinophil mobilization from the bone marrow and primes eosinophils for chemotaxis.

Heinemann A, Schuligoi R, Sabroe I, Hartnell A, Peskar BA.

Department of Experimental and Clinical Pharmacology, Karl Franzens University, Graz, Austria. akos.heinemann@uni-graz.at

PGD(2), a major mast cell mediator, is a potent eosinophil chemoattractant and is thought to be involved in eosinophil recruitment to sites of allergic inflammation. In plasma, PGD(2) is rapidly transformed into its major metabolite

delta(12)-PGJ(2), the effect of which on eosinophil migration has not yet been characterized. In this study we found that delta(12)-PGJ(2) was a highly effective chemoattractant and inducer of respiratory burst in human eosinophils, with the same efficacy as PGD(2), PGJ(2), or 15-deoxy-delta(12,14)-PGJ(2). Moreover, pretreatment of eosinophils with delta(12)-PGJ(2) markedly enhanced the chemotactic response to eotaxin, and in this respect delta(12)-PGJ(2) was more effective than PGD(2). delta(12)-PGJ(2)-induced facilitation of eosinophil migration toward eotaxin was not altered by specific inhibitors of intracellular signaling pathways relevant to the chemotactic response, phosphatidylinositol 3-kinase (LY-294002), mitogen-activated protein kinase/extracellular signal-regulated kinase kinase (U-0126), or p38 mitogen-activated protein kinase (SB-202190). Desensitization studies using calcium flux suggested that delta(12)-PGJ(2) signaled through the same receptor, CRTH2, as PGD(2). Finally, delta(12)-PGJ(2) was able to mobilize mature eosinophils from the bone marrow of the guinea pig isolated perfused hind limb. Given that delta(12)-PGJ(2) is present in the systemic circulation at relevant levels, a role for this PGD(2) metabolite in eosinophil release from the bone marrow and in driving eosinophil recruitment to sites of inflammation appears conceivable.

PMID: 12707356 [PubMed - indexed for MEDLINE]

1351. J Immunol. 2003 May 1;170(9):4601-11.

Influenza A virus infection inhibits the efficient recruitment of Th2 cells into the airways and the development of airway eosinophilia.

Wohlleben G, Müller J, Tatsch U, Hambrecht C, Herz U, Renz H, Schmitt E, Moll H, Erb KJ.

Center for Infectious Diseases and. Department of Pathology, University of Würzburg, Würzburg, Germany.

Most infections with respiratory viruses induce Th1 responses characterized by the generation of Th1 and CD8(+) T cells secreting IFN-gamma, which in turn have been shown to inhibit the development of Th2 cells. Therefore, it could be expected that respiratory viral infections mediate protection against asthma. However, the opposite seems to be true, because viral infections are often associated with the exacerbation of asthma. For this reason, we investigated what effect an influenza A (flu) virus infection has on the development of asthma. We found that flu infection 1, 3, 6, or 9 wk before allergen airway challenge resulted in a strong suppression of allergen-induced airway eosinophilia. This effect was associated with strongly reduced numbers of Th2 cells in the airways and was not observed in IFN-gamma- or IL-12 p35-deficient mice. Mice infected with flu virus and immunized with OVA showed decreased IL-5 and increased IFN-gamma, eotaxin/CC chemokine ligand (CCL)11, RANTES/CCL5, and monocyte chemoattractant protein-1/CCL2 levels in the bronchoalveolar lavage fluid, and increased airway hyperreactivity compared with OVA-immunized mice. These results suggest that the flu virus infection reduced airway eosinophilia by inducing Th1 responses, which lead to the inefficient recruitment of Th2 cells into the airways. However, OVA-specific IgE and IgG1 serum levels, blood eosinophilia, and goblet cell metaplasia in the lung were not reduced by the flu infection. Flu virus infection also directly induced AHR and goblet cell metaplasia. Taken together, our results show that flu virus infections can induce, exacerbate, and suppress features of asthmatic disease in mice.

PMID: 12707338 [PubMed - indexed for MEDLINE]

1352. J Allergy Clin Immunol. 2003 Apr;111(4):869-74.

Atopy patch test reactions show a rapid influx of inflammatory dendritic epidermal cells in patients with extrinsic atopic dermatitis and patients with intrinsic atopic dermatitis.

Kerschenlohr K, Decard S, Przybilla B, Wollenberg A.

Department of Dermatology and Allergy, Ludwig-Maximilians-University, Munich, Germany.

BACKGROUND: Normal human skin harbors a single epidermal dendritic cell (DC) population, the CD1a(+++)CD11b(-) Langerhans cells. In many chronic inflammatory skin diseases, the epidermal DC pool bears a second population, the CD1a(+)CD11b(+++) inflammatory dendritic epidermal cells (IDECs). Immunophenotypic, ultrastructural, and functional aspects of IDECs have been investigated in chronic untreated skin lesions of intrinsic and extrinsic atopic dermatitis (AD), contact dermatitis (CD), and psoriasis, but little is known about freshly induced early skin lesions.
OBJECTIVE: We sought to characterize enumerative and immunophenotypic changes in the epidermal DC pool during the development of eczematous skin lesions.
METHODS: The atopy patch test with aeroallergens and food-protein allergens and a conventional patch test with standard-series haptens were performed as models for early skin lesions of extrinsic and intrinsic AD and CD, respectively. After 72 hours, epidermal cell suspensions were prepared, analyzed in a standardized flow cytometric technique, and compared with the results obtained from chronic lesions.
RESULTS: The migration of IDECs into the epidermis occurs within 72 hours and is thus an early event. It continues in chronic AD, but not in chronic CD, lesions. The specific upregulation of FcepsilonRI, especially on IDECs, occurs later during formation of extrinsic but not intrinsic AD lesions. LCs were negative for Cd36 in patch test lesions, whereas in chronic skin lesions, LCs expressed Cd36.
CONCLUSION: The DC alteration during skin lesion formation can be subdivided into early and late events, with the influx of IDECs as an early event and the alteration of the DC phenotype as a late event.

PMID: 12704371 [PubMed - indexed for MEDLINE]

1353. Infect Immun. 2003 May;71(5):2607-14.

Changes in pulmonary function and parasite burden in rats infected with Strongyloides venezuelensis concomitant with induction of allergic airway inflammation.

Negrão-Corrêa D, Silveira MR, Borges CM, Souza DG, Teixeira MM.

Departamento de Parasitologia, Instituto de Ciências Biológicas, Universidade Federal de Minas Gerais, Belo Horizonte, Minas Gerais, Brazil.
denegra@icb.ufmg.br

The prevalence of allergic diseases such as asthma has increased markedly over the past few decades. To evaluate the possible mutual influence of helminth infection and allergy, the combined effects of experimental allergic airway inflammation and infection with Strongyloides venezuelensis on various parasitological and inflammatory indices were evaluated in the rat. A challenge of immunized rats with aerosolized ovalbumin (OVA) resulted in eosinophilic inflammation that peaked 48 h after the challenge and was accompanied by airway hyperresponsiveness (AHR) to an intravenous acetylcholine challenge. S. venezuelensis infection concomitant with an OVA challenge of immunized rats resulted in prolonged pulmonary inflammation with increased eosinophil

infiltration in bronchoalveolar lavage fluid but not in the lung tissue. These rats also showed a significant parasite burden reduction, especially during parasite migration through the lungs. However, the fecundity rates of worms that reached the intestine were similar in allergic and nonallergic animals. Despite airway inflammation, the increased responsiveness of the airways in the experimental asthma model was suppressed during parasite migration through the lungs (2 days). In contrast, parasite-induced AHR was unchanged 5 days after infection in immunized and challenged rats. In conclusion, infection with S. venezuelensis interfered with the onset of AHR following an antigen challenge of immunized rats. The ability of parasites to switch off functional airway responses is therapeutically relevant because we may learn from parasites how to modulate lung function and, hence, the AHR characteristic of asthmatic patients.

PMCID: PMC153262
PMID: 12704135 [PubMed - indexed for MEDLINE]

1354. J Immunol. 2003 Apr 15;170(8):4325-34.

L-selectin or ICAM-1 deficiency reduces an immediate-type hypersensitivity response by preventing mast cell recruitment in repeated elicitation of contact hypersensitivity.

Shimada Y, Hasegawa M, Kaburagi Y, Hamaguchi Y, Komura K, Saito E, Takehara K, Steeber DA, Tedder TF, Sato S.

Department of Dermatology, Kanazawa University Graduate School of Medical Science, Kanazawa, Japan.

Repeated Ag exposure results in a shift in the time course of contact hypersensitivity (CH) from a typical delayed-type to an immediate-type response followed by a late phase reaction. Chronic CH responses are clinically relevant to human skin allergic diseases, such as atopic dermatitis, that are usually caused by repeated stimulation with environmental Ags. Chronic inflammatory responses result in part from infiltrating leukocytes. To determine the role of leukocyte adhesion molecules in chronic inflammation, chronic CH responses were assessed in mice lacking L-selectin, ICAM-1, or both adhesion molecules. Following repeated hapten sensitization for 24 days at 2-day intervals, wild-type littermates developed an immediate-type response at 30 min after elicitation, followed by a late phase reaction. By contrast, loss of ICAM-1, L-selectin, or both, eliminated the immediate-type response and inhibited the late phase reaction. Similar results were obtained when wild-type littermates repeatedly exposed to hapten for 22 days were treated with mAbs to L-selectin and/or ICAM-1 before the elicitation on day 24. The lack of an immediate-type response on day 24 paralleled a lack of mast cell accumulation after 30 min of elicitation and decreased serum IgE production. Repeated Ag exposure in wild-type littermates resulted in increased levels of serum L-selectin, a finding also observed in atopic dermatitis patients. The current study demonstrates that L-selectin and ICAM-1 cooperatively regulate the induction of the immediate-type response by mediating mast cell accumulation into inflammatory sites and suggests that L-selectin and ICAM-1 are potential therapeutic targets for regulating human allergic reactions.

PMID: 12682269 [PubMed - indexed for MEDLINE]

1355. FEBS Lett. 2003 Apr 10;540(1-3):7-14.

The unfolding tale of PECAM-1.

Jackson DE.

Kronheimer Building, Austin Research Institute, A&RMC, Studley Road, Heidelberg, Vic 3084, Australia. d.jackson@ari.unimelb.edu.au

Platelet endothelial cell adhesion molecule-1 (PECAM-1/CD31) is a member of the immunoglobulin (Ig) superfamily that has distinctive features of an immunoreceptor based upon its genomic structure and the presence of intrinsic immunoreceptor tyrosine inhibitory motifs (ITIMs) in its ligand binding polypeptide. This has lead to its subclassification into the Ig-ITIM superfamily. Its amino-terminal Ig-like domain of PECAM-1 is necessary for its homophilic binding, which plays an important role in cell-cell interactions. Its intracellular ITIMs serve as scaffolds for recruitment of signalling molecules including protein-tyrosine phosphatases to mediate its inhibitory co-receptor activity. Increasing evidence has implicated PECAM-1 in a plethora of biological phenomena, including modulation of integrin-mediated cell adhesion, transendothelial migration, angiogenesis, apoptosis, cell migration, negative regulation of immune cell signalling, autoimmunity, macrophage phagocytosis, IgE-mediated anaphylaxis and thrombosis. In this review, we discuss some of the new developments attributed to this molecule and its unique roles in biology.

Copyright 2003 Published by Elsevier Science B.V. on behalf of the Federation of European Biochemical Societies

PMID: 12681475 [PubMed - indexed for MEDLINE]

1356. Clin Exp Allergy. 2003 Apr;33(4):449-54.

Onset of allergy and asthma symptoms in extra-European immigrants to Milan, Italy: possible role of environmental factors.

Tedeschi A, Barcella M, Bo GA, Miadonna A.

First Division of Internal Medicine, IRCCS Ospedale Maggiore Policlinico, Milan, Italy.

BACKGROUND: Allergy and asthma are typical disorders of the affluent societies. Migrants from developing to industrialized countries seem to be at increased risk of allergy and asthma development.
OBJECTIVE: To evaluate time of onset, spectrum of sensitization and clinical features in a population of extra-European immigrants to Milan, Italy, complaining of allergy and asthma symptoms.
METHODS: Data regarding 243 extra-European immigrants checked at an allergy clinic from 1994 to 2000 were collected retrospectively. The demographic data were compared with those of the extra-European immigrants living in Milan at the end of 1999.
RESULTS: The patients were complaining of asthma (63.7%), rhinoconjunctivitis (56.7%), rhinitis alone (21%) or urticaria (3%). One hundred and eighty-seven out of 222 patients (84.3%) declared they were healthy before migrating and allergy/asthma symptoms started to appear after their arrival in Italy, namely after an average period of 4 years and 7 months. The proportion of male patients was lower than the proportion of men in the extra-European immigrant population (48% vs. 55%), suggesting that in adult immigrants allergy and asthma are more common in women than in men. Furthermore, there was an over-representation of Central-South Americans attending the clinic, which seemed to be due to a genetic predisposition to allergy/asthma development. When data were analysed for single countries, a trend towards an increased risk of allergy and asthma was found in immigrants from all Central-South American countries. A skin test positivity for at least one inhalant allergen was found in 196 out of 232 patients (81%), and

the spectrum of allergic sensitization was similar to that of the Italian population living in the North of Italy.
CONCLUSION: Most extra-European immigrants declared that they were healthy at home and that allergy and asthma symptoms had appeared after immigration to Milan; lifestyle and environmental factors in a western industrialized city seem indeed to facilitate allergy/asthma onset in immigrants from developing countries. Allergy/asthma risk seems to be different in different ethnic groups.

PMID: 12680859 [PubMed - indexed for MEDLINE]

1357. Eur J Immunol. 2003 Apr;33(4):861-9.

Critical role for OX40 ligand in the development of pathogenic Th2 cells in a murine model of asthma.

Hoshino A, Tanaka Y, Akiba H, Asakura Y, Mita Y, Sakurai T, Takaoka A, Nakaike S, Ishii N, Sugamura K, Yagita H, Okumura K.

Medicinal Research Laboratories, Taisho Pharmaceutical Co., Ltd., Saitama, Japan. a.hoshino@po.rd.taisho.co.jp

Bronchial asthma is characterized by massive infiltration of eosinophils and airway hyperreactivity (AHR), which are caused by overproduction of Th2 cytokines (IL-4, IL-5, and IL-13) by allergen-specific T cells. We recently demonstrated a critical contribution of OX40 ligand (OX40L) to the development of Th2-mediated experimental leishmaniasis. In this study, we have examined the role of OX40L in the development of Th2-mediated pulmonary inflammation by utilizing OX40L-deficient mice and a neutralizing anti-OX40L mAb in a murine model of asthma. Sensitization and airway challenge with ovalbumin in wild-type BALB/c mice induced a typical allergic asthma characterized by AHR, accumulation of eosinophils, increased mucus production, and high levels of Th2 cytokines in the lung. All these asthmatic responses were not induced in OX40L-deficient BALB/c mice. Administration of neutralizing anti-OX40L mAb in wild-type BALB/c mice during the sensitization period also abolished the induction of asthmatic responses. In contrast, administration of anti-OX40L mAb during the challenge period did not inhibit the asthmatic responses. These results indicate a critical role for OX40L in the induction phase, which leads to the development of pathogenic Th2 cells, but not in the effector phase, which includes migration and activation of pathogenic Th2 cells in the lung.

PMID: 12672051 [PubMed - indexed for MEDLINE]

1358. Adv Exp Med Biol. 2002;507:211-6.

Inhibition of allergen-induced eosinophil migration by lipoxin (LX)A4 and aspirin-triggered 15-epi-LXA4.

Bandeira-Melo C, Diaz BL, Cordeiro RS, Jose PJ, Serhan CN, Martins MA, Bozza PT.

Department of Physiology and Pharmacodynamics, Oswaldo Cruz Institute, FIOCRUZ, Av. Brasil 4365, Rio de Janeiro-RJ, Brazil 21045-900.

PMID: 12664587 [PubMed - indexed for MEDLINE]

1359. Am J Respir Cell Mol Biol. 2003 Oct;29(4):439-48. Epub 2003 Mar 27.

Acute allergic responses induce a prompt luminal entry of airway tissue

eosinophils.

Erjefalt JS, Korsgren M, Malm-Erjefalt M, Conroy DM, Williams TJ, Persson CG.

Assoc. Prof. Department of Physiological Sciences, BMC F10, Lund University Hospital, 221 84, Lund, Sweden. jonas.erjefalt@mphy.lu.se

Traditionally, traffic and activation of eosinophils in asthmatic airways are thought to take place during the late-phase allergic reaction. The present study tests the hypothesis that when eosinophils are present in the tissue before allergen exposure, as in chronically inflamed asthmatic airways, acute anaphylactic reactions initiate an eosinophil response. Using a guinea-pig allergic model, where eosinophilia is present at baseline conditions, the traffic of resident eosinophils was examined in vivo immediately after allergen challenge. By 2 min after challenge, eosinophils had moved up to apical epithelial positions. Within 10 min, a marked migration of eosinophils into the airway lumen was demonstrated. Along with the allergen-induced egression of eosinophils, acute luminal entry of plasma proteins and eotaxin occurred. Eosinophil egression was effectively inhibited by the antiexudative drug formoterol, whereas the proexudative drug bradykinin could in naive animals evoke a prompt luminal entry of eosinophils. In conclusion, the present study demonstrates that acute allergic reactions initiate a prompt transepithelial migration of resident eosinophils. Our data further suggest that this response in part is initiated by the plasma exudation response, which may alter the transepithelial gradient of eosinophil chemoattractants including eotaxin. We propose that prompt eosinophil response is a significant component of the acute phase of allergic reactions when occurring in airways where these cells are already present in the mucosa.

PMID: 12663331 [PubMed - indexed for MEDLINE]

1360. Glia. 2003 Apr 15;42(2):139-48.

Role of the alpha-chemokine stromal cell-derived factor (SDF-1) in the developing and mature central nervous system.

Lazarini F, Tham TN, Casanova P, Arenzana-Seisdedos F, Dubois-Dalcq M.

Unité de Neurovirologie et Régénération du Système Nerveux, Institut Pasteur, Paris, France. lazarini@pasteur.fr

alpha-chemokines, which control the activation and directed migration of leukocytes, participate in the inflammatory processes in host defense response. One of the alpha-chemokines, CXCL12 or stromal cell-derived factor 1 (SDF-1), not only regulates cell growth and migration of hematopoietic stem cells but may also play a central role in brain development as we discuss here. SDF-1 indeed activates the CXCR4 receptor expressed in a variety of neural cells, and this signaling results in diverse biological effects. It enhances migration and proliferation of cerebellar granule cells, chemoattracts microglia, and stimulates cytokine production and glutamate release by astrocytes. Moreover, it elicits postsynaptic currents in Purkinje cells, triggers migration of cortical neuron progenitors, and produces pain by directly exciting nociceptive neurons. By modulating cell signaling and survival during neuroinflammation, SDF-1 may also play a role in the pathogenesis of brain tumors, experimental allergic encephalitis, and the nervous system dysfunction associated with acquired immunodeficiency syndrome.

Copyright 2003 Wiley-Liss, Inc.

PMID: 12655598 [PubMed - indexed for MEDLINE]

1361. Electrophoresis. 2003 Jan;24(1-2):276-85.

Activation of platelet-derived growth factor pathway in human asthmatic pulmonary-derived mesenchymal cells.

Malmström J, Tufvesson E, Löfdahl CG, Hansson L, Marko-Varga G, Westergren-Thorsson G.

Department of Cell & Molecular Biology, BMCC13, Lund University, Sweden.

Cell cultures of mesenchymal type were obtained from biopsies taken after bronchoscopy from patients with asthma. It was possible to achieve outgrowth of fibroblast-like cells from these lung biopsies, which stained for alpha-smooth actin indicating that they were of myofibroblast type. Morphologically, two types of myofibroblasts could be observed: one intermediate form with more stretched cell shape and lamellipodia protrusions, and one more differentiated compact form of myofibroblast. The intermediate form was the most dominant type in these patients, indicating an active ongoing remodelling process. Further studies showed that platelet-derived growth factor (PDGF) might be the factor that stimulates the formation of the intermediate type of myofibroblasts, since it enhance migration of normal human lung fibroblasts 4-fold compared to control through an induced formation of stress fibers and lamellipodia protrusions. Additionally, intracellular signalling pathways involved in migration, such as RhoA and MAPkinase were stimulated 1.5-fold and 3.5-fold, respectively. By using two-dimensional (2-D) gel electrophoresis and protein identification by peptide mass finger printing matrix assisted laser desporption/ionization - time of flight - mass spectrometry (MALDI-TOF-MS) it was possible to confirm that PDGF affected the synthesis of proteins involved in the remodelling process, such as collagen VI and post-translational forms thereof. PDGF also stimulated the production of FK506 binding protein of 65 kDa, a protein involved in smooth muscle differentiation, and proteins involved in the rearrangement of the cytoskeleton connected to migration such as the actin related protein ARP3, the T-complex protein and the heat shock protein 60. We demonstrate that PDGF has a potential pathological role in asthma and formation of subepithelial fibrosis by inducing changes in the proteome.

PMID: 12652599 [PubMed - indexed for MEDLINE]

1362. J Pharmacol Exp Ther. 2003 Apr;305(1):347-52.

An orally bioavailable small molecule antagonist of CRTH2, ramatroban (BAY u3405), inhibits prostaglandin D2-induced eosinophil migration in vitro.

Sugimoto H, Shichijo M, Iino T, Manabe Y, Watanabe A, Shimazaki M, Gantner F, Bacon KB.

Respiratory Diseases Research, and Medicinal Chemistry, Bayer Yakuhin, Ltd., Kyoto, Japan.

Ramatroban (Baynas, BAY u3405), a thromboxane A(2) (TxA(2)) antagonist marketed for allergic rhinitis, has been shown to partially attenuate prostaglandin (PG)D(2)-induced bronchial hyperresponsiveness in humans, as well as reduce antigen-induced early- and late-phase inflammatory responses in mice, guinea pigs, and rats. PGD(2) is known to induce eosinophilia following intranasal administration, and to induce eosinophil activation in vitro. In addition to the TxA(2) receptor, PGD(2) is known as a ligand for the PGD(2) receptor, and the

newly identified G-protein-coupled chemoattractant receptor-homologous molecule expressed on Th2 cells (CRTH2). To fully characterize PGD(2)-mediated inflammatory responses relevant to eosinophil activation, further analysis of the mechanism of action of ramatroban has now been performed. PGD(2)-stimulated human eosinophil migration was shown to be mediated exclusively through activation of CRTH2, and surprisingly, these effects were completely inhibited by ramatroban. This is also the first report detailing an orally bioavailable small molecule CRTH2 antagonist. Our findings suggest that clinical efficacy of ramatroban may be in part mediated through its action on this Th2-, eosinophil-, and basophil-specific chemoattractant receptor.

PMID: 12649388 [PubMed - indexed for MEDLINE]

1363. J Clin Immunol. 2003 Jan;23(1):34-45.

Production of TARC and MDC by naive T cells in asthmatic patients.

Hirata H, Arima M, Cheng G, Honda K, Fukushima F, Yoshida N, Eda F, Fukuda T.

Department of Pulmonary Medicine and Clinical Immunology, Dokkyo University School of Medicine, Mibu-machi, Shimotsuga-gun, Tochigi, 321-0293, Japan.

The helper (Th)2 cell-attracting chemokines thymus and activation-regulated chemokine (TARC) and macrophage-derived chemokine (MDC) are ligands for the chemokine receptor CCR4. A number of cellular sources of TARC and MDC have been identified, including not only macrophages, dendritic cells, and natural killer cells, but also bronchial epithelial cells. Recent studies report that TARC and MDC may serve as pivotal chemokines for the development of Th2-dominated experimental allergen-induced asthma. This study was designed to assess TARC and MDC production by CD4+ T cells, including naive T cells and memory/effector T cells, purified from peripheral blood mononuclear cells in patients with asthma. Asthmatic subjects included in this study had mild asthmatic symptoms, positive skin test responses to house dust mite allergen, and elevated level of Dermatophagoides farinae immunoglobulin E in the sera. CD4+ T cells--CD45RA+ CD4+ T cells--as naive T cells and CD45RO+ CD4+ T cells--as memory/effector T cells--were purified by negative selection from peripheral blood mononuclear cells obtained from asthmatic patients (n = 6) and healthy controls (n = 6). These cells and established Th1/Th2 cell lines were then cultured in the presence of both anti-CD3 and -CD28 antibodies. After 48 hr of incubation, concentrations of TARC, MDC, interleukin (IL)-4, IL-5, and interferon-gamma in the supernatants were measured by enzyme-linked immunoadsorbent assay. Reverse transcriptase-polymerase chain reaction was performed to analyze mRNA expression of TARC and MDC. Our results clearly showed that TARC and MDC were produced by activated CD45RA+ CD4+ T cells rather than by activated CD45RO+ CD4+ T cells, and the levels of these chemokines in the asthmatic patients were higher than those in the healthy controls. Furthermore, these chemokines production by Th2 cell lines were greater than those by Th1 cell lines, but the level were smaller than those by naive T cells. Our studies suggest that TARC and MDC are produced by naive T cells rather than by memory/effector T cells, including Th2 cells, in asthmatic patients, and these chemokines were produced at modest levels in any T-cell populations from healthy controls. Taken together, naive T cells in asthma have a peculiar function to produce TRAC and MDC, which contribute to local migration of Th2 cells into lung and lymphoid tissues, along with a function as precursor for memory/effector T cell. This novel function of naive T cells may be implicated in the development of asthma.

PMID: 12645858 [PubMed - indexed for MEDLINE]

1364. J Leukoc Biol. 2003 Mar;73(3):356-62.

The role of CCL22 (MDC) for the recruitment of eosinophils during allergic pleurisy in mice.

Pinho V, Oliveira SH, Souza DG, Vasconcelos D, Alessandri AL, Lukacs NW, Teixeira MM.

Immunopharmacology, Departamento de Bioquímica e Imunologia, ICB, Universidade Federal de Minas Gerais, Belo Horizonte, Brazil.

Eosinophils are important inflammatory cells in allergic diseases. In the present study, we have investigated the effects of CCL22 on the recruitment of eosinophils in vivo and in vitro. CCL22 induced a dose- and time-dependent recruitment of eosinophils into the pleural cavity of mice, and this was dependent on the release of platelet-activating factor (PAF) and subsequent generation of CCL11. However, in an allergic pleurisy model, an anti-CCL22 polyclonal antibody given during sensitization or before challenge had no significant effect on eosinophil recruitment. CCL22 did not induce eosinophil chemotaxis in vitro but was able to induce eosinophil degranulation in vitro and in vivo. In conclusion, we show that although exogenously added CCL22 may induce eosinophil migration in vivo via release of PAF and CCL11 (eotaxin), endogenous production of CCL22 does not drive eosinophil migration during allergic inflammation. However, CCL22 may be an important activator of eosinophils once these cells have migrated into tissue.

PMID: 12629149 [PubMed - indexed for MEDLINE]

1365. J Immunol. 2003 Mar 15;170(6):3306-14.

IL-4 down-regulates anaphylatoxin receptors in monocytes and dendritic cells and impairs anaphylatoxin-induced migration in vivo.

Soruri A, Kiafard Z, Dettmer C, Riggert J, Köhl J, Zwirner J.

Department of Immunology, Georg August University Gottingen, Gottingen, Germany.

Anaphylatoxins mobilize leukocytes to the sites of inflammation. In the present study we investigated the impact of GM-CSF, IL-4, and IFN-gamma on anaphylatoxin receptor expression in monocytes and dendritic cells (DC). IL-4 was identified as the strongest down-regulator of the receptors for C5a and C3a in monocytes and monocyte-derived DC (MoDC). To study the impact of IL-4 on anaphylatoxin-induced chemotaxis, an in vivo migration model was established. For this purpose, human monocytes and MoDC were injected i.v. into SCID mice that at the same time received anaphylatoxins into the peritoneal cavity. A peritoneal influx of human monocytes could be demonstrated by 4 h after injections of C5a and C3a. In line with receptor down-regulation, IL-4 treatment inhibited in vivo mobilization of human monocytes and MoDC in response to C5a and C3a. In addition to its effects on human cells, IL-4 reduced C5a receptors in murine bone marrow-derived DC and impaired recruitment of labeled bone marrow-derived DC in syngeneic BALB/c mice to i.p. injected C5a. Overall, these data suggest that inhibition of a rapid anaphylatoxin-induced mobilization of monocytes and DC to inflamed tissues represents an important anti-inflammatory activity of the Th2 cytokine IL-4.

PMID: 12626590 [PubMed - indexed for MEDLINE]

1366. Am J Respir Crit Care Med. 2003 Jun 1;167(11):1504-8. Epub 2003 Mar 5.

Neural expression and increased lavage fluid levels of secretoneurin in seasonal allergic rhinitis.

Korsgren M, Erjefält JS, Hinterholzl J, Fischer-Colbrie R, Emanuelsson CA, Andersson M, Persson CG, Mackay-Sim A, Sundler F, Greiff L.

Department of Clinical Pharmacology, University Hospital, SE-221 85 Lund, Sweden. magnus.korsgren@klinfarm.lu.se

Secretoneurin is a neuropeptide potentially involved in migration of eosinophils, monocytes, and dendritic cells. Whether secretoneurin is present in the human airway mucosa and whether it is released at ongoing allergic airway inflammation is currently unknown. In patients with allergic rhinitis, we have explored the occurrence of secretoneurin in nasal mucosal biopsies and lavage fluids before and during natural allergen exposure. Immunohistochemical analysis revealed an abundance of nerves displaying secretoneurin immunoreactivity, which were distributed predominantly around blood vessels and submucosal glands. A majority of nerve fibers containing vesicular acetylcholine transporter, tyrosine hydroxylase, calcitonin gene-related peptide, and vasoactive intestinal peptide were also secretoneurin-immunoreactive, indicating a localization of secretoneurin in cholinergic, adrenergic, and sensory nerves. Lavage fluid levels of secretoneurin were increased at allergen exposure ($p < 0.01$-0.05). Levels of secretoneurin did not correlate with eosinophil cationic protein ($rho = 0.1$, $p = 0.7$). We conclude that secretoneurin has a widespread occurrence in nasal mucosal nerves of patients with seasonal allergic rhinitis and that increased nasal lavage fluid levels of secretoneurin may characterize ongoing allergen exposure. These data favor a role of secretoneurin in the local traffic of immune cells in human airway mucosa.

PMID: 12626352 [PubMed - indexed for MEDLINE]

1367. Int J Cardiovasc Intervent. 2003;5(1):13-6.

Biodegradable stents as a platform to drug loading.

Tsuji T, Tamai H, Igaki K, Kyo E, Kosuga K, Hata T, Nakamura T, Fujita S, Takeda S, Motohara S, Uehata H.

Department of Cardiology, Shiga Medical Center for Adults, Shiga, Japan. tamai@cct.gr.jp

Despite technical and mechanical improvement in coronary stents the incidence of restenosis caused by in-stent neointimal hyperplasia remains high. Oral administration of numerous pharmacological agents has failed to reduce restenosis after coronary stenting in humans, possibly owing to insufficient local drug concentration. Therefore, drug-eluting stents were developed as a vehicle for local drug administration. The authors developed a new drug-eluting polymer stent that is made of poly-l-lactic acid polymer mixed with tranilast, an anti-allergic drug that inhibits the migration and proliferation of vascular smooth muscle cells induced by platelet-derived growth factor and transforming growth factor->1. Polymer stents might be superior to polymer-coated metallic stents as local drug delivery stents in terms of biodegradation and the amount of loaded drug. Drug-mixed polymer stents can be loaded with a larger amount of drug than can drug-coated metallic stents because the polymer stent struts can contain the drug. Clinical application is required to assess the safety and efficacy of drug-eluting polymer stents against stent restenosis.

PMID: 12623560 [PubMed - indexed for MEDLINE]

1368. Nat Med. 2003 Apr;9(4):431-8. Epub 2003 Mar 3.

F(ab)'2-mediated neutralization of C3a and C5a anaphylatoxins: a novel effector function of immunoglobulins.

Basta M, Van Goor F, Luccioli S, Billings EM, Vortmeyer AO, Baranyi L, Szebeni J, Alving CR, Carroll MC, Berkower I, Stojilkovic SS, Metcalfe DD.

Neuronal Excitability Section, National Institute for Neurological Disorders and Stroke, National Institutes of Health, Bethesda, Maryland, USA. mb29r@nih.gov

High-dose intravenous immunoglobulin (IVIG) prevents immune damage by scavenging complement fragments C3b and C4b. We tested the hypothesis that exogenous immunoglobulin molecules also bind anaphylatoxins C3a and C5a, thereby neutralizing their pro-inflammatory effects. Single-cell calcium measurements in HMC-1 human mast cells showed that a rise in intracellular calcium caused by C3a and C5a was inhibited in a concentration-dependent manner by IVIG, F(ab)2-IVIG and irrelevant human monoclonal antibody. C3a- and C5a-induced thromboxane (TXB2) generation and histamine release from HMC-1 cells and whole-blood basophils were also suppressed by exogenous immunoglobulins. In a mouse model of asthma, immunoglobulin treatment reduced cellular migration to the lung. Lethal C5a-mediated circulatory collapse in pigs was prevented by pretreatment with F(ab)2-IVIG. Molecular modeling, surface plasmon resonance (SPR) and western blot analyses suggested a physical association between anaphylatoxins and the constant region of F(ab)2. This binding could interfere with the role of C3a and C5a in inflammation.

PMID: 12612546 [PubMed - indexed for MEDLINE]

1369. Biochem J. 2003 Jun 1;372(Pt 2):453-63.

Direct binding of a fragment of the Wiskott-Aldrich syndrome protein to the C-terminal end of the anaphylatoxin C5a receptor.

Tardif M, Brouchon L, Rabiet MJ, Boulay F.

Laboratoire de Biochimie et Biophysique des Systèmes Intégrés, Commissariat à l'Energie Atomique (CEA)/Centre National de la Recherche Scientifique (CNRS)/Université Joseph Fourier, CEA-Grenoble, France. tardif@dsvsud.cea.fr

Migration of myeloid cells towards a source of chemoattractant, such as the C5a anaphylatoxin, is triggered by the activation of a G-protein-coupled receptor. In the present study, we have used a yeast two-hybrid approach to find unknown partners of the C5a receptor (C5aR). Using the cytosolic C-terminal region of C5aR as bait to screen a human leucocyte cDNA library, we identified the Wiskott-Aldrich syndrome protein (WASP) as a potential partner of C5aR. WASP is known to have an essential function in regulating actin dynamics at the cell leading edge. The interaction was detected with both the fragment of WASP containing amino acids 1-321 (WASP.321) and WASP with its actin-nucleation-promoting domain [verprolin-like, central and acidic (VCA) domain] deleted. The interaction between C5aR and the WASP.321 was supported further by an in vitro binding assay between a radiolabelled WASP.321 fragment and a receptor C-terminus glutathione S-transferase (GST) fusion protein, as well as by GST pull-down, co-immunoprecipitation and immunofluorescence experiments. In the yeast two-hybrid assay, full-length WASP showed no ability to interact with the C-terminal domain of C5aR. This is most probably due to an auto-inhibited conformation imposed by the VCA domain. In HEK-293T cells co-transfected with full-length WASP and C5aR, only a small amount of WASP was

co-precipitated with the receptor. However, in the presence of the active form of the GTPase Cdc42 (Cdc42V12), which is thought to switch WASP to an active 'open conformation', the amount of WASP associated with the receptor was markedly increased. We hypothesize that a transient interaction between C5aR and WASP occurs following the stimulation of C5aR and Cdc42 activation. This might be one mechanism by which WASP is targeted to the plasma membrane and by which actin assembly is spatially controlled in cells moving in a gradient of C5a.

PMCID: PMC1223397
PMID: 12600272 [PubMed - indexed for MEDLINE]

1370. Bull Soc Pathol Exot. 2002 Nov;95(4):229-37.

[Visceral lesions in mammals and birds exposed to agents of human cercarial dermatitis].

[Article in French]

Bayssade-Dufour Ch, Vuong PN, René M, Martin-Loehr C, Martins C.

Muséum national d'histoire naturelle, biologie parasitaire, 61, rue Buffon, 75005 Paris, France. trematod@mnhn.fr

Over the past few years, the cercarial dermatitis has become a new problem of public health, obviously linked to the prolonged stay of migrant birds on our territory. This is a skin affection characterized by pruriginous and papulous eruptions caused by penetration of avian bilharzian larvae under the skin. These larvae are emitted by molluscs, mostly limneids. In aquatic birds, especially in migrating Anatidae, these larvae reach the visceral vessels, become adults in a few weeks, lay eggs, then degenerate. Corresponding miracidia contaminate new limneids. Since 1993, the total number of annual cases of cercarial dermatitis has increased from only ten to thousands in France and the affection rages in pools where limneids, migrating water birds and swimmers gather together. Fever, respiratory and/or digestive allergic symptoms appear in some cases. This clinical pattern has encouraged to undertake research on the future of these bilharzian larvae in mammals organism. A preliminary investigation on a rodent model showed that, once the skin barrier had been crossed, the schistosomulae migrated into the lungs of the host; there they survived a week and induced lesions. The goal of this study is to carry on the research, over a longer period, after exposure to cercariae, simultaneously in mammals and birds, with two species of bilharziae present in France. The selected models are the gerbil Meriones unguiculatus for mammals, and the ducks Anas platyrhynchos and Cairina moschata, for birds. 5 M. unguiculatus and 2 A. platyrhynchos were exposed to cercariae emitted by Radix auricularia; 2 gerbils and 5 A. platyryhnchos to larvae of R. peregra, 3 C. moschata to larvae emitted by two species of molluscs: 70-230 from R. auricularia and 330-585 from R. peregra. 5 gerbils died between 2 and 5 weeks after exposure, 2 gerbils sacrificed early, served as control animals for skin manifestations. Eight ducks were sacrificed between 2 and 4 weeks after; the 2 last ones, exposed several times, were sacrificed respectively 7 and 13 weeks after the first exposure. Visceral and skin samples were submitted to histological study. The control gerbils developed skin dermatitis. In ducks, R. auricularia was the vector of Trichobilharzia franki, whose selective dwelling site was the mesentery; R. peregra was the vector of an indeterminate species found in the lungs and nose. This species is called Bilharzia sp. in this study. The ducks, exposed to two kinds of larvae, displayed worms in these two main locations. In gerbils, T. franki induced lesions in the mesenteric veins and the peritoneum. Bilharzia sp. gave rise to lesions in lung arteries, pleura and liver veins. Vascular changes encompassed endothelitis and lymphocytic vasculitis, while serosa displayed mesothelial hyperplasia. The types of lesions observed in

gerbils were noticed in ducks, and, according to the species of bilharzia, in the homologous viscera. Additional foreign body granulomas centred on worm's debris or their eggs, and vascular thromboses were present, too. In addition, ducks displayed lesions involving several other viscera including the intestine, the kidneys and the peripheral nerves. These changes were multiple and diffuse in C. moschata exposed to two species of bilharziae. They were observed mainly in mesenteric and intestinal vessels, pulmonary arteries and hepatic veins. In gerbils, the lesions persisted 2 to 5 weeks after exposure, but worms were not identified in the neighbouring tissues near the damaged vessels. In ducks, lesions were important between 2 and 7 weeks after exposure; they co-existed with live or dead worms, sometimes paired, with or without eggs. The hepatic lesions regressed 13 weeks, after exposure. In mammals and birds, young worms could migrate into the same visceral vessels, and stimulating formation of persistent lesions. In individuals exposed to the same cercariae, development of similar lesions would be probable.

PMID: 12596366 [PubMed - indexed for MEDLINE]

1371. Curr Opin Allergy Clin Immunol. 2003 Feb;3(1):1-6.

Nasal polyposis: an update: editorial review.

Pawankar R.

PURPOSE OF REVIEW: Nasal polyposis is a chronic inflammatory disease of the upper airway characterized histologically by the infiltration of inflammatory cells like eosinophils or neutrophils. Several hypotheses have been put forward regarding the underlying mechanisms including chronic infection, aspirin intolerance, alteration in aerodynamics with trapping of pollutants, epithelial disruptions, epithelial cell defects/gene deletions (CFTR gene), inhalant or food allergies. The present review is an update on the pathomechanisms of nasal polyposis.
RECENT FINDINGS: In the majority of nasal polyps, eosinophils comprise more than 60% of the cell population. Besides eosinophils, mast cells and activated T cells are also increased. An increased production of cytokines/chemokines like granulocyte/macrophage colony-stimulating factor, IL-5, RANTES and eotaxin contribute to eosinophil migration and survival. Increased levels of IL-8 can induce neutrophil infiltration. Increased expression of vascular endothelial growth factor and its upregulation by transforming growth factor-beta can contribute to the edema and increased angiogenesis in nasal polyps. Again, transforming growth factor-beta can modulate fibroblast function and thus contribute to eosinophil infiltration and stromal fibrosis. Other mediators like albumin, histamine and immunoglobulins IgE and IgG are also increased in nasal polyps. In addition, the local production of IgE in nasal polyps can contribute to the increased recurrence of nasal polyps via the IgE-mast cell-FcepsilonRI cascade. Finally, mast cell/T cell-epithelial cell/fibroblast interactions can contribute to the persistent eosinophilic inflammation seen in polyps.
SUMMARY: Thus although nasal polyposis is a multifactorial disease with several different etiological factors, chronic persistent inflammation is undoubtedly a major factor irrespective of the etiology.

PMID: 12582307 [PubMed - indexed for MEDLINE]

1372. Clin Exp Allergy. 2003 Feb;33(2):241-8.

Tumour necrosis factor-alpha-induced expression of intercellular adhesion molecule-1 on human eosinophilic leukaemia EoL-1 cells is mediated by the activation of nuclear factor-kappaB pathway.

Ip WK, Wong CK, Lam CW.

Department of Chemical Pathology, The Chinese University of Hong Kong, Prince of Wales Hospital, Shatin, N.T., Hong Kong.

BACKGROUND: Intercellular adhesion molecule-1 (ICAM-1) has been shown to mediate the adhesion and migration of eosinophils to the site of allergic inflammation. However, molecular mechanisms regulating the expression of ICAM-1 in eosinophils are still being elucidated. We investigated the effect of tumour necrosis factor-alpha (TNF-alpha) on ICAM-1 expression of eosinophils.
METHODS: The surface expression of ICAM-1 on a human eosinophilic leukaemic cell line, EoL-1, was assessed by immunocytochemical staining. The phosphorylation of inhibitor kappa B-alpha (IkappaB-alpha) and p38 mitogen-activated protein kinase (MAPK) was detected by Western blot. Nuclear factor kappa-B (NF-kappaB) pathway-related genes were evaluated by the cDNA expression array system, whereas the activity of NF-kappaB was measured by electrophoretic mobility shift assay (EMSA).
RESULTS: TNF-alpha was found to induce the cell surface expression of ICAM-1. A specific proteasome inhibitor N-cbz-Leu-Leu-leucinal (MG-132), but not a p38 MAPK inhibitor (SB 203580), was found to suppress the TNF-alpha-induced expression of ICAM-1 on EoL-1 cells. The gene expressions of ICAM-1, NF-kappaB and IkappaBalpha were up-regulated after the stimulation with TNF-alpha. Further, TNF-alpha was shown to induce IkappaB-alpha phosphorylation and degradation, thereby indicating the activation of NF-kappaB. In EMSA, there was a shifted NF-kappaB band on TNF-alpha-treated cells with or without SB 203580, but no shifted band was observed on MG-132-treated cells.
CONCLUSION: In vitro studies of EoL-1 cells, an eosinophilic leukaemic cell line, confirmed that NF-kappaB plays an important role in the expression of ICAM-1 and recruitment of eosinophils in allergic inflammation.

PMID: 12580918 [PubMed - indexed for MEDLINE]

1373. J Immunol. 2003 Feb 15;170(4):2138-46.

Absence of CCR8 does not impair the response to ovalbumin-induced allergic airway disease.

Goya I, Villares R, Zaballos A, Gutiérrez J, Kremer L, Gonzalo JA, Varona R, Carramolino L, Serrano A, Pallarés P, Criado LM, Kolbeck R, Torres M, Coyle AJ, Gutiérrez-Ramos JC, Martínez-A C, Márquez G.

Departamento de Inmunología y Oncología, Centro Nacional de Biotecnología/Consejo Superior de Investigaciones Cientificas, Universidad Autónoma de Madrid, Cantoblanco, 28040-Madrid, Spain.

Interaction of chemokines with their specific receptors results in tight control of leukocyte migration and positioning. CCR8 is a chemokine receptor expressed mainly in CD4(+) single-positive thymocytes and Th2 cells. We generated CCR8-deficient mice (CCR8(-/-)) to study the in vivo role of this receptor, and describe in this study the CCR8(-/-) mouse response in OVA-induced allergic airway disease using several models, including an adoptive transfer model and receptor-blocking experiments. All CCR8(-/-) mice developed a pathological response similar to that of wild-type animals with respect to bronchoalveolar lavage cell composition, peripheral blood and bone marrow eosinophilia, lung infiltrates, and Th2 cytokine levels in lung and serum. The results contrast with a recent report using one of the OVA-induced asthma models studied here. Similar immune responses were also observed in CCR8(-/-) and wild-type animals in a different model of ragweed allergen-induced peritoneal eosinophilic inflammation,

with an equivalent number of eosinophils and analogous increased levels of Th2 cytokines in peritoneum and peripheral blood. Our results show that allergic diseases course without critical CCR8 participation, and suggest that further work is needed to unravel the in vivo role of CCR8 in Th2-mediated pathologies.

PMID: 12574386 [PubMed - indexed for MEDLINE]

1374. Environ Health Perspect. 2003 Feb;111(2):A92-9.

Give me shelter: the global housing crisis.

Brown VJ.

In both developed and developing countries around the world, the health of significant numbers of people is adversely affected by a lack of adequate housing. Large-scale migrations into already crowded developing nation cities compound existing health problems associated with poor indoor air quality, contaminated drinking water, and limited sanitation infrastructure. In the developed world, lead exposure, indoor air quality, and asthma are among the most serious and costly housing-related health risks.

PMCID: PMC1241368
PMID: 12573924 [PubMed - indexed for MEDLINE]

1375. Blood. 2003 Jun 15;101(12):4725-32. Epub 2003 Jan 30.

Genetic evidence for convergence of c-Kit- and alpha4 integrin-mediated signals on class IA PI-3kinase and the Rac pathway in regulating integrin-directed migration in mast cells.

Tan BL, Yazicioglu MN, Ingram D, McCarthy J, Borneo J, Williams DA, Kapur R.

Section of Neonatal-Perinatal Medicine, Department of Pediatrics, Indiana University School of Medicine, Indianapolis 46202, USA.

Mast cells play a critical role in host defense against a number of pathogens. Increased mast cell infiltration has been described in allergic asthma, in rheumatoid arthritis, and during helminthes infection. Despite the importance of mast cells in allergic disease and defense against infection, little is known about the mechanisms by which mast cells migrate to various tissues under steady state conditions or during infection or inflammation. Here, we show that activation of c-Kit by its ligand, stem cell factor (SCF), cooperates with alpha4 integrin in inducing directed migration of mast cells on fibronectin. A reduction in migration and activation of a small G protein, Rac, was observed in mast cells derived from class IA phosphoinositide-3 kinase (PI-3kinase)-deficient mice in response to SCF stimulation and in mast cells expressing the dominant-negative Rac (RacN17), as well as in mast cells deficient in the hematopoietic-specific small G protein, Rac2. In addition, a PI-3kinase inhibitor inhibited alpha4- as well as SCF-induced migration in a dose-dependent fashion. In contrast, a mitogen-activated protein kinase (MAPK) inhibitor had little effect. Consistent with the pharmacologic results, abrogating the binding of the p85alpha subunit of class IA PI-3kinase to c-Kit also resulted in inhibition of SCF-induced migration on fibronectin. These genetic and biochemical data demonstrate that both c-Kit and alpha4 integrin signaling are linked to class IA PI-3kinase and Rac pathways and regulate integrin-directed (haptotactic) migration in mast cells.

PMID: 12560232 [PubMed - indexed for MEDLINE]

1376. Eur J Immunol. 2002 Nov;32(11):3171-80.

Targeting CLA/E-selectin interactions prevents CCR4-mediated recruitment of human Th2 memory cells to human skin in vivo.

Biedermann T, Schwärzler C, Lametschwandtner G, Thoma G, Carballido-Perrig N, Kund J, de Vries JE, Rot A, Carballido JM.

Novartis Research Institute, Vienna, Austria.

Naive Th cells, bearing receptors for cutaneous antigens, become activated in skin-draining lymph nodes and express cutaneous lymphocyte antigen (CLA), which confers to these cells the capacity to migrate into the skin to exert their normal effector functions. In the case of atopic dermatitis (AD), allergen-specific Th2 cells generate exacerbated responses and induce skin inflammation. In such a situation, interfering with the specific mechanism of skin homing would provide a therapeutic benefit. Here we report that CLA+ Th2 memory cells, derived from skin lesions of AD patients, selectively migrate to human skin grafts transplanted onto SCID mice in response to CCR4 but not CCR3, CCR8 or CXCR3 ligands. Skin homing of human CCR4+ Th2 memory cells was Pertussis toxin sensitive and restricted to the CLA+ subset. Furthermore, treatment of these mice with anti-E-selectin monoclonal antibody was sufficient to prevent CCL22-mediated Th2 cell migration to human skin, which both, validates the model and highlights the importance of CLA/E-selectin interactions in the homing process of Th2 cells to the skin. Using this mechanistic model we demonstrate that skin homing of human Th2 memory cells can be efficiently suppressed using a low molecular weight E-selectin antagonist, which is of clinical relevance for the treatment of inflammatory skin diseases, including AD.

PMID: 12555662 [PubMed - indexed for MEDLINE]

1377. Exp Lung Res. 2003 Mar;29(2):59-77.

Differential regulation of aminopeptidase N (CD13) by transendothelial migration and cytokines on human eosinophils.

Braun RK, Foerster M, Workalemahu G, Haefner D, Kroegel C, Walker C.

Department of Pneumology, Allergy, and Immunology, Medical Clinic IV, Friedrich Schiller University, Jena, Germany.

Aminopeptidase N (CD13) is a cell surface metalloprotease involved in growth regulation, tumor invasion, and down-regulation of regulatory peptides. CD13 expression on eosinophils in bronchoalveolar lavage (BAL) of asthmatics 10 minutes and 18 hours after segmental allergen provocation was significantly increased (+225% to +294%) compared to blood eosinophils. In vitro CD13 expression could be induced on blood eosinophils by transendothelial migration of the cells across interlenkin (IL) 1beta-activated human umbilical cord vein endothelial cells (HUVECs) as well as by the exposure to the cytokines IL-3, IL-5, and granulocyte-macrophage colony-stimulating factor (GM-CSF). The cytokines GM-CSF and IL-5 were significantly less effective in inducing CD13 compared to IL-3. The IL-3-induced expression of CD13 was decreased in the presence of the protein-synthesis inhibitor cycloheximide (-8.8%). Moreover, blocking of CD13 by the protease inhibitors actinonin and bestatin significantly enhanced migration (+40.0% to +80.0%) of eosinophils across HUVEC monolayers. In summary, the data suggest that CD13 is regulated both by the process of transmigration and by the cytokine IL-3. Further, CD13 itself seems to be involved in the process of eosinophil transmigration. aminopeptidase Nendothelial

cellseosinophilsinterleukin-3interleukin-5transendothelial migration

PMID: 12554354 [PubMed - indexed for MEDLINE]

1378. Comp Biochem Physiol A Mol Integr Physiol. 2003 Feb;134(2):377-86.

A complement component C3a-like peptide stimulates chemotaxis by hemocytes from an invertebrate chordate-the tunicate, Pyura stolonifera.

Raftos DA, Robbins J, Newton RA, Nair SV.

Department of Biological Sciences, Macquarie University, NSW 2109, North Ryde, Australia. draftos@rna.bio.mp.edu.au

Recent evidence suggests that the complement system evolved as a critical host defence mechanism among invertebrates, long before the origin among vertebrates of adaptive immune responses mediated by somatically re-arranging antibodies. The current study supports that contention by identifying a complement component C3a-like peptide in the tunicate, Pyura stolonifera. Activation of P. stolonifera serum with common inflammatory elicitors (lipopolysaccharide and zymosan) resulted in the proteolytic generation of an 8.5 kDa peptide, and concomitantly conferred chemoattractant activity on the serum. The 8.5 kDa peptide shares substantial amino acid sequence homology with a previously characterised tunicate complement component C3-like protein (72% amino acid identity in an 18 amino acid overlap). It is also recognised by an anti-C3 antiserum that is known to cross react with tunicate C3 homologues. Hemocyte migration assays performed with the 8.5 kDa peptide that had been partially purified by gel filtration confirmed that the molecule acts as a powerful chemotactic agent. This suggests that the proteolytic activation of tunicate C3-like molecules can initiate inflammatory responses involving cellular recruitment by liberating a pro-inflammatory peptide akin to the vertebrate anaphylatoxin, C3a.

PMID: 12547267 [PubMed - indexed for MEDLINE]

1379. Biochem Soc Trans. 2003 Feb;31(Pt 1):275-80.

Phosphoinositide 3-kinase gamma: a key modulator in inflammation and allergy.

Wymann MP, Björklöf K, Calvez R, Finan P, Thomast M, Trifilieff A, Barbier M, Altruda F, Hirsch E, Laffargue M.

Department of Medicine, University of Fribourg, Rue du Musée 5, CH-1700 Fribourg, Switzerland. Matthias.Wymann@UniFR.ch

Chronic inflammation and allergy involve the activation of tissue-resident cells and, later on, the invasion of effector cells. We have previously shown that the loss of phosphoinositide 3-kinase (PI3K) gamma impairs chemokine-dependent migration of neutrophils and macrophages both in vitro and in vivo. On the other hand, PI3K gamma is not required either during phagocytic processes or in the activation of bactericidal activities like granule secretion and particle-mediated respiratory burst in neutrophils. Tissue mast cells are key regulators in allergy and inflammation and release histamine upon clustering of their IgE receptors. We have demonstrated that murine mast cell responses are exacerbated in vitro and in vivo by autocrine signals, and require functional PI3K gamma. Adenosine, acting through the A(3) adenosine receptor, as well as other agonists of G(alpha i)-coupled receptors, transiently increased PtdIns(3,4,5) P (3) exclusively via PI3K gamma. PI3K gamma-derived PtdIns(3,4,5) P (3) was instrumental for initiation of a sustained influx of external Ca(2+)

and degranulation. Mice that lacked PI3K gamma did not form oedema when challenged by passive systemic anaphylaxis. PI3K gamma thus relays inflammatory signals through various GPCRs, and is thus central to mast cell function. Taken together, this suggests that pharmaceutical targeting of PI3K gamma might alleviate inflammation at both early and late stages of the allergic response.

PMID: 12546701 [PubMed - indexed for MEDLINE]

1380. Lin Chuang Er Bi Yan Hou Ke Za Zhi. 2001 May;15(5):197-8.

[Expression of TNF alpha and VCAM-1 in nasal polyps and relation with eosinophil infiltration].

[Article in Chinese]

Wang C, Dong Z, Guan G, Yang Z.

Department of Otolaryngology-Head and Neck Surgery, Third Affiliated Hospital, Bethune Faculty of Medical Sciences, Jilin University, Changchun 130031.

OBJECTIVE: To study the expression of TNF alpha and VCAM-1 in nasal polyps tissue and its significance.
METHOD: Paraffin sections of nasal polyps and chronic rhinitis samples were studied with immunohistochemical technique.
RESULT: Expression of TNF alpha and VCAM-1 were stranger in nasal polyps tissues than in controls. There was positive relationship between the expression of TNF alpha and that of VCAM-1(r = 0.833), and expression of VCAM-1 coincide with eosinophil infiltration(r = 0.746).
CONCLUSION: It is suggested that TNF alpha may up-regulate the expression of VCAM-1 in vessel endothelium, and prompt adhesion and migration of eosinophils, TNF alpha and VCAM-1 may play important role in the pathogenesis of nasal polyps.

PMID: 12541759 [PubMed - indexed for MEDLINE]

1381. J Immunol Methods. 2003 Feb;273(1-2):125-35.

The SCID-hu Skin mouse as a model to investigate selective chemokine mediated homing of human T-lymphocytes to the skin in vivo.

Carballido JM, Biedermann T, Schwärzler C, de Vries JE.

Novartis Research Institute, Brunner Strasse 59, Vienna A-1235, Austria.

Here we report the establishment of an in vivo mouse model that allows monitoring of human T cell migration into human skin. This model is based on the use of severe combined immunodeficiency (SCID) mice transplanted with human skin (SCID-hu Skin mice). Adoptively transferred human T helper (Th)2 cells obtained from atopic dermatitis skin lesions or peripheral blood T cells selectively migrate to the human skin grafts of these SCID mice in response to defined chemokines locally injected in the human skin grafts. Homing of human T cells into the human skin on SCID-hu Skin mice is a specific process since it only occurs in response to chemokine ligands that are specific for the chemokine receptors expressed on the migrating T cells. This mechanistic model allows analysis of the relevant steps involved in human T-lymphocyte migration into inflamed skin. In addition, it is successfully used for preclinical testing of drug candidates that are highly selective for human target molecules associated with the different steps of T cell migration in an environment that resembles the physiologic or pathologic conditions occurring in man.

Copyright 2002 Elsevier Science B.V.

PMID: 12535803 [PubMed - indexed for MEDLINE]

1382. J Immunol Methods. 2003 Feb;273(1-2):3-13.

Chemokine receptors in inflammation: an overview.

D'Ambrosio D, Panina-Bordignon P, Sinigaglia F.

BioXell, Via Olgettina 58, Milan 20132, Italy. daniele.dambrosio@bioxell.com

Chemokine receptors play a key role in directing the migration of inflammatory cells into various injured or infected organs. However, migration of inflammatory cells into tissues can in itself be a cause and amplifier of tissue damage and disease, particularly in chronic autoimmune or allergic disorders. On this basis, much effort is currently devoted at the identification of molecular signals regulating the recruitment of inflammatory cells into tissues and at developing novel strategies to inhibit discrete pathways in this process. Great progress has recently been made in identification of a number of chemokine receptors involved in the process of leukocyte migration. The challenge is now to elucidate the specific contribution and involvement of the different receptors in distinct inflammatory processes and diseases and to prove that interference with any of these pathways may lead to development of novel therapeutics.

Copyright 2002 Elsevier Science B.V.

PMID: 12535793 [PubMed - indexed for MEDLINE]

1383. J Allergy Clin Immunol. 2003 Jan;111(1):113-6.

Myosin light chain kinase mediates eosinophil chemotaxis in a mitogen-activated protein kinase-dependent manner.

Adachi T, Stafford S, Kayaba H, Chihara J, Alam R.

Department of Clinical and Laboratory Medicine, Akita University School of Medicine, Akita, Japan.

BACKGROUND: Eosinophil migration in the tissue is one characteristic feature of allergic diseases. The CC chemokine eotaxin plays a pivotal role in local accumulation of eosinophils. Myosin light chain kinase (MLCK) is known to regulate cytoskeletal rearrangement and cell motility by means of phosphorylation of myosin light chain (MLC).
OBJECTIVE: We have previously shown that mitogen-activated protein (MAP) kinases are important for eosinophil migration. In the present study we hypothesized that MLCK is downstream of MAP kinases, thereby linking the MAP kinase pathway to the activation of cytoskeletal components required for eosinophil chemotaxis.
METHODS: Blood eosinophils were purified by using Percoll and anti-CD16 antibody-coated magnetic beads. We investigated the phosphorylation of MLCK and MLC by using the phosphorous 32-orthophosphates-labeled eosinophils. The kinase activity of MLCK was determined by measuring the phosphotransferase activity for the MLCK-specific peptide substrate. The chemotaxis assay was performed in a 48-well Boyden microchamber.
RESULTS: The phosphotransferase activity of MLCK for a substrate peptide was enhanced in eotaxin-stimulated eosinophils. We also found that eotaxin induced phosphorylation of MLCK in vivo in phosphorous 32-orthophosphate-labeled

eosinophils. PD98059 (MAP/extracellular signal-regulated kinase inhibitor) or SB202190 (p38 MAP kinase inhibitor) abrogated the eotaxin-induced phosphorylation of MLCK. The phosphorylation of MLC was upregulated by eotaxin. Eosinophil chemotaxis was inhibited by means of pretreatment of the MLCK inhibitor ML-7. CONCLUSION: These results suggest that eotaxin regulates MLCK through both extracellular signal-regulated kinase 1/2 and p38 MAP kinase. MLCK activation is a critical step in the cytoskeletal rearrangements leading to eosinophil migration.

PMID: 12532105 [PubMed - indexed for MEDLINE]

1384. Immunology. 2003 Jan;108(1):98-108.

gammadelta T cells contribute to the systemic immunoglobulin E response and local B-cell reactivity in allergic eosinophilic airway inflammation.

Svensson L, Lilliehöök B, Larsson R, Bucht A.

Division of NBC Defence, Swedish Defence Research Agency, Umeå, Sweden.

Allergic airway inflammation induced in mice is T-cell dependent and recruitment of eosinophils to airspaces requires both alphabeta and gammadelta T cells. From previous studies it is evident that alphabeta T cells are essential for the allergic T helper type 2 (Th2)-like response, while the mechanistic contribution of gammadelta T cells is still unclear. In this study, we have investigated the role of gammadelta T cells in allergic airway eosinophilia induced by ovalbumin hypersensitivity. By comparing the responsiveness to sensitizing allergen of wild-type mice with that of T-cell receptor gammadelta knockout mice (TCRgammadelta KO) we demonstrated that mice lacking gammadelta T cells are defective in the systemic ovalbumin-specific immunoglobulin E (IgE) response. Furthermore, after aerosol challenge with allergen, gammadelta T-cell deficient mice exhibited a significantly decreased migration of B cells and natural killer cells to airways and reduced levels of allergen-specific IgG and IgA in bronchoalveolar lavage fluid. The role for B cells in the airway inflammation was indicated by the impaired ability of mice lacking functional B cells to evoke an eosinophilic response. The diminished eosinophilia in TCRgammadelta KO mice could not be explained by a defective Th2 activation since these mice displayed a normal IgG response in serum and an unaffected IG2b/IgG1 ratio in airways. Analysis of immunoregulatory cytokines in isolated lung tissue, thoracic lymph nodes and spleen further supported the notion that these mice are able to evoke a sufficient activation of T helper cells and that gammadelta T cells are not required for maintaining the Th2 profile. These results indicate that gammadelta T cells contribute to allergic airway inflammation by pathways separate from classical Th2 immune activation.

PMCID: PMC1782860
PMID: 12519308 [PubMed - indexed for MEDLINE]

1385. Blood. 2003 May 15;101(10):3784-93. Epub 2003 Jan 2.

Functional receptor for C3a anaphylatoxin is expressed by normal hematopoietic stem/progenitor cells, and C3a enhances their homing-related responses to SDF-1.

Reca R, Mastellos D, Majka M, Marquez L, Ratajczak J, Franchini S, Glodek A, Honczarenko M, Spruce LA, Janowska-Wieczorek A, Lambris JD, Ratajczak MZ.

Stem Cell Biology Program, University of Louisville, Louisville, KY 40202, USA.

Complement has recently been implicated in developmental pathways and noninflammatory processes. The expression of various complement components and receptors has been shown in a wide range of circulating myeloid and lymphoid cells, but their role in normal hematopoiesis and stem cell homing has not yet been investigated. We report that normal human CD34(+) cells and lineage-differentiated hematopoietic progenitors express the complement anaphylatoxin C3a receptor (C3aR) and respond to C3a. Moreover, C3a, but not the biologically inactive desArg-C3a, induces calcium flux in these cells. Furthermore, we found that C3 is secreted by bone marrow stroma and that, although C3a does not influence directly the proliferation/survival of hematopoietic progenitors, it (1) potentiates the stromal cell-derived factor 1 (SDF-1)-dependent chemotaxis of human CD34(+) cells and lineage-committed myeloid, erythroid, and megakaryocytic progenitors; (2) primes SDF-1-dependent trans-Matrigel migration; and (3) stimulates matrix metalloproteinase-9 secretion and very late antigen 4 (VLA-4)-mediated adhesion to vascular cell adhesion molecule 1 (VCAM-1). Furthermore, we found that murine Sca-1(+) cells primed by C3a engrafted faster in lethally irradiated animals. These results indicate that normal human hematopoietic stem and progenitor cells express functional C3aR and that the C3aR-C3a axis sensitizes the responses of these cells to SDF-1 and thus may be involved in promoting their homing into the bone marrow via cross talk with the SDF-CXC chemokine receptor-4 (CXCR4) signaling axis. C3a is the first positive regulator of this axis to be identified.

PMID: 12511407 [PubMed - indexed for MEDLINE]

1386. Vet Rec. 2002 Dec 7;151(23):691-3.

Role of the chemokine eotaxin in the pathogenesis of equine sweet itch.

Benarafa C, Collins ME, Hamblin AS, Cunningham FM.

Department of Pathology and Infectious Diseases, The Royal Veterinary College, Hawkshead Lane, North Mymms, Hertfordshire AL9 7TA.

The chemokine eotaxin is involved in the recruitment of eosinophils and T helper 2 lymphocytes in human allergic diseases, and drugs that block its activity, including eotaxin receptor (CCR3) antagonists, are being developed. The authors have recently cloned the horse ortholog of eotaxin and shown that it can induce equine eosinophil migration and activation in vitro. Moreover, eotaxin mRNA expression was upregulated in cultured horse dermal fibroblasts exposed to equine interleukin-4, suggesting a possible source of this eosinophil chemoattractant in equine skin. The results of this study show that eotaxin and monocyte chemoattractant protein (MCP) 1, but not MCP-2 or MCP-4, mRNA expression is upregulated in skin biopsies of sweet itch lesions when eosinophils are present, when compared with clinically normal skin from the same ponies.

PMID: 12503787 [PubMed - indexed for MEDLINE]

1387. J Immunol. 2003 Jan 1;170(1):581-7.

CCR8 is not essential for the development of inflammation in a mouse model of allergic airway disease.

Chung CD, Kuo F, Kumer J, Motani AS, Lawrence CE, Henderson WR Jr, Venkataraman C.

Tularik, Inc., South San Francisco, CA 94080, USA.

Chemokine receptors play an important role in the trafficking of various immune cell types to sites of inflammation. Several chemokine receptors are differentially expressed in Th1 and Th2 effector populations. Th2 cells selectively express CCR3, CCR4, and CCR8, which could direct their trafficking to sites of allergic inflammation. Additionally, increased expression of the CCR8 ligand, TCA-3, has been detected in affected lungs in a mouse model of asthma. In this study, CCR8-deficient mice were generated to address the biological role of CCR8 in a model of allergic airway disease. Using two different protocols of allergen challenge, we demonstrate that absence of CCR8 does not affect the development of pulmonary eosinophilia and Th2 cytokine responses. In addition, administration of anti-TCA-3-neutralizing Ab during allergen sensitization and rechallenge failed to inhibit airway allergic inflammation. These results suggest that CCR8 does not play an essential role in the pathogenesis of inflammation in this mouse model of allergic airway disease.

PMID: 12496446 [PubMed - indexed for MEDLINE]

1388. Allergy. 2002;57 Suppl 75:8-12.

Introduction to nasal and pulmonary allergy cascade.

Canonica GW.

Allergy and Respiratory Diseases, Department of Internal Medicine, Genoa University, Genova, Italy.

The early phase of an IgE-dependent allergic reaction is followed by the activation of a complex network of inflammatory phenomena - T lymphocytes, cytokines, mediators, and adhesion molecules - that mediate late and ongoing allergic symptoms. The kinetics of respiratory inflammation following allergen exposure involve the migration of inflammatory cells to the mucosa within about 30 min, increased inflammatory infiltration over the following hours, and then slow subsidence. A relationship between asthma and allergic rhinitis is supported by epidemiological, histological, physiological, and immunopathological data, and by the response of asthma symptoms in rhinitic patients to intranasal corticosteroids and antihistamines. For example, there is no morphological difference between the bronchial inflammatory response following allergen-specific challenge in patients suffering from asthma alone or rhinitis alone. It is the allergen dose that makes the difference in the airway response to allergen in allergic rhinitis and asthma. Recognition of the relationship between asthma and allergic rhinitis has led to the introduction of new diagnostic terminology and treatment recommendations: 1) patients with persistent rhinitis should be evaluated for asthma; 2) patients with persistent asthma should be evaluated for rhinitis; and 3) a strategy should combine the treatment of upper and lower airways in terms of efficacy and safety.

PMID: 12492723 [PubMed - indexed for MEDLINE]

1389. Recenti Prog Med. 2002 Nov;93(11):642-54.

[Chemokines and tumors].

[Article in Italian]

Pellegrino A, Vacca A, Scavelli C, Dammacco F.

Dipartimento di Scienze Biomediche e Oncologia Umana, Università, Bari.
antoniopellegrino@hotmail.com

Chemokines are cytokines which induce chemotaxis on many cell types, thus regulating cell migration within inflammatory and allergic sites, and leucocyte homing. Also, they play a crucial role in inflammatory and tumor-associated angiogenesis, as well as in tumor progression. Chemokines are grouped into: 1) alpha or CXC; 2) beta or CC; 3) gamma or C; 4) delta or CX3C molecules. Each of them recognizes one or more cell surface receptors, named CXCR, CCR, XCR, CX3CR respectively, according to the corresponding subfamily. Many chemokines have been identified within tumor tissues, as a secretory product of tumor cells and/or inflammatory cells. The CXC chemokines (such as IL-8, IP10, Mig, SDF-1 alpha) or CC chemokines (such as MCP-1, MIP-1 alpha, eotaxin, RANTES) have been frequently harvested from tumor tissues or the biological fluids of patients. Some chemokines inhibit tumor growth and progression by activating immunocompetent cytolytic cells or inhibiting tumor-associated angiogenesis. In contrast, other chemokines induce tumor progression by interacting with the specific receptor expressed on the tumor cells and hence by activating chemotaxis and secretion of proteolytic enzymes, or by inducing angiogenesis and metastatic spreading. Sometimes neoplastic cells express chemokine receptors which are not expressed on their normal counterpart. Data from this lab show the CXCR3 expression by cells from lymphoproliferative diseases, such as multiple myeloma and lymphoma, and the stimulation of an invasive phenotype following interaction with specific chemokines.

PMID: 12489485 [PubMed - indexed for MEDLINE]

1390. Int Arch Allergy Immunol. 2002 Dec;129(4):327-40.

Identification of highly expressed genes in peripheral blood T cells from patients with atopic dermatitis.

Matsumoto Y, Oshida T, Obayashi I, Imai Y, Matsui K, Yoshida NL, Nagata N, Ogawa K, Obayashi M, Kashiwabara T, Gunji S, Nagasu T, Sugita Y, Tanaka T, Tsujimoto G, Katsunuma T, Akasawa A, Saito H.

Genox Research, Inc., Teikyo University Biotech Center, Kawasaki, Kanagawa, Japan.

BACKGROUND: Analysis of genes that are differentially expressed in patients with atopic dermatitis (AD) and normal individuals will provide important information on the underlying molecular pathogenetic mechanisms of AD.
METHODS: Transcript of freshly isolated peripheral blood T cells from 59 individuals were analyzed with a fluorescent differential display (FDD) method. Ninety-two differentially expressed genes were identified in this manner. Additionally, real-time quantitative RT-PCR was employed to investigate the expression of the FDD-selected genes and also genes related to T cell function.
RESULTS: A number of genes, including CC chemokine receptor 4, T cell-specific tyrosine kinase (Emt/Itk), integrin beta1, integrin alpha6, IQGAP1 and MAR/SAR DNA-binding protein (SATB1), were shown to be more highly expressed in patients with moderate and/or severe AD than in controls or patients with mild AD. Because the products of these upregulated genes influence chemotaxis, adhesion, migration and Th2 polarization, it is suggested that in more severe AD, circulating T cells may function differently in this regard. Several other genes, the role of which in T cell function is currently unknown, were also found to be differentially expressed in AD. These included the heat shock protein 40 and vasopressin-activated calcium-mobilizing receptor 1.
CONCLUSION: The upregulated genes identified in this work may serve as useful markers for moderate to severe AD as opposed to normal or mild AD and also as markers indicating progression to more severe AD. Further functional characterization will provide a better understanding of the pathophysiology of

circulating T cells in AD.

Copyright 2002 S. Karger AG, Basel

PMID: 12483038 [PubMed - indexed for MEDLINE]

1391. Ophthalmologe. 2002 Dec;99(12):949-59.

[Autologous serum-eye-drops for ocular surface disorders. A literature review and recommendations for their application].

[Article in German]

Geerling G, Hartwig D.

Institute of Ophthalmology,Bath Street, London EC1 V 9EL, UK.
ggerling@ophtha.mu-luebeck.de

The natural tear film has mechanical, optical, antimicrobial and nutritional properties. Tear film components, such as EGF, fibronectin and vitamin A, play a vital role in the proliferation, migration and differentiation of the corneal and conjunctival epithelium. In ocular surface disease, such as severe dry eye, the epithelia may be depleted of these nutritional factors. Replacing the aqueous component of tears alone,by using pharmaceutical tear substitutes,often has little effect on the ocular surface.Eye-drops prepared from autologous serum are a new treatment option for severe ocular surface disease. They can be produced according to the regulations on drug use as an unpreserved blood preparation. Autologous serum eye-drops are non-allergenic and their biomechanical and biochemical properties are similar to normal tears. In cell culture experiments, serum was found to be superior to preserved or unpreserved pharmaceutical products in the maintenance of human keratinocyte morphology and function. It supports the migration of corneal epithelial cells and the differentiation of conjunctival epithelial cells. The first clinical cohort studies report its successful use for severe dry eyes and persistent epithelial defects. In these studies, however, varying methods for the preparation and different concentrations of autologous serum eye-drops were used. These methodological variations determine the biochemical properties and thus the epitheliotrophic effect of serum eye-drops. In this review we summarise the currently available clinical evidence, discuss relevant legislatory restrictions and describe a standard operating protocol for the use of serum eye drops. This has to be evaluated and optimised in more detail before any meaningful, randomised, controlled trial can attempt to establish the role of serum eye-drops in the management of severe ocular surface disease.

PMID: 12478384 [PubMed - indexed for MEDLINE]

1392. Curr Opin Pulm Med. 2003 Jan;9(1):34-41.

The other cells in asthma: dendritic cell and epithelial cell crosstalk.

Lambrecht BN, Hammad H.

Department of Pulmonary Medicine, Erasmus Medical Center, Rotterdam, the Netherlands. lambrecht@lond.azr.nl

Dendritic cells (DCs) and epithelial cells are the first cells to encounter inhaled allergens. The response of these cell types to allergens is fundamentally different in asthmatics compared with nonasthmatics. DCs and epithelial cells

interact through cell-cell interactions and through release of soluble mediators. The response of epithelial cells to allergens can profoundly modify the behavior of intramucosal DCs. Upon migration to the draining nodes, mucosal DCs undergo functional maturation and induce proliferation in naive T cells and primed Th2 cells. The implications of the DC-epithelial interaction for the pathogenesis of asthma is becoming increasingly clear by the use of mouse models and culture systems of human cells.

PMID: 12476082 [PubMed - indexed for MEDLINE]

1393. Curr Opin Pulm Med. 2003 Jan;9(1):28-33.

Role of matrix metalloproteinases in asthma.

Kelly EA, Jarjour NN.

The Pulmonary and Critical Care Section of the Department of Medicine, University of Wisconsin, Madison, 53972, USA.

Airway inflammation and remodeling are key features of asthma. Matrix metalloproteinases (MMPs) and their inhibitors, tissue inhibitors of metalloproteinases (TIMPs) are thought to contribute to the pathogenesis of asthma via their influence on the function and migration of inflammatory cells as well as matrix deposition and degradation. TIMPs bind MMPs in a 1:1 fashion. Thus, an increase in the molar ratio of MMP/TIMP may favor tissue injury, while the reverse could be associated with increased fibrosis. MMP-9 is the predominant MMP in asthma, and its expression is enhanced when patients have spontaneous exacerbations or in response to local instillation of allergen in the airway. As acute inflammation resolves, MMP-9 levels return toward normal. Interestingly, corticosteroids downregulate MMP and enhance TIMPs. Even though it is clear that enhanced airway inflammation in asthma is associated with increased expression of MMPs, whether specific inhibitors of MMP could reduce airway injury and facilitate orderly healing in asthma is still unknown.

PMID: 12476081 [PubMed - indexed for MEDLINE]

1394. Chest. 2002 Dec;122(6 Suppl):275S-278S.

Fibronectin matrix deposition and cell contractility: implications for airway remodeling in asthma.

Hocking DC.

Department of Pharmacology and Physiology, University of Rochester Medical Center, Rochester, NY 14642, USA. denise_hocking@urmc.rochester.edu

The adhesion of cells to the extracellular matrix (ECM) protein, fibronectin, is important in the regulation and coordination of such complex processes as cell growth, migration, differentiation, and ECM organization. The deposition of fibronectin into the ECM is a cell-dependent process that is normally tightly regulated to ensure controlled matrix deposition. Increased deposition of fibronectin and collagen into the subepithelial space of the airways is observed in all forms of asthma and occurs early in the progression of the disease. Experimental evidence suggests a model in which fibronectin matrix accumulation contributes to the progression of asthma by altering both the structural properties of the airways and the functional properties of cells of the airway wall.

PMID: 12475798 [PubMed - indexed for MEDLINE]

1395. J Immunol. 2002 Dec 15;169(12):7063-70.

An aspirin-triggered lipoxin A4 stable analog displays a unique topical anti-inflammatory profile.

Schottelius AJ, Giesen C, Asadullah K, Fierro IM, Colgan SP, Bauman J, Guilford W, Perez HD, Parkinson JF.

Research Business Area Dermatology, Research Laboratories, Schering AG, Berlin, Germany.

Lipoxins and 15-epi-lipoxins are counter-regulatory lipid mediators that modulate leukocyte trafficking and promote the resolution of inflammation. To assess the potential of lipoxins as novel anti-inflammatory agents, a stable 15-epi-lipoxin A(4) analog, 15-epi-16-p-fluorophenoxy-lipoxin A(4) methyl ester (ATLa), was synthesized by total organic synthesis and examined for efficacy relative to a potent leukotriene B(4) (LTB(4)) receptor antagonist (LTB(4)R-Ant) and the clinically used topical glucocorticoid methylprednisolone aceponate. In vitro, ATLa was 100-fold more potent than LTB(4)R-Ant for inhibiting neutrophil chemotaxis and trans-epithelial cell migration induced by fMLP, but was approximately 10-fold less potent than the LTB(4)R-Ant in blocking responses to LTB(4). A broad panel of cutaneous inflammation models that display pathological aspects of psoriasis, atopic dermatitis, and allergic contact dermatitis was used to directly compare the topical efficacy of ATLa with that of LTB(4)R-Ant and methylprednisolone aceponate. ATLa was efficacious in all models tested: LTB(4)/Iloprost-, calcium ionophore-, croton oil-, and mezerein-induced inflammation and trimellitic anhydride-induced allergic delayed-type hypersensitivity. ATLa was efficacious in mouse and guinea pig skin inflammation models, exhibiting dose-dependent effects on edema, neutrophil or eosinophil infiltration, and epidermal hyperproliferation. We conclude that the LXA(4) and aspirin-triggered LXA(4) pathways play key anti-inflammatory roles in vivo. Moreover, these results suggest that ATLa and related LXA(4) analogs may have broad therapeutic potential in inflammatory disorders and could provide an alternative to corticosteroids in certain clinical settings.

PMID: 12471142 [PubMed - indexed for MEDLINE]

1396. Paediatr Respir Rev. 2002 Dec;3(4):278-84.

Eosinophilic lung diseases.

Ribeiro JD, Fischer GB.

Center for Investigation in Pediatrics-CIPED, State University of Campinas (Unicamp), PO Box 6111, Campinas, SP, CEP 13081-970, Brazil. dirceu@fcm.unicamp.br

In the last 30 years studies have shown that the lungs have been the target of eosinophilic migration producing eosinophilic lung diseases (ELD) secondary to allergens, irritants, parasites, infections, antibodies and drugs. Specific diagnoses can be made by: (1) Peripheral blood eosinophilia and chest X-ray infiltrates. (2) Lung biopsy. (3) Bronchoalveolar lavage (BAL). In developing countries ELD are most frequently associated with parasitic infection. This paper defines, classifies and presents the clinical characteristics and treatment of these diseases with emphasis on parasitic lung problems.

PMID: 12457596 [PubMed - indexed for MEDLINE]

1397. Zh Mikrobiol Epidemiol Immunobiol. 2002 Jul-Aug;(4):43-7.

[In vitro migration activity of human peripheral blood neutrophils in the normal state and in immunopathology].

[Article in Russian]

Kozlov IG, Saĭgitov RT, Mitiaseva SA, Cheredeev AN, Koval'chuk LV.

State Medical University, Moscow, Russia.

Methodological approaches to evaluation of the migration activity of human peripheral blood neutrophils into a collagen matrix were worked out. The migration of neutrophils in healthy donors and in patients with severe bronchial asthma was studied. In the normal state there was practically no migration of intact neutrophils into the collagen matrix (1.1 +/- 0.4%). Following their stimulation by formyi peptide about a quarter of their population was drawn into the matrix in avalanche (22.0 +/- 5.9%). In the acute phase of severe bronchial asthma an increase in both spontaneous (3.3 +/- 1.5%, P < 0.01) and stimulated (35.6 +/- 4.6%, P < 0.001) cell migration occurred. Changes in the migration characteristics of the neutrophils of patients and those of the cells of healthy donors, treated with the polycytokine preparation at concentrations exceeding 100 g/ml, followed similar trends. In case of the standard asthma treatment along with positive disease dynamics further increase in spontaneous neutrophil migration (5.8 +/- 2.9%, P < 0.001) in combination with deficiency in cells reaction to formyi peptide (11.8 +/- 3.8%, P < 0.01) was registered. At the same time dexamethasone did not change the character of the in vitro migration of neutrophils into the collagen matrix. Thus the dynamics of the peripheral blood neutrophil migration during treatment of severe bronchial asthma was demonstrated; this dynamics could be indicative of the pathogenetic role of neutrophils in the development of this pathology.

PMID: 12449698 [PubMed - indexed for MEDLINE]

1398. Eur Respir J. 2002 Nov;20(5):1162-6.

Sex-related interactive effect of smoking and household pets on asthma incidence.

Chen Y, Dales R, Tang M, Krewski D.

Dept of Epidemiology and Community Medicine, Faculty of Medicine, University of Ottawa, Ottawa, Ontario, Canada. ychen@uottawa.ca

The authors examined the interactive effect of smoking and pets at home on the incidence of asthma and the difference between sexes. The longitudinal data from the first two cycles of the National Population Health Survey, conducted in Canada, were used. A total of 12,636 subjects who reported no asthma at baseline were included in the analysis. The 2-yr cumulative incidence of asthma was higher in females than in males. Female sex and household pets demonstrated a significant interaction in the development of asthma. After adjustment for age, immigration and history of allergy, the odds ratio for smoking in relation to the asthma incidence was 2.50 (95% confidence interval: 1.24-5.05) for females who had pets at home and close to unity for those who had no pets. The incidence of asthma was not associated with smoking status and household pets in males. These results indicate that smoking, having pets at home and other environmental factors can partly explain asthma morbidity among female Canadians.

PMID: 12449169 [PubMed - indexed for MEDLINE]

1399. Ital J Anat Embryol. 2002 Jul-Sep;107(3):209-14.

Bio-alcamid: a novelty for reconstructive and cosmetic surgery.

Pacini S, Ruggiero M, Morucci G, Cammarota N, Protopapa C, Gulisano M.

Department of Human Anatomy, Histology and Forensic Medicine, University of Firenze, Italy.

Research for new biocompatible and easily implantable materials continously proposes new molecules and new substances with biological, chemical and physical characteristics more and more adapted to aesthetic and reconstructive surgery. Bio-Alcamid (Polymekon, Italy) is a non reabsorbable polymeric material composed of alkylimide-amide groups. This cosmetic agent has been used for the treatment of 2000 patients by a multicentric trial led by different Hospitals and University in Italy and abroad. Very serious aesthetic defects such as pectus excavatum, Poland Syndrome, postoperative traumas, in addition to common aesthetic defects such as lips, cheek-bone and chin hypovolumetry and relaxing of nasolabial sulcus, have been treated by surgical implant of Bio-Alcamid. Aesthetic results were excellent; tissues felt soft and the implants were uniformly distributed. No migration or dislocation of the implants, no granulomas, no allergic response and no kind of intolerance were observed. Only 12/2000 patients had post-operative complications (Staphylococcus infections) and only 3/12 cases could be directly ascribed to the implanted material. For its characteristics Bio-Alcamid can be considered a novelty in the aesthetic and reconstructive surgery; it is absolutely biocompatible, non toxic, non allergenic, easily injectable and quickly removable. Bio-Alcamid can be defined a sort of "endoprosthetis", perfectly suitable for soft tissue augmentation and for the correction of different tissue deficiences, with a long-term safety and efficiency.

PMID: 12437147 [PubMed - indexed for MEDLINE]

1400. Rev Neurol. 2002 Nov 16-30;35(10):985-93.

[Intercellular adhesion molecules and chemotaxic factors in the pathogenesis of multiple sclerosis].

[Article in Spanish]

González-Amaro R, Sánchez-Madrid F.

Departamento de Inmunología. Facultad de Medicina. Universidad Autónoma de San Luis Potosí. México.

INTRODUCTION: Multiple sclerosis (MS) is an inflammatory immune disorder of the central nervous system characterized by the destruction of myelin sheaths and the cells which make them, the oligodendrocytes. Experimental allergic encephalitis (EAE) is an autoimmune condition mainly induced by the myelin basic protein (MBP) that is a very useful model for the study of demyelinating inflammatory diseases, particularly MS.
METHOD: Cellular adhesion molecules are a wide group of membrane receptors which mediate adhesion processes, both cell to cell and between cells and the extracellular matrix. These molecules play an essential role in inflammatory phenomena, including EAE/MS. Integrins of the b1 subfamily (mainly a4b1), as well

as leukocyte integrins and adhesion receptors of the immunoglobulin superfamily (ICAM 1, VCAM 1) are the main molecules involved. Chemokines also have an important role in MS, since they are able to attract and activate leukocytes, essential phenomena in the inflammatory reaction. An increased expression of chemokines CC or beta (e.g., RANTES, MIP 1a and b, MCP 1, etc.) has been found in EAE/MS, and it is very likely that they are involved in the migration of lymphocytes and monocytes towards the MS inflammatory lesions.
CONCLUSION: The pharmacological blockade of adhesion molecules and chemokines is a promising and novel therapeutic approach in MS.

PMID: 12436404 [PubMed - indexed for MEDLINE]

1401. Crit Rev Immunol. 2002;22(2):105-14.

Regulation of macrophage-derived chemokine (MDC, CCL22) production.

Yamashita U, Kuroda E.

Department of Immunology, University of Occupational and Environmental Health, School of Medicine, Kitakyushu, Japan. yama-uki@med.uoeh-u.ac.jp

Macrophage-derived chemokine (MDC, CCL22) is a member of the CC-chemokine family and is composed of 69 amino acid residues. MDC is mainly produced by macrophages and dendritic cells upon the stimulation with microbial products, or anti-CD40 antibody, and is upregulated by TH2-type cytokines, such as IL-4 and -5, but is downregulated by TH1-type cytokines, such as IFN-gamma. MDC-production is also upregulated by prostaglandin and cyclic AMP-elevating agents. MDC causes chemotactic migration of dendritic cells and TH2 cells. Furthermore, MDC is highly-expressed in the lesions of TH2-related diseases, such as airway hypersensitivity and atopic dermatitis. Thus, MDC plays an important role in the recruitment of TH2 cells into the inflammatory sites and the regulation of TH2-related immune responses.

PMID: 12433129 [PubMed - indexed for MEDLINE]

1402. Eur J Pediatr. 2002 Nov;161(11):600-3. Epub 2002 Sep 5.

Glucagon therapy as a possible cause of erythema necrolyticum migrans in two neonates with persistent hyperinsulinaemic hypoglycaemia.

Wald M, Lawrenz K, Luckner D, Seimann R, Mohnike K, Schober E.

University Children's Hospital, University of Vienna, Währinger Gürtel 18-20, 1090 Vienna, Austria.

Erythema necrolyticum migrans (ENM) usually presents as a cutaneous paraneoplastic phenomenon which is in most cases associated with a glucagon-producing tumour. Here it is for the first time described as a side-effect of glucagon treatment in persistent hyperinsulinaemic hypoglycaemia of infancy (PHHI). In both patients, the skin lesions disappeared after discontinuation of glucagon administration. In the first child the erythema resolved without scarring within 10 days after glucagon was substituted with other medication while in the second patient healing followed subtotal pancreatectomy which rendered glucagon infusion unnecessary. Initially the clinical resemblance to atopic dermatitis is prone to cause diagnostic errors, especially in this age group. CONCLUSION: erythema necrolyticum migrans should be considered as a differential diagnosis in patients who develop erythematosquamous skin lesions under glucagon treatment.

PMID: 12424585 [PubMed - indexed for MEDLINE]

1403. J Allergy Clin Immunol. 2002 Nov;110(5):752-6.

Effects of inflammatory cytokines on the permeability of human lung microvascular endothelial cell monolayers and differential eosinophil transmigration.

Sedgwick JB, Menon I, Gern JE, Busse WW.

Division of Allergy and Immunology, Department of Medicine, University of Wisconsin, Madison, USA.

BACKGROUND: Rhinovirus (RV) infections can result in asthma exacerbations in both adults and children. Respiratory epithelium, the primary site of RV replication, responds to the viral infection by generating a variety of cytokines and chemokines capable of promoting airway inflammation and hence might increase asthma severity. Some of these mediators might also affect the permeability of underlying vascular endothelium.
OBJECTIVE: We hypothesized that RV infections can promote airway inflammation and thus asthma by enhancing local vascular permeability.
METHODS: Confluent human lung microvascular endothelial cell (HMVEC-L) monolayers were used as an in vitro model of vascular endothelium to determine whether cytokines associated with RV-induced infections are capable of modulating endothelial cell permeability as measured by means of transendothelial electrical resistance. Recombinant cytokines and chemokines were added to confluent HMVEC-L monolayers cultured on Transwell filters, and permeability was measured as decreased electrical resistance over time. Eosinophil transendothelial migration was assessed under the same experimental conditions.
RESULTS: TNF- alpha, IL-1 beta, and IFN- gamma significantly increased HMVEC-L permeability. In contrast, GM-CSF, G-CSF, IL-8, IL-6, and RANTES had no effect. Although incubation of HMVEC-L monolayers with either TNF-alpha or IL-1beta promoted eosinophil migration, IFN-gamma had no effect, indicating that enhanced permeability alone was not sufficient for eosinophil infiltration.
CONCLUSION: Select cytokines, generated in response to RV infection, can increase vascular permeability and might provide a mechanism by which RV infection can lead to edema, cellular infiltration, and inflammation and thus compromised airflow.

PMID: 12417884 [PubMed - indexed for MEDLINE]

1404. Regul Pept. 2002 Nov 15;109(1-3):199-208.

Immunoeffector and immunoregulatory activities of vasoactive intestinal peptide.

Voice JK, Dorsam G, Chan RC, Grinninger C, Kong Y, Goetzl EJ.

Department of Medicine, University of California, San Francisco, CA 94143, USA.

Vasoactive intestinal peptide (VIP) and its two G protein-coupled receptors, VPAC1R and VPAC2R, are prominent in the immune system and potently affect T cells and macrophages. VPAC1Rs are expressed constitutively by blood and tissue T cells, with an order of prevalence of Th2>Th1>>Ts, and transmit signals suppressive for migration, proliferation and cytokine production. Immune activation of T cells downregulates VPAC1Rs and upregulates VPAC2Rs. VPAC2Rs mediate T cell chemotaxis, stimulation of some Th2-type cytokines, and inhibition of some Th1-type cytokines. A tentative hypothesis that the VIP-VPAC2R axis is the major neuroregulator of Th2/Th1 balance has been confirmed by finding an

increased ratio in CD4 T cells of transgenic (TG) mice, expressing high levels of VPAC2Rs, and a decreased ratio in CD4 T cells of VPAC2R-null (K/O) mice. VPAC2R TG mice exhibit an allergic phenotype, whereas the K/O mice are hypoallergic and have heightened delayed-type hypersensitivity. The mechanisms of VIP-VPAC2R effects include decreased Th2 apoptosis, increased Th2-type cytokine production, and greater generation of Th2 memory cells. VPAC2R antagonists are being developed to alleviate allergic diseases and strengthen effector Th1 cell-mediated immunoprotection.

PMID: 12409234 [PubMed - indexed for MEDLINE]

1405. Int Arch Allergy Immunol. 2002 Oct;129(2):119-28.

Modification of acute and late-phase allergic responses to ovalbumin with lipopolysaccharide.

Tulic MK, Holt PG, Sly PD.

Division of Clinical Sciences, TVW Telethon Institute for Child Health Research and Centre for Child Health Research, University of Western Australia, Perth, W.A., Australia. meri.tulic@mcgill.ca

BACKGROUND: We have previously shown that lipopolysaccharide (LPS) exposure in sensitised animals 18 h after ovalbumin (OVA) challenge inhibits OVA-induced airway hyper-responsiveness (AHR). In the present study, we investigated the effect of LPS on OVA-induced acute and late-phase allergic responses in sensitised rats when challenged with OVA.
METHODS: Rats were sensitised with OVA and 11 days later challenged with 1% OVA in the presence or absence of LPS (0.5-50 microg/ml) given in the same nebulizer. Acute responses to OVA were measured each minute for 30 min after challenge. In a separate group of animals, late-phase responses to OVA were determined at 24 h. At the end of each study, Evans blue dye was injected and animals sacrificed 30 min later. Bronchoalveolar lavage was obtained to monitor inflammatory cell migration and microvascular leakage.
RESULTS: OVA challenge in sensitised animals produced an acute response with changes in lung mechanics peaking 10.0 +/- 0.9 min after OVA and returning to baseline within 30 min. This was followed 24 h later by increased responses to methacholine chloride (MCh), inflammatory cell influx and increased Evans blue leakage into the lungs. Presence of 5 or 50 microg/ml LPS in the nebulizer during OVA challenge altered the kinetics of the acute-phase response, with an immediate decrease in lung function (time to peak decreased from 10.3 +/- 1.2 to 1.8 +/- 0.2 and 2.2 +/- 0.3 min, respectively: p < 0.001, n = 6) and a dose-dependent attenuation of late-phase AHR, cellular influx (n = 5, p < 0.001) and Evans blue leakage (n = 5, p < 0.001) at 24 h.
CONCLUSIONS: In summary, co-administration of OVA with LPS modifies both the acute and late-phase responses to the allergen, inducing an earlier acute change in lung function and a dose-dependent inhibition of late-phase responses to the allergen.

Copyright 2002 S. Karger AG, Basel

PMID: 12403929 [PubMed - indexed for MEDLINE]

1406. J Mol Med (Berl). 2002 Oct;80(10):629-38. Epub 2002 Sep 7.

Rho/Rho-kinase mediated signaling in physiology and pathophysiology.

Wettschureck N, Offermanns S.

Institute of Pharmacology, University of Heidelberg, Im Neuenheimer Feld 366, 69120 Heidelberg, Germany.

The small GTPase Rho is implicated in many cellular functions such as cell adhesion, cell motility and migration, growth control, cell contraction, and cytokinesis. One of its main effectors, Rho-kinase, appears to play a key role in the regulation of force and velocity of actomyosin crossbridging in smooth muscle and nonmuscle cells by inhibiting myosin phosphatase-mediated dephosphorylation of the regulatory chain of myosin II. Abnormal activation of the Rho/Rho-kinase pathway has been shown to play a role in diseases such as hypertension and bronchial asthma. This review summarizes the current knowledge on the physiological and pathophysiological function of the Rho/Rho-kinase mediated pathway in various tissues with a focus on its possible role as a target for therapeutic interventions.

PMID: 12395147 [PubMed - indexed for MEDLINE]

1407. Blood. 2002 Nov 15;100(10):3663-71. Epub 2002 Jul 12.

Allergen-induced accumulation of airway dendritic cells is supported by an increase in CD31(hi)Ly-6C(neg) bone marrow precursors in a mouse model of asthma.

van Rijt LS, Prins JB, Leenen PJ, Thielemans K, de Vries VC, Hoogsteden HC, Lambrecht BN.

Department of Pulmonary and Critical Care Medicine, Erasmus University Medical Center, Rotterdam, The Netherlands. vanrijt@longz.fgg.eur.nl

Airway dendritic cells (DCs) are held responsible for inducing sensitization to inhaled antigen, leading to eosinophilic airway inflammation, typical of asthma. However, less information is available about the role of these cells in ongoing inflammation. In a mouse model of asthma, sensitization to ovalbumin (OVA) was induced by intratracheal injection of myeloid OVA-pulsed DCs. Upon OVA aerosol challenge and induction of eosinophilic airway inflammation in sensitized mice, there was a time-dependent and almost 100-fold increase in the number of MHCII(+) CD11b(+) CD11c(+) endogenous airway DCs as well as CD11b(+) blood DCs. The mechanism of this increase was studied. Adoptive transfer experiments demonstrated that accumulation of airway DCs was not due to reduced migration to the mediastinal lymph nodes. Rather, the massive increase in airway and lymph node DCs was supported by an almost 3-fold expansion of myeloid CD31(hi)Ly-6C(neg) hematopoietic precursor cells in the bone marrow (BM). There was no change in any of the other 5 populations revealed by CD31/Ly-6C staining. When these CD31(hi)Ly-6C(neg) BM precursors were sorted and grown in granulocyte macrophage-colony-stimulating factor, they differentiated into MHCII(+) CD11c(+) DCs. The same CD31(hi)Ly-6C(neg) precursors also expressed the eotaxin receptor CCR3 and differentiated into eosinophils when grown in interleukin 5. Serum levels of eotaxin were doubled in mice with inflammation. These findings in an animal model of asthma suggest that the BM increases its output of myeloid precursors to meet the enhanced demand for DCs and eosinophils in inflamed airways.

PMID: 12393720 [PubMed - indexed for MEDLINE]

1408. J Neuropathol Exp Neurol. 2002 Oct;61(10):914-25.

Vascular endothelial growth factor is expressed in multiple sclerosis plaques and can induce inflammatory lesions in experimental allergic encephalomyelitis rats.

Proescholdt MA, Jacobson S, Tresser N, Oldfield EH, Merrill MJ.

Surgical Neurology Branch, National Institute of Neurological Disorders and Stroke, National Institutes of Health, Bethesda, Maryland 20892-1414, USA.

The active lesions in multiple sclerosis (MS) are characterized by blood-brain-barrier (BBB) breakdown, upregulation of adhesion molecules on capillary endothelial cells, and perivascular inflammation, suggesting that altered vessel permeability and activated endothelial cells are involved in the pathogenesis of the disease. Vascular endothelial growth factor (VEGF) mediates multiple aspects of blood vessel physiology, including regulation of growth, permeability, and inflammation. To investigate a possible relationship between VEGF expression and CNS autoimmune disease, we examined VEGF expression in MS plaques compared to normal white matter by immunohistochemistry and in situ hybridization. VEGF expression was consistently upregulated in both acute and chronic MS plaques. We also examined VEGF expression during the course of experimental allergic encephalomyelitis (EAE) in rats. VEGF-positive cells with astrocytic morphology increased in the spinal cord during the development of EAE and were found in association with inflammatory cells. Furthermore, intracerebral infusion of VEGF in animals previously immunized with myelin basic protein induced an inflammatory response in the brain, whereas infusion of vehicle, or infusion of VEGF in naive animals, did not. These results suggest that overexpression of VEGF may exacerbate the inflammatory response in autoimmune diseases of the CNS by inducing focal BBB breakdown and migration of inflammatory cells into the lesions.

PMID: 12387457 [PubMed - indexed for MEDLINE]

1409. Infect Immun. 2002 Nov;70(11):6263-72.

Infection with Strongyloides venezuelensis induces transient airway eosinophilic inflammation, an increase in immunoglobulin E, and hyperresponsiveness in rats.

Silveira MR, Nunes KP, Cara DC, Souza DG, Corrêa A Jr, Teixeira MM, Negrão-Corrêa D.

Department of Bioquímica e Imunologia, Instituto de Ciências Biológicas, Universidade Federal de Minas Gerais, Belo Horizonte, Minas Gerais, Brazil.

Infection by nematode parasites with a pulmonary migration in their life cycle and allergic asthma are two highly prevalent diseases in humans; therefore, one may expect both may occur concomitantly. There is a predominant and essential role of Th2 lymphocytes in the mechanisms underlying the control of parasite elimination as well as in the pathology observed in the asthmatic lung. The consequences of such situations have been explored, with controversial results, justifying the development of experimental models in which the relationship between allergic airway inflammation and helminth infection might be evaluated. The present work describes the inflammatory, humoral, and functional changes that occur in the lung of rats after single (subcutaneous inoculation of 1,500 L3 larvae) or multiple (five weekly subcutaneous inoculations of 1,500 L3 larvae) Strongyloides venezuelensis infections. The results show that the migration of S. venezuelensis larvae through the lungs of infected rats induces a local eosinophilic inflammation process which is mostly focal and parenchymal for rats infected a single time and which is peribronchial after multiple infections. The inflammatory process is accompanied by mucus hypersecretion, thickening of bronchial epithelial and muscle layers, and local increase in immunoglobulin E concentrations that peak after 5 to 7 days and are resolved after 12 days of single or multiple infections. The peak of lung immunopathologic changes observed

in infected rats coincides with lung airway hyperresponsiveness (AHR), a key functional alteration in asthma. We propose that this experimental model is ideal to carry out further studies on immunoprotection against nematode infection versus immunopathology of allergic airway inflammation.

PMCID: PMC130296
PMID: 12379705 [PubMed - indexed for MEDLINE]

1410. J Allergy Clin Immunol. 2002 Oct;110(4):553-64.

The role of transcription factors in allergic inflammation.

Escoubet-Lozach L, Glass CK, Wasserman SI.

Division of Cellular and Molecular Medicine, Department of Medicine, University of California at San Diego, La Jolla, CA 92093-0637, USA.

The induction of allergic inflammation and the expression of allergic disorders are dependent on the coordinated regulation of numerous genes. The products of these genes determine lymphocyte phenotype, immunologic responsiveness, eosinophil and mast cell development, activation, migration and life span, adhesion molecule expression, cytokine synthesis, cell-surface receptor display, and processes governing fibrosis and tissue repair. Although the expression of gene products involved in these processes is regulated at multiple levels (eg, transcription, mRNA processing, translation, phosphorylation, and degradation), transcription represents an essential and often the most important determinant of their contribution to cellular function. Signal-dependent and cell type-specific regulation of gene expression is generally achieved by means of combinatorial interactions between sequence-specific transcription factors that recruit chromatin remodeling machinery and general transcription factors to promoter and enhancer regions of RNA polymerase II-dependent genes. As targets of signal-transduction pathways, transcription factors integrate the response of the cell to the myriad of inputs it receives. This integration can be accomplished by the effect of signaling cascades on the activation status or subcellular locus of transcription factors or by transcription factor dimerization induced by means of ligand binding. This review will identify the major families of transcription factors important in allergic mechanisms and discuss their interactions, their mechanisms of action, and their interrelated and competitive actions, as well as implications for therapy of allergic disorders.

PMID: 12373260 [PubMed - indexed for MEDLINE]

1411. Int Arch Allergy Immunol. 2002 Sep;129(1):67-75.

A functional study on CysLT(1) receptors in human eosinophils.

Ohshima N, Nagase H, Koshino T, Miyamasu M, Yamaguchi M, Hirai K, Yamamoto K, Fujisawa T, Nakagawa N, Kishikawa K, Morita Y.

Department of Respiratory Medicine, University of Tokyo Graduate School of Medicine, Tokyo, Japan.

BACKGROUND: The cysteinyl leukotrienes (CysLTs) mediate their biological actions through two receptors: CysLT(1) receptor and CysLT(2) receptor.
OBJECTIVE: This study was undertaken to examine the direct effects of CysLTs on eosinophils, such as chemotaxis and degranulation, focusing on CysLT(1).
METHODS: Eosinophils were isolated from venous blood from normal volunteers who had no history of allergy (purity >99%). They were subjected to reverse

transcription-PCR analysis and flow-cytometric analysis for CysLT(1). Binding assays were performed with [(3)H]LTD(4). Purified eosinophils loaded with Fura-2 acetoxymethyl ester were stimulated with CysLTs, and Ca(2+) influx was measured. Eosinophil migration in response to CysLTs was measured using a 96-well multiwell Boyden chamber. Eosinophils were treated with LTD(4) at 10(-6) M for 60 min followed by incubation for 4 h at 37 degrees C in the presence or absence of IL-5 and eosinophil-derived neurotoxin (EDN) release was evaluated.
RESULTS: The expression of the mRNA and protein of CysLT(1) on eosinophils and [(3)H]LTD(4)-specific binding to eosinophils were observed. Neither Th1 cytokine (IFN-gamma) nor Th2 cytokines (IL-4 or IL-5) affected CysLT(1) expression in eosinophils. CysLTs induced an increase in intracellular free Ca(2+) in eosinophils via CysLT(1), as suggested by the efficient inhibition by a CysLT(1) antagonist, pranlukast, in addition to the rank order of potency being LTD(4), LTC(4) and LTE(4). LTD(4) stimulated eosinophils to migrate at 10(-6) M via CysLT(1). LTE(4) also induced significant eosinophil migration at 10(-6) M. LTD(4) enhanced EDN release induced by IL-5 via CysLT(1).
CONCLUSION: CysLTs induce migration and enhance degranulation in eosinophils via CysLT(1). Accordingly, interaction of CysLTs and CysLT(1) on eosinophils has the potential to play a prominent role in the pathophysiology of asthma.

Copyright 2002 S. Karger AG, Basel

PMID: 12373000 [PubMed - indexed for MEDLINE]

1412. Nephron. 2002;92(3):728-9.

Hypersensitivity to paraoxybenzoic Acid esters (parabens) in a dialysis patient.

Sato K, Kazama JJ, Wada Y, Maruyama H, Narita I, Gejyo F, Sugiyama K, Satoh H.

Division of Clinical Nephrology and Rheumatology, Niigata University Graduate School of Medical and Dental Sciences, Niigata, Japan.

A 24-year-old woman undergoing chronic dialysis therapy who had been diagnosed as having 'heparin allergy' presented acute allergic reaction after the intracatheter injection of a heparin agent. Further investigation revealed that mild eosinophilia had persisted for more than 3 years before this incidence despite avoiding the use of heparin agents. The leukocyte migration inhibition test (LMIT) showed that heparin agent A which was used for the patient showed positive reaction while heparin agent B which was not used for the patient showed a negative reaction. Paraoxybenzoic acid esters (parabens) are contained in heparin agent A but not in heparin agent B. Parabens showed positive reactions on LMIT. Parabens are the most common preservatives in drugs, foods, and cosmetics and they sometimes induce allergic reactions through percutaneous and possibly ingestive sensitization. However, they cause more severe allergic reactions when used intravenously. We concluded that the allergen in this patient was not heparin but parabens. Hypereosinophilia of unknown origin often occurs in dialysis patients. Such patients may be hypersensitive to parabens.

Copyright 2002 S. Karger AG, Basel

PMID: 12372968 [PubMed - indexed for MEDLINE]

1413. Clin Exp Allergy. 2002 Oct;32(10):1527-34.

The involvement of matrix metalloproteinases in basement membrane injury in a murine model of acute allergic airway inflammation.

Kumagai K, Ohno I, Imai K, Nawata J, Hayashi K, Okada S, Senoo H, Hattori T, Shirato K.

Department of Cardiovascular Medicine, Tohoku University Graduate School of Medicine, Sendai, Japan.

BACKGROUND: Airway remodelling in asthma such as subepithelial fibrosis is thought to be the repair process that follows the continuing injury as of chronic airway inflammation. However, how acute allergic inflammation causes tissue injury in the epithelial basement membrane in asthmatic airways remains unclear. Matrix metalloproteinases (MMPs) capable of degrading almost all of the extracellular matrix components have been demonstrated to be involved in cell migration through the basement membrane in vivo and in vitro.
OBJECTIVE: We investigated the alterations of matrix construction and the role of MMPs in matrix degradation in the subepithelium during acute allergic airway inflammation.
METHODS: Airway inflammation, the ultrastructure of the subepithelium and injury of types III and IV collagen in tracheal tissues from ovalbumin (OVA)-sensitized mice after OVA inhalation with or without the administration of tissue inhibitor of metalloproteinase-2 (TIMP-2) and dexamethasone were evaluated by cell counting in bronchoalveolar lavage (BAL) fluids, electron microscopy and immunohistochemistry, respectively.
RESULTS: The disruption of the lamina densa and matrix construction and the decrease of the immunoreactivity for type IV collagen in subepithelium were observed in association with the accumulation of inflammatory cells in airways 3 days after OVA inhalation. This disorganization of the matrix components in the subepithelium, as well the cellular accumulation, was abolished by the administration of TIMP-2 and dexamethasone. The immunoreactivity for type IV collagen in the subepithelium in OVA-inhaled mice returned to the level of that in saline-inhaled mice 10 days after inhalation in association with a decrease of the cell numbers in the BAL fluid. The immunoreactivity for type III collagen was changed neither 3 nor 10 days after OVA inhalation.
CONCLUSION: These results suggest that epithelial basement membrane gets injured by, at least in part, MMPs as a consequence of cell transmigration through the membrane during acute allergic airway inflammation.

PMID: 12372136 [PubMed - indexed for MEDLINE]

1414. J Comput Aided Mol Des. 2002 Mar;16(3):201-11.

3D QSAR (COMFA) of a series of potent and highly selective VLA-4 antagonists.

Singh J, van Vlijmen H, Lee WC, Liao Y, Lin KC, Ateeq H, Cuervo J, Zimmerman C, Hammond C, Karpusas M, Palmer R, Chattopadhyay T, Adams SP.

Biogen Inc, Cambridge MA 02142, USA.

The integrin VLA-4 (alpha4,beta1) is involved in the migration of white blood cells to sites of inflammation, and is implicated in the pathology of a variety of diseases including asthma and multiple sclerosis. We report the structure-activity relationships of a series of VLA-4 antagonists that were based upon the integrin-binding sequence of the connecting segment peptide of fibronectin (Leu-Asp-Val), and of VCAM-1 (Ile-Asp-Ser), both natural ligands of VLA-4. We explore variation in the ligand derived peptide portion of these antagonists and also in the novel N-terminal cap, which have discovered through chemical optimization, and which confers high affinity and selectivity. Using the X-ray derived conformation of the Ile-Asp-Ser region of VCAM-1, we rationalize the structure-activity relationships of these antagonists using 3D QSAR (COMFA). The COMFA model was found to be highly predictive with a cross-validated R2CV of

0.7 and a PRESS of 0.49. The robustness of the model was confirmed by testing the influence of various parameters, including grid size, column filtering, as well as the role of orientation of the aligned molecules. Our results suggest that the VCAM-1 structure is useful in generating highly predictive models of our VLA-4 antagonists. The COMFA model coupled with the knowledge that the peptide amides are tolerant to methylation should prove useful in future peptidomimetic design studies.

PMID: 12363218 [PubMed - indexed for MEDLINE]

1415. Allergy. 2002 Nov;57(11):1036-43.

In allergic asthma experimental exposure to allergens is associated with depletion of blood eosinophils overexpressing LFA-1.

Lantero S, Spallarossa D, Silvestri M, Sabatini F, Scarso L, Crimi E, Rossi GA.

Pulmonary Division, G. Gaslini Institute, Largo G. Gaslini 5, 16147 Genoa, Italy.

BACKGROUND: In atopic individuals, exposure to allergens is followed by recruitment of blood eosinophils in the target tissue. We investigated whether allergen inhalation challenge could result in depletion of blood eosinophils overexpressing adhesion molecules involved in eosinophil migration.
METHODS: Blood eosinophils were isolated from seven atopic asthmatic patients and seven control subjects and the "at baseline" expression of lymphocyte function-associated antigen-1 (LFA-1), macrophage antigen-1 (Mac-1) and very late antigen-4 (VLA-4) was assessed by monoclonal antibody staining and flow cytometry analysis. Asthmatic patients underwent allergen challenge and the expression of LFA-1, Mac-1 and VLA-4 by blood eosinophils was again evaluated 3 h and 24 h after allergen challenge.
RESULTS: As compared to controls, eosinophils from atopics showed at baseline enhanced LFA-1 expression ($P=0.0012$), but similar Mac-1 or VLA-4 expression ($P > 0.1$, each comparison). In atopics, the percentage and absolute number of blood eosinophils were significantly decreased 3 h after allergen challenge ($P=0.001$ and $P=0.022$, respectively) but returned to similar values to prechallenge values after an additional 21 h ($P > 0.1$). Allergen challenge was also followed by a significant decrease in LFA-1 expression by eosinophils, at 3 h ($P=0.002$) and at 24 h ($P=0.038$), while no changes in Mac-1 and VLA-4 were observed. A significant correlation between postchallenge decrease in LFA-1 expression and in blood eosinophilia, both expressed as percentage ($r=0.88$; $P < 0.01$) or absolute number ($r=0.87$; $P < 0.01$) was demonstrated at 3 h ($r=0.88$; $P < 0.01$) but not at 24 h ($r=0.64$, $P > 0.05$ and $r=0.11$; $P > 0.05$, respectively).
CONCLUSION: In allergic asthma, an early recruitment of blood eosinophils overexpressing LFA-1 occurs in the first hours after allergen challenge.

PMID: 12359000 [PubMed - indexed for MEDLINE]

1416. Allergy. 2002 Nov;57(11):1008-12.

Hemin, a heme oxygenase substrate analog, both inhibits and enhances neutrophil random migration and chemotaxis.

Andersson JA, Uddman R, Cardell LO.

Department of Otorhinolaryngology, Malmö University Hospital, Malmö 205 02, Sweden.

BACKGROUND: Carbon monoxide (CO), is an endogenously produced gas, generated by

the rate-limiting enzyme heme oxygenase (HO), present in man throughout the respiratory tract. CO can elicit important physiological responses like bronchial relaxation and vasodilation. Both HO expression and CO levels in the airways increase in response to hypoxic challenge and to a wide variety of inflammatory stimuli, such as intermittent allergic rhinitis, asthma and upper respiratory tract infections. A role for CO in airway regulation and inflammation has therefore been suggested. However, information about CO-induced effects on cells involved in airway inflammation is scarce. The present study was designed to investigate if the HO substrate analog hemin could affect neutrophil random migration, and N-formyl-L-methionyl-L-leucyl-L-phenylalanine (fMLP) induced chemotaxis.
METHODS: Hemin was added to and incubated with whole blood and the effects of the anticipated CO production were then evaluated on isolated neutrophils using a chemotaxis chamber.
RESULTS: A biphasic dose-response curve emerged for both the neutrophil spontaneous random migration and the fMLP-induced chemotaxis. Low concentrations of hemin (10(-11) m to 10(-9) m) enhanced the migratory response, whereas higher concentrations (10(-7) m and 10(-5) m) inhibited migration. The inhibition induced by hemin on fMLP-induced migration was abolished after pre-treatment with Rp-8Br-cyclicGMPS, an inhibitor of cyclicGMP.
CONCLUSIONS: The present data indicate that endogenously produced CO can affect both spontaneous and stimulated neutrophil migration, partly via a cyclicGMP-related process, hence strengthening the idea of a role for CO in airway inflammation.

PMID: 12358996 [PubMed - indexed for MEDLINE]

1417. Am J Respir Cell Mol Biol. 2002 Oct;27(4):396-405.

Role of tumor necrosis factor in toluene diisocyanate asthma.

Matheson JM, Lemus R, Lange RW, Karol MH, Luster MI.

Toxicology and Molecular Biology Branch, Health Effects Laboratory Division, National Institute for Occupational Safety and Health, Morgantown, West Virginia 26505, USA. yzm9@cdc.gov

Nearly 9 million workers are exposed to chemical agents associated with occupational asthma, with isocyanates representing the chemical class most responsible. Isocyanate-induced asthma has been difficult to diagnose and control, in part because the biologic mechanisms responsible for the disease and the determinants of exposure have not been well defined. Isocyanate-induced asthma is characterized by airway inflammation, and we hypothesized that inflammation is a prerequisite of isocyanate-induced asthma, with tumor necrosis factor (TNF)-alpha being critical to this process. To explore this hypothesis, wild-type mice, athymic mice, TNF-alpha receptor knockout (TNFR), and anti-TNF-alpha antibody-treated mice were sensitized by subcutaneous injection (20 micro l on Day 1; 5 micro l, Days 4 and 11), and challenged 7 d later by inhalation (100 ppb; Days 20, 22, and 24) with toluene diisocyanate (TDI). Airway inflammation, goblet cell metaplasia, epithelial cell damage, and nonspecific airway reactivity to methacholine challenge, measured 24 h following the last challenge, were reduced to baseline levels in TNF-alpha null mice and athymic mice. TNF-alpha deficiency also markedly abrogated TDI-induced Th2 cytokines in airway tissues, indicating a role in the development of Th2 responses. Despite abrogation of all indicators of asthma pathology, TNF-alpha neutralization had no effect on serum IgE levels or IgG-specific TDI antibodies, suggesting the lack of importance of a humoral response in the manifestation of TDI-induced asthma. Instillation studies with fluorescein-conjugated isothiocyanate and TDI suggested that TNF-alpha deficiency also resulted in a significant reduction in the

migration of airway dendritic cells to the draining lymph nodes. Taken together, these results suggest that, unlike protein antigens, TNF-alpha has multiple and central roles in TDI-induced asthma, influencing both nonspecific inflammatory processes and specific immune events.

PMID: 12356572 [PubMed - indexed for MEDLINE]

1418. Nihon Kokyuki Gakkai Zasshi. 2002 Jun;40(6):494-8.

[A case of toxocariasis with eosinophil-rich pleural effusion].

[Article in Japanese]

Sakai K, Hirasawa Y, Hashimoto A.

Department of Respiratory Medicine, Kitaishikai Uchiyama Hospital, 275-1, Shiromawari, Uchiko-cho, Kita-gun, Ehime, 791-3310, Japan.

A 68-yr-old man presented with fever, cough, and appetite loss. On admission, clinical examination revealed pleural effusion (with eosinophils accounting for 25% of the cellular component), eosinophilia, and mildly elevated liver enzymes. A diagnosis of toxocariasis was reached on the basis of a positive enzyme-linked immunosorbent assay for Toxocara canis, and the efficacy of steroid and tiabendazole. Typical thoracic involvement of toxocariasis causes cough, wheezing, transient infiltration shadows on the radiographs, and other symptoms of Löffler's syndrome. The infiltration shadows reflect the migration of the larvae through the lung, which involves their breaking through the alveolar walls. We report a rare case of toxocariasis with thoracic and pleural involvement without transient pulmonary infiltrates.

PMID: 12325335 [PubMed - indexed for MEDLINE]

1419. Biochem Biophys Res Commun. 2002 Sep 27;297(3):480-5.

Selective CCL5/RANTES-induced mast cell migration through interactions with chemokine receptors CCR1 and CCR4.

Juremalm M, Olsson N, Nilsson G.

Research Group on Mast Cell Biology, Department of Genetics and Pathology, The Rudbeck Laboratory, Uppsala University, SE-751 85 Uppsala, Sweden.

Mast cells (MCs) accumulate at sites of allergic mucosal inflammation where they act as central effector and regulatory cells. Because chemokines are of vital importance in directing inflammatory leukocytes to the sites of inflammations, we have investigated the expression and function of CC-chemokine receptor (CCR) on human MCs. Two previously unrecognized MC-chemokine receptors, CCR1 and CCR4, could be identified on cord blood-derived MCs (CBMCs). CCR1 and CCR4 expressed on CBMCs exhibited a unique response profile. Of seven CCR1 and CCR4 agonists tested, only CCL5/RANTES act as an agonist inducing chemotaxis. The migration could be partially blocked by specific antibodies against CCR1 or CCR4, while a complete inhibition was achieved when both CCR1 and CCR4 were blocked. These results demonstrate that both CCR1 and CCR4 are functional receptors on human mast cells with capacity to mediate migration towards CCL5.

PMID: 12270118 [PubMed - indexed for MEDLINE]

1420. J Pharmacol Exp Ther. 2002 Oct;303(1):300-7.

Histamine h(4) and h(2) receptors control histamine-induced interleukin-16 release from human CD8(+) T cells.

Gantner F, Sakai K, Tusche MW, Cruikshank WW, Center DM, Bacon KB.

Bayer Yakuhin, Ltd., Research Center Kyoto, Therapeutic Research Area Asthma, Japan. florian.gantner.fg@bayer.co.jp

Histamine is known to trigger the release of interleukin (IL)-16 from human CD8(+) cells. However, the individual roles of the presently known histamine receptor subtypes (H(1)-H(4)) in this inflammatory response have not been fully characterized. Histamine stimulation of human CD8(+) T lymphocytes purified from peripheral blood led to a 5- to 8-fold increase in the basal release of IL-16 within 24 h, and this increase was significantly blocked by the H(2)-selective antagonist, cimetidine, or by thioperamide, an antagonist of H(3) and H(4) receptors, respectively. The H(1) antagonist pyrilamine showed limited effects. Agonists selective for H(2) (dimaprit), H(3/4) (R-(-)-alpha-methylhistamine), and H(4) (clobenpropit) were capable of inducing the release of bioactive IL-16 because CD8(+) cell supernatants induced CD4(+) cell migration, which was abrogated by an anti-IL-16 antibody. Furthermore, preincubation of lymphocytes with pertussis toxin abolished IL-16 release triggered by activation of the G(i/o)-coupled H(4) receptor but not by the H(2) receptor. Messenger RNA expression studies confirmed H(4), H(2), and H(1) expression in human CD8(+) lymphocytes, whereas H(3) mRNA was completely absent. All leukocyte populations investigated expressed mRNA for H(4), with highest levels found in eosinophils, dendritic cells, and tonsil B cells. H(4) expression was also detected in human lung, trachea, and various cells of human lung origin, such as fibroblasts, bronchial smooth muscle cells, epithelial, and endothelial cells. Since many of those are known sources of IL-16, immune cell- and lung cell-expressed H(4) receptors may have a general role in the control of this mediator of inflammatory disorders such as asthma.

PMID: 12235264 [PubMed - indexed for MEDLINE]

1421. Eur Cytokine Netw. 2002 Jul-Sep;13(3):340-9.

Bronchopulmonary hyperreactivity and lung eosinophil sequestration but not their migration to the alveolar compartment are independent of interleukin-5 in allergic mice.

Proust B, Ruffié C, Lefort J, Vargaftig BB.

Unité de Pharmacologie Cellulaire, Unité Associée Institut Pasteur-INSERM U.485, 25, rue du Docteur-Roux, 75724 Paris Cedex 15, France.

IL-5 is present in the lung and in the circulation following allergenic challenges in humans and in animals, but its role in bronchopulmonary hyperreactivity (BHR) and lung and bronchoalveolar lavage fluid (BALF) eosinophilia remains unclear. Because compartmentalization of IL-5 is recognized, the anti-IL-5 monoclonal antibody TRFK-5 or its isotype control GL113 were delivered selectively intranasally (i.n.) and/or intravenously (i.v.) before the prior i.n. challenge with 10 mug OVA in BALB/c and BP2 "Biozzi" mice immunized according to optimized protocols with read-outs taken 24 h later. IL-5 in the BALF was suppressed by i.n. TRFK-5, whereas its production persisted in the serum. Conversely, i.v. TRFK-5 suppressed IL-5 in the serum but not in the BALF. IL-5 was suppressed in conditioned medium from lung explants from mice treated with i.n. TRFK-5, which did not affect the other Th2 cytokines, IL-4 and IL-13.

IL-5 is thus present in the alveolar, pulmonary and circulatory compartments following an i.n. allergenic challenge. When specific anti-IL-5 antibodies were delivered by the same i.n. route, BALF eosinophilia was markedly reduced, whereas BHR and lung eosinophil sequestration persisted totally or mostly, in both strains. The passage of eosinophils from lungs to alveoli depends on IL-5 released into the BALF, but not into circulation, whereas their lung sequestration and BHR are mostly IL-5-independent. IL-5 alone does not account for the complexities of BHR or of eosinophil tissue trapping, and lung-targeted immunobiologicals should be delivered into the appropriate compartment in order to assess the role of specific mediators in experimental airways/lung allergy.

PMID: 12231478 [PubMed - indexed for MEDLINE]

1422. J Invest Dermatol. 2002 Sep;119(3):609-16.

Association of a new-type prostaglandin D2 receptor CRTH2 with circulating T helper 2 cells in patients with atopic dermatitis.

Iwasaki M, Nagata K, Takano S, Takahashi K, Ishii N, Ikezawa Z.

Department of Molecular Biology, Toho University School of Medicine, Tokyo, Japan.

Prostaglandin D2 is known to be the major prostanoid produced by allergen-activated mast cells, but its role in the formation of allergic diseases is not well established because of complexity of its receptor system and lack of appropriate inhibitors. We have recently identified a new-type prostaglandin D2 receptor, named CRTH2. Studies with normal subjects have shown that CRTH2 appears to be selectively expressed by T helper 2 cells but not T helper 1 cells among circulating CD4+ lymphocytes. The exact correlation between CRTH2 and T helper 2 cells in various disease settings and the impact of CRTH2-mediated prostaglandin D2 activities on various T helper 2 responses in vivo still remain to be elucidated, however. In this study, we investigated the correlation between CRTH2 and T helper 2 cells among circulating CD4+ lymphocytes in normal adults and patients with atopic dermatitis, a T-helper-2-involving disease. The results showed that virtually all CRTH2+CD4+ lymphocytes had a pure T helper 2 phenotype and formed not all but a large proportion of circulating T helper 2 cells for both normal and atopic dermatitis subjects. In chemotaxis assays, peripheral blood CRTH2+CD4+ lymphocytes were significantly attracted by prostaglandin D2 as well as by a typical T-helper-2-attracting chemokine, thymus and activation regulated chemokine, whereas they showed little chemotactic migration toward typical T-helper-1-attracting chemokines, macrophage inflammatory protein 1beta and interferon-gamma-inducible protein 10. Furthermore, in atopic dermatitis patients, a preferential increase of CRTH2+ cells was noted within the disease-related cutaneous lymphocyte-associated antigen-positive, but not the cutaneous lymphocyte-associated antigen-negative, CD4+ lymphocyte compartment. Our results suggest the involvement of the prostaglandin D2/CRTH2 system in both normal and pathogenic T helper 2 responses.

PMID: 12230502 [PubMed - indexed for MEDLINE]

1423. Gene Ther. 2002 Sep;9(18):1221-9.

Prevention and regression of atopic dermatitis by ointment containing NF-kB decoy oligodeoxynucleotides in NC/Nga atopic mouse model.

Nakamura H, Aoki M, Tamai K, Oishi M, Ogihara T, Kaneda Y, Morishita R.

Division of Gene Therapy Science, Graduate School of Medicine, Osaka University, Suita, Japan.

Atopic dermatitis, a chronic inflammatory skin disease characterized by relapsing eczema and intense prurigo, requires effective and safe pharmacological therapy. In this study, we examined the efficacy of ointment containing NF-kB decoy oligodeoxynucleotides (ODN) on atopic dermatitis lesions in NC/Nga mice, which are characterized by the spontaneous onset of atopic dermatitis in conventional conditions. Topical administration of NF-kB decoy ODN twice a month resulted in a significant reduction in clinical skin condition score and marked improvement of histological findings. Reduction of the atopic skin condition by NF-kB decoy ODN was accompanied by a significant decrease in migration of mast cells into the dermis and an increase in apoptotic cells. Here, we demonstrated the successful treatment of atopic dermatitis with ointment containing NF-kB decoy ODN in a mouse model, promising new therapy for atopic dermatitis.

PMID: 12215889 [PubMed - indexed for MEDLINE]

1424. Am J Respir Cell Mol Biol. 2002 Sep;27(3):329-35.

The role of mitogen-activated protein kinases in eotaxin-induced cytokine production from bronchial epithelial cells.

Cui CH, Adachi T, Oyamada H, Kamada Y, Kuwasaki T, Yamada Y, Saito N, Kayaba H, Chihara J.

Department of Clinical and Laboratory Medicine, Akita University School of Medicine, Akita, Japan.

Eotaxin is a critical chemokine eliciting migration of eosinophils and basophils in the pathogenesis of bronchial asthma. Recent studies have shown that the specific receptor for eotaxin, CCR3, is expressed in bronchial epithelial cells. Although mitogen-activated protein (MAP) kinases are involved in diverse cell functions of bronchial epithelial cells, their role in eotaxin signaling is unknown. In this study, we studied the activation and functional relevance of MAP kinases in bronchial epithelial cells stimulated with eotaxin. Eotaxin (1-100 nM) induced tyrosine/threonine phosphorylation and activation of extracellular regulated kinase (ERK) 1/2 and p38 in NCI-H(292) cells and normal human bronchial epithelial cells. The phosphorylation of these MAP kinases was detectable after 30 s, and peaked at 5 min. Eotaxin stimulated production of interleukin-8 and granulocyte macrophage colony-stimulating factor. Pretreatment of Compound X (a specific CCR3 antagonist), pertussis toxin, genistein, and wortmannin reduced the MAP kinase phosphorylation and cytokine production. The eotaxin-induced cytokine production was inhibited by specific inhibitors for MAP/ERK kinase (PD98059) and p38 MAP kinase (SB202190). These results suggest that both ERK1/2 and p38 MAP kinase activated by eotaxin have a critical role in the pathogenesis of asthma.

PMID: 12204895 [PubMed - indexed for MEDLINE]

1425. Cytokine. 2002 Jul 7;19(1):27-30.

Characterisation of the biological activity of recombinant equine eotaxin in vitro.

Benarafa C, Collins ME, Hamblin AS, Sabroe I, Cunningham FM.

Department of Pathology and Infectious Diseases, The Royal Veterinary College, University of London, Hawkshead Campus, Hertfordshire, AL9 7TA, UK.

The chemokine eotaxin (CCL11) is a key player in the trafficking of eosinophils to normal tissues and in the tissue eosinophilia associated with human allergic disease. We have recently cloned equine eotaxin and here we report the production of rEq eotaxin, with and without a C-terminal fusion peptide, in a novel expression system utilising stably transfected insect cells. rEq eotaxin induced equine eosinophil migration and superoxide production in vitro. A shape change in human eosinophils that could be blocked by 7B11, a monoclonal antibody against human CCR3, was also observed. Biological activity was not dependent on an intact eotaxin C-terminus. These results suggest that equine eotaxin, like its human ortholog, may play a role in eosinophil accumulation and activation in the horse.

PMID: 12200110 [PubMed - indexed for MEDLINE]

1426. Z Geburtshilfe Neonatol. 2002 Jul-Aug;206(4):131-7.

[Syphilis in pregnancy].

[Article in German]

Enders M, Hagedorn HJ.

Labor Enders und Partner, Institut für Virologie, Infektiologie und Epidemiologie, Stuttgart, Germany.

Syphilis, a sexually transmitted infection, has a major impact on the disease burden worldwide. Globally, an estimated 12 million new cases of sexually acquired syphilis occurred in 1997. Developing countries in Africa, Southeast Asia and regions of the former Soviet Union are mainly affected. With rising numbers of human immunodeficiency virus-infected pregnant women and an increase in gonorrhoea in some areas, the incidence of syphilis is expected to increase again. As a consequence of migration from Eastern bloc countries to Europe after the breakdown of the former Soviet Union, the resurgence of syphilis will also affect Germany. Therefore, we present the clinical picture of syphilis as well as review the current recommendations of the German STD Society, the Centers of Disease Control (CDC), USA, and the Clinical Effectiveness Group (CEG), England, for diagnosis and treatment of syphilis with special emphasis on pregnancy. Considering the current epidemiological situation, physicians should include syphilis in their differential diagnosis. Although recommended therapy regimens differ, penicillin is the treatment of choice. Pregnant patients who are allergic to penicillin should be desensitized and treated with penicillin. Early recognition and timely treatment of syphilis are essential to prevent or treat potentially fatal fetal infection.

PMID: 12198589 [PubMed - indexed for MEDLINE]

1427. Hepatol Res. 2002 Aug;23(4):306.

Eosionophilic pseudotumor of the liver due to Ascaris suum infection.

Kim S, Maekawa Y, Matsuoka T, Imoto S, Ando K, Mita K, Kim H, Nakajima T, Ku K, Koterazawa T, Fukuda K, Yano Y, Nakaji M, Kudo M, Kim K, Hirai M, Hayashi Y.

Department of Gastroenterology, Kobe Asahi Hospital, 3-5-25 Bouoji-cho, Nagata-ku, 653-0801, Kobe, Japan

A case of eosinophilic pseudotumor of the liver due to Ascaris (A) suum is described in a 34-year-old-man with a high serum level of immunoglobulin E and

hypereosinophilia ascribed to a history of atopic dermatitis since childhood. Multiple hepatic hypoechoic nodules detected by ultrasound were confirmed as low-density nodules on computed tomography (CT), and as low and high signal intensity lesions on T1-and T2-weighted magnetic resonance imaging (MRI), respectively. CT during arteriography (CTA) and arterial portography revealed multiple nodules with ring-shaped enhancement and perfusion defect, respectively. Biopsied liver tissue specimens did not contain tumor cells or atypical cells; instead, they showed marked infiltration of eosinophils with necrosis and Charcot-Leyden crystals in the portal tracts and hepatic sinusoides, suggesting parasitic infection, although neither larvae nor eggs were detected. The diagnosis of visceral larva migrans (VLM) due to A. suum was based on immunoserological tests. The patient was a habitual consumer of raw bovine liver, which may explain the A. suum infection. After drug therapy with albendazole, the hypoechoic nodules disappeared. Differential diagnoses and the possible transfection route of A. suum are discussed.

PMID: 12191679 [PubMed - as supplied by publisher]

1428. J Invest Dermatol. 2002 Aug;119(2):370-6.

IL-2-IgG2b fusion protein suppresses murine contact hypersensitivity in vivo.

Rückert R, Brandt K, Hofmann U, Bulfone-Paus S, Paus R.

Department of Immunology and Cell Biology, Research Center Borstel, Borstel, Germany.

Interleukin-15 shares several functional properties with interleukin-2, and signals through the beta and gamma chain of the interleukin-2 receptor as well as through its own high affinity alpha chain. In agreement with the concept that interleukin-2 plays a key role in type IV immune responses, we have recently shown that an IL-2-IgG2b fusion protein potently suppresses Th1-type delayed type hypersensitivity reaction and Th2-type allergic sensitization in mice. We have now compared the in vivo effects of IL-2-IgG2b fusion protein with those of IL-15-IgG2b fusion protein in a murine model of Th1-type contact hypersensitivity reaction. Daily systemic injections of IL-2-IgG2b fusion protein during the sensitization phase or application of IL-2-IgG2b fusion protein just 2 h before and 10 h after antigen challenge significantly inhibited the contact hypersensitivity ear swelling response, and this without any overt signs of associated toxicity. Even local injection of IL-2-IgG2b fusion protein into the earlobe around the time of antigen challenge inhibited the ear swelling reaction significantly. In contrast, neither systemic nor local injection of the IL-15-IgG2b fusion protein modulated the contact hypersensitivity reaction significantly. IL-2-IgG2b but not IL-15-IgG2b fusion protein reduced migration of antigen-presenting cells from the skin to local lymph nodes, inhibited the expression of CD80 and CD86, and induced a significant higher number of CD4+CD25+ T cells. Therefore, the IL-2-IgGb fusion protein offers a powerful tool for suppressing and/or preventing T-cell-mediated hypersensitivity reaction in vivo.

PMID: 12190859 [PubMed - indexed for MEDLINE]

1429. Radiol Clin North Am. 2002 Jul;40(4):799-833.

Imaging of aortic stent-grafts and endoleaks.

Thurnher S, Cejna M.

Department of Radiology, University Hospital Vienna, Austria.

siegfried.thurnher@univie.ac.at

Although the technical success of stent-graft implantation is established and relatively safe, data on the long-term safety and efficacy of endovascular repair are just emerging. Because several late complications of aortic stent-graft placement have been observed, life-long follow-up remains essential. Imaging methods form an integral part of every stage of endovascular aortic aneurysm repair. The current imaging strategy should include initial plain films, CT angiography, and color-coded Duplex sonography. Plain films are an excellent means to detect migration, angulation, kinking, and structural changes of the stent mesh, including material fatigue, at follow-up. Helical CT angiography is considered a potentially revolutionary method for the noninvasive complete postprocedural assessment of aortic sten-grafting. Current data justify the use of biphasic C angiography as the postprocedural imaging technique of choice in most patients [118]. Ultrasound offers the advantages of low cost and lack of radiation exposure. High-quality ultrasound reliably excludes endoleaks in patients after stent-grafting of AAAs. There is a substantial variability, however, in measuring the diameter of aneurysm sacs; thus, confirmation using an alternative study is prudent in cases that demonstrate a significant change in size during follow-up. MR angiography serves as an attractive alternative to CT angiography in patients with impaired renal function or known allergic reaction to iodinated contrast media. With current techniques, the visualization of aortic stent-grafts (with the exception of stainless-steel-based devices) is sufficient with MR angiography. There is evidence that MR imaging is superior to CT angiography in detecting small type 2 endoleaks or for excluding retrograde perfusion in patients with suspected endotension. The role of diagnostic catheter angiography is limited to assessment of vascular pathways in equivocal cases or for suspected endotension. Currently, a consensus view about postprocedural management after aortic stent-graft implantation is lacking. The authors propose performing a baseline CT angiography at discharge and a biphasic CT angiography and Duplex ultrasound scan at three months. In patients with no evidence of an endoleak, CT angiography, plain film and Duplex sonography (abdomen) should be repeated every year after endovascular repair. If an endoleak is present at follow-up, immediate appropriate treatment should be initiated.

PMID: 12171186 [PubMed - indexed for MEDLINE]

1430. Am J Physiol Lung Cell Mol Physiol. 2002 Sep;283(3):L503-9.

Cellular biomechanics in the lung.

Waters CM, Sporn PH, Liu M, Fredberg JJ.

Department of Physiology, College of Medicine, University of Tennessee Health Science Center, Memphis 38163, USA. cwaters@physio1.utmem.edu

Mechanical forces affect both the function and phenotype of cells in the lung. In this symposium, recent studies were presented that examined several aspects of biomechanics in lung cells and their relationship to disease. Wound healing and recovery from injury in the airways involve epithelial cell spreading and migration on a substrate that undergoes cyclic mechanical deformation; enhanced green fluorescent protein-actin was used in a stable cell line to examine cytoskeletal changes in airway epithelial cells during wound healing. Eosinophils migrate into the airways during asthmatic attacks and can also be exposed to cyclic mechanical deformation; cyclic mechanical stretch caused a decrease in leukotriene C(4) synthesis that may be dependent on mechanotransduction mechanisms involving the production of reactive oxygen species. Recent studies have suggested that proinflammatory cytokines are increased in ventilator-induced lung injury and may be elevated by overdistention of the lung tissue; microarray

analysis of human lung epithelial cells demonstrated that cyclic mechanical stretch alone profoundly affects gene expression. Finally, airway hyperresponsiveness is a basic feature of asthma, but the relationship between airway hyperresponsiveness and changes in airway smooth muscle (ASM) function remain unclear. New analysis of the behavior of the ASM cytoskeleton (CSK) suggests, however, that the CSK may behave as a glassy material and that glassy behavior may account for the extensive ASM plasticity and remodeling that contribute to airway hyperresponsiveness. Together, the presentations at this symposium demonstrated the remarkable and varied roles that mechanical forces may play in both normal lung physiology as well as pathophysiology.

PMID: 12169567 [PubMed - indexed for MEDLINE]

1431. Eur J Cell Biol. 2002 Jul;81(7):383-9.

Relationship between expression of matrix metalloproteinases and migration of epidermal and in vitro generated Langerhans cells.

Noirey N, Staquet MJ, Gariazzo MJ, Serres M, André C, Schmitt D, Vincent C.

INSERM Unité 346, Laboratoire de Recherche Peau Humaine et Immunité, Hĵpital Edouard Herriot, Lyon, France.

Langerhans cells (LC) are dendritic cells that capture foreign antigens and migrate with them to the regional lymph nodes where they are presented to naive T cells. The possible role of matrix metalloproteinase-9 (MMP-9) in migration was suggested following experiments in a mouse model and in human skin explants. Using in vitro generated LC (iLC) derived from CD34+ cord blood cells and epidermal LC (eLC), we investigated the correlation between MMP-9 and other MMPs production and cell migration. Cells were activated by Bandrowski's base (BB), a chemical allergen, or by recombinant birch pollen allergen 1 (rBetv 1). Contact with allergens triggered migration of these cells, with a maximum rate being reached after 24 h. Migration was preceded by production of MMP-2 and MMP-9; part of the molecules were recovered as pro-MMPs in cell culture supernatant and part were associated with cell membrane proteins. At the cellular level, membrane-type 1 (MT1) and MT3-MMP were also identified. Addition of tumor necrosis factor-alpha (TNF-alpha) initiated pro-MMP-2 and pro-MMP-9 production followed by cell migration in a dose-dependent manner. These data imply that TNF-alpha is a key molecule for MMP production and cell migration. Furthermore, activation of iLC with BB or rBet v 1 induced synthesis of TNF-a and expression of TNF RII on the cell membrane, suggesting an autocrine loop. In conclusion, membrane-associated MMP-2 and-9 rather than soluble MMPs appear to be involved in cell migration.

PMID: 12160146 [PubMed - indexed for MEDLINE]

1432. Am J Reprod Immunol. 2002 May;47(5):295-302.

Prostaglandin D2 and reproduction.

Saito S, Tsuda H, Michimata T.

Department of Obstetrics and Gynecology, Toyama Medical and Pharmaceutical University, Toyama-shi, Japan. s30saito@ms.toyama-mpu.ac.jp

This review highlights recent studies investigating the role of prostaglandin (PG)D2 in reproduction. PGD2 induces sleep, allergic responses, inhibition of platelet aggregation, and relaxation of vascular and non-vascular smooth muscle, and has some roles in reproduction. Two types of PGD2 synthase are known.

Lipocalin-type PGD synthase is present in cerebrospinal fluid, seminal plasma and may play an important role in male reproduction. Another PGD synthase, hematopoietic PGD synthase is present in the spleen, fallopian tube, endometrial gland cells, extravillous trophoblasts and villous trophoblasts, and perhaps plays an important role in female reproduction. Recent studies demonstrate that PGD2 is probably involved in multiple aspects of inflammation through its dual receptor systems, DP and CRTH2. CRTH2 but not DP is a chemo-attractant receptor for PGD2. Interestingly, CRTH2 is a most reliable marker for the detection of human T helper type 2 (Th2) and T cytotoxic type 2 (Tc2) cells, and the percentages of CRTH expressing CD4+-T cells and CD8+-T cells were significantly higher in the decidua especially at the implantation site, suggesting that Th2 and Tc2 cells recruit into the materno-fetal interface, in a PGD2-mediated manner. PGD2 has a very unique effect to inhibit antigen presentation by inhibition of dendritic cell (DC) migration through DP but not CRTH2. PGD2 might appear to contribute to the maintenance of pregnancy by controlling the Th1/Th2 balance and antigen presentation by DCs through its dual receptor systems, CRTH2 and DP.

PMID: 12148545 [PubMed - indexed for MEDLINE]

1433. Toxicol Appl Pharmacol. 2002 Jul 15;182(2):126-35.

Differential regulation of epidermal langerhans cell migration by interleukins (IL)-1alpha and IL-1beta during irritant- and allergen-induced cutaneous immune responses.

Cumberbatch M, Dearman RJ, Groves RW, Antonopoulos C, Kimber I.

Syngenta Central Toxicology Laboratory, Alderley Park, Macclesfield, Cheshire, SK10 4TJ, United Kingdom. Marie.Cumberbatch@Syngenta.com

Tumor necrosis factor-alpha (TNF-alpha), interleukin (IL)-1beta, and IL-18 are all known to contribute to the regulation of epidermal Langerhans cells (LC) migration and the subsequent accumulation of dendritic cells (DC) in draining lymph nodes following skin sensitization. However, the cytokine signals that control these responses following skin irritation have yet to be defined. We demonstrate that IL-1alpha, a cytokine associated with skin injury and inflammation, is able to stimulate the activation and migration from the epidermis of LC and their subsequent accumulation in skin-draining lymph nodes. Stimulation of these responses by IL-1alpha required the local availability of TNF-alpha. Using specific neutralizing antibodies, LC migration induced following skin sensitization with oxazolone (Ox) was found to be dependent upon IL-1beta and independent of a requirement for IL-1alpha. However, the converse was true following stimulation of responses with the nonsensitizing skin irritant sodium lauryl sulfate (SLS). Here, the loss of LC from the epidermis and the accumulation of DC in draining lymph nodes required IL-1alpha and not IL-1beta. Despite utilizing different IL-1 isoforms for LC mobilization, the phenotypic characteristics of DC arriving in draining lymph nodes in response to Ox and SLS were similar with respect to the membrane determinants MHC class II, B7-1, B7-2, and intercellular adhesion molecule-1. These data suggest that contact sensitization and skin irritation employ subtly different cytokine networks in the regulation of LC migration, both involving TNF-alpha but demonstrating differential requirements for IL-1 cytokines. The proposal is that different forms of cutaneous trauma may achieve LC migration through distinct molecular mechanisms.

PMID: 12140176 [PubMed - indexed for MEDLINE]

1434. J Immunol. 2002 Aug 1;169(3):1524-34.

Monocyte-derived dendritic cells induce a house dust mite-specific Th2 allergic inflammation in the lung of humanized SCID mice: involvement of CCR7.

Hammad H, Lambrecht BN, Pochard P, Gosset P, Marquillies P, Tonnel AB, Pestel J.

Institut National de la Santé et de la Recherche Médicale, Unité 416, Institut Pasteur de Lille, Lille, France.

In rodents, airway dendritic cells (DCs) capture inhaled Ag, undergo maturation, and migrate to the draining mediastinal lymph nodes (MLN) to initiate the Ag-specific T cell response. However, the role of human DCs in the pathogenesis of the Th2 cell-mediated disease asthma remains to be clarified. Here, by using SCID mice engrafted with T cells from either house dust mite (HDM)-allergic patients or healthy donors, we show that DCs pulsed with Der p 1, one of the major allergens of HDM, and injected intratracheally into naive animals migrated into the MLN. In the MLN, Der p 1-pulsed DCs from allergic patients induced the proliferation of IL-4-producing CD4(+) T cells, whereas those from healthy donors induced IFN-gamma-secreting cells. In reconstituted human PBMC-reconstituted SCID mice primed with pulsed DCs from allergic patients, repeated exposure to aerosols of HDM induced 1) a strong pulmonary inflammatory reaction rich in T cells and eosinophils, 2) an increase in IL-4 and IL-5 production in the lung lavage fluid, and 3) increased IgE production compared with that in mice primed with unpulsed DCs. All these effects were reduced following in vivo neutralization of the CCR7 ligand secondary lymphoid tissue chemokine. These data in human PBMC-reconstituted SCID mice show that monocyte-derived DCs might play a key role in the pathogenesis of the pulmonary allergic response by inducing Th2 effector function following migration to the MLN.

PMID: 12133980 [PubMed - indexed for MEDLINE]

1435. Allergy. 2002 Aug;57(8):718-22.

Hemin, a heme oxygenase substrate analog, inhibits the cell surface expression of CD11b and CD66b on human neutrophils.

Andersson JA, Egesten A, Cardell LO.

Department of Otorhinolaryngology, Malmö University Hospital, Sweden.

BACKGROUND: Neutrophils are signaled to sites of infection and inflammation by different chemotactic stimuli. In order to reach the airways they have to adhere to, and then migrate through, the endothelium of pulmonary vessels. Carbon monoxide (CO) is a gaseous mediator, endogenously produced in the human airways. Increased CO production has been demonstrated during airway inflammation and CO as well as hemin, a substrate for CO producing enzymes, has been shown to affect neutrophil migration. Our objective was to investigate if the neutrophil cell surface expression of CD11b, CD66b and CD63 was changed during intermittent allergic rhinitis and to establish whether CO could affect the expression of these markers of cellular activation.
METHODS: Blood from 10 healthy volunteers was drawn and incubated with different concentrations of hemin. Blood from 12 other healthy volunteers and from 12 patients with intermittent allergic rhinitis was also drawn during grass pollen season. Neutrophils were then isolated from all these three sets, and their expression of CD antigens measured using flow cytometry.
RESULTS: Patients with symptomatic intermittent allergic rhinitis exhibited lower levels of CD11b and CD66b on the neutrophil cell surface. Incubation with hemin decreased the expression of CD11b and CD66b. CD63 was generally weakly expressed

and not significantly affected by hemin incubation.
CONCLUSION: Our results demonstrate that expressions of neutrophil cell surface glycoproteins are changed during the season in patents with intermittent allergic rhinitis and that hemin, a substrate for CO production, may act as an inhibitor of neutrophil activation. This indicates a possible role for CO in the immune defense system.

PMID: 12121191 [PubMed - indexed for MEDLINE]

1436. Int Arch Allergy Immunol. 2002 Jul;128(3):229-34.

Chymase inhibitor improves dermatitis in NC/Nga mice.

Watanabe N, Tomimori Y, Saito K, Miura K, Wada A, Tsudzuki M, Fukuda Y.

Department of Tropical Medicine, Jikei University School of Medicine, Tokyo, Japan.

BACKGROUND: Mast cell chymase is thought to participate in allergic inflammation, but its precise role remains undetermined. Inbred NC/Nga mice develop skin lesions similar to atopic dermatitis (AD) when they grow up in a conventional environment. To elucidate the possible role of chymase in AD, we examined the effect of a chymase inhibitor on skin lesions of NC/Nga mice.
METHODS: NC/Nga mice were given the chymase inhibitor SUN-C8257 daily at 150 mg/kg/day with drinking water, and the severity of the dermatitis was evaluated on day 35 of the experiment. The role of chymase in dermatitis was further investigated in vitro and in vivo using recombinant mouse mast cell protease-4 (mMCP-4).
RESULTS: Administration of SUN-C8257 significantly reduced the clinical skin and histological score in NC/Nga mice. SUN-C8257 also inhibited the accumulation of inflammatory cells, such as eosinophils and mast cells, in the affected lesions in this model. mMCP-4 stimulated eosinophil migration in vitro, and intradermal injection of the enzyme resulted in a significant accumulation of inflammatory cells, including eosinophils, at the injection site. Thus amelioration of the skin lesions in NC/Nga mice by SUN-C8257 might be, at least in part, due to the suppression of cell infiltration in the lesions.
CONCLUSION: Mast cell chymase may contribute to the pathogenesis of AD, and SUN-C8257 will be beneficial to the treatment of the skin disorder.

Copyright 2002 S. Karger AG, Basel

PMID: 12119505 [PubMed - indexed for MEDLINE]

1437. Lab Invest. 2002 Jul;82(7):929-39.

CCR3-blocking antibody inhibits allergen-induced eosinophil recruitment in human skin xenografts from allergic patients.

Sénéchal S, Fahy O, Gentina T, Vorng H, Capron M, Walls AF, McEuen AR, Buckley MG, Hamid Q, Wallaert B, Tonnel AB, Tsicopoulos A.

INSERM U416, Institut Pasteur de Lille, France.

Eosinophil, basophil, and T helper 2 (TH2) cell recruitment into tissues is a characteristic feature of allergic diseases. These cells have in common the expression of the chemokine receptor CCR3, which may represent a specific pathway for their accumulation in vivo. Although animal models of allergic reactions are available, findings cannot always be extrapolated to man. To overcome these

limitations, we have developed a humanized mouse model of allergic cutaneous reaction using severe combined immunodeficiency mice engrafted with skin and autologous peripheral blood mononuclear cells from allergic donors. Intradermal injection of the relevant allergen into human skin xenografts from allergic individuals induced a significant recruitment of human CD4(+) T cells, basophils, and TH2-type cytokine mRNA-expressing cells, as well as murine eosinophils. Human skin xenografts, atopic status, and autologous peripheral blood mononuclear cell reconstitution were all mandatory to induce the allergic reaction. Next, we addressed the role of CCR3 in the endogenous mechanisms involved in the inflammatory cell recruitment in this experimental model of allergic cutaneous reaction. In vivo administration of an anti-human CCR3-blocking antibody selectively reduced accumulation of eosinophils but not that of CD4(+) cells, basophils, or cells expressing mRNA for TH2-type cytokines. These findings establish a new in vivo model of humanized allergic reaction and suggest that eosinophil migration is mediated mainly through CCR3. Finally, these results suggest that this model might be useful to test human-specific antiallergic modulators.

PMID: 12118095 [PubMed - indexed for MEDLINE]

1438. MMW Fortschr Med. 2002 May 30;144(22):24-8.

[Early diagnosis of Lyme borreliosis. Do not look only for erythema migrans].

[Article in German]

Hofmann H.

Klinik für Dermatologie und Allergologie am Biederstein, Technische Universität München. FuH.Hofmann@lrz.tumuenchen.de

Although the typical erythema migrans is a relatively easy-to-recognize manifestation of Lyme borreliosis, it is by no means the sole cutaneous manifestation of infection with borreliae. In particular the early stages of the disease, with their paucity of symptoms or wide variability of the clinical symptomatology, often present a diagnostic challenge. This means that in the event of unclear unspecific cutaneous lesions indicating, for example, erysipelas or urticaria, Lyme borreliosis should also receive differential diagnostic consideration. Culture of the organism, DNA-based confirmation of B. burgdorferi or, where indicated, a serological investigation can confirm the diagnosis at an early stage. The treatment of choice compromises an antibiotic administered for an adequate duration and at an adequate dose, e.g. doxycycline 2 x 100 mg over three weeks). If treatment is initiated in a local or disseminated early stage, healing rates of more than 85% can be achieved.

PMID: 12116567 [PubMed - indexed for MEDLINE]

1439. J Immunol. 2002 Jul 15;169(2):651-5.

Cutting edge: Th2 cell trafficking into the allergic lung is dependent on chemoattractant receptor signaling.

Mathew A, Medoff BD, Carafone AD, Luster AD.

Center for Immunology and Inflammatory Diseases, Division of Rheumatology, Allergy and Immunology, Massachusetts General Hospital, Harvard Medical School, Boston, MA 02114, USA.

Th2 cells are recruited to the lung where they mediate the asthma phenotype. Since the molecular mechanisms regulating Th2 cell trafficking remain unknown, we sought to determine whether trafficking of Th2 cells into the lung is mediated by G alpha i-coupled chemoattractant receptors. We show here that in contrast to untreated Th2 cells, pertussis toxin-treated Th2 cells were unable to traffic into the lung, airways, or lymph nodes following Ag challenge and therefore were unable to induce allergic inflammation in vivo. Pertussis toxin-treated Th2 cells were functional cells, however, and when directly instilled into the airways of mice, bypassing their need to traffic to the lung, were able to induce airway eosinophilic inflammation. These studies conclusively demonstrate that trafficking of Th2 cells into the lung is an active process dependent on chemoattractant receptors.

PMID: 12097366 [PubMed - indexed for MEDLINE]

1440. JAMA. 2002 Jul 3;288(1):82-90.

The health of Latino children: urgent priorities, unanswered questions, and a research agenda.

Flores G, Fuentes-Afflick E, Barbot O, Carter-Pokras O, Claudio L, Lara M, McLaurin JA, Pachter L, Ramos-Gomez FJ, Mendoza F, Valdez RB, Villarruel AM, Zambrana RE, Greenberg R, Weitzman M.

Department of Pediatrics, Boston University Schools of Medicine and Public Health, Boston Medical Center, Boston, MA 02118, USA. glenn.flores@bmc.org

Erratum in
    JAMA. 2003 Aug 13;290(6):756. Gomez, Francisco R [corrected to Ramos-Gomez, Francisco J].

Comment in
    JAMA. 2002 Oct 23-30;288(16):1981; author reply 1983.
    JAMA. 2002 Oct 23-30;288(16):1981-2; author reply 1983.
    JAMA. 2002 Oct 23-30;288(16):1982-3; author reply 1983.
    JAMA. 2002 Oct 23-30;288(16):1983; author reply 1983.

Latinos recently became the largest racial/ethnic minority group of US children. The Latino Consortium of the American Academy of Pediatrics Center for Child Health Research, consisting of 13 expert panelists, identified the most important urgent priorities and unanswered questions in Latino child health. Conclusions were drawn when consensus was reached among members, with refinement through multiple iterations. A consensus statement with supporting references was drafted and revised. This article summarizes the key issues, including lack of validated research instruments, frequent unjustified exclusion from studies, and failure to analyze data by pertinent subgroups. Latino children are at high risk for behavioral and developmental disorders, and there are many unanswered questions about their mental health needs and use of services. The prevalence of dental caries is disproportionately higher for Latino children, but the reasons for this disparity are unclear. Culture and language can profoundly affect Latino children's health, but not enough cultural competency training of health care professionals and provision of linguistically appropriate care occur. Latinos are underrepresented at every level of the health care professions. Latino children are at high risk for school dropout, environmental hazards, obesity, diabetes mellitus, asthma, lack of health insurance, nonfinancial barriers to health care access, and impaired quality of care, but many key questions in these areas remain unanswered. This article suggests areas in which more research is needed and ways to improve research and care of Latino children.

PMID: 12090866 [PubMed - indexed for MEDLINE]

1441. J Med Chem. 2002 Jul 4;45(14):2988-93.

Identification of potent and novel alpha4beta1 antagonists using in silico screening.

Singh J, Van Vlijmen H, Liao Y, Lee WC, Cornebise M, Harris M, Shu IH, Gill A, Cuervo JH, Abraham WM, Adams SP.

Department of Drug Design and Evaluation, Biogen Inc., 12 Cambridge Center, Cambridge, MA 02142, USA. Juswinder_Singh@biogen.com

The antigen alpha4beta1 (very late antigen-4, VLA-4) plays an important role in the migration of white blood cells to sites of inflammation. It has been implicated in the pathology of a variety of diseases including asthma, multiple sclerosis, and rheumatoid arthritis. We describe a series of potent inhibitors of alpha4beta1 that were discovered using computational screening for replacements of the peptide region of an existing tetrapeptide-based alpha4beta1 inhibitor (1; 4-[N'-(2-methylphenyl)ureido]phenylacetyl-Leu-Asp-Val) derived from fibronectin. The search query was constructed using a model of 1 that was based upon the X-ray conformation of the related integrin-binding region of vascular cell adhesion molecule-1 (VCAM-1). The 3D search query consisted of the N-terminal cap and the carboxyl side chain of 1 because, upon the basis of existing structure-activity data on this series, these were known to be critical for high-affinity binding to alpha4beta1. The computational screen identified 12 reagents from a virtual library of 8624 molecules as satisfying the model and our synthetic filters. All of the synthesized compounds tested inhibit alpha4beta1 association with VCAM-1, with the most potent compound having an IC(50) of 1 nM, comparable to the starting compound. Using CATALYST, a 3D QSAR was generated that rationalizes the variation in activities of these alpha4beta1 antagonists. The most potent compound was evaluated in a sheep model of asthma, and a 30 mg nebulized dose was able to inhibit early and late airway responses in allergic sheep following antigen challenge and prevented the development of nonspecific airway hyperresponsiveness to carbachol. Our results demonstrate that it is possible to rapidly identify nonpeptidic replacements of integrin peptide antagonists. This approach should be useful in identification of nonpeptidic alpha4beta1 inhibitors with improved pharmacokinetic properties relative to their peptidic counterparts.

PMID: 12086484 [PubMed - indexed for MEDLINE]

1442. Recenti Prog Med. 2002 Jun;93(6):346-50.

[Role of chemokine receptors in allergic inflammation and new potential of treatment of bronchial asthma].

[Article in Italian]

D'Ambrosio D.

BioXell S.p.A. Via Olgettina, 58 20132 Milano. daniele.dambrosio@bioxell.com

The recruitment of T lymphocytes into pulmonary tissue is a critical component of the host response to infections. Migration of T cells into the lung also orchestrates inflammation, tissue injury and remodelling of tissue architecture associated with the principal chronic inflammatory pulmonary diseases such as asthma. Chemotactic cytokines, named chemokines, play a major role in regulating localization of Th2 cells and other leukocytes into the lung during an asthmatic

attack. Various proinflammatory functions have been ascribed to chemokines going from chemotaxis, activation and degranulation of distinct leukocyte subsets to remodelling of inflammed pulmonary tissue. This paper focuses on recent data either from clinical observations or animal models that have highlighted the importance of chemokines in pulmonary allergic inflammation. The recent advances in this field may now lead to the development of novel therapies for the allergic pulmonary diseases.

PMID: 12085712 [PubMed - indexed for MEDLINE]

1443. Int J Hyg Environ Health. 2002 May;205(4):269-79.

Fungal flora in groundwater-derived public drinking water.

Göttlich E, van der Lubbe W, Lange B, Fiedler S, Melchert I, Reifenrath M, Flemming HC, de Hoog S.

IWW Rheinisch Westfälisches Institut für Wasserforschung, Mülheim an der Ruhr, Germany. goettlich.elke@gmx.net

In order to assess the dissemination of hygienically relevant fungi via the public drinking water distribution system, a 12-month survey was performed on groundwater-derived drinking water from 29 water supplies in North Rhine-Westphalia, Germany. Frequencies of contaminated water samples, and the prevalent species and patterns of occurrence in raw water, waterworks, the network and house installations were studied on the basis of 2657 water samples. Results were obtained by long-term incubation of 1 ml aliquots of water samples on agar-based culture media, following bacteriological procedures documented in the German drinking water regulations (Anon, 1990). No correlation with standard hygiene indicators, such as E. coli or other coliform bacteria was observed. Common opportunistic and allergenic Aspergillus species were encountered only rarely. The fungal flora was dominated by a limited number of species of Acremonium, Exophiala, Penicillium and particularly Phialophora; some of them occurred throughout the entire drinking water system and are thought to constitute a resident fungal flora. Phialophora sp. nov., to be described as a new species elsewhere, was ubiquitous; it was found in 26.6% of the samples positive for fungi (7.5% of 2657). Fungal diversity in the network itself was significantly lower than in raw water and house installations, indicating that not all fungi gaining access to the system are capable of surviving for longer periods. For species such as Verticillium lecanii, found exclusively after the introduction of newly buried pipes and remaining localized at those sites, introduction via arthropod vectors is likely. The resident species of Phialophora, Exophiala and Acremonium are particularly significant as they are shown to be disseminated efficiently by public drinking water.

PMID: 12068746 [PubMed - indexed for MEDLINE]

1444. Int Arch Allergy Immunol. 2002;128 Suppl 1:3-11.

Effect of extracellular matrix proteins on platelet-activating factor-induced eosinophil chemotaxis.

Yoshikawa M, Matsumoto K, Iida M, Akasawa A, Moriyama H, Saito H.

Department of Allergy and Immunology, National Center for Child Health and Development Research Institute, Tokyo, Japan.

BACKGROUND: Eosinophils accumulate in tissues during various allergic

inflammatory processes. Selective eosinophil recruitment is the result of orchestrated events involving cell adhesion molecules and chemoattractants. However, the exact mechanism of the regulation of integrin avidity during interstitial migration is poorly understood.
OBJECTIVE: The purpose of this study was to investigate whether extracellular matrix proteins might activate human eosinophil chemotaxis and, if so, to clarify the mechanism in terms of integrin avidity.
METHOD: Eosinophils were purified from the peripheral blood of healthy donors. Eosinophil migration was measured using Boyden chambers with filter membranes coated with fibronectin (Fn), vitronectin (Vn), laminin (Ln), hyaluronic acid, collagen type IV, or bovine serum albumin (BSA) overnight. Platelet-activating factor (PAF) was introduced into the lower chamber of each well. Eosinophils were placed in the upper chamber after incubation with IL-5 for 15 min. The number of eosinophils that transmigrated into the lower chamber was calculated by measuring the eosinophil peroxidase activity.
RESULTS: Fn, Ln and BSA enhanced PAF-induced chemotaxis of eosinophils. Inhibition experiments using blocking monoclonal antibodies showed that in the early phase of chemotaxis, Fn and Ln facilitated eosinophil chemotaxis that was mediated by alpha4 and alpha6 integrins, respectively. In the late phase of chemotaxis, BSA, but not other matrix proteins, facilitated both chemokinesis and chemotaxis that was mediated by beta2 integrin.
CONCLUSION: Our data strongly suggest that during chemotaxis, matrix proteins might activate eosinophils via binding with integrins to facilitate PAF-induced chemotaxis, and that such a mechanism might participate in allergic inflammatory processes.

Copyright 2002 S. Karger AG, Basel

PMID: 12065903 [PubMed - indexed for MEDLINE]

1445. Lab Invest. 2002 Jun;82(6):789-94.

Chymase participates in chronic dermatitis by inducing eosinophil infiltration.

Tomimori Y, Muto T, Fukami H, Saito K, Horikawa C, Tsuruoka N, Saito M, Sugiura N, Yamashiro K, Sumida M, Kakutani S, Fukuda Y.

Suntory Biomedical Research Limited, Wakayamadai, Shimamoto-cho, Mishima-gun, Osaka, Japan.

An epicutaneous application of 2,4-dinitrofluorobenzene (DNFB) to a mouse ear caused a transient skin swelling, and the repetition of the challenge enlarged the contact dermatitis. The repeated challenge with DNFB also induced eosinophil infiltration on the application site. Administration of a chymase inhibitor significantly inhibited the ear swelling as well as eosinophil accumulation. An intradermal injection of human chymase to the mouse ear also elicited transient skin swelling and eosinophil infiltration, both of which were augmented in proportion to the number of injections. Human serum albumin and heat-inactivated chymase failed to induce such skin reactions, suggesting the participation of proteolytic activity of the enzyme. In addition, chymase stimulated eosinophil migration in vitro in a concentration-dependent manner. Taken together, these observations suggest that mast cell chymase may contribute to development of the DNFB-induced dermatitis, probably by promoting eosinophil infiltration. It is therefore possible that chymase plays a role in pathogenesis of chronic dermatitis such as atopic dermatitis.

PMID: 12065690 [PubMed - indexed for MEDLINE]

1446. J Invest Dermatol. 2002 Jun;118(6):1052-8.

Nickel-specific CD4(+) and CD8(+) T cells display distinct migratory responses to chemokines produced during allergic contact dermatitis.

Sebastiani S, Albanesi C, Nasorri F, Girolomoni G, Cavani A.

Laboratory of Immunology, Istituto Dermopatico dell'Immacolata, IRCCS, Rome, Italy.

Development of allergic contact dermatitis to haptens depends upon a balance between CD8(+) T lymphocytes with pathogenic activity and CD4(+) T cells, which comprise both effector and regulatory cells. Thus, differential recruitment of CD8(+) and CD4(+) lymphocytes to sites of hapten challenge may have considerable impact on disease expression. Here the migration of cutaneous lymphocyte-associated antigen+, nickel-specific CD8(+) and CD4(+) T cell lines were compared with a panel of chemokines produced in the skin during allergic contact dermatitis. CCL17/TARC and CCL22/MDC induced a 3-fold higher migration of CD4(+) compared with CD8(+) lymphocytes. In contrast, CXCL10/IP-10 was 2-fold more potent in attracting CD8(+) cells. These findings were consistent with the higher expression of CCR4 and CXCR3 on CD4(+) and CD8(+) T cell lines, respectively. Moreover, CCR4 expression was high on nickel-specific T helper 2, intermediate on T helper 1 and T cytotoxic 2, and almost undetectable on T cytotoxic 1 clones. On the contrary, CXCR3 was expressed by T cytotoxic 1 and 2 and T helper 1, but not T helper 2 clones. Reverse transcription-polymerase chain reaction analysis of the skin before and after hapten challenge revealed the constitutive presence of TARC, and the early appearance of CCL2/MCP-1, followed by IP-10, CCL4/MIP-1beta, and MDC mRNA. Supernatants from activated keratinocytes induced a strong migration of CD8(+) lymphocytes, which was blocked by neutralization of IP-10. Conversely, supernatants from immature and mature dendritic cells attracted mostly CD4(+) lymphocytes in a TARC- and MDC-dependent manner. Our data indicate that distinct chemokines and cell types control the accumulation of CD8(+) and CD4(+) T cells within inflamed skin.

PMID: 12060402 [PubMed - indexed for MEDLINE]

1447. Nat Immunol. 2002 Jul;3(7):673-80. Epub 2002 Jun 10.

Human epithelial cells trigger dendritic cell mediated allergic inflammation by producing TSLP.

Soumelis V, Reche PA, Kanzler H, Yuan W, Edward G, Homey B, Gilliet M, Ho S, Antonenko S, Lauerma A, Smith K, Gorman D, Zurawski S, Abrams J, Menon S, McClanahan T, de Waal-Malefyt Rd R, Bazan F, Kastelein RA, Liu YJ.

DNAX, 901 California Avenue, Palo Alto, CA 94304, USA.

Comment in
    Nat Immunol. 2002 Jul;3(7):605-7.

Whether epithelial cells play a role in triggering the immune cascade leading to T helper 2 (T(H)2)-type allergic inflammation is not known. We show here that human thymic stromal lymphopoietin (TSLP) potently activated CD11c(+) dendritic cells (DCs) and induced production of the T(H)2-attracting chemokines TARC (thymus and activation-regulated chemokine; also known as CCL17) and MDC (macrophage-derived chemokine; CCL22). TSLP-activated DCs primed naïve T(H) cells to produce the proallergic cytokines interleukin 4 (IL-4), IL-5, IL-13 and tumor necrosis factor-alpha, while down-regulating IL-10 and interferon-gamma. TSLP was highly expressed by epithelial cells, especially keratinocytes from patients with

atopic dermatitis. TSLP expression was associated with Langerhans cell migration and activation in situ. These findings shed new light on the function of human TSLP and the role played by epithelial cells and DCs in initiating allergic inflammation.

PMID: 12055625 [PubMed - indexed for MEDLINE]

1448. Curr Pharm Des. 2002;8(14):1297-317.

Macrophage migration inhibitory factor and the discovery of tautomerase inhibitors.

Orita M, Yamamoto S, Katayama N, Fujita S.

Yamanouchi Pharmaceutical Co. Ltd., Tsukuba Science City 305-8585, Japan. orita@yamanouchi.co.jp

Macrophage migration inhibitory factor (MIF) is a pro-inflammatory cytokine released from T-cells and macrophages, and is a key molecule in inflammation. Although a detailed understanding of the biological functions of MIF has not yet been found, it is known that MIF catalyzes the tautomerization of phenylpyruvate and a non-physiological molecule, D-dopachrome. A potent tautomerase inhibitor would be expected, as a validation tool, to shed light on role of MIF activity and the relationship between its biological and enzymatic activity. Such tautomerase inhibitors would be useful in the treatment of MIF-related diseases, such as sepsis, acute respiratory distress syndrome (ARDS), asthma, atopic dermatitis, rheumatoid arthritis (RA), nephropathy and tumors. In this review, we have focused on (1) the biological and enzymatic activities of MIF, (2) the discovery of novel, drug-like tautomerase inhibitors of MIF using a structure-based computer-assisted search, and (3) a crystallographic and molecular modeling study of the MIF-tautomerase inhibitor complexes (A review with 133 references).

PMID: 12052220 [PubMed - indexed for MEDLINE]

1449. J Leukoc Biol. 2002 Jun;71(6):1019-25.

Increased responsiveness of murine eosinophils to MIP-1beta (CCL4) and TCA-3 (CCL1) is mediated by their specific receptors, CCR5 and CCR8.

Oliveira SH, Lira S, Martinez-A C, Wiekowski M, Sullivan L, Lukacs NW.

University of Michigan Medical School, Department of Pathology, Ann Arbor, MI 48109, USA.

In the present study, we investigated the regulation of chemokine-mediated responses and receptor expression on eosinophils from mice. MIP-1alpha (CCL3) and eotaxin (CCL11) induced a significant and only partially overlapping intracellular calcium flux in antigen-elicited and peripheral blood eosinophils, and MCP-1 (CCL2), MDC (CCL22), MIP-1beta (CCL4), and TCA-3 (CCL1) did not. To demonstrate functional use of the specific receptors, we examined chemotactic responses. Peripheral blood eosinophils migrated toward MIP-1alpha (CCL3) and eotaxin (CCL11) but not MCP-1 (CCL2), MDC (CCL22), MIP-1beta (CCL4), and TCA-3 (CCL1). Antigen-elicited eosinophils migrated toward MIP-1alpha (CCL3) and eotaxin (CCL11), but also migrated in response to MIP-1beta (CCL4) and TCA-3 (CCL1), suggesting the up-regulation of additional chemokine receptors on antigen-elicited eosinophils. The up-regulation of the additional chemokine-receptor responses appeared to be in part because of cytokine

activation, because TNF-alpha and/or IL-4 were able to up-regulate CCR1, -3, -5, and -8 mRNA expression in eosinophils as well as migration responses to the appropriate ligands. Using antibodies specific for CCR5 and CCR8, the chemotactic response to MIP-1beta and TCA-3, respectively, was reduced significantly. Finally, the expression of these new receptors appears to have an effect on activation and degranulation because MIP-1beta (CCL4) and TCA-3 (CCL1) induce significant levels of LTC4 from elicited eosinophils. These results suggest that eosinophils may up-regulate and use additional chemokine receptors during progression of inflammatory, allergic responses for migration and activation.

PMID: 12050188 [PubMed - indexed for MEDLINE]

1450. Clin Exp Allergy. 2002 Jun;32(6):889-97.

Airway epithelial cells promote transmigration of eosinophils in a new three-dimensional chemotaxis model.

Kato Y, Fujisawa T, Shibano M, Saito T, Gatto W, Kamiya H, Hirai K, Sumida M, Yoshie O.

Department of Psychiatry, University of Mie School of Medicine, Tsu, Japan.

Comment in
    Clin Exp Allergy. 2002 Jun;32(6):811-3.

BACKGROUND: Prominent infiltration of eosinophils in airway mucosa is the pathognomonic sign of asthma. The role of airway epithelial cells in eosinophil infiltration, however, has not been fully elucidated.
OBJECTIVE: The aim of this study is to develop a new in vitro transmigration system composed of airway epithelial cells and extracellular matrix, and to investigate the role of airway epithelial cells in eosinophil infiltration.
METHODS: A layer of type I collagen gel was formed in Netwell, and BEAS-2B bronchial epithelial cells were cultured on the gel. Then the wells covered with epithelial monolayer were filled with medium, inverted, and new upper chambers were constructed on the gel side by applying a ring cap. After further incubation with or without exogenous cytokines for 48 h, eosinophils or neutrophils were loaded in upper chambers (the gel side) and cells transmigrated to lower chambers (the epithelial cell side) were counted. Immunohistochemical analyses were also performed.
RESULTS: While a simple collagen gel hardly promoted eosinophil migration even in the presence of eotaxin or RANTES, significant numbers of eosinophils migrated to lower chambers in the presence of the epithelial cells. Replacement of medium in the lower chamber (the epithelial cell side) with fresh medium, addition of exogenous eotaxin or RANTES in the upper chamber (the gel side), or pre-treatment of eosinophils with anti-CCR3 all inhibited transmigration. We found that the epithelial cells produced and deposited extracellular matrix proteins such as type IV collagen onto the type I collagen gel. Separately, we found that type IV collagen itself was capable of enhancing eotaxin-induced eosinophil migration in a standard chemotaxis assay. Neutrophils also efficiently migrated in the present transmigration system. Pre-treatment of epithelial cells with TNF-alpha and IL-4 enhanced eosinophil transmigration, while that of neutrophils was enhanced by TNF-alpha but suppressed by IL-4.
CONCLUSION: By utilizing a new in vitro transmigration system mimicking the airway mucosa, we have demonstrated that airway epithelial cells play an essential role in transmigration of eosinophils and that multiple factors such as chemokines, extracellular matrix proteins and exogenous inflammatory cytokines are involved in efficient transmigration.

PMID: 12047436 [PubMed - indexed for MEDLINE]

1451. Curr Opin Allergy Clin Immunol. 2002 Jun;2(3):239-48.

Experimental analysis of eosinophil-associated gastrointestinal diseases.

Hogan SP, Foster PS, Rothenberg ME.

Division of Biochemistry and Molecular Biology, John Curtin School of Medical Research, Australian National University, Canberra, Australia.
simon.hogan@anu.edu.au

Eosinophil infiltration into the gastrointestinal tract occurs in a wide range of diseases. However, the underlying cellular and molecular mechanisms involved in eosinophil migration and the role of eosinophils in disease pathogenesis are largely unknown. Recent studies using experimental models of eosinophil-associated gastrointestinal allergy have revealed differential roles for IL-5 and eotaxin in the modulation of eosinophil accumulation into various regions of the gastrointestinal tract. Furthermore, such studies have revealed a possible role for eosinophils in the pathogenesis of gastrointestinal disorders. The present review describes the clinical manifestations of various eosinophil-associated gastrointestinal disorders and the current understanding of the role of IL-5 and eotaxin in the allergic inflammatory response, and the participation of the eosinophilic granulocyte in the expression of disease.

PMID: 12045421 [PubMed - indexed for MEDLINE]

1452. J Neuroimmunol. 2002 Jun;127(1-2):69-79.

Interferon-beta directly influences monocyte infiltration into the central nervous system.

Floris S, Ruuls SR, Wierinckx A, van der Pol SM, Döpp E, van der Meide PH, Dijkstra CD, De Vries HE.

Department of Molecular Cell Biology, VU Medical Center, Van der Boechorststraat 7, 1081 BT Amsterdam, The Netherlands.

Interferon-beta (IFN-beta) has beneficial effects on the clinical symptoms of multiple sclerosis (MS) patients, but its exact mechanism of action is yet unknown. We here suggest that IFN-beta directly modulates inflammatory events at the level of cerebral endothelium. IFN-beta treatment resulted in a marked reduction of perivascular infiltrates in acute experimental allergic encephalomyelitis (EAE), the rat model for MS, which was coupled to a major decrease in the expression of the adhesion molecules ICAM-1 and VCAM-1 on brain capillaries. In vitro, IFN-beta reduced the mRNA levels and protein expression of adhesion molecules of brain endothelial cell cultures and diminished monocyte transendothelial migration. Monocyte adhesion and subsequent migration was found to be predominantly regulated by VCAM-1. These data indicate that IFN-beta exerts direct antiinflammatory effects on brain endothelial cells thereby contributing to reduced lesion formation as observed in MS patients.

PMID: 12044977 [PubMed - indexed for MEDLINE]

1453. Eur Respir J. 2002 May;19(5):980-1; author reply 981.

Symptoms of asthma, bronchial responsiveness and atopy in immigrants and emigrants in Europe.

Kalyoncu AF.

Comment on
    Eur Respir J. 2001 Sep;18(3):459-65.

PMID: 12030742 [PubMed - indexed for MEDLINE]

1454. J Immunol. 2002 Jun 1;168(11):5832-9.

Signal-regulatory protein alpha-CD47 interactions are required for the transmigration of monocytes across cerebral endothelium.

de Vries HE, Hendriks JJ, Honing H, De Lavalette CR, van der Pol SM, Hooijberg E, Dijkstra CD, van den Berg TK.

Department of Molecular Cell Biology, Vrije Universiteit Medical Center, Amsterdam, The Netherlands. HE.de_Vries.Cell@med.vu.nl

Monocyte infiltration into inflamed tissue requires their initial arrest onto the endothelial cells (ECs), followed by firm adhesion and subsequent transmigration. Although several pairs of adhesion molecules have been shown to play a role in the initial adhesion of monocytes to ECs, the mechanism of transendothelial migration is poorly defined. In this study, we have investigated the role of signal-regulatory protein (SIRP)alpha-CD47 interactions in monocyte transmigration across brain ECs. CD47 expression was observed in vivo on cerebral endothelium of both control animals and animals suffering from experimental allergic encephalomyelitis. To investigate whether SIRPalpha-CD47 interactions are instrumental in the trafficking of monocytes across cerebral EC monolayers, in vitro assays were conducted in which the migration of monocytes, but not adhesion, was found to be effectively diminished by blocking SIRPalpha and CD47 on monocytes and ECs, respectively. In this process, SIRPalpha was found to interact solely with its counterligand CD47 on ECs. Overexpression of the CD47 molecule on brain ECs significantly enhanced monocytic transmigration, but did not affect adhesion. SIRPalpha-CD47-mediated transendothelial migration involved Gi protein activity, a known signaling component of CD47. Finally, cross-linking of CD47 on brain ECs induced cytoskeletal reorganization of the endothelium, a process that was Gi protein independent. These data provide the first evidence that the interaction of CD47 with its monocytic counterligand SIRPalpha is of importance in the final step of monocyte trafficking into the brain, a critical event in the development of neuroinflammatory diseases.

PMID: 12023387 [PubMed - indexed for MEDLINE]

1455. Eur J Pharmacol. 2002 May 3;442(1-2):155-62.

Expression of nitric oxide synthases and in vitro migration of eosinophils from allergic rhinitis subjects.

Ferreira HH, Lodo ML, Martins AR, Kandratavicius L, Salaroli AF, Conran N, Antunes E, De Nucci G.

Clinical Pharmacology and Gastroenterology Unit (USF), USF, Bragança Paulista (SP), Brazil. heloisaferreira@saofrancisco.edu.br

The expression of nitric oxide (NO) synthases and the role of the NO cyclic GMP pathway on the migration of eosinophils from untreated patients with allergic rhinitis were investigated. Inducible NO synthase was strongly expressed in

eosinophils from healthy individuals, but not in eosinophils from allergic rhinitis patients. The neuronal isoform was observed in eosinophils from each group studied, whereas no staining for the endothelial isoform was detected in either group. The chemotaxis to N-formyl-methionyl-leucyl-phenylalanine (fMLP, 5 x 10(-7) M) and eotaxin (100 ng/ml) was significantly potentiated in allergic rhinitis eosinophils. In both groups, N(omega)-nitro-L-arginine methyl ester (L-NAME, 1.0 mM) or 1H(1,2,4)-oxadiazolo(4,3,-a)quinoxalin-1-one (ODQ, 0.2 mM) markedly reduced the chemotaxis. The selective iNOS inhibitor N-(3-(aminomethyl)benzyl)acetamidine (1400 W, 0.1-1.0 mM) significantly reduced the chemotaxis of eosinophils from healthy but not from allergic rhinitis subjects. The inhibition by L-NAME was restored by 3-morpholinosydnonimine (SIN-1) and S-nitroso-N-acetyl-penicillamine, whereas the inhibition by ODQ was restored by dibutyryl cyclic GMP. In conclusion, both endothelial and inducible NO synthase isoforms are absent in allergic rhinitis eosinophils, suggesting that the NO cyclic GMP pathway in this cell type is maintained through the activity of a neuronal isoform.

PMID: 12020693 [PubMed - indexed for MEDLINE]

1456. Am J Clin Dermatol. 2002;3(4):229-38.

Cellular and molecular pathophysiology of cutaneous drug reactions.

Pichler WJ, Yawalkar N, Britschgi M, Depta J, Strasser I, Schmid S, Kuechler P, Naisbitt D.

Division of Allergology, Clinic for Rheumatology and Clinical Immunology/Allergology, Inselspital, University of Bern, Bern, Switzerland. werner.pichler@insel.ch

Hypersensitivity reactions to drugs can cause a variety of skin diseases like maculopapular, bullous and pustular eruptions. In recent years increasing evidence indicates the important role of T cells in these drug-induced skin diseases. Analysis of such drug-specific T cell clones has revealed that drugs can be recognized by alpha beta-T cell receptors, not only if bound covalently to peptides, but also if the drug binds in a rather labile way to the presenting major histocompatibility complex (MHC)-peptide. This presentation is sufficient to stimulate T cells. In maculopapular exanthema (MPE), histopathological analysis typically shows a dominant T cell infiltration together with a vacuolar interface dermatitis. Immunohistochemical studies demonstrate the presence of cytotoxic CD4+ and to a lesser degree of CD8+ T cells, which contain perforin and granzyme B. They are close to keratinocytes that show signs of cell destruction. Expression of Fas ligand is barely detectable, suggesting that cytotoxic granule exocytosis may be the dominant pathway leading to keratinocyte cell damage. While in MPE, the killing of cells seems to be predominantly mediated by CD4+ T cells, patients with bullous skin disease show a strong CD8+ T cell migration to the epidermis. This is probably due to a preferential presentation of the drug by MHC class I molecules, and a more extensive killing of cells that present drugs on MHC class I molecules. This might lead to bullous skin diseases. In addition to the presence of cytotoxic T cells, drug-specific T cells also orchestrate the inflammatory skin reaction through the release and induction of various cytokines [i.e. interleukin (IL)-5, IL-6, tumor necrosis factor-alpha and interferon-gamma] and chemokines (RANTES, eotaxin or IL-8). The increased expression of these mediators seems to contribute to the generation of tissue and blood eosinophilia, a hallmark of many drug-induced allergic reactions. However, in acute generalized exanthematous pustulosis (a peculiar form of drug allergy), neutrophils represent the predominant cell type within pustules, probably due to their recruitment by IL-8 secreting drug specific T cells and keratinocytes.

PMID: 12010068 [PubMed - indexed for MEDLINE]

1457. Eur Respir J. 2002 Apr;19(4):684-9.

Prostaglandin D2 inhibits fibroblast migration.

Kohyama T, Liu XD, Wen FQ, Kim HJ, Takizawa H, Rennard SI.

Pulmonary and Critical Care Medicine Section, University of Nebraska Medical Centre, Omaha 68198-5125, USA.

Fibroblasts play an important role in the repair and remodelling processes following injury. Prostaglandin D2 (PGD2) is a potent mediator in inflammatory processes. In this study, the effect of the PGD2 on human foetal lung fibroblasts (HFL-1) chemotaxis induced by human plasma fibronectin (HFn) was investigated using the blindwell chamber technique. PGD2 inhibited HFL-1 chemotaxis to HFn (20 microg x mL(-1)) by 20.8 +/- 3.8% (p<0.05). Checkerboard analysis of HFn-directed migration confirmed that PGD2 inhibited both chemotaxis and chemokinesis. The effect of PGD2 was concentration-dependent and the inhibitory effect diminished with time. The PGD2 receptor (DP) agonist BW245C (500 nM) had a similar effect, inhibiting chemotaxis to 39.4 +/- 6.3%. The inhibitory effects of both PGD2 and BW245C on HFL-1 chemotaxis were blocked by the DP receptor antagonist AH6809 (2 microM). The inhibitory effect of PGD2 on fibroblast chemotaxis was also blocked by the cyclic adenosine monophosphate (cAMP)-dependent protein kinase (PKA) inhibitor, KT5720, suggesting a DP receptor-initiated, cAMP-dependent effect mediated by PKA. Prostaglandin D2 appears to inhibit fibroblast chemotaxis, perhaps by modulating the rate of fibroblast migration. Such an effect may contribute to regulation of the wound healing response following injury in asthma patients.

PMID: 11998998 [PubMed - indexed for MEDLINE]

1458. J Allergy Clin Immunol. 2002 May;109(5):831-8.

Lipid mediators from pollen act as chemoattractants and activators of polymorphonuclear granulocytes.

Traidl-Hoffmann C, Kasche A, Jakob T, Huger M, Plötz S, Feussner I, Ring J, Behrendt H.

Division of Environmental Dermatology and Allergy TUM/GSF, Neuherberg, Germany.

BACKGROUND: Under natural exposure conditions, pollen grains function as allergen carriers that release allergens from internal binding sites on contact with the aqueous phase of mucosa membranes. In addition, we recently demonstrated that pollen are a rich source of eicosanoid-like mediators, which are rapidly released on contact with the aqueous phase.
OBJECTIVE: The current study was designed to characterize the biochemical nature of pollen-derived lipid mediators in more detail and to delineate their biologic activity on polymorphonuclear granulocytes (PMNs).
METHODS: Aqueous and lipid extracts from Phleum pratense L and Betula alba L pollen were analyzed by means of HPLC. PMNs were exposed to aqueous extracts or lipid fractions from pollen or to HPLC-purified lipid mediators identified in pollen extracts. Effects on PMNs were tested with transwell migration, calcium mobilization, and surface expression of CD11b.
RESULTS: Aqueous pollen extracts (APEs) contained predominantly monohydroxylated products derived of linoleic acid and linolenic acid. In chemotaxis assays PMNs displayed significant migration to APEs. Lipid extracts from pollen and the HPLC

fraction containing 13-hydroxy-octadecadienoic acid/hydroxy-linoleic acid and 13-hydroxy-octadecatrienoic acid/hydroxy-linolenic acid induced migratory responses, although to a lesser degree than the APEs. In addition, APE, as well as lipid, extracts induced PMN activation, as documented by means of calcium mobilization and upregulation of CD11b.
CONCLUSION: Pollen grains release mediators that recruit and activate PMNs in vitro. Similar mechanisms may be effective in vivo, suggesting that pollen-derived lipid mediators may act as adjuvants in the elicitation phase of allergic reactions.

PMID: 11994708 [PubMed - indexed for MEDLINE]

1459. Exp Dermatol. 2002 Apr;11(2):135-42.

Expression of the C-C chemokine MIP-3 alpha/CCL20 in human epidermis with impaired permeability barrier function.

Schmuth M, Neyer S, Rainer C, Grassegger A, Fritsch P, Romani N, Heufler C.

Departments of Dermatology, University of Innsbruck, Innsbruck, Austria.
matthias.schmuth@uibk.ac.at

External assault to the skin is followed by an epidermal response including synthesis of DNA, lipids, cytokines and migration of antigen presenting cells. MIP-3 alpha (CCL20, LARC, Exodus-1, Scya20) is a recently described C-C chemokine, predominantly expressed in extralymphoid tissue, which is known to direct migration of dendritic cell precursors and memory lymphocytes to sites of antigen invasion. We assessed the expression of MIP-3 alpha in human skin using semi-quantitative polymerase chain reaction. In vivo, MIP-3 alpha mRNA was constitutively expressed at low levels in untreated human epidermis. After acute disruption of the epidermal permeability barrier MIP-3 alpha mRNA was upregulated in the epidermal fraction, whereas dermal MIP-3 alpha mRNA levels remained unchanged. In vitro, MIP-3 alpha was increased in cultured keratinocytes treated with IL-1 alpha and TNF-alpha and was present in immature and mature dendritic cells, THP-1 monocytic cells and activated T cells. Finally, skin biopsies from patients with psoriasis, contact dermatitis and mycosis fungoides showed abundant expression. In biopsies from atopic dermatitis and graft vs. host disease a weak signal was present, whereas no expression was found in scleroderma and toxic epidermal necrolysis. We conclude that regulation of MIP-3 alpha mRNA is part of the epidermal response to external assault. Its upregulation may represent a danger signal for increased immunosurveillance in barrier disrupted skin and inflammatory skin conditions with impaired barrier function to counteract potential antigen invasion.

PMID: 11994140 [PubMed - indexed for MEDLINE]

1460. Immunol Invest. 2002 Feb;31(1):29-40.

Differences in the sensitisation to ragweed pollen and occurrence of late summer allergic symptoms between native and immigrant workers of the nuclear power plant of Hungary.

Dervaderics M, Fust G, Otos M, Barok J, Pataky G.

3rd Department of Internal Medicine, Faculty of Medicine, Semmelweis University, Budapest, Hungary.

Regular medical screening including allergic symptoms of workers of the Nuclear

Power Plant in Paks, Hungary, offered a unique opportunity to study the influence of the environmental factors on the development of allergy. The city Paks has been one the areas of the country most heavily exposed to ragweed pollen allergens. The occurrence and extent of sensitisation assessed by skin prick test to common allergens and prevalence of seasonal and perennial allergic symptoms were compared among 880 workers (695 immigrants, 185 natives) with self-reported allergic symptoms. The percentage of sensitised people against common allergens (ragweed, grass, D. pteronyssinus) was almost the same in the natives and immigrants. When, however, the strength of sensitivity to ragweed was determined by quantitative skin prick test and specific IgE determination, significantly (P<0.0001) more immigrants (69%) than native workers (20%) exhibited high sensitivity to ragweed and the titres of specific IgE antibodies was also significantly (p < 0.0001) higher in the former group. Similar differences were found in the occurrence and type of allergic symptoms. Seasonal symptoms alone occurred in 69% and 38% of the immigrant and native workers (p < 0.0001). This difference was mostly due to those with late summer symptoms characteristic to ragweed allergy. These symptoms occurred in 57% of the immigrant workers, much more frequently (p < 0.0001) than in the natives (18%). Our findings indicate that the length of exposure to an inhalant allergen does affect the extent of sensitisation to ragweed allergen and markedly influences the clinical symptoms that develop in the ragweed allergic patients upon allergen exposure. It can be assumed that the natives who have been living with ragweed pollen for a long time (that is were exposed to natural immunotherapy), developed a natural tolerance to it.

PMID: 11990461 [PubMed - indexed for MEDLINE]

1461. Immunology. 2002 Apr;105(4):466-77.

Influence of ageing on Langerhans cell migration in mice: identification of a putative deficiency of epidermal interleukin-1beta.

Cumberbatch M, Dearman RJ, Kimber I.

Syngenta Central Toxicology Laboratory, Alderley Park, Macclesfield, Cheshire, SK10 4TJ, UK. marie.cumberbatch@syngenta.com

Previous studies in mice have reported a decrease in epidermal Langerhans cell (LC) density in aged skin, however, the impact of this reduction on LC function and cutaneous immune responses is unclear. In the present series of experiments, the frequency of major histocompatibility complex class II+ LC in the epidermis of older (6-month-old) mice was found to be reduced significantly compared with that observed for young (6-8-week-old) mice. LC mobilization and the subsequent accumulation of dendritic cells (DC) in regional lymph nodes in response to topical challenge with a chemical allergen were found to be less vigorous in older mice. Flow cytometric analyses of DC derived from the draining lymph nodes of fluorescein isothiocyanate (FITC)-sensitized mice revealed that the frequency of FITC+-DC arriving in draining lymph nodes was also reduced in older mice but that the fluorescence intensity was comparable. Control and allergen-treated-older mice also displayed decreased total lymph node cellularity. Contact hypersensitivity responses were found not to be compromised in older mice. However, the cytokine regulation of LC migration in the two age groups of mice did differ. LC migration provoked by intradermal injection of tumour necrosis factor-alpha (TNF-alpha) was reduced in older animals, whereas, the percentage of LC that migrated in response to exogenous interleukin-1beta (IL-1beta) was comparable for both young and aged mice. Since both allergen- and TNF-alpha-induced LC responses are known to require receipt by LC of a signal from IL-1beta for effective migration, the suggestion is that impaired LC migration in older mice may be due to a reduced availability of epidermal

IL-1beta.

PMCID: PMC1782675
PMID: 11985667 [PubMed - indexed for MEDLINE]

1462. Clin Exp Allergy. 2002 Apr;32(4):619-26.

Cellular kinetics of an allergic-type response in a sheep mammary gland model of inflammation.

Bischof RJ, Meeusen EN.

Centre for Animal Biotechnology, School of Veterinary Science, The University of Melbourne, Melbourne, Victoria 3010, Australia. rbischof@unimelb.edu.au

BACKGROUND: Tissue recruitment of eosinophils and activated lymphocytes is a characteristic feature of allergic reactions. However, little is known about the involvement of specific adhesion molecules in the traffic of leucocytes during the allergic response.
OBJECTIVE: To use a sheep mammary infusion model to characterize the kinetics of cell recruitment and expression of cellular adhesion molecules and activation markers on eosinophils and lymphocytes involved in an allergic-type response.
METHODS: Mature non-lactating ewes were primed and challenged by direct infusion of the mammary glands with nematode larvae. Using a non-invasive method of saline infusion and 'milking' of the glands, large numbers of inflammatory cells were repeatedly sampled over 10 to 96 h following their migration into the mammary gland lumen, and analyzed by 2-colour flow cytometry.
RESULTS: Leucocyte recruitment into the mammary lumen was characterized by two separate phases involving an acute neutrophilic response at 10 h post-challenge, followed by a dramatic reduction in neutrophils and appearance of eosinophils and activated lymphocytes. From 48 h post-challenge, eosinophils were predominant and represented 40 to 65% of leucocytes in the mammary lavage (MAL). Increases in activated CD4+ T cells and gammadelta+ T cells were also observed at this time-point. The kinetics of expression of cell surface molecules on eosinophils and lymphocytes in blood and MAL were compared during the course of the allergic-type reaction. Adhesion molecule expression on lymphocytes was modulated following allergen challenge and an activation of MAL vs. blood lymphocytes was seen during the later stages of the allergic response. Eosinophil expression of VLA-4 and l-selectin was down-regulated compared with blood at all time-points examined. There were high levels of expression of CD11b and CD44 on eosinophils during the early compared to the late-phase of the allergic reaction.
CONCLUSION: These results indicate the existence of two separate mechanisms of eosinophil recruitment during the allergic inflammatory response.

PMID: 11972611 [PubMed - indexed for MEDLINE]

1463. J Immunol. 2002 May 1;168(9):4293-300.

Attenuation of experimental allergic encephalomyelitis in complement component 6-deficient rats is associated with reduced complement C9 deposition, P-selectin expression, and cellular infiltrate in spinal cords.

Tran GT, Hodgkinson SJ, Carter N, Killingsworth M, Spicer ST, Hall BM.

Department of Medicine, University of New South Wales, Liverpool Hospital, Liverpool, New South Wales, Australia.

The role of Ab deposition and complement activation, especially the membrane

attack complex (MAC), in the mediation of injury in experimental allergic encephalomyelitis (EAE) is not resolved. The course of active EAE in normal PVG rats was compared with that in PVG rats deficient in the C6 component of complement (PVG/C6(-)) that are unable to form MAC. Following immunization with myelin basic protein, PVG/C6(-) rats developed significantly milder EAE than PVG/C rats. The anti-myelin basic protein response was similar in both strains, as was deposition of C3 in spinal cord. C9 was detected in PVG/C rats but not in PVG/C6(-), consistent with their lack of C6 and inability to form MAC. In PVG/C6(-) rats, the T cell and macrophage infiltrate in the spinal cord was also significantly less than in normal PVG/C rats. There was also reduced expression of P-selectin on endothelial cells, which may have contributed to the reduced cellular infiltrate by limiting migration from the circulation. Assay of cytokine mRNA by RT-PCR in the spinal cords showed no differences in the profile of Th1 or Th2 cytokines between PVG/C and PVG/C6(-) rats. PVG/C rats also had a greater increase in peripheral blood white blood cell, neutrophil, and basophil counts than was observed in the PVG/C6(-). These findings suggest that the MAC may have a role in the pathogenesis of EAE, not only by Ig-activated MAC injury but also via induction of P-selectin on vascular endothelium to promote infiltration of T cells and macrophages into the spinal cord.

PMID: 11970970 [PubMed - indexed for MEDLINE]

1464. Curr Opin Allergy Clin Immunol. 2002 Feb;2(1):47-51.

Factors controlling smooth muscle proliferation and airway remodelling.

Black JL, Johnson PR.

Department of Pharmacology, University of Sydney, New South Wales, Australia 2006. judblack@pharmacol.usyd.edu.au

It is clear that airway smooth muscle plays an important role in the hyperresponsiveness and remodelling that occur in the asthmatic airway. This is by virtue of its roles as a contractile cell, a cell that undergoes proliferation as part of the inflammatory response, a cell that actively participates in the inflammatory response via the production of cytokines and chemokines, and perhaps as a cell that undergoes migration. Now that airway smooth muscle cells cultured from asthmatic patients have been studied in vitro, it is apparent that there is an abnormality in the growth of these cells such that they grow more rapidly than cells derived from nonasthmatic patients. This raises the possibility of identifying the exact point(s) in the signal transduction pathways at which this abnormality occurs. To do this it is necessary to define precisely the mitogenic pathways that lead to proliferation in the airway smooth muscle cell, and this information is accumulating rapidly. The possibility is raised for new therapeutic targets that are aimed specifically at the airway smooth muscle, leading to an effective method for reversing or preventing the airway remodelling that accompanies chronic severe asthma.

PMID: 11964750 [PubMed - indexed for MEDLINE]

1465. Virchows Arch. 2002 Feb;440(2):172-80.

Expression of adhesion molecules in allergic lung diseases.

Popper HH, Pailer S, Wurzinger G, Feldner H, Hesse C, Eber E.

Institute of Pathology, Laboratory of Environmental and Respiratory Pathology, University of Graz, Austria. popper@uni-graz.at

Endothelial adherence and migration of leukocytes into tissue is mediated by different sets of adhesion molecules. The expression of these sets might not only preselect the types of leukocytes that enter the inflammatory sites, but also activate these leukocytes, induce adherence to epithelial cells, and cause the release of cytokines. Atopic asthma, extrinsic allergic alveolitis, and sarcoidosis as examples of immunologic lung diseases were investigated for the expression of adhesion molecules. Bronchial biopsies in chronic obstructive lung disease (COPD) and resected lung tissue of juvenile emphysema were chosen for controls. Immunohistochemistry was done on sections from bronchial and transbronchial biopsies and on smears from bronchoalveolar lavage cells. In all three types of immune disorders, lymphocytes expressed the integrins alpha4/beta1 (VLA4) and ICAM3, whereas lymphocytes in COPD bronchitis and in emphysema controls were unreactive. Eosinophils in atopic asthma bronchitis in contrast to COPD bronchitis also expressed both VLA4 and ICAM3. The expression of VCAM1 on endothelial cells was only seen in atopic asthma and was related to disease activity. The expression of other adhesion molecules was nonspecific. Expression of VCAM1 on endothelial cells and its ligand VLA4 on lymphocytes and eosinophils seems to be a specific event in atopic asthma. Expression of VLA4 and ICAM3 on lymphocytes, however, might be a specific event in all three immune reactions.

PMID: 11964048 [PubMed - indexed for MEDLINE]

1466. Drugs Exp Clin Res. 2001;27(5-6):165-75.

Mucopolysaccharides from psyllium involved in wound healing.

Westerhof W, Das PK, Middelkoop E, Verschoor J, Storey L, Regnier C.

Department of Dermatology, Academic Medical Center, University of Amsterdam, Meibergdreef 9, 1005AZ Amsterdam, The Netherlands. w.westerhof@amc.uva.nl

Mucopolysaccharides derived from the husk of psyllium (Plantago ovata) have properties beneficial for wound cleansing and wound healing. Recent studies indicate that these mucopolysaccharides also limit scar formation. Our in vitro and in vivo studies aimed to investigate the mechanisms involved, e.g., fluid absorption, bacterial adherence and in vitro stimulatory effects on macrophages, which are pivotal in wound healing. The mucopolysaccharides contained in a sachet (Askina Cavity) or in a hydrocolloid mixture (Askina Hydro) were found to have a gradual and sustained absorbency over a period of 7 days, amounting to 4-6 times their weight in water. The swelling index was 9 mm after 312 h. Adherence of wound bacteria to the mucopolysaccharides started after 2 h and was more pronounced after 3 h. Semiquantitative measurements of bacterial adherence used centrifugation and subsequent optical density determinations of supernatant. These confirmed the strong adherence potential of psyllium particles. Lactic acid dehydrogenase staining of pretreated cultured human skin explants did not reveal toxicity of the mucopolysaccharides derived from psyllium husk. Langerhans' cell migration from the epidermis was negligible and interleukin-1 beta expression in the explants was not significant, supporting the very low allergenic potential of psyllium. The characteristics of mucopolysaccharide granulate derived from psyllium husk in Askina Cavity and Askina Hydro related to fluid absorption, bacterial adherence, biocompatibility, stimulation of macrophages, irritancy response and allergenicity showed an optimal profile, supporting the good clinical performance of wound healing products containing psyllium husk.

PMID: 11951574 [PubMed - indexed for MEDLINE]

1467. Diabetes Metab. 2002 Feb;28(1):51-4.

Measurement of accidental urinary insulin loss from a dislocated intraperitoneal insulin catheter.

Kühn-Velten WN, Genesius H, Wiefels KJ.

Clinical Department, German Diabetes Research Institute, Heinrich-Heine-University, D-40225 Düsseldorf, Germany.

We report the case of a type-2 diabetic woman who received continuous intraperitoneal insulin infusion and developed deterioration of metabolic control by accidental insulin loss into urine (54 U per day) as a consequence of catheter migration which probably resulted in bladder wall injury. Due to iodine allergy of this patient, an analyte addition procedure for insulin quantification in urine had to be applied to allow proof of insulin loss from the catheter tip before as well as reversal to zero insulin excretion after implantation of a new intraperitoneal port and a shorter catheter. The lost fraction of insulin accounted nearly completely for the difference between pre- and postoperatively required insulin doses (146 versus 88 U per day).

PMID: 11938029 [PubMed - indexed for MEDLINE]

1468. J Immunol. 2002 Apr 15;168(8):3707-11.

Cutting edge: anti-inflammatory properties of low levels of IFN-gamma.

Flaishon L, Topilski I, Shoseyov D, Hershkoviz R, Fireman E, Levo Y, Marmor S, Shachar I.

Department of Immunology, Weizmann Institute of Science, and Sourasky Medical Center, Tel-Aviv, Israel.

Activation of naive T and B cells occurs only within the context of organized lymphoid tissue. Thus, the continuous recirculation of mature lymphocytes is crucial for the development of primary immune response to foreign Ags. We have previously shown that low levels of IFN-gamma inhibit homing of B cells to the secondary lymphoid organs. In this study, we demonstrate that similarly low doses of IFN-gamma down-regulate integrin-mediated adhesion and migration of naive T and Th2 cells, and have a profound effect on the in vivo homing of naive T cells to the lymph nodes. Moreover, we show that these low doses of IFN-gamma have anti-inflammatory effects in an in vivo asthma model. Thus, in contrast to the proinflammatory effects of IFN-gamma at relatively high concentrations, low dose IFN-gamma appears to exert global suppressory effects on T cell trafficking and may have clinical application as an anti-inflammatory agent.

PMID: 11937520 [PubMed - indexed for MEDLINE]

1469. Eur J Pharmacol. 2002 Mar 29;439(1-3):159-69.

No involvement of interleukin-5 or eosinophils in experimental allergic rhinitis in guinea pigs.

Yamasaki M, Mizutani N, Sasaki K, Nabe T, Kohno S.

Department of Pharmacology, Kyoto Pharmaceutical University, 5 Nakauchi, Misasagi, Yamashina, 607-8414, Kyoto, Japan. Yamasaki_Masashi@takeda.co.jp

The aim of this study is to evaluate whether nasal airway eosinophilia is a true

pathogenetic component of allergic rhinitis. We investigated the effects of TRFK5, an anti-interleukin-5 antibody, not only on leukocyte mobilization from the bone marrow, but also on the development of nasal symptoms and hyperresponsiveness in a guinea pig model of allergic rhinitis. Intranasally sensitized animals were repetitively challenged by exposure to Japanese cedar pollen as antigen. TRFK5 (100 microg/kg, i.p.) given 12 h before the final antigen challenge selectively prevented the antigen-induced eosinophilia in blood and the nasal airway, and suppressed the corresponding decrease in the number of cells in bone marrow; however, it failed to inhibit the immediate development of sneezing, early and late nasal blockage responses, goblet cell degranulation and nasal hyperresponsiveness to histamine. Furthermore, TRFK5 did not significantly affect the production of thromboxane A(2) and cysteinyl leukotrienes in the nasal airway during the late response. These results strongly suggest that while interleukin-5 is essential for eosinophil migration from the bone marrow to the nasal airway, neither interleukin-5 nor eosinophils are required for the development of the nasal symptoms and nasal hyperresponsiveness of allergic rhinitis.

PMID: 11937106 [PubMed - indexed for MEDLINE]

1470. Trends Mol Med. 2002 Apr;8(4):162-7.

Interleukin-5 and eosinophils as therapeutic targets for asthma.

Foster PS, Hogan SP, Yang M, Mattes J, Young IG, Matthaei KI, Kumar RK, Mahalingam S, Webb DC.

Division of Biochemistry and Molecular Biology, John Curtin School of Medical Research, Australian National University, Canberra, ACT, 0200, Australia. Paul.Foster@anu.edu.au

Extensive clinical investigations have implicated eosinophils in the pathogenesis of asthma. In a recent clinical trial, humanized monoclonal antibody to interleukin (IL)-5 significantly limited eosinophil migration to the lung. However, treatment did not affect the development of the late-phase response or airways hyperresponsiveness in experimental asthma. Although IL-5 is a key regulator of eosinophilia and attenuation of its actions without signs of clinical improvement raises questions about the contribution of these cells to disease, further studies are warranted to define the effects of anti-IL-5 in the processes that lead to chronic asthma. Furthermore, eosinophil accumulation into allergic tissues should not be viewed as a process that is exclusively regulated by IL-5 but one in which IL-5 greatly contributes. Indeed, data on anti-IL-5 treatments (human and animal models) are confounded by the failure of this approach to completely resolve tissue eosinophilia and the belief that IL-5 alone is the critical molecular switch for eosinophil development and migration. The contribution of these IL-5-independent pathways should be considered when assessing the role of eosinophils in disease processes.

PMID: 11927273 [PubMed - indexed for MEDLINE]

1471. J Assoc Physicians India. 2002 Mar;50:415-22.

Human strongyloidiasis in AIDS era: its zoonotic importance.

Singh S.

Department of Laboratory Medicine, All India Institute of Medical Sciences, New Delhi.

Human strongyloidiasis is caused by a nematode Strongyloides stercoralis. Many species cause strongyloidiasis in animals. The parasite has predilection to one host only but the host specificity is not strict. When animal species infects humans there is intense allergic reaction in the form of cutaneous larva currens and larva migrans. Therefore, strongyloidiasis in strict terms is a zoonotic disease. The strongyloides species have three stages. The parasitic form inside the host, the free form stage in the soil or water that moults to infective third stage. The later infects the host through skin and migrate to the heart and lung and finally swallowed back to cause intestinal infection. However, in some cases intense pulmonary manifestations may take place. The Strongyloides stercoralis has unique feature of moulting from parasitic form to infective stage within the body, rather than coming out and forming free living stage and causing autoinfection. This may lead to latent infection for indefinite period in an immunocompetant person but fatal hyper or disseminated infection in immunocompromised person like patients of AIDS, organ transplant recipients, cancer and other patients put on immunosuppressive therapy, in whom it can involve any organ of the body. Because this group of patients in last few years have increased tremendously in Africa and South-East Asia, more and more cases of strongyloidiasis are being reported in english literature. The diagnosis of intestinal strongyloidiasis is made by repeated stool smear examinations and in extraintestinal strongyloidiasis the appropriate specimen is examined for the rhabditiform larvae. Recently serological tests have also been developed that can be used for epidemiological purposes. The drug of choice for the treatment of strongyloidiasis remains thiabendazole but due to its unacceptable side effects other medicines like albendazole and ivermectine are being used more frequently. The prevention of the infection is possible by adopting good personal hygiene and safe drinking water supply.

PMID: 11922234 [PubMed - indexed for MEDLINE]

1472. Biochem Cell Biol. 2002;80(1):103-7.

Lactoferrin: influences on Langerhans cells, epidermal cytokines, and cutaneous inflammation.

Kimber I, Cumberbatch M, Dearman RJ, Headon DR, Bhushan M, Griffiths CE.

Syngenta Central Toxicology Laboratory, Macclesfield, Cheshire, UK.
ian.kimber@syngenta.com

It has been suggested previously that, in addition to other biological roles, lactoferrin (LF) may display antiinflammatory properties secondary to the regulation of cytokine expression. To explore this concept further, we have here examined in human volunteers the influence of recombinant homologous LF on the migration of epidermal Langerhans cells (LC), a process that is known to be dependent upon the local availability of certain proinflammatory cytokines including tumor necrosis factor alpha (TNF-alpha) and interleukin 1beta (IL-1beta). In common with previous studies in mice, it was found that topical administration of LF prior to exposure at the same site to the contact sensitizer diphenylcyclopropenone resulted in a significant reduction of allergen-induced LC migration from the epidermis (measured as a function of the frequency of CD1a+ or HLA-DR+ LC found in epidermal sheets prepared from punch biopsies of the treated skin sites). However, under the same conditions of exposure, LF was unable to influence migration of LC induced by the intradermal administration of TNF-alpha data consistent with the hypothesis that one action of LF in the skin is to regulate the local production of this cytokine. Further support for this hypothesis was derived from experiments conducted with IL-1beta. This cytokine is also able to induce the mobilization of LC following intradermal injection,

although in this case, migration is known to be dependent upon the de novo production of TNF-alpha. We observed that prior exposure to LF resulted in a substantial inhibition of IL-1beta-induced LC migration, data again consistent with the regulation of TNF-alpha production by LF. Collectively, these results support the view that LF is able to influence cutaneous immune and inflammatory processes secondary to regulation of the production of TNF-alpha and possibly other cytokines.

PMID: 11911118 [PubMed - indexed for MEDLINE]

1473. J Immunol. 2002 Apr 1;168(7):3595-600.

Contribution of vascular endothelial growth factor to airway hyperresponsiveness and inflammation in a murine model of toluene diisocyanate-induced asthma.

Lee YC, Kwak YG, Song CH.

Department of Internal Medicine, Research Institute of Clinical Medicine, Chonbuk National University Medical School, Chonju, South Korea. leeyc@moak.chonbuk.ac.kr

Isocyanate chemicals, including toluene diisocyanate (TDI), are currently the most common causes of occupational asthma. Although considerable controversy remains regarding its pathogenesis, TDI-induced asthma is characterized by hyperresponsiveness and inflammation of the airways. One of the histological hallmarks of inflammation is angiogenesis, but the possible role of vascular endothelial growth factor (VEGF), a potent angiogenic cytokine, in TDI-induced asthma is unknown. We developed a murine model to investigate TDI-induced asthma by performing two courses of sensitization with 3% TDI and one challenge with 1% TDI using ultrasonic nebulization to examine the potential involvement of VEGF in that disease. These mice develop the following typical pathophysiological features: airway hyperresponsiveness, airway inflammation, and increased VEGF levels in the airway. Administration of VEGFR inhibitors reduced all these pathophysiological symptoms. These results suggest that VEGF is one of the major determinants of TDI-induced asthma and that the inhibition of VEGF may be a good therapeutic strategy.

PMID: 11907124 [PubMed - indexed for MEDLINE]

1474. Nat Rev Immunol. 2001 Nov;1(2):108-16.

Role of chemokines in the pathogenesis of asthma.

Lukacs NW.

University of Michigan Medical School, Department of Pathology, Ann Arbor, Michigan 48109-0602, USA. nlukacs@umich.edu

The prevalence of asthma has risen drastically in the last two decades, with a worldwide impact on health care systems. Although several factors contribute to the development of asthma, inflammation seems to be a common factor that leads to the most severe asthmatic responses. In the past decade, researchers have characterized a large group of chemotactic cytokines, also known as chemokines, which are implicated in asthmatic inflammation. These chemokines control and direct the migration and activation of various leukocyte populations. Targeting chemokines should lead to new ways of controlling the inflammatory asthmatic response.

PMID: 11905818 [PubMed - indexed for MEDLINE]

1475. Curr Allergy Asthma Rep. 2001 Mar;1(2):108-15.

Airway remodeling in the pathogenesis of asthma.

Vignola AM, Gagliardo R, Siena A, Chiappara G, Bonsignore MR, Bousquet J, Bonsignore G.

Istituto di Fisiopatologia Respiratoria, CNR, Clinica Malattie Respiratorie, University of Palermo, Palermo, Italy. vignola.am@iol.it

Asthma is characterized by a chronic inflammatory process of the airways followed by healing, the end result of which is an altered structure referred to as airway remodeling. Although the mechanisms responsible for such structural alterations appear to be heterogeneous, it is likely that abnormal airway cell dedifferentiation, migration, and redifferentiation, together with changes in connective tissue deposition, contribute to the altered restitution of airway structure and function. This altered restitution is often seen as fibrosis and increased smooth muscle, mucus gland mass, and vessel area. As a consequence of these structural changes, the airway wall in asthma is usually characterized by increased thickness and markedly and permanently reduced airway caliber. These features may result in increased airflow resistance, particularly when there is bronchial contraction and bronchial hyperresponsiveness. The effect on airflow is compounded by increased mucus secretion and inflammatory exudate, which not only block the airway passages but also cause increased surface tension favoring airway closure.

PMID: 11899292 [PubMed - indexed for MEDLINE]

1476. Arch Dermatol Res. 2002 Jan;293(11):552-9.

The role of chemokines in allergic contact dermatitis.

Sebastiani S, Albanesi C, De PO, Puddu P, Cavani A, Girolomoni G.

Istituto Dermopatico dell'Immacolata, IRCCS, Roma, Italy.

Chemokines are important mediators of immune-mediated skin diseases. Allergic contact dermatitis (ACD) is the most thoroughly investigated T cell-mediated disorder because of the ability to easily reproduce the lesions in humans and the availability of an excellent mouse model. Migration of dendritic cells from the skin to lymph nodes is absolutely required for induction of hapten sensitization, and depends upon expression of CCR7 by mature dendritic cells and SLC in the lymph nodes. During expression of ACD, recruitment of T lymphocytes is driven by chemokines exposed on the surface of endothelial cells or released by activated resident skin cells such as mast cells, fibroblasts and keratinocytes. Chemokines are produced in a coordinated and sequential manner, with IL-8 and RANTES induced by TNF-alpha during early stages, and MCP-1, IP-10, Mig, I-TAC, I-309 and MDC induced by IFN-gamma during later stages. Infiltrating monocytes, dendritic cells and T cells are additional sources of chemokines for further leukocyte accumulation. Distinct T cell subsets express different chemokine receptors, with type 2 cells mostly attracted by eotaxin, MDC, TARC and I-309, and type 1 cells sensitive to IP-10, Mig, I-TAC, RANTES and MIP-1beta. MCP-1 is effective on both subsets. T regulatory cells, which inhibit dendritic cell function and are probably involved in the termination of ACD, are sensitive to MCP-1, MIPs and TARC, but express high levels of CCR8 and are more specifically attracted by I-309. Targeting chemokines and chemokine receptors may offer new opportunities for therapeutic interventions in ACD and other chronic inflammatory skin

diseases.

PMID: 11876523 [PubMed - indexed for MEDLINE]

1477. Eur Respir J. 2002 Feb;19(2):350-5.

Chemokines, innate and adaptive immunity, and respiratory disease.

Sabroe I, Lloyd CM, Whyte MK, Dower SK, Williams TJ, Pease JE.

Division of Genomic Medicine, University of Sheffield, UK.

Selective leukocyte trafficking and recruitment is primarily regulated by a specific family of small proteins called "chemokines". This extended family shepherds and guides leukocytes through their lives, facilitating their development, regulating their interactions with other leukocyte types, and guiding their recruitment to sites of inflammation. Through the actions of chemokines, allergen sensitization is regulated in atopic asthma, through the controlled migration of dendritic cells, T- and B-lymphocytes, mast cells and basophils. Subsequently, atopic inflammation is driven by chemokine-directed recruitment of eosinophils, basophils and lymphocytes. Diseases from cancer to chronic obstructive pulmonary disease to interstitial fibrosis are all potential targets for chemokine receptor antagonism. Innate immunity (the early pattern-recognition responses to stimuli such as lipopolysaccharide, viral proteins and bacterial DNA) needs to bridge the gap to specific immunity and antibody production and immunological memory. Again, chemokines are likely to be fundamental mediators of these responses. Chemokines are fundamental regulators of leukocyte homeostasis and inflammation, and their antagonism by small molecule chemokine receptor antagonists may be of enormous importance in the future treatment of human respiratory disease.

PMCID: PMC3428840
PMID: 11871367 [PubMed - indexed for MEDLINE]

1478. Immunity. 2002 Feb;16(2):271-83.

A restricted subset of dendritic cells captures airborne antigens and remains able to activate specific T cells long after antigen exposure.

Julia V, Hessel EM, Malherbe L, Glaichenhaus N, O'Garra A, Coffman RL.

DNAX Research Institute, Immunology Department, 901 California Avenue, Palo Alto, CA 94304, USA.

Mice sensitized for a Th2 response to Leishmania LACK antigen developed allergic airway inflammation upon exposure to LACK aerosol. Using multimers of I-A(d) molecules bound to a LACK peptide as probes, we tracked the migration of LACK-specific Th2 cells to the airways. Elevated numbers of LACK-specific Th2 cells remained in the airways for 5 weeks after the last aerosol. Substantial numbers of DC presenting LACK peptides were found in the airways, but not in other compartments, for up to 8 weeks after antigen exposure. These LACK-presenting airway DC expressed CD11c and CD11b as well as high levels of surface molecules involved in uptake and costimulation. Taken together, our results may explain the chronic Th2 airway inflammation characteristic of allergic asthma.

PMID: 11869687 [PubMed - indexed for MEDLINE]

1479. Lab Invest. 2002 Feb;82(2):147-58.

Galectin-1 is overexpressed in nasal polyps under budesonide and inhibits eosinophil migration.

Delbrouck C, Doyen I, Belot N, Decaestecker C, Ghanooni R, de Lavareille A, Kaltner H, Choufani G, Danguy A, Vandenhoven G, Gabius HJ, Hassid S, Kiss R.

E.N.T. Department, Erasmus University Hospital, Brussels, Belgium.

Because of the importance of galectins for various cellular activities, the influence of the glucocorticoid budesonide on the level of expression of galectins-1 and -3 was investigated in human nasal polyposis. Ten nasal polyps obtained from surgical resection were maintained for 24 hours in the presence of various concentrations of budesonide. As quantitatively demonstrated by means of computer-assisted microscopy, 250 ng/ml (the highest dose tested) induced a pronounced increase of galectin-1 expression. This feature was observed in nasal polyps from allergic patients but not in those from nonallergic patients. Since eosinophils represent the main inflammatory cell population in nasal polyps, we investigated the effect of galectin-1 on their migration levels by means of quantitative phase-contrast computer-assisted videomicroscopy. Our results show that galectin-1 (coated on plastic supports) markedly reduced the migration levels of eosinophils in comparison to P-selectin. On the cellular level, marked modifications in the polymerization/depolymerization dynamics of the actin cytoskeleton (as revealed by means of computer-assisted fluorescence microscopy) and, to a much lesser extent, an increase in the adhesiveness of eosinophils to tested substrata were detectable. The present study therefore reveals a new galectin-1-mediated mechanism of action for glucocorticoid-mediated anti-inflammatory effects.

PMID: 11850528 [PubMed - indexed for MEDLINE]

1480. Presse Med. 2001 Dec 22-29;30(39-40 Pt 2):19-25.

[Inflammation and acute otitis media].

[Article in French]

Dubreuil C.

1. EPITHELIUM OF THE MIDDLE EAR: The middle ear has a respiratory type epithelium. The mucus is produced by mucosal and by submucosal cells in the Eustachian tube. 2. LYMPHOID TISSUE: The defensive barrier of the mucosa is potentialized by lymphoid tissue in the middle ear that produces a local specific immune response. Bacterial and viral antigens can induce an inflammatory reaction. 3. TUBOTYMAPNIC CAVITIES: The humoral immune system (B lymphocytes) secreting IgG, IgA and IgM) and cell mediated response (T lymphocytes) are active in the tubotympanic cavities. 4. MAST CELLS: Present in contact with vessels and nervous fibers of the tubotympanic chorion, mast cells are activated by IgE linkage to antigens, leading to local allergic inflammatory phenomena with production of histamine, C leukotrienes, platelet activating factor (PAF) and cytokines. 5. MAINTAINING OPEN CAVITIES: During the inflammatory reaction, mucus-secreting cells are stimulated and their glycoprotein secretions are modified. Since the middle ear epithelium has a water-absorption action contributing to aeration of the middle ear cavities. 6. CYTOKINE RESPONSE: During acute middle ear otitis, cytokines secreted in response to bacterial and viral lipopolysaccharides include tumor necrosis factor alpha (TNFa) which plays a crucial role: induction of fibroblast proliferation, activation of

polymorphonuclears, inhibition of vascular endothelial tissue and B lymphocytes. It facilitates healing of the middle ear infection, but an also induced pathological lesions in case of incomplete repair. 7. BACTERIAL FLORA: Haemophilus influence, Branhamella catarrhalis, and Streptococcus pneumoniae colonize the respiratory epithelium of the middle ear via the Eustachian tube, generally after viral infection. 8. GLUCOCORTICOIDS: Administered before injection of a bacterial endotoxin, glucocorticoids significantly reduce inflammatory phenomena in acute otitis induced in rat models. In acute middle ear models in the guinea pig, corticosteroids reduce lipoperoxidation; free radicals are the cause of the persistence of inflammation in the acute middle ear. 9. NEUTROPHIL MIGRATION: Antibodies blocking cell adhesion molecules (CAM), or instance antiCD11 B and antiCT 18, inhibit polymorphonuclear migration, and could be very useful for the treatment of acute middle ear infection. Use of prostaglandin inhibitors does not significantly reduce the risk of residual effusion at 10 and 30 days after the acute episode. 10. CLINICAL TRIALS: There is a significant body of scientific evidence proving the efficacy of combining antiinflammatory drugs and antibiotics for first line treatment of middle ear infection to prevent seromucosal otitis.

PMID: 11819908 [PubMed - indexed for MEDLINE]

1481. Eur J Immunol. 2002 Feb;32(2):404-12.

Chemokine stimulation of monocyte matrix metalloproteinase-9 requires endogenous TNF-alpha.

Robinson SC, Scott KA, Balkwill FR.

ICRF Translational Oncology Laboratory, Bart's and the London Queen Mary School of Dentistry, Charterhouse Square, London, EC1M 6BQ, GB.
s.c.robinson@icrf.icnet.uk

Leukocyte extravasation into tissues is a multi-step process culminating in the migration of cells through the basement membrane. This requires the production of matrix-degrading enzymes, in particular matrix metalloproteinases (MMP). We investigated the role of chemokines in regulating MMP production in the monocytic cell line THP-1 and in peripheral blood monocytes (PBM). The CC chemokines CCL2 (MCP-1), CCL3 (MIP-1alpha), and CCL5 (RANTES) stimulated the release of monocyte MMP-9 protein in a bell-shaped dose-dependent manner. The increase in MMP-9 protein detected at 24 h was due to de novo synthesis, confirmed by Northern blotting, with MMP-9 mRNA detectable at 6-8 h. Autocrine TNF-alpha was necessary for chemokine stimulation of MMP-9. Chemokines increased TNF-alpha mRNA levels and protein release in monocytes and THP-1 cells, and neutralizing anti-TNF-alpha antibodies inhibited CCL2-induced MMP-9 release. Furthermore, the broad spectrum MMP inhibitor BB 2516, which inhibits TNF-alpha release, abrogated CCL2- and CCL5-induced MMP-9 release in both THP-1 cells and freshly isolated monocytes. Monocyte production of MMP is of major importance in the pathology of cancer, asthma, and rheumatoid arthritis. An understanding of the mechanisms by which these MMP are produced may lead to novel therapies to modulate extravasation of leukocytes in disease.

PMID: 11813159 [PubMed - indexed for MEDLINE]

1482. Eur J Immunol. 2002 Feb;32(2):393-403.

Potential role of IL-8, platelet-activating factor and TNF-alpha in the sequestration of neutrophils in the lung: effects on neutrophil deformability, adhesion receptor expression, and chemotaxis.

Drost EM, MacNee W.

Edinburgh Lung and Environmental Group Initiative (ELEGI)/Colt Laboratories, Department of Medical and Radiological Sciences, Wilkie Building, The Medical School, University of Edinburgh, Teviot Place, Edinburgh EH8 9AG, Scotland, UK.

The microvasculature of the normal lung contains a pool of sequestered neutrophils, which is markedly enhanced in acute lung inflammation. Lung neutrophil sequestration is determined by the cells' deformability and adhesivity to capillary endothelium, and is a pre-requisite for emigration into the airspaces. We assessed the effect of several pro-inflammatory mediators associated with acute lung inflammation on these factors. Platelet-activating factor, IL-8 and formyl-Met-Leu-Phe (fMLP) induced a marked, but transient reduction in neutrophil deformability. Also, increased surface expression of the beta(2)-integrin and CD11b, and shedding of L-selectin (CD62L) was observed for these stimuli. TNF-alpha in contrast caused a small decrease in cell deformability only after 30 min, and shedding of L-selectin, but no change in CD11b levels. However, TNF-alpha-pretreatment markedly enhanced the fMLP response for cell deformability, CD11b expression and CD62L loss. Moreover, all pre-treatments were found to induce chemokinesis, and all except fMLP, enhanced fMLP-directed chemotaxis. We were able to demonstrate, using specific TNF-alpha receptor antagonists, that the TNF-alpha-induced changes in chemotaxis were mediated through the 55-kDa receptor. Also, inhibitors of the mitogen activated protein (MAP) kinase signaling pathway showed that the p38 MAP kinase pathway was involved for fMLP-directed chemotaxis of TNF-pretreated neutrophils, although activation of the extracellular signal-regulated kinase (ERK) pathway was also seen. These data demonstrate the differential role of pro-inflammatory mediators in controlling neutrophil sequestration and migration, which may orchestrate the severity of the inflammatory response in such respiratory diseases as chronic obstructive pulmonary disease and asthma.

PMID: 11813158 [PubMed - indexed for MEDLINE]

1483. Blood. 2002 Feb 1;99(3):946-56.

Immature mouse dendritic cells enter inflamed tissue, a process that requires E- and P-selectin, but not P-selectin glycoprotein ligand 1.

Pendl GG, Robert C, Steinert M, Thanos R, Eytner R, Borges E, Wild MK, Lowe JB, Fuhlbrigge RC, Kupper TS, Vestweber D, Grabbe S.

Institute of Cell Biology, ZMBE, University of Münster, Von-Esmarch Strasse 56, D-48149 Münster, Germany.

Inflammatory processes are associated with the rapid migration of dendritic cells (DCs) to regional lymph nodes and depletion of these potent antigen-presenting cells (APCs) from the inflamed tissue. This study examined whether sites of cutaneous inflammation can be repopulated with DCs from a pool of immature DCs circulating in the blood. In adoptive transfer experiments with ex vivo-generated radioactively labeled primary bone marrow-derived DCs injected into mice challenged by an allergic contact dermatitis reaction, immature DCs were actively recruited from the blood to sites of cutaneous inflammation, whereas mature DCs were not. Immature, but not mature, DCs were able to adhere specifically to immobilized recombinant E- and P-selectin under static as well as under flow conditions. P-selectin-dependent adhesion of immature DCs correlates with their higher level of expression of the carbohydrate epitope cutaneous lymphocyte-associated antigen (CLA) and is blocked by a novel inhibitory antibody against mouse P-selectin glycoprotein ligand 1 (PSGL-1). Surprisingly, however,

emigration of immature DCs into inflamed skin is retained in the presence of this anti-PSGL-1 antibody and is also normal when immature DCs are generated from fucosyltransferase (Fuc-T) Fuc-TVII-deficient mice. By contrast, emigration of wild-type immature DCs is reduced by adhesion-blocking anti-E- and P-selectin antibodies, and immature DCs generated ex vivo from Fuc-TVII/Fuc-TIV double-deficient mice emigrate poorly. Thus, fucosylated ligands of the endothelial selectins, determined in part by Fuc-TIV, and independent of PSGL-1, are required for extravasation of DCs into sites of cutaneous inflammation.

PMID: 11806998 [PubMed - indexed for MEDLINE]

1484. J Clin Invest. 2002 Jan;109(2):183-92.

Beta2 integrins are required for skin homing of primed T cells but not for priming naive T cells.

Grabbe S, Varga G, Beissert S, Steinert M, Pendl G, Seeliger S, Bloch W, Peters T, Schwarz T, Sunderkötter C, Scharffetter-Kochanek K.

Department of Dermatology, University of Münster, Münster, Germany.
grabbe@uni-muenster.de

Beta2 integrins are of critical importance for leukocyte extravasation through vascular endothelia and for T cell activation. To elucidate the role of beta2 integrins in T cell-mediated immune responses, allergic contact dermatitis (ACD), irritant dermatitis, and delayed-type hypersensitivity (DTH) were assessed in mice lacking the beta2 integrin subunit, CD18. ACD and DTH responses, but not edema formation, were severely suppressed in CD18(-/-) mice. Extravasation of CD18(-/-) T cells into eczematous skin lesions was greatly impaired, whereas migration of Langerhans cell precursors and dendritic cells was normal in CD18(-/-) mice. CD18(-/-)lymph nodes (LNs) contained an abnormal population of CD3(-)CD44(high) lymphocytes and showed evidence of widespread T cell activation. T cells from regional LNs of sensitized CD18(-/-) mice proliferated in response to hapten challenge, and subcutaneous injection of sensitized syngeneic LN cells directly into ears of hapten-challenged naive recipients restored the defective ACD in CD18(-/-) mice, suggesting that CD18 is not required for priming of naive T cells but is indispensable for T cell extravasation. Thus, a dysfunction of T cells, in addition to granulocytes, may contribute to the pathophysiology of leukocyte adhesion deficiency type I, which arises from mutations in the human CD18 gene.

PMCID: PMC150832
PMID: 11805130 [PubMed - indexed for MEDLINE]

1485. Rom J Physiol. 1999 Jul-Dec;36(3-4):195-204.

Aspects of immunity in the treatment of bronchial asthma.

Olinescu A.

Cantacuzino Institute, Bucharest.

The two subsets of CD4+ lymphocytes, Th1 and Th 2 secrete two groups of antagonistic cytokines: IL-2/IFNgamma, secreted by the Th1, and IL -4/IL-5, secreted by the Th2 cells. In addition to its antiviral effect, IFNgamma activates phagocytosis, enhances expression of Fc gamma R and MHC antigens on different cell types, stimulates synthesis of IgG molecules, etc. Interleukin-4 induces expression of Fc epsilon R, activates synthesis of IgE antibodies, while

IL-5 stimulates the migration of eosinophils towards the lung. Eosinophils and IgE molecules are considered the main factors inducing bronchial asthma. In order to down-regulate IL-4 and IL-5 synthesis and to block the asthmatic phenomena, treatment of patients by inoculation of IFNgamma instead of glucocorticoids seems to be more reasonable.

PMID: 11797935 [PubMed - indexed for MEDLINE]

1486. Am J Chin Med. 2001;29(3-4):547-58.

Predicting traditional Chinese medicine's use and the marginalization of medical care in Hong Kong.

Lau JT, Leung EM, Tsui HY.

Centre for Clinical Trials and Epidemiological Research, Faculty of Medicine, The Chinese University of Hong Kong, Prince of Wales Hospital, Shatin, NT.

The study examined the importance of socio-demographic factors, health conditions, health beliefs and health seeking behaviors in predicting the use of Traditional Chinese Medicine (TCM) in Hong Kong. A sample of 4,339 respondents was randomly selected and interviewed. Among the 1,651 respondents who had consulted a doctor in the three months prior to the survey, 8.6% consulted a TCM doctor. Besides, 13.5% of the entire sample reported that they had been using TCM drugs frequently or occasionally. Socio-demographic factors, health conditions, health beliefs and health seeking behaviors were all found predictive of the use of TCM. In particular, those who were older, female, new immigrants, unemployed, retired, had chronic disease such as rheumatism, bronchitis, asthma, and those taking non-prescribed medication and not seeking treatment when falling ill were more likely to use TCM. Perceived difficulty in obtaining medical services and high medical cost also predicted TCM use. In sum, the findings suggest that TCM users are likely to be those who have been marginalized in obtaining medical care.

PMID: 11789598 [PubMed - indexed for MEDLINE]

1487. Peptides. 2001 Dec;22(12):2151-4.

Pituitary adenylate cyclase-activating peptide inhibits neutrophil chemotaxis.

Kinhult J, Uddman R, Laan M, Lindén A, Cardell LO.

Allergy laboratory, Department of Otorhinolaryngology, Malmö University Hospital, Malmö, Sweden.

Pituitary adenylate cyclase-activating peptide 38 (PACAP 38) is a neuropeptide that displays several biological effects of interest in the context of airway diseases such as asthma and chronic obstructive pulmonary disease. These effects include inhibition of airway and vascular smooth muscle tone as well as modulation of inflammatory cell activity. However, little is known about the effect of PACAP on granulocytes. The present study was designed to investigate if PACAP and the closely related peptide vasoactive intestinal peptide (VIP) could affect neutrophil migration. A standard 48 well chemotaxis chamber was used to assess the effects of PACAP on N-Formyl-L-methionyl-L-leucyl-L-phenylalanine (fMLP)-induced neutrophil chemotaxis and spontaneous random migration. PACAP 38 and VIP inhibited fMLP-induced human neutrophil chemotaxis. Furthermore, both peptides also exhibited a dose-related trend toward inhibiting the spontaneous, unstimulated migration of neutrophils. Since enhanced cell migration in cell

chamber systems is reported to correlate with increased invasive properties in vivo, the presented inhibitory effects of PACAP 38 on neutrophil chemotaxis, supports the idea of an anti-inflammatory role for PACAP. This together with the well documented bronchodilatory capacity of PACAP might indicate a role for PACAP-agonists in future treatment of asthma and other inflammatory airway diseases.

PMID: 11786203 [PubMed - indexed for MEDLINE]

1488. Physiol Rev. 2002 Jan;82(1):97-130.

Dendritic cells: immune regulators in health and disease.

Lipscomb MF, Masten BJ.

Department of Pathology, University of New Mexico School of Medicine, Albuquerque, New Mexico 87131-5301, USA. mlipscomb@salud.unm.edu

Dendritic cells (DCs) are bone marrow-derived cells of both lymphoid and myeloid stem cell origin that populate all lymphoid organs including the thymus, spleen, and lymph nodes, as well as nearly all nonlymphoid tissues and organs. Although DCs are a moderately diverse set of cells, they all have potent antigen-presenting capacity for stimulating naive, memory, and effector T cells. DCs are members of the innate immune system in that they can respond to dangers in the host environment by immediately generating protective cytokines. Most important, immature DCs respond to danger signals in the microenvironment by maturing, i.e., differentiating, and acquiring the capacity to direct the development of primary immune responses appropriate to the type of danger perceived. The powerful adjuvant activity that DCs possess in stimulating specific CD4 and CD8 T cell responses has made them targets in vaccine development strategies for the prevention and treatment of infections, allograft reactions, allergic and autoimmune diseases, and cancer. This review addresses the origins and migration of DCs to their sites of activity, their basic biology as antigen-presenting cells, their roles in important human diseases and, finally, selected strategies being pursued to harness their potent antigen-stimulating activity.

PMID: 11773610 [PubMed - indexed for MEDLINE]

1489. Int J Circumpolar Health. 2001 Nov;60(4):676-84.

Zoonotic diseases: health aspects of Canadian geese.

Dieter RA Jr, Dieter RS, Dieter RA 3rd, Gulliver G.

The Center for Surgery, Naperville, IL 60563, USA.

OBJECTIVE: Review zoonotic diseases associated with Canadian geese.
STUDY DESIGN: Review article: A review of the multiple physical, microbiologic and safety concerns, and methods used in controlling this potential problem.
RESULTS: Over the last decade the Canadian goose population (protected by international treaties and protection acts) has increased rapidly such that in many cities they have become a pest rather than an admired wild bird. Their increasing numbers have caused a number of potential healthcare concerns including: physical, bacterial, parasitic, allergic and viral potential problems. The Canadian goose fecal droppings of one per minute have caused falls and the flying geese have caused air traffic accidents. Bacterial concerns, including botulism, salmonella and E. coli have all been reviewed and presented concerns.

The viral Newcastle disease may be detected with hemagglutination studies and the Giardia psittaci parasites have been repeatedly found in their droppings. The Cryptosporidium parvum oocytes have been present on stool study.
CONCLUSIONS: Definite links to human infectious diseases have been difficult to prove. Revision of the current laws and new control programs must be developed.

PMID: 11768449 [PubMed - indexed for MEDLINE]

1490. Int J Circumpolar Health. 2001 Nov;60(4):593-603.

Metabolism, endocrine status, and allergy in the extreme north.

Hasnulin PV, Seliatitskaya VG.

Scientific Center of Clinical and Experimental Medicine SB RAMS.

The problem of allergic diseases has become more and more pressing in recent years, owing to a broad prevalence and constant morbidity growth in whole world, especially in high latitude regions. The present allergy situation in polar regions, especially in active industrial assimilation centres, undoubtedly stipulates necessity of a careful analysis of its forming mechanism and a search for effective methods of prevention and correction of allergic disorders. Our studies have revealed the significant influence of ecologically stipulated endocrine status modification on frequency of allergic disorders in the north. However, the character of the immune system and liver function in the case of blood cortisol increase has not allowed us to link allergic reactions with corticosteroid production by activation of the adrenal glands. Allergies in the north are mostly dependent on the influence of insulin and thyroid hormones. As it appears, allergies occur mainly among persons with a higher insulin level. Combination of high blood insulin concentration with functional disorders of the digestive system increases the allergy frequency. A similar picture of allergy frequency dependence also emerges in thyroid hormones blood level evaluation. Generally our investigations allow us to make the conclusion that allergic disorders in migrants to northern territories are an indication of the exhaustion of the organism's reserve abilities and are mainly connected with disorders of the function of the gastrointestinal tract and liver, which are stipulated by extreme climate-geophysical conditions of the north including technogenous industrial contamination. This could probably be connected either to a reduction of gastrointestinal tract barrier function for exogenous allergens or to a decrease of metabolism velocity and the clearing of antigen substances by liver.

PMID: 11768439 [PubMed - indexed for MEDLINE]

1491. Eur J Pharmacol. 2001 Dec 21;433(2-3):217-23.

Effects of wortmannin on airways inflammation induced by allergen in actively sensitised Brown Norway rats.

Tigani B, Hannon JP, Mazzoni L, Fozard JR.

Research Department, Novartis Pharma AG, WSJ-386.510, CH-4002 Basel, Switzerland.

We have investigated the effect of wortmannin, a potent and selective inhibitor of phosphatidylinositol-3-kinase, on the immediate-type allergic response and the late phase pulmonary inflammation induced by allergen challenge in the ovalbumin-sensitised Brown Norway rat. Intratracheal (i.t.) instillation of ovalbumin induced dose-related bronchoconstrictor responses. Administration of wortmannin (1, 10 or 100 microg kg(-1) i.t., 1 h prior to challenge) induced a

marked and dose-dependent inhibition of ovalbumin-induced bronchospasm (ED(50) ca. 5 microg kg(-1) i.t.). At similar doses, wortmannin also suppressed the bronchoconstrictor responses to 5-hydroxytryptamine and methacholine but the degree of blockade of these spasmogens (1.4-1.9-fold) was less than that of ovalbumin (>20-fold). Wortmannin, given intratracheally 1 h prior to allergen challenge, also suppressed the increases in bronchoalveolar lavage fluid leukocyte numbers and eosinophil peroxidase activity measured 24 h post challenge. However, relatively high doses were necessary (ED(50) ca. 100 microg kg(-1) i.t.). The potency of wortmannin was increased when dosed 1 h prior to and 24 h after allergen challenge and the readout was 48 h after challenge (ED(50) 3-5 microg kg(-1) i.t.). Thus, wortmannin is a potent inhibitor of the bronchoconstrictor response induced by allergen in the airways of actively sensitised Brown Norway rats. Inhibition of phosphatidylinositol-3-kinase, an obligatory step in mast cell activation in response to allergen, is the presumed mechanism of action. The fact that similar doses of wortmannin do not suppress the late response to allergen suggests a minimal role for the mast cell in generating the late response to allergen in this model. The striking increase in potency to inhibit the late response when dosed 1 h prior to and 24 h after allergen challenge with the readout taken at 48 h may represent an effect of wortmannin to suppress the migration of leukocytes.

PMID: 11755155 [PubMed - indexed for MEDLINE]

1492. Ann Agric Environ Med. 2001;8(2):255-9.

Young farmers with cellular reactivity to airborne microbes suffer more frequently from work-related skin symptoms and allergic dermatitis.

Spiewak R, Skórska C, Góra A, Horoch A, Dutkiewicz J.

Department of Occupational Biohazards, Institute of Agricultural Medicine, Lublin, Poland. spiewak@galen.imw.lublin.pl

75 farming students (49 males and 26 females aged 16-23 years) underwent dermatological, laryngological and pulmonary examination, skin prick tests with common and farm allergens, Phadiatop and total IgE measurement. After that, the migration inhibition tests with antigens of airborne microbes typical for farm environment (Saccharopolyspora rectivirgula, Pantoea agglomerans, and Aspergillus fumigatus) were carried out. Possible differences between students with positive results and those non-reactive were sought. RESULTS: 10 students reacted to at least one microbial antigen in the migration inhibition test. There were no significant differences in distribution of atopy, prick test results, total IgE, and Phadiatop between the reactive students and their classmates. Only one case of asthma was found, hence a further statistical analysis was not feasible. Allergic rhinitis has been found in 30% of the reactive and in 9.2% of non-reactive students; the difference, however, was not statistically significant (p=0.06). Significant differences were found with respect to the frequency of allergic skin diseases (40% reactive versus 9.2% non-reactive, p = 0.009); no other triggering factors than microbial antigens could be identified in 2 out of 4 reactive students with dermatitis. Work-related symptoms were present in all reactive students (100% versus 27.7%, p=0.001); 8 out of 10 reactive students did not show any other specific sensitisation. Antigens of airborne microbes are commonly associated with lung diseases. Our results, however, suggest that the skin may be affected as well. Relatively strong association between cellular reactivity to airborne microbes and skin diseases deserves further studies.

PMID: 11748885 [PubMed - indexed for MEDLINE]

1493. J Clin Invest. 2001 Dec;108(12):1817-24.

IL-13 alters mucociliary differentiation and ciliary beating of human respiratory epithelial cells.

Laoukili J, Perret E, Willems T, Minty A, Parthoens E, Houcine O, Coste A, Jorissen M, Marano F, Caput D, Tournier F.

Laboratoire de Cytophysiologie et Toxicologie Cellulaire, Université Paris 7, Paris, France.

In animal models of asthma, interleukin-13 (IL-13) induces goblet cell metaplasia, eosinophil infiltration of the bronchial mucosa, and bronchial hyperreactivity, but the basis of its effects on airway epithelia remain unknown. Lesions of the epithelial barrier, frequently observed in asthma and other chronic lung inflammatory diseases, are repaired through proliferation, migration, and differentiation of epithelial cells. An inflammatory process may then, therefore, influence epithelial regeneration. We have thus investigated the effect of IL-13 on mucociliary differentiation of human nasal epithelial cells in primary culture. We show that IL-13 alters ciliated cell differentiation and increases the proportion of secretory cells. IL-13 downregulates the actin-binding protein ezrin and other cytoskeletal components. IL-13 also impairs lateral cell contacts and interferes with the apical localization of ezrin seen in differentiated ciliated cells. In addition, an IL-4 antagonistic mutant protein (Y124D), which binds to the IL-4 receptor alpha subunit, a common chain of IL-4 and IL-13 receptors, inhibits IL-13's effects. IL-13 also decreases ciliary beat frequency in a time- and dose-dependent manner. These results suggest that, in human allergic asthmatic responses, IL-13 affects both ciliated and secretory cell differentiation, leading to airway damage and obstruction.

PMCID: PMC209466
PMID: 11748265 [PubMed - indexed for MEDLINE]

1494. Pharmacol Res. 2001 Dec;44(6):451-4.

Anti-inflammatory capabilities of macrolides.

Zalewska-Kaszubska J, Górska D.

Department of Pharmacodynamics, Medical University, Muszyńskiego 1, 90-145 Lódz, Poland.

Macrolide antibiotics play a significant role in clinical practise due not only to their antibacterial activity, but also to their accompanying anti-inflammatory effect that is independent of their antibiotic action. Several studies reported in literature show that macrolides affect several inflammatory processes, such as migration of neutrophils, the oxidative burst in phagocytes and production of pro-inflammatory cytokines, although the precise mechanisms are not clear. They also inhibit eosinophilic inflammation and may be useful in the treatment of patients with steroid-dependent asthma. Macrolides are also effective in diffuse panbronchiolitis, chronic sinusitis and inflammatory skin diseases.

Copyright 2001 Academic Press.

PMID: 11735349 [PubMed - indexed for MEDLINE]

1495. Am J Respir Crit Care Med. 2001 Nov 15;164(10 Pt 2):S63-6.

Mechanisms of airway remodeling. Airway smooth muscle.

Black JL, Roth M, Lee J, Carlin S, Johnson PR.

Department of Pharmacology, and Institute of Respiratory Medicine, University of Sydney, Sydney, Australia. judblack@pharmacol.usyd.edu.au

The airway smooth muscle cell can contract; relax; participate in allergic and inflammatory responses by expressing adhesion molecules, releasing cytokines, and producing matrix proteins and proteases; and, as has been reported, undergo migration. These properties enable the muscle cell to be a key component in the airway wall remodeling that accompanies persistent asthma. Evidence is emerging that identifies the pivotal steps in the signal transduction pathways that lead to the excessive proliferation of the muscle observed in vitro in airway smooth muscle cells from subjects with asthma. The contractile, biochemical, and growth characteristics of muscle from allergic subjects are different from those of nonallergic subjects. In addition, the allergic response impacts on the extracellular matrix in which the muscle is embedded, by altering the profile of matrix proteins released. Once the relationships between allergy and inflammation of the smooth muscle and its extracellular matrix are better defined, opportunities to prevent or reverse airway remodeling will become available.

PMID: 11734469 [PubMed - indexed for MEDLINE]

1496. Am J Respir Cell Mol Biol. 2001 Dec;25(6):707-16.

LPS induces eosinophil migration via CCR3 signaling through a mechanism independent of RANTES and Eotaxin.

Penido C, Castro-Faria-Neto HC, Vieira-de-Abreu A, Figueiredo RT, Pelled A, Martins MA, Jose PJ, Williams TJ, Bozza PT.

Laboratório de Imunofarmacologia e Laboratório de Inflamação, Departamento de Fisiologia e Farmacodinâmica, Instituto Oswaldo Cruz, Fundação Oswaldo Cruz, Rio de Janeiro, Brazil.

Comment in
    Am J Respir Cell Mol Biol. 2001 Dec;25(6):673-5.

Mounting evidence suggests that lipopolysaccharide (LPS) modulates bronchoconstriction and eosinophil function in asthma. We have investigated the role of different chemokines in the eosinophil influx to the pleural cavity after LPS stimulation. Expression of mRNA for eotaxin, regulated on activation, normal T cells expressed and secreted (RANTES), macrophage inflammatory protein (MIP)-1alpha, MIP-1beta, MIP-2, and monocyte chemotactic protein (MCP)-1 was increased in cells recovered from the mouse pleural cavity 6 h after LPS administration. Eotaxin and RANTES, but not MIP-1alpha, protein levels were also increased in cell-free pleural washes recovered 6 h after LPS stimulation (LPW). Antimurine eotaxin and antimurine RANTES antibodies (Abs) failed to inhibit LPS-induced eosinophil influx into mouse pleural cavity in vivo. Pertussis toxin inhibited LPW-induced eosinophil shape change in vitro, suggesting the involvement of G protein-coupled receptors in LPW signaling. Blockade of CCR3 receptors diminished eosinophil shape change induced by LPW fractions in vitro and LPS-induced eosinophil accumulation in vivo. To investigate further contribution of CC chemokines, we administered a 35-kD CC chemokine neutralizing protein (vCKBP) in vivo. vCKBP inhibited the eosinophil accumulation induced by eotaxin and ovalbumin, but did not block that induced by LPS or LPW. Our data suggest that LPS-induced eosinophil accumulation depends on G protein-coupled CCR3 receptor activation, through a mechanism independent of eotaxin, RANTES, or

other vCKBP-inhibitable CC chemokines.

PMID: 11726396 [PubMed - indexed for MEDLINE]

1497. Int Immunol. 2001 Dec;13(12):1471-8.

IL-1 alpha, but not IL-1 beta, is required for contact-allergen-specific T cell activation during the sensitization phase in contact hypersensitivity.

Nakae S, Naruse-Nakajima C, Sudo K, Horai R, Asano M, Iwakura Y.

Center for Experimental Medicine, Institute of Medical Science, University of Tokyo, 4-6-1 Shirokanedai, Minato-ku, Tokyo 108-8639, Japan.

Contact hypersensitivity (CHS) is a T cell-mediated cellular immune response caused by epicutaneous exposure to contact allergens. In this reaction, after the first epicutaneous allergen sensitization, Langerhans cells (LC) catch allergens and migrate from the skin to draining lymph nodes (LN) and activate naive T cells. Although IL-1 is suggested to be involved in these processes, the mechanisms have not been elucidated completely. In this report, to elucidate roles of IL-1alpha and IL-1beta in CHS, we analyzed ear swelling in 2,4,6-trinitrochlorobenzene (TNCB)-induced CHS using gene-targeted mice. We found that ear swelling was suppressed in IL-1alpha-deficient (IL-1alpha(-/-)) mice but not in IL-1beta(-/-) mice. LC migration from the skin into LN was delayed in both IL-1alpha(-/-) and IL-1beta(-/-) mice, suggesting that this defect was not the direct cause for the reduced CHS in these mice. However, we found that the proliferative response of trinitrophenyl (TNP)-specific T cells after sensitization with TNCB was specifically reduced in IL-1alpha(-/-) mice. Furthermore, adoptive transfer of TNP-conjugated IL-1-deficient epidermal cells (EC) into wild-type mice indicated that only IL-1alpha, but not IL-1beta, produced by antigen-presenting cells in EC could prime allergen-specific T cells. These observations indicate that IL-1alpha, but not IL-1beta, plays a crucial role in TNCB-induced CHS by sensitizing TNP-specific T cells.

PMID: 11717188 [PubMed - indexed for MEDLINE]

1498. Curr Opin Pharmacol. 2001 Jun;1(3):248-53.

Eotaxin and asthma.

Pease JE, Williams TJ.

Division of Biomedical Sciences, Imperial College School of Medicine, London, UK. j.pease@ic.ac.uk

Eotaxin is a small protein that is produced in the lungs of asthmatic patients and is a potent chemoattractant for eosinophils. Eotaxin, a CC chemokine, stimulates the migration of eosinophils from the small blood vessels in the lungs by acting on the CC chemokine receptor CCR3, which is located on the leukocyte cell surface. In the past year, three low molecular weight compounds have been developed that can block this receptor. Such compounds may be developed into orally available drugs aimed at preventing eosinophil recruitment and, hence, the pathogenesis associated with the activation of these cells within the lung tissue.

PMID: 11712747 [PubMed - indexed for MEDLINE]

1499. Semin Nucl Med. 2001 Oct;31(4):342-9.

The impact of recent advances in immunology and cancer therapy on nuclear medicine.

McAfee JG, MacVittie TJ.

Department of Nuclear Medicine, The National Institutes of Health, Bethesda, MD 20892-1180, USA.

The explosive expansion of knowledge in immunology in recent decades has already affected the research and practice of nuclear medicine in several ways. New hematopoietic cells have been isolated and their functions discovered, including hematopoietic stem cells and dendritic cells (DCs). Many new humeral factors have been found that have potent effects on cells, including cytokines, growth factors, and specialized proteins. Radiolabeled compounds are needed to follow the pharmacodynamics of the humeral factors and to follow the migration of mobile cells in animals and humans. In this article, only DCs, cytokines, and growth factors used clinically are discussed. DCs are essential for defense against infectious diseases. Autologous DCs cultured for a week and pulsed with tumor antigens have already proved highly immunogenic compared with other methods for activating cytotoxic T cells, and preliminary studies suggest that DCs are more potent for tumor cell killing than monoclonal antibodies. DCs, unfortunately, also play an important role in causing certain human diseases. In allograft transplants, residual donor DCs are responsible for the cellular rejection; if they could be eliminated, rejection could be prevented. These cells are also detrimental in rheumatoid arthritis, other autoimmune diseases, asthma, and chronic obstructive pulmonary disease. Cytokines such as interleukin-2 and such growth factors as granulocyte-macrophage colony-stimulating factor and granulocyte colony-stimulating factor, administered to patients with malignancies to alleviate the leukopenia of chemotherapy agents, frequently alter the tissue distribution of radiopharmaceuticals; these alterations may be confused with disease.

PMID: 11710776 [PubMed - indexed for MEDLINE]

1500. Am J Respir Crit Care Med. 2001 Oct 15;164(8 Pt 1):1495-500.

Inhaled leukotriene E(4), but not leukotriene D(4), increased airway inflammatory cells in subjects with atopic asthma.

Gauvreau GM, Parameswaran KN, Watson RM, O'Byrne PM.

Asthma Research Group, St. Joseph's Healthcare, Hamilton, Ontario, Canada.

Allergen-induced late airway responses are associated with increased numbers of airway eosinophils and basophils. The purpose of this study was to compare and contrast the effects of inhaled cysteinyl leukotrienes LTD(4) and LTE(4), which are released during allergen- induced airway responses, and allergen, on airway inflammatory cells. Fifteen subjects with atopic, mild asthma inhaled diluent, LTD(4), LTE(4), and allergen. Spirometry was performed for 7 h, and sputum inflammatory cells were measured before, 7 h, and 24 h after challenges. The maximum early percent fall in FEV(1) was 23.6 +/- 1.4%, 21.6 +/- 2.3%, 29.3 +/- 2.4%, and 4.0 +/- 1.1% after LTD(4), LTE(4), allergen, and diluent, respectively. Only inhaled LTE(4) and allergen significantly increased sputum eosinophils at 7 h and 24 h, and sputum basophils at 7 h. Six additional subjects underwent airway biopsies 4 h after inhalation. There were significantly more eosinophils in the lamina propria after inhalation of LTE(4) compared with LTD(4) and diluent ($p < 0.05$). These results suggest cysteinyl leukotrienes play a role in eosinophil

migration into the airways in allergic asthma, and for the same degree of bronchoconstriction, inhaled LTE(4) causes more tissue and airway eosinophilia than LTD(4).

PMID: 11704602 [PubMed - indexed for MEDLINE]

1501. J Pineal Res. 2001 Nov;31(4):363-9.

Melatonin modulates allergic lung inflammation.

Martins E Jr, Ligeiro de Oliveira AP, Fialho de Araujo AM, Tavares de Lima W, Cipolla-Neto J, Costa Rosa LF.

Department of Physiology and Biophysics, the Institute of Biomedical Sciences, University of São Paulo, Av. Prof. Lineu Prestes 1524, 05508-900, São Paulo, SP, Brazil.

Asthma is an inflammatory lung disease characterized by cell migration, bronchoconstriction and hyperresponsiveness, and can be induced, as an experimental model, by ovalbumin sensitization followed by a challenge. In addition to the well-known immunostimulatory effects of melatonin, research has identified some of its anti-inflammatory properties. In this study, we evaluated the influence of pinealectomy and melatonin administration on cell migration in an experimental model of allergic airway inflammation. We evaluated, in pinealectomized rats treated or not with melatonin, cell migration into the bronchoalveolar fluid, the number of cells and their proliferative activity in the bone marrow, and plasma corticosterone levels. Pinealectomy reduces, 24 hr after the challenge, the total cell number count in the lung and bone marrow cell proliferation, without changing the number of cells in the bone marrow or in the peripheral blood. This fact suggests that melatonin is important in the control of cell recruitment from the bone marrow and the migration of those cells to the lung. Melatonin administration to pinealectomized rats seems to restore the ability of cells to migrate from the bone marrow to the bronchoalveolar fluid. So, the development of specific inhibitors of melatonin would benefit patients with asthma.

PMID: 11703567 [PubMed - indexed for MEDLINE]

1502. Clin Exp Med. 2001 Jun;1(2):75-80.

In vitro interferon gamma regulation of CCR-3 mRNA expression in peripheral blood leukocytes from atopic asthmatics.

Bello I, Rizo MR, Badel CB, Blanco E, Valenzuela C, del Rosario M, Quiroga MR, Bayard RB, Saldivar JC, Lopez-Saura P.

Centro de Investigaciones Biológicas, Clinical Trials Division, La Habana, Cuba. iraldo.bello@cigb.edu.cu

Interferon gamma (IFN-gamma) exerts major pro-inflammatory, regulatory, and anti-inflammatory actions in immune defense responses. In asthma the infiltration of eosinophils, neutrophils, and lymphocytes is a critical event. Chemokines stimulate the migration of the susceptible subset of inflammatory cells. The chemokine receptors CCR-3 are mainly expressed in eosinophils, basophils, and Th2 cells. More recently it has been demonstrated that the IFN-gamma downregulates the expression of some chemokine receptors. IgE determinations were performed using an ELISA for total IgE Peripheral blood leukocytes from patients and controls were isolated by Ficoll-Hypaque gradient. The cells were incubated in

the absence or presence of 500 IU/ml of recombinant human IFN-gamma for different times. After incubation the cells were washed and lyzed for reverse transcription-polymerase chain reaction (RT-PCR) analysis. RT-PCR was performed using a Perkin-Elmer kit. The amplified bands were run in 2% agarose gels and quantified. The basal levels of CCR-3 in asthmatic patients with IgE > 150 IU/ml tend to be higher than in controls. IFN-gamma down-regulates the expression of CCR-3 in peripheral blood leukocytes from asthmatics with IgE >150 IU/ml, when compared with the basal levels of expression. In conclusions, through the modification of the expression of CCR-3 in peripheral blood leukocytes from atopic asthmatics, IFN-gamma could exert a beneficial effect in patients with asthma, regulating the migration of some inflammatory cells involved in the pathogenesis of the disease.

PMID: 11699731 [PubMed - indexed for MEDLINE]

1503. Am J Physiol Cell Physiol. 2001 Dec;281(6):C2029-38.

Lung epithelial barrier function and wound healing are decreased by IL-4 and IL-13 and enhanced by IFN-gamma.

Ahdieh M, Vandenbos T, Youakim A.

Department of Biomolecular Screening, Immunex Corporation, Seattle, Washington 98101, USA.

To understand the effects of cytokines on epithelial cells in asthma, we have investigated the effects of interleukin (IL)-4, IL-13, and interferon (IFN)-gamma on barrier function and wound healing in Calu-3 human lung epithelial cells. IL-4 and IL-13 treatment of Calu-3 cells grown on Transwell filters resulted in a 70-75% decrease in barrier function as assessed by electrophysiological and [(14)C]mannitol flux measurements. In contrast, IFN-gamma enhanced barrier function threefold using these same parameters. Cells treated concurrently with IFN-gamma and IL-4 or IL-13 showed an initial decline in barrier function that was reversed within 2 days, resulting in barrier levels comparable to control cells. Analysis of the tight junction-associated proteins ZO-1 and occludin showed that IL-4 and IL-13 significantly reduced ZO-1 expression and modestly decreased occludin expression compared with controls. IFN-gamma, quite unexpectedly given its enhancing effect on barrier function, reduced expression of ZO-1 and occludin to almost undetectable levels compared with controls. In wound-healing assays of cells grown on collagen I, IL-4 and IL-13 decreased migration, whereas IFN-gamma treatment enhanced migration, compared with control cells. Addition of IFN-gamma, in combination with IL-4 or IL-13, restored migration of cells to control levels. Migration differences observed between the various cytokine treatments was correlated with expression of the collagen I-binding alpha(2)beta(1)-integrin at the leading edge of cells at the wound front; alpha(2)beta(1)-integrin expression was decreased in IFN-gamma-treated cells compared with controls, whereas it was highest in IL-4- and IL-13-treated cells. These results demonstrate that IL-4 and IL-13 diminish the capacity of Calu-3 cells to maintain barrier function and repair wounds, whereas IFN-gamma promotes epithelial restitution by enhancing barrier function and wound healing.

PMID: 11698262 [PubMed - indexed for MEDLINE]

1504. Eur J Pharmacol. 2001 Oct 26;430(1):123-33.

Suppressive effects of F-1322 on the antigen-induced late asthmatic response and pulmonary eosinophilia in guinea pigs.

Mochizuki A, Tamura N, Yatabe Y, Onodera S, Hiruma T, Inaba N, Kusunoki J, Tomioka H.

Pharmaceuticals Research Laboratories, FujiREBIO Inc., 51 Komiya-cho, Tokyo 192-0031, Hachioji, Japan. aki.mochizuki@nifty.com

We investigated the effects of F-1322 (N-[2-[4-(benzhydryloxy)piperidino]ethyl]-3-hydroxy-5-(3-pyridylmethoxy)-2-naphthamide), a new compound that inhibits both thromboxane A2 synthetase and 5-lipoxygenase and that functions as a histamine antagonist, on the Ascaris antigen-induced late asthmatic response and pulmonary eosinophilia in guinea pigs. Oral administration of F-1322 (10-100 mg/kg) inhibited the antigen-induced late asthmatic response in a dose-dependent manner. Histological analysis revealed that F-1322 prevented the accumulation of eosinophils in the airways and this was paralleled by a decrease in the number of eosinophils and lymphocytes recovered in bronchoalveolar lavage fluid. F-1322 (0.1-10 microM) inhibited eotaxin-induced chemotaxis and actin polymerization of eosinophils in vitro in a concentration-dependent manner, while oral administration of F-1322 dose-dependently suppressed the migration of eosinophils into the airways in vivo in response to infusion of interleukin 5 and eotaxin in combination. F-1322 may, thus, improve the late asthmatic response in this model, in part, by preventing the accumulation of eosinophils in the airways. The pharmacological profile of F-1322 indicates that this drug is likely to be useful in the treatment of allergic diseases such as asthma.

PMID: 11698072 [PubMed - indexed for MEDLINE]

1505. Eur J Pharmacol. 2001 Oct 19;429(1-3):161-76.

Kinin receptors in pain and inflammation.

Couture R, Harrisson M, Vianna RM, Cloutier F.

Department of Physiology, Faculty of Medicine, Université de Montréal, C.P. 6128, Succursale centre-ville, Montréal, Québec, Canada H3C 3J7. couturer@physio.umontreal.ca

Kinins are among the most potent autacoids involved in inflammatory, vascular and pain processes. These short-lived peptides, including bradykinin, kallidin and T-kinin, are generated during tissue injury and noxious stimulation. However, emerging evidence also suggests that kinins are stored in neuronal elements of the central nervous system (CNS) where they are thought to play a role as neuromediators in various cerebral functions, particularly in the control of nociceptive information. Kinins exert their biological effects through the activation of two transmembrane G-protein-coupled receptors, denoted bradykinin B(1) and B(2). Whereas the B(2) receptor is constitutive and activated by the parent molecules, the B(1) receptor is generally underexpressed in normal tissues and is activated by kinins deprived of the C-terminal Arg (des-Arg(9)-kinins). The induction and increased expression of B(1) receptor occur following tissue injury or after treatment with bacterial endotoxins or cytokines such as interleukin-1 beta and tumor necrosis factor-alpha. This review summarizes the most recent data from various animal models which convey support for a role of B(2) receptors in the acute phase of the inflammatory and pain response, and for a role of B(1) receptors in the chronic phase of the response. The B(1) receptor may exert a strategic role in inflammatory diseases with an immune component (diabetes, asthma, rheumatoid arthritis and multiple sclerosis). New information is provided regarding the role of sensory mechanisms subserving spinal hyperalgesia and intrapleural neutrophil migration that occur upon B(1) receptor activation in streptozotocin-treated rats, a model of insulin-dependent diabetes

mellitus in which the B(1) receptor seems to be rapidly overexpressed. Although it is widely accepted that the blockade of kinin receptors with specific antagonists could be of benefit in the treatment of somatic and visceral inflammation and pain, recent molecular and functional evidence suggests that the activation of B(1) receptors with an agonist may afford a novel therapeutic approach in the CNS inflammatory demyelinating disorder encountered in multiple sclerosis by reducing immune cell infiltration (T-lymphocytes) into the brain. Hence, the B(1) receptor may exert either a protective or detrimental effect depending on the inflammatory disease. This dual function of the B(1) receptor deserves to be investigated further.

PMID: 11698039 [PubMed - indexed for MEDLINE]

1506. J Exp Med. 2001 Nov 5;194(9):1243-52.

Intestinal mast cell progenitors require CD49dbeta7 (alpha4beta7 integrin) for tissue-specific homing.

Gurish MF, Tao H, Abonia JP, Arya A, Friend DS, Parker CM, Austen KF.

Department of Medicine, Harvard Medical School, Boston, MA 02115, USA. mgurish@rics.bwh.harvard.edu

Mast cells (MCs) are centrally important in allergic inflammation of the airways, as well as in the intestinal immune response to helminth infection. A single lineage of bone marrow (BM)-derived progenitors emigrates from the circulation and matures into phenotypically distinct MCs in different tissues. Because the mechanisms of MC progenitor (MCp) homing to peripheral tissues have not been evaluated, we used limiting dilution analysis to measure the concentration of MCp in various tissues of mice deficient for candidate homing molecules. MCp were almost completely absent in the small intestine but were present in the lung, spleen, BM, and large intestine of beta7 integrin-deficient mice (on the C57BL/6 background), indicating that a beta7 integrin is critical for homing of these cells to the small intestine. MCp concentrations were not altered in the tissues of mice deficient in the alphaE integrin (CD103), the beta2 integrin (CD18), or the recombination activating gene (RAG)-2 gene either alone or in combination with the interleukin (IL)-receptor common gamma chain. Therefore, it is the alpha4beta7 integrin and not the alphaEbeta7 integrin that is critical, and lymphocytes and natural killer cells play no role in directing MCp migration under basal conditions. When MCp in BALB/c mice were eliminated with sublethal doses of gamma-radiation and then reconstituted with syngeneic BM, the administration of anti-alpha4beta7 integrin, anti-alpha4 integrin, anti-beta7 integrin, or anti-MAdCAM-1 monoclonal antibodies (mAbs) blocked the recovery of MCp in the small intestine. The blocking mAbs could be administered as late as 4 d after BM reconstitution with optimal inhibition, implying that the MCp must arise first in the BM, circulate in the vasculature, and then translocate into the intestine. Inasmuch as MCp are preserved in the lungs of beta7 integrin-deficient and anti-alpha4beta7 integrin-treated mice but not in the small intestine, alpha4beta7 integrin is critical for tissue specific extravasation for localization of MCp in the small intestine, but not the lungs.

PMCID: PMC2195984
PMID: 11696590 [PubMed - indexed for MEDLINE]

1507. J Exp Med. 2001 Nov 5;194(9):1219-29.

Osteopontin is involved in the initiation of cutaneous contact hypersensitivity by inducing Langerhans and dendritic cell migration to lymph nodes.

Weiss JM, Renkl AC, Maier CS, Kimmig M, Liaw L, Ahrens T, Kon S, Maeda M, Hotta H, Uede T, Simon JC.

Department of Dermatology, University of Freiburg, D-79104 Freiburg, Germany. weiss@haut.ukl.uni-freiburg.de

Osteopontin (OPN) is a chemotactic protein that attracts immune cells, to inflammatory sites. The sensitization phase of allergic cutaneous contact hypersensitivity (CHS) requires the migration of Langerhans cells/dendritic cells (LCs/DCs) from skin to draining lymph nodes. Characterizing OPN function for LC/DC migration we found upregulated OPN expression in hapten sensitized skin and draining lymph nodes. OPN induces chemotactic LC/DC migration, initiates their emigration from the epidermis, and attracts LCs/DCs to draining lymph nodes by interacting with CD44 and alphav integrin. Furthermore, OPN-deficient mice have a significantly reduced CHS response that correlates with an impaired ability of OPN-deficient mice to attract LCs/DCs to draining lymph nodes. In conclusion, OPN is an important factor in the initiation of CHS by guiding LCs/DCs from skin into lymphatic organs.

PMCID: PMC2195976
PMID: 11696588 [PubMed - indexed for MEDLINE]

1508. J Med Invest. 2001 Aug;48(3-4):133-41.

Protease-induced leukocyte chemotaxis and activation: roles in host defense and inflammation.

Tani K, Ogushi F, Shimizu T, Sone S.

Third Department of Internal Medicine, University of Tokushima School of Medicine, Kuramoto-cho, Tokushima 770-8503, Japan.

The migration of leukocytes such as neutrophils, monocytes and lymphocytes into inflamed lesions is one of the critical events of inflammation. Although the traditional function of neutrophil-derived antimicrobial proteases is to ingest and kill bacteria, some neutrophil serine proteases have been shown to induce leukocyte migration and activation. Mast cell-derived chymase also has the chemotactic activity for leukocytes. During the acute phase of inflammatory and allergic diseases, the predominantly migrated cells are neutrophils and mast cells, respectively, and in the subsequent chronic phase, monocytes and lymphocytes are mainly migrated. The chemotactic activity for monocytes and lymphocytes of neutrophil-derived serine proteases and mast cell-derived chymase may have a role in switching acute inflammation to chronic inflammation and delayed-type hypersensitivity. Recently, aminopeptidase N and endothelin were shown to induce chemotactic migration of leukocytes. Thus, protease-induced leukocyte chemotaxis and activation may play an important role in immunologic events of inflammatory and allergic diseases.

PMID: 11694952 [PubMed - indexed for MEDLINE]

1509. Am J Respir Cell Mol Biol. 2001 Oct;25(4):425-33.

Microarray analysis of eosinophils reveals a number of candidate survival and apoptosis genes.

Temple R, Allen E, Fordham J, Phipps S, Schneider HC, Lindauer K, Hayes I, Lockey J, Pollock K, Jupp R.

Aventis Pharmaceuticals, Inc., Route 202-206, Bridgewater, NJ 08807, USA.

Comment in
    Am J Respir Cell Mol Biol. 2001 Oct;25(4):405-8.

The increase in eosinophils at the site of antigen challenge has been used as evidence to suggest that this cell type plays a role in the pathophysiology of asthma. Aberrant production of several different cytokines, particularly interleukin (IL)-5, has been shown to result in eosinophilia. IL-5 influences the development and maturation of eosinophils in a number of different ways. Of note is the ability of IL-5 to act as a survival factor for eosinophils specifically inhibiting apoptosis. The precise mechanism by which IL-5 exerts its effect remains obscure. We used microarray technologies to investigate the changes in the messenger RNA expression profile of eosinophils after treatment with IL-5. Using the Affymetrix Hu6800 chip, a total of 80 genes were observed to be regulated by 2-fold or greater. Many of the genes previously identified as regulated by IL-5 were regulated in our microarray experiments. Of the 73 genes found to be upregulated, many were shown to play a role in adhesion, migration, activation, or survival of eosinophils or hematopoietic cells, whereas the function of others was unknown. To facilitate the identification of genes that govern the apoptosis and survivability of eosinophils, we used an alternative cellular model, TF1.8 cells, whose survival was also dependent on IL-5. Comparison of these models identified four genes, Pim-1, DSP-5 (hVH3, B23), CD24, and SLP-76, whose regulation was similarly coordinated in both systems. Identification of Pim-1 and SLP-76 as regulated by IL-5 led us to suggest a direct role for these proteins in the IL-5 signaling pathway in eosinophils. The tissue distribution of these genes demonstrated that Pim-1 and SLP-76 were relatively restricted to the eosinophil compared with their expression in brain, bone marrow, kidney, liver, and lung. By contrast, DSP-5 and CD24 were confirmed as ubiquitous in their expression by microarray.

PMID: 11694447 [PubMed - indexed for MEDLINE]

1510. Am J Respir Cell Mol Biol. 2001 Oct;25(4):418-24.

Trefoil factor family-peptides promote migration of human bronchial epithelial cells: synergistic effect with epidermal growth factor.

Oertel M, Graness A, Thim L, Bühling F, Kalbacher H, Hoffmann W.

Institut für Molekularbiologie und Medizinische Chemie, Otto-von-Guericke-Universität, Leipziger Strasse 44, D-39120 Magdeburg, Germany.

Comment in
    Am J Respir Cell Mol Biol. 2001 Oct;25(4):401-4.

A process termed "restitution" enables rapid repair of the respiratory epithelium by migration of neighbouring cells. Mucin-associated TFF-peptides (formerly P-domain peptides or trefoil factors) are typical motogens enhancing migration of cells in various in vitro models mimicking restitution of the intestine. The human bronchial epithelial cell line BEAS-2B was used as a model system of airway restitution. The motogenic activities of recombinant human TFF2 as well as porcine TFF2 were demonstrated by in vitro wound healing assays of BEAS-2B cells. TFF2 did not induce phosphorylation of the epidermal growth factor (EGF) receptor. EGF was capable of enhancing the motogenic effect of human TFF2 at a concentration of 3 x 10(-10) M whereas EGF itself (i.e., in the absence of TFF2) did not stimulate migration at this low concentration. Furthermore, TFF2 as well as monomeric and dimeric forms of TFF3 enhanced migration of BEAS-2B cells in

Boyden chambers. Motogenic activity of TFF2 was also shown for normal human bronchial epithelial (NHBE) cells in Boyden chambers. These results suggest that TFF-peptides act as motogens in the human respiratory epithelium triggering rapid repair of damaged mucosa in the course of airway diseases such as asthma.

PMID: 11694446 [PubMed - indexed for MEDLINE]

1511. J Allergy Clin Immunol. 2001 Nov;108(5):688-96.

Multistep navigation of Langerhans/dendritic cells in and out of the skin.

Jakob T, Ring J, Udey MC.

Division of Environmental Dermatology and Allergy GSF/TUM, GSF National Research Center for Environment and Health, Neuherberg-Munich, Germany.

Langerhans cells (LCs) are specialized antigen-presenting cells that reside in the epidermis as sentinels of the immune system. LCs constantly monitor the epidermal microenvironment by taking up antigen and processing it into fragments that can be recognized by cells of the adaptive immune response. Because of their unique migratory ability, LCs can transport antigen from the epidermis to regional lymph nodes, where they can initiate systemic immune responses. The mechanisms of LC trafficking thus seem to be of particular relevance for the induction and maintenance of cutaneous immunity. LCs or their putative precursors express surface molecules that allow them to home to skin and localize in the epidermis for prolonged periods of time. Tissue injury, microbial infection, and other perturbants of epidermal homeostasis (eg, contact allergens) provide danger signals, leading to a local production of proinflammatory cytokines that induce LC mobilization to the lymphoid tissue. At the same time, signals are generated that recruit LC precursors into the skin to maintain the epidermal LC population. Distinct pairs of chemokines and their receptors control the migration from blood to epidermis and from there to the regional lymphatics. In addition, trafficking is controlled at the level of cell adhesion, where LCs downregulate some adhesion molecules to exit the epidermis and upregulate others to migrate across the extracellular matrix and home to T-cell areas of regional lymphoid tissue. The improved understanding of mechanisms that regulate LC trafficking might offer new opportunities for therapeutic interventions to suppress, stimulate, or deviate cutaneous immune responses.

PMID: 11692090 [PubMed - indexed for MEDLINE]

1512. Int J Epidemiol. 2001 Oct;30(5):966-73.

Temporal trends and ethnic variations in amenable mortality in Singapore 1965-1994: the impact of health care in transition.

Niti M, Ng TP.

Department of Community, Occupational and Family Medicine, National University of Singapore, Singapore. cofngtp@nus.edu.sg

Comment in
    Int J Epidemiol. 2001 Oct;30(5):973-5.

BACKGROUND: Amenable mortality is used to assess the effects of health care services on gains in mortality outcomes. Possibly differing patterns of trends in amenable mortality may be expected in economically less developed countries, which have undergone rapid epidemiological transition and recent reforms in

health care systems, but such studies are scarce. This study was set up to examine the trends in amenable mortality in Singapore from 1965 to 1994; to estimate the relative impact of medical care and primary preventive policy measures in terms of gains in mortality outcomes; to examine ethnic differences in amenable mortality among Chinese, Malays and Indians.
METHODS: Age-standardized mortality rates were calculated for 16 amenable causes of death in Singapore for six 5-year periods (1965-1969,..., 1990-1994), and for each of the three main ethnic groups for three periods (1989-1991, 1992-1994, 1995- 1997). Amenable mortality rates were divided into those which can be reduced by timely therapeutic care for 'treatable' conditions (e.g. asthma and appendicitis), or by primary preventive measures for 'preventable' conditions (e.g. lung cancer and motor vehicle injury).
RESULTS: Amenable mortality was higher in males (age-standardized rate 109.7 per 100 000 population) than in females (age-standardized rate 60.7 per 100 000 population). Amenable mortality declined by 1.77% a year in males and 1.72% a year in females. By comparison, the average yearly decline in non-amenable mortality was 0.91% in males and 1.17% in females. The decline in amenable mortality was largely due to 'treatable' causes rather than a decline in mortality due to 'preventable' causes of death. Amenable mortality was lowest for Chinese and highest for Malays. Over the recent 9-year period from 1989 to 1997, amenable mortality declined more in Chinese than in Malays and Indians. However, Indian females showed by far the sharpest decline, whereas Indian males, by contrast, showed an increase in amenable mortality, due to both treatable and preventable causes.
CONCLUSIONS: In line with findings from European countries, amenable mortality in Singapore declined more than non-amenable mortality. There were more significant gains in mortality outcomes from medical care interventions than from primary preventive policy measures. Gender and ethnic differences in amenable mortality were also observed, highlighting issues of socioeconomic equities to be addressed in the financing and delivery of health care.

PMID: 11689505 [PubMed - indexed for MEDLINE]

1513. Clin Exp Allergy. 2001 Oct;31(10):1623-30.

The involvement of matrix metalloproteinase-9 in airway inflammation of patients with acute asthma.

Lee YC, Lee HB, Rhee YK, Song CH.

Department of Internal Medicine, Research Institute of Clinical Medicine, Chonbuk National University Medical School, Chonju, South Korea. leeyc@moak.chonbuk.ac.kr

BACKGROUND: Bronchial asthma is an inflammatory disease of the airway characterized by airway remodelling, and is due at least in part to an excess of extracellular matrix (ECM) deposition in the airway wall, which leads to subepithelial collagen deposition. Matrix metalloproteinase-9 (MMP-9) is the major proteolytic enzyme that induces bronchial remodelling in asthma. MMP-9 is also important in the migration of inflammatory cells through basement membrane components.
OBJECTIVES: We evaluated whether airway inflammatory cells correlated with levels of MMP-9 in acute asthma and we examined the time course of sputum levels of MMP-9 activity in patients with spontaneous asthma exacerbation.
METHODS: We performed zymographic analysis and checked levels of MMP-9 by means of enzyme immunoassay. MMP-9 levels were also evaluated during a spontaneous attack of asthma.
RESULTS: Pro-MMP-9 activities and concentrations of MMP-9 in asthmatic patients significantly exceeded those of control subjects ($P < 0.01$). The activities of pro-MMP-9 were significantly higher in acute asthmatic patients than in stable

asthmatic patients (P < 0.01). The elevated MMP-9 activities significantly decreased after 7 and 28 days of therapy. In acute asthmatic patients, the levels of sputum MMP-9 significantly correlated with the total macrophage + neutrophil + eosinophil cell numbers.
CONCLUSION: These data suggest that airway inflammation after asthma exacerbation correlates with the overproduction of MMP-9, which then leads to airway remodelling.

PMID: 11678864 [PubMed - indexed for MEDLINE]

1514. J Immunol. 2001 Nov 1;167(9):5348-54.

A Brugia malayi homolog of macrophage migration inhibitory factor reveals an important link between macrophages and eosinophil recruitment during nematode infection.

Falcone FH, Loke P, Zang X, MacDonald AS, Maizels RM, Allen JE.

Institute of Cell, Animal and Population Biology, University of Edinburgh, Edinburgh, United Kingdom.

Infections with the helminth parasite Brugia malayi share many key features with Th2-mediated allergic diseases, including recruitment of eosinophils. We have investigated the dynamics of inflammatory cell recruitment under type 2 cytokine conditions in mice infected with B. malayi. Among the cells recruited to the site of infection is a novel population of "alternatively activated" macrophages that ablate cell proliferation and enhance Th2 differentiation. By profiling gene expression in this macrophage population, we found a dramatic up-regulation of a recently described eosinophil chemotactic factor, eosinophil chemotactic factor-L/Ym1, representing over 9% of clones randomly selected from a cDNA library. Because B. malayi is known to secrete homologs (Bm macrophage migration inhibitory factor (MIF)-1 and -2) of the human cytokine MIF, we chose to investigate the role this cytokine mimic may play in the development of the novel macrophage phenotype observed during infection. Strikingly, administration of soluble recombinant Bm-MIF-1 was able to reproduce the effects of live parasites, leading both to the up-regulation of Ym1 by macrophages and a marked recruitment of eosinophils in vivo. Because activity of Bm-MIF-1 is dependent upon an amino-terminal proline, this residue was mutated to glycine; the resultant recombinant (Bm-MIF-1G) was unable to induce Ym1 transcription in macrophages or to mediate the recruitment of eosinophils. These data suggest that macrophages may provide a crucial link between helminth parasites, their active cytokine mimics, and the recruitment of eosinophils in infection.

PMID: 11673551 [PubMed - indexed for MEDLINE]

1515. J Asthma. 2001 Sep;38(6):469-76.

Seasonal variations of T-cell cytokine pattern in peripheral blood from atopic subjects.

Majori M, Rossi G, Caminati A, Bosoni A, Corradi M, Pesci A.

Istituto di Clinica delle Malattie dell'Apparato Respiratorio, dell'Università degli Studi di Parma, Italy. stmed50@nemo.unipr.it

Previous studies on the effect of seasonal exposure to the sensitizing antigen on T-cell cytokine pattern from atopic subjects evaluated T-cell cytokine production by titration in the serum or culture supernatants. The purpose of this study was

to determine the seasonal variations of T-cell cytokine pattern from atopic subjects at the single-cell level. We examined the interleukin-4 (IL-4) and interferon-gamma expression in peripheral blood CD4 + and CD8 + T cells from 11 subjects with grass-pollen-sensitive allergy before and during the 1999 grass pollen season using a flow cytometric method of intracellular cytokine detection. Eight healthy volunteers served as the control group. Flow cytometric analysis of peripheral blood lymphocytes showed no seasonal variations of IL-4- and interferon-gamma-producing T cells in atopic subjects. However, there was a decreased percentage of IL-4-producing cells among peripheral blood CD4+ and CD8+ T cells from the atopic subjects both during and outside the pollen season in comparison to the controLs. We did not find seasonal variations of T-cell cytokine pattern in peripheral blood from atopic subjects. However, we observed a decreased percentage of IL-4-producing T cells in peripheral blood from these subjects in comparison to healthy controls. These data add to the view of a continuous migration of T helper 2 (TH2) cells from the blood to the tissues of primary allergen exposure.

PMID: 11642413 [PubMed - indexed for MEDLINE]

1516. J Vet Med Sci. 2001 Sep;63(9):1035-8.

Molecular cloning of canine thymus and activation-regulated chemokine (TARC) gene and its expression in various tissues.

Maeda S, Mizuno T, Yamashita K, Kurata K, Masuda K, Ohno K, Tsujimoto H.

Department of Veterinary Internal Medicine, Graduate School of Agricultural and Life Sciences, The University of Tokyo, Japan.

Thymus and activation-regulated chemokine (TARC) is known as a functional ligand for CC chemokine receptor 4 (CCR4), which is selectively expressed on Th2 lymphocytes and induces selective migration of the cells to allergic lesions. In this study, we cloned canine TARC cDNA from canine thymus by RT-PCR with rapid amplification of cDNA ends (RACE) method. The canine TARC clone contained a full-length open reading frame encoding 99 amino acids and included four cysteine residues characteristic to CC chemokine family. The canine TARC cDNA showed 77.5%, 67.4%, and 68.5% amino acid sequence similarity with human, mouse and rat homologues, respectively. Expression of TARC mRNA was detected not only in thymus but also in spleen, lymph node, lung and heart of the various normal dog tissues examined. TARC cDNA clone obtained in this study will be useful for further investigation on allergic diseases in dogs.

PMID: 11642275 [PubMed - indexed for MEDLINE]

1517. Exp Lung Res. 2001 Oct-Nov;27(7):617-35.

TNF-alpha enhanced allergic sensitization to house dust mite in brown Norway rats.

Lambert AL, Selgrade MK, Winsett DW, Gilmour MI.

University of North Carolina at Chapel Hill, USA.

We have recently demonstrated that pulmonary exposure to residual oil fly ash (ROFA) resulted in enhanced sensitization to house dust mite (HDM) and augmented the development of allergic lung disease after allergen challenge. This effect was associated with increased tumor necrosis factor alpha (TNF-alpha), a macrophage- and epithelial cell-derived cytokine that promotes granulocyte

migration to the lung. The present study examined whether exogenous administration of TNF-alpha enhances sensitization to HDM. One day prior to pulmonary sensitization with 10 microg HDM (5 microg each on days 1 and 3), female Brown Norway rats were instilled via the trachea with either 2.0 microg recombinant rat TNF-alpha, 2.0 microg bovine serum albumin (BSA), or 1,000 microg ROFA, and were challenged with 10 microg HDM 14 days later. Antigen-induced immediate bronchoconstriction responses, antigen-specific immunoglobulin E (IgE) titers, lymphocyte proliferation, (cytokines (TNF-alpha and interleukin [IL]-13), and eosinophils were elevated in rats treated with ROFA or TNF-alpha compared with BSA-treated controls after HDM challenge. Intratracheal administration of anti-TNF-alpha monoclonal antibody during ROFA exposure did not reduce ROFA-enhanced lymphocyte proliferation or IgE titers, but had a trend for reduced pulmonary inflammation. This study demonstrates that TNF-alpha has similar adjuvant activity as ROFA, but other factors may fulfill this function when TNF-alpha activity is blocked.

PMID: 11597121 [PubMed - indexed for MEDLINE]

1518. Eur J Immunol. 2001 Oct;31(10):3085-93.

The role of ICAM-1 molecule in the migration of Langerhans cells in the skin and regional lymph node.

Xu H, Guan H, Zu G, Bullard D, Hanson J, Slater M, Elmets CA.

Department of Dermatology, University of Alabama at Birmingham, Birmingham 35294, USA. xuhui@uab.edu

ICAM-1 (CD54) plays an important role in the cell-cell interaction and migration of leukocytes. Previous studies have shown that ICAM-1 is involved in inflammatory reactions and that a defect in ICAM-1 gene inhibits allergic contact hypersensitivity. This study indicates that the migration of hapten presenting Langerhans cells into the regional lymph nodes was significantly reduced in ICAM-1-deficient mice compared to wild-type C57BL/6 mice. The reduced number of dendritic cells in regional lymph nodes did not result from abnormal migration of Langerhans cells into the skin of ICAM-1-deficient mice. The concentration and distribution of Langerhans cells in the naïve skin of ICAM-1-deficient mice was equal to that of wild-type mice. Following hapten sensitization, Langerhans cell migration out of the skin and recruitment of fresh Langerhans cells back to the epidermis was not affected in ICAM-1-deficient mice. Further experiments demonstrated that ICAM-1 deficiency on lymphatic endothelium rather than on dendritic cells was responsible for the reduced migration of Langerhans cells into draining lymph nodes. This study indicates that ICAM-1 regulates the migration of dendritic cells into regional lymph nodes but not into or out of the skin.

PMID: 11592085 [PubMed - indexed for MEDLINE]

1519. Clin Exp Allergy. 2001 Sep;31(9):1378-84.

Cetirizine inhibits skin reactions but not mediator release in immediate and developing late-phase allergic cutaneous reactions. A double-blind, placebo-controlled study.

Nielsen PN, Skov PS, Poulsen LK, Schmelz M, Petersen LJ.

The Allergy Unit, National University Hospital, University of Copenhagen, Copenhagen, Denmark.

BACKGROUND: Recent reports have indicated cetirizine, a potent H(1)-receptor antagonist, to possess a number of anti-inflammatory effects, e.g. inhibition of mast cell degranulation and inhibition of leucocyte migration and activation.
OBJECTIVE: The aim of this study was to compare the effects of cetirizine on skin responses and mediator release in intact skin in immediate and developing late-phase allergic reactions by microdialysis technique.
METHODS: Cetirizine 10 mg once daily or matching placebo were administered to 10 atopic subjects for 6 days followed by a 2-week washout in a randomized, double-blind, placebo-controlled, cross-over trial. Immediate skin test responses to allergen, codeine, and histamine and late-phase reactions to allergen were assessed. The time course of extracellular levels of inflammatory mediators in intact skin were monitored by microdialysis techniques using 2 kDa and 3 MDa cut-off fibers, respectively.
RESULTS: Cetirizine significantly reduced immediate weal and flare reactions to allergen, codeine, and histamine. Injection of allergen, but not buffer controls, induced a significant release of histamine, tryptase, prostaglandin D(2), total protein, and eosinophilic cationic protein. No significant increase of leukotriene B(4) and myeloperoxidase was observed. Cetirizine inhibited early total protein extravasation by 40%, but this did not reach a significant level. None of the inflammatory mediators were significantly inhibited by cetirizine. Cetirizine significantly reduced the late-phase skin induration to allergen by approximately 30%.
CONCLUSION: Cetirizine potently reduced skin responses in immediate allergic reactions without inhibition of early mediators. These data indicate cetirizine to be a potent H1-receptor antagonist with no effect on mast cell activation. It did not inhibit any of the late-phase mediators, but it reduced the late skin reaction. These data suggest that mediators other than those actually measured may play a significant role in the clinical late-phase reaction.

PMID: 11591187 [PubMed - indexed for MEDLINE]

1520. Eur Respir J. 2001 Sep;18(3):459-65.

Symptoms of asthma, bronchial responsiveness and atopy in immigrants and emigrants in Europe. European Community Respiratory Health Survey.

Tobias A, Soriano JB, Chinn S, Anto JM, Sunyer J, Burney P; European Community Respiratory Health Survey.

Respiratory and Environmental Health Research Unit, IMIM, Barcelona, Spain.

Comment in
    Eur Respir J. 2002 May;19(5):980-1; author reply 981.

Migration studies on asthma may provide information on its environmental causes. The European Community Respiratory Health Survey has potential advantages due to the number of countries involved, standardized collection of information, assessment of directionality of migration, and availability of physiological data on bronchial responsiveness and atopy. Prevalence rates of symptoms associated with asthma were compared for immigrants, emigrants and nonmigrants living in centres mostly in western Europe. Similar analyses were carried out for bronchial responsiveness (provocative concentration causing a 20% fall in forced expiratory volume in one second and slope) and atopy. Medication and use of health services were also explored. Overall, 1,678 (8.6%) of 19,516 participants were immigrants in the 18 countries participating in the study, of whom 581 were emigrants from one of the participating countries. Rates of asthma symptoms were higher in immigrants (odds ratio (OR): 1.21, 95% confidence interval (CI): 1.00-1.51) and emigrants (OR: 1.31, 95% CI: 0.96-1.51) compared to nonmigrants after controlling

for area, sex, age and smoking status. However, bronchial responsiveness and atopy were equally distributed between immigrants, emigrants and nonmigrants. Use of health services was observed to be similar in migrants and nonmigrants with asthma. In the European Community Respiratory Health Survey, migrants reported more asthma symptoms, but had similar bronchial responsiveness, atopy, and use of health services when compared with the nonmigrant population.

PMID: 11589342 [PubMed - indexed for MEDLINE]

1521. J Med Chem. 2001 Oct 11;44(21):3469-87.

Selective inhibition of ICAM-1 and E-selectin expression in human endothelial cells. 2. Aryl modifications of 4-(aryloxy)thieno[2,3-c]pyridines with fine-tuning at C-2 carbamides.

Zhu GD, Arendsen DL, Gunawardana IW, Boyd SA, Stewart AO, Fry DG, Cool BL, Kifle L, Schaefer V, Meuth J, Marsh KC, Kempf-Grote AJ, Kilgannon P, Gallatin WM, Okasinski GF.

Metabolic Diseases Research, Pharmaceutical Products Division, Abbott Laboratories, Department 04MJ, Building AP10, 100 Abbott Park Road, Abbott Park, Illinois 60064-6101, USA. Gui-Dong.Zhu@abbott.com

The elevated expression of cell adhesion molecules (CAMs) on the lumenal surface of vascular endothelial cells is a critical early event in the complex inflammatory process. The adhesive interactions of these CAMs that include E-selectin, ICAM-1, and VCAM-1 with their counter-receptors on leukocytes, such as integrins of the alpha(L)beta(2) family, result in migration of the leukocytes to the site of inflammation and cause tissue injury. Pharmaceutical agents that could suppress the induced expression of one or more of these cell adhesion molecules would provide a novel mechanism to attenuate the inflammatory responses associated with chronic inflammatory diseases. A-205804 (1), a potent and selective inhibitor of the induced expression of E-selectin and ICAM-1 over VCAM-1, was further modified with emphasis at the C-4 and C-2 positions to identify a more potent drug candidate with a good pharmacokinetic profile and physical properties. Replacement of the C-4 sulfur linkage in 1 with an oxygen atom eliminated one of the two major metabolites for this lead molecule. The para-position of the 4-phenoxy group of the thieno[2,3-c]pyridine lead is found to be very critical for a higher in vitro potency and selectivity of E-selectin and ICAM-1 over VCAM-1 expression. This position is presumably close to the solvent-accessible region of the target protein-inhibitor complex. An attempt to install a water-solubilizing group at the para-position of the phenoxy group to increase the aqueous solubility of this lead series through various linkages failed to provide an ideal inhibitor. Only small substituents such as fluorine are tolerated at the meta- and ortho-positions of the 4-phenoxy to retain a good in vitro potency. Bromo, trifluoromethyl, pyrazol-1-yl, and imidazol-1-yl are among the better substituents at the para-position. With fine-tuning at the C-2 position we discovered a series of very potent (IC(50) < 5 nM for ICAM-1) and selective (>200-fold vs VCAM-1) inhibitors with a good pharmacokinetic profile. Demonstrated efficacy in a rat rheumatoid arthritis model and in a mice asthma model with selected compounds is also reported.

PMID: 11585452 [PubMed - indexed for MEDLINE]

1522. Int Arch Allergy Immunol. 2001 Aug;125(4):335-43.

Theophylline inhibits TNF-alpha-induced CD4 expression on human eosinophils and CD4+ eosinophil migration.

Tsukadaira A, Okubo Y, Horie S, Koyama S.

First Department of Internal Medicine, Shinshu University School of Medicine, Asahi 3-1-1, Matsumoto, 390-8621 Japan.

BACKGROUND: Increasing evidence regarding asthma suggests that CD4+ cells are preferentially recruited to sites of bronchial inflammation. Interleukin (IL)-16 has been reported as playing an important role in the accumulation of CD4+ cells. We have shown that the CD4 molecule is expressed on normal human eosinophils by tumor necrosis factor (TNF)-alpha stimulation.
METHODS: We evaluated the effects of theophylline, KF19514 [a selective phosphodiesterase (PDE) IV inhibitor] and dexamethasone on CD4 expression on eosinophils and eosinophil migration in response to IL-16, a natural soluble ligand of the CD4 molecule.
RESULTS: The maximum eosinophil migration was observed when eosinophils were cultured with TNF-alpha at 10 ng/ml for 18 h and the concentration of IL-16 was 10 pg/ml. CD4+ eosinophil migration in response to IL-16 was mostly, if not fully, chemokinetic and this migration was significantly inhibited by Fab of anti-CD4 monoclonal antibody. Theophylline (10(-4)-10(-3) M), KF19514 (10(-7)-10(-6) M) and dexamethasone (10(-8)- 10(-6) M) significantly inhibited CD4 expression on eosinophils induced by TNF-alpha. Theophylline (10(-3) M) and KF19514 (10(-6) M) inhibited CD4+ eosinophil migratory responses induced by IL-16, but 10(-6) M dexamethasone did not. Theophylline and KF19514 augmented the intracellular adenosine-3',5'-cyclic monophosphate (cAMP) concentration in eosinophils, suggesting modulation by cAMP of CD4 expression and eosinophil migration.
CONCLUSIONS: These data suggest that TNF-alpha-induced CD4+ eosinophils may contribute to eosinophil migratory responses induced by IL-16. Theophylline and selective PDE IV inhibitor may prevent airway inflammation by downregulating CD4 expression on eosinophils and inhibiting eosinophil migration through CD4 and IL-16 interaction.

Copyright 2001 S. Karger AG, Basel

PMID: 11574756 [PubMed - indexed for MEDLINE]

1523. Br J Pharmacol. 2001 Sep;134(2):283-94.

Modulation of eotaxin formation and eosinophil migration by selective inhibitors of phosphodiesterase type 4 isoenzyme.

Silva PM, Alves AC, Serra MF, Pires AL, Silva JP, Barreto EO, Cordeiro RS, Jose PJ, Teixeira MM, Lagente V, Martins MA.

Departmento de Fisiologia e Farmacodinâmica, Instituto Oswaldo Cruz, FIOCRUZ, Av. Brasil. patmar@ioc.fiocruz.br

1. This study was undertaken to investigate the possible contribution of the blockade of eotaxin generation to the anti-eosinophilotactic effect of phosphodiesterase (PDE) type 4 inhibitors. In some experiments, the putative synergistic interaction between PDE type 4 inhibitors and the beta2-agonist salbutamol was also assessed. 2. Sensitized guinea-pigs aerosolized with antigen (5% ovalbumin, OVA) responded with a significant increase in eotaxin and eosinophil levels in the bronchoalveolar lavage fluid (BALF) at 6 h. Eosinophil recruitment was inhibited by both PDE type 4 inhibitors rolipram (5 mg kg(-1), i.p.) and RP 73401 (5 mg kg(-1), i.p.) treatments. In contrast, only rolipram inhibited eotaxin production. 3. Sensitized rats intrapleurally challenged (i.pl.) with antigen (OVA, 12 microg cavity(-1)) showed a marked eosinophil

infiltration at 24 h, preceded by eotaxin generation at 6 h. Intravenous administration of a rabbit anti-mouse eotaxin antibody (0.5 mg kg(-1)) significantly reduced allergen-evoked eosinophilia in this model. 4. Local pretreatment with rolipram (40 microg cavity(-1)) or RP 73401 (40 microg cavity(-1)) 1 h before challenge reduced eosinophil accumulation evaluated in the rat pleural effluent, but only the former was active against eotaxin generation. The inhibitors of PDE type 3 (SK&F 94836) and type 5 (zaprinast) failed to alter allergen-evoked eosinophil recruitment in rats. 5. Local injection of beta2-agonist salbutamol (20 microg cavity(-1)) inhibited both eosinophil accumulation and eotaxin production following pleurisy. The former was better inhibited when salbutamol and rolipram were administered in combination. 6. Treatment with rolipram and RP 73401 dose-dependently inhibited eosinophil adhesion and migration in vitro. These effects were clearly potentiated by salbutamol at concentrations that had no effect alone. 7. Our findings indicate that although rolipram and RP 73401 are equally effective in inhibiting allergen-induced eosinophil infiltration only the former prevents eotaxin formation, indicating that PDE 4 inhibitors impair eosinophil accumulation by mechanisms independent of eotaxin production blockade.

PMCID: PMC1572944
PMID: 11564646 [PubMed - indexed for MEDLINE]

1524. Inflamm Res. 2001 Aug;50(8):400-8.

Therapeutic effects of cysteine protease inhibition in allergic lung inflammation: inhibition of allergen-specific T lymphocyte migration.

Layton GT, Harris SJ, Bland FA, Lee SR, Fearn S, Kaleta J, Wood ML, Bond A, Ward G.

British Biotech Pharmaceuticals plc, Cowley, Oxford, UK. layton@britbio.co.uk

OBJECTIVE AND DESIGN: We have evaluated the effects of the broad-spectrum cysteine protease inhibitor E64 on allergic lung inflammation in the mouse ovalbumin model of human asthma. We have also characterised membrane-associated cathepsin enzyme activity on a range of cell types.
MATERIALS: Balb/C mice, E64 and CA074, various cell lines.
TREATMENT: E64 was administered by subcutaneous minipump into ovalbumin-sensitised mice prior to intranasal ovalbumin challenge. The effect of E64 on ovalbumin-induced inflammation in vivo and ovalbumin-specific T cell proliferation in vitro and ex vivo was examined. Membrane-associated cathepsin activity on various cell types was measured.
RESULTS: E64 treatment (0.36-0.48 mg/day) led to a significant reduction in eosinophil numbers and lung weights in the mouse model. Histological examination of lungs confirmed the anti-inflammatory effect. E64 greatly reduced ovalbumin-specific T cell numbers in the lymph nodes draining the lung following intranasal challenge whilst an accumulation of these T cells was found in the 'priming' lymph nodes. An analysis of various cells involved in lymphocyte priming and migration revealed that monocytes, dendritic cells and endothelial cells express high levels of membrane-associated cathepsin B activity.
CONCLUSIONS: Since E64 is not cell permeable and does not inhibit antigen-induced T cell proliferation in vitro or in vivo, the data indicate that membrane-associated cysteine proteases, possibly cathepsin B, may regulate T lymphocyte migration in vivo.

PMID: 11556520 [PubMed - indexed for MEDLINE]

1525. Rev Med Chil. 2001 Jul;129(7):780-5.

[Toxocariasis in an adult manifested as hypereosinophilic syndrome with predominant neurological involvement. Clinical case].

[Article in Spanish]

Ardiles A, Chanqueo L, Reyes V, Araya L.

Policlínico de Medicina Integral, Servicio de Medicina, Hospital San Juan de Dios, Huérfanos 3255, Santiago.

Hypereosinophilic syndrome is characterized by persistent hypereosinophilia and signs or symptoms due to organ involvement, specially nervous system, heart and skin. It can be primary or secondary to allergies, parasites or cancer. Toxocariasis is an uncommon parasitic disease in adults. There is a variant, called visceral larva migrans, that can involve different organs, and among those, the central nervous system. We report a 61 years old male, with a cerebrovascular disease. There were focalizing symptoms, the CAT scan showed multiple ischemic lesions and a peripheral eosinophilia of 12,152 cells/mm3 was present. Anti toxocara IgG antibody titers were 1/1000. The patient was treated with albendazole for 14 days. After a 2 years follow up the patients is in good conditions and, for the first time, his eosinophil count is within normal limits.

PMID: 11552447 [PubMed - indexed for MEDLINE]

1526. Arch Dermatol Res. 2001 Jul;293(7):350-5.

Enhanced production of CC-chemokines (RANTES, MCP-1, MIP-1alpha, MIP-1beta, and eotaxin) in patients with atopic dermatitis.

Kaburagi Y, Shimada Y, Nagaoka T, Hasegawa M, Takehara K, Sato S.

Department of Dermatology, Kanazawa University School of Medicine, Ishikawa, Japan.

CC-chemokines are potent molecules that direct the migration of leukocytes to inflammatory foci. To determine their role in inflammation associated with atopic dermatitis (AD), we determined serum levels and spontaneous production of CC-chemokines by peripheral blood mononuclear cells (PBMC) in AD patients using an ELISA. Serum levels of RANTES, MCP-1, MIP-1beta, and eotaxin were increased in AD patients (n = 52) compared with normal controls (n = 22). Serum levels of RANTES, MCP-1, and MIP-1beta were increased in AD patients with severe disease (n = 19) compared with normal controls (n = 22). Spontaneous production of RANTES, MCP-1, MIP-1alpha and MIP-1beta by PBMC was augmented in AD patients (n = 39) and in patients with severe AD (n = 14) compared with normal controls (n = 20). Serum RANTES levels correlated with total serum IgE levels, eosinophil numbers, and serum lactate dehydrogenase levels. Our results suggest that augmented production of CC-chemokines correlates with inflammation associated with AD.

PMID: 11550808 [PubMed - indexed for MEDLINE]

1527. Environ Health Perspect. 2001 Aug;109 Suppl 4:553-7.

Allergen-induced generation of mediators in the mucosa.

Mattoli S.

Avail Biomedical Research Institute, Basel, Switzerland.

smattoli@avail-research.com

The inhalation of antigens does not normally lead to allergic inflammation, but airway resident cells and their products may affect the outcome of antigen exposure. It is therefore important to elucidate how potential allergens interact with airway epithelial cells and other cells located within and below the epithelium. Some studies have indicated that certain antigens, particularly the major house dust mite antigen Der p1, penetrate the airway epithelium by intracellular transportation or paracellular passage, depending on their concentrations, time of exposure, and ability of the cells to inactivate them. If an antigen possesses proteolytic activity, such as Der p1, and it reaches high concentrations or the exposure is prolonged, the disruption of the tight junction can also favor the transepithelial passage of other antigens. In this way, the antigens can easily encounter the effector cells located between epithelial cells and below the basement membrane. The magnitude of this phenomenon may be more prominent in the airways of asthmatic patients, as their epithelium is more permeable to Der p1 than the epithelium of nonasthmatic patients and releases cytokines after exposure to very low concentrations of this antigen for brief periods. Epithelial cell activation may facilitate the development of allergic mucosal sensitization to Der p1 and contribute to the antigen-induced inflammatory response by affecting the migration and function of dendritic cells, mast cells, and eosinophils. Also, there might be a secondary release of interleukin-6 and endothelin-1, which can have a detrimental effect on the cardiovascular function.

PMCID: PMC1240580
PMID: 11544162 [PubMed - indexed for MEDLINE]

1528. J Gravit Physiol. 1998 Jul;5(1):P139-40.

Phagocyte system under spaceflight conditions.

Meshkov D, Rykova M, Antropova E, Vdovin A, Biziukin A, Nesvizhsky I.

State Research Center of Russian Federation, Institute for BioMedical Problems.

The spaceflight conditions lead to disturbances in immune system and cause the changes in microbial and chemical environment that create preconditions for immunodeficiency and allergic disease development. With the spaceflights lengthening the problem of crewmembers immunodeficiency and the probability of allergic disease manifestation became actual. The higher risk of various pathological conditions noted in cosmonauts during space flight due to lowered immunological resistance and unnatural biological and chemical environment (autoimmune reactions, bacterial and viral autoinfections, possible allergic events etc.) proves the need of studying the mechanisms of these disturbances and determination the most labile links between the immune system and antigen environment. In this case phagocytes seems to be one of the most important cells that can influence both induction and effector stage of immune reactions and also take part in the regulation of the immune response. The goal of the investigation was to conduct studies of one of the of the phagocytes metabolic and migration activity that are closely connected with functional activity of the cells.

PMID: 11542326 [PubMed - indexed for MEDLINE]

1529. Eur J Immunol. 2001 Sep;31(9):2669-79.

Dynamics of antigen-specific helper T cells at the initiation of airway eosinophilic inflammation.

Kaminuma O, Fujimura H, Fushimi K, Nakata A, Sakai A, Chishima S, Ogawa K, Kikuchi M, Kikkawa H, Akiyama K, Mori A.

Discovery Research Laboratory, Tanabe Seiyaku Co., Ltd., Saitama, Japan. kaminuma@rinshoken.or.jp

Bronchial asthma is characterized by chronic eosinophilic inflammation of the bronchial mucosa in which Th2 cells play crucial roles. Ovalbumin-reactive Th2 clones were labeled with a fluorescent-probe then infused into unprimed mice to elucidate the dynamics of antigen-specific T cells involved in allergic inflammation. Infiltration of not only labeled antigen-specific T cells, but also unlabeled nonspecific CD4(+) T cells into the bronchial mucosa following inhaled antigen challenge was detectable under confocal microscopy and flow cytometry. Accordingly, labeled T cells in the spleen were decreased, whereas those in hilar lymph nodes were increased upon antigen challenge. Approximately 45% of antigen-specific T cells that migrated into the lungs bore CD25, while another early activation marker, CD69, was expressed on 80% of the migrated T cells. Accordingly, antigen challenge to the mice induced in situ proliferation of antigen-specific T cells as well as bronchial epithelial cells in the lungs. Expression of vascular cell adhesion molecule (VCAM)-1, but not intercellular adhesion molecule (ICAM)-1, on the vascular endothelium in the lungs was enhanced following antigen challenge. Nevertheless, treatment with anti-VCAM-1 antibody, and also anti-ICAM-1 antibody strongly suppressed the accumulation of T cells, suggesting that both VCAM-1 and ICAM-1 are essential for antigen-stimulated T cell mobilization into peripheral tissues. Our current study visualized the kinetics and the mechanism of antigen-specific T cell migration in response to local challenge with a protein antigen.

PMID: 11536165 [PubMed - indexed for MEDLINE]

1530. Gastroenterol Clin Biol. 2001 May;25(5):546-8.

[Repetitive acute pancreatitis in a late-diagnosed cystic fibrosis: prevention of relapses by octreotide in the long term].

[Article in French]

Weber A, Queneau PE, Polio JC, Koch S, Fellmann F, Sauve G, Carayon P.

Service de Gastroentérologie, Hôpital J.Minjoz, Cedex, France. audrey.weber@wanadoo.fr

We report on the case of a 35 year-old woman who was initially admitted for acute pancreatitis in october 1995. The patient was suffering from asthma (since childhood) and diffuse abdominal pain (since adolescence). The diagnosis of cystic fibrosis was made fortuitously during a sterility evaluation. After extensive etiological screening the acute pancreatitis was considered to be a manifestation of the cystic fibrosis. Despite therapy with pancreatic enzymes, the patient continued to suffer from chronic abdominal pain. High intake of analgesics was required. Until December 1995, the patient was repeatedly admitted for episodes of acute pancreatitis. In January 1996, we initiated a preventive treatment with subcutaneous octreotide between 100 and 200 microgram, three time a day. Thereafter, there were fewer episodes of pancreatitis and the consumption of analgesics decreased. Side effects of octreotide were intermittent diarrhea and development of cholelithiasis that was complicated by biliary migration in November 1998. In June 1999, the prolonged-release form of the molecule was given without modification of the efficacy.

PMID: 11521109 [PubMed - indexed for MEDLINE]

1531. Am J Respir Cell Mol Biol. 2001 Aug;25(2):170-7.

The role of Mac-1 (CD11b/CD18) in antigen-induced airway eosinophilia in mice.

Kanwar S, Smith CW, Shardonofsky FR, Burns AR.

Speros P. Martel Laboratory of Leukocyte Biology, Department of Pediatrics, Baylor College of Medicine, Houston, Texas 77030, USA. skanwar@bcm.tmc.edu

Mac-1 (CD11b/CD18) is an important adhesion molecule involved in the migration of leukocytes, cell signaling, and subsequent secretory responses. Its precise role in eosinophil recruitment and activation in vivo is not entirely clear. We wished to directly examine the role of Mac-1 in eosinophil migration in a murine model of allergic pulmonary inflammation. Briefly, wild-type (C57Bl/6) and Mac-1-deficient/knockout (Mac-1 KO) mice were intraperitoneally sensitized with ovalbumin (OVA) and alum (AlOH) on Days 0 and 14, and intranasally challenged with OVA either once on Day 14 or five times on Days 14 and 25 through 28. Control animals were challenged with saline. Bronchial hyperresponsiveness was measured, bronchoalveolar lavage (BAL) fluid was collected, and lungs were harvested for histology 24 h after the last challenge. The data demonstrate that wild-type (WT) mice do not respond to one OVA challenge but do develop bronchial hyperreactivity and airway and tissue eosinophilia after five OVA challenges. Conversely, Mac-1 KO mice develop significant airway eosinophilia after one OVA challenge, and the degree of airway inflammation is comparable to that observed in allergic WT mice after five challenges. In Mac-1 KO mice, after five challenges, bronchial hyperreactivity and airway inflammation was significantly enhanced compared with their wild-type counterparts. Administration of an anti-Mac-1 antibody to WT mice, before each of five intranasal OVA challenges, significantly reduces the airway eosinophilia but has no effect on tissue eosinophilia or bronchial hyperresponsiveness. Intravenous injection of interleukin-5 induced a significant blood eosinophilia in both WT and Mac-1 KO mice. Intranasal eotaxin administration induced similar levels of eosinophil migration into the lung tissues and airways of both WT and Mac-1 KO mice. In conclusion, Mac-1-deficient mice develop enhanced eosinophilic inflammation in the lung in response to allergic antigen challenge.

PMID: 11509326 [PubMed - indexed for MEDLINE]

1532. J Allergy Clin Immunol. 2001 Aug;108(2):250-7.

Preclinical efficacy and safety of mepolizumab (SB-240563), a humanized monoclonal antibody to IL-5, in cynomolgus monkeys.

Hart TK, Cook RM, Zia-Amirhosseini P, Minthorn E, Sellers TS, Maleeff BE, Eustis S, Schwartz LW, Tsui P, Appelbaum ER, Martin EC, Bugelski PJ, Herzyk DJ.

Department of Safety Assessment, GlaxoSmithKline Pharmaceuticals, King of Prussia, PA 19406, USA.

BACKGROUND: Allergic respiratory diseases are characterized by large numbers of eosinophils and their reactive products in airways and blood; these are believed to be involved in progressive airway damage and remodeling. IL-5 is the principal cytokine for eosinophil maturation, differentiation, and survival. Mepolizumab (SB-240563), a humanized monoclonal antibody (mAb) specific for human IL-5, is currently in clinical trials for treatment of asthma.
OBJECTIVE: The purpose of this study was to characterize the pharmacologic

activity and long-term safety profile of an anti--human IL-5 mAb to support clinical trials in asthmatic patients.
METHODS: Naive and Ascaris suum -sensitive cynomolgus monkeys received various dose levels of mepolizumab and were monitored for acute and chronic pharmacologic and toxic responses.
RESULTS: To support preclinical safety assessment, cynomolgus monkey IL-5 was cloned, expressed, and characterized. Although monkey IL-5 differs from human IL-5 by 2 amino acids (Ala27Gly and Asn40His), mepolizumab has comparable inhibitory activity against both monkey IL-5 and human IL-5. In A suum--sensitive monkeys, single doses of mepolizumab significantly reduced blood eosinophilia, eosinophil migration into lung airways, and levels of RANTES and IL-6 in lungs for 6 weeks. However, mepolizumab did not affect acute bronchoconstrictive responses to inhaled A suum. In an IL-2--induced eosinophilia model (up to 50% blood eosinophilia), 0.5 mg/kg mepolizumab blocked eosinophilia by >80%. Single-dose and chronic (6 monthly doses) intravenous and subcutaneous toxicity studies in naive monkeys found no target organ toxicity or immunotoxicity up to 300 mg/kg. Monkeys did not generate anti-human IgG antibodies. Monthly mepolizumab doses greater than 5 mg/kg caused an 80% to 100% decrease in blood and bronchoalveolar lavage eosinophils lasting 2 months after dosing, and there was no effect on eosinophil precursors in bone marrow after 6 months of treatment. Eosinophil decreases correlated with mepolizumab plasma concentrations (half-life = 13 days).
CONCLUSION: These studies demonstrate that chronic antagonism of IL-5 by mepolizumab in monkeys is safe and has the potential, through long-term reductions in circulating and tissue-resident eosinophils, to be beneficial therapy for chronic inflammatory respiratory diseases.

PMID: 11496242 [PubMed - indexed for MEDLINE]

1533. J Immunol. 2001 Aug 15;167(4):2298-304.

Sensitized mast cells migrate toward the antigen: a response regulated by p38 mitogen-activated protein kinase and Rho-associated coiled-coil-forming protein kinase.

Ishizuka T, Okajima F, Ishiwara M, Iizuka K, Ichimonji I, Kawata T, Tsukagoshi H, Dobashi K, Nakazawa T, Mori M.

First Department of Internal Medicine, Gunma University School of Medicine, Faculty of Health Sciences, Institute for Molecular and Cellular Regulation, Gunma University, Maebashi, Japan. tamotsui@showa.gunma-u.ac.jp

Erratum in
J Immunol 2001 Oct 15;167(8):following 4770.

Although mast cells accumulate within the mucosal epithelial layer of patients with allergic rhinitis and bronchial asthma, the responsible chemotactic factors are undefined. We investigated whether mast cells sensitized with Ag-specific IgE migrate toward the Ag. MC/9 mast cells sensitized with anti-DNP IgE migrated toward DNP-conjugated human serum albumin. This migration was directional, and the degree was stronger than that induced by stem cell factor. IL-3 and stem cell factor-dependent cultured mast cells derived from mouse bone marrow also migrated toward the Ag. Subsequent migration mediated by the Fc(epsilon)RI was significantly inhibited by incubating the cells with Y-27632, a Rho-associated coiled-coil-forming protein kinase inhibitor, or with SB203580, a p38 mitogen-activated protein kinase (MAPK) inhibitor. Both p38 MAPK and MAPK-activated protein kinase (MAPKAPK)2 were activated following Fc(epsilon)RI aggregation, and activation of MAPKAPK2 was almost completely inhibited by 10 microM SB203580. Wortmannin or a low concentration of SB203580 partially

inhibited MAPKAPK2, but did not block mast cell migration. In contrast, Y-27632 did not affect the activation of MAPKAPK2. These results indicate that Ag works not only as a stimulant for allergic mediators from IgE-sensitized mast cells, but also as a chemotactic factor for mast cells. Both p38 MAPK activation and Rho-dependent activation of Rho-associated coiled-coil-forming protein kinase may be required for Fc(epsilon)RI-mediated cell migration.

PMID: 11490018 [PubMed - indexed for MEDLINE]

1534. J Immunol. 2001 Aug 15;167(4):2292-7.

Eosinophil tissue recruitment to sites of allergic inflammation in the lung is platelet endothelial cell adhesion molecule independent.

Miller M, Sung KL, Muller WA, Cho JY, Roman M, Castaneda D, Nayar J, Condon T, Kim J, Sriramarao P, Broide DH.

Department of Medicine, University of California at San Diego, La Jolla, CA 92093, USA.

Platelet endothelial cell adhesion molecule (PECAM or CD31) is a cell adhesion molecule expressed on circulating leukocytes and endothelial cells that plays an important role in mediating neutrophil and monocyte transendothelial migration in vivo. In this study, we investigated whether eosinophils, like neutrophils and monocytes, utilize PECAM for tissue recruitment to sites of allergic inflammation in vivo. Eosinophils express similar levels of PECAM as neutrophils as assessed by FACS analysis. RT-PCR studies demonstrate that eosinophils like neutrophils express the six extracellular domains of PECAM. Eosinophils exhibit homophilic binding to recombinant PECAM as assessed in a single-cell micropipette adhesion assay able to measure the biophysical strength of adhesion of eosinophils to recombinant PECAM. The strength of eosinophil adhesion to recombinant PECAM is the same as that of neutrophil binding to recombinant PECAM and can be inhibited with an anti-PECAM Ab. Although eosinophils express functional PECAM, anti-PECAM Abs did not inhibit bronchoalveolar lavage eosinophilia, lung eosinophilia, and airway hyperreactivity to methacholine in a mouse model of OVA-induced asthma in vivo. Thus, in contrast to studies that have demonstrated that neutrophil and monocyte tissue recruitment is PECAM dependent, these studies demonstrate that eosinophil tissue recruitment in vivo in this model is PECAM independent.

PMID: 11490017 [PubMed - indexed for MEDLINE]

1535. Cell Biol Int. 2001;25(8):795-803.

Morphological alteration of peritoneal mast cells and macrophages in the mouse peritoneal cavity during the early phases of an allergic inflammatory reaction.

Oliani SM, Lim LH, Christian HC, Pell K, Das AM, Perretti M.

Department of Biology, IBILCE-UNESP, São José do Rio Preto, SP, Brazil.

We investigated the presence of mast cell granules in macrophages following an in vivo model of an allergic reaction. Injection of ovalbumin (100 microg) into the peritoneal cavity of sensitised mice produced a rapid (within 2 h) influx of neutrophils followed by a slower (after >4 h) eosinophil migration. Ovalbumin treatment induced a high incidence (approximately 50%) of mast cell degranulation compared to control phosphated-buffered saline-treated mice. The majority (approximately 90%) of peritoneal macrophages contained mast cell granules as early as 2 h post-ovalbumin, with lower values at later time-points, as

determined by staining with Toluidine blue and Berberine sulphate. This was confirmed by electron microscopy which enabled us to identify the complex mast cell granule sub-structural components in macrophage phagosomes. In conclusion, we used histochemical and ultrastructural analyses to show that mast cell granules become internalised with macrophages during the early stages of an experimental allergic reaction.

Copyright 2001 Academic Press.

PMID: 11482903 [PubMed - indexed for MEDLINE]

1536. Inflamm Res. 2001 Jun;50(6):309-16.

Mechanisms of allergen- and LPS-induced bone marrow eosinophil mobilization and eosinophil accumulation into the pleural cavity: a role for CD11b/CD18 complex.

Larangeira AP, Silva AR, Gomes RN, Penido C, Henriques MG, Castro-Faria-Neto HC, Bozza PT.

Departamento de Fisiologia e Farmacodinâmica, IOC, Fundação Oswaldo Cruz, Rio de Janeiro, RJ, Brazil.

OBJECTIVE: The mechanisms involved in bone marrow eosinophil emigration and recruitment to inflammatory sites are not fully understood. The involvement of CD11b/CD18 in marrow eosinophil release induced by lipopolysaccharide (LPS) or allergen was investigated in mice.
METHODS: Eosinophil and neutrophil counts in the pleural cavity, blood and bone marrow were performed at different time intervals after the intrathoracic injection of LPS (250 ng/cavity) or ovalbumin (OVA, 12 microg/cavity; into actively sensitized mice) and compared to anti-CD11b/CD 18 (5C6, 1 mg/mouse) or anti-IL-5 (TRFK-5, 500 microg/kg) treated mice.
RESULTS: LPS induced local eosinophil influx, that peaked within 24 h and that was preceded by a decrease in marrow eosinophils at 4 h. Antigenic challenge induced a decrease in marrow eosinophils within 4 h, followed by a long lasting pleural eosinophil accumulation and a persistent increase in marrow eosinophil numbers. Pretreatment with anti-CD11b/CD18 abolished LPS-induced neutrophil and eosinophil accumulation in the pleural cavity at 4 and 24 h, respectively. This pretreatment failed to modify neutrophil emigration from bone marrow, but significantly inhibited marrow eosinophil release at 4 h post-LPS or OVA challenge. Anti-IL-5 pretreatment failed to inhibit LPS-induced pleural eosinophil accumulation and mobilization from bone marrow, but it abolished allergen-induced effects, indicating a role for IL-5 in marrow eosinophil mobilization induced by antigen, but not by LPS challenge.
CONCLUSIONS: Our results suggest that eosinophil migration induced by antigen or LPS into the pleural cavity is preceded by bone marrow eosinophil release through a mechanism that depends on CD11b/CD18.

PMID: 11475332 [PubMed - indexed for MEDLINE]

1537. Pulm Pharmacol Ther. 2001;14(3):203-10.

Important roles for L-selectin and ICAM-1 in the development of allergic airway inflammation in asthma.

Tang ML, Fiscus LC.

Department of Immunology, Murdoch Children's Research Institute, Royal Children's Hospital, Melbourne, Australia. tangm@cryptic.rch.unimeld.edu.au

Airway inflammation and airway hyperresponsiveness (AHR) are fundamental features of asthma. Migration of inflammatory cells from the circulation into the lungs is dependent upon adhesion molecule interactions. The cell surface adhesion molecules L-selectin and intercellular adhesion molecule (ICAM)-1 have been demonstrated to mediate leukocyte rolling on inflamed pulmonary endothelium, and ICAM-1 has also been shown to mediate capillary sequestration in inflamed lung. However, their roles in the development of airway inflammation and AHR in asthma have not been directly examined. We have characterised the roles of L-selectin and ICAM-1 in the recruitment of inflammatory cells to the lung and in the development of airway hyperresponsiveness using an ovalbumin (OVA)-induced allergic airway disease model of asthma and adhesion molecule-deficient mice. OVA-sensitized/challenged ICAM-1-deficient mice have dramatically reduced inflammatory influx into the airway/lung and a corresponding attenuation of AHR as compared to wild-type controls. OVA-sensitized/challenged L-selectin-deficient mice demonstrate significantly reduced numbers of CD3(+)lymphocytes and increased numbers of B220(+)lymphocytes in BAL as compared to wild-type mice (P< 0.05). However, other parameters of airway/lung inflammation in OVA-sensitized/challenged L-selectin-deficient mice were equivalent to wild-type control mice. Remarkably, despite a fulminant inflammatory response in the airway/lung, AHR was completely abrogated in OVA-sensitized/challenged L-selectin-deficient mice. These findings suggest a crucial role for ICAM-1 in the development of airway inflammation and AHR in asthma. In contrast, L-selectin plays a more selective role in the development of airway hyperresponsiveness but not allergic inflammation in this animal model of asthma. Thus, L-selectin and ICAM-1 represent potential targets for novel asthma therapies specifically aimed at controlling airway inflammation and/or airway hyperresponsiveness.

Copyright Academic Press.

PMID: 11448147 [PubMed - indexed for MEDLINE]

1538. Pulm Pharmacol Ther. 2001;14(3):193-202.

Chemokine receptors in airway disease: which receptors to target?

Owen C.

Novartis Horsham Research Centre, Wimblehurst Road, Horsham, West Sussex, United Kingdom, RH12 5AB.

Many disease states within the airway result in the co-ordinated infiltration of key inflammatory cells. The cellular influx is choreographed through the temporal and spatially-regulated expression of chemokines, which potentiate the migration of cells along gradients of chemotactic ligands. Chemokines act as ligands for the chemokine receptors; a distinct class of G-protein-coupled receptor. Over 40 chemokine ligands and 18 chemokine receptors have been identified on human cells. Chemokine receptors are divided into several classes; the two most prominent of which are the CC- and CXC-chemokine receptors, classified through the spatial arrangement of two conserved cysteine residues. The role of chemokine receptors such as CCR2, CCR3, CCR4, CCR8 and the CXC chemokine receptors; CXCR1 and CXCR2 on cell types of relevance to respiratory diseases such as asthma, chronic obstructive pulmonary disease (COPD) and chronic bronchitis will be explored in this review. Chemokines have proven to be amenable drug targets for the development of low molecular weight antagonists by the pharmaceutical industry. So far, no chemokine receptor antagonist has entered the clinic in trials for respiratory disease, but over the next few years it is expected that many will do so, at which time the potential of these exciting new targets will be fully realised.

Copyright Academic Press.

PMID: 11448146 [PubMed - indexed for MEDLINE]

1539. Clin Med. 2001 May-Jun;1(3):214-8.

Eosinophil trafficking in asthma.

Wardlaw AJ.

Division of Respiratory Medicine, Leicester-Warwick Medical School, Glenfield Hospital.

Asthma is characterised by a 50-fold increase in the number of eosinophils relative to neutrophils in the bronchial mucosa. This is the result of the cumulative and sequential effects of several, approximately fourfold, increases in selective eosinophil versus neutrophil migration occurring at a number of stages in the life cycle of the eosinophil. These events, which are integrated and directed by allergen-specific T helper 2 lymphocytes through the generation of interleukin (IL)-5, IL-4 and IL-13, include: effects on the bone marrow, mediated principally by IL-5, which result in a fourfold increase in circulating eosinophils selective tethering of eosinophils to venular endothelium through the combined effects of P-selectin/P-selectin glycoprotein ligand (PSGL)-1 and very late activation antigen (VLA)-4/vascular cell adhesion molecule-1, which has the potential for an up to tenfold increase in eosinophil versus neutrophil adhesion selective chemotaxis under the influence of CC chemokines prolonged survival, again mediated by IL-5. The implications of this multistep process are that antagonists of IL-5, VLA-4, PSGL-1 and CC chemokine receptor 3, as well as IL-4 and IL-13, each have the potential markedly to inhibit eosinophil recruitment in asthma.

PMID: 11446618 [PubMed - indexed for MEDLINE]

1540. J Cutan Laser Ther. 2000 Mar;2(1):37-9.

Successful removal by ruby laser of darkened ink after ruby laser treatment of mismatched tattoos for acne scars.

Rüdlinger R.

Cosmetic tattoos are becoming increasingly popular. Elimination of cosmetic tattoos is sought because of misplacement or migration of tattoo pigment, allergic reactions to the various pigments or dissatisfaction of the customer for various reasons. Removal of unwanted pigment is a domain of laser surgery using various Q-switched laser systems, such as the ruby, alexandrite, pulsed dye and Nd:YAG lasers. Dark colours are easily removed by these lasers, whereas red, pink and skin-toned pigment may turn black if exposed to Q-switched laser light. This ink-blackening occurs because Q-switched lasers heat up the pigments, which consist of ferric oxide, and reduce them into ferrous oxide, which is black. Laser-induced black ink is not always readily removed. A successful ruby laser-removal of laser-induced blackened cosmetic tattoos for acne scar camouflage is reported. The advantageous outcome in this case contrasts with other published cases where laser-darkened pigment had to be removed by other measures, or was permanent. Test site treatment can limit the problem to some degree but, in addition to test-treating, some kind of 'tattoo identification card' could help to prevent problems in this field and allow 'in vitro' test treatment.

PMID: 11446091 [PubMed - indexed for MEDLINE]

1541. J Vet Pharmacol Ther. 2001 Jun;24(3):209-14.

Inflammatory mediators induce endothelium-dependent adherence of equine eosinophils to cultured endothelial cells.

Bailey SR, Cunningham FM.

Department of Veterinary Basic Sciences, The Royal Veterinary College, Hawkshead Campus, North Mymms, Hatfield, Hertfordshire AL9 7TA, UK.

Accumulation of equine eosinophils at sites of parasite infestation or allergic inflammation depends upon their adherence to vascular endothelial cells and subsequent migration through the endothelium and extracellular matrix. This study has examined whether cytokines, which cause endothelial cell-dependent eosinophil adherence in other species, and histamine and substance P, which increase adherence of equine eosinophils to protein coated plastic, induce equine eosinophil adherence to cultured equine digital vein endothelial cell (EDVEC) monolayers. The EDVEC monolayers were stimulated with recombinant human (rh) interleukin (IL)-1beta, rhTNFalpha, substance P or histamine for different times and with a range of concentrations of mediators and the adherence of blood eosinophils from normal horses examined. All four mediators caused time- and concentration-dependent increases in adherence. However, neither the response to substance P, nor that to histamine, reached a maximum at the highest concentration tested (10-3 M: 10.6 +/- 2.6% and 4.5 +/- 0.6% adherent cells vs. background adherence of 1.9 +/- 0.4% and 1.1 +/- 0.2%; values for substance P and histamine, respectively, expressed as a percentage of total cells added initially; n=4). These data suggest that, as in other species, cytokines induce endothelial cell-dependent eosinophil adherence and mediators released during allergic inflammation may play a role in eosinophil recruitment by this mechanism.

PMID: 11442800 [PubMed - indexed for MEDLINE]

1542. Eur J Pharmacol. 2001 Jun 29;423(1):85-92.

Role of cysteinyl leukotrienes in nociceptive and inflammatory conditions in experimental animals.

Jain NK, Kulkarni SK, Singh A.

Pharmacology Division, University Institute of Pharmaceutical Sciences, Panjab University, Chandigarh-160 014, India.

The leukotrienes are potent inflammatory mediators, which may have a role in inflammatory diseases such as allergic rhinitis, inflammatory bowl disease and asthma. Zafirlukast, a cysteinyl leukotriene receptor antagonist, is claimed to be effective in asthma. However, it is not known whether these leukotrienes are involved in nociceptive and peripheral inflammation. The present study aimed to assess the role of cysteinyl leukotrienes in nociceptive and inflammatory conditions in experimental animals. Central nociception was assessed with tail-flick and hot-plate methods and peripheral nociception was assessed by acetic acid-induced chemonociception in mice. Local administration (intraplantar) of carrageenan-induced hyperalgesia and inflammation, measured by paw withdrawal latency and paw volumes, respectively. Zafirlukast (2.5--20 mg/kg, p.o.) produced a significant and dose-dependent antinociceptive and antiinflammatory effect

against acetic acid-induced chemonociception in mice and carrageenan-induced paw oedema in rats, respectively. Zafirlukast (2.5 and 5.0 mg/kg, p.o.) also attenuated the carrageenan-provoked hyperalgesia but did not alter the pain threshold in central nociception up to 20 mg/kg. Zafirlukast (5 and 10 mg/kg) significantly inhibited exudate formation and migration of polymorphonuclear leukocytes in carrageenan-induced pleurisy. Further, zafirlukast (5 mg/kg) also reduced myeloperoxidase activity in carrageenan-treated paw. When nimesulide (2 mg/kg, p.o.) was co-administered with zafirlukast, the antinociceptive, antihyperalgesic and antiinflammatory effects of nimesulide were significantly increased as compared to the per se effect. The results indicate that cysteinyl leukotrienes are involved in nociceptive/inflammatory conditions. It is expected that combination of cysteinyl leukotriene receptor antagonist with cyclooxygenase inhibitor would prove to be a novel approach to treat complex inflammatory conditions.

PMID: 11438310 [PubMed - indexed for MEDLINE]

1543. Medicina (B Aires). 2000;60(6):940-6.

[Isoforms modulation of CD44 adhesion molecule in a murine model of ischemia and intestinal reperfusion].

[Article in Spanish]

Cabrera PV, Blanco G, Argibay P, Hajos SE.

Instituto de Estudios de La Inmunidad Humoral (IDEHU), Facultad de Farmacia y Bioquímica, Universidad de Buenos Aires, Junín 956, 1113 Buenos Aires, Argentina.

Gut ischemia-reperfusion (G-IR) induces a systemic inflammatory response, in which leukocyte contribution to this injury in distant organs is important. ICAM-1 as well as CD11/CD18 have been involved in leukocyte infiltration in liver and lungs. CD44 adhesion molecule plays an essential role in other inflammatory processes such as rheumatoid arthritis and allergic contact dermatitis, however its implication in G-IR has not been described. In order to establish a possible role of CD44 in the development of systemic inflammation by G-IR, we have studied CD44 mRNA expression by RT-PCR in a murine model of gut ischemia reperfusion. Animals subjected to G-IR showed an increased number of CD44 variable isoforms expressed in liver and spleen compared to non-treated animals or animals subjected to laparotomy. This finding indicates that G-IR specifically induces the expression of different CD44 variable isoforms. Liver CD44 upregulation in animals subjected to G-IR suggests a contribution of this molecule to lymphocyte activation and migration to this injured organ. Moreover, increased isoform expression in spleen may be induced by the proinflammatory environment resulting from a systemic depuration activity.

PMID: 11436705 [PubMed - indexed for MEDLINE]

1544. Emerg Med J. 2001 Jul;18(4):312-3.

Extraluminal migration of a coin in the oesophagus of a child misdiagnosed as asthma.

Persaud RA, Sudhakaran N, Ong CC, Bowdler DA, Dykes E.

Department of Ear, Nose and Throat Surgery, University Hospital Lewisham, London Department of Paediatric Surgery, University Hospital Lewisham, UK.
RandM.Persaud@ukgateway.net

Ingestion of a foreign body, the commonest being a coin, is a common problem in children. In most cases the coin will pass uneventfully through the gastrointestinal tract. However, on rare occasions it may become lodged in the oesophagus with subsequent extraluminal migration with the potential for serious complications such as vascular fistula or chronic suppurative infection. A case is presented of extraluminal migration of a coin in the oesophageal associated with abscess formation in a 15 month old boy. This case is particularly important because the presenting symptom of wheezing led to the erroneous diagnosis of asthma, which resulted in a three month delay in investigation and treatment. In addition, it raises the issue of whether to perform chest radiography on newly diagnosed asthmatic patients to rule out the presence of a foreign body and thereby prevent serious complications.

PMCID: PMC1725611
PMID: 11435378 [PubMed - indexed for MEDLINE]

1545. J Adv Nurs. 2001 Apr;34(2):171-8.

The feasibility of a nurse practitioner-led primary health care clinic in a school setting: a community needs analysis.

Clendon J, White G.

School of Health Sciences, Massey University at Albany, Auckland, New Zealand. jclendon@xtra.co.nz

AIM: The aim of this New Zealand study was to determine the feasibility of establishing a nurse practitioner-led, family focused, primary health care clinic within a primary school environment as a means of addressing the health needs of children and families. A secondary aim was to ascertain whether public health nurses (PHNs) were the most appropriate nurses to lead such a clinic.
METHOD: Utilizing a community needs analysis method, data were collected from three sources --known demographic data, 17 key informant interviews and two focus group interviews. Questions were asked regarding the health needs of the community, the perceptions of participants regarding the role of the PHN, and the practicalities of establishing a clinic including the services participants would expect a clinic to provide. Analysis was exploratory and descriptive.
RESULTS: Findings included the identification of a wide range of health issues. These included asthma management and control issues, the need to address poor parenting, and specific problems of the refugee and migrant population. Findings also demonstrated that participant understanding of the role of the PHN was less than anticipated and that community expectations were such that for a PHN to lead a primary health care clinic it would be likely that further skills would be required. Outcomes from investigating the practicalities of establishing a nurse practitioner-led clinic resulted in the preparation of a community-developed plan that would serve to address the health needs of children and families in the area the study was undertaken. Services that participants identified as being appropriate included health information, health education, health assessment and referral.
CONCLUSION: Overall findings indicated that the establishment of a nurse practitioner-led, family focused, primary health care clinic in a primary school environment was feasible. While a PHN may fulfil the role of the nurse practitioner, it was established that preparation to an advanced level of practice would be required.

PMID: 11430278 [PubMed - indexed for MEDLINE]

1546. Allergy Asthma Proc. 2001 May-Jun;22(3):133-7.

MCP-1 and RANTES are mediators of acute and chronic inflammation.

Conti P, DiGioacchino M.

Immunology Division, Department of Oncology and Neuroscience, University of Chieti, School of Medicine, Via dei Vestini, 66013 Chieti, Italy.

Regulation of leukocyte migration and activation by chemokines are recognized as potentially important functions in the induction of acute and chronic inflammatory reactions. Regulated upon activation normal T cell expressed and presumably secreted (RANTES), monocyte chemotactic protein-1 (MCP-1), and related molecules constitute the C-C class of the beta chemokine supergene family with inflammatory properties. Here we report that in experimental studies RANTES and MCP-1 provoke mast cell activation and increase histidine decarboxylase mRNA expression in a dose-dependent manner. Moreover, injections of RANTES and MCP-1 in the rat skin cause mast cell, eosinophil, and macrophage recruitment, and prostaglandin E2 (PGE2) generation. In a chronic inflammatory model MCP-1 was found to mediate the recruitment of mononuclear cells in calcified granulomas. In addition, MCP-1 mediated parasitic infections caused by Trichinella spiralis. In accordance with other studies, RANTES and MCP-1 were found to play an important role in the lung allergic inflammation, lung leukocyte infiltration, bronchial hyperresponsiveness, and the recruitment of eosinophils in the pathogenesis of asthma. Here for the first time we propose a new mechanism of pulmonary airway inflammation where RANTES and MCP-1 are deeply involved. We also studied the apparent role played by RANTES in the pathogenesis of relapsing-remitting multiple sclerosis enhancing the inflammatory response within the nervous system.

PMID: 11424873 [PubMed - indexed for MEDLINE]

1547. J Am Acad Dermatol. 2001 Jul;45(1 Suppl):S44-8.

Atopic dermatitis: the role of environmental and social factors, the European experience.

Diepgen TL.

Department of Social Medicine, Center of Dermato-Epidemiology, University Hospital Heidelberg, Germany. thomas_diepgen@med.uni-heidelberg.de

PMID: 11423873 [PubMed - indexed for MEDLINE]

1548. Clin Exp Allergy. 2001 Jun;31(6):836-44.

Effect of a specific cysteinyl leukotriene-receptor 1-antagonist (montelukast) on the transmigration of eosinophils across human umbilical vein endothelial cells.

Virchow JC Jr, Faehndrich S, Nassenstein C, Bock S, Matthys H, Luttmann W.

Abteilung für Pneumologie, Universitätsklinikum Freiburg, Freiburg, Germany. Virchow@med1.UKL.UNI-FREIBURG.DE

Comment in
    Clin Exp Allergy. 2001 Jun;31(6):806-7.

BACKGROUND: Leukotrienes have been implicated in the selective infiltration of eosinophils into the bronchial mucosa in asthma.

OBJECTIVE: We studied whether eosinophil transmigration through cultured human umbilical vein endothelial cells (HUVECs) can be blocked by a specific cysteinyl LT1-receptor-antagonist.
METHODS: Unstimulated and stimulated eosinophils from patients with asthma and normal controls were subjected to confluent human umbilical vein endothelial cell (HUVEC) monolayers separating the upper and lower chamber of Transwell culture plates. Unstimulated eosinophils or cells pre-incubated in the presence of the eosinophil activating cytokines GM-CSF or IL-13 were placed in the upper chambers while PAF, a potent chemoattractant factor for eosinophils, was added to the lower chamber. Migration of eosinophils was quantified by a beta-glucuronidase assay.
RESULTS: The assumption that eosinophils express CysLT1 (cysteinyl-leukotriene 1)-receptors was based on our demonstration of mRNA-expression for the CysLT-1-receptor by polymerase chain reaction on purified eosinophils. The chemotactic response to PAF was significantly reduced when eosinophils were pre-incubated with montelukast for 15 min. When eosinophils were pre-incubated with GM-CSF and/or IL-13, the migratory response to PAF was also significantly reduced by montelukast.
CONCLUSION: From these data we conclude that the specific cysteinyl LT1-receptor antagonist montelukast can inhibit PAF-induced eosinophil transmigration through cultured HUVEC monolayers.

PMID: 11422147 [PubMed - indexed for MEDLINE]

1549. Br J Dermatol. 2001 Jun;144(6):1148-53.

Serum levels of soluble stem cell factor and soluble KIT are elevated in patients with atopic dermatitis and correlate with the disease severity.

Kanbe T, Soma Y, Kawa Y, Kashima M, Mizoguchi M.

Department of Dermatology, St. Marianna University School of Medicine, 2-16-1, Sugao, Miyamae-ku, Kawasaki 216-8511, Japan.

BACKGROUND: Mast cell infiltration in skin lesions of atopic dermatitis (AD) is considered to play an important role in the pathogenesis of the disease. The most common factor that stimulates mast cell growth, migration and differentiation is stem cell factor (SCF), and the interaction of SCF and its receptor, KIT (tyrosine kinase transmembrane receptor), appears to be the key event in the recruitment and proliferation of mast cells.
OBJECTIVES: To determine whether any altered metabolism of SCF and/or KIT is present in patients with AD.
METHODS: We measured serum levels of soluble SCF (sSCF) and soluble KIT (sKIT) using enzyme-linked immunosorbent assay in 54 patients with AD, five patients with erythrodermic psoriasis vulgaris and 64 healthy individuals.
RESULTS: Serum levels of both peptides in AD patients were significantly higher than those in healthy individuals, whereas patients with psoriasis vulgaris did not show any difference from healthy controls. Both sSCF and sKIT levels were positively correlated with the disease severity in AD patients, and decreased after effective treatment with topical corticosteroids. Conclusion Serum levels of sSCF and sKIT may be useful indicators for evaluation of the activity and severity of AD.

PMID: 11422034 [PubMed - indexed for MEDLINE]

1550. Br J Dermatol. 2001 Jun;144(6):1105-13.

Cellular localization of fractalkine at sites of inflammation: antigen-presenting

cells in psoriasis express high levels of fractalkine.

Raychaudhuri SP, Jiang WY, Farber EM.

Psoriasis Research Institute, 600 Town & Country Village, Palo Alto, CA 94301, USA.

BACKGROUND: Chemokines play a key role in cell trafficking at sites of inflammation. The fractalkine CX3C chemokine is unique in several aspects. Fractalkine is expressed on activated endothelial cells and exists in two forms, either membrane anchored or in a soluble form. The soluble form is a potent chemotactic agent for T cells/monocytes and the anchored form functions as an adhesion molecule. In view of these specific functions fractalkine is capable of controlling the key regulatory mechanisms of cell trafficking at sites of inflammation.
OBJECTIVES: Little is known about the significance of this important molecule in inflammatory diseases. We undertook this study to elucidate the role of fractalkine in inflammatory diseases of the skin.
METHODS: We used a polyclonal antifractalkine antibody (immunoperoxidase and immunofluorescence stainings) in cryosections obtained from tissues of normal skin and that of selected cutaneous inflammatory diseases (psoriasis, lichen planus, eczema).
RESULTS: Increased expression of fractalkine was observed in the dermal blood vessels of lichen planus, eczema and psoriasis tissues. The most striking finding was that the dermal dendrocytes in the papillary dermis of psoriasis tissues expressed high levels of fractalkine. Compared with 186.64 +/- 51.69 fractalkine positive dermal dendrocytes per mm2 of the upper dermis of psoriatic tissue, the number of positive cells in lichen planus, eczema, and normal skin were 17.29 +/- 12.50, 12.50 +/- 6.75 and 5.93 +/- 3.53, respectively. We also performed double label immunofluorescence staining with nerve growth factor receptor (NGF-R) antibody and fractalkine antibody. NGF-R-positive terminal cutaneous nerves were in close contact with the fractalkine-positive dermal dendrocytes in psoriatic lesions.
CONCLUSIONS: The results of this study confirm that fractalkine is upregulated at sites of inflammation. Thus, it is likely that this molecule plays a key part in cell trafficking. An increased expression of fractalkine at the dermal papillae provides a plausible explanation for the migration and accumulation of T cells at these sites in psoriasis. Earlier studies have reported an increased number of dermal dendrocytes in psoriatic tissue; however, the functional role of these cells in the pathogenesis of psoriasis is largely unknown. Expression of fractalkine on the surface of dermal dendrocytes suggests an active role for these cells in localization and activation of lesional T cells.

PMID: 11422028 [PubMed - indexed for MEDLINE]

1551. Immunology. 2001 Jun;103(2):244-54.

The pan-chemokine inhibitor NR58-3.14.3 abolishes tumour necrosis factor-alpha accumulation and leucocyte recruitment induced by lipopolysaccharide in vivo.

Reckless J, Tatalick LM, Grainger DJ.

Department of Medicine, University of Cambridge, Addenbrooke's Hospital, Cambridge, UK. jr219@mole.bio.cam.ac.uk

Chemokines participate in the regulation of leucocyte recruitment in a wide variety of inflammatory processes, including host defence and diseases such as asthma, atherosclerosis and autoimmune disorders. We have previously described the properties of Peptide 3, the first broad-specificity chemokine inhibitor in

vitro. Here, we report the properties of NR58-3.14.3, a retroinverso analogue of Peptide 3. NR58-3.14.3 inhibited leucocyte migration induced by a range of chemokines, including monocyte chemoattractant protein-1 (MCP-1) (2.5 nM), macrophage inflammatory protein-1alpha (MIP-1alpha) (5 nM), regulated on activation, normal T-cell expressed and presumably secreted (RANTES) (20 nM), stromal cell-derived factor-1alpha (SDF-1alpha) (25 nM) and interleukin-8 (IL-8) (30 nM), but did not affect migration induced by N-formyl-methionyl-leucyl-phenylalanine (FMLP) or complement C5a (> 100 microM). NR58-3.14.3 is therefore approximately 1000-fold more potent than Peptide 3 but retains the broad-spectrum chemokine inhibitory activity of the parent peptide. In vivo, pretreatment with a systemic dose of 10 mg of NR58-3.14.3, but not the inactive derivative NR58-3.14.4, abolished leucocyte recruitment in response to intradermal injection of 500 ng of MCP-1 into rat skin. This suggests that NR58-3.14.3 is a functional chemokine inhibitor in vivo as well as in vitro. We utilized NR58-3.14.3 as a tool to investigate the role of chemokine activity during leucocyte recruitment in response to lipopolysaccharide (LPS) in vivo. NR58-3.14.3, but not NR58-3.14.4, abolished leucocyte recruitment in response to intradermal injection of 50 ng of LPS into rat skin. Furthermore, NR58-3.14.3 completely inhibited LPS-induced accumulation of tumour necrosis factor-alpha (TNF-alpha). This data is consistent with a model in which multiple chemokines act in parallel upstream of TNF-alpha. NR58-3.14.3 is therefore a powerful anti-inflammatory agent in vivo, suppressing proinflammatory cytokine production and leucocyte recruitment in response to endotoxin stimulus in rat skin.

PMCID: PMC1783230
PMID: 11412312 [PubMed - indexed for MEDLINE]

1552. Int Arch Allergy Immunol. 2001;125 Suppl 1:33-7.

Expression of VLA-4 on eosinophils decreases in patients with eosinophilia.

Kayaba H, Yamada Y, Cui CH, Saito N, Honda K, Kobayashi Y, Urayama O, Chihara J.

Central Clinical Laboratory, Akita University Hospital, Akita, Japan.
kayaba@yukari.hos.akita-u.ac.jp

BACKGROUND: It is assumed that the very late antigen-4 (VLA-4) plays a key role in selective migration and accumulation of eosinophils to the allergic inflammatory focus. The regulatory mechanism for VLA-4 expression is poorly understood, as is its relationship between other adhesion molecules.
OBJECTIVE: The aim of the study was to elucidate the relationship between VLA-4 expression and the activation of eosinophils.
METHODS: The surface expression of VLA-4, Mac-1, ICAM-1, CD4, CD25, CD69, CD89, IL-5 receptor and GM-CSF receptor on eosinophils isolated from the peripheral blood of 15 patients with eosinophilia and 16 healthy volunteers was measured.
RESULTS: The surface expression of VLA-4 presented in mean fluorescent intensity by flow-cytometric analysis showed a significant decrease in the patients with eosinophilia (>700 eosinophils/microl) compared to that of the subjects without eosinophilia. On the other hand, the surface expression of Mac-1 was significantly increased in the patients with eosinophilia. There was an inverse correlation between the expression of VLA-4 and that of Mac-1 (r = -0.81) on the eosinophils obtained from the patients with eosinophilia.
CONCLUSION: The changes on the surface expressions of Mac-1 and VLA-4 may be indicating the activation of eosinophils in the patients with eosinophilia and may contribute to their migration to the allergic inflammatory focus.

Copyright 2001 S. Karger AG, Basel

PMID: 11408770 [PubMed - indexed for MEDLINE]

1553. Int Arch Allergy Immunol. 2001;125 Suppl 1:29-32.

Regulation of chemokine receptor expression in eosinophils.

Nagase H, Miyamasu M, Yamaguchi M, Fujisawa T, Kawasaki H, Ohta K, Yamamoto K, Morita Y, Hirai K.

Department of Respiratory Medicine, University of Tokyo Graduate School of Medicine, Tokyo, Japan.

Signals via chemokine receptors play an important role in the accumulation of eosinophils at allergic inflammatory sites. Eosinophils constitutively express CC chemokine receptor 3 (CCR3) and, to a lesser extent, CCR1. CCR3 is mainly responsible for migration of resting eosinophils, and its specific ligand, eotaxin, represents the most potent chemoattractant for eosinophils. Some reports also suggest the expression of CXC chemokine receptor 1 (CXCR1) and/or CXCR2 in eosinophils. In addition, we recently reported the functional expression of CXCR4. The ligand of CXCR4, stromal cell-derived factor-1 (SDF-1), was able to induce a strong migratory response comparable to that by eotaxin. In contrast to the CCR3/eotaxin system which is mainly regulated at the level of ligand production, the CXCR4/SDF-1 system is regulated at the level of receptor expression. CXCR4 expression was completely attenuated by IL-4 and IL-5 and upregulated by IFN-gamma and dexamethasone, while CCR3 expression was only marginally affected. The balance between the biological effects of these chemokine systems may affect the distribution and migration of eosinophils.

Copyright 2001 S. Karger AG, Basel

PMID: 11408769 [PubMed - indexed for MEDLINE]

1554. Int Arch Allergy Immunol. 2001;125 Suppl 1:7-11.

Eosinophil transmigration across VCAM-1-expressing endothelial cells is upregulated by antigen-stimulated mononuclear cells.

Nagata M, Yamamoto H, Tabe K, Sakamoto Y.

Pulmonary Division, Second Department of Internal Medicine, Saitama Medical School, Iruma-gun, Saitama, Japan. favre4mn@saitama-med.ac.jp

BACKGROUND: Adhesion to and transmigration across endothelial cells expressing vascular cell adhesion molecule 1 (VCAM-1) may be key steps in the development of selective eosinophil accumulation at the allergic inflammation sites. There is evidence that cytokines/chemokines produced by CD4+ T cells play a prominent role in these processes.
OBJECTIVE: The objective of this study was to evaluate whether eosinophil migration across human pulmonary microvascular endothelial cells (HPMEC) expressing VCAM-1 is modulated by the supernatants of antigen-stimulated mononuclear cells obtained from atopic asthmatics.
METHODS: Peripheral blood mononuclear cells (PBMC) were isolated from Dermatophagoides farinae (Df)-sensitive asthmatic subjects and cultured for 96 h at 37 degrees C in the presence or absence of 1 microg/ml Df antigen. Eosinophils were isolated from blood of healthy subjects and placed on the HPMEC monolayers cultured on a transwell filter (3-microm pore size) stimulated with IL-4 plus TNF-alpha (both at 100 pM, 24 h) The supernatants of PBMC were then applied to the lower compartment and the transmigration of eosinophils was examined.
RESULTS: The supernatants of PBMC stimulated with Df significantly enhanced the

eosinophil transmigration across VCAM-1-expressing HPMEC (% migration: 7.6 +/- 0.6 by the supernatants of PBMC cultured without Df vs. 12.3 +/- 1.2 by the PBMC cultured with Df, p < 0.01, n = 8). The enhanced migration, but not spontaneous migration, was blocked by the anti-alpha4 integrin antibody. Moreover, the enhanced transmigration was blocked by anti-CCR3 antibody.
CONCLUSION: The antigen-stimulated PBMC from atopic asthmatics produce an activity to induce eosinophil migration across VCAM-1-expressing endothelial cells. This activity appears to involve CCR3 as an essential molecule.

Copyright 2001 S. Karger AG, Basel

PMID: 11408764 [PubMed - indexed for MEDLINE]

1555. Ther Umsch. 2001 May;58(5):253-8.

[Epidemiology of allergies in Switzerland].

[Article in German]

Wüthrich B.

Allergiestation, Dermatologische Universitätsklinik, Zürich.
wuethric@derm.unizh.ch

The Swiss Study on Air Pollution and Lung Diseases in Adults (SAPALDIA) was carried out during 1991-1993 in eight Swiss areas with different environmental characteristics. The cross-sectional examination included 9651 adults, aged 18-60 years, who all participated in a detailed interview. In 8357 subjects complete allergy skin and in-vitro tests were available in addition. The prevalence of atopic sensitization (positive skin prick test to any of the tested inhalant allergens and/or a positive Phadiatop as an in-vitro screening test for atopy) was 32.3%, with a higher prevalence in males (35.7%) than in females (28.8%). Skin sensitization was predominantly caused by grass pollen (12.7%), followed by house dust mite (8.9%), silver birch pollen (7.9%) and cat epithelia (3.8%). 11.1% suffered from current hay fever, 6.8% from asthma, 4.5% from atopic asthma. Smokers had statistically significant (p < 0.001) higher mean serum IgE concentrations (geometric mean 39.7 kU/l) than nonsmokers (27.2 kU/l), In Phadiatop positive subjects, the IgE levels were highest, with a mean of 104.3 kU/l (99.0-109.8). The SCARPOL Study (Swiss Study on Childhood Allergy and Respiratory Symptoms with respect to Air Pollution and Climate) ist based on a sample of 4470 children from 10 different areas who completed parenteral questionnaire. 35.7% of the 2879 children who underwent skin prick testing were sensitized to at least one tested aeroallergen, 22.5% to gras pollen, 12.4% to house dust mites, 11.4% to birch pollen and 6.4% to cat epithelia. 17% of the 13- to 15-year-old (8th grade) suffered from hayfever. The prevalence of asthma (ever) for the whole sample was 9%, without differences between the age groups. The lifetime prevalence of atopic dermatitis was 13% and the current prevalence 8%. The risk of eczema was higher in Swiss children than in children of immigrants, in infants with a birthweight below 2500 g, in children with a positive family history of atopic dermatitis, and in children from higher socioeconomic classes. Farm children (n = 133) living in a rural area suffer less frequently from pollinosis (2.4%) and bronchial asthma (1.6%) than children (n = 966) with no direct contact to agriculture, but living in the same area (prevalence of hayfever 18.3%, of asthma 9.1%). This figures are similar to results from former East and Western Germany and from the former USSR and Baltic areas. These large Swiss epidemiologic studies confirmed both, the high prevalence of atopy and atopic diseases, and the health impact of moderate air pollution levels and of factors associated with the 'western lifestyle'.

PMID: 11407223 [PubMed - indexed for MEDLINE]

1556. J Allergy Clin Immunol. 2001 Jun;107(6):1019-24.

L-Selectin is required for the development of airway hyperresponsiveness but not airway inflammation in a murine model of asthma.

Fiscus LC, Van Herpen J, Steeber DA, Tedder TF, Tang ML.

University of North Carolina at Chapel Hill School of Medicine, Chapel Hill, NC, USA.

BACKGROUND: Airway inflammation and airway hyperresponsiveness (AHR) are fundamental features of asthma. Migration of inflammatory cells from the circulation into the lungs is dependent on adhesion molecule interactions. The cell surface adhesion molecule L-selectin has been demonstrated to mediate leukocyte rolling on inflamed and noninflamed pulmonary endothelium. However, its role in the development of airway inflammation and AHR in asthma has not been examined.
OBJECTIVE: We sought to characterize the role of L-selectin in the recruitment of inflammatory cells to the airway-lung and the development of AHR in a murine model of asthma.
METHODS: An ovalbumin (OVA)-induced allergic airway disease model of asthma was applied to L-selectin-deficient (LKO) mice and C57BL/6 wild-type (WT) control mice. The development of airway inflammation was assessed by examining leukocyte influx into bronchoalveolar lavage (BAL) fluid and the lung. Total and differential BAL leukocyte counts were determined, and the immunophenotype of BAL lymphocytes was assessed by means of flow cytometry. The development of AHR was assessed by means of whole-body plethysmography.
RESULTS: Airway-lung inflammation was equivalent in LKO and WT mice sensitized-challenged with OVA, as measured by total and differential BAL cell counts and histologic analysis of lung tissue. Numbers of eosinophils, neutrophils, lymphocytes, and monocytes in BAL fluid were equivalent in LKO and WT mice. However, phenotypic analysis of BAL lymphocytes demonstrated significantly reduced CD3(+) populations and increased B220(+) populations in LKO compared with WT mice (P <.05). Remarkably, despite a fulminant inflammatory response in the airway-lung in LKO mice sensitized-challenged with OVA, AHR was completely abrogated.
CONCLUSION: L-selectin plays a crucial role in the development of AHR but not allergic inflammation in an animal model of asthma. L-selectin represents a potential target for novel asthma therapies specifically aimed at controlling AHR.

PMID: 11398079 [PubMed - indexed for MEDLINE]

1557. Crit Rev Immunol. 2000;20(6):477-96.

Mechanisms of IgE elevation in HIV-1 infection.

Marone G, Florio G, Triggiani M, Petraroli A, de Paulis A.

Division of Clinical Immunology and Allergy, University of Naples Federico II, Italy.

Serum IgE levels are high in adults and children with HIV-1 infection and could be a marker of poor prognosis. Allergic reactions and adverse reactions to drugs also tend to increase in HIV-1-infected individuals. An imbalance between a "T(H)1-like" and a "T(H)2-like" cytokine profile has been documented in HIV-1

infection. We have demonstrated that HIV-1 gp 120 from different clades is a stimulus for histamine and cytokine (IL-4 and IL-13) release from basophils. Gp 120 acts as a viral superantigen, interacting with the V(H)3 region of IgE to induce mediator release from human Fc epsilonRI+ cells. Human basophils and mast cells express the chemokine receptor CCR3, which binds the chemokines eotaxin and RANTES. By interacting with the CCR3 receptor on Fc epsilonRI+ cells, HIV-I Tat protein is a potent chemoattractant for human basophils and lung mast cells. Preincubation of basophils with Tat protein upregulates mRNA CCR3 and the surface expression of this chemokine receptor. Tat also induces IL-4 and IL-13 release from basophils. Extracellular Tat can influence the directional migration of human Fc epsilonRI+ cells, the expression of chemokine receptor CCR3, and the release of T(H)2 cytokines. Our results indicate two novel mechanisms by which two HIV-1 proteins, gp120 and Tat, trigger the release of cytokines critical for T(H)2 polarization from human Fc epsilonRI+ cells.

PMID: 11396683 [PubMed - indexed for MEDLINE]

1558. Rinsho Byori. 2001 Apr;49(4):370-5.

[Eosinophils and related chemokines].

[Article in Japanese]

Kayaba H, Chihara J.

Central Clinical Laboratory and Department of Clinical and Laboratory Medicine, Akita University School of Medicine, Akita, 010-8543.

Chemokines such as RANTES, eotaxin, MIP-1 and MCP-4 are considered to be involved in the pathophysiology of allergic inflammation because of their ability to drive eosinophils through their binding sites, chemokine receptors, expressed on eosinophils. Among those chemokines, RANTES and eotaxin are considered to play important roles in the process of the maturation, migration and activation of eosinophils. An overview of the effect of chemokines on eosinophils throughout their migration from bone marrow to the inflammatory focus is described in this paper. Furthermore, our observations on the effects of chemokines on eosinophils such as adherence through beta-2 integrin, the production of reactive oxygen species, intracellular EG2 content and production of RANTES by eosinophils are reported.

PMID: 11391951 [PubMed - indexed for MEDLINE]

1559. Int J Cancer. 2001 Jul 1;93(1):53-61.

N-[3,4-dimethoxycinnamoyl]-anthranilic acid (tranilast) inhibits transforming growth factor-beta relesase and reduces migration and invasiveness of human malignant glioma cells.

Platten M, Wild-Bode C, Wick W, Leitlein J, Dichgans J, Weller M.

Laboratory of Molecular Neuro-Oncology, Department of Neurology, University of Tübingen, School of Medicine, Tübingen, Germany.

Extensive infiltration of normal brain tissue and suppression of anti-tumor immune surveillance mediated by molecules such as transforming growth factor-beta (TGF-beta) are key biological features that contribute to the malignant phenotype of human gliomas. Tranilast (N-[3,4-dimethoxycinnamoyl]-anthranilic acid) is an anti-allergic compound used clinically to control atopic and fibrotic disorders.

These effects are attributed to the suppression of TGF-beta1 synthesis and interference with growth factor-mediated proliferation and migration of fibroblasts and vascular smooth muscle cells. Here, we show that tranilast inhibits DNA synthesis and proliferation of human malignant glioma cells and promotes p21 accumulation in the absence of cytotoxicity. Further, tranilast reduces the release of TGF-beta1 and TGF-beta2 by glioma cells and inhibits migration, chemotactic responses and invasiveness. These effects are not associated with a reduction of alpha(v)beta(3) integrin expression at the cell surface but appear to involve inhibition of matrix metalloproteinase-2 expression and activity. Neither the tranilast-mediated inhibition of proliferation nor the inhibition of migration was counteracted by supplementation with exogenous TGF-beta. Finally, tranilast administered orally inhibited the growth of experimental 9L rat gliomas and reduced expression of TGF-beta2 in vivo. We conclude that tranilast might be a useful therapeutic agent for the treatment of human malignant glioma because of a TGF-beta-independent abrogation of the malignant phenotype of proliferation, migration and invasiveness and because of the antagonism of TGF-beta-associated immunosuppression.

Copyright 2001 Wiley-Liss, Inc.

PMID: 11391621 [PubMed - indexed for MEDLINE]

1560. J Exp Med. 2001 May 21;193(10):1135-47.

Role of the parasite-derived prostaglandin D2 in the inhibition of epidermal Langerhans cell migration during schistosomiasis infection.

Angeli V, Faveeuw C, Roye O, Fontaine J, Teissier E, Capron A, Wolowczuk I, Capron M, Trottein F.

Centre d'Immunologie et de Biologie Parasitaire, Institut National de la Santé et de la Recherche Médicale (INSERM) U547, Lille, France.

Epidermal Langerhans cells (LCs) play a key role in immune defense mechanisms and in numerous immunological disorders. In this report, we show that percutaneous infection of C57BL/6 mice with the helminth parasite Schistosoma mansoni leads to the activation of LCs but, surprisingly, to their retention in the epidermis. Moreover, using an experimental model of LC migration induced by tumor necrosis factor (TNF)-alpha, we show that parasites transiently impair the departure of LCs from the epidermis and their subsequent accumulation as dendritic cells in the draining lymph nodes. The inhibitory effect is mediated by soluble lipophilic factors released by the parasites and not by host-derived antiinflammatory cytokines, such as interleukin-10. We find that prostaglandin (PG)D2, but not the other major eicosanoids produced by the parasites, specifically impedes the TNF-alpha-triggered migration of LCs through the adenylate cyclase-coupled PGD2 receptor (DP receptor). Moreover, the potent DP receptor antagonist BW A868C restores LC migration in infected mice. Finally, in a model of contact allergen-induced LC migration, we show that activation of the DP receptor not only inhibits LC emigration but also dramatically reduces the contact hypersensitivity responses after challenge. Taken together, we propose that the inhibition of LC migration could represent an additional stratagem for the schistosomes to escape the host immune system and that PGD2 may play a key role in the control of cutaneous immune responses.

PMCID: PMC2193325
PMID: 11369785 [PubMed - indexed for MEDLINE]

1561. Blood. 2001 Jun 1;97(11):3433-40.

Chemotaxis and transendothelial migration of CD34(+) hematopoietic progenitor cells induced by the inflammatory mediator leukotriene D4 are mediated by the 7-transmembrane receptor CysLT1.

Bautz F, Denzlinger C, Kanz L, Möhle R.

Department of Medicine II, University of Tübingen, 72076 Tübingen, Germany.

Recent studies suggest that bone marrow (BM)-derived chemotactic mediators such as chemokines play key roles in hematopoietic stem cell trafficking. Lipid mediators, particularly leukotrienes, are involved in leukocyte chemotaxis during inflammation but have also been detected in the normal BM. Therefore, the effects of leukotrienes on hematopoietic progenitor cells were analyzed. Cysteinyl leukotrienes, particularly leukotriene D4 (LTD4), induced strong intracellular calcium fluxes and actin polymerization in mobilized and BM CD34(+) progenitors. Chemotaxis and in vitro transendothelial migration of CD34(+) and more primitive CD34(+)/CD38(-) cells were 2-fold increased by LTD4 at an optimum concentration of 25 to 50 nM. Accordingly, CD34(+) cells expressed the 7-transmembrane LTD4 receptor CysLT1 by reverse transcriptase-polymerase chain reaction and Western blot. Effects of LTD4 were suppressed by the CysLT1 receptor antagonist MK-571 and reduced by pertussis toxin. In contrast, LTB4 induced strong responses only in mature granulocytes. LTD4-induced calcium fluxes were also observed in granulocytes but were not reduced by MK-571, suggesting that these effects were mediated by other receptors (eg, CysLT2) rather than by CysLT1. In addition, expression of 5-lipoxygenase, the key enzyme of leukotriene biosynthesis, was detected in both hematopoietic progenitor cells and mature leukocytes. The study concludes that the functionally active LTD4 receptor CysLT1 is preferentially expressed in immature hematopoietic progenitor cells. LTD4 released in the BM might regulate progenitor cell trafficking and could also act as an autocrine mediator of hematopoiesis. This would be a first physiologic effect of cysteinyl leukotrienes apart from the many known pathophysiologic actions related to allergy and inflammation. (Blood. 2001;97:3433-3440)

PMID: 11369634 [PubMed - indexed for MEDLINE]

1562. Exp Biol Med (Maywood). 2001 Mar;226(3):177-84.

Effect of L-tryptophan supplementation on eosinophils and eotaxin in guinea pigs.

Stahl JL, Cook EB, Pariza MA, Cook ME, Graziano FM.

Department of Medicine, University of Wisconsin-Madison, 53706, USA.

Eosinophilia Myalgia Syndrome is a hypereosinophilic disorder that appears to result from the ingestion of the dietary supplement L-tryptophan by susceptible individuals. It is unclear if this disease results from tryptophan, contaminants found in tryptophan, individual predisposition (such as immune status and allergies), or some combination of effects. To evaluate effects of L-tryptophan on eosinophil migration, guinea pigs were compared with or without supplemental tryptophan (0.4 g/kg/day), with or without immune sensitization, and with or without immune challenge. Eosinophil counts were obtained from bone marrow, blood, lung, and bronchial alveolar lavage fluid (BAL). Lung cells were obtained to measure eotaxin concentrations in supernates and lysates with or without antigen and calcium ionophore challenge using direct ELISA. Skin biopsies were taken from both non-injected and antigen injection sites. The tryptophan supplemented, antigen-sensitized/antigen-challenged guinea pigs showed a significant decrease in blood eosinophils, compared to control (cellulose) supplemented antigen-sensitized/antigen-challenged guinea pigs [(0.086 +/- 0.023)

x 10(6) vs (0.147 +/- 0.021) x 10(6) eosinophils/ml recovered, respectively] with a significant increase in BAL eosinophils [(0.052 +/- 0.008) x 10(6) vs (0.033 +/- 0.005) x 10(6) eosinophils/ml recovered, respectively]. Unchallenged lung cell lysates from tryptophan-supplemented guinea pigs contained significantly less eotaxin compared to cellulose-supplemented guinea pigs regardless of whether they were sensitized (0.006 +/- 0.002 vs 0.027 +/- 0.008 ng/10(6) cells, respectively). No differences were observed in skin biopsies between cellulose and tryptophan groups. These results suggest that L-tryptophan-supplemented guinea pigs have altered eotaxin regulation, a potential mechanism by which human overconsumption of tryptophan dietary supplements could lead to hypereosinophilic disorders in susceptible individuals.

PMID: 11361035 [PubMed - indexed for MEDLINE]

1563. J Invest Dermatol. 2001 May;116(5):641-9.

Interleukin-16 supports the migration of Langerhans cells, partly in a CD4-independent way.

Stoitzner P, Ratzinger G, Koch F, Janke K, Schöller T, Kaser A, Tilg H, Cruikshank WW, Fritsch P, Romani N.

Department of Dermatology, University of Innsbruck, Innsbruck, Austria. patrizia.stoitzner@uibk.ac.at

Migration of cutaneous dendritic cells is essential for the induction of primary immune responses. Chemotaxis plays an important part in guiding migrating cells through the skin. Therefore, we investigated the influence of interleukin-16, a potent chemoattractant, on the migratory properties of cutaneous dendritic cells. Interleukin-16 added to murine and human skin explant cultures, enhanced emigration of Langerhans cells as well as dermal dendritic cells out of the skin. In contrast to tumor necrosis factor-alpha, intradermally injected interleukin-16 did not reduce the density of Langerhans cells suggesting a chemotactic rather than a mechanistic migration-inducing effect of interleukin-16. In support of these findings, the known migration-promoting effect of tumor necrosis factor-alpha in skin explant cultures could be neutralized by anti-interleukin-16 antibody and vice versa, indicating different but cooperative ways of action for both cytokines. In whole skin explant cultures blocking of the interleukin-16 effect was also achieved with a monoclonal antibody against CD4, the receptor for interleukin-16. In contrast, in cultures of murine epidermis alone no blocking by anti-CD4 became obvious and in CD4-deficient mice Langerhans cell migration in response to interleukin-16 was maintained. This suggests that another receptor for interleukin-16 might be operative for Langerhans cells in the mouse epidermis. Finally, we detected interleukin-16-positive cells in the dermis of skin explants, tumor necrosis factor-alpha-treated and contact allergen-treated skin. Taken together, it seems likely that locally secreted interleukin-16 might serve to enhance the migration of cutaneous dendritic cells and optimize the response to foreign antigen encountering the skin.

PMID: 11348450 [PubMed - indexed for MEDLINE]

1564. Int Arch Allergy Immunol. 2001 Apr;124(4):432-46.

Dendritic cells as regulators of the immune response to inhaled allergen: recent findings in animal models of asthma.

Lambrecht BN, Hoogsteden HC, Pauwels RA.

Department of Pulmonary and Critical Care Medicine, Erasmus Medical Center
Rotterdam, Rotterdam, The Netherlands. lambrecht@lond.azr.nl

Antigen-presenting dendritic cells are essential for the recognition and presentation of allergens to the cells of the immune system. Airway dendritic cells capture allergen in the mucosa and present it to naive T cells after migration into the draining lymph nodes. In this review article, we discuss the most recent findings from animal models of asthma, which highlight an essential role for these cells in the induction and maintenance of eosinophilic airway inflammation. This increasing knowledge might lead to the identification of new targets for the prevention and therapy of asthma.

Copyright 2001 S. Karger AG, Basel

PMID: 11340326 [PubMed - indexed for MEDLINE]

1565. Immunol Res. 2000;22(2-3):271-9.

Function and regulation of chemoattractant receptors.

Haribabu B, Richardson RM, Verghese MW, Barr AJ, Zhelev DV, Snyderman R.

Department of Medicine, Duke University Medical Center, Durham, NC 27710, USA.

Phagocyte migration and activation at sites of inflammation is mediated through chemoattractant receptors that are coupled to G-proteins. Early studies from our laboratory demonstrated G-protein-mediated phospholipase C activation by chemoattractants. Recently, this laboratory developed cellular and animal models to allow biochemical, cell biological and molecular genetic approaches to be used in determining the mechanisms of chemoattractant receptor function, regulation, and cross regulation. These studies provided evidence that chemoattractant receptors activate distinct pathways for chemotaxis and exocytosis and cross-regulate each other's function at multiple levels. A major site of regulation is through phosphorylation of receptors by G-protein-coupled receptor kinases and by protein kinase C. In addition, the activation of phospholipase C by chemoattractants is also regulated at additional sites distal to receptor phosphorylation. These may include modulation of G-protein activation by regulators of G-protein signaling (RGS) and modification of phospholipase C. Phosphorylation of phospholipase Cbeta3 by both protein kinase A and protein kinase C has been demonstrated. The function and regulation of chemoattractant receptors are also being examined in mouse models. In these studies, mice deficient in leukotriene B4 receptors have been generated by targeted gene disruption. These mice displayed reduced neutrophil accumulation in certain inflammation models and sex-related differences in platelet-activating-factor induced anaphylaxis.

PMID: 11339362 [PubMed - indexed for MEDLINE]

1566. Expert Opin Pharmacother. 2001 Feb;2(2):267-75.

Chemotherapy of enterobiasis (oxyuriasis).

St Georgiev V.

National Institute of Allergy and Infectious, Disease, NIH, 6700-B Rockledge
Drive, Room 2102, Bethesda, Maryland 20892, USA. vg8q@nih.gov

Enterobius vermicularis (syn. Oxyurus vermicularis), also known as pinworm or

seatworm, is the causative agent of human enterobiasis (oxyuriasis). The disease is more prevalent in temperate regions and is facilitated by factors such as overcrowding in schools and family groupings, as well as inadequate personal and community hygiene. Although the infection is more likely to occur in lower socioeconomic groups, enterobiasis has been reported to affect virtually every level of the general population and especially children. In the great majority of cases, enterobiasis is asymptomatic. One common symptom is intense pruritus ani that in some patients can lead to insomnia, restlessness and irritability. Scratching may cause skin irritation, and in more serious cases, eczematous dermatitis, haemorrhage or secondary bacterial infections. Ectopic migration of E. vermicularis often results in pinworm infestation of the female genital tract often causing granulomas of the uterus, ovary and the fallopian tubes and pelvic peritoneum. Anthelmintic therapies for enterobiasis are successful and include mebendazole, albendazole and pyrantel pamoate. Mass medication of affected groups reduced symptoms rapidly, progressively and in a cost-effective way.

PMID: 11336585 [PubMed - indexed for MEDLINE]

1567. Isr Med Assoc J. 2001 Mar;3(3):190-3.

Prevalence of self-reported allergic conditions in an adult population in Israel.

Shahar E, Lorber M.

Institute of Allergy, Clinical Immunology and AIDS, Rambam Medical Center and Rappaport Faculty of Medicine, Technion-Israel Institute of Technology, Haifa, Israel.

BACKGROUND: Asthma, allergic rhinitis, and atopic dermatitis are leading causes of chronic diseases in developed countries, with at least one allergic condition troubling 10 to 20% of the general population. The few studies performed in Israel determined the prevalence of allergic conditions in selected populations (schoolchildren and soldiers); no study representative of the general population has previously been done.
OBJECTIVES: To determine the prevalence of allergic conditions in the general population in Israel and the differences between ethnic and socioeconomic groups.
METHOD: Using a computer-assisted telephone interview, a telephone questionnaire was conducted in a representative sample of the general Israeli population.
RESULTS: Of the population studied, 14% claimed to have bronchial asthma, 14% allergic rhinitis, and 6% other allergic conditions. Prevalence rates were higher in the Israeli Arab population and in those with low income and low education levels. Of those with allergic conditions, 58% were treated by a primary physician, 32% were not treated at all, and only 10% were treated by a different specialist physician.
CONCLUSIONS: The prevalence of allergic conditions in this study concurs with that found by other studies in developed countries. Allergic conditions are higher in the Israeli Arab population and in those with low income and low education level.

PMID: 11303377 [PubMed - indexed for MEDLINE]

1568. Immunology. 2001 Mar;102(3):323-30.

Interleukin (IL)-18 induces Langerhans cell migration by a tumour necrosis factor-alpha- and IL-1beta-dependent mechanism.

Cumberbatch M, Dearman RJ, Antonopoulos C, Groves RW, Kimber I.

Syngenta Central Toxicology Laboratory, Alderley Park, Macclesfield, Cheshire, UK and Centre for Dermatology, University College London, UK.
Marie.Cumberbatch@CTL.syngenta.com

Following skin sensitization a proportion of epidermal Langerhans cells (LC) are stimulated to leave the skin and to migrate, via afferent lymphatics, to draining lymph nodes where they accumulate as immunostimulatory dendritic cells (DC). It has been demonstrated previously that tumour necrosis factor-alpha (TNF-alpha), an inducible product of epidermal keratinocytes, and interleukin (IL)-1beta, produced exclusively by LC in murine epidermis, provide important signals for the initiation of this response. Recently, it has been demonstrated that IL-18, a cytokine produced by both LC and keratinocytes within the epidermis, may also participate in immune responses induced following skin sensitization. In the present investigations, the ability of IL-18 to contribute to the regulation of LC migration and the accumulation of DC in draining lymph nodes has been examined. It was found that, like IL-1beta, IL-18 administered intradermally to mice resulted in a significant reduction in epidermal major histocompatibility complex (MHC) class II+ LC densities and a marked increase in lymph node DC numbers. Using neutralizing anti-TNF-alpha and blocking anti-type I IL-1 receptor (IL-1RI) antibodies, it was shown also that the induction by IL-18 of both LC mobilization and DC accumulation in regional lymph nodes was dependent upon availability of TNF-alpha and the integrity of IL-1RI signalling. Furthermore, using IL-1beta converting enzyme (caspase-1) knockout mice, IL-18-induced LC migration was found to have a mandatory requirement for active IL-1beta. Importantly, not only was IL-18 able to contribute to the regulation of LC migration, it was found to be essential for the manifestation of these processes in response to topical sensitization with the contact allergen oxazolone.

PMCID: PMC1783183
PMID: 11298831 [PubMed - indexed for MEDLINE]

1569. Br J Dermatol. 2001 Apr;144(4):715-25.

Exogenous topical lactoferrin inhibits allergen-induced Langerhans cell migration and cutaneous inflammation in humans.

Griffiths CE, Cumberbatch M, Tucker SC, Dearman RJ, Andrew S, Headon DR, Kimber I.

Dermatology Centre, University of Manchester, and Department of Pathology, Hope Hospital, Salford, Manchester M6 8HD, UK. cgriffiths@fs1.ho.man.ac.uk

BACKGROUND: Lactoferrin (LF), an iron-binding protein found in exocrine secretions, is known to possess antibacterial properties. It has recently been proposed that LF may also influence inflammatory reactions.
OBJECTIVES: To characterize in humans the ability of recombinant homologous LF to inhibit the induced migration of epidermal Langerhans cells (LCs) from the skin, a process known to be dependent upon the proinflammatory cytokines tumour necrosis factor (TNF)-alpha and interleukin 1beta and to influence cutaneous inflammatory reactions.
METHODS: We investigated the anti-inflammatory properties of LF in human volunteers.
RESULTS: Topical exposure to LF 2 h prior to sensitization caused a significant reduction in contact allergen (diphenylcyclopropenone, DPC)-induced LC migration from the epidermis as judged by the altered frequency of cells expressing either HLA-DR or CD1a determinants. That this reduction was secondary to an inhibition of TNF-alpha production was indicated by the fact that LF failed to influence LC migration induced by intradermal injection of this cytokine. In approximately 50% of those volunteers who displayed local inflammation in response to DPC, LF was

found to cause a discernible reduction in the clinical severity of the reaction, associated with reduced infiltration of inflammatory cells.
CONCLUSIONS: These data demonstrate that LF is able to influence cutaneous immune and inflammatory responses, possibly because of an impaired production of local proinflammatory cytokines.

PMID: 11298528 [PubMed - indexed for MEDLINE]

1570. J Allergy Clin Immunol. 2001 Apr;107(4):734-8.

L-selectin and intercellular adhesion molecule 1 mediate lymphocyte migration to the inflamed airway/lung during an allergic inflammatory response in an animal model of asthma.

Keramidaris E, Merson TD, Steeber DA, Tedder TF, Tang ML.

Department of Immunology, Royal Children's Hospital, Flemington Rd., Parkville, VIC 3052, Australia.

T lymphocytes play a critical role in the development of allergic inflammation in asthma. Early in the allergic response, T lymphocytes migrate from the circulation into the lung to initiate and propagate airway inflammation. The adhesion molecules that mediate lymphocyte entry into inflamed lung have not been defined. This study directly examined the roles of L-selectin and intercellular adhesion molecule 1 (ICAM-1) in lymphocyte migration to the lung during an allergic inflammatory response in an animal model of asthma. Short-term (1 hour) in vivo migration assays and various combinations of adhesion molecule-deficient and wild-type mice were used. Migration of in vivo activated lymphocytes into inflamed lung was significantly greater than entry of resting lymphocytes into noninflamed lung (24.5% +/- 2.7% vs 9.5% +/- 1.3%, P =.001). Migration of activated lymphocytes into inflamed lung was inhibited by 30% in the absence of L-selectin (17.3% +/- 1.3%, P =.04), 47% in the absence of cell surface ICAM-1 (13.0% +/- 2.5%, P =.01), and 47% in the absence of endothelial ICAM-1 (13.0% +/- 2.5%, P =.01). Loss of ICAM-1 on both lymphocytes and lung endothelium inhibited lymphocyte migration by 60% (9.8% +/- 1.8%, P =.002). These findings demonstrate clear roles for both L-selectin and ICAM-1 in lymphocyte migration to the lung during an allergic inflammatory response, with ICAM-1 playing a greater role.

PMID: 11295667 [PubMed - indexed for MEDLINE]

1571. Immunol Rev. 2001 Feb;179:5-15.

Mast cells, basophils, and eosinophils: distinct but overlapping pathways for recruitment.

Bochner BS, Schleimer RP.

Department of Medicine, The Johns Hopkins University School of Medicine, Baltimore, MD 21224, USA. bbochner@jhmi.edu

Mast cells, basophils and eosinophils are bone marrow-derived cells that contribute to a variety of allergic and other immune responses. For example, they are relatively abundant at mucosal sites where allergic inflammation is occurring, and their activation and release of preformed and newly-generated mediators at these sites is considered central to the pathophysiology of allergic diseases. Given their involvement in allergic and other diseases, it is important to understand how these cells are selectively recruited into tissues. These cells share many phenotypic features, including those involved in adhesion and

migration, yet their localization within a given tissue can be quite distinct. In addition, there are examples of selective recruitment of one cell type without the others. From studies with human cells, it is now clear that mast cells, basophils and eosinophils share a number of recruitment pathways with one another and with other cells, but that each possesses unique adhesion and migration responses that can contribute to their preferential accumulation. This review will focus on cell surface structures implicated in adhesion and migration responses of human mast cells, basophils and eosinophils. Both shared and selective expression of these molecules will be highlighted, as well as differences in their relative levels of expression. Cell type-specific stimuli that alter adhesion and migration responses will also be considered.

PMID: 11292027 [PubMed - indexed for MEDLINE]

1572. Eur Cytokine Netw. 2001 Mar;12(1):45-50.

Development of allergic contact dermatitis requires activation of both tumor necrosis factor-receptors.

Becke FM, Hehlgans T, Brockhoff G, Männel DN.

Institute of Pathology, Tumor Immunology, University of Regensburg, Franz-Josef-Strauss-Allee 11, D-93042 Regensburg, Germany.

We investigated the role of the TNF receptors, type I (p55TNFR) and type II (p75TNFR), in a mouse model of contact hypersensitivity, i.e., a model of a delayed type hypersensitivity (DTH) allergic reaction. Mice deficient for p55TNFR or p75TNFR were used to investigate the functions of these receptors in development of the DTH reaction. We show that both TNF receptors have a strong influence on the overall outcome of the DTH reaction, with the two TNF receptors exerting distinct functions. Dendritic cells of mice lacking p55TNFR had a defect in allergen uptake but showed normal migration into regional lymph nodes. In contrast, dendritic cells of p75TNFR-deficient mice showed diminished migration into regional lymph nodes after allergen contact, whereas the allergen uptake was independent of the p75TNFR. Thus, both TNF receptors are required for the development of a complete DTH reaction.

PMID: 11282545 [PubMed - indexed for MEDLINE]

1573. J Bone Joint Surg Am. 2001 Mar;83-A(3):428-36.

Metal sensitivity in patients with orthopaedic implants.

Hallab N, Merritt K, Jacobs JJ.

Department of Orthopaedic Surgery, Rush-Presbyterian-St. Luke's Medical Center, Chicago, IL 60612, USA. nhallab@rush.edu

All metals in contact with biological systems undergo corrosion. This electrochemical process leads to the formation of metal ions, which may activate the immune system by forming complexes with endogenous proteins. Implant degradation products have been shown to be associated with dermatitis, urticaria, and vasculitis. If cutaneous signs of an allergic response appear after implantation of a metal device, metal sensitivity should be considered. Currently, there is no generally accepted test for the clinical determination of metal hypersensitivity to implanted devices. The prevalence of dermal sensitivity in patients with a joint replacement device, particularly those with a failed implant, is substantially higher than that in the general population. Until the

roles of delayed hypersensitivity and humoral immune responses to metallic orthopaedic implants are more clearly defined, the risk to patients may be considered minimal. It is currently unclear whether metal sensitivity is a contributing factor to implant failure.

PMID: 11263649 [PubMed - indexed for MEDLINE]

1574. Cas Lek Cesk. 2000 Dec;139 Suppl 1:42-5.

[Significance of schistosome lectins in the host allergic reaction evoked by penetration of cercaria].

[Article in Czech]

Kolárová L, Horák P.

Oddělení tropické medicíny III. kliniky infekcních a tropických nemocí LF UK a FN Bulovka, Praha. lkolar@lf1.cuni.cz

Cercarial dermatitis is a skin allergic response caused by larval stages (cercariae) of the trematode family Schistosomatidae. In the Czech Republic the main causative agents of the disease are bird schistosomes of the genus Trichobilharzia. In the past it was supposed that cercariae of animal schistosomes die soon after the penetration into the human skin. However, we observed a migration and partial development of T. szidati cercariae within a nonspecific (mouse) host. The initial development of parasites in mice was similar to that observed in a specific host (duck) as well as to that described in human schistosomes causing schistosomiasis. After penetration, the transformation of T. szidati cercaria to schistosomulum is characterised by ultrastructural and biochemical changes, resulting in formation of a new parasite surface as well as in differences in specific binding of various lectins and host immunoglobulins to parasite surface components. It seems that the transformation of parasites represents a part of their immune evasion within the infected host. It was observed that T. szidati cercariae possess lectins localized on the parasite surface and in postacetabular penetration glands. It is supposed that, after the penetration into the skin, when glycocalyx disappears and gland material is expelled, the parasite surface may serve as an activator of the alternative complement pathway. The postacetabular gland components have probably a lytic function and facilitate migration of parasites through the skin. Moreover, the gland content is considered to play a role in shedding of surface antigens and in changes of parasite tegumental architecture.

PMID: 11262902 [PubMed - indexed for MEDLINE]

1575. J Leukoc Biol. 2001 Mar;69(3):353-60.

Eosinophil recruitment into sites of delayed-type hypersensitivity reactions in mice.

Teixeira MM, Talvani A, Tafuri WL, Lukacs NW, Hellewell PG.

Department of Biochemistry and Immunology, Instituto de Ciências Biológicas, Universidade Federal de Minas Gerais, Belo Horizonte, Brazil. mmtex@icb.ufmg.br

The selective accumulation of eosinophils in tissue is a characteristic feature of allergic diseases where there is a predominance of lymphocytes expressing a Th2 phenotype. In an attempt to define factors determining specific eosinophil accumulation in vivo, we have used a radiolabeled technique to assess the

occurrence and the mechanisms underlying (111)In-eosinophil recruitment into Th1- and Th2-predominant, delayed-type hypersensitivity (DTH) reactions. Eosinophils were purified from the blood of IL-5 transgenic mice, labeled with (111)In and injected into nontransgenic CBA/Ca mice. Th1- and Th2-predominant, DTH reactions were induced in mice by immunization with methylated bovine serum albumin (MBSA) in Freund's complete adjuvant or with Schistosoma mansoni eggs, respectively. In these animals, (111)In-eosinophils were recruited in skin sites in an antigen-, time-, and concentration-dependent manner. Depletion of CD4+ lymphocytes abrogated (111)In-eosinophil recruitment in both reactions. Pretreatment of animals with anti-IFN-gamma mAb abrogated (111)In-eosinophil recruitment in MBSA-immunized and -challenged animals, whereas anti-IL-4 inhibited (111)In-eosinophil recruitment in both models. Local pretreatment with an anti-eotaxin polyclonal antibody inhibited the MBSA and SEA reactions by 51% and 39%, respectively. These results demonstrate that, although eosinophilia is not a feature of Th1-predominant, DTH reactions, these reactions produce the necessary chemoattractants and express the necessary cell adhesion molecules for eosinophil migration. The control of the circulating levels of eosinophils appears to be a most important strategy in determining tissue eosinophilia.

PMID: 11261781 [PubMed - indexed for MEDLINE]

1576. Contact Dermatitis. 2001 Apr;44(4):235-9.

Occupational airborne contact dermatitis caused by thyme dust.

Spiewak R, Skorska C, Dutkiewicz J.

Department of Occupational Biohazards, Institute of Agricultural Medicine, ul. Jaczewskiego 2, 20-090 Lublin, Poland.

The aim of the study was to assess occupational hazards to the farmer's skin associated with processing thyme (Thymus vulgaris L.). 46 farmers were studied during the threshing of dried thyme. They were questioned about work-related skin problems and examined before and after work. In all persons, serum thyme-specific IgE was measured. Skin prick tests, the Ouchterlony test and the migration inhibition test were carried out with allergens of airborne bacteria and fungi present in the working environment. Of the 46 farmers studied, 4 showed skin symptoms after 5-30 min of exposure to thyme dust. Thyme-specific IgE was found in 1 person with work-related symptoms, but also in 2 asymptomatic farmers. Therefore, the importance of IgE seems to be questionable in eczema related to thyme dust. Skin and blood tests with microbial allergens also showed no significant differences between the symptomatic and asymptomatic farmers. To our knowledge, this is the 1st description of occupational airborne contact dermatitis caused by thyme dust. The etiology of thyme-related skin symptoms remains obscure, although an irritant mechanism seems probable.

PMID: 11260240 [PubMed - indexed for MEDLINE]

1577. Clin Exp Allergy. 2001 Feb;31(2):249-58.

Interleukin-2 inhibits eosinophil migration but is counteracted by IL-5 priming.

Lampinen M, Håkansson L, Venge P.

Asthma Research Centre, University Hospital, S-751 85 Uppsala, Sweden. maria.lampinen@medsci.uu.se

Conflicting data on the role of interleukin-2 in the recruitment of eosinophil

granulocytes (EOS) to sites of inflammation have been presented. The objective of the present study was to investigate the effect of recombinant human IL-2 and anti-IL-2 on the migration of purified blood EOS. Neutralizing antibodies to IL-2 were added to a cytokine mixture with significant eosinophil chemotactic activity (ECA), and afterwards the ECA was tested on EOS from both normal and allergic donors. EOS migration was measured by a modification of the Boyden technique, using a 48-well microchemotaxis chamber. Recombinant human IL-2 was either added to the lower compartment of the chemotaxis chamber, or to the EOS for a pre-incubation period of 20 min, before migration assays towards the chemotaxins were performed. Anti-IL-2 caused a significant increase of EOS migration towards the cytokine mixture. Pre-incubation of the EOS with rhIL-2 inhibited the chemotaxis towards RANTES, PAF, IL-8 and eotaxin, and EOS migration towards IL-2 was lower than that towards buffer. These effects were more pronounced on EOS from normal than from allergic donors. Priming of the EOS with IL-5 prevented the inhibitory effect of IL-2. We hypothesize that IL-2 acts as an autocrine regulator of EOS migration, and that this inhibitory effect may be downregulated in allergy, allowing an increased migration of EOS towards chemotactic factors.

PMID: 11251626 [PubMed - indexed for MEDLINE]

1578. Am J Contact Dermat. 2001 Mar;12(1):6-17.

Dermal exposure to cinnamaldehyde alters lymphocyte subpopulations, number of interferon-gamma-producing cells, and expression of B7 costimulatory molecules and cytokine messenger RNAs in auricular lymph nodes of B6C3F1 mice.

Karrow NA, Leffel EK, Guo TL, Zhang LX, McCay JA, Germolec DR, White KL Jr.

Department of Pharmacology and Toxicology, Virginia Commonwealth University, Richmond, VA 23298-6013, USA.

BACKGROUND: Although the Murine Local Lymph Node Assay (LLNA) is efficient in identifying chemicals with sensitizing potential, there is increasing need for alternative end points. Cinnamaldehyde (CIN) was chosen for evaluation based on its moderate potency and extensive use in fragrance materials.
OBJECTIVES: The purpose of the present studies is to incorporate some alternative end points, such as phenotypic analysis and cytokine production, into a modified LLNA/irritancy assay (IA) to evaluate the sensitization of female B6C3F1 mice to CIN.
METHODS: Several nontraditional end points, including the analysis of lymphocyte subpopulations, B7 costimulatory molecule and cytokine messenger RNA (mRNA) expression, and intracellular interferon-gamma (IFN-gamma) levels, were incorporated into a modified murine local lymph node (LLNA)/irritancy assay (IA) to evaluate the sensitization of female B6C3F1 mice to cinnamaldehyde (CIN).
RESULTS: The alternate end points used in these studies support the classification of CIN as a moderately potent sensitizer. Dermal treatment with CIN resulted in an increase in the percentage of B cells in the auricular lymph nodes (ALNs) and expression of the costimulatory molecule, B7-2, on B cells. Lymph node cells also showed increased transforming growth factor-beta1, migration-inhibition factor, and mild increases in IFN-gamma and interleukin-2 cytokine mRNA expression. Although the increase in IFN-gamma mRNA expression did not translate into increased intracellular IFN-gamma levels, the absolute number of T cells producing IFN-gamma in the ALNs increased. Conversely, the MEST did not classify CIN as a contact allergen.
CONCLUSION: The nontraditional end points used in the LLNA/IA were not as sensitive as the traditional radioisotope method used to assess cell proliferation. However, they may help identify compounds inappropriately classified as sensitizers or nonsensitizers by the LLNA and MEST.

Copyright 2001 by W.B. Saunders Company

PMID: 11244134 [PubMed - indexed for MEDLINE]

1579. Cell Immunol. 2001 Feb 1;207(2):75-80.

Interleukin (IL)-4/IL-9 and exogenous IL-16 induce IL-16 production by BEAS-2B cells, a bronchial epithelial cell line.

Yoshida N, Arima M, Cheng G, Eda F, Hirata H, Honda K, Fukushima F, Fukuda T.

Department of Pulmonary Medicine and Clinical Immunology, Dokkyo University School of Medicine, Tochigi, 321-0293, Japan.

Previous studies have suggested that bronchial epithelial cells may perpetuate airway inflammation. We have reported that the bronchial epithelial cell line BEAS-2B can produce interleukin (IL)-16, a potent chemoattractant for CD4+ T cells. IL-16 is thought to regulate airway inflammation in asthmatics. Recent studies showed that IL-4 induces inflammatory cytokines in bronchial epithelial cells and that IL-9 is a candidate gene for development of asthma. The present study demonstrated that BEAS-2B cells produced specifically IL-16 by synergistic effects of IL-4 + IL-16, or IL-9 + IL-16, and that the synthesized IL-16 induced migration of CD4+ T cells. This study is a first report indicating that IL-16 production may be maintained by an autocrine machinery by epithelial cell-derived IL-16 with IL-4 and IL-9 in asthma.

Copyright 2001 Academic Press.

PMID: 11243696 [PubMed - indexed for MEDLINE]

1580. J Allergy Clin Immunol. 2001 Mar;107(3):469-76.

Nasal allergen provocation induces adhesion molecule expression and tissue eosinophilia in upper and lower airways.

Braunstahl GJ, Overbeek SE, Kleinjan A, Prins JB, Hoogsteden HC, Fokkens WJ.

Departments of Otorhinolaryngology and Pulmonary Medicine, Erasmus University Medical Center, Rotterdam, The Netherlands.

BACKGROUND: Allergic rhinitis (AR) and asthma are characterized by means of a similar inflammatory process in which eosinophils are important effector cells. The migration of eosinophils from the blood into the tissues is dependent on adhesion molecules.
OBJECTIVE: To analyze the aspects of nasobronchial cross-talk, we studied the expression of adhesion molecules in nasal and bronchial mucosa after nasal allergen provocation (NP).
METHODS: Nine nonasthmatic subjects with seasonal AR and 9 healthy control subjects underwent NP out of season. Bronchial and nasal biopsy specimens were taken before (T(0)) and 24 hours after NP (T(24)). Mucosal sections were analyzed for the presence of eosinophils, IL-5, eotaxin, intercellular adhesion molecule 1 (ICAM-1), vascular cell adhesion molecule 1 (VCAM-1), E-selectin, and human endothelium (CD31).
RESULTS: At T(24), an influx of eosinophils was detected in nasal epithelium (P =.01) and lamina propria (P <.01), as well as in bronchial epithelium (P =.05) and lamina propria (P <.05), of the patients with AR. At T(24), increased expression of ICAM-1, as well as increased percentages of ICAM-1+, VCAM-1+, and E-selectin+ vessels, were seen in nasal and bronchial tissue of patients with AR.

The number of mucosal eosinophils correlated with the local expression of ICAM-1, E-selectin, and VCAM-1 in patients with AR.
CONCLUSION: This study shows that NP in patients with AR results in generalized airway inflammation through upregulation of adhesion molecules.

PMID: 11240947 [PubMed - indexed for MEDLINE]

1581. J Immunol. 2001 Mar 15;166(6):3672-7.

Functional caspase-1 is required for Langerhans cell migration and optimal contact sensitization in mice.

Antonopoulos C, Cumberbatch M, Dearman RJ, Daniel RJ, Kimber I, Groves RW.

Center for Dermatology, Department of Medicine, University College London, London, United Kingdom.

Langerhans cell (LC) migration from epidermis to draining lymph node is a critical first step in cutaneous immune responses. Both TNF-alpha and IL-1 beta are important signals governing this process, but the potential regulatory role of IL-1 alpha processing by caspase-1 is unknown. In wild-type (WT) mice, application of the contact allergens 2,4-dinitrofluorobenzine and oxazolone lead to a marked reduction in epidermal LC numbers, but in caspase-1-deficient mice this reduction was not observed. Moreover, although intradermal injection of TNF-alpha (50 ng) induced epidermal LC migration in WT mice, this cytokine failed to induce LC migration in caspase-1-deficient mice. Intradermal IL-1 beta (50 ng) caused a similar reduction in epidermal LC numbers in both WT and caspase-1-deficient mice, indicating that, given an appropriate signal, caspase-1-deficient epidermal LC are capable of migration. Contact hypersensitivity to both 2,4-dinitrofluorobenzine and oxazolone was inhibited in caspase-1-deficient mice, indicating a functional consequence of the LC migration defect. In organ culture the caspase-1 inhibitor Ac-YVAD-cmk, but not control peptide, potently inhibited the epidermal LC migration that occurs in this system, and reduced spontaneous migration of LC was observed in skin derived from caspase-1-deficient mice. Moreover, Ac-YVAD-cmk applied to BALB/c mouse skin before application of contact sensitizers inhibited LC migration and contact hypersensitivity in vivo. Taken together, these data indicate that caspase-1 may play a central role in the regulation of LC migration and suggest that the activity of this enzyme is amenable to control by specific inhibitors both in vivo and in vitro.

PMID: 11238606 [PubMed - indexed for MEDLINE]

1582. Clin Exp Pharmacol Physiol. 2001 Mar;28(3):233-6.

Role of human airway smooth muscle in altered extracellular matrix production in asthma.

Johnson PR.

Department of Pharmacology, University of Sydney, New South Wales, Australia. pjohno@pharmacol.usyd.edu.au

1. The underlying abnormality in asthma is not fully understood; however, inflammation, airway remodelling and bronchial hyperresponsiveness are key factors. The plasma exudate from the microvascular leakage plays a significant role in remodelling, which includes extracellular matrix (ECM) protein deposition/breakdown and airway smooth muscle (ASM) hyperplasia/hypertrophy. 2.

The ECM is an intricate network of macromolecules that forms the 'scaffolding' of the airways. This scaffolding not only acts as mechanical support that plays a crucial role in the maintenance of airway function and structure, but it is also a dynamic and complex network that has the potential to influence cellular function, including migration, differentiation and proliferation of a number of cell types. 3. In asthmatic airways, the profile of ECM proteins is altered. The deposition of collagen I, III, V, fibronectin, tenascin, hyaluronan, versican and laminin alpha2/beta2 is increased, whereas the deposition of collagen IV and elastin is decreased. 4. This imbalance in the ECM profile within the asthmatic airway could be due to: (i) increased de novo synthesis of ECM proteins; (ii) decreased activity of its degrading enzymes, namely matrix metalloproteinases (MMP); or (iii) upregulation of the tissue-specific inhibitors of metalloproteinases (TIMP). 5. One of the characteristic features of asthma is an increase in the amount of ASM within the airways. The ECM proteins/MMP/TIMP in and around the smooth muscle may play a contributory role in this increased growth. 6. The role of current asthma treatments in the prevention or reversal of airway ECM changes is an area that has only recently become of interest, with the majority of the in vivo work focusing on the effects of corticosteroids. 7. The evidence presented in this review indicates that the ASM may influence its own environment/proliferation through the production of ECM proteins, MMP and TIMP. Further studies are needed to fully understand the role of the ASM in the production of ECM proteins, MMP and TIMP andtheir potential influence in the mechanisms underlying asthma.

PMID: 11236132 [PubMed - indexed for MEDLINE]

1583. J Cataract Refract Surg. 2001 Feb;27(2):337-8.

Intraocular lens subluxation in a patient with facial atopic dermatitis.

Yamazaki S, Nakamura K, Kurosaka D.

Department of Ophthalmology, Keio University School of Medicine, Tokyo, Japan.

A 66-year-old Japanese man presented with subluxation of a posterior chamber intraocular lens (IOL) caused by a rupture of part of Zinn's zonule but no retinal break 2 years after phacoemulsification with IOL implantation. He had a history of atopic dermatitis since infancy. This case presents a rare ocular complication of scratching and rubbing the face and eyelids because of itching related to atopic dermatitis.

PMID: 11226805 [PubMed - indexed for MEDLINE]

1584. Bratisl Lek Listy. 2000;101(11):614-6.

[Skin manifestations of Lyme borreliosis--occurrence, diagnosis, therapy].

[Article in Slovak]

Svecova D, Buchvald J.

Dermatovenerologická klinika LFUK v Bratislave. svecova@faneba.sk

Eight genotypes of Borrelia burgdorferi are known currently. In Slovakia (Carpathian Euroregion) the most frequent genotypes are B. garini, B. afzelii, as well as B. valaisiana and B. lusitaniae. Infestation of the vector Ixodes ricinus is 3-30%. The most frequent early skin manifestation is erythema migrans (60-70%). Borrelia burgdorferi is suggested to be the causative agent in

sclerodermia circumscripta, lichen sclerosus et atrophicus, maybe also in urticaria chronica, granuloma anulare, erythema anulare, erythema nodosum. It can be the causative agent also in neurological diagnoses as e.g. chronic oligosymptomatic encephalopathy, "sclerosis multiplex-like" syndrome and fatigue syndrome, arthralgia, myalgia, seronegative indifferentiated oligoarthritis and fibromyalgies. The serological diagnosis has to be coincide with clinical findings. Used serological examinations are ELISA, Immunoblot, indirect immunofluorescence examination. PCR is an important contribution in examination of synovial fluid (85% detection) and cerebrospinal liquor (24-100%). The importance of PCR is stressed in cases with mixed infections by several borrrelia genotypes. The first line treatment includes doxyciclin, amoxicilin, and erythromycin. The second line includes macrolides, cephalosporines. New perspectives are ascribed to active immunisation with recombined antigen OsA (LYMErix, ImuLyme).

PMID: 11218959 [PubMed - indexed for MEDLINE]

1585. Cell Mol Life Sci. 1999 Oct 30;56(5-6):427-41.

Integrin antagonists.

Curley GP, Blum H, Humphries MJ.

School of Biological Sciences, University of Manchester, UK.

Integrins are a family of cell surface glycoproteins that mediate numerous cell-cell and cell-matrix interactions and are involved in biological processes such as tissue morphogenesis, leukocyte recirculation and migration, wound healing, blood clotting and immune response. Aberrant cell adhesion has been implicated in the pathogenesis of several diseases, including a number of inflammatory disorders such as rheumatoid arthritis, inflammatory bowel disease and asthma, as well as cancer and coronary heart disease. As such integrins are seen as excellent targets for the development of therapeutic agents. This report begins with an examination of the structure of integrin molecules and their ligands and then goes on to review the current state of development of antiintegrin antagonists.

PMID: 11212296 [PubMed - indexed for MEDLINE]

1586. Endod Dent Traumatol. 2000 Aug;16(4):184-7.

Immunologic evaluation of dental patient with history of hypersensitivity reaction to sodium hypochlorite.

Dandakis C, Lambrianidis T, Boura P.

Department of Endodontology, School of Dentistry, Aristotle University of Thessaloniki, Greece.

A 12-year-old girl, with a previous history of bronchial reaction and contact dermatitis to sodium hypochlorite, was referred for root canal treatment. Complete immunologic evaluation revealed a mild hypersensitivity condition, as it was assessed by the RAST investigation to different allergens and the DTH reactivity expressed though migration inhibition test. The absence of a serious immunologic disregulation in the patient's immunologic profile justified the term 'non-allergic hypersensitivity' to sodium hypochlorite to describe the condition.

PMID: 11202880 [PubMed - indexed for MEDLINE]

1587. Arch Immunol Ther Exp (Warsz). 2000;48(6):451-6.

T cell chemokine receptor expression in human Th1- and Th2-associated diseases.

Campbell JD, HayGlass KT.

Department of Immunology, University of Manitoba, Winnipeg, Canada.

The interaction between chemokines and their receptors is an important step in the control of leukocyte migration into sites of inflammation. Chemokines also mediate a variety of effects independent of chemotaxis, including induction and enhancement of Th1- and Th2-associated cytokine responses. Recent studies have shown that human Th1 and Th2 clones, activated under polarizing conditions with polyclonal stimuli in vitro, display distinct patterns of chemokine receptor expression: Th1 clones preferentially express CCR5 and CXCR3, while many Th2 clones express CCR4, CCR8 and, to a lesser extent, CCR3. These differential patterns of chemokine receptor expression suggest a mechanism for selective induction of migration and activation of Th1- and Th2-type cells during inflammation and, perhaps, normal immune homoeostasis. Studies have begun to examine T cell chemokine receptor expression in vivo to determine the relevance of these in vitro observations to human Th1- and Th2-associated diseases. In this review, we critically examine recent reports of T cell chemokine receptor expression in human autoimmune disorders (multiple sclerosis and rheumatoid arthritis) and atopic disorders (allergic rhinitis and asthma) which are believed to arise from inappropriate Th1- and Th2-dominated responses, respectively.

PMID: 11197598 [PubMed - indexed for MEDLINE]

1588. J Exp Med. 2001 Jan 15;193(2):255-61.

Prostaglandin D2 selectively induces chemotaxis in T helper type 2 cells, eosinophils, and basophils via seven-transmembrane receptor CRTH2.

Hirai H, Tanaka K, Yoshie O, Ogawa K, Kenmotsu K, Takamori Y, Ichimasa M, Sugamura K, Nakamura M, Takano S, Nagata K.

R&D Center, BML, Saitama 350-1101, Japan.

Prostaglandin (PG)D2, which has long been implicated in allergic diseases, is currently considered to elicit its biological actions through the DP receptor (DP). Involvement of DP in the formation of allergic asthma was recently demonstrated with DP-deficient mice. However, proinflammatory functions of PGD2 cannot be explained by DP alone. We show here that a seven-transmembrane receptor, CRTH2, which is preferentially expressed in T helper type 2 (Th2) cells, eosinophils, and basophils in humans, serves as the novel receptor for PGD2. In response to PGD2, CRTH2 induces intracellular Ca^{2+}mobilization and chemotaxis in Th2 cells in a Galphai-dependent manner. In addition, CRTH2, but not DP, mediates PGD2-dependent cell migration of blood eosinophils and basophils. Thus, PGD2 is likely involved in multiple aspects of allergic inflammation through its dual receptor systems, DP and CRTH2.

PMCID: PMC2193345
PMID: 11208866 [PubMed - indexed for MEDLINE]

1589. J Allergy Clin Immunol. 2001 Feb;107(2):353-8.

CCR4 memory CD4+ T lymphocytes are increased in peripheral blood and lesional skin from patients with atopic dermatitis.

Nakatani T, Kaburagi Y, Shimada Y, Inaoki M, Takehara K, Mukaida N, Sato S.

Department of Dermatology, Cancer Research Institute, Kanazawa University School of Medicine, Kanazawa, Japan.

BACKGROUND: Recent studies have reported that TH1 and TH2 cells express CXCR3 and CCR4, respectively.
OBJECTIVE: Our goal was to assess the association of CCR4 and CXCR3 expression with TH2 and TH1 cells and association of CCR4 and CXCR3 expression with inflammation in patients with atopic dermatitis (AD).
METHODS: Intracellular cytokine production and chemokine receptor expression in blood T cells were examined by flow cytometry. Immunohistochemical expression of chemokine receptors was also investigated in chronically lesional skin.
RESULTS: CCR4+ and CXCR3+ CD4+ T cells predominantly produced IL-4 and IFN-gamma, respectively. Although the frequency of CXCR3+ cells among CD4+ CD45RO+ T cells was similar for patients with AD (n = 29) and healthy control subjects (n = 19), patients with severe AD (n = 14) had a reduced frequency of CXCR3+ cells. In contrast, the frequency of CCR4+ cells and the CCR4/CXCR3 ratio were higher in patients with AD (n = 22) than healthy control subjects (n = 16) and correlated with disease severity of AD. The frequency of CCR4+ cells correlated positively with eosinophil numbers and serum IgE levels, whereas the frequency of CXCR3+ cells correlated inversely with eosinophil numbers. The frequency of CCR4+ or CXCR3+ cells was similar in patients with psoriasis (n = 6) and healthy control subjects. Immunohistochemical analysis showed that the frequency of CCR4+ cells among CD4+ T cells in chronically lesional skin of patients with AD (n = 9) was higher than that of patients with psoriasis (n = 4).
CONCLUSION: Our data suggest the association of CCR4 expression with TH2 cells, the predominance of CCR4+ cells in blood from patients with AD, and an important role of CCR4 in the migration of TH2 cells from blood into AD lesional skin.

PMID: 11174204 [PubMed - indexed for MEDLINE]

1590. J Allergy Clin Immunol. 2001 Feb;107(2):211-8.

Eosinophil recruitment to the airway nerves.

Jacoby DB, Costello RM, Fryer AD.

Division of Pulmonary and Critical Care Medicine, Johns Hopkins Asthma and Allergy Center, Johns Hopkins University, Baltimore, Md 21205, USA.

Increased vagal reflexes contribute to bronchoconstriction in asthma. Antigen challenge of sensitized animals induces vagal hyperresponsiveness. This review will discuss the evidence that eosinophils increase release of acetylcholine from the parasympathetic nerves. After antigen challenge, eosinophils are actively recruited to the airway nerves, possibly through expression of chemotactic substances and adhesion molecules by the nerves. Tachykinins acting on neurokinin 1 receptors activate the eosinophils. Activated eosinophils release eosinophil major basic protein (MBP), which is an endogenous antagonist for M2 muscarinic receptors. The M2 muscarinic receptors on the parasympathetic nerves in the lungs normally inhibit release of acetylcholine. When M2 receptors are blocked by MBP, acetylcholine release is increased, resulting in hyperresponsiveness. Neutralization of MBP with polyanionic substances restores M2 receptor function and eliminates hyperresponsiveness. Antibodies to MBP prevent M2 receptor dysfunction and hyperresponsiveness, as do antibodies to the adhesion molecule very late antigen 4, which prevent eosinophil migration. A low dose of

dexamethasone, which does not affect total eosinophil influx into the lungs and airways, prevents eosinophils from clustering around the nerves and prevents antigen-induced M2 dysfunction and hyperresponsiveness. Furthermore, animal studies show that viral infections, which are important precipitants of asthma attacks, and exposure to air pollutants such as ozone can also activate airway eosinophils, leading to a chain of events similar to that seen after antigen challenge. Finally, a similar clustering of eosinophils around airway nerves, as well as release of MBP onto the nerves, is seen in fatal asthma, suggesting that similar mechanisms may be involved in human airway hyperresponsiveness.

PMID: 11174183 [PubMed - indexed for MEDLINE]

1591. Am J Pathol. 2001 Feb;158(2):431-40.

Differential and sequential expression of multiple chemokines during elicitation of allergic contact hypersensitivity.

Goebeler M, Trautmann A, Voss A, Bröcker EV, Toksoy A, Gillitzer R.

Department of Dermatology, University of Würzburg Medical School, Würzburg, Germany.

Regulation of chemokine-mediated leukocyte migration within inflammatory tissues is a complex event that cannot be mimicked and analyzed in vitro. We therefore investigated the role of macrophage- and T-lymphocyte-specific chemoattractants involved in the positioning of immune effector cells during the elicitation phase of contact hypersensitivity, a prototype of a T-lymphocyte-mediated immune reaction. Serial sections of skin biopsies obtained from sensitized individuals at distinct time intervals after epicutaneous application of allergens were hybridized with anti-sense probes of a large panel of chemokines or immunohistologically labeled with leukocyte-specific antibodies. Multifocal expression of monocyte chemoattractant protein-1 (MCP-1) was already detected after 6 hours in basal keratinocytes clearly preceding the infiltration of monocytes and T cells. Increasing basal expression of MCP-1 and, in addition, of regulated upon activation, normal T-cell expressed and secreted (RANTES) after 12 hours was accompanied by dermal expression of MCP-1, macrophage-derived chemoattractant (MDC), and RANTES and paralleled by infiltration of mononuclear cells into dermis and epidermis. Expression of the T-lymphocyte-specific chemokines IP-10 and MIG in epidermis and dermis and of MDC, pulmonary and activation-regulated chemokine (PARC), and thymus and activation-regulated chemokine (TARC) exclusively in the dermis started after 12 hours reaching maximum levels at 72 hours and was associated with infiltration of T cells into the epidermal compartment. Our data provide evidence that migrating effector cells encounter multiple chemoattractant signals in a complex spatial and temporal pattern. In particular, keratinocytes contribute to the vigorous immigration by sequential expression of MCP-1, RANTES, and interferon-inducible protein-10 (IP-10) monokine induced by gamma interferon (MIG), indicating that chemokine-mediated nonimmunological mechanisms precede and corroborate antigen-specific mechanisms during elicitation of contact hypersensitivity.

PMCID: PMC1850305
PMID: 11159181 [PubMed - indexed for MEDLINE]

1592. Am J Physiol Lung Cell Mol Physiol. 2001 Mar;280(3):L421-31.

Prostaglandin E(2) regulates wound closure in airway epithelium.

Savla U, Appel HJ, Sporn PH, Waters CM.

Department of Biomedical Engineering, Illinois 60611, USA.

Repair of the airway epithelium after injury is critical for the maintenance of barrier function and the limitation of airway hyperreactivity. Airway epithelial cells (AECs) metabolize arachidonic acid to biologically active eicosanoids via the enzyme cyclooxygenase (COX). We investigated whether stimulating or inhibiting COX metabolites would affect wound closure in monolayers of cultured AECs. Inhibiting COX with indomethacin resulted in a dose-dependent inhibition of wound closure in human and feline AECs. Specific inhibitors for both COX-1 and COX-2 isoforms impaired wound healing. Inhibitors of 5-lipoxygenase did not affect wound closure in these cells. The addition of prostaglandin E(2) (PGE(2)) eliminated the inhibition due to indomethacin treatment, and the exogenous application of PGE(2) stimulated wound closure in a dose-dependent manner. Inhibition of COX with indomethacin only at initial time points resulted in a sustained inhibition of wound closure, indicating that prostanoids are involved in early wound repair processes such as spreading and migration. These differences in wound closure may be important if arachidonic acid metabolism and eicosanoid concentrations are altered in disease states such as asthma.

PMID: 11159024 [PubMed - indexed for MEDLINE]

1593. J Immunol. 2000 Apr 15;164(8):4271-6.

Stem cell factor plays a major role in the recruitment of eosinophils in allergic pleurisy in mice via the production of leukotriene B4.

Klein A, Talvani A, Cara DC, Gomes KL, Lukacs NW, Teixeira MM.

Immunopharmacology Laboratory, Departamento de Farmacologia and Departmento de Patologia, Instituto de Ciencias Biologicas, Universidade Federal de Minas Gerais, Belo Horizonte, Brazil.

The understanding of the mechanisms underlying eosinophil migration into tissue is an essential step in the development of novel therapies aimed at treating allergic diseases where eosinophil recruitment and activation are thought to play an essential role. In this study, we have examined the effects of the in vivo administration of stem cell factor (SCF) on eosinophil recruitment and tested whether endogenous SCF was involved in mediating eosinophil recruitment in response to Ag challenge in sensitized mice. The intrapleural injection of SCF induced a time- and concentration-dependent recruitment of eosinophils in mice. In allergic mice, SCF message was expressed early after Ag challenge and returned to baseline levels after 8 h. In agreement with the ability of SCF to induce eosinophil recruitment and its expression in the allergic reaction, an anti-SCF polyclonal Ab abrogated eosinophil recruitment when given before Ag challenge. SCF increased the levels of leukotriene B4 (LTB4) in the pleural cavity of mice and an LTB4 receptor antagonist, CP105,696, abrogated the effects of SCF on eosinophil recruitment. Similarly, recruitment of eosinophils in the allergic reaction was virtually abolished by CP105,696. Together, our data favor the hypothesis that the local release of SCF following Ag challenge may activate and/or prime mast cells for IgE-mediated release of inflammatory mediators, especially LTB4. The mediators released in turn drive the recruitment of eosinophils. Inhibition of the function of SCF in vivo may reduce the migration of eosinophils to sites of allergic inflammation and may, thus, be a relevant principle in the treatment of allergic diseases.

PMID: 10754325 [PubMed - indexed for MEDLINE]

1594. Eur Respir J. 2000 Nov;16(5):940-6.

Stimulation of eosinophil IgE low-affinity receptor leads to increased adhesion molecule expression and cell migration.

Lantero S, Alessandri G, Spallarossa D, Scarso L, Rossi GA.

Pulmonary Division, National Cancer Research Institute, Genoa, Italy.

Immunoglobulin binding on eosinophil surface receptors results in activation of these cells. Evaluating blood eosinophils from atopic subjects, it was investigated whether ligation of immunoglobulin E low-affinity receptor (FcepsilonRII/ CD23) with specific monoclonal antibodies (Mabs) resulted in enhanced eosinophil migration and adhesion molecule expression. Eosinophils from 20 subjects with allergic asthma (atopic individuals) and nine nonatopic normal individuals (controls) were purified using Percoll gradients. The effect of antihuman CD23 Mabs on: 1) eosinophil migration through human umbilical vein endothelial cells (HUVECs); and 2) eosinophil expression of the adhesion molecules leukocyte function-associated antigen-1 (LFA-1, CD11a/CD18), macrophage antigen-1 (Mac-1, CD11b/CD18) and very late activation antigen-1 (VLA-4, CD49d/CD29) was evaluated by specific Mab staining and flow cytometric analysis. As compared to controls, freshly isolated eosinophils from atopic individuals showed enhanced migration through HUVECs (p<0.05) and increased LFA-1 expression (p<0.01), but similar Mac-1 and VLA-4 expression (p>0.1 for both). In both controls and atopic individuals, eosinophil incubation with antihuman CD23 Mabs induced a dose-dependent increase in cell migration through HUVECs, significant at antihuman CD23 Mab concentrations of 5 microg x mL(-1) (p>0.05 for all). Similarly, incubation of the cells with antihuman CD23 Mabs induced dose-dependent upregulation of LFA-1 and Mac-1 expression, whereas no changes in VLA-4 expression were observed (p>0.1). Finally, the enhanced eosinophil migration induced by antihuman CD23 Mab stimulation was significantly inhibited by antihuman LFA-1 (84+/-14% (mean+/-SEM); p<0.01) and VLA-4 Mabs (47+/-15%; p<0.05) but not by antihuman Mac-1 Mabs (p>0.1). In both atopic and control subjects, immunoglobulin E, low-affinity receptor stimulation induces functional changes in eosinophils characterized by increased eosinophil migration associated with enhanced late function antigen-1 and Mac-1 expression.

PMID: 11153596 [PubMed - indexed for MEDLINE]

1595. Ann Agric Environ Med. 2000;7(2):111-8.

Effects of exposure to flax dust in Polish farmers: work-related symptoms and immunologic response to microbial antigens associated with dust.

Skorska C, Mackiewicz B, Dutkiewicz J.

Department of Occupational Biohazards, Institute of Agricultural Medicine, Jaczewskiego 2, 20-090 Lublin, Poland.

Medical examinations were performed in a group of 51 Polish farmers heavily exposed to flax dust during harvesting and scutching (threshing) and in a group of 50 healthy urban dwellers not exposed to organic dusts (controls). The examinations included: interview concerning the occurrence of respiratory disorders and work-related symptoms, physical examination, X-ray examination of chest, lung function tests, oxymetric examinations, determination of the concentration of cytokines (IL-1alpha IL-6, TNFalpha) in blood serum and allergological tests with microbial antigens associated with organic dust, comprising: skin prick test with 4 antigens, agar-gel precipitation test with 12 antigens and test for specific inhibition of leukocyte migration with 4 antigens.

As many as 32 farmers (62.7%) reported the occurrence of work-related symptoms during harvesting, transporting and scutching of flax. The most common complaint was general weakness reported by 15 farmers (29.4%), followed by headache reported by 14 (27.5%), blocking of the nose - by 11 (21.6%), dry cough, shivering, and eyes itching - each by 10 (19.8%), chest tightness and hoarseness - each by 9 (17.6%). No control subjects reported these work-related symptoms. The mean spirometric values in the examined group of farmers were within a normal range and did not show a significant post-shift decline. In contrast, a significant post-shift decline of oxymetric values was found among flax farmers. The farmers showed a frequency of the positive early skin reactions to environmental allergens in the range of 0-19.6%, a frequency of positive precipitin reactions in a range of 0-56.9%, and frequency of positive reactions of leukocyte migration inhibition in a range of 7.8-21.6%. The members of the control group responded to the majority of allergens with a significantly lower frequency of positive results compared to the farmers. Elevated concentrations of IL-1alpha and IL-6, but not TNFalpha, were found in blood sera of flax farmers. In conclusion, farmers engaged in harvesting and scutching of flax represent a group of elevated professional risk because of high incidence of work-related symptoms and high frequency of allergic reactions to bacteria and fungi associated with organic dust.

PMID: 11153040 [PubMed - indexed for MEDLINE]

1596. J Immunol Methods. 2001 Jan 1;247(1-2):73-82.

An automated method for the quantification of immunostained human Langerhans cells.

Jacobs JJ, Lehé C, Cammans KD, Yoneda K, Das PK, Elliott GR.

Department of Pharmacology, TNO-PML, P.O. Box 45, Rijswijk, The Netherlands.

Allergic contact dermatitis is a frequent and increasing health problem. For ethical reasons, the current animal tests used to screen for contact sensitizers should be replaced by in vitro alternatives. Contact sensitizers have been shown to accelerate Langerhans cell (LC) migration from human organotypic skin explant cultures (hOSECs) more rapidly than non-sensitizers and it has been proposed that the hOSEC model could be used to screen for sensitizers. However, chemically induced decreases in epidermal LC numbers need to be accurately quantified if the alterations in epidermal LC numbers are to form the basis of an alternative system for screening contact sensitizers in vitro. As manual counting of LCs is labour intensive and subject to intra- and inter-personal variation we developed an image analysis routine, using the Leica QWin image analysis software, to quantify LCs in situ using immunohistochemically stained skin sections. LCs can be identified using antibodies against the membrane molecule CD1a or the Lag antibody, which recognises cytoplasmic Birbeck granules. Quantification of epidermal LC number using the image analysis software had a much lower inter-person variation than when the same specimens were counted manually, using both the anti-Lag and CD1a antibodies. The software-aided quantification of epidermal LCs provides an accurate method for measuring chemically-induced changes in LC numbers.

PMID: 11150538 [PubMed - indexed for MEDLINE]

1597. Arch Soc Esp Oftalmol. 2000 Sep;75(9):605-9.

[Polytetrafluoroethylene in scleral buckling surgery].

[Article in Spanish]

Roldán Pallarés M, Awad-El Susi S.

Departamento de Oftalmología, Hospital Universitario San Carlos, UCM, Madrid, España.

PURPOSE: To evaluate the tolerance of PTFEe (second generation) for scleral buckling (SB) surgery of rhegmatogenous retinal detachment (RD).
METHODS: We have used a new material: wide porous expanded polytetrafluoroethylene (PTFEe) for SB of 32 cases with rhegmatogenous RD of extention </= 2 quadrants of retina, without PVR o with PVR </= C(2) and always as first surgical procedure. Follow-up has been between 6 and 26 months (average 14 months). This material is a smooth, unstretchable, strong, not carcinogenical and not allergenic inert vitreous teflon alloplast with a high biocompatibility. It is close to Gore-tex but differs from it for a higher porosity (over 90%) and a wider diameter of its pores (over 50 mu).
RESULTS: We have not found any complications such as migration, infection, erosion, extrusion, intrusion, etc, with this material used as SB in 32 cases with rhegmatogenous RD. It demonstrated excellent tolerance and biocompatibility over all follow-up period (average 14 months, maximum 26 months).
CONCLUSIONS: The first results of this series are very encouraging. With short-term follow-up (maximun 26 months) the tolerance has been better than with other SB biomaterials. Long follow-up will detect other possible side effects.

PMID: 11151232 [PubMed - indexed for MEDLINE]

1598. J Allergy Clin Immunol. 2001 Jan;107(1):22-30.

Dysregulation of the IgE/Fc epsilon RI network in HIV-1 infection.

Marone G, Florio G, Petraroli A, de Paulis A.

Division of Clinical Immunology and Allergy, University of Naples Federico II, Naples, Italy.

Serum IgE levels are increased in adults and children with HIV-1 infection and could be a marker of poor prognosis. Allergic reactions and adverse reactions to drugs are also increased in HIV-1-infected individuals. An imbalance between a T(H)1-like and a T(H)2-like cytokine profile has been documented in HIV-1 infection. We have found that HIV-1 gp120 from different clades is a potent stimulus for histamine and cytokine (IL-4 and IL-13) release from basophils. Gp120 acts as a viral superantigen, interacting with the V(H)3 region of IgE to induce mediator release from human Fc epsilon RI(+) cells. Human basophils and mast cells express the chemokine receptor CCR3, which binds the chemokines eotaxin and RANTES. By interacting with the CCR3 receptor on Fc epsilon RI(+) cells, HIV-1 Tat protein is a potent chemoattractant for human basophils and lung mast cells. Tat protein also induced IL-4 and IL-13 release from basophils. Preincubation of basophils with Tat protein upregulated the surface expression of the CCR3 receptor. Extracellular Tat can influence the directional migration of human Fc epsilon RI(+) cells, the expression of chemokine receptor CCR3, and the release of T(H)2 cytokines. Because Tat protein is actively released by HIV-1-infected cells, our results indicate a novel mechanism by which Fc epsilon RI(+) cells are rendered more susceptible to infection with CCR3-tropic HIV-1 isolates; that is, two HIV-1 proteins, gp120 and Tat, trigger the release of cytokines critical for T(H)2 polarization from Fc epsilon RI(+) cells, and Tat upregulates beta-chemokine receptor CCR3 on these cells.

PMID: 11149986 [PubMed - indexed for MEDLINE]

1599. Ann Pathol. 2000 Dec;20(6):564-9.

[Evaluation of histological criteria for bullous pemphigoid. Correlation with antigens recognized by immunoblotting of anti-epidermal autoantibodies].

[Article in French]

Courville P, Kupfer I, Gilbert D, Thomine E, Metayer J, Joly P.

Service d'Anatomie Pathologique, Unité INSERM U519, CHU Charles Nicolle, Rouen. Anatomie-Pathologie@chu-rouen.fr

The aim of this study was to evaluate the histological findings observed in patients with bullous pemphigoid in whom the diagnosis of bullous pemphigoid could be confirmed by direct immunofluorescence and immunoblot serum analysis. Seven histological criteria were considered for selection of skin biopsy specimens: 1) cleavage of dermal epidermal junction; 2) migration of eosinophils along dermal epidermal junction; 3) presence of intra epidermal eosinophils (with or without associated spongiosis); 4) absence of keratinocyte necrosis; 5) absence of acantholysis; 6) absence of dermal fibrosis; 7) absence of vasculitis. Depending on the number of criteria observed the histological picture was considered as: highly suggestive, suggestive or poorly suggestive of bullous pemphigoid. The histological picture was considered as highly suggestive in 50% of cases, suggestive or poorly suggestive in 37% and 13% of cases respectively. Migration of eosinophils along dermal epidermal junction was observed in 23 biopsy specimens (50%). Histological findings considered as poorly suggestive of bullous pemphigoid consisted of a prurigo-like or an eczematous-like or a drug induced-like picture or no specific cutaneous erosions. An histological picture highly suggestive of bullous pemphigoid was observed in 67% of patients whose serum contained anti-BPAG2 antibodies and in only 36% patients of without anti-BPAG2 antibodies (p=0,04). On the contrary, only one bullous pemphigoid patient (4%) with circulating anti-BPAG2 antibodies had a histological picture poorly suggestive of bullous pemphigoid. These findings are in accordance with the pathogenic properties of anti-BPAG2 antibodies demonstrated in animal models. This study showed that: 1) typical histological findings of bullous pemphigoid are only observed in 50% of skin biopsy specimens. 2) The diagnosis of bullous pemphigoid should be considered in elderly patients even when a poorly specific prurigo-like or eczematous-like histological picture is observed. Moreover, it underlines the usefulness of direct immunofluorescence of skin biopsy specimens and indirect immunofluorescence and immunoblot analysis of serum in such atypical cases of bullous pemphigoid.

PMID: 11148352 [PubMed - indexed for MEDLINE]

1600. J Immunol. 2001 Jan 15;166(2):1233-40.

Rapid up-regulation of CXC chemokines in the airways after Ag-specific CD4+ T cell activation.

Knott PG, Gater PR, Dunford PJ, Fuentes ME, Bertrand CP.

Inflammatory Diseases Unit, Roche Bioscience, Palo Alto, CA 94304, USA.

Ag-specific activation of CD4(+) T cells is known to be causative for the cytokine production associated with lung allergy. Chemokine-induced leukocyte recruitment potentially represents a critical early event in Ag-induced lung inflammation. Whether Ag-specific, lung CD4(+) T cell activation is important in

lung chemokine production is currently not clear. Using alphabeta-TCR transgenic BALB/c DO11.10 mice, we investigated the ability of Ag-specific CD4(+) T cell activation to induce lung chemokine production and leukocyte recruitment. Within 1 h of exposure of DO11. 10 mice to OVA aerosol, lung mRNA and protein for the neutrophil chemokines KC and macrophage inflammatory protein (MIP)-2 were greatly increased. Accordingly, neutrophils in the airways increased by >50-fold, and KC and MIP-2 proved to be functional because their neutralization significantly reduced airway neutrophilia. CD4(+) T cell activation was critical because CD4(+) but not CD8(+) T cell depletion reduced KC production, which correlated well with the previously observed inhibition of neutrophil influx after CD4(+) T cell depletion. In vitro studies confirmed that OVA-induced KC and MIP-2 production was conditional upon the interaction of CD4(+) T cells with APCs. A likely secondary mediator was TNF-alpha, and a probable source of these chemokines in the lung was alveolar macrophages. Thus, Ag-specific CD4(+) T cell activation in the lung leads to rapid up-regulation of neutrophil chemokines and the recruitment of neutrophils to the site of Ag exposure. This may be a key early event in the pathogenesis of Ag-induced lung inflammation.

PMID: 11145706 [PubMed - indexed for MEDLINE]

1601. J Immunol. 2001 Jan 15;166(2):996-1002.

Chemokine receptor expression and function in CD4+ T lymphocytes with regulatory activity.

Sebastiani S, Allavena P, Albanesi C, Nasorri F, Bianchi G, Traidl C, Sozzani S, Girolomoni G, Cavani A.

Laboratory of Immunology, Istituto Dermopatico dell'Immacolata, IRCCS, Rome, Italy.

We have investigated the chemokine receptor expression and migratory behavior of a new subset of nickel-specific skin-homing regulatory CD4(+) T cells (Th(IL-10)) releasing high levels of IL-10, low IFN-gamma, and undetectable IL-4. These cells inhibit in a IL-10-dependent manner the capacity of dendritic cells to activate nickel-specific Tc1 and Th1 lymphocytes. RNase protection assay and FACS analysis revealed the expression of a vast repertoire of chemokine receptors on resting Th(IL-10), including the Th1-associated CXCR3 and CCR5, and the Th2-associated CCR3, CCR4, and CCR8, the latter at higher levels compared with Th2 cells. The most active chemokines for resting Th(IL-10), in terms of calcium mobilization and in vitro migration, were in order of potency: CCL2 (monocyte chemoattractant protein-1, CCR2 ligand), CCL4 (macrophage-inflammatory protein-1beta, CCR5 ligand), CCL3 (macrophage-inflammatory protein-1alpha, CCR1/5 ligand), CCL17 (thymus and activation-regulated chemokine, CCR4 ligand), CCL1 (I-309, CCR8 ligand), CXCL12 (stromal-derived factor-1, CXCR4), and CCL11 (eotaxin, CCR3 ligand). Consistent with receptor expression down-regulation, activated Th(IL-10) exhibited a reduced or absent response to most chemokines, but retained a significant migratory capacity to I-309, monocyte chemoattractant protein-1, and thymus and activation-regulated chemokine. I-309, which was ineffective on Th1 lymphocytes, attracted more efficiently Th(IL-10) than Th2 cells. I-309 and CCR8 mRNAs were not detected in unaffected skin and were up-regulated at the skin site of nickel-allergic reaction, with an earlier expression kinetics compared with IL-10 and IL-4. Results indicate that skin-homing regulatory Th(IL-10) lymphocytes coexpress functional Th1- and Th2-associated chemokine receptors, and that CCR8/I-309-driven recruitment of both resting and activated Th(IL-10) cells may be critically involved in the regulation of Th1-mediated skin allergic disorders.

PMID: 11145678 [PubMed - indexed for MEDLINE]

1602. Pol Merkur Lekarski. 2000 Oct;9(52):649-52.

[Expression of adhesion molecules LFA-1 (CD11a and ICAM-1 (CD54) on lymphocytes and chemokines IL-8 and MCP-1 concentrations in bronchoalveolar lavage of patients with asthma or chronic obstructive pulmonary disease].

[Article in Polish]

Jahnz-Rózyk K, Chciałowski A, Pirozyńska E, Rogalewska A.

Kliniki Chorób Wewnetrznych, Pneumonologii i Alergologii.

Chemokines and cellular adhesion molecules are crucial determinants of the migration of immune effector cells to the tissues asthma and chronic obstructive pulmonary disease (COPD) are a complex of conditions, which have airflow limitation in common. The aim of this study was to determine the numbers and percentages of lymphocytes expressing adhesion molecules: LFA-1, ICAM-1 together with assessment of chemokines concentrations: IL-8 and MCP-1 in bronchoalveolar lavage fluid (BAL) of patients with asthma or chronic obstructive pulmonary disease (COPD). 12 patients with asthma, 14 patients with COPD, and 6 subjects of control group took part in this study. The expression of LFA-1 and ICAM-1 was assessed on lymphocytes by using immunohistochemistry (streptavidyn-biotin, DAKO, Denmark). ELISA test was used to measure IL-8 and MCP-1 concentrations in BAL (kits from R&D, USA). The percentage of lymphocytes expressing LFA-1 and ICAM-1 were: 33.9 +/- 23.8% and 25.8 +/- 12.2% in COPD patients, 23.9 +/- 12.1% and 15.3 +/- 4.42% in asthma patients, and 14.2 +/- 10% and 5.2 +/- 1.6% in the control group respectively. There was observed significant difference between the percentage of lymphocytes expressing LFA-1 and ICAM-1 of COPD and the control group. The concentrations of IL-8 were: 2306 +/- 1501 pg/ml in COPD, 233 +/- 27.3 pg/ml in asthma and 64 +/- 28.7 in the control group ($p < 0.05$). The concentrations of MCP-1 were: 768.9 +/- 668.1 pg/ml in COPD, 126.8 +/- 30.8 pg/ml in asthma, and 83.0 +/- 16.4 pg/ml in the control group ($p < 0.05$). There was observed correlation between lymphocytes expressing LFA-1 and IL-8 concentration ($r = +0.5$, $p < 0.05$) and between lymphocytes expressing LFA-1 and MCP-1 concentration ($r = +0.5$, $p < 0.05$), and between lymphocytes expressing ICAM-1 and MCP-1 concentration ($r = +0.4$, $p < 0.05$) only in COPD patients. Our data suggest that LFA-1 and ICAM-1 are important molecules in the recruitment of leukocytes and together with IL-8 and MCP-1 may have a role in pathomechanism of inflammation in asthma and especially in COPD.

PMID: 11144049 [PubMed - indexed for MEDLINE]

1603. Int Immunol. 2001 Jan;13(1):95-103.

Inducible expression of a CC chemokine liver- and activation-regulated chemokine (LARC)/macrophage inflammatory protein (MIP)-3 alpha/CCL20 by epidermal keratinocytes and its role in atopic dermatitis.

Nakayama T, Fujisawa R, Yamada H, Horikawa T, Kawasaki H, Hieshima K, Izawa D, Fujiie S, Tezuka T, Yoshie O.

Department of Bacteriology, Kinki University School of Medicine, Osaka-Sayama 589-8511, Japan.

Liver-and activation-regulated chemokine (LARC)/macrophage inflammatory protein (MIP)-3alpha/CCL20 is a CC chemokine which is constitutively expressed by follicle-associated epithelial cells in the mucosa, and attracts cells expressing

CCR6 such as immature dendritic cells and alpha(4)beta(7)(high) intestine-seeking memory T cells. Here, we examine LARC/CCL20 expression in the skin. LARC/CCL20 mRNA and protein were induced in primary human keratinocytes upon stimulation with proinflammatory cytokines such as IL-1alpha and tumor necrosis factor (TNF)-alpha. In mice, intradermal injection of IL-1alpha and TNF-alpha rapidly induced a local accumulation of transcripts for LARC/CCL20 and its receptor CCR6 with a lag of several hours in the latter. In humans, immunostaining of LARC/CCL20 was weak if any in normal skin tissues but strongly augmented in lesional skin tissues with atopic dermatitis. Furthermore, massive infiltration of cells with markers such as CD1a, CD3 or HLA-DR was present in atopic skin lesions. Many infiltrating cells were also found to be CCR6(+) by a newly generated monoclonal anti-CCR6. However, Langerhans cells residing within the epidermis were hardly stained by anti-CCR6 in normal and atopic skin tissues. Furthermore, plasma levels of LARC/CCL20 were found to be elevated in patients with atopic dermatitis. Collectively, our results suggest that epidermal keratinocytes produce LARC/CCL20 upon stimulation with proinflammatory cytokines such as IL-1alpha and TNF-alpha, and attract CCR6-expressing immature dendritic cells and memory/effector T cells into the dermis of inflamed skin such as atopic dermatitis. LARC/CCL20 may not, however, play a major role in homeostatic migration of Langerhans cells into the skin.

PMID: 11133838 [PubMed - indexed for MEDLINE]

1604. Intensive Care Med. 2000 Oct;26(10):1578.

Severe anaphylactic shock in a patient with a cystic liver lesion.

Wellhoener P, Weitz G, Bechstein W, Djonlagic H, Dodt C.

PMID: 11126277 [PubMed - indexed for MEDLINE]

1605. Immunology. 2000 Dec;101(4):442-51.

Role of CD4(+) T helper 2-type cells in cutaneous inflammatory responses induced by fluorescein isothiocyanate.

Dearman RJ, Kimber I.

Zeneca Central Toxicology Laboratory, Alderley Park, Macclesfield, Cheshire, UK.

Owing to its skin-sensitizing and fluorochromatic properties, fluorescein isothiocyanate (FITC) is employed frequently as an experimental hapten in mechanistic studies of contact allergy, particularly in the context of the role of migration and activation of Langerhans' cells. In this study we demonstrated that topical exposure of mice to FITC results in the selective development of activated lymph node cells (LNC) expressing a preferential type 2 cytokine-secretion profile, with high levels of interleukin (IL)-4 and IL-10, but low levels of interferon-gamma (IFN-gamma). Negative selection (complement depletion) identified CD4(+) T helper (Th)2-type cells as the primary source in activated LNC of the type 2 cytokines IL-4 and IL-10, whereas the low levels of IFN-gamma produced were derived exclusively from CD8(+) T cytotoxic (Tc) 1-type cells. A biphasic pattern of cutaneous inflammatory reactions was elicited by exposure to FITC, the early phase of which could be transferred passively with serum (presumably immunoglobulin E [IgE] antibody), whereas adoptive transfer experiments demonstrated that Th2-type CD4(+) cells were responsible for the delayed-type component of the dermal hypersensitivity reaction. In contrast with contact allergic reactions induced by other sensitizing haptens, which are considered to be largely Th1/Tc1-mediated immune processes regulated by Th2-type

cells, these results suggest therefore that the skin lesions provoked in mice by FITC are primarily a result of the activation of Th2-type cells.

PMCID: PMC2327104
PMID: 11122447 [PubMed - indexed for MEDLINE]

1606. Clin Exp Dermatol. 2000 Oct;25(7):522-9.

Epidemiology of atopic dermatitis.

Williams HC.

Department of Dermatology, University Hospital, Queen's Medical Centre, Nottingham, UK. hywel.williams@nottingham.ac.uk

Although research into atopic dermatitis (AD) has been dominated by the study of cells and chemical mechanisms over the last 40 years, the last 7 years has witnessed a respectable growth within the field of AD epidemiology. Significant advances include valid disease definitions that can be used in epidemiological studies, global prevalence studies, and studies which quantify the morbidity and economic cost of the disease. These have all helped to argue the case for more research into AD. Epidemiological studies demonstrating that AD is commoner in wealthier families, linkage with small family size, increased prevalence in migrant groups, and the increasing prevalence of the disease all argue strongly towards an important role for the environment in determining disease expression. Future research gaps include evaluation of gene-environment interactions, better studies of the natural history of AD, and better clinical trials that answer questions that are important to physicians and their patients.

PMID: 11122223 [PubMed - indexed for MEDLINE]

1607. J Immunol. 2000 Dec 15;165(12):6743-7.

Dendritic cell migration controlled by alpha 1b-adrenergic receptors.

Maestroni GJ.

Center for Experimental Pathology, Istituto Cantonale di Patologia, Locarno, Switzerland. icpcps@guest.cscs.ch

Dendritic cells (DC) bring Ags into lymphoid organs via lymphatic vessels. In this study, we investigated the possibility that the sympathetic neurotransmitter norepinephrine (NE) influences DC migration. Murine epidermal Langerhans cells mobilization is enhanced by systemic treatment with the alpha(2)-adrenergic antagonist yohimbine and inhibited by local treatment with the specific alpha(1)-adrenergic antagonist prazosin (PRA). Consistently, NE enhances spontaneous emigration of DC from ear skin explants, and PRA inhibits this effect. In addition, local treatment with PRA during sensitization with FITC inhibits the contact hypersensitivity response 6 days later. In vitro, bone marrow-derived immature, but not CD40-stimulated mature DC migrate in response to NE, and this effect is neutralized by PRA. NE seems to exert both a chemotactic and chemokinetic activity on immature DC. Coherently, immature, but not mature DC, express mRNA coding for the alpha(1b)-adrenergic receptor subtype. Inactivation of this adrenergic receptor by the specific and irreversible antagonist chloroethylclonidine hinders the migration of injected DC from the footpad to regional lymph nodes. Thus, besides regulating lymph flow, the sympathetic innervation of lymphatic vessels may participate in directing DC migration from the site of inflammation to regional lymph nodes. Alternatively,

the chemokinetic activity of NE may enhance the ability of DC to sample local Ags, and hence increase the number of DC migrating to the draining lymph nodes. This finding might improve our understanding of the biological basis of skin diseases and allergic reactions, and opens new pharmacological possibilities to modulate the immune response.

PMID: 11120793 [PubMed - indexed for MEDLINE]

1608. Urology. 2000 Dec 4;56(6 Suppl 1):32-40.

Injectable agents in the treatment of stress urinary incontinence in women: where are we now?

Dmochowski RR, Appell RA.

North Texas Center for Urinary Control, (RRD), Fort Worth, Texas, USA.

Periurethral bulking agents have been used for decades. The only currently available agents (in the United States) include glutaraldehyde cross-linked collagen, autologous fat, and carbon bead technology. Initial subjective cure rates with collagen are acceptable, but with the majority of women requiring reinjection. The risk of allergic phenomena complicates collagen use. Autologous fat injection is initially effective in >50% of women, but resorption and fibrous replacement hamper the stability of the transplanted graft. Polytetrafluoroethylene and silicone are not currently approved by the US Food and Drug Administration because of particle migration. Materials in development include biologic agents such as allogeneic human collagen and autologous cartilage. Developmental synthetic agents include microballoon technology, hyaluronic acid with or without microsphere technology, hydroxylapatite, and a variety of polymeric technologies. Patient selection and material characteristics influence the optimal choice for injectable agent.

PMID: 11114561 [PubMed - indexed for MEDLINE]

1609. Eur J Immunol. 2000 Dec;30(12):3533-41.

Expression of cutaneous lymphocyte-associated antigen on human CD4(+) and CD8(+) Th2 cells.

Akdis M, Klunker S, Schliz M, Blaser K, Akdis CA.

Swiss Institute of Allergy and Asthma Research (SIAF), Davos, Switzerland. akdism@siaf.unizh.ch

The cutaneous lymphocyte-associated antigen (CLA) represents the homing receptor involved in selective migration of memory/effector T cells to the skin. Numerous reports demonstrated distinct CLA expression on Th1 cells. However, T cells isolated from skin lesions and CLA(+) T cells circulating in peripheral blood of atopic dermatitis patients expressed high IL-5 and IL-13. Accordingly, we investigated the regulation of CLA on human type 1 and type 2 T cells. CLA was induced on freshly generated Th1 and Tc1 cells only, but not on those of type 2. Anti-CD3 stimulation was sufficient to induce CLA on Th2 cells in the absence of serum in the culture medium. In serum containing medium, IL-4 inhibited CLA and related alpha-fucosyltransferase mRNA expression. IL-12 and/or staphylococcal enterotoxin B (SEB) stimulation up-regulated CLA expression on either Th2 and Tc2 cells. On stimulation with IL-12, CLA was expressed on the surface of bee venom phospholipase A(2)-specific Th1, Th2, Th0 and T regulatory 1 clones, representing non-skin-related antigen-specific T cells. In addition, CLA could be re-induced

on T cells that had lost CLA expression upon resting. These results suggest that skin-selective homing is not restricted to functional and phenotypic T cell subsets.

PMID: 11093173 [PubMed - indexed for MEDLINE]

1610. Minerva Med. 2000 May-Jun;91(5-6):113-6.

Moderate-dose intravenous immunoglobulin treatment of Job's syndrome. Case report.

Bilora F, Petrobelli F, Boccioletti V, Pomerri F.

Department of Surgery and Medicine, Padua University.

Job's syndrome (or hyperimmunoglobulinemia E syndrome) is a rare genetic disease characterized by skin eczema, pyogenic "cold" abscesses, sinopulmonary recidivous infections and high IgE plasma concentrations. Job's syndrome treatment is not satisfactory and cases studied are still limited. To describe the effects of IVIG therapy in a 37-year-old woman with hyper IgE syndrome and pneumonia. We measured IgE serum by immuno-fluorometric test and neutrophil chemotaxis by migration in a Boyden chamber before and after IVIG therapy. A moderate dose of IVIG resolved the clinical-radiological signs of the S. aureus bronchopneumonia and improved cytologic and biohumoral parameters. Intravenous immunoglobulins represent a useful treatment for acute pneumonia in Job's syndrome.

PMID: 11084845 [PubMed - indexed for MEDLINE]

1611. Ann N Y Acad Sci. 2000;919:205-13.

New technologies to prevent and treat contact hypersensitivity responses.

Takashima A, Mummert M, Kitajima T, Matsue H.

Department of Dermatology, University of Texas Southwestern Medical Center, Dallas 75235, USA. atakas@mednet.swmed.edu

Allergic contact dermatitis is a common inflammatory skin disease caused by T cells that recognize environmental and industrial allergens (i.e., haptens). Langerhans' cells (LC), which are skin-specific and "immature" members of the dendritic cell (DC) family of antigen-presenting cells, play crucial roles in the induction of contact hypersensitivity (CH) responses. Upon exposure to haptens, LC migrate from the epidermis to draining lymph nodes, mature into T cell-stimulatory DC, and activate hapten-reactive T cells. Therefore, CH responses should be preventable at the sensitization phase by interfering with one of these changes that occur in LC. Our objective is to develop new technologies for the prevention and treatment of allergic contact dermatitis. In this article, we will introduce three technologies that we have recently developed. First, using a phage display strategy, we have identified a 12-mer peptide (termed "peptide 1") that binds and blocks the function of hyaluronan (HA), which is known to serve as an adhesive substrate for LC migration. Local injection of peptide 1 in mice before topical application of DNFB blocked almost completely the emigration of LC from the epidermis to the draining lymph node, where antigen presentation takes place. Peptide 1 represents a new strategy that is designed to inhibit the initial event of CH. Second, we have established an in vitro experimental system to study the terminal maturation of LC during antigen-specific interaction with T cells. This experimental system, which employs a long-term LC line and T cell clones, should provide a unique tool for

the identification of new immunosuppressive agents that block LC terminal maturation selectively. Finally, under the hypothesis that LC, which are engineered to overexpress a death ligand, would deliver apoptotic signals instead of activation signals to T cells, we created a "killer" LC clone by introducing CD95L cDNA into our long-term LC line XS106. In vivo administration of DNFB-pulsed killer LC into mice, either before or after sensitization, resulted in marked suppression of CH responses to DNFB. The killer LC technology represents an entirely new immunosuppressive therapy that is designed to eliminate only the pathogenic T cells.

PMID: 11083110 [PubMed - indexed for MEDLINE]

1612. J Allergy Clin Immunol. 2000 Nov;106(5 Suppl):S264-9.

Migration of helper T-lymphocyte subsets into inflamed tissues.

Lukacs NW.

Department of Pathology, University of Michigan Medical School, Ann Arbor, 48109-0602, USA.

The localization of lymphocytes to specific tissues is a finely regulated event that has key implications in the development of chronic allergic inflammation that is associated with allergic rhinitis, atopic dermatitis, and asthma. Key players in the tissue localization of lymphocytes and other allergic effector cells include cellular adhesion molecules and chemokines. The expression or activation pattern of these proinflammatory mediators appears to depend, in part, on the local cytokine milieu. For instance, the T(H)1 phenotype is associated with the upregulation of intercellular adhesion molecule-1 and RANTES, whereas the T(H)2 phenotype is associated with the upregulation of vascular cell adhesion molecule and P-selectin. Notably, the recruitment of certain cell populations, such as eosinophils (hallmark of chronic allergic inflammation), into inflamed tissue sites is dependent on the preferential expression of adhesion molecules, chemokines, and associated receptors. The potential mechanisms that underlie cell migration into inflamed tissue as currently understood are reviewed.

PMID: 11080742 [PubMed - indexed for MEDLINE]

1613. J Allergy Clin Immunol. 2000 Nov;106(5):918-24.

Expression and function of P-selectin glycoprotein ligand 1 (CD162) on human basophils.

Taylor ML, Brummet ME, Hudson SA, Miura K, Bochner BS.

Department of Medicine, Division of Clinical Immunology, The Johns Hopkins University School of Medicine, Johns Hopkins Asthma and Allergy Center, Baltimore, MD 21224-6801, USA.

BACKGROUND: The endothelial cell adhesion molecule P-selectin may contribute to selective leukocyte migration in allergic diseases by binding to its ligand, P-selectin glycoprotein ligand 1 (PSGL-1), on eosinophils and other leukocytes. Although expression of PSGL-1 on basophils has been detected in leukocyte typing workshops, its function on basophils has not been explored.
OBJECTIVE: We sought to characterize the expression and function of PSGL-1 on human basophils and a basophil-like cell line (KU812) and to compare these characteristics with those for PSGL-1 on eosinophils and neutrophils.
METHODS: Basophils, eosinophils, and neutrophils were enriched from peripheral

blood by using density gradient centrifugation and immunomagnetic negative selection. KU812 cells were cultured by using standard techniques. Indirect immunofluorescence and flow cytometry were used to determine surface PSGL-1 expression under various conditions, and Western blotting was used to analyze the molecular forms of PSGL-1 on each cell type. Static adhesion assays were performed by using immobilized recombinant P-selectin and relevant blocking antibodies. Histamine release assays were done by using adherent and nonadherent basophils to determine whether adhesion by means of PSGL-1 altered basophil releasability.
RESULTS: The expression of PSGL-1 on basophils was similar to that on neutrophils but was approximately 30% less bright than levels on eosinophils. Levels on basophils were 10-fold higher than on KU812 cells. Basophil activation by means of IgE cross-linking resulted in reductions in surface expression of PSGL-1 and L-selectin, as well as increased CD11b expression. Western blot analysis of PSGL-1 revealed that the molecular weights of the bands for neutrophils and basophils were similar, whereas those for eosinophils were of greater molecular weights. Static adhesion assays demonstrated that basophils bound well to P-selectin, whereas KU812 cells bound poorly. Adhesion of basophils to P-selectin was completely blocked by antibodies to either P-selectin or PSGL-1. Finally, adhesion to P-selectin did not alter the magnitude or kinetics of anti-IgE-induced histamine release.
CONCLUSION: Expression of PSGL-1 on basophils is more similar to that on neutrophils than that on eosinophils. KU812 cells express much lower levels of this molecule but, like basophils and other cells, bind to P-selectin by means of PSGL-1. P-selectin expression at sites of allergic inflammation is likely to play an important role in human basophil recruitment, but adhesion by means of PSGL-1 does not alter IgE-dependent basophil histamine release.

PMID: 11080715 [PubMed - indexed for MEDLINE]

1614. Blood. 2000 Nov 15;96(10):3601-9.

VCAM-1 has a tissue-specific role in mediating interleukin-4-induced eosinophil accumulation in rat models: evidence for a dissociation between endothelial-cell VCAM-1 expression and a functional role in eosinophil migration.

Larbi KY, Allen AR, Tam FW, Haskard DO, Lobb RR, Silva PM, Nourshargh S.

BHF Cardiovascular Medicine Unit, Imperial College School of Medicine at the National Heart and Lung Institute, London, United Kingdom.

Eosinophil accumulation has been associated with the pathogenesis of numerous allergic inflammatory disorders. Despite the great interest in this response, many aspects of eosinophil accumulation remain unknown. This is particularly true with respect to tissue-specific mechanisms that may regulate the accumulation of eosinophils in different organs. This study addressed this issue by investigating and comparing the roles of alpha(4)-integrins and vascular cell adhesion molecule 1 (VCAM-1) adhesion pathways in interleukin 4 (IL-4)-induced eosinophil accumulation in 2 different rat models of inflammation, namely pleural and cutaneous inflammation. Similar to our previous findings in studies in rat skin, locally administered IL-4 induced a time- and dose-dependent accumulation of eosinophils in rat pleural cavities, a response that was associated with generation of the chemokine eotaxin. The IL-4-induced eosinophil accumulation in skin and pleural cavities was totally inhibited by an antirat alpha(4)-integrins monoclonal antibody (mAb) (TA-2). In contrast, whereas an antirat VCAM-1 mAb (5F10) totally blocked the response in skin, IL-4-induced eosinophil accumulation in rat pleural cavities was not affected by VCAM-1 blockade. A radiolabeled mAb technique demonstrated that endothelial-cell VCAM-1 expression was induced in response to IL-4 in both skin and pleural membrane. The results indicate that

although endothelial-cell VCAM-1 is present in skin and pleura, a functional role for it in IL-4-induced eosinophil accumulation was evident only in skin. These findings suggest the existence of tissue-specific adhesive mechanisms in regulating leukocyte migration in vivo and demonstrate a dissociation between VCAM-1 expression and eosinophil accumulation.

PMID: 11071660 [PubMed - indexed for MEDLINE]

1615. Morphologie. 2000 Jun;84(265):45-9.

[Implication of extracellular matrix metalloproteinases in the course of chronic inflammatory airway diseases].

[Article in French]

Lechapt-Zalcman E, Escudier E.

INSERM U492, Faculté de Médecine, Université Paris XII, France.
emmanuele.lechapt@hmn.ap-hop-paris.fr

Matrix metalloproteinases (MMPs) are major proteolytic enzymes that are involved in extracellular matrix (ECM) turn over. MMP-2 (gelatinase A) and MMP-9 (gelatinase B) cleave type IV collagen, which is an important constituent of basement membrane. These enzymes play an important role in normal tissue homeostasis, but imbalance between MMPs and their tissue inhibitors (TIMPs) is thought to be a critical factor in regulating tissue remodeling. MMP-2 is produced by fibroblasts, endothelial, and epithelial cells, while MMP-9 is mainly produced by inflammatory cells. The role of MMPs was investigated through biochemical analysis or in situ expression, in the pathogenesis of two chronic inflammatory airway diseases, asthma and nasal polyposis. Both are characterized with the accumulation of active inflammatory cells, matrix remodeling and epithelial changes. Increased levels of MMP-9 and TIMP-1 were found in asthmatic subjects and NP. In NP, MMP-9 expression was detected in epithelial, endothelial and inflammatory cells. In this setting, MMP-9 could play a crucial role in the transmigration of basement membrane components by inflammatory cells leading to inflammatory cell accumulation and maintenance of inflammation in airway. Moreover, MMP-9 may contribute to cell migration, an important mechanism involved in the repair of the respiratory epithelium.

PMID: 11048298 [PubMed - indexed for MEDLINE]

1616. Am J Respir Crit Care Med. 2000 Oct;162(4 Pt 2):S179-84.

Biotherapeutic targets for the treatment of allergic airway disease.

Coyle AJ, Lloyd CM, Gutierrez-Ramos JC.

Millennium Pharmaceuticals, Inc., Cambridge, Massachusetts, USA. Coyle@mpi.com

T cells are critical mediators of inflammation and as such, their migration to inflammatory sites is a tightly controlled process involving a complex series of molecules expressed by a variety of cell types. As our appreciation of the mechanisms governing T cell surveillance, activation, differentiation, and subsequent homing to sites of inflammation has advanced, the opportunity to develop novel therapeutic agents that modulate the immune system has increased. Importantly, the possibility of specifically targetting subpopulations of effector cells raises the exciting potential for the development of novel agents that selectively modify the immune response to allergens, without resulting in

generalized immune suppression.

PMID: 11029391 [PubMed - indexed for MEDLINE]

1617. Physiol Rev. 2000 Oct;80(4):1669-99.

Role of platelet-activating factor in cardiovascular pathophysiology.

Montrucchio G, Alloatti G, Camussi G.

Laboratorio di Immunopatologia Renale, Dipartimento di Medicina Interna, Dipartimento di Biologia Animale e dell'Uomo e Istituto Nazionale di Fisica della Materia, Università di Torino, Torino, Italy.

Platelet-activating factor (PAF) is a phospholipid mediator that belongs to a family of biologically active, structurally related alkyl phosphoglycerides. PAF acts via a specific receptor that is coupled with a G protein, which activates a phosphatidylinositol-specific phospholipase C. In this review we focus on the aspects that are more relevant for the cell biology of the cardiovascular system. The in vitro studies provided evidence for a role of PAF both as intercellular and intracellular messenger involved in cell-to-cell communication. In the cardiovascular system, PAF may have a role in embryogenesis because it stimulates endothelial cell migration and angiogenesis and may affect cardiac function because it exhibits mechanical and electrophysiological actions on cardiomyocytes. Moreover, PAF may contribute to modulation of blood pressure mainly by affecting the renal vascular circulation. In pathological conditions, PAF has been involved in the hypotension and cardiac dysfunctions occurring in various cardiovascular stress situations such as cardiac anaphylaxis and hemorrhagic, traumatic, and septic shock syndromes. In addition, experimental studies indicate that PAF has a critical role in the development of myocardial ischemia-reperfusion injury. Indeed, PAF cooperates in the recruitment of leukocytes in inflamed tissue by promoting adhesion to the endothelium and extravascular transmigration of leukocytes. The finding that human heart can produce PAF, expresses PAF receptor, and is sensitive to the negative inotropic action of PAF suggests that this mediator may have a role also in human cardiovascular pathophysiology.

PMID: 11015622 [PubMed - indexed for MEDLINE]

1618. Immunology. 2000 Sep;101(1):68-75.

In vitro treatment of human transforming growth factor-beta1-treated monocyte-derived dendritic cells with haptens can induce the phenotypic and functional changes similar to epidermal Langerhans cells in the initiation phase of allergic contact sensitivity reaction.

Aiba S, Manome H, Yoshino Y, Tagami H.

Department of Dermatology, Tohoku University School of Medicine, Sendai, Japan.

Human monocyte-derived dendritic cells (MoDCs) obtained from peripheral blood monocytes (PBMC) cultured with granulocyte-macrophage colony-stimulating factor (GM-CSF) and interleukin-4 (IL-4) can be activated in vitro by a variety of simple chemicals such as haptens and several metals. Recently, it has been demonstrated that transforming growth factor-beta1 (TGF-beta1) can induce further differentiation of MoDCs to the cells that share some characteristics with epidermal Langerhans cells, i.e. they contain Birbeck granules and express E-cadherin. In this study, using such TGF-beta1-treated dendritic cells

(TGF-beta1+ DCs), we examined the in vitro effects of representative haptens, i.e. NiCl2 and dinitrochlorobenzene (DNCB), on their phenotypic and functional characteristics, comparing with those reported in vivo in epidermal Langerhans cells during the sensitization phase of a contact sensitivity reaction. Treatment of TGF-beta1+ DCs with NiCl2 increased their expression of the molecules related to antigen presentation such as CD86, major histocompatibility complex class I and class II, and CD83, although weakly, in addition to that of those essential for their migration to the regional lymph nodes, such as CD49e, CD44 and its variant 6, while it down-regulated the expression of the molecules required for homing to the skin and staying in the epidermis, such as cutaneous leucocyte antigen (CLA) and E-cadherin. It also increased the production of tumour necrosis factor-alpha, but not that of IL-1beta or IL-12. DNCB also increased their CD86 expression and down-regulated E-cadherin and CLA, but did not affect other phenotypic changes that were observed in TGF-beta1+ DCs treated with NiCl2. TGF-beta1+ DCs treated with either NiCl2 or DNCB increased their allogeneic T-cell stimulatory function. In addition, reverse transcribed polymerase chain reaction revealed augmented expression of chemokine receptor 7 mRNA by TGF-beta1+ DCs when treated with either NiCl2 or DNCB. Moreover, consistent with this data, TGF-beta1+ DCs treated with these chemicals chemotactically responded to macrophage inflammatory protein-3beta. These data suggest the possibility that TGF-beta1+ DCs present a good in vitro model to study the biology of epidermal Langerhans cells.

PMCID: PMC2327054
PMID: 11012755 [PubMed - indexed for MEDLINE]

1619. Histol Histopathol. 2000 Oct;15(4):1169-76.

Integrin activation by chemokines: relevance to inflammatory adhesion cascade during T cell migration.

Tanaka Y.

The First Department of Internal Medicine, University of Occupational and Environmental Health, School of Medicine, Kitakyushu, Japan.
tanaka@med.uoech-u.ac.jp

The adhesive function of integrins is regulated through cytoplasmic signaling induced by several stimuli, whose process is designated "inside-out signaling". A large number of leukocytes are rapidly recruited to the sites of inflammation where they form an essential component of the response to infection, injury, autoimmune disorders, allergy, tumor invasion, atherosclerosis and so on. The recruitment of leukocytes into tissue is regulated by a sequence of interactions between the circulating leukocytes and the endothelial cells. Leukocyte integrins play a pivotal role in leukocyte adhesion to endothelial cells. During the process, the activation of integrins by various chemoattractants, especially chemokines, is essential for integrin-mediated adhesion in which a signal transduced to the leukocyte converts the functionally inactive integrin to an active adhesive configuration. We have proposed that H-Ras-sensitive activation of phosphoinositide 3 (PI 3)-kinase and subsequent profilin-mediated actin polymerization, can be involved in chemokine-induced integrin-dependent adhesion of T cells. The present review documents the relevance of cytoplasmic signaling and cytoskeletal assembly to integrin-mediated adhesion induced by chemoattractants including chemokines during inflammatory processes. In contrast, various adhesion molecules are known to transduce extracellular information into cytoplasm, which leads to T cell activation and cytokine production from the cells, designated "outside-in signaling". Such a bi-directional "cross-talking" among adhesion molecules and cytokines is most relevant to inflammatory processes by augmenting immune cell migration from circulation into inflamed tissue such as

rheumatoid arthritis, tumor invasion, Behçet's disease and atherosclerosis.

PMID: 11005242 [PubMed - indexed for MEDLINE]

1620. Am J Respir Crit Care Med. 2000 Sep;162(3 Pt 1):1016-22.

Soluble intercellular adhesion molecule-1 (sICAM-1) and interferon-gamma in bronchoalveolar lavage fluid from children with airway diseases.

Marguet C, Dean TP, Warner JO.

Pediatric Respiratory Disease Unit, Hôpital Ch. Nicolle, Rouen, France.

We have previously described that in bronchoalveolar lavage fluid (BALF), eosinophils characterize asthma and neutrophils are more prominent in infantile wheeze. In this study, we hypothesized that intercellular adhesion molecule 1 (ICAM-1) and interferon-gamma (IFN-gamma) would have a role in promoting migration of both cell types into the airway. To investigate this, we measured soluble (s) ICAM-1 in 68 BALFs from infants and young children with various respiratory problems. Children with asthma were characterized by significantly raised sICAM compared with those with chronic cough without wheeze (p = 0.05) or control subjects with no lower airway pathology (p = 0.045). The levels correlated with disease severity (evaluated with a symptom score) and with lymphocyte numbers. IFN-gamma levels were also raised in children with asthma compared with those with chronic cough (p = 0.05), but there was no correlation with disease activity. Infantile wheeze was characterized by a linear correlation between sICAM-1 and IFN-gamma (r = 0.55; p = 0.002). sICAM-1 levels in infantile wheeze correlated with the severity of the disease and lymphocyte numbers. IFN-gamma levels were elevated in the wheezers treated with inhaled steroids compared with untreated infants (p = 0.03). Although sICAM-1 levels were increased in those with severe cough, no characteristic inflammatory profile was found in the group with chronic cough. Our study suggests that ICAM-1 and IFN-gamma play a role in the activity of the inflammatory process in asthma in childhood and possibly in some infant wheezers, in whom IFN-gamma may be one of the factors increasing the expression of ICAM-1. The role of IFN-gamma, a T helper-1 cytokine, in children with asthma remains to be fully understood.

PMID: 10988123 [PubMed - indexed for MEDLINE]

1621. J Biomed Mater Res. 2000 Sep;53(5):480-9.

A triple assay technique for the evaluation of metal-induced, delayed-type hypersensitivity responses in patients with or receiving total joint arthroplasty.

Hallab NJ, Mikecz K, Jacobs JJ.

Department of Orthopedic Surgery, Rush-Presbyterian-St. Luke's Medical Center, Chicago, IL 60612, USA. nhallab@rush.edu

The determination of biocompatibility has been dominated historically by the characterization of candidate materials based upon the observation of adverse host responses. However, some adverse responses are subtle in clinical settings and continue to foster debate and investigation. One of these responses is "metal allergy" or hypersensitivity to metallic biomaterials. Current methods used to diagnose hypersensitivity reactions, such as dermal patch testing and migration inhibition assays, are not well accepted in orthopedic practice as a means for the characterization of hypersensitivity to metallic joint-replacement

components. An increasing need to resolve whether metal sensitivity may be a significant and/or predisposing factor for eliciting an over-aggressive immune response in patients with metallic implant components requires improved and standardized widespread study. Here we present three in vitro methodologies: (1) a proliferation assay, (2) cytokine analysis using ELISA, and (3) a migration inhibition assay. When in conjunction with one another, these assays may be used to more comprehensively quantify metal-induced hypersensitivity responses. Therefore, these methodologies are detailed with the intent of facilitating multi-center large-scale studies. In the following cases, a multi-assay approach for measuring the prevalence of delayed-type hypersensitivity in orthopedic patients shows the propensity to yield a more comprehensive and, therefore, more conclusive determination than currently employed patch testing or single assay techniques.

Copyright 2000 John Wiley & Sons, Inc.

PMID: 10984695 [PubMed - indexed for MEDLINE]

1622. Clin Exp Allergy. 2000 Sep;30(9):1244-9.

Macrophage migration inhibitory factor (MIF) in bronchial asthma.

Yamaguchi E, Nishihira J, Shimizu T, Takahashi T, Kitashiro N, Hizawa N, Kamishima K, Kawakami Y.

The First Department of Medicine, School of Medicine, Hokkaido University, Japan.

Comment in
    Clin Exp Allergy. 2000 Sep;30(9):1194-6.

BACKGROUND: Macrophage migration inhibitory factor (MIF) is a pro-inflammatory cytokine favouring the secretion of TNFalpha and IL-8 and counteracts anti-inflammatory effects of corticosteroids. Airways inflammation is a central feature of bronchial asthma and is characterized by the accumulation of eosinophils.
OBJECTIVE: The aim of this study was to investigate whether MIF is related to asthma symptoms and eosinophil accumulation in the airways.
METHODS: Serum MIF levels were measured by an enzyme-linked immunosorbent assay in 44 healthy subjects and 44 asthmatics. Levels of MIF in induced sputum were measured in 10 healthy subjects and 15 asthmatics. Levels of eosinophil cationic protein (ECP) in induced sputum were measured by a radioimmunosorbent assay. Fluorescence double immunostaining was conducted to examine cellular source and localization of MIF.
RESULTS: Serum MIF levels were significantly increased in asthmatic patients compared with age and sex-matched control subjects. Symptomatic patients had a higher MIF level than asymptomatic patients. Induced sputum obtained from asthmatics contained higher levels of MIF than those from control subjects. MIF levels in induced sputum were correlated with ECP levels in induced sputum. MIF was colocalized with eosinophil peroxidase staining in the cytoplasm of sputum cells.
CONCLUSION: Increased MIF levels are associated with asthma symptoms and one of the cellular sources of MIF in the airways are eosinophils.

PMID: 10971470 [PubMed - indexed for MEDLINE]

1623. Clin Exp Allergy. 2000 Sep;30(9):1194-6.

Asthma and MIF: innately Th1 and Th2.

Sabroe I, Pease JE, Williams TJ.

Comment on
    Clin Exp Allergy. 2000 Sep;30(9):1244-9.

PMID: 10971462 [PubMed - indexed for MEDLINE]

1624. Am J Respir Cell Mol Biol. 2000 Sep;23(3):379-88.

The effect of transendothelial migration on eosinophil function.

Yamamoto H, Sedgwick JB, Vrtis RF, Busse WW.

Pulmonary Division, Second Department of Internal Medicine, Saitama Medical School, Saitama, Japan.

In bronchial asthma, eosinophils found in the airways have an enhanced inflammatory capacity. We hypothesized that, at least in part, changes in functional phenotype are due to the effect of transendothelial migration. To model in vivo eosinophil trafficking to the lung, we cultured human pulmonary microvascular endothelial cell (HPMEC) monolayers on Transwell filters. The HPMECs were activated with interleukin (IL)-1beta to increase cell expression of intercellular adhesion molecule (ICAM)-1 and, hence, eosinophil transmigration. Peripheral blood eosinophils from allergic patients were added to HPMEC-covered Transwell filters and incubated for 3 h at 37 degrees C. The eosinophils were collected from below (migrated cells) and above (nonmigrated cells) the HPMEC monolayer to determine surface receptor expression, in vitro survival, and oxidative burst. Eosinophils never exposed to HPMECs were used as controls. Eosinophil cell surface expression of CD69, human leukocyte-associated antigen-DR (HLA-DR), and CD54 (ICAM-1) was significantly increased after transendothelial migration through IL-1beta-treated HPMECs compared with control cells (CD69: P<0.0005; HLA-DR and CD54: P<0.05) and nonmigrated eosinophils (CD69 and HLA-DR: P<0.05). Moreover, the percent in vitro survival (48 h) of migrated eosinophils was also significantly greater (P<0.0001 by trypan blue exclusion, P< 0.05 by flow cytometry) than that of control or nonmigrated eosinophils. Prolonged survival of migrated eosinophils was inhibited by addition of anti-granulocyte macrophage colony-stimulating factor (GM-CSF) antibodies (P<0.05) to the 48-h survival culture, suggesting that autocrine production of GM-CSF was, at least partially, responsible for increased eosinophil survival. Although GM-CSF protein was not measurable in survival culture supernates, GM-CSF messenger RNA (mRNA) was expressed in both nonmigrated and migrated eosinophils but not in control cells. Similarly, the eosinophils' oxidative burst induced by platelet-activating factor, formylmethionyl leucylphenylalanine, or phorbol myristate acetate was equally, and significantly, increased in both nonmigrated and migrated eosinophils (P<0.05 versus control). Therefore, whereas exposure of eosinophils to cytokine-activated HPMECs can increase surface receptor expression, in vitro survival, GM-CSF mRNA, and the respiratory burst, transendothelial migration can further potentiate receptor expression and survival in migrated cells. These results suggest that the process of transendothelial migration selectively participates in determining the eventual phenotype of airway eosinophils.

PMID: 10970830 [PubMed - indexed for MEDLINE]

1625. J Biol Chem. 2000 Nov 24;275(47):36626-31.

Identification of potent, selective non-peptide CC chemokine receptor-3 antagonist that inhibits eotaxin-, eotaxin-2-, and monocyte chemotactic

protein-4-induced eosinophil migration.

White JR, Lee JM, Dede K, Imburgia CS, Jurewicz AJ, Chan G, Fornwald JA, Dhanak D, Christmann LT, Darcy MG, Widdowson KL, Foley JJ, Schmidt DB, Sarau HM.

Departments of Immunology, Biomolecular Discovery, Pulmonary Biology, Gene Expression Sciences, and Medicinal Chemistry, SmithKline Beecham Pharmaceuticals, King of Prussia, Pennsylvania 19406, USA. John_R_White@SBPHRD.COM

Eosinophils have been implicated in the pathogenesis of asthma and other allergic diseases. Several CC chemokines including eotaxin (CCL-11), eotaxin-2 (CCL-24), RANTES (CCL-5), and monocyte chemotactic protein-3 (MCP-3, CCL-7) and 4 (MCP-4, CCL-13) are potent eosinophil chemotactic and activating peptides acting through CC chemokine receptor-3 (CCR3). Thus, antagonism of CCR3 could have a therapeutic role in asthma and other eosinophil-mediated diseases. A high throughput, cellular functional screen was configured using RBL-2H3 cells stably expressing CCR3 (RBL-2H3-CCR3) to identify non-peptide receptor antagonists. A small molecule CCR3 antagonist was identified, SK&F 45523, and chemical optimization led to the generation of a number of highly potent, selective CCR3 antagonists including SB-297006 and SB-328437. These compounds were further characterized in vitro and demonstrated high affinity, competitive inhibition of (125)I-eotaxin and (125)I-MCP-4 binding to human eosinophils. The compounds were potent inhibitors of eotaxin- and MCP-4-induced Ca(2+) mobilization in RBL-2H3-CCR3 cells and eosinophils. Additionally, SB-328437 inhibited eosinophil chemotaxis induced by three ligands that activate CCR3 with similar potencies. Selectivity was affirmed using a panel of 10 seven-transmembrane receptors. This is the first description of a non-peptide CCR3 antagonist, which should be useful in further elucidating the pathophysiological role of CCR3 in allergic inflammatory diseases.

PMID: 10969084 [PubMed - indexed for MEDLINE]

1626. Postgrad Med. 2000 Sep 15;108(4 Suppl):45-53. doi: 10.3810/pgm.09.15.2000.suppl7.43.

Treating asthma in the new millennium.

Corren J.

University of California, Los Angeles, UCLA School of Medicine, USA. joncorren@hotmail.com

As the pathophysiologic process of asthma has become better defined, new molecular targets for treating asthma have emerged. Resident airway cells, circulating leukocytes, and various cell-derived mediators and cytokines contribute to the inflammatory events of asthma. New therapeutic approaches to moderate to severe asthma currently in clinical development include antibodies to IgE and interleukin-5, a soluble receptor that would sequester interleukin-4, cytokine and chemokine receptor antagonists, and phosphodiesterase type 4 inhibitors. Adhesion molecules, which are involved in the migration of inflammatory cells into lung tissue, are also important targets for new antiasthma therapies. Because of researchers' focus on specific pathologic processes and molecular targets, the adverse effects of new agents may be minimized. Also, the longer duration of action of some of the new agents allows weekly to monthly dosing, which may well enhance patient compliance.

PMID: 19667533 [PubMed - in process]

1627. Histol Histopathol. 2000 Jul;15(3):899-920.

Mechanisms underlying eosinophil trafficking and their relevance in vivo.

Cara DC, Negrao-Correa D, Teixeira MM.

Department of Pathology, Federal University of Minas Gerais, Belo Horizonte, Brazil.

After their formation in the bone marrow, eosinophils circulate with a short half-life and are distributed throughout the body, especially in mucosal and sub-mucosal regions. Although a small amount of these cells are normally seen in healthy tissue, blood and tissue eosinophilia is a hallmark of helminthic and allergic diseases. The role of eosinophils in the normal physiology of mucosal tissues is not understood, but there is good evidence to demonstrate that these cells protect the host at least against some intestinal helminths, specially those with a lung cycle. In addition, there are now many data that support a role for eosinophils in the pathophysiology of allergic diseases, such as asthma. Because helminthic diseases have been largely controlled in developed countries, there has been much interest in the development of drugs which affect eosinophil migration and/or activation in the tissue and which may, thus, be useful in the treatment of allergic conditions. The understanding of the mechanisms controlling eosinophil trafficking and/or activation are essential in the development of anti-eosinophil-based therapeutic strategies. The present paper reviews aspects of eosinophil biology with emphasis on the role of eosinophils in parasitic infections and allergy, the basic mechanisms underlying the trafficking of eosinophils into tissue and how these can be modulated pharmacologically.

PMID: 10963133 [PubMed - indexed for MEDLINE]

1628. Ann Otol Rhinol Laryngol. 2000 Aug;109(8 Pt 1):726-30.

Ultrastructural changes in platelet activating factor-induced epithelial damage in rabbit maxillary sinus mucosa.

Jeon SY, Jeong PS, Rhee CK, Min YG.

Department of Otolaryngology, GyeongSang National University, Chinju, Korea.

Platelet activating factor (PAF), a potent chemical mediator in inflammation and allergic reaction, has been thought to induce mucociliary inhibition and epithelial damage in the airway mucosa. However, several recent papers have reported that PAF may not readily damage the airway epithelium. The aim of this study was to elucidate the pathogenesis of PAF-induced epithelial damage in terms of ultrastructural changes. Sixteen micrograms of PAF (1 mL of 16 microg/mL) was administered into the maxillary sinuses of rabbits. The rabbits were divided into 2 groups according to time intervals, and the antral mucosa was taken 1 and 3 days after administration of PAF. The tissue was processed for routine transmission electron microscopy. No epithelial degeneration was observed other than platelet aggregation, red blood cell stasis, and swelling of the endothelial cells 1 day after administration of PAF. Migration of inflammatory cells into the perivascular connective tissue, infiltration of eosinophils into the subepithelial and intraepithelial spaces, and vacuolar degeneration of the epithelial cells with focal loss of cilia were seen 3 days after administration of PAF. In conclusion, PAF induced infiltration of eosinophils into the epithelium, and resulted in epithelial degeneration that varied according to the time interval. Our findings suggest that PAF may cause epithelial damage through a series of secondary events, probably due to cytotoxicity of eosinophils infiltrating the epithelium.

PMID: 10961804 [PubMed - indexed for MEDLINE]

1629. Allergy Asthma Proc. 2000 Jul-Aug;21(4):215-20.

Immunomodulation and allergy.

Knopf PM.

Department of Molecular Microbiology and Immunology, Brown University, Providence, Rhode Island 02912, USA.

A major function of the immune system is to protect the body from infection and the diseases caused by infectious agents. The immune system also provides protection against cancer cells, for once they arise, cancers can essentially behave as "foreign" cells capable of causing pathology. In contrast, allergy is a manifestation of the immune response to certain environmental cells or molecules that are usually neither a threat for infection nor cancer. Allergic reactions are generally an annoynance, even life-threatening. I will focus on type I allergy, characterized in part by induction of IgE antibody responses to allergens. It should be noted that not all IgE responses cause allergic symptoms. There is even evidence that IgE responses to tropical helminthic parasites offer a degree of immunity to reinfection. I have three objectives: (1) review T cell differentiation leading to the Th1/Th2 paradigm; (2) evaluate the increased prevalence of atopy, including asthma, as a consequence of a Th2-dominated immune system; (3) relate the high prevalence of asthma in inner city United States black children to the relatively recent migration of their ancestors from tropical regions of Africa, where genetically biased Th2-dependent IgE responses may be important in protection against high burdens of parasitic worms.

PMID: 10951887 [PubMed - indexed for MEDLINE]

1630. Thorax. 2000 Sep;55(9):775-9.

Diet and childhood asthma in a society in transition: a study in urban and rural Saudi Arabia.

Hijazi N, Abalkhail B, Seaton A.

Department of Community Medicine and Primary Health Care, Faculty of Medicine and Allied Sciences, King Abdulaziz University, Jeddah, Saudi Arabia.

BACKGROUND: The causes of the worldwide increases in asthma and allergic diseases in childhood, which seem to relate to increasing prosperity, are unknown. We have previously hypothesised that a reduction in the antioxidant component of the diet is an important factor. An investigation was undertaken of dietary and other risk factors for asthma in Saudi Arabia where major lifestyle differences and prevalences of allergic disease are found in different communities.
METHODS: From a cross sectional study of 1444 children with a mean age of 12 (SD 1) years in Jeddah and a group of rural Saudi villages, we selected 114 cases with a history of asthma and wheeze in the last 12 months and 202 controls who had never complained of wheeze or asthma, as recorded on the ISAAC questionnaire. Risk factors for asthma and allergies (family history, social class, infections, immunisations, family size, and diet) were ascertained by questionnaire. Atopy was assessed by skin prick testing.
RESULTS: In univariate analyses, family history, atopy, and eating at fast food outlets were significant risk factors for wheezy illness, as were the lowest intakes of milk and vegetables and of fibre, vitamin E, calcium, magnesium,

sodium, and potassium. These differences were present also in the urban children considered separately. Sex, family size, social class, infections, and parental smoking showed no relationship to risk. In multiple logistic regression analysis, urban residence, positive skin tests, family history of allergic disease, and the lowest intakes of vitamin E, magnesium and sodium related significantly and independently to risk. The lowest tertile of intake of vitamin E was associated with a threefold (95% CI 1.38 to 6.50) increase in risk when adjusted for the other factors. Intake of milk and vegetables both showed inverse linear relationships to being a case.
CONCLUSIONS: This study suggests that dietary factors during childhood are an important influence in determining the expression of wheezy illness, after allowing for urban/rural residence, sex, family history, and atopy. The findings are consistent with previous studies in adults and with the hypothesis that change in diet has been a determinant of the worldwide increases in asthma and allergies.

PMCID: PMC1745853
PMID: 10950897 [PubMed - indexed for MEDLINE]

1631. Pol Arch Med Wewn. 1999 Oct;102(4):865-9.

[Low molecular weight heparin (LMWH) modulates migration of peripheral blood mononuclear cells and neutrophils of asthmatics].

[Article in Polish]

Krasnowska M, Zak-Nejmark T, Krasnowski R.

Katedra i Klinika Chorób Wewnetrznych i Alergologii AM we Wrocławiu.

Evidence has now accumulated that heparin can significantly affect immune response including allergic inflammation. Cell migration is supposed to be very crucial in this process. Thus the aim of that study was to investigate whether low molecular weight heparin-nadroparine is chemoattractant for some inflammatory cells in asthmatics. Peripheral blood mononuclear cells (PBMC) and neutrophils from 15 asthmatics were obtained by gradient centrifugation. Chemotaxis was compared with that induced with known chemoattractants-fMLP and IL-8. We found that nadroparine caused significant and dose dependent chemotaxis of PBMC and comparable with influence of fMLP and IL-8. Nadroparine amplified chemotaxis of neutrophils but not significantly. Chemotaxis induced by fMLP and IL-8 was diminished when blood cells were incubated earlier with 50 mg of nadroparine.

PMID: 10948710 [PubMed - indexed for MEDLINE]

1632. Immunol Cell Biol. 2000 Aug;78(4):415-22.

Chemokine and cytokine cooperativity: eosinophil migration in the asthmatic response.

Simson L, Foster PS.

Division of Biochemistry and Molecular Biology, John Curtin School of Medical Research, Australian National University, Canberra, Australian Capital Territory.

Eosinophils play a central role in the pathophysiology of allergic disease. The mechanisms that regulate eosinophil migration are complex; however, chemokines and cytokines produced in both the early and late phases of the asthmatic response appear to cooperate in eosinophil recruitment. In particular, there

exists a unique synergy between eotaxin and IL-5. The role of chemokine/cytokine cooperativity has been investigated in the extracellular matrix, adhesion molecule/integrin interactions, receptor polarization and aggregation and the convergence and divergence of intracellular signalling pathways. Understanding the mechanisms whereby eosinophils migrate will allow the development of specific therapeutic strategies aimed at attenuating specific components of the allergic response.

PMID: 10947867 [PubMed - indexed for MEDLINE]

1633. Pol Merkur Lekarski. 2000 May;8(47):341-6.

[The role of heparin in allergic inflammation].

[Article in Polish]

Jerzyńska J, Stelmach I, Kuna P.

Oddziału Interny Dzieciecej i Alergologii Wojewódzkiego Szpitala Specjalistycznego w Zgierzu.

Heparin is a glycosaminoglican used in prophylactic and treatment of thrombosis. Heparin possesses also non-anticoagulant properties, including modulation of various proteases, anticomplement activity, and anti-inflammatory actions. Inhaled heparin has been shown to reduce early phase of asthmatic reaction and suppress allergen induced rise in bronchial hyperreactivity. Heparin inhibits the acute cutaneous reaction due to allergens. Moreover, inhaled heparin prevents exercise-induced asthma. The exact mechanism of heparin action in bronchial asthma remains obscure. It has been observed that heparin acts as a specific blocker of IP3 receptors and inhibits IP3-mediated calcium release in various cell types, including vascular smooth muscle and airway smooth muscle. In this mechanism heparin inhibits allergen induced mast cell degranulation and prevents subsequent development of reaction cascade leading to inflammation, bronchial hyperreactivity and asthma. It also modulates migration of proinflammatory cells, eosinophils and neutrophils, into the site of allergic reaction. Furthermore, heparin inhibits the increased vascular permeability induced by a wide range of agonists acting via specific receptors located on the vascular endothelial cells. The cationic peroxidases, such as major basic protein and eosinophil peroxidase, are neutralized by the highly anionic heparin; thus heparin inhibits the epithelial damage induced by some of these cationic proteins. The mechanism involved in the control of bronchial hyperreactivity by heparin has been studied little and is yet poorly understood. Heparin deserves further investigations in large number of subjects to provide further insight into the pathophysiology of asthma. Heparin may also be of clinical importance and may form the basis of novel therapeutic approaches.

PMID: 10944959 [PubMed - indexed for MEDLINE]

1634. Arerugi. 2000 Jul;49(7):600-3.

[Inhibitory effect of suplatast tosilate on eosinophil migration].

[Article in Japanese]

Kobayashi Y, Nagata M, Yamamoto H, Sakamoto Y.

Second Department of Internal Medicine, Saitama Medical School.

Suplatast tosilate has been shown to inhibit generation of Th2-type cytokines in vitro and used in the treatment of allergic diseases. Although the exact mechanism remains unknown, this compound attenuates the eosinophil infiltration in the asthmatic airway. In the present study, we examined whether suplatast tosilate directly modulates eosinophil migration in response to inflammatory mediators in vitro. Suplatast did not modify the spontaneous migration of eosinophils. On the other hand, eosinophil migration in response to PAF (1 microM) was significantly inhibited by 1 microM suplatast. Similarly, IL-5 (100 pM)-induced eosinophil migration was inhibited by 1 microM suplatast. These results suggest that suplatast tosilate inhibits eosinophil locomotion in response to PAF or IL-5 and thereby attenuates infiltration of the cells in allergic inflammation.

PMID: 10944827 [PubMed - indexed for MEDLINE]

1635. Clin Exp Immunol. 2000 Aug;121(2):339-45.

Expression of intercellular adhesion molecule-1 (ICAM-1) in nasal epithelial cells of atopic subjects: a mechanism for increased rhinovirus infection?

Bianco A, Whiteman SC, Sethi SK, Allen JT, Knight RA, Spiteri MA.

Lung Injury and Inflammation Research Group, Centre for Cell and Molecular Medicine, Keele University, and Department of Respiratory Medicine, North Staffordshire Hospital Trust, Stoke-on-Trent, UK.

Since clinical experimental studies indicate that upper respiratory tract viral infections may exacerbate acute asthma symptoms in atopic/asthmatic individuals, we have investigated the expression and modulation of ICAM-1 on human nasal epithelial cells (HNEC) from normal and atopic subjects. ICAM-1 is the attachment molecule for the majority of serotypes of human rhinovirus (HRV), including HRV-14, and is also critical for the migration and activation of immune effector cells. Basal ICAM-1 expression was significantly higher in HNEC obtained by brushings from atopic compared with non-atopic subjects (P = 0.031), and was also significantly increased on atopic HNEC harvested in season compared with out of season (P < 0.05). Atopic HNEC showed further up-regulation in ICAM-1 expression when cultured with clinically relevant allergen (P = 0.032). ICAM-1 levels on normal HNEC were also increased by infection with HRV-14 (P < 0.05). Basal expression of ICAM-1 on atopic nasal polyp epithelial cells (EC) was significantly higher than on both normal and atopic nasal HNEC. This elevated nasal polyp ICAM-1 level was not increased further by allergen, although HRV infection resulted in a small significant increase. Recovered viral titres from HRV-infected nasal polyp EC were 1.5-fold higher than from infected normal nasal HNEC. The data are consistent with the hypothesis that allergen, by enhancing expression of the HRV attachment target on host cells, facilitates viral infection in atopic subjects; simultaneously HRV-induced increases in ICAM-1 levels would favour migration and activation of immune effector cells to the airway, resulting in enhanced atopic inflammation.

PMCID: PMC1905704
PMID: 10931151 [PubMed - indexed for MEDLINE]

1636. Vestn Oftalmol. 2000 May-Jun;116(3):37-9.

[Clinical course of isolated larval infestation of orbit in children].

[Article in Russian]

Dubovskaia LA, Tumol'skaia NI, Sidorenko NI, Kotiasheva GI, Odoshashvili ED, Gorbunov AV.

Clinical course of an isolated infestation of orbital tissues by larvae of helminths parasitizing in dogs (Toxocara canis) has been followed up in 5 patients aged 6-13 years. The process ran a wave-like course for 3-8 months and was characterized by cyclic inflammatory changes in the orbit, presenting by toxic allergic tenonitis, regional lymphadenitis, optic nerve perineuritis with formation of parasitic granuloma detected by computer-aided rhoentgenotomography of the orbit. No clinical or laboratory signs of common inflammatory and allergic reaction in the presence of Toxocara antigen sensitization were observed in any case, which was confirmed by detection of specific antibodies (IgG and IgE). Specific therapy with anti-nematode drug albendazol was effective.

PMID: 10918851 [PubMed - indexed for MEDLINE]

1637. Nihon Kokyuki Gakkai Zasshi. 2000 May;38(5):385-90.

[Levofloxacin-induced eosinophilic pneumonia complicated by bronchial asthma].

[Article in Japanese]

Fujimori K, Shimatsu Y, Suzuki E, Arakawa M, Gejyo F.

Department of Internal Medicine, Niigata Prefectural Shibata Hospital, Shibata, Japan.

A 76-year-old woman who had complained of cough and productive sputum since mid-January, 1999, was admitted to our hospital with fever and dyspnea on February 4, 1999. She had been treated with levofloxacin at an outpatient clinic. On admission, she had orthopnea, and auscultation revealed coarse crackles and wheeze in the bilateral lung fields. Chest x-ray and CT films showed non-segmental infiltration in bilateral lung fields. Laboratory data revealed eosinophilia in peripheral blood (= 24%) and sputum (= 10%), airflow limitation, hypoxemia (PaO2: 46 Torr), and increased airway responsiveness to methacholine (Dmin: 0.127 units). A bronchoalveolar lavage (BAL) fluid showed increased total cells and a 55% increase in eosinophils, and CD4/CD8 ratio was decreased to 0.8. In addition, IL-5 was increased in BAL fluid. Transbronchial lung biopsy specimens revealed infiltrations of eosinophils in the alveolar and interstitial compartments. Histological features of the bronchial biopsy specimens included increased eosinophils in the submucosa and goblet cell metaplasia. The woman was diagnosed with eosinophilic pneumonia complicated by bronchial asthma. She was given theophylline, pranlukast hydrate, and an inhaled beta 2 receptor agonist (procaterol hydrochloride), and pre-admission drugs including Levofloxacin were discontinued. Her symptoms were improved, peak expiratory flow rate and PaO2 increased, airway responsiveness to methacholine decreased (Dmin: 0.615 units), and radiographic abnormalities disappeared without steroid therapy. A leukocyte migration test for levofloxacin was weakly positive. An environmental provocation test in the patient's home gave negative results. A challenge test for levofloxacin was not performed due to a lack of informed consent. Based on these findings, we diagnosed this case as levofloxacin-induced lung injury manifesting as eosinophilic pneumonia complicated by bronchial asthma. Levofloxacin should be added to the list of agents that can produce eosinophilic pneumonia.

PMID: 10921286 [PubMed - indexed for MEDLINE]

1638. Am J Respir Cell Mol Biol. 2000 Aug;23(2):182-7.

Inhaled particulate matter causes expression of nuclear factor (NF)-kappaB-related genes and oxidant-dependent NF-kappaB activation in vitro.

Shukla A, Timblin C, BeruBe K, Gordon T, McKinney W, Driscoll K, Vacek P, Mossman BT.

Departments of Pathology and Biostatistics, University of Vermont, Burlington, Vermont 05405, USA.

High levels of ambient air pollution are associated with exacerbation of asthma and respiratory morbidity, yet little is known concerning the mechanisms of inflammation and toxicity by components of inhaled particulate matter (PM). Brief inhalation of PM(2.5) (particles of an aerodynamic diameter of < 2.5 microns) (300 microg/m(3) air for 6 h followed by a period of 24 h in clean air) by either C3H/HeJ or C57/BL6 mice caused significant (P </= 0.05) increases in steady-state messenger RNA (mRNA) levels of a number of nuclear factor (NF)-kappaB-associated and/ or -regulated genes, including tumor necrosis factor-alpha and -beta, interleukin-6, interferon-gamma, and transforming growth factor-beta. Lung mRNA levels of lymphotoxin-beta and macrophage migration inhibitory factor were unchanged. In murine C10 alveolar cells and an NF-kappaB-luciferase reporter cell line, exposure to PM(2.5) at noncytotoxic concentrations resulted in increases in transcriptional activation of NF-kappaB-dependent gene expression which were inhibited in the presence of catalase. Early and persistent increases in intracellular oxidants, as measured by flow cytometry and cell imaging using the oxidant probe 2'-7'-dichlorofluoroscin diacetate, were observed in epithelial cells exposed to PM(2.5) and ultrafine carbon black particles. Studies here are the first to show NF-kappaB-related inflammatory and cytokine gene expression after inhalation of PM(2.5) and oxidant-dependent induction of NF-kappaB activity by PM(2.5) in pulmonary epithelial cells.

PMID: 10919984 [PubMed - indexed for MEDLINE]

1639. J Immunol. 2000 Aug 1;165(3):1395-402.

IL-4 enhances keratinocyte expression of CXCR3 agonistic chemokines.

Albanesi C, Scarponi C, Sebastiani S, Cavani A, Federici M, De Pità O, Puddu P, Girolomoni G.

Istituto Dermopatico dell'Immacolata, IRCCS, Rome, Italy. c.albanesi@iti.it

IFN-induced protein of 10 kDa (IP-10), monokine induced by IFN-gamma (Mig), and IFN-inducible T-cell alpha-chemoattractant (I-TAC) belong to the non-glutamate-leucine-arginine motif CXC chemokine family and act solely through the CXCR3 receptor for potent attraction of T lymphocytes. In this study, we evaluated the capacity of the T cell-derived cytokines IL-4, IL-10, and IL-17 to modulate IP-10, Mig, and I-TAC in cultured human keratinocytes and CXCR3 expression in T cells from allergic contact dermatitis (ACD). IL-4, but not IL-10 or IL-17, significantly up-regulated IFN-gamma- or TNF-alpha-induced IP-10, Mig, and I-TAC mRNA accumulation in keratinocytes and increased the levels of IP-10 and Mig in keratinocyte supernatants. Immunohistochemistry of skin affected by ACD revealed that >70% of infiltrating cells were reactive for CXCR3 and that CXCR3 staining colocalized in CD4+ and CD8+ T cells. Nickel-specific CD4+ and CD8+ T cell lines established from ACD skin produced IFN-gamma and IL-4 and expressed moderate to high levels of CXCR3. Finally, CXCR3 agonistic chemokines released by stimulated keratinocytes triggered calcium mobilization in skin-derived nickel-specific CD4+ T cells and promoted their migration, with supernatant from keratinocyte cultures stimulated with IFN-gamma and IL-4 attracting more efficaciously than supernatant from keratinocytes activated with

IFN-gamma alone. In conclusion, IL-4 exerts a proinflammatory function on keratinocytes by potentiating IFN-gamma and TNF-alpha induction of IP-10, Mig, and I-TAC, which in turn may determine a prominent recruitment of CXCR3+ T lymphocytes at inflammatory reaction sites.

PMID: 10903743 [PubMed - indexed for MEDLINE]

1640. Parasitol Res. 2000 Jun;86(6):480-5.

Inhibition of the protective IgA response by ketotifen is related to the inflammatory reaction in the peritoneal cavity and intestinal mucosa of BALB/c mice infected with Trichinella spiralis.

Doligalska M, Laskowska M.

Department of Parasitology, Institute of Zoology, University of Warsaw, Poland. mdolig@eco1.biol.uw.edu.pl

Interleukin-5 (IL-5) production, eosinophilia, and IgA responses of BALB/c mice infected with Trichinella spiralis were measured in the peritoneal cavity and intestinal mucosa. Ketotifen, an anti-allergic compound, was used to control the inflammatory response. IgA responses differed against adult and muscle stages of the parasite and between the intestine and the peritoneal cavity. IL-5 and eosinophil production also differed between the intestine and the peritoneal cavity. The occurrence of parasite-specific IgA antibodies in the peritoneal cavity may reflect the migration of cells from the lamina propria. Following ketotifen treatment there was a reduction in the IL-5, eosinophilic, and IgA responses; these responses affected both the size of worms and the number of worms.

PMID: 10894474 [PubMed - indexed for MEDLINE]

1641. Allergy Asthma Proc. 2000 May-Jun;21(3):129-33.

Antihistamines and epithelial cells.

Graziano FM, Cook EB, Stahl JL.

University of Wisconsin, Madison 53792-3244, USA.

Antihistamines have long been utilized in the symptomatic management (antihistaminic effects) of allergic rhinitis and conjunctivitis. Investigation into the nonsedating second-generation antihistamines suggests that they also possess antiinflammatory activity, and may be useful in the management of inflammation associated with allergic airway disease. In vitro studies have shown that these antihistamines decrease the migration and activation of eosinophils and diminish the release of pro-inflammatory mediators from mast cells and basophils after induction by immunological and nonimmunological stimuli. In vivo studies have also demonstrated that these antihistamines decrease inflammatory cell infiltration in allergic airway disease, and mediator release from mast cells and basophils. Epithelial cells, due to their spatial arrangement and predominance in the airways, play a pivotal role in the etiology of airway disease. There is evidence that antihistamines may modulate airway inflammation by influencing the activity of these airway epithelial cells. Studies have shown that expression of adhesion molecules on epithelial cells is decreased by second-generation antihistamines. Collectively, these studies suggest that second-generation H1-histamine receptor antagonists have potential use either as safe antiinflammatory alternatives to corticosteroids or as rescue medication in

combination with corticosteroids for the management of severe airway disease.

PMID: 10892513 [PubMed - indexed for MEDLINE]

1642. Mediators Inflamm. 2000;9(1):15-23.

Role of macrophage migration inhibitory factor (MIF) in allergic and endotoxin-induced airway inflammation in mice.

Korsgren M, Källström L, Uller L, Bjerke T, Sundler F, Persson CG, Korsgren O.

Department of Physiological Sciences, Lund University Hospital, Sweden. Magnus.Korsgren@mphy.lu.se

Macrophage migration inhibitory factor (MIF) has recently been forwarded as a critical regulator of inflammatory conditions, and it has been hypothesized that MIF may have a role in the pathogenesis of asthma and chronic obstructive pulmonary disease (COPD). Hence, we examined effects of MIF immunoneutralization on the development of allergen-induced eosinophilic inflammation as well as on lipopolysaccharide (LPS)-induced neutrophilic inflammation in lungs of mice. Anti-MIF serum validated with respect to MIF neutralizing capacity or normal rabbit serum (NRS) was administered i.p. repeatedly during allergen aerosol exposure of ovalbumin (OVA)-immunized mice in an established model of allergic asthma, or once before instillation of a minimal dose of LPS into the airways of mice, a tentative model of COPD. Anti-MIF treatment did not affect the induced lung tissue eosinophilia or the cellular composition of bronchoalveolar lavage fluid (BALF) in the asthma model. Likewise, anti-MIF treatment did not affect the LPS-induced neutrophilia in lung tissue, BALF, or blood, nor did it reduce BALF levels of tumor necrosis factor-alpha (TNF-alpha) and macrophage inflammatory protein-1alpha (MIP-1alpha). The present data suggest that MIF is not critically important for allergen-induced eosinophilic, and LPS-induced neutrophilic responses in lungs of mice. These findings do not support a role of MIF inhibition in the treatment of inflammatory respiratory diseases.

PMCID: PMC1781742
PMID: 10877450 [PubMed - indexed for MEDLINE]

1643. Ann Allergy Asthma Immunol. 2000 Jun;84(6):623-7.

Grain dust induces IL-8 production from bronchial epithelial cells: effect on neutrophil recruitment.

Park HS, Suh JH, Kim SS, Kwon OJ.

Department of Allergy and Clinical Immunology, Ajou University School of Medicine, Suwon, Korea.

BACKGROUND: There have been several investigations suggesting an involvement of activated neutrophils in the development of grain dust (GD)-induced occupational asthma. Interleukin-8 in the sputa from GD-induced asthmatic patients increased significantly after the exposure to GD.
OBJECTIVE: To confirm IL-8 production from bronchial epithelial cells when exposed to GD, and to evaluate the role of IL-8 on neutrophil recruitment.
MATERIALS AND METHOD: We cultured Beas-2B, a bronchial epithelial cell line. To observe GD-induced responses, four different concentrations ranging from 1 to 200 microg/mL of GD were incubated for 24 hours and compared with those without incubation of GD. To evaluate the effect of pro-inflammatory cytokines on IL-8 production and neutrophil chemotaxis, epithelial cells were incubated with

peripheral blood mononuclear cell (PBMC) culture supernatant derived from subjects with GD-induced asthma exposed to 10 microg/mL of GD, and then compared with those without addition of PBMC supernatant. The level of released IL-8 in the supernatant was measured by enzyme-linked immunosorbent assay. Neutrophil chemotactic activity of the culture supernatant was determined by modified Boyden chamber method.
RESULTS: Interleukin-8 production and neutrophil chemotactic activity from bronchial epithelial cells significantly increased with additions of GD in a dose-dependent manner (P < .05, respectively), and were significantly augmented with additions of PBMC supernatant (P < .05, respectively) at each concentration. Close correlation was noted between neutrophil chemotactic activity and IL-8 level (r = 0.87, P < .05). Compared with the untreated sample, pre-treatment of anti-IL-8 antibody induced a significant suppression (up to 67.2%) of neutrophil chemotactic activity in a dose-dependent manner.
CONCLUSION: These results suggest that IL-8 produced from bronchial epithelial cells may be a major cytokine, which induces neutrophil migration into the airways when exposed to GD.

PMID: 10875492 [PubMed - indexed for MEDLINE]

1644. Ann Allergy Asthma Immunol. 2000 Jun;84(6):569-79; quiz 579-81.

Are chemokines essential or secondary participants in allergic responses?

Gangur V, Oppenheim JJ.

Laboratory of Molecular Immunoregulation, Division of Basic Sciences, National Cancer Institute-Frederick Cancer Research and Development Center, Maryland, USA.

OBJECTIVES: This review will provide a concise and critical overview of the rapidly evolving concepts in chemokine biology with a special relevance to allergic responses. The article is intended for clinicians with little or no expertise in chemokine biology.
DATA SOURCES: A detailed literature search was performed through MEDLINE (PubMed). Those reports considered important and relevant to the topic were critically reviewed and their conclusions included.
RESULTS: Chemokines are a group of structurally related small proteins with a common biological activity of inducing directional migration (chemotaxis) of various cell types. Chemokines such as eotaxins and MCP-4 play a key role in selective eosinophil recruitment to sites of inflammation in allergies and asthma. Several other chemokine activities relevant to allergic responses are: activation of basophils and eosinophils to release inflammatory mediators, regulation of IgE responses, and Th1/Th2-type cytokine balance. A number of therapeutic strategies aimed at inhibiting chemokine function are being tested in animal models of allergies and asthma.
CONCLUSIONS: Chemokines have been widely viewed as pathogenic mediators of acute and chronic inflammation and tissue damage in allergies and asthma. On the other hand, recent evidence suggests that endogenous production of certain chemokines might be beneficial to the host in preventing allergic response. Met-RANTES, a modified antagonist of RANTES, and eotaxin receptor (CCR3) antagonists, represent promising novel therapeutic agents potentially useful in atopic disorders. Thus, suppression of chemokines may interrupt the sequence of signals culminating in an allergic response. Whether chemokines are actually essential for an allergic response awaits confirmation with gene knockout animal experiments.

PMID: 10875484 [PubMed - indexed for MEDLINE]

1645. Int Arch Allergy Immunol. 2000 May;122 Suppl 1:15-9.

Eosinophil-adhesion-inducing activity produced by antigen-stimulated mononuclear cells involves GM-CSF.

Nagata M, Yamamoto H, Tabe K, Sakamoto Y, Matsuo H.

Pulmonary Division, Second Department of Internal Medicine, Saitama Medical School, Saitama, Japan. favre4mn@saitama-med.ac.jp

BACKGROUND: The initial step of eosinophil accumulation in allergic inflammation is adhesion of circulating eosinophils to vascular endothelial cells (EC). There is evidence that the adhesive property of circulating eosinophils is upregulated following antigen exposure. Although the exact mechanism remains to be established, cytokine(s) produced by antigen-stimulated mononuclear cells is (are) likely key factor(s).
OBJECTIVE: The objective of this study was to examine the factor(s) responsible for eosinophil adhesion and migration induced by the antigen-stimulated mononuclear cells obtained from atopic asthmatics.
METHODS: Peripheral blood mononuclear cells (PBMC) isolated from house-dust-mite-sensitive bronchial asthmatics were cultured for 96 h in the presence or absence of 1 microg/ml Dermatophagoides farinae (Df) antigen. Eosinophils were isolated from peripheral blood of healthy subjects. Eosinophil-adhesion-inducing activity in the culture supernatants of PBMC was examined by the ability to modify the adhesion of eosinophils to human pulmonary microvascular endothelial cells (HPMEC) in the presence or absence of anti-cytokine/chemokine antibodies. Eosinophil migration induced by the supernatants was also examined. Results: Eosinophil adhesion to HPMEC was significantly augmented by the supernatants of Df-stimulated PBMC, which was significantly inhibited by anti-GM-CSF, but not by anti-IL-5, anti-RANTES, or isotype-matched controls. On the other hand, eosinophil migration induced by the supernatants was inhibited by anti-GM-CSF and partly by anti-RANTES.
CONCLUSION: Both eosinophil adhesion and migration induced by the antigen-stimulated PBMC involve GM-CSF. In contrast, RANTES is involved only in the eosinophil migration. These molecules may participate in the development of eosinophil accumulation at the allergic inflammation sites.

Copyright 2000 S. Karger AG, Basel.

PMID: 10867501 [PubMed - indexed for MEDLINE]

1646. Allergol Immunopathol (Madr). 2000 May-Jun;28(3):110-5.

[Adhesion molecules and asthma].

[Article in Spanish]

Alvaro M.

Sección de Alergia e Inmunología Clínica, Unidad Integrada de Pediatría, Hospital Sant Joan de Déu, Hospital Clínic, Universitat de Barcelona, Barcelona, España.

The inflammation markers in asthma can help the prognosis, diagnosis and monitoring of the respiratory illness. The adhesion molecules (AM) are membrane glycoproteins that intervene in the contact between the two cells or between the cell and the extracellular matrix. Their aim is to maintain the contact between two cells for the time that is necessary in order to establish a communication between them and so that the function indicated takes place. The most important functions of the AM's are to facilitate the adhesion of the circulating leukocytes to the vascular endothelium with the posterior transendothelial

migration that contribute, by doing so, to the perpetuation of the inflammatory reaction in bronchial asthma. The AM's can act as receptors or as specific ligands for these receptors and are expressed both in leukocytes and endothelial cells as well as in epithelial cells. There are several AM families that allow for these interactions and that collaborate in the specificity of the cellular recognition in the immunologic reaction. The most known are the selectines, the super family of the immunoglobulins and the integrins, that act in a cascading manner. In bronchial asthma, the inflammatory reaction consists of three sequential processes: the recognition-activation phase, the inflammation phase and the solution phase. The AM's act in the second or the inflammation phase. The positive control of the E-selectine, followed by that of the ICAM-1 on the surface of the endothelium cell, leads in sequence to the adhesion, initially of the neutrophils and then the lymphocytes and monocytes, as a response to the antigenic stimulus. The initial loose adhesion of the leukocytes to the vascular endothelium, activated by contact or by cytokines, takes place by means of the selectines. In the microvasculature the strength of the flow pushes the adhered leukocytes, causing the disappearance of the selectine-ligand interactions which form againg quickly in descending direction as the leukocyte moves. The result is the rolling of the spherical leukocyte along the surface of the endothelium. This leukocyte becomes activated and its cytoskeleton is rearranged, and now has a flat shape and the affinity of its integrins is increased by the endothelial ligands. This leukocyte can die within a few days, can activate itself or exit via the lymphatic vessels. The activated monocytes that are located in the extravascular tissues are differentiated from the histiocytes, which are the final cellular effectors of the inflammatory reaction. The importance of the knowledge of the AM structure and the functions, is due to the fact that there is a possible pharmacological action on them. The data obtained from clinical studies, as well as the in vitro results, confirm the importance of the AM modulation as well as therapeutic approach to bronchial asthma.

PMID: 10867380 [PubMed - indexed for MEDLINE]

1647. J Immunol. 2000 Jul 1;165(1):221-5.

The polarization defect of Wiskott-Aldrich syndrome macrophages is linked to dislocalization of the Arp2/3 complex.

Linder S, Higgs H, Hüfner K, Schwarz K, Pannicke U, Aepfelbacher M.

Institut für Prophylaxe und Epidemiologie der Kreislaufkrankheiten and Max von Pettenkofer-Institut für Medizinische Mikrobiologie, Ludwig-Maximilians-Universität, München, Germany.

Wiskott-Aldrich syndrome (WAS) is an X-linked recessive disorder originally characterized by the clinical triad eczema, thrombocytopenia, and severe immunodeficieny, with recurrent bacterial and viral infections, indicating a profound immune cell defect. Such altered immune cells include monocytes, macrophages, and dendritic cells, which were reported to display disturbed cell polarization or chemotaxis. WAS is caused by mutations in the WAS protein (WASp), which is thought to organize the actin cytoskeleton through the Arp2/3 complex. Here we show that the Arp2/3 complex is an integral part of podosomes, actin-rich adhesion structures of macrophages, and that WAS macrophages fail to organize the Arp2/3 complex into podosomes. We also demonstrate that microinjection of a C-terminal acidic stretch of WASp into normal macrophages displaces Arp2/3 from podosomes and, in combination with chemoattractant stimulation of cells, induces a phenotype resembling the polarization-defective phenotype of stimulated WAS macrophages. These findings point to an important role of the Arp2/3 complex in polarization and migration of immune cells.

PMID: 10861055 [PubMed - indexed for MEDLINE]

1648. J Allergy Clin Immunol. 2000 Jun;105(6 Pt 1):1041-53.

Tissue remodeling as a feature of persistent asthma.

Vignola AM, Kips J, Bousquet J.

Istituto di Fisiopatologia Respiratoria, Palermo, Italy.

A chronic inflammatory process is almost invariably associated with tissue damage and healing. Healing results in repair and replacement of dead or damaged cells by viable cells. Repair usually involves 2 distinct processes: regeneration, which is the replacement of injured tissue by parenchymal cells of the same type, and replacement by connective tissue and its eventual maturation into scar tissue. In many instances both processes contribute to the healing response. Chronic inflammatory disease can therefore lead to a wide variety of consequences, from complete or partial restitution of organ structure and function to fibrosis. Asthma is characterized by a chronic inflammatory process of the airways. The ensuing healing process results in structural alterations referred to as a remodeling of the airways. The mechanisms underlying these structural alterations are still largely unknown. They are likely to be heterogeneous, leading-through the highly dynamic process of cell de-differentiation, migration, differentiation, and maturation-to changes in connective tissue deposition and to the altered restitution of airways structure, resulting in mucus gland hyperplasia, neovascularization, fibrosis, and an increase in smooth muscle mass.

PMID: 10856134 [PubMed - indexed for MEDLINE]

1649. Eur Respir J. 2000 May;15(5):870-7.

Differential effects of nitric oxide synthase inhibitors in an in vivo allergic rat model.

Tulić MK, Wale JL, Holt PG, Sly PD.

TVW Telethon Institute for Child Health Research and Dept of Paediatrics, University of Western Australia, Perth, Australia.

The in vivo role of nitric oxide in inflammatory cell migration, vascular permeability and the development of hyperresponsiveness to methacholine (MCh) was studied in rats 24 h following ovalbumin (OVA) challenge. The NO synthase (NOS) inhibitors N(G)-mono-methyl-L-arginine (L-NMMA; nonselective), aminoguanidine (two-fold inducible NOS-selective), N(omega)-nitro-L-arginine methyl ester (L-NAME; 2000-fold endothelial cell NOS-selective) or S-methyl-L-thiocitrulline (100-fold neuronal NOS-selective) were administered (100 mg x kg(-1) s.c.) to OVA-sensitized Piebald-Virol-Glaxo rats on 3 consecutive days during which they were challenged with allergen (1% OVA). Responses to inhaled MCh were measured in anaesthetized animals 24 h after OVA challenge. Cellular inflammation and vascular permeability were assessed using bronchoalveolar lavage (BAL) fluid collected 30 min after administration of Evans blue (50 mg x kg(-1) i.v.). OVA challenge in sensitized animals induced hyperresponsiveness to MCh, inflammatory cell influx and increased leakage of Evans blue into the BAL fluid (n=9, p<0.001). Aminoguanidine was effective in inhibiting the allergen-induced cellular influx and microvascular leakage (n=9, p<0.001) without altering responses to MCh. This effect was reserved by L-arginine. L-NAME (n=5, p<0.01) and S-methyl-L-thiocitrulline (n=6, p<0.001) further potentiated the

allergen-induced hyperresponsiveness without altering cellular inflammation. L-NMMA attenuated both the OVA-induced cellular influx and Evans blue leakage (n=8, p<0.001) as well as further potentiating the hyperresponsiveness to MCh (p<0.05). From these studies, it is suggested that, in allergic Piebald-Virol-Glaxo rats, nitric oxide production by inducible nitric oxide synthase plays a role in the migration of inflammatory cells and increase in vascular permeability following allergen challenge, whereas nitric oxide produced by the constitutively expressed neuronal nitric oxide synthase limits hyperresponsiveness to methacholine.

PMID: 10853851 [PubMed - indexed for MEDLINE]

1650. Clin Exp Allergy. 2000 Jun;30 Suppl 1:37-41.

Epithelial damage and response.

Holgate ST.

Southampton General Hospital, UK.

Epithelial damage is a characteristic feature of asthma. The epithelium is not merely a passive barrier but can generate a range of mediators that may play a role in the inflammatory and remodelling responses that occur in the lungs in asthma. For example, the cytokine granulocyte macrophage colony-stimulating factor (GM-CSF), whose principal source is the epithelium, can prolong eosinophil survival while transforming growth factor is a potent profibrogenic cytokine. Deposition of collagen in the epithelial subbasement membrane is a characteristic feature of the remodelling response in asthma. This may be due to abnormal associations between myofibroblasts and epithelium, both of which are involved in early lung development (epithelial-mesenchymal trophic unit). In asthma, there may be a primary defect in the epithelium such that it responds abnormally to various stimuli and cannot undergo the normal repair response. Epidermal growth factor (EGF) appears to be a key factor in bronchial epithelial repair; it stimulates epithelial cell proliferation and migration. The 3v isoform of the adhesion molecule CD44 is overexpressed in damaged epithelium and seems to regulate the repair response by presenting EGF more efficiently to its receptor. Although EGF receptor expression is increased in asthma, it does not lead to an appropriate proliferative response and restitution of normal epithelium. Other factors such as transforming growth factor (TGF)beta which are generated by inflammatory cells and epithelium are also upregulated in asthma. An epithelial/fibroblast co-culture system has shown that following epithelial damage various growth factors are released from the underlying myofibroblasts and are responsible for the proliferative response. The TGFbeta family are most likely responsible for collagen production. In an in vitro study, an EGF receptor inhibitor slowed epithelial repair but enhanced TGFbeta production by the slowly repairing epithelial cells. In conclusion, the interaction between epithelial cells and myofibroblasts, i.e. reactivation of the epithelial-mesenchymal trophic unit appears to be central to the airway wall remodelling response.

PMID: 10849473 [PubMed - indexed for MEDLINE]

1651. Clin Exp Allergy. 2000 Jun;30(6):807-17.

Transepithelial migration of activated eosinophils induces a decrease of E-cadherin expression in cultured human nasal epithelial cells.

Kobayashi N, Terada N, Hamano N, Numata T, Konno A.

Department of Otorhinolaryngology, School of Medicine, Chiba University, Chiba City, Chiba, Japan.

BACKGROUND: The damage of respiratory epithelium in allergic diseases has a close correlation with the extent of eosinophil infiltration. It seems to be a good possibility that eosinophil infiltration could induce the changes in the expression of the epithelial cell adhesion molecules, which play a key role in the maintenance of structural and functional rigidity of epithelium.
OBJECTIVE: We observed the expression of E-cadherin in cultured human nasal epithelial cells (HNECs) to study whether could it be affected by transepithelial migration of inflammatory cells, especially eosinophils.
METHODS: In vitro study of the transmigration assay was designed using various types of inflammatory cells and HNEC monolayers. Various assays of each experimental group were done under the stimulation of interleukin-5 (IL-5) and/or platelet activating factor (PAF). Subsequently immunohistochemistry for E-cadherin was performed in the HNECs. The intensity of immunofluorescence of E-cadherin was quantified using confocal laser scanning microscopy (CLSM) system and compared before and after the transmigration.
RESULTS: The mean intensity of immunofluorescence for E-cadherin decreased significantly after the transmigration of any types of inflammatory cells. Above all, the migration of eosinophils treated with IL-5 and PAF had an eminent effect on the decrease, whereas the degranulation extracts derived from eosinophils activated by IL-5 and secretory IgA (sIgA) did not affect the intensity.
CONCLUSION: This work suggests that transepithelial migration of inflammatory cells can directly induce the decrease in epithelial E-cadherin expression. Furthermore, the most prominent change was induced by transmigration of activated eosinophils, which might be caused by some mechanisms independent of the eosinophil contents. The decrease in E-cadherin expression may trigger the damage of epithelial barrier, which contributes to the pathogenesis of allergic diseases.

PMID: 10848899 [PubMed - indexed for MEDLINE]

1652. Am J Public Health. 2000 Jun;90(6):929-35.

Asthma and Latino cultures: different prevalence reported among groups sharing the same environment.

Ledogar RJ, Penchaszadeh A, Garden CC, Iglesias Garden.

CIETinternational, New York, NY 10011, USA. rledogar@ciet.org

OBJECTIVES: This 1999 study measured asthma prevalence among Latinos of different cultural traditions who live on the same streets and in the same buildings.
METHODS: Health promoters from El Puente in North Brooklyn, New York City, surveyed 3015 people in 946 households, asking standard asthma prevalence questions.
RESULTS: Some 46% of households identified themselves as Dominican, 42% as Puerto Rican, 6% as other Latino, and 6% as other. Reported asthma period prevalence was 5.3% (93 of 1749) among Dominicans and other Latinos, compared with 13.2% (147 of 1115) among Puerto Ricans (odds ratio = 0.37; 95% confidence interval = 0.28, 0.49), a difference not explained by location (cluster or building), household size, use of home remedies, educational attainment, or country where education was completed. Differences were least detectable among 13- to 24-year-olds of both sexes and sharpest among women aged 45 years and older and girls from birth to 12 years.
CONCLUSIONS: Further research on gene-environment interactions is needed among Puerto Ricans and Dominicans, but asthma's associations with low income and unhealthy environment, which more recent immigrants seem better able to

withstand, should not be overlooked.

PMCID: PMC1446273
PMID: 10846511 [PubMed - indexed for MEDLINE]

1653. Int Arch Allergy Immunol. 2000 Apr;121(4):284-91.

Purification and immunobiochemical characterization of folding variants of the recombinant major wasp allergen Ves v 5 (antigen 5).

Suck R, Weber B, Kahlert H, Hagen S, Cromwell O, Fiebig H.

Allergopharma Joachim Ganzer KG, Reinbek, Germany.

BACKGROUND: Antigen 5 is one of three major allergens in wasp venoms, but unlike phospholipase A(1) and hyaluronidase, both of which are enzymes, its biological function is unknown. The cDNA coding for this allergen has been isolated and used for recombinant expression. Thorough analysis of the expression product is essential in order to evaluate the usefulness for in vivo or in vitro application.
OBJECTIVE: In this study, folding variants of the recombinant major allergen Ves v 5 from Vespula vulgaris were immunologically and biochemically investigated in order to determine their possible applicability for diagnostic or therapeutic purposes.
METHOD: The cDNA encoding Ves v 5 was cloned into the expression vector pSE420 which generates recombinant products lacking a tag sequence. After expression, inclusion bodies were purified, subsequently denatured and dialyzed against different solutions. The structural properties of soluble proteins were analyzed by size exclusion chromatography, non-reducing SDS-PAGE, native PAGE, N-terminal sequencing, proteolytic digestion and ion exchange chromatography. Immunological investigations were performed by using different monoclonal antibodies (mAbs) specific for Ves v 5 and IgE from patients allergic to wasp venom allergens.
RESULTS: After dialysis, soluble monomeric recombinant Ves v 5 was more than 95% pure in each case. Using different dialysis solutions, clearly distinguishable folding variants were obtained. In one case, the recombinant allergen was comparable with the natural counterpart in respect of migration in non-reducing SDS-PAGE, native PAGE and IgE reactivity. This variant reacted with two different Ves v 5-specific mAbs and produced a stable fragment after proteolytic digestion. Elution from a cation exchange chromatography column was achieved with 320 mM NaCl. In two other cases, folding variants exhibited a different migration behavior in SDS-PAGE and native PAGE compared with the natural allergen. Also, the mAb 1E11 recognized none of these variants since it presumably detected a conformational epitope. Moreover, the IgE reactivity was clearly reduced and proteolytic digestion effected almost complete degradation. These variants eluted from the cation exchange column with 400 mM NaCl.
CONCLUSION: Defined folding strategies resulted in both soluble misfolded variants with reduced IgE reactivity, potentially suitable for immunotherapy, and natural-like folded variants for diagnosis.

Copyright 2000 S. Karger AG, Basel

PMID: 10828718 [PubMed - indexed for MEDLINE]

1654. Photodermatol Photoimmunol Photomed. 2000 Apr;16(2):67-73.

Timecourse study of UVB-induced cytokine induction in whole mouse skin.

Scordi IA, Vincek V.

Department of Microbiology and Immunology, University of Miami School of Medicine, Florida, USA.

Comment in
    Photodermatol Photoimmunol Photomed. 2001 Dec;17(6):284-5.

Ever since the skin was recognized as a site of immunologic activity, a number of laboratories have studied the production of cytokines by skin cells and the effects of chemicals, allergens, contact irritants and UVB radiation on their production. Most research to date has been carried out using either purified populations of primary cells, or established cell lines. Cytokines, however, do not function in isolation but they appear in human tissues within the context of other cytokines that can, in turn, strongly influence the final biological outcome. Therefore, in vivo studies using whole skin are more physiologically relevant since all cell types are present and interactions among them are allowed to proceed. We report here the results of a detailed timecourse study using whole mouse skin, consisting of both dermis and epidermis, irradiated with either a low or high dose of UVB and analyzed using a Multi-probe RNase protection assay system. The results show that in whole skin the kinetics of cytokine induction are different than what was previously observed in tissue culture. In addition to already known skin-associated cytokines, we report here the presence and UVB induction of cytokines not previously reported.

PMID: 10823315 [PubMed - indexed for MEDLINE]

1655. J Immunol. 2000 Jun 1;164(11):5935-43.

Expression of CXCR4 in eosinophils: functional analyses and cytokine-mediated regulation.

Nagase H, Miyamasu M, Yamaguchi M, Fujisawa T, Ohta K, Yamamoto K, Morita Y, Hirai K.

Departments of Respiratory Medicine, Allergy and Rheumatology, and Bioregulatory Function, University of Tokyo Graduate School of Medicine, Tokyo, Japan.

We examined the expression of transcripts of a panel of chemokine receptors in human eosinophils and found intense constitutive expression of CXCR4 mRNA. Although surface CXCR4 protein was hardly detectable in the peripheral blood or freshly isolated eosinophils, surface expression of CXCR4 became gradually apparent during incubation at 37 degrees C. In contrast, the level of CCR3 expression was virtually unchanged during the incubation. Stromal cell-derived factor-1alpha (SDF-1alpha), the natural ligand of CXCR4, elicited an apparent Ca2+ influx in these cells and induced a strong migratory response comparable to that by eotaxin. The surface expression of CXCR4 in eosinophils was up-regulated by IFN-gamma, TNF-alpha, and TGF-beta while it was down-regulated by IL-4 and eosinophil-directed hemopoietins such as IL-5. The CXCR4 expression did not always parallel the apoptotic changes in cytokine-treated eosinophils. In contrast to IL-4 and IFN-gamma, IL-5 potently reduced the level of CXCR4 mRNA. It seems unlikely that CXCR4 is fundamentally involved in the pathogenesis of allergic disorders by inducing the migration of eosinophils toward inflammatory sites, because a Th2-dominant state down-regulates eosinophil CXCR4 expression. However, CXCR4 may affect the size of the mobilizable pool by holding eosinophils at noninflamed tissues. Th2-dominant state may favor the liberation of eosinophils by down-regulating CXCR4 expression. The interplay between CXCR4 and SDF-1alpha in eosinophils potentially plays an important role in the accumulation of these cells at the allergic inflammatory sites.

PMID: 10820276 [PubMed - indexed for MEDLINE]

1656. Clin Infect Dis. 2000 May;30(5):811-4. Epub 2000 May 18.

Treatment of cutaneous larva migrans.

Caumes E.

Service des Maladies Infectieuses et Tropicales, Hôpital Pitié-Salpêtrière, Paris, France. egcaumes@cybercable.fr

Cutaneous larva migrans caused by the larvae of animal hookworms is the most frequent skin disease among travelers returning from tropical countries. Complications (impetigo and allergic reactions), together with the intense pruritus and the significant duration of the disease, make treatment mandatory. Freezing the leading edge of the skin track rarely works. Topical treatment of the affected area with 10%-15% thiabendazole solution or ointment has limited value for multiple lesions and hookworm folliculitis, and requires applications 3 times a day for at least 15 days. Oral thiabendazole is poorly effective when given as a single dose (cure rate, 68%-84%) and is less well tolerated than either albendazole or ivermectin. Treatment with a single 400-mg oral dose of albendazole gives cure rates of 46%-100%; a single 12-mg oral dose of ivermectin gives cure rates of 81%-100%.

PMID: 10816151 [PubMed - indexed for MEDLINE]

1657. J Neuroimmunol. 2000 Jul 1;106(1-2):60-8.

A role for alpha4-integrin in the pathology following Semliki Forest virus infection.

Smith JP, Morris-Downes M, Brennan FR, Wallace GJ, Amor S.

Immunology Department, Rayne Institute, United Medical and Dental School of Guy's and St. Thomas' Hospital, London, UK.

Migration of cells into the central nervous system (CNS) is a pivotal step in the pathogenesis of immune-mediated diseases such as multiple sclerosis (MS), experimental allergic encephalomyelitis (EAE) and virus-induced demyelinating diseases. Such migration is dependent on expression of adhesion molecules. The expression of adhesion molecules in the CNS was studied in Biozzi ABH mice infected with Semliki Forest virus (SFV) A7(74) - an important demyelinating model of MS. Expression of LFA-1alpha/CD11a, LFA-1beta/CD18 and ICAM-1/CD56 were rapidly elevated and remained high whereas MAC-1, CD44 and VCAM-1/CD106 were less widely expressed. The alpha4-integrin VLA-4/CD49d was more specifically associated with CNS lesions. To identify the importance of VLA-4, CD44, ICAM-1 and MAC-1 in the pathogenesis of SFV infection, monoclonal antibodies that block these adhesion molecules were administered in vivo during infection. Anti-VLA-4 treatment dramatically reduced the cellular infiltrates and demyelination within the CNS but did not affect the clearance of virus while antibodies to CD44, ICAM and MAC-1 antibody treatment had no effect. This study demonstrates that SFV infection induces the expression of adhesion molecules within the CNS and that VLA-4 plays an important role in the development of inflammation and demyelination in the CNS following SFV infection.

PMID: 10814783 [PubMed - indexed for MEDLINE]

1658. Immunology. 2000 May;100(1):21-8.

Regulation of epidermal Langerhans cell migration by lactoferrin.

Cumberbatch M, Dearman RJ, Uribe-Luna S, Headon DR, Ward PP, Conneely OM, Kimber I.

Zeneca Central Toxicology Laboratory, Macclesfield, Cheshire, UK.

Lactoferrin (LF) is a member of the transferrin family of iron-binding glycoproteins to which several anti-inflammatory functions have been ascribed. LF has been shown to down-regulate expression of the pro-inflammatory cytokine tumour necrosis factor-alpha (TNF-alpha), although the possibility has been raised that the activity of LF in this regard was indirect and secondary to its ability to bind to and inactivate the bacterial lipopolysaccharide (LPS) used to induce cytokine production. However, the identification of putative membrane receptors for LF raises the possibility that the interaction of LF with its receptor may be one important route through which this protein exerts anti-inflammatory activity. In the present investigations the biological properties of LF have been examined in a model of cutaneous immune function where the allergen-induced migration of epidermal Langerhans cells (LC) from the skin and their subsequent accumulation as dendritic cells (DC) in skin-draining lymph nodes are known to be dependent upon the de novo synthesis of TNF-alpha, but independent of exogenous LPS. Consistent with the protein having direct anti-inflammatory properties, it was found that the intradermal injection of recombinant murine LF (either iron-saturated or iron-depleted LF) inhibited significantly allergen (oxazolone) -induced LC migration and DC accumulation. That these inhibitory effects were secondary to the inhibition of local TNF-alpha synthesis was suggested by the findings that first, LF was unable to inhibit LC migration induced by intradermal injection of TNF-alpha itself, and second, that migration stimulated by local administration of another epidermal cytokine, interleukin 1beta, which is also dependent upon TNF-alpha production, was impaired significantly by prior treatment with LF. Finally, immunohistochemical analyses demonstrated the presence of LF in skin, associated primarily with keratinocytes. Collectively these data support the possession by LF of direct immunomodulatory and/or anti-inflammatory activity, probably associated in this case with inhibition of cytokine production. Furthermore, the results suggest that as a constituent of normal skin, LF may play a role in homeostatic regulation of cutaneous immune function.

PMCID: PMC2326987
PMID: 10809955 [PubMed - indexed for MEDLINE]

1659. J Allergy Clin Immunol. 2000 May;105(5):967-74.

Deficient cytokine response of human allergen-specific T lymphocytes from humanized SCID mice and reconstitution by professional antigen-presenting cells.

Jarman ER, Perschke K, Montermann E, Herz U, Renz H, Knop J, Reske-Kunz AB.

Clinical Research Unit, Department of Dermatology, Johannes Gutenberg-University, Mainz, Germany.

BACKGROUND: Hu-PBL-SCID mice generated by the transfer of PBMCs from atopic individuals may provide a physiologic in vivo model for investigating human responses to allergens and potential approaches toward immunotherapy.
OBJECTIVE: This study was undertaken to investigate the functional activity and cytokine profile of human allergen-reactive T lymphocytes isolated from hu-PBL-SCID mice.

METHODS: PBMCs from allergic individuals were coinjected with allergen into SCID mice. Human lymphocyte migration and phenotype were established by reverse transcription-PCR and immunohistochemistry, IgE levels in sera were determined, and the frequency of allergen-reactive cytokine-producing T lymphocytes was established.
RESULTS: After immunization with allergen, specific IgE levels in hu-PBL-SCID sera were comparable with levels in donor sera. Although the majority of lymphocytes remained in the peritoneum, significant numbers of T lymphocytes were located in the spleen, where human IL-4, IL-5, and IFN-gamma messenger RNA expression was detected after stimulation with PHA and phorbol myristate acetate. Failure to induce cytokine production by human T lymphocytes isolated from the peritoneum and spleen of hu-PBL-SCID mice by allergen was reversed by stimulating with allergen in the presence of exogenously added IL-2 and antigen-presenting cells (APC), particularly CD14(+) monocytes. Under these conditions, allergen-reactive T cells expressed a T(H)2-like phenotype.
CONCLUSIONS: These data suggest that, after initial activation and induction of antibody production, human T lymphocytes enter a state of unresponsiveness, arising from a loss of human professional APC, in hu-PBL-SCID mice. The use of hu-PBL-SCID mouse models in studies on therapeutic approaches for allergy may benefit from the additional transfer of human professional APC.

PMID: 10808178 [PubMed - indexed for MEDLINE]

1660. Nat Genet. 2000 May;25(1):96-101.

Transcript imaging of the development of human T helper cells using oligonucleotide arrays.

Rogge L, Bianchi E, Biffi M, Bono E, Chang SY, Alexander H, Santini C, Ferrari G, Sinigaglia L, Seiler M, Neeb M, Mous J, Sinigaglia F, Certa U.

Roche Milano Ricerche, Milano, Italy. lars.rogge@roche.com

Many pathological processes, including those causing allergies and autoimmune diseases, are associated with the presence of specialized subsets of T helper cells at the site of inflammation. Understanding the genetic program that controls the functional properties of T helper type 1 (Th1) versus T helper type 2 (Th2) cells may provide insight into the pathophysiology of inflammatory diseases. We compared the gene-expression profiles of human Th1 and Th2 cells using high-density oligonucleotide arrays with the capacity to display transcript levels of 6,000 human genes. Here we analyse the data sets derived from five independent experiments using statistical algorithms. This approach resulted in the identification of 215 differentially expressed genes, encoding proteins involved in transcriptional regulation, apoptosis, proteolysis, and cell adhesion and migration. A subset of these genes was further upregulated by exposure of differentiated Th1 cells to interleukin-12 (IL-12), as confirmed by kinetic PCR analysis, indicating that IL-12 modulates the effector functions of Th1 cells in the absence of antigenic stimulation. Functional assays and in vivo expression of selected genes have validated the biological relevance of our study. Our results provide new insight into the transcriptional program controlling the functional diversity of subsets of T helper cells.

PMID: 10802665 [PubMed - indexed for MEDLINE]

1661. Med Hypotheses. 2000 Feb;54(2):193-7.

Cell phenotype as a target of drug therapy in chronic inflammatory diseases.

Shiels IA, Taylor SM, Fairlie DP.

Department of Physiology and Pharmacology, University of Queensland, Brisbane, Queensland, Australia. shiels@plpk.uq.edu.au

Many diseases share common pathological changes which could in principle be targets for new drugs. Vascular leakage of plasma and migration of cells into perivascular tissues are common to chronic inflammatory diseases such as asthma, atherosclerosis, arthritis, and proliferative nephropathy as well as some non-inflammatory proliferative disorders such as diabetes mellitus. Individual components of plasma have been shown to stimulate cellular proliferation, matrix deposition and phenotypic change, leading to tissue-damaging structural changes. Whereas most anti-inflammatory drugs either downregulate expression of inflammatory mediators or inhibit their actions on cells, there are alternate potential therapeutic strategies described here based on moderating vascular leakage or its consequences in chronic diseases. The hypothesis is that drugs that can modify a cell's phenotype could be used to limit structural changes which accompany inflammation and thus reduce permanent debility resulting from these diseases. Such drugs include the differentiating agents being developed for cancer therapy.

Copyright 2000 Harcourt Publishers Ltd.

PMID: 10790751 [PubMed - indexed for MEDLINE]

1662. Biol Pharm Bull. 2000 Apr;23(4):506-10.

Stereoselective internal acyl migration of 1beta-O-acyl glucuronides of enantiomeric 2-phenylpropionic acids.

Akira K, Hasegawa H, Shinohara Y, Imachi M, Hashimoto T.

School of Pharmacy, Tokyo University of Pharmacy and Life Science, Hachioji, Japan.

Internal acyl migration reactions of 1beta-O-acyl glucuronides of 2-arylpropionic acids (profens) are of interest because of their possible role in covalent binding to serum proteins and consequent allergic reactions. The stereoselective degradation of 1beta-O-acyl glucuronides of enantiomeric 2-phenylpropionic acids (PAs), the basic structures of profens, in phosphate buffer (pH 7.4) at 37 degrees C, has been investigated using HPLC. Apparent first-order degradation of 1beta-O-acyl glucuronide and the sequential appearance of 2-, 3- and 4-O-acyl isomers were observed for each enantiomer. Acyl migration was observed to predominate over hydrolysis as in the other profen glucuronides. All the positional isomers and anomers were characterized using NMR and HPLC-NMR. The overall degradation half-life of (R)- and (S)-PA glucuronides was 1.8 and 3.3 h, respectively. These results suggest that (R)-PA glucuronide could be more susceptible to covalent binding to proteins via acyl migration than the corresponding antipode. The lability of the (R)-diastereomer over the antipode is consistent with previous reports on other profen glucuronides. Thus, the diastereomeric PA glucuronides are considered to be the best model compounds for the computation of structural physicochemical parameters to control the stereoselective internal acyl migration of profen glucuronides because PA has the simplest chemical structure of all the profens.

PMID: 10784437 [PubMed - indexed for MEDLINE]

1663. Allergy. 2000 Apr;55(4):363-8.

Glucocorticosteroids rapidly inhibit allergen-induced expression of E-selectin in vitro in a mucosal model of allergic rhinitis.

Rudack C, Bachert C.

ENT Department, University Hospital WWU Münster, Germany.

BACKGROUND: Transendothelial migration of cells to sites of inflammation is a hallmark of the allergic reaction. The adhesion cascade involves the initial expression of the adhesion molecule E-selectin on endothelial cells. The aim of the study was to determine the efficacy of a 30-min preincubation of the glucocorticosteroids (GCS) fluticasone, prednisolone, and fluocortin butyl on allergen- and interleukin (IL)-1beta-induced E-selectin expression in allergic rhinitis.
METHODS: Freshly taken nasal inferior turbinate mucosa of 19 subjects with allergic rhinitis was cut into small cubes and preincubated for 30 min with prednisolone (n = 6), fluticasone (n = 5), and fluocortin butyl (n = 3) in different concentrations, followed by allergen exposure at a concentration of 1000 BU/ml for 1 and 2 h. Additionally, fluticasone-preincubated tissues were exposed to recombinant human rhIL-1beta (n = 5) at a concentration of 2 pg/ml. The expression of E-selectin was assessed by immunohistochemistry (APAAP technique) and computerized image evaluation.
RESULTS: In this model, E-selectin expression was significantly upregulated by allergen and rhIL- 1beta within 1 and 2 h. After 30-min preincubation with prednisolone and fluocortin butyl at drug concentrations of 10-8 mol/1, we found a significant (> or = 50%) reduction of the E-selectin expression after 1 and 2 h. Allergen-induced E-selectin expression was nearly abolished at concentrations of 10-5 (prednisolone) and 10-4 mol/l (fluocortin butyl). Fluticasone significantly inhibited E-selectin expression by > or = 50% at concentrations of 10-14 and 10-12 mol/l after 1 and 2 h, and abolished E-selectin induction at concentrations of 10-12 and 10-10 mol/l, respectively. Exposure of mucosal cubes to rhIL-lbeta (n = 5) also induced rapid upregulation of E-selectin expression, an effect which could be only partially suppressed by fluticasone preincubation at concentrations of 10-l0 mol/l.
CONCLUSIONS: Allergen-induced E-selectin expression is significantly and rapidly inhibited by GCS preincubation, fluticasone being more potent than prednisolone and fluocortin butyl. We suggest that this rapid effect is mainly indirect, possibly by inhibition of allergen-induced cytokine release.

PMID: 10782521 [PubMed - indexed for MEDLINE]

1664. J Leukoc Biol. 2000 Apr;67(4):585-9.

Chymase is a potent chemoattractant for human monocytes and neutrophils.

Tani K, Ogushi F, Kido H, Kawano T, Kunori Y, Kamimura T, Cui P, Sone S.

Third Department of Internal Medicine, School of Medicine, Institute for Enzyme Research, The University of Tokushima, Japan.

Chymase is a major chymotrypsin-like serine protease expressed in the secretory granules of mast cells in many mammalian species. In this study, we revealed the chemotactic activity of chymase for human mononuclear cells and neutrophils with a 48-well microchemotaxis chamber technique. Human chymase showed the potent chemotactic activity for monocytes and neutrophils dose-dependently in a concentration range from 0.1 to 10 microg/mL, corresponding to about 4-400 microM. The activity was as potent as that of N-formyl-methionyl-leucyl-phenylalanine. Chymase also stimulated cell migration

of lymphocytes and purified T cells, but checkerboard analysis revealed that the effect was chemokinetic rather than chemotactic. Inhibition of chymase activities with chymase inhibitors, such as antileukoprotease and Bowman-Birk soybean trypsin inhibitor, significantly inhibited the chemotactic activity of chymase, suggesting that the proteolytic activity of chymase participates in the chemotactic activity. Our results suggest that mast cell chymase acts as a chemoattractant, and may play a role in the accumulation of inflammatory cells in development of the chronic inflammatory responses of allergic and nonallergic diseases.

PMID: 10770293 [PubMed - indexed for MEDLINE]

1665. Rev Mal Respir. 2000 Feb;17(1):99-102.

[A rare cause of asthma exacerbation: systemic anguilluliasis].

[Article in French]

Rivals A, Rouquet RM, Recco P, Linas MD, Leophonte P, Didier A.

Service de Pneumologie-Allergologie, CHR Rangueil, Toulouse.

Strongyloides is an helminthic infection that may induce bronchospasm during systemic migration of larvae. We report a case of a 60 years old man originating from Caribbean who had corti-codependent asthma since 30 years. He was hospitalized for severe exacerbation that worsen when steroid dosage was increase. Sputum examination revealed the presence in great number of Larvae of Strongyloides stercoralis. A good clinical evolution was achieved after specific anti-helminthic treatment. Later on, it was even possible to stop completely steroid treatment. This clinical case demonstrates the interest to look for Strongyloides superinfection in asthmatic patients that worsen when receiving increasing dose of steroids. This is particularly important for patients who have resided, even many years earlier, in areas where intestinal helminthic infection are endemic.

PMID: 10756561 [PubMed - indexed for MEDLINE]

1666. J Biol Chem. 2000 Jun 23;275(25):19000-8.

Identification and characterization of a potent, selective, and orally active antagonist of the CC chemokine receptor-1.

Liang M, Mallari C, Rosser M, Ng HP, May K, Monahan S, Bauman JG, Islam I, Ghannam A, Buckman B, Shaw K, Wei GP, Xu W, Zhao Z, Ho E, Shen J, Oanh H, Subramanyam B, Vergona R, Taub D, Dunning L, Harvey S, Snider RM, Hesselgesser J, Morrissey MM, Perez HD.

Departments of Discovery Research, Biological Research, Pharmacology, and Immunology, Berlex Biosciences, Richmond, Calfornia 94804, USA.
meina_lang@berlex.com

The CC chemokine receptor-1 (CCR1) is a prime therapeutic target for treating autoimmune diseases. Through high capacity screening followed by chemical optimization, we identified a novel non-peptide CCR1 antagonist, R-N-[5-chloro-2-[2-[4-[(4-fluorophenyl)methyl]-2-methyl-1-piperazinyl]-2-oxoethoxy]phenyl]urea hydrochloric acid salt (BX 471). Competition binding studies revealed that BX 471 was able to displace the CCR1 ligands macrophage inflammatory protein-1alpha (MIP-1alpha), RANTES, and monocyte chemotactic

protein-3 (MCP-3) with high affinity (K(i) ranged from 1 nm to 5.5 nm). BX 471 was a potent functional antagonist based on its ability to inhibit a number of CCR1-mediated effects including Ca(2+) mobilization, increase in extracellular acidification rate, CD11b expression, and leukocyte migration. BX 471 demonstrated a greater than 10,000-fold selectivity for CCR1 compared with 28 G-protein-coupled receptors. Pharmacokinetic studies demonstrated that BX 471 was orally active with a bioavailability of 60% in dogs. Furthermore, BX 471 effectively reduces disease in a rat experimental allergic encephalomyelitis model of multiple sclerosis. This study is the first to demonstrate that a non-peptide chemokine receptor antagonist is efficacious in an animal model of an autoimmune disease. In summary, we have identified a potent, selective, and orally available CCR1 antagonist that may be useful in the treatment of chronic inflammatory diseases.

PMID: 10748002 [PubMed - indexed for MEDLINE]

1667. Indian J Otolaryngol Head Neck Surg. 2000 Apr;52(2):118-21. doi: 10.1007/BF03000326.

Putative histogenesis of post nasal angiofibroma.

Mishra SC.

E.N.T. Department, K.G. Medical College, 226 003 Lucknow.

Inspite of the histological resemblance, these tumefactions do not behave like true neoplasms. Harma (1959) believed their histogenesis from angioblasls. Taxy (1977) regards the fihroblasts as the main stroma cell which has resemblences like those of the granulation tissues, and hence appear to be hybrid fihroblasts. Vascular and fibrous element could be produced by any mesodermal "stem cell" (Shenoi. 1989). Therefore both fihroblasts and angioblasts may be inter-convertible.The cavernous element of tumour suggest it to be the hamartoma. The posterior part of nose and turbinates have very vascular and cavernous mucosa. The hamartomas are influenced by endocrine factors and growth hormones. The mesodermal element of this tumour also appear to have androgen receptors (Lee et al, 1980). These endocrine factors could be the initial triggering mechanisms, for their development appear most rapidly at the parapubertal age. The tumour is also influenced by peri-and apocrine factors. The eytogenic growth factors are locally available. The neoangiogenesit is, perhaps, influenced by these factors, which also induce destruction of basement membrane of vessels, migration and mitosis of angioblasts and formation of new blood vessels of capillary and larger sizes. The maturation of tumour appears to he heralded by reduction in endocrine factor tike growth hormone and establishment of sex hormones beyond the puberty. Further it is accelerated by local peri-and apocrine factors contributed by macrophages, lymphocytes etc. Those cases with heavy infiltration, therefore, have more often the signs of maturity like collagenisation, encapsulation, obliteration of vascular elements and necrobiotic phenomenon. These are exceptional tumours estntially observed amongst para-puberal males, having easy bleeding tendency and multidirectional extensions. They destroy the hones of skull and occupy adjoining cavities including cranial extensions in almost 20% cases (Ward et al, 1974). There is high recurrence rate (20 to 50% after initial excision). There are different views regarding their aetiopathogenesis, but none is able to explain all its features. These are considered to be inflammatory or allergic in origin (Willis, 1953), hyperplastic tissue reactions (Harma, 1959), due to androgen deficiency (Martin et al., 1948), vascular malformation (Osborn,1959) and hamartomas (Mishra & Bhatia.1964). Lately, micro-histological and marker techniques and better understanding of oncogenesis, have been available which give more insight in explaining its behaviour. In view of controversies, a retrospective study of more than 300 cases of angiofibromas is

carried out to formulate a putative pathogenesis of this tumour and its peculiar behaviour.

PMCID: PMC3451275
PMID: 23119647 [PubMed]

1668. Immunopharmacology. 2000 Mar;46(3):209-22.

C3a and C5a enhance granulocyte adhesion to endothelial and epithelial cell monolayers: epithelial and endothelial priming is required for C3a-induced eosinophil adhesion.

Jagels MA, Daffern PJ, Hugli TE.

Department of Immunology, The Scripps Research Institute, La Jolla, CA 92037 USA.

Effects of the anaphylatoxins C3a and C5a on eosinophil and neutrophil adhesion to HUVEC and to primary culture human bronchial epithelial cells (HBEC) were investigated. Activities on both leukocytes and on structural cells were examined. C3a upregulated beta2 integrin expression and caused shedding of L-selectin on eosinophils, but had no effect on neutrophil adhesion molecule expression. C5a upregulated beta2 integrins and caused shedding of L-selectin on both eosinophils and neutrophils. The potency of C5a was equivalent on both cell types; however, the magnitude of the changes in each of these adhesion molecules was significantly greater in neutrophils than eosinophils. Neither C3a nor C5a altered expression of ICAM-1, VCAM-1, E-selectin or P-selectin on either HUVEC or HBEC. C5a induced adhesion of both neutrophils and eosinophils to unstimulated HUVEC or HBEC, and adhesion was further enhanced when HUVEC and HBEC were "primed" with TNF-alpha and IFN-gamma, respectively. C3a failed to enhance adhesion of either eosinophils or neutrophils to unprimed HUVEC or HBEC, and enhanced only eosinophil adhesion to cytokine-primed HUVEC or HBEC. Similar to C3a, C3a(desArg) and a C3a-analog peptide E7 also enhanced eosinophil adhesion only to cytokine-primed HUVEC and HBEC. These results support the traditional view of anaphylatoxins as leukocyte-specific mediators. The specificity of C3a for eosinophils implicates this molecule as a potential participant in allergic inflammation. The pro-adhesive effects of C3a(desArg) suggest that this molecule, previously characterized as a spasmogenically inactive derivative of C3a, may also alter leukocyte dynamics and migration. Finally, activation of endothelium may represent an important control mechanism for C3a-mediated adhesion preventing unchecked eosinophil adhesion to uninflamed systemic vasculature.

PMID: 10741901 [PubMed - indexed for MEDLINE]

1669. Respirology. 2000 Mar;5(1):1-10.

The interaction of neutrophils with respiratory epithelial cells in viral infection.

Wang SZ, Forsyth KD.

Department of Paediatrics, Flinders Medical Centre, Bedford Park, South Australia, Australia.

Viral respiratory infection is very common. Respiratory syncytial virus (RSV) infects almost all children during the first 2 years of life. Respiratory syncytial virus is the most frequent cause of bronchiolitis, which is strongly linked with asthma. However, the pathophysiology of RSV bronchiolitis is unclear. Neutrophils are the predominant airway leucocytes in RSV bronchiolitis and other

viral infections. Neutrophils and their products are likely to play an important role in viral infection. Current evidence indicates that: (i) viral infection of epithelial cells increases the production of neutrophil chemoattractants or chemokines, which induce neutrophil migration into the inflammatory sites; (ii) the expression of adhesion molecules on neutrophils and epithelial cells is up-regulated in viral infection, and neutrophil-epithelial adhesion is increased; (iii) neutrophils augment epithelial damage and detachment induced by viral infection and contribute to the pathophysiology of viral disease; (iv) neutrophil apoptosis is up-regulated in RSV infection, which may be an in vivo mechanism to limit neutrophil-induced epithelial damage; (v) inhibitors of chemokines, adhesion molecules or neutrophil proteases may be useful in prevention of neutrophil-induced epithelial damage. In conclusion, neutrophils play an important role in viral infection, and intervention to prevent neutrophil-induced epithelial damage may be a potential clinical therapy.

PMID: 10728725 [PubMed - indexed for MEDLINE]

1670. J Immunol. 2000 Apr 1;164(7):3847-54.

Migration of eosinophils across endothelial cell monolayers: interactions among IL-5, endothelial-activating cytokines, and C-C chemokines.

Shahabuddin S, Ponath P, Schleimer RP.

Department of Medicine, Division of Clinical Immunology, Johns Hopkins Asthma and Allergy Center, Johns Hopkins University School of Medicine, Baltimore, MD 21224, USA.

Eosinophils are the predominant cell type recruited in inflammatory reactions in response to allergen challenge. The mechanisms of selective eosinophil recruitment in allergic reactions are not fully elucidated. In this study, the ability of several C-C chemokines to induce transendothelial migration (TEM) of eosinophils in vitro was assessed. Eotaxin, eotaxin-2, monocyte chemotactic protein (MCP)-4, and RANTES induced eosinophil TEM across unstimulated human umbilical vein endothelial cells (HUVEC) in a concentration-dependent manner with the following rank order of potency: eotaxin approximately eotaxin-2 > MCP-4 approximately RANTES. The maximal response induced by eotaxin or eotaxin-2 exceeded that of RANTES or MCP-4. Preincubation of eosinophils with anti-CCR3 Ab (7B11) completely blocked eosinophil TEM induced by eotaxin, MCP-4, and RANTES. Activation of endothelial cells with IL-1beta or TNF-alpha induced concentration-dependent migration of eosinophils, which was enhanced synergistically in the presence of eotaxin and RANTES. Anti-CCR3 also inhibited eotaxin-induced eosinophil TEM across TNF-alpha-stimulated HUVEC. The ability of eosinophil-active cytokines to potentiate eosinophil TEM was assessed by investigating eotaxin or RANTES-induced eosinophil TEM across resting and IL-1beta-stimulated HUVEC in the presence or absence of IL-5. The results showed synergy between IL-5 and the chemokines but not between IL-5 and the endothelial activator IL-1beta. Our data suggest that eotaxin, eotaxin-2, MCP-4, and RANTES induce eosinophil TEM via CCR3 with varied potency and efficacy. Activation of HUVEC by IL-1beta or TNF-alpha or priming of eosinophils by IL-5 both promote CCR3-dependent migration of eosinophils from the vasculature in conjunction with CCR3-active chemokines.

PMID: 10725746 [PubMed - indexed for MEDLINE]

1671. Pediatr Allergy Immunol. 1999 Nov;10(4):253-7.

Cutaneous lymphocyte-associated antigen expression in children with atopic

dermatitis and non-atopic healthy children.

Campbell DE, Kemp AS.

Department of Immunology, Royal Children's Hospital, Melbourne, Parkville, Victoria, Australia.

Cutaneous lymphocyte-associated antigen (CLA) is a cell surface glycoprotein which has been implicated in the homing of lymphocytes to cutaneous sites. It is postulated to play an important role in T-cell migration to skin in atopic dermatitis; however, the expression of CLA in both normal children and children with atopic dermatitis has not been extensively studied. If CLA expression on T cells were important in the traffic of lymphocytes to atopic dermatitis skin lesions, it might be expected that the proportion of CLA+ T cells in unstimulated peripheral blood from children with atopic dermatitis would be elevated. We have examined the proportion of CLA+ T cells in children with atopic dermatitis and non-atopic age-matched controls. The proportion of CLA+ T cells in non-atopic children was highly associated with and increased with increasing age ($r = 0.88$, $p < 0.001$). There was no difference between the proportion of T cells expressing CLA in the unstimulated peripheral blood mononuclear cells from children with severe ($p = 0.18$) or with mild/moderate ($p = 0.3$) atopic dermatitis and age-matched non-atopic controls. Despite this, children with atopic dermatitis did show evidence of perturbation of CLA expression, as unlike the non-atopic children the proportion of CLA+ T cells in the atopic children did not correlate with age. These findings suggest that while CLA expression may play a role in atopic dermatitis, other as yet undefined surface markers are likely to principally determine the migration of T cells to skin in atopic dermatitis.

PMID: 10678721 [PubMed - indexed for MEDLINE]

1672. Pulm Pharmacol Ther. 2000;13(1):31-8.

Inhibition of eosinophil transepithelial migration and downregulation of adhesion molecule expression on eosinophils and airway epithelial cells induced by budesonide.

Gonzalez Rodriguez R, Silvestri M, Cordone A, Salami A, Rossi GA.

Pulmonary Division, G, Gaslini Institute, Genoa, Italy.

In asthma, eosinophil migration through the bronchial mucosa is mediated by the expression of surface molecules on eosinophils and airway epithelial cells. To characterize the activity of budesonide on eosinophil transepithelial migration, blood eosinophils were isolated from atopic asthmatic subjects and human bronchial epithelial cells (HBECs) from surgically resected bronchi. In the presence of different concentrations of budesonide (0.1-100 nM), we tested: a) eosinophil migration induced by C5a through HBEC monolayers; b) ICAM-1 expression on HBECs, stimulated with C5a and c) LFA-1 and Mac-1 expression on eosinophils, stimulated with C5a or with ah-CD23 mabs plus GM-CSF. Eosinophils showed a remarkable chemotactic response to C5a ($P<0.001$), that was effectively down-regulated by the presence in the chemotactic chambers of budesonide at all the concentrations tested ($P<0.05$). A weaker, but still present, inhibitory activity on cell locomotion was observed when HBECs or eosinophils were preincubated with budesonide before the chemotaxis assay, which was performed in absence of the drug. Preincubation of the cells with different concentrations of budesonide was also effective in down-regulating the C5a-induced ICAM-1 expression on HBECs and the ah-CD23 and GM-CSF-induced LFA-1 and Mac-1 expression on eosinophils. Thus, budesonide-induced down-regulation of eosinophil migration through airway epithelial cells is associated with, and possibly partially

dependent on inhibition of adhesion molecule expression on both cell types.

Copyright 2000 Academic Press.

PMID: 10718988 [PubMed - indexed for MEDLINE]

1673. Clin Exp Allergy. 2000 Apr;30(4):469-75.

Clara cell secretory protein (CC16): characteristics and perspectives as lung peripheral biomarker.

Broeckaert F, Bernard A.

Industrial Toxicology and Occupational Medicine Unit, Faculty of Medicine, School of Public Health, Catholic University of Louvain, Brussels, Belgium.

Clara cell protein (CC16) is a 15.8-kDa homodimeric protein secreted in large amounts in airways by the non-ciliated bronchiolar Clara cells. This protein increasingly appears to protect the respiratory tract against oxidative stress and inflammation. In vitro, CC16 has been shown to modulate the production and/or the activity of various mediators of the inflammatory response including PLA2, interferon-gamma and tumour necrosis factor-alpha. CC16 has also been found to inhibit fibroblast migration or to bind various endogenous or exogenous substances such as polychlorobiphenyls (PCBs). This protective role is confirmed by studies on transgenic mice, showing that CC16 deficiency is associated with an increased susceptibility of the lung to viral infections and oxidative stress. In humans, a polymorphism of the CC16 gene, localized to a region linked to airway diseases, has recently been discovered in association with an increased risk of developing childhood asthma. Finally, CC16 also presents a major interest as a peripheral marker for assessing the integrity of the lung epithelium. The determination of CC16 in serum is a new non-invasive test to detect Clara cell damage or an increased epithelial permeability in various acute and chronic lung disorders.

PMID: 10718843 [PubMed - indexed for MEDLINE]

1674. Blood. 2000 Mar 15;95(6):2052-8.

Nerve growth factor functions as a chemoattractant for mast cells through both mitogen-activated protein kinase and phosphatidylinositol 3-kinase signaling pathways.

Sawada J, Itakura A, Tanaka A, Furusaka T, Matsuda H.

Department of Veterinary Clinic, Faculty of Agriculture, Tokyo University of Agriculture and Technology, Fuchu, Tokyo, Japan.

Despite being a well-characterized neurotrophic factor, nerve growth factor (NGF) influences survival, differentiation, and functions of mast cells. We investigated whether NGF was able to induce directional migration of rat peritoneal mast cells (PMCs). NGF clearly induced chemotactic movement of PMCs in a dose-dependent manner with the drastic morphological change and distribution of F-actin, which was completely blocked by pretreatment with Clostridium botulinum C(2) toxin, an actin-polymerization inhibitor. Because PMCs constitutively express the NGF high-affinity receptor (TrkA) with a tyrosine kinase domain, we focused on downstream effectors in signaling cascades following the TrkA. NGF rapidly activated both mitogen-activated protein kinase (MAPK) and phosphatidylinositol 3-kinase (PI3K), and the addition of inhibitors specific for

MAPK kinase and PI3K suppressed cell migration and these signals. In the coculture system with PMCs and fibroblasts, which produce biologically active NGF, directional migration of PMCs to fibroblasts was observed, and the addition of anti-NGF polyclonal antibodies significantly suppressed the migration of PMCs. These findings suggested that NGF initiated chemotactic movement of PMCs through both MAPK and PI3K signaling pathways following TrkA activation. Thus, locally produced NGF may play an important role in mast cell accumulation in allergic and nonallergic inflammatory conditions. (Blood. 2000;95:2052-2058)

PMID: 10706874 [PubMed - indexed for MEDLINE]

1675. Mult Scler. 2000 Feb;6(1):3-13.

Chemokines and chemokine receptors in the pathogenesis of multiple sclerosis.

Zhang GX, Baker CM, Kolson DL, Rostami AM.

Department of Neurology, University of Pennsylvania Medical School, Philadelphia, PA 19104-4283, USA.

In recent years we have seen growing evidence for the role of chemokines in the pathogenesis of several infectious and non-infectious inflammatory CNS disease states, including Multiple Sclerosis (MS) and its animal model, experimental allergic encephalomyelitis (EAE). An increase in proinflammatory chemokines has been associated with demyelinating lesions and clinical neurological dysfunction in patients with MS; these chemokines could be potential targets for MS therapy. Besides a clearly defined role in mediating leukocyte migration, these and other chemokines may act as immunoregulatory molecules in the driving to Th1/Th2 responses, switch of cytokine profiles, and the induction of tolerance. Since chemokine receptors have now been identified on macrophages, microglia, astrocytes, and endothelial cells as well as neurons in the CNS, chemokine/receptor interactions may mediate functional responses in a variety of CNS cell types during the course of inflammatory disease states. Therefore, clarification of the roles of chemokines and their receptors in the pathogenesis of EAE and MS will be useful in establishing immunotherapeutic strategies for these neurological autoimmune disorders.

PMID: 10694839 [PubMed - indexed for MEDLINE]

1676. J Immunol. 2000 Mar 1;164(5):2267-71.

Cutting edge: lipoxin (LX) A4 and aspirin-triggered 15-epi-LXA4 block allergen-induced eosinophil trafficking.

Bandeira-Melo C, Bozza PT, Diaz BL, Cordeiro RS, Jose PJ, Martins MA, Serhan CN.

Department of Physiology and Pharmacodynamics, Oswaldo Cruz Institute, Fundaçao Oswaldo Cruz, Rio de Janeiro, Brazil.

Tissue eosinophilia prevention represents one of the primary targets to new anti-allergic therapies. As lipoxin A4 (LXA4) and aspirin-triggered 15-epi-LXA4 (ATL) are emerging as endogenous "stop signals" produced in distinct pathologies including some eosinophil-related pulmonary disorders, we evaluated the impact of in situ LXA4/ATL metabolically stable analogues on allergen-induced eosinophilic pleurisy in sensitized rats. LXA4/ATL analogues dramatically blocked allergic pleural eosinophil influx, while concurrently increasing circulating eosinophilia, inhibiting the earlier edema and neutrophilia associated with allergic reaction. The mechanisms underlying this LXA4/ATL-driven allergic

eosinophilia blockade was independent of mast cell degranulation and involved LXA4/ATL inhibition of both IL-5 and eotaxin generation, as well as platelet activating factor action. These findings reveal LXA4/ATL as a novel class of endogenous anti-allergic mediators, capable of preventing local eosinophilia.

PMID: 10679058 [PubMed - indexed for MEDLINE]

1677. Int Arch Allergy Immunol. 2000 Jan;121(1):25-33.

The effect of antigen stimulation on alpha(4), beta(1) and beta(7) chain integrin expression and function in CD4+ cells.

Tarkowski M, Pacheco KA, Rosenwasser LJ.

Institute of Occupational Medicine, Department of Occupational Diseases, Lodz, Poland.

BACKGROUND: The alpha(4) integrin, as alpha(4)beta(1) (VLA-4) or alpha(4)beta(7), is critical for T cell migration and proliferation, although its functional modulation remains poorly understood. We hypothesized that increased receptor density, based on new receptor chain synthesis, was one such mechanism. We examined the surface receptor density of the alpha(4) and beta(1) chains on CD4+CD45RO+ cells, and the mRNA expression of these and the beta(7) chain in response to allergen and nonallergen antigen stimulation.
METHODS: Flow-cytometric analyses for CD49d, CD29, and CD45RO were performed on T cell lines specific for timothy, tetanus, and Candida from atopic and nonatopic donors. RNA was extracted from cells sorted to select CD4+/CD49d-positive cells before and after stimulation. Equivalent amounts of cDNA for beta-actin, alpha(4), beta(1) and beta(7) were used in PCR, and the products were quantified using phosphoimaging.
RESULTS: CD49d expression is heterogeneous on T cell lines and is upregulated by antigen stimulation on CD4+ T cells. The surface expression on CD4+CD45RO+ timothy allergen or tetanus toxoid T cell lines is at least double that found on CD45RO- cells. Antigen stimulation upregulated CD49d expression on the CD4+CD45RO+ subpopulation of both cell lines although it was not as significant as in the case of all CD4+ T cells. CD29 surface expression behaves similarly. Candida had no effect on CD49d or CD29. Messenger RNA expression for the alpha(4) chain (CD49d) is significantly upregulated 48 h following the addition of timothy or tetanus. beta(7) chain expression also rises significantly on both cell lines. beta(1) chain expression increases, but not significantly.
CONCLUSIONS: The surface expression of the CD49d is heterogeneous and much higher on CD4+CD45RO+ cells than on CD4+RO- T cells. The CD49d integrin chain on CD4+ T cells is upregulated following antigen exposure. However, the CD4+CD45RO+ subpopulation is only partially responsible for this increase suggesting other T cells to have this receptor expression upregulated. CD29 expression behaves similarly. Messenger RNA expression increases coordinately for alpha(4), beta(7), and not significantly for beta(1) in these cells. These observations provide a potential mechanism for the selective accumulation of T cells at sites of inflammation, and suggest an important point of intervention for allergic and inflammatory disease.

Copyright 2000 S. Karger AG, Basel

PMID: 10686506 [PubMed - indexed for MEDLINE]

1678. Am J Respir Crit Care Med. 2000 Feb;161(2 Pt 1):558-66.

Lymphocytes migrate from the blood into the bronchoalveolar lavage and lung

parenchyma in the asthma model of the brown Norway rat.

Schuster M, Tschernig T, Krug N, Pabst R.

Centers of Anatomy, Anesthesiology and Pneumology, Medical School of Hanover, Hanover, Germany.

Lymphocyte migration from the blood into the lung has been suggested as being responsible for the increase of lymphocytes, in particular CD4 T cells, in the bronchoalveolar lavage (BAL) and bronchial mucosa in human asthma, but so far there has been no direct proof. We studied lymphocyte immigration and lymphocyte subpopulations in three lung compartments in ovalbumin (OVA)-sensitized and -challenged brown Norway (BN) rats. Increased numbers of CD4 and interleukin 2 (IL-2) receptor-positive T cells were found in the BAL and lung parenchyma in treated animals, but also increased numbers of CD8 T cells, B cells, and natural killer (NK) cells. For direct proof of lymphocyte migration from the blood into the lung, leukocytes were labeled with a fluorescent dye, 5- (and 6-) carboxyfluorescein-diacetate-succinimidyl-ester (CFSE), and injected intravenously immediately prior to OVA aerosol challenge. One day after challenge the number of CFSE(+), i.e., newly immigrated lymphocytes, was determined by flow cytometry gated on the lymphocyte cluster. A 15 times (1.5 times) higher number of CFSE(+) lymphocytes was found in the BAL (the lung parenchyma) of treated animals in comparison with control rats. In the BAL 51.8% of CFSE(+) cells were CD4-positive (parenchyma 72.7%) and 29.4% IL-2 receptor-positive (parenchyma 34.2%). There was no difference whether the leukocytes for labeling and injection were obtained from untreated or from OVA-sensitized donor animals. Our data show that lymphocyte immigration is at least in part responsible for the increase in lymphocyte numbers in the BAL and lung parenchyma in this animal asthma model.

PMID: 10673200 [PubMed - indexed for MEDLINE]

1679. J Leukoc Biol. 2000 Feb;67(2):189-95.

Role of endothelins on lymphocyte accumulation in allergic pleurisy.

Sampaio AL, Rae GA, Henriques MM.

Laboratory of Applied Pharmacology, FarManguinhos, FIOCRUZ, Rio de Janeiro, Brazil.

Endothelins participate in different aspects of inflammatory reactions, including edema formation and eosinophil accumulation in allergic reaction. In this study, we demonstrated a role for endogenous endothelins in eosinophil and T lymphocyte recruitment and cytokine secretion in a murine model of allergic inflammation. Intrathoracic stimulation with endothelin-1 triggered a neutrophil accumulation at 4 h, concomitant with an increase of CD4+ and CD8+ T lymphocyte populations. Antigen challenge in sensitized animals leads to an increase in eosinophil and mononuclear cell numbers at 24 h. Treatment with ETA receptor antagonist (BQ123) inhibited antigen-induced eosinophil and mononuclear cell migration, whereas the selective ETB receptor antagonist BQ-788 was ineffective. The latter effect of BQ-123 was due to inhibition of CD4+ and CD8+ T lymphocytes. Treatment with BQ-123 also inhibited interleukin-5 levels in the exudate and plasma as well as intracellular staining of interleukin-4, interleukin-5, and interferon-gamma in CD4+ lymphocytes. These findings suggest that endogenous endothelins contribute to allergic inflammation by modulating lymphocyte recruitment and cytokine production.

PMID: 10670579 [PubMed - indexed for MEDLINE]

1680. J Allergy Clin Immunol. 2000 Feb;105(2 Pt 2):S514-7.

Structural consequences of airway inflammation in asthma.

Vignola AM, Chanez P, Bonsignore G, Godard P, Bousquet J.

Istituto di Fisiopatologia Respiratoria, Palermo, Italy.

Asthma represents a chronic inflammatory process of the airways followed by healing, the end-result of which is an altered structure referred to as a remodeling of the airways. Repair usually involves 2 distinct processes: regeneration (which is the replacement of injured tissue by parenchymal cells of the same type) and replacement by connective tissue and its eventual maturation into scar tissue. In many instances both processes contribute to the healing response and inflammation. In asthma the processes of cell dedifferentiation, migration, differentiation, and maturation and connective tissue deposition can be followed either by complete or altered restitution of airway structure and function, the latter often seen as fibrosis and increase in smooth muscle and mucus gland mass. These features result in an increased resistance to airflow, particularly when there is bronchial contraction and bronchial hyperresponsiveness. The effect on airflow is compounded by the presence of increased mucous secretion and inflammatory exudate, which not only blocks the airway passages but also causes an increased surface tension that favors airway closure.

PMID: 10669534 [PubMed - indexed for MEDLINE]

1681. J Immunol. 2000 Feb 15;164(4):2142-50.

The effect of IL-5 and eotaxin expression in the lung on eosinophil trafficking and degranulation and the induction of bronchial hyperreactivity.

Mould AW, Ramsay AJ, Matthaei KI, Young IG, Rothenberg ME, Foster PS.

Division of Biochemistry, John Curtin School of Medical Research, Australian National University, Canberra, Australia.

The mechanisms regulating the selective migration and degranulation of eosinophils in the asthmatic lung and the subsequent development of airways hyperreactivity (AHR) have not been fully delineated. In this investigation, we have employed a novel transgene model to facilitate the dissection of the contributions of IL-5 and/or eotaxin to eosinophil function in the absence of complex tissue signals derived from the allergic lung. Gene transfer of IL-5 and/or eotaxin to the lungs of naive mice induced a pronounced and selective airways eosinophilia, but did not result in eosinophil degranulation or AHR. Airways eosinophilia occurred independently of the induction of a blood eosinophilia, but was markedly augmented by the coexpression of both cytokines and/or by the transient mobilization of eosinophils from the bone marrow by the administration of i.v. IL-5. However, for eosinophil degranulation and AHR to occur, the inhalation of Ag was required in association with IL-5 and eotaxin expression. Investigations in IL-5-deficient mice linked eosinophilia, and not solely IL-5 and eotaxin, with the induction of AHR. Furthermore, eosinophil degranulation and AHR were dependent on CD4+ T cells. Importantly, this investigation shows that IL-5 regulates eosinophilia within the lung as well as in the circulation and also amplifies eotaxin-induced chemotaxis in the airway compartment. Moreover, the interplay between these cytokines, CD4+ T cells, and factors generated by Ag inhalation provides fundamental signals for eosinophil degranulation and the induction of AHR.

PMID: 10657668 [PubMed - indexed for MEDLINE]

1682. Respir Med. 1999 Dec;93(12):919-22.

Early house moves, indoor air, heating methods and asthma.

Jones RC, Hughes CR, Wright D, Baumer JH.

The Roborough Surgery 1, Eastcote Close, UK.

OBJECTIVES: To assess whether house moves or certain housing conditions are a risk factor for the development of childhood asthma.
DESIGN: A case-control study of asthmatic and non-atopic children aged 4-16 years.
SUBJECTS: One hundred children with confirmed asthma in a group general practice of 11000 patients in Plymouth, U.K. Each was matched by age and gender with a child with no history of wheeze, eczema or hay fever. Main outcome measures: House moves and main heating methods, prior to the age of onset of asthma in cases and controls.
RESULTS: There was a non-significant association between early house moves and the subsequent development of asthma. No association was found with heating methods, except for ducted-air heating which, because of the small numbers involved could have occurred by chance. None of the other factors studied affecting indoor air showed an association.
CONCLUSION: Moving house at an early age may increase the risk of developing asthma, or may be associated with other more important risk factors, such as increased general mobility and hence, exposure to viral infections. Heating methods or other factors likely to affect the indoor air quality in early life were not useful predictors of subsequent asthma in children.

PMID: 10653056 [PubMed - indexed for MEDLINE]

1683. J Immunol. 2000 Feb 1;164(3):1488-97.

C-C chemokine receptor 3 antagonism by the beta-chemokine macrophage inflammatory protein 4, a property strongly enhanced by an amino-terminal alanine-methionine swap.

Nibbs RJ, Salcedo TW, Campbell JD, Yao XT, Li Y, Nardelli B, Olsen HS, Morris TS, Proudfoot AE, Patel VP, Graham GJ.

Cancer Research Campaign Laboratories, Beatson Institute for Cancer Research, Glasgow, United Kingdom. r.nibbs@beatson.gla.ac.uk

Allergic reactions are characterized by the infiltration of tissues by activated eosinophils, Th2 lymphocytes, and basophils. The beta-chemokine receptor CCR3, which recognizes the ligands eotaxin, eotaxin-2, monocyte chemotactic protein (MCP) 3, MCP4, and RANTES, plays a central role in this process, and antagonists to this receptor could have potential therapeutic use in the treatment of allergy. We describe here a potent and specific CCR3 antagonist, called Met-chemokine beta 7 (Ckbeta7), that prevents signaling through this receptor and, at concentrations as low as 1 nM, can block eosinophil chemotaxis induced by the most potent CCR3 ligands. Met-Ckbeta7 is a more potent CCR3 antagonist than Met- and aminooxypentane (AOP)-RANTES and, unlike these proteins, exhibits no partial agonist activity and is highly specific for CCR3. Thus, this antagonist may be of use in ameliorating leukocyte infiltration associated with allergic inflammation. Met-Ckbeta7 is a modified form of the beta-chemokine macrophage

inflammatory protein (MIP) 4 (alternatively called pulmonary and activation-regulated chemokine (PARC), alternative macrophage activation-associated C-C chemokine (AMAC) 1, or dendritic cell-derived C-C chemokine (DCCK) 1). Surprisingly, the unmodified MIP4 protein, which is known to act as a T cell chemoattractant, also exhibits this CCR3 antagonistic activity, although to a lesser extent than Met-Ckbeta7, but to a level that may be of physiological relevance. MIP4 may therefore use chemokine receptor agonism and antagonism to control leukocyte movement in vivo. The enhanced activity of Met-Ckbeta7 is due to the alteration of the extreme N-terminal residue from an alanine to a methionine.

PMID: 10640766 [PubMed - indexed for MEDLINE]

1684. J Allergy Clin Immunol. 2000 Jan;105(1 Pt 1):58-64.

Intranasal administration of eotaxin increases nasal eosinophils and nitric oxide in patients with allergic rhinitis.

Hanazawa T, Antuni JD, Kharitonov SA, Barnes PJ.

Department of Thoracic Medicine, Imperial College School of Medicine at the National Heart and Lung Institute, London, United Kingdom.

BACKGROUND: Nitric oxide (NO) plays an important role as an inflammatory mediator in the airways. Inducible NO synthase in the nasal mucosa is upregulated in perennial allergic rhinitis, and nasal NO is reduced after treatment with topical corticosteroids. A previous study has suggested that there is a significant correlation between exhaled NO and sputum eosinophils in patients with asthma.
OBJECTIVE: We investigated the ability of intranasal administration of eotaxin, a potent chemoattractant for eosinophils, to induce eosinophil accumulation and the relationship between eosinophil recruitment in the nasal mucosa and nasal NO production in patients with allergic rhinitis.
METHODS: Nine patients with allergic rhinitis were studied. Eotaxin or diluent was delivered intranasally in patients by using a metered spray pump. Nasal NO, symptom scores, and the influx of inflammatory cells in nasal lavage fluid were assessed before and after the challenge. Immunoreactivity for inducible NO synthase and nitrotyrosine was evaluated in nasal lavage cells.
RESULTS: Eotaxin induced a significant influx of eosinophils ($P < .05$) with mild symptoms of rhinitis. There was neither significant migration of lymphocytes, basophils, and macrophages into nasal lavage fluid nor a shedding of nasal epithelial cells after eotaxin challenge. Nasal NO was increased significantly ($P < .05$) 8 hours after eotaxin challenge compared with diluent challenge. Nitrotyrosine immunoreactivity was moderately elevated in nasal epithelial cells after the challenge.
CONCLUSION: We have shown that eotaxin causes chemotaxis of eosinophils with a clinically symptomatic inflammatory response in the nasal mucosa and that eosinophil recruitment accompanies an increase in nasal NO, contributing to oxidative stress.

PMID: 10629453 [PubMed - indexed for MEDLINE]

1685. QJM. 1999 Aug;92(8):463-71.

CCR7 (EBI1) receptor down-regulation in asthma: differential gene expression in human CD4+ T lymphocytes.

Syed F, Blakemore SJ, Wallace DM, Trower MK, Johnson M, Markham AF, Morrison JF.

Molecular Medicine Unit, University of Leeds, St James's University Hospital, UK.
rmrfs@stjames.leeds.ac.uk

Asthma is an inflammatory disorder, and the CD4+ T lymphocyte plays a key role in mediating the inflammatory response. We used a high-density grid, hybridization-based, differential gene expression technology to analyse molecular mechanisms underlying in vivo CD4+ T-cell activation in both steroid-resistant asthma (SRA) and steroid-sensitive asthma (SSA). Hybridization of radioactively-labelled first-strand cDNAs prepared from different biological samples, to identical high-density gridded arrays of PCR amplicons derived from cDNA clone inserts immobilized on nylon membranes, was compared by phosphorimaging. Hybridization data were captured and processed using image analysis software that can identify the location and signal intensity of each hybridized cDNA. This produces a hierarchy of signals of differing intensities between the two grids, representing differential gene expression in the two different RNA samples. CCR7 (EBI1), a lymphocyte-specific G-protein-coupled receptor, was down-regulated in the CD4+ T cells of SRA and SSA non-atopic, compared to non-asthmatic non-atopic individuals. This observation is intriguing given that CCR7 and its ligand EBI1-Ligand Chemokine (ELC), may play a role in the migration and homing of normal lymphocytes. Also, TNFR2 is up-regulated in both SSA non-atopic and SRA atopic as compared to non-asthmatic controls. LAMR1 is down-regulated in CD4+ T cells of SRA compared to non-asthmatic individuals, irrespective of their atopic status. These could be general phenomena resulting from cytokine release.

PMID: 10627863 [PubMed - indexed for MEDLINE]

1686. J Invest Dermatol. 2000 Jan;114(1):135-41.

Imiquimod, a topical immune response modifier, induces migration of Langerhans cells.

Suzuki H, Wang B, Shivji GM, Toto P, Amerio P, Tomai MA, Miller RL, Sauder DN.

Division of Dermatology, Sunnybrook Health Science Center, University of Toronto, Ontario, Canada.

Langerhans cells are bone marrow derived dendritic cells that represent the major antigen-presenting cells in the skin. Langerhans cells take up and process antigen within the epidermis and present processed antigen to T lymphocyte in the regional lymph nodes and thus form an integral part of the cutaneous immune response. The cutaneous immune response can be modified by a number of pharmacologic agents, including corticosteroids, cyclosporine, and retinoids as well as physical agents, such as ultraviolet light. For the most part these agents act by suppressing immune function. A topical immune response modifier, imiquimod has been shown to enhance the cutaneous immune response. Imiquimod has anti-viral and anti-tumor effects in animal models and has been approved for the topical treatment of external genital and perianal warts in humans. The biologic activity of imiquimod in part is due to its effect as a cytokine inducer. Preliminary data suggested that imiquimod could have an effect on Langerhans cells. In order to clarify this effect on Langerhans cells, we examined Langerhans cell morphology and migration in imiquimod-treated skin. The density of Ia + cells decreased 2 d after treatment, falling to approximately 43% by day 10. The Ia positive in cells remaining in the skin appeared larger and more dendritic suggesting an activated state. ATPase staining of epidermal sheet confirmed the decreased number of Langerhans cells. To clarify status of Langerhans cells, the activation of B7 was examined. Activation of B7-1 or B7-2 was not detected. Imiquimod, however, did enhance Langerhans cell migration from skin to draining lymph nodes. This enhanced Langerhans cell migration was also

associated with an enhanced allergic contact hypersensitivity. These results suggest that the mechanism of modulation of immune response by imiquimod is in part due to effects on Langerhans cells.

PMID: 10620129 [PubMed - indexed for MEDLINE]

1687. J Ethnopharmacol. 1999 Nov 30;67(3):259-67.

Effect of a herbal protein, CI-1, isolated from Cajanus indicus on immune response of control and stressed mice.

Datta S, Sinha S, Bhattacharyya P.

Department of Chemistry, Bose Institute, Calcutta, India.

The effect of a herbal protein, CI-1, purified from the leaves of Cajanus indicus was evaluated as a probable immunomodulator on immune response in control and stressed mice. In mice, sensitized with sheep red blood cells (SRBC), CI-1 enhanced IgG level by 28% over control ($P < 0.05$). Primary and secondary antibody response to SRBC and bovine serum albumin (BSA) were significantly increased in CI-1 treated group. Furthermore CI-1 facilitated the delayed type of hypersensitivity (DTH) response to SRBC in sensitized mice and also enhanced leucocyte and macrophage migration inhibition response in immunized mice. A fewer mice displayed symptoms of anaphylactic shock after CI-1 administration at a dose of 6.0 mg/kg body wt. in BSA sensitized mice. The immediate effect of CI-1 on anaphylactic shock was not seen when 150 microg of CI-1 was injected in combination with BSA in the shocking injection. These results suggest that CI-1 influences both humoral and cell-mediated immune response.

PMID: 10617060 [PubMed - indexed for MEDLINE]

1688. Methods Mol Med. 2000;44:67-80. doi: 10.1385/1-59259-072-1:67.

Flow cytometric analysis of blood monocytes and alveolar macrophages.

Taylor ML, Bochner BS.

Department of Medicine, Johns Hopkins Allergy and Asthma Center, Johns Hopkins University Medical School, Baltimore, MD.

Pulmonary monocytes and macrophages are believed to function in a wide range of biological roles, including host defense against foreign organisms, maintenance of immunological homeostasis in the lung, presentation of antigen to lymphocytes, and migration to sites of tissue injury and inflammation (1). There is also mounting evidence that recruited blood monocytes and resident alveolar macrophages (AM) in lung disease express an activated phenotype, suggesting that they may play important roles in chronic respiratory diseases, including asthma and interstitial lung diseases (2-6).

PMID: 21312121 [PubMed]

1689. Forum (Genova). 1999 Oct-Dec;9(4):315-24.

Structure and function of the CC chemokine receptor (CCR) 8.

Napolitano M, Santoni A.

Laboratorio di Patologia Vascolare, Istituto Dermopatico dell'Immacolata-IRCCS, Roma, Italy.

The CC chemokine receptor (CCR) 8 belongs to the seven transmembrane-spanning receptor family and functionally responds to the eukaryotic CC chemokines I-309, thymus and activation-regulated chemokine, macrophage inflammatory protein-1 beta (MIP-1b) and to the products viral MIP-I and viral MIP-II of the Kaposi-associated herpesvirus (HHV-8). Although it has not yet been fully characterised, its restricted expression to lymphoid tissues, i.e. thymus, spleen and lymph nodes, and its abundant up-regulation in Th2 lymphocytes suggest a potential role in lymphocyte activation, migration and differentiation and in allergic diseases. In this article we review the data known up to now related to CCR8 from cloning to protein structure, expression patterns and functional activation by its agonists.

PMID: 10611408 [PubMed - indexed for MEDLINE]

1690. Ann Agric Environ Med. 1999;6(2):167-70.

Allergic alveolitis due to herb dust exposure.

Mackiewicz B, Skórska C, Dutkiewicz J, Michnar M, Milanowski J, Prazmo Z, Krysinska-Traczyk E, Cisak E.

Clinic of Lung Diseases, Medical Academy, Jaczewskiego 8, 20-950 Lublin, Poland.

We report an episode of allergic alveolitis in a female farmer due to massive exposure to organic dust contaminated with microorganisms during threshing of herbs (thyme). The patient's medical history, the results of exposure test, inhalation challenge, and bronchoalveolar lavage suggested the diagnosis of allergic alveolitis

PMID: 10607999 [PubMed - indexed for MEDLINE]

1691. Ann Pharm Fr. 1999 Nov;57(6):429-34.

[Principal cellular and molecular mechanisms of xenobiotic-induced hypersensitivity reactions].

[Article in French]

Lebrec H, Kerdine S, Gaspard I, Pallardy M.

Laboratoire de Toxicologie, Faculté de Pharmacie Paris Sud, Châtenay-Malabry.

Sensitization to xenobiotic first involves an interaction between the compound and antigen presenting cells such as Langerhans cells within the epidermis. This leads to the transdermal migration of this presenting cells towards draining lymph nodes. A second step consists in activation, proliferation and differenciation of CD4+ T cells, and CD8+ T lymphocytes involved in delayed hypersensitivity reactions such as contact dermatitis. T helper lymphocytes can differenciate into Th1 (producing interferon-gamma and interleukin (IL)-2) or Th2 (producing IL-4, IL-5, IL-10, IL-13) lymphocytes. Some experimental models allowed to demonstrate a link between Th1- or Th2-type responses and different hypersensitivity reactions. Specific antibody production also plays a key role in xenobiotic-induced allergy, especially IgE production involved in mastocytes degranulation and immediate hypersensitivity reactions.

PMID: 10605147 [PubMed - indexed for MEDLINE]

1692. Respir Med. 1999 Nov;93(11):779-87.

Current and future medical costs of asthma and chronic obstructive pulmonary disease in The Netherlands.

Rutten-van Mölken MP, Postma MJ, Joore MA, Van Genugten ML, Leidl R, Jager JC.

Institute for Medical Technology Assessment, Erasmus University Rotterdam, The Netherlands. m.p.rutten@econ.bmg.eur.nl

The aim of this study was to estimate the healthcare costs of asthma and chronic obstructive pulmonary disease (COPD), in the Netherlands, in 1993. Also studied was the future development of these costs, as a result of ageing and possible changes in smoking behavior. A prevalence-based cost-of-illness approach was used to estimate direct medical costs. Age- and gender-specific data were obtained from representative national registries and large, representative surveys. To model future costs, cost estimates were linked to an epidemiological model based on a dynamic multi-state lifetable. It describes 1 yr changes, from one state to another, that result from ageing, birth, migration, incidence, recovery from asthma and death due to asthma, COPD or other causes, and starting or quitting smoking. Three different scenarios were modelled: 1) a reference scenario which primarily predicts the impact of ageing. 2) an 'attainable' smoking reduction scenario and 3) an 'extreme' smoking reduction scenario. Direct medical costs were estimated to be $US 346 million in 1993. With increasing age, the relative importance of asthma in total asthma and COPD costs decreased from 91% to less than 4%. Annual costs per patient were estimated to be $US 499 for asthma and $US 876 for COPD. The breakdown of costs differed considerably between asthma and COPD. The reference scenario predicted the costs to increase by 60% to reach $US 555 million by 2010, COPD prevention as modelled in the second and the third scenario reduced the projected cost increase from 60%, to 57% and 48%, respectively. Together, the direct costs of asthma and COPD represent 1.3% of the Dutch health care budget. The breakdown of the costs shows different patterns for asthma and COPD. The costs of these diseases are expected to increase by 60% in the near future. In the short run the impact of smoking reduction on reducing this increase is relatively small, but it will be greater in the long run.

PMID: 10603626 [PubMed - indexed for MEDLINE]

1693. Rev Esp Enferm Dig. 1999 Oct;91(10):684-92.

Diagnosis and percutaneous treatment of gastrointestinal hemorrhage. Long-term experience.

[Article in English, Spanish]

Carreira JM, Reyes R, Pulido-Duque JM, Travieso MM, Górriz E, Pardo MD, Maynar M.

Laboratorio de Radiología, Facultad de Medicina, Santiago de Compostela, La Coruña, 15704, España.

OBJECTIVE: to report our experience in the diagnosis and treatment of gastrointestinal hemorrhage.
METHOD: from April 1987 to April 1997, 196 patients with gastrointestinal hemorrhage (134 men and 62 women) were studied. 165 (84%) were diagnosed as presenting upper gastrointestinal hemorrhage, and 31 (16%) presented lower gastrointestinal hemorrhage. The patients were studied with endoscopy and

arteriography, and embolization was prescribed in 131 (67%). Patients with bleeding from esophageal varices were excluded from this study.
RESULTS: a bleeding point was identified angiographically in 33% (n = 65) patients. 131 (67%) patients were treated with therapeutic embolization, which was successful in 89% (n = 116) patients. The bleeding was resolved in 80% (n = 93) of the patients. Complications included arterial spasm (n = 12), pain (n = 24), coil migration (n = 8), allergic reaction (n = 2) and celiac trunk dissection (n = 2). During follow-up 16 patients presented rebleeding that stopped after reembolization in 9 cases, whereas in 7 cases surgery was needed.
CONCLUSIONS: in our experience, diagnostic angiography and percutaneous therapeutic embolization are effective, less aggressive methods that lead to few complications. Both methods have become indispensable tools in managing patients with gastrointestinal hemorrhage that does not respond to conservative therapy. Even in patients with no evidence of angiographic bleeding, embolization in selected patients is successful.

PMID: 10601757 [PubMed - indexed for MEDLINE]

1694. Methods Find Exp Clin Pharmacol. 1999 Oct;21(8):541-7.

Effect of anti-ICAM-1 on bronchial response: bronchoalveolar lavage fluid (BALF) and ultrastructural changes of bronchial epithelium in guinea pigs with dual phase bronchial response.

Tohda Y, Kubo H, Nakajima S, Fukuoka M.

Fourth Department of the Internal Medicine, Kinki University School of Medicine, Osaka, Japan.

Eosinophils play an important role in the development of bronchial asthma, and the association between ICAM-1 and activation and migration of local eosinophils is attracting attention. Using an asthmatic model of dual phase bronchial response, the effects of anti-ICAM-1 antibody on the airway resistance, cell composition in the bronchoalveolar lavage fluid (BALF) and ultrastructure of bronchial ciliated epithelium were examined under the provoked response by inhalation of the antigen. By administration of anti-ICAM-1 antibody, the late asthmatic response (LAR) was suppressed. In the examination of bronchoalveolar lavage fluid, a significant decrease in eosinophils was found in LAR. In examining transmission and scanning electron microscopies, no difference was found in the immediate asthmatic response, but marked suppression of deciduation of bronchial ciliated epithelium was observed in LAR. These results indicated that anti-ICAM-1 antibody suppressed bronchial asthmatic attack, mainly in LAR, by controlling differentiation and migration of eosinophils.

PMID: 10599053 [PubMed - indexed for MEDLINE]

1695. Eur Respir J. 1999 Oct;14(4):767-75.

Procaterol inhibits IL-1beta- and TNF-alpha-mediated epithelial cell eosinophil chemotactic activity.

Koyama S, Sato E, Masubuchi T, Takamizawa A, Kubo K, Nagai S, Isumi T.

The First Dept of Internal Medicine, Shinshu University School of Medicine, Matsumoto, Japan.

Theophylline inhibits eosinophilic infiltration into the bronchial wall. It is unknown whether this is mediated by a cyclic adenosine monophosphate

(c-AMP)-dependent reduction in eosinophil chemotactic activity (ECA) from bronchial epithelial cells (BEC). Therefore the effect of a beta2-agonist, procaterol and theophylline on the release of ECA from a BEC line, BEAS-2B was evaluated in response to interleukin (IL)-1beta and tumour necrosis factor-alpha (TNF-alpha). ECA was assessed using a blind-well chemotactic chamber, and the release and gene expression of cytokines were evaluated by means of enzyme-linked immunosorbent assay and reverse transcriptase polymerase chain reaction. IL-1beta and TNF-alpha stimulated the release of ECA from BEAS-2B cells in a dose- and time-dependent manner. Procaterol and theophylline directly inhibited eosinophil migration to IL-1beta and TNF-alpha-conditioned medium. The pretreatment of BEAS-2B cells with the same concentrations of procaterol inhibited the release of ECA in a dose-dependent fashion. Anti-IL-8, anti-regulated on activation, normal T-cell expressed and secreted (RANTES), and anti-granulocyte-macrophage colony-stimulating factor (GM-CSF) inhibited ECA. Procaterol inhibited the release of RANTES, GM-CSF and IL-8 in a dose-dependent fashion. The effect of theophylline was less potent. Procaterol augmented cAMP levels in BEAS-2B cells in a time- and dose-dependent manner. The expression of IL-8, RANTES, and GM-CSF messenger ribonucleic acid was not inhibited by procaterol and theophylline. These data indicate that procaterol and theophylline may directly inhibit eosinophil migration and that procaterol may further inhibit the release of eosinophil chemotactic activity from BEAS-2B cells via a cyclic adenosine monophosphate-dependent mechanism. This warrants further studies on the involvement of bronchial epithelial cells in the anti-inflammatory effects of procaterol and theophylline in patients with asthma.

PMID: 10573218 [PubMed - indexed for MEDLINE]

1696. Rhinology. 1999 Sep;37(3):97-103.

An update on the pathophysiology of rhinovirus upper respiratory tract infections.

van Kempen M, Bachert C, Van Cauwenberge P.

Dept. of Otorhinolaryngology, University Hospital of Ghent, Belgium.

Upper respiratory tract infections are one of the most common infectious diseases in man and are characterized by relatively mild symptoms. However, complications of bacterial super-infection or asthma exacerbations are not seldomly seen. Most upper respiratory tract infections are caused by rhinoviruses. The rhinovirus is a non-enveloped 30 nm RNA-virus with over 100 serotypes that belongs to the Picornaviridae family and only replicates in primates. It is characterized by a single positive stranded genome acting not only as a template for RNA synthesis, but also encoding for a single polypeptide necessary for viral replication. The viral capsid has an icosahedral symmetry and demonstrates deep canyons, with a receptor-binding domain. Rhinoviruses are transmitted mainly via direct- or indirect contact with infected secretions and invade their host by binding to the ICAM-1 receptor on the nasal epithelium. Typical for rhinovirus upper respiratory tract infections are isolated scattered foci of infected epithelium, not showing any striking damage or cytopathic alterations, between large areas of normal epithelium. Today there is still little detailed knowledge on the pathophysiology of common cold, especially on the aspect of cellular migration and defense. A better understanding in mechanisms underlying this cellular response would not only have therapeutical consequences, but may also explain the relationship between viral infectious rhinitis and asthma or atopy. During a rhinovirus infection, a selective neutrophil and monocyte recruitment is observed. In vitro and in vivo data have demonstrated a time-limited, rhinovirus-induced increase in bradykinin, cytokine, chemokine and sICAM-1 concentrations. Epithelial derived proinflammatory cytokines initiate an adhesion cascade and activate T lymphocytes

that create a TH1-type cytokine environment within the infected tissue, necessary to eradicate the virus infection. The selective recruitment of neutrophils seems linked to increased concentrations of the chemokine IL-8 and common cold symptoms. It is doubtful that the cytokine-regulated-production of specific neutralising immunoglobulins is necessary for recovery from viral illnesses and presumably only contributes to a late and temporary protection against rhinovirus reinfection. These observations confirm the crucial role that cytokines and mediators play in the pathogenesis of a rhinovirus infection by mediating chemotaxis, transmigration and activation of inflammatory- and immunocompetent cells.

PMID: 10567986 [PubMed - indexed for MEDLINE]

1697. Pediatr Allergy Immunol. 1999 Aug;10(3):209-15.

Migration and atopic disorder in Swedish conscripts.

Hjern A, Rasmussen F, Johansson M, Aberg N.

Department of Clinical Sciences, Huddinge University Hospital, Karolinska Institutet, and Center for Epidemiology, Swedish National Board of Health and Welfare, Stockholm. anders.hjern@sos.se

We have studied asthma and allergic rhinitis prevalence in Swedish conscripts born 1973-1977 according to the military service conscription register in relation to the socio-economic status and country of birth of the conscripts and their parents, and age when granted residency in Sweden. There was an increase in prevalence of asthma and allergic rhinitis over time in all groups irrespective of country of birth or ethnic origin. Conscripts who themselves were born in Africa, Asia, Latin America and the Mediterranean had a significantly lower risk for asthma and allergic rhinitis than Swedish-born conscripts. The risk of atopic disorder among the foreign-born conscripts increased with time of residency in Sweden. Conscripts with mothers from Latin America, Asia and Africa were identified as having the highest risk for atopic disorder among Swedish-born conscripts with high socio-economic status; the adjusted risk ratio (RR) for asthma was 2.6 (95% CI 1.7-4.0) and that for allergic rhinitis was 2.0 (1.5-2.6). The conscripts with mothers from the Mediterranean had the lowest risk for atopic disorders of the Swedish-born conscripts with low socio-economic status; the RR for asthma was 0.43 (0.34-0.56) and that for allergic rhinitis was 0.84 (0.76-0.93). This study demonstrates that factors related to migration and ethnicity are important determinants of atopic disorder among Swedish conscripts.

PMID: 10565562 [PubMed - indexed for MEDLINE]

1698. Acta Paediatr. 1999 Oct;88(10):1107-12.

Social adversity, migration and hospital admissions for childhood asthma in Sweden.

Hjern A, Haglund B, Bremberg S, Ringbäck-Weitoft G.

Department of Clinical Sciences, Huddinge University Hospital, Karolinska Institutet, Stockholm, Sweden. anders.hjern@sos.se

The number of children admitted to hospitals because of asthma has been reported to decrease in Sweden in recent decades despite an increasing prevalence of childhood asthma. This decrease has been explained by improved maintenance therapy of children with severe asthma. In this study we used data on hospital

admissions for asthma from the Swedish National Patient Discharge Registers in Stockholm, Malmö and Gothenburg 1990-1994 to identify social and ethnic characteristics of children 2-18-y-old in need of improvement in disease management. Children in families on social welfare (adjusted odds ratios (OR): 1.3 and 1.5) and children in single-parent households (adjusted OR: 1.3 and 1.4) were more often admitted to hospital because of asthma at least once during a calendar year in the 2-6- and 7-18-y-old groups. Children in families on social welfare had a particularly high risk of being admitted more than once during a calendar year (adjusted OR: 1.6 in the younger age group and 2.9 in the older group). Exposure to smoking during pregnancy was more common in socially disadvantaged families and increased the risk of hospital admission in children below 3 y of age. Children born outside Western Europe, the USA and Australia were less commonly admitted to hospital because of asthma than other children in the population (adjusted OR: 0.1-0.5). Swedish-born children with mothers who were born in Eastern and Southern Europe were also at lower risk for admission to hospital with a diagnosis of asthma (adjusted OR: 0.2-0.6). This probably indicates a lower prevalence of asthma in these ethnic groups. Further studies are needed to identify factors that can explain these ethnic differences in childhood asthma.

PMID: 10565458 [PubMed - indexed for MEDLINE]

1699. Am J Respir Crit Care Med. 1999 Nov;160(5 Pt 1):1516-24.

Relationship of cellular transmigration and airway response after allergen challenge.

Ndukwu IM, Naureckas ET, Maxwell C, Waldman M, Leff AR.

Section of Pulmonary and Critical Care Medicine, Committees on Cellular Physiology, Division of the Biological Sciences, The University of Chicago, Chicago, Illinois, USA. mndukwu@medicine.bsd.uchicago.edu

We examined the relationship between eosinophil migration into the bronchoalveolar space and change in FEV(1) after endobronchial allergen challenge (EBAC) in atopic asthmatic (AA) and atopic nonasthmatic (ANA) subjects. The purpose of this study was to obtain continuous, intrasubject controlled assessment of the relationship between cell migration in control and allergen-challenged segments in the same individuals over 96 h. In AA subjects, the eosinophil (Eos) count in the bronchoalveolar lavage fluid (BALF) increased from a baseline of 7,896 +/- 3,865 to 416,476 +/- 231,012 Eos/ml by 72 h (p = 0.001) in the challenged segment post-EBAC. For ANA subjects, the postsegmental challenge count was 29,874 +/- 474 Eos/ml (p = 0.03 versus baseline and p < 0.05 AA peak versus ANA peak). In both groups, there was a comparable decrease in peripheral blood eosinophil count beginning 5 h after challenge, which resolved at 24 h. In AA subjects, 416,476 +/- 231,012 Eos/ml was obtained from the allergen-challenged segment and 23,522 +/- 8,298 Eos/ml was obtained from the sham-challenged segment (p < 0.001) at 72 h. In contrast, there was no difference in the Eos count obtained from the BALF between the antigen- and sham-challenged segments of ANA subjects. We also found that increased airway neutrophils were present in equal numbers in allergen-challenged and sham-challenged segments in both AA and ANA subjects. We conclude that augmented eosinophil migration after EBAC is a characteristic of atopic asthma and is not present in atopic subjects who do not have asthma. We find that BAL eosinophilia in ANA patients as well as neutrophilia in both ANA and AA subjects are nonspecific consequences of bronchoscopy. Finally, we find no relationship between specific airway eosinophil migration into the BALF and FEV(1) < 72 h after challenge; however, at 96 h, there is a substantial decrease in FEV(1) that accompanies BALF eosinophilia.

PMID: 10556114 [PubMed - indexed for MEDLINE]

1700. J Allergy Clin Immunol. 1999 Nov;104(5):917-26.

Molecular basis for selective eosinophil trafficking in asthma: A multistep paradigm.

Wardlaw AJ.

Division of Respiratory Medicine, Leicester University Medical School, Leicester, United Kingdom.

Asthma is characterized by a 50- to 100-fold increase in the number of eosinophils relative to neutrophils in the bronchial mucosa. This increase is not the result of a single molecular event but of the cumulative and sequential effects of several approximately 4-fold increases in selective eosinophil versus neutrophil migration, occurring at a number of stages in the life cycle of the eosinophil. These steps include (1) effects on the bone marrow, mediated principally by IL-5, which result in a 4-fold increase in circulating eosinophils, (2) selective tethering of eosinophils to venular endothelium through the combined effects of P-selectin/P-selectin glycoprotein ligand 1 and very late activation antigen-4/vascular cell adhesion molecule-1, which has the potential for an up to 10-fold increase in eosinophil versus neutrophil adhesion, (3) selective chemotaxis under the influence of CC chemokines, and (4) prolonged survival, again mediated by IL-5. These events are integrated and directed by allergen-specific T(H)2 lymphocytes through the generation of IL-5, IL-4, and IL-13. The implications of this multistep process are that antagonists of IL-5, very late activation antigen-4, P-selectin glycoprotein ligand 1, and CCR3 as well as IL-4 and IL-13 each have the potential to markedly inhibit eosinophil recruitment in asthma.

PMID: 10550733 [PubMed - indexed for MEDLINE]

1701. Protein Sci. 1999 Oct;8(10):2041-54.

Backbone dynamics of the human CC chemokine eotaxin: fast motions, slow motions, and implications for receptor binding.

Crump MP, Spyracopoulos L, Lavigne P, Kim KS, Clark-lewis I, Sykes BD.

The Protein Engineering Network of Centres of Excellence and Department of Biochemistry, University of Alberta, Edmonton, Canada.

Eotaxin is a member of the chemokine family of about 40 proteins that induce cell migration. Eotaxin binds the CC chemokine receptor CCR3 that is highly expressed by eosinophils, and it is considered important in the pathology of chronic respiratory disorders such as asthma. The high resolution structure of eotaxin is known. The 74 amino acid protein has two disulfide bridges and shows a typical chemokine fold comprised of a core of three antiparallel beta-strands and an overlying alpha-helix. In this paper, we report the backbone dynamics of eotaxin determined through 15N-T1, T2, and [1H]-15N nuclear Overhauser effect heteronuclear multidimensional NMR experiments. This is the first extensive study of the dynamics of a chemokine derived from 600, 500, and 300 MHz NMR field strengths. From the T1, T2, and NOE relaxation data, parameters that describe the internal motions of eotaxin were derived using the Lipari-Szabo model free analysis. The most ordered regions of the protein correspond to the known secondary structure elements. However, surrounding the core, the regions known to be functionally important in chemokines show a range of motions on varying

timescales. These include extensive subnanosecond to picosecond motions in the N-terminus, C-terminus, and the N-loop succeeding the disulfides. Analysis of rotational diffusion anisotropy of eotaxin and chemical exchange terms at multiple fields also allowed the confident identification of slow conformational exchange through the "30s" loop, disulfides, and adjacent residues. In addition, we show that these motions may be attenuated in the dimeric form of a synthetic eotaxin. The structure and dynamical basis for eotaxin receptor binding is discussed in light of the dynamics data.

PMCID: PMC2144122
PMID: 10548050 [PubMed - indexed for MEDLINE]

1702. Rev Invest Clin. 1999 Jul-Aug;51(4):255-68.

[Chemokines, a new family of cytokines in inflammatory cell recruitment].

[Article in Spanish]

Martínez Martínez CM, Hernández Pando R.

Departamento de Patología, Instituto Nacional de la Nutrición Salvador Zubirán, México, D.F.

Cell recruitment is a crucial event in the establishment of both acute and chronic inflammatory responses, including acute and delayed type hypersensitivity reactions. Among other significant factors like adhesion molecules, chemokines and its receptors are crucial elements that lead leukocyte migration to the tissues. Chemokines are a large group of peptidic cytokines which have a conserved motif of 4 cisteins. These cistein residues form pairs which permit to classify them in two groups, the alpha and beta subfamilies. In general terms, alpha subfamily has preferential chemotactic activity on granulocytes, and beta subfamily attracts mainly lymphocytes and macrophages. Besides their chemotactic activity, chemokines also participate in some other important biological processes like hematopoiesis, angiogenesis, and anti-tumoral activity. Chemokines also play an important role in certain pathological conditions, for instance in some allergic processes they have an essential role in the pathogenesis. In autoimmune and infectious diseases, this cytokine family is also important as is suggested by the presence of chemokine receptors in rheumatoid arthritis inflammed synovia or the HIV receptor activity that chemokine receptors display which apparently play a significant role in the natural resistance against this infectious agent. Preferential leukocyte recruitment mediated by chemokines is a potential target for pharmacological modulation, which in turn may lead to a novel and efficient types of therapeutic control of inflammatory diseases with diverse etiology.

PMID: 10546507 [PubMed - indexed for MEDLINE]

1703. Int Arch Allergy Immunol. 1999 Oct;120(2):126-34.

Murine allergic respiratory responses to the major house dust mite allergen Der p 1.

Clarke AH, Thomas WR, Rolland JM, Dow C, O'Brien RM.

Medicine and Pathology, Western Hospital Footscray, Perth, Australia.

BACKGROUND: Although many studies have examined chronic asthma, limited data exist on acute immunopathogenic events induced by allergens. The aim of the study

was to investigate the acute cellular, serologic and histopathologic events in airway inflammation produced by intranasal challenge of mice sensitised to the major house dust mite allergen Der p 1.
METHODS: C57BL/6 mice were immunised subcutaneously with Der p 1 in alum. Mice were bled and challenged intranasally with Der p 1 on day 14 and killed on day 17. Lungs were fixed in situ, processed and stained with haematoxylin and eosin. The degree of inflammation and eosinophil infiltration was quantified by image analysis. Specific IgE was determined by passive cutaneous anaphylaxis. Cells from spleen and draining lymph nodes were cultured for 24 h with Der p 1, and IL-3/GM-CSF released into supernatants was measured by bioassay.
RESULTS: Intranasal challenge of sensitised mice induced eosinophilic influx into the large and small airways and the alveolar regions of the lung, mucus plugging and in severe cases numerous Charcot-Leyden crystals. The quantitation of the inflammation induced by different sensitisation and challenge doses showed that optimal inflammation could be produced using only 1 microg of allergen for both sensitisation and challenge. The degree of inflammation was not related to the titre of IgE antibody and was indeed produced in its absence. T cell reactivity of spleen cells to the allergen was decreased suggesting cell migration or inactivation.
CONCLUSIONS: Mice sensitised and challenged intranasally with as little as 1 microg of Der p 1 produced an extensive pulmonary eosinophilic inflammation which shared many of the features of the inflammation found in asthma. The small amount of allergens required and the use of intranasal challenge should provide a useful model.

PMID: 10545766 [PubMed - indexed for MEDLINE]

1704. Laryngorhinootologie. 1999 Sep;78(9):481-90.

[Cytokines and chemokines in paranasal sinus diseases].

[Article in German]

Rudack C, Bachert C.

HNO-Klinik der westfälischen Wilhelmsuniversität Münster.

BACKGROUND: New insights into inflammatory processes became possible by investigating the pattern of cytokines and chemokines as well as adhesion molecules in different acute and chronic sinus diseases since the last decade. This review aims to update and discuss findings of in vitro and in vivo studies concerning the role of cytokines and chemokines in inflammatory sinus diseases during the last years.
RESULTS: Discrepancies in research findings may be due to the small available databases today, the use of different techniques for investigation, and the lack of a valuable classification of sinus diseases. Despite this discrepancies, there is evidence that in acute bacterial and viral sinusitis, proinflammatory cytokines play a dominant role in initiating and sustaining the inflammation, which is especially characterized by neutrophil tissue infiltration. In chronic sinusitis IL-3 dominates the cytokine profile, giving support to a variety of inflammatory cells. IL-3 may also contribute to fibrosis and constant thickening of the mucosa leading to an obstruction of the ostiomeatal complex. In most bilateral nasal polyps, tissue eosinophilia is a striking finding which is believed to play a central role in pathogenesis, as it does in asthma. Eosinophilia may be explained by increased migration and prolonged eosinophil survival. Activation and survival of eosinophils in nasal polyps are thought to be regulated by autocrine stimulation by IL-5. Therefore, IL-5 represents the main target for future therapy in nasal polyposis.
CONCLUSIONS: Defining cytokine and chemokine patterns in inflammatory sinus

diseases leads to a better understanding of immunologic processes in nasal mucosa. Results of cytokine and chemokine research are of paramount interest in developing new therapeutic approaches.

PMID: 10535064 [PubMed - indexed for MEDLINE]

1705. Int Arch Allergy Immunol. 1999;120 Suppl 1:45-7.

CCR3 mRNA expression in bronchial epithelial cells and various cells in allergic inflammation.

Oyamada H, Kamada Y, Kuwasaki T, Yamada Y, Kobayashi Y, Cui C, Honda K, Kayaba H, Saito N, Chihara J.

Division of Central Laboratory, Akita University Hospital, Akita, Japan.

BACKGROUND: RANTES and eotaxin are important chemokines involved in the activation and migration of eosinophils and are considered to play a major role in allergic inflammation.
METHODS: In this study, we used RT-PCR to investigate the kinds of cells that express mRNA for CCR3, a common receptor of these chemokines, and eotaxin, a ligand for CCR3.
RESULTS: CCR3 mRNA was expressed in eosinophils, peripheral mononuclear cells, an eosinophilic cell line (EoL-1), a bronchial epithelial cell line (NCI-H(292)), human endothelial cells and nasal washings from patients with allergic rhinitis.
CONCLUSION: These results suggest that the CCR3-eotaxin system plays an important role in generating inflammation, since these substances are expressed not only in cells implicated in activation or migration of eosinophils but also in various other cells involved in allergic inflammation.

PMID: 10529603 [PubMed - indexed for MEDLINE]

1706. Int Arch Allergy Immunol. 1999;120 Suppl 1:24-6.

Regulatory mechanisms of eosinophil adhesion to and transmigration across endothelial cells by alpha4 and beta2 integrins.

Yamamoto H, Nagata M.

Pulmonary Division, Second Department of Internal Medicine, Saitama Medical School, Irumagun, Saitama, Japan.

To participate in allergic airway inflammation, it is necessary for blood eosinophils (Eos) to adhere to and migrate across pulmonary vascular endothelial cells (EC). Accumulating evidence has established that Eos adhesion molecules, such as alpha4 and beta2 integrins, play central roles in these processes. We have reported that Eos spontaneously adhere to recombinant human (rh)-VCAM-1, while adhesion to rh-ICAM-1 requires a second stimulus such as GM-CSF. Furthermore, our study employing human pulmonary microvascular endothelial cells (HPMEC) revealed that although the alpha4 integrin/VCAM-1 pathway is crucial for the firm adhesion of Eos, subsequent transendothelial migration occurred dependent on the beta2 integrin/ICAM-1 pathway. We also discuss secretagogues that would affect Eos recruitment to the airways via regulation of alpha4 and beta2 integrins.

PMID: 10529598 [PubMed - indexed for MEDLINE]

1707. Biochem Biophys Res Commun. 1999 Oct 14;264(1):163-70.

Novel association of the src family kinases, hck and c-fgr, with CCR3 receptor stimulation: A possible mechanism for eotaxin-induced human eosinophil chemotaxis.

El-Shazly A, Yamaguchi N, Masuyama K, Suda T, Ishikawa T.

Department of Otorhinolaryngology and Head and Neck Surgery, Kumamoto University School of Medicine, Honjo 1-1-1, Kumamoto, 860, Japan. 106726.2140@compuserve.com

The chemokine eotaxin is a potent and relatively eosinophil-specific chemoattractant implicated in the cell migration to inflammatory sites in allergic diseases. Eotaxin exerts its activity solely through the CCR3 receptor, but the signaling pathways are poorly defined. In this study, we show that eotaxin induces an increase in tyrosine phosphorylation of multiple cellular proteins in normal human eosinophils. Eotaxin-dependent tyrosine phosphorylation was detected 1 min after stimulation and increased for at least 15 min with kinetics similar to those of eotaxin-induced cell shape changes. Herbimycin A, a tyrosine kinase inhibitor, blocked both eotaxin-induced tyrosine phosphorylation and cell shape changes as well as chemotaxis. Immunofluorescence microscopy analyses showed that eotaxin-induced cell shape changes were accompanied by redistribution of tyrosine-phosphorylated proteins and F-actin reorganization that were sensitive to herbimycin A. Coimmunoprecipitation studies revealed that binding of eotaxin to CCR3 greatly enhanced association of the Src family kinases, Hck and c-Fgr, with CCR3 after internalization of CCR3. These results may indicate that recruitment of Hck and c-Fgr to CCR3 in a compartment triggers tyrosine phosphorylation, leading to rapid cell shape changes required for cell migration.

Copyright 1999 Academic Press.

PMID: 10527858 [PubMed - indexed for MEDLINE]

1708. Clin Immunol. 1999 Nov;93(2):107-13.

Blocking ICAM-1 (CD54) and LFA-1 (CD11a) inhibits experimental allergic conjunctivitis.

Whitcup SM, Chan CC, Kozhich AT, Magone MT.

National Eye Institute, National Institutes of Health, Bethesda, Maryland 20892-1863, USA.

Cell adhesion molecules are critical for the homing and migration of leukocytes into inflamed tissues. We investigated the role of ICAM-1 and LFA-1 in a previously described experimental model of ragweed (Rw)-induced allergic conjunctivitis. SWR/J mice were treated intraperitoneally 6 and 1 h prior to topical challenge with Rw with injections of anti-ICAM-1 monoclonal antibody (mAb), anti-LFA-1 mAb, both anti-ICAM-1 and anti-LFA-mAbs, or rat IgG. Blocking ICAM-1 or LFA-1 reduced the clinical signs of allergic conjunctivitis. Treatment with anti-ICAM-1 or anti-LFA-1 mAbs also significantly inhibited cellular infiltration into the conjunctiva. The greatest inhibitory effect was achieved with the combination of antibodies against both cell adhesion molecules. Since antibodies against ICAM-1 and LFA-1 significantly inhibit the development of the clinical and histologic signs of allergic conjunctivitis, they may be useful for treating patients with ocular allergy.

PMID: 10527686 [PubMed - indexed for MEDLINE]

1709. J Allergy Clin Immunol. 1999 Oct;104(4 Pt 1):852-6.

Reducing relative humidity to control the house dust mite Dermatophagoides farinae.

Arlian LG, Neal JS, Vyszenski-Moher DL.

Department of Biological Sciences, Wright State University, Dayton, Ohio 45435-0001, USA.

BACKGROUND: Indoor relative humidity (RH) is the key factor that determines the survival and population development of the house dust mite Dermatophagoides farinae. Maintaining RH below 50% is one recommendation in a comprehensive plan to reduce house dust mites and mite allergen levels in homes. Even when mean daily RH is reduced below 50%, RH may rise above 50% intermittently for brief periods because of activities in the home (eg, cooking, bathing, and ventilation).
OBJECTIVE: The purpose of this study was to determine how brief daily periods of moist air alternating with long spells of low ambient RH (0% or 35%) influence population survival and growth of D farinae.
METHODS: Population growth was determined for D farinae at daily RH regimens of 2, 4, 6, and 8 hours at 75% or 85% RH alternating with 22, 20, 18, and 16 hours at 0% or 35% RH.
RESULTS: D farinae populations declined at daily regimens of 2 hours at 75% or 85% RH alternating with 22 hours at 0% or 35% RH. Daily regimens of 4, 6, and 8 hours at 75% RH alternating with 20, 18, and 16 hours, respectively, at 35% RH provided sufficient moisture for small growths in population size. These growths after 10 weeks were reduced by 98.2%, 98.0%, and 97.3% for daily regimens of 4, 6, and 8 hours, respectively, at 75% RH (with the remainder of the day at 35% RH) compared with the growth of populations continuously exposed to 75% RH. Continuous exposure to 85% RH inhibited population growth, but alternating daily regimens of 16, 18, and 20 hours at 35% RH allowed small populations to develop, although they were reduced by 99.4%, 98.8%, and 99.1% compared with population growth at a continuous 75% RH.
CONCLUSION: This study indicates that maintaining mean daily RH below 50%, even when RH rises above 50% for 2 to 8 hours daily, effectively restricts population growth of these mites and thus the production of allergen. To completely prevent population growth of D farinae, RH must be maintained below 35% for at least 22 hours per day when the daily RH is 75% or 85% for the remainder of the day.

PMID: 10518832 [PubMed - indexed for MEDLINE]

1710. Am J Pathol. 1999 Oct;155(4):1195-204.

Tryptase-chymase double-positive human mast cells express the eotaxin receptor CCR3 and are attracted by CCR3-binding chemokines.

Romagnani P, De Paulis A, Beltrame C, Annunziato F, Dente V, Maggi E, Romagnani S, Marone G.

Department of Physiopathology, Section of Clinical Immunology, University of Florence, Florence, Italy.

Eosinophils, basophils, and Th2 cells express the chemokine receptor CCR3, which binds eotaxin, RANTES, and some other chemokines. Using immunohistochemistry and flow cytometry, we demonstrate that CCR3 is also expressed by a variable proportion of human mast cells in gut, skin, and lung tissue. By contrast, with

the same anti-CCR3 antibody (B711), CCR3 was poorly if at all detectable on human Th2 cells in vitro and in vivo. Eotaxin neither induced histamine release from purified human mast cells nor increased anti-IgE-stimulated histamine secretion. However, both eotaxin and RANTES elicited mast cell migration in vitro with a similar efficacy. High percentages of CCR3-expressing mast cells were present in the skin and in the intestinal submucosa; much lower percentages were found in the intestinal mucosa and in lung interstitium. Double immunostaining with anti-CCR3 and anti-chymase antibody showed that the vast majority of CCR3-expressing mast cells in the various tissues examined were tryptase-chymase double-positive. Therefore, tryptase-chymase double-positive mast cells express CCR3 and are attracted by CCR3-binding chemokines, eotaxin, and RANTES. Our findings indicate that these chemokines may play an important role in the differentiation and/or migration of this mast cell subset in connective tissues, as well as in sites of allergic inflammation.

PMCID: PMC1867029
PMID: 10514402 [PubMed - indexed for MEDLINE]

1711. Allergy. 1999 Sep;54(9):944-50.

Eosinophil chemotaxis by chemokines: a study by a simple photometric assay.

Nagase H, Yamaguchi M, Jibiki S, Yamada H, Ohta K, Kawasaki H, Yoshie O, Yamamoto K, Morita Y, Hirai K.

Department of Respiratory Medicine, University of Tokyo Graduate School of Medicine, Japan.

BACKGROUND: The effects of a panel of 15 chemokines on eosinophil chemotaxis were studied by a new photometric assay which is both less tedious and less laborious than the conventional manual counting methods. Approximately 40 chemokines have been identified to date, but there is little information on the eosinophil migration-inducing ability of chemokines other than CC chemokine receptor (CCR) 3 ligands.
METHODS: Eosinophil migration was measured by the Boyden chamber technique with a 96-well multiwell chamber and polycarbonate membrane filter. Eosinophil migration was assessed by determination of the eosinophil peroxidase (EPO) activity, and photometric measurement was performed with a microtiter plate reader.
RESULTS: The assay was sensitive enough to detect 200 eosinophils, and the time required was within 4 h. CCR3 ligands, i.e., regulated on activation normal T-cell expressed and secreted (RANTES), eotaxin, eotaxin-2, and monocyte chemoattractant protein (MCP)-3, induced significant migration, while other chemokines showed no significant migration-inducing ability. Although the chemotaxis induction by these chemokines was efficiently inhibited by anti-CCR3 mAb, anti-CCR1 mAb failed to show any inhibitory effects.
CONCLUSIONS: The photometric assay is suitable for analyzing a large number of samples. CCR3 ligands are the most important chemokines inducing eosinophil chemotaxis; thus, CCR3 represents a possible therapeutic target for the treatment of allergic diseases.

PMID: 10505457 [PubMed - indexed for MEDLINE]

1712. J Neuroimmunol. 1999 Sep 1;99(1):1-12.

Calpain activity and expression are increased in splenic inflammatory cells associated with experimental allergic encephalomyelitis.

Shields DC, Schaecher KE, Goust JM, Banik NL.

Department of Neurology, Medical University of South Carolina, Charleston 29425, USA.

Since calcium-activated neutral proteinase (calpain) activity and expression are significantly increased in activated glial/inflammatory cells in the central nervous system of animals with autoimmune demyelinating diseases, this enzyme may also play a role in peripheral organ systems in these diseases. In this study, the activity and expression of calpain and the endogenous inhibitor, calpastatin, were evaluated at transcriptional and translational levels in spleens of Lewis rats with acute experimental allergic encephalomyelitis (EAE) prior to the onset of clinical symptoms. Calpain activity and translational expression were increased by 475.5% and 44.3% respectively, on day 4 post-induction in adjuvant controls and animals with EAE. These levels remained elevated compared to normal controls on days 8 and 12. Calpastatin translational expression was similarly increased at these time points although transcriptional expression was not significantly altered at any time following induction of EAE. Likewise, transcriptional expression of mu-calpain was unchanged following induction, while small increases in m-calpain transcriptional expression were observed on days 2 and 8. Most calpain expression was observed in activated splenic macrophages at day 8 post-induction even though activated T cells were also calpain positive. In spinal cords of animals with EAE, calpain expression was significantly increased in rats with severe disease compared to those exhibiting only mild symptoms at day 12 post-induction. Thus, prior to symptomatic EAE, increased calpain activity and expression in peripheral lymphoid organs may play an important role in T cell migration and subsequent disease progression.

PMID: 10496171 [PubMed - indexed for MEDLINE]

1713. Pneumonol Alergol Pol. 1999;67(3-4):118-25.

[The effect of theophylline on neutrophil and lymphocyte chemotaxis stimulated with fMLP in health subjects and asthma patients].

[Article in Polish]

Kraus-Filarska M, Zak-Nejmark T, Małolepszy J, Barg W, Filarski J.

Katedry i Kliniki Chorób Wewnetrznych i Alergologii, Akademii Medycznej we Wrocławiu.

Theophylline, for many years used as a bronchodilatator, is also known to have some anti-inflammatory properties. The aim of this study was to investigate the effect of theophylline on random locomotion and chemotaxis of neutrophils and lymphocytes from 12 patients with mild bronchial asthma and 12 healthy volunteers. fMLP in concentration 10(-8) M was used as a chemoattractant. The experiment was performed using the modified Boyden method. The cells were incubated with theophylline in therapeutic concentrations (from 5 to 20 micrograms/ml) at 37 degrees C for 30 minutes. Motility of neutrophils and lymphocytes was determined as the migration distance of the cell leading front inside the filter (micron). We found no statistically significant differences in both spontaneous and fMLP-stimulated cell motility between asthmatics and healthy subjects. fMLP-stimulated chemotaxis was significantly inhibited after neutrophil preincubation with theophylline in therapeutic concentrations (5-20 micrograms/ml) in both groups. Random locomotion of lymphocytes was slightly but significantly higher in healthy subjects comparing to asthmatics. Preincubation with theophylline at concentrations 10 to 20 micrograms/ml resulted in significant inhibition of fMLP-stimulated chemotaxis in both groups. Our data show the inhibitory action of theophylline on fMLP-induced chemotaxis of both

neutrophils and lymphocytes indicating the anti-inflammatory activity of the drug.

PMID: 10497444 [PubMed - indexed for MEDLINE]

1714. Infect Immun. 1999 Oct;67(10):5315-23.

Trapping and immobilization of Nippostrongylus brasiliensis larvae at the site of inoculation in primary infections of interleukin-5 transgenic mice.

Daly CM, Mayrhofer G, Dent LA.

Department of Microbiology and Immunology, The University of Adelaide, Adelaide, South Australia, Australia 5005.

Interleukin-5 (IL-5) transgenic mice are highly resistant to primary infections with the intestinal nematode Nippostrongylus brasiliensis; few parasites are found in the intestines of infected animals, and egg production is minimal. While adult worms may be damaged in the intestine, larval migration, development, and viability may also be impaired in other tissues. This study addresses the migration of N. brasiliensis larvae through the skin and lungs and associated cellular responses in primary infections of IL-5 transgenic mice. Although some larvae may have been trapped and killed in the lungs of IL-5 transgenic mice, most apparently failed to reach this site. Two or more hours after infection of IL-5 transgenic mice, eosinophils were a major component of the cellular infiltrate at the subcutaneous site of injection, and localized eosinophil degranulation was extensive. Seventy-five to ninety-five percent of the larvae injected into subcutaneous air pouches in IL-5 transgenic mice were retained there for at least 24 h. In contrast, in nontransgenic mice, less than 20% of larvae could be recovered from the skin 2 or more h postinjection, and eosinophil activity was modest at all times. The data strongly suggest that eosinophils can restrict the movement of N. brasiliensis larvae in the first few hours of a primary infection and that this has profound effects on later stages of parasite development. Preexisting eosinophilia, due either to allergy or to infection with tissue-invasive helminth species, may therefore confer some degree of immediate and nonspecific resistance in primary infections with parasitic worms.

PMCID: PMC96886
PMID: 10496911 [PubMed - indexed for MEDLINE]

1715. Arch Dis Child. 1999 Aug;81(2):159-62.

Respiratory symptoms and duration of residence in immigrant teenagers living in Melbourne, Australia.

Powell CV, Nolan TM, Carlin JB, Bennett CM, Johnson PD.

Department of Emergency Medicine, University of Melbourne, Royal Children's Hospital, Melbourne, Parkville 3032, Victoria, Australia.

OBJECTIVE: Examination of the relation between respiratory symptoms and time since arrival in Australia in immigrant teenagers living in Melbourne.
DESIGN: Two stage, stratified, cross sectional survey.
SETTING: High schools (n = 51).
SUBJECTS: 9794 people aged 13-19 years.
MAIN OUTCOME MEASURES: Prevalence of wheeze during a 12 month period, region of birth, duration of residence in Australia.
RESULTS: The estimated population 12 month period prevalence of wheeze was 18.9%

(95% confidence interval (CI), 18.0 to 19.9). In subjects born outside Australia, residence for five to nine years in Australia was associated with a 2.1-fold (CI, 1.1 to 4.0) increase in the odds of self reported wheeze; after 10-14 years, this risk increased 3.4-fold (CI, 1.8 to 6.7). There was no difference in severity of wheeze, measured by reported frequency of attacks, between Australian born and non-Australian born subjects.
CONCLUSIONS: The notion of a continued secular increase in the prevalence of wheezing is not supported. There is a time dose effect on the prevalence of symptoms in subjects born outside Australia and now living in Melbourne, which is independent of age and country of birth.

PMCID: PMC1718023
PMID: 10490527 [PubMed - indexed for MEDLINE]

1716. Allergy. 1999 Aug;54(8):771-83.

The epidermal growth factor receptor and its ligand family: their potential role in repair and remodelling in asthma.

Davies DE, Polosa R, Puddicombe SM, Richter A, Holgate ST.

University Medicine, Southampton General Hospital, UK.

The bronchial epithelium acts as a physical barrier to the ingress of airborne irritants and plays an active role in the airways immune response, reacting to a range of environmental stimuli to enable interaction with immune and inflammatory cells. It is well established that mucosal damage and epithelial cell shedding are important features of inflammatory airways diseases such as asthma. Evidence now suggests that the repairing epithelium has the potential to contribute to disease chronicity through production of an array of soluble mediators and adhesion molecules. However, the biochemical mechanisms that control epithelial maintenance and repair are poorly understood, even though avoiding or reversing the changes in the airways which are likely to result from chronic inflammation and epithelial restitution remains a significant challenge in asthma therapy. The purpose of this review is to highlight the potential of the epidermal growth factor receptor (EGFR/c-erbB) and its ligands in restitution of the bronchial epithelium. This receptor tyrosine kinase plays a pivotal role in regulation of epithelial cell behaviour, having the capacity to elicit a broad spectrum of cellular responses ranging from migration or proliferation to differentiation and enhanced survival; it also has the ability to regulate expression of various inflammatory mediators, mucins, adhesion molecules, matrix proteins, and metalloproteinases which are relevant to the chronic disease phenotype.

PMID: 10485380 [PubMed - indexed for MEDLINE]

1717. Arch Immunol Ther Exp (Warsz). 1999;47(4):245-9.

Heparin modulates migration of human peripheral blood mononuclear cells and neutrophils.

Zak-Nejmark T, Krasnowska M, Jankowska R, Jutel M.

Department of Internal Medicine and Allergology, University Medical School, Wrocław, Poland.

Evidence has now accumulated that heparin can significantly affect immune response including allergic inflammation. Cell motility is supposed to be very crucial in this process. Thus the aim of our study was to investigate whether

heparin is a chemoattractant for some inflammatory cells and is also capable of influencing chemotaxis induced by typical chemoattractants. Peripheral blood mononuclear cells (PBMCs) and neutrophils from 10 healthy subjects were obtained by gradient centrifugation. Chemotaxis assays towards either heparin (molecular weight 16 kDa) or low molecular weight heparin--fraxiparine (molecular weight 5 kDa) were performed in Boyden chambers. We found that both heparin molecules are chemoattractants for both PBMCs and neutrophils in the wide concentration range (0.1-2000 microg/ml). However, maximal chemotaxis was observed at concentrations 50-100 microg/ml (fraxiparine) and 1-50 microg/ml (heparin). We also found that fraxiparine was able to significantly increase chemokinesis and decrease chemotaxis in the gradient of both fMLP and IL-8. These results indicate that heparin is a potent regulator of cell migration.

PMID: 10483873 [PubMed - indexed for MEDLINE]

1718. J Allergy Clin Immunol. 1999 Sep;104(3 Pt 1):659-64.

Increased production of macrophage migration inhibitory factor by PBMCs of atopic dermatitis.

Shimizu T, Abe R, Ohkawara A, Nishihira J.

Department of Dermatology and the Central Research Institute, Hokkaido University School of Medicine, Sapporo, Japan.

BACKGROUND: Atopic dermatitis (AD) is a chronic pruritic inflammatory skin disorder. The underlying cause of AD is multifactorial, and several cytokines are considered to be involved in this severe inflammatory skin disease. Macrophage migration inhibitory factor (MIF) is an immunoregulatory cytokine essential for T-cell activation and delayed-type hypersensitivity. Recently we demonstrated that serum MIF content was significantly elevated in patients with AD. Consistent with this, expression of MIF messenger RNA in keratinocytes of the eczematous skin lesion was up-regulated.
OBJECTIVE AND METHOD: Although keratinocytes are considered to be a potential source of increased serum MIF content in AD, precise evaluation has not been carried out in other tissues. MIF is ubiquitously expressed in various cells, including T cells and macrophages. In this study we examined MIF production and its messenger RNA level of PBMCs from patients with AD to investigate the contribution of these cells to elevated serum MIF content and to its pathologic characteristics.
RESULTS: Consistent with our previous findings, the serum MIF content of patients with AD was significantly elevated compared with nonatopic healthy control subjects and patients with chronic urticaria without eczema. As for the MIF productivity of unstimulated PBMCs, the MIF content in the culture medium of PBMCs obtained from patients with AD (40.4 +/- 8.4 ng/mL) (mean +/- SEM) was significantly increased compared with that from healthy control subjects (6.6 +/- 1.1 ng/mL) and patients with chronic urticaria (8.5 +/- 1.4 ng/ml) (P <.0001). When PBMCs were stimulated by concanavalin A, MIF production by PBMCs of patients with AD was more enhanced than in control subjects or patients with chronic urticaria. The increased ratio of MIF production by PBMCs in response to concanavalin A was significantly correlated with the severity of clinical features of AD. Supporting these results, the level of MIF mRNA in PMBCs of patients with AD was significantly higher than in nonatopic healthy control subjects.
CONCLUSIONS: The current results showed that PBMCs should be an important source of increased serum MIF in AD. Because MIF has the potential to induce local and systemic inflammatory and immune responses, it is conceivable that MIF produced by PBMCs may affect local and systemic pathologic features in AD.

PMID: 10482843 [PubMed - indexed for MEDLINE]

1719. J Allergy Clin Immunol. 1999 Sep;104(3 Pt 1):517-23.

Mast cell-T cell interactions.

Mekori YA, Metcalfe DD.

Allergy Clinical-Immunology Unit, Department of Medicine, Meir General Hospital, Kfar-Saba, Israel.

In addition to being a major effector cell in the elicitation of allergic inflammation, mast cells have been found to be activated in various T cell-mediated inflammatory processes and to reside in close physical proximity to T cells. Such observations and the wide spectrum of mediators produced and secreted by mast cells have led investigators to propose a functional relationship between these 2 cell populations. Indeed, mast cell activation has been reported to induce T-cell migration either directly by the release of chemotactic factors, such as lymphotactin or IL-16, or indirectly by the induction of adhesion molecule expression on endothelial cells. Mast cells are also able to present antigens to T cells, resulting in their activation in either an MHC class I- or class II-restricted and costimulatory molecule-dependent fashion. Adhesion molecule-dependent intercellular contact or MHC class II cognate interactions between T cells and mast cells result in the release of both granule-associated mediators and cytokines from the latter. Also, T cell-derived mediators, such as beta-chemokines, directly induce mast cell degranulation. On the other hand, mast cell-derived cytokines, such as IL-4, have been found to polarize T cells to preferentially differentiate into the T(H2) subset. Thus T cell-mast cell interactions are bidirectional, fulfilling regulatory and/or modulatory roles affecting various aspects of the immune response.

PMID: 10482820 [PubMed - indexed for MEDLINE]

1720. Pediatr Allergy Immunol. 1999 May;10(2):101-6.

Age at adoption, ethnicity and atopic disorder: a study of internationally adopted young men in Sweden.

Hjern A, Rasmussen F, Hedlin G.

Department of Clinical Sciences, Huddinge University Hospital, Karolinska Instituet, Stockholm, Sweden. anders.hjern@sos.se

Epidemiological and laboratory studies have implied that the environment during early childhood is important for the risk of developing atopic disorders. In this study we analyzed the prevalence of asthma, hayfever and eczema among 1901 internationally adopted young men at the military-induction medical examination in relation to indicators of their early childhood environment. The adopted young men who came to Sweden before 2 years of age suffered from asthma, hayfever and eczema significantly more often than those who came to Sweden between 2 and 6 years of age; the risk ratios (RR) were 1.6, 2.5 and 2.1, respectively. The young men who were born in the Far East were identified as being particularly susceptible to the development of hayfever and eczema, with RRs of 1.3 and 1.7. This study demonstrates that the environment during the first 6 years of life has a profound influence on the risk of suffering from atopic disorders as young adults.

PMID: 10478611 [PubMed - indexed for MEDLINE]

1721. Respir Med. 1999 Jan;93(1):16-20.

Prevalence of asthma and 'probable' asthma in the Asian population in Blackburn, U.K.

Ormerod LP, Myers P, Prescott RJ.

Chest Clinic, Blackburn Royal Infirmary, U.K.

Local research had shown increased asthma admission rates in the Asian ethnic group in Blackburn, U.K. Patients also reported that asthma seemed to develop some years after first arrival in the U.K. A community prevalence survey of respiratory symptoms and asthma was undertaken in three practices with no special asthma interest. The questionnaire was administered by a Health Visitor and language link worker. Of the Asian patients in the practices, 96.6% were studied. The age distribution was similar to that of the local 1991 census. Of the patients, 181/1783 (10.2%) had diagnosed asthma but positive responses to individual questions suggested underdiagnosis of asthma. Asthma prevalence was higher in males up to age 20 (14.6% vs. 8.2%), and aged over 50 (16.5% vs. 10.5%), but higher in females aged 20-49 (5.6% vs. 9.2%). There were no correlations with social class or Jarman index, and no effect of country of origin or duration in the U.K. by multivariate analysis. The prevalence of diagnosed asthma at ages 5-9 and 10-14 was higher than in previous studies. Diagnosed asthma prevalence rates fell in the 20-49 age band but rose again in the over-50s. In all age groups the prevalence of asthma is probably underestimated. Asthma prevalence was not related to social factors. The data show that those born in the U.K. are more likely to describe regular symptoms and to be on regular treatment, but that for those born abroad there was an increasing rate of symptoms and medication use with increasing duration in the country. These observations confirm patient views but are explained by the age/sex distribution of those born in the U.K. compared to immigrants.

PMID: 10464843 [PubMed - indexed for MEDLINE]

1722. Int Immunol. 1999 Sep;11(9):1451-62.

TCR-mediated activation of allergen-specific CD45RO(+) memory T lymphocytes results in down-regulation of cell-surface CXCR4 expression and a strongly reduced capacity to migrate in response to stromal cell-derived factor-1.

Abbal C, Jourdan P, Hori T, Bousquet J, Yssel H, Pène J.

INSERM U454, Hôpital Arnaud de Villeneuve, 375 Avenue Doyen Gaston Giraud, 34295 Montpellier Cedex 5, France.

The selective migration of functional T(h) lymphocyte subsets with different cytokine production profiles into inflamed tissue is likely to depend on the state of activation of the cells, as well as on the differential expression of various adhesion molecules and chemokine receptors. In this study, we have analyzed the effect of allergen-specific activation on the expression of the chemokine receptor CXCR4 on T lymphocytes. We show that stimulation of peripheral blood mononuclear cells from atopic patients with the allergen Der p results in down-regulation of CXCR4 surface expression on Der p-activated CD25(+)CD45RO(+) antigen-specific memory cells which was caused by a decrease in CXCR4 gene transcription and did not seem to be mediated by endogenous cytokines, such as IFN-gamma. In contrast, however, CXCR4 surface expression was enhanced on naive CD25(-)CD45RO(-) and resting CD25(-)CD45RO(+) memory T cells, as a result of the

presence of endogenous IL-4, most likely produced by Der p-activated memory T cells. Antigen-specific CD25(+)CD45RO(+) T lymphocytes, purified 7 days after stimulation with Der p, had a strongly reduced capacity to migrate in response to stimulation with stromal cell-derived factor (SDF)-1, the ligand for CXCR4. Together, these results suggest that differential expression of CXCR4 on activated and resting T cells is due to the counteracting effects of TCR-mediated down-regulation and IL-4-mediated up-regulation of this chemokine receptor respectively, and furthermore indicate that antigen-activated memory T cells are unlikely to migrate into inflamed tissue in response to SDF-1.

PMID: 10464166 [PubMed - indexed for MEDLINE]

1723. Food Chem Toxicol. 1999 May;37(5):553-60.

Thresholds in contact sensitization: theoretical and practical considerations.

Kimber I, Gerberick GF, Basketter DA.

Zeneca Central Toxicology Laboratory, Alderley Park, Macclesfield, Cheshire, UK.

The existence of thresholds for both the induction and the elicitation of contact sensitization is an important issue for hazard assessment in this area of toxicology. In this paper, the evidence for such thresholds has been reviewed from both theoretical and practical perspectives. When the mechanisms of skin sensitization are considered, the existence of thresholds can be shown to occur at several stages. They are both quantitative, for example in terms of the degree of protein haptenation and in the sufficiency of the signals for Langerhans cell migration and maturation, as well as qualitative, in terms of the type of immune response that is engaged. Such considerations are evidenced by a substantial body of practical observation. In humans and in animal models of skin sensitization there is abundant evidence for the existence of thresholds for both the induction and the elicitation of reactions. In addition to, and in distinction from, the experimental situation, in the general human population there is extensive evidence for threshold effects. This evidence arises directly from the observation that only a proportion of those who are exposed become sensitized (i.e. are patch test positive), and of that latter group only a proportion develop allergic contact dermatitis.

PMID: 10456684 [PubMed - indexed for MEDLINE]

1724. Clin Exp Immunol. 1999 Aug;117(2):223-9.

Contrasting roles for RANTES and macrophage inflammatory protein-1 alpha (MIP-1 alpha) in a murine model of allergic peritonitis.

Das AM, Ajuebor MN, Flower RJ, Perretti M, McColl SR.

Department of Biochemical Pharmacology, The William Harvey Research Institute, St Bartholomew's and the Royal London School of Medicine and Dentistry, London, UK.

Cell accumulation and CC chemokine production were assessed in the peritoneal cavity of ovalbumin (OVA)-sensitized mice following antigen challenge. Intraperitoneal challenge with OVA induced a significant eosinophil influx from 6 h post-challenge with increased numbers persisting at 24 h. At 6 h there was also a marked presence of neutrophils. Messenger RNA expression and protein levels for the chemokines RANTES and MIP-1 alpha were measured in the cell pellets and supernatants, respectively, from peritoneal washes following OVA challenge. RANTES mRNA was detected from 2 h to 4 h following OVA injection, whereas mRNA

for MIP-1 alpha was only detectable at 4 h. RANTES protein was first detected from 4 h after OVA injection and by 24 h the protein levels had increased further. Basal levels of MIP-1 alpha were detected in peritoneal washes. These levels peaked at 2 h after OVA challenge and rapidly declined to basal levels by 6 h. A functional role for the chemokines was assessed using neutralizing polyclonal antibodies. Co-injection of OVA with anti-RANTES antibodies resulted in a significant inhibition of eosinophil infiltration into the cavity at 6 h and 24 h (63% and 52% inhibition, respectively) without significantly influencing the number of neutrophils present. In contrast, injection of anti-MIP-1 alpha antibodies only inhibited neutrophil migration at the 6 h time point by 44% without significantly affecting the accumulation of eosinophils. These results demonstrate an important role for RANTES in mediating eosinophil influx in allergic inflammation and a contrasting role for MIP-1 alpha in mediating neutrophil recruitment.

PMCID: PMC1905339
PMID: 10444251 [PubMed - indexed for MEDLINE]

1725. Clin Exp Allergy. 1999 Jul;29 Suppl 3:64-8; discussion 69-71.

Effect of antihistamines on epithelial cells.

Devalia JL, Davies RJ.

Academic Department of Respiratory Medicine, St Bartholomews and the Royal London School of Medicine and Dentistry, University of London, London, UK.

Antihistamines have mostly been used in the management of allergic rhinitis, primarily for their symptomatic relief. Recent studies, however, have suggested that the non-sedating second-generation antihistamines also possess anti-inflammatory activity, and consequently may be useful in the management of inflammation in allergic airways disease. Several in vivo studies have demonstrated that antihistamines decrease inflammatory cell infiltration in allergic disease, mediator release from mast/basophil cells, and the expression of adhesion molecules on epithelial cells. Similarly, in vitro studies have demonstrated that antihistamines decrease the migration and activation of eosinophils and the release of pro-inflammatory mediators from mast/basophil cells, induced by immunological and non-immunological stimuli. More recent evidence suggests that the antihistamines may modulate airway inflammation by influencing the activity of airway epithelial cells, which due to their spatial arrangement and predominance in the airways, are thought to play a pivotal role in the aetiology of airway disease. We and others have demonstrated that antihistamines attenuate allergen- or chemical-induced expression and/or release of mediators which influence the activity of inflammatory cells, such as eosinophils, mast cells, basophils and lymphocytes, known to be involved in the pathogenesis of allergic airway diseases. Collectively, these studies suggest that second-generation H1-histamine receptor antagonists may have potential use either as safe anti-inflammatory alternatives to corticosteroids, or as rescue medication in combination with corticosteroids, for the management of severe airway disease.

PMID: 10444215 [PubMed - indexed for MEDLINE]

1726. J Immunol. 1999 Aug 15;163(4):1827-32.

Identification of domains in IL-16 critical for biological activity.

Nicoll J, Cruikshank WW, Brazer W, Liu Y, Center DM, Kornfeld H.

The Pulmonary Center, Boston University School of Medicine, MA 02118, USA.

IL-16 is a proinflammatory cytokine implicated in the pathogenesis of asthma and other conditions characterized by recruitment of CD4+ T cells to sites of disease. It is postulated that CD4 is an IL-16 receptor, although other receptors or coreceptors may exist. Among several known functions, IL-16 is a chemoattractant factor for CD4+ T cells and it inhibits MLR. We previously reported that an oligopeptide corresponding to the 16 C-terminal residues of human IL-16 inhibits chemoattractant activity. To identify functional domains with greater precision, shorter oligonucleotides containing native or mutated C-terminal IL-16 sequences were tested for IL-16 inhibition. Within the 16 C-terminal residues, the minimal peptide RRKS (corresponding to Arg106 to Ser109) was shown to mediate inhibition of IL-16 chemoattractant activity. Inhibition was lost when either arginine was substituted with alanine. Point mutations in IL-16 revealed that Arg107 is critical for chemoattractant activity, but MLR inhibition was unaffected by mutation of Arg107 or even deletion of the C-terminal tail through Arg106. Deletion of 12 or 22 N-terminal residues of IL-16 had no impact on chemoattractant activity, but MLR inhibition was reduced. Deletion of 16 C-terminal plus 12 N-terminal residues abolished both chemoattractant and MLR-inhibitory activity of IL-16. These data indicate that receptor interactions with IL-16 that activate T cell migration are not identical with those required for MLR inhibition, and suggest that both N-terminal and C-terminal domains in IL-16 participate in receptor binding or activation.

PMID: 10438915 [PubMed - indexed for MEDLINE]

1727. Am J Pathol. 1999 Aug;155(2):591-8.

Effects of reactive oxygen and nitrogen metabolites on RANTES- and IL-5-induced eosinophil chemotactic activity in vitro.

Sato E, Simpson KL, Grisham MB, Koyama S, Robbins RA.

Research Services, Tucson and Overton Brooks VA Medical Centers, Department of Medicine, University of Arizona, Tucson, Arizona, USA.

Eosinophils and increased production of nitric oxide (NO) and superoxide, components of peroxynitrite, have been implicated in the pathogenesis of a number of allergic disorders including asthma. Peroxynitrite induced protein nitration may compromise enzyme and protein function. We hypothesized that peroxynitrite may modulate eosinophil migration by modulating chemotactic cytokines. To test this hypothesis, the eosinophil chemotactic responses of regulated on activation, normal T cell expressed and secreted (RANTES) and interleukin (IL)-5 incubated with and without peroxynitrite were evaluated. Peroxynitrite-attenuated RANTES and IL-5 induced eosinophil chemotactic activity (ECA) in a dose-dependent manner ($P < 0.05$) but did not attenuate leukotriene B4 or complement-activated serum ECA. The reducing agents deferoxamine and dithiothreitol reversed the ECA inhibition by peroxynitrite, and exogenous L-tyrosine abrogated the inhibition by peroxynitrite. PAPA-NONOate, a NO donor, or superoxide generated by lumazine or xanthine and xanthine oxidase, did not show an inhibitory effect on ECA. The peroxynitrite generator, 3-morpholinosydnonimine, caused a concentration-dependent inhibition of ECA. Peroxynitrite reduced RANTES and IL-5 binding to eosinophils and resulted in nitrotyrosine formation. These findings are consistent with nitration of tyrosine by peroxynitrite with subsequent inhibition of RANTES and IL-5 binding to eosinophils and suggest that peroxynitrite may play a role in regulation of eosinophil chemotaxis.

PMCID: PMC1866862

PMID: 10433951 [PubMed - indexed for MEDLINE]

1728. J Ethnopharmacol. 1999 Jul;66(1):19-24.

Studies on the anti-allergic activity of Mikania glomerata.

Fierro IM, da Silva AC, Lopes Cda S, de Moura RS, Barja-Fidalgo C.

Departamento de Farmacologia e Psicobiologia, Instituto de Biologia, Universidade do Estado do Rio de Janeiro, Rio de Janeiro, Brazil. fierro@openlink.com.br

A fraction (MG1) obtained from the ethanolic extract of Mikania glomerata Sprengel (Compositae), popularly known as 'guaco' and used as 'an' anti-allergic and anti-inflammatory agent, was evaluated for these properties on ovalbumin-induced allergic pleurisy and in models of local inflammation induced by biogenic amines, carrageenan and PAF. Plasma exudation as well as neutrophil and eosinophil infiltration evoked by the intrapleural injection of the antigen were significantly reduced by the fraction. Likewise, PAF-induced pleural neutrophil migration was inhibited by the treatment with MG1. On the other hand, pre-treatment of the animals with MG1 failed to modify the pleurisy induced by histamine, serotonin or carrageenan. These results suggest that MG1 is effective in inhibiting immunologic inflammation but did not affect acute inflammatory response caused by other agents.

PMID: 10432203 [PubMed - indexed for MEDLINE]

1729. Int Urogynecol J Pelvic Floor Dysfunct. 1999;10(3):200-6.

Injection therapy for stress incontinence in women.

Su TH, Hsu CY, Chen JC.

Mackay Memorial Hospital and Taipei Medical College, Taiwan.

Periurethral bulking agents have been used to treat intrinsic sphincter deficiency for decades. Currently available agents include polytetrafluoroethylene, glutaraldehyde cross-linked collagen, autologous fat and silicone microimplants. Polytetrafluoroethylene has never been approved by the United States Food and Drug Administration for periurethral injection because of the risk of particle migration. Early results have reported subjective cure rates of up to 95% with collagen injections. However, collagen may cause allergic reactions and long-term results are still awaited. Periurethral fat injection has a reported success rate ranging from 23% to 65%. It is simple and avoids the use of foreign materials, but the main disadvantage relates to the variability of resorption and connective tissue replacement. Silicone microimplants need further study to evaluate their efficacy and safety. The best material has not yet been defined. Long-term studies are still necessary to define the optimal injectable agent.

PMID: 10430015 [PubMed - indexed for MEDLINE]

1730. Immunopharmacology. 1999 Apr;41(3):235-43.

Inhibition by dexamethasone of Langerhans cell migration: influence of epidermal cytokine signals.

Cumberbatch M, Dearman RJ, Kimber I.

Zeneca Central Toxicology Laboratory, Macclesfield, Cheshire, UK.
marie.cumberbatch@ctl.zeneca.com

The influence of dexamethasone (DEX), a synthetic glucocorticoid, on the induction in mice of Langerhans cell (LC) migration has been investigated. Systemic treatment of mice with DEX was found to inhibit significantly the ability of a topically applied contact allergen (oxazolone) to induce the migration of LC from the epidermis and their subsequent accumulation as dendritic cells (DC) in draining lymph nodes. The stimulation of LC migration during skin sensitization is dependent upon signals provided by the epidermal cytokines tumour necrosis factor alpha (TNF-alpha) and interleukin 1beta (IL-1beta). It was found that treatment with DEX was unable to inhibit either LC migration or DC accumulation induced by the intradermal injection of TNF-alpha. In contrast, LC migration provoked by similar exposure of mice to IL-1beta (the action of which is dependent upon the de novo synthesis of TNF-alpha) was inhibited by DEX as was the arrival of DC in draining lymph nodes induced by this cytokine. Taken together, the data reported here indicate that DEX is able to inhibit very markedly the stimulation of LC migration during skin sensitization and it is proposed that such inhibition may represent an important aspect of the immunosuppressive properties of glucocorticoids and of their proven utility in the treatment of cutaneous inflammatory disorders. The results also indicate that DEX does not inhibit LC migration secondary to direct effects on cell motility. The proposal is that impaired LC migration results from the regulation by DEX of the de novo synthesis and/or release of TNF-alpha, an inducible epidermal cytokine that provides one important signal for LC to traffic from the skin.

PMID: 10428652 [PubMed - indexed for MEDLINE]

1731. Allergy. 1999;54 Suppl 49:35-41.

Choosing the right outcomes.

Silverman M.

Department of Child Health, University of Leicester, UK.

Outcome measures are used to monitor the effects of interventions in clinical practice or in formal clinical trials. They may also be used to assess changes within populations either spontaneously or as a result of public-health measures. They are used to monitor the course of illness as part of a management plan or, for larger groups, to identify changes brought about, for instance, by migration or immunization. The choice of outcome measure depends on the age of the child, the complexity of the outcome (for instance, whether its application is to individuals or populations), and the time scale over which it is necessary to detect changes in outcome. The most commonly used outcome measures are clinical symptoms, which are often compiled into scoring systems. Surprisingly, these are often the least well-validated measures of outcome. Physiologic measures, although well validated, are difficult to apply to infants and pre-school children. The role of inflammatory markers is currently limited to research rather than clinical practice. Other outcome measures such as quality of life, impact-of-asthma instruments, and measures to determine the health-economic aspects of asthma are poorly developed in childhood. The right outcome depends on the question being asked, the age of the subjects, and the time scale and complexity over which measurement is required.

PMID: 10422746 [PubMed - indexed for MEDLINE]

1732. Oftalmologia. 1998;44(3):20-6.

[Corneal ulcerative lesions in type-I immediate hypersensitivity].

[Article in Romanian]

Giuri S.

Policlinica Deta, Spitalul judeţean, Timişoara.

The vernal keratoconjunctivitis (KCV) is included within the category of the hypersensitiveness diseases, the immunopathological mechanism which causes the disease being represented by a type-I hypersensibility reaction. The mechanism which determines the appearance of the corneal lesions isn't entirely cleared up, but there are however some pathogenic links which have been already deciphered. The type I hypersensibility reaction is taking place within two stages: stage I the stage of the sensitizing contact and stage II the stage of the unleashing contact. During the first stage, the Langerhans cells take over and process the allergen, exhibiting on their surface only the antigenic part. The Langerhans cells interact with the T helper native cells (Tho), cells from which there will result the predominantly differentiated Th2 subtype. The Thz cells will activate, by means of the interleukines, the B cells (which produce the IgE), the mast cells and the eosinophilic cells. During the second stage, the allergen is coming into contact with the IgE specific antibodies, which are fastened on the mast cells membrane, generating the opening of their granules. The result of this evolution is represented by the unleash of vasoactive mediators, own enzymes, chemical mediators (among which there is also the eosinophilic chemotactic factor ECFA). The latter contributes to the infiltration of the epithelial and of the subepithelial tissue with eosinophilic cells. The major basic protein (PBM), one of the proteins released from the eosinophilic cells' big granules, plays a major pathogenic role in the production of the corneal ulcer, by means of its direct cytotoxic effect and also by means of inhibiting the migration of the epithelial corneal cells. The role of the mast cells and also the role of the neutrophile cells within the framework of the pathogenesis of the ulcer is disputable, because some specific enzymes tryptase, respectively elastase--have been found within the debris of the corneal ulcer. The allergic keratoconjunctivitis (KCA) represents the ocular manifestation of the systemic hypersensitiveness. In the beginning the immunopathogenic mechanism which causes the lesions is represented by a type-I hypersensibility reaction, but during its evolution, the characteristic histopathological changes (chronic granulomas, perivascularitis, subendothelial fibrosis) are suggesting a complex immunoregulatory disfunction.

PMID: 10418608 [PubMed - indexed for MEDLINE]

1733. Ann N Y Acad Sci. 1999 Jun 30;878:12-24.

Role of matrix metalloproteinases and their inhibition in cutaneous wound healing and allergic contact hypersensitivity.

Pilcher BK, Wang M, Qin XJ, Parks WC, Senior RM, Welgus HG.

Department of Medicine, Washington University School of Medicine, St. Louis, Missouri 63110, USA.

Normal wounds can heal by secondary intention (epidermal migration to cover a denuded surface) or by approximation of the wound edges (e.g., suturing). In healing by secondary intention, epidermis-derived MMPs are important. Keratinocyte migration begins within 3-6 hr post injury, as basal cells detach from underlying basal lamina and encounter a dermal substratum rich in type I

collagen. Cell contact with type I collagen in vitro stimulates collagenase-1 expression, which is mediated by the alpha 2 beta 1 integrin, the major keratinocyte collagen-binding receptor. Collagenase-1 activity alone is necessary and sufficient for keratinocyte migration over a collagen subsurface. Stromelysins-1 and -2 are also found in the epidermis of normal acute wounds. Stromelysin-2 co-localizes with collagenase-1 and may facilitate cell migration over non-collagenous matrices of the dermis. In contrast, stromelysin-1 is expressed by keratinocytes behind the migrating front and which remain on basal lamina, i.e., the proliferative cell population. Studies with stromelysin-1-deficient mice that suggest this MMP plays a role in keratinocyte detachment from underlying basement membrane to initiate cell migration. In chronic ulcers, MMP levels are markedly elevated, in contrast to their precise temporal and spatial expression in acute wounds. Both collagenase-1 and stromelysin-1 are found in fibroblasts underlying the nonhealing epithelium, and stromelysin-1 expression is especially prominent. Two key questions underlie the use of MMP inhibitors and wound healing: (1) will these agents impair normal reepithelialization in wounds that heal by secondary intention; and (2) can MMP inhibitors be effective therapy for chronic ulcers? The answer to neither is known. Batimastat and marimastat appear not to interfere with normal wound healing, but only in sutured surgical wounds, a situation in which MMP expression has practically no role. We also show the first example of an in vivo immune response, contact hypersensitivity, which is dependent upon MMP activity. Using gene-deficient mice, we demonstrate that stromylysin-1 (MMP-3) is required for sensitization, whereas gelatinase B (MMP-9) is required for timely resolution of the reaction to antigenic challenge.

PMID: 10415717 [PubMed - indexed for MEDLINE]

1734. J Immunol. 1999 Aug 1;163(3):1611-8.

Activation of mitogen-activated protein kinase regulates eotaxin-induced eosinophil migration.

Boehme SA, Sullivan SK, Crowe PD, Santos M, Conlon PJ, Sriramarao P, Bacon KB.

Neurocrine Biosciences, Inc., San Diego, CA 92121, USA. sboehme@neurocrine.com

Eotaxin is a potent eosinophil chemoattractant that plays an important role in regulating eosinophil tissue levels both in healthy individuals and in diseases associated with significant eosinophil infiltrates, such as the allergic inflammation observed in asthma. Here, we demonstrate that treatment of eosinophils with eotaxin induces the phosphorylation of the mitogen-activated protein kinases (MAPKs) p42 and p44, leading to kinase activation. Blockade of MAPK activation by the MAPK kinase inhibitor PD98059 leads to a dramatic decrease in eotaxin-induced eosinophil rolling in vivo and chemotaxis in vitro. This blockade in the leukocyte migration process is consistent with the observed inhibition of actin polymerization and rearrangement within the eosinophil following treatment with MAPK inhibitor. It is suggested, therefore, that the intrinsic mechanism of eotaxin-induced eosinophil rolling and migration involves activation of the p42/p44 MAPK, possibly through regulation of the cytoskeletal rearrangements necessary for chemotaxis.

PMID: 10415066 [PubMed - indexed for MEDLINE]

1735. Med Trop (Mars). 1998;58(4 Suppl):433-6.

[Eosinophils: receptors, mediators, functions].

[Article in French]

Prin L, Dubucquoi S.

Service d'Immunologie, Faculté de Médecine, CHRU de Lille, France.
prin@univ-lille2.fr

Eosinophils have been studied using various models. Resulting data indicate that these polynuclear cells may have good and bad effects on the host. Eosinophils have been shown to destroy parasites in vitro but also to contribute to inflammation especially in association with bronchial asthma. These findings illustrate the functional versatility of a cell with the ability to interpret messages from its environment including chemokines, cytokines, growth factors, lipid mediators, and neuropeptides. In succession, these signals can stimulate eosinophils to express a range of regulated (homeostasis) or unregulated (pathogenesis) activity. Some signals cause selective migration into target tissues, especially submucosa in contact with the environment. Other signals determine local function. Understanding these mechanisms will be crucial to development of effective management of persistent hypereosinophilia syndromes.

PMID: 10410361 [PubMed - indexed for MEDLINE]

1736. J Neuroimmunol. 1999 Jun 1;97(1-2):51-9.

Chemokine mRNA expression in the cauda equina of Lewis rats with experimental allergic neuritis.

Fujioka T, Purev E, Rostami A.

Department of Neurology, University of Pennsylvania School of Medicine, Philadelphia 19104-4283, USA.

Chemokines play an important role in the migration of leukocytes to inflammatory sites. In this study, using the quantitative competitive reverse transcriptase PCR method, we analyzed sequential expression of certain chemokine mRNAs in the cauda equina (CE) of rats with experimental allergic neuritis (EAN). Interferon-gamma-inducible protein (IP)-10, monocyte chemotactic protein (MCP)-1, macrophage inflammatory protein (MIP)-1alpha, the regulated upon activation normal T cell expressed and secreted chemokine (RANTES), and lymphotactin were analyzed on days 0 (pre-immunization), 7 (preclinical stage), 10 (disease onset), 13 (clinical progression), 17 (disease peak), as well as on days 20, 24, and 34 post-immunization (p.i.) (recovery). MCP-1 message increased at the preclinical stage and peaked at day 17 p.i. The increase in the early stage was not detected in other tissues, indicating peripheral nerve-specific upregulation. MIP-1alpha and IP-10 messages surged at day 13, then returned to low in the recovery stage. RANTES message increased at day 13 and peaked at day 17 p.i.; however, unlike other chemokines, it showed a second peak of expression on day 24. Lymphotactin message was undetectable at any time point. MCP-1 protein was detected immunohistologically in endothelial cells at day 7 p.i. The sequential expression of these chemokines in relation to the inflammatory process in the nerve leading to demyelination is discussed.

PMID: 10408979 [PubMed - indexed for MEDLINE]

1737. Ann Allergy Asthma Immunol. 1999 Jun;82(6):579-85.

Expression of ICAM-1 on conjunctival epithelium and ECP in tears and serum from children with allergic conjunctivitis.

Oh JW, Shin JC, Jang SJ, Lee HB.

Department of Pediatrics, Hanyang University, College of Medicine, Seoul, Korea.

BACKGROUND: Conjunctival eosinophilia may be considered to be an indicator of conjunctival allergic disease. The absence of eosinophils on conjunctival scraping, however, cannot rule out the diagnosis of allergic conjunctivitis because eosinophil infiltration may be deeper in conjunctival tissue. Eosinophil cationic protein (ECP) is a toxic product secreted by activated eosinophil as a marker of eosinophil activation. Eosinophil cationic protein concentrations in body fluids correlate with the severity of some allergic diseases. ICAM-1 promotes adhesion of leukocytes to epithelium, endothelium, and upregulates inflammation. Expression of adhesion can be modified by many extracellular and intracellular variables such as proinflammatory cytokines, extracellular matrix proteins, and viral infection.
OBJECTIVE: We investigated whether local eosinophils are only activated in conjunctival epithelium or circulating activated eosinophils are involved in peripheral blood during allergic reaction of the eye. We also demonstrated the possible expression of ICAM-1 on epithelial cells from conjunctival scraping and compared them with soluble ICAM-1 values of serum and tears in children with allergic conjunctivitis and healthy children.
METHODS: Seventeen subjects were selected on the basis of clinical manifestations, history, skin prick test, and total serum IgE. A microcapillary tube was used to collect the tears from the inner canthus. Conjunctival epithelia were obtained by scraping the upper tarsal conjunctiva. The level of ECP was measured by the CAP system, soluble ICAM-1 was measured by ELISA, and ICAM-1 on conjunctival epithelial cells were expressed by the avidine-biotin peroxide complex procedure.
RESULTS: Serum IgE and the eosinophil count were increased in 10 out of 17 patients, positive skin prick tests were positive in 11 patients (Dermatophagoides pternyssinus; 9, Dermatophagoides farinae: 8), and eosinophilia in conjunctival epithelium was in 11 patients (4 patients: >3/HPF, 7 patients: 1-3/HPF). The ECP levels in tears were significantly increased in the patient group (12.0+/-8.0 versus 3.9+/-3.8 microg/mL, P = .01), but not in serum (52.5+/-43.1 versus 28.3+/-25.9 microg/mL). There is significant correlation between the eosinophil count in peripheral blood and on conjunctival epithelium (P = .007, r = .62; n = 25). The ICAM-1 expression score on conjunctival epithelial cells was significantly different between the patient group and controls (patient group: 1.77+/-1.25 versus control: 0.13+/-0.35 ng/mL, P = .002). There was a significant correlation between ICAM-1 expression on conjunctival epithelial cells and the ECP levels of tears (P = .01, r = .58; n = 25). Soluble ICAM-1 levels in serum and tears showed no significant difference between the patient group and controls, and also, there was no correlation between sICAM-1 levels in the serum and tears.
CONCLUSION: Eosinophil cationic protein in tears and ICAM-1 expression scores on conjunctival epithelium showed a significant difference between children with allergic conjunctivitis and the healthy controls, but circulating ECP and sICAM-1 in serum were not significantly different between the two groups. These results may suggest that ICAM-1 is locally upregulated in inflammation, mediating eosinophil activation and migration to conjunctival epithelium, but is not involved as inflammatory mediators in peripheral blood during allergic response in children with allergic conjunctivitis.

PMID: 10400487 [PubMed - indexed for MEDLINE]

1738. Methods Find Exp Clin Pharmacol. 1999 May;21(4):303-8.

Effects of Sho-seiryu-to on experimental allergic rhinitis in guinea pigs.

Sakaguchi M, Mase A, Ikeda Y, Iizuka A, Imamura S, Komatsu Y, Ukai K, Sakakura Y.

Central Research Laboratories, Tsumura & Co., Ibaraki, Japan.
hirokoma@po.iijnet.or.jp

The effects of Sho-seiryu-to, an antiallergic Kampo medicine, on experimental allergic rhinitis were investigated in actively sensitized guinea pigs. The number of sneezes and scratches by the animals after a topical antigen challenge was significantly inhibited by pretreatment with Sho-seiryu-to (1000 mg/kg per os p.o.). The antigen-induced eosinophil infiltration in the nasal mucosa was significantly inhibited by Sho-seiryu-to (1000 mg/kg p.o.). Sho-seiryu-to (100 mg/kg p.o.) also reduced the increase in dye leakage to the nasal cavity induced by the antigen challenge and the antigen-induced decrease in volume of the nasal cavity was inhibited. Moreover, Sho-seiryu-to (1000 mg/kg p.o.) suppressed the volume change in the nasal cavity induced by leukotriene D4. These results demonstrate that Sho-seiryu-to inhibits experimental allergic rhinitis in guinea pigs, confirming that the agent may be beneficial for the treatment of allergic rhinitis.

PMID: 10399140 [PubMed - indexed for MEDLINE]

1739. Blood. 1999 Jul 15;94(2):509-18.

The thrombocytopenia of Wiskott Aldrich syndrome is not related to a defect in proplatelet formation.

Haddad E, Cramer E, Rivière C, Rameau P, Louache F, Guichard J, Nelson DL, Fischer A, Vainchenker W, Debili N.

INSERM U 362, PR1, Institut Gustave Roussy, Villejuif, France.

The Wiskott-Aldrich syndrome (WAS) is an X-linked hereditary disease characterized by thrombocytopenia with small platelet size, eczema, and increased susceptibility to infections. The gene responsible for WAS was recently cloned. Although the precise function of WAS protein (WASP) is unknown, it appears to play a critical role in the regulation of cytoskeletal organization. The platelet defect, resulting in thombocytopenia and small platelet size, is a consistent finding in patients with mutations in the WASP gene. However, its exact mechanism is unknown. Regarding WASP function in cytoskeletal organization, we investigated whether these platelet abnormalities could be due to a defect in proplatelet formation or in megakaryocyte (MK) migration. CD34(+) cells were isolated from blood and/or marrow of 14 WAS patients and five patients with hereditary X-linked thrombocytopenia (XLT) and cultured in serum-free liquid medium containing recombinant human Mpl-L (PEG-rHuMGDF) and stem-cell factor (SCF) to study in vitro megakaryocytopoiesis. In all cases, under an inverted microscope, normal MK differentiation and proplatelet formation were observed. At the ultrastructural level, there was also no abnormality in MK maturation, and normal filamentous MK were present. Moreover, the in vitro produced platelets had a normal size, while peripheral blood platelets of the same patients exhibited an abnormally small size. However, despite this normal platelet production, we observed that F-actin distribution was abnormal in MKs from WAS patients. Indeed, F-actin was regularly and linearly distributed under the cytoplasmic membrane in normal MKs, but it was found concentrated in the center of the WAS MKs. After adhesion, normal MKs extended very long filopodia in which WASP could be detected. In contrast, MKs from WAS patients showed shorter and less numerous filopodia. However, despite this abnormal filopodia formation, MKs from WAS patients normally migrated in response to stroma-derived factor-1alpha (SDF-1alpha), and actin normally polymerized after SDF-1alpha or thrombin stimulation. These results suggest that

the platelet defect in WAS patients is not due to abnormal platelet production, but instead to cytoskeletal changes occuring in platelets during circulation.

PMID: 10397718 [PubMed - indexed for MEDLINE]

1740. J Immunol. 1999 Jul 15;163(2):970-7.

Chemotaxis of rat mast cells toward adenine nucleotides.

McCloskey MA, Fan Y, Luther S.

Department of Zoology and Genetics, Iowa State University, Ames 50011, USA.
drmike@iastate.edu

Rat mucosal mast cells express P2 purinoceptors, occupation of which mobilizes cytosolic Ca2+ and activates a potassium conductance. The primary function of this P2 system in mast cell biology remains unknown. Here, we show that extracellular ADP causes morphological changes in rat bone marrow-cultured mast cells (BMMC) typical of those occurring in cells stimulated by chemotaxins, and that the nucleotides ADP, ATP, and UTP are effective chemoattractants for rat BMMC. ADP was also a chemotaxin for murine J774 monocytes. The nucleotide selectivity and pertussis toxin sensitivity of the rat BMMC migratory response suggest the involvement of P2U receptors. Poorly hydrolyzable derivatives of ADP and ATP were effective chemotaxins, obviating a role for adenosine receptors. Buffering of external Ca2+ at 100 nM or reduction of the electrical gradient driving Ca2+ entry (by elevating external K+) blocked ADP-driven chemotaxis, suggesting a role for Ca2+ influx in this process. Anaphylatoxin C5a was a potent chemotaxin (EC50 approximately 0.5 nM) for J774 monocytes, but it was inactive on rat BMMC in the presence or absence of laminin. Ca2+ removal or elevated [K+] had modest effects on C5a-driven chemotaxis of J774 cells, implicating markedly different requirements for Ca2+ signaling in C5a- vs ADP-mediated chemotaxis. This is supported by the observation that depletion of Ca2+ stores with thapsigargin completely blocked migration induced by ADP but not C5a. These findings suggest that adenine nucleotides liberated from parasite-infested tissue could participate in the recruitment of mast cells by intestinal mucosa.

PMID: 10395694 [PubMed - indexed for MEDLINE]

1741. Inflammation. 1999 Jun;23(3):287-304.

Selective inflammatory response induced by intratracheal and intravenous administration of poly-L-arginine in guinea pig lungs.

Arseneault D, Maghni K, Sirois P.

Institut de Pharmacologie de Sherbrooke, Faculté de Medecine, Université de Sherbrooke, PQ Canada.

Major basic protein (MBP) is a cationic protein found in eosinophil granules that was postulated to participate in the pathogenesis of bronchial asthma. Recently, it has been demonstrated that MBP level in serum or bronchoalveolar lavage (BAL) fluid was correlated with bronchial hyperresponsiveness (BHR) in asthmatics. A number a studies have established that MBP actions could be mimicked by synthetic polycations as poly-L-arginine. In this study, we investigated the effects of intratracheal and intravenous administration of poly-L-arginine on lung inflammatory response development. The intratracheal injection of poly-L-arginine at the doses of 1, 10, 100 nmol/animal increased the number of eosinophils (up to 3.2 fold) and neutrophils (up to 12 fold) in BAL fluid. Eosinophil and neutrophil

infiltration was reversed by 88% and 67% respectively following low molecular weight heparin treatment (500 microg/animal). The intravenous injection of increasing doses of poly-L-arginine (1, 10, 100, 500 nmol/animal) increased the number of eosinophils (up to 2.7 fold) but not neutrophil infiltration in guinea pig lungs. Eosinophil infiltration was reversed by 87% following low molecular weight heparin treatment (1.5 mg/animal). Intratracheal treatment with poly-L-arginine (100 nmol/animal) produced an important increase of beta-glucuronidase, histamine, eosinophil peroxidase (EPO) and albumin levels in BAL fluid, whereas the intravenous treatment (500 nmol/animal) did not. These results show that the route of administration of poly-L-arginine greatly influences its effect on inflammatory cell recruitment since both administration routes elicited eosinophil migration but only the intratracheal route stimulated the migration of neutrophils. Moreover, poly-L-arginine appeared to induce other inflammatory responses since it increased beta-glucuronidase, histamine, EPO and albumin levels in BAL fluid following intratracheal treatment. These results also showed that low molecular weight heparin significantly blocks the inflammatory responses elicited by poly-L-arginine.

PMID: 10392762 [PubMed - indexed for MEDLINE]

1742. Br J Pharmacol. 1999 May;127(2):537-45.

Tranilast inhibits protein kinase C-dependent signalling pathway linked to angiogenic activities and gene expression of retinal microcapillary endothelial cells.

Koyama S, Takagi H, Otani A, Suzuma K, Nishimura K, Honda Y.

Department of Ophthalmology and Visual Sciences, Kyoto University Graduate School of Medicine, Japan.

1. Tranilast, first developed as an anti-allergic drug, has been reported to inhibit vascular endothelial growth factor (VEGF)-induced angiogenesis and vasopermeability. To further clarify the inhibitory mechanism, we investigated the effects of tranilast on VEGF binding and subsequent intracellular signalling pathway linked to angiogenic activities and gene expression of bovine retinal microcapillary endothelial cells. 2. Tranilast significantly (P<0.01) inhibited VEGF, basic fibroblast growth factor (bFGF), and hypoxia conditioned media-induced BREC proliferation in a dose dependent manner with IC50's of 22, 82 and 10 microM, respectively. 3. VEGF-induced migration was also inhibited by tranilast in a dose dependent manner, with IC50 of 18 microM, and complete inhibition was observed at 300 microM (P<0.01). Tranilast suppressed VEGF-induced tube formation in a dose dependent manner with maximum (46%) inhibition observed at 300 microM (P<0.05). 4. Tranilast inhibited phorbol myristate acetate (PMA)-dependent stimulation of [3H]-thymidine incorporation and VEGF- and PMA-induced gene expression of integrin alpha v and c-fos in BREC. 5. Tranilast suppressed VEGF- and PMA-stimulated PKC activity in BREC. 6. Tranilast did not affect VEGF binding or VEGF-induced phosphorylation of tyrosine residues of VEGF receptor- and phospholipase Cgamma and their associated proteins. 7. These data suggest that tranilast might prove an effective inhibitor to prevent retinal neovascularization in ischaemic retinal diseases, and that its inhibitory effect might be through suppression of PKC-dependent signal transduction in BREC.

PMCID: PMC1566036
PMID: 10385256 [PubMed - indexed for MEDLINE]

1743. J Immunol. 1999 Jul 1;163(1):403-11.

Mouse monocyte-derived chemokine is involved in airway hyperreactivity and lung inflammation.

Gonzalo JA, Pan Y, Lloyd CM, Jia GQ, Yu G, Dussault B, Powers CA, Proudfoot AE, Coyle AJ, Gearing D, Gutierrez-Ramos JC.

Millennium Pharmaceuticals, Inc., Cambridge, MA 02139, USA.

The cloning, expression, and function of the murine (m) homologue of human (h) monocyte-derived chemokine (MDC) is reported here. Like hMDC, mMDC is able to elicit the chemotactic migration in vitro of activated lymphocytes and monocytes. Among activated lymphocytes, Th2 cells were induced to migrate most efficiently. mMDC mRNA and protein expression is modulated during the course of an allergic reaction in the lung. Neutralization of mMDC with specific Abs in a model of lung inflammation resulted in prevention of airway hyperreactivity and significant reduction of eosinophils in the lung interstitium but not in the airway lumen. These data suggest that mMDC is essential in the transit/retention of leukocytes in the lung tissue rather than in their extravasation from the blood vessel or during their transepithelial migration into the airways. These results also highlight the relevance of factors, such as mMDC, that regulate the migration and accumulation of leukocytes within the tissue during the development of the key physiological endpoint of asthma, airway hyperreactivity.

PMID: 10384142 [PubMed - indexed for MEDLINE]

1744. Lung. 1999;177(4):219-28.

Budesonide down-regulates eosinophil locomotion but has no effects on ECP release or on H2O2 production.

Lantero S, Oddera S, Silvestri M, Gonzalez Rodriguez R, Morelli MC, Rossi GA.

Pulmonary Division, G. Gaslini Institute, Genoa, Italy.

Treatment of allergic asthma with inhaled corticosteroids results in local down-regulation of proinflammatory cytokine synthesis and in marked decrease in tissue eosinophilia. Blood concentrations of inhaled corticosteroids, although significantly lower than those measured in the lung, may still have antiinflammatory effects on circulating eosinophils, reducing their ability to migrate. The aim of our study was to evaluate in vitro the activity of budesonide on blood eosinophils by measuring their chemotactic response, eosinophil cationic protein (ECP) release, and hydrogen peroxide (H2O2) production in the presence of different drug concentrations similar to those obtained at airway level (10(-8) and 10(-7) M) and at blood level (10(-10) and 10(-9) M). Partially purified blood eosinophils, isolated from 23 asthmatic subjects, were used to evaluate the activity of budesonide on: (1) chemotaxis toward the activated fifth component of complement (C5a, 0.1 microg/ml) or recombinant human (rh) interleukin (IL)-5 (200 pg/ml), (2) ECP release by cells stimulated with tetradecanoylphorbol acetate (TPA) and (3) H2O2 production by TPA-activated cells. The chemotactic response to C5a was down-regulated significantly by budesonide only by the highest concentrations tested (10(-8) and 10(-7) M); differently, budesonide was effective in inhibiting eosinophil migration toward rhIL-5, at all concentrations tested ($p < 0.01$, each comparison). By contrast, no drug-induced modifications were observed in ECP release or in H2O2 production ($p > 0.05$, each comparison). We conclude that concentrations of budesonide similar to those obtained in vivo are effective in inhibiting eosinophil locomotion but not in down-regulating the release of reactive oxygen species and granule-associated proteins.

PMID: 10384060 [PubMed - indexed for MEDLINE]

1745. Exp Lung Res. 1999 Jun;25(4):303-16.

A rat model presenting eosinophilia in the airways, lung eosinophil activation, and pulmonary hyperreactivity.

Carvalho C, Jancar S, Mariano M, Sirois P.

Department of Pharmacology, Medical School, University of Sherbrooke, P.Q., Canada.

The aim of this study was to examine antigen-induced lung cell migration, eosinophil activation, and pulmonary reactivity of Wistar rats exposed to a new sensitization technique. The animals were sensitized with a single subcutaneous implant of a fragment of heat coagulated hen egg white and challenged 21 days later with an intratracheal injection of heat-aggregated ovalbumin (EWI). For comparison, another group of rats were sensitized by an intraperitoneal injection of ovalbumin in alum as adjuvant, with one booster on day 14 and challenge on day 21 post immunization (OVA/AL). Twenty-four hours after antigen challenge, the EWI group presented a higher number of eosinophils in the bronchoalveolar lavage (BAL) (4.85 +/- 1.43 x 10(6)) than the OVA/AL group (0.2 +/- 0.06 x 10(6)) or the control group, where the level of eosinophils were essentially undetectable. Levels of eosinophil peroxidase activity were increased in the cell-free BAL and homogenates of lung tissue in the EWI group (12.10 +/- 2.97 mg/mL and 36.14 +/- 7.21 ng/mg, respectively), but not in the OVA/AL group (4.83 +/- 1.4 ng/mL and 11.95 +/- 2.54 ng/mg, respectively), as compared with controls (5.16 +/- 1.65 ng/mL and 12.13 +/- 1.74 ng/mg, respectively). Thromboxane B2 levels were also increased in the BAL of EWI group (2.89 +/- 0.54 ng/mL) but not the OVA/AL group (1.13 +/- 0.23 ng/mL) as compared with controls (1.14 +/- 0.19 ng/mL). In contrast, the levels of prostaglandin E2 in the BAL were increased in both groups (456.4 +/- 11.8 pg/mL in the EWI group and 303.5 +/- 31.7 pg/mL in the OVA/AL group) as compared with controls (205.7 +/- 29.7 ng/mL). Moreover, only the EWI group developed increased pulmonary reactivity to serotonin (around two-fold), 24 hours after antigen challenge. The extent of lung eosinophil migration and activation and the pulmonary hyperreactivity induced by this novel sensitization procedure without adjuvants represents a significant improvement over existing experimental models of asthma.

PMID: 10378102 [PubMed - indexed for MEDLINE]

1746. Infect Immun. 1999 Jul;67(7):3444-51.

Development of antibody isotype responses to Schistosoma mansoni in an immunologically naive immigrant population: influence of infection duration, infection intensity, and host age.

Naus CW, Kimani G, Ouma JH, Fulford AJ, Webster M, van Dam GJ, Deelder AM, Butterworth AE, Dunne DW.

Division of Microbiology and Parasitology, Department of Pathology, University of Cambridge, Cambridge, United Kingdom.

We have identified the influence of host and parasite factors that give rise to characteristic antibody isotype profiles with age seen in human populations living in different areas of schistosomiasis endemicity. This is important in the immunobiology of this disease. It is also of interest in the context of human responses to chronic antigen stimulation, vaccines, allergens, and other pathogens. In populations exposed to endemic schistosomiasis, factors such as

intensity and duration of infection are age dependent. They therefore confound the influence of host age on antiparasite responses. Here, we resolved these confounding factors by comparing the developing antibody responses of an immunologically naive immigrant population as they acquired the infection for the first time with those of chronically infected resident inhabitants of the same region of Schistosoma mansoni endemicity in Kenya. Recent arrival in the area strongly favored immunoglobulin G3 (IgG3) responses against the parasite. The antibody isotype responses associated with human susceptibility to reinfection after chemotherapy were elevated in those suffering high intensities of infection (IgG4 responses against worm and egg antigens) or were characteristic responses of young children irrespective of the intensity or duration of infection (IgG2 responses against egg antigen). IgE responses against the adult worm, a response associated with resistance to reinfection after chemotherapy, increased with the ages of infected individuals and were also favored in those currently suffering higher intensities of infection.

PIP: This paper examines the influence of infection duration, infection intensity, and host age on specific antibody responses to Schistosoma mansoni in Masongaleni, Kenya. The serum levels of a circulating worm antigen, circulating anodic antigen, were measured to obtain accurate estimates of intensities of infection synchronous with antibody isotype levels measured in the same sera. Recent arrival in the area strongly favored immunoglobulin G3 (IgG3) responses against the parasites. The antibody isotype responses associated with human susceptibility to reinfection after chemotherapy were elevated in those suffering high intensities of infection (IgG4 responses against worm and egg antigens) or were characteristic responses of young children irrespective of the intensity or duration of infection (IgG2 responses against egg antigen). IgE responses against the adult worm increased with age of infected individuals and were also favored in currently suffering higher intensities of infection. In summary, specific IgG1 and IgG4 responses against worm antigen, as well as IgG4 responses against egg antigen, were strongly associated with intensity of infection, while specific IgG1 and IgG2 responses against egg antigen decreased with age. Finally, IgG3 responses were related to duration of exposure and showed no association with either infection intensity or age.
PMCID: PMC116530
PMID: 10377125 [PubMed - indexed for MEDLINE]

1747. Exp Eye Res. 1999 Jun;68(6):739-46.

Histamine effects on conjunctival fibroblasts from patients with vernal conjunctivitis.

Leonardi A, Radice M, Fregona IA, Plebani M, Abatangelo G, Secchi AG.

Department of Ophthalmology, Institute of Physiopathological Optics, University of Padova, Italy.

Histamine, an important mast cell mediator in allergic disorders, may affect extracellular matrix production and cell growth in vernal keratoconjunctivitis (VKC). In the present study, the histamine reactivity of conjunctival fibroblasts derived from VKC patients was investigated in vitro. Conjunctival fibroblast cultures were derived from biopses of 8 tarsal VKC patients and 5 normal subjects. These cells were maintained in vitro and stimulated with different concentrations of histamine with and without H1 (clorpheniramine) and H2 (cimetidine) receptor antagonists. Comparisons were made to fibroblasts grown in the same media without histamine and to fibroblasts stimulated with just antihistamine. The effects of histamine were evaluated by: (1) the MTT test to assess cell proliferation; (2) an in vitro wound model for cell migration and (3) the measurement of procollagen I (PIP) and procollagen III (PIIIP) in

supernatants for collagen production. Results showed: (1) While VKC-derived fibroblasts proliferated at a faster rate than normal cells in unstimulated media, after histamine stimulation, VKC and normal cells grew at a similar rate. Both H1 and H2 antagonists significantly inhibited (P<0.05) histamine-induced cell proliferation. (2) Histamine enhanced cell migration after wounding; this effect was inhibited only by H2 antagonism. (3) When stimulated with histamine, VKC fibroblasts produced significantly more PIP than those in control media. Furthermore, VKC-derived fibroblasts were more sensitive to histamine challenge, producing significantly more PIP than normal fibroblasts. H1 and H2 antagonists did not modify histamine-stimulated PIP production. The enhanced proliferative and productive capacity of VKC fibroblasts may be the result of a selective overgrowth of one or more fibroblast subpopulations in a chronically inflamed tissue. Histamine increased proliferation, migration and collagen production in both normal and VKC fibroblasts. Since H2 antagonism modulated both cell growth and migration, but not histamine-induced collagen production, the latter may be mediated by a different receptor. These results showed that histamine is at least partially responsible for fibroblast stimulation.

Copyright 1999 Academic Press.

PMID: 10375437 [PubMed - indexed for MEDLINE]

1748. Allergy. 1999 Apr;54(4):338-45.

Cellular infiltration and cytokine mRNA expression in perennial allergic rhinitis.

Varga EM, Jacobson MR, Till SJ, Masuyama K, O'Brien F, Rak S, Lund V, Scadding GK, Hamid QA, Durham SR.

Upper Respiratory Medicine, Imperial College School of Medicine at the National Heart and Lung Institute, London, UK.

BACKGROUND: Allergen challenge in allergic rhinitis patients leads to local eosinophilia and Th2-type cytokine expression. Natural exposure to grass pollen is additionally characterized by epithelial mast-cell infiltration. We hypothesized that perennial allergic rhinitis is also associated with T-cell and eosinophil infiltration of the nasal mucosa, local Th2-type cytokine expression, and increased numbers of nasal epithelial mast cells.
METHODS: Nasal biopsies from perennial allergic rhinitis patients and controls were analysed by immunocytochemistry for different cell populations and in situ hybridization for cytokine mRNA-expressing cells.
RESULTS: Perennial allergic rhinitis was associated with increased numbers of submucosal CD3+ T cells (P=0.05), EG2+ activated eosinophils (P=0.01), and CD68+ macrophages (P=0.01) compared to controls. Epithelial, but not submucosal, tryptase-positive mast cells were also elevated in rhinitics compared to controls (P=0.01). The numbers of cells expressing interleukin (IL)-5 were higher (P=0.01) and the numbers of cells expressing IL-2 were lower (P=0.04) in rhinitic patients than controls. There were no significant differences for either IL-4 or interferon-gamma between the groups.
CONCLUSIONS: Perennial allergic rhinitis is characterized by mast-cell migration into the epithelium; submucosal infiltration by T cells, eosinophils, and macrophages; and an imbalance in local T-cell cytokine production in favour of enhanced IL-5 and reduced IL-2 expression.

PMID: 10371092 [PubMed - indexed for MEDLINE]

1749. Life Sci. 1999;64(17):1485-91.

Systemic effects of ingested nickel on the immune system of nickel sensitised women.

Boscolo P, Andreassi M, Sabbioni E, Reale M, Conti P, Amerio P, Di Gioacchino M.

Department of Medicine and Sciences of Aging, Centre of Occupational Medicine and Ergophthalmology, University G. D'Annunzio, Chieti, Italy.

This study evaluates the immune response to ingestion of 10 mg of nickel (Ni) (as Ni sulphate) in 19 young non-atopic Ni-sensitised or 9 non-allergic women (group A). After Ni ingestion at 8 a.m, non-allergic and 12 Ni-sensitised women (group B) were non-symptomatic, while 7 Ni-sensitised women (group C) showed a flare up of urticaria and/or eczema. Serum and urine Ni were greatly lower before Ni administration than after 4 and 24 hours, without difference among the 3 groups. Before treatment, group B and C showed higher values of blood CD19+ and CD5--CD19+ cells than group A, while group C showed higher serum interleukin (IL) 2 and lower serum IL-5. Four hours after Ni ingestion, group C showed significant increase in serum IL-5. Twenty-four hours after treatment, group A showed a significant reduction in blood CD4+-CD45RO- "virgin" cells and an increase of CD8+ lymphocytes, while group C showed a marked decrease in total blood lymphocytes and CD3+, CD4+-CD45RO-, CD4+-CD45RO+, CD8+, CD19+ and CD5--CD19+ cell subsets. These data may be explained with migration of lymphocytes in tissues with a Th0-like immune response, as shown by the elevated serum IL-2 and the increase of serum IL-5 during the test.

PMID: 10353613 [PubMed - indexed for MEDLINE]

1750. J Leukoc Biol. 1999 May;65(5):649-57.

Selective antibody blockade of lymphocyte migration to mucosal sites and mast cell adhesion.

Palecanda A, Marshall JS, Li X, Briskin MJ, Issekutz TB.

Department of Pediatrics, Dalhousie University, Halifax, Nova Scotia, Canada.

The integrins alpha4beta7 and alpha4beta1 mediate adhesion to the mucosal addressin cell adhesion molecule-1 (MAdCAM-1) and the vascular cell adhesion molecule-1 (VCAM-1) and are important in T cell and allergic inflammatory reactions in the rat. The relative contributions of alpha4beta7 and alpha4beta1 in these reactions is unknown. To examine the role of alpha4beta7 in the rat a new mAb, TA-6, was developed. TA-6 inhibited adhesion to MAdCAM-1 but not to VCAM-1, a characteristic of alpha4beta7 adhesion, and immunofluorescence and immunoprecipitation studies were compatible with binding to alpha4beta7. TA-6 blocked rat lymphocyte adhesion to mesenteric lymph nodes and T cell migration to mucosal lymphoid tissues and it bound to rat mucosal mast cells. TA-6 did not inhibit lymphocyte adhesion to peripheral lymph nodes and T cell migration to peripheral lymphoid tissues or cutaneous inflammatory sites, and was not expressed on connective tissue mast cells.

PMID: 10331494 [PubMed - indexed for MEDLINE]

1751. J Allergy Clin Immunol. 1999 May;103(5 Pt 1):931-6.

The mucosal adhesion receptor alpha4beta7 integrin is selectively increased in lymphocytes stimulated with beta-lactoglobulin in children allergic to cow's milk.

Eigenmann PA, Tropia L, Hauser C.

Department of Pediatrics, and the Division of Immunology and Allergy, University of Geneva School of Medicine, Geneva, Switzerland.

BACKGROUND: It has been shown in mice that the integrin alpha4beta7 directs the migration of memory T cells into the gut-associated lymphoid tissue. However, little is known about T-cell homing mechanisms in children with food allergies.
OBJECTIVE: We investigated the expression of this and other integrins in children with different manifestations of cow's milk allergy (urticaria, atopic dermatitis, and wheezing).
METHODS: PBMCs were stimulated with beta-lactoglobulin, 1 of the major allergenic proteins in cow's milk, and tetanus toxoid. Integrin expression was studied by flow cytometric analysis after 1 week of culture.
RESULTS: We found significantly higher expression of the alpha4beta7 integrin in cells from patients compared with control subjects with no allergies (P =. 005) when beta-lactoglobulin was used to stimulate the cells. alpha4beta7 integrin was also expressed at significantly higher levels in beta-lactoglobulin-stimulated cells than in tetanus toxoid-stimulated cells (P =.005). The alphaEbeta7 and the alpha4beta1 integrins were not upregulated by allergen stimulation. Most alpha4beta7 integrin-expressing cells were identified as CD4(+) T cells.
CONCLUSION: These results show that alpha4beta7 integrin expression after stimulation with beta-lactoglobulin correlates with the presumptive site of cow's milk sensitization (ie, the gut-associated lymphoid tissue but not with the site of symptoms of cow's milk allergy).

PMID: 10329831 [PubMed - indexed for MEDLINE]

1752. J Am Acad Dermatol. 1999 May;40(5 Pt 1):708-13.

Allergic contact dermatitis: correlation of in vivo confocal imaging to routine histology.

González S, González E, White WM, Rajadhyaksha M, Anderson RR.

Wellman Laboratories of Photomedicine, Boston, Massachusetts 02114, USA.

BACKGROUND: Allergic contact dermatitis (ACD) is a common and often challenging clinical problem. In vivo near-infrared confocal reflectance microscopy (CM) is a new vital microscopy technique.
OBJECTIVE: CM was used to evaluate acute ACD.
METHODS: Patch testing by means of Finn Chambers technique was performed in 5 subjects to induce an acute allergic skin reaction. Noninvasive CM images from normal and eczematous skin were sequentially recorded before and after removal of the Finn Chambers.
RESULTS: The epidermis and papillary dermis were clearly seen in high resolution. Retention of nuclei in stratum corneum, epidermal edema with microvesicle formation, and transepidermal migration of inflammatory cells were observed in vivo. Isolated dendritic cells were present in the ACD sites of 2 subjects, with morphology, size, and location consistent with Langerhans cells. Dermal vasodilation was observed as well.
CONCLUSION: CM is a useful tool to study ACD and may be able to track Langerhans cell activation.

PMID: 10321598 [PubMed - indexed for MEDLINE]

1753. Mund Kiefer Gesichtschir. 1999 Mar;3(2):98-101.

[Risk of oral piercing].

[Article in German]

Bethke G, Reichart PA.

Universitätsklinikum Charité, Berlin.

Oral and facial piercing with different kinds of body art are being observed more frequently in medical and dental practices. Principally, piercing is not a new form of body art and is traditional in different geographical areas. Various materials are used. Besides tongue and lip piercing, different locations of the face such as the eyebrows and the nose are anatomical areas of piercing. The aim of this article is to demonstrate different forms of oral piercing, illustrated by own observations. The piercing procedure is briefly described. Piercing is usually performed without local anaesthesia and stepwise. In a literature review, the possible risks and complications are summarised. Postprocedural complications are oedema, haemorrhage and infection. Other complications are foreign body granulomas or allergies, particularly against nickel. Dentists, and oral- and maxillofacial surgeons should be in a position to advise patients with oral or facial piercings or those who plan to have this type of body art performed.

PMID: 10234960 [PubMed - indexed for MEDLINE]

1754. Immunology. 1999 Feb;96(2):176-83.

Human eotaxin induces eosinophil extravasation through rat mesenteric venules: role of alpha4 integrins and vascular cell adhesion molecule-1.

Nagai K, Larkin S, Hartnell A, Larbi K, Razi Aghakhani M, Windley C, Davies D, Lobb RR, Williams TJ, Nourshargh S.

Applied Pharmacology, Imperial College School of Medicine at the National Heart & Lung Institute, London, UK.

Eotaxin is a potent eosinophil-specific CC-chemokine, which has been shown to play a role in the selective induction of eosinophil accumulation in a number of allergic models of inflammation. Many aspects of the mechanism by which eotaxin induces eosinophil accumulation in vivo remain unresolved. In the present study, we investigated the direct effect of synthetic human eotaxin on leucocyte/endothelial cell interactions within rat mesenteric venules, as quantified by intravital microscopy. Topical eotaxin (30 pmol) induced rapid firm adhesion and extravasation of leucocytes within the rat mesentery, the extravasated leucocytes all being eosinophils, as determined by histological analysis. Whilst eotaxin was unable to stimulate the interaction of rat eosinophils with vascular cell adhesion molecule-1 (VCAM-1) under static conditions in vitro, eotaxin-induced responses in vivo were significantly suppressed by anti-alpha4 integrin and anti-VCAM-1 monoclonal antibodies (mAbs). The anti-alpha4 integrin mAb, HP2/1 (3.5 mg/kg), inhibited the eotaxin-induced firm adhesion and extravasation, 60 min postapplication of the chemokine, by 89% and 84%, respectively. In the same set of experiments, the anti-VCAM-1 mAb, 5F10 (3.5 mg/kg), inhibited leucocyte adhesion and extravasation by 61% and 63%, respectively. These results demonstrate that eotaxin-induced migration of eosinophils through rat mesenteric venules in vivo is dependent on an alpha4 integrin/VCAM-1 adhesion pathway, the significance of which may only be evident under flow conditions and/or following the ligation of other adhesion molecules expressed on eosinophils.

PMCID: PMC2326743
PMID: 10233693 [PubMed - indexed for MEDLINE]

1755. Eur Respir J. 1999 Mar;13(3):577-82.

Lower prevalence of asthma and atopy in Turkish children living in Germany.

Kabesch M, Schaal W, Nicolai T, von Mutius E.

University Children's Hospital, Munich, Germany.

Comment in
    Eur Respir J. 2000 Sep;16(3):576-7.

Ethnic origin has been reported to affect the prevalence of atopic diseases in several studies in different parts of the world. However, little is known about the prevalence of asthma and atopy in immigrants living in Europe. The objective of this study was to evaluate the prevalence of asthma and atopy in Turkish children living in Germany and to investigate the role of ethnic origin on the development of asthma and atopy in this population. In a cross-sectional survey the prevalence of physician-diagnosed asthma, atopy, skin-prick tests and bronchial hyperresponsiveness (BHR) to cold dry air challenge was assessed in 7,445 school children aged 9-11 yrs, living in Munich, south Germany. Questionnaires were distributed to the parents for self-completion and children underwent skin prick tests and cold air hyperventilation challenge. The Turkish children showed a significantly lower prevalence of asthma (5.3 versus 9.4%, p<0.05) than their German peers. Furthermore, atopy, as assessed by skin prick tests (24.7 versus 36.7%, p<0.001) and BHR (3.9 versus 7.7%, p<0.001), was less common in Turkish children. In multivariate regression models controlling for potential explanatory factors, Turkish origin still showed a significantly lower risk of developing asthma, atopic sensitization and BHR. The prevalence of childhood asthma was therefore shown to be lower in Turkish children living in Germany than in Turkey. These findings suggest that the lower prevalence of asthma and allergy in Turkish children living in Germany might be attributable to a selection bias affecting the parents of these children, as healthy individuals may have decided to come to Germany for work.

PMID: 10232429 [PubMed - indexed for MEDLINE]

1756. Immunol Lett. 1999 Apr 1;67(2):141-5.

C3a(desArg) does not bind to and signal through the human C3a receptor.

Wilken HC, Götze O, Werfel T, Zwirner J.

Department of Immunology, University of Göttingen, Germany.

Contradictory results have been published in the past regarding the functional responses of different cell types to the anaphylatoxin C3a and its natural catabolite C3a(desArg). To elucidate the interaction of the C3a receptor (C3aR) with its ligand(s) we studied the binding of human recombinant C3a (rC3a) and rC3a(desArg) to RBL-2H3 transfectants which express the C3aR. As the addition of 11 aminoterminal amino acids did not alter the functional activity of the recombinant C3a as compared to serum-derived C3a the specific binding of rC3a and rC3a(desArg) to the transfectants could be determined by flow cytometry using a monoclonal antibody (mab) against their N-terminal histidine tag. Recombinant C3a bound to the C3aR with a half maximal concentration of about 3 nM whereas no evidence for a binding of rC3a(desArg) could be obtained. Furthermore,

rC3a(desArg) did not signal through the C3aR. Neither the release of lysosomal N-acetyl-beta-D-glucosaminidase nor the directional migration of C3aR-expressing RBL-2H3 transfectants could be detected in response to rC3a(desArg) whereas rC3a was highly active in both assays. Our data demonstrate a defined ligand specificity of the C3aR for the anaphylatoxin C3a. Its natural catabolite C3a(desArg), however, does not signal through the C3aR. Modulating effects of C3a(desArg) on the synthesis of cytokines in human monocytes and B lymphocytes may therefore be induced by receptor-independent mechanisms while their in vivo relevance remains as yet undefined.

PMID: 10232396 [PubMed - indexed for MEDLINE]

1757. Jpn J Pharmacol. 1999 Mar;79(3):351-7.

Adhesive explant culture of allergic nasal mucosa: effect of emedastine difumarate, an anti-allergic drug.

Okubo K, Pawankar R, Gotoh M, Yagi T.

Department of Otolaryngology, Nippon Medical School, Tokyo, Japan.

Allergic reaction of the nose comprises of an immediate and a late reaction. To evaluate nasal allergic reactions, many experiments have been performed by investigators. In this study, we performed a new tissue culture technique (adhesive explant culture) to analyze the migration of cells into the culture medium from the cultured allergic nasal mucosa in response to an allergen. Basophilic cells (mast cells and basophils) and eosinophils, which were released into the culture medium after the allergen challenge, were evaluated by the analysis of histamine and eosinophil cationic protein (ECP) content in the culture medium. Histamine and basophilic cells in the culture medium were more abundant in the immediate phase (within 30 min) after challenge than in the late phase (from 30 min to 10 hr). On the other hand, ECP and eosinophils in the culture medium were more abundant in the late phase than in the immediate phase. The increase of histamine content in both phases were not inhibited by pre-treatment of emedastine difumarate (EME), an anti-allergic drug. However, the increase of ECP in the late phase was inhibited by pre-treatment with EME. Moreover, the number of EG2-positive cells was also decreased by pre-treatment with EME. These results suggest that EME might lower the activation of eosinophils in the late phase of the allergic reaction. The present study also indicates that this adhesive explant culture system is useful model for studying the cellular allergic responses to drugs ex vivo.

PMID: 10230864 [PubMed - indexed for MEDLINE]

1758. Eur J Immunol. 1999 Apr;29(4):1324-33.

P- and L-selectin mediate binding of T cells to chronically inflamed human airway endothelium.

Symon FA, McNulty CA, Wardlaw AJ.

Division of Respiratory Medicine, Leicester University Medical School, Glenfield Hospital, GB.

The inflammatory process that underlies allergic diseases such as asthma is characterized by tissue infiltration of eosinophils and T cells. We have used the Stamper-Woodruff frozen-section assay to characterize the receptors involved in adhesion of human peripheral blood T cells to nasal polyp endothelium (NPE) as a

model of T cell migration in allergic disease. T cells bound specifically to NPE in a temperature-, cell concentration- and shear stress-dependent fashion. Adhesion was inhibited by approximately 70% by antibodies against P-selectin and its counter-receptor P-selectin glycoprotein-1 (PSGL-1). In addition, a blocking monoclonal antibody (mAb) against L-selectin caused significant although lesser inhibition. Cells adhering to NPE were primarily of the CD45RO+ memory subset. Although only a minority subset of peripheral blood T cells expressed functional PSGL-1, as determined by binding of a P-selectin Fc chimera, the majority of the P-selectin chimera-binding cells were found to be CD45RO+. This is consistent with the observation that memory T cells bind to NPE via P-selectin. Using blocking mAb we also investigated which integrins and their counter-structures were involved in T cell binding. A combination of anti-beta1 and beta2 mAb was able to inhibit adhesion by almost 50%. An antibody against intercellular adhesion molecule (ICAM)-2 gave an inhibition similar to that by anti-CD18 mAb, suggesting ICAM-2 was the major counter-receptor involved for the beta2 integrin component. This study suggests that P-selectin, and to a lesser extent L-selectin, may be acting as specific homing receptors for the airway mucosa in the context of chronic allergic disease.

PMID: 10229100 [PubMed - indexed for MEDLINE]

1759. Int Arch Allergy Immunol. 1999 Feb-Apr;118(2-4):429-32.

Matrix metalloproteinase-9 in myeloid cells: implications for allergic inflammation.

Hayashi M, Schellenberg RR, Tsang S, Roberts CR.

University of British Columbia Pulmonary Research Laboratory, St. Paul's Hospital, Vancouver, Canada.

Matrix metalloproteinase-9 (MMP-9; 92 kDa gelatinase) is utilized by myeloid and lymphoid cells for migration across basement membranes. Although eosinophils are commonly seen infiltrating asthmatic airways, the role of basophils in allergic inflammation is debated. This study was undertaken to evaluate the content of MMP-9 in purified basophils compared with eosinophils and neutrophils. Peripheral blood basophils were isolated to greater than 95% purity using negative selection with antibody-coated magnetic beads using a 12-antibody cocktail. Eosinophils of greater than 98% purity were obtained by negative selection and neutrophils by positive selection using anti-CD16 magnetic beads. MMP-9 activity was assessed by gelatin zymography of cell lysates. Under parallel conditions, neutrophils contained 1,000-fold more MMP-9 than eosinophils. No activity was detected from 2x10(5) basophils. Immunocytochemistry with an anti-MMP-9 antibody showed bright staining of all neutrophils, lesser staining of eosinophils and no detectable staining of basophils. The failure to find MMP-9 in basophils may explain their paucity in asthmatic airway inflammation or suggest they secrete other enzymes capable of degrading type IV collagen.

PMID: 10224466 [PubMed - indexed for MEDLINE]

1760. Atherosclerosis. 1999 Apr;143(2):307-13.

Tranilast inhibits the proliferation of human coronary smooth muscle cell through the activation of p21waf1.

Kusama H, Kikuchi S, Tazawa S, Katsuno K, Baba Y, Zhai YL, Nikaido T, Fujii S.

Discovery Research Laboratories, Kissei Pharmaceutical Co. Ltd., Minamiazumi,

Nagano, Japan. hiroshi_kusama@pharm.kissei.co.jp

Restenosis after percutaneous transluminal coronary angioplasty (PTCA) occurs due to vascular smooth muscle cell proliferation and migration. Recently, tranilast, an anti-allergic drug, has been used for the prevention of restenosis after PTCA. To determine the molecular mechanism involved, the effect of tranilast on the proliferation of human coronary smooth muscle cells (SMCs) was investigated. Tranilast arrested the proliferation of human coronary SMCs at the G0/G1 phase of the cell cycle. In association with this inhibitory effect, tranilast increased p21waf1 and p53 tumor suppressor factor, and decreased cyclin-dependent kinase 2 (CDK2) activity. These results suggest that tranilast inhibits the proliferation of human coronary SMCs during restenosis after PTCA via an induction of p21waf1 and p53. Tranilast may thus allow us to prevent restenosis after PTCA by interfering with this mechanism.

PMID: 10217359 [PubMed - indexed for MEDLINE]

1761. Fam Med. 1999 Apr;31(4):276-9.

An unusually high prevalence of asthma in Ethiopian immigrants to Israel.

Rosenberg R, Vinker S, Zakut H, Kizner F, Nakar S, Kitai E.

Department of Family Medicine, Tel Aviv University, Israel.

Comment in
Fam Med. 1999 Apr;31(4):280-2.

BACKGROUND AND OBJECTIVES: The past 20 years have seen a large immigration of Ethiopian Jews to Israel, primarily in 2 airlifts, one in 1984-1985 and one in 1991. Infectious and parasitic diseases were the dominant health problems in the early years. Recently, we noticed changing health patterns in this population, particularly an increase in clinic visits for asthma, which contrasted with asthma rates of 2.5% reported among Ethiopian Jews at the time of immigration to Israel. This study evaluated the prevalence and characteristics of asthma in a population of Jews of Ethiopian origin who had been in Israel for 8-17 years.
METHODS: We audited 302 files of adult patients of Ethiopian origin and 604 files of age- and gender-matched patients of non-Ethiopian origin. Each file was reviewed by 2 physicians. Asthma was defined by published clinical criteria as found in the patient file. Data on allergies and eosinophilia were collected as well.
RESULTS: The average age of the 2 groups of asthmatics was 44.1 +/- 16.2 and 42.4 +/- 20.7 years, respectively. The prevalence of asthma in the patients of Ethiopian origin was 51/302 (17%), compared with 35/604 (5.8%) in the control group. Thirty-three percent of the Ethiopian asthmatics and 37% of the control group suffered from various allergic diseases. Among the patients of Ethiopian origin, the prevalence of eosinophilia was 44%, with no significant difference between asthmatics and non-asthmatic patients (49% versus 43%). Eosinophilia was found in 8.4% of the control group. Asthma was more prevalent among patients with eosinophilia (22%) than without eosinophilia (6.4%).
CONCLUSIONS: Asthma is 3 times as prevalent in adults of Ethiopian origin, compared with the general population in our clinics, and markedly increased above the rate reported for newly immigrated Ethiopian Jews. We conclude that the move from the rural hills of Ethiopia to the more urban and industrialized setting of Israel has resulted in an increased prevalence of asthma in this population, but the specific cause is uncertain.

PMID: 10212770 [PubMed - indexed for MEDLINE]

1762. Eur J Dermatol. 1999 Apr-May;9(3):185-90.

Contact sensitizers decrease 33D1 expression on mature Langerhans cells.

Herouet C, Cottin M, Galanaud P, Leclaire J, Rousset F.

L'Oréal Advanced Research, Life Sciences Research, 1, avenue Eugène-Schueller, BP 22, 93601 Aulnay-sous-Bois Cedex, France.

Langerhans cells play a critical role in allergic contact hypersensitivity. In vivo, these cells capture xenobiotics that penetrate the skin and transport them through the lymphatic vessels into regional lymph nodes for presentation to T cells. During this migration step, Langerhans cells become mature dendritic cells according to their phenotype and their high immunostimulatory capacity. In vitro, when isolated from the skin and cultured for 3 days, Langerhans cells undergo similar phenotypic and functional maturation. In this study, the capacity of sensitizers, irritants and neutral chemicals to modulate the surface marker expression and morphology of pure mature murine Langerhans cells in vitro was examined. Contact with 4 sensitizers (2,4-dinitrobenzenesulfate, 4-ethoxymethylene-2-phenyl-2-oxazolin-5-one, p-phenylenediamine, mercaptobenzo-thiazole) resulted in a rapid, specific, marked fall in 33D1 expression, a murine specific dendritic cell marker. No effect was observed with 2 neutral chemicals (sodium chloride, methyl nicotinate) or 2 irritants (dimethyl sulfoxide, benzalkonium chloride). Nevertheless, sodium lauryl sulfate, a very irritant detergent, altered morphology and down-regulated all membrane markers. These preliminary data suggest that in vitro modulation of 33D1 expression by strong sensitizers may be an approach to the development of an in vitro model for the identification of chemicals that have the potential to cause skin sensitization and to distinguish them as far as possible from irritants.

PMID: 10210782 [PubMed - indexed for MEDLINE]

1763. Clin Exp Allergy. 1999 Mar;29(3):314-22.

The role of interleukin-5, interleukin-8 and RANTES in the chemotactic attraction of eosinophils to the allergic lung.

Lampinen M, Rak S, Venge P.

Asthma Research Centre, University Hospital, Uppsala, Sweden.

Comment in
    Clin Exp Allergy. 1999 Mar;29(3):287-90.

BACKGROUND: Bronchoalveolar lavage (BAL) fluid from patients with birch-pollen allergy lavaged during the season showed an elevated chemotactic activity for eosinophils compared with BAL fluid from the same patients before the start of the season.
AIM: The aim of this study was to identify the eosinophil chemotactic agents in the BAL fluid, to compare these findings with in vitro studies on selected cytokines, and to investigate the interactions between these cytokines.
METHODS: Neutralizing antibodies for interleukins (IL) -2, -5 and -8, RANTES and leukaemia inhibitory factor (LIF) were added to the BAL fluid, and the chemotactic activity was tested with eosinophils from allergic donors. Eosinophils from healthy donors were preincubated with IL-5 in order to mimic the primed state of eosinophils from allergics, and the migration towards recombinant IL-5, IL-8, and RANTES in different combinations was measured. Eosinophils from allergic donors were also used.

RESULTS: Anti-IL-5, anti-IL-8 and anti-RANTES inhibited the chemotactic activity in the BAL fluid. Recombinant RANTES induced migration, which was enhanced by preincubation of the cells with IL-5. Only eosinophils from symptomatic allergics responded to IL-8, and IL-5 was not sufficient to prime normal eosinophils in vitro to an IL-8 response. A negative correlation was found between the level of in vivo activation of the cells and their response to IL-5, and a positive correlation with the response to RANTES.
CONCLUSION: IL-8 and RANTES are important for eosinophil accumulation to the lung of pollen-allergic asthmatics. IL-5 alone may not be responsible for the priming of eosinophils in vivo, but is an essential cofactor for the other chemoattractants.

PMID: 10202337 [PubMed - indexed for MEDLINE]

1764. J Immunol. 1999 Apr 1;162(7):4285-92.

Eotaxin activates T cells to chemotaxis and adhesion only if induced to express CCR3 by IL-2 together with IL-4.

Jinquan T, Quan S, Feili G, Larsen CG, Thestrup-Pedersen K.

Department of Dermatology, University Marselisborg Hospital, Aarhus University, Denmark. TAN@RH.DK

The transmigration and adherence of T lymphocytes through microvascular endothelium are essential events for their recruitment into inflammatory sites. In the present study, we investigated the expression of CC chemokine receptor CCR3 on T lymphocytes and the capacities of the CC chemokine eotaxin to induce chemotaxis and adhesion in T lymphocytes. We have observed a novel phenomenon that IL-2 and IL-4 induce the expression of CCR3 on T lymphocytes. We also report that CC chemokine eotaxin is a potent chemoattractant for IL-2- and IL-4-stimulated T lymphocytes, but not for freshly isolated T lymphocytes. Eotaxin attracts T lymphocytes via CCR3, documented by the fact that anti-CCR3 mAb blocks eotaxin-mediated T lymphocyte chemotaxis. In combination with IL-2 and IL-4, eotaxin enhances the expression of adhesion molecules such as ICAM-1 and several integrins (CD29, CD49a, and CD49b) on T lymphocytes and thus promotes adhesion and aggregation of T lymphocytes. The eotaxin-induced T lymphocyte adhesion could be selectively blocked by a specific cAMP-dependent protein kinase inhibitor, H-89, indicating that eotaxin activates T lymphocytes via a special cAMP-signaling pathway. Our new findings all point toward the fact that eotaxin, in association with the Th1-derived cytokine IL-2 and the Th2-derived cytokine IL-4, is an important T lymphocyte activator, stimulating the directional migration, adhesion, accumulation, and recruitment of T lymphocytes, and paralleled the accumulation of eosinophils and basophils during the process of certain types of inflammation such as allergy.

PMID: 10201960 [PubMed - indexed for MEDLINE]

1765. J Immunol. 1999 Apr 1;162(7):4212-9.

Inhibition of matrix metalloproteinases prevents allergen-induced airway inflammation in a murine model of asthma.

Kumagai K, Ohno I, Okada S, Ohkawara Y, Suzuki K, Shinya T, Nagase H, Iwata K, Shirato K.

First Department of Internal Medicine, Tohoku University School of Medicine, Sendai, Japan.

Although matrix metalloproteinases (MMPs) have been reported to play crucial roles in the migration of inflammatory cells through basement membrane components in vitro, the role of MMPs in the in vivo accumulation of the cells to the site of inflammation in bronchial asthma is still obscure. In this study, we investigated the role of MMPs in the pathogenesis of bronchial asthma, using a murine model of allergic asthma. In this model, we observed the increase of the release of MMP-2 and MMP-9 in bronchoalveolar lavage fluids after Ag inhalation in the mice sensitized with OVA, which was accompanied by the infiltration of lymphocytes and eosinophils. Administration of tissue inhibitor of metalloproteinase-2 to airways inhibited the Ag-induced infiltration of lymphocytes and eosinophils to airway wall and lumen, reduced Ag-induced airway hyperresponsiveness, and increased the numbers of eosinophils and lymphocytes in peripheral blood. The inhibition of cellular infiltration to airway lumen was observed also with tissue inhibitor of metalloproteinase-1 and a synthetic matrix metalloproteinase inhibitor. These data suggest that MMPs, especially MMP-2 and MMP-9, are crucial for the infiltration of inflammatory cells and the induction of airway hyperresponsiveness, which are pathophysiologic features of bronchial asthma, and further raise the possibility of the inhibition of MMPs as a therapeutic strategy of bronchial asthma.

PMID: 10201949 [PubMed - indexed for MEDLINE]

1766. Patol Fiziol Eksp Ter. 1999 Jan-Mar;(1):11-4.

[Increased sensitivity to cockroaches of the species Blattella germanica in patients with allergic diseases].

[Article in Russian]

Gerbazieva VB, Zhirova SN, Sveranovskaia VV.

Sensitization to Blattella germanica (B.g.) was studied in 290 allergic patients having increased sensitivity to house dust (HD). The study carried out by ELISA techniques with the use of allergen especially developed by the authors, revealed that the sera of adults and children with bronchial asthma (BA) contained high levels of IgE antibodies (Ab) to B.g. in 34 and 63.5% of cases, respectively. The presence of pronounced IgE-linked sensitization of target cells of BA patients was confirmed in leukocyte alteration test and in the natural leukocyte migration inhibition test. Increased sensitization to B.g. was shown to be the second in the total spectrum of sensitization to different arthropod species in house dust, and the presence of mixed sensitization to B.g. and HD mites was not due to cross reactions caused by main mite allergens. The content of IgG and IgG4 Ab to B.g. in BA patients exceeds that in healthy subjects, the tendency towards reverse correlation (r = -0.4) between the content of specific IgE and IgG Ab being revealed in the former. An essential role of sensitization to cockroaches in pathogenesis of BA is emphasized.

PMID: 10199166 [PubMed - indexed for MEDLINE]

1767. Allergy. 1999 Jan;54(1):57-63.

Soluble E-selectin correlates with disease activity in cyclosporin A-treated patients with atopic dermatitis.

Kägi MK, Joller-Jemelka H, Wüthrich B.

Department of Dermatology, University Hospital Bern, Inselspital, Switzerland.

BACKGROUND: The expression of adhesion molecules on endothelial cells regulates leukocyte migration. The level of soluble adhesion molecules which are shed into the circulation is known to reflect the degree of inflammation, and this level can therefore be used as an indicator of disease activity. The objective of this study was first to investigate the relationship between sE-selectin levels and disease activity parameters (scores of extent, severity, itch, and sleep) in atopic dermatitis (AD) patients, and second to determine the effect of therapy with an immunosuppressive drug (cyclosporin A) on sE-selectin levels.
METHODS: Fourteen patients with severe AD and 41 healthy controls were studied. sE-selectin was measured by ELISA both 2 weeks before therapy with cyclosporin A and after 16 weeks of treatment.
RESULTS: At baseline, the level of sE-selectin was significantly higher in patients with AD than in healthy control subjects (P<0.0001). After treatment of AD with cyclosporin A, there was a significant reduction of the sE-selectin levels (P<0.0001). In addition, changes in sE-selectin levels significantly correlated with changes in disease activity parameters such as severity (P<0.002) and extent of disease (P<0.049).
CONCLUSIONS: Soluble E-selectin is a new serologic marker in AD which reflects disease activity. Therefore, soluble E-selectin may be a useful parameter in the monitoring of this disease.

PMID: 10195358 [PubMed - indexed for MEDLINE]

1768. Arch Pediatr. 1999;6 Suppl 1:105S-107S.

[Corticoids and allergy].

[Article in French]

Delacourt C.

Service de pneumologie et de pathologie professionnelle, centre hospitalier intercommunal de Créteil, France.

Inflammation is constantly observed in allergic reactions. Corticosteroids are most effective in preventing the late phase of allergic reaction. The action of glucocorticosteroids is mediated through glucocorticoid receptors present in the cellular cytoplasm. When activated, glucocorticoid receptors form a dimer and bind to DNA after migration into the nucleus. Interaction to DNA induces changes in the transcription rate, leading to either gene induction or gene repression. Glucocorticoid receptors are also able to interact with transcriptional factors such as AP-1 (activator protein-1) of NF-kappa B (nuclear factor-kappa B). Through these actions glucocorticosteroids are susceptible to modify functions of cells involved in the allergic inflammatory response. They are in particular able to inhibit most of the pro-inflammatory functions of the eosinophils.

PMID: 10191934 [PubMed - indexed for MEDLINE]

1769. Am J Respir Cell Mol Biol. 1999 Apr;20(4):787-96.

Role of very late adhesion integrins in mediating repair of human airway epithelial cell monolayers after mechanical injury.

White SR, Dorscheid DR, Rabe KF, Wojcik KR, Hamann KJ.

Section of Pulmonary and Critical Care Medicine, Department of Medicine, Division of Biological Sciences, University of Chicago, Chicago, Illinois, USA.

swhite@medicine.bsd.uchicago.edu

Repair of the airway epithelium after injury requires that processes such as adhesion and cell migration occur in a defined order. Both of these processes depend on interactions between extracellular matrix (ECM) proteins and appropriate integrins. To study these interactions, we examined monolayer wound repair in a cultured human airway epithelial cell line, 16HBE14o-. Wounds created in confluent monolayers grown on either collagen-IV, laminin-1, or laminin-2 matrix closed quickly in response to 15 ng/ml epidermal growth factor (EGF). Concurrent treatment of cells grown on each matrix protein with EGF and a monoclonal antibody (mAb) to beta1-integrin inhibited wound closure. Treatment with a mAb to alpha2-, alpha3-, and alpha6-integrin blocked wound repair in monolayers grown on collagen-IV but did not do so in monolayers grown either on laminin-1 or laminin-2. Inhibition was not due to cell detachment or apoptosis. These data demonstrate that integrins expressed by airway epithelial cells mediate wound closure on different constitutive ECM proteins. These data suggest that beta1-integrin subunit function is required to permit migration and spreading of epithelial cells, and that alpha-integrin subunits alone do not mediate migration of epithelial cells grown on either laminin-1 or laminin-2. These differences may become important if the matrix protein composition of airway basement membrane changes in disease states such as asthma.

PMID: 10101012 [PubMed - indexed for MEDLINE]

1770. J Epidemiol. 1999 Feb;9(1):20-6.

Process and current status of the epidemiologic studies on cedar pollinosis in Japan.

Tanihara S, Oki I, Ojima T, Nakamura Y, Yanagawa H.

Department of Public Health, Jichi Medical School, Tochigi, Japan.

This paper reviews the present situation and future aspects of epidemiologic studies on Japanese cedar pollinosis. Increase of allergic rhinitis patients is observed in both the Patient Survey and the Reports on the Surveys of Social Medical Care Insurance Services, however, these surveys are conducted when cedar pollens do not pollute the air. Many have reported on the prevalence of pollinosis in limited areas but only a few nationwide epidemiologic surveys have been conducted. Most of the studies were conducted at special medical facilities such as university hospitals. There is a high possibility that patients who visit the specific facilities do not exactly represent the actual number of patients and epidemiologic pictures of pollinosis in Japan. The rapid advances in laboratory test methods may change the diagnostic criteria and increase the number of reported patients. Therefore, the prevalence of Japanese cedar pollinosis in Japan has not been determined yet. Determination of the prevalence of cedar pollinosis and description of the epidemiologic pictures constitute the essential steps toward the control of this clinical entity. Thus it is necessary to conduct an epidemiologic survey on Japanese representative samples with a standardized survey form with clear and concise diagnostic criteria.

PMID: 10098349 [PubMed - indexed for MEDLINE]

1771. Allergy. 1998;53(48 Suppl):41-6.

Reflections on the control of mites and mite allergens.

de Boer R.

Section of Population Biology (ISP), University of Amsterdam, The Netherlands.
boer@bio.uva.nl

In cool climates, the humidity circumstances in a home during the summer months are more important for allergen accumulation than those in the winter months. For the same reasons, a reduction of humidity will have a much greater impact during the summer months. Not only the relative humidity, but also the removal of the dust mites' food by cleaning can be an important factor in determining the ultimate level of mite allergens. These theoretic deductions remain to be experimentally tested. An understanding of the dynamics of mite populations and allergen concentrations in homes and public buildings is still hampered by gaps in our knowledge. These gaps mainly concern the food availability and food requirements of dust mites and the kinetics of mite allergens.

PMID: 10096806 [PubMed - indexed for MEDLINE]

1772. Ann Allergy Asthma Immunol. 1999 Mar;82(3):287-92.

Trends in asthma mortality in young people in southern Brazil.

Chatkin JM, Barreto SM, Fonseca NA, Gutiérrez CA, Sears MR.

The Medical School, Catholic University of Rio Grande do Sul, Porto Alegre, Brazil.

Comment in
Ann Allergy Asthma Immunol. 1999 Dec;83(6 Pt 1):572-3.

BACKGROUND: Mortality from asthma increased and is now declining in some countries, but little is known about these trends in South America.
OBJECTIVE: We aimed to assess trends in mortality from asthma in southern Brazil in children and young adults.
METHODS: Death certificates of 425 people in the state of Rio Grande do Sul aged between 5 and 39 years in whom asthma was reported to be the underlying cause of death during the period 1970 to 1992 were reviewed. Population data were available in 10-year age groups. Testing for trends in mortality rates was conducted using linear and log-linear regression procedures.
RESULTS: Asthma mortality rates in the age groups 5 to 19 and 20 to 39 years ranged between 0.04 and 0.39/100,000 and 0.28 to 0.75/100,000, respectively, and were nonuniformly distributed over the study period. The mean annual increase in rate in 5- to 19-year olds was +0.01 (95% CI 0.003 to 0.016), an average annual percentage increase of +6.8% (95% CI 3% to 11%), with a total increase of 352% between 1970 and 1992. This increase was not due to a shift in labeling from bronchitis to asthma. In the 20 to 39-year age group, asthma and bronchitis mortality rates showed no trend to increase or decrease.
CONCLUSIONS: Asthma mortality in southern Brazil is low, but rose significantly between 1970 and 1992 in the 5 to 19-year age group. This trend differs from that found in other states of Brazil and several other Latin American countries. Reasons for this difference remain unclear.

PIP: Levels of mortality due to asthma are declining in some countries. To measure trends in mortality from asthma in southern Brazil among children and young adults, the death certificates of 425 people in the state of Rio Grande do Sul aged 5-39 years in whom asthma was reported to be the underlying cause of death during the period 1970-92 were reviewed. Asthma mortality rates among people aged 5-19 and 20-39 years were 0.04-0.39/100,000 and 0.28-0.75/100,000, respectively, and were nonuniformly distributed over the study period. The mean annual increase in mortality rate among 5-19 year olds was 0.01, an average

annual percentage increase of 6.8%, with a total increase of 352% during 1970-92. This increase was not due to a shift in labeling from bronchitis to asthma. Among people aged 20-39 years, asthma and bronchitis mortality rates showed no trend of increase or decrease. Reasons for the dramatic increase in asthma-related mortality among 5-19 year olds are unclear.
PMID: 10094220 [PubMed - indexed for MEDLINE]

1773. Plast Reconstr Surg. 1999 Apr;103(4):1277-9.

Expanded polytetrafluoroethylene threads for lip augmentation induce foreign body granulomatous reaction.

Hubmer MG, Hoffmann C, Popper H, Scharnagl E.

Department of Plastic Surgery, Institute of Pathology, University Hospital Graz, Austria.

Especially in cases of facial aesthetic surgery, the demands of the patients are high. The formation of a visible and painful nodule will cause not only discomfort but also dissatisfaction. When alloplastic materials are used for facial and lip augmentation, the possibility of migration, allergic reaction, and formation of a foreign body granuloma is always present. Although e-PTFE is the most bioinert alloplastic material available, the authors could show the formation of a foreign body granuloma. When using e-PTFE threads for facial augmentation, the surgeon should keep in mind and inform the patients that the threads can induce a foreign body granulomatous reaction.

PMID: 10088521 [PubMed - indexed for MEDLINE]

1774. Acta Derm Venereol. 1999 Jan;79(1):18-22.

In vitro release of interferon-gamma and macrophage migration inhibition factor in drug-induced urticaria and angioedema.

Livni E, Lapidoth M, Halevy S.

Transplant Laboratory, Rabin Medical Center, Beilinson Campus, Petah Tiqva , Israel.

T-cells are involved in the pathogenesis of cutaneous drug reactions. T-cell phenotype and cytokine release pattern in rivo and in vitro might correlate with the type of immune response involved in cutaneous drug reactions. In vitro release of interferon-gamma and macrophage migration inhibition factor (MIF) from peripheral blood lymphocytes, following in vitro challenge with the suspected unmodified drugs, was studied in 12 patients with drug-induced urticaria and/or angioedema and in two group-matched controls. The occurrence of positive interferon-gamma and MIF responses was significantly higher in patients with drug-induced urticaria and/or angioedema than in controls. The sensitivity and specificity of the interferon-gamma test (50% and 92%, respectively) were similar to that of the MIF test (58% and 96%, respectively). Percentage agreement between both tests was 80.9 (kappa = 0.76). In vitro release of interferon-gamma and MIF in drug-induced urticaria and/or angioedema suggests a drug-specific immune response, and may implicate the drug as a possible inducer of the reaction.

PMID: 10086852 [PubMed - indexed for MEDLINE]

1775. Acta Derm Venereol. 1999 Jan;79(1):9-11.

Upregulation of RANTES in psoriatic keratinocytes: a possible pathogenic mechanism for psoriasis.

Raychaudhuri SP, Jiang WY, Farber EM, Schall TJ, Ruff MR, Pert CB.

Psoriasis Research Institute, Palo Alto, CA 94301, USA.

Intraepidermal collections of neutrophils and lymphocytes are unique features of the inflammatory reaction of psoriasis. Migration of leukocytes from dermis to the epidermis suggests a role for chemotactic agent(s). In recent years, increased levels of chemokines such as IL-8 , GRO-a and MCP-1 have been reported in the keratinocytes of psoriatic tissue. IL-8 and GRO-alpha belong to a subfamily (C x C) class and MCP-1 is a beta chemokine. In this study, we investigated RANTES, which is a beta chemokine (C-C class); RANTES has been found to be associated with various cell-mediated hypersensitive disorders. We obtained eight skin biopsies from chronic psoriatic plaques, and five biopsies each from non-lesional psoriatic skin, lichen planus, eczematous dermatitis and skin from healthy controls. Snap-frozen samples were cut into 7 microm cryosections and stained with 6 mg/ml of monoclonal anti-RANTES mouse IgG (DNAX, Palo Alto, CA). Standard immunohistochemistry techniques were applied. RANTES was detected only in the keratinocytes. The number of keratinocytes in per mm2 of epidermis stained for RANTES were 116.79+/-98.42 in psoriatic tissues compared to 32.00+/-46.05 (p<0.05), 6.39+/-3.59 (p<0.01), 2.64 +/-1.15 (p<0.01) and 3.53+/-5.26 (p<0.01), respectively, in the non-lesional, lichen planus, eczematous lesions and normal skin. This is the first study to report that the keratinocytes of psoriatic tissue express high levels of RANTES compared to the controls. IL-8 and related molecules (C x C class) are predominantly chemotactic for neutrophils and MCP-1 is a strong chemotactic factor for monocytes. In contrast, RANTES is chemotactic for memory T cells and activated naive T cells. Increased amounts of RANTES as reported here provide an explanation for migration of the activated T cells to the epidermis of the psoriatic lesions. In addition, RANTES activates T cells. These results suggest that RANTES may have a significant role in the inflammatory process of psoriasis. Our findings further substantiate a regulatory role for keratinocytes in the inflammatory process of psoriasis.

PMID: 10086850 [PubMed - indexed for MEDLINE]

1776. Immunopharmacol Immunotoxicol. 1999 Feb;21(1):125-40.

Inhibition of eosinophil infiltration into the mouse peritoneal cavity by a traditional Chinese medicine, Bu-zhong-yi-qi-tang (Japanese name: Hochu-ekki-to).

Kaneko M, Kawakita T, Nomoto K.

Kampo (Traditional Chinese Medicine) Research Laboratories, Kanebo Co., Ltd., Osaka, Japan.

Our previous study showed that the serum level of antigen-specific IgE antibodies in primary response was decreased by a traditional Chinese medicine, Bu-zhong-yi-qi-tang (Japanese name; Hochu-ekki-to, HOT). In this study, we examined inhibition of secondary IgE response and of eosinophil infiltration by HOT. BALB/c mice were intraperitoneally immunized with aluminum hydroxide adsorbed with DNP-KLH (DNP-KLH + alum) on day -14 and on day 0. In mice treated with HOT daily from day -14, the serum level of antigen-specific IgE antibodies after the secondary immunization was significantly decreased compared to that in mice not treated with HOT. Eosinophils increased in number after 6 and 24 hr, and CD4+ T cells in the peritoneal cavity increased in number 24 hr after the secondary immunization. HOT suppressed accumulation of eosinophils and CD4+ T

cells in the peritoneal cavity. Furthermore, HOT suppressed the numbers of IL-4- and IL-5-producing cells 24 hr after the secondary immunization, but did not inhibit the number of IFN-gamma-producing cells. HOT also suppressed IL-5 mRNA expression. Furthermore, HOT also inhibited antigen-induced late-phase reaction (LPR) in the skin. These results suggested that HOT exhibited anti-allergic effects mainly by inhibiting Th2 cell responses.

PMID: 10084334 [PubMed - indexed for MEDLINE]

1777. J Allergy Clin Immunol. 1999 Mar;103(3 Pt 2):S378-81.

Mediators of inflammation and the inflammatory process.

White M.

Institute of Allergy and Asthma, Washington Hospital Center, Washington, DC 20010, USA.

A complex interplay of inflammatory cells and chemical mediators is responsible for allergic inflammation. It is now understood that the allergic reaction consists of an early-phase response involving mast cell degranulation with the release of histamine and a late-phase response characterized by the migration of inflammatory cells. This review provides a summary of the early- and late-phase events associated with allergic inflammation and an overview of the principal chemical mediators involved in the inflammatory process.

PMID: 10069896 [PubMed - indexed for MEDLINE]

1778. Int Immunol. 1999 Jan;11(1):1-10.

Selective eosinophil transendothelial migration triggered by eotaxin via modulation of Mac-1/ICAM-1 and VLA-4/VCAM-1 interactions.

Jia GQ, Gonzalo JA, Hidalgo A, Wagner D, Cybulsky M, Gutierrez-Ramos JC.

The Center for Blood Research, Inc, Boston, MA 02115, USA.

We have recently cloned eotaxin, a highly efficacious eosinophilic chemokine involved in the development of lung eosinophilia during allergic inflammatory reactions. To understand more precisely how eotaxin facilitates the specific migration of eosinophils, we have studied which adhesion receptors are essential for eotaxin action both in vivo and in vitro. Experiments using mice genetically deficient in adhesion receptors demonstrated that molecules previously reported to be involved in both leukocyte tethering/rolling (P-selectin and E-selectin) and in sticking/ transmigration (ICAM-1 and VCAM-1) are required for eotaxin action in vivo. To further elucidate the mechanism(s) involved in this process, we have used an in vitro transendothelial chemotaxis model. mAb neutralization studies performed in this system suggest that the integrins Mac-1 (CD11b/18), VLA-4 (alpha4beta1) and LFA-1 (CD11a/18) are involved in the transendothelial chemotaxis of eosinophils to eotaxin. Accordingly, the expression of these integrins on eosinophils is elevated by direct action of this chemokine in a concentration-dependent manner. Taken together, our results suggest that eotaxin-induced eosinophil transendothelial migration in vivo and in vitro relies on Mac-1/ICAM-1 and VLA-4NCAM-1 interactions, the latter ones becoming more relevant at later time points of the eotaxin-induced recruitment process.

PMID: 10050668 [PubMed - indexed for MEDLINE]

1779. Orv Hetil. 1999 Jan 24;140(4):191-4.

[Cutaneous manifestations of strongyloidosis].

[Article in Hungarian]

Károlyi Z, Erös N, Kriston R.

Miskolc Város Onkormányzat Semmelweis Kórház-Rendelöintézet Börgyógyászati Osztály.

Strongyloidiasis is a parasitic disease, caused by Strongyloides stercoralis, an intestinal nematode, which is mainly endemic in tropical and subtropical regions. It can be sporadically found in the temperate zone, especially in closed communities and among people living under bad social conditions. Gastrointestinal, pulmonary and cutaneous symptoms may arise during the migration of the larvae. The infections are chronic and poor in symptoms among immunocompetent patients. Sometimes the cutaneous manifestation is the only symptom of the disease besides the distinct eosinophilia. Intense itching, erythematosus papule and petechiae develop at the site of the skin infection. Rapidly progressing linear, serpiginous, urticarial streaks are the pathognomic cutaneous manifestations that are called larva currents. The appearance of erythematosus, linear stripes are due to the migrating larvae in the skin. The most common nonspecific symptoms are urticaria, maculopapular exanthema, localized or generalized pruritus and prurigo. The parasite is uniquely able to carry out its whole life cycle inside the human body, so in immunocompromised patients the disease can lead to a hyperinfection syndrome with high mortality, due to the accelerated endogenous autoinfection. Authors present all possible skin manifestations of the strongyloidiasis, based on the case history of three brothers and sisters and that of a female patient suffering from hyperinfection syndrome.

PMID: 10047707 [PubMed - indexed for MEDLINE]

1780. Am J Respir Cell Mol Biol. 1999 Mar;20(3):448-57.

The role of alpha4 (CD49d) and beta2 (CD18) integrins in eosinophil and neutrophil migration to allergic lung inflammation in the Brown Norway rat.

Schneider T, Issekutz TB, Issekutz AC.

Department of Pediatrics, Dalhousie University, Halifax, Nova Scotia, Canada.

We investigated the role of beta2 (CD18) and alpha4 (CD49d) integrins in eosinophil and neutrophil recruitment to lung parenchyma and bronchoalveolar lavage fluid (BALF) of allergen-challenged Brown Norway (BN) rats. Challenge of sensitized BN rats with ovalbumin induced an eosinophil- and neutrophil-rich infiltrate in BALF at 24 h, accompanied by an increase in BALF protein content. Treatment with either the TA-2 monoclonal antibody (mAb) against alpha4 (as an F[ab']2 fragment) or the WT.3 mAb against beta2 integrin significantly reduced eosinophil and neutrophil accumulation in BALF by 54 to 66% and eosinophil accumulation in the parenchyma by 48%. A significant difference in effect was observed between mAb TA-2 in intact immunoglobulin G or F(ab)2 form. Combined treatment with mAbs WT.3 plus TA-2 (F[ab]2) virtually abolished eosinophil accumulation in BALF and in the parenchyma, and reduced neutrophil accumulation in BALF by 91%. In contrast, neutrophil accumulation in the lung was not inhibited by these mAb treatments. The increase in BALF protein concentration was significantly inhibited by TA-2 (by 40%) and by WT.3 plus TA-2 in combination

(71% inhibition). We conclude that eosinophil and neutrophil migration into the air space in allergic lung inflammation is partially CD18 (beta2)- and CD49d (alpha4)- dependent and that alpha4 integrins mediate essentially all of the CD18-independent migration. Similarly, eosinophil accumulation in the parenchyma is completely alpha4 and CD18 (beta2) integrin-dependent. In marked contrast, neutrophil accumulation in the lung in this allergen model can occur independently of both alpha4 and beta2 integrins.

PMID: 10030843 [PubMed - indexed for MEDLINE]

1781. Arerugi. 1998 Dec;47(12):1264-72.

[A study of clinical significance of leucocyte migration test in drug eruption].

[Article in Japanese]

Abe M, Uno K.

Suibarago Hospital.

In 202 patients suspected of drug eruption, the identification of the allergenic drugs were performed by leucocyte migration test (LMT). Leucocyte migration activating factor (LMAF) was detected in 94 cases (46.5%) and Leucocyte migration inhibitory factor (LMIF) in 93 cases (46.0%) for tests either without patients' serum or with patient's serum. Either LMAF or LMIF was detected in 158 cases (78.2%). LMAF was detected in 46 cases (22.8%) for tests only without patient's serum, and in 68 cases (33.7%) for tests only with patient's serum. Accordingly, LMAF was found significantly more frequently with patient's serum than without patient's serum ($p < 0.01$, chi 2-test). The drugs showing either LMAF-positive or LMIF-positive were detected in 193 of all 647 suspected drugs, in which 53 drugs (27.5%) were beta-lactam antibiotics and 36 (28.0%) were non-steroidal antiinflammatory drugs. LMAF was detected significantly higher than LMIF in beta-lactam antibiotics-induced eruptions ($p < 0.01$, chi 2-test), which LMIF was detected significantly higher than LMAF in non-steroidal antiinflammatory drugs-induced eruptions ($p < 0.01$, chi 2-test). Our finding indicate that both LMAF and LMIF may be involved in the pathogenesis of drug eruptions, that the detection of either LMAF or LMIF may be valuable to identify the allergenic drugs in drug eruption by means of LMT and that the production of LMAF may be enhanced in the presence of patients' sera. Furthermore, the pathogenic mechanism of beta-lactam antibiotics-induced eruption may be different from that of non-steroidal antiinflammatory drugs-induced eruption.

PMID: 10028721 [PubMed - indexed for MEDLINE]

1782. Scand J Immunol. 1999 Jan;49(1):67-72.

Interleukin-13 and human immunoglobulin E production in severe combined immunodeficiency mice transplanted with human peripheral blood lymphocytes.

Steinsvik TE, Aaberge IS, Gaarder PI, Løvik M.

Department of Environmental Medicine, National Institute of Public Health, Oslo, Norway.

As normal mice do not respond to interleukin-13 (IL-13), we have used mice with severe combined immunodeficiency transplanted with human peripheral blood lymphocytes (hu-PBL-SCID mice) as an in vivo model for studying human IL-13. PBL from three donors (two allergic and one non-allergic) were prestimulated with

IL-13 in vitro and thereafter transplanted into SCID mice. As evidenced by flow cytometry, IL-13 in the in vitro cell cultures was physiologically active and suppressed CD14 expression, while it enhanced the expression of CD23 on human monocytes. In the in vivo experiments, SCID mice transplanted with cells from both allergic donors produced twice as high maximum levels of IgE when the cells were preincubated with IL-13 in vitro before transplantation, as compared with mice receiving cells that had not been preincubated with IL-13. Two succeeding intraperitoneal (i.p.) injections of IL-13 resulted in a further increase of maximum IgE levels. Using cells from the non-allergic donor, no enhancing effect of IL-13 was observed. Transplanted human cells from one allergic donor examined were shown to migrate to the spleen and lungs of the recipient mice, while cells from the non-allergic donor were found only in the peritoneal cavity. Altogether, our results indicate that IL-13 enhances human IgE production in vivo and suggest that lymphocytes in allergic individuals are hyper-reactive to this cytokine. Furthermore, the allergic status of the cell donor may affect migration and engraftment of cells transplanted into SCID mice.

PMID: 10023859 [PubMed - indexed for MEDLINE]

1783. Pacing Clin Electrophysiol. 1999 Jan;22(1 Pt 2):206-11.

Complications of third-generation implantable cardioverter defibrillator therapy.

Grimm W, Menz V, Hoffmann J, Timmann U, Funck R, Moosdorf R, Maisch B.

Department of Cardiology, Hospital of the Philipps-University of Marburg, Germany.

To determine the incidence of complications of third-generation implantable cardioverter defibrillator (ICD) therapy, 144 patients were prospectively studied who underwent first implant of third-generation devices (i.e., ICD systems with biphasic shocks, ECG storage capability, and nonthoracotomy lead systems). During 21 +/- 15 months of follow-up, 41 (28%) patients had one or more complications. No patient died perioperatively (30 days) and no ICD infection was observed during follow-up. Complications included bleeding or pocket hematoma (hemoglobin drop > 2 g/dL) in 5 (3%) patients, prolonged reversible ischemic neurological deficit in 1 (1%) patient, postoperative deep venous thrombosis of leg in 1 (1%) patient, pneumothorax in 2 (1%) patients, difficulty to defibrillate ventricular fibrillation intraoperatively in 2 (1%) patients, generator malfunction in 1 (1%) patient, arthritis of the shoulder in 3 (2%) patients, and allergic reaction to prophylactic antibiotics in 2 (1%) patients. A total of seven lead related complications were observed in six (4%) patients including endocardial lead migration in four (3%) patients. Twenty-three (16%) patients received inappropriate shocks for supraventricular tachyarrhythmias (n = 13), non-sustained ventricular tachycardia (VT) (n = 7), or myopotential oversensing (n = 3). We conclude that serious complications such as perioperative death or ICD infection are rare in patients with third-generation ICDs. Lead-related problems and inappropriate shocks during follow-up are the most frequent complications of third-generation ICD therapy. Recognition of these complications should promote advances in ICD technology and management strategies to avoid their recurrence.

PMID: 9990632 [PubMed - indexed for MEDLINE]

1784. Int Arch Allergy Immunol. 1999 Jan;118(1):51-8.

111In-labelled leukocyte migration to the lungs of ovalbumin-sensitized guinea-pigs after aerosol challenge with ovalbumin monitored by gamma

scintigraphy.

Lewis CA, Warren SJ, Broadley KJ.

Pharmacology, Welsh School of Pharmacy, Cardiff University, Cardiff, UK.

Erratum in
Int Arch Allergy Immunol 1999 May;119(1):73-4.

BACKGROUND: To determine whether antigen challenge is associated with increased accumulation of leukocytes in the lungs, the pulmonary accumulation of 111In-labelled neutrophils and eosinophils was monitored by gamma scintigraphy.
METHODS: Guinea-pigs were sensitized with ovalbumin (OA) 14-21 days before challenge with aerosolized OA (10 mg/ml) for 2 min, and protected against fatal anaphylaxis by mepyramine (30 mg/kg). Comparisons were made with OA-sensitized guinea-pigs challenged with saline. 5 or 24 h following the OA challenge, guinea-pigs were anaesthetized and the jugular vein and right carotid artery were cannulated, with the contralateral artery tied off. 2MBq 99mTc macroaggregated albumin (MAA) was injected intravenously to create a pulmonary perfusion image as a template for the lungs. 111In-labelled neutrophils or eosinophils were then injected via the carotid artery and gamma scintigraphic images obtained. Activity in the lung region of each animal was determined by superimposing the 99mTcMAA image of the lungs on the whole body image.
RESULTS: A significant increase in activity (p<0.05) in the lung region was observed after injection of 111In-labelled neutrophils at 5 h after the OA challenge compared with saline challenge. 24 h after OA challenge there was a significant increase in activity (p<0.05) in the lung region after injection of 111In-labelled eosinophils, but no change in activity after injection of labelled neutrophils compared with the saline-challenged animals.
CONCLUSION: This technique models the migration of leukocyte to the lungs seen in guinea-pigs in our previous studies, namely an eosinophilia at 24 h and a neutrophilia at 5 h after OA challenge. It will therefore be useful for investigating anti-inflammatory drugs on the airways.

PMID: 9925963 [PubMed - indexed for MEDLINE]

1785. Am J Respir Cell Mol Biol. 1999 Feb;20(2):248-55.

Effects of chronic anti-interleukin-5 monoclonal antibody treatment in a murine model of pulmonary inflammation.

Garlisi CG, Kung TT, Wang P, Minnicozzi M, Umland SP, Chapman RW, Stelts D, Crawley Y, Falcone A, Myers JG, Jones H, Billah MM, Kreutner W, Egan RW.

Allergy and Immunology, Schering-Plough Research Institute, Kenilworth, New Jersey 07033-0539, USA. charles.garlisi@spcorp.com

The maturation of eosinophils in bone marrow, their migration to pulmonary tissue, and their subsequent degranulation and release of toxic granule proteins contributes to the pathophysiology observed in asthma. Interleukin-5 (IL-5) is essential for these processes to occur. Therefore, much emphasis has been placed on attempts to inhibit the production or activity of IL-5 in order to attenuate the inflammatory aspect of asthma. In this report, the immunological consequences of long-term exposure to an antibody recognizing IL-5 (TRFK-5) were studied in a murine pulmonary inflammation model. A single dose of TRFK-5 (1 mg/ kg, intraperitoneally) reversibly inhibited antigen-dependent lung eosinophilia in mice for at least 12 wk and inhibited the release of eosinophils from bone marrow for at least 8 wk. Normal responses to aerosol challenge were attained after 24 wk. In mice treated acutely with antibody (2 h before challenge), 50% inhibition

of pulmonary eosinophilia occurred when 0. 06 mg/kg TRFK-5 was administered (intraperitoneally; ED50), resulting in 230 ng/ml (IC50) in serum. In mice treated with one dose of TRFK-5 (1 mg/kg) and rested before challenge, the antibody exhibited a half-life of 2.4 wk. After 18 to 19 wk, antigen challenge-induced eosinophilia was inhibited by 50% and serum levels of TRFK-5 were 25 ng/ml. TRFK-5 remaining in mice 8 wk after a single injection of TRFK-5 was sufficient to inhibit at least 50% of the eosinophilia induced in blood 3 h after injection of recombinant murine IL-5 (10 microg/kg, intravenously). To assess the biologic effect of long-term exposure of mice to antibody, several parameters of immune-cell function were measured. Throughout the extended period of activity of TRFK-5 (>/= 12 wk) there were no gross effects on antigen-dependent increases in T-cell recruitment into bronchoalveolar fluid (BALF), in IL-4 and IL-5 steady-state mRNA levels in lung tissue, or in immunoglobulin E (IgE) and IgG levels in serum. There was a small increase in IL-5 steady-state mRNA production in TRFK-5-treated mice after 2 h or 2 wk, but this was not observed at other times examined. In untreated mice, IL-5 steady-state mRNA production in response to antigen challenge decreased > 6-fold with age, although at all time points there was an increase in mRNA levels following challenge. Therefore, at later times, 25 ng/ml rather than 230 ng/ml of TRFK-5 inhibited BALF eosinophilia, probably because of reduced IL-5 levels. Twenty-four weeks after treatment with TRFK-5, when challenge-induced eosinophilia was restored, there was an excess of CD4(+) T cells in BALF from challenged mice. However, these T cells had no measurable effects on other responses to challenge, including cytokine production, B-cell accumulation, and immunoglobulin production in serum. Thus, the biologic duration of TRFK-5 was several months, and its activity was due to the presence of antibody above a therapeutic threshold rather than to any profound effect on the immune system.

PMID: 9922215 [PubMed - indexed for MEDLINE]

1786. J Immunol. 1999 Jan 15;162(2):1127-36.

A comparison of C3a and C5a-mediated stable adhesion of rolling eosinophils in postcapillary venules and transendothelial migration in vitro and in vivo.

DiScipio RG, Daffern PJ, Jagels MA, Broide DH, Sriramarao P.

Laboratory of Immunology and Vascular Biology, La Jolla Institute for Experimental Medicine, CA 92037, USA.

The comparative ability of the complement anaphylatoxins C3a and C5a to mediate leukocyte adhesion and transendothelial migration in vivo and in vitro was investigated. Superfusion of IL-1beta-stimulated rabbit mesentery with C3a resulted in a rapid and stable adhesion of rolling eosinophils, but not neutrophils, to postcapillary venules. However, C3a failed to evoke subsequent transmigration of the adherent eosinophils. In contrast, C5a induced both the rapid activation-dependent firm adhesion and transmigration of eosinophils and neutrophils through venular endothelium. C3a induced selective shedding of L-selectin and an increase in alphaMbeta2 integrin expression on eosinophils but not neutrophils, while C5a induced shedding of L-selectin and up-regulation of alphaMbeta2 integrin on both eosinophils and neutrophils. Both C3a- and C5a-dependent adhesion to venular endothelium was blocked by ex vivo treatment of eosinophils with anti-alpha4 and anti-beta2 integrin mAbs. In vitro, both C3a (but not C3a(desArg)) and C5a (including C5a(desArg))-dependent transmigration of eosinophils across IL-1beta-stimulated endothelial monolayer was mediated by alpha4beta1 and alphaMbeta2 integrins. Overall these studies suggest that C3a is eosinophil-specific chemotactic mediator that influences selectively eosinophil adhesion but not transmigration in vivo. C5a in contrast is a complete activator of integrin-dependent adhesion as well as transmigration of eosinophils and

neutrophils.

PMID: 9916743 [PubMed - indexed for MEDLINE]

1787. Curr Opin Immunol. 1998 Dec;10(6):614-9.

Langerhans cells and chemical allergy.

Kimber I, Dearman RJ, Cumberbatch M, Huby RJ.

Zeneca Central Toxicology Laboratory Alderley Park Macclesfield Cheshire SK10 4TJ UK. Ian.Kimber@CTL.ZENECA.com

Epidermal Langerhans cells (LCs) play a pivotal role in the induction of cutaneous immune responses, including those provoked by chemical allergens. The delivery by LCs of allergen to draining lymph nodes requires cell migration from the skin, a process that is dependent upon the availability of epidermal cytokines -particularly TNF-alpha and IL-1beta. Here we consider the ways in which these cytokines interact with LCs to both induce and regulate their mobilization in response to skin sensitization. In addition, the effects of these cytokines on both the selectivity of LC migration from the skin and protection of LCs from cell death are considered. Finally, the possible counter-regulatory activity of other cutaneous cytokines and the influence of LCs on the development of selective T lymphocyte responses are explored.

PMID: 9914225 [PubMed - indexed for MEDLINE]

1788. Arerugi. 1998 Nov;47(11):1198-204.

[Correlations of leucocyte migration activating factor with interleukin-1 alpha, interleukin-1 beta and tumor necrosis factor-alpha in drug allergy].

[Article in Japanese]

Sano N, Uno K.

Suibarago Hospital.

The pathogenic mechanism of drug allergy was investigated by determining leucocyte migration activating factor (LMAF), interleukin-1 alpha (IL 1 alpha) and 1 beta (IL-1 beta), and tumor necrosis factor-alpha (TNF-alpha) levels in 13 patients with suspected hypersensitivity to drugs, following with the relevant agents. LMAF was detected in 10 out of 11 patients in the absence of serum and in 8 out of 9 patients in the presence of serum by means of the leucocyte migration inhibition test (LMIT). The drug-stimulated group had a significantly higher level of IL-1 alpha production than a non-stimulated group, both without serum ($p < 0.05$) and with serum ($p < 0.05$), among patients positive for LMAF. Moreover, the LMAF-positive group had a significantly higher level of IL-1 alpha production than the LMIT-negative group, both without serum ($p < 0.05$) and with serum ($p < 0.05$). In contrast, the level of IL-1 beta production showed no significant difference, either without or with serum, between drug-stimulated and non-drug-stimulated patients who were positive for LMAF. The production of TNF-alpha in the LMAF-positive group was significantly greater in drug-stimulated patients than in non-drug-stimulated patients, but only in the presence of serum ($p < 0.05$). However, the level of TNF-alpha production showed no significant difference, either without or with serum, between the LMAF-positive group and the control group. Our findings suggest that IL-1 alpha may be prominently involved in the production of LMAF in allergic reactions to drugs and that the production

of TNF-alpha may be enhanced in the presence of serum.

PMID: 9893337 [PubMed - indexed for MEDLINE]

1789. Br J Dermatol. 1998 Oct;139(4):598-604.

Culture of human epidermal Langerhans cells in a skin equivalent.

Fransson J, Heffler LC, Tengvall Linder M, Scheynius A.

Departments of Dermatology and Laboratory Medicine, Karolinska Hospital and Institute, S-171 76 Stockholm, Sweden.

Langerhans cells (LCs) have been cultured in a skin equivalent (SE). Seventy-two SEs were produced by inserting skin biopsies from nine subjects into dermal equivalents consisting of fibroblasts in a collagen matrix. The SEs were cultured in a serum-free medium containing 2-mercaptoethanol with or without 5 ng/mL granulocyte-monocyte colony-stimulating factor (GM-CSF). The SEs were cultured for 12 or 15 days. In the latter case, 0, 1 or 10 microg/mL cyclosporin A (CyA) was added for the last 3 days. The SEs were then snap frozen for immunohistochemistry. The migration of LCs was evaluated by measuring the distances from the inserted skin biopsy in the SEs to the HLA-DR + and CD1a+ dendritic cells localized at the longest distance from the biopsy in the epidermal outgrowth on both sides of the biopsy. The density of these cells was estimated in 15-day-old SEs by counting them on both sides of the inserted skin biopsy and dividing the number of positive cells by the migrated distances. All epidermal outgrowths (range 0.6-3.7 mm) were well differentiated and displayed HLA-DR+, CD1a+ and Lag+ dendritic cells. Only occasionally were CD83+ cells observed. In the 15-day-old SEs cultured with GM-CSF, a few CD86+ cells were seen in the epidermal outgrowths and occasionally CD80+ cells. The median (n = 4) density of CD1a+ and HLA-DR+ cells in the epidermal outgrowths at day 15 was 5.2 and 9.1 cells/mm, respectively. GM-CSF did not influence migration in 12-day-old SEs, but there was a tendency to increased migration of HLA-DR+ dendritic cells in 15-day-old SEs. CyA did not affect migration or density. We conclude that LCs can be cultured with an in vivo-like density in a SE. They express the phenotype of immature antigen-presenting cells efficient in capturing and processing antigen. This model may be suitable for studies of the initial phase of contact allergic reactions.

PMID: 9892902 [PubMed - indexed for MEDLINE]

1790. J Immunol. 1999 Jan 1;162(1):277-83.

Enhanced epidermal Langerhans cell migration in IL-10 knockout mice.

Wang B, Zhuang L, Fujisawa H, Shinder GA, Feliciani C, Shivji GM, Suzuki H, Amerio P, Toto P, Sauder DN.

Division of Dermatology, Sunnybrook Health Science Center, University of Toronto, Ontario, Canada.

The migration of epidermal Langerhans cells (LC) to lymph nodes (LN) is critical in the initiation of contact hypersensitivity (CHS) responses. Studies suggest that contact allergen-induced epidermal proinflammatory cytokines, including IL-1 and TNF-alpha, play important roles in promoting LC migration. Contact allergens also induce epidermal anti-inflammatory cytokines such as IL-10. Since IL-10 down-regulates proinflammatory cytokine production and inhibits CHS, we hypothesized that IL-10 might inhibit LC migration. To test this hypothesis,

IL-10 knockout (KO) mice were epicutaneously sensitized with the hapten, FITC, and 24 h later hapten-bearing cells in the draining LN were examined. The number of hapten-bearing cells in the LN was significantly greater in IL-10 KO mice than in wild-type mice. The mutant mice also had an exaggerated CHS to FITC. Pretreatment with anti-TNF-alpha Ab or IL-1R antagonist significantly reduced the number of hapten-bearing cells in the LN, suggesting that IL-10 modulation of LC migration involves IL-1 and TNF-alpha. Moreover, IL-10 KO mice demonstrated a greater increase in TNF-alpha, IL-1alpha, and IL-1beta mRNAs in the allergen-exposed epidermis, and keratinocytes derived from the mutant mice were able to produce higher amounts of TNF-alpha and IL-1alpha protein. These data suggest that IL-10 plays an inhibitory role in LC migration and that this effect may occur via the down-regulation of TNF-alpha and IL-1 production.

PMID: 9886396 [PubMed - indexed for MEDLINE]

1791. Eur Respir J. 1998 Nov;12(5):1094-8.

LFA-1 expression by blood eosinophils is increased in atopic asthmatic children and is involved in eosinophil locomotion.

Lantero S, Alessandri G, Spallarossa D, Scarso L, Rossi GA.

Pulmonary Division, G. Gaslini Institute, Genoa, Italy.

Allergic asthma is characterized by eosinophil migration in the airways, which is strictly dependent on the expression of adhesion molecules. This study investigated whether the expression of adhesion molecules on eosinophils is increased and associated with disease activity in allergic asthma. Twenty atopic asthmatic (AA) subjects and nine controls were studied and the expression of lymphocyte function-associated antigen-1 (LFA-1; CD11a/CD18), Mac-1 (CD11b/CD18) and very late antigen-4 (VLA-4; CD49d/CD29) on blood eosinophils was evaluated by specific monoclonal antibody (Mab) staining and flow-cytometric analysis. Compared with controls, eosinophils from AA showed increased expression of LFA-1 ($p<0.005$), but not of Mac-1 or VLA-4 ($p>0.1$). In addition, LFA-1 expression correlated positively with blood eosinophil number ($r=0.792$, $p<0.05$), while no correlations were observed between Mac-1 or VLA-4 expression and blood eosinophil number. The migration of eosinophils through human umbilical vein endothelial cells with or without anti-LFA-1, Mac-1 and VLA-4-blocking Mab was studied. Compared with controls, eosinophils from AA showed increased migration toward C5a ($p<0.01$). Cell migration was totally inhibited by preincubating eosinophils with anti-LFA-1 ($p<0.05$), while anti-Mac-1 had no effect ($p>0.1$). Thus, the expression of lymphocyte function-associated antigen-1 by blood eosinophils is increased in atopic asthmatics and seems to modulate the enhanced eosinophil migration observed in allergic asthma.

PMID: 9864003 [PubMed - indexed for MEDLINE]

1792. Srp Arh Celok Lek. 1998 Mar-Apr;126(3-4):138-44.

[The role of endothelial cells in allergic inflammation reactions].

[Article in Serbian]

Stefanović Lj, Bogić M, Balaban J, Mitrović O, Stosović R, Andrejević S.

Department of Allergology and Clinical Immunology, Clinical Centre of Serbia, Belgrade.

Inflammatory response in tissue results from a complex network of interactions between inflammatory cells (mast cells, eosinophils, basophils, macrophages) and resident cells belonging to the lung structure (like endothelial cells, fibroblasts, epithelial cells). Among structural cells, endothelial cells play a critical role. The important role of endothelium is also reflected in the fact that it occupies an area exceeding 1000 m2. Thus, endothelium is the largest and the most active paracrine organ in the body, producing potent vasoactive, procoagulant, anticoagulant, and proinflammatory substances. Endothelial cells have four key functions that alter in the process of inflammation: 1 a) Regulation and control of leukocyte traffic through the expression of adhesion molecules (selectins E and P, molecules of immunoglobulin superfamily ICAM-1, ICAM-2, VCAM); 1 b) They are also able to amplify leukocyte activation through the production of proinflammatory cytokines like IL-1, IL-6 and chemokines like IL-8 and RANTES molecules; 2) Regulation of vascular tone by production of PGI-2, EDRF/NO and elements of local renin-angiotensin system; 3) Regulation of local coagulation by controlling the production of t-PA and PAI-1; 4) Regulation of the vascular permeability. In the states of acute inflammation, the endothelial cell takes on a proinflammatory phenotype and as such becomes chemoattractant, facilitating leukocyte adhesion, activation and migration, becomes prothrombotic and demonstrates enhanced vascular permeability.

PMID: 9863370 [PubMed - indexed for MEDLINE]

1793. Srp Arh Celok Lek. 1998 Mar-Apr;126(3-4):130-7.

[The role of eosinophilic leukocytes in allergic inflammation].

[Article in Serbian]

Stosović R, Bogić M.

Institute of Allergology and Immunology, Clinical Centre of Serbia, Belgrade.

Eosinophilic leukocytes are tissue cells of granulocytic structure and secretory nature. They are produced in the bone marrow and transported to the targeted tissue via the blood where they are present in concentrations hundred times higher than in peripheral circulation. Eosinophilic leukocytes are the essential effector of allergic inflammation, which is a pathophysiological basis of allergic diseases. These diseases are characterized by disturbed distribution of eosinophilic leukocytes, i.e., peripheral eosinophilia and/or infiltration of the affected organs. Migration of these cells from the peripheral circulation into the targeted tissues, i.e., affected with the allergic inflammation, is influenced by helper T2 cells-dependent cytokines, and other mediators of inflammation. Subsequent to their activation, eosinophilic leukocytes release numerous made and newly produced mediators of inflammation and also present antigens which define their effector function in allergic inflammation. In this way, eosinophilic leukocytes participate in numerous pathological and pathophysiological disorders characteristic of allergic diseases which clearly confirm the active role of these cells in their production.

PMID: 9863369 [PubMed - indexed for MEDLINE]

1794. Ned Tijdschr Geneeskd. 1998 Sep 19;142(38):2105-9.

[Migrants in family practice: their symptoms and diagnoses differ from the Dutch].

[Article in Dutch]

Weide MG, Foets M.

Nederlands instituut voor onderzoek van de gezondheidszorg (NIVEL), Utrecht.

OBJECTIVE: To determine to what extent Turks, Moroccans and Surinamese differ from the Dutch in health problems, while taking the impact of relevant background characteristics into account.
DESIGN: Secondary analysis.
SETTING: Dutch Institute for health care research, Utrecht, the Netherlands.
PATIENTS AND METHODS: In 1987/'88, 161 general practitioners and their assistants in 103 general practices recorded all patient contacts during three months in four consecutive periods for the Dutch National Survey on Morbidity and Interventions in General Practice. Data on 1165 Turkish, 853 Moroccan, 1355 Surinamese and 1471 Dutch persons (a 1% sample) between 18 and 65 years of age were used. Differences in health problems (complaints and diagnoses) between groups were tested by means of logistic regression analysis. Sex, age, educational level, working situation, marital status, health insurance, region and urbanisation were included in the analyses as covariates, for which the data were corrected.
RESULTS: The pattern of complaints and diagnoses of ethnic minorities agreed with that of the Dutch in many respects, but there were also clear differences. Surinamese differed the most. Digestive problems, acute and chronic, eye problems, acute musculoskeletal problems, especially muscle pain or fibrositis, respiratory infections and eczema occurred more often in all three minority groups. Surinamese had more diagnoses in the categories of blood and endocrine/metabolic disorders (diabetes mellitus), Moroccanshad fewer diagnoses of the circulatory system (hypertension). Turks and Surinamese had more general and social problems, while Surinamese also had more psychological problems.
CONCLUSION: In some areas ethnic minorities have more health problems than Dutch patients who are comparable with them in background characteristics. This clearly suggests an 'ethnic' factor.

PMID: 9856225 [PubMed - indexed for MEDLINE]

1795. Ned Tijdschr Geneeskd. 1998 Sep 5;142(36):2009-13.

[Migrane treatment in the Netherlands in the early twentieth century].

[Article in Dutch]

Koehler PJ, Bruyn GW.

Ziekenhuis De Wever en Gregorius, afd. Neurologie, Heerlen.

The treatment of migraine in the Netherlands underwent important changes in the beginning of the 20th century. Several factors played a role, including the development of analgesics (acetylsalicylic acid, phenacetin, and fampridine++) at the end of the 19th century. The introduction of phenobarbital for the treatment of epilepsy in 1912 resulted in prescription of this drug for migraine, because of the supposed similarities between both afflictions. Although ergotamine had been reported for the treatment of headache at the end of the 19th century and been recommended again during the 1920s, it appeared in the Dutch literature only at the end of the 1930s. Research abroad showed this drug to have vasoconstrictive properties in migraine, again confirming the vasogenic origin of the affliction. This is striking as vasodilating drugs had been prescribed for several years. The non-medical treatment largely remained as previously, notably the prescription of diets. The relation between migraine and anaphylaxis, based on research by the French school, was also investigated in the Netherlands and

resulted in the prescription of diets to immunize against the proteins involved.

PMID: 9856202 [PubMed - indexed for MEDLINE]

1796. Int J Mol Med. 1998 Jul;2(1):17-28.

Novel pathophysiological aspects of macrophage migration inhibitory factor (review).

Nishihira J.

Central Research Institute, Hokkaido University School of Medicine, Sapporo 060, Japan.

Macrophage migration inhibitory factor (MIF) was the first T-cell-derived soluble lymphokine to be identified. It was originally found to inhibit the migration of macrophages and activate them at inflammatory loci. During the past few years, however, previously unrecognized properties of MIF have been discovered. It also functions, for example, as a pituitary hormone, glucocorticoid-induced immunomodulator and isomerase. We cloned rat MIF cDNA and reported that the nucleotide sequence of the cDNA predicts a protein consisting of 114 amino acids. Northern blot analysis indicated that the MIF mRNA was expressed in a wide variety of organs, including the brain, kidney, and liver. Following this, we demonstrated definitively that MIF was expressed in a variety of cells, suggesting its involvement in various biological events such as wound healing, atopic dermatitis, and, possibly, diabetes/obesity. Furthermore, we elucidated its physicochemical properties, including the tertiary structures of both human and rat MIF. These tertiary structures showed that this protein forms a homotrimer with each monomer consisting of two beta/alpha/beta motifs, thus resembling 5-carboxymethyl-2-hydroxymuconate isomerase and d-dopachrome tautomerase. From the available data on MIF, including ours, it is considered that the protein is associated not only with immune responses but also with cell growth and differentiation during wound repair and carcinogenesis. Thus, MIF could become a major target protein in a variety of pathophysiological states and anti-MIF antibodies and antagonists could be applied therapeutically in the clinical situation for treatment of various diseases. Bearing this in mind, this review discusses the role of MIF, considering its gene and protein structures as well as its pathophysiological functions in various organs and disease states, finally considering perspectives for the future.

PMID: 9854138 [PubMed - indexed for MEDLINE]

1797. Ann Agric Environ Med. 1998;5(1):31-43.

Allergic alveolitis among agricultural workers in eastern Poland: a study of twenty cases.

Milanowski J, Dutkiewicz J, Potoczna H, Kuś L, Urbanowicz B.

Clinic of Lung Diseases, Medical Academy, Jaczewskiego 8, 20-090 Lublin, Poland.

The aim of this study was to identify the specific agents which caused extrinsic allergic alveolitis (EAA) in the selected group of 20 agricultural workers from eastern Poland. The microbiological analysis of the samples of plant materials or dusts reported by the patients as causing symptoms has been carried out, followed by allergological tests (inhalation challenge, agar-gel precipitation test, inhibition of leukocyte migration, skin test) with extrinsic microbial antigens. It was found that the causative agents of allergic alveolitis in the examined

group of patients were mesophilic, non-branching bacteria associated with grain dust, mostly Pantoea agglomerans (synonyms: Erwinia herbicola, Enterobacter agglomerans) and Arthrobacter globiformis (each in eight cases). The remaining agents were Alcaligenes faecalis (in two cases), and Brevibacterium linens and Staphylococcus epidermidis (in one case each). On the basis of the clinical picture, the bronchoalveolar lavage (BAL) and allergological tests, the diagnosis of the chronic form of the disease was stated in 14 patients and an acute form - in 6 patients. EAA patients demonstrated in the BAL fluid a typical lymphocytic alveolitis both in terms of percentage and absolute number of lymphocytes. Also, the numbers of eosinophils and neutrophils were significantly higher in EAA patients.

PMID: 9852490 [PubMed - indexed for MEDLINE]

1798. Lik Sprava. 1998 Aug;(6):94-6.

[The possibility for the intravital diagnosis of a dissecting aortic aneurysm].

[Article in Russian]

Chernykh SN, Golubchenko OK, Radugina GS, Galalu VV.

On the basis of experience gained in vital diagnosis of dissecting aneurysm, its thoracic portion, in 3 patients the authors note that accurate diagnosis of this pathology cannot be made without analysis of a complex of diagnostic studies that rely on a correct assessment of clinical manifestations of the disease. The following items should be noted: particular features of the pain syndrome, migration of the pain zone and relapsing character of pain; lack of a clear-cut interrelation between intensity and duration of pain and frequency of development of cardiogenic shock and cardial asthma; correlation between changes in blood count (leukocytosis) and blood levels of transaminases; absence of the typical ECG features of myocardial infarction, and character of time-related changes.

PMID: 9844886 [PubMed - indexed for MEDLINE]

1799. J Immunol Methods. 1998 Nov 1;220(1-2):1-17.

Chemokines and their role in tumor growth and metastasis.

Wang JM, Deng X, Gong W, Su S.

Laboratory of Molecular Immunoregulation, Division of Basic Sciences, National Cancer Institute-Frederick Cancer Research and Development Center, MD 21702, USA.

Chemokines are a superfamily of pro-inflammatory polypeptide cytokines that selectively attract and activate different cell types. Many patho-physiological conditions require the participation of chemokines, including inflammation, infection, tissue injury, allergy, cardiovascular diseases, as well as malignant tumors. Chemokines activate cells through their binding to shared or unique cell surface receptors which belong to the seven-transmembrane, G-protein-coupled Rhodopsin superfamily. The role of chemokines in malignant tumors is complex: while some chemokines may enhance innate or specific host immunity against tumor implantation, others may favor tumor growth and metastasis by promoting tumor cell proliferation, migration or neovascularization in tumor tissue. In this review, the authors summarize some of the recent advances in chemokine research and emphasis is made on the effect of chemokines in tumor growth and metastasis.

PMID: 9839921 [PubMed - indexed for MEDLINE]

1800. Inflamm Res. 1998 Oct;47(10):409-15.

Sulochrin inhibits eosinophil activation and chemotaxis.

Ohashi H, Motegi Y, Kita H, Gleich GJ, Miura T, Ishikawa M, Kawai H, Fukamachi H.

Pharmaceutical Research Laboratory, Kirin Brewery Co., Ltd., Takasaki, Gunma, Japan. hohashi@kirin.co.jp

OBJECTIVE: Because eosinophils likely play important roles in the pathophysiology of allergic diseases, specific inhibitors of eosinophils may be desirable to treat such diseases. To evaluate the capacity of a novel compound, sulochrin, as an inhibitor of eosinophilic inflammation, we examined the effects of this compound on various effector functions of eosinophils.
MATERIALS AND METHODS: We examined the effects of sulochrin on degranulation of human eosinophils stimulated with platelet-activating factor (PAF) or Sepharose 4B beads coated with secretory IgA (sIgA) or IgG. The effects of sulochrin on other effector functions of human eosinophils, including superoxide anion (O2-) production, leukotriene (LT) C4 release, and interleukin (IL)-8 production induced by sIgA-beads were also studied. Finally, using PAF and LTB4 as chemoattractants, we evaluated the potency of sulochrin to inhibit eosinophil migration in vitro and in vivo.
RESULTS: Sulochrin inhibited EDN release by eosinophils stimulated with sIgA-beads. IgG-beads and PAF in a concentration-dependent manner; IC50 values were 0.75 microM, 0.30 microM and 0.03 microM. Eosinophil O2- production, LTC4 release, and IL-8 production were also inhibited by sulochrin. Furthermore, PAF-induced chemotaxis of human eosinophils and LTB4-induced chemotaxis of guinea pig eosinophils were abolished by 1 microM of sulochrin. Finally, sulochrin potently inhibited LTB4-induced infiltration of eosinophils into the skin of guinea-pig in vivo.
CONCLUSIONS: These results suggest that sulochrin is a potent inhibitor of various effector functions of eosinophils. Sulochrin and its derivatives may be useful in the development of therapeutic approaches for patients with allergic diseases.

PMID: 9831326 [PubMed - indexed for MEDLINE]

1801. Med Hypotheses. 1998 Oct;51(4):281-4.

High rate of asthma among immigrants.

Ballin A, Somekh E, Geva D, Meytes D.

Pediatric Hematology Unit, Edith Wolfson Medical Center, Holon, Israel.

Articles dealing with the epidemiological aspects of asthma were carefully reviewed in order to support or reject our clinical impression of increased rate of asthma among immigrants. Particular emphasis was put on data on very high or very low rates of asthma. The proposed theories to explain these differences were critically examined. The prevalence of asthma in China and in Africa is 1-2% and 0.5-5%, respectively. The prevalence of the disease in other indigenous populations ranges between 0.5% and 12%. On the other hand, asthma is much more frequently seen in Australia and in New Zealand (approximately 20-25%), where peoples' ancestors immigrated from distant areas. Statistical meta-analysis found a significant difference between the rates of asthma in the two groups of populations (P < 0.001). Immunoglobulin E levels of immigrants in Sweden are higher than those of native Swedes. Similarly, cord blood immunoglobulin E

concentrations are more elevated in neonates whose mothers emigrated to Germany from Eastern countries than in those of native German mothers. There is an increased rate of IgE-mediated asthma among immigrant populations.

PMID: 9824830 [PubMed - indexed for MEDLINE]

1802. Exp Clin Immunogenet. 1998;15(3):144-53.

Seasonal variations in T-lymphocyte response to grass pollen allergens from pollen-allergic patients and healthy controls.

Jepsen KF, Nielsen L, Olsen OT, Bendtzen K, Poulsen LK.

Laboratory of Medical Allergology, Allergy Unit, 7542, RHIMA Center, National University Hospital, Copenhagen, Denmark. kfjepsen@rh.dk

Previous studies have demonstrated the presence of grass pollen-specific T cells in grass pollen-allergic patients (GPA) as well as nonallergic subjects (NA). In order to elucidate a possible seasonal variation in proliferation and cytokine production of peripheral blood mononuclear cells (PBMC), PBMC from 13 GPA and 11 NA were stimulated with extracts of Phleum pratense and tetanus toxoid before and during two grass pollen seasons. IL-4, IL-5 and interferon-gamma were determined by ELISAs. PBMC from GPA demonstrated a decreased proliferative response to grass pollen allergens during the pollen season as compared to NA ($p < 0.05$), but no difference was found in the response to tetanus toxoid. Cells from GPA produced higher amounts of IL-4 and IL-5 than NA ($p < 0.05$) and the seasonal variation in GPA proliferation was paralleled by the grass pollen-induced production of both IL-4 and IL-5 ($p < 0.05$). We conclude that during the grass pollen season PBMC from GPA have a reduced ability to proliferate and to produce Th2-type cytokines. This may be due to seasonal migration of the grass pollen-specific T cells from the blood to the tissues of primary allergen exposure.

PMID: 9813412 [PubMed - indexed for MEDLINE]

1803. Rev Pneumol Clin. 1998 Sep;54(4):225-7.

[Larva migrans syndrome: a rare differential asthma diagnosis].

[Article in French]

Nesme P, Deniaud F, Perol M, Guérin JC.

We report a case of sudden-onset bronchospasm which developed in a 68-year-old patient. Peripheral eosinophila suggested several possible diagnoses. Positive serology for Toxocara canis and clinical and radiological regression without treatment were particularly noteworthy.

PMID: 9805754 [PubMed - indexed for MEDLINE]

1804. Pediatr Res. 1998 Nov;44(5):791-7.

Chronic oral antigen exposure induces lymphocyte migration in anaphylactic mouse intestine.

Ohtsuka Y, Suzuki R, Nagata S, Oguchi S, Shimizu T, Yamashiro Y, Okumura K, Ra C.

Department of Pediatrics, Juntendo University School of Medicine, Tokyo, Japan.

Persistent diarrhea, vomiting, and dehydration are symptoms often seen in patients suffering from food allergy after chronic antigen exposure; however, the precise mechanisms involved have not been well defined. In an effort to clarify the mechanisms of the chronic intestinal changes attributable to genuine IgE-mediated anaphylactic reactions induced by orally administered antigen, a mouse model was established by s.c. implantation of a murine hybridoma capable of producing monoclonal anti-trinitrophenyl IgE antibody, and the morphologic and immunologic changes occurring in the intestine upon chronic antigen exposure were investigated. In the early stage after ingestion of the antigen, diarrhea and noticeable infiltration of mast cells as well as eosinophils into the lamina propria were observed. A substantial increase in serum histamine levels as well as an increase in leukotriene C4 synthesis in the jejunal mucosa were observed 1 h after antigen challenge. Also, the synthesis of leukotriene B4 was significantly elevated for up to 9 h after antigen challenge. The expression of both intercellular adhesion molecule-1 (ICAM-1) on mucosal vascular endothelial cells and IAd on epithelial cells was markedly enhanced, and noticeable infiltration of eosinophils and lymphocytes was also confirmed in the mouse model after chronic antigen exposure. These findings suggest that oral antigen exposure induces anaphylactic reactions in the intestine mediated by mast cells and eosinophils in response to the IgE-antigen complex in the early phase, and also induces lymphocyte migration after chronic antigen exposure.

PMID: 9803464 [PubMed - indexed for MEDLINE]

1805. J Exp Med. 1998 Nov 2;188(9):1621-32.

Mechanisms of acute eosinophil mobilization from the bone marrow stimulated by interleukin 5: the role of specific adhesion molecules and phosphatidylinositol 3-kinase.

Palframan RT, Collins PD, Severs NJ, Rothery S, Williams TJ, Rankin SM.

Leukocyte Biology, Division of Biomedical Sciences, Imperial College School of Medicine, Sir Alexander Fleming Building, South Kensington, London SW7 2AZ, United Kingdom.

Mobilization of bone marrow eosinophils is a critical early step in their trafficking to the lung during allergic inflammatory reactions. We have shown previously that the cytokine interleukin (IL)-5, generated during an allergic inflammatory reaction in the guinea pig, acts systemically to mobilize eosinophils from the bone marrow. Here, we have investigated the mechanisms underlying this release process. Examination by light and electron microscopy revealed the rapid migration of eosinophils from the hematopoietic compartment and across the bone marrow sinus endothelium in response to IL-5. Using an in situ perfusion system of the guinea pig hind limb, we showed that IL-5 stimulated a dose-dependent selective release of eosinophils from the bone marrow. Eosinophils released from the bone marrow in response to IL-5 expressed increased levels of beta2 integrin and a decrease in L-selectin, but no change in alpha4 integrin levels. A beta2 integrin-blocking antibody markedly inhibited the mobilization of eosinophils from the bone marrow stimulated by IL-5. In contrast, an alpha4 integrin blocking antibody increased the rate of eosinophil mobilization induced by IL-5. In vitro we demonstrated that IL-5 stimulates the selective chemokinesis of bone marrow eosinophils, a process markedly inhibited by two structurally distinct inhibitors of phosphatidylinositol 3-kinase, wortmannin and LY294002. Wortmannin was also shown to block eosinophil release induced by IL-5 in the perfused bone marrow system. The parallel observations on the bone marrow eosinophil release process and responses in isolated eosinophils in vitro suggest that eosinophil chemokinesis is the driving force for release in

vivo and that this release process is regulated by alpha4 and beta2 integrins acting in opposite directions.

PMCID: PMC2212511
PMID: 9802974 [PubMed - indexed for MEDLINE]

1806. J Clin Invest. 1998 Nov 1;102(9):1704-14.

GM-CSF transgene expression in the airway allows aerosolized ovalbumin to induce allergic sensitization in mice.

Stämpfli MR, Wiley RE, Neigh GS, Gajewska BU, Lei XF, Snider DP, Xing Z, Jordana M.

Department of Pathology and Molecular Medicine, Immunology and Infection Programme, and Centre for Gene Therapeutics, McMaster University, Hamilton, Ontario, Canada L8N 3Z5.

The purpose of this study was to explore whether repeated exposure to aerosolized ovalbumin (OVA) in the context of local expression of GM-CSF can initiate a Th2-driven, eosinophilic inflammation in the airways. On day -1, Balb/c mice were infected intranasally with an adenovirus construct expressing GM-CSF (Ad/GM-CSF). From day 0 to day 9 mice were exposed daily to an OVA aerosol. Mice exposed to OVA alone did not show any evidence of airway inflammation. Mice receiving both Ad/GM-CSF and aerosolized OVA exhibited marked airway inflammation characterized by eosinophilia and goblet cell hyperplasia. Migration of eosinophils into the airway was preceded by a rise in IL-5 and IL-4. Both IL-5 and class II MHC were critically required to generate airway eosinophilia. After resolution, airway eosinophilia was reconstituted after a single OVA exposure. Flow cytometric analysis of dispersed lung cells revealed an increase in macrophages and dendritic cells expressing B7.1 and B7.2, and expansion of activated (CD69-expressing) CD4 and CD8 T cells in mice exposed to OVA and Ad/GM-CSF. Our data indicate that expression of GM-CSF in the airway compartment increases local antigen presentation capacity, and concomitantly facilitates the development of an antigen-specific, eosinophilic inflammatory response to an otherwise innocuous antigen.

PMCID: PMC509118
PMID: 9802884 [PubMed - indexed for MEDLINE]

1807. Nihon Rinsho. 1998 Sep;56(9):2228-34.

[Mucosal immune system of the intestine].

[Article in Japanese]

Miura S, Fujimori H, Koseki S, Hokari R.

Second Department of Internal Medicine, National Defense Medical College.

Gut-associated lymphoid tissue is the major inductive site of mucosal immune system, functionally independent of the systemic immune system. Particulate antigens are mainly uptaken from M cell of Peyer's patches, inducing IgA production in the intestinal mucosa. Lymphocytes are continuously recirculating through the intestinal mucosa to facilitate intestinal immune response. Dysregulation of lymphocyte migration and cytokine imbalance in the intestinal mucosa may be largely involved in the pathogenesis of inflammatory bowel diseases including intestinal allergy and Crohn's disease. There is also a possibility

that dietary components especially long chain fatty acid could influence immune cell function of the intestinal mucosa. Because dietary components are closely associated with immunological function of intestinal mucosa, the importance of dietary manipulation for the management of inflammatory bowel diseases should be concerned.

PMID: 9780697 [PubMed - indexed for MEDLINE]

1808. Vestn Oftalmol. 1998 Jul-Aug;114(4):43-4.

[Alomide eyedrops in the treatment of allergic conjunctivitis and keratoconjunctivitis].

[Article in Russian]

Maĭchuk IuF, Khaitova KN, Grishakova MB.

New drug lodoxamine (alomide) opens new vistas in the treatment of allergic diseases of the eyes highly prevalent both in adults and children. This drug prevents release of mast cell mediators and delays eosinophil migration to conjunctival and corneal tissue, thus exerting a spectrum of antiallergic effects. Clinical studies carried out in 170 patients demonstrated a high efficacy of alomide in the treatment of subacute and chronic pollenosis conjunctivitis, spring keratoconjunctivitis, multiple and toxic allergic keratitis, and other allergic conjunctivities. Alomide can be used as a preventive drug in patients with allergies under high-risk conditions and in patients wearing contact lenses. It is effective in combined therapy of keratitis and keratouveitis. Alomide eye drops are well tolerated.

PMID: 9771090 [PubMed - indexed for MEDLINE]

1809. Eur Respir J. 1998 Sep;12(3):585-91.

Effect of theophylline on CD11b and L-selectin expression and density of eosinophils and neutrophils in vitro.

Spoelstra FM, Berends C, Dijkhuizen B, de Monchy JG, Kauffman HF.

Dept of Allergology, University Hospital Groningen, The Netherlands.

The nonspecific phosphodiesterase inhibitor theophylline, widely used in asthma therapy, may cause a decrease in inflammatory responses of airways. In asthma, eosinophils migrate to the airway wall and become activated. Activated eosinophils are characterized by low cell density, as well as increased expression of CD11b and reduced expression of L-selectin, two adhesion molecules involved in transendothelial migration. To study the anti-inflammatory effect of theophylline on granulocyte adhesion molecules in vitro, the platelet-activating factor (PAF)-induced density shift was determined by density centrifugation and the modulation of CD11b and L-selectin expression by flow cytometry on eosinophils and neutrophils in human whole blood. A relatively high concentration of theophylline (10(-3) M) inhibited the increase in the percentage of hypodense eosinophils and neutrophils in whole-blood samples after PAF stimulation in vitro. A more pharmacological concentration (10(-4) M) inhibited the CD11b upregulation and L-selectin shedding induced by PAF (10(-7) M) on both eosinophils and neutrophils. The effect of isoproterenol on the inhibitory effect of theophylline was mainly additive, but a small synergistic effect could not be excluded. In conclusion theophylline can attenuate eosinophil and neutrophil activation in vitro at the level of adhesion molecule expression and changes in

cell density. This may have implications for transendothelial migration of these cells in asthma.

PMID: 9762784 [PubMed - indexed for MEDLINE]

1810. Clin Exp Allergy. 1998 Aug;28 Suppl 3:47-52.

Theophylline inhibits the release of eosinophil survival cytokines--is Raf-1 the protein kinase A target?

Shute JK, Tenor H, Church MK, Holgate ST.

Immunopharmacology Group, University Medicine, Southampton General Hospital, UK.

Increased numbers of activated eosinophils in bronchial tissue is a feature of asthma and may, in part, be attributed to the prolonged cytokine-dependent survival of eosinophils within the inflamed microenvironment. Low-dose oral theophylline was previously shown to reduce the number of activated eosinophils within the sub-mucosa following allergen exposure. A number of inhibitory actions of theophylline have been described which relate to eosinophil recruitment and activation, including inhibition of cell migration and release of granule basic proteins. In this study we investigated the ability of theophylline to inhibit the release of preformed GM-CSF and IL-8 from eosinophils in vitro, as these cytokines may serve an autocrine function in eosinophil survival in vivo. Eosinophils rapidly released GM-CSF and IL-8 spontaneously, and release was further enhanced in response to sIgA-coated beads. Theophylline inhibited the stimulated, but not the spontaneous, release of both cytokines. We previously reported the role of protein kinase A in inhibition of arachidonic acid mobilization and LTC4 synthesis. Therefore we speculate that cAMP-dependent activation of protein kinase A following theophylline treatment of eosinophils resulted in inhibition of Raf-1 and MAPK/MAPKK dependent activation of phospholipase A2 and consequently inhibition of degranulation and cytokine release.

PMID: 9756186 [PubMed - indexed for MEDLINE]

1811. Orv Hetil. 1998 Aug 30;139(35):2065-9.

[Food allergy in patients with respiratory allergy. Diagnostic possibilities and problems].

[Article in Hungarian]

Kiss F, Jármay K, Kadocsa E, Husz S, Judák R, Lencse G, Wittmann T, Lonovics J.

Belgyógyászati Klinika, Szent-Györgyi Albert Orvostudományi Egyetem, Szeged.

In the past two years the authors examined 28 patients with abdominal complaints and allergic respiratory symptoms. Detailed internal, gastroenterological, allergological examinations were made.METHODS: 1 skin Prick-test (SPT) with inhalative and nutritive panel 2. measuring of food-specific (gliadin, alpha-lactalbumin beta-lactoglobulin, ovalbumin) IgG-antibody level detecting with ELISA method, 3. leukocyte migration inhibition (LMI) test against the same foodstuffs, 4. histological examination of the stomach and the duodenum especially for mucosal mastocytes (MMCs).
RESULTS: 1. SPT was positive in 23/28 patients for inhalative, but in the 5 cases we did not identify any inhalative allergen. The SPT for the main foodstuffs were positive in 18 patients while in 3 other patients there was urtica only for the

other antigens. 2. The food-specific IgG-antibody level was increased in 18/27 patients against one or more antigens. The SPTs and the antibody determination showed identity in 8/18 cases. 3. The LMI tests were positive against one or more main food-products in 23/27 cases. There was common positivity in respect of antigens (between LMI test and antibody identification) in 17 cases. Pathological immunological reactions were presented against the same main foodstuffs with at least two methods for flour in 11, for egg in 10 and for milk in 12 patients. Endoscopic examinations were performed in 27 cases. The number of the MMCs were increased in 22/27 patients. After a specific elimination diet open-food challenges were performed and they confirmed the results of the in vitro and in vivo examinations.
CONCLUSION: It is common that the respiratory allergic symptoms in atopic patients accompanied with food allergy for the main foodstuffs caused not only more severe respiratory symptoms, but abdominal complaints too. In respect to the many positive LMI tests the late-type hypersensitivity have important pathogenetical role in it. This three methods together define well the main food-products, which can be antigens as well. The examination of the MMCs supports the local disturbance in the immunoregulatory system.

PMID: 9755625 [PubMed - indexed for MEDLINE]

1812. Eur J Immunol. 1998 Sep;28(9):2770-9.

Acquired thymic tolerance and experimental allergic encephalomyelitis in the rat. I. Parameters and analysis of possible mechanisms.

Wilson DB, Wilson DH, Schroder K.

Torrey Pines Institute for Molecular Studies, San Diego 92121, USA.
dbwilson@tpims.org

Intrathymic injection of guinea pig myelin basic protein (MBP) or the immunodominant, encephalitogenic fragment of MPB, 68-86, without otherwise compromising the peripheral lymphocyte pool in adult LEW rats, dramatically inhibits onset of experimental allergic encephalomyelitis (EAE) caused by the usual peripheral inoculation with MBP in complete Freund's adjuvant. This surprising finding demonstrates that interaction of antigen and one or more components of an intact thymus can down-regulate systemic responses by mature T cells already existing in the peripheral lymphocyte pool. How this happens is not known. In studies designed to explore possible mechanisms: (a) adult thymectomized animals remain susceptible to active EAE, thus EAE cannot be attributed solely to recent thymic emigrants that might be inactivated by antigen deposited in the thymus; (b) heterotopic isografts of injected thymic lobes transfer thymic tolerance to secondary recipients, thus the tolerance effect is dominant over an intact, non-treated thymus; (c) T cells from made thymic tolerant but not immunized donors are less effective in causing EAE following adoptive transfer into, and active immunization of, secondary, irradiated recipients; and (d) animals resistant to active EAE as a consequence of thymic tolerance are fully vulnerable to adoptive EAE caused by already activated MBP-specific T cell subpopulations. These results rule out a possible mechanism previously proposed for acquired thymic tolerance, i. e., that potentially pathogenic T cells traffic to the antigen-injected thymus where they are inactivated or eliminated.

PMID: 9754564 [PubMed - indexed for MEDLINE]

1813. Eur J Immunol. 1998 Sep;28(9):2640-7.

Eosinophils are not required to induce airway hyperresponsiveness after nematode infection.

Coyle AJ, Köhler G, Tsuyuki S, Brombacher F, Kopf M.

Department of Biology, Millenium Pharmaceuticals, Cambridge, Boston, USA.

Eosinophilic inflammation of the airways is believed to play a central role in the pathogenesis of bronchial asthma. Inoculation of mice with the nematode Nippostrongylus brasiliensis induces pulmonary inflammation, characterized by a marked infiltration of eosinophils, subsequent to the migration of parasites through the lungs. Infection is associated with polarized Th2 responses in different strains of mice tested. Thus, this model may be useful to determine the relationship between established pulmonary eosinophilic inflammation, Th2 immune responses and airway changes in a nonallergic background. In the present study, we have used IL-5-deficient mice to evaluate the role of IL-5 in eosinophilic lung inflammation and airway hyperresponsiveness (AHR). In wild-type C57B/6 mice, infection with N. brasiliensis resulted in eosinophil accumulation, associated with extensive lung damage characterized by hemorrhage and alveolar wall destruction, and a strong AHR following methacholine treatment. In IL-5-deficient mice, eosinophil infiltration and the associated lung damage was abrogated. Nonetheless, AHR was unimpaired. Our results suggest that eosinophil accumulation plays a central role in lung damage but is not responsible for the induction of airway constriction following N. brasiliensis infection.

PMID: 9754552 [PubMed - indexed for MEDLINE]

1814. Can Respir J. 1998 Jul-Aug;5 Suppl A:31A-5A.

Eosinophilia in antigen-induced airways inflammation.

Stämpfli MR, Jordana M.

McMaster University, Hamilton, Canada.

Airways eosinophilia is a hallmark of asthmatic inflammation. Accumulation of eosinophils in the airways of asthmatic patients is a terminal event of a process that involves complex cellular and molecular interactions. In the past five years, murine models of experimental asthmatic inflammation have provided insight into its complexity and regulation. The three main steps involved in the development of airways eosinophilia are discussed. The first step is the elicitation of an allergen-specific immune response whereby the allergen, captured and processed by antigen-presenting cells, is presented to T lymphocytes to initiate a specific immunological response. In addition to the class II major histocompatibility T cell receptor interaction, this process critically requires costimulation through two independent pathways. The second step is eosinopoiesis in the bone marrow. Under normal conditions, eosinophils represent only 1% to 3% of the white blood cell pool. Therefore, an eosinopoietic event must precede peripheral blood and tissue eosinophilia. The third step is the recruitment of eosinophils from the vascular compartment into the airways. Migration through the endothelium is an active process that involves a number of molecules such as integrins and adhesion molecules. Understanding these three key steps in the development of airways eosinophilia will help to identify new targets and unveil novel strategies for an even more effective treatment for asthma.

PMID: 9753514 [PubMed - indexed for MEDLINE]

1815. J Immunol. 1998 Sep 15;161(6):3064-70.

Triple role of platelet-activating factor in eosinophil migration across monolayers of lung epithelial cells: eosinophil chemoattractant and priming agent and epithelial cell activator.

Liu L, Zuurbier AE, Mul FP, Verhoeven AJ, Lutter R, Knol EF, Roos D.

Central Laboratory of The Netherlands Red Cross Blood Transfusion Service, Amsterdam.

Infiltration of eosinophils into the lung lumen is a hallmark of allergic asthmatic inflammation. To reach the lung lumen, eosinophils must migrate across the vascular endothelium, through the interstitial matrix, and across the lung epithelium. The regulation of this process is obscure. In this study, we investigated the migration of human eosinophils across confluent monolayers of either human lung H292 epithelial cells or primary human bronchial epithelial cells. Established eosinophil chemoattractants (IL-8, RANTES, platelet-activating factor (PAF), leukotriene B4, and complement fragment 5a (C5a)) or activation of the epithelial cells with IL-1beta induced little eosinophil transmigration (<7% in 2 h). In contrast, addition of PAF in combination with C5a induced extensive (>20%) transepithelial migration of unprimed and IL-5-primed eosinophils. Eosinophil migration assessed in a Boyden chamber assay, i.e., without an epithelial monolayer, was only slightly increased upon addition of PAF and C5a. Preincubation of eosinophils with the PAF receptor antagonist WEB 2086 only inhibited migration of unprimed eosinophils toward PAF and C5a, whereas preincubation of epithelial cells with WEB 2086 abolished migration of both IL-5-primed and unprimed eosinophils. This latter result indicated the presence of PAF receptors on epithelial cells. Indeed, addition of PAF to epithelial cells induced an increase in cytosolic free Ca2+, which was blocked by the PAF receptor antagonists WEB 2086 and TCV-309. Our results show that PAF induces permissive changes in epithelial cells, and that PAF acts as a chemoattractant and priming agent for the eosinophils.

PMID: 9743372 [PubMed - indexed for MEDLINE]

1816. Ann Allergy Asthma Immunol. 1998 Aug;81(2):176-80.

Soluble adhesion molecules and cytokines in perennial allergic rhinitis.

Liu CM, Shun CT, Cheng YK.

Department of Otolaryngology, College of Medicine, National Taiwan University, Taipei.

BACKGROUND: Increasing evidence suggests adhesion molecules and cytokines in patients with inflammatory airway diseases are involved in steps of entrapment and migration of inflammatory cells. Recently, soluble forms of adhesion molecules and cytokines have been detected in the sera and other body fluids of patients with various diseases.
OBJECTIVE: Eosinophilia in nasal mucosa is characteristic of allergic rhinitis. Vascular adhesion molecules expressed on the endothelium are essential for eosinophils to move from the peripheral blood into the sites of inflammation. Herein, soluble forms of vascular adhesion molecules and eosinophil-activating cytokines are measured to investigate the significance of their appearance in the sera with eosinophil infiltration in the nasal mucosa of perennial allergic rhinitis.
METHODS: With the quantitative sandwich enzyme immunoassay technique, the sera of 36 patients of perennial allergic rhinitis and 20 nonatopic subjects were used to measure the levels of soluble intercellular adhesion molecule-1 (sICAM-1),

vascular cell adhesion molecule-1 (sVCAM-1), E-selectin (endothelial leukocyte adhesion molecule-1, sELAM-1), interleukin-3 (IL-3), and interleukin-5 (IL-5). RESULTS: No significant differences in the levels of soluble vascular adhesion molecules were noted between the two groups. Eosinophil-activating cytokines, IL-3 and IL-5, were significantly increased in the group with perennial allergic rhinitis, and were correlated with eosinophil infiltration in nasal scrapings. CONCLUSION: Although the vascular adhesion molecules expressed on the endothelium are necessary for eosinophils to appear in allergic tissues, eosinophil-activating cytokines as IL-3 and IL-5 are likely to be essential for eosinophils to function in tissues. The elevated concentrations of IL-3 and IL-5 in allergic rhinitis may reflect the inflammatory response occurring in the T cell activation and in relation to manifestation of eosinophils.

PMID: 9723565 [PubMed - indexed for MEDLINE]

1817. J Egypt Soc Parasitol. 1998 Aug;28(2):365-72.

Toxocariasis as a possible cause of allergic diseases in children.

Oteifa NM, Moustafa MA, Elgozamy BM.

Department of Parasitology, Faculty of Medicine, Ain Shams University, Cairo, Egypt.

Allergic diseases are common in young children yet the cause of allergy remains unknown in most of them. The association of Toxocara infection with two common allergic diseases in children was investigated. Sera from 60 children (average age 4-6y) suffering from idiopathic bronchial asthma or chronic urticaria were tested for toxocariasis using ELISA test and compared to 30 apparently healthy age matched children . Seropositive children were investigated for evidence of sensitization by measuring serum specific IgE concentration and eosinophil count. The frequency of Toxocara seropositivity was 26.6%, 13.3% and 30% in asthmatic, chronic urticarial and control children respectively. The seropositive allergic children showed significant increase in IgE and eosinophil levels when compared to controls. A contribution of toxocariasis to the allergic sensitization was suggested.

PMID: 9707665 [PubMed - indexed for MEDLINE]

1818. Arch Dermatol Res. 1998 Jun;290(6):291-7.

Increased expression of platelet-derived growth factor receptor alpha and beta and vascular endothelial growth factor in the skin of patients with chronic venous insufficiency.

Peschen M, Grenz H, Brand-Saberi B, Bunaes M, Simon JC, Schöpf E, Vanscheidt W.

Department of Dermatology, University of Freiburg, Germany.

Growth factors produced by a variety of cells act as signalling peptides through specific cell surface receptor pathways. Functions such as cell proliferation, migration and differentiation have been assigned to each of them. Here, we report alterations of platelet-derived growth factor receptor alpha (PDGFR-alpha) and beta (PDGFR-beta) and vascular endothelial growth factor (VEGF) expression patterns in the progressive clinical stages of chronic venous insufficiency (CVI). A total of 30 punch biopsies were taken from patients with CVI, and VEGF and PDGFR were detected by indirect immunofluorescence and immunoperoxidase techniques. PDGFR-alpha and PDGFR-beta expression was strongly increased in

endothelial cells of capillaries, pericapillary cells and connective tissue cells in the stroma of the skin of venous eczema and venous leg ulcer patients, and to a smaller extend in the dermis of those with lipodermatosclerosis. VEGF staining showed a similar expression pattern in the progressive CVI stages. However, staining of vessels in particular might simply reflect binding of VEGF, secreted by keratinocytes or fibroblasts, to its receptors. Growth factor and receptor expression in specimens from telangiectases and reticular veins, and from pigmented areas, resembled that of normal skin. We conclude that PDGFR-alpha, PDGFR-beta and VEGF play an important role in mediating inflammation and epithelial hyperproliferation in venous eczema, inducing connective tissue sclerosis in lipodermatosclerosis, and causing the reduced reepithelialization tendency in venous ulcers. We speculate that endothelial proliferation with chronic venous hypertension might be mediated by these growth factors.

PMID: 9705159 [PubMed - indexed for MEDLINE]

1819. Eur Respir J. 1998 Jul;12(1):41-4.

Asthma and respiratory symptoms in urban and rural Saudi Arabia.

Hijazi N, Abalkhail B, Seaton A.

Dept of Community Medicine and Primary Health Care, Faculty of Medicine and Allied Science, King Abdulaziz University, Jeddah, Saudi Arabia.

The aim of this study was to contrast the prevalence of allergic symptoms in children living in urban and rural areas of Saudi Arabia and to investigate factors associated with any differences found. A cross-sectional questionnaire survey was conducted of a social class-stratified sample of 1,020 urban and 424 rural 12 yr old children, recording symptoms of current and past allergic disease and doctors' diagnoses, together with nationality and the fathers' educational level and occupations. A significantly greater prevalence of allergic symptoms was found in urban than in rural children and in Saudi than in non-Saudi Arab children. Males were more likely to have some respiratory symptoms and females had more eye and skin symptoms. Educational level and occupation of the father did not influence the likelihood of having symptoms. Logistic regression analyses showed that urban residence and Saudi nationality were the two main risk factors associated with asthmatic symptoms. There is likely to have been a recent increase in the prevalence of allergic disease in Saudi children associated with increased affluence, which has not affected non-Saudi migrants moving into the same environment to the same extent. This is consistent with the hypothesis that the environment, possibly through changes in lifestyle and patterns of infection, influences the expression of allergic disease.

PMID: 9701412 [PubMed - indexed for MEDLINE]

1820. Allergy. 1998 Jul;53(7):690-7.

The potential role of interleukin-13 in eosinophilic inflammation in nasal mucosa.

Terada N, Hamano N, Hohki G, Ikeda T, Sai M, Yamashita T, Konno A.

Department of Otorhinolaryngology, Chiba University, School of Medicine, Chiba City, Japan.

BACKGROUND: Recent studies have revealed that interleukin (IL)-13, as well as IL-4, causes de novo surface expression of vascular cell adhesion molecule-1

(VCAM-1) on endothelial cells of the umbilical vein and accelerates selective eosinophil migration. However, its role in allergic rhinitis remains to be clarified. Of particular interest is whether IL-13 upregulates VCAM-1 expression in human mucosal microvascular endothelial cells (HMMECs), to which eosinophils adhere in nasal mucosa.
METHODS: To understand the potential role of IL-13 in eosinophilic inflammation in nasal mucosa, we examined the effects of IL-13 on the adhesiveness between HMMECs and eosinophils.
RESULTS: IL-13 increased VCAM-1 expression in HMMECs, the adhesiveness of endothelial cells to eosinophils, and the transendothelial migration. On the other hand, IL-13 decreased the adhesiveness of eosinophils to HMMECs, and, as a result, accelerated eosinophil infiltration. Those effects are more potent than was those of IL-4. In addition, we also report that the amount of IL-13 in nasal mucosa was higher than that of IL-4.
CONCLUSIONS: These results strongly indicate that IL-13, as well as IL-4, may be important in eosinophilic inflammation in the nasal mucosa.

PMID: 9700038 [PubMed - indexed for MEDLINE]

1821. Mol Immunol. 1998 Feb;35(3):137-48.

Cloning, expression, sequence determination, and chromosome localization of the mouse complement C3a anaphylatoxin receptor gene.

Hollmann TJ, Haviland DL, Kildsgaard J, Watts K, Wetsel RA.

Institute of Molecular Medicine for the Prevention of Human Diseases, Research Center for Immunology and Autoimmune Diseases, Houston, Texas, USA.

The complement C3a anaphylatoxin receptor (C3aR) is a seven-transmembrane G-protein coupled chemoattractant receptor that on binding the C3a peptide ligand mediates numerous cellular responses, including histamine release from mast cells. smooth muscle contraction, and the directed migration of eosinophils. To delineate the murine C3aR coding sequence, gene structure, 5'-flanking region, and chromosome location, cDNA and genomic clones encoding the mouse C3a receptor were isolated, characterized, and used in fluorescence in situ hybridization experiments. The results from this study indicate that the murine C3a receptor structural gene is a single copy gene of approximately 8 kb comprised of 2 exons which are separated by a large intervening intron of 4724 bp. The first exon encodes 97 bp of 5'-untranslated sequence. Exon 2 encodes the remaining 8 bp of 5'-untranslated sequence and the entire coding and 3'-untranslated sequences. This genomic organization is typical of most other chemoattractant receptor genes in that the entire coding sequence is contained on a single exon. The human and mouse C3a receptor genes were localized to syntenic chromosomal bands 12q13.2-3 and 6F1, respectively. No other seven-transmembrane receptor genes, to date, have been localized to these chromosomal regions. Primer extension experiments using mouse macrophage RNA indicated a single transcriptional initiation site. Sequence analysis 5' of the transcriptional site indicated a TATA-less promoter with possible cis-acting motifs that may regulate C3a receptor gene expression. These included the recognition sequence for the nuclear transcription factor SP1 and the phorbol ester response sequence which binds the Fos/Jun heteromeric transcription factor AP1.

PMID: 9694514 [PubMed - indexed for MEDLINE]

1822. Eur J Immunol. 1998 Jul;28(7):2152-8.

Eotaxin-2 activates chemotaxis-related events and release of reactive oxygen

species via pertussis toxin-sensitive G proteins in human eosinophils.

Elsner J, Petering H, Kluthe C, Kimmig D, Smolarski R, Ponath P, Kapp A.

Hannover Medical University, Department of Dermatology and Allergology, Germany.
jelsner@compuserve.com

Eosinophils play an important role in allergic and autoimmune diseases. They are activated by distinct chemokines, leading to the immigration into the inflamed tissue, and mediate tissue damage by releasing reactive oxygen species. Recently, eotaxin was found to have the broadest spectrum of activities of all eosinophil-activating CC chemokines. In this study we investigated the effect of the novel CC chemokine, eotaxin-2, on eosinophil effector functions and compared its activity with eotaxin. Using nitrobenzoxadiazole-phallacidin staining and flow cytometry, we show that eotaxin-2 induced rapid and transient actin polymerization, a prerequisite for cell migration and modulation of the respiratory burst, in eosinophils in the same range of efficacy as observed for eotaxin. Eotaxin-2 induced the release of reactive oxygen species in a dose-dependent manner; half maximal and maximal release were found at 50 ng/ml and 500 ng/ml, respectively. Surprisingly, the efficacy of eotaxin-2 was comparable to that of eotaxin and C5a. Release of reactive oxygen species was inhibited by pertussis toxin, indicating the involvement of Gi proteins in the signaling of eotaxin-2. Moreover, the anti-CC chemokine receptor 3 (CCR3) monoclonal antibody, 7B11, was able to inhibit transient rise in the cytosolic Ca2+ concentration and the release of reactive oxygen species following stimulation with eotaxin-2. Therefore, eotaxin-2 represents a potent CC chemokine for human eosinophils activating chemotaxis-related events, such as actin polymerization, and the respiratory burst via the CCR3. Moreover, the efficacy of eotaxin-2 seems to be in the same range as that of eotaxin which might re-evaluate the recent profile of activity of CC chemokines in the activation of human eosinophils.

PMID: 9692884 [PubMed - indexed for MEDLINE]

1823. Med Parazitol (Mosk). 1998 Apr-Jun;(2):54-9.

[The interrelation of Toxocara invasion with bronchial asthma].

[Article in Russian]

Mazmanian MV, Tumol'skaia NI, Chervinskaia TA.

PMID: 9691624 [PubMed - indexed for MEDLINE]

1824. Eur J Dermatol. 1998 Jul-Aug;8(5):334-8.

Patterns of epidermal growth factor receptor, basic fibroblast growth factor and transforming growth factor-beta3 expression in skin with chronic venous insufficiency.

Peschen M, Grenz H, Grothe C, Schöpf E, Vanscheidt W.

Department of Dermatology, University of Freiburg, Hauptstr.7, D-79104 Freiburg, Germany. Peschen@haut.ukl.uni-freiburg.de

Growth factors which act as signalling peptides through specific cell surface receptors are involved in functions such as cell proliferation, migration, and differentiation. Here, we report on alterations of the epidermal growth factor

receptor (EGFR), basic fibroblast growth factor (bFGF) and transforming growth factor beta3 (TGF-beta3) expression patterns in the skin at various stages of chronic venous insufficiency (CVI). Thirty punch biopsies were taken from patients with CVI and growth factors or the growth factor receptor were detected by indirect immunofluorescence and immunoperoxidase techniques. EGFR, bFGF, and TGF-beta3 expression is strongly increased in the stroma of venous eczema and in leg ulcer skin, and to a lesser extent in the dermis of patients with lipodermatosclerosis. Venous eczema and lipodermatosclerosis epidermis show an elevated EGFR and bFGF synthesis throughout all strata. In the different CVI stages, telangiectases and reticular veins and pigmentation EGFR and bFGF staining are limited to the basal layer. We conclude that the alterations in the expression of EGFR, bFGF and TGF-beta3 precede changes in the affected skin within progressing stages of CVI. The exact mechanisms of growth factor involvement in the pathogenesis of venous ulceration remain to be resolved.

PMID: 9683865 [PubMed - indexed for MEDLINE]

1825. Respiration. 1998;65(3):214-8.

Diffuse pulmonary hemosiderosis after exposure to pesticides. A case report.

Kayser K, Plodziszewska M, Waitr E, Slodkowska J, Altiner M, Gabius HJ.

Department of Pathology, Thoraxklinik, Heidelberg, Germany.

This report describes the clinical, radiological, microscopical and ligandohistochemical findings in a 17-year-old woman who suffered from an acute onset of pulmonary hemosiderosis after inhalation of pesticides used for the cultivation of strawberries. She complained of headache, dyspnea, rhinitis, weakness and recurrent severe hemoptysis. Chest radiographs revealed bilateral patchy infiltrates, predominantly in the lower parts of both lungs. The consecutive severe anemia was treated by multiple blood transfusions which were repeated every 4-5 days. Open lung biopsies displayed signs of diffuse hemorrhage with hemosiderin-loaded macrophages, some hyaline membranes, focal fibroid deposits with intermingled histiocytes, mild interstitial fibrosis and focal intra-alveolar calcified bodies surrounded by foreign body giant cells. Analysis of endogenous lectins failed to demonstrate expression of binding capacities for maltose, fucose, mannose, lactose and sialic acid, Neither binding capacities for the macrophage-migration-inhibitory factor nor its presence, as analyzed by labeled sarcolectin, could be detected histochemically. The light microscopical findings are consistent with a longer-lasting diffuse pulmonary hemosiderosis; the presence of calcified bodies and foreign body giant cells (including the ligandohistochemical data) argues for a causal role of inhaled substances. The patient's clinical course improved after cyclophosphamide treatment, which restored her ability to work and released her from the need for recurrent blood transfusions.

PMID: 9670307 [PubMed - indexed for MEDLINE]

1826. J Immunol. 1998 Jul 15;161(2):1026-33.

Monocytes from Wiskott-Aldrich patients display reduced chemotaxis and lack of cell polarization in response to monocyte chemoattractant protein-1 and formyl-methionyl-leucyl-phenylalanine.

Badolato R, Sozzani S, Malacarne F, Bresciani S, Fiorini M, Borsatti A, Albertini A, Mantovani A, Ugazio AG, Notarangelo LD.

Clinica Pediatrica, Universita' di Brescia, Italy. badolato@master.cci.unibs.it

Wiskott-Aldrich syndrome (WAS) is an X-linked disorder characterized by trombocytopenia, eczema, and progressive decline of the immune function. In addition, lymphocytes and platelets from WAS patients have morphologic abnormalities. Since chemokines may induce morphologic changes and migration of leukocytes, we investigated the monocyte response to chemoattractants in cells from WAS patients with an identified mutation in the WAS protein gene. Here, we report that monocytes derived from four patients with molecularly defined typical WAS have a severely impaired migration in response to FMLP and to the chemokines monocyte chemoattractant protein-1 (MCP-1) and macrophage inflammatory protein-1alpha compared with normal donors. Conversely, neither MCP-1 binding to monocytes nor induction of the respiratory burst by MCP-1 and FMLP is significantly different between WAS patients and normal donors. Within a few minutes of stimulation, monocytes respond to chemokines with increased expression of adhesion molecules and with morphologic changes such as cell polarization. Although up-regulation of CD11b/CD18 expression following stimulation with FMLP or MCP-1 is preserved in WAS patients, cell polarization is dramatically decreased. Staining of F-actin by FITC-phalloidin in monocytes stimulated with chemoattractants shows F-actin to have a rounded shape in WAS patients, as opposed to the polymorphic distribution of F-actin in the polarized monocytes from healthy donors. These results suggest that WAS protein is involved in the monocyte response to the chemokines MCP-1 and macrophage inflammatory protein-1alpha.

PMID: 9670984 [PubMed - indexed for MEDLINE]

1827. J Immunol. 1998 Jul 15;161(2):971-7.

Differential regulation of eosinophil adhesion and transmigration by pulmonary microvascular endothelial cells.

Yamamoto H, Sedgwick JB, Busse WW.

Department of Medicine, University of Wisconsin, Madison 53792, USA.

In bronchial asthma, eosinophils (EOS) adhere to, and migrate across, the lung microvasculature to exert their effector functions in the airways. This study was conducted to determine the effect of cytokines on adhesion molecule expression on human pulmonary microvascular endothelial cells (HPMEC) and the influence of these molecules on EOS adhesion and transmigration in vitro. Unlike ICAM-1 expression (>80% positive cytokine-treated HPMEC by flow cytometry), VCAM-1 expression varied with the cytokine(s) pretreatment; the order of potency was: TNF-alpha + IL-4 (82.2 +/- 4.2% positive cells) > TNF-alpha (41.8 +/- 5.1%) > IL-1beta (20.8 +/- 4.7%). IL-4 alone had no effect on either ICAM-1 or VCAM-1 expression. EOS adhesion to cytokine-treated HPMEC followed the same order as that observed for VCAM-1 expression. Interestingly, EOS migration across cytokine-treated HPMEC varied inversely with VCAM-1 expression on, and EOS adhesion to, HPMEC; IL-1beta (21.2 +/- 1.4% migration) > TNF-alpha (12.6 +/- 2.6%) > TNF-alpha + IL-4 (9.1 +/- 2.0%). EOS adhesion was greatest with TNF-alpha + IL-4-treated HPMEC, was dependent on VCAM-1, and inhibited with anti-alpha4 integrin mAb (67.7 +/- 7.5% inhibition, $p < 0.0005$). In contrast, the highest EOS migration occurred across IL-1beta-treated HPMEC and was inhibited by anti-beta2 integrin mAb (40.4 +/- 2.5% inhibition, $p < 0.005$). Viable HPMEC were required for EOS migration but not adhesion. Our results suggest that EOS adhesion and transmigration are differentially regulated by VCAM-1 and ICAM-1 expression and the interaction of these adhesion proteins with their respective counterligands, i.e., alpha4 and beta2 integrins on EOS.

PMID: 9670977 [PubMed - indexed for MEDLINE]

1828. Nihon Yakurigaku Zasshi. 1998 May;111(5):897-302.

[A novel photochemical model of intimal thickening in the rat femoral artery and evaluation of anti-proliferative drugs].

[Article in Japanese]

Kondo K.

Department of Pharmacology, Hamamatsu University School of Medicine, Japan.

We have established a novel photochemical model of intimal thickening in the rat femoral artery. The endothelium was injured by the photochemical reaction between rose bengal and green light, which was followed by thrombotic occlusion, vascular smooth muscle cells (VSMC) migration and proliferation. The neointima was formed by proliferated VSMC and the extracellular matrix, reaching to maximal thickness within 3 weeks after the endothelial injury. Using this model, we have investigated the effect of several anti-proliferative drugs, anti-allergic drugs, angiotensin converting enzyme inhibitors, prostaglandin E1, anti-thrombotics, or leukotrienes receptor antagonists, on intimal thickening. This model has two major advantages in comparison with other methods: one is that the media is free from mechanical stress, and the model is expected to represent pathological changes close to clinical atherosclerosis. Another advantage is that this method is also applicable to small animals such as mice, including transgenic mice. These advantages are very helpful for investigating the mechanism of atherogenesis.

PMID: 9666484 [PubMed - indexed for MEDLINE]

1829. Eur J Epidemiol. 1998 Apr;14(3):247-52.

Increasing risk of asthma without other atopic diseases in school children: a repeated cross-sectional study after 13 years.

Nystad W, Magnus P, Gulsvik A.

Section of Epidemiology, National Institute of Public Health, Oslo, Norway.

Some children develop asthma and other atopic diseases, others asthma without atopic diseases. To better understand secular trends, we estimated the relative increase in asthma in children with (atopy related asthma) and without (non-atopy related asthma) other atopic diseases (eczema or hay fever) in two samples of school children born, 1965-1975 (n = 1674) and 1978-1988 (n = 2188). By analysing the samples as historical cohorts, age-specific prevalence rates were estimated and incidence rates were calculated (number of new cases by 1000 person years under risk). Cox regression was used to estimate the relative risk (RR) of asthma by year of birth. The point prevalence of asthma was 1.9% (95% CI: 1.4-2.4) in the 1965-1975 cohort and 4.6% (95% CI: 3.8-5.4) in the 1978-1988 cohort for three-year old children, and remained fairly constant throughout childhood. The age-specific prevalence of non-atopy related asthma increased relatively more from 1965-1975 to 1978-1988 compared to atopy related asthma. The age-specific incidence rates of asthma showed that the RRs comparing the two cohorts tended at all ages to be highest for non-atopy related asthma. The relative risks of non-atopy related asthma by gender and birth cohort, showed that the effect of cohort was higher for non-atopy related asthma, aRR: 4.0 (95 % CI: 2.5-6.5), than for atopy-related asthma aRR: 2.0 (95% CI: 1.3-3.2). Children without other

atopic diseases have a higher relative risk of being diagnosed with asthma than children with other atopic diseases across all ages comparing two samples of school children born 1965-1975 and 1978-1988.

PMID: 9663517 [PubMed - indexed for MEDLINE]

1830. Eur J Epidemiol. 1998 Apr;14(3):239-45.

Is the prevalence of atopic diseases in East and West Germany already converging?

Heinrich J, Richter K, Magnussen H, Wichmann HE.

GSF-Forschungszentrum für Umwelt und Gesundheit, Institut für Epidemiologie, Oberschleissheim, Germany. Joachim.Heinrich@GSF.DE

Studies comparing respiratory health of residents in the areas of former East and West Germany have shown higher rates of asthma and allergies in children and young adults in former West Germany. It has been speculated that some factors associated with western lifestyle may be related to higher rates of atopic diseases among residents of former West Germany. We examined if the prevalence rates of self-reported asthma and nasal allergies in adults converged between the areas of former East and West Germany five years after re-unification. During the years 1990-1992 and 1994-1995 two independently drawn random samples of more than 3,000 subjects between the ages of 20 to 44 years answered a screening questionnaire of the European Community Respiratory Health Survey in Erfurt (East Germany) and in Hamburg (West Germany). The prevalence rates of asthma attacks, asthma medication use, allergic rhinitis, and wheezing remained stable in Hamburg but increased significantly in Erfurt approaching those of Hamburg. The data indicate that there is a tendency for the prevalence rates of self-reported allergic rhinitis and asthma-related respiratory symptoms in the eastern part of Germany to increase to West-German levels. It is not yet clear if this is due to a true increase in morbidity or only to a higher awareness for these diseases among doctors and the public.

PMID: 9663516 [PubMed - indexed for MEDLINE]

1831. J R Soc Med. 1998 Mar;91(3):133-4.

Exacerbation of atopic dermatitis after bacillus Calmette-Guérin vaccination.

Dalton SJ, Haeney MR, Patel L, David TJ.

Department of Child Health, University of Manchester, Booth Hall Children's Hospital, Blackley, UK.

In two children with atopic dermatitis, routine vaccination with bacillus Calmette-Guérin (BCG) was followed by severe exacerbation of skin disease. If the sequence is cause and effect, a possible mechanism is stimulation of a Th2 lymphocyte cytokine profile by the vaccine, with migration of activated lymphocytes to inflamed skin. In children with active atopic dermatitis, BCG vaccination is best deferred until remission.

PMCID: PMC1296561
PMID: 9659324 [PubMed - indexed for MEDLINE]

1832. Respirology. 1998 Mar;3(1):13-20.

The effect of inhaled budesonide on the diurnal variation in airway mechanics, airway responsiveness and serum neutrophil chemotactic activity in Asian patients with predominant nocturnal asthma.

Tan WC, Koh TH, Hay CS, Taylor E.

Department of Medicine, National University of Singapore, Singapore.

The effectiveness of inhaled corticosteroids in the control of daytime symptoms in asthma is well established, but the specific use against nocturnal asthma has not been systematically studied in Asian patients. This study examined the effect of treatment with inhaled budesonide on the nocturnal variation in measurements of airway calibre, bronchial hyperresponsiveness to inhaled histamine and circulating neutrophil chemotactic activity in Asian patients with nocturnal asthma. Thirty patients, with nocturnal asthma, were randomized into a 2-month, double-blind, parallel group study. Twice as many subjects were allocated to the group who received two consecutive months of inhaled budesonide 1600 microg daily as to the group who received placebo followed by budesonide. Spirometry, lung mechanics, bronchial hyperresponsiveness and serum neutrophil chemotactic factor (NCA) were measured at 16.00 h, 22.00 h and at 04.00 h on 3 days and nights, 4 weeks apart before and after either placebo or budesonide. The combined measurements for the two groups at 04.00 h before and after treatment with budesonide were: forced expiratory volume in 1 s (FEV1) mean (SEM) litres 1.34 (0.17) before, 2.00 (0.19) after; thoracic gas volume (TGV) litres 3.05 (0.32) before, 2.25 (0.14) after; specific airway conductance (sGaw) (cmH20.0 sec)(-1) 0.39 (0.07) before, 1.16 (0.17) after; PD20 microg geometric mean 1.16 before, 44.74 after; neutrophil chemotactic activity (NCA) in units of graduations of migration 98.8 (4.2) before, 101 (14.2) after. The data showed that short and intermediate term high dose inhaled budesonide is an effective specific treatment for nocturnal asthma in Asian patients, resulting in marked improvements in symptoms and in lung mechanics, and reductions in the diurnal variations in bronchial hyperresponsiveness, before any change could be demonstrated in a circulating marker of airway inflammation.

PMID: 9657654 [PubMed - indexed for MEDLINE]

1833. J Neuroimmunol. 1998 Jun 1;86(1):1-12.

Chemokine receptor expression in cultured glia and rat experimental allergic encephalomyelitis.

Jiang Y, Salafranca MN, Adhikari S, Xia Y, Feng L, Sonntag MK, deFiebre CM, Pennell NA, Streit WJ, Harrison JK.

Department of Pharmacology and Therapeutics, College of Medicine, University of Florida, Gainesville 32610-0267, USA.

Chemokines are a group of pro-inflammatory peptides that mediate leukocyte migration and activation. Several members of the chemokine family have been shown to be synthesized by cells of the central nervous system (CNS). To begin to address the role of chemokine receptors in CNS physiology, we identified, by molecular cloning techniques, the rat orthologs of the chemokine receptors, CCR2, CCR3, CCR5, and CXCR4. CCR2 and CCR5 expression was detected in rat spleen, lung, kidney, thymus and macrophages; CCR5 mRNA was also detected in rat brain. Primary cultures of rat microglia expressed CCR5 mRNA that was regulated by IFN-gamma, while both cultured astrocytes and microglia were found to contain mRNA for CXCR4 and CX3CR1. Induction of experimental allergic encephalomyelitis (EAE) in the rat was accompanied by increased levels of CCR2, CCR5, CXCR4, and CX3CR1 mRNAs in the lumbar spinal cords of animals displaying clinical signs of the disease. These

data identify the rat orthologs of chemokine receptors and demonstrate that brain, spinal cord, and cultured glial cells express chemokine receptors that can be regulated both in vitro and in vivo.

PMID: 9655467 [PubMed - indexed for MEDLINE]

1834. J Allergy Clin Immunol. 1998 Jun;101(6 Pt 1):793-806.

Ultrastructural analysis of human skin biopsy specimens from patients receiving recombinant human stem cell factor: subcutaneous injection of rhSCF induces dermal mast cell degranulation and granulocyte recruitment at the injection site.

Dvorak AM, Costa JJ, Monahan-Earley RA, Fox P, Galli SJ.

Department of Pathology, Beth Israel Deaconess Medical Center and Harvard Medical School, Boston, Mass 02215, USA.

We performed an ultrastructural analysis of 10 skin biopsy specimens that had been obtained from three women who were undergoing daily subcutaneous dosing with recombinant methionyl-human stem cell factor (rhSCF) as part of a phase I clinical trial. The biopsy specimens were obtained at sites of subcutaneous administration of rhSCF, within approximately 1 to 2 hours of rhSCF injection, and, at the same time, at contralateral control sites that had not been directly injected with rhSCF. We previously reported that subcutaneous dosing with rhSCF in these subjects induced the local development of a wheal and flare response, which was associated with evidence of mast cell degranulation, as well as a systemic increase in numbers of cutaneous mast cells. The present electron microscopic analysis revealed that all biopsies of swollen, erythematous rhSCF-injected sites exhibited anaphylactic degranulation of both mature and immature mast cells, an acute inflammatory response characterized by the migration of neutrophils, basophils (some of which exhibited evidence of piecemeal degranulation), and eosinophils through blood vessel walls into the perivascular and extravascular spaces, and edema and fibrin deposition within the interstitium. By contrast, the control biopsies contained no evidence of mast cell degranulation or acute inflammation. However, both control and rhSCF-injected sites exhibited mast cells that were undergoing granule building and maturation. Thus at the doses tested in these subjects, subcutaneous injection of rhSCF induced anaphylactic-type degranulation of dermal mast cells at the injection site, with an acute inflammatory response that was associated with the recruitment of granulocytes. By contrast, mast cells at sites distant from those directly injected with rhSCF exhibited no evidence of enhanced secretion.

PMID: 9648707 [PubMed - indexed for MEDLINE]

1835. Environ Health Perspect. 1998 Jun;106 Suppl 3:787-94.

Children's health and the environment: a new agenda for prevention research.

Landrigan PJ, Carlson JE, Bearer CF, Cranmer JS, Bullard RD, Etzel RA, Groopman J, McLachlan JA, Perera FP, Reigart JR, Robison L, Schell L, Suk WA.

Department of Community Medicine, Mount Sinai School of Medicine, New York, NY 10029-6574, USA. plandrigan@smtplink.mssm.edu

Patterns of illness in American children have changed dramatically in this century. The ancient infectious diseases have largely been controlled. The major diseases confronting children now are chronic and disabling conditions termed the

"new pediatric morbidity"--asthma mortality has doubled; leukemia and brain cancer have increased in incidence; neurodevelopmental dysfunction is widespread; hypospadias incidence has doubled. Chemical toxicants in the environment as well as poverty, racism, and inequitable access to medical care are factors known and suspected to contribute to causation of these pediatric diseases. Children are at risk of exposure to over 15,000 high-production-volume synthetic chemicals, nearly all of them developed in the past 50 years. These chemicals are used widely in consumer products and are dispersed in the environment. More than half are untested for toxicity. Children appear uniquely vulnerable to chemical toxicants because of their disproportionately heavy exposures and their inherent biological susceptibility. To prevent disease of environmental origin in America's children, the Children's Environmental Health Network (CEHN) calls for a comprehensive, national, child-centered agenda. This agenda must recognize children's vulnerabilities to environmental toxicants. It must encompass a) a new prevention-oriented research focus; b) a new child-centered paradigm for health risk assessment and policy formulation; and c) a campaign to educate the public, health professionals, and policy makers that environmental disease is caused by preventable exposures and is therefore avoidable. To anchor the agenda, CEHN calls for long-term, stable investment and for creation of a national network of pediatric environmental health research and prevention centers.

PMCID: PMC1533065
PMID: 9646038 [PubMed - indexed for MEDLINE]

1836. Clin Exp Allergy. 1998 May;28(5):606-15.

CD11b and L-selectin expression on eosinophils and neutrophils in blood and induced sputum of patients with asthma compared with normal subjects.

in 't Veen JC, Grootendorst DC, Bel EH, Smits HH, Van Der Keur M, Sterk PJ, Hiemstra PS.

Department of Pulmonology, Leiden University Medical Centre, The Netherlands.

BACKGROUND: Patients with asthma show altered surface expression of the adhesion molecules CD11b and L-selectin on airway granulocytes compared with blood granulocytes.
OBJECTIVE: To investigate whether this modulation is related to disease activity or due to transendothelial migration, we compared the CD11b and L-selectin expression on blood and induced sputum eosinophils and neutrophils between patients with asthma and normal subjects.
METHODS: Eleven normal subjects (21-43 years), nine patients (21-34 years) with mild atopic asthma and 10 patients (20-47 years) with moderate to severe atopic asthma on regular treatment with inhaled steroids underwent sputum induction by inhalation of nebulized hypertonic saline (4.5%). CD11b and L-selectin expression on granulocytes from blood and DTT-homogenized sputum were analysed by flow cytometry. Eosinophils could be discriminated from neutrophils by using depolarized light scatter. Disease activity was assessed by baseline FEV1 and airway responsiveness to histamine (PC20).
RESULTS: Sputum eosinophils showed higher expression of CD11b ($P<0.001$) and lower expression of L-selectin ($P<0.001$) compared with peripheral blood eosinophils. CD11b and L-selectin expression on eosinophils from blood or sputum did not differ between the three groups. Similar results were obtained for neutrophils. The PC20 in the patients with moderate-to-severe asthma was related to CD11b expression on blood ($R=-0.92$, $P=0.001$) and sputum eosinophils ($R=0.75$, $P=0.02$).
CONCLUSIONS: Flow cytometry of induced sputum granulocytes from asthmatic as well as normal subjects is feasible. We conclude that the modulated expression of CD11b and L-selectin on airway granulocytes is not specific for asthmatic airway inflammation, but is probably the result of tissue migration per sé. This implies

that CD11b and L-selectin expression on granulocytes in induced sputum cannot be used as marker of disease activity.

PMID: 9645598 [PubMed - indexed for MEDLINE]

1837. Am J Respir Cell Mol Biol. 1998 Jun;18(6):777-85.

Reduction of antigen-induced airway hyperreactivity and eosinophilia in ICAM-1-deficient mice.

Wolyniec WW, De Sanctis GT, Nabozny G, Torcellini C, Haynes N, Joetham A, Gelfand EW, Drazen JM, Noonan TC.

Department of Immunological Diseases, Research and Development Center, Boehringer Ingelheim Pharmaceuticals Inc., Ridgefield, Connecticut 06877, USA. wwolyniec@BI_pharm.com

A murine model of asthma is described in which we examined the role of intercellular adhesion molecule-1 (ICAM-1) in the pathogenesis of airway reactivity, pulmonary eosinophilia, and inflammation. We sensitized wild-type control [C57BL/6J, (+/+)] and ICAM-1 knockout [C57BL/6J-ICAM-1, (-/-)] mice to ovalbumin (OVA), and challenged them with OVA delivered by aerosol (OVA-OVA) to induce a phenotype consistent with an asthmatic response. Bronchial responsiveness to methacholine and counts of cell numbers and measurements of eosinophil content and cytokine levels in bronchoalveolar lavage fluid (BALF) were significantly attenuated in ICAM-1(-/-) mice as compared with (+/+) mice. We also showed that the absence of ICAM-1 had no significant affects on the production of serum IgE antibody, but did have an effect on ex vivo lymphocyte proliferation. Additionally, immunohistochemistry: (1) revealed increased staining for vascular cell adhesion molecule-1 (VCAM-1) after antigen challenge in the ICAM-1(-/-) mice but not in the ICAM-1(+/+) controls; and (2) confirmed the presence of alternatively spliced forms of ICAM-1 in the lungs of ICAM-1(-/-) mice. Thus, despite the availability of alternate adhesion pathways in ICAM-1(-/-) mice, the absence of ICAM-1 prevented eosinophils from entering the airways. In summary, we found that the ICAM-1 knockout mice exhibited a significantly inhibited response to aerosol antigen challenge for most of the parameters examined, and conclude that ICAM-1 is an important ligand mediating T-cell proliferation in response to antigen, eosinophil migration into the airways, and the development of airway hyperreactivity (AHR) in allergen-sensitized and -challenged mice.

PMID: 9618382 [PubMed - indexed for MEDLINE]

1838. J Clin Invest. 1998 Jun 15;101(12):2869-74.

Human circulating eosinophils secrete macrophage migration inhibitory factor (MIF). Potential role in asthma.

Rossi AG, Haslett C, Hirani N, Greening AP, Rahman I, Metz CN, Bucala R, Donnelly SC.

Respiratory Medicine Unit, Department of Medicine (RIE), Rayne Laboratory, University of Edinburgh Medical School, Edinburgh, United Kingdom.

Macrophage migration inhibitory factor (MIF) is a potent proinflammatory mediator that has been shown to potentiate lethal endotoxemia and to play a potentially important regulatory role in human acute respiratory distress syndrome (ARDS). We have investigated whether eosinophils are an important source of MIF and whether

MIF may be involved in the pathophysiology of asthma. Unstimulated human circulating eosinophils were found to contain preformed MIF. Stimulation of human eosinophils with phorbol myristate acetate in vitro yielded significant release of MIF protein. For example, eosinophils stimulated with phorbol myristate acetate (100 nM, 8 h, 37 degreesC) released 1,539+/-435 pg/10(6) cells of MIF, whereas unstimulated cells released barely detectable levels (< 142 pg/10(6) cells, mean+/-SEM, n = 8). This stimulated release was shown to be (a) concentration- and time-dependent, (b) partially blocked by the protein synthesis inhibitor cycloheximide, and (c) significantly inhibited by the protein kinase C inhibitor Ro-31,8220. In addition, we show that the physiological stimuli C5a and IL-5 also cause significant MIF release. Furthermore, bronchoalveolar lavage fluid obtained from asthmatic patients contains significantly elevated levels of MIF as compared to nonatopic normal volunteers (asthmatic, 797.5+/-92 pg/ml; controls, 274+/-91 pg/ml). These results highlight the potential importance of MIF in asthma and other eosinophil-dependent inflammatory disorders.

PMCID: PMC508878
PMID: 9637721 [PubMed - indexed for MEDLINE]

1839. Int J Immunopharmacol. 1997 Sep-Oct;19(9-10):517-27.

Airway hyperresponsiveness: first eosinophils and then neuropeptides.

Kraneveld AD, Folkerts G, Van Oosterhout AJ, Nijkamp FP.

Department of Pharmacology and Pathophysiology, Utrecht Institute for Pharmaceutical Sciences, Utrecht University, The Netherlands.
A.D.Kraneveld@FAR.RUU.NL

Airway hyperreactivity to bronchoconstrictor mediators is a main characteristic in the majority of asthmatic patients and correlates well with the severity of the disease. The airways of asthmatic patients are characterized by an inflammatory state resulting in activation of lung tissue cells and attraction and infiltration of leukocytes from the blood. The accumulation of eosinophilic leukocytes is a prominent feature of inflammatory reactions that occurs in allergic asthma. The increase in number of eosinophils is important since it correlates in time with an increase in bronchial hyperresponsiveness. Viral respiratory infections can also induce eosinophilia and airway hyperresponsiveness in humans and animals and can worsen asthmatic reactions. This report reviews current opinions on the relationship between inflammation-induced eosinophil accumulation/activation and the development of airway hyperresponsiveness and the possible role for sensory neuropeptides in this process. Firstly, CC chemokines play an important role in allergic airway inflammation and respiratory viral infections leading to eosinophil recruitment. Secondly, it can be concluded that IL5 is involved in the development in airway hyperresponsiveness. IL5 has profound effects on eosinophils as promoter of growth, differentiation and proliferation, chemoattractant, activator and primer. However, it is conceivable that in animal models for allergic asthma besides IL5 other regulatory mediators may be involved in eosinophil migration and activation in the lung, which in turn will lead to airway hyperresponsiveness. Recent data support the possible role of eotaxin and its eosinophil-specific receptor CCR-3 in eosinophil chemotaxis and activation in allergic asthma. Moreover, it is suggested that the development of airway eosinophilia in vivo involves a two-step mechanism, elicited by eotaxin and IL5. The precise mechanism by which eosinophils induce bronchial hyperresponsiveness is at present unknown. Sensory neuropeptides could be important mediators in this process, since it has been demonstrated that airway nerves are surrounded by and infiltrated with eosinophils after antigen challenge. Sensory neuropeptides could be the final, more downstream, common pathway after eosinophil infiltration and activation in

inducing airway hyperresponsiveness due to allergen inhalation or respiratory viral infections. In conclusion, in the process of the development of airway hyperresponsiveness observed during viral infections or in allergic asthma, the IL5/eotaxin-induced infiltration and activation of eosinophils in the airways is evident. Following this step, eosinophil-derived inflammatory mediators will induce the release of sensory neuropeptides (possibly NK2-receptor activating tachykinins) which in turn will lead to airway hyperresponsiveness.

PMID: 9637348 [PubMed - indexed for MEDLINE]

1840. J Exp Med. 1998 Jun 15;187(12):1927-40.

Patterns for RANTES secretion and intercellular adhesion molecule 1 expression mediate transepithelial T cell traffic based on analyses in vitro and in vivo.

Taguchi M, Sampath D, Koga T, Castro M, Look DC, Nakajima S, Holtzman MJ.

Department of Internal Medicine, Washington University School of Medicine, St. Louis, Missouri 63110, USA.

Immune cell migration into and through mucosal barrier sites in general and airway sites in particular is a critical feature of immune and inflammatory responses, but the determinants of transepithelial (unlike transendothelial) immune cell traffic are poorly defined. Accordingly, we used primary culture airway epithelial cells and peripheral blood mononuclear cells to develop a cell monolayer system that allows for apical-to-basal and basal-to-apical T cell transmigration that can be monitored with quantitative immunofluorescence flow cytometry. In this system, T cell adhesion and subsequent transmigration were blocked in both directions by monoclonal antibodies (mAbs) against lymphocyte function-associated antigen 1 (LFA-1) or intercellular adhesion molecule 1 (ICAM-1) (induced by interferon gamma [IFN-gamma] treatment of epithelial cells). The total number of adherent plus transmigrated T cells was also similar in both directions, and this pattern fit with uniform presentation of ICAM-1 along the apical and basolateral cell surfaces. However, the relative number of transmigrated to adherent T cells (i.e., the efficiency of transmigration) was increased in the basal-to-apical relative to the apical-to-basal direction, so an additional mechanism was needed to mediate directional movement towards the apical surface. Screening for epithelial-derived beta-chemokines indicated that IFN-gamma treatment caused selective expression of RANTES (regulated upon activation, normal T cell expressed and secreted), and the functional significance of this finding was demonstrated by inhibition of epithelial-T cell adhesion and transepithelial migration by anti-RANTES mAbs. In addition, we found that epithelial (but not endothelial) cells preferentially secreted RANTES through the apical cell surface thereby establishing a chemical gradient for chemotaxis across the epithelium to a site where they may be retained by high levels of RANTES and apical ICAM-1. These patterns for epithelial presentation of ICAM-1 and secretion of RANTES appear preserved in airway epithelial tissue studied either ex vivo with expression induced by IFN-gamma treatment or in vivo with endogenous expression induced by inflammatory disease (i.e., asthma). Taken together, the results define how the patterns for uniform presentation of ICAM-1 along the cell surface and specific apical sorting of RANTES may serve to mediate the level and directionality of T cell traffic through epithelium (distinct from endothelium) and provide a basis for how this process is precisely coordinated to route immune cells to the mucosal surface and maintain them there under normal and stimulated conditions.

PMCID: PMC2212358
PMID: 9625753 [PubMed - indexed for MEDLINE]

1841. Drug Metab Dispos. 1998 May;26(5):457-64.

Studies on the stereoselective internal acyl migration of ketoprofen glucuronides using 13C labeling and nuclear magnetic resonance spectroscopy.

Akira K, Taira T, Hasegawa H, Sakuma C, Shinohara Y.

School of Pharmacy, Tokyo University of Pharmacy and Life Science, Tokyo, Japan.

Internal acyl migration reactions of drug 1beta-O-acyl glucuronides are of interest because of their possible role in covalent binding to serum proteins and consequent allergic reactions as well as their influence on drug disposition. An approach using 13C labeling and nuclear magnetic resonance (NMR) spectroscopy has been used to investigate in situ the kinetics of acyl migration and hydrolysis of 1beta-O-acyl glucuronides of enantiomeric ketoprofens (KPs) in phosphate buffer solutions at 37 degrees C. Apparent first-order degradation of the 1beta-O-acyl glucuronide labeled in the ester carbonyl carbon and the sequential appearance of 2-, 3-, and 4-O-acyl isomers as both alpha- and beta-anomeric forms were observed for each enantiomer. All of the positional isomers and anomers were characterized using two-dimensional NMR spectroscopy (heteronuclear multiple bond correlation, correlated spectroscopy, totally correlated spectroscopy) of the reaction mixtures. The overall degradation rate constants (hr-1) of (R)- and (S)-KP glucuronides were 1.07 +/- 0.154 and 0.55 +/- 0.034, respectively. To evaluate in detail the stereoselective reactivity, a kinetic model describing the rearrangement reactions was constructed, and the kinetics were simulated using a theoretical approach. Only the acyl migration, 1beta-->2beta, was found to have significant stereoselectivity. The rate constants (hr-1) for 1beta-->2beta migration of (R)- and (S)-KP glucuronides were 1.04 +/- 0.158 and 0. 52 +/- 0.029, respectively. The results may suggest that (R)-KP glucuronide could be more susceptible to covalent binding to proteins via acyl migration than the corresponding antipode. This stereoselective reactivity may be responsible for the stereoselective pharmacokinetics of KP. The direct approach using 13C labeling and NMR spectroscopy could also provide insight into the reactivities of other labile drug acyl glucuronides and their isomeric glucuronides.

PMID: 9571227 [PubMed - indexed for MEDLINE]

1842. Chest. 1998 Jun;113(6):1673-9.

Tropical pulmonary eosinophilia.

Ong RK, Doyle RL.

Department of Respiratory and Critical Care Medicine, Singapore General Hospital.

Tropical pulmonary eosinophilia (TPE) usually affects people living in the tropics, especially those in Southeast Asia, India, and certain parts of China and Africa. However, owing to the rising frequency of world-wide travel and the migration between continents, this disease is increasingly seen in the West, where the diagnosis can be easily missed since it is rarely encountered and can mimic many other conditions. Cases of TPE have typically been reported to masquerade as acute or refractory bronchial asthma. TPE results from a hypersensitivity reaction to lymphatic filarial parasites found in endemic regions. There is evidence that it is more likely to occur in nonimmune individuals, ie, visitors to endemic regions, than in individuals of endemic populations who have developed immunity to filarial infections. Clinical features include paroxysmal cough, wheezing and dyspnea, and systemic manifestations such as fever and weight loss. A history of residence in a filarial endemic region and

a finding of peripheral eosinophilia >3,000/mm3 should initiate a consideration of this disease. Other criteria for the diagnosis of TPE include absence of microfilariae in the blood, high titers of antifilarial antibodies, raised serum total IgE >1,000 U/mL, and a favorable response to the antifilarial, diethylcarbamazine, which is the recommended treatment. This disease, if left untreated or treated late, may lead to long-term sequelae of pulmonary fibrosis or chronic bronchitis with chronic respiratory failure. Herein lies the importance of early diagnosis and treatment of TPE.

PMID: 9631810 [PubMed - indexed for MEDLINE]

1843. Klin Padiatr. 1998 May-Jun;210(3):139-43.

[Toxocariasis--a neglected ubiquitous helminthiasis in children and adolescents].

[Article in German]

Hohenschild S.

More than 40 years ago, Toxocara ssp. was identified as the cause of larva migrans visceralis and ocular larva migrans, which mainly affect infants and children. Although widespread in most parts of the world, the parasitic disease is rarely diagnosed in Germany. Focusing on clinical and pathophysiological similarities with allergic asthma of childhood, the main epidemiological, clinical, therapeutical, and pathophysiological aspects of toxocariasis will be reviewed in the following survey.

PMID: 9629550 [PubMed - indexed for MEDLINE]

1844. Klin Padiatr. 1998 May-Jun;210(3):128-31.

[Toxocariasis in a 5-year-old boy--manifesting as bronchial asthma and behavioral disorder].

[Article in German]

Varga EM, Auer H, Zach M.

Klinische Abteilung für Pulmonologie, Univ.-Klinik für Kinder- und Jugendheilkunde Graz.

We report on a five year old boy who was admitted to hospital because of obstructive airway disease; initially, findings were interpreted to indicate bronchial asthma. In addition, the patient presented with a behavioural abnormality of aggressiveness and hyperactivity. Laboratory examinations showed an elevated IgE level and eosinophilia, chest x-ray revealed infiltrations in both lungs. After excluding a spectrum of chronic lung disorders by relevant investigations, serological testing for parasitosis revealed massively elevated toxocara IgG antibodies. The diagnosis of a "covert form" of toxocarosis was established and an antihelminthic therapy with albendazole was initiated. Chronic respiratory symptoms in childhood can also indicate the presence of a parasitic infestation.

PMID: 9629547 [PubMed - indexed for MEDLINE]

1845. Dtsch Med Wochenschr. 1998 May 15;123(20):626-30.

[Ocular toxocariasis in Austria].

[Article in German]

Dietrich A, Auer H, Tittl M, Barisani-Asenbauer T.

Universitätsklinik für Augenheilkunde und Optometrie, Universität Wien.

HISTORY AND CLINICAL FINDINGS: Two women (aged 21 and 44 years) were referred because of a suspect retinal lesion. An ophthalmological examination in both revealed prominent retinal granulomatous foci, probably ocular toxocariasis. Both women were otherwise well; both reported close contact with dogs.
INVESTIGATIONS: Among a full array of laboratory tests the only major pathologic findings were high antibody titres against Toxocara canis (patient 1: 70 antibody units [AU]; patient 2: > 100 AU), specific antibodies in the ELISA and Western blot tests confirming the diagnosis of T. canis infection.
DIAGNOSIS, TREATMENT AND COURSE: Both patients were treated with prednisolone (initially 75 mg/d, gradually decreasing over 4 months) and albendazole (2 x 800 mg/d for 6 days), with complete healing of the chorioretinal foci.
CONCLUSION: General physicians as well as ophthalmologists should more often include Toxocara canis infection in the differential diagnosis, because the larvae, in their migration through the body, can infest various organs where they can cause inflammatory or allergic reactions.

PMID: 9627571 [PubMed - indexed for MEDLINE]

1846. Am J Pathol. 1998 Jun;152(6):1463-76.

Endothelial gaps and adherent leukocytes in allergen-induced early- and late-phase plasma leakage in rat airways.

Baluk P, Bolton P, Hirata A, Thurston G, McDonald DM.

Cardiovascular Research Institute, University of California, San Francisco 94143-0130, USA. pbaluk@itsa.ucsf.edu

Exposure of sensitized individuals to antigen can induce allergic responses in the respiratory tract, manifested by early and late phases of vasodilatation, plasma leakage, leukocyte influx, and bronchoconstriction. Similar responses can occur in the skin, eye, and gastrointestinal tract. The early-phase response involves mast cell mediators and the late-phase response is leukocyte dependent, but the mechanism of leakage is not understood. We sought to identify the leaky blood vessels, to determine whether these vessels contained endothelial gaps, and to analyze the relationship of the gaps to adherent leukocytes, using biotinylated lectins or silver nitrate to stain the cells in situ and Monastral blue as a tracer to quantify plasma leakage. Most of the leakage occurred in postcapillary venules (< 40-microns diameter), whereas most of the leukocyte migration (predominantly neutrophils) occurred in collecting venules. Capillaries and arterioles did not leak. Endothelial gaps were found in the leaky venules, both by silver nitrate staining and by scanning electron microscopy, and 94% of the gaps were distinct from sites of leukocyte adhesion or migration. We conclude that endothelial gaps contribute to both early and late phases of plasma leakage induced by antigen, but most leakage occurs upstream to sites of leukocyte adhesion.

PMCID: PMC1858452
PMID: 9626051 [PubMed - indexed for MEDLINE]

1847. Ned Tijdschr Geneeskd. 1998 Mar 7;142(10):532-5.

[Kimura's disease].

[Article in Dutch]

Wagenaar M, Marres HA, Verhofstad A, de Wilde P.

Academisch Ziekenhuis, Nijmegen.

Comment in
    Ned Tijdschr Geneeskd. 1998 Apr 25;142(17):985.

An Asian man aged 42 had a pre-auricular swelling on the left with local skin itching. Owing to the highly probably benign nature at examination, surgical treatment was refrained from. One year later, the swelling had increased while the itching was unchanged, so that surgical excision was performed. Morbid-anatomical examination revealed angiolymphoid hyperplasia with eosinophilia, compatible with Kimura's disease. Kimura's disease is a chronic inflammatory disorder which is very rare in Europe, but which is frequently diagnosed in Asian countries and in immigrants from these countries. Tumours in the head and neck region, enlarged lymph nodes and increased eosinophil counts are typical signs. The IgE level is increased. The cause of this disease is so far not clear, although there are indications that an inflammatory reaction to an unknown allergen plays a part. It is essential that this disease should be distinguished in the diagnostic work-up from presence of a malignancy. Fine-needle aspiration cytology is often inconclusive; as a rule the diagnosis can only be made after surgical excision of the tumour. There is no consensus on the treatment of this disease, but symptomatic therapy usually suffices.

PMID: 9623102 [PubMed - indexed for MEDLINE]

1848. J Exp Med. 1998 Jun 1;187(11):1779-88.

Impaired anaphylactic responses with intact sensitivity to endotoxin in mice lacking a platelet-activating factor receptor.

Ishii S, Kuwaki T, Nagase T, Maki K, Tashiro F, Sunaga S, Cao WH, Kume K, Fukuchi Y, Ikuta K, Miyazaki J, Kumada M, Shimizu T.

Department of Biochemistry and Molecular Biology, Faculty of Medicine, The University of Tokyo, Tokyo 113, Japan.

Platelet-activating factor (PAF) is a potent phospholipid mediator with diverse biological activities in addition to its well-known ability to stimulate platelet aggregation. Pharmacologic studies had suggested a role for PAF in pregnancy, neuronal cell migration, anaphylaxis, and endotoxic shock. Here we show that disruption of the PAF receptor gene in mice caused a marked reduction in systemic anaphylactic symptoms. Unexpectedly, however, the PAF receptor-deficient mice developed normally, were fertile, and remained sensitive to bacterial endotoxin. These mutant mice clearly show that PAF plays a dominant role in eliciting anaphylaxis, but that it is not essential for reproduction, brain development, or endotoxic shock.

PMCID: PMC2212308
PMID: 9607919 [PubMed - indexed for MEDLINE]

1849. Lung. 1998;176(4):237-47.

Airway eosinophilic inflammation and bronchial hyperresponsiveness after allergen inhalation challenge in asthma.

Oddera S, Silvestri M, Penna R, Galeazzi G, Crimi E, Rossi GA.

Divisione di Pneumologia, Istituto G. Gaslini, Genova, Italy.

Allergen exposure in atopic asthmatic patients is associated with recruitment and activation of eosinophils in the airways. Once activated, eosinophils release toxic products, including the eosinophil cationic protein (ECP), able to damage bronchial structures and to increase bronchial hyperresponsiveness. With this background, the present study was designed to evaluate whether ECP levels in bronchoalveolar lavage (BAL) fluid could reflect, better than BAL eosinophil counts, the cellular activation that follows allergen exposure in atopic asthmatics. Twenty-two atopic patients attended the laboratory on two separate days. On the 1st day, they underwent methacholine (MCh) inhalation challenge to detect the degree of nonspecific bronchial hyperresponsiveness. On the 2nd day, they underwent fiberoptic bronchoscopy and BAL, at baseline or 4-6 h after allergen inhalation challenge. In this latter patient group, MCh challenge was repeated 3-5 h after allergen challenge, 1 h before fiberoptic bronchoscopy. The analysis of the mean baseline FEV1 values and the degree of bronchial reactivity to MCh (MCh Pd20) on the 1st study day did not demonstrate differences between the two patient groups ($p > 0.1$, each comparison). In addition, in the allergen-challenged group, MCh Pd20 was decreased significantly after allergen challenge (151.4 micrograms/ml and 67.6 micrograms/ml, respectively, before and after challenge; $p < 0.05$). Evaluation of the different BAL cell types demonstrated that the proportions of eosinophils and epithelial cells were increased significantly in the allergen-challenged group compared with the group evaluated at baseline ($p < 0.01$ and $p < 0.05$, respectively). Moreover, ECP levels, corrected by the correspondent albumin levels (ECP/Alb), were higher in the allergen-challenged group compared with the group evaluated at baseline ($p < 0.05$). In addition, although a positive correlation was demonstrated between BAL eosinophil percentages and ECP/Alb values ($r = 0.72$, $p < 0.05$) in the group evaluated at baseline, no links were found between these parameters in the allergen-challenged group ($p > 0.1$). However, in this latter group, a weak positive correlation was demonstrated between eosinophil percentages and delta Mch, i.e., the increased non-specific bronchial reactivity, which is observed after allergen challenge ($r = 0.55$; $p < 0.05$). Thus, in stable asthmatic patients an ongoing activation of eosinophils parallels their migration, but this eosinophilic inflammation is not strictly related to bronchial reactivity to Mch. By contrast, after allergen inhalation challenge, eosinophil recruitment and activation seem to follow different temporal kinetics, and eosinophilic inflammation may be partially associated with the degree of airway hyperresponsiveness.

PMID: 9617740 [PubMed - indexed for MEDLINE]

1850. Annu Rev Immunol. 1998;16:645-70.

Positive versus negative signaling by lymphocyte antigen receptors.

Healy JI, Goodnow CC.

Department of Microbiology and Immunology, Stanford University School of Medicine, California 94305, USA. Healy@cmgm.stanford.edu

Antigen receptors on lymphocytes play a central role in immune regulation by transmitting signals that positively or negatively regulate lymphocyte survival,

migration, growth, and differentiation. This review focuses on how opposing positive or negative cellular responses are brought about by antigen receptor signaling. Four types of extracellular inputs shape the response to antigen: (a) the concentration of antigen; (b) the avidity with which antigen is bound; (c) the timing and duration of antigen encounter; and (d) the association of antigen with costimuli from pathogens, the innate immune system, or other lymphocytes. Intracellular signaling by antigen receptors is not an all-or-none event, and these external variables alter both the quantity and quality of signaling. Recent findings in B lymphocytes have clearly illustrated that these external inputs affect the magnitude and duration of the intracellular calcium response, which in turn contributes to differential triggering of the transcriptional regulators NF kappa B, JNK, NFAT, and ERK. The regulation of calcium responses involves a network of tyrosine kinases (e.g. lyn, syk), tyrosine or lipid phosphatases (CD45, SHP-1, SHIP), and accessory molecules (CD21/CD19, CD22, FcR gamma 2b). Understanding the biochemistry and logic behind these integrative processes will allow development of more selective and efficient pharmaceuticals that suppress, modify, or augment immune responses in autoimmunity, transplantation, allergy, vaccines, and cancer.

PMID: 9597145 [PubMed - indexed for MEDLINE]

1851. Allergy. 1998 Mar;53(3):249-54.

Prevalence of allergy in children in relation to prior BCG vaccination and infection with atypical mycobacteria.

Strannegård IL, Larsson LO, Wennergren G, Strannegård O.

Department of Pediatrics, University of Göteborg, Sweden.

Comment in
    Allergy. 1999 Mar;54(3):285-6.

By influence on the Th1/Th2 cell balance, infectious agents may affect the development of atopic allergy. In this study, we investigated whether previous BCG vaccination or infection with atypical mycobacteria might be related to the development of atopic disease. The study, which involved skin testing with mycobacteria and answers to a questionnaire for more than 6000 children in Sweden, revealed a low prevalence of allergy among BCG-vaccinated children who were immigrants or adopted from other countries. Vaccinated children born in Sweden, however, did not have significantly lower allergy prevalence than age-matched, unvaccinated children. Furthermore, the overall frequencies of skin-test reactivity to the atypical mycobacteria M. avium and M. scrofulaceum were higher rather than lower in allergic than in nonallergic children. By contrast, there was a tendency toward a lower frequency of more strongly positive skin reactions (> or = 10 mm) to mycobacteria in allergic than in nonallergic children. These findings do not support the hypothesis that early mycobacterial infections have a suppressive effect on the development of atopic disease. Earlier findings of an apparent association between atopy and lack of previous mycobacterial infection may possibly be explained by a relatively decreased ability of atopic patients to mount strong Th1 cell-mediated immune responses.

PMID: 9542604 [PubMed - indexed for MEDLINE]

1852. Acta Otolaryngol. 1998 Mar;118(2):235-9.

Capsaicin desensitization of the nasal mucosa reduces symptoms upon allergen challenge in patients with allergic rhinitis.

Stjärne P, Rinder J, Hedén-Blomquist E, Cardell LO, Lundberg J, Zetterström O, Anggård A.

Department of Oto-Rhino-Laryngology, Karolinska Institute, Stockholm, Sweden.

Patients with birch pollen allergic rhinitis were treated locally, out of season, in the nasal cavity with capsaicin (30 microM) or saline. The capsaicin treatment resulted in a statistically significant reduction of symptoms upon allergen challenge, which lasted for 2 months. Saline had no effect on the symptom score upon allergen challenge. Neither capsaicin nor saline treatment had any effect on allergen challenge-induced nasal mucosal swelling monitored by acoustic rhinometry. Allergen challenge-induced eosinophil migration to the nasal mucosa was affected by neither capsaicin nor the saline treatment. The finding that capsaicin treatment reduces allergic symptoms indicates that selective, non-peptide neurokinin receptor antagonists may be an alternative in the future in the treatment of nasal allergy. However, owing to the pain involved in local capsaicin treatment this treatment is unlikely to be of clinical use.

PMID: 9583792 [PubMed - indexed for MEDLINE]

1853. Cytokine. 1998 Mar;10(3):213-9.

Production and in situ localization of cutaneous tumour necrosis factor alpha (TNF-alpha) and interleukin 6 (IL-6) following skin sensitization.

Flint MS, Dearman RJ, Kimber I, Hotchkiss SA.

Department of Molecular Toxicology, Imperial College School of Medicine at St. Mary's, London, UK.

The induction of contact sensitization and other cutaneous immune responses is dependent upon the activity of epidermal cytokines. One such, keratinocyte-derived tumour necrosis factor alpha (TNF-alpha), is thought to provide the stimulus for the migration of Langerhans cells from the epidermis and their accumulation as immunocompetent dendritic cells in draining lymph nodes. In these investigations we have examined the stimulation by allergen of cutaneous TNF-alpha production and the induced epidermal expression of mRNA for TNF-alpha. Topical exposure of mice to oxazolone, a skin-sensitizing chemical, resulted in cutaneous TNF-alpha protein production that was maximal 2-h following treatment and then declined markedly. The same treatment resulted in highly localized and transient expression of epidermal TNF-alpha mRNA as judged by in situ hybridization. Epidermal mRNA for TNF-alpha was apparent 10 min following exposure to oxazolone, but was no longer detectable at 20 min. A similar pattern of TNF-alpha mRNA expression in the epidermis was provoked by intradermal exposure to interleukin 1 beta, a cytokine shown previously to induce TNF-alpha. Such rigorous regulation of temporal and spatial expression was shown not to be a characteristic of all epidermal cytokines induced by chemical allergen. Exposure to oxazolone under the same conditions resulted in a more widespread and more persistent expression of epidermal mRNA for interleukin 6. These data demonstrate that during skin sensitization the induced expression of epidermal TNF-alpha is finely controlled in space and time. It is proposed that such regulation facilitates the initiation of cutaneous immune responses while preventing excessive inflammation that would result from more persistent TNF-alpha production.

PMID: 9576067 [PubMed - indexed for MEDLINE]

1854. Eur J Pharmacol. 1998 Feb 19;343(2-3):233-7.

Inhibitory effect of F-1322 on allergic eosinophil infiltration in airways.

Mochizuki A, Lee B, Shibata M, Kasai M, Tanaka T, Iwamoto I.

Pharmaceuticals Research Laboratories, Fujirebio Inc., Hachioji, Tokyo, Japan.

The effects of F-1322
(N-[2-[4-(benzhydryloxy)piperidino]ethyl]-3-hydroxy-5-(3-pyridy
lmethoxy)-2-naphthamide) on antigen-induced eosinophil infiltration and interleukin-5 production in the airways and on in vitro eosinophil migration were investigated. F-1322 (10-30 mg/kg, p.o.) inhibited antigen-induced eosinophil infiltration and interleukin-5 production in the airways of sensitized mice in a dose-dependent manner. Furthermore, F-1322 (0.1-10 microM) prevented the in vitro migration of eosinophils from guinea-pigs and humans induced by recombinant human interleukin-5, platelet-activating factor, and leukotriene B4 in a concentration-dependent manner. These results indicate that F-1322 has an inhibitory effect on allergic eosinophil infiltration of the airways by preventing both eosinophil migration and interleukin-5 production. These pharmacological profiles suggest that F-1322 will be a useful therapeutic for allergic diseases, especially asthma.

PMID: 9570472 [PubMed - indexed for MEDLINE]

1855. J Allergy Clin Immunol. 1998 Apr;101(4 Pt 2):S434-9.

Development of fluticasone propionate and comparison with other inhaled corticosteroids.

Johnson M.

International Medical Affairs, Respiratory, Glaxo Wellcome Research and Development, Uxbridge, Middlesex, United Kingdom.

Fluticasone propionate (FP) is a trifluorinated glucocorticoid based on the androstane nucleus. It was selected for development from structure-activity relationships (topical anti-inflammatory, cutaneous vasoconstriction, and hypothalamic-pituitary-adrenal axis suppression) of a series of 17beta-carbothioates. FP is 3-, 300-, and 1000-fold more lipophilic than beclomethasone dipropionate, budesonide, and triamcinolone acetonide, respectively. FP has an absolute affinity (KD) for the glucocorticoid receptor of 0.5 nmol/L and a relative receptor affinity 1.5-fold higher than beclomethasone-17-monopropionate (17-BMP) and mometasone furoate, 3-fold higher than budesonide, and 20-fold higher than flunisolide and triamcinolone acetonide. The rate of association of FP with the receptor is faster and the rate of dissociation slower than other corticosteroids. The resulting half-life of the FP active steroid-receptor complex is >10 hours, compared with approximately 5, 7.5, and 4 hours for budesonide, 17-BMP, and triamcinolone acetonide, respectively. FP has high selectivity for the glucocorticoid receptor, with little or no activity at other steroid receptors. FP is more potent than beclomethasone dipropionate, budesonide, triamcinolone acetonide, and mometasone furoate in inhibiting human T-cell migration and proliferation, inhibiting CD4+ T-cell cytokine and basophil histamine release, attenuating adhesion molecule expression, stimulating inflammatory cell apoptosis, and inducing cellular antiprotease release. In asthma patients, FP decreases the number of CD3+, CD4+, CD8+, and CD25+ T cells, mast cells, and eosinophils in bronchial biopsies, in addition to suppressing CD1a-dendritic and IgE+ cells and HLA-DR. FP, therefore, has a good pharmacologic profile for a topical steroid with increased intrinsic glucocorticoid potency and

potent anti-inflammatory activity.

PMID: 9563368 [PubMed - indexed for MEDLINE]

1856. Inflammation. 1998 Apr;22(2):229-42.

Expression of adhesion molecules on granulocytes and monocytes from patients with asthma stimulated in vitro with interleukin-8 and monocyte chemotactic protein-1.

Verdegaal EM, Zegveld ST, Blokland I, Beekhuizen H, Bakker W, Willems LN, van Furth R.

Department of Infectious Diseases, University Hospital Leiden, The Netherlands. VERDEGAAL@RULLF2.LeidenUniv.NL

Upregulation of adhesion molecule expression on endothelial cells (EC) and circulating leukocytes, by locally produced inflammatory mediators, may result in the enhanced infiltration of leukocytes into tissue, e.g. the airways of asthma patients. The present study investigates whether the expression of adhesion molecules on granulocytes and monocytes from asthma patients is affected by chemotactic factors, i.e. interleukin-8 (IL-8) and monocyte chemotactic protein-1 (MCP-1). Flow cytometric analysis showed that the intrinsic expression of the various adhesion molecules on peripheral blood phagocytes from asthma patients was not different from that of healthy individuals. However, stimulation of monocytes with MCP-1 resulted only in upregulation of the expression of CD14 on monocytes from symptomatic asthma patients but not on monocytes from asymptomatic asthma patients and healthy individuals. Stimulation of granulocytes with IL-8 did not change the expression of the various beta 1- and beta 2-integrin molecules, such as VLA-4, LFA-1, CR3 and p150,95. Since earlier studies have shown that CD14 on monocytes mediates monocyte adhesion to activated vascular EC the present findings suggest that during the active phase of asthma upregulation of CD14 on monocytes by MCP-1 may lead to an increased adhesion of monocytes to vascular endothelium and their subsequent transendothelial migration into the tissue of the airways.

PMID: 9561931 [PubMed - indexed for MEDLINE]

1857. J Immunol. 1998 Jan 15;160(2):624-33.

Blockade of chemokine activity by a soluble chemokine binding protein from vaccinia virus.

Alcamí A, Symons JA, Collins PD, Williams TJ, Smith GL.

Sir William Dunn School of Pathology, University of Oxford, United Kingdom.

Chemokines direct migration of immune cells into sites of inflammation and infection. Chemokine receptors are seven-transmembrane domain proteins that, in contrast to other cytokine receptors, cannot be easily engineered as soluble chemokine inhibitors. Poxviruses encode several soluble cytokine receptors to evade immune surveillance, providing new strategies for immune modulation. Here we show that vaccinia virus and other orthopoxviruses (cowpox and camelpox) express a secreted 35-kDa chemokine binding protein (vCKBP) with no sequence similarity to known cellular chemokine receptors. The vCKBP binds CC, but not CXC or C, chemokines with high affinity (Kd = 0.1-15 nM for different CC chemokines), blocks the interaction of chemokines with cellular receptors, and inhibits chemokine-induced elevation of intracellular calcium levels and cell migration in vitro, thus representing a soluble inhibitor that binds and sequesters

chemokines. The potential of vCKBP as a therapeutic agent in vivo was illustrated in a guinea pig skin model by the blockade of eotaxin-induced eosinophil infiltration. a feature of allergic inflammatory reactions. Furthermore, vCKBP may enable the rational design of antagonists to neutralize pathogens that use chemokine receptors to initiate infection, such as HIV or the malarial parasite.

PMID: 9551896 [PubMed - indexed for MEDLINE]

1858. Eur Respir J. 1998 Feb;11(2):299-305.

Interleukin-8 plays a significant role in IgE-mediated lung inflammation.

Erger RA, Casale TB.

VA Medical Center and Department of Internal Medicine, University of Iowa College of Medicine, Iowa City, USA.

Interleukin (IL)-8 is a potentially important cytokine in allergic respiratory responses since it is released by many resident lung cells, and it is a potent granulocyte chemoattractant. Therefore, we induced an immunoglobulin (Ig)E-mediated response in human lung samples and studied whether IL-8 was produced in sufficient quantities to promote human neutrophil and eosinophil migration across naked filters and endothelial and pulmonary epithelial monolayers cultured on these filters. Fresh human lung fragments from 16 thoracotomy specimens were treated with either a 1:100 dilution of anti-IgE or buffer (control) for 30 min. All anti-IgE treated lung samples had significant release of histamine and neutrophil and eosinophil chemotactic activity. Fourteen of the 16 lung samples had a significant increase in IL-8 subsequent to anti-IgE treatment (p<0.01). Anti-IL-8 antibody (4 microg x mL[-1]) inhibited 42% and 53% of neutrophil and eosinophil chemotactic activity respectively, contained in supernatants from anti-IgE-treated lung samples. Finally, we found that IL-8 at a concentration near that measured after anti-IgE treatment of lung samples (2,000 pg x mL[-1]) induced neutrophil and eosinophil migration through naked filters and endothelial and pulmonary epithelial cell monolayers. Thus, human lung IgE-mediated responses in vitro results in the rapid release of interleukin-8 in amounts sufficient to affect a biological response, granulocyte transcellular migration, indicating that interleukin-8 may play a significant role in allergic respiratory diseases.

PMID: 9551728 [PubMed - indexed for MEDLINE]

1859. J Immunol. 1997 Dec 15;159(12):6148-55.

Depressed Langerhans cell migration and reduced contact hypersensitivity response in mice lacking TNF receptor p75.

Wang B, Fujisawa H, Zhuang L, Kondo S, Shivji GM, Kim CS, Mak TW, Sauder DN.

Division of Dermatology, Sunnybrook Health Science Centre, University of Toronto, ON, Canada.

Epidermal Langerhans cells (LC) belong to the dendritic cell family and represent the major APC within the skin. LC capture epicutaneous Ag, migrate into regional lymph nodes, and present Ag to T cells, thereby initiating primary immune response. The migratory properties of LC are an essential component of their function. The molecular mechanisms responsible for LC migration are far less defined. However, evidence has been accumulating to suggest that TNF-alpha, a major proinflammatory cytokine, plays an important role in promoting DC

migration. To confirm the role of TNF-alpha in LC migration and to examine which type of TNF receptor signaling is involved in such an event, we utilized gene-targeted knockout mice lacking TNF receptor p55 or p75. The migration of LC was assessed by examining the frequency of hapten-bearing cells in draining lymph nodes following hapten FITC painting, and the accumulation of dendritic cells in draining lymph nodes after intradermal injection of TNF-alpha. While LC migration was normal in p55-deficient mice, the migration was markedly depressed in p75-deficient mice. Receptor p75-deficient mice also demonstrated a hyporesponsiveness in allergen-induced contact dermatitis, but a normal responsiveness in irritant-induced contact dermatitis. These results suggest that p75-dependent signaling plays a crucial role in the migration of LC and in the initiation of cutaneous immune responses.

PMID: 9550416 [PubMed - indexed for MEDLINE]

1860. J Invest Dermatol. 1998 Apr;110(4):441-8.

Entry into afferent lymphatics and maturation in situ of migrating murine cutaneous dendritic cells.

Weinlich G, Heine M, Stössel H, Zanella M, Stoitzner P, Ortner U, Smolle J, Koch F, Sepp NT, Schuler G, Romani N.

Department of Dermatology, University of Innsbruck, Austria.

An important property of dendritic cells (DC), which contributes crucially to their strong immunogenic function, is their capacity to migrate from sites of antigen capture to the draining lymphoid organs. Here we studied in detail the migratory pathway and the differentiation of DC during migration in a skin organ culture model and, for comparison, in the conventional contact hypersensitivity system. We report several observations on the capacity of cutaneous DC to migrate in mouse ear skin. (i) Upon application of contact allergens in vivo the density of Langerhans cells in epidermal sheets decreased, as determined by immunostaining for major histocompatibility complex class II, ADPase, F4/80, CD11b, CD32, NLDC-145/DEC-205, and the cytoskeleton protein vimentin. Evaluation was performed by computer assisted morphometry. (ii) Chemically related nonsensitizing or tolerizing compounds left the density of Langerhans cells unchanged. (iii) Immunohistochemical double-staining of dermal sheets from skin organ cultures for major histocompatibility complex class II and CD54 excluded blood vessels as a cutaneous pathway of DC migration. (iv) Electron microscopy of organ cultures revealed dermal accumulations of DC (including Birbeck granule containing Langerhans cells) within typical lymphatic vessels. (v) Populations of migrating DC in organ cultures upregulated markers of maturity (the antigen recognized by monoclonal antibody 2A1, CD86), but retained indicators of immaturity (invariant chain, residual antigen processing function). These data provide additional evidence that during both the induction of contact hypersensitivity and in skin organ culture, Langerhans cells physically leave the epidermis. Both Langerhans cells and dermal DC enter lymphatic vessels. DC mature while they migrate through the skin.

PMID: 9540989 [PubMed - indexed for MEDLINE]

1861. Soc Sci Med. 1998 Mar;46(6):729-41.

The problem of atopic eczema: aetiological clues from the environment and lifestyles.

McNally NJ, Phillips DR, Williams HC.

Department of Geography, University of Nottingham, UK.

Atopic eczema is the most common inflammatory skin disease in children, affecting around 10% of children in the developed world. It can be a distressing condition, influencing children's well-being, personal and educational development, and family life, and it has huge economic implications for health services and individual budgets. Like other atopic diseases such as asthma and hay fever, the prevalence of atopic eczema has increased substantially over the last 30 years, for reasons largely unknown. Although a genetic predisposition to the disease has been implicated, evidence from a range of sources suggests that environmental factors play a crucial role in the disease expression. This paper reviews the epidemiology of atopic eczema, with particular attention to potential environmental aetiological factors and draws evidence from studies in the UK and internationally. First, atopic eczema has been found to vary socially and to be more prevalent in the UK among social class I and II families than among other socio-economic groups. Second, it has been suggested that cross infection from other siblings in large families may have a protective role in atopic disease expression. Third, it has been proposed that an increased risk of atopic eczema may result from decreases in helminthic infestation. Fourth, studies of migrant groups have shown large increases in disease prevalence compared with migrants' country of origin, suggesting clues as to the importance of socio-economic and environmental changes such as those associated with industrialization. Finally, a distinct and consistent geographical pattern of eczema has been observed in the UK which cannot be explained by social class distribution. The various types of study have attempted to identify reasons for differences in prevalence but, to date, no definitive causation has been identified. In some cases, specific risk factors have been suggested and include house dust mites, dietary allergens and irritants. It is argued here that the aetiology is unlikely to be simple or uni-causal and that an understanding of the relationships between the disease and behaviour, lifestyle, home and external environmental factors is crucial. This paper reports the preliminary stages of an interdisciplinary research project involving dermatologists, epidemiologists and health geographers, and calls for investigation into associations between atopic eczema and possible environmental and lifestyle factors. These include behavioural factors, microenvironment factors and macroenvironments.

PMID: 9522432 [PubMed - indexed for MEDLINE]

1862. Am J Respir Crit Care Med. 1998 Mar;157(3 Pt 1):899-906.

Intranasal beclomethasone reduces allergen-induced symptoms and superficial mucosal eosinophilia without affecting submucosal inflammation.

Baroody FM, Rouadi P, Driscoll PV, Bochner BS, Naclerio RM.

Section of Otolaryngology/Head and Neck Surgery, The University of Chicago, Illinois 60637, USA. fbaroody@surgery.bsd.uchicago.edu.

Previous investigations have suggested that nasal secretions, obtained by lavage or scraping, and the nasal submucosa, sampled by biopsy, are two distinct compartments. We investigated the effect of intranasal corticosteroids on antigen-induced eosinophil influx into both compartments. We performed a double-blind, placebo-controlled study in 15 patients with seasonal allergic rhinitis. Beclomethasone dipropionate, 84 microg twice a day, was delivered to one nostril while the other nostril received placebo for 1 wk. Subjects were then challenged with grass or ragweed extracts on each inferior turbinate. Nasal scrapings from both inferior turbinates were obtained before and 24 h after challenge, and bilateral inferior turbinate biopsies were obtained 24 h after

challenge, with the subjects still receiving treatment. Intranasal steroids led to a significant reduction in sneezes and eosinophil influx in nasal secretions without affecting the number of eosinophils in the submucosa. Furthermore, intranasal steroids had no effect on the numbers of submucosal EG2+ (activated eosinophils) or CD25+ (IL-2-receptor-bearing) cells, nor did they decrease the endothelial expression of vascular cell adhesion molecule-1 (VCAM-1). These data show that pretreatment with intranasal steroids successfully inhibited the clinical response to allergen and reduced eosinophils in the superficial compartment of the nasal mucosa, but it had no effect on inflammation in the deeper compartment. This might be related to a different distribution of the active medication and antigen into the nasal mucosa or to a specific effect of the active medication on the epithelium resulting in inhibited migration of eosinophils across this layer.

PMID: 9517609 [PubMed - indexed for MEDLINE]

1863. Am J Respir Crit Care Med. 1998 Mar;157(3 Pt 1):873-9.

RANTES induces nasal mucosal inflammation rich in eosinophils, basophils, and lymphocytes in vivo.

Kuna P, Alam R, Ruta U, Gorski P.

Department of Occupational Medicine, Institute of Occupational Medicine, Lodz, Poland.

RANTES is a CC chemokine that causes chemotaxis of eosinophils, basophils, and lymphocytes in vitro. The objective of this study was to investigate the effect of RANTES on the influx of inflammatory cells into the nasal mucosa of 12 allergic patients. In the first phase, each patient was challenged with RANTES or diluent on two subsequent days. RANTES caused a significant ($p < 0.05$) influx of eosinophils as compared with the diluent. The number of eosinophils were 5,548 +/- 1,532/ml and 462 +/- 206/ml after RANTES and diluent challenge, respectively, at the peak of the response at 2 h. There was also a significant influx of metachromatic cells and lymphocytes, but not monocytes, neutrophils, or epithelial cells after RANTES challenge. In the second phase, the patients were first challenged with an allergen and 24 h later, challenged with RANTES or diluent. In the allergen-primed mucosa RANTES induced a significantly higher influx of eosinophils, basophils, and lymphocytes. Further, RANTES caused migration of monocytes and neutrophils, and shedding of epithelial cells. The influx of the inflammatory cells was associated with symptoms of rhinitis. We conclude that RANTES induces a clinically symptomatic inflammatory response in vivo by causing chemotaxis of eosinophils, basophils, and mononuclear cells.

PMID: 9517605 [PubMed - indexed for MEDLINE]

1864. Am J Rhinol. 1998 Jan-Feb;12(1):3-8.

Cytokines and adhesion molecules in allergic rhinitis.

Bachert C, Wagenmann M, Holtappels G.

ENT Department, University Hospital Düsseldorf, Germany.

This review summarizes our current knowledge of nasal allergic inflammation based on studies of cytokines, chemokines, and adhesion molecules in allergic rhinitis. The article also includes some aspects of viral rhinitis. Due to artificial or natural allergen exposure, an increase in the number of eosinophils and

basophils, mast cells, IgE-positive cells, macrophages, monocyte-like cells, Langerhans cells, and activated T-cells can be observed within the mucosa and on the mucosal surface. Mediators are known to be released in response to allergens, but do not seem to be adequate to initiate the cell recruitment. After antigen challenge, the release of proinflammatory and regulatory cytokines could be demonstrated, and TH2-type cytokine mRNA upregulation in allergic mucosa has been shown. Proinflammatory cytokines initiate an adhesion cascade and activate T-cells that create an "atopic" cytokine environment within the tissue, which also may be linked to the long-term selective recruitment of eosinophils. However, the acute selective migration of eosinophils after allergen challenge is not fully understood, nor is the role of chemokines in allergic and viral rhinitis. Allergic rhinitis clearly represents an inflammatory reaction.

PMID: 9513652 [PubMed - indexed for MEDLINE]

1865. Brain Behav Immun. 1997 Dec;11(4):245-63.

Inflammatory events at the blood brain barrier: regulation of adhesion molecules, cytokines, and chemokines by reactive nitrogen and oxygen species.

Merrill JE, Murphy SP.

Department of Immunology, Berlex Biosciences, Richmond, California 94804, USA. jean_merrill@berlex.com

Recruitment of inflammatory cells into the CNS during pathological processes associated with neurodegeneration, trauma, autoimmune disease, and infection involves the generation of signaling molecules that are both cell-associated and soluble. Alteration in the permeability of the blood brain barrier, adhesion of blood-borne leukocytes to cerebral vessels, activation of chemoattractants and their receptors, and migration of inflammatory cells into the CNS are events that have been proposed to be regulated by cytokines and reactive oxygen and nitrogen species. In this review we propose associative connections between these events and the molecules involved as they may relate to CNS inflammation, placing illustrative emphasis on multiple sclerosis and the animal model for MS, experimental allergic encephalomyelitis.

PMID: 9512813 [PubMed - indexed for MEDLINE]

1866. J Immunol. 1998 Mar 15;160(6):2998-3005.

Involvement of IL-16 in the induction of airway hyper-responsiveness and up-regulation of IgE in a murine model of allergic asthma.

Hessel EM, Cruikshank WW, Van Ark I, De Bie JJ, Van Esch B, Hofman G, Nijkamp FP, Center DM, Van Oosterhout AJ.

Department of Pharmacology and Pathophysiology, Utrecht Institute for Pharmaceutical Sciences, Utrecht University, The Netherlands. E.M.Hessel@FAR.RUU.NL

Experiments were designed to investigate the role of IL-16 in a mouse model of allergic asthma. OVA-sensitized mice were repeatedly exposed to OVA or saline aerosols. Bronchoalveolar lavage fluid (BALF) was collected after the last aerosol, and the presence of IL-16 was evaluated using a migration assay with human lymphocytes. Migration of lymphocytes was significantly increased in the presence of cell-free BALF from OVA-challenged mice compared with BALF from saline-challenged controls. This response was significantly inhibited after

addition of antibodies to IL-16, demonstrating the presence of IL-16 in BALF of OVA-challenged animals. Immunohistochemistry was performed and revealed IL-16 immunoreactivity particularly in airway epithelial cells but also in cellular infiltrates in OVA-challenged mice. IL-16 immunoreactivity was absent in nonsensitized animals; however, some reactivity was detected in epithelial cells of sensitized but saline-challenged mice, suggesting that sensitization induced IL-16 expression in airway epithelium. Treatment of mice with antibodies to IL-16 during the challenge period significantly suppressed up-regulation of OVA-specific IgE in OVA-challenged animals. Furthermore, antibodies to IL-16 significantly inhibited the development of airway hyper-responsiveness after repeated OVA inhalations, whereas the number of eosinophils in bronchoalveolar lavage or airway tissue was not affected. In conclusion, IL-16 immunoreactivity is present in the airways after sensitization. After repeated OVA inhalation, IL-16 immunoreactivity is markedly increased and IL-16 is detectable in BALF. Furthermore, IL-16 plays an important role in airway hyper-responsiveness and up-regulation of IgE but is not important for eosinophil accumulation in a mouse model of allergic asthma.

PMID: 9510204 [PubMed - indexed for MEDLINE]

1867. J Allergy Clin Immunol. 1998 Feb;101(2 Pt 1):265-73.

Ragweed-specific antibodies in bronchoalveolar lavage fluids and serum before and after segmental lung challenge: IgE and IgA associated with eosinophil degranulation.

Peebles RS Jr, Liu MC, Adkinson NF Jr, Lichtenstein LM, Hamilton RG.

Department of Medicine, Johns Hopkins University School of Medicine, Baltimore, Md., USA.

BACKGROUND: Migration of eosinophils and release of eosinophil degranulation products into bronchoalveolar lavage fluid is a consistent finding in studies of late responses to allergen challenge in the lung. However, the mechanism of eosinophil activation and release of eosinophil products in vivo is unclear.
OBJECTIVE: We investigated the hypothesis that antigen-specific IgG, IgA, secretory IgA, or IgE is responsible for the eosinophil activation observed in the late-phase pulmonary reaction.
METHODS: Ragweed-specific IgE, IgA, secretory IgA, and IgG were measured by monoclonal antibody-based immunoassays in bronchoalveolar lavage (BAL) fluid and in serum from 19 asthmatic subjects allergic to ragweed and six healthy nonallergic control subjects before and 20 hours after segmental lung challenge with ragweed extract. Eosinophil cationic protein (ECP) was also measured in BAL fluid as a marker of eosinophil activation.
RESULTS: Most allergic asthmatic subjects had detectable levels of ragweed-specific IgE, IgA, and IgG in their serum and BAL fluid, whereas normal subjects had ragweed-specific IgA with no ragweed-specific IgE and little ragweed-specific IgG. IgA was the dominant ragweed-specific antibody isotype in BAL fluids. Ragweed-specific sIgA (r[s] = 0.52, p = 0.02) and IgA (r[s] = 0.50, p = 0.03) in BAL fluid after segmental lung challenge were significantly correlated with ECP. Ragweed-specific IgE and IgA in serum also correlated with ECP (r[s] = 0.74, p < 0.001 and r[s] = 0.48, p = 0.04, respectively).
CONCLUSIONS: The correlation of allergen-specific IgA and IgE antibody levels with ECP as a marker of eosinophil degranulation suggests an important role for IgE antibodies in allergic pulmonary inflammation and a potential role for antigen-specific IgA in eosinophil degranulation in the lung after antigen challenge.

PMID: 9500761 [PubMed - indexed for MEDLINE]

1868. Br J Pharmacol. 1998 Feb;123(3):538-44.

The role of lipocortin-1 in the inhibitory action of dexamethasone on eosinophil trafficking in cutaneous inflammatory reactions in the mouse.

Teixeira MM, Das AM, Miotla JM, Perretti M, Hellewell PG.

Applied Pharmacology, Imperial College School of Medicine at the National Heart and Lung Institute, London.

1. The ability of glucocorticosteroids to inhibit tissue eosinophilia may be an important feature of their anti-inflammatory action in allergic diseases. Our previous work showed that an effect of dexamethasone on the release of eosinophils from the bone marrow could explain its inhibitory action on eosinophil accumulation in a mouse air-pouch model. Thus, it was unclear from that study whether dexamethasone could interfere with the process of eosinophil trafficking. In the present study, therefore, we used a newly developed mouse model to evaluate the effects of systemic treatment with dexamethasone on the recruitment of (111)In-labelled blood eosinophils to sites of cutaneous inflammation in the mouse and whether lipocortin-1 (LC-1) was involved. 2. The i.d. injection of ovalbumin (OVA) in sensitized mice induced a dose-dependent recruitment of (111)In-labelled blood eosinophils which peaked at 4 to 8 h after antigen challenge. Systemic treatment with dexamethasone (50 microg per mouse, 3 h after antigen) effectively inhibited (111)In-eosinophil recruitment in this reaction by 70 to 85%. Similarly, a 1 h pretreatment with dexamethasone significantly suppressed (111)In-eosinophil induced by platelet-activating factor (PAF), leukotriene B4(LTB4) and the chemokine macrophage inflammatory protein-1alpha (MIP-1alpha) by 40 to 70%. 3. Two experimental approaches were used to evaluate the role of LC-1: treatment with LC-1 fragment Ac2-26 and use of an anti-LC-1 antiserum. LC-1 fragment Ac2-26 (100 microg per mouse) failed to affect (111)In-eosinophil recruitment. Moreover, pretreatment of animals with an anti-LC-1 antiserum failed to reverse the inhibitory effects of dexamethasone on (111)In-eosinophil recruitment induced by MIP-1alpha and by antigen in sensitized mice. 4. In contrast, the LC-1 fragment significantly inhibited glycogen-induced neutrophil recruitment into the peritoneal cavity of mice. Furthermore, the anti-LC-1 antiserum reversed the inhibitory effects of dexamethasone on the glycogen-induced neutrophil recruitment. 5. Thus, our results suggest that dexamethasone can inhibit the recruitment of eosinophils in mouse skin independent of an action on the bone marrow. However, by use of two different approaches, we showed that LC-1 does not play a role in mediating the inhibitory action of dexamethasone on eosinophil migration into cutaneous inflammatory reactions in the mouse. These data add further support to a LC-1-independent action of dexamethasone on eosinophils in vivo.

PMCID: PMC1565186
PMID: 9504395 [PubMed - indexed for MEDLINE]

1869. Scand J Immunol. 1998 Feb;47(2):110-5.

Allergen-induced migration of human cells in allergic severe combined immunodeficiency mice.

Duez C, Akoum H, Marquillies P, Cesbron JY, Tonnel AB, Pestel J.

INSERM U 416, Lille, France.

Recently, we have shown that severe combined immunodeficiency (SCID) mice,

intraperitoneally reconstituted with peripheral blood mononuclear cells (PBMC) from Dermatophagoides pteronyssinus (Dpt)-sensitive patients, produced human IgE and developed a pulmonary inflammatory-type reaction after exposure to allergen aerosol. In order to understand the potential mechanisms involved in the human cell migration in SCID mice, we analysed their phenotypic profile in the lungs, spleen and thymus, 2 months after Dpt inhalation. The human cell recruitment in these organs was found to be allergen-dependent as CD45+ human cells were only detected in hu-SCID mice after Dpt exposure. The composition of the pulmonary human T-cell infiltrate, preferentially memory (CD45RO), activated (human leucocyte antigen (HLA)-DR) and CD4+ cells, was similar to that described in asthmatic patients. However, CD20+ B cells were predominately recruited in the spleen and thymus and may be IgE-producing cells in the spleen. In the lungs, the percentage of human leucocytes expressing the alpha-chain of the lymphocyte function-associated antigen-1 (LFA-1) (CD11a) was higher than those of CD49d+ or CD54+ cells, in contrast to the spleen and thymus, suggesting a potential role of LFA-1 in the human cell migration towards SCID mice lung. In conclusion, this model could be useful in the study of factors implicated in the cellular migration towards the lymphoid organs during an allergic reaction.

PMID: 9496684 [PubMed - indexed for MEDLINE]

1870. Br J Pharmacol. 1998 Jan;123(2):281-91.

Characterization of the receptor and the mechanisms underlying the inflammatory response induced by des-Arg9-BK in mouse pleurisy.

Vianna RM, Calixto JB.

Department of Pharmacology, Centre of Biological Sciences, Universidade Federal de Santa Catarina, Florianópolis, Brazil.

1 The characterization of the B1 kinin receptor, and some mediators involved in the inflammatory response elicited by intrathoracic (i.t.) administration of des-Arg9-bradykinin (BK) in the mouse model of pleurisy, was investigated. 2 An i.t. injection of des-Arg9-BK (10-100 nmol per site), a selective B1 agonist, caused a significant and dose-related increase in the vascular permeability observed after 5 min, which peaked at 1 h, associated with an increase in cell influx, mainly neutrophils, and, to a lesser extent, mononuclear cell influx, peaking at 4 h and lasting for up to 48 h. The increase in fluid leakage caused by des-Arg9-BK was completely resolved 4 h after peptide injection. I.t. injection of Lys-des-Arg9-BK (30 nmol per site) caused a similar inflammatory response. 3 Both the exudation and the neutrophil influx elicited by i.t. injection of des-Arg9-BK were significantly antagonized (P<0.01) by an i.t. injection of the selective B1 antagonists des-Arg9-[Leu8]-BK (60 and 100 nmol per site) or des-Arg9-NPC 17731 (5 nmol per site), administered in association with des-Arg9-BK (P<0.01), or 30 and 60 min before the cellular peak, respectively. In contrast, an i.t. injection of the B2 bradykinin selective receptor antagonist Hoe 140 (30 nmol per site), at a dose which consistently antagonized bradykinin (10 nmol per site)-induced pleurisy, had no significant effect on des-Arg9-BK-induced pleurisy. 4 An i.t. injection of the selective tachykinin receptor antagonists (NK1) FK 888 (1 nmol per site), (NK2) SR 48968 (20 nmol per site) or (NK3) SR 142801 (10 nmol per site), administered 5 min before pleurisy induction, significantly antagonized neutrophil migration caused by i.t. injection of des-Arg9-BK. In addition, FK 888 and SR 142801, but not SR 48968, also prevented the influx of mononuclear cells in response to i.t. injection of des-Arg9-BK (P<0.01). However, the NK3 receptor antagonist SR 142801 (10 nmol per site) also significantly inhibited des-Arg9-BK-induced plasma extravasation. An i.t. injection of the calcitonin gene-related peptide (CGRP) receptor antagonist CGRP8-37 (1 nmol per site), administered 5 min before pleurisy induction,

inhibited des-Arg9-BK-induced plasma extravasation (P<0.01), without significantly affecting the total and differential cell migration. 5 The nitric oxide synthase inhibitors L-NOARG and L-NAME (1 pmol per site), administered 30 min beforehand, almost completely prevented des-Arg9-BK (i.t.)-induced neutrophil cell migration (P<0.01), and, to a lesser extent, mononuclear cell migration (P<0.01). The D-enantiomer D-NAME had no effect on des-Arg9-BK-induced pleurisy. At the same dose range, L-NOARG and L-NAME inhibited the total cell migration (P<0.01). L-NAME, but not L-NOARG caused significant inhibition of des-Arg9-BK-induced fluid leakage. Indomethacin (1 mg kg(-1), i.p.), administered 1 h before des-Arg9-BK (30 nmol per site), inhibited the mononuclear cell migration (P<0.05), but, surprisingly, increased the neutrophil migration at 4 h without interfering with plasma extravasation. The administration of terfenadine (50 mg kg(-1), i.p.), 30 min before des-Arg9-BK (30 nmol per site), did not interfere significantly with the total cell migration or with the plasma extravasation in the mouse pleurisy caused by i.t. injection of des-Arg9-BK. 6 Pretreatment of animals with the lipopolysaccharide of E. coli (LPS; 10 microg per animal, i.v.) for 24 h did not result in any significant change of the inflammatory response induced by i.t. injection of des-Arg9-BK compared with the saline treated group. However, the identical treatment of mice with LPS resulted in a marked enhancement of des-Arg9-BK induced paw oedema (P<0.01). 7 In conclusion, we have demonstrated that the inflammatory response induced by i.t. injection of desArg9-BK, in a murine model of pleurisy, is mediated by stimulation of constitutive B1 receptors. (These responses are largely mediated by release of neuropeptides such as substanceP or CGRP and also by NO, but products derived from cyclo-oxygenase pathway and histamine seem not to be involved. Therefore, these results further support the notion that the B1 kinin receptor has an important role in modulating inflammatory responses, and it is suggested that selective B1 antagonists may provide therapeutic benefit in the treatment of inflammatory and allergic conditions.

PMCID: PMC1565152
PMID: 9489617 [PubMed - indexed for MEDLINE]

1871. Toxicol Lett. 1997 Dec;93(2-3):185-94.

TDI inhalation in guinea-pigs involves migration of dendritic cells.

Ban M, Hettich D, Goutet M, Bonnet P.

Institut National de Recherche et de Securite, Vandoeuvre, France.

Toluene diisocyanate (TDI) can cause occupational asthma, but the mechanism underlying sensitization to this chemical compound remains controversial. The present study aims to investigate whether tumor necrosis factor-alpha (TNF-alpha) and interleukin-6 (IL-6) liberated in the lungs after TDI inhalation can contribute to the migration of dendritic cells from respiratory airways towards lung associated lymph nodes for presentation of TDI hapten. Exposure was studied in two modes: (1) acute exposure (experiment no. 1, 2 and 3) where animals were exposed to 2.962, 1.060, and 1.076 ppm TDI for 1, 4, and two periods of 4 h, respectively; (2) subacute exposure (experiment no. 4, 5 and 6) where animals were exposed to 0.066, 0.110, and 0.999 ppm TDI for 48 h for the two lower doses and 5 days for the highest dose. Depending on the modes of exposure, two to four post exposure times were selected. After acute exposure to 2.962 ppm TDI for 1 h, the increase in TNF-alpha and IL-6 levels in bronchoalveolar lavage (BAL) fluid was observed immediately at the end of inhalation exposure, whereas the maximum number of dendritic cells and total cells occurred at post exposure times of 48 h and 5 days, respectively. In two other acute exposures, the peak increases in TNF-alpha, IL-6 and total cell numbers were observed at 48 h post exposure time, whereas the peak increase in dendritic cells occurred at 24 h. After subacute

exposure to 48 h TDI, where TDI concentrations were relatively low (0.006 or 0.110 ppm), a parallel increase in TNF-alpha and IL-6 levels, dendritic and total cell numbers were observed at 0 h post exposure time. This phenomenon was also apparent at 24 h post exposure time when the animals had been exposed to 1.999 ppm TDI for 5 days. From these results, we can conclude that dendritic cells could play a key role as antigen presenting cells in the development of TDI-induced respiratory sensitization, and that their migration toward lung-associated lymph nodes is probably conditioned by cytokine release in their micro-environment. Future work must delineate whether TNF-alpha and IL-6 are solely responsible for the migration of dendritic cells after TDI inhalation, for example by using antibodies to neutralize these cytokines.

PMID: 9486955 [PubMed - indexed for MEDLINE]

1872. Immunology. 1997 Nov;92(3):388-95.

Langerhans cells require signals from both tumour necrosis factor-alpha and interleukin-1 beta for migration.

Cumberbatch M, Dearman RJ, Kimber I.

Zeneca Central Toxicology Laboratory, Macclesfield, Cheshire, UK.

The induction phase of contact sensitization is associated with the movement of epidermal Langerhans cells (LC) from the skin and their migration, via afferent lymphatics, to draining lymph nodes where they accumulate as immunostimulatory dendritic cells (DC). It has been demonstrated previously that tumour necrosis factor-alpha (TNF-alpha) provides an important signal for LC migration and that in the absence of this cytokine, movement of LC from the epidermis to regional lymph nodes is inhibited. Recent evidence indicates that interleukin-1 beta (IL-1 beta), a cytokine produced in murine epidermis exclusively by LC, may also play a role in LC migration. The purpose of the investigations described here was to clarify, using relevant neutralizing anti-cytokine antibodies, the contributions made by TNF-alpha and IL-1 beta to the migration of LC from the epidermis. It was found that like anti-TNF-alpha, anti-IL-1 beta administered systemically to mice (by intraperitoneal injection), prior to skin sensitization with the contact allergen oxazolone, resulted in a marked inhibition of DC accumulation in draining lymph nodes. It was shown also that anti-IL-1 beta inhibited TNF-alpha-induced LC migration and DC accumulation and that; in similar fashion, the stimulation of LC migration and DC accumulation induced by IL-1 beta was compromised by prior treatment with anti-TNF-alpha. Based upon these data it is proposed that the stimulation of LC migration in response to skin sensitization requires the receipt by LC of two independent signals, one provided by TNF-alpha and the other by IL-1 beta. Morphological analyses of LC in epidermal sheets prepared from animals exposed to these cytokines with or without prior systemic treatment with anti-cytokine antibody suggested that the changes induced in LC by TNF-alpha and IL-1 beta may include the altered expression of adhesion molecules and acquisition of the ability to interact with and pass through the basement membrane.

PMCID: PMC1363801
PMID: 9486113 [PubMed - indexed for MEDLINE]

1873. Neurochem Res. 1998 Mar;23(3):329-40.

Inflammation in traumatic brain injury: role of cytokines and chemokines.

Ghirnikar RS, Lee YL, Eng LF.

VAPA Health Care System, Palo Alto, CA 94304, USA.

A traumatic injury to the adult mammalian central nervous system (CNS), such as a stab wound lesion, results in reactive astrogliosis and the migration of hematogenous cells into the damaged neural tissue. The roles of cytokines and growth factors released locally by the damaged endogenous cells are recognized in controlling the cellular changes that occur following CNS injury. However, the role of chemokines, a novel class of chemoattractant cytokines, is only recently being studied in regulating inflammatory cell invasion in the injured/diseased CNS (1). The mRNAs for several chemokines have been shown to be upregulated in experimental allergic encephalomyelitis (EAE), an inflammatory demyelinating disease of the CNS, but chemokine expression in traumatic brain injury has not been studied in detail. Astrocytes have been demonstrated to participate in numerous processes that occur following injury to the CNS. In particular, astrocytic expression of cytokines and growth factors in the injured CNS has been well reviewed (2). Recently a few studies have detected the presence of chemokines in astrocytes following traumatic brain injury (3,4). These studies have suggested that chemokines may represent a promising target for future therapy of inflammatory conditions. This review summarizes the events that occur in traumatic brain injury and discusses the roles of resident and non-resident cells in the expression of growth factors, cytokines and chemokines in the injured CNS.

PMID: 9482245 [PubMed - indexed for MEDLINE]

1874. Srp Arh Celok Lek. 1997 Nov-Dec;125(11-12):363-9.

[Mast cells and basophilic leukocytes in allergic inflammation].

[Article in Serbian]

Bukilica M, Bogić M, Andrejević S, Nikolić B.

Institute of Allergology and Immunology, Clinical Centre of Serbia, Belgrade.

The human mast-cell population is composed of a heterogenous group of cells with respect to structure and function. Mast-cells can no longer be regarded simply as cells that initiate acute allergic reactions through the release of rapidly metabolized mediators, such as histamine and products of arachidonic acid oxidation. The production of a wide range of cytokines by mast-cells in the centre of the allergic inflammation. These cytokines influence migration and activation of other different cells including basophils and eosinophils and also lymphocytes, thus perpetuating allergic inflammation. Mediators release from human basophils are considered to contribute to the late phase of allergic response.

PMID: 9480572 [PubMed - indexed for MEDLINE]

1875. Am J Respir Cell Mol Biol. 1998 Feb;18(2):286-93.

CD49d expression and function on allergen-stimulated T cells from blood and airway.

Pacheco KA, Tarkowski M, Klemm J, Rosenwasser LJ.

Department of Medicine, National Jewish Medical and Research Center, Denver, Colorado 80206, USA.

The alpha4 chain (CD49d), which constitutes one of the chains of alpha4beta1 (very late activating antigen-4 [VLA-4]) and alpha4beta7 integrins, mediates migration of T cells to extravascular spaces. The interaction between VLA-4 and vascular cell adhesion molecule-1 (VCAM-1) has been shown to be the critical pathway for the selective accumulation of eosinophils and basophils at sites of allergic inflammation. T lymphocytes are also specifically recruited into allergic sites, including the allergic asthmatic airway. Increased numbers of activated CD4+ cells expressing the DR antigen subset of the human leukocyte antigens (HLA-DR) appear in the allergic lung 48 h after allergen inhalation. The mechanisms by which these cells localize into the lung are still unknown. We report that stimulation of allergen-specific T cells with allergen in vitro resulted in enhanced expression of alpha4 chain (CD49d) as measured by receptor density on allergen-specific T-cell lines and T-cell clones. Kinetic studies showed that CD49d density was enhanced over a 24- to 48-h period in a time-dependent fashion, and was coordinately upregulated with HLA-DR expression. We also demonstrated that increased expression of CD49d on T-cell lines 24 h and 48 h after stimulation correlated with increased adhesion to the CS-1 fragment of fibronectin. In contrast, lymphocyte function-associated antigen-1b (LFA-1b) (CD11b), LFA-3 (CD58), and intercellular adhesion molecule-1 (ICAM-1) (CD54) expression did not change with allergen stimulation. We also showed that CD49d receptor density on T cells obtained by bronchoalveolar lavage (BAL) of allergic patients before and 48 h after allergen challenge was significantly higher than that on T cells taken from BAL of normal subjects and from controls with other inflammatory lung diseases. Taken together, these findings indicate that allergen stimulation activates allergen-specific T cells and coordinately induces increased CD49d receptor expression and binding to counterligands. We postulate that allergen-driven upregulation of CD49d, which together with the beta1 chain constitutes VLA-4 integrin, may be responsible for the selective accumulation of T cells in the allergic asthmatic lung.

PMID: 9476917 [PubMed - indexed for MEDLINE]

1876. Arch Intern Med. 1998 Feb 9;158(3):294-6.

Cushing syndrome due to surreptitious glucocorticoid administration.

Quddusi S, Browne P, Toivola B, Hirsch IB.

Department of Medicine, University of Washington, Seattle, USA.

We report a case of subtle Cushing syndrome in a Pakistani man who self-treated his asthma with a potent long-acting oral glucocorticoid (betamethasone disodium phosphate [Bentelan]) for more than 30 years. He presented with cushingoid features, insulin resistance, and refractory hypertension. Laboratory evaluation revealed undetectable cortisol levels and suppression of the hypothalamic-pituitary-adrenal axis. The patient obtained the drug from his country of origin, with no understanding of the potential adverse effects imposed by long-term use of steroids. He is now being slowly weaned off the drug. The apparent widespread availability, access, and abuse of such potent steroids are a cause of concern in developing countries. We suggest that physicians in the United States be aware of the potential abuse of such potent drugs in all populations, including immigrants.

PMID: 9472211 [PubMed - indexed for MEDLINE]

1877. Jpn J Pharmacol. 1997 Dec;75(4):355-62.

Cysteinyl leukotrienes induce nasal symptoms of allergic rhinitis via a receptor-mediated mechanism in guinea pigs.

Fujita M, Nakagawa N, Yonetomi Y, Takeda H, Kawabata K, Ohno H.

Minase Research Institute, Ono Pharmaceutical Co., Ltd., Mishima-gun, Osaka, Japan.

To examine whether cysteinyl leukotrienes (cysLTs: LTC4, LTD4 and LTE4) induce symptoms of allergic rhinitis via their receptors, we studied the following: i) the specific binding of radiolabeled cysLTs to guinea pig nasal mucosa membrane and ii) effects of nasal LTD4 challenge in normal guinea pigs. The binding study indicated that there was a single population of binding sites for LTC4, LTD4 and LTE4 with Kd and Bmax values of 34.9+/-2.0, 0.252+/-0.015 and 0.589+/-0.039 nM and 10, 140+/-490, 122+/-11 and 306+/-23 fmol/mg protein, respectively. The in vivo study showed that topical nasal challenge of LTD4 (0.1-30 microg/nose) increased nasal secretion, nasal airway resistance and nasal eosinophil infiltration without inducing sneezing. While the increases in nasal secretion and nasal airway resistance were transient, peaking 10 to 20 min after LTD4 challenge, nasal eosinophil infiltration persisted at least until 24 hr post-challenge. These nasal symptoms were dose-dependently suppressed by oral administrations of pranlukast (0.3-3 mg/kg). The results suggest that cysLTs cause not only early-phase symptoms but also nasal eosinophil migration, a characteristic associated with the late-phase symptom of allergic rhinitis, via a receptor-mediated mechanism. Cysteinyl leukotrienes, thus, may be important mediators in allergic rhinitis.

PMID: 9469641 [PubMed - indexed for MEDLINE]

1878. J Exp Med. 1998 Jan 5;187(1):129-34.

Differential expression of chemokine receptors and chemotactic responsiveness of type 1 T helper cells (Th1s) and Th2s.

Bonecchi R, Bianchi G, Bordignon PP, D'Ambrosio D, Lang R, Borsatti A, Sozzani S, Allavena P, Gray PA, Mantovani A, Sinigaglia F.

Istituto di Ricerche Farmacologiche "Mario Negri", I-20157 Milan, Italy.

T helper cells type 1 (Th1s) that produce interferon-gamma predominantly mediate cellular immune responses and are involved in the development of chronic inflammatory conditions, whereas Th2s which produce large amounts of IL-4 and IL-5 upregulate IgE production and are prominent in the pathogenesis of allergic diseases. The precise factors determining whether Th1- or Th2-mediated immune responses preferentially occur at a peripheral site of antigen exposure are largely unknown. Chemokines, a superfamily of polypeptide mediators, are a key component of the leukocyte recruitment process. Here we report that among four CXC (CXCR1-4) and five CC (CCR1-5) chemokine receptors analyzed, CXCR3 and CCR5 are preferentially expressed in human Th1s. In contrast, Th2s preferentially express CCR4 and, to a lesser extent, CCR3. In agreement with the differential chemokine receptor expression, Th1s and Th2s selectively migrate in response to the corresponding chemokines. The differential expression of chemokine receptors may dictate, to a large extent, the migration and tissue homing of Th1s and Th2s. It may also determine different susceptibility of Th1s and Th2s to human immunodeficiency virus strains using different fusion coreceptors.

PMCID: PMC2199181
PMID: 9419219 [PubMed - indexed for MEDLINE]

1879. Clin Exp Allergy. 1997 Dec;27(12):1412-6.

Mutation screening of interferon-gamma (IFNgamma) as a candidate gene for asthma.

Hayden C, Pereira E, Rye P, Palmer L, Gibson N, Palenque M, Hagel I, Lynch N, Goldblatt J, Lesouëf P.

Department of Paediatrics, University of Western Australia, Perth.

BACKGROUND: Reduced levels of interferon gamma (IFNgamma) mRNA and protein have been detected in the bronchoalveolar lavage fluid of atopic asthmatics. IFNgamma is secreted by TH1 cells while IL-4 and IL-5 are secreted by TH2 cells and an imbalance in the TH1/TH2 response may be responsible for atopic asthma. The gene for IFNgamma is located on chromosome 12; a region of the genome which has been shown in linkage studies to be associated with asthma.
OBJECTIVE: To determine if there are any mutations present in the coding exons and 5' flanking region of the IFNgamma gene in atopic asthmatic subjects compared with controls to explain the lower levels of this cytokine as an inherited, rather than acquired, factor in the asthmatic subjects.
METHODS: The four exons and 5' flanking region of the IFNgamma gene were amplified by polymerase chain reaction (PCR) from genomic DNA of 265 individuals from a Western Australian and a Venezuelan population. The PCR products were examined by single strand conformational polymorphism and heteroduplex analyses to see if there were any changes in the DNA migration patterns which would suggest the presence of a sequence variation.
RESULTS: The four exons and the 5' flanking region of the IFNgamma gene were amplified from 265 individuals from two populations. Single strand conformational polymorphism and heteroduplex analyses did not reveal any mutations in the regions examined.
CONCLUSION: The gene for IFNgamma appears to be highly conserved as no sequence variations were detected in 265 individuals. These results suggest that mutations of the IFNgamma gene are unlikely to be a significant cause of an inherited asthma diathesis.

PMID: 9433936 [PubMed - indexed for MEDLINE]

1880. J Pediatr Surg. 1997 Oct;32(10):1429-31.

Pearls and perils in the management of prolonged, peculiar, penetrating esophageal foreign bodies in children.

Gilchrist BF, Valerie EP, Nguyen M, Coren C, Klotz D, Ramenofsky ML.

Division of Pediatric Surgery, State University of New York-Health Science Center at Brooklyn, 11201, USA.

BACKGROUND/PURPOSE: Most retained esophageal foreign bodies (FB) are identified soon after ingestion and are easily extracted. A minority of FB ingestions are not identified for weeks to years and present significant problems for retrieval. The purpose of this study was to describe the diagnostic and therapeutic strategies needed to care for children who have chronic esophageal FBs.
METHODS: Five children were identified as having retained esophageal FBs 2 months to 2 years after ingestion. During the same 3-year period, 100 children who had acute FBs were identified and had their foreign bodies removed endoscopically. The average age of the children was 3 years (range, 2.4 to 3.5).
RESULTS: The average age of the five children identified in this study was 3 years. The items ingested included coins, a heart pendant, a clothespin spring, and a toy soldier. Complications from chronically retained foreign bodies were

bronchoesophageal fistula, mediastinitis, esophageal diverticulum, and lobar atelectasis. One patient died from an aortoesophageal fistula. In all children, endoscopic removal was attempted. Barium esophagram was then performed, and foreign bodies were eventually removed via right thoracotomy.
CONCLUSIONS: Long-retained esophageal FBs are extremely morbid and life threatening. History most often identifies excess salivation, new onset asthma, and/or recurrent upper respiratory infections. Three diagnostic adjuncts are helpful in identifying the presence of a long retained FB: (1) Chest x-ray (PA and lateral), (2) barium swallow, and (3) esophagoscopy. Indications for thoracotomy for removal of foreign body include (1) Poor endoscopic visualization of FB because of inflammatory tissue and (2) Herald bleeding during endoscopy.

PMID: 9349761 [PubMed - indexed for MEDLINE]

1881. Am J Respir Cell Mol Biol. 1997 Dec;17(6):702-12.

Kinetics and quantitation of eosinophil and neutrophil recruitment to allergic lung inflammation in a brown Norway rat model.

Schneider T, van Velzen D, Moqbel R, Issekutz AC.

Department of Pediatrics, Dalhousie University, Halifax, Nova Scotia, Canada.

We quantitated neutrophil and eosinophil migration into lung parenchyma using specific peroxidase enzyme assays, and into the bronchoalveolar compartment by bronchoalveolar lavage (BALF), in sensitized brown Norway (BN), Fischer, and Lewis rats and also assessed the lungs by histopathology. Fourteen days after sensitization with ovalbumin (OA in alum [given subcutaneously] and OA with Bordetella pertussis [given intraperitoneally]), rats were challenged with an OA aerosol for 1 h. In BN rats, there was marked perivascular and peribronchial edema, focal hemorrhages, and increase in lung wet weight and BALF protein content, accompanied by neutrophilic infiltration at 3-14 h postchallenge. Few eosinophils were seen at 14 h in lung tissue or in BALF. Neutrophils peaked at 24 h in parenchyma ([94 +/- 7] x 10[6]) and in BALF ([2.7 +/- 0.4] x 10[6]) and declined rapidly thereafter. Marked eosinophil infiltration into parenchyma was apparent by 24 h. Eosinophil accumulation peaked at 48 h in parenchyma ([127 +/- 18] x 10[6]) and at 72 h in BALF ([10 +/- 2.4] x 10[6]), comprising up to 85% of lavage cells at this time. Lung eosinophilia persisted for at least 6 d with only a slow decline or clearance, not approximating baseline until day 13 after challenge. Histopathology showed peribronchial and interstitial eosinophilic pneumonia, most severe on day 3. In contrast to the BN rats, essentially no pulmonary inflammation was observed in Lewis and Fischer rats. This model in the BN rat, and the specific peroxidase assays for quantitating tissue eosinophils and neutrophils, should be useful for investigating the regulation of allergen-induced eosinophil and neutrophil migration into and clearance from the lung.

PMID: 9409557 [PubMed - indexed for MEDLINE]

1882. Am J Respir Crit Care Med. 1997 Nov;156(5):1628-32.

The GM-CSF analogue E21R induces apoptosis of normal and activated eosinophils.

Iversen PO, Robinson D, Ying S, Meng Q, Kay AB, Clark-Lewis I, Lopez AF.

Division of Human Immunology, Hanson Centre for Cancer Research, Adelaide, Australia.

There is evidence that eosinophils have an important role in the pathogenesis of allergy and asthma. These cells are regulated by two classes of polypeptides, the colony-stimulating factors, such as granulocyte-macrophage colony-stimulating factor (GM-CSF), and the chemokines, such as RANTES and eotaxin. GM-CSF is involved in the production, survival, and functional activation of eosinophils. RANTES and eotaxin regulate the migration of eosinophils to inflammatory sites, but any effect of these chemokines on eosinophil survival is not known. In this study we demonstrate that although GM-CSF promoted eosinophil survival, the specific GM-CSF analogue E21R induced apoptosis of eosinophils. Apoptosis was observed with unstimulated as well as with chemokine (RANTES and eotaxin)-activated eosinophils. Neither RANTES nor eotaxin supported eosinophil survival, and a RANTES antagonist did not affect either cell survival or apoptosis. E21R also induced apoptosis of eosinophils from asthmatic patients. These findings suggest that the GM-CSF receptor may actively control the death as well as the survival of eosinophils, and thus precisely regulate their numbers and activities. Our data also indicate that chemokines are not involved in regulating the lifespan of eosinophils. The introduction of the GM-CSF analogue E21R may offer a novel therapy in inflammatory diseases associated with eosinophil infiltration of different etiologies.

PMID: 9372686 [PubMed - indexed for MEDLINE]

1883. J Exp Med. 1997 Nov 17;186(10):1725-35.

Alpha 6 integrins are required for Langerhans cell migration from the epidermis.

Price AA, Cumberbatch M, Kimber I, Ager A.

Division of Cellular Immunology, National Institute for Medical Research, London, United Kingdom.

Topical exposure of mice to chemical allergens results in the migration of epidermal Langerhans cells (LCs) from the skin and their accumulation as immunostimulatory dendritic cells (DCs) in draining lymph nodes. Epidermal cell-derived cytokines have been implicated in the maturation and migration of LCs, but the adhesion molecules that regulate LC migration have not been studied. We hypothesized that integrin-mediated interactions with extracellular matrix components of the skin and lymph node may regulate LC/DC migration. We found that alpha 6 integrins and alpha 4 integrins were differentially expressed by epidermal LCs and lymph node DCs. A majority of LCs (70%) expressed the alpha 6 integrin subunit, whereas DCs did not express alpha 6 integrins. In contrast, the alpha 4 integrin subunit was expressed at high levels on DCs but at much lower levels on LCs. The anti-alpha 6 integrin antibody, GoH3, which blocks binding to laminin, completely prevented the spontaneous migration of LCs from skin explants in vitro and the rapid migration of LCs from mouse ear skin induced after intradermal administration of TNF-alpha in vivo. GoH3 also reduced the accumulation of DCs in draining lymph nodes by a maximum of 70% after topical administration of the chemical allergen oxazolone. LCs remaining in the epidermis in the presence of GoH3 adopted a rounded morphology, rather than the interdigitating appearance typical of LCs in naive skin, suggesting that the cells had detached from neighboring keratinocytes and withdrawn cellular processes in preparation for migration, but were unable to leave the epidermis. The anti-alpha 4 integrin antibody PS/2, which blocks binding to fibronectin, had no effect on LC migration from the epidermis either in vitro or in vivo, or on the accumulation of DCs in draining lymph nodes after oxazolone application. RGD-containing peptides were also without effect on LC migration from skin explants. These results identify an important role for alpha 6 integrins in the migration of LC from the epidermis to the draining lymph node by regulating access across the epidermal basement membrane. In contrast, alpha 4 integrins, or

other integrin-dependent interactions with fibronectin that are mediated by the RGD recognition sequence, did not influence LC migration from the epidermis. In addition, alpha 4 integrins did not affect the accumulation of LCs as DCs in draining lymph nodes.

PMCID: PMC2199129
PMID: 9362532 [PubMed - indexed for MEDLINE]

1884. J Allergy Clin Immunol. 1997 Nov;100(5):669-78.

Recruitment of circulating allergen-specific T lymphocytes to the lung on allergen challenge in asthma.

Borgonovo B, Casorati G, Frittoli E, Gaffi D, Crimi E, Burastero SE.

Department of Biotechnology, San Raffaele Scientific Institute, Milan, Italy.

BACKGROUND: In allergic subjects with asthma, the migration of CD4+ T cells to the lungs in the hours after allergen exposure may contribute to allergic inflammation in the target organ.
OBJECTIVE: We studied allergen-specific T cells from the peripheral blood and lungs of allergic subjects with asthma at baseline and after allergen challenge.
METHODS: In each patient, blood samples were taken 10 minutes before and 24 hours after the inhalation of a major sensitizing allergen. In vitro proliferation of peripheral blood CD4+ T cells specific for the same allergen used in the in vivo challenge was assessed. In one patient two Dermatophagoides pteronyssinus-specific T-cell clones (TCCs) were derived from peripheral blood, and their T-cell receptors were sequenced to determine their clonotypic determinants on the beta chains. The T-cell receptor determinants of the allergen-specific TCCs were sought in blood and bronchoalveolar lavage samples taken from this patient.
RESULTS: We found that allergen inhalation is followed by a decrement in the specific proliferation of peripheral CD4+ T cells to the same allergen used for bronchial provocation. In one patient the clonotypic determinants of two allergen-specific TCCs diminished in the peripheral blood, whereas they were simultaneously expanded in the lower respiratory tract.
CONCLUSION: Our data suggest that allergen-specific T cells are recruited from the peripheral blood to the bronchial lumen after allergen challenge.

PMID: 9389298 [PubMed - indexed for MEDLINE]

1885. Environ Toxicol Pharmacol. 1997 Dec;4(3-4):209-17.

Acquired risk factors and susceptibility to environmental toxicants.

Grassman J.

National Institute of Environmental Health Sciences, PO 12233, MC C4-05, Research Triangle Park, NC 17709, USA.

Susceptibility to illness after exposure to environmental toxicants is determined by the interaction of numerous factors involving both constitutive and acquired traits. Constitutive susceptibility (risk) factors are the intrinsic traits determined by developmental stage, gender, and genetic makeup. Within a population, changes in constitutive risk factors tend to occur slowly, through aging, alterations in the birth or death rate, or by migration in or out of the population. Often overlooked is the effect of acquired susceptibility factors on susceptibility to environmental toxicants. Acquired susceptibility factors, which

are related to the effects of living conditions, psychosocial factors, diet, behavior and access to medical care, may modify the effect of constitutive factors. Three examples demonstrate the interaction of acquired susceptibility factors with exposure and constitutive factors. The increased prevalence of asthma in children is suspected of having a strong environmental component but the underlying acquired susceptibility factors, if any, are difficult to identify because of the multifactorial nature of asthma and the use of surrogate risk factors such as parent's education. β-Carotene is a dietary component which may modify acquired susceptibility. While numerous observational studies find that dietary β-carotene reduces the risk of lung cancer in cigarette smokers, intervention studies do not support this role. Hepatitis B is an example of an infectious agent functioning as an acquired susceptibility factor. Hepatitis B synergistically increases the risk of hepatocellular carcinoma when accompanied by exposure to aflatoxin, a relationship that may be modified by constitutive risk factors, such as epoxide hydrolase capabilities. Acquired risk factors have the potential to greatly influence risk and their impact should be included in future studies of the health effects of environmental toxicants.

PMID: 21781823 [PubMed]

1886. Arzneimittelforschung. 1997 Oct;47(10):1112-6.

Eosinophil chemotaxis induced by several biologically active substances and the effects of apafant on it in vitro.

Nabe T, Yamamura H, Kohno S.

Department of Pharmacology, Kyoto Pharmaceutical University, Japan.

Chemotaxis of guinea pig eosinophils induced by various stimuli in use of a modified Boyden chamber technique in vitro and the effect of a platelet-activating factor (PAF) antagonist, apafant (CAS 105219-56-5, WEB 2086 BS), on it were examined. The eosinophils were obtained by bronchoalveolar lavage from the animals treated by i.v. injection with Sephadex G-200 and purified by Percoll density gradient centrifugation. PAF significantly and potently induced the chemotaxis at a broad range of 10(-17) to 10(-7) mol/l, where no concentration-dependency was observed. Leukotriene B4 also induced the chemotaxis in a concentration-dependent manner at 10(-14) to 10(-12) mol/l and the enhanced migration was not declined until 10(-7) mol/l. Interleukin-5 (IL-5), IL-8 and regulated on activation normal T expressed and secreted (RANTES) only modestly enhanced the chemotaxis in some concentrations at 10(-13) to 10(-7) mol/l with or without significance and with no concentration-dependency while formyl-methionyl-leucyl-phenylalanine (FMLP), a known chemoattractant, increased the migration at 10(-7) to 10(-5) mol/l. Apafant at 10(-8) to 10(-6) mol/l strongly and concentration-dependently inhibited 10(-8) mol/l PAF-induced chemotaxis. However, the drug showed nominal or no influences on their chemotaxis stimulated by the other agonists, at the concentrations of which the enhanced migration was observed. From these results, it is concluded that IL-5, IL-8 and RANTES, different from PAF and LTB4, are not potent stimuli for the eosinophil chemotaxis and that apafant is a selective antagonist of PAF, which is expected to be therapeutically effective for PAF-associated diseases including bronchial asthma.

PMID: 9368704 [PubMed - indexed for MEDLINE]

1887. Biochem Biophys Res Commun. 1997 Nov 7;240(1):173-8.

Macrophage migration inhibitory factor is an essential immunoregulatory cytokine

in atopic dermatitis.

Shimizu T, Abe R, Ohkawara A, Mizue Y, Nishihira J.

Department of Dermatology, Hokkaido University School of Medicine, Sapporo, Japan.

Macrophage migration inhibitory factor (MIF) is one of the immunoregulatory cytokines involved in T-cell activation and delayed-type hypersensitivity. To elucidate involvement of this cytokine in the pathogenesis of atopic dermatitis (AD), we examined serum MIF concentrations of patients with AD and non-atopic normal healthy individuals. The mean serum MIF concentration of the AD patients (n = 36) was 36.4 +/- 3.7 ng/ml (mean +/- SEM), whereas that of the non-atopic dermatitis patients (n = 17) or healthy individuals (n = 61) were 13.1 +/- 1.8 or 6.5 +/- 0.45 ng/ml, respectively. Accordingly, immunohistochemistry of the inflammatory skin lesion of an AD patient demonstrated that MIF protein was diffusely expressed throughout the whole epidermal layer. After 4-week steroid ointment treatment, the MIF concentration decreased as clinical symptoms improved. The serum level of TNF-alpha was also decreased in parallel with that of MIF. Considering the T-cell dysfunction and disordered cytokine-network reported in AD, it was strongly suggested that MIF was a critical protein for immunoregulation in the pathophysiological mechanism of AD. In this context, MIF may become a useful laboratory parameter to comprehend the clinical course of the disease.

PMID: 9367905 [PubMed - indexed for MEDLINE]

1888. J Biol Chem. 1997 Nov 7;272(45):28206-9.

Chemokine receptor CCR3 function is highly dependent on local pH and ionic strength.

Dairaghi DJ, Oldham ER, Bacon KB, Schall TJ.

Department of Immunobiology, DNAX Research Institute, Palo Alto, California 94304, USA.

The CC chemokine receptor 3 (CCR3) plays an important role in the regulation of the migration of eosinophils, a leukocyte population involved in many inflammatory pathologies including asthma. CCR3 binds to the CC chemokine eotaxin, a promigratory cytokine originally isolated as the key component in a model of eosinophil-induced airway inflammation. We show here that eotaxin/CCR3 binding interactions exhibit a marked sensitivity to relatively small changes in the extracellular environment. In particular, modest variations in the pH and the level of sodium chloride over a range of physiologic and near physiologic conditions had dramatic effects on eotaxin binding and CCR3-mediated cytoplasmic Ca^{2+} mobilization. These biochemical indices were reflected at the functional level as well; small changes in pH and salt also resulted in striking changes in the migration of primary human eosinophils in vitro. These results reveal that relatively small perturbations in extracellular buffer conditions can yield widely disparate interpretations of CCR3 ligand binding and affinities and suggest that modulation of the tissue microenvironment might be utilized to control the affinity and efficacy of chemokine-mediated cell migration.

PMID: 9353270 [PubMed - indexed for MEDLINE]

1889. Inflammation. 1997 Oct;21(5):489-99.

Retrograde migration of starch in the genital tract of rabbits.

Edelstam GA, Sjösten AC, Ellis H.

Department of Obstetrics and Gynaecology, SOS, Karolinska Institute, Sweden.

This study in a rabbit model simulates contamination with glove powder in association with a routine gynaecological examination. Large individual variations of powder contamination were found and there were no overall statistically significant differences between the control and experimental animals. The findings are supported by the observation that some but not all women develop adhesions after gynaecological surgery. Analyzes of variances indicate differences in the migration of starch particles in the genital tract with the highest amount of particles found three days after starch contamination of the vagina. Since no adhesions were observed, there would probably need to be an ongoing post surgical or post infectious inflammation in the tissue, when the starch particles are added. Starch powder from latex gloves can cause adhesions and increase the risk of latex allergy in healthcare workers. Retrograde migration in the genital tract cannot be excluded, powdered examination products should be eliminated from the gynecologicla examination room.

PMID: 9343747 [PubMed - indexed for MEDLINE]

1890. J Med Chem. 1997 Oct 10;40(21):3359-68.

Potent alpha 4 beta 1 peptide antagonists as potential anti-inflammatory agents.

Jackson DY, Quan C, Artis DR, Rawson T, Blackburn B, Struble M, Fitzgerald G, Chan K, Mullins S, Burnier JP, Fairbrother WJ, Clark K, Berisini M, Chui H, Renz M, Jones S, Fong S.

Department of Bioorganic Chemistry, Genentech Inc., South San Francisco, California 94080, USA.

The migration, adhesion, and subsequent extravasation of leukocytes into inflamed tissues contribute to the pathogenesis of a variety of inflammatory diseases including asthma, rheumatoid arthritis, inflammatory bowel disease, and multiple sclerosis. The integrin adhesion receptor alpha 4 beta 1 expressed on leukocytes binds to the extracellular matrix protein fibronectin and to the cytokine inducible vascular cell adhesion molecule-1 (VCAM-1) at inflamed sites. Binding of alpha 4 beta 1 to VCAM-1 initiates firm adhesion of the leukocyte to the vascular endothelium followed by extravasation into the tissue. Monoclonal antibodies generated against either alpha 4 beta 1 or VCAM-1 can moderate this inflammatory response in a variety of animal models. Recently peptides containing a consensus LDV sequence based on the connecting segment-1 (CS-1) of fibronectin and cyclic peptides containing an RCD motif have shown promise in modulating leukocyte migration and inflammation presumably by blocking the interaction of alpha 4 beta 1 with VCAM-1. Here we describe novel, highly potent, cyclic peptides that competitively inhibit alpha 4 beta 1 binding to VCAM-1 and fibronectin at sub nanomolar concentrations. The structure of a representative analog was determined via NMR spectroscopy and used to facilitate optimization of peptide leads. The peptides discussed here utilize similar functional groups as the binding epitope of VCAM-1, inhibit lymphocyte migration in vivo, and are highly selective for alpha 4 beta 1. Furthermore the structure--activity relationships described here have provided a template for the structure-based design of small molecule antagonists of alpha 4 beta 1-mediated cell adhesion processes.

PMID: 9341911 [PubMed - indexed for MEDLINE]

1891. Allergy Asthma Proc. 1997 Sep-Oct;18(5):287-92.

Update on mast cells and mast cell precursors and hypersensitivity responses.

Castells M.

Department of Medicine, Harvard Medical School, Brigham and Women's Hospital, Boston, Massachusetts, USA.

Mast cells are important effector cells providing granule and membrane mediators as well as cytokines in allergic and inflammatory diseases. The study of surface molecules such as immunoglobulin receptors and adhesion molecules has greatly expanded the functional implications of mast cells. An active role for mast cells in antigen presentation to T cells has recently been shown, and direct interaction between mast cells and B cells providing signals for specific IgE production has been demonstrated. Functional receptors other than the high affinity IgE (Fc epsilon RI) have been implicated in the anaphylactic response of IgE-deficient mice, suggesting that IgG receptors present in mast cells may be involved in immediate hypersensitivity reactions. Although metachromatic mast cells are easily recognized in peripheral tissues, little is known about the phenotype of mast cell precursors, their fate from the bone marrow to the tissues, migration and homing processes, and factors and adhesion molecules that affect those processes. This review will describe the most recent studies in mouse and human mast cell biology and ontogeny.

PMID: 9337422 [PubMed - indexed for MEDLINE]

1892. J Clin Invest. 1997 Oct 1;100(7):1657-66.

Chemokine-induced eosinophil recruitment. Evidence of a role for endogenous eotaxin in an in vivo allergy model in mouse skin.

Teixeira MM, Wells TN, Lukacs NW, Proudfoot AE, Kunkel SL, Williams TJ, Hellewell PG.

Applied Pharmacology, Imperial College School of Medicine at the National Heart and Lung Institute, London, SW3 6LY, United Kingdom. mauro.teixeira@ic.ac.uk

Selective eosinophil recruitment into tissues is a characteristic feature of allergic diseases. Chemokines are effective leukocyte chemoattractants and may play an important role in mediating eosinophil recruitment in various allergic conditions in man. Here, we describe a novel mouse model of eosinophil recruitment in which we have compared the in vivo chemoattractant activity of different C-C chemokines. Furthermore, we describe the use of antibodies to chemokines and receptor blockade to address the endogenous mechanisms involved in eosinophil recruitment in a late-phase allergic reaction in mouse skin. Intradermal injection of mEotaxin and mMIP-1alpha, but not mMCP-1, mRANTES, mMCP-5, or mMIP-1beta, induced significant 111In-eosinophil recruitment in mouse skin. Significant 111In-eosinophil recruitment was also observed in an active cutaneous anaphylactic reaction. Pretreatment of skin sites with antieotaxin antiserum, but not an antiMIP-1alpha antibody, suppressed 111In-eosinophil recruitment in this delayed-onset allergic reaction. Similarly, desensitization of the eosinophil eotaxin receptor CCR3 with mEotaxin, or blockade of the receptor with metRANTES, significantly inhibited 111In-eosinophil recruitment in the allergic reaction. These results demonstrate an important role for endogenous eotaxin in mediating the 111In-eosinophil recruitment in allergic inflammation, and suggest that blockade of the CCR3 receptor is a valid strategy to inhibit

eosinophil migration in vivo.

PMCID: PMC508348
PMID: 9312163 [PubMed - indexed for MEDLINE]

1893. J Psychosom Res. 1997 Sep;43(3):259-69.

Stress and sensitization in children: a controlled prospective psychophysiological study of children exposed to international relocation.

Anderzén I, Arnetz BB, Söderström T, Söderman E.

Karolinska Institute, National Institute for Psychosocial Factors and Health, Stockholm, Sweden.

This controlled prospective study investigated the development of sensitization as a result of international relocation in children, using the analyzing system Phadiatop. The effects of climate and predisposition to allergy were also measured. Children were examined prior to and during their first year of living abroad. A control group living at home was also examined during the same period. Participants answered a questionnaire before and after 1 year abroad, and blood samples were collected to determine sensitization. Before going abroad, there were no significant differences in atopic sensitization between groups nor in other key variables. After 1 year abroad, the proportion of children showing sensitization had increased significantly as compared with the control group at home. The exposed group reported an increase in skin symptoms during the year abroad. This study suggests that unidentified factors associated with foreign relocation increase the risk of sensitization in predisposed children. Stress might be one factor.

PMID: 9304552 [PubMed - indexed for MEDLINE]

1894. J Clin Invest. 1997 Sep 1;100(5):1137-43.

High expression of the chemokine receptor CCR3 in human blood basophils. Role in activation by eotaxin, MCP-4, and other chemokines.

Uguccioni M, Mackay CR, Ochensberger B, Loetscher P, Rhis S, LaRosa GJ, Rao P, Ponath PD, Baggiolini M, Dahinden CA.

Theodor Kocher Institute, University of Bern, CH-3000 Bern 9, Switzerland.

Eosinophil leukocytes express high numbers of the chemokine receptor CCR3 which binds eotaxin, monocyte chemotactic protein (MCP)-4, and some other CC chemokines. In this paper we show that CCR3 is also highly expressed on human blood basophils, as indicated by Northern blotting and flow cytometry, and mediates mainly chemotaxis. Eotaxin and MCP-4 elicited basophil migration in vitro with similar efficacy as regulated upon activation normal T cells expressed and secreted (RANTES) and MCP-3. They also induced the release of histamine and leukotrienes in IL-3-primed basophils, but their efficacy was lower than that of MCP-1 and MCP-3, which were the most potent stimuli of exocytosis. Pretreatment of the basophils with a CCR3-blocking antibody abrogated the migration induced by eotaxin, RANTES, and by low to optimal concentrations of MCP-4, but decreased only minimally the response to MCP-3. The CCR3-blocking antibody also affected exocytosis: it abrogated histamine and leukotriene release induced by eotaxin, and partially inhibited the response to RANTES and MCP-4. In contrast, the antibody did not affect the responses induced by MCP-1, MCP-3, and macrophage inflammatory protein-1alpha, which may depend on CCR1 and CCR2, two additional

receptors detected by Northern blotting with basophil RNA. This study demonstrates that CCR3 is the major receptor for eotaxin, RANTES, and MCP-4 in human basophils, and suggests that basophils and eosinophils, which are the characteristic effector cells of allergic inflammation, depend largely on CCR3 for migration towards different chemokines into inflamed tissues.

PMCID: PMC508288
PMID: 9276730 [PubMed - indexed for MEDLINE]

1895. J Exp Med. 1997 Aug 29;186(5):749-56.

Impaired inflammatory responses in the reverse arthus reaction through genetic deletion of the C5a receptor.

Höpken UE, Lu B, Gerard NP, Gerard C.

Ina Sue Perlmutter Cystic Fibrosis Laboratory, Children's Hospital, Department of Medicine, Beth Israel Hospital, Harvard Medical School, Boston, Massachusetts 02115, USA.

We recently demonstrated that gene-targeted disruption of the C5a anaphylatoxin receptor prevented lung injury in immune complex-mediated inflammation. In this study, we compare the effect of C5aR deficiency in immune complex-induced inflammation in the peritoneal cavity and skin with the results derived from our immune complex alveolitis model. C5aR- deficient mice exhibit decreased migration of neutrophils and decreased levels of TNF-alpha and interleukin 6 in the peritoneal reverse passive Arthus reaction compared to their wild-type littermates. In the reverse passive Arthus reaction in the skin the C5aR was also required for the full expression of neutrophil influx and edema formation; C5aR-deficient mice showed reduced neutrophil migration and microvascular permeability changes. In contrast to our studies in immune complex-induced lung inflammation, C5aR deficiency does not completely prevent injury in the peritoneal cavity and skin. These data indicate a dominant role for the C5aR and its ligand in the reverse passive Arthus reaction in the lung and a synergistic role together with other inflammatory mediators in immune complex-mediated peritonitis and skin injury.

PMCID: PMC2199021
PMID: 9271590 [PubMed - indexed for MEDLINE]

1896. Am J Respir Crit Care Med. 1997 Aug;156(2 Pt 1):367-74.

Role for neurokinin-2 receptor in interleukin-5-induced airway hyperresponsiveness but not eosinophilia in guinea pigs.

Kraneveld AD, Nijkamp FP, Van Oosterhout AJ.

Department of Pharmacology, Utrecht Institute of Pharmaceutical Sciences, Utrecht University, The Netherlands. A.D.Kraneverd@FAR.RUU.NL

In the guinea pig, interleukin-5 (IL-5) has been shown to induce airway hyperresponsiveness as well as eosinophilia, which are important symptoms in asthma. IL-5 seems to be a critical cytokine since it selectively affects eosinophil functions. The mechanism of action by which IL-5 leads to airway hyperresponsiveness may be important for our understanding of the pathogenesis of asthma. Neurogenic inflammation, which is mediated by nonadrenergic noncholinergic nerves (NANC), may play a role in the IL-5-induced effects in guinea pig airways. In this study, the role of neuropeptides in the IL-5-induced

airway hyperresponsiveness and eosinophilia in the guinea pig was examined using selective neurokinin receptor antagonists. Intra-airway application of IL-5 (1 microgram, twice) induces a selective eosinophil migration (control: 12 [8-22] x 10(5) cells and IL-5: 90 [67-187] x 10(5) cells, $p < 0.05$) and activation (control: 6.3 +/- 0.9 ng eosinophil peroxidase [EPO]/ml bronchoalveolar lavage [BAL] fluid and IL-5: 29.3 +/- 4.9 ng EPO/ml BAL fluid, $p < 0.05$) and a pronounced airway hyperresponsiveness in vivo. The maximal responses to histamine are increased by 160 +/- 16% ($p < 0.05$) after IL-5. Treatment of guinea pigs with either the nonselective neurokinin (NK)-receptor antagonist, FK224, or the selective NK2-receptor antagonist, SR48968, results in a complete inhibition of the in vivo hyperresponsiveness found after application of IL-5. Vice versa, intra-airway administration of substance P (10 micrograms, twice) results in an airway hyperresponsiveness (increased maximal response after substance P: 166 +/- 15% [$p < 0.05$]) without inducing migration or activation of eosinophils. All examined NK-receptor antagonists do not influence the IL-5-induced eosinophil accumulation. In addition, no effect of the NK-receptor antagonists is observed on the IL-5-induced eosinophil activation, as determined by BAL fluid EPO levels. The release of NK2-receptor active tachykinins plays an important role in the development of IL-5-induced airway hyperresponsiveness. This feature appears to be a step following eosinophil infiltration and activation since there are no effects on eosinophil function by pretreatment of the used NK-receptor antagonists.

PMID: 9279211 [PubMed - indexed for MEDLINE]

1897. Chest. 1997 Aug;112(2):505-10.

Airway inflammation in COPD assessed by sputum levels of interleukin-8.

Yamamoto C, Yoneda T, Yoshikawa M, Fu A, Tokuyama T, Tsukaguchi K, Narita N.

Second Department of Internal Medicine, Nara Medical University, Japan.

STUDY OBJECTIVE: To assess the characteristics of airway inflammation in patients with COPD.
METHODS: We measured the sputum concentration of interleukin-8 (IL-8), a chemokine involved in the migration and activation of neutrophils and eosinophils. We also measured myeloperoxidase (MPO) as a parameter of neutrophil activity and eosinophil cationic protein (ECP) as a parameter of eosinophil activity. Spontaneous sputum samples were obtained from 33 patients with stable COPD and 30 patients with asthma. Induced sputum samples were obtained from 12 normal control subjects.
RESULTS: The sputum concentration of IL-8 was significantly higher in the patients with COPD than in the patients with asthma or in the control subjects ($p<0.0001$). Concentrations of MPO and ECP were significantly higher in the patients with COPD than in the control subjects but did not differ significantly between the patients with COPD and those with asthma. In the patients with COPD, the sputum concentration of IL-8 was significantly correlated with the concentration of MPO ($r=0.55$, $p<0.001$) and of ECP ($r=0.53$, $p<0.01$). The sputum concentration of IL-8 was negatively correlated with FEV1/FVC ($r=-0.78$, $p<0.0001$) in the COPD group.
CONCLUSIONS: Results suggest the activation of both neutrophils and eosinophils in the airways of patients with COPD. It appears that IL-8 plays a primary role in this activation. The sputum concentration of IL-8 appeared to be closely associated with the degree of airflow obstruction in patients with COPD and may serve as a marker in evaluating the severity of airway inflammation, which is a risk factor for COPD.

PMID: 9266891 [PubMed - indexed for MEDLINE]

1898. Am J Dermatopathol. 1997 Aug;19(4):391-5.

Expression of intercellular adhesion molecule 3 (CDw50) on endothelial cells in cutaneous lymphomas. A comparative study between nodal and cutaneous lymphomas.

Dommann SN, Dommann-Scherrer CC, Ziegler T, Meyer J, Trüeb RM, Kündig T, Panizzon R, Burg G.

Department of Dermatology, University Hospital of Zurich, Switzerland.

Advances in the molecular definition of surface proteins (adhesion molecules) involved in tumor metastasis may help to explain the invasive behavior of malignant tumors, that is, the migration of tumor cells involving reversible adhesive contacts, their release in the circulation, and their extravasation into distant sites. Intercellular adhesion molecule-3 (ICAM-3), the third receptor for the lymphocyte function-associated antigen molecule-1 (LFA-1) was recently characterized. We investigated fresh frozen skin biopsies from 10 patients with mycosis fungoides, four with pleomorphic T-cell lymphoma, six with Sézary syndrome, 10 with primary cutaneous B-cell lymphoma, and 10 with eczematous lesions as controls. The biopsies were compared with lymph node biopsies of five patients with known cutaneous T-cell lymphoma (CTCL), 10 with primary nodal B-cell lymphoma, and 11 with lymph-node specimens showing dermatopathic lymphadenopathy as controls. The specimens were stained with ICAM-3 antibody (Bender Medical Science) using the alkaline phosphatase antialkaline phosphatase method. Using cytomorphologic criteria, neoplastic lymphocytes could be differentiated from smaller reactive cells. Staining intensities were classified semiquantitatively as follows: 4, strong expression in 75 to 100% of the tumor cells; 3, 50 to 75%; 2, 25 to 50%; 1, 5 to 25%; and 0 fewer than 5% of the tumor cells. The endothelial cells in skin biopsies of seven of 30 primary cutaneous lymphomas expressed ICAM-3. In contrast, no expression of ICAM-3 could be demonstrated on endothelial cells in lymph nodes infiltrated with tumor cells of CTCL. Finally, endothelial cells of lymph nodes infiltrated with primary nodal B-cell lymphomas showed expression of ICAM-3 in three of 10 patients. The endothelial cells in the 11 control patients presenting with both eczematous lesions and dermatopathic lymphadenopathy showed no staining for ICAM-3. Every patient who expressed ICAM-3 on endothelial cells showed systemic spread of this disease. The findings suggest that ICAM-3 expression may be induced on endothelial cells in late-stage cutaneous lymphomas, probably by a cytokine-mediated mechanism.

PMID: 9261475 [PubMed - indexed for MEDLINE]

1899. Int Arch Allergy Immunol. 1997 Aug;113(4):495-504.

Adhesion molecules in atopic dermatitis: upregulation of alpha6 integrin expression in spontaneous lesional skin as well as in atopen, antigen and irritative induced patch test reactions.

Jung K, Imhof BA, Linse R, Wollina U, Neumann C.

Clinic of Dermatology Erfurt, Germany.

Atopic dermatitis is a lymphocyte-mediated skin disease. We studied the expression of the adhesion molecule alpha6 integrin by immunohistochemistry in spontaneous atopic inflammation as well as during the eliciting phase of atopen (Dermatophagoides pteronyssinus), antigen (nickel sulfate) and irritative (anthralin) induced patch test reactions in atopic skin. Results were compared

with nickel sulfate patch test reactions in normal skin. A role of the alpha6 integrin, expressed at the luminal side of blood vessels, for T cell migration in lesional atopic skin was supposed. In normal human skin the alpha6 integrin was weakly expressed by blood vessels and by basal epithelial cells of the epidermis. In acute and chronic lesional skin of patients with atopic dermatitis dramatic upregulation of alpha6 integrin expression was observed on endothelial cells and in the epidermis. The similar pattern of upregulated suprabasal alpha6 integrin expression was established in the patch test reactions 48 h after atopen and antigen application or irritation of the skin without differences in dependence on the eliciting substance. No difference of alpha6 integrin expression was seen between atopic and normal skin. Tumor necrosis factor alpha, interleukin-1, interleukin-4 and interferon gamma play a role in atopic inflammation. Tumor growth factor beta and interleukin-6 are mitogenic/growth factors for keratinocytes. For this reason the effect of these cytokines and of phorbol-12-myristate-13-acetate on the expression level of alpha6 integrin was tested in short-term skin organ culture of normal and atopic skin as well as in keratinocyte cultures. In these assays no cytokines had an effect on alpha6 integrin expression suggesting another mechanism which regulates this integrin. However, the increased expression of alpha6 integrin in the suprabasal epidermis is associated with a T cell influx into the epidermis. We speculate that the alpha6 integrin expression may lead to an epidermotropism of T cells during inflammation.

PMID: 9250597 [PubMed - indexed for MEDLINE]

1900. Allerg Immunol (Paris). 1997 Jul;29 Spec No:25-7.

[Allergy to cow's milk].

[Article in French]

Fourrier E.

Laboratoire d'Immuno-Allergologie-CHU, Angers.

After recalling the medical reluctance as well as the risks that there are in complete elimination of milk in infants, the author presents several clinical pictures and then a classification of the immunological types. Allergic shock of neonates, digestive and extra-digestive (skin and respiratory airways) symptoms finally the rare chronic gastro-enteritis to cow milk. Non-reaginic food allergies: Acute gastro-enteropathy to cow milk, with villous atrophy and Heiner's syndrome, delayed hypersensitivities are studied, of difficult diagnosis that may cover almost all pathologies. They may be found in the digestive system, respiratory, the kidneys and even in the organs of behaviour. Migrane of food origin must be remembered. Development in regressive rules is a function of the type of allergy and the suddenness of the symptoms. Diagnosis is above all by questioning and confirmation or not by skin and in vitro tests. Certainty can only be shown by tests of elimination and re-introduction. The diet, at the same time of both diagnostic and therapeutic value, is based on the replacement of cow milk by foods that contain the same amount of proteins. It is essential, especially in the very small, to have perfect match of food so as to avoid any risk of a dramatic hypoprotinemia, which may happen if the child does not like the suggested diet, or if the parents cannot buy the substitution products. In such conditions great care must be taken to avoid provoking a crisis. Care must be taken to decide: If the elimination of cow milk is always justified each time. If it is, always check that the substituted protein is properly made, the family may change the diet mistakenly. It is better, finally, to keep the eczema, rather than die with it healed.

PMID: 9376082 [PubMed - indexed for MEDLINE]

1901. Klin Monbl Augenheilkd. 1997 Jul;211(1):70-1.

[Intrapalpebral migration of a form stable contact lens: a rare complication in contact lens practice].

[Article in German]

Heine S, Wohlrab TM, Rohrbach JM.

PURPOSE: To demonstrate a rare complication in contactology.
CASE REPORT: A 26-year-old female had "lost" her hard contact lens in the right eye 2 years previously. Afterwards, new contact lenses were not tolerated. The patient had a slight intermittent epiphora but no further complaints. She consulted her ophthalmologist for new spectacles. At presentation, there was a firm tumour without signs of inflammation in the right upper lid area. When the lid was everted a hard contact lens was found within the tarsal plate which could be easily removed in the operating room. Microbiologic investigation disclosed no bacteria. Histology showed a circumscribed papillary reaction and a chronic non-specific inflammation with few eosinophils and no giant cells. Two weeks later the lens-related cavity was only slightly filled up by granulation tissue.
CONCLUSIONS: After "loss" of a contact lens superior dislocation and finally tarsal implantation should be kept in mind. The process of contact lens migration reveals some interesting features: 1. Generally, it causes only minor symptoms though it may last for years. 2. Bacterial contamination rarely occurs. 3. Histologically, the inflammation is often mild or even absent. Eosinophils and giant cells are of minor or no importance indicating that allergy and giant-cell reaction play no significant role. 4. The lens related cavity probably heals slowly, possibly because of a (partial) epithelialization.

PMID: 9340412 [PubMed - indexed for MEDLINE]

1902. Br J Pharmacol. 1997 Jul;121(6):1150-4.

Inhibition of eotaxin-mediated human eosinophil activation and migration by the selective cyclic nucleotide phosphodiesterase type 4 inhibitor rolipram.

Santamaria LF, Palacios JM, Beleta J.

Grupo Farmacéutico Almirall, S. A. Research Center, Barcelona, Spain.

1. The effect of the selective type 4 phosphodiesterase (PDE 4) inhibitor rolipram on human eosinophil activation and migration mediated by eotaxin was investigated. 2. Studies were performed with human freshly isolated eosinophils from peripheral blood of healthy donors by a magnetic cell separation (MACS) technique to a purity > 99%. To test the effect of rolipram, eosinophils were stimulated with recombinant human eotaxin and the cell surface activation markers CD11b and L-selectin were analysed by flow cytometry. Furthermore, eotaxin mediated eosinophil migration was measured in a transendothelial chemotaxis assay. 3. Our results indicate that rolipram inhibited eotaxin-induced CD11b up-regulation up to 60.6 +/- 7.6% at the highest tested dose (10 microM), whereas transendothelial chemotaxis was partially inhibited reaching a plateau of approx. 30% at a rolipram concentration of 0.1 microM. 4. We conclude that the selective PDE 4 inhibitor rolipram decreases eotaxin mediated eosinophil activation, an observation that may contribute to elucidate the mechanism by which PDE 4 inhibitors reduce antigen-induced eosinophil infiltration in different animal models of allergic inflammation.

PMCID: PMC1564801
PMID: 9249251 [PubMed - indexed for MEDLINE]

1903. Eur Respir J. 1997 Jul;10(7):1467-75.

Relationship between allergic manifestations and Toxocara seropositivity: a cross-sectional study among elementary school children.

Buijs J, Borsboom G, Renting M, Hilgersom WJ, van Wieringen JC, Jansen G, Neijens J.

Laboratory for Parasitology and Mycology, National Institute of Public Health and Environment, Bilthoven, The Netherlands.

Toxocara (the cause of visceral larva migrans in humans) and allergy have in common both elevated immunoglobulin E (IgE) levels and eosinophilia. In the present study, we investigated: 1) associations between Toxocara seropositivity and allergic manifestations; 2) risk factors for Toxocara infection; and 3) differences in Toxocara seroprevalence, allergic manifestations and the associations between these two, in children from urban and rural environments. Blood samples from 1,379 Dutch urban and rural elementary schoolchildren, were examined for Toxocara antibodies, eosinophil numbers, total IgE concentrations, and the occurrence of inhaled allergen-specific IgE. Questionnaires investigating respiratory health and putative risk factors for infection were completed. It was found that 8% of the children had Toxocara antibodies, occurring significantly less often in females than in males. The means of total serum IgE levels and blood eosinophils were significantly higher in the Toxocara-seropositive than in the seronegative group. Allergic asthma/recurrent bronchitis was found in 7% of the children, allergic reaction on animal contact in 4%, and IgE to at least one inhaled allergen in 16%. These variables were associated with Toxocara seroprevalence. Inhaled allergen-specific IgE and asthma/recurrent bronchitis occurred significantly less often in rural than in urban areas, and significantly less often among girls than among boys. Furthermore, occurrence of allergen-specific IgE increased significantly with age. No association existed between Toxocara seroprevalence and assumed risks, i.e. contact with pet animals and public playgrounds. In conclusion, our results indicate that allergic manifestations occur more often in Toxocara-seropositive children. A relationship with an already existing allergic condition is plausible.

PMID: 9230232 [PubMed - indexed for MEDLINE]

1904. Allerg Immunol (Paris). 1997 Jun;29(6):165-8.

[Clinical signs of food allergy].

[Article in French]

Le Sellin J.

Laboratoire d'Immuno-Allergologie, CHU, Angers.

To investigate the clinical signs of food allergy is to open new horizons for the understanding of worrying acute symptoms for the patients and too often only symptomatic treatment. It is also possible to take control in a more logical way of illnesses that are classified as chronic and so incurable. The clinical examination requires great attention and much listening. Urticaria, eczema, rhinitis, asthma, colitis are the most frequent symptoms. More rarely, there may

be certain migranes or lipoid nephrosis in children.

PMID: 9254122 [PubMed - indexed for MEDLINE]

1905. Eur J Immunol. 1997 Jun;27(6):1406-12.

Differential expression of binding sites for chemokine RANTES on human T lymphocytes.

Utsunomiya I, Tani K, Gong W, Oppenheim JJ, Wang JM.

Laboratory of Molecular Immunoregulation, National Cancer Institute-Frederick Cancer Research and Development Center, MD 21702, USA.

The C-C chemokine RANTES, a T lymphocyte chemoattractant, is considered an important mediator of inflammation, allergy, and host defense against HIV-1 infection. In this study, we investigated the modulation of binding of RANTES to T lymphocytes. Human peripheral blood CD3+ T cells, when freshly isolated from buffy-coat blood, expressed a considerable number of high-affinity binding sites for RANTES. These cells also showed significant chemotactic migration in response to RANTES in vitro. After 6-15 h incubation at 37 degrees C, the binding of RANTES, but not of macrophage inflammatory protein-1 alpha (MIP-1 alpha) or of monocyte chemotactic protein-3 (MCP-3), consistently increased. Scatchard analyses indicated that the number of binding sites for RANTES increased about threefold by 15 h without any change in the affinity. The increase in RANTES binding was no longer detected by 24 h. This increase in the specific binding was mainly attributable to CD4+ T cells and was not associated with increased chemotactic activity of these cells in response to RANTES. Incubation with anti-CD3 antibody for 15 h markedly reduced the binding capability of T cells for RANTES and was associated with decreased chemotactic activity. On the other hand, when T cells were incubated with interleukin-2 (IL-2) for 1 week, the specific binding for all three C-C chemokines, RANTES, MIP-1 alpha, and MCP-3 was markedly increased in comparison to cells cultured in the absence of IL-2. These results suggest that the expression of binding sites on T cells for RANTES is differentially modulated, indicating the existence of novel receptors for RANTES that do not bind MIP-1 alpha.

PMID: 9209492 [PubMed - indexed for MEDLINE]

1906. Semin Respir Infect. 1997 Jun;12(2):138-48.

Dirofilaria, visceral larva migrans, and tropical pulmonary eosinophilia.

Chitkara RK, Sarinas PS.

Pulmonary and Critical Care Medicine, Stanford University Medical Center, Palo Alto, CA, USA.

Helminthic infections are prevalent worldwide. The intestinal ascarid, Toxocara, the animal filarial parasite, Dirofilaria, and the human filarial parasite, Wuchereria or Brugia, produce an array of pulmonary disease in humans. Infections are common in temperate, tropical, and subtropical regions of the world. Pulmonary dirofilariasis is essentially an asymptomatic disease. Most cases are diagnosed accidentally after thoracotomy for a solitary pulmonary nodule presumed to be lung cancer. Clinical manifestations of toxocariasis or visceral larva migrans (VLM) are the result of allergic and inflammatory responses of the host, and manifest with airway reactivity, acute pneumonia, and persistent eosinophilia. VLM is a self-limited disease and specific treatment is rarely

necessary. In acute cases, a short course of steroids reduces morbidity and mortality but preventive measures are more important in curbing toxocara infection. Tropical pulmonary eosinophilia (TPE) is the result of immunologic hyperresponsiveness to the human filarial antigen and eosinophils play a crucial role in its pathogenesis. Airway hyperreactivity, extreme eosinophilia, and pulmonary physiologic impairment are the characteristic features. Treatment of TPE with diethylcarbamazine results in dramatic amelioration of symptoms. However, low grade inflammation persists in a significant number of patients and can lead to chronic interstitial lung disease. Mass treatment of patients in certain endemic areas has been effective in eliminating TPE.

PMID: 9195679 [PubMed - indexed for MEDLINE]

1907. Semin Respir Infect. 1997 Jun;12(2):130-7.

Ascariasis and hookworm.

Sarinas PS, Chitkara RK.

Dept. of Pulmonary and CCM, Stanford University Medical Center, CA, USA.

Ascariasis and hookworm (ancylostomiasis) remain the most common intestinal nematodes in the world with significant economic, social, and medical impact. An understanding of the transmission and pathogenesis of ascariasis and hookworm are necessary to recognize their clinical manifestations and to manage the pulmonary sequelae of infection. Transmission occurs predominantly in the tropics and rural areas where there is suboptimal sanitation, personal hygiene, and education regarding these parasites. Ascariasis generally occurs through hand-to-mouth ingestion of agricultural products or food contaminated with parasite eggs. Hookworm is transmitted through larval penetration of the skin. Larval pulmonary migration generally is asymptomatic. However, symptomatic pulmonary disease may occur with fever, cough, chest pain, hemoptysis, dyspnea, and wheezing due to (1) Loffler's syndrome, (2) the effects of larval tissue migration, (3) airway reactivity or bronchospasm, (4) infectious bacterial complications from parasitic migration and associated aspiration, and rarely (5) chronic eosinophilic pneumonia, transdiaphragmatic penetration, or symptoms of upper airway obstruction. Clinical evaluation shows pulmonary opacities on chest radiograph, peripheral blood eosinophilia, and larvae in respiratory or gastric secretions. Symptomatic treatment may be necessary with bronchodilators and systemic steroids or antibiotics for bacterial complications. The drug of choice is mebendazole (Vermox) 100 mg twice a day for 3 days. Alternatives include a single dose of pyrantel pamoate (Antiminth) 11 mg/kg (maximum dose, 1 g) or albendazole (Zentel) 400 mg orally once. Invermectin (Mectizan) is available through the World Health Organization, and, in the United States, through the manufacturer on a compassionate-use basis. Ivermectin is as effective as currently available drugs against Ascaris but shows only partial efficacy against hookworms, which infest humans. Preventive measures, improvement of sanitary facilities, education, and school screening may be important in the endemic areas to control these parasitic infections.

PMID: 9195678 [PubMed - indexed for MEDLINE]

1908. J Neuroophthalmol. 1997 Jun;17(2):108-21.

Neuro-ophthalmic manifestations of Lyme disease.

Balcer LJ, Winterkorn JM, Galetta SL.

Department of Neurology, Hospital of the University of Pennsylvania,
Philadelphia, USA.

Lyme disease is a multisystem disorder caused by infection with the Borrelia burgdorferi spirochete. The diagnosis of Lyme disease usually is based on several clinical criteria, with supportive data from laboratory testing. The presence of the bullseye skin lesion, erythema migrans, is the single pathognomonic criterion. In the 20 years since the initial description of Lyme disease in the United States, B. burgdorferi has been implicated as an etiologic agent in numerous ophthalmic and neuro-ophthalmic syndromes, involving most structures from the cornea to the cranial nerves. Neuro-ophthalmic and ocular manifestations of Lyme disease include meningitis with papilledema, cranial neuropathies, follicular conjunctivitis, nummular keratitis, and intraocular inflammation. Although an association with Lyme disease has been purported for numerous other syndromes, a definite causal relationship has not been proved in many cases. During a period of rapidly increasing awareness of Lyme disease, a high index of suspicion and poorly defined criteria for its presence have resulted in over-diagnosis of Lyme disease. In the authors' experience, the incorrect diagnosis of Lyme disease initially has been made in patients with allergic conjunctivitis, keratoconus, morning glory syndrome, craniopharyngioma, meningioma, CNS lymphoma, paraneoplastic syndrome, multiple sclerosis, sarcoid, syphilis, and functional illness. Nevertheless, this treatable infection must be an important consideration in the differential diagnosis of certain ocular or neurologic diseases.

PMID: 9176782 [PubMed - indexed for MEDLINE]

1909. J Immunol. 1997 Jun 1;158(11):5464-70.

Eosinophil adhesion regulates RANTES production in nasal epithelial cells.

Terada N, Maesako K, Hamano N, Houki G, Ikeda T, Sai M, Yamashita T, Fukuda S, Wakita A, Yoshimura K, Konno A.

Department of Otorhinolaryngology, Chiba University School of Medicine, Chiba City, Japan.

Among the many known chemotactic factors for eosinophils, the proinflammatory chemokine RANTES is particularly important, because it is potently and selectively chemotactic for eosinophils. Throughout the process of the migration of eosinophils from the blood vessels into the nasal cavity, eosinophil functions are assumed to be regulated by surface adhesion molecules. Conversely, the messages conferred by the eosinophils to the endothelial and epithelial cells are also of great interest. In the present study, we showed that eosinophil adhesion to human nasal epithelial cells (HNECs) inhibits RANTES production in HNECs. Eosinophils were isolated from peripheral blood obtained from patients with allergic rhinitis. Human mucosal microvascular endothelial cells and HNECs were isolated from human nasal mucosa specimens. After stimulation of the HNECs in the presence of eosinophils, the secretion of RANTES, induced by a combination of TNF-alpha and IFN-gamma, appeared to have decreased. The amount of the decrease was a function of the number of involved eosinophils. On the other hand, the presence of eosinophils did not affect RANTES production by the endothelial cells. After pretreatment of the eosinophils with anti-CD18 mAb or coculture with HNECs in Transwell culture inserts, these cells did not inhibit the TNF-alpha- and IFN-gamma-induced RANTES production. These results were virtually identical with those observed on RANTES mRNA expression. The adhesion of eosinophils to HNECs plays a key role in the inhibition of RANTES production. Our data indicate that a certain established system causes the signal transfer from eosinophils to HNECs to inhibit RANTES production, thus decreasing the eosinophil infiltration.

PMID: 9164969 [PubMed - indexed for MEDLINE]

1910. Scand J Immunol. 1997 May;45(5):471-81.

Human leukaemic (HMC-1) and normal skin mast cells express beta 2-integrins: characterization of beta 2-integrins and ICAM-1 on HMC-1 cells.

Weber S, Babina M, Feller G, Henz BM.

Department of Dermatology, Virchow-Klinikum, Humboldt-Universität zu Berlin, Germany.

Mast cells are bone marrow-derived, ubiquitous connective tissue resident cells. However, their mechanisms of migration, the distribution of immature and mature cells and their interaction with other inflammatory cells are largely unclarified. Possibly, beta 2-integrins play an important role in these processes. In the present investigation, the authors studied the expression and regulation of the beta 2-integrins LFA-1 (CD11a/CD18), Mac-1 (CD11b/CD18), p150,95 (CD11c/CD18) and of the LFA-1/Mac-1 counter-receptor intercellular adhesion molecule-1 (ICAM-1; CD54) on leukaemic (HMC-1 cell subclone 5C6) and on normal mature human skin mast cells. The HMC-1 cells clearly expressed CD11a, CD18 and CD54, while expression of CD11b and CD11c was low. The apparent molecular weights were 180 kDa (CD11a), 95 kDa (CD18) and 90 kDa (CD54) as determined by Western blot analysis. Phorbol myristate acetate (PMA) induced a time- and dose-dependent up-regulation of CD11a, CD11b, CD11c, CD18 and CD54 that was inhibited by cycloheximide, suggesting a dependence on de novo protein synthesis. Enhanced expression of CD11a, CD11b, CD11c and CD18 could also be confirmed at the gene level as demonstrated by semi-quantitative reverse transcription-polymerase chain reaction (RT-PCR). Increased expression of LFA-1/ICAM-1 in response to PMA was accompanied by strong enhancement of homotypic cell aggregation, suggesting that newly synthesized LFA-1/ICAM-1 is functionally active. In order to determine a physiologically relevant way of mast cell beta 2-integrin modulation, several cytokines and chemotactic mediators (interleukin-4, IL-4; nerve growth factor beta, NGF beta; C5a; and leukotriene B4, LTB4) were tested for their influence on adhesion molecule cell surface density. Only LTB4 was shown specifically to up-regulate CD11a and CD18, but not CD11b or CD11c. The presence of CD11a, CD11c and CD18 could be confirmed on a low percentage of normal skin mast cells by immunofluorescence, using a double staining technique. In comparison to normal skin, a significantly higher percentage of CD18+ mast cells was found in inflammatory dermatoses such as psoriasis vulgaris, atopic dermatitis and lichen planus. Therefore, mast cell beta 2-integrins possibly play an important role during homing of immature mast cells as well as during the interaction of activated mast cells with other inflammatory cells.

PMID: 9160089 [PubMed - indexed for MEDLINE]

1911. J Invest Dermatol. 1997 May;108(5):743-7.

Enhanced expression of T-cell activation and natural killer cell antigens indicates systemic anti-tumor response in early primary cutaneous T-cell lymphoma.

Asadullah K, Friedrich M, Döcke WD, Jahn S, Volk HD, Sterry W.

Department of Dermatology, Medical School Charité, Humboldt UniversityBerlin, Germany.

Indolent, primary cutaneous T-cell lymphomas (CTCL) are characterized by hyper-proliferation of malignant T-helper cells in the skin with a favorable prognosis in the early stages. Cytotoxic T cells (CTLs) are believed to be of major importance for tumor surveillance, but there is not yet sufficient evidence for a systemic anti-tumor response in mycosis fungoides (MF). On the contrary, there are hints of systemic immunodepression. We wondered whether signs of a systemic anti-tumor response were demonstrable in peripheral blood of patients with MF and CD30+ pleomorphic T cell lymphoma. Using multiparameter flow cytometry, we investigated blood samples from 39 CTCL patients at different stages and compared them with those from patients with psoriasis, atopic dermatitis, and healthy volunteers. In CTCL patients, an elevated number of lymphocytes expressing natural killer cell markers were found, as well as considerable T-cell activation, indicated by increased percentages of T cells expressing HLA-DR, IL-2 receptor alpha-chain, and transferrin receptor. The CD8+ T cells, which were the most strongly activated T-cell subset, were of polyclonal origin, as shown by their usage of different T-cell receptor families. The enhanced expression of activation antigens was associated with an increased proportion of CD8+ T cells with high expression of the adhesion molecule LFA-1, demonstrating the capacity for migration of these cells. These CD8+ effector cells are suspected to be CTLs and may be responsible for the favorable prognosis of indolent, primary CTCL. Interestingly, a stage-dependent decrease in T-cell activation antigen expression was observed, suggesting the development of a lack in tumor surveillance in advanced MF stages. Further investigations are necessary to verify whether any of the parameters determined are of predictive value for prognosis and response to therapy in CTCL.

PMID: 9129226 [PubMed - indexed for MEDLINE]

1912. J Immunol. 1997 May 1;158(9):4398-404.

Differential recruitment of leukocyte populations and alteration of airway hyperreactivity by C-C family chemokines in allergic airway inflammation.

Lukacs NW, Strieter RM, Warmington K, Lincoln P, Chensue SW, Kunkel SL.

Department of Pathology, University of Michigan Medical School, Veterans Affairs Medical Center, Ann Arbor 48109, USA.

Allergic airway inflammation is characterized by peribronchial leukocyte accumulation within the airway. Subsequent tissue damage leading to airway hyperreactivity is a result of activation of multiple leukocyte populations. Using an established model of allergic airway inflammation induced by intratracheal challenge with parasite (Schistosoma mansoni) egg Ag in presensitized mice, we have examined differential leukocyte recruitment. These studies have identified key chemokines involved in the accumulation of specific subsets of cells and the induction of airway hyperreactivity. In this study we have examined three C-C family chemokines, MCP-1, MIP-1alpha, and RANTES, which promote mononuclear cell- and eosinophil-specific recruitment to the airway. The in vivo neutralization of either MIP-1alpha or RANTES, but not MCP-1, significantly reduced the intensity of the eosinophil recruitment to the lung and airway during the allergic airway response by >50 and >60%, respectively. In contrast, neutralization of MCP-1 significantly reduced total leukocyte migration (>50% reduction), whereas neutralization of RANTES and MIP-1alpha had no significant affect on the overall leukocyte migration. Further examination of the effect of MCP-1 depletion indicated that both CD4+ and CD8+ lymphocyte subsets were decreased. Depletion of MCP-1 significantly reduced the airway hyperreactivity to near control levels, whereas depletion of MIP-1alpha or RANTES did not affect the intensity of airway hyperreactivity. These data indicate that

multiple C-C chemokines are involved in the recruitment of particular leukocyte populations and that neutralization of MCP-1, but not RANTES or MIP-1alpha, significantly reduced airway hyperreactivity.

PMID: 9127004 [PubMed - indexed for MEDLINE]

1913. Proc Natl Acad Sci U S A. 1997 Apr 29;94(9):4610-4.

A cholera toxoid-insulin conjugate as an oral vaccine against spontaneous autoimmune diabetes.

Bergerot I, Ploix C, Petersen J, Moulin V, Rask C, Fabien N, Lindblad M, Mayer A, Czerkinsky C, Holmgren J, Thivolet C.

Institut National de la Santé et de la Recherche Médicale 449, Faculté Alexis Carrel, Lyon, France.

Mucosally induced immunological tolerance is an attractive strategy for preventing or treating illnesses resulting from untoward inflammatory immune reactions against self- or non-self-antigens. Oral administration of relevant autoantigens and allergens has been reported to delay or suppress onset of clinical disease in a number of experimental autoimmune and allergic disorders. However, the approach often requires repeated feeding of large amounts of tolerogens over long periods and is only partly effective in animals already systemically sensitized to the ingested antigen such as in animals already harboring autoreactive T cells, and thus presumably also in humans with an autoimmune disease. We have recently shown that oral administration of microgram amounts of antigen coupled to cholera toxin B subunit (CTB), can effectively suppress systemic T cell reactivity in naive as well as in immune animals. We now report that feeding small amounts (2-20 microg) of human insulin conjugated to CTB can effectively suppress beta cell destruction and clinical diabetes in adult nonobese diabetic (NOD) mice. The protective effect could be transferred by T cells from CTB-insulin-treated animals and was associated with reduced lesions of insulitis. Furthermore, adoptive co-transfer experiments involving injection of Thy-1,2 recipients with diabetogenic T cells from syngeneic mice and T cells from congenic Thy-1,1 mice fed with CTB-insulin demonstrated a selective recruitment of Thy-1,1 donor cells in the peripancreatic lymph nodes concomitant with reduced islet cell infiltration. These results suggest that protection against autoimmune diabetes can be achieved by feeding minute amounts of a pancreas islet cell autoantigen linked to CTB and appears to involve the selective migration and retention of protective T cells into lymphoid tissues draining the site of organ injury.

PMCID: PMC20771
PMID: 9114038 [PubMed - indexed for MEDLINE]

1914. Blood. 1997 Apr 15;89(8):2863-70.

C3a and C5a stimulate chemotaxis of human mast cells.

Hartmann K, Henz BM, Krüger-Krasagakes S, Köhl J, Burger R, Guhl S, Haase I, Lippert U, Zuberbier T.

Department of Dermatology, Virchow Clinics, Humboldt University of Berlin, Germany.

The factors that control migration of mast cells to sites of inflammation and tissue repair remain largely undefined. Whereas several recent studies have

described chemotactic factors that induce migration of murine mast cells, only stem cell factor (SCF) is known to induce migration of human mast cells. We report here that the anaphylatoxins C3a and C5a are chemotactic factors for the human mast cell line HMC-1, human cord blood-derived mast cells (CBMC) and cutaneous mast cells in vitro. The presence of an extracellular matrix protein, laminin, was required for chemotaxis in response to complement peptides. Migration of mast cells towards C3a and C5a was dose-dependent, peaking at 1 microg/mL (100 nmol/L), and was inhibited by specific antibodies. Pretreatment with pertussis toxin inhibited the anaphylatoxin-mediated migration of HMC-1 cells, indicating that Gi proteins are involved in complement-activated signal transduction pathways in human mast cells. Both C3a and C5a also induced a rapid and transient mobilization of intracellular free calcium ([Ca2+]i) in HMC-1 cells. Besides SCF, other chemotactic factors tested, such as interleukin-3, nerve growth factor, transforming growth factor beta, RANTES (regulated upon activation, normal Tcell expressed and secreted), monocyte chemotactic protein-1 (MCP-1), MCP-2, MCP-3, macrophage inflammatory protein-1alpha (MIP-1alpha), and MIP-1beta, failed to stimulate migration of human mast cells. In summary, these findings indicate that C3a and C5a serve as chemotaxins for human mast cells. Anaphylatoxin-mediated recruitment of mast cells might play an important role in hypersensitivity and inflammatory processes.

PMID: 9108406 [PubMed - indexed for MEDLINE]

1915. Berl Munch Tierarztl Wochenschr. 1997 Apr;110(4):128-33.

[Allergenicity and specific protein profiles of mange mites Chorioptes bovis, Psoroptes ovis (Acari: Psoroptidae), Saroptes suis and Notoedres cati (Acari: Sarcoptidae) using SDS-PAGE and immunoblotting].

[Article in German]

Beck W, Hiepe T.

Institut für Parasitologie und Tropenveterinärmedizin (Standort Mitte), Freien Universität Berlin.

A main point of immunoparasitological research in regard to pest arthropod-infestation is biochemical and immunological characterization of antigens. Precondition of own examinations to the specific protein pattern of mange mites were in quality and amount sufficient antigen preparations in mite extract solutions. For mite separation and antigen refinement field strains of Chorioptes bovis, Psoroptes ovis, Sarcoptes suis and Notoedres cati from definitive host animals cattle, sheep, pig and cat have been used. Parasites were isolated in a migration procedure. After having applicated subepidermally a low dose of mite extract solutions in sensitized animals allergic skin changes (Immediate reaction type 1) became apparent. SDS-PAGE exhibited specific protein patterns of 4 pathogen mite species. For Chorioptes bovis 16, Psoroptes ovis 15, Sarcoptes suis 27, and Notoedres cati 36 fractions have been detected. Proteins are antigens or allergen structures to be found in saliva, faecal output or moulting products of developmental stages and other metabolites of the parasites. Protein components were transferred onto nitrocellulosis. Immunoblotting made fractions with antigen activity visible.

PMID: 9182511 [PubMed - indexed for MEDLINE]

1916. Am J Respir Cell Mol Biol. 1997 Apr;16(4):455-63.

Transmigration of eosinophils through basement membrane components in vitro:

synergistic effects of platelet-activating factor and eosinophil-active cytokines.

Okada S, Kita H, George TJ, Gleich GJ, Leiferman KM.

Department of Immunology, Mayo Clinic and Mayo Foundation, Rochester, Minnesota 55905, USA.

Migration of eosinophils through the basement membrane barrier is an important step for their infiltration into tissues. To investigate the mechanism of transmigration, we used a chamber fitted with a Matrigel membrane as a model of the basement membrane. In this model, eosinophils treated with cytokines or chemotactic factors alone did not transmigrate from the upper to the lower chamber. However, platelet-activating factor (PAF) strongly induced transmigration of eosinophils stimulated by interleukin (IL)-5, indicating that both a cytokine and a chemotactic factor are required for eosinophil migration through Matrigel. Granulocyte-macrophage colony-stimulating factor (GM-CSF) and IL-3 also stimulated eosinophil transmigration in the presence of PAF. Of seven eosinophil chemotactic factors tested, leukotriene B4, C5a, RANTES, macrophage inflammatory protein-1alpha, and IL-8 did not induce significant eosinophil transmigration. Only PAF and eotaxin induced transmigration of eosinophils through Matrigel in the presence of IL-5; PAF was more potent than eotaxin at the optimal concentration. In contrast, PAF, eotaxin, and RANTES all potently induced migration of eosinophils through bare membrane in the absence of IL-5. Finally, eosinophil migration through Matrigel was markedly reduced by a combination of anti-CD18 and anti-CD29 monoclonal antibodies, suggesting that it is mediated by beta1- and beta2-integrin adhesion molecules. Our findings demonstrate that eosinophil transmigration through a basement membrane model requires both a specific chemoattractant, such as PAF, and an eosinophil-activating cytokine, such as IL-5. This synergistic effect is likely important in the tissue accumulation of activated eosinophils in allergic and other eosinophil-associated diseases.

PMID: 9115757 [PubMed - indexed for MEDLINE]

1917. Pediatr Med Chir. 1997 Mar-Apr;19(2):141-2.

[Toxocariasis: a case report].

[Article in Italian]

Paolillo F, Migliori C, Fornari M, Belloni C.

Divisione di Pediatria, Ospedale Maggiori di Lodi, (Italia).

The Authors present a case of infestation by Toxocara canis occurring in a eighteen months' child. A first time he was hospitalised for wheezing, reported hypereosinophilia and a thoracic X-ray with a soften congestion in the right lung's base. Two months later he was admitted to hospital for the persistence of high level of eosinocyte. The clinical tests and the patient's history explain the first symptoms like the passage of the Toxocara's larvae in the lungs, but don't justify the anomalous behaviour of eosinocytes. In fact the hypereosinophilia was present two months before the admission in our Department, but two tests, executed during the first stay in hospital, didn't show abnormality. A few months after, another blood's sample showed an high level of eosinocytes and, in the same time, a new control of thoracic X-ray, didn't show any change in the image.

PMID: 9312752 [PubMed - indexed for MEDLINE]

1918. Biull Eksp Biol Med. 1997 Mar;123(3):315-8.

[Changes in clinico-immunological indices of experimental allergic encephalomyelitis after treatment with perftoran].

[Article in Russian]

Zhitnukhin IuL, Litvinenko IV, Bisaga GN, Odinak MM.

PMID: 9162244 [PubMed - indexed for MEDLINE]

1919. J Pediatr Health Care. 1997 Mar-Apr;11(2):51-60.

Health care of the internationally adopted child part 1. Before and at arrival into the adoptive home.

Mitchell MA, Jenista JA.

International adoptees are frequently encountered in pediatric practice. Their health status in the country of origin is quite variable. Once in the United States, there is no mandated medical evaluation for these infants and children. Commonly missed or ignored conditions include vision or hearing loss, infectious diseases, incomplete immunizations, and various allergies and food intolerances. The prepared nurse practitioner can provide invaluable assistance in ensuring the efficient and appropriate evaluation of each internationally adopted child. Part 1 of this series discusses concerns before the child's arrival in the adoptive home and the medical issues most important soon after arrival Part 2 addresses long-term issues including the follow-up of initial assessments, management of chronic medical problems, and concerns about growth, development, and social adjustments.

PMID: 9155348 [PubMed - indexed for MEDLINE]

1920. Intern Med. 1997 Mar;36(3):179-85.

Interleukin-13 but not interleukin-4 prolongs eosinophil survival and induces eosinophil chemotaxis.

Horie S, Okubo Y, Hossain M, Sato E, Nomura H, Koyama S, Suzuki J, Isobe M, Sekiguchi M.

First Department of Internal Medicine, Shinshu University School of Medicine, Nagano.

The effects of recombinant human (rh) interleukin (IL)-4 or rhIL-13 on survival, and chemotactic activity of human eosinophils were examined. Only rhIL-13 prolonged eosinophil survival in a dose-dependent manner above 3 ng/ml. Eosinophil survival induced by rhIL-13 was inhibited by monoclonal antibodies (mAbs) against IL-3 ($p < 0.01$) and granulocyte-macrophage colony-stimulating factor (GM-CSF) ($p < 0.05$), suggesting that rhIL-13 induced IL-3 and GM-CSF production from eosinophils and an autocrine mechanism is responsible for the eosinophil survival. The effects of rhIL-13 on eosinophil chemotactic activity were also examined. rhIL-13 showed chemotactic activity for eosinophils in a dose-dependent manner. Checkerboard analysis revealed that eosinophil migration was dependent on the concentration gradient, confirming that rhIL-13 is a chemotactic factor. rhIL-4 showed no effects. IL-13 may play an important role in

the survival and recruitment of eosinophils in allergic diseases.

in IL-5 deficient mice. The eosinophilic response induced by intravenous eotaxin in wild type mice did not correlate with a significant reduction in the level of bone marrow eosinophils, whereas intravenous IL-5 resulted in depletion of this store. These results suggest the existence of two mechanisms by which eosinophils can be rapidly mobilized in response to intravenous eosinophil chemoattractants; first, mobilization of an IL-5 dependent bone marrow pool, and second, an eotaxin-induced sequestration of eosinophils from tissues into the blood. Subcutaneous injection of eotaxin induced a local tissue eosinophilia in wild type mice but not in IL-5 deficient mice. Furthermore, tissue eosinophilia in wild type mice, but not in IL-5 deficient mice, was enhanced by adoptive transfer of eosinophils or the administration of intravenous IL-5. However, pretreatment of IL-5 deficient mice with intraperitoneal IL-5 for 72 h restored eosinophil homing and tissue accumulation in response to subcutaneous eotaxin. We propose that eotaxin secreted from inflamed tissue may play an important role in initiating both blood and tissue eosinophilia in the early phases of allergic inflammation. Furthermore, IL-5 is not only essential for mobilizing eosinophils from the bone marrow during allergic inflammation, but also plays a critical role in regulating eosinophil homing and migration into tissues in response to eotaxin and possibly other specific chemotactic stimuli.

PMCID: PMC507915
PMID: 9062365 [PubMed - indexed for MEDLINE]

1924. J Clin Invest. 1997 Mar 1;99(5):926-36.

Production of the novel C-C chemokine MCP-4 by airway cells and comparison of its biological activity to other C-C chemokines.

Stellato C, Collins P, Ponath PD, Soler D, Newman W, La Rosa G, Li H, White J, Schwiebert LM, Bickel C, Liu M, Bochner BS, Williams T, Schleimer RP.

Johns Hopkins Asthma and Allergy Center, Division of Clinical Immunology, Baltimore, Maryland 21224, USA.

Monocyte chemotactic protein-4 (MCP-4) is a newly identified C-C chemokine with potent eosinophil chemoattractant properties. We describe studies of its biological activity in vitro to induce chemotaxis of peripheral blood eosinophils and to induce histamine release from IL-3-primed peripheral blood basophils. MCP-4 and eotaxin caused a similar rise in eosinophil intracytoplasmic Ca2+ and complete cross-desensitization. MCP-4 also abolished the eosinophil Ca2+ response to MCP-3 and partially desensitized the response to macrophage inflammatory protein-1alpha. MCP-4 activated cell migration via either CCR2b or CCR3 in mouse lymphoma cells transfected with these chemokine receptors. MCP-4 inhibited binding of 125I-eotaxin to eosinophils and CCR3-transfected cells and inhibited 125I-MCP-1 binding to CCR2b-transfectants. MCP-4 mRNA was found in cells collected in bronchoalveolar lavage of asthmatic and nonasthmatic subjects and was prominently expressed in human lung and heart. MCP-4 mRNA was expressed in several human bronchial epithelial cell lines after cytokine stimulation. Pretreatment of BEAS-2B epithelial cells with the glucocorticoid budesonide inhibited MCP-4 mRNA expression. These features make MCP-4 a candidate for playing a role in eosinophil recruitment during allergic respiratory diseases.

PMCID: PMC507900
PMID: 9062350 [PubMed - indexed for MEDLINE]

1925. J Allergy Clin Immunol. 1997 Mar;99(3):410-6.

Soluble E-selectin as a marker of disease activity in atopic dermatitis.

Yamashita N, Kaneko S, Kouro O, Furue M, Yamamoto S, Sakane T.

Department of Immunology, St. Marrianna University School of Medicine, Kawasaki, Kanagawa, Japan.

BACKGROUND: Augmentation in the expression of adhesion molecules on endothelial cells can regulate leukocyte migration. These molecules are also shed into the circulation. The level of soluble adhesion molecules in the serum is known to reflect the degree of systemic inflammation, and this level can therefore be used as a marker of inflammation.
OBJECTIVE: To elucidate whether soluble adhesion molecules can be used as the marker of disease severity in atopic dermatitis, we examined the levels of soluble E-selectin, soluble intercellular adhesion molecule-1, and soluble vascular cell adhesion molecule-1 and compared these levels with the patients' symptom scores and their total serum IgE levels.
METHODS: Fifty-three patients with atopic dermatitis were studied. Soluble adhesion molecules were measured by ELISA.
RESULTS: The level of soluble E-selectin was higher in patients with atopic dermatitis than in healthy control subjects ($p < 0.01$). Moreover, soluble E-selectin is correlated with disease severity ($p < 0.01$). Soluble E-selectin also reflected the changes in symptom scores. In contrast, there were no differences in soluble intercellular adhesion molecule-1 and soluble vascular cell adhesion molecule-1 between the patients and control subjects.
CONCLUSION: Soluble E-selectin is a good marker of disease severity and of its activity in atopic dermatitis.

PMID: 9058698 [PubMed - indexed for MEDLINE]

1926. J Immunol. 1997 Mar 1;158(5):2500-5.

Dichotomy of blood- and skin-derived IL-4-producing allergen-specific T cells and restricted V beta repertoire in nickel-mediated contact dermatitis.

Werfel T, Hentschel M, Kapp A, Renz H.

Department of Dermatology, Hannover Medical School, Germany.

In this study we compared the phenotype and cytokine patterns of nickel-specific T cell clones (TCC) derived from blood samples and positive patch test reactions. A total of 252 nickel-specific TCC were established from three nonatopic patients with allergic contact dermatitis caused by nickel. All TCC expressed the TCR-alpha beta, and 77% were CD4+ compared with 21% CD8+ TCC. In contrast to blood-derived TCC, the majority of skin-derived CD4+ or CD8+ T lymphocytes produced IL-4 either in combination with IFN-gamma (type 0 cytokine pattern) or IL-4 exclusively (type 2 pattern). Skin-derived nickel-specific TCC of each patient secreted significantly more IL-4 than blood-derived TCC of the same individual. Analysis of TCR-V beta repertoire from two patients indicated that >40% of the tested TCC expressed one of the following V beta elements: V beta 13.1/13.2, V beta 20, V beta 2, V beta 6.7, or V beta 14. Only 20% of unstimulated T cells but >40% of nickel-stimulated T cells derived from peripheral blood of the same individuals expressed these V beta elements, suggesting a selection of certain TCR-V beta elements by nickel sulfate in these patients. In contrast to the compartmentalization of IL-4 production, there were no major differences in the expression of TCR-V beta elements between blood- and skin-derived nickel-specific TCC. These results point to a modulation of the cytokine production pattern of T lymphocytes after their migration from peripheral blood into the skin and a production of the type 2 cytokine IL-4 in acute eczematous lesions in nonatopic individuals.

PMID: 9037002 [PubMed - indexed for MEDLINE]

1927. Biochem Biophys Res Commun. 1997 Feb 13;231(2):365-8.

Eotaxin is a potent chemotaxin for human basophils.

Yamada H, Hirai K, Miyamasu M, Iikura M, Misaki Y, Shoji S, Takaishi T, Kasahara T, Morita Y, Ito K.

Department of Medicine and Physical Therapy, University of Tokyo School of Medicine, Japan.

We studied the effect of eotaxin, a novel eosinophil-active CC chemokine with high target cell specificity, on human basophils. Eotaxin induced higher levels of chemotactic response with a lower ED50 compared with RANTES in basophils; half-maximal migration occurred at a concentration of approximately 3 nM. On the other hand, it exerted only a marginal effect on either histamine release or leukotriene C4 generation. In addition, nested PCR amplification experiments revealed the expression of CC CKR3, a putative receptor for eotaxin, on basophils. Since accumulation of both basophils and eosinophils is an important aspect of allergic inflammation, eotaxin potentially plays a pathogenic role in allergic disorders by inducing migration of both of these cell types.

PMID: 9070280 [PubMed - indexed for MEDLINE]

1928. Chest. 1997 Feb;111(2 Suppl):27S-34S.

Leukotrienes in the pathogenesis of asthma.

O'Byrne PM.

Asthma Research Group, McMaster University, Hamilton, Ontario, Canada.

Asthma is a chronic inflammatory disease that is associated with widespread but variable airflow obstruction. The mechanisms that lead to airflow obstruction in asthma are bronchoconstriction, mucosal edema, increased secretion of mucus, and an inflammatory-cell infiltrate that is rich in eosinophils. Leukotrienes (LTs) B4, C4, D4, and E4 have been shown experimentally to play a role in each of these inflammatory mechanisms and to mimic the pathologic changes seen in asthma. Inhaled LTC4 and LTD4 are the most potent bronchoconstrictors yet studied in human subjects. LTC4 and LTD4 also may cause migration of inflammatory cells into the asthmatic airway. LTs are derived from the 5-lipoxygenase (5-LO) pathway of arachidonic acid metabolism, and increased production of LTs has been demonstrated in patients who have asthma. Leukotriene receptor antagonists and specific inhibitors of the 5-LO pathway hold great promise as new therapies to treat asthma. Because LTC4, LTD4, and LTE4 appear to interact with a common LTD4 receptor, selective LTD4 receptor antagonists (eg, pranlukast [SB205312/ONO-1078], zafirlukast [ICI 204,219], MK-571, and MK-679), as well as zileuton (A-64077, a direct inhibitor of 5-LO) have been developed as antiasthma agents. Clinical and experimental studies have demonstrated the efficacy of these compounds in reducing not only the symptoms of asthma, but use of beta 2-agonists and bronchoconstriction induced by exposure to allergens, exercise, aspirin, and cold air.

PMID: 9042024 [PubMed - indexed for MEDLINE]

1929. Mem Inst Oswaldo Cruz. 1997;92 Suppl 2:211-4.

Description of an in vivo model for the assessment of eosinophil chemoattractants in the mouse.

Teixeira MM, Williams TJ, Hellewell PG.

Imperial College of Medicine, National Heart and Lung Institute, London. mmtex@mono.icb.ufmg.br

Chemokines (chemoattractant cytokines) induce potent and selective chemotaxis of leukocyte subsets in vitro. Here, we review briefly the chemokines shown to induce eosinophil chemotaxis in vitro and describe a novel model for the study of the ability of chemokines to stimulate eosinophil migration in vivo. Eosinophils were purified from the blood of mice over-expressing the IL-5 gene and labelled with 111In. Only the C-C chemokines, eotaxin and MIP-1 alpha, but not RANTES, MCP-1, MCP-3, MCP-4, MIP-1 beta, KC and MIP-2, effectively induced the recruitment of 111 In-eosinophils in mouse skin. We suggest that this mouse model will be useful in assessing the role of endogenously-generated chemokines in mediating eosinophil migration to sites of allergic inflammation in vivo.

PMID: 9698936 [PubMed - indexed for MEDLINE]

1930. Mem Inst Oswaldo Cruz. 1997;92 Suppl 2:193-6.

A comparison of the inhibitory activity of selective PDE4 inhibitors on eosinophil recruitment in guinea pig skin.

Teixeira MM, Miotla JM, Cooper N, Gristwood RW, Hellewell PG.

Imperial College of Medicine, National Heart and Lung Institute, London, UK.

The elevation of intracellular cyclic AMP by phosphodiesterase (PDE)4 inhibitors in eosinophils is associated with inhibition of the activation and recruitment of these cells. We have previously shown that systemic treatment with the PDE4 inhibitor rolipram effectively inhibit eosinophil migration in guinea pig skin. In the present study we compare the oral potency and efficacy of the PDE4 inhibitors rolipram, RP 73401 and CDP 840 on allergic and PAF-induced eosinophil recruitment. Rolipram and RP 73401 were equally effective and potent when given by the oral route and much more active than the PDE4 inhibitor CDP 840. We suggest that this guinea pig model of allergic and mediator-induced eosinophil recruitment is both a sensitive and simple tool to test the efficacy and potency of PDE4 inhibitors in vivo.

PMID: 9698932 [PubMed - indexed for MEDLINE]

1931. Mem Inst Oswaldo Cruz. 1997;92 Suppl 2:183-91.

The role of the eosinophil-selective chemokine, eotaxin, in allergic and non-allergic airways inflammation.

Conroy DM, Humbles AA, Rankin SM, Palframan RT, Collins PD, Griffiths-Johnson DA, Jose PJ, Williams TJ.

Department of Applied Pharmacology, Imperial College School of Medicine, National Heart and Lung Institute, London, U.K.

Blood eosinophilia and tissue infiltration by eosinophils are frequently observed

in allergic inflammation and parasitic infections. This selective accumulation of eosinophils suggested the existence of endogenous eosinophil-selective chemoattractants. We have discovered a novel eosinophil-selective chemoattractant which we called eotaxin in an animal model of allergic airways disease. Eotaxin is generated in both allergic and non-allergic bronchopulmonary inflammation. The early increase in eotaxin paralleled eosinophil infiltration in the lung tissue in both models. An antibody to IL-5 suppressed lung eosinophilia, correlating with an inhibition of eosinophil release from bone marrow, without affecting eotaxin generation. This suggests that endogenous IL-5 is important for eosinophil migration but does not appear to be a stimulus for eotaxin production. Constitutive levels of eotaxin observed in guinea-pig lung may be responsible for the basal lung eosinophilia observed in this species. Allergen-induced eotaxin was present mainly in the epithelium and alveolar macrophages, as detected by immunostaining. In contrast there was no upregulation of eotaxin by the epithelial cells following the injection of sephadex beads and the alveolar macrophage and mononuclear cells surrounding the granuloma were the predominant positive staining cells. Eotaxin and related chemokines acting through the CCR3 receptor may play a major role in eosinophil recruitment in allergic inflammation and parasitic diseases and thus offer and attractive target for therapeutic intervention.

PMID: 9698931 [PubMed - indexed for MEDLINE]

1932. Mem Inst Oswaldo Cruz. 1997;92 Suppl 2:157-64.

Expression and function of beta 1 integrins on human eosinophils.

Seminario MC, Bochner BS.

Department of Medicine, Johns Hopkins Asthma and Allergy Center, Johns Hopkins University, Baltimore, MD 21224-6801, USA.

Eosinophils preferentially accumulate at sites of chronic allergic diseases such as bronchial asthma. The mechanisms by which selective eosinophil migration occurs are not fully understood. However, interactions of cell-surface adhesion molecules on the eosinophil with molecular counterligands on endothelial and epithelial cells, and on extracellular matrix proteins, are likely to be critical during the recruitment process. One possible mechanism for selective eosinophil recruitment involves the alpha-4-beta-1 (VLA-4) integrin which is not expressed on neutrophils. Correlations have been found between infiltration of eosinophils and endothelial expression of VCAM-1, the ligand for VLA-4, in the lungs of asthmatic individuals as well as in late phase reactions in the lungs, nose and skin. Epithelial and endothelial cells respond to the Th2-type cytokines IL-13 with selective de novo expression of VCAM-1, consistent with the possible role of VCAM-1/VLA-4 interactions in eosinophil influx during allergic inflammation. Both beta-1 and beta-2 integrins on eosinophils exist in a state of partial activation. For example, eosinophils can be maximally activated for adhesion to VCAM-1 or fibronectin after exposure to beta-1 integrin-activating antibodies or divalent cations, conditions that do not necessarily affect the total cell surface expression of beta-1 integrins. In contrast, cytokines like IL-5 prevent beta-1 integrin activation while promoting beta-2 integrin function. Furthermore, ligation of integrins can regulate the effector functions of the cell. For example, eosinophil adhesion via beta-1 and/or beta-2 integrins has been shown to alter a variety of functional responses including degranulation and apoptosis. Thus, integrins appear to be important in mediating eosinophil migration and activation in allergic inflammation. Strategies that interfere with these processes may prove to be useful for treatment of allergic diseases.

PMID: 9698928 [PubMed - indexed for MEDLINE]

1933. Dermatology. 1997;195(4):325-8.

Toxocariasis and Wells' syndrome: a causal relationship?

Hurni MA, Gerbig AW, Braathen LR, Hunziker T.

Dermatological Clinic, University of Berne, Inselspital, Berne, Switzerland.

BACKGROUND: The etiology of Wells' syndrome or eosinophilic cellulitis is unknown. Various triggering factors, such as myeloproliferative disorders, lymphoma, infections/infestations, insect bites and drugs have been reported. In 1979, Wells was the first who pointed out some common features of eosinophilic cellulitis and skin lesions in toxocariasis.
OBJECTIVE: We report 2 patients who exhibited the characteristic clinical and histological features of Wells's syndrome together with elevated antibody titers to the excretory-secretory antigen of Toxocara canis.
RESULTS: In both patients, the skin lesions disappeared after oral albendazole treatment and no recurrences were observed. The clinical response was followed by a normalization of the Toxocara antibody titer. In contrast, a patient with eczematoid skin lesions, eosinophilia and an elevated Toxocara antibody titer did not benefit from albendazole treatment despite serological normalization.
CONCLUSION: Taken together, these cases lend support to a causal relationship of Toxocara in selected patients with Wells' syndrome.

PMID: 9529550 [PubMed - indexed for MEDLINE]

1934. Pediatr Pulmonol Suppl. 1997;16:13-4.

Genetic and environmental determinants in the pathogenesis of allergic diseases.

Geller-Bernstein C.

Pediatric Allergy Unit, Kaplan Hospital, Rehovot, Israel.

PMID: 9443176 [PubMed - indexed for MEDLINE]

1935. G Ital Med Lav Ergon. 1997 Jan-Mar;19(1):56-8.

[Allergic contact dermatitis to nickel: modification of receptor expression on peripheral lymphocytes of women after oral provocation tests (preliminary data)].

[Article in Italian]

Di Gioacchino M, Andreassi M, Boscolo P, Cervone M, Bavazzano P, Gravante M, Pavone G, Cavallucci E, Ramondo S, Schiappoli M, Verna N, Masci S, Amerio P, Giuliano G.

Immunologia Clinica ed Allergologia, Centro di Medicina del Lavoro ed Ergoftalmologia, Università G. D'Annunzio. Chieti.

Blood lymphocyte subset evaluation was performed before after oral challenge with 10 mg of Ni, in 9 healthy women and in 15 allergic to Ni. Following challenge, 7 allergic showed a flare up of eczema and/or urticaria. In the controls, CD4+ lymphocytes were modified 24 hours after Ni challenge: CD4+/CD44RO- "virgin" cells were reduced while CD4+/CD45RO+ "memory" cells increased. The allergic women, not sensitive to oral Ni, showed an increase of B lymphocytes after the

test. On the contrary, the oral Ni reacting patients presented a reduction of monocytes 4 hours after Ni ingestion and marked reduction (ranging from 20 to 50%) of T and B lymphocytes after 24 hours. These significant T and B lymphocytes changes suggest a migration of the cells in peripheral tissues, likely skin and GUT mucosa.

PMID: 9377749 [PubMed - indexed for MEDLINE]

1936. Acta Otorrinolaringol Esp. 1997 Jan-Feb;48(1):11-4.

[Determination of intercellular adhesion molecule-1 (ICAM-1) in nasal secretions of patients with rhinitis].

[Article in Spanish]

Bujía J, Rasp G.

Departamento de ORL, Universidad Ludwig-Maximilians de Munich.

Intercellular adhesion molecule-1 (ICAM-1) is an integrin involved in the regulation of cell migration and activation in various tissues. ICAM-1 expression has been shown to be associated closely with cell infiltration in allergic diseases. We analyzed the possible participation of soluble ICAM-1 (sICAM-1) in the pathogenesis of various diseases of the nasal mucosa by measuring sICAM-1 levels in nasal secretions using sandwich ELISA. Soluble ICAM-1 levels in patients with allergic rhinitis and nasal polyposis did not increase with respect to healthy controls, but patients with chronic sinusitis had higher sICAM-1 levels. Soluble ICAM-1 levels also increased in patients with allergic rhinitis after a provocation test with the specific allergen. These results show that ICAM-1 is involved in the pathogenesis of chronic sinusitis and allergic rhinitis.

PMID: 9131919 [PubMed - indexed for MEDLINE]

1937. Pediatr Res. 1997 Jan;41(1):128-31.

Prenatal contact with inhalant allergens.

Van Duren-Schmidt K, Pichler J, Ebner C, Bartmann P, Förster E, Urbanek R, Szépfalusi Z.

Department of Pediatrics, University of Vienna, Austria.

Pollen contact in early infancy may enhance the risk for subsequent pollen allergy. In this study likelihood of a prenatal antigen contact, as a result of inhalation of pollen allergens by the mother, was investigated. Due to the seasonal occurrence of allergens studied, the date of priming can be estimated, and this can supply data about the maturation of the fetal immune system. Proliferative responses of umbilical cord blood mononuclear cells (UCB MNCs) to the recombinant major allergens of birch (rBet v 1) and timothy grass (rPhl p 1) were analyzed throughout the whole year. A positive proliferative response was regarded as the criterion for a prenatal contact of the immune system with the allergen. Prenatal priming with both allergens was observed. Timothy grass pollen displayed considerably higher antigenicity than did birch pollen. The susceptibility of the fetal immune system to be primed by these allergens varies during the gestation period. The majority of positive responses to rPhl p 1 and rBet v 1 were found in UCB samples in which antigen contact (the respective pollen season) took place in the first 6 mo of pregnancy. Our results offer

indirect evidence that, shortly after migration of T cell precursors to the epithelial thymus, T cells are mature enough for priming with antigens. No relationship was found between the susceptibility of the fetal immune system to be primed by these allergens and the clinical history of the family concerning type I allergy.

PMID: 8979301 [PubMed - indexed for MEDLINE]

1938. J Travel Med. 1996 Dec 1;3(4):237-238.

Genital Schistosomiasis After a Missed Diagnosis of Katayama Syndrome.

Landry P, Favrat B, Raeber PA.

University Medical Policlinic, Lausanne.

Schistosomiasis is increasingly reported in travelers to subSaharan Africa.1,2 Bathing in tropical lakes3 or in other fresh waters2,4 is a recognized risk factor for acquiring it. Most cases present with cercarial dermatitis or, 3 to 6 weeks after infection by Schistosoma mansoni1,2 (occasionnally Schistosoma haematobium), with acute schistosomiasis (Katayama syndrome), when the immune response of the body to the larval maturation and migration elicits fever, sweating, arthralgia, urticaria, and digestive or respiratory symptoms. Late and unusual clinical presentations in travelers include features of spinal cord compression5,6 and ectopic dermal or genital localization,3,7 which can result from a missed diagnosis of the early symptoms of the disease. In the following case, a female traveler developed genital schistosomiasis 1 year after a missed diagnosis of Katayama syndrome.

PMID: 9815465 [PubMed - as supplied by publisher]

1939. Nihon Kyobu Shikkan Gakkai Zasshi. 1996 Dec;34(12):1317-25.

[Infiltration of inflammatory cells and expression of adhesion molecules in bronchial mucosa of patients with asthma].

[Article in Japanese]

Nakamura Y.

Second Department of Internal Medicine, Toho University School of Medicine, Tokyo, Japan.

To study the infiltration of inflammatory cells and the expression of adhesion molecules, I examined bronchial tissue from atopic patients with asthma. T lymphocytes, macrophages, neutrophils, eosinophils, and mast cells in samples of bronchial mucosa were stained with monoclonal antibodies. The expression of adhesion molecules (ICAM-1, VCAM-1, ELAM-1, and PADGEM) in epithelium, endothelium, or both, were observed. Samples were obtained by biopsy from patients with asthma and from healthy control subjects. The numbers of mucosal T lymphocytes, eosinophils, and mast cells were significantly higher in tissue from the patients than in tissue from the control subjects. Immunostaining for ICAM-1 was observed in both the epithelium and endothelium, but staining for VCAM-1 ELAM-1, and PADGEM was seen only in the endothelium. Expression of adhesion molecules except ELAM-1 was found to differ significantly between patients and control subjects. Expression of VCAM-1 in endothelium correlated significantly with the number of eosinophils, and expression of ICAM-1 in epithelium correlated significantly with the number of mast cells. These results suggest that

expression of adhesion molecules in bronchial mucosa play an important role in the migration of inflammatory cells in patients with asthma.

PMID: 9022313 [PubMed - indexed for MEDLINE]

1940. J Allergy Clin Immunol. 1996 Dec;98(6 Pt 1):1135.

Is immigration a prognostic factor for oral allergy syndrome in patients with birch pollen hypersensitivity?

Kalyoncu AF.

Comment on
J Allergy Clin Immunol. 1996 Feb;97(2):611-6.

PMID: 8977522 [PubMed - indexed for MEDLINE]

1941. Ann Allergy Asthma Immunol. 1996 Dec;77(6):475-9.

Comparison of histamine-releasing factor recovered from skin and peripheral blood mononuclear cells of patients with chronic idiopathic urticaria.

Claveau J, Lavoie A, Brunet C, Bédard PM, Hébert J.

Division de dermatologie, Université Laval, Sainte-Foy, Québec, Canada.

BACKGROUND: The pathogenesis of chronic idiopathic urticaria is characterized by defective histamine release. Skin mast cells show an increased release of histamine while circulating basophils are less responsive to immunologic stimulus.
OBJECTIVE: The purpose of the study was to examine and compare the production of the histamine-releasing factor in the skin and within the peripheral blood of patients with chronic idiopathic urticaria and normal control subjects, as a possible factor responsible for the difference observed in the releasability of both skin mast cells and basophils.
METHODS: Using the skin chamber technique, histamine-releasing factor production and histamine concentration were assessed in normal-appearing skin of patients with chronic idiopathic urticaria (n = 12) and normal controls (n = 5) over a 2-hour observation period. In both groups, histamine-releasing factor production by peripheral blood mononuclear cells was also measured.
RESULTS: The weighted average of histamine-releasing factor production during the 2-hour observation period was higher in the non-lesional skin of patients with chronic idiopathic urticaria as compared with normal controls (5.6 +/- 1.4% versus 0.7 +/- 0.6%, P < .01). In contrast, less histamine-releasing factor was produced by peripheral blood mononuclear cells in chronic urticaria as opposed to normal controls (17.2 +/- 2.1% versus 25.7 +/- 2.8%, P < .03). Spontaneous histamine concentration was not significantly different in patients with chronic urticaria than in normal controls.
CONCLUSION: Histamine-releasing factor production is increased in the skin, and decreased in the peripheral blood of patients with chronic idiopathic urticaria when compared with nonatopic controls. The lower production of histamine releasing factor in the blood could be explained by the migration of activated T-lymphocytes in the skin.

PMID: 8970437 [PubMed - indexed for MEDLINE]

1942. J Immunol. 1996 Nov 15;157(10):4664-71.

Stimulating properties of 5-oxo-eicosanoids for human monocytes: synergism with monocyte chemotactic protein-1 and -3.

Sozzani S, Zhou D, Locati M, Bernasconi S, Luini W, Mantovani A, O'Flaherty JT.

Mario Negri Institute of Pharmacologic Research, Milan, Italy.

The newly described products of 5-hydroxyeicosanoid dehydrogenase, 5-oxo-6,8,11,14-eicosatetraenoic acid (ETE) and 5-oxo-15(OH)ETE, induced directional migration and actin polymerization of human monocytes in vitro. At peak concentrations, the two eicosanoids had a chemotactic activity of about 40% of that observed in the presence of an optimal concentration of FMLP and twice the activity elicited by the related eicosanoid 5-hydroxy-6,8,11,14-eicosatetraenoic acid (5-HETE). 15-Oxo-ETE showed a very low but detectable chemotactic activity. All of these chemotactic responses were blocked by Bordetella pertussis toxin, but were resistant to LY255283, a leukotriene B4 (LTB4) receptor antagonist. 5-Oxo-ETEs and 5-HETE induced homologous desensitization of chemotactic response, but did not cross-desensitize to other chemotactic agonists (e.g., monocyte chemotactic protein (MCP)-1 and LTB4). 5-Oxo-ETEs increased in a synergistic fashion the monocyte migration to MCP-1 and MCP-3. In the same range of concentrations, 5-oxo-ETE increased MCP-1-induced release of arachidonic acid from labeled monocytes. No synergistic interaction was observed when FMLP was used as chemoattractant. Thus, this study identifies monocytes as cells responsive to 5-oxo-ETEs and shows that monocyte activation by 5-oxo-ETEs occurs through an LTB4 receptor-independent mechanism that associates with pertussis toxin-sensitive G proteins. The synergistic interaction between 5-oxo-ETEs and C-C chemokines, two families of mediators both synthesized by phagocytic cells, may be relevant in vivo for the regulation of monocyte accumulation at sites of allergic and inflammatory reactions.

PMID: 8906847 [PubMed - indexed for MEDLINE]

1943. Laryngorhinootologie. 1996 Nov;75(11):665-70.

[Cell activation markers in rhinitis and rhinosinusitis. 1: Eosinophilic cationic protein (ECP)].

[Article in German]

Klimek L, Rasp G.

HNO-Universitätsklinik Mainz.

BACKGROUND: The quantitative analysis of the migration and activation of inflammatory cells is standard in current diagnosis of inflammatory diseases of the nasal mucosa. Histological and cytological examinations are mostly used for this purpose. Development and validation of assays for specific marker proteins of the different cell populations make similar analyses possible from nasal secretion samples. Myeloperoxidase (MPO) or Elastase are important markers for neutrophil granulocytes as is Tryptase for mast cells, Lysozyme for macrophages/monocytes and Eosinophil Cationic Protein (ECP). Eosinophil Neurotoxin/Eosinophil Protein X (EDN/EPX) or Major Basic Protein (MBP) for eosinophil granulocytes.
METHODS: We performed a prospective study on healthy volunteers and patients with different inflammatory nasal and paranasal sinus diseases and analysed such cell activation markers in nasal secretions. In the healthy volunteers, 183 nasal secretion samples were obtained. A total of 515 samples were obtained in patients with the diagnosis: chronic sinusitis (n = 49), allergic rhinitis to perennial

allergens (n = 94), chronic sinusitis with additional allergic rhinitis to perennial allergens (n = 36), nasal polyps (n = 28), allergic rhinitis to seasonal allergens extraseasonally (n = 131), and allergic rhinitis to seasonal allergens during the pollen season (n = 177).
RESULTS: In part I of this paper we describe the results for the Eosinophil Cationic Protein (ECP). In all patients with active inflammatory reactions significantly higher ECP levels than in the controls were found. Moreover, ECP levels differed between the diseases investigated.
CONCLUSIONS: ECP nasal secretion level seem to be a valuable marker for the assessment of nasal eosinophilic inflammation and might become an adjunct to current diagnostic measures.

PMID: 9063834 [PubMed - indexed for MEDLINE]

1944. Enferm Infecc Microbiol Clin. 1996 Nov;14(9):548-50.

[2 cases of toxocariasis (visceral larva migrans)].

[Article in Spanish]

López-Vélez R, Turrientes MC, Malo Q, Fenoy MS, Guillén JL.

Unidad de Medicina Tropical y Parasitología Clínica, Hospital Ramón y Cajal, Madrid.

BACKGROUND: Different epidemiological studies have demonstrated that specific anti-Toxocara antibodies are detected in the serum of a high percentage of the Spanish population. But very few clinical cases of visceral larva migrans are being confirmed.
METHODS AND RESULTS: Two cases of visceral toxocarosis, in two sisters, are described. In the first, the prevailing clinic was swelling of joints and upper respiratory tract symptoms; and asthma and cutaneous allergic manifestations in the second patient. Both cases presented with an elevated blood eosinophil count, high levels of total IgE and high titlers of anti-Toxocara antibodies. All symptoms disappeared after treatment with diethylcarbamazine and they remain asymptomatic several months after.
CONCLUSIONS: In pediatric population, toxocarosis should be ruled out in every patient with respiratory symptoms, allergic cutaneous manifestations and elevated blood eosinophil count. The anti-Toxocara antibodies assay is of great value in establishing the diagnosis of this parasitic disease.

PMID: 9035713 [PubMed - indexed for MEDLINE]

1945. Thorax. 1996 Nov;51(11):1155-7.

Selectins and their counter receptors: a bitter sweet attraction.

Symon FA, Wardlaw AJ.

Department of Respiratory Medicine, University of Leicester School of Medicine, Glenfield Hospital, UK.

Selectins are adhesion receptors expressed by leucocytes, platelets, and endothelial cells. They mediate the initial binding of leucocytes to vascular endothelium in the post-capillary venules. This is an essential first step in leucocyte migration into tissue. The selectin family of adhesion receptors consists of three C-type lectins (E, P, and L selectin). Their ligands (counter structures) are sialylated and fucosylated carbohydrate molecules which, in most

cases, decorate mucin-like glycoprotein membrane receptors. Studies using blocking monoclonal antibodies have shown that inhibition of selectin function can ameliorate a range of inflammatory processes, offering the possibility that antagonists of selectin function may be useful in the treatment of inflammatory lung diseases such as asthma.

PMCID: PMC1090529
PMID: 8958901 [PubMed - indexed for MEDLINE]

1946. Fundam Appl Toxicol. 1996 Nov;34(1):15-24.

Specific antibody responses to subtilisin Carlsberg (Alcalase) in mice: development of an intranasal exposure model.

Robinson MK, Babcock LS, Horn PA, Kawabata TT.

Procter & Gamble Company, Cincinnati, Ohio 45253, USA.

An intranasal (i.n.) dosing model was developed in mice as a potential alternative to more difficult, time-consuming, and costly guinea pig intratracheal (GPIT) or mouse intratracheal models for assessment of the respiratory immunogenicity of detergent enzymes. Using a benchmark enzyme, Alcalase (protease subtilisin Carlsberg), studies were conducted to standardize the model in terms of mouse strain, dosing and serum harvest regimen, and the primary immunoglobulin endpoint to use. The primary assay endpoint selected was the enzyme-specific IgG1 titer determined by an Alcalase-specific ELISA. This is not the primary allergenic antibody in mice (IgE is); however, IgG1 is coregulated with IgE via the IL-4/TH2 pathway and may have a role in mediating allergic-type responses. BDF1 mice (C57B1/6 x DBA/2) were selected as representative of high responder strains, with high response associated with the H-2b (C57B1/6) parent. The dosing regimen used for most studies incorporated three i.n. exposures (Days 1, 3, and 10) and bleeding of the animals on Day 15. The animals were anesthetized and then immunized by allowing them to inhale 5-microliters aliquots of dosing solution into each nostril at each immunization. Positioning of the animals with their heads down (vs up) may have allowed more of the dosing solution to remain in the nasal region for a slightly longer period of time, but did not change the eventual GI tract migration and excretion of each dose. The presence of a detergent matrix in the enzyme dosing solution enhanced the IgG1 response. Immunizing with enzyme plus detergent gave highly consistent dose-response curves for Alcalase when evaluated over many studies. An enzyme-specific allergic antibody (IgE) response was weak and inconsistent under the dosing regimen used to generate the IgG1 response, but was stronger with longer-term dosing, consistent with the delay in IgE vs IgG1 responses seen in some other studies. Using IgG1 as a surrogate for allergic sensitization, we have preliminary data showing similar differential potencies between Alcalase and other test enzymes as detected in previous GPIT tests. On the basis of these data, we believe the i.n. immunization/IgG1 response model is a robust technique that may be useful in determining the relative immunogenicities of detergent enzymes and other proteins.

PMID: 8937888 [PubMed - indexed for MEDLINE]

1947. Chest. 1996 Nov;110(5):1236-42.

BAL neutrophilia in asthmatic patients. A by-product of eosinophil recruitment?

Frangova V, Sacco O, Silvestri M, Oddera S, Balbo A, Crimi E, Rossi GA.

Divisione di Pneumologia, Istituto G. Gaslini, Genoa, Italy.

Comment in
    Chest. 1996 Nov;110(5):1138-9.

Although neutrophil number may be increased in the airways of patients with asthma, its pathogenetic role in this disorder remains unclear. We evaluated BAL of 8 normal control subjects, 30 +/- 2 years of age, and 24 patients with mild asthma: 17 patients with allergic asthma, 24 +/- 1 years of age, and 7 patients with nonallergic asthma, 30 +/- 1 years of age. The BAL of asthmatic patients showed increased numbers of neutrophils ($p < 0.01$), eosinophils ($p < 0.01$), and ciliated epithelial cells ($p < 0.05$) and increased concentrations of myeloperoxidase (MPO) ($p < 0.01$) compared with control subjects. Positive correlations were observed between the number of BAL neutrophils and eosinophils (Rs = 0.780, $p < 0.0001$) and between BAL neutrophil numbers and BAL MPO levels (Rs = 0.40, $p < 0.05$). No correlations were found between the following: (1) BAL eosinophils or neutrophils and BAL epithelial cells ($p > 0.05$, each comparison); (2) BAL neutrophils or eosinophils and log Pd15 methacholine (MCh) ($p > 0.05$, each comparison); or (3) BAL epithelial cells or log Pd15 MCh and BAL MPO ($p > 0.05$, each comparison). Dividing the patient population into two groups, allergic asthmatics and nonallergic asthmatics, similar BAL neutrophil, eosinophil, and epithelial cell numbers and similar MPO levels were found ($p > 0.05$, each comparison). In addition, the correlations between BAL neutrophils and eosinophils showed similar significance in the two patient subgroups ($p > 0.05$, each comparison). These results suggest that, both in allergic and nonallergic asthma, airway recruitment and activation of neutrophils occur as does parallel eosinophil migration. However, airway neutrophils do not seem to contribute significantly to epithelial cell injury or to airway hyperresponsiveness in the steady state.

PMID: 8915227 [PubMed - indexed for MEDLINE]

1948. Am J Respir Crit Care Med. 1996 Nov;154(5):1267-71.

Effects of inhaled budesonide on allergen-induced airway responses and airway inflammation.

Gauvreau GM, Doctor J, Watson RM, Jordana M, O'Byrne PM.

Department of Medicine, McMaster University, Hamilton, Ontario, Canada.

Allergen inhalation by sensitized subjects results in acute bronchoconstriction, which can be followed by a later bronchoconstrictor response, allergen-induced airway hyperresponsiveness, and increases in airway inflammatory cells. Treatment with inhaled glucocorticosteroids attenuates allergen-induced asthmatic airway responses. The purpose of this study was to determine whether a 1-wk pretreatment with inhaled budesonide influences allergen-induced changes in inflammatory cells in blood and induced sputum. Seven subjects with mild atopic asthma were treated in a double-blind, placebo-controlled, randomized, crossover fashion with either inhaled budesonide 400 microg/d, or placebo for 7 d. Allergen challenges were carried out the morning after treatment was discontinued and sputum samples were obtained 7 h after allergen inhalation. Methacholine airway responsiveness was measured, and blood and sputum samples were obtained 24 h post-allergen. Budesonide treatment attenuated the magnitude of both the early and the late asthmatic response, reduced allergen-induced methacholine airway hyperresponsiveness, and attenuated allergen-induced increases in total eosinophils and activated eosinophils. These results suggest that the effects of inhaled glucocorticosteroids on allergen-induced airway responses may be mediated through their inhibition of allergen-induced eosinophil migration and activation.

PMID: 8912734 [PubMed - indexed for MEDLINE]

1949. Ann N Y Acad Sci. 1996 Oct 31;796:124-37.

Adhesion interactions involved in eosinophil migration through vascular endothelium.

Wardlaw AJ, Walsh GM, Symon FA.

Department of Respiratory Medicine, Leicester University Medical School, Glenfield Hospital, UK.

Considerable progress has been made in our understanding of the molecular mechanisms involved in eosinophil migration into sites of allergic inflammation. A number of differences between eosinophils and neutrophils have been observed in their pattern of adhesion interactions. These include expression of alpha 4 beta 1, alpha 4 beta 7, and alpha 6 beta 1 by eosinophils but not neutrophils and structural differences between eosinophil and neutrophil PSGL-1 associated with increased eosinophil binding to P-selectin. On the basis of current evidence the various receptors and mediators involved are summarized in FIGURE 1. Once in the tissues eosinophils may persist for several days or weeks, surviving under the influence of locally generated cytokines and this persistence may also partly explain selective tissue accumulation of eosinophils. Understanding the molecular mechanisms involved in leucocyte migration offers the possibility of selective and effective antagonists to treat allergic disease by preventing cell migration. Results in a number of animal models already offer the hope that this approach may be successful. The development of drugs that can be tested in the clinic are awaited with considerable interest.

PMID: 8906219 [PubMed - indexed for MEDLINE]

1950. J Laryngol Otol. 1996 Oct;110(10):929-33.

Increase in epithelial mast cell numbers in the nasal mucosa of patients with perennial allergic rhinitis.

Slater A, Smallman LA, Drake-Lee AB.

Department of Pathology, Medical School, University of Birmingham, UK.

The aim of the study was to compare the numbers and distribution of mast cells in the nasal mucosa of perennial allergic rhinitis (PAR) patients and controls, as demonstrated by different staining methods for light microscopy. Biopsies of inferior turbinate mucosa were taken from 10 patients with PAR and 10 patients undergoing septoplasty or septorhinoplasty (control group). Sections for light microscopy were stained with azure A. chloroacetate esterase and an ABC immunohistochemical technique using antibody to tryptase. Three times more mast cells were found in the epithelium of PAR patients compared to controls using the immunohistochemical technique ($p = 0.0074$). This method demonstrated considerably more mast cells than the other stains. The increase in epithelial mast cells is consistent with the migration of mast cells seen in seasonal allergic rhinitis, and this may be important in the phenomenon of nasal priming seen after repeated antigen exposure.

PMID: 8977855 [PubMed - indexed for MEDLINE]

1951. Immunology. 1996 Oct;89(2):250-5.

The role of CD4 molecules in the induction phase of contact hypersensitivity cytokine profiles in the skin and lymph nodes.

Fujisawa H, Kondo S, Wang B, Shivji GM, Sauder DN.

Division of Dermatology, University of Toronto, Ontario, Canada.

We have previously demonstrated that CD4 gene-targeted mutant mice (CD4- mice) demonstrate hyporesposiveness in contact hypersensitivity (CHS) suggesting that CD4 molecules are required for optimal induction of CHS. In the present study, we wished to examine the mechanisms of this hyporesponsiveness, in particular, we examined whether cytokines were altered in the skin and lymph nodes of CD4- mice following exposure to the contact allergen dinitrofluorobenzene (DNFB). Cytokine mRNA in the ear skin and draining lymph nodes (DLN) were examined by reverse transcription-polymerase chain reaction (RT-PCR) at various times after sensitization. Skin cytokine patterns revealed that in normal mice, interleukin (IL)-2, interferon (IFN)-gamma and tumour necrosis factor (TNF)-alpha mRNA levels increased at 12 hr sensitization, whereas these cytokines were below the level of detection in CD4- mice. In the DLN of normal mice following the hapten application, sequential upregulation of cytokine mRNA including IL-1 alpha, IL-1 beta, IL-2, IL-4, IL-10, IFN-gamma and TNF-alpha was found. No change was seen for IL-1 alpha, IL-1 beta, IL-10 and TNF-alpha and IL-2, IL-4 and IFN-gamma mRNA levels were below the level of detection in DLN from CD4- mice following the hapten application. However, IL-1 beta, IL-2 and TNF-alpha mRNA levels of lymph node cells from CD4- mice could be upregulated by phorbol myristate acetate in vitro. Flow cytometry study has revealed that the number of Langerhans' cells (LC) in DLN of CD4- mice was similar to that of normal mice, thus, inferring that the alterations of cytokine milieu in the ear skin did not have a significant effect on LC migration to DLN. These results suggest that CD4 molecules are crucial for the induction of certain cytokines in the skin and in inducing sequential cytokine signals in DLN required for optimal development of CHS, but that these changes in cytokines do not effect LC migration.

PMCID: PMC1456479
PMID: 8943722 [PubMed - indexed for MEDLINE]

1952. Clin Exp Allergy. 1996 Oct;26(10):1220.

Possible explanation for the different trends of asthma and allergy in East and West Germany and immigrants.

Kalyoncu AF.

Comment on
    Clin Exp Allergy. 1996 Jun;26(6):621-3.

PMID: 8911710 [PubMed - indexed for MEDLINE]

1953. Am J Respir Crit Care Med. 1996 Oct;154(4 Pt 1):850-7.

Persistent airway eosinophilia after leukotriene (LT) D4 administration in the guinea pig: modulation by the LTD4 receptor antagonist, pranlukast, or an interleukin-5 monoclonal antibody.

Underwood DC, Osborn RR, Newsholme SJ, Torphy TJ, Hay DW.

Department of Pulmonary Pharmacology and Toxicology, SmithKline Beecham Pharmaceuticals, King of Prussia, Pennsylvania, USA.

Aerosolized cysteinyl leukotrienes (CysLTs) elicit migration of eosinophils into guinea pig lungs and the airways of patients with asthma. The present studies were designed to analyze the concentration-response relationship, time course, and pharmacologic and histologic characteristics of leukotriene D4 (LTD4)-induced eosinophil influx into the airways of conscious guinea pigs. Animals were exposed to aerosols of 0.3 to 30 microg/ml LTD4 for 1 min, during which specific airway conductance (sGaw) was monitored. Bronchoalveolar lavages (BALs) of guinea pig airways were conducted at selected times from 4 h to 4 wk after LTD4 challenge. LTD4 produced maximal decreases in sGaw (70 to 90% reduction) at all concentrations tested and concentration-related increases in eosinophil levels in BALs, assessed 24 h after challenge. Increased numbers of eosinophils in the bronchial epithelium and subepithelium were confirmed histologically. Significant eosinophilia was maintained for up to 4 wk postchallenge. Pretreatment with the LTD4 receptor antagonist, pranlukast (ONO-1078, SB 205312) (20 mg/kg, intragastrically), significantly inhibited both the bronchoconstriction and the eosinophilia at 24 h, whereas the cyclooxygenase inhibitor, meclofenamic acid (5 mg/kg, intragastrically), had no effect on either parameter. Histologic observations were consistent with BAL results. Pretreatment with the rat anti-mouse antibody to interleukin-5 (IL-5), TRFK-5 (10-300 microg, intraperitoneally), produced dose-related inhibition of LTD4-induced eosinophilia, measured in 24 h or 3 wk BAL, but did not affect the acute bronchoconstriction. These results indicate that LTD4 elicits airway eosinophil influx in guinea pigs which persists as long as 4 wk after a single exposure, and provide the first evidence that IL-5 may have a role in LTD4-induced airways inflammation. This and other previously reported proinflammatory effects of LTD4 may contribute significantly to its overall influential role in the pathophysiology of asthma, and may underlie the therapeutic benefit of CysLT receptor antagonists, such as pranlukast, in this disorder.

PMID: 8887574 [PubMed - indexed for MEDLINE]

1954. Am J Respir Cell Mol Biol. 1996 Oct;15(4):443-50.

Eosinophil infiltration in nonallergic chronic hyperplastic sinusitis with nasal polyposis (CHS/NP) is associated with endothelial VCAM-1 upregulation and expression of TNF-alpha.

Hamilos DL, Leung DY, Wood R, Bean DK, Song YL, Schotman E, Hamid Q.

Department of Medicine, Washington University School of Medicine, St. Louis, Missouri 63110, USA.

We studied potential mechanisms of eosinophil accumulation in nonallergic chronic hyperplastic sinusitis with nasal polyposis (CHS/NP). We measured expression of endothelial vascular cell adhesion molecule-1 (VCAM-1), which mediates selective eosinophil transendothelial migration, the cytokines interleukin (IL)-1 beta, TNF-alpha and IL-13 which upregulate VCAM-1 expression, and the chemokine RANTES which mediates lymphocyte, monocyte, and eosinophil chemotaxis in chronic hyperplastic sinusitis with nasal polyposis (CHS/NP) nasal polyps (nonallergic versus allergic) and middle turbinate biopsies from normal controls. By immunohistochemical staining, the density of EG2+ eosinophils was increased in both the nonallergic and allergic CHS/NP subgroups compared to normal controls. VCAM-1 expression was significantly increased in CHS/NP subjects compared to normal controls (P = 0.0005), with the highest intensity seen in nonallergic CHS/NP. By in situ hybridization, the densities of IL-1 beta, TNF-alpha, IL-13, and RANTES mRNA+ cells were all increased in nonallergic CHS/NP compared to

normal controls (P = 0.009, 0.0005, 0.0005, and 0.001, respectively). In comparison to allergic CHS/NP, nonallergic CHS/NP had a significantly higher tissue density of TNF-alpha (P = 0.04) and a lower density of IL-13 (P = 0.005) mRNA+ cells. In general, VCAM-1 expression correlated strongly in CHS/NP with the density of TNF-alpha (R = .91, P = 0.0005) but not the density of IL-1 beta, IL-13, or RANTES mRNA+ cells. We conclude that upregulation of VCAM-1 and elaboration of RANTES may contribute to the marked accumulation of eosinophils in nonallergic CHS/NP. TNF-alpha may play a critical role in VCAM-1 upregulation in this nonallergic eosinophilic disorder.

PMID: 8879177 [PubMed - indexed for MEDLINE]

1955. Proc Natl Acad Sci U S A. 1996 Oct 1;93(20):10939-44.

Differential regulation of beta 1 and beta 2 integrin avidity by chemoattractants in eosinophils.

Weber C, Kitayama J, Springer TA.

Center for Blood Research, Harvard Medical School, Boston, MA 02115, USA.

The CC chemokines regulated on activation normal T expressed and secreted (RANTES) and monocyte chemotactic protein 3 (MCP-3), and the anaphylatoxin C5a, induce activation, degranulation, chemotaxis, and transendothelial migration of eosinophils. Adhesion assays on purified ligands showed differential regulation of beta 1 and beta 2 integrin avidity in eosinophils. Adhesiveness of VLA-4 (alpha 4 beta 1, CD29/CD49d) for vascular cell adhesion molecule 1 or fibronectin was rapidly increased but subsequently reduced by RANTES, MCP-3, or C5a. The deactivation of VLA-4 lead to cell detachment, whereas phorbol 12-myristate 13-acetate induced sustained activation of VLA-4. In contrast, chemoattractants stimulated a prolonged increase in the adhesiveness of Mac-1 (alpha M beta 2, CD11b/CD18) for intercellular adhesion molecule 1. Inhibition by pertussis toxin confirmed signaling via G protein-coupled receptors. Chemoattractants induced transient, while phorbol 12-myristate 13-acetate induced sustained actin polymerization. Disruption of actin filaments by cytochalasins inhibited increases in avidity of VLA-4 but not of Mac-1. Chemoattractants did not upregulate a Mn2+-inducible beta 1 neoepitope defined by the mAb 9EG7, but induced prolonged expression of a Mac-1 activation epitope recognized by the mAb CBRM1/5. This mAb inhibited chemoattractant-stimulated adhesion of eosinophils to intercellular adhesion molecule 1. Thus, regulation of VLA-4 was dependent on the actin cytoskeleton, whereas conformational changes appeared to be crucial for activation of Mac-1. To our knowledge, this is the first demonstration that physiological agonists, such as chemoattractants, can differentially regulate the avidity of a beta 1 and a beta 2 integrin expressed on the same leukocyte.

PMCID: PMC38262
PMID: 8855287 [PubMed - indexed for MEDLINE]

1956. Clin Immunol Immunopathol. 1996 Oct;81(1):48-54.

Human C1q induces eosinophil migration.

Kuna P, Iyer M, Peerschke EI, Kaplan AP, Reid KB, Ghebrehiwet B.

Department of Medicine, SUNY-Stony Brook, New York 11794-8161, USA.

Eosinophils (Eo) play a significant role in allergic inflammation and the host's immunity to parasitic infections. Although the presence of C1q-binding cell

surface molecule(s) (C1q-R) on Eo had been previously implicated by the ability of C1q to augment IgG-dependent, Eo-mediated killing of schistosomula, little is known about the structure or the function of this receptor. The present studies were therefore undertaken to immunochemically demonstrate and to examine the biology of Eo C1q-R. Eo were purified to homogeneity (>90%) and viability (>98%) from hypereosinophilic donors by Percoll density gradient. Western blot analysis using antibodies to cC1q-R and gC1q-R showed distinct bands corresponding to cC1q-R (60 kDa) and gC1q-R (33 kDa) when immunoblotted with their respective antibodies. The Eo C1q-R was tested for its ability to induce chemokinesis and/or chemotaxis as assessed by the modified Boyden microchamber assay utilizing 5-micrometer-pore polycarbonate membranes and using C1q, cC1q, or gC1q (10 micrograms/ml) as agonists. The known chemotactic factors C5a and RANTES (10(-8)M) were used as positive controls. The results showed that at this concentration, cC1q was most efficient in its ability to induce Eo migration (20 +/- SEM 12, n = 4) followed by C1q (107 +/- SEM 7, n=7) and gC1q (77 +/- SEM 10, n = 10). When checkerboard analysis was performed, the data indicated that the observed phenomenon was likely to be due largely to chemokinesis. As expected, C5a (145 +/- SEM 15, n = 7) and RANTES (145 +/- SEM 43, n = 7) were both chemotactic. Furthermore, incubation of Eo with 50 micrograms of either C1q, gC1q, or cC1q (1 hr, 37 degrees C) did not cause release of eosinophil cationic protein as measured by RIA, nor did it enhance the expression of CD11b or CD29 as assessed by FACS analysis. The data presented in this paper show that Eo express both cC1q-R and gC1q-R and may participate in Eo function by providing a primary signal for locomotion.

PMID: 8808641 [PubMed - indexed for MEDLINE]

1957. Ned Tijdschr Geneeskd. 1996 Sep 14;140(37):1872-4.

[Congenital syphilis as an imported disease].

[Article in Dutch]

Hendriks T, Calkoen AH, Schulpen TW, Oranje AP.

Ziekenhuis Overvecht, afd. Kindergeneeskunde, Utrecht.

Two infants, a two-month-old boy and a two-month-old girl adopted from Sri Lanka, were diagnosed as having congenital syphilis and treated accordingly. The girl presented with only skin symptoms and a developing pseudoparalysis of Parrot. The boy was in quite a bad condition, having rhinitis, an oedematous appearance, skin symptoms, severe anaemia and hepatosplenomegaly. In both patients serological blood tests and cerebrospinal fluid tests for lues were positive. Both children showed osteochondritis and periostitis of the long bones on X-rays. This disease is rare in the Netherlands, but it is important to diagnose it early, because early adequate treatment may result in complete cure.

PMID: 8927160 [PubMed - indexed for MEDLINE]

1958. Community Dent Health. 1996 Sep;13 Suppl 2:69-71.

Does fluoridation harm immune function?

Challacombe SJ.

Department of Oral Medicine and Pathology, UMDS, Guy's Hospital, London, UK.

Comment in

Community Dent Health. 1998 Jun;15(2):121.

There have been suggestions, mainly in the lay press, that fluoridation might affect immunity. Careful examination of various studies on fluoride and immune function do not support this suggestion. Whilst fluoride at high concentrations can have inhibitory effects on lymphocyte and polymorphonuclear leucocyte function, these concentrations are many times higher than levels which would be expected from fluoridation. Fluoride can act as an immunological adjuvant. There is no evidence of any deleterious effect on specific immunity following fluoridation nor any confirmed reports of allergic reactions.

PMID: 8897755 [PubMed - indexed for MEDLINE]

1959. J Invest Dermatol. 1996 Sep;107(3):419-22.

High-dose ultraviolet A1 (UVA1), but not UVA/UVB therapy, decreases IgE-binding cells in lesional skin of patients with atopic eczema.

Grabbe J, Welker P, Humke S, Grewe M, Schöpf E, Henz BM, Krutmann J.

Department of Dermatology, Virchow Clinics, Humboldt University, Berlin, Germany.

In order to further elucidate the mechanisms by which high-dose ultraviolet A1 (UVA1) therapy leads to improvement in patients with atopic eczema, we assessed skin sections from patients before and after high-dose UVA1 therapy (n = 5) or conventional UVA/UVB therapy (n = 4) for changes in Langerhans cells and mast cells expressing the high-affinity IgE receptor Fc epsilon RI and in surface-bound IgE by histochemical and immunohistochemical techniques. The two treatment groups exhibited different patterns of changes in the number of Fc epsilon RI+, CD1a+, and mast cells within the dermis: The density of both Langerhans cells and mast cells was decreased after high-dose UVA1 therapy, but not after UVA/UVB therapy. High-dose UVA1 and UVA/UVB therapy significantly increased the number of CD1a+ cells within the epidermis, but only high-dose UVA1 reduced the relative number of IgE+ intraepidermal Langerhans cells typically found in atopic eczema. Reduction of numbers of dermal Langerhans cells and mast cells, as well as relative numbers of intraepidermal IgE+ Langerhans cells, was closely linked to significant clinical improvement by high-dose UVA1, but not UVA/UVB therapy. These studies support the notion that IgE-binding cutaneous cells are involved in the pathogenesis of atopic eczema. We propose that UVA1 radiation exerts its effects in atopic eczema, at least in part, by inhibiting Langerhans cell migration out of the epidermis and, in particular, by reducing the number of IgE-bearing Langerhans cells and mast cells in the dermis.

PMID: 8751980 [PubMed - indexed for MEDLINE]

1960. J Immunol. 1996 Aug 15;157(4):1711-9.

Functional and structural characterization of the eosinophil P-selectin ligand.

Symon FA, Lawrence MB, Williamson ML, Walsh GM, Watson SR, Wardlaw AJ.

Department of Respiratory Medicine, Leicester University Medical School, Glenfield Hospital, United Kingdom.

Our recent studies have indicated an important role for P-selectin in eosinophil adhesion. We have therefore compared eosinophil and neutrophil binding with nasal polyp endothelium as well as purified P-selectin. We have also compared the structure and expression of the eosinophil and neutrophil P-selectin ligands.

Using the frozen section assay, eosinophils bound to 2-fold more blood vessels within the nasal polyp tissue than neutrophils. Up to 10-fold more eosinophils than neutrophils bound per unit length of endothelium. Neutrophil and eosinophil binding was inhibited by a mAb against P-selectin and a P-selectin chimera which binds to the P-selectin ligand. Eosinophils bound with approximately 2-fold greater avidity to purified P-selectin under flow conditions. Using SDS-PAGE we characterized the eosinophil P-selectin ligand as a sialylated, homodimeric glycoprotein consistent with the known structure of PSGL-1. However, expression of PSGL-1 by eosinophils was significantly greater than on neutrophils. The eosinophil ligand had a calculated molecular mass by SDS-PAGE of approximately 10 kDa greater than the neutrophil ligand, which was not due to differences in N-glycosylation. Eosinophils expressed the 15-decapeptide repeat form of PSGL-1 compared with neutrophils that have the 16-decapeptide repeat form. The increased binding of eosinophils, compared with neutrophils, to P-selectin in both an ex-vivo and in vitro assay suggests that P-selectin may have a role directing the specific migration of eosinophils in diseases such as asthma. The increased avidity may be due to increased expression of PSGL-1 by eosinophils, differences in the peptide backbone, or post-translational modifications.

PMID: 8759760 [PubMed - indexed for MEDLINE]

1961. J Immunol. 1996 Aug 15;157(4):1693-8.

C3a and C5a are chemotaxins for human mast cells and act through distinct receptors via a pertussis toxin-sensitive signal transduction pathway.

Nilsson G, Johnell M, Hammer CH, Tiffany HL, Nilsson K, Metcalfe DD, Siegbahn A, Murphy PM.

Department of Pathology, University Hospital, University of Uppsala, Sweden.

Mast cells are known to accumulate at sites of inflammation, however, the chemotaxins involved are undefined. Since most natural leukocyte secretagogues also induce cell migration, and since the anaphylatoxins C3a and C5a are mast cell secretagogues, we hypothesized that both C3a and C5a are also mast cell chemotaxins. Here we report that C3a and C5a are, in fact, potent chemotaxins for the human mast cell line HMC-1. The optimal concentrations, half-maximal effective concentrations (a measure of agonist potency) and the efficacy (response at the optimal concentration) compared with medium control were, for C3a: 10 nM, 0.5 nM, and 256%, respectively; for C5a: 1 nM, 10 pM and 145%. Chemotaxis of HMC-1 cells to both C3a and C5a was blocked by pertussis toxin, suggesting that Gi-coupled receptors are involved in signal transduction. C3a and C5a also induced transient pertussis toxin-inhibitable increases in [Ca2+]i (ED50 = 1 nM for both) that could be homologously but not heterologously desensitized, suggesting that the receptors for C3a and C5a are distinct. These results make C3a the most effective mast cell chemotaxin identified to date. The chemotactic potency described here for C3a is also 100- to 1000-fold greater than for all of its previously described cellular actions. Direct chemoattraction of mast cells by C3a and C5a may help explain the rapid accumulation of mast cells at sites of inflammation.

PMID: 8759757 [PubMed - indexed for MEDLINE]

1962. Eur J Immunol. 1996 Aug;26(8):1919-25.

Human eotaxin represents a potent activator of the respiratory burst of human eosinophils.

Elsner J, Höchstetter R, Kimmig D, Kapp A.

Hannover Medical School, Department of Dermatology, Germany.
101346.3021@compuserve.com

Increased numbers of eosinophils are found in parasitic infections, autoimmune diseases and allergic diseases such as allergic asthma. They are activated by distinct cytokines and chemokines leading to the immigration in the inflamed tissue and mediate tissue damage by releasing reactive oxygen species. Here, the effect of the recently cloned CC chemokine human eotaxin was investigated for its ability to affect different eosinophil effector functions and compared to the CC chemokines MCP-3 and RANTES. Human eotaxin induced chemotaxis of human eosinophils in a dose-dependent manner. The range of efficacy of the CC chemokines compared to the well-known chemotaxin C5a was eotaxin = RANTES > MCP-3 = C5a. In addition, eotaxin induced rapid and transient actin polymerization, a prerequisite for cell migration, in eosinophils in the same range of efficacy as observed for chemotaxis. To investigate whether eotaxin was able to activate the respiratory burst of eosinophils, release of reactive oxygen species was measured by lucigenin-dependent chemiluminescence. Eotaxin induced production of significantly high amounts of reactive oxygen species at a concentration between 10 ng/ml and 500 ng/ml. Surprisingly, the effect of eotaxin was comparable to the well-known eosinophil activator C5a. The range of efficacy of the CC chemokines compared to C5a in the activation of the respiratory burst was eotaxin = C5a > MCP-3 > RANTES. Production of reactive oxygen species was inhibited by pertussis toxin, staurosporin, genestein and wortmannin. Furthermore, eotaxin induced transient increases in intracellular calcium concentration ([Ca2+]i) in human eosinophils. Therefore, pertussis toxin-sensitive Gi-proteins, protein kinase C, tyrosine kinase, phosphatidylinositol-3-kinase and transient increases in [Ca2+]i are involved in the signal transduction of eosinophils following stimulation with eotaxin. In summary, this study reveals the importance of the CC chemokine eotaxin as a potent activator of the respiratory burst, actin polymerization and chemotaxis. Eotaxin, therefore, plays an important role not only by attracting eosinophils to the site of inflammation but also by damaging tissue by its capacity to induce the release of reactive oxygen species.

PMID: 8765040 [PubMed - indexed for MEDLINE]

1963. Allergol Immunopathol (Madr). 1996 Jul-Aug;24(4):177-83.

Antiallergic properties of antihistamines.

Negro-Alvarez JM, Funes E, García Cánovas A, Hernández J, García-Sellés FJ, Pagán JA, López-Sánchez JD.

Allergology Section, H. U. Virgen de la Arrixaca, El Palmar, Murcia.

Histamine is a major mediator of the allergic reaction, and histamine H1-receptor antagonists have a long history of clinical efficacy in a variety of allergic disorders. The pathogenesis of allergic disease is complex, involving not only histamine and mast cell-derived tryptase, but also eosinophil and neutrophil derived mediators, cytokines, and intercellular adhesion molecules (ICAM-1). A number of "in vitro" and "in vivo" studies have been performed to assess the clinical effectiveness of antihistamines in inhibiting the allergen-induced inflammatory process in the skin and mucosa. In vitro human studies have shown that high concentration of second generation antihistamines can block inflammatory mediator release from basophils and mast cells, and reduce ICAM-1 expression in epithelial cell lines. In vivo studies have also shown an effect on the allergen-induced inflammatory reaction; both oral and intranasal antihistamines cause a reduction in nasal symptoms and inflammatory cell influx.

Analysis of secretory fluids and tissues after challenge indicates that antihistamines interfere with mediator release. Recruitment of inflammatory cells to the site of the allergic insult is also disturbed by antihistamines of second-generation, suggesting that these drugs may inhibit upregulation of molecules involved in cell adhesion and migration, and perhaps they may interfere with the cytokine cascade through their ability of stabilizing mast cells and of limiting the incursion of inflammatory cells. This article reviews available human data on the antiallergic effects of antihistamines.

PMID: 8939275 [PubMed - indexed for MEDLINE]

1964. Biochem Mol Biol Int. 1996 Jul;39(4):729-40.

Isolation of toxin TsTX-VI from Tityus serrulatus scorpion venom. Effects on the release of neurotransmitters from synaptosomes.

Sampaio SV, Coutinho-Netto J, Arantes EC, Marangoni S, Oliveira B, Giglio JR.

Departamento de Análises Clínicas, Toxicológicas e Bromatológicas, Faculdade de Ciências Farmacêuticas, Universidade de São Paulo, Brazil.

A detailed procedure for the purification of Tityustoxin-VI, TsTX-VI, from Tityus serrulatus scorpion venom is described. For comparative purposes, a second toxin, CM-VI, obtained from the same fractionation procedure, was analyzed in parallel. Typical biochemical parameters, such as electrophoretic migration, mol.weight, amino acid composition and N-terminal sequence (first 42 amino acid residues out of a total of approx. 60) were determined for both. Our data showed that CM-VI is identical or extremely homologous to gamma-toxin (TsTX-I), the highly toxic major toxin from T. serrulatus venom. TsTX-VI was less toxic, although still effective at inducing an allergic reaction, lacrymation and contraction of the hind legs of mice. Both toxins produced a dose dependent release of the neurotransmitters glutamic acid and gamma aminobutyric acid from rat brain synaptosomes, this effect being blocked by tetrodotoxin.

PMID: 8843341 [PubMed - indexed for MEDLINE]

1965. Acta Otolaryngol. 1996 Jul;116(4):604-10.

Positive identification in situ of mRNA expression of IL-6, and IL-12, and the chemotactic cytokine RANTES in patients with chronic sinusitis and polypoid disease. Clinical relevance and relation to allergy.

Davidsson A, Danielsen A, Viale G, Olofsson J, Dell'Orto P, Pellegrini C, Karlsson MG, Hellquist HB.

Department of Otorhinolaryngology, Medical Center Hospital, Orebro, Sweden.

Interleukins 6 (IL-6) and 12 (IL-12), and the chemoattractant chemokine RANTES were studied in ethmoidal mucosa, using reverse transcriptase polymerase chain reaction. The 49 patients had chronic sinusitis or nasal/paranasal polyposis, and some also allergy. To the best of our knowledge, this is the first study that demonstrates RANTES and IL-12 on mRNA level in human sinonasal mucosa in situ. mRNA for IL-6, IL-12 and RANTES were detected in 2, 8 and 6 patients with chronic sinusitis, respectively, and in mucosa from patients with polyposis a positive expression was observed in 4, 14 and 10 cases. There were no statistically significant differences. Analysing the entire group of 49 patients, disregarding type of mucosal disease, the number of patients with positive RANTES was significantly higher than that for IL-6. Similarly, IL-12 positivity was more

frequently expressed than IL-6. mRNA for IL-6 was expressed in only 2 of the allergic patients. The cytokine production studied thus seems to be unrelated to the clinically defined entities. There is thus a local production in human diseased sinonasal mucosa of RANTES, as well as of IL-6 and IL-12. The local production of RANTES is an important prerequisite for recruitment and migration of inflammatory cells into the tissue. IL-12 is a co-stimulator of antigen-specific responses of established T helper 1 (Th1) clones, and regulates the responsiveness of the clones to a number of T cell growth factors. The study supports a shift towards Th1 cells in these disease entities.

PMID: 8831850 [PubMed - indexed for MEDLINE]

1966. J Am Anim Hosp Assoc. 1996 Jul-Aug;32(4):353-7.

A fatal case of intrathoracic cuterebriasis in a cat.

Fitzgerald SD, Johnson CA, Peck EJ.

Department of Pathology, College of Veterinary Medicine, Michigan State University, East Lansing 48824, USA.

A seven-year-old, castrated male domestic shorthair was presented with lethargy, fever, and sneezing. In spite of intensive therapy, the cat's condition progressively worsened to severe dyspnea and death. At necropsy, a single, second instar larval stage of Cuterebra sp. was found in the trachea. This represents an unusual site for Cuterebra migration in an aberrant host. The cause of death was attributed to a combination of local tissue damage and anaphylaxis. Veterinarians should include Cuterebra migration in their differential diagnosis list for dyspnea.

PMID: 8784727 [PubMed - indexed for MEDLINE]

1967. Am J Respir Cell Mol Biol. 1996 Jul;15(1):35-44.

TNF alpha is important in human lung allergic reactions.

Casale TB, Costa JJ, Galli SJ.

Department of Internal Medicine, VA Medical Center, Iowa City, Iowa, USA.

Tumor necrosis factor alpha (TNF alpha) is a potentially important cytokine in allergic respiratory reactions since it is released by mast cells and eosinophils, and it can promote mediator and cytokine release, adhesion molecule expression, and granulocyte migration. Therefore, we induced an IgE-mediated response in human lung samples and studied: (1) whether TNF alpha was produced in sufficient quantities to promote granulocyte migration; and (2) which cells expressed mRNA for TNF alpha using in situ hybridization. Lung fragments (from thoracotomy) were treated for 30 min with either anti-IgE, 1:100 dilution, or buffer (control). Anti-IgE treatment of 16 lungs resulted in greater than 4-fold increase in histamine release and the significant production of chemotactic activity. The chemotactic activity generated induced dose-responsive neutrophil and eosinophil migration through naked filters and endothelial and pulmonary epithelial monolayers. Fourteen of 16 samples had a significant increase in TNF alpha subsequent to anti-IgE treatment ($P < 0.05$). Anti-TNF alpha antibody (4 micrograms/ml) inhibited about 25% of the neutrophil chemotactic activity in supernatants from anti-IgE treated lungs. TNF alpha at a concentration measured after anti-IgE treatment of lung samples (50 pg/ml) induced neutrophil transendothelial migration. Finally, we found that anti-IgE treatment led to an

increase in TNF alpha mRNA-positive cells by in situ hybridization (1.6/ mm2 experimental versus 0.5/mm2 control), some of which were eosinophils. Thus, human lung IgE-mediated responses in vitro results in: (1) release of TNF alpha in amounts sufficient to effect a biologic response, granulocyte chemotaxis: and (2) upregulation of mRNA for TNF alpha in eosinophils and other cells. These findings suggest that TNF alpha is an important effector molecule in the pathogenesis of allergic respiratory reactions.

PMID: 8679220 [PubMed - indexed for MEDLINE]

1968. Respirology. 1996 Jun;1(2):123-6.

Asthma and migration.

Leung R.

Department of Medicine, Prince of Wales Hospital, Shatin, Hong Kong.

The dramatic influx of immigrants from South-East Asian countries into Australia over the past 20 years was associated with an increase is asthma and allergic disease amongst these immigrants. Epidemiological data showed that the prevalence rates of asthma and allergic disease increased with the duration of residence in Australia so that after 10 years in Australia, up to 60% of South-East Asian immigrants developed hayfever while 15% had symptoms of asthma. Many immigrants developed these conditions for the first time after arrival in Australia suggesting that the environment plays a important role in the pathogenesis of asthma and allergy. While sensitization to inhalant allergens such as house dust mite and grass pollen are apparently important in some, other yet undefined local factors are likely to contribute significantly to the overall increase in prevalence. A prospective study designed to follow a group of migrants in areas where asthma and allergic disease are common such as Australia, may reveal the pathogenic role of the environment and provide valuable information that may explain the global distribution and increasing trend of asthma and allergies.

PMID: 9434327 [PubMed - indexed for MEDLINE]

1969. Br J Pharmacol. 1996 Jun;118(3):811-9.

Anti-inflammatory effects of theophylline, cromolyn and salbutamol in a murine model of pleurisy.

Saleh TS, Calixto JB, Medeiros YS.

Department of Pharmacology, Centre of Biological Sciences, Universidade Federal de Santa Catarina, Brazil.

1. The aim of this study was to examine the effect of theophylline, cromolyn and salbutamol, three well-known anti-asthmatic drugs, on the early (4 h) and late (48 h) phases of cell migration and fluid leakage induced by carrageenin in the pleural cavity of mice. 2. In the first set of experiments, animals were pretreated (30 min) with different doses of theophylline (0.5-50 mg kg-1, i.p.), cromolyn (0.02-0.2 mg per pleural cavity) or salbutamol (0.05-50 mg kg-1, i.p.); the total and differential cell content, and also the exudate were analysed 4 h after carrageenin (1%) administration. Afterwards, in order to evaluate the time course effects of these drugs on both phases of the inflammatory reaction, one dose employed in the above protocol was chosen, to pretreat (0.5-24 h) different groups of animals. The studied parameters were evaluated 4 and 48 h after pleurisy induction. 3. Acute administration of theophylline (1-50 mg kg-1, i.p.)

cromolyn (0.02-0.2 mg per pleural cavity) and salbutamol (0.5-50 mg kg-1, i.p.), 30 min prior to carrageenin, caused significant inhibition of total cell and fluid leakage in the pleural cavity at 4 h ($P < 0.01$). All drugs exerted a long-lasting inhibitory effect on both exudation and cell migration ($P < 0.01$) when administered 0.5-8 h before pleurisy induction. However, the temporal profile of the inhibitory effect induced by these drugs on the first phase of the inflammatory reaction was clearly different. Thus, the inhibitory effect induced by theophylline and cromolyn on exudation was significantly longer (up to 24 h) in comparison to their effects on cell migration (only up to 8 h). In contrast, although salbutamol when administered 30 min before pleurisy induction abolished fluid leakage ($P < 0.01$), this effect was not sustained in the groups pretreated for 4-8 h. In these latter groups, a significant but much smaller reduction of exudation was observed ($P < 0.01$), whereas the magnitude of cell migration inhibition did not vary. 4. The second phase (48 h) of the inflammatory reaction induced by carrageenin (1%) was significantly inhibited by cromolyn (0.02 mg per pleural cavity) when this drug was administered 0.5-24 h before pleurisy induction ($P < 0.01$). Similar results were observed when theophylline (50 mg kg-1, i.p.) was administered 0.5-4 h before the injection of the phlogistic agent ($P < 0.01$). Treatment of the animals with salbutamol (5 mg kg-1, i.p.), 0.5-24 h before pleurisy induction, did not inhibit either cell migration or fluid leakage. In this condition, a significant increase of these parameters was observed in the group pretreated with salbutamol 8-24 h before pleurisy induction ($P < 0.01$). 5. These results indicate that theophylline and cromolyn were able to inhibit the early (4 h) and late (48 h) phases of the inflammatory reaction induced by carrageenin in a murine model of pleurisy. Salbutamol was effective only against the early phase. The inhibitory effects of theophylline, cromolyn and salbutamol on the early phase of this inflammatory reaction were long-lasting, although a distinct profile of inhibition was observed among them. These findings confirm and extend previous results described in other models of asthma and support both clinical and experimental evidence suggesting that these anti-asthmatic agents exhibit marked anti-inflammatory properties.

PMCID: PMC1909745
PMID: 8762112 [PubMed - indexed for MEDLINE]

1970. Int Arch Allergy Immunol. 1996 Jun;110(2):124-31.

Specific high affinity binding of platelet activating factor to intact human blood neutrophils and eosinophils.

Korth RM.

Forschung in der Allgemeinmedizin, FIDA, Munich, Germany.

Neutrophils and eosinophils are involved in various inflammatory reactions such as leukocyte migration, adherence and phagocytosis. A regulation of platelet-activating factor (PAF) receptors in intact human blood neutrophils and eosinophils is clinically important. Intact human blood neutrophils and eosinophils prepared under sterile conditions specifically bound [3H]PAF in the presence of fatty acid-free serum albumin (0.25% BSA). Excess unlabeled PAF (500 nM) or the specific PAF receptor antagonist WEB 2086 (1 microM) inhibited the [3H]PAF binding. PAF receptors on the surface of intact blood neutrophils and eosinophils had high affinity Kd values of 0.55 and 2.3 nM at 4 degrees C. The Bmax values were 200 fmol/2.5 x 10(6) neutrophils and 26 fmol/2.5 x 10(5) eosinophils. PAF receptors on the outer plasma membranes were functionally relevant as high dose PAF displaced WEB 2086 after 3 min preincubation mediating maximal cytosolic [Ca2+]i flux. High doses of PAF or phorbol myristate acetate (PMA) downregulated neutrophils and a low dose PAF decreased the specific [3H]PAF binding to eosinophils determined with WEB 2086 at 20 degrees C. Only neutrophils

were significantly upregulated by low dose PAF (5 nM), lyso PAF or low dose PMA (1 nM). Up- and downregulation by PAF itself of neutrophil and eosinophil PAF receptors might explain their desensitization and some clinical controversy concerning the role of PAF in inflammatory and allergic diseases. The latter hypothesis would lead to a novel combination of antagonists against PAF receptors and PAF production.

PMID: 8645989 [PubMed - indexed for MEDLINE]

1971. Hepatogastroenterology. 1996 May-Jun;43(9):627-36.

Surgical treatment of hydatid disease of the liver: an experience from outside the endemic area.

Aeberhard P, Fuhrimann R, Strahm P, Thommen A.

Department of Surgery, Cantonal Hospital, Aarau, Switzerland.

BACKGROUND/AIMS: Hydatid disease is quite rare in European countries outside the endemic area around the Mediterranean Sea. Most of the cases observed in Central and Northern Europe occur in emigrants from the endemic area, whose number has been increasing over the last decade. In Switzerland about twenty-five new cases are being diagnosed per year, an incidence of about 0.33 cases per 10(5) inhabitants. Surgery remains the principal treatment modality of hydatid liver disease. There is still debate about conservative surgery as opposed to radical surgical treatment in which the cyst is totally removed including the pericyst by total cystoperi-cystectomy, partial hepatectomy or a combination of both. Surgeons working inside the endemic area tend to favor conservative methods, whereas those outside the endemic area have the tendency to favor radical surgery. This article reviews the results of surgery for liver hydatid disease obtained in a country outside the endemic area.
PATIENTS AND METHODS: In our institution 24 patients (12 female, 12 male) have been treated for liver hydatid disease from 6/1983 to 2/1995. Twenty-two patients were immigrants from the endemic area. Surgery indication was primary liver hydatid disease in 23 patients, and recurrent disease in one.
RESULTS: Twenty-one patients underwent radical procedures, and three were treated by cystectomy, unroofing and omentoplasty. Radical procedures were pericystectomy in 11 patients, partial hepatectomy in five and pericystectomy combined with partial hepatectomy in five. There was no operative mortality in 23 patients operated on for primary disease, but the only patient operated upon for recurrence died from anaphylactic shock. Eighteen of the 23 surviving patients could be followed up for a median time of 6.5 years (eight months to 12.5 years). Sixteen of 18 patients have remained free of recurrence. One has been reoperated for a retrocaval recurrence four years after right hepatectomy, and one patient is being observed for suspected recurrence after unroofing and omentoplasty.
CONCLUSIONS: The policy of applying radical surgery whenever feasible can be followed with acceptable morbidity and near zero mortality. Radical surgery has, however, to be applied judiciously, and there is still an important role for conservative surgery.

PMID: 8799407 [PubMed - indexed for MEDLINE]

1972. J Am Board Fam Pract. 1996 May-Jun;9(3):182-90.

Asthma as an inflammatory disease: implications for management.

Dolen WK.

Department of Pediatrics, Medical College of Georgia, Augusta 30912, USA.

BACKGROUND: Eosinophilic inflammation plays a central role in the pathogenesis of asthma. Striking inflammatory changes are present in the airways of patients with all levels of disease severity. The degree of airway inflammation correlates with airway hyperresponsiveness, the primary physiologic abnormality of asthma. Inflammation is typically initiated by immunologic events (including allergy) and is driven by mediators released by various cells of the immune system, particularly eosinophils, monocytes and macrophages, lymphocytes, and mast cells.
METHODS: Literature on asthma and the inflammatory response was drawn from recent articles presented and reviewed in journal clubs and from selected articles from the National Library of Medicine.
RESULTS AND CONCLUSIONS: The inflammatory process can be divided into six steps: triggering, signaling, migration, inflammatory cell activation, tissue damage, and resolution. Recognition of the importance of inflammation in the pathogenesis of asthma and the progression of the disease has shifted research efforts and the development of new therapeutic agents toward reduction of airway inflammation. Anti-inflammatory therapy, which can be directed against specific steps in the inflammatory process, actually reduces bronchial hyperresponsiveness. Although anti-inflammatory management has assumed a primary role in asthma therapy, short acting beta 2-adrenergic receptor agonists are needed for treatment of acute symptoms, and some patients require regular beta 2-agonist therapy despite apparently adequate anti-inflammatory therapy.

PMID: 8743231 [PubMed - indexed for MEDLINE]

1973. Am J Respir Cell Mol Biol. 1996 May;14(5):425-38.

Lung inflammation and epithelial changes in a murine model of atopic asthma.

Blyth DI, Pedrick MS, Savage TJ, Hessel EM, Fattah D.

Cellular Science Department, Glaxo Research Ltd., Greenford, Middlesex, United Kingdom.

A murine model of allergen-induced airway inflammation and epithelial phenotypic change, and the time-courses of these events, are described. Mice were sensitized to ovalbumin using an adjuvant-free protocol, and challenged by multiple intratracheal instillations of ovalbumin by a non-surgical technique. Many of the characteristic features of human atopic asthma were seen in the mice. A marked eosinophilic infiltration of lung tissue and airways followed allergen challenge, and its severity increased with each challenge, as did the number of eosinophils in the blood. Lymphocytes, neutrophils, and monocytes also invaded the lungs. Airway macrophages showed signs of activation, their appearance resembling those recovered from antigen-challenged human asthmatic airways. The airway epithelium was thickened and displayed a marked goblet cell hyperplasia in terminal bronchioles and larger airways. After repeated challenges, the reticular layer beneath the basement membrane of the airway epithelium showed fibrosis, reproducing a commonly observed histologic feature of human asthma. Goblet cell hyperplasia began to appear before eosinophils or lymphocytes had migrated across the airway epithelium, and persisted for at least 11 days after the third intratracheal challenge with ovalbumin, despite the number of inflammatory cells in the lungs and airways having decreased to near-normal levels by 4 days. Plugs of mucus occluded some of the airways. These results indicate that some of the phenotypic changes in airway epithelium that follow an allergic response in the lung can be initiated before the migration of eosinophils or lymphocytes across the epithelial layer.

PMID: 8624247 [PubMed - indexed for MEDLINE]

1974. Clin Orthop Relat Res. 1996 May;(326):71-9.

Immune response to synthetic materials. Sensitization of patients receiving orthopaedic implants.

Merritt K, Rodrigo JJ.

Metallic orthopaedic devices are composed of elements that are known to be skin sensitizers in the general population. There is concern about the possibility of sensitivity reactions in patients bearing these implants. Blood samples were drawn from 22 patients having primary total joint replacement and who had no known prior metal allergies or exposure. Repeat blood samples were drawn 3 months to 1 year later. All preoperative blood samples showed no immune reactions against titanium, cobalt, chromium, or nickel ion solutions in a leukocyte migration inhibition test. Thirty two percent (7 of 22) of the patients developed sensitivity to at least 1 of the antigens, but only 5 percent (1 of 22) developed a severe reaction. Review of the literature and these studies has indicated that such reactions can occur. However, the incidence seems to be very low.

PMID: 8620661 [PubMed - indexed for MEDLINE]

1975. Orv Hetil. 1996 Apr 7;137(14):739-41.

[Food allergy in patients with chronic urticaria].

[Article in Hungarian]

Husz S, Kiss Z, Judák R, Molnár K, Kiss M, Dobozy A.

Szent-Györgyi Albert Orvostudományi Egyetem Börgyógyászati Klinika, Szeged.

Basic food allergens (bread, milk and egg) were investigated in patients with idiopathic chronic urticaria. In 29 of 43 patients, sensitivity could be shown against one or more antigens. The results suggest not only the anaphylactic IgE-mediated reaction in same patients, but also an antibody or cell-mediated mechanisms in the development of the disease. Besides the Prick test, therefore, testing of other circulating antibodies by means of the ELISA technique and the leukocyte migration test is recommended to establish the causative agents in patients with chronic urticaria.

PMID: 8927319 [PubMed - indexed for MEDLINE]

1976. J Chir (Paris). 1996 Apr;133(2):65-71.

["500 consecutive cases of laparoscopic cholecystectomy". Argument for the association to endoscopic sphincterotomy: analysis].

[Article in French]

Lagrange M.

C.H. de Nevers.

Our series of 500 consecutive laparoscopic cholecystectomies has drawn attention to several factors. Results would favor endoscopic sphincterotomy in cases with associated treatment of gall stones in the main bile duct. history taking should

search for past history of laparoscopic surgery, especially in men with an extensive pillosity, work-up should include ultrasonography, liver function tests and intravenous cholangiography (in all patients excepting cases of allergy), it is important to use an identical material in a given hospital facility for identical procedures in order to avoid equipment-related conversions, an interesting alternative in emergency situations would be echo-guided transcutaneous transperietal cholecystotomy which allows time for safe opacification, safety is of prime importance and rapide conversion should be made when there is any doubt, especially concerning the main duct, morbidity and mortality in this series were nearly identical to those previously reported large series, for endoscopic sphincterotomy proposed as complementary therapy for cases with associated lithiasis in the main bile duct, 2/3 were performed peroperatively and 1/3 postoperatively. Considering all sphincterotomies, 2/3 were positive with extraction of a stone and demonstration of an enlarged bile duct evidencing recent migration (no failure or iatrogenic event), the relationship between the different elements should allow rapid indications in emergency situations and identify complications immediately (mean hospitalization less than 48 hours) or later. Finally, first intention laparoscopic cholecystectomy can be proposed for patients with signs of biliary distress with lithiasis depite other, sometimes contradictory, conclusions (ANDEM, CPAM, consensus conference). First intention laparoscopic cholecystectomy should eliminate in the future most of the major biliary-pancreatic abdominal syndromes.

PMID: 8763563 [PubMed - indexed for MEDLINE]

1977. J Emerg Nurs. 1996 Apr;22(2):158-9.

A 52-year-old man with wheezing, fever, and abdominal pain.

Molitor L.

PMID: 8716313 [PubMed - indexed for MEDLINE]

1978. J Exp Med. 1996 Apr 1;183(4):1893-8.

Inhibition of leukotriene B4-receptor interaction suppresses eosinophil infiltration and disease pathology in a murine model of experimental allergic encephalomyelitis.

Gladue RP, Carroll LA, Milici AJ, Scampoli DN, Stukenbrok HA, Pettipher ER, Salter ED, Contillo L, Showell HJ.

Central Research Division, Pfizer, Inc., Groton, Connecticut 06340, USA.

Leukotriene B4 (LTB4) is a chemotactic and cell-activating factor present at inflammatory sites in a variety of autoimmune diseases including multiple sclerosis (MS). In this study, we used a murine model of MS, experimental allergic encephalomyelitis (EAE), to assess the potential role of LTB4 on cell infiltration and paralysis. Injection of encephalogenic T cells into naive animals induced paralysis and weight loss that was completely inhibited by treatment with the selective LTB4 receptor antagonist CP-105,696 (ED50= 8.6 mg/kg orally). Although migration of lymphocytes into the central nervous system was unaffected, the efficacious effects of CP-105,696 correlated with up to a 97% decrease in eosinophil infiltration into the lower spinal cord as determined by light and electron microscopy and quantitated by levels of the specific enzyme marker eosinophil peroxidase. These results demonstrate that eosinophil recruitment in EAE is dependent on LTB4 receptor ligation and further reveal a previously unrecognized role for eosinophils in the pathogenesis of this disease.

PMCID: PMC2192487
PMID: 8666945 [PubMed - indexed for MEDLINE]

1979. Eur J Immunol. 1996 Apr;26(4):825-33.

Lymphocyte binding to vascular endothelium in inflamed skin revisited: a central role for vascular adhesion protein-1 (VAP-1).

Arvilommi AM, Salmi M, Kalimo K, Jalkanen S.

National Public Health Institute, Finland. anna-maija.arvilommi@utu.fi

The binding of leukocytes to vascular endothelium and their migration into tissues is mediated by adhesion molecules on the endothelial cells and leukocytes. Vascular adhesion protein-1 (VAP-1) is a 170-180/90-kDa endothelial molecule expressed most prominently in high endothelial venules in peripheral lymph node (PLN) type lymphatic tissues. VAP-1 mediates lymphocyte binding to PLN, tonsil and synovium. The expression of VAP-1 is induced in inflammatory diseases such as arthritis and gut inflammation. We examined the expression, structure and function of VAP-1 in normal and inflamed skin and compared it to those of other adhesion molecules implicated in skin homing. In psoriasis lichen ruber planus, pemphigoid and allergic lesions, VAP-1 was markedly upregulated. The expression of VAP-1 was also increased in biopsies of healthy skin of the patients. The VAP-1 molecule induced in skin is decorated with abundant sialic acids. VAP-1 inflamed skin is functional, since inhibition with anti-VAP-1 monoclonal antibodies caused a 60% reduction in lymphocytes adhesion to vascular endothelium. Antibodies against E-selectin, which has been regarded as the major vascular addressin directing cutaneous lymphocyte traffic, and, surprisingly, against peripheral lymph node addressin (PNAd), caused inhibitions of 30% and 60%, respectively, in the frozen section adhesion assay. These findings suggest important roles also for VAP-1 and PNAd in lymphocyte homing into inflamed skin.

PMID: 8625974 [PubMed - indexed for MEDLINE]

1980. Acta Otolaryngol. 1996 Mar;116(2):149-59.

Immune functions and immunopathology of the mucosa of the upper respiratory pathways.

Brandtzaeg P, Jahnsen FL, Farstad IN.

Laboratory for Immunohistochemistry and Immunopathology (LIIPAT), University of Oslo, National Hospital, Rikshospitalet, Norway.

The specific defence of airway mucosae depends primarily on secretory immunity. The B cells involved are initially stimulated in organized mucosa-associated lymphoid tissue, apparently including the tonsils and adenoid. From these inductive sites, memory cells migrate to secretory effector sites where they differentiate terminally to immunoglobulin (Ig)-producing plasma cells. Locally produced Ig consists mainly of J chain-containing dimers and larger polymers of IgA (pIgA) that are selectively transported through glandular cells by an epithelial receptor called secretory component or the pIg receptor. IgG can participate in immune exclusion because it reaches the secretions by passive diffusion. However, its proinflammatory properties render IgG antibodies of local immunopathological importance when elimination of penetrating antigens is unsuccessful. T helper (Th) cells activated in this process may by a Th2 cytokine profile promote persistent inflammation with extravasation and priming of

eosinophils. This development appears to be part of the late-phase allergic reaction, perhaps initially driven by interleukin-4 (IL-4) released from mast cells that are subjected to IgE-mediated activation, and subsequently also by Th2 cell activation. Eosinophils are potentially tissue-damaging, particularly after priming with IL-5. Various cytokines up-regulate adhesion molecules on endothelial and epithelial cells, thereby enhancing migration of eosinophils into the mucosa, and perhaps in addition causing aberrant immune regulation within the epithelium. Soluble antigens bombarding the epithelial surfaces normally seem to induce several immunosuppressive mechanisms, but mucosal homeostasis appears less patent in the airways than oral tolerance to dietary antigens operating in the gut.

PMID: 8725503 [PubMed - indexed for MEDLINE]

1981. Int J Clin Pharmacol Ther. 1996 Mar;34(3):96-100.

The effect of a 1-week administration of cetirizine on the chemotaxis and superoxide anion production of neutrophils from healthy volunteers.

Condino-Neto A, Muscara MN, Bozza PT, Castro-Faria-Neto HC, De Nucci G.

Department of Pediatrics, Faculty of Medical Sciences, State University of Campinas, Brazil.

The aim of the present investigation was to examine the effect of a 1-week oral administration of the anti-allergic drug cetirizine on healthy volunteer neutrophil chemotaxis and superoxide anion (O2-) production. Eight male volunteers were selected after clinical examination and laboratory tests. Neutrophils were isolated from peripheral blood using a discontinuous density gradient. Spontaneous migration and platelet activating factor (PAF, 10(-6) and 10(-8) M) or zymosan-activated plasma (ZAP)-induced chemotaxis were studied in a 48-well microchemotaxis chamber. Basal and phorbol 12-myristate 13-acetate (PMA, 30 nM) stimulated O2- production were measured spectro-photometrically. Cetirizine (10 mg per day) was given orally during 1 week. Both neutrophil chemotaxis and O2- production were assessed before and 2 h, 24 h, and 1 week after orally administered cetirizine. Plasma cetirizine levels were monitored by high performance liquid chromatography (HPLC) with ultraviolet detection. Spontaneous neutrophil migration and PAF-or ZAP-induced chemotaxis showed no significant variation before or at various intervals after the initiation of treatment with cetirizine. Basal and PMA-stimulated neutrophil O2-production was also not affected by cetirizine. The maximum concentration attained by cetirizine (Cmax) was 293 +/- 38 ng/ml and generally peaked (Tmax) within 1.9 +/- 0.7 h. We conclude that the administration of cetirizine for 1 week does not alter human neutrophil chemotaxis and O2- production.

PMID: 8705094 [PubMed - indexed for MEDLINE]

1982. J Leukoc Biol. 1996 Mar;59(3):347-56.

Mechanisms involved in eosinophil migration. Platelet-activating factor-induced chemotaxis and interleukin-5-induced chemokinesis are mediated by different signals.

Schweizer RC, van Kessel-Welmers BA, Warringa RA, Maikoe T, Raaijmakers JA, Lammers JW, Koenderman L.

Department of Pulmonary Diseases, University Hospital Utrecht, The Netherlands.

Eosinophils play an important role in the pathogenesis of allergic diseases such as allergic asthma. Eosinophil migration in vitro can be divided into directed migration, or chemotaxis, and random migration, or chemokinesis. Here, we studied intracellular signals involved in eosinophil migration in vitro induced by platelet-activating factor (PAF) and interleukin-5 (IL-5), applying a Boyden chamber assay. Migration induced by PAF (10(-11)-10(-6) M) largely consisted of chemotaxis with some chemokinesis, whereas IL-5 (10(-12)-10(-8) M) induced chemokinesis only. Eosinophils were depleted from intracellular and extracellular Ca2+ to study the role of Ca2+ as a second messenger. Ca2+ depletion did not change PAF-induced chemotaxis, however, IL-5-induced chemokinesis was inhibited. Interestingly, PAF, but not IL-5, induced changes in [Ca2+]i. This rise originated mainly from internal stores. Inhibition of protein kinase A by H-89 and protein kinase C by GF 109203X had no effect on both forms of eosinophil migration. Addition of the protein kinase inhibitor staurosporine significantly inhibited IL-5-induced chemokinesis. Inhibition of tyrosine kinases by herbimycin A completely blocked IL-5-induced chemokinesis. PAF and IL-5-induced actin polymerization was studied to compare migratory responses with a migration-associated intracellular response. Ca2+ depletion significantly enhanced PAF-induced (10(-8) M) actin polymerization, whereas IL-5-induced actin polymerization was not influenced. Addition of staurosporine led to an increase in F-actin. Subsequent addition of PAF or IL-5 resulted in an additive increase in F-actin content. In summary, both forms of eosinophil migration are protein kinase A and protein kinase C independent. In contrast to PAF-induced chemotaxis, Il-5-induced chemokinesis was found to be completely Ca2+ and tyrosine kinase dependent.

PMID: 8604012 [PubMed - indexed for MEDLINE]

1983. BMJ. 1996 Feb 3;312(7026):315.

Moving house unlikely to pose substantial risk of childhood asthma.

Strachan DP, Butland BK, Carey IM, Anderson HR.

Comment on
    BMJ. 1995 Oct 21;311(7012):1069-70.

PMCID: PMC2349863
PMID: 8611814 [PubMed - indexed for MEDLINE]

1984. Inflamm Res. 1996 Feb;45(2):89-95.

Cytokines induce selective granulocyte chemotactic responses.

Bittleman DB, Erger RA, Casale TB.

Department of Internal Medicine, VA Medical Center, Iowa City 52242, USA.

Neutrophils, eosinophils and cytokines are important in allergic airway inflammatory responses. However, it is unclear how cytokines selectively influence neutrophils versus eosinophils to migrate to an inflammatory site. The cytokines, transforming growth factor-beta1 (TGF-beta1), interleukin (IL)-1alpha, IL-5, IL-8, granulocyte macrophage-colony stimulating factor (GM-CSF) and tumor necrosis factor-alpha (TNF-alpha), are released subsequent to allergic reactions and affect both neutrophil and eosinophil functions. We studied whether these cytokines differed in capacity to induce human neutrophil versus eosinophil migration through naked filters and human umbilical vein endothelial cell (HUVEC) and human pulmonary type II-like epithelial (A549) cell monolayers grown on

filters. Dose-response experiments using all barriers were performed for each granulocyte and cytokine. TGF-beta1 did not induce granulocyte migration. IL-5 induced eosinophil migration only through naked filters. IL-1alpha stimulated neutrophil migration through cellular barriers, but not through naked filters. TNF-alpha and GM-CSF induced neutrophil and eosinophil migration through filters, but only neutrophil migration through cellular monolayers. Only IL-8 induced significant neutrophil and eosinophil migration; however, there were clear-cut differences between the neutrophilotactic and eosinophilotactic responses through all barriers employed. Thus, our data show that these cytokines induce distinct chemotactic responses for neutrophils versus eosinophils. Moreover, by using relevant cellular barriers versus naked filters, our data better examines the capability of these cytokines to induce selective granulocyte migration to an inflammatory site in lung diseases such as asthma.

PMID: 8907590 [PubMed - indexed for MEDLINE]

1985. Nihon Jibiinkoka Gakkai Kaiho. 1996 Feb;99(2):292-8.

[The effect of histamine on the adhesion of endothelial cells to eosinophils].

[Article in Japanese]

Terada N, Hamano N, Hiruma K, Konno A, Sirotori K, Isikawa K, Togawa K.

Department of Otorhinolaryngology, Chiba University, School of Medicine.

Recent studies have revealed that eosinophils and eosinophil-derived mediators strongly contribute to the onset of nasal swelling and nasal hyperreactivity. The effect of histamine on the adhesion of endothelial cells to 35S-labeled eosinophils and on eosinophil transendothelial migration was investigated. Human microvascular endothelial cells were isolated and cultured from the mucosa of the inferior turbinates of patients with nasal allergy. Histamine caused dose-related enhancement of adhesiveness to eosinophils. Incubation of endothelial cells treated with 10(-5)M and 10(-4)M histamine increased adhesion to eosinophils by a mean of 56.4% ($p < 0.05$) and 66.0% ($p < 0.05$), respectively. When eosinophils were incubated with histamine, they did not induce any increase in adhesion to endothelial cells. Preincubation of endothelial cells with anti-ELAM-1 significantly inhibited histamine-induced adhesion, whereas anti-ICAM-1 and anti-VCAM-1 had no inhibitory effect. Histamine did not increase eosinophil transendothelial migration. Histamine is known to be vasoactive, mediating vasodilation and plasma extravasation. In addition, the results of this study raise the possibility that histamine promotes eosinophil adhesion to endothelial cells by increasing ELAM-1 molecules on endothelial cells and promotes nasal inflammatory and allergic reactions.

PMID: 8851334 [PubMed - indexed for MEDLINE]

1986. Lab Invest. 1996 Feb;74(2):422-36.

Effects of contact allergens on human Langerhans cells in skin organ culture: migration, modulation of cell surface molecules, and early expression of interleukin-1 beta protein.

Rambukkana A, Pistoor FH, Bos JD, Kapsenberg ML, Das PK.

Department of Dermatology, Academic Medical Center, University of Amsterdam, The Netherlands.

Epidermal Langerhans cells (LC) and cytokines play a critical role in the initiation phase of contact hypersensitivity reactions in the skin. Most of the studies of these aspects have been performed in animal models and relatively little is known about the human system. Short-term human skin organ cultures, in which LC preserved their characteristics and distribution within the epidermis, were used to examine the time course effects of contact allergens on human LC in situ and whether these effects are mediated by cytokines. Epicutaneous application of nontoxic concentrations of contact allergens 2,4-dinitrofluorobenzene, 2,4-dinitrochlorobenzene, and nickel sulphate, but not the irritants sodium dodecylsuphaye and croton oil or the tolerogen 2,4-dichloronitrobenzene, significantly reduced the total number of LC in the epidermis: remaining LC were localized along the epidermal-dermal junction, suggesting a migration of LC within and out of the epidermis. LC that are migrated to the epidermal-dermal junction showed a decreased expression of CD1a+ and MHC-II and an upregulation of ICAM-I. While these effects were observed after 24 hours, the expression of IL-1 beta protein was induced exclusively by LC as early as 4 hours after skin challenge with contact allergens alone. After 24 hours, contact allergens not only increased the expression of IL-1 beta but also induced the expression of IL-1 alpha, TNF-alpha, GM-CSF, and IL-6 proteins mainly by suprabasal keratinocytes. In an attempt to study the possible relation between allergen-induced epidermal cytokines and the migration and phenotypic changes of LC, skin explants were incubated with corresponding human recombinant (hr) cytokines. After 12 hours, hr IL-1 beta, but not other hr cytokines (IL-1 alpha, TNF-alpha, GM-CSF, and IL-6), induced the migration within and out of the epidermis and decreased the expression of CD1a+ and MHC-II on remaining epidermal LC similar to that caused by contact allergens. Pre-incubation of skin explants with neutralizing IL-1 beta antibodies, but not antibodies to IL-1 alpha, TNF-alpha, or GM-CSF, significantly prevented the allergen-induced migration of LC. This study showed that contact allergens preferentially induced the migration of LC within and out of the epidermis and modulated the expression of cell surface molecules on migrated LC as well as induced the early expression of LC-derived IL-1 beta. We also provide evidence that IL-1 beta is critically involved in contact allergen-induced changes on human epidermal LC and suggest that IL-1 beta plays a role in the initiation of contact hypersensitivity in human skin in vivo.

PMID: 8780161 [PubMed - indexed for MEDLINE]

1987. Trends Biotechnol. 1996 Feb;14(2):46-51.

Chemokines: progress toward identifying molecular targets for therapeutic agents.

Howard OM, Ben-Baruch A, Oppenheim JJ.

SAIC Frederick, National Cancer Institute-Frederick Cancer Research and Development Center, MD 21702-1201, USA.

Leukocyte migration towards injury sites is directed by the interaction of chemokines with their receptors. The stages of migration are closely regulated events that involve chemokine-induced leukocyte adhesion, diapedesis and homing. Current research suggests a pathophysiological role for chemokines in diverse inflammatory states arising from viral, bacterial and parasitic infection, allergic and asthmatic reactions, atherosclerosis and arthritis. A role for chemokines in tumor immunity and angiogenesis has recently been demonstrated. A basis for the rational design of chemokine antagonists is emerging from a knowledge of tertiary structures and mutational analysis of chemokine ligands and receptors. Here, we discuss advances in knowledge about chemokine structure and function, with emphasis on potential therapeutic agents.

PMID: 8746116 [PubMed - indexed for MEDLINE]

1988. Allerg Immunol (Paris). 1996 Feb;28(2):34-8.

Adhesion molecules in allergy.

Passalacqua G, Montagna P, Pesce G, Canonica GW, Bagnasco M.

Dept of Internal Medicine, Genoa University, Italy.

Cell adhesion molecules have been recognized to play a major role in a variety of physiological and pathophysiological phenomena, such as embryogenesis, maintenance of tissue architecture and tissue damage repair, recruitment of leukocytes into tissues, immunological reactions requiring cell-to-cell contact. Their role in inflammatory reactions is crucial, in particular their involvement in allergic inflammation has been extensively studied. It has become apparent that the expression patterns and cell localization of certain adhesion molecules are related to the dynamic of allergic inflammation, and specifically to selective migration of eosinophils and interactions with epithelial cells of the target organs. In some instances, cell adhesion molecules by themselves may be regarded as reliable markers for clinical purposes. In addition, the pharmacological modulation of expression and release of adhesion molecules may provide new insight about the mechanisms of action and effectiveness of antiallergic drugs.

PMID: 8703306 [PubMed - indexed for MEDLINE]

1989. Exp Dermatol. 1996 Feb;5(1):12-9.

Expression of adhesion molecules and their ligands in contact allergy.

Wahbi A, Marcusson JA, Sundqvist KG.

Department of Dermatology, Huddinge University Hospital, Sweden.

Sequential biopsies from skin lesions induced by nickel sulphate and sodium lauryl sulphate, respectively, were investigated with respect to expression of extracellular matrix proteins and adhesion molecules on lymphocytes, endothelial cells, and keratinocytes. The majority of the infiltrating lymphocytes expressed VLA-4, LFA-1, CD44 and ICAM-1, a variable fraction expressed Leu-8 and VLA-5, and few or no cells were positive for VLA-1, VLA-2 and VLA-6. Noteworthy, was that the infiltrating cells showed a substantial amount of fibronectin but relatively small or negligible presence of laminin, collagen type IV, IgG, IgA, IgM, and albumin. The fibronectin was associated with cell bodies as well as the area surrounding infiltrating cells. The number of infiltrating cells was larger in biopsies from nickel-sulphate induced lesions and the infiltrates contained more fibronectin than biopsies from lesions induced by sodium lauryl sulphate. However, at the single-cell level, the expression of VLA antigens, LFA-1, CD44 and ICAM-1 was similar in both groups. The endothelial cells of skin biopsies from nickel-sulphate-induced lesions showed a stronger expression of VCAM-1, ELAM-1 and ICAM-1 compared to biopsies from sodium lauryl sulphate-induced lesions. In the biopsies from nickel sulphate-induced lesions, the keratinocytes showed a tendency to less VLA-6 expression. These results suggest that fibronectin plays a role in lymphocyte extravasation or extravascular lymphocyte migration.

PMID: 8624606 [PubMed - indexed for MEDLINE]

1990. Int Arch Allergy Immunol. 1996 Feb;109(2):97-109.

Chemokines as mediators of allergic inflammation.

Bacon KB, Schall TJ.

DNAX Research Institute of Molecular and Cellular Biology, Inc., Palo Alto, CA 94303-1104, USA.

The selective distribution of reactive leukocytes to foci of inflammation or lymphoid organs is thought to rely on the generation of highly specific 'attractive' forces which can enhance or subvert the physiological trafficking process. It is becoming increasingly apparent that the selective trafficking of leukocytes is governed by both the release of soluble mediators, or chemoattractants, as well as the matrix upon or through which the cells must traverse. A balance exists between endogenous cellular adhesion receptors (as well as extracellular matrix proteins) and other inducible adhesion receptors which can be up-regulated on this 'docking station'. This dynamic environment provides a prominent signal for leukocyte extravasation from the blood or lymph vessel lumenal surface through to the tissue space. This report reviews current thinking on the delicate interplay between a superfamily of chemoattractant cytokines, the chemokines, and the various classes of cellular adhesion molecules. In it we highlight the idea that the balance between basal and inducible regulators of cell adhesion and migration is critical. Should it be disrupted, the signals responsible for induction and maintenance of an inflammatory response and those responsible for its resolution become disregulated, resulting in inflammatory pathology.

PMID: 8563497 [PubMed - indexed for MEDLINE]

1991. Biochem Biophys Res Commun. 1996 Jan 5;218(1):54-60.

Phosphorylation of Rho GDI stabilizes the Rho A-Rho GDI complex in neutrophil cytosol.

Bourmeyster N, Vignais PV.

Laboratoire de Biochimie, URA 1130 CNRS, Départment de Biologie Moléculaire et Structurale, CEA/Grenoble, France.

The GDP dissociation inhibitor Rho GDI from bovine neutrophil cytosol was purified in association with prenylated Rho A. Upon treatment of this complex with alkaline phosphatase, the Rho A and Rho GDI components were released to their free forms. Following migration in 2D-PAGE and specific immunodetection, the shape of the spot of Rho GDI was found to depend markedly on whether Rho GDI subjected to electrophoresis was present in a Rho A-Rho GDI complex or in a free form. In the first case Rho GDI focused as an elongated spot between pI 5.2 and pI 4.6 whereas in the later case it focused at a pI of 5.0-5.2 as a round spot. Activation of neutrophils by anaphylatoxin C5a in a [32Pi] supplemented medium resulted in radiolabeling of Rho GDI. In vitro incubation of Rho GDI with a neutrophil homogenate in the presence of [gamma 32P] ATP led also to radiolabeling of Rho GDI. Taken together these results suggest that Rho GDI in the Rho A-Rho GDI complex is phosphorylated and that the stability of the complex depends on the phosphorylation state of Rho GDI.

PMID: 8573175 [PubMed - indexed for MEDLINE]

1992. World Watch. 1996 Jan-Feb;9(1):22-3.

Russia's population sink.

Nelson T.

PIP: Russia's public health problems, which are a result in part of uncontrolled development, are a lesson for developing countries. Trends in births and deaths in Russia indicate that as socioeconomic conditions declined in recent years, the death rate increased. During 1992-93 the death rate increased from 12.1 per 1000 population to 14.5, with 75% of the increase due to cardiovascular disease, accidents, murder, suicide, and alcohol poisoning. Quality of health care was given as one reason for the high cardiovascular disease rate that included deaths due to even mild heart attacks. 20-30% of deaths are attributed to pollution. 75% of rivers and lakes in the former Soviet Union are considered unfit for drinking, and 50% of tap water is unsanitary. An estimated 15% of Russia's land area is considered to be an ecological disaster zone. Births declined from a peak of 2.5 million in 1987 to 1.4 million in 1994. During this same period deaths increased from 1.5 million to 2.3 million. In 1994 deaths exceeded births by 880,000. Life expectancy declined from 65 to 57 years for men and from 75 years to 71 years for women. Infant mortality is rising. 11% of newborns had birth defects, and 60% showed evidence of allergies or vitamin D deficiencies. The death rate during pregnancy was 50 per 1000 births, and 75% of Russian women experienced complications during pregnancy. Women's health in the reproductive years was compromised by gynecological infections. A survey in 1992 revealed that 75% of Russian women gave insufficient income as a reason for reduced childbearing. The social conditions in Russia and the former Soviet republics reflect a lack of confidence in the future. Demographic trends are affected by a complex set of factors including economic collapse, economic change and uncertainty, inadequate health care, and poor environmental conditions. These changes occurred during the mid-1980s and before the collapse of the Soviet Union in 1991.
PMID: 12347136 [PubMed - indexed for MEDLINE]

1993. Ter Arkh. 1996;68(12):25-8.

[The in-vivo test of the inhibition of leukocyte natural migration with aspirin and analgin in the specific diagnosis of the asthmatic triad].

[Article in Russian]

Kanchurina NA, Poroshina IuA, Prasolova NI, Zemskov VM.

Asthmatic triad (AT) is a clinical syndrome incorporating bronchial asthma (BA), recurrent nasal and sinus polyps, intolerance of aspirin, derivatives of pirasolone and other nonsteroid antiinflammatory drugs. Specific diagnosis of aspirin and analgin intolerance in patients with AT was made using the test of natural leukocyte emigration inhibition in vivo in the oral cavity. The test was performed in 22 patients with AT and 13 patients with BA with pollenosis without aspirin and analgin intolerance. In all the patients the test provided positive results, in control patients these were negative. High significance, safety and simplicity of the test made it applicable for specific diagnosis of AT in allergological and pulmonological departments.

PMID: 9054032 [PubMed - indexed for MEDLINE]

1994. Ter Arkh. 1996;68(8):28-31.

[Absolute diet therapy and antibiotic tolerance in bronchial asthma patients].

[Article in Russian]

Fedoseev GB, Smirnov AIu, Zukheĭr A, Ivanova OA, Smirnova OI, Alekseeva EF, Totolian AA.

In 10 patients with bronchial asthma (BA) treated conventionally (control group), 10 BA patients on absolute diet therapy (group 1) and 10 BA patients in absolute diet therapy combined with wheat herb juice (group 2) tolerance to 12 antibiotics of different classes and some immunity factors were determined using a complex of diagnostic methods. The latter implies: case history, humoral and cell immunity defense system tests and special tests (chemical erythrograms and leukocyte migration inhibition test in plane capillary tubes with tested drugs). The data obtained evidence for increased tolerance of the antibiotics in groups 1 and 2 as well as for changes in humoral defense system, especially in immunoglobulin E. The drug tolerance may be a therapeutic criterion of absolute diet therapy in bronchial asthma patients.

PMID: 9019827 [PubMed - indexed for MEDLINE]

1995. Pneumonol Alergol Pol. 1996;64(7-8):379-85.

[Chemotactic activity of neutrophils from atopic and non-atopic subjects--effect of sodium cromoglycate (DSCG)].

[Article in Polish]

Szkudlińska B, Kowalski ML, Grzegorczyk J, Pierzchała A.

Katedry i Zakładu Immunologii Klinicznej A. M. w Lodzi.

It has been well documented, that sodium cromoglycate (DSCG) is capable in inhibiting activity of several inflammatory cells putatively involved in allergic and non-allergic asthmatic inflammation. The goal of this study was to compare the effect of DSCG on random locomotion and chemotaxis of neutrophils to several stimuli in atopic and non-atopic subjects. In 10 seasonal asthmatic (SA), and 10 healthy subjects (HS) chemotactic responses of neutrophils were examined using modified Boyden's microchamber (Neuroprobe) technique. Neutrophils isolated from both HS and SA demonstrated similar spontaneous migration and dose dependent chemotactic responses to FMLP (10(-12) - 10(-5M)), PAF (10(-7) - 10(-5M)) and ZAS (2.5% - 50%). DSCG in concentration range 10(-7) - 10(-9M) expressed a dose-dependent inhibition of both random migration and chemotactic responses to all stimuli tested, with maximal inhibition ranging from 58%-89% and 67%-75% for HS and SA, respectively. Our results confirm potent anti-inflammatory activity of DSCG in vitro, and demonstrate, that this activity is similar in atopic asthmatics, and in healthy subjects.

PMID: 8983437 [PubMed - indexed for MEDLINE]

1996. J Asthma. 1996;33(6):353-66.

Anti-leukotriene agents: a new direction in asthma therapy.

Nathan RA.

University of Colorado Health Sciences Center Denver, USA.

Comment in

J Asthma. 1996;33(6):351-2.

A role for the leukotrienes in asthma has been postulated for many years. These mediators induce potent bronchoconstriction, stimulate mucous secretion and decrease mucus transport, increase vascular permeability (thereby promoting edema formation), and induce migration of eosinophils into the lung. Recent studies with both leukotriene receptor antagonists and leukotriene synthesis inhibitors have demonstrated that these new agents can be effective in asthma induced by exercise, aspirin, and allergen challenges. Further, in patients with mild-to-moderate asthma, these drugs improve pulmonary function, decrease symptoms, and reduce the need for "rescue" bronchodilators. Anti-leukotrienes thus represent an important step forward in asthma management.

PMID: 8968291 [PubMed - indexed for MEDLINE]

1997. Can J Physiol Pharmacol. 1996 Jan;74(1):80-4.

Inhibitory effects of tranilast on proliferation, migration, and collagen synthesis of human vascular smooth muscle cells.

Fukuyama J, Miyazawa K, Hamano S, Ujiie A.

Central Research Laboratories, Kissei Pharmaceutical Co. Ltd., Nagano, Japan.

The aim of this study was to examine the effects of tranilast (anti-allergic drug) on proliferation, migration, and collagen synthesis in cultures of human vascular smooth muscle cells. Tranilast at 100 and 300 microM had several inhibitory effects. One is the effect on vascular smooth muscle cell proliferation induced by fetal bovine serum and platelet-derived growth factor (PDGF)-BB. Second is the effect on PDGF-BB-induced migration. Third is the effect on c-myc expression after PDGF-BB stimulation. Lastly, tranilast reduced the spontaneous collagen synthesis without reducing total protein synthesis. These results suggest that tranilast may prevent restenosis after percutaneous transluminal coronary angioplasty via the inhibitory effects on proliferation, migration, c-myc gene expression, and collagen synthesis of vascular smooth muscle cells.

PMID: 8963955 [PubMed - indexed for MEDLINE]

1998. Int Arch Allergy Immunol. 1996;111 Suppl 1:26-8.

Comparison of surface antigens on eosinophils from patients with eosinophilia.

Sakamoto S, Oki K, Takahashi H, Arakawa Y, Sugita H, Hirano H, Takeuchi K, Tomichi N, Sakuma T, Futai K, Kawabata Y.

Department of Respiratory Medicine, Iwate Prefectural Central Hospital, Morioka, Japan.

Recent studies have suggested that there may be heterogeneity among human eosinophils. To study this further, surface antigens on blood eosinophils from patients with eosinophilia (23 bronchial asthma, 6 eosinophilic pneumonia, 1 Kimura's disease and 1 adult T-cell leukemia) and from 8 control subjects were examined using a new direct method for fluorescence detection of eosinophils. HLA-DR+ and CD4+ eosinophil counts were higher in patients with bronchial asthma and adult T-cell leukemia (ATL) than in patients from other groups and in control subjects. CD11b+ eosinophil counts in Kimura's disease and ATL were smaller than those in the other groups. CD45RO+ eosinophil counts in bronchial asthma and

eosinophilic pneumonia were significantly higher ($p < 0.05$) compared with Kimura's disease, ATL and control subjects. CD44+ eosinophil counts in eosinophilic pneumonia were significantly higher ($p < 0.05$) compared with the other groups and control subjects. These results suggest the existence of functional heterogeneity in the different eosinophilic diseases, with eosinophils in bronchial asthma and eosinophilic pneumonia being more highly activated in migration, activation and immunoregulation. On the other hand, eosinophils in Kimura's disease and ATL might be functionally down-regulated. This heterogeneity of eosinophils may reflect differences in the pathogenesis of various eosinophilic diseases.

PMID: 8906108 [PubMed - indexed for MEDLINE]

1999. Int Arch Allergy Immunol. 1996;111 Suppl 1:22-5.

Heterogeneous chemotactic response of eosinophils from patients with atopic dermatitis to eosinophil chemotactic factors.

Yoshida T, Fujita K, Nishimoto M, Takaiwa T, Hirashima M.

Department of Dermatology, Kagawa Medical School, Japan.

The chemotactic response of eosinophils from 16 patients with atopic dermatitis (AD) to 5 eosinophil chemotactic factors (ECFs) were examined to clarify whether the response is associated with the clinical severity of AD. The factors included ECF-P15, -P16, -P17, -P18 and -P19 and were derived from a T cell line, STO-2. The patients were divided into 2 groups according to the percentage migration of eosinophils produced by the ECFs: a high-responding group (migration > 40%), and a low-responding group (migration < 30%). In a statistical analysis, eosinophils from patients with AD and atopic respiratory diseases (ARD) were found to be high-responding and those from patients with AD alone low-responding ($p < 0.01$). In a comparison of the chemotactic response of eosinophils from patients with AD alone at remission and at exacerbation, the percentage migrations in response to ECF-P15 and ECF-P16 at exacerbation were significantly higher than that at remission ($p < 0.05$). It is thus suggested that this type of heterogeneous response of eosinophils to STO-2-derived ECFs could provide a useful tool for evaluation of disease severity in patients with AD.

PMID: 8906107 [PubMed - indexed for MEDLINE]

2000. Arch Immunol Ther Exp (Warsz). 1996;44(1):77-80.

Spontaneous motility and chemotaxis of neutrophils is influenced by glycocorticosteroid therapy.

Zak-Nejmark T, Jankowska R, Małolepszy J, Jutel M, Kraus-Filarska M, Nadobna G.

Department of Internal Medicine and Allergology, Medical Academy, Traugutta, Wrocław, Poland.

The migration of neutrophils is an important part of the allergic inflammatory response. The aim of our study was to investigate the effect of glucocorticosteroids (GCS) on the stimulated and unstimulated migration of neutrophils. The study comprised 103 asthmatics including 44 subjects under GCS therapy (20 GCS resistant and 24 GCS sensitive) as well as 96 healthy control individuals. Unstimulated (random) motility as well as chemotactic response towards fMLP (10-8 mol/l) were determined after Boyden method. Neutrophil motility was determined by the distance of the leading front in filter. In both

resistant and sensitive asthmatics under GCS therapy we observed significantly increased unstimulated motility as compared to normal controls ($p < 0.001$). However, this effect was not demonstrated in the GCS untreated group. Neutrophil chemotaxis towards fMLP was increased in GCS untreated group as compared to healthy controls ($p < 0.05$). In GCS sensitive subjects the chemotatic activity was decreased. In GCS resistant asthmatics it was moderately increased. We conclude that increased unstimulated neutrophil motility might be one of the immunosuppressive mechanisms of GCS by preventing the cell accumulation at the sites of inflammation.

PMID: 8874774 [PubMed - indexed for MEDLINE]

2001. J Mol Med (Berl). 1996 Jan;74(1):13-33.

Intercellular adhesion molecule-1.

van de Stolpe A, van der Saag PT.

Department of Hematology, University Hospital Nijmegen, Netherlands.

The intercellular adhesion molecule (ICAM) 1 is an Ig-like cell adhesion molecule expressed by several cell types, including leukocytes and endothelial cells. It can be induced in a cell-specific manner by several cytokines, for example, tumor necrosis factor-alpha, interleukin-1, and interferon-gamma, and inhibited by glucocorticoids. Its ligands are the membrane-bound integrin receptors LFA-1 and Mac-1 on leukocytes, CD43, the soluble molecule fibrinogen, the matrix factor hyaluronan, rhinoviruses, and Plasmodium falciparum malaria-infected erythrocytes. ICAM-1 expression is predominantly transcriptionally regulated. The ICAM-1 promoter contains several enhancer elements, among them a novel kappa B element which mediates effects of 12-O-tetradecanoylphorbol-13-acetate, interleukin-1, lipopolysaccharide, tumor necrosis factor-alpha, and glucocorticoids. Expression regulation is cell specific and depends on the availability of cytokine/hormone receptors, signal transduction pathways, transcription factors, and posttranscriptional modification. ICAM-1 plays a role in inflammatory processes and in the T-cell mediated host defense system. It functions as a costimulatory molecule on antigen-presenting cells to activate MHC class II restricted T-cells, and on other cell types in association with MHC class I to activate cytotoxic T-cells. ICAM-1 on endothelium plays an important role in migration of (activated) leukocytes to sites of inflammation. ICAM-1 is shed by the cell and detected in plasma as sICAM-1. Regulation and significance of sICAM-1 are as yet unclear, but sICAM-1 is increased in many pathological conditions. ICAM-1 may play a pathogenetic role in rhinovirus infections. Derangement of ICAM-1 expression probably contributes to the clinical manifestations of a variety of diseases, predominantly by interfering with normal immune function. Among these are malignancies (e.g., melanoma and lymphomas), many inflammatory disorders (e.g., asthma and autoimmune disorders), atherosclerosis, ischemia, certain neurological disorders, and allogeneic organ transplantation. Interference with ICAM-1 leukocyte interaction using mAbs, soluble ICAM-1, antisense ICAM-1 RNA, and in the case of melanoma mAb-coupled immunotoxin, may offer therapeutic possibilities in the future. Integration of knowledge concerning membrane-bound and soluble ICAM-1 into a single functional system is likely to contribute to elucidating the immunoregulatory function of ICAM-1 and its pathophysiological significance in various disease entities.

PMID: 8834767 [PubMed - indexed for MEDLINE]

2002. Respiration. 1996;63(1):55-8.

Tropical pulmonary eosinophilia masquerading as acute bronchial asthma.

Jiva TM, Israel RH, Poe RH.

University of Rochester School of Medicine and Dentistry, N.Y., USA.

With the influx of immigrants from developing countries, deployment of American troops on foreign soil, and wide-ranging travel patterns of some United States citizens, one should expect an increase in the frequency of parasitic pulmonary diseases. We report a case of tropical pulmonary eosinophilia in a recent immigrant to Upstate New York from India. Tropical pulmonary eosinophilia is unfamiliar to most physicians practicing in North America, but should be included in the differential diagnosis of asthmatic bronchitis with hypereosinophilia when there is a history of recent travel to or residence in endemic areas. Furthermore, knowledge of this entity should also help in the differential diagnosis of other hypereosinophilic syndromes.

PMID: 8833995 [PubMed - indexed for MEDLINE]

2003. Allergy Asthma Proc. 1996 Jan-Feb;17(1):3-4.

Basophils, cytokines, and the allergic inflammatory response.

DuBuske LM.

Harvard Medical School, Boston, MA, USA.

Immediate allergic response has long been recognized to be related to the activation of mast cells by antigen. The role of the mast cells as producers of cytokines, however, has only more recently been extensively studied. The effect of TH2 lymphocytes in the inflammatory process is now well recognized in animal models. The central role of cytokines in the allergic inflammatory response is currently an area of intense clinical investigation. Cytokines influence production, migration, and activation of basophils. A wide array of cytokines is produced by mast cells upon initiation of the immediate allergic response. These cytokines influence a number of other different cells including basophils and eosinophils, and also activate lymphocytes, thus perpetuating allergic inflammation.

PMID: 8814937 [PubMed - indexed for MEDLINE]

2004. Am J Ind Med. 1996 Jan;29(1):23-32.

Respiratory health of Hispanic migrant farm workers in Indiana.

Garcia JG, Matheny Dresser KS, Zerr AD.

Department of Medicine, Indiana University School of Medicine, Indianapolis, USA.

The prevalence of respiratory disease in a Midwest Hispanic (mostly Mexican) migrant farm worker population was investigated. Chronic respiratory symptoms (cough, wheezing, sputum production) in adult workers (n = 354) were elevated (8.5%, 6.2%, 6.5%, respectively) and were accompanied by physiologic abnormalities as determined by pulmonary function testing. Over 15% of the adult cohort exhibited a FEV1/FVC < 75, and over 14% had FEF25-75 values which were less than 60% of predicted. The observed airflow obstruction of both large and small airways was not explained by cigarette usage (43%) in the adult cohort (current/past smokers). Tuberculin skin tests (TST) were positive (> or = 10 mm)

in 55/195 men and 35/123 women for a total prevalence of 28.3%. No case of active tuberculosis (TB) was identified by either chest X-ray (CXR) or sputum cultures (in selected cases). In contrast to adult farm workers, who were predominantly born in Mexico (70%), only 36% of adolescent workers (age 11-18 years, n = 107) were born in Mexico with only 7.5% exhibiting TST positivity. Airflow obstruction of large airways (5.8%) and small airways (12.9%) were also less common in adolescents than adults. In summary, these studies document respiratory dysfunction in Hispanic migrant farm workers in Indiana and highlight the need to closely monitor the respiratory health of this high-risk population.

PMID: 8808039 [PubMed - indexed for MEDLINE]

2005. Przegl Lek. 1996;53(1):12-9.

[Bronchial asthma--a chronic inflammatory disorder].

[Article in Polish]

Adamek-Guzik T, Czerniawska-Mysik G, Guzik T.

Z Katedry i Kliniki Chorób Wewnetrznych i Medycyny Wsi, w Szpitalu Specjalistycznym im. J. Dietla w Krakowie.

Inflammation is a major process in the pathogenesis of bronchial asthma. Pivotal role in the induction of the inflammation in atopic subjects is played by mast cells and eosinophils and their mediators. Lymphocytes T and macrophages modulate this process. Although the pathogenesis of asthma in non-atopic subjects is not totally clear the inflammation has similar course as in asthma with IgE overproduction. Current research emphasise e.g. the role of adhesion molecules, bronchial epithelial cells and nitric oxide in the pathogenesis of asthma. Selectin E, ICAM-1, VCAM-1 take part in the migration of inflammatory cells in the asthmatic lung. Expression of these molecules is included by IL-1, TNF-alpha, and IL-4. Inflammation leads to the bronchial epithelial damage and release of proinflammatory cytokines, which augment the above process. The epithelial damage causes exposure of nerve endings, which can lead to the activation of axon reflexes. The concentration of NO in the exhaled air of asthmatics is much higher than in healthy subjects. It may be produced by inflammatory cells and may augment the inflammation as well as cause bronchial hyperresponsiveness. The better understanding of inflammatory patterns of bronchial asthma has major influence on the therapeutic approach. Inhaled anti-inflammatory drugs are of the first choice in pharmacotherapy of even mild forms of asthma.

PMID: 8711169 [PubMed - indexed for MEDLINE]

2006. Am J Respir Cell Mol Biol. 1996 Jan;14(1):84-94.

Role of interleukin-4 and vascular cell adhesion molecule-1 in selective eosinophil migration into the airways in allergic asthma.

Fukuda T, Fukushima Y, Numao T, Ando N, Arima M, Nakajima H, Sagara H, Adachi T, Motojima S, Makino S.

Department of Internal Medicine and Clinical Immunology, Dokkyo University School of Medicine, Tochigi, Japan.

Recent in vitro studies have suggested that interleukin-4 (IL-4) may be involved in the preferential migration of eosinophils into the airways in allergic asthma through its capacity to selectively increase vascular cell adhesion molecule-1

(VCAM-1) expression on vessels. To test this hypothesis, we studied the expression of VCAM-1, E-selectin, and intercellular adhesion molecule-1 (ICAM-1) on vascular endothelium in bronchial mucosal biopsies from 20 allergic asthmatics using an immunohistochemistry technique and related the observations to IL-4 levels in bronchoalveolar lavage (BAL) fluid simultaneously obtained and to eosinophil infiltration in the bronchial mucosa. IL-4 was detectable in BAL fluid from nine subjects (range, 15.1 to 110 pg/ml in 20-fold concentrated BAL fluid) (IL-4-positive asthmatics) but unmeasurable in the remaining 11 subjects (IL-4-negative asthmatics). The IL-4-positive asthmatics showed a significantly increased expression of VCAM-1 but not E-selectin and ICAM-1 on vessels as compared with both IL-4-negative asthmatics ($P < 0.001$) and diseased control subjects ($P < 0.001$). In asthmatics, VCAM-1 expression was positively correlated with BAL IL-4 levels ($rs = 0.89$; $P < 0.0001$). Moreover, there was a significant correlation between the endothelial expression of VCAM-1 and the number of eosinophils, but not neutrophils, in the bronchial submucosa ($r2 = 0.76$; $P < 0.001$). A significant correlation was also found between BAL IL-4 levels and the number of eosinophils. These results suggest that IL-4 is a VCAM-1-selective activator also in human airways and the VCAM-1-dependent pathways play a role in selective migration of eosinophils into the airways in allergic asthma, and support the hypothesis described above.

PMID: 8534490 [PubMed - indexed for MEDLINE]

2007. Proc Natl Acad Sci U S A. 1995 Dec 19;92(26):12290-4.

Eosinophil recruitment into the respiratory epithelium following antigenic challenge in hyper-IgE mice is accompanied by interleukin 5-dependent bronchial hyperresponsiveness.

Eum SY, Hailé S, Lefort J, Huerre M, Vargaftig BB.

Unité de Pharmacologie Cellulaire, Unité Associée Institut Pasteur-Institut National de la Santé et de la Recherche Médicale 285, Paris, France.

A murine model for antigen-induced bronchial hyperreactivity (BHR) and airway eosinophilia, two hallmarks of asthma, was developed using ovalbumin-immunized mice, which produce large amounts of IgE (named BP2, "Bons Producteurs 2," for High Line of Selection 2). A single intranasal ovalbumin challenge failed to modify the bronchial responses, despite the intense eosinophil recruitment into the bronchoalveolar lavage fluid and airways. When mice were challenged twice a day for 2 days or once a day for 10 days, BHR in response to i.v. 5-hydroxytryptamine or to inhaled methacholine was induced in BP2 mice but not in BALB/c mice. Histological examination showed that eosinophils reached the respiratory epithelium after multiple ovalbumin challenges in BP2 mice but remained in the bronchial submucosa in BALB/c mice. Total IgE titers in serum were augmented significantly with immunization in both strains, but much more so in BP2 mice. Interleukin 5 (IL-5) titers in serum and bronchoalveolar lavage fluid of BP2 mice were augmented by the antigenic provocation, and a specific anti-IL5 neutralizing antibody suppressed altogether airway eosinophilia and BHR, indicating a participation of IL-5 in its development. Our results indicate that the recruitment of eosinophils to the airways alone does not induce BHR in mice and that the selective effect on BP2 mice is related to their increased IgE titers associated with antigen-driven eosinophil migration to the epithelium, following formation and secretion of IL-5.

PMCID: PMC40342
PMID: 8618887 [PubMed - indexed for MEDLINE]

2008. Arerugi. 1995 Dec;44(12):1401-9.

[A study of clinical significance of leukocyte migration inhibition test in drug-induced hypersensitivity pneumonitis].

[Article in Japanese]

Uno K, Kondo A.

Pharmacy, Suibarago Hospital.

In 71 patients suspected of drug-induced pneumonitis, the identification of the allergenic drugs were studied by leukocyte migration inhibition test (LMIT). The LMIT was positive in 61 cases (85.9%). Leukocyte migration activating factor (LMAF) was detected in 22 cases (30.9%), and leukocyte migration inhibitory factor (LMIF) in 39 cases (54.9%), which was found significantly higher than LMAF ($p < 0.05$). There was no considerable difference in the LMIT-positive rate between interstial and eosinophilic pneumonia. The LMIT-positive drugs were detected in 66 of all 180 suspected drugs, in which 33 drugs (50%) were antibacterial agents and 11 were Kampo prescriptions. beta-Lactam antibiotics accounted for about half the number (16 drugs) in antibacterial agents. LMAF was detected more frequently in beta-lactam antibiotics-induced pneumonitis, which LMIF was detected more often in Kampo prescriptions-induced pneumonitis ($p < 0.005$). Furthermore, the latent period from drug initial to the onset of pneumonitis were about 10 days in beta-lactam antibiotics-induced pneumonitis and a few months in Kampo prescriptions-induced pneumonitis ($p < 0.001$). Our findings indicate that LMIT is valuable to identify the allergenic drugs in drug-induced hypersensitivity pneumonitis and that delayed-type hypersensitivity (DTH), with which LMIF is related closely, plays a major role in the pathogenesis of this lung lesion. Furthermore, the pathogenic mechanism of beta-lactam antibiotics-induced pneumonitis may be different from that of Kampo prescriptions-induced pneumonitis.

PMID: 8871295 [PubMed - indexed for MEDLINE]

2009. Atherosclerosis. 1995 Dec;118(2):213-21.

Inhibition of PDGF- and TGF-beta 1-induced collagen synthesis, migration and proliferation by tranilast in vascular smooth muscle cells from spontaneously hypertensive rats.

Miyazawa K, Kikuchi S, Fukuyama J, Hamano S, Ujiie A.

Pharmacological Laboratories, Kissei Pharmaceutical Co. Ltd., Nagano, Japan.

Vascular smooth muscle cells (VSMC) from spontaneously hypertensive rats (SHR) proliferate faster and are more sensitive to transforming growth factor-beta 1 (TGF-beta 1) than those of normotensive Wistar-Kyoto rats. We studied the in vitro effects of tranilast, an anti-allergic drug, on the proliferation, migration and extracellular matrix synthesis in the SHR-VSMC. There were many inhibitory effects of tranilast (30-300 microM) on SHR-VSMC. One is the effect on the proliferation stimulated with fetal bovine serum (FBS), TGF-beta 1 and platelet-derived growth factor-BB (PDGF-BB). Another is the effect on the PDGF-BB-induced migration. Lastly, tranilast exhibited inhibitory effects on spontaneous collagen synthesis and TGF-beta 1-induced collagen and glycosaminoglycan synthesis. On the other hand, collagen induced the VSMC migration concentration-dependently. These results suggest that tranilast may prevent restenosis after percutaneous transluminal coronary angioplasty.

PMID: 8770315 [PubMed - indexed for MEDLINE]

2010. Toxicol Lett. 1995 Dec;82-83:491-6.

Cytokine regulation of chemical sensitization.

Kimber I, Holliday MR, Dearman RJ.

Zeneca Central Toxicology Laboratory, Alderley Park, Macclesfield, Cheshire, UK.

The skin is an immunologically active tissue. Epidermal cells, both keratinocytes and Langerhans cells (LC), produce constitutively or can be stimulated to produce a variety of cytokines, many of which play important roles in the induction and regulation of allergic responses to sensitizing chemicals. Tumor necrosis factor alpha (TNF-alpha) provides the signal for LC migration from the skin and granulocyte/macrophage colony-stimulating factor (GM-CSF), interleukin 1 (IL-1) and other cytokines effect the functional maturation of LC and their acquisition of immunostimulatory potential. The initial stimulus for induced or increased epidermal cytokine production derives from chemical exposure, or some other form of skin trauma. However, some epidermal cytokines are regulated in paracrine or autocrine fashion by other cytokines produced locally. The availability of epidermal cytokines has a major impact on the induction of sensitization and on the characteristics of immune responses to chemical allergens.

PMID: 8597100 [PubMed - indexed for MEDLINE]

2011. Tuber Lung Dis. 1995 Dec;76(6):493-504.

International union against tuberculosis and lung disease (IUATLD): initiatives in non-tuberculous lung disease.

Becklake MR.

International Respiratory Disease Research Unit, McGill University, Montreal, Quebec, Canada.

IUATLD initiatives in non-tuberculous lung disease developed in the late 1970s, coincident with improving tuberculosis control, and have targeted acute respiratory infections in children and chronic airways disease in adults and in children. The focus has been on methodology and the tools required to document the distribution and determinants of disease, and is illustrated in data gathered in African populations. Instruments developed include a simplified method of measuring bronchial hyper-reactivity and an asthma questionnaire Non-standard methods of questionnaire administration have also been validated, methods which are appropriate for use in the burgeoning urban communities and workforces of sub-Saharan Africa made up of rural migrants from different tribes and language groups. In addition, a review of reference values available for interpreting lung function in sub-Saharan African populations indicates a need to take into account a secular trend over the last two decades towards higher spirometric values. In the published data from Africa, not inconsiderable between-country differences are evident in the prevalence of chronic bronchitis in adults and of asthma in children. In addition, rates for childhood asthma were consistently higher in urban vs rural communities, with environmental factors playing an important role as well as being locally specific. Not only does the burden of morbidity attributable to both the chronic airway diseases reviewed justify past IUATLD initiatives in non-tuberculous lung disease, but it also argues that future initiatives should focus on investigating between- and within-country differences using a standardized methodology, with a view to identifying local environmental

determinants susceptible to intervention and control. Curbing tobacco use is clearly important, not only to benefit the health of adult smokers for whom the ill-health consequences have long been recognized, but, and more important, to protect the health of children, born and unborn, with whom they share the environment.

PMID: 8593369 [PubMed - indexed for MEDLINE]

2012. Allergy. 1995 Nov;50(11):878-83.

Platelet-derived growth factor in asthma.

Chanez P, Vignola M, Stenger R, Vic P, Michel FB, Bousquet J.

Clinique des Maladies Respiratoires, Hôpital Arnaud de Villeneuve, Centre Hospitalier Universitaire, Montpellier, France.

Platelet-derived growth factor (PDGF) controls cellular growth, migration, and differentiation. It is secreted by various cell types, including macrophages, and participates in tissue repair and epithelial regeneration. PDGF may therefore be involved in airway remodeling in asthma. This study compared the immunoreactivity of PDGF and its receptors (R alpha and R beta) in bronchial biopsies and the levels of PDGF in bronchoalveolar lavage (BAL) fluid of asthmatics and control subjects. Bronchial biopsies were done in a subsegmental bronchus of 11 asthmatics and 11 control subjects by flexible bronchoscope. PDGF AA and BB, and PDGF receptors R alpha and R beta were studied with monoclonal antibodies and revealed by immunoperoxidase staining. The percentage of subjects presenting positive staining with PDGFs and its receptors was studied in the epithelium and submucosa. PDGF AA, AB, and BB were measured in BAL fluid of 18 asthmatics and 10 controls by specific ELISA. In biopsies, there was no significant difference between asthmatics and controls for PDGF AA, BB, PDGF-R alpha and R beta (Fisher's exact test and Bonferroni's correction). Moreover, the levels of PDGF, AA, AB, and BB were similar in asthmatics and controls. This study does not support a role for PDGF in the repair processes of asthma.

PMID: 8748719 [PubMed - indexed for MEDLINE]

2013. Exp Lung Res. 1995 Nov-Dec;21(6):889-99.

Effect of elastase on the directional migration of lung fibroblasts within a three-dimensional collagen matrix.

Chetty A, Davis P, Infeld M.

Department of Pediatrics, Medical College of Wisconsin, Milwaukee, USA.

Interactions between airway epithelial cells and bronchial fibroblasts often require close proximity between these cells. Previous studies have demonstrated that airway epithelial cells direct the migration of lung fibroblasts, but the factors that regulate this process during airway injury are not clear. We hypothesized that exposure of culture substrates to proteolytic enzymes, like those present in the inflamed airway, would increase fibroblast recruitment. We also postulated that elastase might affect the epithelium's ability to attract fibroblasts. We used an in vitro model with fibroblasts embedded between two layers of collagen gel to investigate their migration. Embedded fibroblasts exposed to culture medium alone (baseline) had a slight downward migration (migration directed to the upper gel layer expressed as a percentage of total migration was -2.8 +/- 1.4), but medium supplemented with porcine pancreatic

elastase (PPE) resulted in a slight upward migration (2.0 +/- 1.4). When airway epithelial cells were cultured on the upper gel surface, the index of directed migration toward them was 15.9 +/- 1.3. Addition of PPE to the culture medium resulted in a significant increase to 22.3 +/- 1.5 (p < .05). Human neutrophil elastase (HNE) produced similar results, and these effects were inhibited by alpha 1-proteinase inhibitor. Similarly, total fibroblasts per 20 high-powered fields were counted in all conditions, suggesting that mitogenic interactions were not important in this system. The percentage of the total fibroblasts migrating at least 5 microns in any direction was also similar in all groups, suggesting chemokinetic mechanisms were not involved. These data suggest that elastase exposure in a model of the human airway increases directed fibroblast migration through the extracellular matrix. This phenomenon may play a role in the development of subepithelial fibrosis seen in inflammatory airway diseases like asthma.

PMID: 8591792 [PubMed - indexed for MEDLINE]

2014. BMJ. 1995 Oct 21;311(7012):1069-70.

Moving house: a risk factor for the development of childhood asthma?

Hughes CH, Baumer JH.

Health Centre, Estover, Plymouth.

Comment in
    BMJ. 1996 Feb 3;312(7026):315.

PMCID: PMC2551371
PMID: 7580667 [PubMed - indexed for MEDLINE]

2015. Allerg Immunol (Paris). 1995 Oct;27(8):284-91.

[Toxocariasis. A cosmopolitan parasitic zoonosis].

[Article in French]

Humbert P, Buchet S, Barde T.

Service Dermatologie, CHU Saint-Jacques, Besançon.

The infection by Toxocara canis transmitted by dogs (30% of them are infected in our countries) and less frequently by cats lead to larva migrans visceral syndrome with neurological manifestations, ophtalmological affection and various cutaneous manifestations observed in 24% of the extra-ocular infections: chronic urticaria often associated with asthmatic manifestations and chronic rhinitis, angio-oedema or local oedema reaching particularly the eyclid, chronic pruritus associated with lesions due to scratching or to nodular prurigo. An hypereosinophilia is an argument in favour of a progressive infection. High total IgE is an hallmark of visceral infections by parasites and total IgE level is well correlated with the presence of intra-tissular larva. The serological diagnosis is based on the determination of specific IgG by ELISA which appears also to be interesting for the patient's follow up. The western blot method seems to be more specific than the other methods and so is useful to confirm a diagnosis. The treatment given as early as possible is based on the use of diethylcarbamazine but also of thiabendazole, albendazole and mebendazole. Prophylaxis of toxocara infection includes the prohibition of dog access to children games areas but also a frequent turn over of the sand in public parks.

PMID: 8851035 [PubMed - indexed for MEDLINE]

2016. Arerugi. 1995 Oct;44(10):1216-22.

[Generation of tumor necrosis factor alpha by human nasal epithelial cells and inhibition by fluticasone propionate].

[Article in Japanese]

Ohnishi M, Takizawa R, Yokoshima K, Okubo K, Okuda M, Yagi T.

Department of Otorhinolaryngology, Nippon Medical School.

Accumulation of mast cells and eosinophils in the nasal epithelial layer occurs in nasal allergic reaction, However, the mechanism of accumulation of these cells has not yet been well clarified. We hypothesized that cytokines generated from the nasal epithelial cells contributed to the accumulation of these cells in the nasal epithelial layer. Recently tumor necrosis factor (TNF) was shown to promote polymorphonuclear neutrophils and eosinophils migration. And also TNF increased eosinophil binding to vascular endothelial cells. In this in vitro study we examined whether or not nasal epithelial cells can produce TNF-alpha and also whether or not glucocorticosteroid fluticasone propionate (FP) can modulate TNF-alpha production from nasal epithelial cells. Nasal epithelial cells constitutively produce TNF-alpha in accordance with the nasal epithelial cells' number and this was substantially increased in the state of nasal epithelial cell's proliferating. FP significantly reduced the level of TNF-alpha in the supernatant of cultured nasal epithelial cells for a period of 6 days. In addition, preincubation of nasal epithelial cells with FP for 6 days caused significant reduction of TNF-alpha level in the supernatant of cultured nasal epithelial cells during a further period of 6 days without FP. These data support the concept that structural cells play an active role in the control of allergic and related inflammatory processes.

PMID: 8579483 [PubMed - indexed for MEDLINE]

2017. Environ Health Perspect. 1995 Oct;103(10):936-41.

Health effects in a casual sample of immigrants to Israel from areas contaminated by the Chernobyl explosion.

Kordysh EA, Goldsmith JR, Quastel MR, Poljak S, Merkin L, Cohen R, Gorodischer R.

Epidemiology and Health Services Evaluation Unit, Soroka Medical Center, Beer Sheva, Israel.

We analyzed questionnaire and physician examination data for 1560 new immigrants from the former USSR divided into three groups by potential exposure to Chernobyl radiation. Two groups were chosen according to soil contamination by cesium-137 at former residences, as confirmed by our findings in a 137Cs body burden study. The third group consisted of "liquidators," persons who worked at the Chernobyl site after the disaster. Liquidators had greater self-reported incidences of symptoms commonly accepted as acute effects of radiation exposure, increases in prevalence of hypertension, and more health complaints. Excesses of bronchial asthma and health complaints were reported in children from the more exposed communities. Asthma prevalence in children potentially exposed in utero appears to be increased eightfold. Older adults from more exposed areas had more hypertension as assessed by history and measurements. These findings suggest the

possible association of radiation exposure with several nonmalignant effects.

PMCID: PMC1519167
PMID: 8529590 [PubMed - indexed for MEDLINE]

2018. Immunology. 1995 Oct;86(2):296-303.

Migration of human antigen-presenting cells in a human skin graft onto nude mice model after contact sensitization.

Hoefakker S, Balk HP, Boersma WJ, van Joost T, Notten WR, Claassen E.

Division of Immunological and Infectious Diseases, TNO Prevention and Health, Leiden, The Netherlands.

Fluorescent contact chemical allergens provoke sensitization after application on both syngeneic and allogeneic skin grafts in mice. We attempted to determine whether the functional activity in a contact sensitization response of human skin graft was affected at the level of antigen uptake and migration. After xenogeneic skin transplantation, we examined the effect of topical exposure of the graft to rhodamine B isothiocyanate (RITC). This paper describes the migration of RITC-carrying cells and human major histocompatibility complex (MHC) class II DR (HLA-DR)+ cells, from the graft to mouse draining lymph nodes. As demonstrated by immunohistochemistry, grafting resulted in a time-dependent decrease of human HLA-DR+ and CD1a+ cells, and an increase of mouse MHC class II (Ia)+ cells within the graft. Application of RITC on a 3-week-old human skin graft showed optimal migration capability compared to 6- or 9-week-old grafts. In addition, the time-dependent increase of frequencies of RITC+ and HLA-DR+ cells in the draining lymph nodes, and the time-dependent decrease of HLA-DR+ cells in the 3-week-old human skin graft, were concurrent. Supporting these data, human cytokine interleukin-1 alpha (IL-1 alpha), IL-1 beta and tumour necrosis factor-alpha (TNF-alpha), analysis in situ revealed that cytokine production by keratinocytes, a property associated with dendritic cell migration, was preserved in the human skin graft. Thus, like dendritic cells in contact sensitization in allografted skin, dendritic cells from human xenografted skin onto nude mice are capable of migration to mouse draining lymph nodes after allergen application. Induction of contact hypersensitivity is possible in a human skin graft onto nude mice model, although the use of this ex vivo model to analyze contact sensitivity is probably limited to 3 weeks after transplantation.

PMCID: PMC1384009
PMID: 7490132 [PubMed - indexed for MEDLINE]

2019. Am J Respir Cell Mol Biol. 1995 Sep;13(3):360-5.

Involvement of IL-5 in a murine model of allergic pulmonary inflammation: prophylactic and therapeutic effect of an anti-IL-5 antibody.

Kung TT, Stelts DM, Zurcher JA, Adams GK 3rd, Egan RW, Kreutner W, Watnick AS, Jones H, Chapman RW.

Schering-Plough Research Institute, Kenilworth, NJ 07033-0539, USA.

Interleukin-5 (IL-5) is important in the control of differentiation, migration, and activation of eosinophils. In order to study the role of IL-5 in the development of eosinophilic inflammation of the airways, we have used a monoclonal antibody to murine IL-5 (TRFK-5) in a murine model of allergic pulmonary inflammation. B6D2F1 mice were sensitized with alum-precipitated

ovalbumin and were challenged with aerosolized ovalbumin on day 12 after sensitization. Samples of bronchoalveolar lavage (BAL) fluid, lung tissue, blood, and bone marrow aspirate were collected at different times after ovalbumin challenge. Twenty-four hours after challenge there were significant increases in the number of eosinophils in the BAL fluid, lung tissue, and blood while bone marrow eosinophils were decreased. Treatment of sensitized mice with TRFK-5 (0.01-1 mg/kg, i.p.) 2 h before ovalbumin challenge reduced the numbers of eosinophils in the BAL fluid and lung tissue and prevented the decrease in bone marrow eosinophils in a dose-dependent fashion. The number of eosinophils in the BAL fluid, peribronchial and alveolar regions of the lung was also reduced when TRFK-5 (2 mg/kg, i.p.) was given up to 5 d after ovalbumin challenge. Furthermore, there was no evidence of increased epithelial damage, edema, or the presence of mucus that could have resulted from eosinophil apoptosis and release of toxic proteins after neutralization of IL-5. These results demonstrate an important role for IL-5 in the development of eosinophilic inflammation of the airways and for the migration of eosinophils from the bone marrow into blood in response to antigen challenge.(ABSTRACT TRUNCATED AT 250 WORDS)

PMID: 7654390 [PubMed - indexed for MEDLINE]

2020. Am J Respir Cell Mol Biol. 1995 Sep;13(3):323-9.

Interleukin-8 mediates interleukin-1 alpha-induced neutrophil transcellular migration.

Bittleman DB, Casale TB.

Department of Internal Medicine, VA Medical Center, Iowa City, Iowa, USA.

Interleukin-1 alpha (IL-1 alpha) is a cytokine with a myriad of potent proinflammatory effects. Neutrophils are important immune effector cells in allergic and inflammatory lung diseases. We examined the effects of IL-1 alpha on human neutrophil migration across naked filters and human umbilical vein endothelial (HUVE) cell and type II-like pulmonary epithelial cell (A549) monolayers cultured on these filters. IL-1 alpha from 10(-13) to 10(-9) M induced dose-dependent neutrophil migration through both HUVE and A549 cellular monolayers but not through naked filters. Neutrophil migration was consistently greater through A549 monolayers compared with HUVE monolayers. IL-1 alpha-induced neutrophil migration was also time dependent, and the kinetics of neutrophil migration through HUVE and A549 monolayers were similar. Significant migration through either monolayer was not observed until 2 h, and maximal migration occurred at 3 h through A549 and 5 h though HUVE cellular monolayers. Supernatants of IL-1 alpha (10(-11) M)-stimulated HUVE and A549 monolayers induced significantly more migration of neutrophils across naked filters than 10(-11) M IL-1 alpha itself, suggesting the release of soluble secondary chemotactic factor(s). Pretreatment of HUVE and A549 monolayers with actinomycin D inhibited both IL-1 alpha-induced production of soluble chemotactic factor(s) and transcellular migration by > 90%. Supernatants from IL-1 alpha-treated HUVE and A549 cells contained significant concentrations of interleukin 8 (IL-8), and coincubation of these supernatants with anti-IL-8 inhibited approximately 50% of supernatant-induced chemotaxis.(ABSTRACT TRUNCATED AT 250 WORDS)

PMID: 7654388 [PubMed - indexed for MEDLINE]

2021. Br J Dermatol. 1995 Aug;133(2):157-67.

Down-regulation of Langerhans cell protein kinase C-beta isoenzyme expression in inflammatory and hyperplastic dermatoses.

Reynolds NJ, Yi JY, Fisher GJ, Cooper KD, Voorhees JJ, Griffiths CE.

Department of Dermatology, University of Michigan Medical Center, Ann Arbor, USA.

The family of protein kinase C (PKC) isoenzymes plays a fundamental part in signal transduction, and thereby regulates important cellular functions, including growth, differentiation, cytokine production and adhesion molecule expression. In lesional psoriatic skin, Ca(2+)-dependent PKC activity, PKC-beta protein and epidermal Langerhans cell (LC) PKC-beta immunostaining are significantly decreased, indicating activation and subsequent down-regulation of PKC. Whether these changes occur in other inflammatory/hyperplastic dermatoses is, however, unknown. We examined PKC-alpha and PKC-beta expression in normal skin, psoriasis, cutaneous T-cell lymphoma (CTCL), lamellar ichthyosis, non-bullous ichthyosiform erythroderma, atopic dermatitis, urushiol-induced allergic contact dermatitis, and sodium lauryl sulphate (SLS)-induced irritant contact dermatitis. Cryostat sections were stained for PKC-alpha and PKC-beta, and the LC marker CD1a, using an immunoperoxidase technique and specific monoclonal antibodies. Double-labelling studies, in normal skin, revealed co-expression of PKC-beta and CD1a by epidermal LCs. Analysis of the number of PKC-beta+ and CD1a+ epidermal LCs, in diseased compared with normal skin, revealed three categories: (i) in psoriasis and CTCL, the PKC-beta+ epidermal LC number was significantly reduced, whereas the CD1a+ epidermal LC number was unchanged; (ii) in allergic and irritant contact dermatitis, both PKC-beta+ and CD1a+ epidermal LCs were significantly reduced in number; and (iii) in atopic dermatitis, the PKC-beta+ epidermal LC number was normal, and CD1a+ epidermal LCs were significantly increased in number. Moreover, the ratio of epidermal LC PKC+/CD1a+ was reduced in all the dermatoses studied, suggesting activation of PKC-beta, with subsequent down-regulation. Within the dermis, increased PKC-beta staining of infiltrating cells was observed in all the conditions studied except lamellar ichthyosis and non-bullous ichthyosiform erythroderma. These data indicate that: (i) down-regulation of LC PKC-beta occurs in a variety of inflammatory and hyperplastic skin disorders, and is not unique to psoriasis, and (ii) the pattern of epidermal LC PKC-beta and CD1a expression varies among the diseases studied. In mice, PKC activation induces LC migration. Thus, down-regulation of epidermal LC PKC-beta associated with reduced CD1a+ epidermal LCs in allergic and irritant contact dermatitis suggests that PKC-beta may transduce the signal for migration of LCs from human epidermis.

PMID: 7547380 [PubMed - indexed for MEDLINE]

2022. J Invest Dermatol. 1995 Jul;105(1 Suppl):2S-7S.

Maturation and migration of cutaneous dendritic cells.

Steinman R, Hoffman L, Pope M.

Laboratory of Cellular Physiology and Immunology, Rockefeller University, New York, NY 10021, USA.

Dendritic cells have been isolated from the epidermis, dermis, and lymphatics of skin. Cells from each cutaneous compartment can exhibit the distinct morphology, surface phenotype, and strong T-cell-stimulating activity of dendritic cells that are isolated from other organs. Of importance are the mechanisms by which the maturation and movement of dendritic cells are regulated within intact tissues. Epidermal dendritic cells turn over slowly in the steady state. Stimuli, including contact allergens and transplantation, perhaps by inducing a release of cytokines such as granulocyte macrophage-colony-stimulating factor, mobilize these dendritic cells into the dermis and lymph. This migration is accompanied by

the maturation of dendritic cell functions; e.g., antigen-presenting major histocompatibility complex molecules and B7 costimulators increase markedly. On the other hand, there is a sizable, steady-state flux of dendritic cells in afferent lymph draining the skin, which suggests a constant traffic through the dermis that is independent of sessile epidermal dendritic cells. When explants of skin are placed in organ culture, dendritic cells emigrate into the medium for 1-3 d. The dendritic cells are mature and can bind tightly to small memory T cells that also migrate in these cultures. The emigrated mixtures of dendritic cells and T cells should be useful in the study of many clinical states. This is illustrated by recent experiments showing that migratory skin cells are readily infected with human immunodeficiency virus (HIV)-1. A strong productive infection takes place in the absence of exogenous cytokines, foreign sera, or mitogens or antigens. The dendritic cell-T-cell conjugates are the essential site for infection. This cellular milieu may model events during the sexual transmission of HIV-1, where relevant mucosal surfaces are covered by skin-like epithelia. The capture of CD4+ memory T cells by dendritic cells may explain the chronic drain of immune memory in HIV infection.

PMID: 7615992 [PubMed - indexed for MEDLINE]

2023. J Immunol. 1995 Jul 1;155(1):325-32.

TGF-beta 2 decreases migration of lymphocytes in vitro and homing of cells into the central nervous system in vivo.

Fabry Z, Topham DJ, Fee D, Herlein J, Carlino JA, Hart MN, Sriram S.

Department of Pathology, University of Iowa College of Medicine, Iowa City 52242, USA.

Migration of leukocytes through an in vitro, cell culture model of the blood-brain barrier (BBB) composed of murine brain microvessel endothelial (En) cells and astrocytes, and in vivo in experimental allergic encephalomyelitis (EAE), was investigated. We have recently shown that the adhesiveness of cultured murine brain microvascular endothelial cells for lymphocytes can be increased significantly by pretreatment with IL-1 beta, TNF-alpha, IFN-gamma, and LPS. In the present study, we investigated the role of TGF-beta 2 on the migration of leukocytes through the BBB. In vitro migration was assessed by measuring the percentage of 51Cr-labeled leukocytes migrating through the En/astrocyte monolayers. The basal level of migration was up-regulated significantly by treating the En/astrocyte monolayers with IL-1 alpha, IFN-gamma, TNF-alpha, and LPS. The ability of these cytokines to modulate migration was dose-dependent. Treatment of En cell/astrocyte monolayers with TGF-beta 2 down-regulated the level of leukocyte migration up-regulated by IL-1 alpha, IFN-gamma, and TNF-alpha in vitro in a dose-dependent manner. TGF-beta 2 also inhibited the migration of lymphocytes into the central nervous system (CNS) in vivo in a dose-dependent fashion. Taken together, these findings strongly suggest that TGF-beta plays an important role in the reduction of lymphocyte infiltration into the CNS in inflammatory demyelinating diseases such as EAE.

PMID: 7602108 [PubMed - indexed for MEDLINE]

2024. Thorax. 1995 Jul;50(7):785-92.

Effects of topical budesonide on epithelial restitution in vivo in guinea pig trachea.

Erjefält JS, Erjefält I, Sundler F, Persson CG.

Department of Medical Cell Research, University of Lund, Sweden.

BACKGROUND: Continuous epithelial shedding and restitution processes may characterise the airways in diseases such as asthma. Epithelial restitution involves several humoral and cellular mechanisms that may potentially be affected by inhaled anti-asthma drugs. The present study examines the effect of a topical steroid on epithelial restitution in vivo in the guinea pig.
METHODS: The airway epithelium was mechanically removed from well defined areas of guinea pig trachea without surgery and without damage to the basement membrane or bleeding. An anti-inflammatory dose of budesonide (1 mg) was administered repeatedly to the tracheal surface by local superfusion 24 hours before, at (0 hours), and 24 hours after the denudation. Migration of epithelial cells, formation of a plasma exudation-derived gel, and appearance of luminal leucocytes were recorded by scanning electron microscopy. Cell proliferation was visualised by bromodeoxyuridine immunohistochemistry and tissue neutrophils and eosinophils by enzyme histochemistry.
RESULTS: Immediately after creation of the denuded zone ciliated and secretory cells on its border dedifferentiated, flattened out, and migrated speedily (mean (SE) 2.3 (0.3) micron/min) over the basement membrane. After 48 hours the entire denuded zone (800 microns wide) was covered by a tightly sealed epithelium; at this time increased proliferation was observed in new and old epithelium and subepithelial cells. Budesonide had no detectable effect on epithelial dedifferentiation, migration, sealing, or proliferation. Immediately after denudation and continuously during the migration phase plasma was extravasated creating a fibrinous gel rich in leucocytes, particularly neutrophils, over the denuded area. Budesonide had no effect on either the gel or the leucocyte density.
CONCLUSIONS: These observations suggest that topical glucocorticoids may not interfere with a fast and efficient restitution of the epithelium in the airways.

PMCID: PMC474655
PMID: 7570417 [PubMed - indexed for MEDLINE]

2025. Br J Dermatol. 1995 Jul;133(1):48-53.

Beta 2-integrins in different forms of urticaria.

Haas N, Iwen W, Grabbe J, Hamann K, Czarnetzki BM.

Department of Dermatology, Clinical Immunology and Asthma, Rudolf Virchow Hospital, Free University Berlin, Germany.

As urticarial lesions involve tissue invasion by inflammatory cells, and as beta 2-integrins play a central part in adhesion of leucocytes to endothelia, allowing their migration into the tissues, we have explored the distribution and sequential expression of these molecules in tissue sections from different forms of urticaria. Prick test weals (of 10 min duration) to common inhalant allergens showed only a minor increase of CD18, whereas in a case of cold urticaria CD11b and CD18 molecules were increasingly upregulated within the first 30 min after elicitation of the lesions. Skin test sites in delayed pressure urticaria, and urticarial lesions (> 6 h duration) of acute and chronic recurrent urticaria also showed marked upregulation of CD11b and CD18, and to a lesser extent of CD11a, but this did not strongly correlate with the intensity of the mixed cellular infiltrate. Non-lesional skin showed expression of beta 2-integrins in chronic urticaria, delayed pressure urticaria, and less so in acute urticaria, suggesting generalized leucocyte activation. This analysis of integrins thus suggests an early and extensive involvement of these molecules in the pathological events associated with the evolution of urticarial lesions.

PMID: 7545421 [PubMed - indexed for MEDLINE]

2026. Clin Exp Immunol. 1995 Jul;101(1):164-71.

Allergen-stimulated T lymphocytes from allergic patients induce vascular cell adhesion molecule-1 (VCAM-1) expression and IL-6 production by endothelial cells.

Delneste Y, Jeannin P, Gosset P, Lassalle P, Cardot E, Tillie-Leblond I, Joseph M, Pestel J, Tonnel AB.

Unité INSERM U 416, Institut Pasteur, Lille, France.

Adhesion of inflammatory cells to endothelium is a critical step for their transvascular migration to inflammatory sites. To evaluate the relationship between T lymphocytes (TL) and vascular endothelium, supernatants from allergen-stimulated TL obtained from patients sensitive to Dermatophagoides pteronyssinus (Dpt) versus healthy subjects were added to endothelial cell (EC) cultures. TL were stimulated by autologous-activated antigen-presenting cells (APC) previously fixed in paraformaldehyde to prevent monokine secretion. Two parameters were measured: the expression of adhesion molecule and the production of IL-6. Related allergen-stimulated TL supernatants from allergic patients induced an increase of VCAM-1 and intercellular adhesion molecule-1 (ICAM-1) expression when supernatants of the control groups (TL exposed to an unrelated allergen or not stimulated or TL obtained from healthy subjects) did not. E-selectin expression was not modulated whatever the supernatant added to EC culture. IL-6 production by EC was significantly enhanced after activation with related allergen-stimulated TL supernatants from allergics compared with control supernatants. Induction of VCAM-1 expression was inhibited by adding neutralizing antibodies against IL-4, whereas IL-6 production and ICAM-1 expression were inhibited by anti-interferon-gamma (IFN-gamma) antibodies. Enhanced production of IL-4 and IFN-gamma was detected in related allergen-stimulated TL supernatants from allergic subjects compared with the different supernatants. These data suggest that allergen-specific TL present in the peripheral blood of allergic patients are of Th1 and Th2 subtypes. Their stimulation in allergic patients may lead to the activation of endothelial cells and thereby participate in leucocyte recruitment towards the inflammatory site.

PMCID: PMC1553285
PMID: 7542574 [PubMed - indexed for MEDLINE]

2027. Int J Gynaecol Obstet. 1995 Jun;49(3):323-4.

Echinococcosis of the liver during pregnancy.

van Vliet W, Scheele F, Sibinga-Mulder L, Dekker GA.

Department of Obstetrics and Gynecology, Free University Hospital, Amsterdam, The Netherlands.

A 20-year-old Turkish woman with three huge echinococcus cysts of the liver was admitted in the third trimester of pregnancy. During pregnancy she received albendazole and during vaginal delivery she received both albendazole and medication aimed at preventing anaphylactic reaction. We believe that the presence of large hydatid cysts during pregnancy should be managed conservatively with courses of albendazole after the first trimester of pregnancy.

PMID: 9764873 [PubMed - indexed for MEDLINE]

2028. Am J Respir Cell Mol Biol. 1995 Jun;12(6):691-6.

C5a-induced migration of human monocytes is primed by dexamethasone.

Pieters WR, Houben LA, Koenderman L, Raaijmakers JA.

Department of Pulmonary Diseases, University Hospital Utrecht, The Netherlands.

Allergic inflammation in the lung is characteristic of allergic asthma. This inflammatory process is inhibited by treatment with glucocorticoids. One of the cell types involved in the inflammatory process, the monocyte, is found in enhanced numbers in mucosal lung biopsies of asthmatic patients. Little is known about the mechanisms that lead to increased numbers of monocytes in lung tissue. We studied one of the processes involved, chemotaxis, in a modified Boyden Chamber assay. The effect of the antiinflammatory drug dexamethasone was tested on monocyte chemotactic responses to complement fragment C5a. Human monocytes from peripheral blood of normal human volunteers were purified by centrifugal elutriation. The monocytes showed a reproducible chemotactic response toward C5a with an optimum at a concentration of 10(-9) M. After culture of the monocytes overnight, the monocyte responses were clearly impaired. It is interesting that upon culture, dexamethasone increased monocyte chemotaxis in a dose-dependent manner. Analysis of the filters with an image analyzer showed that the effect was not through modulation of a subpopulation of monocytes. This steroid effect was specific and modulated via steroid receptors, because the introduction of RU 38486, a steroid receptor antagonist, completely inhibited the effect of dexamethasone. These findings are a further illustration of the complex mechanisms involved in the orchestration of the inflammatory response in asthma.

PMID: 7766432 [PubMed - indexed for MEDLINE]

2029. Am J Respir Cell Mol Biol. 1995 Jun;12(6):613-23.

Phenotypic characterization of T lymphocytes emigrating into lung tissue and the airway lumen after antigen inhalation in sensitized mice.

Kennedy JD, Hatfield CA, Fidler SF, Winterrowd GE, Haas JV, Chin JE, Richards IM.

Upjohn Laboratories, Kalamazoo, Michigan 49001, USA.

Cytokines released from CD4+ T lymphocytes contribute to the pathogenesis of asthma by influencing the differentiation and function of eosinophils, the primary effector cells that cause airway epithelial damage. Using a model of ovalbumin (OA)-induced, eosinophil-rich chronic lung inflammation in sensitized mice, we have defined the role of T lymphocytes further by using three-color flow cytometry to characterize the adhesion and activation antigens that may be associated with the migration of these cells into the lung and airway lumen. OA inhalation in OA-sensitized C57BL/6 mice resulted in an early (6 to 24 h) influx of neutrophils into the bronchial lumen as enumerated by bronchoalveolar lavage (BAL), which was followed by a marked accumulation of lymphocytes and eosinophils between 24 to 72 h. Phenotypic analysis of BAL or lung tissue T cells showed that most Thy-1 CD3+ T cells were CD4+ (CD4: CD8 ratio of 3 to 4:1). The majority (90%) of the T cells in lung or BAL fluid expressed alpha beta T-cell receptors (TCR). Only 3 to 7% of the T cells were gamma delta TCR+ even though almost 25% of the T cells were CD4- CD8-. There were very few natural killer (NK) or B cells in BAL fluid compared with 15% B cells in dissagregated lung tissue. In contrast to T cells in spleen, almost all the lung and BAL T cells were of the memory phenotype, as ascertained by the expression of high levels of CD44 and by the

absence of L-selectin and CD45RB on the cell surface. Fifty to ninety percent of lung and BAL T cells from vehicle-sensitized or OA-sensitized and challenged mice expressed the adhesion molecules CD11a (LFA-1), CD54 (ICAM-1), and CD49d (VLA-4). The early T-cell activation marker CD69 was upregulated on 30% of the lung and BAL T cells in OA-sensitized mice after antigen inhalation. When BAL fluid T cells from OA-sensitized and challenged mice were analyzed for their coexpression of adhesion and/or activation molecules, 75% of the cells that expressed one of three adhesion molecules, CD54, CD49d, or CD11a, also expressed at least one of the other two antigens. At least 15% of BAL T cells had all three of these molecules on their cell surfaces. The OA-dependent, temporally regulated emigration of T cells into the bronchial lumen after exposure to aerosolized antigen may be correlated with the accumulation of cells that express the memory phenotype with enhanced expression of adhesion molecules.

PMID: 7766426 [PubMed - indexed for MEDLINE]

2030. Allerg Immunol (Paris). 1995 Jun;27(6):178-81.

Cytokines and adhesion molecules in respiratory allergy.

Palma-Carlos AG, Palma-Carlos ML, Santos MC, Melo A.

Centro de Hematologia e Imunologia, Faculdade de Medicina de Lisboa, Portugal.

Cytokines and adhesion molecules play a central role in the inflammatory process of respiratory allergy. Cytokines like IL4 acts on IgE synthesis and expression of low affinity CD23 IgE receptors, IL-5 on eosinophil differentiation and activation and IL-2 on T cell activation and on the expression of CD25 IL-2 receptors. IL-2, IL-4 and IL-2 soluble receptor have been studied in pollen sensitive patients before, during and after pollen season. IL-2 serum levels initially increase and decrease at the end of allergen exposition. IL-4 serum level do not significantly changes during pollen season. Adhesion molecules are essential for recruitment and migration of inflammatory cells to tissues. CD45RO T memory cells expressing generally the adhesion molecule CD29 have also been studied in a group of pollen sensitive patients. During the peak of antigen exposition CD45RO/CD29 cells significantly decrease a turnover between CD45RA naive cells and memory cells being observed. The study of cytokines and adhesion molecules could add new data on the comprehension of inflammation in respiratory allergy.

PMID: 7626191 [PubMed - indexed for MEDLINE]

2031. Br J Med Psychol. 1995 Jun;68 (Pt 2):135-42.

Migration, traumatic bereavement and transcultural aspects of psychological healing: loss and grief of a refugee woman from Begameder county in Ethiopia.

Schreiber S.

Department of Psychiatry, Hadassah University Hospital, Jerusalem, Israel.

Post-traumatic stress disorder (PTSD), grief and bereavement may be manifested in a variety of ways in different populations and cultures. Following is the description of an Ethiopian immigrant woman from the county of Begameder whose baby died during the long exodus from Ethiopia and who, because of the different environmental conditions in 'the new land', could not undergo traditional purification rituals. Subsequently she suffered various cultural signs and symptoms of PTSD due to complicated bereavement and, on top of that, was seen as

'impure' by both her family and herself. For two years her symptoms were attributed to a severe form of bronchial asthma and she did not respond to treatment. When eventually brought to psychiatric attention, she was erroneously diagnosed as suffering from psychosis and treated inappropriately. Accurate anamnesis, combined with adequate counselling, provided the correct diagnosis, and a combination of supportive psychotherapy, traditional healing and purification rituals resulted in a resolution of the syndrome. Thirty months of follow-up showed that the results of the treatment were stable and satisfactory. The specific aspects of cultural manifestations of grief and mourning, the meaning of hallucinations not in the context of psychosis, purification rituals, the role of traditional healing among immigrants from a totally different culture, and the difficulties that helpers may have interpreting and making sense of the immigrants' behaviour and complaints are discussed.

PMID: 7547610 [PubMed - indexed for MEDLINE]

2032. J Allergy Clin Immunol. 1995 May;95(5 Pt 1):979-86.

Cetirizine inhibits the in vitro and ex vivo chemotactic response of T lymphocytes and monocytes.

Jinquan T, Reimert CM, Deleuran B, Zachariae C, Simonsen C, Thestrup-Pedersen K.

Department of Dermatology, Marselisborg Hospital, Aarhus C., Denmark.

We have studied the effect of a nonsedating antihistamine, cetirizine dihydrochloride, on the in vitro chemotaxis of leukocytes from human peripheral blood. We observed that 0.25 microgram/ml of cetirizine dihydrochloride in vitro significantly inhibited the chemotaxis of monocytes toward N-formyl-methionyl-leucyl-phenylalanine and leukotriene B4. Higher concentrations of cetirizine, 1.0 and 2.5 micrograms/ml, completely inhibited monocyte chemotaxis without affecting cell viability. T-lymphocyte migration was also significantly depressed but not abolished. Pyrilamine (mepyramine) was not inhibitory in equimolar concentrations. According to these in vitro observations, we extended our studies to measure monocyte and T-lymphocyte chemotaxis in an open study, where four healthy volunteers and six patients with atopic dermatitis took 10 and 20 mg/day cetirizine 3 days. We observed a reduction in ex vivo monocyte and T-lymphocyte chemotaxis toward N-formyl-methionyl-leucyl-phenylalanine and leukotriene B4 without a reduction of the blood cell count. The results were confirmed in an ensuing double-blind, placebo-controlled study of 16 healthy subjects and 14 adult patients with atopic dermatitis, where ex vivo monocyte chemotaxis was reduced or abolished during cetirizine therapy. Serum levels of the two eosinophil-derived granule proteins, eosinophilcationic protein P and eosinophil protein X, were not changed during the treatment period of 7 days. The results show that cetirizine dihydrochloride has an inhibitory effect on monocytes and T lymphocytes in vitro and ex vivo. Our findings support the clinical observations that cetirizine dihydrochloride has an antiinflammatory effect besides its H1-blocking activity.

PMID: 7751519 [PubMed - indexed for MEDLINE]

2033. Int Arch Allergy Immunol. 1995 May-Jun;107(1-3):359-62.

Allergen specificity and endothelial transmigration of T cells in allergic contact dermatitis and atopic dermatitis are associated with the cutaneous lymphocyte antigen.

Santamaria LF, Perez Soler MT, Hauser C, Blaser K.

Swiss Institute of Allergy and Asthma Research (SIAF), Davos Platz.

Recent investigations have indicated a role for antigen-specific T lymphocytes in the local skin immunity. The cutaneous lymphocyte antigen (CLA) is supposed to represent a skin-homing receptor for T cells. Inhibition experiments with specific monoclonal antibody demonstrate that CLA participates in selective transendothelial migration of memory/effector T cells in vitro by interaction with E-selectin on endothelial cell layers after activation with proinflammatory cytokines. In addition, the receptor-ligand pairs VLA-4/VCAM-1 and LFA-1/ICAM-1 are involved in this process. Moreover, only CLA+, CD45RO+ (memory/effector) T cells freshly isolated from peripheral blood of patients with allergic contact dermatitis or atopic dermatitis specifically proliferate in response to the respective allergen. CLA-, CD45RO- T cells from these patients do not respond to the allergens. In contrast, memory T cells from asthmatic individuals and patients with both asthma and atopic dermatitis express the allergen specificity in both T cell subsets. Tetanus toxoid, a systemically acting antigen, also induces a proliferative response in both CLA+ and CLA- memory/effector T cell subsets. These results strongly support the selective role of CLA in homing T cells to the cutaneous tissues and therefore playing a role in the local immunity and inflammatory reactions of the skin.

PMID: 7613172 [PubMed - indexed for MEDLINE]

2034. J Immunol. 1995 Apr 15;154(8):3742-52.

Regulation of human T lymphocyte chemotaxis in vitro by T cell-derived cytokines IL-2, IFN-gamma, IL-4, IL-10, and IL-13.

Tan J, Deleuran B, Gesser B, Maare H, Deleuran M, Larsen CG, Thestrup-Pedersen K.

Department of Dermatology, Aarhus University Hospital, Denmark.

There has been a number of conflicting reports regarding the T lymphocyte chemotactic activities of several cytokines. IL-2 and IFN-gamma are known to promote augmentation of immune inflammation, whereas IL-4, IL-10, and IL-13 display immunomodulatory effects on inflammatory cells including inhibition of cytokine production. Their effects on chemotaxis of inflammatory cells are unknown. We observed that IL-1 alpha could induce chemotaxis both in overnight cultured and anti-CD3 mAb-activated T lymphocytes and that overnight culture and anti-CD3 activation increase the number of IL-1R on T lymphocytes. In contrast, IL-8 selectively attracts freshly isolated T lymphocytes. Staurosporine inhibits freshly isolated T lymphocyte chemotaxis toward IL-8, whereas tyrphostin 23 inhibits chemotaxis of overnight cultured and anti-CD3-activated T lymphocytes toward IL-1 alpha. We have found that IL-2 and IL-13 inhibit the chemotactic migration of both CD4+ and CD8+ T lymphocytes toward IL-8, and RANTES. IL-4 inhibits only CD8+ T lymphocyte chemotaxis toward RANTES, IL-8 and IL-10. IL-10 inhibits only CD4+ T lymphocytes in their chemotactic response toward RANTES and IL-8. IFN-gamma does on the other hand augment the sensitivity of human T lymphocytes to chemotactic stimuli. Thus, our results demonstrate that different proinflammatory cytokines will induce chemotactic migration of T lymphocytes under different circumstances acting through different signaling pathways. The T cell-derived cytokines IL-2, IL-4, IL-10, and IL-13 are able to block further T lymphocyte chemotaxis, thus leading to a focusing of T lymphocytes in an area of T lymphocyte activation. These mechanisms seem relevant in our understanding of the specific and continuous localization of T lymphocytes in allergic and autoimmune disorders.

PMID: 7535813 [PubMed - indexed for MEDLINE]

2035. Biochem Biophys Res Commun. 1995 Apr 6;209(1):316-21.

Mast cell chemotactic activity of RANTES.

Mattoli S, Ackerman V, Vittori E, Marini M.

Institute of Experimental Medicine, Milano, Italy.

RANTES is a cytokine produced by activated T-lymphocytes that has been shown to exert chemotactic activity for memory-type CD4 T-lymphocytes and eosinophils. In this study, RANTES caused directional migration of human mast cells. When compared to other potential chemoattractants of the same cells, RANTES was found to be more potent than fibronectin and the c-kit receptor ligand, on a molar basis. This cytokine may be a common mechanism in allergic reactions which culminate in the selective migration of memory CD4 T-lymphocytes, eosinophils and mast cells at the tissue site. Asthma and allergic rhinitis may represent possible clinical examples.

PMID: 7537040 [PubMed - indexed for MEDLINE]

2036. Allerg Immunol (Paris). 1995 Apr;27(4):111-5.

[From IgE to the cell adhesion molecule].

[Article in French]

Stain JP.

C.H.U. de Rouen.

The aim of this work is to contribute to increasing our knowledge of asthma. The IgE can't alone be imputed in the anaphylactic reaction. Asthma is a bronchial inflammatory disease. The secretion of lymphokines increases the inflammatory response. The eosinophils cells damage the respiratory epithelium. The mast cells and the basophils cells release the chemical mediators and also the cytokines. The adhesion molecules situated in the cell membrane permit leukocyte cells to integrate with extracellular matrix during intra-tissue migration. Adhesion molecules may play a primary role in the pathogenesis of inflammatory response. The key role of the CD4 + T lymphocytes subset appeared in the last years.

PMID: 7772245 [PubMed - indexed for MEDLINE]

2037. Int Arch Allergy Immunol. 1995 Apr;106(4):335-44.

Adhesion of lymphocytes to endothelial cells in experimental allergic encephalomyelitis before and after treatment with endotoxin lipopolysaccharide.

Matsuda M, Tsukada N, Miyagi K, Yanagisawa N.

Department of Medicine (Neurology), Shinshu University School of Medicine, Matsumoto, Japan.

We investigated the in vitro adhesion of 51Cr-labeled lymphocytes to cultured brain endothelial cells and the in vivo expression of intercellular adhesion molecule-1 (ICAM-1) on cerebral endothelial cells in a rat model of experimental allergic encephalomyelitis (EAE) before and after treatment with

lipopolysaccharide (LPS). Adhesion of lymphocytes to cerebral endothelial cells was significantly increased in EAE compared with controls ($p < 0.01$), and was significantly correlated with the percentage of major histocompatibility complex class II antigen-positive cells in lymph node cells ($p < 0.001$). LPS enhanced ICAM-1 expression on endothelial cells and lymphocyte adhesion to those cells, and caused a significant increase in the in vivo expression of ICAM-1 compared with controls ($p < 0.001$). Lymphocyte adhesion to endothelial cells was significantly blocked by monoclonal antibodies against ICAM-1, lymphocyte function-associated antigen-1, or very late activation antigen-4. Our findings suggest that lymphocyte adhesion to brain endothelial cells may contribute to lymphocyte migration across the blood-brain barrier in EAE and that LPS may cause progression of EAE lesions.

PMID: 7719150 [PubMed - indexed for MEDLINE]

2038. Am J Respir Crit Care Med. 1995 Apr;151(4):1244-9.

Antagonism of ICAM-1 attenuates airway and tissue responses to antigen in sensitized rats.

Nagase T, Fukuchi Y, Matsuse T, Sudo E, Matsui H, Orimo H.

Department of Geriatrics, Faculty of Medicine, University of Tokyo, Japan.

Airway inflammation is involved in the pathogenesis of bronchial asthma. Intercellular adhesion molecule-1 (ICAM-1) is a ligand for lymphocyte function-associated antigen-1 alpha (LFA-1 alpha) and has been shown to be required for leukocyte migration into inflamed area. The purpose of this report was to investigate the role of ICAM-1/LFA-1 alpha pathway in a rat model of extrinsic asthma using monoclonal antibodies (mAbs). We chose to study ovalbumin (OA)-sensitized Brown-Norway rats, an animal model in which there is a high prevalence of both early (ER) and late responses (LR) after antigen challenge. We measured tracheal and alveolar pressure using alveolar capsules in open-chested, mechanically ventilated animals to calculate resistance of lung (RL), tissue (Rti), and airway (Raw). In the OA group, both ER (RL, Rti, Raw = 263 +/- 16, 235 +/- 10, 309 +/- 38% baseline) and LR (RL, Rti, Raw = 265 +/- 26, 238 +/- 13, 316 +/- 55% baseline) were observed. The administration of mAbs to ICAM-1 and LFA-1 alpha significantly attenuated the ER (RL, Rti, Raw = 146 +/- 9, 141 +/- 11, 156 +/- 8% baseline) and LR (RL, Rti, Raw = 128 +/- 8, 124 +/- 5, 137 +/- 1% baseline), indicating that both airway and lung tissues were involved in this mechanism. The current observations suggest that ICAM-1/LFA-1 alpha pathway is involved in both the early and late responses in a rat model of allergic asthma. The antagonism of ICAM-1 and LFA-1 alpha may provide a potential therapeutic approach to the early and late responses of bronchial asthma.

PMID: 7697260 [PubMed - indexed for MEDLINE]

2039. Aust N Z J Med. 1995 Apr;25(2):177.

Prevalence of asthma and allergic diseases related with emigration.

Kalyoncu AF.

Comment on
    Aust N Z J Med. 1994 Jun;24(3):255-7.

PMID: 7605306 [PubMed - indexed for MEDLINE]

2040. Inflammation. 1995 Apr;19(2):193-205.

Antiinflammatory effects of second-generation leukotriene B4 receptor antagonist, SC-53228: impact upon leukotriene B4- and 12(R)-HETE-mediated events.

Fretland DJ, Anglin CP, Bremer M, Isakson P, Widomski DL, Paulson SK, Docter SH, Djuric SW, Penning TD, Yu S, et al.

Department of Inflammatory Diseases Research, Searle Research and Development, Chesterfield, Missouri, USA.

Leukotriene B4 (LTB4) and 12(R)-hydroxyeicosatetraenoic acid [12(R)-HETE] are proinflammatory products of arachidonic acid metabolism that have been implicated as mediators in a number of inflammatory diseases. When injected intradermally into the guinea pig. LTB4 and 12(R)-HETE elicit a dose-dependent migration (chemotaxis) of neutrophils (PMNs) into the injection sites as assessed by the presence of a neutrophil marker enzyme myeloperoxidase. SC-41930 (7-[3-(4-acetyl-3-methoxy-2-propylphenoxy)propoxyl]-3,4-dihy dro-8-propyl-2H -1-benzopyran-2-carboxylic acid), a first-generation LTB4 receptor antagonist, inhibited the chemotactic actions of LTB4 when given orally with an ED50 value of 1.7 mg/kg. The second-generation LTB4 receptor antagonist, SC-53228 [(+)-(S)-7-(3-(2-(cyclopropylmethyl)-3-methoxy-4-[(methylamino)carbonyl]phenoxy)propoxy)-3,4-dihydro-8-propyl-2H-1-benzopyran-2-propanoic acid], inhibited LTB4-induced chemotaxis when given intragastrically with an ED50 value of 0.07 mg/kg. Furthermore, SC-53228 inhibited 12(R)-HETE-induced granulocyte chemotaxis with an oral ED50 value of 5.8 mg/kg. When dosed orally over a range of 0.03-100 mg/kg, SC-53228 gave Cmax plasma concentrations of 0.015-41.1 micrograms/ml. SC-53228 inhibited LTB4-primed membrane depolarization of human neutrophils with an IC50 value of 34 nM. As a potent LTB4 receptor antagonist, SC-53228 may well have application in the medical management of disease states such as asthma, rheumatoid arthritis, inflammatory bowel disease, contact dermatitis, and psoriasis, in which LTB4 and/or 12(R)-HETE are implicated as inflammatory mediators.

PMID: 7601505 [PubMed - indexed for MEDLINE]

2041. Am J Respir Cell Mol Biol. 1995 Mar;12(3):260-7.

5-Hydroxyeicosatetraenoic acid (HETE)-induced neutrophil transcellular migration is dependent upon enantiomeric structure.

Bittleman DB, Casale TB.

Department of Internal Medicine, VA Medical Center, Iowa City, Iowa.

Comment in
    Am J Respir Cell Mol Biol. 1995 Mar;12(3):251-3.

The 5(R) and 5(S) hydroxyeicosatetraenoic acids (5[R]-HETE, 5[S]-HETE) are important inflammatory mediators in lung diseases: they increase mucus, induce airway contraction, and potentiate neutrophil chemotaxis. Neutrophils are important cells in allergic and inflammatory lung diseases. Therefore, we examined the effects of both 5(R)-HETE and 5(S)-HETE on human neutrophil migration across naked filters and human umbilical vein endothelial (HUVE) cell and human type II-like pulmonary epithelial cell (A549) monolayers cultured on these filters. Time courses for both 5(R)-HETE and 5(S)-HETE show significant neutrophil migration at 40 min and maximal migration at 60 to 90 min through all three barriers. Checkerboard analysis showed that migration was chemotactic.

Dose-response curves for both isomers through cellular monolayers had the same shapes, but 5(R)-HETE was more potent than 5(S)-HETE. There was greater migration through cellular barriers than through naked filters. Actinomycin D pretreatment of the cellular monolayers slightly inhibited the neutrophil transcellular chemotactic response to both 5-HETEs equally. Enhanced transcellular migration was not due to the production of a soluble chemotactic factor. Thus, although both isomers of 5-HETE were potent chemotactic agents, 5(R)-HETE was slightly more potent. Moreover, relevant endothelial and epithelial monolayers enhance both dose- and time-dependent neutrophil migration stimulated by 5(R)-HETE and 5(S)-HETE. These data indicate that (1) both 5(R)-HETE and 5(S)-HETE are important in mediating lung inflammatory processes, and (2) 5(R)-HETE may play a more important role in neutrophil-rich lung inflammatory responses than 5(S)-HETE because it is a more potent inducer of neutrophil migration through endothelial and epithelial barriers.

PMID: 7873191 [PubMed - indexed for MEDLINE]

2042. Clin Infect Dis. 1995 Mar;20(3):542-8.

Dermatoses associated with travel to tropical countries: a prospective study of the diagnosis and management of 269 patients presenting to a tropical disease unit.

Caumes E, Carrière J, Guermonprez G, Bricaire F, Danis M, Gentilini M.

Département des Maladies Infectieuses, Parasitaires, Tropicales et Santé Publique, Hôpital de la Salpêtrière, Paris, France.

The full spectrum of skin diseases related to travel in tropical areas is unknown. We prospectively studied 269 consecutive patients with travel-associated dermatosis who presented to our tropical disease unit in Paris during a 2-year period. The median age of these patients was 30 years; 137 patients were male; 76% of the patients were tourists; 38% had visited sub-Saharan Africa; and 85% had been appropriately vaccinated against tetanus. Cutaneous lesions appeared while the patient was still abroad in 61% of cases and after the patient's return to France in 39%. The diagnosis was definite in 260 cases; 137 of these cases (53%) involved an imported tropical disease. The most common diagnoses were cutaneous larva migrans (25%); pyodermas (18%); pruritic arthropod-reactive dermatitis (10%); myiasis (9%); tungiasis (6%); urticaria (5%); fever and rash (4%); and cutaneous leishmaniasis (3%). Hospitalization was necessary in 27 cases (10%), with a median duration of 5 days (range, 2-21 days). Travelers should be advised on how to avoid exposure to the agents and vectors of infectious dermatoses. Travel first-aid kits should include insect repellents and antibiotics effective against bacterial skin infections.

PMID: 7756473 [PubMed - indexed for MEDLINE]

2043. Thorax. 1995 Mar;50(3):265-9.

Effect of platelet activating factor (PAF) on the migration of human lymphocytes.

McFadden RG, Bishop MA, Caveney AN, Fraher LJ.

Lawson Research Institute of St. Joseph's Health Centre, London, Ontario, Canada.

BACKGROUND: There is growing evidence to suggest the importance of the lymphocyte in the pathogenesis of asthma, particularly in the late phase reactions and ongoing bronchial hyperreactivity. Platelet activating factor (PAF) has also been

identified as a potentially important mediator in asthma.
METHODS: The migration of human peripheral blood lymphocytes obtained from normal volunteers in response to PAF and the effect of PAF antagonists was studied in a well standardised in vitro assay using nitrocellulose micropore filters in a microchemotaxis chamber.
RESULTS: PAF is a potent stimulus to in vitro human lymphocyte migration; at an optimal concentration of 1 nM it augmented lymphocyte chemokinesis to 310% (SE 33%) of control values. The response to PAF appears to be specific since lyso-PAF and other related membrane phospholipids had no effect. PAF-induced migration could be abrogated by specific PAF receptor antagonists such as WEB 2086 (100 nM), and was partially blocked by the cyclooxygenase inhibitor flurbiprofen at a concentration of 1 microM.
CONCLUSIONS: PAF stimulates the in vitro migration of human lymphocytes through a specific PAF receptor. Part of the response may be due to the generation of cyclooxygenase products. PAF may play a part in the recruitment of lymphocytes to asthmatic airways.

PMCID: PMC1021190
PMID: 7660340 [PubMed - indexed for MEDLINE]

2044. J Immunol. 1995 Mar 1;154(5):2393-402.

Bone marrow-derived murine mast cells migrate, but do not degranulate, in response to chemokines.

Taub D, Dastych J, Inamura N, Upton J, Kelvin D, Metcalfe D, Oppenheim J.

Clinical Services Program, National Cancer Institute-Frederick Cancer Research and Development Center, MD 21702.

We have determined that several chemokines induce mast cell migration in vitro. This directed migration is dependent on the presence of particular extracellular matrix proteins and the activation status of the cells. Mast cell haptotactic responses were observed in response to various chemokines on vitronectin-, laminin-, and fibronectin-coated filters. Unstimulated mast cells were chemoattracted only by monocyte chemotactic protein-1 and RANTES on vitronectin-coated and, to a lesser extent, laminin-coated filters, whereas IgE-activated mast cells migrated in response to monocyte chemotactic protein-1, regulated on activation normal T expressed and secreted, platelet factor-4, and macrophage inflammatory protein-1 alpha on all three matrix proteins. No significant migration was observed on collagen type IV-coated or uncoated filters. Mast cell migration in response to chemokines on extracellular matrices and its enhancement by IgE-dependent activation provide a mechanism by which cells may be drawn to sites of inflammation. Chemokine-induced mast cell recruitment may be particularly relevant in host defense responses to parasitic infections, allergic reactions, Jones-Mote reactions, and in wound healing.

PMID: 7532669 [PubMed - indexed for MEDLINE]

2045. Eur J Pharmacol. 1995 Feb 24;275(1):75-82.

Phosphodiesterase inhibitors reduce bronchial hyperreactivity and airway inflammation in unrestrained guinea pigs.

Santing RE, Olymulder CG, Van der Molen K, Meurs H, Zaagsma J.

Groningen/Utrecht Institute for Drug Exploration, Department of Medicinal Chemistry, University of Groningen, Netherlands.

A new guinea pig model of allergic asthma was used to investigate the effects of low doses of the phosphodiesterase inhibitors, rolipram (phosphodiesterase IV selective), ORG 20241 (N-hydroxy-4-(3,4-dimethoxyphenyl)-thiazole-2-carboximidamide; dual phosphodiesterase III/IV inhibitor with some selectivity for the phosphodiesterase IV isoenzyme), and of theophylline (non-selective) on allergen-induced early and late phase asthmatic reactions, bronchial hyperreactivity to histamine inhalation, and airway inflammation. Theophylline (25 mg/kg i.p.) and ORG 20241 (5 mg/kg i.p.) did not affect histamine-induced bronchoconstriction, whereas rolipram (75 micrograms/kg i.p.) only slightly reduced the response to histamine at 7 h after administration. However, bronchial hyperreactivity after the early and after the late reaction was significantly reduced by theophylline, rolipram and ORG 20241, while bronchoalveolar lavage studies revealed a selective inhibition of airway inflammation by the phosphodiesterase inhibitors. Theophylline significantly reduced the number of eosinophils, neutrophils and macrophages; rolipram reduced the number of neutrophils and lymphocytes, and ORG 20241, the number of eosinophils and macrophages. None of the compounds at the dosage indicated reduced the early and late reaction when administered i.p. 1 h before allergen exposure to defined dual responding animals. These results indicate that non-bronchodilator doses of these phosphodiesterase inhibitors markedly reduce the allergen-induced development of bronchial hyperreactivity as well as airway inflammation, presumably by selectively inhibiting cellular migration. The results suggest that an orchestrated series of cellular interactions is involved in the development of bronchial hyperreactivity. It is concluded that phosphodiesterase inhibitors may be very useful in the treatment of bronchial asthma.

PMID: 7774665 [PubMed - indexed for MEDLINE]

2046. Int Immunol. 1995 Feb;7(2):251-8.

Expression and function of fibronectin binding integrins on rat mast cells.

Yasuda M, Hasunuma Y, Adachi H, Sekine C, Sakanishi T, Hashimoto H, Ra C, Yagita H, Okumura K.

Department of Immunology, Juntendo University School of Medicine, Tokyo, Japan.

Adhesion molecules of the integrin family are implicated not only in leukocyte migration but also in leukocyte activation. Here we characterize the expression and function of fibronectin receptor integrins on rat mast cells. A rat basophilic leukemia cell line (RBL-2H3) and phorbol ester-stimulated rat peritoneal mast cells adhered to fibronectin (FN), vitronectin and fibrinogen. These mast cells expressed fibronectin receptor integrins, including very late antigen (VLA)-4, VLA-5 and vitronectin receptor (VNR), as estimated by immunofluorescent staining and inhibition of FN adherence by newly established mAbs reactive with the rat alpha 4 (MR alpha 4-1), alpha 5 (HM alpha 5-1) or beta 3 (HM beta 3-1) chains of the integrin molecules. The beta-hexosaminidase release, a marker for mast cell degranulation, triggered by high affinity IgE receptor (Fc epsilon RI)-mediated stimulation, was enhanced by adhesion of RBL-2H3 cells to either immobilized FN, MR alpha 4-1, HM alpha 5-1 or HM beta 3-1. This FN enhancement of beta-hexosaminidase release was inhibited by soluble MR alpha 4-1, HM alpha 5-1 and HM beta 3-1 as well as by GRGDSP and DELPQLVTLPHPNHLGPEILDVPST peptides which abrogate VLA-5/VNR and VLA-4 binding to FN respectively. In vivo, passive cutaneous anaphylaxis induced by IgE anti-DNP and DNP-BSA was inhibited by concurrent s.c. injection of MR alpha 4-1, HM alpha 5-1 and HM beta 3-1. These results demonstrate that FN receptor integrins expressed on rat mast cells play an important role in regulating mast cell

activation both in vitro and in vivo.

PMID: 7734420 [PubMed - indexed for MEDLINE]

2047. J Clin Invest. 1995 Feb;95(2):913-8.

Milk-induced eczema is associated with the expansion of T cells expressing cutaneous lymphocyte antigen.

Abernathy-Carver KJ, Sampson HA, Picker LJ, Leung DY.

Department of Pediatrics, National Jewish Center for Immunology and Respiratory Medicine, Denver, Colorado 80206.

The extravasation of T cells at sites of inflammation is critically dependent on the activity of homing receptors (HR) involved in endothelial cell recognition and binding. Two such HR (the cutaneous lymphocyte antigen [CLA] and L-selectin) have been shown to be selectively involved in T cell migration to skin and peripheral lymph nodes, respectively. This study was designed to assess the relationship between the organ specificity of an allergic reaction to food and the expression of HR on T cells activated in vitro by the relevant food allergen. Peripheral blood mononuclear cells were isolated from seven milk allergic children with a history of eczema when exposed to milk. All patients had a positive prick skin test and double-blind placebo-controlled food challenge to milk. 10 children with either allergic eosinophilic gastroenteritis or milk-induced enterocolitis and 8 nonatopic adults served as controls. Five-parameter flow cytometry using monoclonal antibodies was used for detection of the specific HR on freshly isolated T cells versus T cell blasts induced by a 6-d incubation with casein, as compared with Candida albicans. After in vitro stimulation with casein, but not C. albicans, patients with milk allergy and atopic dermatitis had a significantly greater percentage of CLA+ T cells ($P < 0.01$) than controls with milk-induced enterocolitis, allergic eosinophilic gastroenteritis, or nonatopic healthy controls. In contrast, the percentage of L-selectin-expressing T cells did not differ significantly between these groups. These data suggest that after casein stimulation allergic patients with milk-induced skin disease have an expanded population of CLA+ T cells, as compared with nonatopics or allergic patients without skin involvement. We postulate that heterogeneity in the regulation of HR expression on antigen-specific T cells may play a role in determining sites of involvement in tissue-directed allergic responses.

PMCID: PMC295586
PMID: 7532192 [PubMed - indexed for MEDLINE]

2048. Otolaryngol Pol. 1995;49 Suppl 23:161-3.

[The influence of phleum pratense and secale cercale allergens on selected functions of PMNL granulocytes].

[Article in Polish]

Grzybowski A, Zalewski P, Jeziorski A, Olszewska-Ziaber A, Stefaniak T, Stawska-Drzymala V.

Zakładu Medycyny Zapobiegawczej Instytutu OOZW WAM w Lodzi.

The evaluation of granulocytes function (PMNL) at patients with pollinosis. There were 59 people examined and divided into: 30 patients with pollinosis, 29 healthy

people. There were evaluated: absorption of isotope (phagocytic index) in our modification migration in vivo and the bacteriotoxic index. The function of granulocytes (postpreincubation) of allergens Phlenum pratens and Secale cercale in patients with pollinosis were changed more in comparison with the healthy people.

PMID: 9499888 [PubMed - indexed for MEDLINE]

2049. Immunol Res. 1995;14(4):317-24.

Skin-homing T cells in human cutaneous allergic inflammation.

Santamaria Babi LF, Perez Soler MT, Hauser C, Blaser K.

Swiss Institute of Allergy and Asthma Research (SIAF), Davos, Switzerland.

The cutaneous lymphocyte-associated antigen (CLA) is a carbohydrate epitope present on memory/effector T cells that infiltrate inflamed skin. E-selectin is the ligand for CLA and is induced under inflammation on endothelial cells. CLA was originally postulated as a phenotype marker for skin-associated T cells. We studied the specific in vitro response to skin-associated allergens of CLA+ and CLA-CD45RO+ T cells in atopic dermatitis (AD) and contact dermatitis (CD), which represent two well-characterized T cell-mediated cutaneous allergic inflammations. Whereas CLA+ T cells from AD patients preferentially responded to house dust mite (HDM) and CLA+ T cells from nickel CD patients showed an increased response to nickel, CLA-T cells showed very little response in both cases. In contrast, tetanus toxoid, a systemically acting antigen, induced a proliferative response in both CLA+ and CLA- cells. Interestingly the response to HDM in patients with asthma +/- AD was preferentially found in the CLA- subset indicating the involvement of different homing receptors for mucosal tissues. Moreover, CLA+ T cells showed enhanced migration through activated human umbilical vein endothelial cell monolayers compared to CLA- T cells. The CLA binding to E-selectin is initially responsible for the extravasation that also involves VLA-4/VCAM-1 and LFA-1/ICAM-1 interactions. We have recently identified IL-8 as an endothelial cell-derived chemokine and the IL-8 receptor type B which control CLA+ T cell migration. Such a CLA-mediated migration would localize memory/effector T cells that respond to antigens and reach the body through inflamed skin. Our data support the existence of a regionalization of the immune system and in particular of the skin immune system. It may allow an efficient distribution of the immune defense to different sites of the body.

PMID: 8722046 [PubMed - indexed for MEDLINE]

2050. Nord Med. 1995;110(3):79-81.

[Cytokines and skin diseases].

[Article in Danish]

Thestrup-Pedersen K.

Dermatologisk afd, Marselisborg Hospital/Aarhus Universitetshospital.

Many skin diseases such as eczema and psoriasis are characterised by a chronic inflammatory skin condition. In this respect they resemble other chronic diseases such as rheumatoid arthritis, bronchial asthma, ulcerous colitis and Crohn's disease. A persistent accumulation, predominantly of T-lymphocytes constitutes the central pathophysiological feature of such diseases. The past 15-20 years

have witnessed the characterisation of an extensive series of peptides known as cytokines. These are soluble, relatively low molecular weight peptides which at low concentrations mediate regulation of cellular receptors, new phenotype expression, secretion and migration. Many cytokines have been found to be present in conjunction with skin diseases, and it is suggested that they are involved in the development of inflammation.

PMID: 7892122 [PubMed - indexed for MEDLINE]

2051. Immunology. 1995 Jan;84(1):31-5.

Tumour necrosis factor-alpha is required for accumulation of dendritic cells in draining lymph nodes and for optimal contact sensitization.

Cumberbatch M, Kimber I.

Zeneca Central Toxicology Laboratory, Macclesfield, Cheshire, UK.

Following skin sensitization epidermal Langerhans' cells (LC), many of which bear antigen, are stimulated to migrate from the skin and traffic via afferent lymphatics to lymph nodes draining the site of exposure. It has been proposed previously that tumour necrosis factor-alpha (TNF-alpha), a keratinocyte-derived epidermal cytokine (the expression of which is augmented following cutaneous sensitization), provides one signal for LC migration. In the experiments described here the influence of systemically administered neutralizing anti-TNF-alpha antibody on dendritic cell (DC) accumulation in draining lymph nodes has been investigated. Treatment with anti-TNF-alpha inhibited markedly the frequency of DC in draining nodes measured 18 hr following exposure to the skin allergens oxazolone and fluorescein isothiocyanate or to the non-sensitizing skin irritant sodium lauryl sulphate. Similar treatment with anti-TNF-alpha 2 hr prior to primary exposure to oxazolone impaired significantly the efficiency of skin sensitization measured 5 days later as a function of challenge-induced increases in ear thickness. The same antibody administered 18 hr following initial exposure to oxazolone was without effect on skin sensitization. These data confirm the importance of TNF-alpha for the migration of LC from the skin to draining lymph nodes and demonstrate that this cytokine is required for optimal contact sensitization.

PMCID: PMC1415198
PMID: 7890303 [PubMed - indexed for MEDLINE]

2052. Thorax. 1995 Jan;50(1):67-73.

Reduction in leukotriene B4 generation by bronchoalveolar lavage cells in asthma.

Restrick LJ, Sampson AP, Piper PJ, Costello JF.

Department of Thoracic Medicine, King's College School of Medicine and Dentistry, London.

BACKGROUND: Leukotrienes are inflammatory mediators implicated in the pathogenesis of asthma. The capacity of inflammatory cells within the airways to generate leukotrienes may be altered in asthma. This hypothesis was tested using bronchoalveolar lavage (BAL) to sample cells within the airways from atopic asthmatic and normal subjects, and by measuring their capacity to generate leukotriene B4 (LTB4) and leukotriene C4 (LTC4) in response to A23187, a potent stimulus of leukotriene generation.
METHODS: Bronchoalveolar lavage was performed in 12 mild asymptomatic atopic

asthmatic patients and 12 normal subjects. Mixed BAL cell aliquots (approximately 80% alveolar macrophages) were incubated with 0-20 microM A23187 for 10 minutes and with 4 microM A23187 for 0-30 minutes, and leukotrienes were measured by radioimmunoassay and high performance liquid chromatography.
RESULTS: Mixed BAL cells from asthmatic subjects generated less LTB4 than cells from normal subjects in dose response and time course experiments (area under the curve 81.5 (0.0-228.5) ng.min.10(-6) cells in asthmatic subjects and 197.9 (13.9-935.6) ng.min.10(-6) cells in normal subjects. There were no differences in LTC4 generation between BAL cells from asthmatic and normal subjects.
CONCLUSIONS: Generation of LTB4 by BAL cells from atopic asthmatic subjects in response to A23187 was reduced. As the alveolar macrophage is the major source of LTB4 in BAL cells, these results probably reflect reduced generation of LTB4 by alveolar macrophages from asthmatic patients. This may be a consequence of monocyte migration into the lung, or altered alveolar macrophage function in asthma, or both.

PMCID: PMC473713
PMID: 7886653 [PubMed - indexed for MEDLINE]

2053. Am J Physiol. 1995 Jan;268(1 Pt 1):L117-22.

Interleukin-8 is a potent mediator of eosinophil chemotaxis through endothelium and epithelium.

Erger RA, Casale TB.

Department of Internal Medicine, Veterans Affairs Medical Center, Iowa City, Iowa 52242.

Interleukin-8 (IL-8), a potent pro-inflammatory cytokine, has been shown to have chemotactic activity for neutrophils, lymphocytes, and basophils. Effects of IL-8 on eosinophil chemotaxis are unresolved. Because eosinophils accumulate at the site of allergic inflammation and may play a role in the pathogenesis of asthma, we investigated the eosinophilotactic capacity of IL-8. We examined the ability of IL-8 to induce human eosinophil migration across 3-microns pore naked filters, and human umbilical vein endothelial cell and human pulmonary type II-like epithelial cell (A549) monolayers cultured on these filters. IL-8 induced similar dose-related eosinophil migration through all three barriers. Kinetic experiments indicated more rapid migration through noncellular barriers but equivalent migration through all barriers by 3 h. Chemotactic/chemokinetic data show that IL-8-induced eosinophil migration is chemotactic. We also determined that the ability of IL-8 to induce transcellular migration was unique in comparison with other cytokines and was not dependent on the use of fresh vs. passaged monolayer cells as barriers. Therefore our data indicate that IL-8 may play a significant role in tissue eosinophilia observed in allergic respiratory diseases.

PMID: 7840216 [PubMed - indexed for MEDLINE]

2054. Am J Respir Cell Mol Biol. 1995 Jan;12(1):65-70.

Comparative studies indicate that platelet-activating factor is a relatively weak eosinophilotactic mediator.

Erger RA, Casale TB.

Department of Internal Medicine, VA Medical Center, Iowa City, Iowa.

Eosinophils are important immune effector cells in a variety of allergic

responses and inflammatory lung diseases. Bacterial products and inflammatory mediators have been implicated in inducing an influx of eosinophils into the respiratory tract subsequent to an acute inflammatory response. Therefore, to better understand the role of eosinophils in lung inflammation, we compared the ability of three known chemoattractants, formylmethionylleucylphenylalanine (FMLP), leukotriene B4 (LTB4), and platelet-activating factor (PAF), to induce human eosinophils to migrate across 3.0-microns-pore naked filters and human umbilical vein endothelial cells (HUVEC) and A549 human pulmonary type II-like epithelial (A549) cells cultured in monolayers on these filters. Kinetic experiments indicated that eosinophil migration through all three barriers occurred by 60 min and plateaued by 2 h. Each of these chemoattractants induced eosinophil migration in dose-responsive fashion across all three barriers. Although similar maximal eosinophil migration was observed, the doses at which this occurred varied, indicating that the rank order of potency through naked filters is FMLP > PAF > or = LTB4. However, their relative chemotactic potency through cellular barriers was different, with FMLP > LTB4 > PAF. In contrast to previous studies with neutrophils, the rank order of potency of the three chemoattractants was not influenced by the barrier through which the eosinophil migrated. Thus, these and previous data show that FMLP, LTB4, and PAF are eosinophil and neutrophil chemoattractants. Therefore, it is likely that these three agents are important mediators of granulocytic inflammatory responses in the lung, albeit with different potency profiles.(ABSTRACT TRUNCATED AT 250 WORDS)

PMID: 7811471 [PubMed - indexed for MEDLINE]

2055. Allergy. 1995;50(22 Suppl):13-21.

The airway inflammatory response in allergic asthma and its relationship to clinical disease.

Howarth PH.

Southampton General Hospital, U.K.

Endobronchial biopsy and lavage studies have revealed the presence of mast cell, eosinophil, T-lymphocyte and epithelial cell activation in asthma, along with the structural changes of tissue eosinophil infiltration, loss of superficial columnar ciliated epithelial cells and enhanced collagen deposition in the laminar reticularis. As these cellular and structural changes underlie the clinical features of asthma, i.e., symptom expression, variable airflow obstruction and bronchial hyperresponsiveness, and understanding of their induction and regulation is essential to the understanding of the asthmatic process. The acute airway response to allergen has been studied by the technique of local endobronchial allergen challenge with direct airway sampling in asthma. These studies identify allergen-mast cell interaction as the initial airway event, with mediator release inducing bronchoconstriction and enhancing vascular permeability. As preformed cytokines are present in mast cells, cytokine release from this cell population is likely to initiate the process of endothelial cell activation, with upregulation of cell adhesion molecules, and tissue cell recruitment. Subsequent cytokine elaboration from airway macrophages and T-lymphocytes will perpetuate this response while in chronic clinical disease T-lymphocytes, mast cells, matrix tissue, epithelial cells and eosinophils themselves are all likely to contribute to the cytokine pool within the airways and thus to the regulation of inflammatory cell migration and activation.

PMID: 7793534 [PubMed - indexed for MEDLINE]

2056. Allergy Proc. 1995 Jan-Feb;16(1):13-21.

Eosinophils and allergy in asthma.

Makino S, Fukuda T.

Department of Medicine and Clinical Immunology, Dokkyo University School of Medicine, Tochigi, Japan.

Eosinophils are recruited to the site of IgE-mediated allergic reaction in the airway in asthma. Major eosinophil-chemotactic factors released from mast cells are platelet activating factor and Leukotriene B4. In addition, T cells and bronchial epithelial cells produce eosinophil chemotactic cytokines. Cytokines including IL-5, IL-3, and GM-CSF, which are released mainly from CD4+ T cells and possibly Th2, activates eosinophils for migration, tissue damage, and survival. Adhesion molecules on eosinophils and constituent structures of the airway participate in the process of eosinophil migration. Among a variety of adhesion molecules, VLA-4 and VCAM-1 are unique to the interaction between eosinophils and endothelial cells. A major role of recruited eosinophils in the airway in asthma is considered to be damage to the bronchial epithelium caused by eosinophil specific granules proteins, in addition to production of lipid mediators, production of cytokines, antigen-presenting cell function, and possible induction of basement membrane thickening in the airway.

PMID: 7768455 [PubMed - indexed for MEDLINE]

2057. Eur Arch Otorhinolaryngol. 1995;252 Suppl 1:S8-21.

Immunocompetent cells of the upper airway: functions in normal and diseased mucosa.

Brandtzaeg P.

Laboratory for Immunohistochemistry and Immunopathology LIIPAT, University of Oslo, National Hospital, Norway.

Secretory immunity is central in primary defense of the airway mucosa. B cells involved in this local immune system are initially stimulated in mucosa-associated lymphoid tissue, including tonsils and adenoids, and then migrate to secretory effector sites where they become immunoglobulin (Ig)-producing plasma cells. Locally produced Ig consists mainly of J-chain-containing dimers and larger polymers of IgA (pIgA) that are selectively transported through glandular cells by an epithelial receptor called secretory component or pIgR. Secretory antibodies perform surface protection by immune exclusion of soluble antigens as well as infectious agents. IgG can also participate in this primary defense because it reaches secretions by passive diffusion similar to IgE. However, the inflammatory properties of antibodies belonging to the latter two classes explain their involvement in mucosal immunopathology when elimination of penetrating antigens is unsuccessful. T helper (Th) cells activated in this process may by a Th2 profile of cytokines promote persistent inflammation with extravasation and priming of eosinophils. This mechanism appears to occur in the late-phase allergic reaction, perhaps driven mainly by interleukin-4 (IL-4) released from mast cells subjected to IgE-mediated degranulation. Eosinophils are potentially tissue-destructive cells, particularly after priming with IL-5. Cytokines also up-regulate adhesion molecules on vascular endothelium and epithelium, thereby enhancing migration of eosinophils and other leukocytes into the mucosa.(ABSTRACT TRUNCATED AT 250 WORDS)

PMID: 7734979 [PubMed - indexed for MEDLINE]

2058. Ann Allergy Asthma Immunol. 1995 Jan;74(1):17-22.

Asthma, hypereosinophilia and peripheral neuropathy in a migrant farm worker.

Frazier TC, Stafford CT.

Allergy-Immunology Section, Medical College of Georgia, Augusta, USA.

PMID: 7719876 [PubMed - indexed for MEDLINE]

2059. Occup Environ Med. 1995 Jan;52(1):33-42.

Respiratory health of young shipyard welders and other tradesmen studied cross sectionally and longitudinally.

Chinn DJ, Cotes JE, el Gamal FM, Wollaston JF.

Department of Environmental and Occupational Medicine, University, Newcastle upon Tyne.

OBJECTIVE: To assess the effectiveness of current measures for protecting shipyard welders and caulker/burners (WCBs) from the respiratory effects of fumes.
METHODS: Shipyard tradesmen born after 1953 (cohort 1), and 181 older men, subjects of a previous study (cohort 2), were assessed, then followed up after an average interval of 6.7 years. The respiratory associations with shipyard trades were assessed cross sectionally and longitudinally and an estimate made of the likely effects of selection bias. Cohort 1 comprised 90% of the 462 eligible WCBs and 239 other tradesmen; there were 31 exclusions. At follow up 139 of 146 men still in the shipyard and 43% of those who had left were reassessed. The lapses were mainly due to migration. All members of cohort 2 were followed up for respiratory symptoms (from MRC questionnaire), were recorded, and indices reflecting all aspects of lung function were measured.
RESULTS: At the initial assessment and independent of smoking, trade as a WCB was associated with increased prevalences of chronic cough, phlegm, and wheeze, a reduced transfer factor, and an enhanced age related deterioration in peak expiratory flow (measured cross sectionally). Continued work as a WCB was associated with enhanced deterioration in lung function despite some amelioration of respiratory symptoms; the deterioration was influenced by whether or not exhaust ventilation had been used for every weld. The effects of fume on forced expiratory volume, flow-volume curvilinearity, mean transit time, and moment ratio were independent of and at least as large as those due to smoking. Enhanced deterioration in peak expiratory flow was confined to WCBs who smoked. These effects of trade, but not those of smoking, were nearly independent of atopy.
CONCLUSION: In WCBs the working practices over the period of the study did not prevent the development of mild respiratory impairment. In WCBs who used exhaust ventilation at all times, the impairment seemed to reverse by discontinuation of exposure. Thus existing hygiene measures should be applied rigorously. The biological effectiveness of these and any other necessary supplementary measures should be assessed by long term monitoring of forced expiratory volume and peak expiratory flow.

PMCID: PMC1128147
PMID: 7697138 [PubMed - indexed for MEDLINE]

2060. Ciba Found Symp. 1995;189:91-101; discussion 101-6.

Adhesion molecules in cutaneous inflammation.

Barker JN.

St John's Institute of Dermatology, United Medical School, St Thomas' Hospital, London, UK.

As in other organs, leukocyte adhesion molecules and their ligands play a major role in cutaneous inflammatory events both by directing leukocyte trafficking and by their effects on antigen presentation. Skin biopsies of inflamed skin from patients with diseases such as as psoriasis or atopic dermatitis reveal up-regulation of endothelial cell expression of P- and E-selectin, vascular cell adhesion molecule 1 and intercellular adhesion molecule 1. Studies of evolving lesions following UVB irradiation, Mantoux reaction or application of contact allergen, demonstrate that expression of these adhesion molecules parallels leukocyte infiltration into skin. When cutaneous inflammation is widespread (e.g. in erythroderma), soluble forms of these molecules are detectable in serum. In vitro studies predict that peptide mediators are important regulatory factors for endothelial adhesion molecules. Intradermal injection of the cytokines interleukin 1, tumour necrosis factor alpha and interferon gamma into normal human skin leads to induction of endothelial adhesion molecules with concomitant infiltration of leukocytes. In addition, neuropeptides rapidly induce P-selectin translocation to the cell membrane and expression of E-selectin. Adhesion molecules also play a crucial role as accessory molecules in the presentation of antigen to T lymphocytes by Langerhans' cells. Expression of selectin ligands by Langerhans' cells is up-regulated by various inflammatory stimuli, suggesting that adhesion molecules may be important in Langerhans' cell migration. The skin, because of its accessibility, is an ideal organ in which to study expression of adhesion molecules and their relationship to inflammatory events. Inflammatory skin diseases are common and inhibition of lymphocyte accumulation in skin is likely to prove of great therapeutic benefit.

PMID: 7587640 [PubMed - indexed for MEDLINE]

2061. J Asthma. 1995;32(2):125-30.

A beta 2-agonist, procaterol, inhibits basophil migration.

Yamaguchi M, Hirai K, Ohta K, Ito K, Morita Y.

Department of Medicine and Physical Therapy, University of Tokyo School of Medicine, Japan.

Beta 2-receptor agonists have recently been reported to be effective on allergen-induced late-phase reaction (LPR) in addition to their inhibitory effect on immediate-phase reaction, although the precise mechanism is not fully understood. In this study, we tested the effect of a selective beta 2-agonist, procaterol, on human basophil migration, which may be an important characteristic of LPR. Procaterol inhibited IL-8- and C5a-induced basophil migration in a dose-dependent fashion; 10(-7) M of procaterol reduced 30% of migration induced by both factors. The action of procaterol was rapid since the inhibition of migration was detected without preincubation and was not via the toxic effect on basophils as assessed by trypan blue test. The results of this study extend the repertoire of anti-inflammatory actions of beta 2-agonists.

PMID: 7559262 [PubMed - indexed for MEDLINE]

2062. Eur Arch Otorhinolaryngol. 1995;252 Suppl 1:S44-9.

Proinflammatory cytokines in allergic rhinitis.

Bachert C, Hauser U, Prem B, Rudack C, Ganzer U.

ENT Department, University of Düsseldorf, Germany.

Allergic diseases such as allergen-induced rhinitis represent an inflammatory reaction that is characterized by the chemotaxis and activation of various cell populations. A high degree of cell-to-cell communication is needed to orchestrate this inflammatory immune response. A variety of cytokines and adhesion receptors seem to play an important role in the allergic late phase reaction. Here we demonstrate that proinflammatory cytokines such as interleukin(IL)-1, IL-8 and TNF-alpha (tumor necrosis factor-alpha) can be detected in nasal secretions and mucosa by enzyme-linked immunosorbent assay and immunohistochemistry. The increased expression of adhesion receptors in mucosa specimens of patients with seasonal allergic rhinitis points to their role in regulating the cellular migration and probably represents a key event in allergic inflammation. We established an in vitro model using freshly taken nasal mucosa to study the induction of adhesion receptors by proinflammatory cytokines. E-selectin, an endothelial receptor, was strongly upregulated by IL-1 beta, TNF-alpha and allergen. The induction due to allergen exposure of the mucosa was markedly inhibited by soluble cytokine receptors (sIL-1R, TNF-BP) or by a receptor antagonist (IL-1ra) and prednisolone, These findings indicate that proinflammatory cytokines may be key factors for the upregulation of adhesion processes in human nasal mucosa and the activation of various cell populations involved in the allergic inflammation. They therefore represent a main target for new therapeutic strategies.

PMID: 7537566 [PubMed - indexed for MEDLINE]

2063. Int Arch Allergy Immunol. 1995 Jan;106(1):69-77.

Expression of E-selectin, ICAM-1 and VCAM-1 on bronchial biopsies from allergic and non-allergic asthmatic patients.

Gosset P, Tillie-Leblond I, Janin A, Marquette CH, Copin MC, Wallaert B, Tonnel AB.

INSERM U416, Institut Pasteur, Lille, France.

The bronchial mucosa of asthmatic patients is characterized by a large influx of eosinophils, monocytes and lymphocytes. Leucocyte migration and accumulation is thought to involve adhesion molecules, as shown in an animal of allergic asthma and in humans with other allergic diseases. The aim of this study was to evaluate the expression of E-selectin, intercellular adhesion molecule-1 (ICAM-1) and vascular cellular adhesion molecule-1 (VCAM-1) on endothelium and epithelium in bronchial biopsies obtained from patients with allergic (n = 17) and non-allergic asthma (n = 18). Bronchial biopsies were taken in asthmatic patients and control subjects (n = 10) by fiberoptic bronchoscopy and embedded in paraffin. The cellular infiltrate was evaluated by May-Grünwald-Giemsa staining. Adhesion molecule expression was analyzed by immunohistochemistry using mouse monoclonal antibodies; the results were expressed as the percentage of positive cells. For each patient, we evaluated the severity of asthma as defined by the AAS score and the treatment. In controls, low expression of ICAM-1 was observed on the epithelium and endothelium (9.6 +/- 2.7 and 11.2 +/- 4.1%, respectively), while E-selectin and VCAM-1 were not expressed. In patients with allergic asthma, a

significant increase of ICAM-1 expression was observed on epithelium and endothelium (28 +/- 5.3 and 35.6 +/- 5%, respectively), whereas E-selectin (17.4 +/- 4.8%) and VCAM-1 (12.8 +/- 3.6%) were overexpressed only on endothelium. In allergic asthmatic patients, adhesion molecule expression on endothelium was correlated with eosinophil and total leucocyte infiltrate ($p < 0.05$). In contrast, adhesion molecule expression in biopsies from patients with non-allergic asthma (14.1 +/- 5.2 and 15.3 +/- 3.6% for ICAM-1 expression on epithelium and endothelium, respectively) was not significantly different from the control subjects.(ABSTRACT TRUNCATED AT 250 WORDS)

PMID: 7529075 [PubMed - indexed for MEDLINE]

2064. Am J Respir Cell Mol Biol. 1995 Jan;12(1):4-12.

In situ expression of the cell adhesion molecules in bronchial tissues from asthmatics with air flow limitation: in vivo evidence of VCAM-1/VLA-4 interaction in selective eosinophil infiltration.

Ohkawara Y, Yamauchi K, Maruyama N, Hoshi H, Ohno I, Honma M, Tanno Y, Tamura G, Shirato K, Ohtani H.

First Department of Internal Medicine, Tohoku University School of Medicine, Sendai, Japan.

Eosinophils play a critical role in the pathogenesis of bronchial asthma by releasing various mediators. To understand the mechanisms of eosinophil migration to the site of inflammation, we examined the expression of adhesion molecules in the bronchial tissues of asthmatic subjects with air flow limitation. By immunohistochemical analysis, Mac-1, LFA-1, and VLA-4 were strongly positive in eosinophils and mononuclear cells infiltrated in the bronchial mucosa and submucosa. Their number was significantly increased compared with those in control tissue. Immunolocalization for ICAM-1, the ligand of Mac-1 and LFA-1, was detected in the endothelial cells of capillaries and venules, in the mononuclear cells in submucosa, and in the basal layer of the epithelium. Endothelial cells in capillaries and venules were also strongly positive for VCAM-1, the ligand of VLA-4. Immunolocalization for E-selectin was detected in some endothelial cells in capillaries and venules in bronchial submucosa, whereas there were very few positive cells in the bronchial tissues from control subjects. In situ hybridization demonstrated ICAM-1 mRNA expression in the endothelial cells and mononuclear cells in bronchial submucosa. Immunoelectron microscopy for ICAM-1, VCAM-1, and E-selectin demonstrated de novo synthesis of these molecules and their expression along the luminal cell membrane of endothelial cells. These results suggested that ICAM-1, VCAM-1, and E-selectin were newly synthesized prior to spontaneous asthma attacks, and that their expression, particularly that of VCAM-1, may play a key role in eosinophil infiltration into the airway.

PMID: 7529029 [PubMed - indexed for MEDLINE]

2065. J Invest Dermatol. 1995 Jan;104(1):101-6.

Human dermal endothelial cells express membrane-associated mast cell growth factor.

Weiss RR, Whitaker-Menezes D, Longley J, Bender J, Murphy GF.

Department of Dermatology, University of Pennsylvania School of Medicine, Philadelphia.

Mast cell growth factor (MGF), a molecule that serves as a ligand for the receptor tyrosine kinase c-kit, is important in mast cell differentiation, migration, and activation. Previous studies of paraffin-embedded human skin using antibody to murine MGF and reverse transcription-polymerase chain reaction have demonstrated MGF protein and mRNA expression in keratinocytes and isolated dermal cells. We utilized a monoclonal antibody to human MGF to further define patterns of immunoreactivity in frozen specimens of neonatal and adult skin from normal individuals and from patients with urticaria pigmentosa. In addition to keratinocytes and isolated dermal cells in normal and urticaria pigmentosa skin, MGF was detected in cells lining superficial and mid-dermal vessels. Co-expression of MGF and the vascular antigen CD31, and immunoelectron microscopy, identified MGF-positive cells as endothelial cells. Patterns of endothelial MGF expression were not influenced by mast cell degranulation and endothelial E-selectin induction in vitro. By ultrastructure, unfixed specimens demonstrated MGF expression both within the endothelial cytoplasm and in association with lumenal, but not ablumenal, surfaces. Specimens fixed with Nakane's solution had diminished endothelial cytoplasmic MGF reactivity, but lumenal expression was maintained, suggesting persistence of a membrane-associated reactivity. MGF mRNA was also detected in cultured dermal microvascular endothelial cells using reverse transcription-polymerase chain reaction. These data establish human dermal endothelial cells as sites of MGF production and expression in human skin. Mast cell precursors must home to skin via vascular channels and differentiate in the immediate perivascular space. Thus, endothelial MGF may be an important determinant of adhesion and differentiation of mast cell progenitors expressing receptors for MGF.

PMID: 7528242 [PubMed - indexed for MEDLINE]

2066. Phytomedicine. 1994 Dec;1(3):209-11. doi: 10.1016/S0944-7113(11)80067-X.

Antiinflammatory effects of Tremulacin, a Salicin-related substance isolated from Populus tomentosa Carr. leaves.

Cheng GF, Liu DP, Yang DX, He KQ, Bai JY, Zhu XY.

Institute of Materia Medica, Chinese Academy of Medical Sceinces, Beijing, China.

Tremulacin was shown to inhibit carrageenan-induced paw edema in rats and croton oil-induced ear edema in mice. It was also found to inhibit peritoneal leucocyte migration in rats and acetic acid-induced writhing responses in mice. Experiments with isolated longitudinal muscle strips of sensitized guinea pig ileum showed that tremulacin decreased the biosynthesis of Slow Reaction Substance of Anaphylaxis. Tremulacin exerted inhibitory effects on leukotriene B4 biosynthesis in intrapleural leucocytes. These results suggest that the mechanism of antiinflammatory actions of tremulacin is relevant to inhibition of 5-lipoxygenase activity. This is quite different from non-steroid antiinflammatory drugs, such as aspirin, which inhibits prostaglandin synthesis and being a cyclooxygenase inhibitor.

Copyright © 1994 Gustav Fischer Verlag, Stuttgart · Jena · New York. Published by Elsevier GmbH.. All rights reserved.

PMID: 23195941 [PubMed]

2067. Clin Immunol Immunopathol. 1994 Dec;73(3):305-11.

Antigen-induced recruitment of eosinophils: importance of CD4+ T cells, IL5, and mast cells.

Hom JT, Estridge T.

Lilly Research Laboratories, Indianapolis, Indiana 46285.

Eosinophils of sensitized mice readily recruit to the site of antigen challenge. In the present study, experiments were performed to determine the involvement of different cell types in the antigen-induced recruitment of eosinophils. We demonstrated that a single treatment with anti-L3T4 monoclonal antibody (mAb) on the day of allergen challenge significantly decreased antigen-induced recruitment of eosinophils. Treatments with anti-L3T4 mAb during the sensitization period also caused a substantial reduction in the migration of eosinophils into the site of challenge with antigen. Thus, it appears that both stages of eosinophil recruitment, sensitization and antigen-challenge, are dependent upon the presence of L3T4+ T cells. Moreover, while treatments with anti-IL5 mAb blocked eosinophil migration, anti-IL2 mAb failed to alter the antigen-induced recruitment of eosinophils. In addition, significant numbers of eosinophils from the mast-cell-deficient mice were found to migrate into the peritoneal cavities upon allergen challenge. Eosinophil migration was also observed in several mouse strains of different H-2 haplotypes. The present findings suggest that CD4+ T cells and IL5 but not IL2 may play important roles in modulating the recruitment of eosinophils. Moreover, the involvement of mast cells does not appear to be essential for eosinophil migration. Finally, the development of antigen-induced recruitment of eosinophils is probably not under the immunogenetic regulation by genes within the H-2 complex.

PMID: 7955558 [PubMed - indexed for MEDLINE]

2068. Allergy. 1994 Dec;49(10):797-807.

Mechanisms of eosinophil and basophil migration.

Wardlaw AJ, Walsh GM, Symon FA.

Department of Respiratory Medicine, Leicester University School of Medicine, Glenfield Hospital, UK.

Considerable progress has been made in our understanding of the molecular mechanisms involved in eosinophil and basophil migration into sites of allergic inflammation. It is clearly a staged process, each stage offering a level of control over the cell specificity and degree of migration. On the basis of current evidence, the various receptors and mediators involved are summarized in Table 4. Once in the tissues, eosinophils may persist for several days or weeks, surviving under the influence of locally generated cytokines, and this persistence may also partly explain the selective tissue accumulation of eosinophils and basophils. Understanding the molecular mechanisms involved in leukocyte migration may lead to the discovery of selective and effective antagonists to treat allergic disease by preventing cell migration. Results in a number of animal models already suggest that this approach may be successful. The development of drugs that can be tested in the clinic is awaited with much interest.

PMID: 7709988 [PubMed - indexed for MEDLINE]

2069. J Allergy Clin Immunol. 1994 Dec;94(6 Pt 1):1006-16.

Nasal challenge with allergen upregulates the local expression of vascular endothelial adhesion molecules.

Lee BJ, Naclerio RM, Bochner BS, Taylor RM, Lim MC, Baroody FM.

Department of Medicine (Division of Clinical Immunology), Johns Hopkins University School of Medicine, Baltimore, MD.

To understand the events involved in selective eosinophil migration into allergic inflammatory sites, we studied the expression of vascular endothelial adhesion molecules in the nasal mucosa. Ten subjects with asymptomatic seasonal allergic rhinitis and 13 nonallergic subjects underwent localized allergen challenge of one inferior turbinate. Twenty-four hours later, biopsy specimens were obtained from the inferior turbinates, bilaterally in the seasonally allergic subjects and unilaterally in the nonallergic control subjects. The specimens were divided, sectioned, and either stained for identification of eosinophils or analyzed immunohistochemically for intercellular adhesion molecule-1, E-selectin, vascular cell adhesion molecule-1 (VCAM-1), and von Willebrand's factor. Intercellular adhesion molecule-1 expression was observed in all mucosal specimens, with no significant difference among groups. E-selectin showed minimal baseline expression, and low levels were significantly induced on the challenged mucosa of the allergic compared with nonallergic subjects ($p < 0.05$). VCAM-1 was expressed basally and was significantly upregulated by allergen challenge, compared with the nonchallenged side and nonallergic control subjects ($p < 0.05$). Submucosal eosinophils increased significantly in allergic subjects 24 hours after antigen challenge, compared with nonallergic control subjects and weakly correlated with VCAM-1 expression ($r_s = 0.33$, $p = 0.06$). Our results suggest that endothelial activation accompanies allergic inflammation. Furthermore, because the counterligand for VCAM-1, very late activation antigen-4, is present on eosinophils, VCAM-1 upregulation may contribute to the selective recruitment of these cells to the nasal mucosa.

PMID: 7528231 [PubMed - indexed for MEDLINE]

2070. Br J Pharmacol. 1994 Nov;113(3):749-56.

Role of interleukin-5 in enhanced migration of eosinophils from airways of immunized guinea-pigs.

Coëffier E, Joseph D, Vargaftig BB.

Unité de Pharmacologie Cellulaire, Unité Associée Institut Pasteur-INSERM no. 285, Paris, France.

1. Platelet activating factor (PAF), leukotriene B4 (LTB4) and interleukin-5 (IL-5) are potent chemoattractants for guinea-pig eosinophils, which may be involved in eosinophil recruitment and up-regulation in allergic diseases. Eosinophils from the bronchoalveolar lavage fluid (BALF) of ovalbumin-sensitized guinea-pigs were collected 24 h after antigen provocation and migration induced by PAF, LTB4 and rhIL-5 was studied. 2. Total BALF content and distribution of eosinophils were greater in immunized, ovalbumin-challenged guinea-pigs (5.0 +/- 0.8 x 10(6)/guinea-pig; 12 +/- 1%) than in immunized, saline-challenged animals (3.0 +/- 0.7 x 10(6)/guinea-pig; 7 +/- 1%). 3. The chemoattraction of eosinophils isolated on a metrizamide gradient was studied in micro-Boyden chambers, results being expressed as the number of migrating cells (mean +/- s.e. mean). PAF and LTB4-induced migration of eosinophils from immunized and OA-challenged guinea-pigs were significantly enhanced, as compared to immunized and saline-challenged animals (170 +/- 36 vs 35 +/- 9 migrating eosinophils for 10 nM PAF; 271 +/- 60 vs 110 +/- 19 for 1 nM LTB4). 4. The IL-5 antibody TRFK-5, in vivo, reduced eosinophil recruitment in BALF of antigen-challenged immunized animals as well as the enhanced responsiveness of eosinophils from the challenged

animals, suggesting a role for IL-5 in the priming of eosinophils in vivo. 5. In contrast to TRFK-5, nedocromil sodium reduced to a similar extent eosinophil, macrophage and lymphocyte recruitment into the BALF of antigen-challenged, but failed to down-regulate the enhanced responsiveness of eosinophils from the challenged animals. 6. The increased eosinophil content in lungs from antigen-challenged guinea-pigs is thus selectively reduced by the anti-IL-5 antibody, which also attenuates the concomitant enhancement of the eosinophil responsiveness, supporting the concept that IL-5 is essential for recruitment and priming of eosinophils in vivo. In contrast, nedocromil sodium reduced non-selectively the total cell recruitment to the airways,but failed to attenuate the enhanced responsiveness of those eosinophils which migrated, indicating that its effects involve a different target.

PMCID: PMC1510407
PMID: 7858864 [PubMed - indexed for MEDLINE]

2071. Rinsho Shinkeigaku. 1994 Nov;34(11):1148-52.

[Eosinophilic meningo-encephalo-myelitis due to Toxocara canis].

[Article in Japanese]

Ota S, Komiyama A, Johkura K, Hasegawa O, Kondo K.

Department of Neurology, Yokohama City University School of Medicine.

A 21-year-old woman was admitted to our hospital because of frontal headache, low-grade fever and convulsion. The patient had long been in a close contact with a dog. Neurologic examination revealed meningeal irritation signs and cerebellar ataxia. Slight leukocytosis with an increased rate of eosinophils (23.2%) was present. A lumbar puncture yielded 330 leukocytes/microliters with 30% of eosinophils; protein, 55 mg/dl; and increased IgG synthesis, 43.6 mg/day. The antibody titer against Toxocara canis was positive both in serum and in CSF by the use of immunoblotting assay, Ouchterlony, indirect immunofluorescence and ELISA. Cranial MRI showed a number of lesions located mainly cortically or subcortically, which had a hyperintense appearance on T2-weighted images and were clearly enhanced with Gd-DTPA. A diagnosis of eosinophilic meningoencephalo-myelitis due to Toxocara canis was made. In spite of the treatment with diethylcarbamazine and prednisolone, other lesions including cervical cord and optic nerves developed. Although CSF antibodies against Toxocara canis were reduced in titer, neurologic symptoms relapsed, raising the possibility that some allergic mechanisms may, at least in part, be responsible for this neurologic complication.

PMID: 7729097 [PubMed - indexed for MEDLINE]

2072. Lab Invest. 1994 Nov;71(5):671-9.

Experimental allergic encephalomyelitis. T cell trafficking to the central nervous system in a resistant Thy-1 congenic mouse strain.

Skundric DS, Huston K, Shaw M, Tse HY, Raine CS.

Department of Pathology (Neuropathology), Albert Einstein College of Medicine, Bronx, New York.

BACKGROUND: The understanding of recognition events that underlie the migration of antigen-specific T cells to a target organ during immune-mediated damage will

be integral to the therapy of a number of human conditions of proven or suspected autoimmune etiology. In experimental allergic encephalomyelitis (EAE), the laboratory model of the human demyelinating disease, multiple sclerosis, previous studies have concentrated on susceptible strains and have shown that myelin-specific T cells play an early, key role in central nervous system (CNS), lesion formation. Not known in this model is whether in EAE-resistant strains, similar antigen-specific T cells possess the ability to recognize CNS endothelium and infiltrate the CNS.
EXPERIMENTAL DESIGN: Myelin basic protein (MBP)-responsive T cells derived from mice of the C57BL/6 strain (bearing the Thy-1.2 allele) were adoptively transferred to the Thy-1.1 congenic strain C57BL/Ka. Some recipients were given a subsequent challenge with MBP in adjuvant, a protocol recently shown to break resistance in this strain and cause EAE. On the basis of the difference in Thy-1 allele, T cell trafficking was followed in this EAE-resistant congenic strain following the different sensitization protocols.
RESULTS: In C57BL/Ka mice receiving adoptively transferred C57BL/6 cells followed by MBP challenge, donor MBP-responsive Thy-1.2+ lymphocytes were detected by immunocytochemistry in the Thy-1.1 host CNS and also in peripheral lymphoid organs. In mice given MBP-sensitized cells without additional antigen challenge, although Thy-1.2+ cells were found in the spleen and lymph nodes, similar cells could not be found in the CNS, and animals displayed neither clinical nor pathologic signs of EAE. Donor T lymphocytes appeared in the host CNS with clinical onset, 10 to 14 days after challenge. When mice went into remission, Thy-1.2+ lymphocytes could not be found in the CNS, but were still present in peripheral lymphoid organs up to 3 months after challenge. From the total number of infiltrating cells, T cell receptor-alpha beta+ cells constituted 27% in perivascular cuffs, 15% in meninges, and 13% in the parenchymal infiltrates in the spinal cord. Thy-1.2+ cells contributed up to about 40% of total T cell receptor-alpha beta+ lymphocytes. Approximately 60% of all infiltrating T cells expressed L3T4 (helper/inducer), whereas 18% expressed Lyt-2 (suppressor/cytotoxic). The majority of infiltrating cells were memory and activated cells expressing on their surface Pgp-1 and CD 25. Immunostaining for cytokines showed that the majority of infiltrating cells belonged to the TH1 subset and contained interferon-gamma and tumor necrosis factor-alpha, while a minority were positive for interleukin-4.
CONCLUSIONS: These results suggest that: (a) T lymphocytes from an EAE-resistant strain of mouse are capable of homing to the CNS; (b) T lymphocytes from an EAE-resistant strain express phenotypic characteristics, activation, memory, and cytokine profiles similar to infiltrating cells derived from susceptible strains; and (c) the presence of donor T cells in the recipient CNS correlates with clinical and histopathologic signs of EAE.

PMID: 7526038 [PubMed - indexed for MEDLINE]

2073. J Immunol. 1994 Oct 15;153(8):3717-23.

Stem cell factor is a chemotactic factor for human mast cells.

Nilsson G, Butterfield JH, Nilsson K, Siegbahn A.

Department of Pathology, University Hospital, University of Uppsala, Sweden.

The mast cell is one of the major effector cells in inflammatory reactions and can be found in most tissues throughout the body. During inflammation, an increase in the number of mast cells can be seen, e.g., in the intraepithelial cell layer after a provoked allergic reaction. Such accumulation probably requires directed migration of mature mast cells or their precursors. To study the migration of human mast cells we used as a model the human mast cell line, HMC-1, and stem cell factor-dependent (also referred to as mast cell growth

factor or Kit ligand) cord blood-derived mast cells. The results show that stem cell factor is a potent chemotactic factor for human mast cells in vitro. The chemotactic response to SCF was found to be dose dependent, reaching a maximum at 50 ng/ml. The activity of SCF could be blocked by anti-SCF Abs. We also tested the effect of different intercrines, i.e., IL-8, MIP-1 alpha, MIP-1 beta, RANTES, and MCAF (also referred to as monocyte chemotactic protein 1), on human mast cell migration. Only RANTES was chemotactic for in vitro-developed mast cells. None of the tested intercrines induced migration of HMC-1 cells. For migration, the mast cells were dependent on binding to an extracellular matrix protein. Thus, coating of the filters with fibronectin was required, whereas collagen or laminin did not promote migration. Adhesion of HMC-1 cells to fibronectin could also be shown in an adhesion assay. In addition, expression of receptors for fibronectin could be detected on the surface of the mast cells. These results show that SCF is not only a growth and differentiation factor for human mast cells in vitro but also a potent chemoattractant for such cells.

PMID: 7523504 [PubMed - indexed for MEDLINE]

2074. Med J Aust. 1994 Oct 3;161(7):418-25.

Asthma, allergy and atopy in Asian immigrants in Melbourne.

Leung RC, Carlin JB, Burdon JG, Czarny D.

St Vincent's Hospital, Fitzroy, Vic.

OBJECTIVES: To compare the prevalence of asthma, hay fever and atopy in Asian immigrants in Melbourne with that in Australian-born non-Asians and Australian-born Asians, and to investigate the association of these conditions with atopic status, length of stay in Australia and IgE levels in Asian immigrants.
DESIGN: We performed a cross-sectional study by telephone interviews, using standard questionnaire items on respiratory and allergic symptoms. A random sample of 636 recent Asian immigrants of ethnic Chinese origin, 109 Australian-born Asians and 424 Australian-born non-Asians were selected from the 1991 Melbourne Telephone Directory, using a presumptive surname list. Skin tests to determine atopic status were performed on 269 Asian immigrants and 167 of these also had serum levels of total and specific IgE estimated.
RESULTS: In the under 20 years age group the prevalence of wheeze or asthma ever was higher in Australian-born non-Asians and Australian-born Asians than in Asian immigrants ($P < 0.001$), and the prevalence of hay fever was higher in Asian immigrants and Australian-born Asians than in Australian-born non-Asians. In those older than 20 years, hay fever was almost twice as common in Asian immigrants as in Australian-born non-Asians ($P < 0.001$ for 20-40 years age group; $P < 0.01$ for > 40 years). The prevalence of hay fever and, to a lesser degree, asthma in Asian immigrants increased significantly with length of stay in Australia, independent of age at arrival, sex and atopic status (trend test: $P < 0.001$ for hay fever; $P = 0.05$ for asthma). Atopy was more common in Asian immigrants and Australian-born Asians than in Australian-born non-Asians ($P < 0.001$) and was very strongly associated with both hay fever and asthma, irrespective of length of stay. Pollen and mite sensitivities were more common in Asian subjects (twice as common for Asian-born and 1.5 times for Australian-born) than non-Asian subjects ($P < 0.01$). Among Asian immigrants, elevated total IgE level (> 100 IU/mL) was strongly associated with a history of hay fever ($P < 0.01$) and wheeze or asthma ever ($P < 0.05$), atopy ($P < 0.001$) and the presence of specific IgE antibodies to grass pollen, dust mite, cockroach and Ascaris antigens ($P < 0.05$ for all).
CONCLUSION: We found substantial differences in the prevalence of asthma, hay fever and atopy between Asian immigrants, Australian-born Asians and non-Asians.

The prevalence of hay fever and asthma in Asian immigrants was strongly associated with length of stay in Australia, suggesting that environmental factors are important in the pathogenesis of these diseases.

PMID: 7935095 [PubMed - indexed for MEDLINE]

2075. Pathol Biol (Paris). 1994 Oct;42(8):767-74.

[Current data on epidermal Langerhans cells].

[Article in French]

Dezutter-Dambuyant C.

Unité INSERM 346, Clinique Dermatologique, Pavillon R, Hôpital Edouard Herriot, Lyon, France.

The skin may be considered as well as a target and an initiator of self immune reactions. Two to 5% of the epidermal cells are Langerhans cells which are the only cells to specifically take, process and present the antigens to lymphocytes in order to induce an immune response. Such an ability and the location of the Langerhans cells enhance the role that they may play in antigenic stimulations or immuno-allergic situations. TNF alpha was shown to potentiate the effect of GM-CSF for the generation of Langerhans cells from their CD34-positive precursors found in the cord blood. Originated from the bone marrow, the Langerhans cells colonize skin and mucosa where they act as antigen presenting cells (APC) by taking, processing antigens, and migrating to lymph nodes in order to sensitize lymphocytes. Such a migration was shown, for the first in humans, in an induced irritant contact dermatitis. In vitro incubation of isolated Langerhans cells induces morphological, phenotypical and functional modifications which make Langerhans cells similar to interdigitating cells. Such an observation suggests that in vivo Langerhans cells could undergo a maturation when they migrate to lymph nodes. Many points remain to be explored in order to clarify the conditions which may stimulate or make langerhans migrate and play their immune function. For example, cellular interactions or positive/negative effects due to soluble mediators (cytokines, neuropeptides) may modulate their role as antigen presenting cells. In vitro model of T-cell sensitization confirmed their in vivo role of cutaneous surveillance in the recognition and elimination of exogenous antigens.(ABSTRACT TRUNCATED AT 250 WORDS)

PMID: 7724248 [PubMed - indexed for MEDLINE]

2076. Chest. 1994 Sep;106(3):762-6.

The prevalence and response to therapy of Strongyloides stercoralis in patients with asthma from endemic areas.

Wehner JH, Kirsch CM, Kagawa FT, Jensen WA, Campagna AC, Wilson M.

Santa Clara Valley Medical Center, Division of Respiratory and Critical Care Medicine, San Jose, Calif 95128.

STUDY OBJECTIVE: To evaluate the prevalence and response to therapy of Strongyloides stercoralis infection in immigrant patients with asthma from areas endemic for Strongyloides.
DESIGN AND INTERVENTIONS: In all patients, we performed a complete history and physical examination, complete blood cell counts (CBC), S stercoralis serologic tests, spirometry, and evaluated three stool samples for ova and parasites.

Patients treated for S stercoralis infection had follow-up CBC, spirometry, serologic tests, and at least three additional stool examinations to confirm eradication of the parasite.
SETTING: Ambulatory and hospitalized patients who were referred to the respiratory medicine clinic of a general hospital for the evaluation and treatment of asthma.
PATIENTS: Forty-five asthmatic adults, representing 12 endemic countries, ranging in age from 20 to 76 years, were prospectively evaluated.
RESULTS: Six of 45 patients were infected with S stercoralis, which yielded a prevalence of 13 percent. The patients with asthma and S stercoralis infection had higher blood eosinophil counts ($p = 0.006$) and were younger ($p = 0.006$) compared with patients with only asthma. There was no difference in the duration of asthma, spirometry, or steroid use between the two groups. Patients with S stercoralis and asthma tended to be more recent immigrants ($p = 0.05$). Five of the six patients with S stercoralis agreed to be treated with thiabendazole but only four returned for follow-up evaluation. All four patients had eradication of S stercoralis infection confirmed by negative stool examinations and a decline in S stercoralis serology (160 +/- 25 percent vs 13 +/- 13 percent, $p = 0.03$). All four patients had a decline in total blood eosinophil counts (2,476 +/- 832 cells per cubic millimeter vs 551 +/- 138 cells per cubic millimeter, $p = 0.03$) without a clinical improvement in asthma.
CONCLUSIONS: Our data suggest that patients with asthma from areas endemic for S stercoralis, who have elevated peripheral blood eosinophil counts, should be screened for S stercoralis infection. Successful eradication of S stercoralis, however, may not result in a clinical improvement of asthma.

PMID: 8082356 [PubMed - indexed for MEDLINE]

2077. Minerva Cardioangiol. 1994 Sep;42(9):435-41.

[Calcitonin-induced anaphylactic shock. Case report and review of the literature].

[Article in Italian]

Piccone U, Pala M, Caprari M.

Divisione di Cardiologia, Ospedale S. Carlo Borromeo, Milano.

A case of anaphylactic shock determined by intramuscular administration of a dose of synthetic calcitonin in a 64-years-old man is described. The patient had not suffered significant cardiovascular events in the past; he smoked twenty cigarettes a day and he was treated with calcitonin for osteoporosis and polyarthrosis. Allergy to diclofenac was demonstrated in the past while preceding administrations of spray calcification didn't provoke side-effects in the patient. Nevertheless after the second i.m. administration of the drug he suddenly fainted. Dyspnea, severe hypotension and maculo-papular erythema were present at the moment of admission to our hospital. The continuous electrocardiogram monitoring showed a characteristic "migrant" ST elevation at first in the anterior leads, then in inferior and septal leads, and premature ventricular and atrial beats. The echocardiographic transtoracic examination proved an apical and septal akinesia which completely disappeared after one hour at a second echocardiographic examination. In spite of intensive medical treatment (lignocaine and hydrocortisone e.v.) the patient had a sustained ventricular tachycardia that quickly degenerated into ventricular fibrillation. After one DC shock at 300 joules we observed spontaneous spontaneous restoration of the normal sinus rhythm. The following clinical evolution was good and no other arrhythmias or cardiovascular symptoms were observed. In order to estimate the reasons of the clinical picture the patient was submitted to serial blood

examinations, serial electrocardiograms, exercise stress test, echodypiridamole stress test and serial echocardiograms. The blood examinations showed a relative eosynophilia (3%), the increase of IgE serum level (316 UI) and transient ipokalemia (2.3 mEq/l). None pathological findings were observed in the other examinations.(ABSTRACT TRUNCATED AT 250 WORDS)

PMID: 7991163 [PubMed - indexed for MEDLINE]

2078. Allergol Immunopathol (Madr). 1994 Sep-Oct;22(5):204-8.

Increased chemotactic responses of neutrophils in intrinsic and mixed asthmatic patients.

Sin B, Misirligil Z, Demirel YS, Gürbuz L, Bavbek S, Acican T.

University of Ankara, Faculty of Medicine, Department of Allergic Diseases, Turkey.

Neutrophils are thought to contribute actively in the pathogenesis of asthma since they infiltrate into the lung tissue. The aim of this study is to compare the chemotactic responses of neutrophil granulocytes from 10 intrinsic, 13 extrinsic and 10 mixed type asthmatic patients with each other and chemotactic response of neutrophil granulocytes from 26 healthy individuals. All patients were free of infection and not receiving systemic corticosteroids. Significant differences were not found in random migration between all of the groups. However, chemotactic activity with zymosan activated serum was significantly elevated in intrinsic and mixed asthmatic patients ($p < 0.001$). In conclusion, the present investigation demonstrated increased chemotactic responses of neutrophils from asthmatic patients except extrinsic type.

PMID: 7840021 [PubMed - indexed for MEDLINE]

2079. Immunology. 1994 Sep;83(1):128-32.

Dendritic cells of the oral mucosa and the induction of oral tolerance. A local affair.

Van Wilsem EJ, Van Hoogstraten IM, Brevé J, Scheper RJ, Kraal G.

Department of Cell Biology & Immunology, Vrije Universiteit, Amsterdam, The Netherlands.

The oral mucosa is an important site to induce immunological tolerance to protein antigens. Previously we have established that oral contacts to allergen can lead to systemic tolerance in both humans and experimental animals. Because of the importance of tolerance induction as a possible way to modulate allergic reactivity, we wished to study the mechanisms involved in efficient tolerance induction via the oral mucosa. Dendritic Langerhans' cells in both skin and oral epithelium are the first cells to encounter antigen. Therefore, possible functional differences between Langerhans' cells from skin and oral mucosa were studied by migration and transfer experiments. It was found that dendritic cells derived from the oral mucosa were not able to transfer tolerance, but that they acted as antigen-presenting cells in sensu stricto irrespective of the source and route of antigen administration.

PMCID: PMC1415022
PMID: 7821957 [PubMed - indexed for MEDLINE]

2080. J Immunol. 1994 Sep 1;153(5):2153-60.

Eosinophil transendothelial migration induced by cytokines. III. Effect of the chemokine RANTES.

Ebisawa M, Yamada T, Bickel C, Klunk D, Schleimer RP.

Department of Medicine, Johns Hopkins University School of Medicine, Johns Hopkins Asthma and Allergy Center, Baltimore, MD 21224.

Selective eosinophil recruitment occurs after experimental Ag challenge and in tissue sites of allergic diseases. The mechanisms of selective eosinophil migration are still unknown. In our study, we examined the ability of chemokines to induce transendothelial migration (TEM) of eosinophils in vitro. Among the chemokines tested, only RANTES induced eosinophil TEM. RANTES failed to induce TEM of neutrophils. Interestingly, IL-8 induced neutrophil TEM and had no effect on eosinophil TEM. RANTES-induced TEM was concentration-dependent and was inhibited by Abs directed against the beta 2 integrin CD18. When IL-1-activated endothelial cells were utilized, RANTES-induced TEM also involved the eosinophil beta 1 integrin VLA-4. RANTES did not increase eosinophil adhesion to either resting or IL-1-activated endothelial cells, nor did the chemokine increase CD11b or decrease L-selectin expression. A gradient of RANTES appears to be required for eosinophil TEM. Pre-exposure of eosinophils to IL-5 dramatically potentiated the TEM response to RANTES. These findings suggest that the chemokine RANTES is a potent and selective inducer of eosinophil TEM. Because RANTES appears to be produced in vivo during allergic reactions or in allergic diseases, we speculate that these findings may have some direct relevance to the mechanism of selective eosinophil recruitment in vivo in humans.

PMID: 7519642 [PubMed - indexed for MEDLINE]

2081. Toxicol In Vitro. 1994 Aug;8(4):685-7.

Epidermal cytokines and skin sensitization hazard.

Cumberbatch M, Kimber I.

Zeneca Central Toxicology Laboratory, Alderley Park, Macclesfield, Cheshire SK10 4TJ, UK.

The induction phase of skin sensitization is associated with the passage of antigen-bearing Langerhans cells (LC) from the epidermis to the draining lymph nodes. Recent investigations have revealed that the induction of LC migration following topical sensitization is dependent on tumour necrosis factor alpha (TNF-alpha), an epidermal cytokine. While in transit to the lymph nodes LC are subject to both phenotypic and functional maturation which, by analogy with in vitro studies, is also effected by epidermal cytokines (granulocyte/macrophage colony-stimulating factor, GM-CSF and interleukin-1, IL-1). It is now apparent that Langerhans cell function, the induction of cutaneous immune responses and effective sensitization are dependent on the availability of such cytokines and that contact allergens are able to provoke their production by keratinocytes and by Langerhans cells themselves. The development of screening strategies for the evaluation of skin sensitization potential as a function of epidermal cytokine production is discussed.

PMID: 20692987 [PubMed]

2082. Allergol Immunopathol (Madr). 1994 Jul-Aug;22(4):179-83.

Quantification of eosinophil cationic protein and eosinophils in nasal secretions of allergen-induced nasal inflammation.

Wang DY, Clement P, Smitz J, De Waele M, Derde MP.

Department of Otorhinolaryngology, University Hospital, Free University Brussels, Belgium.

By using the microsuction technique, a quantitative determination of eosinophil cationic protein (ECP) in nasal secretions was performed in 18 atopic patients and 10 healthy volunteers. The percentage of the eosinophils was counted by simple microscopic examination. In the control group, results showed that the percentage of the eosinophils was almost 0 (range: 0-0.7%), and the median baseline ECP concentration was measured at 105 ng/g (range: 2-281 ng/g). No significant changes in either eosinophils or ECP were observed in this group. In the patient group, there existed a significantly higher baseline percentage of eosinophils (median: 2.7%, range: 0-45%) than in the control group but not for baseline ECP concentration (median: 160 ng/g, range: 6-1475 ng/g). One hour after nasal allergen challenge (NAC), a significant ($P < 0.01$) increase was seen in the percentage of eosinophils of the nasal secretions and this was remained for 10 hours (median: 5.5-13.3%). However, the difference that occurred was that ECP reached its highest concentration (median: 401 ng/g, range: 32-2298 ng/g) only 24 hours after NAC, while the percentage of the eosinophils decreased. Our findings again support a role of migration and activation of eosinophils in the pathogenesis of nasal allergic reaction, especially during the late phase.

PMID: 7976971 [PubMed - indexed for MEDLINE]

2083. J Investig Allergol Clin Immunol. 1994 Jul-Aug;4(4):192-6.

Atopic sensitization in children of Somali immigrants in Italy.

Asseyr AF, Businco L.

Division of Pediatric Allergy and Clinical Immunology, University of Rome, La Sapienza, Italy.

Children of immigrants are known to be at greater risk for developing allergic manifestations than the population in general. In this study we observed children of Somali origin living in Italy in order to find plausible explanations for the high risk of allergic disease in immigrants. Fifty-two children aged 0-14 years (mean = 6.7 years) were examined during the spring season. The mothers of the children were asked to fill in a questionnaire on symptoms and signs of atopic diseases and the family history of atopy. In addition, a physical examination and skin tests were performed. All families used mattresses, pillows and/or blankets made of wool, known to favor the growth of mites. In the majority of these children's families we found at least five people cohabitating in the same room. Skin prick tests were most frequently positive for Dermatophagoides pteronyssinus. Seventy-four percent (14/19) of those with symptoms were positive to D. pteronyssinus. Only 14% (2/14) of those positive to D. pteronyssinus were positive to Lolium perenne. Thirty-seven percent (19/52) had atopic symptoms and 15% (5/33) of those without symptoms were positive to D. pteronyssinus ($p < 0.0001$ compared to those with symptoms). Sensitization to food allergens occurred less frequently as compared to common inhalant allergens ($p < 0.0001$). A high prevalence of atopic diseases among children of Somali immigrants in Italy might be attributed to contact with the new environment and poor socioeconomic conditions that promote, for example, the growth of mites. However, further

studies are needed to document these differences.

PMID: 7850033 [PubMed - indexed for MEDLINE]

2084. Stomatologiia (Mosk). 1994 Jul-Sep;73(3):37-40.

[The immunotropic and allergenic activity of hydroxyapatite with an ultrahigh degree of dispersion].

[Article in Russian]

Pankratov AS, Zuev VP, Ivanov NG, Chernousov AD.

Hydroxyapatite is a well-known biologic material intended for intraosseous implantation which is widely used in practical dentistry. The agent causes no inflammatory reaction at the site of administration and is not characterized by acute or chronic toxicity. The possibility of development of specific contraindications against hydroxyapatite has not yet been studied; such contraindications might be connected with its possible immunotropism and allergenic activity which are to this or that measure present in all materials used for implantation into the bone. Since hydroxyapatite is known as surfactant, we used in our studies its modification characterized by the highest specific surface known at present, and therefore the most biochemically active. "Cold", that is, not exposed to high-temperature treatment ceramics was used in order to prevent reduction of hydroxyapatite reaction capacity. Complex of animal experiments showed that hydroxyapatite cannot possess immunotropic or allergenic characteristics.

PMID: 7846711 [PubMed - indexed for MEDLINE]

2085. Immunology. 1994 Jul;82(3):473-81.

Interleukin-8 and RANTES induce the adhesion of the human basophilic cell line KU-812 to human endothelial cell monolayers.

Bacon KB, Flores-Romo L, Aubry JP, Wells TN, Power CA.

Glaxo Institute for Molecular Biology, Geneva, Switzerland.

Basophils are implicated in the pathogenesis of the late-phase allergic reaction, but the mechanisms by which circulating basophils adhere to vascular endothelium and migrate to lesional sites remain unclear. In order to assess the biological similarity of the basophilic cell line KU-812 to normal human basophils, we have compared the adhesion response of this cell line and normal basophils, following challenge with interleukin-8 (IL-8) and RANTES. We demonstrate here that IL-8 and RANTES are able to stimulate the adherence of the basophilic cell line, KU-812, to cytokine-activated human umbilical vein endothelium (HUVEC). The chemokine-induced increase in adhesion was dose-related and was maximal after prior priming with IL-5. The stimulation of adhesion was partially inhibited by co-incubation with anti-CD18 and anti-CD11c antibodies and antibodies to the beta 1-integrins. In comparison, the chemokine-induced adhesion of normal human basophils was only inhibited by the beta 2-integrins. These chemokines were also able to induce the migration of KU-812 in a dose-dependent manner, but only after prior treatment with phorbol myristate acetate (PMA) or IL-5. In all cases tested, IL-8 was more potent and efficacious than RANTES. We conclude from these studies that these members of the chemokine superfamily may play an important role in the recruitment of reactive leukocytes in allergic inflammation, by stimulating their adhesion and subsequent migration from the vasculature into the

inflammatory sites. However, it is apparent that KU-812 is not an adequate substitute for normal human basophils in order to investigate chemokine biology.

PMCID: PMC1414888
PMID: 7525460 [PubMed - indexed for MEDLINE]

2086. J Exp Med. 1994 Jul 1;180(1):371-6.

Eosinophil adhesion to nasal polyp endothelium is P-selectin-dependent.

Symon FA, Walsh GM, Watson SR, Wardlaw AJ.

Department of Respiratory Medicine, University of Leicester Medical School, Glenfield General Hospital, United Kingdom.

Tissue eosinophilia is a characteristic feature of a number of inflammatory diseases including asthma and nasal polyposis. Eosinophil migration into tissues is controlled in part by interactions between eosinophil adhesion receptors and counter-structures on the vascular endothelium. To determine the receptors used by eosinophils to adhere to vascular endothelium in allergic inflammation we have adapted the Stamper-Woodruff frozen section assay (FSA) to study eosinophil adhesion to nasal polyp endothelium. Immunohistology indicated that intercellular adhesion molecule 1 (ICAM-1), E-selectin and P-selectin were well expressed by nasal polyp endothelium, whereas expression of vascular cell adhesion molecule 1 (VCAM-1) was weak or absent. Unstimulated human peripheral blood eosinophils adhered specifically to nasal polyp endothelium. Adherence was temperature and divalent cation-dependent and saturable at cell densities > 5 x 10(6) cells/ml. Eosinophil adhesion was almost completely inhibited by a monoclonal antibody (mAb) against P-selectin and by a chimeric molecule consisting of the Fc portion of human IgG and the lectin binding domain of P-selectin, which binds to the P-selectin ligand on leucocytes. Anti-Mac-1 mAb partially inhibited eosinophil adhesion whereas mAb against E-selectin, L-selectin, ICAM-1, VCAM-1, very late activation antigen 4, and lymphocyte function-associated antigen 1 had no effect. P-selectin is stored in intracellular granules within the endothelial cell and in vitro is only transiently expressed. To determine if P-selectin was expressed on the membrane of the nasal polyp endothelium we compared P-selectin expression in normal skin and nasal polyps after acetone fixation, which permeabilizes cells, and paraformaldehyde, which only allows staining of membrane expressed receptors. In the skin, good expression was seen with acetone fixation but no expression was seen after paraformaldehyde treatment, whereas in nasal polyps, similar expression was observed with both fixatives. In addition immunofluorescence with confocal microscopy demonstrated lumenal staining of nasal polyp endothelium indicating that P-selectin was located on the surface of endothelial cells while in skin only an intracellular granular distribution was apparent. Lastly, whereas eosinophils bound consistently to nasal polyp endothelium, no binding was observed to blood vessels in normal skin further supporting the idea that eosinophils were binding to membrane expressed and not intracellular P-selectin. The importance of P-selectin in eosinophil adhesion to nasal polyp endothelium suggests that P-selectin antagonists may be effective at inhibiting eosinophil accumulation at sites of allergic inflammation.

PMCID: PMC2191567
PMID: 7516413 [PubMed - indexed for MEDLINE]

2087. J Invest Dermatol. 1994 Jun;102(6):843-6.

IL-4 induces chemotaxis of blood eosinophils from atopic dermatitis patients, but not from normal individuals.

Dubois GR, Bruijnzeel-Koomen CA, Bruijnzeel PL.

Department of Dermatology/Allergology, University Hospital Utrecht, The Netherlands.

T lymphocytes present in allergically inflamed tissue synthesize and secrete the cytokines interleukin (IL)-3, IL-4, IL-5, and granulocyte/macrophage colony-stimulating factor (GM-CSF). IL-3, IL-5, and GM-CSF, but also IL-4, may act as a chemotaxin on eosinophils. In contrast to the former cytokines, IL-4 is only chemotactic for eosinophils from the peripheral blood of patients with atopic dermatitis and not for eosinophils from normal individuals. IL-4 has the same chemotactic potency as the other cytokines. The optimal chemotactic potency is reached at a concentration of 10 nM. In contrast, neutrophils do not respond chemotactically to IL-4. Checkerboard analysis, inhibition studies with monoclonal anti-IL-4 antibodies, and desensitization experiments indicated specific interaction of IL-4 with eosinophils. In eosinophils from normal individuals, IL-4 responsiveness could be induced by pretreatment of the cells with IL-5 and GM-CSF. In addition to the fact that IL-4 may be responsible for selective eosinophil transendothelial migration, IL-4 may exert an important modulatory mode of action on eosinophil migration and function within allergically inflamed tissue. Our findings suggest the presence of a functional IL-4R on eosinophils from atopic dermatitis patients.

PMID: 8006446 [PubMed - indexed for MEDLINE]

2088. Aust N Z J Med. 1994 Jun;24(3):255-7.

Asthma, allergy and atopy in South-east Asian immigrants in Australia.

Leung R.

Comment in
    Aust N Z J Med. 1995 Apr;25(2):177.

PMID: 7980206 [PubMed - indexed for MEDLINE]

2089. Ann Pharmacother. 1994 Jun;28(6):730-1.

Erythema multiforme and hypersensitivity myocarditis caused by ampicillin.

Garty BZ, Offer I, Livni E, Danon YL.

Kipper Institute of Allergy and Immunology Children's Medical Center of Israel, Beilinson Medical Campus, Petah Tiqva.

OBJECTIVE: To report a case of erythema multiforme and hypersensitivity myocarditis caused by ampicillin.
CASE SUMMARY: A 13-year-old boy was treated with ampicillin and gentamicin because of suspected septicemia. Medications were discontinued when erythema multiforme and congestive heart failure caused by myocarditis occurred. The patient was treated with methylprednisolone and gradually improved. Macrophage-migration inhibition (MIF) test with ampicillin was positive.
DISCUSSION: After most infections causing erythema multiforme and myocarditis were ruled out, a drug-induced allergic reaction was suspected. Positive MIF test for ampicillin showed sensitization of the patient's lymphocytes to ampicillin.
CONCLUSIONS: Hypersensitivity myocarditis is a rare and dangerous manifestation of allergy to penicillins.

PMID: 7919560 [PubMed - indexed for MEDLINE]

2090. Atherosclerosis. 1994 Jun;107(2):179-85.

Prominent inhibitory effects of tranilast on migration and proliferation of and collagen synthesis by vascular smooth muscle cells.

Tanaka K, Honda M, Kuramochi T, Morioka S.

4th Department of Internal Medicine, Shimane Medical University, Japan.

To obtain some ideas about prevention of restenosis after percutaneous transluminal coronary angioplasty (PTCA), we examined the effects of transilast (anti-allergic agent) on migration and proliferation of, and collagen synthesis by, cultured vascular smooth muscle cells (VSMC) from the thoracic aorta of WKY rats. Tranilast was added to culture medium containing 10% fetal calf serum (FCS). The cultures were pulse-labeled with 3H-thymidine (TdR) or 3H-proline (Pro). TdR and Pro uptake into VSMC were measured. The effect of tranilast on migration of VSMC was examined by using culture dishes of an original design. We also examined the inhibitory effects of various drugs, such as a Ca antagonist, an angiotensin converting enzyme (ACE) inhibitor, a phosphodiesterase inhibitor, elastase, colchicine, and mitomycin C, on proliferation and migration of VSMC. Our data showed that the inhibitory effects of tranilast on migration and proliferation of, and collagen synthesis by, VSMC were prominent. Maximal percentage inhibition of proliferation, migration and collagen synthesis was 60.8 +/- 2.3%, 52.7 +/- 14.7% and 62.1 +/- 8.1%, respectively. On the other hand, the inhibitory effects of other drugs, with the exception of colchicine and mitomycin C, on proliferation and/or migration of VSMC were not very strong. Although the inhibitory effects of colchicine and mitomycin C were strong in vitro, their clinical usefulness may be limited by systemic side-effects. These results indicate the potential usefulness of tranilast for prevention of restenosis of coronary arteries after PTCA.

PMID: 7526874 [PubMed - indexed for MEDLINE]

2091. Ann N Y Acad Sci. 1994 May 28;725:268-73.

IL-4-induced migration of eosinophils in allergic inflammation.

Dubois GR, Bruijnzeel PL.

Department of Dermato-Allergology, University Hospital Utrecht, The Netherlands.

PMID: 8030998 [PubMed - indexed for MEDLINE]

2092. J Immunol. 1994 May 1;152(9):4590-6.

Eosinophil transendothelial migration induced by cytokines. II. Potentiation of eosinophil transendothelial migration by eosinophil-active cytokines.

Ebisawa M, Liu MC, Yamada T, Kato M, Lichtenstein LM, Bochner BS, Schleimer RP.

Department of Medicine, Johns Hopkins University School of Medicine, Johns Hopkins Asthma and Allergy Center, Baltimore, MD 21224.

Activation of HUVEC monolayers by IL-1 beta or TNF-alpha induces migration of

eosinophils across the endothelial monolayer (i.e., transendothelial migration) in vitro. In the present study, we demonstrate that culture of freshly isolated eosinophils in the presence of IL-5 for 24 to 48 h before use in the assay dramatically potentiated CD18-dependent eosinophil transendothelial migration through unstimulated endothelial monolayers. Granulocyte macrophage (GM)-CSF induced eosinophil transendothelial migration but did not induce neutrophil transendothelial migration. When IL-1 beta-activated endothelial cells were used, GM-CSF, IL-3, or IL-5 caused only modest potentiation of eosinophil transendothelial migration. Since activated endothelial cells are known to produce GM-CSF, we hypothesized that endothelial-derived GM-CSF might play a role in the process of IL-1 beta-induced eosinophil transendothelial migration. IL-1 beta-activated endothelial monolayers grown on the permeable supports produced 0.3 +/- 0.1 ng/ml of GM-CSF during a 4-h incubation and neutralizing Ab against GM-CSF inhibited eosinophil transendothelial migration by 48%, suggesting that endothelial-derived GM-CSF may participate in the response. Eosinophils purified from bronchoalveolar lavage 18 to 20 h after experimental Ag challenge in the lungs of allergic volunteers showed enhanced transendothelial migration, indicating that the cells may have undergone cytokine activation in vivo. Eosinophil-active cytokines may contribute to the preferential accumulation of eosinophils in vivo in part via potentiation of eosinophil transendothelial migration.

PMID: 8157972 [PubMed - indexed for MEDLINE]

2093. Allergy. 1994 May;49(5):371-5.

Dexamethasone inhibits basophil migration.

Yamaguchi M, Hirai K, Nakajima K, Ohtoshi T, Takaishi T, Ohta K, Morita Y, Ito K.

Department of Medicine and Physical Therapy, University of Tokyo School of Medicine, Japan.

Glucocorticoids have been shown to inhibit the local accumulation of basophils during the allergen-induced late-phase reaction (LPR). Since migration is an essential step in the recruitment of basophils from the circulation, we examined whether the widely used glucocorticoid, dexamethasone (DEX), directly acts on basophils to inhibit the migration caused by C5a, interleukin (IL)-3, and IL-8. When purified basophils were preincubated with various concentrations of DEX, a dose-dependent inhibition was observed; DEX at concentrations as low as 1 nM reduced the number of migrated basophils by 30-40%; at higher concentrations, it showed a slightly stronger inhibitory effect. There was no significant difference in the effect of DEX on the migration caused by the three chemoattractants. The action of DEX took place rapidly; apparent inhibition was observed even when migration was initiated without preincubation. Although the inhibitory effect of this agent was not reversed when DEX was removed by washing, the inhibition was not mediated by the toxicity as measured by the trypan-blue exclusion test. These results indicate that the in vivo blocking effect of glucocorticoids on basophil accumulation during LPR is mediated in part by direct action to inhibit the migration of basophils.

PMID: 8092436 [PubMed - indexed for MEDLINE]

2094. Am J Respir Crit Care Med. 1994 May;149(5):1149-52.

Circulating adhesion molecules in asthma.

Montefort S, Lai CK, Kapahi P, Leung J, Lai KN, Chan HS, Haskard DO, Howarth PH,

Holgate ST.

Department of Immunopharmacology, University of Southampton, Southampton General Hospital, United Kingdom.

There is increasing evidence that leukocyte-endothelial adhesion molecules are important in inflammatory airway disease because of their involvement in the primary steps of entrapment and migration of leukocytes to the site of inflammation. Recently, circulating forms of these adhesion molecules have been described, although their origin, fate, and function are still unknown. We have used an antigen capture ELISA to measure the concentrations of circulating intercellular adhesion molecule-1 (cICAM-1), E-selectin (cE-selectin), and vascular cell adhesion molecule-1 (cVCAM-1) in the peripheral blood of 13 atopic and 16 non-atopic normal subjects, 29 patients with stable asthma, and inpatients with acute asthma on Day 1 (n = 38), Day 3 (n = 29), and Day 28 (n = 13) of an asthmatic episode. Circulating ICAM-1 and E-selectin levels were significantly raised in acute asthma on all three study days when compared with those observed in stable asthma, atopic normal, or nonatopic normal volunteers with no significant differences among the latter three groups. Circulating VCAM-1 was not significantly increased in any of the groups studied. There were no correlations among the concentrations of these three circulating adhesion molecules. The elevated concentrations of cICAM-1 and cE-selectin in acute asthma may reflect the extensive inflammatory response occurring in the airways during acute exacerbations of the disease with airway obstruction. It is possible that the cytokine and mediator profiles in acute asthma lead to the preferential synthesis and expression of these two circulating adhesion molecules in comparison with cVCAM-1.

PMID: 7513593 [PubMed - indexed for MEDLINE]

2095. Ned Tijdschr Geneeskd. 1994 Feb 12;138(7):367-70.

[International and intercultural aspects of pediatrics and adolescent health care].

[Article in Dutch]

Schulpen TW.

Wilhelmina Kinderziekenhuis, afd. Sociale Pediatrie, Utrecht.

PIP: Population statistics of Amsterdam between the 17th and 19th centuries indicate that 20-30% of young married people had been born in foreign lands. At the present time, 6% of the country's population, nearly 1 million people, are direct descendants of foreign parents: 240,000 Surinamese, 210,000 Turks, 170,000 Moroccans, and 80,000 from the Antilles. 40% of foreigners live in the four large cities, and there they make up about 15% of the population; 30-50% of children in these cities have foreign born parents. Among health concerns affecting these people are parasitic diseases, tuberculosis, salmonellosis, and the importation of infections such as viral B hepatitis, which so far has been successfully controlled. About 4% of the foreigners (30,000 people) carry a defective gene, and when two such people marry, in 25% of cases a child can be born with a severe defect as well as thalassemia major (mainly children of Moroccans and Turks) and sickle cell anemia (Surinamese and Antillans). 20-40% of children from tropical or subtropical areas also have lactase enzyme deficiency, which gives them stomach complaints because of incomplete metabolism of milk sugar. In recent years it has been reported that asthma and respiratory infections with longer hospitalizations occur more frequently among foreign children. Infant mortality is also 2-3 times higher among them. Intercultural aspects affecting Turkish and

Moroccans immigrants include communication problems, primarily those of the first generation, which should be facilitated by language centers and educational materials. Generation conflicts arise from contrasts between homelife and the outside world as well as from the fact that many of the parents are illiterate. Cultural difference are rooted in Islam, which requires loyalty to the group with traditional role patterns. Other problems pertain to the social isolation of the mother and the lower position of women, and the uncertain legal position of foreigners, which can result in sometimes unwarranted feelings of discrimination.
PMID: 8121529 [PubMed - indexed for MEDLINE]

2096. Chest. 1994 Feb;105(2):520-3.

Erythromycin does not directly affect neutrophil functions.

Hojo M, Fujita I, Hamasaki Y, Miyazaki M, Miyazaki S.

Department of Pediatrics, Saga Medical School, Japan.

To determine whether erythromycin could affect neutrophil functions, we measured N-formyl-methionyl-leucyl-phenylalanine (FMLP)-induced chemotaxis and superoxide generation of neutrophils in the presence of erythromycin at various concentrations. Erythromycin had no effect on either of them. We further confirmed that intracellular free calcium concentration ([Ca2+]i) was not influenced by FMLP stimulation in the presence of erythromycin. Our results indicate that erythromycin has no direct effects on neutrophil functions in vitro, although it is reported that erythromycin inhibits the local migration of neutrophils in the small airways of subjects with asthma.

PMID: 8306756 [PubMed - indexed for MEDLINE]

2097. Am J Respir Crit Care Med. 1994 Feb;149(2 Pt 1):345-51.

Immunophenotyping of eosinophils recovered from blood and BAL of allergic asthmatics.

Mengelers HJ, Maikoe T, Brinkman L, Hooibrink B, Lammers JW, Koenderman L.

Department of Pulmonary Diseases, University Hospital, Utrecht, The Netherlands.

Studies of bronchoalveolar lavage (BAL) fluid from patients with allergic asthma have demonstrated active migration of eosinophils into the bronchial lumen after allergen challenge. The mechanisms mediating this eosinophil infiltration and cell activation are largely unexplained. The expression of several cell-surface molecules was measured on eosinophils derived from blood and BAL fluid 4 h after an allergen-induced early asthmatic reaction in order to find indications for a role of these molecules during extravasation to and activation in the bronchial compartment. Nine patients with allergic asthma participated in the study. An eosinophil-specific, high-depolarization signal enabled us to measure expression on eosinophils in a fluorescence activated cell sorter (FACS) analysis without isolation of these cells. Eosinophils recovered from BAL showed a different phenotype than blood eosinophils; upregulation of CR-3, p150/95, CD67, and CD63, and downregulation of L-selectin indicate that the cells are activated in terms of degranulation. Up-regulation of intercellular adhesion molecule-1 (ICAM-1), LFA-3, and human leukocyte antigen II (HLA-II) might enable cell-cell contact between T-lymphocytes and eosinophils, probably leading to immunomodulation and cell activation. The finding that eosinophils in BAL are activated and can interact with T cells is further evidence for the proinflammatory role of these cells in allergic asthma.

PMID: 8306028 [PubMed - indexed for MEDLINE]

2098. Immunology. 1994 Feb;81(2):303-8.

Langerhans' cell expression of the selectin ligand, sialyl Lewis x.

Ross EL, Barker JN, Allen MH, Chu AC, Groves RW, MacDonald DM.

Laboratory of Applied Dermatopathology, St John's Institute of Dermatology, UMDS, Guy's Hospital, London, U.K.

Cellular adhesion molecules play a central role in leucocyte migration through peripheral blood and tissues. A crucial stage in these events in selectin-mediated adhesion involving E-selectin expressed on activated endothelium interacting with a range of carbohydrate ligands expressed by specific subpopulations of leucocytes. As such mechanisms may be relevant to bone marrow-derived dendritic epidermal Langerhans' cell (LC) migration, expression of these carbohydrate ligands was assessed immunocytochemically in whole skin biopsies and in epidermal cell suspensions obtained from adult humans. Double-labelling experiments revealed that sialyl Lewis x, recognized by the monoclonal antibody CSLEX1, was expressed on epidermal LC (n = 9). Furthermore, expression was enhanced at 24 hr following epicutaneous application of antigen and in the inflammatory disorder psoriasis (n = 10). E-selectin was concomitantly strongly expressed on dermal endothelium in psoriasis and allergic contact dermatitis. Intradermal injection of the T-cell-derived cytokine interferon-gamma (IFN-gamma) led to increased LC expression of sialyl Lewis x. In epidermal cell suspensions, in contrast to keratinocytes, CD1a+ cells expressed sialyl Lewis x, intensity of which was enhanced after 4 days in culture. CSLEX1 staining could be abolished and CD15 (non-sialated Lewis x) expression induced by saponification and treatment with neuraminidase. Expression of other selectin ligands was also examined. While the cutaneous lymphocyte antigen defined by the monoclonal antibody HECA-452 reacted with a small minority of LC, sialyl Lewis a and sulphatide were not expressed under any experimental conditions. These studies indicate that E-selectin-sialyl Lewis x interactions are potentially important in LC migration, both into and out of skin.

PMCID: PMC1422301
PMID: 7512530 [PubMed - indexed for MEDLINE]

2099. Rev Mal Respir. 1994;11(2):161-70.

[Mechanisms of bronchial hyperreactivity. Bronchial edema, mechanical and vascular factors].

[Article in French]

Regnard J, Dinh-Xuan AT, Matran R.

Laboratoire de Physiologie, Faculté de Médecine, Besançon.

Hindrance to gas flow in the bronchi is affected not only by airway smooth muscle tone but also by airway circulation. Congestion and oedema increase airway wall thickness and act in series with airway smooth muscle contraction to reduce airway calibre, an effect which is more marked in small and intermediate bronchi. Many mediators, neuromediators, paracrine mediators produced by resident (epithelium) or migrant (inflammatory cells) cells share bronchomotor and vascular effects. In addition, contraction of airway smooth muscle and vascular

phenomena are mechanically coupled. Contraction of airway smooth muscle facilitates vascular congestion and oedema because the diameter of the muscle ring is more reduced than the external diameter of the airways. In addition, a negative intrathoracic pressure, e.g. in asthma, increases the mechanical loading of both ventricles, thereby facilitating pulmonary and bronchial oedema. The effects of this mechanical coupling are enhanced by airway inflammation that facilitates both vascular congestion and leakage. Stimuli such as exercise and hyperventilation cause airway vasodilatation which, in turn, facilitates and, possibly, triggers the post-exercise asthma attack. Conversely, congestion and vasodilatation may have a protective effect through an increase in the clearance of bronchoconstrictor substances, or in reducing the amplitude of airway cooling and water loss in exercise-induced asthma. The relative role in bronchial hyperresponsiveness of airway smooth muscle contraction and vascular phenomena probably depends upon individual factors such as, for instance, both intensity and nature of inflammation of the airway walls.

PMID: 8202606 [PubMed - indexed for MEDLINE]

2100. Int Arch Allergy Immunol. 1994;104 Suppl 1(1):21-3.

Chemotactic heterogeneity of eosinophils in Kimura's disease.

Saita N, Ueno M, Yoshida M, Kimura T, Ando M, Hirashima M.

Department of Internal Medicine, Kumamoto University Medical School, Japan.

We evaluated the chemotactic heterogeneity of eosinophils in Kimura's disease. Patients with Kimura's disease were divided into two groups according to their clinical findings: one group had no other symptoms (KD), and another was accompanied with atopic dermatitis (KD + AD). The chemotactic response of eosinophils from two groups to 5 eosinophil chemotactic factors (ECF) derived from STO-2, an established T cell line. Eosinophils from KD were attracted only by ECF-PI5 and PI6 but not by ECF-PI7, PI8 and PI9. On the other hand, eosinophils from KD + AD responded to all 5 ECF. Eosinophils were further fractionated into normodense and hypodense eosinophils, and assessed for their chemotactic response. We thus found that there was little essential difference in their chemotactic responses to STO-2-derived ECF except ECF-PI9, though random migration of hypodense eosinophils was enhanced. The hypothesis that hypodense eosinophils are in the activated form was not always true, especially in the chemotactic response to ECF.

PMID: 8155998 [PubMed - indexed for MEDLINE]

2101. Parasite Immunol. 1994 Jan;16(1):1-9.

Toxocara canis-induced murine pulmonary inflammation: analysis of cells and proteins in lung tissue and bronchoalveolar lavage fluid.

Buijs J, Lokhorst WH, Robinson J, Nijkamp FP.

Laboratory of Parasitology and Mycology, National Institute of Public Health and Environmental Protection, Bilthoven, The Netherlands.

The pulmonary immuno-inflammatory reaction and its effect on microvascular integrity was studied in Toxocara canis infected BALB/c mice. The investigation aimed to compare changes in lung histology and composition of bronchoalveolar lavage fluid (BALF) caused by T. canis infection with those described to occur in allergic asthma. Groups of (non)-infected mice (1000 ova) were investigated until

90 days post infection (p.i.). Migration of the larvae through the lungs was followed by a rapidly progressing multifocal interstitial and alveolar inflammation. Eosinophils and lymphocytes formed perivascular and partially peribronchial mixed cellular infiltrates. Lymphocytes with plasma cell morphology staining intracellularly for either alpha, epsilon or gamma immunoglobulins were demonstrated. BALF, collected from mice infected with either 250, 500 or 1000 ova was analysed at 14 and 28 days p.i. A dose-related increase in cell numbers and in albumin and IgA concentration was observed. IgE increase was independent of the infective dose. Peak values were measured at 14 days p.i. Albumin increase in lung homogenate was highest at 28 days p.i. 30% of the lymphocytes consisted of T cells carrying Thy-1,2 and L3T4 surface antigens. It is concluded that T. canis-induced pulmonary inflammation affects the permeability of the microvasculature. This is expressed by interstitial oedema and plasma exudation in the airway lumen. Both phenomena occur also in allergic asthma. It is suggested that increased permeability of the microvasculature is mediated by T cells and eosinophils.

PMID: 8152829 [PubMed - indexed for MEDLINE]

2102. Int Arch Allergy Immunol. 1994;103(4):384-90.

Effect of cetirizine on human eosinophil superoxide generation, eosinophil chemotaxis and eosinophil peroxidase in vitro.

Okada C, Eda R, Miyagawa H, Sugiyama H, Hopp RJ, Bewtra AK, Townley RG.

Allergic Disease Center, Creighton University School of Medicine, Omaha, NB 68178.

Cetirizine, a potent H1-antagonist, has been reported to inhibit eosinophil migration into human skin. We, therefore, further evaluated the effect of cetirizine on eosinophil function, including superoxide anion generation, chemotaxis, and eosinophil peroxidase (EP) release. In allergic subjects, superoxide anion generation 60 min after platelet-activating factor (PAF) activation was inhibited by concentrations of cetirizine ranging from 0.01 to 1 microgram/ml (2.612 x 10(-8) to 2.612 x 10(-6) M). No significant inhibition was observed in normal subjects. PAF (10(-6) M)-induced eosinophil chemotaxis was also inhibited by cetirizine. In allergic subjects, percent inhibitions were 47.5 +/- 6.1% at 0.01 microgram/ml, 50.8 +/- 5.1% at 0.1 microgram/ml and 58.9 +/- 6.4% at 1 microgram/ml of cetirizine. In allergic subjects, N-formyl-methionyl-lencyl-phenylalanine induced eosinophil chemotaxis was inhibited by cetirizine, although EP release was not. These results suggest cetirizine has effects on eosinophils which can not be explained by H1-blockade alone.

PMID: 8130652 [PubMed - indexed for MEDLINE]

2103. Rev Alerg Mex. 1994 Jan-Feb;41(1):19-24.

[Insulin allergy in type II diabetes: rapid desensitization].

[Article in Spanish]

Ruíz Barrera E, Martínez-Cario Cueto S.

Departamento de inmunología clínica y alergia, Hospital de Especialidades, Centro Médico Nacional Siglo XXI, México.

Eight patients (7 female, 1 male) diagnosed as being allergic to insulin, between the ages of 49 and 68, were evaluated. In seven patients there was a previous history of allergies to other drugs: 4 to penicillin, two to sulfurs and one to nalidixic acid. The clinical manifestations were predominantly systemic: ras, six patients; angioedema, 3 patients; hypotension, three patients; bronchial spasm, 2 patients; generalized eruption, one patient. All 8 patients were subjected to skin tests which were positive, one patient showed a positive local skin reaction and the other seven proved to be positive. All reactions were favorable in all cases which allowed for the continuation of the administration of insulin without adverse reactions.

PMID: 8087231 [PubMed - indexed for MEDLINE]

2104. J Investig Allergol Clin Immunol. 1994 Jan-Feb;4(1):18-22.

The migration, phagocytosis and reduction ability of granulocytes from skin infiltrations of patients with atopic asthma.

Grzybowski A, Matusiewicz R, Grzybowska K.

Department of Medicine Prevention, Military Medical Academy, Lódź, Poland.

The migration, phagocytosis and reduction ability of granulocytes from the skin of atopic asthmatics (n = 30) and healthy control subjects (n = 20) were evaluated. In all subjects, granulocyte migration was evaluated in vitro using the Clausen test and in vivo using the Matusiewicz and Brzezińska test, and Park's tests of phagocytosis of neutral latex particles and the reduction of nitroblue tetrazolium (NBT) were also performed. It was demonstrated that patients with atopic asthma had a defect in skin granulocyte migration. Our studies also showed that the ability of granulocytes from skin infiltrations to reduce NBT was increased. The ability of granulocytes from skin infiltrations to migrate was inversely proportional to the degree of oxidative burst in patients with atopic asthma.

PMID: 8081536 [PubMed - indexed for MEDLINE]

2105. Allergy. 1994;49(18 Suppl):15-20.

The effects of patterns in climate and pollen abundance on allergy.

Emberlin J.

Pollen Research Unit, London, UK.

Recent climatic trends in Europe have been examined in the context of longer term variations and vegetation zonation. The most recent computer models of future climatic changes resulting from increasing carbon dioxide emissions are discussed in relation to the possible impacts that the predicted climatic shifts may have on the distribution and abundance of the main allergenic pollen types. The probable repercussions of these changes on the patterns and incidences of seasonal allergic rhinitis are considered. This study focuses on pollen from Betula, the Poaceae, Ambrosia, Parietaria and Olea, but also examines the implications of climatic change for other aeroallergens, including those from various crops such as Brassica species (oil-seed rape) and Helianthemum (sunflowers). In the case of natural distributions, the response of the plants to climatic changes are viewed in relation to the potential migration rates of the species. For agricultural crops, shifts in the location of productive areas involve agricultural economics both at the national and European community

levels. In addition to broad regional impacts, this study examines the influence of increasing ultraviolet radiation on pollen production over various areas of Europe, and considers the effect of changing airflow trajectories on the long-range transport of pollen with pollution from central and northern Europe into Scandinavia.

PMID: 8053536 [PubMed - indexed for MEDLINE]

2106. Klin Lab Diagn. 1994;(1):13-5.

[Clinical and laboratory assessment of interactions between poly- and mononuclear phagocytes in inflammatory diseases of the skin].

[Article in Russian]

Adamenko GP, Kozin VM.

Interactions between polymorphonuclear leukocytes (PMNL) and monocytes are changed in inflammatory diseases of the skin; these changes manifest by enhanced stimulation of PMNL migration under the effect of monocytes in psoriasis and by depression of this migration in eczema. In psoriasis HLA-DR-antigens and endogenic monocyte prostaglandins participate in this modulation of cooperative interactions between phagocytic cells, whereas in eczema PMNL superoxide anion takes part in this process. These data may be useful in the diagnosis of inflammatory diseases of the skin and disclose new aspects in the pathogenesis of psoriasis and eczema connected with phagocytic system cells.

PMID: 8032720 [PubMed - indexed for MEDLINE]

2107. Dermatology. 1994;189(1):58-61.

Patch test reactions to the acetone-soluble fraction of human dander in atopic dermatitis.

Sasaki K, Sugiura H, Uehara M.

Department of Dermatology, Shiga University of Medical Science, Otsu, Japan.

An acetone-soluble fraction of human dander provoked positive patch test reactions in 45 (38%) of 120 patients with atopic dermatitis. The positive patch tests were rare in normal controls and clinical controls. The positive patch test reactions histologically showed spongiosis and mononuclear cell migration in the epidermis, edema and mononuclear cell infiltrate in the upper dermis. Scratch tests with the acetone-soluble human dander fraction were positive in only 2 (2%) of the 120 patients examined. It was suggested that in a considerable number of patients with atopic dermatitis, an acetone-soluble fraction of human dander provokes a delayed-type skin reaction without inducing an immediate-type skin reaction.

PMID: 8003789 [PubMed - indexed for MEDLINE]

2108. Ter Arkh. 1994;66(7):81-3.

[The manifestation of immune reactions and the chromium content of the blood in patients with occupational allergic dermatoses].

[Article in Russian]

Pavlovskaia NA, Somov BA, Iatsyna IV.

One of the mechanisms responsible for occupational dermatoses of constructors in contact with Cr-containing substances is supposed to be antibodies production as a result of Cr ion migration in the blood. The authors' measurements led them to the conclusions that no significant rise in blood Cr content occurs with an increase in the service duration, there is a close relationship between Cr blood concentrations and specific skin tests as well as between blood Cr and allergic manifestations. In patients with initial and established forms of occupational dermatosis the number of positive responses to Cr skin tests and frequency, intensity of allergic reactions grow with elevation of Cr concentrations in the blood. It is inferred that the test for blood chromium may serve an additional criterium of early diagnosis of occupational dermatosis.

PMID: 7985140 [PubMed - indexed for MEDLINE]

2109. Pneumonol Alergol Pol. 1994;62 Suppl 2:16-20.

[Activation and migration of eosinophils in patients with digestive tract ulcers sensitized to food allergens].

[Article in Polish]

Bartuzi Z, Zbikowska-Gotz M, Staszyńska M, Romański B.

Katedry i Kliniki Alergologii i Chorób Wewnetrznych, Akademii Medycznej, Bydgoszczy.

PMID: 7894363 [PubMed - indexed for MEDLINE]

2110. Rev Mal Respir. 1994;11(6):593-5.

[Acute eosinophilic pneumonia and the larva migrans syndrome: apropos of a case in an adult].

[Article in French]

Bouchard O, Arbib F, Paramelle B, Brambilla C.

Clinique de Pneumologie, CHU de Grenoble.

Toxocariasis is a frequent disease in children, but the severe clinical manifestations are rare in the literature (diffuse interstitial pneumonia with hypoxaemia and acute severe asthma). The diagnosis is made thanks to the reliability of serological techniques (the ELISA test and using antigen excretion-secretion tests of the larvae of Toxocara canis). The authors report a case of acute severe eosinophilic pneumonia whose outcome was rapidly favourable following steroid therapy; the existence of positive Toxocara canis serology with a contamination risk of the patient in the domestic environment leads us to integrate the clinical picture into the larva migrans syndrome.

PMID: 7831510 [PubMed - indexed for MEDLINE]

2111. Eur J Obstet Gynecol Reprod Biol. 1993 Dec 30;52(3):217-8.

Allergic vasculitis induced by Decapeptyl: confirmation by macrophage migration

inhibition factor (MIF) test.

Amichai B, Grunwald MH, Halevy S.

Department of Dermatology, Soroka Medical Center of Kupat Holim, Ben-Gurion University of the Negev, Beer-Sheva, Israel.

A case of allergic vasculitis of the skin following treatment with Decapeptyl (D-Trp6-LHRH) for in vitro fertilization is described. The possible role of an allergic mechanism in this reaction has been suggested by a positive macrophage migration inhibition factor (MIF) test toward the drug.

PMID: 8163039 [PubMed - indexed for MEDLINE]

2112. Clin Exp Allergy. 1993 Dec;23(12):1027-36.

Interleukin-8 is a chemo-attractant for eosinophils purified from subjects with a blood eosinophilia but not from normal healthy subjects.

Sehmi R, Cromwell O, Wardlaw AJ, Moqbel R, Kay AB.

Department of Allergy and Clinical Immunology, National Heart and Lung Institute, London, UK.

Interleukin-8 (IL-8), a pro-inflammatory cytokine with potent neutrophil chemotactic activity, was studied for its effect on eosinophil migration responses, in vitro. Normal density eosinophils were isolated from healthy, non-atopic subjects (<0.35 x 10(9) eosinophils/l) and individuals with various diseases associated with a blood eosinophilia (range 0.56 x 10(9)-12.2 x 10(9) eosinophils/l). IL-8 produced a dose-dependent migrational response for eosinophils from subjects with an eosinophilia, optimal at 10(-8) M (P < 0.01) and the major component of the migrational response was chemokinesis. On a molar basis, IL-8 (EC50 approximately 10(10) M) was 100-fold more potent than platelet activating factor (PAF), although a comparison of the migrational responses showed that at optimal concentrations IL-8 (10(-8) M) produced only 30% maximal responses stimulated by PAF (10(-6) M). In contrast, IL-8 tested over a wide concentration range had a negligible effect on eosinophils from normal subjects. A direct correlation between the total blood eosinophil counts for all subjects and the absolute magnitude of the migrational response to IL-8 (r = 0.727, P < 0.01 at 10(-8) M), PAF (r = 0.551, P < 0.03 at 10(-6) M) and N-formyl-methionyl-leucyl-phenylalanine (fMLP) (r = 0.689, P < 0.02 at 10(-8) M), suggested that heightened eosinophil migrational responses to inflammatory mediators may arise as a consequence of in vivo priming mechanism(s) associated with the development of an eosinophilia. In this regard, eosinophils derived from human cord blood mononuclear cells cultured in the presence of eosinophilopoietic cytokines IL-3 and IL-5, produced migrational responses to IL-8 and PAF, that were comparable with that of eosinophils from eosinophilic subjects. Furthermore, incubation of eosinophils from normal donors with IL-5 (optimal concentration 10(-9) M), significantly enhanced the subsequent migrational responses to both IL-8 (10(-8) M, P < 0.01) and PAF (10(-8) M, P < 0.05). Therefore, the increased responsiveness of eosinophils from eosinophilic subjects may reflect in vivo priming by IL-5 and this phenomenon may contribute partly to the mechanism(s) by which eosinophils preferentially accumulate at sites of allergic inflammation.

PMID: 10779297 [PubMed - indexed for MEDLINE]

2113. Laryngorhinootologie. 1993 Dec;72(12):585-9.

[The role of pro-inflammatory cytokines in recruiting inflammatory cells in the nose].

[Article in German]

Bachert C, Ganzer U.

Hals-Nasen-Ohrenklinik, Medizinische Einrichtungen, Universität Düsseldorf.

Cytokines and cell adhesion receptors play a pivotal role in the recruitment of cells from the peripheral blood into inflamed tissue. Allergic rhinitis has previously been described as an inflammatory reaction characterised by the migration of granulocytes into the nasal mucosa. Using this model, we investigated the release of proinflammatory cytokines (interleukin IL-1 beta, IL-6, IL-8 and tumour necrosis factor-alpha TNF-alpha) and the expression of cell adhesion molecules (ELAM-1, ICAM-1 and LFA-1) in two studies involving biopsies as well as lavage and brush techniques. IL-1 beta and TNF-alpha can be found rapidly after allergen exposure and seem to initiate the cellular infiltration. The release of the chemokine IL-8 correlates with the continuously increasing number of granulocytes on the mucosal surface. Allergic rhinitis subjects showed significantly increased secretion levels of the proinflammatory cytokines IL-1 beta and IL-6 and of the chemokine IL-8. These findings correspond to a higher expression of the adhesion receptors ELAM-1, ICAM-1 and LFA-1 in allergic mucosa. We conclude that proinflammatory cytokines regulate the cell infiltration by the induction of adhesion receptor expression.

PMID: 7511383 [PubMed - indexed for MEDLINE]

2114. Arzneimittelforschung. 1993 Dec;43(12):1331-7.

Antiallergic profile of the novel H1-antihistaminic compound levocabastine.

Tasaka K, Kamei C, Akagi M, Mio M, Shirasaka T, Chokki M.

Department of Pharmacology, Faculty of Pharmaceutical Sciences, Okyama University, Japan.

Levocabastine hydrochloride (R50 547, CAS79516-68-0) caused no inhibitory effect on the histamine release from rat peritoneal mast cells induced by compound 48/80, A23187 and concanavalin A. However, the drug inhibited histamine release from passively sensitized mast cells and passive peritoneal anaphylaxis in rats, though higher concentrations or doses were required. Moreover, levocabastine provided a relatively potent inhibitory effect on histamine release from lung pieces of actively sensitized guinea pigs exposed to antigen, and simultaneously the drug prevented a decrease in the cyclic AMP (cAMP) content. Levocabastine potently inhibited histamine-induced cutaneous reactions in rats and the drug also prevented histamine-induced contraction of isolated guinea pig ileum. Levocabastine did not induce any significant changes in platelet aggregation or in the contraction of guinea pig ileum induced by platelet activating factor (PAF). However, the drug inhibited eosinophil migration induced by PAF. The chemotaxis of neutrophils induced by N-formyl-methionyl-leucylphenylalanine (fMLP) was also inhibited by levocabastine in a dose-dependent fashion. Levocabastine has no influence on the order parameter tested with liposomes, suggesting that the drug provides no significant effect on the membrane fluidity of lipid bilayer. These results seem to indicate that the antiallergic effect of levocabastine is mainly dependent on its potent antihistaminic activity.

PMID: 7511378 [PubMed - indexed for MEDLINE]

2115. Am Rev Respir Dis. 1993 Dec;148(6 Pt 2):S75-8.

Adhesion molecules in allergic inflammation.

Smith CH, Barker JN, Lee TH.

Department of Dermatology, Guy's Hospital, London, United Kingdom.

Allergic inflammation is characterized by recruitment of specific leukocyte subpopulations from blood into tissue and requires a series of cell adhesion-molecule-mediated interactions between postcapillary vascular endothelium and the leukocyte cell surface. Three major groups are involved: selectins, integrins, and the immunoglobulin gene superfamily. P- and E-selectin mediate initial leukocyte adhesion, whereas beta 2-integrin/ICAM-1 and VLA-4/VCAM-1 pathways mediate leukocyte arrest and transendothelial migration. Because VLA-4 expression is restricted to eosinophils and lymphocytes, VCAM-1 has been implicated in selective eosinophil recruitment characterizing allergic inflammation. However, additional factors such as profile of cytokine release are likely to operate since tissue eosinophilia has been observed in the absence of VCAM-1 expression. Recent use of monoclonal antibodies against functional epitopes on various cell adhesion molecules in animal models of extrinsic allergic asthma offers new possibilities in management of allergic disease.

PMID: 7504897 [PubMed - indexed for MEDLINE]

2116. J Immunol. 1993 Nov 15;151(10):5624-30.

Regulation of eosinophil migration by adult T cell leukemia-derived factor.

Hori K, Hirashima M, Ueno M, Matsuda M, Waga S, Tsurufuji S, Yodoi J.

Institute for Virus Research, Kyoto University, Japan.

Adult T cell leukemia-derived factor (ADF), originally defined as an IL-2 receptor alpha-chain (IL-2R alpha)/p55 (Tac) inducer, is a human thioredoxin homologue and has many cytokine-like activities. In this study, we examined the regulatory effect of ADF on eosinophil migration using human eosinophils and an eosinophilic subline of HL-60 human promyelocytic leukemia cells, YY-1. rADF induced migration of eosinophils from patients with hypereosinophilia, although rADF exhibited little activity on eosinophils from healthy donors. When human eosinophils were incubated with rADF (0.1-10 micrograms/ml) at 37 degrees C for 24 h, both chemotactic and chemokinetic activity of the complement anaphylatoxin peptide C5a on eosinophil migration was markedly enhanced in a dose-dependent manner. Similarly, this enhancing effect of rADF was observed in the migration assay using YY-1 cells. In contrast, rADF showed no modulation of migratory behavior of human eosinophils and YY-1 cells by IL-3, IL-5, nor granulocyte-macrophage colony-stimulating factor. Scatchard analysis of C5a receptors on YY-1 cells using 125I-C5a showed that rADF modulated neither the density nor the affinity of the cell membrane significantly. Furthermore, mutant ADF (mADF), which had no reducing activity, had no enhancing effect on C5a-induced eosinophil migration. These results indicate a possible involvement of ADF in the recruitment of eosinophils through redox regulation by a dithiol reductase activity.

PMID: 8228251 [PubMed - indexed for MEDLINE]

2117. J Lab Clin Med. 1993 Nov;122(5):567-75.

Interleukin-4 induces tissue eosinophilia in mice: correlation with its in vitro capacity to stimulate the endothelial cell-dependent selective transmigration of human eosinophils.

Moser R, Groscurth P, Carballido JM, Bruijnzeel PL, Blaser K, Heusser CH, Fehr J.

Department of Medicine, University of Zurich, Switzerland.

Interleukin-4 (IL-4) has been shown to play a crucial role in the pathogenesis of allergic disease. In this study, intraperitoneal administration of IL-4 in mice led to selective accumulation of eosinophils, and intradermal injection induced marked eosinophil infiltration. However, IL-4 had no detectable effect on neutrophil accumulation. Preincubation of mouse IL-4 with the neutralizing mAb 11B11 abolished this peritoneal and dermal eosinophilia. These in vivo data correlate with the in vitro capacity of IL-4 to specifically promote the selective transendothelial migration of eosinophils. Supernatants of antigen-stimulated T cell clones derived from hypersensitized individuals induced significant eosinophil transmigration that was inhibited by the neutralizing mAb 8F12 against human IL-4. These experiments impressively demonstrate a link between specific antigenic recognition and the selective recruitment of eosinophils by the endothelial barrier. Furthermore, data are presented supporting our previous evidence that eosinophils need initial priming to transmigrate across IL-4-activated monolayers. Whereas freshly isolated eosinophils from nonallergic individuals failed to transmigrate, the eosinophils from a group of patients with allergic asthma showed spontaneous layer penetration. These data further support the evidence that eosinophils from allergic patients undergo in vivo priming and are functionally different with respect to their capacity to transmigrate.

PMID: 8228575 [PubMed - indexed for MEDLINE]

2118. J Investig Allergol Clin Immunol. 1993 Nov-Dec;3(6):321-9.

The effect of granulocyte factor and grass pollen allergen on T-lymphocytes from atopic patients in vitro.

Kocur E, Zeman K, Tchorzewski H.

Department of Pathophysiology & Immunology, Institute of Basic Medical Sciences, Military Medical Academy, Lódź, Poland.

In allergy the immune response is significantly modified by inflammatory processes. Polymorphonuclear leukocytes (PMNLs) are involved in inflammatory processes. Activated PMNLs release many substances, including granulocyte factor (GF), which exerts immunomodulating effects. The present study was performed to determine the effects of allergens and/or GF on the expression of lymphocyte differentiation antigens in short-term cultures and to evaluate the production of migration inhibitory factor (MIF) under the influence of these substances. The studies were carried out on peripheral blood mononuclear cells isolated from patients with type I hypersensitivity, before and after the grass pollen season, and from healthy subjects. GF and allergens were found to increase the CD8 cell number, particularly in 7-day cultures and in patients before exposure to allergens, which correlated with MIF release in these patients under the influence of these factors. The results suggest that the PMNLs may participate in allergic inflammatory reactions.

PMID: 8012650 [PubMed - indexed for MEDLINE]

2119. J Immunol. 1993 Oct 15;151(8):4371-82.

Cytokines and adhesion molecules contribute to the ability of myelin proteolipid protein-specific T cell clones to mediate experimental allergic encephalomyelitis.

Kuchroo VK, Martin CA, Greer JM, Ju ST, Sobel RA, Dorf ME.

Department of Pathology, Harvard Medical School, Boston, MA.

We have derived a panel of CD4+, TCR-alpha/beta + T cell clones from SJL (H-2s) mice specific for an encephalitogenic determinant of myelin proteolipid protein (PLP) 139-151 (HSLGKWLGHPDKF). All the clones are Ag specific and IAs restricted, but they show heterogeneity in their ability to induce experimental allergic encephalomyelitis (EAE), i.e., one group induces EAE in naive mice, a second group induces disease only in mice that are pretreated with pertussis and irradiation, whereas a third group is essentially nonencephalitogenic. To determine the basis for this functional heterogeneity, the clones were tested for the expression of adhesion molecules and cytokines and for Ag-specific cytolytic activity. All of the clones expressed comparable levels of LFA-1 and CD44 but lacked expression of Mel 14. However, those clones that induced EAE only in irradiation- and pertussis-treated recipients did not express VLA4. Because pretreatment with pertussis has been suggested to increase permeability of the blood-brain barrier and facilitate migration of T cells into the central nervous system, the absence of VLA4 on this group of clones may account for the need for pretreatment to induce EAE. The nonencephalitogenic clones expressed all of the adhesion molecules tested but were not cytolytic in vitro and failed to produce one or more of the proinflammatory cytokines after Ag-specific stimulation. One nonencephalitogenic clone that did not produce many cytokines on activation with specific Ag, however, could be activated with Con A to express mRNA for most cytokines and this was accompanied by a concomitant change in the encephalitogenic potency of this clone. These results suggest that adhesion molecules and cytokines both play a critical role in the encephalitogenicity of PLP peptide-specific T cell clones. Furthermore, the nonencephalitogenicity of some clones may be related to a defect in Ag-mediated activation.

PMID: 7691946 [PubMed - indexed for MEDLINE]

2120. Brain. 1993 Oct;116 (Pt 5):1043-58.

Suppression of experimental allergic neuritis by an antibody to the intracellular adhesion molecule ICAM-1.

Archelos JJ, Mäurer M, Jung S, Toyka KV, Hartung HP.

Department of Neurology, Julius-Maximilians-Universität Würzburg, Germany.

Experimental allergic (autoimmune) neuritis (EAN) was induced in Lewis rats either by inoculation with bovine spinal root myelin or injection of neuritogenic P2-specific T cells. Injection of a purified monoclonal antibody (1A-29) to the intercellular adhesion molecule-1 (ICAM-1) prevented or transiently suppressed myelin-induced EAN depending on the timing of antibody application. Administration of 1A-29 suppressed moderate adoptive transfer EAN (AT-EAN) but not severe AT-EAN. In contrast, treatment with phosphate buffered saline or an unrelated IgG1 had no effect on the course of the disease. Histological sections of the peripheral nervous system (PNS) showed a marked reduction of inflammatory infiltrates and perivascular demyelination in rats injected with 1A-29. The effect of 1A-29 on the concanavalin A (Con A)- and P2-dependent proliferation of

neuritogenic P2-specific T cells was studied in vitro. Our data suggest that antibodies to ICAM-1 act on the induction and effector phase of the immune response by inhibiting both early interactions between immunocompetent cells after exposure to foreign antigen and transendothelial migration of primed T cells into the peripheral nerve. Treatment with antibodies to leucocyte adhesion molecules could be a useful therapeutic approach to autoimmune disease of the PNS.

PMID: 7693297 [PubMed - indexed for MEDLINE]

2121. J Allergy Clin Immunol. 1993 Sep;92(3):412-24.

Bronchial epithelial cells of patients with asthma release chemoattractant factors for T lymphocytes.

Bellini A, Yoshimura H, Vittori E, Marini M, Mattoli S.

Diagnostic Center for Respiratory and Allergic Diseases, Institute of Experimental Medicine, Milan, Italy.

BACKGROUND: T lymphocytes may orchestrate the inflammatory response in atopic asthma, but the mechanisms that promote T-cell accumulation in asthmatic airways are still unclear. In this study, we tested the hypothesis that bronchial epithelial cells of patients with atopic asthma release chemoattractant factors for T lymphocytes.
METHODS: Sixteen patients with atopic asthma and eight healthy control subjects were selected for this study. Bronchial epithelial cells were isolated from biopsy specimens obtained by means of bronchoscopy and cultured for 48 hours in serum- and hormone-free medium, with or without 10(-6) mol/L histamine.
RESULTS: Only the supernatants of cells from donors with asthma showed chemotactic activity for T lymphocytes, and this was significantly increased ($p < 0.025$) by exposure to histamine. Chemotactic activity was in part mediated by interleukin-8 (IL-8), because an antibody against human IL-8 significantly reduced it ($p < 0.05$) and the cell supernatants contained appreciable amounts of immunoreactive IL-8 (0.89 +/- 0.39 ng/ml). Both the residual chemotactic activity of unstimulated epithelial cells and the increased activity caused by histamine were mediated by a single protease-sensitive substance with an apparent molecular weight of 56,000 d and an estimated isoelectric point of 8.8 to 9.1. The partially purified chemoattractant specifically enhanced the migration of CD4+ T lymphocytes, and its activity was inhibited by the univalent Fab fragment of a monoclonal antibody against CD4.
CONCLUSION: These results extend our previous observations, indicating an important effector role of bronchial epithelium in asthma.

PMID: 8360392 [PubMed - indexed for MEDLINE]

2122. J Pediatr Surg. 1993 Sep;28(9):1140-3.

Giant hydatid lung cysts in the Canadian northwest: outcome of conservative treatment in three children.

Lamy AL, Cameron BH, LeBlanc JG, Culham JA, Blair GK, Taylor GP.

Department of Cardiovascular and Thoracic Surgery, British Columbia Children's Hospital, Vancouver, Canada.

Hydatid lung disease due to Echinococcus granulosus in the Canadian northwest and Alaska is often asymptomatic and usually benign. We reviewed the course and

outcome of three children with giant hydatid lung cyst seen over a 2-year period. All were North American Indian children aged 9 to 12 years who presented with cough, fever, and chest pain. One had a rash. There was a history of exposure to domestic dogs who had been fed moose entrails in each case. Chest x-rays showed solitary lung cysts with air-fluid levels, from 6 cm to 12 cm in diameter. Aspiration of each cyst demonstrated Echinococcus hooklets and protoscolices. Serology was unhelpful, being negative in two cases. Transient pneumonitis and pneumothorax were seen as complications of needle aspiration. Two cysts gradually resolved over the following 6 months. One child returned after 9 months with a lung abscess due to superimposed infection of the cyst remnant with Haemophilus influenzae, and eventually required lobectomy. The existence of an endemic benign variant of E granulosus in Canada is not widely known, and it is important to distinguish it from the more aggressive pastoral form of the disease seen in immigrants from sheep-rearing countries. The native Canadian disease usually resolves spontaneously, does not cause anaphylaxis, and does not implant daughter cysts if spilled. Surgical treatment should be avoided except for complications such as secondary bacterial infection.

PMID: 8308679 [PubMed - indexed for MEDLINE]

2123. J Investig Allergol Clin Immunol. 1993 Sep-Oct;3(5):259-64.

Granulocyte migration in vivo and in vitro in healthy children of parents with aspirin-sensitive asthma.

Matusiewicz R, Grzybowska K, Grzybowski A, Urbankowska B.

First Department of Internal Diseases, Grochow Hospital, Warsaw, Poland.

The test of granulocyte migration in vitro and in vivo was carried out in 30 healthy children who had at least one parent with aspirin-sensitive asthma. A defect in granulocyte migration was demonstrated in children with both parents having aspirin-sensitive asthma.

PMID: 8298750 [PubMed - indexed for MEDLINE]

2124. J Investig Allergol Clin Immunol. 1993 Sep-Oct;3(5):237-44.

Abnormal migratory activity of peripheral neutrophils from asthmatic patients and its modulation by inhaled glucocorticoids.

Paggiaro PL, Dente FL, Azzarà A, Bancalari L, Bernard P, Bacci E, Carrara M, Di Franco A, Giannini D, Vagaggini B, et al.

2nd Institute of Internal Medicine, University of Pisa, Italy.

In order to investigate if bronchial asthma is associated with enhanced markers of activation in peripheral neutrophils, the migratory capacity of neutrophils in venous blood was measured by means of the Boyden chamber technique in 29 subjects with bronchial asthma of differing severity. Random migration (random motility), but not locomotion toward 10% Escherichia coli supernatant as chemoattractant (chemotaxis), was increased in asthmatic subjects with respect to 11 normal subjects (98 +/- 20 microns vs. 85 +/- 6 microns; $p < 0.05$). When asthmatic subjects were subdivided into groups of different disease severity, subjects with mild and mild to moderate asthma showed significantly higher values for random motility and chemotaxis than normal subjects; on the other hand, subjects with more severe disease showed the lowest values for migratory activity. No correlation was found between migratory activity and clinical findings of asthma,

except for baseline FEV1 (% of the predicted value), which showed a slight but significant positive correlation with chemotaxis ($r = 0.44$, $p < 0.05$). Subjects with atopic or occupational asthma had higher values for migratory activity than subjects with nonallergic asthma. Thirteen asthmatic subjects repeated all evaluations after 1 month of treatment with high doses of inhaled glucocorticoids (beclomethasone dipropionate 1500 micrograms/day). Random motility (95 +/- 24 microns vs. 75 +/- 15 microns; $p < 0.05$) and chemotaxis (130 +/- 22 microns vs. 105 +/- 25 microns; $p < 0.05$) were significantly reduced after treatment, as well as the symptom score; on the other hand, symptom score but not bronchial hyperresponsiveness to methacholine challenge significantly changed.(ABSTRACT TRUNCATED AT 250 WORDS)

PMID: 8298747 [PubMed - indexed for MEDLINE]

2125. Infect Dis Clin North Am. 1993 Sep;7(3):717-32.

Zoonotic roundworm infections.

Glickman LT, Magnaval JF.

Department of Veterinary Pathobiology, Purdue University, West Lafayette, Indiana.

The larval stage of several animal parasites can infect humans and produce severe disease. Visceral and ocular larval migrans caused by the common dog roundworm, Toxocara canis, are two well-recognized clinical syndromes. With the wider availability of serodiagnostic tests for toxocaral infection, other syndromes characterized by neuropsychologic deficits, epilepsy, asthma, abdominal distress, and chronic allergy have been described. Treatment with corticosteroids in conjunction with anthelminthic drugs may be life- or sight-saving. Recognition of the risk factors for infection is key to prevention, reinfection, and more serious illness.

PMID: 8254168 [PubMed - indexed for MEDLINE]

2126. Cell Adhes Commun. 1993 Sep;1(2):161-76.

Eosinophil adhesion and maturation is modulated by laminin.

Tourkin A, Anderson T, LeRoy EC, Hoffman S.

Medical University of South Carolina, Division of Rheumatology, Charleston 29425.

Eosinophils (Eo) participate in the inflammatory response to parasites, allergins, toxins, and epitopes recognized by autoimmune antibodies. Nonetheless, little attention has heretofore been paid to the interactions of Eo with extracellular matrix (ECM) proteins during their migration through the subendothelial basement membrane and into the surrounding tissue. Therefore, we have studied the adhesion of Eo to specific ECM proteins and the effect of this adhesion on Eo viability and maturation. Control Eo (from normal donors) adhere no better to substrates coated with laminin (LM), fibronectin (FN), cytotactin (CT), or collagen types I or IV (Col IV) than they do to human serum albumin coated substrates. In contrast, Eo activated in vitro with IL-5 or in vivo in patients with eosinophilia bind well to LM, FN and Col IV. LM is by far the most avid ligand among these molecules. For example, 43% of input cells bind to a substrate bearing 200 fmol/cm2 of LM; a similar level of adhesion to FN requires 30 times as much absorbed protein. Antibody inhibition experiments suggest that the alpha 6 beta 1 integrin heterodimer is the predominant LM receptor on these

cells. Flow cytometry showed similar levels of these subunits on control and activated Eo, suggesting that Eo adhesion to LM is not regulated simply by cell surface integrin concentration. The effects of ECM proteins on Eo behavior were also examined. A LM-coated substrate (with no added cytokine) was found to be almost as effective as IL-5 in maintaining Eo viability while an equally adhesive FN-coated substrate had much less effect. Normally, even in the presence of 10% serum, no Eo survive a 5-day incubation in vitro unless IL-3, IL-5, or GM-CSF is added to the medium. Conditions that inhibit adhesion to LM (anti-integrin antibodies in the medium or CT on the substrate) and certain anti-cytokine antibodies inhibited the promotion of Eo viability by LM. During incubation on LM, Eo become hypodense, as they do in the presence of IL-5, indicating that they have become activated. These observations suggest that the interactions of Eo and ECM proteins may be important both for their potential to direct Eo migration and for their ability to regulate Eo viability, cytokine production, and maturation.

PMID: 8081878 [PubMed - indexed for MEDLINE]

2127. Nihon Rinsho. 1993 Aug;51(8):1993-7.

[Platelet functions in atherosclerosis].

[Article in Japanese]

Matsumoto W, Maruyama I.

Department of Internal Medicine (III), Kagoshima University School of Medicine.

Recent advances on platelet functions involved in atherosclerosis and subsequent thromboembolic diseases are reviewed. Platelets show multiple roles in hemostasis/thrombosis, wound healing, allergy, inflammation, metastasis of malignant cells and vasospasms through aggregation/secretion reaction. In atheromatous plaque, migration and proliferation of vascular smooth muscle cells and macrophages are the most prominent findings. In this pathological state, platelet may play as an enhancer by the secretion of bioactive substances including platelet-derived growth factor, serotonin and platelet factor 4. Investigation on platelet dynamics must be carried out not only for monitoring but also for the assessment of progressive factors in atherosclerotic disease.

PMID: 8411662 [PubMed - indexed for MEDLINE]

2128. Behring Inst Mitt. 1993 Aug;(92):178-83.

Eosinophil adhesion receptors.

Wardlaw AJ.

Department of Respiratory Medicine, Glenfield General Hospital, Leicester, Great Britain.

Eosinophilic inflammation is often characterized by a paucity of tissue neutrophils. A possible explanation of this selective accumulation is utilization of different adhesion pathways by the two cell types. Eosinophil adhesion in vitro to unstimulated HUVEC is selectively enhanced by IL-5 and IL-3. This pathway appears to be Mac-1 dependent. At sites of chronic inflammation an array of adhesion molecules are likely to be induced on venular endothelium. Eosinophils, like other leukocytes, can potentially utilize all three selectin adhesion receptors as well as the immunoglobulin family member ICAM-1. In addition, unlike neutrophils, eosinophils express VLA-4 and can use the

VLA-4/VCAM-1 pathway. In vitro IL-4 selectively upregulates VCAM-1 on HUVEC and promotes eosinophil transmigration via VCAM-1. However, bronchial biopsies of both asthmatics and controls revealed strong expression of E-selectin and ICAM-1 with very weak expression of VCAM-1. This would suggest that, despite the in vitro findings, VCAM-1 is not involved in eosinophil migration into the airways in chronic asthma.

PMID: 8250810 [PubMed - indexed for MEDLINE]

2129. J Immunol Methods. 1993 Jul 6;163(1):49-58.

A rapid biochemical method for measuring antigen-induced pulmonary eosinophil margination in allergic guinea pigs.

Tagari P, Black C, Marshall S, Ford-Hutchinson AW.

Department of Pharmacology, Merck Frosst Centre for Therapeutic Research, Pointe Claire-Dorval, Quebec, Canada.

The ability of purified guinea pig peritoneal eosinophils (EOS) to oxidise 3,3',5,5'-tetramethylbenzidine (TMB) was assessed in the presence/absence of Br- (3 mM), and compared with that of unpurified elicited peritoneal polymorphonuclear leukocytes (PMN). Br- selectively stimulated EOS peroxidase activity in a cell number-dependent manner, which was not significantly affected by the presence of diluted lung homogenate. By comparison with the peroxidase activity of added purified EOS, lung parenchyma homogenate from naive guinea pigs was estimated to contain 1.04 +/- 0.18 x 10(5) cells/mg wet tissue (n = 6), a value comparable to those calculated from published histological analyses. This was not significantly increased by ovalbumin (OA) allergen inhalation in unsensitised guinea pigs (1.4 x 10(5) EOS/mg), but was increased two-fold over the latter control to 3.0 +/- 0.18 x 10(5) cells/mg after 17 h in animals sensitised by a single injection of OA and subsequently exposed to an aerosol of bronchoactive allergen (n = 13, $p < 0.05$). Similar results were obtained in a parallel study using bronchoalveolar lavage (saline challenge, 20.2 +/- 2.2% EOS in lavage fluid; OA challenge, 47.1 +/- 3.6% EOS; n = 6, $p < 0.05$). In animals that had been doubly sensitised (two injections) to OA, the pulmonary eosinophilic response measured biochemically was more pronounced (4.9 +/- 0.2 x 10(5) cells/mg) and was significantly greater than both a non-specific protein inhalation in this sensitisation group, and OA inhalation in singly sensitised animals (n = 12, $p < 0.05$). Sera from the latter group was shown to contain five times less specific anti-OA IgG than the doubly sensitised animals, suggesting that EOS margination in guinea pigs is proportionate to the animals' immune status for a defined immunological challenge. These data demonstrate that in vivo EOS migration into the whole guinea pig lung can be rapidly determined by biochemical methods, and thus facilitate the in vivo assessment of novel therapeutic agents against the eosinophilic inflammation characteristic of human allergic asthma.

PMID: 8335959 [PubMed - indexed for MEDLINE]

2130. Toxicol In Vitro. 1993 Jul;7(4):295-8.

Epidermal cytokines in contact hypersensitivity: Immunological roles and practical applications.

Kimber I.

Zeneca Central Toxicology Laboratory, Alderley Park, Macclesfield, Cheshire SK10

4TJ, UK.

Local trauma and topical exposure to sensitizing chemicals induces the production by epidermal cells of a variety of cytokines including interleukins 1 (alpha and beta), 6, 8 and 10 (IL-1alpha, IL-1beta, IL-6, IL-8 and IL-10), granulocyte/macrophage colony-stimulating factor and tumour necrosis factor alpha. There is mounting evidence that keratinocyte-derived cytokines play a role of some importance in cutaneous immune responses and the development of contact sensitization. The induction phase of contact sensitization is dependent upon the action of epidermal Langerhans cells (LC). Following epicutaneous exposure to skin sensitizing chemicals LC are induced to migrate via afferent lymphatics to the draining lymph nodes. During migration, or shortly following arrival in the nodes, LC acquire the characteristics of lymphoid dendritic cells. This functional maturation is accompanied by elevated expression of MHC class II (Ia) antigens and of intercellular adhesion molecule-1 (ICAM-1). Increased expression of ICAM-1 and Ia facilitates the interaction with, and stimulation of, T lymphocytes by dendritic cells. The role epidermal cytokines play in this process is described. Characterization of the role epidermal cytokines play in regulating LC function may allow not only clarification of the molecular events which initiate skin sensitization, but also provide opportunities to investigate in vitro the consequences of the interaction of chemical allergens with the skin.

PMID: 20732204 [PubMed]

2131. J Allergy Clin Immunol. 1993 Jul;92(1 Pt 2):159-64.

Effects of nedocromil sodium on in vitro induced migration, activation, and mediator release from human granulocytes.

Bruijnzeel PL, Warringa RA, Kok PT, Hamelink ML, Kreukniet H, Koenderman L.

Department of Pulmonary Disease, State University Hospital Utrecht, The Netherlands.

Using the allergen-induced late-phase asthmatic reaction as a working model, we studied the activity of certain inflammatory cells and their reaction to nedocromil sodium. The processes that were examined in vitro included the following: the chemotaxis of purified neutrophils and eosinophils, the early steps of neutrophil and eosinophil activation, and the release of mediators from these cells. Nedocromil sodium strongly inhibited neutrophil mobilization caused by four chemotactic factors (zymosan activated serum, N-formyl-methionyl-leucyl-phenylalanine platelet-activating factor [PAF], and leukotriene B4 [LTB4] and eosinophil mobilization caused by two factors (PAF and LTB4). In vitro treatment of eosinophils from normal subjects with picomolar concentrations of interleukin-3, interleukin-5, or granulocyte-macrophage colony stimulating factor increased the chemotactic responsiveness toward PAF and LTB4 and induced a chemotactic responsiveness toward N-formyl-methionyl-leucyl-phenylalanine and neutrophil activating factor/interleukin-8. The zymosan activated serum-induced chemotactic responsiveness remained unaltered. Nedocromil sodium inhibited the cytokine-primed chemotactic responsiveness to the various chemotaxins, not the influence of the cytokines on the cells. Activation of granulocytes, as measured by Ca^{2+} influx, was not inhibited by nedocromil sodium. Mediator formation in eosinophils was modified only slightly. These results suggest that inhibiting the mobilization of inflammatory cells in the lung tissue may be an important action of nedocromil sodium. Therefore these effects may be relevant to the treatment of asthma given the role of airway inflammation in this disease process.

PMID: 8393021 [PubMed - indexed for MEDLINE]

2132. Hautarzt. 1993 Jul;44(7):466-9.

["Baboon syndrome". A particular manifestation of hematogenous contact reaction].

[Article in German]

Herfs H, Schirren CG, Przybilla B, Plewig G.

Dermatologische Klinik und Poliklinik, Ludwig-Maximilians-Universität, München.

We report on a 52-year-old patient with a typical manifestation of a haematogenous contact reaction. The characteristic distribution of light-red erythema predominantly located in the major flexural areas of the extremities and on the buttocks was consistent with the diagnosis of "baboon syndrome." The term derives from the skin lesions, which are compared to the red gluteal region of the baboon. An allergic type-IV reaction to systemically administered allergens probably underlies lesions of this type. In our case the baboon syndrome had been induced by amoxicillin. So far, mercury, nickel, ampicillin, and heparin have been reported as causes of baboon syndrome.

PMID: 8365882 [PubMed - indexed for MEDLINE]

2133. Eur Respir J. 1993 Jul;6(7):1044-54.

Leucocyte-endothelial adhesion molecules and their role in bronchial asthma and allergic rhinitis.

Montefort S, Holgate ST, Howarth PH.

Immunopharmacology Group, Southampton General Hospital, UK.

Leucocyte-endothelial adhesion molecules are involved in the initial stages of the recruitment and migration of inflammatory leucocytes from the circulation to sites of inflammation. There is accumulating evidence for their involvement in the pathophysiology of airway mucosal allergic inflammation, such as that found in asthma and rhinitis. The best characterized adhesion molecule families are the integrins, the immunoglobulin supergene family and the selectins. This review article describes some of the characteristics and properties of these families. We also discuss the situations in which these adhesion molecules might be involved in inflammatory airway diseases, and how evidence for this role might lead to future modes of therapy for these common conditions.

PMID: 7690333 [PubMed - indexed for MEDLINE]

2134. Clin Exp Immunol. 1993 Jun;92(3):487-93.

Ultrastructural studies bearing on the mechanism of UVB-impaired induction of contact hypersensitivity to DNCB in man.

Mommaas AM, Mulder AA, Vermeer M, Boom BW, Tseng C, Taylor JR, Streilein JW.

Department of Dermatology, University Hospital Leiden, The Netherlands.

In both murine and human experimental systems, acute, low dose exposure of skin to ultraviolet B light (UVB) impairs the induction of allergic contact dermatitis (ACD) by haptens such as dinitrochlorobenzene (DNCB) in a significant proportion

of individuals. By light microscopy, epidermal Langerhans cells (LC) have been reported to be depleted by UVB exposure as well as by epicutaneous hapten application, implying that LC may be the locus of action of the effects of both UVB and DNCB. However, light microscopy can not readily distinguish cell density changes secondary to LC necrosis from changes resulting from down-modulation of expression of LC surface molecules. Using a highly sensitive immunogold electron microscopic approach, we have evaluated the differential effects of UVB and/or DNCB on human epidermal LC. The results reveal that DNCB alone caused significant up-regulation of cell surface HLA class II expression on a very small number of LC, the major fraction of LC expressing normal levels of HLA class II. Furthermore, DNCB alone caused a modest reduction in the density of LC at the treated sites without evidence of cell necrosis. Treatment with UVB alone or UVB exposure followed by DNCB resulted in a reduction in the density of LC, with widespread evidence of LC necrosis. However, the few remaining intact LC were all intensely HLA class II-positive after UVB exposure followed by DNCB, whereas treatment with UVB alone did not result in changes in LC HLA class II expression. The findings that after DNCB painting only a small proportion of the LC were strongly HLA class II-positive, but after UVB exposure followed by DNCB all intact LC displayed significant up-regulation of cell surface HLA class II expression, imply that UVB exposure inhibits the migration of epidermal LC. This is consistent with the view that DNCB fails to induce ACD when hapten is painted on UVB-exposed skin because insufficient LC are available to initiate T cell activation in the draining lymph node.

PMCID: PMC1554789
PMID: 8513580 [PubMed - indexed for MEDLINE]

2135. Inflammation. 1993 Jun;17(3):353-60.

Leukotriene B4-induced granulocyte trafficking in guinea pig dermis. Effect of second-generation leukotriene B4 receptor antagonists, SC-50605 and SC-51146.

Fretland DJ, Widomski DL, Anglin CP, Penning TD, Yu S, Djuric SW.

Department of ImmunoInflammatory Diseases Research, Skokie, Illinois 60077.

Leukotriene B4 (LTB4) is a proinflammatory product of arachidonic acid metabolism that has been implicated as a mediator in a number of inflammatory diseases. When injected intradermally into the guinea pig, LTB4 elicits a dose-dependent migration (chemotaxis) of neutrophils (PMNs) into the injection sites as assessed by the presence of a neutrophil marker enzyme myeloperoxidase. SC-41930 (7-[3-(4-acetyl-3-methoxy-2-propylphenoxy)propoxy]-3,4-dihydro-8-p ropyl-2H-1-benzopyran-2-carboxylic acid), a first-generation LTB4 receptor antagonist inhibited the chemotactic actions of LTB4 when coadministered into the dermal site and when given orally with ED50 values of 340 ng and 1.7 mg/kg, respectively. The second-generation LTB4 receptor antagonists SC-50605 (7-[3-[2(cyclopropylmethyl)-3-methoxy-4-(4-thiazolyl)phenoxy]propoxy]-3,4-dihydro-8-propyl-2H-1-benzopyran-2-carboxylic acid) and SC-51146 (7-[3-[2(cyclopropylmethyl)-3-methoxy-4-[(methylamino)carbonyl] phenoxy]propoxy]-3,4-dihydro-8-propyl-2H-1-benzopyran-2-propanoic acid) inhibited LTB4-induced chemotaxis when coadministered with ED50 values of 70 ng and 32 ng, respectively, and when given intragastrically with ED50 values of 0.10 and 0.09 mg/kg, respectively. SC-41930, SC-50605, and SC-51146 had oral durations of action of 5.5, 15, and 21 h, respectively. These potent, LTB4 receptor antagonists may well have application in the medical management of disease states such as asthma, rheumatoid arthritis, inflammatory bowel disease, contact dermatitis, and psoriasis, where LTB4 is implicated as an inflammatory mediator.

PMID: 8392493 [PubMed - indexed for MEDLINE]

2136. J Invest Dermatol. 1993 May;100(5):717-20.

Further evidence for Borrelia burgdorferi infection in morphea and lichen sclerosus et atrophicus confirmed by DNA amplification.

Schempp C, Bocklage H, Lange R, Kölmel HW, Orfanos CE, Gollnick H.

Department of Dermatology, University Medical Center Steglitz, Free University of Berlin, Germany.

We present further evidence in support of the notion that Borrelia burgdorferi is possibly involved in the pathogenesis of morphea and lichen sclerosus et atrophicus (LSA). Running a nested polymerase chain reaction (PCR) with a primer set specific for the flagellin gene of B. burgdorferi enabled us to demonstrate the presence of Borrelia DNA in skin biopsies of patients with morphea (nine of nine) of LSA (six of six). Biopsy specimens obtained from patients with erythema chronicum migrans (two patients, four of four samples) and acrodermatitis chronica atrophicans (one patient, one of one sample) also showed positive PCR results. By contrast, there was no amplification of Borrelia DNA in control biopsies either from patients with chronic eczema (three of three) or psoriasis (two of two) or from normal skin (three of three). Antibodies directed against B. burgdorferi were only detected in the serum of patients with erythema chronicum migrans (two of two) and acrodermatitis chronica atrophicans (one of one) but were not present in cases of morphea (five of five), LSA (three of three), or in control subjects (three of three). These data suggest that B. burgdorferi may play a role in the pathogenesis of both morphea and LSA. Furthermore, we conclude that PCR analysis provides an important diagnostic tool, even in seronegative Borrelia infections.

PMID: 8491994 [PubMed - indexed for MEDLINE]

2137. J Immunol. 1993 May 1;150(9):4116-24.

Selective and nonselective stages in homing of T lymphocytes to the central nervous system during experimental allergic encephalomyelitis.

Karin N, Szafer F, Mitchell D, Gold DP, Steinman L.

Department of Neurology and Neurological Sciences, Stanford University School of Medicine, CA 94305-5235.

The diversity of Ag-specific receptors on T cells homing to an inflammatory infiltrate in the central nervous system has been analyzed. Experimental autoimmune encephalomyelitis, a T cell-mediated inflammatory disease of the central nervous system, was induced in Lewis rats with a CD4+, CD8- T cell line specific for peptide 68-86 of myelin basic protein. Within the line a wide array of TCR V beta genes was transcribed including the V beta 8, V beta 10, V beta 15, V beta 16, and V beta 19 families. Accumulation of T cells at the site of inflammation was determined by using RNA-polymerase chain reaction amplification of rearranged TCR V beta transcripts derived from brain. By 8 to 10 h after i.p. infusion of the pathogenic T cell line, TCR V beta transcripts, including mainly V beta families that were predominantly rearranged by the line, could be identified in brains. Restricted TCR V gene transcripts with predominance of the V beta 8 family were identified in brain 48 h after injection, before onset of disease. Paralysis was apparent by 4 to 5 days after injection. At this time diverse V beta gene transcripts were detected in brain, reaching a maximum by day 9, when paralyzed rats have recovered. By day 14 a second stage of limited

heterogeneity in the T cell infiltrate could be identified with predominant expression of V beta 8, V beta 9, V beta 10, and V beta 19. Interestingly, three out of these four V beta families were predominantly expressed within the encephalitogenic line. Thus, T cell migration to brain in experimental autoimmune encephalomyelitis is characterized by a rapid penetration of T cells followed by a selective trapping of T cells before the clinical manifestations of disease. When clinical disease was present the T cell infiltrate was diverse, whereas in the post-acute phase of disease the T cells in the central nervous system had limited heterogeneity with selective accumulation of T cells transcribing the same V regions that were detected in the line that incited disease.

PMID: 8386207 [PubMed - indexed for MEDLINE]

2138. Arch Dermatol. 1993 May;129(5):600-4.

Vitiliginous vs pigmented skin response to intradermal administration of interferon gamma.

Gilhar A, Aizen E, Ohana N, Etzioni A.

Skin Research Laboratory, Bruce Rappaport Faculty of Medicine, Technion-Israel Institute of Technology, Haifa.

Comment in
    Arch Dermatol. 1994 Mar;130(3):387-8.

BACKGROUND AND DESIGN: Decreased sensitization and elicitation of contact allergens in vitiliginous skin has been described. This may be related to altered epidermal Langerhans cell migration with bound hapten to dermis and draining lymph nodes. The aim of the present study was to detect the potential ability of vitiliginous skin to respond to an in vivo immunologic stimulus such as intradermal injections of interferon gamma (IFN-gamma). Vitiliginous and normal pigmented skin of each patient was injected intradermally with 10 micrograms of recombinant IFN-gamma diluted in 0.1 mL of sterile water for 3 consecutive days. On day 5, punch biopsy specimens were obtained from the injected sites. Histologic and immunohistochemical staining was performed on all sections. The cryostat sections were stained with adenosine triphosphatase as well as with the indirect immunoperoxidase technique employing murine monoclonal antibodies to HLA-DR, ICAM-1, CD1, CD11a, and CD18.
RESULTS: HLA-DR and ICAM-1 expression by epidermal cells, combined with perivascular accumulation of mononuclear cells with CD11a and CD18 expression, was observed in all sites injected with IFN-gamma. However, absence of an effect on the epidermal Langerhans cell population was noted only on the vitiliginous skin.
CONCLUSION: The reactivity of depigmented and pigmented skin was found to be different after IFN-gamma administration, with fewer CD1-positive cells in the depigmented skin. As adenosine triphosphatase staining also showed fewer positive cells, it may be concluded that no effect on the migration of epidermal Langerhans cells was noted in the involved skin. This may shed light on the immunologic aberration seen in vitiliginous skin.

PMID: 8097622 [PubMed - indexed for MEDLINE]

2139. Sangyo Igaku. 1993 Mar;35(2):75-87.

[Occupational diseases caused by exposure to sensitizing metals].

[Article in Japanese]

Kusaka Y.

Department of Environmental Health, Jichi Medical School.

Diseases caused by occupational exposure to sensitizing metals including platinum (Pt), rhodium (Rh), nickel (Ni), chromium (Cr), cobalt (Co), gold (Au), mercury (Hg), zirconium (Zr) and beryllium (Be) are reviewed. Allergic reactions induced by the metals are described according to the classification by Coombs and Gell. Metals with unproven sensitizing potential are not discussed if reports on these are either very rare or devoid of convincing evidence for allergic involvement. The sensitizing metals are haptens which are not themselves able to act as antigens. There is evidence that combination of the metals with circulating or tissue protein gives rise to new antigens. An alternative hypothesis is that these metals interfere with the antigen recognition step of the immune response. Immunomodulatory effects or immunotoxicity of the metals may be also involved in metal-induced hypersensitivity. Occupational exposure to Pt, Rh, Ni, Cr, and Co causes allergic asthma via type I allergic reaction in which serum from affected individuals shows specific IgE antibodies against mental-human serum albumin conjugates. Some rheumatoid arthritis patients treated with gold salt therapy develop glomerulonephritis, thrombocytopenia, or agranulocytosis, which arise from type II and/or type III allergic reactions. Occupational exposure to mercury causes glomerulonephritis in which involvement of type III reaction is suggested. Type IV hypersensitivity reaction of the skin also takes place following exposure to the metals: allergic contact dermatitis is evoked by exposure to Ni, Cr, Co, Rh, and Hg; cutaneous granuloma is formed by contact with Zr and Be. Be is also a sensitizer of the lungs, resulting in granulomatous disease. Diagnosis of metal-induced allergic diseases is made on the basis of allergological tests with metal antigens including skin tests, radioallergosorbent test for specific antibody, lymphocyte transformation test, macrophage migration inhibition test, and provocation test. Atopy is a predisposing factor and smoking is a risk factor for developing metal-induced asthma. Evidence for genetic factors in the development of metal contact dermatitis is conflicting, although animal models implicate genetic factors in skin sensitization with some metals and respiratory sensitization with Be. Skin irritation, forearm injury, complication with atopic dermatitis and concomitant sensitization to other agents are determinants for prognosis of the dermatitis. Epidemiological reports of occupational diseases from allergic reactions to metals in industries are reviewed with respect to prevalence and allergic manifestations. There is a report on a clinical trial of hyposensitization with Pt in a platinum asthma patient. Predictive methods for evaluating sensitization potential of metals have been developed and new methods, which quantify potential more objectively, are sought.

PMID: 8510347 [PubMed - indexed for MEDLINE]

2140. Arerugi. 1993 Mar;42(3 Pt 1):236-42.

[Effect of capsaicin on the migration of eosinophils into the bronchi of guinea pigs].

[Article in Japanese]

Sagara H, Yukawa T, Arima M, Makino S.

Department of Medicine and Clinical Immunology, Dokkyo University School of Medicine.

Substance P (SP), a potent neuropeptide, which is localized in the sensory nerves and released by many physiological stimuli has been implicated in airway

neurogenic inflammation. We have studied the effects of capsaicin (CAP), which releases tachykinins (TK) from the sensory nerves, on eosinophil (EOS) recruitment in the airway in guinea pigs in vivo. Male guinea pigs were used. The respiratory resistance (Rrs) of the guinea pigs were measured by an oscillation technique and histological studies of the right main bronchus were carried out. Exposure to inhaled CAP resulted in a significant increase in Rrs with PC200 CAP of 0.97 +/- 0.25 (x 10(-6) M) (n = 5). This stimulation also provoked striking eosinophilia in the right bronchus in a dose-dependent manner. A neutral endopeptidase (NEP) inhibitor, phosphoramidon, potentiated CAP-induced EOS infiltration. By contrast, pretreatment with [D-Pro2, D-Trp7,9]-SP, an analogue of SP and its receptor antagonist, diminished the response. We conclude that CAP-induced tachykinin release is capable of causing striking eosinophilia in the lung in vivo. This mechanism may contribute to airway inflammation in patients with asthma. This would provide further support for a link between tachykinin and bronchial eosinophilia in asthma.

PMID: 8498895 [PubMed - indexed for MEDLINE]

2141. Nihon Rinsho. 1993 Mar;51(3):795-9.

[Episodic angioedema with eosinophilia].

[Article in Japanese]

Tomoe S, Iwamoto I.

Second Department of Internal Medicine, Chiba University School of Medicine.

In 1984, Gleich et al. originally reported a novel syndrome of episodic angioedema with eosinophilia. The syndrome is characterized by recurrent angioedema, urticaria, weight gain, elevated IgM levels, marked blood eosinophilia, and eosinophil infiltrates in the dermis. It has also been observed that activated CD4+ T cells are present in the dermis of patients with this syndrome, suggesting that T cell-derived cytokines especially IL-5 might be involved in the migration and activation of eosinophils in the skin. Indeed, it has recently been reported that serum IL-5 levels elevate during clinical exacerbation of this syndrome. However, it still remains to be elucidated what antigen periodically activates CD4+ T cells, what factor(s) determine selective migration of eosinophils into the skin, and so on.

PMID: 8492460 [PubMed - indexed for MEDLINE]

2142. Nihon Rinsho. 1993 Mar;51(3):593-7.

[Expression of surface CD11b antigen and eosinophil activation].

[Article in Japanese]

Yachie A, Toma T, Miyawaki T, Taniguchi N.

Department of Pediatrics, School of Medicine, Kanazawa University.

Recent evidences suggest that the expression of adhesion molecules on eosinophils and endothelial surface play pivotal roles in the migration of activated eosinophils to the site of allergic inflammation. Among these, CD11b/ICAM-1 system is known to be responsible for at least part of the eosinophil/endothelial adhesion. CD11b expression on the surface of circulating eosinophils is significantly elevated in various allergic disorders, including atopic dermatitis

and bronchial asthma. Various eosinophilopoietic cytokines, including IL-3, IL-5 and GM-CSF induced rapid upregulation of CD11b expression on eosinophils in vitro. These observations suggest that surface CD11b may serve as a useful parameter of eosinophil activation and may reflect the in vivo level of eosinophilopoietic cytokines.

PMID: 8098378 [PubMed - indexed for MEDLINE]

2143. Clin Exp Allergy. 1993 Mar;23(3):196-204.

Down modulation of L-Selectin expression on eosinophils recovered from bronchoalveolar lavage fluid after allergen provocation.

Mengelers HJ, Maikoe T, Hooibrink B, Kuypers TW, Kreukniet J, Lammers JW, Koenderman L.

Department of Pulmonary Diseases, University Hospital, Utrecht, The Netherlands.

In allergic asthma eosinophils infiltrate into the lung after allergen challenge. The mechanism of this cellular infiltration is not fully understood. L-Selectin is involved in leucocyte-endothelial cell recognition and participates in homing of leucocytes into sites of inflammation. To find indications for a role of L-Selectin in the migration of eosinophils to the bronchoalveolar space we measured L-Selectin expression on eosinophils in peripheral blood and bronchoalveolar lavage fluid (BAL) 4 hr after the early allergic reaction after allergen challenge. Nine patients with allergic asthma participated in the study. An eosinophil specific high depolarization signal enabled us to measure L-Selectin expression on eosinophils in a FACS analysis without isolation of these cells. Eosinophils recovered from BAL showed a strong decrease of L-Selectin expression compared to blood eosinophils. This decrease in L-Selectin expression can be induced in vitro by activation of eosinophils with PMA or FMLP whereas priming of eosinophils during several hours with GM-CSF did not influence L-Selectin expression. Our results are a first indication that L-Selectin may play a role during homing of eosinophils in the lung in asthma after allergen challenge. Moreover, the low expression of L-Selectin on eosinophils in the lung is a further indication that these cells exhibit an activated phenotype.

PMID: 7682471 [PubMed - indexed for MEDLINE]

2144. J Invest Dermatol. 1993 Feb;100(2):137-42.

Eosinophil migration in atopic dermatitis. I: Increased migratory responses to N-formyl-methionyl-leucyl-phenylalanine, neutrophil-activating factor, platelet-activating factor, and platelet factor 4.

Bruijnzeel PL, Kuijper PH, Rihs S, Betz S, Warringa RA, Koenderman L.

Swiss Institute of Allergy and Asthma Research, Davos-Platz.

Eosinophil granular protein deposits have been demonstrated in lesional atopic dermatitis skin. This suggests active tissue infiltration of eosinophils. To find an explanation for the tissue influx of eosinophils, eosinophil migration was studied in vitro by means of a microchemotaxis assay. Eosinophils from the circulation of patients with atopic dermatitis showed an altered capacity to respond to chemotactic stimuli in vitro compared with eosinophils from healthy donors. Eosinophils from patients with atopic dermatitis had significantly increased migratory responses toward dose ranges of N-formyl-methionyl-leucyl-phenylalanine, neutrophil-activating factor,

platelet-activating factor, and platelet factor 4. Eosinophils from normal individuals did not respond to N-formyl-methionyl-leucyl-phenylalanine and neutrophil-activating factor and responded only slightly to platelet factor 4. The migratory responses toward tumor necrosis factor-alpha and complement factor C5a were identical in both groups. Interleukin-5, an eosinophil-selective cytokine, is a strong modulator of the migratory responses to these chemotaxins in eosinophils from normal donors. A migratory response toward N-formyl-methionyl-leucyl-phenylalanine and neutrophil-activating factor was induced by interleukin-5, whereas the migratory response toward platelet-activating factor and platelet factor 4 was markedly potentiated. In contrast, the response to complement fragment C5a was only slightly influenced. Our findings indicate that the increased migratory responsiveness of eosinophils from patients with atopic dermatitis to various chemotaxins reflects in vivo "priming" of eosinophils, presumably by circulating cytokines such as interleukin-5. This in vivo "priming" is not optimal because it can be further potentiated by renewed contact with interleukin-5.

PMID: 8429236 [PubMed - indexed for MEDLINE]

2145. Arch Dermatol. 1993 Jan;129(1):74-80.

Quantification of chemotactic peptides (C5a anaphylatoxin and IL-8) in psoriatic lesional skin.

Takematsu H, Tagami H.

Department of Dermatology, Tohoku University School of Medicine, Sendai, Japan.

BACKGROUND AND DESIGN: Psoriatic scale extracts contain a unique chemotactic peptide fraction that is likely to be involved in the induction of rhythmic transepidermal leukocyte chemotaxis. Recent studies have identified the presence of two unrelated chemotactic peptides in this fraction, ie, C5a/C5a des Arg and interleukin 8 (IL-8), and its related cytokines. To investigate their relative contribution to the transepidermal leukocyte migration as well as their interrelationship in psoriatic lesions, we have quantified concentrations of immunoreactive C5a/C5a des Arg and IL-8 in psoriatic lesional scale extracts and those from related sterile pustular dermatoses such as subcorneal pustular dermatosis and pustulosis palmaris et plantaris.
RESULTS: The concentrations of C5a/C5a des Arg and IL-8 were more significantly increased in the horny-tissue extracts from lesional skin than in those from noninflammatory orthokeratotic skin ($P < .01$). The increase of C5a/C5a des Arg concentration was specific to the lesional scale extracts, but showed a rather wide range of variation. By contrast, IL-8 concentration, although consistently increased in the lesional scale extracts, was also moderately increased even in noninflammatory scale extracts prepared from ichthyosis vulgaris. The elevation of IL-8 levels in psoriatic lesions was also confirmed by measuring their levels in cutaneous tissue fluid samples collected from suction blisters. However, unexpectedly, some control samples obtained from normal skin also showed a moderate increase in the IL-8 level. Neutrophil chemotactic activity correlated significantly only with the levels of C5a/C5a des Arg in the scales ($P < .05$). No such significant correlation was found between chemotactic activity and IL-8 or between C5a/C5a des Arg and IL-8.
CONCLUSION: Based on these results, we speculate that, although IL-8 may exert a synergistic effect with C5a/C5a des Arg in the induction of transepidermal leukocyte chemotaxis, it constitutes a proinflammatory cytokine that is involved in the production of the persistent inflammatory changes characterized by a T-lymphocyte infiltration. In contrast, C5a/C5a des Arg seems to be generated only in the inflammatory lesional skin under specific circumstances that preferentially favor complement activation and also seems to play a major role in

the induction of cyclic transepidermal leukocyte chemotaxis from "squirting papillae."

PMID: 8420495 [PubMed - indexed for MEDLINE]

2146. Am J Respir Cell Mol Biol. 1993 Jan;8(1):77-82.

Platelet-activating factor-induced human eosinophil transendothelial migration: evidence for a dynamic role of the endothelium.

Casale TB, Erger RA, Little MM.

Department of Internal Medicine, University of Iowa College of Medicine, Iowa City 52242-1009.

Stimulated migration of eosinophils out of the bloodstream and into the lung is key in the development of tissue eosinophilia and inflammation in asthma. Platelet-activating factor (PAF) has been implicated as an important inflammatory mediator in asthma pathogenesis in part because of its chemotactic capacity. We therefore studied the ability of PAF to induce human peripheral blood eosinophil migration through naked filters and human umbilical vein endothelial cells (HUVECs) cultured on these filters. PAF induced eosinophil migration through both barriers in a time-dependent fashion, with maximal eosinophil migration occurring at 180 min. Significant eosinophil migration was observed at PAF concentration > or = 0.1 microM and was dose dependent up to 10.0 microM. No significant differences in eosinophil chemotactic responses were noted between naked filter and HUVEC barriers. The PAF receptor antagonist, WEB 2086, inhibited (> 85%) eosinophil transendothelial migration when co-incubated with PAF or when used as a pretreatment of either the eosinophils or HUVECs. However, WEB 2086 pretreatment of HUVECs did not inhibit PAF-induced neutrophil transendothelial migration, nor did it affect leukotriene B4-induced neutrophil or eosinophil transendothelial migration. Thus, the data indicate that the endothelial cell plays an important role in PAF-induced eosinophil inflammatory processes. Moreover, these data suggest that PAF's pathogenic role in asthma may in part be due to its ability to stimulate eosinophil migration across endothelial barriers and into the airways.

PMID: 8380250 [PubMed - indexed for MEDLINE]

2147. Agents Actions Suppl. 1993;43:163-72.

Adhesion proteins on airway eosinophils in allergy and asthma.

Sedgwick JB, Busse WW.

Department of Medicine, University of Wisconsin, Madison 53792.

Adhesion to, and interaction with, airway endothelium, interstitial matrix and epithelium during migration to the allergen-challenged airways of allergic rhinitis and asthma patients may account for the observed functional upregulation (priming) of airway eosinophils compared with corresponding blood eosinophils.

PMID: 8368160 [PubMed - indexed for MEDLINE]

2148. Int Arch Allergy Immunol. 1993;101(4):425-30.

In vitro interleukin-5 production of peripheral blood mononuclear cells is

increased in patients with asthma.

Endo H, Iwamoto I, Nakajima H, Yoshida S.

Second Department of Internal Medicine, Chiba University School of Medicine, Japan.

To determine whether the capacity of interleukin-5 (IL-5) production is increased in patients with asthma, we studied in vitro IL-5 production from peripheral blood mononuclear cells (PBMC) in 27 asthmatics (16 allergic asthmatics and 11 nonallergic asthmatics) and 10 normal subjects. IL-5 production of phytohemagglutinin (PHA)-stimulated PBMC was significantly greater in asthmatics than in normal subjects ($p < 0.02$). IL-5 production of PBMC by IL-2 stimulation was also significantly increased in asthmatics compared with that of normal subjects ($p < 0.05$). In contrast, IL-2 production of PHA-stimulated PBMC did not significantly differ between asthmatics and normal subjects. In addition, the number of CD4+ and CD8+ T cells in PBMC or CD4+/CD8+ ratio did not significantly differ between asthmatics and normal subjects, whereas CD25+ T cells were significantly increased in asthmatics compared with those of normal subjects ($p < 0.02$). Finally, there was no significant correlation between the in vitro IL-5 production of PBMC and blood eosinophil counts in asthmatics and normal subjects. Our results indicate that the capacity of IL-5 production, but not of IL-2 production, is increased in asthmatics. The increased capacity of IL-5 production might be involved in the migration and activation of eosinophils in the airways of asthmatics.

PMID: 8353463 [PubMed - indexed for MEDLINE]

2149. Allergol Immunopathol (Madr). 1993 Jan-Feb;21(1):11-4.

Survey on the allergic status in a Turkish population in Sweden.

Kalyoncu AF, Stålenheim G.

Hacettepe University, School of Medicine, Dept. of Chest Diseases, Ankara, Turkey.

A limited survey on the allergic status and total IgE was performed on 205 persons of Turkish origin aged between 16 and 76 years living in Stockholm, Uppsala, Sweden in 1990. A questionnaire was mailed to 205 persons and was returned by 92%. 71 persons were randomly selected among those who returned the questionnaire, and were examined after an interview, skin-prick test (SPT) and total IgE levels were also measured. The atopy prevalence of the randomly selected group of Turks was found 32.4%. Clinical symptoms were significantly associated with positive SPT reactivity. IgE levels in the atopic group were significantly higher than those of non atopic group (78.2 ku/l vs 28.4 ku/l respectively). However, the difference in IgE levels were significant between non atopic and atopic groups residing in Sweden longer than 10.5 years, but not significant between those who resided less than 4.5 years. The allergic spectrum changed with residence time spent in Sweden in Turks. Skin test positivity to birch, cat, and dog increased with time. This increase may be related to the change in life styles and habits, such as indoor contact with pets, and intensive environmental birch pollen exposure. In conclusion, our data indicate that immunologic status of persons is influenced by a new millieu. Within a few years the allergic status of Turkish immigrants adapted to the new environment.

PMID: 8328351 [PubMed - indexed for MEDLINE]

2150. Med Tr Prom Ekol. 1993;(1):43-5.

[Diagnostic value of leukocyte migration test in vivo in patients with occupational bronchial asthma (a case report)].

[Article in Russian]

Stasenkova TIu.

PMID: 8075939 [PubMed - indexed for MEDLINE]

2151. Acta Haematol. 1993;89(4):213-5.

Induction of normal human eosinophil migration in vitro by substance P.

Wiedermann FJ, Kähler CM, Reinisch N, Wiedermann CJ.

Department of Internal Medicine, Faculty of Medicine, University of Innsbruck, Austria.

To investigate the role of tachykinins in allergic inflammation, the effect of substance P (SP) on normal human eosinophil migration was examined in Boyden chamber type assays. SP stimulated eosinophil migration in vitro with an EC50 of about 1-10 pM and maximal effects were seen at 100 pM. A carboxy-terminal fragment of SP possessed appreciable activity whereas an amino-terminal fragment was inactive. Data represented in this paper and previous studies suggest that eosinophils are attracted by SP and activated to enhanced mediator release and cytotoxic activity.

PMID: 7692691 [PubMed - indexed for MEDLINE]

2152. Laryngorhinootologie. 1992 Dec;71(12):644-8.

[Chronic otitis externa from the dermatologic viewpoint].

[Article in German]

Krahl D.

Universitäts-Hautklinik Heidelberg.

Chronic external otitis may be divided into several diagnostic categories. Disposition for psoriasis, seborrhoeic and atopic eczema are main endogenous reasons. Exogenous pathogens for external otitis are microbes and allergens. There are numerous interrelations by coincidence of dispositional diseases, e.g. psoriasis and atopic eczema and by combination of exogenous and endogenous pathogens. This holds good for the yeast Pityrosporum ovale vs. orbiculare in seborrhoeic eczema and for the susceptibility to contact (type IV) and respiratory (type I) allergy in atopic individuals as well. Mycotic and bacterial, especially gram negative external otitis are linked to predisposing factors like eczema, long-term microbicidal therapy, hot and humid environment. Contact allergic external otitis may occur during long lasting local therapy with various substances including vehicles, the most common allergen being neomycin. Mucosal allergic reactions (Type I) in the upper respiratory tract may impair ventilation of the Eustachian tube and middle ear and therefore epithelial migration, as a drainage mechanism of the auditory canal. Examination should include functional assessment of the Eustachian tube and middle ear and allergy testing (patch, prick test). Preparations for local therapy should contain a

limited number of constituents and avoid common allergens. Surgical procedures to reestablish ventilation of the middle ear are also a therapy for chronic external otitis.

PMID: 1492891 [PubMed - indexed for MEDLINE]

2153. Toxicol Appl Pharmacol. 1992 Dec;117(2):137-46.

Dendritic cells and cutaneous immune responses to chemical allergens.

Kimber I, Cumberbatch M.

ICI Central Toxicology Laboratory, Macclesfield, Cheshire, United Kingdom.

This article reviews the role of epidermal Langerhans cells (LC) in the development of cutaneous immune responses to chemical allergens. Following topical exposure to sensitizing chemicals, LC, many of which bear allergen, are induced to migrate from the skin, via the afferent lymphatics, to the draining lymph nodes. The phenotypic and functional changes to which LC are subject during this process and their development into active immunostimulatory cells closely resembling lymphoid dendritic cells is discussed. The migration and maturation of LC following skin sensitization is of critical importance to the effective presentation of chemical allergens to T lymphocytes and the induction of allergic responses. Evidence is reviewed which suggests that these events are initiated and regulated by epidermal cytokines. The conclusion drawn is that an early event during the induction of skin sensitization is the production by keratinocytes of cytokines which stimulate the migration of LC from the skin and which also result in the functional maturation of LC into potent antigen-presenting cells.

PMID: 1471146 [PubMed - indexed for MEDLINE]

2154. J Exp Med. 1992 Dec 1;176(6):1489-95.

RANTES and macrophage inflammatory protein 1 alpha induce the migration and activation of normal human eosinophil granulocytes.

Rot A, Krieger M, Brunner T, Bischoff SC, Schall TJ, Dahinden CA.

Institute of Clinical Immunology, Bern, Switzerland.

The cellular infiltrates of certain inflammatory processes found in parasitic infection or in allergic diseases consist predominantly of eosinophilic granulocytes, often in association with activated T cells. This suggests the existence of chemotactic agonists specific for eosinophils and lymphocyte subsets devoid of neutrophil-activating properties. We therefore examined four members of the intercrine/chemokine superfamily of cytokines (monocyte chemotactic peptide 1 [MCP-1], RANTES, macrophage inflammatory protein 1 alpha [MIP-1 alpha], and MIP-1 beta), which do not activate neutrophils, for their ability to affect different eosinophil effector functions. RANTES strongly attracted normal human eosinophils by a chemotactic rather than a chemokinetic mechanism with a similar efficacy as the most potent chemotactic myeloid cell agonist, C5a. MIP-1 alpha also induced eosinophil migration, however, with lower efficacy. RANTES and MIP-1 alpha induced eosinophil cationic protein release in cytochalasin B-treated eosinophils, but did not promote leukotriene C4 formation by eosinophils, even after preincubation with interleukin 3 (IL-3), in contrast to other chemotactic agonists such as C5a and formyl-methionyl-leucyl-phenylalanine (FMLP). RANTES, but not MIP-1 alpha, induced a biphasic chemiluminescence response, however, of lower magnitude than C5a. RANTES and MIP-1 alpha both promoted identical

transient changes in intracellular free calcium concentration ([Ca2+]i), with kinetics similar to those induced by chemotactic peptides known to interact with G protein-coupled receptors. No cross-desensitization towards other peptide agonists (e.g., C5a, IL-8, FMLP) was observed, suggesting the presence of specific receptors. Despite its weaker eosinophil-activating properties, MIP-1 alpha was at least 10 times more potent on a molar basis than RANTES at inducing [Ca2+]i changes. Interestingly, RANTES deactivated the MIP-1 alpha-induced [Ca2+]i changes, while the RANTES response was preserved after MIP-1 alpha stimulation. MCP-1, a potent monocyte chemoattractant and basophil agonist, as well as MIP-1 beta, a peptide with pronounced homology to MIP-1 alpha, did not activate the eosinophil functions tested. Our results indicate that RANTES and MIP-1 alpha are crucial mediators of inflammatory processes in which eosinophils predominate.

PMCID: PMC2119467
PMID: 1281207 [PubMed - indexed for MEDLINE]

2155. Clin Exp Allergy. 1992 Nov;22(11):1020-5.

Effects of cytokines on human basophil chemotaxis.

Tanimoto Y, Takahashi K, Kimura I.

Second Department of Internal Medicine, Okayama University Medical School, Japan.

Basophil chemotactic activity (BCA) of eight recombinant human (rh) cytokines was examined. Highly purified basophils were obtained by Percoll discontinuous gradients, followed by negative selection using flow cytometry. Then BCA was measured by means of modified Boyden chamber method. Both interleukin (IL)-3 and granulocyte-macrophage colony-stimulating factor (GM-CSF) had much more potent BCA than complement C5a, leukotriene B4 and platelet activating factor, well known as granulocyte chemotactic factors. Chemotaxis rather than chemokinesis was shown in chequerboard analysis of basophil migration induced by IL-3 and GM-CSF. Relatively high concentrations of IL-5 also induced basophil migration, although predominantly chemokinetic. IL-8 had apparent BCA, which was not so high as that of C5a. In contrast, IL-2, IL-4, interferon(IFN)-gamma and granulocyte colony-stimulating factor (G-CSF) had no significant BCA. These findings suggest that IL-3, IL-5, GM-CSF and, perhaps, IL-8 have an effect on basophil migration as well as modulation of basophil mediator release and may provide some insight into the basophil accumulation observed in late-phase allergic responses.

PMID: 1334781 [PubMed - indexed for MEDLINE]

2156. J Allergy Clin Immunol. 1992 Oct;90(4 Pt 2):720-5.

Antiallergic properties of the second-generation H1 antihistamines during the early and late reactions to antigen.

Townley RG.

Department of Medicine, Creighton University School of Medicine, Omaha, Neb. 68178.

Some of the second-generation H1 antihistamines reduce the bronchoconstrictor response after exercise and antigen challenge. For example, terfenadine causes a slight but significant increase in forced expiratory volume after 1 second. At doses of 120 and 240 mg, terfenadine has a protective effect against asthma induced by ultrasonic nebulized distilled water and cold air hyperventilation

challenge. Certain other newer antihistamines, such as ketotifen, azelastine, and cetirizine, have additional antiallergy properties. These effects include inhibition of eosinophil, basophil, and neutrophil migration and platelet-activating factor-induced eosinophil accumulation in skin. The ability of cetirizine (and perhaps other antihistamines) to inhibit these responses at usual therapeutic doses may be useful in investigating the late allergic reaction.

PMID: 1383311 [PubMed - indexed for MEDLINE]

2157. Ann Allergy. 1992 Oct;69(4):323-8.

Effects of nedocromil sodium and WEB 2086 on chemoattractant-stimulated neutrophil migration through cellular and noncellular barriers.

Carolan EJ, Casale TB.

Department of Internal Medicine, University of Iowa College of Medicine, Iowa City.

Nedocromil sodium (Tilade) is an effective therapeutic agent against asthma and has been shown to exhibit antiinflammatory activity in vitro; however, its mode of action is yet to be described fully. Using an in vitro assay designed to mimic the extravasation of neutrophils from the peripheral circulation through cellular barriers to sites of inflammation, the effect of nedocromil sodium on chemoattractant-stimulated neutrophil migration was examined. We also examined the effects of WEB 2086, a platelet-activating factor (PAF) receptor antagonist, in parallel. Neutrophils and the cellular barrier were pretreated and/or co-incubated with nedocromil or WEB 2086 and the effects on neutrophil chemotaxis measured. In all treatments, nedocromil did not significantly affect chemotaxis through cellular or noncellular barriers to N-formyl-methionyl-leucyl-phenylalanine (FMLP), leukotriene B4 (LTB4), or PAF. In contrast, WEB 2086 inhibited PAF-induced neutrophil migration through both naked filters and endothelial and epithelial monolayers cultured on these filters. We conclude that while nedocromil has been shown to have inhibitory effects on neutrophils and is an effective therapeutic agent for asthma and inflammatory conditions, its activity is not primarily mediated by inhibition of neutrophil chemotaxis. Platelet-activating factor antagonists may partially be effective in asthma through inhibitory effects on neutrophil chemotaxis.

PMID: 1329582 [PubMed - indexed for MEDLINE]

2158. Semin Respir Infect. 1992 Sep;7(3):179-92.

Allergic bronchopulmonary mycosis complicating cystic fibrosis.

Knutsen A, Slavin RG.

Department of Pediatrics, St. Louis University Medical Center, MO 63104.

Allergic bronchopulmonary aspergillosis (ABPA) is a hypersensitivity lung disease caused by bronchial colonization with Aspergillus fumigatus that affects approximately 10% of patients with cystic fibrosis (CF). The diagnosis in CF patients is difficult because the cardinal symptoms of ABPA occur frequently in CF, ie, pulmonary infiltrates and wheezing, as well as the frequent colonization with A fumigatus that leads to humoral reactivity. If left untreated, ABPA leads to bronchiectasis and pulmonary fibrosis. The pathogenesis of ABPA seems to be a prolonged asthmatic late-phase reaction orchestrated by CD4+ Th2-like T cells in

response to persistent pulmonary A fumigatus allergen exposure. Thus, polyclonal and A fumigatus-specific IgE antibodies (and IgA and IgG) and blood pulmonary eosinophilia are stimulated by Th2-derived cytokines such as IL-4 and IL-5. In addition, IL-4 would also promote pulmonary transendothelial migration of eosinophils, basophils, and lymphocytes via induction of cell adhesion molecules and their ligands. IgE mast cell interactions would also contribute to the bronchial reactivity and inflammation. Recent advances have begun to identify immunodominant A fumigatus allergens. Evaluation of the quantity of IgE antibodies (and IgA and IgG) and T-cell cytokine responses to specific A fumigatus allergens should aid in the diagnosis and immunopathogenesis of ABPA, especially in CF patients.

PMID: 1475542 [PubMed - indexed for MEDLINE]

2159. Rhinology. 1992 Sep;30(3):161-8.

NARES: a model of inflammation caused by activated eosinophils?

Moneret-Vautrin DA, Jankowski R, Bene MC, Kanny G, Hsieh V, Faure G, Wayoff M.

Service de Médecine D, Allergologie et Immunologie Clinique, CHU de Brabois, Vandoeuvre-les-Nancy, France.

Twenty patients were selected on the basis of perennial rhinitis, the absence of allergy and with an eosinophil count higher than 20% of total leucocytes in nasal secretions (NARES). Nasal endoscopy with biopsies from the middle turbinate and sinus CT were performed. Biopsies were processed for histological examination and for immunofluorescence. The clinical progress during treatment was scrutinized. An acute congestive aspect of the nasal mucosa was noted in 4 cases, and micropolyposis in 9 cases. Sinus CT showed opacity of the ethmoidal cells in 87% of cases (maxillary sinuses: 75%; frontal sinus: 46%; sphenoidal sinus: 31%). An eosinophilic infiltrate of the nasal mucosa was constituted in 9 cases: In 6 cases, the cells expressed the Fc epsilon RII receptor, recognized by the monoclonal antibody Bb10. Anti-H1 drugs usually failed to result in a clinical improvement and local eosinophilia was not changed. Local corticoids were more effective but not sufficient in some cases, so that oral corticotherapy was needed. Ethmoidectomy was performed in three cases. NARES seems to evolve in three stages: (1) migration of eosinophils from the vessels to the secretions; (2) retention of eosinophils in the mucosa which might be linked to activation of unknown origin; (3) nasal polyposis. Numerous interactions between irritation of the epithelium, release of substance P, and eosinophils, lead to the hypothesis of a neurogenic origin of NARES.

PMID: 1448672 [PubMed - indexed for MEDLINE]

2160. J Am Acad Dermatol. 1992 Sep;27(3):383-8.

Epidermal dendritic cells in psoriasis possess a phenotype associated with antigen presentation: in situ expression of beta 2-integrins.

McGregor JM, Barker JN, Ross EL, MacDonald DM.

Laboratory of Applied Dermatopathology, United Medical School, London, U.K.

BACKGROUND: Epidermal dendritic cells (DCs) isolated from psoriasis possess greatly enhanced T lymphocyte-activating properties compared with DCs from normal skin, suggesting that DCs in psoriasis express surface antigens crucial for antigen presentation. These include beta 2-integrins and intercellular adhesion

molecule (ICAM)-1.
OBJECTIVE: Our purpose was to determine DC phenotype in psoriatic compared with normal epidermis with respect to these molecules.
METHODS: Tissue sections were single labeled with a peroxidase antiperoxidase (PAP) immunohistochemical technique and double labeled where necessary with a combination of a PAP and an alkaline phosphatase-anti-alkaline phosphatase technique.
RESULTS: In psoriatic compared with normal skin, decreased numbers of DCs expressed CD1a (p less than 0.05), whereas increased numbers of DCs expressed class II major histocompatibility antigens (p less than 0.05). In normal skin positive staining for CD18 was not observed, whereas in psoriasis both CD1a+ and CD1a- DCs expressed beta 2-integrins, LFA-1 (CD11a/CD18), and gp 150/95 (CD11c/CD18). DCs in atopic dermatitis and lichen planus were also found to express beta 2-integrins. Neither MAC 1 (CD11b/CD18) nor ICAM-1 was observed on DCs.
CONCLUSION: These data are consistent with either migration of dendritic antigen-presenting cells into the epidermis or in situ cytokine modulation of Langerhans cell phenotype in inflamed skin. Furthermore, they indicate that epidermal DCs in psoriasis and other cutaneous inflammatory diseases express molecules that are known to be crucial for Langerhans cell-driven T-cell activation in vitro.

PMID: 1401271 [PubMed - indexed for MEDLINE]

2161. J Pharmacol Exp Ther. 1992 Sep;262(3):964-70.

Effect of H1 receptor blockade on the early and late response to cutaneous allergen challenge.

Charlesworth EN, Massey WA, Kagey-Sobotka A, Norman PS, Lichtenstein LM.

Johns Hopkins University, Department of Medicine, Johns Hopkins Asthma and Allergy Center, Baltimore, Maryland.

We investigated whether cutaneous antigen-induced inflammatory cell infiltration and mediator release were modified by H1 receptor antagonists. Three chemically unrelated antihistamines (cetirizine, promethazine and chlorpheniramine) were tested in three groups of allergic subjects in a double-blind, crossover design. Chamber fluids were collected for 12 hr and histamine release, prostaglandin D2 production and cellular infiltration were quantified. Cetirizine significantly decreased late leukocyte migration into antigen-challenged chambers: eosinophils by 68% (P less than .04), basophils by 64% (P less than .04) and neutrophils by 72% (P less than .04), whereas mononuclear cells were not significantly affected. No alteration in the numbers of peripheral blood leukocytes or eosinophils occurred while on cetirizine treatment, suggesting that the decrease in inflammatory cells during the late phase reaction in the skin is not secondary to alterations in the peripheral leukocyte pool. In contrast, neither promethazine nor chlorpheniramine induced any significant alteration in inflammatory cell infiltration. All three antihistamines caused significant inhibition of the immediate reaction to antigen without any significant alteration in late phase reaction cutaneous reactivity. None of the three antihistamines caused any significant alteration in histamine or prostaglandin D2 levels. Thus, cetirizine may be an antihistamine uniquely capable of downregulating the late phase reaction inflammatory cell milieu without altering either early or late mediator production. The mechanisms involved and the clinical relevance of these findings remain to be explored.

PMID: 1382132 [PubMed - indexed for MEDLINE]

2162. J Allergy Clin Immunol. 1992 Sep;90(3 Pt 2):521-33.

Sinusitis and asthma: an animal model.

Irvin CG.

Department of Medicine, National Jewish Center for Immunology and Respiratory Medicine, Denver, Colo.

We have developed an animal model in which nonspecific lower airways hyperresponsiveness to inhaled histamine was elicited in rabbits after complement-induced maxillary sinusitis. The most likely mechanism to explain this occurrence is the direct passage ("postnasal drip") of inflammatory mediators from the upper to the lower respiratory tract. The contribution of other potential mechanisms, such as the blood-borne delivery of inflammatory mediators, nasobronchial reflexes, and passage of cells with the induction of a secondary inflammatory process, could not be demonstrated. Rather, the most likely explanation for the current finding is the passage of mediators elaborated from activated inflammatory cells into the lower airways. Whether these findings explain the common clinical association of upper airways disease to lower airways dysfunction in sinusitis and asthma remains to be determined. These results suggest that even small numbers of granulocytes, when activated, can exert significant effect on lower airways function. It is perhaps appropriate to speculate at this point about the anecdotal but dramatic improvement in the asthma of patients with sinusitis who undergo surgery. The current results cause us to suggest that this success is due to the removal of the source of inflammatory products that drip into the lung. More important, these current results may have an important implication in the diagnosis of asthma. Finally, there is the clear conclusion that airways dysfunction can be caused by a mechanism that is associated with inflammation but without evidence of cell migration into the airways.

PMID: 1382085 [PubMed - indexed for MEDLINE]

2163. J Neuroimmunol. 1992 Sep;40(1):57-69.

Direct demonstration of the infiltration of murine central nervous system by Pgp-1/CD44high CD45RB(low) CD4+ T cells that induce experimental allergic encephalomyelitis.

Zeine R, Owens T.

Department of Medicine, Montreal Neurological Institute, McGill University, Quebec, Canada.

In experimental allergic encephalomyelitis (EAE), autoimmune T cells infiltrate the central nervous system (CNS) and initiate demyelinating pathology. We have used flow cytometry to directly analyse the migration to the CNS of MBP-reactive CD4+ T cells labelled with a lipophilic fluorescent dye (PKH2), in SJL/J mice with passively transferred EAE. Labelled cells constituted about 45% of the CNS CD4+ population at the time of EAE onset. Almost all (greater than 90%) of the PKH2-labelled CD4+ T cells from EAE CNS were blasts and were alpha/beta T cell receptor (TCR)+, CD44(Pgp-1)high, and the majority were CD45RB(low). By contrast, most PKH2-labelled CD4+ T cells in lymph nodes, although CD44high, were CD45RBhigh cells. The cells that were transferred to induce EAE were essentially similar to antigen-primed lymph node cell populations, containing less than 15% CD44high cells, and most of them were CD45RBhigh. The CD44high CD45RB(low) phenotype is characteristic of memory/effector T cells that have been activated

by antigen recognition. The difference in CD45RB expression between CNS and LN could therefore reflect differential exposure and/or response to antigen. Consistent with this, PKH2-labelled CD4+ cells isolated from the CNS were responsive to MBP in vitro, whereas PKH2+ CD4+ cells from lymph nodes showed almost undetectable responses. In control experiments in which ovalbumin (OVA)-reactive T cells were transferred, a small number of fluorescent-labelled CD4+ T cells were also detected in CNS, but there were very few blasts, and these remained CD45RBhigh. These results argue for induction of the memory/effector phenotype of CD4+ T cells, and their selective retention in the CNS, as a consequence of antigen recognition.

PMID: 1381382 [PubMed - indexed for MEDLINE]

2164. J Investig Allergol Clin Immunol. 1992 Sep-Oct;2(5):274-7.

Granulocyte migration in vivo and in vitro in healthy children of parents with infectious asthma.

Matusiewicz R, Grzybowski A, Urbankowska B.

First Department of Internal Diseases, Grochow Hospital, Warsaw, Poland.

Our study was carried out on 28 healthy children aged 6-11 years (mean = 9 years) with at least one parent having infectious asthma, and 13 children aged 7-14 years (mean = 9 years), both of whose parents had infectious asthma. The age of the affected parents was 26-45 years (mean = 36 years). The control group comprised 20 healthy subjects aged 25-37 years (mean = 32 years) and 20 healthy children aged 6-14 years (mean = 9 years) with no family history of atopic diseases. In all subjects, the test of granulocyte migration in vitro was carried out using the method of Clausen, modified by us. The in vivo test was performed according to the Southam method. The results of our investigations demonstrate a defect of granulocyte migration in patients with infectious bronchial asthma. In healthy children, both of whose parents had infection, the migration of the granulocytes was impaired.

PMID: 1342910 [PubMed - indexed for MEDLINE]

2165. Res Vet Sci. 1992 Sep;53(2):223-9.

Platelet activating factor is a mediator of equine neutrophil and eosinophil migration in vitro.

Foster AP, Lees P, Cunningham FM.

Department of Veterinary Basic Sciences, Royal Veterinary College, North Mymms, Hertfordshire.

Platelet activating factor (PAF) is known to be a chemoattractant for equine neutrophils in vivo and in vitro. In this study the in vitro migratory response of equine eosinophils and neutrophils to PAF has been examined and compared with that to leukotriene (LT)B4. PAF (10(-8) to 10(-5) M), but not lyso-PAF (10(-6) M), caused dose related migration of both equine eosinophils and neutrophils, maximal responses occurring at 10(-6) M. Responses to PAF were inhibited by the receptor antagonist WEB 2086. LTB4 (10(-8) to 10(-6) M) also induced migration of both cell types, although the maximum effect was observed with a 10-fold lower concentration. Moreover, the maximum response of equine eosinophils to LTB4 was significantly greater than to PAF. It is concluded that LTB4 and PAF, if released in vivo at sites of allergic or inflammatory reactions, could mediate the

recruitment of leucocytes to the involved tissue.

PMID: 1332153 [PubMed - indexed for MEDLINE]

2166. Clin Exp Dermatol. 1992 Sep;17(5):316-20.

Epidermal proliferation and keratinization following standardized elicitation with diphenylcyclopropenone.

van der Steen PH, van Baar HM, Boezeman JB, van Erp PE, van Vlijmen IM, van de Kerkhof PC.

Department of Dermatology, University of Nijmegen, The Netherlands.

Diphenylcyclopropenone (DCP) was applied to the upper arms of five alopecia areata patients using 10% of the concentration that had been applied previously to the scalp during topical immunotherapy. DCP applied in this concentration evoked a mild eczematous reaction. Biopsies were taken before DCP application and after 24, 48 and 96 h. A large increase in T-lymphocytes and CD14-positive cells in the dermis was seen after 24 h. Migration of these cells into the epidermis was mainly observed during the first 48 h. This was followed by epidermal proliferation as assessed by the number of Ki-67-positive nuclei and the degree of Ks8.12-binding. Both showed their main increase after 48 h; but after 24 h the increase of Ki-67-positive nuclei was significant ($P < 0.04$). Involucrin and filaggrin showed a gradual increase which became substantial after 96 h (both $P < 0.04$). As the invasion of inflammatory cells into the epidermis preceded the main increase in epidermal proliferation, cytokines are suggested as possible mediators for the initial phase of the proliferative response after DCP application.

PMID: 1281054 [PubMed - indexed for MEDLINE]

2167. J Immunol. 1992 Aug 15;149(4):1432-8.

IL-4 controls the selective endothelium-driven transmigration of eosinophils from allergic individuals.

Moser R, Fehr J, Bruijnzeel PL.

Department of Medicine, University Hospital, Zurich, Switzerland.

The mechanism leading to selective accumulation of eosinophils in allergic inflamed tissue is still unknown. In this article, transendothelial migration of circulating eosinophils from normal and allergic individuals is characterized by means of human umbilical vein endothelial cells cultivated on extracellular matrix from human fibroblasts. IL-4 pretreatment of these vascular constructs induced adherence and impressive layer penetration of eosinophils but not of neutrophils. For layer penetration, blood eosinophils from nonallergic donors needed in vitro priming by granulocyte/macrophage-CSF, IL-3, or IL-5. In contrast, freshly isolated blood eosinophils from a group of patients with atopic dermatitis spontaneously penetrated IL-4-activated vascular constructs. The here described selective pathway of eosinophil transmigration was 1) specifically induced by IL-4; 2) inhibited by the IL-4 specific, neutralizing mAb 8F12; and 3) dependent upon endothelial mRNA synthesis. Both eosinophil adherence and transmigration were present at an IL-4 concentration of 1 U/ml. The effect of endothelial preincubation with IL-4 culminated at 16 h and persisted up to 48 h. A linear increase of subendothelial accumulating eosinophils was observed within 2 h, reaching almost 100% after 4 h of coincubation. From inhibition experiments

using different mAb, we conclude that the integrins CD11a/CD18, CD11b/CD18, and very late Ag-4 (CDw49d/CD29) are involved in this selective pathway of eosinophil transmigration. Taken together, this study demonstrates a novel mechanism which allows in vitro or in vivo primed eosinophils to leave the vascular compartment without influencing emigration of neutrophils.

PMID: 1354235 [PubMed - indexed for MEDLINE]

2168. Allergy. 1992 Aug;47(4 Pt 1):277-80.

Serum IgE levels and allergic spectra in immigrants to Sweden.

Kalyoncu AF, Stålenheim G.

Department of Chest Diseases, Hacettepe University, School of Medicine, Ankara, Turkey.

We evaluated the allergy status of 134 immigrants from Asia, Africa, the Middle East and South America, who were referred to our clinic during the past 10 years. Fifty Swedish patients were used for comparison. When the atopy state was not taken into account, no significant difference was found between the two groups with respect to total IgE levels. However, IgE levels of non-atopic immigrants were significantly higher than the IgE levels of non-atopic Swedes. While there was no significant difference in IgE levels between atopic and non-atopic immigrants, this difference was significant in Swedish patients. In general, IgE levels of immigrants showed a decline with time and reached approximately the same levels as for the Swedish patients in 10.5 years. In the immigrant group atopic women had a considerably lower IgE level than the atopic men. Among the atopics there were no differences between sexes. In Swedes and immigrants pollen was the most common group of allergens. The spectrum of allergy in the immigrant group changed with time in Sweden, and gradually became more similar to the Swedish spectrum. Skin and/or RAST positivity to birch increased from 16% within 2.5 years to 53% after more than 10.5 years in Sweden. Our data indicate that environmental factors rather than hereditary differences determine the IgE state. Within a few years the immunologic status of immigrants adapts to the new environment.

PMID: 1443445 [PubMed - indexed for MEDLINE]

2169. J Laryngol Otol. 1992 Aug;106(8):704-8.

Is injectable collagen truly safe?

Takayama E, Ikeda M, Tsuru S, Ogura M, Kitahara S, Inouye T, Healy GB.

Department of Otolaryngology, National Defense Medical College, Saitama, Japan.

Most patients have no response to injectable collagen or silicone, but some cases may have positive or 'undersea' (= clinically negative but immunologically positive) response to collagen. From the results of the Macrophage migration inhibition test, the relative immunogenicity was augmented most when we used implants with the following combination. The first immunization was collagen and the second one was collagen with silicone. The augmented antigenicity might be enough to cause an allergic reaction to the patients who had no response to each implant alone.

PMID: 1402361 [PubMed - indexed for MEDLINE]

2170. J Exp Med. 1992 Aug 1;176(2):587-92.

Cytokine RANTES released by thrombin-stimulated platelets is a potent attractant for human eosinophils.

Kameyoshi Y, Dörschner A, Mallet AI, Christophers E, Schröder JM.

Department of Dermatology, University of Kiel, Germany.

Thrombin stimulation of human platelets results in the release of a preformed proteinaceous human eosinophil (Eo)-chemotactic activity. By the use of different high-performance liquid chromatography techniques, two Eo-chemotactic polypeptides (EoCPs), tentatively termed EoCP-1 and EoCP-2, were purified to homogeneity. Upon SDS-PAGE analysis, these chemotaxins showed molecular masses near 8 kD. NH2-terminal amino acid sequence analysis revealed identical sequences for both EoCP-1 and EoCP-2, which are also identical to that of RANTES, a cytokine that structurally belongs to the interleukin 8 superfamily of leukocyte selective attractants, and that is known to be a "memory-type" T lymphocyte-selective attractant. In the major Eo chemotaxin, EoCP-1, the residues 4 and 5, which in EoCP-2 were found to be serine residues, could not be identified. Electrospray mass spectrometry (ESP-MS) of EoCPs revealed for EoCP-2 a molecular mass of 7,862.8 +/- 1.1 daltons, which is 15.8 mass units higher than the calculated value of RANTES, indicating that EoCP-2 is identical to the full-length cytokine, and oxygenation, probably at methionine residue number 64, has taken place. Upon ESP-MS, EoCP-1 showed an average molecular mass of 8,355 +/- 10 daltons, suggesting O-glycosylation at these serine residues. Both natural forms of RANTES showed strong Eo-chemotactic activity (ED50 = 2 nM) with optimal chemotactic migration at concentrations near 10 nM, however, there were no significant migratory responses with human neutrophils. Chemotactic activity of RANTES for human Eos could be confirmed using recombinant material, which has been found to be as active as the natural forms. Since RANTES gene expression has been detected in activated T lymphocytes, and recombinant RANTES was shown to be a "memory" T lymphocyte-selective attractant, it is now tempting to speculate about an important role of RANTES in clinical situations such as allergene-induced late-phase skin reactions in atopic subjects or asthma, where in affected tissues both memory T cells and Eos are characteristic.

PMCID: PMC2119329
PMID: 1380064 [PubMed - indexed for MEDLINE]

2171. ICCW News Bull. 1992 Jul-Dec;40(3-4):53-9.

Children at health risks.

Sekar HR.

PIP: In India, 69% of the children of the working class die, most of whom are child laborers. Economic pressure forces parents to make their children work. Employers want child workers because they can manipulate them and pay them low wages, thereby ensuring their viability. The caste system induces social inequality, inheritance invokes cultural inequality, and patriarchal socialization is responsible for gender inequality, all of which perpetuates exploitation of children by employers. In Sivakasi, an estimated 125,000 children make up the child labor force, comprising 30% of the entire labor force. 75% are from the lowest castes. 90% of child workers are girls because they are more obedient and accept even lower wages than boys, and girls need to save for their dowry. Girls often suffer verbal and physical abuse. Like their parents who were also child workers, child workers are illiterate and work long hours. A small

rich elite in Sivakasi controls most of the trading and industrial capital, educational institutions, and voluntary organizations. Employers' agents give parents a loan and use their children's labor as security. Each day, they bring child workers to Sivakasi in factory buses from villages to work at least 12 hour days. They work under hazardous conditions, e.g., working with toxic chemicals. Coughing, sore throat, dizziness, methemoglobinemia, and anemia are common effects of ingestion or inhalation of chlorate dust. Inhalation of sulphur dust causes respiratory infections, eye infections, and chronic lung diseases (e.g., asthma). Fires and explosions are common risks for working children. Factory management seldom undertake fire prevention measures. An extensive survey of the problem of child labor is needed in Sivakasi before systematic planning to protect children could be done. Overall development, especially agricultural development, is needed. Parents, employers, enforcement authorities, trade unions, and social groups need to be sensitized to the abomination of child labor. The government should provide monetary incentives to employers that do not use child labor and disincentives to those that do.
PMID: 12318359 [PubMed - indexed for MEDLINE]

2172. ZPG Report. 1992 Jul;24(3):1-2.

Building a new urban order.

Merrill J.

PIP: In the early 1900s, 10% of the population lived in rural areas, but today almost 50% live in cities. Many people move to cities in hopes of finding employment. An overloaded infrastructure, rapid population growth, rural-urban migration, and pollution do not allow cities to support their citizens, however. Even though these problems are more serious for cities in developing countries, they also exist in the US. For example, inadequate sewer systems spill billions of gallons of untreated waste into streams annually. Some US cities lose up to 30% of their daily drinking water through pipes in disrepair. In developed countries, much of destruction of cities is a result of planning or lack of planning centered around the automobile, e.g., rapid suburbanization. Environmental pollution in cities adversely affect the health of residents, e.g., exacerbating asthma in developed countries and diarrhea in developing countries. Since the 1970s, Western European planners have incorporated compact development into their urban plans. They contain suburbanization by revitalizing inner cities and diverting growth into fully functioning satellite towns. Urban planning should emphasize intensive land use, mass transit, conservation of resources, and energy efficiency. Some economists believe that urban poverty will be the most significant problem in the 2000s. In developing nations, the poor live in shantytowns with no running water, sanitation, urban transport, or adequate shelter. In these urban areas, high birth rates play a bigger role in urban growth than does rural-urban migration. US federal policies during the 1980s have resulted in considerable decay inner cities. Recent riots in Los Angeles have alerted policymakers to the costs of neglect of inner cities. US citizens must discuss what needs to be done to transforms into vital living and cultural areas. Revitalization of the cities is a must.
PMID: 12286284 [PubMed - indexed for MEDLINE]

2173. Clin Exp Allergy. 1992 Jul;22(7):701-10.

Dynamics of mast cells in the nasal mucosa of patients with allergic rhinitis and non-allergic controls: a biopsy study.

Fokkens WJ, Godthelp T, Holm AF, Blom H, Mulder PG, Vroom TM, Rijntjes E.

Department of Otorhinolaryngology, Erasmus University, Rotterdam, The Netherlands.

Mast cell degranulation is thought to be an important component of the pathogenesis of allergic rhinitis. Quantitative studies on mast cells in nasal mucosa after allergen exposure have given widely divergent results, ranging from an overall decrease via redistribution to an overall increase. We investigated this problem by employing a combination of anti-IgE and toluidine blue staining of biopsy specimens. In allergic patients anti-IgE was found to identify all mast cells and toluidine blue to detect mast cells that were not (totally) degranulated. The study was composed of two parts done in different patient groups. In the first part of the study biopsies were performed in 23 patients with isolated grass-pollen allergy, once during natural provocation in the summer and once in the winter. Biopsies were also performed in 12 controls. Non-allergic controls were found to have the same number of mast cells in the lamina propria as asymptomatic allergic patients. The controls seldom have mast cells in the epithelium. The patients with isolated grass-pollen allergy showed an increase in the numbers of mast cells in the lamina propria during natural provocation and the same seemed to occur in the epithelium as well. During natural provocation almost all of the mast cells in the epithelium and half of those in the lamina propria were degranulated. In the second part of the study 17 patients with isolated grass-pollen allergy and four controls were challenged daily with allergen extract during a 2-week period in the winter. During this period biopsies were performed at eight different occasions, i.e. once before, six occasions during and once after the provocation period. The results of this part of the study showed that during provocation mast cells migrate to the surface of the nasal mucosa, where they become degranulated, and that the pool of mast cells in the lamina propria was apparently replenished by migration of mast cells from the vessels in the lamina propria. The total number of mast cells in the lamina propria remained approximately the same while the mast cells residing in an increasingly thick layer measured from the basal membrane into the lamina propria became degranulated. After 2 weeks, 82% of the mast cells in the lamina propria was degranulated and it was only in the deepest layers that some toluidine blue positive cells were found.(ABSTRACT TRUNCATED AT 400 WORDS)

PMID: 1504893 [PubMed - indexed for MEDLINE]

2174. Arerugi. 1992 Jul;41(7):778-86.

[Inhibitory effects of cyclosporin A and FK-506 on eosinophil chemotactic factor activity in culture supernatants of mononuclear cells from asthmatics].

[Article in Japanese]

Akutsu I, Fukuda T, Makino S.

Department of Medicine and Clinical Immunology, Dokkyo University School of Medicine.

We have previously reported that the 5-day culture supernatants of peripheral blood mononuclear cells (PBMC) from Dermatophagides farinae (DF) sensitive asthmatics stimulated with 10 ng/ml DF antigen contain eosinophil chemotactic activity (ECA) with an apparent molecular weight greater than 30000 Da. In the present study, we examined the effects of CyA and FK on the ECA. ECA was tested using modified Boyden chamber method. We found that when CyA or FK was added to the culture throughout the experiment, the production of the factors with ECA by PBMC was inhibited in a dose-dependent manner. These inhibitory effects were unchanged by the addition of a sufficient dose of IL-2 to the culture medium. Isoelectrofocusing of the PBMC culture supernatants consistently yielded a major

ECF activity at pH 7.0-7.5. The addition of CyA inhibited this major peak. In conclusion, these results suggest that mononuclear cells stimulated with related antigen produce substances which possess ECA and that CyA and FK can block the production of this substance. Therefore, there is a possibility that an immunosuppressive agent may be useful in bronchial asthma therapy by inhibiting the migration of eosinophils.

PMID: 1280086 [PubMed - indexed for MEDLINE]

2175. Blood. 1992 Jun 1;79(11):2937-45.

Migration of primed human eosinophils across cytokine-activated endothelial cell monolayers.

Moser R, Fehr J, Olgiati L, Bruijnzeel PL.

Department of Medicine, University Hospital, Zurich, Switzerland.

Eosinophils are known to adhere to cytokine-activated endothelium. Whereas transendothelial migration for neutrophils is an inevitable consequence of this endothelial-dependent adherence, this has not yet been shown for eosinophils. By means of human umbilical vein endothelial cells (HUVE) grown to confluence on microporous filters as an in vitro model of leukocytic migration across postcapillary venules, we have characterized the conditions leading to endothelium-driven transmigration of blood eosinophils from normals and from patients with allergic asthma. Freshly isolated eosinophils from nonallergic donors adhered to interleukin-1 (IL-1) and tumor necrosis factor-activated HUVE, but did not penetrate these monolayers. In contrast, eosinophils from allergic asthma patients showed an increased adherence and transmigration capacity. This increased functional competence was not caused by a difference in density phenotype, because the eosinophils from both groups showed a comparable density distribution over discontinuous Percoll gradients. Moreover, no difference existed within one group among eosinophils harvested from the Percoll density bands 1.080, 1.085, and 1.090 g/mL in terms of transendothelial migration. In vitro cultivation of freshly isolated eosinophils from nonallergic individuals in the presence of granulocyte-macrophage colony-stimulating factor (GM-CSF) and IL-3 induced a stepwise decrease of the density distribution over such gradients. In contrast, eosinophils from patients with allergic asthma directly shifted to a final density of 1.075 g/mL within 24 hours of culture. Notwithstanding the kinetics of density changes, eosinophils from nonallergic donors already expressed the capacity to transmigrate IL-1-activated HUVE monolayers 20 hours after cultivation with different combinations of GM-CSF, IL-3, and IL-5. Inhibition studies with monoclonal antibodies showed that endothelium-driven transmigration of eosinophils predominantly implicates CD11/CD18 structures on the eosinophil surface, whereas no significant inhibition was found with the anti-VLA-4 monoclonal antibody HP2/1. From cytofluorometric studies, we conclude that spontaneous transmigration of eosinophils from allergic asthma patients is not accompanied by quantitative upregulation of these antigens. Taken together, these results allow the conclusion that blood eosinophils from allergic asthma patients have undergone in vivo priming, mimicked in vitro by cytokines such as GM-CSF, IL-3, and IL-5, leading to induction of the capacity to migrate across cytokine-activated HUVE monolayers.

PMID: 1586739 [PubMed - indexed for MEDLINE]

2176. J Lipid Mediat. 1992 Jun-Jul;5(2):121-6.

Sensitization primes platelet-activating factor (PAF)-induced accumulation of

eosinophils in mouse skin lesions: contribution of cytokines to the response.

Yukawa T, Terashi K, Terashi Y, Arima M, Sagara H, Motojima S, Fukuda T, Makino S.

Department of Medicine and Clinical Immunology, Dokkyo University School of Medicine, Tochigi, Japan.

We have examined the hypothesis that cytokines mediate the enhanced responsiveness of eosinophils to PAF in sensitized mouse skin. PAF (10 ng per site) resulted in a considerable degree of eosinophil accumulation in ovalbumin (OA)-sensitized mice but not in non-sensitized mice. Intradermal preadministration of cytokines (IL-5, IL-3 and GM-CSF) also significantly enhanced PAF-induced migration of eosinophils in a dose-dependent manner. The relative potency with which these cytokines primed cell migration was IL-5 greater than IL-3 greater than GM-CSF, however, each cytokine alone showed no direct effect. We conclude that the sensitization or the exogenous application of cytokines is capable of augmenting PAF-induced eosinophil migration in mice in vivo, and the cytokines thus elicited by sensitization may contribute to the extensive recruitment of inflammatory cells in allergic diseases.

PMID: 1525351 [PubMed - indexed for MEDLINE]

2177. Ann Intern Med. 1992 May 15;116(10):838-40.

Visceral larva migrans associated with the hypereosinophilic syndrome and the onset of severe asthma.

Feldman GJ, Parker HW.

Dartmouth-Hitchcock Medical Center, Lebanon, New Hampshire.

PMID: 1567098 [PubMed - indexed for MEDLINE]

2178. Am J Respir Cell Mol Biol. 1992 May;6(5):557-66.

Selective eosinophil leukocyte recruitment by transendothelial migration and not by leukocyte-endothelial cell adhesion.

Morland CM, Wilson SJ, Holgate ST, Roche WR.

Department of Pathology, University of Southampton, United Kingdom.

Eosinophil infiltration is the hallmark of allergic inflammatory events. However, the mechanisms governing the influx of eosinophils into the tissue at a site of an allergic reaction remains unclear. We have examined the interactions of eosinophils and neutrophils isolated from the same atopic donor with cultured human umbilical vein endothelial cell (EC) monolayers in the search for a mechanism for this selective eosinophil recruitment. First, the adherence of eosinophils and neutrophils to ECs stimulated with lipopolysaccharide, interleukin (IL)-1 alpha, and tumor necrosis factor-alpha were compared. Each mediator induced a similar dose-dependent enhancement of eosinophil adhesiveness for both eosinophils and neutrophils. Thus, although cytokine activation of ECs in the vasculature adjacent to an inflammatory site probably serves as an important focusing mechanism for the extravasation of inflammatory cells at this site, there does not appear to be any selective EC-dependent mechanism for eosinophil recruitment. Little or no effect on eosinophil and neutrophil adherence was observed with IL-3, IL-5, granulocyte/macrophage colony-stimulating

factor, platelet-activating factor (PAF), leukotriene B4, or histamine. Second, the migration of eosinophils and neutrophils through an EC monolayer in response to chemoattractants was examined. PAF was found to selectively enhance eosinophil transendothelial migration at doses of 10(-7) to 10(-10) M, with optimal effect at 10(-8) M. This effect was gradient dependent and could be inhibited by WEB 2086, a specific PAF inhibitor. These results suggest that localized production of PAF may be a prime factor in the events leading to eosinophil accumulation at allergic inflammatory sites, and that selectivity for eosinophil recruitment occurs at the stage of transendothelial cell migration under the influence of cell-specific chemoattractants.

PMID: 1316135 [PubMed - indexed for MEDLINE]

2179. Schweiz Med Wochenschr. 1992 Feb 29;122(9):298-301.

Early activation or "priming" of eosinophils in asthma.

Bruijnzeel PL, Rihs S, Virchow JC Jr, Warringa RA, Moser R, Walker C.

Swiss institute of allergy and asthma research, Davos Platz.

Increased numbers of blood and tissue eosinophils are regularly observed in subjects suffering from bronchial asthma. The eosinophil number in the diseased organ is normally closely associated with the presence of clinical symptoms. Not only the cell number, but also the concentration of eosinophil-granule derived mediators is increased in the diseased organ. In particular toxic proteins released by the eosinophil may be responsible for the allergic inflammatory reaction and the concomitant tissue damage. Our recent investigations have shown that eosinophilic granulocytes from asthmatic individuals have the same phenotype as eosinophils from normal individuals (i.e. with respect to their density distribution pattern and surface receptor expression). In contrast, eosinophils from asthmatic individuals do possess increased metabolic activity (i.e. increased leukotriene C4 (LTC4) generating capacity and migration capacity). This increased metabolic activity is due to the presence of circulating factors, i.e. the cytokines interleukin 3 (IL-3), interleukin 5 (IL-5) and granulocyte-macrophage colony stimulating factor (GM-CSF). These cytokines are demonstrable in the circulation of asthmatic, but not normal individuals; they are synthetized by activated T-lymphocytes. This early activation, called "priming", should be the goal of future pharmacological endeavours in order to achieve more efficient treatment of asthma.

PMID: 1546279 [PubMed - indexed for MEDLINE]

2180. Clin Exp Allergy. 1992 Mar;22(3):379-83.

Haemopoietic growth factors induce human basophil migration in vitro.

Yamaguchi M, Hirai K, Shoji S, Takaishi T, Ohta K, Morita Y, Suzuki S, Ito K.

Department of Medicine and Physical Therapy, University of Tokyo School of Medicine, Japan.

Accumulation of basophils in inflammatory sites is an important aspect of the late-phase allergic reaction involving skin and upper and lower airways, suggesting the existence of mechanisms for basophil migration. Because haemopoietic growth factors have been shown to stimulate various functions of human basophils, we tested the ability of haemopoietic growth factors to migrate basophils in vitro. Both IL-3 and granulocyte-macrophage colony-stimulating

factor (GM-CSF) induced migration of purified normal basophils (purity c. 80%) in a dose-dependent fashion at picomolar concentrations, while granulocyte (G)-CSF, macrophage (M)-CSF, and IL-4 had no effect at all. Chequerboard analyses indicate that migratory activity of both factors are chemokinetic. These results suggest that local production of both factors during allergic reactions might potentially play an initial role in the recruitment of basophils from the circulation to sites of inflammatory reactions.

PMID: 1586877 [PubMed - indexed for MEDLINE]

2181. J Am Mosq Control Assoc. 1992 Mar;8(1):65-8.

Comparison of people who request mosquito control services and their non-requesting neighbors.

Morris CD, Clanton KB.

Florida Medical Entomology Laboratory, IFAS-University of Florida, Vero Beach 32962.

The personal profiles of people who called to request mosquito control services were compared with their neighbors who did not call. Demographically, callers were generally representative of their neighbors. Callers considered the mosquito problem to be greater than did their non-calling neighbors, tended to do more things outdoors, tended to be home more, and considered themselves more attractive to mosquitoes and more allergic to mosquito bites than non-callers. There were also more newer (less than 1 year) residents among the callers than non-callers. Callers were almost exclusively from year-round-resident homeowners in long-term-resident neighborhoods.

PMID: 1583492 [PubMed - indexed for MEDLINE]

2182. Arerugi. 1992 Mar;41(3):418-27.

[Studies on the pathogenic mechanism of beta-lactam hypersensitivity by the detection of leucocyte migration activating factor and leucocyte migration inhibitory factor--structural correlations with allergic symptoms due to beta-lactam antibiotics].

[Article in Japanese]

Uno K.

Suibarago Hospital.

Structural correlations between allergic symptoms and beta-lactam antibiotics were investigated by the use of leucocyte migration inhibition tests on 147 patients in whom allergy to the drugs was detected from among 193 patients suspected of having beta-lactam hypersensitivity. No significant difference was found in the allergic symptoms between the mother nucleus structure of beta-lactam antibiotics or the C-3 side chain structure of cephem antibiotics. But, in both beta-lactam and cephem antibiotics, those with an aminothiazolyl group in the acylamido in their side chain structure induced fever or hepatopathy significantly more often than skin eruption. In contrast, drugs with a benzyl group induced skin eruptions significantly more often than hepatopathy. Leucocyte migration activating factor (LMAF) was found significantly more often than leucocyte inhibitory factor in patients with fever or hepatopathy, and cephem antibiotics with an aminothiazolyl group in the 7-acylamido side chain produced

LMAF at a very high rate (80%). Our findings indicate that the specificity of the chemical structure of beta-lactam antibiotics and allergic symptoms is dependent on the acylamido side chain structure, and an aminothiazolyl group structure has a high capability of inducing fever or hepatopathy. Moreover, the high LMAF-inducing ability of the aminothiazolyl group structure is involved in this pathogenic mechanism.

PMID: 1575639 [PubMed - indexed for MEDLINE]

2183. Ann Allergy. 1992 Mar;68(3):286-90.

Eosinophils in asthma.

Busse WW, Sedgwick JB.

University of Wisconsin Medical School, Madison.

Eosinophils, a prominent feature of asthma, are found in increased numbers in the circulation and sputum, usually in relation to the severity of asthma. As a consequence of these clinical observations, investigators now speculate that the eosinophil has a central role in the pathogenesis of asthma. Recent evidence has begun to confirm these speculations. The allergic reaction of the airway to antigen and the development of the late asthmatic reaction have provided a clinical model to study asthma and the contribution of eosinophils to bronchial reactivity. In the late asthmatic reaction, airway eosinophilia occurs. Through a series of independent observations, the following eosinophil-related events have been noted with the development of late asthmatic reactions. With either laboratory or natural exposure to antigen, eosinophilic chemotactic factors are released. Although the sources of eosinophil chemotaxis are multicellular, this is an early step in the attraction of eosinophils to the airway. As this process is initiated, a series of events occurs to cause eosinophils to arrive in the airway and promote obstruction, injury, and bronchial hyperresponsiveness. These steps include eosinophil migration through the vascular endothelium, upregulation of eosinophils (characterized by a change in cell density), adhesion of eosinophils to airway epithelium, and release of eosinophil toxic products. This presentation will review some of the eosinophil-dependent factors that can cause asthma. Furthermore, the eosinophil may be a good target for future therapeutic interventions.

PMID: 1546825 [PubMed - indexed for MEDLINE]

2184. Orv Hetil. 1992 Feb 9;133(6):359-61.

[Helicobacter pylori allergy].

[Article in Hungarian]

Varga L, Löcsei Z, Döbrönte Z, Lakatos F, Brózik M, Merétey K.

Vas Megyei Markusovszky Kórház, I. Belgyógyászati Osztály.

A case of a 44 year old woman with antrum gastritis and H. pylori infection was reported. After unsuccessful treatment of the disorder with bismuth and tinidazole, an auto-vaccine was prepared from the bacterium in order to eliminate the infection. After the first injection of the vaccine a generalised urticaria was observed. In the development of the skin eruptions a type I, and a type IV allergic reaction could be demonstrated using the H. pylori specific RAST-test and leukocyte migration inhibition respectively. After eradication of the

bacterium by amoxycillin treatment, the clinical signs of both the gastrointestinal and allergic diseases disappeared.

PMID: 1741153 [PubMed - indexed for MEDLINE]

2185. Ann Allergy. 1992 Feb;68(2):190-6.

Use of cetirizine to investigate non-H1 effects of second-generation antihistamines.

Townley RG, Okada C.

Department of Medicine, Creighton University School of Medicine, Omaha, Nebraska.

In addition to their increased potency as H1 blockers and their nonsedating effects, the second-generation antihistamines have other unusual and potentially beneficial properties. Evidence is accumulating from several laboratories that at least one of these agents under investigation, cetirizine, may be effective in inhibiting the late reaction. The Johns Hopkins group showed that during the cutaneous late phase response (LPR), histamine release was not altered by cetirizine, 20 mg, pretreatment. The most dramatic effect of cetirizine was attenuation of inflammatory cell migration into the chamber. Eosinophils, neutrophils, and basophils were reduced by about 75% during hours 6 to 8. It can be concluded that cetirizine influences the LPR by causing a reduction in the inflammatory cell infiltrate. Cetirizine, 10 mg, orally once a day also induced a significant decrease in the wheal and flare skin reactions caused by pollen, histamine, and compound 48/80. Cetirizine inhibited eosinophil recruitment and platelet-activating factor (PAF) in skin chambers 24 hours after pollen challenge. We and others have studied the mechanisms of this effect. The release of eosinophil peroxidase induced by PAF and formyl-methionyleucyl/phenylalanine was not attenuated by cetirizine. At therapeutic concentrations, however, cetirizine has a potent inhibitory action in vitro on eosinophil chemotaxis induced either by formyl-methionyleucyl/phenylalanine or PAF and also on IgE-dependent stimulation of platelets. In a separate study in patients with chronic urticaria, cetirizine markedly reduced both the immediate wheal and flare induced by PAF and the delayed reaction at six hours. These results suggest that cetirizine acts on eosinophil migration to inhibit the late reaction.

PMID: 1346737 [PubMed - indexed for MEDLINE]

2186. Acta Derm Venereol. 1992;72(3):169-71.

Expression of beta-2 integrin molecules on human keratinocytes in cytokine-mediated skin diseases.

Simon M Jr, Hunyadi J, Dobozy A.

Department of Dermatology, University of Erlangen-Nürnberg, FRG.

Integrins are cell surface molecules of importance in a wide variety of cellular functions, including morphogenesis, cell migration and cell matrix interactions. The beta-2 (B2) integrin (leukocyte integrin, CD11/CD18) subfamily comprising three members, each consisting of a shared beta subunit (CD18) non-covalently associated with unique alpha subunits (CD11a, CD11b, CD11c). In the present study, we have analysed the expression pattern of B2 integrins on the surface of human keratinocytes (HKs) in biopsies obtained from healthy volunteers, from positive tuberculin skin tests and from patients with acute urticaria (AU), lichen planus (LP), psoriasis vulgaris (PV), mycosis fungoides (MF) or purpura

pigmentosa chronica (PPC). In biopsies obtained from positive tuberculin tests and from the clinically involved skin of patients with LP, PV, MF or PPC, a multifocally occurring, suprabasal peroxidase-positive reaction was observed on the membranes of the HKs when the monoclonal antibodies (MABs) Dako CD11a, Dako-p150, 95 or Dako CD18 were used. In contrast, no specific staining of the HKs was observed with the same MABs in biopsies from healthy volunteers, from patients with AU and in the uninvolved skin specimens obtained from the other patients. The HKs from PV, LP, MF, PPC and AU patients and those from the healthy subjects failed to give a positive reaction when the MAB against CD11b (OKM1) was used. Our present findings provide further evidence that HKs may be actively involved in cell adhesion processes.

PMID: 1357849 [PubMed - indexed for MEDLINE]

2187. Allergy Proc. 1992 Jan-Feb;13(1):7-10.

The Africanized honey bee.

McKenna WR.

The Africanized honey bee (AHB), "the bee with an attitude problem," is described as more defensive, more likely to defend its nest in large numbers, and therefore cause multiple stings compared with the European honey bee (EHB) with which we are familiar. We can expect a greater number of toxic reactions related to multiple stings in addition to the more familiar allergic (IgE-mediated) reactions. The title "Killer Bees" is largely media derived. The first identified colony arriving by natural northward migration arrived in the Lower Rio Grande Valley in October 1990. Additional colonies have been trapped, identified, and destroyed by now. Because of extensive media coverage, the facts become very important because patients and possibly media may be contacting you as specialists in stinging insect allergy. Those living in the Southern United States are most likely to be involved. State and federal government agencies as well as certain industries have recognized this situation as important with a potentially very serious impact on agricultural interest. Some of these impacts will be discussed as well as local involvement in the Lower Rio Grande Valley of the Texas AHB Management Plan and medical plans.

PMID: 1577266 [PubMed - indexed for MEDLINE]

2188. Adv Immunol. 1992;51:323-82.

The pathobiology of bronchial asthma.

Arm JP, Lee TH.

Department of Allergy and Allied Respiratory Disorders, U.M.D.S., Guy's Hospital, London, England.

Early studies of patients dying from status asthmaticus revealed marked inflammation of the bronchial tree. Subsequent histological studies of the airways and examination of bronchoalveolar lavage fluid of subjects with mild asthma have confirmed the presence of airway inflammation in life. There is epithelial edema and desquamation, subepithelial deposition of collagen and fibronectin, and an inflammatory cell infiltrate in the mucosa. There are increased numbers of activated eosinophils, CD25-positive T lymphocytes, and immature macrophages with the phenotypic characteristics of blood monocytes. An increased expression of HLA class II is present on epithelium, macrophages, and other infiltrating cells. The severity of clinical asthma correlates with several

measurements of the severity of the inflammatory response, suggesting a crucial role for airway inflammation in the pathophysiology of the disease. There is considerable interest and research into the mechanisms underlying the pathogenesis and maintenance of the inflammatory response in asthma. The development and maintenance of the inflammatory response in asthma is likely to be a consequence of a complicated interaction between various cells and the mediators they generate. The characterization of an ever-increasing number of cytokines is of particular interest. Interleukin-3, interleukin-5, and granulocyte-macrophage colony-stimulating factor are hematopoietic growth factors that increase the survival of eosinophils in culture and enhance certain eosinophil functions, such as mediator generation and toxicity. Alveolar macrophages derived from asthmatic subjects produce twofold to threefold more GM-CSF than do those from normal control subjects. Using in situ hybridization, the presence of IL-5 mRNA has been demonstrated in bronchial biopsies from asthmatic subjects. Thus IL-3, IL-5, and GM-CSF influence eosinophil function and survival, and may be generated by T lymphocytes and/or alveolar macrophages within the airways in asthma. In addition to these three cytokines, IL-4 and interferon-gamma may be crucial to the regulation of IgE biosynthesis. TNF-alpha and IL-1 are potentially important in the up-regulation of endothelial adhesion molecules. An important step in the recruitment of leukocytes to an inflammatory focus is margination to the vascular endothelium. Our understanding of the molecular events involved in migration of leukocytes to an inflammatory focus has been advanced by the discovery and characterization of a variety of cell adhesion molecules. The potential role of ELAM-1 and ICAM-1 in allergic inflammation is suggested by their up-regulation on vascular endothelium in association with late cutaneous responses to allergen and by their role in certain primate models of asthma.(ABSTRACT TRUNCATED AT 400 WORDS)

PMID: 1502977 [PubMed - indexed for MEDLINE]

2189. Trop Geogr Med. 1992 Jan;44(1-2):109-12.

Visceral larva migrans in seven members of one family in Trinidad.

Bratt DE, Tikasingh ES.

Department of Child Health, University of the West Indies, Port of Spain, Trinidad.

The occurrence of the first seven cases of visceral larva migrans in Trinidad and Tobago is described. All cases occurred in children of the same family. The ELISA test was used to confirm the clinical diagnosis.

PIP: Visceral larva migrans is a syndrome caused by the extraintestinal migration of larval nematodes of wild and domestic animals through human tissues. Most cases occur in children under 5 years of age, with the common dog round worm apparently being the primary causative agent. This paper describes the occurrence of the first 7 cases of visceral larva migrans in Trinidad and Tobago. Cases occurred among siblings in the same family and were confirmed using the ELISA test. A mentally retarded 10-year old East Indian male was admitted to the Port of Spain General Hospital with a strong history of pica, recurrent wheezing, and epilepsy. Five siblings and one cousin were found living under extremely unsanitary conditions and in dire poverty. All had pica and Toxocara canis eggs were found in the dirt surrounding the dwelling. These eggs are most probably the common source of infection. Infection with Toxocara species is probably far more common than reported in Trinidad, given the large stray dog population and areas of primitive sanitary conditions. ELISA tests could be used to reveal additional infections in the country.
PMID: 1496701 [PubMed - indexed for MEDLINE]

2190. Lung. 1992;170(4):221-33.

Expression of the CD11/CD18 cell surface adhesion glycoprotein family and MHC class II antigen on blood monocytes and alveolar macrophages in interstitial lung diseases.

Hoogsteden HC, van Hal PT, Wijkhuijs JM, Hop W, Hilvering C.

Department of Pulmonary Medicine, University Hospital Dijkzigt, The Netherlands.

The expression of molecules of the CD11/CD18 cell surface adhesion glycoprotein family and HLA/DR antigen was studied on peripheral blood monocytes (PBM) and alveolar macrophages (AM) in bronchoalveolar lavage (BAL) fluid from patients with sarcoidosis, idiopathic pulmonary fibrosis (IPF), and extrinsic allergic alveolitis (EAA). Patients with these interstitial lung diseases showed increased numbers of macrophages in BAL fluid. This was probably caused by an increased influx of PBM to the alveoli since the numbers of cells with a monocytic morphology were also significantly increased in BAL samples from patients with interstitial lung disease, most prominently in IPF and EAA. The increased influx of PBM into the alveoli in patients with interstitial lung diseases was not reflected by an increased expression of the CD11/CD18 leukocyte function antigens on PBM. In healthy volunteers as well as in those with sarcoidosis, IPF, and EAA, the percentages of AM positive for CD11b (the C3bi complement receptor) and CD11c were lower than among PBM. This indicates that the expression of these cell surface adhesion molecules is downregulated during maturation and migration of PBM to the alveoli. The absolute numbers of AM positive for CD11b were increased in BAL fluid of IPF and EAA patients compared to healthy volunteers. EAA patients also showed increased absolute numbers of AM positive for CD11a and CD11c. This differentially increased expression of these leukocyte function antigens on AM suggests the influence of locally produced cytokines.

PMID: 1355796 [PubMed - indexed for MEDLINE]

2191. Med Pregl. 1992;45(9-10):365-8.

[Skin and laboratory tests: comparison of the epicutaneous patch test with the TTL and LIF tests in the diagnosis of medicamentous allergic contact dermatitis].

[Article in Croatian]

Jovanović M, Poljacki M, Milakov J, Duran V.

Klinika za infektivne o dermatovenerologske bolesti, Hajduk Veljkova, Medicinski fakultet, Novi Sad.

We investigated 158 patients from 15 to 82 years of age with clinically evident contact dermatitis, diagnosed at the Department for Allergic Diseases Investigations-Clinic for Dermatovenerological Diseases, Medical Faculty in Novi Sad, during the period of 1 year. We performed patch-epicutaneous test, lymphocyte transformation test (TTL) and leukocyte migration inhibition test (LIF). Among the 130 (82.2%) patients suffering from contact dermatitis, with the positive patch tests to commercial or standard battery epicutaneous allergens, 26 (20%) had at least one positive patch test to the medicament. In these cases contact allergic medicamentous dermatitis (SAMD) was proved by positive clinical and allergic investigations. In only one case, patch test was negative, with booth the TTL and LIF test positive. Among the medicaments TTL and LIF tested, antibiotics were the most frequent in 9 (34.61%) cases, analgetics were found in

6 (23.08%). Professional contact allergy to medicaments has been estimated in 5 patients (19.23%). According to the obtained results and statistic findings, relationships between the two variables-TTL and patch test, and LIF and patch test, were estimated due to the contact allergy to medicaments. Both of them were low and negative without statistic significance. Patch test remains to be the irreplaceable test with regard to CAMD.

PMID: 1344475 [PubMed - indexed for MEDLINE]

2192. Arch Immunol Ther Exp (Warsz). 1992;40(5-6):271-4.

The migration of granulocytes from allergic and nonallergic infiltrations in patients with atopic asthma.

Matusiewicz R, Grzybowska K, Urbankowska B, Kowalczyk M.

First Department of Internal Diseases, Grochow Hospital, Warsaw, Poland.

The granulocyte migration inhibition tests both in vivo and in vitro from allergic skin infiltrations were carried out in 30 patients with atopic asthma and in 20 healthy people used as a control. It was found that the migration of leukocytes from allergic skin infiltrations was defective with the lowest ability to migrate. Neutrophils comprised about 80% cells found in the cutaneous allergic infiltrations.

PMID: 1340181 [PubMed - indexed for MEDLINE]

2193. Pneumonol Alergol Pol. 1992;60 Suppl 2:92-3.

Granulocyte migration abnormality in patients suffering from allergic rhinitis.

Samolińska U, Zawisza E, Jakubas-Przewocka J.

Allergy Department, Warsaw Medical School.

PMID: 1305514 [PubMed - indexed for MEDLINE]

2194. Br J Clin Pharmacol. 1991 Nov;32(5):557-61.

Theophylline therapy inhibits neutrophil and mononuclear cell chemotaxis from chronic asthmatic children.

Condino-Neto A, Vilela MM, Cambiucci EC, Ribeiro JD, Guglielmi AA, Magna LA, De Nucci G.

Department of Pediatrics, Faculty of Medical Sciences, UNICAMP, SP, Brazil.

1. Theophylline is commonly used to relieve symptoms of chronic asthma. Since neutrophil and mononuclear cell activation are associated with late phase asthmatic reactions, effects of theophylline on these cells may be of importance. 2. In the present investigation we compared neutrophil and mononuclear cell chemotaxis from chronic asthmatic children during and after theophylline therapy. 3. Thirty patients were recruited for the study. Each patient received theophylline orally for 10 days. The theophylline dose was 20 mg kg-1 day-1 given in four divided doses. On the tenth day, blood was collected into heparinized (100 u ml-1) and siliconized tubes 2 h after the last theophylline dose for chemotactic assays, cAMP and theophylline plasma determinations. When clinical

conditions allowed, theophylline was discontinued for 7 days and the chemotactic assays, cAMP and theophylline plasma concentrations repeated. Serum complement and IgE levels were also determined. 4. Theophylline therapy clearly inhibited both spontaneous and stimulated neutrophil and mononuclear cell chemotaxis. Twenty-seven patients had therapeutic plasma concentrations of theophylline (5-20 micrograms ml-1). Discontinuation of theophylline therapy caused a significant decrease in plasma cAMP levels (44 and 31 pmol ml-1 respectively during and after treatment, n = 30, P less than 0.001). 5. The inhibition of neutrophil and mononuclear cell migration by theophylline therapy in chronic asthmatic children may be beneficial for the control of the inflammatory response observed in these patients.

PMCID: PMC1368630
PMID: 1659436 [PubMed - indexed for MEDLINE]

2195. J Immunol. 1991 Oct 15;147(8):2595-602.

Activation of guinea pig eosinophils by human recombinant IL-5. Selective priming to platelet-activating factor-acether and interference of its antagonists.

Coëffier E, Joseph D, Vargaftig BB.

Unité de Pharmacologie Cellulaire, Unité Associée Institut Pasteur-INSERM no. 285, Paris, France.

The potential role of platelet-activating factor (PAF)-acether and of IL-5 as an eosinophil-proliferating, activating, and/or recruiting mediator in asthma led us to study the effects of human (h) rIL-5 (hrIL-5) and PAF-acether, alone or combined, on isolated guinea pig eosinophils. Two populations of eosinophils were separated from peritoneal lavages of polymyxin B-treated guinea pigs upon a discontinuous metrizamide gradient: one of low density (between 20 and 22% of metrizamide, purity: 63 +/- 3%, n = 27) and another of normal density (between 22 and 24% of metrizamide, purity: 87 +/- 2%, n = 16). Chemotactic activity was evaluated on a micro-Boyden chamber, results being expressed as the number of migrating eosinophils (mean +/- SEM) at 40 microns through a cellulose nitrate filter (3 microns pore size) in the presence of the agonist or of the solvent alone. hrIL-5 dose-dependently stimulated normodense eosinophil chemotaxis, reaching a peak at 500 ng/ml (98 +/- 21 migrating eosinophils, n = 5, p less than 0.05). These eosinophils also responded to PAF-acether and to LTB4 and not to FMLP, hrTNF alpha, and LPS. Eosinophil preincubation with hrIL-5 increased significantly the migration by PAF-acether (173 +/- 23 migrating eosinophils with PAF-acether 10 nM after preincubation with hrIL-5 500 ng/ml vs 69 +/- 10 after preincubation with buffer alone, p less than 0.01) and failed to enhance migration by LTB4 or to uncover an activity for FMLP. Migration by PAF-acether was antagonized when the cells were preincubated with the antagonists BN 52021 and WEB 2086, which also inhibited migration by hrIL-5. Eosinophils were auto-desensitized by and to PAF-acether or LTB4, but were not cross-desensitized to each other. Eosinophils desensitized to PAF-acether failed to migrate with hrIL-5, but those desensitized to LTB4 responded to hrIL-5 as controls. hrIL-5 failed to induce the elevation of intracellular free calcium concentration and superoxide anion generation from basal values, whereas preincubation of eosinophils with hrIL-5 induced a significant increase in the rise in intracellular free calcium concentration and in superoxide anion generation by 10 nM PAF-acether but not by LTB4. In conclusion, the in vivo eosinophil migration in allergy may involve hrIL-5, particularly associated to PAF-acether.

PMID: 1655895 [PubMed - indexed for MEDLINE]

2196. Arch Dermatol. 1991 Oct;127(10):1558-63.

Recent developments in the pathogenesis of allergic contact dermatitis.

Kalish RS.

Department of Dermatology, University of Minnesota, Minneapolis.

Allergic contact dermatitis is both an important clinical problem and a model system for lymphocyte-mediated pathologic changes. Elicitation of allergic contact dermatitis requires interaction of antigen with epidermal Langerhans cells, followed by migration of the Langerhans cells to the lymph nodes to present antigen to T lymphocytes. These activated T lymphocytes must then home to the antigen-exposed skin. Adhesion molecules such as LFA-1 and ICAM-1 have a role in this homing. Only a small proportion of the T lymphocytes in the skin lesion are specific for the inducing antigen. Studies of poison ivy (urushiol dermatitis) have determined this fraction to be less than one per 100 infiltrating lymphocytes. By a variety of amplification mechanisms, it is possible for this small number of antigen-specific T lymphocytes to induce the pathologic changes of allergic contact dermatitis. Improved understanding of this condition should result in increased knowledge of the pathogenesis of a variety of T lymphocyte-mediated skin conditions.

PMID: 1929465 [PubMed - indexed for MEDLINE]

2197. Clin Immunol Immunopathol. 1991 Sep;60(3):333-48.

Leukocyte adhesion in host defense and tissue injury.

Patarroyo M.

Department of Immunology, Karolinska Institute, Stockholm, Sweden.

During their life span, leukocytes adhere transiently to one another, to other cell types, such as vascular endothelial cells, and to extracellular matrix proteins. This adhesiveness is mediated by families of specific cell surface adhesion molecules, namely, integrins, immunoglobulin superfamily molecules, and selectins. Adhesion is required for leukocyte-mediated cytotoxicity, phagocytosis, chemotaxis, and induction of lymphocyte proliferation and maturation. It also participates in recirculation and homing of lymphocytes into lymphoid organs and in leukocyte migration from the vascular compartment to extravascular tissues. Adhesion underlies the beneficial or detrimental role of leukocytes in immune and inflammatory responses. In animals, blocking monoclonal antibodies to adhesion molecules dramatically reduce vascular and tissue injury in several organs following ischemia-reperfusion, and delay renal allograft rejection. Moreover, expression of particular adhesion molecules is induced or increased in cells which are targets for allergic or autoimmune reactions and in inflamed tissues. On the other hand, a congenital deficiency of the CD11/CD18 integrins (Leu-CAMs) leads to recurrent, and sometimes fatal, bacterial infections, and lack of particular cell-adhesion molecules on Burkitt's lymphoma cells may enable these cells to escape immunosurveillance.

PMID: 1830830 [PubMed - indexed for MEDLINE]

2198. Am Rev Respir Dis. 1991 Sep;144(3 Pt 1):714-7.

Effects of a long-standing challenge on pulmonary neuroendocrine cells of actively sensitized guinea pigs.

Bousbaa H, Fleury-Feith J.

Faculté de Médecine, Université Paris XII, Créteil, France.

Histologic studies using the silver stain method have implicated pulmonary neuroendocrine cells (NEC) in asthma by demonstrating an increase in their number in the bronchi of guinea pigs actively sensitized with ovalbumin and 10 min after challenge. We verified the same data and completed them by a study of the long-standing effects of a challenge on NEC number in guinea pig bronchi. Actively sensitized animals were killed 2, 6, 24, 48, 72, and 144 h after being challenged by an aerosolized solution of ovalbumin. This study was completed by the evaluation of eosinophilic infiltration of bronchi to test the recently proposed hypothesis of the possible eosinophil recruitment by NEC product. Our results confirmed the increase in NEC number in the bronchial wall after sensitization. Originally we demonstrated that, 24 h after challenge, the NEC number decreased significantly, compared to sensitized only animals, suggesting possible product release. Eosinophilic migration was observed in sensitized animals and, more importantly, in all sensitized plus challenged animals. We suggest that bronchial NEC may play a role in immunoallergic events that take place in the lung after challenge, probably by releasing mediators that may influence, among other effects, eosinophilic recruitment.

PMID: 1679982 [PubMed - indexed for MEDLINE]

2199. J Allergy Clin Immunol. 1991 Aug;88(2):187-92.

Comparison of platelet-activating factor-induced chemotaxis of normodense and hypodense eosinophils.

Little MM, Casale TB.

Department of Internal Medicine, Veterans Administration Medical Center, Iowa City, Iowa.

Platelet-activating factor (PAF)-induced eosinophil (EOS) migration is an important event in the development of tissue eosinophilia and allergic inflammation. EOSs are heterogeneous cells in that different states of activation have been ascribed to EOSs of varying densities. We therefore studied the ability of PAF to induce hypodense and normodense EOS chemotaxis. Both hypodense and normodense EOSs were isolated in pure form from seven subjects and studied concurrently. Dose-response and time-course experiments indicated no significant differences in PAF-induced hypodense versus normodense EOS chemotactic responses. Hypodense and normodense cells achieved maximal chemotaxis in response to 1 mumol/L of PAF, and maximal chemotaxis was achieved at 2 hours. However, marked differences in PAF-induced EOS chemotactic responses existed between patients. We conclude that PAF is a potent EOS chemoattractant, and despite reported differences in metabolic activity, normodense and hypodense EOSs exhibit similar chemotactic responsiveness to PAF.

PMID: 1880319 [PubMed - indexed for MEDLINE]

2200. Afr Health. 1991 Jul;13(5):10-1.

Persistent diarrhoea syndrome.

Behrens R.

PIP: Persistent diarrhea (PD) is 3 or more stools/day which lasts nonstop for 14 days. Some small intestine disorders impede its diagnosis. PD follows 3-20% of acute diarrhea cases. It is more difficult to treat than acute diarrhea and often brings about nutritional and metabolic complications, e.g., growth failure. Skin infection, systemic infection, and micronutrient deficiency often accompany PD so it is often referred to PD syndrome (PDS). PDS patients often have more frequent recurrences of diarrhea although not of PD. Deaths of hospitalized PDS patients range from 10-12% and most occur within the 1st 48 hours. Physicians should immediately follow the guidelines for managing sepsis dehydration, fever, hypoglycemia, and malnutrition when 1st treating a hospitalized PDS patient. They should then start broad spectrum antibiotics. Once stable, nutrition management can begin. This includes maintaining breast feeding or using expressed breast milk, a digestible balanced diet free of allergenic proteins, and additional micronutrients and vitamins. Upon arrival at home, the child should eat a high energy high protein diet. PDS most often occurs in young infants, e.g., peaking at 7 months in Bangladesh. Other risk factors include nonbreast feeding, recent antibiotic therapy, history of bloody diarrhea, vitamin A deficiency, and malnutrition. Giardia lamblia and aggregative, enterotoxigenic Escherichia coli in the stool have been associated with PDS, but have not yet been identified as causative agents. Scientists surmise that PDS is caused by an insult to the intestine which allows the passage of proteins, especially dietary proteins, through the mucosa thereby inducing a hypersensitive reaction which causes more mucosal damage. Excess bacterial growth plays a role in production of an irritant product which contributes to fluid loss.
PMID: 12316913 [PubMed - indexed for MEDLINE]

2201. Rev Alerg. 1991 Jul-Aug;38(4):105-9.

[Leukocyte migration inhibitory factor and basophil degranulation in drug reactions].

[Article in Spanish]

Rangel H, Montero P, Espinosa F, Castillo FJ.

Departamento de alergia e inmunología, Hospital de Especialidades Centro Médico La Raza, Instituto Mexicano del Seguro Social.

We performed a prospective study in patients with a medical history of adverse reaction to drugs with the purpose of rule out allergy. We included 31 patients who were attended in the Allergy Service. We compared the sensibility and specificity of the test of inhibition factor of leucocytes migration and degranulation basophil against the exposition. After the statistical analysis, we concluded: the laboratory test, we have already mentioned, have little sensibility and specificity so the exposition test in the quickest, useful, and more simple method to determine drugs allergy, but more dangerous.

PMID: 1724705 [PubMed - indexed for MEDLINE]

2202. J Am Acad Dermatol. 1991 Jun;24(6 Pt 2):1094-6.

Eosinophil infiltration: effects of H1 antihistamines.

Fadel R, David B, Rassemont R, Herpin-Richard N, Borgnon A, Rihoux JP.

Unité d'Immuno-Allergie, Institut Pasteur, Paris, France.

This article reviews the contribution of cell-mediated inflammatory responses to

the immediate immunoglobulin E-dependent allergic reaction. Apparently eosinophils play an important part in the pathogenesis of allergic reactions. Some new H1 antihistamines may also have non-H1-mediated antiinflammatory properties. In two double-blind, placebo-controlled, crossover studies of allergic and normal subjects, we showed that oral cetirizine, at dosages of 10 and 20 mg/day, significantly inhibited wheal-and-erythema reactions induced by grass pollen, 48/80, histamine, platelet-activating factor acether, and N-formyl-methionyl-leucyl-phenylalanine. In the first study, cutaneous eosinophil migration was significantly inhibited by cetirizine at pollen and 48/80 skin test sites (61%, p less than 0.01, and 53%, p less than 0.01, respectively), although no change was observed at histamine skin test sites. Inhibition of neutrophil accumulation was also observed at pollen and 48/80 sites (41%, p less than 0.1, and 31%, p less than 0.1, respectively). Monocyte accumulation was not affected by cetirizine. In the second study, cetirizine suppressed the eosinophil influx induced by pollen, platelet-activating factor, 400 ng, and platelet-activating factor, 40 ng (63%, p less than 0.001; 58.5%, p less than 0.001; and 57.8%, p less than 0.01, respectively). This inhibition was effective 2 hours after challenge and persisted through hours 4, 8, and 24. N-Formyl-methionyl-leucyl-phenylalanine induced a weak eosinophil accumulation that was inhibited by cetirizine.

PMID: 1677013 [PubMed - indexed for MEDLINE]

2203. J Immunol. 1991 May 15;146(10):3419-23.

Human eosinophil, but not neutrophil, adherence to IL-1-stimulated human umbilical vascular endothelial cells is alpha 4 beta 1 (very late antigen-4) dependent.

Walsh GM, Mermod JJ, Hartnell A, Kay AB, Wardlaw AJ.

Department of Allergy and Clinical Immunology, National Heart and Lung Institute, London, England.

Eosinophils, through their ability to generate an array of potent mediators, are thought to be the major effector cells in a number of conditions, including parasitic infection, asthma, and other allergic diseases. The mechanism(s) by which eosinophils, as opposed to neutrophils, accumulate at inflammatory sites is unknown. One possible mechanism would be an eosinophil-specific pathway of adhesion to vascular endothelium. In this study we have demonstrated that human eosinophils, but not neutrophils, constitutively express alpha 4 beta 1 (CD49d/CD29). Expression was not increased on low density eosinophils or normal density cells stimulated with platelet-activating factor. Eosinophils, but not neutrophils, specifically adhered to COS cells transfected with vascular adhesion molecule-1 in a alpha 4 beta 1-dependent manner. Eosinophil, but not neutrophil, adhesion to IL-1 stimulated human umbilical vascular endothelial cells was significantly inhibited by alpha 4 beta 1 mAb at both 5 h (p less than 0.05) and 20 h (p less than 0.001). Inhibition of both resting and platelet-activating factor-(10(-7) M) stimulated eosinophil adhesion was observed. We conclude that the alpha 4 beta 1/vascular adhesion molecule-1 adhesion pathway may be involved in specific eosinophil, as opposed to neutrophil, migration into sites of eosinophilic inflammation.

PMID: 1709195 [PubMed - indexed for MEDLINE]

2204. Cell Immunol. 1991 May;134(2):505-10.

Cytokines in the central nervous system of mice during chronic relapsing

experimental allergic encephalomyelitis.

Baker D, O'Neill JK, Turk JL.

Department of Pathology, Royal College of Surgeons of England, London, United Kingdom.

Clinical disease phases of chronic relapsing experimental allergic encephalomyelitis (CREAE) in the Biozzi AB/H mouse model are associated with extensive cellular infiltration of the central nervous system, principally the spinal cord. The activation of these cells is further suggested by the immunocytochemical demonstration of cytokines (migration inhibition factor, interferon-gamma, tumour necrosis factor-alpha, and interleukins 1, 2, and 3) within these infiltrates. The in vitro functions attributed to these cytokines indicate their potential role in cell recruitment, activation, and differentiation of the ongoing immune response which could contribute to the pathogenesis of disease.

PMID: 1902401 [PubMed - indexed for MEDLINE]

2205. J Clin Invest. 1991 May;87(5):1805-9.

Expression of endothelial-leukocyte adhesion molecule-1 in elicited late phase allergic reactions.

Leung DY, Pober JS, Cotran RS.

Division of Pediatric Allergy-Immunology, National Jewish Center for Immunology and Respiratory Medicine, Denver, Colorado 80206.

To better understand the events involved in the local migration of inflammatory cells into sites of allergic reactions, we studied expression of the cytokine inducible endothelial cell (EC) neutrophil adhesion molecule, endothelial-leukocyte adhesion molecule (ELAM-1), in sequential skin biopsies from patients with respiratory allergy during the late phase reaction (LPR) between 20 min and until 24 h after intradermal allergen (ragweed or dust mites) injection. In 7 of 7 atopic patients but in only 1 of 4 apparently normal controls, allergen induced appearance of ELAM-1 on EC. ELAM-1 expression occurred concurrently with the development of inflammatory cell infiltrates by 3-4 h after intradermal injection. Saline injected sites in all subjects were negative. Skin organ cultures demonstrated that allergen could produce the same EC changes in vitro whether allergen was injected in vivo 20 min before culture or added during skin culture. These EC changes in organ culture were inhibited by the presence of combined anti-sera to both TNF-alpha and IL-1, but not by antisera to either cytokine alone. We conclude that EC activation occurs in elicited LPR and suggest that cytokine-induced EC activation may play a role in the migration of inflammatory cells into allergic skin reactions. Furthermore, resident cells in the skin rather than infiltrating leukocytes appear to be the source of the cytokines that mediate endothelial activation.

PMCID: PMC295299
PMID: 1708785 [PubMed - indexed for MEDLINE]

2206. Schweiz Med Wochenschr. 1991 Apr 6;121(14):501-8.

[Bronchial asthma and kitchen salt].

[Article in German]

Medici TC, Vetter W.

Departement für Innere Medizin der Universität Zürich.

Epidemiological studies show considerable geographic differences in asthma prevalence and mortality. The regions with high prevalence and mortality are countries with Western-type culture and a high degree of technological progress. They differ from less technically developed countries in a number of ways, including their higher salt intake. Air pollution is often given as the cause of the high prevalence of asthma in the industrialized countries. Against this, it must be pointed out that in the urban agglomerations of the developing countries and in rural areas where heating is by means of open fireplaces (indoor pollution), there is also considerable air pollution. Migration studies from New Zealand and South Africa, where asthma prevalence increases parallel to salt intake, provide evidence that other factors arising from westernization and urbanization play a role. In the industrialized countries England and USA there is also a clear connection between salt intake and asthma: the greater the salt consumption, the higher the asthma prevalence and mortality. On the basis of these observations, the following questions were investigated: (1.) Does salt loading worsen the clinical and functional findings in asthmatics? (2.) Is the sodium or the chloride in salt the more important? To answer these questions, the effect of salt loading (+6.1 +/- 2.8 g NaCl/d = 105 +/- 48 mmol Na), salt restriction, and loading with sodium citrate in equimolar concentrations (+140 +/- 40 ml Shohl's solution = 120 +/- 34 mmol Na) was investigated in 14 asthmatics in a controlled crossover study. Statistical analysis showed that salt intake worsened symptoms (p = 0.06) and increased the use of inhaled steroids (p less than or equal to 0.05).(ABSTRACT TRUNCATED AT 250 WORDS)

PMID: 2035006 [PubMed - indexed for MEDLINE]

2207. Am J Physiol. 1991 Apr;260(4 Pt 1):L189-206.

Inflammation and cell-cell interactions in airway hyperresponsiveness.

Leff AR, Hamann KJ, Wegner CD.

Department of Medicine, University of Chicago, Illinois 60637.

Airway hyperresponsiveness results from the conversion of normally reactive airways to a state of augmented responsiveness to constrictor stimuli. Although the mechanism accounting for the induction of airway hyperresponsiveness remains elusive, recent investigations have suggested that inflammation may be a sine qua non for human asthma. Numerous experimental models have demonstrated the necessity of circulating granulocytes as mediators of augmented bronchoconstriction during immune challenge. It is not known how granulocytes are targeted for selective migration to the conducting airways of the lung during hyperresponsive states; however, recent evidence implicates the upregulation of granulocyte adhesion molecules on both the endothelial and epithelial surfaces of the airway. There is evidence that during migration diapedesis, granulocytes interact with epithelial and endothelial cells to produce regionally secreted mediators that upregulate the responsiveness of adjacent airway smooth muscle and/or cause lumenal edema, thus augmenting the effect of constrictor stimuli. Most evidence suggests that the eosinophil is the most important granulocyte in these responses and that eosinophilic infiltration and activation may account for the unique, spasmodic, and cyclic nature of hyperreactive airways. The molecular biology of the eosinophil granule proteins has characterized four distinct substances, each of which exerts potential cytotoxic effects on airway epithelium by different mechanism. In addition, at least one of these proteins, the major

basic protein, appears to cause direct, noncytotoxic stimulation of epithelial secretion that upregulates nonspecifically the response of airway smooth muscle to contractile stimuli. The recognition of inflammation as the essential component to airway hyperresponsiveness provides a fresh approach to a difficult problem and suggests a host of novel therapies for human asthma.

PMID: 2018143 [PubMed - indexed for MEDLINE]

2208. Photodermatol Photoimmunol Photomed. 1991 Apr;8(2):62-4.

A comparison of cetirizine and terfenadine in the management of solar urticaria.

Bilsland D, Ferguson J.

Department of Dermatology, Ninewells Hospital and Medical School, Dundee, United Kingdom.

Many solar urticaria patients may benefit from the use of antihistamines. Historically, the value of such therapy was limited by sedation. Newer agents such as terfenadine and cetirizine that are relatively non-sedating appear to be better tolerated by patients. The latter drug, in addition to its antihistamine effect, also appears to inhibit eosinophil migration, which terfenadine and other potent H1 antagonists do not significantly affect. Eosinophils have been reported as early migrating cells in induced solar urticaria, raising the possibility that the dual action of cetirizine may provide a greater potential benefit in the management of solar urticaria. Six patients with idiopathic solar urticaria were entered into a double-blind, phototest study to compare cetirizine and terfenadine. Using the minimal urticarial dose as a phototest end-point, both drugs were equally effective in raising the threshold of sensitivity in 4 patients. Two patients failed to respond to either therapy, which is in keeping with the known variable response to histamine blockade in solar urticaria. At the dosage used, cetirizine therapy appears to be no more effective than terfenadine.

PMID: 1684515 [PubMed - indexed for MEDLINE]

2209. Int Arch Allergy Appl Immunol. 1991;95(2-3):191-4.

Appearance of macrophage migration inhibition factor in patients with systemic reactions to bee venom.

Goldberg A, Livni E, Mekori YA.

Unit of Allergy and Clinical Immunology, Meir General Hospital, Kfar Saba, Israel.

Cellular responses in bee venom (BV) allergy is a controversial issue. Previous studies could not reach an agreement whether this mechanism is activated as a result of allergic sensitization to bee venom. All previous works have used lymphocyte proliferation as their method to analyze cell-mediated immunity. In the present work, we tried to explore whether the production of macrophage migration inhibition factor (MIF), which is another in vitro correlate with cellular responses, is increased in these patients. We also examined which of the major antigenic components of BV played a significant role in the cellular response. Peripheral blood lymphocytes from 10 patients with systemic allergic reactions to bee sting and 9 healthy volunteers were examined for their ability to induce positive MIF responses. Macrophage inhibition was significantly increased in allergic patients when tested with BV, phospholipase A2 (PLA2) and with melittin. Positive MIF responses to other components were also more common

in allergic patients than in the control group. Our results indicate that cellular response to BV is expressed in patients with systemic allergic reaction to BV. When major antigenic components of BV are examined, PLA2 seems to play the major role in inducing this response.

PMID: 1937921 [PubMed - indexed for MEDLINE]

2210. Pediatriia. 1991;(5):82-4.

[Diagnosis of allergy to mold fungi in children with bronchial asthma].

[Article in Russian]

Sukovatykh TN, Sukheil'D.

As many as 360 children with bronchial asthma were examined for fungal sensitization. In addition to intracutaneous tests, use was made of the natural leukocyte migration inhibition test and leukocyte lysis test under the action of a specific allergen. The data obtained attest to a high portion (23.3%) of fungal sensitization among children suffering from bronchial asthma in the BSSR, high specificity of the leukocyte migration inhibition test and leukocyte lysis in the diagnosis of fungal sensitization.

PMID: 1866246 [PubMed - indexed for MEDLINE]

2211. J Leukoc Biol. 1991 Jan;49(1):58-64.

Inhibition of human neutrophil activation by the allergic mediator release inhibitor, CI-949.

Wright CD, Stewart SF, Kuipers PJ, Hoffman MD, Thueson DO, Conroy MC.

Department of Experimental Therapy, Parke-Davis Pharmaceutical Research Division, Warner-Lambert Company, Ann Arbor, Michigan 48105.

The allergic mediator release inhibitor Cl-949 [5-methoxy-3-(1-methylethoxy)-1-phenyl-N-1H-tetrazol-5-yl-1H -indole-2-carboxamide, L-arginine salt] was evaluated for its effects on human neutrophil functions. Cl-949 (100 microM) inhibited spontaneous migration and chemotaxis toward f-met-leu-phe (FMLP) by 49.1% and 45.8%, respectively. At the same concentration, Cl-949 inhibited the phagocytosis of serum-opsonized zymosan (SOZ) by 39.0%. Cl-949 inhibited leukotriene B4 and thromboxane B2 release in response to SOZ with IC50s of 2.0 microM and 3.3 microM, while inhibiting the response to FMLP with IC50s of 1.7 and 2.0 microM. Cl-949 also inhibited myeloperoxidase release from primary lysosomal granules in response to the following stimuli with the respective IC50s (microM): C5a (40.3); FMLP (34.4): SOZ (21.4); concanavalin A (Con A) 3.9); and calcium ionophore A23187 (91.2). In contrast, Cl-949 inhibited lysozyme release from secondary granules in response to SOZ and Con A with IC50s of 99.3 and 56.1 microM, while inhibiting the response to C5a, FMLP, and A23187 by 41.2%, 52.4%, and 10.0%, respectively, at 100 microM. Cl-949 (100 microM) had no inhibitory effect against lysozyme release in response to L-alpha-1,2 dioctanoylglycerol (DiC8), or phorbol 12-myristate 13-acetate (PMA). Cl-949 inhibited superoxide anion generation stimulated by FMLP and Con A with IC50s of 33.9 and 25.8 microM, while inhibiting the response to C5a, SOZ, and A23187 by 36.6%, 24.8%, and 14.1% and having no effect on the response to DiC8 or PMA at 100 microM. These results demonstrate preferential inhibition of arachidonic acid metabolism and degranulation of primary lysosomal granules by Cl-949 with selectivity for stimuli which promote intracellular

calcium mobilization or calcium influx.

PMID: 1845811 [PubMed - indexed for MEDLINE]

2212. Pneumonol Alergol Pol. 1991;59(11-12):31-4.

[Effect of platelet activating factor (PAF) on granulocyte migration in vivo in patients with atopic asthma].

[Article in Polish]

Matusiewicz R, Urbankowska B, Kowalczyk M.

Oddział Chorób Wewnetrznych Szpitala Grochowskiego, Warszawie.

The study was carried out in 40 patients with atopic asthma, age 16-31 years (mean 26 years) and 30 healthy volunteers, age 19-25 years (mean 20 years). In vivo granulocyte migration tests were carried out simultaneously using two methods: Southam's and the authors' original (Matusiewicz, Brzezińska). The above mentioned tests were carried out twice: the first time in December/January, the second time February/March after subcutaneous administration of PAF. The authors have demonstrated that in patients with atopic asthma a defect in migration of tissue granulocytes is present. They have also shown that PAF does not effect significantly granulocyte migration in atopic patients.

PMID: 1843897 [PubMed - indexed for MEDLINE]

2213. Ann Dermatol Venereol. 1991;118(12):939-40.

[Changes in the flora and cross contact allergy to plants].

[Article in French]

Ducombs G.

Clinique Dermatologique, Hôpital Pellegrin, Bordeaux.

PMID: 1799244 [PubMed - indexed for MEDLINE]

2214. Lab Delo. 1991;(12):38-40.

[A modification of the leukocyte migration inhibition test in vivo].

[Article in Russian]

Tarasov AV, Zherdev AV, Shuvalov LP.

Estimation of the degree of leukocyte migration inhibition in the oral cavity, following exposure to allergen solution, is a test widely used to assess a subject's allergy to drugs. To simplify this test and make it more effective, the authors suggest placing the samples in plate wells and drying them there; optically transparent polystyrene plates for enzyme immunoassay are employed. Effective adsorption of the cells on the carrier and the possibility of analyzing the samples with the help of multiple magnification permit a reliable detection of the migration inhibition. The method was tried in examinations of workers engaged in the manufacture of an x-ray contrast agent triambrin. Reduced migration levels were detected in 8 of the 12 examinees.

PMID: 1724487 [PubMed - indexed for MEDLINE]

2215. Agents Actions Suppl. 1991;34:269-93.

Cetirizine: more than an antihistamine?

Bernheim J, Arendt C, de Vos C.

UCB Pharmaceutical Sector, R & D, Chemin du Foriest, Braine l'Alleud, Belgium.

Cetirizine, a metabolite of hydroxyzine, is an antihistamine with as distinguishing features: 1) exquisite anti-H1 specificity: cetirizine appears unique in being devoid of action on receptors other than the H1 receptor; 2) potency: at unit dose it is the most potent antihistamine in the skin and the lung; 3) absence of metabolism. These three characteristics suggest that cetirizine be considered the choice H1 antagonist for experiments on the immediate allergic reaction. Cetirizine additionally in vitro inhibits the migration of eosinophils, and in vivo, in the skin, the infiltration by eosinophils that is characteristic for the late phase allergic reaction. Other antihistamines are less active or inactive with respect to this property. According to several lines of evidence, the effect of cetirizine on eosinophils is unlikely to be due to H1 antagonism, but is more likely a novel property of the compound.

PMID: 1686525 [PubMed - indexed for MEDLINE]

2216. Clin Ther. 1991 Jan-Feb;13(1):92-9.

Cetirizine: a unique second-generation antihistamine for treatment of rhinitis and chronic urticaria.

Pierson WE.

Department of Pediatrics, University of Washington, Seattle.

The recent development of selective H1-antagonists that minimally cross the blood-brain barrier has greatly improved the management of allergic rhinitis and chronic urticaria. These new agents have much reduced anticholinergic and sedative side effects, which were the major drawbacks of the classic H1-antihistamines. Cetirizine, a new second-generation H1-antagonist, offers several properties that may further improve the treatment of allergic rhinitis and chronic urticaria. Cetirizine is the only antihistamine known to possess activity against both the histamine-mediated early phase of the allergic response and the late-phase response of immediate hypersensitivity characterized by migration of inflammatory cells to the site of the reaction. Its efficacy has been demonstrated in clinical trials of patients with seasonal rhinitis and urticaria. The most common side effects associated with cetirizine, such as sedation, are similar to those of other second-generation antihistamines. These properties, combined with a once-daily dosage regimen, should help improve patient compliance and optimize antihistamine therapy.

PMID: 1674232 [PubMed - indexed for MEDLINE]

2217. Ann N Y Acad Sci. 1991;629:274-87.

Contribution of leukotriene B4 to airway inflammation and the effect of

antagonists.

Richards IM, Sun FF, Taylor BM, Shields SK, Griffin RL, Morris J, Wishka DG, Smith HW, Johnson RA, Dunn CJ.

Hypersensitivity Diseases Research, Upjohn Company, Kalamazoo, Michigan 49004.

Inhalation of aerosols of ovalbumin in sensitized guinea pigs produced a marked, bronchoalveolar eosinophilia 24 hr after challenge. The lung eosinophilia was not prevented by the cyclooxygenase inhibitors, indomethacin or PAF antagonists (WEB-2086 and L-652731) but was inhibited by methylprednisolone, the 5-LO inhibitor, U-66858 and a series of structural analogs of LTB4, U-75302, U-77692, U-75485 and U-78489. The effectiveness of LTB4 antagonists but not PAF antagonists in vivo was consistent with in vitro studies in which LTB4 was shown to be far more chemotactic than PAF for guinea pig eosinophils. LTB4 elicited maximal directional migration of guinea pig eosinophils at concentrations from 10(-7) M to 10(-9) M while PAF showed no effect over the same concentration range. The structural analogs of LTB4 were shown to inhibit LTB4 induced chemotaxis of guinea pig eosinophils and produced a dose-related inhibition of binding of LTB4 to guinea pig eosinophil membranes. To add further proof to the hypothesis that LTB4 contributed to the antigen-induced lung eosinophilia we attempted to measure LTB4 release into BAL fluid immediately after and at various time points up to 24 hr after antigen inhalation. However, using a sensitive radioimmunoassay (detection limit 10 pg/ml) very low levels of LTB4 (24.9-67.9 pg/ml) or its metabolite, 20-OH LTB4 (24.9-98.2 pg/ml) were detected in BAL fluid and these levels did not increase significantly following antigen provocation. Inhalation of LTB4 aerosols in unsensitized Brown-Norway rats or inhalation of aerosols of ovalbumin in sensitized Brown-Norway rats also produced a marked "late-phase" eosinophil-rich influx of inflammatory cells into the lungs. The lung eosinophilia in the rat was prevented by two structurally unrelated leukotriene B4 (LTB4) antagonists, U-75302 and Ly255283. These data implicate LTB4 as a mediator of allergen-induced bronchopulmonary eosinophilia. Leukotriene B4 antagonists may provide leads for the development of compounds which inhibit the chronic airway inflammation associated with asthma in man.

PMID: 1659282 [PubMed - indexed for MEDLINE]

2218. Arerugi. 1990 Dec;39(12):1605-11.

[Studies on the pathogenic mechanism of beta-lactam hypersensitivity by the detection of leucocyte migration activating factor and leucocyte migration inhibitory factor].

[Article in Japanese]

Uno K.

Pharmacy, Suibarago Hospital.

In 145 suspected cases of beta-lactam hypersensitivity, the identities of the allergenic drugs were performed by leucocyte migration inhibition test (LMIT). The involvement of cell-mediated immunity using leucocyte migration activating factor (LMAF) and leucocyte migration inhibitory factor (LMIF) was investigated for each allergic symptom. The proportion of LMIT positives was 74% of all 145 cases, and more than 70% for each allergic symptom except for anaphylactic shock, and in particular more than 90% for fever and eosinophilia. LMAF and LMIF were detected 39% and 35% of 145 cases, respectively, however LMAF was more frequently detected than LMIF in cases with hepatopathy. LMAF was more frequently detected than LMIF when the latent period (the duration of sensitization) of skin eruption

or fever was less than 10 days, whereas LMIF was more frequently detected than LMAF when it was more than 10 days. The same result also was obtained at the boundary between 14 days of the latent period in hepatopathy. These results indicate that in the pathogenesis of beta-lactam hypersensitivity, cell-mediated immunity plays a major role. Both LMAF and LMIF are involved, and their production is dependent on the duration of allergenic drug-sensitization, LMAF is produced during the early period of sensitization, whereas LMIF is produced during the late period of sensitization.

PMID: 2096806 [PubMed - indexed for MEDLINE]

2219. Eur Respir J Suppl. 1990 Dec;12:595s-600s; discussion 600s-601s.

The hamster cheek pouch as a model in microcirculation research.

Svensjö E.

Dept of Exploratory Pharmacology, Draco, Lund, Sweden.

Hamster cheek pouches are bilateral invaginations of the oral mucosa and can easily be everted with their blood flow intact and are, therefore, well suited for intravital microscopy. The preparation has been used extensively for studies of inflammation, tumour growth, vascular smooth muscle function, blood flow regulation, and cellular behaviour at the microcirculatory level. The introduction and use of fluorescein-labelled dextrans (FITC-dextran) as tracers of macromolecular permeability changes have provided evidence that macromolecular permeability is subject to physiological and pharmacological regulation by gap formation between endothelial cells in the postcapillary venules. Studies with clinically used asthma drugs like isoprenaline, terbutaline, budesonide, theophylline and cromoglycate have shown that they all counteract histamine-induced permeability increase in postcapillary venules and, furthermore, that budesonide and terbutaline inhibit increased permeability caused by bradykinin, LTB4 and phorbol-dibutyrate, tertiary butylhydroperoxide and ischaemia. Pharmacological studies on the inhibition of mediator-induced leakage and migration of neutrophils in the cheek pouch could be of value not only for a better understanding of the inflammatory process in general but also for the characterization of asthma drugs having an anti-inflammatory action on the airways.

PMID: 2076153 [PubMed - indexed for MEDLINE]

2220. Prostaglandins Leukot Essent Fatty Acids. 1990 Oct;41(2):131-8.

Enhanced arachidonic acid metabolism and human neutrophil migration in asthma.

Radeau T, Chavis C, Damon M, Michel FB, Crastes de Paulet A, Godard PH.

INSERM U 58, Montpellier, France.

In stable state asthmatic patients (AP) without any airway obstruction, the capacity of peripheral blood polymorphonuclear neutrophils (PMN) to produce 5-lipoxygenase metabolites and to migrate, was investigated and compared with the response in healthy subjects (HS). After calcium-ionophore A23187 stimulation, PMN from AP and HS produced LTB4, its hydroxylated derivatives: omega-OH-and omega-CO2H-LTB4) (omega-LTB4, i.e 6-trans-LTB4 and 5,6-diHETE isomers, and 5-HETE. We found an increase in LTB4 (+59%), omega-LTB4 (+39%), 6-trans-LTB4 (+128%), and free 5-HETE (+63%) generation of AP as compared with HS. Unstimulated migration was enhanced in AP (122 +/- 27 PMN/10 high power fields

(hpf) in AP versus 74 +/- 25 PMN/10 hpf in HS, p less than 0.025) and suggested a greater capacity of PMN from AP to migrate. This was confirmed by the PAF-induced chemotaxis studies which showed, in AP, a greater PAF-sensitivity of PMN (10(-6) M versus 10(-5) M in HS) and a greater chemotaxis response (600 +/- 50 PMN versus 200 +/- 35 PMN in HS). In AP, we compared the capacity of PMN to generate LTB4 and 5-HETE with their capacity to migrate. We found an inverse correlation (r = 0.86, p less than 0.007) of intracellular free 5-HETE with chemotaxis to PAF.

PMID: 2125732 [PubMed - indexed for MEDLINE]

2221. Med Interne. 1990 Oct-Dec;28(4):295-303.

Applicability of the leukocyte migration inhibition test in the clinical practice.

Stanciu L, Dumitrescu D, Radu D.

Department of Cellular Immunology, Institute of Hygiene and Public Health, Cluj-Napoca, Romania.

The leukocyte migration inhibition test reveals in vitro the presence of lymphocyte sensitivity and, consequently, of cell-mediated immunity, to a given antigen. Applied in a variety of immune and allergic cases it proved to be useful for the positive diagnosis of the disease and/or for the detection of cell-mediated immune deficiency. The results obtained recommend the leukocyte migration inhibition test in the clinical practice.

PMID: 2100874 [PubMed - indexed for MEDLINE]

2222. J Allergy Clin Immunol. 1990 Oct;86(4 Pt 2):666-9.

The role of antihistamines in atopic dermatitis.

Hanifin JM.

Oregon Health Sciences University, Portland 97201.

Although several lines of evidence support a role for histamine in the pathogenesis of atopic dermatitis, antihistamines have generally offered only marginal therapeutic benefit. The efficacy of the classic antihistamines has been severely limited by sedative effects, demonstrating the need for improved, nonsedating agents. Multifunctional antihistamines, or third-generation "antiallergic" drugs, appear to offer a variety of advantages beyond their ability to inhibit histamine release, such as inhibition of mediator release and interference with eosinophil migration. Double-blind studies of high-dose regimens are needed to help clarify the therapeutic efficacy of these antiallergic drugs.

PMID: 1699988 [PubMed - indexed for MEDLINE]

2223. J Epidemiol Community Health. 1990 Sep;44(3):231-6.

Regional variations in wheezing illness in British children: effect of migration during early childhood.

Strachan DP, Golding J, Anderson HR.

Department of Epidemiology and Population Sciences, London School of Hygiene and Tropical Medicine.

STUDY OBJECTIVE: The aim was to examine the regional distribution of wheezing illness among British children, and the age at which geographical differences may be determined.
DESIGN: Cross sectional analyses and study of interregional migrants were used.
SUBJECTS: The subjects were national cohorts of British children born in 1958 and 1970.
MEASUREMENTS AND MAIN RESULTS: The regional distribution of wheezing illness showed significant heterogeneity at age 5 (1970 cohort) and 7 (1958 cohort). In both cohorts, children in Scotland had a low prevalence of wheeze, which could not be attributed to underreporting of mild cases. There was a less consistent tendency for high prevalence in Wales, and in the South Western and Midlands regions of England. In the 1958 cohort, the regional differentials diminished progressively with age and were negligible at age 23. There was a poor correlation between the regional distribution of childhood asthma and the common geographical pattern shown by eczema in infancy and hay fever at age 23. Analysis of interregional migrants suggested that the regional variation in each cohort at age 5-7 was primarily related to the region of current residence, and not to the region of birth.
CONCLUSIONS: Genetic constitution, perinatal exposures, or early childhood experiences are unlikely to account for the regional variation in wheezing illness. Although local patterns of symptom reporting or disease labelling may be acquired by parents who move to a new region, environmental factors operating at a regional level probably determine the prevalence of asthma in primary school children. These influences do not appear to have long lasting effects upon the tendency to wheeze in adolescence and early adulthood.

PMCID: PMC1060648
PMID: 2273362 [PubMed - indexed for MEDLINE]

2224. Rhinology. 1990 Sep;28(3):149-58.

IgE-positive mast cells on the human nasal mucosal surface in response to allergen exposure.

Bachert C, Prohaska P, Pipkorn U.

Dept. of O.R.L., University of Heidelberg, Klinikum Mannheim, F.R.G.

IgE-bearing mediator cells are suggested to be the effector cells in type I allergic rhinitis. These cells can be demonstrated by their granular constituents or by the surface-bound IgE antibodies. We developed immunohistochemical techniques in order to stain the cell-bound IgE using polyclonal or monoclonal anti-human IgE antibodies. These techniques can be applied to nasal biopsies as shown previously or to cytospin specimen harvested by a brush method. They deliver excellent staining results with well-preserved morphological details. Brush samples were taken from 24 grass pollen allergic subjects before season, after a nasal allergen provocation and two weeks after the onset of season. There was a statistically significant increase in toluidine blue positive and IgE-positive cells 24 hours after nasal provocation (app. 12-fold, p less than 0.05) and more pronounced within the season (app. 58-fold, p less than 0.001) compared to preseasonal values. These cells appeared to be mast cells rather than blood basophils judged by morphological criteria. There was a striking correlation between the number of toluidine blue cells and that of IgE-positive cells (r = 0.98). The number of eosinophils also increased due to the seasonal allergen exposure (p less than 0.001), but less pronouncedly compared to the mast cells. These data re-emphasize the migration of IgE-bearing mast cells and

eosinophils into the epithelial lining of the nasal mucosa due to allergen interaction and point to a possible role of mast cells as a carrier for IgE-molecules.

PMID: 2251464 [PubMed - indexed for MEDLINE]

2225. J Allergy Clin Immunol. 1990 Sep;86(3 Pt 1):314-20.

In vivo effects of cetirizine on cutaneous reactivity and eosinophil migration induced by platelet-activating factor (PAF-acether) in man.

Fadel R, David B, Herpin-Richard N, Borgnon A, Rassemont R, Rihoux JP.

Unité d'Emmuno-Allergie, Institut Pasteur, Paris, France.

The aim of the study was to determine the effect of cetirizine, a new potent H1 antihistamine, on acute cutaneous inflammatory response and eosinophil accumulation induced in vivo by platelet-activating-factor (PAF-acether) and allergen. In a double-blind, crossover study, seven subjects allergic to grass pollen and three nonallergic control subjects received orally either cetirizine, 20 mg/day, or placebo for 4 days. On day 4, the subjects were skin tested with grass pollen and PAF-acether (400 and 40 ng per site). After the challenge, an evaluation of time-course cutaneous eosinophil infiltrations by a skin window technique was performed. Cetirizine pretreatment reduced skin wheal and erythema elicited by allergen and PAF, 400 and 40 ng, by 74.6% (p less than 0.001), 53.9% (p less than 0.001), and 47% (p less than 0.01), respectively. Skin reactivity induced by PAF-acether was also significantly reduced by cetirizine in nonallergic subjects. Cetirizine reduced at hour 24 eosinophil infiltrations induced by allergen and PAF, 400 and 40 ng, by 63% (p less than 0.001), 58.5% (p less than 0.001), and 57.8% (p less than 0.01), respectively. This inhibitory effect of cetirizine on allergen and PAF-induced eosinophil infiltration was already effective 2 hours after the challenge. PAF induced a nonsignificant eosinophil influx in all nonallergic subjects. In conclusion, cetirizine inhibited both the immediate cutaneous response and the eosinophil influx induced by allergen and by a potent eosinophil chemotactic factor, such as PAF-acether. Therefore, cetirizine, besides its anti-H1 effect, has the potential to modulate the allergic inflammatory response.

PMID: 1976664 [PubMed - indexed for MEDLINE]

2226. Vet Pathol. 1990 Jul;27(4):217-22.

Pulmonary eosinophilia and granulomatous pulmonary arteritis induced in rats by intravenous Sephadex.

Sorden SD, Lemanske RF Jr, Castleman WL.

Department of Pathobiological Sciences, School of Veterinary Medicine, University of Wisconsin-Madison.

Airway eosinophilia has been proposed as a major pathogenetic event in bronchial asthma and airway hyperresponsiveness. Intravenous injection of Sephadex G200 in rats induces pulmonary and blood eosinophilia and alters pulmonary responsiveness to 5-hydroxytryptamine. To characterize the early pulmonary inflammatory responses following Sephadex administration, and to determine the timing of the onset of pulmonary eosinophilia relative to blood eosinophilia, Sprague-Dawley rats were injected intravenously with Sephadex G200 beads. Lungs and other tissues were examined by light and transmission electron microscopy at 4, 12, 24,

48, 72, and 96 hours after injection. Blood eosinophil counts were determined at 0, 24, and 96 hours after injection. Sephadex beads were trapped initially in small caliber muscular pulmonary arteries associated with terminal bronchioles and in intra-acinar locations. There was marked infiltration of eosinophils and macrophages around the beads and into arterial walls and edematous periarterial and peribronchiolar connective tissue as early as 4 hours after injection. Periarterial-peribronchiolar eosinophil aggregates peaked in density at 24 and 48 hours. Macrophages and multinucleated cells dominated the inflammatory cell responses in arteries immediately surrounding partially degraded Sephadex beads from 24 to 96 hours. Bone marrow eosinophilopoiesis and blood eosinophilia were not detected until 96 hours. We conclude that Sephadex induces pulmonary eosinophilia prior to blood eosinophilia and suggest that Sephadex may induce pulmonary release of one or more eosinophil chemotactic substance(s). This model may prove useful in the study of factors that influence eosinophil migration into the lung in various disease states.

PMID: 1698322 [PubMed - indexed for MEDLINE]

2227. Vrach Delo. 1990 Jul;(7):99-101.

[Circulating immune complexes in workers in glass fiber-reinforced plastics manufacture].

[Article in Russian]

Vitrishchak VIa, Frolov VM, Peresadin NA.

The authors examined 182 workers engaged in manufacturing of glass-reinforced plastics and revealed an increase of the level of circulating immune complexes and their content middle-molecular immune complexes. This makes it possible to suggest the pathogenetic role of circulating immune complexes in the development of allergic lesions in workers contacting with epoxide compounds.

PMID: 2238615 [PubMed - indexed for MEDLINE]

2228. Gastroenterology. 1990 Jun;98(6):1558-66.

Intestinal anaphylaxis in the rat. Effect of chronic antigen exposure.

Curtis GH, Patrick MK, Catto-Smith AG, Gall DG.

Intestinal Disease Research Unit, University of Calgary, Alberta, Canada.

The effect of chronic dietary antigen challenge on the intestine was examined in sensitized rats. Three groups of Hooded-Lister rats were studied: animals sensitized to egg albumin; sham-sensitized animals; and unmanipulated controls. In sensitized rats, serum immunoglobulin E titers to egg albumin were greater than or equal to 1:64, whereas control and pair-fed rats showed no response. Sensitized rats received egg albumin 1 mg/ml in drinking water and rat chow ad libitum. Pair-fed animals also received egg albumin but were pair-fed with sensitized animals. Controls received water and rat chow ad libitum. Chronic antigen challenge resulted in reduced food intake and weight gain in sensitized animals. When the rats were killed after 9 days of antigen exposure, proximal intestine from experimental animals showed decreased disaccharidase activity, brush-border microvillus surface, area, and villus height. Crypt depth and enterocyte migration rate were increased. Mucosal mast cell involvement was suggested by mast cell proliferation, evidence of mast cell degranulation, and increased serum rat mast cell protease II levels. At the time of death, only

sensitized jejunum demonstrated an increase in short-circuit current in Ussing chambers in response to antigen challenge. The findings indicate that chronic antigen exposure leads to intestinal injury, reduced food intake, and diminished weight gain.

PMID: 2186952 [PubMed - indexed for MEDLINE]

2229. J Invest Dermatol. 1990 Jun;94(6):786-92.

Lymphocyte migration into the skin: the role of lymphocyte homing receptor (CD44) and endothelial cell antigen (HECA-452).

Jalkanen S, Saari S, Kalimo H, Lammintausta K, Vainio E, Leino R, Duijvestijn AM, Kalimo K.

Department of Medical Microbiology, Turku University, Finland.

Lymphocyte migration into the lymphoid organs and sites of inflammation is controlled by lymphocyte-endothelial cell interaction at sites where lymphocytes exit from the blood. Expression of Hermes-defined CD44 class of lymphocyte homing receptor and HECA-452 antigen specific for high-endothelium-mediating physiologic lymphocyte extravasation was studied in dermatitis herpetiformis, celiac disease, psoriasis, mycosis fungoides, lymphocytosis cutis, atopic dermatitis, and allergic contact dermatitis. Also, duodenal biopsies of patients suffering from dermatitis herpetiformis or celiac disease were studied for existence of these antigens. Infiltrating lymphocytes in the skin and in the duodenal area expressed homing receptor molecules when studied with monoclonal antibodies, Hermes-1 and Hermes-3, that recognize the CD44 class of molecules involved in lymphocyte binding to high endothelial venules in peripheral lymph nodes, mucosa-associated lymphatic tissues, and inflamed synovium. However, the HECA-452 antigen was not detected on the venules, neither in the skin nor in the duodenum. Even the venules possessing high endothelium morphologically were HECA-452 negative. These findings suggest the CD44 class of lymphocyte homing receptor(s) is also involved in lymphocyte homing to inflamed skin and the duodenal area of the gut. However, on the basis of HECA-452 staining, high endothelial venules in inflamed skin and duodenum are not antigenically identical with high endothelial venules in organized lymphoid tissues. This finding indirectly supports the idea that molecules and/or mechanisms mediating lymphocyte extravasation might be distinct in these organs.

PMID: 1693939 [PubMed - indexed for MEDLINE]

2230. Inflammation. 1990 Apr;14(2):223-37.

Desensitization of rabbit skin by repeated exposure to UV-visible light of sites injected with Rose Bengal.

Ranadive NS, Kumar JR, Menon IA.

Department of Pathology, University of Toronto, Ontario, Canada.

We have shown in a previous paper that irradiation of rabbit skin sites injected with Rose Bengal (RB) produces immediate increase in vascular permeability and accumulation of PMNs. Studies on the development of temporary tolerance and the biological parameters related to the development of such tolerant state by repeated exposure to light of RB-injected sites are reported here. The increase in VP and PMN migration induced by RB (10 nmol) are of an immediate nature, i.e., occur within the first 3 h of irradiation, and the reaction subsides gradually

after 24 h. When such moderate insult is repeated, the skin becomes tolerant to subsequent exposure to light in the presence of RB. This tolerant state is temporary, i.e., the desensitized sites are fully recovered in 72 h. The loss of responsiveness of RB-injected sites previously exposed to light was not due to diffusion of the injected dye from the sites since reinjected sites also showed reduced response and the sites injected three days before but not irradiated showed normal response. The sites that were made tolerant to RB-induced phototoxic reactions, when injected with compound 48/80, an agent known to degranulate mast cells, did not show an increase in VP. This suggests that either the mast cells were depleted from the sites or the mast cells in the sites were rendered refractory by previous exposure to light. It was also found that the sites made tolerant to RB plus light were unresponsive to exogenously injected histamine. The sites tolerant to RB plus light when injected with zymosan-activated serum (ZAS) did not stimulate the migration of PMNs. This loss of chemotactic response to ZAS may have relevance to photodamage of vascular endothelium. These observations are discussed in relation to the development of the tolerant state by repeated exposures to subthreshold doses of light in solar urticaria.

PMID: 2323811 [PubMed - indexed for MEDLINE]

2231. Fiziol Zh. 1990 Mar-Apr;36(2):31-4.

[The role of peritoneal mast cells in the development of anaphylactic shock].

[Article in Russian]

Bashmakov IuK, Briuzgina TS.

An increase in the content of mast cells and macrophages in the bronchoalveolar lavage, liberation of arachidonic acid from the alveolar surfactant, formerly blocked by the caused deficiency of peritoneal mast cells have been observed under conditions of the experiment excluding the possibility of the allergen-specific hyperplasia of mastocytes in respiratory organs--anaphylactoid response of rats to the intrauterine introduction of the egg-white. A conclusion is drawn as to the possibility of interregional migration of mast cells whose regulating function with regards to the surfactant phospholipids is likely to be accomplished in cooperation with alveolar macrophages.

PMID: 2361552 [PubMed - indexed for MEDLINE]

2232. Clin Exp Allergy. 1990 Mar;20(2):131-6.

Atopic dermatitis: a defect of intracellular secondary messenger systems?

Holden CA.

Skin Laboratory, St Helier Hospital, Carshalton, Surrey, U.K.

The clinical manifestations of atopic dermatitis comprise a complex mixture of pharmacological, physiological and immunological responses. Circumstantial evidence suggests that atopic disease may arise consequent upon the migration of bone marrow-derived cells into the target tissue of skin or respiratory mucosa. Mediator release from such cells has been shown to be abnormal in atopic dermatitis, and itch, the hallmark of the disease, may be the result of chronic inflammatory mediator release into the skin. Abnormal release of mediators has been shown to correlate with inadequate nucleotide control of cell function. In particular, elevated cyclic AMP-specific PDE activity causing cyclic AMP

hyporesponsiveness has been found in peripheral blood mononuclear leucocytes in atopic dermatitis. Investigation of this pathway has led to the discovery of additional abnormalities of other secondary messenger systems, including abnormalities of protein kinase C activity and of inositol activation. The biochemical abnormalities may be a consequence of down-regulation of the second messenger systems because of chronic exposure to low levels of inflammatory mediators, but may themselves subsequently permit further mediator release. They may provide a biochemical mechanism for many of the immunological abnormalities seen in atopic dermatitis. In particular, they offer a biochemical explanation for the paradox of increased type 1-mediated immunity and diminished cell-mediated immunity commonly observed in this complex disease.

PMID: 2162724 [PubMed - indexed for MEDLINE]

2233. J Pediatr Gastroenterol Nutr. 1990 Feb;10(2):253-6.

Hypersensitivity reaction in an infant fed hydrolyzed lactalbumin contained in a semielemental formula.

Heyman MB, Stoker TW, Rudolph CD, Frick OL.

Department of Pediatrics, University of California, San Francisco 94143-0136.

Comment in
    J Pediatr Gastroenterol Nutr. 1991 Nov;13(4):429.

Following introduction of milk protein formula feedings, a 6-month-old male developed profuse, watery diarrhea progressing to shock, requiring cardiopulmonary resuscitation. Reinstitution of enteral feedings with a formula containing hydrolyzed lactalbumin (Travasorb STD) resulted in recurrence of diarrhea with fever. Intestinal and rectal biopsies showed only nonspecific inflammatory changes. He was discharged on an elemental formula (Vivonex). Twenty-three months later, while admitted for evaluation of hypophosphatemic rickets, immunologic testing using the lymphocyte migration inhibition factor (LIF) test demonstrated positive reactions, especially to alpha-lactalbumin (56% inhibition) and whole cow's milk (22%, normal of less than 20% inhibition). Skin tests revealed sensitivity to cow's milk and eggs. Soy formula also produced diarrhea and bloody stools. Protein hydrolysate formulas, touted as hypoallergenic diets, are useful in infants with intolerance to milk protein. This is the first documented case of an immunological reaction to the hydrolyzed whey protein, lactalbumin. Although protein hydrolysate formulas are effective treatment in most infants with milk protein intolerance, allergic reactions are possible. Caution and close observation should be exercised in immunologically sensitized infants rechallenged with any formula.

PMID: 2135732 [PubMed - indexed for MEDLINE]

2234. Science. 1990 Jan 26;247(4941):456-9.

Intercellular adhesion molecule-1 (ICAM-1) in the pathogenesis of asthma.

Wegner CD, Gundel RH, Reilly P, Haynes N, Letts LG, Rothlein R.

Department of Pharmacology, Boehringer Ingelheim Pharmaceuticals, Inc., Ridgefield, CT 06877.

Airway eosinophilia, epithelial desquamation, and hyperresponsiveness are characteristics of the airway inflammation underlying bronchial asthma. The

contribution of intercellular adhesion molecule-1 (ICAM-1) to eosinophil migration and airway responsiveness was studied. ICAM-1 partially mediated eosinophil adhesion to to endothelium in vitro and was upregulated on inflamed bronchial endothelium in vivo. ICAM-1 expression was also upregulated on inflamed airway epithelium in vitro and in vivo. In a primate model of asthma, a monoclonal antibody to ICAM-1 attenuated airway eosinophilia and hyperresponsiveness. Thus, antagonism of ICAM-1 may provide a therapeutic approach to reducing airway inflammation, hyperresponsiveness, and asthma symptoms.

PMID: 1967851 [PubMed - indexed for MEDLINE]

2235. Toxicol In Vitro. 1990;4(4-5):246-51.

In vitro tests with sensitized lymphocytes-Relevance for predictive allergenicity testing.

von Blomberg-van der Flier BM, Scheper RJ.

Department of Pathology, Free University Hospital, De Boelelaan 1117, 1081 HV, Amsterdam, The Netherlands.

The main obstacle to routine in vitro tests to identify contact sensitizers is the inappropriate application of the allergens in vitro, even when sufficient antigen-presenting cells are available. Some allergens may need the skin tissue for adequate conjugation with carrier molecules, whereas others may be poorly solubilized or toxic for the cultures. Methods, therefore, which make use of the in vivo system for allergen binding and presentation, but which assess in vitro the subsequent lymphocyte activation are most promising. For this reason, the local lymph node assay deserves more attention as a predictive assay in allergenicity testing and should be validated in more laboratories in the near future. The value of allergen-stimulated in vitro tests for predictive allergenicity screening will remain limited. This type of in vitro test is, however, invaluable for mechanistic studies on contact sensitivity in well defined allergen models. Also for clinical diagnosis of allergic contact sensitivity to selected allergens, the lymphocyte transformation test and macrophage migration inhibition test may be useful, particularly when skin tests fail to be conclusive.

PMID: 20702172 [PubMed]

2236. Rev Esp Geriatr Gerontol. 1990 Jan-Feb;25(1):4-10.

[Comparison of mortality in Spain and France, following the method of "potential years of life lost"].

[Article in Spanish]

Calatayud Sarthou A, Sabater Pons A, Alfonso Sanchez JL, Hernandez Galve A, Cortina Greus P.

PIP: Mortality trends in Spain from 13 major causes of death are analyzed for the period 1972-1982 and compared with trends for the same period in France. Increases in mortality in Spain are noted for three causes--malignant tumors, cardiac diseases, and suicides and homicides--whereas significant declines in mortality are noted for pneumonia, influenza, chronic bronchitis, and asthma. Excess mortality for males is common to both countries. (SUMMARY IN ENG)
PMID: 12342982 [PubMed - indexed for MEDLINE]

2237. Dermatol Clin. 1990 Jan;8(1):189-92.

Pressure dermatitis from an implanted pacemaker.

Wilkerson MG, Jordan WP Jr.

Department of Dermatology, Medical College of Virginia, Richmond.

In this eleventh report of dermatitis conforming to the outline of an underlying pacemaker, the authors suggest that most of these reactions are isomorphic responses to expansion of the subcutaneous tissues by the hard device. Allergic contact dermatitis has been documented in a few cases.

PMID: 2406058 [PubMed - indexed for MEDLINE]

2238. Int Arch Allergy Appl Immunol. 1990;91(3):270-7.

Antigen-induced mucosal damage and restitution in the small intestine of the immunized rat.

D'Inca R, Ramage JK, Hunt RH, Perdue MH.

Department of Pathology, McMaster University, Hamilton, Canada.

Intestinal mucosal damage and restitution were examined following antigen-induced systemic anaphylaxis in Nippostrongylus brasiliensis immunized rats. The rats were injected intravenously with N. brasiliensis antigen or saline. At 60 min, morphological and biochemical parameters were determined in jejunum and ileum, and the epithelial permeability was assessed by measuring recovery of 51Cr-ethylenediaminetetraacetic acid in the blood after injecting it into a ligated segment. Antigen challenge resulted in significant abnormalities: (1) villus damage with sloughing of enterocytes; (2) decreased activities of brush border enzymes; (3) decreased levels of mucosal histamine and rat mast cell protease II (mast cell mediators), and (4) increased uptake of 51Cr-ethylenediaminetetraacetic acid. Progression of the injury was examined by taking consecutive biopsies at 15-min intervals for 60 min and then at 5 h. At 15 min, an abnormality was present in all sections which ranged from minor oedema and enterocyte detachment at villus tips to virtual complete destruction of the apical region. Restitution occurred by villus contraction with migration of the epithelium over the damaged regions. At 5 h, the epithelium had resealed, but the villi were significantly reduced in height.

PMID: 2354870 [PubMed - indexed for MEDLINE]

2239. Nauchnye Doki Vyss Shkoly Biol Nauki. 1990;(10):37-45.

[Seasonal changes in the population count of allergenic mites (Acariformes, Pyroglyphidae) in the dust of living quarters in the city of Moscow].

[Article in Russian]

Petrova AD, Zhëltikova TM.

The seasonal dynamics of allergenic mite quantity in house dust of three model apartments in Moscow has been observed during three years. Two periods of increase in mites' quantity have been revealed: wintry and summer-autumnal. Mite

breeding takes place both in winter and in summer-autumnal period. The quantity variations of allergenic mites are cyclic and have individual traits peculiar to each population of these mites.

PMID: 2275945 [PubMed - indexed for MEDLINE]

2240. Arch Dermatol Res. 1990;282(6):374-8.

Detection of migration inhibitory factor (MIF) by a monoclonal antibody in the microvasculature of inflamed skin.

Gómez RS, Diepgen TL, Neumann C, Sorg C.

Department of Dermatology, University of Erlangen-Nürnberg, Federal Republic of Germany.

Macrophage migration inhibitory factor (MIF) is known as a mediator of cellular immunity with specific effects on the differentiation of mononuclear phagocytes. There is little information on the production of MIF in vivo and its role in the pathophysiology of inflammation. We studied the distribution of MIF in various tissues with a monoclonal antibody against human MIF (1C5/B) using the indirect immunoperoxidase method. Here, we investigate the expression of MIF on endothelial cells of dermal vessels. Our results show that dermal vessels may constitutively express MIF and can be strongly activated to express MIF in acute inflammations such as eczema and psoriasis in contrast to the chronically infiltrated skin from patients with pseudolymphomas and sarcoidosis. In these cases a possibly MIF defective state of vessels and a restriction of positive vessels to distinct anatomical sites of the inflamed skin was detected. The significance of the described association of MIF with vascular endothelium is still a matter of speculation. MIF expression on endothelium may provide an important differentiogenic signal for mononuclear phagocytes on their way to the tissue site.

PMID: 2260882 [PubMed - indexed for MEDLINE]

2241. Adv Ophthalmic Plast Reconstr Surg. 1990;8:46-52.

Mobility implants: a review.

Danz W Sr.

We present a brief review of mobility implants, their contribution, and the experiences derived after almost 40 years since the new concepts of full mobility implants were introduced. In early 1940, experiments with a new material for the making of plastic artificial eyes was also being considered for the making of orbital implants. Methyl-methacrylate (MMA) had proven inert and satisfactory for dental products. The Surgeon Generals office of the Armed Services encouraged further research and experimental work in the development of plastic eyes. The success of the new material sponsored the beginning of great expansion with new concepts for orbital implants. Through a period of more than a decade, the design and types of implants went through three stages. First, the buried implant was introduced, then the exposed integrated followed, and the buried integrated subsequently followed. The path of progress was not smooth. Theoretically correct designs and surgical procedures met unexpected practical difficulties for the ophthalmic surgeon, the patient, and the eye maker. Surgical and technical efforts were carefully reviewed to eliminate the problems encountered, only to have further unforeseen complications arise. Infections, extrusions, and migration of the implant were not uncommon. The exposed integrated implant was

eventually abandoned. However, there were some extraordinary successes of mobility. A new era introduced fully buried mobility implants that were more successful. However, this procedure also produced some problems, causing infection (or allergy), extrusion, and migration. Tantalum mesh and gauze gave great promise with the inception of their use. Orbital tissue grew into the material in an astonishing way, making it possible to secure the extraocular muscles and tenons.(ABSTRACT TRUNCATED AT 250 WORDS)

PMID: 2248721 [PubMed - indexed for MEDLINE]

2242. Pediatriia. 1990;(5):14-8.

[Increased sensitivity to Candida in patients with bronchial asthma].

[Article in Russian]

Pronina EV, Karaev ZO, Alferov VP.

As many as 86 children aged 3 to 14 years with bronchial asthma were examined using mycological, immunological and allergological methods including the prick-test, basophil degranulation test (direct Shelley's test) and leukocyte migration inhibition test with Candida antigen. As far as the patients living in the vicinity of a microbiological factory are concerned, hypersensitivity to Candida was detected in 83.3% of cases, primarily that of the delayed type (37%). In children with verified candidiasis, hypersensitivity to Candida fungi was also detectable in 83.3% of cases, but in the majority of patients (47%), it was of the immediate type. In the control group, allergic candidiasis was diagnosed in 20% of cases. Clinically, bronchial asthma associated with allergic candidiasis was characterized by refractory, lingering obstructive bronchitis amenable to antimycotic drugs.

PMID: 2204874 [PubMed - indexed for MEDLINE]

2243. Ann Dermatol Venereol. 1990;117(3):185-93.

[Langerhans cells in the physiopathology of atopic dermatitis].

[Article in French]

Bieber T, Bruijnzeel-Koomen C.

Clinique et Policlinique Dermatologique, l'Université Ludwig-Maximilian, Munich, RFA.

INTRODUCTION. Atopic dermatitis (AD), allergic rhino-conjunctivities and allergic asthma constitute the classical triad of atopic diathesis attended, in many cases, by high serum IgE levels. While the pathophysiology of IgE-mediated allergic respiratory diseases is now better understood, the pathophysiological significance of atopic phenomena in the genesis and control of AD is still far from being clear. Numerous clinical and laboratory data point to a pathophysiological relation between IgE-mediated reactions and AD, but no one yet knows by which mechanism this interaction takes place. Some recent studies suggest that Langerhans cells might well be the missing link. THE LANGERHANS CELLS. Langerhans cells (LC) are dendritic epidermal cells originating in the bone marrow and supposedly belonging to the monocyte lineage. Their circulating precursors, the mechanism of their migration into the epidermis and their relationship with other dendritic cells, such as the interdigitating follicular cells, are controverted. LC express numerous surface markers, such as class I and

II HLA, CD1a, CD4 and receptors for complement and IgE Fc fragments. Under normal conditions, LC do not express IgE receptors. Ultrastructurally, LC are characterized by the presence of Birbeck granules in their cytoplasm. Among the presumed functions of LC in the skin, the best documented is the presentation of antigens to T lymphocytes in allergic contact dermatitis. LANGERHANS CELLS IN ATOPIC DERMATITIS. Quantitative studies. Modern immunohistological methods based on the reactivity of monoclonal anti-CD1a antibodies have given results that are sometimes conflicting due to differences in the quantification techniques utilized. However, morphometric enumeration of LC on cryostat sections have shown that their number is about the same in AD and in normal skin. PRESENCE OF IgE BEARING LANGERHANS CELLS IN ATOPIC DERMATITIS. The presence of IgE molecules on the LC surface has been demonstrated in subjects with AD. It must be noted that in atopic subjects IgE bearing Lc are only found in patients with high serum IgE levels. They are absent in asthma patients without eczema, irrespective of their serum IgE levels. Daily applications of corticosteroids on AD lesions result in a decrease of anti-IgE markers on LC after one week and in their complete disappearance after 2 weeks. IN ATOPIC DERMATITIS LANGERHANS CELLS EXPRESS A RECEPTOR SPECIFIC TO Fc FRAGMENTS OF IgE. The exact nature of the receptor for IgE expressed in situ in AD patients is still conjectural. Some authors have been able to demonstrate that the binding of IgE molecules by LC isolated from the skin of atopic patients is inhibited by a monoclonal antibody directed against the low affinity receptor (Fc epsilon R2) of eosinophils and macrophages. This strongly suggests that certain factors induce the expression by LC of an Fc epsilon R2 receptor. IN VITRO INDUCTION OF IgE RECEPTORS ON NORMAL LANGERHANS CELLS...

PMID: 2193589 [PubMed - indexed for MEDLINE]

2244. Med Dosw Mikrobiol. 1990;42(1-2):95-9.

[Various immunochemical properties of nonspecific bacterial vaccines].

[Article in Polish]

Aleksandrowicz J, Janaszek W, Fiejka M, Matusiewicz R, Dzielska D.

Zakład Badania Surowic i Szczepionek PZH w Warszawie.

After critical evaluation of the composition and technology of preparation of nonspecific bacterial vaccines (nsb) some of them were withdrawn from the production (Neoflaminum, Neurovaccinum) and replaced with a new one (Polyvaccinum submite). Part of them were modified (media) and modernised (Panodinum, vaccine according to Delbet). Panodine produced at the present is free from bovine bile, which according to the results of the studies is strongly haemolytic for human and animal red blood cells. Thus it was justified to withdraw this component from Neoflaminum and Panodinum preparations. Purity and degree of lysis of these preparations were evaluated on the basis of ratio between nucleic acid absorbance and protein absorbance A260/A280. This allowed to determine if nsb tested are partially lysed and if their production process is reproducible. Our pilot studies on five patients suffering from infectious asthma indicate that a restoration of phagocytic activity of granulocytes and an increase of leukocytes migration after subsequent Panodine injections take place.

PMID: 2087137 [PubMed - indexed for MEDLINE]

2245. Int Arch Allergy Appl Immunol. 1990;93(1):47-53.

Correlation between lymphocyte proliferative responses and dendritic cell

migration in regional lymph nodes following skin painting with contact-sensitizing agents.

Kimber I, Kinnaird A, Peters SW, Mitchell JA.

ICI Central Toxicology Laboratory, Alderley Park, Macclesfield, UK.

We have investigated whether there exists a correlation between the induction of draining lymph node cell (LNC) proliferation in contact allergy and the accumulation of dendritic cells (DC) within such lymph nodes. CBA/Ca mice, which compared with mice of BALB/c strain, mount a more vigorous lymphocyte proliferative response following sensitization, also exhibited a more marked accumulation of DC in draining lymph nodes 24 h following skin painting. Moreover, studies with the skin-sensitizing fluorochromes fluorescein isothiocyanate (FITC) and rhodamine B isothiocyanate (RITC) revealed that DC-enriched fractions of draining LNC prepared from CBA/Ca mice contained a higher percentage of antigen-bearing cells than did those from BALB/c mice. A relationship between DC migration into lymph nodes and the magnitude of the induced LNC proliferative response was also indicated by experiments performed in BALB/c mice with a variety of contact allergens. It was observed that there was a direct correlation between the vigour of the proliferative response measured 3 days following exposure and the frequency of DC in draining nodes at 24 h. Collectively these data suggest that following skin sensitization the migration of DC into the draining lymph nodes influences quantitatively the primary immune response and the development of contact allergy.

PMID: 2086486 [PubMed - indexed for MEDLINE]

2246. Int Arch Allergy Appl Immunol. 1990;92(4):356-60.

Differences in migration inhibitory factor production by C57Bl/6 and BALB/c mice in allergic and irritant contact dermatitis.

Malorny U, Goebeler M, Gutwald J, Roth J, Sorg C.

Institute of Experimental Dermatology, University of Münster, FRG.

Two strains of mice, BALB/c and C57Bl/6, which are known to differ in their inflammatory responsiveness to allergens, were analyzed regarding their expression of macrophage migration inhibitory factor (MIF). Allergic contact dermatitis to 2,4-dinitro-1-fluorobenzene and irritant contact dermatitis to croton oil were studied immunohistologically at designated time intervals after elicitation. BALB/c mice presented a significantly more intense ear swelling response than C57Bl/6 mice and showed a strong endothelial MIF expression in the early phase of inflammation in both allergic and irritant contact dermatitis. Endothelial MIF expression was much weaker in C57Bl/6 mice. Furthermore, the infiltration rate of inflammatory cells (MIF+ and BM8+ macrophages, Lyt2+ and L3T4+ T cells) was significantly higher in BALB/c than in C57Bl/6 mice. We conclude that genetically determined differences of susceptibility to allergens and irritants in BALB/c and C57Bl/6 mice are reflected by the intensity of MIF expression in the microvascular endothelium and immigrating inflammatory cells. MIF seems to appear as first molecular equivalent of developing inflammation and probably determines the degree of cellular infiltration.

PMID: 2083971 [PubMed - indexed for MEDLINE]

2247. Int Arch Allergy Appl Immunol. 1990;93(4):294-9.

Expression of intercellular adhesion molecule-1 in murine allergic contact dermatitis.

Goebeler M, Gutwald J, Roth J, Meinardus-Hager G, Sorg C.

Institute of Experimental Dermatology, University of Münster, FRG.

Intercellular adhesion molecule-1 (ICAM-1) plays an important role in the interaction of immunocompetent cells in inflammation. It contributes to lymphocyte recruitment, antigen presentation and T-cell activation. Recently, the murine homologue of ICAM-1, originally designated as MALA-2, has been identified. The monoclonal antibody YN1/1.7 for murine ICAM-1 enables the analysis of ICAM-1 expression in murine allergic contact dermatitis, a prototype of cutaneous T-cell-mediated inflammatory response. At 0, 1, 8, 24, 48, 72 h, and 7 and 14 days after challenge with 2,4-dinitrofluoro-1-benzene (DNFB) cryostat sections of ear skin were immunostained for ICAM-1, I-A and mononuclear cells (L3T4, Lyt-2, BM8). In normal skin ICAM-1 labeling was restricted to endothelial cells and dermal dendritic cells/macrophages; keratinocytes (KCs) did not express ICAM-1. The dermal cellular infiltrate increased progressively from 8 to 72 h after DNFB challenge. The majority of infiltrating cells were BM8+ macrophages (75%) and L3T4+ (10%) or Lyt-2+ T cells (10%); maximally 30% of those stained positive for ICAM-1. At 24 h, focal ICAM-1 expression on KCs developed, reached a maximum at 72 h and faded thereafter. Migration of T cells into the epidermal layer started at 48 h at sites which had already expressed ICAM-1. Our data provide evidence that expression of ICAM-1 by epidermal cells precedes infiltration of the epidermis by T lymphocytes as shown before in human cutaneous disorders. Thus, a mouse model may be useful to investigate the role of ICAM-1 in inflammation further.

PMID: 1983170 [PubMed - indexed for MEDLINE]

2248. J Allergy Clin Immunol. 1989 Nov;84(5 Pt 1):768-72.

The relationships between atopy, bronchial hyperresponsiveness, and a family history of asthma: a cross-sectional study of migrant Tokelauan children in New Zealand.

Crane J, O'Donnell TV, Prior IA, Waite DA.

Department of Medicine, Wellington School of Medicine, New Zealand.

We have examined age-related changes in the association between nonspecific bronchial hyperresponsiveness (BHR) and atopy in 494 second-generation Polynesian migrant children, aged 5 to 15 years. BHR (provocative dose of methacholine, less than or equal to 7.8 mumol, causing a 20% fall in FEV1) was present in 125 children (25.3%). Atopy (skin wheal, greater than or equal to 4 mm diameter) was present in 157 children (32%). BHR associated with atopy demonstrated a constant age-related frequency in the 7- to 15-year-old children that was influenced by a family history (FH) of asthma (FH, 50%; no FH, 34%; p = 0.051). BHR not associated with atopy demonstrated a marked decreasing frequency with age from 25% in 5- to 7-year-old children to 3% in 13- to 15-year-old children and was uninfluenced by an FH of asthma. We conclude that the differences in the frequency of BHR with age, together with the genetic influence on BHR associated with atopy, compared with the findings in nonatopic children, indicate distinct heterogeneity in the pathogenesis of BHR in these Tokelauan children. These differences may be important for understanding the relationships between nonspecific BHR, atopy, and asthma in children.

PMID: 2809026 [PubMed - indexed for MEDLINE]

2249. Food Chem Toxicol. 1989 Nov;27(11):755-62.

Aspects of the immune response to contact allergens: opportunities for the development and modification of predictive test methods.

Kimber I.

ICI Central Toxicology Laboratory, Alderley Park, Macclesfield, Cheshire, UK.

A variety of guinea-pig tests are currently employed to assess the skin-sensitizing potential of chemicals. Although some such tests, in particular the guinea-pig maximization test and the occluded patch test of Buehler, have become well established, widely applied and are of proven value in the safety evaluation of chemicals, they have certain limitations. It is the purpose of this review to examine various aspects of the immune response to contact allergens and the way in which an understanding of the molecular and cellular events that characterize the induction and elicitation of contact sensitivity may be applied to the development and modification of predictive test methods. Attention is focused on the role of dendritic cell migration and T lymphocyte activation during the induction phase of skin allergy and the association of acute-phase proteins and vasoactive amines with the elicitation reaction.

PMID: 2693285 [PubMed - indexed for MEDLINE]

2250. Agents Actions. 1989 Nov;28(3-4):198-203.

Effect of ketotifen on the distribution and degranulation of uterine eosinophils in estrogen-treated rats.

Soto J, Tchernitchin AN, Poloni P, Voigt G, Caro B, Agurto M.

Department of Experimental Medicine, University of Chile Medical School, Santiago.

Eosinophil leukocytes migrate from the blood to the uterus under estrogen stimulation, redistribute through uterine extravascular compartment, degranulate in the organ, and release agents that are involved in several parameters of estrogen action. Agents that induce blood eosinopenia, block their migration to the uterus, interfere with their redistribution within the organ or modify their degranulation, selectively interfere with eosinophil-mediated responses to estrogen. The present study investigated whether ketotifen, an antiallergic agent that inhibits allergen-induced eosinophil degranulation, interferes with estrogen-induced eosinophil migration to the uterus and their subsequent degranulation. Ketotifen does not interfere with estrogen-induced eosinophil accumulation in the uterus, but decreases the proportion of eosinophils located in endometrium and inhibits their degranulation. These results suggest that neither histamine, calcium or slow reacting substance of anaphilaxis are involved in eosinophil migration to the uterus. The inhibition by ketotifen of eosinophil degranulation may diminish eosinophil migration through extravascular compartment via a decrease in the release from degranulating eosinophils of enzymes required for this migration. It is possible that the inhibition by ketotifen of both, eosinophil degranulation and eosinophil motility through uterine extravascular compartment, interfere with eosinophil-mediated responses to estrogen or with other functions of these cells.

PMID: 2596371 [PubMed - indexed for MEDLINE]

2251. Ugeskr Laeger. 1989 Oct 23;151(43):2791-4.

[Treatment of hydatidosis with albendazol].

[Article in Danish]

Magnussen P, Gelletlie R, Bygbjerg IC.

Eight patients with hydatidosis treated with albendazol in daily doses of 10 mg/kg daily in courses of 28 days (4-6 courses) were analysed. The patients came from Morocco, Spain, Turkey and Yugoslavia. Seven patients had a cyst (or cysts) in the liver and one had also cysts in the kidneys. One patient had cysts in the muscles of the extremities. As assessed by ultrasonic scanning, computed tomography of the cysts, general condition, serology and the presence of hydatid antigen in the serum, treatment was effective in six patients. One patient developed an allergic reaction to albendazol. All of the patients had varying degrees of liver involvement which were reversible. Neutropenia did not occur. Various parameters for assessing the therapeutic effect are mentioned. Albendazol appears to be effective in the treatment of non-operable hydatid disease and to prevent recurrence after surgery.

PMID: 2588356 [PubMed - indexed for MEDLINE]

2252. J Invest Dermatol. 1989 Oct;93(4):471-4.

Immunohistochemical demonstration of migration inhibitory factor in different types of urticaria.

Czarnetzki BM, Zwadlo-Klarwasser G, Bröcker EB, Sorg C.

Department of Clinical, University Clinics, Münster, Federal Republic of Germany.

Because urticarial lesions can persist for extended periods of time, we have investigated the histochemical expression of an antibody against the cytokine macrophage inhibitory factor in 23 patients with different types of urticaria. Positive staining of upper and middermal dendritic cells was noted in sections from all three biopsy specimens of acute urticaria, eight of chronic urticaria, and all six of urticaria pigmentosa lesions. In all but one biopsy specimen, endothelial cells reacted as well. In three sections (two chronic urticaria, one urticaria pigmentosa), luminal lining cells of sweat glands were also noted to stain positively. In contrast, lesional skin from all eight patients with pressure urticaria was negative, as was the clinically normal skin of all patients, with the exception of one patient with urticaria pigmentosa. The data suggest that cytokines may be involved in lesions of acute type immunologic processes and that they need not be expressed in delayed type reactions.

PMID: 2476510 [PubMed - indexed for MEDLINE]

2253. Rev Infect Dis. 1989 Sep-Oct;11 Suppl 6:S1475-81.

Dermatologic manifestations of Lyme disease.

Berger BW.

Department of Dermatology, New York University School of Medicine, New York.

Erythema migrams (EM), the distinctive cutaneous lesion of Lyme disease, has a

variable clinical appearance, but at some point presents as a centrifugally expanding, usually erythematous, annular patch. Of 237 patients with this condition, 201 (85%) were examined initially from May through September. Thirty-four (14%) remembered having been bitten by a deer tick. The median interval from the bite to the appearance of EM was 9 days (range, 1-36 days). Forty-one (17%) of the patients had multiple EM lesions. Of the 237 patients, 128 (54%) manifested major extracutaneous signs and symptoms. Although EM also has a variable histologic picture, the presence of a deep and superficial perivascular and interstitial lymphohistiocytic infiltrate containing plasma cells is diagnostic. Spirochetes can be demonstrated with Warthin-Starry staining in approximately 40% of the biopsy specimens. Concomitant cutaneous lesions appeared on some patients before and during antibiotic therapy. Nine patients with serologic evidence of Borrelia burgdorferi infection had cutaneous lesions other than EM, including granuloma annulare (three), erythema nodosum (two), papular urticaria (two), Henoch-Schönlein-like purpura (one), and morphea (one). Whether these entities are cutaneous markers of Lyme disease or are coincidental findings is yet to be determined.

PMID: 2814169 [PubMed - indexed for MEDLINE]

2254. Allergol Immunopathol (Madr). 1989 Sep-Oct;17(5):241-4.

Granulocyte migration ability in subjects hypersensitive to aspirin.

Matusiewicz R, Lebiedowski K, Czajkowski M, Wacek W.

I Department of Internal Diseases. Grochow Hospital, Warsaw, Poland.

The reported study was carried out on 32 patients with atopic asthma. Twenty-seven patients had atopic asthma and were hypersensitive to aspirin. Twenty patients were hypersensitive only to aspirin. At the time of the investigation the patients were in the remission period at the beginning of the investigations and the lowest aspirin dose producing hypersensitivity symptoms was established in each case. It ranged from 0.05 to 0.5 g. In the group of patients hypersensitive to aspirin were included those in whom spirometric measurements demonstrated a FEV1 fall below 20% of the initial level. In all the patients and controls studied the "in vitro" granulocyte migration test was done by Clausen's method and "in vivo" by Southam's method. As shown in the presented results, patients with aspirin hypersensitivity have a defect in the migration of granulocytes of the vascular and tissue pools.

PMID: 2610186 [PubMed - indexed for MEDLINE]

2255. Bratisl Lek Listy. 1989 Sep;90(9):695-9.

[Larval toxocariasis--a severe course of the manifest infection].

[Article in Slovak]

Redhammer R, Boca M, Sobota K.

The case of a 17 year old patient with severe course of toxocariasis is reported. Over a period of 6 months the patient developed signs of serious systemic condition with fever, respiratory infections, diarrhea, urticaria, weight loss, and muscular atrophies. The most remarkable organ derangements involved bilateral exudative neuroretinitis, severe degree of peripheral motoneuron derangement, and grave kidney damage with developing polyuria, hypokalemia, metabolic alkalosis and therapeutically hardly tractable hypertension. The most important laboratory

findings were high erythrocyte sedimentation, absolute and relative eosinophilia, and hypergammaglobulinemia. Serological examination exhibited weak larval toxocariasis positivity. Treatment with Mintezol and subsequent administration of prednisone resulted in complete restoration of the clinical state, including organ and laboratory manifestations. The reported case documents the occurrence of larval toxocariasis in our population as well as the possibility of a very severe course of this parasitic infection in man. The therapeutic effect is remarkable since literary data have so far reported mostly unsatisfactory results of toxocariasis treatment.

PMID: 2590855 [PubMed - indexed for MEDLINE]

2256. J Antimicrob Chemother. 1989 Aug;24(2):251-64.

Structural correlations with cross-reactivity of beta-lactam antibiotics in delayed type hypersensitivity. Cross-allergenicity in hypersensitivity to cephems with a tetrazolyl group in the C-3 side chain.

Uno K, Yamasaku F.

Department of Internal Medicine, Suibarago Hospital, Niigata, Japan.

Cross-reactivity associated with delayed type hypersensitivity (DTH) arising from cephem antibiotics with a tetrazolyl group in the C-3 side chain was investigated by clinical testing and animal experiments. Clinical cross-reaction testing was performed with the leucocyte migration inhibition test (LMIT) with respect to sixteen patients with hypersensitivity induced by cephems with a tetrazolyl group in the C-3 side chain. The proportion of positive LMIT cross-reactions to cephems with a tetrazolyl group in the C-3 side chain was 78% (25/32), to cephems without a tetrazolyl group, 5% (1/22), and to penams, 0% (0/12). The proportion of positives in tests performed with methyl-tetrazolethiol (MTT) and hydroxyethyl-tetrazolethiol (HTT), which essentially represent the structures of the C-3 side-chains in the allergenic drugs, was 29% (4/14), while the corresponding proportion for 7-aminocephalosporanic acid (7ACA), which represents the skeleton structure of the cephem antibiotics, was 33% (1/3). The animal experiments were performed with guinea pigs, with latamoxef, cefoperazone and MTT as the sensitizing agents and testing for cross-reactivity by means of delayed type intradermal reactions as well as the LMIT. The results of intradermal testing and the LMIT agreed almost completely, thus providing strong support for the clinical results. Latamoxef and cefoperazone displayed the same cross-reactivity, manifesting cross-reactions with MTT, HTT and 7ACA as well as with cephems having a tetrazolyl group in the C-3 side chain. Moreover, DTH induced by MTT displayed cross-reactivity with cephems possessing tetrazolyl groups in the C-3 side chain. The above results indicate that free MTT radicals, as well as the skeleton ring structure represented by 7ACA, are strongly involved in DTH arising from cephem antibiotics with a tetrazolyl group in the C-3 side chain.

PMID: 2793648 [PubMed - indexed for MEDLINE]

2257. J Antimicrob Chemother. 1989 Aug;24(2):241-50.

Application of leucocyte migration tests to detection of allergenic drugs in patients with hypersensitivity to beta-lactam antibiotics.

Uno K, Yamasaku F.

Department of Internal Medicine, Suibarago Hospital, Niigata, Japan.

In 90 suspected cases of beta-lactam hypersensitivity manifesting exanthema, pyrexia and hepatopathic symptoms, the identities of the allergenic drugs were investigated by immediate type skin reactions, sensitized erythrocyte agglutination tests and leucocyte migration inhibition tests (LMIT). In addition 30 control patients were tested for hypersensitivity to ampicillin, cephalexin and latamoxef. The control patients manifested the same symptoms as the patients with suspected hypersensitivity to beta-lactam but had not been treated with beta-lactam antibiotics. In the immediate type skin reaction, all the control cases as well as all the suspected beta-lactam hypersensitive patients displayed negative reactions to each of the antibiotics tested. In the sensitized erythrocyte agglutination tests, no antibodies to any of the three drugs were detected in the control group, while antibodies with a titre of 1:2 or more were detected in only 7% (6/90) of the suspected beta-lactam hypersensitive patients. On the other hand, in the LMIT, only 4% (4/90) of the control patients displayed positive reactions to the three test antibiotics, whereas the suspected sensitizing drugs elicited a high rate of positive responses (68/90; 76%) in the group of suspected beta-lactam hypersensitive patients. Broken down into symptomatic categories, response to the LMIT was positive in 41 of 58 cases of suspected drug rash (71%), 26 of 26 cases of suspected drug fever (96%), and 20 of 24 cases of suspected drug-induced hepatopathy (83%). Thus, the pyrexial group exhibited a particularly high ratio of positive reactions, and in fact exanthemic cases with concomitant symptoms (eosinophilia, pyrexia, hepatopathy, etc.) also displayed a notably high ratio of positives (19/20; 95%). The overall results indicated that delayed type hypersensitivity (DTH) is largely involved in exanthema, pyrexia and hepatopathy induced by beta-lactam antibiotics, and that the LMIT constitutes an effective means of detecting allergenic drugs in patients with beta-lactam hypersensitivity.

PMID: 2793647 [PubMed - indexed for MEDLINE]

2258. Warasan Prachakon Lae Sangkhom. 1989 Jul;2(1):63-90.

[Health problems and illness of female workers in textile industries].

[Article in Thai]

Soonthorndhada K.

PIP: This paper examines 3 major health-related issues: 1) existing health problems and illnesses resulting from physical environmental conditions at workplaces; 2) female workers' perception on illness and health protection; and 3) the relationship between illness and risk factors. The study area is textile factories in Bangkok and its peripheries. Data are drawn from the 1987 Survey of Occupational Health and Textile Industrial Development in Thailand: Effect on Health and Socioeconomics of Female Migrant Workers. This study shows that about 20% of female workers have ill-health problems and illness after a period of working mainly due to high levels of dust and noise, and inadequate light. These conditions are hazardous to the respiratory system (resulting in cough and chest tightness), the hearing system (pains as well as impaired and hearing loss), eye systems (irritation, reduced visual capacity) and skin allergy. Such illnesses are intensified in the long- run. The analysis of variances reveals that education, section of work, perception (particularly mask and ear plug) significantly affect these illnesses. This study concludes that health education and occupational health should be provided in factories with emphasis on health prevention and promotion.
PMID: 12316145 [PubMed - indexed for MEDLINE]

2259. Med Hypotheses. 1989 Jul;29(3):167-9.

A mechanism of peripheral spread or localization of inflammatory reactions--role of the localized ground substance adaptive phenomenon.

Stone OJ.

It is known that connective tissue-active peptides (CTAP) are released at sites of inflammation. Some of this material diffuses to immediately adjacent tissue and increases ground substance viscosity and fibroblast proliferation. This contributes to host protection against spread of infections and tumors. In a person with normal inflammatory reactivity, it should prevent spread of mediators and products of local inflammation. However, the host with an increased reactivity in sites of increased ground substance viscosity or who is highly reactive to dilution of tissue fluid would respond with more inflammation. A non-infectious, non-malignant process in a host with a highly reactive inflammatory or immune response could end up with peripheral spread. This could occur in any tissue but it occurs with great vigor in the skin. It could present as a peripheral extension of a local disease process, such as psoriasis, or the migration of cyclic lesions with clearing of the central area. There are over a dozen variants of peripherally spreading, ringed lesions described in the dermatologic literature. This includes erythema marginatum of rheumatic fever, erythema gyratum repens associated with cancer, and erythema annulare centrificum associated with allergic reactions to fungi. Many of the ringed dermatologic lesions have an immunologic component. They tend to be associated with inflammatory immune reactions at distant sites. Dermatologists have been gathering information on the ringed phenomenon at least since Hebra in 1854. The acute localized ground substance adaptive phenomenon is a broadly beneficial biologic response.(ABSTRACT TRUNCATED AT 250 WORDS)

PMID: 2779476 [PubMed - indexed for MEDLINE]

2260. Zhongguo Yao Li Xue Bao. 1989 Jul;10(4):360-5.

[Anti-inflammatory and anti-allergic action of aloperine].

[Article in Chinese]

Zhou CC, Gao HB, Sun XB, Shi HB, Liu W, Yuan HN, Wang ZX.

Aloperine, an alkaloid isolated from Sophora alopecuroides L, showed a marked suppressive effect on the swelling of the rat's hind paw induced by injecting carrageenin, macostatin, PGE2, histamine, 5-HT, on the rat's scald oedema induced by scalding its hind paw, and on the increased permeability of capillaries caused by histamine and the leukocytic migratory response. The swelling induced by injecting carrageenin into the hind paw of adrenolectomized rats was still significantly inhibited. Noticeably, aloperine reduced the content of PGE and histamine in the exudate formed after injecting carrageenin and dextran into the rat's hind paw, and increased the stability of red cell membranes, the activity of catalase (CAT) in hepatic tissue of mice, and reduced the content of malondialdehyde (MDA) in hepatic tissue of intoxicated mice. It had no apparent effect either on the activity of superoxide dismutase (SOD) in mice serum or on the phagocytosis of the monocyte-macrophage system, or on Forssman cutaneous vasculitis and the content of immune complex in serum of rats with Arthus reaction. But it had certain inhibitory effect on the PCA reaction and significant inhibitory effect upon such allergic reaction as Arthus reaction, reversible passive Arthus reaction, the delayed hypersensitivity reaction induced by tuberculin in rats, and adjuvant arthritis.

PMID: 2533795 [PubMed - indexed for MEDLINE]

2261. N Z Med J. 1989 May 10;102(867):211-3.

Attendance failure at Middlemore Hospital asthma clinic.

McClellan VE, Garrett JE.

Green Lane Hospital, Auckland.

A population of 138 outpatients' sociodemographic and sociobehavioural characteristics and clinic organisational factors were studied in response to a concern about poor attendance rates at Middlemore Hospital's asthma clinic. Low and nonattendance was found to be higher among Maoris (44.7%) and Pacific Islanders (31.6%), outpatients of lower socioeconomic status (SES group 5 and 6, 55.3%), and those referred from the accident and emergency (A & E) department (66%). Migration out of the south Auckland region was common among the group of low attending outpatients (23.9%). Since neither the clinic nor the outpatients' general practitioners were aware of this group's migrational movement there exists a large population who do not have regular medical care and are therefore at risk of significant morbidity or mortality from their asthma.

PMID: 2717101 [PubMed - indexed for MEDLINE]

2262. Ugeskr Laeger. 1989 May 1;151(18):1125-6.

[Anaphylactic shock caused by rupture of hydatid cysts].

[Article in Danish]

Magnussen P, Thorsen S.

A case of anaphylactic shock in a Maroccan man with hydatid cysts in the liver is described. Attention is drawn to the increased prevalence of hydatid disease among immigrants from South European countries, the Middle East and Africa. Medicinal treatment of hydatosis in cases with risk of spread is mentioned.

PMID: 2734882 [PubMed - indexed for MEDLINE]

2263. Stomatologiia (Mosk). 1989 May-Jun;68(3):46-8.

[The prevention of allergy to metal dentures].

[Article in Russian]

Doĭnikov AI, Demner DL.

Investigation of 124 patients visiting the clinic for incompatibility with metal dentures revealed the risk factors for allergic response in the oral cavity. Reliable and easily applied techniques were developed to diagnose allergic responses in an outpatient dentistry units which allowed to preselect the optimal alloy to manufacture dentures for each individual patient. Clinical signs of allergy to several metals were clarified.

PMID: 2672420 [PubMed - indexed for MEDLINE]

2264. Clin Exp Allergy. 1989 May;19(3):327-34.

Reduction of increased serum neutrophil chemotactic activity following effective hyposensitization in house dust mite allergy.

McHugh SM, Ewan PW.

Department of Immunology, St Mary's Hospital Medical School, London.

Changes in the level of serum neutrophil chemotactic activity (S-NCA) were investigated in 20 subjects with allergic rhinitis, with or without asthma, undergoing clinically effective hyposensitization to house dust mite with Pharmalgen Dermatophagoides pteronyssinus. Two control groups were studied: 28 subjects with allergic rhinitis, with or without asthma, receiving placebo injections for 1 yr in a double-blind controlled trial with Pharmalgen D. pteronyssinus (from whom the actively treated group in this study were recruited), and eight non-atopic asymptomatic controls. S-NCA and serum IgE specific to D. pteronyssinus were measured in the subjects before, during (3-6 months) and 12 months after treatment, and once in the non-atopic controls. The mean S-NCA was significantly higher (0.01 greater than P greater than 0.001) in subjects before treatment (mean +/- s.e. = 63.8 +/- 3.6 arbitrary units of migration (AUM] compared with the non-atopic controls (48.5 +/- 3.7 AUM), but had fallen to normal levels after 6 months (46.8 +/- 4.0 AUM) and 12 months treatment (45.2 +/- 3.8 AUM). The levels of S-NCA in the placebo treated group were significantly higher than normal at the start of treatment (69.2 +/- 4.1) and remained raised throughout the 12 months treatment. In the actively treated group, the level of S-NCA had fallen in 18 out of 19 subjects after 12 months immunotherapy, and was unaltered in one. Mean levels of D. pteronyssinus IgE rose during the first 6 months and declined to initial levels by the end of the treatment.(ABSTRACT TRUNCATED AT 250 WORDS)

PMID: 2660970 [PubMed - indexed for MEDLINE]

2265. Ann Allergy. 1989 May;62(5):461-9.

Patch tests demonstrating immune (antibody and cell-mediated) reactions to foods.

Breneman JC, Sweeney M, Robert A.

Suburban Medical Clinic, Galesburg, MI 49053.

For decades food allergists have sought a simple, inexpensive test for food allergy. Intradermal tests of aqueous material have not proved reliable. When positive, they are probably only 10% accurate. The newer laboratory tests for food allergy are expensive and highly sophisticated. They are impractical for use in the laboratories of practicing allergists with whom individuals with a potential food allergy are most liable to consult. The RAST is capable of identifying only Type I of the Gell-Coombs' reaction classification. Since 1980, a patch test of individual foods suspended in dimethylsulfoxide has been used as a screen for sensitivity to foods. Controlled clinical studies suggest this might prove to be a valuable test for food allergy. Immune studies appear to confirm the accuracy and reliability of this inexpensive test. No systemic reactions have been observed in the 400 patients tested indicating it to be a safe procedure.

PMID: 2470275 [PubMed - indexed for MEDLINE]

2266. J Allergy Clin Immunol. 1989 May;83(5):905-12.

Effect of cetirizine on mast cell-mediator release and cellular traffic during the cutaneous late-phase reaction.

Charlesworth EN, Kagey-Sobotka A, Norman PS, Lichtenstein LM.

Department of Medicine, Johns Hopkins University School of Medicine, Good Samaritan Hospital, Baltimore, Md 21239.

A new H1 antihistamine, cetirizine, was studied to determine its effects on mediators and cellular infiltration during the cutaneous late-phase response (LPR). Ten ragweed-allergic subjects, who had previously demonstrated a cutaneous LPR, were examined in a double-blind, crossover study. Either cetirizine, 20 mg, or placebo was administered orally once daily for 2 days before and the morning of placement of a skin chamber overlying an unroofed heat/suction-induced blister to which was added antigen or buffer. Skin test erythema was significantly reduced by cetirizine at 15 minutes, 2 hours, and 4 hours by 56%, 40%, and 39%, respectively (all, p less than or equal to 0.01), but by 6 and at 8 hours, the cutaneous erythema was not significantly lessened. Histamine release was not altered by cetirizine treatment, but prostaglandin D2 (PGD2) production, which peaked at 3 to 5 hours, was clearly reduced by cetirizine treatment, being lower at all time points during the reaction; this was significant by analysis of variance (p less than or equal to 0.04). The inhibition was most marked during the fifth hour of the reaction when there was a 50% suppression of the PGD2 level by cetirizine (0.193 ng/ml to 0.075 ng/ml [p less than or equal to 0.03]). The most dramatic effect of cetirizine was attenuation of the inflammatory cell migration into the chamber. Eosinophil infiltration was decreased by about 75% during hours 6, 7, and 8 (p less than or equal to 0.04), whereas the number of neutrophils was reduced by the same magnitude at the same times (p less than or equal to 0.04).(ABSTRACT TRUNCATED AT 250 WORDS)

PMID: 2469708 [PubMed - indexed for MEDLINE]

2267. Dermatol Clin. 1989 Apr;7(2):291-300.

Schistosomiasis, cercarial dermatitis, and marine dermatitis.

González E.

Harvard Medical School, Massachusetts General Hospital, Boston.

The three anthropophilic species of schistosomes produce local or systemic reactions that are a reflection of host interaction with the stage of the parasite. Dermatitis schistosomica is a transient, local irritant or hypersensitivity reaction during the penetration phase of the cercariae. Early nonspecific reactions are characterized by a severe, transient, systemic, immune-complex disease referred to as Katayama disease, which occurs during migration of the juvenile worm in the bloodstream. Late nonspecific reactions are characterized by a mild, transient hypersensitivity reaction during oviposition by adult worms. Specific late cutaneous manifestations are rare and are produced by the ectopic migration of ova or worms into the skin, with asymptomatic or symptomatic, chronic, granulomatous, and fibrotic mucocutaneous lesions with variable morphologic characteristics. The avian schistosomes produce a cercarial dermatitis that is a more severe form of dermatitis schistosomica but lacks systemic complications and reflects the host reaction to the destruction of the cercariae in the skin. Marine dermatitis or "sea bather's eruption" is probably an irritant or toxic transient reaction of unknown origin with no systemic implication, whereas "Dogger Bank itch" is an allergic contact eczematous dermatitis caused by a metabolite produced by a marine organism.

PMID: 2670374 [PubMed - indexed for MEDLINE]

2268. Allerg Immunol (Paris). 1989 Mar;21(3):103-6.

[Epidemiologic study of adult bronchial asthma in a Benin hospital environment].

[Article in French]

Monteiro B, Gninafon M, Agbodjogbe A.

Cas du Centre National Hospitalier et Universitaire (C.N.H.U.) Cotonou.

This work is a contribution to the epidemiological study of bronchial asthma in adults in a hospital environment at Cotonou. It is a question of the necessity of admission to hospital of clinical cases. The study has emphasized the following points: Separation according to age has shown that 52% of patients are less than 35 years old, young subjects are in this group. The two sexes are equally involved, with a sex ratio of 1. The different socio-professional groups involved have shown a predominance of those without profession. The main allergic factors identified by questioning are dominated by housedust, which suggests, failing allergological tests, that the role of mites is probably preponderant. There does not seem to be any particular influence of meteorological factors (rain or dryness) on the frequency of crises. Finally, it should be emphasized that technical difficulties make it very difficult to study asthma in Benin.

PMID: 2713042 [PubMed - indexed for MEDLINE]

2269. J Neurol Sci. 1989 Mar;90(1):77-85.

Inhibition of experimental allergic encephalomyelitis by the alpha-glucosidase inhibitor castanospermine.

Willenborg DO, Parish CR, Cowden WB.

Neurosciences Research Unit, Royal Canberra Hospital, Australian National University.

The alkaloid castanospermine is a potent inhibitor of oligosaccharide processing in vitro. Our recent findings indicating the importance of carbohydrate moieties in some critical step of the neuro-immunologic inflammatory process of allergic encephalomyelitis prompted us to investigate the effect of castanospermine on this disease process. The alkaloid inhibited passively induced allergic encephalomyelitis in a dose-dependent manner when administered continuously for 7 days beginning at the time of lymphocyte transfer. Although clinical disease was totally inhibited, treated animals did have inflammatory lesions in the central nervous system. These lesions were qualitatively different from those seen in untreated animals in that the inflammatory cells were tightly packed around the vessels and showed little migration into surrounding tissues. Castanospermine also effectively inhibited clinical disease in recipient animals which had had a previous episode of allergic encephalomyelitis. Castanospermine did not alter the disease when treatment was started after the onset of clinical symptoms.

PMID: 2656918 [PubMed - indexed for MEDLINE]

2270. N Z Med J. 1989 Feb 8;102(861):36-8.

Symptoms of asthma, methacholine airway responsiveness and atopy in migrant

Tokelauan children.

Crane J, O'Donnell TV, Prior IA, Waite DA.

Epidemiology Unit, Wellington Hospital.

503 migrant Tokelauan children between five and 15 years resident predominantly in Porirua and the Hutt Valley were surveyed as part of a study of asthma prevalence in a recently migrant population. The survey consisted of domiciliary interview with parents, physical examination, assessment of bronchial hyperresponsiveness and atopy, by allergen skin prick testing. Forty-three children (8%) had a history of wheezy breathing or asthma. 160 children (32%) had evidence of increased airway responsiveness defined as a PD20 (provocative dose of methacholine causing a less than or equal to 20% fall in FEV1, of less than or equal to 12.2 mumoL methacholine). Of the 43 children with a history of asthma, 40 (93%) had evidence of bronchial hyperresponsiveness, 36 (84%) were atopic and 35 (81%) had both bronchial hyperresponsiveness and atopy. Forty-five children (9%) were found to be wheezing on the day of examination only 16 of these had a history of wheezing. Twenty seven of the wheezing children demonstrated bronchial hyperresponsiveness and 22 of these were atopic. Of the 18 children wheezing but with no evidence of bronchial hyperresponsiveness only six were atopic. These contrasting findings suggest differences in the cause of symptoms among the children. Regional differences were observed for the prevalence of symptoms and signs of asthma, bronchial hyperresponsiveness and atopy. Hutt Valley Tokelauan children exhibited a higher prevalence than the Porirua children. Migrants to the Hutt Valley and Porirua are from different atolls, and these differences raise the possibility of a genetic influence on the development of asthma.

PMID: 2662064 [PubMed - indexed for MEDLINE]

2271. Otolaryngol Pol. 1989;43(1):40-4.

[Cytology of nasal mucous membrane in pollen allergy].

[Article in Polish]

Miszke A, Sanokowska E, Chomiak-Stachura E, Rybarska I.

In 33 patients with pollen allergy the cytograms were made by means of half-quantity method during the acute phase and remission. In comparison with healthy mucous membrane there were a large number of migration cells with eosinophils of 46%. In some cases there were also basophils. In superficial layer prevailed the cuboid cells (mean X = 43%) and the lower number of parabasal cells. During the remission as the deviation from the norm there were only the large number of migration cells with eosinophils.

PMID: 2812796 [PubMed - indexed for MEDLINE]

2272. Bull Soc Pathol Exot Filiales. 1989;82(4):581-3.

[Loa loa filariasis: cause of severe eosinophilia].

[Article in French]

Carme B, Nkoua JL.

Laboratoire de Parasitologie et Mycologie, Centre Hospitalier et Universitaire de Brazzaville, R. P. du Congo.

Two cases of very high hypereosinophilia (28,160 and 11,232/mm3) observed in congolese patients are presented. Although microfilaraemia was not detectable, loiasis was diagnosed, given the clinical manifestations, epidemiological data, history of sub-conjunctival migration of the adult worm (in one case), spectacular recovery (clinical and biological) after treatment with diethylcarbamazine. This "allergic form" of filariasis is often considered unusual in indigenous subjects.

PMID: 2805187 [PubMed - indexed for MEDLINE]

2273. Vestn Otorinolaringol. 1989 Jan-Feb;(1):46-9.

[Indicators of the activity of the immune system during laser therapy of vasomotor rhinitis].

[Article in Russian]

Tulebaev RK, Sadykov ShB, Romanov VA, Khalitova GKh.

Helium-neon laser LH-75 was used to treat 60 inbred mice and 62 patients with vasomotor rhinitis. The patients were given 10 laser sessions. In animals, all responses of T-lymphocytes were modified. Cyclic changes that developed during laser irradiation were accompanied by immunity suppression or activation. In patients, the count of T- and B-lymphocytes and the leucocyte migration inhibition reaction were measured. The patients with vasomotor rhinitis showed a significant increase of T-lymphocytes and a higher capacity of T-cells to form the migration inhibition factor. The efficacy of laser therapy of vasomotor rhinitis became optimal on irradiation day 6 or 7 and reached a maximum on day 10.

PMID: 2785311 [PubMed - indexed for MEDLINE]

2274. Int Arch Allergy Appl Immunol. 1989;89(2-3):314-7.

Inhibition by histamine of platelet-activating-factor-induced neutrophil chemotaxis in bronchial asthma.

Rabier M, Damon M, Chanez P, Mencia-Huerta JM, Braquet P, Bousquet J, Michel FB, Godard P.

INSERM U-58, Montpellier, France.

Circulating human polymorphonuclear neutrophils are involved in asthma after their migration into the lung by local chemotactic factors. Investigation of the locomotion of neutrophils in Boyden chambers, showed that the chemotactic intensity of the platelet-activating factor (PAF) was similar in cells from healthy subjects and allergic asthmatics, although the optimal effect of the mediator was observed at 10(-6) M and 10(-8) M, respectively. Histamine had no direct chemoattractant effect on neutrophils but inhibited PAF-induced chemotaxis of neutrophils from healthy subjects and allergic asthmatics. This study provides additional evidence that neutrophils are involved in asthma, and points out the interaction between PAF and histamine in the migration of neutrophils to the lung.

PMID: 2759720 [PubMed - indexed for MEDLINE]

2275. Dermatologica. 1989;179 Suppl 1:1-8.

Inflammation and immunity in dermatophytosis.

Tagami H, Kudoh K, Takematsu H.

Department of Dermatology, Tohoku University School of Medicine, Sendai, Japan.

Infections by dermatophytes (dermatophytosis) naturally stimulate the immune system as in those by other microorganisms to induce various immunological phenomena. However, differing from other infections, the infecting organisms cannot become a direct target of antibody response or phagocytosis because they reside only in the barrier membrane of the body surface, i.e. in the stratum corneum. Thus, the immunity against dermatophytes is relative as compared with the absolute one noted in other infections such as measles and mumps. In dermatophytosis, a unique behavior of the epidermis as noted in contact dermatitis plays an important role in the defense against infection. Dermatitic changes induced by fungal products, particularly those due to contact sensitivity to a fungal antigen, trichophytin, enhance epidermopoiesis, which leads to increased turnover of the epidermis with their resultant elimination from the skin surface. Furthermore, the dermatophytes in the stratum corneum provoke transepidermal leukocyte chemotaxis by generating chemotactic C5a anaphylatoxin in exudating serum via alternative complement pathway activation in addition to a release of low molecular weight chemotactic factors. Such neutrophilic migration with the formation of subcorneal pustules also enhances epidermal proliferation as in psoriasis.

PMID: 2673850 [PubMed - indexed for MEDLINE]

2276. Int Arch Allergy Appl Immunol. 1989;90(4):331-7.

Passive air-pouch anaphylaxis in rats. I. Induction of anaphylaxis.

Konno-Amano S, Hirao T, Kohno H, Mori H, Sato Y.

Shiseido Basic Research Laboratories, Yokohama, Japan.

Induction of an experimental passive anaphylaxis of the air-pouch type, passive air-pouch anaphylaxis, was carried out in an attempt to induce a reproducible anaphylaxis model suitable for quantitative studies. Rats were injected subcutaneously with 10 ml of air into the dorsal skin to make an air-pouch and with 2 ml of antiserum at an appropriate dilution for passive sensitization, and then 5 ml of air was removed. The challenge with 5 ml of antigen solution into the air-pouch 48 h later provoked mast cell degranulation and increased vascular permeability induced by released histamine. Treatment with monovalent hapten prior to the antigen challenge almost completely inhibited histamine release and plasma exudation to levels similar to those in the nonsensitized group. In this model, mast cell-dependent late-phase allergic reaction, such as leukocyte migration or the increase of plasma exudation following mast cell degeneration, was not observed.

PMID: 2613340 [PubMed - indexed for MEDLINE]

2277. Arch Dermatol Res. 1989;281(7):482-6.

Demonstration of neutrophil chemotactic anaphylatoxins in human dandruff.

Kikuchi T, Horii I, Sakamoto T, Nakayama Y, Tagami H.

Shiseido Basic Research Laboratories, Yokohama, Japan.

In contrast to scales collected from the scalps of nine healthy individuals where a few parakeratotic cells are observable, a large number of parakeratotic cells associated with some infiltrated polymorphonuclear leukocytes (PMNLs) were found in the scales obtained from 11 individuals complaining of dandruff. Therefore, we determined the neutrophil chemotactic properties of the water-soluble extracts of dandruff scales and normal control scalp scales. Aqeous extracts fractionated by Sephadex G-75 showed a potent chemotactic activity only in the fractions of the dandruff patients that eluted with cytochrome C marker (cyt C; molecular weight, 12 kDa). It was comparatively stable to heat but was greatly inhibited by the addition of anti-C5 antiserum. Radioimmunoassay demonstrated that, although small amounts of C5a and C4a anaphylatoxins were demonstratable even in the extracts of normal scalp, they were found in significantly increased amounts in the extracts of dandruff. Moreover, there was a significantly positive correlation between C5a and C4a concentrations in these extracts. These results suggest that classical complement pathway activation with resultant production of C5a anaphylatoxin is involved in the migration of PMNLs into the lesional skin of dandruff.

PMID: 2610519 [PubMed - indexed for MEDLINE]

2278. Acta Derm Venereol. 1989;69(3):253-6.

Prostaglandin E1 and prostaglandin F2 alpha in exudate in nickel allergy.

Lerche A, Bisgaard H, Kassis V, Christensen JD, Søndergaard J.

Department of Dermatology, Bispebjerg Hospital, Copenhagen, Denmark.

Ten nickel-allergic patients and 5 healthy control subjects participated in a study of the kinetics of the flux and concentration of migrated leukocytes and extracellular PGE1 and PGF2 alpha during a 48 h period, using a skin chamber technique. The patients were provided with two skin chambers, one with and one without nickel challenge. A higher flux of leukocytes, PGE1 and PGF2 alpha was observed during the second day of allergen exposure, while the concentrations probably due to dilution were unchanged or diminished, indicating an unspecific role of the prostaglandins during the contact allergic reaction. No correlations were found within the groups between the migration of leukocytes and the prostaglandin content.

PMID: 2566235 [PubMed - indexed for MEDLINE]

2279. Pediatriia. 1989;(9):38-42.

[Characteristics of antiviral immunity in children with the atopic form of bronchial asthma].

[Article in Russian]

Nisevich LL, Romanova LA, Shigina OM, Sominina AA.

In atopic bronchial asthma, an earlier "encounter" of children with viruses that cause acute respiratory diseases was established as was mass infection of asthma children with respiratory viruses. Disorders of antiviral immunity in children suffering from atopic asthma primarily manifested themselves in overproduction of antibodies among viruses. The leukocyte migration inhibition test has demonstrated that atopic asthma children are sensitized with respiratory viruses.

Sensitization to viruses rises with age whereas the presence of the foci of chronic infection promotes ever higher sensitization of the body with respiratory viruses, bringing about inhibition of leukocyte function.

PMID: 2555767 [PubMed - indexed for MEDLINE]

2280. Dermatologica. 1989;179 Suppl 1:54-9.

From eosinophil chemotactic factor of anaphylaxis to leukotriene B4--chemistry, biology and functional significance of eosinophil chemotactic leukotrienes in dermatology.

Czarnetzki BM, Rosenbach T.

Universitäts-Hautklinik, Münster, FRG.

The present paper examines evidence for the identity of low molecular weight eosinophil chemotactic factor (ECF) and of leukotriene B4 (LTB4) and its metabolites. Total congruity between the two entities is found regarding (1) cells of origin; (2) conditions for in vitro generation and pharmacological modulation; (3) physiochemical properties; (4) in vitro chemotaxis towards human monocytes, fibroblasts and guinea pig eosinophils; (5) in vivo activities in humans, and (6) occurrence of the factors in various dermatological diseases. Quantitative differences were observed only for in vitro neutrophil migration which may be due to neutrophil chemotactic mono-HETEs and possibly platelet activating factor in the ECF preparations. The name ECF should therefore be replaced by LTB4 and its metabolites, as has happened for SRS which is now called LTC4/D4.

PMID: 2550288 [PubMed - indexed for MEDLINE]

2281. Drugs. 1989;37 Suppl 1:9-18; discussion 69-77.

Inhibitory effects of nedocromil sodium on the in vitro induced migration and leukotriene formation of human granulocytes.

Bruijnzeel PL, Warringa RA, Kok PT, Hamelink ML, Kreukniet J.

Department of Pulmonary Disease, State University Hospital Utrecht, The Netherlands.

Inflammatory cells, such as neutrophils and eosinophils, are thought to actively contribute to the pathogenesis of asthma since they infiltrate into the lung tissue and may be activated locally to release bronchoconstrictor mediators. In this study we provide evidence that nedocromil sodium is capable of effectively inhibiting the platelet-activating factor (PAF) and zymosan-activated serum (ZAS)-induced chemotaxis of polymorphonuclear granulocytes (PMN) [IC50 approximately 1 nmol/L and 0.1 mumol/L respectively]. The same inhibitory potency was obtained with sodium cromoglycate. Thus, nedocromil sodium may effectively inhibit the mobilisation of inflammatory cells in the lung. Furthermore, nedocromil sodium is capable of inhibiting the formation of the bronchoconstrictor mediator leukotriene-C4 (LTC4) by eosinophils in a concentration-dependent way [IC30 for A23187: 5.6 10(-5) mol/L; IC30 for opsonised zymosan (OZ): 6.3 10(-5) mol/L], whereas this drug is not capable of inhibiting leukotriene-B4 (LTB4) formation by neutrophils. These findings indicate that nedocromil sodium inhibits the release of bronchoconstrictor mediators not only from mast cells but also from eosinophils.

PMID: 2547570 [PubMed - indexed for MEDLINE]

2282. Vestn Dermatol Venerol. 1989;(2):66-7.

[Allergic dermatitis caused by pyrogenic silica (aerosol)].

[Article in Russian]

Liashenko IN, Lutsiuk NB, Otkalenko AK, Odnorogov IuV.

A case of allergic dermatitis developing after a contact exposure of the skin to aerosil is described. The authors suppose that violated intactness of the skin integument is largely responsible for the allergic reaction. The C-reactive protein, Hoigne's, and leucocyte migration inhibition tests have been all markedly positive. It is recommended that types of aerosil other than powder-forming be utilized and that means protecting the skin and the upper respiratory tract be used.

PMID: 2543155 [PubMed - indexed for MEDLINE]

2283. Adv Prostaglandin Thromboxane Leukot Res. 1989;19:539-42.

Dual action of prostaglandin E2 in allergic inflammation.

Hedqvist P, Raud J, Dahlén SE.

Department of Physiology, Karolinska Institutet, Stockholm, Sweden.

The findings reported here demonstrate that PGE2 can exert both anti- and pro-inflammatory activities in one and the same tissue, as exemplified by inhibition of mediator release and enhancement of mediator action. Furthermore, the complete reversal of indomethacin potentiation of allergic inflammation by addition of PGE2 in low concentration advocates a regulatory function of the endogenous and locally formed PGE2. In the present in vivo model for mast cell-dependent inflammation the influence of PGE2 was mainly on the release of mediators. However, factors such as the degree of local blood flow, or the state of the tissue and the site of prostaglandin production, may in other instances shift the Yin-Yang balance in favour of PGE2 action at the target level for released mediators. Finally, it was noted that the cheek pouch mast cells, in addition to their pivotal role in the initiation of inflammatory reactions, have a predominant periarteriolar distribution that promotes oriented and, in terms of covered area, efficient migration of recruited leukocytes. Whether this previously unrecognized organization is specific for the hamster cheek pouch or exemplifies a more general phenomenon is presently not known.

PMID: 2526534 [PubMed - indexed for MEDLINE]

2284. Vestn Dermatol Venerol. 1989;(1):52-7.

[The immune status of patients with foot mycosis and eczema].

[Article in Russian]

Iutskovskiĭ AD.

Thirty-five patients with squamous-hyperkeratotic mycoses and onychomycoses were examined, as were 68 ones with exudative mycoses of the soles and 97 ones with

disseminated eczemas. The following tests have been employed in the study: direct and indirect leucocyte migration inhibition, investigation of the lymphocyte functional activity, skin test with fungal antigen, quantitation of the absolute counts of lymphocytes, neutrophils and phagocytizing neutrophils, T and B rosette forming cells. Patients with mycoses of the soles with involvement of the nail plates, as well as those suffering from eczemas combined with mycoses developed a most marked reduction of the activity of the leukocyte migration inhibition factor (LMIF) and of the T lymphocyte mediator activity in the presence of the fungal antigen. The studies have detected the pattern of the leukocyte phagocytic reaction disturbances in the patients with mycoses and eczemas of the soles. No inhibiting effect of LMIF on the leukocytes in these patients appears to be due to the defect of the target cells and not of the producer cells. The direct and indirect leukocyte migration inhibition tests are recommended to be included in the complex of immunologic tests used in examinations of patients with exudative mycoses and eczemas of the soles and with the transitional and combined forms of these two conditions.

PMID: 2524143 [PubMed - indexed for MEDLINE]

2285. Gig Tr Prof Zabol. 1989;(4):22-4.

[Histamine-related reactions in patients with byssinosis].

[Article in Russian]

Grishina TI, Aĭzina NL, Babok AL, Alekseeva OG.

The purpose of the study was to analyze the mechanism of histamine action in histamine-dependent reactions of neutrophils and lymphocytes in patients with byssinosis and chronic asthmatic bronchitis under cotton and flax dust effect. The appraisal of histamine content in blood serum, receptor/histamine distribution of lymphocyte and neutrophil subpopulations (rosette-forming double and triple reactions), determination of histamine modeling effect on lymphocyte-neutrophil cooperation in the inhibition reaction of leukocyte migration revealed that under cotton dust effect neutrophils and the complement system were involved into the histamine liberation process in byssinosis patients, lymphocytes were most likely not involved into the process. Flax dust-affected histamine reactions were not so distinct: lymphocyte and neutrophil reactivity in byssinosis patients did not exceed the standards. Patients with chronic asthmatic bronchitis had high blood concentration of histamine and experienced some changes in cells' migration characteristics. It was assumed that primarily nonimmune mechanisms of histamine liberation and activation of the complement system were involved into byssinosis pathological process in patients exposed to cotton and flax dust effect. In patients with chronic asthmatic bronchitis there occurred all 3 activation mechanisms of biologically active substances, i.e., allergic and nonantigenic ways of histamine liberation and activation of the complement system.

PMID: 2473011 [PubMed - indexed for MEDLINE]

2286. Immunol Invest. 1988 Nov-Dec;17(8-9):679-705.

Leucocytes in asthma.

Kay AB.

Department of Allergy and Clinical Immunology, National Heart & Lung Institute, London, U.K.

A diagrammatic representation of the interactions between mediators of
hypersensitivity and leucocytes in early-, late-phase, and ongoing asthma is shown in Fig. 1. Early-phase or immediate reactions are largely the result of bronchoconstriction consequent to the release of mediators such as histamine, PGD2, LTC4/D4 and PAF. The principal mediator cell (MC) is the mast cell (although other IgE receptor-bearing cells such as the macrophage, eosinophil and platelet might also be involved in this immediate response). The stimulus for mediator cell activation may be either immunologic (IgE-dependent) or non-immunologic (i.e. changes in osmolarity as a result of the respiratory water loss associated with exercise-induced asthma). Late-phase reactions appear to be a consequence of infiltration with neutrophils (N), eosinophils (E) and macrophages (MO). These cells are recruited and activated either by mast cell-associated chemotactic factors [such as LTB4, PAF, the eosinophil chemotactic factor of anaphylaxis (ECF-A) or high molecular weight neutrophil chemotactic activity (NCA (HMW]] and/or "lymphokines" derived from T helper cells (TH) which have been stimulated by antigen processed by the antigen processing cells (APC). These mononuclear cell interactions are under the control of regulatory T cells [T suppressor (TS) cells] and it is speculated that the availability of these subsets may determine the magnitude of the late-phase response. Lymphokines and monokines which selectively activate neutrophils, eosinophils and monocytes include LIF, EAF and INF-gamma respectively. Macrophage-derived tumour necrosis factor (TNF) also amplifies the inflammatory response by its capacity to enhance eosinophil cytotoxicity. Eosinophil-derived agents such as PAF, LTC4, MBP and ECP might be responsible for submucosal oedema and non-specific bronchial hyperreactivity which are characteristic features of late-phase reactions. T cell-derived lymphokines such as EDF (IL-5), together with GM-CSF, might lead to eosinophilopoiesis and account for the prolonged eosinophilia of ongoing chronic asthma. The T cell is prominent in the pathology of chronic asthma and is possibly "chronically activated". Thus lymphocytes, driven by as yet undetermined "antigens" (possibly viral), may perpetuate the inflammatory response in and around the bronchi. IL-5-like products from these putative activated lymphocytes might perpetuate (a) eosinophil production by the bone marrow, (b) its release into the circulation, (c) its migration into bronchial tissue and (d) activation to release PAF, LTC4, MBP, etc.(ABSTRACT TRUNCATED AT 400 WORDS)

PMID: 3068126 [PubMed - indexed for MEDLINE]

2287. Clin Allergy. 1988 Sep;18(5):469-79.

Clinical manifestations of cows' milk allergy in childhood. I. Associations with in-vitro cellular immune responses.

Hill DJ, Ball G, Hosking CS.

Royal Children's Hospital, Melbourne, Australia.

In this study 51 children who presented with symptoms of cows' milk allergy (CMA) were categorized clinically by their response to cows' milk challenge. Forty-two patients had unequivocal evidence of CMA and nine were milk tolerant. Of the patients with CMA two groups were identified. The first, made up of 32 patients, had immediate-type hypersensitivity reactions to milk associated with both positive skin-prick test (SPT) and RAST. The second group of 10 late reacting patients developed symptoms of CMA over several hours or days; significant increases in irritability, frequency of bowel actions, and rhinitis following milk ingestion were noted in this group. Leucocyte inhibition factor (LIF) produced in response to alpha-lactalbumin, beta-lactoglobulin and alpha-casein was assessed in the immediate and late reacting CMA patients as well as in the

milk-tolerant group. There was no difference in LIF production between the milk-tolerant group and those with immediate reactions. However, these two groups produced less LIF than the late reacting patients for alpha-lactalbumin (P = 0.02), alpha-casein (P = 0.03) and beta-lactoglobulin (P = 0.05). A clinical dairy score card was found to be a useful instrument to assess the response of non-immediate reactions to milk ingestion.

PMID: 3069237 [PubMed - indexed for MEDLINE]

2288. Chem Res Toxicol. 1988 Sep-Oct;1(5):281-7.

Intra- and intermolecular reactions of 4,4'-diisocyanatodiphenylmethane with human serum albumin.

Jin RZ, Karol MH.

Jilin Provincial Institute of Industrial Health and Occupational Disease, Changchun, Jilin Province, People's Republic of China.

Diisocyanates are highly reactive industrial chemicals that have been shown to possess toxic activity, including potential for allergic sensitization. To assist in diagnosis of sensitization, immunoassays for diisocyanate-specific antibodies are performed; such assays require preparation of diisocyanate-containing hapten-protein conjugates. Conditions were investigated for formation of conjugates yielding varying degrees of hapten binding. Relative concentrations of haptens and proteins were varied as were pH, temperature, and time of reaction. Quantitation of 4,4'-diisocyanatodiphenylmethane (MDI) binding with human serum albumin (HSA) was assessed by absorbance of the isolated conjugates at 250 nm after determination of the molar extinction coefficient for MDI. At pH 7.4 and 37 degrees C, the binding reaction was found to be biphasic with binding of 5-6 mol of MDI groups/mol of HSA within the first minute, followed by incorporation at a rate of 0.16 mol/min during the next 2 h. Evaluation of reaction products using SDS-PAGE revealed extensive inter- and intramolecular cross-linking of HSA by MDI. Intramolecular cross-linking was accompanied by an increased migration of conjugates from an initial molecular mass of 66 kDa, typical of HSA, to a molecular mass of 44 kDa. The change in migration was also produced by using disuccinimidyl tartarate (DST) as hapten and was eliminated when DST was cleaved with sodium periodate. It was attributed to altered protein shape. Conditions that favored binding of MDI with HSA were a high relative concentration of MDI:HSA, a pH of 9.4, and a temperature of 37 degrees C. Under such conditions it was calculated that 53 mol of MDI were bound per mole of HSA after 24 h.(ABSTRACT TRUNCATED AT 250 WORDS)

PMID: 2979744 [PubMed - indexed for MEDLINE]

2289. Ann Allergy. 1988 Jun;60(6):508-12.

Migration of neutrophils in experimental asthma.

Taki F, Iwata M, Sugiyama S, Takagi K, Satake T, Ozawa T.

Department of Internal Medicine and Biomedical Chemistry, Faculty of Medicine, University of Nagoya, Japan.

Ovalbumin (OA) aerosol exposure caused an increase in phospholipase (PLase) activity in guinea pig lung membranes and in leukotriene B4 (LTB4) in lung lavages with subsequent neutrophil influx into lung lavages. These results suggest that activation of PLase after exposure to OA aerosol triggers an

increase in LTB4 synthesis resulting in neutrophil influx into the airways.

PMID: 2837926 [PubMed - indexed for MEDLINE]

2290. Allergol Immunopathol (Madr). 1988 May-Jun;16(3):167-70.

In vitro granulocyte migration from bone marrow, tissues and blood vessels in atopic patients.

Matusiewicz R, Rusiecka-Matusiewicz K.

First Department of Internal Medicine, Grochow Hospital, Warsaw, Poland.

An examination of the migration ability of pool tissue and vascular granulocytes was performed in 60 patients and in 9 patients, the migration ability of bone marrow granulocytes. The test for granulocyte migration by Clausen, Bendixen and Søborg was performed with each granulocyte pool separately. It was proved that in patients with atopic asthma there is a significant statistical reduction in bone marrow and peripheral blood granulocyte migration during the asymptomatic period. During dyspnea, a decrease in migration of granulocytes from tissue and peripheral blood is seen.

PMID: 3177153 [PubMed - indexed for MEDLINE]

2291. Biochim Biophys Acta. 1988 Apr 15;959(3):332-42.

Characterization of CGS 8515 as a selective 5-lipoxygenase inhibitor using in vitro and in vivo models.

Ku EC, Raychaudhuri A, Ghai G, Kimble EF, Lee WH, Colombo C, Dotson R, Oglesby TD, Wasley JW.

Research Department, Ciba-Geigy Corporation Summit, NJ 07901.

CGS 8515 inhibited 5-hydroxyeicosatetraenoic acid (5-HETE) and leukotriene B4 synthesis in guinea pig leukocytes (IC50 = 0.1 microM). The compound did not appreciably affect cyclooxygenase (sheep seminal vesicles), 12-lipoxygenase (human platelets), 15-lipoxygenase (human leukocytes) and thromboxane synthetase (human platelets) at concentrations up to 100 microM. CGS 8515 inhibited A23187-induced formation of leukotriene products in whole blood (IC50 values of 0.8 and 4 microM, respectively, for human and rat) and in isolated rat lung (IC50 less than 1 microM) in vitro. The selectivity of the compound as a 5-lipoxygenase inhibitor was confirmed in rat whole blood by the 20-70-fold separation of inhibitory effects on the formation of leukotriene from prostaglandin products. Ex vivo and in vivo studies with rats showed that CGS 8515, at an oral dose of 2-50 mg/kg, significantly inhibited A23187-induced production of leukotrienes in whole blood and in the lung. The effect persisted for at least 6 h in the ex vivo whole blood model. CGS 8515, at oral doses as low as 5 mg/kg, significantly suppressed exudate volume and leukocyte migration in the carrageenan-induced pleurisy and sponge models in the rat. Inhibitory effects of the compound on inflammatory responses and leukotriene production in leukocytes and target organs are important parameters suggestive of its therapeutic potential in asthma, psoriasis and inflammatory conditions.

PMID: 2833314 [PubMed - indexed for MEDLINE]

2292. Clin Exp Immunol. 1988 Apr;72(1):136-40.

Effect of cyclosporin A on rat mucosal mast cells and the associated protease RMCPII.

Cummins AG, Munro GH, Ferguson A.

Gastro-Intestinal Unit, University of Edinburgh, Scotland, UK.

The effects of Cyclosporin A (CyA) on rat mucosal mast cells (MMC) have been investigated by cell counts in the jejunal mucosa and assays of the MMC-specific granule protease RMCPII in tissues and serum. CyA was administered by subcutaneous injection; for the majority of experiments the rats received 50 mg/kg daily for 3 days as a loading dose, then 50 mg/kg on alternate days. Treatment with this drug has two actions on MMC, a gradual reduction in the number of MMC and in the tissue content of RMCPII in the jejunum; and a rapid fall in the serum concentration of RMCPII, detectable 3 h after i.v. administration of CyA, 50 mg/kg. These phenomena were demonstrated in normal rats and in animals with an expanded jejunal MMC population due to graft vs host reaction or recent helminth infection. The functional relevance of the MMC depletion was demonstrated in immune rats given CyA for 3 days prior to induction of systemic anaphylaxis; intestinal permeability to i.v. Evan's blue was significantly reduced by CyA treatment. We suggest that CyA depletes intestinal MMC by suppression of T-cell-mediated regulatory stimuli to proliferation of mast cell precursors and/or their migration. The effects of the drug on serum RMCPII, evident before there were changes in the number of intestinal MMC, indicate that it also suppresses the secretion of granule mediators by MMC, probably indirectly via effects on mucosal T cells.

PMCID: PMC1541508
PMID: 3165062 [PubMed - indexed for MEDLINE]

2293. Lancet. 1988 Mar 26;1(8587):692-5.

The expanded spectrum of toxocaral disease.

Taylor MR, Keane CT, O'Connor P, Mulvihill E, Holland C.

National Children's Hospital, Dublin.

Among 137 members of 30 families, 6% (and 8% of those aged under 15 years) were seropositive for toxocara antibodies. In these seropositive subjects and in 84 patients known to have raised toxocara titres the commonest clinical features were abdominal pain, hepatomegaly, anorexia, nausea, vomiting, lethargy, sleep and behaviour disturbances, pneumonia, cough, wheeze, pharyngitis, cervical adenitis, headache, limb pains, and fever. 61% of patients with raised toxocara titres had recurrent abdominal pain. Eosinophilia was in many cases associated with a raised toxocara titre, but 27% of patients with high titres had normal eosinophil counts. Toxocariasis is common, especially in children, and is associated with clinical features that are generally regarded as non-specific but together form a recognisable symptom complex. Toxocariasis should be considered in the differential diagnosis of such symptoms and especially in recurrent abdominal pain, which might otherwise be labelled as idiopathic. The absence of eosinophilia does not exclude toxocariasis.

PMID: 2895221 [PubMed - indexed for MEDLINE]

2294. Auris Nasus Larynx. 1988;15(1):33-42.

Transepithelial migration of eosinophils in experimental nasal allergy in guinea pigs.

Samejima Y, Masuyama K, Ishikawa T.

Department of Otolaryngology, Kumamoto University, School of Medicine, Japan.

We examined eosinophil-migration through the nasal epithelium into nasal cavity in a guinea pig model exhibiting 8-day passive cutaneous anaphylaxis antibody-dependent nasal allergy. Transmission electron microscopic observation of this process revealed that eosinophils traversed the epithelium through the intercellular space and split the tight-junctions of epithelial lining cells. Freeze-fracture studies of this process showed that the morphology of tight-junction was not changed after eosinophil-migration. These observations may indicate that tight-junctions close after eosinophil-migration.

PMID: 3421864 [PubMed - indexed for MEDLINE]

2295. Hautarzt. 1988 Jan;39(1):34-7.

[Prurigo and further diagnostically significant skin symptoms in strongyloidosis].

[Article in German]

Böckers M, Bork K.

Hautklinik der Johannes Gutenberg-Universität Mainz.

An increasing incidence of strongyloidosis must be expected in European countries as a result of the increasing numbers of immigrants, as well as holiday-makers returning from tropical regions. In addition to gastrointestinal symptoms, dermatological complaints are predominant. Only rarely are cutaneous symptoms the only clinical manifestation of disease. The penetration of filariform larvae may cause "ground itch." In cases of chronic disease, larva currens is the most obvious sign and consists of linear urticarial wheals evoked by larva migration. The most common non-specific symptoms are rashes, pruritus and urticaria. A further symptom of strongyloidosis, intensely itching prurigo, is described in a 20-year-old female Thai. Remission was achieved following tiabendazole therapy.

PMID: 3356553 [PubMed - indexed for MEDLINE]

2296. Clin Exp Immunol. 1988 Jan;71(1):164-70.

Immunohistochemical demonstration of migration inhibitory factor (MIF) in experimental allergic contact dermatitis.

Malorny U, Knop J, Burmeister G, Sorg C.

Department of Experimental Dermatology, University of Münster, FRG.

The kinetics of appearance of MIF+ cells was investigated in experimental contact dermatitis using a monoclonal antibody (7D10) against murine MIF which was reacted with cryostat sections of tissues and detected by the indirect immunoperoxidase test. Four groups of BALB/c mice were investigated: (1) sensitized with 2,4-dinitrofluorobenzene (DNFB); (2) unsensitized controls; (3) tolerized; (4) unsensitized. A challenge dose of DNFB was applied to the ear of animals of groups 1-3 and of croton oil to those of group 4. Three phases could

be distinguished in group 1: (a) an initial vascular and exudative reaction; (b) an early cellular phase; and (c) a late cellular phase. At zero time rarely any T lymphocytes (Lyt 1+; Lyt 2+) were seen in all four groups. Within less than 30 min venous endothelial cells became strongly MIF+. This was followed by an influx of monocytes/macrophages reaching a maximum of 72 h in group 1 and a slight peak at 12 h in groups 2 and 3. At 16-24 h in all groups the endothelial reaction weakened while many 7D10+ macrophages appeared in group 1. By double-labelling it was shown that lymphocytes were 7D10-. The influx of lymphocytes, part of which carried the T cell receptor, began at 12 h, reaching a maximum at 72 h in group 1. In groups 2 and 3 only a weak lymphocytic infiltrate developed which declined at 24 h. Group 4 developed an inflammatory reaction after the initial phase with similar kinetics as in group 1. The data suggest that an immune inflammatory reaction is preceded by a nonspecific reaction of the vascular endothelium and the mononuclear phagocytic system and that MIF is playing a central role in these events.

PMCID: PMC1541650
PMID: 3280179 [PubMed - indexed for MEDLINE]

2297. Pol Arch Weter. 1988;28(3-4):5-16.

Early immunization of calves with an inactivated vaccine against trichophytosis.

Wawrzkiewicz K, Wawrzkiewicz J.

Department of Microbiology, Institute of Infectious and Invasive Diseases, Agricultural Academy, Lublin.

The investigations demonstrated that the inactivated vaccine against trichophytosis, elaborated by the authors, induced immune response in calves, aged 5-8 days. The state of immunity was assessed in vitro by the leukocyte migration inhibition test, and in vivo by the allergic test and the challenge test. In all vaccinated calves (10 animals) there occurred delayed hypersensitivity and in six cases also a significant leukocyte migration inhibition. Vaccinated animals exposed to challenge were to a large extent resistant to experimental infection with the virulent strain of T. verrucosum.

PMID: 3272000 [PubMed - indexed for MEDLINE]

2298. Acta Otolaryngol Suppl. 1988;458:181-9.

Eosinophils in nasal secretion.

Masuyama K, Samejima Y, Ishikawa T.

Department of Otorhinolaryngology, Kumamoto University, School of Medicine, Japan.

In order to understand the migration mechanism and character of eosinophils in the nose, the eosinophils in nasal mucosa and secretion were observed morphologically and physicochemically. In our electron microscopic observation of animal and human nasal mucosa, eosinophils migrated through the intercellular space, projecting pseudopods into the apical region of the space and splitting the junctions between epithelial cells. However, a freeze-fracture study in the experimental animals showed that the morphology of tight junction was not significantly changed except for slight decrease in the number of strands in antigen challenged animals. Eosinophilotactic activity was clearly demonstrated in nasal secretion of patients with allergic rhinitis. The factor in nasal

secretion was a molecule smaller than 10,000 and sensitive to heating in higher degree. Additionally, the eosinophils in nasal secretion were hypodense, implying that they may be in activated state.

PMID: 3245428 [PubMed - indexed for MEDLINE]

2299. Ter Arkh. 1988;60(10):82-7.

[Clinico-immunologic characteristics of acute toxic-allergic reactions to drugs and current methods for their treatment].

[Article in Russian]

Latysheva TV, Gushchin IS, Poroshina IuA, Leskov VP, Prozorovskiĭ NS.

The paper is concerned with clinicoimmunological and allergological characterization of 29 cases of acute toxic allergic reactions (ATAR) to drugs. Four degrees of severity of disease were identified; the most severe form was toxic epidermal necrolysis caused by the use of sulfanilamides and pyrazolones in patients with acute respiratory virus and bacterial infections. Blood analysis in patients with III-IV degree of severity showed a sharp decrease in Ctot and C3 up to 0, an increase in the level of circulating immunocomplexes and "average molecules". Cellular immunity in III degree of severity was decreased. The main principles of therapy using extracorporeal immunocorrective methods were worked out. A leukocyte natural migration inhibition test was used for specific diagnosis of intolerance to medication.

PMID: 3222755 [PubMed - indexed for MEDLINE]

2300. Drugs. 1988;36 Suppl 5:43-8, discussion 49-50.

The present status of anti-inflammatory agents in dermatology.

Stüttgen G.

Hautklinik Freie Universität Berlin, Rudolf-Virchow-Krankenhaus, West Germany.

Many classes of drugs exert anti-inflammatory activity through mechanisms which affect all or part of the inflammatory process. Some of these agents are beneficial in the practice of dermatology, while others, such as penicillamine, mast cell blockers and serotonin antagonists, find little or no application. Corticosteroids, for example, are nonspecific in their anti-inflammatory effects and remain a mainstay of therapy, despite their side effect profile. Other drugs, such as the non-steroidal anti-inflammatory agents or gold, can be used in the treatment of diseases associated with rheumatic or autoimmune states. Moreover, antihistamines play an important role in the control of itching, but are mainly indicated in controlling non-dermatological allergic sequelae. Interestingly, chloroquine and dapsone, which were originally developed for use in malaria prophylaxis and leprosy, respectively, have value in treating a wide range of dermatological conditions via mechanisms which include the inhibition of P-450 isoenzymes. In diseases characterised by disturbed cornification (e.g. psoriasis pustulosa), retinoids are of particular value. These drugs are thought to act by inhibition of collagenases, proteases and granulocyte migration. Undoubtedly, further investigation of drug classes such as oxygen radical controllers and immunomodulators will clarify their mechanisms and establish their therapeutic usefulness among the anti-inflammatory agents now available for dermatological use.

PMID: 3076131 [PubMed - indexed for MEDLINE]

2301. Arch Immunol Ther Exp (Warsz). 1988;36(3):351-60.

The influence of aminophylline on human neutrophils--possible protection of lung from proteolytic injury.

Nowak D, Rozniecki J, Ruta U, Bednarowicz A, Izdebski J.

Clinic of Pneumonology and Allergology, Medical Academy, Lódź.

Protease-antiprotease imbalance in the lung is considered to be a likely pathogenetic mechanism in the development of lung injury--particularly emphysema. Aminophylline is often used in bronchitis, bronchial asthma and emphysema. To assess, whether aminophylline indeed affects this mechanism we evaluated in vitro its influence at therapeutical concentrations (12 and 20 micrograms/ml) on phagocytosis, release of total protein and lysosomal enzymes after phagocytosis, spontaneous migration and chemotaxis of human neutrophils to zymosan-activated serum. There were no significant differences in phagocytosis, release of leukoprotease and acid phosphatase between neutrophils with and without aminophylline at both concentrations. However, the release of total protein was different (p less than 0.02, 12 micrograms/ml) and lower (p less than 0.02, 20 micrograms/ml) than the control. The mean decrease in protein release was 13.5 +/- 6% of the control and aminophylline inhibited the release of the protein with molecular weight below 35.000 daltons. Significant migration inhibition was found in 22% cases (12 micrograms/ml, n = 9) and in 53% (20 micrograms cm-3, n = 13). Neutrophil chemotaxis was different (p less than 0.02, 12 micrograms/ml) and lower (p less than 0.05, 20 micrograms/ml) than the control. The obtained results suggest that high doses, of aminophylline may diminish inflammatory recruitment of neutrophils--a rich source of elastase to the lung, and thus diminish proteolytic pulmonary injury.

PMID: 3074741 [PubMed - indexed for MEDLINE]

2302. Int Ophthalmol Clin. 1988 Winter;28(4):332-7.

New directions in therapy for ocular allergy.

Wiens JJ, Jackson WB.

Department of Ophthalmology, McGill University, Royal Victoria Hospital, Montreal, Quebec.

PMID: 3053495 [PubMed - indexed for MEDLINE]

2303. Arch Dermatol Res. 1988;280(6):354-7.

Epidermal lymphocyte chemotactic factor specifically attracts OKT4-positive lymphocytes.

Zachariae C, Ternowitz T, Larsen CG, Nielsen V, Thestrup-Pedersen K.

Department of Dermatology, Marselisborg Hospital, Aarhus, Denmark.

Epidermal lymphocyte chemotactic factor (ELCF) from skin overlying a positive tuberculin reaction was compared with the chemoattractants leukotriene B4 (LTB4), N-formyl-methionyl-leukyl-phenylalanine (FMLP), and complement split product C5a

(C5a). The chemotactic assay used is a modified Boyden chamber technique. The lymphocytes were subsets of T lymphocytes from healthy young individuals first separated by flotation of E rosettes on Isopaque Ficoll followed by incubation of T cells with anti-CD4 and anti-CD8 monoclonal antibodies and further separation using fluorescence-activated cell sorting. ELCF specifically attracted OKT4+ lymphocytes, while LTB4, FMLP, and C5a induced significant migration in both OKT4+ and OKT8+ lymphocytes without any clear difference between the various chemoattractants or cell populations. We found no blocking of the chemotactic capacity of ELCF when we added antibodies towards IL-1 alpha and IL-1 beta to the chemotactic assay. Further recombinant IL-1 alpha and Il-1 beta did not induce any chemotactic response. Our observation may be of significance in explaining the predominance of OKT4+ cells in allergic contact dermatitis and certain other skin diseases.

PMID: 2903723 [PubMed - indexed for MEDLINE]

2304. Infect Immun. 1987 Dec;55(12):2922-7.

Allergenic and blastogenic reactivity of three antigens from Mycobacterium tuberculosis in sensitized guinea pigs.

Worsaae A, Ljungqvist L, Hasløv K, Heron I, Bennedsen J.

Mycobacteria Department, Statens Seruminstitut, Copenhagen, Denmark.

Three antigens from a culture filtrate of Mycobacterium tuberculosis H37Rv were purified by affinity chromatography, using monoclonal antibodies. The molecular weights of the purified antigens are 17,000 to 19,000, 32,000 to 33,000, and 39,000, respectively, and by their migration patterns in sodium dodecyl sulfate-polyacrylamide gel electrophoresis under reducing and nonreducing conditions, they all appeared to be single-chain polypeptides. Western blot and enzyme-linked immunosorbent assay analyses indicated that the antigens are non-cross-reactive. All antigens generated an intermediate to strong skin reaction when tested in guinea pigs previously immunized with a live M. bovis BCG vaccine or with an oil emulsion preparation of phenol-or heat-killed M. tuberculosis. Lymphocytes isolated from peripheral blood or lymph nodes of similarly immunized guinea pigs could be stimulated by purified protein derivative and the purified antigens. Qualitative differences in stimulatory capacity between the preparations were demonstrated. The antigens may prove useful in further studies of the immunology and pathogenesis of tuberculosis.

PMCID: PMC260007
PMID: 3119491 [PubMed - indexed for MEDLINE]

2305. Allergy. 1987 Nov;42(8):612-8.

Leukocyte migration inhibition test in children with cow milk allergy.

Vanto T, Smogorzewska EM, Viander M, Kalimo K, Koivikko A.

Department of Paediatrics, University of Turku, Finland.

The usefulness of the leukocyte migration inhibition factor (LIF) test to detect cow milk (CM) hypersensitivity was studied in 40 children with suspected allergy to CM. Hypersensitivity was carefully investigated by oral milk challenges, which gave a final confirmation of cow milk hypersensitivity in 12 subjects, and excluded it in the remaining 28 subjects. Leukocyte migration inhibition was measured using beta-lactoglobulin (BLG), alpha-lactalbumin (ALA), alpha-casein

(ACA) and bovine serum albumin (BSA) as antigens. IgA and IgG antibodies to these antigens were measured by an enzyme-linked immunosorbent assay, and IgE antibodies to these antigens and to CM by radioallergosorbent test (RAST). Skin prick test with CM was performed in 38 subjects, and with BLG, ALA, ACA and BSA in 29 subjects. Leukocyte migration was more often inhibited by cow milk antigens in the CM challenge positive (CM+) subjects than in the challenge negative (CM-) subjects. Of the specific milk antigens, ALA was the most potent inhibitor, and gave a positive LIF test result in all CM+ subjects, and significantly (P less than 0.02) less often (15/24) in CM- subjects. Also in the skin prick test and RAST, ALA gave positive results more often than the other milk antigens. BLG, ACA and BSA had an inhibiting effect on leukocyte migration, but the difference between the CM+ and CM- subjects was not statistically significant. Two of the 12 CM+ subjects had no demonstrable IgE antibodies to CM proteins; both of them, however, had a positive LIF test with at least one of the CM antigens.(ABSTRACT TRUNCATED AT 250 WORDS)

PMID: 3425860 [PubMed - indexed for MEDLINE]

2306. Eur J Pharmacol. 1987 Oct 13;142(2):245-51.

Effects of proglumetacin maleate and its major metabolites on allergic air pouch inflammation in rats.

Ono N, Sunami A, Yamasaki Y, Yamamoto N, Miyake H.

Tokushima Research Institute, Taiho Pharmaceutical Co., Ltd., Japan.

The mechanism of the anti-inflammatory effect of proglumetacin maleate, a novel indomethacin derivative, was examined in vivo in an allergic air pouch inflammation model in rats. Proglumetacin maleate did not affect the volume of pouch exudate 6 h after immunological challenge, irrespective of whether it was administered orally or locally, but it caused dose-dependent inhibition 24 h after challenge. It also caused dose-dependent reduction of leukocyte migration into the pouch exudate both 6 and 24 h after challenge. It markedly decreased the prostaglandin E2 content of the pouch exudate, but tended to increase the leukotriene B4 content. The main metabolites of proglumetacin maleate, desproglumideproglumetacin maleate and indomethacin, had effects similar to those of proglumetacin maleate on these four parameters on an equimolar dose basis. Unlike these three drugs, dexamethasone decreased the leukotriene B4 content of the pouch exudate. These results suggest that the action of proglumetacin maleate is qualitatively the same as that of indomethacin in vivo; that is, it inhibits cyclo-oxygenase in inflammatory sites.

PMID: 2826180 [PubMed - indexed for MEDLINE]

2307. Age Ageing. 1987 Jul;16(4):249-55.

Elderly immigrants--a disadvantaged group?

Ebrahim S, Smith C, Giggs J.

Asian, Caribbean and British born Nottingham hospital discharge rates of people of pensionable age for a variety of diagnoses and elective surgery were compared. No evidence of under-utilization of services by immigrants was found, but marked excess discharges were observed for tuberculosis, diabetes, asthma, gastrointestinal bleeding and cataract surgery. Increased hospital use for most diagnoses probably reflects true differences in disease risk between immigrants and the indigenous population.

PMID: 3630849 [PubMed - indexed for MEDLINE]

2308. Rev Alerg Mex. 1987 Jul-Sep;34(3):55-62.

[Evaluation of the immunologic system in patients with allergic bronchial asthma].

[Article in Spanish]

Aguilar Angeles D, Serrano E, Tenorio S, Estrada Parra S.

PMID: 3318537 [PubMed - indexed for MEDLINE]

2309. Clin Allergy. 1987 Jul;17(4):373-9.

Inhibitory effect of cetirizine 2HCl on eosinophil migration in vivo.

Fadel R, Herpin-Richard N, Rihoux JP, Henocq E.

The effect of a potent antihistamine, cetirizine, was studied on allergic patients and normal subjects by means of an in-vivo 'skin window' technique. All subjects showed significant inhibition of skin-test responses to grass pollen, compound 48/80, histamine and methacholine, after administration of a single dose (10 mg) of cetirizine. Compared to placebo, cetirizine significantly decreased the eosinophils attraction at skin sites challenged with grass pollen and compound 48/80. In allergic patients no change in eosinophil migration pattern was noted with histamine and methacholine skin-tested sites. In normal subjects, compound 48/80 and histamine did not induce eosinophil accumulation and cetirizine did not modify cellular patterns as compared to placebo. These results suggest that cetirizine acts on eosinophil migration by inhibiting the release of mast cell mediators or inhibiting the eosinophilotactic mediators themselves.

PMID: 2887304 [PubMed - indexed for MEDLINE]

2310. J Immunol. 1987 Jun 15;138(12):4169-74.

Identification of a shrimp-derived allergen as tRNA.

Nagpal S, Metcalfe DD, Rao PV.

During an attempt to isolate shrimp allergens, evidence was obtained that shrimp ribonucleic acid was capable of eliciting a specific IgE response in man and an experimental animal model system. The shrimp ribonucleic acid was extracted from boiled whole shrimp (Peneaus indicus), and was isolated by salt precipitation and sequential chromatography over DEAE-Sephacel and BioGel P-100. The allergenic material was identified as a ribonucleic acid based on the following criteria: a maximal absorption at 258 nm, failure to stain positively with Coomassie Brilliant Blue on slab gel electrophoresis, positive staining with ethidium bromide, co-migration with yeast tRNA on submerged gel electrophoresis in 1.5% Agarose M, and sensitivity to ribonuclease T2 and 0.3 M NaOH. Treatment with protease did not alter its allergenic activity. The RNA was capable of binding allergen-specific IgE in sera from two shrimp-sensitive patients, as demonstrated by microELISA and solid-phase radioimmunoassay (SPRIA) using antigen-coated nitrocellulose filter paper discs and purified 125I-labeled goat anti-human IgE. RNA isolated from shrimp by a conventional tRNA isolation procedure also had the ability to specifically bind IgE in the sera of shrimp-sensitive patients. IgE

antibodies to shrimp RNA did not recognize yeast tRNA or salmon testes DNA, and were not detected in sera of other subjects. The shrimp-derived RNA was further able to induce a reaginic response in mice. A combination of in vitro aminoacylation of shrimp tRNA and SPRIA resulted in the identification of the allergenic tRNA as tRNA(Tyr) and tRNA(Arg). Thus, shrimp tRNA is capable of inducing a specific IgE response in man.

PMID: 3584974 [PubMed - indexed for MEDLINE]

2311. Am J Epidemiol. 1987 Jun;125(6):1019-34.

Visceral larva migrans in French adults: a new disease syndrome?

Glickman LT, Magnaval JF, Domanski LM, Shofer FS, Lauria SS, Gottstein B, Brochier B.

Visceral larva migrans is apparently an endemic disease among adults in southwest France. Thirty-seven adults living in the Midi-Pyrenees region of France were confirmed as having visceral larva migrans based on an increased specific antibody titer to Toxocara canis as detected by enzyme-linked immunosorbent assay (ELISA) and by the Western blot method. The disease was characterized clinically by weakness, pruritus, rash, difficulty breathing, abdominal pain, and pathologically by allergic manifestations including eosinophilia and increased serum immunoglobulin (Ig) E levels. Conditional logistic regression analysis using a control group of 37 hospital patients with other conditions who were individually matched to patients with visceral larva migrans by age and sex revealed an increased risk for visceral larva migrans associated with hunting or living in a household with a hunter (odds ratio (OR) = 9.0, p = 0.02) and with living in a village of less than 500 persons (OR = 5.7, p = 0.04). Owning two or more dogs compared with owning one or no dogs increased the risk of visceral larva migrans for hunting or living in a household with a hunter (OR = 9.6 vs. OR = 4.5) and for persons living in nonhunting households (OR = 2.1 vs. OR = 1.0). These findings, however, could not be duplicated when 60 age- and sex-matched neighbors were used as a second control group.

PMID: 3578244 [PubMed - indexed for MEDLINE]

2312. Ann Allergy. 1987 Jun;58(6):425-8.

In vivo and in vitro granulocyte migration in patients with extrinsic and intrinsic bronchial asthma.

Matusiewicz R, Rusiecka-Matusiewicz K.

Sixty-five patients with atopic asthma, 54 with infectious asthma, and 30 healthy controls were evaluated by in vitro granulocyte migration and by the in vivo skin window method of Southam. Quantitative IgM, IgG, IgA, IgE serum concentrations were measured. In vivo migration of granulocytes was decreased in patients with atopic asthma and elevated levels of IgE as well as in patients with infectious asthma and elevated levels of IgG. In vivo migration of granulocytes showed significant decreases in both asthmatic groups only during asthmatic attacks.

PMID: 3296868 [PubMed - indexed for MEDLINE]

2313. Pneumonol Pol. 1987 May;55(5):206-10.

[The leukocyte migration inhibition test and skin tests using bacterial

suspensions in patients with infectious bronchial asthma].

[Article in Polish]

Kraus-Filarska M, Małolepszy J, Stankiewicz M.

PMID: 3658795 [PubMed - indexed for MEDLINE]

2314. Arzneimittelforschung. 1987 May;37(5A):606-13.

[The effect of benzydamine on various functions of human granulocytes and their interaction with endothelial cells].

[Article in German]

Damerau B, Otte G, Haller Y, Löffler BM.

The influence of the non-steroidal antiinflammatory drug benzydamine (Tantum) was studied on several functions of human polymorphonuclear leukocytes, namely their adhesion to endothelial cells, the leukocyte auto-aggregation and their locomotion into cellulose nitrate filters or on glass surfaces. The granulocytes were stimulated either by the synthetic oligopeptide N-formyl-methionyl-leucyl-phenylalanine (FMLP) or the physiologically important complement anaphylatoxins C3a and C5a-desArg. The experiments showed that benzydamine reduces effectively the attachment of granulocytes to endothelium of isolated guinea pig aortic strips (IC50 3-4 X 10(-6) mol/l). This effect seems to be exclusively due to the inhibition of granulocyte adhesiveness and cannot be washed out. Benzydamine also diminishes leukocyte aggregation induced by either the complement peptides C3a, C5a-desArg or FMLP, and in addition causes deaggregation of already formed leukocyte aggregates. However, benzydamine is inhibitory only at 1-3 X 10(-4) mol/l. Likewise, C5a-desArg-induced leukotaxis and phagocyte polarization on glass surfaces as well as spontaneous migration of unstimulated granulocytes in Boyden chambers are decreased only at 10(-4) mol/l. By contrast, benzydamine usually augments chemotaxis in Boyden chambers induced by concentration gradients of the stimuli. This effect might be explained by the prevention of the known auto-oxidative inhibition of phagocytes exerted by benzydamine. Regarding the therapeutic significance, inhibition of the leukocyte-endothelial interaction appears to be of considerable pharmacologic relevance to explain the antiphlogistic properties of benzydamine in vivo.

PMID: 3619981 [PubMed - indexed for MEDLINE]

2315. Zh Mikrobiol Epidemiol Immunobiol. 1987 May;(5):80-3.

[Comparative characteristics of the diagnostic properties of staphylococcal allergens in an experiment].

[Article in Russian]

Spiricheva NKh, Beklemishev ND, Ermekova RK, Molotilov BA.

The diagnostic value of five staphylococcal allergens prepared from a single S. aureus strain by different methods and in different institutions has been tested on the experimental models of delayed, immediate and mixed (immediate and delayed) hypersensitivity in guinea pigs. The advantages of the allergens prepared in Kazan (USSR) for the detection of delayed hypersensitivity and the ultrasonicated allergen, as well as the allergen made in Czechoslovakia, for the detection of immediate hypersensitivity have been noted.

PMID: 3618026 [PubMed - indexed for MEDLINE]

2316. N Engl Reg Allergy Proc. 1987 May-Jun;8(3):173-7.

Allergy and pulmonary impairment in Iowa veterinarians.

Will LA, Nassif EG, Engen RL, Patterson RA, Zimmerman D.

Department of Veterinary Microbiology and Preventive Medicine, College of Veterinary Medicine, Iowa State University, Ames 50011.

If small animal practice exposure, including the laboratory animal situations encountered in academic and other research pursuits, is more detrimental to veterinarians than large animal practice exposure for induction of allergic respiratory disease, then preventive measures such as increased ventilation, use of high efficiency particulate filters, and wearing of masks should be encouraged to reduce allergen exposures. Migration from large animal practice, likewise, should be discouraged. Failure to migrate to low occupational allergy risk situations early enough in a veterinary career can have severe and even fatal results. If the observed respiratory disease in veterinarians is in fact due to exposure, then unfortunately, it may in some cases be progressive and not just chronic. Data which could provide criteria for predicting occupational allergy and possible related respiratory disease outcome is scant at this time and career counselling is difficult. If the veterinary occupational animal allergy data should be proven correct such results can be used to help others.

PMID: 3477686 [PubMed - indexed for MEDLINE]

2317. J Neuroimmunol. 1987 May;15(1):85-95.

Lymphocytic activation in peripheral blood and cerebrospinal fluid during the course of chronic relapsing experimental allergic encephalomyelitis.

Suckling AJ, Baron PW, Symons JA, Rumsby MG.

A monoclonal antibody against the human interleukin-2 receptor (anti-Tac) has been found to cross-react with an antigen on the surface of guinea pig leucocytes. Cells marking with anti-Tac and with an anti-pan T cell monoclonal antibody have been quantitated in the peripheral blood and cerebrospinal fluid (CSF) of guinea pigs with chronic relapsing experimental allergic encephalomyelitis (CR-EAE). T cells account for about 90% of peripheral blood leucocytes in all animals whilst in the CSF, T cells are the major contributor only when there is a pleocytosis. The proportion of T cells marking with anti-Tac, a measure of T cell activation, in blood and CSF of control animals is 12%, rising to 23% in blood in the post-acute phase of the disease. However, a fall in the blood Tac/T ratio to 13% occurs during the first 10 days of relapse with a subsequent rise to 30-35%. This change is related to the time after onset of relapse irrespective of the subsequent course of the disease. From first relapse onwards CSF lymphocytes show a greater level of activation than lymphocytes from paired peripheral blood samples but the proportion of Tac+ cells in CSF does not increase with increasing CSF pleocytosis. The data is consistent with migration of activated T cells from blood to CSF at the onset of relapse.

PMID: 3106414 [PubMed - indexed for MEDLINE]

2318. J Invest Dermatol. 1987 Apr;88(4):362-8.

In vitro studies in nickel allergy: diagnostic value of a dual parameter analysis.

von Blomberg-van der Flier M, van der Burg CK, Pos O, van de Plassche-Boers EM, Bruynzeel DP, Garotta G, Scheper RJ.

A comparison was made between the diagnostic value of assaying nickel-induced lymphocyte proliferation (lymphocyte transformation test, LTT) and migration inhibition factor (MIF) production in nickel contact sensitivity. Although lymphocyte proliferation was significantly increased in the group of patients with skin test reactivity to nickel, positive LTT were also frequently found in skin test-negative subjects: in 63% of subjects with and in 30% of subjects without a history of metal allergy. This would limit the value of the LTT as an in vitro correlate of skin test reactivity. However, in certain patients positive lymphocyte transformation may reveal nickel sensitization at a time of undetectable skin reactivity. Data obtained with the macrophage migration inhibition test (MMIT) showed a good correlation with nickel patch test reactions. Accurate determination of MIF became feasible by using cells from the human monocytoid cell line U937 as target cells in a microdroplet agarose assay. Using this MMIT, positive reactions occurred in 13% of the healthy controls and false-negative reactions were found in 26% of patients with positive skin test reactivity to nickel. As LTT and MMIT data appeared to be only weakly correlated in the individuals tested, a dual parameter analysis was performed. An excellent correlation [p = 1.8 (10(-8]] was found between skin test and in vitro reactivity for individuals with matching in vitro results (60% of all individuals tested). In those individuals with discordant in vitro data, skin testing will remain indispensable for diagnosing nickel allergy.

PMID: 3549912 [PubMed - indexed for MEDLINE]

2319. Mater Med Pol. 1987 Apr-Jun;19(2):133-6.

Atopic asthma in children. Possible role of delayed hypersensitivity.

Wartenberg J, Lewandowska J, Marcinkowska W.

PMID: 3448419 [PubMed - indexed for MEDLINE]

2320. Allergy. 1987 Apr;42(3):173-6.

Granulocyte migration in vivo and in vitro in healthy children of parents with atopic asthma.

Matusiewicz R, Rusiecka-Matusiewicz K.

Healthy children of atopically asthmatic parents received in vivo and in vitro granulocyte migration tests. Eleven children whose parents were both asthmatic showed statistically significant inhibition of granulocyte migration compared with controls.

PMID: 3296842 [PubMed - indexed for MEDLINE]

2321. J Invest Dermatol. 1987 Mar;88(3 Suppl):18s-23s.

Psoriasis and leukocyte chemotaxis.

Tagami H, Iwatsuki K, Takematsu H.

Transepidermal migration of leukocytes, with resultant formation of microscopic or macroscopic sterile subcorneal pustules is a phenomenon characteristically noted in psoriasis and related sterile pustular dermatoses. It is natural to assume the presence of potent neutrophil chemotactic substances in the subcorneal portion of the lesional epidermis, because this location is the target of the in vivo leukocyte chemotaxis. In fact, crude psoriasis scale extracts show remarkably high neutrophil chemotactic and activating properties as compared with those of other non-psoriatic inflammatory dermatoses. We isolated a psoriatic leukotactic factor (PLF) having a molecular mass of around 12 kD, distinct from those common to other inflammatory changes involving the skin or those released by bacteria. Further analysis of PLF identified C5 cleavage fragments, together with other chemotactic peptides, such as those derived from monocytes. Likewise, potent low-molecular-mass chemotactic factors, including cell membrane lipid derived chemotactic factor, e.g. leukotriene B4, are also increased in psoriatic lesions, as in other nonpsoriatic inflammatory dermatoses. However, their activity to stimulate the generation of oxygen radicals in neutrophils was found to be much weaker than that of PLF. The peripheral blood leukocytes from active psoriatic patients show enhanced function in chemotaxis, phagocytosis, active oxygen production, and enzyme release; patients' sera contain substances such as anaphylatoxins that activate leukocyte function. Further research is required for clearer understanding of the series of events resulting in the leukocyte chemotaxis, as well as for the elucidation of the background immunoregulatory mechanisms.

PMID: 3493302 [PubMed - indexed for MEDLINE]

2322. Br Med J (Clin Res Ed). 1987 Feb 14;294(6569):423-4.

Immunisation of children by a nurse without a doctor present.

Jefferson N, Sleight G, Macfarlane A.

Over 16 months 148 children were referred by health visitors and general practitioners to a specially trained nurse for failing to complete courses of immunisation. A further 91 children of travellers' families were identified as needing immunisation. The nurse carried out 810 immunisations on 237 of these children in their homes without a doctor being present. There were only two refusals, and one child suffered a mild anaphylactic shock. The cost per immunisation, in nurse's salary and travel expenses, was pounds 8. This is an effective and fairly inexpensive way of achieving uptake of immunisation in such groups of children, and there seems no reason why trained nurses should not give immunisations either in a child health clinic or at home, without a doctor present.

PMCID: PMC1245421
PMID: 3101907 [PubMed - indexed for MEDLINE]

2323. Presse Med. 1987 Feb 7;16(4):151-4.

[New elements in the symptomatology of visceral larva migrans].

[Article in French]

Magnaval JF.

A retrospective study of 92 cases of visceral larva migrans (VLM) seen in

Toulouse demonstrated the high prevalence of the disease in the Midi-Pyrénées region (southern France). The patient population was homogeneous, of middle age and predominantly female. The principal symptoms were chronic asthenia of one year duration on average, associated with cutaneous signs of allergy (pruritus, rashes) and with pain in the right hypochondrium. High total IgE level with presence of anti-VLM IgE was more frequent than eosinophilia, which was moderate. In one-third of the cases VLM also seemed to be responsible for an increase in serum gamma GT. VLM is a disease that is often unrecognized. The classical descriptions apply, in reality, to its caricatural forms which are exceptional.

PMID: 2950444 [PubMed - indexed for MEDLINE]

2324. Int Arch Allergy Appl Immunol. 1987;84(2):123-8.

Nasal mucosal mast cells and histamine in hay fever. Effect of topical glucocorticoid treatment.

Pipkorn U, Enerbäck L.

Department of Pathology, Sahlgrenska Hospital, Gothenburg University, Göteborg, Sweden.

Symptomatic seasonal allergic rhinitis has previously been found to be associated with a redistribution of mast cells from the subepithelial stroma to the epithelial lining and the surface of the nasal mucosa. The present study was designed in order to elucidate the interaction between topical glucocorticosteroids, effective in the treatment of allergic rhinitis, and the migration of mast cells described earlier. Six patients treated prophylactically in the nose with budesonide were studied. Imprints and biopsies from the nasal mucosa were taken 2-3 weeks before and 2-3 weeks into the birch pollen season. The biopsies were used for light microscopy and tissue histamine determination. The morphologic studies showed, also in the actively treated patients, an increased number of metachromatically stained cells on the nasal mucosal surface of the same order of magnitude as previously reported for untreated patients. We did, however, find a decrease in the histamine content of the nasal mucosa, which was not associated with a decrease in the number of mast cells. Together with similar previous findings in the unstimulated allergic nasal mucosa these results suggest that glucocorticosteroids induce a decrease in the mast cell histamine pool, possibly due to an inhibition of the intracellular synthesis of histamine. This effect might contribute to the clinically beneficial effect of topical glucocorticosteroids in the treatment of hay fever.

PMID: 3653999 [PubMed - indexed for MEDLINE]

2325. J Allergy Clin Immunol. 1987 Jan;79(1):16-24.

Longitudinal changes in allergen skin test reactivity in a community population sample.

Barbee RA, Kaltenborn W, Lebowitz MD, Burrows B.

A cohort of 1333 subjects, aged 3 years and older, was followed for a mean of 8.1 years to assess changes in allergen skin test reactivity. The overall prevalence of reactivity to the five antigen mixtures was 39.1% during the initial survey and 50.7% after the follow-up period. The greatest increase in prevalence occurred among children and teenagers (22.2% and 19.5%) with only minimal increases after the age of 65 years (6.0%). No difference in prevalence between male and female subjects was apparent, either initially or at the end of the

follow-up period. In-migration to the Tucson area was a major factor in determining changes in reaction prevalence. Among subjects more than 35 years of age, recent in-migrants accounted for most of the increased prevalence. Comparisons of atopy among consistent smoking groups confirmed the previous observation that smokers are less atopic than either nonsmokers or exsmokers, probably because of a self-selection process. In contrast, exsmokers were generally the most atopic, both initially and at the end of the longitudinal observation period. The high overall prevalence of allergen reactivity in this population is believed to be due in large measure to high year-round concentrations of multiple aeroallergens in the Tucson environment.

PMID: 3492524 [PubMed - indexed for MEDLINE]

2326. Arch Immunol Ther Exp (Warsz). 1987;35(3):303-6.

The in vitro migration of peripheral leukocytes of atopic asthmatic patients in relation to the level of serum immunoglobulin E (IgE).

Matusiewicz R, Stempniak M.

1st Department of Internal Diseases Grochów Hospital, Warsaw.

In 50 patients with atopic asthma aged 18-32 (average 25) the level of serum IgE was measured radioimmunologically and the peripheral leukocyte migration in agar plates was determined. The control group consisted of 20 healthy people aged 20-31 (average 26). An increased level of IgE was found in 39 atopic patients. Statistically significant inhibition of peripheral leukocyte migration was noted. In each patient the degree of inhibition of migration was reciprocal to the concentration of serum IgE.

PMID: 3446086 [PubMed - indexed for MEDLINE]

2327. Int J Immunopharmacol. 1987;9(2):243-53.

Inhibition of delayed hypersensitivity reactions by a new agent, cis-1-methyl-4-isohexylcyclohexane carboxylic acid (IG-10)--II. The mechanism regarding the action on lymphokines.

Nakatomi I, Nakamura K, Furukawa K, Koda A.

A newly synthesized anti-allergic agent, cis-1-methyl-4-isohexylcyclohexane carboxylic acid (IG-10), has the capacity to inhibit the effector phase of delayed hypersensitivity reactions. In the present paper, the effect of IG-10 was studied on the generation of superoxide anion (O2) from macrophages, on macrophage chemotaxis, and on the activity of lymphokines such as skin reactive factor (SRF), macrophage migration inhibitory factor (MIF) and monocyte/macrophage chemotactic factor (MCF) in guinea pigs. Oral administration of IG-10 (50-200 mg/kg) inhibited SRF-induced skin erythema in a dose-dependent manner. In vitro, this agent (10(-7) -10(-5) g/ml) did not inhibit the generation of O2- from macrophages. The agent (10(-7) -10(-6) g/ml) significantly inhibited the activity of MIF and this inhibition was not due to the facilitation of normal migration. For macrophage chemotaxis, IG-10 (10(-8) -10(-6) g/ml) significantly inhibited MCF-induced chemotaxis. The agent also depressed the macrophage chemotaxis induced by N-formyl-methionyl-leucyl-phenylalanine but not the chemotaxis induced by E. coli culture filtrate. The inhibitory action of IG-10 on MCF activity was not influenced by antiglucocorticoid agents such as 17 alpha-methyltestosterone and androstenedione which reverse significantly the inhibitory action of glucocorticoids. The inhibitory action of IG-10 was

relatively dependent on exogenous Ca2+ and Mg2+, and was antagonized by dbc-GMP.

PMID: 3034812 [PubMed - indexed for MEDLINE]

2328. Dermatologica. 1987;174(6):285-9.

Quinidine-induced lichenoid and eczematous photodermatitis.

Wolf R, Dorfman B, Krakowski A.

We report 6 cases of photodistributed rashes due to oral administration of quinidine: 4 cases with a lichen-planus-like eruption and 2 with an eczematous dermatitis. The casual relationship between the drug and the eruption was established mainly by means of circumstantial evidence. It was further strengthened by an in vitro challenge test in 4 cases and by a rechallenge in 2 patients. The in vitro challenge test used was the macrophage migration inhibition test, which can be of diagnostic aid whenever a rechallenge of the patient is contraindicated. Quinidine-induced lichenoid eruption in a photodistribution seems to be more common than previously reported. Physicians should be aware of this type of drug eruption.

PMID: 2957249 [PubMed - indexed for MEDLINE]

2329. Int Arch Allergy Appl Immunol. 1987;82(3-4):249-55.

Mucosal mast cells in the rat and in man.

Enerbäck L.

The proteoglycan structure of mucosal mast cells (MMC) of the two species has been analyzed with histochemical in situ techniques. The findings indicate that human MMC, like human mast cells of several other sites, contain a heparin proteoglycan, unlike rat MMC which lack heparin but contain an oversulphated chondroitin sulphate. However, the dye-binding of the human MMC proteoglycan, like that of the rat, is highly susceptible to blocking by formaldehyde. Human MMC also exhibit a lower critical electrolyte concentration (CEC) of dye-binding than mast cells of other connective tissue sites, suggesting a relatively lower charge density and/or molecular weight of the glycosaminoglycan of the MMC. These findings thus suggest that the human MMC like those of the rat have a distinctive proteoglycan structure. Recent findings of another group indicate that the human MMC like those of the rat have also a distinctive proteinase composition. Finally, the mast cell response of the nasal mucosa during birch pollen allergy shows fundamental similarities to the nematode response of the rat intestinal mucosa. During both conditions mast cells are redistributed from the lamina propria into the epithelium, probably as a result of migration of mast cells or mast cell precursors. Taken together, these findings suggest the existence of a distinctive MMC phenotype also in man.

PMID: 2437041 [PubMed - indexed for MEDLINE]

2330. Scand J Work Environ Health. 1986 Dec;12(6):538-44.

Mortality and incidence of cancer among Swedish rubber workers, 1952-1981.

Gustavsson P, Hogstedt C, Holmberg B.

The mortality and incidence of cancer was studied among 8,734 workers from two

Swedish rubber manufacturing companies. Mortality was investigated from 1952 to 1981 and cancer incidence from 1959 to 1980. The expected numbers of deaths were calculated from national statistics. No significant risk excesses were detected when the cohort was analyzed without consideration of employment time or latency period. However, the mortality from coronary heart disease and the incidence of lung cancer were increased when the study period was limited to greater than or equal 40 years since first employment. The standardized mortality ratio for coronary heart disease correlated positively with employment duration. The mortality from asthma, bronchitis, and emphysema was nonsignificantly increased. The incidence of bladder cancer was increased among individuals with heavy and long-term exposure in the weighing and mixing departments. Twenty-five percent of the individuals in the cohort were not Swedish citizens at the time of employment, and an analysis of the mortality and cancer incidence in this group showed a markedly increased lung cancer incidence for certain immigrant groups, probably mainly due to ethnic factors. The results indicate that ethnic factors must be considered in the analysis of occupational groups when a high proportion of the workers are immigrants.

PMID: 3823802 [PubMed - indexed for MEDLINE]

2331. J Chir (Paris). 1986 Dec;123(12):733-8.

[Liver abscess with pleuropulmonary fistula following hepatobiliary ascariasis in a 18-month-old infant. Apropos of a case with recovery for over 10 years].

[Article in French]

Bouasakao N.

This study deals solely with the problem of intra-hepatic collected suppuration due to an ascaris migration through the liver ducts. It is due to the infestation of the intestine germs carried away by the worm but not by the ascaris itself which can die calcified inside the liver without hearing of it, provided no mechanical complication has happened, or if it has returned to the intestine environment. When the illness starts with hepatic colic, a pyrexy with a painful right hypochondria and a painful liver-swelling, if the positive diagnosis, that can have been made from the cutaneous allergy test, the R.A.S.T. [Radioallergosorbent Test]. the sonographic diagnosis, the cholangiography and the C.T. scan, can be proved only by curative aiming surgery, mainly in on-endemic countries. The pre and post-operative anti-helmintics are strongly advised to avoid a reinfestation of the illness.

PMID: 3805184 [PubMed - indexed for MEDLINE]

2332. Zentralbl Bakteriol Mikrobiol Hyg A. 1986 Dec;263(1-2):209-28.

Clinical features of early erythema migrans disease and related disorders.

Weber K, Neubert U.

104 patients with erythema migrans disease (EMD), 7 patients with borrelia lymphocytoma (BL), and 21 patients with acrodermatitis chronica atrophicans (ACA) were prospectively followed for a median of 20, 14, and 12 months, respectively. 99 patients with EMD and 6 with BL were treated with antibiotics early for their illness. 72 patients with EMD had 1 to 10 constitutional symptoms besides the erythema migrans, 32 had erythema migrans alone, and a child with BL had urticaria. Out of 39 patients with EMD, 23 acquired arthralgia, 18 signs and symptoms consistent with neurologic manifestations and 8 with cardiac involvement

before or after therapy. 4 patients with EMD and 1 with BL had up to 10 multiple erythema migrans lesions. 3 patients with EMD experienced a reinfection and 1 with ACA a relapse. Several patients with ACA developed signs and symptoms consistent with neurologic, cardiac and joint involvement, and 2 had a history of EMD. Elevated antibody titers against Borrelia burgdorferi were present in 48% of 69 patients with EMD, 5 with BL and 11 with ACA, and in 93% of another group with EMD, 2 with BL and 10 with ACA, when tested against other borreliae. Increased values of the ESR, IgG, IgA and IgM were found in more patients with ACA than with EMD. The median of IgA and IgM was significantly higher in ACA than in EMD. Borreliae were found in brain and liver of a newborn. Early EMD appears to be quite similar to early Lyme disease.

PMID: 3577481 [PubMed - indexed for MEDLINE]

2333. J Immunol. 1986 Dec 1;137(11):3428-35.

Interaction between myelin basic protein-sensitized T lymphocytes and murine cerebral vascular endothelial cells.

McCarron RM, Spatz M, Kempski O, Hogan RN, Muehl L, McFarlin DE.

Lymph node cells from SJL mice immunized with guinea pig myelin basic protein proliferate in vitro to the same antigen. This proliferative response is abolished by depletion of macrophages-monocytes, but can be reconstituted by the addition of cerebral vascular endothelial cells (EC) freshly isolated from syngeneic mice with adoptively transferred acute experimental allergic encephalomyelitis (EAE). Reconstitution by EC from mice with EAE can be blocked by pretreatment of EC with syngeneic anti-I-A antisera. Freshly isolated EC from normal syngeneic mice do not restore responsiveness, but can be induced to present antigen by culture with murine recombinant immune interferon-gamma or supernatants from a variety of immune cell cultures. These findings are consistent with the hypothesis that immune cells release interferon and/or other soluble factors which induce I-A molecules on EC, which subsequently acquire the capacity to present antigen. The implications of these findings relate to the migration of cells across the blood-brain-barrier into the central nervous system, and are of importance in the understanding of the pathogenesis of several neurologic disorders.

PMID: 2431034 [PubMed - indexed for MEDLINE]

2334. Isr J Med Sci. 1986 Nov;22(11):761-5.

Pulmonary functions and respiratory symptoms and diseases among adult Israelis. Variations by country of origin.

Goren AI, Bruderman I.

A study group of 1,299 adult Israelis aged 30 to 65 years was chosen from persons referred for evaluation of possible pulmonary diseases in two outpatient chest clinics. They were interviewed using the ATS-NHLI (American Thoracic Society-National Heart and Lung Institute) health questionnaire and underwent the pulmonary function test (PFT), which included the following parameters: forced vital capacity (FVC), forced expiratory volume in 1st sec (FEV1), FEV1/FVC, peak expiratory flow (PEF), FEF50 and FEF25 (forced expiratory flow at 50 and 25% of FVC, respectively). The effect of the country of origin of the subjects on the distribution of respiratory symptoms, pulmonary diseases and PFT was analyzed. The lowest PFT values and an excess of reported respiratory symptoms and chronic obstructive airways diseases--especially asthma--among subjects and their parents

were found among immigrants from Iraq-Iran. In immigrants from Morocco, reported respiratory symptoms, pulmonary diseases and impaired PFT were relatively uncommon. The different distribution of reported respiratory symptoms, pulmonary diseases and impaired PFT by country of origin could not be explained by environmental factors, such as smoking habits and socioeconomic background. The high prevalence of reported asthma among immigrants from Iraq-Iran is most probably due to a genetic factor.

PMID: 3793434 [PubMed - indexed for MEDLINE]

2335. Am J Gastroenterol. 1986 Nov;81(11):1084-6.

Use of macrophage inhibition factor and mast-cell degranulation tests for diagnosis of cloxacillin-induced cholestasis.

Aderka D, Livni E, Salamon F, Weinberger A, Pinkhas J.

Cloxacillin-induced cholestasis was diagnosed with the help of the macrophage inhibition factor and mast-cell degranulation tests. The simultaneous occurrence of both immediate type as well as cell-mediated hypersensitivity to the drug suggested that a defect in the histamine-induced suppressor cells may underly this cloxacillin-induced allergic reaction.

PMID: 3776960 [PubMed - indexed for MEDLINE]

2336. Clin Allergy. 1986 Nov;16(6):527-33.

Cell-mediated immune responses to artificial food additives in chronic urticaria.

Warrington RJ, Sauder PJ, McPhillips S.

In some cases of chronic urticaria it is suspected that food additives such as tartrazine and sodium benzoate or salicylates may play a role in the pathogenesis of the condition. Since, at times, chronic urticaria may appear histologically similar to a mild cell-mediated immune response, the release of the T cell-derived lymphokine leucocyte inhibitory factor (LIF), in response to incubation with these additives and with acetylsalicylic acid (ASA), was measured in vitro using cells from normal controls, from patients with chronic urticaria with or without clinically associated additive sensitivity and from patients with asthma with or without associated ASA sensitivity. It was found that significant production of LIF occurred in response to tartrazine and sodium benzoate in those individuals with chronic additive induced urticaria. In addition, tartrazine caused LIF release from mononuclear cells of ASA-sensitive asthmatics. These results may indicate a possible role for additive-induced cell-mediated immune responses in the pathogenesis of some cases of chronic urticaria and suggest a potential diagnostic test for this condition.

PMID: 3539408 [PubMed - indexed for MEDLINE]

2337. Allerg Immunol (Paris). 1986 Nov;18(9):23-7.

[What problems does childhood toxocariasis currently pose? Apropos of 6 clinical cases].

[Article in French]

Lelong M, Wattré P, Vaudour G, Bras C, Bouvier C, Drain JP.

Pavillon de l'Enfance Centre Hospitalier, Lens.

About six observations of toxocariasis (visceral larva migrans syndrome). We relate six observations of toxocariasis among children. In one case, an ocular localization is probable. For other five patients, they are inapparent forms. The allergologist pediatrician may be consulted because of a major hypereosinophilia (greater than 10,000/mm3) and an elevation of total IgE (greater than 2,000 UI/ml). Allergic and current parasitologic assays are negative and diagnostic key is given by toxocara serology. We insist on interest and reliability of passive hemagglutination test with a purified antigen (titer greater than or equal to 1/320). Treatment now is preferably flubendazole (50 mg/kg/day for six days) eventually renewed.

PMID: 3331109 [PubMed - indexed for MEDLINE]

2338. Acta Otorhinolaryngol Ital. 1986 Nov-Dec;6(6):613-8.

[Production of LIF and gamma interferon in patients with ragweed-induced allergic rhinosinusitis].

[Article in Italian]

Sartoris A, Beatrice F, Giordano C, Succo G, Vico F, Sorrentino R, Beatrice C.

PMID: 3109204 [PubMed - indexed for MEDLINE]

2339. J Invest Dermatol. 1986 Sep;87(3):358-61.

Different in situ distribution patterns of dendritic cells having Langerhans (T6+) and interdigitating (RFD1+) cell immunophenotype in psoriasis, atopic dermatitis, and other inflammatory dermatoses.

Bos JD, van Garderen ID, Krieg SR, Poulter LW.

Dendritic cells bearing Langerhans cell (OKT6+) or interdigitating cell (RFD1+) immunophenotype may be regularly detected within the dermis of chronic skin diseases characterized by a lymphohistiocytic (lymphoreticular) infiltrate. These 2 subsets of antigen-presenting cells within the dermis of lesions of exacerbating chronic plaque psoriasis, exacerbating nummular dermatitis (discoid eczema), atopic dermatitis, allergic contact dermatitis, pityriasis rosea, lichen ruber planus, and cutaneous lupus erythematosus were quantified using computer-assisted morphometry. The mean dendrite length per dermal dendritic cell was significantly higher for RFD1 than for OKT6 (74.4 +/- 0.98 microns vs 70.0 +/- 1.26 microns: p = 0.0023). The mean dendrite length per dermal dendritic cell was remarkably constant for each marker in the various diagnostic categories studied. Disease-specific patterns of total dendrite length and number (expressed per 100 infiltrating mononuclear cells) of these 2 dendritic cell types within the subepidermal infiltrates were obtained. Pityriasis rosea was characterized by its unique high percentage of OKT6+ Langerhans cells. Atopic dermatitis and psoriasis had relatively high percentages of both RFD1+ interdigitating cells and OKT6+ Langerhans cells. Nummular dermatitis had an intermediate number and total dendrite length for OKT6, but was relatively low in RFD1+ cells. Allergic contact dermatitis, lichen planus, and lupus erythematosus had low numbers and dendrite lengths for both dendritic cell subsets. It is suggested that pityriasis rosea is characterized by an abnormal migration pattern of Langerhans cells. Psoriasis and atopic dermatitis may be examples of diseases in which skin-localized antigen-presenting and T-cell-inducing events are continuously taking place. The

other diseases may reflect inflammatory processes in which local antigen presentation is less relevant to the tissue reaction.

PMID: 3734488 [PubMed - indexed for MEDLINE]

2340. J Immunol. 1986 Aug 15;137(4):1286-93.

Noncytolytic terminal complement complexes may serve as calcium gates to elicit leukotriene B4 generation in human polymorphonuclear leukocytes.

Seeger W, Suttorp N, Hellwig A, Bhakdi S.

Complement effects on human polymorphonuclear leukocytes (PMN) have generally been ascribed to the anaphylatoxin C5a, which induces degranulation, superoxide anion generation, migration, and cell aggregation via interaction with membrane receptors. We here report that complement activation on the surface of antibody-sensitized human PMN provokes generation of the potent lipid mediator leukotriene B4 (LTB4) in strict dependence on complement component C8, but in the absence of detectable C9. The kinetics of LT generation are rapid, comparable with those observed after challenge with the calcium-ionophore A23187. LTB4 release is a distinct event that is dissociable from cytotoxicity as assessed by lactate dehydrogenase (LDH) release (dependent on C9) and from superoxide generation (independent of C8 and C9). It is dose dependent on extracellular calcium and is not observed in the absence of calcium. It is inhibited by substances interfering with calcium-calmodulin function (trifluoperazine and W7), but not by blockers of physiologic calcium channels (nimodipine, verapamil, and D 888). Addition of purified C8 to cells bearing C5b-7 induces a severalfold increase in their passive permeability to 45calcium. Sieving experiments with the use of marker molecules of different sizes collectively indicate the existence of small hydrophilic channels consisting exclusively or predominantly of C5b-8 complexes, which allow passive transmembrane flux of small molecules with Mr less than 200. Thus, noncytolytic terminal complement complexes may serve as a biological bypass gate for calcium in PMN membranes, triggering the arachidonic acid cascade with generation of LTB4 at doses well below the threshold required to invoke overt cell damage.

PMID: 2426360 [PubMed - indexed for MEDLINE]

2341. Br J Dis Chest. 1986 Jul;80(3):242-8.

Acute asthma in Asian patients: hospital admissions and duration of stay in a district with a high immigrant population.

Ayres JG.

Acute asthma admissions to a District General Hospital in the East District of Birmingham from 1972 to 1982 show that 20% of admissions are in Asian patients who comprise only 9.7% of the population. Annual admissions for acute asthma have risen from 240 to 377 (+57%) for non-Asians and from 60 to 100 (+67%) for Asians in that time although the proportion of readmissions has remained unchanged. For 1981, admission rates for acute asthma per 100,000 population, assuming equal asthma prevalence rates, was 198 for Asians compared to 79 for non-Asians, a relative risk of 2.5. Mean duration of hospital stay has fallen from 13.6 days to 6.7 days but there is no difference when comparing Asian to non-Asian populations at each age level, duration of stay rising with increasing age. The reasons for the higher admission rates for Asians are probably multifactorial but poor asthma education due to language problems and poor compliance may be significant causes, although this remains to be confirmed.

PMID: 3790414 [PubMed - indexed for MEDLINE]

2342. Immunology. 1986 Jul;58(3):411-6.

Proliferation and transepithelial migration of mucosal mast cells in interstitial cystitis.

Aldenborg F, Fall M, Enerbäck L.

The distribution and abundance of mast cells, as well as their fixation, staining and ultrastructural properties, were studied in the urinary bladders of 16 patients with interstitial cystitis (IC) and in 14 normal subjects. Tissues were fixed in both standard formaldehyde solution and a special fixative, IFAA, optimized for the preservation of mucosal mast cells. An expansion of two distinct mast cell populations was observed in IC. One of these, comprising formaldehyde-sensitive cells, was found only in the mucosa underlying lesions of IC. They were most numerous in the lamina propria but were also frequent in the epithelial layer as well as in the bladder washings, indicating a migratory capacity for these cells. The other mast cell population was visualized equally well irrespective of fixation and staining procedure. In control subjects, such cells were found both in the lamina propria and detrusor muscle, but not in the epithelium nor in bladder washings. In lesions of IC they were increased in the detrusor muscle only. Both types of mast cell contained granules with the highly characteristic lamellar arrays and scrolls, distinguishing human mast cell granules from those of blood basophils. The proliferation and intraepithelial distribution of mucosal mast cells are unusual findings, but prominent features of helminth responses and human mucosal allergic reactions. These findings thus suggest that the mucosal mast cell-IgE system may be involved in the pathogenesis and/or aetiology of IC.

PMCID: PMC1453481
PMID: 3733146 [PubMed - indexed for MEDLINE]

2343. Br Med J (Clin Res Ed). 1986 Jun 7;292(6534):1483-6.

Response to inhaled histamine and 24 hour sodium excretion.

Burney PG, Britton JR, Chinn S, Tattersfield AE, Platt HS, Papacosta AO, Kelson MC.

A relation between the prevalence of asthma and economic development has been suggested by studies in migrants and other surveys in developing countries. That this correlation might be partially explained by an increased intake of salt in the diet is supported by the observation that sales of table salt in the different regions of England and Wales are independently correlated with mortality from asthma for men and for children. As part of a wider survey of asthma 138 men living in two Hampshire villages and aged 18-64 were given a bronchial histamine challenge test and had their 24 hour urinary excretion of sodium measured. Bronchial reactivity was strongly related to 24 hour excretion of sodium after allowing for the effects of age, atopy, and cigarette smoking, there being on average a 10-fold difference in reactivity over the 95% range of sodium excretion recorded in the study. The data suggest that a high sodium diet may potentiate bronchial reactivity.

PMCID: PMC1340493
PMID: 3087486 [PubMed - indexed for MEDLINE]

2344. Inflammation. 1986 Jun;10(2):99-108.

Decreased extracellular release of granule enzymes from in vitro-stimulated polymorphonuclear leukocytes in guttate psoriasis.

Glinski W, Tigalonowa M, Jablonska S, Janczura E.

In vitro degranulation of polymorphonuclear leukocytes, which were stimulated either with synthetic chemotactic peptide (N-formyl-methionyl-leucyl-phenylalanine, FMLP) or with C3b-opsonized zymosan as a promotor of phagocytosis, was studied in 66 patients with psoriasis, 18 lesion-free psoriatics, 18 healthy subjects, and 14 other dermatological disorder controls. Stimulated release of lysozyme (from specific granules and azurophil granules) and beta-glucuronidase (from azurophil granules) in the presence of both FMLP and serum-activated zymosan was markedly reduced in patients with actively spreading guttate psoriatic lesions, in whom relapse of lesions lasted for less than 1 month and papules involved about 13-25% of skin surface. In contrast, stimulated degranulation was within normal range in active plaque psoriasis, stationary plaque psoriasis, symptomless psoriatics, and patients with disseminated eczema. Spontaneous release of lysozyme and beta-glucuronidase (background) was found to be not different in all groups studied; however, patients with active guttate psoriasis had significantly lower total lysozyme activity than those with active and stationary plaque psoriasis as well as psoriatics in the remission. These data are in favor of in vivo activation of neutrophils in active guttate psoriasis by some factors related to the early relapse of the lesions. This results in a possible combination of the following phenomena: (1) in vivo partial degranulation of neutrophils; (2) induction of "unresponsiveness state" of these cells to subsequent in vitro stimulation; and/or (3) migration of highly responsive neutrophils to skin lesions, which leaves in the circulation the subpopulation less reactive to chemotactic and phagocytic stimuli.

PMID: 3710563 [PubMed - indexed for MEDLINE]

2345. Zh Mikrobiol Epidemiol Immunobiol. 1986 Jun;(6):59-62.

[Sensitizing properties of soluble Haemophilus influenzae antigens].

[Article in Russian]

Dobritsa VP, Sukhodoeva GS, Beklemishev ND.

The sensitizing properties of different doses of the antigenic preparation obtained from H. influenzae by ultrasonic treatment have been studied. Small doses of the antigen have been found to induce immunoallergic transformations of type IV in sensitized guinea pigs. A considerable increase in the sensitizing dose of the antigen has been found to lead to the appearance of allergic reactions of type I. The regularities of the development of allergy to H. influenzae are discussed.

PMID: 3489339 [PubMed - indexed for MEDLINE]

2346. Ann Allergy. 1986 May;56(5):402-5.

Lichenoid eruption due to hydrochlorothiazide. Diagnostic aid of macrophage migration inhibition factor (MIF) test.

Halevy S, Grunwald MH, Feuerman EJ, Livni E.

The case of a lichenoid photosensitive eruption induced by hydrochlorothiazide is described. The macrophage migration inhibition factor test identified hydrochlorothiazide as the offending drug out of the several suspected drugs taken by the patient. It is suggested that an allergic reaction towards hydrochlorothiazide was involved in the development of the photosensitive lichenoid eruption in this case.

PMID: 3518549 [PubMed - indexed for MEDLINE]

2347. Ann Allergy. 1986 Apr;56(4):341-4.

The migration inhibition factor test for identification of hypersensitivity reactions to drugs.

Aderka D, Livni E, Sharon C, Pinkhas J.

The migration inhibition factor (MIF) test detects the in vitro release of lymphokine from lymphocytes in in vitro contact with a drug that had sensitized them in vivo. The specificity and sensitivity of the MIF test in identifying a drug inducing an allergic reaction is presented. The MIF test detected the drugs responsible for 20 out of 21 allergic episodes (95.2%) while the basophil degranulation test detected only eight of them (P less than .001). The sensitivity of a positive MIF test was 95.2% and its specificity was 76.9%. The specificity of a negative MIF test was 94.7%. The positive MIF test assisted the physician in indicating the drugs responsible for an allergic reaction in half of the patients. The drugs for which the MIF test was negative could be considered innocent in 95% of the cases. It is concluded that although the results of the present studies are encouraging, the clinical utility of the MIF test is still limited and improvement of the test specificity is required.

PMID: 3963526 [PubMed - indexed for MEDLINE]

2348. Acta Paediatr Scand. 1986 Mar;75(2):308-12.

Leucocyte migration inhibition in cow's milk protein intolerance.

Khoshoo V, Bhan MK, Arora NK, Sood D, Kumar R, Stintzing G.

The leucocyte migration inhibition (LMI) was determined in an assay after in vitro challenge with beta-lactoglobulin. The assay was considered positive when migration inhibition index was greater than 20% (mean +3 SD of healthy infants). Ninety-eight infants with protracted diarrhoea and failure to thrive, 16 healthy, 12 malnourished, and 16 infants suffering from acute gastroenteritis were studied. Of the 98 patients with protracted diarrhoea, 12 fulfilled Goldman's criteria for cow's milk protein intolerance, 63 had lactose malabsorption, and in 15 no associated causative factor was identified. The mean index of migration inhibition in the cow's milk allergic group (58.83 +/- 11.98) was higher than in healthy controls (8.25 +/- 3.91), the difference being statistically significant (p less than 0.05). The test was positive in all patients with cow's milk protein intolerance. The assay was also positive in four other patients suffering from protracted diarrhoea, two of whom had lactose malabsorption. All the infants with acute gastroenteritis and malnutrition had values within the normal range. The migration inhibition index in five patients with cow's milk intolerance had declined to 24.74 +/- 4.87 in assays performed 1-6 weeks after return of clinical tolerance to cow's milk (p less than 0.05) but the test was still within the positive range in three of the five infants. These results suggest that this cell

mediated immune assay is a sensitive test for the diagnosis of cow's milk protein intolerance in infants. The specificity needs to be reassessed in the light of more objective criteria for the diagnosis of cow's milk protein intolerance.

PMID: 3962662 [PubMed - indexed for MEDLINE]

2349. Vet Parasitol. 1986 Mar;20(1-3):103-16.

A review of eosinophil chemotaxis and function in Taenia taeniaeformis infections in the laboratory rat.

Potter K, Leid RW.

The eosinophil has long been associated with diseases of acute hypersensitivity and with parasite infections, but its exact role in the pathogenesis of these conditions remains uncertain. Characterization of factors associated with migration of eosinophils into tissues has helped to elucidate eosinophil function. Eosinophil chemotactic factors associated with acute hypersensitivity reactions include the eosinophil chemotactic factors of anaphylaxis, histamine, and arachidonic acid metabolites, all of which are released from mast cells, and the lymphokine eosinophil stimulation promoter (ESP). Eosinophilotaxins associated with parasitic diseases include the lymphokine ESP and the low molecular weight factor ECF-G, both associated with schistosome infection in mice. In addition, in several parasite infections parasite-derived protein eosinophil chemotactic factors have been identified and characterized. The proteins associated with Ascaris, Anisakis, and Schistosoma infections appear to be distinct from one another. We have recently partially characterized a protein from Taenia taeniaeformis larvae which has marked chemotactic activity for eosinophils. In addition we have demonstrated eosinophil chemotactic activity associated with metabolism of arachidonic acid by T. taeniaeformis metacestodes. The results of studies in taeniasis and other parasite infections, therefore, indicate that parasite-derived factors may directly influence migration of eosinophils.

PMID: 3518212 [PubMed - indexed for MEDLINE]

2350. J Clin Invest. 1986 Mar;77(3):1014-9.

Effects of fibrinogen derivatives upon the inflammatory response. Studies with human fibrinopeptide B.

Senior RM, Skogen WF, Griffin GL, Wilner GD.

Fibrin formation and turnover are intimately associated with inflammation and wound healing. To explore whether fibrin(ogen)-derived peptides exert direct effects upon cells involved in inflammation and tissue repair we examined the capacity of human fibrinopeptide B (hFpB), a thrombin-derived proteolytic cleavage product of the fibrinogen B beta-chain, to stimulate neutrophils (PMN), monocytes, and fibroblasts. hFpB caused directed cell migration of PMN and fibroblasts that was optimal at approximately 10(-8) M. This chemotactic activity was blocked by preincubating hFpB with antiserum to hFpB. hFpB was not chemotactic for monocytes. The chemotactic potency of hFpB for PMN was equivalent to that of anaphylatoxin from the fifth component of human complement (C5a), leukotriene B4 (LTB4), and formyl-methionyl-leucyl-phenylalanine (fMLP), and for fibroblasts its chemotactic activity was comparable to that of platelet-derived growth factor. hFpB did not interact with PMN receptors for C5a, LTB4, or fMLP as (a) desensitization with 10(-7) M hFpB abolished chemotaxis to hFpB but had no effect upon chemotaxis to C5a, LTB4, or fMLP and (b) induction of chemotactic

responses to fMLP and LTB4 in neutrophilic leukemic cells (HL-60 cells) by incubation with dimethylsulfoxide did not extend to hFpB. Like fMLP, hFpB caused a rapid, dose-dependent increase in PMN cytoskeletal associated actin, but unlike fMLP, hFpB did not cause PMN aggregation, release of lysosomal enzymes (lysozyme and beta-glucuronidase), or the production of superoxide anion. These results suggest that hFpB may have a role in recruiting PMN and fibroblasts at sites of fibrin deposition and turnover. The capacity of hFpB to cause PMN chemotaxis without causing concurrent release of lysosomal enzymes or the production of superoxide anion is further evidence for the complexity of PMN responses to chemotactic agents.

PMCID: PMC423507
PMID: 3005361 [PubMed - indexed for MEDLINE]

2351. Nurse Pract. 1986 Feb;11(2):54-8, 60, 65.

Providing for the health needs of migrant children.

Schneider B.

Migrant children frequently have health needs that go unmet due to fragmented care caused by their mobility, poverty, lack of medical and financial resources, language barriers, superstitions and poor education. Their common health problems primarily fall into four categories: 1) Diseases and conditions caused by overcrowded and poor living conditions and frequent moves to new climatic areas with different water supplies and native viruses; 2) nutritional problems; 3) congenital anomalies, inherited conditions and allergies; and 4) neglect and lack of adequate medical treatment. Migrant children present nurse practitioners with a unique challenge to provide the children and their families with comprehensive health care which includes 1) diagnosis and treatment of common illnesses, infections and infestations within the family's meager economic means; 2) referrals for congenital anomalies, chronic conditions and those conditions requiring additional or specialized health services; and 3) the adaptation of teaching programs for the child and his/her parents (including health education in hygiene, immunization status, growth and development, stimulation, nutrition, etc.). This article discusses migrant health problems and makes recommendations for providing health care and referrals for migrant children.

PMID: 3945421 [PubMed - indexed for MEDLINE]

2352. Arch Dis Child. 1986 Feb;61(2):194-6.

Pancytopenia caused by iron-dextran.

Hurvitz H, Kerem E, Gross-Kieselstein E, Brand A, Branski D.

Pancytopenia after intramuscular iron-dextran treatment occurred in an infant with Down's syndrome. Haematological abnormalities recurred on subsequent challenge. Positive migration inhibiting factor and mast cell degranulation tests support an allergic pathogenesis for the pancytopenia. These side effects have not been reported previously.

PMCID: PMC1777573
PMID: 2937372 [PubMed - indexed for MEDLINE]

2353. Int Arch Allergy Appl Immunol. 1986;80(1):44-51.

Intraepithelial migration of nasal mucosal mast cells in hay fever.

Enerbäck L, Pipkorn U, Granerus G.

Mast cells were studied by light microscopy in mucosal imprints and in biopsies of nasal mucosa of 12 birch pollen allergic individuals before and during the pollen season, using techniques optimized for the demonstration of mucosal mast cells. We also measured the histamine content of nasal mucosa, whole blood and plasma, and counted the numbers of circulating blood basophils. Before the pollen season the nasal mucosa was found to contain many mast cells located in the mucosal connective tissue stroma, and very few cells with basophilic and metachromatic granules were found in mucosal imprints. During the pollen season there was a redistribution of mast cells into the epithelium, many such cells now being recovered in mucosal imprints. The total number of mucosal mast cells counted in tissue sections did not change significantly with the onset of the pollen season, suggesting a redistribution of mucosal mast cells by migration. Judged by morphologic appearance and naphthol-AS-D chloroacetate esterase activity, the intraepithelial mast cells found in tissue sections had rather the properties of tissue mast cells than of blood basophils, and only a few of the basophilic cells of the imprints had a morphology compatible with blood basophils. The histamine content of the mucosa, as well as histamine levels of whole blood and plasma, and circulating blood basophil numbers did not change significantly in relation to the pollen season. These findings suggest that an intraepithelial migration of mucosal mast cells is part of the allergic mucosal response.(ABSTRACT TRUNCATED AT 250 WORDS)

PMID: 3957446 [PubMed - indexed for MEDLINE]

2354. Arch Dermatol Res. 1986;278(6):454-9.

Defective monocyte and polymorphonuclear leukocyte chemotaxis and clinical characteristics in atopic dermatitis.

Ternowitz T, Herlin T.

Using highly purified cell suspensions, monocyte (MO) and polymorphonuclear leukocyte (PMN) chemotaxis was measured by the 51Cr-labeled cells technique in 30 adult patients with atopic dermatitis (AD). MO chemotaxis was depressed in 60% of the patients; in one-third both MO and PMN chemotaxis was impaired. All patients with normal MO chemotaxis had normal PMN chemotaxis. The defective chemotaxis was related to the presence of cutaneous infection and to the activity of the disease. Cutaneous infection was observed in 70% of the patients with low MO and PMN chemotaxis. We found no relation between the chemotaxis defects and serum IgE levels. Presence of asthma in addition to AD did not influence the results. Preincubation of normal leukocytes with AD plasma did not alter the chemotactic responses. Plasma from atopics had a lower capacity for inducing migration than normal plasma using leukocytes from healthy subjects as test cells.

PMID: 3789804 [PubMed - indexed for MEDLINE]

2355. Int Arch Allergy Appl Immunol. 1986;81(4):289-97.

Intraepithelial migration of mucosal mast cells in hay fever: ultrastructural observations.

Enerbäck L, Pipkorn U, Olofsson A.

Evidence has been presented suggesting that a migration of nasal mast cells from

the mucosal connective tissue stroma into the epithelium is part of the mucosal response in birch pollen allergy. In a previous study, the identification of these intraepithelial cells as tissue mast cells rather than blood basophils was based on light microscopical morphology and histochemistry. We have now studied the ultrastructure of these cells in mucosal biopsies taken before and during the birch pollen season. Intraepithelial cells with basophil or metachromatic granules were only observed in biopsies taken during the season. Some of these cells had the ultrastructural appearance of tissue mast cells, including cytoplasmic lipid droplets and a granular substructure composed of multilamellar arrays and scrolls, serving to distinguish human mast cells from blood basophils. The ultrastructural traits of the remaining cells were heterogeneous, some reminiscent of human blood basophils, others of globule leucocytes of other species, but entirely typical blood basophils could not be identified. The results thus support our previous suggestion that a migration of mucosal mast cells from the connective tissue stroma into the epithelium is part of the human allergic mucosal response. It cannot be determined whether the ultrastructural heterogeneity of these cells is the result of an adaptation to the intraepithelial environment of one single mast cell type or to the existence of an ultrastructurally distinct mucosal mast cell.

PMID: 3781638 [PubMed - indexed for MEDLINE]

2356. Allergol Immunopathol (Madr). 1986 Jan-Feb;14(1):15-20.

Laboratory diagnosis in thyroid auto-allergic diseases.

Suárez-Chacón R, Sánchez M.

Different parameters of humoral and cellular immunologic sensitization have been described in thyroid autoallergic disease, however, the percutaneous biopsy of the gland remains as one of the principal parameters of diagnostic confirmation, wherein the possible error of the sample being taken in an area not affected by the disease. The present study assesses two different parameters of immunologic sensitization, humoral and cellular as they are determination of thyroid antimicrosomal antibodies by hemagglutination and leukocyte inhibition migration tests against the same microsomal antigen. Both types of sensitization are proposed as being relevant, and can be found present alone or combined in the same patient, which suggests that the determination of antibodies is the most useful proof with diagnostic motives, but this should be followed by the determination of leukocyte inhibition migration when the result is negative.

PMID: 3754382 [PubMed - indexed for MEDLINE]

2357. Zh Mikrobiol Epidemiol Immunobiol. 1986 Jan;(1):81-5.

[Dependence of the migration activity of blood granulocytes from healthy donors and from bronchial asthma patients on the presence of cellular mediators].

[Article in Russian]

Totolian AA, Freĭdlin IS.

The migration activity of granulocytes in human blood has been shown to depend both on the locomotor properties of these cells and on the presence of cell mediators secreted by blood mononuclears and by granulocytes themselves. Granulocytes in the blood of patients with the atopic form of bronchial asthma differ from granulocytes in healthy donors by sharply decreased spontaneous migration activity. Granulocytes and mononuclears in the blood of patients with

the atopic form of bronchial asthma differ from the corresponding cells of donor blood in the activity of cell mediators secreted by them and influencing the migration of granulocytes in the donors.

PMID: 3705808 [PubMed - indexed for MEDLINE]

2358. Jpn J Antibiot. 1986 Jan;39(1):141-53.

[Investigation on effectiveness and safety of fosfomycin in treatment of patients with allergy induced by antibacterial agents].

[Article in Japanese]

Suzuki K, Tamai H.

Twenty-four patients with urinary tract infections were treated with fosfomycin (FOM) to evaluate its effectiveness and safety. They all had shown allergic reactions, mainly to beta-lactams. Lymphocyte stimulation test (LST), leukocyte migration inhibition test (LMT), passive cutaneous anaphylaxis (PCA) and the precipitin reaction were performed to test whether FOM would also cause an allergic reaction. FOM was judged by the physician-in-charge to be effective in all 6 patients (100%) with acute simple cystitis and 8 (72.7%) of 11 patients with chronic complicated urinary tract infections. The drug was also effective in 15 (83.3%) of 18 patients with epididymitis, etc. Regarding the usefulness of FOM, it was judged to be useful in 21 (91.3%) of 23 patients. FOM was very useful in 8 (34.8%) of these 23. In patients tested for LST, the value was lower in cases given FOM than in cases given ampicillin (ABPC), cefazolin (CEZ) or latamoxef (LMOX); there was an especially significant (p less than 0.01) difference with ABPC. All the drugs tested were negative for the LMT, PCA and precipitin reaction tests. No subjective or objective abnormalities were attributed to the FOM treatment, and there were also no abnormal laboratory test values. FOM was evaluated to be an effective and safe antibacterial agent without in vitro or clinical allergic reactions.

PMID: 3702056 [PubMed - indexed for MEDLINE]

2359. Polim Med. 1986;16(1-2):55-61.

[The search for an "ideal" surgical dressing].

[Article in Polish]

Kleczyński S, Niedźwiecki T, Brzeziński M.

Trials of a new occlusive dressing, Op-site (Smith Nephew), were conducted on a group of patients. Op-site is a fine, transparent, elastic, self-adhesive polyurethan film. Although non-porous and therefore water- and bacteria-proof, it is permeable to gases. The existing dressings fulfil only a few of the criteria of an "ideal" dressing and in some cases actually interfere with the healthy process. The main disadvantages are: the disturbance of newly formed epithelium, when many dressings are removed, their fibres become embedded in the new tissues and cause inflammation and delayed healing. Few dressings are true bacterial barriers and the hazard of infection of the wound is always present. Recent studies of the mechanism of wound healing have indicated that a moist, not dry surrounding provides the optimum conditions for wound repair. Healing under Op-site is said to be quicker because the serous exudate permits unhindered migration of new cells across the wound bed and prevents cellular dehydration. In contrast, under dry conditions healing is delayed because the new skin cells must

first cleave a path through dehydrated dermis before migrating across the wound. The Op-site wound dressing can be readily applied over the joints and allows complete freedom of movement. The skin remains dry and the wound moist, providing the ideal environment for rapid healing. The film does not adhere to the moist wound and can therefore be removed without damage to the newly formed epidermis. The adhesive is low allergic. Finally, the wound can be assessed without removing the transparent Op-site.(ABSTRACT TRUNCATED AT 250 WORDS)

PMID: 3540915 [PubMed - indexed for MEDLINE]

2360. Soc Sci Med. 1986;22(11):1105-13.

Unemployment-related lifestyle changes and health disturbances in adolescents and children in the western countries.

Olafsson O, Svensson PG.

According to official statistics 11 million under 25's, thereof 5-6 million under 20's in the 12 OECD member states are unemployed at any given time. In depth studies show that this figure is at least 40-50% higher. In many countries a systematic under-reporting exists in the registration of unemployed. Surveys used to show more relevant figures. Unemployment hits mainly adolescents, school leavers, young adults (unskilled male and female) immigrants and then, indirectly, those who are in need of familial and social support, i.e. the frail, sick, disabled children and old people. In many cities in Europe 40-50% of 18-25 year olds are unemployed and figures as high as 90% have been reported (inner cities). Unemployment is endangering the socio-economic status of people, in spite of short-time unemployment benefits and is creating inequalities in health and serious social misfits. Loss of job or the mere prospect of becoming jobless have, in follow-up studies on an individual level, been found to cause elevated blood pressure and serum cholesterol, increased concentration of blood catecholamine and elimination of noradrenaline, an increase in the frequency of stress and psychosomatic diseases. After regaining employment, these values have normalized. Unemployment is therefore considered by many as a real source of stress. Chronic stress is now considered as a major contributor to cardiovascular diseases, ulcers, asthma and some other diseases. According to several well designed and controlled studies on the individual level in the developed countries, the majority of young people do not learn to cope with unemployment. It fosters isolation, loss of self-esteem, frustration and hopelessness.(ABSTRACT TRUNCATED AT 250 WORDS)

PMID: 3529413 [PubMed - indexed for MEDLINE]

2361. Acta Otolaryngol Suppl. 1986;430:39-48.

Mast cell degranulation in nasal polyps.

Takasaka T, Kaku Y, Hozawa K.

Light- and electronmicroscopic observations as well as immunohistochemical studies were made on nasal polyps from 15 patients. The patients included 2 cases of aspirin intolerance (AA), 6 cases of allergic rhinitis (NA) and 7 cases of chronic rhinitis (CS) with negative skin tests against major inhalant allergens. Nasal polyps commonly contained many inflammatory cells such as neutrophils (PMN), eosinophils, plasma cells, mast cells, lymphocytes and macrophages. Two morphological features were conspicuous in our study: 1) PMN migration and attachment to the basal lamina, 2) accelerated degranulation of mast cells. Mean values of degranulated granules were 0.532473/micron2 in AA, 0.492615/micron2 in

NA and 0.253591/micron2 in CS. These results indicate that mast cell degranulation in CS is much less than that in AA and NA. Immunohistochemical investigations revealed very few IgE-positive cells in both AA and NA, and none in CS. On the other hand IgG and IgA were frequently observed in all cases of nasal polyps. The present study suggests that mast cell degranulation plays an important role in the formation of nasal polyps, but it may not only be an IgE-dependent mechanism. To elucidate other possibilities, more extensive immunological studies will be required.

PMID: 3526808 [PubMed - indexed for MEDLINE]

2362. Contact Dermatitis. 1986 Jan;14(1):26-31.

Expression of OKT6 antigen by Langerhans cells in patch test reactions.

Christensen OB, Daniels TE, Maibach HI.

Previous studies in humans and guinea pigs indicate that Langerhans cells (LC) participate in elicitation of allergic contact dermatitis. The number and morphology of LC was determined in acute and healed allergic patch test sites, control (petrolatum) sites and normal skin in nickel-sensitive individuals using OKT6 monoclonal antibody and avidin-biotin/peroxidase labeling. Compared to normal skin, the staining intensity and number of epidermal LC was significantly increased in allergic (p less than 0.02) and petrolatum control (p less than 0.02) sites, and in 6- to 8-week old allergic patch test sites (p less than 0.006). The in situ changes in LC induced by petrolatum alone may be a result of the occlusive patch test, or may suggest that petrolatum is not as neutral as previously believed. The nickel-induced increase in LC may indicate changes in the LC present in the epithelium at the time of testing, or migration of additional LC into the epithelium, which can then remain in situ for weeks after the antigenic challenge. The specificity of the LC increase in allergic contact dermatitis is questioned on the basis of the increase noted with petrolatum.

PMID: 3485029 [PubMed - indexed for MEDLINE]

2363. Int Arch Allergy Appl Immunol. 1986;79(1):38-44.

Role of leukotrienes in rat reversed passive Arthus pleurisy and the effect of AA-861, a 5-lipoxygenase inhibitor.

Makino H, Ashida Y, Saijo T, Kuriki H, Terao S, Maki Y.

In studies of the role of leukotrienes in inflammatory reactions, the induction of rat reversed passive Arthus pleurisy (a type III allergic reaction) was employed. Increases of exudate volume, vascular permeability, and migration of inflammatory cells in the pleural cavity were observed. The vascular permeability was enhanced biphasically during 0-30 min (early response) and during 3-6 h (late response) after induction of the pleurisy. The infiltration of inflammatory cells, mainly polymorphonuclear leukocytes, into the cavity increased and reached a maximum 6 h after the pleurisy was induced. Leukotriene B4 (LTB4), 5-monohydroxyeicosatetraenoic acid (5-HETE), and slow-reacting substance of anaphylaxis (SRS-A), consisting of LTC4, LTD4 and LTE4, were detected in the exudate by reversed-phase high-performance liquid chromatography during the early response. The contents of LTC4 reached a maximum 10 min after the challenge, followed by a rapid decrease within 1 h. The rise and decay of LTC4 correlated with the increase in vascular permeability during the early phase. AA-861, a 5-lipoxygenase inhibitor, given intrapleurally inhibited the increase in vascular permeability, cell migration, and generation of leukotrienes during the early

phase of the pleurisy. These results indicate that products of the 5-lipoxygenase pathway, such as LTC4 and LTB4, may play an important role as chemical mediators in the inflammatory reaction.

PMID: 3000949 [PubMed - indexed for MEDLINE]

2364. Ter Arkh. 1986;58(10):76-8.

[Clinico-immunological and allergological characteristics of urticaria caused by pyrazolone derivatives].

[Article in Russian]

Poroshina IuA, Luss LV, Gervazieva VG.

The authors describe the results of clinical, allergological and immunological examination of 35 patients with urticaria caused by pyrazolone derivatives. Clinically, the patients with pyrazolone-induced urticaria were marked by chronic diseases requiring the prolonged and frequent intake of the analgesics, pyrazolone derivatives. The allergological examination of the 35 patients with pyrazolone-induced urticaria showed that only one of the patients had pollenosis, 6 patients had IgG-mediated reactions to egg protein and one patient to penicillin. For specific diagnosis of drug allergy use was made of the natural leukocyte migration test in vivo according to A. D. Ado. The test appeared positive with analgin in all the 35 patients suffering from pyrazolone-induced urticaria. It represents a simple and accessible method for specific diagnosis of drug allergy both in inpatients and in those visiting allergological rooms at the polyclinics. The immunological examination made with the aid of the histograms demonstrated an appreciable reduction in the content of D-phagocytosing neutrophils. The latter fact might explain the presence of multiple chronic foci of infection in patients with pyrazolone-induced urticaria. Such patients manifested a decrease in C3 that might be related to immediate activation of the alternative pathway of complement by pyrazolone derivatives.

PMID: 2948292 [PubMed - indexed for MEDLINE]

2365. Acta Derm Venereol. 1986;66(2):93-7.

Presence of chemotactic peptides other than C5a anaphylatoxin in scales of psoriasis and sterile pustular dermatoses.

Takematsu H, Terui T, Ohkohchi K, Tagami H, Suzuki R, Kumagai K.

A unique leukocyte chemotactic factor of around 12 kD molecular weight has been suggested to be responsible for the mechanisms involved in transepidermal migration of leukocytes observed in psoriasis and related sterile pustular dermatoses. Although we confirmed with radioimmunoassay the presence of a C5-cleavage product in the chemotactic fractions from psoriatic scale extract eluted by gel filtration HPLC, the neutrophil chemotactic activity demonstrated in the peak fraction was only partially inhibited by rabbit antiserum to human C5a, whereas that noted in the peak fraction, prepared from zymosan activated serum, was totally abrogated by the same treatment. Similarly the chemotactic fraction prepared from the scale extracts of other related pustular dermatoses were only partially inhibited with anti-C5a antiserum. These findings suggest that chemotactic peptides other than C5a also play a role in the transepidermal migration of leukocytes in these dermatoses.

PMID: 2424251 [PubMed - indexed for MEDLINE]

2366. Zh Vopr Neirokhir Im N N Burdenko. 1985 Nov-Dec;(6):15-9.

[Changes in immunologic indices in patients with brain tumors].

[Article in Russian]

Lisianyĭ NI, Mamytov MM.

Patients with tumors of the brain underwent complex immunological examination. Anticerebral antibodies were discovered and the IgM and IgG levels were found to be diminished. Reduction of the T lymphocyte content was paralleled by the compensatory increase of the B lymphocyte level in the blood. It is shown that according to the findings of some allergic tests, neurosensitization develops; the tests employed were the leukocyte injury, leukocyte agglomeration, and leukocyte migration inhibition. The immune disorders and the autoimmune shifts correlate with the stage of the organism compensation, the degree of tumor malignancy, and the efficacy of the operation.

PMID: 4090848 [PubMed - indexed for MEDLINE]

2367. Nihon Yakurigaku Zasshi. 1985 Nov;86(5):329-40.

[Effect of traxanox sodium on inflammatory response].

[Article in Japanese]

Terasawa M, Imayoshi T, Maruyama Y, Abe C.

Traxanox was inactive against classic acute and subacute inflammation models such as carrageenin paw edema, UV erythema, 6-hr Evans blue-carrageenin (E-C) pleurisy and cotton pellet granuloma formation, and it failed to inhibit the production of prostaglandin E2 and a slow reacting substance from rat peritoneal leucocytes which phagocytize killed bacteria in vitro. On the other hand, traxanox inhibited the anaphylactoid reaction and decreased the pleural fluid in 24-hr E-C pleurisy. Traxanox (100 mg/kg, p.o.) also showed a tendency to suppress dextran edema and cotton pellet granuloma formation in adjuvant arthritis (AA) in rats. In experimental models of delayed type hypersensitivity (DTH), traxanox (100 mg/kg, p.o.) inhibited the accumulation of the exudate and the leucocyte migration in B. pertussis-induced pleurisy in rats. Traxanox (50 mg/kg) did not show any effect on AA in Lewis rats when administered orally for 21 days after the adjuvant inoculation, but the combined administration of traxanox with hydrocortisone (10 mg/kg, p.o.) or indomethacin (0.25 mg/kg, p.o.) resulted in a synergistic inhibition of AA. When the administration of traxanox was started 21 days before the adjuvant inoculation, it inhibited AA in a dose-dependent manner (50-100 mg/kg, p.o.). On the other hand, traxanox (100 mg/kg, p.o.) enhanced the concanavalin A-induced DTH-like skin reaction in guinea pigs. These results indicate that the mode of action of traxanox on inflammatory responses resembles that of D-penicillamine or levamisole, so that it may prove to be clinically effective in treating rheumatoid arthritis.

PMID: 2867959 [PubMed - indexed for MEDLINE]

2368. Clin Allergy. 1985 Nov;15(6):509-15.

Dynamics of mast cell degranulation in human allergic nasal epithelium after provocation with allergen.

Kawabori S, Okuda M, Unno T, Nakamura A.

The histamine content in the nasal epithelial layer of twenty-five patients with nasal allergy was measured before, 10 min after and 1 hr after nasal provocation with allergen. A decrease in histamine content was observed 10 min after provocation compared to the values obtained before provocation (P less than 0.05). There was a tendency for an increase in the histamine content of the nasal epithelium one hour after provocation when compared with the amounts present 10 min after provocation (P less than 0.1). Mast cells in the nasal epithelial layer of a further five patients were studied by electron microscopy 10 min and 1 hr after provocation. The rate of mast cell degranulation appeared to decrease 1 hr after provocation when compared with 10 min. Our study suggests that some mast cells commence their migration to the nasal epithelial layer over a short time period and that they may play a role in the onset of the allergic nasal reaction in patients with allergic nasal symptoms.

PMID: 2416488 [PubMed - indexed for MEDLINE]

2369. Rhinology. 1985 Sep;23(3):187-90.

Mast cells on the surface of the mucous membrane--a general feature of inflammatory reactions in the nose?

Melén I, Pipkorn S, Pipkorn U.

A redistribution of mast cells towards the epithelial lining of the nasal mucous membrane has been shown to be a part of the allergic inflammatory reaction in hay fever. This results in an increased number of metachromatically stained cells on the surface of the mucous membrane. The involvement of mast cells in other inflammatory reactions in the human nose is not clarified and this may partly be due to methodological difficulties. Utilizing a recently developed imprint technique, specimens were taken from patients with infectious rhinosinusitis in acute and chronic stages. The total number of mast cells on 2 cm2 of the imprint area were counted. Mast cells in extremely low numbers were found in 5 out of 26 patients. Our results indicate that mast cell migration is not present in patients with infectious inflammatory reactions of the nasal mucous membrane.

PMID: 4059803 [PubMed - indexed for MEDLINE]

2370. G Ital Dermatol Venereol. 1985 Sep-Oct;120(5):317-23.

[Leukocyte migration inhibition test in allergic eczema due to contact with nickel salts].

[Article in Italian]

Cacciuttolo R, Sertoli A, Giorgini S, Francalanci S, Cencioni R.

PMID: 3908296 [PubMed - indexed for MEDLINE]

2371. Contact Dermatitis. 1985 Sep;13(3):166-74.

Lymphocyte subsets and Langerhans cells in allergic and irritant patch test reactions: histometric studies.

Ferguson J, Gibbs JH, Beck JS.

This study has attempted to distinguish between allergic and irritant reactions to patch tests by semiquantitative histological methods. The extent of perivascular chronic inflammatory infiltrate at 72 h in irritant patch test reactions to sodium lauryl sulphate was shown to be small and very consistent, whereas in allergic reactions to nickel sulphate it was generally larger and more variable in size (p less than 0.02). The two major lymphocyte subsets (T4 and T8) were randomly intermixed in both types of reaction and formed the major component of both the perivascular and diffuse dermal infiltrate, without any evidence of selective migration. The T4:T8 ratios were similar in focal and diffuse infiltrates. The number of T6 dendritic (putative Langerhans) cells in the epidermis (per mm inner epidermal length) was usually greatly reduced in irritant reactions (5-16 mm-1, mean 10 mm-1) but remained within normal limits in allergic reactions (6-33 mm-1, mean 21 mm-1) (p less than 0.001). Comparable results were seen with other irritants (mercuric chloride and benzalkonium chloride) and other allergens (neomycin sulphate, ethylene diamine and potassium dichromate). In additional experiments, pairs of biopsies were taken from the reaction and from adjacent unaffected skin. The T6 cell density in the epidermis did not significantly differ between allergic reactions and control skin. By contrast, the irritant reactions had fewer T6 cells than the control skin (p less than 0.001).

PMID: 2932284 [PubMed - indexed for MEDLINE]

2372. Alergia. 1985 Jul-Sep;32(3):65-7.

[Allergic rhinitis and cellular immunity].

[Article in Spanish]

Lara MC, Montes y Montes J, Carro C, Torres S.

PMID: 4073405 [PubMed - indexed for MEDLINE]

2373. J Clin Lab Immunol. 1985 May;17(1):21-4.

Polymorphonuclear dysfunction in bronchopulmonary diseases in human adults.

Laharrague P, Corberand J, Fillola G, Leophonte P, Gleizes B, Fontanilles AM, Gyrard E.

Polymorphonuclear (PMN) functions were assessed in 55 patients with asthma or bronchial bacterial infection to evaluate the systemic phagocyte capability of patients with bronchopulmonary diseases. Random migration, nitroblue tetrazolium dye reduction, and Candida killing activity were markedly decreased in the 2 types of patients studied. PMN dysfunction was more pronounced in the most affected and heavily treated patients. Considering both the rare occurrence of congenital polymorphonuclear defects and the age of the patients studied we concluded that the PMN abnormalities observed were secondary to the onset of respiratory disease. This impairment of circulating phagocytes may contribute to the rise of a systemic susceptibility to infection able to aggravate the underlying bronchopulmonary disease.

PMID: 4045995 [PubMed - indexed for MEDLINE]

2374. Zh Mikrobiol Epidemiol Immunobiol. 1985 May;(5):83-6.

[Use of a Brucella protein-polysaccharide antigen for the allergen diagnosis of brucellosis].

[Article in Russian]

Dzhasybaeva TS, SUkhodoeva GS, Vershilova PA, Dranovskaia EA, Kasymova KhA.

The experimental and clinical study of the allergen diagnostic properties of new Brucella protein-polysaccharide antigen in comparison with brucellin has been made in the leukocyte migration inhibition test, and its working doses have been determined. The results thus obtained suggest that Brucella protein-polysaccharide antigen has considerable diagnostic advantages over commercial brucellin, which presents vast possibilities of using this antigen for the allergen diagnosis of brucellosis in the leukocyte migration inhibition test.

PMID: 3929503 [PubMed - indexed for MEDLINE]

2375. Gac Med Mex. 1985 May-Jun;121(5-6):195-8.

[Tobacco addiction and the immune system: II. Production of the cellular migration inhibitory factor in the presence of tobacco extract as an antigen, in patients with extrinsic bronchial asthma].

[Article in Spanish]

Bernal-Madrazo MA, Casales-Ortiz G, Ham-Carrillo MS.

PMID: 3899838 [PubMed - indexed for MEDLINE]

2376. Allergol Immunopathol (Madr). 1985 May-Jun;13(3):187-92.

[Behavior of leukocyte chemotaxis in various clinico-immunological situations].

[Article in Spanish]

Suárez Saro JM, Laguna Martínez R, Callol Sánchez L, Caro de Miguel C, Gómez de Terrenos Sánchez FJ.

Chemotaxis is a property common to all free cells or unicellular microorganisms. It is not a simple spontaneous cellular migration but one which is directed towards the source or nucleus, producer of the chemotactic substance. One of the first phenomenon which is established as a defense mechanism of the organism is the attraction of polymorphonuclears. In 1955 Rebuck and Crowley described a method, "skin window" for the study of in vivo leukocyte chemotaxis. The aim of this work was to go deeper into the study of this test and to establish its clinical use. Two hundred and seventy patients from both sexes were studied and divided into five groups: Group I - 60 healthy subjects as control. Group II - 60 patients with pathologic leukocyte response: 10 cirrhotics, 15 Hodgkin's disease, 15 chronic renal insufficiency, 2 drepanocytosis and 3 sarcoidosis. Group III - 60 patients with no theoretical alterations in the leukocyte chemotaxis: 22 bronchial asthma, 23 nonlymphoid neoplasm, 13 iritis and 2 histiocytosis X. Group IV - 40 active tuberculosis patients. Group V - 30 patients with bacterial pneumonia non-tuberculosis. The Rebuck test was carried out on all patients. As lymphocyte markers, E rosettes, superficial immunoglobulins and the lymphoblast transformation test against PHA were performed on all the groups of patients. As to the results obtained, the positive responses for Groups I, II, III, IV and V were 87%, 28%, 83.3%, 45% and 63.3%, respectively. These results were evaluated in relation to the Mantoux reaction. The modified Rebuck test is useful for

leukocyte chemotactic study. This was found to be altered in 13% of the healthy population.(ABSTRACT TRUNCATED AT 250 WORDS)

PMID: 3898789 [PubMed - indexed for MEDLINE]

2377. Mater Med Pol. 1985 Apr-Jun;17(2):107-10.

Inhibition of leucocyte migration by the Clausen agarose method (LMAT) in chromium eczema.

Wasik F, Miklaszewska M, Blizanowska A, Sierawska M.

PMID: 4079467 [PubMed - indexed for MEDLINE]

2378. J Clin Microbiol. 1985 Apr;21(4):649-51.

Evaluation of cellular immune response during chronic schistosomiasis in humans by the leukocyte aggregation test and the leukocyte migration inhibition test.

Rouveix B, Derouin F, Levacher M.

Cellular immune response was evaluated in 31 patients with chronic Schistosoma haematobium and Schistosoma mansoni infections and in 15 healthy normal persons by using S. mansoni soluble worm and egg antigens. Although the leukocyte migration inhibition test demonstrated false-positive reactions, the specificity of the leukocyte aggregation test was confirmed by the negativity of all of the controls. Among the patients, only 10% were positive for the leukocyte aggregation test. This low cellular reactivity was in contrast to markedly elevated specific humoral response determined by an enzyme-linked immunosorbent assay for immunoglobulin G and paper allergosorbent test for immunoglobulin E with soluble worm antigen. These results confirm that the cellular immune reactivity to schistosome antigen as demonstrated by the leukocyte aggregation test is either minimal or absent in chronically infected patients.

PMCID: PMC271742
PMID: 3988906 [PubMed - indexed for MEDLINE]

2379. Z Gesamte Inn Med. 1985 Mar 15;40(6):165-72.

[Biochemistry and significance of prostaglandins, prostacyclins, thromboxanes and leukotrienes and the modification of their biosynthesis by essential fatty acids].

[Article in German]

Kolb E.

A survey is given of the biochemistry and importance of the prostaglandins, the thromboxanes, the prostacyclins and the leukotrienes. The formation of these compounds takes place from poly-unsaturated fatty acids and is regulated: here the phospholipase A2 plays a role. Among others the prostaglandins take part in the regulation of the blood supply of various tissues (heart, kidneys), in the regulation of the reproduction and in the evokation of labour. The thromboxanes and prostacyclins influence the aggregation and the desaggregation of the thrombocytes, respectively. The leukotrienes take part in the regulation of the permeability of the capillaries, in the migration of the leucocytes, the formation of inflammatory processes and in the evokation of asthma bronchiale.

The insufficient intake leads to functional disturbances in various tissues, which partly are to be traced back to a decrease of the formation of the tissue hormones mentioned. An intake transgressing the demand has a favourable effect in various diseases: thus the inclination to aggregation of the thrombocytes is decreased and the development of arteriosclerotic changes is inhibited.

PMID: 2988221 [PubMed - indexed for MEDLINE]

2380. Clin Allergy. 1985 Mar;15(2):117-30.

Asthma and urticaria induced by seminal plasma in a woman with IgE antibody and T-lymphocyte responsiveness to a seminal plasma antigen.

Blair H, Parish WE.

A patient with asthma urticaria and angioedema induced by allergy to seminal plasma was examined at intervals for 10 years. Before treatment her anaphylactic susceptibility to seminal plasma was manifested by very strong prick-test responses, IgE antibody to an allergenic fraction of seminal plasma determined by RAST, and by antigen-induced histamine release from her blood leucocytes. The skin test and in vitro lymphocyte tests indicated concomitant delayed hypersensitivity to the same allergen. The patient's lymphocytes treated with seminal plasma allergen fraction showed much increased incorporation of thymidine, and also synthesis of a product (NIF) that inhibited migration of neutrophils from a normal donor. The allergen fraction of seminal plasma had about five components in the range of 20 000-40 000 daltons molecular weight; the major fraction binding IgE appeared to be a glycoprotein. The patient was successfully desensitized by injections of her husband's seminal plasma. Desensitization was not associated with persistent amounts of antigen-specific IgG antibodies.

PMID: 2581721 [PubMed - indexed for MEDLINE]

2381. Ter Arkh. 1985;57(11):113-6.

[Clinico-immunological characteristics of bronchial asthma with opisthorchiasis].

[Article in Russian]

Volkov VT.

PMID: 4082008 [PubMed - indexed for MEDLINE]

2382. Dermatologica. 1985;171(5):366-7.

Larva currens following systemic steroid therapy in a case of strongyloidiasis.

Orecchia G, Pazzaglia A, Scaglia M, Rabbiosi G.

The authors report a case of larva currens following systemic steroid administration for acute contact eczema. The patient was found affected with subclinical strongyloidiasis. Strongyloides is not very common in Northern Italy; it is occasionally diagnosed from stool samples from patients complaining of persisting itching.

PMID: 4076506 [PubMed - indexed for MEDLINE]

2383. Int Arch Allergy Appl Immunol. 1985;76(1):52-7.

Relation between components of the immune response in rats sensitized by aerosol and subcutaneous injections.

Ahlstedt S, Björkstén B, Holgersson M, Nygren H, Smedegård G.

Relations between the appearance of various components of the immune response were analyzed in two groups of rats sensitized by aerosol and subcutaneous injections in the neck region, respectively. The relations were expressed by Spearman rank correlation coefficient and studied by cluster analysis. In the aerosol-sensitized animals, there was a close association between IgA and IgG antibody levels in bronchial fluid and these in turn were related to IgG levels in serum and more loosely to IgE levels in bronchial fluid. There was an apparent association between IgE antibody formation and mast cell maturation in cultures of regional lymph node cells and the appearance of mucous cells in the lungs. These variables seemed associated with spontaneous cell proliferation in vitro and the numbers of mast cells in the lungs. This indicates that local stimulation with antigen induces local immune responses and immune-mediated migration of cells. In subcutaneously sensitized animals, formation of IgG antibodies in vitro seemed related to the stimulated proliferation of regional lymph node cells. The levels of IgG and IgE antibodies in bronchial fluid and in serum also appeared to be related. Unlike the findings in aerosol-sensitized animals there was no apparent relation between the differentiation of mast cells and mucous cells. This was possibly due to lack of immune-mediated antigen-induced cell migration. The different immune response patterns in aerosol and subcutaneously sensitized rats should be considered when studies are designed aiming to explore the pathogenesis of allergic inflammatory diseases. The findings also indicate that the various parameters of immunity are more closely related in aerosol than in subcutaneously immunized animals.

PMID: 3967932 [PubMed - indexed for MEDLINE]

2384. Int Arch Allergy Appl Immunol. 1985;78(2):139-44.

Characterization of allergens and patient sera by a nitrocellulose immunoprint technique.

Bengtsson A, Rolfsen W, Einarsson R.

A rapid and convenient protein-gel blot technique for qualitative detection of antigens/allergens in pollen allergen extracts and IgE/IgG antibodies in patient sera has been developed. The antigens were separated by isoelectric focusing in agarose gel and transferred to nitrocellulose by capillary migration. After incubation of the nitrocellulose strips with serum from allergic patients, the binding of the patient's specific IgE or IgG antibodies was analyzed by using isotope-labelled or enzyme-labelled anti-IgE or anti-IgG. The time needed for detection with isotope-labelled antibody was approximately 20 h and with enzyme-labelled antibody 2 h. The immunoprint technique is easy to use, which renders it suitable for routine use in allergy research and quality control.

PMID: 3899946 [PubMed - indexed for MEDLINE]

2385. Int Arch Allergy Appl Immunol. 1985;77(4):384-9.

Effects of a single oral dose of dinitrochlorobenzene on T lymphocyte distribution and migration in the gut.

Jeurissen SH, Schmidt ED, Sminia T, Kraal G.

In the present study the effects of a large oral dose of the contact allergen dinitrochlorobenzene (DNCB) on the distribution of T-cell subsets in the small intestines and the emigration pattern of lymphocytes from Peyer's patches were investigated. Apart from the inflammatory effects, DNCB administration resulted in an influx on the T-helper cells in the villi. Precise quantification of T lymphocytes showed a decrease in Peyer's patches and an increase in mesenteric lymph node cell suspensions. However, no differences could be found in the emigration rate of T-cell subsets from Peyer's patches into mesenteric lymph nodes, suggesting that the increase of T cells in mesenteric lymph nodes results from T-helper cells directly immigrating from the villi.

PMID: 3160666 [PubMed - indexed for MEDLINE]

2386. Jpn J Pharmacol. 1985 Jan;37(1):83-90.

Pharmacological studies of N-(2-mercapto-2-methylpropanoyl)-L-cysteine (SA96) (3). Effects of SA96 on experimental allergic reactions.

Nakata K, Suda H, Yamauchi H, Iso T.

The effects of an antirheumatic agent, N-(2-mercapto-2-methylpropanoyl)-L-cysteine (SA96), were investigated on allergic reactions in rats and guinea pigs. The effects of SA96 were compared with those of D-penicillamine (D-Pc). SA96 given twice orally at the doses of 10 to 50 mg/kg significantly caused inhibitions of 28%, 29% and 44% against passive cutaneous anaphylaxis (PCA), reversed cutaneous anaphylaxis (RCA) and reversed passive Arthus (RPA) reactions, which are classified as Type I, Type II and Type III allergic reactions, respectively. D-Pc also showed inhibitions of 30%, 23% and 18% on Type I, Type II and Type III reactions, respectively, and inhibitions on Type II and Type III reactions were not significant. On the other hand, SA96 (10 to 50 mg/kg twice) had no influence on the Type IV allergic reaction, delayed hypersensitivity, while D-Pc (20 mg/kg twice) showed an enhancement of 27% on the Type IV reaction. In the in vitro study, SA96 inhibited the hemolytic complement activity at 10(-4) to 10(-2) M and the macrophage migration at 1 X 10(-4) to 5 X 10(-3) M in a dose-dependent manner. These in vitro activities of SA96 were more potent than those of D-Pc. These results showed that SA96 had some different immunopharmacological properties on experimental allergic reactions as compared with those of D-Pc.

PMID: 3157817 [PubMed - indexed for MEDLINE]

2387. J Immunol. 1984 Dec;133(6):3037-42.

Redistribution of Lyt-bearing T cells in acute murine experimental allergic encephalomyelitis: selective migration of Lyt-1 cells to the central nervous system is associated with a transient depletion of Lyt-1 cells in peripheral blood.

Hauser SL, Bahn AK, Che M, Gilles F, Weiner HL.

Experimental allergic encephalomyelitis (EAE) was induced in SJL/J mice by using two injections of spinal cord homogenate in incomplete Freund's adjuvant supplemented with mycobacteria. Analysis of circulating Lyt-bearing subsets by indirect immunofluorescence during the course of acute EAE revealed the following: 1) during the pre-clinical phase of EAE (1 to 2 days before the onset

of paralysis), there was a decrease in the percentage of Lyt-1- but not of Lyt-2-bearing cells in peripheral blood, and of both Lyt-1- and Lyt-2-bearing cells in spleen; 2) with the onset of clinically evident EAE, there was a decrease in both Lyt-1 and Lyt-2 cells in peripheral blood and an increase in the percentage of Lyt-1-bearing cells in pooled inguinal and axillary lymph node; and 3) after these early changes, there was a rapid reconstitution of the percentages of total Lyt-bearing cells and of both Lyt-1- and Lyt-2-bearing cells in peripheral blood. Immunohistochemical analysis of the central nervous system infiltrate revealed that the earliest lesions consisted predominantly of Lyt-1 T lymphocytes, with few Lyt-2 cells present. These results demonstrate that the influx of cells of the Lyt-1 inducer subset to the central nervous system in acute EAE is accompanied by a transient decrease in Lyt-1 cells in peripheral blood.

PMID: 6333452 [PubMed - indexed for MEDLINE]

2388. J Exp Med. 1984 Nov 1;160(5):1532-43.

Inhibition of autoimmune neuropathological process by treatment with an iron-chelating agent.

Bowern N, Ramshaw IA, Clark IA, Doherty PC.

Lewis rats that are primed with guinea pig spinal cord homogenized in complete Freund's adjuvant (GPSCH-CFA) develop overt symptoms of experimental allergic encephalomyelitis (EAE). Treatment with the iron-chelating agent, desferrioxamine B mesylate (DFOM), at various times before the onset of EAE, dramatically suppressed both the severity and duration of disease. When DFOM was administered to rats soon after the development of neurological signs, a rapid recovery occurred, though mild, transient symptoms could be seen approximately 1 wk after withdrawal of the drug. Treatment with DFOM was always accompanied by a diminution of T cell responsiveness on the part of the delayed-type hypersensitivity/helper subset and, on histological examination, an absence of inflammatory cells from lesions. Iron is believed to influence both the migration and function of immune effector cells. It can also act as a catalyst in the formation of free radicals, which are highly toxic agents causing tissue damage in sites of inflammation. The mechanisms underlying the effect of DFOM on the severity of EAE, and the possible implications for treatment of multiple sclerosis are discussed.

PMCID: PMC2187509
PMID: 6333485 [PubMed - indexed for MEDLINE]

2389. Allergol Immunopathol (Madr). 1984 Nov-Dec;12(6):431-8.

[Induction of immunologic tolerance to transplantation antigens using conditioning of the lymphocyte genome of the recipient with ribonucleic acid from the donor].

[Article in Spanish]

Suárez-Chacón R, Ortega Suárez M.

The phenomenon of specific immunosuppression which we call "immunologic tolerance" constitutes the final result common to diverse processes partially known. The study of the phenomena inter or intra cellular involved in the divese mechanisms of production, however constitutes a field which is quite obscure. The explanation of these phenomena could offer us some fundamental knowledge towards

the better understanding and subsequent manipulation of the process. This present project of investigation proposes to study the possibility of transferring one of these states of tolerance which is better established as is the spontaneous tolerance to the proper antigens of an animal (donor) to a second animal (receptor) genetic and for that reason, antigenically different, through an informational molecule such as the ribonucleo acid (RNA) extracted from the donor animal. This hypothesis is based theoretically on the following points: 1) The state of "self-immunologic tolerance" is probably an active and dynamic phenomenon, in which one can determine the presence of lymphocytes with receptors for proper antigens, without establishing in normal conditions an efferent phase of the immunologic answer, which determines an effect on this antigen. 2) The effective immunologic reactivity (allergic answer) of a determined animal is transferable to a second non-sensitized animal through RNA from the first animal. In this study we present the results of the conditioning of the allogenic reactivity of one strain of mice, through the exposition of the same to RNA extracted from one congenic-resistent strain with respect to first.(ABSTRACT TRUNCATED AT 250 WORDS)

PMID: 6241425 [PubMed - indexed for MEDLINE]

2390. Br Dent J. 1984 Oct 20;157(8):272-5.

Acrylic 'allergy'?

Devlin H, Watts DC.

PMID: 6388609 [PubMed - indexed for MEDLINE]

2391. Med Hypotheses. 1984 Oct;15(2):103-8.

A new approach to contact allergenicity screening.

Bos JD.

A new approach to contact allergenicity screening is proposed. As a first step, an immunogen-Langerhans cell (IC) binding assay (a) is suggested. LC's are isolated from trypsinized epidermis by fluorescence activated cell sorting using monoclonal antibody anti-T6. The chemical or drug under study (pre-incubated in homogenized skin as it may be allergenic only after binding to protein or upon metabolic interaction) is tested for LC binding by a radiometric assay. When binding is not established, the drug is probably inert. When binding occurs its possible effect on LC activity is studied by a LC activation/migration assay (b). When negative, this assay indicates passive binding and the drug or chemical may be expected to be inert. When migration does occur, the activated LC's may be co-cultured with autologous lymphocytes in a lymphocyte blastogenicity assay (c). A positive result in this assay will indicate the drug or chemical's allergenicity. The proposed three-step procedure for contact allergenicity screening is expected to have a high profitability.

PMID: 6513827 [PubMed - indexed for MEDLINE]

2392. Agents Actions. 1984 Oct;15(3-4):264-6.

5-Hydroxytryptamine releasing activity in culture supernatants of guinea pig lymphocytes.

Hagen M, Paegelow I, Karnstedt U.

5-Hydroxytryptamine (5-HT) is not only involved in anaphylactic reactions but also in delayed-type hypersensitivity (DTH) reactions. Supernatants of cultivated guinea pig spleen lymphocytes were investigated for possessing 5-HT releasing activity. Both fractionated supernatants of mixed lymphocytes unspecifically stimulated by Concanavalin A (Con A) and fractionated supernatants of non-stimulated mixed lymphocytes decrease the 5-HT content of rabbit thrombocytes. Fractions of a molecular weight smaller than 12,500 daltons are more effective than fractions of a higher molecular weight range. The 5-HT releasing activity of the supernatants is discussed in comparison to a cell migration stimulating activity.

PMID: 6240929 [PubMed - indexed for MEDLINE]

2393. Gastroenterol Clin Biol. 1984 Aug-Sep;8(8-9):604-8.

[Duodenal complications of rheumatoid purpura. Endoscopic aspects].

[Article in French]

Chapoy P, Guidon MJ, Louchet E.

The aim of this work was to describe the endoscopic features and clinical outcome of the duodenal complications in anaphylactoid purpura. Over a 3-year period, 20 patients were hospitalized in our unit because of purpura rheumatica. Duodenal complications occurred in 5 cases warranting endoscopic assessment. All patients had bilious vomiting and epigastric pain, constantly associated with low-grade purpuric rash. Plasma factor XIII concentrations were always decreased. The duodenal complication was suspected radiologically in 2 cases when "thumbprint" impressions were seen. Petechiae, oedema and intramural hematoma with superficial erosions were present endoscopically in 3 cases. The lesions were severe and extensive, involving the entire duodenum in 3 cases and the jejunum in one case. In one patient, there was a stricture of the upper part of the second duodenum. Treatment consisted of parenteral nutrition (using a central catheter: 3 cases, or a peripheral vein: 2 cases) and cimetidine (30 mg/kg.bw). The clinical outcome was favorable in 4 patients; the symptoms vanished and the endoscopic lesions were reversible (including the stricture) with restitutio ad integrum after 10 days. The last patient died the 8th day of treatment, 3 days after digestive improvement; the cause of death was probably iatrogenic and related to accidental migration of the central catheter. These results suggest that endoscopic examination should be performed in all patients with anaphylactoid purpura presenting with bilious vomiting. Endoscopy seems to be of great value in deciding if parenteral nutrition is indicated--or not--and perhaps in order to contraindicate the use of steroid therapy in the case of ulcerated hematomas.

PMID: 6436133 [PubMed - indexed for MEDLINE]

2394. Pneumonol Pol. 1984 Aug;52(8):381-4.

[In vivo and in vitro migration of peripheral leukocytes of patients with atopic asthma].

[Article in Polish]

Matusiewicz R, Waśniewski J.

PMID: 6393072 [PubMed - indexed for MEDLINE]

2395. J Neuroimmunol. 1984 Jul;6(4):265-81.

IgM and IgG responses during chronic relapsing experimental allergic encephalomyelitis (r-EAE).

Olsson T, Henriksson A, Link H, Kristensson K.

During chronic relapsing experimental allergic encephalomyelitis (r-EAE) in guinea pigs, serum IgM and IgG concentrations increased markedly early in disease. Serum IgM and IgG increased similarly in control animals immunized with Freund's incomplete adjuvant (FIA) and Mycobacterium tuberculosis (MT). In the chronic phase of r-EAE but not in control animals, elevated IgM was also found in central nervous system (CNS) extracts, suggesting intrathecal IgM synthesis. IgG antibodies against myelin and myelin basic protein (MBP) were regularly detected in r-EAE sera from day 21 post inoculation (p.i.), reaching maximum levels in the early chronic phase. IgG antibodies against galactocerebroside (GC) and galactose appeared in some r-EAE sera. Oligoclonal IgG bands were demonstrated in all r-EAE guinea pig sera 21-26 days p.i. The bands in serum decreased in number and strength in the chronic phase. They could be traced to antibodies against MT in 4 of 10 animals, but not to antibodies against myelin, MBP, GC or galactose. Oligoclonal IgG bands were also regularly visualized in r-EAE CNS 124 days p.i., suggesting persistent intrathecal IgG synthesis. They varied in number and migration between different regions of individual CNS. Oligoclonal CNS IgG was related to antibodies against MT in only one of 7 animals, and in no case to antibodies against myelin.

PMID: 6203934 [PubMed - indexed for MEDLINE]

2396. J Immunol. 1984 May;132(5):2402-7.

The immunopathology of experimental allergic encephalomyelitis. II. Endothelial cell Ia increases prior to inflammatory cell infiltration.

Sobel RA, Blanchette BW, Bhan AK, Colvin RB.

Experimental allergic encephalomyelitis (EAE) is a T cell-mediated neuroimmunologic disease model characterized by meningeal and parenchymal mononuclear cell infiltrates (see preceding companion paper). Here we report enhanced staining for Ia in the central nervous system (CNS) microvasculature endothelium in acute EAE in adult strain 13 guinea pigs (GP) sensitized with GP spinal cord homogenate (SC) or with GP myelin basic protein (MBP) in complete Freund's adjuvant (CFA). Cryostat sections of CNS and other tissues were stained with two monoclonal antibodies, 5S2 and 22C4, to GP Ia determinants, and with polyclonal antibody to factor VIII-related antigen (VIII-RA) as an endothelial cell marker. Morphometric techniques were employed on immunoperoxidase counterstained and coded sections to determine the frequency of Ia+ vessels and cells. Rare (approximately 10% of VIII-RA+) vascular endothelial cells were Ia+ in the CNS of normal and CFA-sensitized controls. SC- or MBP-sensitized strain 13 GP sacrificed on day 7, before the onset of neurologic signs (pre-clinical), had no detectable CNS mononuclear cell infiltrates, but had increased (approximately 30% of VIII-RA+) endothelial cell Ia staining over controls (p less than 0.001). The endothelial Ia staining persisted (approximately 35% of VIII-RA+) in vessels as the animals developed paralysis. There were no differences in endothelial cell Ia between SC- and MBP-induced disease. EAE-resistant strain 2 GP sensitized with SC/CFA had no neurologic signs, and had fewer inflammatory foci than strain 13 GP with EAE, but had similar numbers of Ia+ endothelial cells. No differences in endothelial cell Ia staining were found in non-CNS tissues among any GP groups. In EAE, increased endothelial cell Ia is a pre-inflammatory, target

organ-specific alteration that persists during inflammation. The findings suggest that in vivo modulation of endothelial cell Ia may be part of the local immune response. Endothelial cells may play a significant role, in antigen presentation or in promoting T cell migration, in the in situ immune response in the CNS.

PMID: 6425402 [PubMed - indexed for MEDLINE]

2397. J Immunol. 1984 May;132(5):2393-401.

The immunopathology of experimental allergic encephalomyelitis. I. Quantitative analysis of inflammatory cells in situ.

Sobel RA, Blanchette BW, Bhan AK, Colvin RB.

Acute experimental allergic encephalomyelitis (EAE) is a T cell-mediated, neurologic disease that is under immunogenetic control. We systematically analyzed the quantity and distribution of T cells, B cells, and macrophages in the central nervous system (CNS) of susceptible and resistant guinea (GP) with a panel of seven monoclonal antibodies by using the avidin-biotin complex (ABC) immunoperoxidase technique and alpha-naphthyl-butyrate esterase (ANBE) staining. Adult EAE-susceptible strain 13 GP immunized with isogeneic spinal cord homogenate (SC) or with myelin basic protein (MBP) developed clinical signs (paralysis, weight loss, etc.) in 2 to 3 wk. T cells were present in all CNS inflammatory foci and comprised 44% of the perivascular mononuclear cells. T cells diffusely infiltrated the neuropil away from inflammatory cell aggregates. These T cells were judged to be extravascular by the lack of an associated identifiable vessel in counter-stained sections, and by their persistence following exhaustive perfusion of the brains. In routine sections, mononuclear cells could be detected only in perivascular aggregates. IgM+ B cells comprised 9% of the perivascular infiltrates and did not diffusely infiltrate the parenchyma. ANBE+ macrophages comprised the remaining 47% of the identified perivascular cells. SC- and MBP-immunized GP showed equivalent numbers of inflammatory foci, T cells, and macrophages, but SC-immunized GP had more IgM+ cells in the meninges and choroid plexus (p less than 0.001, p less than 0.02, respectively). Virtually all cells in perivascular locations were Ia+. Ia+ mononuclear cells were also present in the neuropil. EAE-resistant strain 2 GP immunized with SC developed no clinical signs. These GP had fewer perivascular foci than strain 13 GP but, when present, the cellular composition, including the density of diffuse parenchymal T cell infiltrates, was indistinguishable. Significantly fewer parenchymal mononuclear cells in the strain 2 GP, however, displayed Ia, both in perivascular and diffuse infiltrates (p less than 0.001). We conclude that T cell migration into the CNS parenchyma is a characteristic feature of acute EAE in the GP, but that T cells can occur in this pattern without clinical signs of disease. The two features that distinguish susceptible and resistant strains were the frequency of perivascular infiltrates and the expression of Ia on parenchymal mononuclear cells, which probably reflects their enhanced immunologic activation in situ.

PMID: 6371137 [PubMed - indexed for MEDLINE]

2398. Agents Actions. 1984 Apr;14(3-4):484-7.

Properties of a high molecular weight neutrophil chemotactic factor, possibly derived from mast cells: evidence for chemokinetic rather than chemotactic activity.

Cundell DR, Moodley I, Davies RJ.

Kay and his colleagues [1] have suggested that the neutrophil high molecular weight chemotactic factor (NCF) found in the serum of patients suffering from a variety of allergic diseases is mainly derived from mast cells and is therefore an indicator of mast cell activation. We have studied some of the properties of NCF obtained from patients with atopic extrinsic asthma and compared it with N-formyl-1-methionyl-1-leucyl-1-phenyl-alanine (FMLP), a chemotactic peptide [2]. A number of differences between FMLP and NCF were observed. In contrast to FMLP, checkerboard analysis showed that NCF caused random migration of neutrophils. In addition microscopic analysis of neutrophil locomotion in response to FMLP demonstrated the characteristic pseudopod formation. Furthermore, it was found that in contrast to FMLP, NCF did not cause the release of lysosomal enzymes from cytochalasin B-treated neutrophils. These results suggest that NCF has chemokinetic rather than chemotactic properties.

PMID: 6375306 [PubMed - indexed for MEDLINE]

2399. Ann Allergy. 1984 Apr;52(4):300-2.

Concurrent cell-mediated immunity and immediate hypersensitivity in animal model.

Wolf R, Livni E, Joshua H.

Drug induced allergic reactions in treated patients are sometimes accompanied by laboratory evidence of a simultaneous drug-specific humoral and cellular immunity. The relationship between the two types of immune response was studied in an experimental model. In the present studies ICR mice were sensitized with horse serum to induce an anaphylactic shock. Macrophage migration inhibition factor (MIF) test, which is an in vitro correlate for cell-mediated immunity (CMI), was performed with the animal's lymphocytes against horse serum. A significant inhibition in the macrophage migration was observed in the sensitized mice as compared to the control group. No significant difference was observed between the migration index of the mice that suffered fatal anaphylactic shock and those with milder symptoms that survived it. The demonstration of a positive MIF in an immediate type allergic reaction does not necessarily indicate a direct involvement of the CMI system in the production of clinical manifestations, although the MIF test may be clinically useful for diagnostic purposes, even in immediate hypersensitivity cases.

PMID: 6370046 [PubMed - indexed for MEDLINE]

2400. Allergy. 1984 Apr;39(3):195-201.

Human leukocyte cyclic AMP and cyclic GMP levels during chemotaxis in delayed type hypersensitivity.

Lerche A, Bisgaard H, Christensen JD, Søndergaard J.

Ten nickel-allergic patients and six healthy control subjects participated in a study of the morphology, kinetics and evolution of the cAMP and cGMP concentrations of migrated leukocytes, using an improved skin chamber technique. Also studied was the effect of nickel exposure in the chamber medium during development of an eczematous reaction in the nickel-allergic patients. Nickel exposure had a specific effect on the morphology, from the 24th hour to the end of the 48 h observation period, with a significant increase in the percentages of basophils, eosinophils and lymphocytes and a decrease of neutrophils. A significantly increased leukocyte migration rate (LMR) was observed from the 27th to 39th hour in six of the allergic patients exposed to nickel. There were no specific permanent changes in cAMP and cGMP concentrations during nickel

exposure. The control chambers of the allergic patients and healthy controls had identical leukocyte morphology, LMR and leukocyte concentrations of cAMP and cGMP. However, no correlations were found between LMR, cAMP and cGMP in the eczema patients throughout the observation period.

PMID: 6324609 [PubMed - indexed for MEDLINE]

2401. Allergol Immunopathol (Madr). 1984 Mar-Apr;12(2):129-34.

The mechanisms of bronchospasm in experimental microbial sensitization. I. Immunological stage.

Beklemishev ND, Belyaev NN, Sukhodoeva GS, Bulvakhter YL.

Immune mechanisms of smooth muscle spastic bronchial reactions were reproduced and studied in isolated guinea pig lungs and found to develop type I, III and IV allergic reactions at sensitization to brucella and staphylococcus. Type I reactions were reproduced in animal lungs with immediate hypersensitivity to soluble microbial antigens. Type III reactions were obtained by transfusion of soluble immune complexes containing microbial antigens into isolated lungs Bronchospastic reactions arose only in the presence of the complement. Antibodies responsible for bronchospasm belonged to the IgG class. Type IV reactions were produced in vitro using supernatants obtained by incubation of lymphoid cells isolated from animals with delayed hypersensitivity with homologic microbial antigens. The supernatants possessed bronchospastic action.

PMID: 6464938 [PubMed - indexed for MEDLINE]

2402. Pneumonol Pol. 1984 Mar;52(3):123-30.

[Leukocyte migration inhibition test in patients with pollen-induced asthma and the results of hyposensitization therapy with Pollinex].

[Article in Polish]

Grzegorczyk J, Rozniecki J, Kałła M.

PMID: 6427758 [PubMed - indexed for MEDLINE]

2403. Int Arch Allergy Appl Immunol. 1984;74(3):211-5.

Granulocyte migration abnormality in patients suffering from seasonal allergic rhinitis: failure of treatment with cimetidine.

Maderazo EG, Albano SD.

Granulocyte locomotory responses in 5 patients with symptomatic seasonal allergic rhinitis were lower compared with similar responses from 27 normal nonallergic controls. In a subsequent controlled, double-blind crossover study, neither cimetidine (histamine H2-receptor blocker) nor placebo improved these responses. In our in vitro study, histamine did not inhibit granulocyte responses to chemotactic attractant. These results indicate that defective granulocyte response in patients with seasonal allergic rhinitis may be due to factors other than or in addition to histamine.

PMID: 6724715 [PubMed - indexed for MEDLINE]

2404. Arch Immunol Ther Exp (Warsz). 1984;32(4):369-73.

Granulocyte migration in vivo in patients with atopic bronchial asthma during specific desensitization.

Matusiewicz R, Sliwiński J.

Examination was made of 40 patients suffering from atopic bronchial asthma during specific desensitization using Rebuck's and Southam's methods for testing phagocyte migration in vivo. It was found that specific allergens during specific desensitization caused an increase in phagocyte migration to the skin test site.

PMID: 6534313 [PubMed - indexed for MEDLINE]

2405. Acta Haematol. 1984;72(4):264-73.

Cytogenetic and functional studies of leukocytes with Pelger-Huët anomaly.

Matsumoto T, Harada Y, Yamaguchi K, Matsuzaki H, Sanada I, Yoshimura T, Honda M, Tanaka R.

A cytogenetic study was undertaken in 15 cases of Pelger-Huët (P-H) anomaly in 3 families. An enlarged short arm of chromosome 22 (22p+) was found in 14 cases, but in these families 4 cases without P-H anomaly did not show 22p+ in the karyotype. In P-H anomaly, delayed skin hypersensitivity reactions, levels of serum IgG, IgM and IgA, lymphocyte subpopulations, and natural killer and antibody-dependent, cell-mediated cytotoxicity activities were within normal range. The level of serum IgE, mitogen responses in peripheral blood lymphocyte and plaque-forming cell counts were also within normal range with the exception of a case with atopic eczema. Enzymatic activities, nitroblue tetrazolium reduction and phagocytic capacities of neutrophils appeared normal. Abnormalities of neutrophils in cases of P-H anomaly, as compared with normal subjects, were also negative in examinations for chemotaxis and spontaneous migration under agarose and in a membrane filter.

PMID: 6438994 [PubMed - indexed for MEDLINE]

2406. Bull World Health Organ. 1984;62(1):7-25.

The mucosal immune system in health and disease, with an emphasis on parasitic infection.

Allardyce RA, Bienenstock J.

This article briefly describes the network of immunity involving selected humoral and cellular elements shared between mucosal surfaces that are both exposed to and remote from antigen challenge. The mechanisms promoting the production, concentration, and secretion of specific antibody isotypes, as well as the migration and localization of various lymphoid cell populations, have been discussed with regard to host mucosal protection against pathogenic agents and other potentially harmful macromolecules.Although certain aspects of the mucosal immune system may be viewed as separate from the systemic immune system, they are not exclusively so. We have drawn attention to their interactions with systemic immune reactants and other, nonimmunological, cellular and humoral constituents of mucosal surfaces and tissues such as the liver. At another level of interaction we have considered the teleological translation of host defence and immunoregulation from one generation to the next through the medium of colostrum

and breast milk.The manipulation of the mucosal immune system in order to enhance host resistance, modulate autoimmune and allergic systemic reactivity, or even modify fertility holds great promise. Achievement of these goals depends on gaining further insight into the mechanisms that contribute to mucosal immunity and their interactions with the systemic immune system. Much of our current knowledge is based upon experimental animal models or human populations living in relative prosperity. However, the results of oral vaccination, for example, are known to differ considerably in populations that suffer from parasitic infestations, lack adequate nutrition, and are very old or very young. We have chosen to focus attention on these groups because they constitute a large proportion of the world's population and because mucosal infections are a common cause of illness and death among them.Lastly, the recent discovery that immune deficiencies due to insufficient dietary zinc may extend to subsequent generations of optimally nourished offspring calls for a re-evaluation of immunization protocols in malnourished populations, and of our current understanding of disease inheritance and susceptibility.

PMCID: PMC2536281
PMID: 6424959 [PubMed - indexed for MEDLINE]

2407. J Pharmacol. 1984;15 Suppl 1:53-68.

[Pharmacology of the leukotrienes].

[Article in French]

Sirois P, Borgeat P.

Leukotrienes are a new family of metabolites of arachidonic acid produced by a C-5 lipoxygenase. As shown on figure 1, leukotriene A4, the key compound in their biosynthesis could either be hydrolysed to form leukotriene B4 or combine with glutathione to form leukotriene C4. Removal of glycine from the glutathione substituent or removal of glycine and gamma-glutamic acid yields the leukotriene D4 and E4 respectively. Leukotriene C4, D4 and E4 are the main bioactive components of the long elusive "Slow Reacting Substance of Anaphylaxis" (SRS-A). They induce powerful contractions of lung parenchyma strips and trachea in vitro (fig. 2) as well as powerful bronchoconstriction in vivo. They also favor mucus production from airways and slow its transport. Leukotrienes exhibit vasoconstrictor activity both on large blood vessels and on the microcirculation and induce marked increases in blood pressure followed by long lasting slight decreases (fig. 3). Injections of leukotriene B4 produce erythema, neutrophil migration and, in association with prostaglandin E2, increase vascular permeability and cause oedema. Leukotriene B4 is a powerful chemoattractant for polymorphonuclear leukocytes, stimulates cellular aggregation, degranulation and the release of lysosomal enzymes. It is also involved in the modulation of the immune response by inducing the formation of suppressor and of cytotoxic cells. The pharmacological actions of leukotriene B4 are mediated by very specific receptors while the actions of leukotriene C4, D4 and E4 are mediated by another type of receptors which are blocked by the selective SRS-A antagonist FPL-55712. The actions of leukotrienes depend partly upon the formation of prostaglandins and thromboxanes in the guinea-pig lungs while in man, their actions appear mostly a direct myotropic effect (fig. 4). Leukotriene formation has been stimulated in lungs and in various leukocyte populations by inflammatory or hypersensitivity reactions and by non specific stimuli such as the ionophore A-23187 and the C5a-anaphylatoxin. Inhibitors of lipoxygenases and corticosteroids could inhibit their release while non-steroid anti-inflammatory drugs such as aspirin may potentiate their formation by rechanneling their substrate from the cyclooxygenase cascade (fig. 5). The activity and potency of leukotrienes, their putative role in various important diseases as well as the

explanations which they may give to old problems are good reasons to justify our interest on these novel compounds.

PMID: 6328128 [PubMed - indexed for MEDLINE]

2408. Ann Dermatol Venereol. 1984;111(1):39-46.

[A syndrome of hyper-IgE and recurrent infections. Developmental variants? A familial study].

[Article in French]

Lorette G, Lebranchu Y, Legrand MF, Bardos P, Despert F, Grojean MF, Larrègue M.

The first patient suffered from a very severe atopic dermatitis with intense pruritus and thickened skin. He had also recurrent infections, particularly related to Staphylococcus coagulase +, and axillary and inguinal lymphodermopathy. The use of tetracosactide given intramuscularly allowed controlling the evolution of his atopic dermatitis. After several months of treatment, the skin became less infiltrated, lymphodermopathy disappeared and no severe infection had happened. The second patient had a less severe atopic dermatitis and recurrent infections without any particular severity. Topical corticosteroids allowed to control the atopic dermatitis. These two patients had high levels of circulating IgE and an important deficiency of polymorphonuclear chemotaxis which was evaluated by migration through boyden room. Study of the family showed atopic manifestations in several members, but with lower levels of IgE. The most characteristic abnormality of this syndrome is the according to considerable increase of IgE. The deficit in polymorphonuclear chemotaxis may vary according to time and even become normal. The prognosis over long periods remains to be determined.

PMID: 6233927 [PubMed - indexed for MEDLINE]

2409. J Neuroimmunol. 1983 Dec;5(3):271-81.

Chronic relapsing experimental allergic encephalomyelitis. The presence in the cerebrospinal fluid of factors chemotactic for monocytes.

Kirby JA, Suckling AJ, Rumsby MG.

Cerebrospinal fluid (CSF) was removed from guinea pigs with chronic relapsing experimental allergic encephalomyelitis (CR-EAE) and control inoculated animals by puncture of the cisterna magna. The fluid from 7/8 animals in relapse and 2/4 animals in remission phases of CR-EAE was found to promote the migration of peripheral blood monocytes through a 5-micron pore diameter polycarbonate membrane filter. Monocytes were also found to orient towards the migration-stimulating CSF in a gradient formed between such fluid and CSF derived from a control animal, thereby indicating the presence of a chemotactic factor. The factor responsible for promoting monocyte migration had a molecular weight of between 50 000 and 300 000 as defined by ultrafiltration. The results are discussed in relation to the known pathohistological features of the chronic relapsing disease.

PMID: 6655049 [PubMed - indexed for MEDLINE]

2410. Cesk Patol. 1983 Nov;19(4):193-8.

[Larval toxocariasis in a 40-year-old man with detection of larvae in a liver biopsy].

[Article in Czech]

Vortel V, Pavelka I, Uhliková M, Hübner J, Zezulka B.

A 40-year old cattle feeder has been suffering from indigestion, leukocytosis and conspicuous eosinophilia for about 4 years. A sample of liver tissue was taken during gastrectomy and multiple allergic granulomas rich in eosinophilia leukocytes were found. Toxocara canis was identified in serial sections outside the granulomas. Larval toxocariasis was confirmed by a high level of specific antibodies which failed to decrease even after administration of Mintezol antithelmintic. In this country, there was serological proof of 287 cases of larval toxocariasis till July 1982. This is the first finding of Toxocara larvae in liver biopsy published in Czechoslovak literature. Another finding of liver Toxocara larvae was made in a 86-year old woman included among 4 cases of allergic granulomatosis of the liver published before.

PMID: 6661784 [PubMed - indexed for MEDLINE]

2411. Pediatr Res. 1983 Oct;17(10):820-4.

Neutrophil and T lymphocyte characteristics of two patients with hyper-IgE syndrome.

Soderberg-Warner M, Rice-Mendoza CA, Mendoza GR, Stiehm ER.

Immunologic parameters including quantitative and qualitative immunoglobulin studies, various T cell functions and neutrophil chemotaxis were evaluated in two patients with the Hyper-IgE syndrome. Both exhibited pruritic dermatitis in locations atypical for atopic dermatitis, marked elevations in serum IgE levels (to 40,000 IU/ml), recurrent staphylococcal abscesses, coarse facial features and variable chemotactic defects characteristic of this syndrome. Both patients responded favorably to courses of trimethoprim-sulfamethoxazole, particularly in helping control the cutaneous infections. We believe that this is a useful therapeutic alternative to anti-staphylococcal antibiotics and prophylactic treatment has permitted therapeutic response. Serum IgG, IgG subclasses, IgM, and IgA were normal for age. Serum IgD was markedly deficient in one patient. Functional IgM was normal with positive isohemagglutinin titers. IgG poliovirus titers were present in both patients; however, tetanus titers were not detectable in either patient, despite repeated immunizations. Despite normal E rosette numbers, subtle T cell abnormalities were noted with variable responses to both in vivo SK-SD, candida, and mumps skin tests and in vitro PHA-, Con A-tetanus-induced lymphocyte proliferation. Lymphocyte production of macrophage inhibitory factor and interferon and responsiveness in a mixed lymphocyte culture were normal in both patients. Considerable Con-A-induced suppressor cell activity was present in one patient, but diminished in the other. In vivo chemotaxis determined by a Rebuck skin window, revealed a markedly delayed PMN migration in both patients during a time when both patients were clinically free of furunculosis or dermatitis.(ABSTRACT TRUNCATED AT 250 WORDS)

PMID: 6195589 [PubMed - indexed for MEDLINE]

2412. Przegl Dermatol. 1983 Sep-Dec;70(5-6):481-5.

[Atopic dermatitis. V. Monocyte chemotaxis].

[Article in Polish]

Silny W.

PMID: 6669718 [PubMed - indexed for MEDLINE]

2413. Przegl Dermatol. 1983 Sep-Dec;70(5-6):475-80.

[Atopic dermatitis. IV. Granulocyte chemotaxis].

[Article in Polish]

Silny W.

PMID: 6669717 [PubMed - indexed for MEDLINE]

2414. Allergol Immunopathol (Madr). 1983 Sep-Oct;11(5):339-45.

Modifying factors on phagocytosis of polymorphonuclear allergic subjects.

Levallois C, Caravano R.

Supernatants from pollen-activated cultures of mononuclear (MN) cells obtained from two subjects allergic to gramineae pollen were tested for their effect on phagocytic activity of human polymorphonuclear leucocytes (PMN). An enhancement of phagocytosis was observed. Similarly prepared supernatants from MN cells of non-allergic control subjects did not affect PMN phagocytic activity. On the basis of these findings it is suggested that in the presence of allergen, cultures of MN cells from pollen-sensitized subjects produce a substance that stimulates PMN phagocytic activity. The direct effect of pollen on PMN was also studied. The allergen caused an inhibition of phagocytosis by PMN from allergic subjects. Pollen had no effect on phagocytosis by PMN from non-allergic control subjects.

PMID: 6362379 [PubMed - indexed for MEDLINE]

2415. J Reprod Fertil. 1983 Sep;69(1):279-88.

Cell-mediated and humoral immune responses to aspermatogenic antigen in experimental allergic orchitis in the guinea-pig.

Meng AL, Tung KS.

Guinea-pigs were immunized with a defined and highly potent aspermatogenic antigen, G75m, and the occurrence of orchitis was correlated with (1) cell-mediated immune response to G75m, determined by lymph node cell proliferation and by secretion of macrophage migration inhibitory factor (MIF) by peritoneal exudate cells, and (2) humoral antibodies to G75m and to cell surface antigens of guinea-pig testicular cells, by radioimmunometric assays. A consistent temporal relationship between cell-mediated immune responses and disease was found: lymph node cell proliferation was positive by Day 4, followed 3 days later by maximum secretion of MIF, and orchitis lesions were manifest on Day 10. In contrast, maximal IgG antibodies to G75m or to the surface antigens of spermatozoa/testicular cells were detected at a time when cell-mediated immune responses and active testicular lesions had subsided. In individual animals, lymph node cell proliferation increased with severity of orchitis, while MIF secretion by peritoneal cells increased with orchitis only late in the disease.

Early in disease, MIF response showed a negative correlation with orchitis. Moreover, peritoneal injection of oil reduced the incidence of early lymph node cell proliferative responses, and delayed the onset of testicular disease. These findings are consistent with competition between different inflammatory sites for recently antigen-activated T lymphocytes. We conclude that (1) the development of orchitis correlates with cell-mediated immune responses to purified aspermatogenic antigens but not with IgG antibody responses, and (2) when the same animal is used to assess different aspects of cellular immunity and autoimmune disease, one study may significantly influence the other.

PMID: 6350573 [PubMed - indexed for MEDLINE]

2416. Afr J Med Med Sci. 1983 Sep-Dec;12(3-4):155-64.

Helminthiasis, the hypereosinophilic syndrome and endomyocardial fibrosis: some observations and an hypothesis.

Andy JJ.

It has been shown that chronic African endomyocardial fibrosis (E.m.f.) is most likely the burnt-out phase of parasite-induced hypereosinophilia. It has also been shown that African E.m.f. and Loffler's heart disease are pathologically identical. The mechanism by which these parasites and/or eosinophilia are associated with endomyocardial damage remains, however, unknown. The parasites which have been associated with induction of eosinophilia in E.m.f. include filariasis; trichinosis; ascariasis and hookworm and schistosomiasis. These parasites are known to produce neurologic, cardiac, pneumonic, hepatic and dermal damage during the migration of their larvae; at which time eosinophilia is usually most severe. The tissue damage induced by larval migration of these parasites appears comparable to findings seen in the hypereosinophilic syndrome. The evidence from our observations and this review suggests that the cardiac damage induced by larval migration, like the neurologic, pneumonic and dermal damage, is allergic in nature. Endomyocardial fibrosis has previously been shown to be an allergic heart disease. It appears reasonable to regard African endomyocardial fibrosis as representing the most intense, non-specific cardiac allergic reaction to helminthic larvae.

PMID: 6326549 [PubMed - indexed for MEDLINE]

2417. Allergol Immunopathol (Madr). 1983 Jul-Aug;11(4):225-8.

Evaluation of nasal and blood eosinophilia in children suffering from perennial allergic rhinitis treated with beclomethasone dipropionate.

Elkhalil M, Fiore L, Bellioni P, Cantani A, Corgiolu M, Businco L.

In 16 children suffering from allergic rhinitis, and treated with intranasal Beclomethasone Dipropionate (BDP) for 3 months, therapy significantly diminished the number of nasal eosinophils, while it did not cause any change in blood eosinophilia. This may confirm the local effect of this drug, given at 300 microgram/die. The reduction of nasal eosinophilia during BDP therapy can be explained by the attenuation of the tissue damage characterized by allergic inflammation. The release of mediators, particularly histamine, increases epithelial permeability and promotes penetration of the high molecular weight allergens which thereby gain access to submucosal mast cells. Further release of mediators makes the mucosa more and more permeable to allergen entry. BDP decreases mucous permeability and allergen absorption, so reducing inflammation and consequently the migration of eosinophils to the sites of the allergic

reaction.

PMID: 6650306 [PubMed - indexed for MEDLINE]

2418. Med Interne. 1983 Jul-Sep;21(3):231-4.

Test of leukocyte migration inhibition. 1. Value of the test in the diagnosis of certain immune diseases.

Urcan S, Stanciu L, Georoceanu A, Popescu S.

The test of leukocyte migration inhibition (LMI) being a specific and sensitive method for the detection of cell-mediated immunity, its ever increasing use in the clinic necessitates a more accurate estimation of its actual value. The technique used in this study is that of leukocyte migration in capillary tubes according to Soborg and Bendixen. The test was performed in three groups of patients: A--with drug intoxication; B--with chronic post viral hepatitis and C--with alcoholic liver disease. The results obtained showed that in the patients with drug allergy the LMI test using the incriminated drugs as antigens was positive in 83% of the cases, in the patients with chronic post viral hepatitis the cellular migration inhibition to the HBsAg was observed in 35% of the cases and in alcoholic liver disease lymphocyte sensitization to ethanol was present in 28.7% of the cases. It is concluded that in drug allergy the LMI test is useful and has diagnostic value being preferable to other in vitro determinations and even, in certain cases, to the skin tests. In chronic liver diseases the value of the test is limited to investigation purposes as it can reveal the degree of involvement of cell-mediated immunity in the pathogenesis of disease.

PMID: 6364312 [PubMed - indexed for MEDLINE]

2419. Nihon Yakurigaku Zasshi. 1983 May;81(5):431-40.

[Action of piroxicam on allergic inflammation].

[Article in Japanese]

Abe N, Tanaka K, Kanaoka K, Egawa M, Watanabe I, Hirai S.

Effects of piroxicam on allergic inflammation were investigated with allergic air pouch inflammation and antigen-induced arthritis in rats. In allergic air pouch inflammation, piroxicam exerted a dose-dependent inhibition (1-10 mg/kg, p.o.) of the exudate production, the migration of leukocytes and the release of lysosomal enzyme into the exudate; and its potency was superior to that of indomethacin and equivalent to that observed with prednisolone. In contrast with this, the suppressive effect of piroxicam on non-allergic air pouch inflammation was as weak as indomethacin. Prednisolone showed a similar effect on both types of air pouch inflammation. In antigen-induced arthritis, piroxicam showed a dose-dependent (0.3-3 mg/kg, p.o.) inhibitory effect on knee joint swelling and an improving action on the functional disorder of the inflamed joint. On this model, piroxicam was 3 to 4 times more active than both indomethacin and prednisolone. In non-allergic joint inflammation induced with croton oil in rats, however, the anti-inflammatory potency of piroxicam was almost equal to those of indomethacin and prednisolone. Piroxicam showed more potent inhibition than indomethacin on heterologous passive cutaneous anaphylaxis in rats, but showed only a slight inhibition on the increased vascular permeability caused by histamine and bradykinin. Piroxicam had no influence upon the plaque-forming cell response and the delayed hypersensitivity reaction in mice; furthermore, the hemolytic activity of complement in guinea-pig serum was scarcely affected by

piroxicam in vitro. These results indicate that piroxicam possesses prominent efficiency on allergic inflammation and may function on several activities of inflammatory cells.

PMID: 6629214 [PubMed - indexed for MEDLINE]

2420. J Invest Dermatol. 1983 May;80(5):398-402.

Antigen-specific T-cell lines in DNCB-contact sensitivity in guinea pigs.

Polak L, Scheper RJ.

Expanded DNP-specific guinea pig T cells exhibit enhanced in vitro and in vivo activity as demonstrated by DNA synthesis and systemic adoptive transfer of contact sensitivity. Moreover, expanded lymphocytes are capable of accomplishing local passive transfer of contact sensitivity and producing interleukin 2, vascular permeability-increasing and macrophage migration-inhibiting factors. Expansion of hapten-specific lymphocytes offers a suitable model for studying eliciting mechanism of allergic contact dermatitis in humans.

PMID: 6601680 [PubMed - indexed for MEDLINE]

2421. Am J Med. 1983 May;74(5):909-14.

Severe delayed inflammatory reactions from injected insulin. Association with cell-mediated immunity.

White WB, DeMartino SA, Yoshida T.

A diabetic patient had severe, delayed cutaneous reactions to insulin accompanied by fever, leukocytosis, and elevated erythrocyte sedimentation rate. Replacement of isophane beef-pork insulin suspension with other commercially available lente beef-pork, single-component beef and pork insulin preparations brought about no improvement. Intradermal testing demonstrated biphasic reactions to a wide variety of insulin preparations except for two recently available purified single-component (less than 1 ppm proinsulin) insulins. Immunologic studies showed increased incorporation of tritiated thymidine by the patient's peripheral blood lymphocytes after incubation with these insulin preparations that incited subcutaneous and intradermal reactions. Additionally, there was significant elaboration of the lymphokine, migration inhibitory factor, in response only to isophane beef-pork insulin. Control lymphocytes from a normal subject, from a patient with non-insulin-dependent diabetes, and from a patient with insulin-dependent diabetes without delayed allergy demonstrated no response to insulin preparations. The data suggest these delayed reactions were associated with a cellular immune response to one or more contaminants in the less purified insulin preparations.

PMID: 6220604 [PubMed - indexed for MEDLINE]

2422. J Toxicol Environ Health. 1983 Apr-Jun;11(4-6):545-53.

Airway cell changes in tracheal lavage of sheep after ozone exposure.

Sielczak MW, Denas SM, Abraham WM.

We were interested in whether ozone (O3) could stimulate the migration of mast cells into the tracheal lumen. To test this we determined the effect of an acute

O3 exposure on the types and relative numbers of cells recovered by tracheal lavage. Seven conscious sheep were intubated with an elongated nasotracheal tube. The trachea between the larynx and the cuff of the tracheal tube (15-20 cm) was lavaged repeatedly with 10-15-ml aliquots (total 350 ml) of 0.9% buffered (pH 7.4 saline, which contained the mast cell-stabilizer disodium cromoglycate (10 micrograms/ml). One hour after a baseline lavage, the sheep were exposed on separate occasions to either air (control) or 0.5 ppm O3 for 2 h. Lavages were repeated 24 h later. Cells were recovered from the lavage effluent by centrifugation across a saline/Ficoll Paque gradient. From part of this material we estimated total cells and total viable cells (with Trypan blue). The rest of the material was recentrifuged at 400 X g for 5 min, and cytological slides were made from the cell pellet. Slides were stained with Polichrome and Wright-Giemsa, and were analyzed by light microscopy. The percentages of epithelial cells, macrophages, lymphocytes, and mast cells were determined from a total count of 500 cells/slide. Differences in cell percentages between pre- and postexposure were calculated both for air and O3 exposures, and these differences were compared. Exposure to O3 resulted in an increased number of mast cells and lymphocytes when compared to the changes observed with air. It seems likely that the increase in number of luminal mast cells and lymphocytes following O3 exposure signals an enhanced inflammatory response and that these changes could contribute to O3-induced increased nonspecific airway hyperresponsiveness and susceptibility to allergic IgE-mediated airway reactions.

PMID: 6620400 [PubMed - indexed for MEDLINE]

2423. J Immunol. 1983 Apr;130(4):1891-5.

Generation of C3a anaphylatoxin from human C3 by human mast cell tryptase.

Schwartz LB, Kawahara MS, Hugli TE, Vik D, Fearon DT, Austen KF.

Tryptase, the dominant neutral protease of human pulmonary mast cell secretory granules, has the capacity in vitro to generate C3a anaphylatoxin from purified human C3. Only the alpha-chain of C3 is cleaved, and major fragments with apparent m.w. of 105,000, 39,500, 34,000, 29,000, and 9000 are detected by sodium dodecyl sulfate (SDS) polyacrylamide gel electrophoresis under reducing conditions. Fragments of 34,000 and 9000 m.w. are detected without reduction. A portion of the 9000 m.w. protein corresponds to C3a by virtue of its co-migration in SDS polyacrylamide gels with purified C3a and with trypsin-generated C3a, by its detection in a radioimmunoassay for C3a, and by its contractile activity on the guinea pig ileum bioassay. In the presence of heparin, another component of the mast cell secretory granule, the rate of appearance and the distribution of C3 cleavage fragments as assessed in SDS polyacrylamide gels are not appreciably changed with the exception that no C3a material can be detected in the SDS polyacrylamide gels or by radioimmunoassay and bioassay of the unresolved reaction mixture. Enhanced catabolism of authentic C3a by tryptase occurs in the presence of heparin and by analogy when C3a is generated from C3 by tryptase in the presence of heparin. Whereas tryptase secreted by activated human mast cells may generate C3a, a potentially important additional mediator of immediate hypersensitivity events, the concomitant release of heparin may serve to down-regulate C3a irrespective of its mechanism of generation.

PMID: 6339618 [PubMed - indexed for MEDLINE]

2424. Int Arch Allergy Appl Immunol. 1983;71(4):368-70.

Correlation of leukocyte accumulation with chemotactic activity in the exudate of an allergic air-pouch inflammation.

Kurihara A, Ohuchi K, Tsurufuji S.

Chemotactic activity at the site of an allergic air-pouch inflammation induced with azobenzene-arsonate-conjugated acetyl bovine serum albumin as an antigen was studied and a close correlation of the chemotactic activity with the intensity of leukocyte migration was demonstrated. In the period of vigorous leukocyte immigration into the fluid in the allergic air-pouch, chemotactic activity of the exudate was maintained at a high level, while no significant activity was detected after the number of leukocytes in the pouch fluid reached maximum.

PMID: 6862665 [PubMed - indexed for MEDLINE]

2425. Adv Exp Med Biol. 1983;156 (Pt B):755-66.

Suppressive effect of bradykinin to cellular immune responses in vivo and in vitro.

Kimura Y, Saiga T, Furuya M, Fujiwara H, Norose Y, Okabe T, Hida M.

Bradykinin, as well as histamine, one of the mediators in IgE mediated immediate type allergic reactions or acute inflammation, may affect the in vitro and in vivo cell-mediated immune reactions of the immunized animals. It was demonstrated in our present experiment that the appearance of delayed type hypersensitivity (DTH) skin reaction in the immunized guinea pig was remarkably suppressed by treatment of bradykinin or histamine and the suppression of cutaneous DTH by bradykinin was inhibited by H-2 antagonist (burimamide) but not by H-1 receptor blocker (chlorpheniramine). It was also clearly demonstrated that bradykinin suppressed the production of antigen-induced macrophage migration inhibitory factor (MIF) of the immune guinea pig peritoneal exudate cells (PECs) and the production of MIF was blocked by H-2 antagonist (burimamide) or H-2 agonist (tolazolin) but not by H-1 antagonist (chlorpheniramine). Antigen-induced lymphocyte proliferation of the immunized mice, one of the indicators of the cellular immune response, was also suppressed by treatment of bradykinin. These results indicate that bradykinin as well as histamine may have some role in the subsequent expression of cellular immune reactions.

PMID: 6858764 [PubMed - indexed for MEDLINE]

2426. J Hyg Epidemiol Microbiol Immunol. 1983;27(1):85-90.

Migration inhibition tests in penicillin allergy.

Suciu I, Surcel D, Gabor S.

The lymphocyte blastic transformation (TTB), leucocyte and macrophage inhibition tests has been compared in 18 hypersensitive to penicillin patients and in 15 patients with hypersensitivity to PPD. It has been shown that TTB was positive in all penicillin allergy cases, due to the constantly LT sensitization, and therefore this test constitutes an efficient method for the in vitro diagnosis of penicillin hypersensitivity. Macrophage inhibitory factor (MIF) was absent in all penicillin allergic subjects. Leucocyte migration inhibition test was positive only when gamma globulin coupled penicillin was used or in the presence of autologous plasma pretreated penicillin. This suggests that leucocyte migration inhibition resulted from the specific penicillin antibodies.

PMID: 6854013 [PubMed - indexed for MEDLINE]

2427. Int Arch Allergy Appl Immunol. 1983;70(1):30-3.

Leukocytes from patients allergic to chromium and nickel examined by the sealed capillary migration technique.

Nordlind K, Sandberg G.

Leukocytes from patients allergic to chromium and nickel were studied by a sealed capillary migration test. The migration indices were determined at 2, 5 and 24 h. The chromium-allergic group could be differentiated from the control group at all investigated times, and especially at 2 and 5 h, by using a potassium dichromate concentration of 1.7 x 10(-5) M in the capillaries. The migration indices in the nickel-allergic group could not be used for discrimination from the controls. The highest chromate (1.1 x 10(-4) M) and nickel sulfate (3.8 x 10(-4) M0 concentrations inhibited the migration of leukocytes from both patients and controls, indicating toxic effects.

PMID: 6848474 [PubMed - indexed for MEDLINE]

2428. Rocz Panstw Zakl Hig. 1983;34(1):7-12.

[Lysosomal enzyme activity as an indicator for the toxicological evaluation of contact allergens. I. Contact allergy].

[Article in Polish]

Kiss E.

PMID: 6612191 [PubMed - indexed for MEDLINE]

2429. Eur J Respir Dis Suppl. 1983;128 (Pt 1):7-15.

Basophil leukocytes and mast cells in the nose.

Okuda M, Ohtsuka H, Kawabori S.

The aim of the present study has been to examine the pathophysiology of allergic rhinitis, and the number, distribution and antigen-reactivity of basophilic cells. Blown nasal secretions, nasal washings, scrapings and excised specimens of nasal mucosa were collected and examined cytologically and histologically in both light and electron microscopes. Antigen-induced histamine release was also studied in these specimens. The results indicate that migration of basophilic cells (mainly basophil leukocytes in the nasal secretion and predominantly mast cells in the epithelium) to the mucosal surface is characteristic for allergy and that the total number and the histamine content of these cells are sufficient to produce the nasal manifestation of allergy. In conclusion, this study provides evidence supporting our concept of the mechanism of nasal allergy, i.e., that allergic reaction to inhaled allergens is initiated on the mucosal surface, and not in the lamina propria.

PMID: 6578087 [PubMed - indexed for MEDLINE]

2430. Med Interne. 1983 Jan-Mar;21(1):19-22.

Some aspects of cellular and humoral immunity in bronchial asthma and allergic rhinitis.

Stanciu L, Urcan S, Părău N, Radu D, Itu I, Georoceanu A.

Blood lymphocyte subpopulations were investigated in 50 patients with allergic diseases (38 with allergic rhinitis and 12 with allergic bronchial asthma). The total T lymphocyte (Tt) count (E rosette test), the active T lymphocyte (Ta) count (active E rosette test) and the B lymphocyte count (EAC rosette test) were determined and the results were correlated with: 1) lymphocyte sensitization in vitro to different allergens and to PPD; 2) circulating immune complexes; 3) serum histaminopexic capacity. In allergic rhinites a decrease of Tt and B lymphocytes and an increase of Ta lymphocytes was observed. In allergic bronchial asthma the Tt lymphocyte were found reduced. Lymphocyte reactivity to PPD in vitro was present in 100% of the cases and in vitro lymphocyte sensitivity to several allergens was observed in most of the cases. The histaminopexic capacity was absent or low in 75% of the patients with allergic rhinitis and in 66.6% of the patients with allergic bronchial asthma. The involvement of cellular mediated immunity in allergic diseases is discussed.

PMID: 6342109 [PubMed - indexed for MEDLINE]

2431. Clin Allergy. 1983 Jan;13(1):21-9.

Diagnosis of penicillin allergy. An evaluation of the leucocyte aggregation test in man.

Levacher M, Rouveix B, Badenoch-Jones P.

A study was made to establish the value of the leucocyte aggregation test (LAT) in drug allergy using penicillin antigen. The antigen-induced human peripheral blood leucocyte aggregation was measured quantitatively. The results obtained have been compared with the leucocyte migration inhibition test (LMIT) in patients with or without delayed penicillin allergy. Among forty-four penicillin-allergic subjects and thirty-six control subjects, LAT was found positive in respectively 70.5 and 30.5% (P less than 0.001) whereas LMIT was found positive in respectively 56.8 and 50% of the patients. These results were confirmed by multiple correspondence analysis (MCA), using a computer. Furthermore, this method, enables a more comprehensive and reliable interpretation of the tests, by the help of various quantitative and qualitative criteria. It is concluded that LAT shows more discrimination than the LMIT in distinguishing a penicillin-allergic population from a non-allergic one. In addition, LAT offers great technical advantages over the LMIT for the diagnosis of drug allergy.

PMID: 6339114 [PubMed - indexed for MEDLINE]

2432. J Allergy Clin Immunol. 1983 Jan;71(1 Pt 1):47-56.

Characterization of the mononuclear cell infiltrate in atopic dermatitis using monoclonal antibodies.

Leung DY, Bhan AK, Schneeberger EE, Geha RS.

Tissue sections from involved and uninvolved skin of nine patients with atopic dermatitis (AD) were investigated by light microscopy, electron microscopy, and an immunoperoxidase method using monoclonal antibodies to cell-surface antigens. Acute lesions were characterized by spongiotic epidermis, with increased numbers of infiltrating mononuclear cells consisting predominantly of lymphocytes. Perivascular dermal infiltrates consisted of lymphocytes and a few

monocytes-macrophages. Capillary endothelial cells were not enlarged. In chronic lesions the epidermis was hyperplastic, with virtually no cellular infiltrate. The perivascular dermal infiltrates consisted primarily of monocytes-macrophages intermixed with lymphocytes. Capillary lumens were narrowed by enlarged endothelial cells. The majority of the infiltrating lymphocytes in all skin biopsy specimens from AD patients were stained with anti-T3, anti-Leu-1, anti-T4, and anti-Leu-3 antibodies, suggesting that most of the infiltrating lymphocytes were T cells possessing the helper/inducer phenotype. In contrast, a smaller number of infiltrating cells reacted with anti-T8 or anti-Leu-2 antibodies, which define the suppressor/cytotoxic T cell population. Langerhans cells, as defined by reactivity with anti-T6 monoclonal antibody, were increased in the diseased skin of AD patients. The presence of increased numbers of Langerhans cells and T cells of the helper/inducer phenotype may reflect increased antigen processing in the diseased skin of patients. In addition, the smaller number of T8+ cells infiltrating into the skin suggests that the depression of circulating T8+ cells observed in the majority of patients with AD is not due to the selective migration of these T8+ cells into the skin.

PMID: 6337197 [PubMed - indexed for MEDLINE]

2433. Morphol Embryol (Bucur). 1983 Jan-Mar;29(1):31-4.

Ultrastructural aspects in skin allergic vasculitis.

Dobrescu G, Dobrescu A, Stoica L.

Twelve cases of skin allergic vasculitis were electronmicroscopically studied. The most striking changes were noticed at the level of capillaries, whose wall components were all affected, especially the endothelial cells. These presented at first adaptive changes, with swelling, microvillosities of plasmalemma protruding into the lumen, and many pinocytotic vesicles showing an intensely active transport. Followed different degrees of degenerative changes of cytoplasms and nuclei, the junctional complexes being interrupted, even discontinuous. The pericytes showed similar changes. The basement membrane was thickened, nonhomogeneous, with a spongy aspect, small discontinuities and some electron-dense depots. The endothelial cell damage and the discontinuity of basement membranes, as well as the alteration of pericytes allowed the migration of blood cells into interstitial spaces, followed by leucocytoclasia. As a consequence, in the perivascular tissue a polymorphous cellular infiltrate developed. The mechanisms and significance of these changes are discussed.

PMID: 6220209 [PubMed - indexed for MEDLINE]

2434. Sem Hop. 1982 Dec 16;58(46):2691-5.

[Severe anaphylactoid complications in anesthesia. Apropos of 35 cases].

[Article in French]

Le Cam B, Tanguy RL, Egreteau JP.

35 cases of severe anaphylactoid accidents during anesthesia are reported. Clinical symptomatology is dominated by cardiovascular collapse and/or bronchospasm and/or cutaneous and mucous manifestations. The course is always favourable. The usual risk factors (drug allergy, atopic background, spasmophilia, multiple anesthesia) are found. Pentothal alone or added to succinylcholine is most incriminated, followed by C-T 1341, curare-like agents, propanidid and macromolecular solutions. The immunoallergic investigation is

incomplete, founded on the inhibition of leukocyte migration test TIML. The principal mechanisms responsible for these accidents and the most frequent causal products are recalled. Laboratory tests are reviewed. In conclusion, the authors propose a curative and preventive action to be taken when faced with this severe and non-exceptional accident of anesthesia.

PMID: 6297067 [PubMed - indexed for MEDLINE]

2435. Unfallchirurgie. 1982 Dec;8(6):347-52.

[Allergy to metal following osteosynthesis].

[Article in German]

Hierholzer S, Hierholzer G.

Corrosions of metallic implants can lead to demonstrable accumulation of implant-specific metals in the tissue surrounding the implant. This can give rise to metal sensitization after activation of cellular immune mechanism of the body. A connection between metal sensitization and bone infection morbidity after insertion of metallic implants is thus conceivable. In our investigations to clarify this question, we were able to obtain the following results using the leukocyte migration inhibition test for cobalt, chromium and nickel on various investigation groups after statistical analysis: 1. Patients bearing metallic implants without infection show the same rate of metal allergy as persons without implants. 2. Patients bearing metallic implants with infection show an increased rate of allergy to cobalt and nickel compared to persons without implants and to patients bearing metallic implants without infection. They are sensitive to one of the three tested metals more than twice as frequently as patients with aseptic osteosynthesis. Furthermore, this study has addressed the question of causality: Does metal allergy cause an inflammatory reaction or does infection cause sensitization, e.g. via increased corrosion of metallic implants following infection.

PMID: 7164188 [PubMed - indexed for MEDLINE]

2436. J Invest Dermatol. 1982 Oct;79(4):213-7.

Cell-mediated immunity in untreated and PUVA treated atopic dermatitis.

Soppi E, Viander M, Soppi AM, Jansén CT.

In 22 adult patients with atopic dermatitis, lymphocyte subpopulation counts, mitogenic responses to several PHA and Con A concentrations, nonspecific mitogen induced, and indomethacin sensitive suppressor cell functions and leukocyte migration inhibitory factor production were investigated. Patients with severe atopic dermatitis had normal PHA and Con A responses, although somewhat lower than matched controls, while mild AD patients equalled controls in this respect. No significant differences between AD patients and controls were detected with respect to lymphocyte subpopulations, suppressor cell function or leukocyte migration inhibitory factor production. Methoxalen plus ultraviolet light (PUVA) therapy induced significant clinical improvement in 9 out of 10 treated patients. A concomitant decrease of the mitogenic responses and increase of the Con A induced nonspecific suppressor cell activity was recorded, while other immunological parameters remained unaffected by the therapy. Thus PUVA therapy induces both local clinical and systemic immunological manifestations. The possibility that the observed immunological changes would be operative in the PUVA induced healing process, however, seems unlikely, as the present study did

not disclose any obvious relationship between immune parameters and severity of atopic dermatitis.

PMID: 6215450 [PubMed - indexed for MEDLINE]

2437. Jpn J Pharmacol. 1982 Oct;32(5):813-22.

Immunopharmacological studies of the aqueous extract of Cinnamomum cassia (CCAq). I. Anti-allergic action.

Nagai H, Shimazawa T, Matsuura N, Koda A.

Effect of the aqueous extract of Cinnamomum Cassia (CCAq) on experimental allergic reaction was investigated. IgE mediated reactions, homologous passive cutaneous anaphylaxis (PCA), degranulation of mast cells, and the release of histamine from sensitized lung tissues classified as the type I reaction by Coombs and Gell were not affected by CCAq. Complement dependent reactions including reversed cutaneous anaphylaxis (RCA), Forssman cutaneous vasculitis (FCV), and nephrotoxic serum (NTS) nephritis classified as type II and the Arthus reaction classified as type III were clearly inhibited by CCAq. However, CCAq did not affect the nephritis caused by the F(ab')2 portion of the nephrotoxic IgG antibody. CCAq in a high concentration inhibited the immunological hemolysis, chemotactic migration of neutrophils in response to complement activated serum, and the generation of chemotactic factors. The type IV reaction, contact dermatitis, was not affected by CCAq. The production of hemolytic plaque forming cells was slightly inhibited by CCAq. These results suggest that CCAq has an anticomplement action and inhibits the complement dependent allergic reaction.

PMID: 6184511 [PubMed - indexed for MEDLINE]

2438. J Exp Med. 1982 Sep 1;156(3):756-65.

Modulation of human lymphocyte function by C3a and C3a(70-77).

Payan DG, Trentham DE, Goetzl EJ.

Human C3a and the synthetic octapeptide C3a (70-77), which retains the activities of an anaphylatoxin, inhibit in a concentration-dependent manner the generation of leukocyte inhibitory factor (LIF) activity by human mononuclear leukocytes and T lymphocytes cultured with the mitogens phytohemagglutinin (PHA) or concanavalin A (Con A) or the antigen streptokinase-streptodornase (SK-SD). The generation of LIF activity was inhibited by 50% by 10(-8) M C3a or C3a(70-77) with PHA or Con A as the stimulus, whereas a more than 10-fold higher concentration of C3a(70-77) than C3a was required to achieve the same level of suppression with SK-SD as the stimulus. Similar concentrations of C3a(70-77) inhibited to the same extent the migration of T lymphocytes stimulated by alpha-thioglycerol of Con A. Neither C3a nor C3a(70-77) altered significantly the uptake of [3H]thymidine by human mononuclear cells exposed to PHA, Con A, or SK-SD. The capacity of C3a(70-77)-Sepharose,m but not Sepharose alone, to adsorb or inactivate mononuclear leukocytes required for the generation of LIF activity established a direct interaction. Analysis of the lymphocytes in the effluent from C3a(70-77)-Sepharose columns, using monoclonal antibodies to surface antigens, showed a selective depletion of the helper/inducer population of lymphocytes. C3a might represent an important mediator of the functionally selective regulation of human T lymphocyte activities by the complement system.

PMCID: PMC2186792
PMID: 7050289 [PubMed - indexed for MEDLINE]

2439. Sangyo Igaku. 1982 Sep;24(5):441-9.

[The mechanism of the development of contact sensitivity and the methods of assessing allergenicity of contact sensitizers].

[Article in Japanese]

Nakano Y, Hara I.

Recent reports on the mechanism of the development of contact sensitivity and on the method of assessing allergenicity both in vivo and in vitro are reviewed. Various cell types and two processes, afferent and efferent limbs, involved in the contact sensitivity are discussed. Conditions for applying contact sensitizers which induce specific unresponsiveness, desensitization and antigenic competition are also discussed. Diagnostic methods for identifying contact sensitizers in vivo, patch testing, and in vitro, migration inhibition test and blastoid transformation are reported. Moreover, methods of assessing the allergenicity of contact sensitizers are reported centering on the guinea pig maximization testing.

PMID: 6984095 [PubMed - indexed for MEDLINE]

2440. Clin Allergy. 1982 Sep;12(5):475-88.

Eosinophilia VI, spontaneous synthesis of chemokinetics, chemotactic, complement receptor-inducing activities for eosinophils by bronchial t lymphocytes of asthmatic-bronchitic patients.

Parish WE, Luckhurst E.

T lymphocytes were separated from the bronchial mucus of five patients with extrinsic asthma and chronic and mild bronchitis and who had numerous eosinophils in the bronchial mucus but no significant blood eosinophilia. The bronchial lymphocytes spontaneously, that is without treatment with antigens or mitogens, released into serum-free synthetic medium one or more substances in the molecular weight range of 30 000 to 60 000 daltons. These substances when tested on eosinophils of normal persons stimulated random movement (chemokinesis), attracted them (chemotaxis), enhanced their chemotactic response to activated complement, and increased the expression of C4 and C3b receptors. Chemokinetic and chemotactic activity for neutrophils was weak. No eosinophil-stimulating activity was found in cultures of bronchial lymphocytes treated with puromycin to inhibit synthesis. The blood lymphocytes did not spontaneously synthesize the substance(s). It is not known if the several eosinophil-stimulating activities are due to one or more substance(s), but the nature of the eosinophil responses, molecular weight and other features, indicate similarities with the 'eosinophil stimulation promoter-chemotactic' factor reported to be released from mouse or human lymphocytes treated with antigen. Eosinophil stimulation resulting in increased expression of specific receptors, and potential for non-specific adherence, by trapping or arresting randomly migrating cells, is believed to mediate accumulation of cells in an affected organ. Prolonged synthesis of such products as from activated lymphocytes in the lung, could account for much of the local eosinophilia.

PMID: 6982790 [PubMed - indexed for MEDLINE]

2441. Rev Infect Dis. 1982 Jul-Aug;4(4):806-14.

Ascariasis: host-pathogen biology.

Pawlowski ZS.

Ascaris lumbricoides is one of the most common intestinal parasites in humans. Daily global contamination of the soil by A. lumbricoides eggs is enormous (approximately 9 x 10(14) eggs/day). Physical factors, particularly temperature and moisture, are critical in determining the maturation of eggs to the infective stage and their survival. Transmission of the infection to humans, on the other hand, depends more on various socioeconomic factors. In theory, ascariasis is preventable; it is indeed on the way to disappearing completely in developed societies where there is a high standard of sanitation. Ascariasis remains a problem in developing countries, however, where methods of disposal of human excreta are inadequate. The intensity of invasion is regulated by specific and nonspecific responses of the host to migrating A. lumbricoides larvae. Whether or not ascariasis becomes symptomatic depends on the intensity of the infection, the nutritional and immunologic status of the host, and the possible complications that may arise. Host responses to A. lumbricoides are brisk during the larval migratory stage in which hypersensitivity reactions may become clinically manifest, whereas people are rather tolerant of intestinal infections with adult worms. The role of ascariasis in the prevalence of allergic asthma still remains unclear. Complications due to migration of adult worms into the biliary duct system and to intestinal obstructions are the major causes of acute morbidity and mortality in ascariasis.

PMID: 6812197 [PubMed - indexed for MEDLINE]

2442. Blood. 1982 Jun;59(6):1259-66.

Metabolic, membrane, and functional responses of human polymorphonuclear leukocytes to platelet-activating factor.

Ingraham LM, Coates TD, Allen JM, Higgins CP, Baehner RL, Boxer LA.

The phospholipid mediator of anaphylaxis, platelet-activating factor (PAF) is chemotactic for polymorphonuclear leukocytes (PMN). We have examined this agent's effects on several other PMN functions. Human PMN were prepared from heparinized venous blood by Ficoll gradient. Metabolic burst was examined by measurement of O2 use and O2.- production in the presence or absence of PAF (10(-6)--10(-9) M). Unless cells were treated with cytochalasin-B (5 micrograms/ml), no significant respiratory burst was demonstrated. However, pretreatment with PAF (10(-7) M) enhanced approximately threefold the O2 utilization found when cells were subsequently stimulated with 10(-7) M FMLP. PAF also stimulated arachidonic acid metabolism in 14C-arachidonic acid-labeled PMN. Thin-layer chromatography analysis of chloroform-methanol extracts showed substances that comigrated with authentic 5-hydroxyeicosatetraenoic acid had a marked increase in radioactivity following PAF stimulation at 10(-7) M. PAF failed to stimulate release of granule enzymes, B-glucuronidase, lysozyme, or myeloperoxidase unless cytochalasin-B were added. PAF from 10(-6) M to 10(-10) M affected PMN surface responses. PMN labeled with the fluorescent dye, chlorotetracycline, showed decreased fluorescence upon addition of PAF, suggesting translocation of membrane-bound cations. Further, the rate of migration of PMN in an electric field was decreased following PAF exposure, a change consistent with reduced cell surface charge. PMN self-aggregation and adherence to endothelial cells were both influenced by PAF (10(-6) M--10(-9) M). Aggregation was markedly stimulated by the compound, and the percent PMN adhering to endothelial cell monolayers increased almost twofold in the presence of 10(-8) M PAF. Thus, PAF promotes a variety of PMN responses: enhances respiratory burst, stimulates arachidonic acid turnover, alters cell

membrane cation content and surface charge, and promotes PMN self-aggregation as well as adherence to endothelial cells.

PMID: 6282362 [PubMed - indexed for MEDLINE]

2443. Eur J Pediatr. 1982 May;138(3):250-3.

Cellular immunity in children with coeliac disease.

Ashkenazi A, Levin S, Idar D, Handzel ZT, Altman Y, Or A, Barzilai N.

The experimental evidence implicating defective cell-mediated immunity in coeliac disease, a condition where symptomatology is believed to be due to immunological reaction to wheat gluten, is often inconsistent and sometimes controversial. Studies of certain parameters of cellular immunity in four groups of pediatric patients were performed: coeliac patients on normal diet; coeliac patients consuming gluten-free diet; children with cow's milk sensitivity. In all these assays no significant differences were found between treated or untreated coeliac children, infants with milk allergy or the gastro-intestinal control groups. On the basis of this study we could find no evidence of impairment of cell-mediated immunity in coeliac children. This conclusion is compatible with the hypothesis that intestinal damage may be due to a subpopulation of lymphocytes sensitive to gluten in persons with normal immune systems. In adults where abnormalities of cell-mediated immunity have sometimes been noted, the reason could be a loss of lymphocytes from the damaged mucosa of the gastrointestinal tract following prolonged antigenic stimulation. This indicated the need for strict adherence to a gluten-free-diet.

PMID: 6749506 [PubMed - indexed for MEDLINE]

2444. Acta Neurol Scand. 1982 May;65(5):432-41.

Allergic reactions to steroids presenting with neurological symptoms.

Behan PO, Thomas M, Behan WM.

PMID: 6287794 [PubMed - indexed for MEDLINE]

2445. Br J Cancer. 1982 May;45(5):754-61.

Cellular immunity to encephalitogenic peptide in tumour-bearing mice.

Yong WK, Halliday WJ.

Mice bearing a methylcholanthrene-induced tumour were tested for their cell mediated reactivity to the experimental allergic encephalomyelitis (EAE) peptide of human myelin basic protein (MBP) in the leucocyte adherence inhibition (LAI) test. Tested over a range of peptide concentrations, peritoneal cells (PC) from tumour-bearing mice exhibited optimal adherence inhibition at 640 ng/ml; PC from normal and parasite-infected mice were unreactive. The EAE peptide also stimulated PC from tumour-bearing mice in the E-rosette augmentation (ERA) test and in the macrophage migration inhibition (MMI) test. MMI appeared to be the most sensitive assay, in that significant reaction at peptide concentrations well below those giving significant LAI and ERA. LAI reactivity to the peptide was detected 5 days after tumour transplantation, and continued to be detectable even with very large tumours. In vitro assays were confirmed by demonstration of EAE peptide recognition in vivo, in tumour-bearing and tumour-excised mice, using the

delayed-type hypersensitivity reaction. The present experiments demonstrate an antigenic determinant in murine tumours, similar to the well-characterized EAE peptide of human MBP, and establish an animal model for study and characterization of common tumour-associated antigens.

PMCID: PMC2011013
PMID: 6177329 [PubMed - indexed for MEDLINE]

2446. Zh Mikrobiol Epidemiol Immunobiol. 1982 Apr;(4):74-7.

[Characteristics of the causative agent and of the immunological status of the body in experimental dormant staphylococcal infection].

[Article in Russian]

Shilov AB, Romanov VA, Malafeeva EV.

Pathogenic staphylococci were found to persist in the focus of dormant infection in guinea pigs till day 100 of the experiment without changing their biological properties and sensitivity to antibiotics. The latent period of dormant staphylococcal infection was characterized by the increasing titers of antibodies to staphylococcal autostrains, by the positive results of the intradermal allergic test and the macrophage migration inhibition test with hemolytic staphylococcal allergen, as well as by the suppression of serum lysozyme activity. No changes in the content of complement and the total bactericidal activity of blood serum were detected.

PMID: 7080764 [PubMed - indexed for MEDLINE]

2447. J Clin Invest. 1982 Apr;69(4):889-99.

Identification and partial characterization of an exercise-induced neutrophil chemotactic factor in bronchial asthma.

Lee TH, Nagy L, Nagakura T, Walport MJ, Kay AB.

A heat-stable neutrophil chemotactic factor (NCF) has been identified in the serum of 13 atopic asthmatic subjects after treadmill exercise. Peak activity was detected at 10 min and returned to prechallenge values by 1 h. No NCF activity was detected in the sera of three nonasthmatic atopic or four normal nonatopic individuals performing the same task. NCF produced by exercise (NCFEX) had a similar time-course of release to NCF provoked by specific antigen (NCFAG). The appearance of circulating NCFEX and NCFAG closely paralleled the fall in peak expiratory flow rate/forced expiratory volume in 1 s (PEFR/FEV1). Histamine challenge in atopic asthmatics at concentrations giving a comparable change in PEFR/FEV1 to that evoked by exercise or inhaled antigen was not associated with the appearance of circulating NCF. In seven subjects NCFEX release was inhibited by prior administration of disodium cromoglycate. NCFEX and NCFAG eluted as single peaks of activity when applied separately to columns of Sephadex G-200, and both were an estimated 750,000 daltons. NCFEX and NCFAG also eluted as single peaks of activity, at between 0.15 and 0.30 M NaCl, following anion exchange chromatography on DEAE-Sephacel (pH 7.8). The isoelectric points of NCFEX and NCFAG were virtually identical (between pH 6.0 and 6.5) as determined by chromatofocusing on Polybuffer Exchanger 94. The activities of NCFEX and NCFAG were substantially reduced, in both a time- and dose-dependent fashion, after incubation with trypsin and chymotrypsin. Partially purified NCFEX and NCFAG promoted both stimulated random migration (chemokinesis) as well as directional migration (chemotaxis). These experiments indicate that NCFEX and NCFAG might be

identical substances and raise the possibility that mediators by hypersensitivity are released during exercise-induced asthma in atopic subjects.

PMCID: PMC370143
PMID: 7076852 [PubMed - indexed for MEDLINE]

2448. Nouv Presse Med. 1982 Mar 6;11(11):843-6.

[Leukocyte aggregation test for assessing cell-mediated immunity: its use in drug allergy (author's transl)].

[Article in French]

Rouveix B.

The author has designed a new quantitative leucocyte aggregation test a measure cell-mediated immunity in man. The test involves continuous recording of light absorbance by stirred leucocyte suspensions. Potentially, measurement of direct leucocyte aggregation offers considerable advantages in terms of sensitivity, reproducibility and simplicity of execution. The technique was initially developed in animals and made it possible to assay very small amounts of lymphokines. The results obtained in man with antigenic drugs, such as penicillin, confirm that the aggregation test is much more sensitive than the migration inhibition test. Its precise significance remains uncertain, but it already promises to constitute a simple and highly reliable means of diagnosing and preventing drug hypersensitivity.

PMID: 7070976 [PubMed - indexed for MEDLINE]

2449. Clin Immunol Immunopathol. 1982 Mar;22(3):375-83.

Macrophage locomotion in experimental allergic thyroiditis of the rat.

Fujiwara H, Sakata M, Tomooka Y, Tanaka M, Torisu M.

PMID: 7049464 [PubMed - indexed for MEDLINE]

2450. Schweiz Med Wochenschr. 1982 Feb 6;112(6):192-7.

[Recent findings on occupational allergic alveolitis].

[Article in French]

Molina C, Bedu M, Jeanneret A, Jouanel P, Motta C, Dastugue B.

Occupational allergic alveolitis may be observed in agricultural or industrial environment. The presence of serum precipitins to specific antigens remains a valuable symptom. But other immunologic tests, such as inhibition of leucocyte migration, may be performed in doubtful cases or for retrospective diagnosis. It shows the role of delayed hypersensitivity in the pathogenesis of the disease. The functional investigation confirms the alveolar involvement but also reveals in some cases (by flow/volume curves and compliance tests) evidence of small airways disease. The broncho-alveolar lavages show the high percentage of lymphocytes (mainly T lymphocytes) in in the cell population recovered and a very typical phospholipid profile in the supernatant (total absence of lecithin whereas the two other fractions phosphatidyl-inositol and phosphatidyl-ethanolamine are considerably enhanced). The tensioactive properties

of the phospholipids were investigated by fluorescence polarisation technique. High values of microviscosity were found in patients. These findings provide a new diagnostic guidance and a new therapeutic approach of these diseases.

PMID: 7079705 [PubMed - indexed for MEDLINE]

2451. Br J Dermatol. 1982 Feb;106(2):161-8.

Hapten conjugation in the leucocyte migration inhibition test in allergic chromate eczema.

Rytter M, Haustein UF.

We studied the in vitro conjugation of chromium salts and found that trivalent chromium binds to protein 10 times more strongly than hexavalent chromium. We also investigated the use of two different lymphocyte migration inhibition tests for the diagnosis of chromium allergy. The capillary method proved superior to the Clausen technique, and potassium dichromate was shown to be a better antigen than chromium chloride. The test results correlated well with standard patch tests and we recommend the capillary method for the investigation of difficult cases of suspected chromium allergy.

PMID: 6174139 [PubMed - indexed for MEDLINE]

2452. Tr Inst Im Pastera. 1982;57:85-8.

[Use of a Candida guilliermondii allergen for diagnostic purposes].

[Article in Russian]

Nikiforov IuF, Karaev ZO, Malevannyĭ IN.

PMID: 7170743 [PubMed - indexed for MEDLINE]

2453. Parasite Immunol. 1982 Jan;4(1):59-76.

Immunological and histopathological reactions of the rat against the tapeworm Hymenolepis diminuta and the effects of anti-thymocyte serum.

Hindsbo O, Andreassen J, Ruitenberg J.

Anti-thymocyte-serum (ATS) treated Wistar rats infected with 100 cysticercoids of the rat intestinal cestode Hymenolepis diminuta showed a delayed destrobilation and expulsion of the worms compared with saline-treated infected rats. This result strengthens previous evidence of an immunological nature of the destrobilation and expulsion in lumen-dwelling cestodes--even in their most susceptible hosts. The migration of the worms in the small intestine during the first 20 days of a primary 100-worm infection is described and the anterior migration of the destrobilated worms to the first 10% of the pylorus is emphasized and compared with similar migrations of the nematode Nippostrongylus brasiliensis in the rat. No serum antibodies were detected using passive cutaneous anaphylaxis and the indirect immunofluorescence test, although the thymus-independent areas of the mesenteric lymph nodes showed an increase in pyroninophilic cells. In the small intestine, no response to the tapeworm infection could be detected in pyroninophilic cells and globule leucocytes, but mast cell and eosinophilic cell numbers were increased in the saline-treated infected rats. Although the host responses to H. diminuta are shown to be

thymus-dependent, the possibility of thymus-independent activity in the host reactions cannot be ruled out.

PMID: 6977126 [PubMed - indexed for MEDLINE]

2454. Int Arch Allergy Appl Immunol. 1982;68(4):326-31.

A possible role of arachidonate metabolism in allergic air pouch inflammation in rats. Anti-inflammatory effect of indomethacin and dexamethasone and the level of prostaglandin E2 in the exudate.

Ohuchi K, Yoshino S, Kanaoka K, Tsurufuji S, Levine L.

The effect of indomethacin and dexamethasone on an allergic inflammation in rats, a novel model of allergic inflammation of an air pouch type, was examined. Indomethacin and dexamethasone exerted a dose-dependent inhibition of both the accumulation of inflammatory exudate and the migration of leukocytes into the exudate. And although prostaglandin E2 levels in the exudate were lowered to the same extent by treatment with indomethacin and dexamethasone, inhibition of both exudate accumulation and and leukocyte migration was more pronounced after treatment with dexamethasone. The difference in the effectiveness of indomethacin and dexamethasone in terms of inhibition of arachidonate metabolism in the allergic air pouch inflammation are discussed.

PMID: 6954133 [PubMed - indexed for MEDLINE]

2455. Int J Vitam Nutr Res Suppl. 1982;23:23-34.

Effects of ascorbate on normal and abnormal leucocyte functions.

Anderson R.

The stimulatory effects of ascorbate on neutrophil motility in vitro and in vivo and lymphocyte transformation to mitogens following ingestion or intravenous injection of ascorbate have been found to be related entirely to inhibition of the autooxidative effect of the myeloperoxidase/hydrogen peroxide/halide system (MPO/H2O2/halide system). Stimulation of neutrophil migration and lymphocyte transformation following a single intravenous injection of 1 g of ascorbate was associated with inhibition of the MPO/H2O2/halide system. The immunostimulatory activity and peroxidase inhibitory activity was related entirely to the serum ascorbate level. The relationship between inhibition of the peroxidase/h2O2/halide system and stimulation of neutrophil motility and lymphocyte mitogen-induced transformation was further established by using the horseradish peroxidase (HRP)/H2O2/halide system in vitro. Neutrophils and lymphocytes, exposed to this system, manifested markedly impaired chemotactic responsiveness and mitogen-induced transformation, respectively. However inclusion of ascorbate with the peroxidative system protected the neutrophils and lymphocytes from these inhibitory effects. Further studies in 3 patients with chronic granulomatous disease (CGD) and 10 patients with bronchial asthma suggested that ascorbate may be of value to improve the primary immunological abnormalities (neutrophil motility and antimicrobial activity) in CGD and the secondary abnormalities (neutrophil motility and lymphocyte transformation) found in some individuals with bronchial asthma.

PMID: 6811483 [PubMed - indexed for MEDLINE]

2456. J Invest Dermatol. 1981 Dec;77(6):464-8.

IgA-associated inhibition of polymorphonuclear leukocyte chemotaxis in neutrophilic dermatoses.

Schröder JM, Szperalski B, Koh CJ, Christophers E.

The chemotactic activity of normal human polymorphonuclear leukocytes (PMNs) confronted with heat inactivated sera from patients with psoriasis as well as various chronic proliferative diseases was determined using modified Boyden chambers. By the addition of phorbol myristate acetate (PMA) at a concentration of 1 ng/ml the chemoattractant activities of the sera were greatly potentiated. However, the chemotactic migration of normal PMNs was strongly inhibited by sera from patients with long standing and wide spread psoriasis, pyoderma gangrenosum, severe acne conglobata, Sweet syndrome, and some patients with chronic arthritis following rheumatoid fever. In acute guttate psoriasis and atopic dermatitis increased migratory activities were seen. The inhibition of chemotaxis correlated with increased serum IgA levels as determined by radial immuno diffusion. Column chromatography (Sephacryl S-300) revealed serum fractions of strong inhibitory potency at a molecular weight near 200,000 Dalton. These inhibitory fractions were seen in patients with long standing neutrophil related diseases and could not be detected in normal control sera. It appears that inhibition of PMN chemotaxis is a secondary phenomenon and may play an autoregulatory role in PMN related inflammation.

PMID: 7310170 [PubMed - indexed for MEDLINE]

2457. J Immunol. 1981 Nov;127(5):1915-9.

Unresponsiveness to experimental allergic encephalomyelitis in mice: replacement of suppressor cells by a soluble factor.

Lando Z, Dori Y, Teitelbamum D, Arnon R.

A soluble suppressor factor has been prepared from cells of mice rendered nonsusceptible to experimental allergic encephalomyelitis (EAE) by treatment with mouse spinal cord homogenate in incomplete Freund's adjuvant. The specific activity of this factor can be augmented by using a cell population enriched on plates coated with anti-mouse Fab and the specific antigen, mouse basic encephalitogen (MBE). The resultant suppressor factor had the same biologic activities as the cells from which it originated. Thus, it suppressed specifically the delayed-type hypersensitivity (DTH) response to MBE in vivo, and blocked in vitro the effector lymphocytes that adoptively transfer the DTH response. The suppressor factor reactivity was manifested also by the capacity to suppress the activity of macrophage migration inhibition factor produced by sensitized lymphocytes in the presence of the specific antigen MBE. The suppressor factor is antigen-specific and can bind the MBE in vitro and thus compete with its antibody binding. The most significant activity of the soluble suppressor factor is its ability to interfere with the induction of clinical EAE.

PMID: 6170680 [PubMed - indexed for MEDLINE]

2458. Zh Mikrobiol Epidemiol Immunobiol. 1981 Sep;(9):42-6.

[Comparative immunochemical and immunoallergic characteristics of staphylococci of different pathogenicity. II. Diagnostic value of extracts of Staphylococcus aureus and Staphylococcus epidermidis in delayed type allergic reactions].

[Article in Russian]

Darkanbaeva ET, Sukhodoeva GS, Bul'vakhter IaL, Shuratova SG, Koval' GP.

A total of 20 different allergens extracted from various staphylococcal strains were studied in the delayed hypersensitivity reactions in experiments on guinea-pigs. The most intensive dermatoallergic reactions were observed after the injection of the cytoplasmic fraction, acidic and alkaline extracts; reactions to the allergen of Ando-Verzhikovsky were less pronounced. The cytoplasmic fraction and the allergen of ando-Verzhikovsky were more active in the reaction of leukocyte migration inhibition in the blood. Both common and individual allergenic components were detected. No correlation between the results of the immunochemical and allergological studies of staphylococcal extracts was revealed.

PMID: 7304031 [PubMed - indexed for MEDLINE]

2459. Vestn Otorinolaringol. 1981 Sep-Oct;(5):20-5.

[Immunological indices as a criterion for evaluating the results of ORL management in bronchial asthma].

[Article in Russian]

Mirgani FA, Savchenko ZI, Kornienko AM, Ovchinnikov IuM.

PMID: 6975527 [PubMed - indexed for MEDLINE]

2460. J Occup Med. 1981 Sep;23(9):610-6.

Diverse profiles of immunoreactivity in toluene diisocyanate (TDI) asthma.

Gallagher JS, Tse CS, Brooks SM, Bernstein IL.

Possible immunoreactivity to chemically well-characterized mono- and diisocyanate protein conjugates was reevaluated in 15 workers with TDI asthma and 17 normal (nonexposed) volunteers. Lymphocytes of nine sensitive workers were incubated with TDI human serum albumin (HSA) conjugates. Leucocyte inhibitory factor (LIF) was produced. Leucocyte inhibitory factor was also induced by hexamethylene diisocyanate (HDI) protein conjugates in four of these workers who had no prior history of exposure to HDI. Disappearance of TDI- and HDI-induced LIF was noted in several sensitive workers who were removed from further TDI exposure. Three LIF-positive workers also demonstrated positive intracutaneous reactivity to TDI-HSA. One workers had a markedly positive RAST (25.5% binding) to a monofunctional (p-tolyl isocyanate) protein reagent. These studies suggest that isocyanates have the potential for eliciting heterogeneous immune responses in certain subpopulations of exposed workers. Continued contact with isocyanates may be necessary for maintenance of specific immunity. Possible cross reactivity between TDI and HDI may be determined by new antigenic sites created by isocyanate protein interactions.

PMID: 6268767 [PubMed - indexed for MEDLINE]

2461. Przegl Dermatol. 1981 Sep-Dec;68(5-6):571-4.

[Allergy to jellyfish and seaweed in the light of allergological studies].

[Article in Polish]

Raszeja-Kotelba B, Bowszyc J, Sarnowska I, Derezińska G, Kaczorowska E.

PMID: 6126915 [PubMed - indexed for MEDLINE]

2462. Wiad Lek. 1981 Aug 1;34(15):1247-9.

[Leukocyte migration inhibition test with food allergens and E. coli 0-14 antigen in patients with ulcerative colitis].

[Article in Polish]

Kuczyńska-Sekieta K.

PMID: 7036540 [PubMed - indexed for MEDLINE]

2463. Morphol Igazsagugyi Orv Sz. 1981 Jul;21(3):161-7.

[Morphology and cytodynamics of oxazolone-induced cellular immune response].

[Article in Hungarian]

Kalász V, Németh A.

Dynamics of lymphoid tissue changes induced by oxazolone, a contact allergen responsible for immune reaction of delayed type was studied. Basing on light-and electron microscopic findings and morphometrical analysis the following components of the reaction may be distinguished: an early active lymphocytic migration, a delayed paracortex proliferation and a following follicular response. In the regulation of the outflow of lymphocytes a primary role may be attributed to the endothelial lining of the postcapillary venules. For the morphological changes of endothelial cells an effect of circulating antigen specific lymphocytes may be responsible.

PMID: 7266549 [PubMed - indexed for MEDLINE]

2464. Immunopharmacology. 1981 Jun;3(2):161-78.

Statistically designed assay of migration-inhibitory activity in lymphokine preparations.

den Hollander FC, van Lieshout JI, Schuurs AH.

The migration inhibiton test in agarose as described by Clausen (Acta Allergol 26:56, 1971) was modified into a statistically designed assay comprising a laboratory standard in each assay, and using buffy coat cells or peripheral polymorphonuclear leukocytes from pigs as target cells. The precision of the assay was improved by combining cells from several pigs. Fifty-5000 microliters of lymphokine-containing test fluid could be used in the assay provided that this fluid contains the essential nutrients. In our hands, the capillary tests was unsuitable for a quantitative assessment of migration inhibition activity.

PMID: 7251343 [PubMed - indexed for MEDLINE]

2465. Hepatogastroenterology. 1981 Jun;28(3):147-50.

Studies on intrahepatic cholestasis in drug-induced allergic hepatitis: intrahepatic cholestasis induced in the rat by the culture supernatant of activated lymphocytes.

Mizoguchi Y, Ohnishi F, Monna T, Yamamoto S, Otani S, Morisawa S.

A marked reduction in bile flow and bile acid excretion was whenever peripheral lymphocytes from patients with drug-induced allergic intrahepatic cholestasis were stimulated with a specific drug in vitro in the presence of a soluble liver-specific antigen fraction, and their culture supernatant injected into the mesenteric vein of rats. A gel filtration study of the active fraction of the supernatant that caused a reduction in bile flow, suggested that the molecular size of this active principle is similar to that of the macrophage migration inhibitory factor (MIF). Histologically, dilated bile canaliculi with decreased microvilli were observed via electron microscopy in rat liver after injection of culture supernatant. No such changes were observed in rats after injection of the supernatant of a lymphocyte culture similarly prepared from normal individuals. These results strongly suggested that sensitized lymphocytes obtained from patients with drug-induced intrahepatic cholestasis produce a factor (of factors) causing cholestasis when stimulated with a specific drug in the presence of liver-specific antigen fractions.

PMID: 7250894 [PubMed - indexed for MEDLINE]

2466. Nihon Hifuka Gakkai Zasshi. 1981 May;91(6):653-7.

[Study on the abnormal skin reactions of atopic dermatitis utilizing the skin window technique: macrophage and neutrophil migration (author's transl)].

[Article in Japanese]

Take M.

PMID: 7031311 [PubMed - indexed for MEDLINE]

2467. Nutr Rev. 1981 Mar;39(3):122-3.

Immunologic assessment of cow's milk allergy.

[No authors listed]

PMID: 7027096 [PubMed - indexed for MEDLINE]

2468. Vopr Virusol. 1981 Mar-Apr;(2):196-200.

[Tests of specific cellular and humoral immunity in herpetic infections of the eyes].

[Article in Russian]

Slepova OS, Murav'eva TV, Zaĭtseva NS.

The ratio of the values of lymphocyte blast transformation and leukocyte migration inhibition to the level of humoral antibodies, detection of HSV antigen in the eye and activity of the clinical process in the time course of disease was studied in 213 patients with ophthalmoherpes. The immunological markers characterizing the features of pathogenesis and prognosis of herpetic eye disease

were determined. In the favourable course of ophthalmoherpes blast transformation of 5-10% and stable migration inhibition throughout the disease were observed. A decrease in the functional activity of lymphocytes determined in the blast transformation test with phytohemagglutinin, the lack of specific blast transformation and migration inhibition, as well as marked increase of blast formation (above 10-15%) are the factors aggravating the course of the disease. The infectious and infectious-allergic period in the course of ophthalmoherpes were identified. The infectious stage was characterized by changes in the values of both cellular and humoral immunity. In the infectious-allergic stage cellular immunity reactions prevail in the presence of stable humoral antibody levels.

PMID: 7023054 [PubMed - indexed for MEDLINE]

2469. Cesk Dermatol. 1981 Feb;56(1):7-10.

[Uses of the indirect migration inhibition test (MIT) in contact allergy to chromium (author's transl)].

[Article in Czech]

Znojemská S, Kalenský J, Janecková V, Pekárek V, Svejcar J.

PMID: 6452228 [PubMed - indexed for MEDLINE]

2470. J Clin Invest. 1981 Feb;67(2):547-52.

Enhancement of basophil chemotaxis in vitro by virus-induced interferon.

Lett-Brown MA, Aelvoet M, Hooks JJ, Georgiades JA, Thueson DO, Grant JA.

It is well established that viral infections may precipitate or worsen attacks of bronchial asthma. Furthermore, in symptomatic atopic subjects, the local accumulation of basophils and the production of a basophil chemotactic factor have been reported. We have investigated the effect of cell-free supernates from viral stimulated cultures of human mononuclear cells on the in vitro migration of human basophils. Our results show the presence of a factor in these culture supernates that enhances the migration of basophils toward two separate chemoattractants, a peptide from C5 and a lymphokine. The enhancing activity, while affecting basophil migration, did not change the response of monocytes. The enhancing activity resembled viral-induced interferon when (a) pH 2 stability, (b) heat resistance, (c) trypsin sensitivity, and (d) species-specificity were compared. Finally, the enhancing activity for basophil chemotaxis and the interferon titer were highly correlated in preparations with a 10(4)-fold difference in interferon specific activity. Our studies show that viral-induced interferon can augment the in vitro chemotactic response of basophils. Because mediators present in basophils may be involved in the pathogenesis of immediate hypersensitivity, the modulation of basophil movement by interferon suggests a possible mechanism for the association between viral infections and atopic disorders.

PMCID: PMC370598
PMID: 6161946 [PubMed - indexed for MEDLINE]

2471. Sem Hop. 1981 Jan 8-15;57(1-2):5-15.

[152 cases of tuberculosis in African Negro immigrants in a Parisian service of internal medicine (author's transl)].

[Article in French]

Piéron R, Blanchi A, Lesobre B, Mafart Y.

This study of 152 cases of tuberculosis in African Negro immigrants seen between 1972 and 1976 showed the predominance of especially pulmonary respiratory lesions and/or hilar ganglio-mediastinal lesions. The other lesions are frequent, i.e. the lesions of lymph nodes, bones, peritoneum, liver, pericardium ; the multiple lesions are likewise frequent. Smears and culture of sputum and/or biopsy of organs or tissues such as pleura, lymph node, liver and peritoneum are the diagnostic procedures. In spite of frequent resistance, treatment with isoniazid, rifampicin and ethambutol gave good results in high risk subjects, who are almost always allergic on arrival in France.

PMID: 6258238 [PubMed - indexed for MEDLINE]

2472. Zentralbl Veterinarmed B. 1981;28(3):210-25.

Ultrastructural study of experimental allergic neuritis in the chicken. I. Cell migration, granuloma formation and demyelination.

Ichijo K, Fujimoto Y, Okada K.

PMID: 7282179 [PubMed - indexed for MEDLINE]

2473. Trans R Soc Trop Med Hyg. 1981;75(1):1-4.

Chironomid midges as a cause of allergy in the Sudan.

Cranston PS, Gad El Rab MO, Kay AB.

Hypersensitivity to Chironomidae (non-biting midges) has been a problem in the Sudan since about 1927 and appears to be due to increased breeding of a single chironomid species, Cladotanytarsus lewisi (Freeman). Mass emergence of the midges in thought to be related to the larval diet of algae and diatoms, the numbers of which are greatly enhanced by the retention of plant nutrients in lacustrine conditions resulting from interruption to the natural flow of the Nile by the construction of dams. Immunological studies in allergic individuals using an allergen extract prepared from C. lewisi indicate that the concentration of specific immunoglobulin E ("allergic antibody") directed against C. lewisi is raised in patients with established hypersensitivity to the midge but not in control subjects. The concentration of specific IgE is also related to the severity of clinical symptoms. These results indicate that this widespread and important "man made" hypersensitivity in the Sudan has the features of well recognized immediate-type allergy commonly associated with pollens and other air-borne allergens.

PMID: 7268834 [PubMed - indexed for MEDLINE]

2474. Z Erkr Atmungsorgane. 1981;156(2):159-66.

[The complex immunological examination of tuberculous patients (author's transl)].

[Article in German]

Tschernuschenko EF, Mamolat AS, Kogosowa LS, Petraschenko AI.

The significance of immunological reactivity in tuberculosis was investigated in 325 patients with various forms of pulmonary tuberculosis (disseminated, destructive, severe, treated, and not yet treated forms) for T-lymphocytes with the aid of the spontaneous rosette formation, the lymphocytes transformation test ot phytohaemagglutinin, and the determination of the lymphocyte sensibility to cortisol; for the B-lymphocytes with the aid of complement rosette formation, the determination of immunoglobulins M, G, A, and the heterophile agglutinins; for the evaluation of the intensity of tuberculin allergy by the cutaneous test, the lymphocyte transformation test, the leucocyte migration inhibition test, the neutrophils toxicity test to tuberculin, and the passive haemagglutination. These investigations revealed a considerable impairment of the immunological reactivity in dependence of disease pattern and duration.

PMID: 7257447 [PubMed - indexed for MEDLINE]

2475. Int Arch Allergy Appl Immunol. 1981;65(2):144-52.

Depression of neutrophil chemotaxis in atopic individuals. An H2 histamine receptor response.

Radermecker M, Maldague MP.

Neutrophil chemotaxis was compared in normal and atopic individuals using a modified Boyden chamber with as chemotactant, autologous serum either unactivated or activated by zymosan or an endotoxin-containing house dust preparation. A high incidence of defective leukotaxis was found in atopics when cells were opposed to activated serum. The cause of this abnormality is not intrinsic to the leukocytes since random migration and chemotaxis towards unactivated serum were comparable in normal and atopic subjects. The defect persisted when atopic leukocytes were opposed to activated normal serum and the chemotactic response of normal neutrophils was not imparied when tested against activated atopic serum. Leukotaxis was significantly depressed by incubating atopic leukocytes with allergen to which they were sensitized, suggesting an inhibitory effect of mediators of anaphylaxis. Histamine inhibited in vitro neutrophil chemotaxis in normal and atopic subjects. This inhibition was dose-related and significantly more pronounced in atopics. Incubation of atopic leukocytes with an H2 antagonist, cimetidine, was capable of enhancing their chemotactic responsiveness towards activated autologous serum to levels observed in normal controls. In the same conditions, an H1 blocker, promethazine, was without effect. These data indicate that the leukotactic dysfunction of atopic individuals results from an abnormal sensitivity of these leukocytes to histamine which, in the chemotactic chamber, may be released from basophils by products of complement activation and, in some experimental conditions, by antigen to which cells are sensitized.

PMID: 7228431 [PubMed - indexed for MEDLINE]

2476. Vopr Onkol. 1981;27(2):39-43.

[Sensitization study of oncological patients to bacterial allergens with the aid of the leukocyte migration inhibition reaction].

[Article in Russian]

Kochetkova VA, Znamenskaia IN.

The results of studying sensitization of 88 patients with cancer (larynx,

esophagus, ovaries) to 7 bacterial allergens (pathogenic and nonpathogenic staphylococcus, hemolytic streptococcus, catarrhal diplococcus, Proteus, Escherichia coli and blue pus bacilli) are reported. These bacilli are most frequently the cause of development of postoperative suppurations. The studies performed (before, during and after the combined therapy) indicated by leucocyte migration inhibition test that during the disease ad therapeutic course there occurs nonspecific sensitization of leucocytes to bacterial allergens which gradually disappears following termination of the therapy. Postoperatively, the sensitization offers grounds for the development of suppurative complications, the latter is associated with the enhanced inhibition of leucocytes migration by an allergen of that microbial species which caused the development of suppurative complications. Therefore, this test with a set of bacterial allergens may be used as a diagnostic adjunct early reveal the postoperative suppurations.

PMID: 7210594 [PubMed - indexed for MEDLINE]

2477. Sud Med Ekspert. 1981 Jan-Mar;24(1):57-8.

[Case of anaphylactic shock complicating the migration phase of superinvasive ascariasis].

[Article in Russian]

Astaf'ev BA, Mititel AV.

PMID: 7210098 [PubMed - indexed for MEDLINE]

2478. Gastroenterol Jpn. 1981;16(3):249-59.

Immunological studies on the drug-induced allergic hepatitis.

Mizoguchi Y, Shiba T, Ohnishi F, Monna T, Yamamoto S, Morisawa S.

The possible involvement of cell-mediated immunity in the pathogenesis of drug-induced allergic hepatitis was investigated in 21 patients; 6 patients with cholestasis, two cases with the hepatitis resembling viral hepatitis and 13 cholestatic hepatitis. The peripheral blood lymphocytes from all these patients showed the positive lymphocyte transformation and MIF production when stimulated by the offending drug in the presence of liver specific lipoprotein. By injection of the culture medium prepared from activated lymphocytes into mesenteric vein of rat, a marked reduction of bile flow and bile acid secretion was observed in 12 cases among 17 patients tested. Active material which caused the reduction of bile flow was fractionated by a gel filtration and was identified to have similar molecular size to MIF. Morphologically, a dilated bile canaliculus with diminution of microvilli and vesicles around the dilated bile canaliculus were observed by an electron microscopy after injection of culture supernatant or their fractionated material into mesenteric vein of rat. No such changes could be seen in rats by administering the supernatant of lymphocytes from normal individuals prepared as above. Macrophage activating factor (MAF), a kind of lymphokines, was also detected in the culture medium of activated lymphocytes from seven patients among eight cases tested. The MAF-activated macrophages were shown to exhibit a cytotoxic effect on the separated liver cells by judging from the inhibition of albumin biosynthesis. Moreover, the antibody-dependent cell-mediated cytotoxic reaction as well as lymphocyte-mediated cytotoxicity were also demonstrated in three cases among nine patients tested. These observations suggest that diverse immune reactions were possible correlated to the pathogenesis of the drug-induced allergic hepatitis although their exact participation or relative significances are remained to be elucidated.

PMID: 7021300 [PubMed - indexed for MEDLINE]

2479. Med Pr. 1981;32(6):423-7.

[Modified leukocyte migration inhibition test with workers of the buildings and metals industries with chromium eczema].

[Article in Polish]

Bowszyc J, Bowszyc J, Hryniewiecka I.

In 59 workers of metal, building and galvanizing industries a test of leucocytes migration inhibition was carried out. We modified this test so that it consists in that the whole blood was used with no isolated leucocytes, with no plexiglass chambers but with very thin capillaries and short incubation. The result of the test is obtained on the day it is carried out (after 6 hours). A comparison with the results of epidermal test using 0.5% of potassium dichromate confirmed the specificity of the test. The sensitivity of the test was however less, compared to epidermal tests and the standard test of leucocytes migration.

PMID: 6210822 [PubMed - indexed for MEDLINE]

2480. Acta Derm Venereol. 1981;61(6):517-23.

Human leukocyte mobilization and morphology in nickel contact allergy using a skin chamber technique.

Lerche A, Bisgaard H, Christensen JD, Søndergaard J.

An improved skin chamber technique has been devised and used for quantitative evaluation of the leukocyte mobilization rate (LMR). The method was applied in 10 nickel-hypersensitive patients exposed to nickel sulphate. Each patient served as his own control and for additional control purpose, 5 healthy individuals without nickel hypersensitivity were studied. The kinetics of the mobilized leukocytes were followed over a 48-hour period. After an initial lag phase of 2-4 hours, maximum migration was observed from the 24th to the 48th hour, with a wide interindividual variability in the number of mobilized cells at the time of maximum LMR response. The median cumulative leukocyte count was 1.412 x 10(6) leukocytes/cm2/48 h. In the same period a statistically significant increase in the basophils for all the nickel allergic patients was observed. In 8 out of 10 patients a statistically significant effect upon the lymphocytes could be demonstrated. Six of these 8 patients had an increased lymphocyte mobilization. Throughout the period the neutrophil granulocytes were the dominant cell type, although the number decreased as the number of basophils and lymphocytes increased. The chamber technique is a valuable means for quantitative evaluation of leukocyte mobilization and morphology in skin exudates during exposure to an allergen in delayed hypersensitivity reactions.

PMID: 6177163 [PubMed - indexed for MEDLINE]

2481. Lab Delo. 1981;(6):362-4.

[Determination of indices of delayed-type autoallergies in children with respiratory allergies].

[Article in Russian]

Osin AIa, Osina TD.

PMID: 6169919 [PubMed - indexed for MEDLINE]

2482. Arch Immunol Ther Exp (Warsz). 1981;29(1):49-56.

The evaluation of immune status in children with the past history of obstructive bronchitis.

Sciślicki A, Rudnik J, Gaweł J, Pryjma J.

Selected parametres reflecting cellular and humoral immunity were analyzed in 98, 10 year-old children, who had been hospitalized during first 2 years of life because of bronchitis. In this group, 46 children suffered from obstructive bronchitis and 52 from other types of bronchitis. Serum immunoglobulins, complement components, and antiproteolytic factor levels, T and B lymphocytes counts, leukocyte migration inhibition, and skin tests to PHA and Polyvaccine were compared and analyzed in relation to bronchial obstruction and allergic symptoms in early childhood. The results obtained did not show any significant difference when the group with obstructive type of bronchitis was compared to the rest of children studied. Some differences (increase of the percentage of EAC-FRC and decrease of the levels of the antiproteolytic factors) were noted in relation to allergic symptoms.

PMID: 6169321 [PubMed - indexed for MEDLINE]

2483. Clin Allergy. 1981 Jan;11(1):37-41.

Supravital observation of in vitro basophils in immunological reactions.

Kimura I, Tanizaki Y, Sato S, Takahashi K, Saito K, Ueda N.

The migration velocity and morphology of basophils in vitro were examined after the addition of anti-immunoglobulin (anti-IgE or anti-IgG). In atopic asthma patients with high serum IgE levels (more than 1000 i.u./ml) basophils showed increased migration velocity and showed pear-shaped and palmate processes after the addition of anti-IgE, but on the addition of anti-IgG, these basophils did not show increased migration velocity or morphological changes. In intractable asthma patients with low serum IgE levels, these changes occurred with anti-IgG but not with anti-IgE. These in vitro findings suggest a basophil reactivity to anti-IgE and anti-IgG in individual patients and support our previous differentiation of asthma patients according to basophil reaction.

PMID: 6163572 [PubMed - indexed for MEDLINE]

2484. Nouv Presse Med. 1980 Dec 6;9(46):3505-8.

[Drug allergy].

[Article in French]

Paupe J.

PMID: 7243578 [PubMed - indexed for MEDLINE]

2485. Immunology. 1980 Dec;41(4):989-95.

The effects of a soluble factor released by sensitized mononuclear cells incubated with S. haematobium ova on eosinophil migration.

Wadee AA, Sher R.

Incubation of sensitized mononuclear cells from patients with schistosomiasis with specific antigen containing a suspension of viable Schistosoma haematobium ova resulted in the release of a soluble factor which was chemotactic for eosinophils. Production of this substance was detectable at 24 h and demonstrated peak chemotactic activity after 2 days in culture. The chemotactic potential was dose-dependent and attracted eosinophils obtained from patients with schistosomiasis or allergic diathesis. Human neutrophil motility was unaffected by this chemoattractant. Preliminary studies demonstrated that the chemotactic factor is a heat-stable substance with peak activity associated with a molecular weight of approximately 42,000. These findings may reflect an in vitro correlate of cell-mediated immunity and may indicate a role played by the lymphocyte in the control of eosinophil function in human biology.

PMCID: PMC1458290
PMID: 7461720 [PubMed - indexed for MEDLINE]

2486. Immunology. 1980 Dec;41(4):981-8.

Suppression of antibody-mediated accumulation of eosinophils in chronic inflammatory lesions by concomitant delayed hypersensitivity reactions.

van den Berg WB, Haasakker TC, Bax J, Scheper RJ.

Studies were carried out in order to explain the often small contribution of eosinophils to immunologically mediated chronic inflammatory reactions. A chronic inflammation model was used in which large numbers of eosinophils accumulated in the peritoneal injections with PPD or ovalbumin (OA). Eosinophil accumulation could be strongly suppressed by pre-immunization with a mycobacteria-containing adjuvant, which induces delayed hypersensitivity to the stimulating antigen. Suppression of OA-induced eosinophil accumulation could be obtained in a non-specific way by concomitant delayed hypersensitivity reactions to PPD. The absence o eosinophil accumulation was not related to a lack of relevant antibodies. Histological studies suggested that similar numbers of basophils were locally available in both non-pre-immunized and pre-immunized groups to allow for the attraction of eosinophils by the expression of basophil anaphylactic reactivity. Skin tests and macrophage migration inhibition measurements provided further support for the hypothesis that local lymphokine release may suppress the accumulation of eosinophils in chronic inflammatory lesions.

PMCID: PMC1458300
PMID: 7461719 [PubMed - indexed for MEDLINE]

2487. Klin Monbl Augenheilkd. 1980 Nov;177(5):584-600.

[Toxocara canis (larva migrans visceralis) from an ophthalmological point of view (author's transl)].

[Article in German]

Huismans H.

Visceral Larva Migrans (Toxocara canis) is usually a relatively benign disease which is caused by infective second-stage larvae of the common cosmopolitan ascarid of dogs, characterized chiefly by sustained eosinophilia, pulmonary symptoms and hepatomegaly. Its severity varies with the number of larvae in the tissue and the immune or allergic state of the infected individual. The most important aspect of the neurotropic larvae perhaps is its potential as a facilitating agent, for instance, for Virus or Toxoplasma gondii invasion of the central nervous system by destroying the blood-brain barrier. Ocular invasion characteristically occurs after primary infestation, seldom bilaterally. Larvae may present three different ocular lesions: a granulomatosis at the posterior pole (solitary granuloma), a chronic endophthalmitis or peripheral retinal lesions with proliferation. Prognosis regarding visual acuity depends on early diagnosis and larval localization. The author reports on successful therapy with a combination of antibiotics, sulfonamides, prednisolone and vermifuge. The microprecipitation test on living larvae is considered to be superior all serological tests at present but a negative result (at first) does not exclude T. canis invasion (compare case report). LMV syndrome should be ruled out if the patient suffers from cerebral spasms whose cause is unclear.

PMID: 7253511 [PubMed - indexed for MEDLINE]

2488. Postgrad Med J. 1980 Oct;56(660):738-41.

Cholestatic jaundice: an immune response to prajmalium bitartrate.

Rotmensch HH, Liron M, Yust I, Klejman A, Livni E, Moalem T, Gefel A.

Cholestatic jaundice associated with chills, pruritus and blood eosinophilia developed in a patient who received prajmalium bitartrate therapy for ventricular arrhythmia following acute myocardial infarction. Discontinuation of the drug resulted in a spontaneous improvement in the clinical and biochemical findings. Challenge by prajmalium bitartrate caused rapid reappearance of the clinical and biochemical features. In immunological studies, deposits of IgG and IgA were detected at the bile canaliculi by fluorescent staining, and the patient's lymphocytes produced macrophage migration inhibition after in vitro incubation with prajmalium bitartrate. Thus, laboratory results support the assumption of an allergic mechanism.

PMCID: PMC2426036
PMID: 7220413 [PubMed - indexed for MEDLINE]

2489. J Allergy Clin Immunol. 1980 Oct;66(4):314-21.

Leukocyte inhibition factor in delayed-onset food allergy.

Minor JD, Tolber SG, Frick OL.

To investigate the possiblity of a cellular immune hypersensitivity reaction in patients who developed allergic symptoms 2 or more hours after ingestion of a particular food, two in vitro tests were employed: leukocyte migration inhibition factor (LIF) and lymphoblastogenesis. Of the children and adults with food allergy, 73% (30 of 41) had a positive LIF test with whole cow's milk or its fractions or corn. Of nonallergic or grass-pollen sensitive controls, 15% (four of 26) had positive LIF. Lymphocyte transformation often correlated with LIF results in food-allergic patients but was also positive in 77% of controls (seven of nine). We suggest that many patients with delayed-onset food-induced allergy symptoms may have a cellular immune component to their sensitivity. Serum IgA, where measured, was absent or low in half of these patients.

PMID: 6999066 [PubMed - indexed for MEDLINE]

2490. Pediatrics. 1980 Sep;66(3):399-402.

In vitro cell-mediated immunologic assay for cow's milk allergy.

Ashkenazi A, Levin S, Idar D, Or A, Rosenberg I, Handzel ZT.

The production of a lymphokine, the leukocyte-migration-inhibition factor (LIF), by peripheral blood lymphocytes in response to an in vitro challenge with bovine beta-lactoglobulin was assayed in infants and children suspected of having allergy to cow's milk protein. Of the patients studied, 24 had cow's milk allergy, 24 were normal control subjects, 18 had recovered from milk allergy, 10 were newborns, and 10 were babies suffering from acute gastroenteritis. All patients with milk allergy demonstrated significant LIF production in response to beta-lactoglobulin (23.5% +/- 6.4%). In the normal control subjects, LIF was 3.1% +/- 4.3% (P < .0005). Only two of the 24 control subjects and two of the ten newborns had high-normal values bordering on the positive. None of the ten babies with acute gastroenteritis gave a positive response. Most of the children who had recovered from milk allergy and were ingesting cow's milk had negative assays. This cell-mediated immune assay is shown to be a reliable test for the diagnosis of sensitivity to milk protein in infants and children, and for determining dietary treatment and when this treatment can be safely terminated. In most cases, its use should eliminate the need for the potentially dangerous and ethically questionable provocation test, as well as the need for repeated intestinal biopsies.

PMID: 6999447 [PubMed - indexed for MEDLINE]

2491. Vestn Dermatol Venerol. 1980 Sep;(9):38-40.

[Leukocyte migration inhibition reaction in Bacto-Agar and its clinical use in occupational allergic dermatoses].

[Article in Russian]

Toktaliev DD.

PMID: 6448523 [PubMed - indexed for MEDLINE]

2492. Immunology. 1980 Aug;40(4):673-81.

Chronic inflammatory reactions in the guinea-pig peritoneal cavity induced by continuous local PPD stimulation. Immune status dependency of the accumulation of lymphocytes and eosinophils.

van den Berg WB, Haasakker TC, van Maarsseveen AC, Scheper RJ.

A chronic inflammation model is described which allows the study of the relationship between the level of specific cell-mediated and humoral immunity to the triggering antigen, and the presence of cells or mediators in the inflammatory exudate. In the present study special attention is paid to the participation of lymphocytes and eosinophils. Normal or FCA-pre-immunized guinea-pigs received repeated intraperitoneal injections with PPD for periods up to 21 weeks. In non-pre-immunized animals the inflammation was characterized by a strong accumulation of eosinophils, whereas only a few lymphocytes were present.

In contrast, the FCA-pre-immunized guinea-pigs showed a strong lymphocytic accumulation in the absence of eosinophils. Both peritoneal inflammations were shown to remain dependent on the continuous PPD administration. Therefore, the participation of either lymphocytes or eosinophils could be directly correlated to differences in the specific immune status. Both experimental groups developed similar strong anti-PPD antibody responses as assessed by haemagglutination and passive cutaneous anaphylaxis. Only the FCA-pre-immunized guinea-pigs, however, showed strong cell-mediated immunity. The development of CMI to the continuously administered antigen appears to be a prerequisite for the accumulation of lymphocytes, while it seems to prevent antibody-mediated eosinophil accumulation.

PMCID: PMC1458090
PMID: 7000691 [PubMed - indexed for MEDLINE]

2493. Aust N Z J Med. 1980 Aug;10(4):449-53.

The role of alpha 1 protease inhibitor (alpha 1 antitrypsin) in the regulation of immunologic and inflammatory reactions.

Breit SN, Penny R.

Twenty-six different alleles have been identified for alpha 1 protease inhibitor (alpha 1 antitrypsin), each designated by a letter of the alphabet. In any individual two alleles codominantly determine the characteristics of alpha 1 protease inhibitor (Pi), including mobility on electrophoresis, serum concentration and acute phase response. Recent evidence has linked some mildly deficient phenotypes of Pi with a variety of chronic immunologic and inflammatory disorders, such as rheumatoid arthritis, juvenile chronic arthritis, systemic lupus erythematosus, ankylosing spondylitis, uveitis, asthma and fibrosing alveolitis, in addition to the well recognised association of severe deficiency with emphysema and chronic liver disease. This disease susceptibility in phenotypes associated with reduced serum levels may be due to alteration in lymphocyte responses, complement activation and leukocyte migration. Pi can also influence the autolytic effects of leukocytic enzymes on tissues and may inhibit some aspects of coagulation and fibrinolysis. Therefore patients with deficient Pi phenotypes are likely to have exaggerated immunologic and inflammatory responses.

PMID: 6968557 [PubMed - indexed for MEDLINE]

2494. Zh Mikrobiol Epidemiol Immunobiol. 1980 Jul;(7):75-9.

[Effect of antigen dose on the course of experimental mycogenic sensitization].

[Article in Russian]

Sardyko NV.

Guinea pigs were sensitized with different doses of homogenous Candida albicans cells introduced by 3 subcutaneous injections at intervals of 2--3 days. The allergic rearrangement in the animal body thus induced was found to depend on the dose of the preparation injected into the animals: the lowest dose of the antigen (0.1 mg) induced immediate allergic reaction, and doses 5--10 times higher induced double rearrangement i.e. immediate and delayed reactions.

PMID: 7001816 [PubMed - indexed for MEDLINE]

2495. Rev Ig Bacteriol Virusol Parazitol Epidemiol Pneumoftiziol Pneumoftiziol. 1980 Jul-Sep;29(3):137-42.

[Significance of immunological investigation in pulmonary pathology].

[Article in Romanian]

Cernuşenko EF, Mamolat AS, Kogosova LS, Lerner CI.

An extensive range of investigations were used in a comparative study of immune reactivity in the tuberculous pulmonary pathology, as well as in sarcoidosis, pneumonia and cancer of the lungs. T and B lymphocyte populations were followed, indexes of leucocyte migration, induced lymphoblastogenesis to phytohaemagglutinin, and the serum levels of immunoglobulins. Also the intensity of the hypersensitivity to tuberculin was investigated, to allergens extracted from staphylococcus and streptococcus strains, as well as against organ antigens (pulmonary tissues). Differences were evidenced, of immunologic reactivity in various nosologic entities investigated. The dynamic follow-up of some of the cases has demonstrated that the immunological reactivity varies in parallelly with severity of cases, deficiences being corrected by adequate therapy. The immunologic status may be considered a criterium for a rational pathogenetic treatment, for assessing the efficiency of such a treatment, as well as for the differential diagnosis of pulmonary diseases.

PMID: 6254126 [PubMed - indexed for MEDLINE]

2496. Ann Allergy. 1980 Jul;45(1):37-42.

A practitioner's guide to interferon.

Warren SL.

The discovery and subsequent development of interferon as an anti-viral, ant-cancer therapeutic agent may well be a major milestone in the history of medical therapeutics. The results of the limited clinical trials available offer great promise for this biological agent. Only time, with large scale clinical trials, will serve to evaluate the true benefit of this modality of therapy. In the interim it is most desirable that the prcticing allergist-immunologist maintain an awareness of the use and mens of production of interferon so that he has available at his descretion a therapeutic modality for possible use in cases that have drastic, very poor or no therapeutic alternatives, remembering that each case add to the compendium of clinical knowledge.

PMID: 6157337 [PubMed - indexed for MEDLINE]

2497. Pol Tyg Lek. 1980 Jun 16;35(24):909-11.

[Leukocyte migration inhibition test in the allergological diagnosis of pollen-induced asthma and evaluation of the results of desensitization treatment].

[Article in Polish]

Grzegorczyk J, Kałła M, Rozniecki J.

PMID: 7413514 [PubMed - indexed for MEDLINE]

2498. Am J Epidemiol. 1980 Jun;111(6):728-35.

Asthma and bronchitis in Sydney schoolchildren. II. The effect of social factors and smoking on prevalence.

Peat JK, Woolcock AJ, Leeder SR, Blackburn CR.

During a study of the prevalence of asthma and bronchitis in Sydney schoolchildren, some social and environmental factors were documented to ascertain if these affected the prevalence of either of these diseases. Socioeconomic status obtained from father's occupation, area of residence, family size, nationality, country of birth and smoking habits were examined. Although small differences were found between groups, no consistent relationship was found between social class and lung disease with the exception of increased prevalence of asthma in boys in social class I and girls in social class II. There were no major associations with area of residence or with family size. Children who were born outside Australia tended to have less asthma and bronchitis than children born inside Australia and migrant children tended to first develop asthma several years after arriving in Australia. Bronchitis was found to be more common in children with an earlier history of bronchitis in infancy and early childhood. The prevalence of recent bronchitis in the group of children who smoked 20 or more cigarettes per week was higher than that in the nonsmoking group of children.

PMID: 7386448 [PubMed - indexed for MEDLINE]

2499. Stomatologiia (Mosk). 1980 Jun;59(3):5-8.

[Leukocyte migration inhibition phenomenon in vivo and in vitro in drug allergy].

[Article in Russian]

Ado AD, Bondareva GP, Chitaeva VG.

PMID: 6930725 [PubMed - indexed for MEDLINE]

2500. Zh Mikrobiol Epidemiol Immunobiol. 1980 May;(5):103-6.

[Use of the basophil degranulation test and the leukocyte migration inhibition test to characterize mycogenic sensitization].

[Article in Russian]

Sardyko NV.

The results of experiments in using the basophil degranulation test and the leukocyte migration inhibition test for the characterization of mycogenous sensitization are presented. The model of mycogenous sensitization was created in guinea pigs by 3 injections of homogenized Candida albicans cells. Immediate allergic reaction is shown to develop earlier, as indicated by the results of the determination of homocytotropic antibody tites in passive skin anaphylaxis and by the results of the basophil degranulation test, than the appearance of delayed hypersensitivity detected by the leukocyte migration inhibition test.

PMID: 6998215 [PubMed - indexed for MEDLINE]

2501. J Laryngol Otol. 1980 May;94(5):507-14.

Altered cell mediated immunity in atrophic rhinitis.

Fouad H, Afifi N, Fatt-Hi A, El-Sheemy N, Iskander I, Abou Saif MN.

Cellular immunity was studied in patients with atrophic rhinitis using the in vitro leucocyte migration and the spontaneous rosette tests. Cellular hypersensitivity to crude nasal homogenate was detected in 90 per cent of our cases as shown by inhibition of the leucocyte migration. The spontaneous rosette test showed a reduction in the number of T-cells forming rosettes which reflects a decrease in the absolute number of T-lymphocytes. This altered cellular reactivity or loss of tolerance to nasal tissues may be precipitated primarily by virus infection, malnutrition and/or immuno-deficiency which trigger a destructive auto-immune process with the release of antigen(s) of nasal mucosa into the circulation.

PMID: 6967505 [PubMed - indexed for MEDLINE]

2502. Am J Surg. 1980 Apr;139(4):477-9.

Metal allergy and the surgical patient.

Mayor MB, Merritt K, Brown SA.

Allergy to metal implants is under study at the Dartmouth-Hitchcock Medical Center using in vitro examination of white blood cell migration. Retrospective data from 121 patients confirm that allergic responses do occur. Prospective data are being gathered to investigate the incidence, prevalence and relation to morbidity.

PMID: 6989280 [PubMed - indexed for MEDLINE]

2503. Ann Allergy. 1980 Apr;44(4):225-7.

Infection and allergy.

Bristow VG.

PMID: 6154435 [PubMed - indexed for MEDLINE]

2504. Environ Health Perspect. 1980 Apr;35:153-64.

The lung mast cell: its physiology and potential relevance to defense of the lung.

Wasserman SI.

The mast cell, located at mucosal surfaces and surrounding venules, is uniquely positioned to respond rapidly to insults to the host by mediating the development of a wide-ranging inflammatory response. Activaton of the mast cell releases preformed granule-associated chemical mediators and generates de novo biologically active materials. The properties of the mast cell mediators permit development of both acute and prolonged inflammatory responses. the immediate response is characterized by edema and the delayed response by leukocyte infiltration and vascular damage. the mast cell mediators responsible for these inflammatory events are characterized functionally. The vasoactive/smooth muscle reactive mediators include preformed histamine and serotonin and newly-generated

platelet activating factor, slow reacting substance of anaphylaxis and prostaglandins. Chemotactic mediators include eosinophil-selective ECF-A and ECF-oligopeptides, neutrophil-selective NCF, and lipid chemotactic mediators with broad specificity. These factors induce directed migration and localization of leukocytes. The mast cell releases the structural proteoglycan, heparin, which is anticoagulant and inhibits complement. Released mast cell enzymes include chymotryptic and tryptic proteases, arylsulfatase, beta-glucuronidase, and hexosaminidase. The proteolytic enzymes may activate inflammatory pathways while the others degrade ground substance. The capacity of the mast cell to enhance vascular permeability, to cause the influx of regulatory or inflammatory leukocytes, and to provide a variety of active enzymes permits regulation of inflammatory events at the site of tissue injury.

PMCID: PMC1568448
PMID: 6105956 [PubMed - indexed for MEDLINE]

2505. Sem Hop. 1980 Mar 8-15;56(9-10):409-13.

[Often unrecognized cutaneous manifestations of strongyloidosis: linear dermatitis or larva currens (author's transl)].

[Article in French]

Lapierre J.

The linear dermatitis of "larva currens" is a typical manifestation of strongyliasis, however is generally too unrecognized. It is observed in about 7% of cases, more frequent in white race. Due to the migration of Strongyloides larvae in epidermis of sensitized person, it appears in form of rectilinear urticaria from 10--30 cm, developed from 12--48 hours and disappeared without leaving any trace. It can appear at any place of the body, sometimes in many places simultaneously. The total duration of the evolution similiar to that of strongyliasis can be developed during a many tenth of years. In reality the differential diagnosis is easy with filariasis (loa-loa) and specially with cutaneous larva migrans (ankylostomiasis, creeping eruption, larbish). The thiabendazole cures the strongyliasis nad its cutaneous manifestations in 85% of cases with a single oral dose of 6 tablets 6 x 250 mg).

PMID: 6244657 [PubMed - indexed for MEDLINE]

2506. Sov Med. 1980;(2):3-5.

[Immunological mechanisms of drug allergy].

[Article in Russian]

Zamotaev IP, Mutina ES, Pol'ner AA, Serova TI, Bulycheva NA.

PMID: 7361181 [PubMed - indexed for MEDLINE]

2507. Int Arch Allergy Appl Immunol. 1980;61(1):111-3.

Seasonal variation in drug action and animal responses in models of inflammation.

Warne PJ, West GB.

During the summer months, aspirin was relatively ineffective in rats in

suppressing leucocyte migration both into the pleural space inflamed by carrageenan and into inert sponges implanted subcutaneously. At this time period, rats were insensitive to intravenous histamine and relatively insensitive to anaphylactic shock, and survival rates after traumatic or tourniquet shock have also been reported to be at peak values. The cause of this resistance has still to be found, though hormonal activation may play a major role.

PMID: 7350121 [PubMed - indexed for MEDLINE]

2508. Glas Srp Akad Nauka Med. 1980;(32):213-6.

[Cellular immunity responses in the allergic manifestations of immediate hypersensitivity].

[Article in Serbian]

Sluzić I, Stanojević-Bakić N, Vucković-Dekić Lj, Marinković M, Sami I.

PMID: 7346334 [PubMed - indexed for MEDLINE]

2509. Women Health. 1980 Fall;5(3):17-37.

Recent trends in sex mortality differentials in the United States.

Verbrugge LM.

In the 1970s, the United States population experienced a notable drop in mortality rates, after several decades of relative stability. Has this increased longevity been enjoyed equally by males and females, so that sex mortality differentials are essentially the same as before? Or has one sex benefited more than the other? This paper focuses on the mortality experience of males and females between 1970 and 1977, by age and by leading causes of death. The 1970-1977 data are compared with two earlier periods (1920-1950 and 1950-1970) to show how recent trends contrast with previous ones. Overall, the recent data suggest a new situation for sex mortality differentials. In prior decades, females' longevity advantage over males increased. This continuing increase appeared for virtually all ages and leading causes. But in the 1970s, the increase slowed. Females' situation relative to males actually worsened for several age groups (under 1, 55-64) and for several leading causes (conditions of early infancy, bronchitis/empysema/asthma, homicide, peptic ulcer). Moreover, the pace of females' gains for heart diseases and cancer has slowed, and relative gains have stopped for cerebrovascular diseases and accidents. Reasons for recent changes in sex mortality differentials and possible future trends are discussed.

PMID: 7245783 [PubMed - indexed for MEDLINE]

2510. Pharmatherapeutica. 1980;2(6):397-400.

Leucocyte migration inhibition test results during desensitization treatment of children with atopic asthma.

Sychłowy A.

A study was carried out in 16 children with perennial allergic asthma to evaluate the use of the leucocyte migration inhibition factor, one of the indicators of cellular change effected by specific antigens, as a measure of the efficacy of desensitization therapy with a tyrosine-adsorbed house dust mite vaccine. Eleven

of the children showed a good response and 5 a poor response to desensitization. The results of the migration inhibition test suggest that during specific desensitization therapy asthmatic children acquire the ability to inhibit leucocyte migration by a specific allergen and this phenomenon appears to be related to an improved response to treatment.

PMID: 7001494 [PubMed - indexed for MEDLINE]

2511. J Clin Lab Immunol. 1980 Jan;3(1):39-43.

Transfer of cell migration inhibition in vitro with xenogeneic RNA in experimental allergic orchitis.

Sztein MB, Satz ML, Serrate SA.

Experimental allergic orchitis (EAO) was induced in rats following the injection of homologous testicular homogenate (THr) emulsified in Freund's complete adjuvant. Immune RNA (iRNA) was extracted from the spleen and lymph nodes. Normal guinea-pig peritoneal exudate cells (GP-PEC), when incubated in vitro with iRNA, were able to specifically recognise and respond to the immunising antigens (THr and PPD) as assessed by the direct migration inhibition reaction (MIR). Positive MIR's were also observed when incubated peritoneal exudate cells were tested against testicular homogenates from mice and guinea-pigs. This would confirm the presence of a common testicular antigen(s) in the three species studied. The reaction would appear to be organ-specific, kidney homogenate being unable to cause migration inhibition. GP-PEC incubated with iRNA which had been pretreated with RNAse did not show antigen-specific migration inhibition. Similarly, GP-PEC treated with RNA extracted from non-immunised donor rats did not respond.

PMID: 6991705 [PubMed - indexed for MEDLINE]

2512. Gastroenterol Jpn. 1980;15(1):14-9.

Immunological studies on drug-induced allergic hepatitis--hepatocellular injury by macrophage-mediated cytotoxicity.

Mizoguchi Y, Shiba T, Ohnishi F, Monna T, Yamamoto S, Otani S, Morisawa S.

When the peripheral blood lymphocytes from patients with drug-induced allergic hepatitis were stimulated with a specific drug in vitro in the presence of a liver cytosol fraction containing liver specific antigen, lymphocyte transformation was seen in eight out of 11 patients. The macrophage activating factor (MAF), a kind of lymphokines, was also detectable in the culture medium of activated lymphocytes from seven out of eight patients who showed positive blastogenesis evaluated the uptake of 3H-glucosamine into macrophages. MAF-activated macrophages exhibited a cytotoxic effect on separated liver cells resulting in a marked inhibition of albumin synthesis. This macrophage-mediated cytotoxicity was also observed in eight out of 11 patients who showed positive lymphocyte transformation. These observations suggest that macrophage-mediated cytotoxicity may play some role in the pathogenesis of drug-induced allergic hepatitis.

PMID: 6987126 [PubMed - indexed for MEDLINE]

2513. Cell Immunol. 1980 Jan;49(1):34-42.

The presence of specific antigen-reactive cells during the induction, recovery,

and resistance phases of experimental allergic encephalomyelitis.

Waxman FJ, Fritz RB, Hinrichs DJ.

PMID: 6153156 [PubMed - indexed for MEDLINE]

2514. Biull Eksp Biol Med. 1979 Dec;88(12):700-2.

[Role of T- and B-lymphocytes in the heterogeneity of human cell-mediated reactions to bacterial antigens].

[Article in Russian]

Novikov DK, Novikova VI.

Leucocytes from 30 patients with allergy to tuberculin and bacterial antigens were treated with antithymus (ATS) and anti-immune globulin (AIGS) sera. The leucocyte migration inhibition test (LMIT) was performed with these antigens. ATS abolished the LMIT induced by tuberculin and sometimes by bacterial antigens (staphylococcal, streptococcal etc.). AIGS frequently abolished the LMIT induced by bacterial antigens, but not by tuberculin. In some cases the treatment with any serum abolished the LMIT induced by the antigens, or, on the contrary, it was abolished only by a successive treatment with both sera. The lymphocyte types (T or B) determining the secondary immune response to the same antigen are different in various patients, as well as they differ in the same patients in relation to diverse antigens. Five types of lymphocyte - antigen interrelation in the LMIT have been distinguished.

PMID: 316338 [PubMed - indexed for MEDLINE]

2515. Pol Tyg Lek. 1979 Nov 12;34(46):1803-4.

[Studies of biological functions of granulocytes with special reference to neutrophils and their role in the pathogenesis of bronchial asthma].

[Article in Polish]

Kowal E.

PMID: 545322 [PubMed - indexed for MEDLINE]

2516. Immunology. 1979 Oct;38(2):311-6.

In vitro transfer of cellular immunity in experimental allergic orchitis by means of immune RNA.

Fainboim L, Sztein MB, Serrate S, Satz L.

Ribonucleic acid (RNA) extracts were obtained from lymph nodes of guinea-pigs that had previously been immunized with a purified testicular antigen emulsified in Freund's complete adjuvant. The RNA extracts were incubated with normal guinea-pig peritoneal exudate cells (NGP-PEC). After treatment, the NGP-PEC cells showed specific inhibition of migration when tested with the specific antigen. No inhibition of migration was observed when cells were tested with an unrelated antigen or when the cells were incubated with RNA obtained from animals immunized with adjuvant alone. Failure of inhibition of migration was also observed when the 'immune' RNA was degraded with RNAse. The appearance of this I-RNA in the

immunized guinea-pigs correlates with the appearance of delayed hypersensitivity in vivo.

PMCID: PMC1457946
PMID: 511220 [PubMed - indexed for MEDLINE]

2517. Am J Pathol. 1979 Oct;97(1):93-110.

Complement fragments, alveolar macrophages, and alveolitis.

Henson PM, McCarthy K, Larsen GL, Webster RO, Giclas PC, Dreisin RB, King TE, Shaw JO.

Mechanisms of neutrophil infiltration into the rabbit alveolus have been investigated. Complement activation in the circulation induced pulmonary vascular margination but not a significant level of alveolar infiltration. Instillation of C5 fragments into the airways, however, attracted neutrophils into the alveolar airspaces. The anaphylatoxin-inactive fragment of C5, C5a des Arg, was found to be much more active in this regard than C5a. Furthermore, these fragments were shown to induce the production of a neutrophil-directed chemoctactic factor from pulmonary macrophages, raising the question of whether the C5a des Arg was acting directly to attract neutrophils or indirectly via the macrophage. To substantiate a possible role for C5 and C5 fragments in alveolitis, active C5 was demonstrated in lavage fluids, and macrophage-derived C5 cleaving enzymes have been described. Finally, a route of neutrophil infiltration via migration through the alveolar capillary wall into the interstitium is proposed, and subsequent penetration of the alveolar epithelium out into the airspace. (Am J Pathol 97:93--110, 1979).

PMCID: PMC2042383
PMID: 495697 [PubMed - indexed for MEDLINE]

2518. Clin Exp Immunol. 1979 Oct;38(1):148-57.

Studies of migration inhibition tests in penicillin hypersensitivity.

Warrington RJ, Sauder PJ, Rutherford WJ.

The release of the migration inhibition factors, leucocyte inhibitory factor (LIF) and macrophage migration inhibition factor (MIF) from stimulated peripheral blood lymphocytes has been compared in patients with immediate (IgE-mediated) penicillin allergy and in patients with delayed hypersensitivity to tuberculin PPD. It has been shown that in these two groups of subjects, a comparable specific proliferative response can occur following stimulation with the appropriate drug (benzylpenicillin) or antigen (PPD). By cell fractionation studies, the proliferation was found to occur in the isolated T cell population in both subject groups. However, the lymphocyte response to benzylpenicillin was rarely associated with the release of LIF or MIF, in contrast to the situation in tuberculin sensitivity where a concomitant release of LIF and MIF was found. In about one third of penicillin allergic subjects, culture supernatants from specifically stimulated lymphocyte cultures induced migration inhibition in the indirect leucocyte migration test, but the inhibitory activity apparently resulted from the presence of penicillin-specific antibody and not from LIF.

PMCID: PMC1537837
PMID: 393437 [PubMed - indexed for MEDLINE]

2519. Gig Tr Prof Zabol. 1979 Sep;(9):9-15.

[Use of immunological study methods in the clinical hygienic verification of the MPEL of industrial allergens (the example of the MPEL of Be in the air of a work area)].

[Article in Russian]

Alekseeva OG, Brekhova NN, Vasil'eva EV, Volkova AP, Grishina TI.

PMID: 535765 [PubMed - indexed for MEDLINE]

2520. Postgrad Med J. 1979 Sep;55(647):608-10.

Failure of treatment in chronic dermatophyte infections.

Hay RJ.

A proportion of dermatophyte infections fail to respond to normally adequate courses of griseofulvin and topical antifungal therapy. The organism Trichophyton rubrum was isolated from 96% of 50 patients studied, but no instances of in vitro resistance were seen. Of these patients, 57% had an underlying condition, commonly hay fever/asthma, atopic eczema, collagen disease or ichthyosis. Defective delayed type hypersensitivity responses and leucocyte migration inhibition to the specific antigen, trichophytin, were demonstrated. Immediate type hypersensitivity was seen in 58% and this was partially suppressible with chlorpheniramine and cimetidine. The relationship between these abnormalities and failure of treatment is discussed.

PMCID: PMC2425655
PMID: 523349 [PubMed - indexed for MEDLINE]

2521. Clin Allergy. 1979 Sep;9(5):489-94.

Asthma in Asian immigrants.

Partridge MR, Gibson GJ, Pride NB.

Adult Asian immigrants to the United Kingdom attending an asthma clinic have been compared with a control group of non-immigrant Caucasian asthmatic patients of similar age distribution. The Asian immigrants had a later age of onset of asthma than their non-immigrant controls. Comparison with studies of asthmatic patients in India suggests that this may be an intrinsic ethnic difference but an effect of migration in early adult life is not excluded. Despite a later age of onset, the frequency of positive skin prick tests to common allergens was similar in the immigrant and control groups and 71% of the Asians had positive reactions to the house dust mite. Most other clinical features, the variability of airways obstruction and the requirements for treatment showed no significant differences between the two groups.

PMID: 498492 [PubMed - indexed for MEDLINE]

2522. Br J Cancer. 1979 Sep;40(3):424-36.

Immunological cross-reactivity between acid extracts of myelin, liver and neoplastic tissues: studies in immunized guinea-pigs.

Flavell DJ, Goepel J, Wilson AP, Potter CW.

Groups of 4 guinea-pigs were immunized with acid extracts prepared from bovine myelin (EF), normal human liver tissue and malignant or benign neoplastic tissues in Freund's complete adjuvant (FCA1. The animals were weighed daily and examined for clinical signs of experimental allergic encephalomyelitis (EAE). All the animals immunized with EF developed clinical symptoms of EAE within 21 days of the initial immunization, whilst some of the animals immunized with certain tumour extracts developed symptoms which closely resembled those of EAE. Control animals immunized with FCA only remained asymptomatic. Cellular immunity to the various extracts in immunized animals was assessed 20 days after immunization by i.d. skin testing, and upon killing at Day 21 with the direct peritoneal-exudate macrophage migration inhibition (MMI) test. Brains and spinal cords were removed at killing, fixed in formalin and processed for histological examination. I.d. skin testing was shown to be most consistent in demonstrating positive delayed hypersensitivity, whilst the MMI test frequently gave negative results in the presence of pronounced skin responses to specific extracts. Thus it was shown that 3/4 animals immunized with basic proteins extracted from an adenocarcinoma of the lung or related hepatic metastases, and 1/2 animals immunized with an extract of a carcinoma of the breast, gave intense erythema and induration responses 5 mm in diameter 24 h after i.d. challenge with EF. No such response was obtained in animals immunized with basic proteins extracted from normal human liver, any of the other neoplastic tissues, or in control animals immunized with FCA only. Examination of brains and spinal cords from animals immunized with EF revealed dense infiltration by mononuclear cells in the ependyma and choroid plexus of levels in the spinal cord. Examination of brains and spinal cords from animals immunized with the lung-tumour extract or related hepatic metastases which showed demonstrable immunological cross-reactivity with EF in immunized animals, revealed a number of inflammatory changes characterized by dense infiltrates of mononuclear cells sub-ependymally, and perivascular cuffing in the cortex. However, no significant lesions were seen in the spinal cords of these animals. Polyacrylamide-gel electrophoresis of the 2 tumour extracts exerting this apparent encephalitogenic effect did not reveal proteins within the mol. wt range of EF. Thus the observed pathological effects and cross-reactivity with EF were probably not due to contamination with nervous-tissue components. It is suggested that these tumour extracts may have contained a component or components other than EF, immunologically cross-reactive with EF, and capable of inducing the observed encephalitis.

PMCID: PMC2010050
PMID: 92328 [PubMed - indexed for MEDLINE]

2523. Ann Allergy. 1979 Aug;43(2):69-72.

The syndrome of asthma, recurrent viral infections and T-cell immunodeficiency: investigations and management.

Khan A.

The syndrome of asthma, recurrent viral infections and T-cell immunodeficiency is discussed. It is suggested that the management of these patients should include correction of the T-cell defects using immunopotentiators such as transfer factor and/or thymosin. Procedures for detecting subtle T cell defects are also described.

PMID: 380414 [PubMed - indexed for MEDLINE]

2524. Kardiologiia. 1979 Jun;19(6):22-7.

[Clinical morphological characteristics of a heart lesion in drug allergy].

[Article in Russian]

Sidorenko MI, Semenovich NI, Kalikshteĭn DB.

Observation of 40 patients with drug myocarditis developing in the presence of acute manifestations of drug allergy, and morphological examination of the myocardium of 15 patients who had died of different manifestations of allergy and drug myocarditis show that drug myocarditis develops quite often in acute manifestations of allergy. This is confirmed by the clinical symptoms, the electrocardiographic findings and the results of immunological study with the use of the leukocyte migration inhibition test with the cardiac antigen. In contrast to persons of the control groups, patients with drug myocarditis showed positive results beginning with the 4th-5th day of the development of the pathological process in the myocardium. The migration index was 40.6% on the average (normal value 80%). The morphological findings indicated the development of delayed and immediate allergic processes in the heart manifested by reaction of the myocardiocytes, stroma and vessels. The degree of severity and character of the morphological changes corresponded to the clinical picture of myocarditis.

PMID: 459226 [PubMed - indexed for MEDLINE]

2525. Arch Dermatol Res. 1979 May 29;265(1):9-14.

Evidence for cell-mediated autoimmunity in patients with pemphigus vulgaris and bullous pemphigoid.

Hunyadi J, Dobozy A, Kenderessy AS.

Lymphoid cells from 4 of 5 patients diagnosed as pemphigus vulgaris (PV) and 6 of 7 patients diagnosed as bullous pemphigoid (BP) demonstrated specific cell-mediated immunity by the production of migration inhibitory factor (MIF) in the presence of autologous epidermal saline extracts. Clinical treatment of these patients with immunosuppressive agents resulted in a state of unresponsiveness of their lymphoid cells to similar concentrations of the antigen. Controls consisted of lymphoid cells from patients with bullous burns or various drug allergies which failed to show significant MIF production in the presence of autologous skin extract. These studies suggest that both PV and BP patients posses cell-mediated immunity (CMI) to their own autologous tissue antigens and this CMI may play a role in the pathogenesis of these diseases.

PMID: 383026 [PubMed - indexed for MEDLINE]

2526. Pol Tyg Lek. 1979 May 21;34(21):833-5.

[Effects of selected factors on in vivo and in vitro leukocyte migration in patients with asthma].

[Article in Polish]

Kowal E, Chyrek-Borowska S.

PMID: 471819 [PubMed - indexed for MEDLINE]

2527. Med Parazitol (Mosk). 1979 May-Jun;48(3):66-9.

[Dynamics of the population density of the house dust mite, Dermatophagoides pteronyssinus Tr., on different culture media and the specific activity of the mite allergen].

[Article in Russian]

Kanchurin AKh, Petrova-Nikitina AD, Berzhets VM, Kryzhanovskaia EA, Muzyleva IL.

PMID: 460055 [PubMed - indexed for MEDLINE]

2528. Zh Mikrobiol Epidemiol Immunobiol. 1979 May;(5):83-7.

[Immunological diagnosis of Mycoplasma pneumonias].

[Article in Russian]

Baĭzhomartov MS, Prozorovskiĭ SV, Vasil'eva VI, Efremova II, Furman MA.

A complex of immunological cell tests with M. pneumoniae antigen (the lymphocyte blast-cell transformation test, the allergic neutrophil alteration test) was carried out in order to establish the correlation between the results of positive seroconversion and the sepcific immunological reactivity of lymphoid cells in pneumonia patients. Mycoplasmic cutireactive allergen, when used for the accelerated diagnosis of mycoplasmic pneumonia in humans, was shown to be specific and safe. Cuti-allergic tests with mycoplasmic allergen allowed to diagnose mycoplasmic pneumonia at early stages (beginning from days 5--7), which ensures the possibility of indicating etiotropic treatment to patients in due time.

PMID: 452781 [PubMed - indexed for MEDLINE]

2529. Clin Pediatr (Phila). 1979 May;18(5):313.

Eosinophilia, fever, hepatosplenomegaly and wheezing.

Ziai M.

PMID: 445935 [PubMed - indexed for MEDLINE]

2530. Pathol Biol (Paris). 1979 May;27(5):274-8.

[Cell mediated immune reactions in allergic extrinsic alveolitis. Studies of broncho-alveolar lavages (author's transl)].

[Article in French]

Jeanneret A, Brun J, Aiache JM, Roche G, Molina C.

PMID: 388299 [PubMed - indexed for MEDLINE]

2531. J Invest Dermatol. 1979 Mar;72(3):128-30.

Leukocyte migration inhibition factor in atopic dermatitis: induction by concanavalin A in vitro.

Horsmanheimo M, Horsmanheimo A.

Assays for the production of leukocyte migration inhibition factor (LMIF) activity by cultured lymphocytes in the presence of the T-cell mitogen concanavalin A (ConA) have been shown to be highly sensitive for detecting decreases in cell-mediated immunity in patients with malignant diseases and primary and secondary immunodeficiency diseases. We have applied the leukocyte migration inhibition test in agarose using supernatants from ConA-stimulated lymphocyte cultures (indirect leukocyte migration inhibition test in agarose for studying patients with atopic dermatitis. Only 5 of 14 lymphocyte cultures from atopic dermatitis patients produced LMIF when stimulated with ConA, as compared with 34 of 34 controls. This difference is highly significant.

PMID: 311368 [PubMed - indexed for MEDLINE]

2532. Rev Fr Mal Respir. 1979 Mar-Apr;7(2):189-90.

[Epidemiologic studies about the allergic diseases (author's transl)].

[Article in French]

Smith JM.

Three large epidemiological studies in Birmingham over a period of 20 years showed a significant increase in the prevalence of asthma in children. Children of immigrants from the West India and Asia had a low prevalence if born abroad, but as high a prevalence as European Children if born in England. The age of onset of asthma and eczema is earlier than that of maximum appearance of skin reactions to common allergens. Among atopic children, 35% have raised levels of IgG4. These children respond poorly to cromoglycate and usually need steroids. Those with eczema have high levels of both IgG4 and IgE. Asthmatic children are more frequently born between May and October compared with the general population. This excess of birth of asthmatic children in Summer is due to mite sensitive children. The excess of births in Summer is not related to hay fever or to pollen sensitivity.

PMID: 115072 [PubMed - indexed for MEDLINE]

2533. Pol Tyg Lek. 1979 Feb 12;34(7):261-4.

[Macrophages and their participation in allergic reactions].

[Article in Polish]

Socha B.

PMID: 375211 [PubMed - indexed for MEDLINE]

2534. Arch Dermatol. 1979 Feb;115(2):161-4.

Cell-mediated immunity in vitro in atopic dermatitis.

Horsmanheimo M, Horsmanheimo A, Banov CH, Ainsworth SK, Fudenberg HH.

The function of T cells in atopic dermatitis was studied by leukocyte migration agarose and lymphocyte transformation tests. We found that phytohemagglutinin (PHA)- and PPD-induced release of leukocyte migration inhibition factor (LIF) from lymphocytes is significantly decreased (P less than .001) in patients with

atopic dermatitis as compared with healthy controls. Blastogenic response of lymphocytes induced by PPD was also decreased in atopic patients as compared with controls (P less than .01). No differences were found in spontaneous blastogenesis or in blastogenic response of lymphocytes in vitro to PHA, concanavalin A (ConA), or streptokinase-streptodornase (SK-SD) between patients with atopic dermatitis and controls.

PMID: 311620 [PubMed - indexed for MEDLINE]

2535. Ann Allergy. 1979 Feb;42(2):95-8.

Bacterial allergy in allergic rhinitis and bronchial asthma.

Bacigaluppi JE, Negroni R, de Severino HM.

Nineteen patients suffering from allergic rhinitis and bronchial asthma were studied for bacterial allergy with Staphylococcus aureus, Kelbsiella pneumoniae and Diplococcus pneumoniae. Allergy skin tests, provocative tests and the Migratory Inhibition Factor were employed. The correlation indicates to the authors that bacterial allergy is more important than bacterial "infection" as a cause of allergic rhinitis and asthma in many instances. This is often overlooked by practicing allergists.

PMID: 33586 [PubMed - indexed for MEDLINE]

2536. Lancet. 1979 Jan 20;1(8108):122-4.

"Insulin" allergy due to zinc.

Feinglos MN, Jegasothy BV.

An investigation of two unrelated patients who had local cutaneous hypersensitivity reactions after injection of any commercially available insulin preparation has shown that the cause of the allergy was zinc. Zinc-insulin and zinc sulphate induced transformation and proliferation of peripheral-blood lymphocytes from these patients; they also induced the production of a specific leucocyte inhibitory factor. Intradermal skin-tests for zinc were positive in both patients. Similar studies carried out in a patient whose cutaneous allergy to insulin was corrected by changing from mixed beef-pork to pure pork insulin were negative. Zinc-free insulin did not produce any allergy in the first patients. The number of patients in whom zinc (which is present in all commercially available insulin preparations) is a cause of "insulin" allergy is unknown. These patients may by identified by intradermal skin-tests. This previously unrecognised allergy should be considered in all patients whose insulin allergy does not respond to conventional therapy.

PMID: 84149 [PubMed - indexed for MEDLINE]

2537. Glas Srp Akad Nauka Med. 1979;(31):37-43.

[Peripheral blood leukocyte migration inhibition in drug allergy].

[Article in Serbian]

Stanojević-Bakić N, Spuzić I, Milosević D, Vucković-Dekić Lj.

PMID: 544340 [PubMed - indexed for MEDLINE]

2538. Glas Srp Akad Nauka Med. 1979;(31):29-36.

[Results of in vitro and in vivo tests in allergic status].

[Article in Serbian]

Spuzić I, Milosević D, Sami I, Stanojević-Bakić N, Marinković M.

PMID: 544339 [PubMed - indexed for MEDLINE]

2539. Glas Srp Akad Nauka Med. 1979;(31):111-5.

[Leukocyte migration inhibition test in contact allergic stomatitis].

[Article in Serbian]

Vucković-Dekić Lj, Stanojević-Bakić N, Milosević D, Sluzić I.

PMID: 397152 [PubMed - indexed for MEDLINE]

2540. Allerg Immunol (Leipz). 1979;25(4):281-90.

[On the suitability of migration inhibition techniques in the in-vitro-diagnostics of chromium allergy (author's transl)].

[Article in German]

Rytter M, Haustein UF, Kipping D, Nuhn P, Dittrich K.

1. Under the conditions of cell culture potassium dichromate is reduced to trivalent chromium (diphenylcarbacid/fotometry). Trivalent chromium reacts with proteins more strongly than the hexavalent chromium (gelchromatography/atom absorption spectrophotometry) and presumably represents the actual hapten. 2. Because of this in-vitro-conjugation chromium salts are suitable in their unconjugated form for applying in migration inhibition tests (capillary/Clausen technique). 3. In migration inhibition tests potassium dichromate showed a better antigenicity than chromium chloride. The capillary method was more sensitive than the Clausen technique when using the same test concentration. 4. Correlations between the degree of the patch test and the value of the migration inhibition did not exist.

PMID: 161950 [PubMed - indexed for MEDLINE]

2541. Allerg Immunol (Leipz). 1979;25(2):116-31.

[The Langerhans cell--its macrophages-analogous function in the triggering of the allergic contact eczema (author's transl)].

[Article in German]

Haustein UF.

1. Langerhans cells represent specific granula-containing dendritic cells which do not have desmosomes, tonofilaments and (pre)melanosomes and which therefore appear as clear cells in the epithelial tissue. 2. They occur in the squamous

epithelium, and also in the corium, lymph node and thymus. They account for 1-1,7% of the whole volumen of the epidermis. 3. They do not represent effete melanocytes, but they originate from the mesenchym. Their migration from the dermis into the epidermis, their identity with histiocytosis X cells, their surface receptors as well as function underline the mesenchymal origin. 4. Probably the Langerhans cells regulate the mitosis and differentiation of the keratinocytes. 5. The antigen-presenting and lymphocyte stimulating functions of Langerhans cells as effector cells in allergic contact eczema are proved. They are able to phagocytize antigens (haptens), to apposite lymphozytes, to proliferate after the challenge by antigens as well as to migrate through the lymph vessels into the regional lymph nodes. 6. At their surface they bear receptors for Fc-IgG and C3 as well as Ia-antigens as immune response genes of the major histocompatibility complex (MHC). 7. With regard to point 5 and 6 they have the same properties as the macrophages. 8. Langerhans cells are damaged and destroyed, respectively, by immune complexes after activation of the complement pathway as well as by killer T-lymphocytes. Thus they are regulated as target cells by humoral and cellular mechanisms. 9. The allergic inflammatory reaction is triggered by mediator substances (of lysosomal origin?) which are liberated by the destruction of the Langerhans cells as well as by lymphokines. The preferently suprabasal occurrence of the Langerhans cells explains the early beginning of the edema and spongiosis as well as their localization in the lower layers of the epidermis. 10. The macrophages-analogous and antigen-presenting functions, which are genetically regulated are essential for the sensitization in allergic contact eczema. The ability to stimulate allogenic T-Lymphocytes in the mixed lymphocyte reaction might be of particular importance for the skin transplantation.

PMID: 159606 [PubMed - indexed for MEDLINE]

2542. Cesk Epidemiol Mikrobiol Imunol. 1979 Jan;28(1):18-23.

[Application of the test of macrophage migration inhibition to assess allergy to 2-phenyl-4,5-dichloro-3-pyridazinone (author's transl)].

[Article in Slovak]

Barlogová S, Stolcová B, Ulrich L.

PMID: 156591 [PubMed - indexed for MEDLINE]

2543. Herz. 1978 Dec;63(36):357-61.

[Complications after pacemaker-implantation and their treatment (author's transl)].

[Article in German]

Klövekorn WP, Struck E, Sasa T.

Of the 1050 pacemaker-implantations in the German Heart Centre in Munich from April 1974 to December 1977 complications arose in 229 cases necessitating a renewed surgical intervention. In accordance with the literature the dislocation of electrodes was the most frequent complication in the first days and weeks after implantation in our patients wil 11,1%. In cases of electrode dislocation the repositioning of the electrode is necessary; in certain cases an epi-myocardial electrode must be substituted for the usual transvenous electrode. Essential causes of complications were disturbances in woundhealing, necrosis of the skin and infection of the pacemaker-system, which were seen in 3,7% of all

cases. In the case of infection of therapy of choice is the explntation of the pacemaker and the implantation of a new system in another position. Technical complications due to material are electrode fractures and battery defects. Complications due to material defects necessitate the exchange of the pacemaker or the electrode. Rare indications for reoperation were increase in threshold, muscle stimulation and allergi reactions caused by pacemakers.

PMID: 400158 [PubMed - indexed for MEDLINE]

2544. Eur J Immunol. 1978 Dec;8(12):905-9.

Cell-mediated immunity to myelin basic protein in Lewis rats made unresponsive to experimental allergic encephalomyelitis.

McGraw TP, Swanborg RH.

Lewis rats with experimental allergic encephalomyelitis (EAE) exhibited cell-mediated immunity to myelin basic protein as determined both with in vivo and in vitro assays. Positive skin test reactions and production of migration inhibitory factor (MIF) were observed before onset and after recovery from EAE. Rats rendered unresponsive to EAE exhibited in vitro cell-mediated immunity to basic protein, although in vivo manifestations were depressed. However, tolerant rats failed to respond to the encephalitogenic determinant; rats with EAE exhibited cell-mediated immunity to this region of the molecule. The results indicate that EAE-unresponsive rats possess lymphocytes capable of responding to basic protein, but that reactivity to the encephalitogenic peptide is suppressed.

PMID: 365547 [PubMed - indexed for MEDLINE]

2545. Arch Ophthalmol. 1978 Nov;96(11):2069-72.

Ascorbate therapy in impaired neutrophil and monocyte chemotaxis. With atopy, hyperimmunoglobulinemia E, and recurrent infection.

Foster CS, Goetzl EJ.

A Candida albicans corneal ulcer developed in a 24-year-old man with a history of eczema, asthma, and multiple bacterial infections since childhood. The infection responded well to oral flucytosine (12 g/day for 15 days) and topical amphotericin B. Positive laboratory findings included eosinophilla, hyperimmunoglobulinemia E, and impaired neutrophil and monocyte spontaneous migration and chemotactic responses. Ascorbic acid corrected the monocyte defect in vitro and in vivo, but had no effect on neutrophil function.

PMID: 718498 [PubMed - indexed for MEDLINE]

2546. Eur J Immunol. 1978 Nov;8(11):771-6.

Experimental allergic encephalitis: study of cellular immunity during disease suppression.

von Muller CS, Spitler LE, LeCocq J.

Administration in complete Freund's adjuvant of encephalitogenic protein (EP), derived from central nervous tissue to guinea pigs, regularly results in the development of experimental allergic encephalitis (EAE) which leads to the death of the animals. Administration of EP in incomplete Freund's adjuvant at an

appropriate time will completely suppress the clinical development of disease. Results reported herein show that animals receiving suppressive injections of EP for 7 days show depression of lymphocyte DNA synthesis and macrophage migration inhibition, but not of skin reactivity, in response to EP immediately following the injections, and subsequently show recovery of lymphocyte reactivity but do not develop clinical manifestations of EAE. Humoral or other factors may prevent the development of disease in these animals. Guinea pigs receiving injections of EP for 14 days show profound and prolonged depression of lymphocyte reactivity to EP and macrophage migration inhibition. Possible mechanisms for these results include a diminished number or function of reactive cells or activity of a population of cells with the capacity to suppress cellular immune responses. Nonspecific suppression of reactivity to an unrelated antigen during the suppressive injections was not observed.

PMID: 82509 [PubMed - indexed for MEDLINE]

2547. J Biol Chem. 1978 Oct 10;253(19):6955-64.

Primary structural analysis of the polypeptide portion of human C5a anaphylatoxin. Polypeptide sequence determination and assignment of the oligosaccharide attachment site in C5a.

Fernandez HN, Hugli TE.

The C5a molecule is one of two spasmogenic fragments (i.e. C3a and C5a) released from serum components C3 and C5 during complement activation. These fragments are called anaphylatoxins because their ability to stimulate mast cell histamine release, smooth muscle contraction, and increased vascular permeability may lead to a fatal reaction resembling anaphylactic shock in experimental animals. In addition, the C5a molecule, which is a glycoprotein, is perhaps the most potent of all humoral chemoattractants for polymorphonuclear leukocytes. Most of the structural analyses in this study were performed on the desArg 74 form of human C5a (C5adesArg). C5adesArg represents a natural form of C5a that is recovered from activated serum when no inhibitors are added to block the action of serum carboxypeptidase. The complete primary structure of the human C5a polypeptide portion is reported here. A partial characterization of intact human C5a has been previously reported (Fernandez, H. N., and Hugli, T. E. (1976) J. Immunol. 117, 1688--1694). The polypeptide portion of C5a contains 74 amino acids, accounting for a molecular weight of 8,200 while the carbohydrate portion accounts for approximately 3,000. The carbohydrate portion of C5a exists as a single complex oligosaccharide unit attached to an asparagine at position 64. An unusual feature of the C5a molecule is its large content of half-cystine, which accounts for more than 9% of its total residues. Two repeating Cys sequences occur in the linear structure and 6 of the 7 half-cystines in C5a are located at nearly identical positions to those in the human C3a molecule. In fact, sequence similarities between C3a and C5a indicate their common genetic ancestry. The role of C5a and C5adesArg as chemotactic factors prompted comparisons of their structural features with those of the chemotactically active formyl-Met peptides (Schiffman E., Corcoran, B. A., and Wahl, S. M. (1975) Proc. Natl. Acad. Sci. U.S.A. 72, 1059--1062). Removal of the COOH-terminal arginyl residue from C5a reduces chemotactic activity; therefore, the terminal portion of this molecule appears to play an active role in stimulating leukocyte migration. Hence the COOH-terminal sequence of C5a was examined for structural similarities to that of the formyl-Met peptides. Since methionine assumes a special functional importance in the formyl-Met peptides, attention is focused on the single methionyl residue in C5a. This methionyl residue, located near the COOH terminus of the molecule, may play an active role in the functional expression of C5a as a chemotactic factor. Although human and pig C3a show a close structural and functional relationship to C5a they lack the ability to excite leukotaxis, and this difference may correlate

with the absence of a methionyl residue near the COOH terminus of C3a.

PMID: 690134 [PubMed - indexed for MEDLINE]

2548. Clin Exp Immunol. 1978 Oct;34(1):92-9.

Transfer of experimental allergic orchitis with immune RNA studies in vivo.

Fainboim L, Sztein MB, Serrate S, Mancini RE.

Ribonucleic acid extracts (RNA) obtained from the lymph nodes and spleens of guinea pigs, which were immunized with testicular antigen emulsified in Freund's complete adjuvant (FCA), were injected intraperitoneally into normal guinea pigs. The transferred guinea pigs developed a delayed hypersensitivity to sperm antigens and testicular lesions which resembled the lesions obtained in the donor RNA guinea pigs. When the transfer was performed with RNA extracted from guinea pigs immunized with FCA alone or with 'immune' RNA treated with Ribonuclease, neither cellular immunity nor testicular lesions were observed.

PMCID: PMC1537468
PMID: 750120 [PubMed - indexed for MEDLINE]

2549. J Neurocytol. 1978 Oct;7(5):541-53.

Glial bridges and Schwann cell migration during chronic demyelination in the C.N.S.

Raine CS, Traugott U, Stone SH.

The formation of fibrotic bridges from subpial astrocytes into the subarachnoid space of the spinal cord and the migration of Schwann cells to the central nervous system (C.N.S.) is appraised in chronically demyelinated C.N.S. lesions. Spinal cord tissue was studied from inbred, Strain 13 guinea pigs with chronic experimental allergic encephalomyelitis (EAE). It has been found that uncommitted Schwann cells are present around remyelinated fibres in nerve root entry zones, between meningeal cells at a distance from the roots and along blood vessels within the spinal cord parenchyma. It is speculated that these cells migrate via the above route to the C.N.S. In the present model, this invasion might be aided by glial fibrosis, a process which leads to surface irregularities in the spinal cord, an extensive extracellular space and possible breaches in the glia limitans through which Schwann cells might penetrate.

PMID: 722315 [PubMed - indexed for MEDLINE]

2550. J Invest Dermatol. 1978 Sep;71(3):209-12.

Physical forces in blister formation. The role of colloid osmotic pressure and of total osmolality in fluid migration into the rising blister.

Bork K.

The physical forces operative in the fluid migration from the interstitial spaces into the blister cleft have not been directly measured until now. The colloid osmotic pressure and the total osmolality were determined in suction blister fluid after mild suction blister production by a modified "Dermovac" and in blister fluid of patients with dermatitis herpetiformis, bullous allergic contact dermatitis and pemphigus vulgaris and in the sera of healthy persons. The colloid

osmotic pressure was measured by means of a recently developed osmometer with a semipermeable membrane between 2 chambers, one of them filled with Ringer solution, the other with the blister fluid or serum sample. The negative pressure in the first chamber was determined. The colloid osmotic pressure of suction blister fluid averages approximately 7 cm H2O, the values reach about 20 cm H2O in bullous diseases and about 38 cm H2O in the normal sera. The blister fluid colloid osmotic pressure has to rise to about 15 cm H2O or more to cause the fluid transport from the interstitial spaces of the surrounding tissue into the blister because of the negative interstitial fluid pressure and the colloid osmotic pressure of the interstitial fluid. Otherwise the blister fluid is reabsorbed back into the interstitial spaces. The total osmolality does not differ in the serum and in the blister fluid. It does not seem to be etiologically connected with the fluid transport into the rising blister.

PMID: 690485 [PubMed - indexed for MEDLINE]

2551. J Immunol. 1978 Sep;121(3):844-50.

Chronic permeability of the central nervous system to mononuclear cells in experimental allergic encephalomyelitis in the Lewis rat.

Stohl W, Gonatas NK.

In order to assess whether experimental allergic encephalomyelitis (EAE), a putative animal model for multiple sclerosis (MS), is an ongoing chronic disorder, we have studied the permeability of spinal cords of Lewis rats with EAE to 3H-uridine- or 3H-thymidine-labeled lymphoid cells obtained from thymuses of naive donors or from draining lymph nodes of donors injected with guinea pig spinal cord + complete Fruend's adjuvant (CFA), guinea pig myelin basic protein + CFA, or with CFA alone. During the acute clinical phase of EAE there is a high-level infiltration of 3H-thymidine- or 3H-uridine-labeled cells into the spinal cords. After clinical recovery from EAE up to 58 days post-inoculation, there is a low-level infiltration of 3H-thymidine-labeled cells into the spinal cords. A similar infiltration into the spinal cords by 3H-uridine-labeled cells was not detected. Donor cells from animals immunized with CFA alone showed similar levels of infiltration into the spinal cords of animals with EAE as donor cells from animals immunized with the encephalitogenic emulsion. Spinal cords from recipients immunized with CFA alone showed no increased permeability to labeled cells. Heat-killed labeled cells did not migrate into the spinal cords of animals with EAE. We conclude that a) EAE is a chronic disease and in this regard is a valid model for MS; and B) in the chronic phase of EAE, recently divided cells (3H-thymidine-labeled cells) show higher levels of migration into the target tissue than 3H-uridine-labeled cells.

PMID: 308523 [PubMed - indexed for MEDLINE]

2552. Z Hautkr. 1978 Aug 1;53(15):531-6.

[Latest studies on chromium and nickel allergy].

[Article in German]

Fritz J, Ludvan M.

PMID: 356444 [PubMed - indexed for MEDLINE]

2553. Parazitologiia. 1978 Jul-Aug;12(4):323-6.

[Importance of cellular immunity factors in the pathogenesis of experimental balantidiasis].

[Article in Russian]

Karapetian AE, Isaakian ZS, Zavgorodniaia AM.

Adult white rats were immunized by numerous subcutaneous injections of antigenes obtained from the cultures of B. coli and B. suis. After the rats were sensibilized they were infected with cultural forms of Balantidium. 75% of infected rats were found to have ciliates in the lumen of the large intestine. In the tissues of the intestinal wall up to the muscular layer there were observed certain pathomorphological changes such as hyperemy, oedema, haemorrhagia and ulcers. By means of the macrophaga migration test it was established that in rats during their immunization and following infection appear lymphocytes which are sensibilized in relation to the balantidial antigene that points to the formation of slow allergy in their organisms.

PMID: 673460 [PubMed - indexed for MEDLINE]

2554. Therapie. 1978 Jul-Aug;33(4):455-61.

[Leukocyte migration test and the allergenicity of a drug].

[Article in French]

Ponvert C, Saurat JH, Tilles G, Galoppin L, Paupe J.

PMID: 32640 [PubMed - indexed for MEDLINE]

2555. Z Hautkr. 1978 May 15;53(10):335-40.

[Methods for the detection of drug allergies].

[Article in German]

Juhlin L.

The following in vitro methods are predominantly discussed: Specific IgE determination (RAST). The test can be used to detect allergies of the immediate type such as anaphylactic shock and urticaria. Antibodies can be detected to a limited number of drugs such as penicillin, ACTH, TSH, insulin, asparaginase and proteins of animal sources. Degranulation of basophil leucocytes and histaminliberation have been used for many years. The practical value of the test has been limited but improved methods for analysis have given the tests hopes for a come-back. Cellular tests like lymphocyteproliferation and macrophage inhibition test (MIF) do not yet give such information which make them helpful as practical tests to detect drug allergy.

PMID: 77590 [PubMed - indexed for MEDLINE]

2556. Pol Arch Med Wewn. 1978 May;59(5):521-6.

[Leukocyte migration inhibition test in studying penicillin allergy].

[Article in Polish]

Karna T, Chyrek-Borowska S.

PMID: 673891 [PubMed - indexed for MEDLINE]

2557. Rev Med Chir Soc Med Nat Iasi. 1978 Apr-Jun;82(2):279-81.

[Immunological studies of contact eczema in experimental animals].

[Article in German]

Günther E, Helmke R, Rietschel L, Meyer J.

PMID: 705126 [PubMed - indexed for MEDLINE]

2558. Antibiotiki. 1978 Mar;23(3):259-64.

[Increased streptomycin sensitivity and its associations with microbial allergy].

[Article in Russian]

Ermekova RK, Tsarevskiĭ LP, Tsoĭ IG, Koval' GP.

Regularities of streptomycin hypersensitivity development and its association with microbial allergy were studied on 75 guinea pigs. A model of retarded allergy was obtained by the animal sensitization with streptomycin in doses of 20 000 gamma per 1 kg of the body weight. Two procedures were used for the animal sensitization, i.e. with the use of the Freund adjuvant or without it. Positive skin-allergic tests to streptomycin (mainly 24-hour) were registered 2 weeks after discontinuation of the sensitization and persisted for the whole observation period (up to 6--8 weeks). The tests for the leucocyte migration were also positive and precipitating antibodies were found in the serum according to the Hoigné method. Simultaneous sensitization with streptomycin and staphylococci resulted in some suppression of the development of retarded hypersensitivity to the microbial antigen. Subsequent sensitization at first with staphylococci and then with streptomycin favoured a mutual increase in the allergic reconstruction to both antigens. The histomorphological studies confirmed the data of the immunoallergological examination.

PMID: 637533 [PubMed - indexed for MEDLINE]

2559. J Neurol Sci. 1978 Mar;36(1):55-61.

Experimental allergic encephalomyelitis--migration of early T cells from the circulation into the central nervous system.

Traugott U, Stone SH, Raine CS.

Study of lymphocytes from the blood of guinea pigs with acute EAE induced by isologous spinal cord in adjuvant reconfirmed that in comparison to normals, the percentage of early (active or high affinity rosetting) T cells decreases dramatically and that these changes can be correlated with clinical signs. In addition, we have investigated matching samples of CNS infiltrating cells recovered by ultrasonication and have found that coinciding with the decrease in early T cells in the circulation, significantly higher levels (P less than 0.001) of these cells appear within the CNS compartment; It is concluded that the decrease of early T cells in the circulation is caused by their migration to the

target organ, the CNS.

PMID: 306421 [PubMed - indexed for MEDLINE]

2560. Kosm Biol Aviakosm Med. 1978 Mar-Apr;12(2):15-9.

[Immunological reactivity and the prognosis for allergic complications in the crew of the 2d Saliut-4 expedition].

[Article in Russian]

Konstantinova IV, Nefedov IuG, Eremin AV, Drozdova VI, Skriabin AS.

Immunological reactivity of P. I. Klimuk and V. I. Sevastyanov was studied before and after their 63-day flight aboard the orbital station Salyut-4. The study used the methods to assay reactivity of T- and B-lymphocytes (PHA-blast-transformation, nonspecific formation of rosettes with sheep red blood cells, immunofluorescence to identify cells carrying immunoglobulin receptors on their surface, serum level of immunoglobulins as a function of Becells). Sensitization of the human body to allergenes of representatives of normal automicroflora of Staphylococcus, Streptococcus, Proteus and E. coli was examined. Specific immunological reactivity which was marked on the 2nd day and tended to return to normal on the 7th day postflight as well as a significant decrease of IgA were noted. Postflight P. I. Klimuk showed sensitization to allergens of Staphylococcus and Streptococcus. Results of immunological examinations of the first and second expeditions aboard the orbital station Salyut-4 were compared.

PMID: 305975 [PubMed - indexed for MEDLINE]

2561. Vet Med Nauki. 1978;15(9):53-8.

[Allergen effect on the number of monocytes in guinea pigs].

[Article in Bulgarian]

Kostov G.

Followed up are the changes in the number of monocytes under the effect of venal injection of an allergen from Salmonella abortus ovis to guinea pigs, sensibilized by live and killed cultures of the same Salmonella. After inoculation of healthy guinea pigs with a live culture, the number of monocytes doubles and remains at this level up to the 11th day after the infection. The killed culture of this Salmonella does not possess a similar action. A venal injection of Salmonella abortus ovis allergen to sensibilized guinea pigs, possessing a dermal-allergic reaction, leads to a marked decrease in the number of monocytes in the blood on the 2nd and 6th hrs after application of the allergen, while on the 24th h their number regains the level prior injection. The "disappearance" of monocytes from the peripheral blood is antigen-specific and the assumption is made that it is the result of the synthesis of the migration-inhibition factor in blood circulation at the contact of the sensibilized "T" lymphocytes with the specific antigen.

PMID: 753035 [PubMed - indexed for MEDLINE]

2562. J Hyg Epidemiol Microbiol Immunol. 1978;22(3):278-83.

Cellular and humoral hypersensitivity reactions during silicosis and silicotuberculosis.

Kitaev MI, Tyurebayeva BN.

Together with the synthesis of specific antibodies and autoantibodies, effects of cellular allergy, such as damage to neutrophils, agglomeration of leukocytes and inhibition of migration of leukocytes with tuberculin and isologous pulmonary antigen were established in patients with silicosis and silicotuberculosis. Autoallergy becomes manifest already in the "pre-roentgenological" stage of development of silicosis; in silicotuberculosis it correlates with the stage of the specific process. In both silicosis and silicotuberculosis, it includes elements of immediate and delayed hypersensitivity. In clinical practice, allergic reactions of leukocytes with tuberculin can be of assistance in determining the stage of the specific process and in differential diagnosis of silicosis and silicotuberculosis.

PMID: 570987 [PubMed - indexed for MEDLINE]

2563. Lab Delo. 1978;(11):680-4.

[Leukocyte migration inhibition test with chemical allergens in occupational allergic dermatitis].

[Article in Russian]

Ado VA, Goriachkina LA, Demicheva NI, Minkov IV, Tomilets VA, Somov BA.

PMID: 82649 [PubMed - indexed for MEDLINE]

2564. Gastroenterol Jpn. 1978;13(4):281-9.

Leukocyte migration inhibition induced by the combination of drug and a liver constituent in patients with drug-induced hepatitis.

Morizane T.

The leukocyte migration inhibition test in agarose medium was performed in 23 cases of clinically diagnosed drug-induced hepatitis. When the test antigen was the combination of soluble phase of a liver homogenate fractionated by Sephadex G-100 which should have contained liver specific antigen and the offending drug the leukocyte migration was inhibited in 86% of cases. Whereas none of 12 cases of drug allergy without hepatic injury showed a positive result with the same combination of antigens. Other organ homogenate-muscle and kidney-never gave positive results when mixed with the offending drugs in cases of drug-induced hepatitis. It was concluded that in hypersensitivity type drug-induced hepatitis cell-mediated immunity might be established to the complex of liver specific antigen and the drug.

PMID: 81789 [PubMed - indexed for MEDLINE]

2565. Arch Dermatol Res. 1977 Dec 27;260(3):217-26.

[Physical forces in blister formation. I. Direct measurement of blister fluid colloid osmotic pressure in suction blisters and in bullous diseases (author's transl)].

[Article in German]

Bork K.

The physical forces operative in the fluid migration from the interstitial spaces into the blister cleft have not been directly measured until now. The colloid osmotic pressure was determined in suction blister fluid after mild suction blister production by a modified "Dermovac" and in blister fluid of patients with dermatitis herpetiformis, bullous allergic contact dermatitis and pemphrigus vulgaris and in the sera of healthy persons. The colloid osmotic pressure was measured by means of a recently developed osmometer with a semipermeable membrane between two chambers, one of them filled with Ringer solution, the other with the blister fluid sample. The negative pressure in the first chamber was determined. The colloid osmotic pressure of suction blister fluid averages approximately 7 cm H2O, the values reach about 20 cm H2O in bullous diseases and about 38 cm H2O in the normal sera. The blister fluid colloid osmotic pressure has to rise to about 15 cm H2O or more to cause the fluid transport from the interstitial spaces of the surrounding tissue into the blister because of the negative interstitial fluid pressure and the colloid osmotic pressure of the interstitial fluid. Otherwise the blister fluid is reabsorbed back into the interstitial spaces.

PMID: 603254 [PubMed - indexed for MEDLINE]

2566. Z Gesamte Inn Med. 1977 Dec 15;32(24):676-80.

[Significance of test results in drug hypersensitivity].

[Article in German]

Wozniak KD.

For the diagnostics of allergic drug reactions in 2,246 patients tests of the skin and in vitro tests were carried out. As causes of the drug rashes analgetics/antipyretics, antibiotics, sulfonamides, local anaesthetics, oral anticonceptive drugs, remedies for the circulation, psychopharmaca and many others have been established. In these cases by means of skin test in 81.5%, by means of the lymphocyte transformation test in 42.9% and by means of the migration inhibition test in 35.9% of the patients a concordant result could be achieved concerning the clinical course of the disease. Relevant to practice from the results must be derived that in sensibilisation proved the avoidance of the pharmacon and of immunochemical related substances is necessary as well as principally in every anamnesis the question for drug tolerances must be asked. The possibility of the development of side effects of pharmaca when these facts are not taken into consideration is emphasized with the help of examples.

PMID: 605634 [PubMed - indexed for MEDLINE]

2567. Wien Med Wochenschr. 1977 Dec 15;127(23):697-702.

[Allergic contact eczema and lymphokines].

[Article in German]

Zelger J.

PMID: 339557 [PubMed - indexed for MEDLINE]

2568. Br J Dermatol. 1977 Nov;97(5):481-6.

Granuloma annulare and sarcoidosis.

Umbert P, Winkelmann RK.

Granuloma annulare (GA) and sarcoidosis are two diseases of unknown cause which involve the skin and whose basic pathology is a mononuclear histiocytic cellular reaction. Biopsy plays the major role in the diagnosis of both diseases, and no other routine laboratory test for either disease is currently available. Sarcoidosis is generally considered to be an allergic or immune granuloma with inconstant defects in cell-mediated immunity (Broom & MacLaurin, 1973). There have been no immunological studies of GA. We recently studied 14 cases and found circulating lymphokines (macrophage migration inhibition factor) in 11 which correlated with circulating macrophage migration inhibition factor in sarcoidosis (9 of 10 cases) (Umbert, Belcher & Winkelmann, 1976). The co-existence of GA and sarcoidosis in 5 patients suggests to us that there are very similar immunological reactions as well as pathology in both diseases and that the elucidation of the pathogenesis of one disease should aid in understanding the other. To our knowledge, there are no prior reports of systemic sarcoidosis coexisting with or manifesting the histological picture of GA. In this report we have demonstrated by clinical and histological criteria that GA and sarcoidosis can co-exist.

PMID: 588462 [PubMed - indexed for MEDLINE]

2569. Clin Allergy. 1977 Nov;7(6):515-25.

Analysis of immunological features of intrinsic and extrinsic asthma and clinical response to DSCG.

Cuevas M, Marmier S, Girard J.

A large range of immunological investigations in patients with extrinsic or intrinsic asthma showed higher leucocyte counts in intrinsic asthma. Blood sputum eosinophilia were almost equally frequent. Immediate skin responses to inhalant antigens are seldom present in intrinsic asthma, but delayed responses to bacterial antigens, endotoxins, Candida albicans ans aspiryl-PPL are frequently positive. This correlates with the frequent presence of precipitins and in vitro stimulation of lymphocytes, and the production of macrophage migration inhibition factor by most of these substances. These results suggest that bacterial factors may play a prominent role as aetiological agents in at least some cases of intrinsic asthma. In intrinsic asthma there was a high incidence of tissue autoantibodies, whereas the incidence was negligible in patients with atopic asthma. The level of serum immunoglobulins was not statistically different between the two groups of patients except for IgE. The patients with intrinsic asthma showed a favorable response to sodium cromoglycate.

PMID: 412613 [PubMed - indexed for MEDLINE]

2570. Probl Tuberk. 1977 Sep;(9):43-8.

[Increase in the diagnostic possibilities of certain cellular phenomena in drug allergy in tuberculosis patients].

[Article in Russian]

Kitaev MI, Tsatskina ES.

PMID: 918046 [PubMed - indexed for MEDLINE]

2571. Pathol Biol (Paris). 1977 Sep;25(7):437-42.

[Induction of autoallergic pulpitis in rabbit (author's transl)].

[Article in French]

Pekovic D, Mascrès C, Adamkiewicz VW.

Six male rabbits were injected on days 0, 6 and 12, subcutaneously, intraperitoneally and subcutaneously with 0.5 ml/kg of a 20% suspension of homologous dental pulp, in complete Freund's adjuvant. Six controls received complete Freund's adjuvant only. Leukocytes were counted regularly. A skin test was performed on day 18. On day 22, a dental test was performed to study the macrophage migration inhibition "in vivo". On day 23, the animals were killed, the teeth and skin were analysed histologically. The following results were observed in the experimental group: 1) inhibition of growth, 2) leukocytosis and eosinophilia, 3) a positive skin test with a strong lymphocytic infiltration, 4) an inhibition of "macrophage migration", 5) pulpal lesions, numbering 1 to 6 per rabbit, with lymphocytic plasmocytic and eosinophilic perivascular infiltration. These results indicate an experimental auto-allergic pulpitis. The mechanisms of human pulpitis ought to be reexamined in the light of these observations.

PMID: 339157 [PubMed - indexed for MEDLINE]

2572. Johns Hopkins Med J. 1977 Sep;141(3):126-34.

The defense of the lung: studies of the role of cell-mediated immunity.

Johnson JE 3rd, Philp JR.

The defense of the lung against infections, toxins and allergens is accomplished by an excretory transport mechanism and by the interaction of cellular and humoral defense systems. Pulmonary alveolar macrophages represent a common effector pathway for both nonspecific cellular phagocytic defenses and for specifically triggered cell-mediated immunity, via T lymphocytes. Nonspecific activation of macrophages is induced by toxic substances. Studies of the immunocytologic system indicate partial compartmentalization and "local" immunity for both cellular and humoral systems. Further studies on pulmonary cell-mediated immunity have characterized an amplification mechanism by which antigen-induced stimulation of T lymphocytes leads to recruitment of nonsensitive lymphocytes through the production of a low-molecular weight "transfer factor." Other lymphocyte-produced mediators (lymphokines) act to attract, aggregate and accumulate macrophages in areas of inflammation. In addition, macrophages are "activated" and show enhanced microbicidal capabilities as well as enhanced resistance to the cytotoxic effects of certain ingested microorganisms. It is postulated that cellular (nonspecific) and cell-mediated (specific) immune defenses play important roles in protection against several categories of microorganisms in a hierarchy of virulence.

PMID: 330913 [PubMed - indexed for MEDLINE]

2573. Pol Tyg Lek. 1977 Aug 15;32(33):1265-7.

[Quantitative evaluation of leukocyte migration in vivo in patients with

endogenous asthma].

[Article in Polish]

Kowal E.

PMID: 905166 [PubMed - indexed for MEDLINE]

2574. Acta Allergol. 1977 Aug;32(4):236-7.

Long-term transfer factor treatment in severe atopic dermatitis.

Thulin H, Ellegaard J, Thestrup-Pedersen K, Zachariae H.

Transfer factor therapy was applied in three patients with severe atopic dermatitis and given at regular intervals for 1 1/2 years. Clinically, slight improvements were seen, attacks of impetigo ceased and admissions to hospital were not necessary. However, IgE concentrations in serum remained constantly high in all cases and the absolute number of T and B lymphocytes was continuously subnormal despite treatment. The in vitro cellular reactivity to PPD as assayed by a leucocyte migration test was not significantly altered in the patients, although a slight increase was found early on in the therapy. Finally, a serum factor inhibiting leucocyte migration and appearing simultaneously with attacks of impetigo disappeared during treatment. In conclusion, no convincing effect of transfer factor therapy was encountered in immune parameters and no major alterations were found in the status of the patients' atopic dermatitis.

PMID: 578361 [PubMed - indexed for MEDLINE]

2575. Dermatol Monatsschr. 1977 Jun;163(6):468-70.

[Detectin of cell-bound proteoglycan antibodies in vasculitis allergica].

[Article in German]

Göring HD, Schramm G, Siegel G.

PMID: 561716 [PubMed - indexed for MEDLINE]

2576. Lab Delo. 1977 Jun;(6):343-6.

[Leukocyte migration inhibition test for the diagnosis of cell-mediated allergy].

[Article in Russian]

Novikov DK, Adamenko GP, Novikova VI.

PMID: 69755 [PubMed - indexed for MEDLINE]

2577. J Neurol Sci. 1977 Jun;32(2):255-63.

Multiple sclerosis: sensitization of a myelin basic protein fragment (peptide T) encephalitogenic to primates. A preliminary report.

Sheremata W, Eylar EH, Cosgrove JB.

Myelin basic A1 protein is the sole antigen of the central nervous system capable of inducing experimental allergic encephalitis (EAE), but sensitization with peptide fragments of the molecule may also induce disease. Using the macrophage migration inhibition factor (MIF) assay we have compared sensitization to portions of the molecule active in inducing EAE in monkeys with results obtained concomitantly using the intact protein. Cellular sensitization to human myelin A1 protein, peptide L (residues 1-116), peptide T (residues 117-170), and petide Y (residues 154-170) was studied using the Thor-Rocklin MIF assay system. Lymphocytes of 10 normal subjects, 10 multiple sclerosis patients 0-3 weeks after onset, 10 4 weeks to 3 months after and 10 6 months or longer after onset of an acute exacerbation were assayed. Results of the investigation reveal evidence of cellular sensitization to myelin basic protein encephalitogenic peptide T occurring during attacks of multiple sclerosis. Peptide L, relatively nonencephalitogenic to primates, failed to induce a significant lymphocyte response, whereas peptide Y which is encephalitogenic gave irregular results.

PMID: 69018 [PubMed - indexed for MEDLINE]

2578. J Oral Pathol. 1977 May;6(3):161-87.

Autoimmune disease antigens.

Hiramoto RN, Ghanta VK.

The first step towards understanding the cellular interaction which results in autoimmune disease is to determine what triggers the recognition between a specific autoimmune antigen determinant and the cellular receptor. In this review, we have focused on the antigen inducing experimental allergic encephalitis (EAE) because the antigen has been characterized and a relatively large body of information on its biological activities has been accumulated. Clearly, a specific allergic encephalitis-producing determinant is present and is represented on a relatively small portion of the molecule. The determinant induces a wide variety of biological reactivities, some of which are classed as cellular mediated. An attempt is made to dissect activities such as blast transformation (BT), migration inhibitory factor (MIF), in vivo delayed type hypersensitivity reaction (DTH) and EAE and to relate them to the structural requirements which the determinants possess. The complexities which arise indicate that subpopulations of cells with different receptor activities may respond selectively and that recognition of the receptor is produced by an EAE determinant consisting of three amino acids in a specific linear sequence. Furthermore, under experimental circumstances the EAE activity can be dissociated from the other activities (BT, MIF, DTH), indicating that while these tests are used generally to follow various human autoimmune disease activities, they may represent the reaction of a broad spectrum of cells.

PMID: 68112 [PubMed - indexed for MEDLINE]

2579. Sem Hop. 1977 Apr 9-16;53(14-15):805-11.

[Tuberculosis of migrant black Africans].

[Article in French]

Pieéron R, Mafart Y, Lesobre B, Hoock J.

A study of 357 cases of tuberculosis in African negro immigrants seen between 1967 and 1971 showed the predominance of respiratory lesions especially pulmonary and/or hilar ganglio-mediastinal lesions compared with lesions elsewhere, e.g.

lymph nodes, bones, joints, pericardium, peritoneum, livers, and the frequency of multiple lesions. Routine detection should be more frequent in high risk subjects, who are almost always allergic on arrival in France. In spite of frequent resistance, above all primary, treatment gave good results in 83% of cases. In future, we will have to take into consideration the possibility of relapses and perhaps more numerous resistances, including to rifamycin and ethambutol.

PMID: 194330 [PubMed - indexed for MEDLINE]

2580. Zh Mikrobiol Epidemiol Immunobiol. 1977 Apr;(4):106-10.

[Detection of a factor suppressing leukocyte migration in the sera of allergy patients following antigen administration].

[Article in Russian]

Gorbach AD, Novikov DK, Gorbach IN.

The effect on donor leukocyte migration of serum obtained from the patients with tuberculosis of the lungs, chronic pneumonia and healthy persons was studied after subcutaneous or intradermal injection of the microbial antigen (PPD, streptococcus and staphylococcus antigen). A factor inhibiting donor leukocyte migration appeared in the blood serum of sensitized individuals after the antigen injection. This factor proved to be localized in the serum fraction III obtained after the gel-filtration of sephadex G-200, and is sorbed by leukocytes.

PMID: 325958 [PubMed - indexed for MEDLINE]

2581. Fed Proc. 1977 Apr;36(5):1742-7.

Immune responses to environmental antigens that act on the skin: the role of lymphokines in contact dermatitis.

de Weck AL.

Immune responses to enviornmental agents affecting the skin may take various clinical forms, among which contact dermatitis is the most prominent representative of delayed-type hypersensitivity. Whereas in industrialized countries a relatively restricted amount of chemical agents is responsible for the majority of contact dermatitis cases, other factors from the environment such as natural flora, seasonal or nutritional factors may also play a role. Like other immune responses, contact dermatitis is strongly influenced by genetic factors and the existence of immune response genes, in part linked to the major histocompatibility complex, has been established in experimental animals. Whereas the formation of conjugates between skin-specific proteins and contactant allergens is held by some to represent an important feature in contact dermatitis, recent experiments suggest that the direct binding of contactants to monocyte and lymphocyte membranes represents the most efficient way in inducing sensitization of the T lymphocytes primarily responsible for contact hypersensitivity. At the effector level, complete inhibition of contact dermatitis and other delayed type hypersensitivity reactions by an antiserum prepared against guinea pig lymphokines (especially migration inhibition factor) offers strong evidence that lymphokines, as products of activated lymphocytes, also play a decisive role in vivo. The properties of antibodies raised against purified lymphokine fractions are reviewed. Localized contact dermatitis reactions, as well as accompanying phenomenons such as flar-up reactions and generalized maculopapular rashes, may, however, still involve other elements than

T lymphocytes and lymphokines. The participation of other secondary cell types and of local antibody formation is briefly discussed.

PMID: 321256 [PubMed - indexed for MEDLINE]

2582. Fortschr Med. 1977 Mar 3;95(9):535-8.

[Pathogenesis of multiple sclerosis. Work-hypotheses and experimental data].

[Article in German]

Neu I.

In spite of a history of more than 100 years, the pathoaetiology of multiple sclerosis is still unknown today. Research is based on three working hypotheses, i.e. on an immunopathological disease origin, on the conception that MS, as an infectious disease, is caused by a specific pathogen (slow virus infection) and on the assumption of a disturbance of basal metabolism or utilisation. The present position of the scientific foundation of the working hypotheses is presented in detail and supplemented by the results of our own investigations. Of particular interest are the geomedical studies which show that MS occurs more frequently in temperate climatic regions. In Europe, a latitude of 46 degrees forms a conspicuous boundary; in the USA this boundary is found at 38 degrees. North of this line there is a morbidity rate of 30 to 60 patients per 100 000 inhabitants, while south of it 15 cases at most per 100 000 inhibitants are found. Asia, especially in China and Japan, and tropical countries, where Multiple Sclerosis is practically unknown in the native populations, are exceptions. The observation that immigrants from areas with a low MS incidence into regions with a high risk of MS fall ill with the disease after years remains also unexplained. These peculiarities have given rise to the consideration whether there is a still unknown factor in the soil of high-risk areas or a specific pathogenic spectrum. In this connection, the question is also discussed whether the risk of MS in northern countries is associated with the excessive consumption of animal fat. The possible therapeutic and prophylactic significance of unsaturated fatty acids is emphasized. Our own results with the Schilling-test, determination of gastric acids, rubella titres in serum and cerebrospinal fluid, the immunofluorescence test of the serum and CSF, determination of tissue antigens (HLA) in families with multiple incidence of Multiple Sclerosis are discussed. On evaluation of a large series of patients, it is striking that Multiple Sclerosis and juvenile diabetes seem to be mutually exclusive (Schrader). Likewise, in MS statistics no other immunopathologic disease such as rheumatic diseases or bronchial asthma was found. Interestingly, also in 400 MS patients examined, hyperuricaemia or gout, which are widespread among the populace, were not found in a single case.

PMID: 844779 [PubMed - indexed for MEDLINE]

2583. Z Gesamte Inn Med. 1977 Mar 1;32(5):123-5.

[Proof of drug hypersensitivity using an in vivo leukocyte migration inhibition test].

[Article in German]

Ruffert K, Wätzig V.

22 chloramphenicol allergics, 10 penicillin allergics and 6 patients with a chromate eczema as well as altogether 34 non-allergic control persons with

healthy skin were examined according to the skin chamber method. After addition of the adequate antigen (chloramphenicol, penicillin, ammonium bichromate) into one of the two simultaneously applied skin chambers in sensitized persons an inhibition of the leucocyte migration in the antigen chamber develops. The polymorphonuclear neutrophilic granulocytes mobilised into the inflammation field were regarded as indicator cells. 5 chloramphenicol allergics with eczematous skin abnormalities and all control persons were negative. The investigation method is simply to be performed and therefore it is particularly suitable for clinical routine work.

PMID: 868183 [PubMed - indexed for MEDLINE]

2584. Z Gesamte Inn Med. 1977 Mar 1;32(5):SUPPL 73-8.

[Current problems in the pathology of the thyroid gland].

[Article in German]

Dhom G.

22 chloramphenicol allergics, 10 penicillin allergics and 6 patients with a chromate eczema as well as altogether 34 non-allergic control persons with healthy skin were examined according to the skin chamber method. After addition of the adequate antigen (chloramphenicol, penicillin, ammonium bichromate) into one of the two simultaneously applied skin chambers in sensitized persons an inhibition of the leucocyte migration in the antigen chamber develops. The polymorphonuclear neutrophilic granulocytes mobilised into the inflammation field were regarded as indicator cells. 5 chloramphenicol allergics with eczematous skin abnormalities and all control persons were negative. The investigation method is simply to be performed and therefore it is particularly suitable for clinical routine work.

PMID: 868179 [PubMed - indexed for MEDLINE]

2585. J Allergy Clin Immunol. 1977 Mar;59(3):254-62.

An approach to diagnosis and treatment in the migrant allergic population.

Schatz M.

An approach to allergy diagnosis and treatment in the migrant population of the United States is derived from a review of selected aspects of pollen aerobiology and the nature and distribution of allergenic plants. Pollen distribution and importance are defined and divided into four relatively homogenous geographic regions of the continental United States. Botanic relationships between pollens are then summarized, especially as they relate to the methodology and results of studies of antigenic relationships between pollens. From this information, 13 mixes of tree pollens, 12 mixes of grass pollens, and 12 mixes of week pollens are proposed which would include the pollens of major and secondary importance found in the four defined geographic regions and which would be appropriate for puncture testing. Further grouping of antigens is described for use in intradermal testing and formulating treatment mixtures. A sample skin test sheet is presented which summarizes this information in a practical format.

PMID: 65374 [PubMed - indexed for MEDLINE]

2586. Drugs. 1977 Feb;13(2):124-36.

Anthelmintics.

Katz M.

This article describes the drugs used in helminthic infections and their therapeutic indications, mode of action, toxicity and other details of each of the recommended drugs, and discusses the nature and treatment of infection by helminths important in human medicine. Infestation due to the roundworms Enterobius vermicularis, Ascaris lumbricoides and the hookworms, Ancylostoma duodenale and Necator americanus can all be treated effectively with pyrantel pamoate. For Enterobius vermicularis, however, a newer drug, mebendazole, is equally as effective. The advantage of these drugs in the indicated circumstances is that they can be administered in a single dose. Unfortunately, pyrantel pamoate is not a panacea and in the case of Necator it is not as effective as in the other roundworms. In that situation the use of tetrachlorethylene is preferable. For treatment of Strongyloides stercoralis, and important human parasite, because it can become disseminated and lead to fatal infections in immunoincompetent hosts, the only effective drug is thiabendazole. In treatment of Trichuris trichiura infection, mebendazole, administered over a period of 3 days, is the most effective available drug. For the roundworms inhabiting tissues--either as aberrant infections of man or as the normal part of their life cycle in man--therapy tends to be largely non-specific. For example, in visceral larva migrans, caused by the dog roundworm Toxocara canis, only palliative therapy with systemic anti-inflammatory agents and corticosteroids may be helpful. Cutaneous larva migrans, caused by the dog hookworms Ancylostoma brasiliensis and Ancylostoma caninum, is also treated primarily with symptomatic measures, but there is a suggestion that thiabendazole may kill the larvae and thus be effective. Trichinella spiralis may cause severe, even fatal infections in man, but only symptomatic therapy can be offered. Therapy for filarial infections is regrettably complicated and not completely effective. Diethylcarbamazine remains the best available drug, but in some of these infections local surgical excision may also be used. It is important to bear in mind that release of antigens from dying or dead worms may cause systemic inflammatory and allergic reactions that may require therapy with corticosteroids. Therapy for Cestodes is achieved most effectively with niclosamide, but the antimicrobial agent paromomycin has also been effective. For the aberrant cestode infections of man, such as echinococcal cysts or Taenia solium cycticerci, treatment is surgical if the affected areas are accessible. Treatment of schistosomal infections is quite toxic and, therefore, it is mandatory to determine viability of the worms before recommending therapy. If therapy is required, then Schistosoma mansoni infections are treated with stibophen and S. japonicum with antimony potassium tartrate, taking care in both of these instances to watch for the early signs of antimony toxicity; therapy of S. haematobium infections is based on administration of niridazole...

PMID: 319991 [PubMed - indexed for MEDLINE]

2587. J Allergy Clin Immunol. 1977 Feb;59(2):115-9.

A hyperimmunoglobulin E syndrome with normal chemotaxis in vitro and defective leukotaxis in vivo.

Weston WL, Humbert JR, August CS, Harnett J, Mass MF, Dean PB, Hagen IM.

A 26-yr-old male with a lifelong history of atopic dermatitis and recurrent severe staphylococcal abscesses was found to have hyperimmunoglobulinemia E. Evaluation of both the humoral and cellular aspects of chemotaxis in vitro showed both neutrophils and monocytes to be normal. However, quantitative neutrophil

migration in vivo was significantly suppressed using the patient's own serum as the attractant. This defective migration in vivo was partially corrected by serum from normal donors as the attractant and also partially corrected following plasma infusion in this patient. Evaluation of quantitative leukocyte migration in vivo may be most useful in patients suspected of defects of leukocyte mobility.

PMID: 299862 [PubMed - indexed for MEDLINE]

2588. Clin Exp Immunol. 1977 Jan;27(1):118-26.

PPD and mitogen responsiveness of lymphocytes from patients with atopic dermatitis.

Thestrup-Pedersen K, Ellegaard J, Thulin H, Zachariare H.

Lymphocytes from twenty-five patients with atopic dermatitis were investigated for their in vitro reactivity to stimulation with tuberculin (PPD), lipopolysaccharide (LPS), phytohaemagglutinin (PHA), concanavalin A (Con A) and pokeweed mitogen (PWM). The response to a low dose of Con A was increased, and the reactivity in unstimulated cultures tended to be lower than similar cultures from the control group. Addition of inactivated autologous plasma to the cultures had an inhibitory effect, when the plasma came from patients with high levels of IgE. The patients' in vitro reactivity to PPD in a leucocyte migration test was equal to that found in normal persons and no effect was observed after addition of autologous serum. The mean percentage of E rosette forming cells was significantly reduced in patients with high levels of IgE. The number of EAC rosette forming cells was within normal range. It is hypothesized that the observations could reflect the existence of suppressor mechanisms in patients where the immune system is strongly stimulated.

PMCID: PMC1540904
PMID: 849645 [PubMed - indexed for MEDLINE]

2589. Int Arch Allergy Appl Immunol. 1977;53(1):29-36.

Different concentrations of chemotactic factors can produce attraction or migration inhibition of leukocytes.

Jungi TW.

Parallel tests were conducted utilizing, the capillary tube migration test and the Boyden chamber assay, in order to determine whether the decrease in leukocyte chemotaxis that occurs if overoptimal cytotaxin concentrations are applied is due to migration inhibition. Overoptimal doses of casein produced decreased chemotactic response and migration inhibition for both rabbit macrophages and neutrophils. However, guinea pig neutrophils exhibited no decrease in chemotaxis despite high casein doses. Overoptimal doses of acid-denatured anaphylatoxin produced a decreased chemotactic response and migration inhibition of neutrophils. In both assays, this agent showed no effect upon macrophages. It is concluded that a chemotactic signal at different concentrations can elicit unidirectional migration or migration inhibition. Accordingly, chemotactic leukocyte attraction could be antagonistically regulated not only by serum-derived and lymphocyte-derived migration inhibitory factors but also by high doses of the chemotactic factor itself. Thus, the Boyden chamber technique can measure both chemotactic migration and migration inhibition phenomena.

PMID: 838513 [PubMed - indexed for MEDLINE]

2590. Blood. 1977 Jan;49(1):3-8.

Acquired granulocyte abnormality during drug allergic reactions: possible role of complement activation.

Bowers TK, Craddock PR, Jacob HS.

A profound defect in granulocyte chemotaxis was documented in an otherwise healthy 21-yr-old man who failed to localize granulocytes to an area of cellulitis during an allergic reaction to cephalothin. During the period of drug allergy, characterized by urticaria, eosinophilia, and profound hypocomplementemia, in vitro migration of the patient's granulocytes in the Boyden chamber was markedly impaired. Although devoid of hemolytic complement activity, the patient's serum possessed supranormal chemotactic activity, even following heat inactivation, suggesting the presence of chemotactically active complement split products. Chemotactic function improved concomitantly with steroid therapy and normalization of serum complement levels, and was entirely normal following clinical recovery and cessation of steroid therapy. The chemotactic abnormality noted in the patient's cells was reproduced in normal granulocytes by preincubation either with patient serum or with cobra venom-activated fresh (but not heated) normal serum, suggesting that in vivo exposure of granulocytes to activated complement was responsible for the patient's abnormal chemotactic response. This mechanism may contribute to the increased infection propensity noted in other conditions characterized by in vivo complement activation, such as rheumatoid arthritis and systemic lupus erythematosis.

PMID: 830375 [PubMed - indexed for MEDLINE]

2591. Ter Arkh. 1977;49(12):101-4.

[Leukocyte migration inhibition reaction as a method of detecting drug allergy].

[Article in Russian]

Semenovich NI, Samoĭlova LN.

PMID: 601685 [PubMed - indexed for MEDLINE]

2592. Dermatol Monatsschr. 1977 Jan;163(1):5-12.

[Leukocyte migration inhibition test performed with the skin window technic. An in vivo proof of chloramphenicol allergy].

[Article in German]

Wätzig V, Ruffert K.

PMID: 320058 [PubMed - indexed for MEDLINE]

2593. Vopr Onkol. 1977;23(11):18-23.

[Cellular immunity indices in osteogenic sarcoma].

[Article in Russian]

Rodionov SV.

Prior to the treatment 39 patients with osteogenic sarcoma were examined for some cell immunity indices by the skin-allergic reaction of delayed hypersensitivity (RDH) and the reaction of suppression of leucocytes migration (RSLM). As an antigen a polysaccharide fraction of osteogenic sarcoma was employed, for the control--normal bone polysaccharide fraction. In 25 patients the course of the disease was followed up for 8--14 months after the primary examination. Both RDH and RSLM were found to show no essential difference in the reactions for polysaccharide antigens isolated from osteogenic sarcoma and normal bone. In patients with a rapid growth of the primary tumor negative RSLM was noted, while RDH indices failed to show such differences. In patients without any signs of the progressing disease during 8--14 months since the moment of the examination, as a rule, positive RSLM and RDH are noted. In patients with a precipitous course of the disease 1--8 months prior to the treatment negative RSLM and RDH are more frequently observed.

PMID: 271419 [PubMed - indexed for MEDLINE]

2594. Rev Ig Bacteriol Virusol Parazitol Epidemiol Pneumoftiziol Pneumoftiziol. 1977 Jan-Mar;26(1):9-17.

[Active-evolutive pulmonary tuberculosis with negative tuberculin reaction].

[Article in Romanian]

Moisescu V, Nicoară O, Rusu L, Popescu M.

In a lot of 452 patients with various forms of tuberculosis, in various locations, the authors have investigated the efficiency of tests currently employed in detecting tuberculin sensitivity (intradermoreaction to PPD, the test to sensitines and the Ustvedt test), in relation with several immunologic tests performed in vitro (index of lymphoblastogenesis to PPD and/or phytohaemagglutinin, leucocyte migration inhibition test, and the rosette test). It was noted that the two groups of investigations give similar results when tuberculin reactions are positive and intense. Under the conventionally admitted threshold of tuberculinic allergy the test of blastic transformation of lymphocytes, and especially the leucocyte migration inhibition tests are still positive and may be considered as a valuable means for measuring infra-tuberculinic allergy, as well as for the diagnosis of latent tuberculous infections.

PMID: 191895 [PubMed - indexed for MEDLINE]

2595. Allerg Immunol (Leipz). 1977;23(4):322-7.

Experimental investigations in guinea pigs on the recognition of the allergic eczematous reaction: A comparison of the macrophage migration inhibition test with the patch test.

Rytter M, Haustein UF.

1. The patch test as an in vivo test has disadvantages. It is our impression that biologically false reactions occur in approximately 5%. 2. The MIT as an in vitro test is very time consuming, but safe for the patient. It is supposed to give up to 20% false reactions. 3. In 76 and 72% of 156 guinea pigs sensitized with potassium bichromate and sulfasol respectively, both the test methods gave

results in agreement. 4. Sensitization by potassium bichromate estimated by patch test only was 30%, and by MIT only also 30%, but the two test methods disagreed in 24%. 5. Sensitization by sulfasol evaluated in the patch test only was 36%, and in the MIT only 48% but the two test methods disagreed in 28%. 6. The differences between the results of the two test methods are not satistically significant and their failure rate does not provide absolutely sure results, but we believe that the patch test is preferrable in recognition of contact allergy because it is in vivo and probably has a lower failure rate in comparison with the MIT in the human.

PMID: 147621 [PubMed - indexed for MEDLINE]

2596. Allerg Immunol (Leipz). 1977;23(2):69-78.

[The detection of chloramphenicol allergy by leucocyte migration inhibition test in the skin chamber (author's transl)].

[Article in German]

Wätzig V, Ruffert K.

In the leucocyte migration inhibition test in the skin chamber under in vivo conditions an immune reaction is induced, which influences the migration of polymorphonuclear neutrophilic leucocytes into the chamber in a characteristic way. An inhibition of the migration of leucocytes above 40 per cent is considered positive. In 16 out of 17 patients with chloramphenicol exanthema the test was positive. Five allergic patients with eczematous skin lesions presented negative results. In 14 controlpatients chloramphenicol caused a negative result, too. The mechanical irritation of the skin and the immune response produce different mechanisms, which promote and inhibit migration. The observed influence of the number of leukocyte in the skin chamber is the result of these mechanisms.

PMID: 145172 [PubMed - indexed for MEDLINE]

2597. J Allergy Clin Immunol. 1976 Dec;58(6):623-34.

Mediator release from basophil granulocytes in chronic myelogenous leukemia.

Clark RA, Gallin JI, Kaplan AP.

Mediator release from the leukocytes of two patients with chronic myelogenous leukemia and basophilia was studied using rabbit antihuman IgE antibody. The release of histamine, slow reacting substance of anaphylaxis (SRS-A), platelet activating factor (PAF), chemotactic activity for neutrophils and eosinophils, and an inhibitor of eosinophil migration was observed. However, the release of SRS-A from the basophils of one patient and the release of chemotactic activity from both patients displayed unusual properties. During acceleration of the disease process, the basophils of one patient released maximal SRS-A activity at progressively lower concentrations of anti-IgE. Both patients released a high molecular weight factor (M.W. greater than 20,000) which enhanced the migration of neutrophils and eosinophils and a low molecular weight chemotactic factor (M.W. less than 500) which selectively attracted eosinophils. A double peak of eosinophil chemotactic activity was routinely observed for the low molecular weight factor; this was shown to represent the eosinophil chemotactic activity of histamine with relative inhibition of migration at the histamine peak. There was little release of the tetrapeptides, ECF-A, in these patients which facilitated demonstration of this eosinophilotactic activity of histamine. These results suggest that the eosinophil chemotactic activity observed in acute allergic

reactions is the net effect of the release of multiple chemotactic factors.

PMID: 62774 [PubMed - indexed for MEDLINE]

2598. J Lab Clin Med. 1976 Nov;88(5):796-806.

Severe staphylococcal disease associated with allergic manifestations, hyperimmunoglobulinemia E, and defective neutrophil chemotaxis.

Hill HR, Estensen RD, Hogan NA, Quie PG.

Neutrophil granulocyte function was determined in three patients with systemic staphylococcal infection, clinical manifestations of generalized allergic disease, and hyperimmunoglobulinemia E. Each of the patients had urticarial skin rashes before or at the time of development of staphylococcal suppurative lymphadenitis, pneumonia, or sepsis. Neutrophil chemotaxis, random migration, phagocytosis, and bactericidal capacity were assessed to determine if an abnormality in these functions might have contributed to the development of severe staphylococcal infections. Each of the three patients with generalized urticaria was found to have a marked defect in neutrophil chemotaxis. The mean chemotactic index of the patients was 12 +/- 4, whereas that of 20 controls was 72 +/- 11. Neutrophil random migration, phagocytosis, and bactericidal capacity were normal in each patient. The serum or plasma of the patients did not inhibit chemotaxis of control neutrophils and did not contain an increased concentration of the chemotactic-factor inactivator found in normal serum. Treatment of the neutrophils of these three patients with the competitive histamine H2 receptor blocking agent, burimamide, produced a significant increase in chemotactic responsiveness. These studies suggest the possibility of pharmacologic modification of neutrophil granulocyte function.

PMID: 978042 [PubMed - indexed for MEDLINE]

2599. Am J Pathol. 1976 Nov;85(2):419-36.

Modulation of human eosinophil polymorphonuclear leukocyte migration and function.

Goetzl EJ.

Eosinophil migration toward a concentration gradient of a chemotactic factor is regulated at four levels. Diverse immunologic pathways generate stimuli with eosinophil chemotactic activity, including the complement products C5a and a fragment of C3a and the peptide products of mast cells and basophils activated by IgE-mediated reactions, such as eosinophil chemotactic factor of anaphylaxis (ECF-A) and other oligopeptides. The intrinsic preferential leukocyte activity of the chemotactic stimuli represents the second level of modulation, with ECF-A and other mast cell-derived peptides exhibiting the most selective action on eosinophils. The third level of control of eosinophil chemotaxis is composed of inactivators and inhibitors of chemotactic stimuli and is exemplified by degradation of C5a by anaphylatoxin inactivator or chemotactic factor inactivator and of ECF-A by carboxypeptidase-A or aminopeptidases. The activity of ECF-A is uniquely suppressed by equimolar quantities of its NH2- terminal tripeptide substituent, presumably by eosinophil membrane receptor competition. Factors comprising the fourth level of regulation, which alter eosinophil responsiveness to chemotactic stimuli, include the chemotactic factors themselves, through deactivation; nonchemotactic inhibitors such as the COOH-terminal tripeptide substituent of ECF-A, the neutrophil-immobilizing factor (NIF), the phagocytosis-enhancing factor Thr-Lys-Pro-Arg, and histamine at concentrations

greater than 400 ng/ml; and nonchemotactic enhancing principles represented by ascorbate and by histamine at concentrations of 30 ng/ml or less. Local concentrations of eosinophils called to and immobilized at the site of a hypersenitivity reaction may express their regulatory functions by degrading the chemical mediators elaborated including histamine, slow-reacting substance of anaphylaxis (SRS-A), and platelet-activating factor (PAF) by way of their content of histaminase, arylsulfatase B, and phospholipase D, respectively. Immunologic pathways may thus provide the capability for early and specific host defense reactions with a later influx of eosinophils preventing irreversible local tissue alterations or distant organ effects.

PMCID: PMC2032558
PMID: 793410 [PubMed - indexed for MEDLINE]

2600. Arch Dermatol. 1976 Oct;112(10):1391-6.

Immunodeficiencies in severe atopic dermatitis. Depressed chemotaxis and lymphocyte transformation.

Rogge JL, Hanifin JM.

Various reports have indicated assorted immune defects in atopic dermatitis, but the prevalence and degree of the defects remain unclear. We assessed various immunological factors in 14 patients with atopic dermatitis to determine whether immunodeficiencies were present consistently and were reflected by the patients' clinical characteristics. A high incidence of cutaneous infection was noted. Cutaneous delayed-hypersensitivity testing showed anergy in eight (67%) patients. Only the seven patients with the most severe condition showed altered leukocyte function, as determined by polymorphonuclear and mononuclear leukocyte chemotaxis and by lymphocyte responsiveness to phytohemaglutinin. All three cell types where shown to be simultaneously dysfunctional during severe atopic flares. Chemotactic studies during clinical remissions disclosed notable improvement in cell migration. Serum IgE levels were elevated in each patient, but did not correlate with the degree of cutaneous anergy or altered leukocyte function.

PMID: 962332 [PubMed - indexed for MEDLINE]

2601. Aust J Exp Biol Med Sci. 1976 Aug;54(4):317-27.

The specificity of nematode allergens in the diagnosis of human visceral larva migrans.

Hogarth-Scott RS, Feery BJ.

Extracts of nematodes have been used as skin test antigens in the diagnosis of nematode infections for many years. Ascaris lumbricoides and Toxocara canis are two nematodes commonly involved in human parasitism, the latter being associated with the clinical condition of Visceral Larva Migrans. In vitro and in vivo experiments reported in this paper confirm experimentally, as well as clinically, the existence of cross-reacting antigens between T. canis and Ascaris spp., and probably between T. canis and other nematodes. These cross-reactions compromise the usefulness of skin tests in the diagnosis of such parasitic infections.

PMID: 65168 [PubMed - indexed for MEDLINE]

2602. Ann Sclavo. 1976 Jul-Aug;18(4):623-9.

[Activity inhibiting leukocyte migration in human sera of healthy subjects and of subjects with delayed hypersensitivity and findings of possible serum factors with antagonistic activity (author's transl)].

[Article in Italian]

Jirillo E, De Rinaldis P, Pasquetto N.

The AA. have tested human sera from subjects with a clinical condition of delayed hypersensitivity (TBC and eczematous contact dermatitis) in order to detect factors affecting leukocyte migration in agarose plates. They found either inhibiting or stimulating factors of cellular migration. According to the most recent data about the same area they emphasyze a sort a regulatory mechanism of LIF or factors with activity LIF-like, due to antagonistic substances present in sera of their patients. Furthermore they suggest a list of factors able to inhibit or to enhance cellular migration.

PMID: 1088062 [PubMed - indexed for MEDLINE]

2603. Klin Med (Mosk). 1976 Jul;54(7):95-100.

[Allergy to cow's milk in the clinical aspects of gastrointestinal diseases].

[Article in Russian]

Martynov SM, Fedorenko TA.

PMID: 979100 [PubMed - indexed for MEDLINE]

2604. Infect Immun. 1976 Jul;14(1):290-7.

In vitro activity of guinea pig transfer factor released into plasma.

Paquet A Jr, Olson GB, Jeter WS.

Plasma fractions and plasma dialysate from 2,4-dinitrochlorobenzene- and tuberculin-sensitive guinea pigs that had been treated with either antilymphocytic serum or normal control serum were analyzed for their ability to transfer lymphocyte transformation, passive cutaneous anaphylaxis, and macrophage migration inhibition, as well as delayed hypersensitivity in vivo. Antilymphocytic serum caused rapid release of material, which has characteristics of transfer factor, into the plasma. It was dialyzable, migrated electrophoretically with the alpha globulins and albumin, possessed a 280/260 (nm) optical density ratio of 0.7, and caused in vitro lymphocyte transformation in the presence of the specific antigen. Passive cutaneous anaphylaxis antibodies were also present in the plasma of sensitive animals, but they were isolated in electrophoretic or dialysis fractions separate from those containing transfer activity.

PMCID: PMC420875
PMID: 947843 [PubMed - indexed for MEDLINE]

2605. Ann Intern Med. 1976 Jul;85(1):39-43.

Recurrent staphylococcal abscesses associated with defective neutrophil chemotaxis and allergic rhinitis.

Hill HR, Williams PB, Krueger GG, Janis B.

Four patients with recurrent staphylococcal furonculosis and deep abscess formation were evaluated to determine if a defect in the host defense mechanism could account for the unusual incidence of infection. Each also suffered repeated allergic rhinitis, often preceding the onset of infection. A marked defect in neutrophil granulocyte chemotaxis occurred when the patients were symptomatic with rhinitis and abscess formation. Their mean chemotactic index (+/- SD) was 16 +/- 6, while that of 25 control subjects was 70 +/- 11. Neutrophil random migration, phagocytosis, bactericidal activity, and lymphocyte T-cell populations were normal, as were serum concentrations of IgA, IgG, IgM, and IgE. Serial neutrophil function tests revealed normal chemotactic responsiveness when the patients were symptom-free of allergic rhinitis and no longer having abscesses. Abnormal function returned, however, when symptoms recurred. These studies suggest that defective neutrophil function associated with allergic phenomena need not be accompanied by hyperimmunoglobulinemia E. Such defects may be intermittent, appearing when allergic symptomatology and infections develop.

PMID: 937922 [PubMed - indexed for MEDLINE]

2606. Rev Stomatol Chir Maxillofac. 1976 Jul-Aug;77(5):713-8.

[Diagnostic methods in drug allergies].

[Article in French]

Saurat JH.

Immunological exploration of a patient following an allergic complication is based on tests in vitro, which are free of danger. At the present time it remains very difficult, on the one hand on the basis of the antigenic radicle responsible, secondly because of the characteristics of the immune response which it induces. The aim of exploration is the objective demonstration, using several techniques, of an immune response directed specifically against the medication. The pathological significance of this response must be analysed in each case.

PMID: 63984 [PubMed - indexed for MEDLINE]

2607. Biull Eksp Biol Med. 1976 Jun;81(6):707-10.

[Determining hypersensitivity to antigens by computing cells migrating from capillary tubes].

[Article in Russian]

Novikov DK, Adamenko GP, Novikova VI.

The test technique of inhibition of leukocyte and macrophage migration from the capillaries in the presence of a specific antigen is described. The method is highly sensitive and permitted to use a small amount of cells and many (up to 600 and more) capillaries. Sodium azide, phitohemagglutinin and incubation at 4 degrees C inhibited the cell migration from the capillaries. Dead cells did not pass from the capillaries into the medium. Tuberculin and BCG vaccine inhibited the migration of macrophages of guinea pigs immunized with BCG, but not with staphylococcus allergen.

PMID: 953309 [PubMed - indexed for MEDLINE]

2608. Fortschr Med. 1976 May 13;94(14):809-14.

[Cellular immunity in poxvirus infections. Demonstration, significance and cellular cross reactivity with brain matter].

[Article in German]

Stickl H.

Its proof, significance, and the cellular cross reactivity to basic encephalitogenic protein of vaccinia-stimulated T-cells. Vaccination against smallpox induces both, a cellular allergy and immunity, and the production of humoral antibodies. Of greater importance for the defense of the organism against infections by orthopox viruses is the cellular immunity. New methods for the proof of cellular immunity and an in vitro-technique for its examination by challange with variola vera virus are described. In close connection with induction of cellular allergy by vaccination an immunological cell-bound reactivity of isolated lymphocytes develops against the basic encephalitogenic protein. In this antigen-stimulated resp.-irritated stage isolated lymphocytes show an enhanced rate of chromosomal aberrations and a high rate of spontaneous transformations. Lymphocytes of patients with Multiple Sclerosis demonstrate a high stimulation rate (3H- resp. 14C-Thymidine-inlay) after challenge with antigen of vaccinia virus. The T cell-mediated cross reactivity between vaccinia and myelin extracts is discussed as an hypothetic pathogenic factor of CNS complications after primary smallpox vaccination.

PMID: 57915 [PubMed - indexed for MEDLINE]

2609. Ann Clin Lab Sci. 1976 May-Jun;6(3):256-62.

Berylliosis as an auto-immune disorder.

Reeves AL.

Exposure to compounds of beryllium can cause dermatitis, acute pneumonitis and chronic pulmonary granulomatosis ("berylliosis") in humans. These syndromes seem to have an allergic-immunologic component in common. Hypersensitivity to beryllium is of the delayed (cell-mediated) type and can be measured as skin reactivity to patch test; lymphocyte blast transformation; and macrophage migration inhibition. There is good correlation between the results of these tests in exposed populations, but the degree of hypersensitivity is not necessarily a measure of either extent of exposure or severity of berylliosis. In animal experiment, inhalation exposure has suppressed a previously established cutaneous hypersensitivity, and degree of hypersensitivity and degree of berylliosis were in significant inverse correlation.

PMID: 942183 [PubMed - indexed for MEDLINE]

2610. Br J Dis Chest. 1976 Apr;70(2):73-7.

The prevalence of asthma and wheezing in children.

Smith JM.

The prevalence of asthma and wheezing in children in Birmingham in 1974-5 was compared with the previous studies in 1968-9 and 1956-7. The frequency of asthma increased over the 20-year period, although the rate of increase had slowed.

Asthma was commoner in boys than in girls. Children of the same racial group were more prone to asthma, and probably to other atopic diseases, if born in England than if born in poor tropical countries. Environmental factors in early life profoundly influence the prevalence of asthma in childhood.

PMID: 952726 [PubMed - indexed for MEDLINE]

2611. Zh Mikrobiol Epidemiol Immunobiol. 1976 Apr;(3):28-32.

[Allergenic fractions of bacteria belonging to the enteric family. VII. Characteristics of the intracellular proteins comprising the allergenic substrate of Escherichia coli using ion exchange chromatography].

[Article in Russian]

Maianskiĭ AN, Molchanova IV, Alatyrtseva IE.

A method of successive chromatography on Sephadex G-50 and G-100 and DEAE-Sephadex A-50 was applied for separation of the allergenic complex of E. coli intracellular proteins into a number of immunochemically different or significantly differing fractions with a definite set of antigens. Results of analysis of the fractions obtained in the tests of increased sensitivity of delayed type (ISDT) in vivo and in vitro corresponded to the view of the immunological polyvalency of the allergenic substrate of enterobacteria. Four groups of antigens were distinguished by the ratio of the activity indices in the skin reactions of ISDT and in the tests of specific inhibition of macrophage migration. Singificance of individual antigens in the formation of the allergenic profile of bacterial cells is determined by their quantitative content and the extent of the allergenic activity.

PMID: 785905 [PubMed - indexed for MEDLINE]

2612. Vestn Khir Im I I Grek. 1976 Apr;116(4):51-5.

[Study of bacterial allergy in acute appendicitis by the method of leukocyte migration inhibition in vitro].

[Article in Russian]

Shturich PI, Novikov DK, Adamenko GP.

Under study was the effect of various bacterial allergens on the migration of blood leucocytes from capillaries in vitro in 23 patients with acute appendicitis and in 18 healthy individuals. Bacterial allergens wound suppress leucocytes migration in all patients. Most frequently, sensibilization to allergens of hemolytic staphylococcus and hemolytic streptococcus was found. In 4 healthy persons this methods indicated sensibilization to some allergens. The interaction of activity sensibilized lymphocytes and passively sensibilized polynuclears of the appendix with bacterial antigens is assumed to tigger the hyperergic reaction in acute appendicitis.

PMID: 785785 [PubMed - indexed for MEDLINE]

2613. Ann Clin Res. 1976 Apr;8(2):74-84.

Immunological mechanisms and diagnostic tests in allergic drug reactions.

Girard JP, Cattin S, Cuevas M.

Current knowledge about the immunological mechanisms involved in drug hypersensitivity is reviewed, with comments on the major conditions which favour the development of reactions to drugs. In spite of the numerous tests available to establish the in vitro diagnosis of allergic reactions to drugs, it is often very difficult to relate the clinical picture to the laboratory findings. The results of various tests of antibody and cell-mediated responses are presented and discussed.

PMID: 59570 [PubMed - indexed for MEDLINE]

2614. Ann Sclavo. 1976 Mar-Apr;18(2):291-8.

[Parasites of the respiratory system: research and significance (author's transl)].

[Article in Italian]

De Carneri I, Trane F.

A review is made of the methods of diagnosing both autochthonous and exotic protozoal and helminthic diseases of the respiratory system. Referring to protozooses, recent findings on respiratory pathology due to amoebae of the genus Acanthamoeba are commented, and modern methods are discussed of checking for Pneumocystis carinii in the patient, not just on autopsy material. Referring to helminthiases, in addition to pulmonary echinococcosis which is of prevalent interest in Italy, attention is also given to the pathology of migrant larvae of nematodes. Finally, the role of some microscopic mites in the pathogenesis of respiratory allergic disease from house dust is discussed.

PMID: 1087869 [PubMed - indexed for MEDLINE]

2615. Vopr Pitan. 1976 Mar-Apr;(2):43-6.

[Intolerance to cow's milk].

[Article in Russian]

Martynov SM, Fedorenko TA.

The authors report results of allergological history, of passive haemagglutination reaction, lymphocytes blast transformation and leucocytes migration inhibition tests in 100 patients suffering from affections involving the gastro-intestinal tract. Control investigations were carried out in 20 practically healthy individuals. From these results the authors conclude that the data of the allergological history and clinical symptoms of the cow milk intolerance are determined, above all, by the immuno-allergic mechanisms, this being confirmed by the results of the "in vitro" methods characterizing the humoral and cellular types of the immunological reactivity.

PMID: 988940 [PubMed - indexed for MEDLINE]

2616. Clin Allergy. 1976 Mar;6(2):131-4.

Allergy and parasites: the measurement of total and specific IgE levels in urban and rural communities in Rhodesia.

Merrett TG, Merrett J, Cookson JB.

Eighty adult asthmatics living in an African city had a significantly higher serum IgE level (799 u/ml) than the control group (350 u/ml). A high proportion (78.7%) of the asthmatics had demonstrable circulating mite-specific IgE antibodies. The rural population of a filariasis endemic region was investigated and although no allergic subjects were identified, the group had a significantly higher IgE level (1613 u/ml) than the asthmatics and also showed a relatively high incidence of grass pollen-specific IgE antibodies (35%). The discrepancy between clinical history and laboratory results supports the mast cell saturation hypothesis and suggests: (a) an explanation for the susceptibility to allergy of African and Asian immigrants to Great Britain, and (b) a practical approach for preventing allergic reactions in vivo.

PMID: 945137 [PubMed - indexed for MEDLINE]

2617. Acta Allergol. 1976 Feb;31(1):1-17.

Lymphocyte transformation, leucocyte migration, specific IgE, IgG and IgM before, during, and after penicillin treatment without adverse reaction. A follow-up study.

Saurat JH, Ponvert C, Burtin C, Soubrane C, Lebel B, Beucher F, Paupe J.

This in vitro follow-up study of the penicillin-induced immune response by a well-tolerated regimen was conducted with an association of four tests: lymphocyte transformation test; leucocyte migration test; passive anaphylaxis on monkey lung tissue; and passive hemagglutination. It showed (1) that previously sensitized subjects (including IgE) tolerated the treatment by pencillin. (2) That the treatment induced either a transient positive conversion of previously negative tests or a negative conversion of previously positive tests. These in vitro findings are in accordance with previously established concepts based upon cutaneous tests and must be taken into consideration when interpretating in vitro diagnosis of penicillin allergy.

PMID: 946571 [PubMed - indexed for MEDLINE]

2618. Tohoku J Exp Med. 1976 Feb;118(2):137-43.

Dissociation of MIF activity and in vitro lymphocyte transformation in the development of experimental allergic thyroiditis in guinea pigs.

Hiramine C, Hojo K.

Macrophage migration inhibitory factor (MIF) activity and in vitro lymphocyte transformation to homologous thyroid antigen were compared in cultures of lymph node cells obtained from 123 guinea pigs at weekly intervals after a single injection of homologous thyroid extracts in complete Freund's adjuvant (CFA). MIF activity was not detectable at the early stage of immunization but became manifest 2 and 3 weeks after sensitization and a high level was maintained during 4-7 weeks. On the other hand, lymphocyte transformation preceded the appearance of MIF activity and attained its maximum 2 weeks after sensitization before the establishment of thyroiditis. It is indicated that MIF activity and lymphocyte transformation predominate separately in the course of immunization. There was a considerable correlation between intensity of MIF activity and severity of thyroiditis in individual animals during the advanced stage (4-6 weeks after sensitization) of the disease. Also, a lower but significant correlation was seen

between lymphocyte transformation and severity of thyroiditis. However, about half of animals in the advanced stage of thyroiditis failed to exhibit antigen-stimulated lymphocyte transformation, despite a positive MIF activity.

PMID: 790649 [PubMed - indexed for MEDLINE]

2619. Pol Tyg Lek. 1976 Jan 5;31(1):13-4.

[Use of leukocyte migration inhibition test in studying allergy to rifampicin].

[Article in Polish]

Janicka G.

PMID: 765975 [PubMed - indexed for MEDLINE]

2620. Int Arch Allergy Appl Immunol. 1976;51(3):338-58.

Heterogeneity of rabbit homologous skin sensitizing antibodies: IgE and a new subclass IgGa.

Stux SV, Ovary Z.

Homologous skin sensitizing antibodies in the rabbit can be differentiated by their requirement for complement in expressing the passive cutaneous anaphylactic reaction as complement dependent (CD) or complement independent (CI). CI antibodies are representative of the IgE class, while the CD antibodies belong to a proposed new subclass of IgG designated IgGa (a = anaphylaxis). The following are characteristics of CI (IgE) antibodies: elution at 0.05 M on DEAE at pH 8.1 migration ahead of 7S in ultracentrifugation, molecular weight 215,000 daltons by Sephadex G-200, fast gamma-electrophoretic mobility, isoelectric point, pI = 4.98 (5.68-4.58), 85-95% heat sensitivity, sensitivity to reduction plus alkylation and option at 0.01 M on DEAE, 7S in ultracentrifugation, MW 138,000 daltons, slow gamma-electrophoretic mobility pI = 6.90 (8.30-5.70), resistance to heating and to reduction plus alkylation and 1-day optimal SP. CI antibodies could be absorbed by anti-Fab, anti-epsilon, the homologous antigen and were not absorbed by anti-gamma, anti-alpha, or heterologous antigens. The CD antibodies were absorbed by anti-Fab, anti-gamma and not absorbed by anti-epsilon, anti-alpha, or heterologous antigens.

PMID: 1279025 [PubMed - indexed for MEDLINE]

2621. Br J Dermatol. 1976 Jan;94(1):65-70.

A study on the clinical application of a direct leukocyte migration test in chromium contact allergy.

Tio D.

A capillary tube leukocyte migration inhibition assay has been adopted as an in vitro method for the demonstration of chromium hypersensitivity in twenty-two subjects with clinically proven or suspected chromium allergy. Two complexes of trivalent chromium and human serum albumin, exerting different migration inhibitory effects, have been prepared and used as the antigen. In the presence of the chromium-albumin complex with strong inhibitory activity, sensitivity to chromium was demonstrated independent of the clinical condition of the skin in all the patch test positive subjects. An additional positive response to the

second chromium-albumin complex was observed only in those patients who were clinically in a state of exacerbation of an allergic contact dermatitis in which chromium allergy was an active causative factor. The results were not influenced by skin allergic reactivity to compounds other than chromium and the method was found to be of practical clinical value for diagnosing chromium allergy.

PMID: 1252340 [PubMed - indexed for MEDLINE]

2622. Br J Dermatol. 1976 Jan;94(1):63-4.

The leukocyte migration inhibition test in allergic nickel contact dermatitis.

Macleod TM, Hutchinson F, Raffle EJ.

No statistical difference was found between the migration indices of nickel sensitive and control subjects using the leukocyte migration inhibition test with two concentrations of nickel sulphate. The results contrast with the specific positive results demonstrated previously in the same subjects with the lymphocyte transformation test.

PMID: 1252339 [PubMed - indexed for MEDLINE]

2623. Mod Probl Ophthalmol. 1976;16:41-50.

Experimental allergic uveitis: induction by retinal rod outer segments and pigment epithelium.

Meyers RL.

EAU was produced in strain 13 guinea pigs after immunization with purified guinea pig retinal rod outer segments and with retinal pigment epithelium in mycobacterial adjuvant emulsion. The lesions of EAU appear as inflammatory infiltrates of the iris and ciliary body followed by choroidal inflammation, often with retinal photoreceptor degeneration. Specific antibodies are detected in the serum of a majority of the animals with clinical disease. Immunohistochemical staining of normal and inflamed eyes with serum from the immunized animals with uveitis demonstrates specific antigens localized in the outer segments. All immunized animals demonstrate delayed-type hypersensitivity characterized by skin reactions of mononuclear cells, by the in vitro inhibition of migration of peritoneal macrophages from the sensitized animals in the presence of specific antigen, and by antigen-specific lymphocyte stimulation. More pronounced delayed-type hypersensitivity reactions occurred during uveitis and indicated that cellular immunity correlates with clinical EAU, whereas no correlation was found with serum antibody. Successful experiments at transferring clinical disease passively with sensitized lymphocytes from animals with uveitis to normal recipients were conducted via the intravenous as well as intravitreal routes. No inflammatory reactions occurred after similar transfer of nonsensitized lymphocytes. The recipients of the passively transferred cells demonstrated both humoral and delayed type hypersensitivity to the retinal antigens. These findings suggest that the retinal rod outer segments and pigment epithelium are the sourve of significant antigens in autoimmune uveitis and retinitis.

PMID: 1250238 [PubMed - indexed for MEDLINE]

2624. Can J Surg. 1976 Jan;19(1):23-5.

[An unusual case of hydatid cyst].

[Article in French]

Dontigny L, Mercier C, Pagé A, Lévy R, Cossette R, Pelletier C, Verdant A.

A hydatid cyst of the spleen developed in a Greek-Canadian woman who had lived on a sheep farm. The cyst extended through the diaphragm to the left lower lobe. Splenectomy and left lower lobectomy and excision of contiguous diaphragm were performed. Histopathologic examination confirmed the presence of hydatid cysts in both spleen and lung. Postoperative course was uneventful. In Canada hydatid disease is rare and its occurrence sporadic. It is commoner among Canadian Indians and immigrants than native Canadians. Treatment is surgical; en bloc excision of tissue eliminates the possibility of anaphylaxis of dissemimination of scolices.

PMID: 1245004 [PubMed - indexed for MEDLINE]

2625. Exp Pathol (Jena). 1976;12(6):295-300.

Experimental allergic encephalomyelitis.-- The effect of dexamethasone on growth and proliferation in the draining lymph node.

Matous-Malbohan I, Holub M, Mares V, Lodin Z.

Dexamethasone, when administered from day 0 to day 9 following the sensitizing injection of encephalitogenic protein in complete Freund's adjuvant, suppressed both the characteristic enlargement of the paracortical area of the draining lymph node and the development of clinical and histopathological signs of experimental allergic encephalomyelitis. Division of cells within the node was not inhibited and therefore the effect of dexamethasone appeared to be a consequence of reduced migration of lymphocytes into the node.

PMID: 1087608 [PubMed - indexed for MEDLINE]

2626. Acta Med Scand. 1976;199(1-2):105-11.

Sequential studies of lymphocytes, neutrophils and serum proteins during prednisone treatment.

Clemmensen O, Andersen V, Hansen NE, Karle H, Koch C, Soborg M, Weeke B.

Seven patients (6 with connective tissue diseases, 1 with bronchial asthma) have been studied before, during, and after prednisone therapy. Maximum dose was 15 mg daily, which was tapered off to zero within three months. All patients showed striking subjective improvement during therapy. The ESR reflected this improvement but the acute phase proteins did not. The serum concentration of prealbumin rose significantly during the period of most intensive steroid treatment. IgE decreased in the patient with bronchial asthma, but otherwise the immunoglobulins did not change, and positive serological tests remained unchanged. Contact sensitization to haptens was induced without impairment during therapy. Prednisone induced rises in blood lymphocyte and neutrophil concentrations. Lymphocyte transformation, both mitogen- and antigen-induced, was not influenced by therapy, but PPD-induced inhibition of leucocyte migration decreased. Neutrophil phagocytosis was unimparied, but bactericidal capacity, stimulated nitroblue tetrazolium reduction, and neutrophil and plasma lysozyme concentrations were all depressed during treatment with prednisone.

PMID: 1082710 [PubMed - indexed for MEDLINE]

2627. Arkh Patol. 1976;38(5):60-6.

[Functional-morphological changes in the lungs in bronchial asthma].

[Article in Russian]

Kodolova IM.

Histological and immunohistochemical studies of 57 bioptic specimens of the mucosa of the main bronchi taken during an attack of bronchial asthma, and those of the lungs of 27 patients who had died on the attack, showed that in the course of the attack of bronchial asthma serous-desquamous allergic inflammation, which proceeded according to the type of hypersensitivity, of the immediate type, developed along the full length of the broncho-vascular barrier. It occurred under the effect of biologically active substances liberated in the reaction of the antigen with IgE. Immunochemically, there was detected luminescence of IgE on the basal membranes of the mucous glands, on the basal membranes of the mucosa, as well as in lymphoid, plasmic, and mast cells infiltrating the mucosa. In allergic inflammation in the bronchi there were noted drastic delatation and increased permeability of vessels of the microcirculatory bed, odema, migration of eosinophils, the mast-cell reaction with degranulation of mast cells, spasm of musculature, elevated permeability of the basal membrane, impregnation of the latter with plasmic protein with fibrin, hypersecretion and desquamation of the epithelium, hypersecretion of mucous glands. As a result of the inflammatory changes in the bronchial system disorders of the drainage function of the bronchi with obstruction of their lumens which were most pronounced in small bronchi and bronchioles, developed.

PMID: 1023853 [PubMed - indexed for MEDLINE]

2628. Ter Arkh. 1976;48(7):20-2.

[Study of sensitization to the allergen of hemolytic streptococcus in patients with chronic glomerulonephritis].

[Article in Russian]

Sochnev AM, Shif BA.

PMID: 996742 [PubMed - indexed for MEDLINE]

2629. Probl Tuberk. 1976;3:59-62.

[Allergic reactions of leukocytes in diagnosis of silicotuberculosis].

[Article in Russian]

Kitaev MI, Tiurebaeva BN.

PMID: 967861 [PubMed - indexed for MEDLINE]

2630. Int Arch Allergy Appl Immunol. 1976;51(5):529-43.

Influence of detergent on aerosol allergic sensitization with enzymes of Bacillus

subtilis.

Markham RJ, Wilkie BN.

An anionic detergent, sodiumdodecylbenzenesulphonate, had an adjuvant effect upon aerosol allergic sensitization with subtilopeptidase A, a proteolytic enzyme of Bacillus subtilis. Both local and systemic antibody responses were accelerated and prolonged by use of the detergent. This adjuvant effect was only seen when an inactive form of the enzyme was used. The detergent, however, was able to potentiate early clinical responses upon initial exposure to the active enzyme.

PMID: 823120 [PubMed - indexed for MEDLINE]

2631. Mod Probl Ophthalmol. 1976;16:125-30.

Prostaglandins and inflammation in the eye.

Nelson EL.

Traumatic injury of the eye promotes the release of prostaglandins E2 and F2alpha from the iris and other tissues. These induce vasodilation, increased capillary permeability and an increase in protein content of the aqueous. They are leukotactic. With an influx of leukocytes PGE1 appears in the aqueous having been synthesized by these cells. Infectious agents also attract leukocytes and sensitized lymphocytes characterize inflammation of both allergic and infectious origin. The cascade of molecular and cellular events seen in ocular inflammation of various origin seem ultimately to result in a reaction largely mediated by prostaglandins. Effective therapy should be directed at preventing their synthesis (synthetase inhibitors), interferring with their action once synthesized (receptor blockers), and inhibiting the migration of leukocytes into the eye.

PMID: 765779 [PubMed - indexed for MEDLINE]

2632. Allerg Immunol (Leipz). 1976;22(1):97-103.

[Leukocyte migration inhibition test in skin chamber--an in-vivo method].

[Article in German]

Wätzig V, Ruffert K, Güldner G.

For the demonstration of a cell-mediated sensitisation, tuberculin allergics, chromate allergics, penicillin allergics, and controls were investigated. The method used was the measurement of leukocyte migration inhibition in a Teflon skin chamber with and without addition of antigen, which approaches to testing under in vivo conditions. In 10 of 11 tuberculin allergics, 6 of 7 chromate allergics, and 7 penicillin allergics, a clear inhibition of leucocyte migration after addition of antigen could be observed (maximum 16 h after addition of antigen). In 16 unsensitised controls no significant migration inhibition appeared. Possible advantages of this method over in vitro methods and intracutaneous tests are discussed.

PMID: 135507 [PubMed - indexed for MEDLINE]

2633. Allerg Immunol (Leipz). 1976;22(1):109-10.

[Cellular immunity in experimental allergic arthritis in rabbits (short communication)].

[Article in German]

Stiehl P, Püschel W, Rosenkranz M, Franke UM.

PMID: 135494 [PubMed - indexed for MEDLINE]

2634. Lab Delo. 1976;(11):674-7.

[Diagnosis of allergy to cow's milk in vitro].

[Article in Russian]

Martynov SM, Fedorenko TA.

PMID: 66355 [PubMed - indexed for MEDLINE]

2635. Lab Delo. 1976;(12):731-4.

[Study of specific allergy in patients with bacillary dysentery by means of the leukocyte migration inhibition test].

[Article in Russian]

Fradkin VA, Lodinova LM, Shchetinina IN.

PMID: 63617 [PubMed - indexed for MEDLINE]

2636. Arch Immunol Ther Exp (Warsz). 1976;24(3):359-66.

Formed blood elements in peritoneal effusion and the macrophage migration inhibition test in healthy guinea pigs and in guinea pigs with experimental allergic encephalomyelitis.

Wróblewski T.

An attempt was made to correlate the percentages of macrophages, lymphocytes and granulocytes in the peritoneal effusion in healthy guinea pigs and guinea pigs with experimental allergic encephalomyelitis (EAE), with the macrophage migration inhibition (MMI) test. Varying percentages of the cells had no influence on values of MMI. Similarly, in guinea pigs with EAE, percentages of formed elements in peritoneal effusion were not correlated with intensity of MMI or with histopathologic lesions in the brain and spinal cord. It is suggested that the observed differences are due to individual immunologic responsiveness of animals and, probably, to other hitherto unknown mechanisms.

PMID: 60984 [PubMed - indexed for MEDLINE]

2637. Ann Anesthesiol Fr. 1976;17(3):259-67.

[Diagnosis of drug allergies related to anesthesia and surgery].

[Article in French]

Girard JP, Cuevas M.

Drug allergies are increasingly common. They may occur in 5-10 p. 100 of patients treated with drugs. From a diagnostic standpoint, the problem is a complex one, many aspects of which are still poorly understood. This is particularly true with regard to the nature of the antigenic determinants and their vectors. Allergic reactions in patients submitted to anaesthesia and surgery represent a very particular case of the situation. A study of the immune status of such patients was undertaken and revealed that T lymphocyte depression, lasting from one to three weeks, often occurs. It is important to take this concept into account when choosing laboratory tests designed to substantiate the diagnosis. Several examples are presented and discussed.

PMID: 10777 [PubMed - indexed for MEDLINE]

2638. Int J Fertil. 1976;21(1):49-54.

A new model for antisperm autoimmunity in guine pigs.

Mazzolli AB, Bustuoabad OD, Barrera C, Mancini RE.

Adult outbread male guinea pigs were autoimmunized without adjuvant. Homogenates were prepared with one of their own testes previously submitted "in vivo" and "in vitro" to thermal injury. Animals received a single or daily repeated intradermal injection without added adjuvant, in one or different skin sites. Guinea pigs daily sensitized in the same site during 30 days showed the presence of: a) dermal granuloma at the site of injection; b) several foci of typical allergic orchitis; c) delayed hypersensitivity detected by inhibition of macrophage migration; d) moderate titres of spermagglutinins and negligible levels of hemagglutinating antibodies. Guinea pigs receiving a single dose in one site only developed delayed hypersensitivity. Animals daily sensitized with the same dose of altered antigen in different sites, or with normal testis antigen either in one or different sites, showed negative results. The correlation among testicular lesion, dermal granuloma and cellular immunity is discussed. It is concluded that testis autosensitization is obtained in the absence of added adjuvant provided that a thermally injured gonad used as antigen is repeatedly injected in the same site.

PMID: 8395 [PubMed - indexed for MEDLINE]

2639. Contact Dermatitis. 1975 Oct;1(5):306-10.

Allergic contact dermatitis to All-trans-retinoic acid; epicutaneous and leukocyte migration inhibition testing.

Jordan WP Jr, Higgins M, Dvorak J.

Two male prison volunteers had severe cutaneous reactions to the initial patch testing of All-trans-retinoic acid containing products being evaluated for irritancy. Muliple patch testing procedures performed throughout one year, along with leukocyte migration inhibition studies, speak strongly for delayed hypersensitivity reactions in these subjects. The failure to document a previous exposure in subjects living in a controlled environment suggests that these responses were cross-reactions to a prior sensitizer, perhaps unique to this environment.

PMID: 1235282 [PubMed - indexed for MEDLINE]

2640. Biull Eksp Biol Med. 1975 Sep;80(9):83-6.

[Reactivity of the neuroglia in guinea pigs sensitized with Mycobacteria tuberculosis].

[Article in Russian]

Kanchurin AKh, Brusina LI.

The changes in the glial cell reactivity depended on the phase and severity of the pathological process in the experimental allergic encephalomyelitis induced in guinea pigs by injection of Mycobacteria tuberculosis in a mixture of arlacel and vaselin. These reactivity changes measured by the method of inhibition of the glial cell migration in the tissue culture was specific of the nervous tissue antigens. The same changes in the reactivity could be induced in the tissue culture of the glial cells from the intact animals by the addition of sera or the lymph node cells from the actively sensitized guinea pigs. The present data did not allow to attribute the changes in the glial cell reactivity to the products of destruction of the nervous tissue only to the action of the humoral or the cell factors (elements) penetrating into the CNS from the blood vessels or the lymphatic tissue.

PMID: 816399 [PubMed - indexed for MEDLINE]

2641. Pol Tyg Lek. 1975 Aug 4-11;30(31-32):1327-8.

[Delayed allergy to indocyanine green (ICG) and bromsulphalein (BSP) studied by means of leukocyte migration inhibition test].

[Article in Polish]

Juszczyk J, Szkaradkiewicz A, Adamek J.

PMID: 1161575 [PubMed - indexed for MEDLINE]

2642. Sov Med. 1975 Aug;(8):31-5.

[In vitro suppression of leukocyte migration reaction in drug allergy].

[Article in Russian]

Novikova VI, Novikov DK.

PMID: 1233758 [PubMed - indexed for MEDLINE]

2643. Sov Med. 1975 Aug;(8):28-31.

[Leukocyte migration inhibition test in the diagnosis of drug allergy].

[Article in Russian]

Kitaev MI, Tsatskina ES.

PMID: 1233756 [PubMed - indexed for MEDLINE]

2644. Am J Epidemiol. 1975 Aug;102(2):153-63.

Tucson epidemiologic study of obstructive lung diseases. II: Effects of in-migration factors on the prevalence of obstructive lung diseases.

Lebowitz MD, Burrows B.

The effects of in-migration factors on respiratory symptoms, chronic health problems, and lung function were examined in the stratified Tucson population as part of a longitudinal epidemiologic study of obstructive lung diseases. Migration to the area specifically for health reasons explained part of the high prevalences of disease found in the study. But natives still had higher rates of disease than those found generally in the United States, especially for asthma and allergic rhinitis; It was found that previous urban residence was related to the prevalence of several conditions, even when controlling for age, sex, and smoking habits. However, the trends were not always clear and the differences were not great enough to explain the Tucson population's much higher rates of these conditions than reported nationally or in similar studies elsewhere.

PMID: 1155444 [PubMed - indexed for MEDLINE]

2645. Leber Magen Darm. 1975 Aug;5(4):123-31.

[Humoral and cellular immune phenomena in an acute viral hepatitis (author's transl)].

[Article in German]

Sodomann CP.

During the course of acute viral hepatitis A and B, several humoral and cellular immune phenomena have been observed, part of which is predominantly or even exclusively associated with hepatitis B: 1. Relative and absolute counts for T-lymphocytes depressed and for "null" -cells elevated; 2. mild elevation of serum globulin levels; 3. IgM augmentation occurring fastly, pronounced, and long lasting in typical cases; 4. IgG augmentation occurring later, less pronounced, and for a shorter period in typical cases; 5. autoantibodies to smooth muscles and mitochondria in low titers in some patients; 6. specific antibodies to "e" -antigen (early) and HB-Ag (later in the course) in part of the cases with hepatitis B; 7. immune complexes including HB-Ag, IgG and probably IgM (and IgA) as well as complement in some cases; 8. depressed levels of the fourth component of complement and - in cases complicated by "allergic" symptoms - of C3, C4, and total complement; 9. occurrence of activated lymphocytes ("virocytes") in peripheral blood; 10. enhanced spontaneous lymphocytic DNA-synthesis; 11. enchanced phytohaemagglutinin stimulation of lymphocytes; 12. mild lymphocyte proliferation to HB-Ag in part of the acute and convalescent cases of hepatitis B; 13. production of migration inhibition factor to liver specific protein (and HB-Ag) or lymphocytes in different percentages of patients with hepatitis B. Origin, diagnostic and prognostic importance, as well as pathogenetic revelance of the described immune phenomena are discussed.

PMID: 772340 [PubMed - indexed for MEDLINE]

2646. Zentralbl Bakteriol Orig A. 1975 Jul;232(2-3):266-86.

[Studies of the toxic components of Erysipelothrix rhusiopathiae. 2. Communication: Detailed characterization of an extracted endotoxin (author's transl)].

[Article in German]

Leimbeck R, Böhm KH, Ehard H, Schulz LC.

The endotoxins of different Erysipelothrix rhusiopathiae strains were examined in various biological systems, since in earlier investigations (Leimbeck and Böhm, 1975) the lethal effect for 10 day old chick embryos had been found to be highly dependent upon the virulence of the strains. The lethal effect for mice and rats was found to be much less. The toxin of the strain T28 proved to be highly pyrogenic for rabbits and inducing acute shock-effects in swine SCHULZ et al. (1961) already suggested that a shock-like pathogenesis existed in the swine erysipelas infection. Furthermore the endotoxic nature was confirmed by the typical course of temperature after i.v. application of the toxin, the ability to cause the Sanarelli-Schwartzman-reaction (both in rabbits), the protection against toxicity by cortisones, and heat stability. An erysipelas antiserum did not neutralize the toxin. It was found to have low antigenic and apparantly no immunogenic properties and an allergic skin-reaction in experimentally infected swine could not be induced. The toxins of various strains were tested by the macrophagemigration-inhibition test. In respect to a preliminary classification the toxin could be identified as a complexed water-soluble and heat-stable glucoproteid with an estimated molecular weight of 31.700. The polysaccharides were found to be the presumable carriers of toxicity.

PMID: 1182046 [PubMed - indexed for MEDLINE]

2647. Rev Gastroenterol Mex. 1975 Jul-Aug;40(4):218-9.

Detection of hypersensitivity to drugs by lymphocyte cultures in allergic drug hepatitis [proceedings].

Yamamoto, Sukeo, Enomoto, Mizoguchi T, Yashuhiro.

PMID: 801199 [PubMed - indexed for MEDLINE]

2648. Infect Immun. 1975 Jun;11(6):1187-94.

Immunochemical studies on the human pathogen Sporothrix schenckii: effects of chemical and enzymatic modification of the antigenic compounds upon immediate and delayed reactions.

Shimonaka H, Noguchi T, Kawai K, Hasegawa I, Nozawa Y, Ito Y.

The rhamnose-containing polysaccharide-peptide compound derived from the cells of the pathogenic fungus Sporothrix schenckii has been shown to contain 87.1% carbohydrate and 12.5% peptide and to give rise to both immediate- and delayed-type reactions in sensitized guinea pigs. The capacity to induce immediate-type reaction, passive cutaneous anaphylaxis, was completely lost by degradation of the carbohydrate moiety by periodate, whereas the ability to induce the delayed-type reactions of migration inhibition (in vitro) and the sporotrichin reaction (in vivo) were only slightly affected by periodate treatment. On the other hand, delayed "reactivities" to the compound were considerably reduced by treatment with papain, whereas the immediate-type reaction remained positive. These results lead to a conclusion that the rhamnose-containing polysaccharide of the polysaccharide-peptide antigenic compound plays an important role in the immediate-type reaction, whereas the peptide is largely responsible for the delayed-type reaction.

PMCID: PMC415198
PMID: 166914 [PubMed - indexed for MEDLINE]

2649. J Allergy Clin Immunol. 1975 Jun;55(6):411-21.

Properties and activities of transfer factor.

Kirkpatrick CH.

Although there is agreement that transfer factor endows skin test-negative subjects with the ability to develop the delayed allergic responses of the transfer factor donors, there is little direct information on the mechanism of this phenomenon or on the nature of the active components (s). This report reviews some of the known effects of transfer factor or immune responses and inflammation. It is concluded that transfer factor has multiple sites of action, including effects on the thymus, on lymphocyte-monocyte and/or lymphocyte-lymphocyte interactions, as well as direct effects on cells in inflammatory sites. It is also suggested that the "specificity" of transfer factor is determined by the immunologic status of the recipient rather than by informational molecules in the dialysates. Finally, it is proposed that many effects of transfer factor may be due to changes in intracellular cyclic nucleotide content, especially accumulation of cGMP, in immunologically reactive cells.

PMID: 48524 [PubMed - indexed for MEDLINE]

2650. Zh Mikrobiol Epidemiol Immunobiol. 1975 May;(5):127-32.

[Immunological polyfunctionality of proteins and its meaning for the analysis of an allergen-active bacterial substrate].

[Article in Russian]

Maianskiĭ AN.

It was shown with the aid of immunosorption of an allergen-active substrate of E. coli 020: K84 (No. 2-rII) that protein substances taking part in the phenomenon of cell hypersensitivity were active in the humoral immunity reactions. The allergenic and immunochemical activity served as functions of the same molecules of bacterial proteins, this substantiating the use of immunochemical analysis for the study of an allergen-active bacterial substrate. By protein denaturing it is possible to obtain immunochemical inert allergen-active preparations capable of detecting the cell hypersensitivity to crude bacterial proteins. The problem of immunological polyfunctionality of proteins is discussed from the aspect of nonhomogeneity of their antigenic determinant groups.

PMID: 1948 [PubMed - indexed for MEDLINE]

2651. Q J Med. 1975 Apr;44(174):325-47.

Bronchial asthma in the Nigerian savanna region. A clinical and laboratory study of 106 patients with a review of the literature on asthma in the tropics.

Warrell DA, Fawcett IW, Harrison BD, Agamah AJ, Ibu JO, Pope HM, Maberly DJ.

One hundred and six asthma patients were studied in Zaria in the Nigerian savanna region. This group resembled hospital attenders in general in containing a

disproportionately large number of immigrants from southern Nigeria and students undergoing higher education. Childhood asthma was rare. Asthma started after the age of 19 years in 69 per cent of patients. Twenty-seven per cent gave a history of rhinitis but none had had eczema. Twenty-two per cent gave a family history of asthma. Cutaneous hypersensitivity to house dust supported by a history of attacks being precipitated by dust was found in 41 per cent of patients. Asthma was worst in the rainy season in 45 per cent of patients. Mites were found in mattress dust samples; the mean count was 243 mites per g dust; Dermatophagoides farinae formed 86-6 per cent of the total mite population. The variability of airways obstruction averaged 50 per cent of maximum values for forced expiratory volume in the first second (FEV1) and peak expiratory flow (PEF). The median severity of airways obstruction measured as FEV1/VC per cent was four standard deviations below predicted normal. Eighty-seven per cent of patients were positive to prick skin tests with one or more allergens. The commonest reactions were to house dust (58 per cent), house dust mite (45 per cent) and Dermatophagoides farinae (44 per cent). Fifty-one per cent of a group of controls were also positive on skin testing but the pattern of responses was different from the asthmatic patients. This high proportion of reactors is explained by high allergen load. Serum IgE levels were lower in the asthmatics than in a group of healthy controls who showed the very high levels characteristic of some African populations. We suggest that the controls were protected from atopic disease by developing high blocking levels of non-specific IgE, perhaps in response to gut helminths. The clinical pattern of asthma in Zaria is compared with other countries in the tropical and temperate zones. The particular problems of treating asthma in developing tropical countries are discussed.

PMID: 1101285 [PubMed - indexed for MEDLINE]

2652. J Immunol. 1975 Apr;114(4):1269-74.

Pathogenesis of experimental allergic uveitis induced by retinal rod outer segments and pigment epithelium.

Meyers RL, Pettit TH.

Experimental allergic unveitis (EAU) was produced in strain 13 guinea pigs after immunization with purified guinea pig retinal rod outer segments and pigment epithelium (PE) in mycobacterial adjuvant emulsion. The lesions of EAU appear as inflammatory infiltrates of the iris, ciliary body, and choroid, often with photorecptor degeneration. Specific antibodies are frequently detected in the serum of some but not of all animals with clinical uveitis. Immunohistochemical staining of normal and inflamed eyes with serum from the immunized animals with clinical disease demonstrated specific antigens localized in the retinal photoreceptor layers, whether or not circulating precipitating antibodies were also present in the serum. All immunized animals demonstrated delayed-type hypersensitivity (DTH) characterized by skin reactions of mononuclear cells and by the vitro inhibition of migration of cells from the sensitized animals in the presence of specific antigen whether or not clinical uveitis occurred. However, stronger DTH reactions were observed during clinical uveitis. Cellular immunity appears to correlate with clinical EAU, whereas, no correlation was found with serum antibody. These findings suggest that the retinal photoreceptor cell and PE are the source of the significant antigens in autoimmune uveitis and retinitis.

PMID: 804008 [PubMed - indexed for MEDLINE]

2653. Pol Tyg Lek. 1975 Feb 3;30(5):185-8.

[Leukocyte migration inhibition test in the diagnosis of allergy to antibiotics].

[Article in Polish]

Karna T, Chyrek-Borowska S.

PMID: 1114112 [PubMed - indexed for MEDLINE]

2654. Zh Mikrobiol Epidemiol Immunobiol. 1975 Feb;(2):12-9.

[Allergenic fractions of Enterobacteriaceae. VI. Gel-chromatographic profile of intracellular protein antigens, forming the allergen-active substrate of enterobacteria].

[Article in Russian]

Maianskiĭ AN, Molchanova IV, Alatyrtseva IE.

Allergeno-active protein substrate of enterobacteria (E. coli and R. rettgeri strains) was divided into 7 fractions with a different molecular weight with the aid of many-stage gel-filtration through Sephadex (G-50, G-75, G-100, G-150, G-200). A study was made of their biochemical composition, antigenic and protein spectrum, allergenic properties in the reactions of hypersensitivity of delayed type in vivo (skin tests) and in vitro (inhibition of macrophage migration). The principal part of allergenic activity was determined by components with a molecular weight of 30-150 and over 800 thousand. The majority of the antigens had a molecular weight of 30-150 thousand. A physico-chemical heterogeneity of immunologically affiliated components was noted. The allergenic substrate of the bacterial cell included a complex of molecular-nonhomogenous "strong" and "weak" protein allergens.

PMID: 804783 [PubMed - indexed for MEDLINE]

2655. J Immunol. 1975 Feb;114(2 Pt 1):546-9.

Experimental allergic encephalomyelitis in resistant and susceptible guinea pigs: in vivo and in vitro correlates.

Lisak RP, Zweiman B, Kies MW, Driscoll B.

Strain 2 guinea pigs develop less severe experimental allergic encephalomyelitis than do strain 13 and Hartley guinea pigs when sensitized with equivalent amounts of homologous myelin basic protein (BP) in complete Freund's adjuvant. In vivo and in vitro correlates of delayed hypersensitivity to myelin basic protein are depressed in the strain 2 guinea pigs relative to the two susceptible strains. The incidence of circulating anti-BP antibodies is also lower in sera from strain 2 guinea pigs than in sera from strain 13 or Hartley guinea pigs. There was no difference among the three strains in their ability to mount delayed hypersensitivity to tuberculin, nor in the response of their cells to PHA in vitro.

PMID: 47353 [PubMed - indexed for MEDLINE]

2656. Gastroenterol Jpn. 1975;10(4):300-6.

Detection of hypersensitivity to drugs by lymphocyte cultures in drug-induced allergic hepatitis.

Mizoguchi Y, Yamada T, Monna T, Yamamoto S, Morisawa S.

Fifty-three out of 70 patients with drug-induced liver injury showed an immunological response to the drug using either the lymphocyte transformation test or the macrophage migration inhibitory test (MI test) or both. Both the separated lymphocyte culture and the microvolume whole blood culture technique are available as assay methods. The latter method is simpler and the results of both methods are comparable. The administration of a synthetic copolymer of polyadenylic and polyuridylic acid (poly A:U) to the culture medium enhances the lymphocyte response to antigen and is therefore very useful to detect weak responses.

PMID: 1234107 [PubMed - indexed for MEDLINE]

2657. Verh Dtsch Ges Inn Med. 1975;81:1504-6.

[Studies on delayed insulin allergy using cell-migration-inhibition technics in diabetics].

[Article in German]

Rehn K, Matthiensen R, Keintzel E, Hunstein W, Uhl N.

PMID: 1227008 [PubMed - indexed for MEDLINE]

2658. Int Arch Allergy Appl Immunol. 1975;48(6):750-5.

In vitro lymphocyte transformation in the development of experimental allergic thyroiditis.

Hiramine C, Hojo K.

In vitro lymphocyte transformation to homologous thyroid antigen has been studied in cultures of lymph node cells obtained from 97 guinea pigs at weekly intervals after a single injection of homologous thyroid extract in complete Freund's adjuvant, using a method for measurement of 3-H-thymidine incorporation. Lymph node cell cultures from animals 1 week after sensitization showed a significant increase in thymidine incorporation after the addition of antigen. Two weeks after sensitization the stimulation was maximal, and then a lower but significant response continued during the next 5 weeks. There was no correlation between lymphocyte transformation and the level of circulating antibodies throughout the post-sensitization period examined. The early lymphocyte transformation contrasted with the later development of macrophage migration inhibitory factor activity.

PMID: 1095496 [PubMed - indexed for MEDLINE]

2659. Int Arch Allergy Appl Immunol. 1975;48(2):276-86.

Subcellular fractions from dermis and epidermis in contact sensitization of guinea pigs to 1-chloro-2,4-dinitrobenzene.

Camm EL, Mitchell JC, McMaster WR, Towers GH.

Subcellular fractions were prepared from the epidermis of guinea pigs which had been painted with 1-chloro-2,4 dinitrobenzene 14 h previously. The microsomal fraction showed less activity than other fractions in the MIF test. Similarly,

the microsomal fraction from the dermis of skin irritated with croton oil before DNCB painting was relatively inactive. However, the microsomal fractions from the epidermis and more especially from the dermis of skin painted with DNCB while undergoing allergic contact dermatitis from pentadecylcatechol showed significant MIF activities.

PMID: 1090544 [PubMed - indexed for MEDLINE]

2660. Int Arch Allergy Appl Immunol. 1975;49(5):585-96.

Skin reaction, inhibition of macrophage migration, and lymphocyte transformation with tuberculin active peptide (TAP) and arabinogalactan obtained from tubercle bacilli.

Niinaka T, Kishimoto S, Aoki T, Ikegami H, Ito F.

Arabinogalactan purified from heat-killed tubercle bacilli failed to elicit a delayed type of skin reaction and had no ability to induce in vitro lymphocyte blast formation in sensitized guinea pigs. It was, however, inhibitory to migration of bronchoalveolar washing cells by the indirect test, but not by the direct test, and capable of eliciting an immediate type of skin reaction and anaphylaxis when injected into sensitized guinea pigs. Tuberculin active peptide (TAP) was active in all of in vitro and in vivo cell-mediated immune responses, but not in immediate responses.

PMID: 809364 [PubMed - indexed for MEDLINE]

2661. Lab Delo. 1975;(6):360-3.

[Leukocyte migration inhibition test as an indicator of viral allergy in systemic lupus erythematosus].

[Article in Russian]

Galenok VA.

PMID: 51126 [PubMed - indexed for MEDLINE]

2662. J Exp Med. 1975 Jan 1;141(1):72-81.

The immune response against myelin basic protein in two strains of rat with different genetic capacity to develop experimental allergic encephalomyelitis.

McFarlin DE, Hsu SC, Slemenda SB, Chou FC, Kibler RF.

After challenge with guiena pig basic protein (GPBP) Lewis (Le) rats, which are homozygous for the immune response experimental allergic encephalomyelitis (Ir-EAE) gene, developed positive delayed skin tests against GPBP and the 43 residue encephalitogenic fragment (EF); in addition, Le rat lymph node cells (LNC) were stimulated and produced migration inhibitory factor (MIF) when incubated in vitro with these antigens. In contrast Brown Norway (BN) rats, which lack the Ir-EAE gene, did not develop delayed skin tests to EF and their LNC were not stimulated and did not produce MIF when incubated in vitro with EF. These observations indicate that the Ir-EAE gene controls a T-cell response against the EF. Le rats produced measurable anti-BP antibody by radioimmunoassay after primary challenge. Although no antibody was detectable in BN rats by radioimmunoassay, radioimmunoelectrophoresis indicated that a small amount of

antibody was formed after primary immunization. After boosting intraperitoneally, both strains of rat exhibited a rise in anti-BP antibody; which was greater in Le rats. In both strains of rat the anti-BP antibody reacted with a portion of the molecule other than the EF. Since EF primarily evokes a T cell response, it is suggested that the EF portion of the BP molecule may contain a helper determinant in antibody production.

PMCID: PMC2190502
PMID: 46914 [PubMed - indexed for MEDLINE]

2663. Cell Immunol. 1975 Jan;15(1):143-51.

Experimental allergic encephalitis: study of cellular immunity to the encephalitogenic determinant.

Spitler LE, von Muller CM, Young JD.

PMID: 45837 [PubMed - indexed for MEDLINE]

2664. Z Hautkr. 1974 Oct 1;49(19):847-51.

[Allergy to sulfanilamides, demonstration using the macrophage migration inhibition test].

[Article in German]

Rytter M, Barth J.

PMID: 4464726 [PubMed - indexed for MEDLINE]

2665. Dermatol Monatsschr. 1974 Oct;160(10):791-7.

[Comparative immunological studies in animal experimental contact eczema].

[Article in German]

Günther E, Helmke R, Meyer J, Reich P, Rietschel L, Schleicher H.

PMID: 4426450 [PubMed - indexed for MEDLINE]

2666. Nouv Presse Med. 1974 Sep 21;3(31):1956.

[Letter: Leukocyte migration test in the diagnosis of drug allergy. A useful test].

[Article in French]

Pupil P, Moneret-Vautrin DA, Savinet H.

PMID: 4444939 [PubMed - indexed for MEDLINE]

2667. Acta Tuberc Pneumol Belg. 1974 Sep-Dec;64(5):397-412.

[BCG-allergy. Immunological basis and antigen choice (author's transl)].

[Article in French]

Depelchin A.

PMID: 4546123 [PubMed - indexed for MEDLINE]

2668. Rev Med Suisse Romande. 1974 Sep;94(9):697-710.

[Diagnosis of drug allergies].

[Article in French]

Girard JP.

PMID: 4140566 [PubMed - indexed for MEDLINE]

2669. J Immunol. 1974 Sep;113(3):993-7.

Migration inhibition test in leukocytes from patients allergic to penicillin.

Ortiz-Ortiz L, Zamacona G, Garmilla C, Arellano MT.

PMID: 4137843 [PubMed - indexed for MEDLINE]

2670. Infect Immun. 1974 Jul;10(1):21-4.

Mycobacterial components responsible for the induction of a chronic immunological inflammatory response in rabbit lungs.

Moore VL, Myrvik QN.

Fractions of the tubercle bacillus were examined for their capacity to produce an allergic pulmonary granulomatous response in rabbits. We found that emulsions containing old tuberculin and even old tuberculin alone in an emulsion could induce a pulmonary inflammatory reaction comparable in intensity to that produced with killed BCG. Emulsions lacking old tuberculin failed to produce an allergic pulmonary inflammatory response. The significance of these results in relation to immunological and nonimmunological chronic inflammation is discussed.

PMCID: PMC414950
PMID: 4210331 [PubMed - indexed for MEDLINE]

2671. Johns Hopkins Med J. 1974 Jun;134(6):356-64.

Corticosteroid allergy in a patient with systemic lupus erythematosus.

von Maur K, Rocklin RE, Stevens MB.

PMID: 4610250 [PubMed - indexed for MEDLINE]

2672. Med Klin. 1974 May 17;69(20):915-20.

[Diagnostic possibilities under suspicion of drug allergy].

[Article in German]

Raab W.

PMID: 4603471 [PubMed - indexed for MEDLINE]

2673. Cell Immunol. 1974 Apr;12(1):85-93.

Experimental allergic encephalomyelitis and cellular immunity in the Lewis rat.

Vandenbark AA, Hinrichs DJ.

PMID: 4142415 [PubMed - indexed for MEDLINE]

2674. Cell Immunol. 1974 Mar 30;11(1-3):212-20.

In vitro and in vivo immune responses to homologous myelin basic protein in experimental allergic encephalomyelitis.

Lisak RP, Zweiman B.

PMID: 4141935 [PubMed - indexed for MEDLINE]

2675. J R Coll Gen Pract. 1974 Mar;24(140):171-180.

Incidence of hay fever in a North London practice.

Coffman DA, Chalmers CP.

A practice in North west London consisting of about 6,000 patients, 30 per cent of whom are immigrant, yielded a sample of 77 patients who could be considered to be suffering from hay fever. These were questioned and skin tested.The disease was found to be more prevalent in the immigrant West Indian section of the practice. These patients had not suffered from the disease before arrival in this country but had developed their symptoms after an interval of up to 14 years with an average of about five years. Most of the patients had arrived in this country as adults, and some differences in the pattern of disease from that encountered in the indigenous population were observed. The tendency is for the disease to begin between the age of five and 15 for the indigenous patient, although extreme cases with ages of onset of two and 74 years were found. However, in the immigrant West Indian group the age of onset tended to be between 25 and 45. In addition, the older an immigrant was on arrival in this country the longer hay fever took to develop. It was found that May was stated to be the month of onset of the disease for the indigenous group whereas June tended to be the month of onset for the immigrant group.The sample proved too small to detect any existing patterns in personal or family history, but sex links were found in both response to grass pollen and a personal history of asthma, in that men showed less tendency to asthma whilst proportionately less women than men responded to grass pollen skin tests only.We suggest that a diagnosis of hay fever should be considered in both the young and the elderly who present with recurrent symptoms occurring only in the summer months, of one or more of the following: sneezing, lacrimation, nasal drip, nasal blockage, wheezing, dry throat, or itchy eyes. The diagnosis can readily be confirmed by simple skin testing.

PMCID: PMC2157282
PMID: 4464353 [PubMed - indexed for MEDLINE]

2676. J Clin Invest. 1974 Mar;53(3):735-44.

Cell-mediated immune response of ragweed-sensitive patients to ragweed antigen E. In vitro lymphocyte transformation and elaboration of lymphocyte mediators.

Rocklin RE, Pence H, Kaplan H, Evans R.

The in vivo and in vitro responses to ragweed antigen E were evaluated in 28 untreated atopic patients with ragweed hayfever. The methods employed included direct skin testing, measurement of total serum IgE, measurement of specific IgE anti-ragweed antibodies, leukocyte histamine release, lymphocyte transformation, and release of lymphocyte mediators (migration inhibitory factor and mitogenic factor). The patients could be divided into sensitive and insensitive groups on the basis of their in vitro reactivity to antigen E. 20 patients in the sensitive group had statistically higher levels of total serum IgE, higher levels of specific IgE anti-ragweed antibodies, and greater leukocyte sensitivity as measured by antigen-induced histamine release than did eight patients in the insensitive group. Lymphocytes from sensitive patients produced greater amounts of migration inhibitory factor and mitogenic factor when challenged by antigen E than did lymphocytes from insensitive patients. A possible role for the lymphocyte in this allergic disease is discussed. The results of this study indicate that the immune response to ragweed antigen is complex and involves components of both immediate and delayed hypersensitivity.

PMCID: PMC333054
PMID: 4130213 [PubMed - indexed for MEDLINE]

2677. Vet Clin North Am. 1974 Feb;4(1):91-105.

Bacterial allergy.

Scherago M.

PMID: 4274407 [PubMed - indexed for MEDLINE]

2678. J Med Liban. 1974;27(6):673-8.

Some factors influencing allergy in Lebanon.

Farah FS.

PMID: 4465458 [PubMed - indexed for MEDLINE]

2679. Int Arch Allergy Appl Immunol. 1974;46(2):161-71.

Contact sensitivity in vitro: activation of actively allergized lymphocytes by a beryllium complex.

Jones JM, Amos HE.

PMID: 4206346 [PubMed - indexed for MEDLINE]

2680. Acta Derm Venereol. 1974;54(4):279-81.

The leukocyte migration test in atopic dermatitis.

Uehara M, Ofuji S.

PMID: 4137281 [PubMed - indexed for MEDLINE]

2681. Pol Tyg Lek. 1973 Oct 1;28(40):1536-8.

[Leukocyte migration inhibition tests in the study of streptomycin allergy].

[Article in Polish]

Chyrek-Borowska S, Karna T, Obrzut D.

PMID: 4760707 [PubMed - indexed for MEDLINE]

2682. Infect Immun. 1973 Sep;8(3):349-54.

Cell-mediated immune response in cattle to Mycoplasma mycoides var. mycoides.

Roberts DH, Windsor RS, Masiga WN, Kariavu CG.

The cell-mediated immune response of cattle to Mycoplasma mycoides var. mycoides was studied. Sensitized lymphocytes in blood leukocyte preparations showed a significant degree of antigen-induced transformation, judged by the uptake of tritiated thymidine. The increase in tritiated thymidine uptake in sensitized lymphocytes in the presence of M. mycoides membrane antigen varied from 2- to 13-fold compared with the controls, and this increase in activity was observed from 3 days after artificial infection. Inhibition of leukocyte migration by M. mycoides membrane antigen commenced between 17 and 30 days after infection, and preliminary observations indicate that this test correlated with the intradermal allergic test. M. mycoides-induced unresponsiveness was demonstrated 23 and 30 days after infection. Unresponsiveness, in that the lymphocytes did not respond to phytohemagglutinin, was very marked in two of three animals and partial in the third animal, whereas the humoral antibody response did not appear to be affected. Antigen-induced transformation was demonstrated in only two out of six cattle vaccinated two months previously with M. mycoides T(1) broth culture vaccine, and óne animal only gave a doubtful intradermal allergic reaction. A further six cattle vaccinated 15 months previously were negative to both the leukocyte migration inhibition test and the intradermal allergic test.

PMCID: PMC422855
PMID: 4581007 [PubMed - indexed for MEDLINE]

2683. Isr J Med Sci. 1973 Aug;9(8):1014-21.

Bronchial asthma in an Israel community. A community survey using primary care resources.

Asch AJ, Rabin DL, Hurwitz A, Medalie JH.

PMID: 4753842 [PubMed - indexed for MEDLINE]

2684. J Neurol Neurosurg Psychiatry. 1973 Feb;36(1):139-45.

Experimental allergic neuritis: a new experimental approach.

Sheremata WA, Behan PO.

The technique of macrophage migration, as a specific measure of delayed or cellular hypersensitivity, was applied to the guinea-pig model of experimental allergic neuritis (EAN) to explore the relevance of hypersensitivity to peripheral and central nervous system antigens. Results indicate that in EAN there is hypersensitivity to central as well as peripheral nervous tissue antigens. In contrast, animals with experimental allergic encephalitis showed hypersensitivity only to the central nervous system antigen used. These studies provide further evidence of the role of hypersensitivity to nervous system antigens in the pathogenesis of EAN and provide evidence of a common antigenic component to both peripheral and central nervous system antigens. A preliminary hypothesis is proposed.

PMCID: PMC494288
PMID: 4691686 [PubMed - indexed for MEDLINE]

2685. Br Med J. 1973 Jan 6;1(5844):15-6.

Lymphocyte sensitization in asthma with special reference to nature and identity of intrinsic form.

Caspary EA, Feinmann EL, Field EJ.

Studies of lymphocyte sensitization in patients with asthma showed that the intrinsic and extrinsic forms of the disease fell into two distinct groups. Intrinsic disease showed a general sensitization to a number of non-specific antigens, while the extrinsic form had only slight elevation above normal values. These findings suggest that intrinsic asthma results from a defect of general immunity, whereas extrinsic asthma is a specific sensitization.

PMCID: PMC1588534
PMID: 4567105 [PubMed - indexed for MEDLINE]

2686. Dermatol Monatsschr. 1973 Jan;159(1):16-25.

[Examination technics for the diagnosis of delayed hypersensitivity as demonstrated on the example of experimental allergic contact eczema].

[Article in German]

Sochor H.

PMID: 4712535 [PubMed - indexed for MEDLINE]

2687. Int Arch Allergy Appl Immunol. 1973;45(1):304-7.

Rosette-forming cells and MIF-producing cells in penicillin allergies.

Glauser MP, Frei PC.

PMID: 4542234 [PubMed - indexed for MEDLINE]

2688. J Clin Invest. 1972 Dec;51(12):3216-24.

The Wiskott-Aldrich syndrome. Results of transfer factor therapy.

Spitler LE, Levin AS, Stites DP, Fudenberg HH, Pirofsky B, August CS, Stiehm ER, Hitzig WH, Gatti RA.

12 patients with Wiskott-Aldrich syndrome were treated with therapeutic doses of transfer factor in an attempt to induce cellular immunity. Clinical improvement was noted after transfer factor therapy in 7 of the 12 patients treated. Because this disease has a variable course and temporary spontaneous improvement can occur, the observed improvement cannot necessarily be attributed to the transfer factor. However, in two patients repeated remissions consistently followed transfer factor administration on repeated occasions. This included freedom from infections, regression of splenomegaly, and clearing of eczema. An unexpected finding was a decrease in bleeding in 3 of the 10 patients who had bleeding. Conversion of skin reactivity was obtained in all seven patients who clinically seemed to respond to transfer factor. In vitro studies performed after the administration of transfer factor demonstrated that the lymphocytes of the patients now produced migration inhibitory factor in response to appropriate test antigens, but did not undergo increased radioactive thymidine incorporation in response to the same antigens. A defect in the monocyte IgG receptors has been found in certain patients with the disease, and the current study shows that all patients with defective monocyte IgG receptors responded to transfer factor, whereas only one patient with normal receptors showed any response. This test may thus prove to be useful in predicting the results of transfer factor therapy in patients with Wiskott-Aldrich syndrome, although evaluation of a larger series of patients will be necessary to confirm this point. We conclude that cellular immunity can be induced, that there appears to be clinical benefit in certain patients with Wiskott-Aldrich syndrome by the use of transfer factor, and that this mode of therapy warrents trial in these patients and others with defects of cellular immunity.

PMCID: PMC333003
PMID: 4640955 [PubMed - indexed for MEDLINE]

2689. Clin Allergy. 1972 Dec;2(4):317-23.

Allergenic and biological activities of melittin from honey bee venom.

Mackler BF, Russell AS, Kreil G.

PMID: 4629300 [PubMed - indexed for MEDLINE]

2690. J Clin Invest. 1972 Nov;51(11):2948-58.

Effect of transfer factor on lymphocyte function in anergic patients.

Kirkpatrick CH, Rich RR, Smith TK.

Dialyzable transfer factor, obtained from frozen-thawed peripheral blood leukocytes from a single donor, was given to five anergic patients with chronic mucocutaneous candidiasis. Studies of immunological responses including delayed cutaneous hypersensitivity, in vitro antigen-induced thymidine incorporation, and production of macrophage migration inhibition factor (MIF) were conducted both before and after injection of transfer factor. Before transfer factor, none of the patients had delayed skin responses to any of the natural antigens studied. Their lymphocytes did not produce MIF after exposure to antigens in vitro and only one patient showed increased thymidine incorporation when his lymphocytes were cultured with candida and streptokinase-streptodornase (SK-SD). After injection of transfer factor, four patients developed delayed skin responses to antigens to which the donor was sensitive; no recipient reacted to an antigen to

which the donor was nonreactive. Lymphocytes from recipients produced MIF when cultured with antigens that evoked positive delayed skin tests. Only one patient developed antigen-induced lymphocyte transformation and this response occurred only intermittently. Attempts to sensitize three of the patients with the contact allergen, chlorodinitrobenzene, both before and after transfer factor, were unsuccessful. The fifth patient, a 9-yr old boy with an immunologic profile similar to the Nezelof syndrome, did not become skin test-reactive or develop positive responses to the in vitro tests. These findings suggest that transfer factor acts on the immunocompetent cells that respond to antigens with lymphokine production, but has little, if any, effect on cells that respond to antigens by blastogenesis. The failure to sensitize the subjects with chlorodinitrobenzene illustrates the specificity of the immunologic effects of transfer factor, and implies that it does not function through nonspecific, adjuvant-like mechanisms. Failure of transfer factor to produce positive skin tests or MIF production in a patient with Nezelof's syndrome may be evidence that lymphokine-producing cells are thymus derived.

PMCID: PMC292445
PMID: 5080419 [PubMed - indexed for MEDLINE]

2691. Przegl Dermatol. 1972 Sep-Oct;59(5):677-82.

[Migration inhibition test and its use in studies on delayed allergy].

[Article in Polish]

Moskalewska K.

PMID: 4564918 [PubMed - indexed for MEDLINE]

2692. J Exp Med. 1972 Jul 1;136(1):156-74.

Experimental allergic encephalitis. Dissociation of cellular immunity to brain protein and disease production.

Spitler LE, Von Muller CM, Fudenberg HH, Eylar EH.

The encephalitogenic determinant of brain protein, a nonapeptide having the amino acid sequence Phe-Ser-Trp-Gly-Ala-Glu-Gly-Gln-Lys, has been characterized and synthesized. In a previous study, analogues of this encephalitogenic peptide were synthesized and some were shown to be encephalitogenic while others were not. Guinea pigs were immunized with encephalitogenic peptides having amino acid sequences different from that in the native protein. These guinea pigs did not show cellular immunity in vivo (skin reactivity) or in vitro (lymphocyte stimulation or macrophage migration inhibition) to the encephalitogenic brain protein (EP) although they did show cellular immunity to the immunizing antigenic peptide. Guinea pigs immunized with an encephalitogenic peptide having the same amino acid sequence as the brain protein, or with a nonencephalitogenic peptide having the same amino acid sequence as the native protein but lacking the terminal lysine, did develop cellular immunity to the EP. Animals immunized with EP showed cellular immunity to this protein, but not to the encephalitogenic peptides. Animals immunized with nonencephalitogenic protein (NEP), prepared by altering the tryptophan residue of EP, did not develop disease but did show cellular immunity in vitro and in vivo to the EP. Animals protected from disease by immunization with NEP similarly showed cellular immunity to EP. Thus, the results suggest a dissociation between cellular immunity to EP and the production of experimental allergic encephalitis (EAE). Animals immunized with the encephalitogenic peptides develop EAE, but do not show cellular immunity to EP,

and animals immunized with NEP show cellular immunity to EP but do not develop EAE. A fresh approach to the examination of the pathogenesis of EAE is now possible through the use of these well-characterized antigens.

PMCID: PMC2139197
PMID: 5033419 [PubMed - indexed for MEDLINE]

2693. Nouv Presse Med. 1972 May 20;1(21):1409-13.

[Streptococcal allergy. Correlation between clinical allergologic exploration and the leukocyte migration inhibition test (L.M.T.). Apropos of 68 cases].

[Article in French]

Saurat JH, Husson JM, Paupe J.

PMID: 5036696 [PubMed - indexed for MEDLINE]

2694. Rev Clin Esp. 1972 Apr 15;125(1):63-8.

[Study of drug allergy by in vitro M.I.T. tests (macrophage migration inhibition test].

[Article in Spanish]

Del Rey Calero J, Gonzălez Rodrĭguez-Sa.

PMID: 5036749 [PubMed - indexed for MEDLINE]

2695. J Clin Invest. 1972 Apr;51(4):955-63.

Naturally occurring human antiglobulins with specificity for E.

Williams RC Jr, Griffiths RW, Emmons JD, Field RC.

Human sera have been examined for antibodies with specific reactivity for gammaE using the tanned cell hemagglutination test. Cells tanned with three different gammaE myeloma proteins provided a reproducible test system. Inhibition of agglutination reactions by gammaE proteins, but not by gammaG, gammaA, gammaM, or gammaD confirmed the specificity of these reactions. 8.5% of 304 serial serum samples obtained from miscellaneous hospitalized patients showed clear-cut anti-gamma-globulins with specificity for gammaE. In most of these instances no definite clinical history of concomitant allergic disorders could be obtained. 53% of 73 patients with well-established allergic disorders (hay fever, extrinsic asthma) showed serum anti-gamma-globulins with reactivity for gammaE. Some patients studied before and after desensitization to Bermuda grass allergen showed an increase in titer or a conversion from negative to positive reactions for anti-gammaE antibodies following several month courses of progressive desensitization. Gradient and gel filtration studies indicated that anti-gammaE globulins were 19S gammaM in all instances. No clear correlation was noted between quantitative serum gammaE levels and titer of anti-gammaE antibodies.19S serum fractions with anti-gammaE antibody activity did not release histamine from normal human peripheral blood leukocytes, whereas specific rabbit anti-gammaE antisera consistently induced leukocytic histamine release. Moreover, macroglobulin fractions with anti-gammaE activity did not block allergen-specific leukocyte histamine release induced by in vitro leukocyte challenge with allergens such as Bermuda grass and leukocytes from allergic donors. In some

instances 19S human serum fractions with anti-gammaE activity appeared to potentiate histamine release when incubated concomitantly with specific allergen and leukocytes from allergic individuals.

PMCID: PMC302209
PMID: 4111367 [PubMed - indexed for MEDLINE]

2696. Ann Genet. 1972 Mar;15(1):55.

[Study of drug allergy by in vitro M.I.T. tests (macrophage migration inhibition test].

Del rey Calero J, González Rodríguez-Sa.

PMID: 4537616 [PubMed - indexed for MEDLINE]

2697. Hautarzt. 1972 Feb;23(2):75-8.

[Comparison of cell migration in the Rebuck test in healthy subjects and patients with contact eczema before and during epicutaneous patch testing].

[Article in German]

Piroth M.

PMID: 4551934 [PubMed - indexed for MEDLINE]

2698. Med Dosw Mikrobiol. 1972;24(3):173-80.

[Macrophage migration inhibition test as a method for detecting tuberculin allergy in mice].

[Article in Polish]

Sankowski A, Kwiek S, Wyszomirska J.

PMID: 4653975 [PubMed - indexed for MEDLINE]

2699. Arch Dermatol Forsch. 1972;244:312-7.

[Immunocyte reactions in vitro in allergic eczema].

[Article in German]

Klaschka F, Nymphius M.

PMID: 4567629 [PubMed - indexed for MEDLINE]

2700. Clin Exp Immunol. 1971 Oct;9(4):449-65.

The production of lymphocyte mitogenic factor and migration-inhibition factor by antigen-stimulated lymphocytes of subjects with grass pollen allergy.

Maini RN, Dumonde DC, Faux JA, Hargreave FE, Pepys J.

PMCID: PMC1713015
PMID: 5123223 [PubMed - indexed for MEDLINE]

2701. Ann Allergy. 1971 Oct;29(10):510-24.

Environmental problems and the allergist.

McKee WD.

PMID: 4106536 [PubMed - indexed for MEDLINE]

2702. Pol Arch Med Wewn. 1971 Sep;47(3):211-6.

[Our modification of leukocyte migration test and attempt at applying it to the diagnosis of drug allergy].

[Article in Polish]

Chyrek-Borowska S, Sydor A, Sydor U.

PMID: 5116353 [PubMed - indexed for MEDLINE]

2703. J Reprod Fertil. 1971 Aug;26(2):161-6.

Demonstration in vitro of delayed hypersensitivity in experimental allergic orchitis in guinea-pigs.

Mazzolli AB.

PMID: 5558404 [PubMed - indexed for MEDLINE]

2704. Bull Soc Pathol Exot Filiales. 1971 Jul-Aug;64(4):486-98.

[Mobile recurrent linear urticaria during strongyloidiasis. Cure with thiabendazole].

[Article in French]

Thomas J, Renambot J, Barbotin M, Rigaud J, Saint-André P.

PMID: 5172372 [PubMed - indexed for MEDLINE]

2705. Immunochemistry. 1971 Mar;8(3):281-7.

Tartrazine: quantitative passive hemagglutination studies on a food-borne allergen of small molecular weight.

Johnson HM, Peeler JT, Smith BG.

PMID: 5150656 [PubMed - indexed for MEDLINE]

2706. Int Arch Allergy Appl Immunol. 1971;41(1):25-35.

Significance of leucocyte chemotaxis in allergic reactions.

Sorkin E, Stecher VJ.

PMID: 5560977 [PubMed - indexed for MEDLINE]

2707. Z Immunitatsforsch Allerg Klin Immunol. 1971;141(2):181-9.

The problem of reproducibility of migration inhibition of peripheral leucocytes in cases of tuberculin-allergy.

Alzer G, Schumacher K, Hirschmann WD, Oerkermann H.

PMID: 4251115 [PubMed - indexed for MEDLINE]

2708. Adv Biol Skin. 1971;11:95-101.

Roles of haptens and carriers in delayed allergies.

Salvin SB.

PMID: 4124146 [PubMed - indexed for MEDLINE]

2709. Rev Sanid Hig Publica (Madr). 1970 Dec;44(12):1307-18.

[Macrophage migration inhibition test (M.I.T) in experimental allergic encephalitis].

[Article in Spanish]

Del rey Calero J, Calbo Torrecillas F, Otero Puime AA, Gonzalez R-Salinas MC.

PMID: 5525220 [PubMed - indexed for MEDLINE]

2710. J Wildl Dis. 1970 Oct;6(4):266-71.

Nematodes transmitted to man by fish and aquatic mammals.

Myers BJ.

Division of Microbiology and Infectious Diseases, Southwest Foundation for Research and Education, San Antonio, Texas, USA.

Zoonotic nematodes may cause disease in man through migrating larva (larva migrans), through direct infection or possibly through allergic responses. The parasitic genera Ancylostoma, Uncinaria, Bunostomum and Toxocara can cause larva migrans. The cod worm (Phocanema decipiens) a parasite found in fish and seals, can infect man, as can Anisakis, Dioctophyme renale and Gnathostoma hispidium larvae obtained from eating raw fish.

PMID: 16512125 [PubMed - indexed for MEDLINE]

2711. Acta Pathol Microbiol Scand A. 1970;78(6):625-48.

Experimental allergic thyroiditis in the guinea-pig. A light, fluorescence and electron microscopic study, with particular reference to the migration of

lymphocytes through the vessel walls.

Kåresen R.

PMID: 5499386 [PubMed - indexed for MEDLINE]

2712. Int Arch Allergy Appl Immunol. 1970;38(3):230-45.

Influence of contact allergy on thymus lymphoid cell migration.

Linna TJ.

PMID: 5416020 [PubMed - indexed for MEDLINE]

2713. Z Immunitatsforsch Exp Klin Immunol. 1970;141(1):14-9.

Delayed hypersensitivity studies in experimental allergic encephalomyelitis; a comparison of macrophage migration inhibition tests and electrophoretic mobility.

Hughes D, Caspary EA, Field EJ.

PMID: 4251430 [PubMed - indexed for MEDLINE]

2714. J Allergy. 1970 Jan;45(1):30-42.

Elevated levels of gamma-E (IgE) in conditions other than classical allergy.

Heiner DC, Rose B.

PMID: 4188613 [PubMed - indexed for MEDLINE]

2715. J Immunol. 1969 Jun;102(6):1431-6.

Inhibition of in vitro cell migration in experimental allergic encephalomyelitis.

Rauch HC, Ferraresi RW, Raffel S, Einstein ER.

PMID: 5785348 [PubMed - indexed for MEDLINE]

2716. Int Arch Allergy Appl Immunol. 1969;35(1):47-57.

Bone marrow cell migration to peripheral lymph nodes and skin in contact allergic guinea pigs.

Lidén S, Linna TJ.

PMID: 5774348 [PubMed - indexed for MEDLINE]

2717. Schweiz Med Wochenschr. 1968 Oct 19;98(42):1655-6.

[Value of the in-vitro lymphoid cell migration inhibition test as applied to the diagnosis of medicamentous allergies].

[Article in French]

Girard JP.

PMID: 5698344 [PubMed - indexed for MEDLINE]

2718. Experientia. 1968 Jun 15;24(6):589-90.

Migration of lymphocytes through the endothelium of venules in experimental allergic neuritis.

Aström KE.

PMID: 5697740 [PubMed - indexed for MEDLINE]

2719. Int Arch Allergy Appl Immunol. 1968;34(3):237-56.

Lymphocyte sensitivity to encephalitogenic factor in guinea pigs with experimental allergic encephalomyelitis as shown by in vitro inhibition of macrophage migration.

Hughes D, Newman SE.

PMID: 5681104 [PubMed - indexed for MEDLINE]

2720. Int Arch Allergy Appl Immunol. 1968;33(1):45-58.

Inhibition of macrophage migration in vitro by brain and encephalitogenic factor in allergic encephalomyelitis.

Hughes D, Field EJ.

PMID: 5637124 [PubMed - indexed for MEDLINE]

2721. Trans St Johns Hosp Dermatol Soc. 1967;53(2):154-9.

Leucocytic migration in vitro as an indicator of allergy in eczematous contact dermatitis.

Nordqvist B, Rorsman H.

PMID: 4231607 [PubMed - indexed for MEDLINE]

2722. Glas Srp Akad Nauka Med. 1966;(19):21-5.

[Allergic manifestations in the population of Zmajevo (of natives and immigrants from Serbia, Monte Negro, Lika, Bosnia and Hercegovina].

[Article in Serbian]

Spuzić V, Mojović M, Ljaljević J, Dordević S, Milosević M, Jancić M.

PMID: 6000021 [PubMed - indexed for MEDLINE]

2723. J Occup Med. 1966 Jan;8(1):21-3.

Prevention of allergic immunization reactions.

Kawchak J.

PMID: 5900460 [PubMed - indexed for MEDLINE]

2724. J Exp Med. 1965 Dec 1;122(6):1161-71.

In vitro demonstration of cellular sensitivity in allergic encephalomyelitis.

David JR, Paterson PY.

Peritoneal cells obtained from animals exhibiting delayed hypersensitivity are inhibited from migrating in vitro by specific sensitizing antigen. This test for the detection of delayed hypersensitivity was applied to the problem of cellular sensitivity in allergic encephalomyelitis (AE). The migration of peritoneal cells obtained from guinea pigs with AE was inhibited specifically by nervous tissue antigens. The specificity of this reaction was further studied. Neonatal rat nervous tissue, which was shown to lack the encephalitogenic antigen, i.e. did not produce AE when injected with complete adjuvant into guinea pigs and rats, did not inhibit the migration of cells from animals with AE. Adult rat nervous tissue, which readily produces AE, and thus contains the encephalitogenic antigen did inhibit the migration of such cells. The finding that cells from animals with AE display hypersensitivity which appears to be directed specifically to the encephalitogenic antigen strongly supports the view that such cells could play an important role in the pathogenesis of this disease.

PMCID: PMC2138099
PMID: 5873682 [PubMed - indexed for MEDLINE]

2725. Minerva Med. 1965 Sep 26;56(77):3283-9.

[Transient and migrant pulmonary atelectasis in the course of bronchial asthma].

[Article in Italian]

Sticotti S.

PMID: 5837028 [PubMed - indexed for MEDLINE]

2726. Z Haut Geschlechtskr. 1965 Jul 15;39(2):83-8.

[Phlebitis migrans and its relation to anthropozoonoses. (A contribution to vascular allergids)].

[Article in German]

Michel E.

PMID: 5891327 [PubMed - indexed for MEDLINE]

2727. Med Interna (Bucur). 1964 Dec;16:1431-7.

[THE ROLE OF INFECTIOUS ALLERGY IN THE GENESIS OF SOME TYPES OF RECURRENT AND MIGRANT PHLEBITIS. THERAPEUTIC IMPORTANCE].

[Article in Romanian]

PAUNESCUPODEANU A, MOISE O.

PMID: 14305635 [PubMed - indexed for MEDLINE]

2728. J Bacteriol. 1964 May;87(5):1114-7.

Metabolism of mannitol by Coccidioides immitis.

Lones GW, Peacock C.

Lones, George W. (National Institute of Allergy and Infectious Diseases, U.S. Public Health Service, Bethesda, Md.), and Carl Peacock. Metabolism of mannitol by Coccidioides immitis. J. Bacteriol. 87:1114-1117. 1964.-Strain M-11 of Coccidioides immitis was found to utilize mannitol for growth in the mycelial form but not in the spherule form. Cell-free extracts of both forms, grown on glucose, were capable of reducing nicotinamide adenine dinucleotide with mannitol-1-PO(4) but not with mannitol. The extracts accomplished a rapid oxidation of reduced nicotinamide adenine dinucleotide by fructose-6-PO(4), the expected product of mannitol-1-PO(4) oxidation. Fructose was inactive. Paper electrophoresis and chromatography with several solvent systems demonstrated a substance in extracts of both mycelium and spherules having a migration consistent with that of mannitol.

PMCID: PMC277154
PMID: 4289442 [PubMed - indexed for MEDLINE]

2729. Klin Monbl Augenheilkd Augenarztl Fortbild. 1962 Dec;141:653-62.

[Phlebitis migrans with ocular involvement (a case report on the corticosteroid treatment of allergic-hyperergic vascular diseases)].

[Article in German]

NEUBAUER H, RODENHAEUSER JH.

PMID: 13938126 [PubMed - indexed for MEDLINE]

2730. JAMA. 1962 May 5;180(5):355-8.

Long-term administration of norethindrone in fertility control.

Rice-wray E, Schulz-contreras M, Guerrero I, Aranda-rosell A.

PIP: Fertility control by cyclic norethindrone (Norlutin), 17 alpha-ethinyl 19-nortestosterone, plus .06 mg 3-methoxy ethinyl estradiol (Ortho-Novum) was studied in 364 women over a period of 32 months for a total of 6062 cycles. No patient who followed the instructions became pregnant. 37 patients stopped the medication for various reasons. The interval between stopping medication and becoming pregnant averaged 1.6 months. 13 of these pregnancies occurred after 11-15 cycles of treatment. Children born to these mothers were normal with no virilization observed. Findings from all Papanicolaou smears and cervical biopsies were normal. The desirable effects of diminishing the menstrual flow, reducing dysmenorrhea and regulating the menstrual cycle, plus the all-important one of contraception, far outweighed minimal and infrequent undesirable side

effects (in order of frequence: chloasma, hot flashes, headache, nausea, acne, abdominal pain, dizziness and urticaria). In only 4.8% of the total 6062 cycles was some complaint made.
PMID: 12275504 [PubMed - indexed for MEDLINE]

2731. J Allergy. 1960 Mar-Apr;31:175-80.

Quantitative measurement of the migration of intracutaneously injected cottonseed allergen in passive transfer studies.

SPIES JR, BERNTON HS, CHAMBERS DC.

PMID: 13833328 [PubMed - indexed for MEDLINE]

2732. Ann Allergy. 1959 Jul-Aug;17:596-601.

The effect of migration to Miami, Florida on allergic respiratory disease.

GITTELSON G.

PMID: 13828233 [PubMed - indexed for MEDLINE]

2733. J Exp Med. 1958 Feb 1;107(2):319-32.

Tissue culture studies on bacterial allergy in experimental brucellosis. I. The effect of Brucella suis whole antigen on cultures of spleen from normal and brucella-infected guinea pigs.

HEILMAN DH, HOWARD DH, CARPENTER CM.

Tissue culture methods have been used to investigate infectious allergy in experimental brucellosis. A study was made of the effect of whole cell Brucella antigen on cultures of spleen from normal and Brucella-infected guinea pigs. The degree of toxicity was based upon the inhibition of migration of wandering cells and upon the morphologic appearance of stained sections of tissue cultures at different periods of incubation. A suspension of heat-killed Br. suis was more toxic for splenic cells from guinea pigs infected with Br. suis than for normal splenic cells. Macrophages were more sensitive than leucocytes to the toxic action of the antigen. The degenerative changes observed in Brucella-sensitive cells exposed to the antigen were similar to the degeneration previously observed in cultures of tuberculin-sensitive cells in the presence of tuberculin. The specific toxicity of the whole Brucella antigen, however, was more marked than that of tuberculin. Preliminary experiments indicate that serum and plasma containing specific antibodies obtained from Brucella-infected guinea pigs reduce the toxic effect of the antigen in cultures of both normal and Brucella-sensitive cells. The protective action of the homologous antiserum was greater for Brucella-sensitive cells than for normal cells.

PMCID: PMC2136791
PMID: 13491765 [PubMed - OLDMEDLINE]

2734. Harefuah. 1954 Feb 15;46(4):78-80.

[Asthma among new immigrants from Iraq].

[Article in Undetermined Language]

SOMEKH E.

PMID: 13162320 [PubMed - OLDMEDLINE]

2735. J Am Med Assoc. 1954 Jan 30;154(5):412.

MIGRATION for relief from allergies.

[No authors listed]

PMID: 13117632 [PubMed - indexed for MEDLINE]

2736. Am J Clin Pathol. 1953 Dec;23(12):1208-15.

Allergic granulomatosis associated with visceral larva migrans; case report with autopsy findings on Toxocara infection in a child.

BRILL R, CHURG J, BEAVER PC.

PMID: 13104359 [PubMed - OLDMEDLINE]

2737. J Exp Med. 1950 Feb;91(2):197-218, pl.

Studies on the virulence of tubercle bacilli; isolation and biological properties of a constituent of virulent organisms.

BLOCH H.

The bacillary cords characteristic for virulent tubercle bacilli are readily disrupted when wet bacilli are suspended in hydrocarbons such as paraffin oil or petroleum ether. The disruption of cords is due to the removal of a material coating the surface of the bacilli and causing them to adhere to each other. This material can be obtained from virulent bacilli by extracting them with petroleum ether. It is a lipid. Avirulent variants of tubercle bacilli do not yield a similar material after extraction in the same manner; only little of it is obtained from BCG bacilli. The following properties of the fraction obtained by petroleum ether extraction are described: (a) It inhibits the migration of leukocytes in vitro. (b) If repeatedly injected in small doses into mice, it is toxic, whereas a single high dose does not give rise to toxic manifestations. (c) The susceptibility of mice to the toxic action of repeated injections parallels to some extent their degree of susceptibility to infection with the strain of tubercle bacilli from which the fraction was obtained. (d) The injection of the extracted material into guinea pigs does not induce a state of allergic reactivity toward tuberculin. Likewise, tuberculin-positive guinea pigs do not show hypersensitivity against injections of the extracted substance. Bacilli extracted with petroleum ether do not lose their viability. They grow out normally in vitro, and they are still pathogenic. However, the removal of the petroleum ether-soluble lipid from the bacilli results in a loss of the ability of the organisms to inhibit the migration of polymorphonuclear leukocytes. Moreover, mice and guinea pigs infected with extracted bacilli may develop tuberculosis considerably slower than animals injected with comparable amounts of unextracted organisms. The significance of these findings is discussed in relation to the problem of the virulence of tubercle bacilli.

PMCID: PMC2135951
PMID: 15404225 [PubMed - indexed for MEDLINE]
